# Application of a joint latent space item response model to clustering stressful life events and the Beck Depression Inventory-II: results from Korean epidemiological survey data

**DOI:** 10.4178/epih.e2022093

**Published:** 2022-10-24

**Authors:** Hyun Joo Kim, Ye Jin Jeon, Hyeon Chang Kim, Ick Hoon Jin, Sun Jae Jung

**Affiliations:** 1 Department of Applied Statistics, Yonsei University, Seoul, Korea; 2 Department of Statistics and Data Science, Yonsei University, Seoul, Korea; 3 Department of Public Health, Graduate School, Yonsei University, Seoul, Korea; 4 Department of Preventive Medicine, Yonsei University College of Medicine, Seoul, Korea; 5 Department of Epidemiology, Harvard T. H. Chan School of Public Health, Boston, MA, USA

**Keywords:** Stressful life event, Depression, Joint latent space item response model

## Abstract

**OBJECTIVES:**

According to previous findings, stressful life events (SLEs) and their subtypes are associated with depressive symptoms. However, few studies have explored potential models for these events and incidental symptoms of depression.

**METHODS:**

Participants (3,966 men; 5,709 women) were recruited from the Cardiovascular and Metabolic Diseases Etiology Research Center cohort. SLEs were measured using a 47-item Life Experiences Survey (LES) with a standardized protocol. Depressive symptoms were assessed using the Beck Depression Inventory-II (BDI-II). Joint latent space item response models were applied by gender and age group (<50 vs. ≥50 years old).

**RESULTS:**

Among the LES items, death or illness of close relatives, legal problems, sexual difficulties, family relationships, and social relationships shared latent positions with major depressive symptoms regardless of gender or age. We also observed a gender-specific domain: occupational and family-related items.

**CONCLUSIONS:**

By projecting LES and BDI-II data onto the same interaction map for each subgroup, we could specify the associations between specific LES items and depressive symptoms.

## GRAPHICAL ABSTRACT

[Fig f3-epih-44-e2022093]

## INTRODUCTION

Depression is a common mental illness that severely limits psychosocial functioning and increases the risk of morbidity after midlife [1]. Depressive disorder in the middle-aged or older population is a critical public health issue from epidemiological, clinical, and social perspectives. Depressive symptoms among members of the public can be assessed with sensitivity using indices developed for the general population level, such as the Beck Depression Inventory-II (BDI-II) [2-4], the Center for Epidemiological Studies Depression Scale [5], and the Patient Health Questionnaire-9 [6].

Stressful life events (SLEs) are risk factors for major depression during one’s lifetime [7,8]; these include physical illness, financial problems, workplace stressors, and changes in family members or job status [9-14]. A greater number of SLEs is associated with a higher prevalence of depressive symptoms and major depressive episodes. A large population-based Chinese study (n= 512,891) showed a strong association between SLEs and major depressive episodes. In the same study, family-related events were particularly strongly associated with depressive symptoms, and the interaction with gender was statistically significant [14]. In a study conducted among Iranian community-dwelling adults (n = 4,763), the social stressors domain (including financial problems, social relations, and personal and job conflicts) was associated with depression [9]. While SLEs are positively associated with depression overall, each dimension of SLE items may be differently associated with distinct subdomains of depression symptoms. Hence, it is necessary to verify the unique associations between SLEs and depression subdomains.

Other considerations regarding SLEs and depressive symptoms include gender [14-17] and age [18,19]. In a previous study, women reported more interpersonal events such as housing problems, difficulties getting along with individuals in their personal networks, and crises involving those individuals, whereas men reported more legal and work-related events [16]. This gender-based difference in SLEs could at least partially explain the greater prevalence of depression in women than in men [20]. Although less epidemiological evidence exists regarding age-based differences, the exposure rate to SLEs is expected to increase with age [21,22].

Here, we used a joint latent space item response model (LSIRM) to project SLEs and depressive symptoms as measured by the BDI-II onto an interaction map. This map exploratively represents the interaction of BDI-II items and underlying traits with each SLE, while accounting for heterogeneity by gender and age group.

### Open practices statement

This research involved data from the Cardiovascular and Metabolic Diseases Etiology Research Center (CMERC) study. The CMERC data are not publicly accessible, but requests for these data can be sent to the corresponding author. The questionnaires used are included in the Supplementary Material 1 associated with this article. The code for the study is not publicly accessible. Preregistration information for the CMERC study can be found at cris.nih.go.kr (study registration No. KCT0001038).

## MATERIALS AND METHODS

### Study participants

The CMERC study is a multicenter study exploring risk factors for cardiovascular and metabolic diseases. The study was designed to include a community-based low-risk population and a hospitalbased high-risk population (CMERC-HI). The community-based low-risk population (n= 8,697) was recruited mainly through advertisements in regional newspapers, promotional posters in public areas, or the acquaintances of other study participants at Yonsei University College of Medicine (in Seoul) and Ajou University School of Medicine (in Suwon). The high-risk cardiovascular patients (aged 20-75 years with hypertension, diabetes mellitus, renal disease with dialysis, asymptomatic peripheral arterial disease, and/or relatives with a history of acute myocardial infarction) were recruited at Severance Hospital (Seoul, n= 3,267) using promotional posters in public areas around the hospital. Participants in the community-based low-risk study met the following criteria: (1) aged 30-64 years; (2) community-dwelling; (3) able to articulate their own opinions regarding study participation; (4) no medical history of cancer (within 2 years), myocardial infarction, stroke, or heart failure; and (5) not pregnant. In accordance with the study protocol, health-related measurements were taken in the following order: anthropometric measurement, fasting blood sample collection, blood pressure measurement, and standardized health questionnaire (including demographic characteristics, socioeconomic status, medical history, health behaviors, and psychological condition).

A total of 11,964 participants were enrolled between December 2013 and June 2018. Details of this cohort study have been described in a previous cohort profile [23]. Of the total population, we excluded young (aged 20-29 years) and older (aged 65+ years) adults as well as those who did not respond to the psychological questionnaires (n= 2,709). We included 9,675 participants in the final analysis.

### Measurements of stressful life events and depression

Depressive symptoms were assessed using the Korean version of the BDI-II, constructed using a 21-item multiple-choice self-report inventory. Each question was marked on a 4-point Likert scale ranging from 0 to 3, with higher scores indicating more severe symptoms. The validity of the Korean version was verified in previous studies (area under the curve, 0.93). A total score of 0 to 13 is interpreted as minimal depression, 14 to 19 as mild, 20 to 28 as moderate, and 29 to 63 as severe [24]. In this study, the reliability of the BDI-II was found to be good (Cronbach alpha, 0.883).

SLEs from the past 6 months were measured using the Korean version of the Life Experiences Survey (LES) questionnaire, which consists of 47 possible life events, including marriage, divorce, death of family members or friends, problems related to housing and economic circumstances, lifestyle changes, and educational or occupational successes and failures [25]. Although no previous validation study has been conducted in a Korean population, the reliability of the LES questionnaire was found to be acceptable in the present study (Cronbach alpha, 0.702).

### Statistical analysis

To present the baseline characteristics according to gender, we used the chi-square test for categorical variables and analysis of variance (F test) for continuous variables (Supplementary Material 1). To confirm heterogeneity between study centers, we also used the chi-square test and the F test (Supplementary Materials 2 and 3). In addition, we applied the joint LSIRM for each subgroup by study center to observe the heterogeneity of item interactions between centers (Supplementary Material 4).

The LSIRM is a network approach model used for analyzing binarized item response data [26]. It was designed to alleviate the conditional independence assumptions for items and respondents and the homogeneity-independence assumption defined in the traditional item response model [27]. We observed the interactions within items of the LES and the BDI-II individually by applying a separate LSIRM to the LES item response data and the BDI-II item response data for all 4 subgroups (Supplementary Material 5). However, it was impossible to observe the interactions between 2 different item sets (LES and BDI-II), even with common respondents. Therefore, to further observe the interactions between LES and BDI-II items answered by common respondents, we developed a new model, termed the joint LSIRM. We applied this model to estimate the dependent structures between respondents and LES items, respondents and BDI-II items, and LES and BDI-II items. The interactions between LES and BDI-II items were embedded in a common interaction map for each subpopulation group. To fit the LSIRM and the joint LSIRM, each BDI-II item was recorded as a dichotomous variable (0 as no symptoms; 1-3 as symptoms present).

The joint LSIRM was extended from the LSIRM for application to 2 different sets of binary item response data answered by common respondents. By embedding the latent positions for 2 different sets of items and respondents in a common interaction map, we can directly observe the interactions between the LES and BDI-II items. The joint LSIRM with 2 different sets of item response data and common respondents is expressed using the formula:


$$\mathrm{logit}\left\{\pi_{ki_{1}}\right\}=\mathrm{logit}\left\{y_{ki_{1}}=1\right\}=\beta_{i1}+\theta_{1k}-||w_{i1}-z_{k}||,\;\mathrm{logit}\left\{\pi_{ki_{2}}\right\}=\mathrm{logit}\left\{y_{ki_{2}}=1\right\}=\beta_{i2}+\theta_{2k}-||w_{i2}-z_{k}||.$$

The likelihood is as follows:


$$L\left(Y_{1},Y_{2}|Z,W_{1},W_{2},\Theta_{1},\Theta_{2},\beta_{1},\beta_{2}\right)=\prod_{k=1}^{n}\left\{\prod_{i_{1}=1}^{p_{1}}\pi_{ki_{1}}^{y_{ki_{1}}}{\left(1-\pi_{ki_{1}}\right)}^{1-y_{ki_{1}}}\right\}\left\{\prod_{i_{2}=1}^{p_{2}}\pi_{ki_{2}}^{y_{ki_{2}}}{\left(1-\pi_{ki_{2}}\right)}^{1-y_{ki_{2}}}\right\}$$

In the formula, *θ*_1_ and *β*_1_ represent the person and item main effects, respectively, on the probability of a positive response for the LES items, and *θ*_2_ and *β*_2_ represent the person and item main effects, respectively, on the probability of a positive response for the BDI-II items. The coefficients of both the LSIRM and the joint LSIRM are interpreted as the tendency to be answered positively for *β* and the tendency to answer the item positively for *θ*. The interpretation of the coefficients can be flexible, according to the specific details of various applications, as long as it is applied to binary item response data. The latent positions for the 2 sets of items and common respondents are estimated by the parameters *w*_1_, *w*_2_, and *z*, respectively, and are embedded in a common interaction map. The details of this approach are described in Supplementary Material 6. After generating the estimated interaction map for each group, we matched the 4 interaction maps using the Procrustes matching algorithm to locate the SLE and BDI-II items in a similar quadrant across groups [28]. The joint LSIRM can accommodate 2 different sets of item response data, and extension is possible for a larger number of sets.

In the main analysis, we conducted subgroup analyses by gender and age group. Since the median value of the age distribution was 50 years, participants were classified into younger (< 50 years) and older (≥ 50 years) groups. The latent positions for the younger men group were chosen as the baseline, and those for the other groups were adjusted accordingly. Then, we applied a *k*-means clustering method to each interaction map to identify which LES and BDI-II items were located close together. For the *k*-means clustering method, the optimal number of clusters was determined using the elbow method and silhouette analysis. The elbow method involves measuring the within-cluster sum of squares of the k number of clusters for each subgroup. In addition, we conducted a silhouette analysis, which involves measuring silhouette scores for the k number of clusters. These scores are the averages of silhouette coefficients, representing the similarities of each data point by comparing its within-cluster to the other clusters [29]. The optimal numbers of clusters were selected as 5 and 4 for the men and women groups, respectively, based on the elbow point and the highest silhouette coefficient. The degree of interaction between the items can be assessed by the Kullback-Leibler divergence of the distance distribution of the corresponding items [30] (Supplementary Material 7). We performed the analysis using SAS version 9.5 (SAS Institute Inc., Cary, NC, USA) and R version 3.6.3 (Rcpp package; R Foundation for Statistical Computing, Vienna, Austria). We used 2-sided p-values with an α= 0.05 threshold indicating statistical significance.

### Ethics statement

All participants provided written informed consent, and the study protocol was approved by the Institutional Review Boards of Severance Hospital, Yonsei University Health System (4-2013-0661, 4-2013-0581), and Ajou University Hospital (AJIRBBMRSUR-13-272).

## RESULTS

Characteristics of the 9,675 study participants are shown in Supplementary Material 1. The participants were predominantly women (59.0%) and people older than 50 years (65.6%). All general characteristics were distributed differently by gender (p<0.001). First, we identified interactions among LES items and BDI-II items separately by individually applying an LSIRM to these sets of items by gender and age (Supplementary Material 5). In the LES interaction maps for all 4 age-based and gender-based subgroups, the items for similar SLEs have similar positions, with 5 clusters in men and 4 clusters in women. In men, the identified clusters were “family/living” (cluster 1), “finance/work” (cluster 2), “separation” (cluster 3), “somatic” (cluster 4), and “other” (cluster 5). The clusters were similar in women, except the men’s clusters 3 and 4 were merged into “separation/somatic” (cluster 3), leaving “other” as cluster 4 for women. In the BDI-II interaction maps for the 4 subgroups, cognitive and somatic depressive symptoms were located close to each other. BDI-II items related to cognitive depressive symptoms include guilty feelings, punishment feelings, and worthlessness, while those related to somatic depressive symptoms are loss of energy, changes in sleep, and changes in appetite.

**Figure 1.**
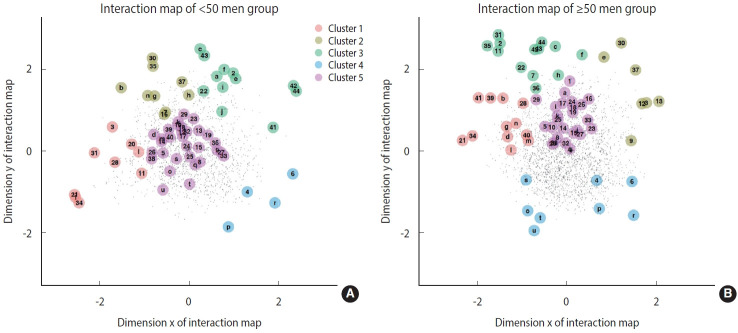
The numbers and alphabet letters represent the latent position of items of stressful life events and Beck Depression Inventory-II, respectively. The dots represent the latent position of respondents. The color of each circle around the numbers and letters represents the corresponding cluster.

By applying the joint LSIRM, we could additionally analyze which LES items related to which specific depressive symptoms by gender and age via each interaction map consisting of both sets of items (Tables 1 and 2). In the younger men group (< 50 years) (Figure 1A), clusters 1, 3, and 5 contained most of the BDI-II items. This implies that changes in working situations and familial and conjugal states have the strongest impact on the state of depression in younger men. We investigated the Euclidean distances between the latent positions of BDI-II items and the centroid positions of each cluster’s SLE items to quantify the interactions. The average distance between the finance/work cluster of the SLE items and the BDI-II items representing self-criticism was shorter than the average distance between pairs of other clusters of BDIII and SLE items, indicating that the interaction between these 2 groups is stronger than the interactions between other group pairs. This implies that older men are more likely to experience depressive feelings when they undergo stressful finance- and work-related events than in other types of situations.

In the older men group (Figure 1B), clusters 1 and 5 contained BDI-II items that represent a strong sense of depression. This implies that changes in familial and living states and separation have the strongest impact on the state of depression in older men. The interactions between BDI-II items representing cognitive symptoms (past failures, punishment feelings, and self-criticalness) and cluster 1 of the SLE items were particularly strong, as indicated by their corresponding distances. This indicates that older men tend to feel more depressive in response to stressful events regarding family/living states and separation than with other situations.

**Figure 2.**
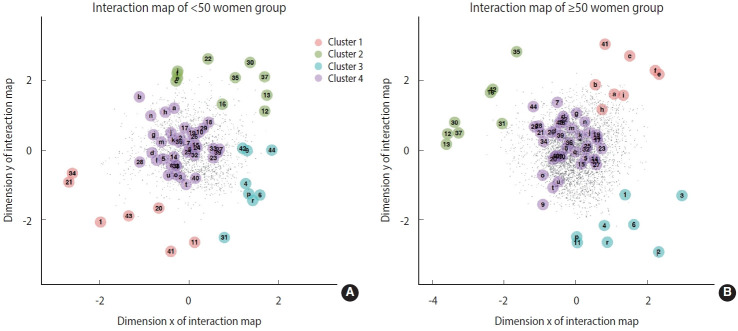
The numbers and alphabet letters represent the latent position of items of stressful life events and Beck Depression Inventory-II, respectively. The dots represent the latent position of respondents. The color of each circle around the numbers and letters represents the corresponding cluster.

In the younger women group (Figure 2A), most of the BDI-II items were located near the center of the interaction map, implying that interactions between depressive symptoms and SLEs are similar by type of SLEs. However, SLE items regarding loss of , illness of, and conflicts with family members, as well as those related to financial states, had particularly strong interactions with the manifestation of depression in younger women.

In the older women group (Figure 2B), most of the BDI-II items were near clusters 1 and 4 and separated from clusters 2 and 3. This implies that changes in familial and conjugal relationships affect depression in older women more than other events, such as changes in one’s working situation. For example, both cognitive and somatic BDI-II items are particularly close to the SLE items related to changes in familial and conjugal states, such as the gain of a new family member and conflict with partners and relatives.

Overall, negative experiences regarding financial state seem to affect depression regardless of gender and age. The SLE items related to financial states, such as seizure of property, were relatively near the BDI-II items in the interaction maps for all 4 groups. Similarly, familial and conjugal states were found to relate to depression to a similar extent regardless of subgroup.

In the 2 men groups, the SLE items farthest from the BDI-II items primarily related to changes in sleep and appetite. The younger and older groups differed in the distance between the working situation-related and BDI-II items. The corresponding items were closer together in the younger men group than in the older group, indicating that the younger men were more likely to experience depression in relation to their working situations than the older men.

In the 2 women groups, the SLE items farthest from the BDI-II items primarily related to changes in working situation, such as getting fired and changing jobs. However, the distance between working situation-related and BDI-II items was shorter in the interaction map for the younger women than in that for the older women. This can be verified by the Kullback-Leibler divergence between the distance distributions of the 2 groups (Supplementary Material 7). This finding implies that although changes in familial and conjugal states most strongly impact depression in women in general, younger women were more likely to experience depression in relation to their working situations than older women.

## DISCUSSION

In this study, we proposed a novel analysis framework to observe the interaction between SLEs and depressive symptoms across gender-based and age-based subgroups. We applied a network approach model, the joint LSIRM, to generate an interaction map. The results indicate that in general, symptoms of depression are closely related to SLEs regarding changes in financial, familial, and conjugal states.

According to previous research on the factor structure for the measurement of depressive symptoms, this factor structure and included items can differ by study population. An earlier study by the present authors was conducted using a subset of the CMERC study (n= 1,273; aged 30-64 years with multiplex markers) and included BDI-II factor analysis. As a result, a 2-factor solution was suggested, and the BDI-II items were divided into somaticaffective and cognitive items [3]. In a meta-analysis of the BDI-II factor structure (n= 20) [2], most of the studies reported the same 3-factor structure under the exploratory/confirmatory factor analysis. The BDI-II items were divided into different dimensions according to the target population of the study, with some studies suggesting 2-factor solutions (cognitive and somatic-affective or cognitive-affective and somatic). Furthermore, most findings were derived from relatively small populations (n< 1,000) with a specific qualification (patients with depression or other mental health disorders, or university students) and did not consider age or gender differences. In a previous United States study including undergraduate students (n= 7,369), the factor loadings were invariant between men and women [31]. However, in our study, we clustered depressive symptoms in large community-based populations that included healthy individuals and patients (n= 8,697) while considering age and gender.

Previous studies have reported an association between the types or factors of SLEs and severe depressive symptoms [7]. In an Iranian study (n= 4,763), among the 2 factors of SLEs described, social stressors (e.g., family, economic, and occupational change) were more strongly associated with depression and psychological problems than personal stressors. In that study, a greater association between social stressors and psychological problems was found in women relative to men. In a Chinese cross-sectional study that enrolled about half a million participants, SLEs were divided into 3 factors: family-related, finance-related, and other. Family-related events showed stronger associations with depression in women than in men [14]. Regardless of the study population and the tools used to assess depressive symptoms or SLEs, the associations between the sub-factors of SLEs and depressive symptoms vary by gender. In our study, women, more so than men, tended to exhibit SLE items related to their spouse or children at the center of the interactive map.

In previous studies, researchers investigated the factor structure of each depressive symptom or SLE, or estimated the association between SLEs and depression; however, in our study, we were able to simultaneously consider the factor structure of SLEs and depressive symptoms using a joint LSIRM. This study has implications in that we derived an interactive map between SLE and BDI-II items by applying a new statistical methodology and exploring differences according to gender and age. Clinically, our findings suggest that life experiences share latent space with individual depressive symptoms. Although future studies are needed to confirm the generalizability of these results, this approach to estimate latent clusters between SLEs and depressive symptoms may help identify individuals who are vulnerable to experiencing a specific life event associated with certain depressive symptoms. For example, finance-oriented and job-oriented SLEs were related to more BDI-II items in younger men and women compared to the older participants, and the included BDI-II items were mostly self-criticism, self-dislike, or feeling of punishment (Tables 1 and 2, Supplementary Material 7). Accordingly, we may be able to estimate depressive symptoms that are relatively likely to arise among participants in their 30s and 40s who experience financial or job-related problems. By taking a history of certain past life experiences, it may be possible to predict subgroup symptoms of or vulnerabilities to depressive disorder. Doing so may aid in the treatment of ongoing mental health problems and the development of guidelines for public mental health intervention.

Limitations, in this study, we extended previous findings by applying a joint LSIRM to explore the SLE clusters that share space with BDI-II items. Relative to the older groups, the interaction maps of younger men and women showed a stronger relationship between depression symptoms and SLEs regarding changes in work situations. However, limitations should be considered when interpreting these results. First, we explored potential responsibility characteristics using data-driven methods, making it difficult to confirm the robustness of our findings or ascertain clinical applicability. Many previous studies on the BDI-II have explored which items explain the principal components of depression and the number of dimensions describing depression symptoms; however, the results depend on the target population. Further investigation to confirm the generalizability and clinical implications is required. Second, because SLEs from the past 6 months were collected using a questionnaire, measurement bias may have been present. The reliability and validity of the Korean version of the LES in the source population needs to be confirmed. Third, the study participants were derived from the CMERC and CMERC-HI populations, and the results were heterogeneous between centers. Since the LSIRM could not be used to adjust the heterogeneity between centers, the validity of the results is limited. Finally, the joint LSIRM can only be applied to binary response types; therefore, ordinal or continuous data should be extended for other response types.

In conclusion, we used latent space item response modeling to project SLEs and depressive symptoms as measured by the BDI-II, thus representing associations of depressive symptoms and underlying traits with each life event by gender and age group. Using such methods, we could efficiently visualize the heterogeneous correlations of each SLE and BDI-II item according to gender and age.

## Figures and Tables

**Figure f3-epih-44-e2022093:**
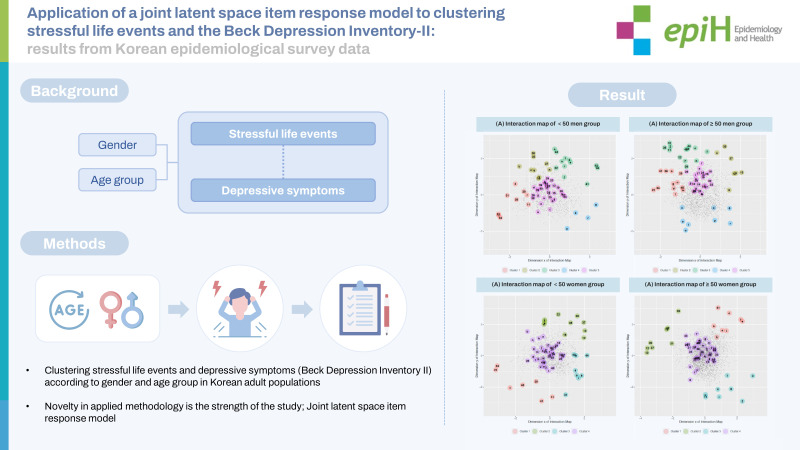


**Table 1.** Stressful life events (SLEs) and Beck Depression Inventory-II (BDI-II) clusters in men

SLEs/BDI-II^1^					
Cluster 1 (left)		Cluster 2 (upper left)	Cluster 3 (upper right)	Cluster 4 (lower right)	Cluster 5 (middle)
Aged <50 yr					
	1) Marriage	7) Foreclosure on mortgage or loan	2) Detention in jail	4) Change in sleeping habits	5) Death of a family member
	3) Death of spouse		22) Marital separation (due to conflict)		8) Death of a close friend
	11) Wife/girlfriend's pregnancy	16) Troubles with the boss/employer		6) Change in eating habits	9) Outstanding personal achievement
			41) Engagement		10) Minor violation of the law
	20) Gain of a new family member	30) Being fired from job	42) Breaking up with boy/girlfriend		12) Changes in work situation
					13) New job
	21) Change in residence	35) Divorce	43) Leaving home for the first time		14) Serious illness of a family member
	28) Borrowing more than $10,000	37) Retirement from work			15) Sexual difficulties
			44) Reconciliation with boy/girlfriend		17) Problems with in-laws
	31) Wife/girlfriend's abortion				23) Change in church activities
	34) Change in living conditions				24) Marital reconciliation
					25) Change in the number of arguments with spouse
					26) Change in wife's work
					27) Change in usual type/amount of recreation
					29) Borrowing less than $10,000
					32) Major personal injury or illness
					33) Change in social activities
					36) Serious illness of a close friend
					38) Son or daughter leaving home
					39) End of formal schooling
					40) Estrangement from spouse
	l) Loss of interest	b) Pessimism	a) Sadness	p) Changes in sleeping pattern	d) Loss of pleasure
		g) Self-dislike	c) Past failure		k) Agitation
		h) Self-criticalness	e) Guilty feelings	r) Changes in appetite	m) Indecisiveness
		n) Worthlessness	f) Punishment feelings		o) Loss of energy
			i) Suicidal thoughts or wishes j) Crying		q) Irritability
					s) Concentration difficulty
					t) Tiredness or fatigue
					u) Loss of interest in gender
Aaged ≥50 yr					
	21) Change in residence	3) Death of spouse	2) Detention in jail	4) Change in sleeping habits	1) Marriage
	28) Borrowing more than $10,000	9) Outstanding personal achievement	7) Foreclosure on mortgage or loan	6) Change in eating habits	5) Death of a family member
	34) Change in living conditions	12) Changes in work situation	11) Wife/girlfriend’s pregnancy		8) Death of a close friend
	39) End of formal schooling	13) New job	22) Marital separation (due to conflict)		10) Minor violation of the law
	40) Estrangement from spouse	30) Being fired from job	31) Wife/girlfriend's abortion		14) Serious illness of a family member
	41) Engagement	37) Retirement from work	35) Divorce		15) Sexual difficulties
			36) Serious illness of a close friend		16) Troubles with the boss/employer
			42) Breaking up with boy/girlfriend		17) Problems with in-laws
			43) Leaving home for the first time		18) Change in financial status
			44) Reconciliation with boy/girlfriend		19) Change in closeness of family
					20) Gain of a new family member
					23) Change in church activities
					24) Marital reconciliation
					25) Change in the number of arguments with spouse
					26) Change in wife's work
					27) Change in usual type/amount of recreation
					29) Borrowing less than $10,000
					32) Major personal injury or illness
					33) Change in social activities
					38) Son or daughter leaving home
	b) Pessimism	e) Guilty feelings	c) Past failure	o) Loss of energy	a) Sadness
	d) Loss of pleasure		f) Punishment feelings	p) Changes in sleeping pattern	i) Suicidal thoughts or wishes
	g) Self-dislike		h) Self-criticalness	r) Changes in appetite	j) Crying
	l) Loss of interest			s) Concentration difficulty	k) Agitation
	m) Indeciveness			t) Tiredness or fatigue	q) Irritability
	n) Worthlessness			u) Loss of interest in gender	

1 SLE items are indexed with numbers; BDI-II items are indexed with letters.

**Table 2.** Stressful life events (SLEs) and Beck Depression Inventory-II (BDI-II) clusters in women

SLEs/BDI-II^1^				
Cluster 1 (lower left)		Cluster 2 (upper right)	Cluster 3 (right)	Cluster 4 (middle)
Aged <50 yr				
	1) Marriage	12) Changes in work situation	4) Change in sleeping habits	2) Detention in jail
	11) Pregnancy	13) New job	6) Change in eating habits	3) Death of spouse
	20) Gain of a new family member	16) Troubles with the boss/employer	9) Outstanding personal achievement	5) Death of a family member
	21) Change in residence	22) Marital separation (due to conflict)	31) Abortion	7) Foreclosure on mortgage or loan
	34) Change in living conditions	30) Being fired from job	42) Breaking up with boy/girlfriend	8) Death of a close friend
	41) Engagement	35) Divorce	44) Reconciliation with boy/girlfriend	10) Minor violation of the law
	43) Leaving home for the first time	37) Retirement from work		14) Serious illness of a family member
				15) Sexual difficulties
				17) Problems with in-laws
				18) Change in financial status
				19) Change in closeness of family members
				23) Change in church activities
				24) Marital reconciliation
				25) Change in the number of arguments with spouse
				26) Change in husband's work
				27) Change in usual type/amount of recreation
				28) Change in husband's work
				29) Borrowing less than $10,000
				32) Major personal injury or illness
				33) Change in social activities
				36) Serious illness of a close friend
				38) Son or daughter leaving home
				39) End of formal schooling
				40) Estrangement from spouse
		c) Past failure	p) Changes in sleeping pattern	a) Sadness
		e) Guilty feelings	r) Changes in appetite	b) Pessimism
		f) Punishment feelings		d) Loss of pleasure
		i) Suicidal thoughts or wishes		g) Self-dislike
				h) Self-criticalness
				j) Crying
				k) Agitation
				l) Loss of interest
				m) Indeciveness
				n) Worthlessness
				o) Loss of energy
				q) Irritability
				s) Concentration difficulty
				t) Tiredness or fatigue
				u) Loss of interest in gender
Aged ≥50 yr				
	41) Engagement	12) Changes in work situation	1) Marriage	5) Death of a family member
		13) New job	2) Detention in jail	7) Foreclosure on mortgage or loan
		16) Troubles with the boss/employer	3) Death of spouse	8) Death of a close friend
		30) Being fired from job	4) Change in sleeping habits	9) Outstanding personal achievement
		31) Abortion	6) Change in eating habits	10) Minor violation of the law
		35) Divorce	11) Pregnancy	14) Serious illness of a family member
		37) Retirement from work		15) Sexual difficulties
		42) Breaking up with boy/girlfriend		17) Problems with in-laws
				18) Change in financial status
				19) Change in closeness of family members
				20) Gain of a new family member
				21) Change in residence
				22) Marital separation (due to conflict)
				23) Change in church activities
				24) Marital reconciliation
				25) Change in the number of arguments with spouse
				26) Change in husband's work
				27) Change in usual type/amount of recreation
				28) Change in husband's work
				29) Borrowing less than $10,000
				32) Major personal injury or illness
				33) Change in social activities
				34) Change in living conditions
				36) Serious illness of a close friend
				38) Son or daughter leaving home
				39) End of formal schooling
				40) Estrangement from spouse
				43) Leaving home for the first time
				44) Reconciliation with boy/girlfriend
	a) Sadness		p) Changes in sleeping pattern	d) Loss of pleasure
	b) Pessimism		r) Changes in appetite	g) Self-dislike
	c) Past failure			j) Crying
	e) Guilty feelings			k) Agitation
	f) Punishment feelings			l) Loss of interest
	h) Self-criticalness			m) Indeciveness
	i) Suicidal thoughts or wishes			n) Worthlessness
				o) Loss of energy
				q) Irritability
				s) Concentration difficulty
				t) Tiredness or fatigue
				u) Loss of interest in gender

SLEs, stressful life events; BDI-II, Beck Depression Inventory-II.

1 SLE items are indexed with numbers; BDI-II items are indexed with letters.

## SUPPLEMENTARY MATERIALS

Supplementary Material 1. General characteristics of study participants (N=9,675)

Supplementary Material 2 General characteristics of study participants (N=9,675)

Supplementary Material 3 Response frequency of each LES event in male and female (N=9,675)

Supplementary Material 4 Twelve interaction maps based on the joint latent space item response model applied to each subgroup by center. (Male group under 50 (Center 1), Male group under 50 (Center 2), Male group under 50 (Center 3), Male group over 50 (Center1), Male group over 50 (Center2), Male group over 50 (Center3), Female group under 50 (Center 1), Female group under 50 (Center 2), Female group under 50 (Center 3), Female group over 50 (Center 1), Female group over 50 (Center 2), Female group over 50 (Center 3)) Der F, *Dermatophagoides farina*; Der P, *Dermatophagoides pteronyssinus*; sIgE, specific immunoglobulin E.

Supplementary Material 5 Four interaction map pairs based on latent space item response model applied to each LES and BDI II items by four subgroups. (Male group under 50, Male group over 50, Female group under 50, Female group over 50)

Supplementary Material 6 Description and Validation of Latent Space Item Response Model & joint Latent Space Item Response Model

Supplementary Material 7 Kullback-Leibler divergence values between distance distributions of two of the four groups (Male group under 50, Male group over 50, Female group under 50, Female group over 50) for every item pair of SLE and BDI II.
