# Supplementary material for: Application of a joint latent space item response model to clustering stressful life events and the Beck Depression Inventory-II: results from Korean epidemiological survey data
Source: Epidemiol Health. 2022 Oct 24;44:e2022093. doi: 10.4178/epih.e2022093 (PMC10185968; doi:10.4178/epih.e2022093)
Supplement: Supplementary Material 2 — General characteristics of study participants (N=9,675) [file epih-44-e2022093-Supplementary-2.docx]

**Supplementary Material 2. General characteristics of study participants (N=9,675)**

| **Variables** | **CMERC - Yonsei** | | **CMERC - Ajou** | | **CMERC-HI - Severance** | | ***P* value** |  |
| --- | --- | --- | --- | --- | --- | --- | --- | --- |
|  | **(N=4,058)** | | **(N=4,037)** | | **(N=1,580)** | |  |  |
|  | Mean, N | (SD, %) | Mean, N | (SD, %) | Mean, N | (SD, %) |  |  |
| Age (years), mean (SD) | 51.31 | (9.35) | 51.53 | (7.99) | 52.85 | (8.19) | <.001 |  |
| Age group, N (%) |  |  |  |  |  |  |  |  |
| <50 years | 1,397 | (34.43) | 1,420 | (35.17) | 510 | (32.28) | 0.1209 |  |
| ≥50 years | 2,661 | (65.57) | 2,617 | (64.83) | 1,070 | (67.72) |  |  |
| Sex |  |  |  |  |  |  |  |  |
| Male | 1,424 | (35.09) | 1,382 | (34.23) | 1,160 | (73.42) | <.001 |  |
| Female | 2,634 | (64.91) | 2,655 | (65.77) | 420 | (26.58) |  |  |
| Education level, N (%) |  |  |  |  |  |  |  |  |
| ≤6 years | 230 | (5.67) | 187 | (4.63) | 133 | (8.42) | <.001 |  |
| 6-9 years | 350 | (8.62) | 430 | (10.65) | 132 | (8.35) |  |  |
| 9-12 years | 1,583 | (39.01) | 1,918 | (47.51) | 537 | (33.99) |  |  |
| 12+ years | 1,895 | (46.70) | 1,502 | (37.21) | 778 | (49.24) |  |  |
| Occupational status, N (%)^b^ |  |  |  |  |  |  |  |  |
| Office worker | 1,513 | (37.28) | 1,263 | (31.29) | 831 | (52.59) | <.001 |  |
| Site worker | 1,191 | (29.35) | 1,483 | (36.74) | 267 | (16.90) |  |  |
| Home maker | 1,147 | (28.27) | 1,139 | (28.21) | 242 | (15.32) |  |  |
| Unemployed | 207 | (5.10) | 152 | (3.77) | 240 | (15.19) |  |  |
| Household income level (year), N (%) |  |  |  |  |  |  |  |  |
| Q1 (<24,000 $) | 998 | (24.59) | 910 | (22.54) | 671 | (42.47) | <.001 |  |
| Q2 (24,000-34,641 $) | 1,078 | (26.56) | 1,084 | (26.85) | 272 | (17.22) |  |  |
| Q3 (34,641-48,299 $) | 998 | (24.59) | 1,095 | (27.12) | 269 | (17.03) |  |  |
| Q4 (≥ 48,299 $) | 984 | (24.25) | 948 | (23.48) | 368 | (23.29) |  |  |
| Marital status, N (%) |  |  |  |  |  |  |  |  |
| Unmarried | 229 | (5.64) | 97 | (2.40) | 149 | (9.43) | <.001 |  |
| Married-death of spouse | 160 | (3.94) | 148 | (3.67) | 30 | (1.90) |  |  |
| Married-separated | 192 | (4.73) | 214 | (5.30) | 88 | (5.57) |  |  |
| Married-living together | 3,477 | (85.68) | 3,578 | (88.63) | 1,313 | (83.10) |  |  |
| Previous chronic disease, N (%)^c^ |  |  |  |  |  |  |  |  |
| Yes | 1,786 | (44.01) | 1,815 | (44.96) | 1,463 | (92.59) | <.001 |  |
| No | 2,272 | (55.99) | 2,222 | (55.04) | 117 | (7.41) |  |  |
| Depressive symptom, N (%) |  |  |  |  |  |  |  |  |
| BDI-II <20 | 3,651 | (89.97) | 3,508 | (86.90) | 1,408 | (89.17) | <.001 |  |
| BDI-II ≥20 | 407 | (10.03) | 529 | (13.10) | 171 | (10.83) |  |  |
| ^a^ CMERC study enrolled participants among community-dwellers without overt CVDs (ever) or cancer (past 2 years) history; CMERC-HI study enrolled participants from patients who are at a high risk of developing ASCVD. | | | | | | | | |
| ^b^ 26 participants excluded due to missing/unknown information | | | | | | | | |
| ^c^ Chronic diseases history included in CMERC study: stroke, transient ischemic attacks, myocardial infarction, angina pectoris, heart failure, chronic kidney disease, hypertension, dyslipidemia, diabetes, thyroid disease, fatty liver, chronic hepatitis, liver cirrhosis, asthma or chronic obstructive pulmonary disease, osteoporosis, arthritis, autoimmune disease, and malignant tumors | | | | | | | | |

(Abbreviation) Standard deviation, SD; CMERC, Cardiovascular and Metabolic Diseases Etiology Research Center; BDI-II, Beck Depression Inventory-II
