# Supplementary material for: Application of a joint latent space item response model to clustering stressful life events and the Beck Depression Inventory-II: results from Korean epidemiological survey data
Source: Epidemiol Health. 2022 Oct 24;44:e2022093. doi: 10.4178/epih.e2022093 (PMC10185968; doi:10.4178/epih.e2022093)
Supplement: Supplementary Material 3 — Response frequency of each LES event in male and female (N=9,675) [file epih-44-e2022093-Supplementary-3.docx]

**Supplementary Material 3. Response frequency of each LES event in male and female (N=9,675)**

| **Item** | | **Male**  **(N=3,966)** | |  | **Female**  **(N=5,709)** | |
| --- | --- | --- | --- | --- | --- | --- |
|  |  | N | (%) |  | N | (%) |
| 1 | Marriage | 25 | (0.63) |  | 27 | (0.47) |
| 2 | Detention in jail or other institution | 6 | (0.15) |  | 2 | (0.04) |
| 3 | Death of spouse | 3 | (0.08) |  | 21 | (0.37) |
| 4 | Major change in sleeping habits | 818 | (20.63) |  | 1,737 | (30.43) |
| 5 | Death of a close family member | 360 | (9.08) |  | 507 | (8.88) |
| 6 | Major change in eating habits | 804 | (20.27) |  | 1,610 | (28.20) |
| 7 | Foreclosure on mortgage or loan | 97 | (2.45) |  | 146 | (2.56) |
| 8 | Death of a close friend | 220 | (5.55) |  | 178 | (3.12) |
| 9 | Outstanding personal achievement | 966 | (24.36) |  | 1,195 | (20.93) |
| 10 | Minor violations of the law | 709 | (17.88) |  | 338 | (5.92) |
| 11 | Male: Wife/girlfriend's pregnancy;  Female: pregnancy ^a^ | 50 | (1.26) |  | 32 | (0.56) |
| 12 | Changes in work situation | 1,000 | (25.21) |  | 946 | (16.57) |
| 13 | New job | 281 | (7.09) |  | 413 | (7.23) |
| 14 | Serious illness of close family member | 600 | (15.13) |  | 993 | (17.39) |
| 15 | Sexual difficulties | 404 | (10.19) |  | 385 | (6.74) |
| 16 | Troubles with the boss/employer | 346 | (8.72) |  | 287 | (5.03) |
| 17 | Problems with in-laws | 290 | (7.31) |  | 287 | (5.03) |
| 18 | Major change in financial status | 530 | (13.36) |  | 646 | (11.32) |
| 19 | Major Change in closeness of family members | 363 | (9.15) |  | 815 | (14.28) |
| 20 | Gaining a new family member | 205 | (5.17) |  | 770 | (13.49) |
| 21 | Changes in residence | 317 | (7.99) |  | 391 | (6.85) |
| 22 | Marital separation from mate (due to conflict) | 31 | (0.78) |  | 40 | (0.70) |
| 23 | Major change in church activities | 185 | (4.66) |  | 445 | (7.79) |
| 24 | Marital reconciliation with mate | 169 | (4.26) |  | 340 | (5.96) |
| 25 | Major change in the number of arguments with spouse | 841 | (21.21) |  | 1,539 | (26.96) |
| 26 | Married male: Change in wife's work outside of home,  Married female: Change in husband's work ^a^ | 212 | (5.35) |  | 406 | (7.11) |
| 27 | Major change in usual type and/or amount of recreation | 433 | (10.92) |  | 670 | (11.74) |
| 28 | Borrowing more than $10,000 | 448 | (11.30) |  | 414 | (7.25) |
| 29 | Borrowing less than $10,000 | 213 | (5.37) |  | 284 | (4.97) |
| 30 | Being fired from job | 37 | (0.93) |  | 48 | (0.84) |
| 31 | Male: Wife/girlfriend having abortion,  Female: Having abortion ^a^ | 16 | (0.40) |  | 25 | (0.44) |
| 32 | Major personal injury or illness | 252 | (6.35) |  | 366 | (6.41) |
| 33 | Major change in social activities | 597 | (15.05) |  | 984 | (17.24) |
| 34 | Major change in living conditions | 291 | (7.34) |  | 428 | (7.50) |
| 35 | Divorce | 12 | (0.30) |  | 21 | (0.37) |
| 36 | Serious illness of close friends | 123 | (3.10) |  | 223 | (3.91) |
| 37 | Retirement from work | 136 | (3.43) |  | 180 | (3.15) |
| 38 | Son or daughter leaving home | 309 | (7.79) |  | 595 | (10.42) |
| 39 | Ending of formal schooling | 53 | (1.34) |  | 81 | (1.42) |
| 40 | Separation from spouse (due to work, travel, etc.) | 176 | (4.44) |  | 303 | (5.31) |
| 41 | Engagement | 10 | (0.25) |  | 8 | (0.14) |
| 42 | Breaking up with boyfriend/girlfriend | 35 | (0.88) |  | 42 | (0.74) |
| 43 | Leaving home for the first time | 32 | (0.81) |  | 44 | (0.77) |
| 44 | Reconciliation (getting back together)  with boyfriend/girlfriend | 20 | (0.50) |  | 27 | (0.47) |

^a^ Responded separately in (married-)male/female
