# Supplementary material for: Application of a joint latent space item response model to clustering stressful life events and the Beck Depression Inventory-II: results from Korean epidemiological survey data
Source: Epidemiol Health. 2022 Oct 24;44:e2022093. doi: 10.4178/epih.e2022093 (PMC10185968; doi:10.4178/epih.e2022093)
Supplement: Supplementary Material 4 — Twelve interaction maps based on the joint latent space item response model applied to each subgroup by center. (Male group under 50 (Center 1), Male group under 50 (Center 2), Male group under 50 (Center 3), Male group over 50 (Center1), Male group over 50 (Center2), Male group over 50 (Center3), Female group under 50 (Center 1), Female group under 50 (Center 2), Female group under 50 (Center 3), Female group over 50 (Center 1), Female group over 50 (Center 2), Female group over 50 (Center 3)) Der F, Dermatophagoides farina; Der P, Dermatophagoides pteronyssinus; sIgE, specific immunoglobulin E. [file epih-44-e2022093-Supplementary-4.pdf]

Supplementary Material 4. Twelve interaction maps based on the joint latent space item response model applied to each subgroup by center. (Male group under 50 (Center 1), Male group under 50 (Center 2), Male group under 50 (Center 3), Male group over 50 (Center1), Male group over 50 (Center2), Male group over 50 (Center3), Female group under 50 (Center 1), Female group under 50 (Center 2), Female group under 50 (Center 3), Female group over 50 (Center 1), Female group over 50 (Center 2), Female group over 50 (Center 3))

We applied a joint LSIRM for each subgroup by center to observe the heterogeneity of item interactions among three centers. In general, the LES and BDI-II items asking similar questions are located closely one another for all subgroups. However, item interactions for each center show some heterogeneous patterns. In the male groups, the interactions among items from center 3 show more even patterns than the other centers. Specifically, the BDI-II items are relatively evenly distributed around the LES items in the interaction map of center 3 while the items for the male groups from center 1 and center 2 show more clear clusters. In the female groups, the items from center 3 show much stronger interactions one another than those from the other centers. The LES and BDI-II items are located closely one another in the interaction map of center 3 while the items for the female groups from center 1 and center 2 show more clear clusters.

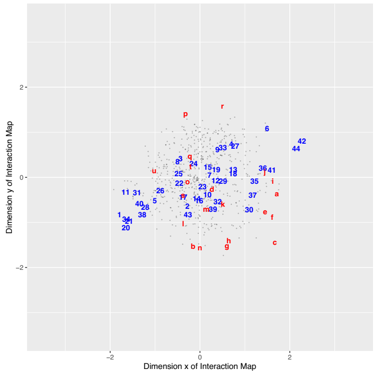

(a) Center 1 of < 50 male group

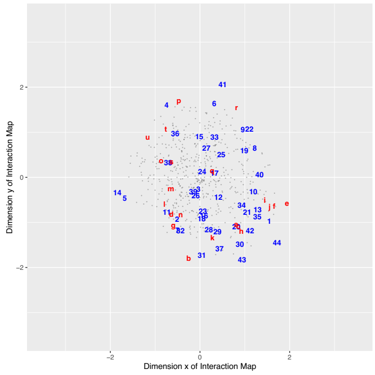

(b) Center 2 of < 50 male group

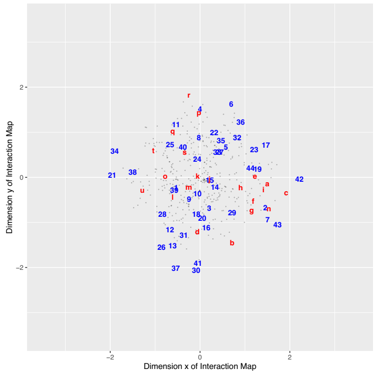

(c) Center 3 of < 50 male group

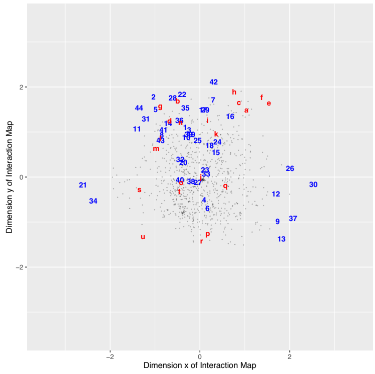

(a) Center 1 of $\geq$ 50 male group

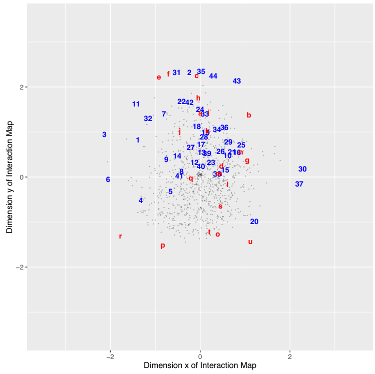

(b) Center 2 of $\geq$ 50 male group

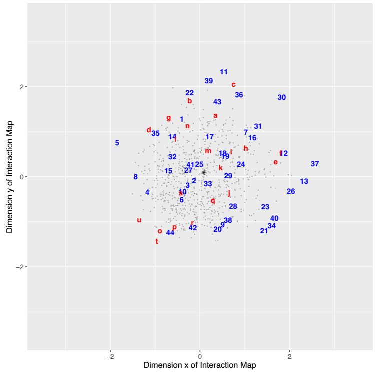

(c) Center 3 of $\geq$ 50 male group

Figure 1: Interaction maps by center of the male groups. The numbers and letters represent the latent positions of LES and BDI-II items, respectively.

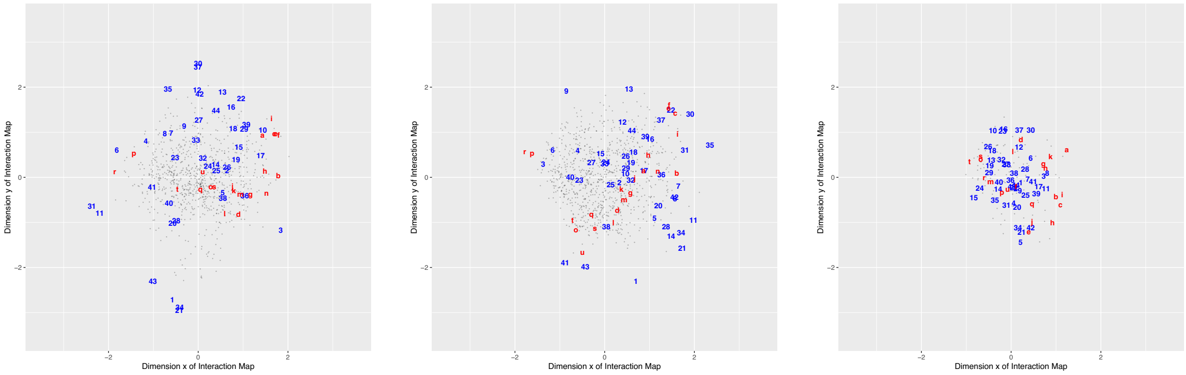

(a) Center 1 of < 50 female group (b) Center 2 of < 50 female group (c) Center 3 of < 50 female group

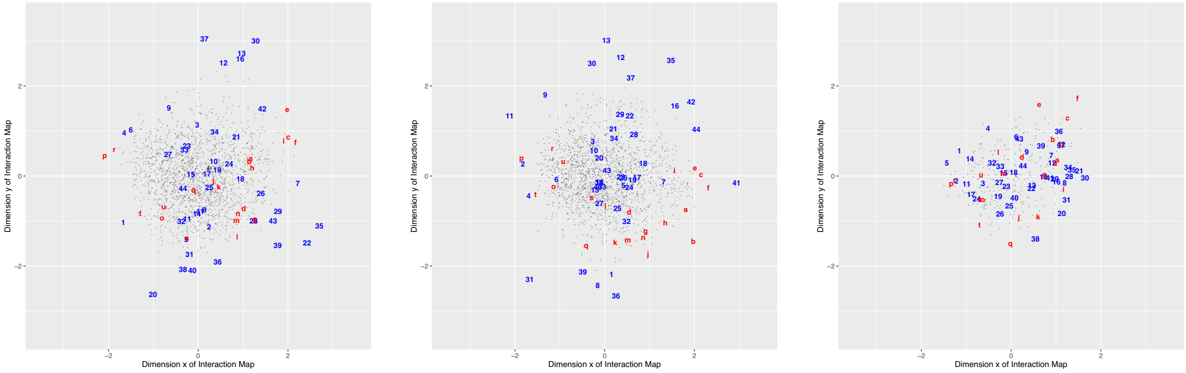

(a) Center 1 of $\geq$ 50 female group (b) Center 2 of $\geq$ 50 female group (c) Center 3 of $\geq$ 50 female group

Figure 2: Interaction maps by center of the female groups. The numbers and letters represent the latent positions of LES and BDI-II items, respectively.
