# Supplementary material for: Application of a joint latent space item response model to clustering stressful life events and the Beck Depression Inventory-II: results from Korean epidemiological survey data
Source: Epidemiol Health. 2022 Oct 24;44:e2022093. doi: 10.4178/epih.e2022093 (PMC10185968; doi:10.4178/epih.e2022093)
Supplement: Supplementary Material 5 — Four interaction map pairs based on latent space item response model applied to each LES and BDI II items by four subgroups. (Male group under 50, Male group over 50, Female group under 50, Female group over 50) [file epih-44-e2022093-Supplementary-5.pdf]

Supplementary Material 5. Four interaction map pairs based on latent space item response model applied to each LES and BDI II items by four subgroups. (Male group under 50, Male group over 50, Female group under 50, Female group over 50)

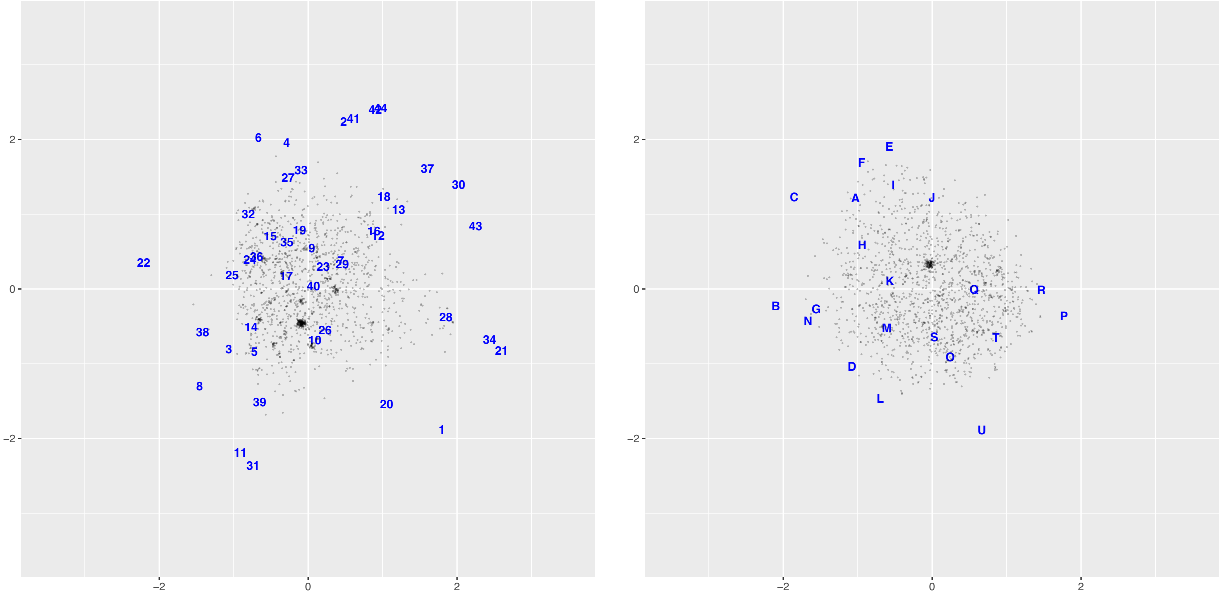

Figure 1: The numbers and alphabet letters in two interaction maps represent the latent positions of items of SLE and BDI II, respectively. The dots represent the latent positions of respondents. Latent space item response model was applied separately to each survey item for $\leq$ 50 male group.

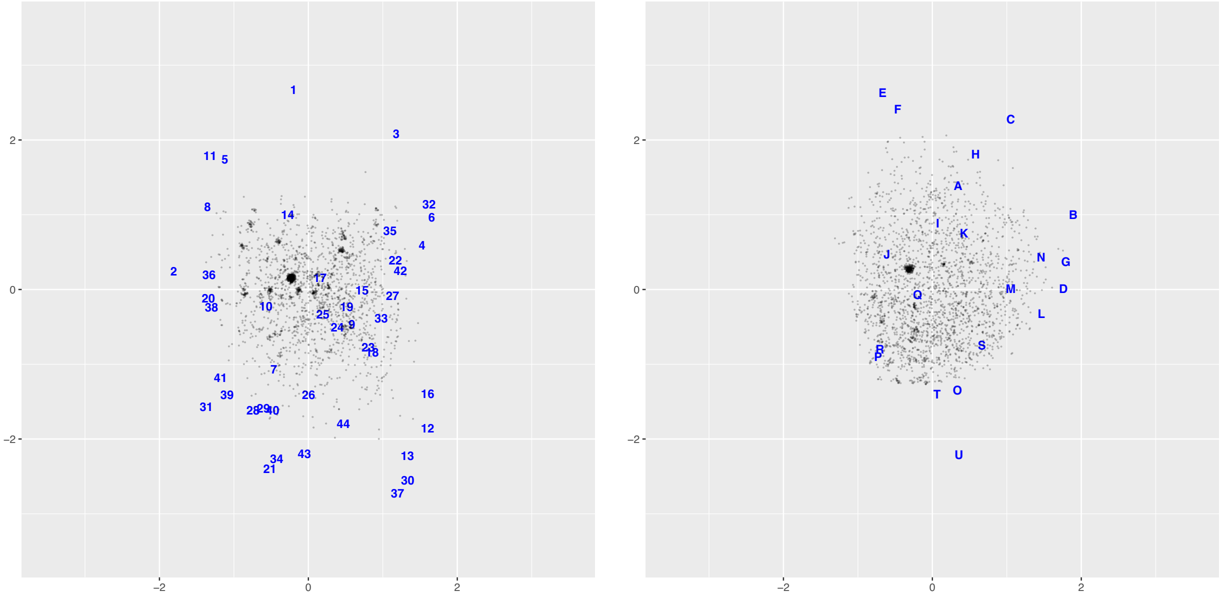

Figure 2: The numbers and alphabet letters in two interaction maps represent the latent positions of items of SLE and BDI II, respectively. The dots represent the latent positions of respondents. Latent space item response model was applied separately to each survey item for $\geq$ 50 male group.

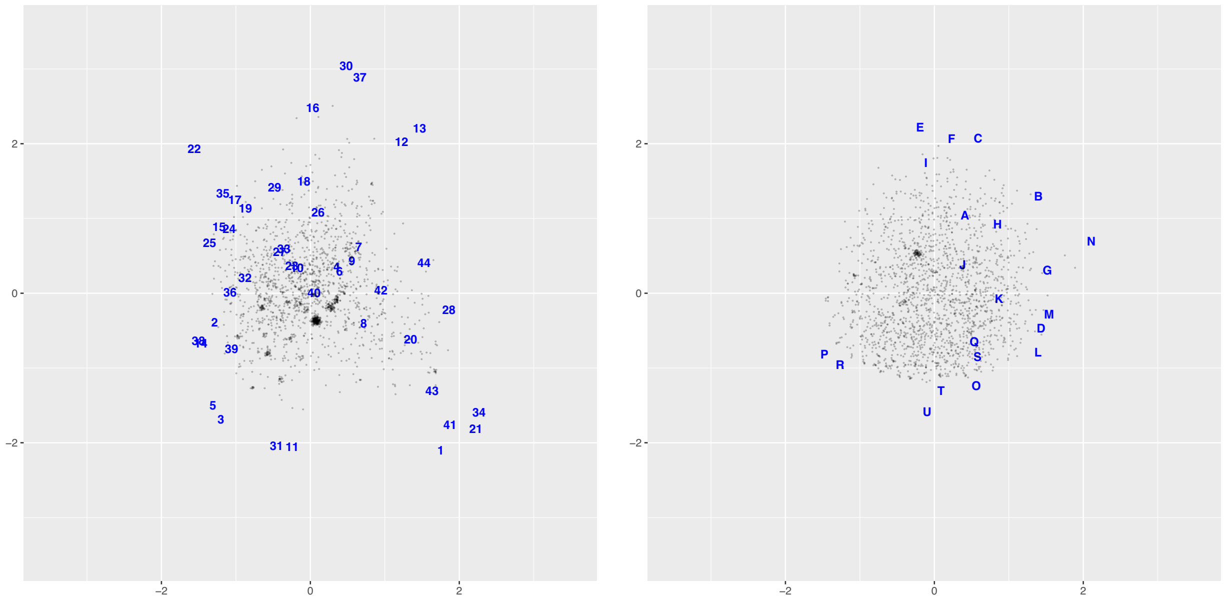

Figure 3: The numbers and alphabet letters in two interaction maps represent the latent positions of items of SLE and BDI II, respectively. The dots represent the latent positions of respondents. Latent space item response model was applied separately to each survey item for ≤ 50 female group.

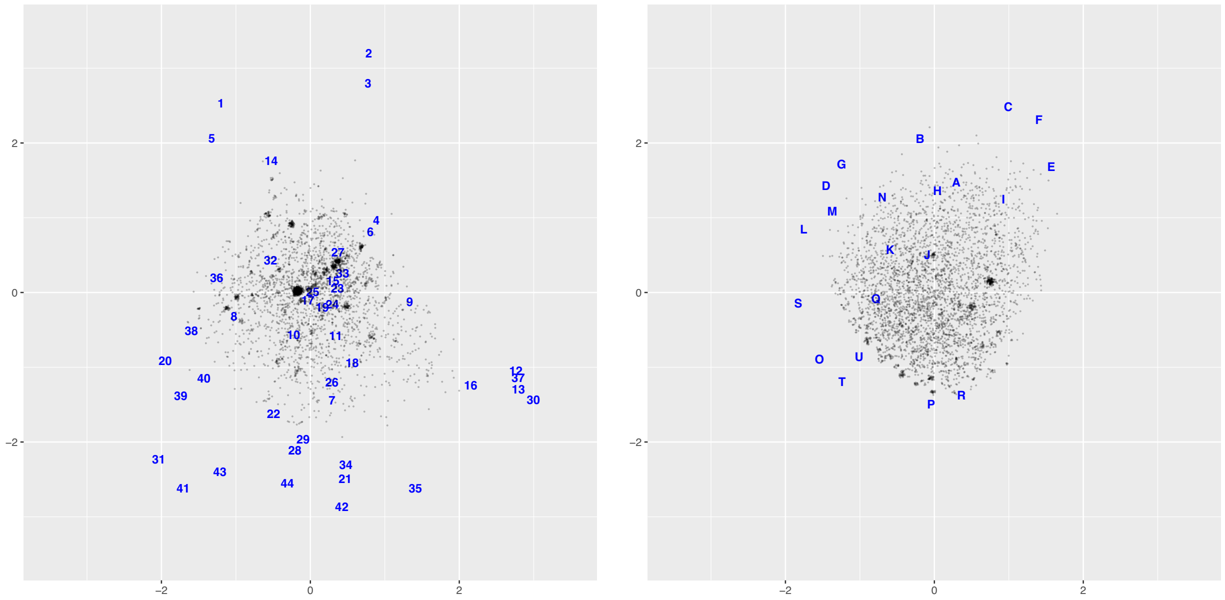

Figure 4: The numbers and alphabet letters in two interaction maps represent the latent positions of items of SLE and BDI II, respectively. The dots represent the latent positions of respondents. Latent space item response model was applied separately to each survey item for ≥ 50 female group.
