# Supplementary material for: Application of a joint latent space item response model to clustering stressful life events and the Beck Depression Inventory-II: results from Korean epidemiological survey data
Source: Epidemiol Health. 2022 Oct 24;44:e2022093. doi: 10.4178/epih.e2022093 (PMC10185968; doi:10.4178/epih.e2022093)
Supplement: Supplementary Material 6 — Description and Validation of Latent Space Item Response Model & joint Latent Space Item Response Model [file epih-44-e2022093-Supplementary-6.docx]

**Supplementary Material 6. Description and Validation of Latent Space Item Response Model & joint Latent Space Item Response Model**

Latent Space Item Response Model is a network-approach model to analyze binarized item-response data. It was designed to alleviate the assumption of independence for items and respondents defined in traditional Item Response Model (Rasch Model). The traditional Item Response Model represents a probability of positive response for a particular item with an item-wise coefficient $\beta_{i}$ and a respondent-wise coefficient $\theta_{k}$. The model assumes that the respondents are independent of each other (unit independence), and so are the item responses within respondent (local independence). Maintaining the elements of Item Response Model, Latent Space Item Response Model provides more flexibility by using latent positions for items and respondents, which is expressed as $w_{i}$, $z_{k}$ respectively. Each item and respondent are assumed to be located in a common latent space called “interaction map” in the form of a latent position determined based on the observed item-response data. Details can be found in previous literature (Jeon *et al.*, 2021). The probability of a positive response in Latent Space Item Response Model is defined as formula (1):

$\mathrm{logit} \left\{ P(y_{\mathrm{ki}}=1) \right\}=\beta_{i}+\theta_{k}-\gamma\left\| w_{i}-z_{k} \right\|$. (1)

$\mathrm{logit} \left\{ P(y_{\mathrm{ki}}=1) \right\}=\beta_{i}+\theta_{k}$. (2)

One can notice that Latent Space Item Response Model written in formula (1) contains the form of traditional Item Response Model written in formula (2). An item-wise coefficient, $\beta$, indicates the extent to which an item is expected to be responded positive and the respondent-wise coefficient, $\theta$, indicates respondent's willingness to respond positive. $w_{i}$ and $z_{k}$ represents the latent position for an item and a respondent, respectively. Latent Space Item Response Model contains a distance term of these two latent positions. $\gamma$ determines how much the distance term affects the overall probability. The full likelihood of Latent Space Item Response Model is given as follows:

$P\left( Y | Z,W,\Theta, \beta\right)= {\prod_{k=1}^{N} \prod_{i=1}^{P} \left( \frac{exp(\beta_{i}+\theta_{k}-\gamma\left\| w_{i}-z_{k} \right\|}{1+exp(\beta_{i}+\theta_{k}-\gamma\left\| w_{i}-z_{k} \right\|} \right)}^{y_{ki}}\left( \frac{1}{1+exp(\beta_{i}+\theta_{k}-\gamma\left\| w_{i}-z_{k} \right\|} \right)^{1-y_{ki}}$ (3)

The distance term in Latent Space Item Response Model represents the Euclidean distance between two latent positions of an item-respondent pair, and it measures their interaction. The latent position of individual item and respondent is determined by the observed elements of item-response data. The binarized element indicates whether a connection exists between a particular item-response pair. When a respondent gave a positive response, that item-respondent pair is considered to have interaction. When another respondent gave a positive response to the same item as well, it means that item-respondent pair has interaction, and similarity exists between the two different respondents. This is called ‘transitivity property’, and it is applied to the case of the items likewise. Based on all elements of item-response data, latent position is determined for each item and respondent. Similar items and respondents would be located relatively close to one another in the latent space. Then, the distance term of corresponding positions would be small. The opposite interpretation can be applied to the item-respondent pairs which are located far from one another. Thus, the distance term enables us to capture the dependence structure across the items and respondents beyond the individual item and respondent’s own effect. In a similar vein, the latent space is also expressed as an "interaction map" as the latent positions of items and respondents reflect the interaction of one another.

Overall importance of the distance term is determined by $\gamma$, which represents the weight of the distance term on the probability of positive response. Moreover, we embody the interaction map in 2-dimension to observe the interaction structure across the items and respondents, which is useful for intuitive visualization and interpretation.

The joint LSIRM with two different item response data and common respondents is expressed by the following formula:

$logit\{\pi_{\mathrm{ki}_{1}}\}=logit \left\{ P\left( y_{\mathrm{ki}_{1}}=1 \right) \right\}=\beta_{i_{1}}+\theta_{1k}-\left\| w_{i_{1}}-z_{k} \right\|$,

${logit\{\pi}_{ki_{2}}\}=logit \left\{ P\left( y_{\mathrm{ki}_{2}}=1 \right) \right\}=\beta_{i_{2}}+\theta_{2k}-\left\| w_{i_{2}}-z_{k} \right\|$.

(4)

The likelihood is as follows:

$$L\left( Y_{1},Y_{2} | Z,W_{1},W_{2},\Theta_{1},\Theta_{2}, \beta_{1},\beta_{2} \right)= \prod_{k=1}^{n} \left\{ \prod_{i_{1}=1}^{p_{1}} \pi_{{ki}_{1}}^{y_{{ki}_{1}}}\left( 1-\pi_{{ki}_{1}} \right)^{1-y_{{ki}_{1}}} \right\}\left\{ \prod_{i_{2}=1}^{p_{2}} \pi_{{ki}_{2}}^{y_{{ki}_{2}}}\left( 1-\pi_{{ki}_{2}} \right)^{1-y_{{ki}_{2}}} \right\}$$

(5)

where $\theta$ and $\beta$ in each LSIRM represent the person and item main effects on the probability of a positive response, respectively. The proposed model can accommodate two different sets of item response data, and the extension is possible for a larger number of sets. In the joint LSIRM, the parameter γ from the LSIRM was removed and set to 1. Since common respondents and different item sets share the corresponding interaction map, the distance term on the probability of a positive response should be identical and have a constant effect. The corresponding coefficients of LSIRM and joint LSIRM are interpreted as “ease of being answered positive” for $\beta$ and “tendency to answer positive for the item” for $\theta$, respectively. The interpretation of coefficients can be flexibly dependent on various applications as long as it is applied to binary item response data (Jeon et al., 2021). The parameters were estimated by a fully Bayesian approach based on Markov chain Monte Carlo (MCMC) sampling. For the attribute model, we specified prior distributions for the model parameters as follows:

$\pi\left( \beta_{i_{1}} \right) \sim N(0,\tau_{\beta_{1}}^{2})$, $\pi\left( \beta_{i_{2}} \right) \sim N(0,\tau_{\beta_{2}}^{2})$

$\pi\left( \theta_{1k}|\sigma^{2} \right) \sim N(0,\sigma^{2})$, $\pi\left( \theta_{2k}|\sigma^{2} \right) \sim N(0,\sigma^{2})$

$$\pi\left( \sigma^{2} \right) \sim\Gamma^{-1}(a_{\sigma},b_{\sigma})$$

$$\pi\left( z_{k} \right) \sim MVN_{d}(0,I_{d})$$

$\pi\left( w_{i_{1}} \right) \sim MVN_{d}\left( 0,I_{d} \right)$, $\pi\left( w_{i_{2}} \right) \sim MVN_{d}\left( 0,I_{d} \right)$

To evaluate the validity of the joint LSIRM, we used the posterior predictive checking based on the generated samples from the posterior predictive distribution (Gelman et al. 2013). For every subgroup, 10,000 response datasets were generated from the probability based on the generated posterior samples. Then, for each item in LES and BDI-II, the proportion of positive responses from the generated datasets was compared to those from the observed dataset. The boxplots representing the predicted proportion per item and the corresponding Bayesian p-values between 0.2 and 0.85 for each subgroup showed little discrepancy between the measures of generated and observed datasets, which indicated that the joint LSIRM fit the data well. The boxplots of the predicted proportion of the four subgroups are presented in Supplementary Figures 1 and 2, as shown below.


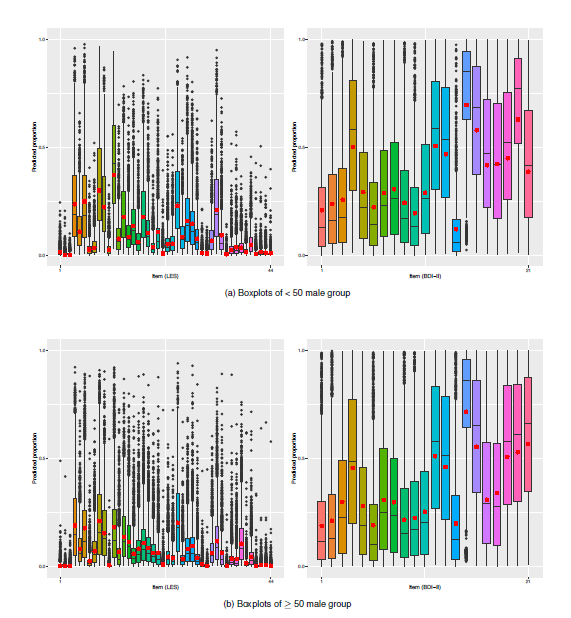


**Supplementary Figure 1.** Predicted proportion of correct responses per item for the male groups.

***(Note)*** Box plots represent the distribution of predicted proportions of correct responses for the predicted samples. The red mark in each box indicates the proportion of correct responses calculated from the observed samples.


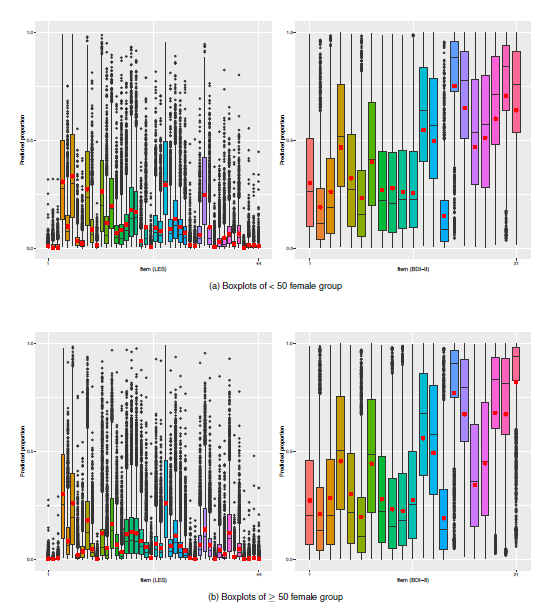


**Supplementary Figure 2.** Predicted proportion of correct responses per item for the female groups.

***(Note)*** Box plots represent the distribution of predicted proportions of correct responses for the predicted samples. The red mark in each box indicates the proportion of correct responses calculated from the observed samples.

**Reference**

1. **Gelman *et al.*** (2013). Bayesian Data Analysis 3^rd^ Edition.
2. **Jeon M *et al.*** (2021). Mapping Unobserved Item–Respondent Interactions: A Latent Space Item Response Model with Interaction Map. *Psychometrika*, 1-26.
