# Supplementary material for: Application of a joint latent space item response model to clustering stressful life events and the Beck Depression Inventory-II: results from Korean epidemiological survey data
Source: Epidemiol Health. 2022 Oct 24;44:e2022093. doi: 10.4178/epih.e2022093 (PMC10185968; doi:10.4178/epih.e2022093)
Supplement: Supplementary Material 7 — Kullback-Leibler divergence values between distance distributions of two of the four groups (Male group under 50, Male group over 50, Female group under 50, Female group over 50) for every item pair of SLE and BDI II. [file epih-44-e2022093-Supplementary-7.pdf]

Supplementary Material 7. Kullback-Leibler divergence values between distance distributions of two of the four groups (Male group under 50, Male group over 50, Female group under 50, Female group over 50) for every item pair of SLE and BDI II.

The distances between items of SLE and BDI II were formed into distributions based on Bayesian estimation. The following plots represent distance distributions of two of the four groups for every item pair of SLE and BDI II. The light blue distributions represent distances from the male group under 50

years old, the dark blue distributions represent distances from the male group over 50 years old, the coral distributions represent distances from the female group under 50 years old, and the pink distributions represent distances from the female group over 50 years old. The numbers written on the upper left of subplots represent corresponding Kullback-Leibler divergence value.

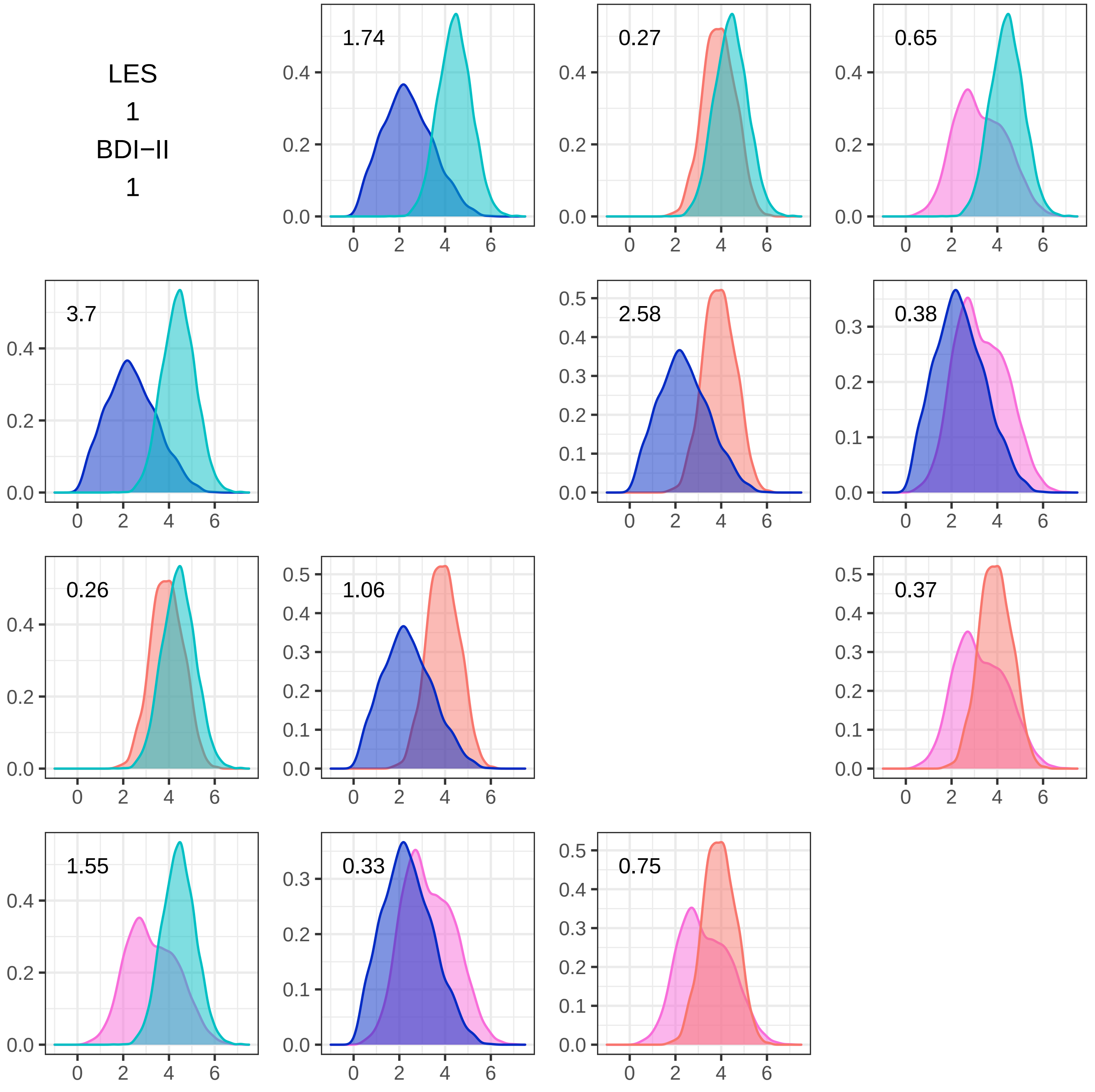

LES
1
BDI−II
1
1.74
0.27
0.65
3.7
2.58
0.38
0.26
1.06
0.37
1.55
0.33
0.75

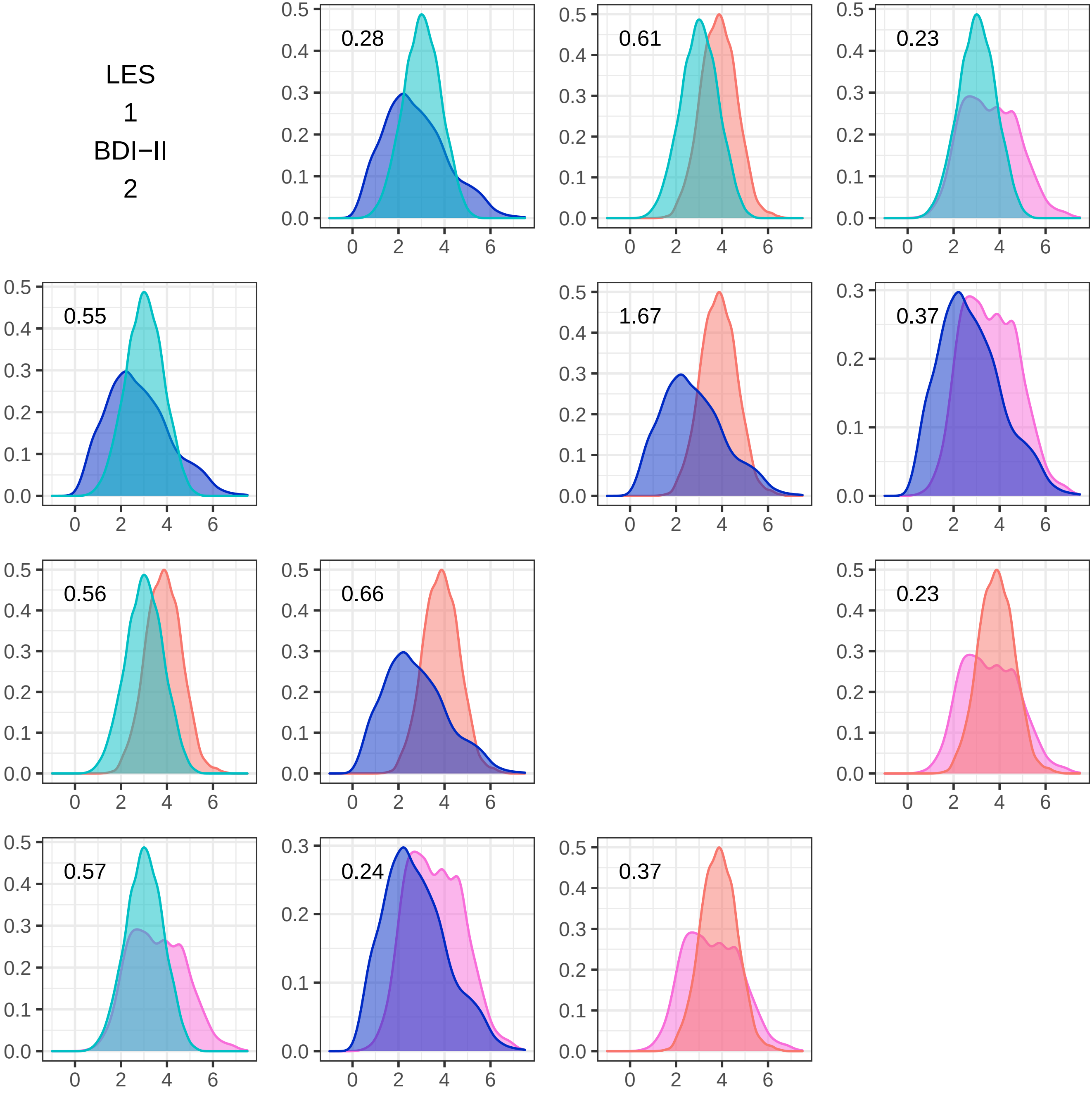
LES
1
BDI−II
2
0.28
0.61
0.23
0.55
1.67
0.37
0.56
0.66
0.23
0.57
0.24
0.37

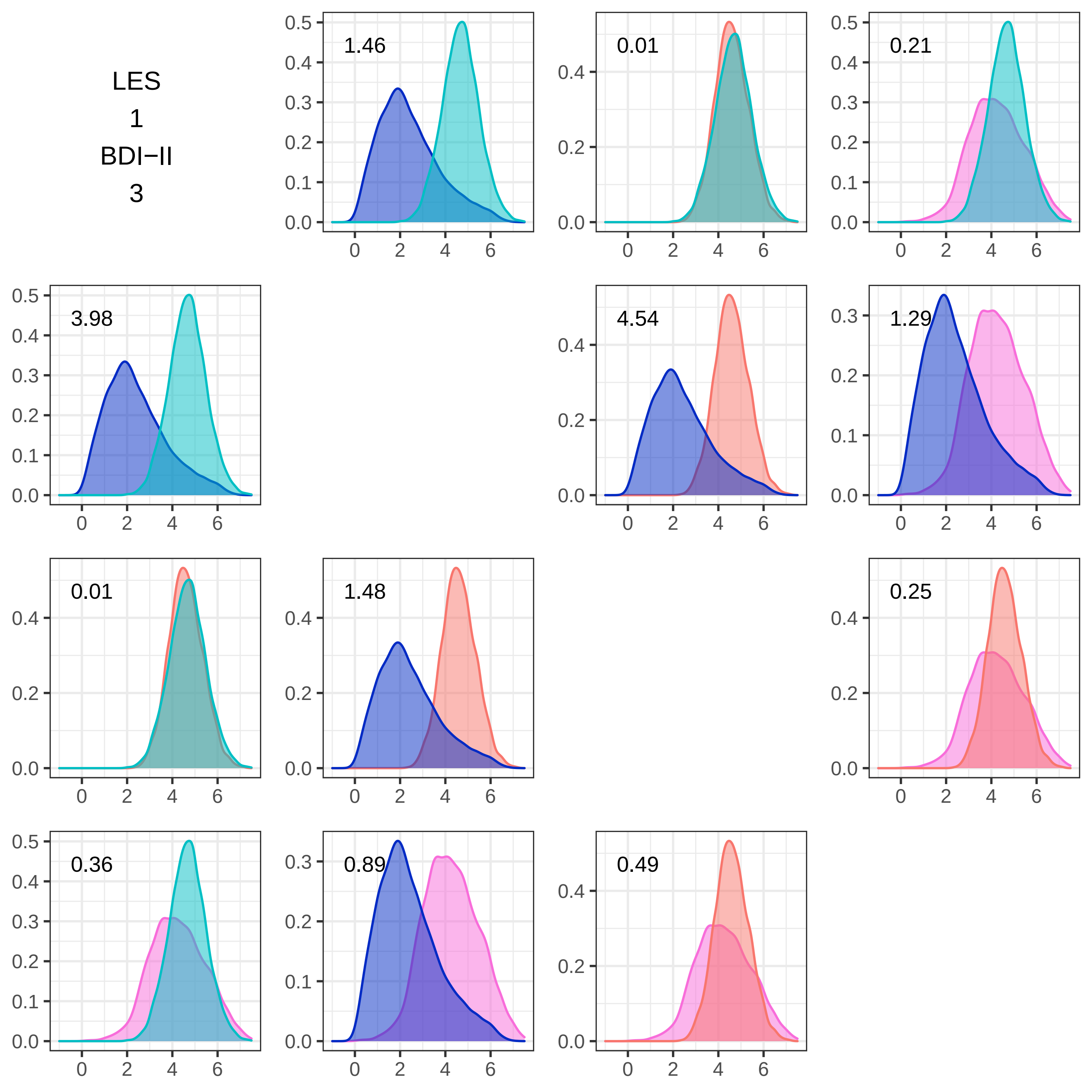

LES
1
BDI−II
3
1.46
0.01
0.21
3.98
4.54
1.29
0.01
1.48
0.25
0.36
0.89
0.49

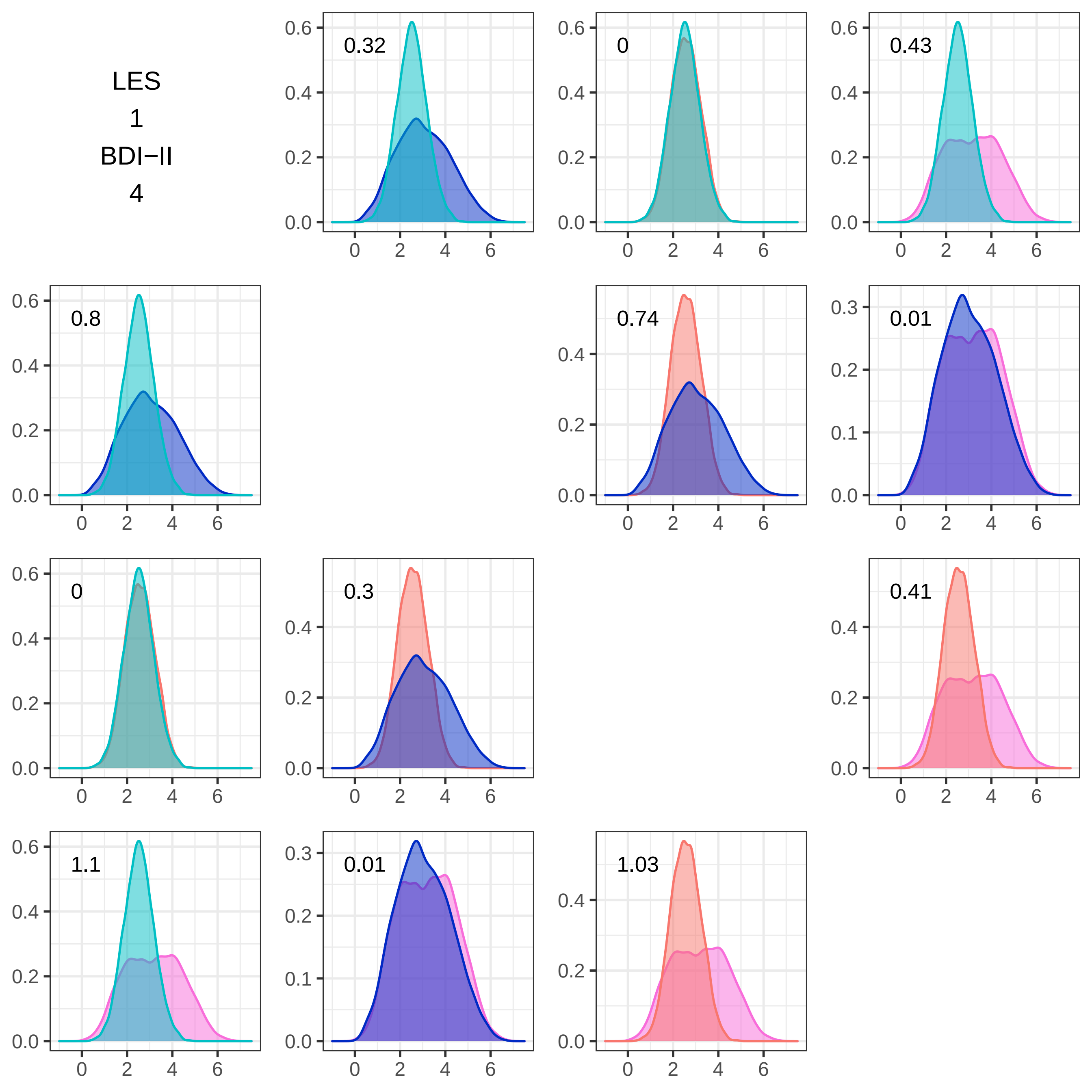
LES
1
BDI−II
4
0.32
0
0.43
0.8
0.74
0.01
0
0.3
0.41
1.1
0.01
1.03

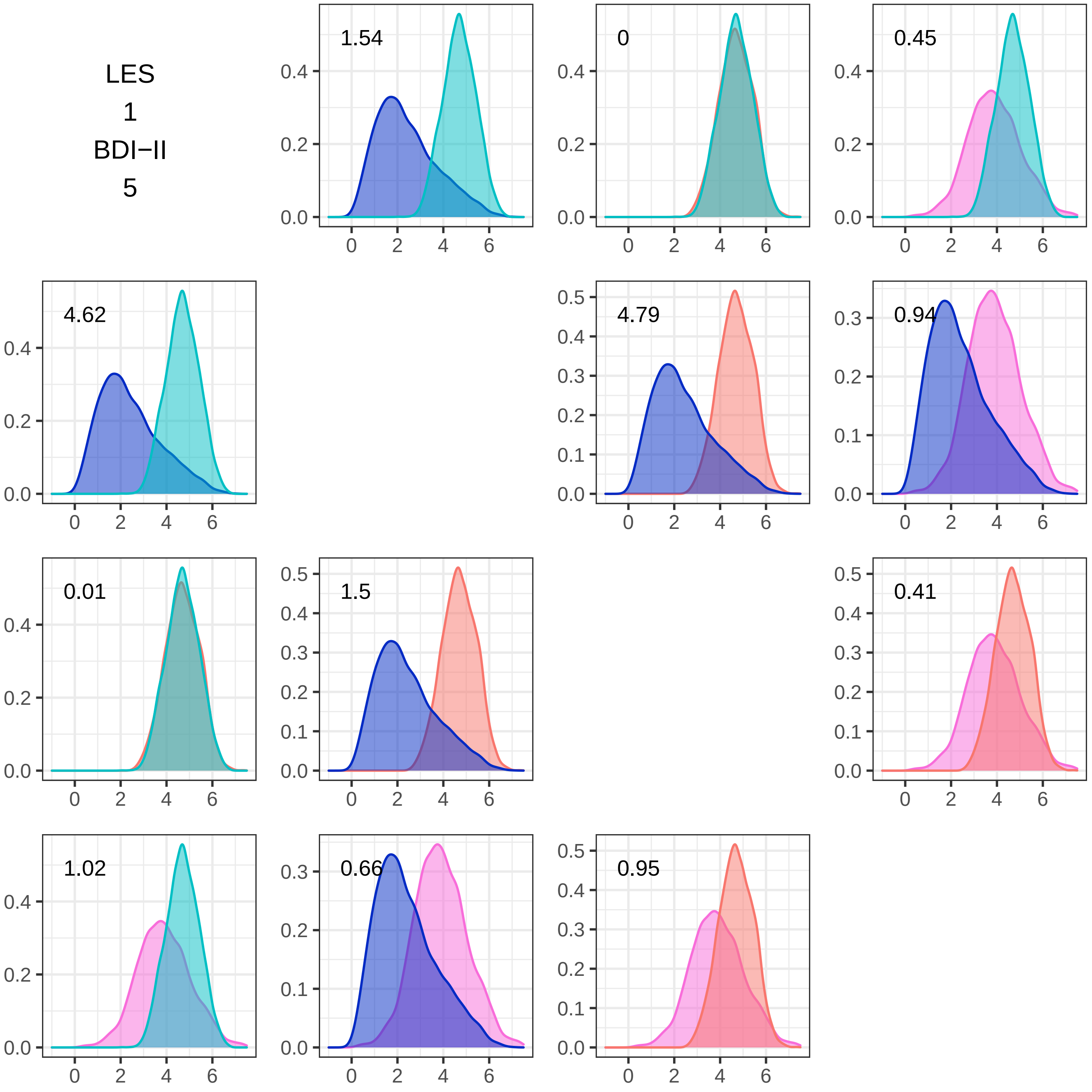
LES
1
BDI−II
5
1.54
0
0.45
4.62
4.79
0.94
0.01
1.5
0.41
1.02
0.66
0.95

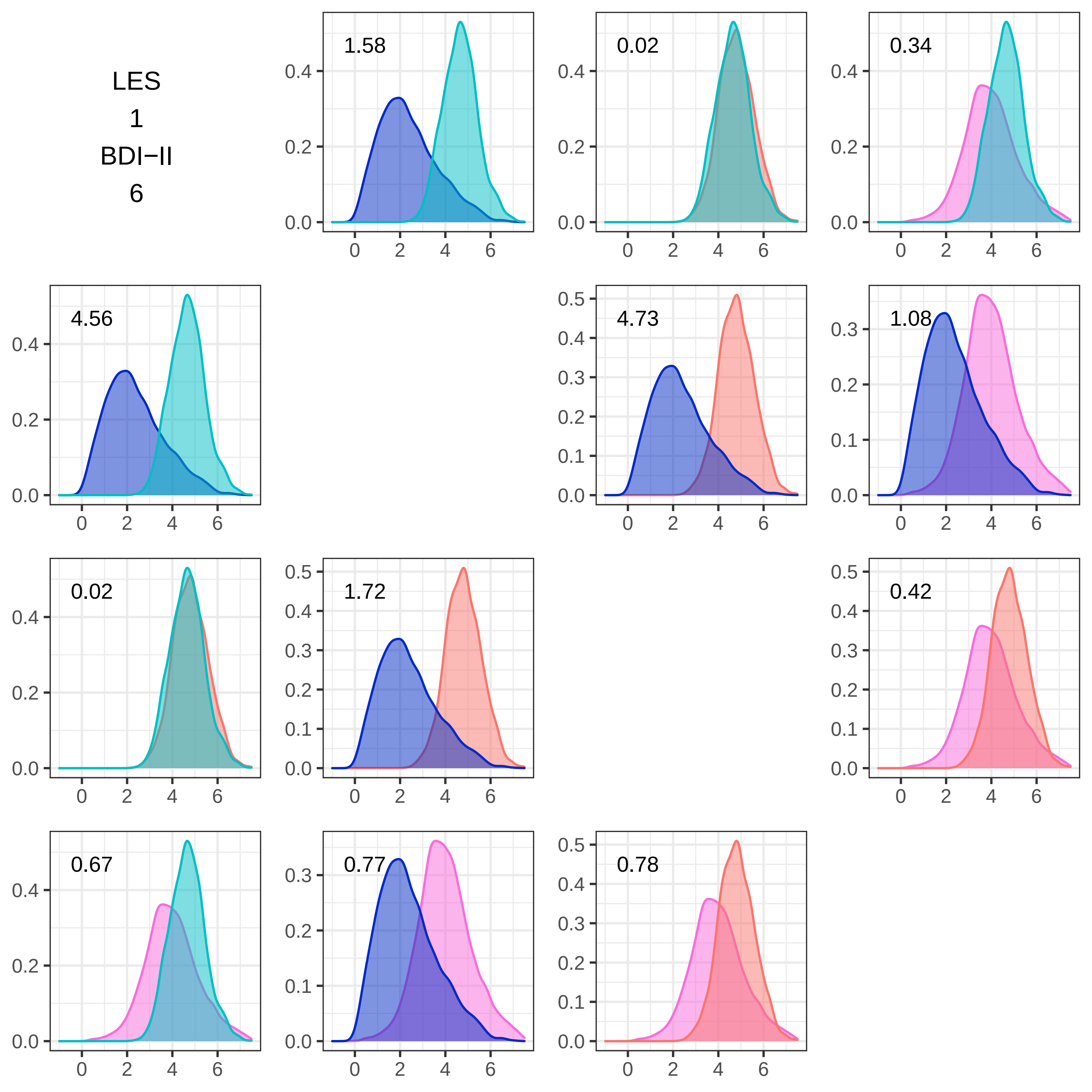
LES
1
BDI−II
6
1.58
0.02
0.34
4.56
4.73
1.08
0.02
1.72
0.42
0.67
0.77
0.78

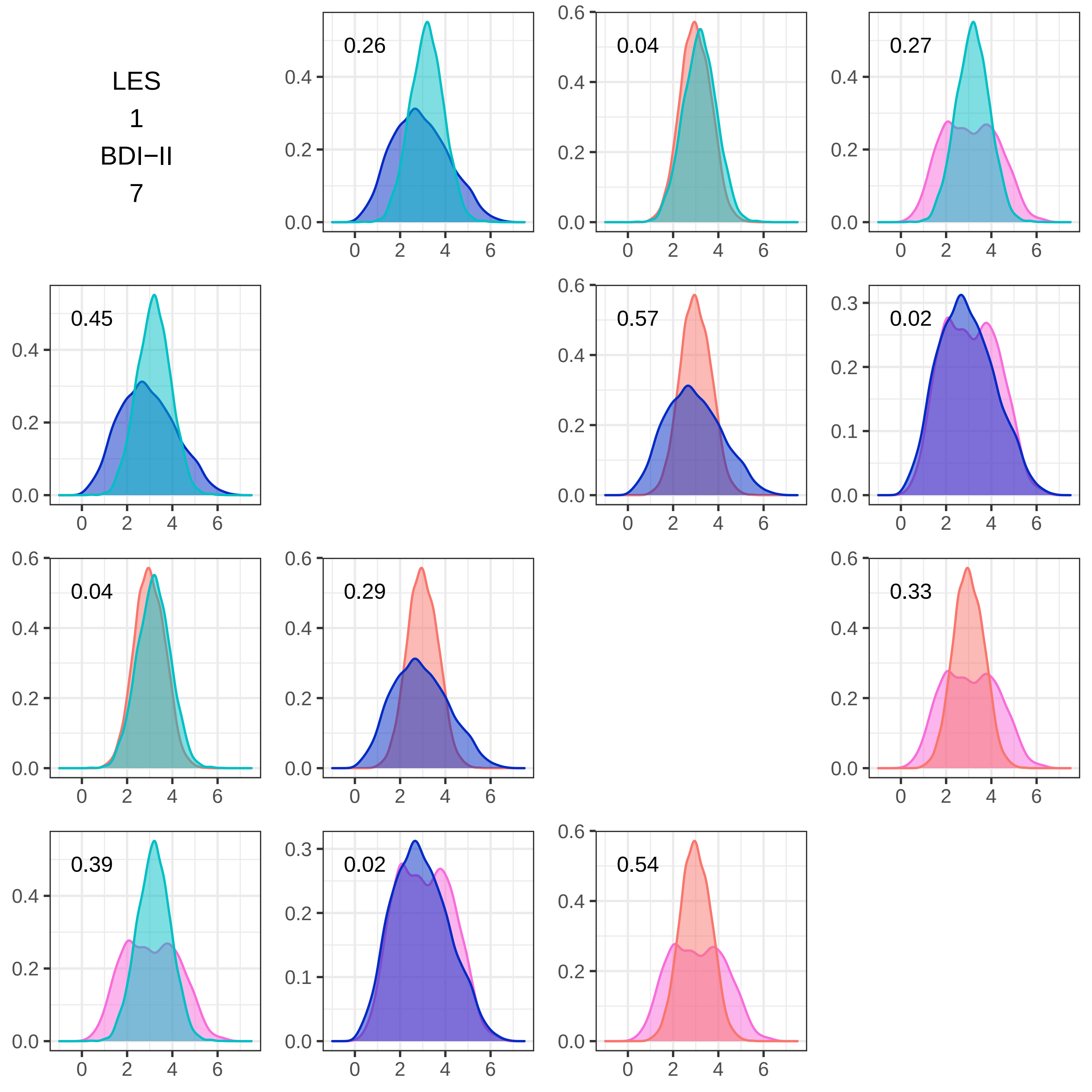

LES
1
BDI−II
7
0.26
0.04
0.27
0.45
0.57
0.02
0.04
0.29
0.33
0.39
0.02
0.54

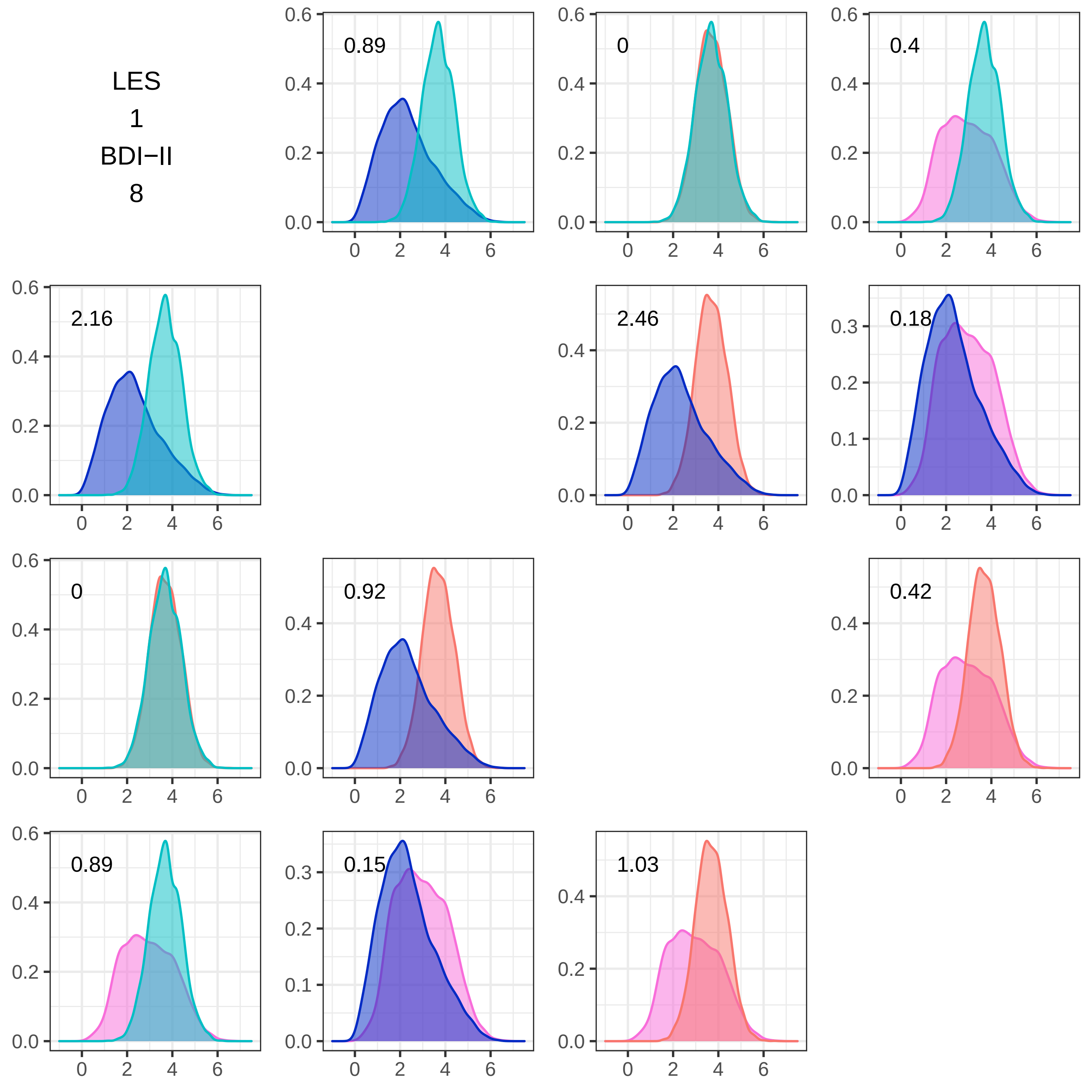
LES
1
BDI−II
8
0.89
0
0.4
2.16
2.46
0.18
0
0.92
0.42
0.89
0.15
1.03

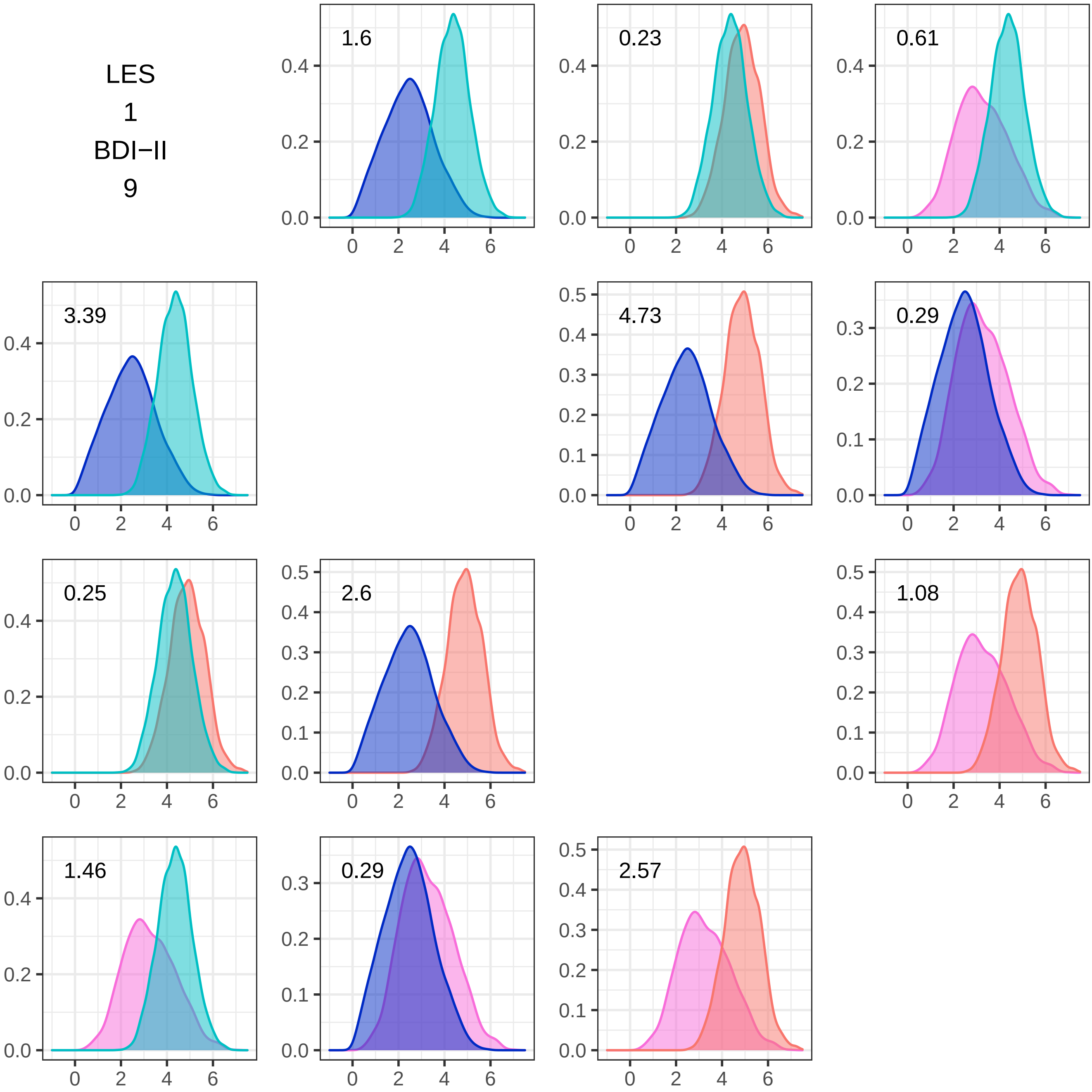

LES
1
BDI−II
9
1.6
0.23
0.61
3.39
4.73
0.29
0.25
2.6
1.08
1.46
0.29
2.57

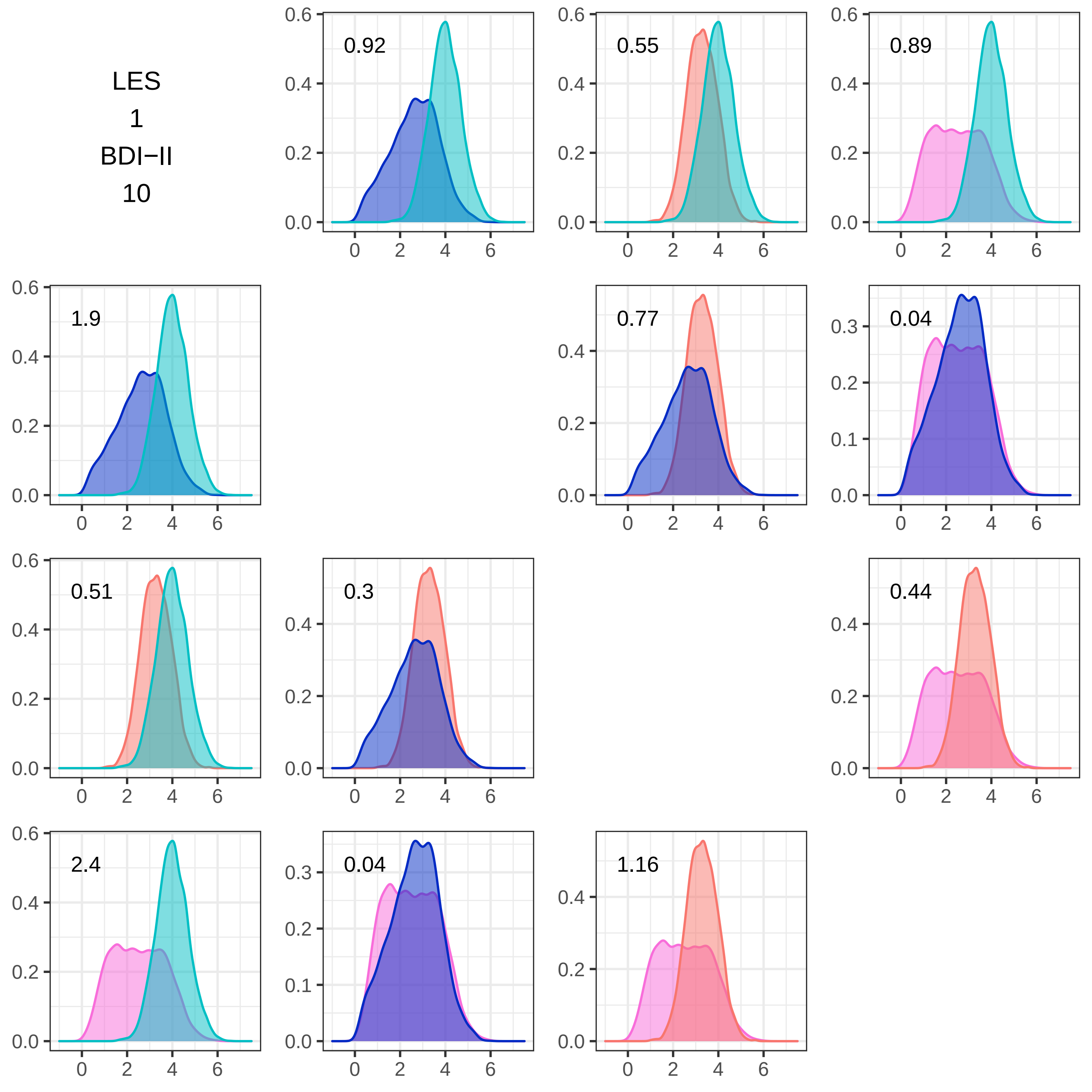
LES
1
BDI−II
10
0.92
0.55
0.89
1.9
0.77
0.04
0.51
0.3
0.44
2.4
0.04
1.16

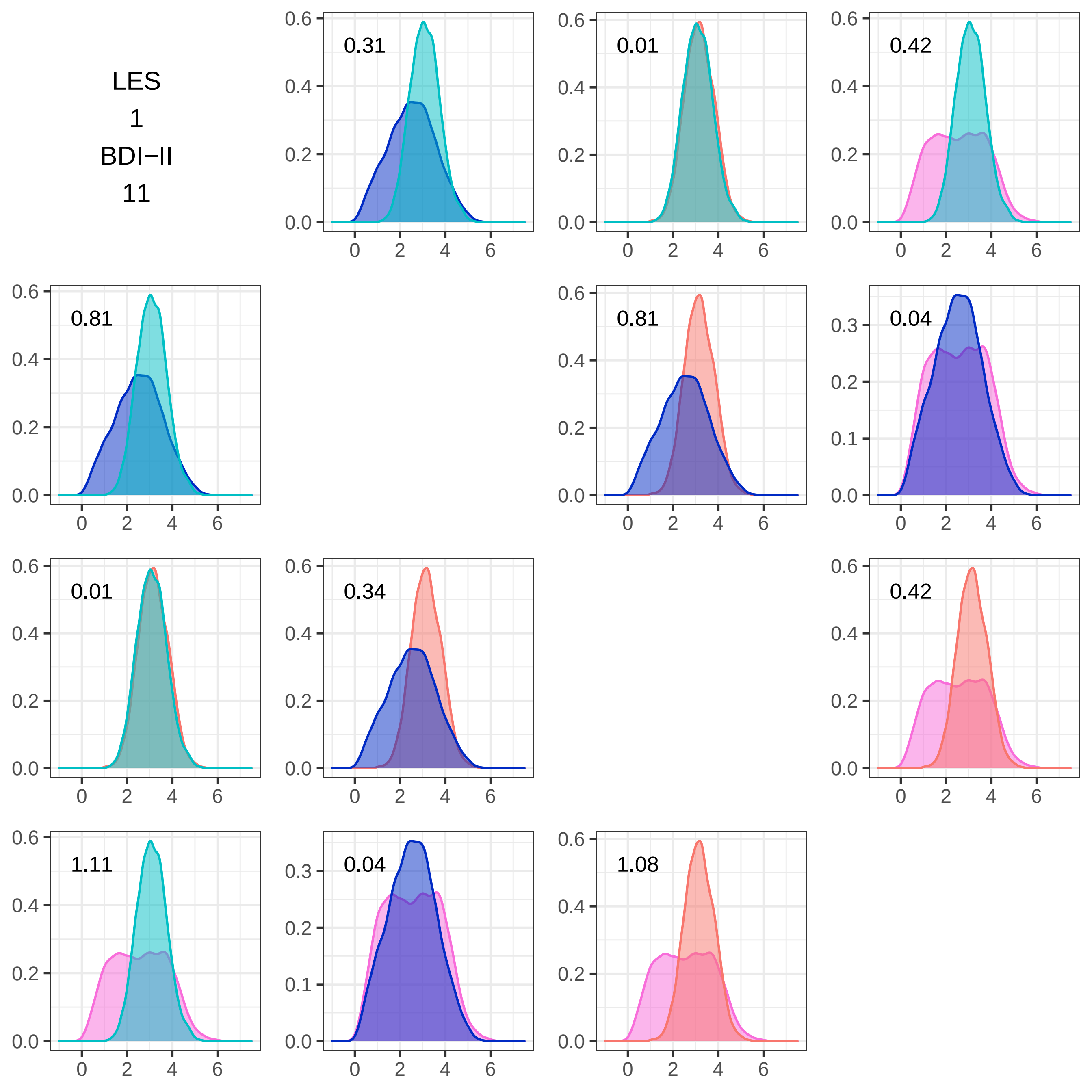
LES
1
BDI−II
11
0.31
0.01
0.42
0.81
0.81
0.04
0.01
0.34
0.42
1.11
0.04
1.08

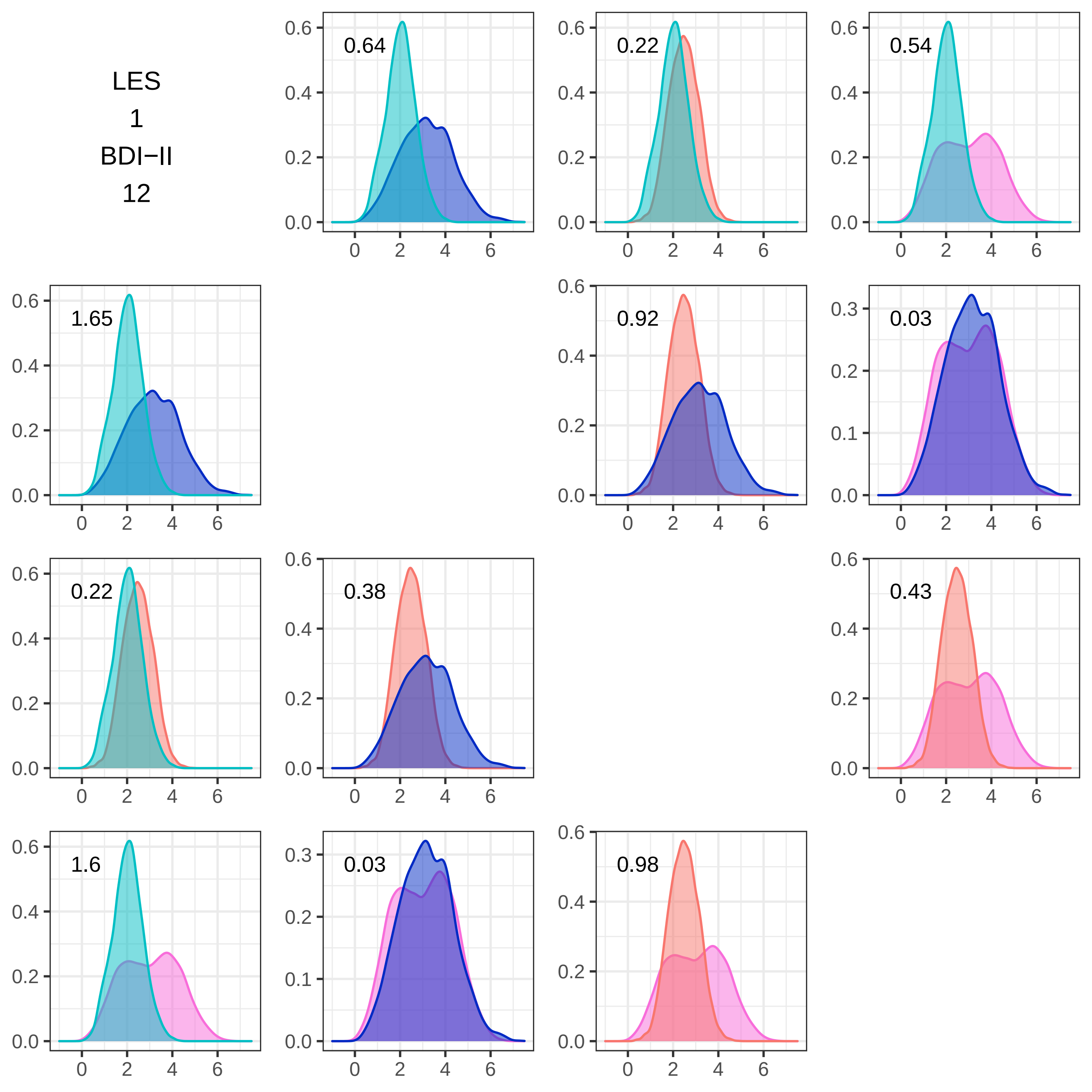
LES
1
BDI−II
12
0.64
0.22
0.54
1.65
0.92
0.03
0.22
0.38
0.43
1.6
0.03
0.98

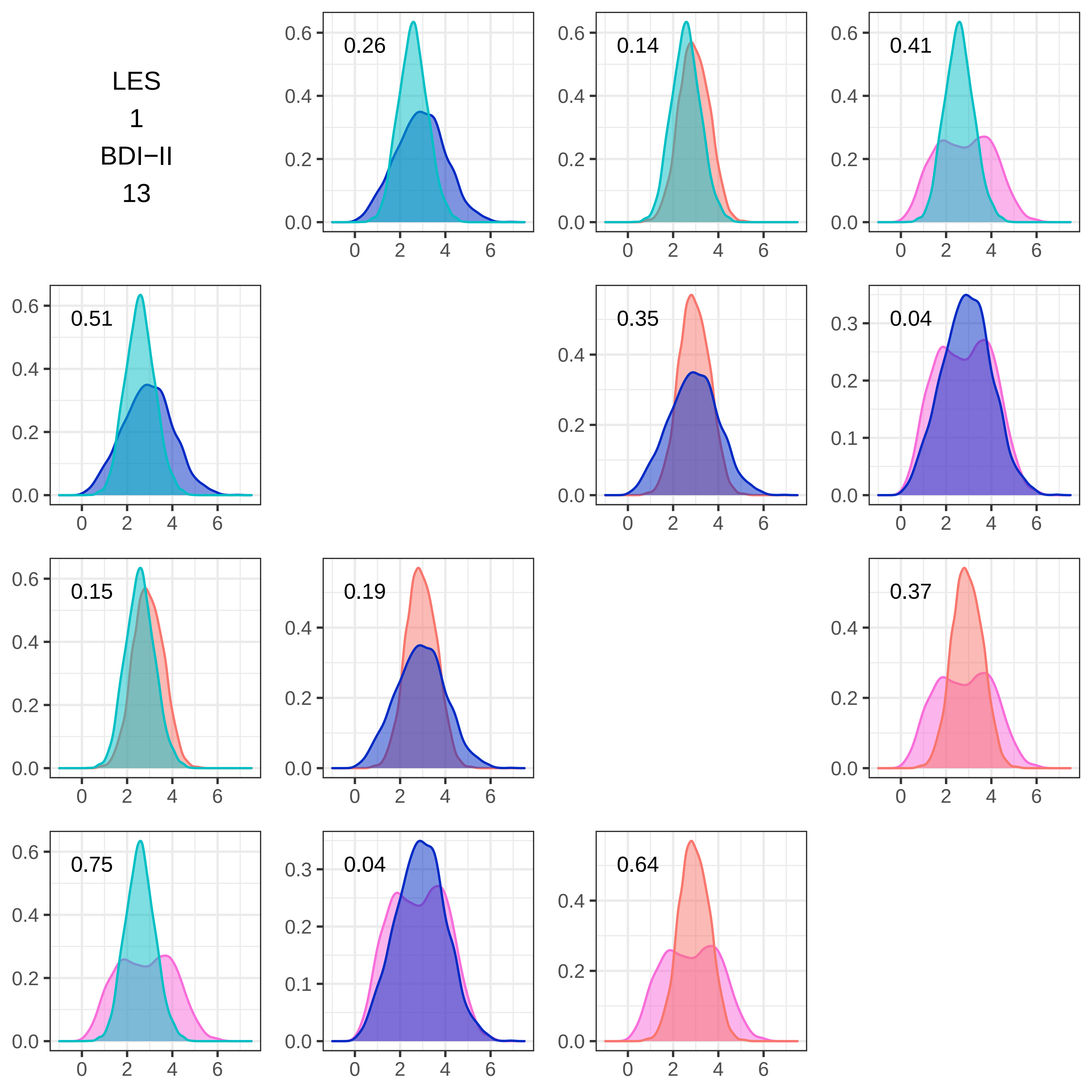
LES
1
BDI−II
13
0.26
0.14
0.41
0.51
0.35
0.04
0.15
0.19
0.37
0.75
0.04
0.64

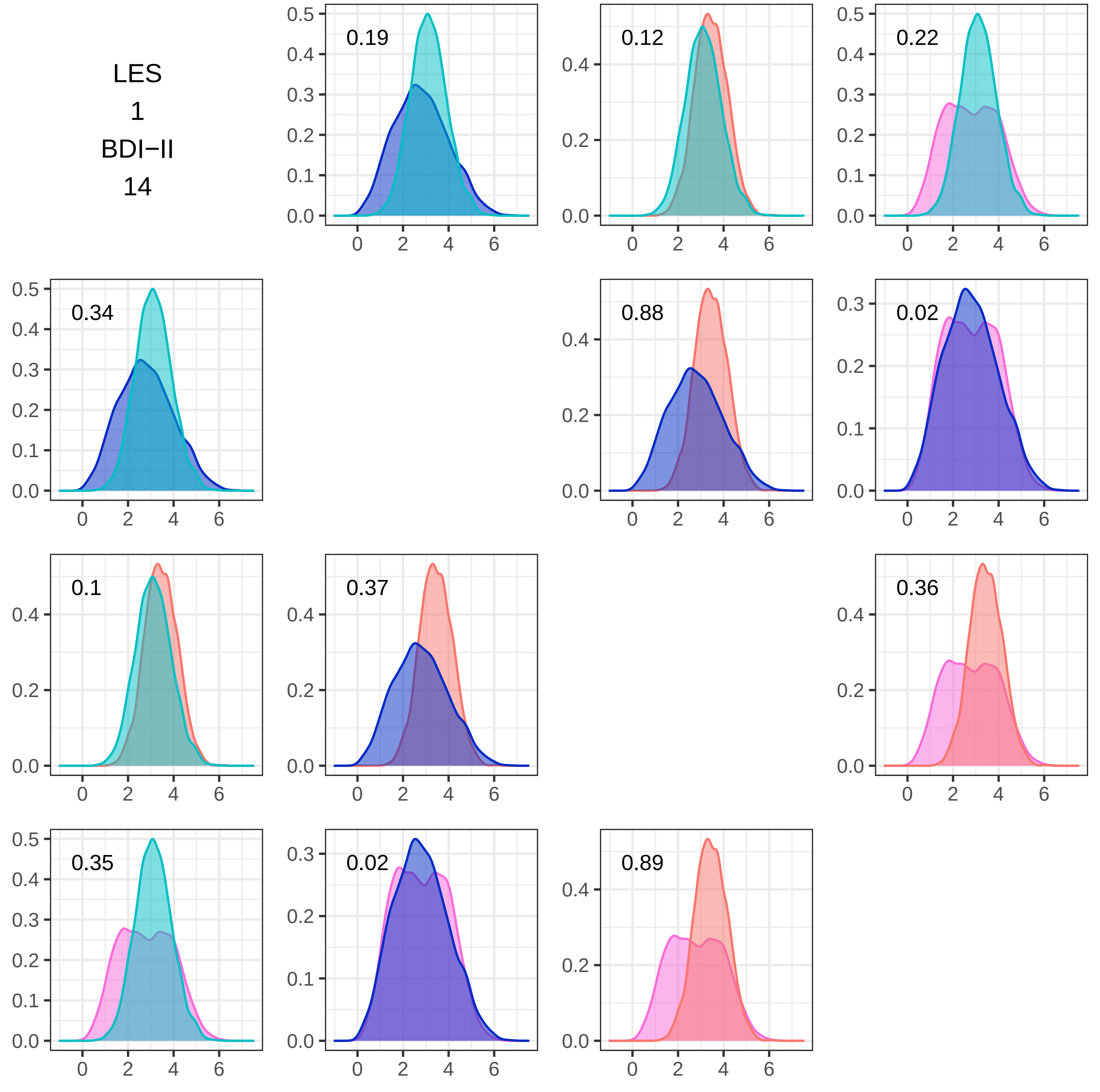

LES
1
BDI−II
14
0.19
0.12
0.22
0.34
0.88
0.02
0.1
0.37
0.36
0.35
0.02
0.89

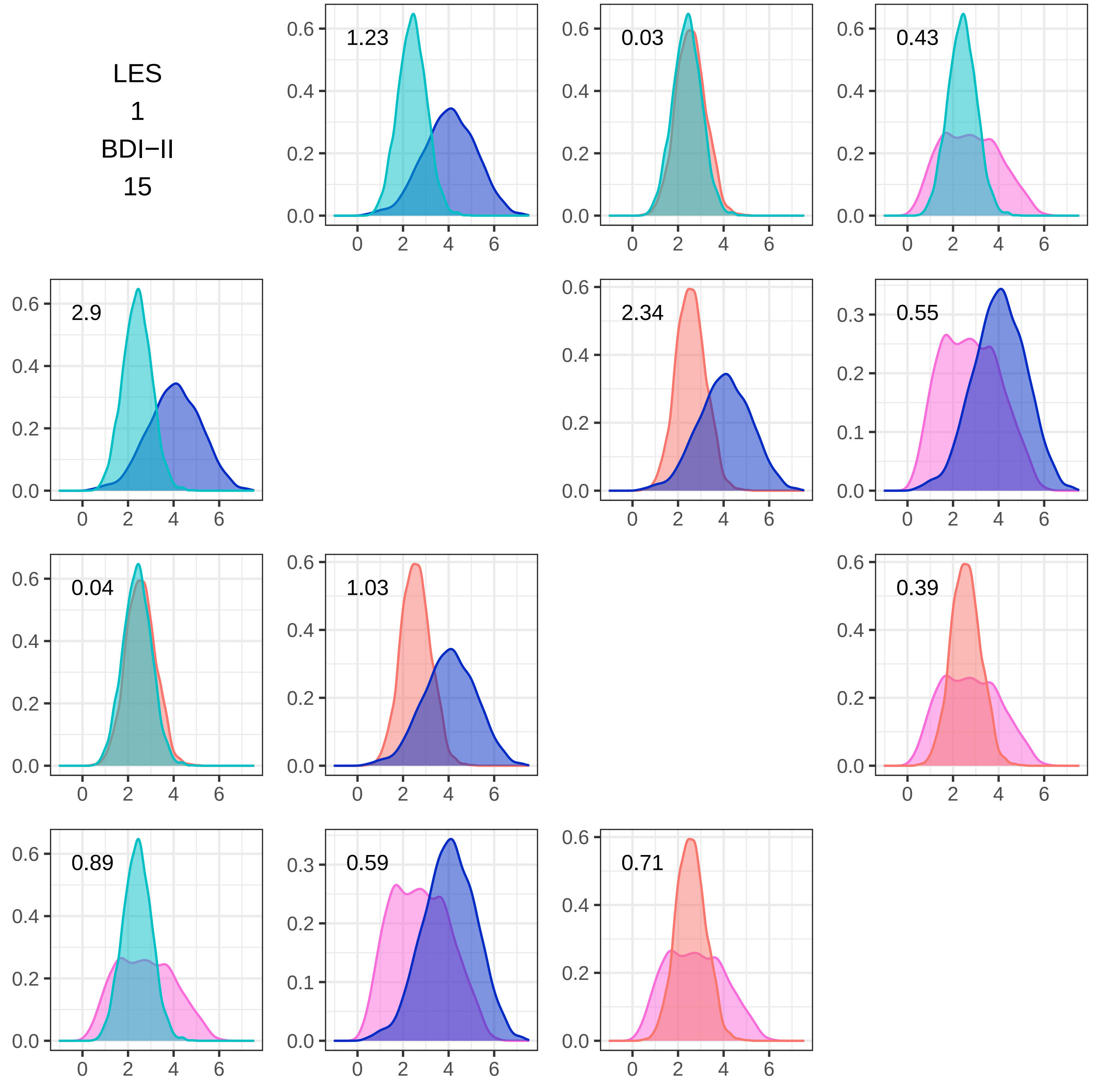
LES
1
BDI−II
15
1.23
0.03
0.43
2.9
2.34
0.55
0.04
1.03
0.39
0.89
0.59
0.71

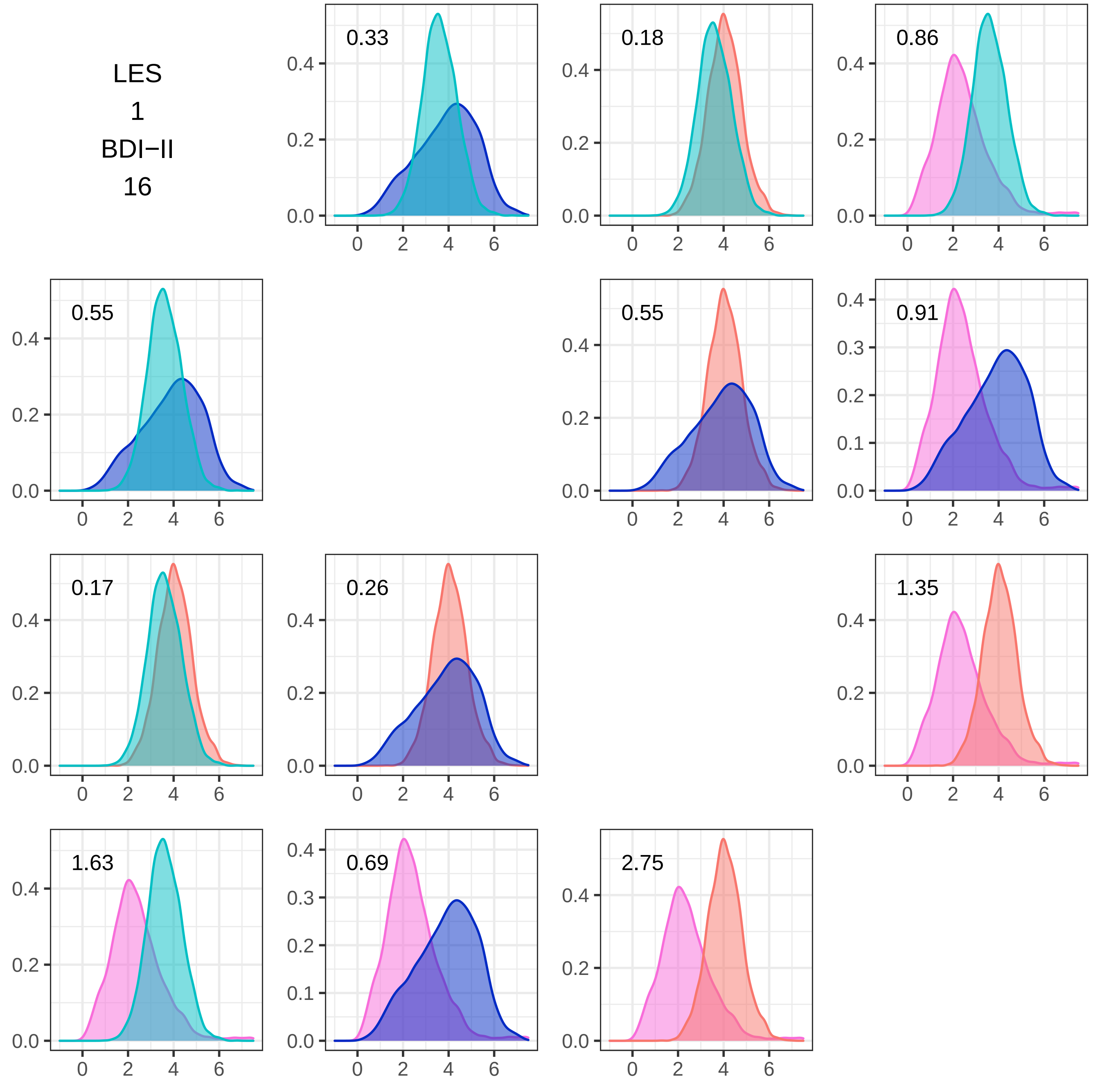

LES
1
BDI−II
16
0.33
0.18
0.86
0.55
0.55
0.91
0.17
0.26
1.35
1.63
0.69
2.75

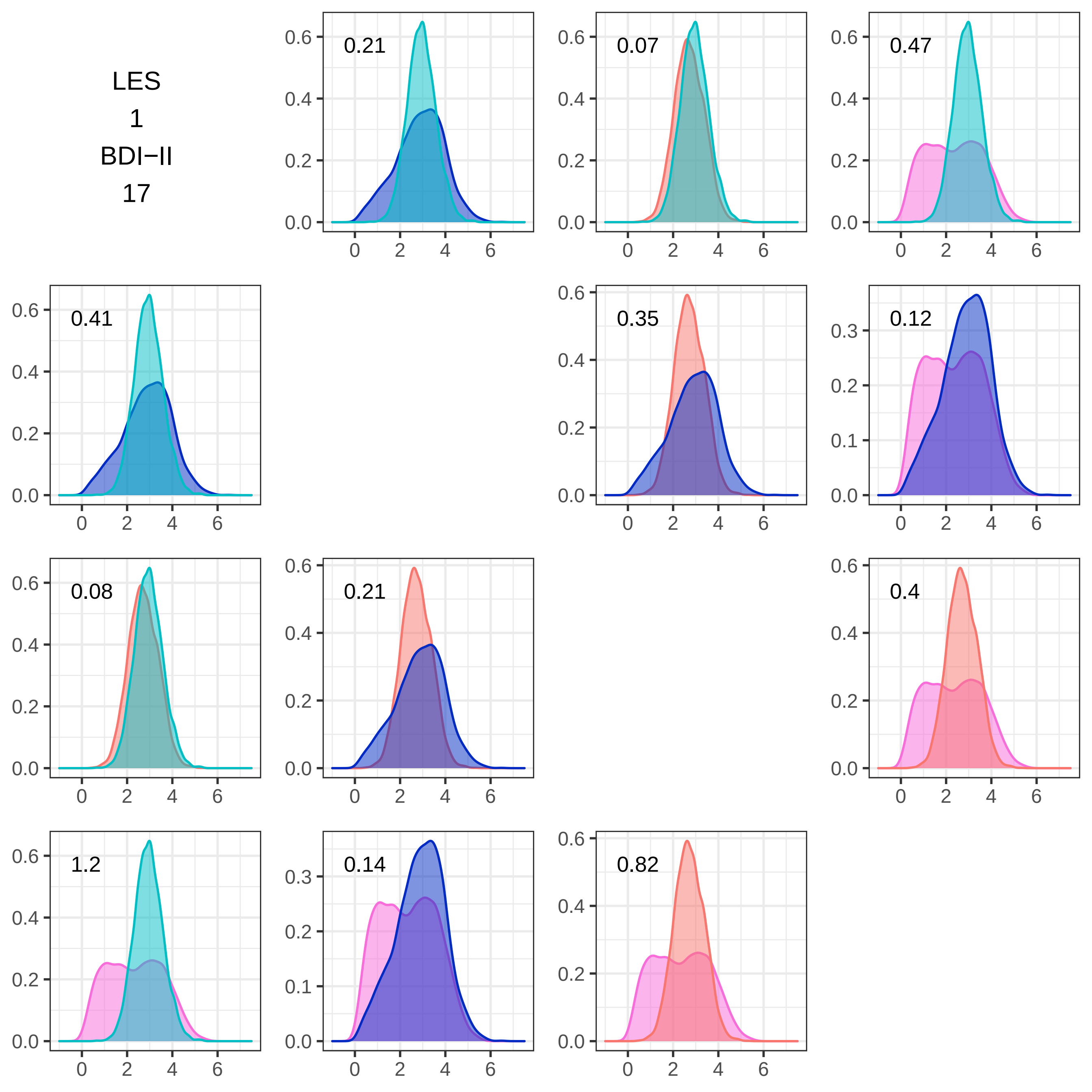
LES
1
BDI−II
17
0.21
0.07
0.47
0.41
0.35
0.12
0.08
0.21
0.4
1.2
0.14
0.82

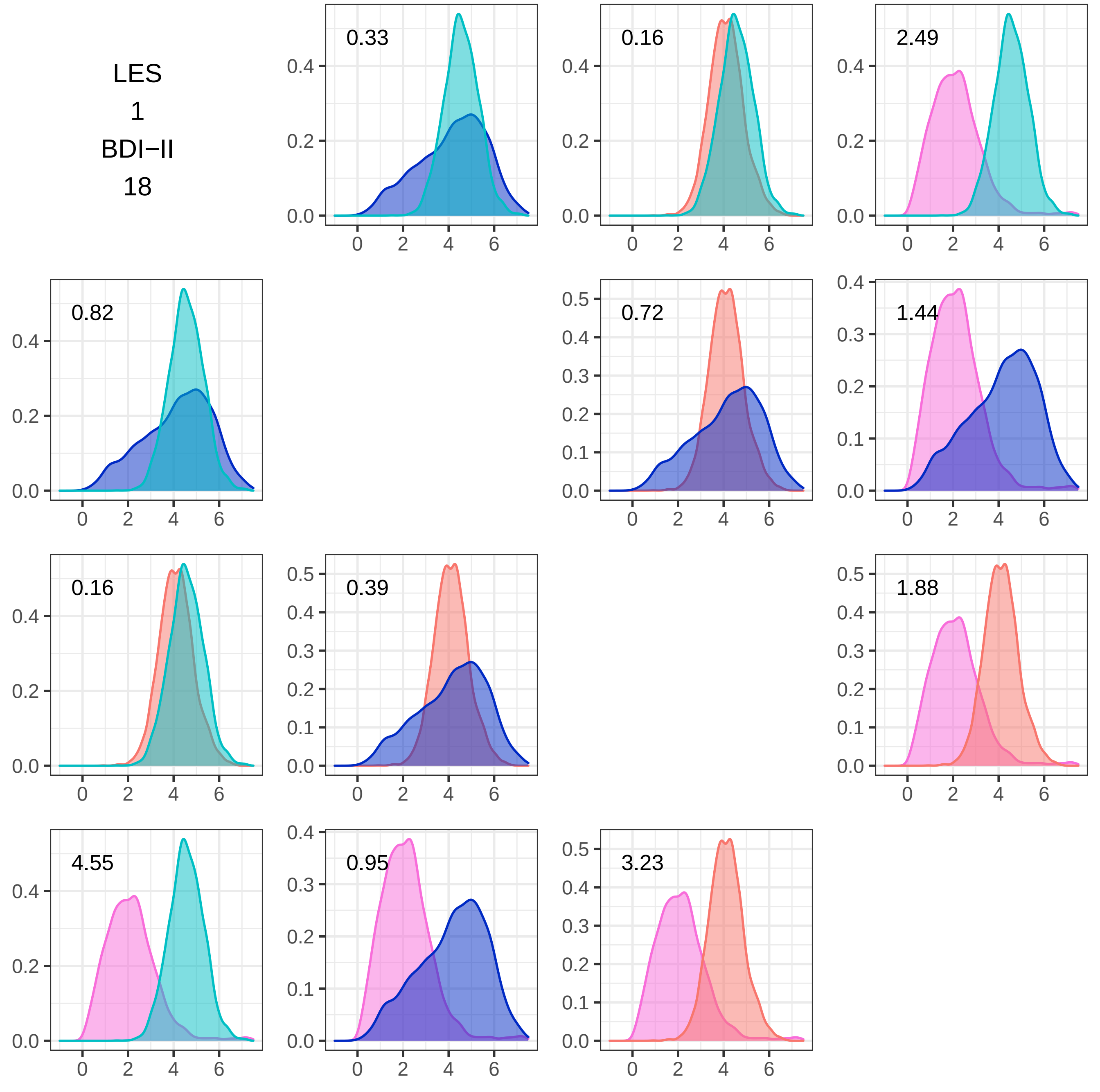
LES
1
BDI−II
18
0.33
0.16
2.49
0.82
0.72
1.44
0.16
0.39
1.88
4.55
0.95
3.23

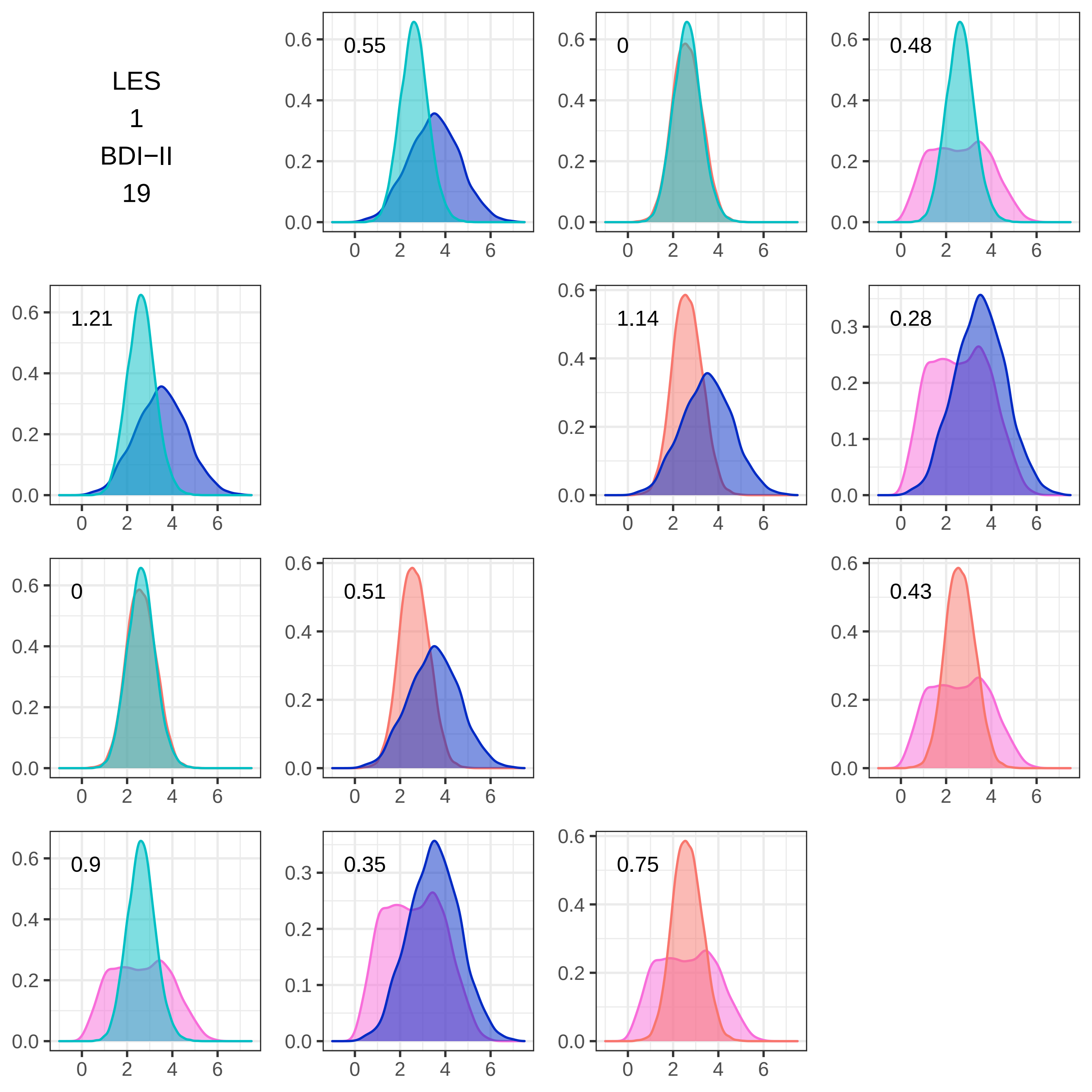
LES
1
BDI−II
19
0.55
0
0.48
1.21
1.14
0.28
0
0.51
0.43
0.9
0.35
0.75

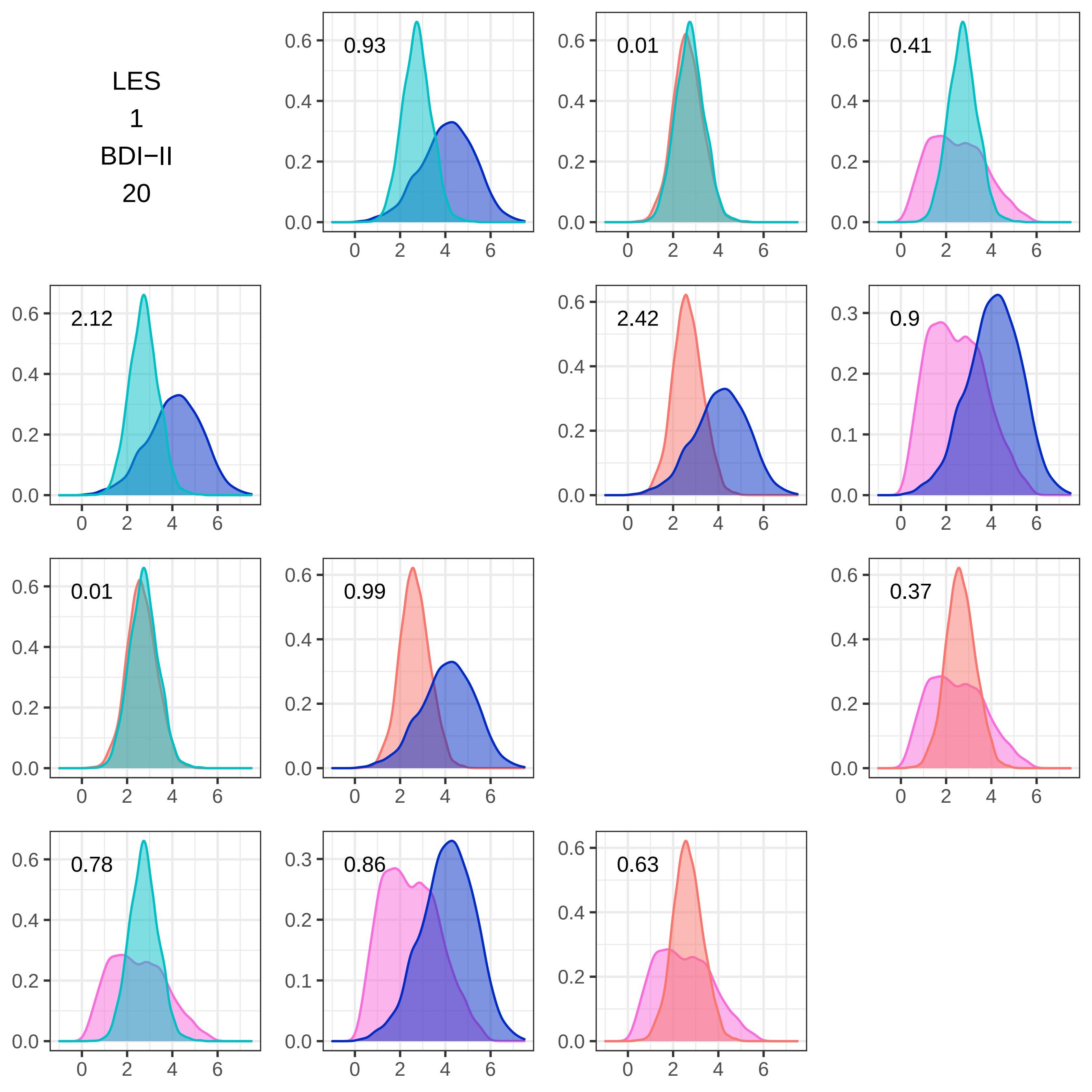
LES
1
BDI−II
20
0.93
0.01
0.41
2.12
2.42
0.9
0.01
0.99
0.37
0.78
0.86
0.63

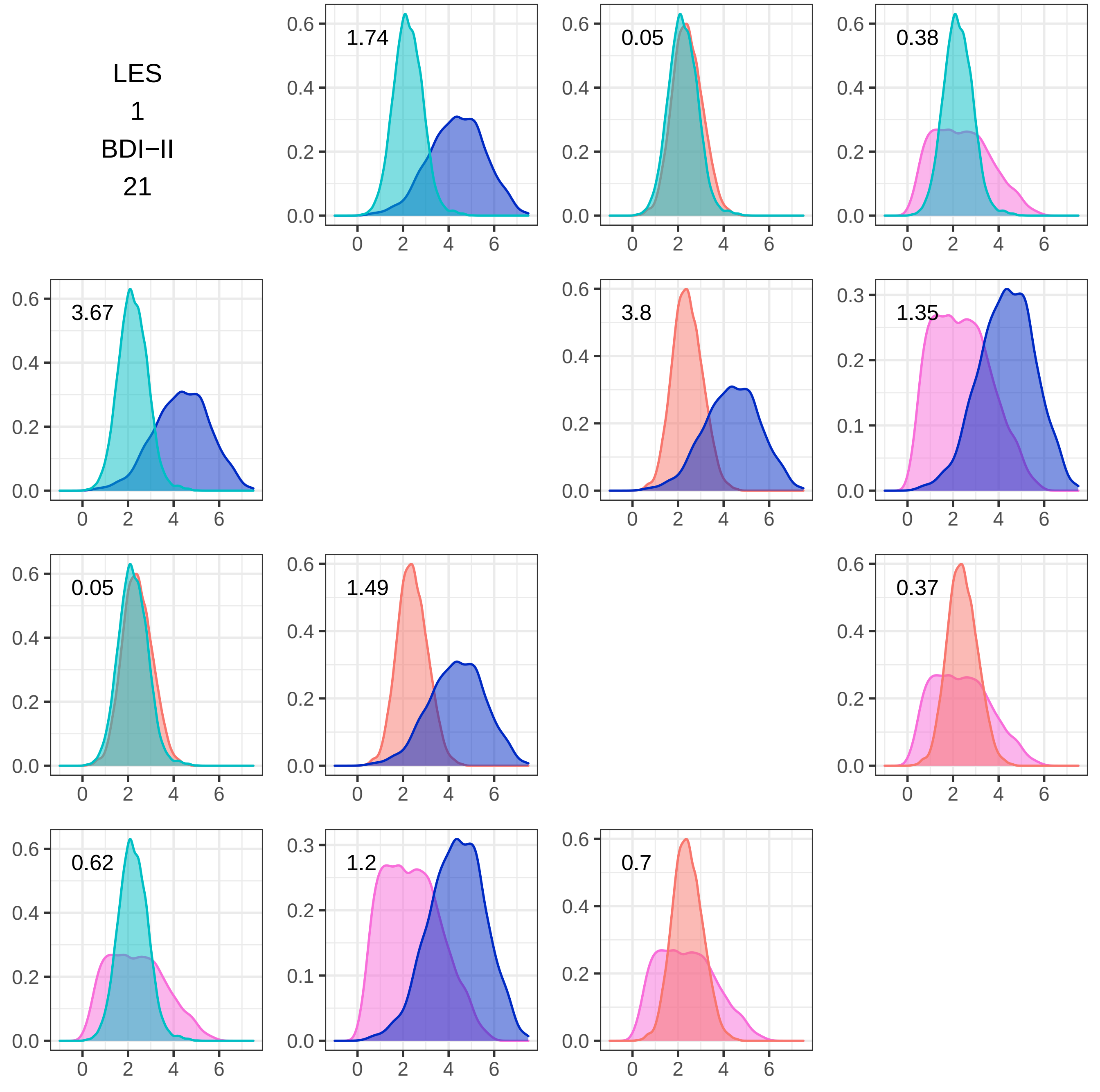

LES
1
BDI−II
21
1.74
0.05
0.38
3.67
3.8
1.35
0.05
1.49
0.37
0.62
1.2
0.7

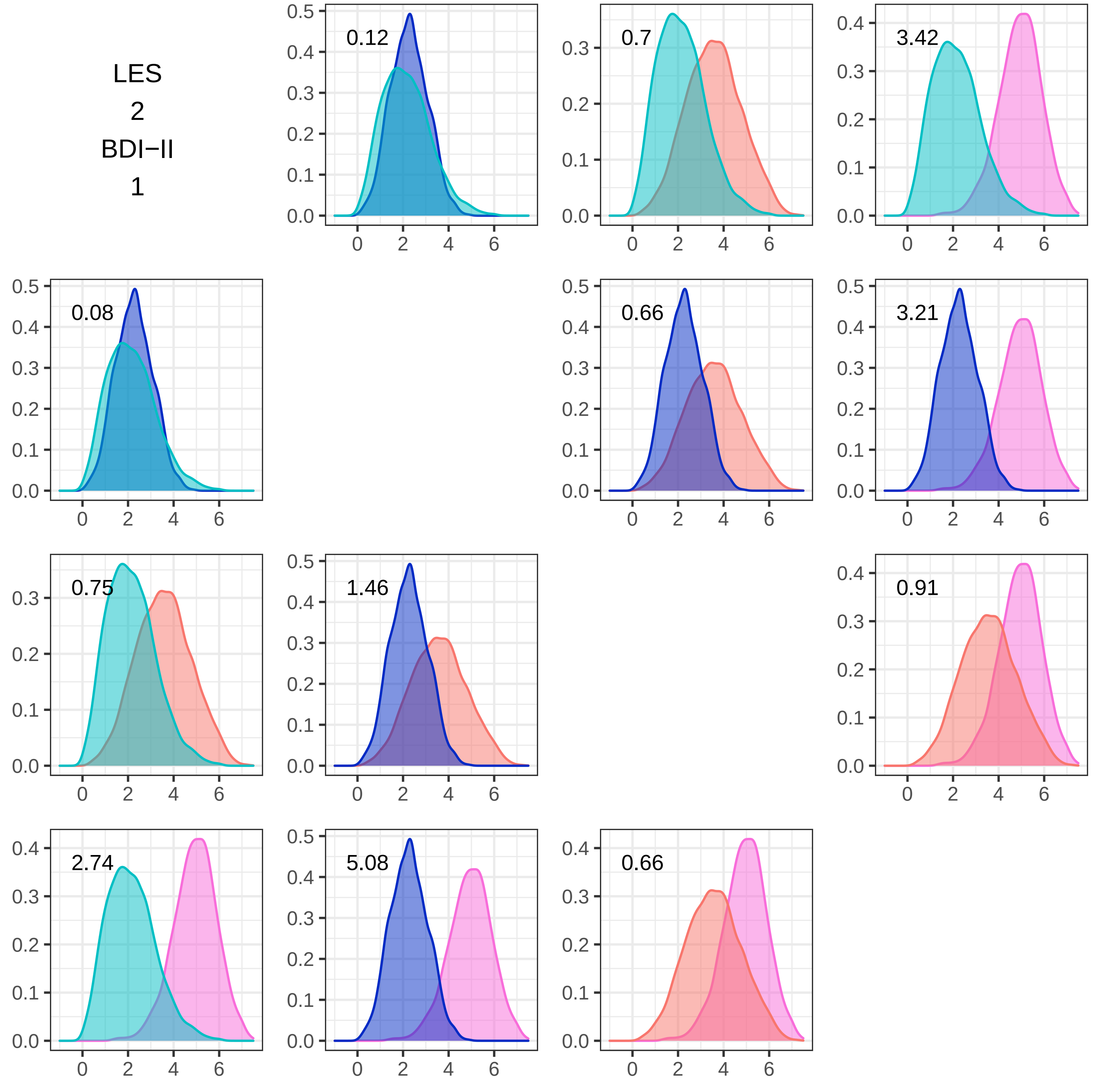
LES
2
BDI−II
1
0.12
0.7
3.42
0.08
0.66
3.21
0.75
1.46
0.91
2.74
5.08
0.66

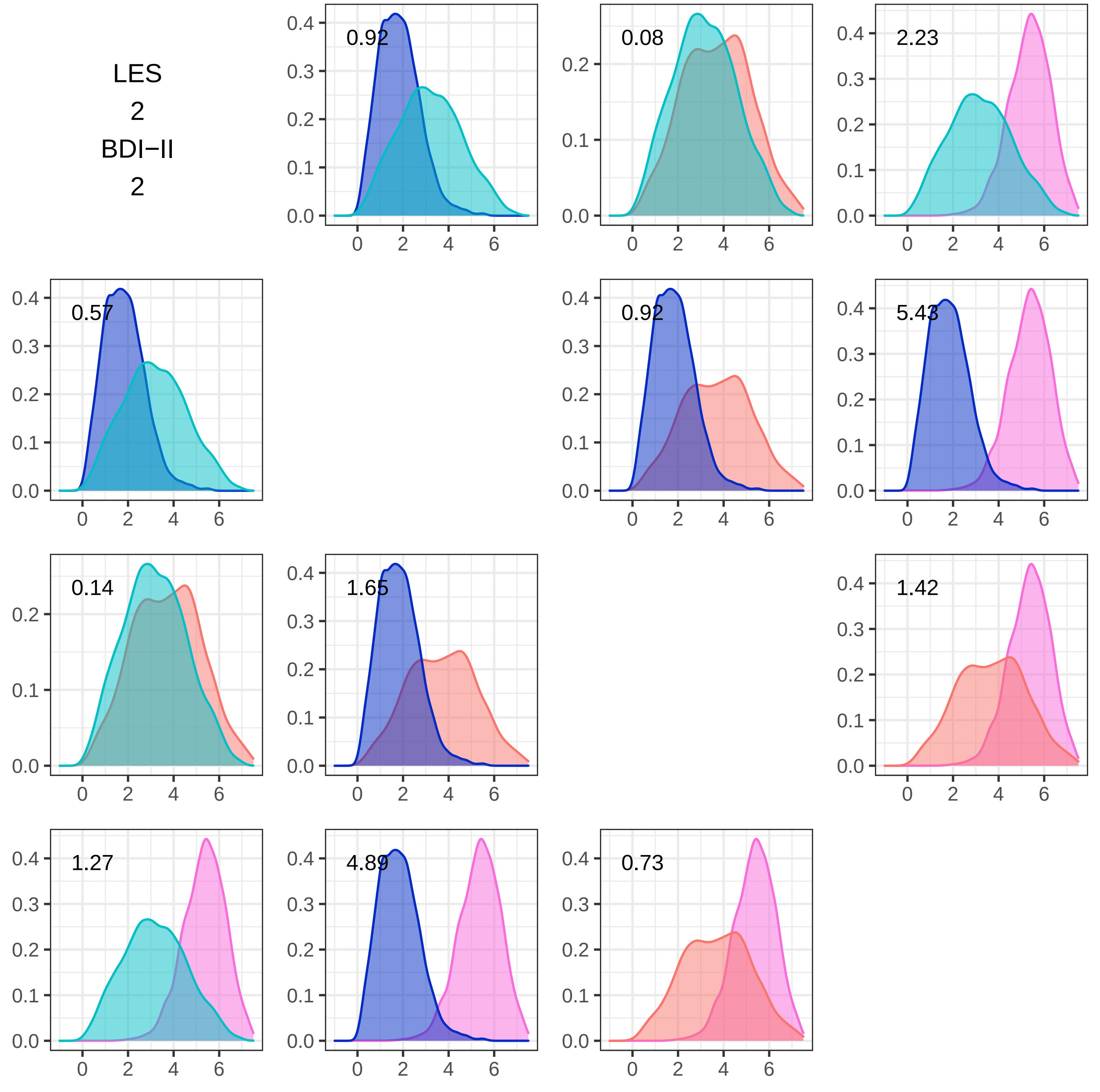
LES
2
BDI−II
2
0.92
0.08
2.23
0.57
0.92
5.43
0.14
1.65
1.42
1.27
4.89
0.73

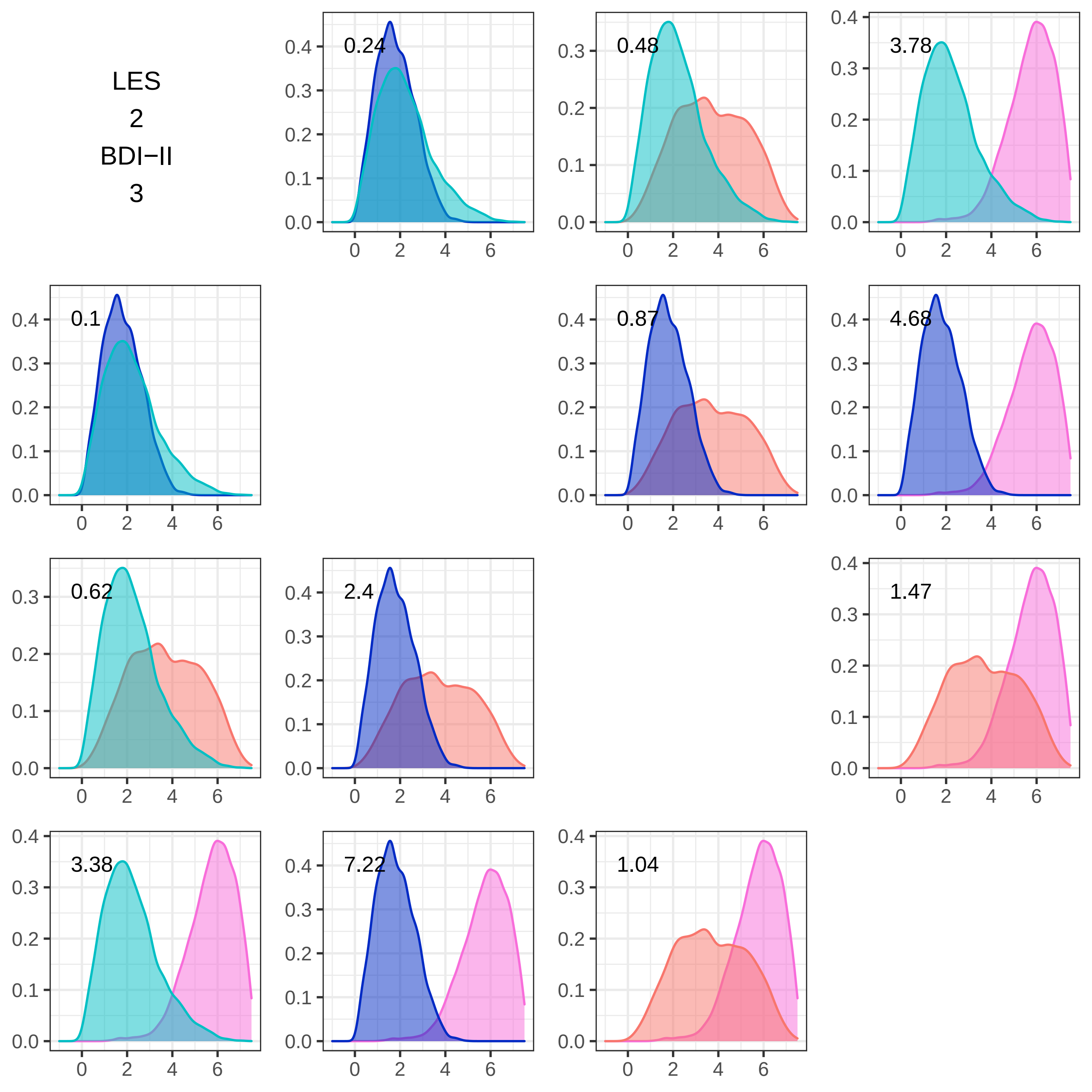
LES
2
BDI−II
3
0.24
0.48
3.78
0.1
0.87
4.68
0.62
2.4
1.47
3.38
7.22
1.04

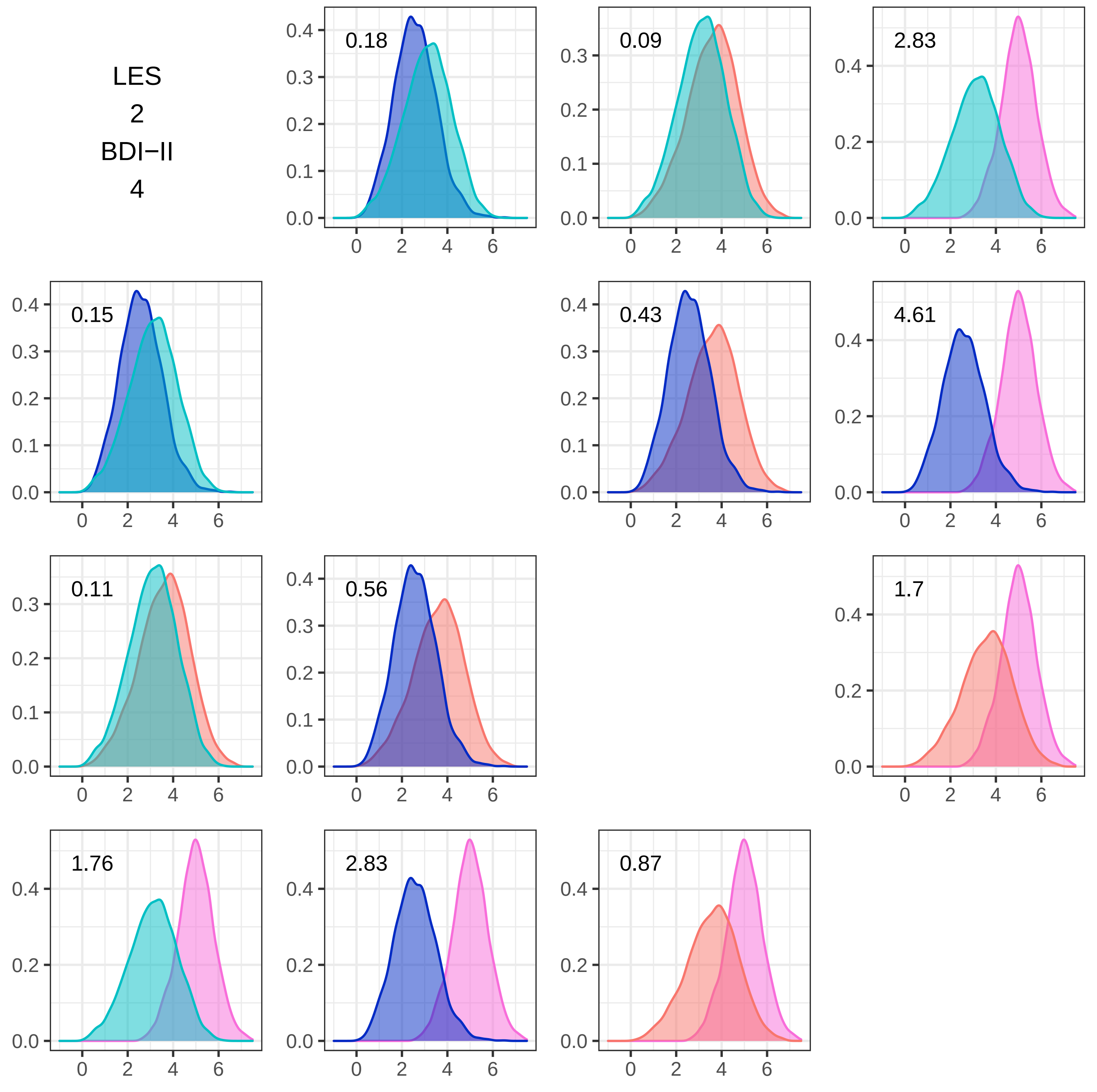
LES
2
BDI−II
4
0.18
0.09
2.83
0.15
0.43
4.61
0.11
0.56
1.7
1.76
2.83
0.87

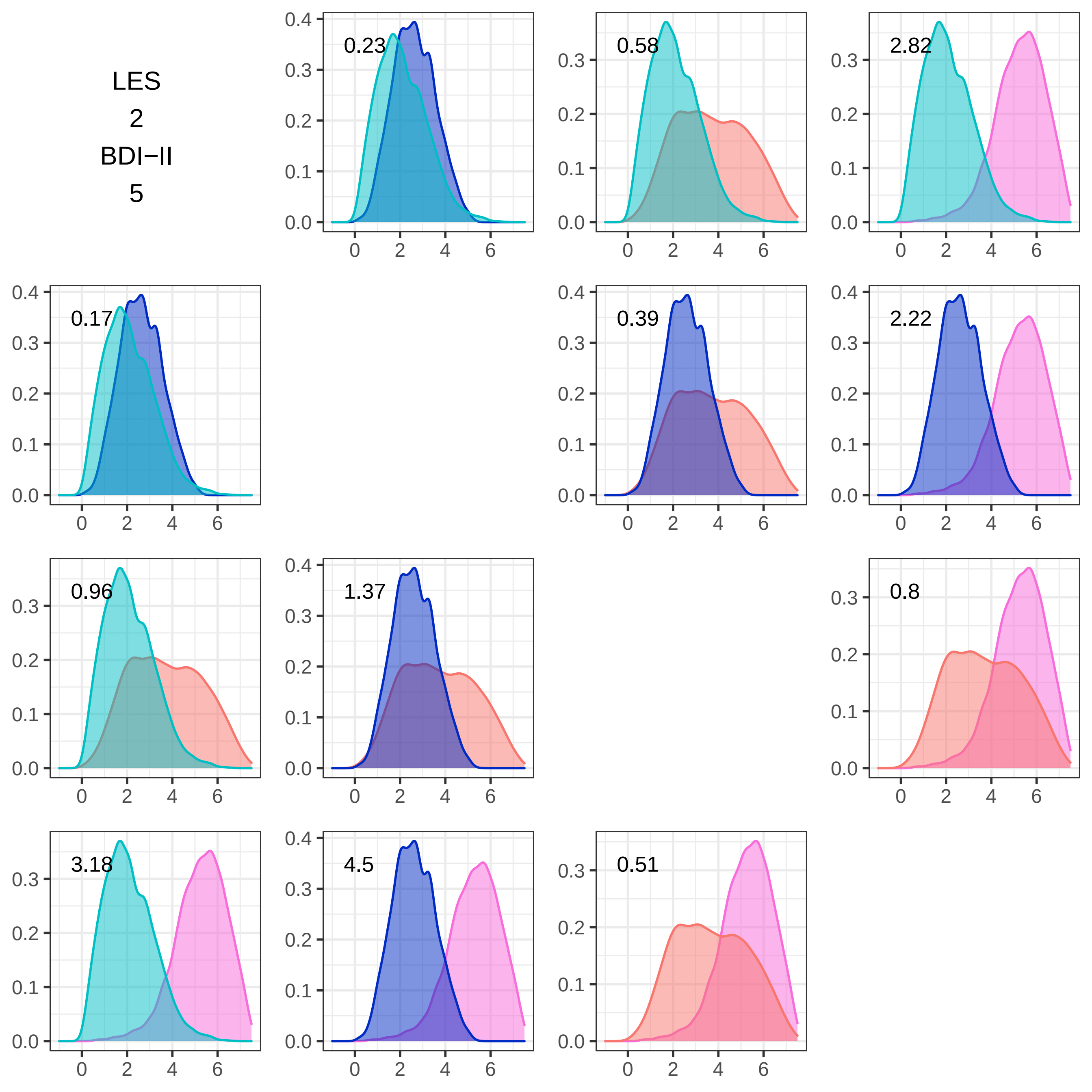

LES
2
BDI−II
5
0.23
0.58
2.82
0.17
0.39
2.22
0.96
1.37
0.8
3.18
4.5
0.51

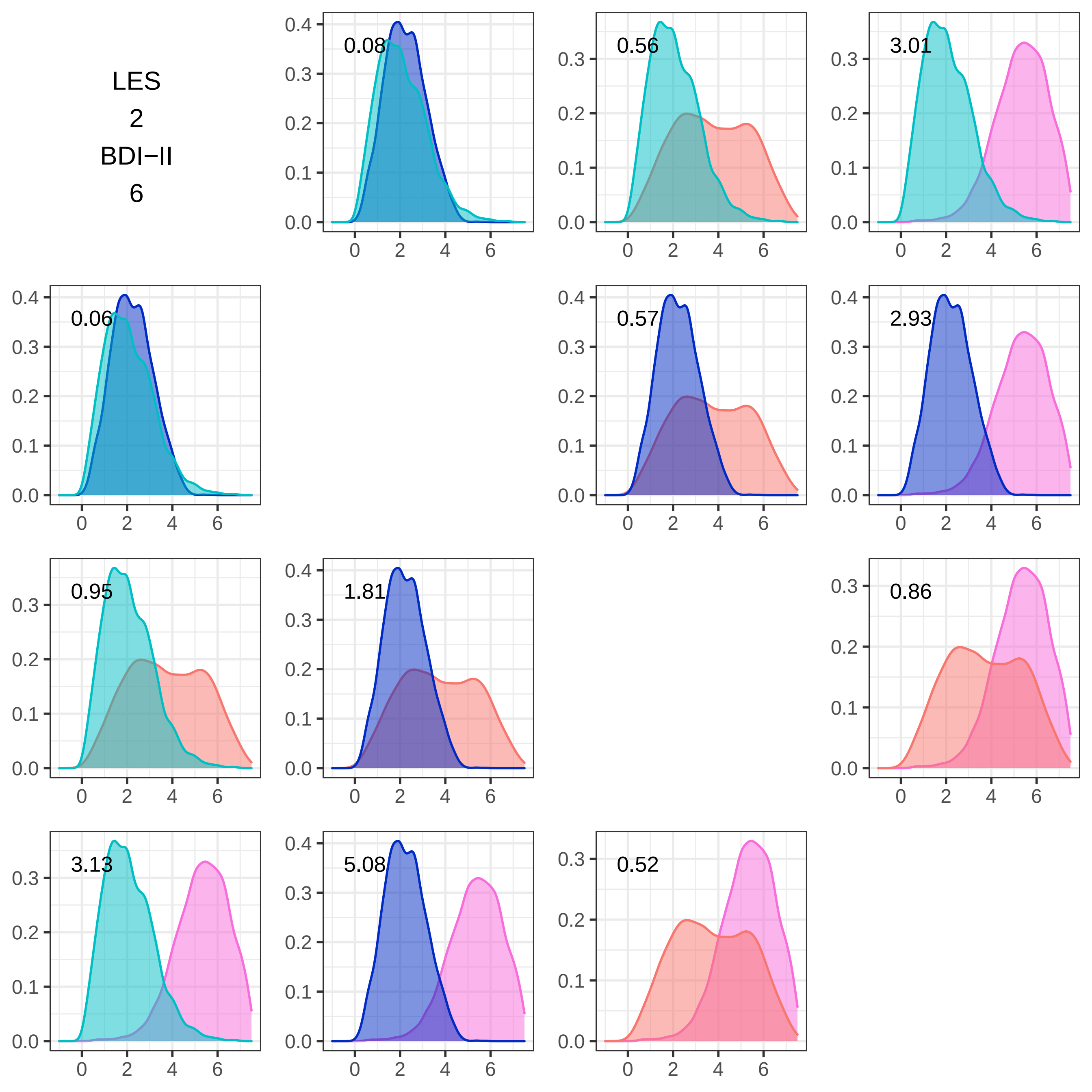
LES
2
BDI−II
6
0.08
0.56
3.01
0.06
0.57
2.93
0.95
1.81
0.86
3.13
5.08
0.52

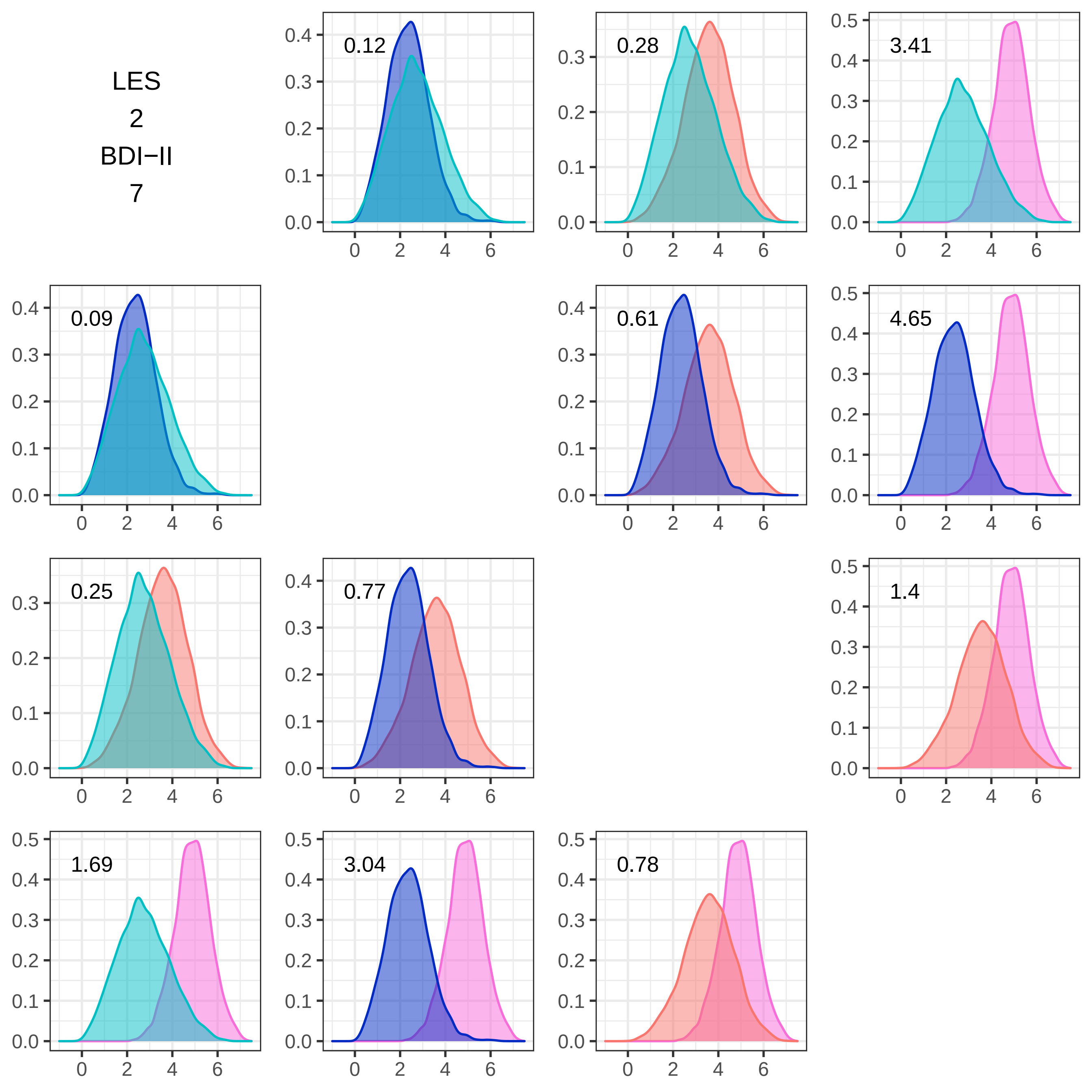

LES
2
BDI−II
7
0.12
0.28
3.41
0.09
0.61
4.65
0.25
0.77
1.4
1.69
3.04
0.78

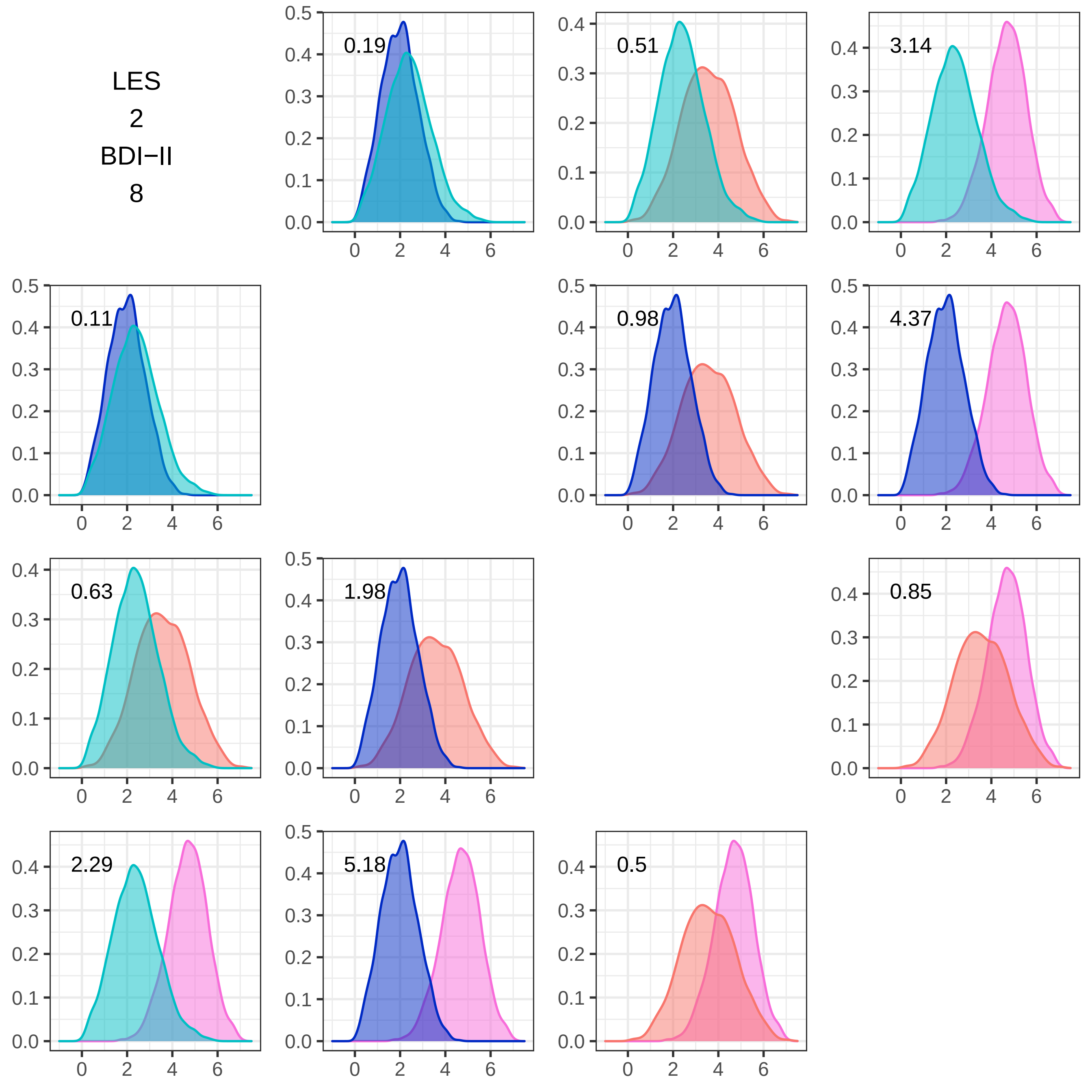
LES
2
BDI−II
8
0.19
0.51
3.14
0.11
0.98
4.37
0.63
1.98
0.85
2.29
5.18
0.5

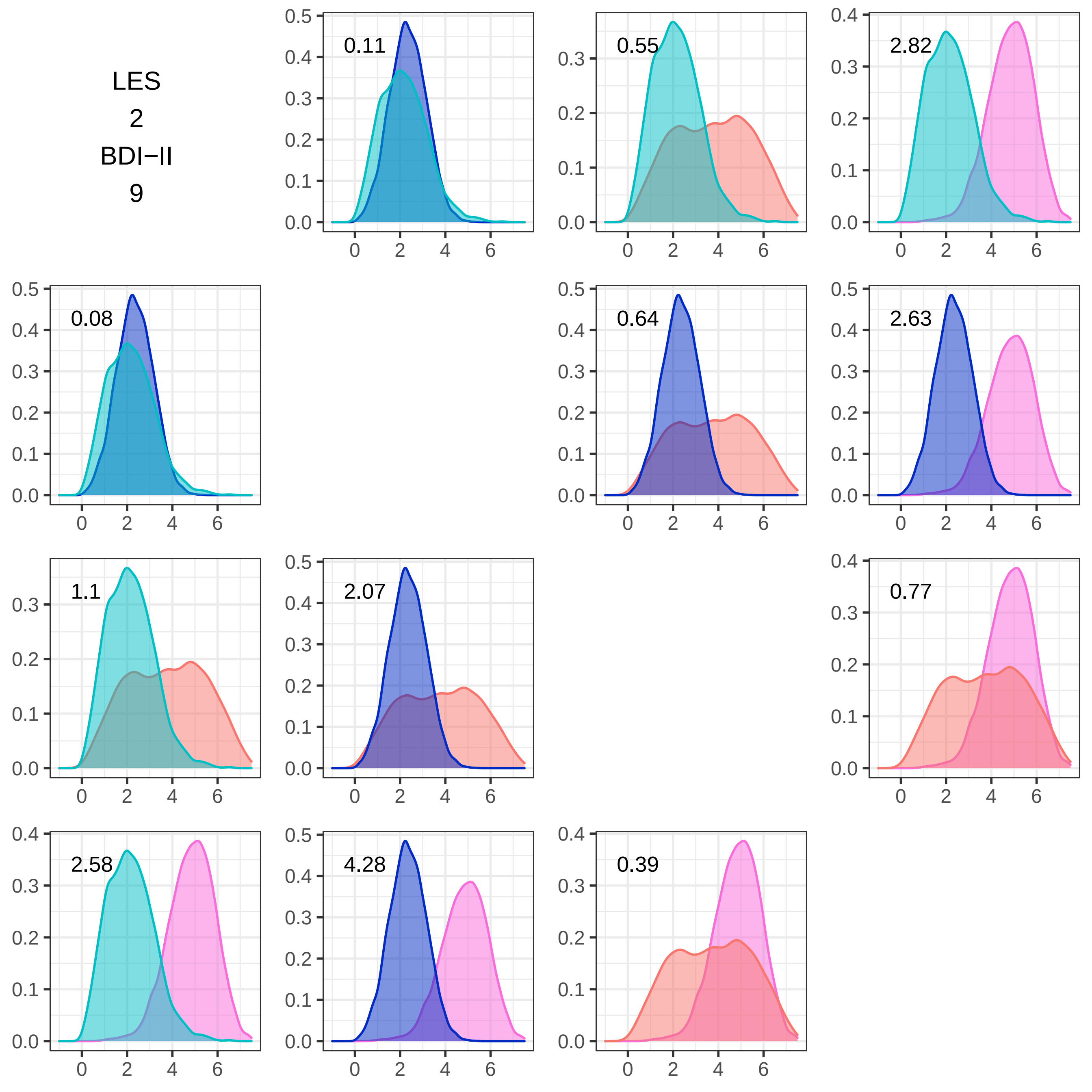
LES
2
BDI−II
9
0.11
0.55
2.82
0.08
0.64
2.63
1.1
2.07
0.77
2.58
4.28
0.39

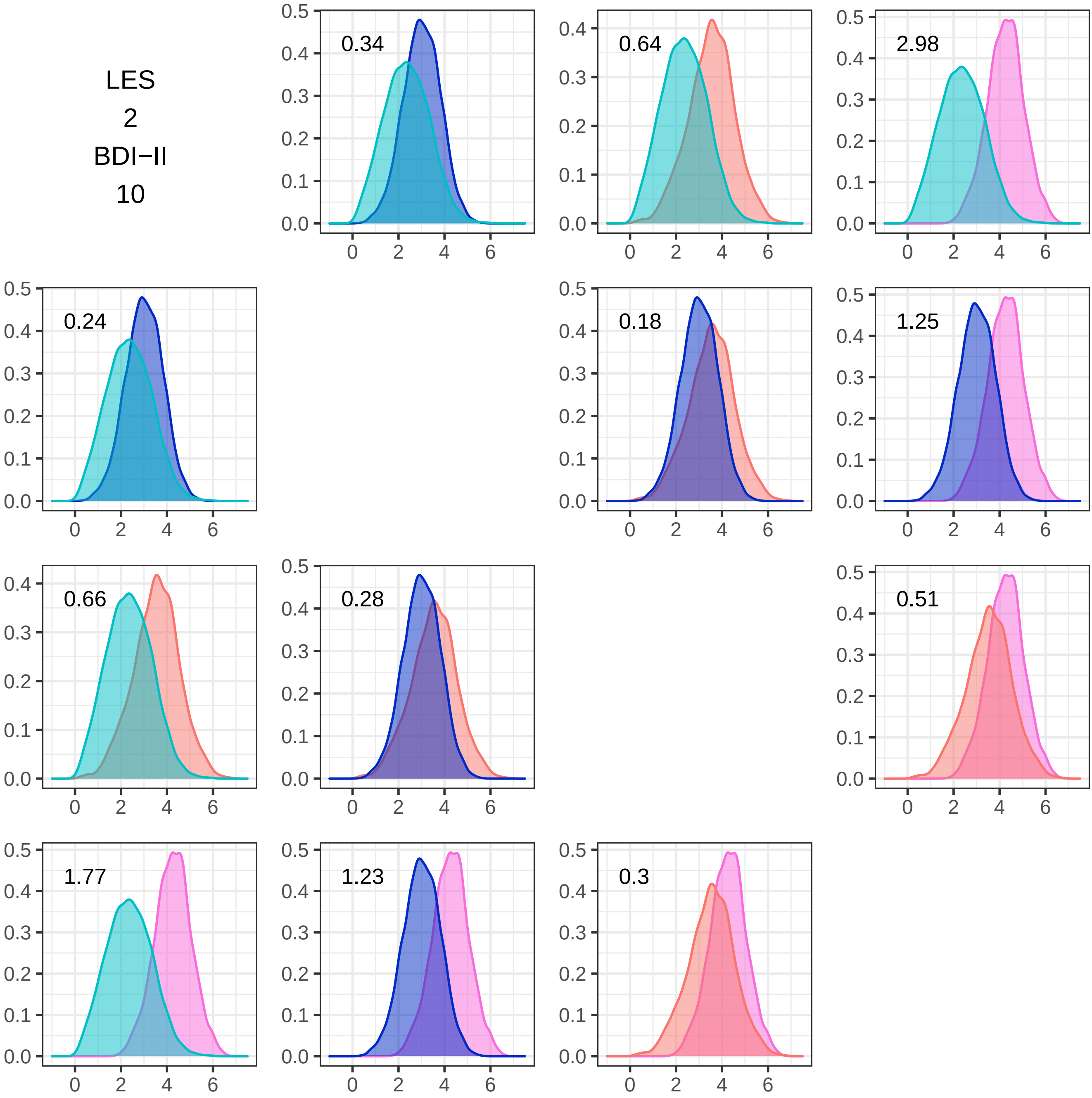
LES
2
BDI−II
10
0.34
0.64
2.98
0.24
0.18
1.25
0.66
0.28
0.51
1.77
1.23
0.3

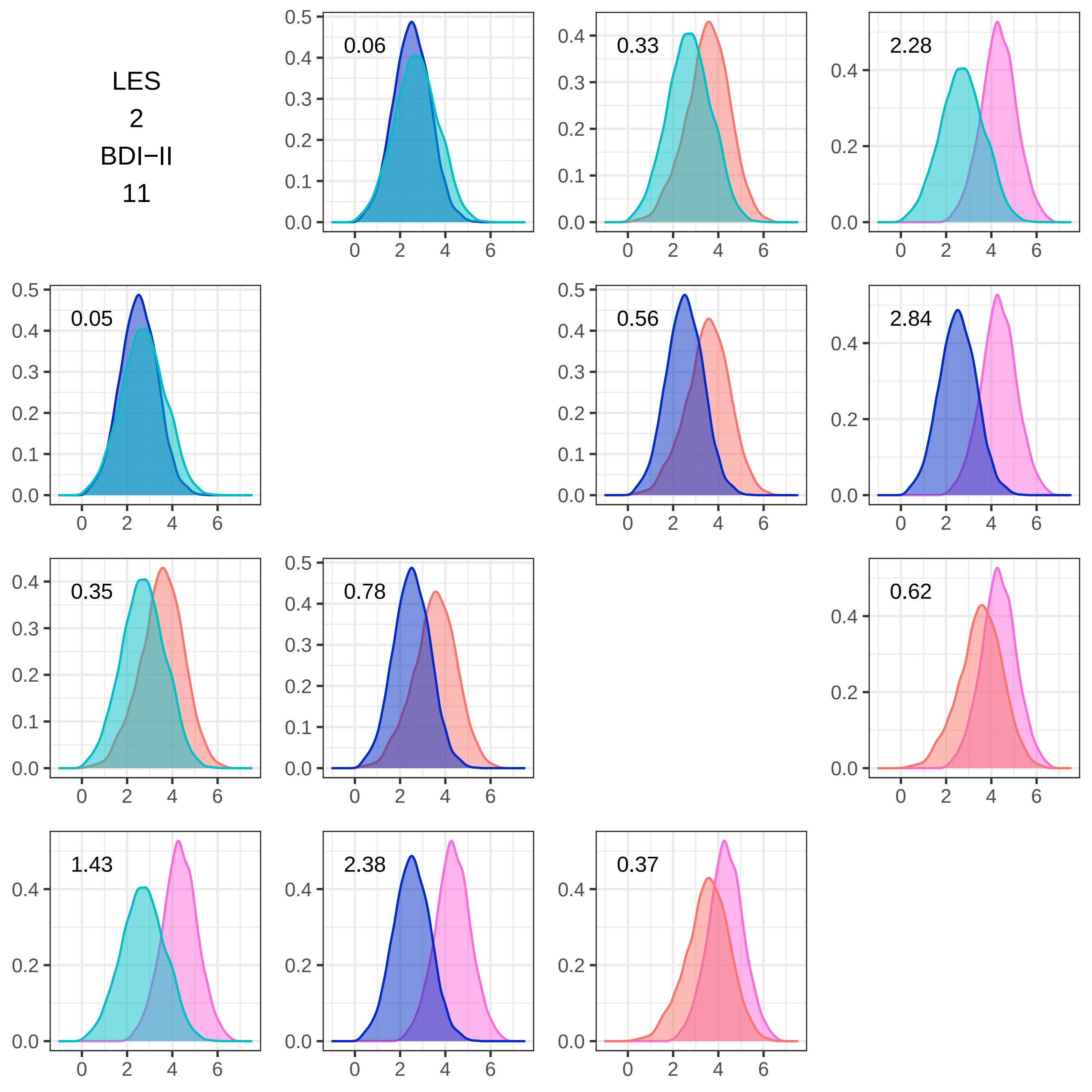
LES
2
BDI−II
11
0.06
0.33
2.28
0.05
0.56
2.84
0.35
0.78
0.62
1.43
2.38
0.37

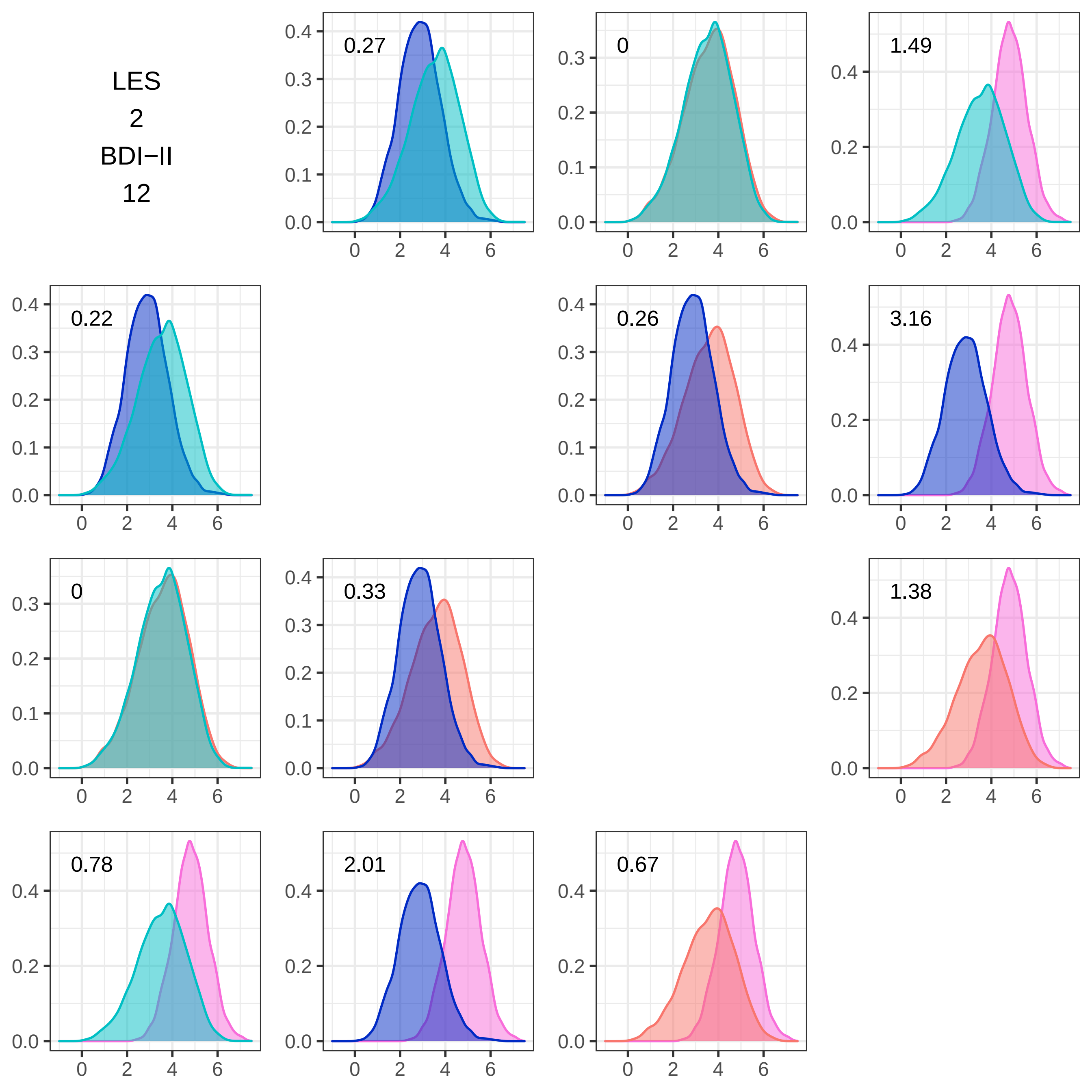
LES
2
BDI−II
12
0.27
0
1.49
0.22
0.26
3.16
0
0.33
1.38
0.78
2.01
0.67

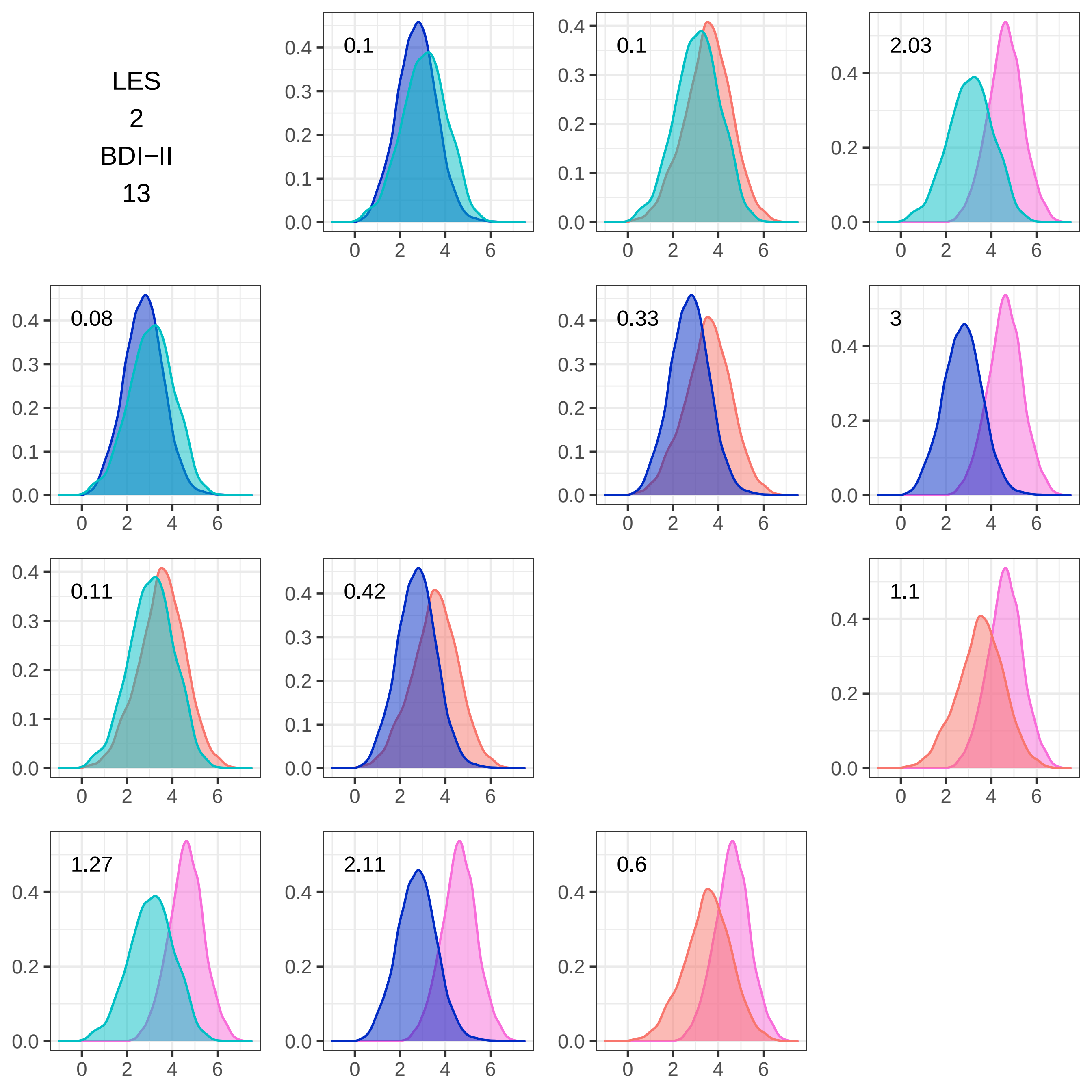
LES
2
BDI−II
13
0.1
0.1
2.03
0.08
0.33
3
0.11
0.42
1.1
1.27
2.11
0.6

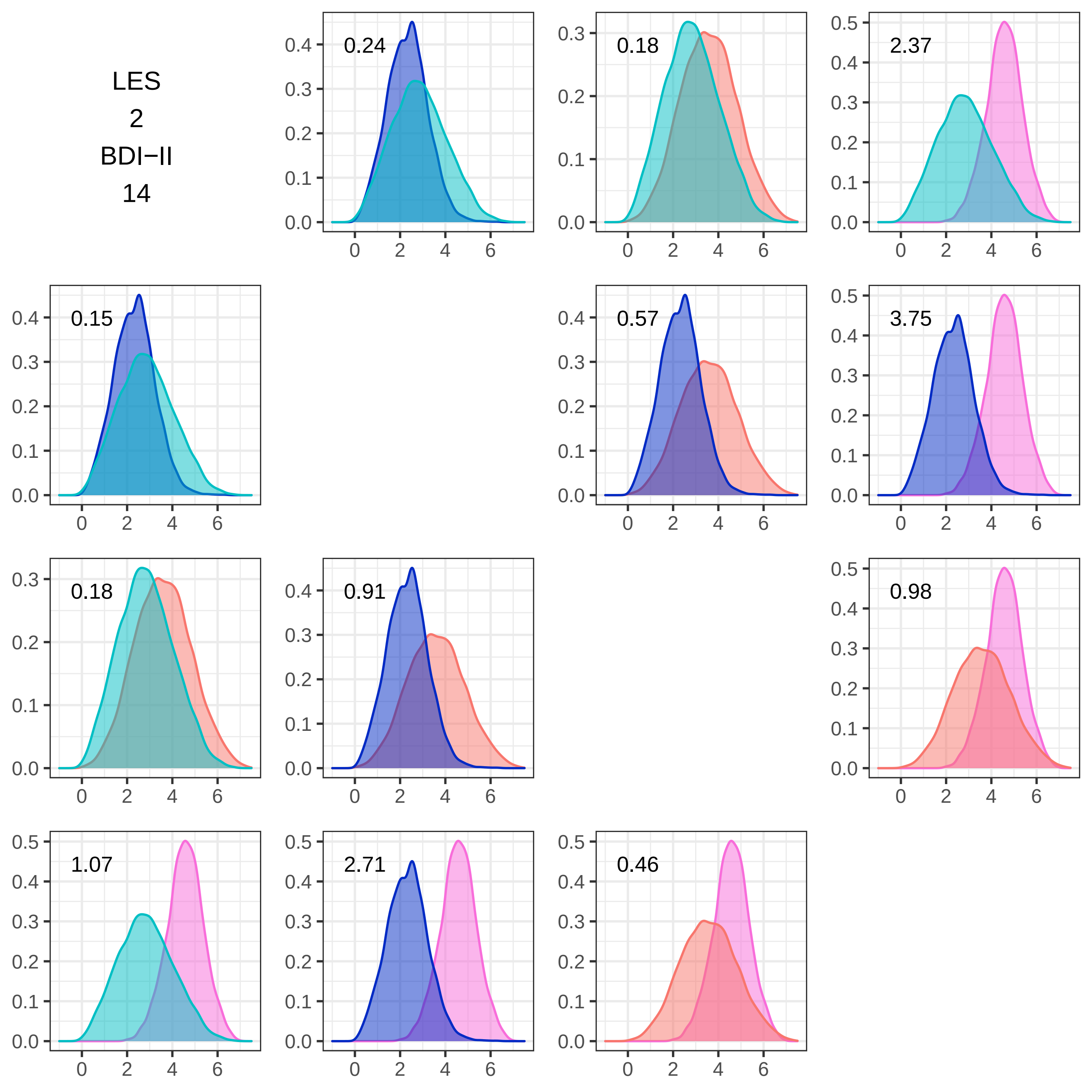
LES
2
BDI−II
14
0.24
0.18
2.37
0.15
0.57
3.75
0.18
0.91
0.98
1.07
2.71
0.46

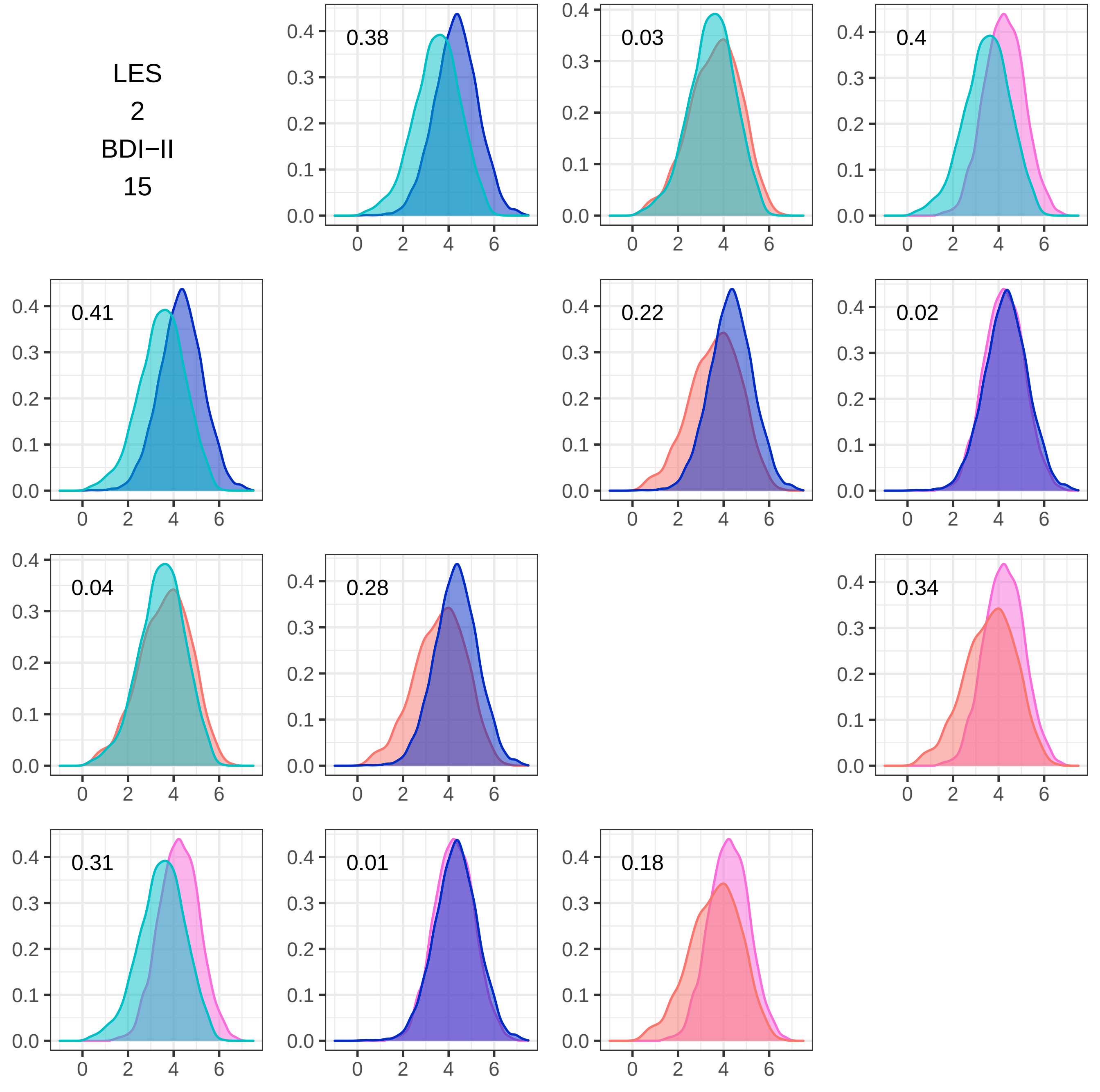

LES
2
BDI−II
15
0.38
0.03
0.4
0.41
0.22
0.02
0.04
0.28
0.34
0.31
0.01
0.18

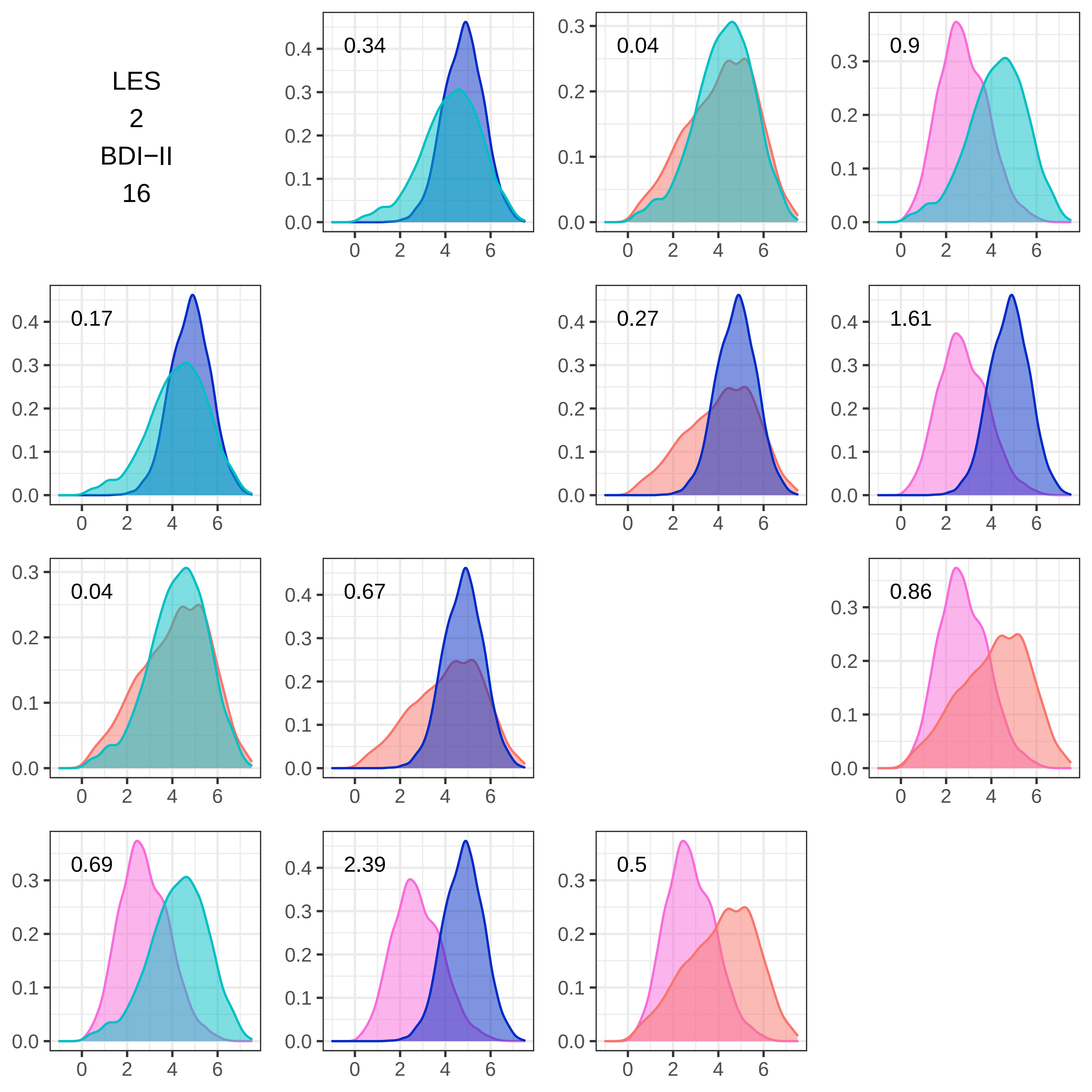
LES
2
BDI−II
16
0.34
0.04
0.9
0.17
0.27
1.61
0.04
0.67
0.86
0.69
2.39
0.5

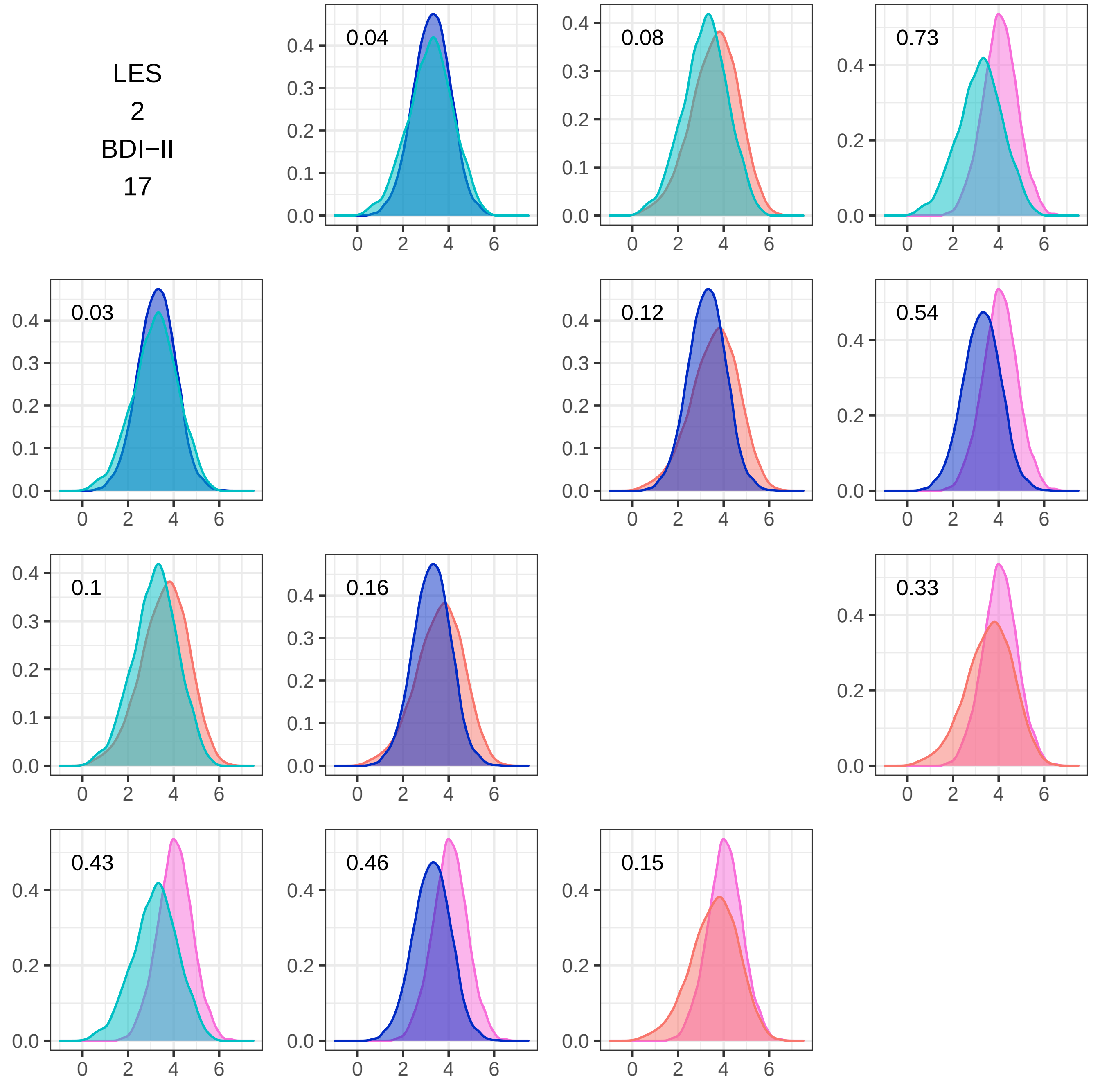

LES
2
BDI−II
17
0.04
0.08
0.73
0.03
0.12
0.54
0.1
0.16
0.33
0.43
0.46
0.15

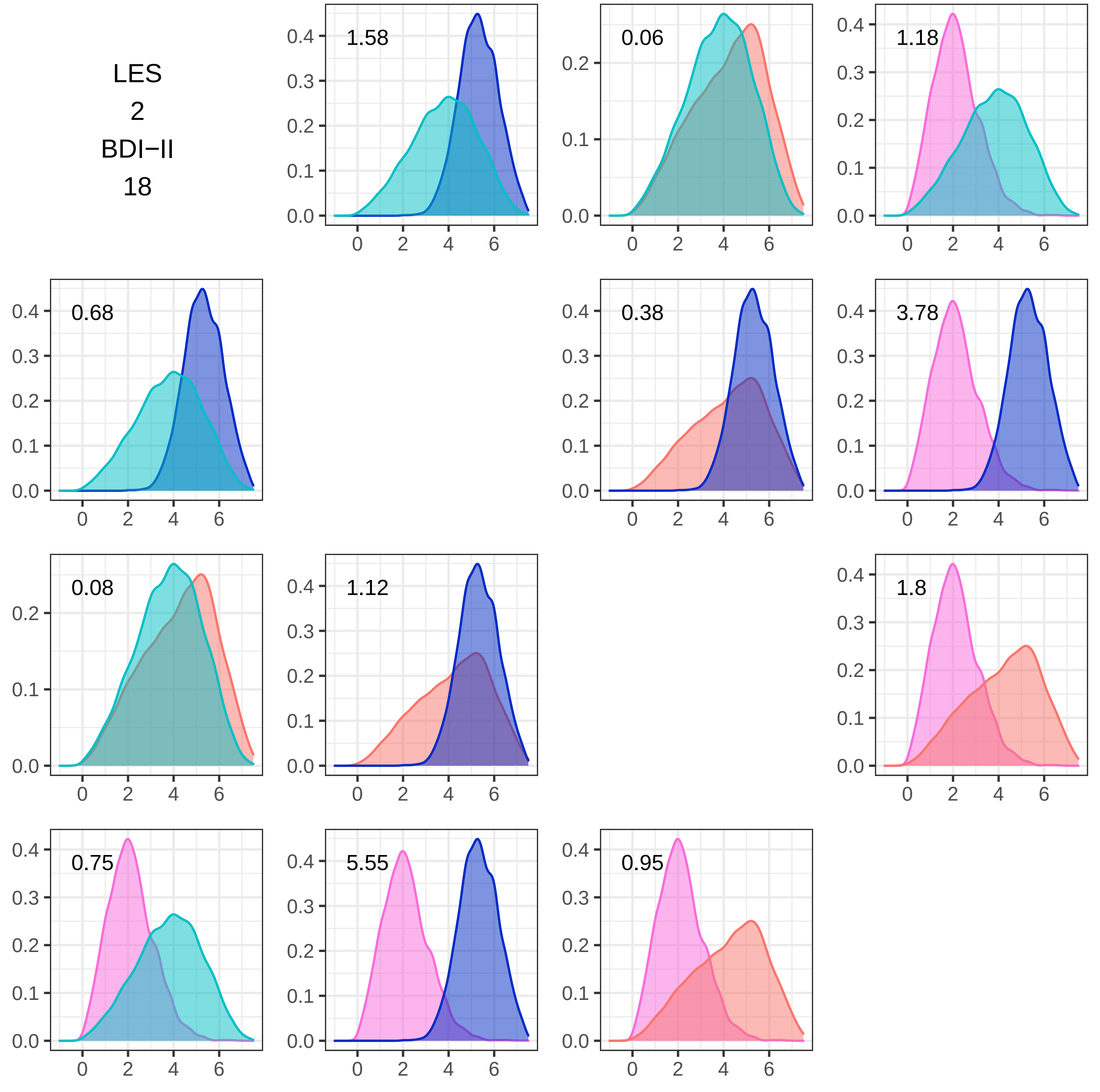

LES
2
BDI−II
18
1.58
0.06
1.18
0.68
0.38
3.78
0.08
1.12
1.8
0.75
5.55
0.95

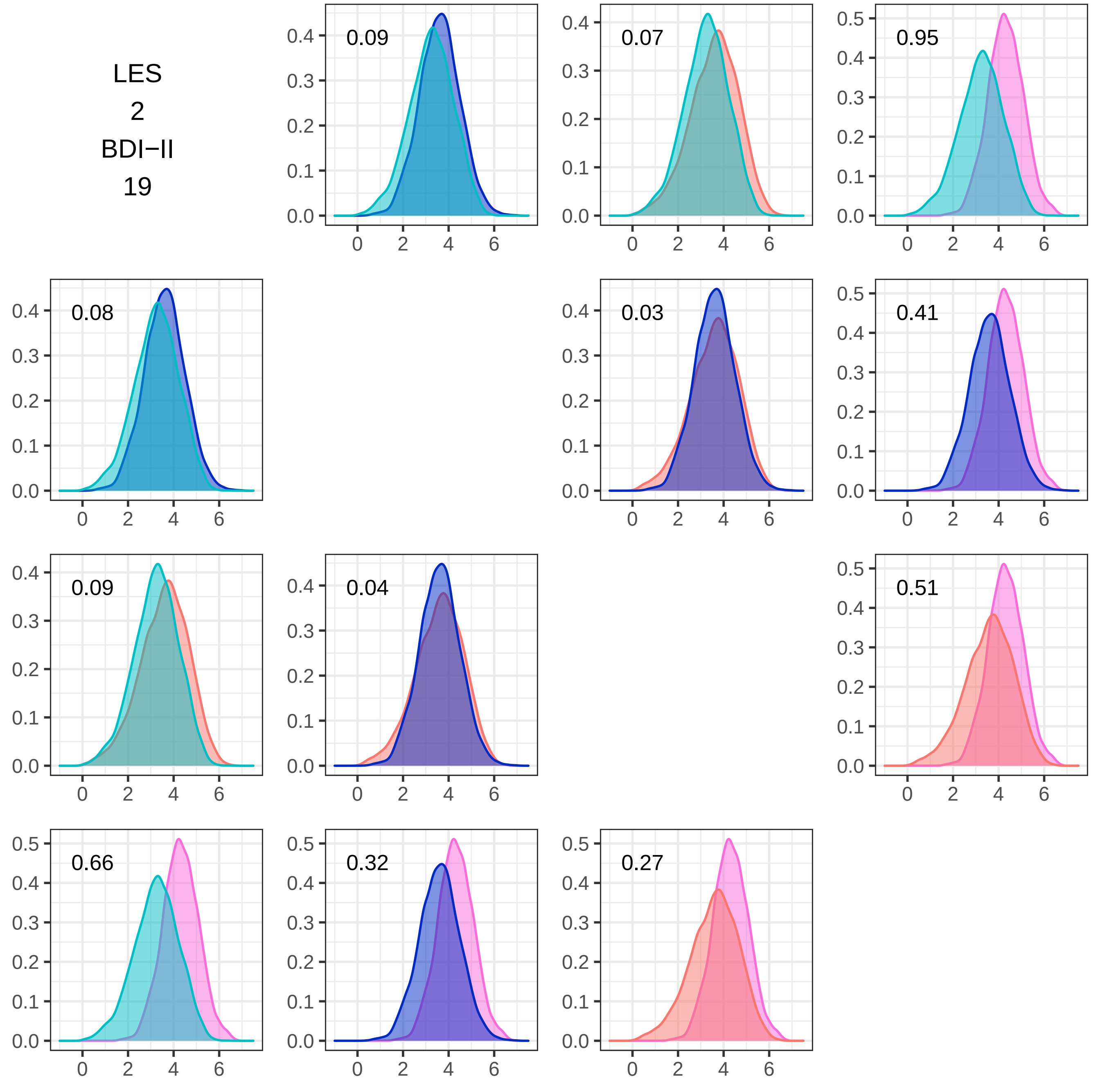
LES
2
BDI−II
19
0.09
0.07
0.95
0.08
0.03
0.41
0.09
0.04
0.51
0.66
0.32
0.27

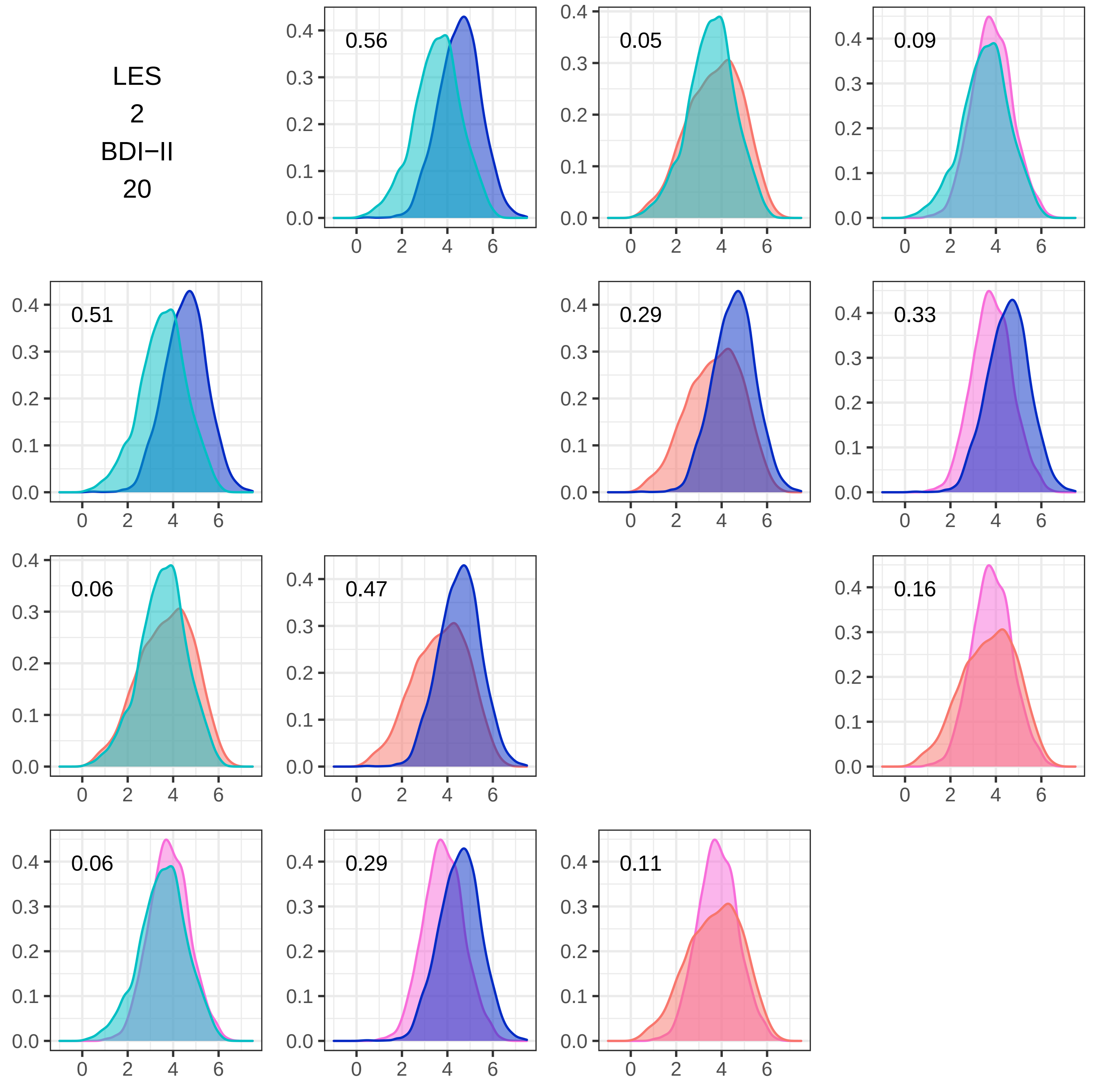
LES
2
BDI−II
20
0.56
0.05
0.09
0.51
0.29
0.33
0.06
0.47
0.16
0.06
0.29
0.11

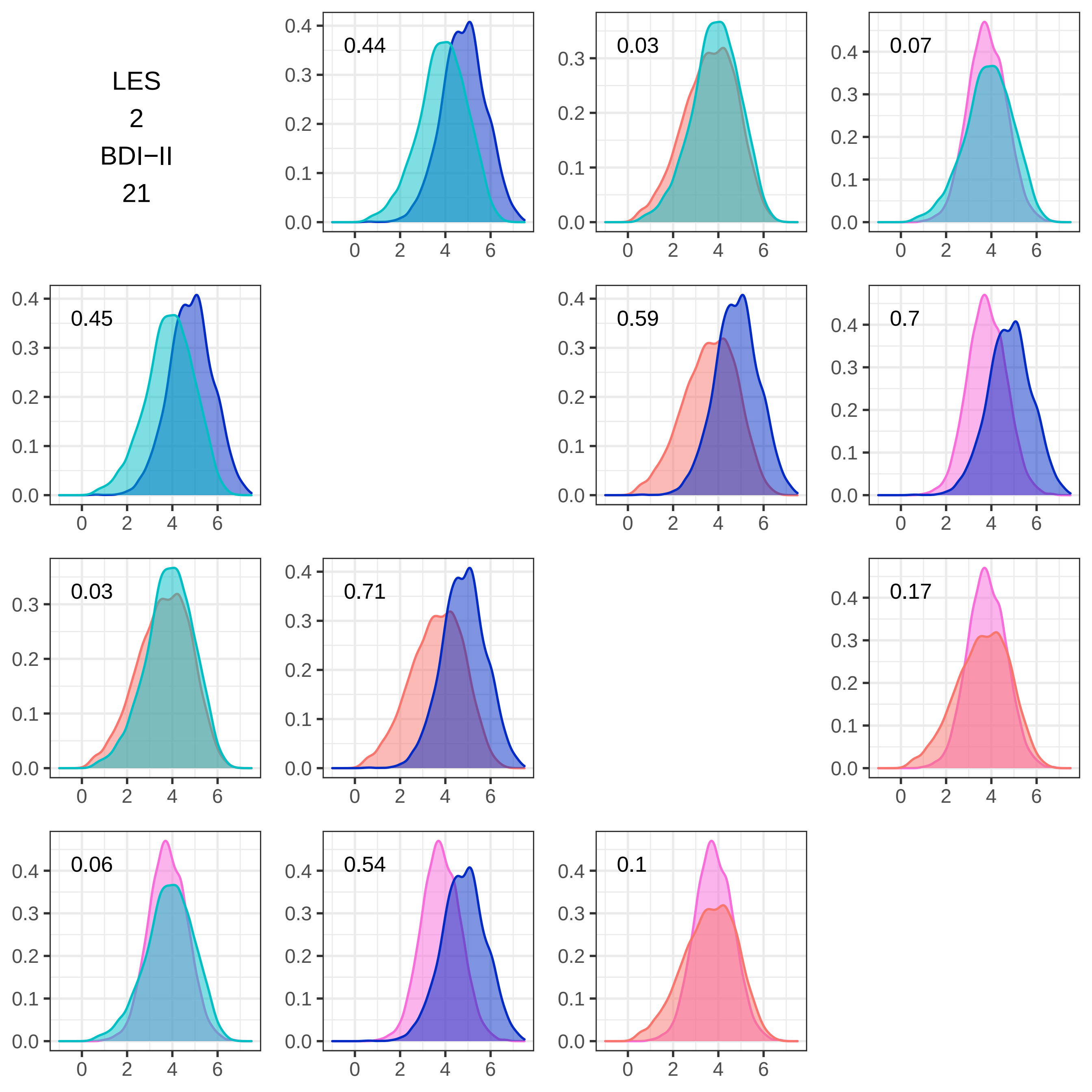

LES
2
BDI−II
21
0.44
0.03
0.07
0.45
0.59
0.7
0.03
0.71
0.17
0.06
0.54
0.1

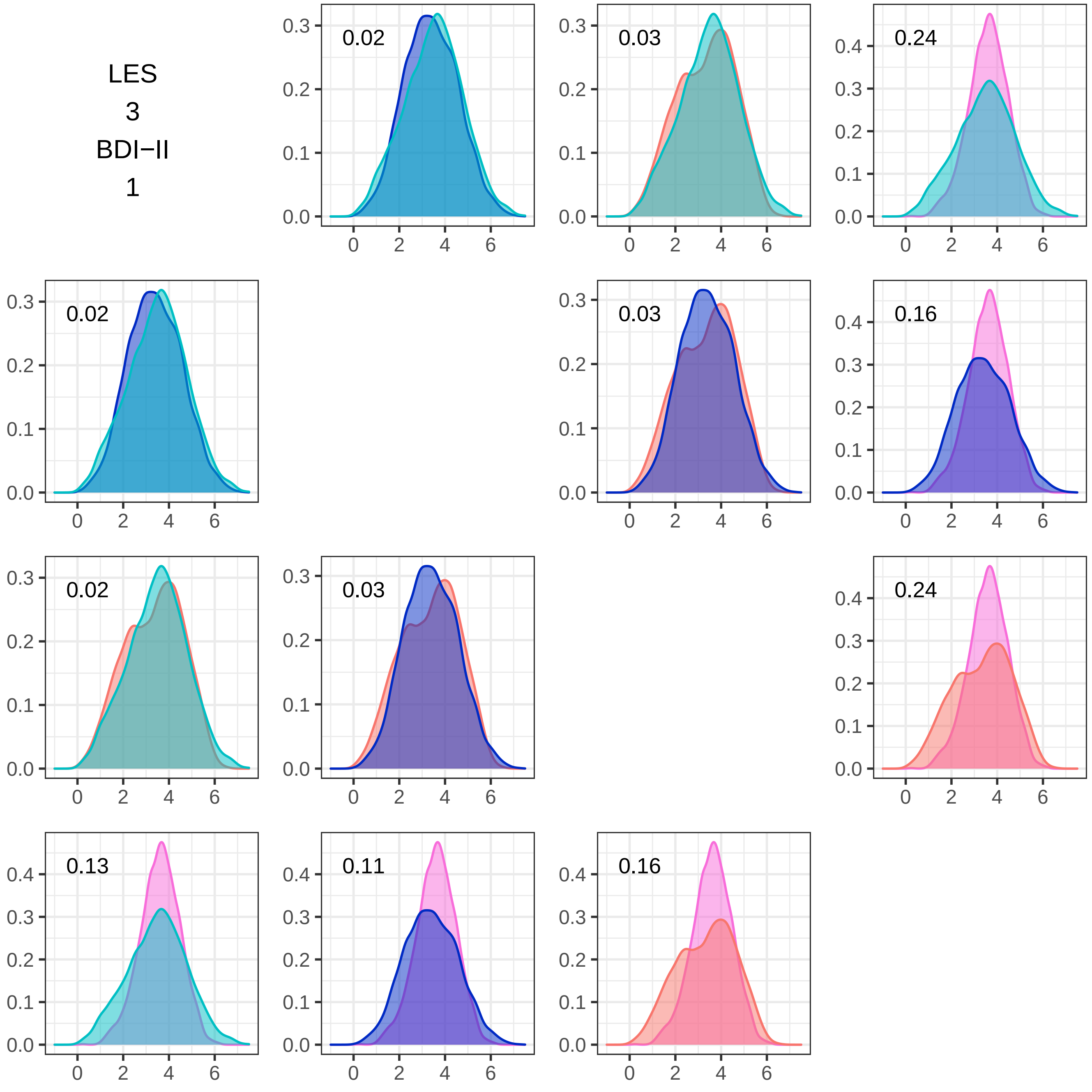
LES
3
BDI−II
1
0.02
0.03
0.24
0.02
0.03
0.16
0.02
0.03
0.24
0.13
0.11
0.16

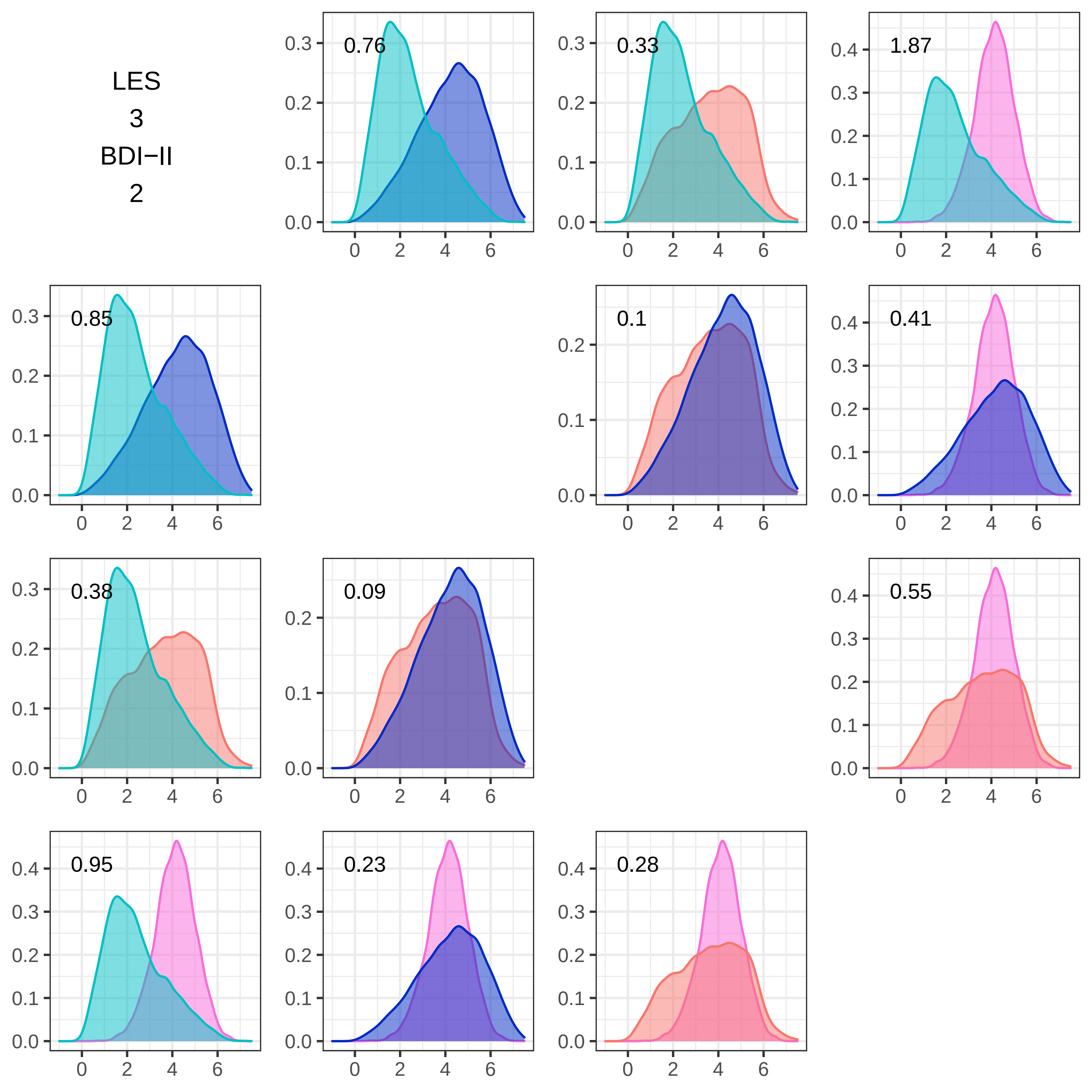

LES
3
BDI−II
2
0.76
0.33
1.87
0.85
0.1
0.41
0.38
0.09
0.55
0.95
0.23
0.28

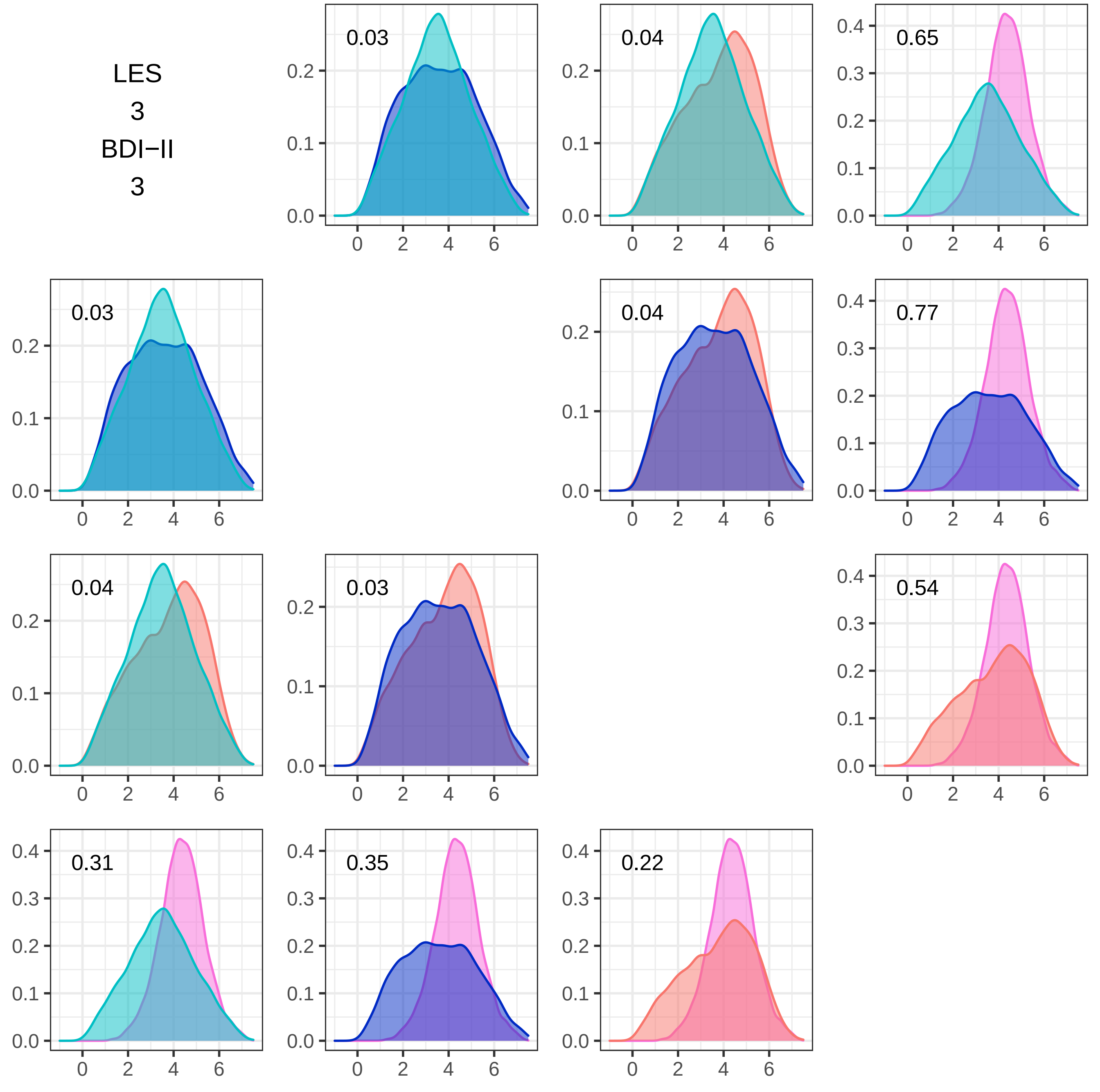
LES
3
BDI−II
3
0.03
0.04
0.65
0.03
0.04
0.77
0.04
0.03
0.54
0.31
0.35
0.22

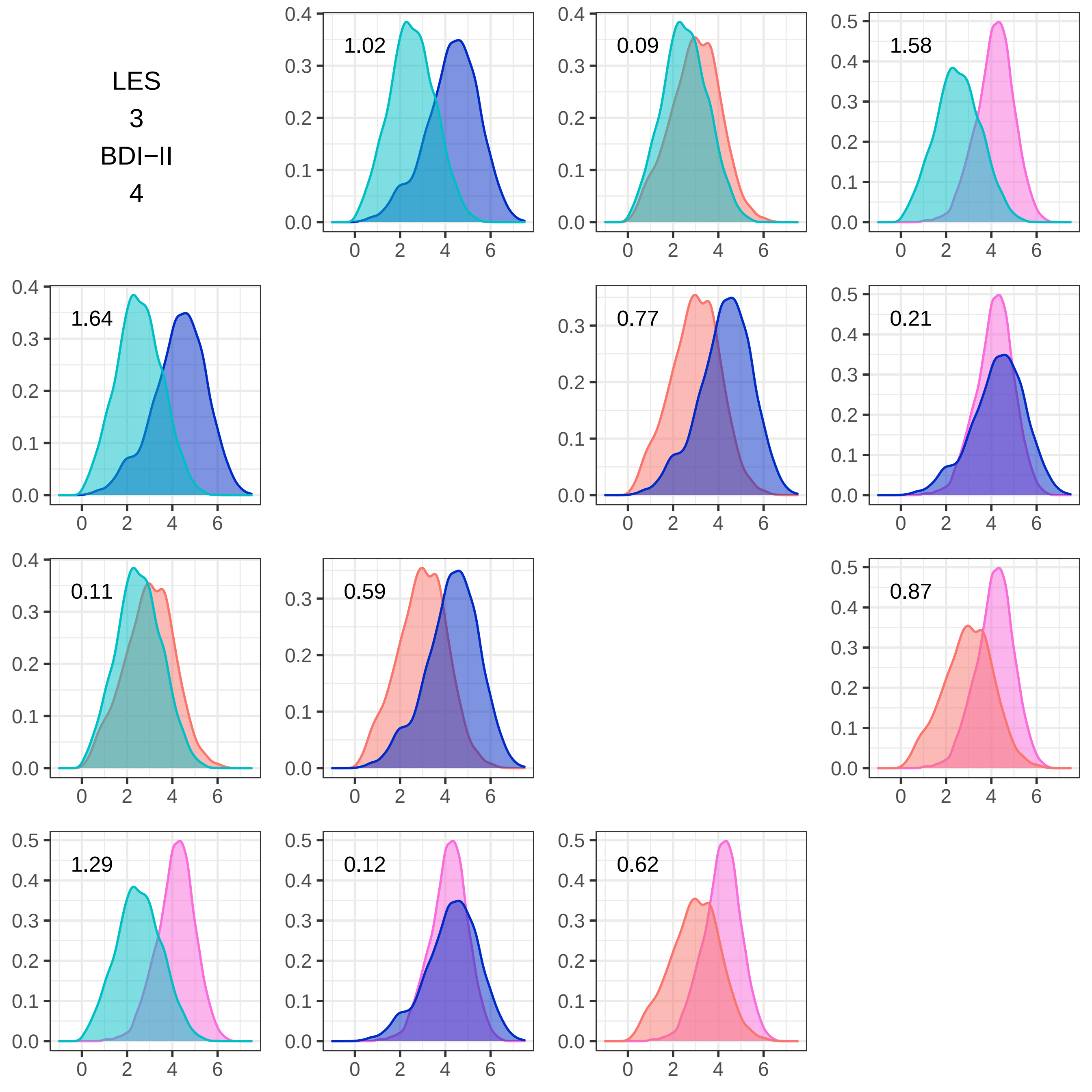
LES
3
BDI−II
4
1.02
0.09
1.58
1.64
0.77
0.21
0.11
0.59
0.87
1.29
0.12
0.62

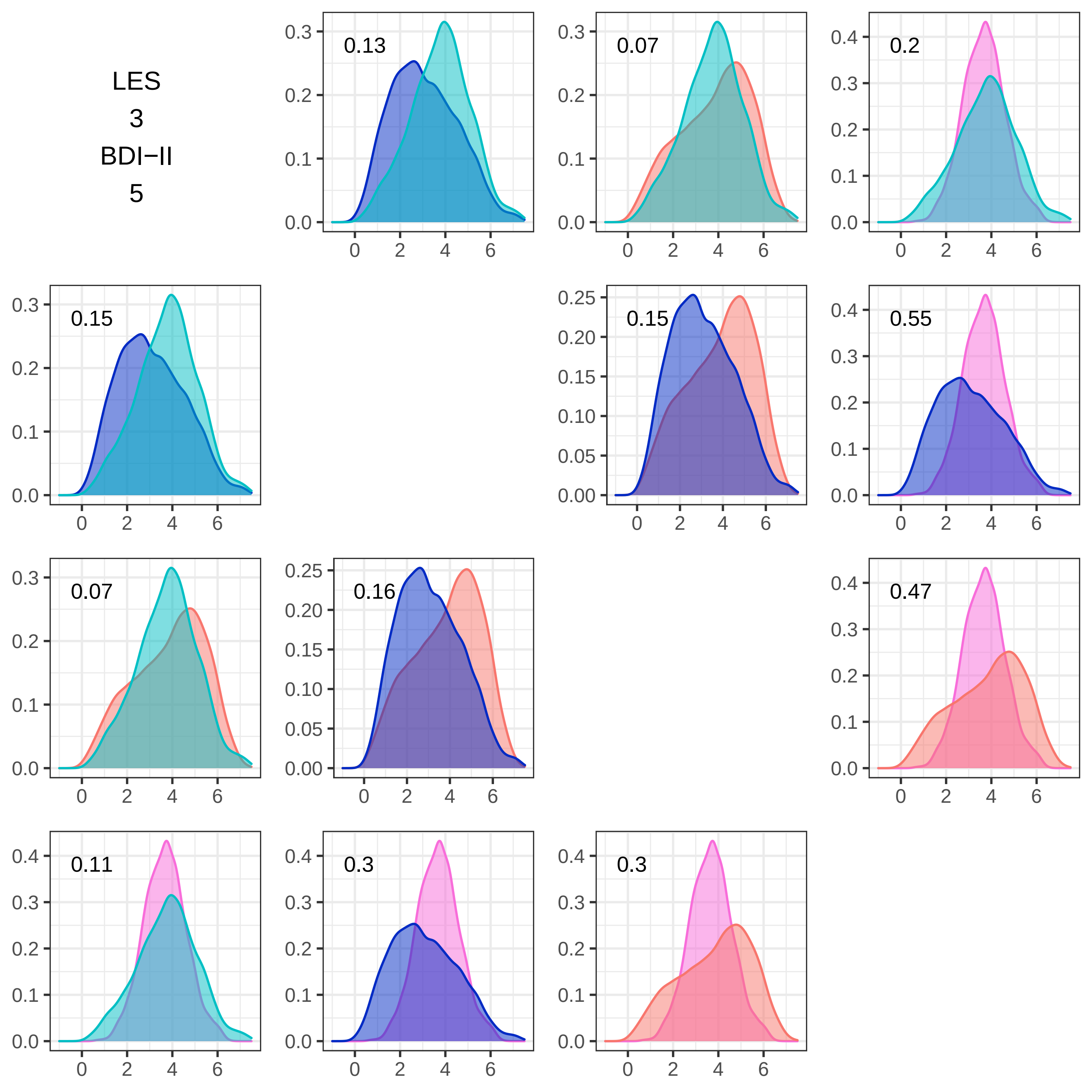
LES
3
BDI−II
5
0.13
0.07
0.2
0.15
0.15
0.55
0.07
0.16
0.47
0.11
0.3
0.3

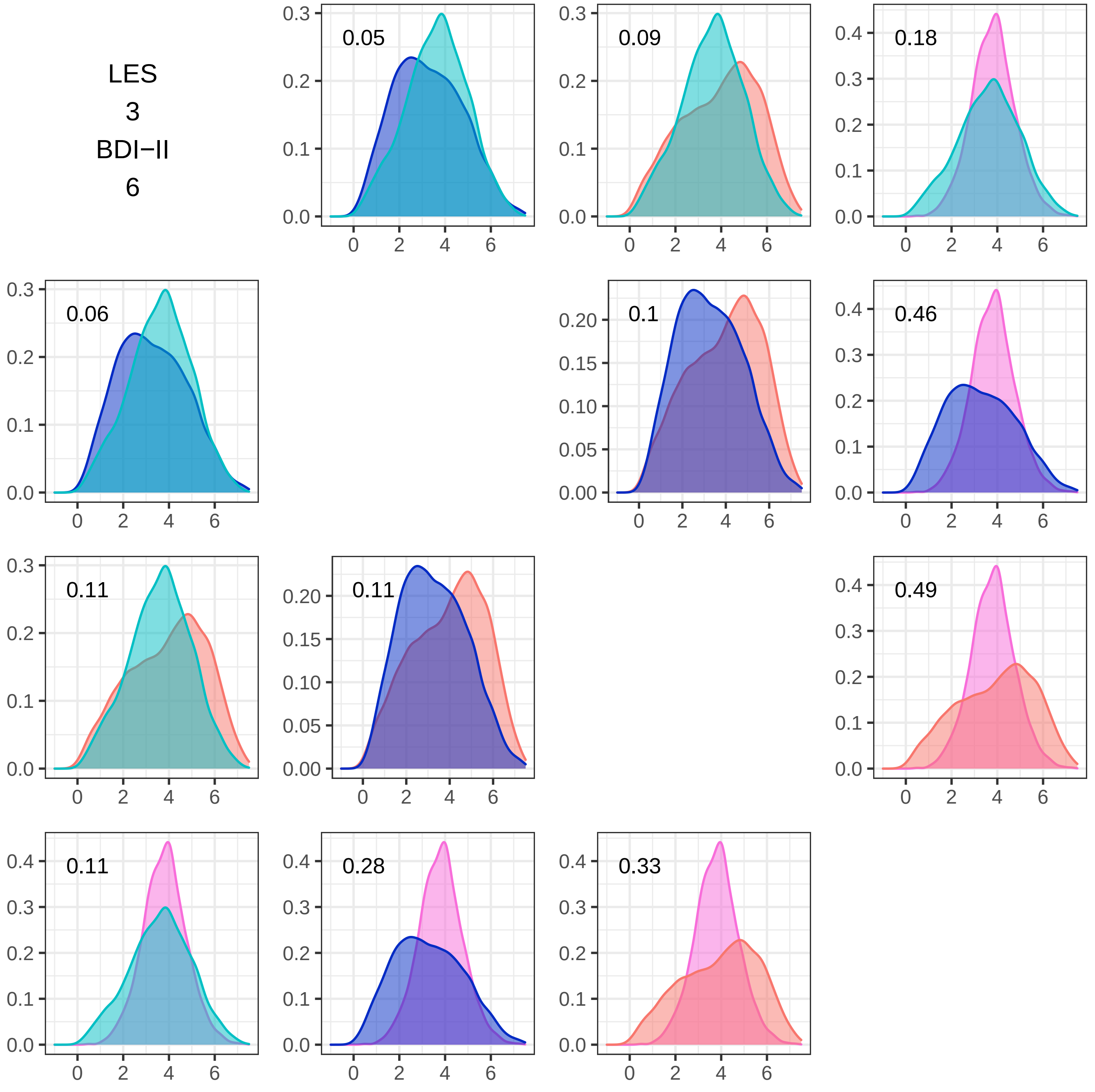
LES
3
BDI−II
6
0.05
0.09
0.18
0.06
0.1
0.46
0.11
0.11
0.49
0.11
0.28
0.33

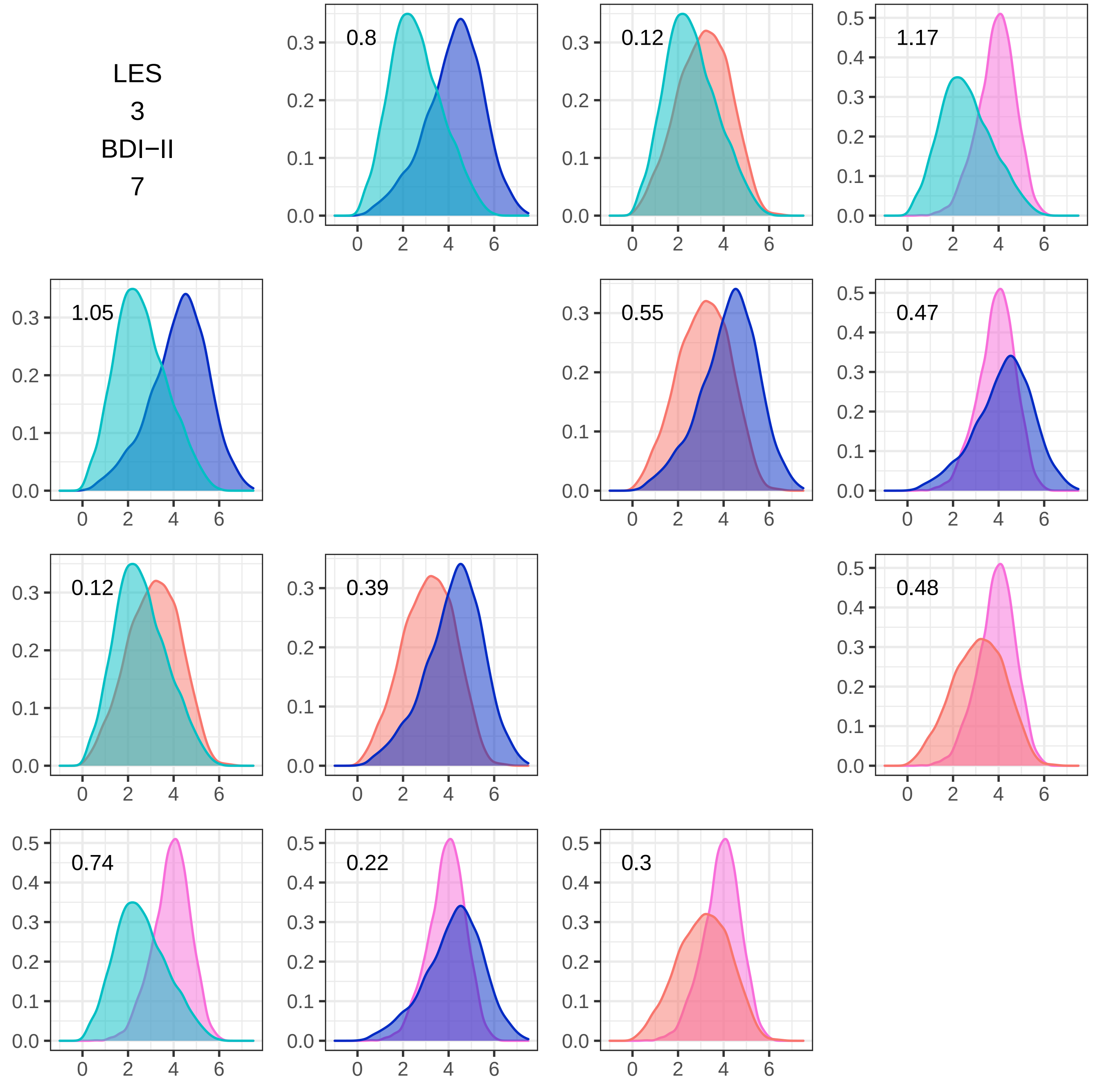

LES
3
BDI−II
7
0.8
0.12
1.17
1.05
0.55
0.47
0.12
0.39
0.48
0.74
0.22
0.3

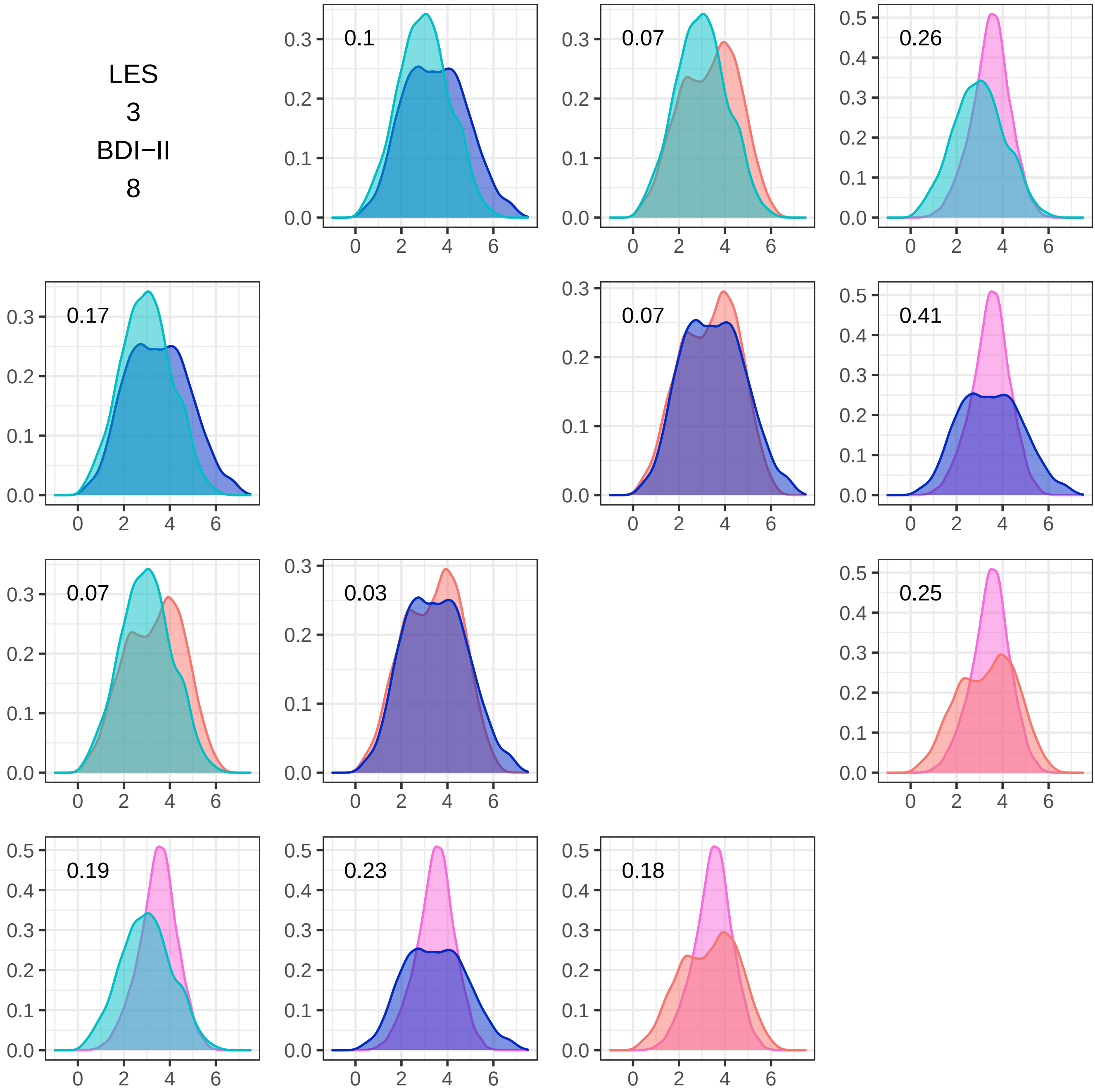
LES
3
BDI−II
8
0.1
0.07
0.26
0.17
0.07
0.41
0.07
0.03
0.25
0.19
0.23
0.18

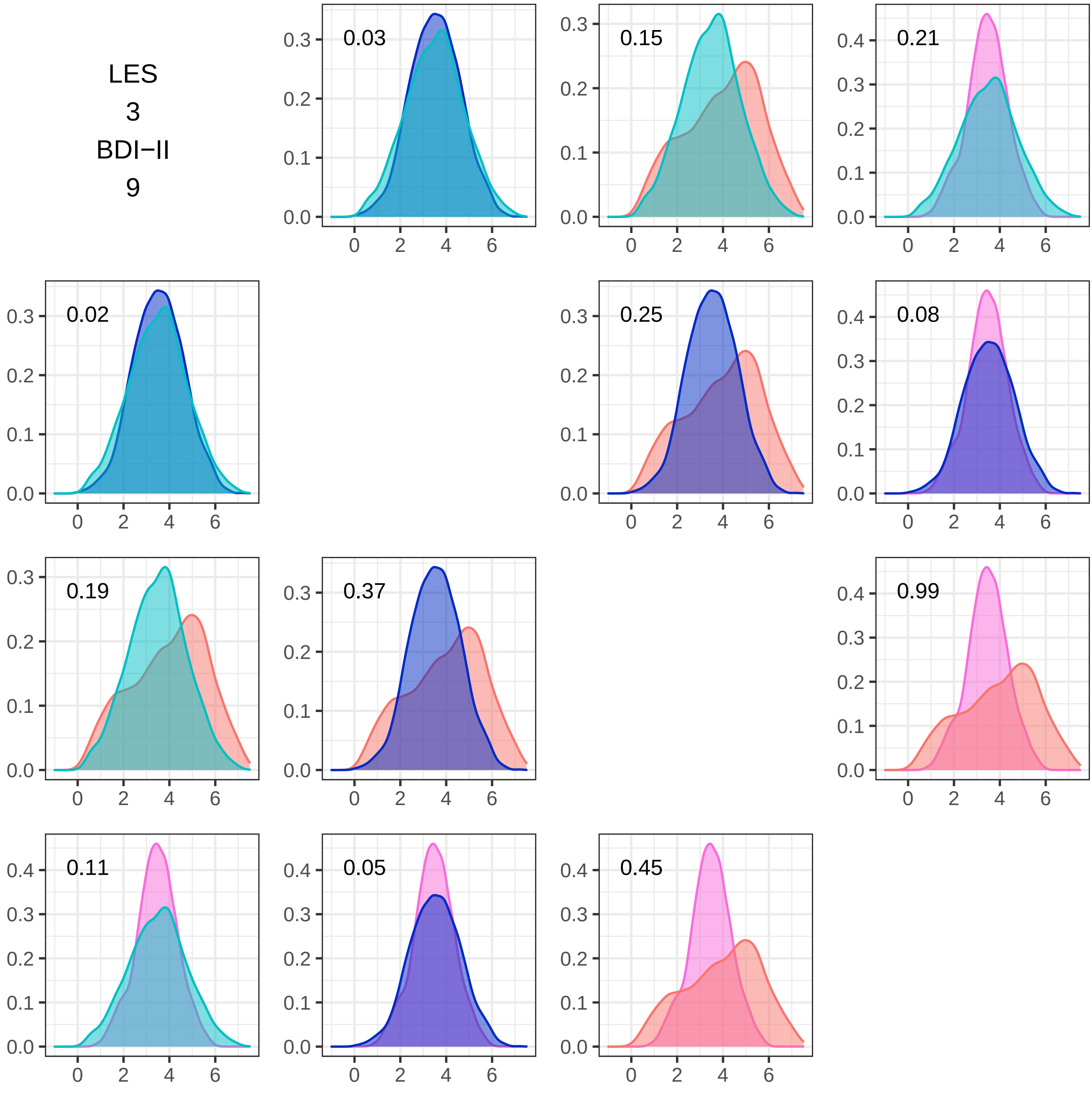
LES
3
BDI−II
9
0.03
0.15
0.21
0.02
0.25
0.08
0.19
0.37
0.99
0.11
0.05
0.45

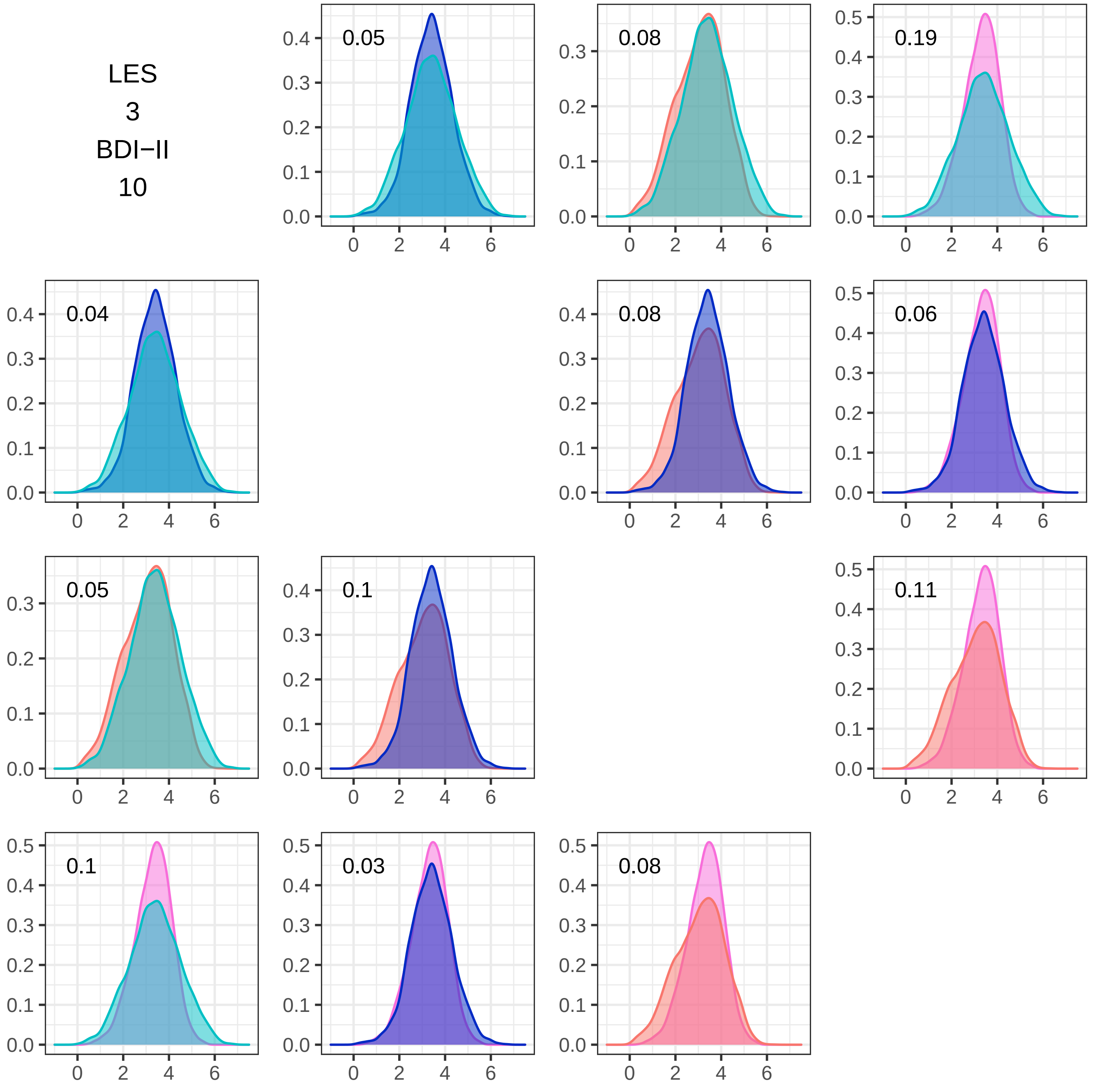
LES
3
BDI−II
10
0.05
0.08
0.19
0.04
0.08
0.06
0.05
0.1
0.11
0.1
0.03
0.08

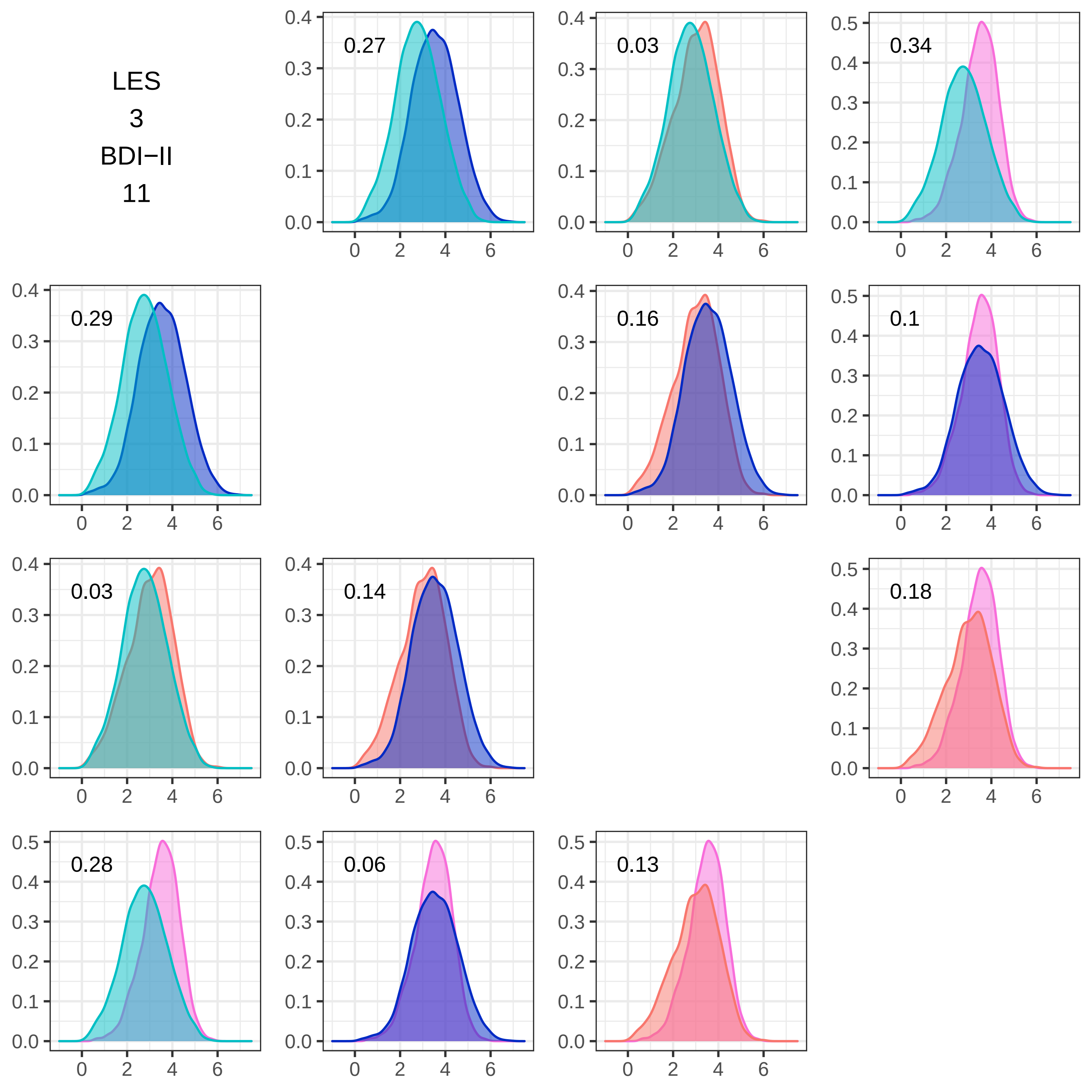
LES
3
BDI−II
11
0.27
0.03
0.34
0.29
0.16
0.1
0.03
0.14
0.18
0.28
0.06
0.13

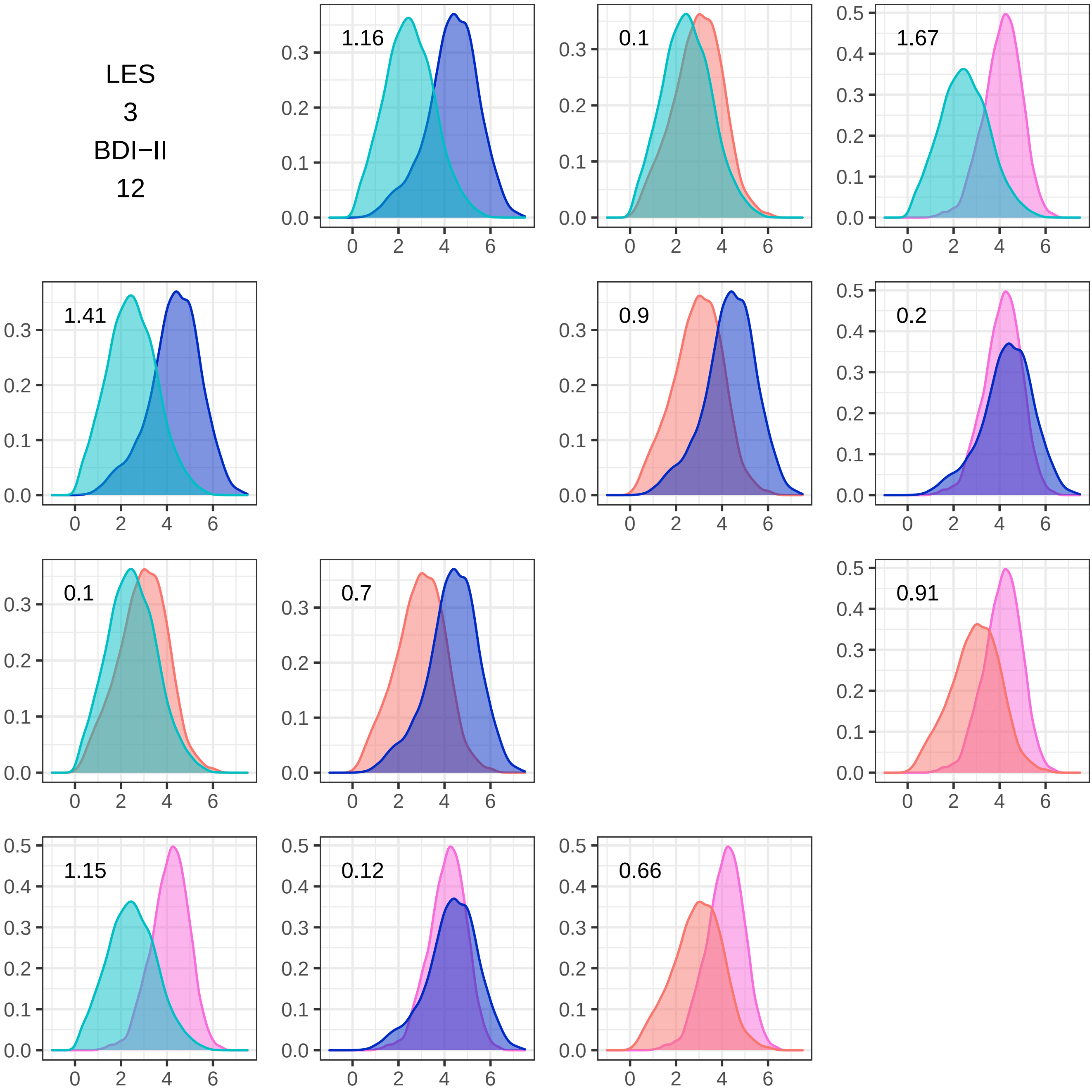

LES
3
BDI−II
12
1.16
0.1
1.67
1.41
0.9
0.2
0.1
0.7
0.91
1.15
0.12
0.66

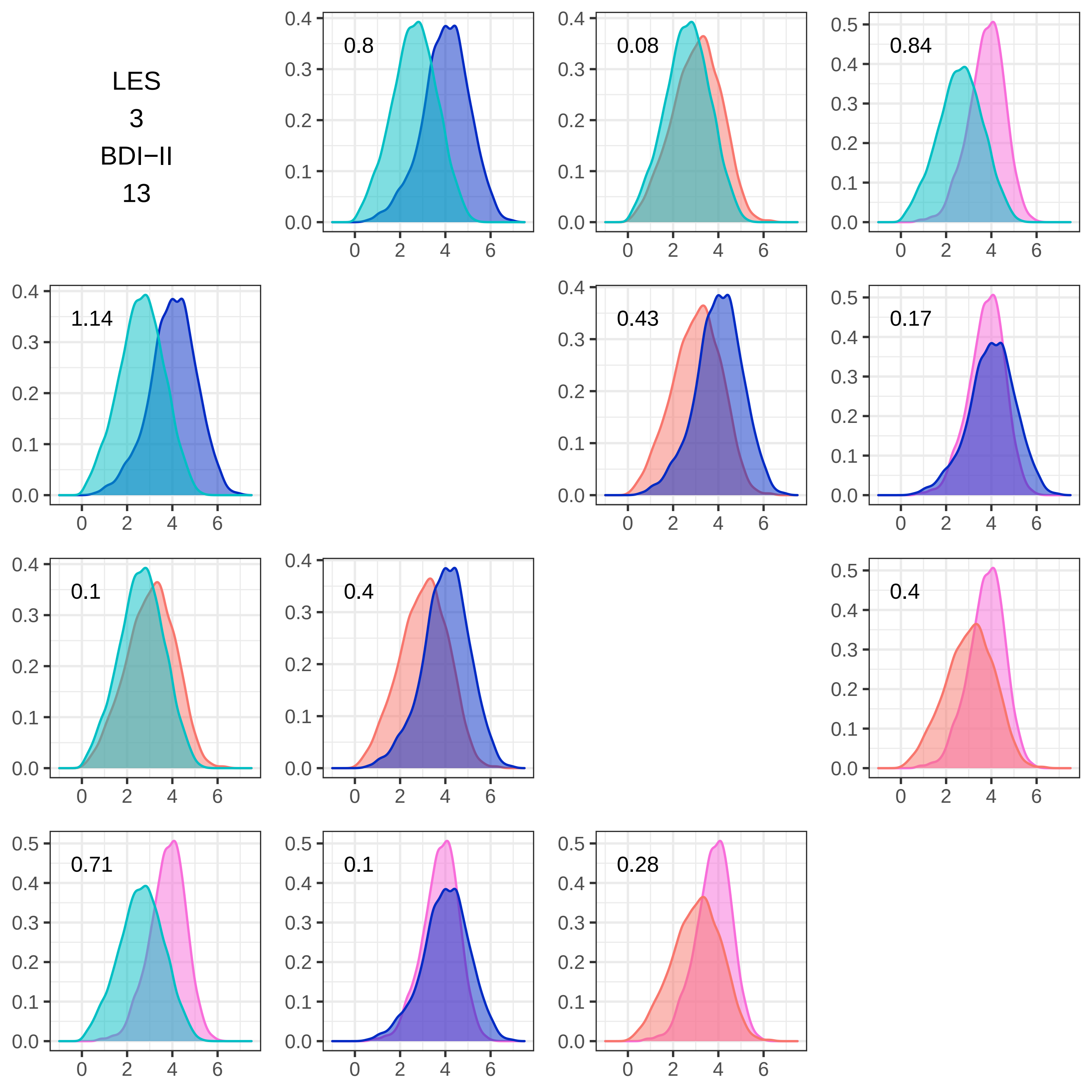

LES
3
BDI−II
13
0.8
0.08
0.84
1.14
0.43
0.17
0.1
0.4
0.4
0.71
0.1
0.28

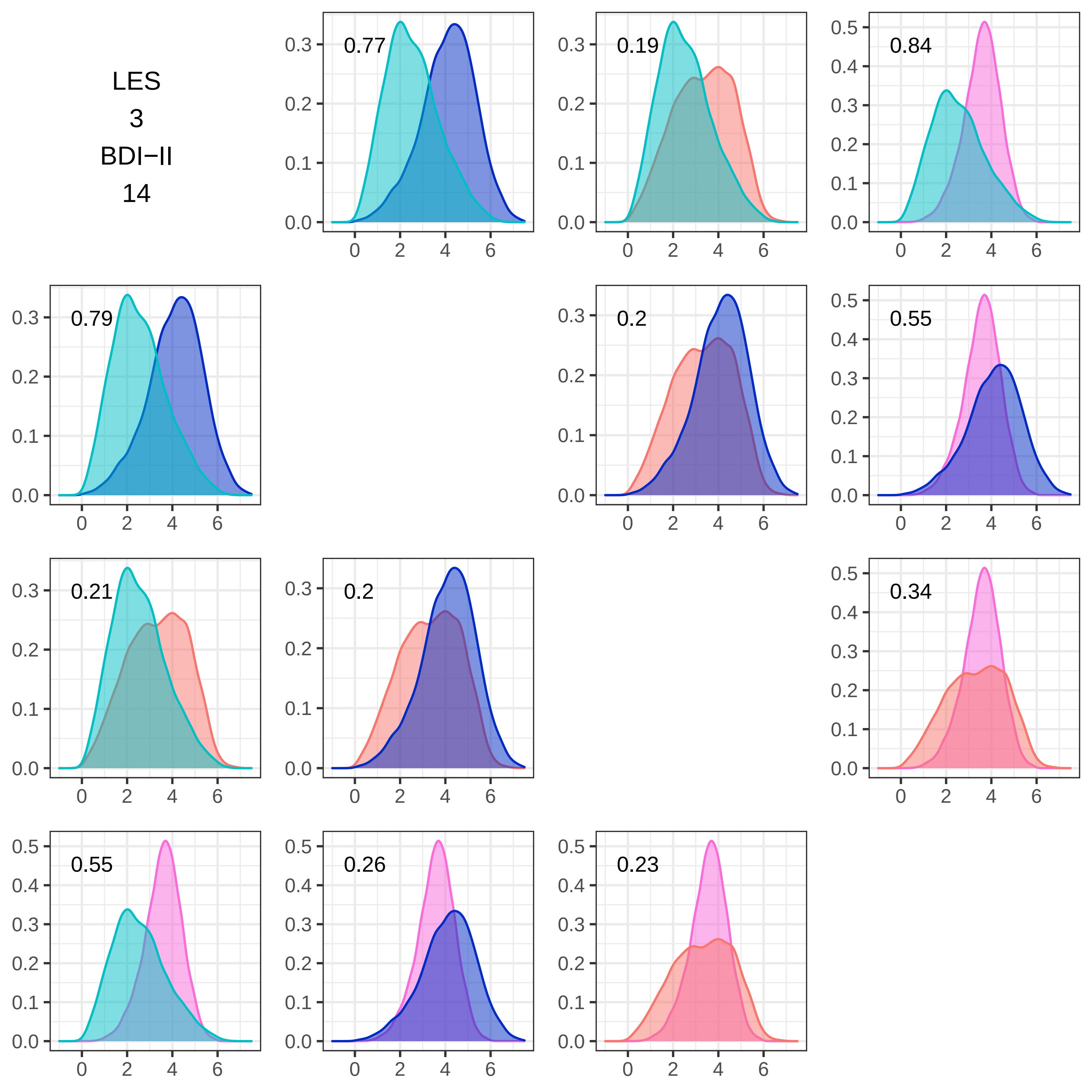

LES
3
BDI−II
14
0.77
0.19
0.84
0.79
0.2
0.55
0.21
0.2
0.34
0.55
0.26
0.23

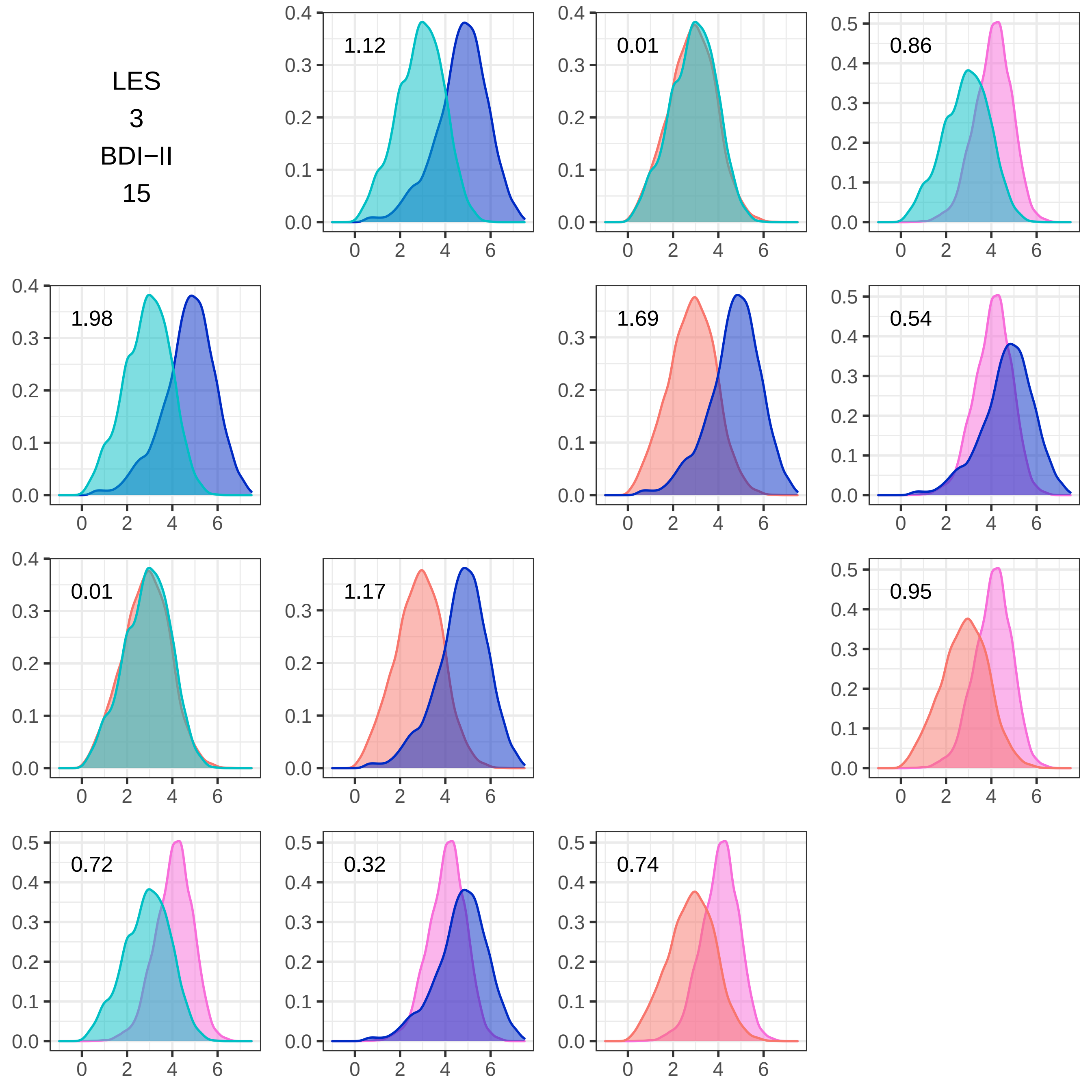
LES
3
BDI−II
15
1.12
0.01
0.86
1.98
1.69
0.54
0.01
1.17
0.95
0.72
0.32
0.74

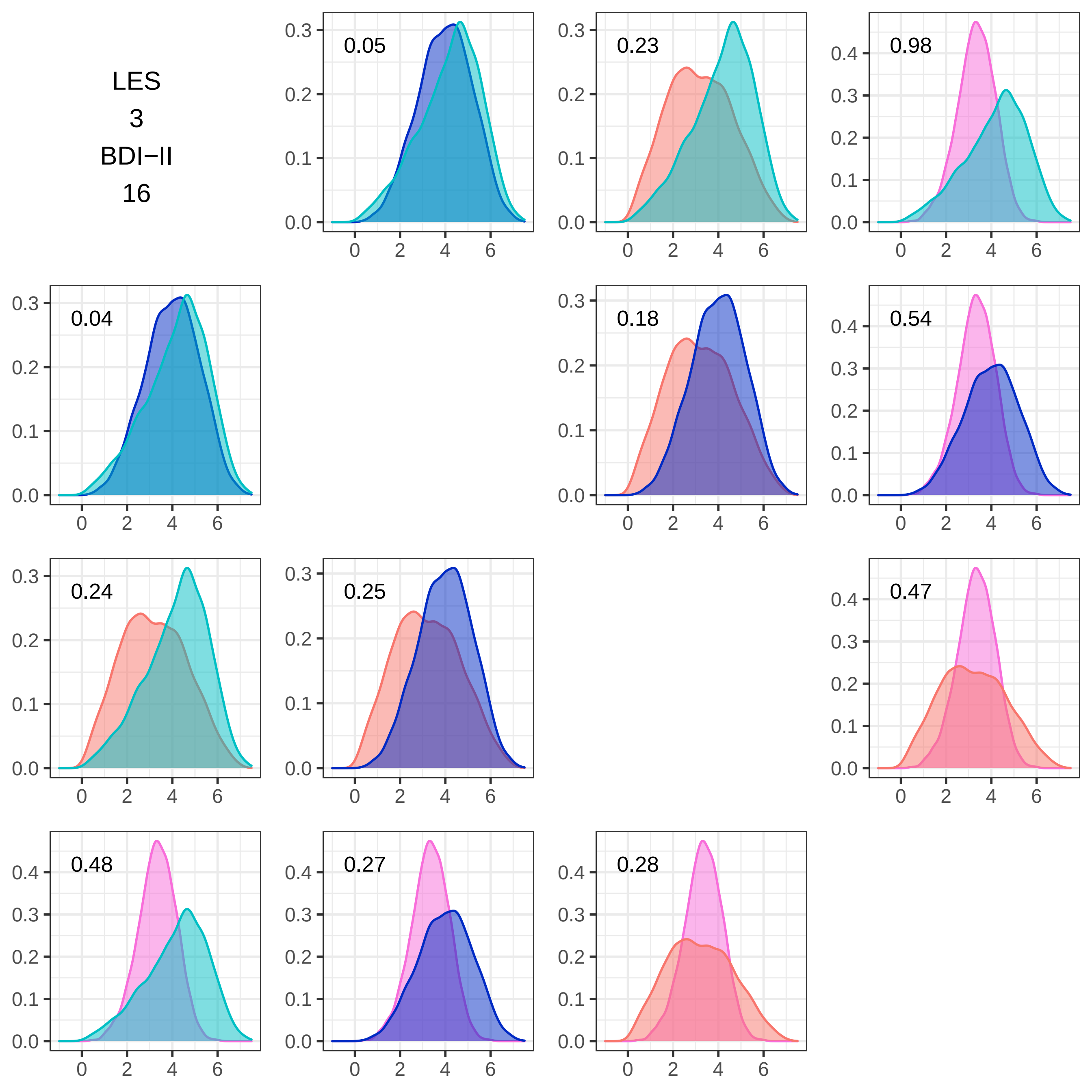

LES
3
BDI−II
16
0.05
0.23
0.98
0.04
0.18
0.54
0.24
0.25
0.47
0.48
0.27
0.28

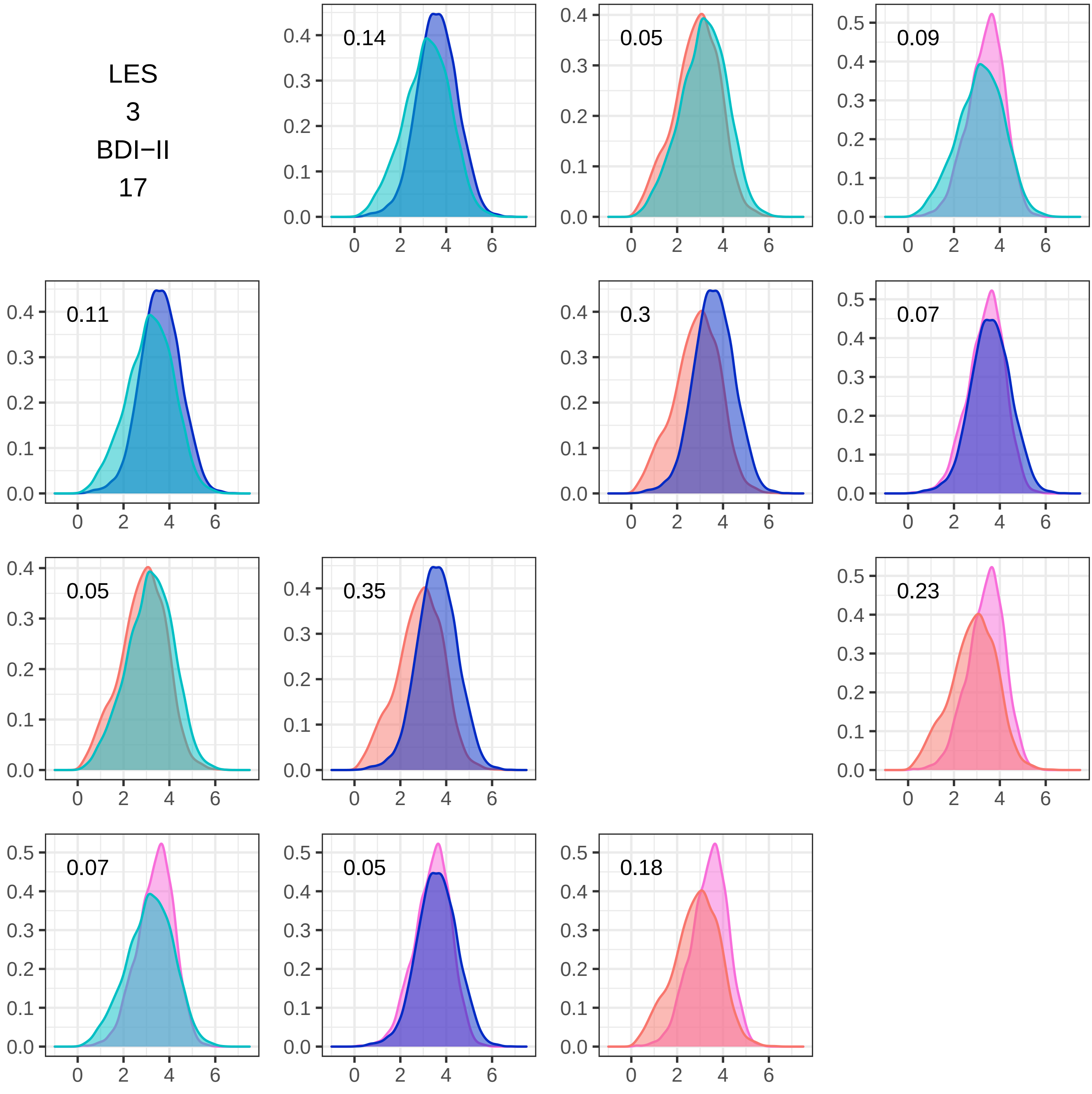
LES
3
BDI−II
17
0.14
0.05
0.09
0.11
0.3
0.07
0.05
0.35
0.23
0.07
0.05
0.18

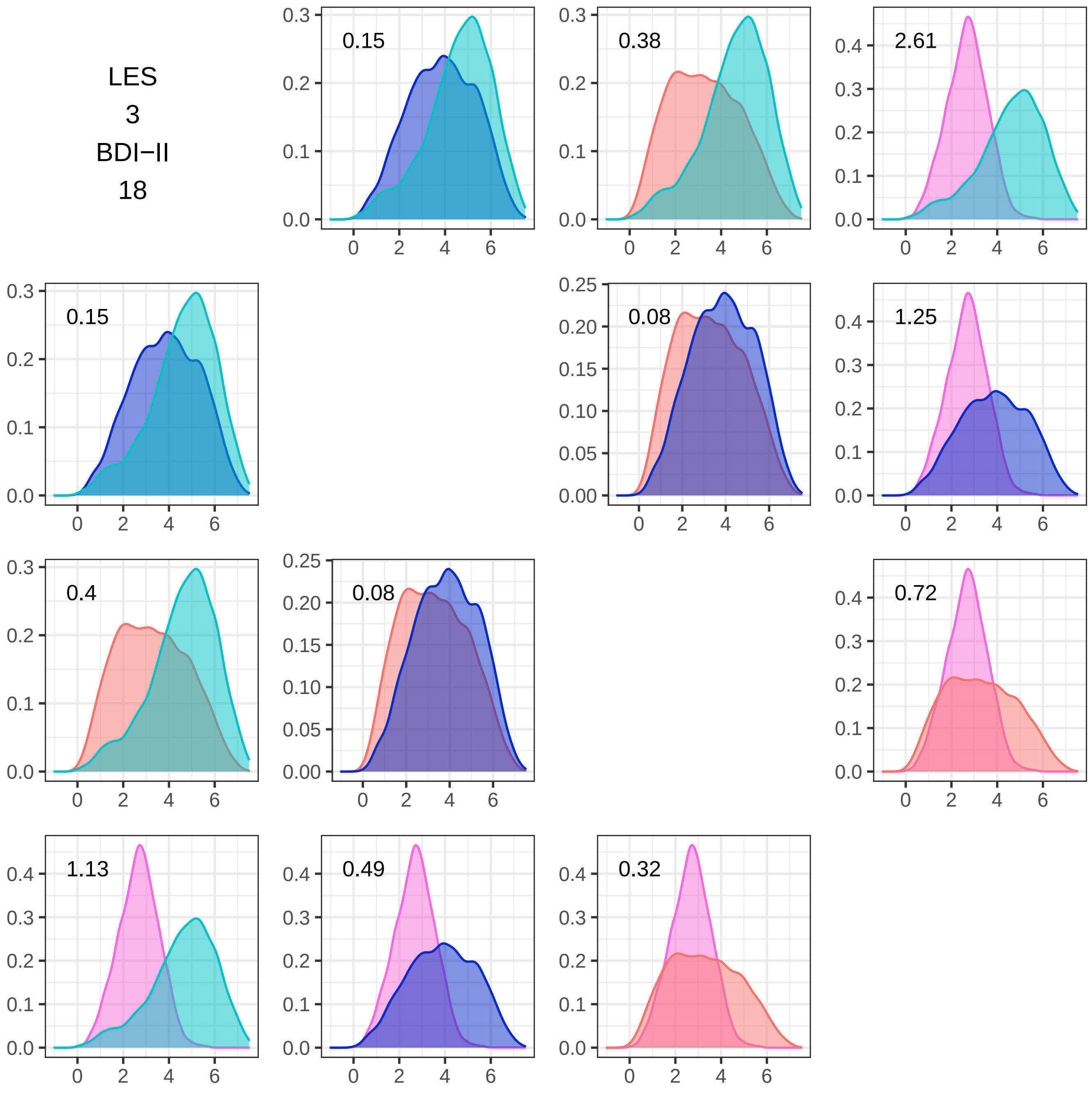

LES
3
BDI−II
18
0.15
0.38
2.61
0.15
0.08
1.25
0.4
0.08
0.72
1.13
0.49
0.32

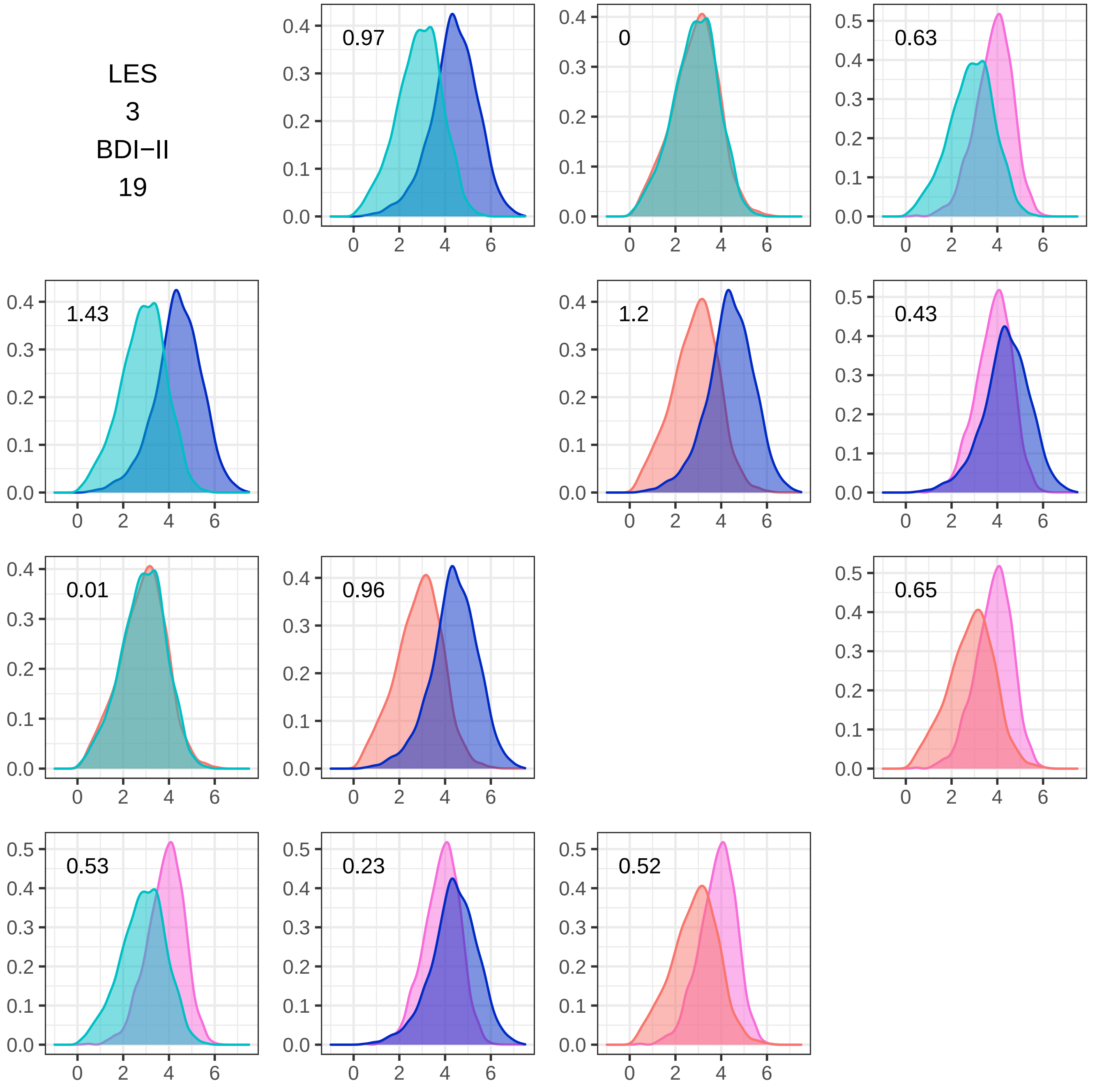
LES
3
BDI−II
19
0.97
0
0.63
1.43
1.2
0.43
0.01
0.96
0.65
0.53
0.23
0.52

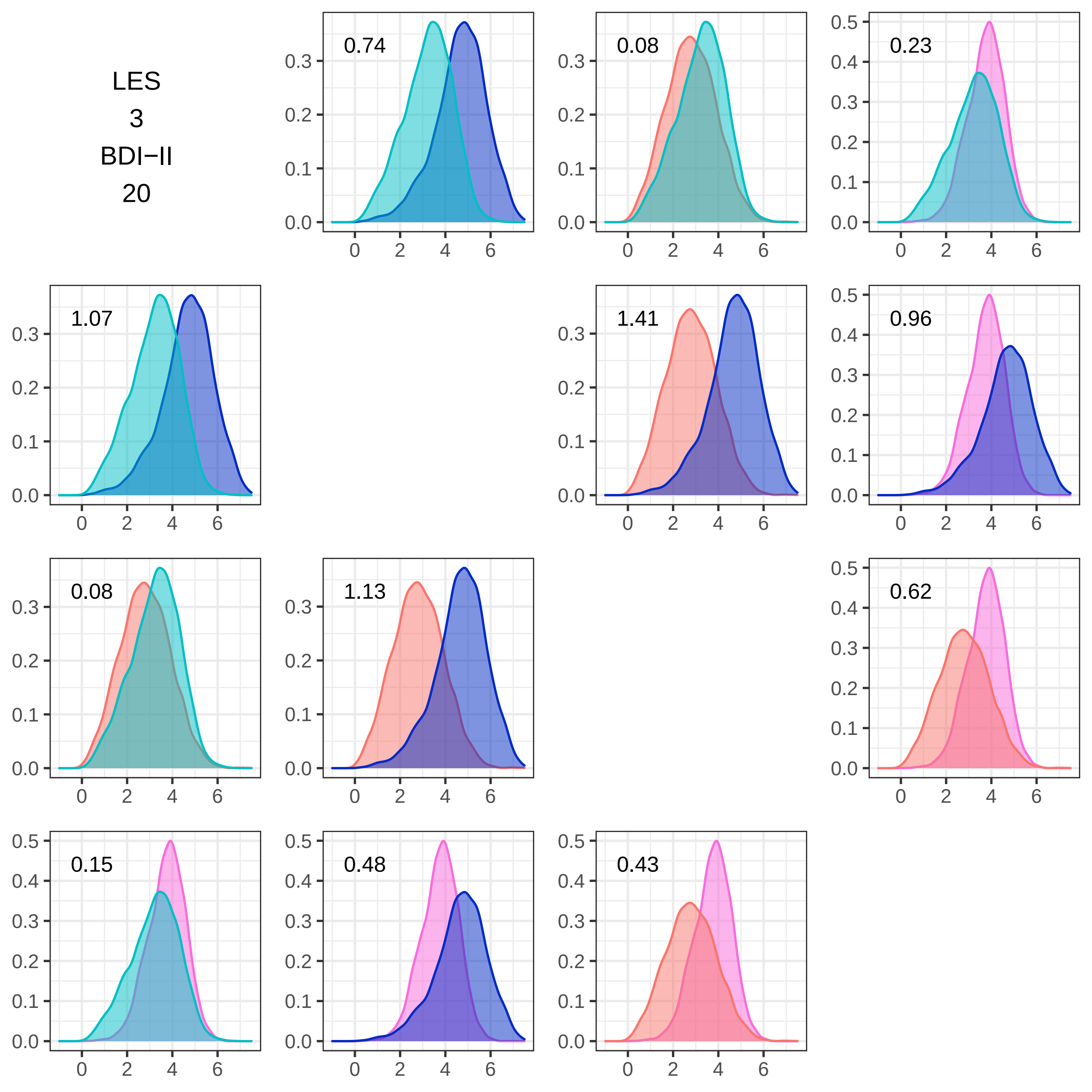
LES
3
BDI−II
20
0.74
0.08
0.23
1.07
1.41
0.96
0.08
1.13
0.62
0.15
0.48
0.43

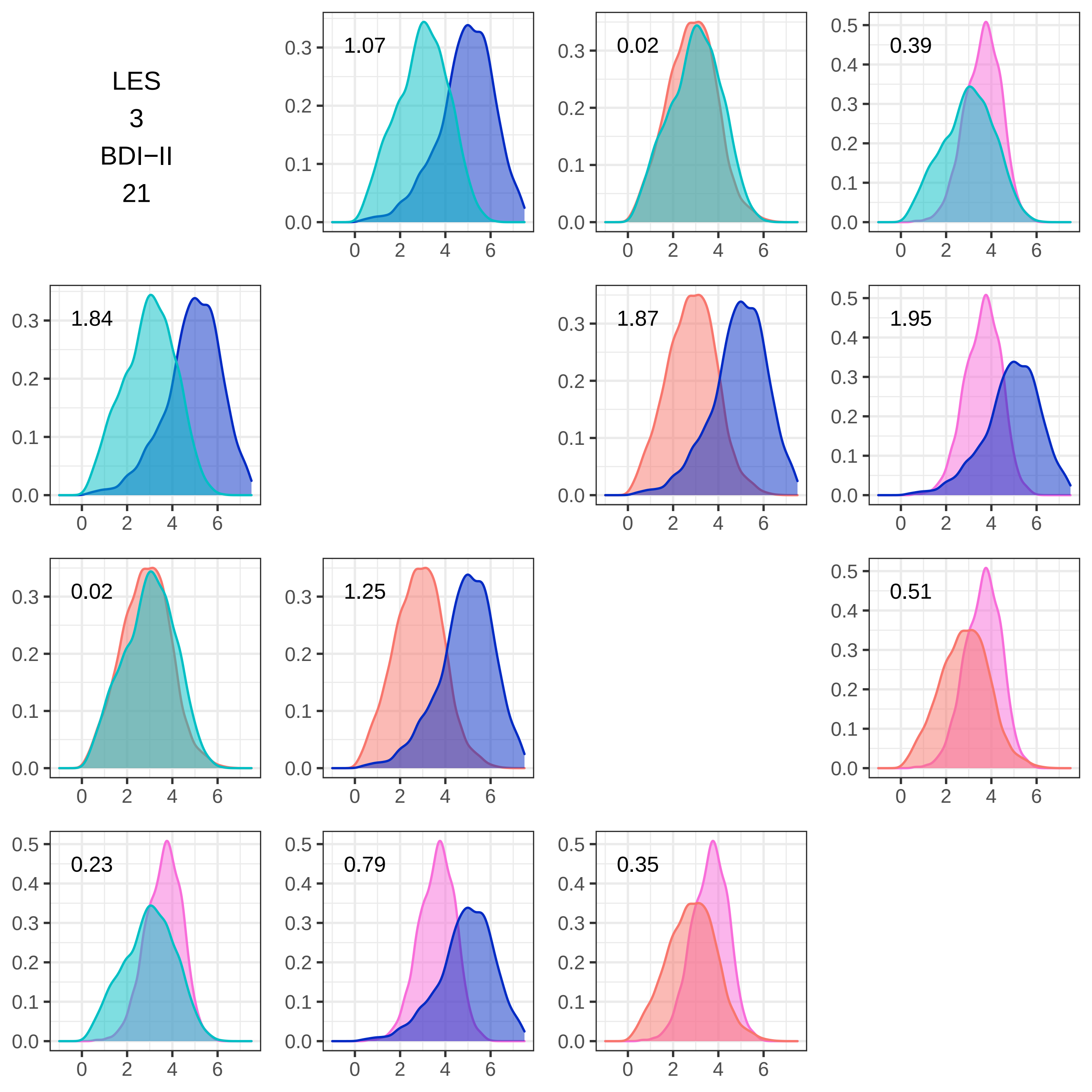
LES
3
BDI−II
21
1.07
0.02
0.39
1.84
1.87
1.95
0.02
1.25
0.51
0.23
0.79
0.35
0.0
0.1
0.2
0.3
0.4
0.5
0
2
4
6

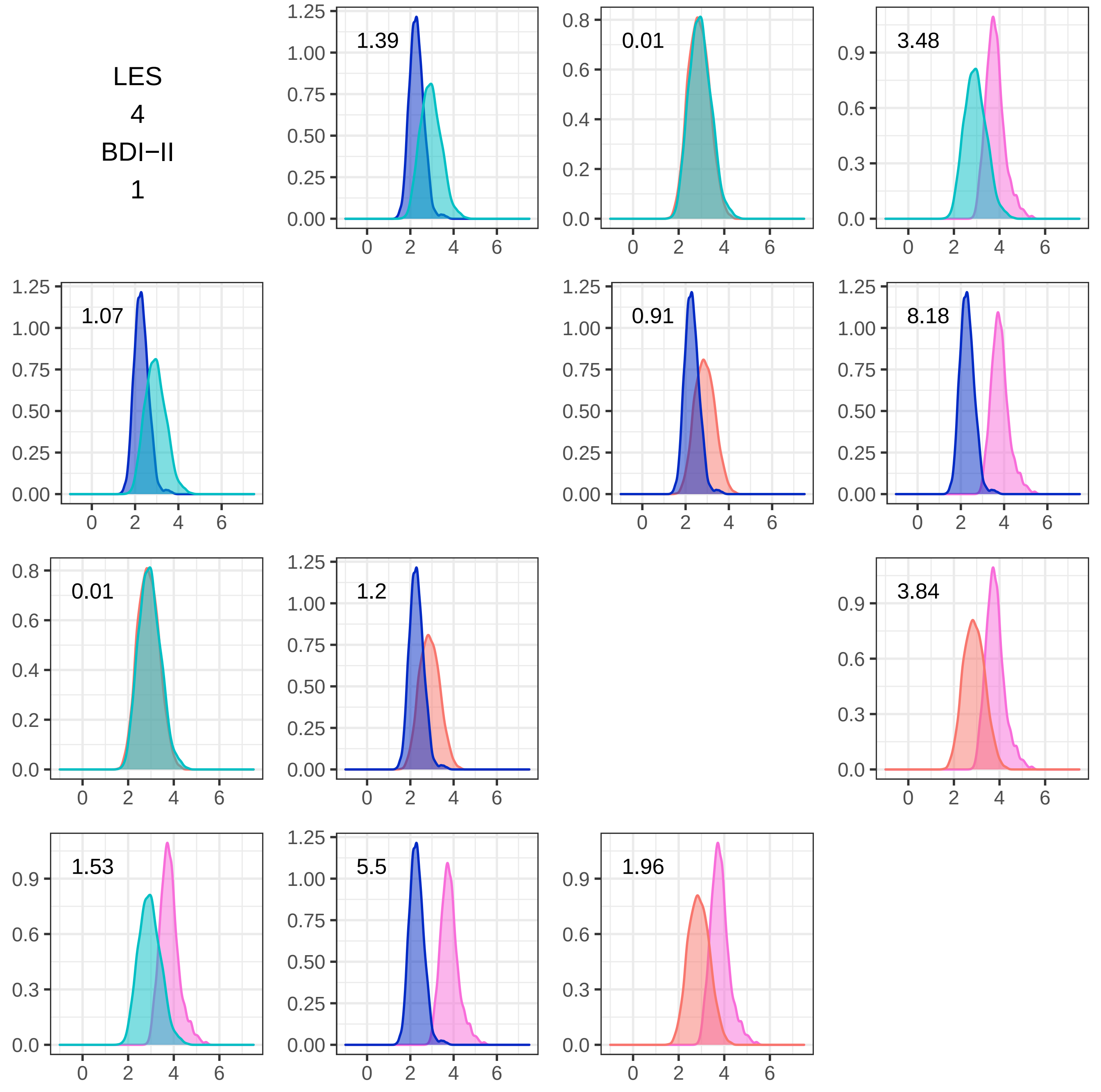
LES
4
BDI−II
1
1.39
0.01
3.48
1.07
0.91
8.18
0.01
1.2
3.84
1.53
5.5
1.96

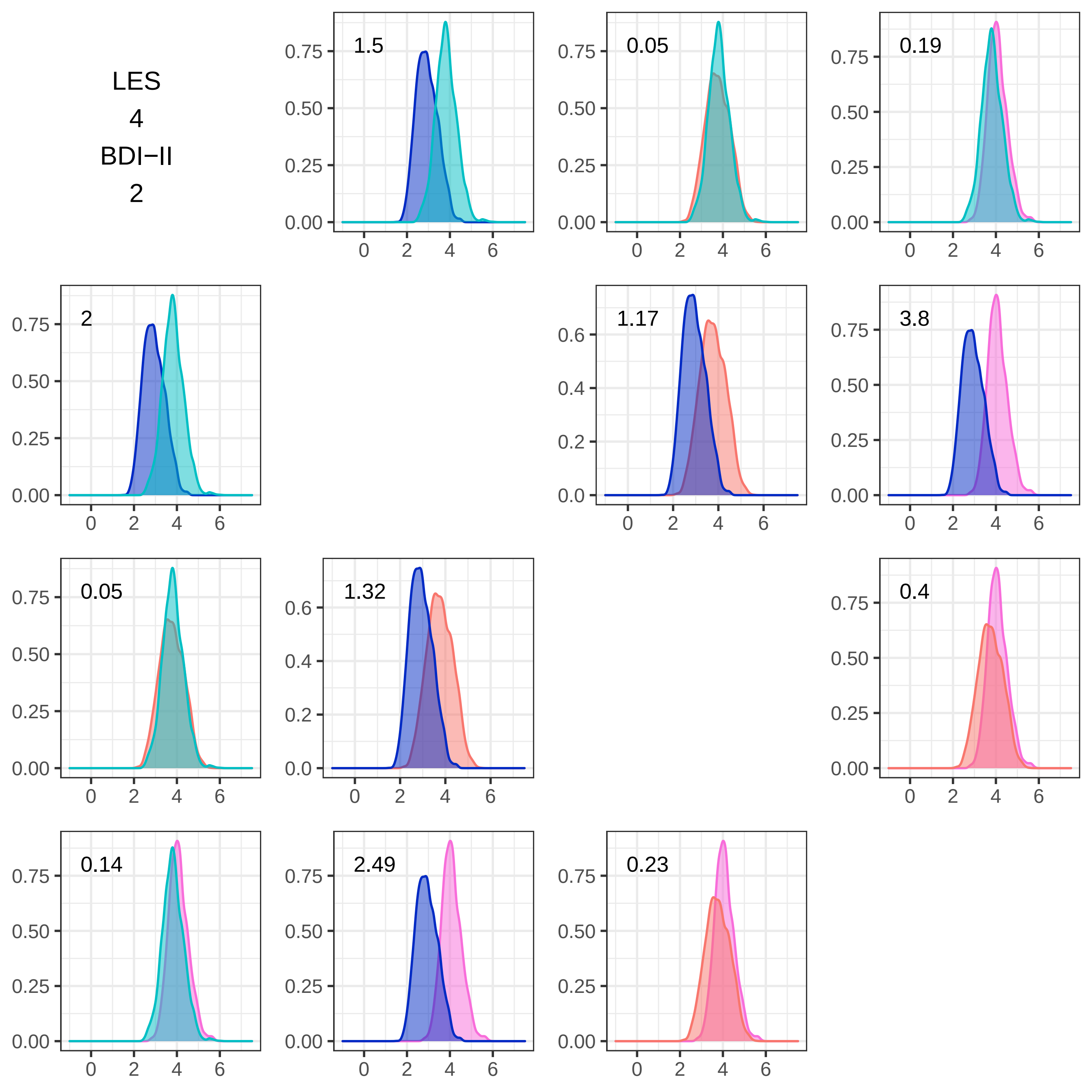
LES
4
BDI−II
2
1.5
0.05
0.19
2
1.17
3.8
0.05
1.32
0.4
0.14
2.49
0.23
0.00
0.25
0.50
0.75
0.0
0.2
0.4
0.6
0
2
4
6

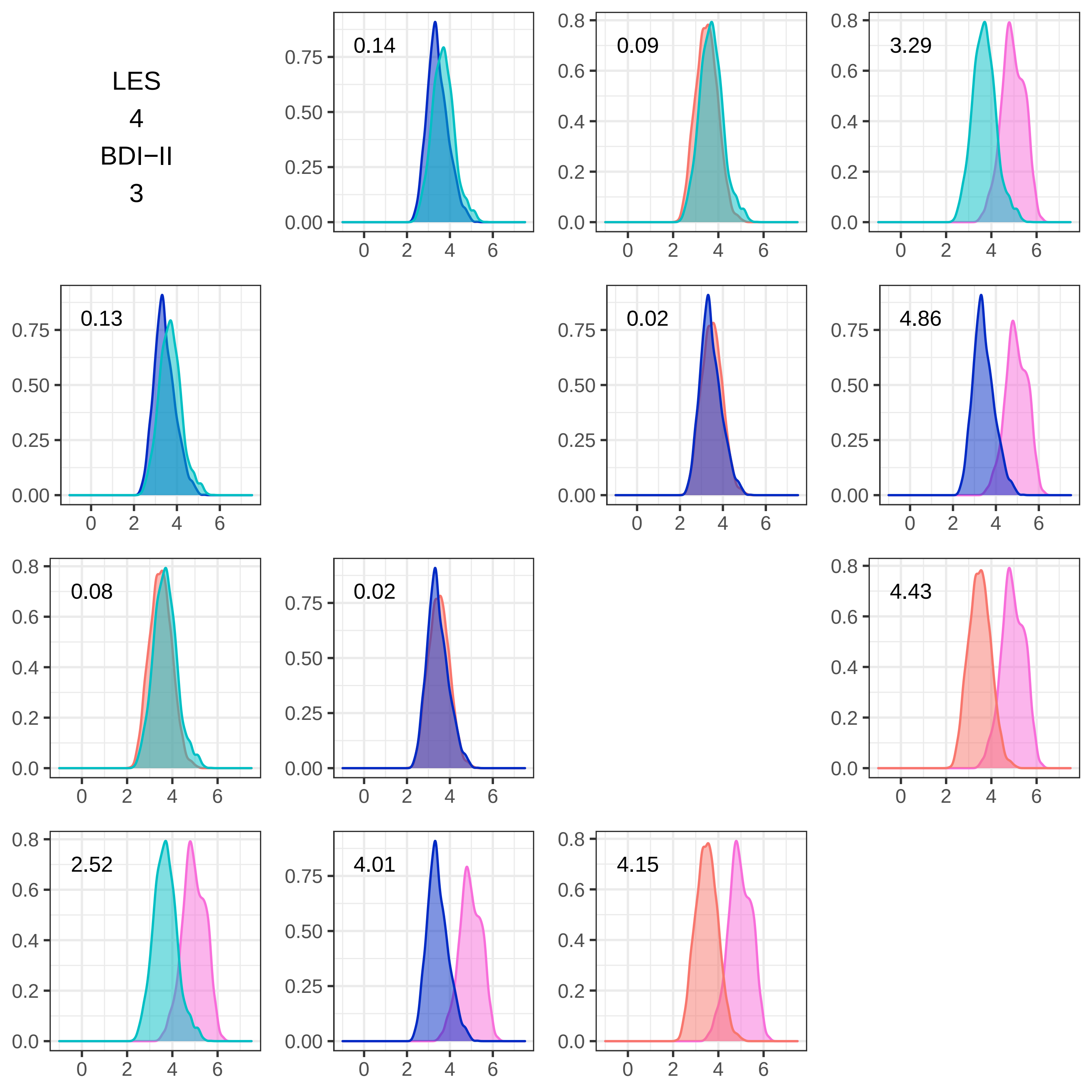
LES
4
BDI−II
3
0.14
0.09
3.29
0.13
0.02
4.86
0.08
0.02
4.43
2.52
4.01
4.15

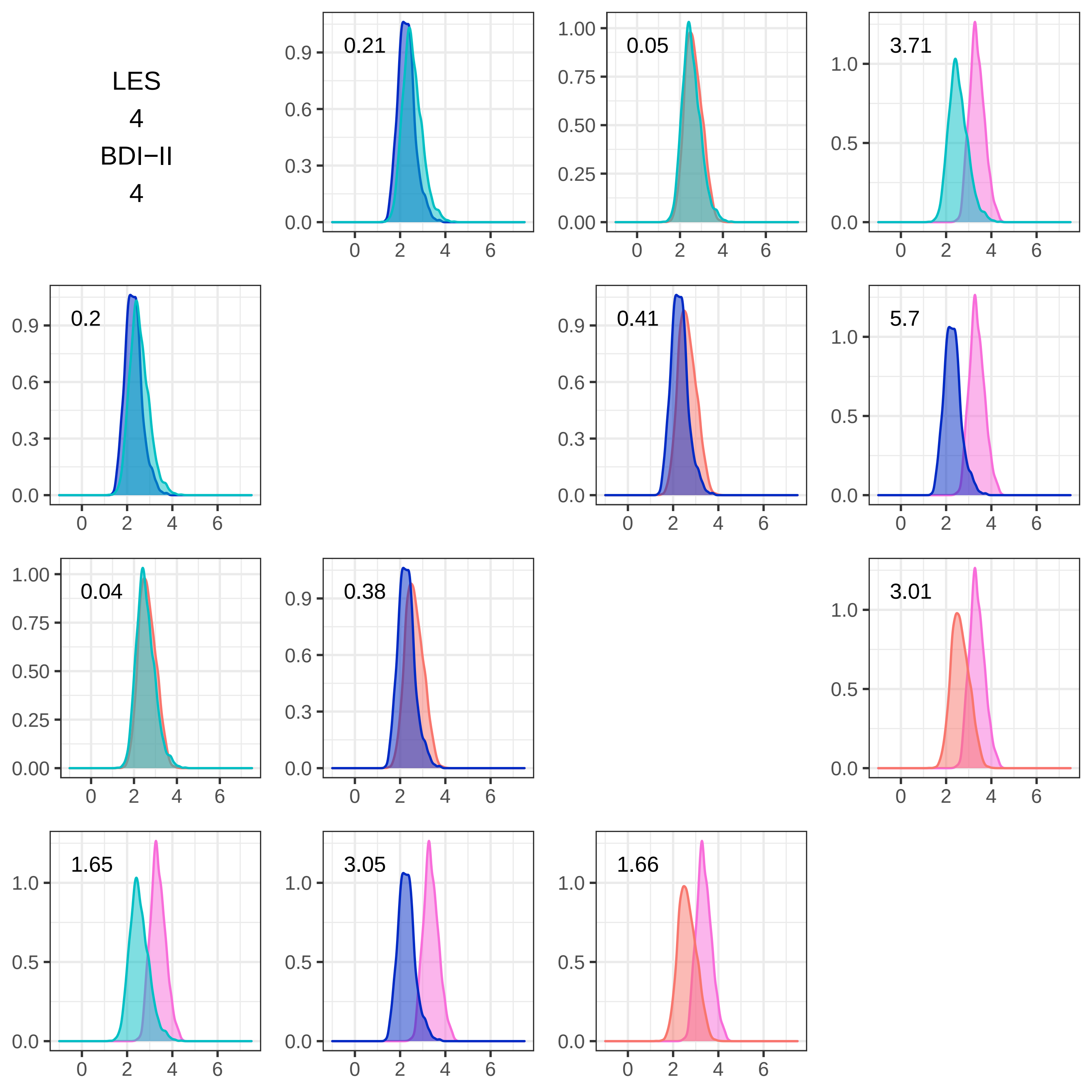
LES
4
BDI−II
4
0.21
0.05
3.71
0.2
0.41
5.7
0.04
0.38
3.01
1.65
3.05
1.66

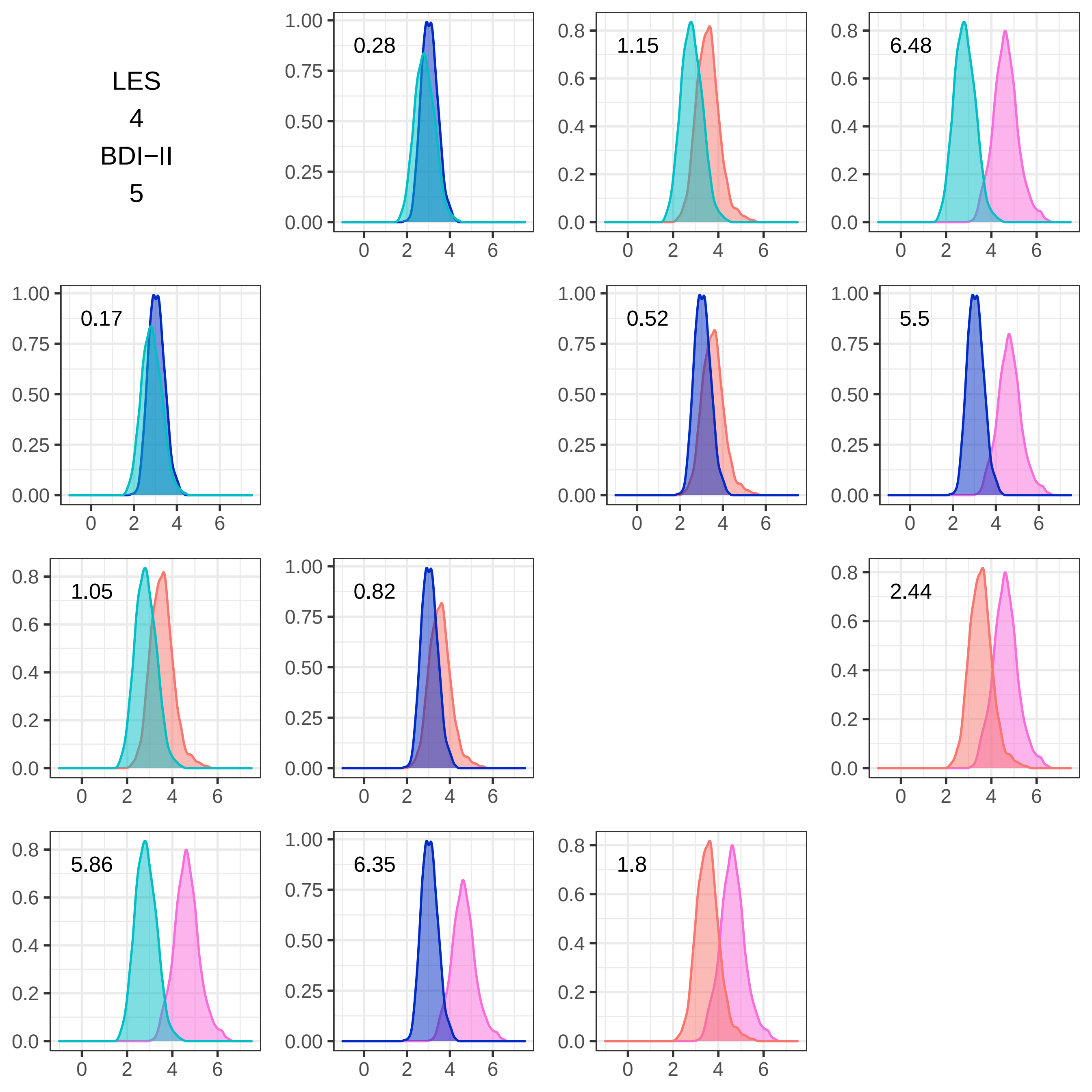

LES
4
BDI−II
5
0.28
1.15
6.48
0.17
0.52
5.5
1.05
0.82
2.44
5.86
6.35
1.8

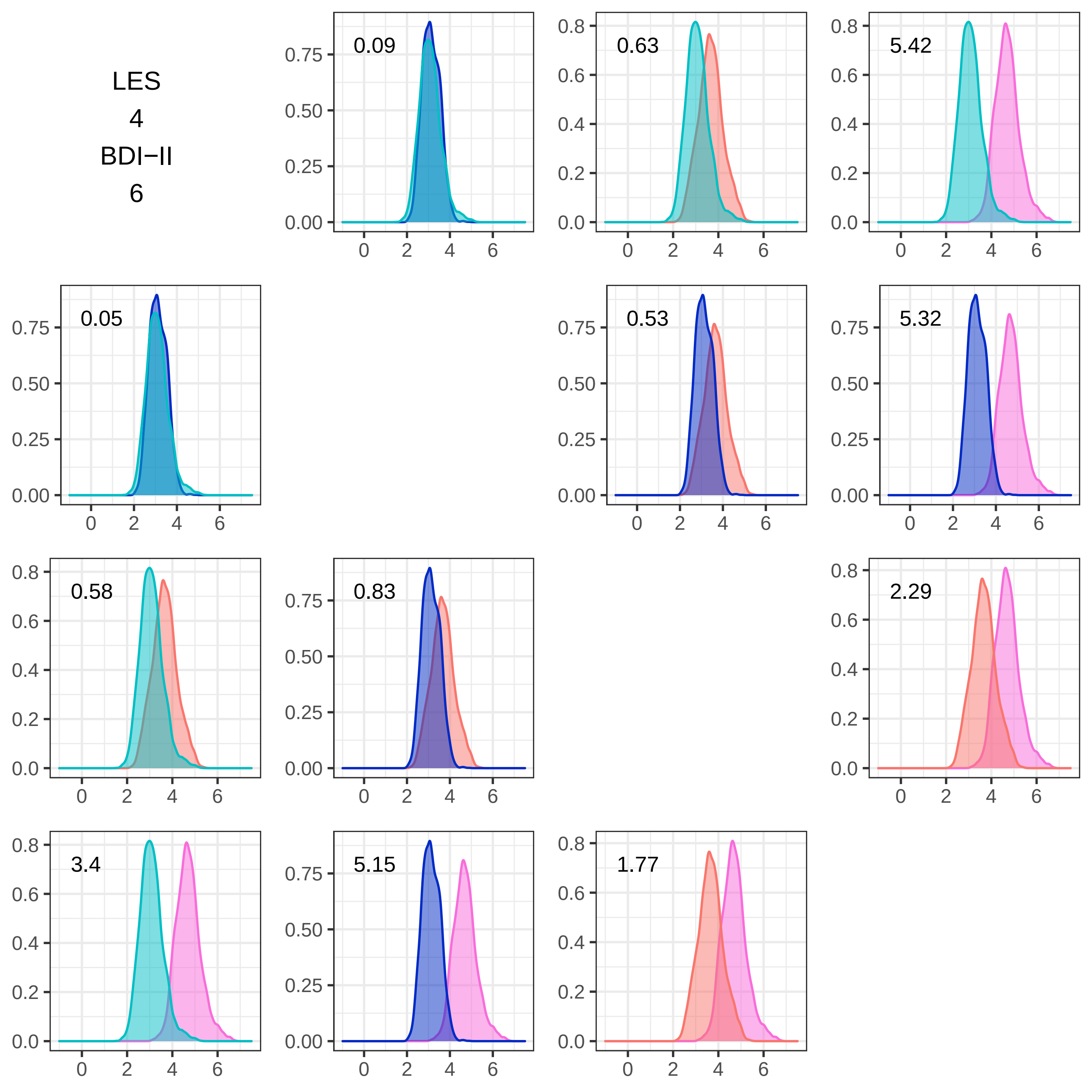

LES
4
BDI−II
6
0.09
0.63
5.42
0.05
0.53
5.32
0.58
0.83
2.29
3.4
5.15
1.77

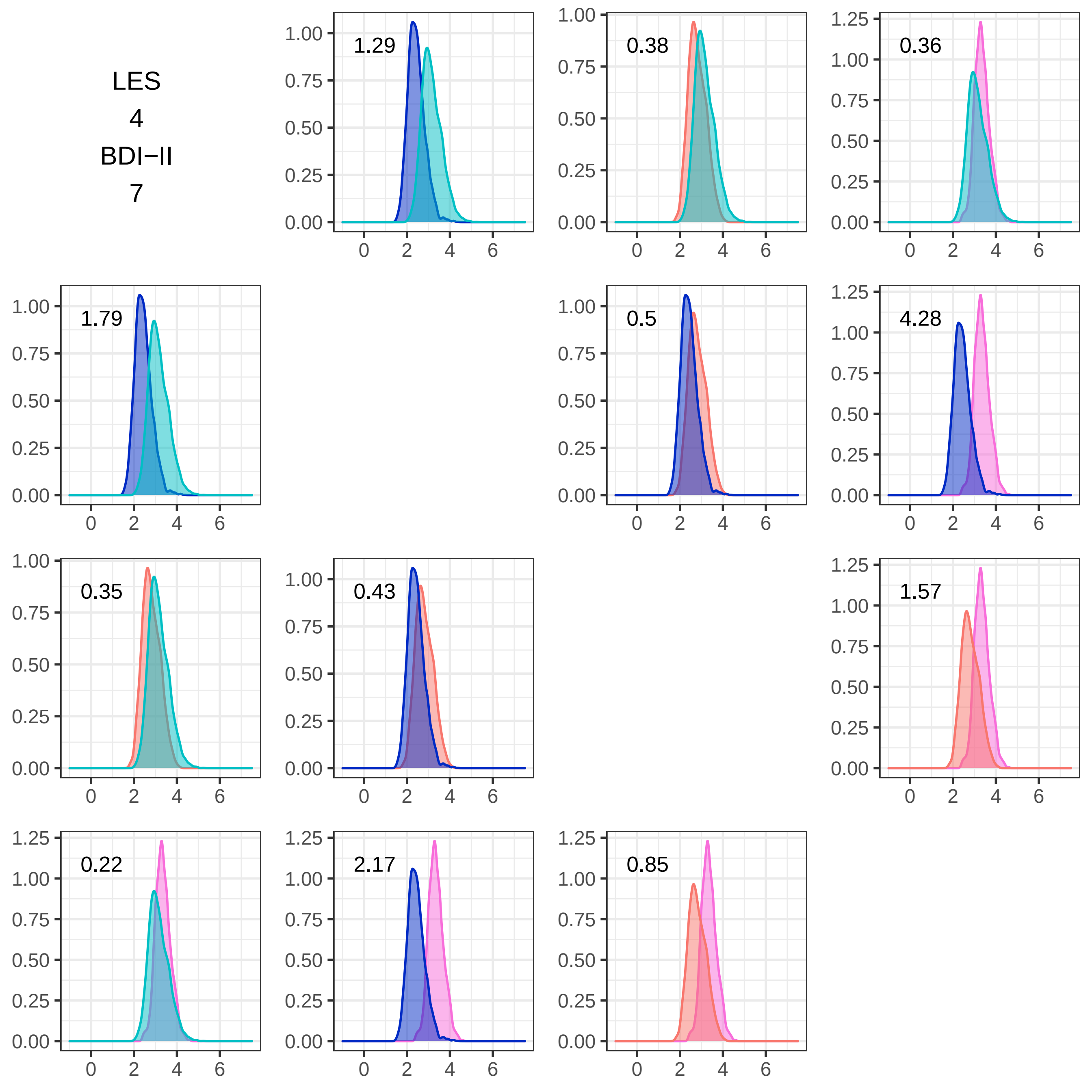

LES
4
BDI−II
7
1.29
0.38
0.36
1.79
0.5
4.28
0.35
0.43
1.57
0.22
2.17
0.85

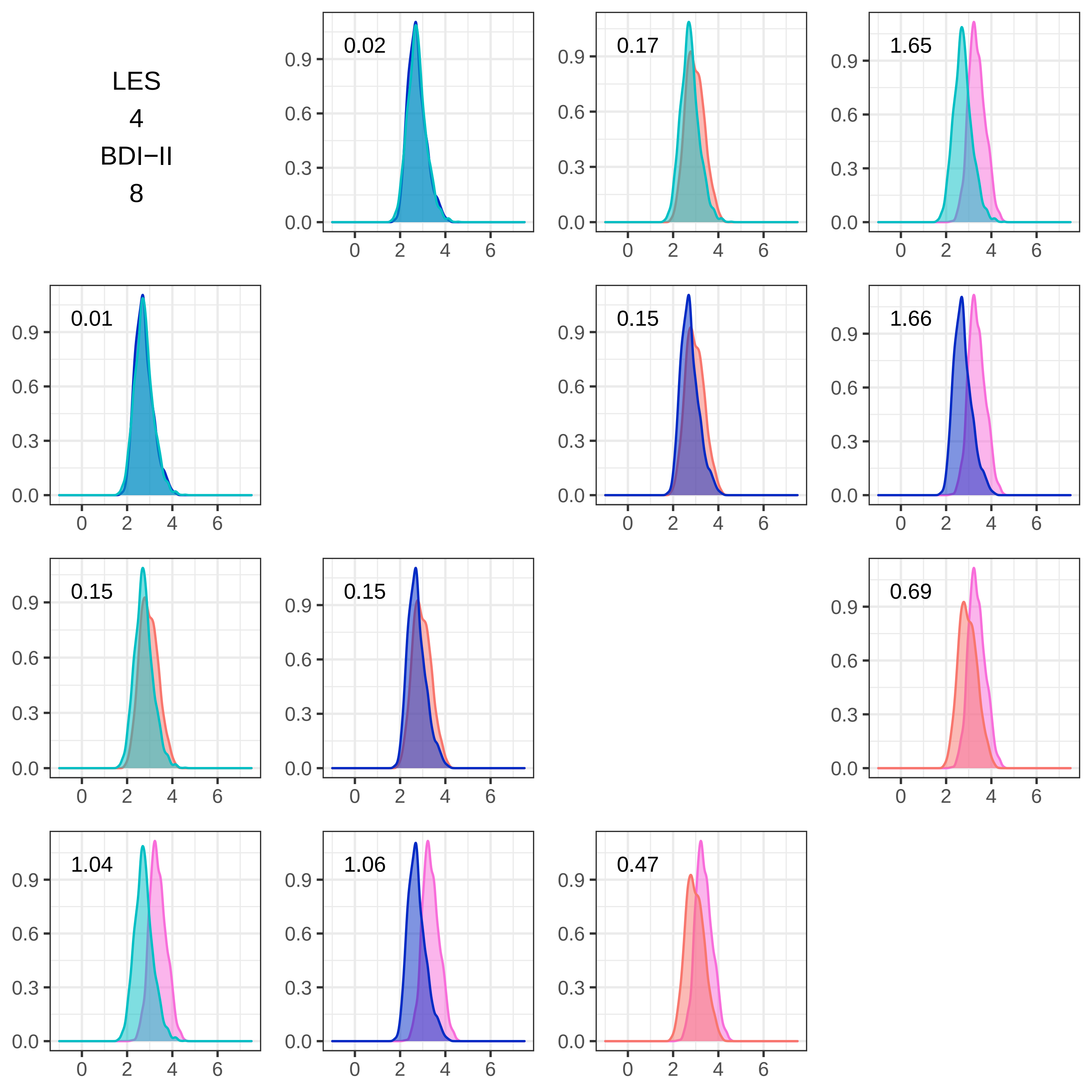
LES
4
BDI−II
8
0.02
0.17
1.65
0.01
0.15
1.66
0.15
0.15
0.69
1.04
1.06
0.47

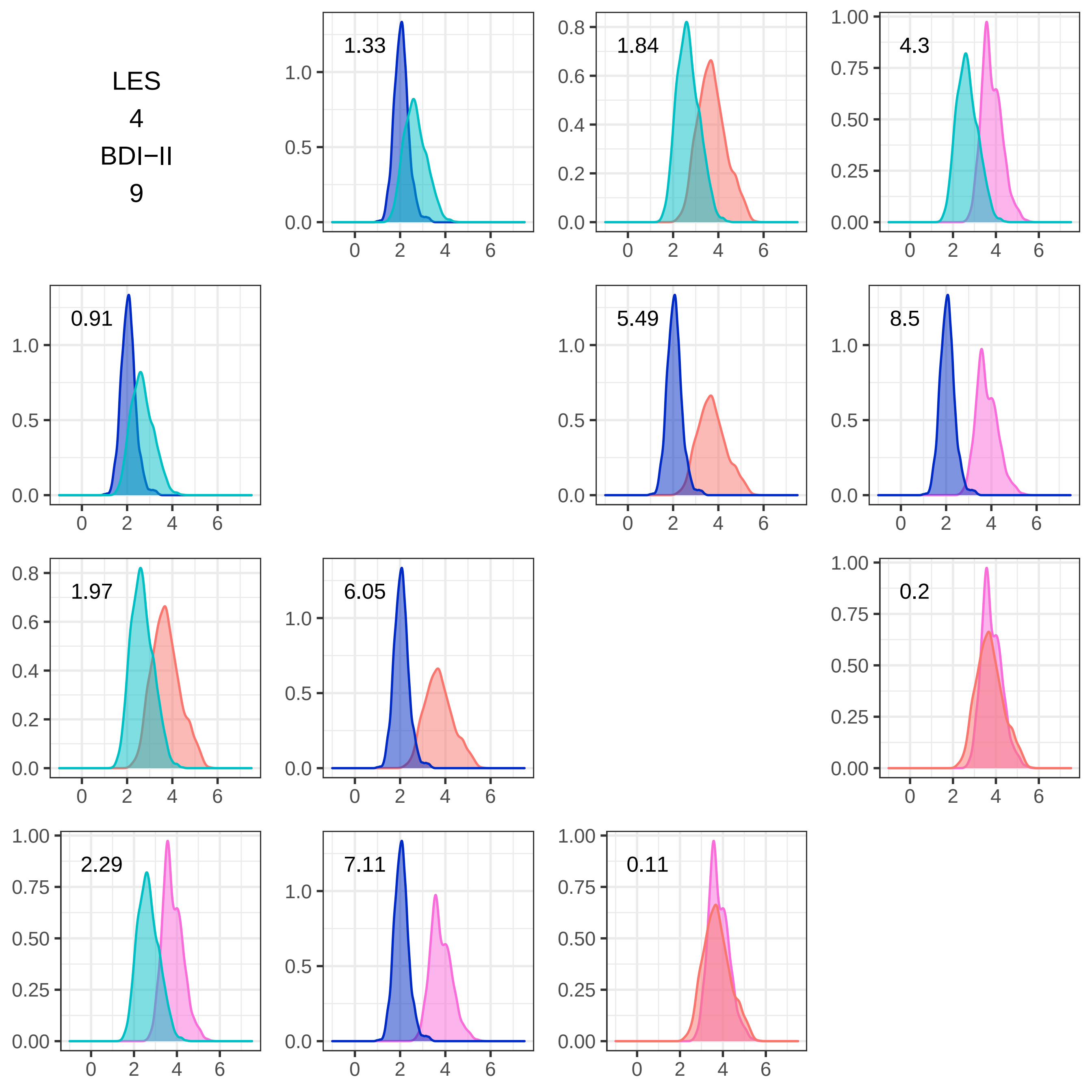
LES
4
BDI−II
9
1.33
1.84
4.3
0.91
5.49
8.5
1.97
6.05
0.2
2.29
7.11
0.11

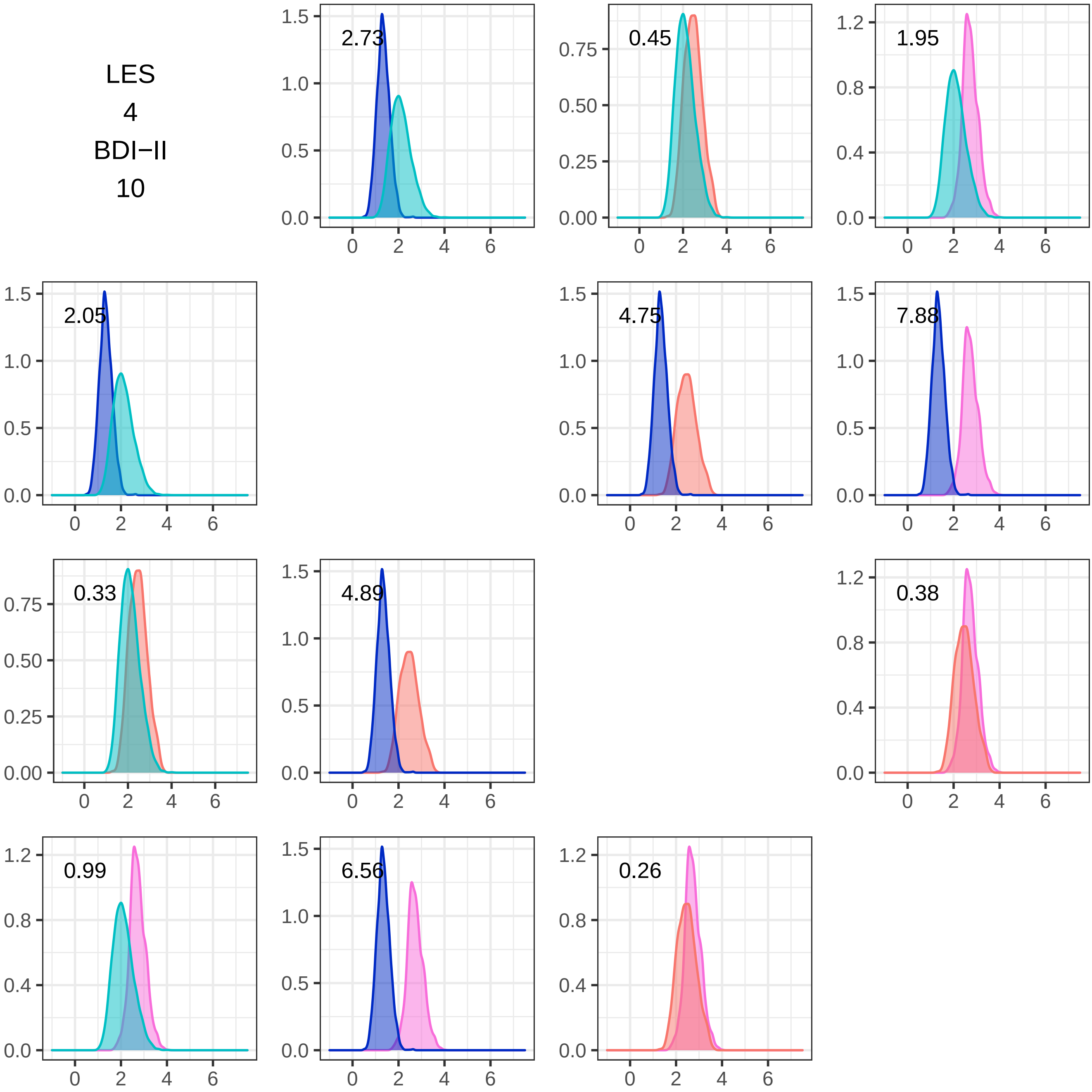
LES
4
BDI−II
10
2.73
0.45
1.95
2.05
4.75
7.88
0.33
4.89
0.38
0.99
6.56
0.26

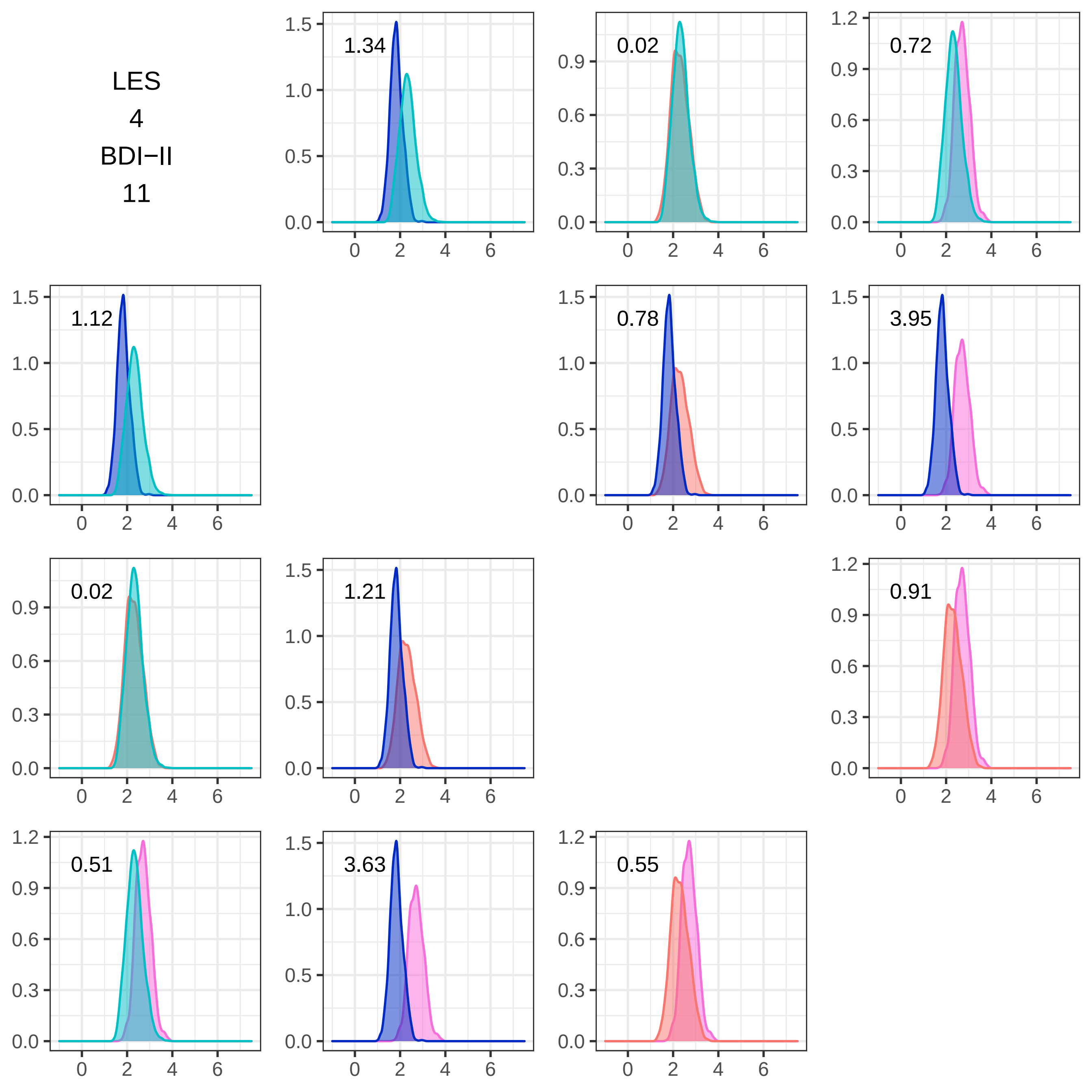
LES
4
BDI−II
11
1.34
0.02
0.72
1.12
0.78
3.95
0.02
1.21
0.91
0.51
3.63
0.55

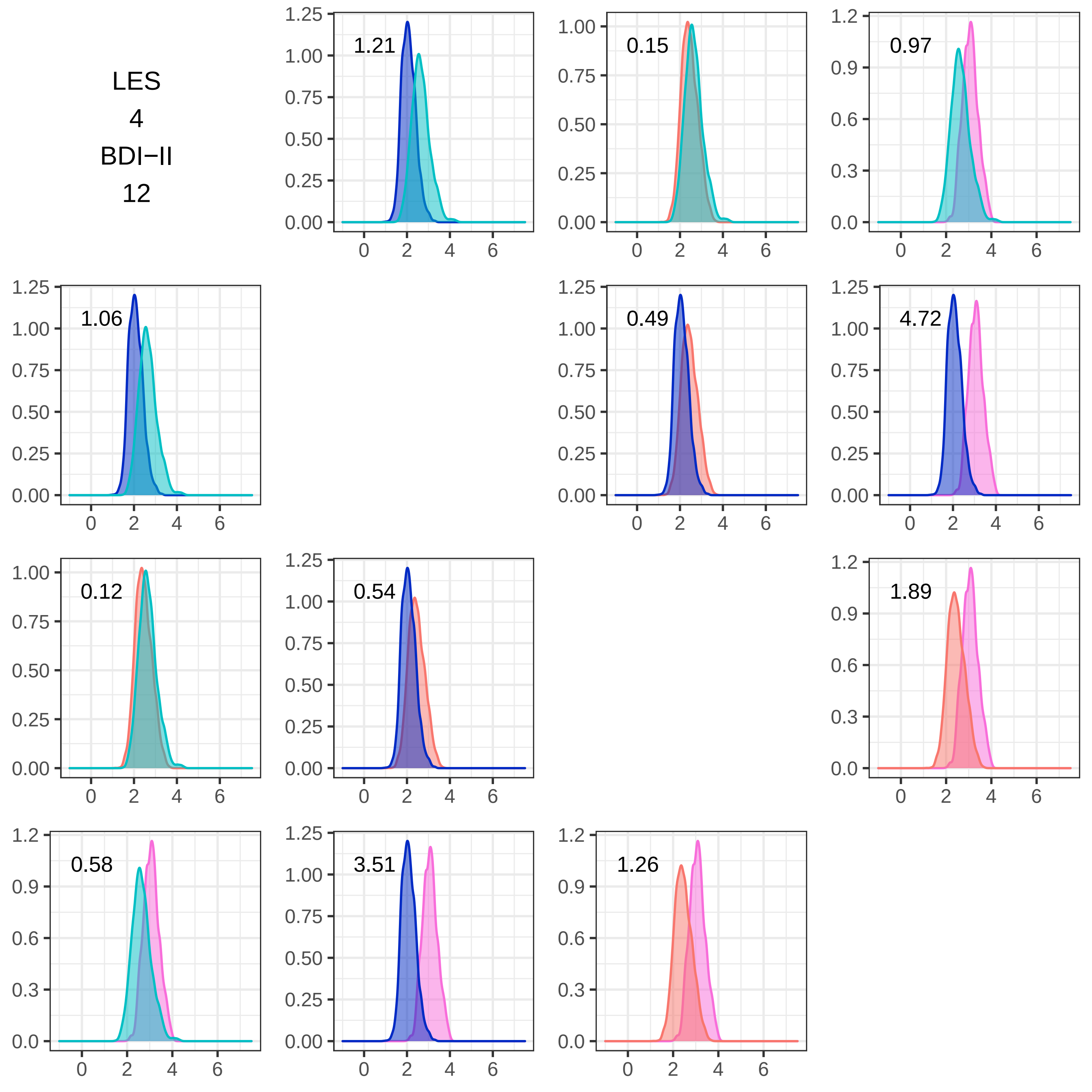

LES
4
BDI−II
12
1.21
0.15
0.97
1.06
0.49
4.72
0.12
0.54
1.89
0.58
3.51
1.26

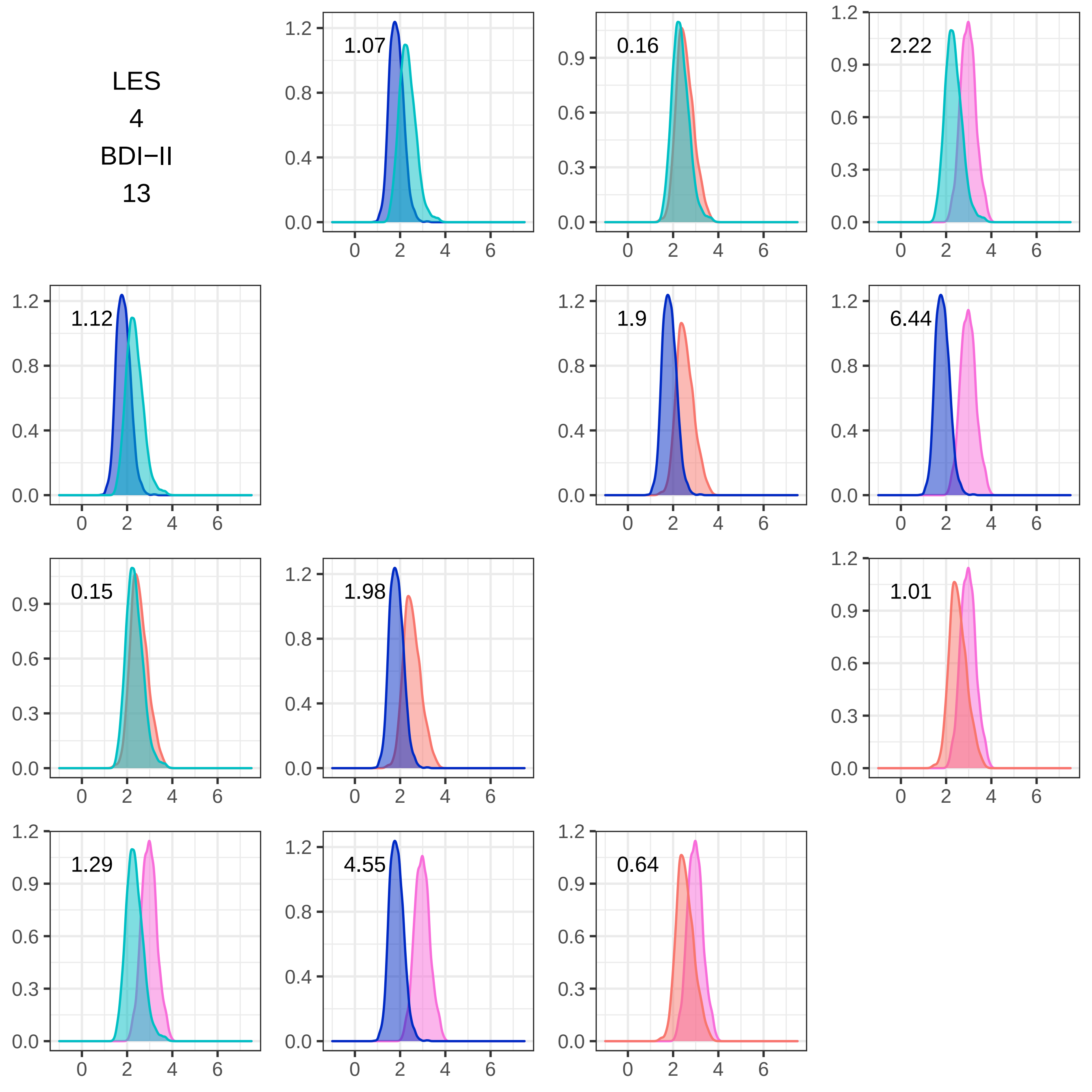
LES
4
BDI−II
13
1.07
0.16
2.22
1.12
1.9
6.44
0.15
1.98
1.01
1.29
4.55
0.64

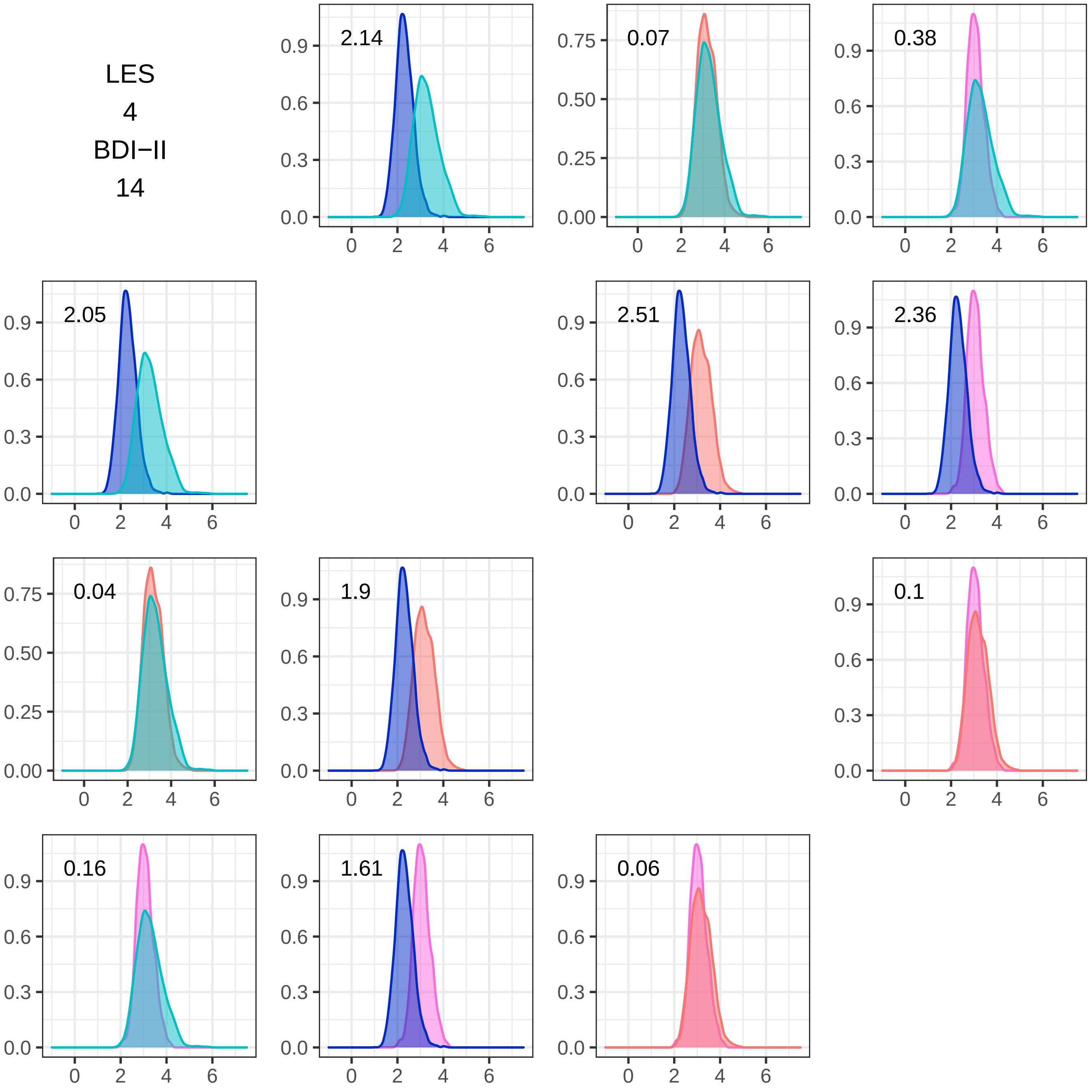

LES
4
BDI−II
14
2.14
0.07
0.38
2.05
2.51
2.36
0.04
1.9
0.1
0.16
1.61
0.06

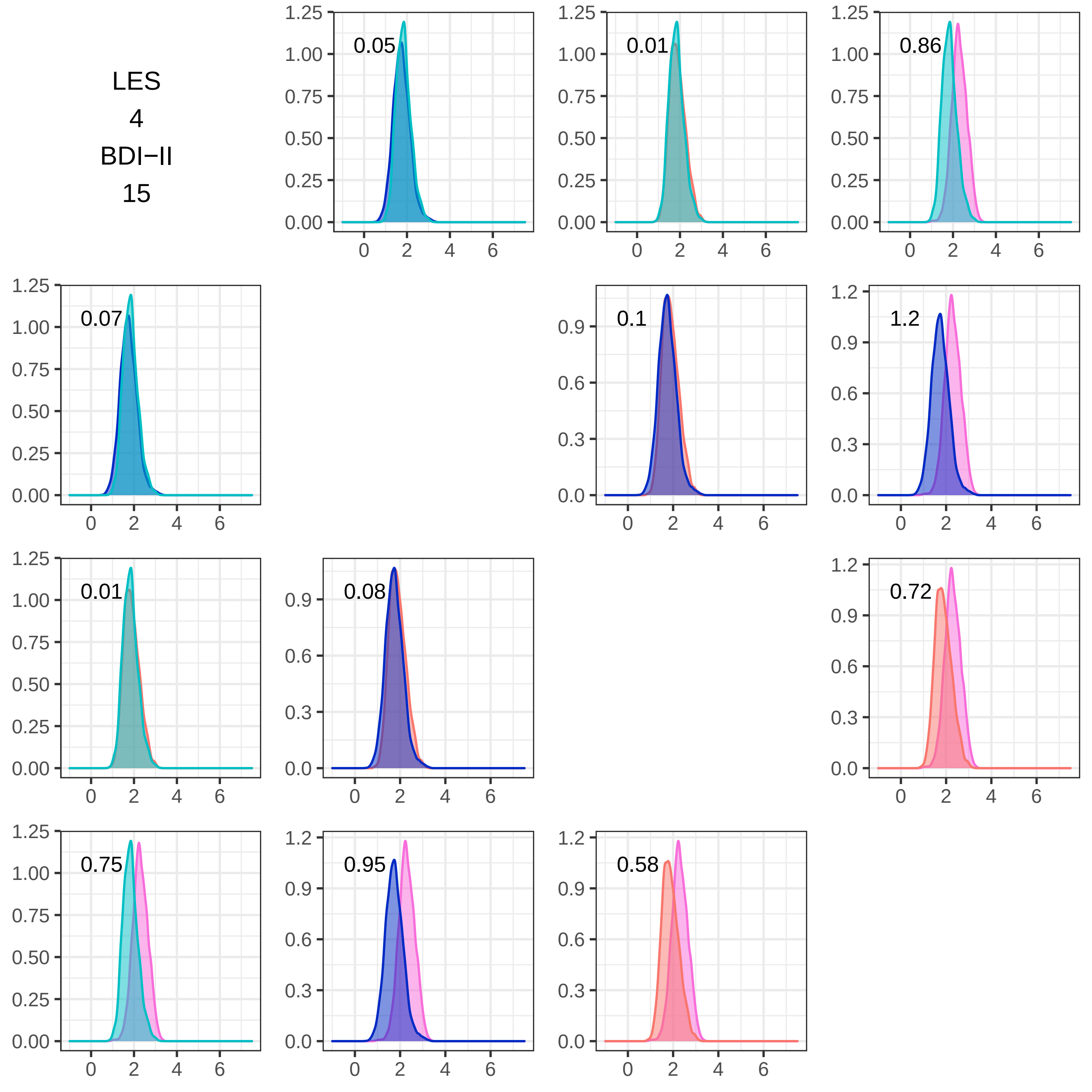
LES
4
BDI−II
15
0.05
0.01
0.86
0.07
0.1
1.2
0.01
0.08
0.72
0.75
0.95
0.58

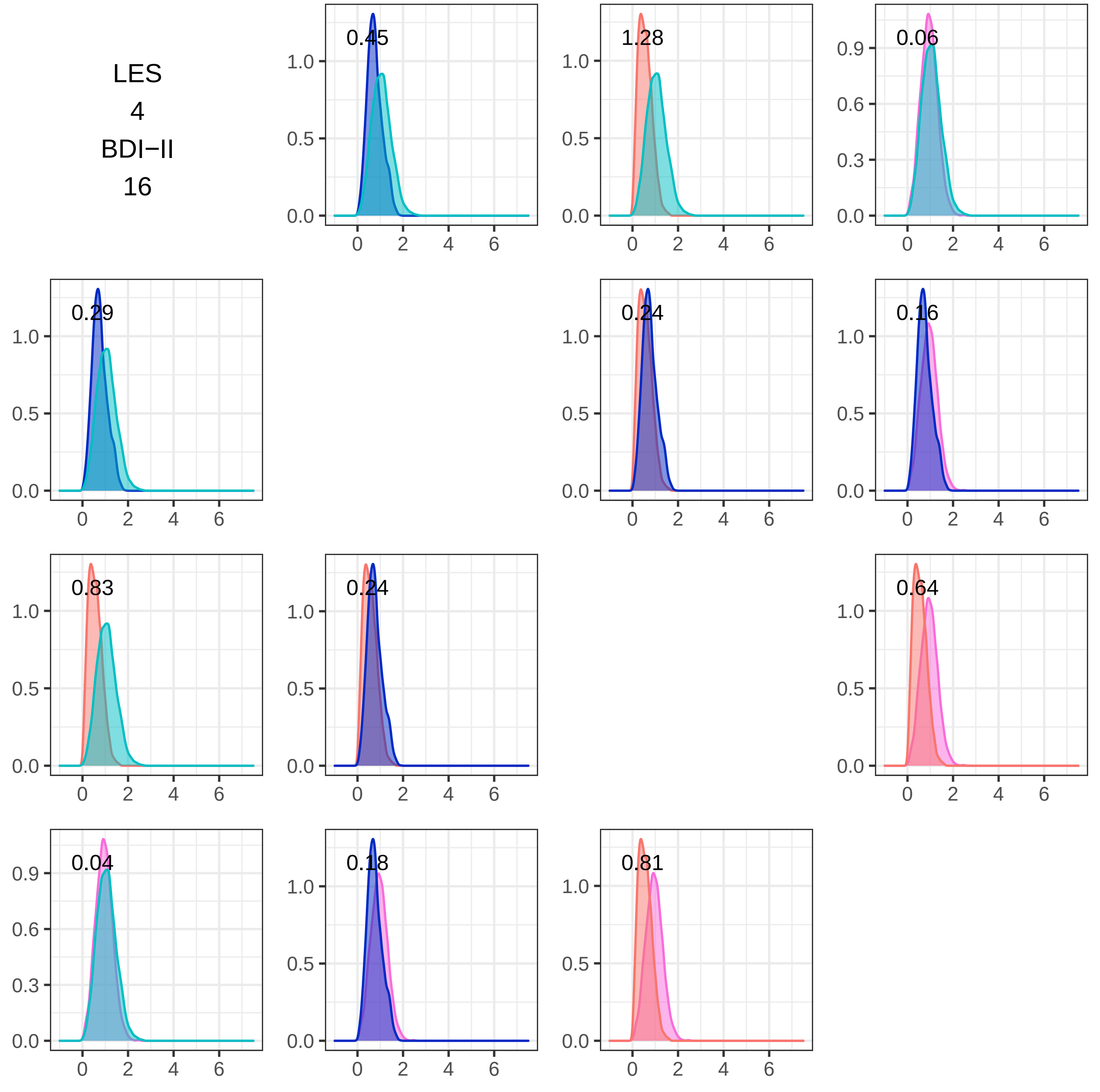
LES
4
BDI−II
16
0.45
1.28
0.06
0.29
0.24
0.16
0.83
0.24
0.64
0.04
0.18
0.81

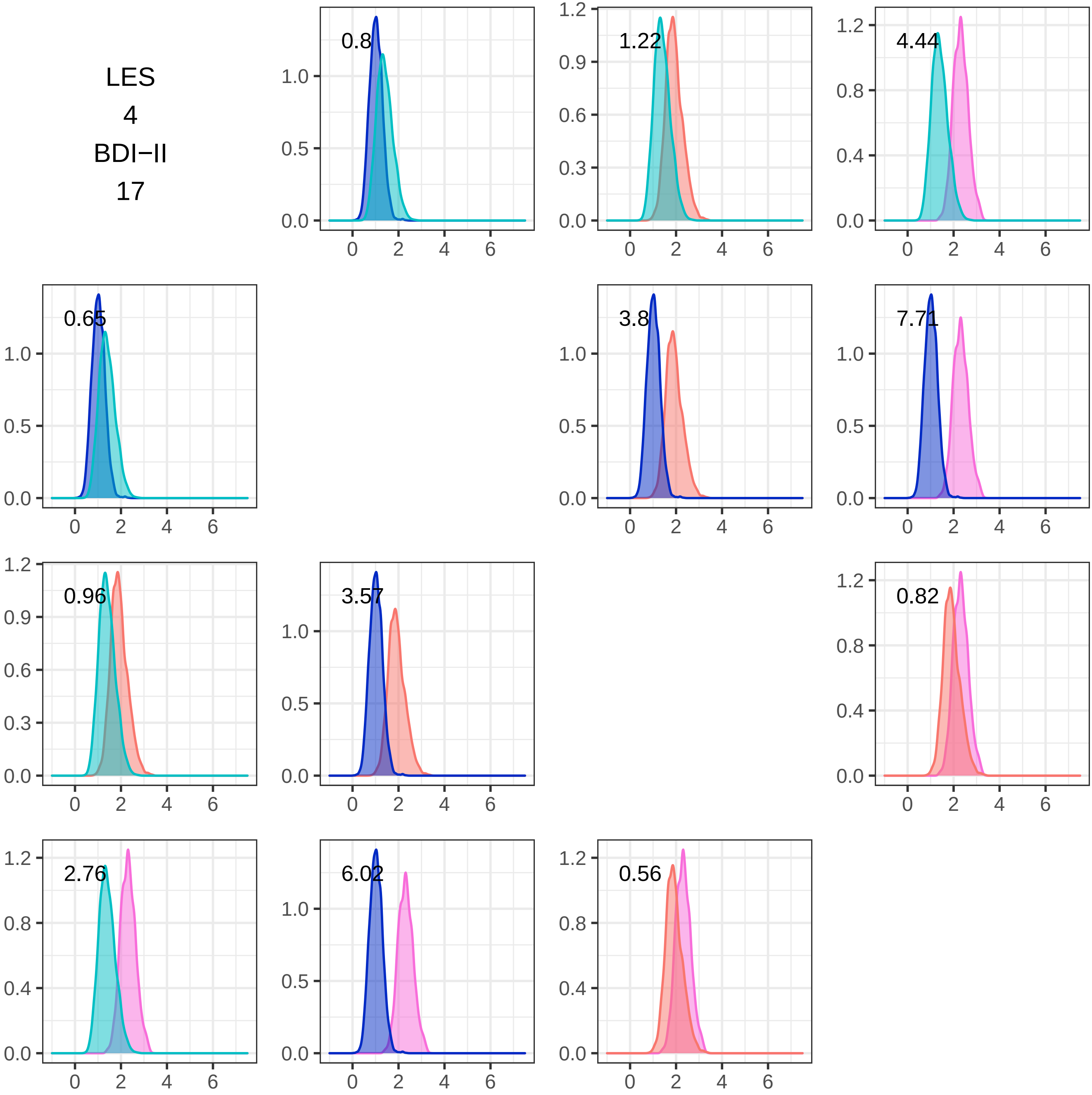

LES
4
BDI−II
17
0.8
1.22
4.44
0.65
3.8
7.71
0.96
3.57
0.82
2.76
6.02
0.56

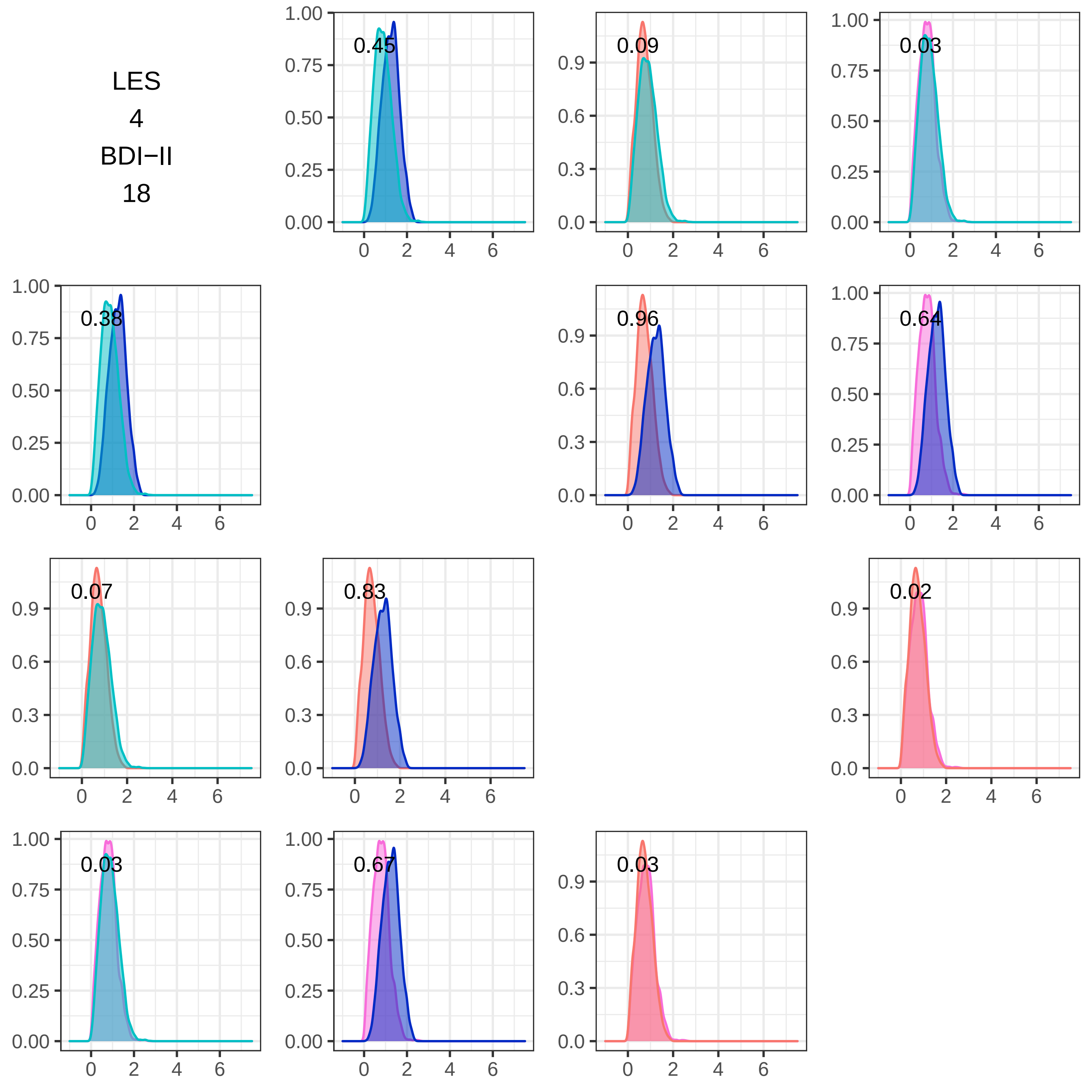

LES
4
BDI−II
18
0.45
0.09
0.03
0.38
0.96
0.64
0.07
0.83
0.02
0.03
0.67
0.03

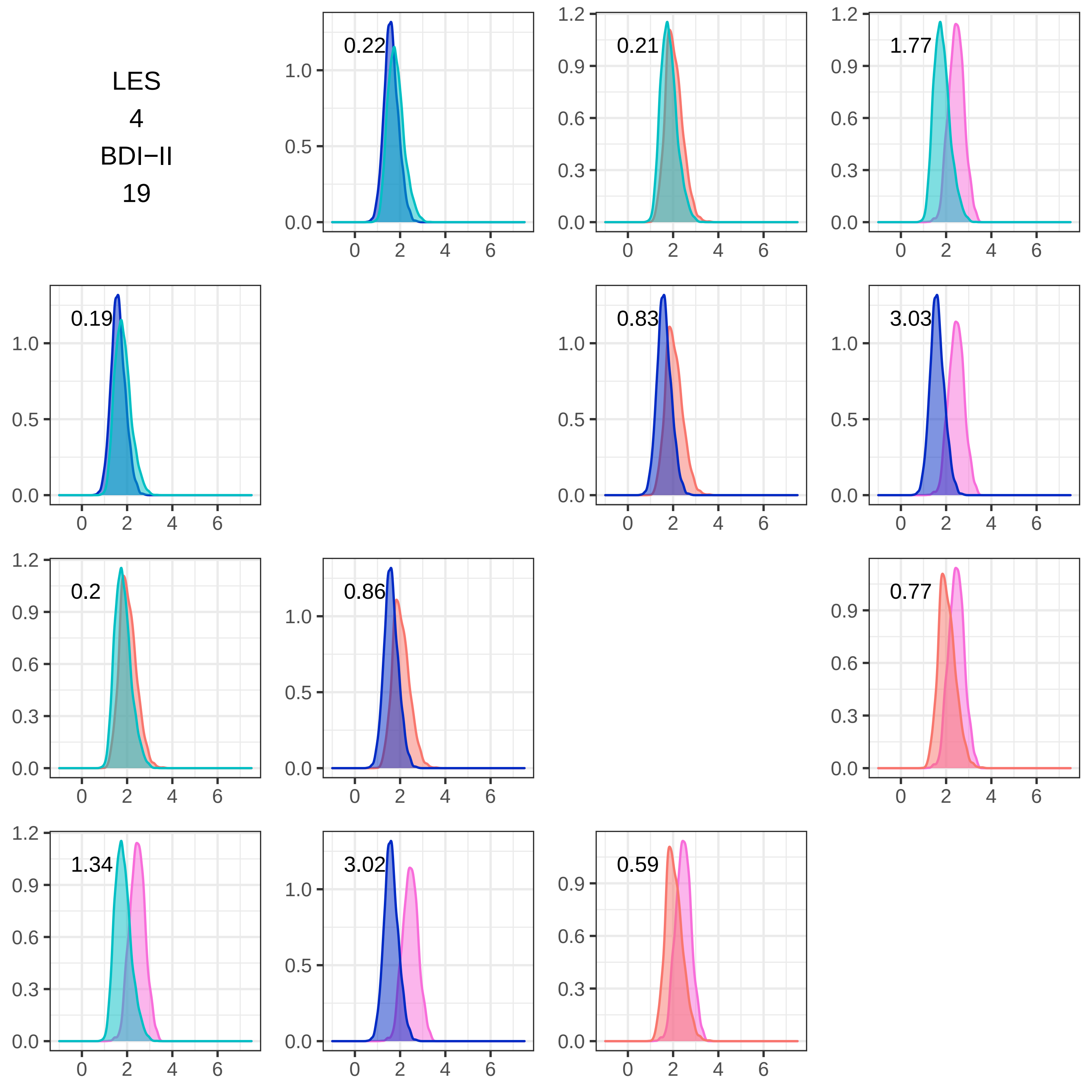
LES
4
BDI−II
19
0.22
0.21
1.77
0.19
0.83
3.03
0.2
0.86
0.77
1.34
3.02
0.59

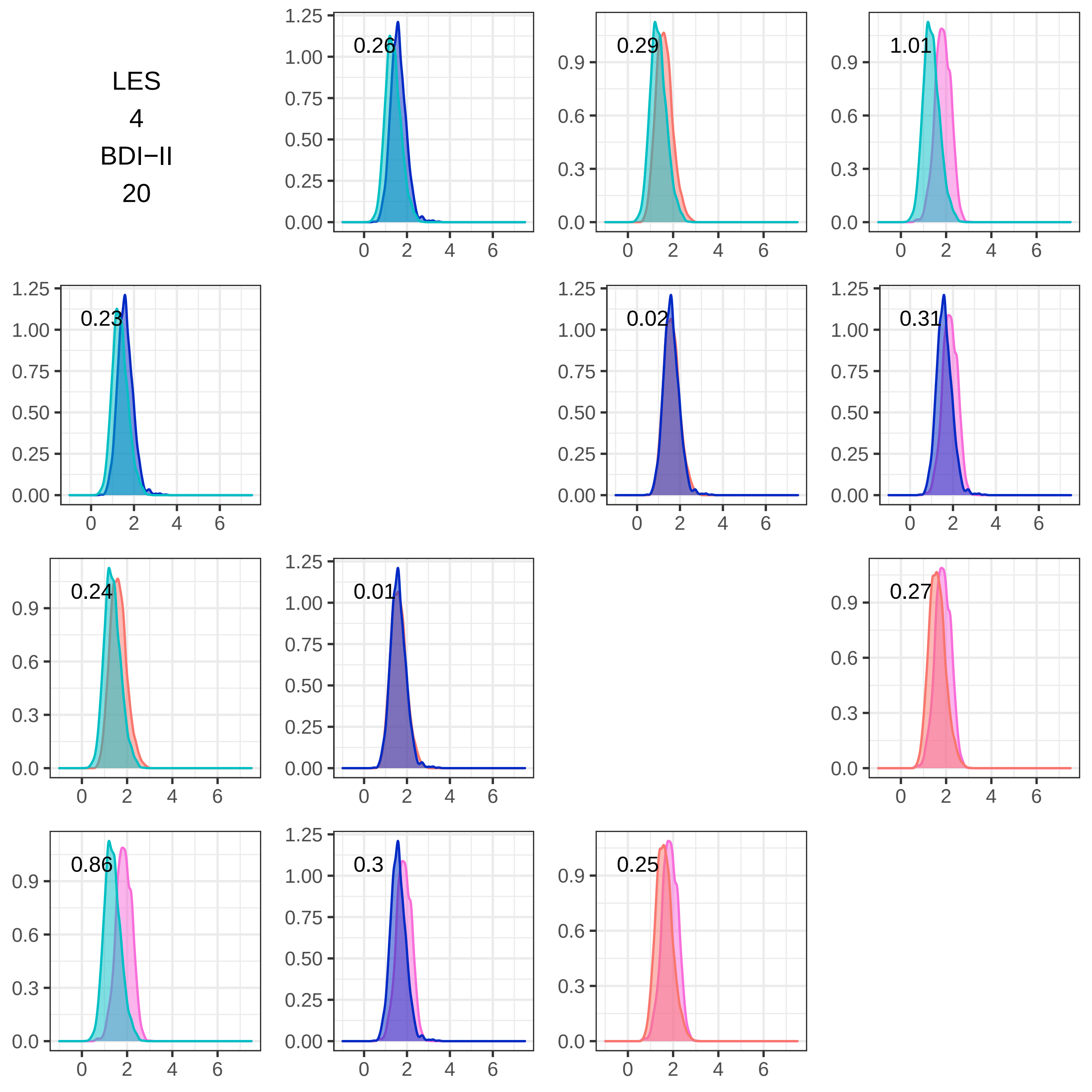

LES
4
BDI−II
20
0.26
0.29
1.01
0.23
0.02
0.31
0.24
0.01
0.27
0.86
0.3
0.25

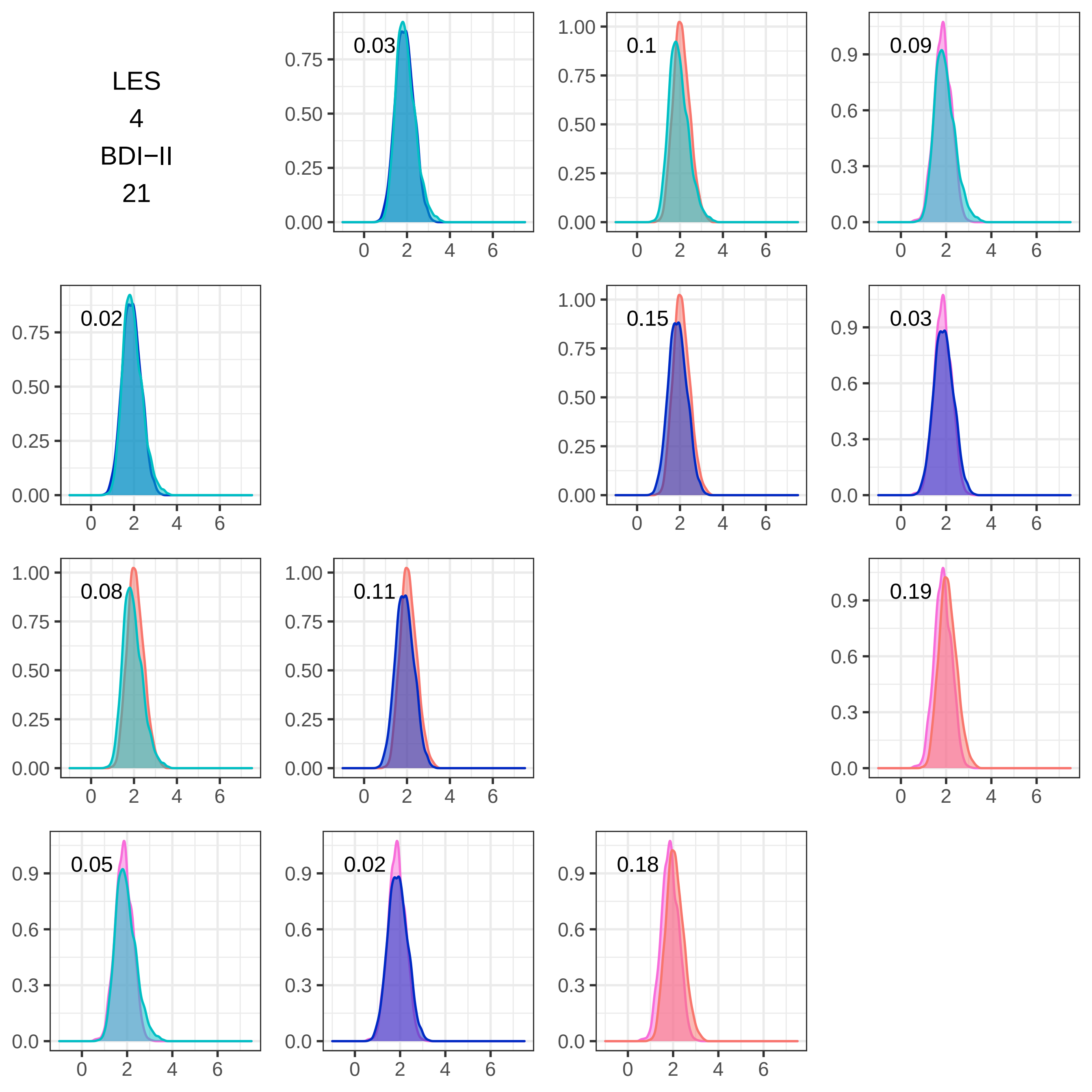

LES
4
BDI−II
21
0.03
0.1
0.09
0.02
0.15
0.03
0.08
0.11
0.19
0.05
0.02
0.18

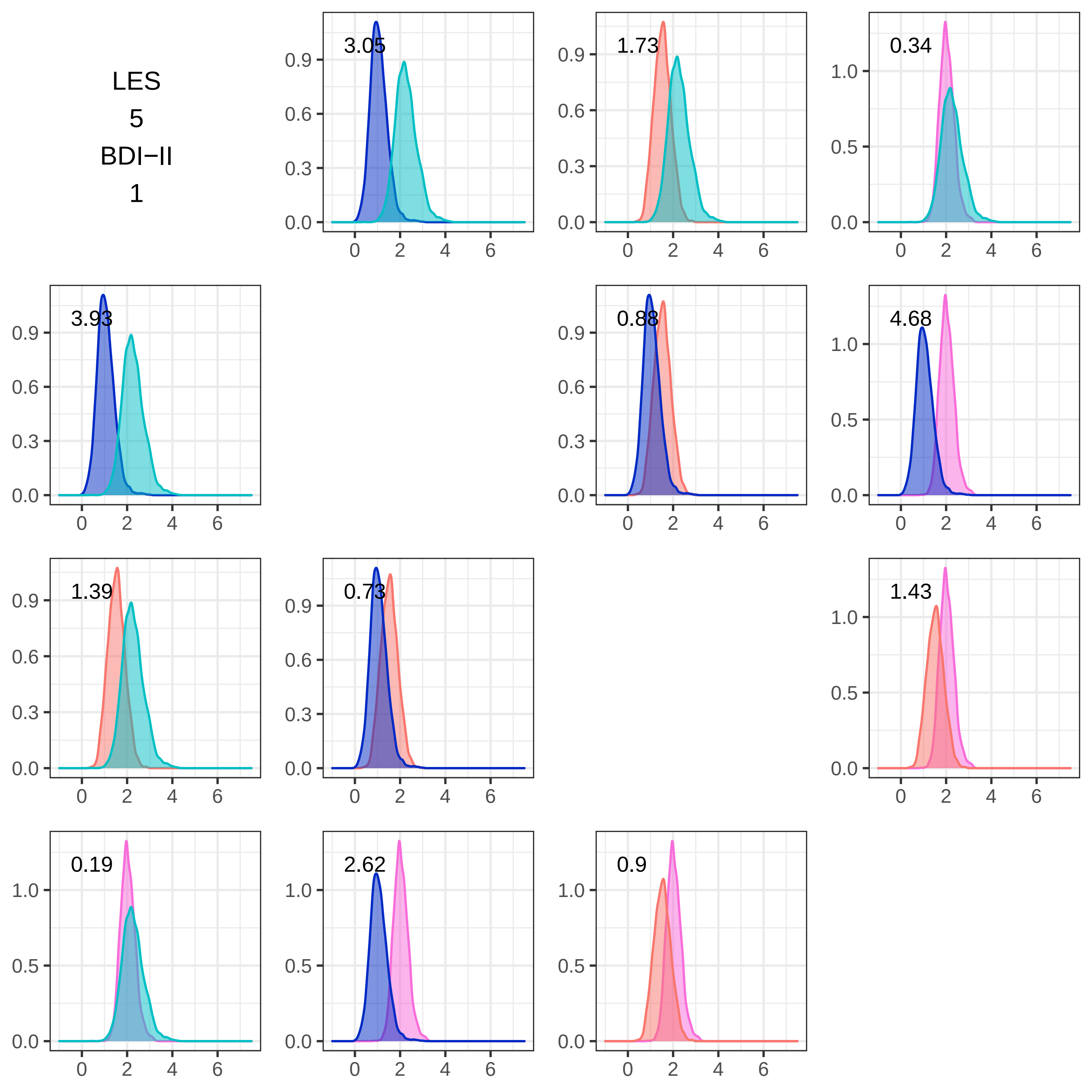
LES
5
BDI−II
1
3.05
1.73
0.34
3.93
0.88
4.68
1.39
0.73
1.43
0.19
2.62
0.9

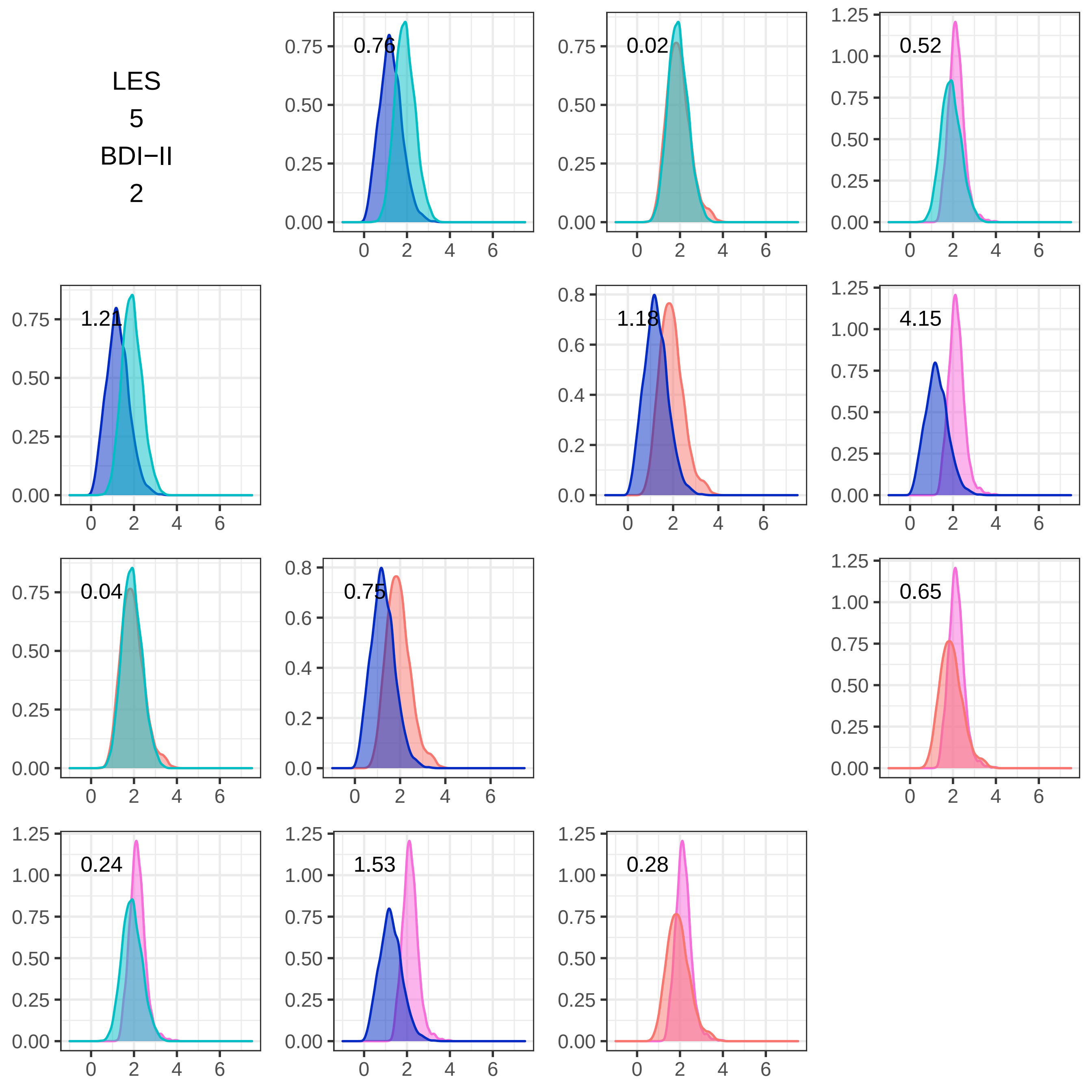

LES
5
BDI−II
2
0.76
0.02
0.52
1.21
1.18
4.15
0.04
0.75
0.65
0.24
1.53
0.28

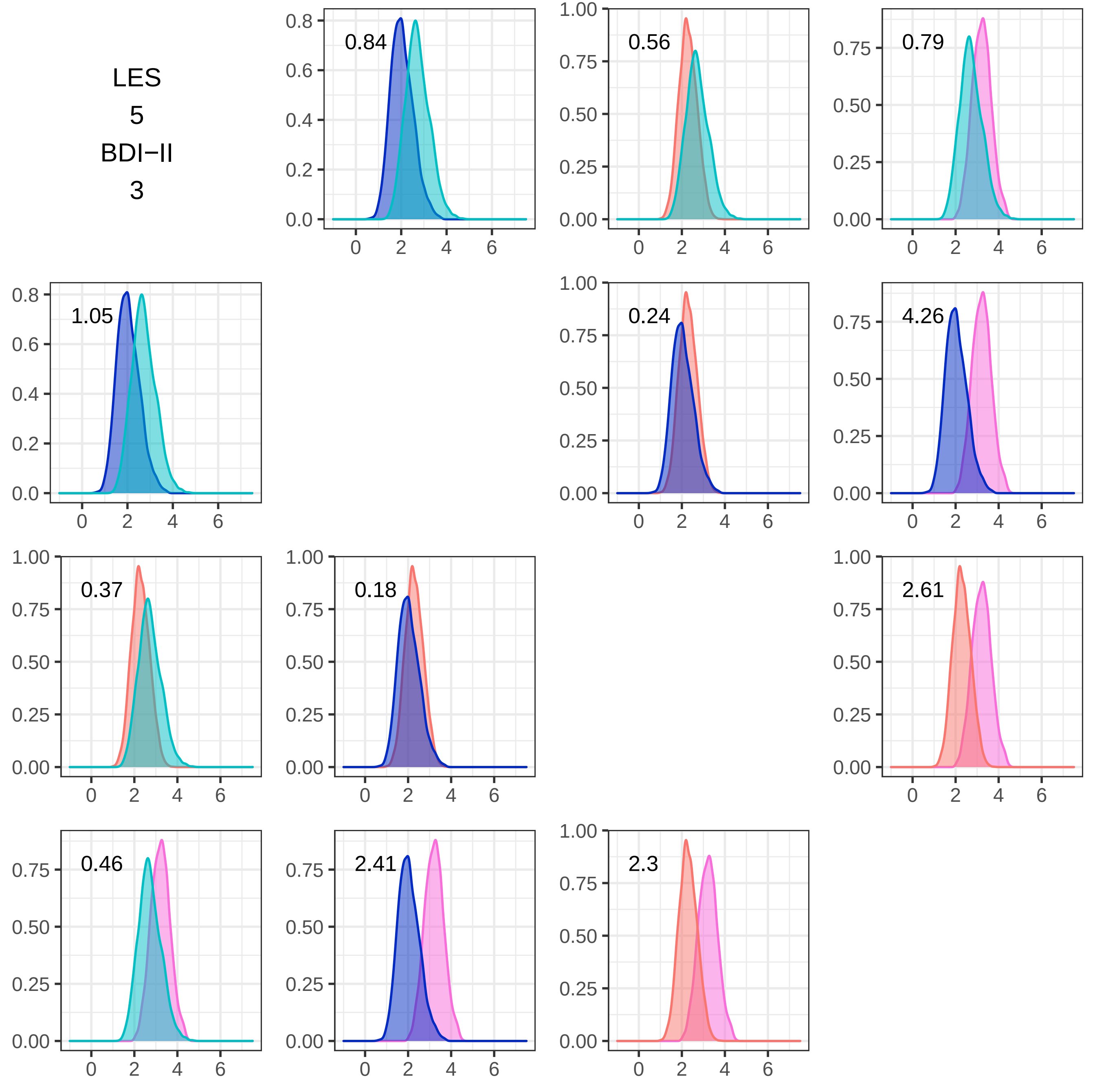

LES
5
BDI−II
3
0.84
0.56
0.79
1.05
0.24
4.26
0.37
0.18
2.61
0.46
2.41
2.3

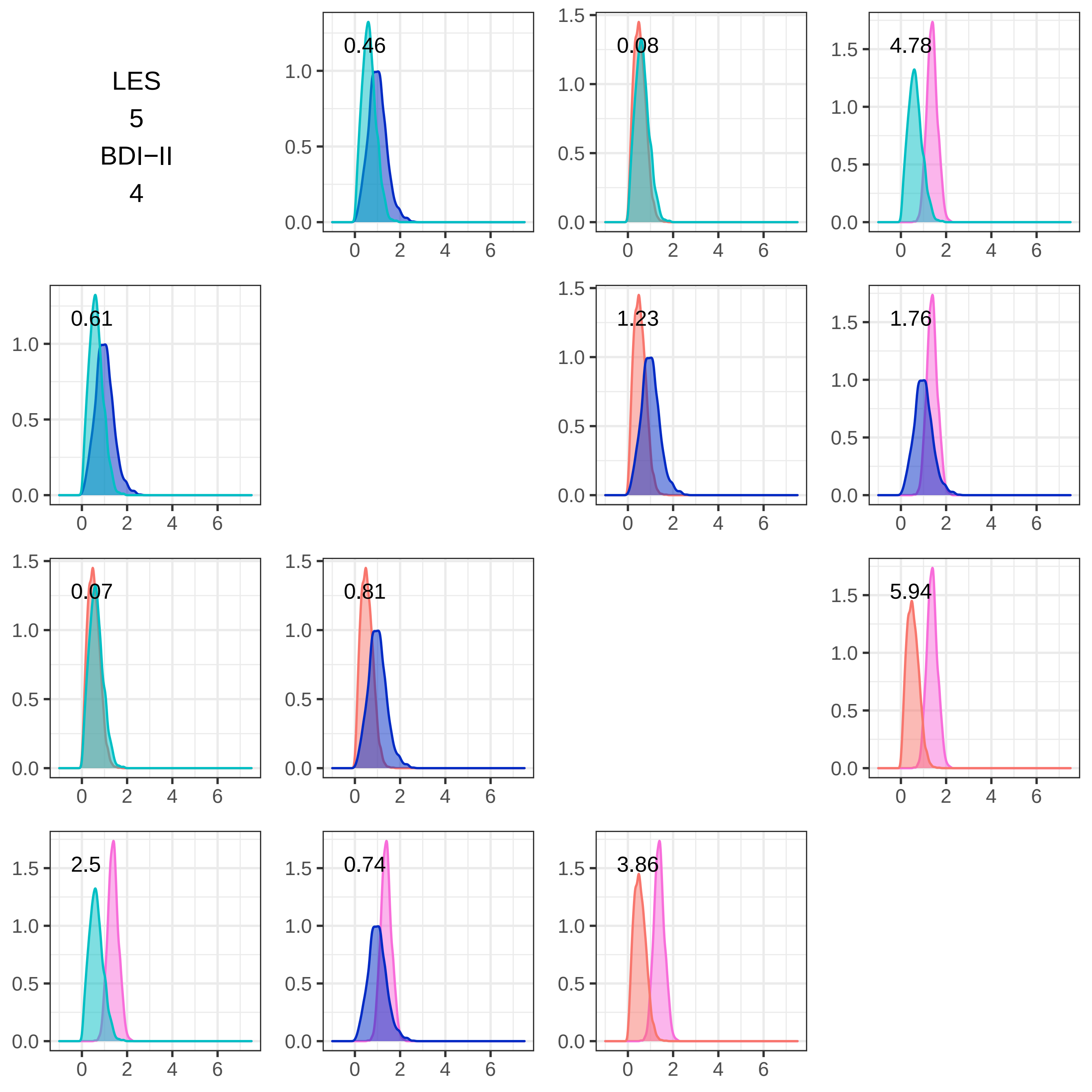
LES
5
BDI−II
4
0.46
0.08
4.78
0.61
1.23
1.76
0.07
0.81
5.94
2.5
0.74
3.86

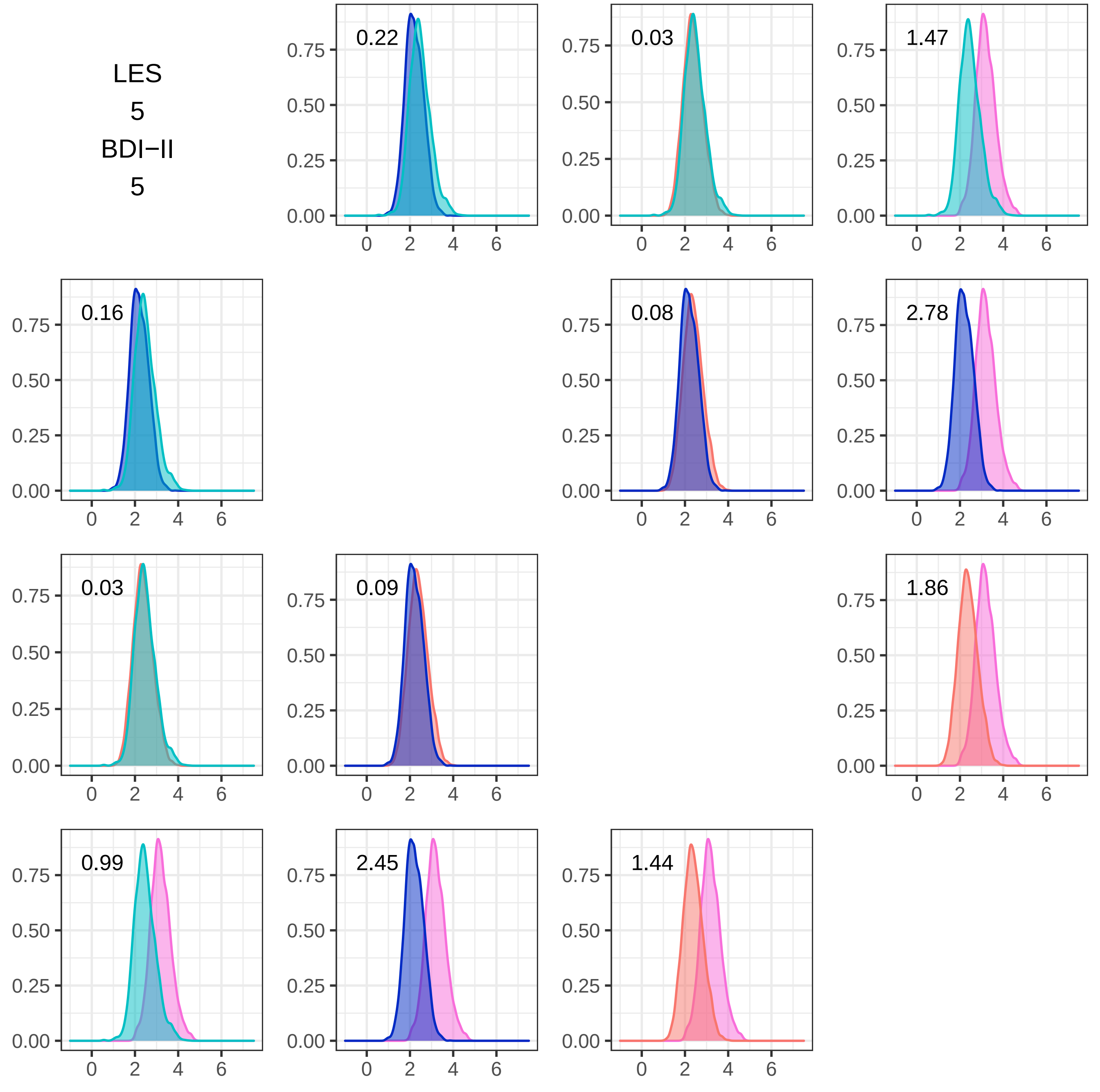

LES
5
BDI−II
5
0.22
0.03
1.47
0.16
0.08
2.78
0.03
0.09
1.86
0.99
2.45
1.44

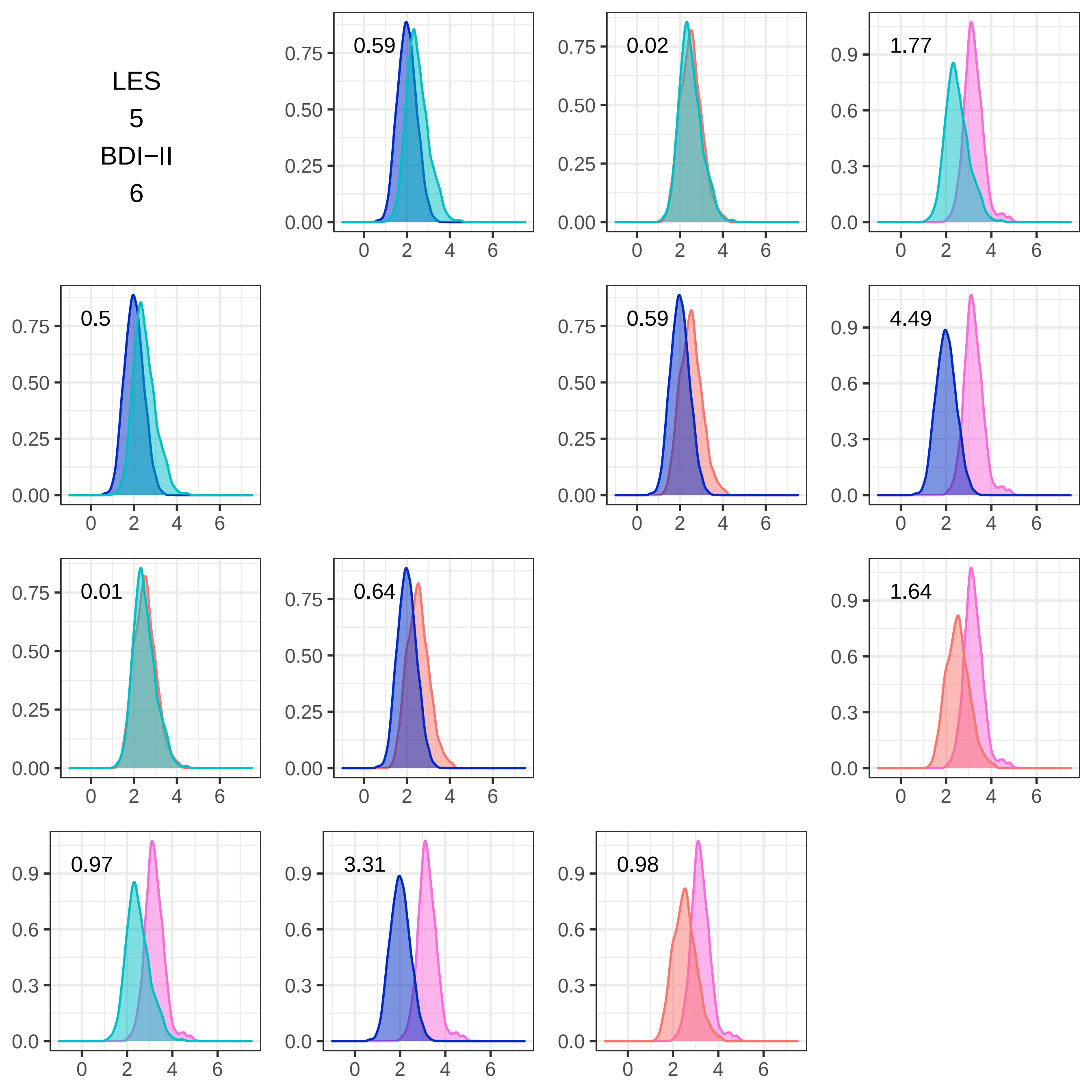
LES
5
BDI−II
6
0.59
0.02
1.77
0.5
0.59
4.49
0.01
0.64
1.64
0.97
3.31
0.98

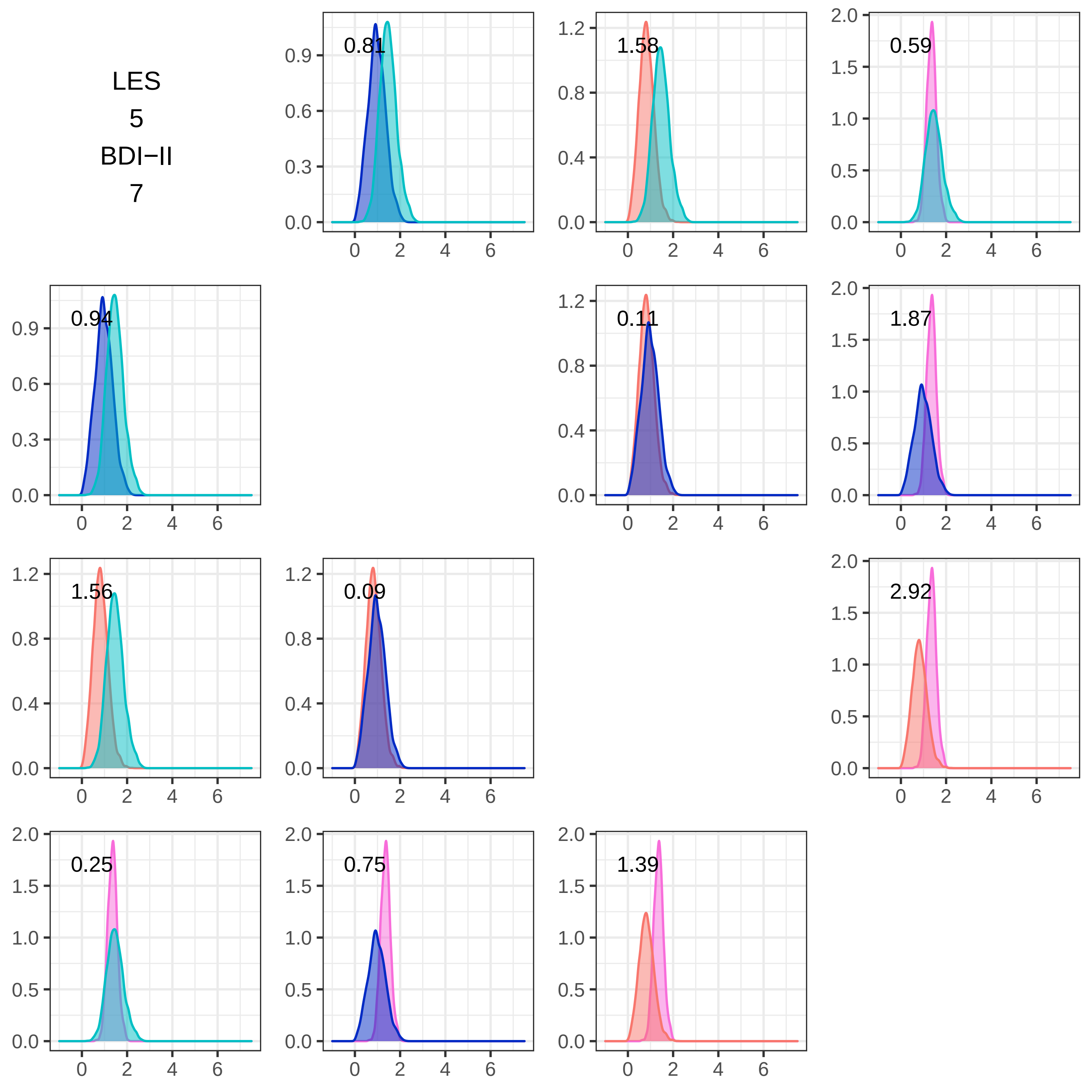
LES
5
BDI−II
7
0.81
1.58
0.59
0.94
0.11
1.87
1.56
0.09
2.92
0.25
0.75
1.39

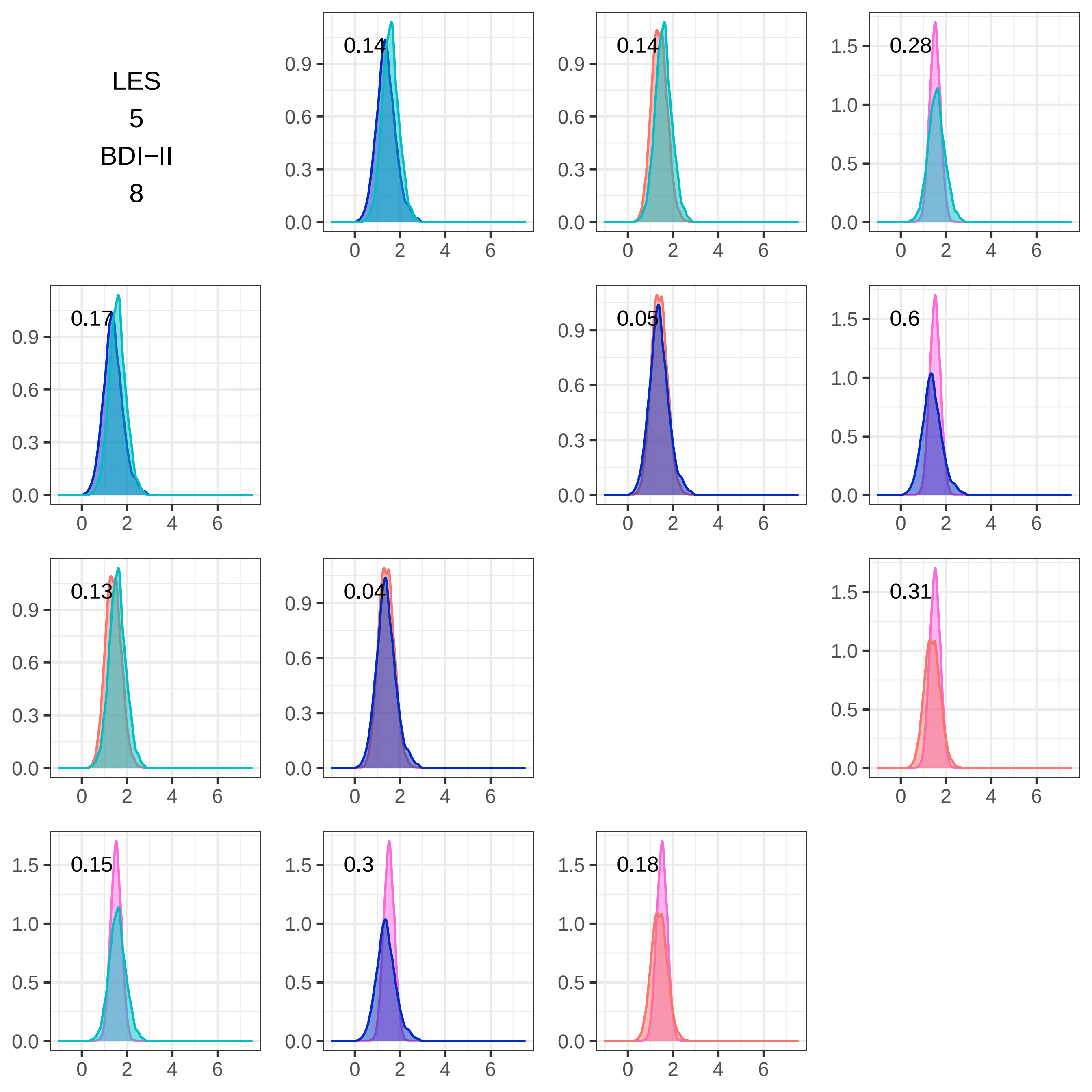

LES
5
BDI−II
8
0.14
0.14
0.28
0.17
0.05
0.6
0.13
0.04
0.31
0.15
0.3
0.18

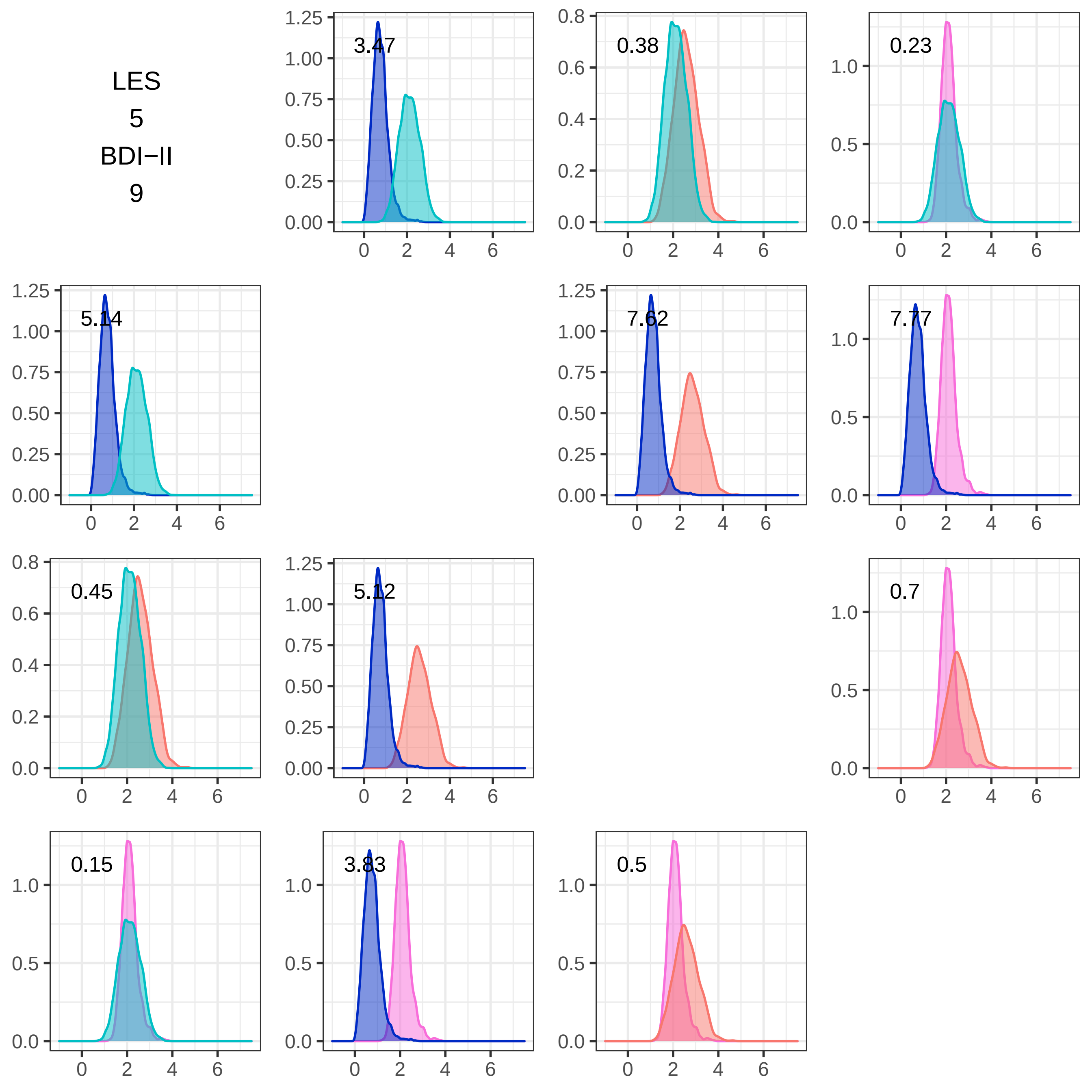

LES
5
BDI−II
9
3.47
0.38
0.23
5.14
7.62
7.77
0.45
5.12
0.7
0.15
3.83
0.5

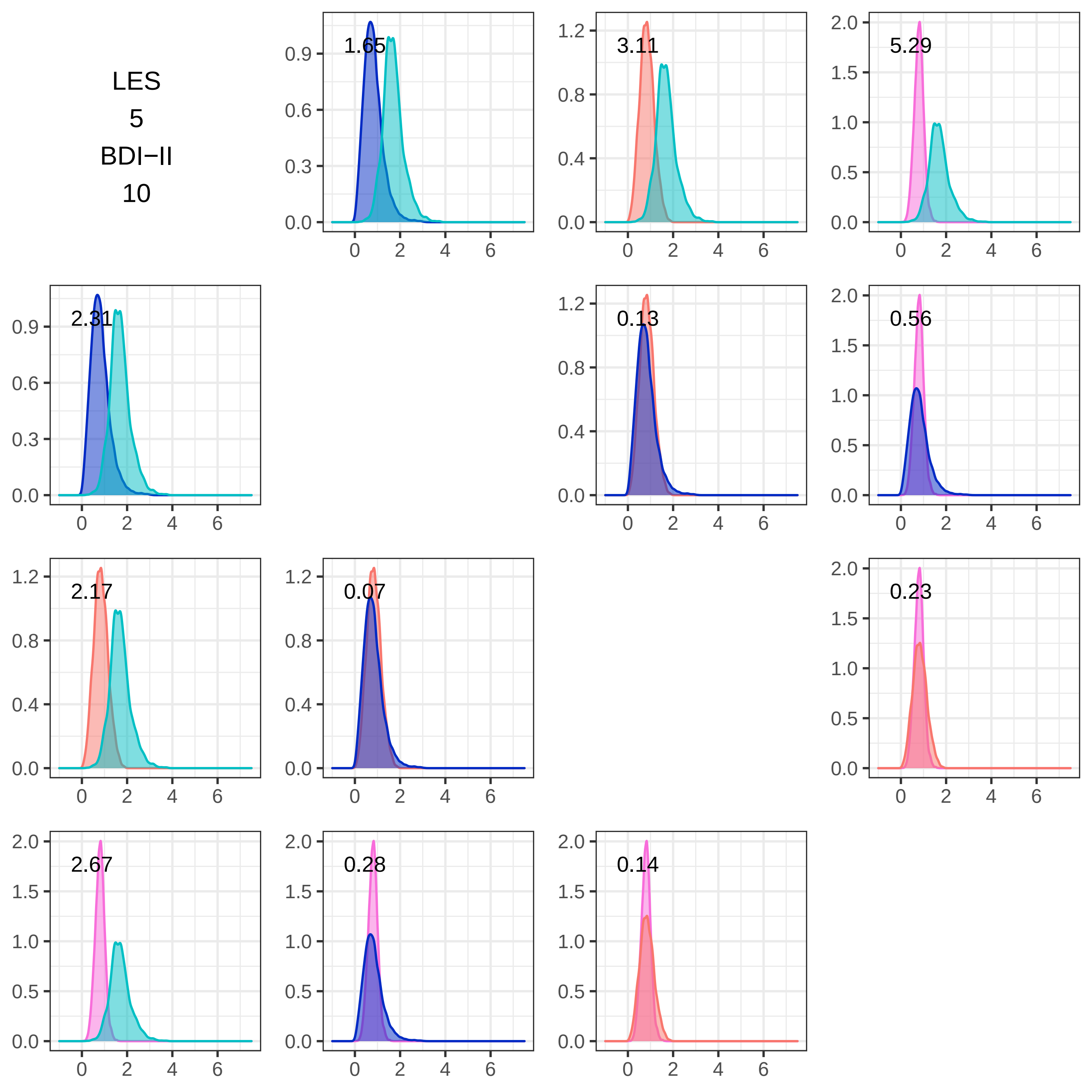
LES
5
BDI−II
10
1.65
3.11
5.29
2.31
0.13
0.56
2.17
0.07
0.23
2.67
0.28
0.14

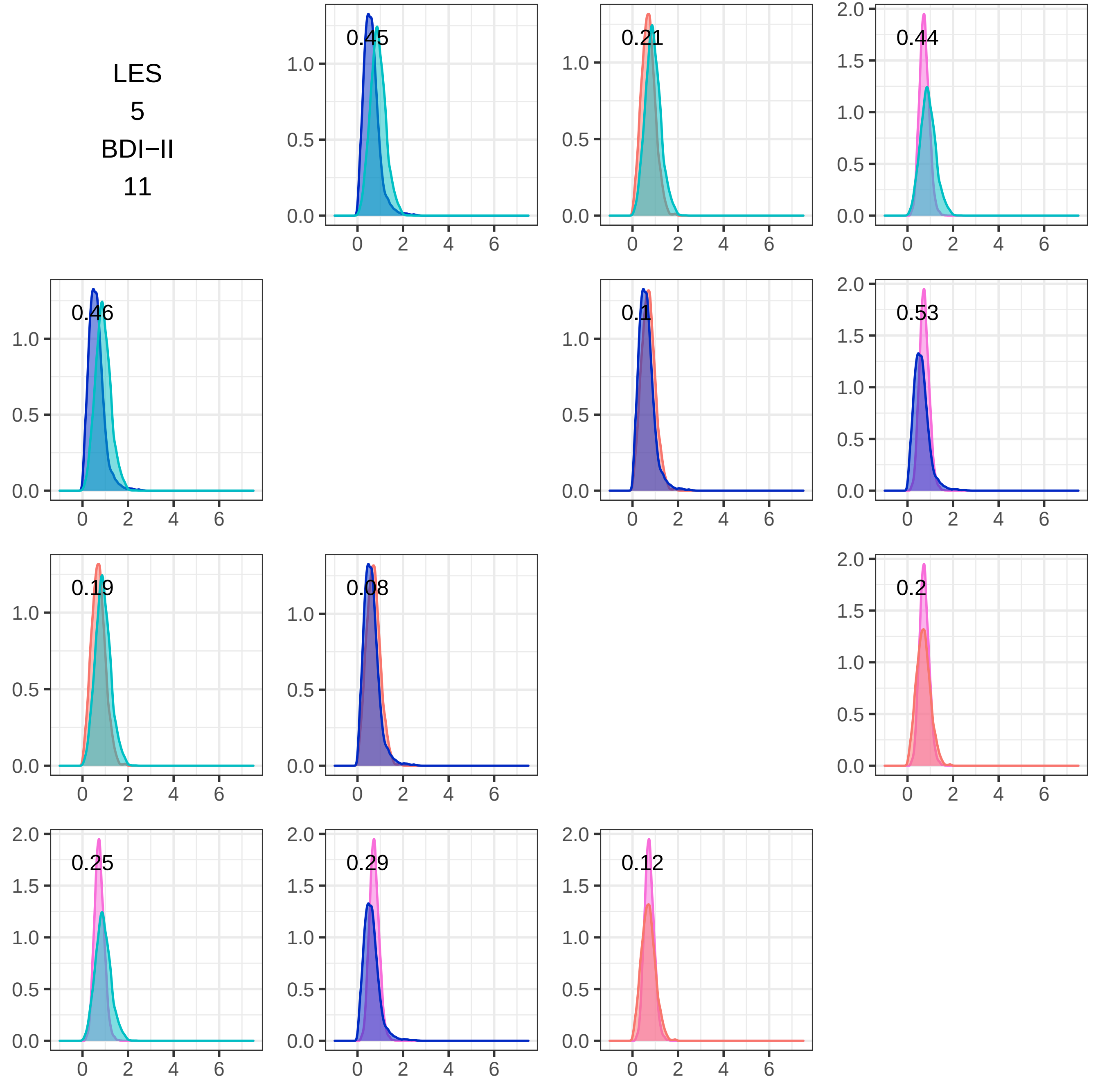
LES
5
BDI−II
11
0.45
0.21
0.44
0.46
0.1
0.53
0.19
0.08
0.2
0.25
0.29
0.12

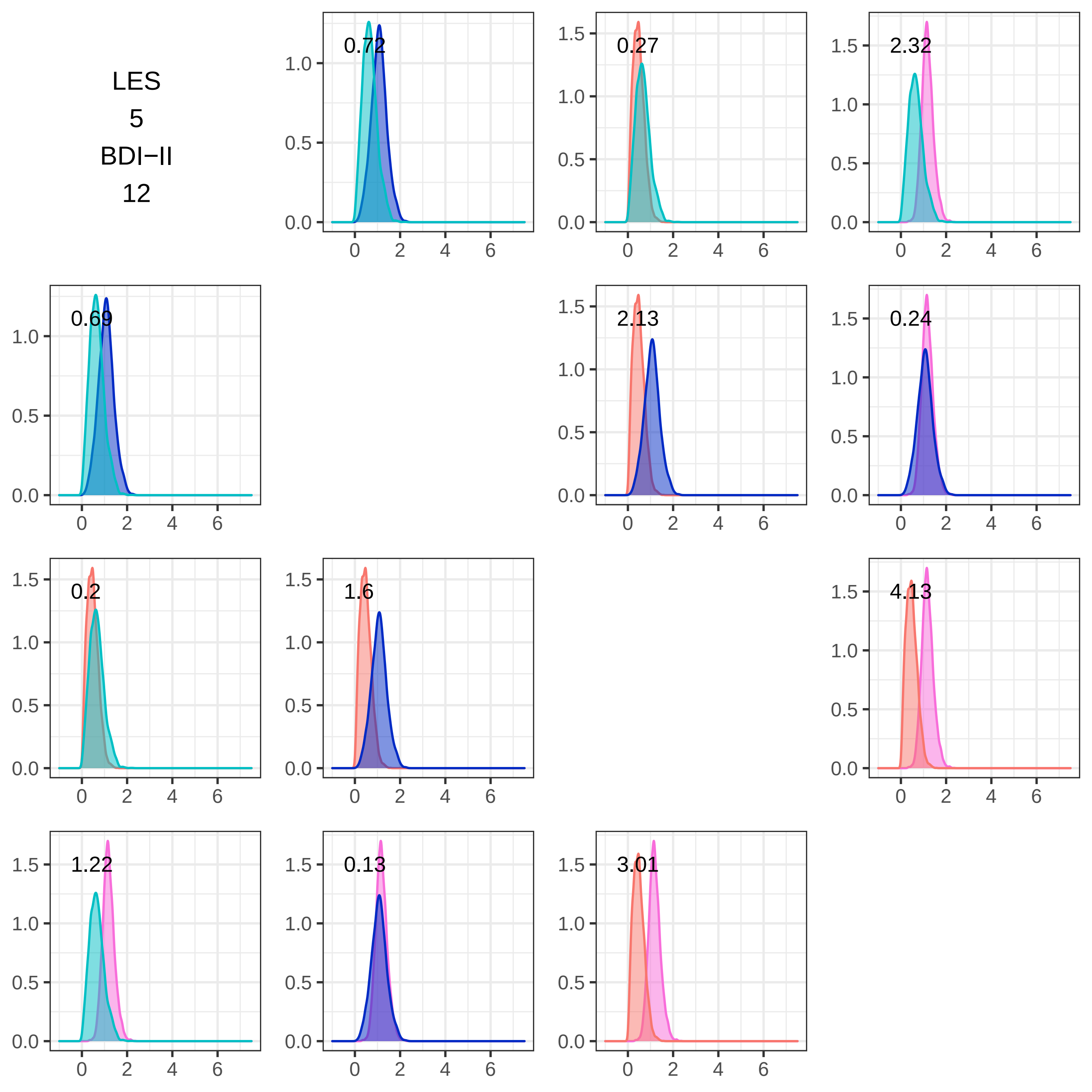
LES
5
BDI−II
12
0.72
0.27
2.32
0.69
2.13
0.24
0.2
1.6
4.13
1.22
0.13
3.01

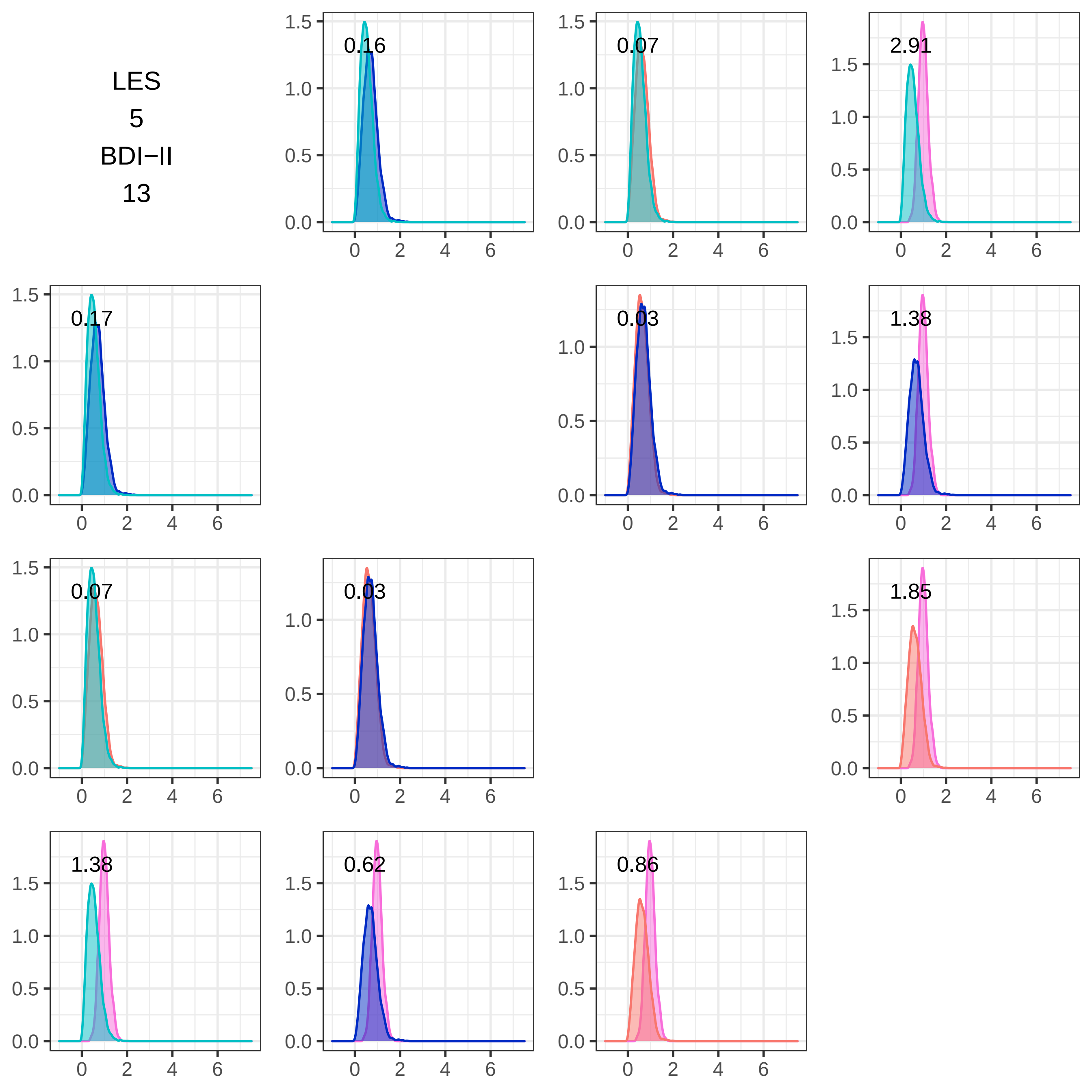

LES
5
BDI−II
13
0.16
0.07
2.91
0.17
0.03
1.38
0.07
0.03
1.85
1.38
0.62
0.86

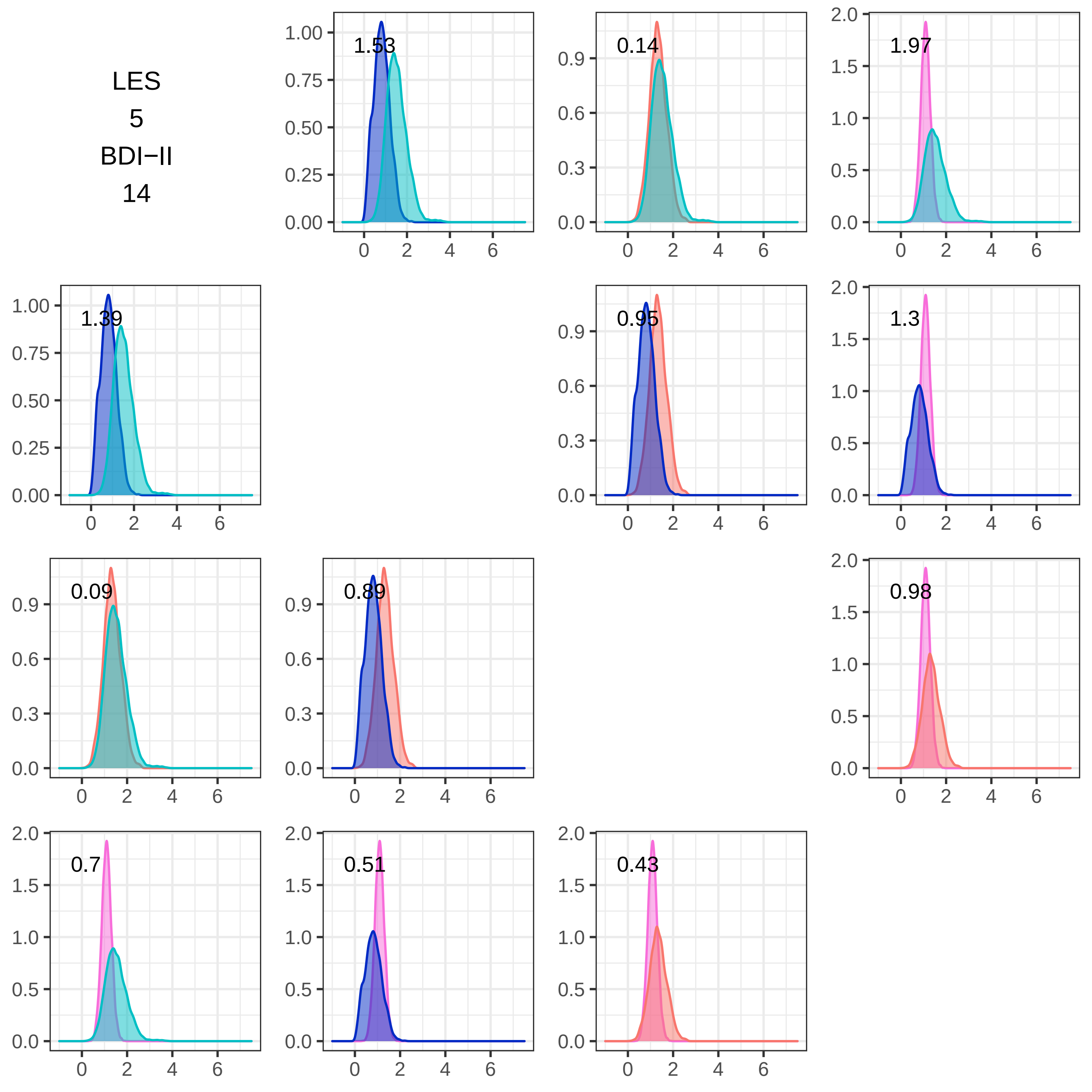
LES
5
BDI−II
14
1.53
0.14
1.97
1.39
0.95
1.3
0.09
0.89
0.98
0.7
0.51
0.43

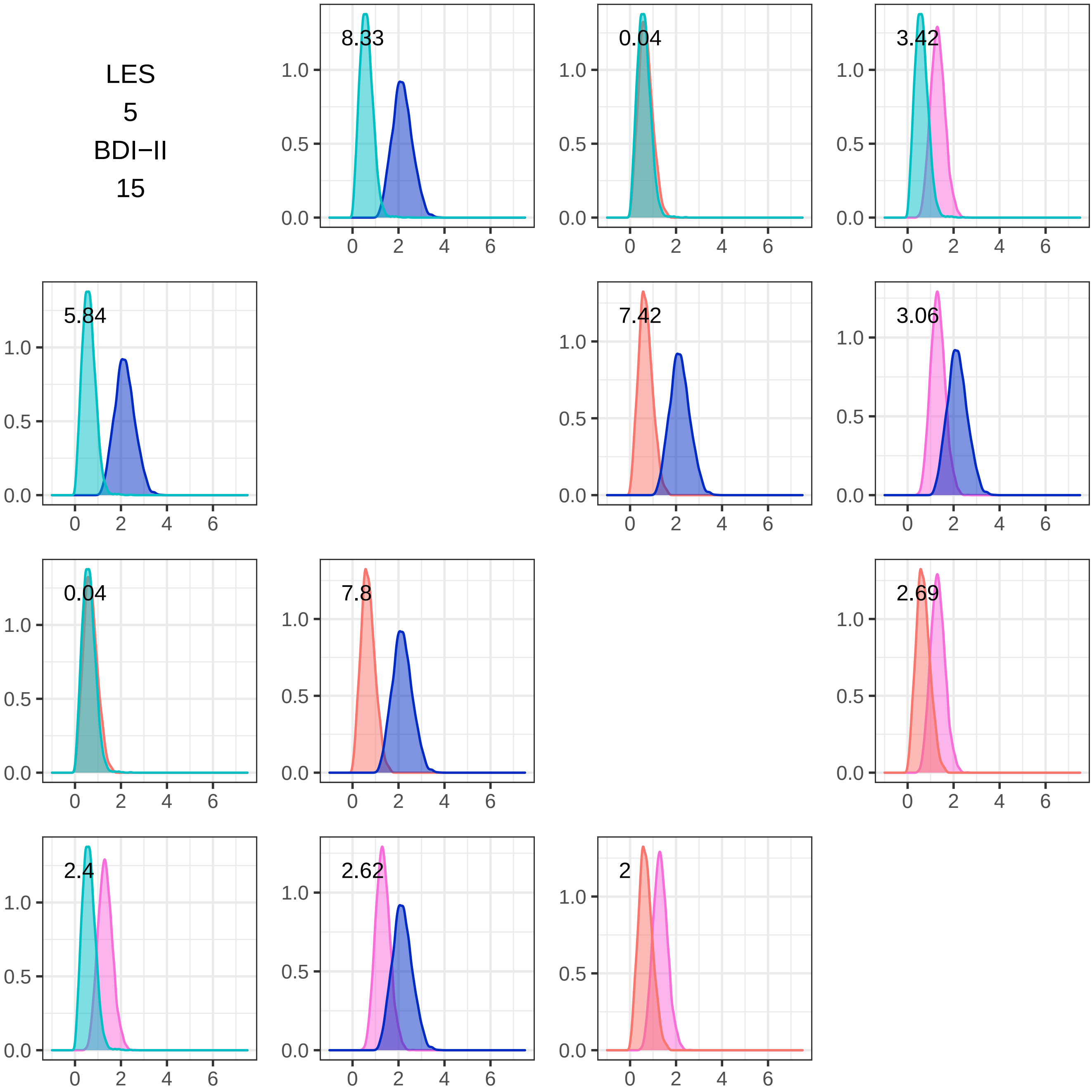
LES
5
BDI−II
15
8.33
0.04
3.42
5.84
7.42
3.06
0.04
7.8
2.69
2.4
2.62
2

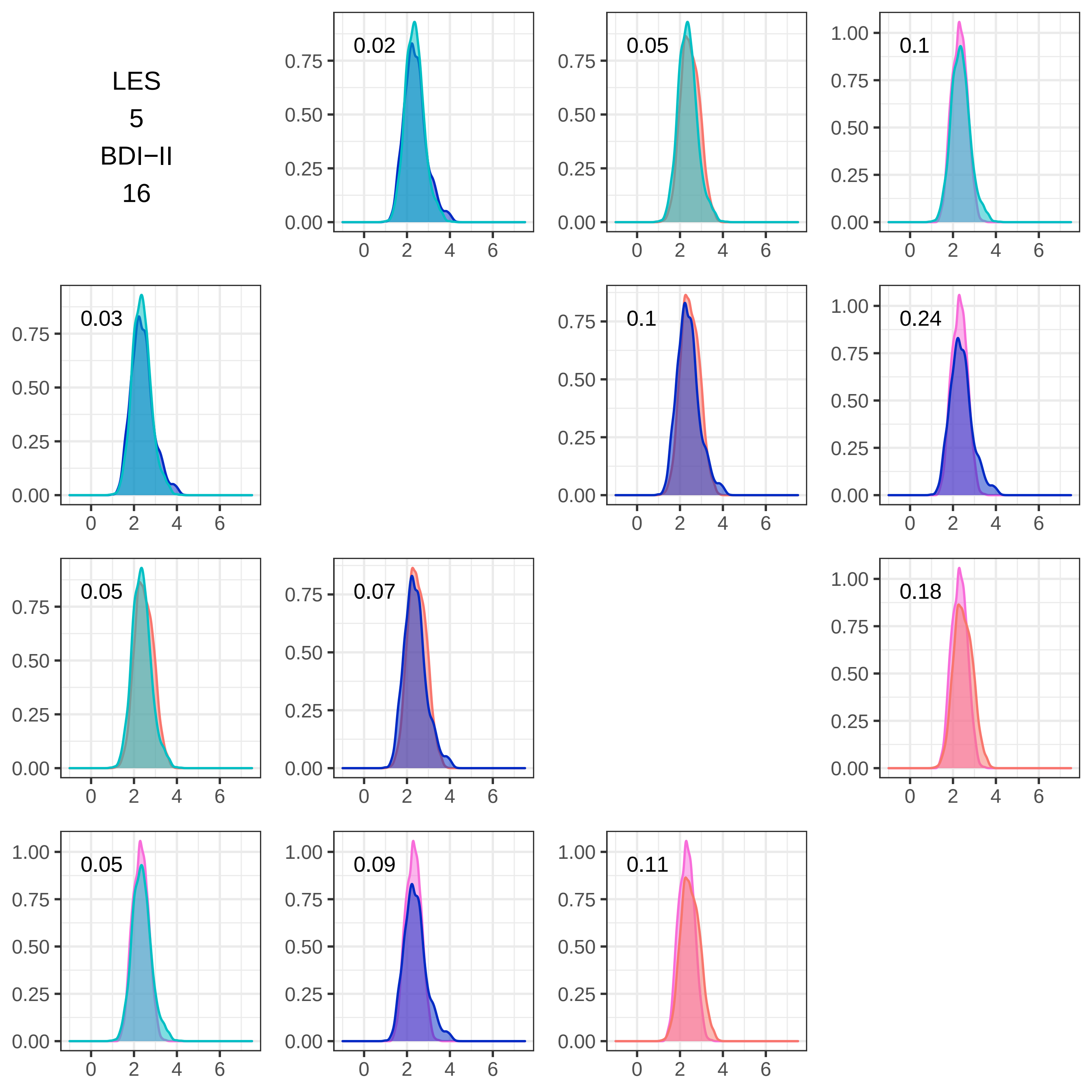
LES
5
BDI−II
16
0.02
0.05
0.1
0.03
0.1
0.24
0.05
0.07
0.18
0.05
0.09
0.11

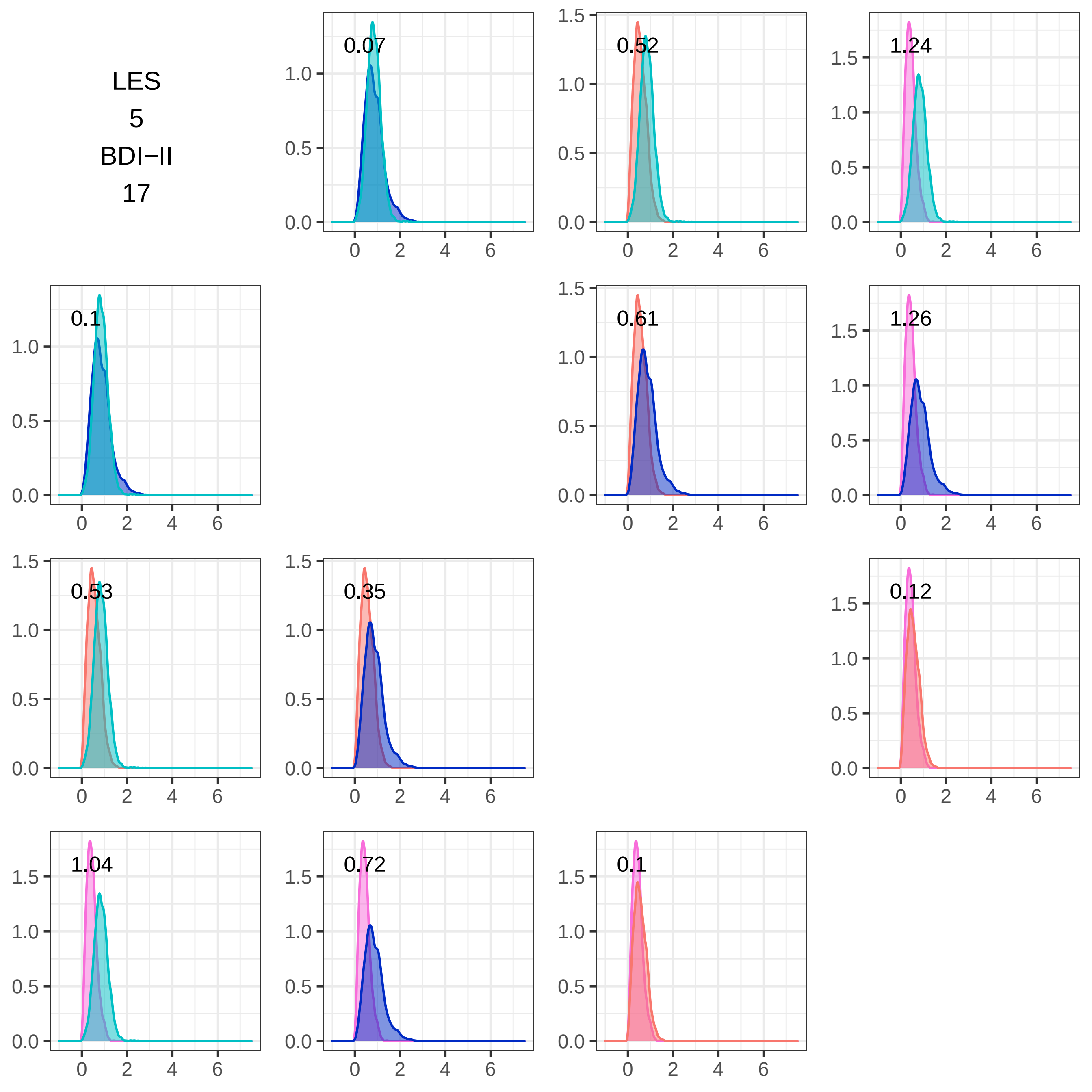
LES
5
BDI−II
17
0.07
0.52
1.24
0.1
0.61
1.26
0.53
0.35
0.12
1.04
0.72
0.1

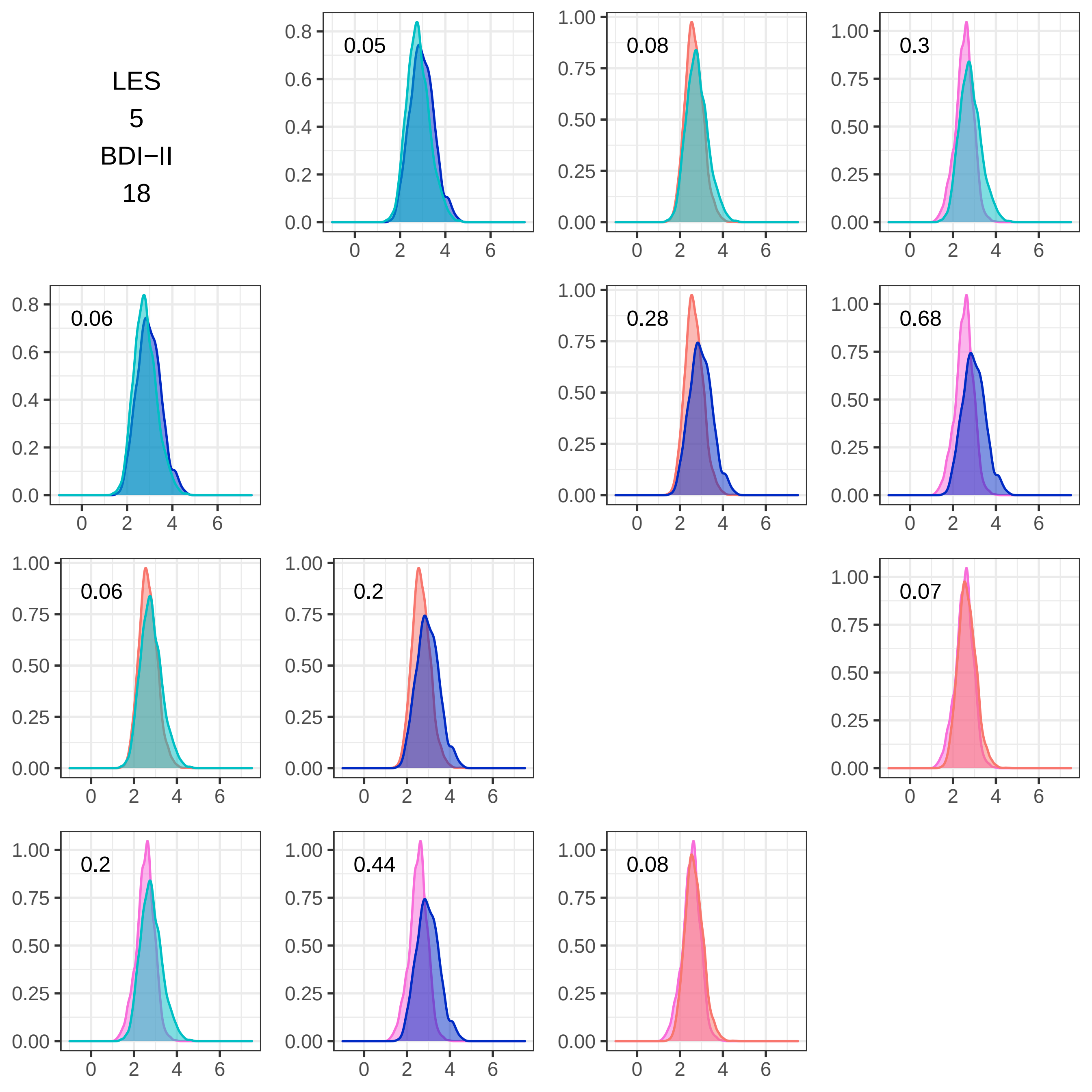
LES
5
BDI−II
18
0.05
0.08
0.3
0.06
0.28
0.68
0.06
0.2
0.07
0.2
0.44
0.08

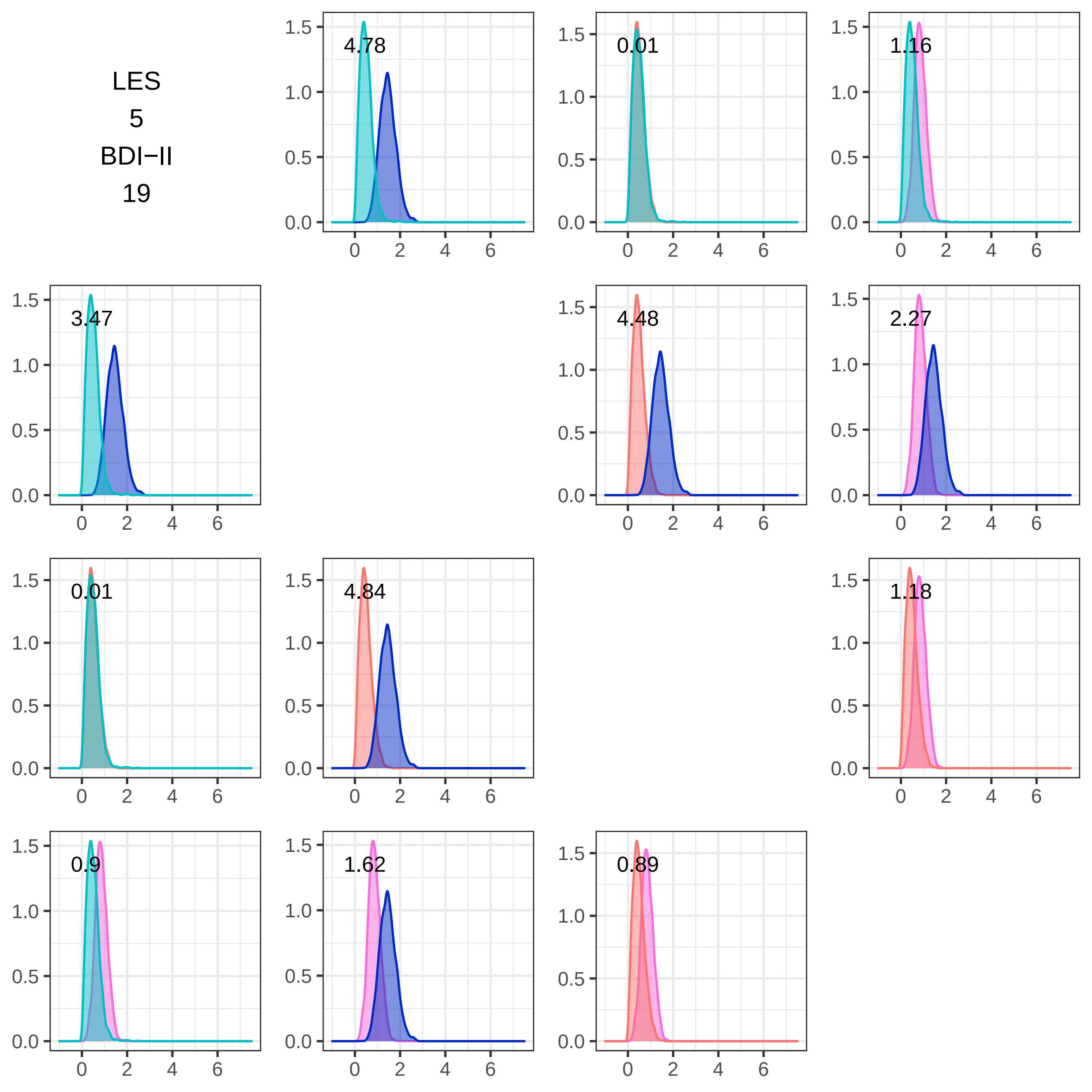

LES
5
BDI−II
19
4.78
0.01
1.16
3.47
4.48
2.27
0.01
4.84
1.18
0.9
1.62
0.89

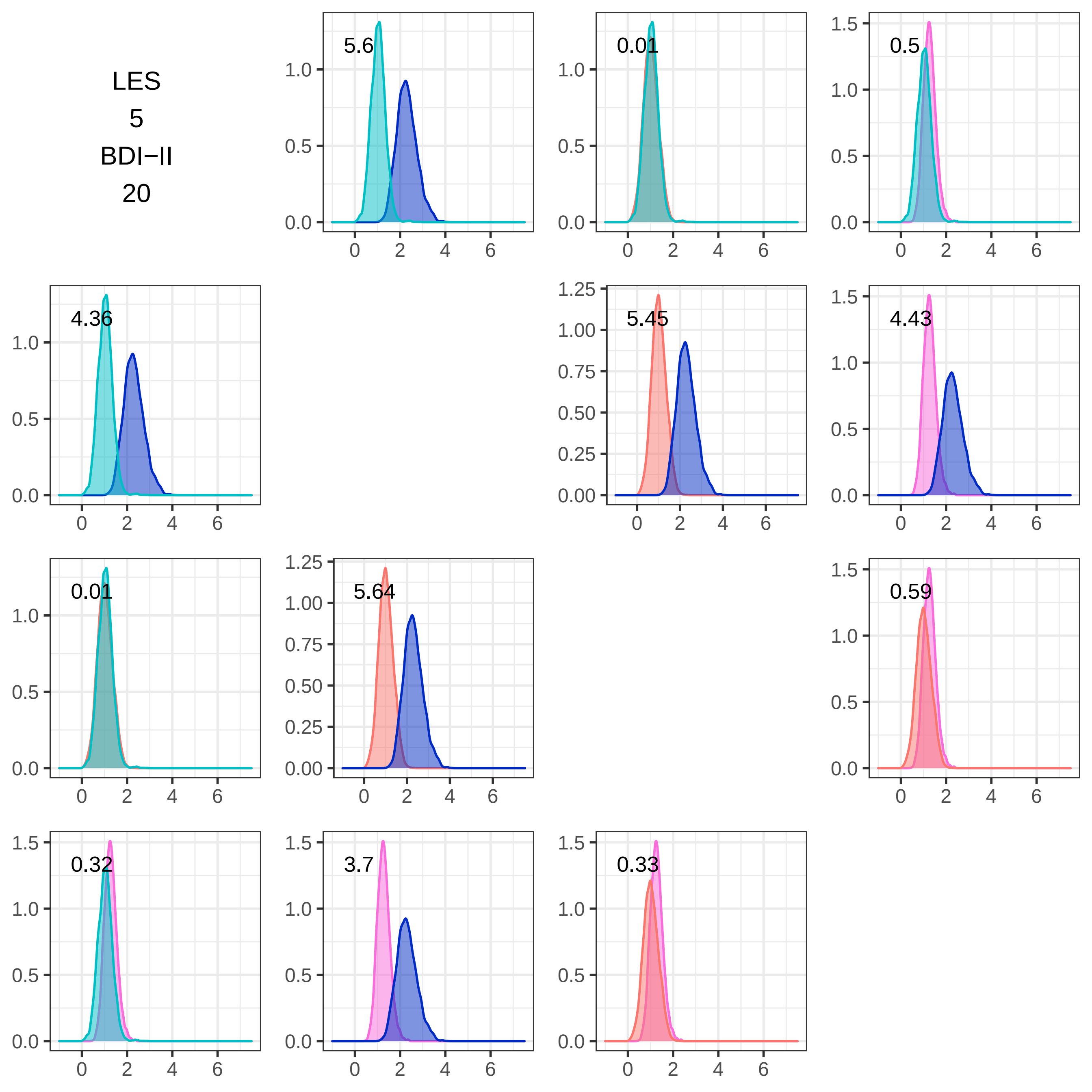

LES
5
BDI−II
20
5.6
0.01
0.5
4.36
5.45
4.43
0.01
5.64
0.59
0.32
3.7
0.33

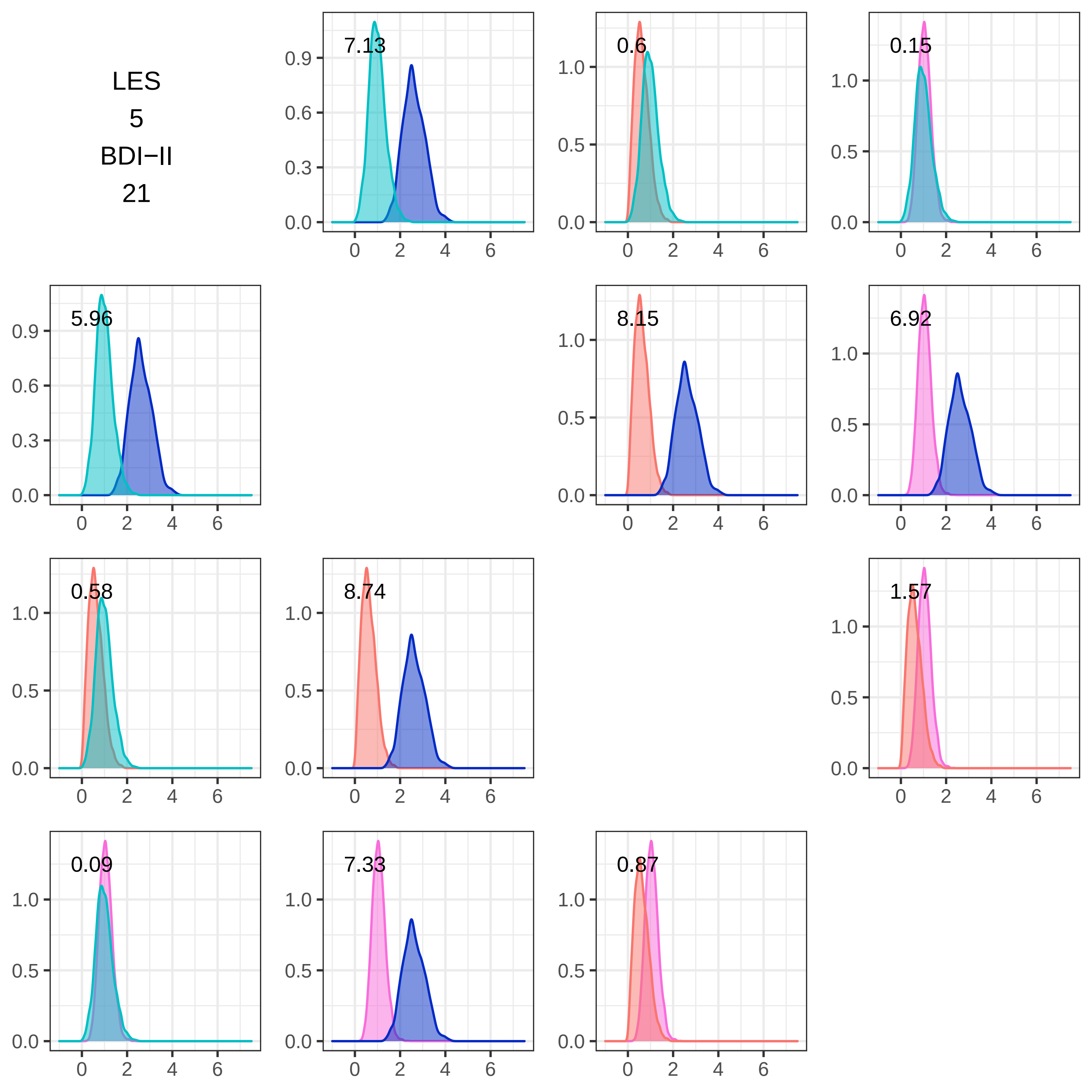
LES
5
BDI−II
21
7.13
0.6
0.15
5.96
8.15
6.92
0.58
8.74
1.57
0.09
7.33
0.87

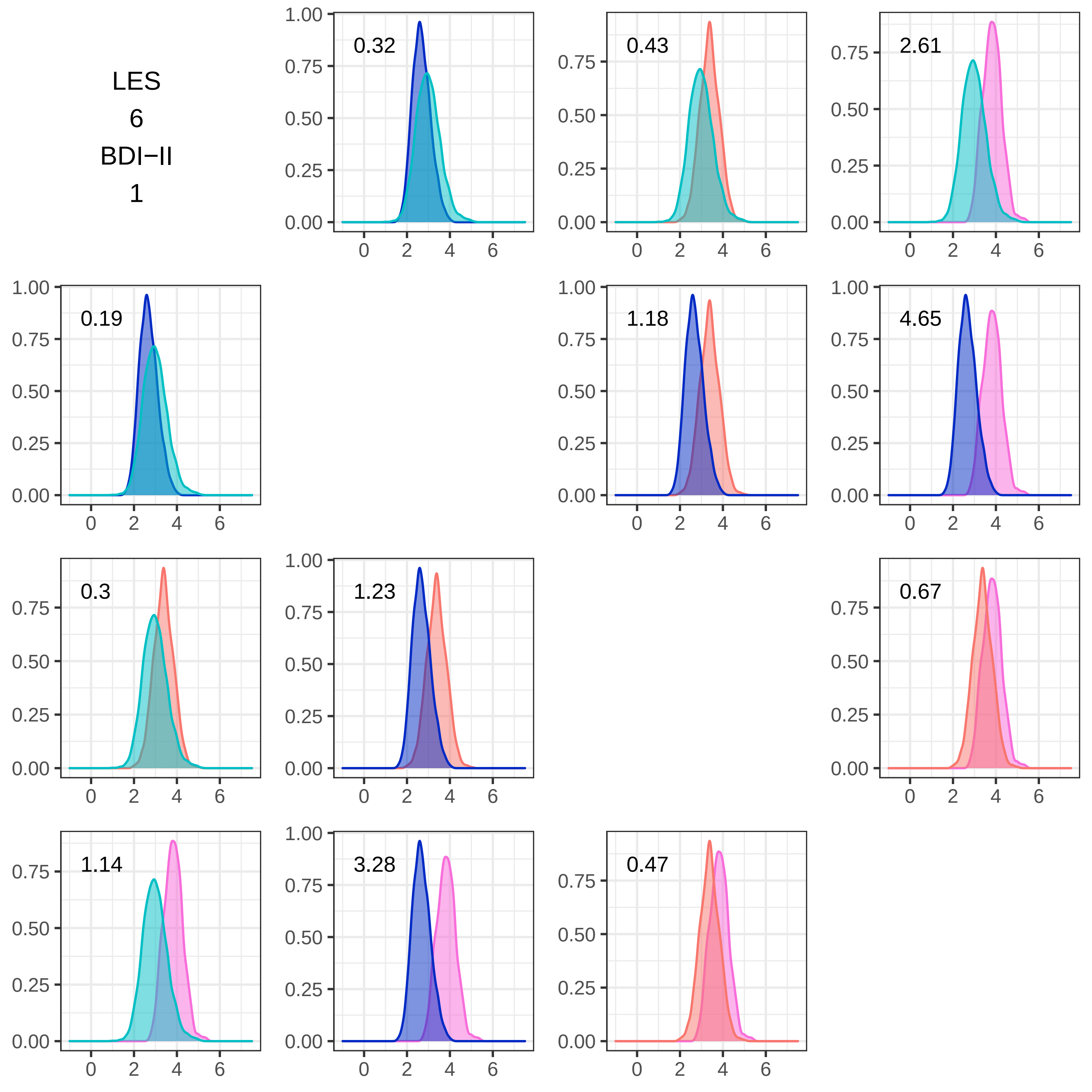

LES
6
BDI−II
1
0.32
0.43
2.61
0.19
1.18
4.65
0.3
1.23
0.67
1.14
3.28
0.47

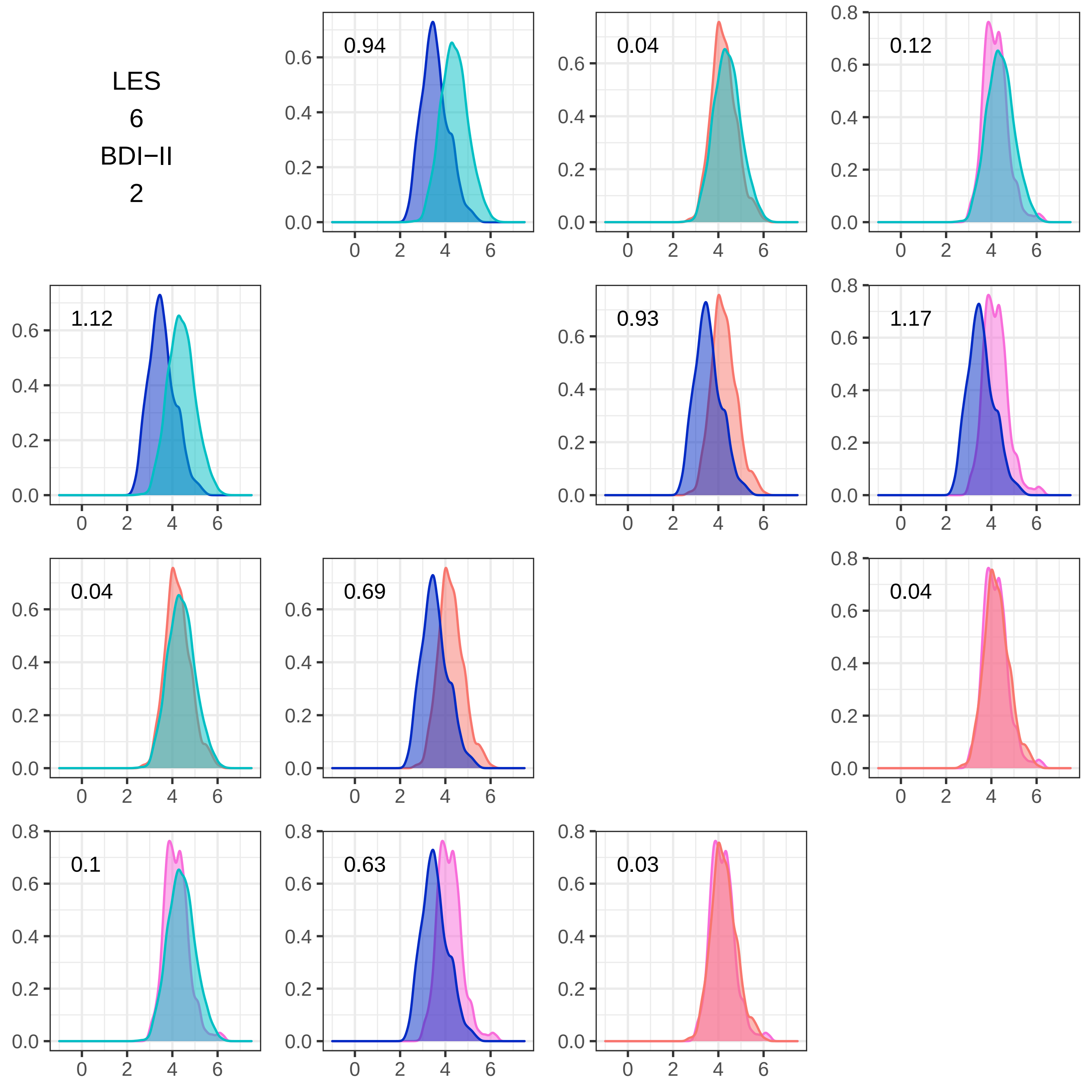
LES
6
BDI−II
2
0.94
0.04
0.12
1.12
0.93
1.17
0.04
0.69
0.04
0.1
0.63
0.03

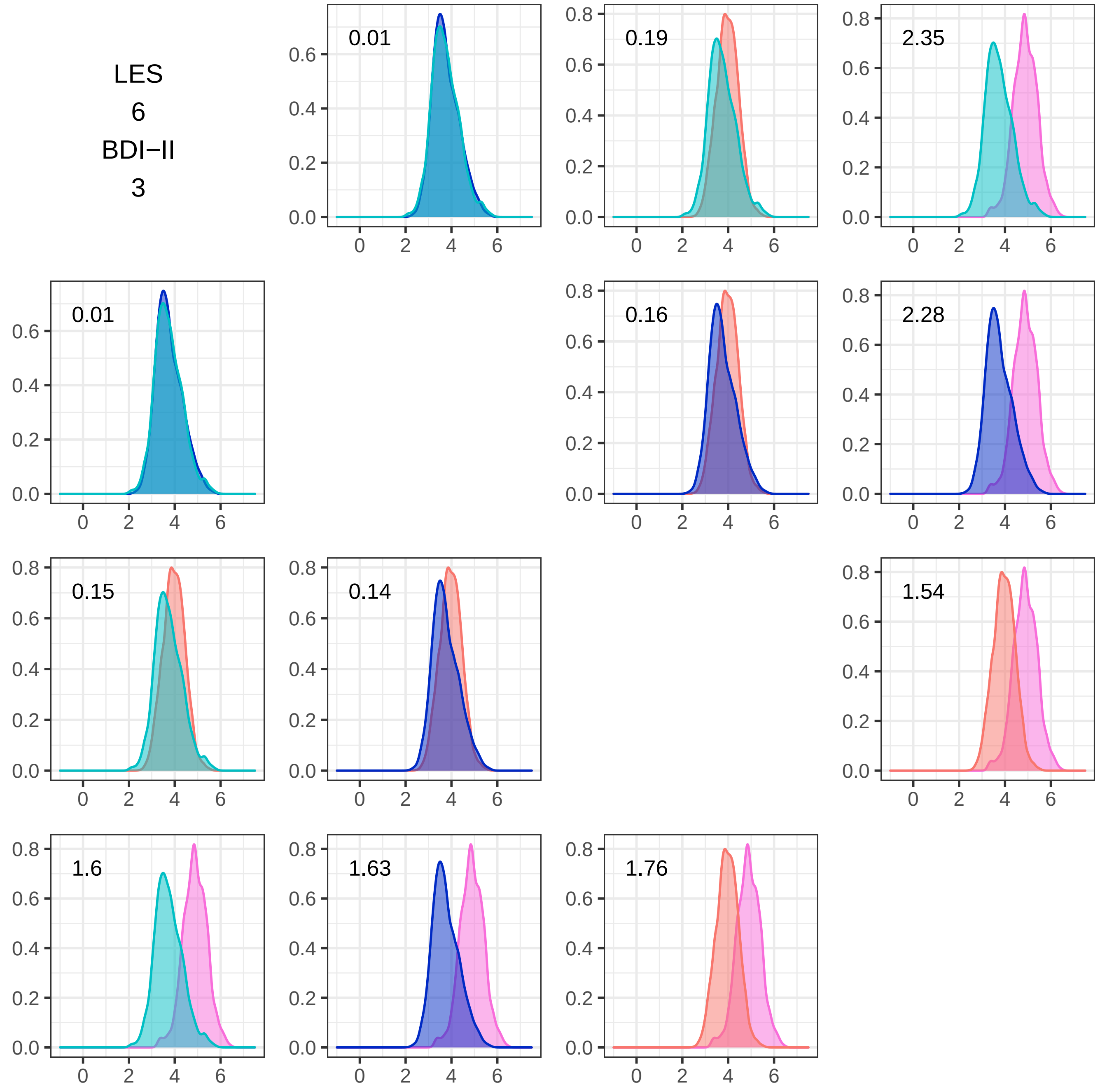
LES
6
BDI−II
3
0.01
0.19
2.35
0.01
0.16
2.28
0.15
0.14
1.54
1.6
1.63
1.76

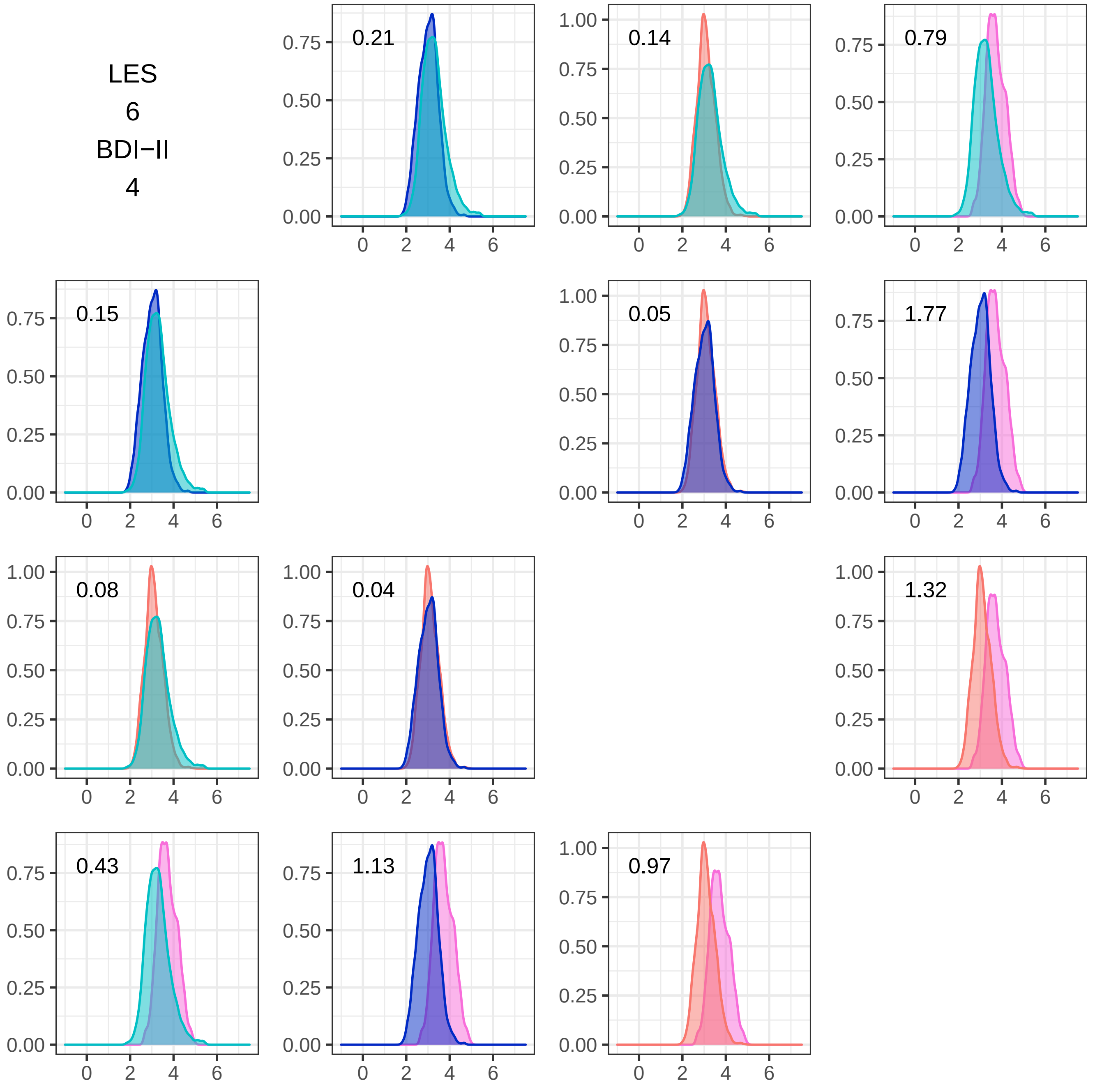
LES
6
BDI−II
4
0.21
0.14
0.79
0.15
0.05
1.77
0.08
0.04
1.32
0.43
1.13
0.97

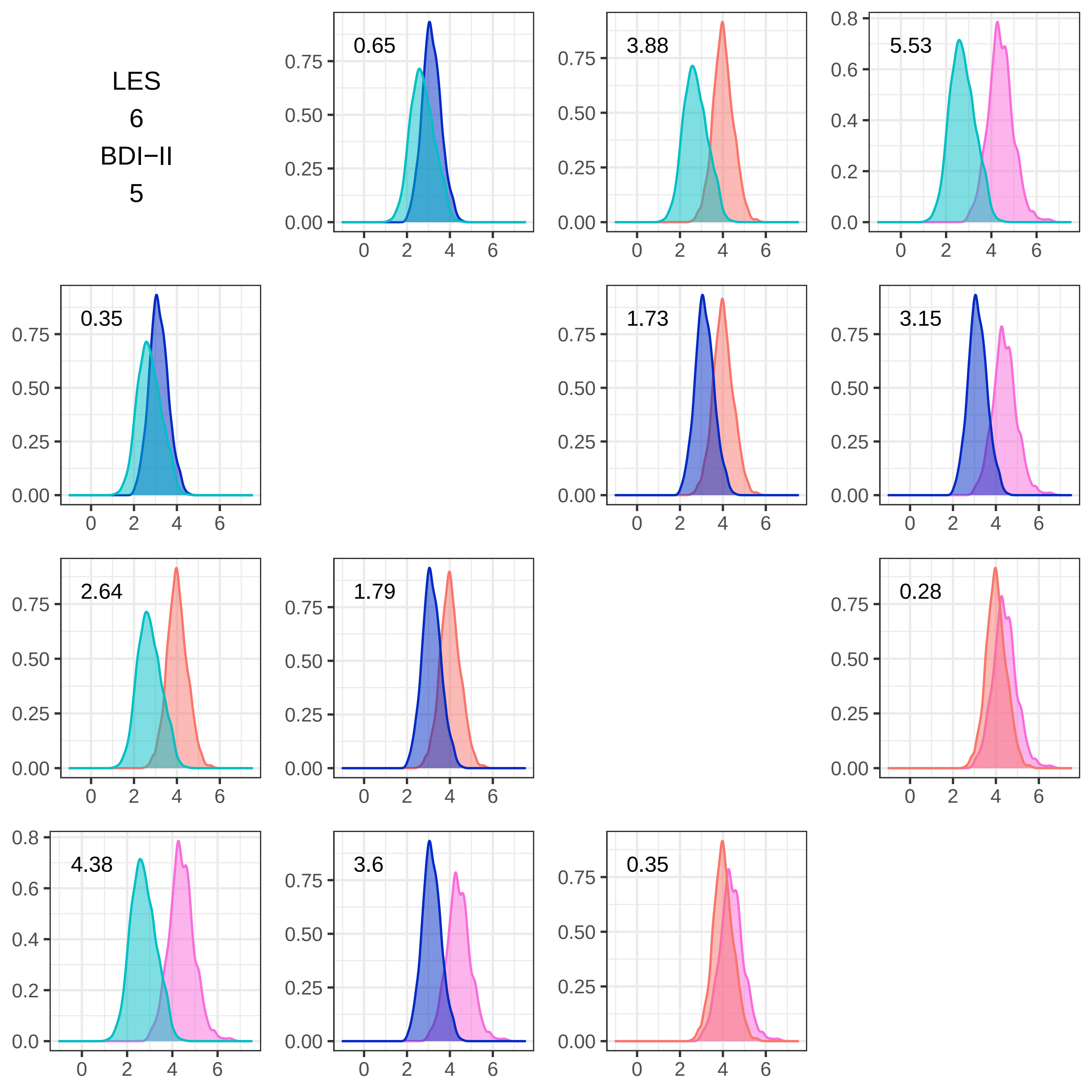

LES
6
BDI−II
5
0.65
3.88
5.53
0.35
1.73
3.15
2.64
1.79
0.28
4.38
3.6
0.35

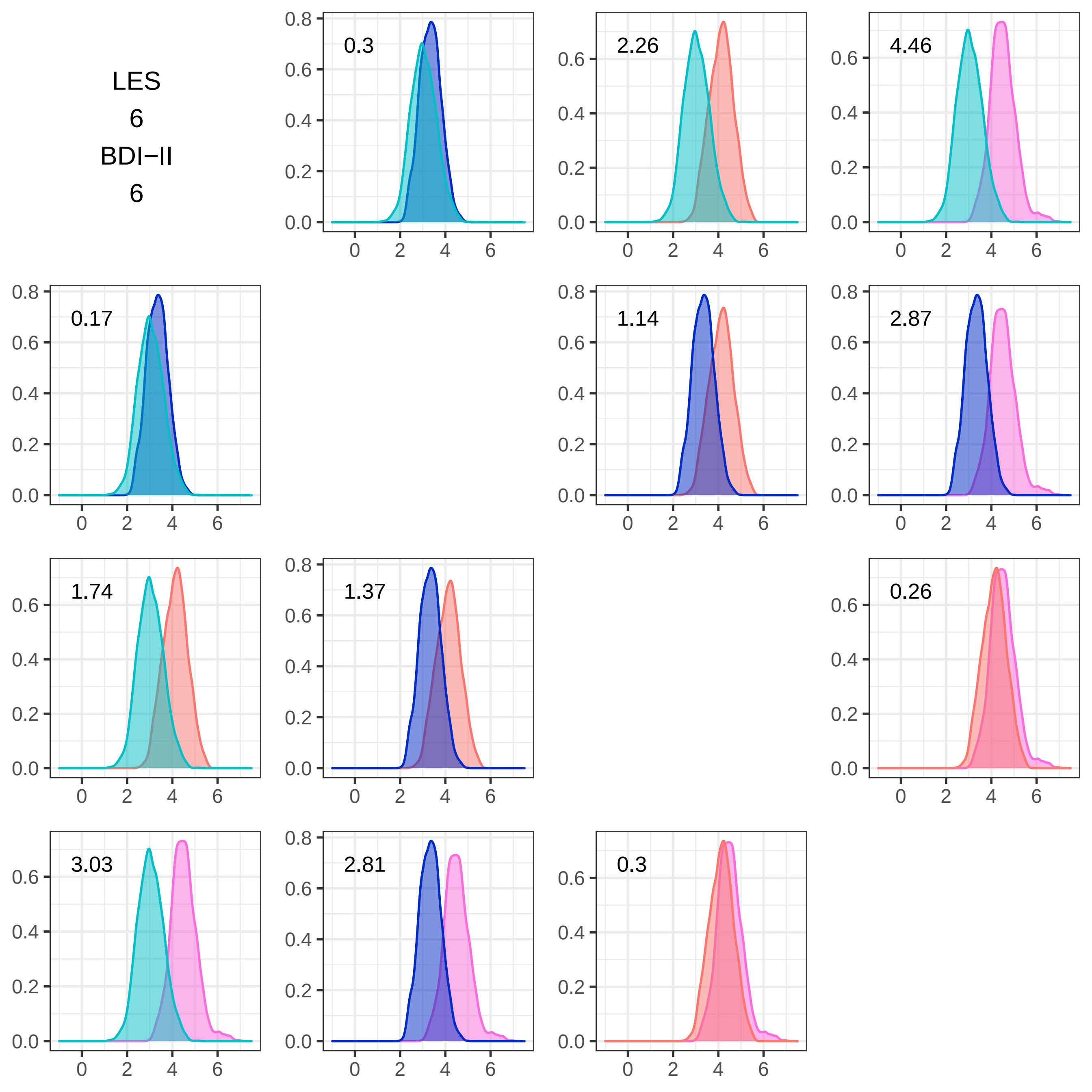
LES
6
BDI−II
6
0.3
2.26
4.46
0.17
1.14
2.87
1.74
1.37
0.26
3.03
2.81
0.3

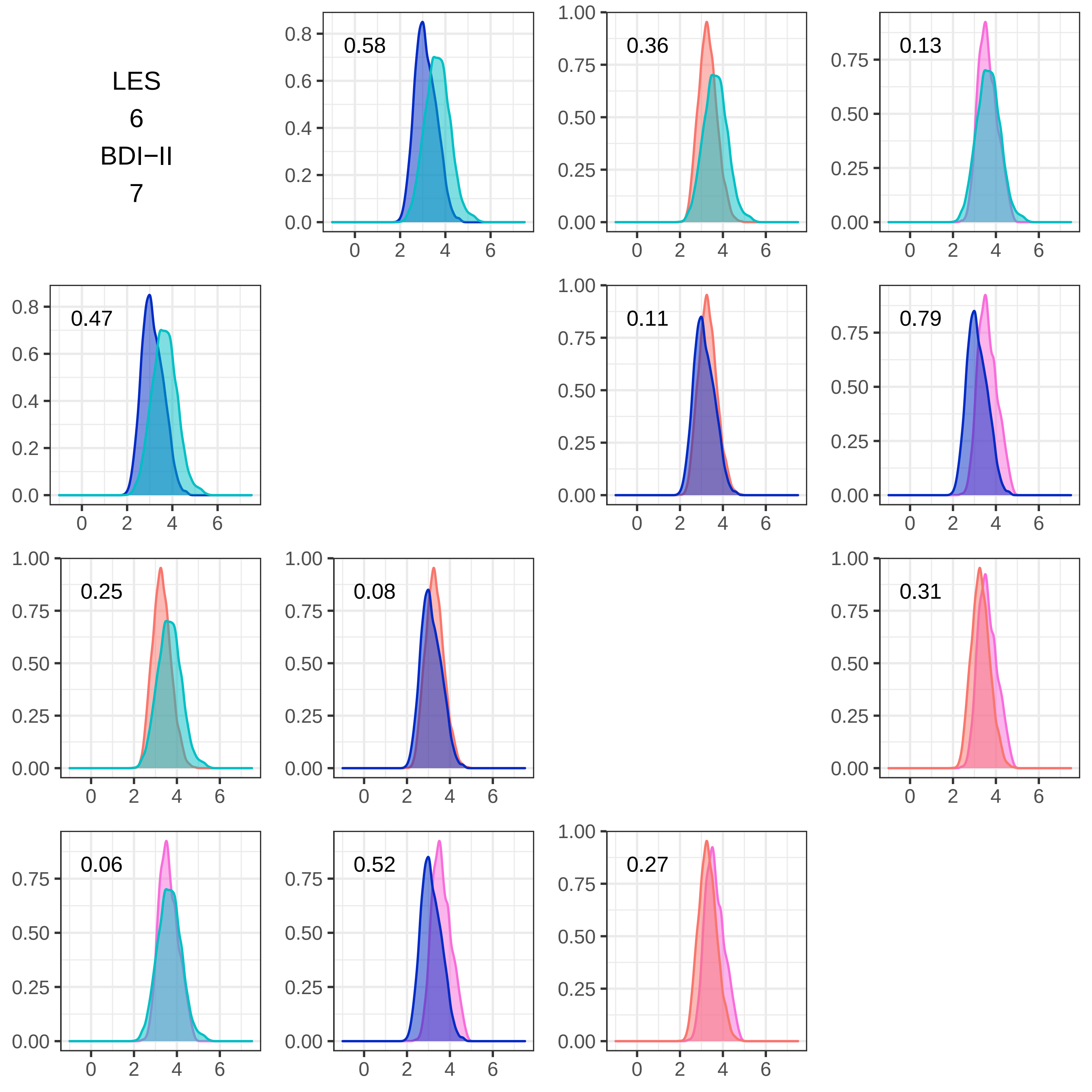
LES
6
BDI−II
7
0.58
0.36
0.13
0.47
0.11
0.79
0.25
0.08
0.31
0.06
0.52
0.27

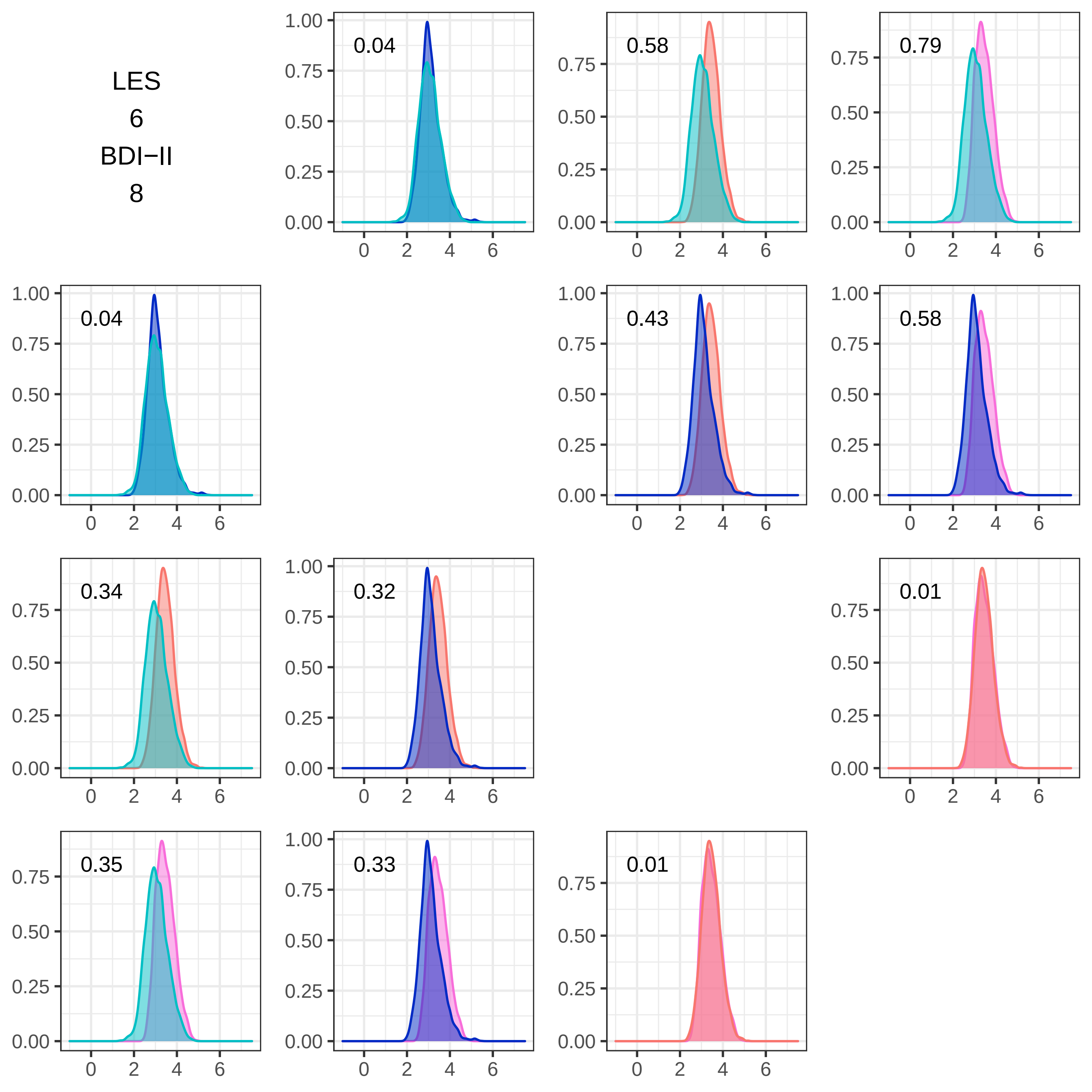
LES
6
BDI−II
8
0.04
0.58
0.79
0.04
0.43
0.58
0.34
0.32
0.01
0.35
0.33
0.01

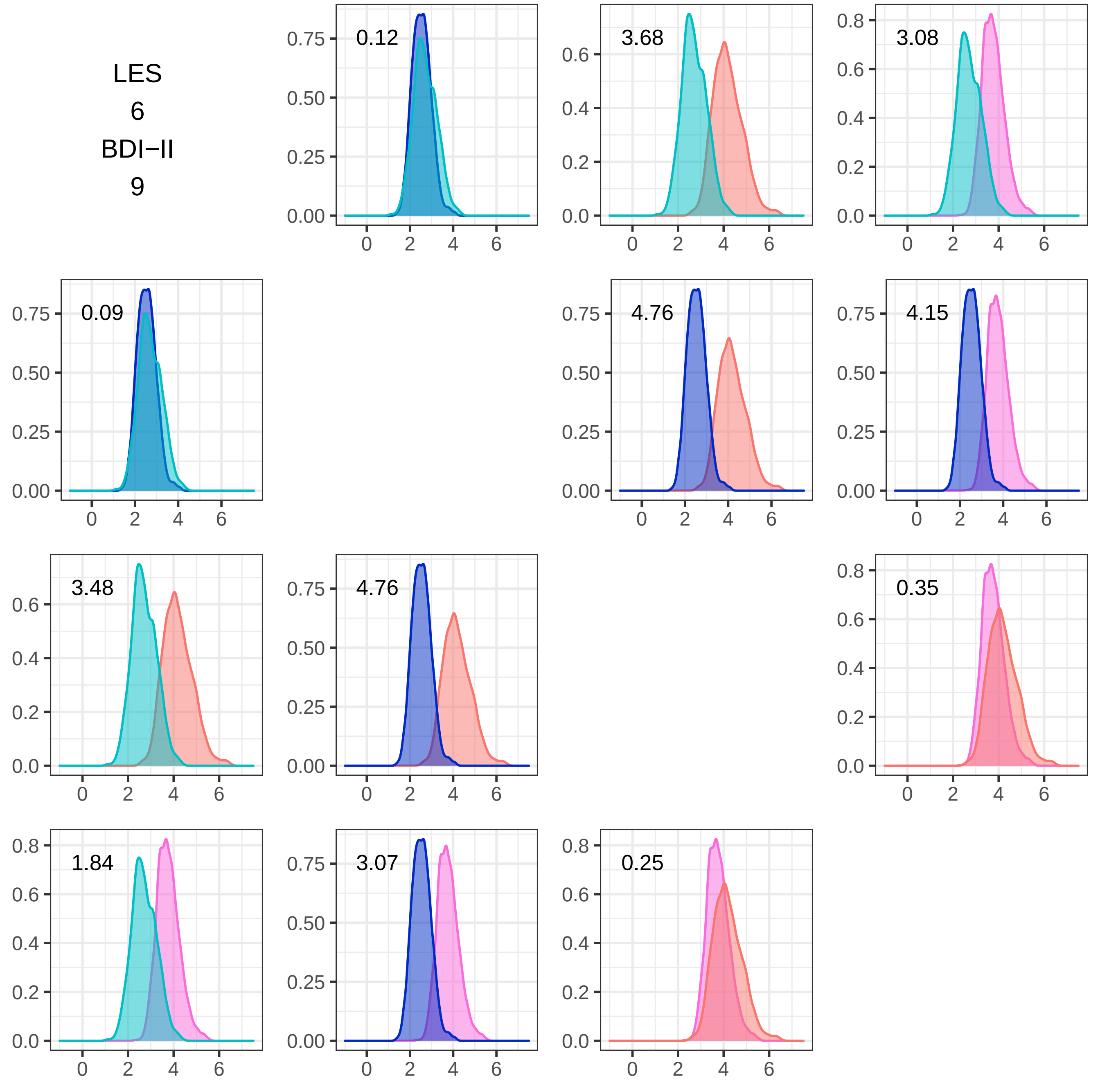

LES
6
BDI−II
9
0.12
3.68
3.08
0.09
4.76
4.15
3.48
4.76
0.35
1.84
3.07
0.25

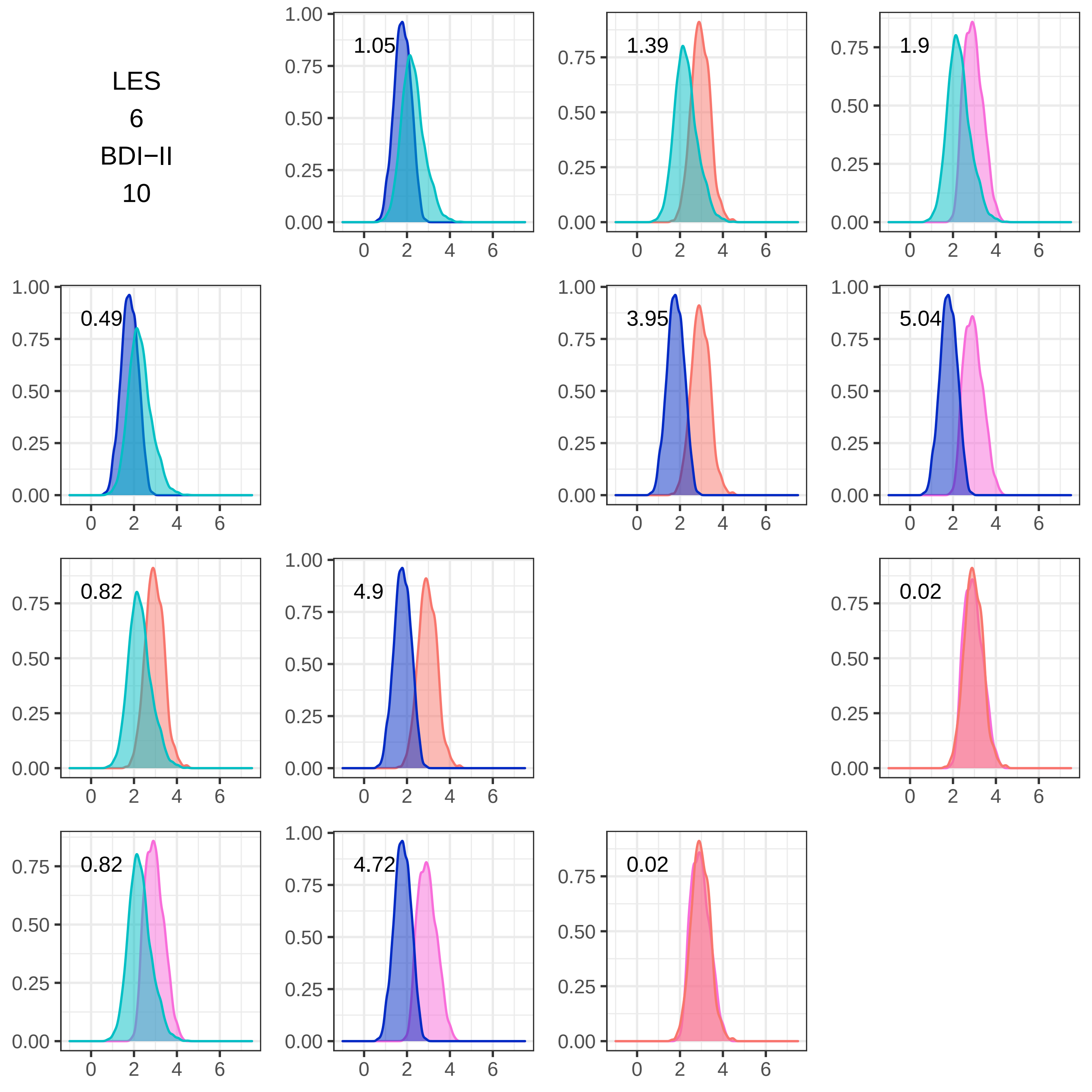

LES
6
BDI−II
10
1.05
1.39
1.9
0.49
3.95
5.04
0.82
4.9
0.02
0.82
4.72
0.02

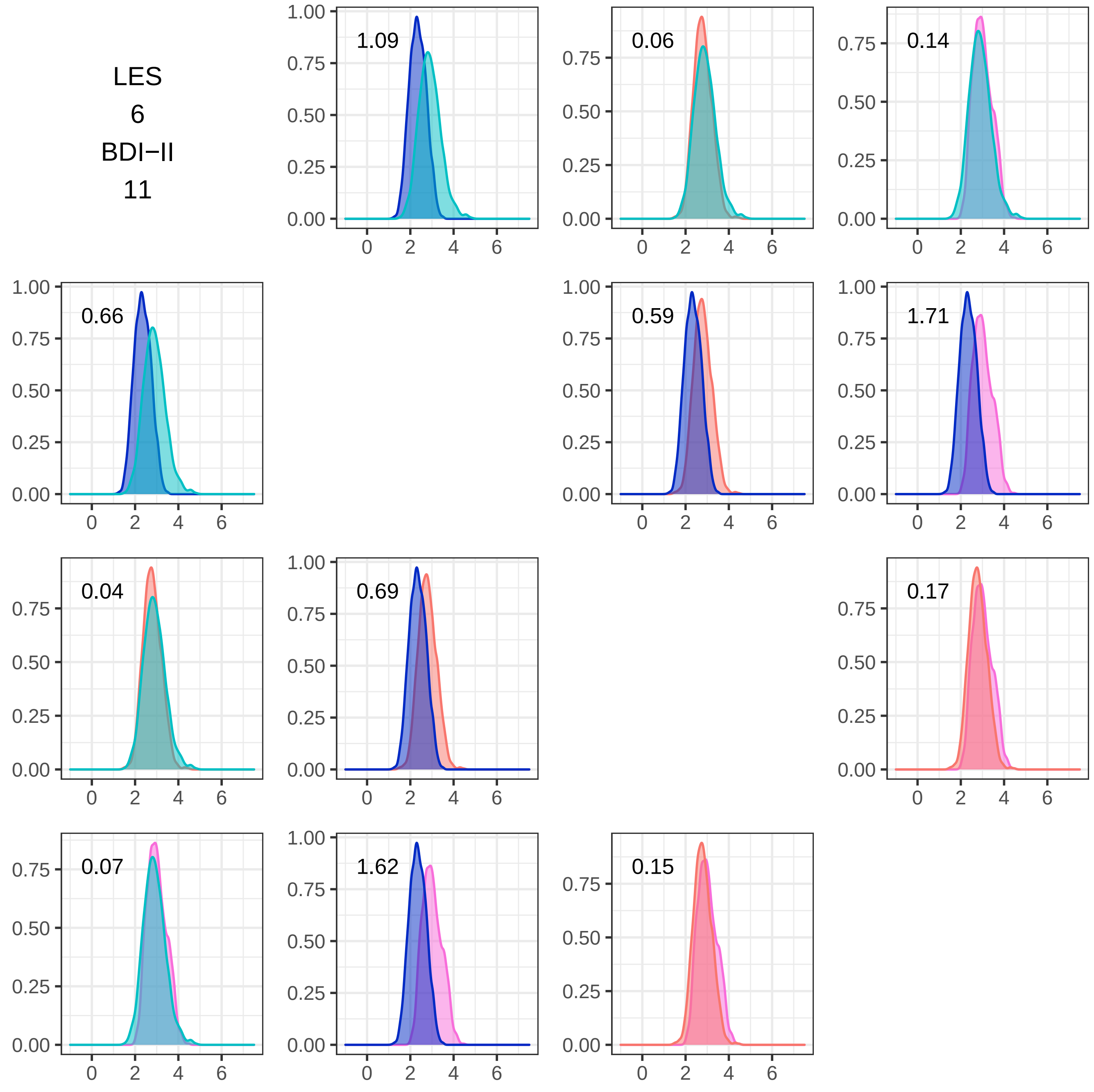
LES
6
BDI−II
11
1.09
0.06
0.14
0.66
0.59
1.71
0.04
0.69
0.17
0.07
1.62
0.15
0.00
0.25
0.50
0.75
1.00
0
2
4
6

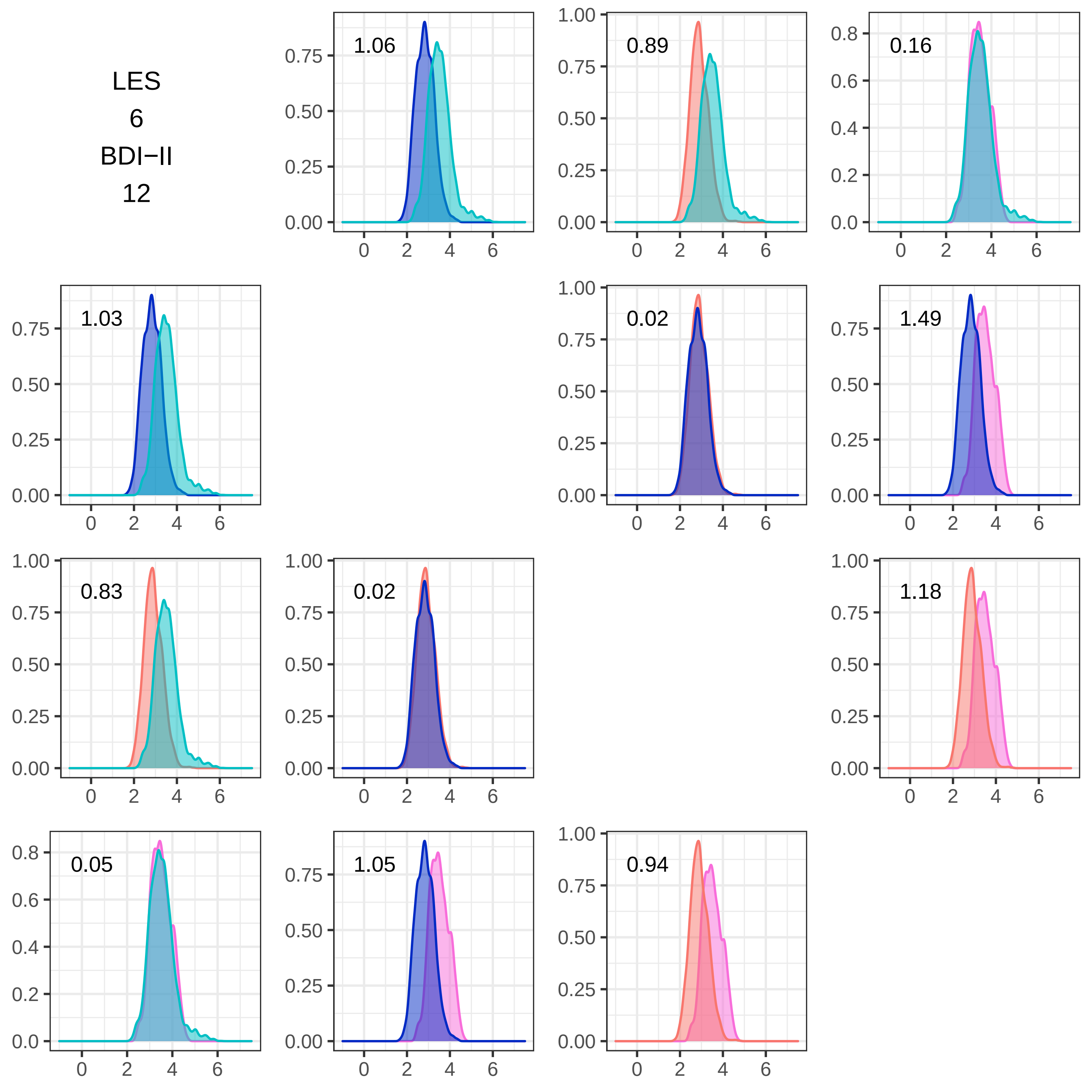

LES
6
BDI−II
12
1.06
0.89
0.16
1.03
0.02
1.49
0.83
0.02
1.18
0.05
1.05
0.94

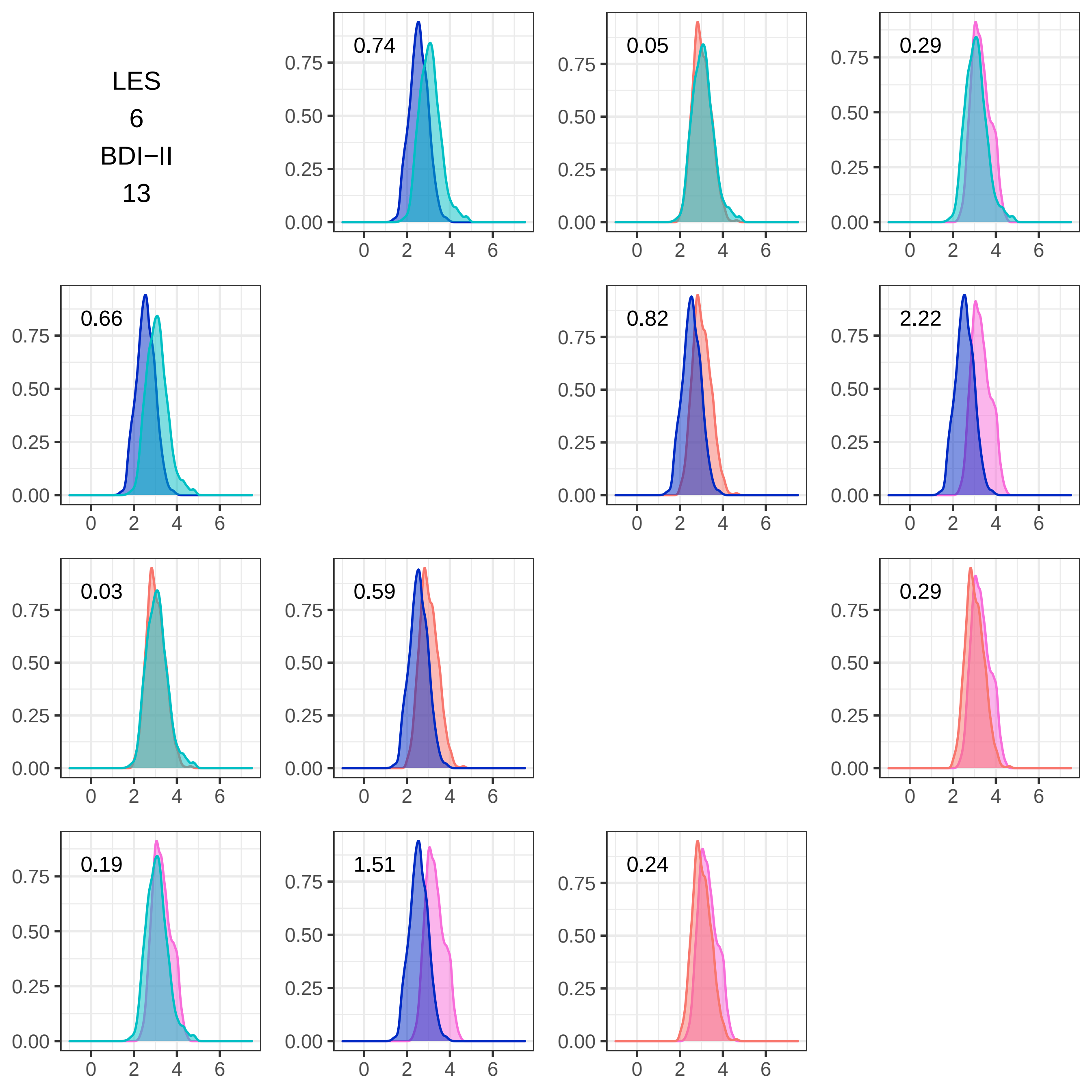

LES
6
BDI−II
13
0.74
0.05
0.29
0.66
0.82
2.22
0.03
0.59
0.29
0.19
1.51
0.24
0.00
0.25
0.50
0.75
0
2
4
6

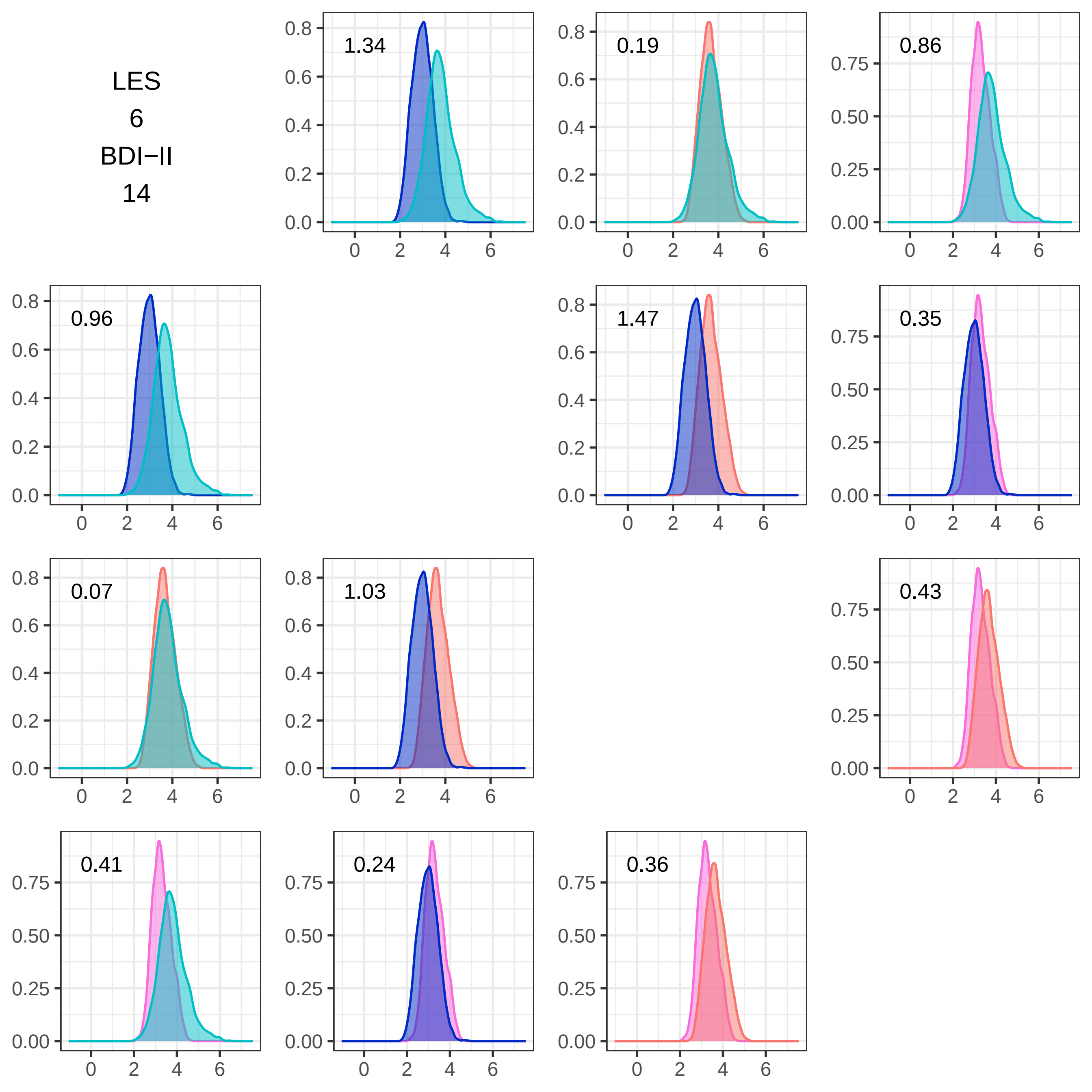
LES
6
BDI−II
14
1.34
0.19
0.86
0.96
1.47
0.35
0.07
1.03
0.43
0.41
0.24
0.36

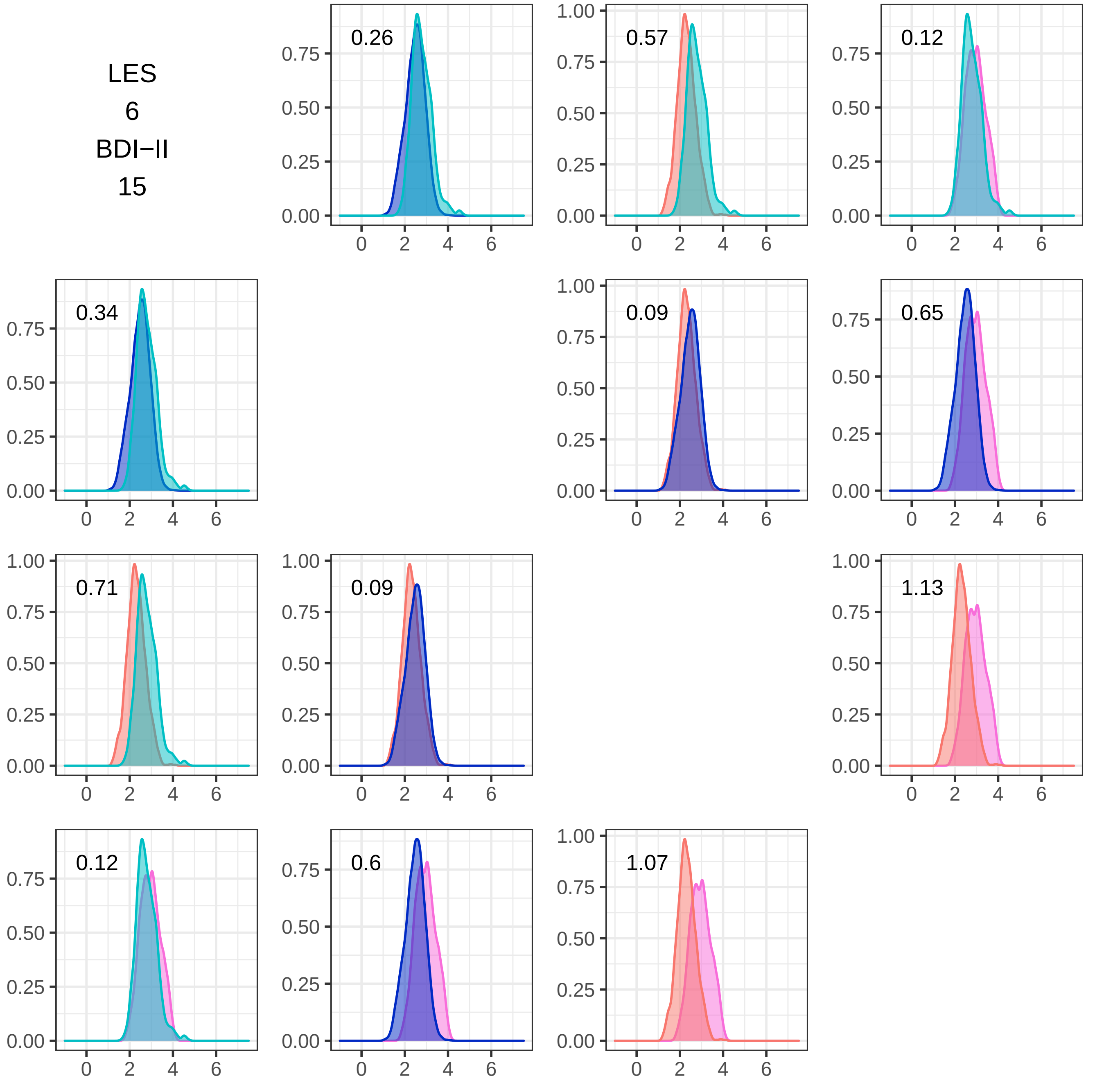

LES
6
BDI−II
15
0.26
0.57
0.12
0.34
0.09
0.65
0.71
0.09
1.13
0.12
0.6
1.07
0.00
0.25
0.50
0.75
1.00
0
2
4
6

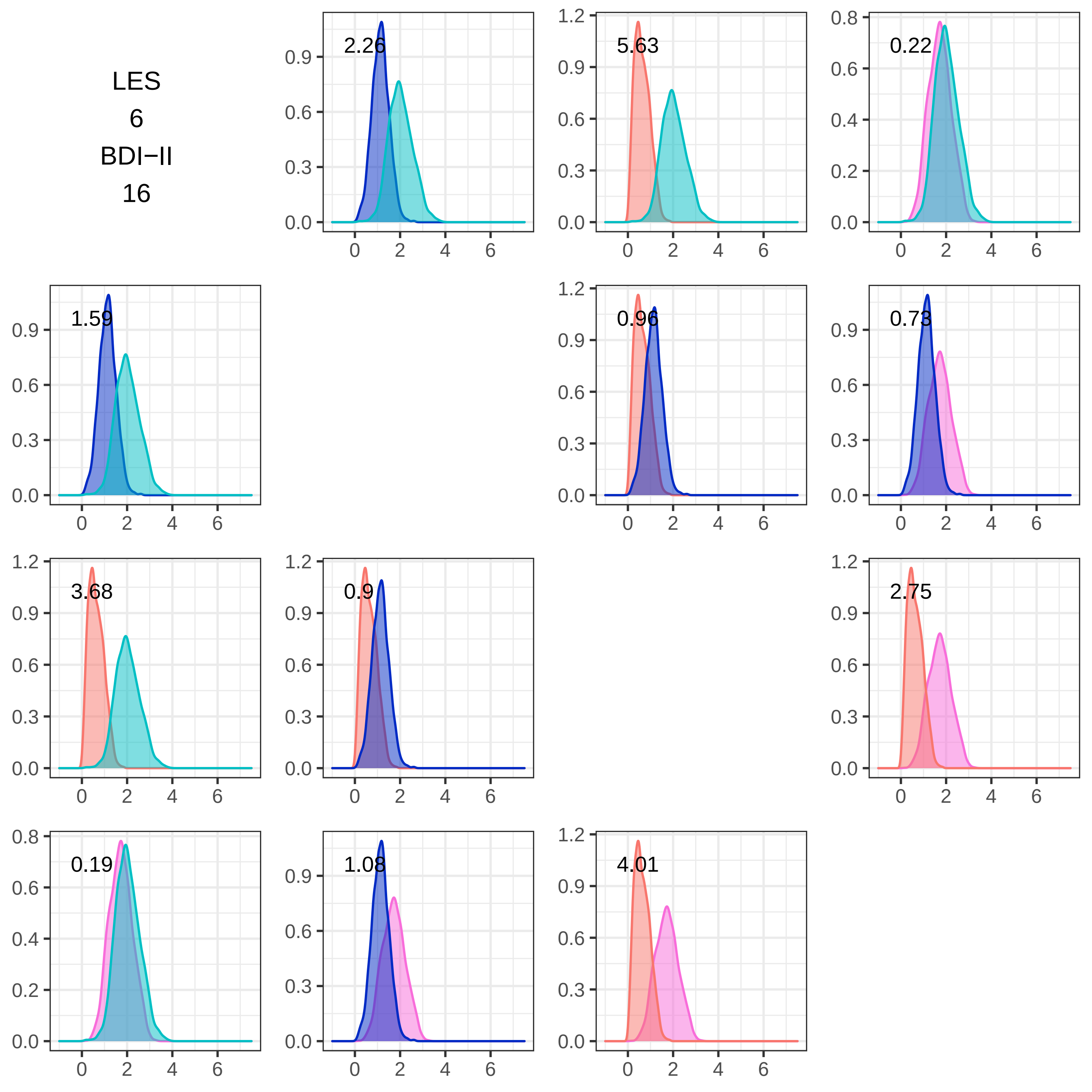
LES
6
BDI−II
16
2.26
5.63
0.22
1.59
0.96
0.73
3.68
0.9
2.75
0.19
1.08
4.01

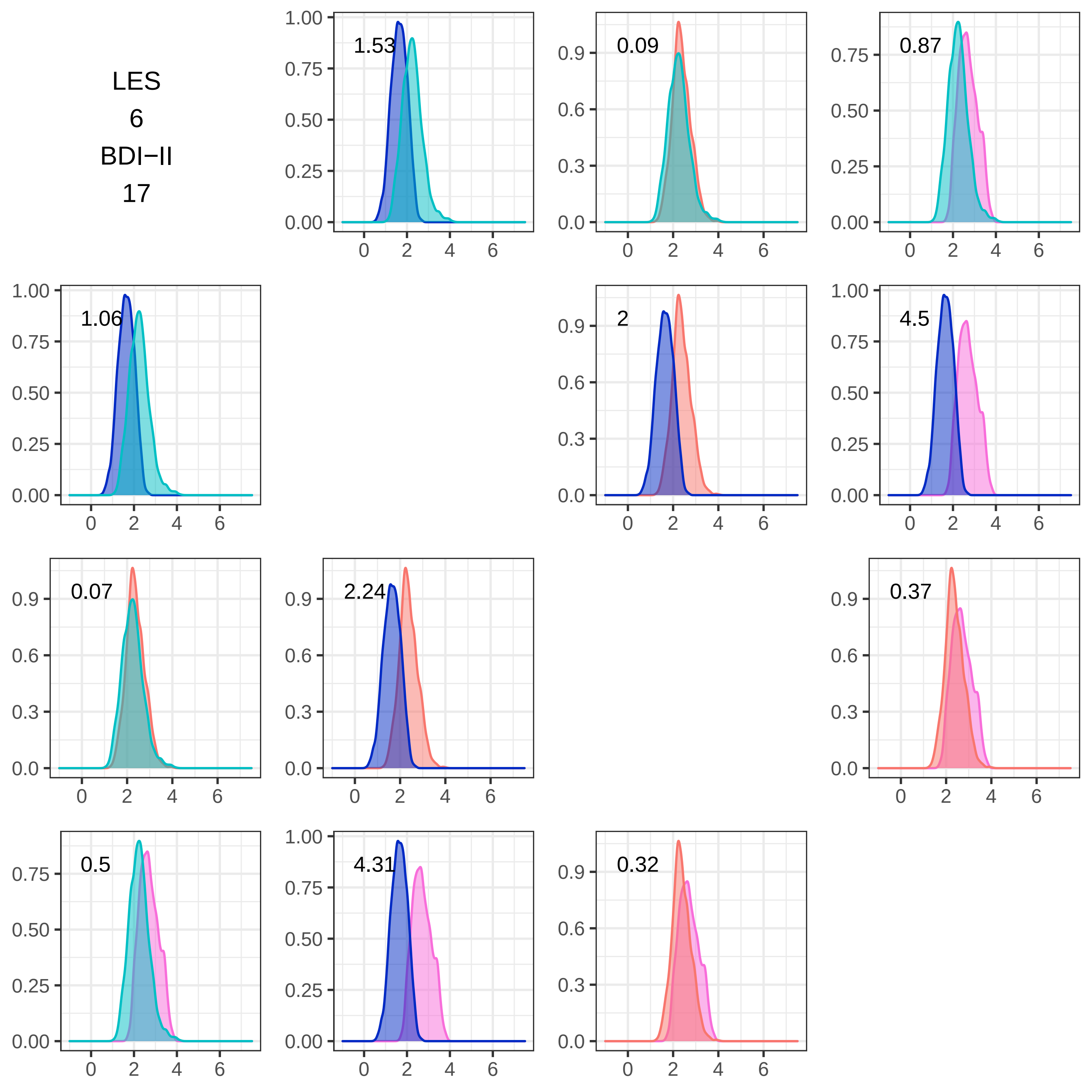

LES
6
BDI−II
17
1.53
0.09
0.87
1.06
2
4.5
0.07
2.24
0.37
0.5
4.31
0.32

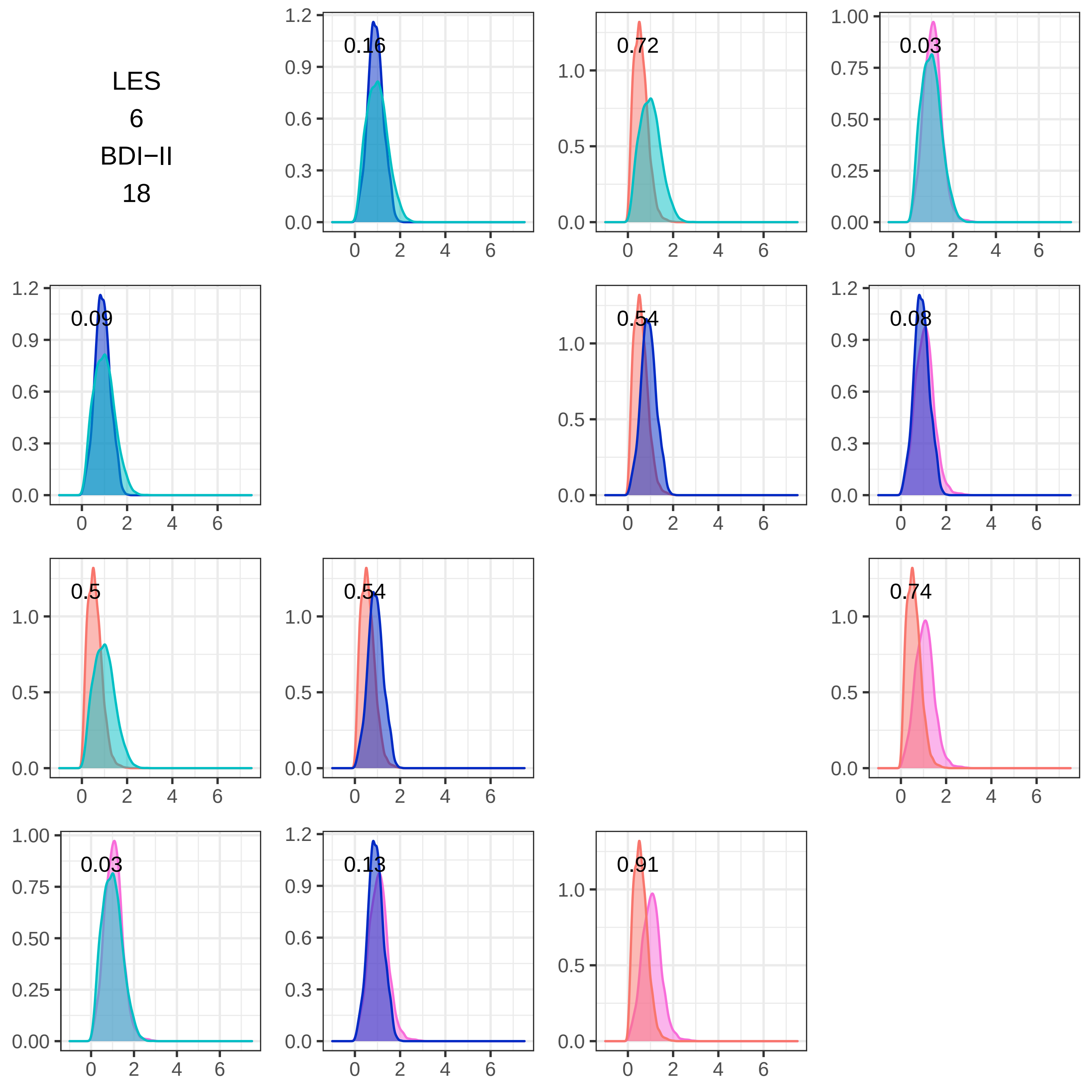

LES
6
BDI−II
18
0.16
0.72
0.03
0.09
0.54
0.08
0.5
0.54
0.74
0.03
0.13
0.91

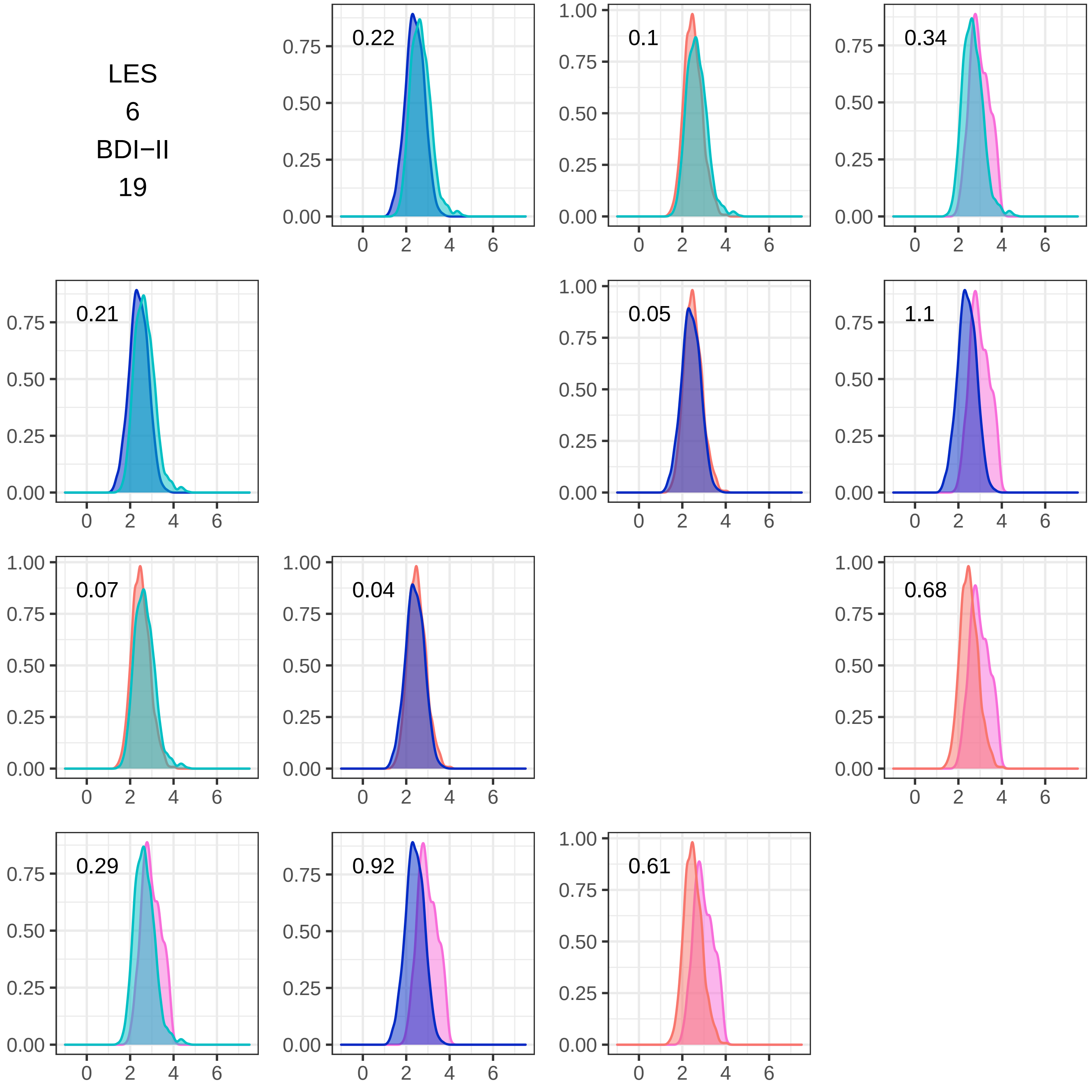
LES
6
BDI−II
19
0.22
0.1
0.34
0.21
0.05
1.1
0.07
0.04
0.68
0.29
0.92
0.61

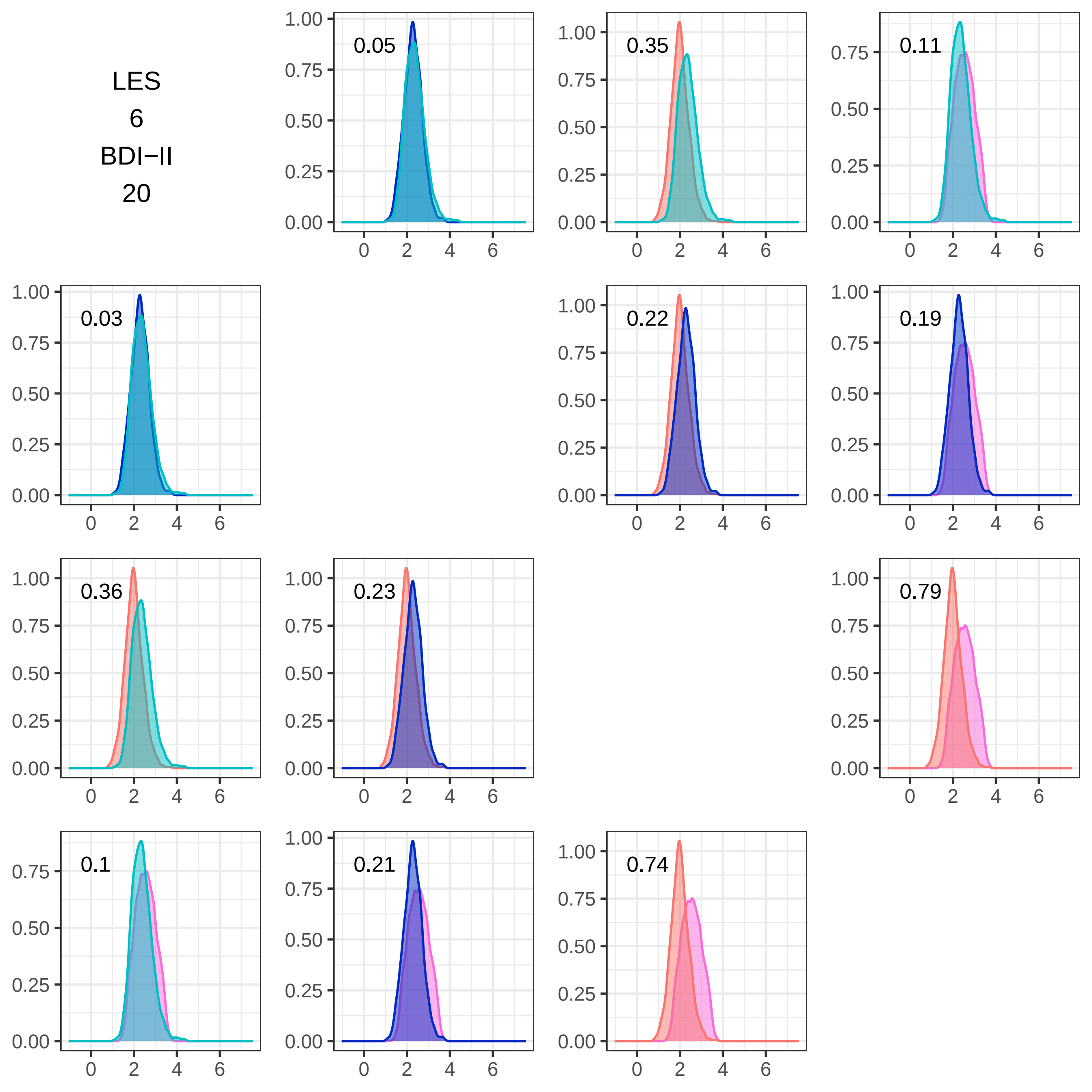
LES
6
BDI−II
20
0.05
0.35
0.11
0.03
0.22
0.19
0.36
0.23
0.79
0.1
0.21
0.74

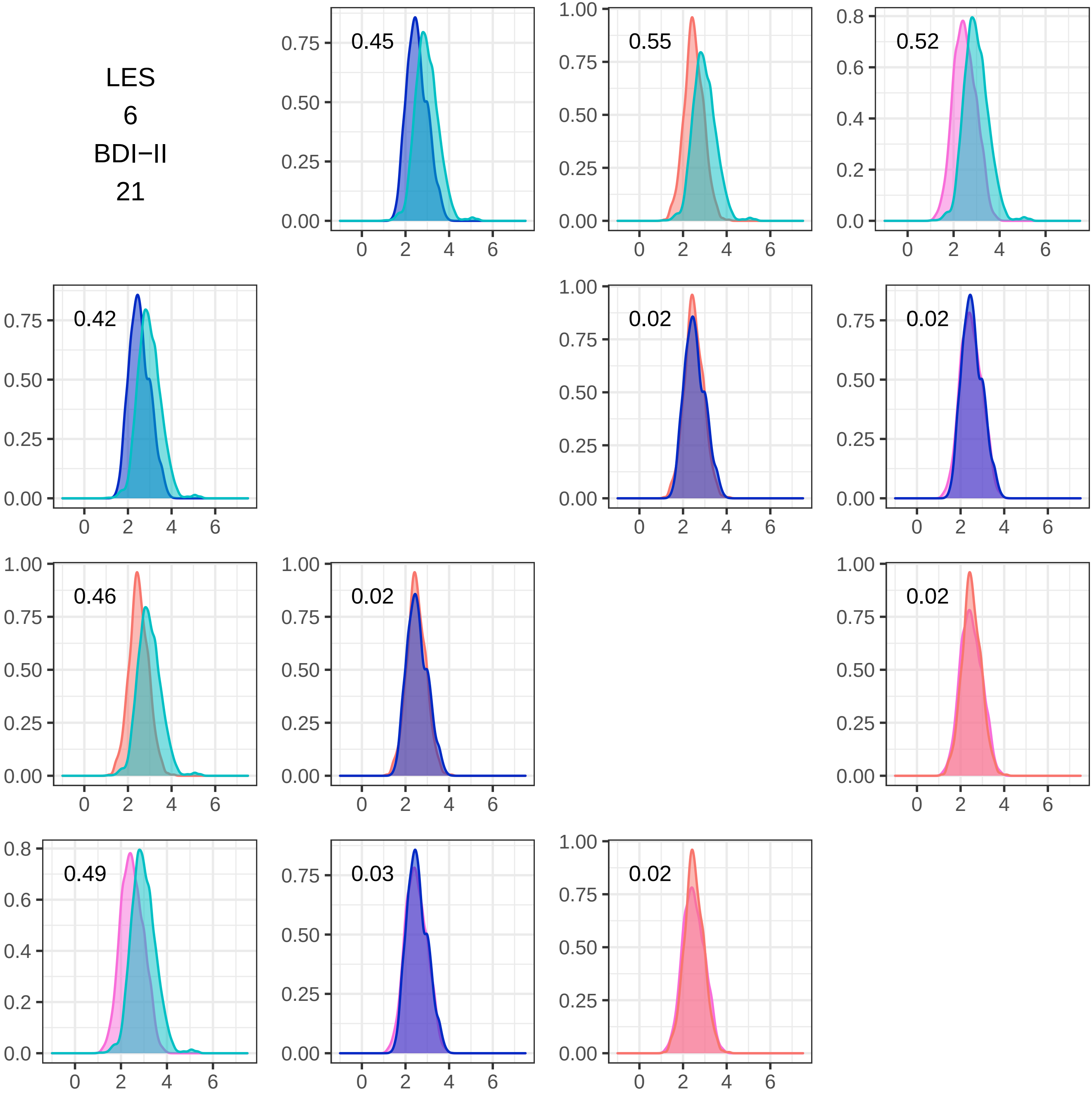
LES
6
BDI−II
21
0.45
0.55
0.52
0.42
0.02
0.02
0.46
0.02
0.02
0.49
0.03
0.02

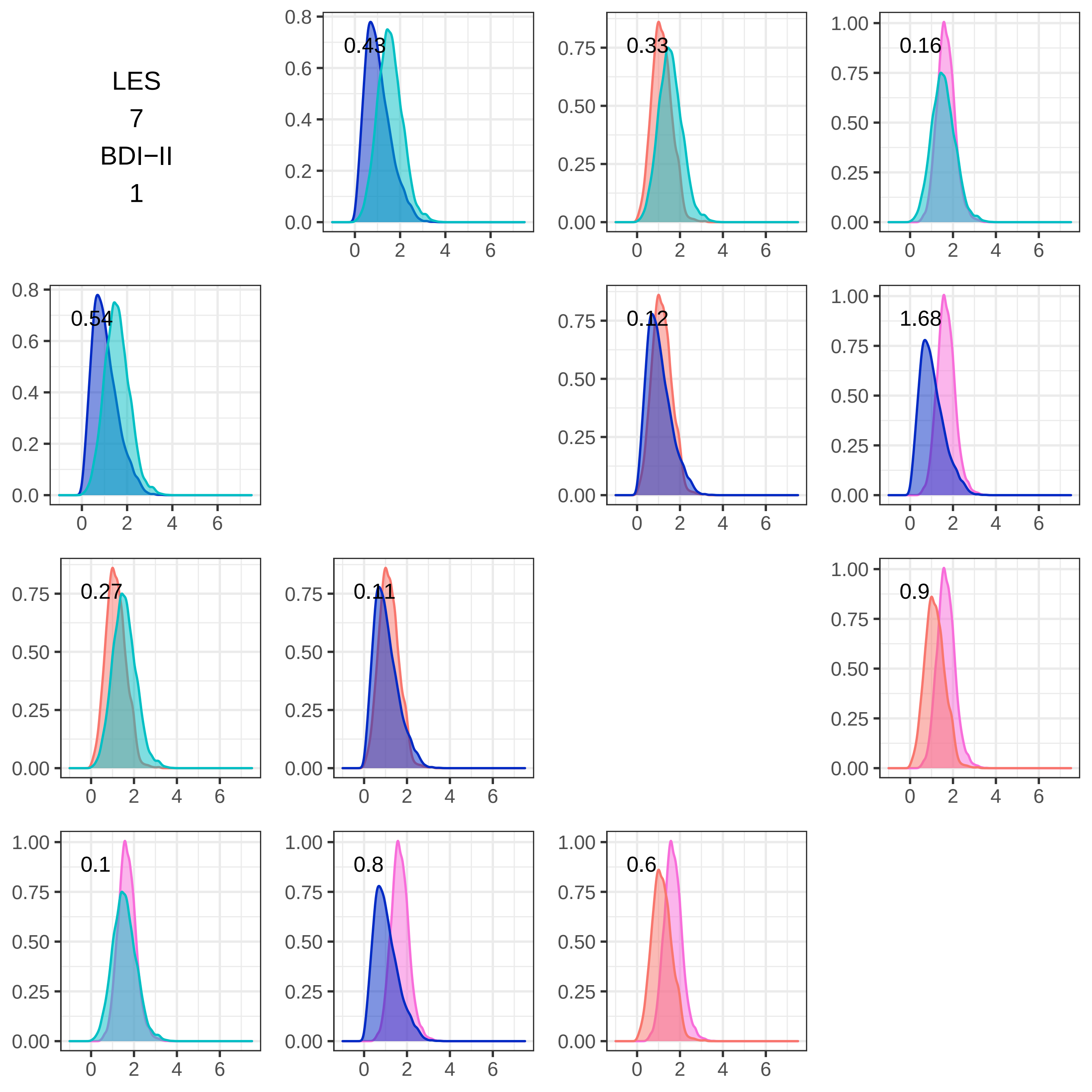
LES
7
BDI−II
1
0.43
0.33
0.16
0.54
0.12
1.68
0.27
0.11
0.9
0.1
0.8
0.6

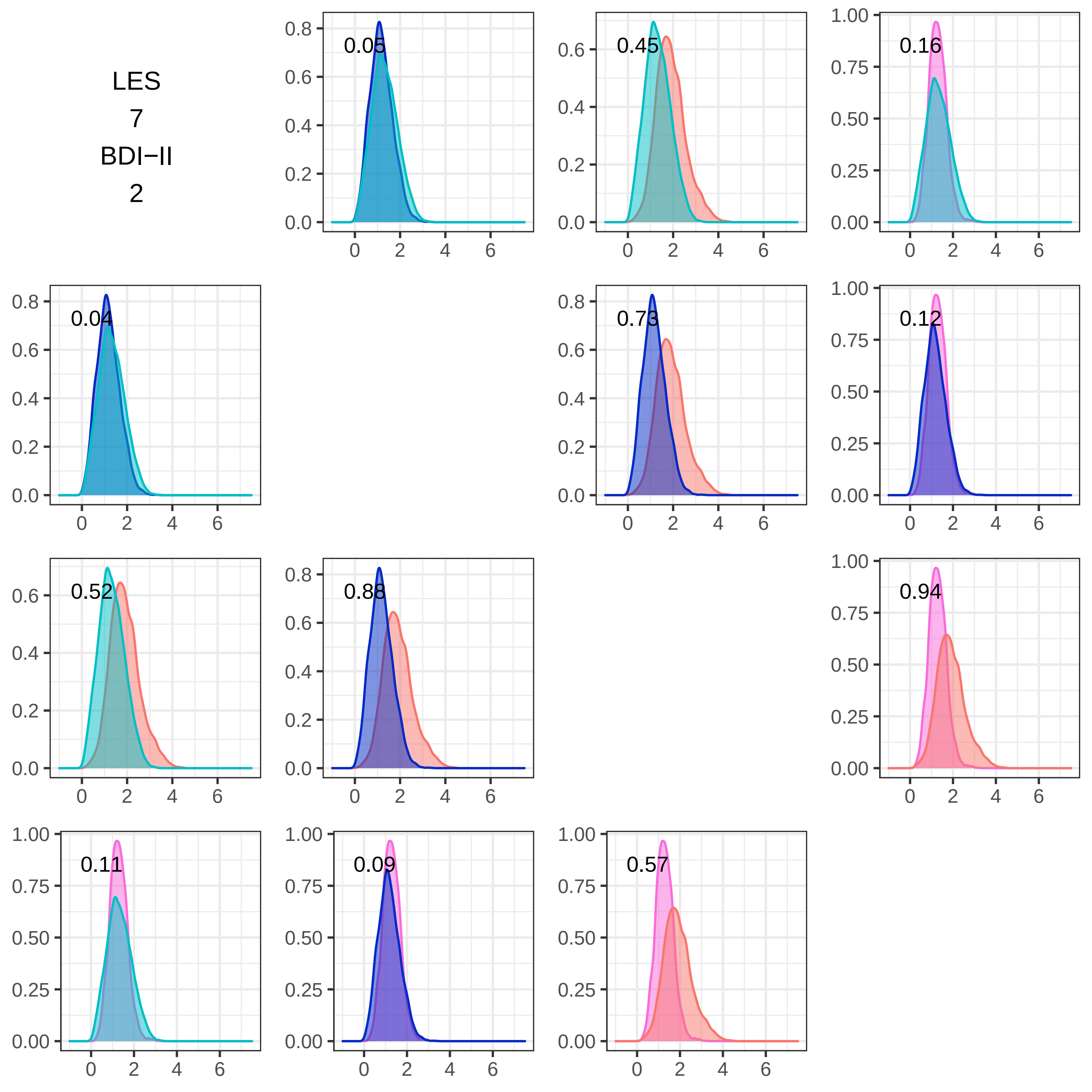
LES
7
BDI−II
2
0.05
0.45
0.16
0.04
0.73
0.12
0.52
0.88
0.94
0.11
0.09
0.57

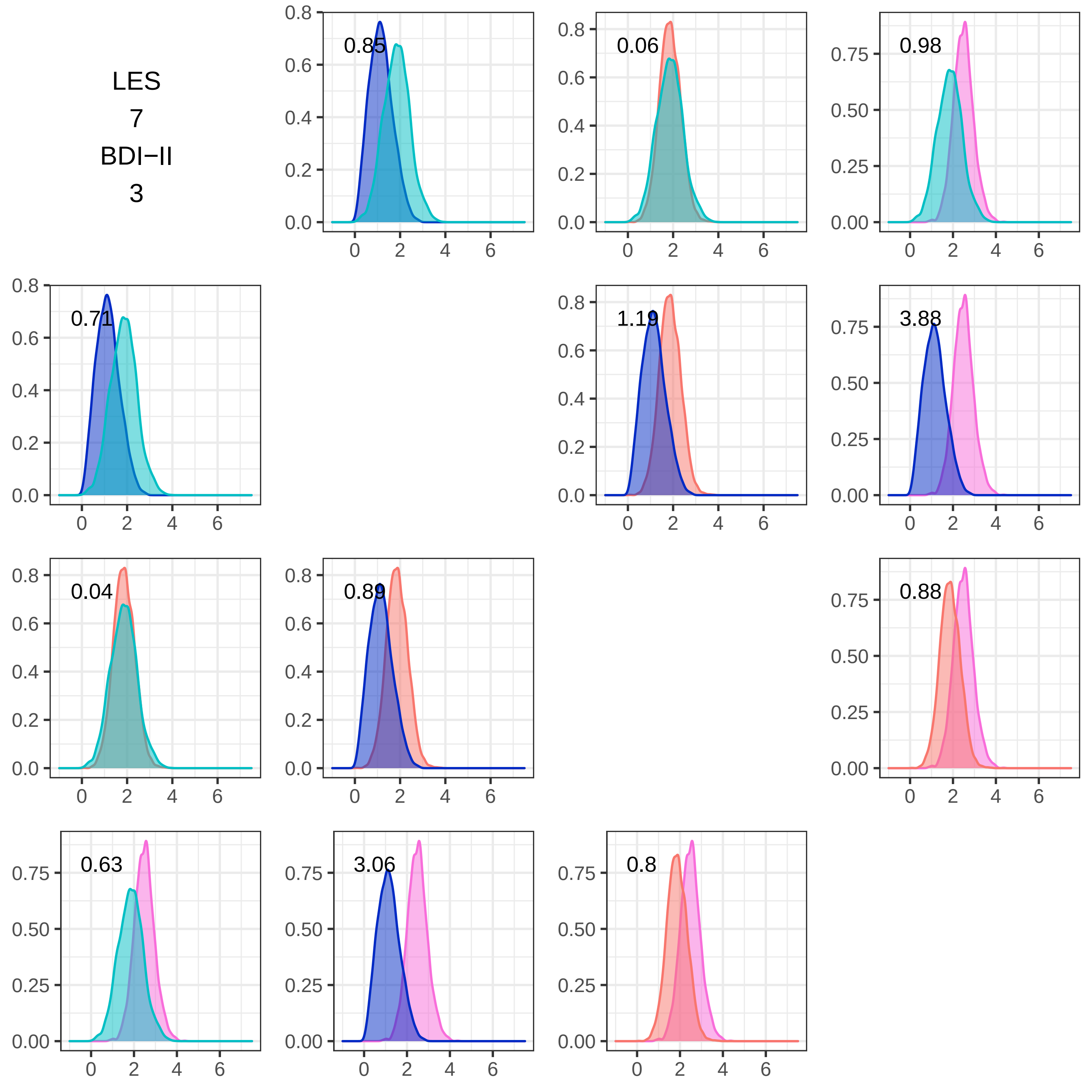

LES
7
BDI−II
3
0.85
0.06
0.98
0.71
1.19
3.88
0.04
0.89
0.88
0.63
3.06
0.8

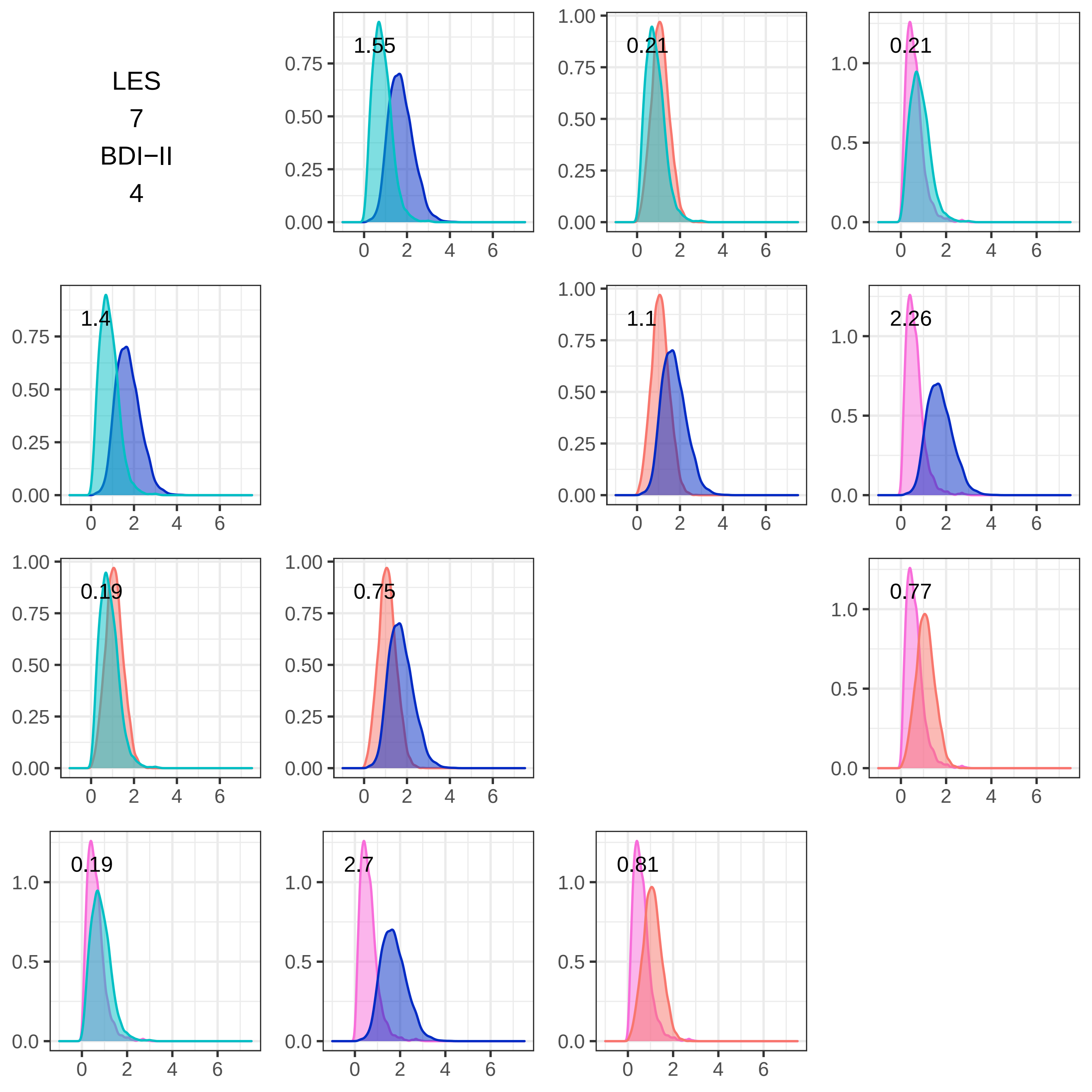
LES
7
BDI−II
4
1.55
0.21
0.21
1.4
1.1
2.26
0.19
0.75
0.77
0.19
2.7
0.81

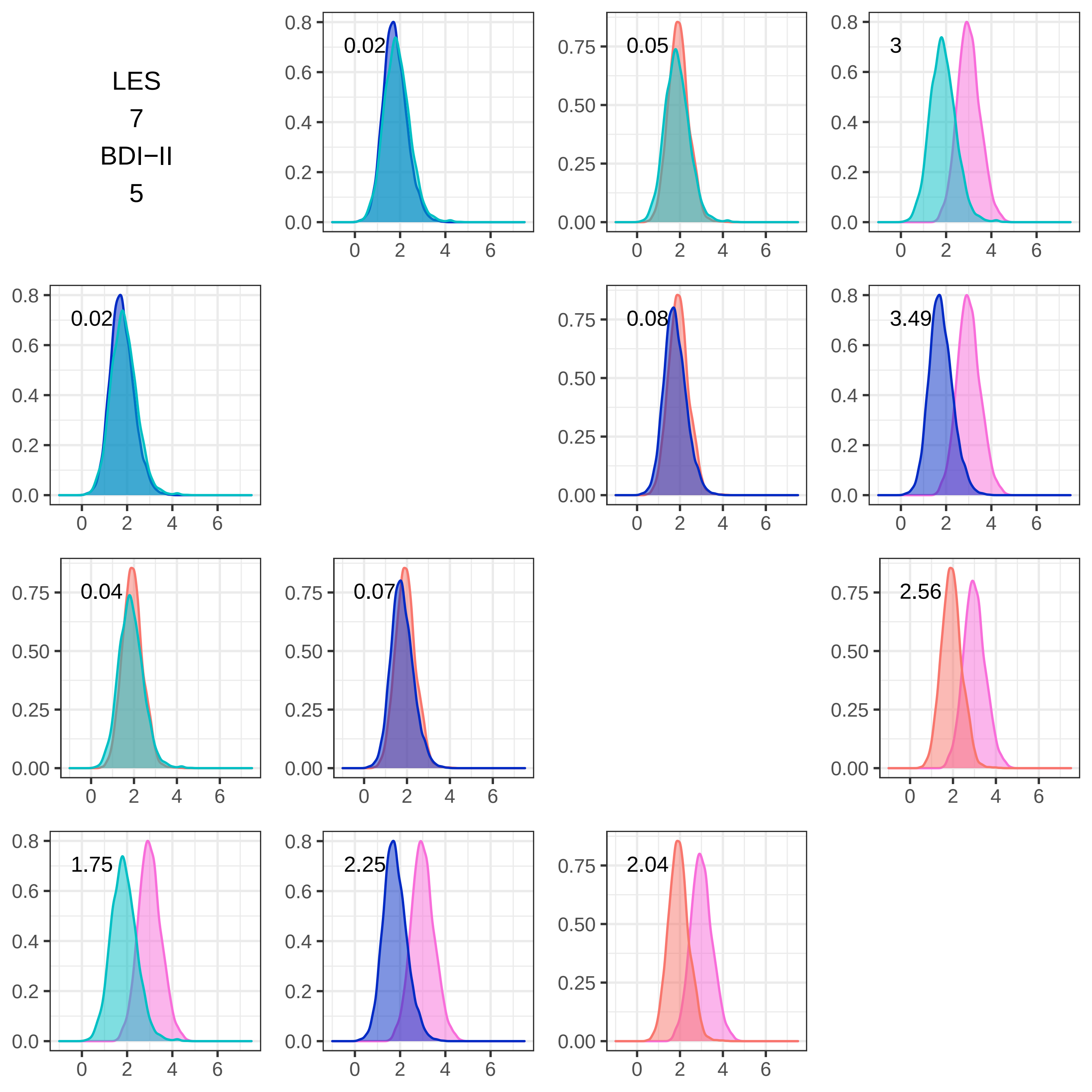

LES
7
BDI−II
5
0.02
0.05
3
0.02
0.08
3.49
0.04
0.07
2.56
1.75
2.25
2.04

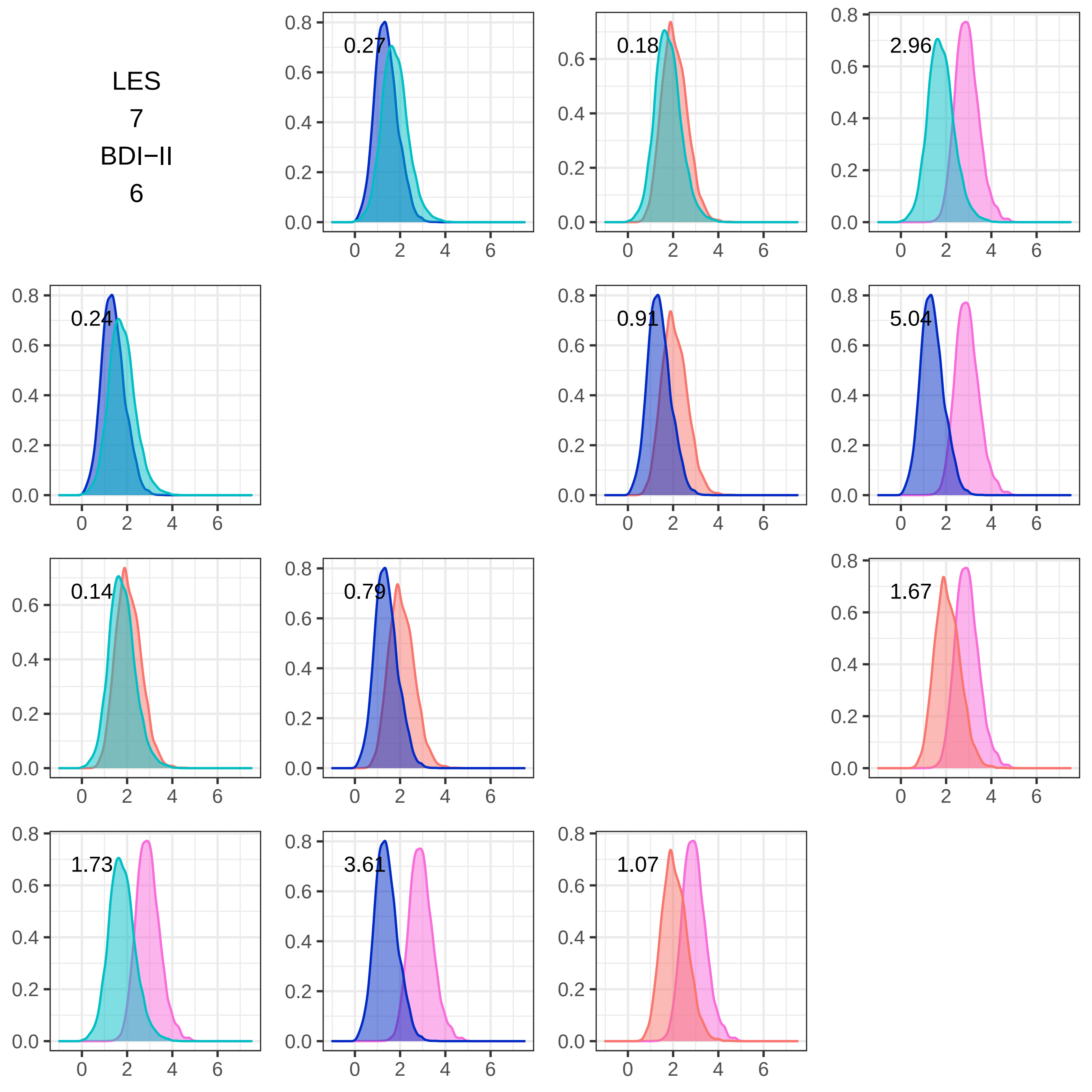

LES
7
BDI−II
6
0.27
0.18
2.96
0.24
0.91
5.04
0.14
0.79
1.67
1.73
3.61
1.07

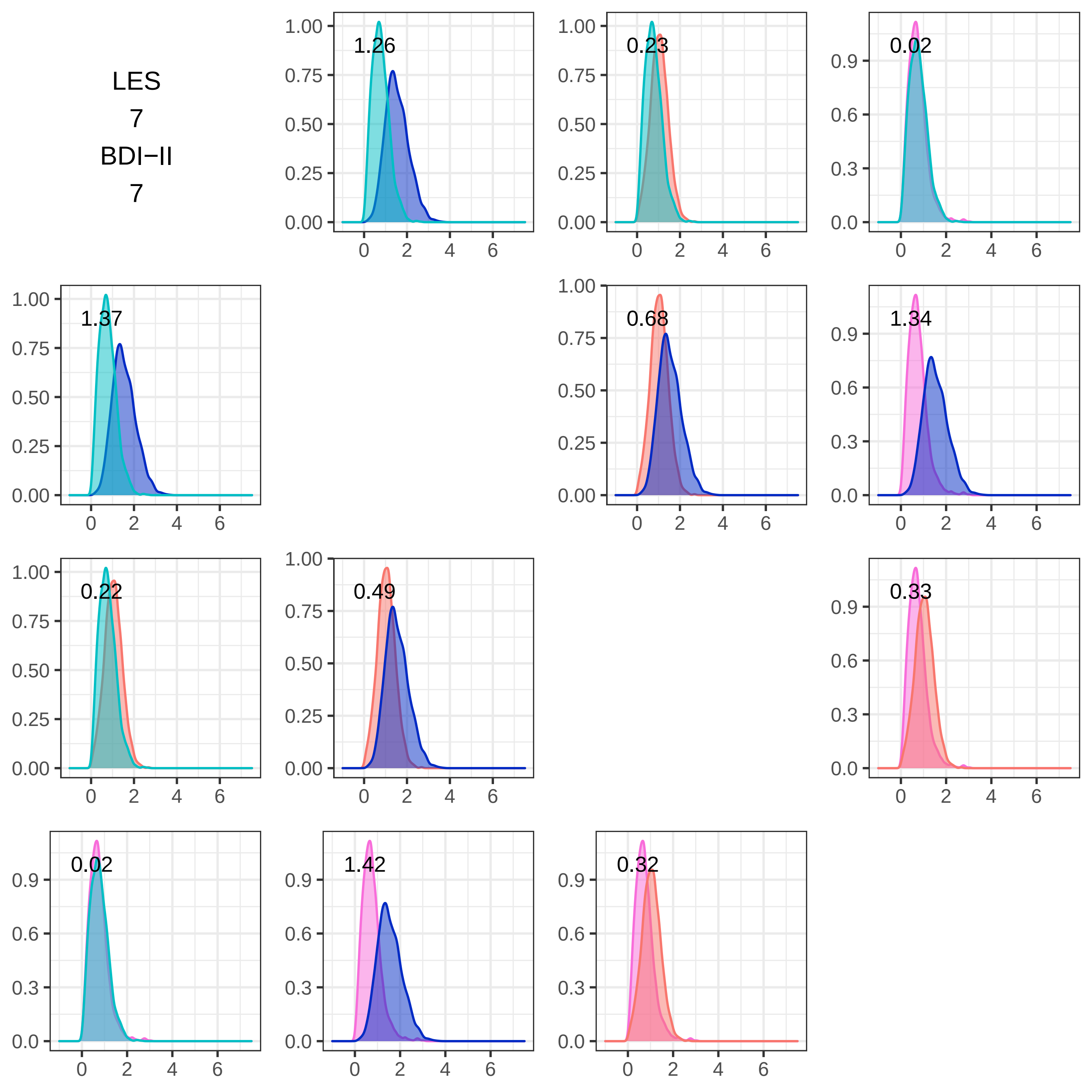
LES
7
BDI−II
7
1.26
0.23
0.02
1.37
0.68
1.34
0.22
0.49
0.33
0.02
1.42
0.32

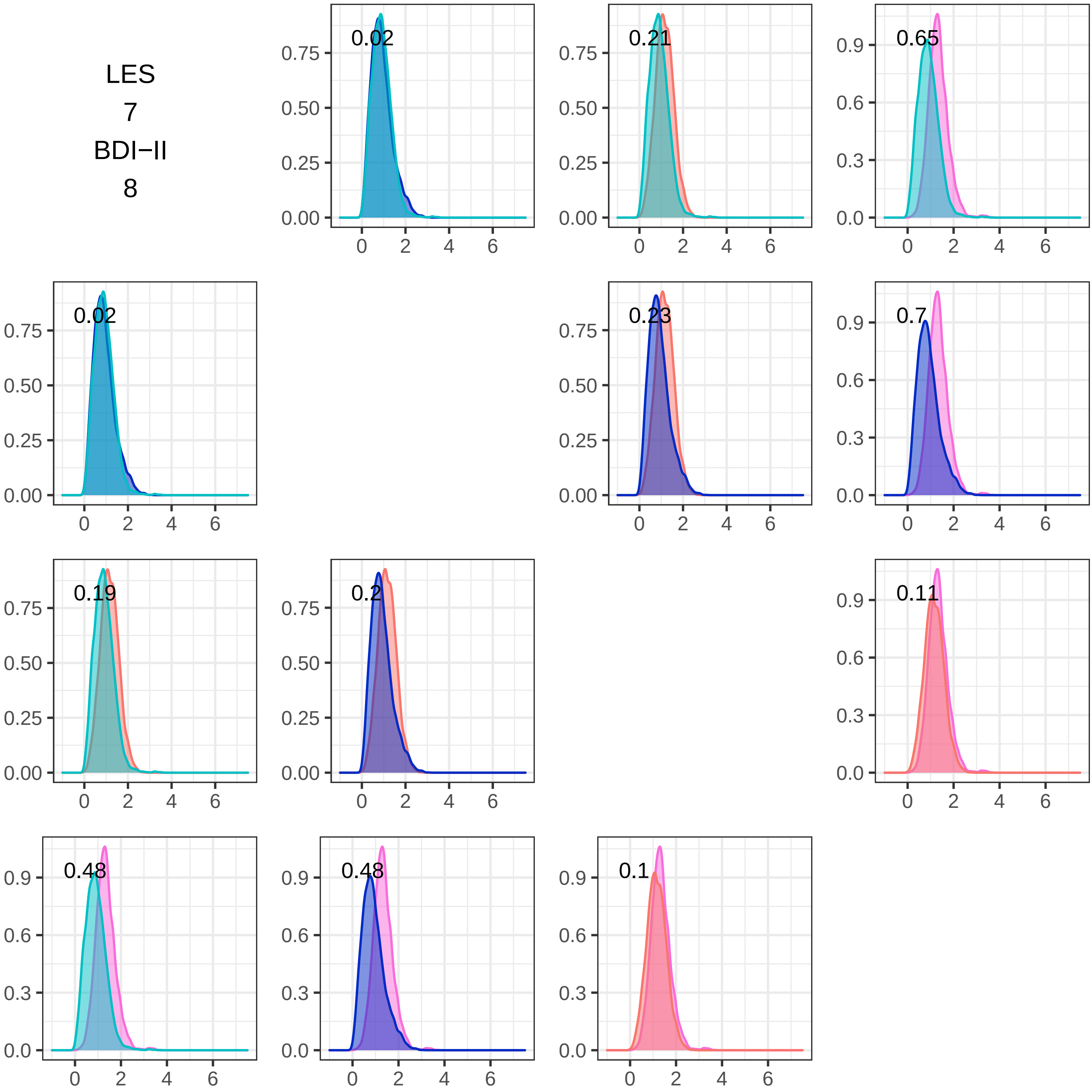
LES
7
BDI−II
8
0.02
0.21
0.65
0.02
0.23
0.7
0.19
0.2
0.11
0.48
0.48
0.1

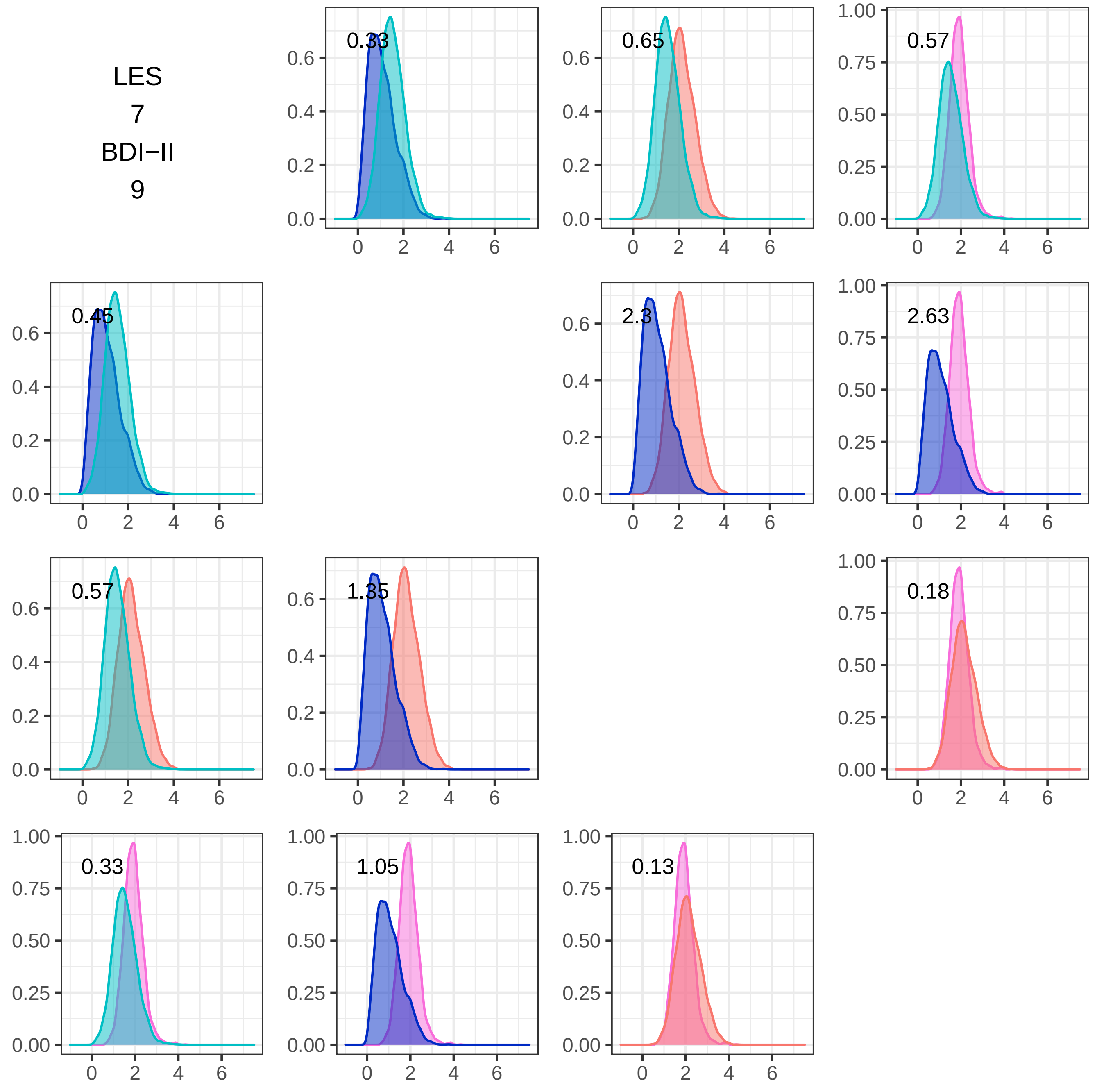

LES
7
BDI−II
9
0.33
0.65
0.57
0.45
2.3
2.63
0.57
1.35
0.18
0.33
1.05
0.13

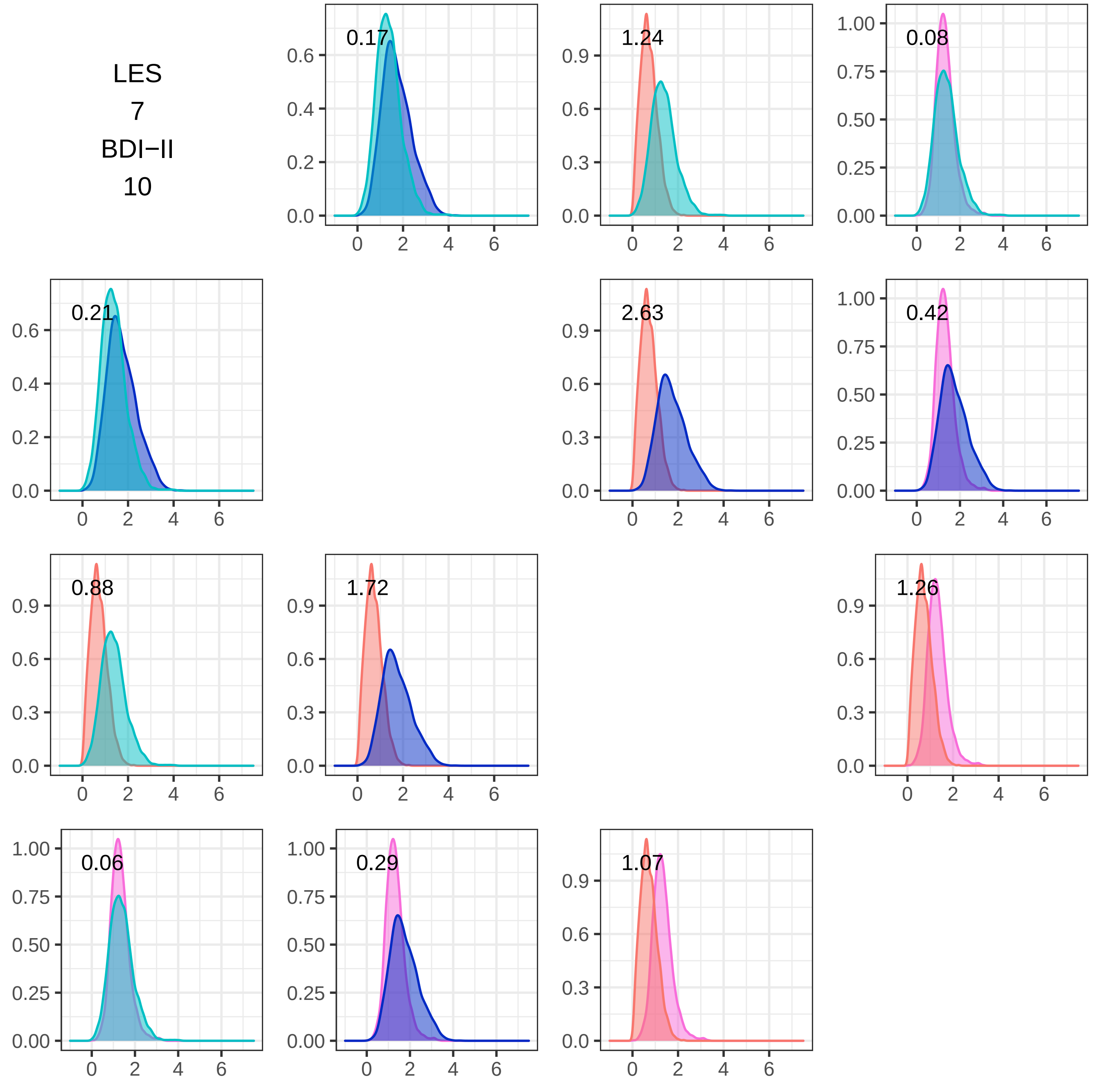

LES
7
BDI−II
10
0.17
1.24
0.08
0.21
2.63
0.42
0.88
1.72
1.26
0.06
0.29
1.07

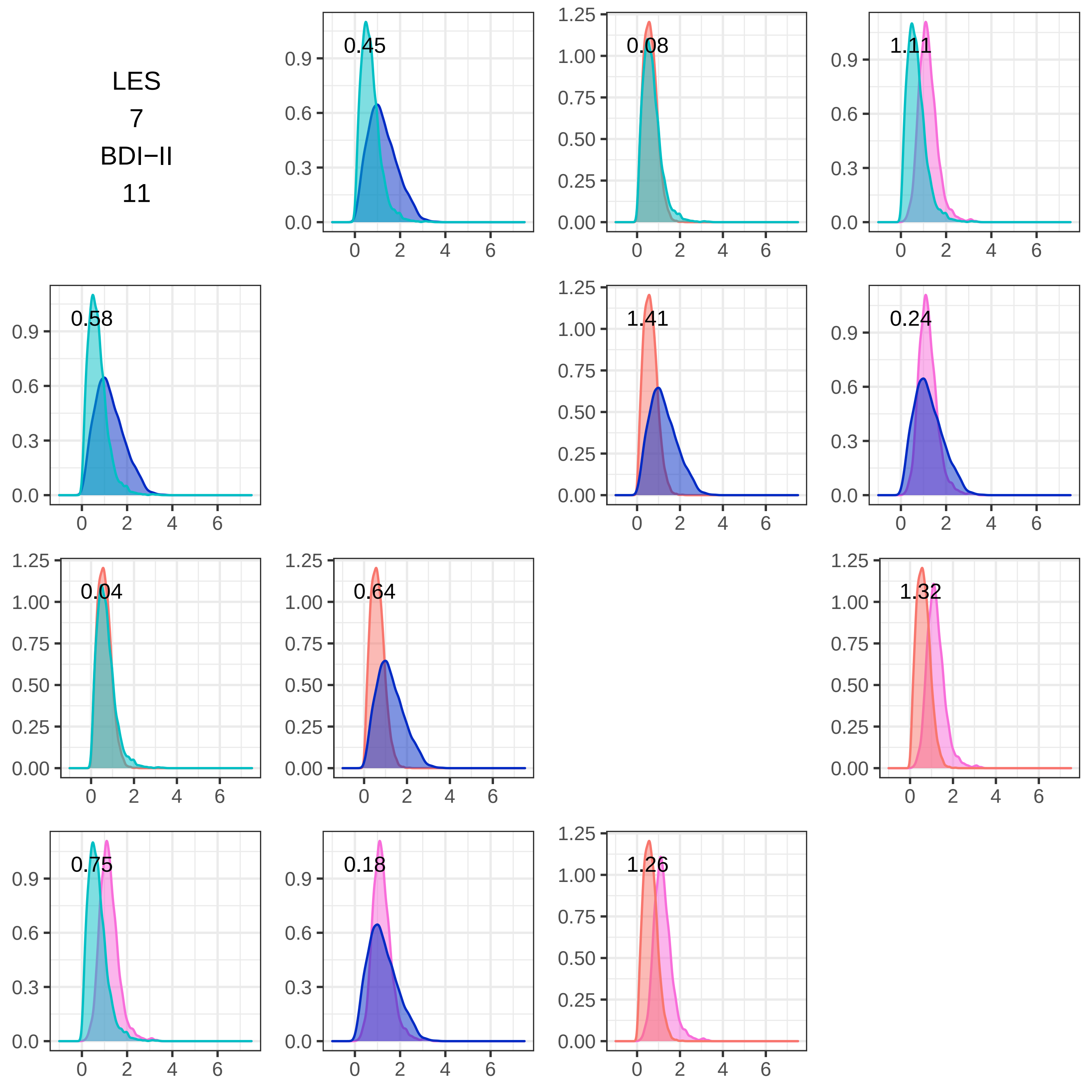
LES
7
BDI−II
11
0.45
0.08
1.11
0.58
1.41
0.24
0.04
0.64
1.32
0.75
0.18
1.26

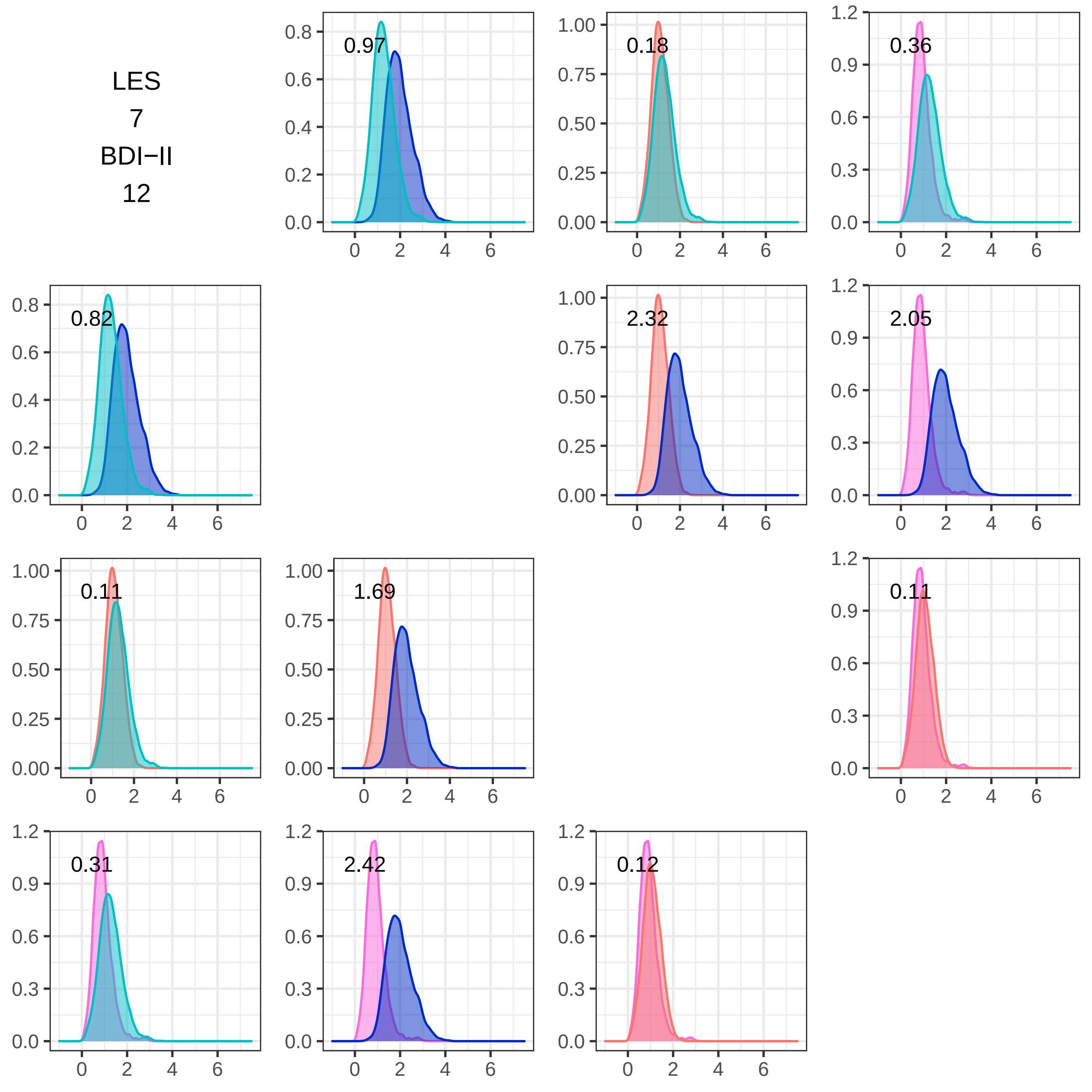
LES
7
BDI−II
12
0.97
0.18
0.36
0.82
2.32
2.05
0.11
1.69
0.11
0.31
2.42
0.12

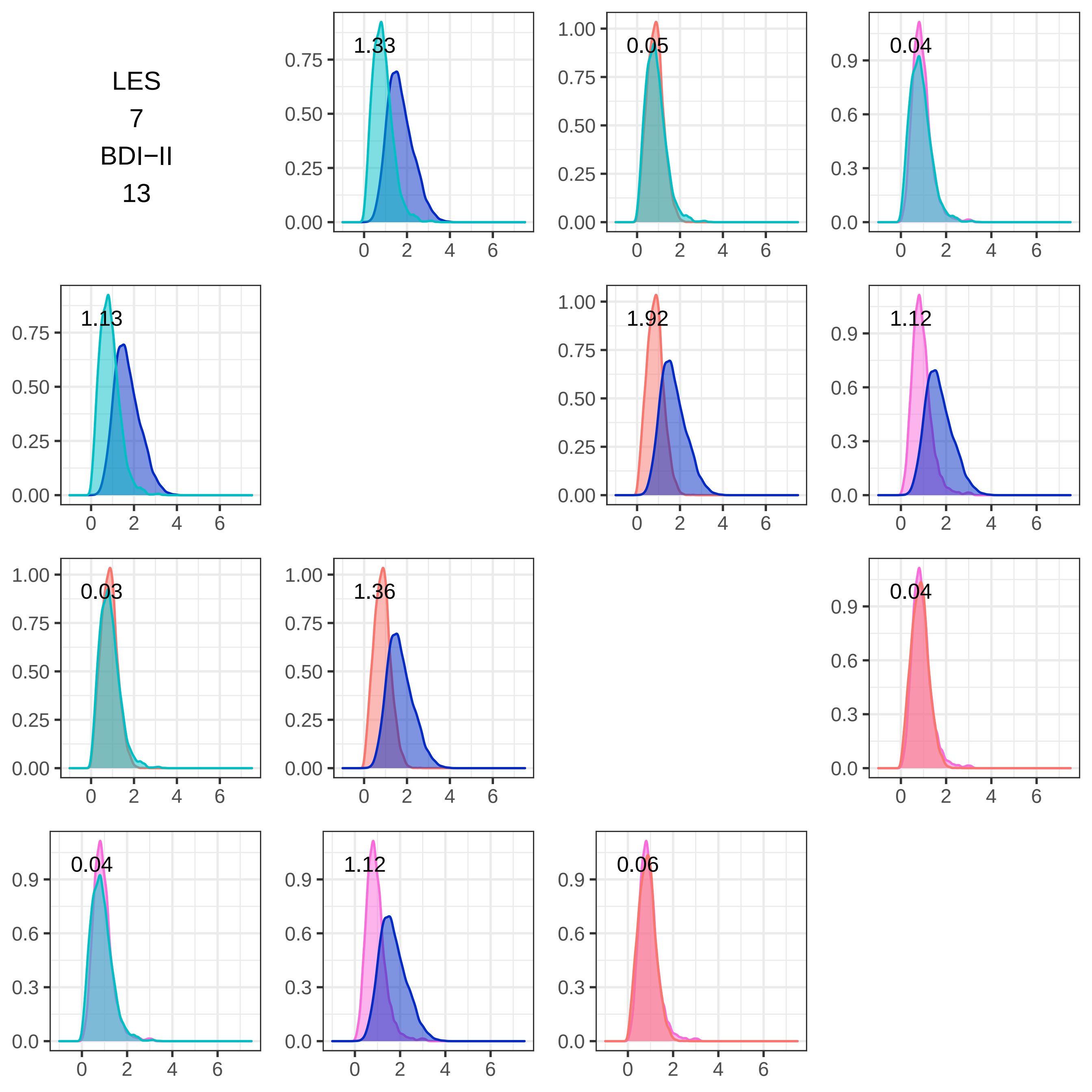
LES
7
BDI−II
13
1.33
0.05
0.04
1.13
1.92
1.12
0.03
1.36
0.04
0.04
1.12
0.06

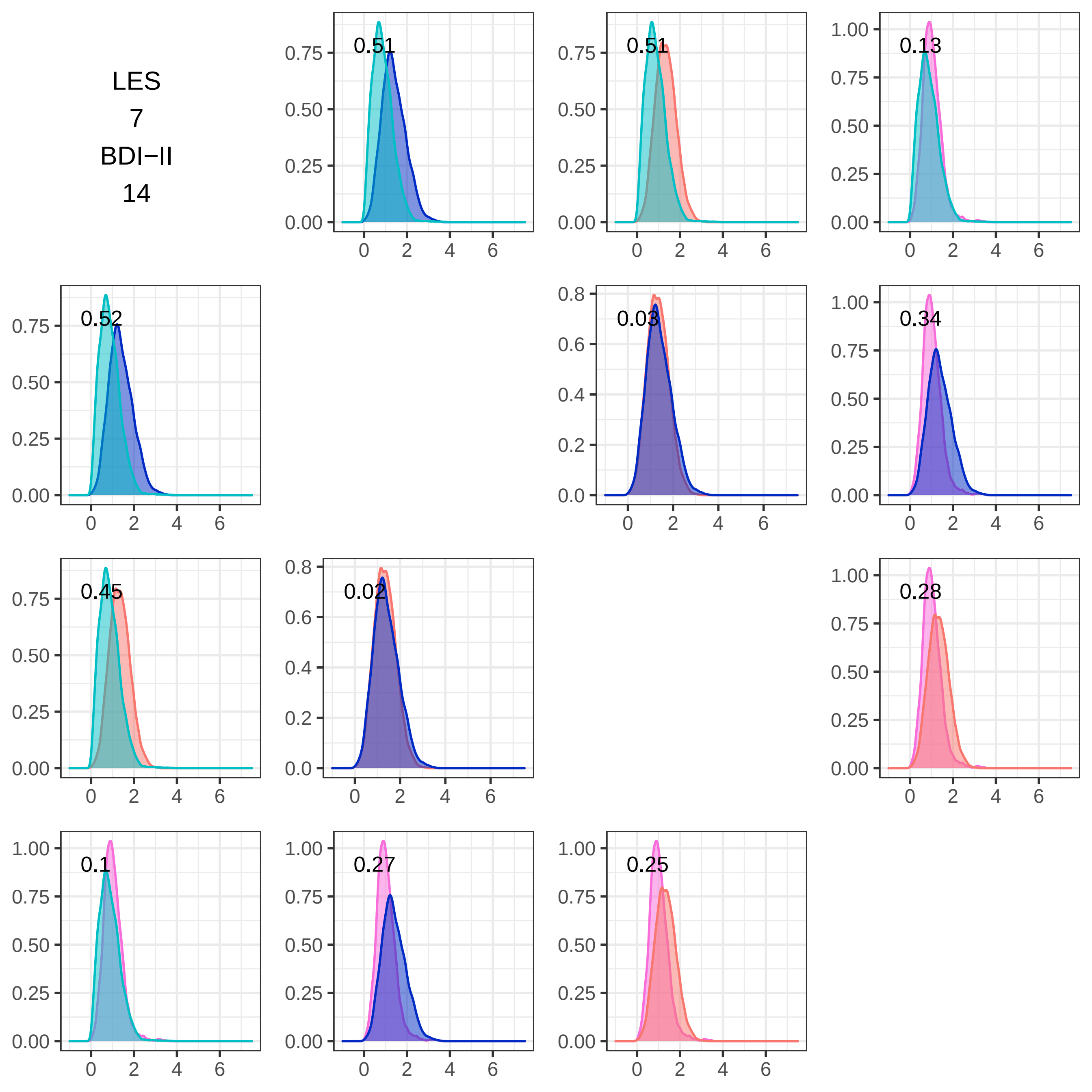
LES
7
BDI−II
14
0.51
0.51
0.13
0.52
0.03
0.34
0.45
0.02
0.28
0.1
0.27
0.25

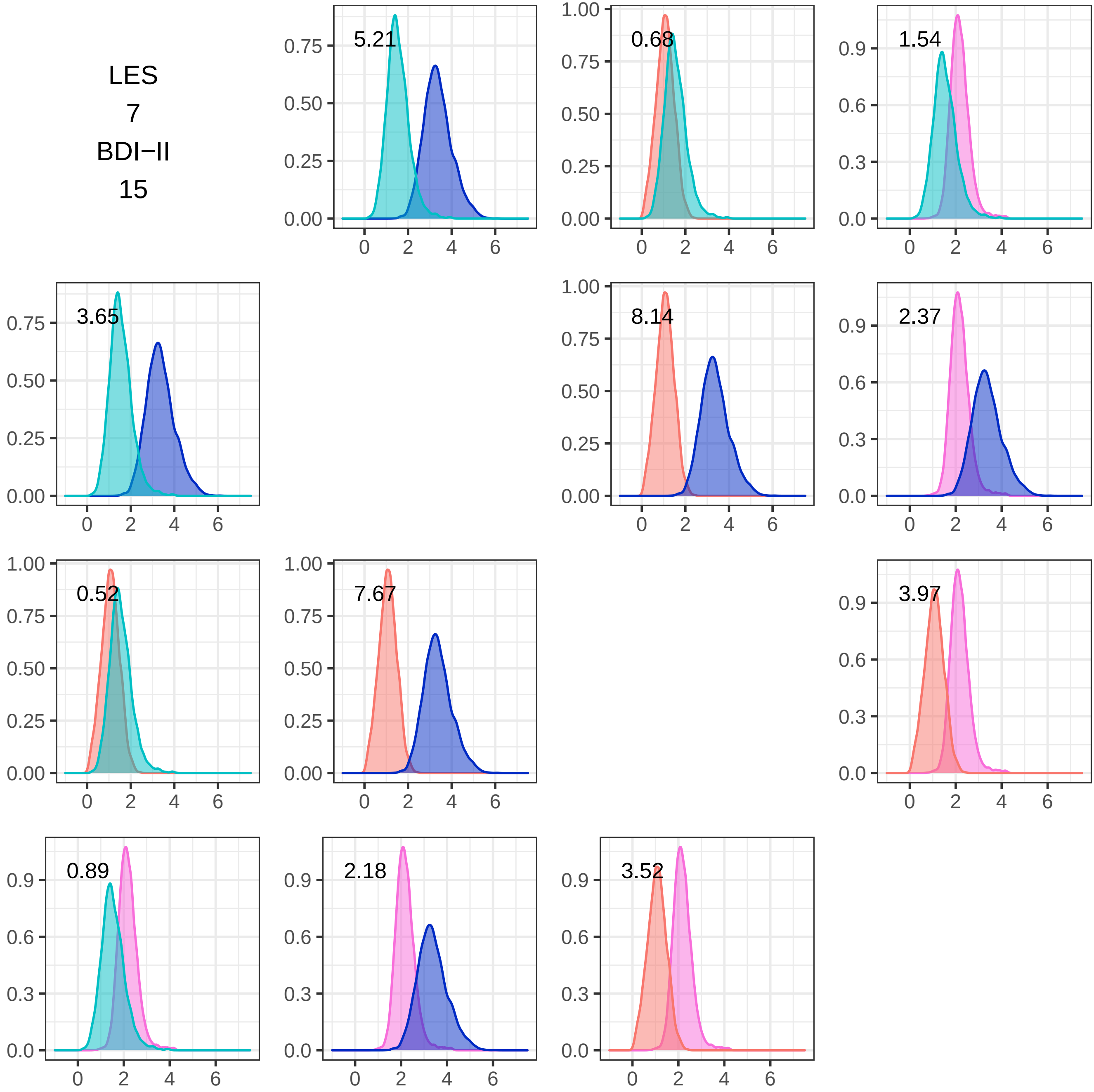
LES
7
BDI−II
15
5.21
0.68
1.54
3.65
8.14
2.37
0.52
7.67
3.97
0.89
2.18
3.52

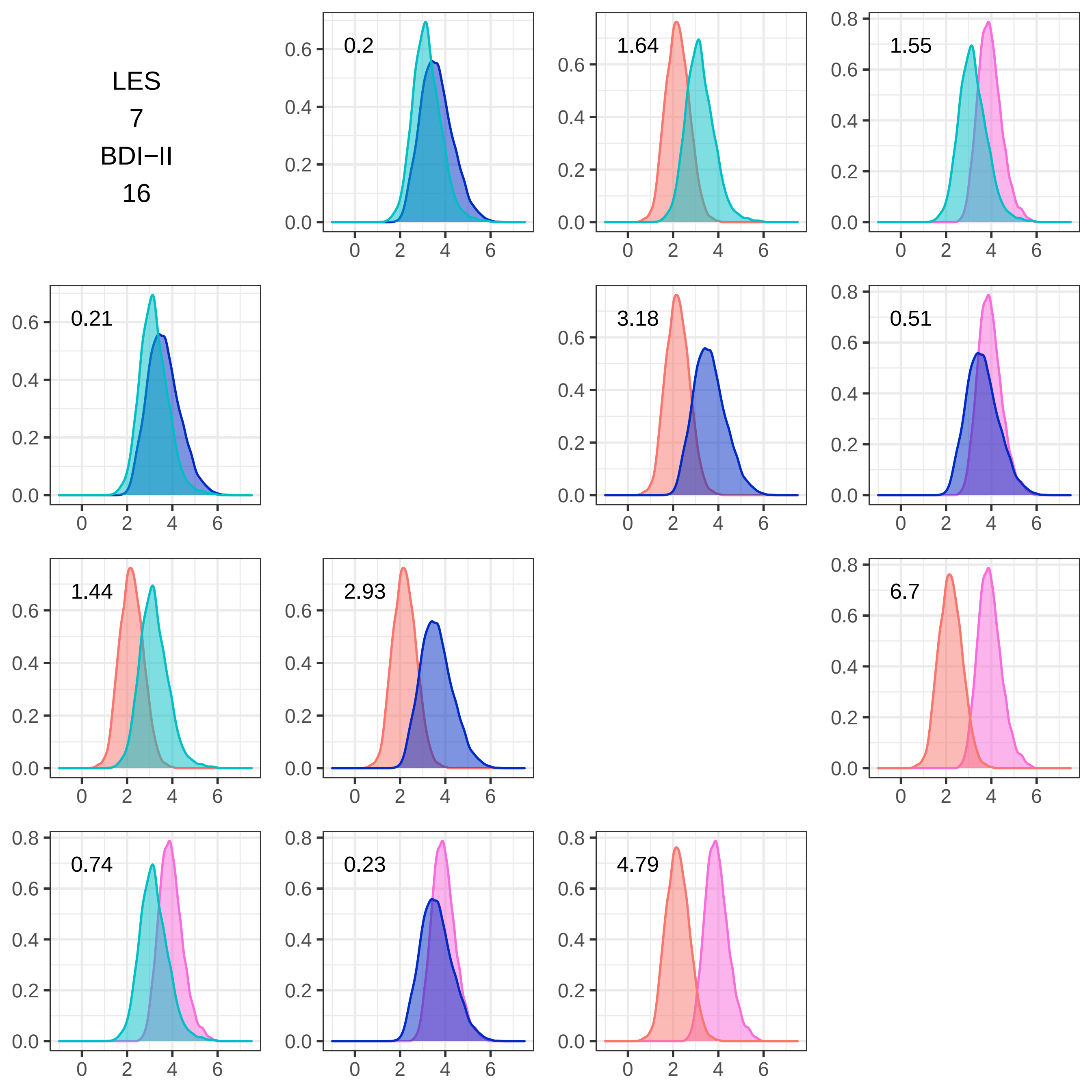
LES
7
BDI−II
16
0.2
1.64
1.55
0.21
3.18
0.51
1.44
2.93
6.7
0.74
0.23
4.79

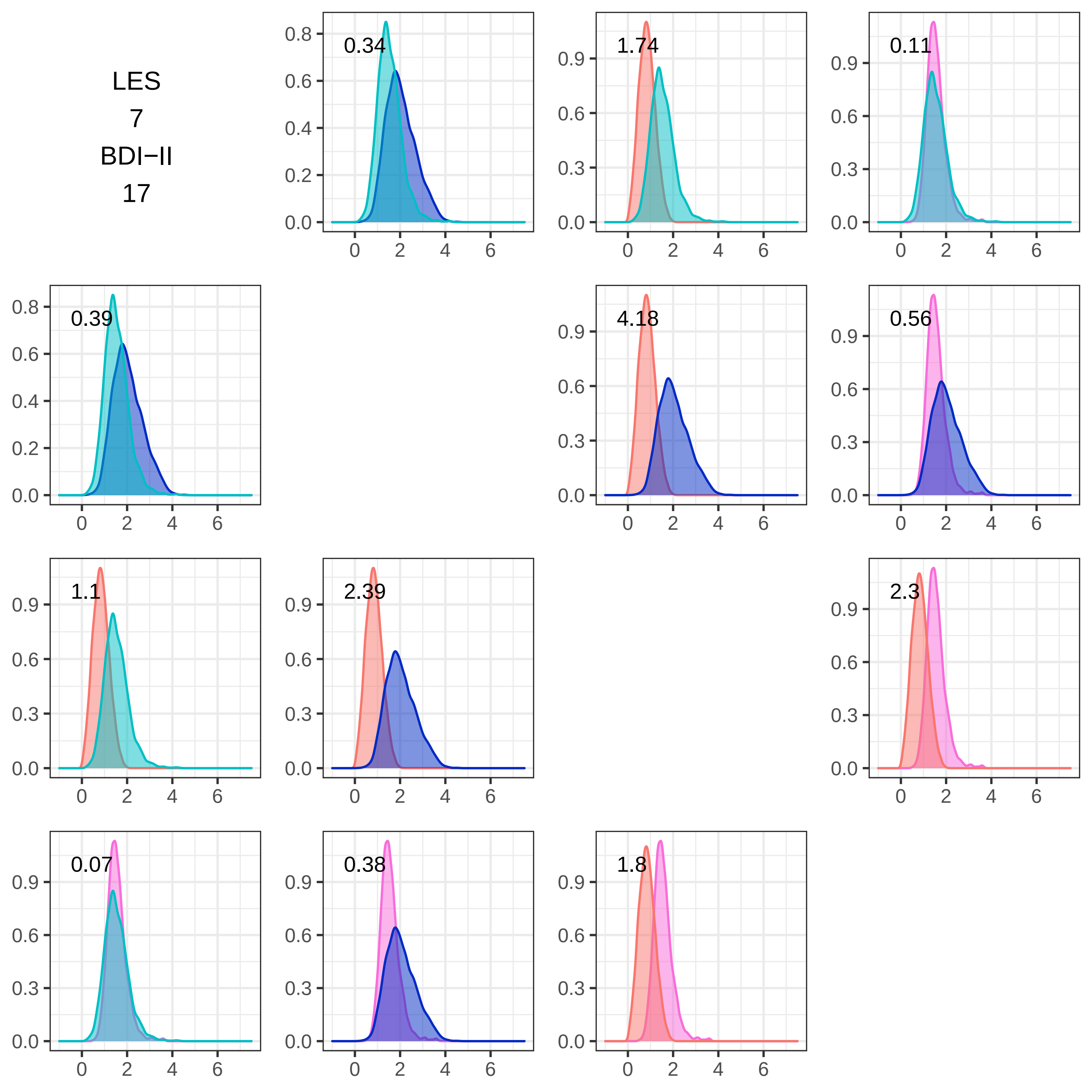
LES
7
BDI−II
17
0.34
1.74
0.11
0.39
4.18
0.56
1.1
2.39
2.3
0.07
0.38
1.8

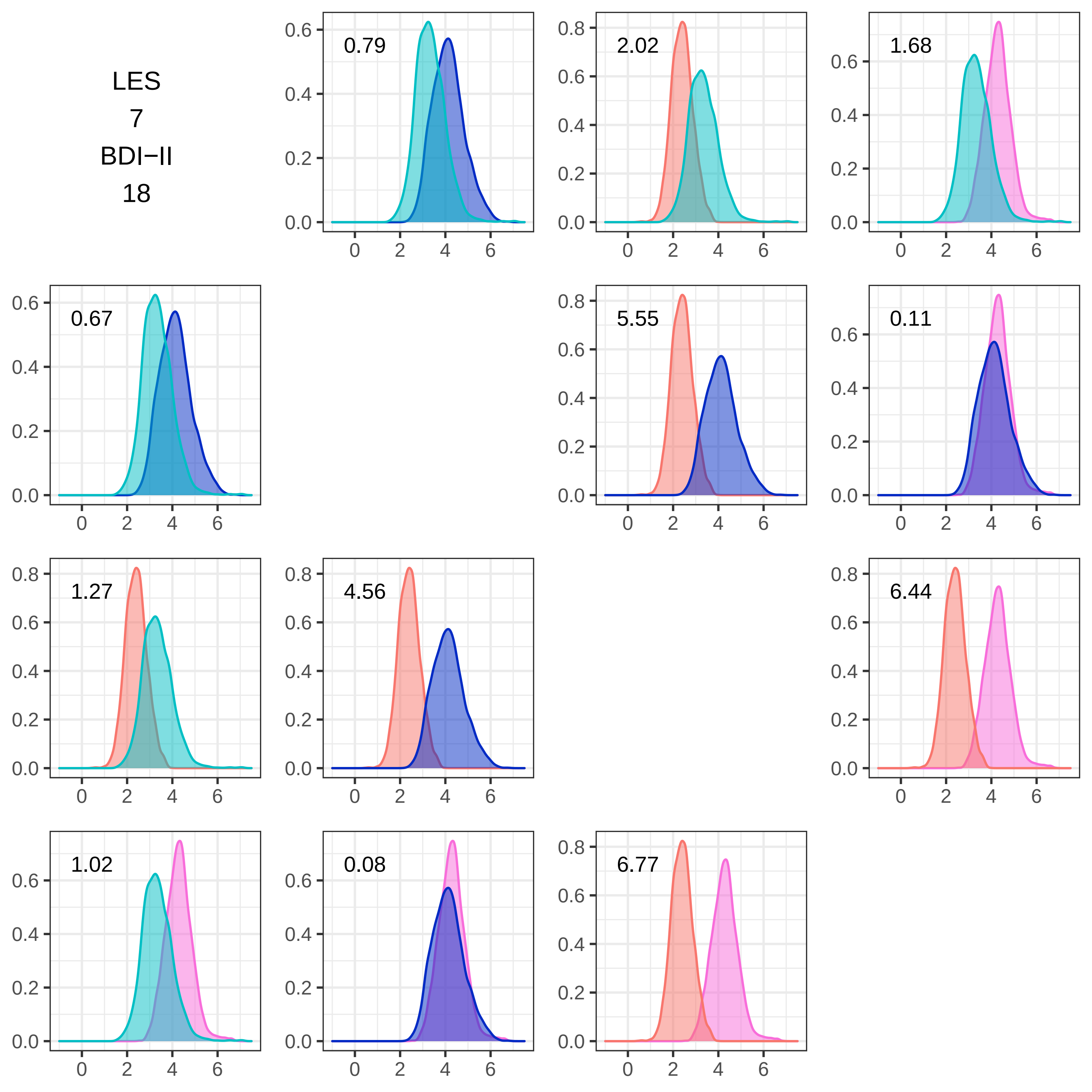

LES
7
BDI−II
18
0.79
2.02
1.68
0.67
5.55
0.11
1.27
4.56
6.44
1.02
0.08
6.77

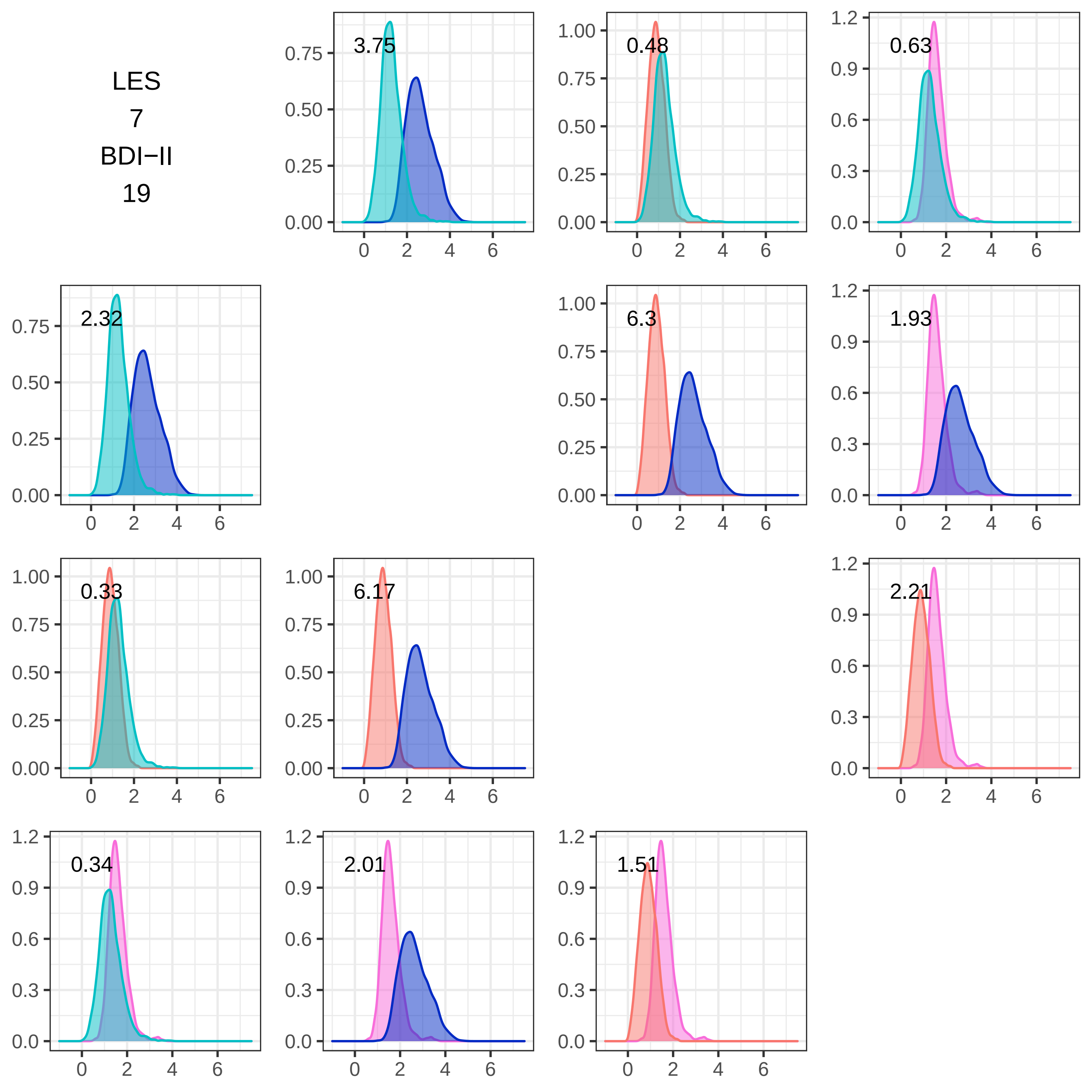

LES
7
BDI−II
19
3.75
0.48
0.63
2.32
6.3
1.93
0.33
6.17
2.21
0.34
2.01
1.51

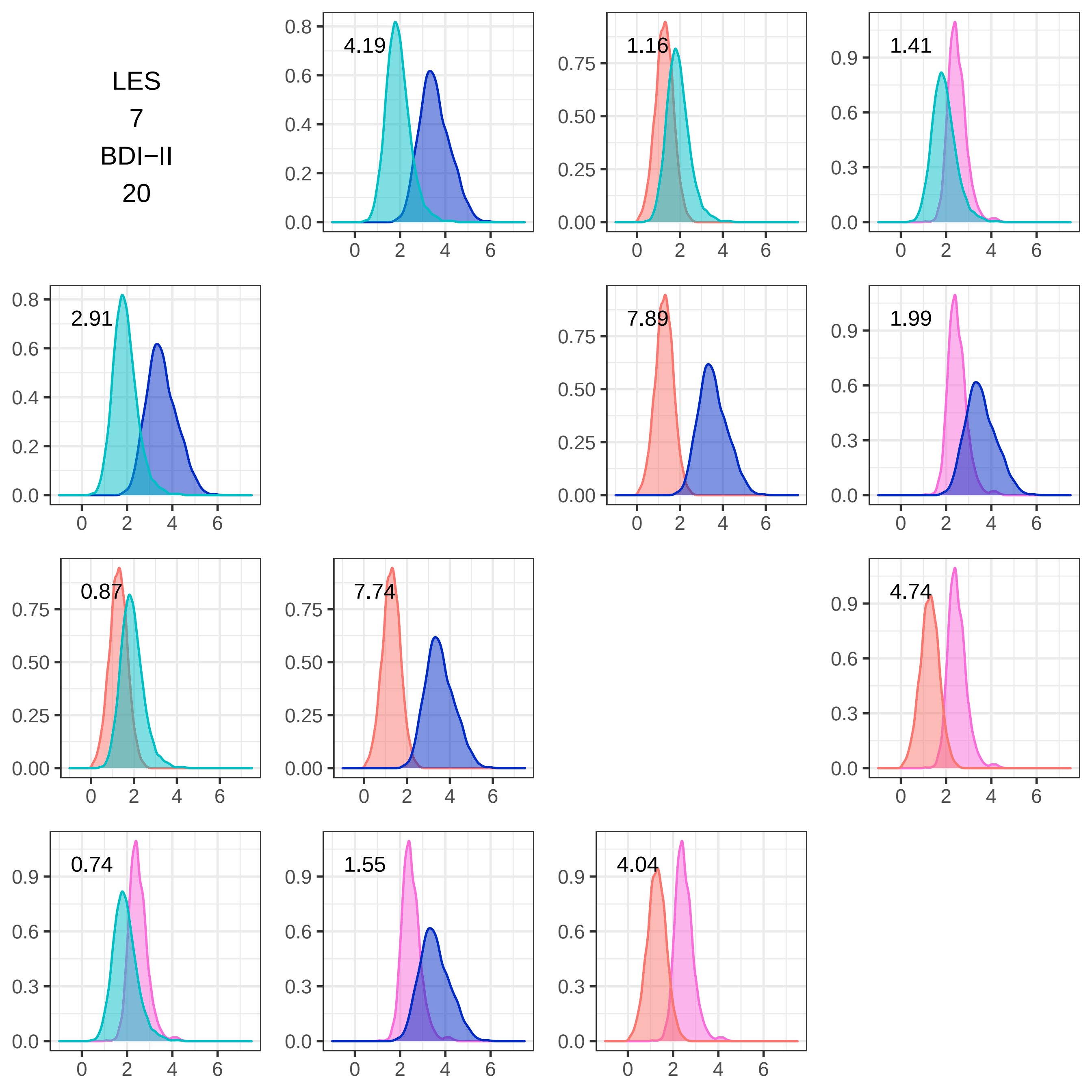

LES
7
BDI−II
20
4.19
1.16
1.41
2.91
7.89
1.99
0.87
7.74
4.74
0.74
1.55
4.04

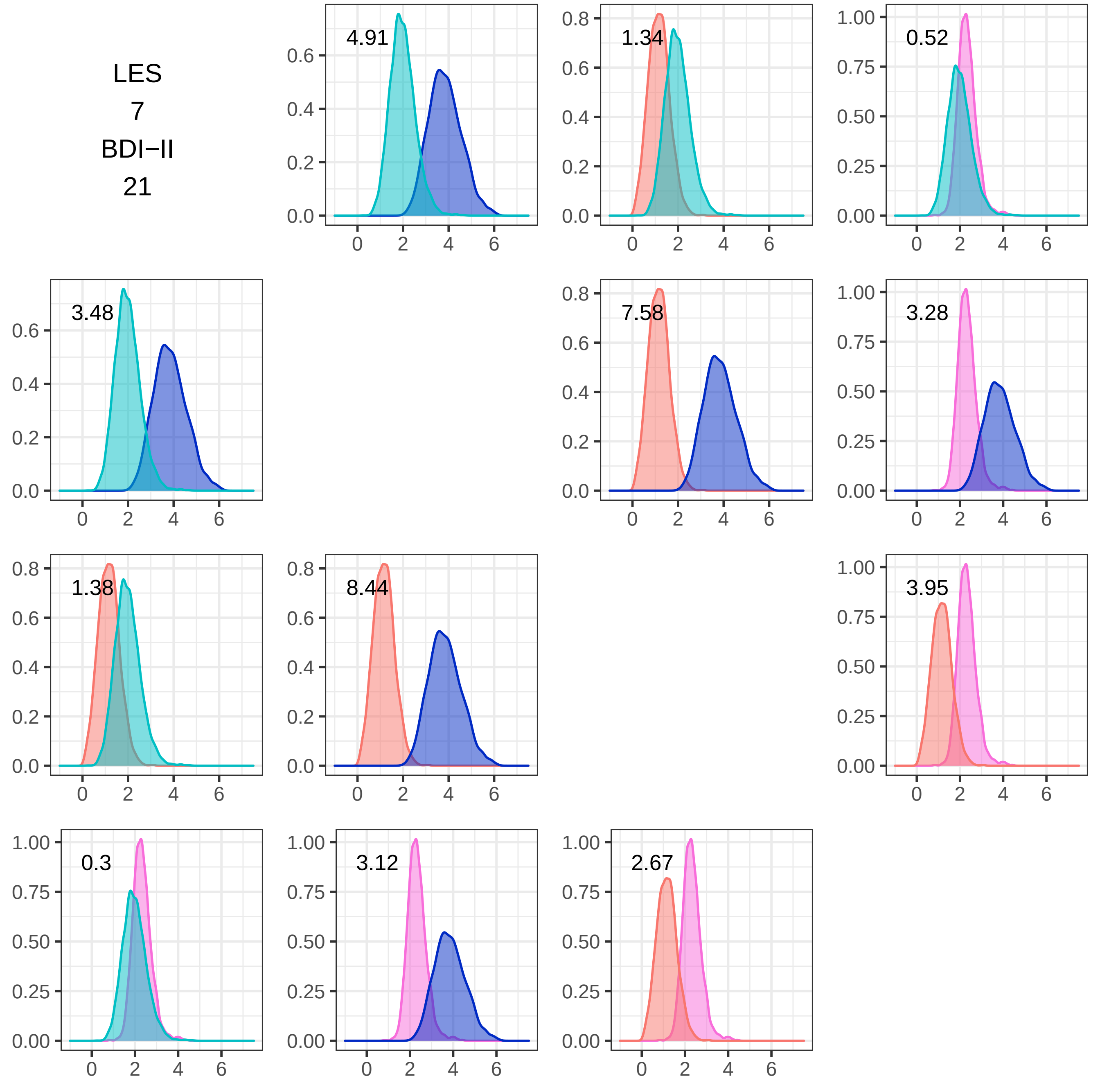

LES
7
BDI−II
21
4.91
1.34
0.52
3.48
7.58
3.28
1.38
8.44
3.95
0.3
3.12
2.67

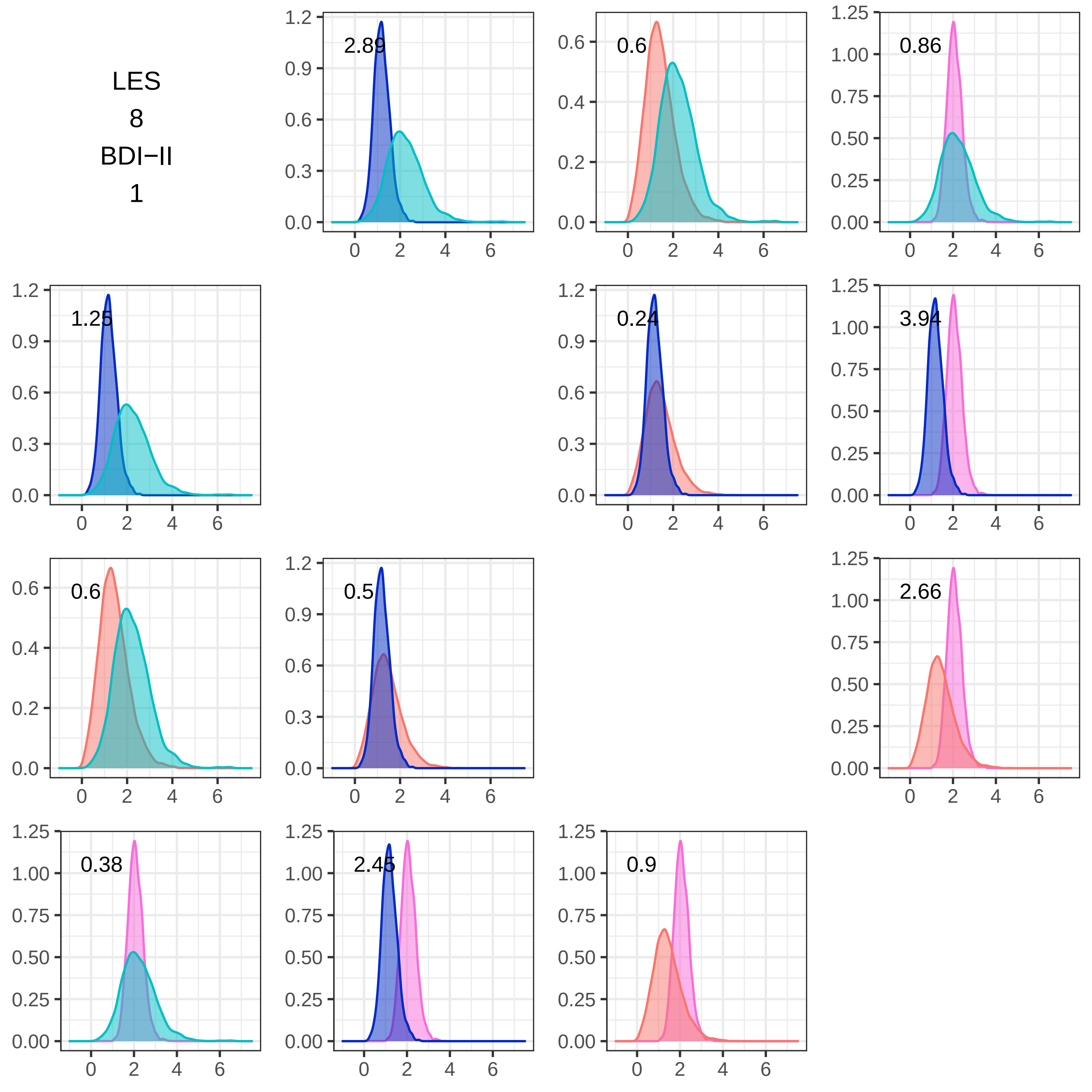
LES
8
BDI−II
1
2.89
0.6
0.86
1.25
0.24
3.94
0.6
0.5
2.66
0.38
2.45
0.9

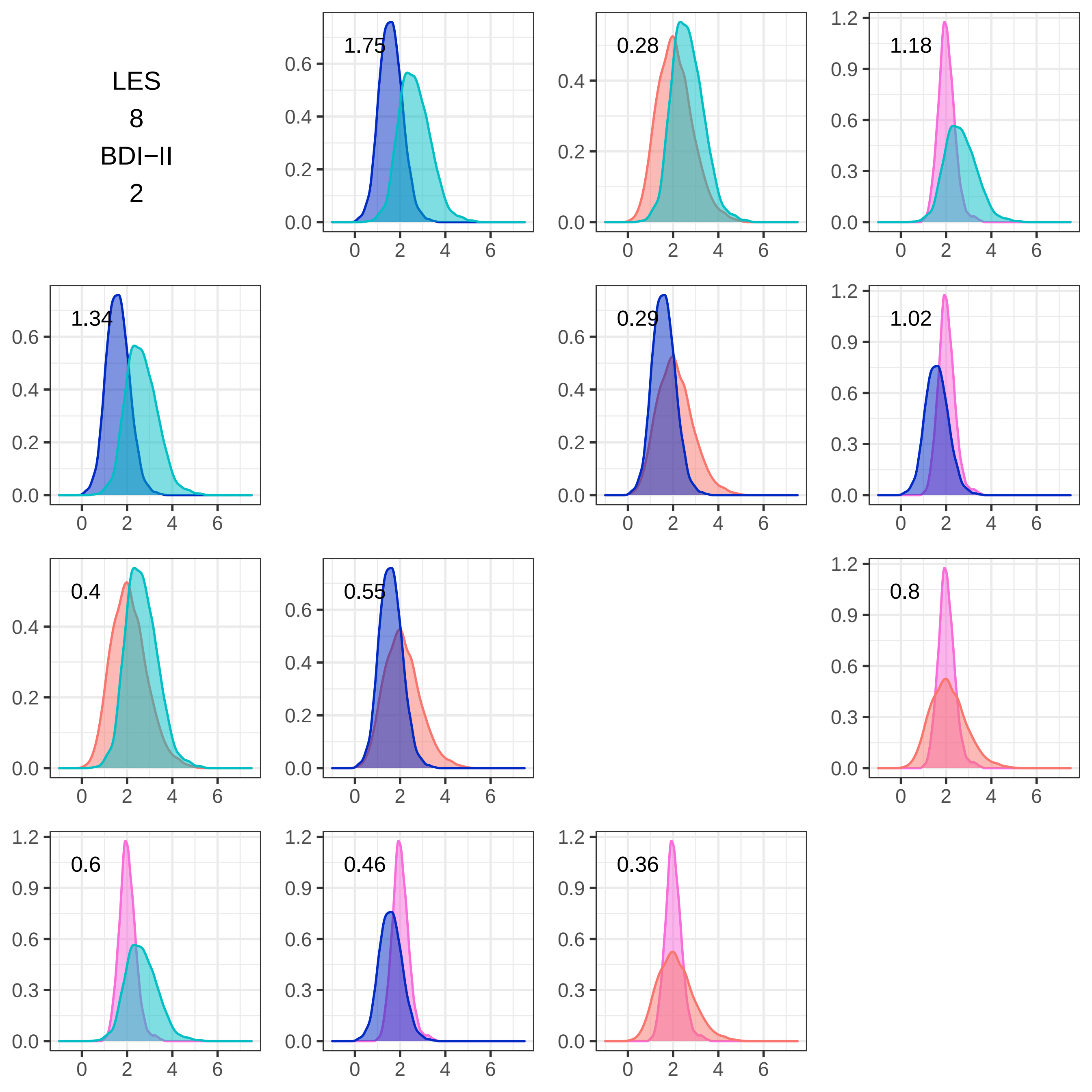

LES
8
BDI−II
2
1.75
0.28
1.18
1.34
0.29
1.02
0.4
0.55
0.8
0.6
0.46
0.36

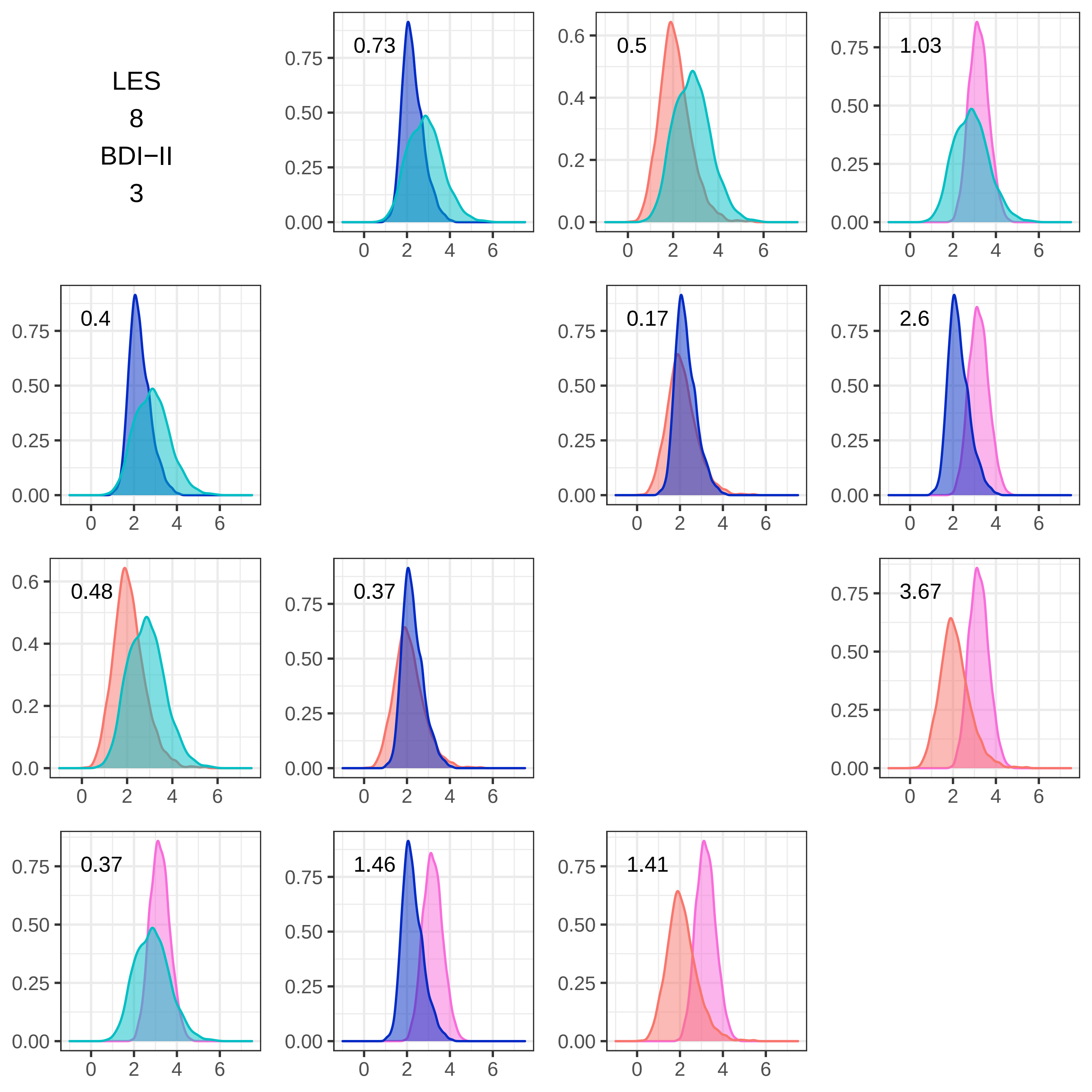

LES
8
BDI−II
3
0.73
0.5
1.03
0.4
0.17
2.6
0.48
0.37
3.67
0.37
1.46
1.41

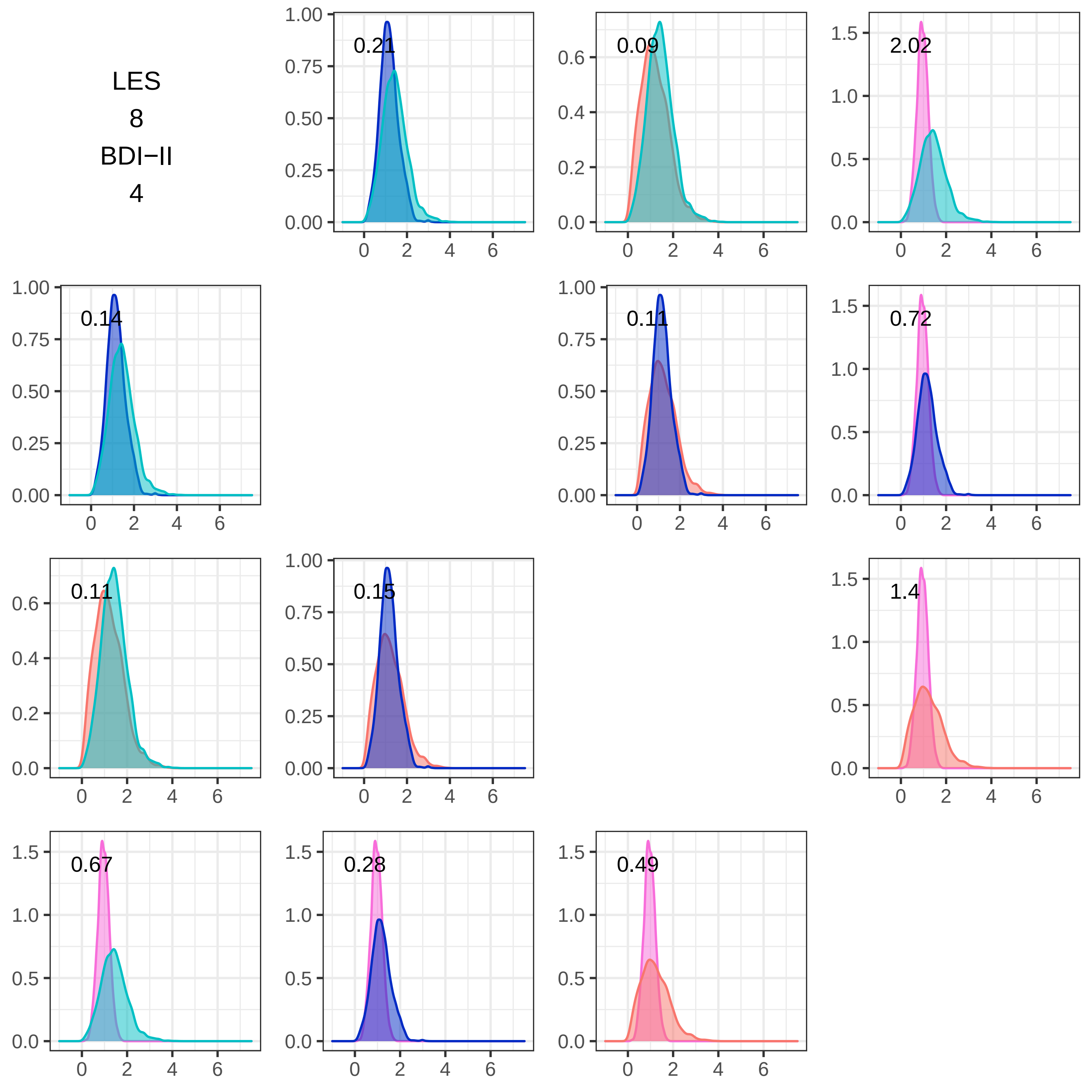

LES
8
BDI−II
4
0.21
0.09
2.02
0.14
0.11
0.72
0.11
0.15
1.4
0.67
0.28
0.49

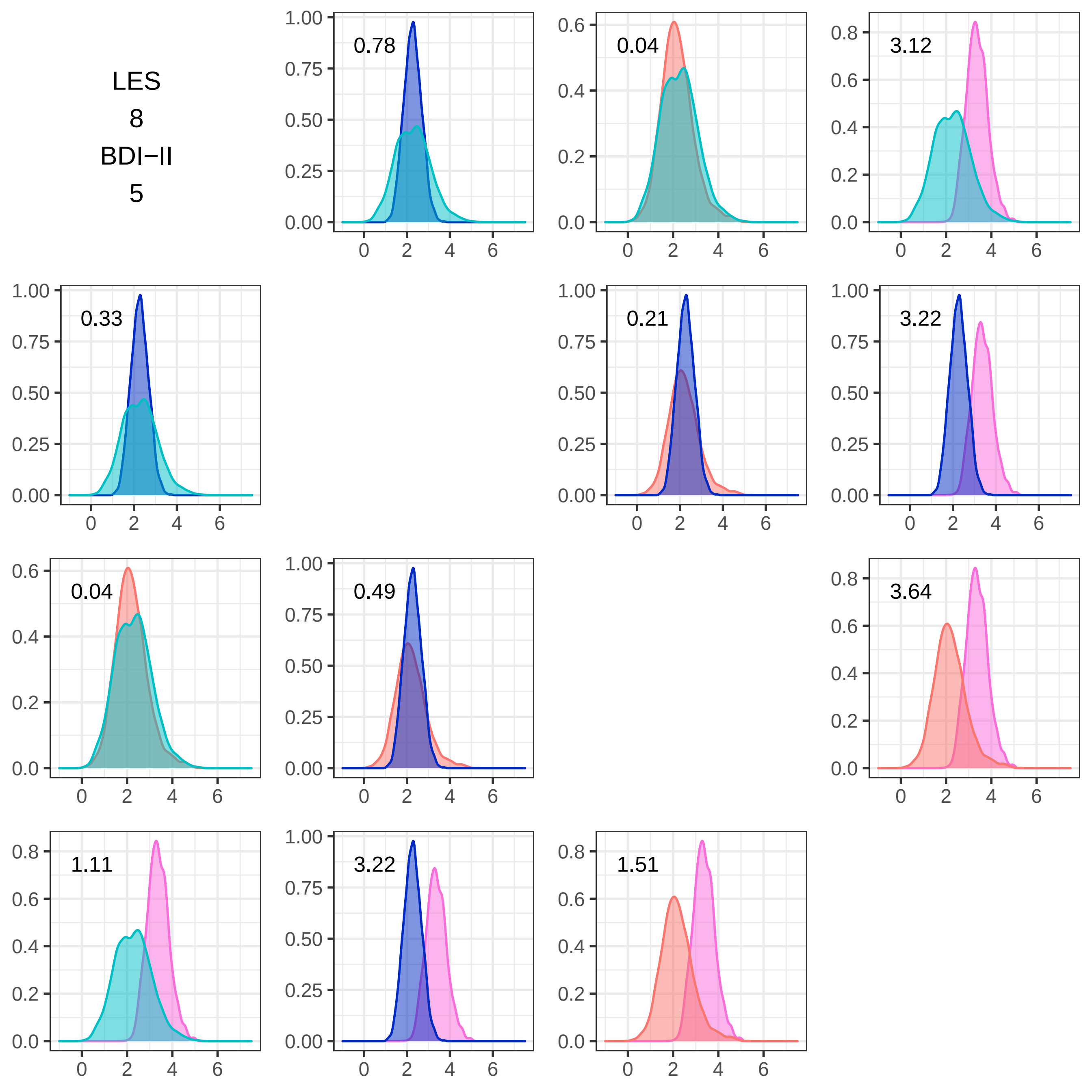
LES
8
BDI−II
5
0.78
0.04
3.12
0.33
0.21
3.22
0.04
0.49
3.64
1.11
3.22
1.51

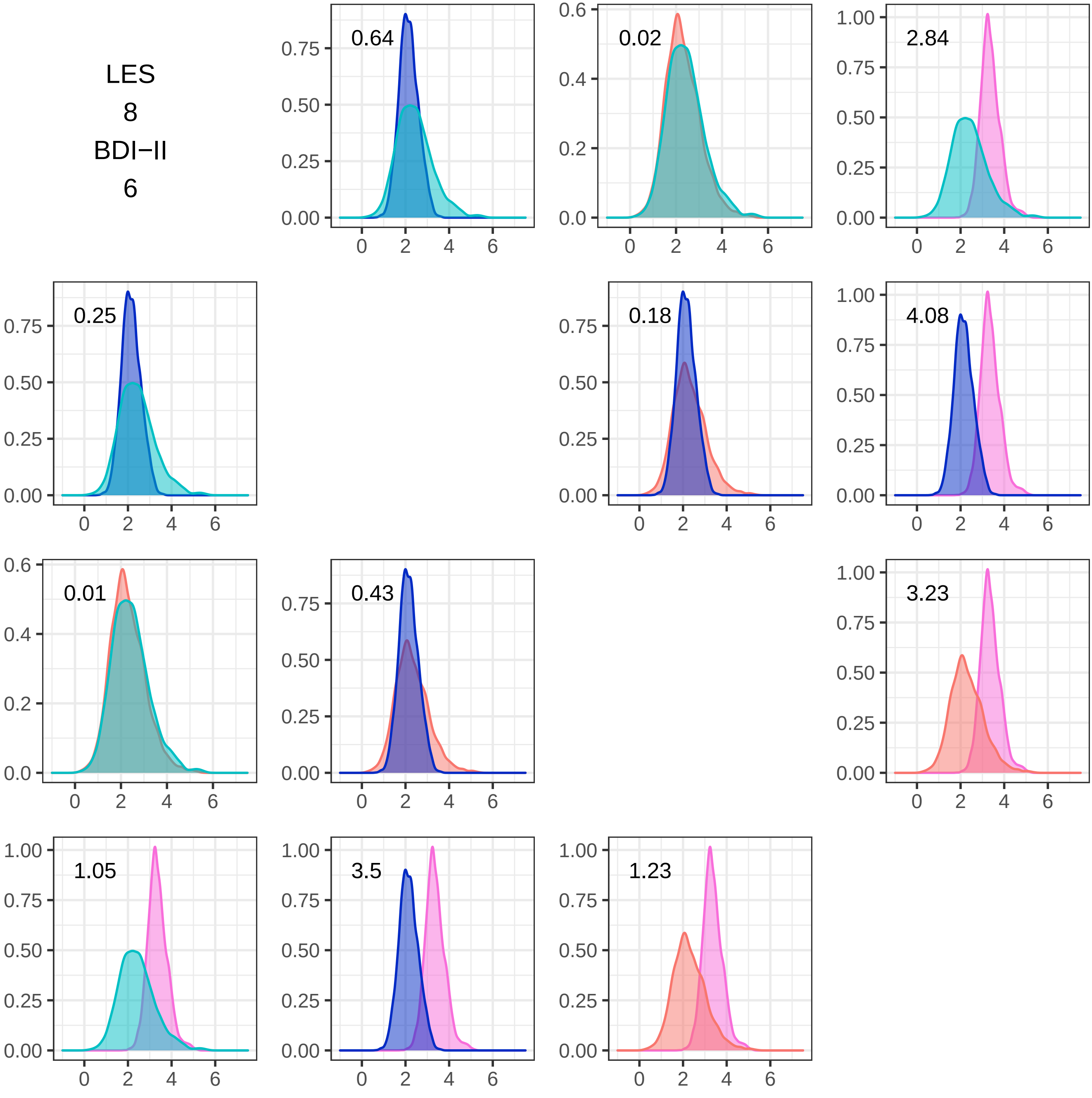

LES
8
BDI−II
6
0.64
0.02
2.84
0.25
0.18
4.08
0.01
0.43
3.23
1.05
3.5
1.23

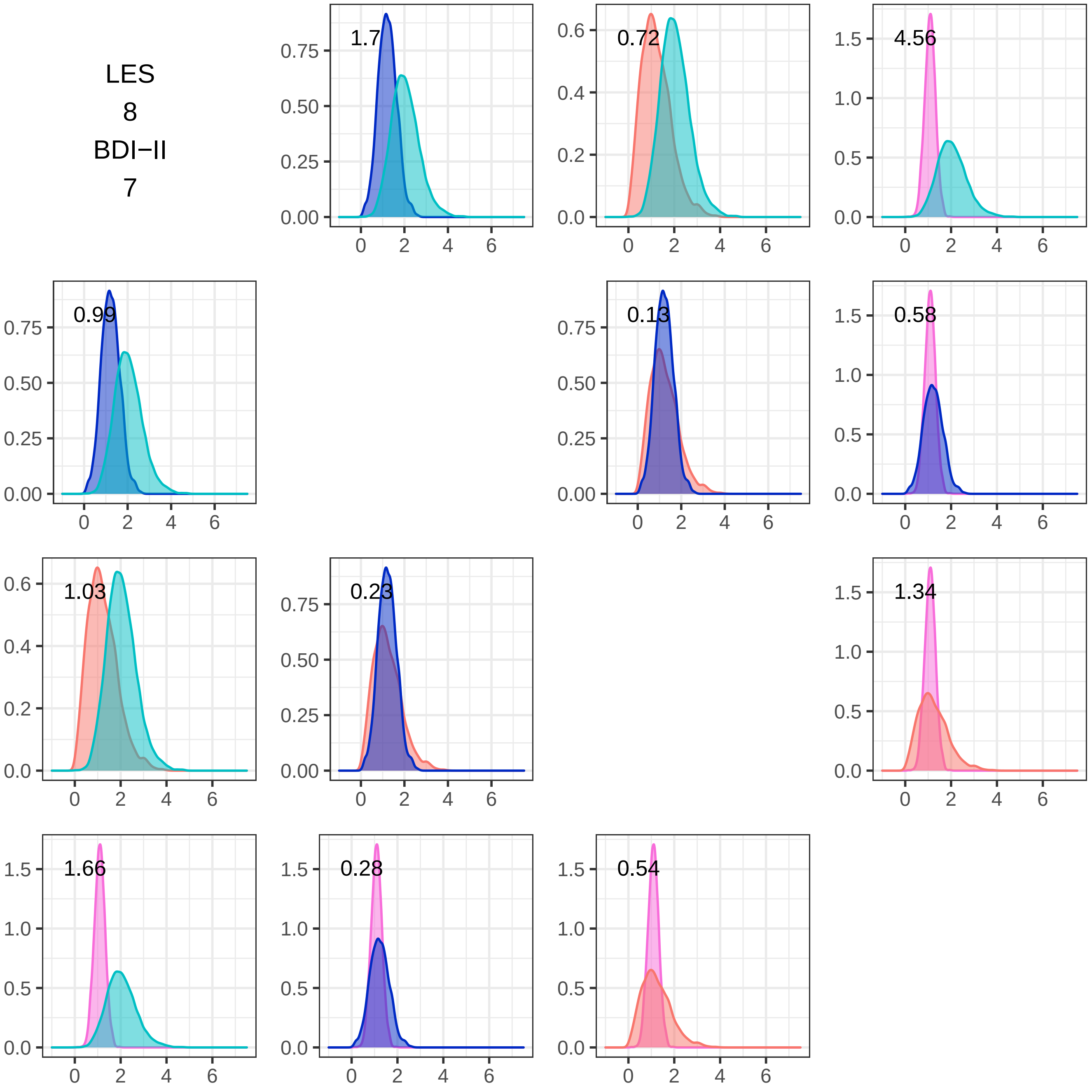

LES
8
BDI−II
7
1.7
0.72
4.56
0.99
0.13
0.58
1.03
0.23
1.34
1.66
0.28
0.54

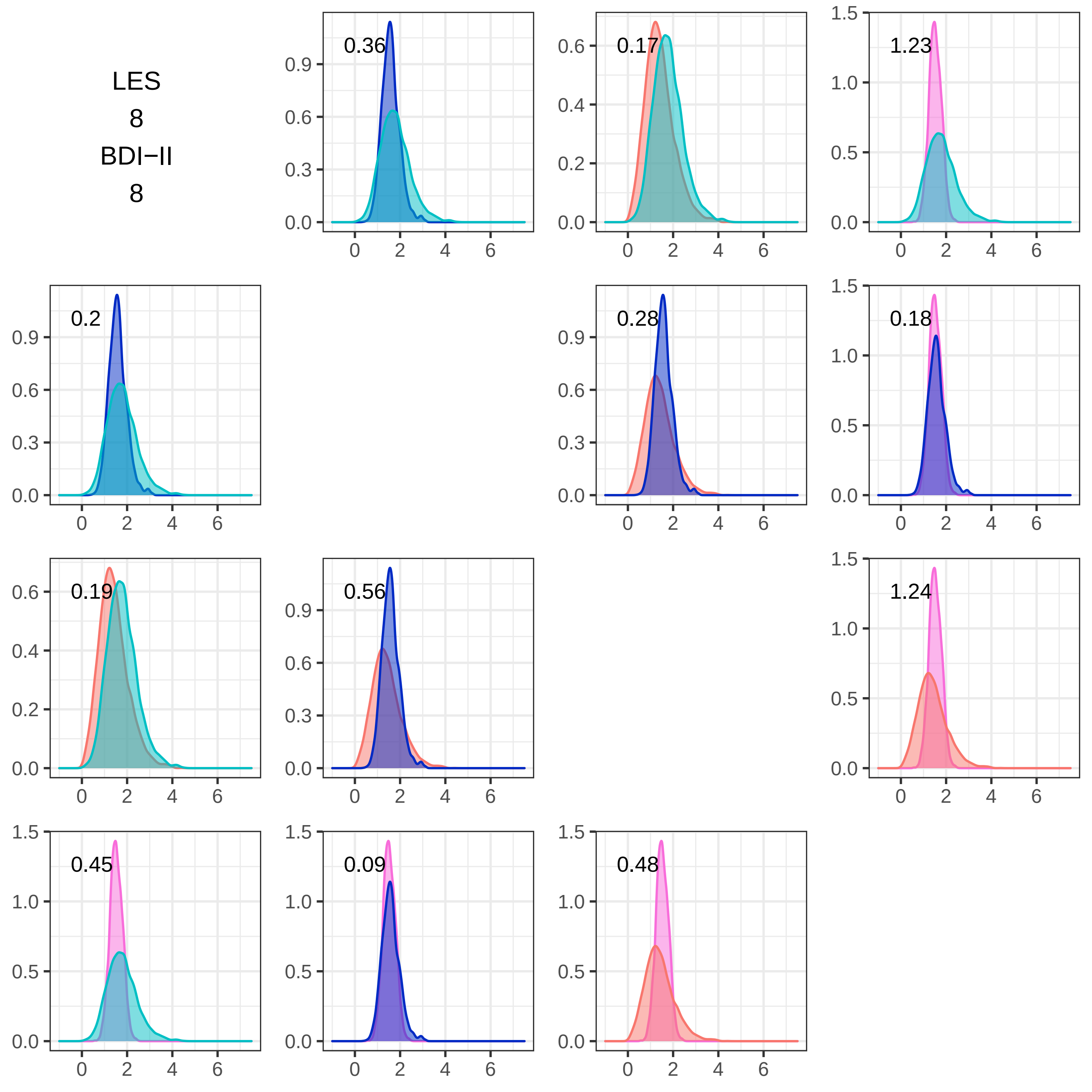

LES
8
BDI−II
8
0.36
0.17
1.23
0.2
0.28
0.18
0.19
0.56
1.24
0.45
0.09
0.48

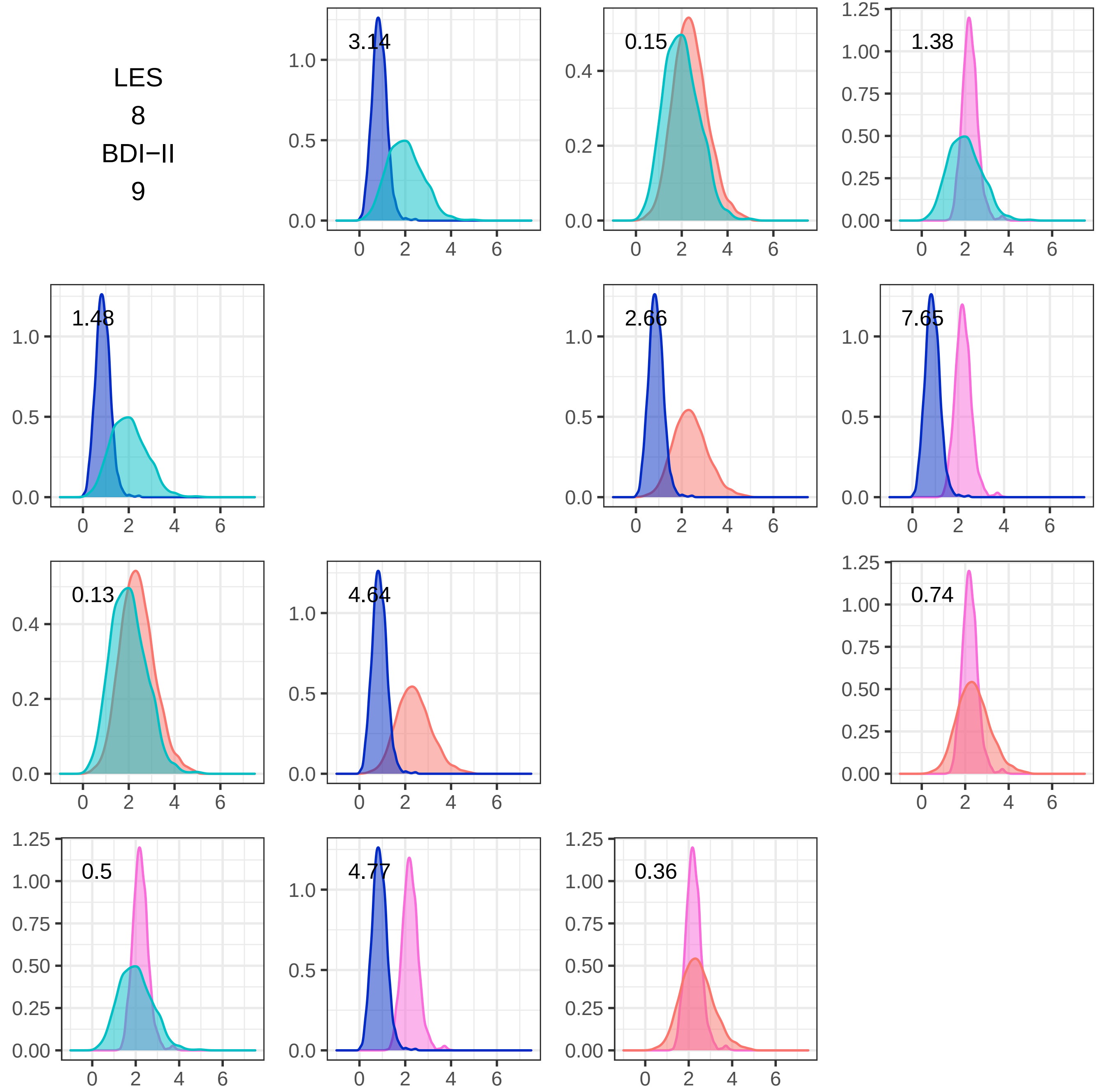

LES
8
BDI−II
9
3.14
0.15
1.38
1.48
2.66
7.65
0.13
4.64
0.74
0.5
4.77
0.36

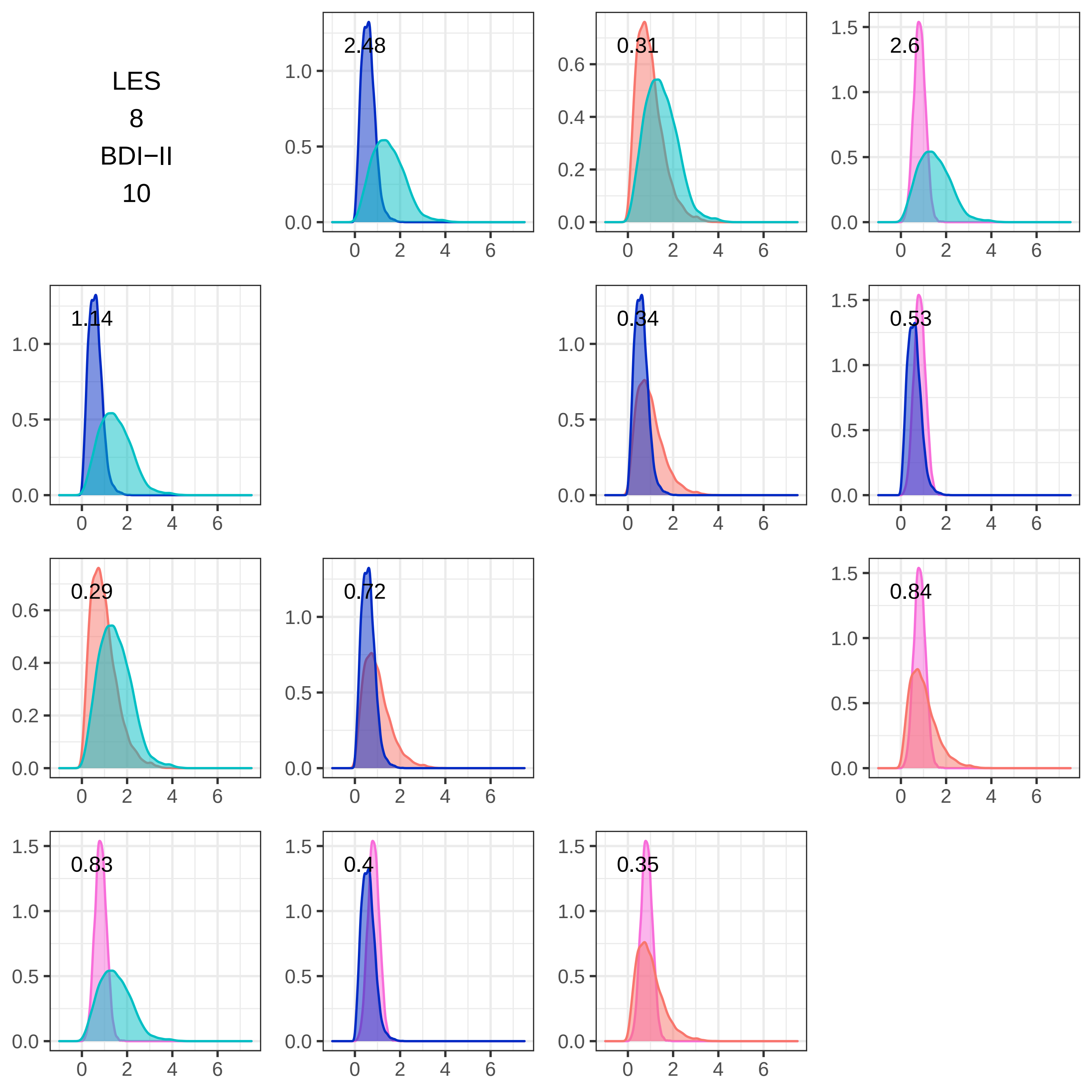

LES
8
BDI−II
10
2.48
0.31
2.6
1.14
0.34
0.53
0.29
0.72
0.84
0.83
0.4
0.35

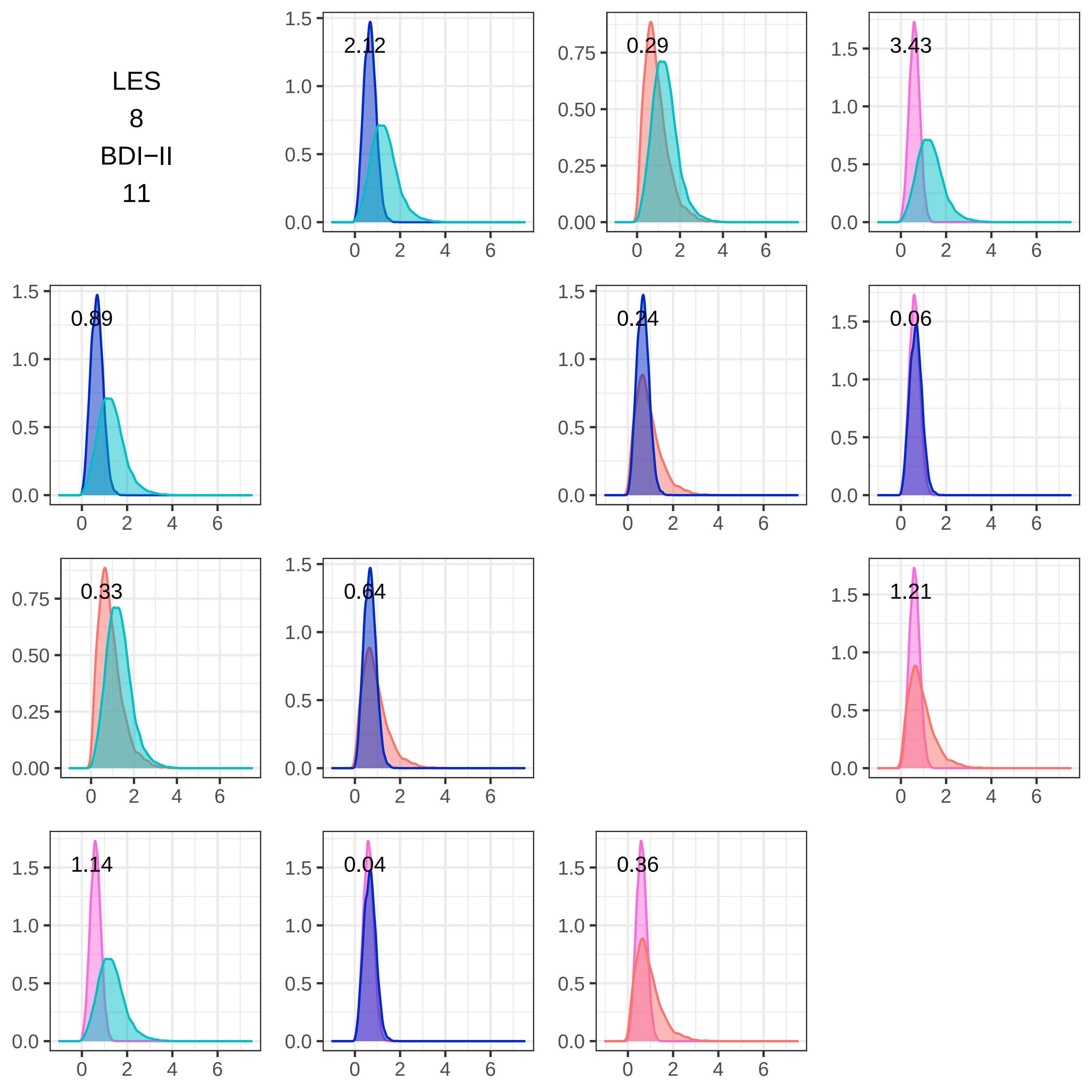
LES
8
BDI−II
11
2.12
0.29
3.43
0.89
0.24
0.06
0.33
0.64
1.21
1.14
0.04
0.36

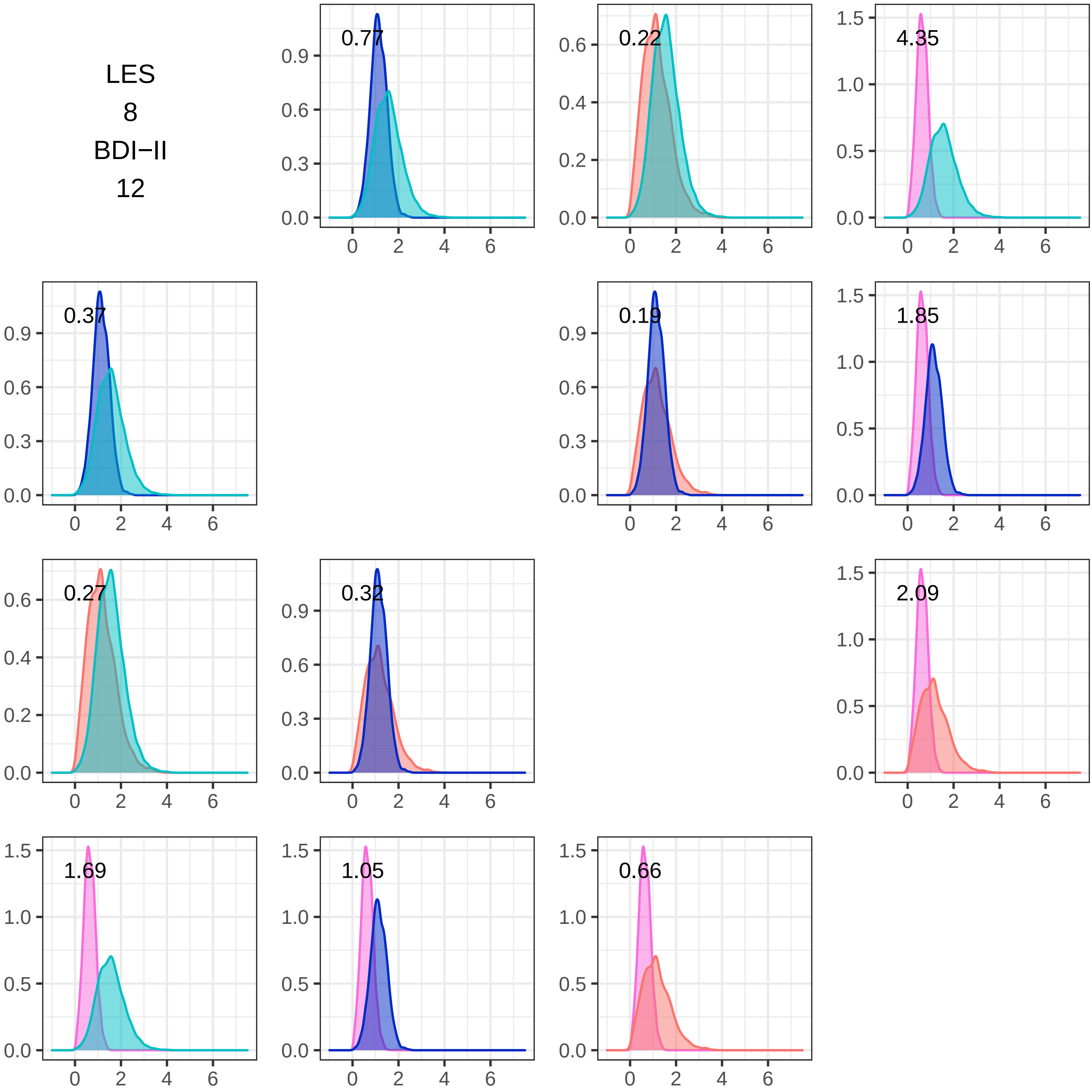
LES
8
BDI−II
12
0.77
0.22
4.35
0.37
0.19
1.85
0.27
0.32
2.09
1.69
1.05
0.66

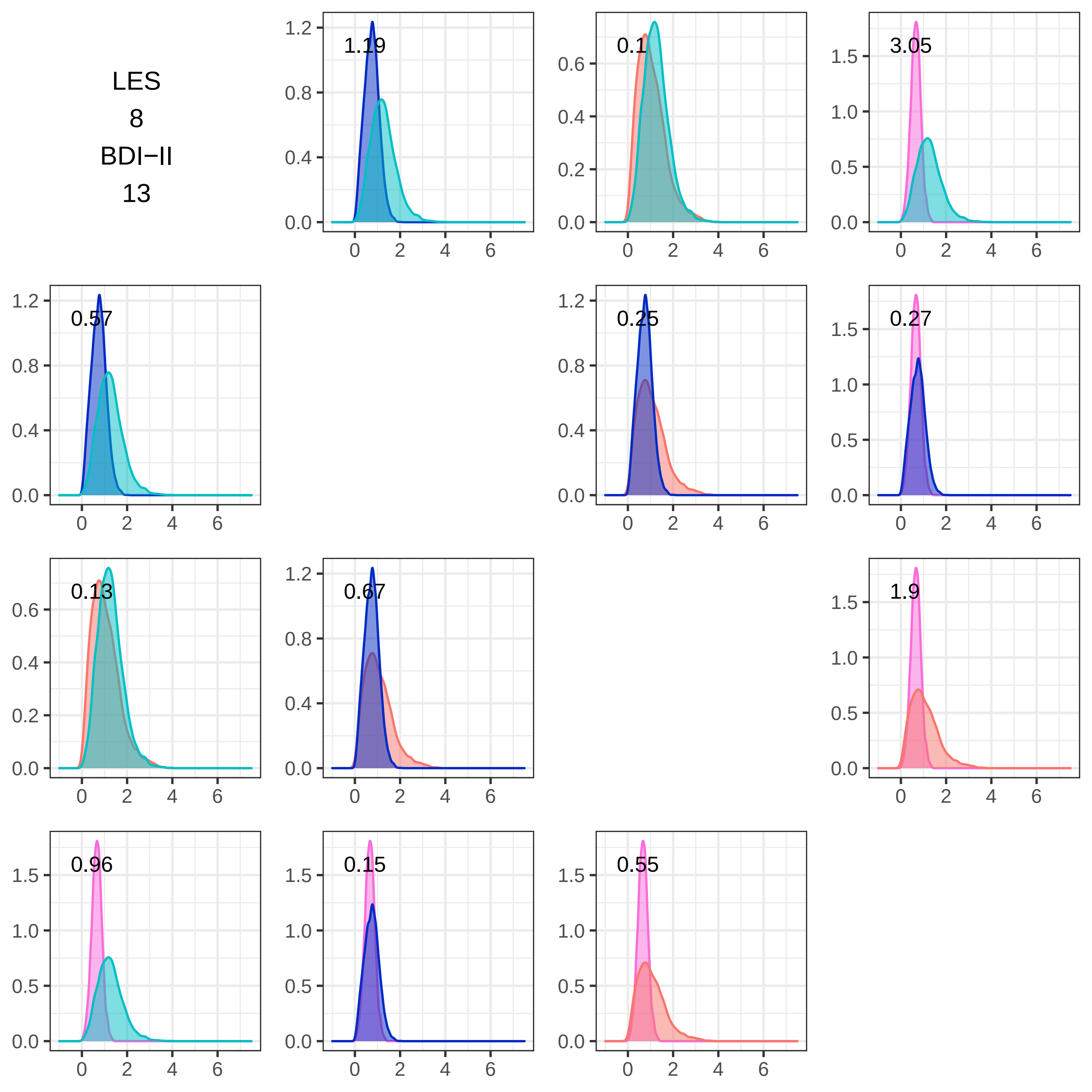

LES
8
BDI−II
13
1.19
0.1
3.05
0.57
0.25
0.27
0.13
0.67
1.9
0.96
0.15
0.55

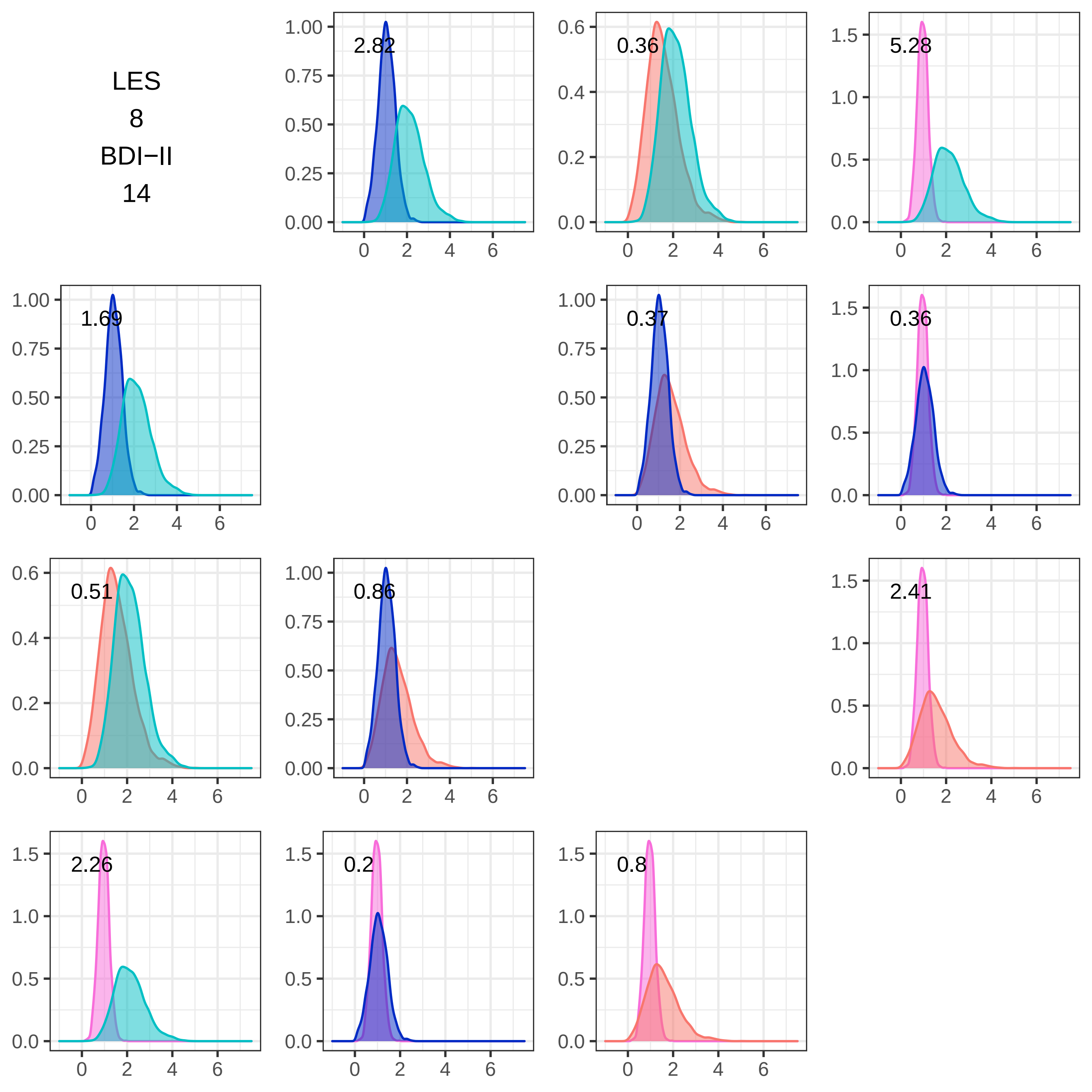
LES
8
BDI−II
14
2.82
0.36
5.28
1.69
0.37
0.36
0.51
0.86
2.41
2.26
0.2
0.8

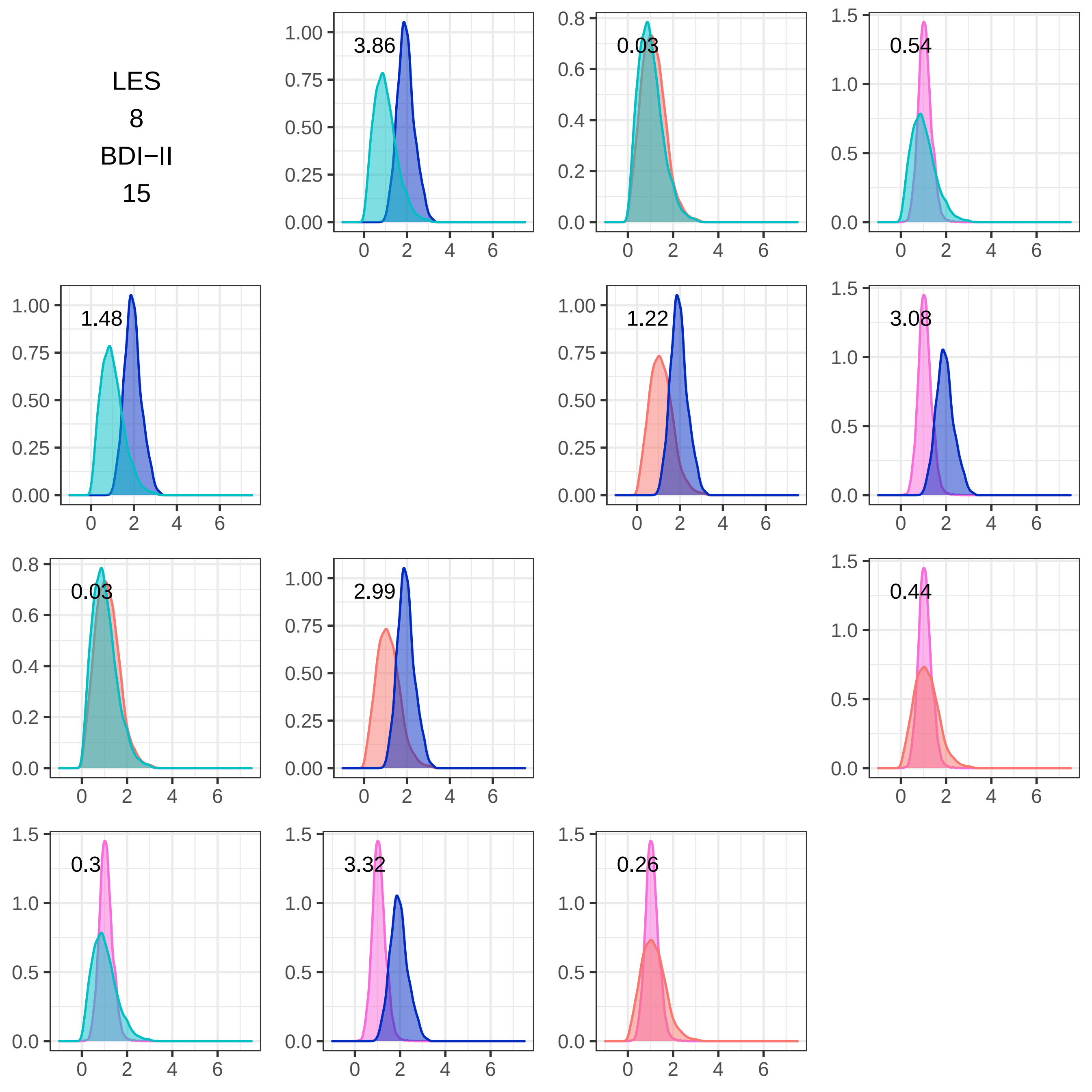
LES
8
BDI−II
15
3.86
0.03
0.54
1.48
1.22
3.08
0.03
2.99
0.44
0.3
3.32
0.26

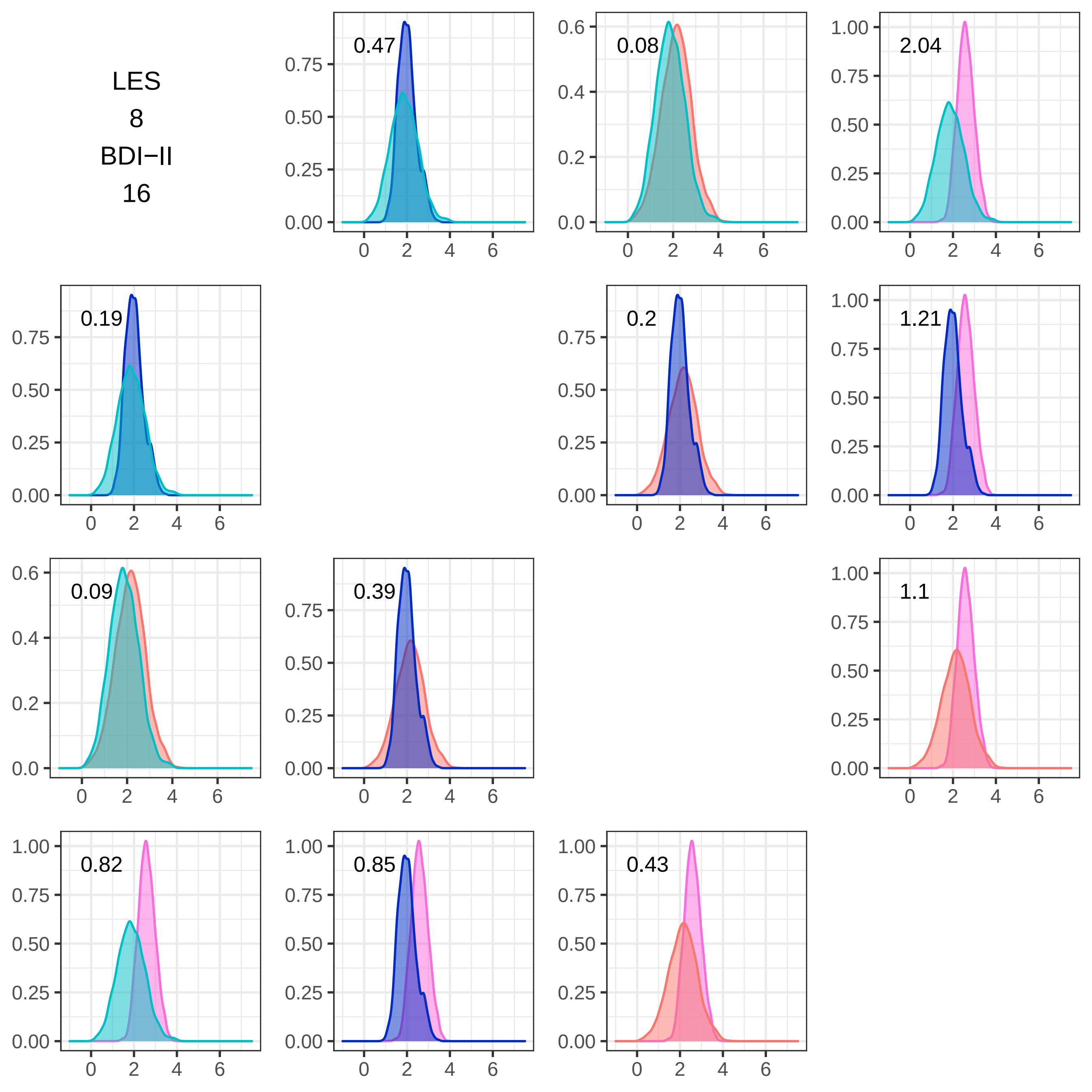
LES
8
BDI−II
16
0.47
0.08
2.04
0.19
0.2
1.21
0.09
0.39
1.1
0.82
0.85
0.43

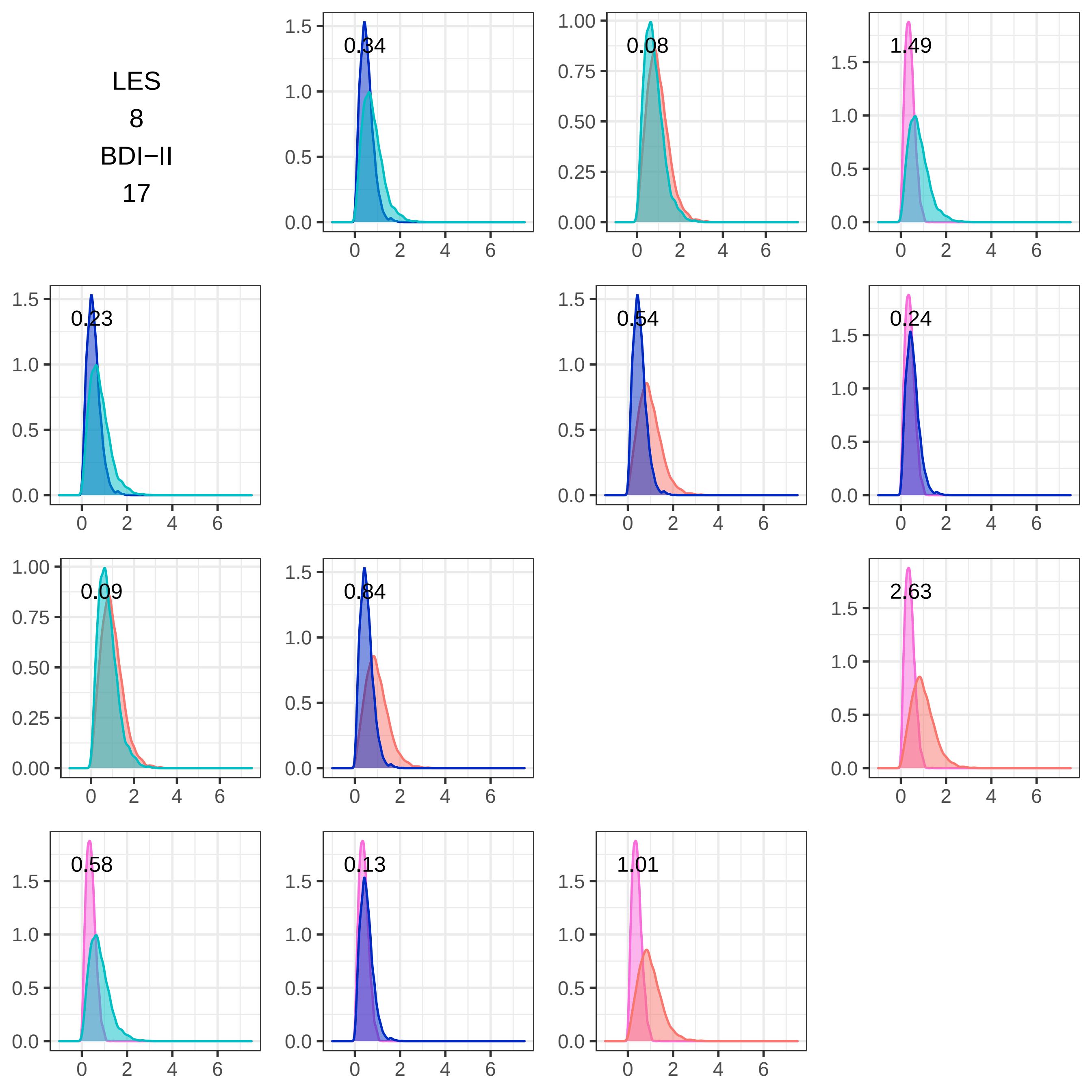

LES
8
BDI−II
17
0.34
0.08
1.49
0.23
0.54
0.24
0.09
0.84
2.63
0.58
0.13
1.01

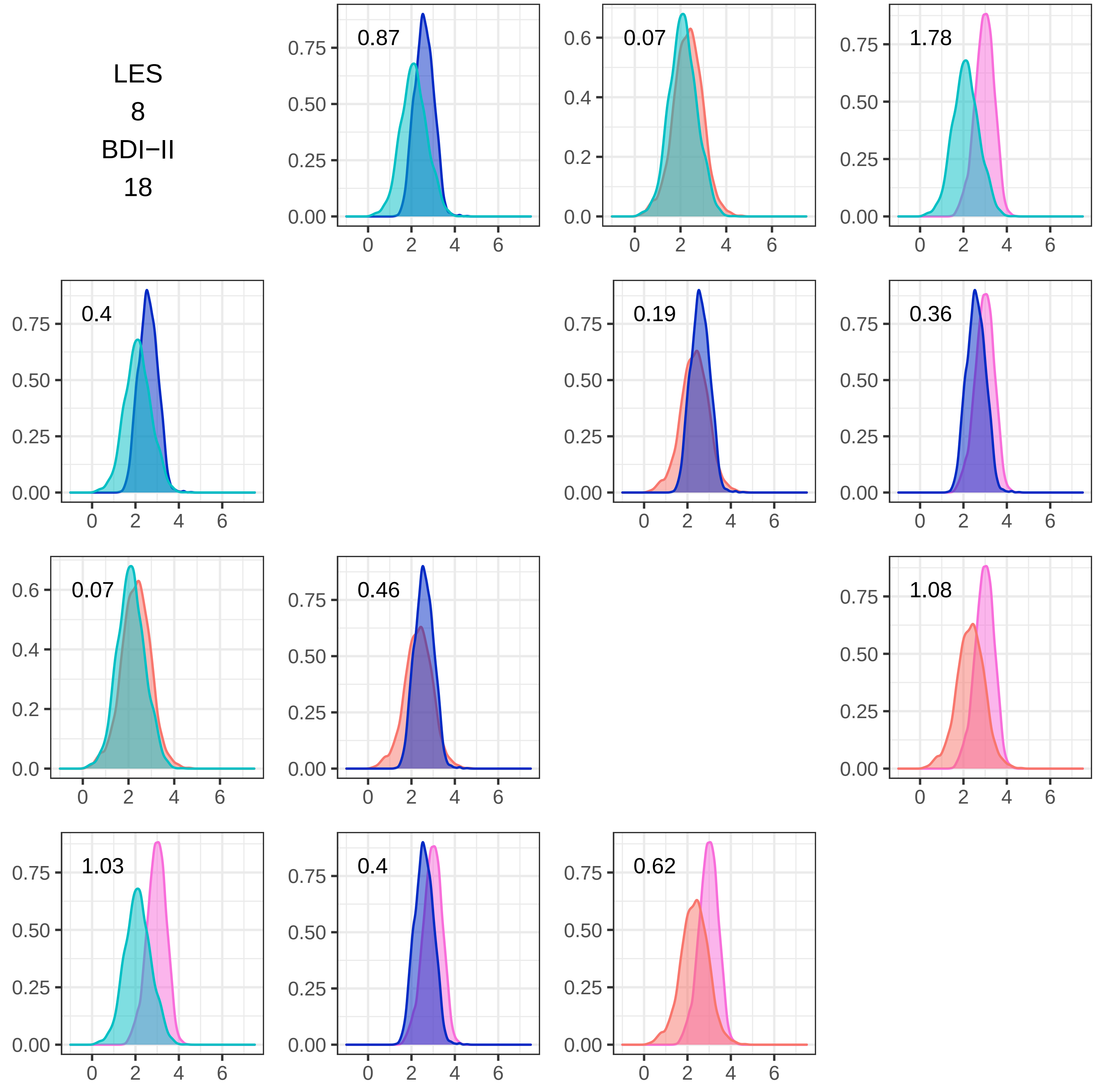
LES
8
BDI−II
18
0.87
0.07
1.78
0.4
0.19
0.36
0.07
0.46
1.08
1.03
0.4
0.62

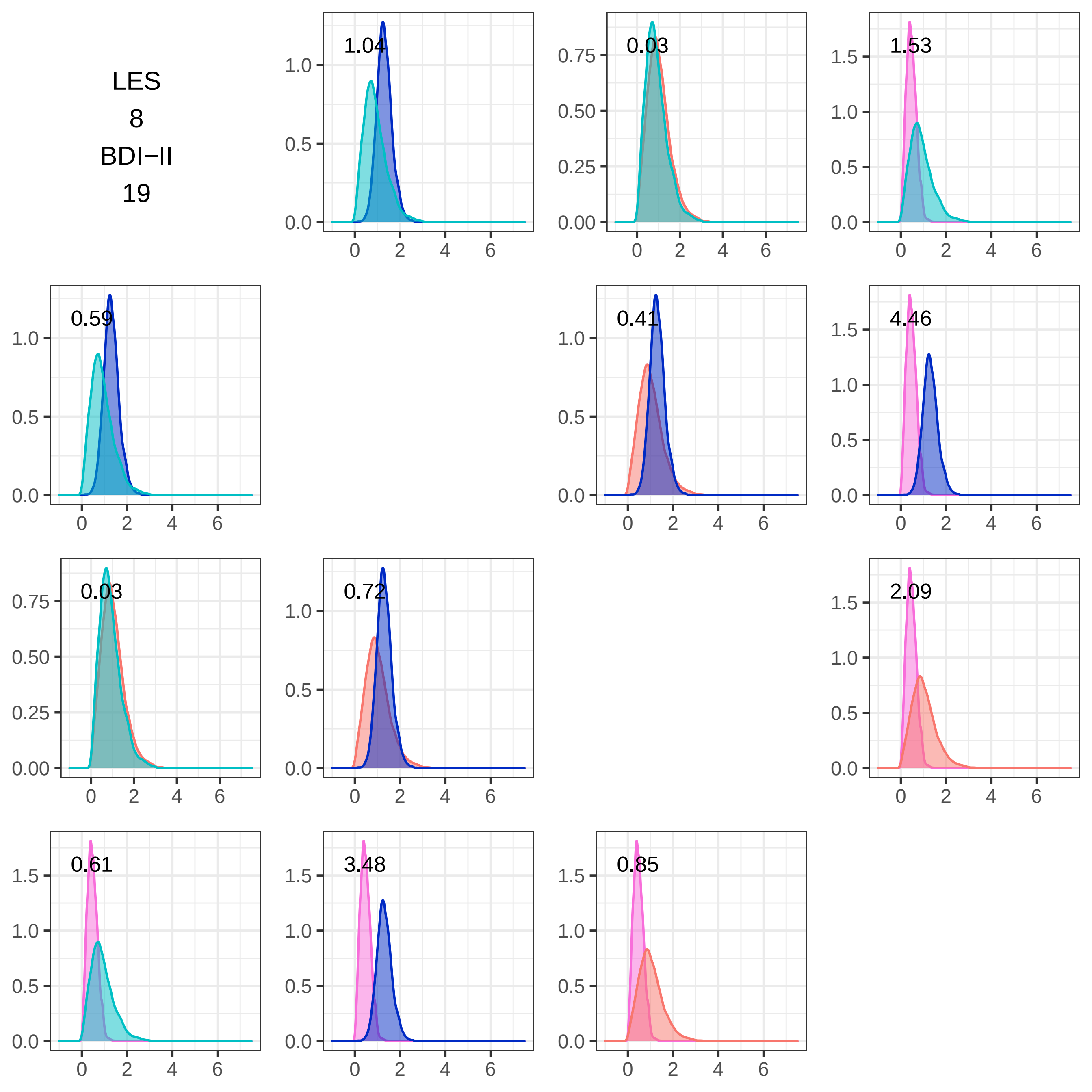

LES
8
BDI−II
19
1.04
0.03
1.53
0.59
0.41
4.46
0.03
0.72
2.09
0.61
3.48
0.85

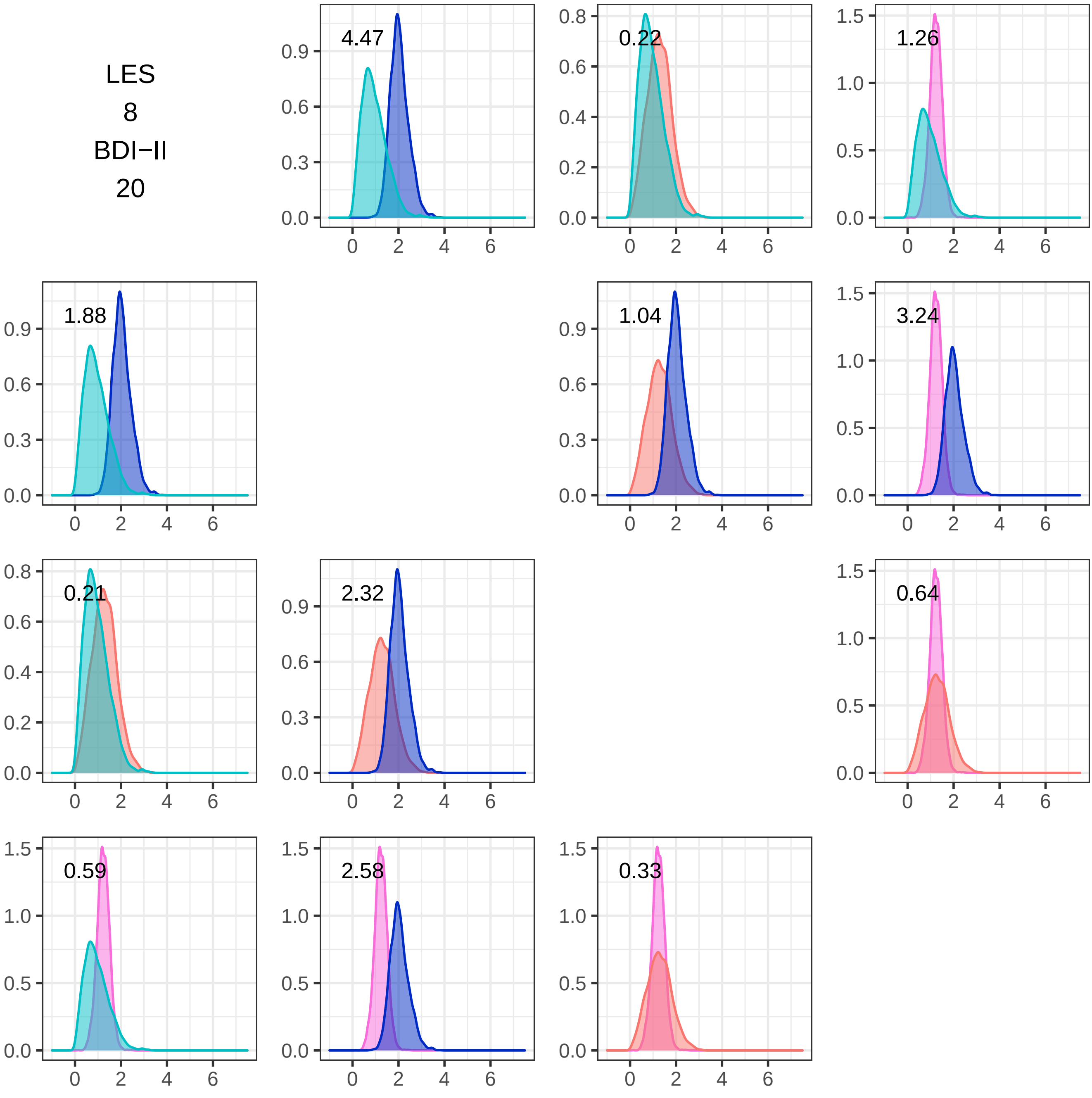

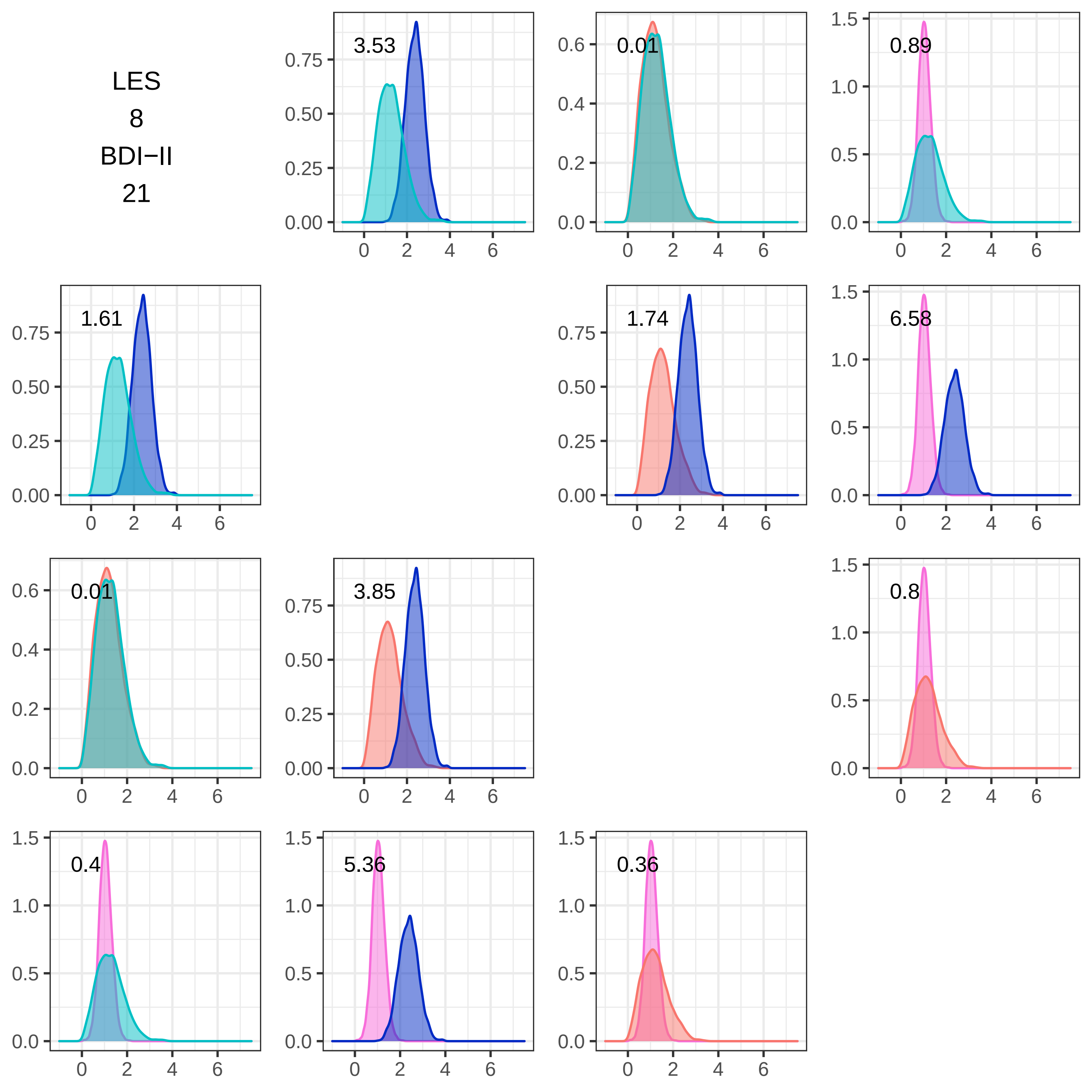

LES
8
BDI−II
21
3.53
0.01
0.89
1.61
1.74
6.58
0.01
3.85
0.8
0.4
5.36
0.36

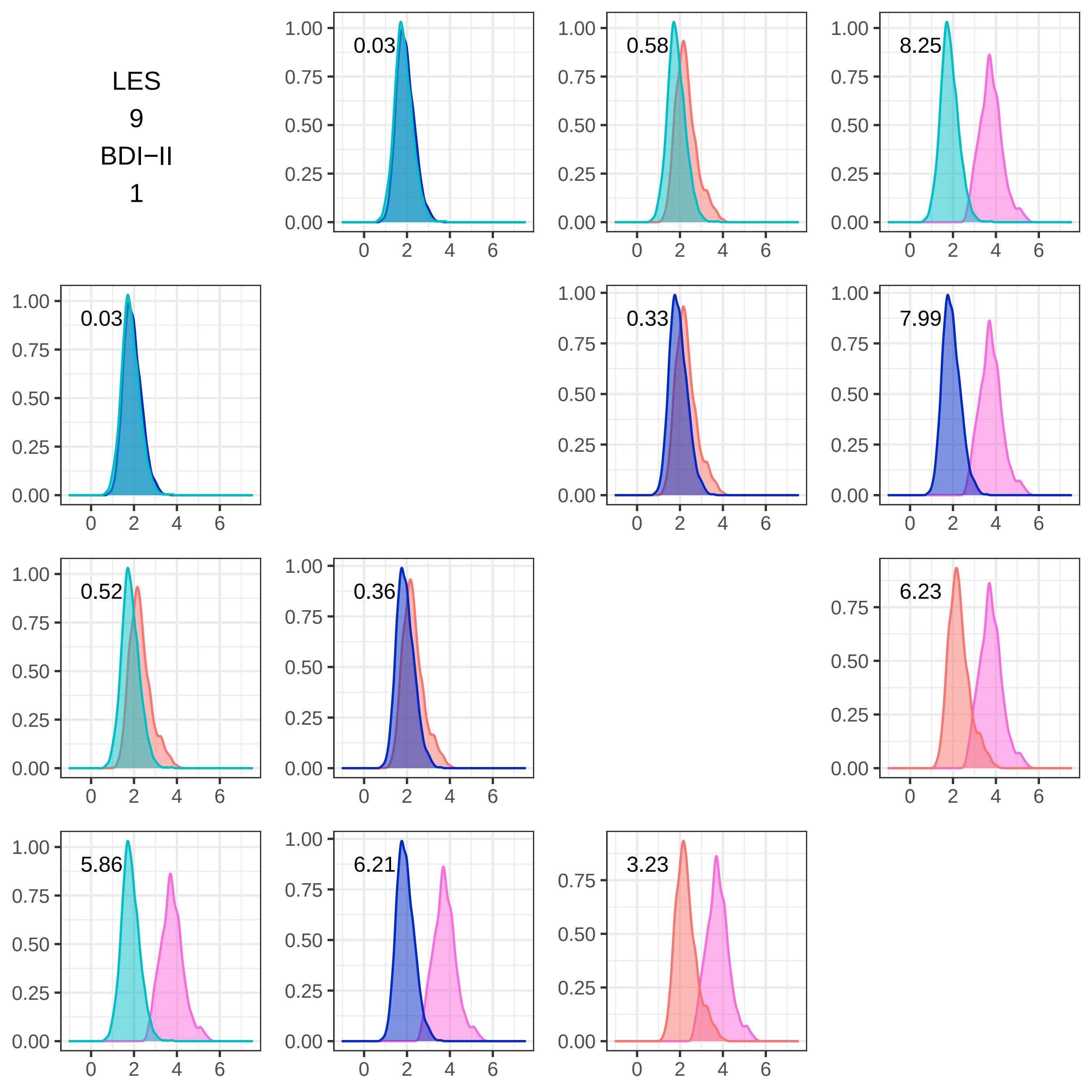
LES
9
BDI−II
1
0.03
0.58
8.25
0.03
0.33
7.99
0.52
0.36
6.23
5.86
6.21
3.23

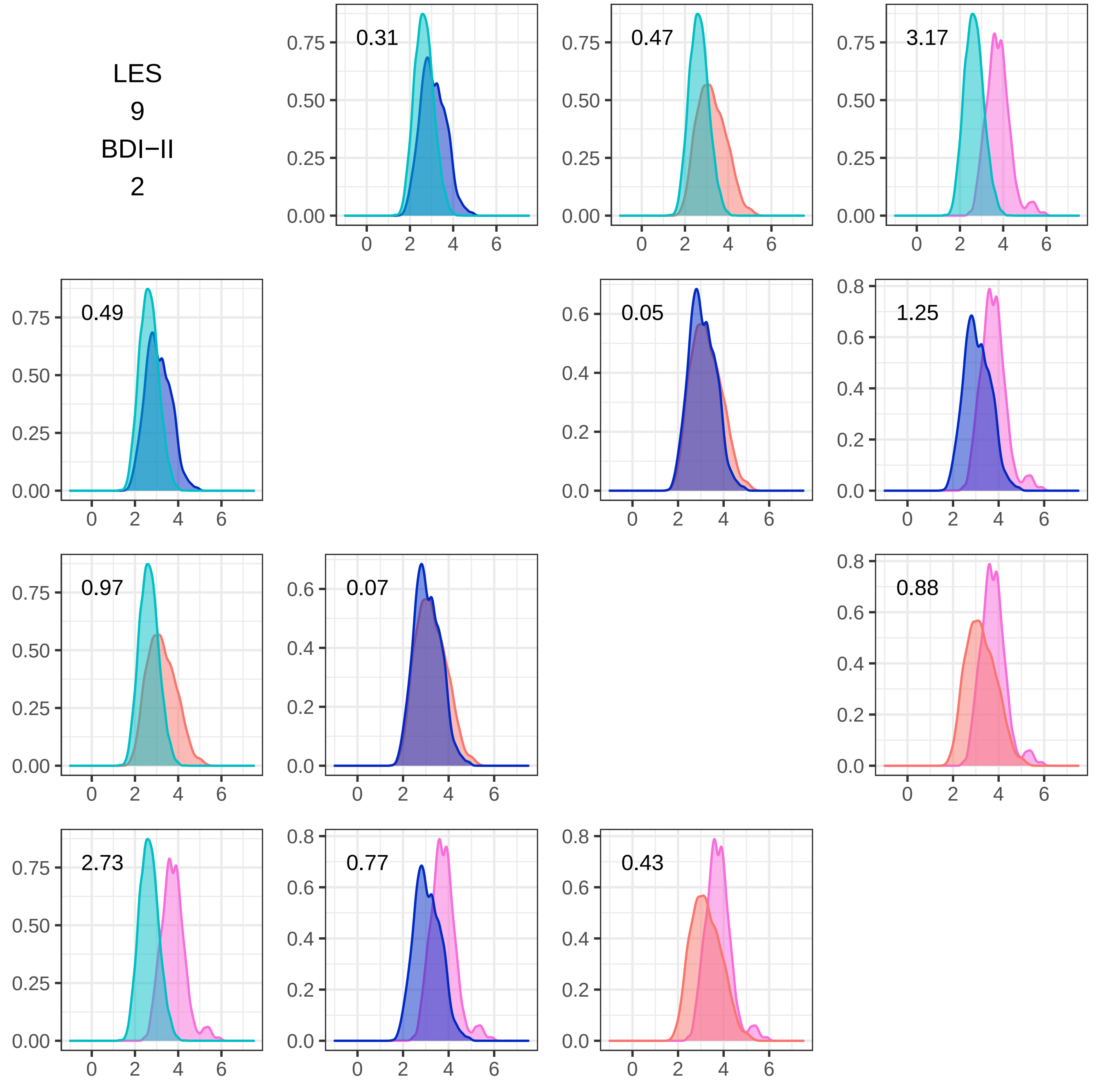
LES
9
BDI−II
2
0.31
0.47
3.17
0.49
0.05
1.25
0.97
0.07
0.88
2.73
0.77
0.43

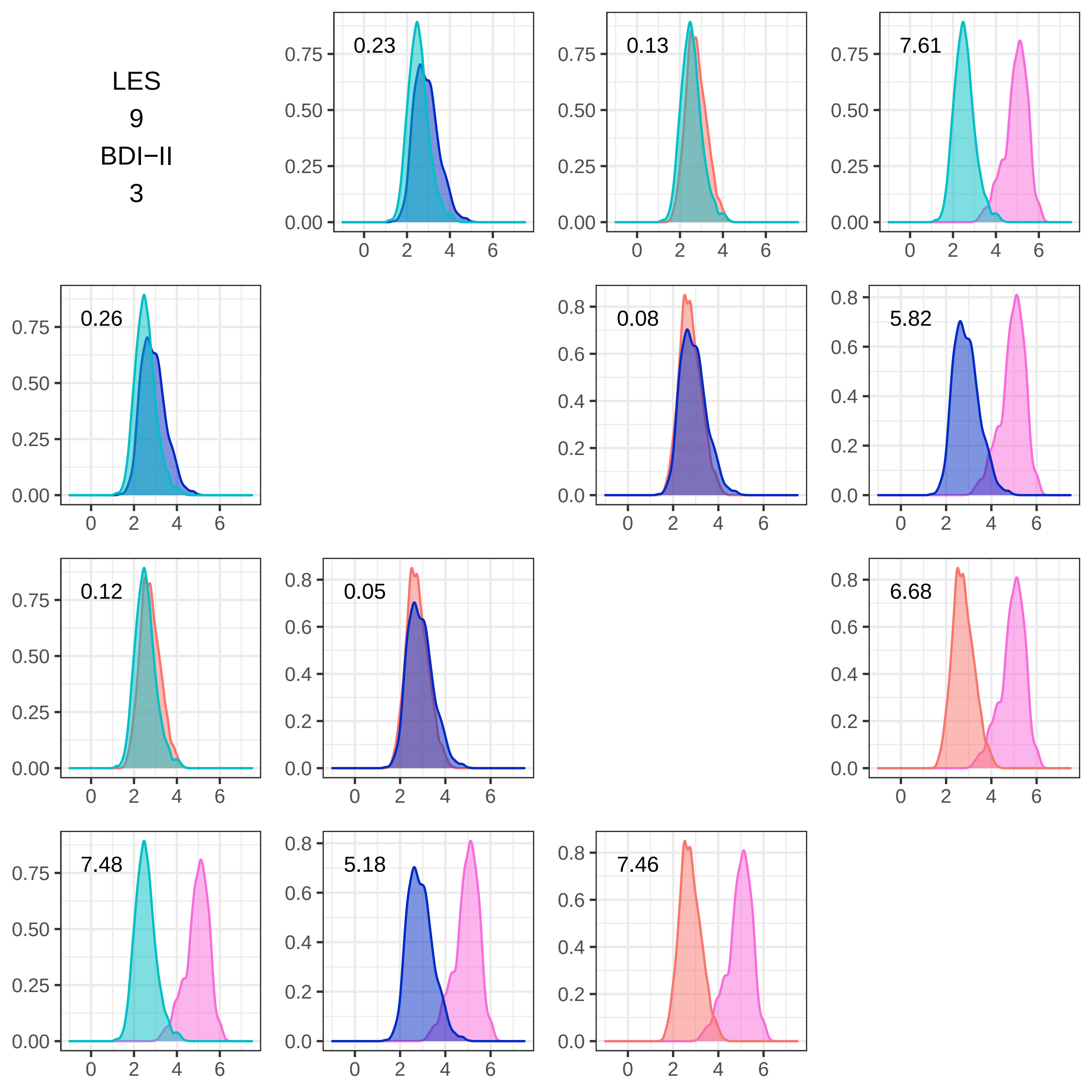

LES
9
BDI−II
3
0.23
0.13
7.61
0.26
0.08
5.82
0.12
0.05
6.68
7.48
5.18
7.46

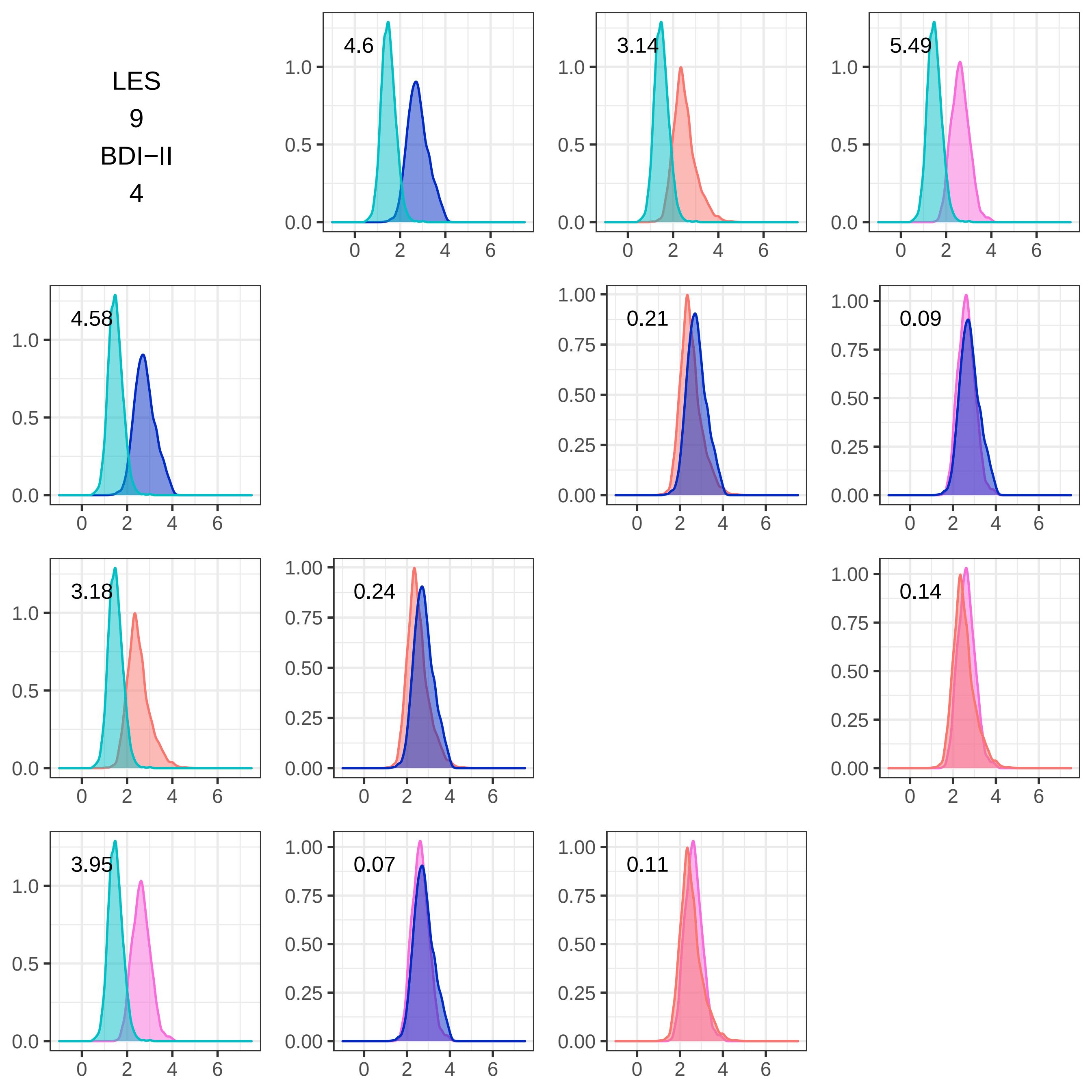

LES
9
BDI−II
4
4.6
3.14
5.49
4.58
0.21
0.09
3.18
0.24
0.14
3.95
0.07
0.11

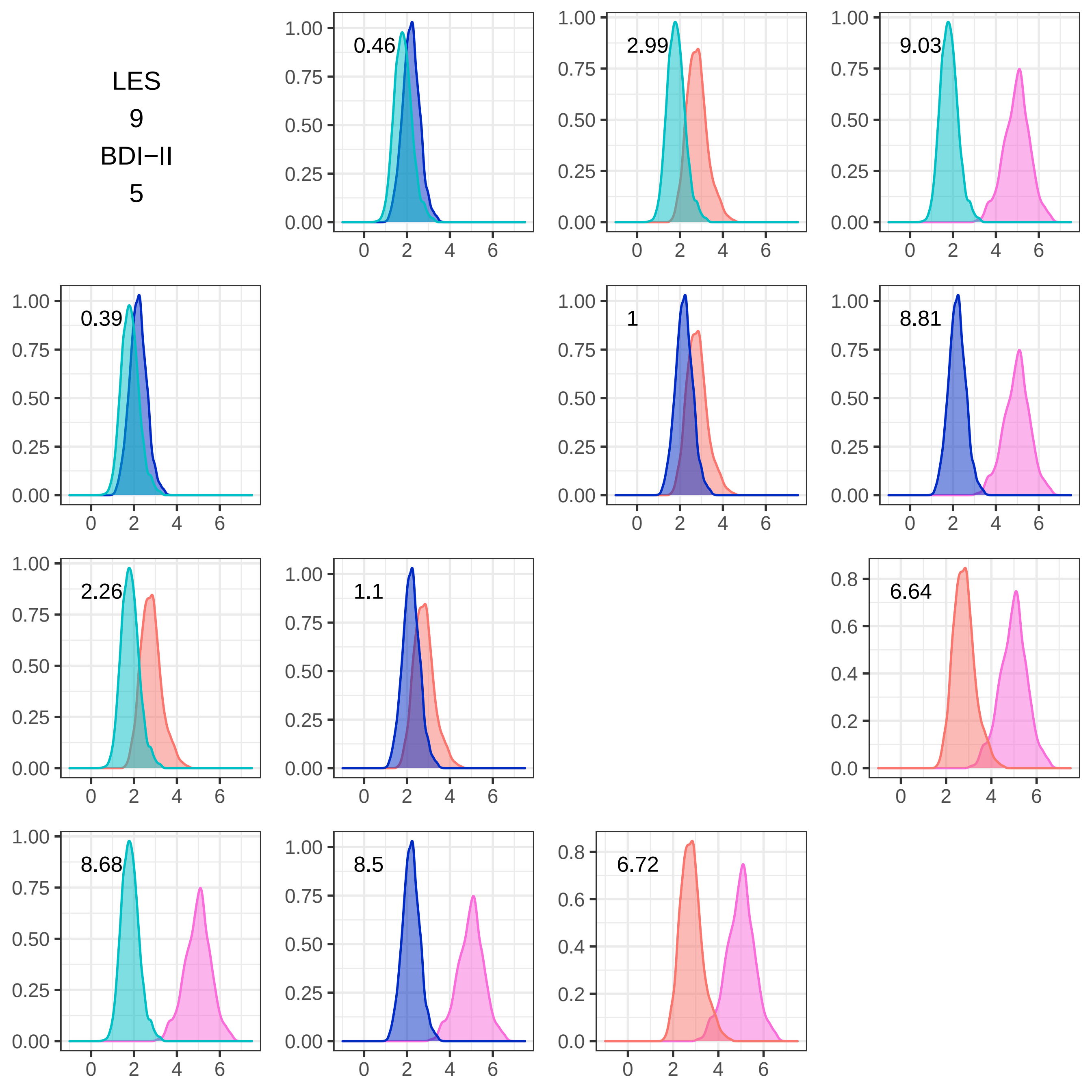
LES
9
BDI−II
5
0.46
2.99
9.03
0.39
1
8.81
2.26
1.1
6.64
8.68
8.5
6.72

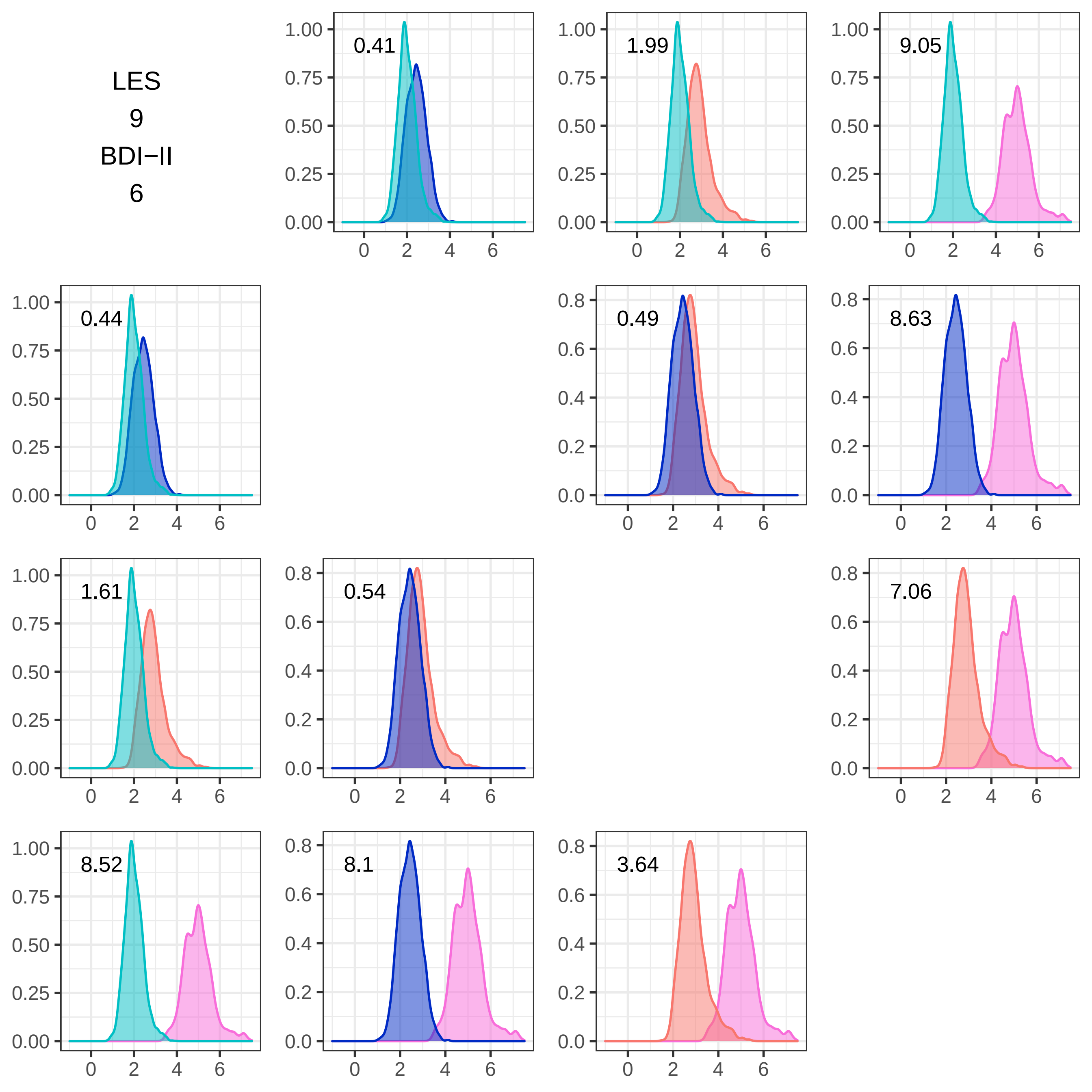
LES
9
BDI−II
6
0.41
1.99
9.05
0.44
0.49
8.63
1.61
0.54
7.06
8.52
8.1
3.64

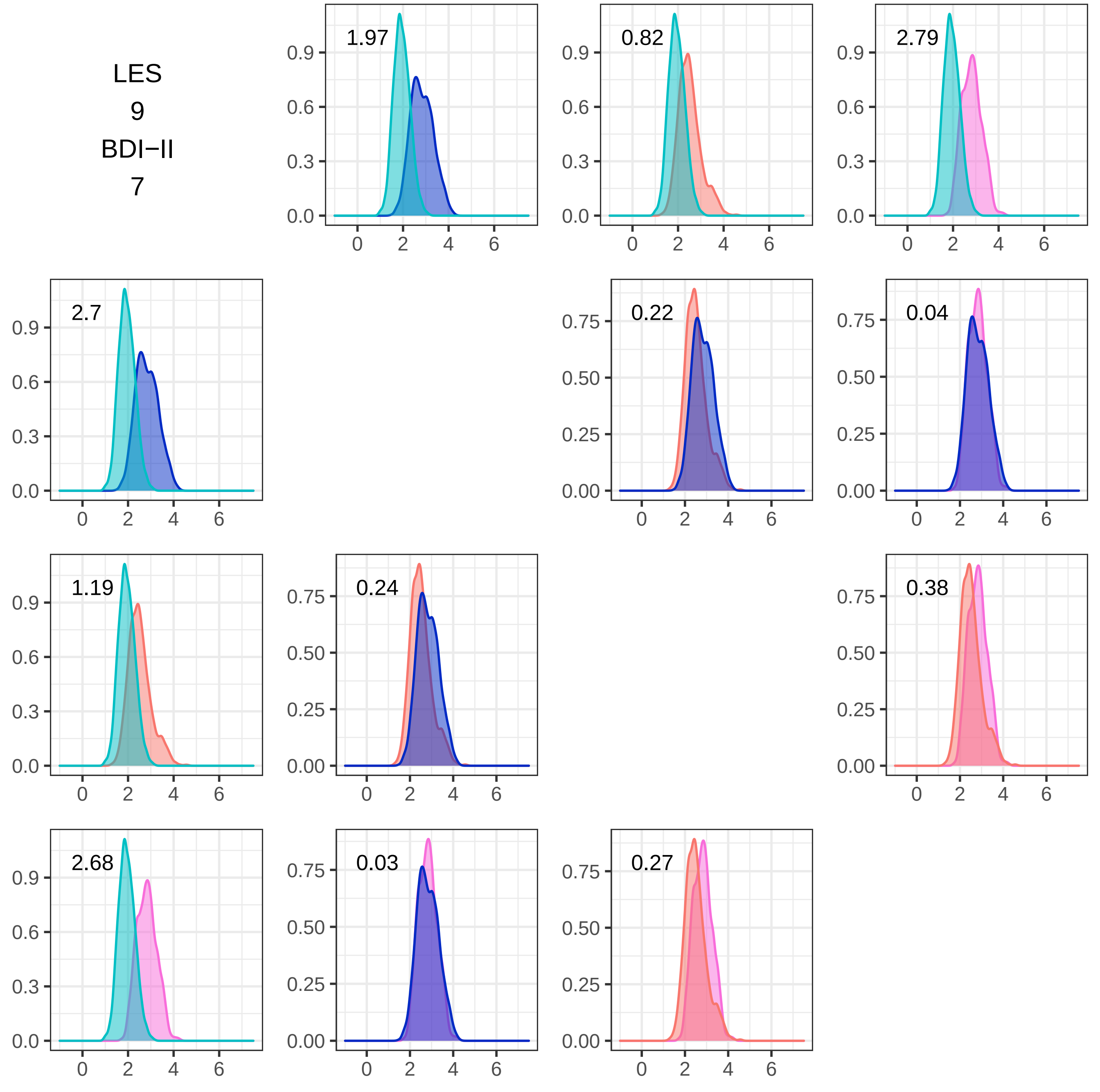

LES
9
BDI−II
7
1.97
0.82
2.79
2.7
0.22
0.04
1.19
0.24
0.38
2.68
0.03
0.27

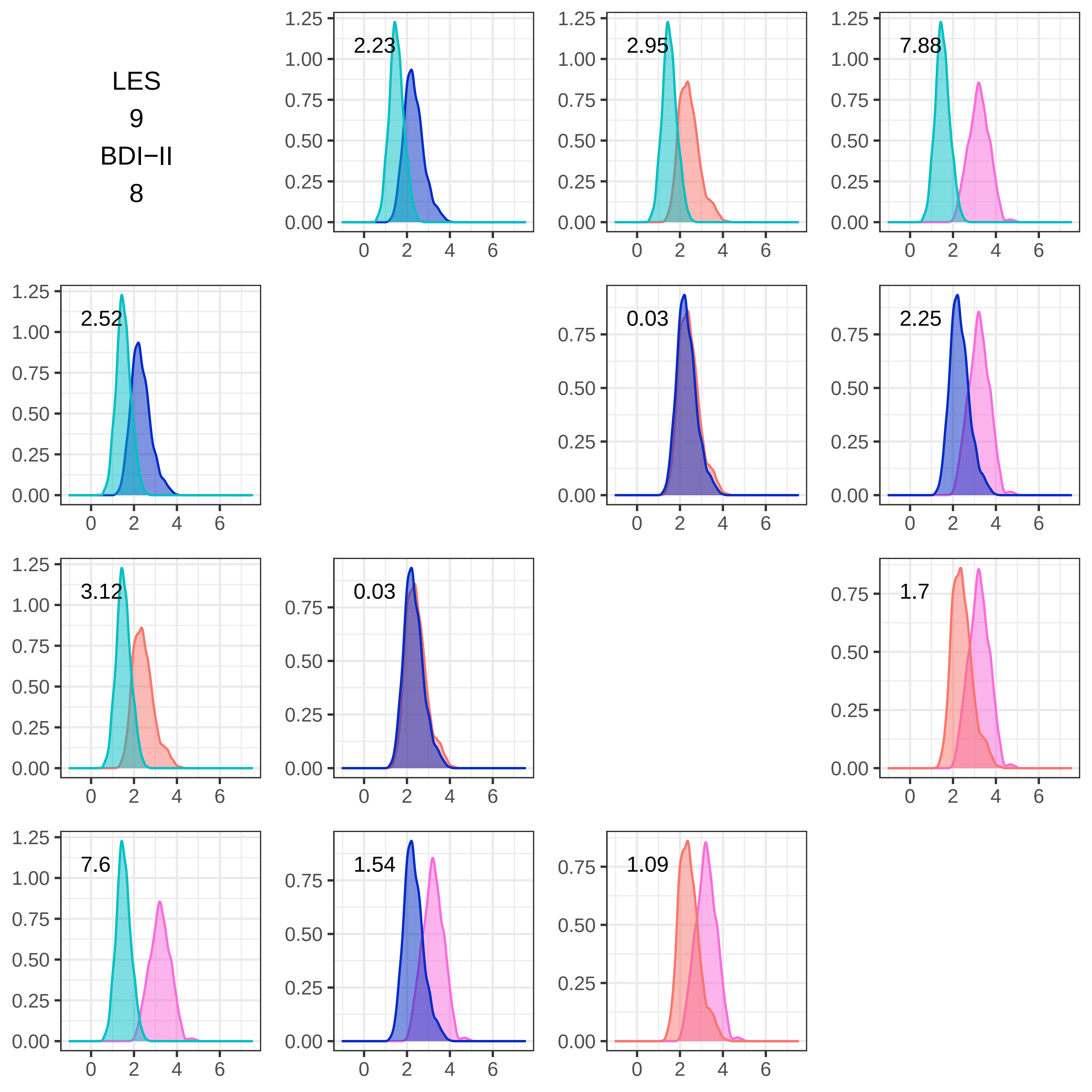
LES
9
BDI−II
8
2.23
2.95
7.88
2.52
0.03
2.25
3.12
0.03
1.7
7.6
1.54
1.09

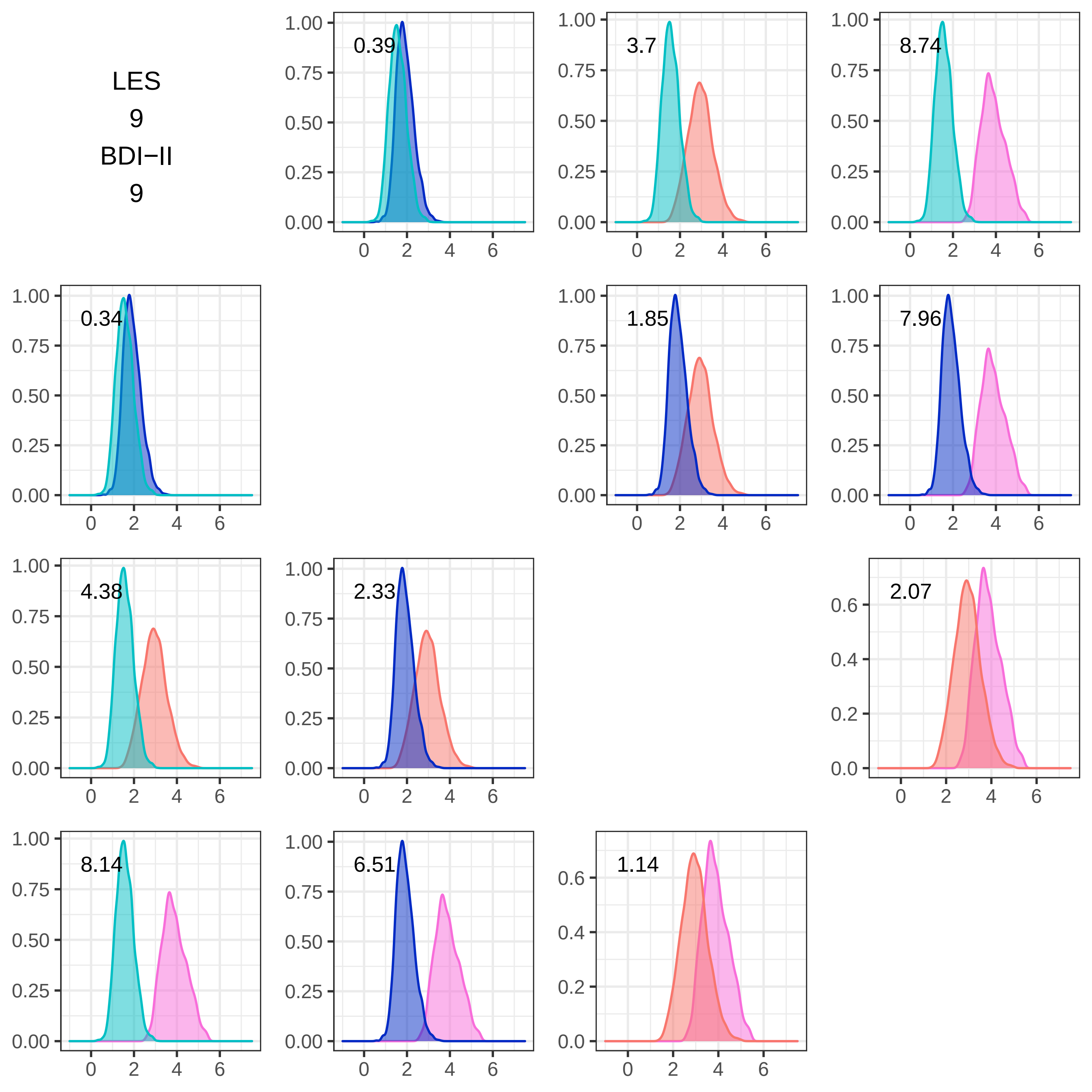
LES
9
BDI−II
9
0.39
3.7
8.74
0.34
1.85
7.96
4.38
2.33
2.07
8.14
6.51
1.14

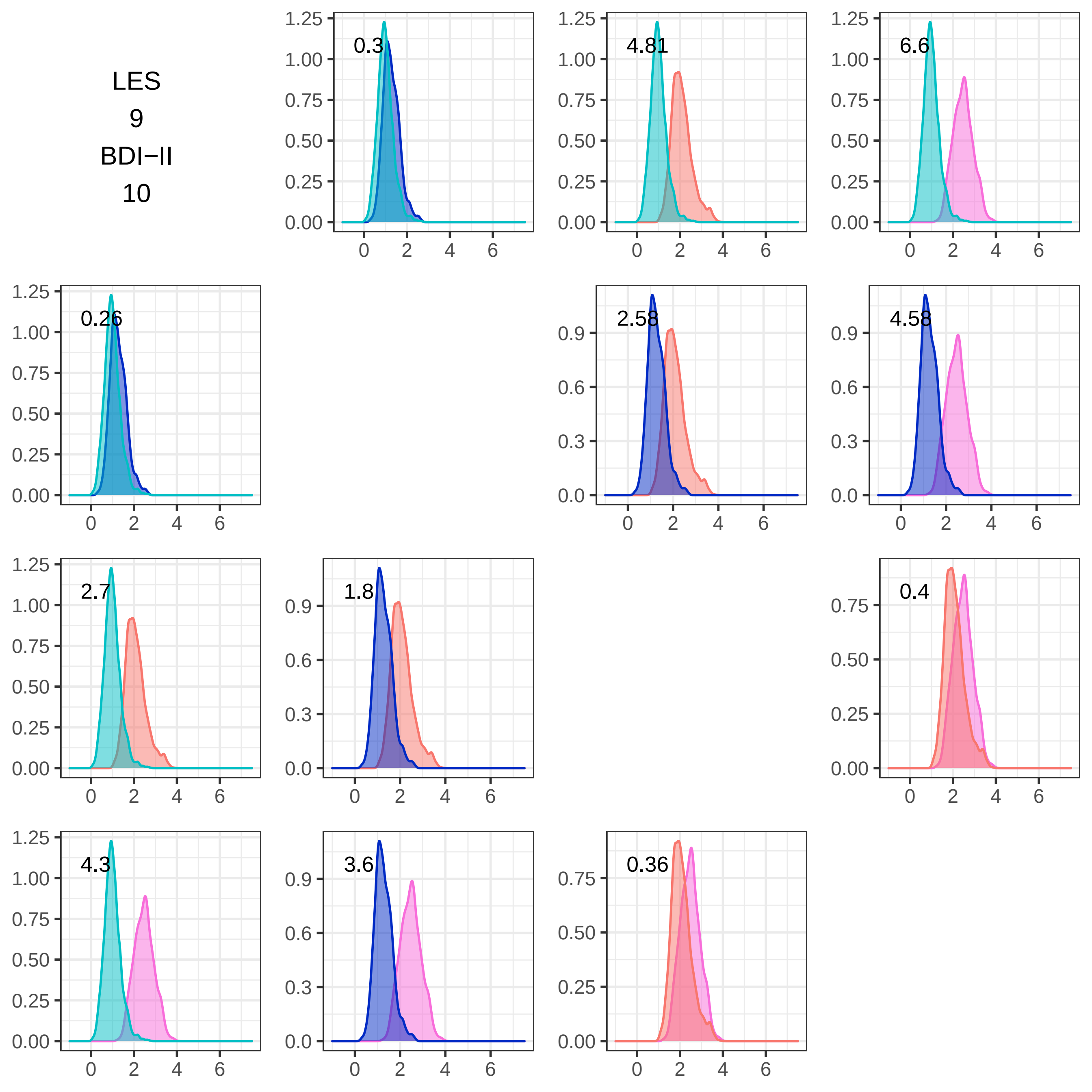
LES
9
BDI−II
10
0.3
4.81
6.6
0.26
2.58
4.58
2.7
1.8
0.4
4.3
3.6
0.36

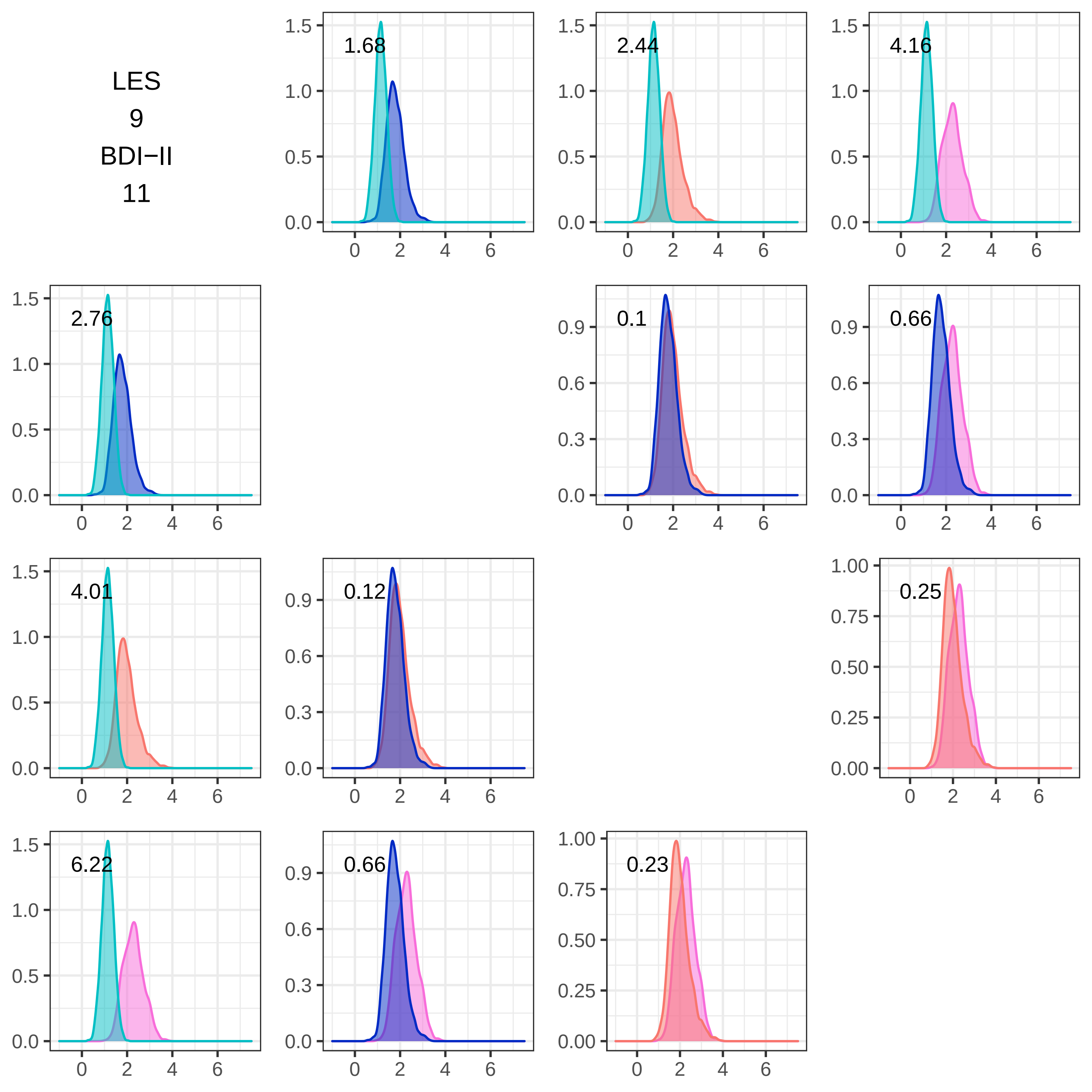

LES
9
BDI−II
11
1.68
2.44
4.16
2.76
0.1
0.66
4.01
0.12
0.25
6.22
0.66
0.23

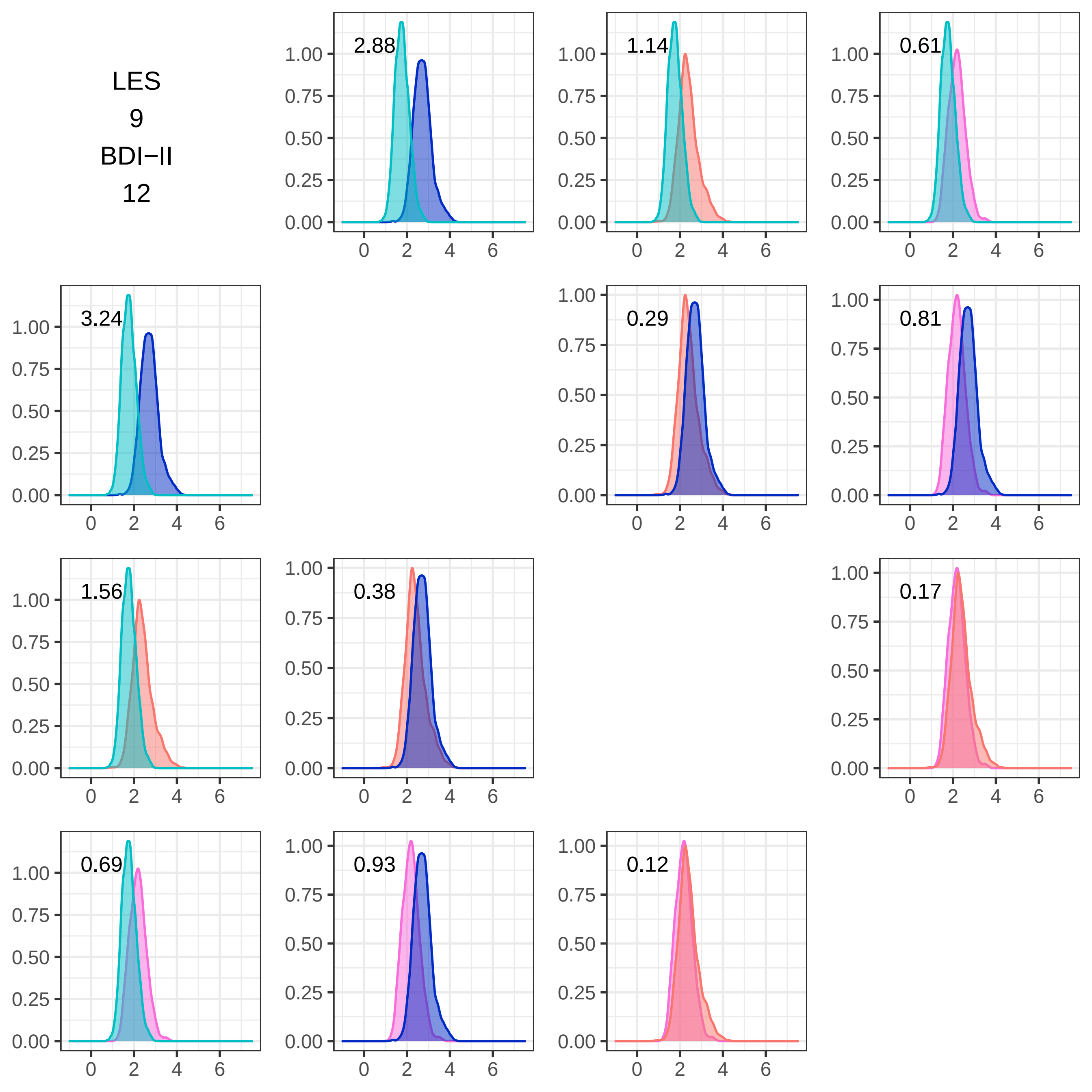
LES
9
BDI−II
12
2.88
1.14
0.61
3.24
0.29
0.81
1.56
0.38
0.17
0.69
0.93
0.12

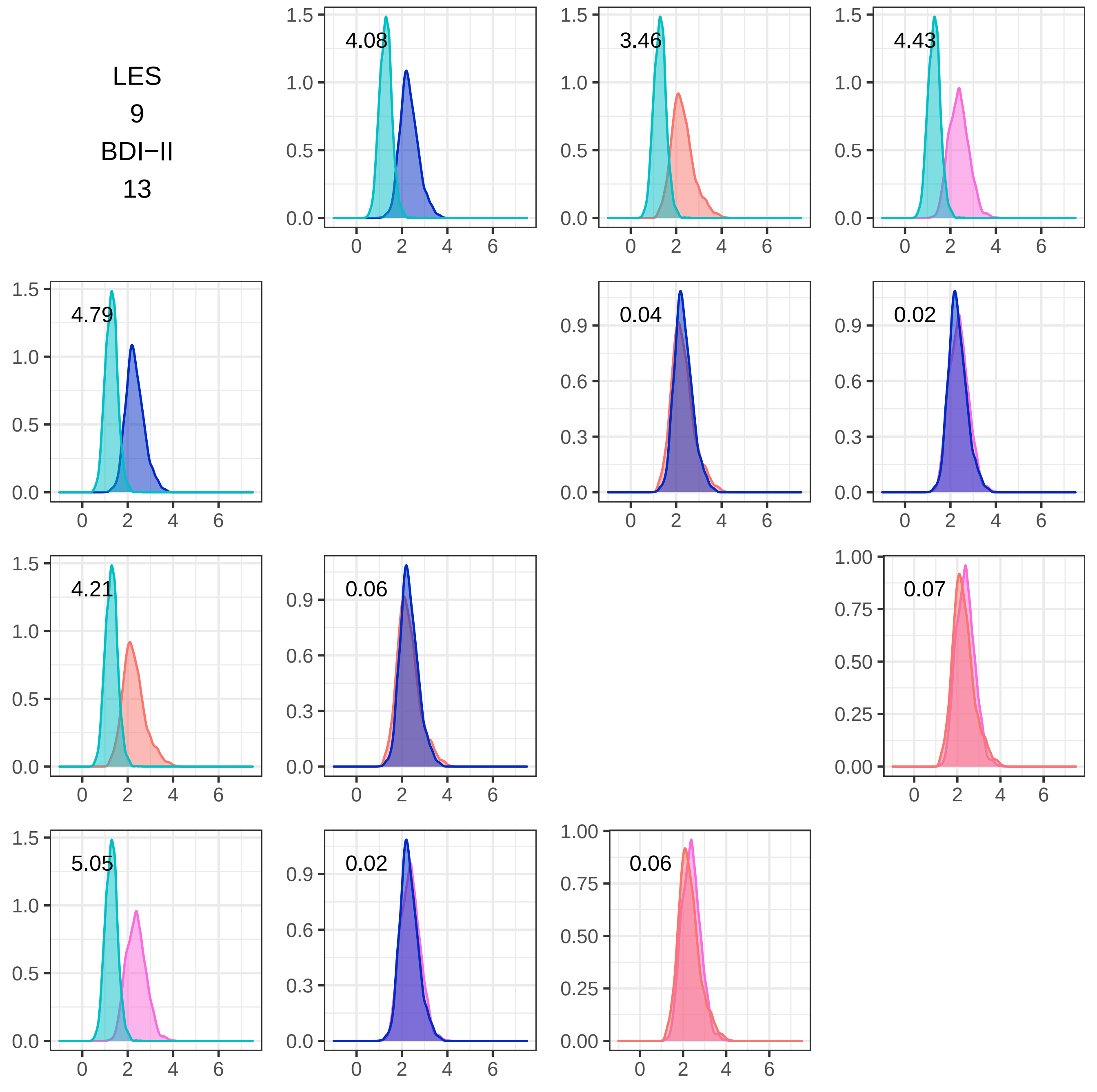

LES
9
BDI−II
13
4.08
3.46
4.43
4.79
0.04
0.02
4.21
0.06
0.07
5.05
0.02
0.06

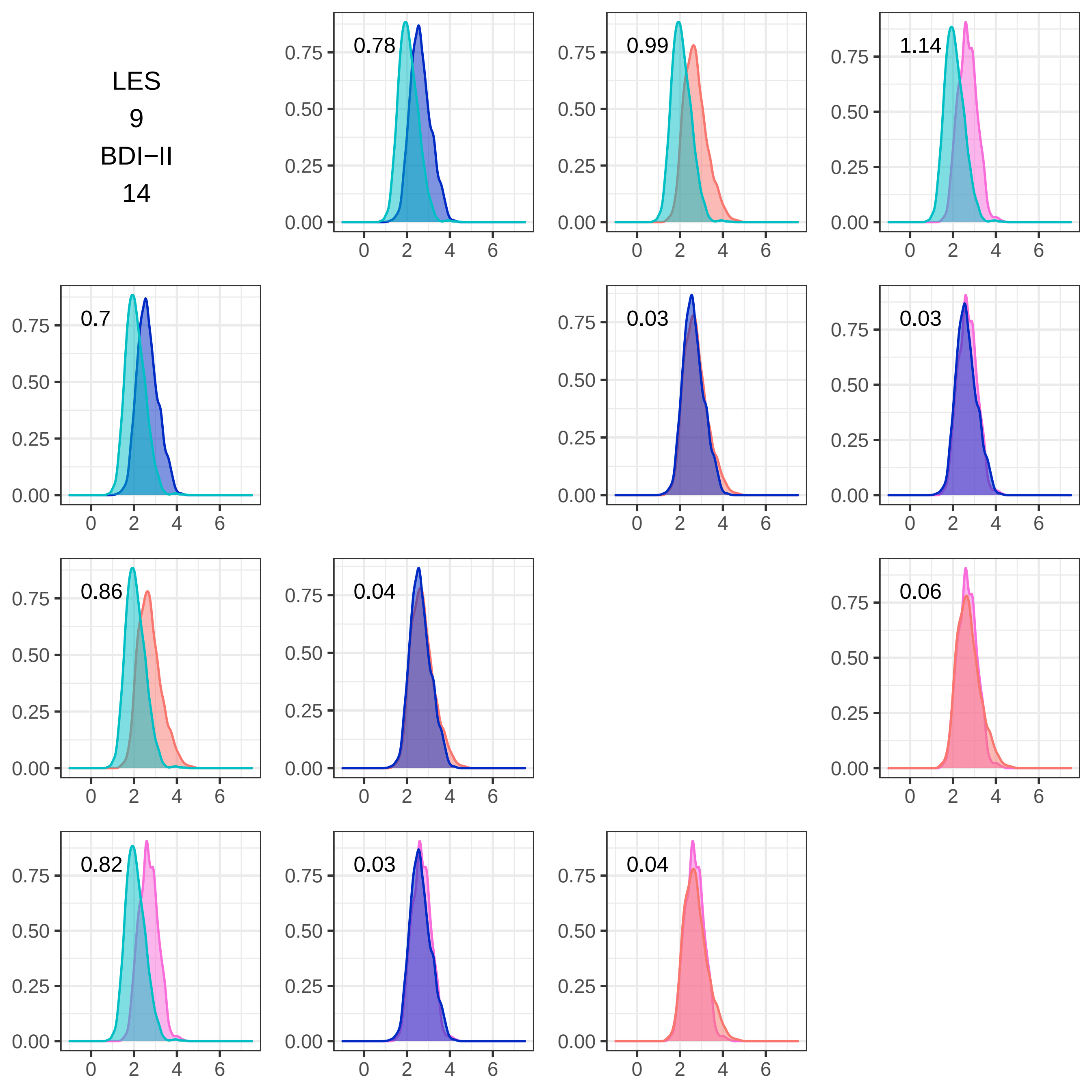
LES
9
BDI−II
14
0.78
0.99
1.14
0.7
0.03
0.03
0.86
0.04
0.06
0.82
0.03
0.04

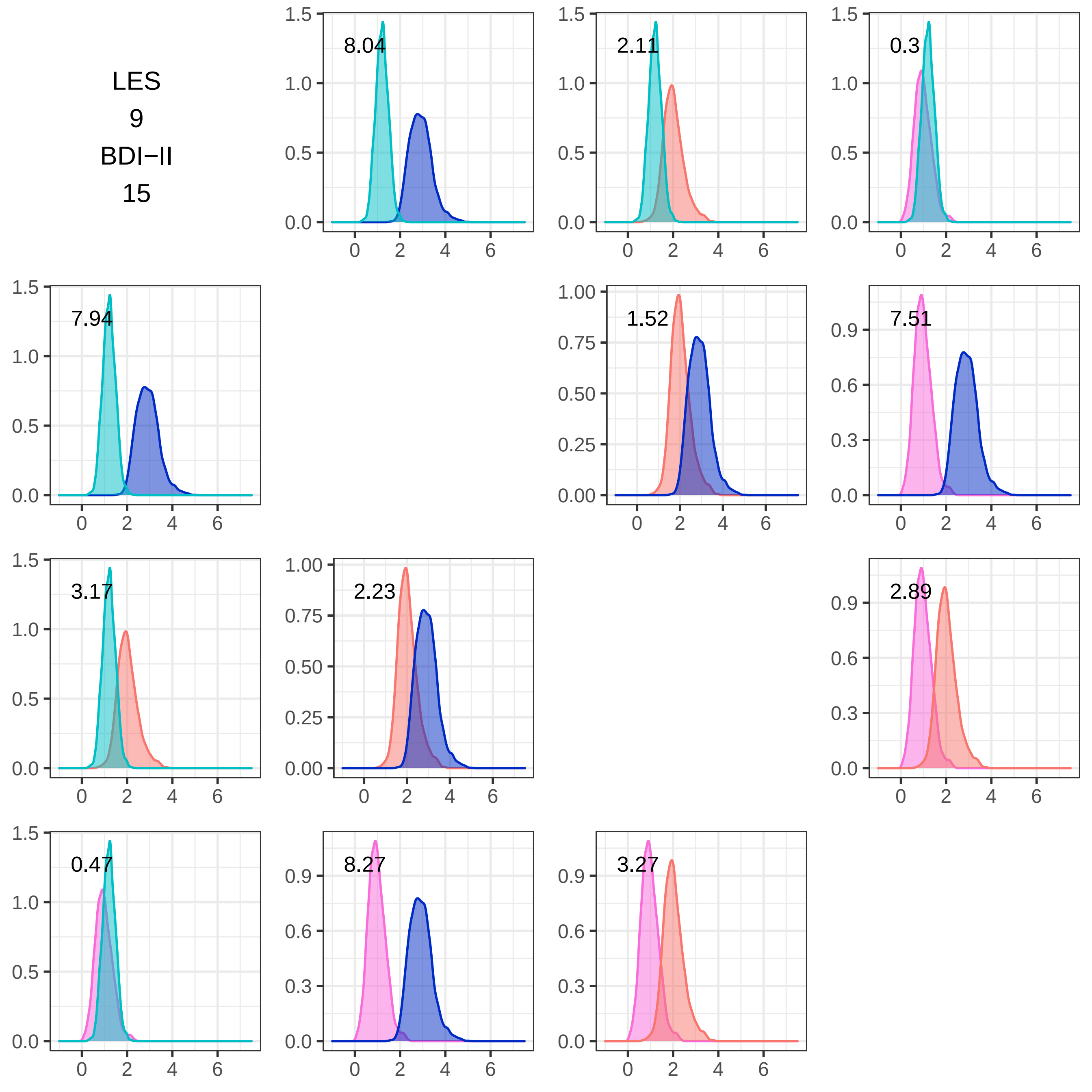
LES
9
BDI−II
15
8.04
2.11
0.3
7.94
1.52
7.51
3.17
2.23
2.89
0.47
8.27
3.27

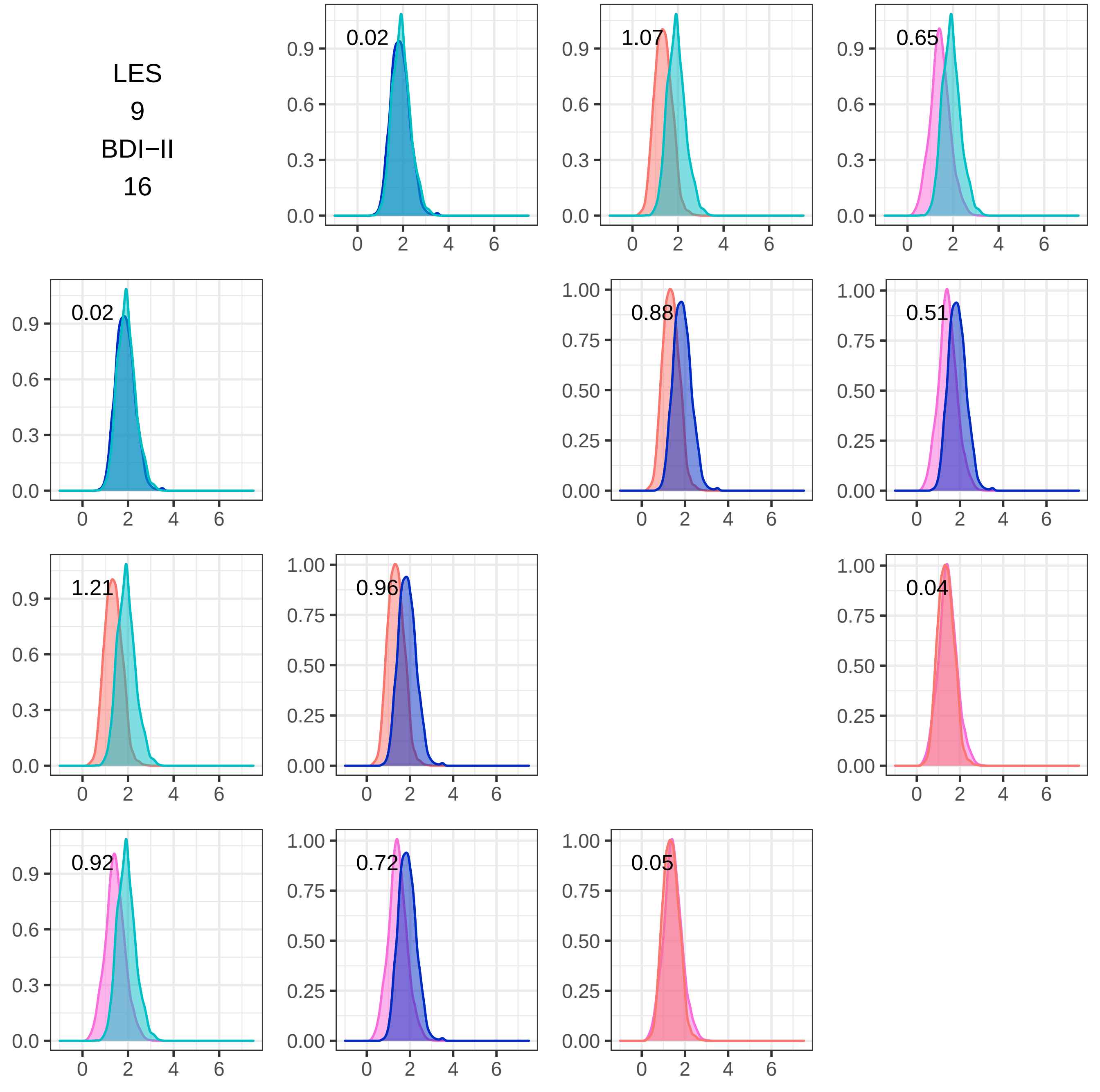

LES
9
BDI−II
16
0.02
1.07
0.65
0.02
0.88
0.51
1.21
0.96
0.04
0.92
0.72
0.05

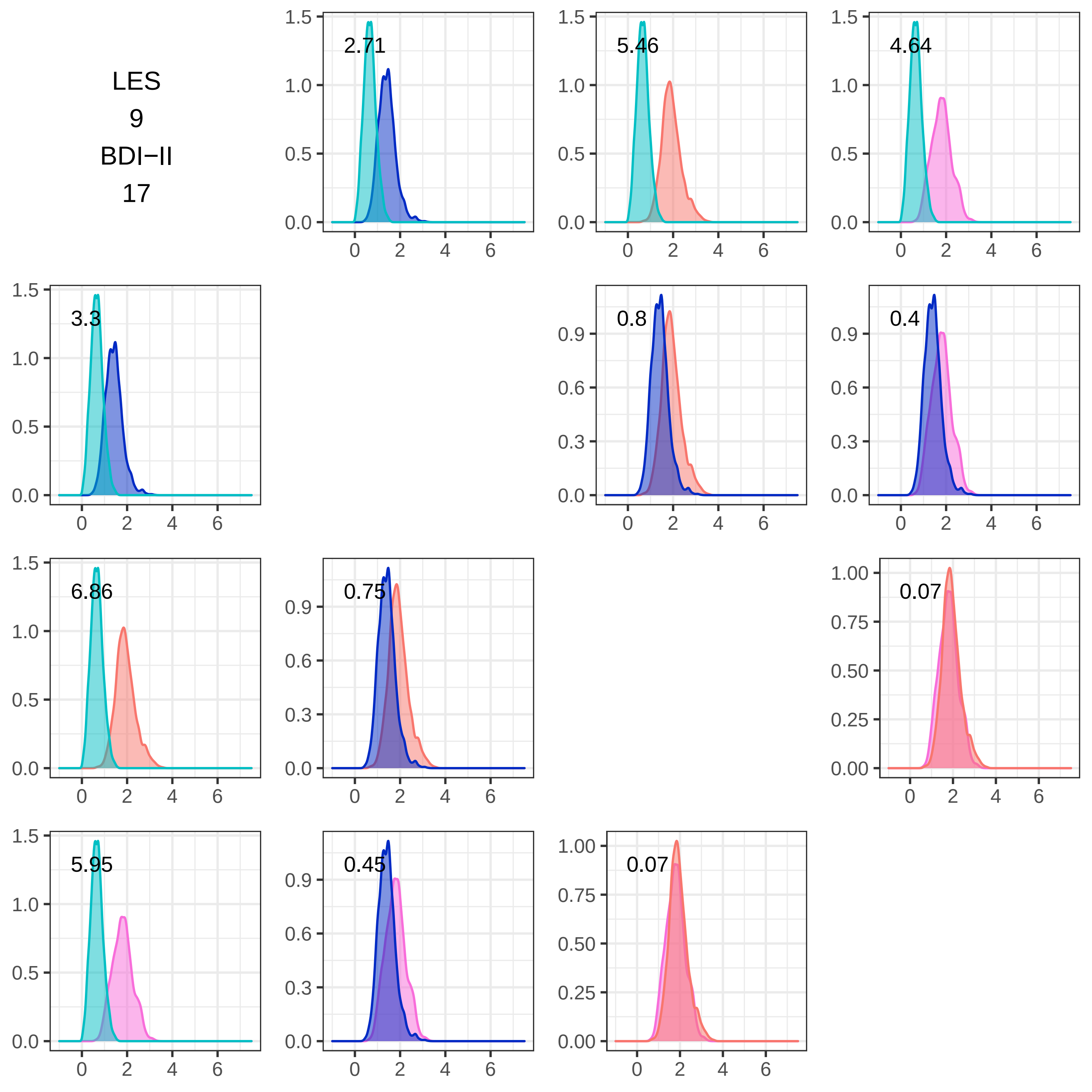

LES
9
BDI−II
17
2.71
5.46
4.64
3.3
0.8
0.4
6.86
0.75
0.07
5.95
0.45
0.07

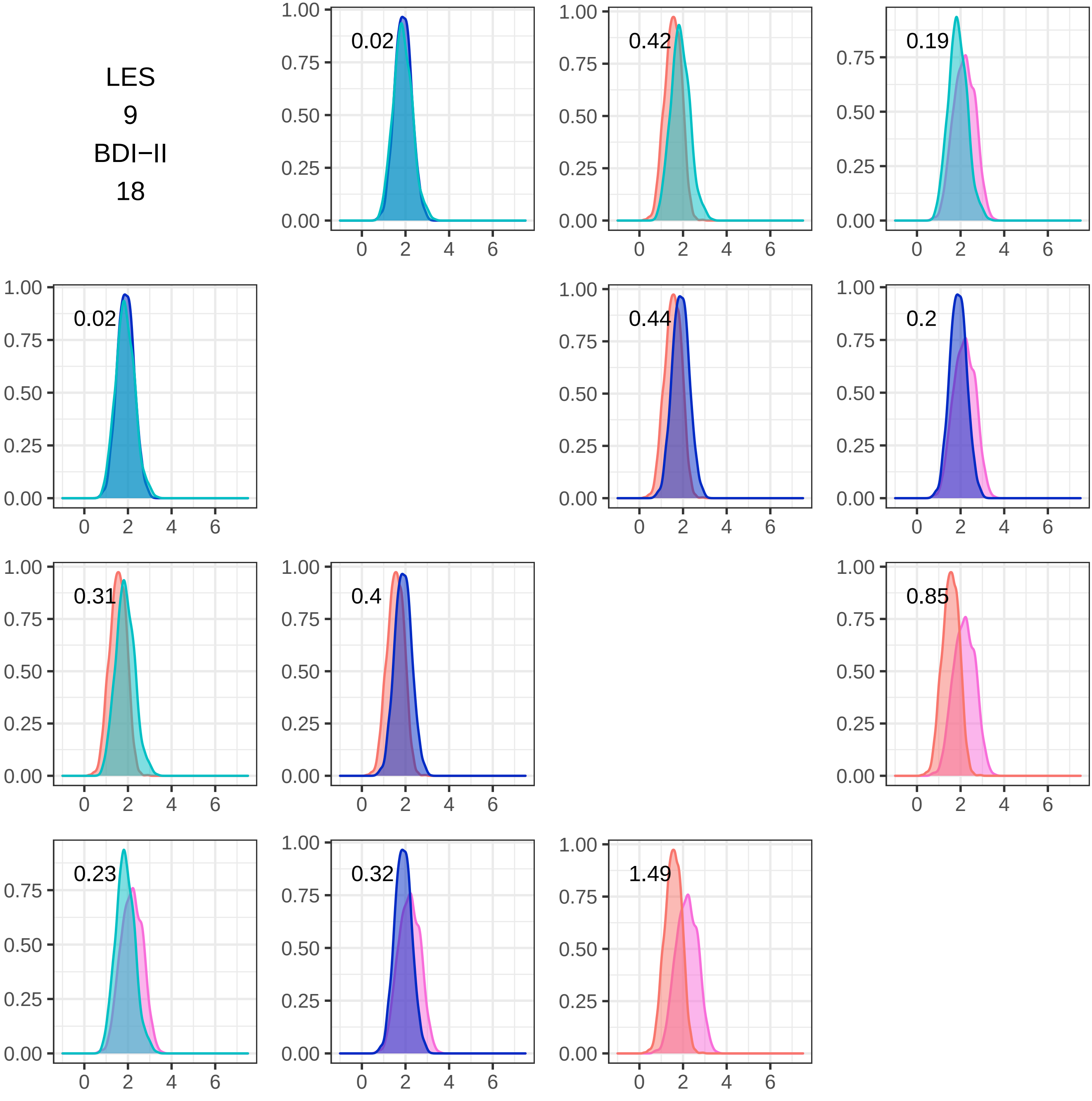
LES
9
BDI−II
18
0.02
0.42
0.19
0.02
0.44
0.2
0.31
0.4
0.85
0.23
0.32
1.49

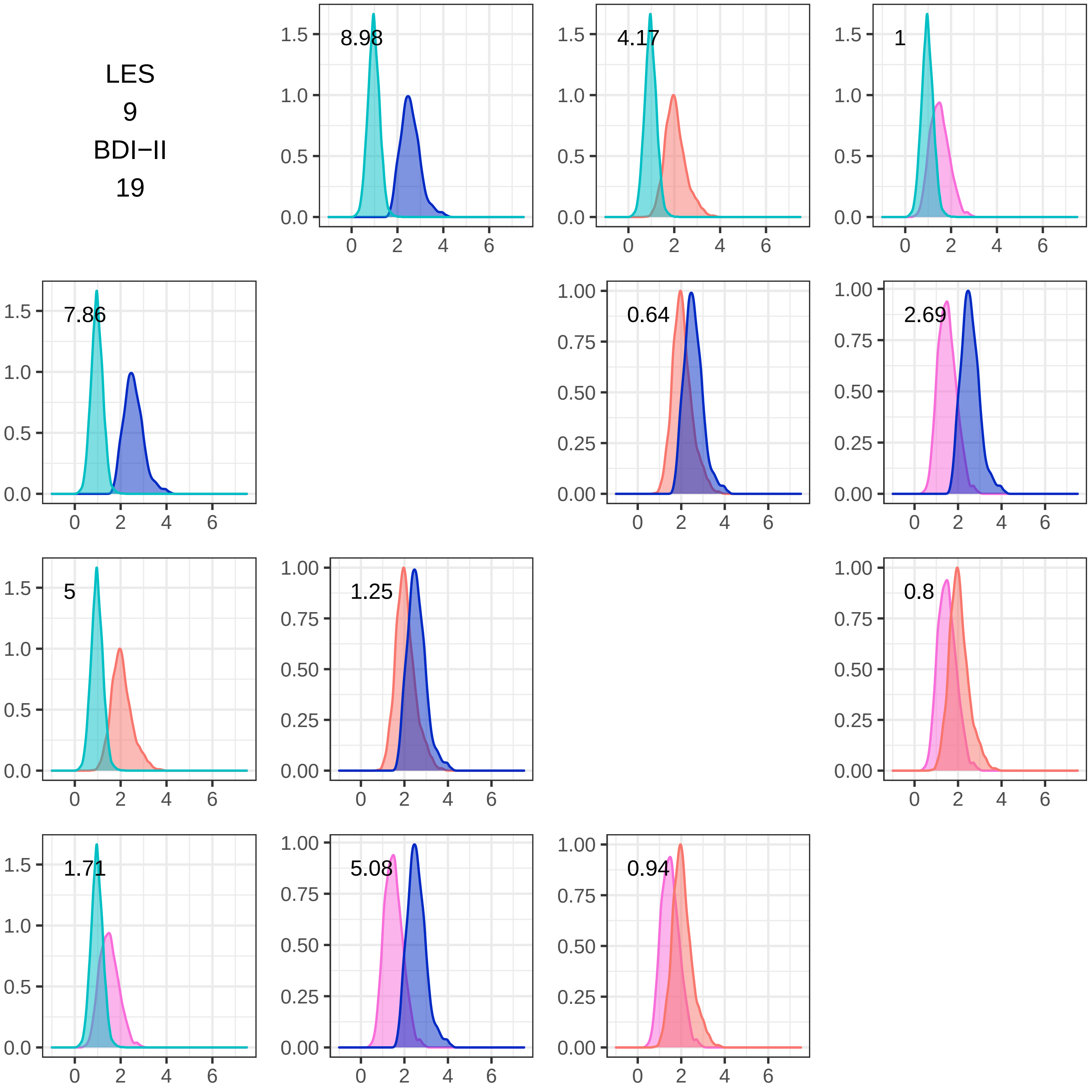
LES
9
BDI−II
19
8.98
4.17
1
7.86
0.64
2.69
5
1.25
0.8
1.71
5.08
0.94

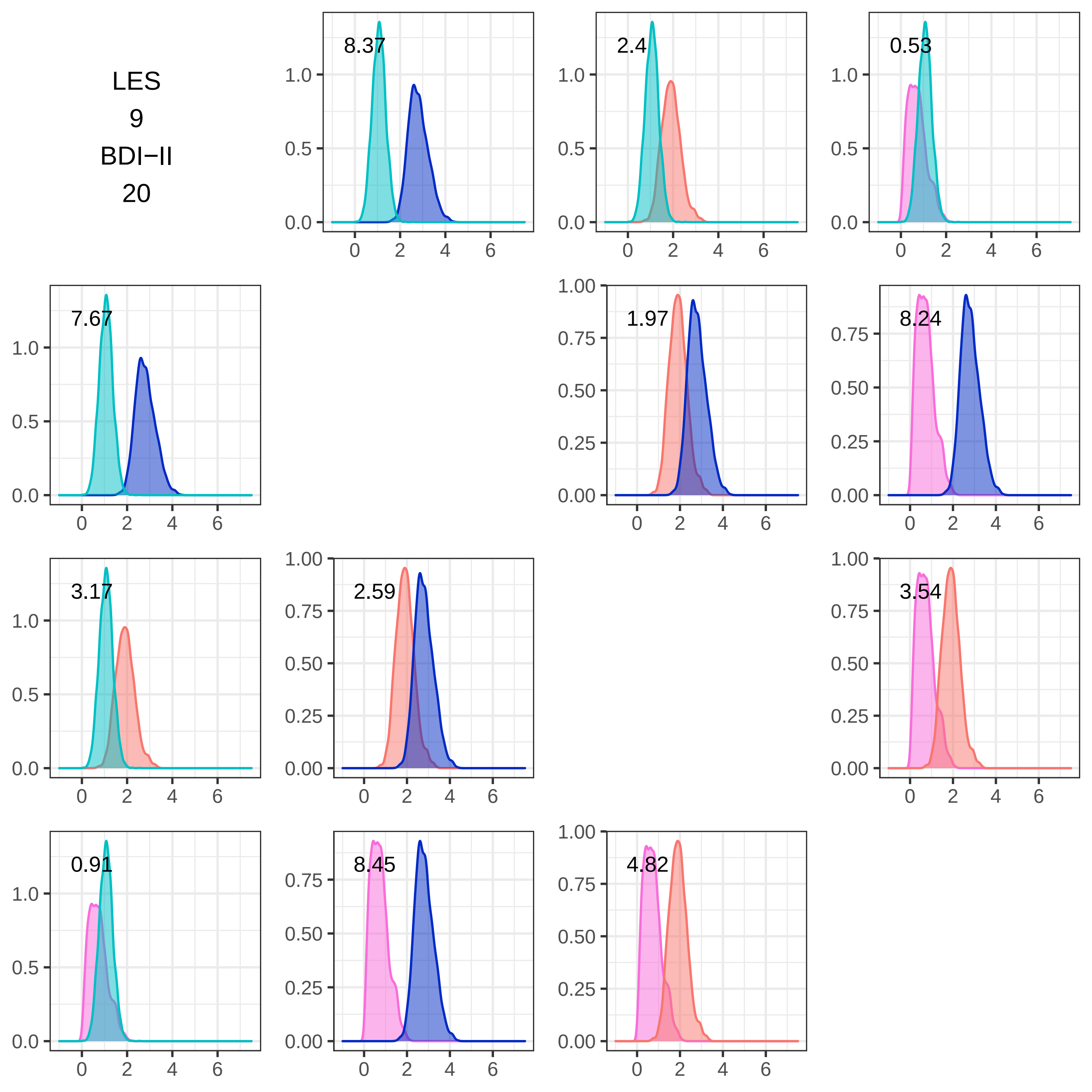
LES
9
BDI−II
20
8.37
2.4
0.53
7.67
1.97
8.24
3.17
2.59
3.54
0.91
8.45
4.82

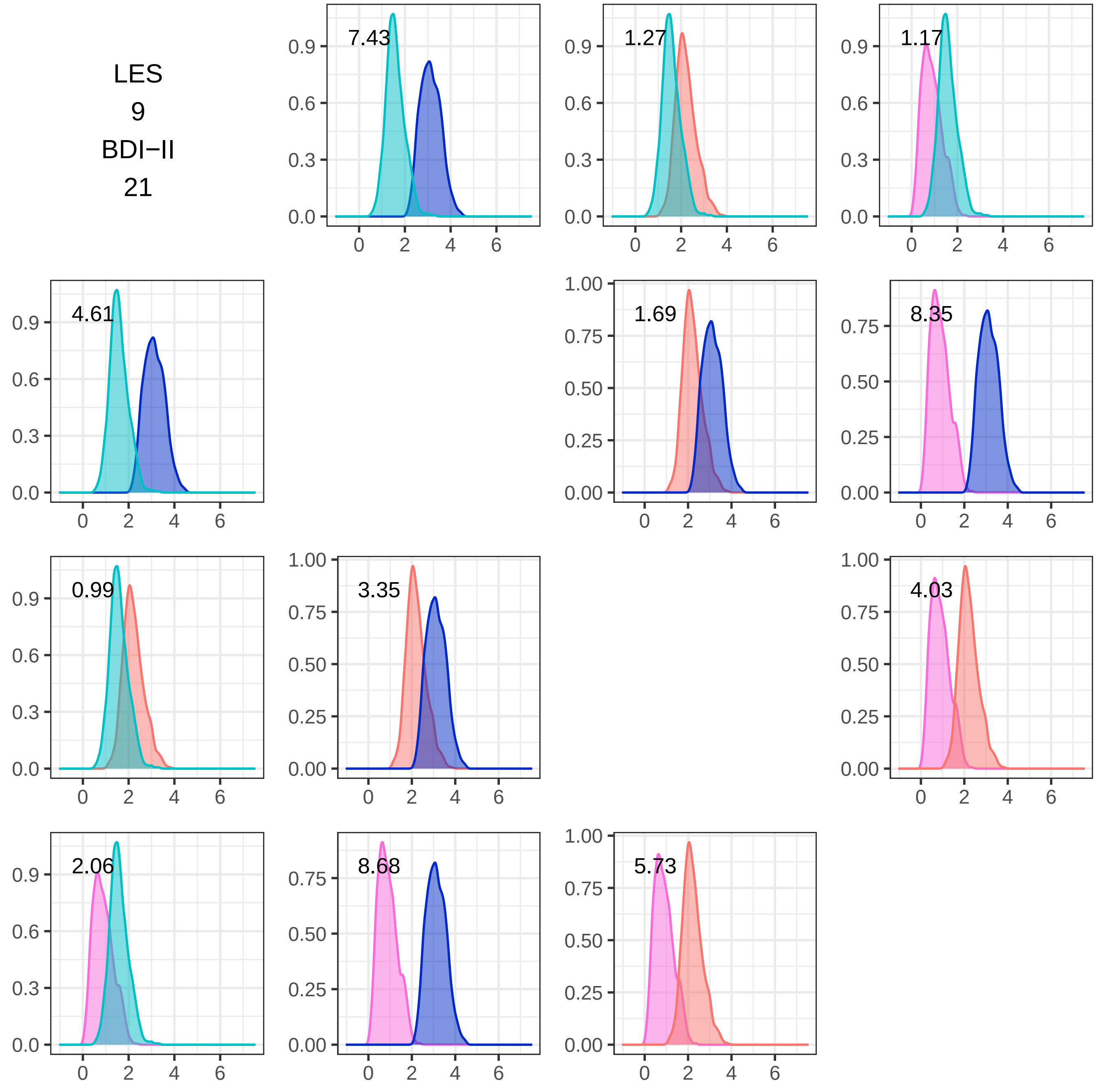
LES
9
BDI−II
21
7.43
1.27
1.17
4.61
1.69
8.35
0.99
3.35
4.03
2.06
8.68
5.73

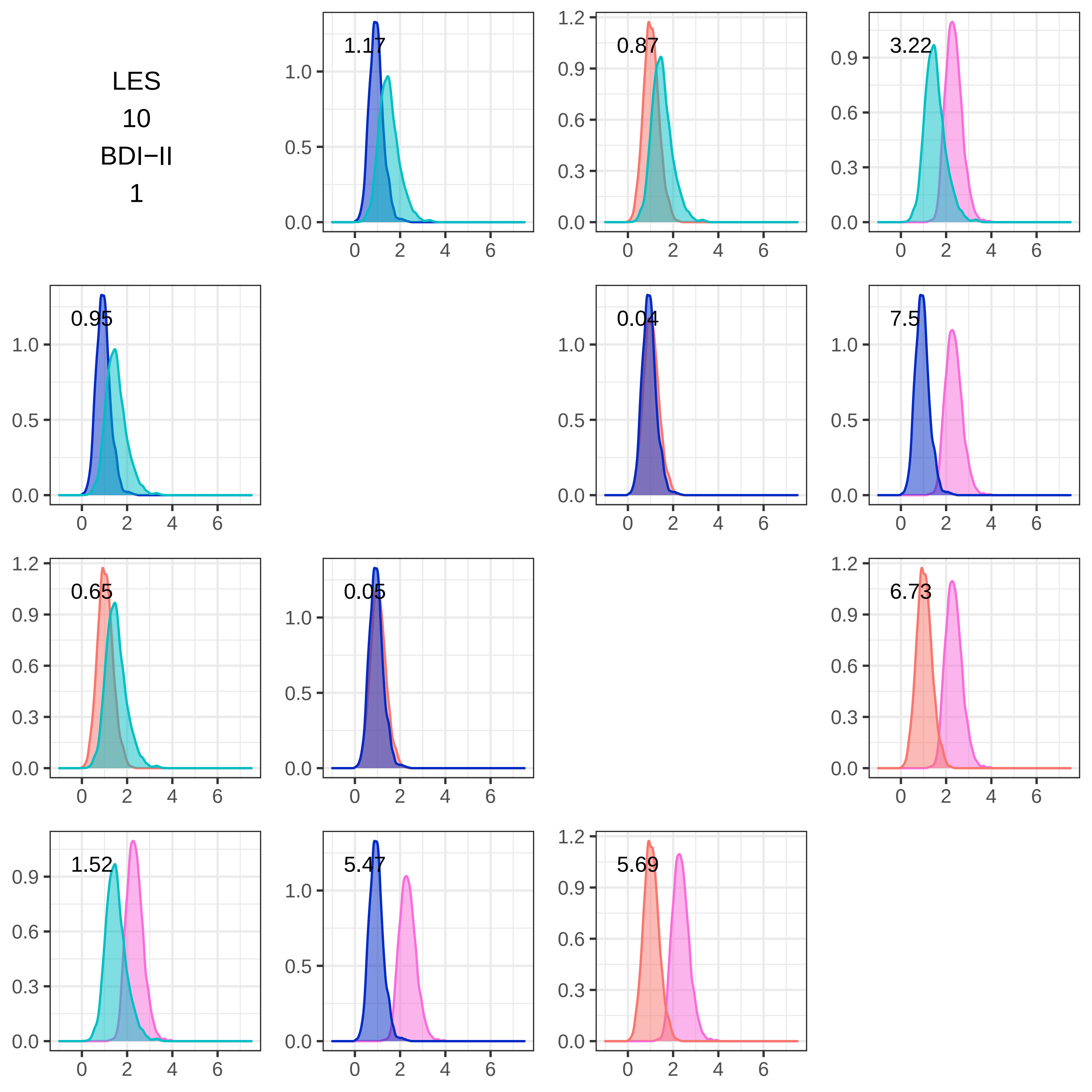
LES
10
BDI−II
1
1.17
0.87
3.22
0.95
0.04
7.5
0.65
0.05
6.73
1.52
5.47
5.69

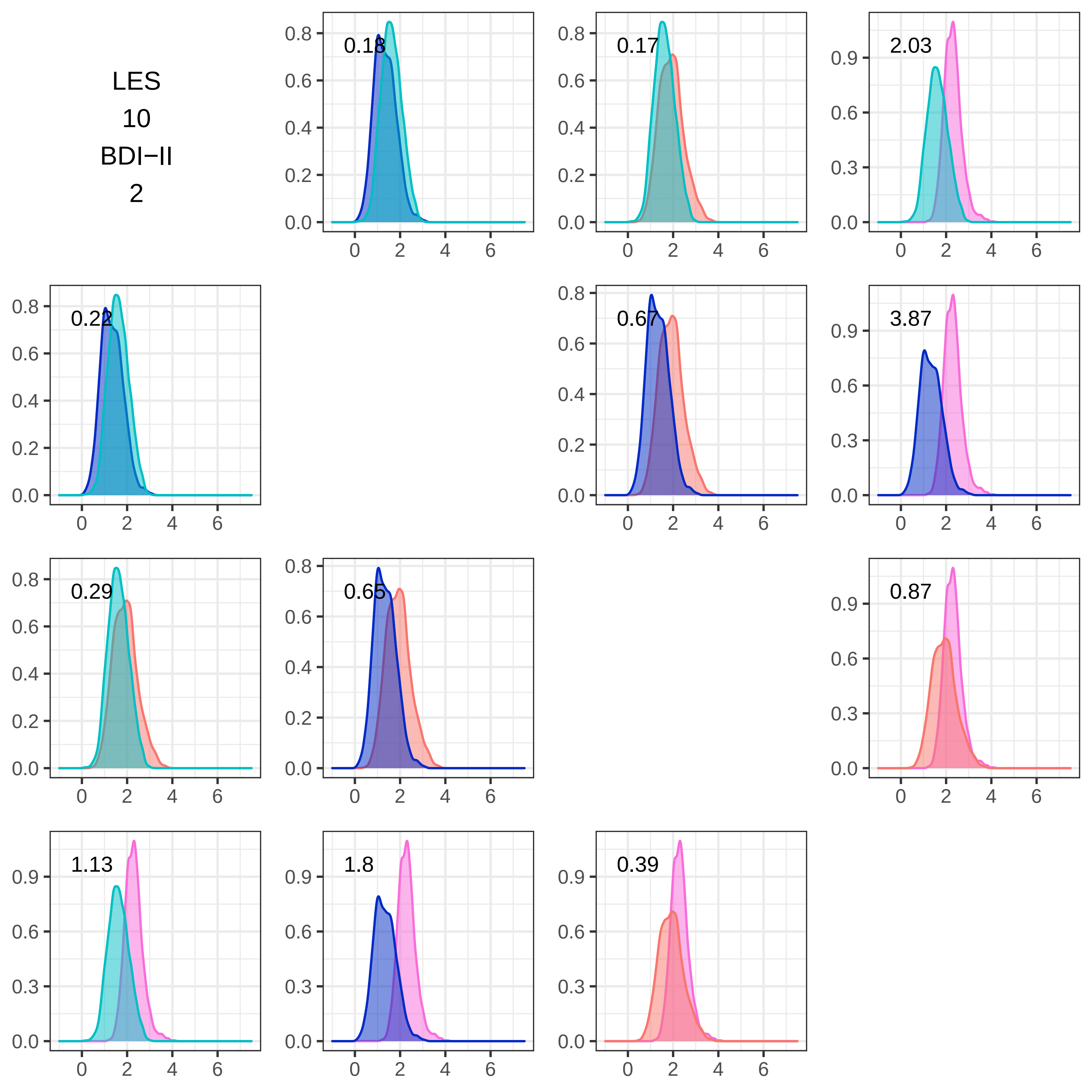
LES
10
BDI−II
2
0.18
0.17
2.03
0.22
0.67
3.87
0.29
0.65
0.87
1.13
1.8
0.39

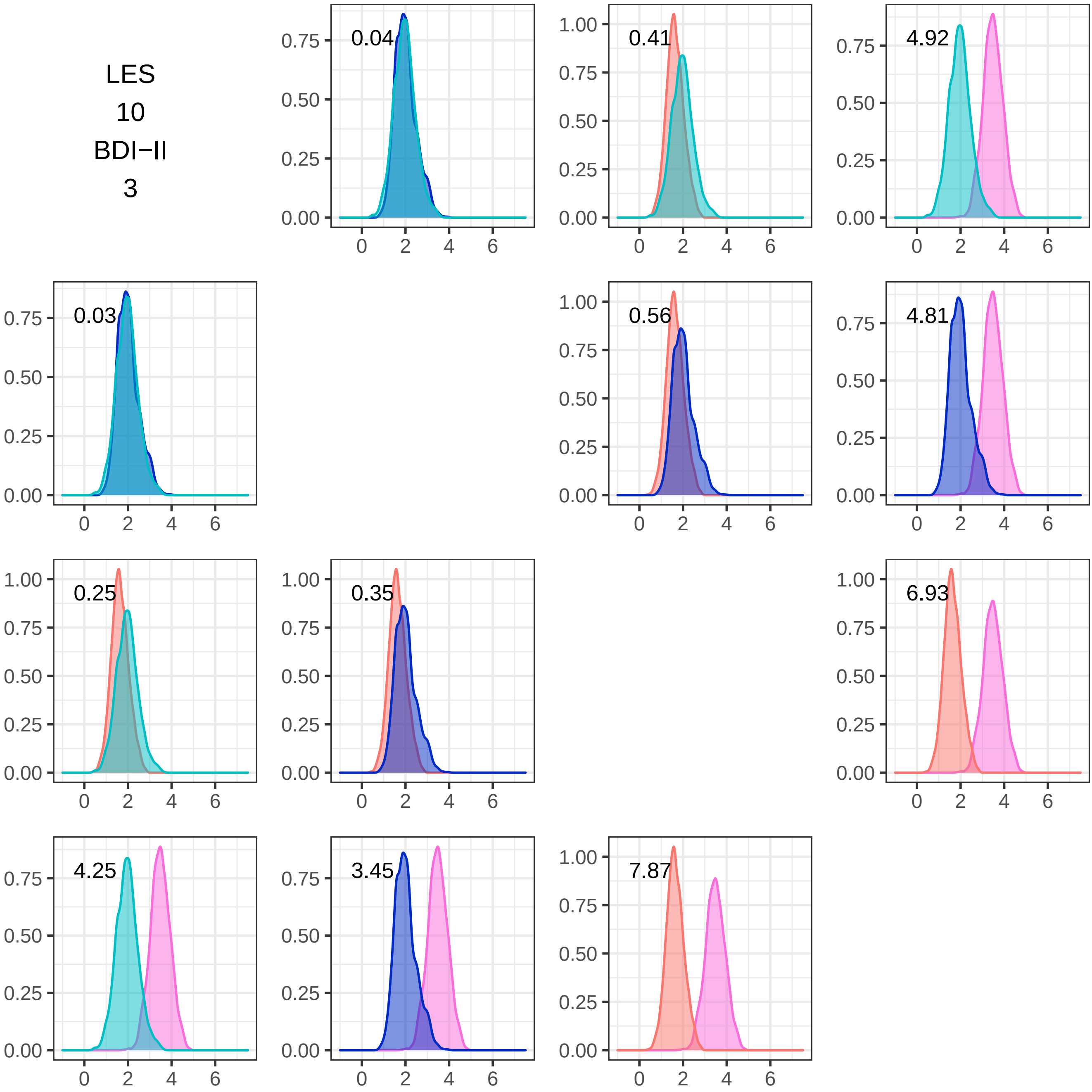
LES
10
BDI−II
3
0.04
0.41
4.92
0.03
0.56
4.81
0.25
0.35
6.93
4.25
3.45
7.87

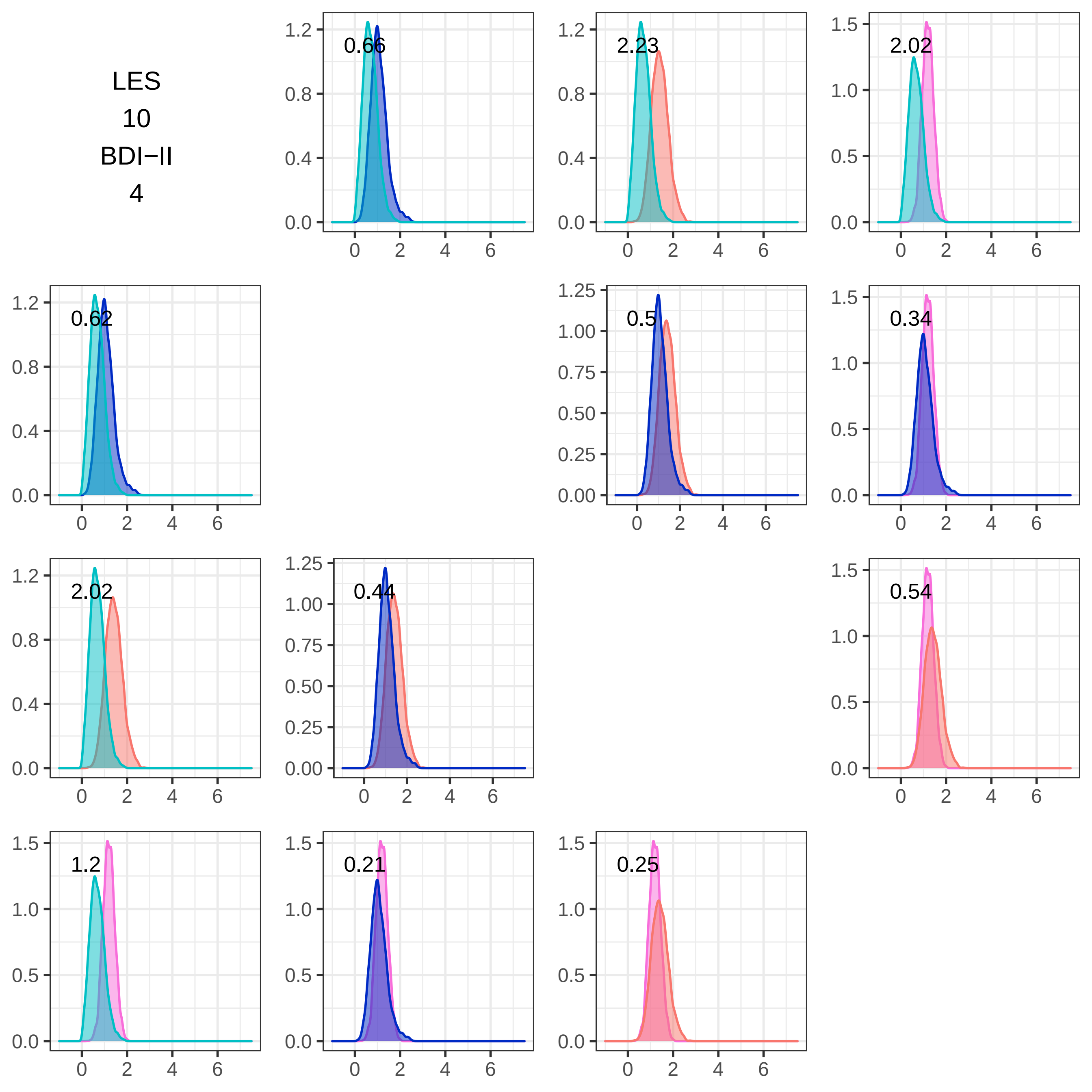

LES
10
BDI−II
4
0.66
2.23
2.02
0.62
0.5
0.34
2.02
0.44
0.54
1.2
0.21
0.25

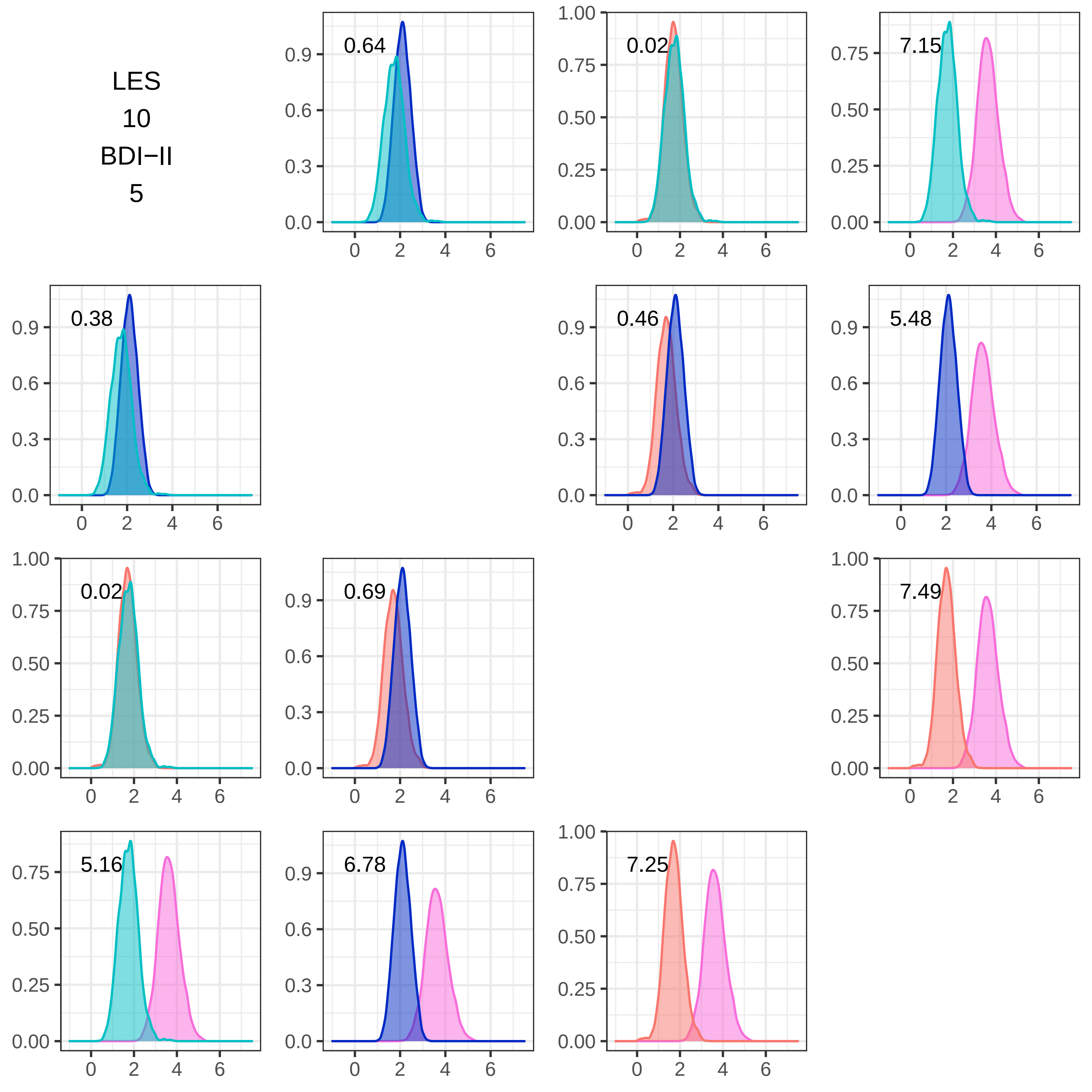

LES
10
BDI−II
5
0.64
0.02
7.15
0.38
0.46
5.48
0.02
0.69
7.49
5.16
6.78
7.25

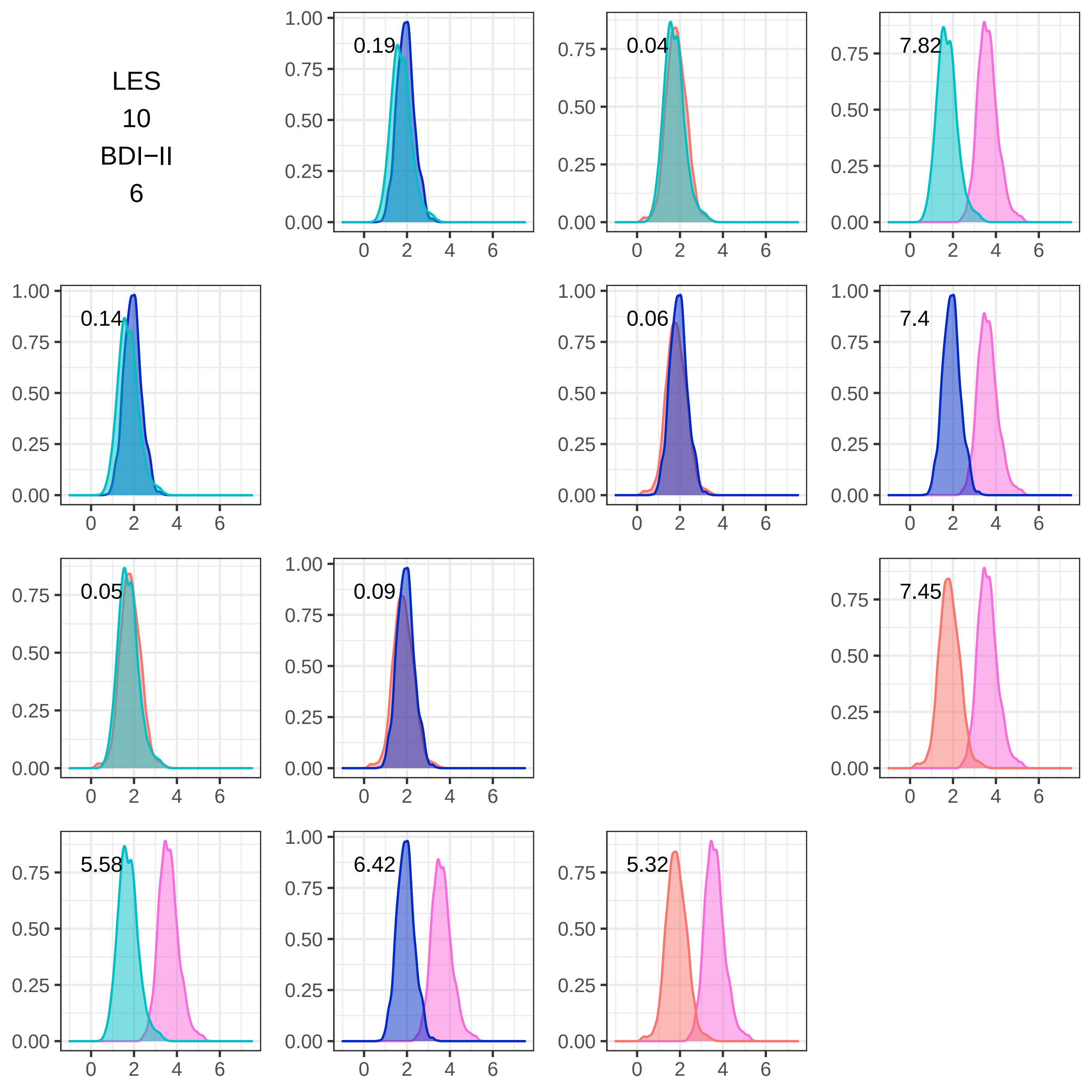
LES
10
BDI−II
6
0.19
0.04
7.82
0.14
0.06
7.4
0.05
0.09
7.45
5.58
6.42
5.32

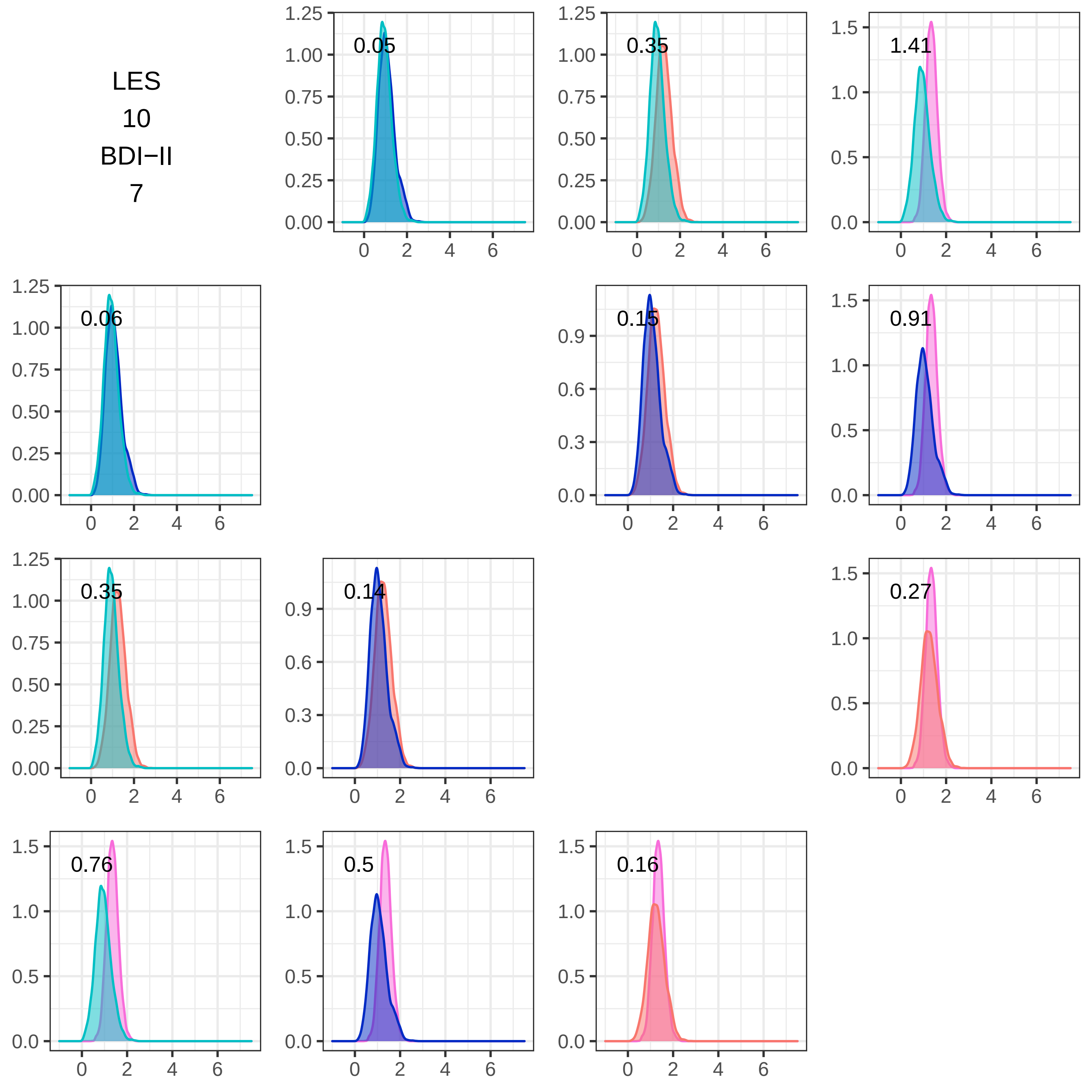
LES
10
BDI−II
7
0.05
0.35
1.41
0.06
0.15
0.91
0.35
0.14
0.27
0.76
0.5
0.16

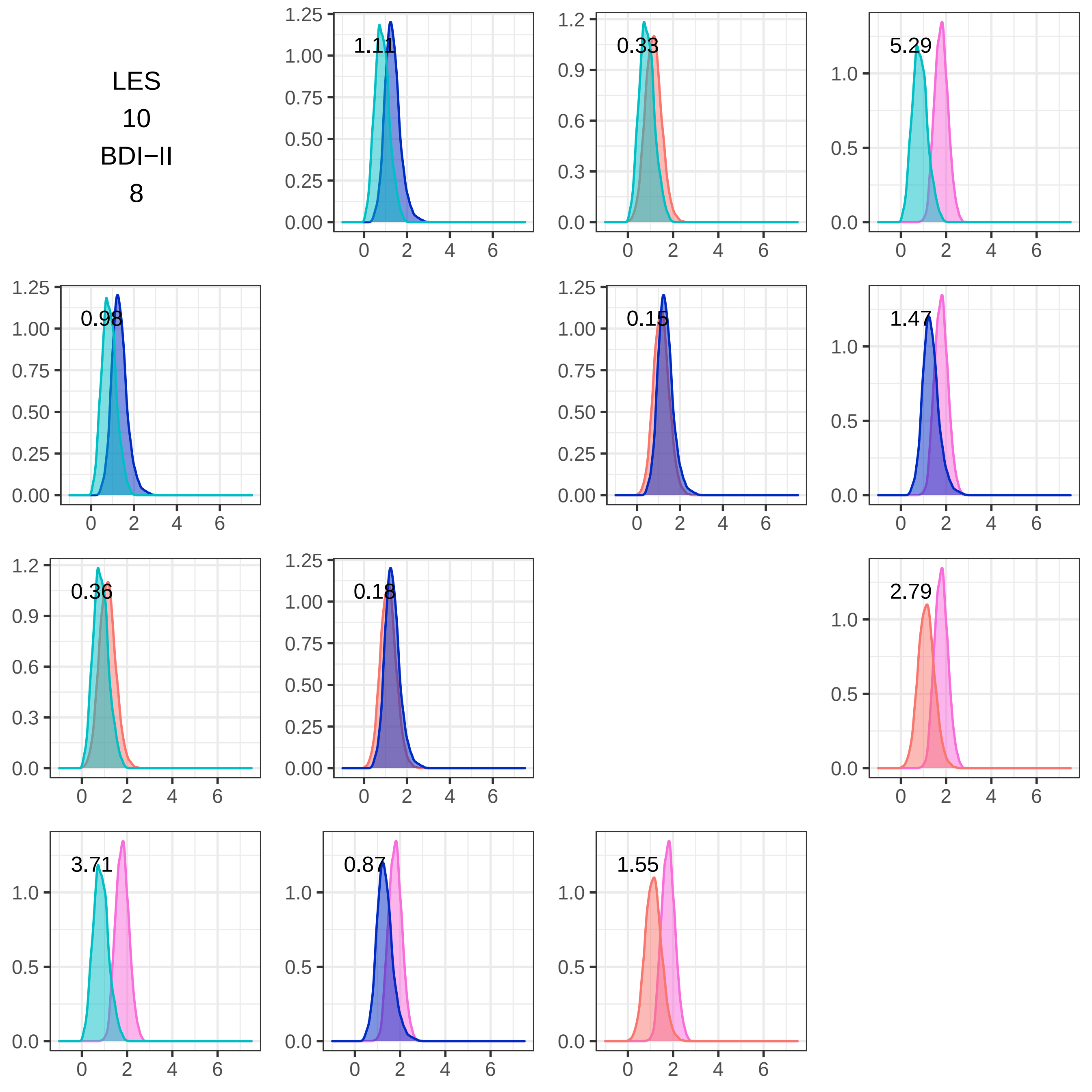
LES
10
BDI−II
8
1.11
0.33
5.29
0.98
0.15
1.47
0.36
0.18
2.79
3.71
0.87
1.55

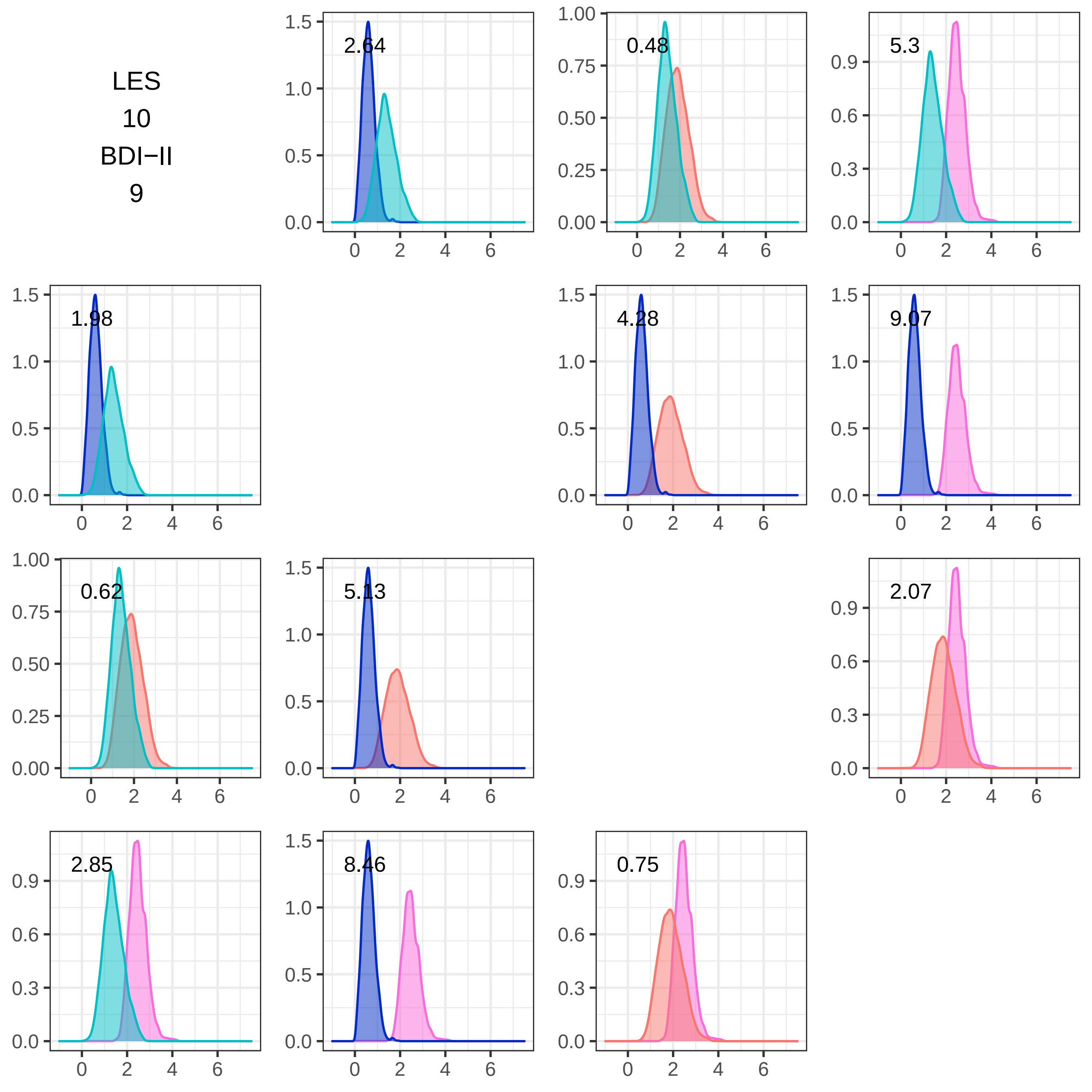

LES
10
BDI−II
9
2.64
0.48
5.3
1.98
4.28
9.07
0.62
5.13
2.07
2.85
8.46
0.75

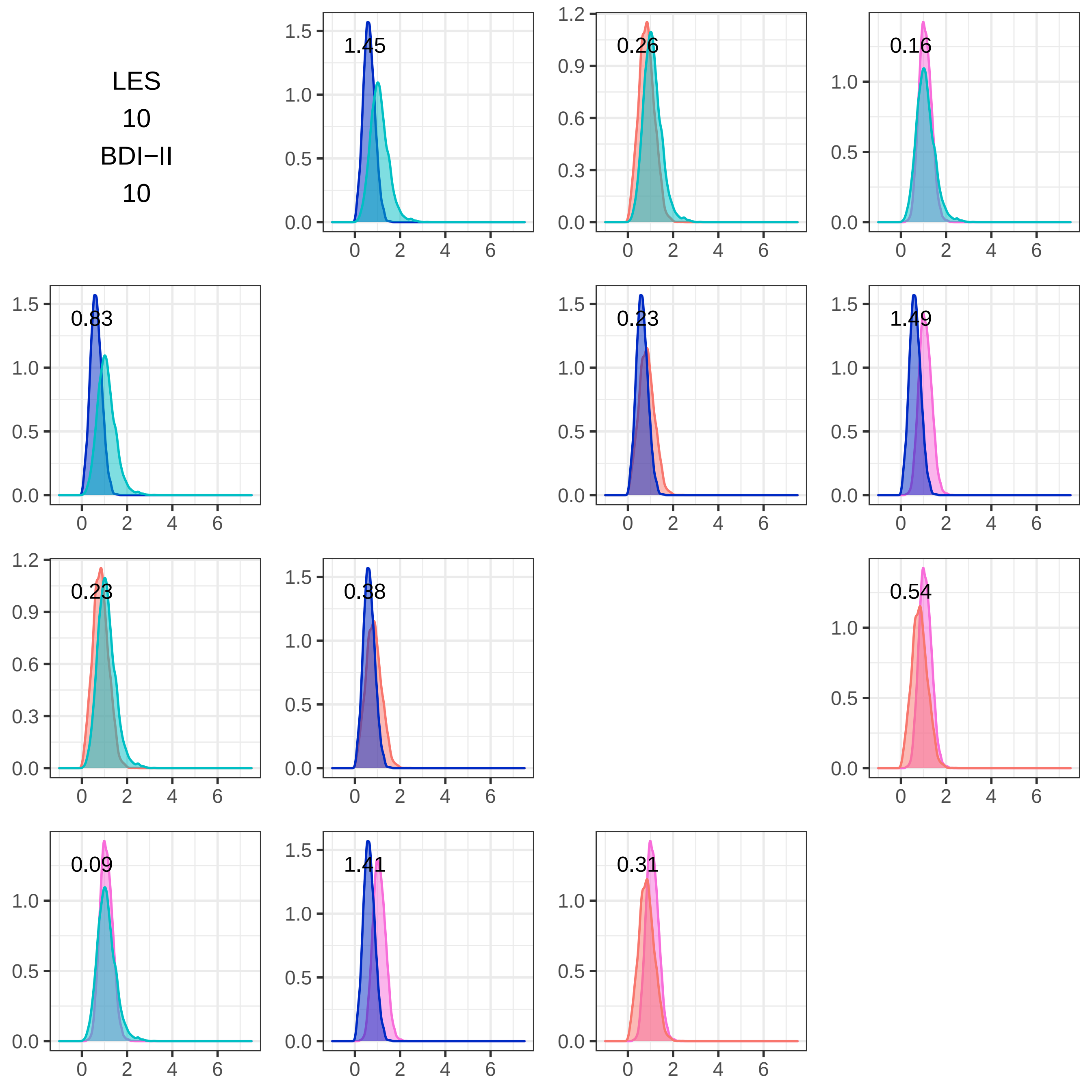

LES
10
BDI−II
10
1.45
0.26
0.16
0.83
0.23
1.49
0.23
0.38
0.54
0.09
1.41
0.31

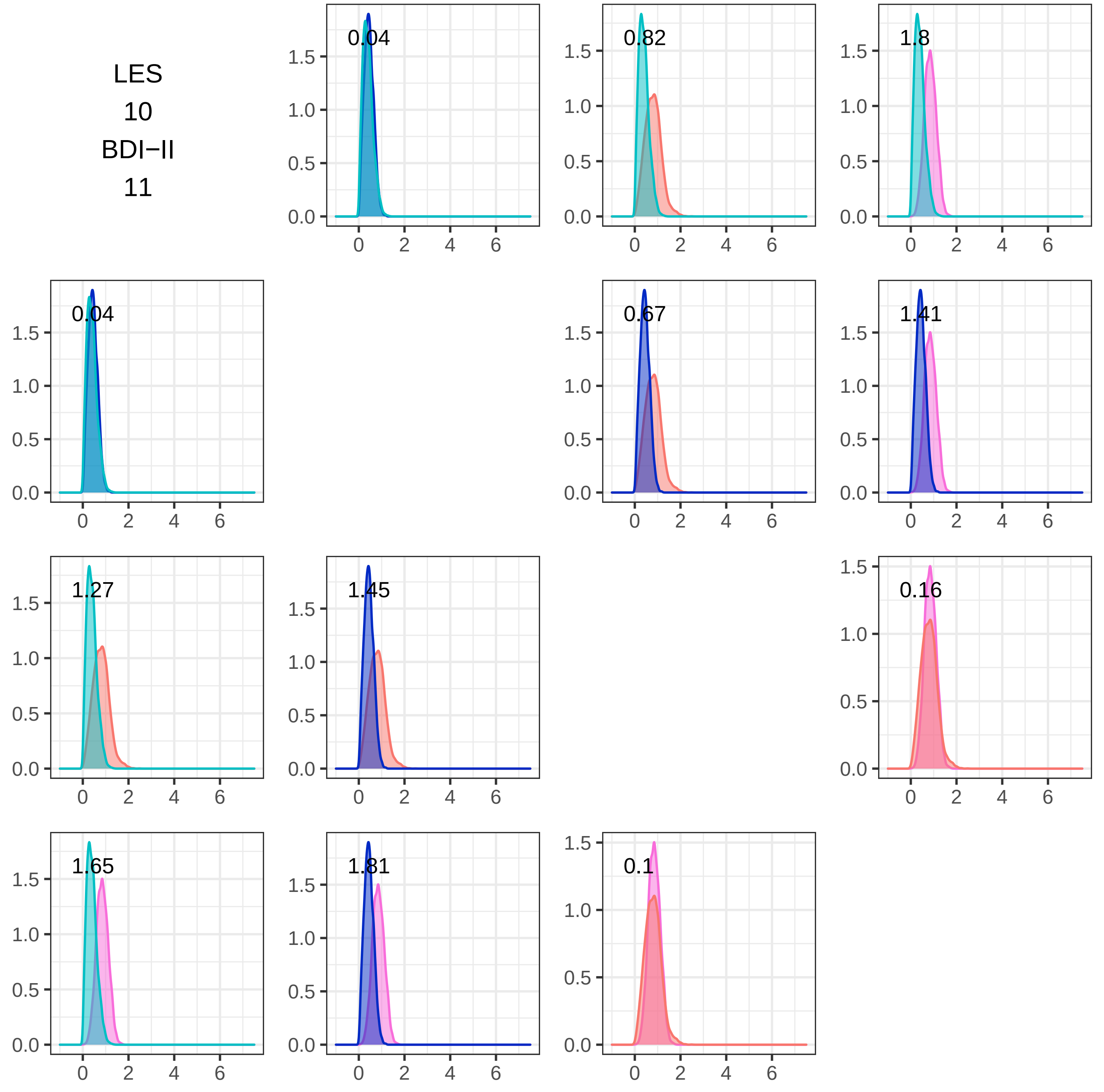
LES
10
BDI−II
11
0.04
0.82
1.8
0.04
0.67
1.41
1.27
1.45
0.16
1.65
1.81
0.1

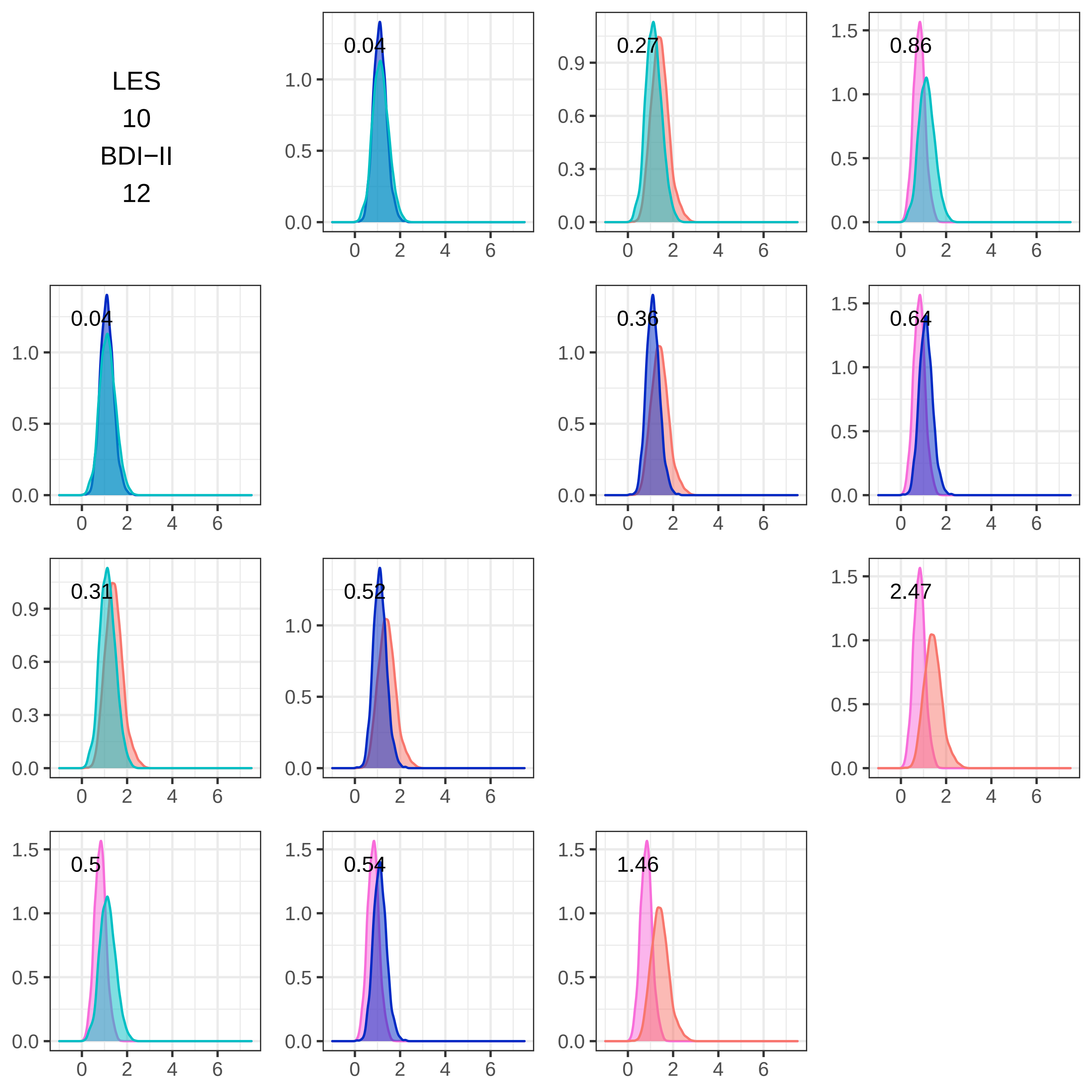

LES
10
BDI−II
12
0.04
0.27
0.86
0.04
0.36
0.64
0.31
0.52
2.47
0.5
0.54
1.46

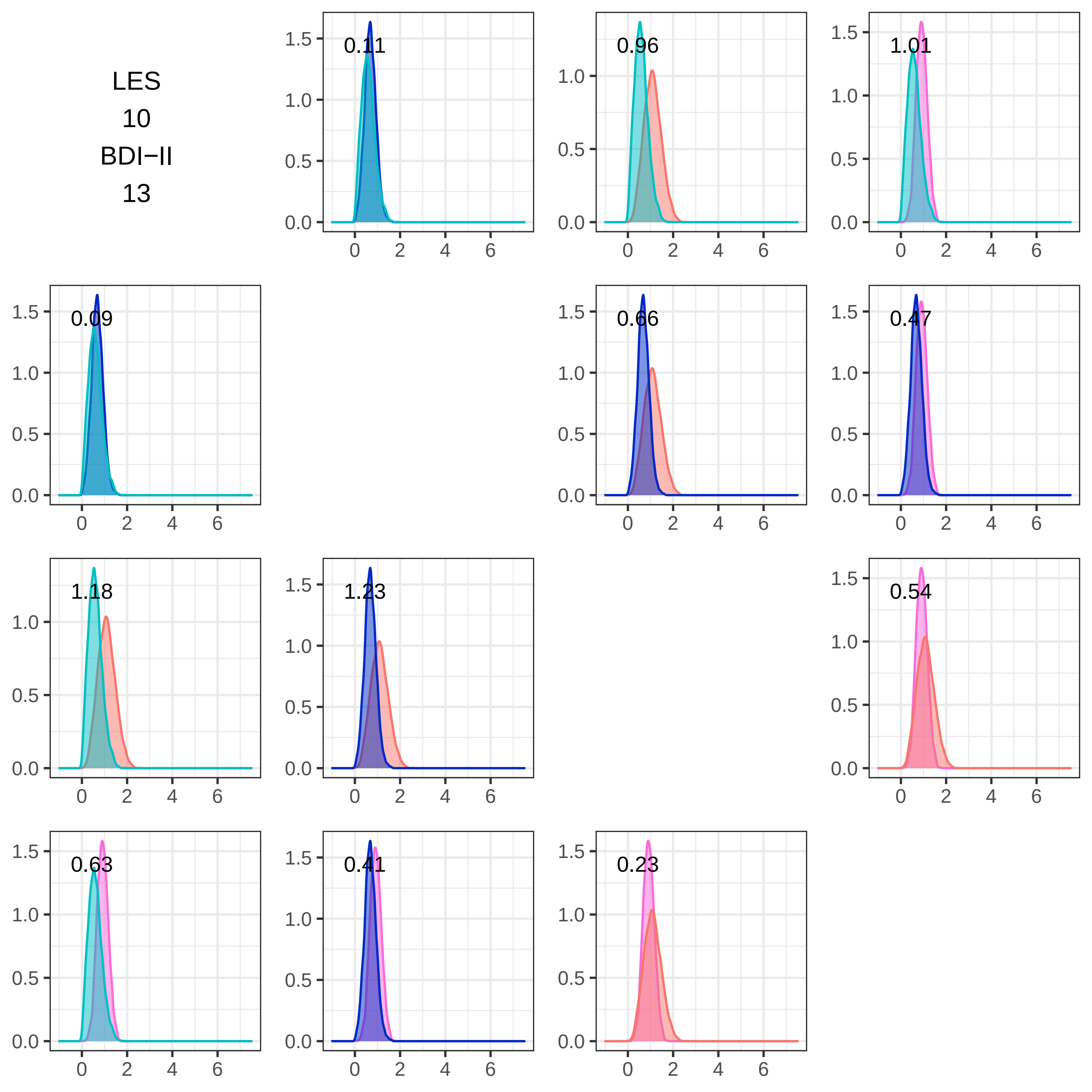
LES
10
BDI−II
13
0.11
0.96
1.01
0.09
0.66
0.47
1.18
1.23
0.54
0.63
0.41
0.23

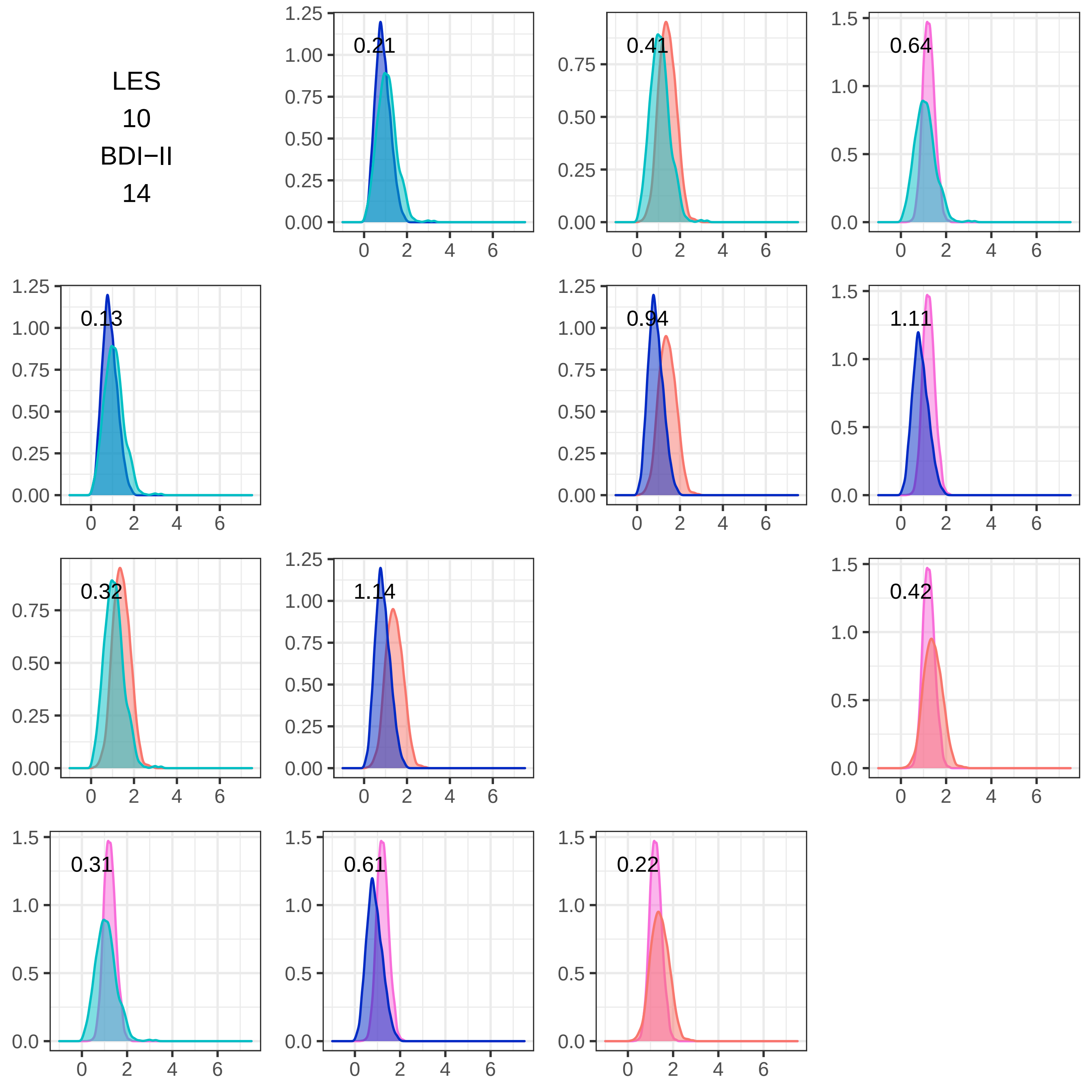
LES
10
BDI−II
14
0.21
0.41
0.64
0.13
0.94
1.11
0.32
1.14
0.42
0.31
0.61
0.22

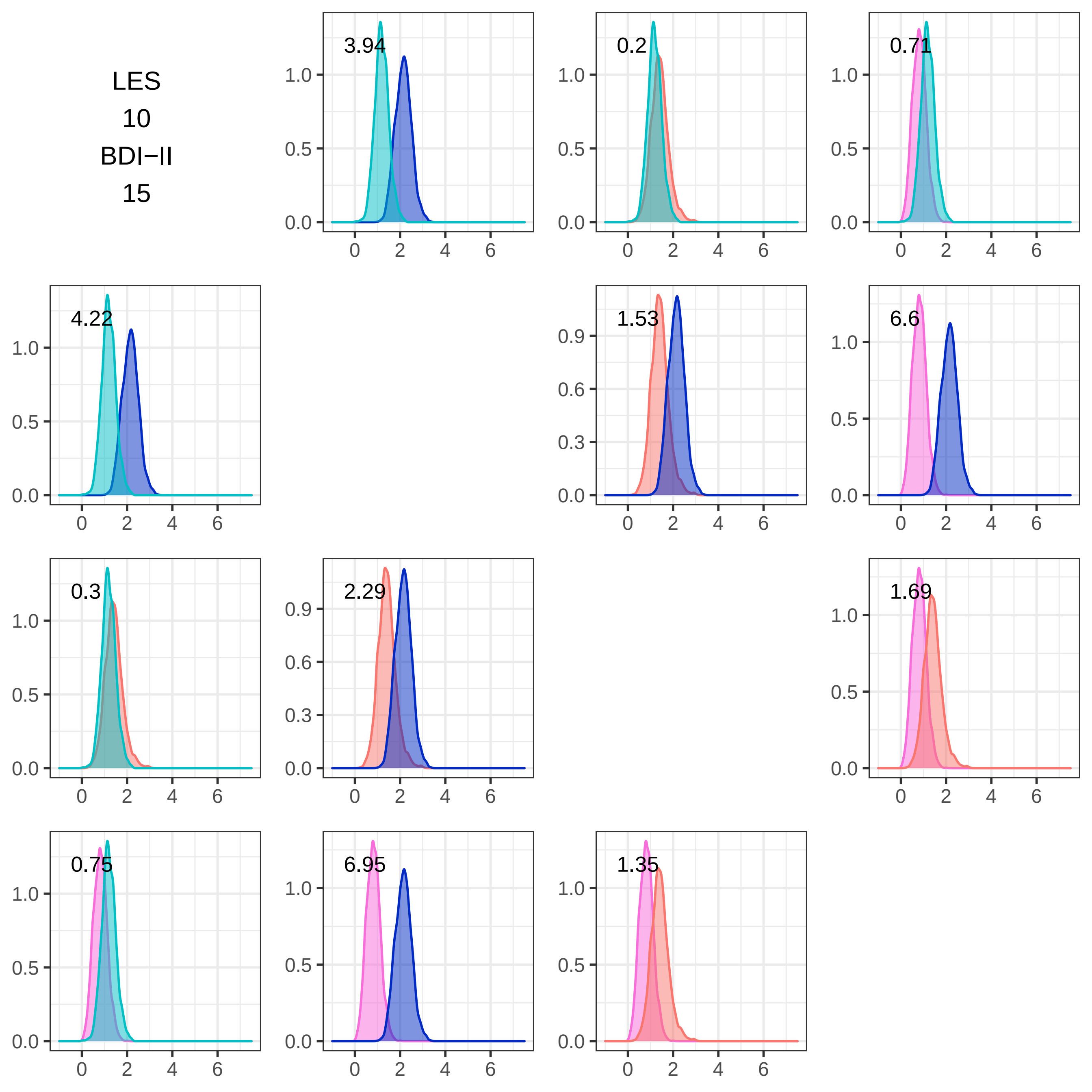
LES
10
BDI−II
15
3.94
0.2
0.71
4.22
1.53
6.6
0.3
2.29
1.69
0.75
6.95
1.35

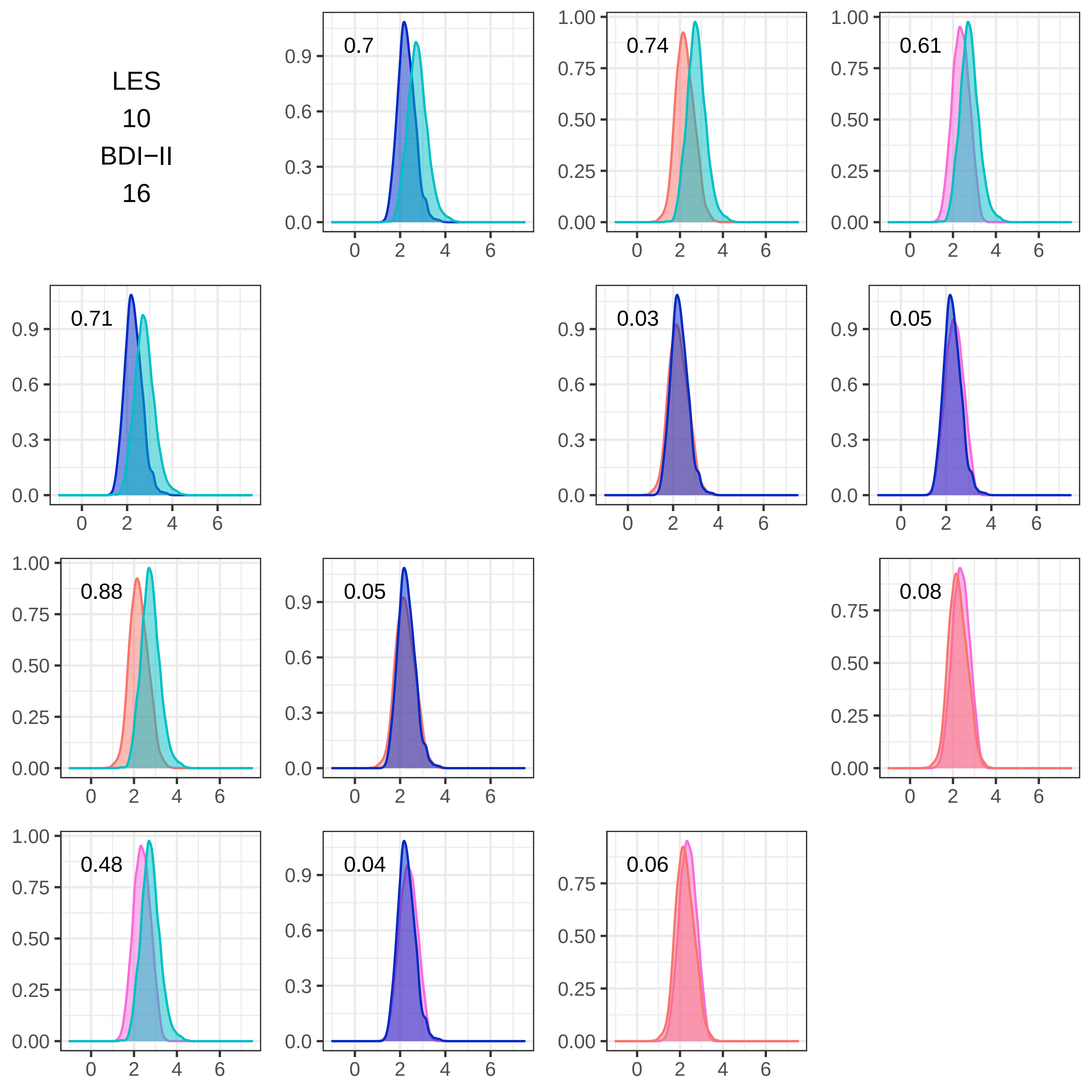

LES
10
BDI−II
16
0.7
0.74
0.61
0.71
0.03
0.05
0.88
0.05
0.08
0.48
0.04
0.06

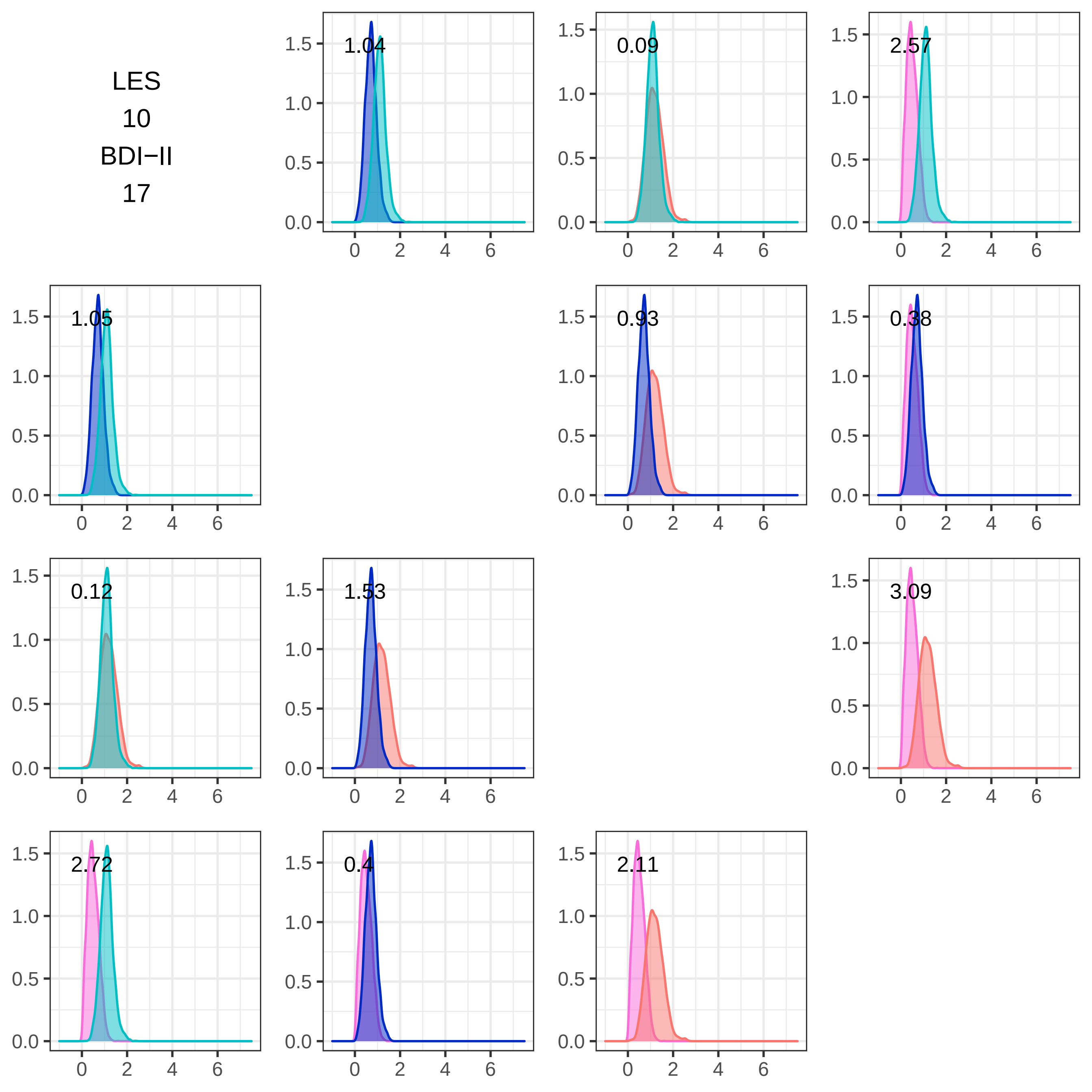

LES
10
BDI−II
17
1.04
0.09
2.57
1.05
0.93
0.38
0.12
1.53
3.09
2.72
0.4
2.11

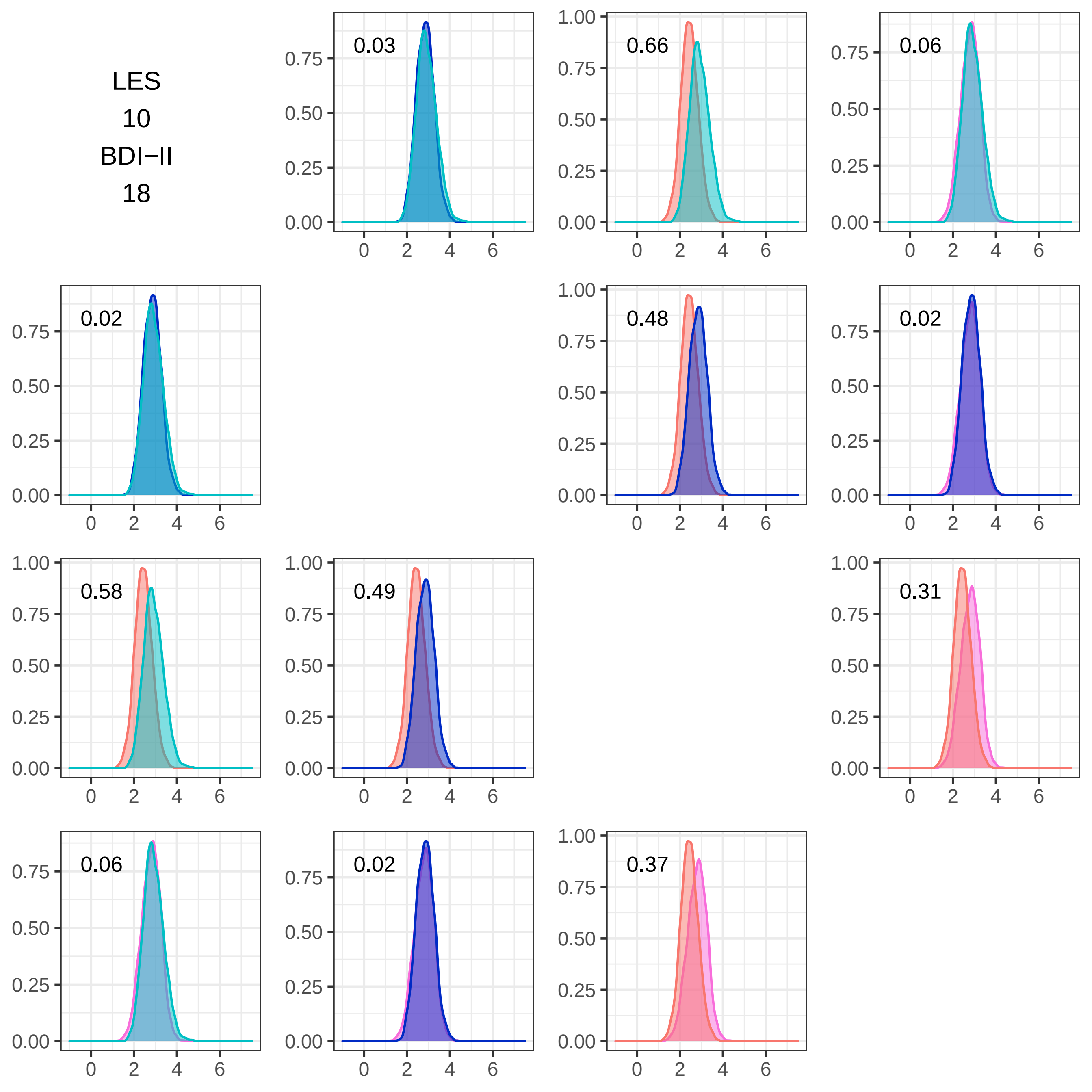
LES
10
BDI−II
18
0.03
0.66
0.06
0.02
0.48
0.02
0.58
0.49
0.31
0.06
0.02
0.37

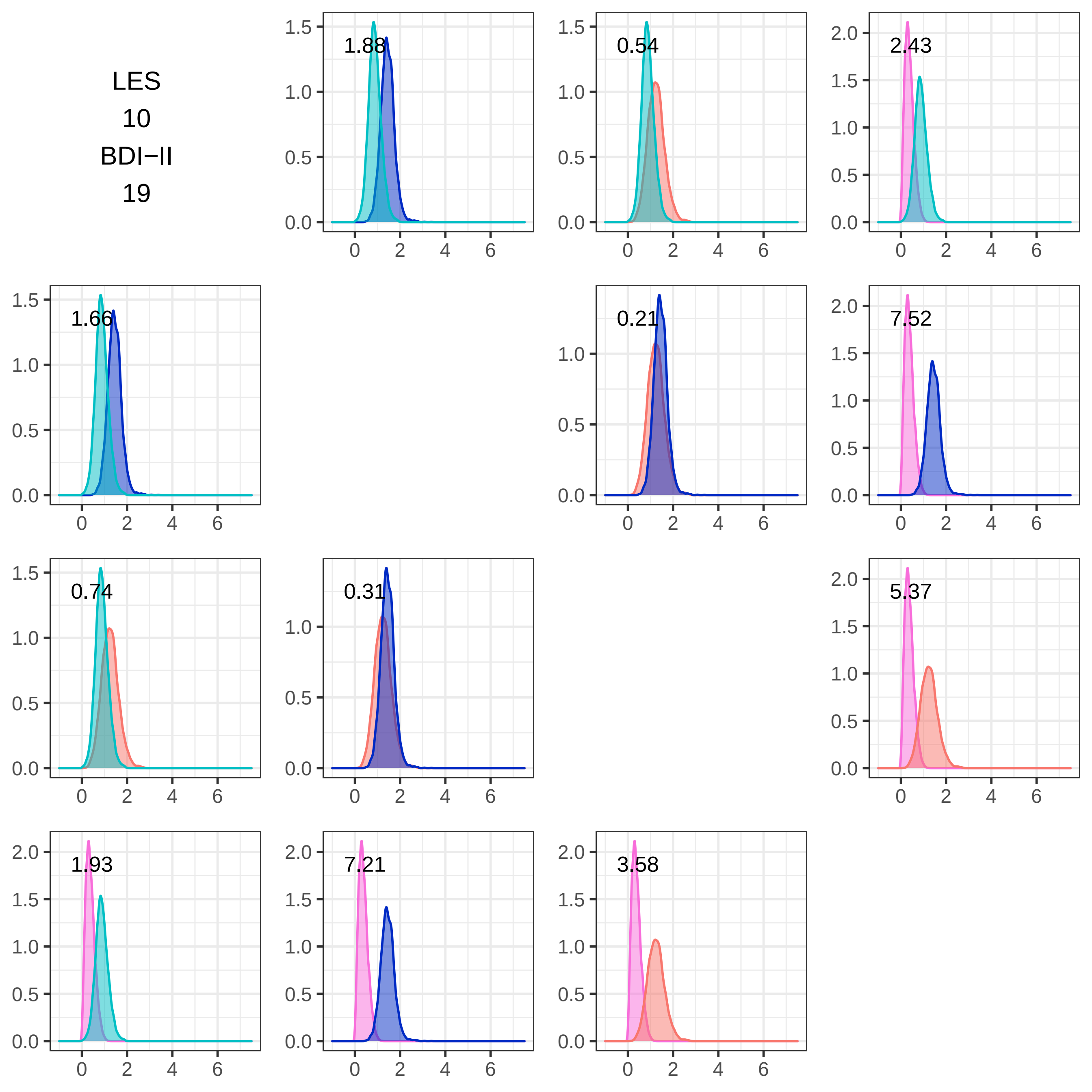

LES
10
BDI−II
19
1.88
0.54
2.43
1.66
0.21
7.52
0.74
0.31
5.37
1.93
7.21
3.58

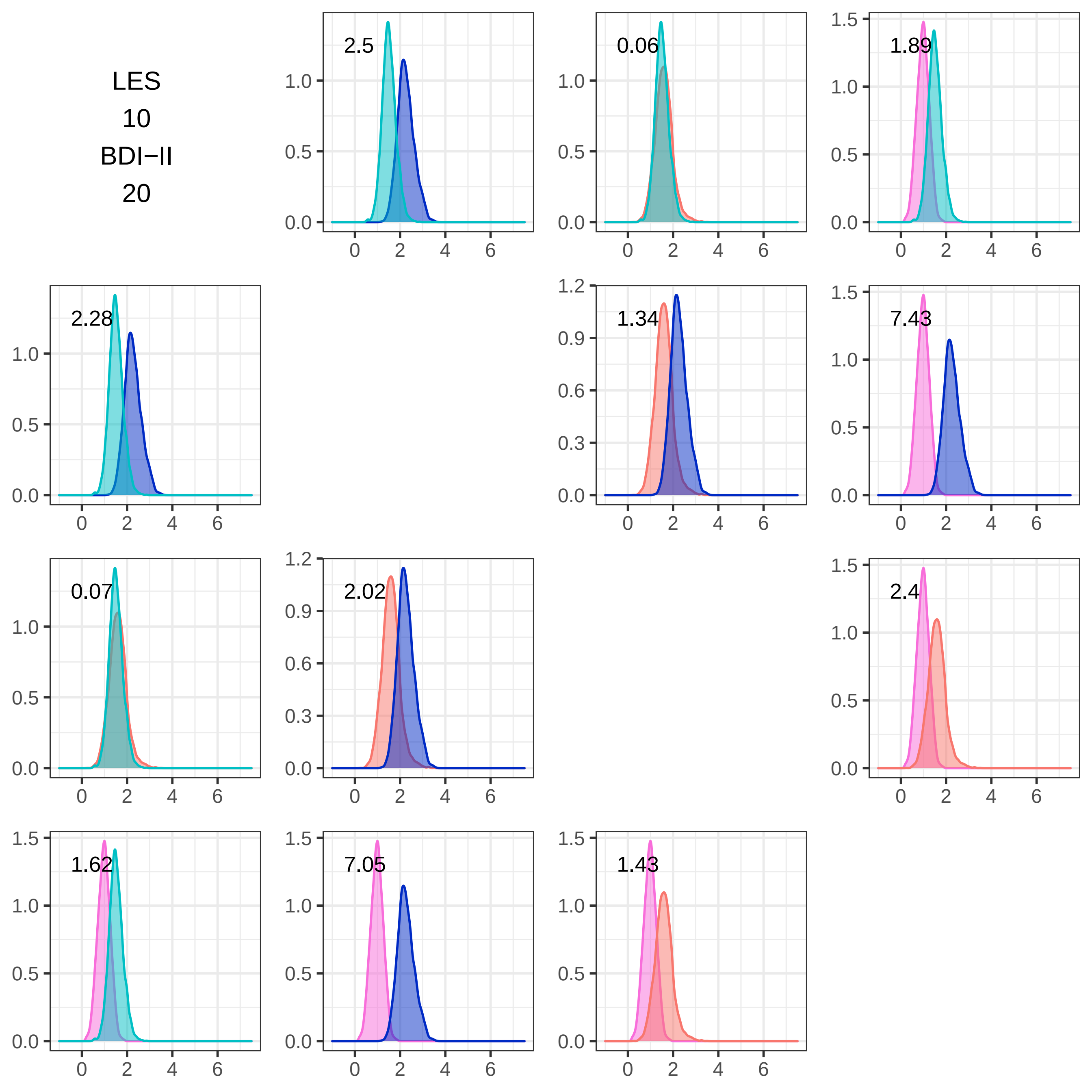
LES
10
BDI−II
20
2.5
0.06
1.89
2.28
1.34
7.43
0.07
2.02
2.4
1.62
7.05
1.43

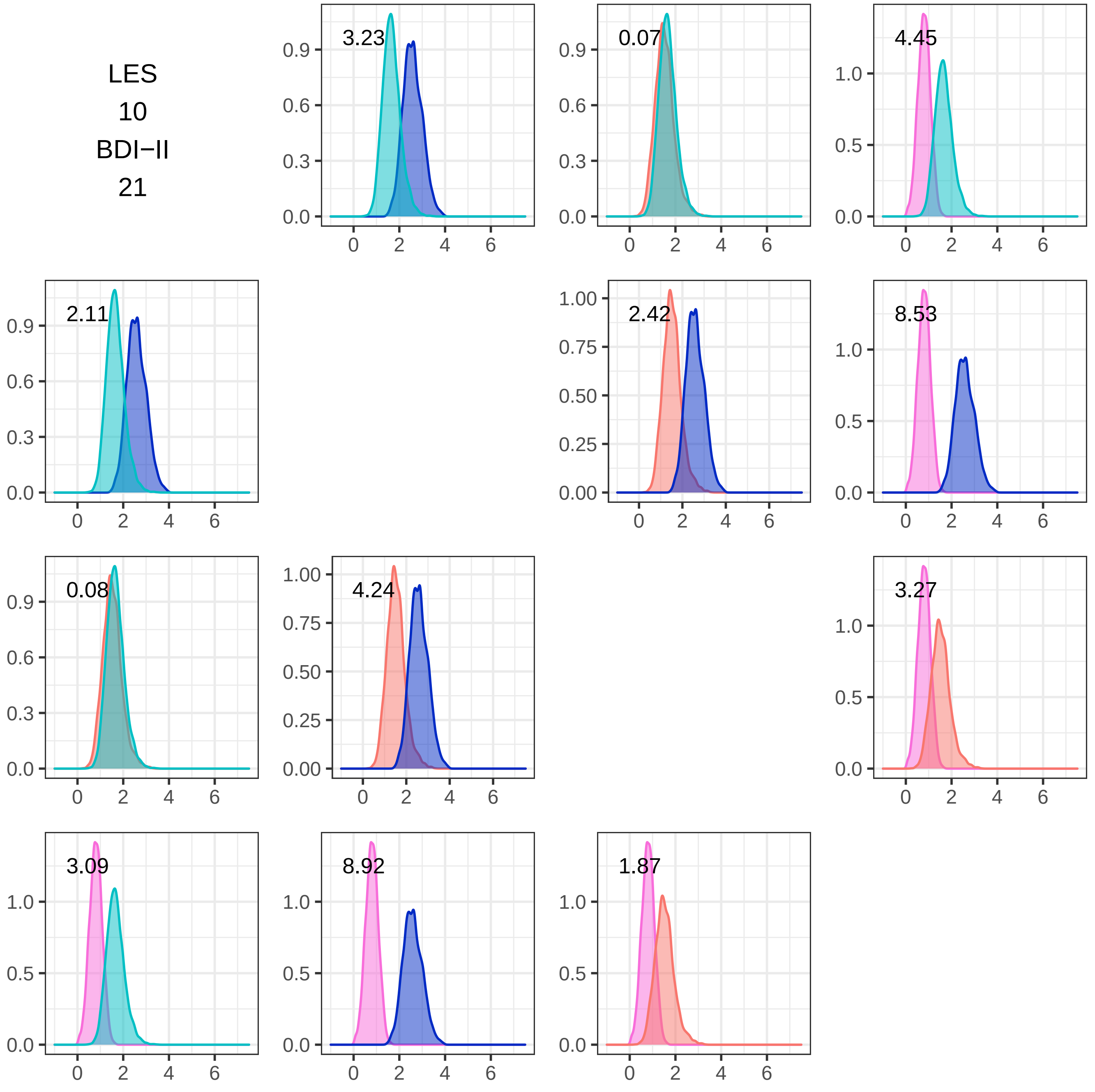
LES
10
BDI−II
21
3.23
0.07
4.45
2.11
2.42
8.53
0.08
4.24
3.27
3.09
8.92
1.87

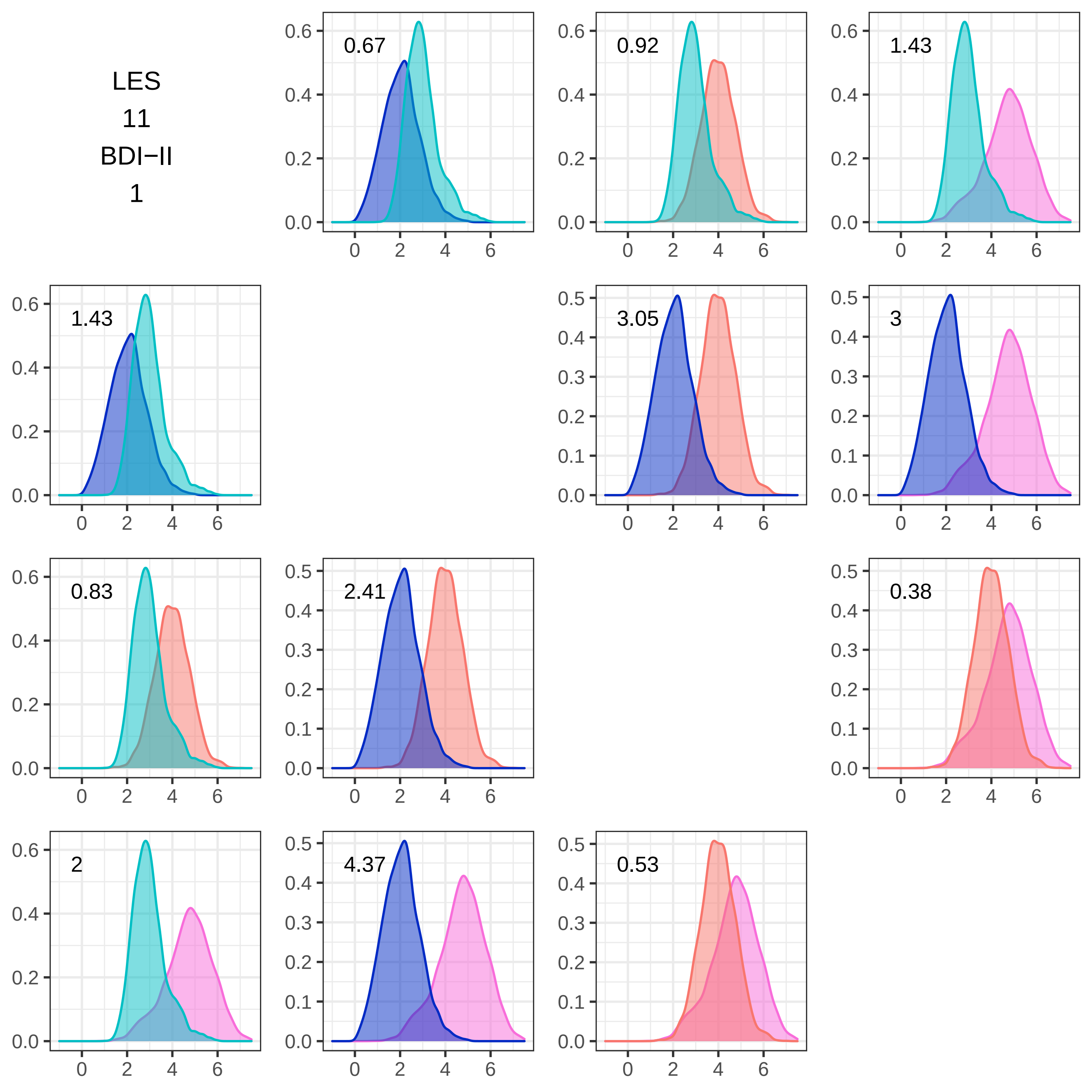
LES
11
BDI−II
1
0.67
0.92
1.43
1.43
3.05
3
0.83
2.41
0.38
2
4.37
0.53

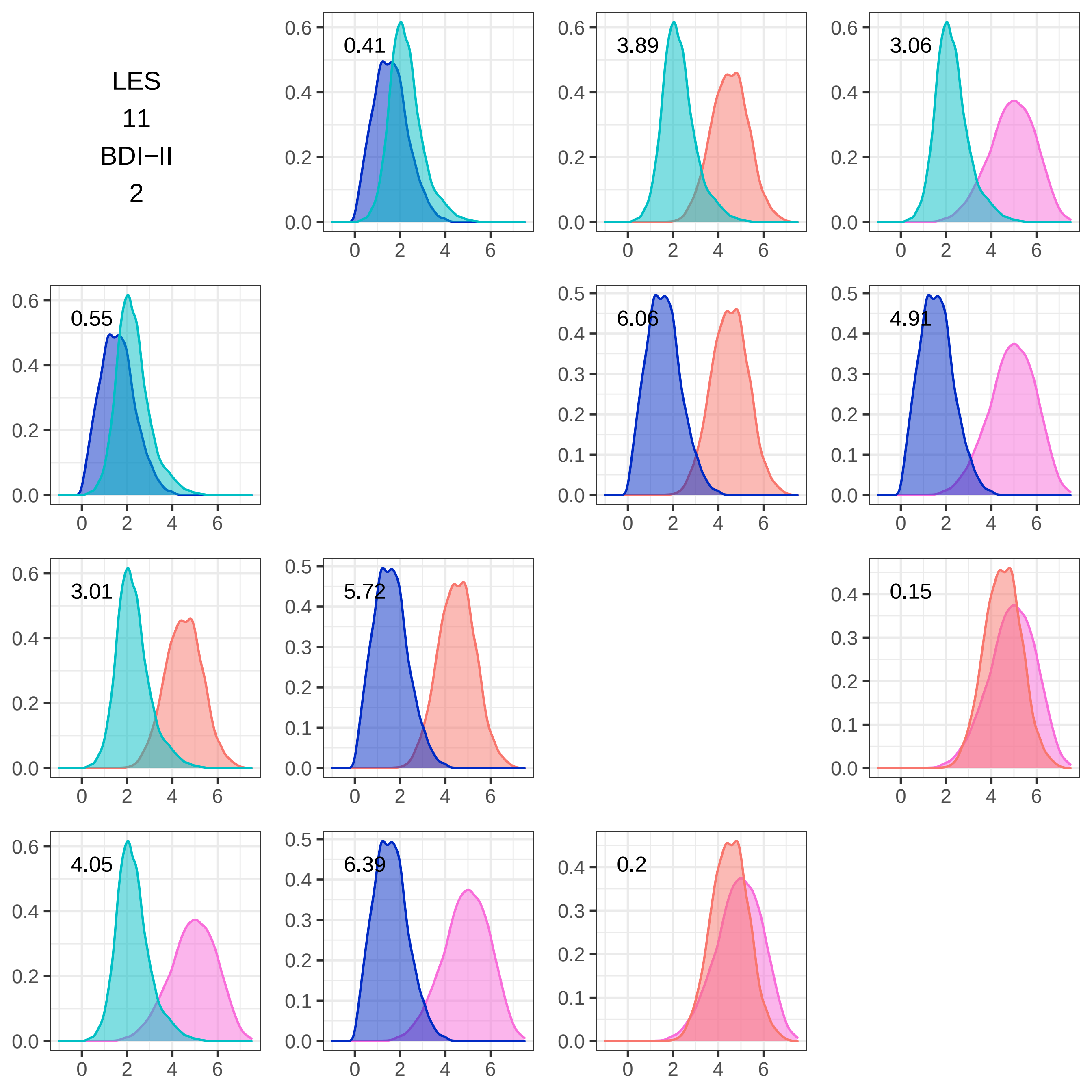
LES
11
BDI−II
2
0.41
3.89
3.06
0.55
6.06
4.91
3.01
5.72
0.15
4.05
6.39
0.2

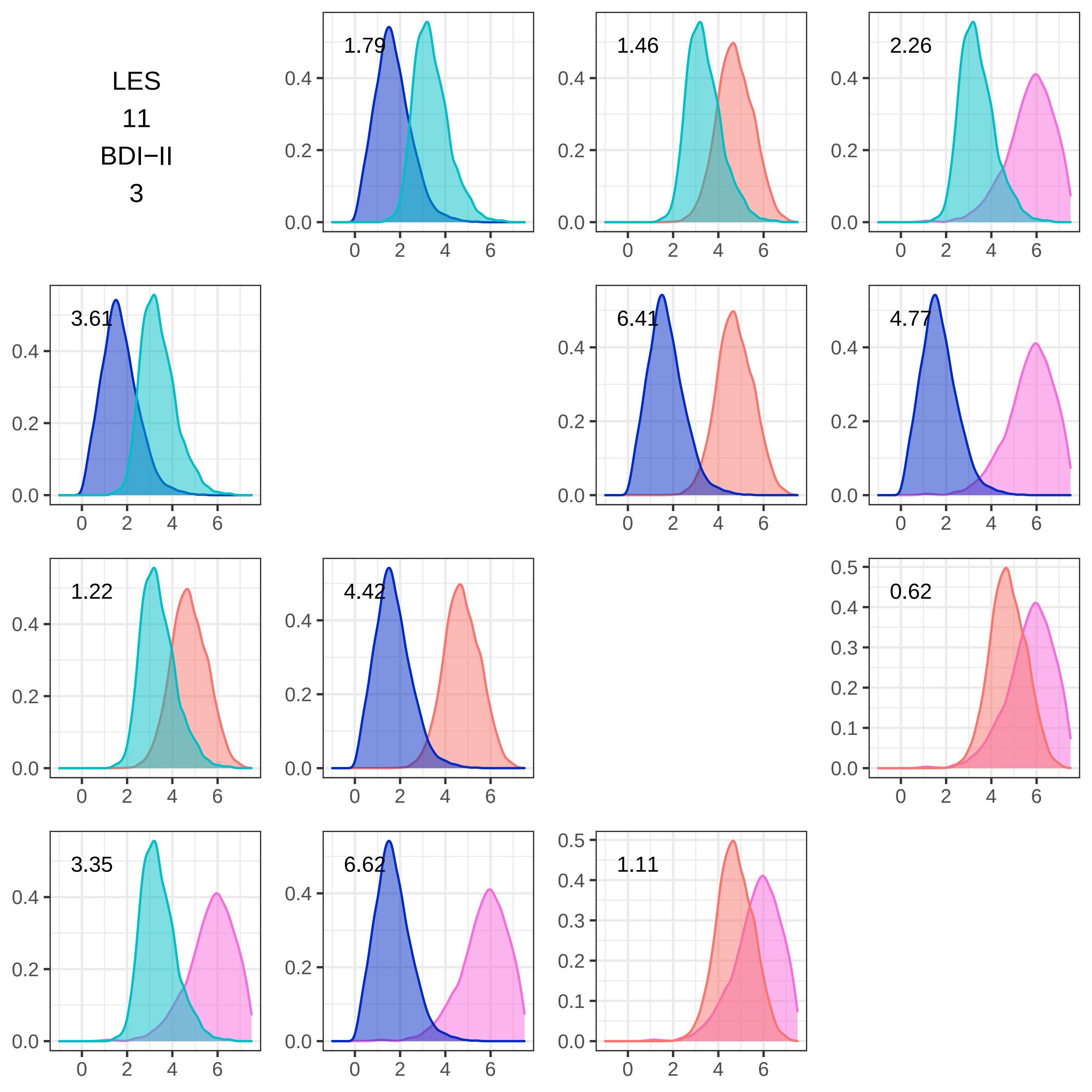
LES
11
BDI−II
3
1.79
1.46
2.26
3.61
6.41
4.77
1.22
4.42
0.62
3.35
6.62
1.11

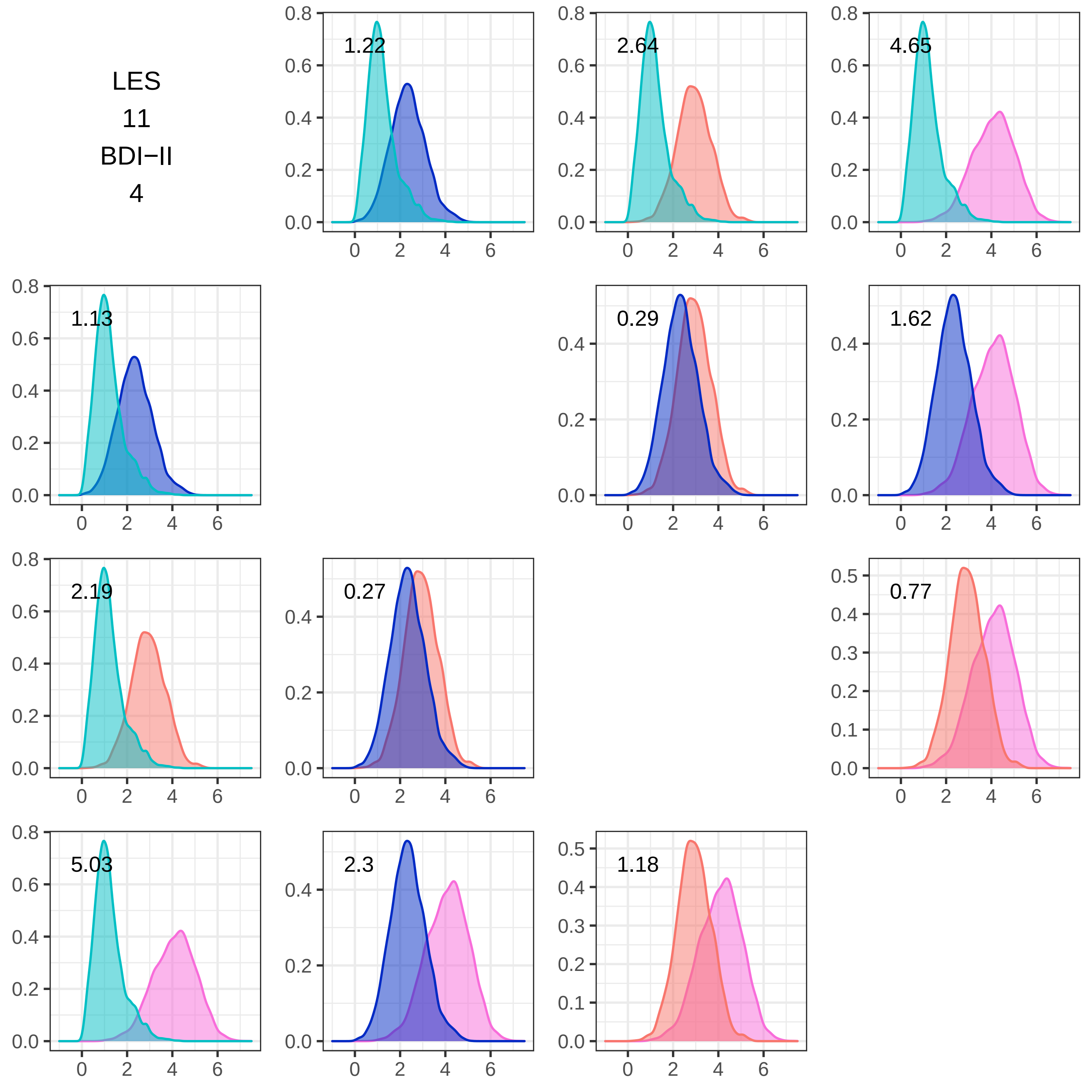
LES
11
BDI−II
4
1.22
2.64
4.65
1.13
0.29
1.62
2.19
0.27
0.77
5.03
2.3
1.18

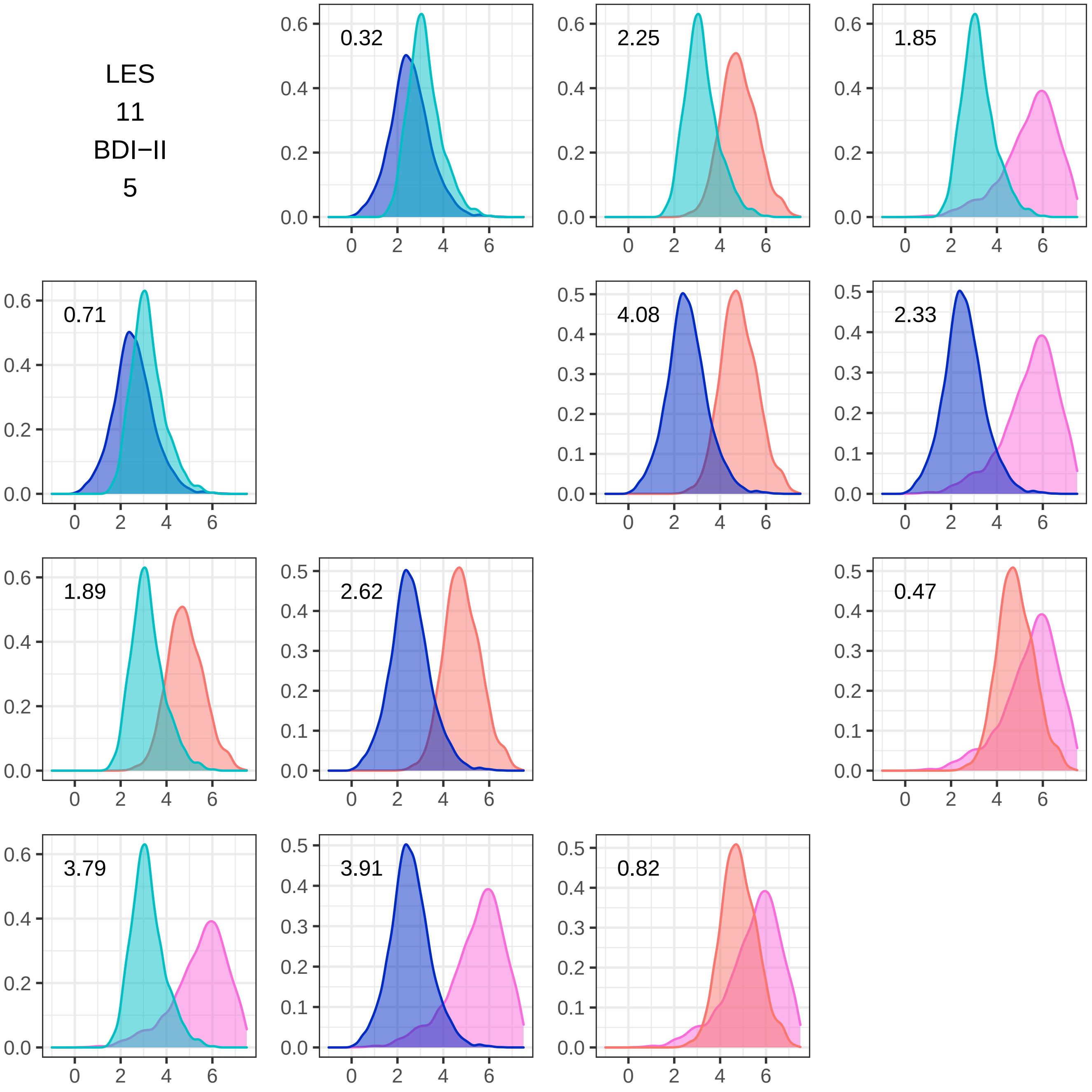
LES
11
BDI−II
5
0.32
2.25
1.85
0.71
4.08
2.33
1.89
2.62
0.47
3.79
3.91
0.82

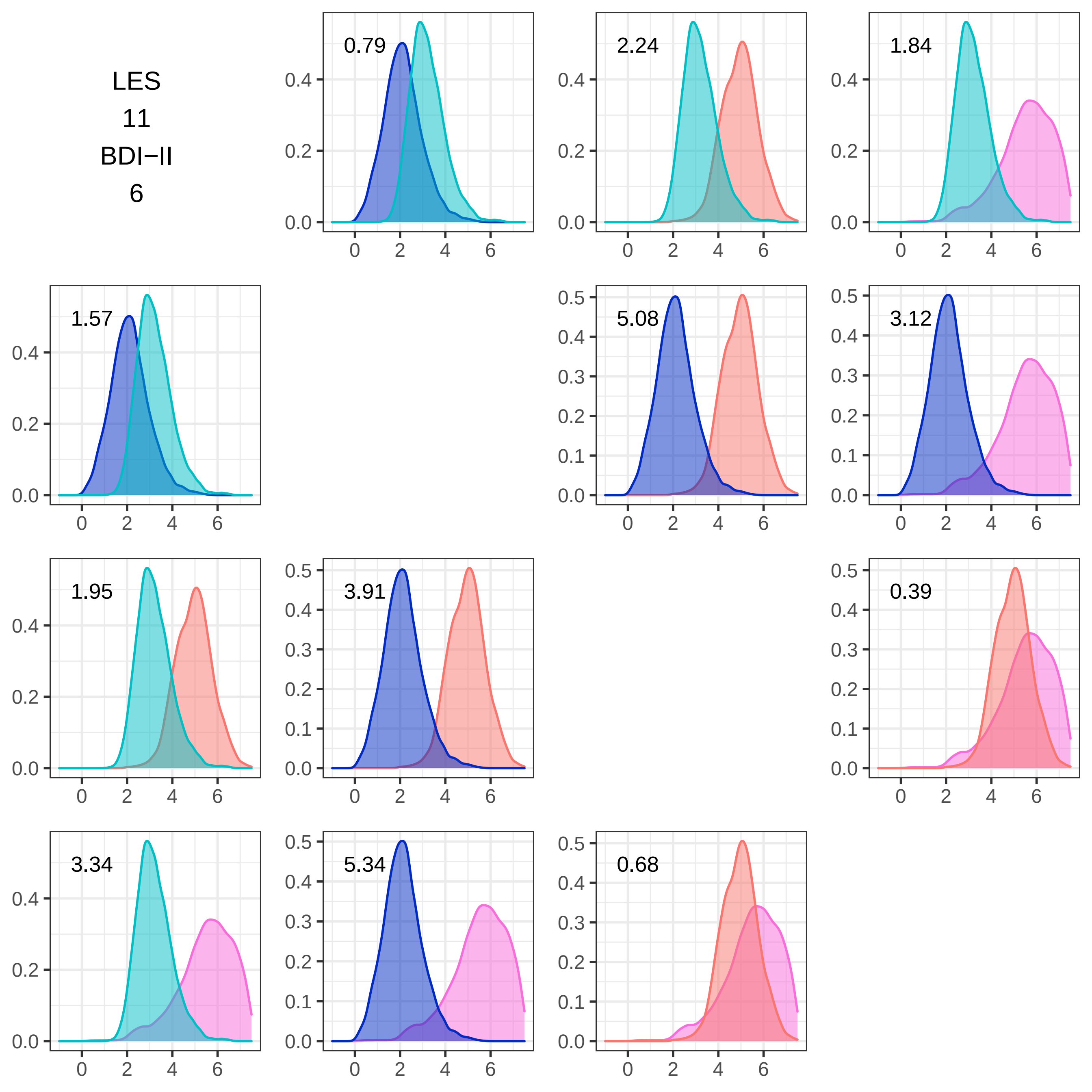

LES
11
BDI−II
6
0.79
2.24
1.84
1.57
5.08
3.12
1.95
3.91
0.39
3.34
5.34
0.68

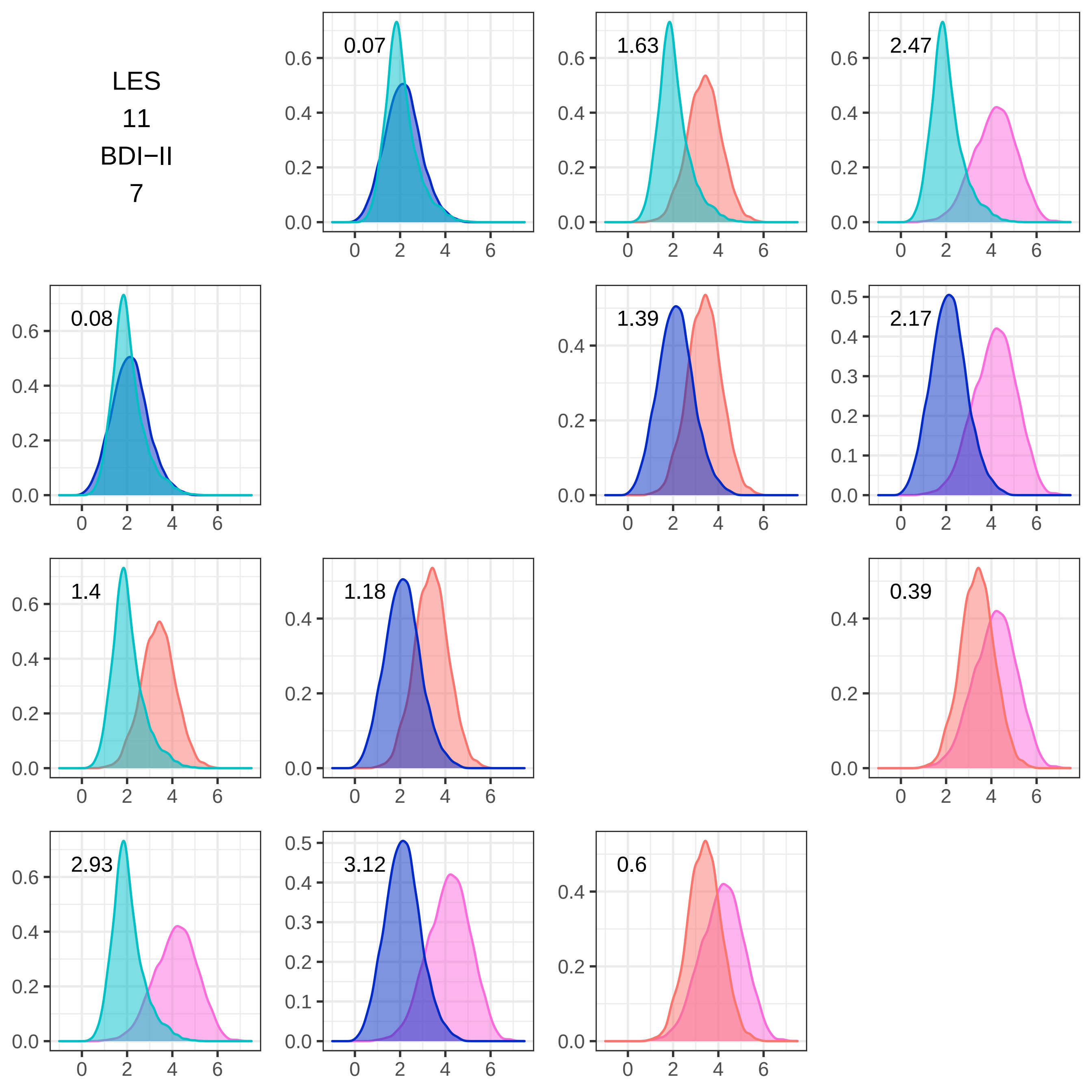
LES
11
BDI−II
7
0.07
1.63
2.47
0.08
1.39
2.17
1.4
1.18
0.39
2.93
3.12
0.6

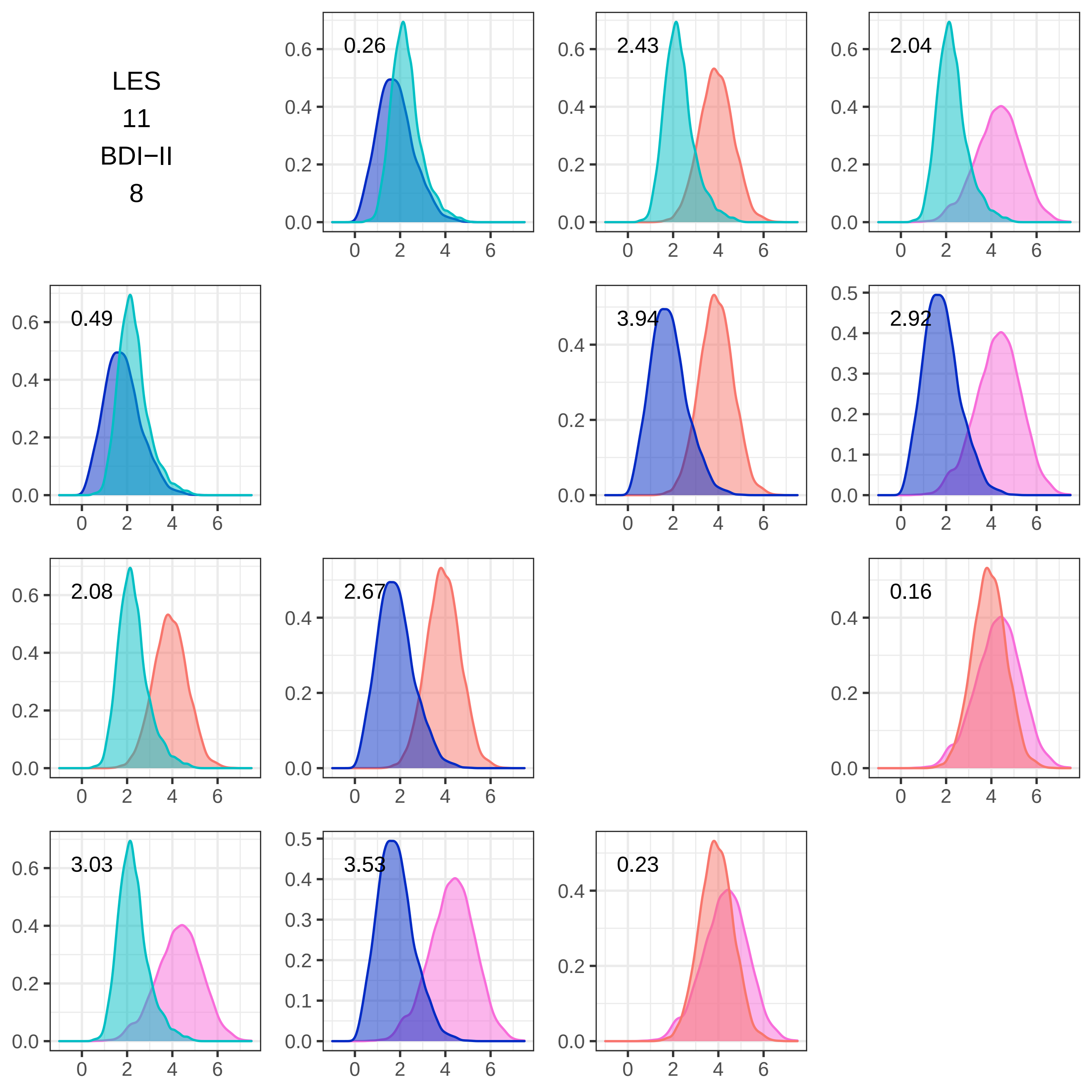
LES
11
BDI−II
8
0.26
2.43
2.04
0.49
3.94
2.92
2.08
2.67
0.16
3.03
3.53
0.23

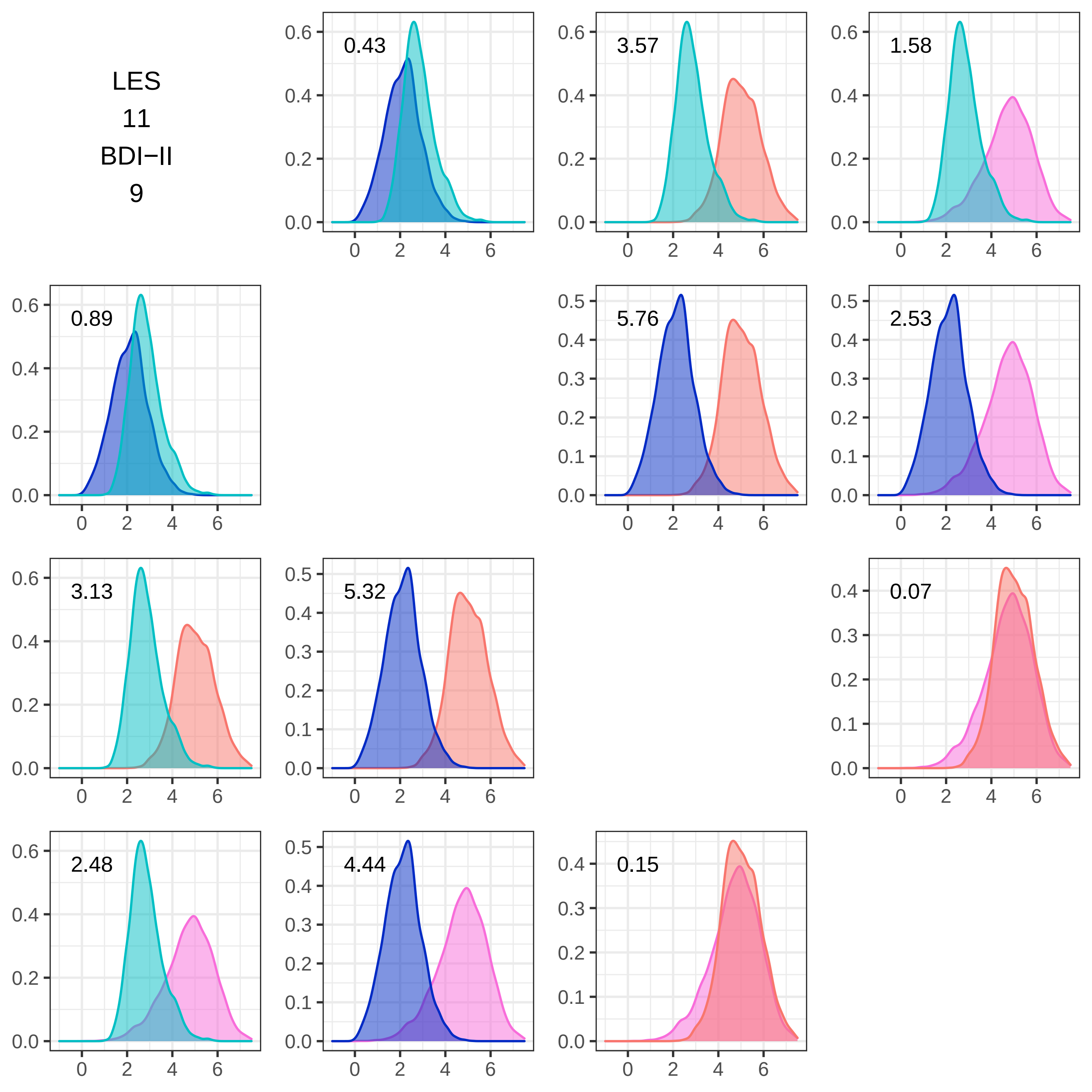
LES
11
BDI−II
9
0.43
3.57
1.58
0.89
5.76
2.53
3.13
5.32
0.07
2.48
4.44
0.15

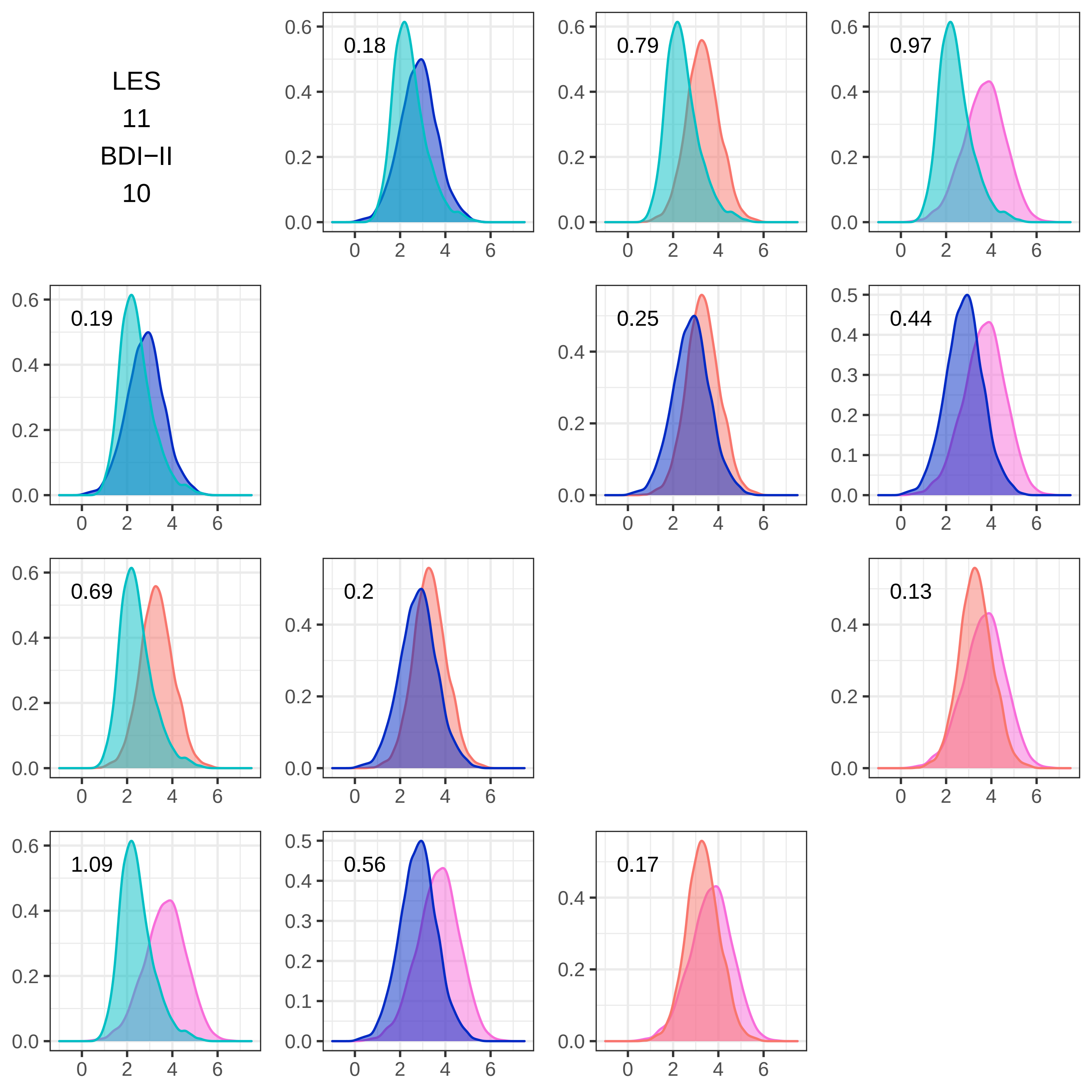
LES
11
BDI−II
10
0.18
0.79
0.97
0.19
0.25
0.44
0.69
0.2
0.13
1.09
0.56
0.17

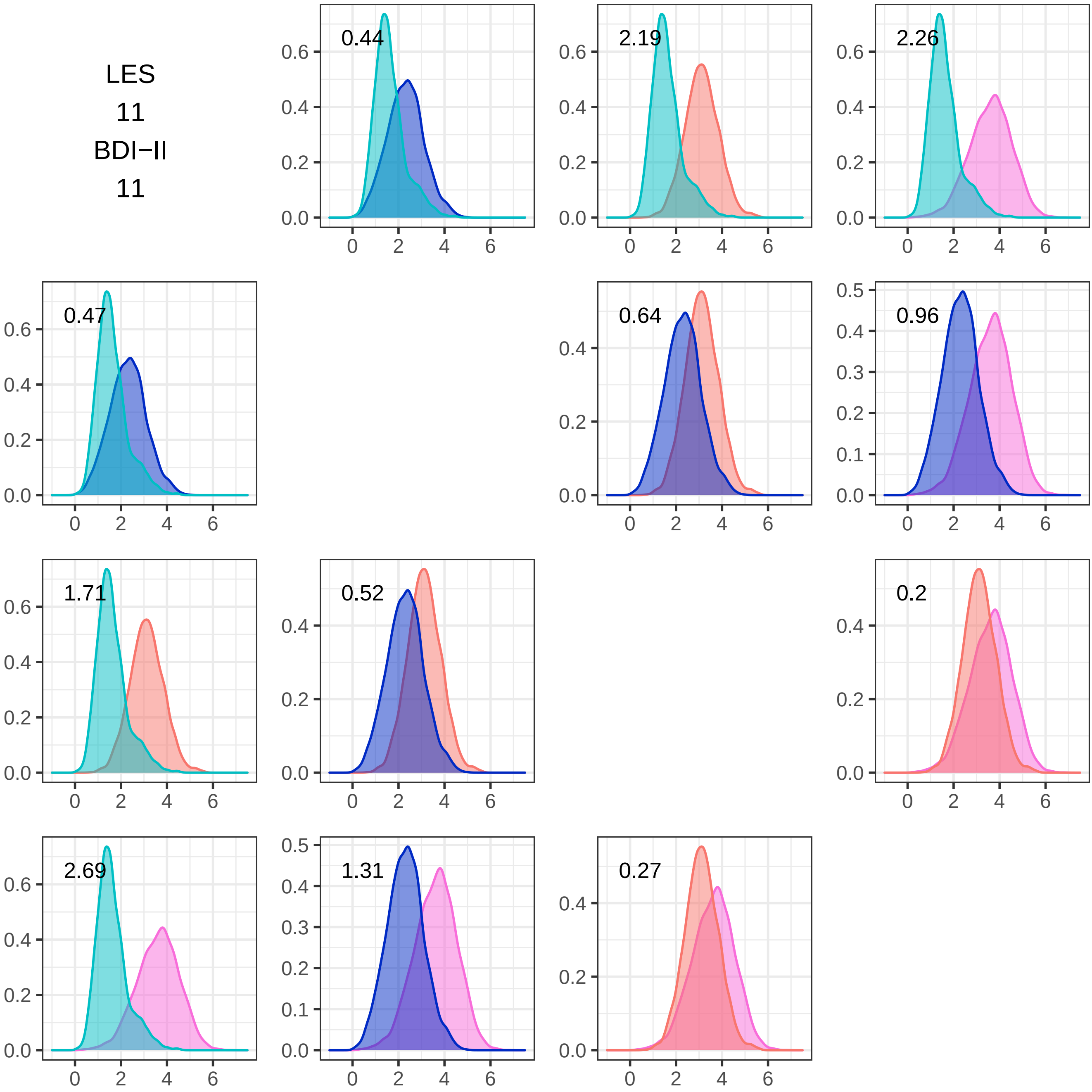
LES
11
BDI−II
11
0.44
2.19
2.26
0.47
0.64
0.96
1.71
0.52
0.2
2.69
1.31
0.27

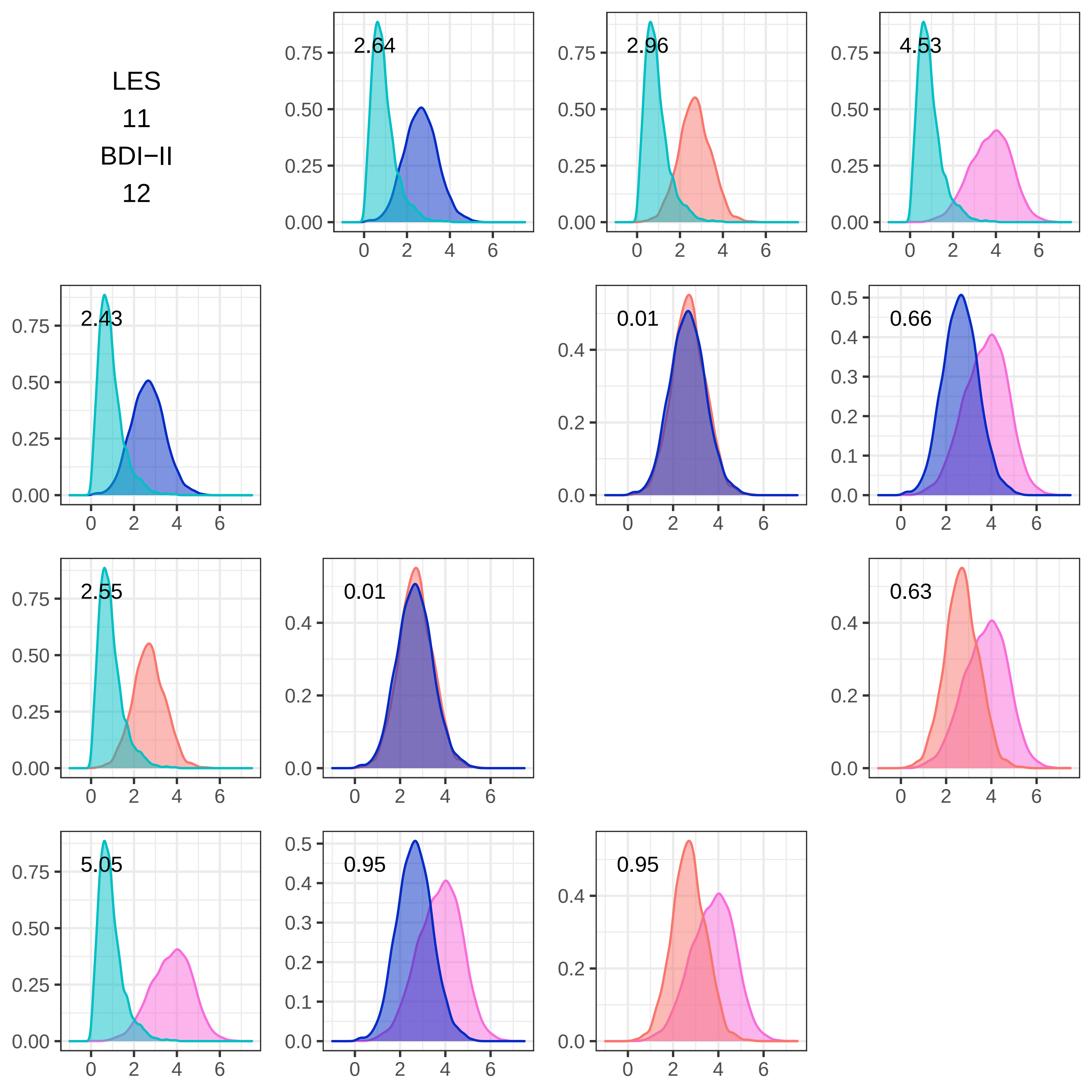

LES
11
BDI−II
12
2.64
2.96
4.53
2.43
0.01
0.66
2.55
0.01
0.63
5.05
0.95
0.95

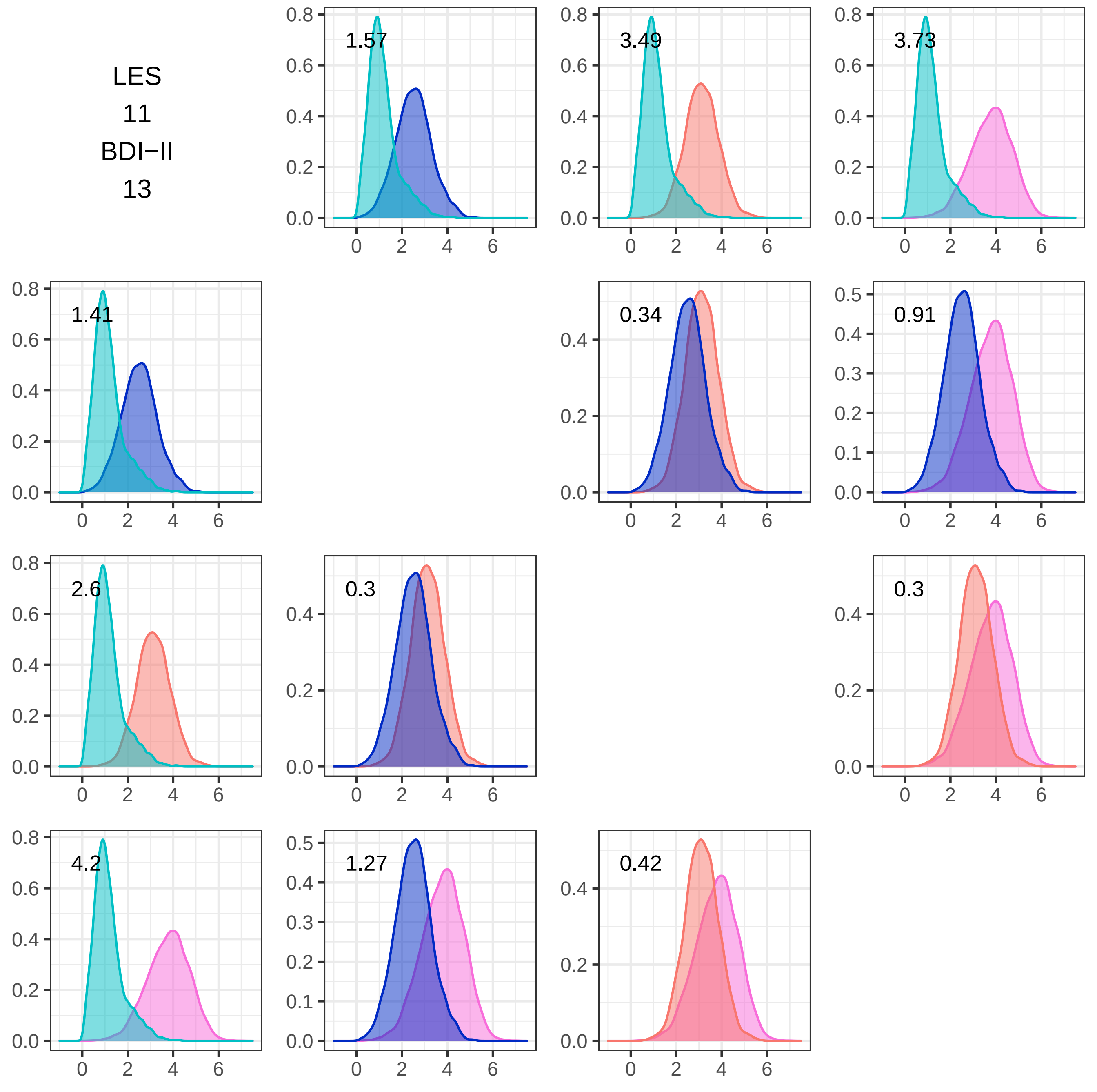
LES
11
BDI−II
13
1.57
3.49
3.73
1.41
0.34
0.91
2.6
0.3
0.3
4.2
1.27
0.42

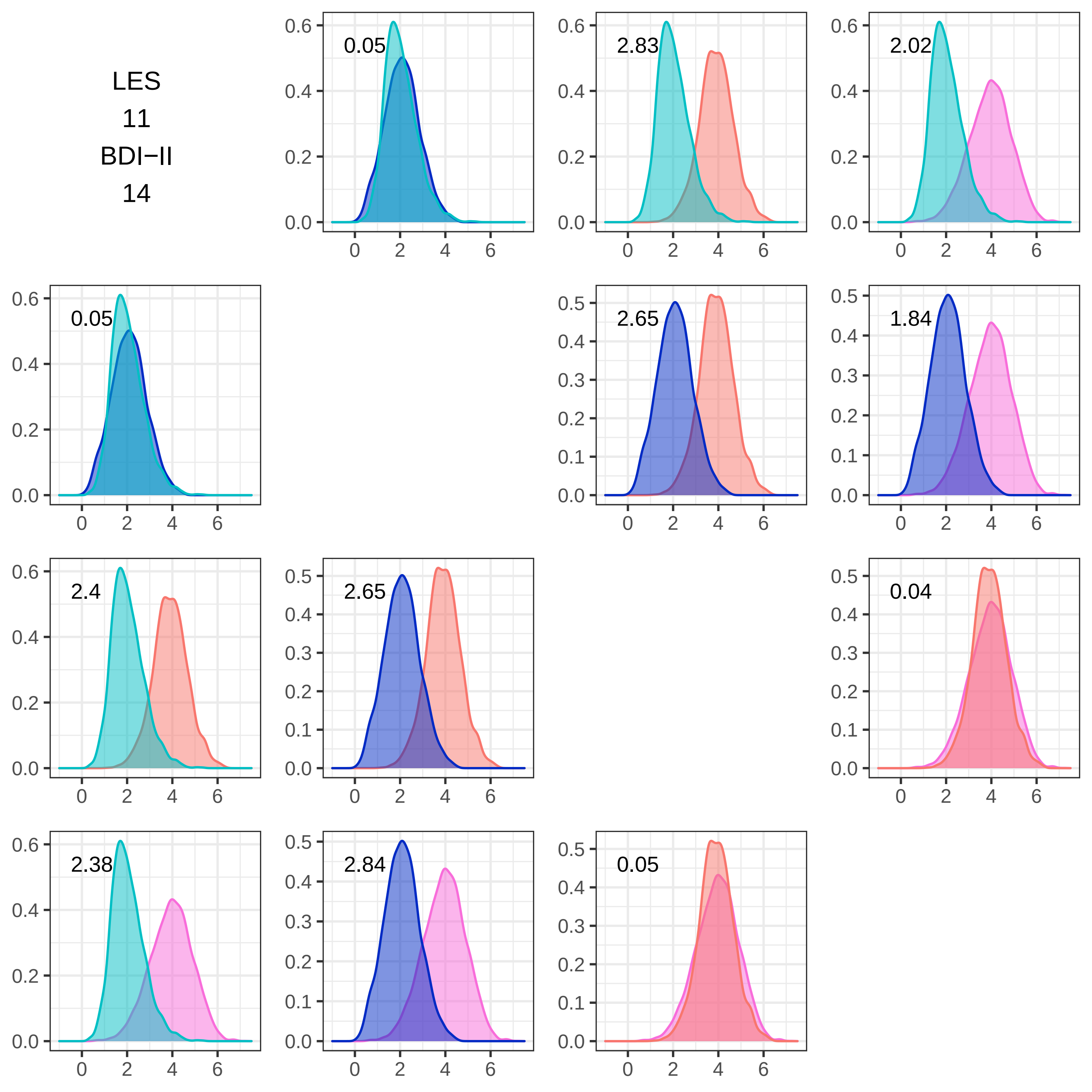

LES
11
BDI−II
14
0.05
2.83
2.02
0.05
2.65
1.84
2.4
2.65
0.04
2.38
2.84
0.05

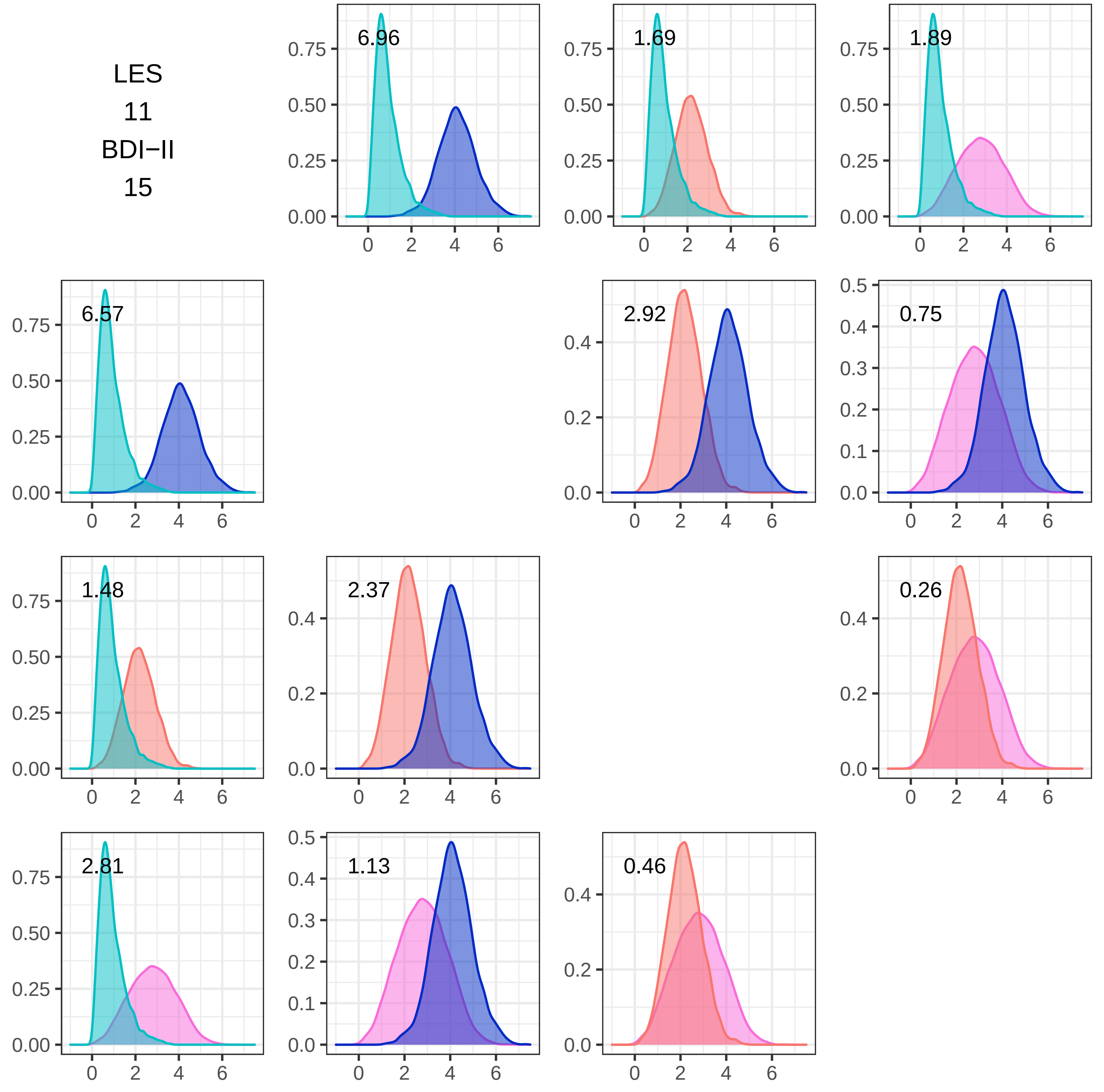

LES
11
BDI−II
15
6.96
1.69
1.89
6.57
2.92
0.75
1.48
2.37
0.26
2.81
1.13
0.46

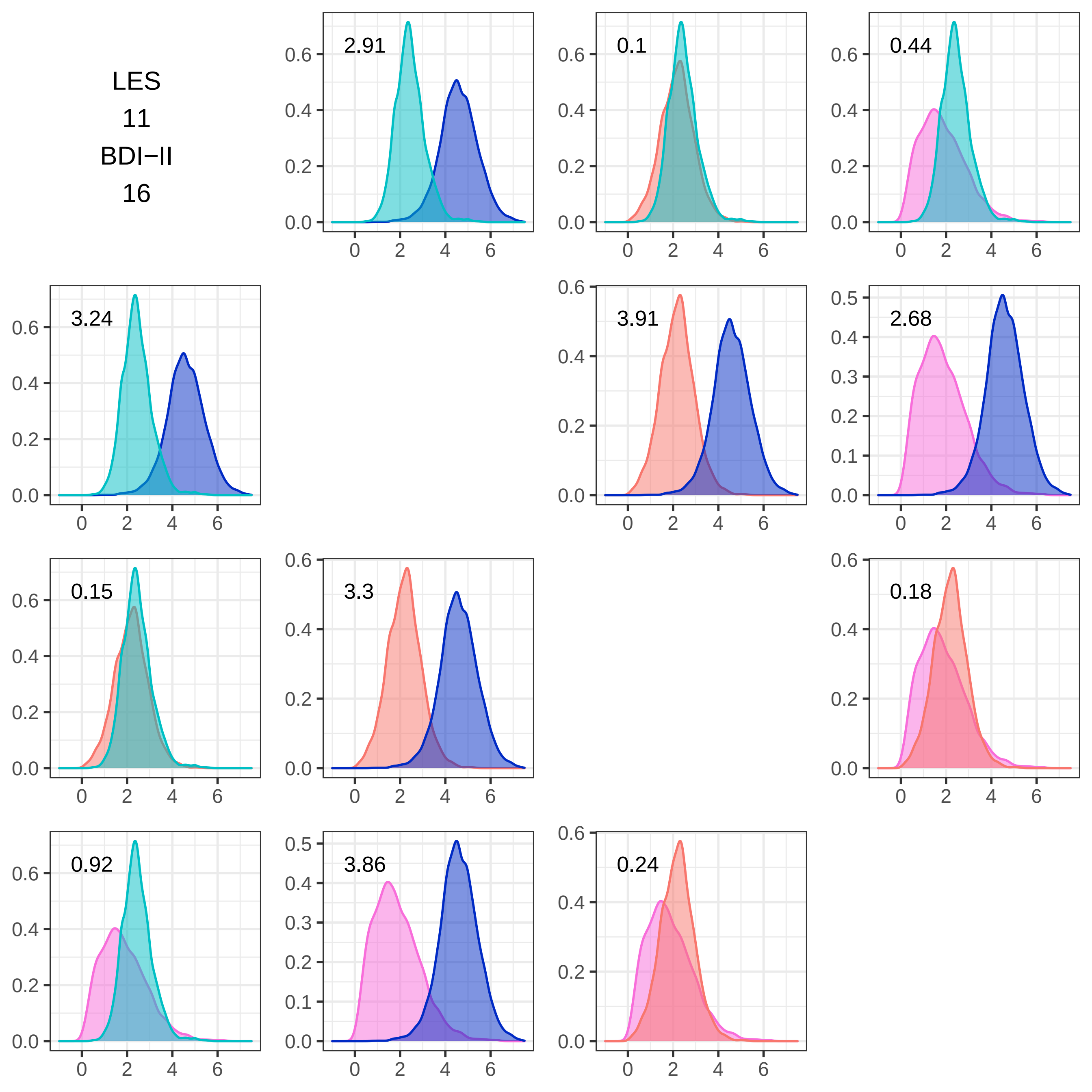
LES
11
BDI−II
16
2.91
0.1
0.44
3.24
3.91
2.68
0.15
3.3
0.18
0.92
3.86
0.24

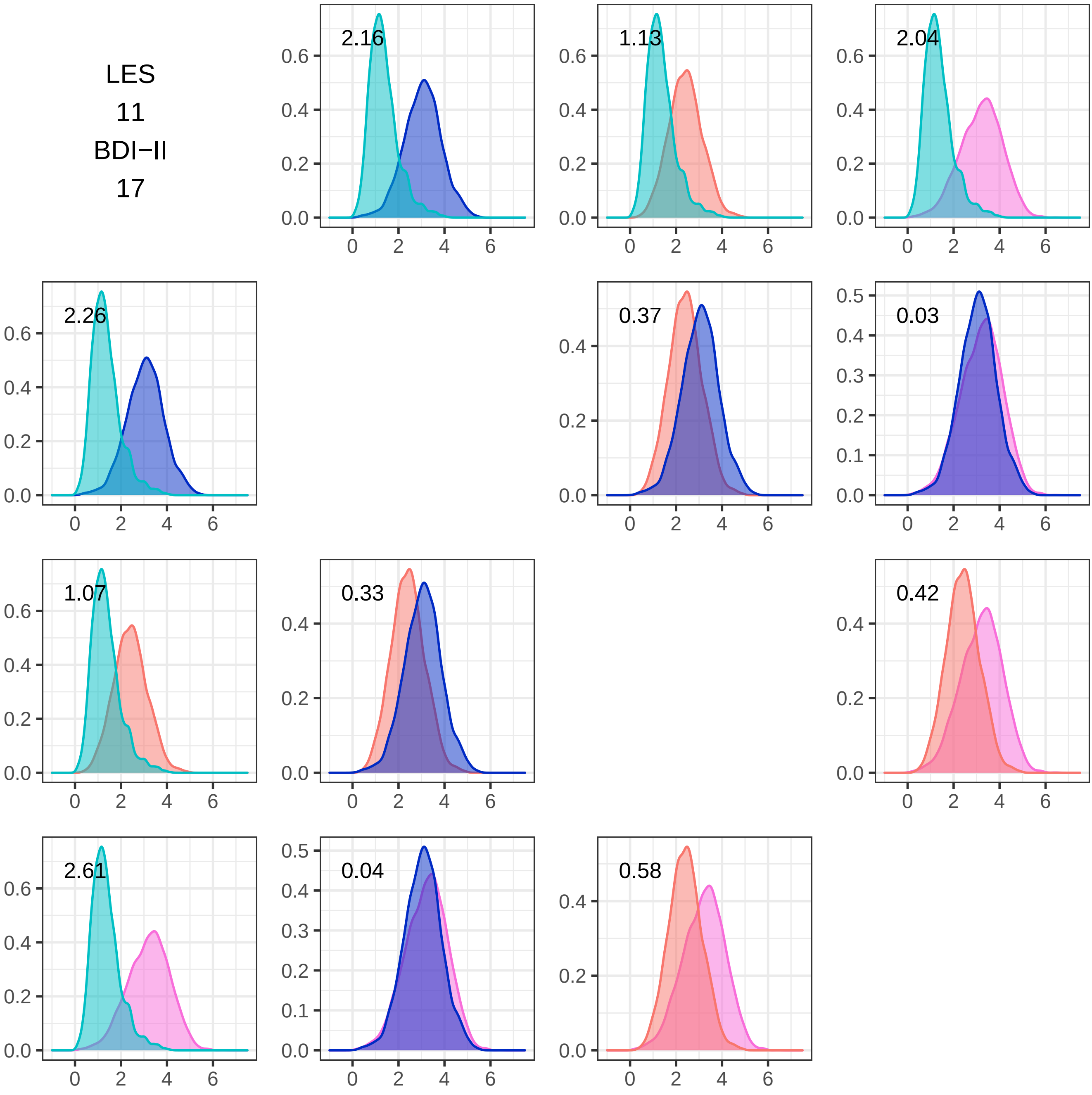
LES
11
BDI−II
17
2.16
1.13
2.04
2.26
0.37
0.03
1.07
0.33
0.42
2.61
0.04
0.58

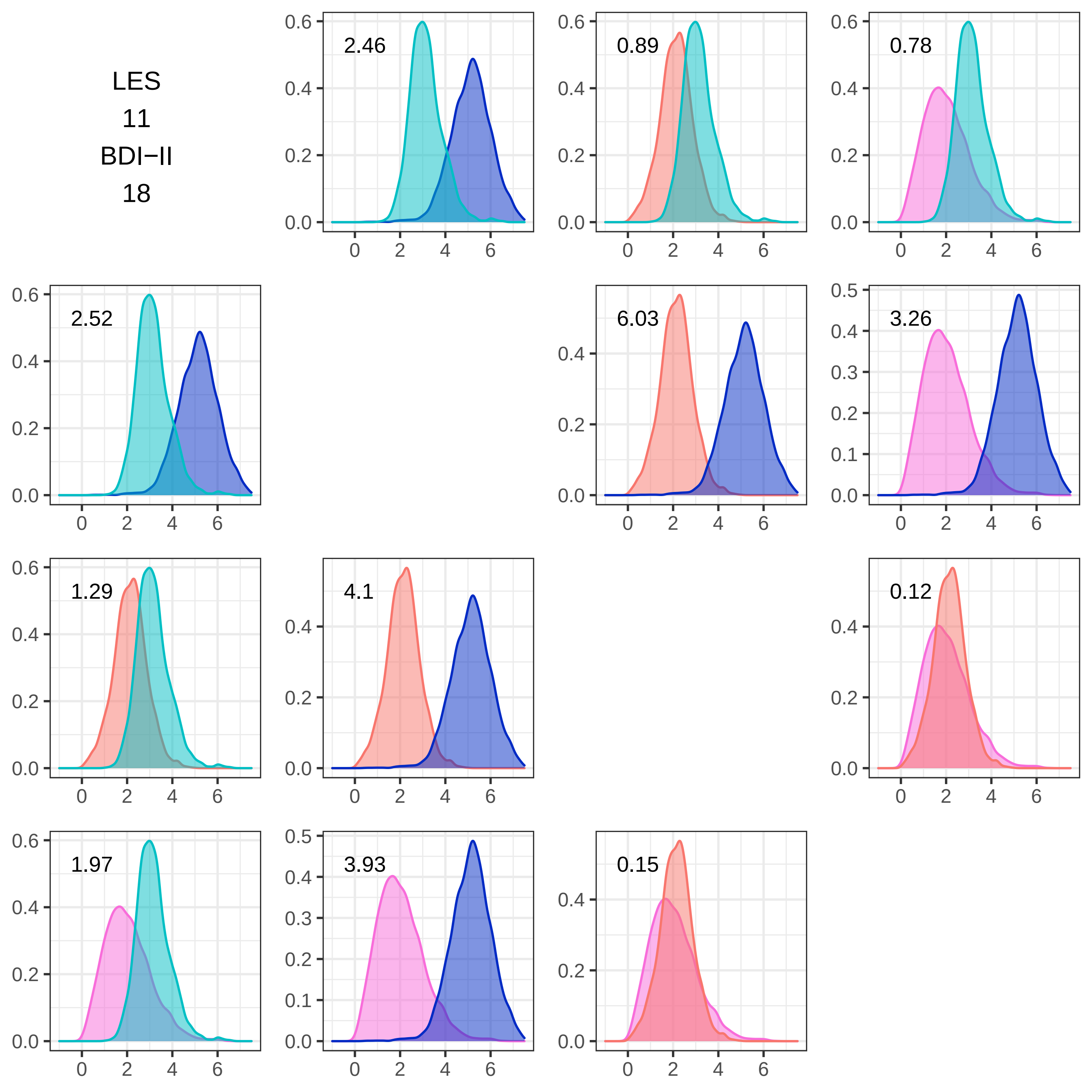

LES
11
BDI−II
18
2.46
0.89
0.78
2.52
6.03
3.26
1.29
4.1
0.12
1.97
3.93
0.15

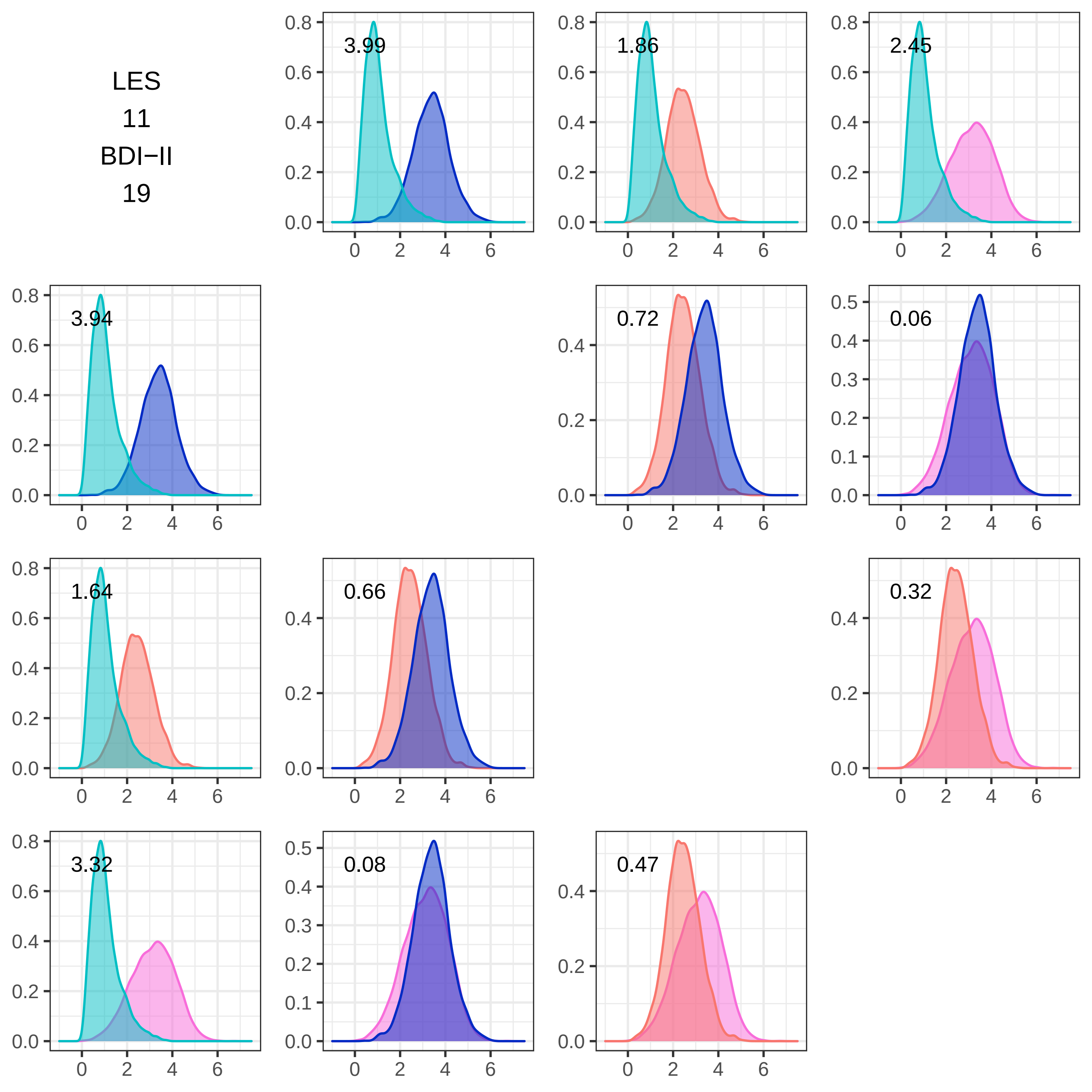

LES
11
BDI−II
19
3.99
1.86
2.45
3.94
0.72
0.06
1.64
0.66
0.32
3.32
0.08
0.47

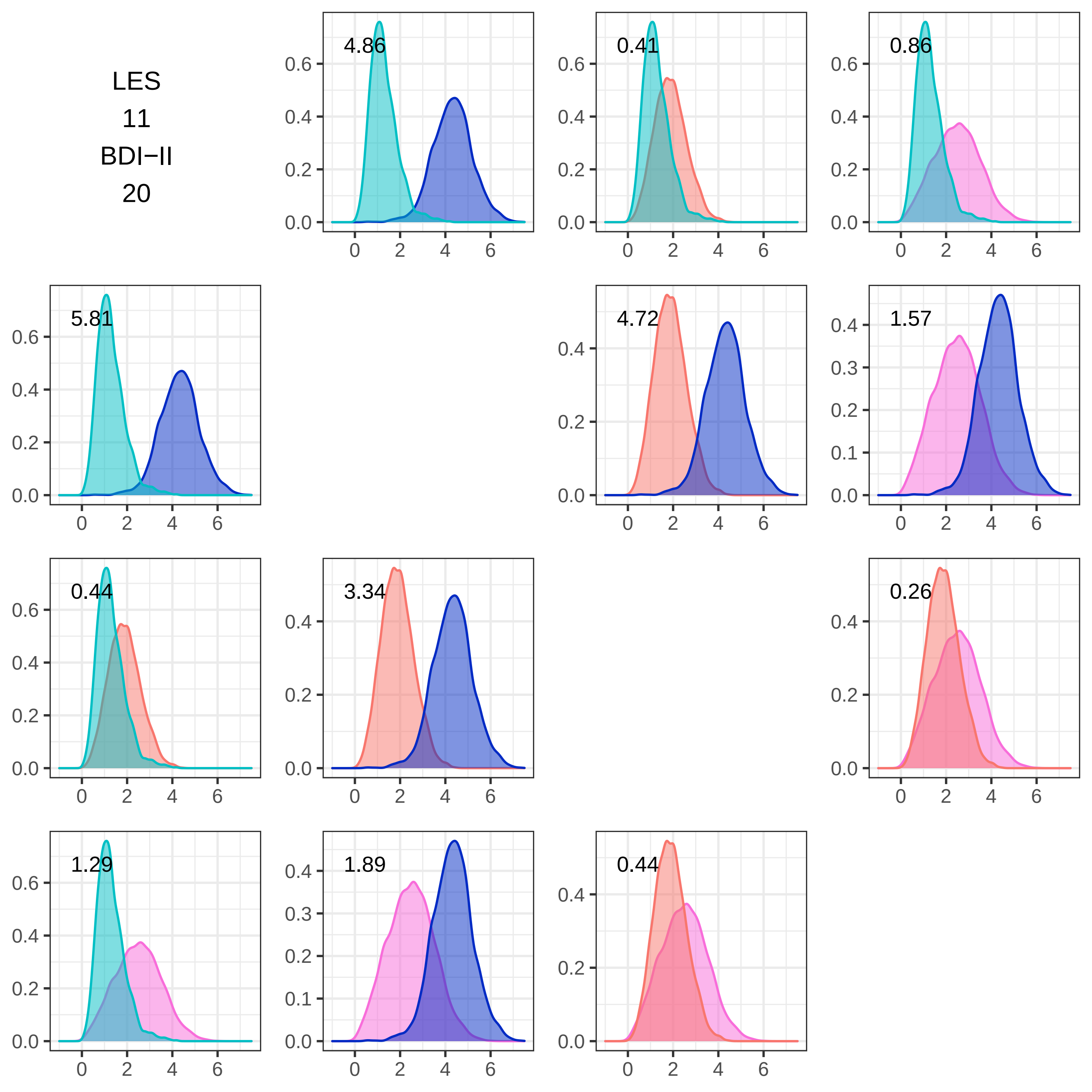

LES
11
BDI−II
20
4.86
0.41
0.86
5.81
4.72
1.57
0.44
3.34
0.26
1.29
1.89
0.44

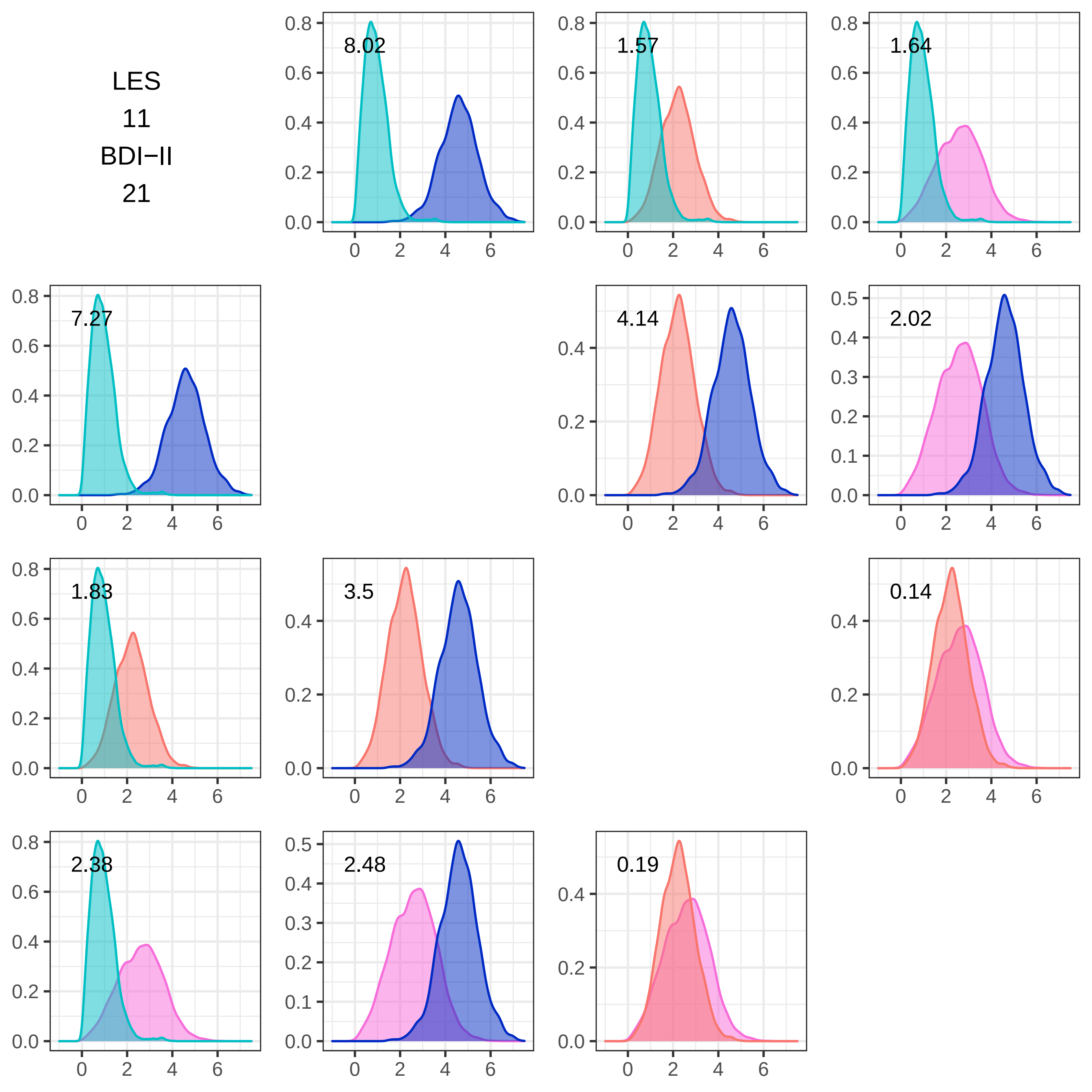
LES
11
BDI−II
21
8.02
1.57
1.64
7.27
4.14
2.02
1.83
3.5
0.14
2.38
2.48
0.19

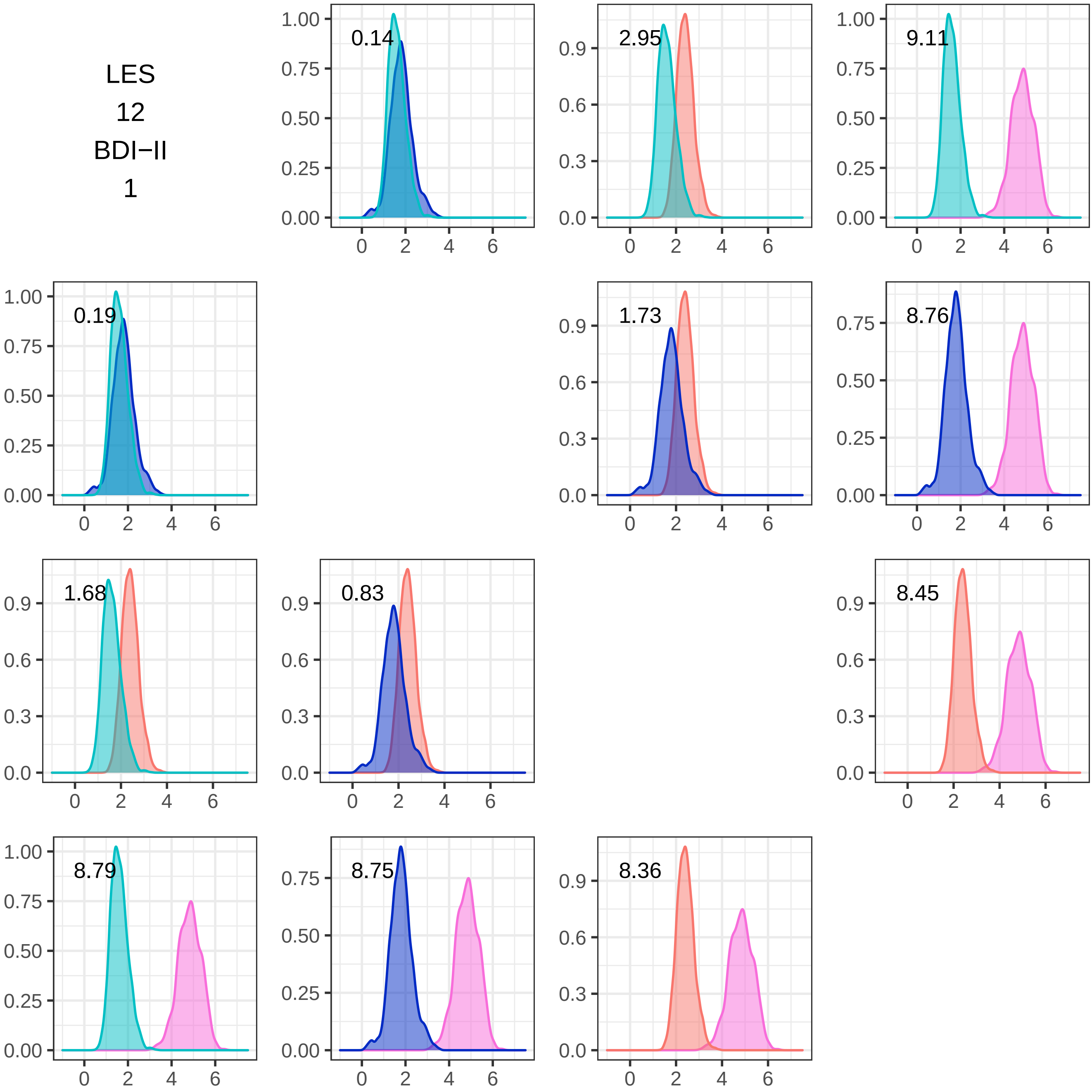
LES
12
BDI−II
1
0.14
2.95
9.11
0.19
1.73
8.76
1.68
0.83
8.45
8.79
8.75
8.36

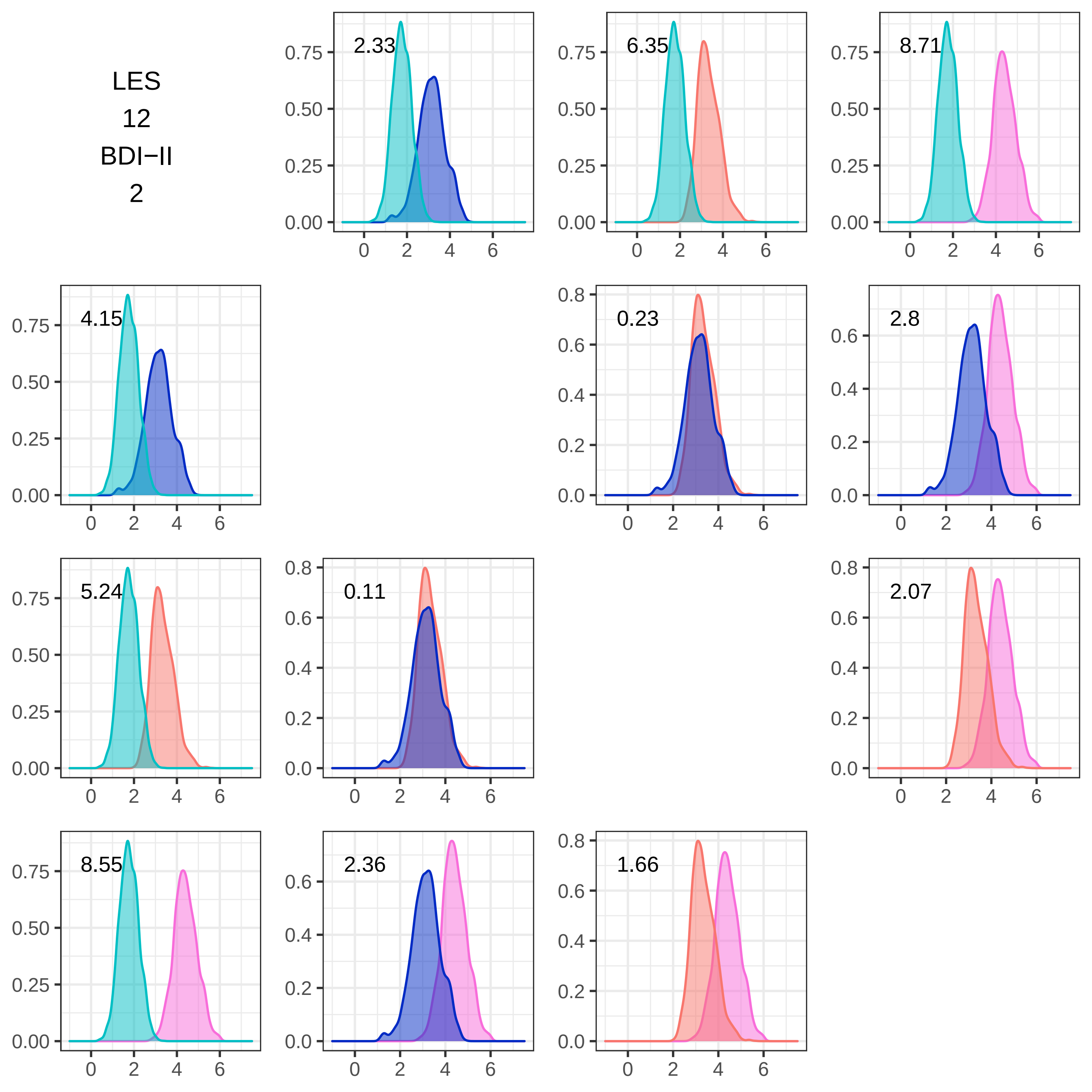

LES
12
BDI−II
2
2.33
6.35
8.71
4.15
0.23
2.8
5.24
0.11
2.07
8.55
2.36
1.66

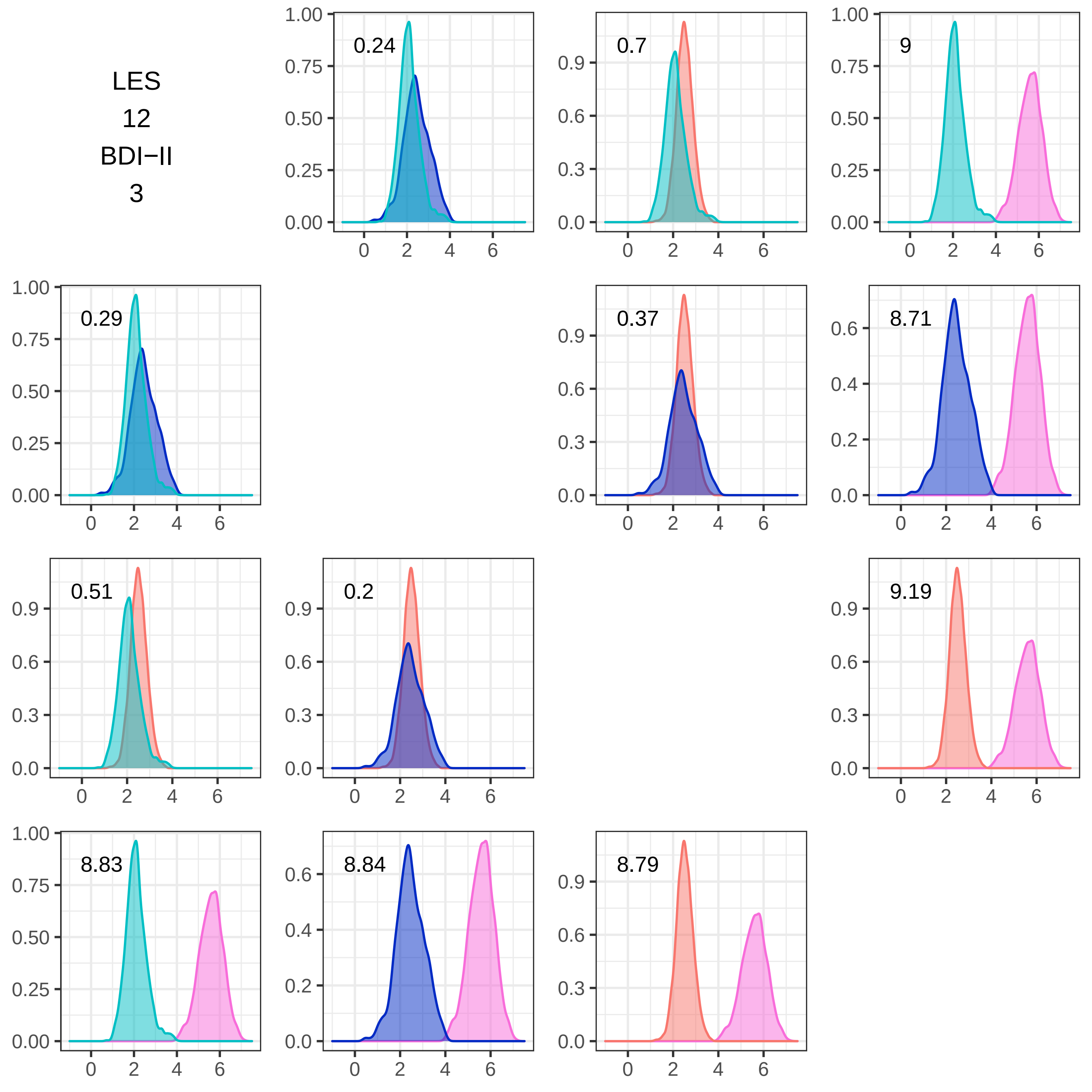

LES
12
BDI−II
3
0.24
0.7
9
0.29
0.37
8.71
0.51
0.2
9.19
8.83
8.84
8.79

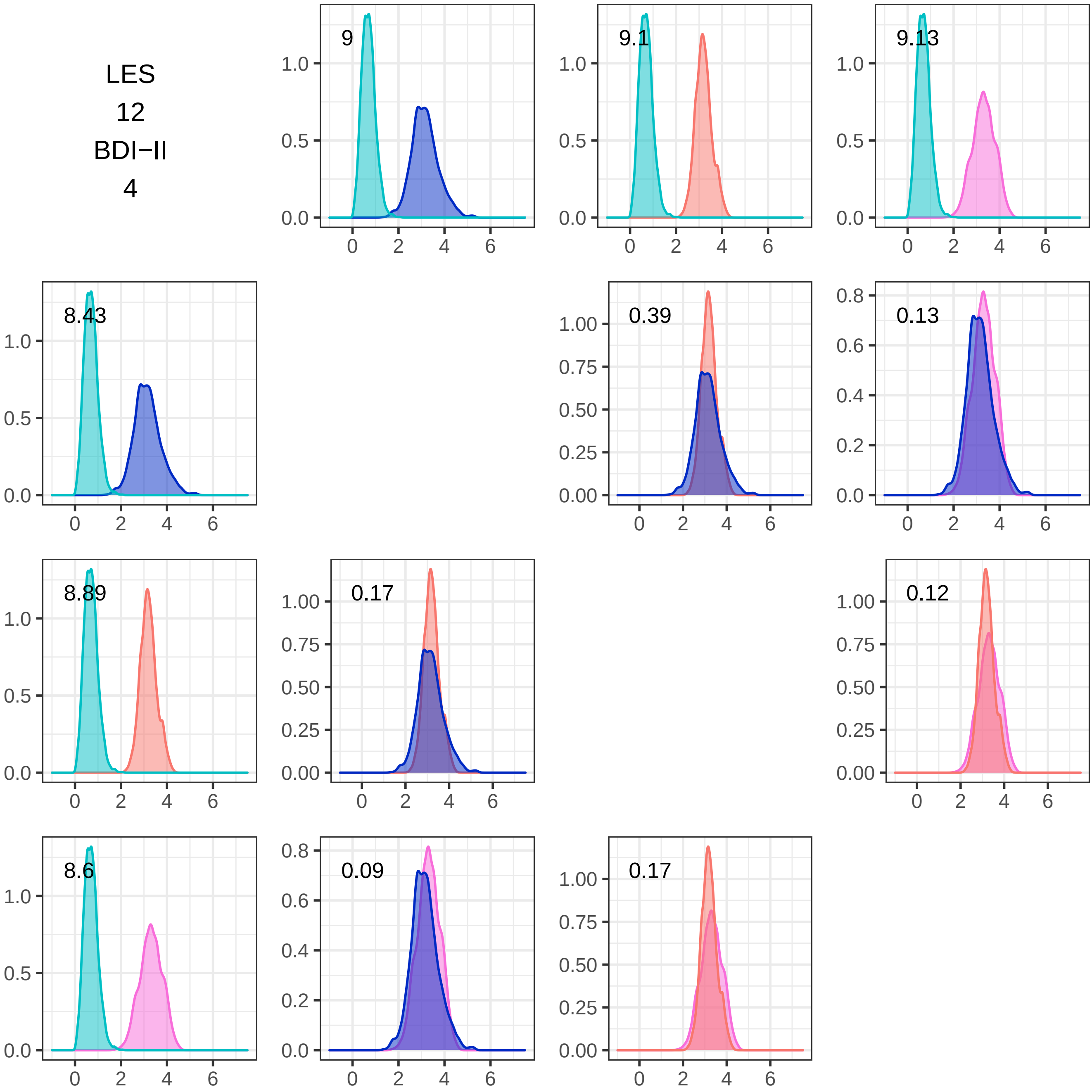
LES
12
BDI−II
4
9
9.1
9.13
8.43
0.39
0.13
8.89
0.17
0.12
8.6
0.09
0.17

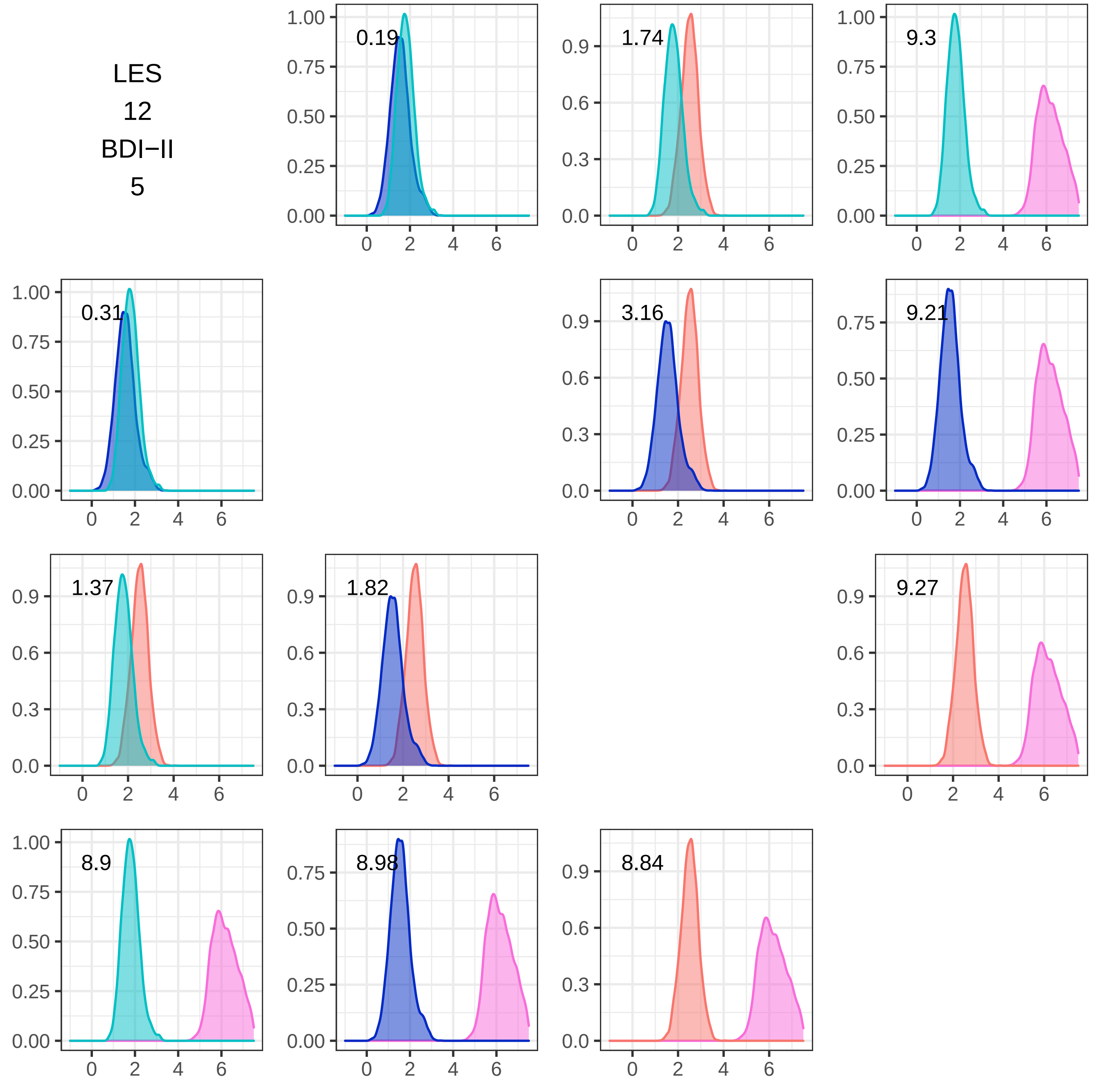
LES
12
BDI−II
5
0.19
1.74
9.3
0.31
3.16
9.21
1.37
1.82
9.27
8.9
8.98
8.84

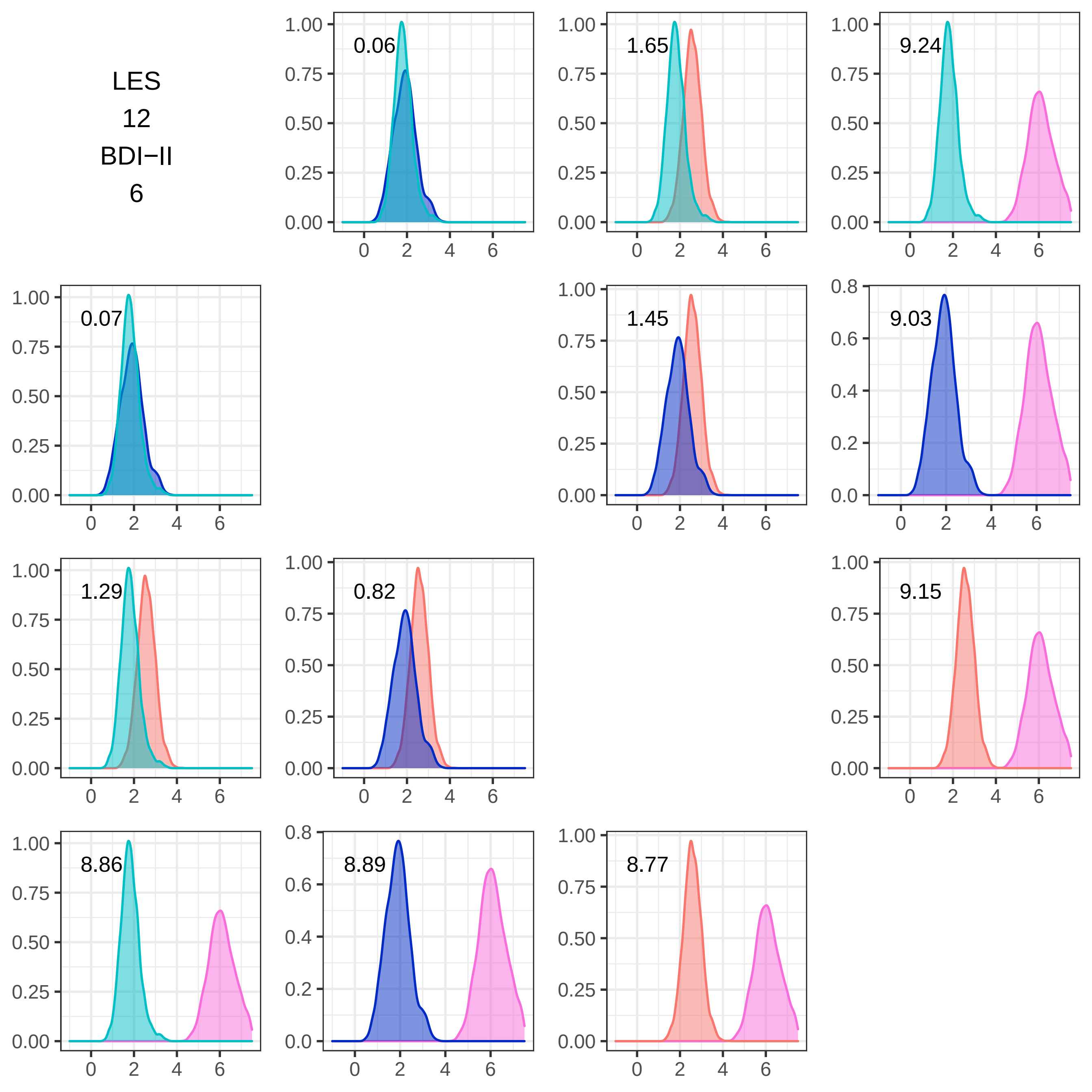
LES
12
BDI−II
6
0.06
1.65
9.24
0.07
1.45
9.03
1.29
0.82
9.15
8.86
8.89
8.77

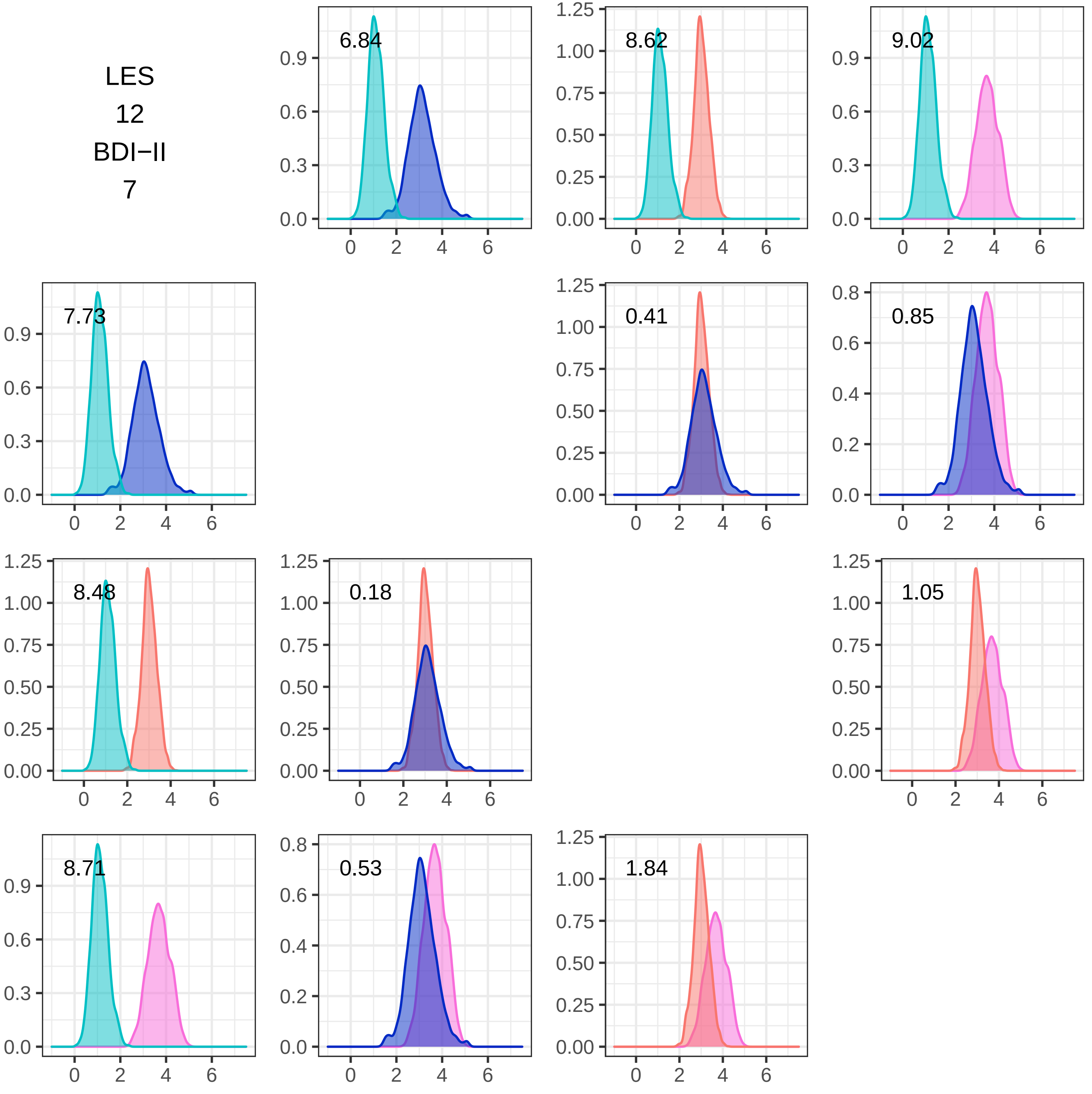
LES
12
BDI−II
7
6.84
8.62
9.02
7.73
0.41
0.85
8.48
0.18
1.05
8.71
0.53
1.84

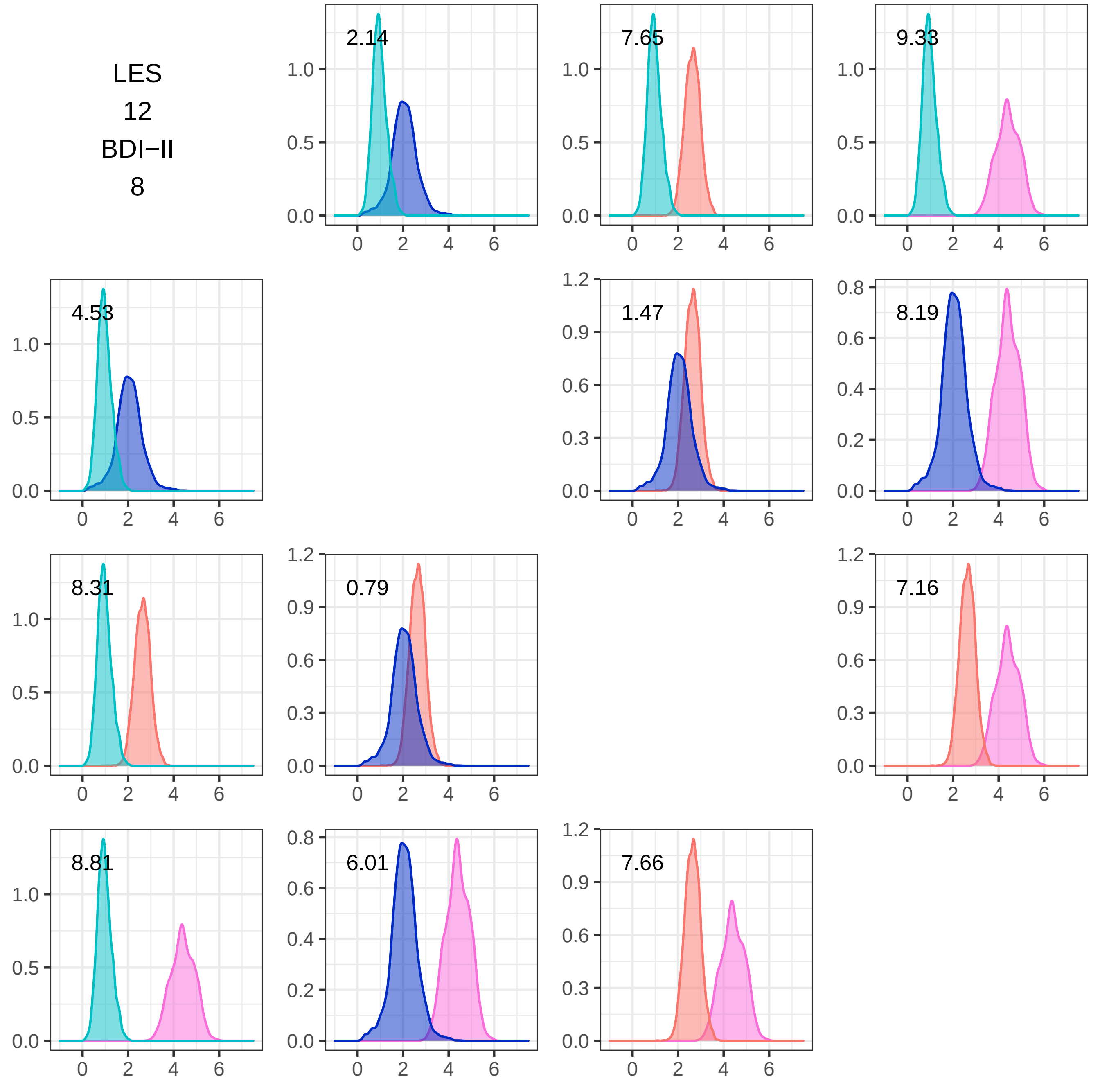
LES
12
BDI−II
8
2.14
7.65
9.33
4.53
1.47
8.19
8.31
0.79
7.16
8.81
6.01
7.66

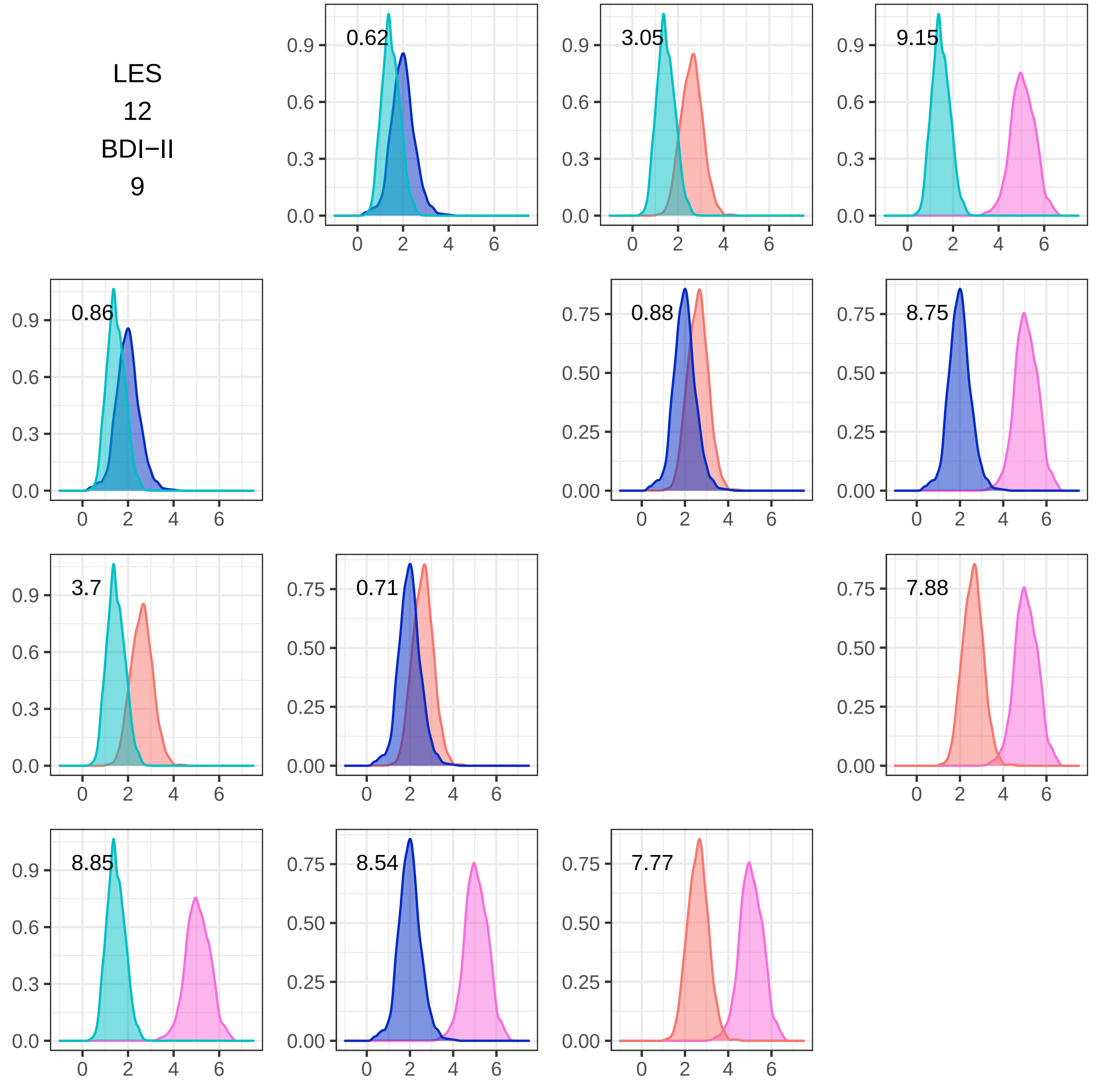
LES
12
BDI−II
9
0.62
3.05
9.15
0.86
0.88
8.75
3.7
0.71
7.88
8.85
8.54
7.77

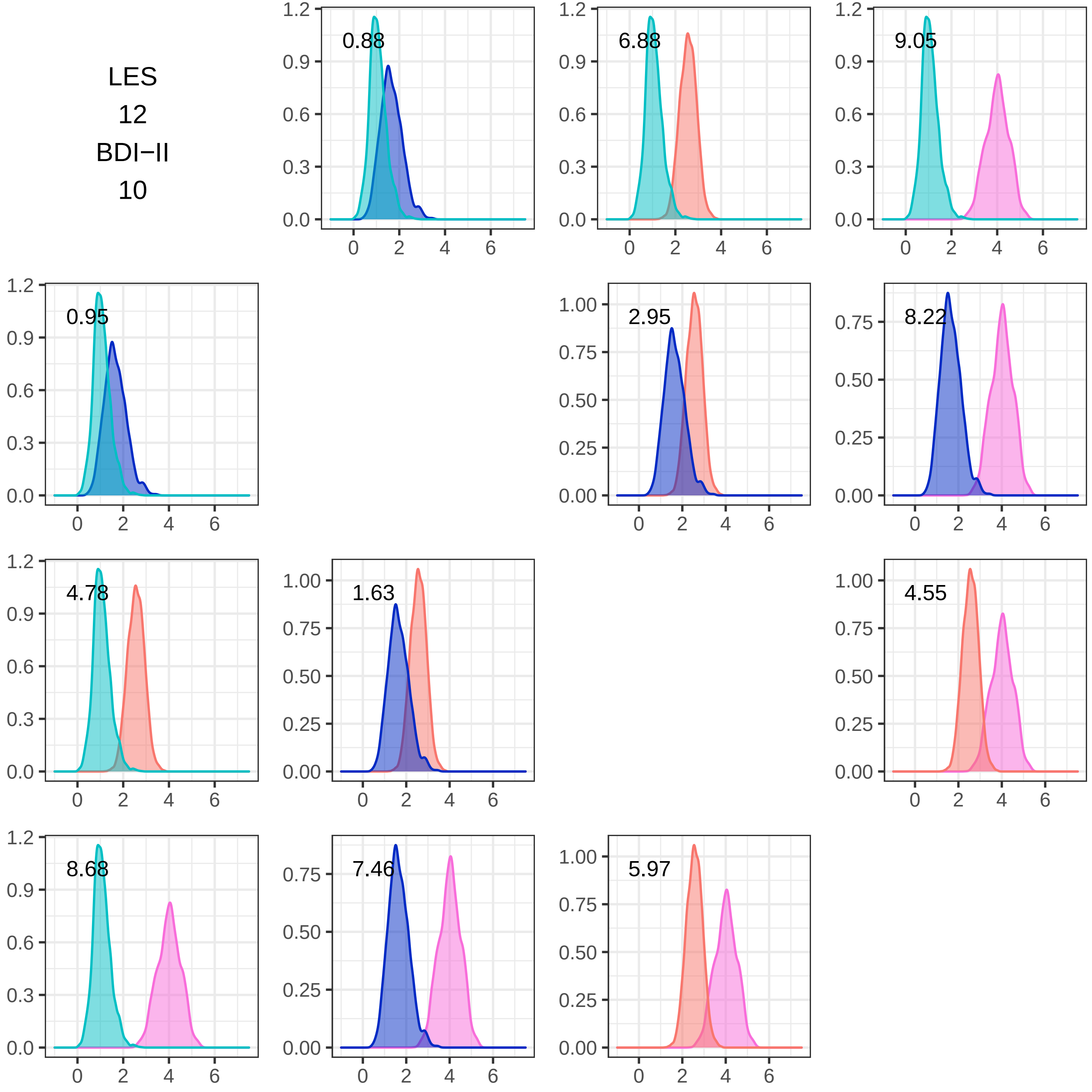

LES
12
BDI−II
10
0.88
6.88
9.05
0.95
2.95
8.22
4.78
1.63
4.55
8.68
7.46
5.97

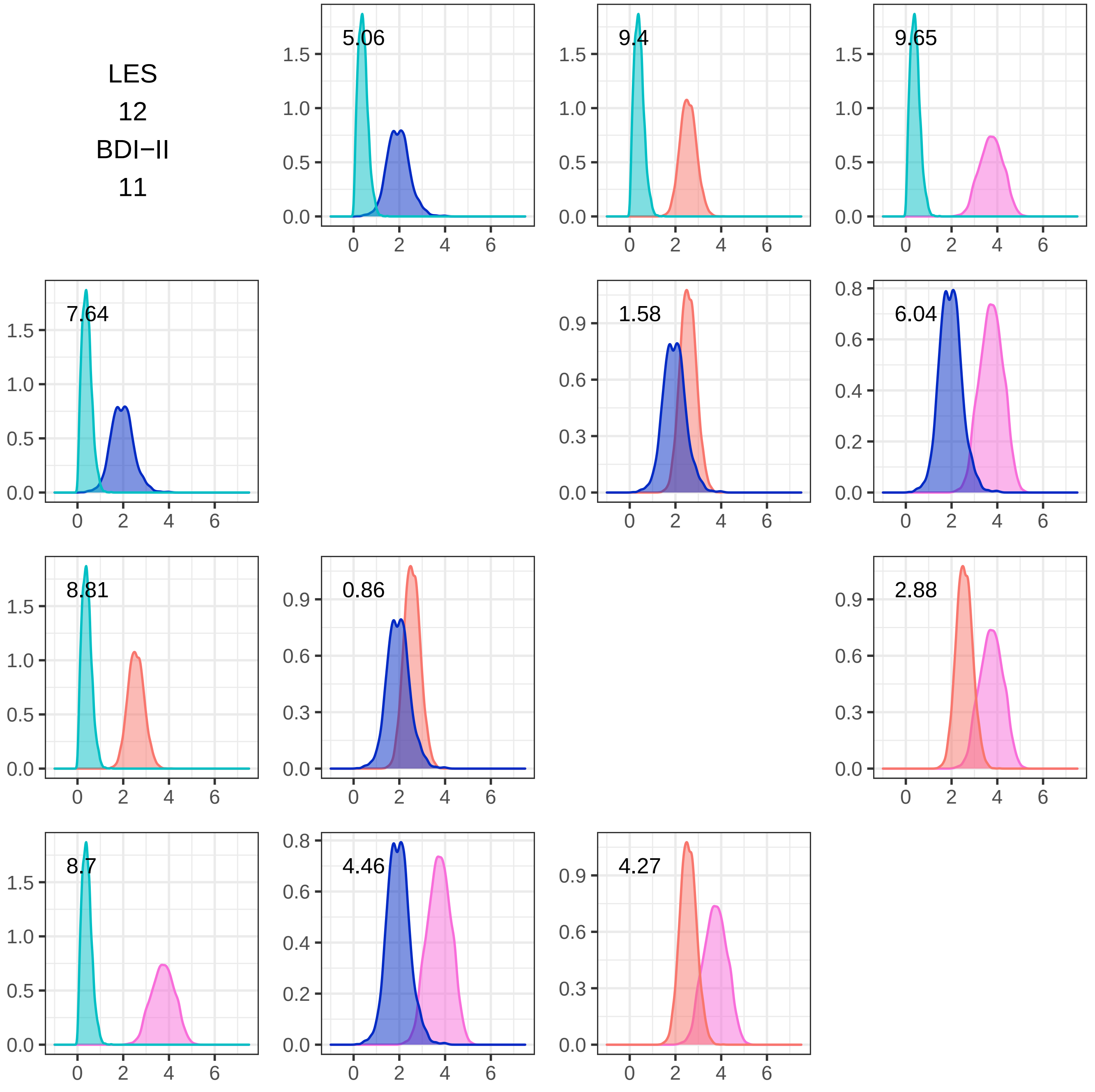

LES
12
BDI−II
11
5.06
9.4
9.65
7.64
1.58
6.04
8.81
0.86
2.88
8.7
4.46
4.27

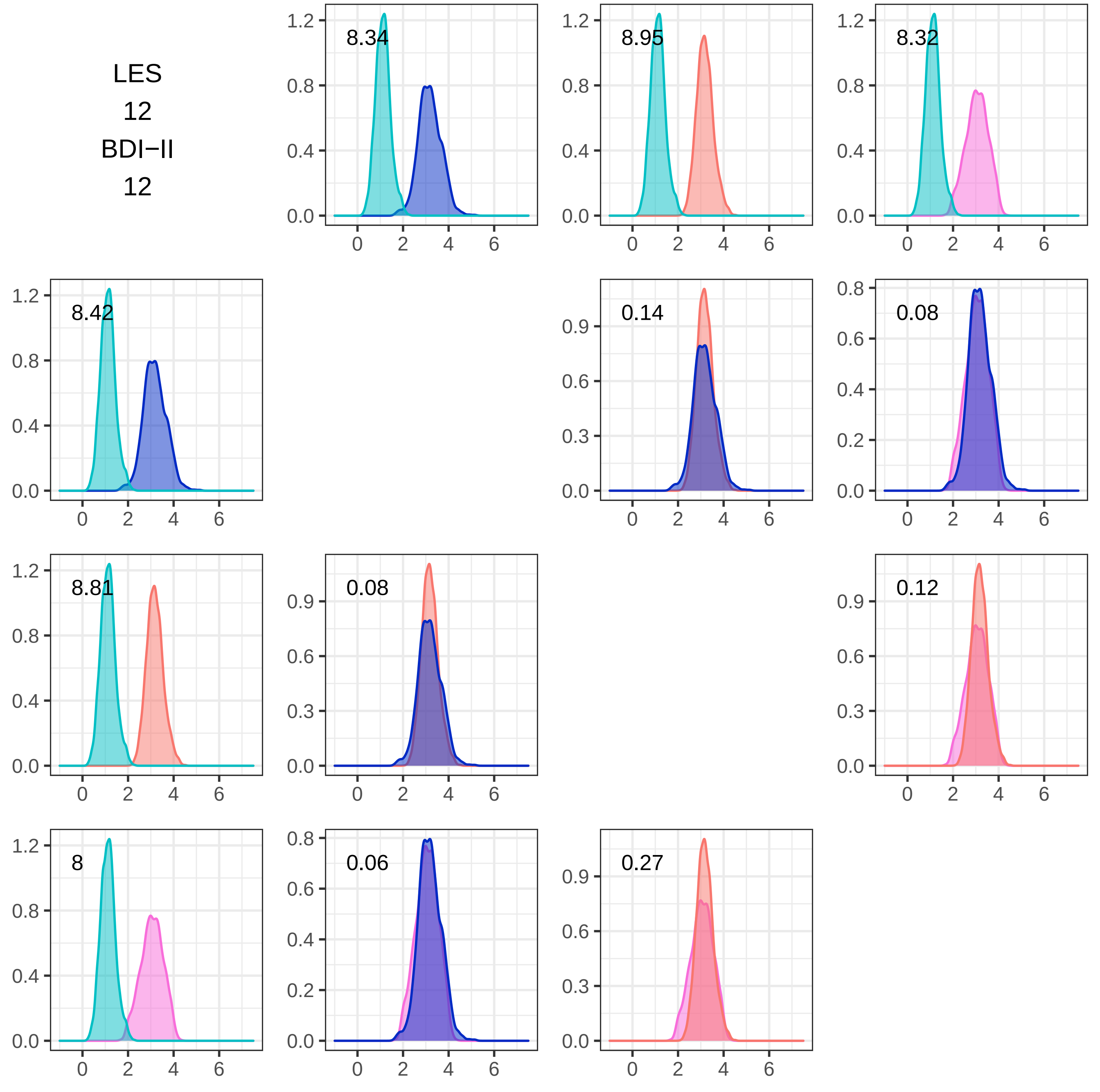

LES
12
BDI−II
12
8.34
8.95
8.32
8.42
0.14
0.08
8.81
0.08
0.12
8
0.06
0.27

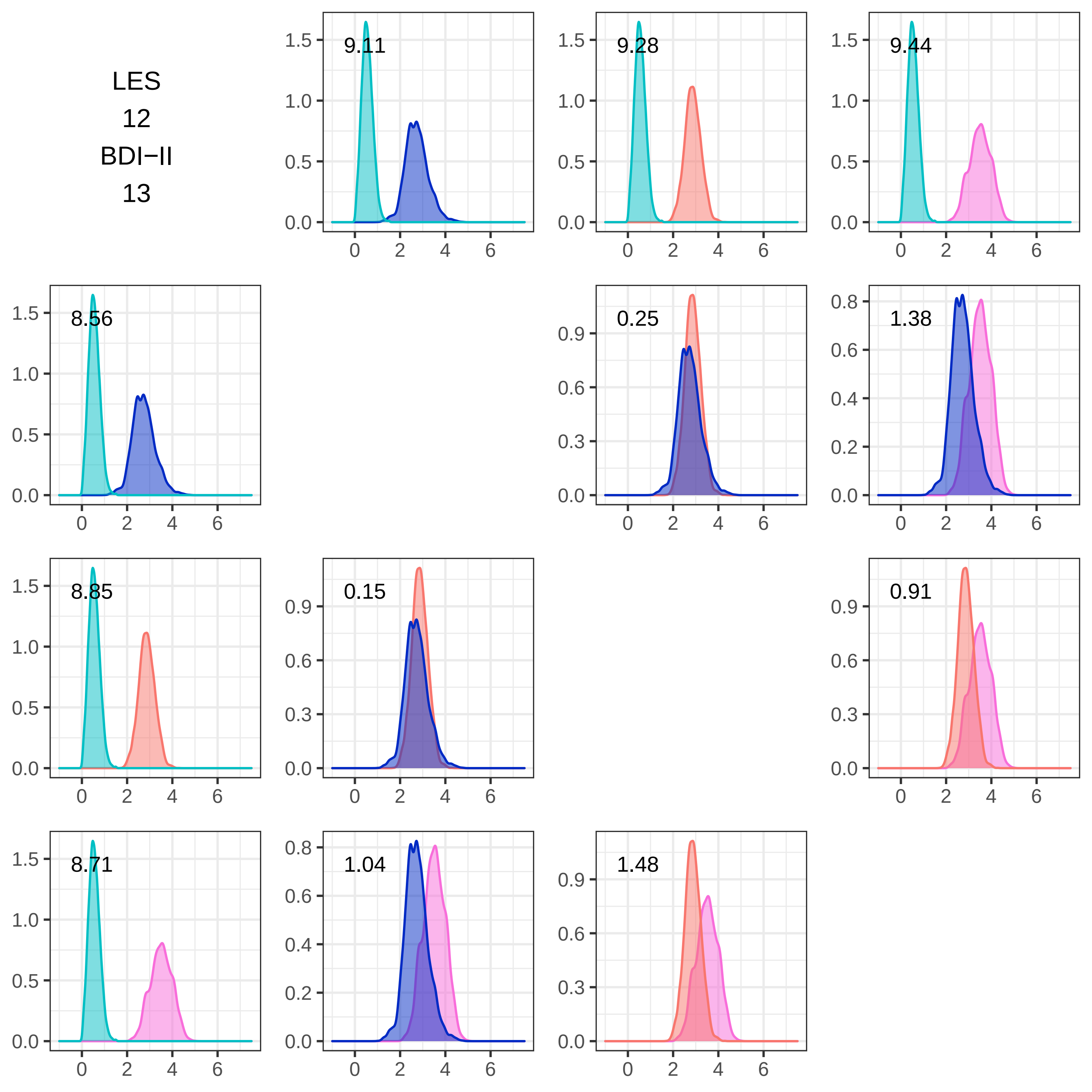

LES
12
BDI−II
13
9.11
9.28
9.44
8.56
0.25
1.38
8.85
0.15
0.91
8.71
1.04
1.48

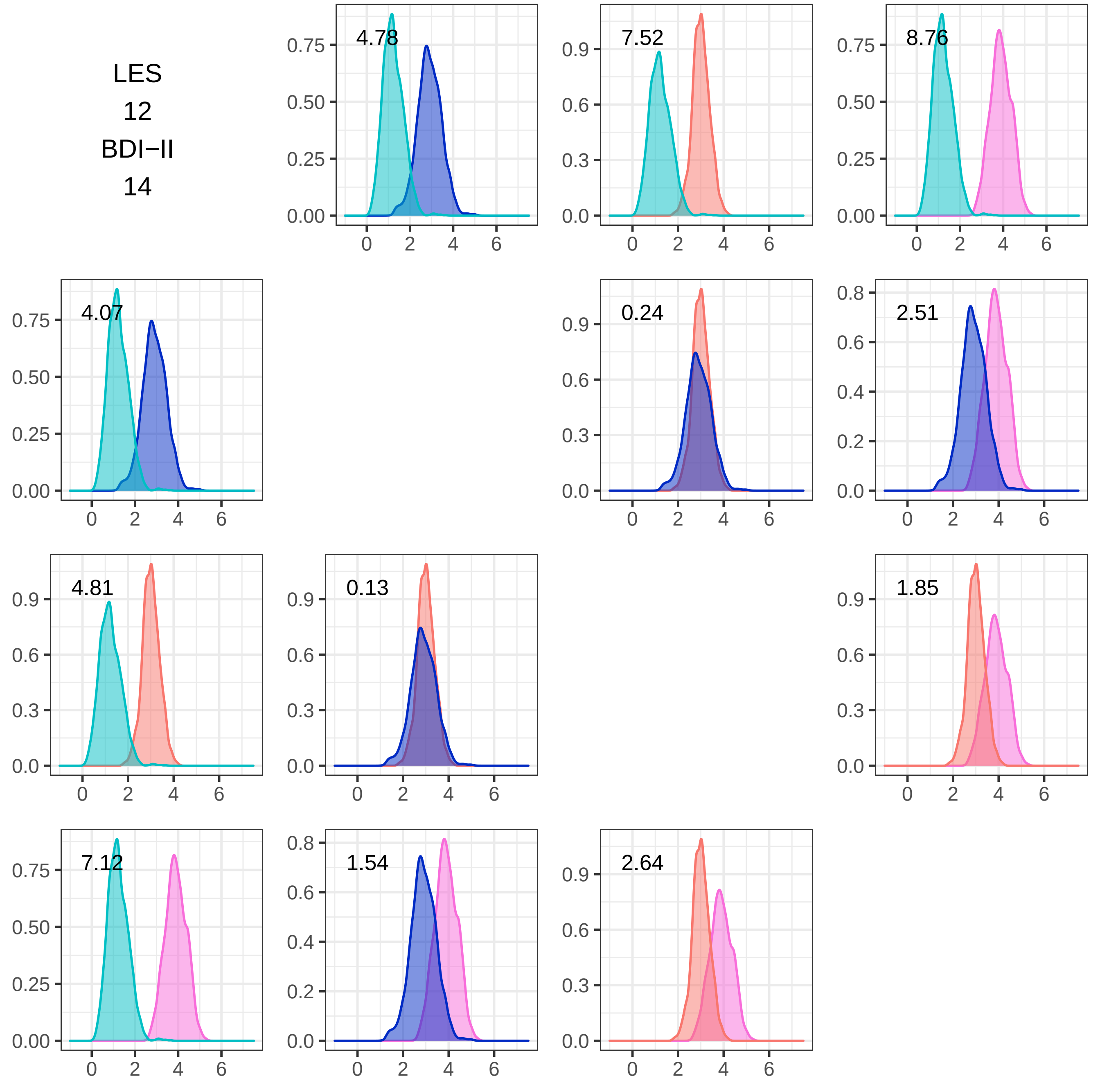
LES
12
BDI−II
14
4.78
7.52
8.76
4.07
0.24
2.51
4.81
0.13
1.85
7.12
1.54
2.64

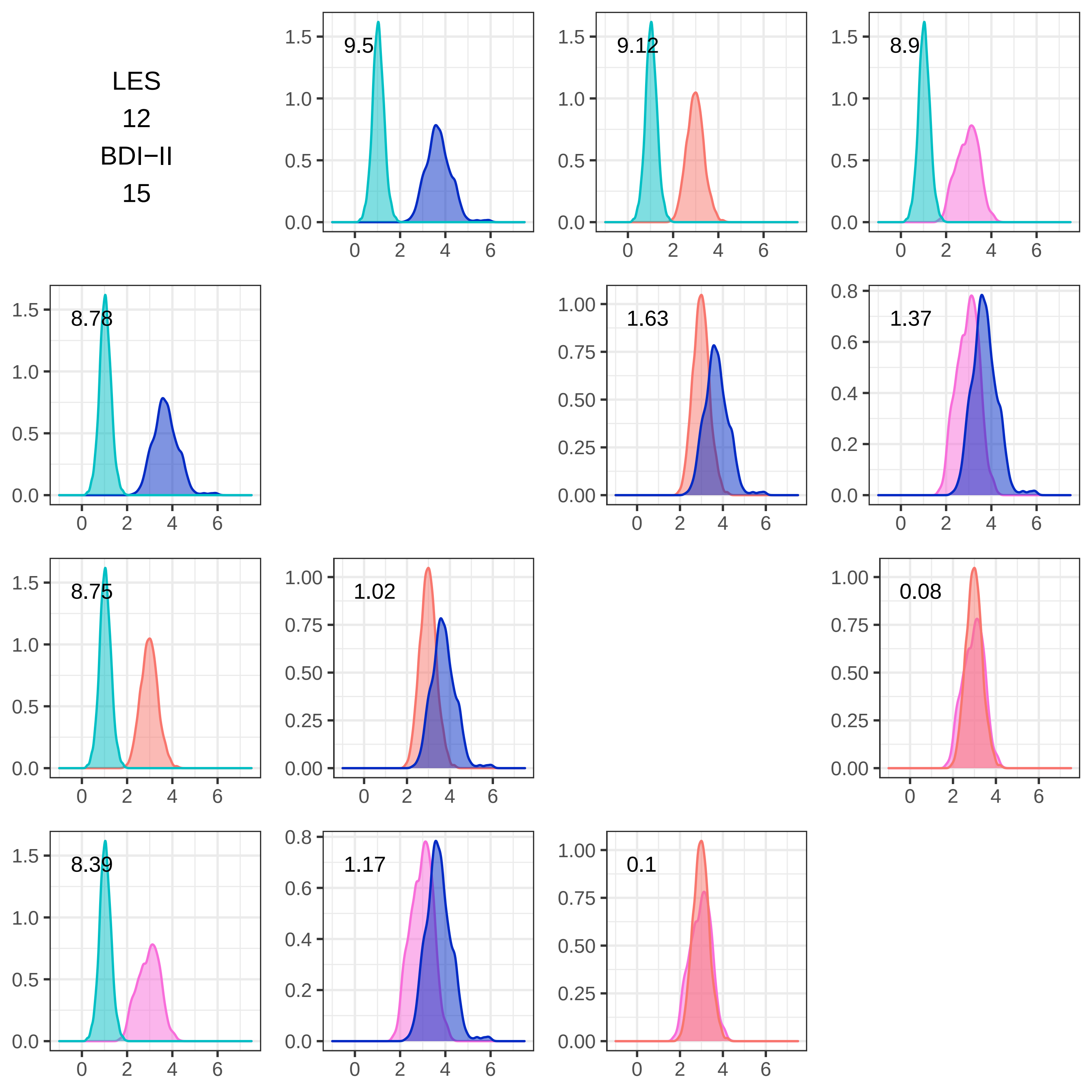
LES
12
BDI−II
15
9.5
9.12
8.9
8.78
1.63
1.37
8.75
1.02
0.08
8.39
1.17
0.1

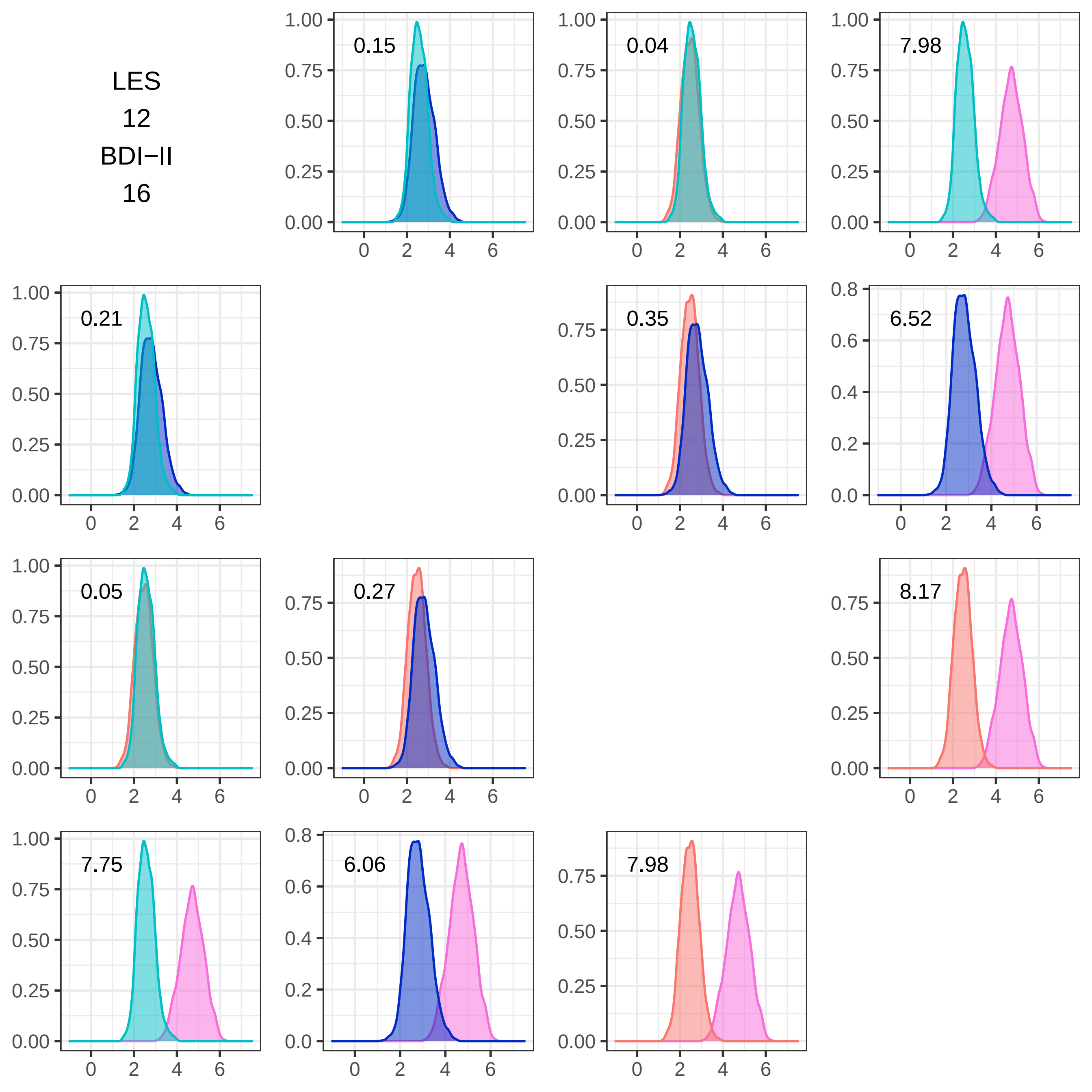
LES
12
BDI−II
16
0.15
0.04
7.98
0.21
0.35
6.52
0.05
0.27
8.17
7.75
6.06
7.98

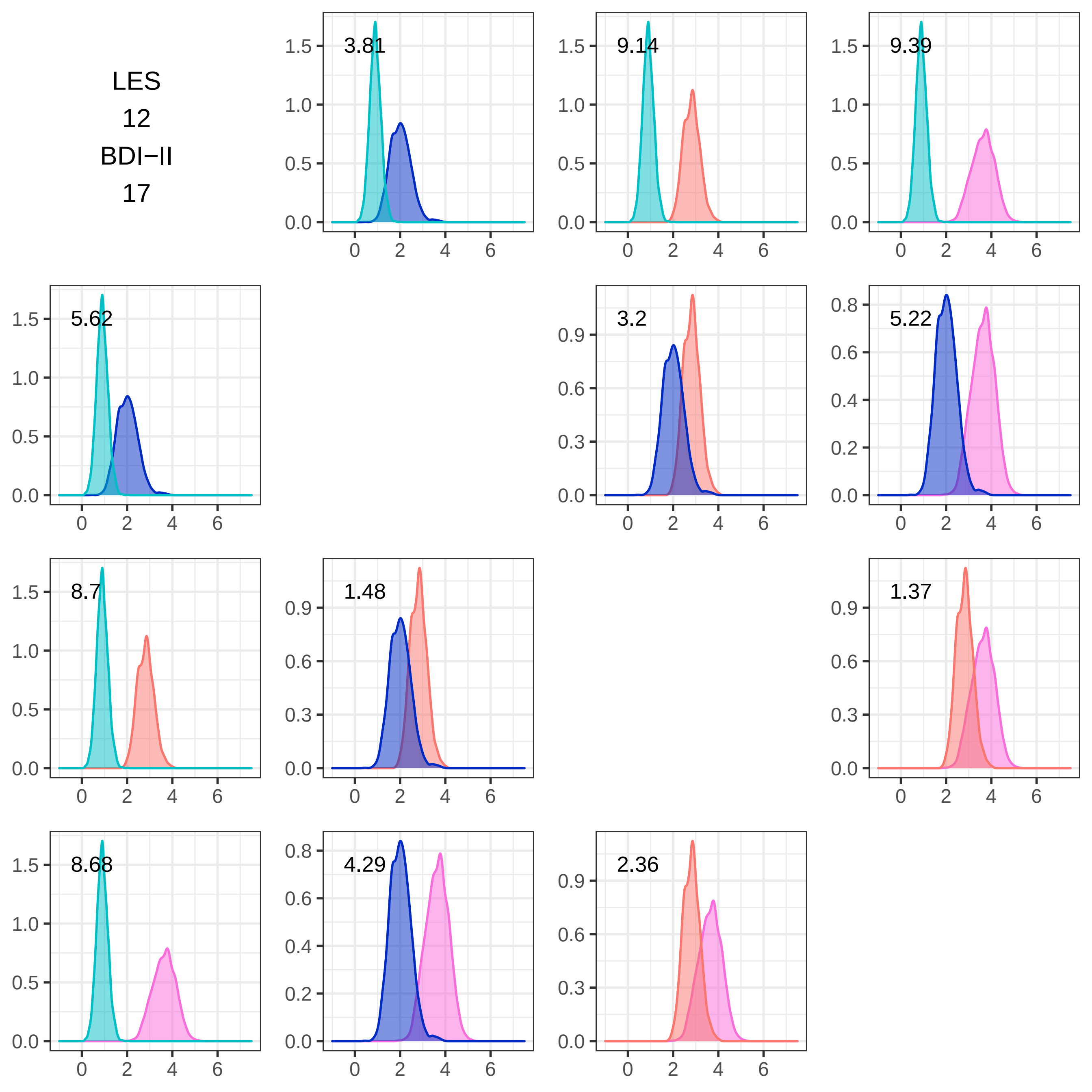

LES
12
BDI−II
17
3.81
9.14
9.39
5.62
3.2
5.22
8.7
1.48
1.37
8.68
4.29
2.36

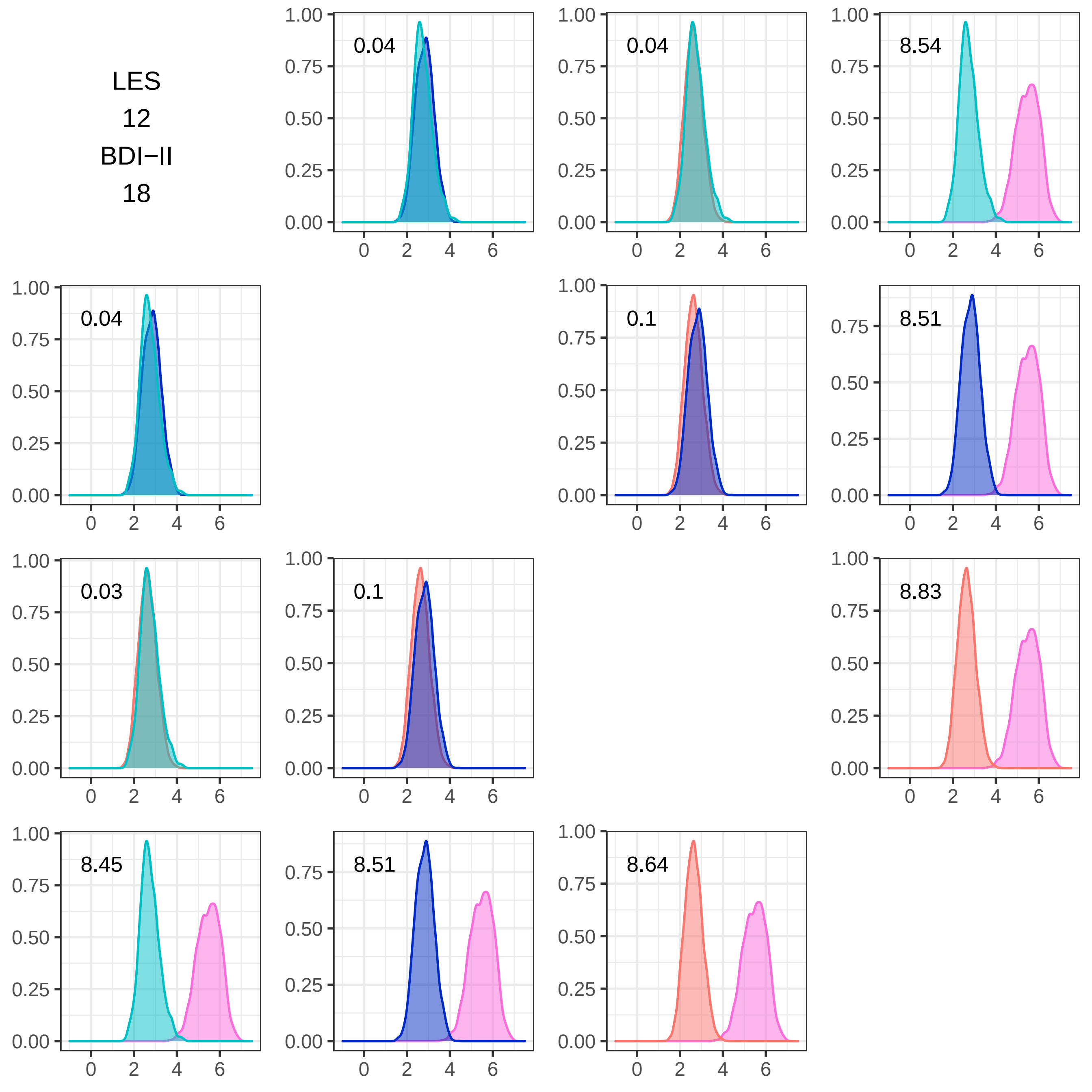
LES
12
BDI−II
18
0.04
0.04
8.54
0.04
0.1
8.51
0.03
0.1
8.83
8.45
8.51
8.64
1.00
0.75
0.50
0.25
0.00
0
2
4
6

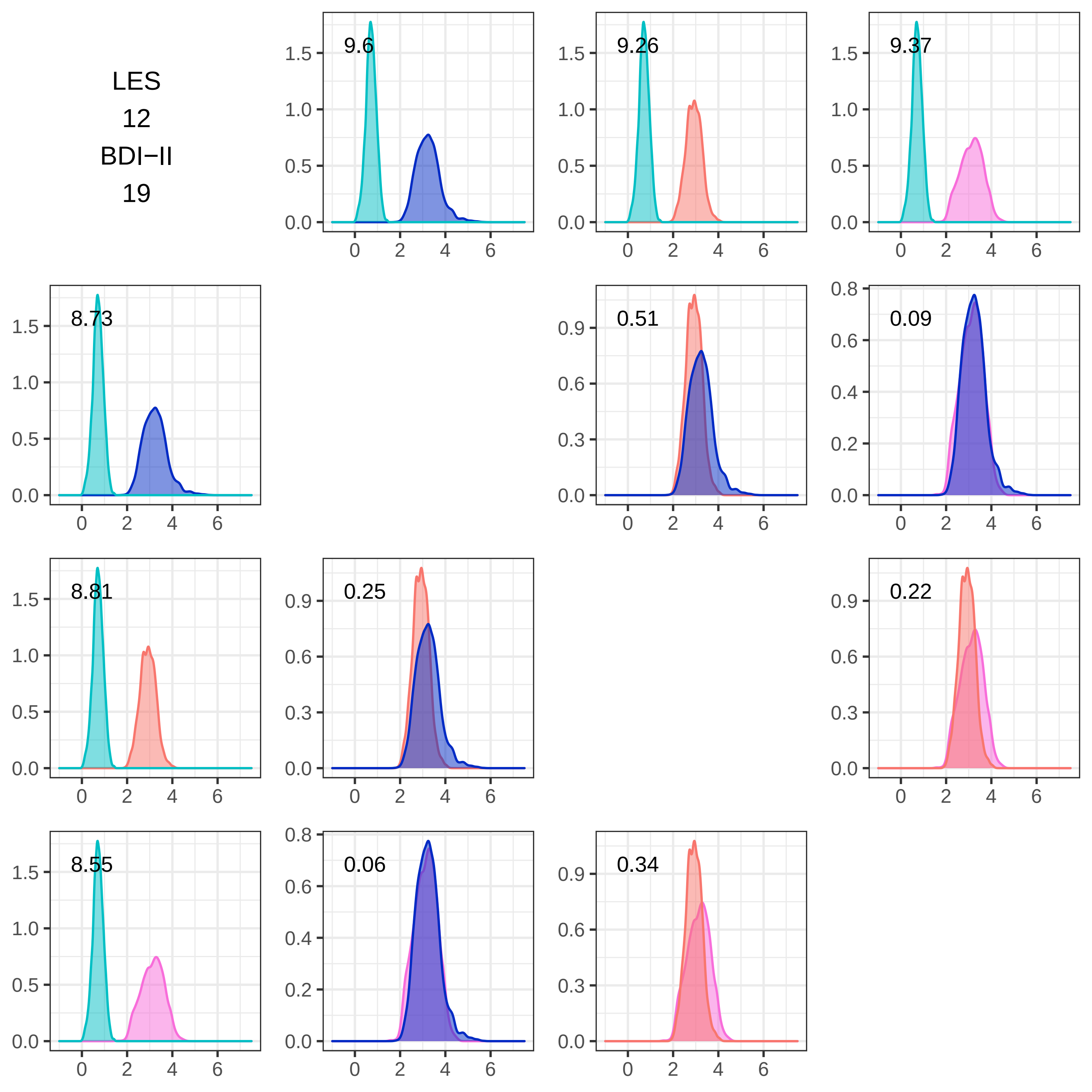

LES
12
BDI−II
19
9.6
9.26
9.37
8.73
0.51
0.09
8.81
0.25
0.22
8.55
0.06
0.34

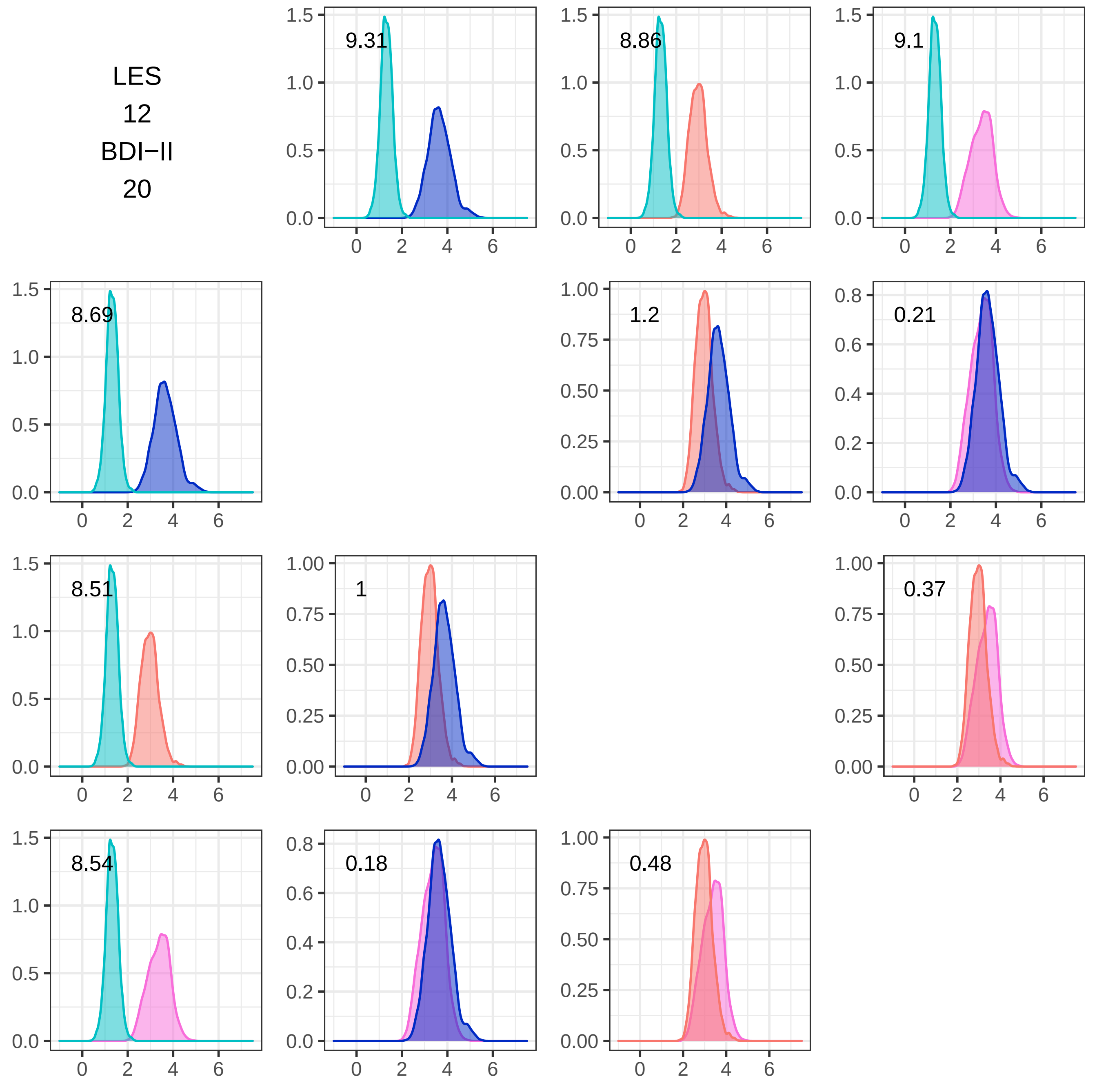
LES
12
BDI−II
20
9.31
8.86
9.1
8.69
1.2
0.21
8.51
1
0.37
8.54
0.18
0.48

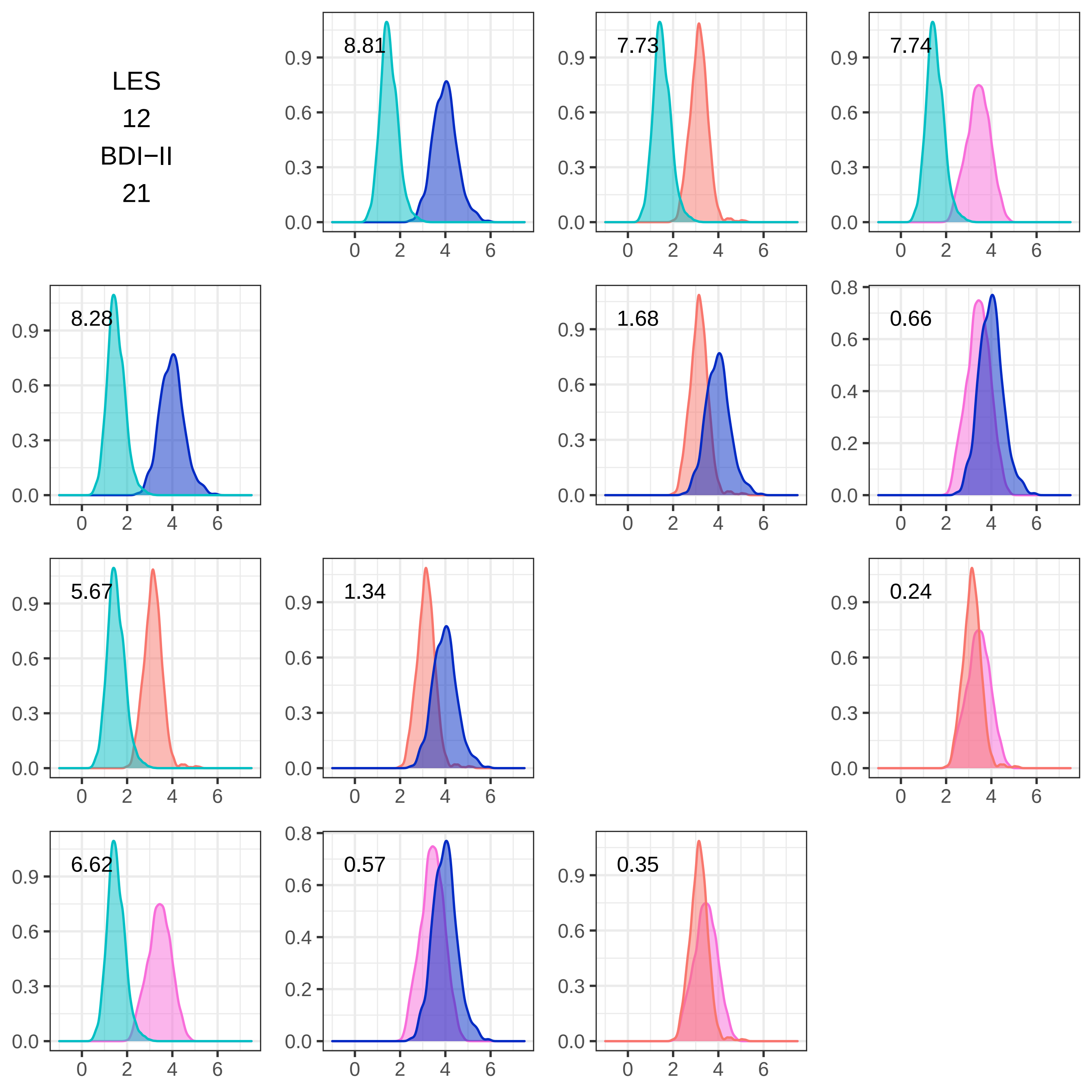

LES
12
BDI−II
21
8.81
7.73
7.74
8.28
1.68
0.66
5.67
1.34
0.24
6.62
0.57
0.35

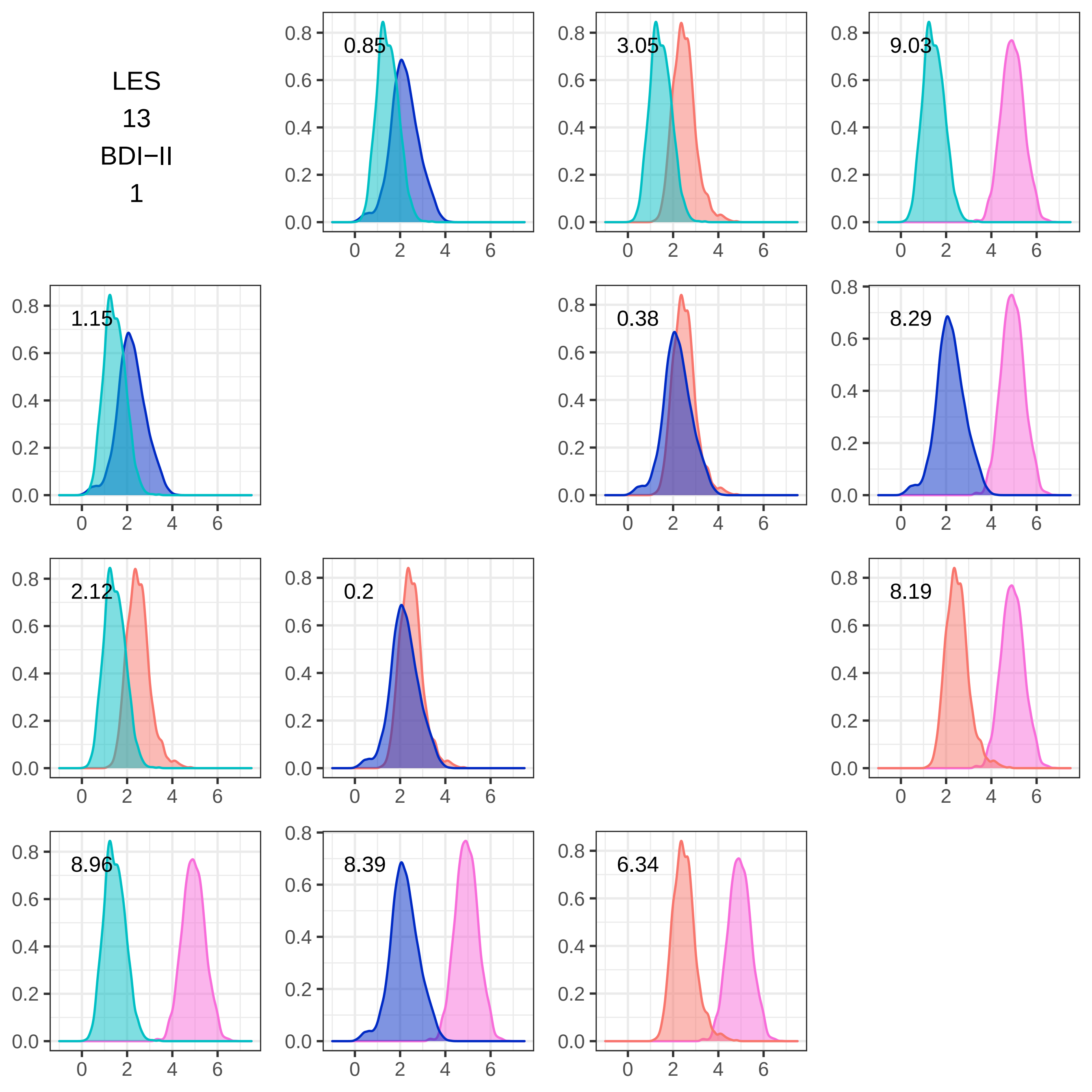
LES
13
BDI−II
1
0.85
3.05
9.03
1.15
0.38
8.29
2.12
0.2
8.19
8.96
8.39
6.34

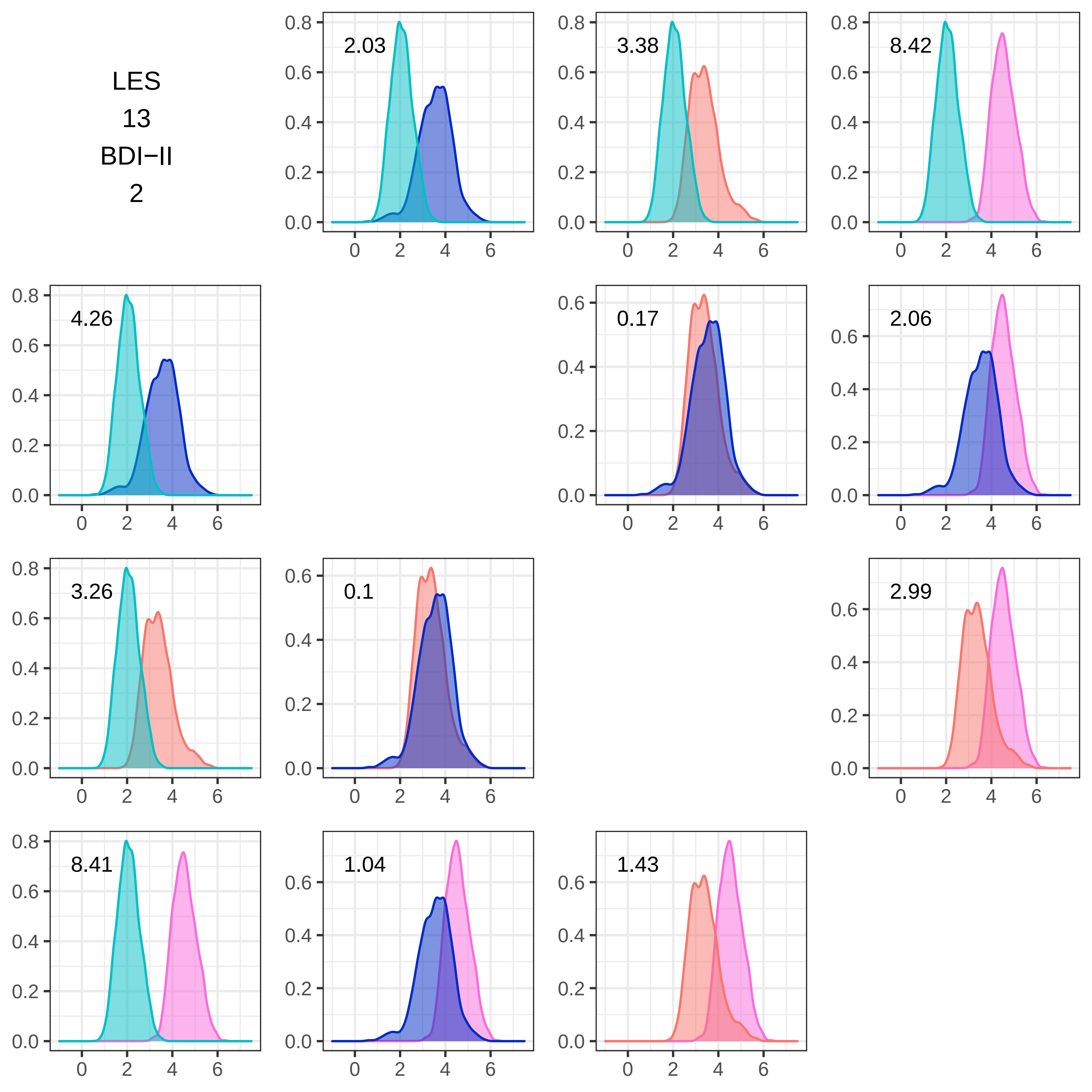

LES
13
BDI−II
2
2.03
3.38
8.42
4.26
0.17
2.06
3.26
0.1
2.99
8.41
1.04
1.43

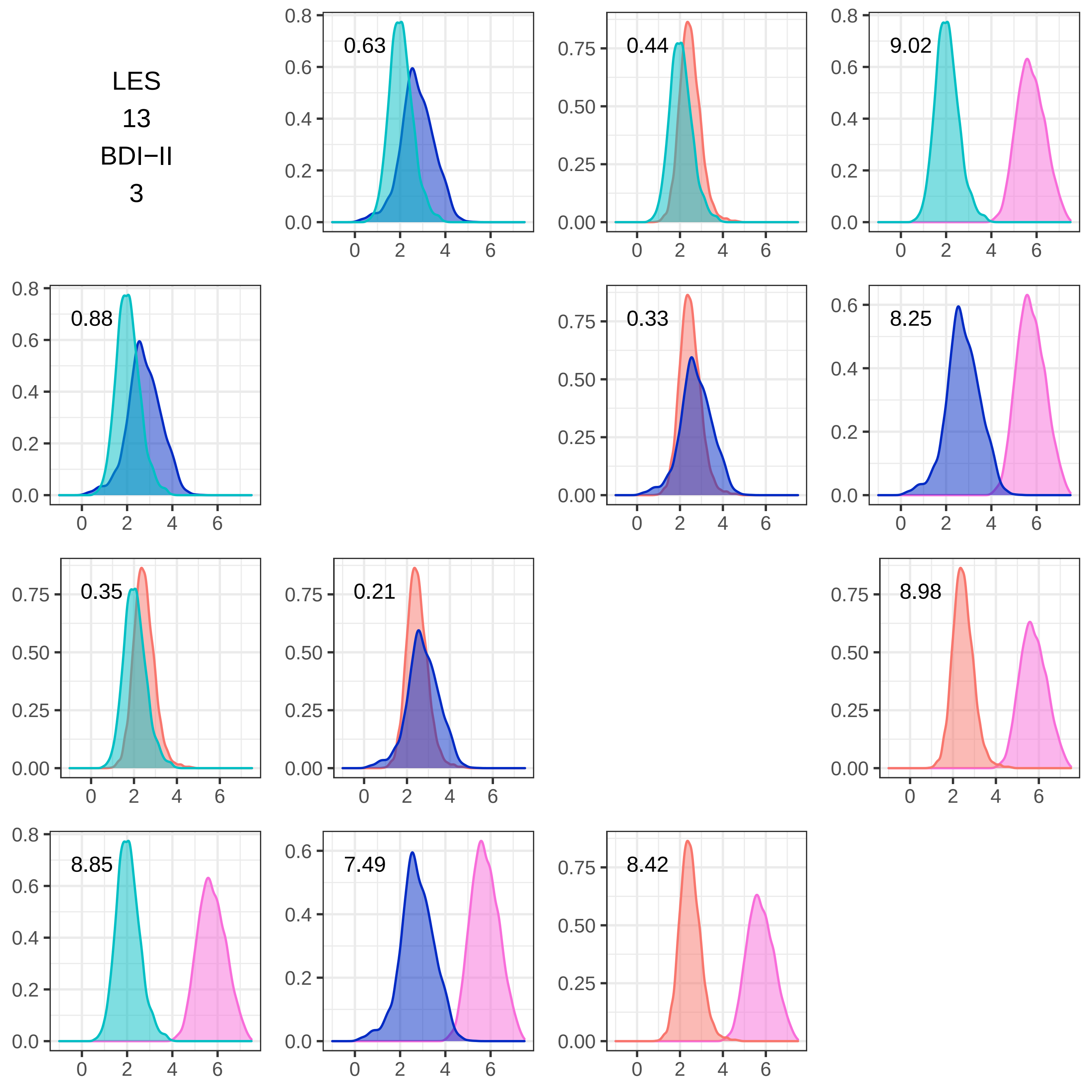

LES
13
BDI−II
3
0.63
0.44
9.02
0.88
0.33
8.25
0.35
0.21
8.98
8.85
7.49
8.42

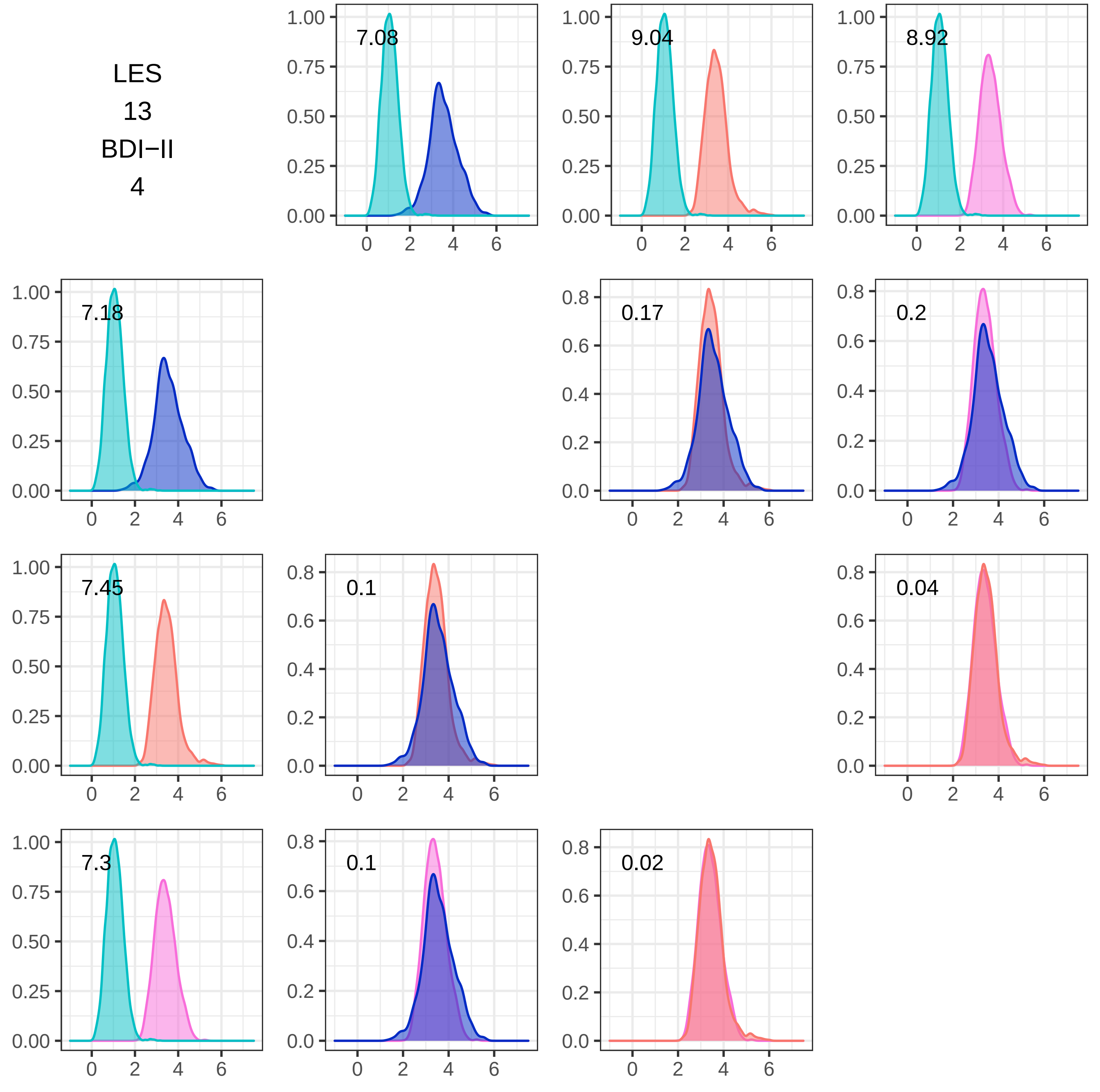
LES
13
BDI−II
4
7.08
9.04
8.92
7.18
0.17
0.2
7.45
0.1
0.04
7.3
0.1
0.02

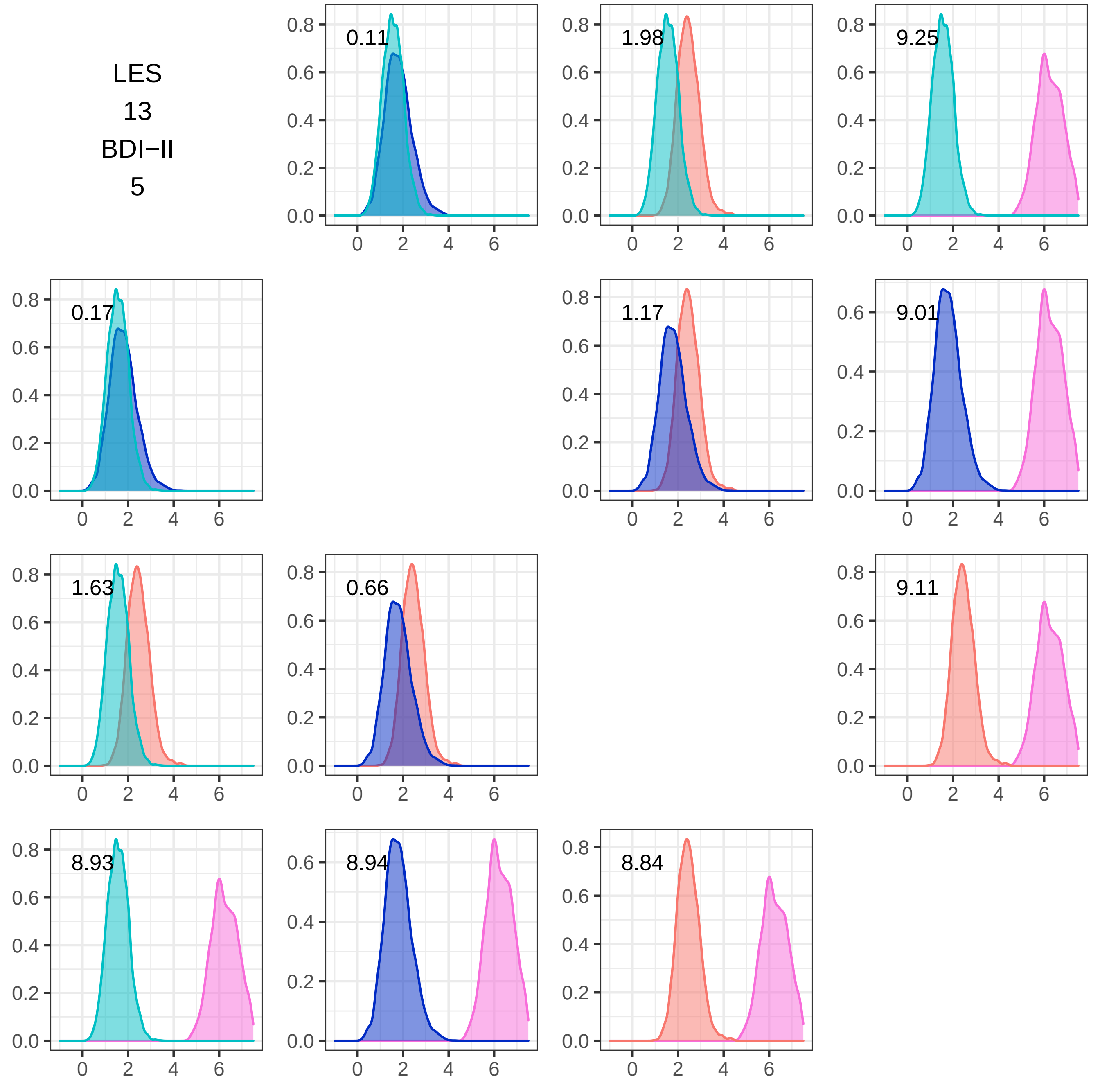

LES
13
BDI−II
5
0.11
1.98
9.25
0.17
1.17
9.01
1.63
0.66
9.11
8.93
8.94
8.84

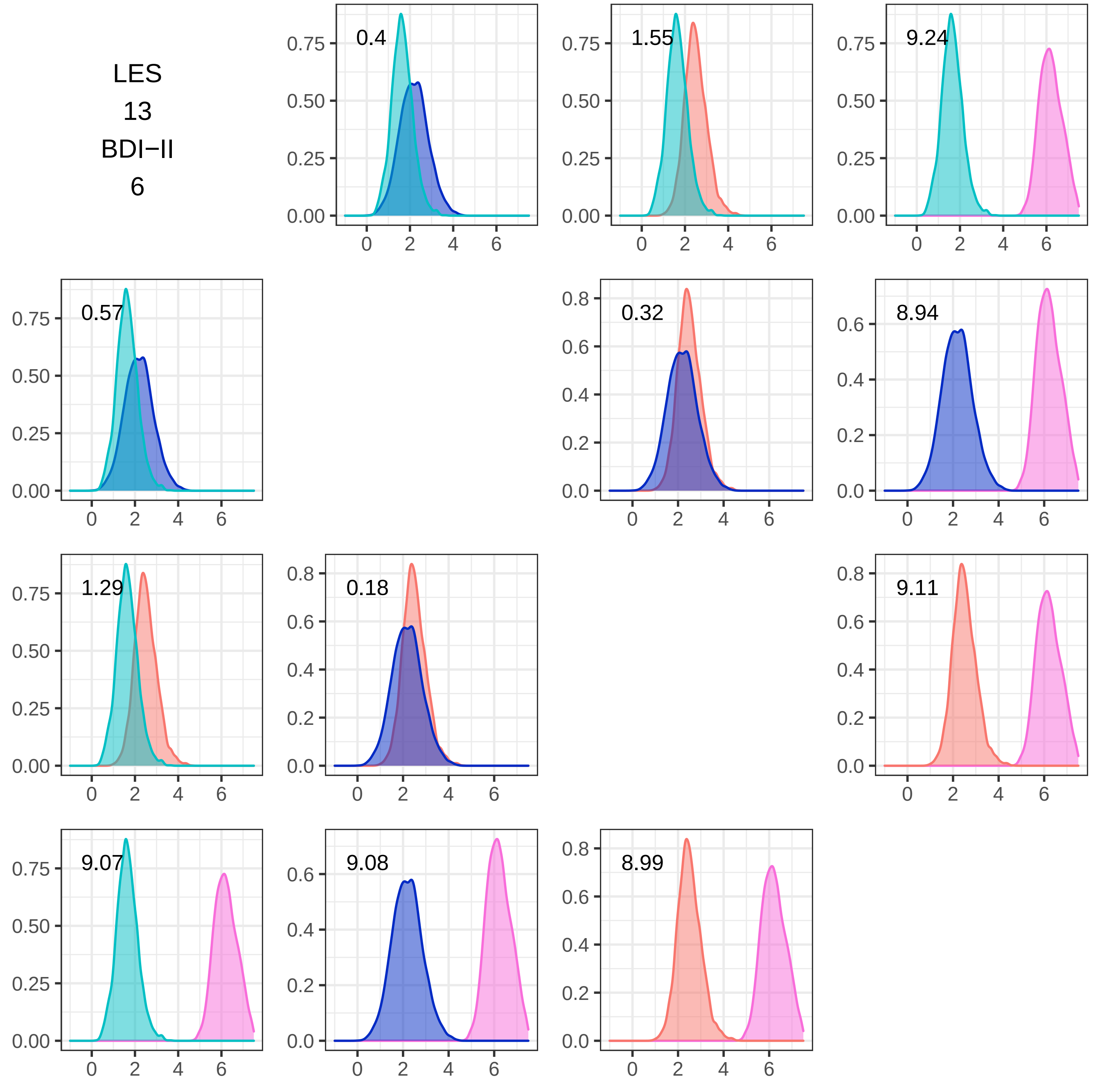

LES
13
BDI−II
6
0.4
1.55
9.24
0.57
0.32
8.94
1.29
0.18
9.11
9.07
9.08
8.99

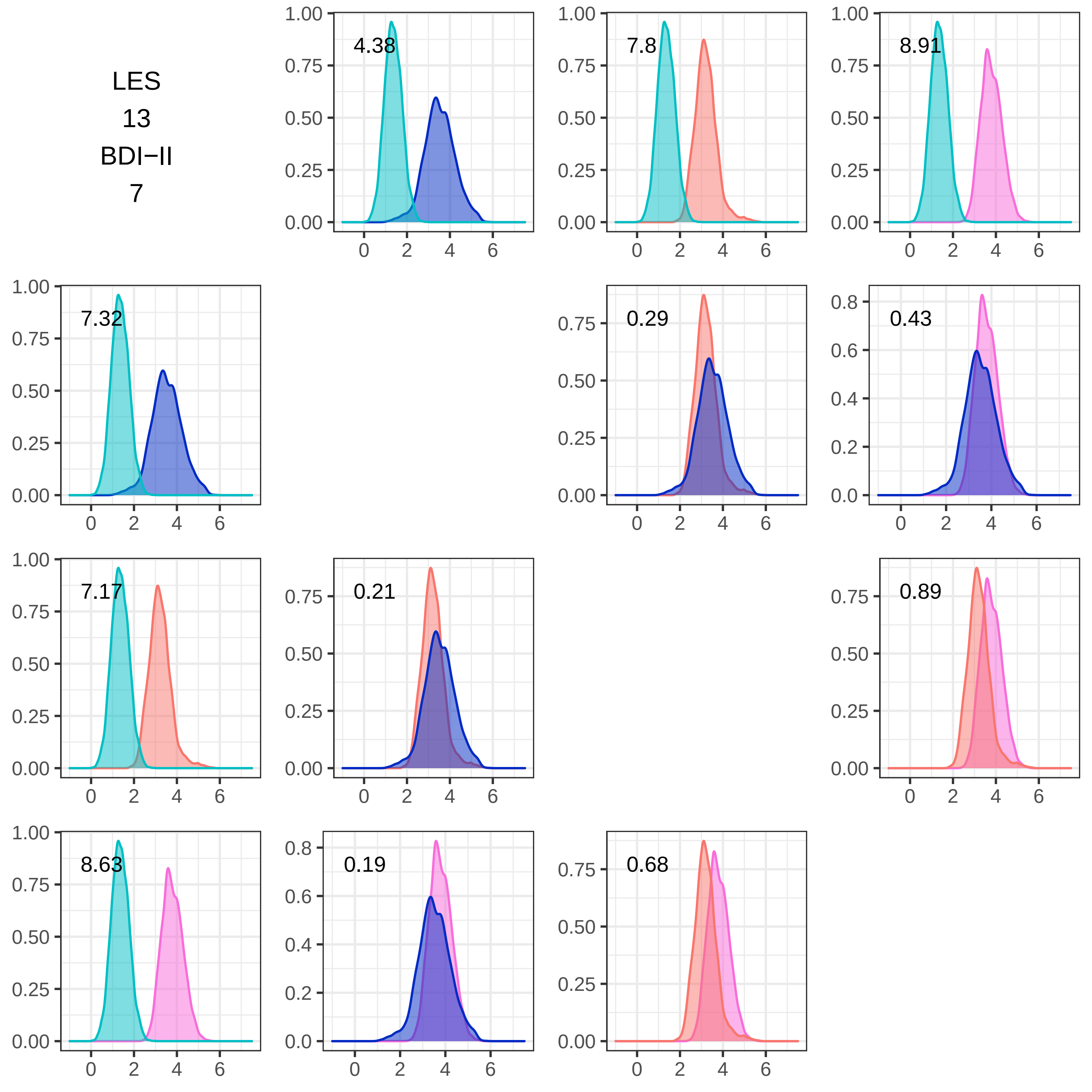
LES
13
BDI−II
7
4.38
7.8
8.91
7.32
0.29
0.43
7.17
0.21
0.89
8.63
0.19
0.68

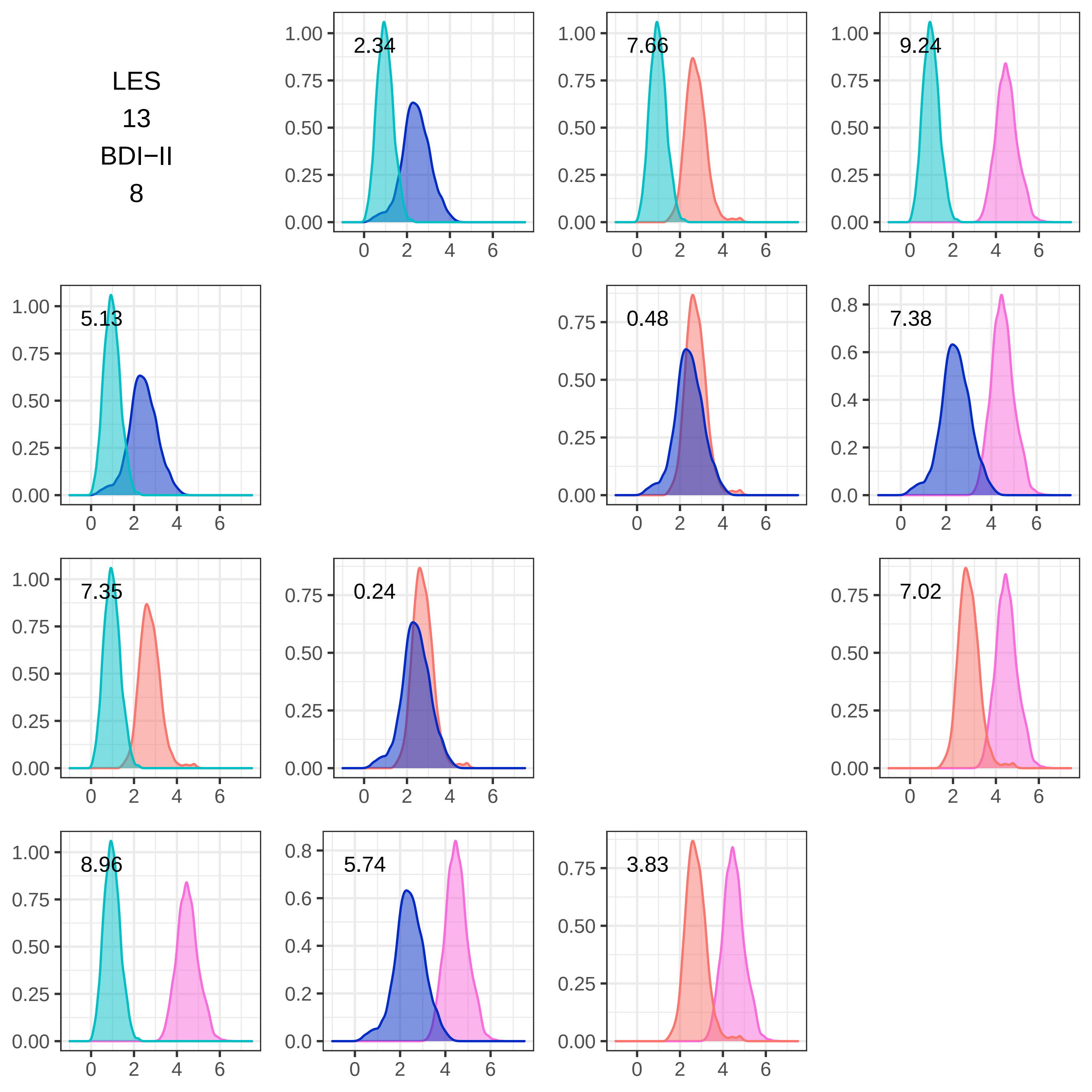

LES
13
BDI−II
8
2.34
7.66
9.24
5.13
0.48
7.38
7.35
0.24
7.02
8.96
5.74
3.83

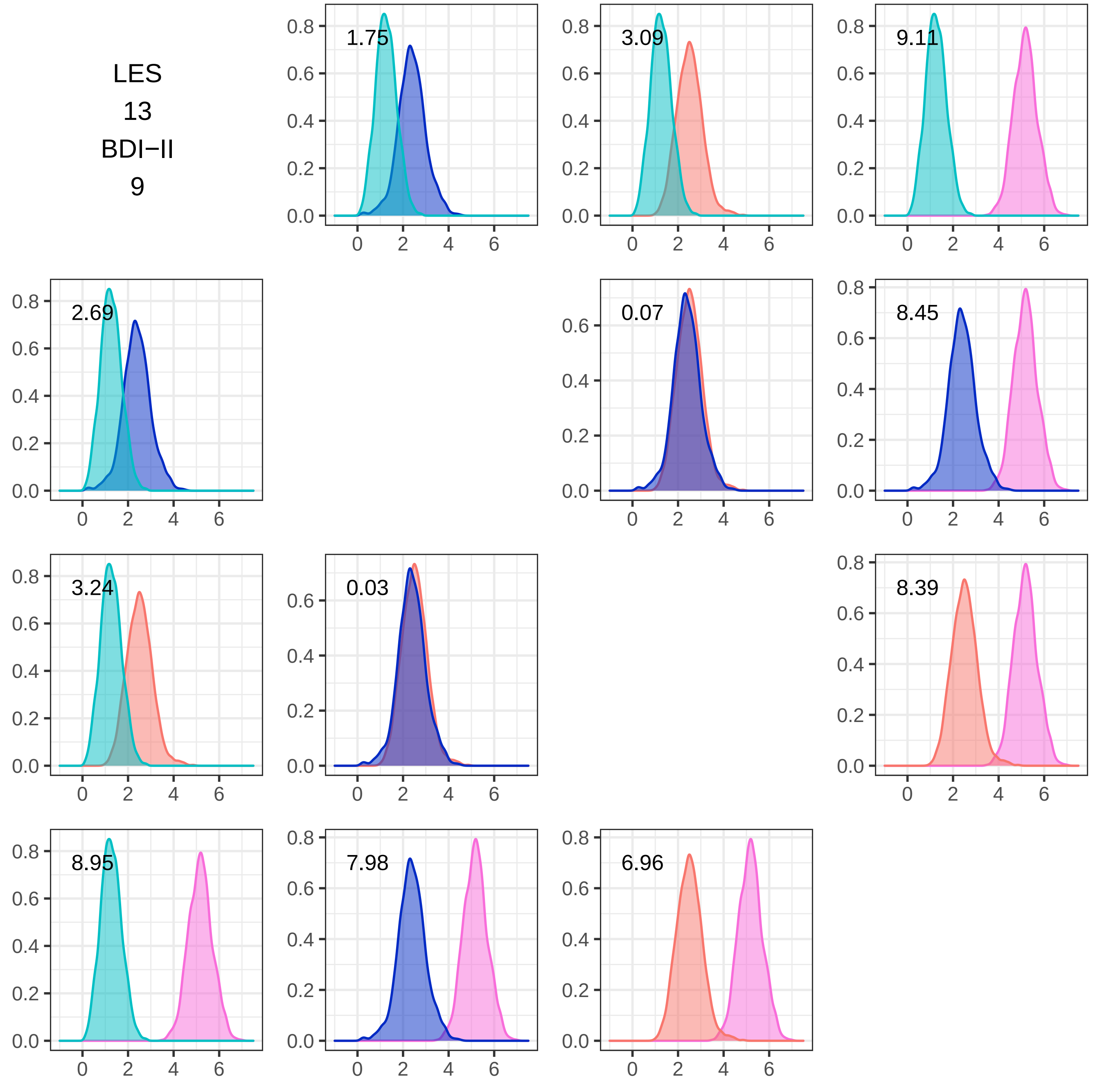

LES
13
BDI−II
9
1.75
3.09
9.11
2.69
0.07
8.45
3.24
0.03
8.39
8.95
7.98
6.96

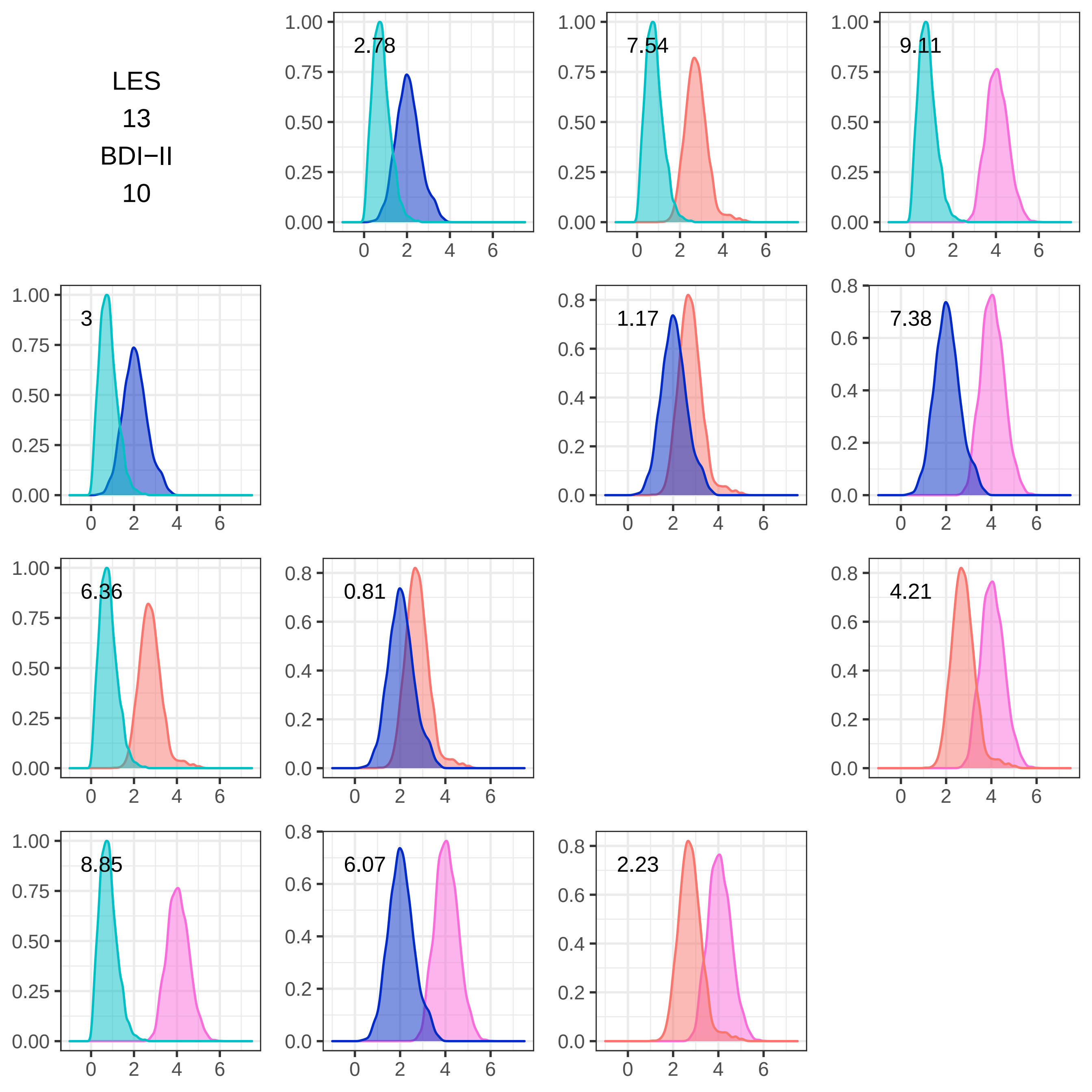
LES
13
BDI−II
10
2.78
7.54
9.11
3
1.17
7.38
6.36
0.81
4.21
8.85
6.07
2.23

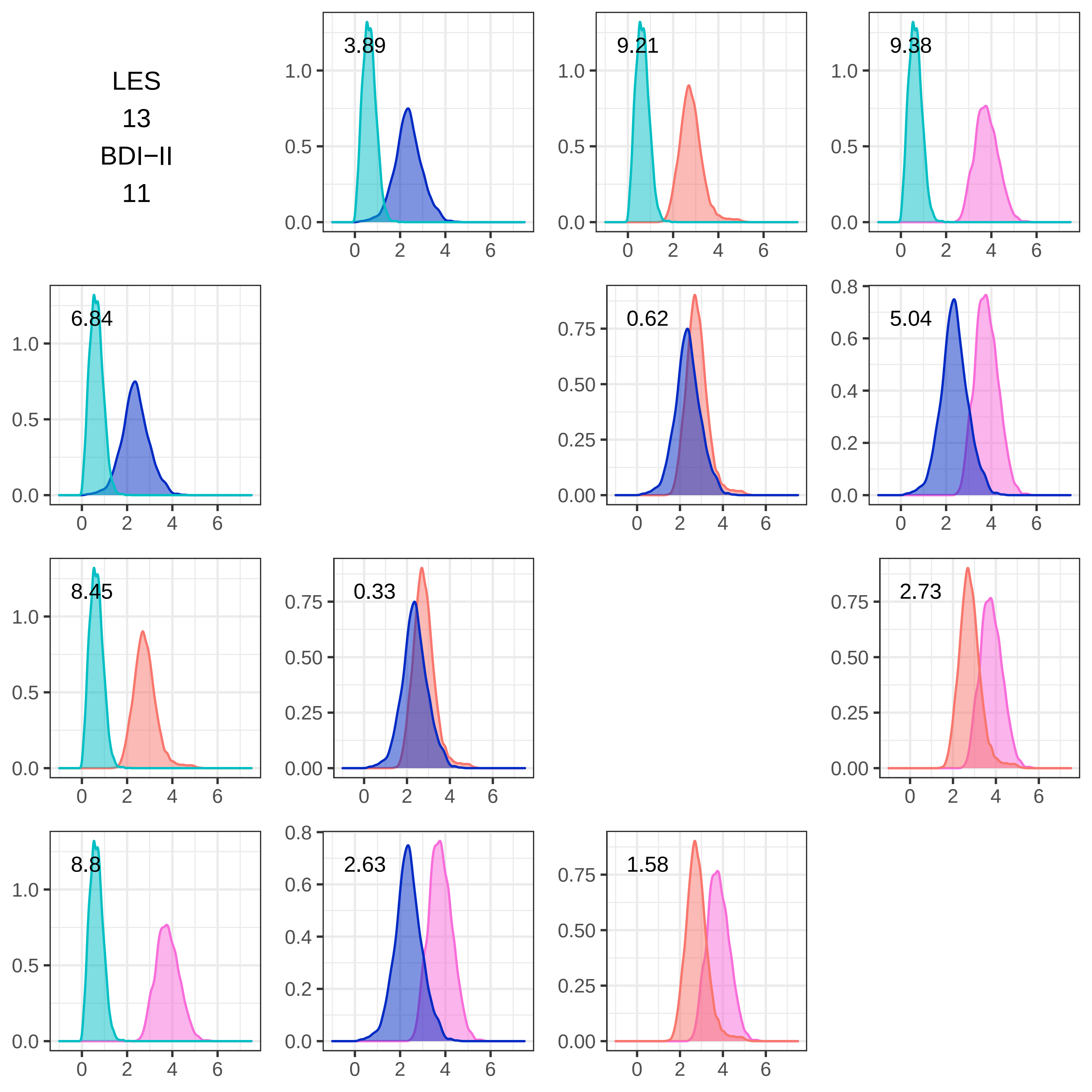
LES
13
BDI−II
11
3.89
9.21
9.38
6.84
0.62
5.04
8.45
0.33
2.73
8.8
2.63
1.58

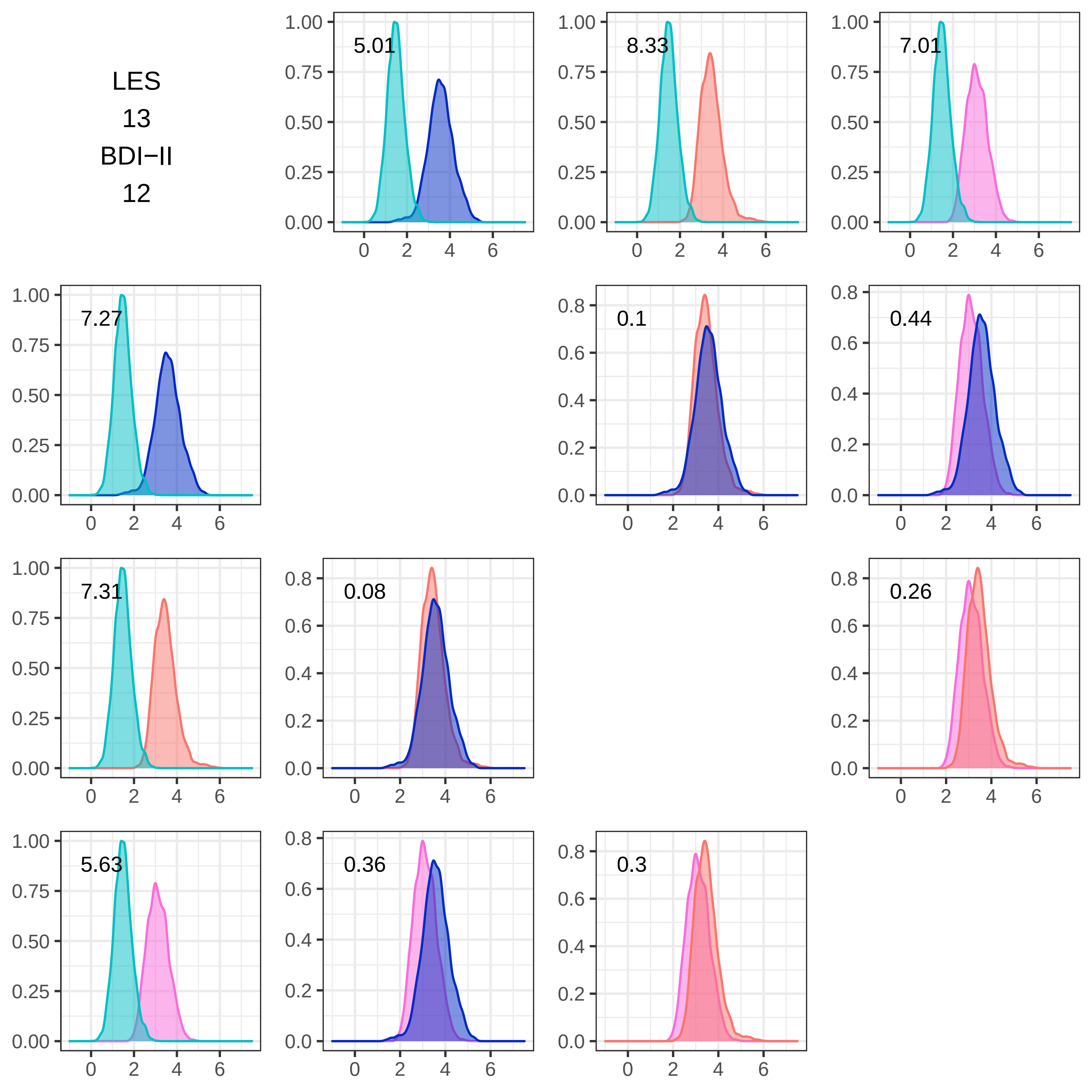
LES
13
BDI−II
12
5.01
8.33
7.01
7.27
0.1
0.44
7.31
0.08
0.26
5.63
0.36
0.3

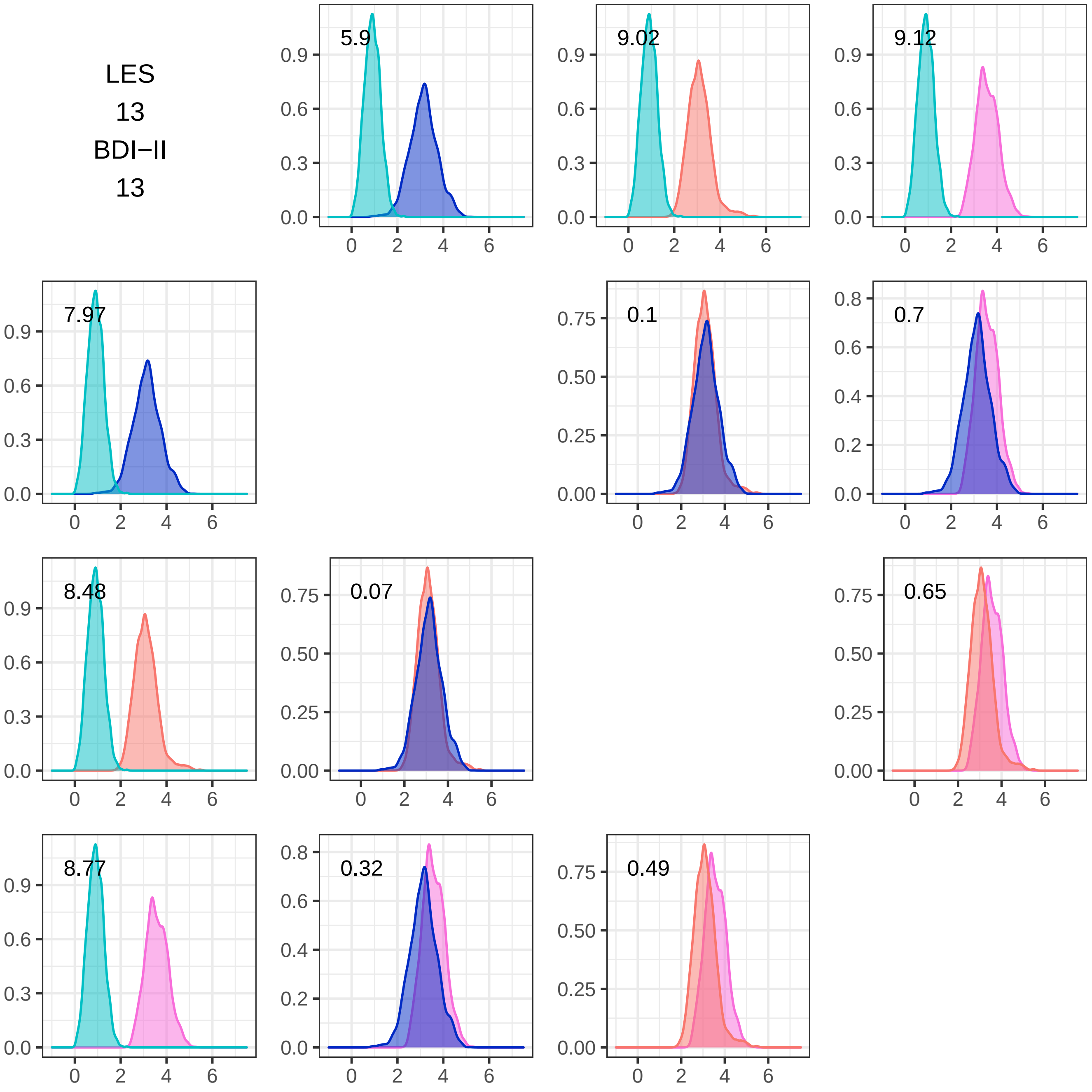
LES
13
BDI−II
13
5.9
9.02
9.12
7.97
0.1
0.7
8.48
0.07
0.65
8.77
0.32
0.49

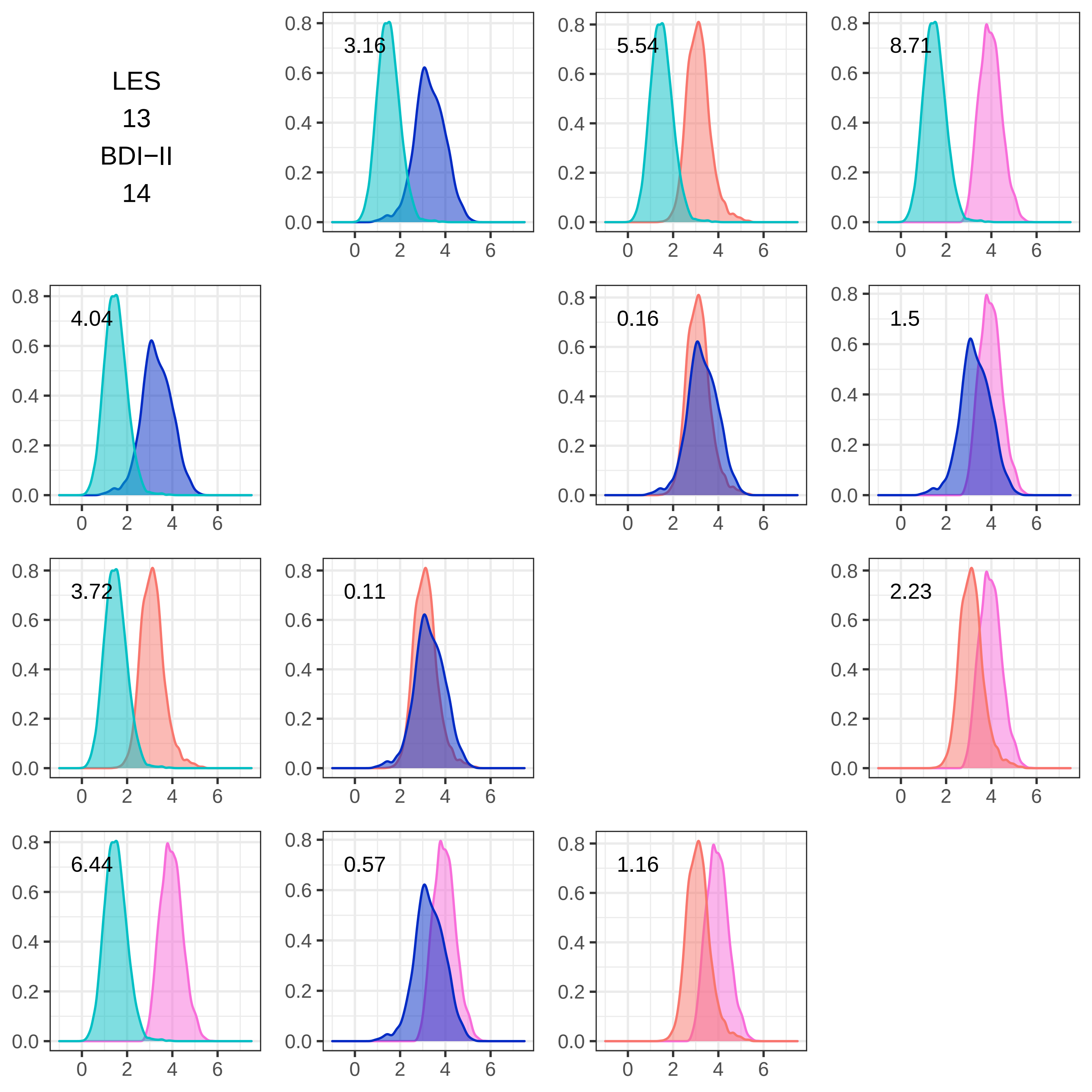
LES
13
BDI−II
14
3.16
5.54
8.71
4.04
0.16
1.5
3.72
0.11
2.23
6.44
0.57
1.16

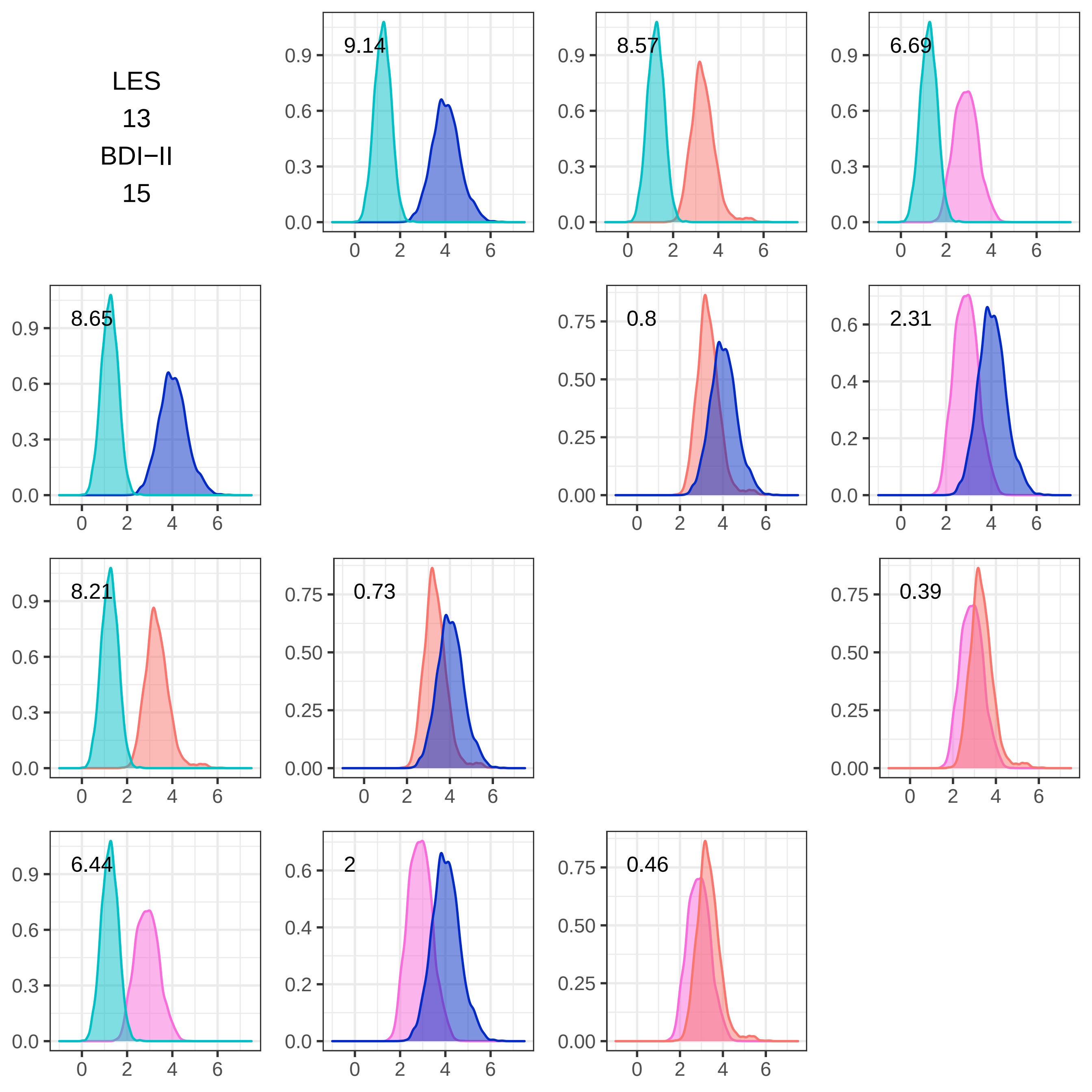
LES
13
BDI−II
15
9.14
8.57
6.69
8.65
0.8
2.31
8.21
0.73
0.39
6.44
2
0.46

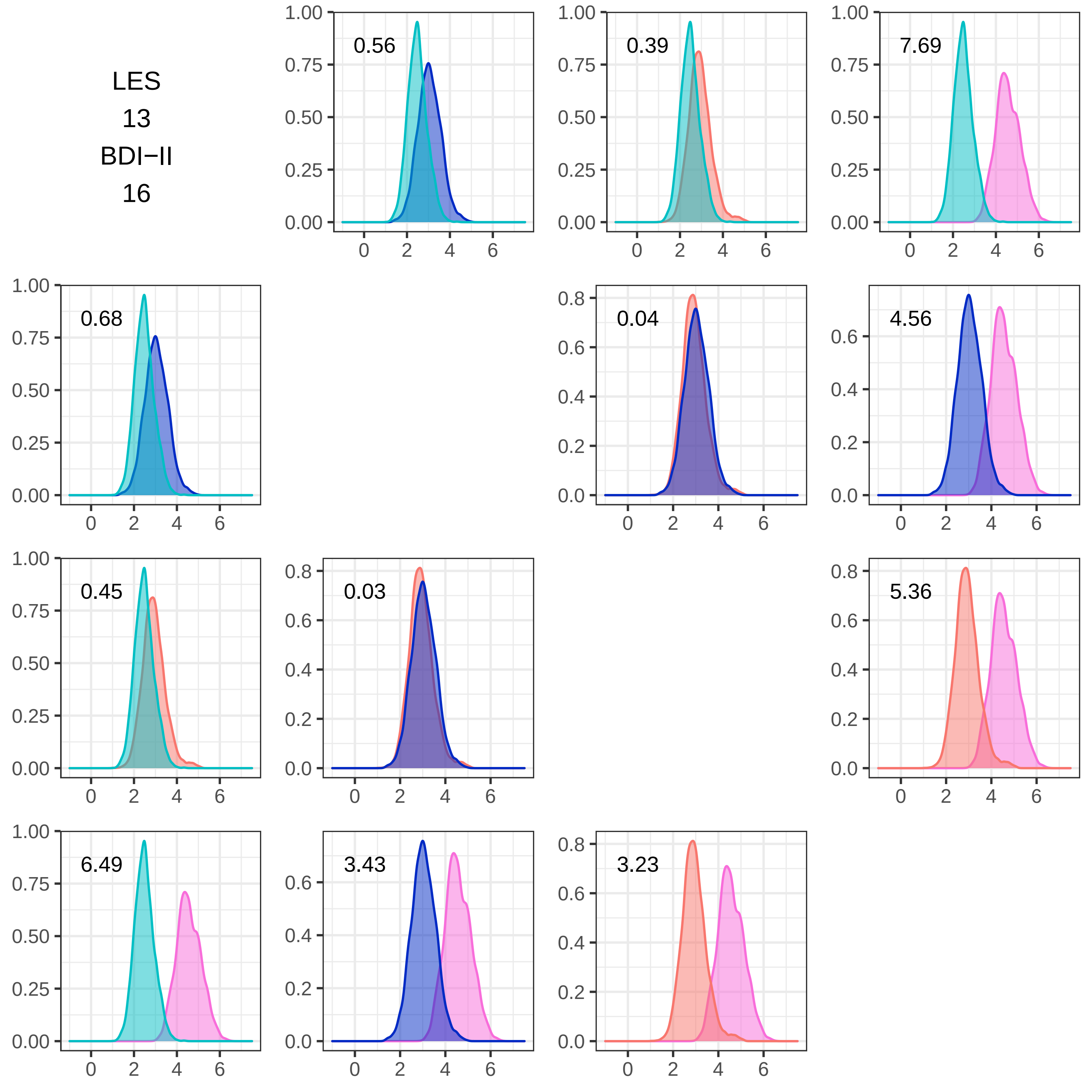

LES
13
BDI−II
16
0.56
0.39
7.69
0.68
0.04
4.56
0.45
0.03
5.36
6.49
3.43
3.23

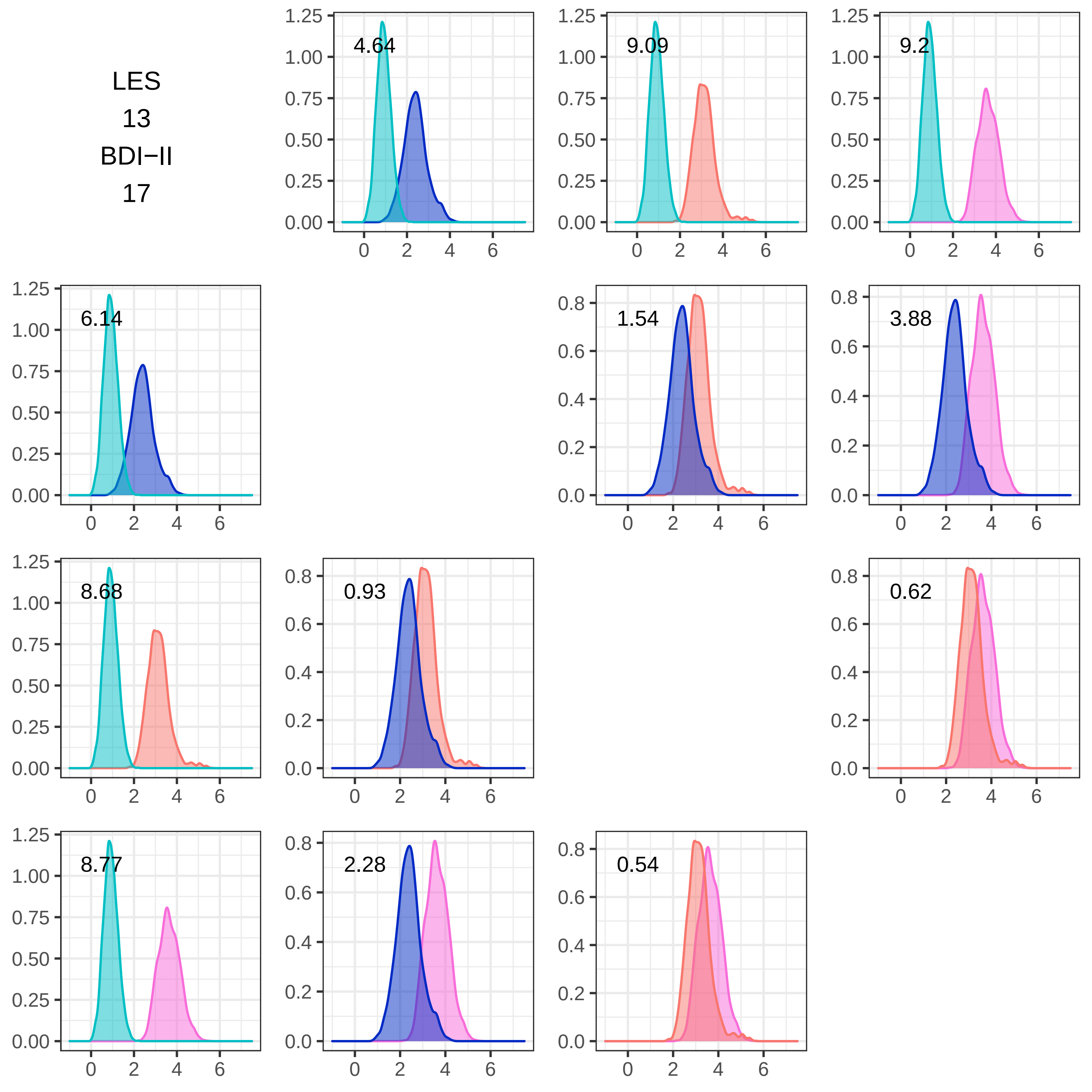

LES
13
BDI−II
17
4.64
9.09
9.2
6.14
1.54
3.88
8.68
0.93
0.62
8.77
2.28
0.54

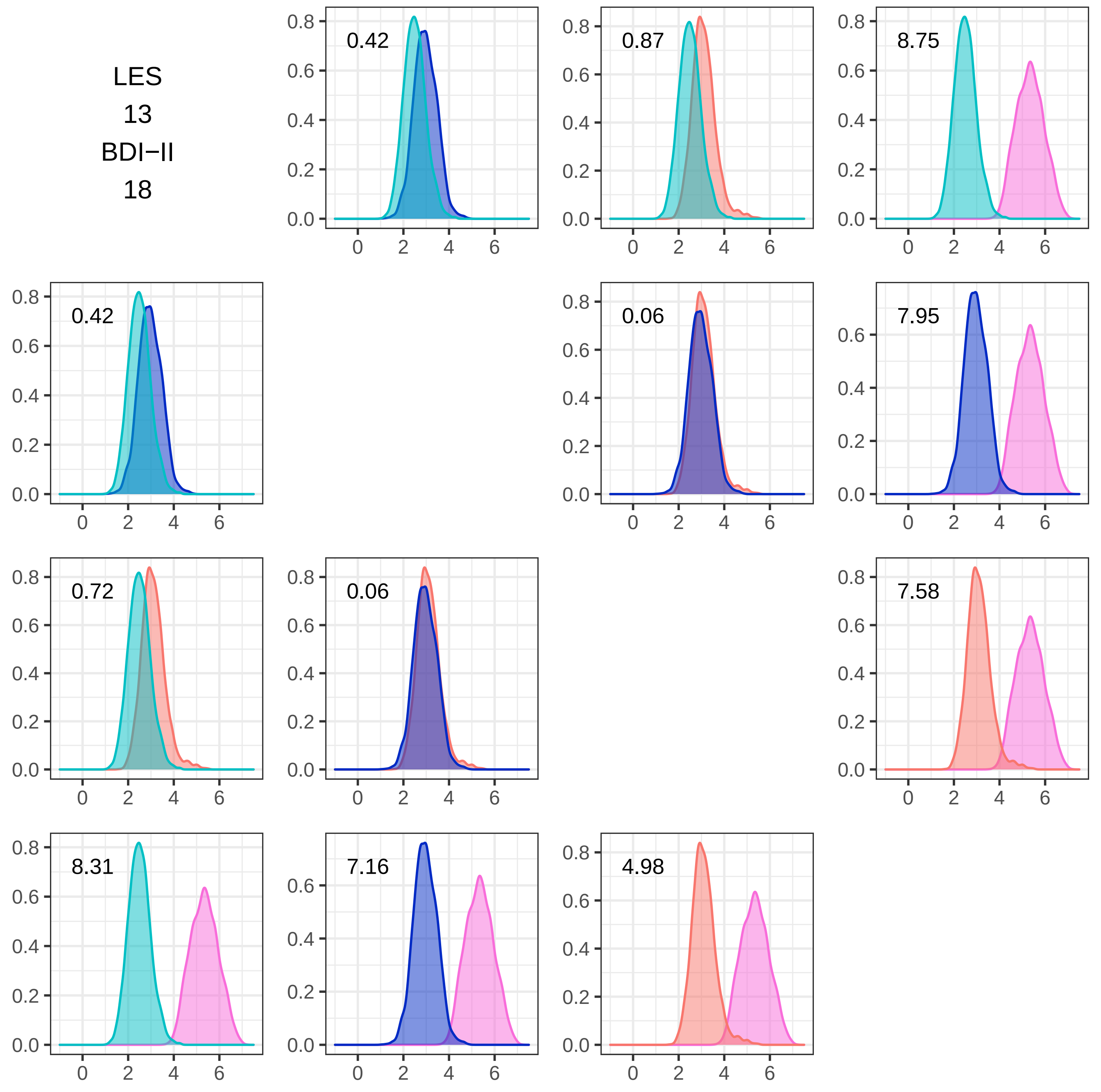
LES
13
BDI−II
18
0.42
0.87
8.75
0.42
0.06
7.95
0.72
0.06
7.58
8.31
7.16
4.98

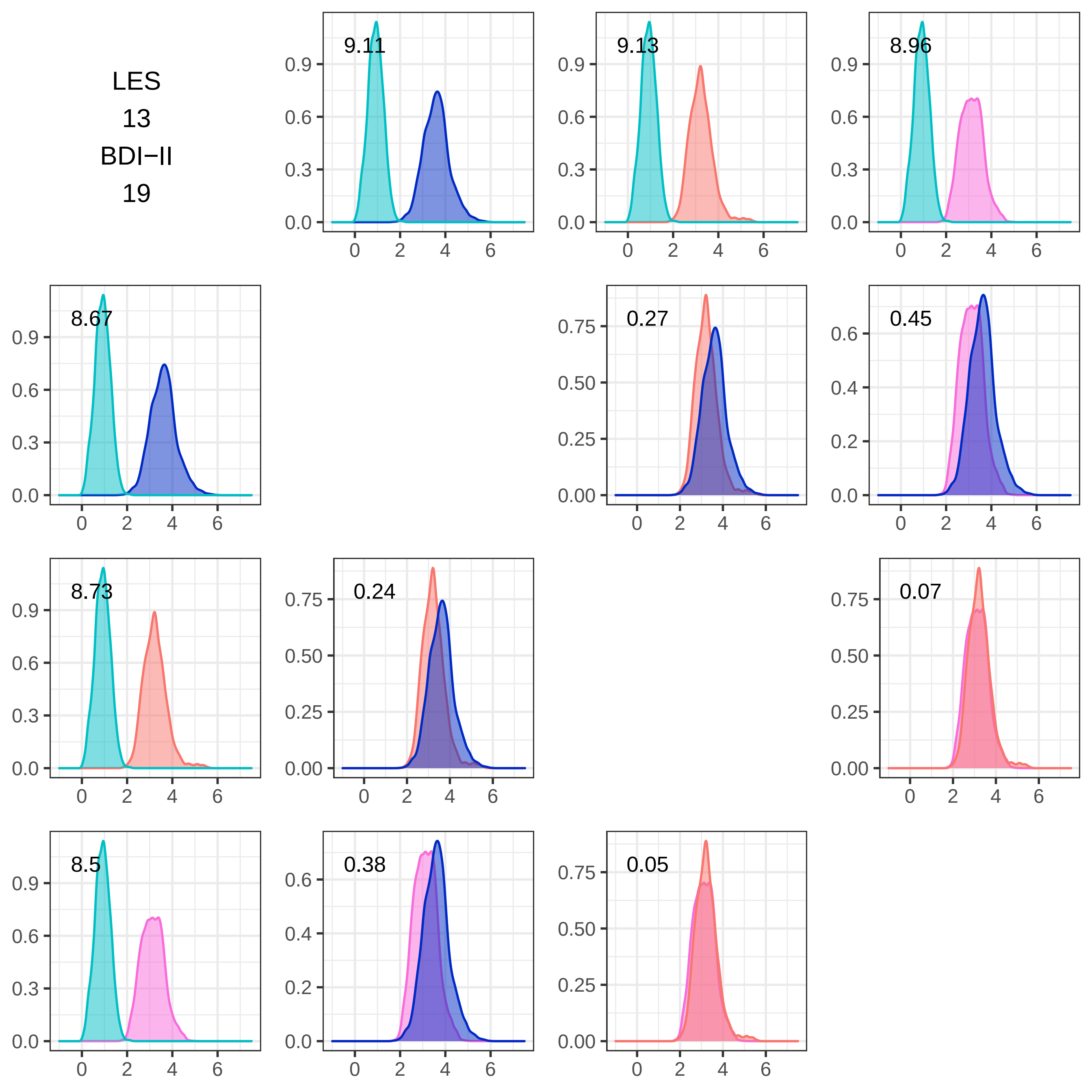

LES
13
BDI−II
19
9.11
9.13
8.96
8.67
0.27
0.45
8.73
0.24
0.07
8.5
0.38
0.05

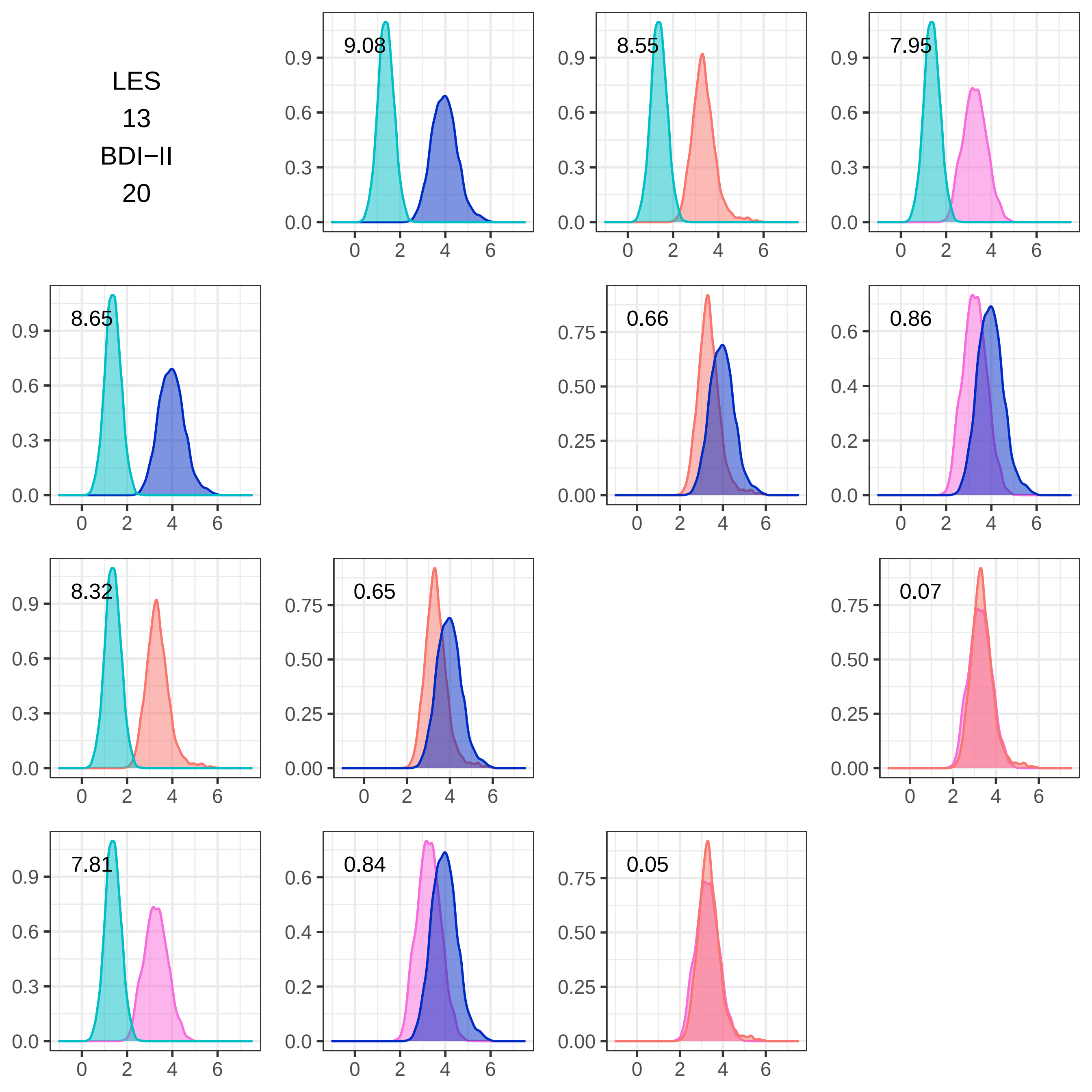

LES
13
BDI−II
20
9.08
8.55
7.95
8.65
0.66
0.86
8.32
0.65
0.07
7.81
0.84
0.05

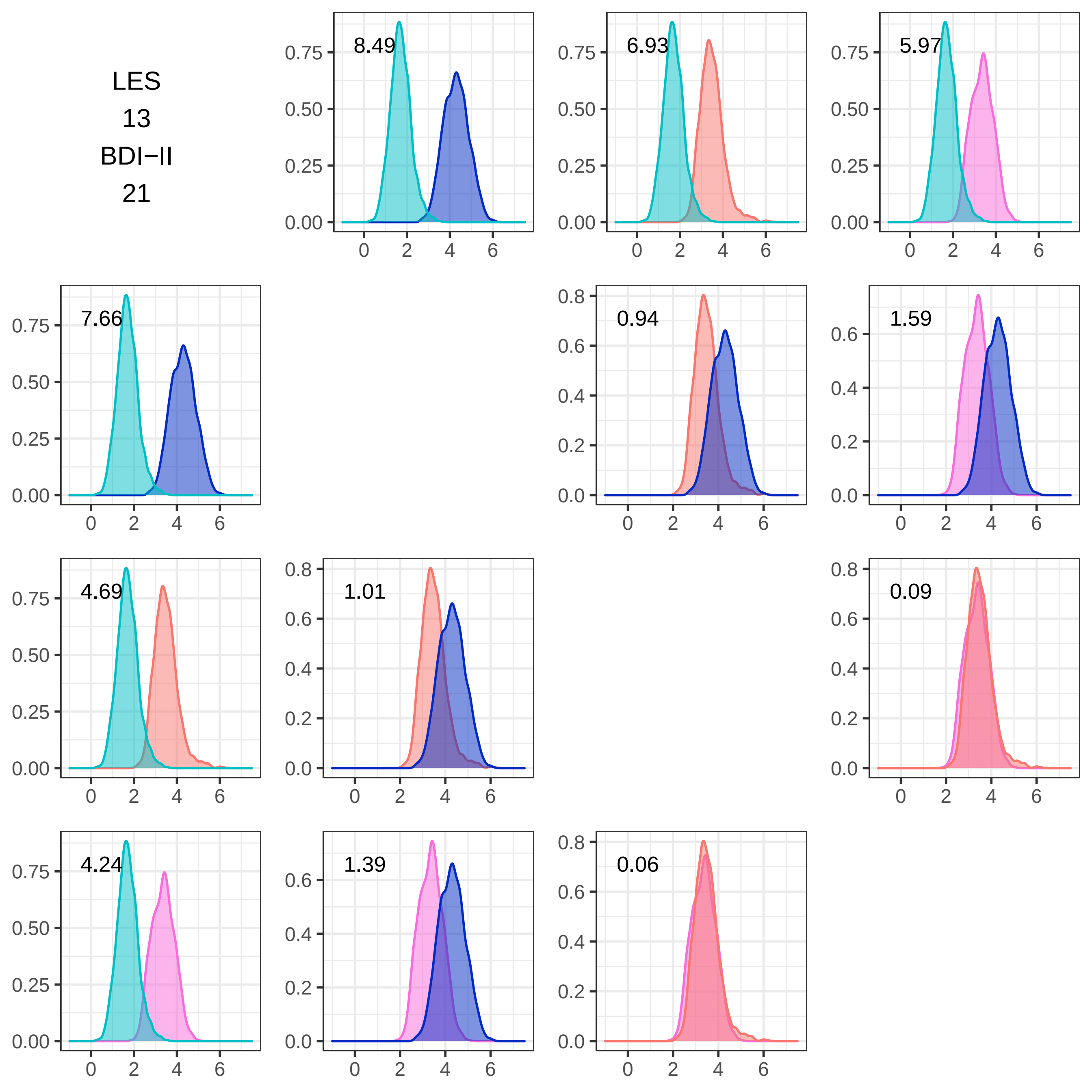
LES
13
BDI−II
21
8.49
6.93
5.97
7.66
0.94
1.59
4.69
1.01
0.09
4.24
1.39
0.06

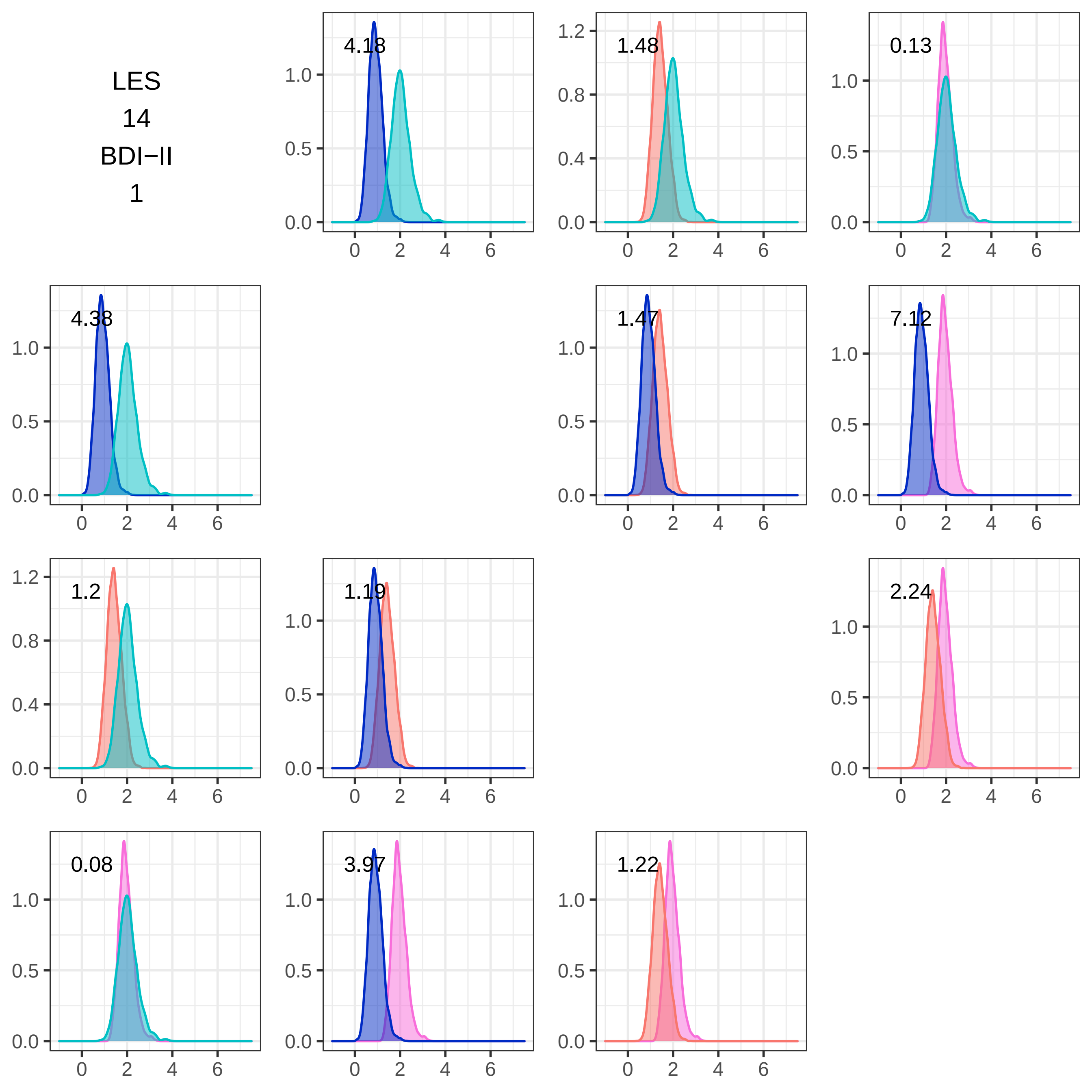
LES
14
BDI−II
1
4.18
1.48
0.13
4.38
1.47
7.12
1.2
1.19
2.24
0.08
3.97
1.22

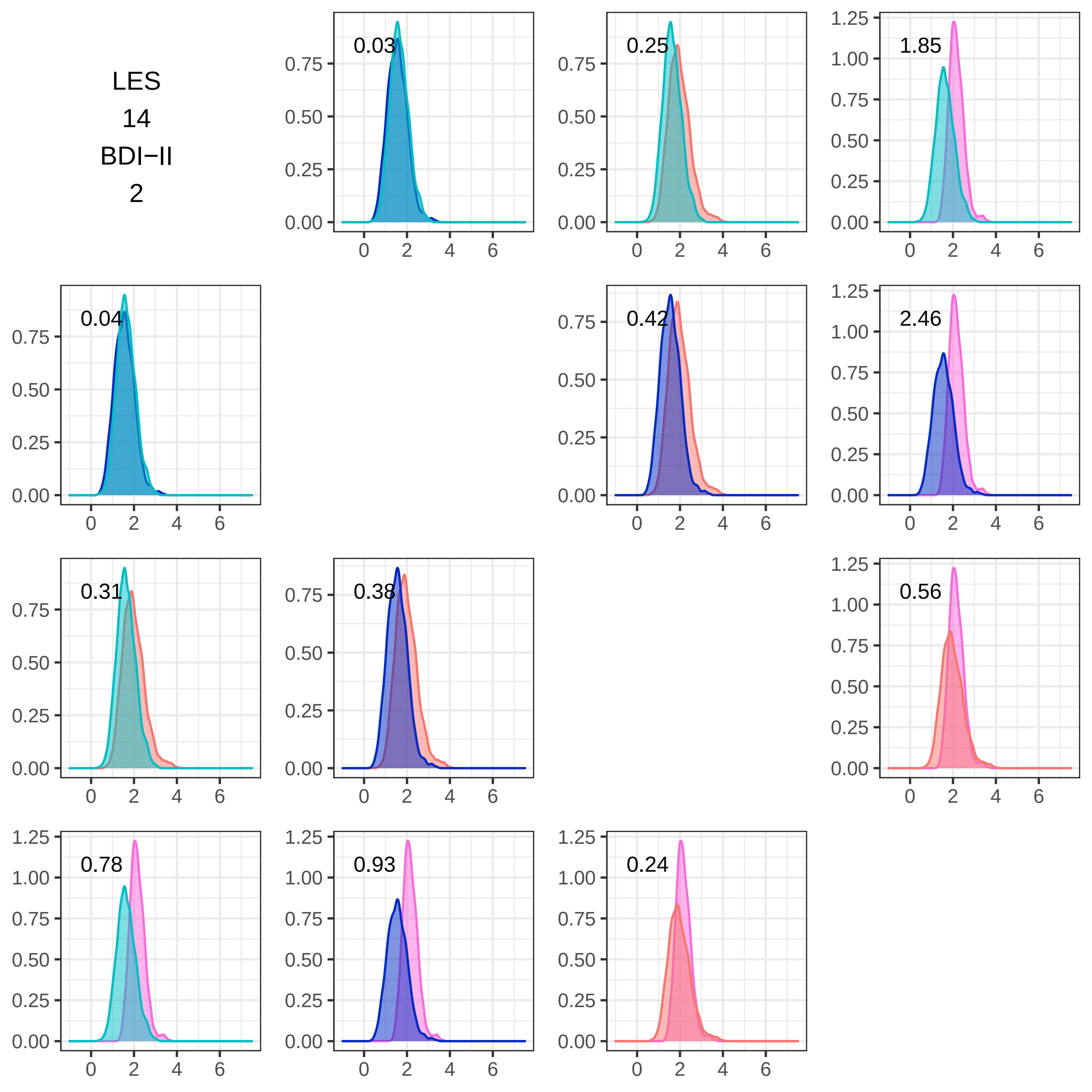
LES
14
BDI−II
2
0.03
0.25
1.85
0.04
0.42
2.46
0.31
0.38
0.56
0.78
0.93
0.24

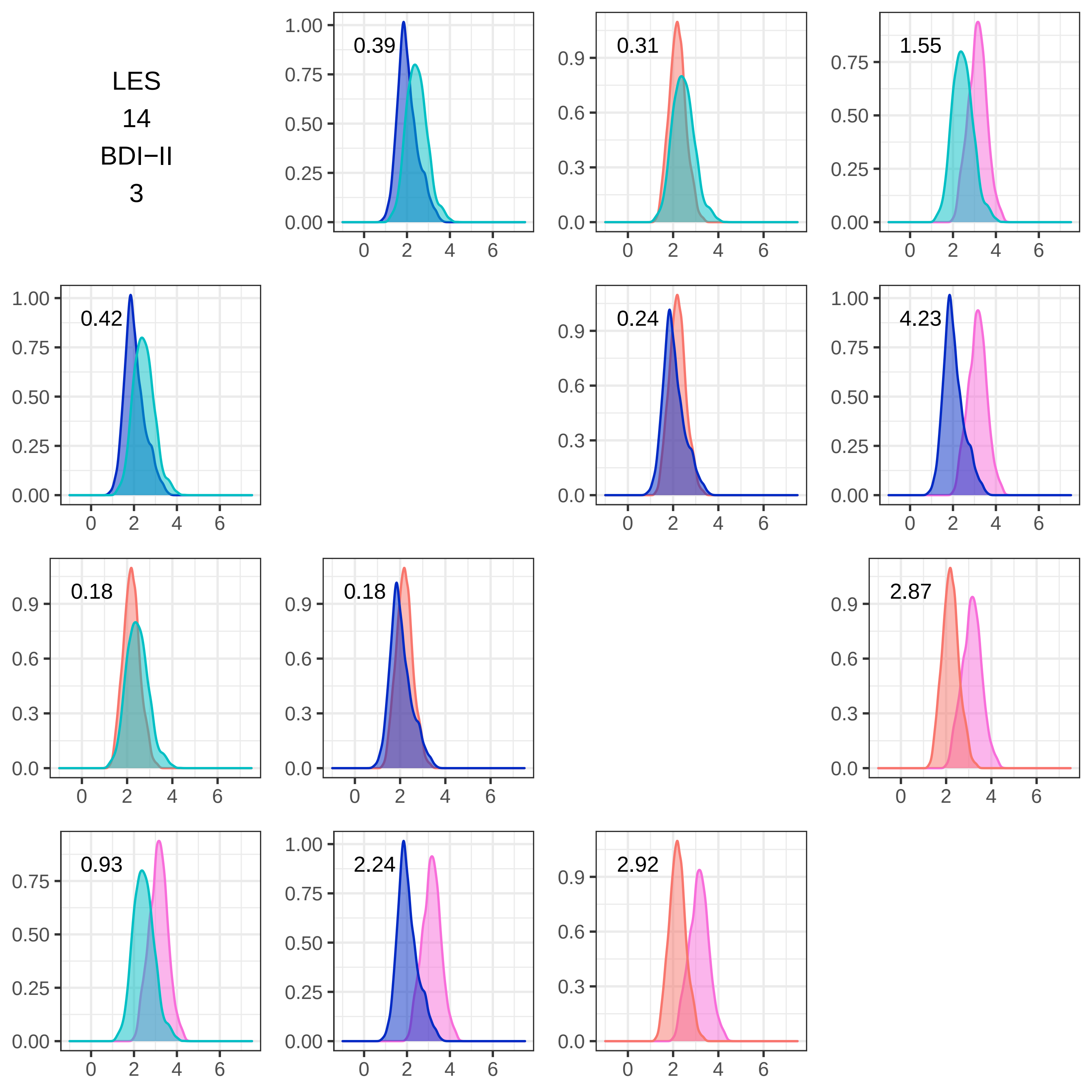
LES
14
BDI−II
3
0.39
0.31
1.55
0.42
0.24
4.23
0.18
0.18
2.87
0.93
2.24
2.92

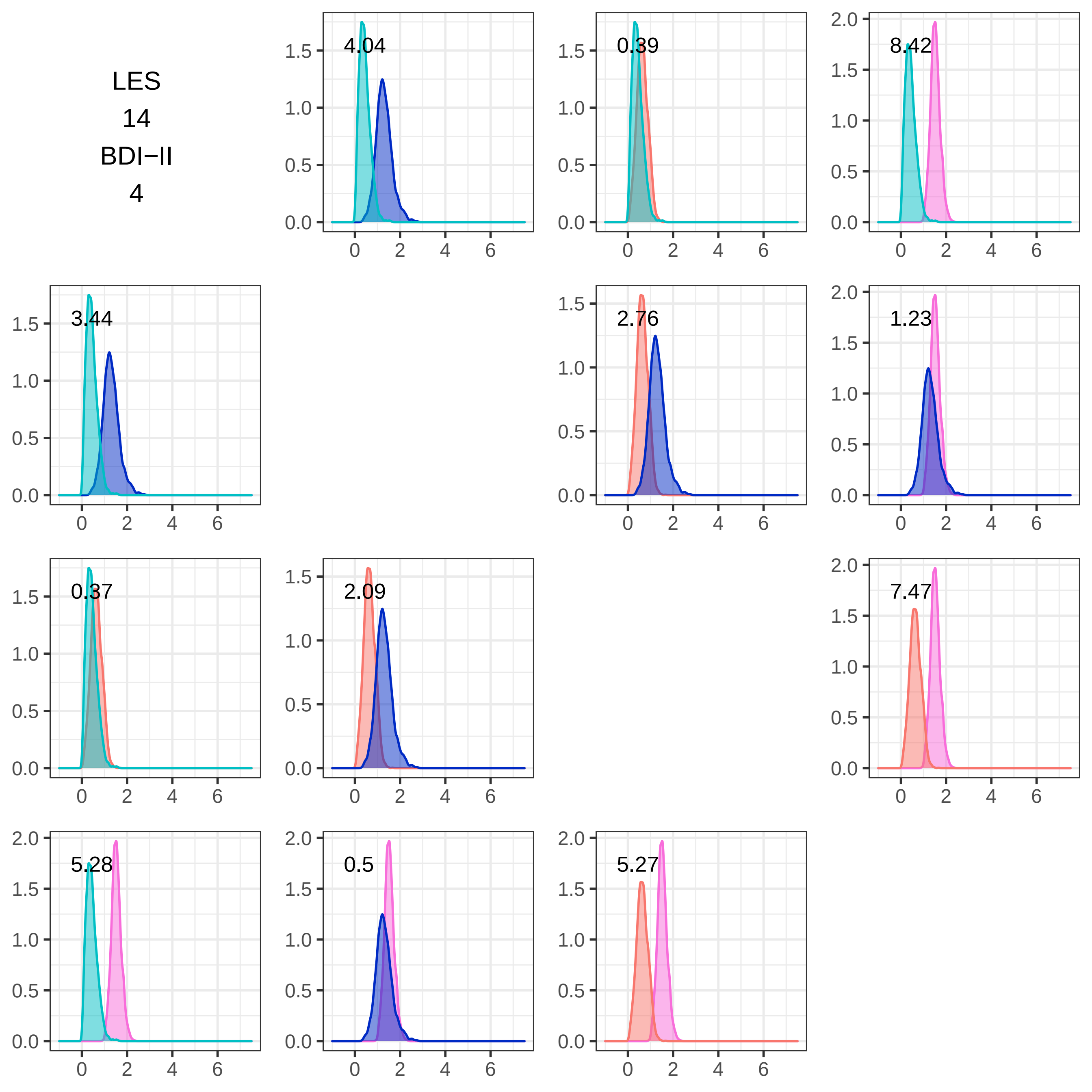

LES
14
BDI−II
4
4.04
0.39
8.42
3.44
2.76
1.23
0.37
2.09
7.47
5.28
0.5
5.27

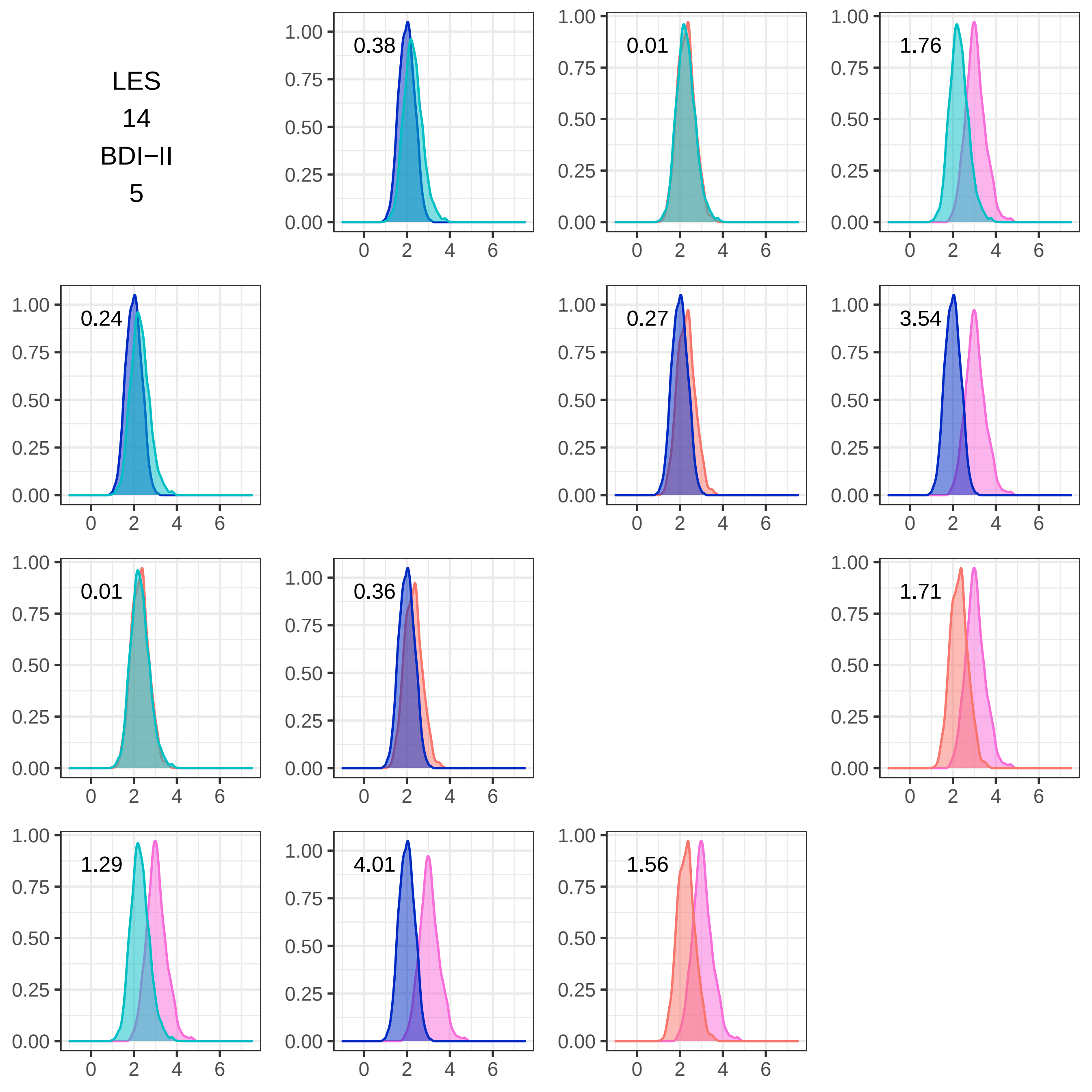

LES
14
BDI−II
5
0.38
0.01
1.76
0.24
0.27
3.54
0.01
0.36
1.71
1.29
4.01
1.56

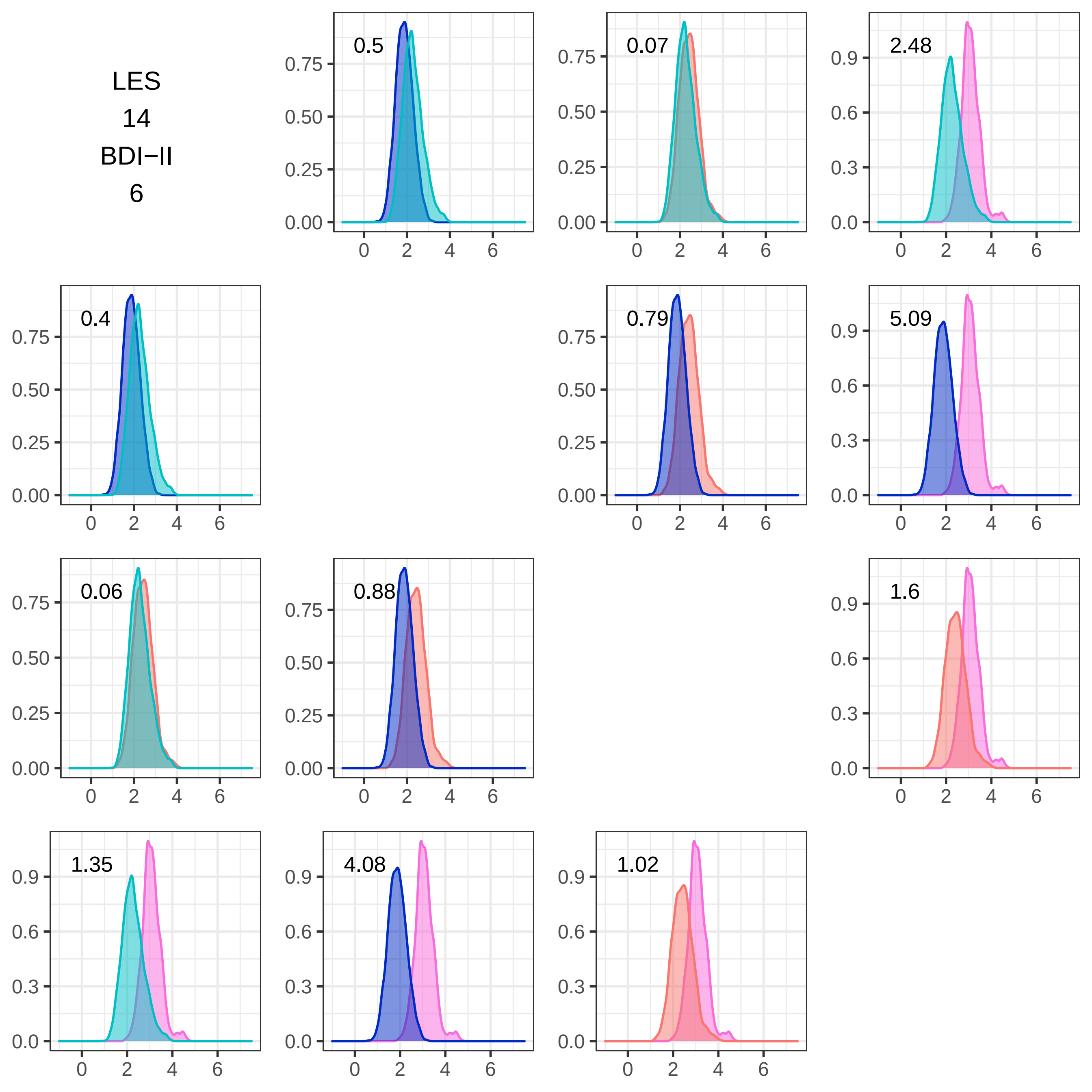
LES
14
BDI−II
6
0.5
0.07
2.48
0.4
0.79
5.09
0.06
0.88
1.6
1.35
4.08
1.02
0.00
0.25
0.50
0.75
0.0
0.3
0.6
0.9
0
2
4
6

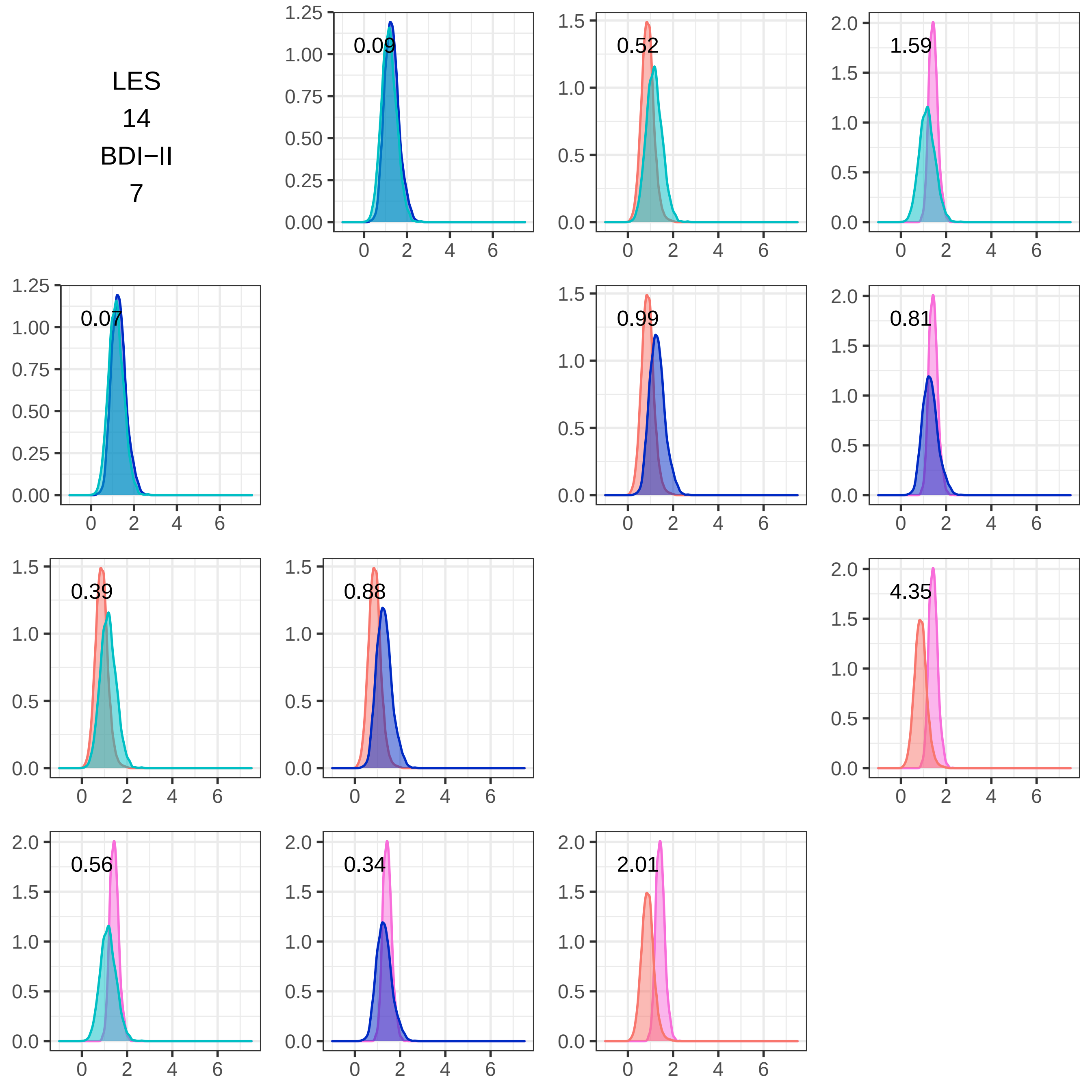
LES
14
BDI−II
7
0.09
0.52
1.59
0.07
0.99
0.81
0.39
0.88
4.35
0.56
0.34
2.01

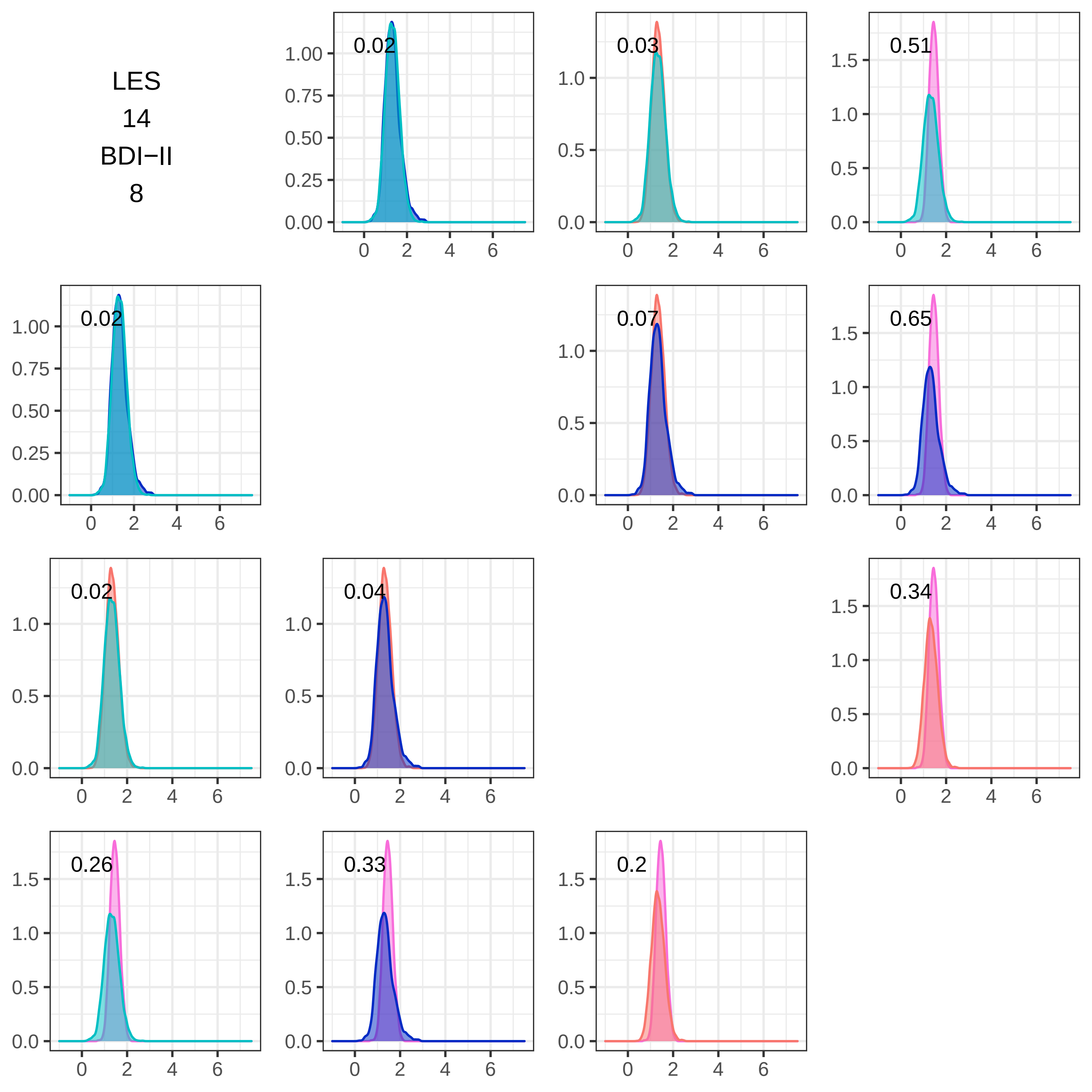

LES
14
BDI−II
8
0.02
0.03
0.51
0.02
0.07
0.65
0.02
0.04
0.34
0.26
0.33
0.2

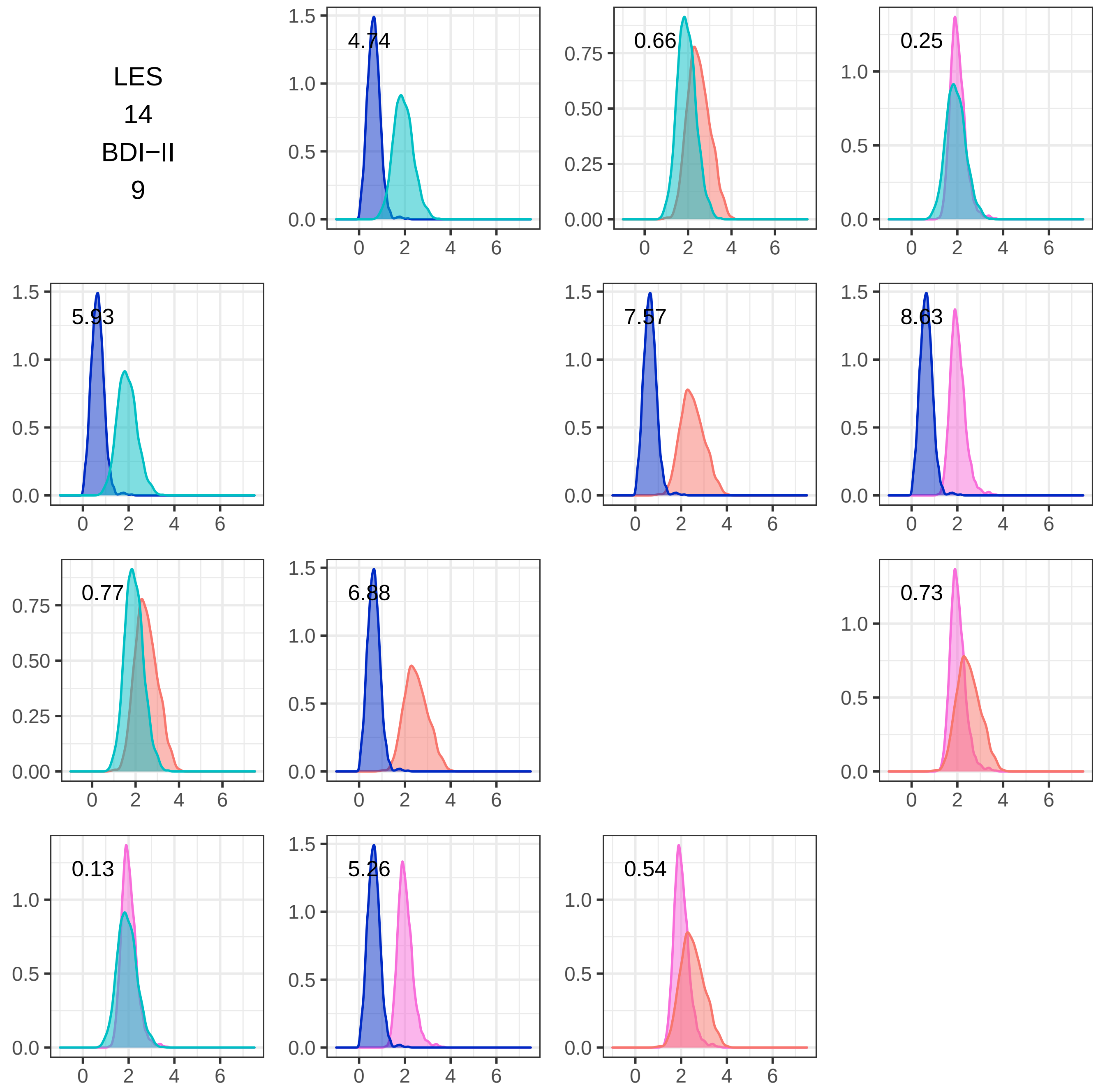
LES
14
BDI−II
9
4.74
0.66
0.25
5.93
7.57
8.63
0.77
6.88
0.73
0.13
5.26
0.54

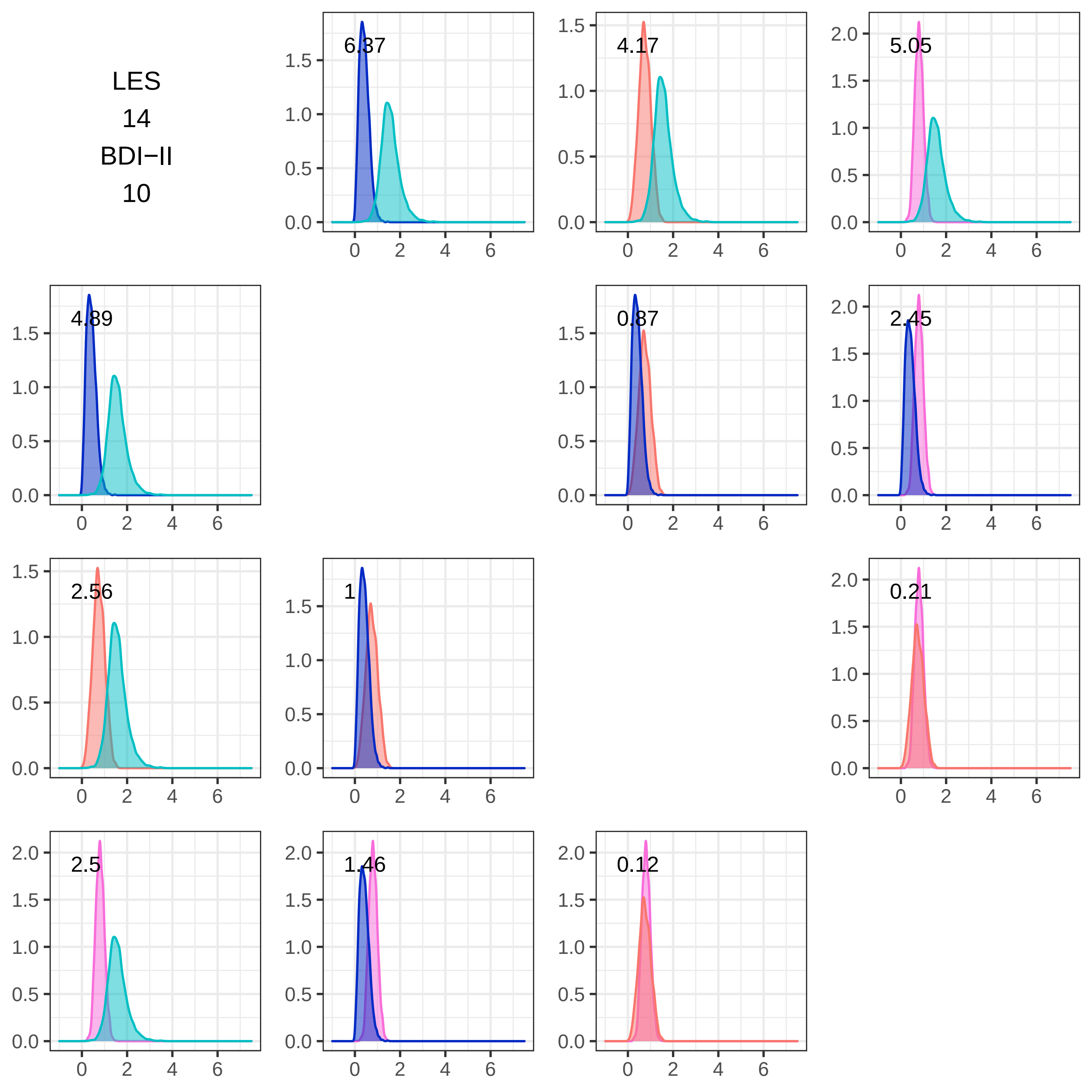

LES
14
BDI−II
10
6.37
4.17
5.05
4.89
0.87
2.45
2.56
1
0.21
2.5
1.46
0.12

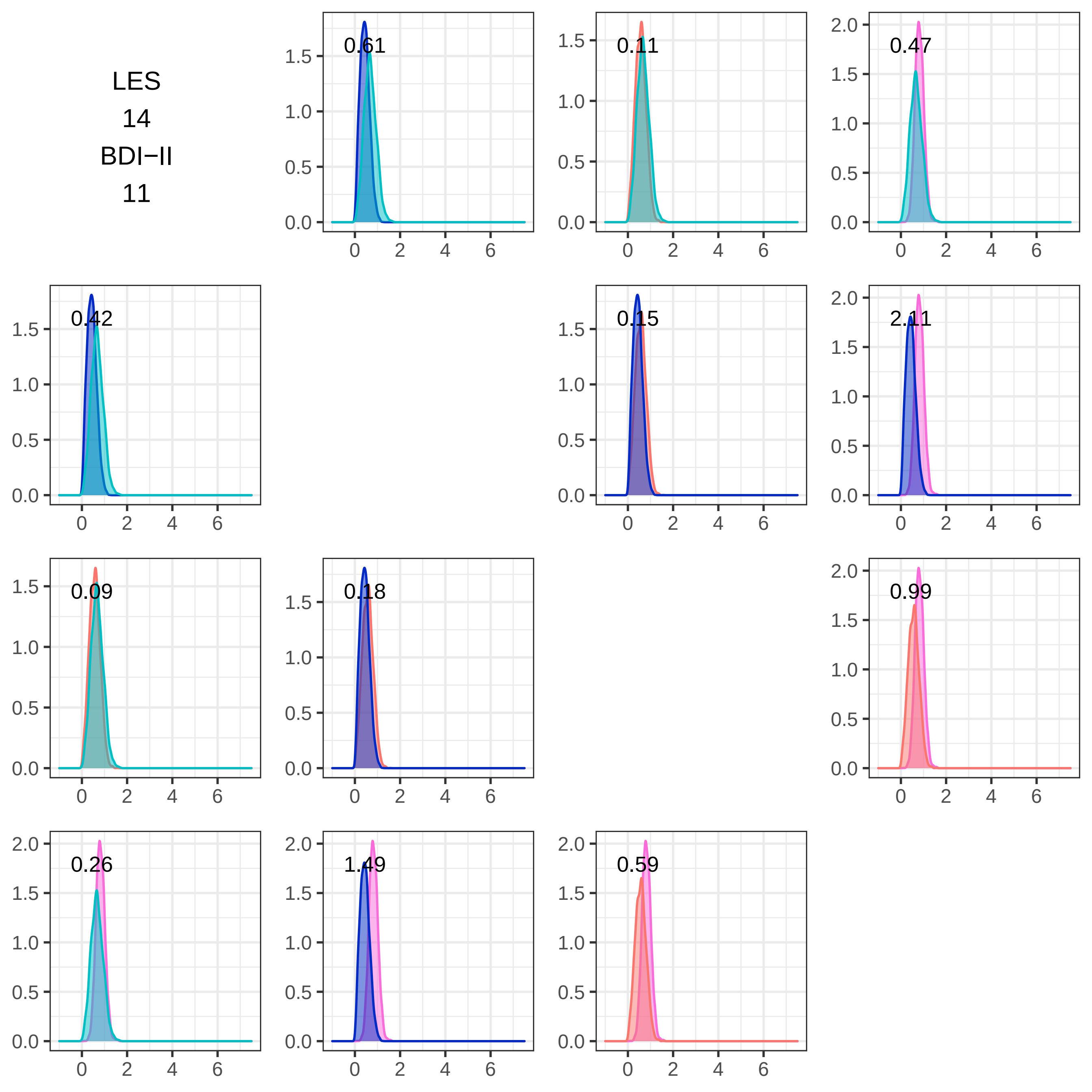
LES
14
BDI−II
11
0.61
0.11
0.47
0.42
0.15
2.11
0.09
0.18
0.99
0.26
1.49
0.59

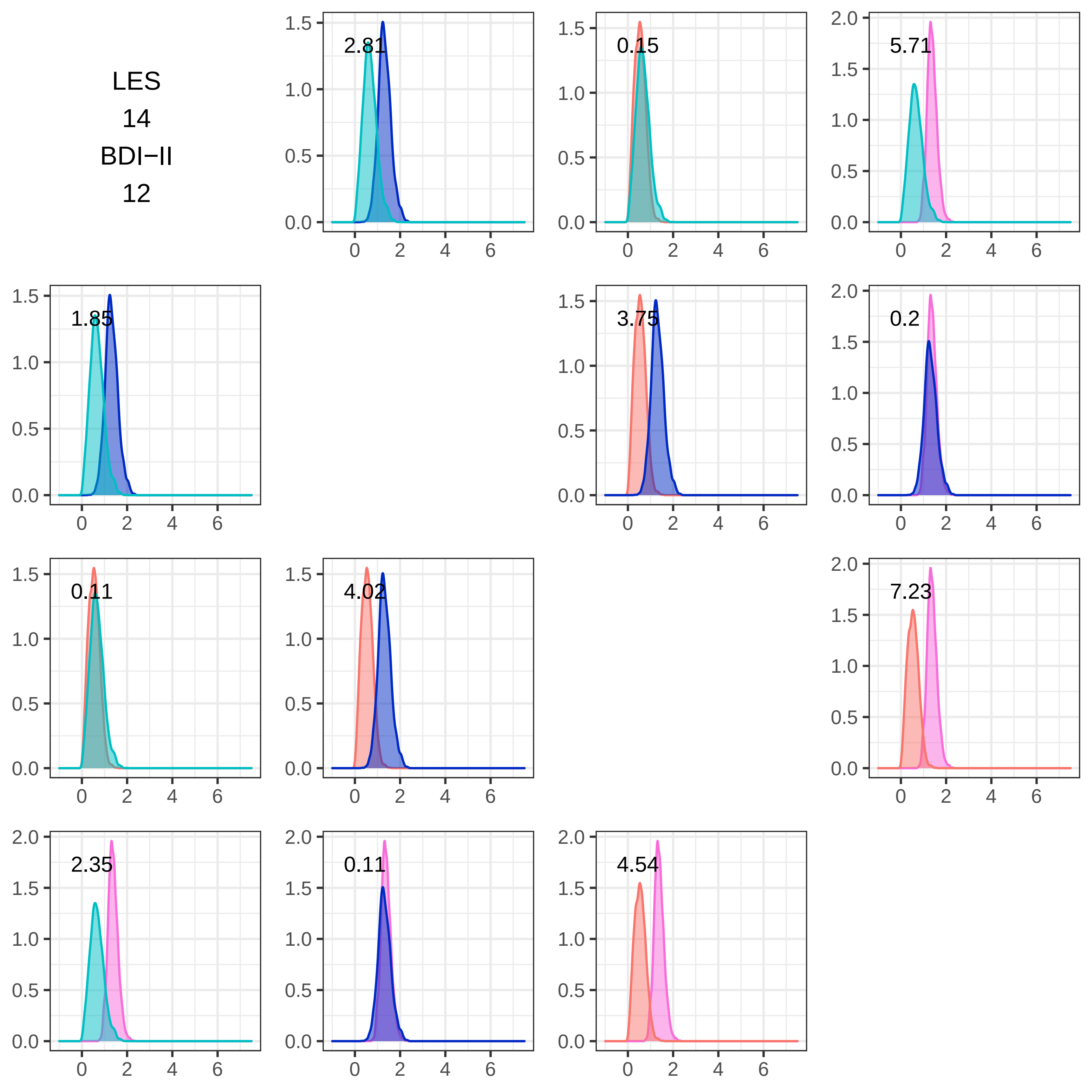

LES
14
BDI−II
12
2.81
0.15
5.71
1.85
3.75
0.2
0.11
4.02
7.23
2.35
0.11
4.54

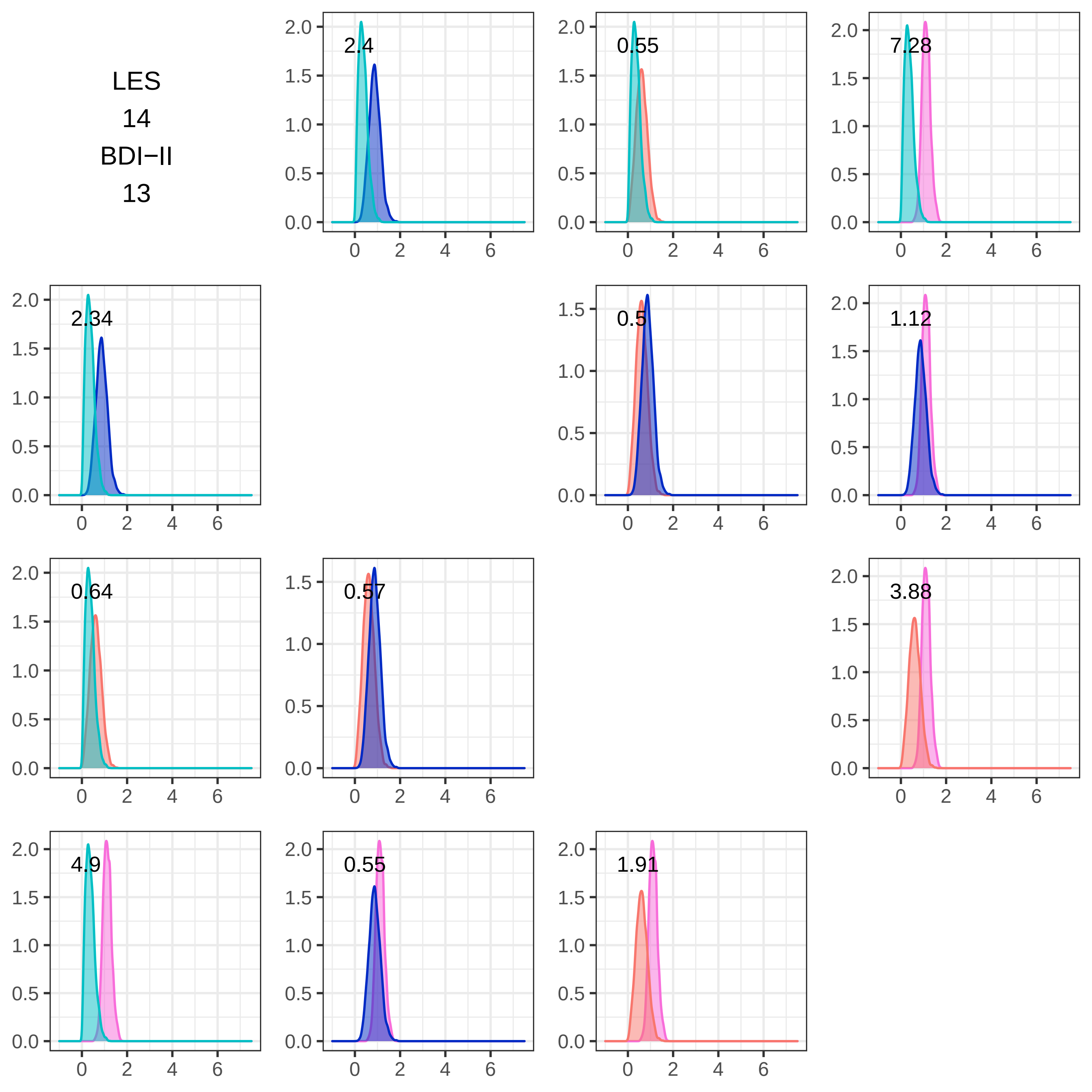
LES
14
BDI−II
13
2.4
0.55
7.28
2.34
0.5
1.12
0.64
0.57
3.88
4.9
0.55
1.91

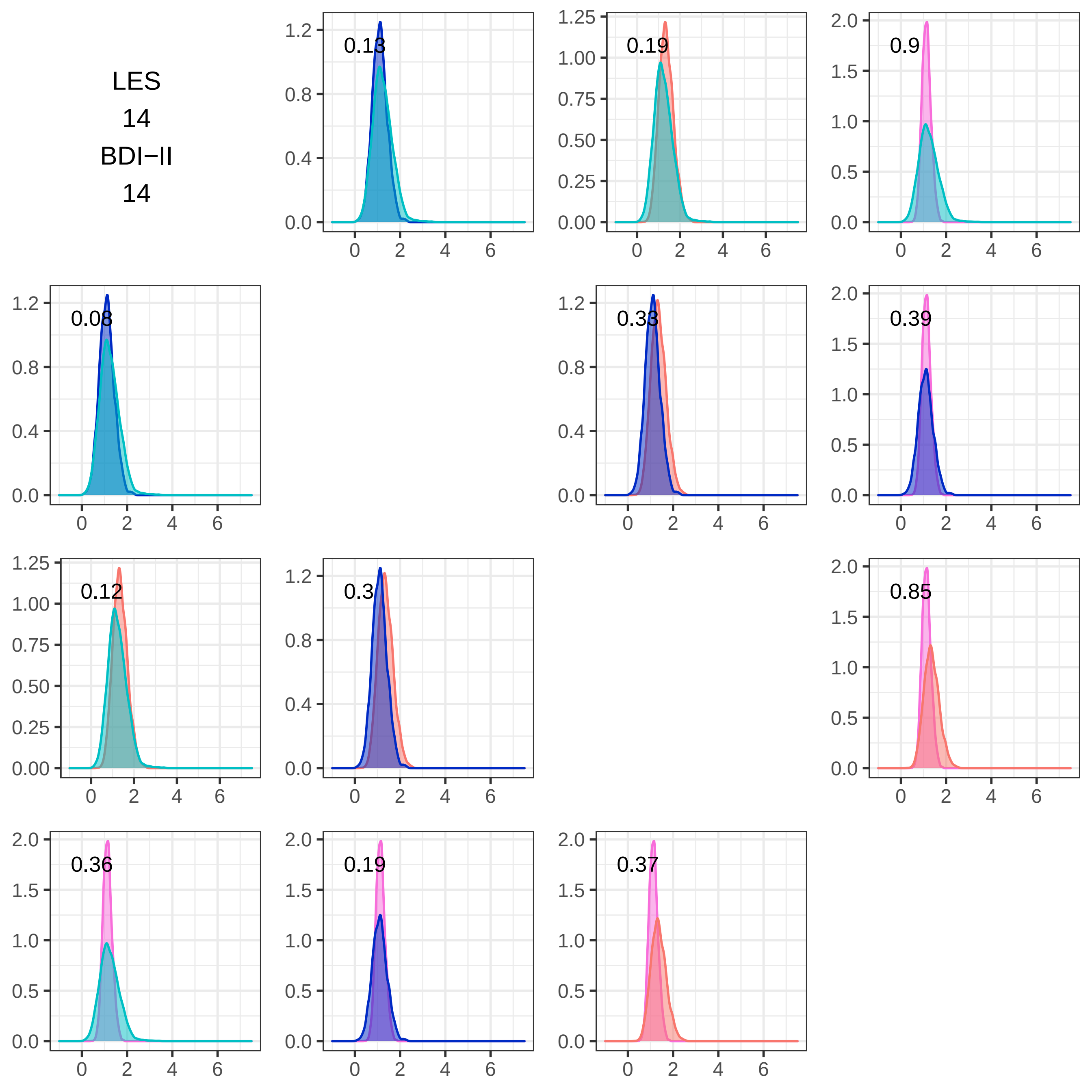

LES
14
BDI−II
14
0.13
0.19
0.9
0.08
0.33
0.39
0.12
0.3
0.85
0.36
0.19
0.37

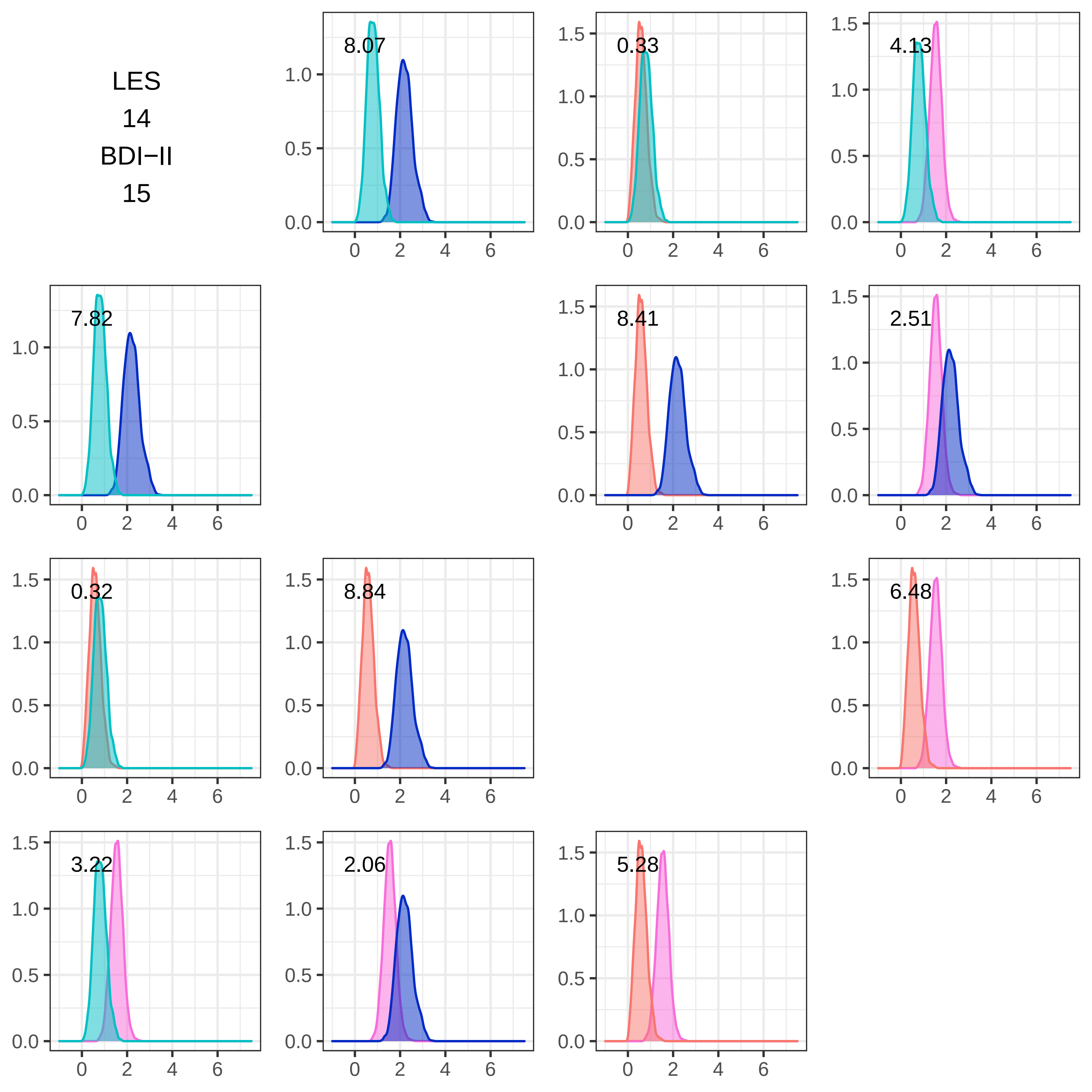

LES
14
BDI−II
15
8.07
0.33
4.13
7.82
8.41
2.51
0.32
8.84
6.48
3.22
2.06
5.28

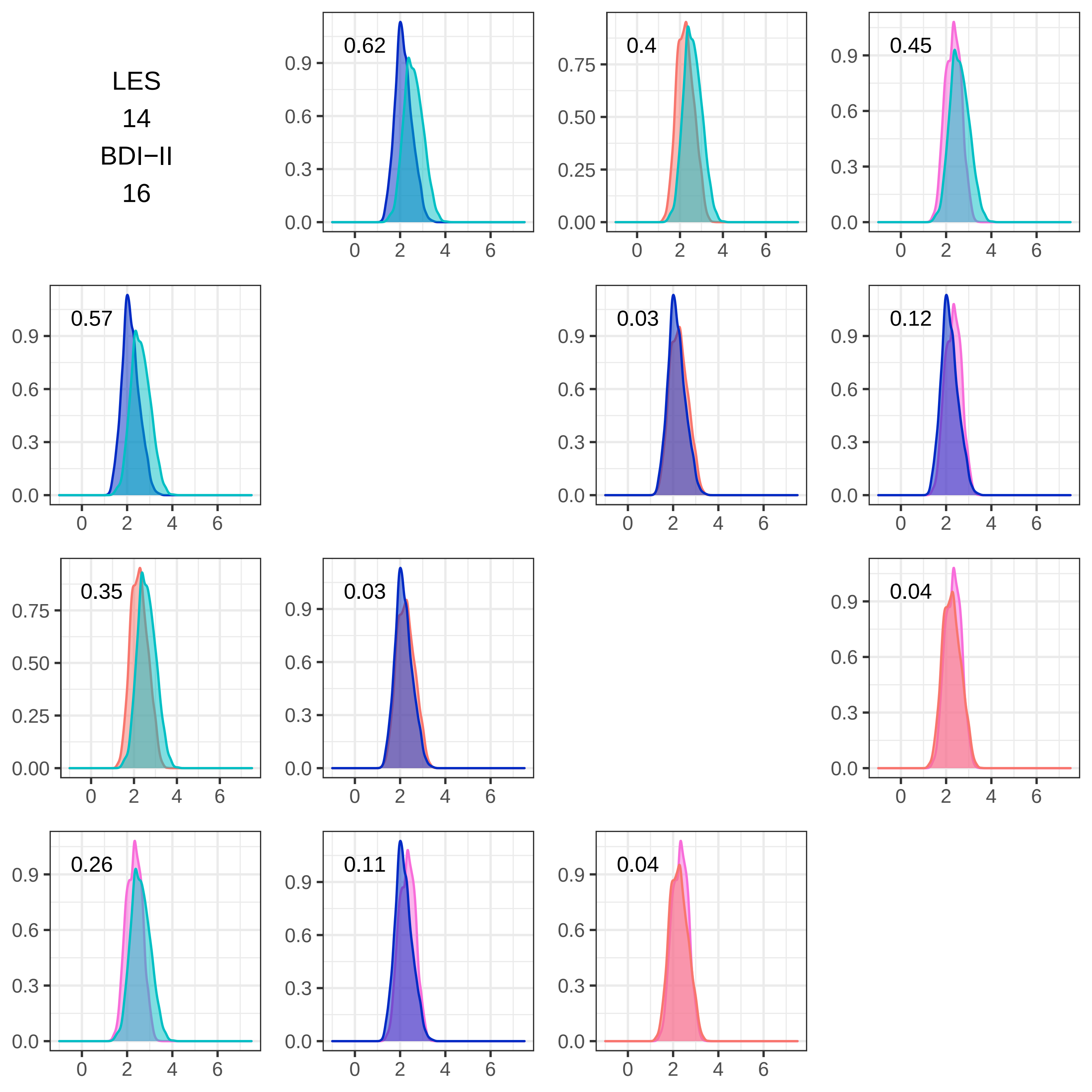

LES
14
BDI−II
16
0.62
0.4
0.45
0.57
0.03
0.12
0.35
0.03
0.04
0.26
0.11
0.04

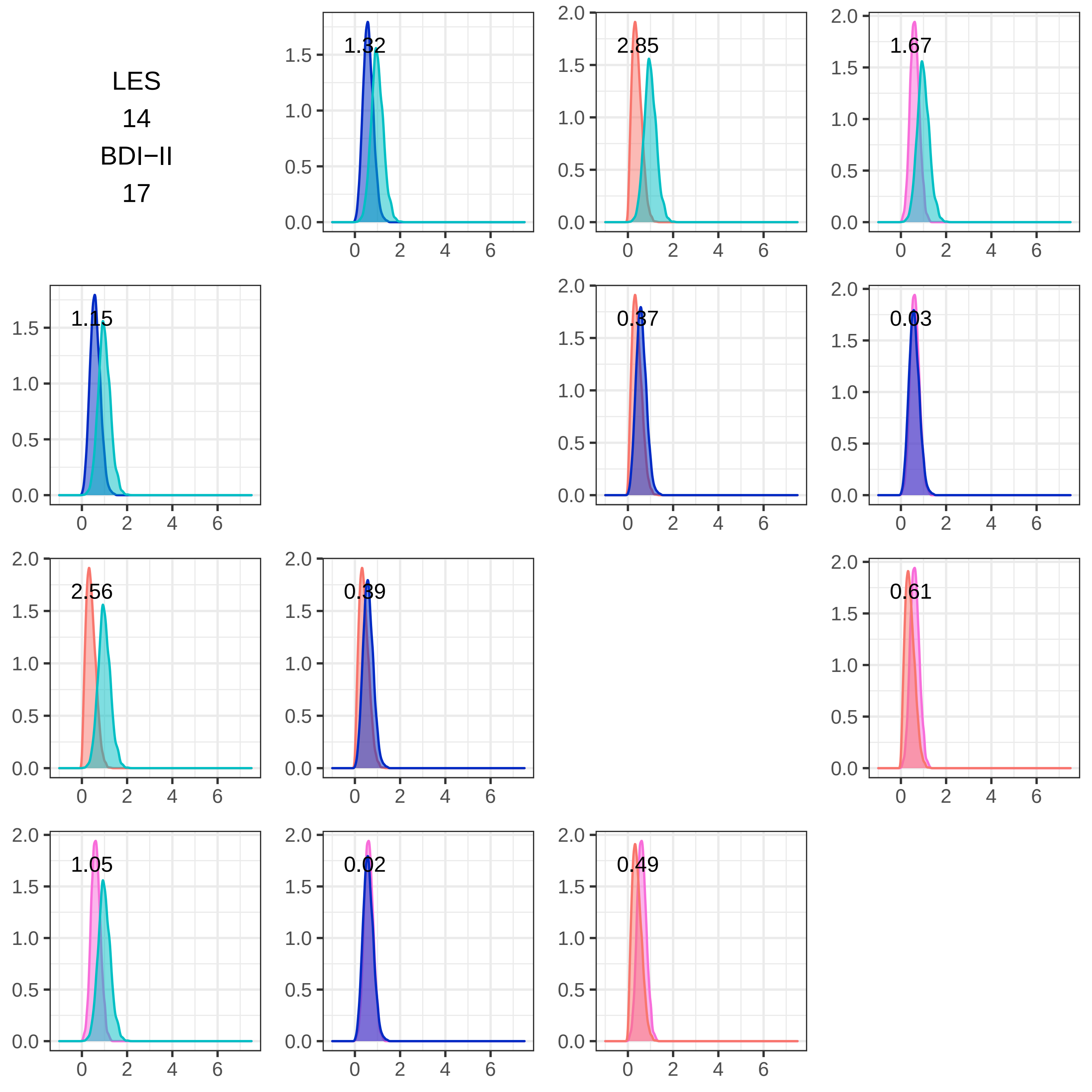

LES
14
BDI−II
17
1.32
2.85
1.67
1.15
0.37
0.03
2.56
0.39
0.61
1.05
0.02
0.49

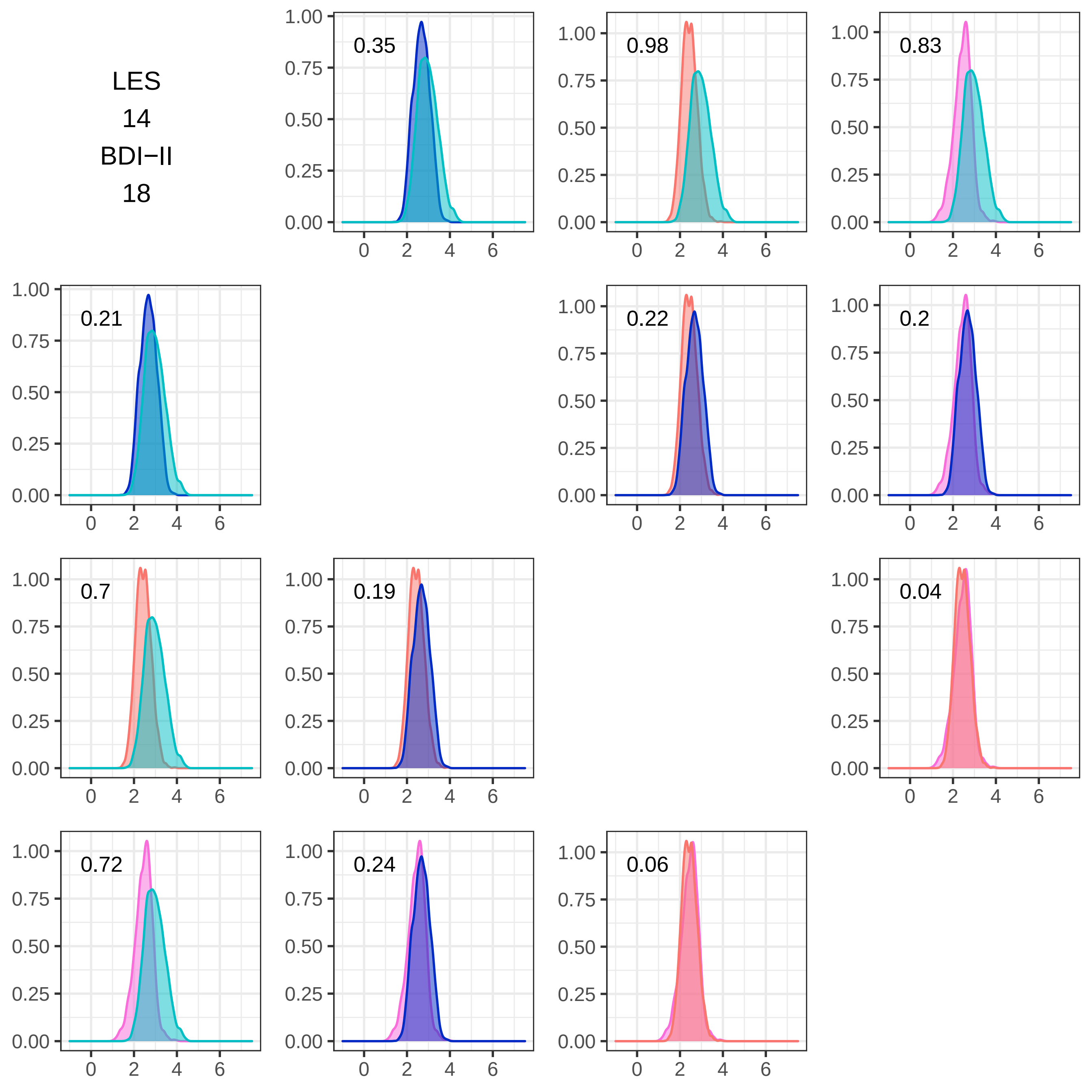

LES
14
BDI−II
18
0.35
0.98
0.83
0.21
0.22
0.2
0.7
0.19
0.04
0.72
0.24
0.06

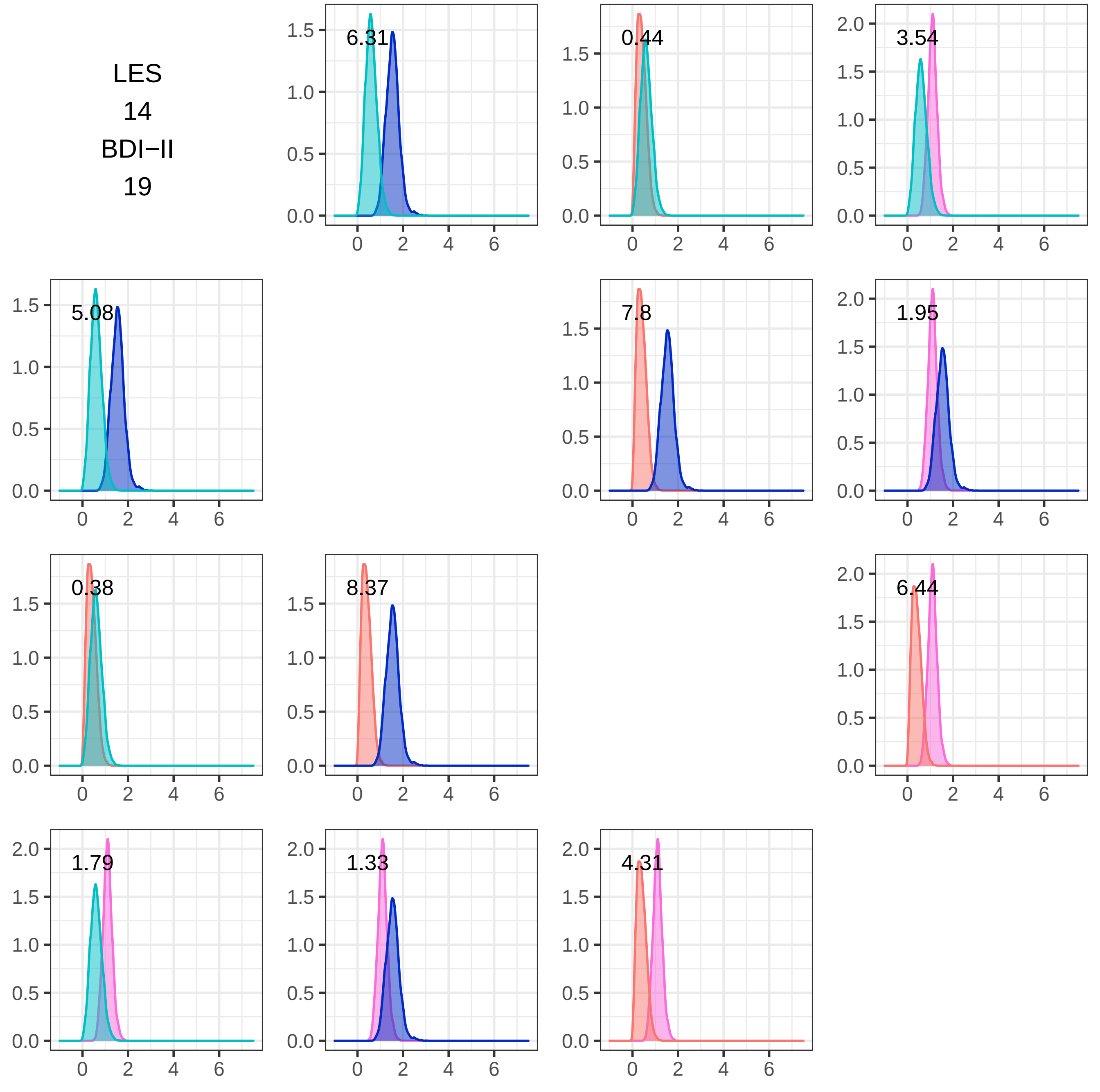
LES
14
BDI−II
19
6.31
0.44
3.54
5.08
7.8
1.95
0.38
8.37
6.44
1.79
1.33
4.31

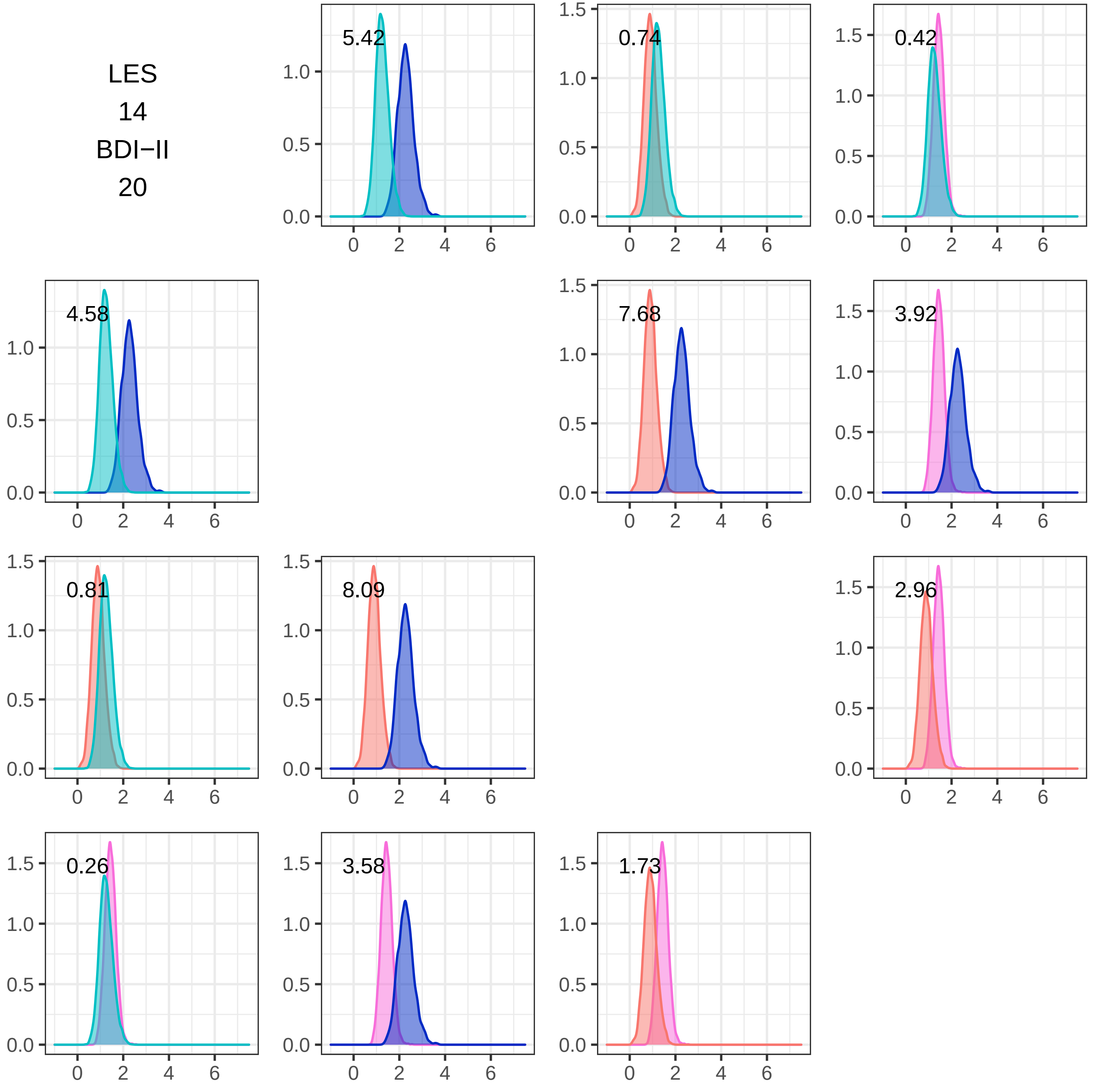
LES
14
BDI−II
20
5.42
0.74
0.42
4.58
7.68
3.92
0.81
8.09
2.96
0.26
3.58
1.73

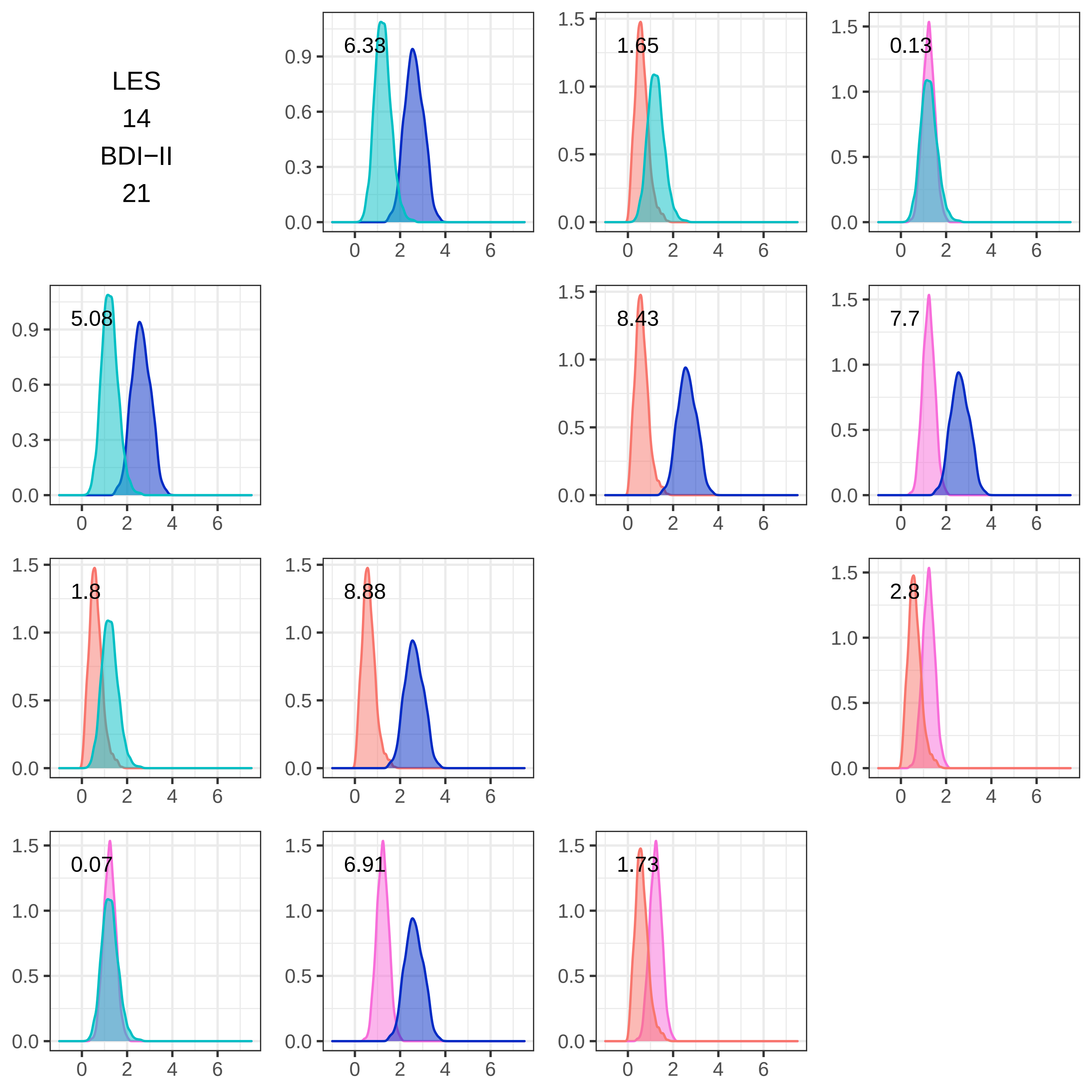

LES
14
BDI−II
21
6.33
1.65
0.13
5.08
8.43
7.7
1.8
8.88
2.8
0.07
6.91
1.73

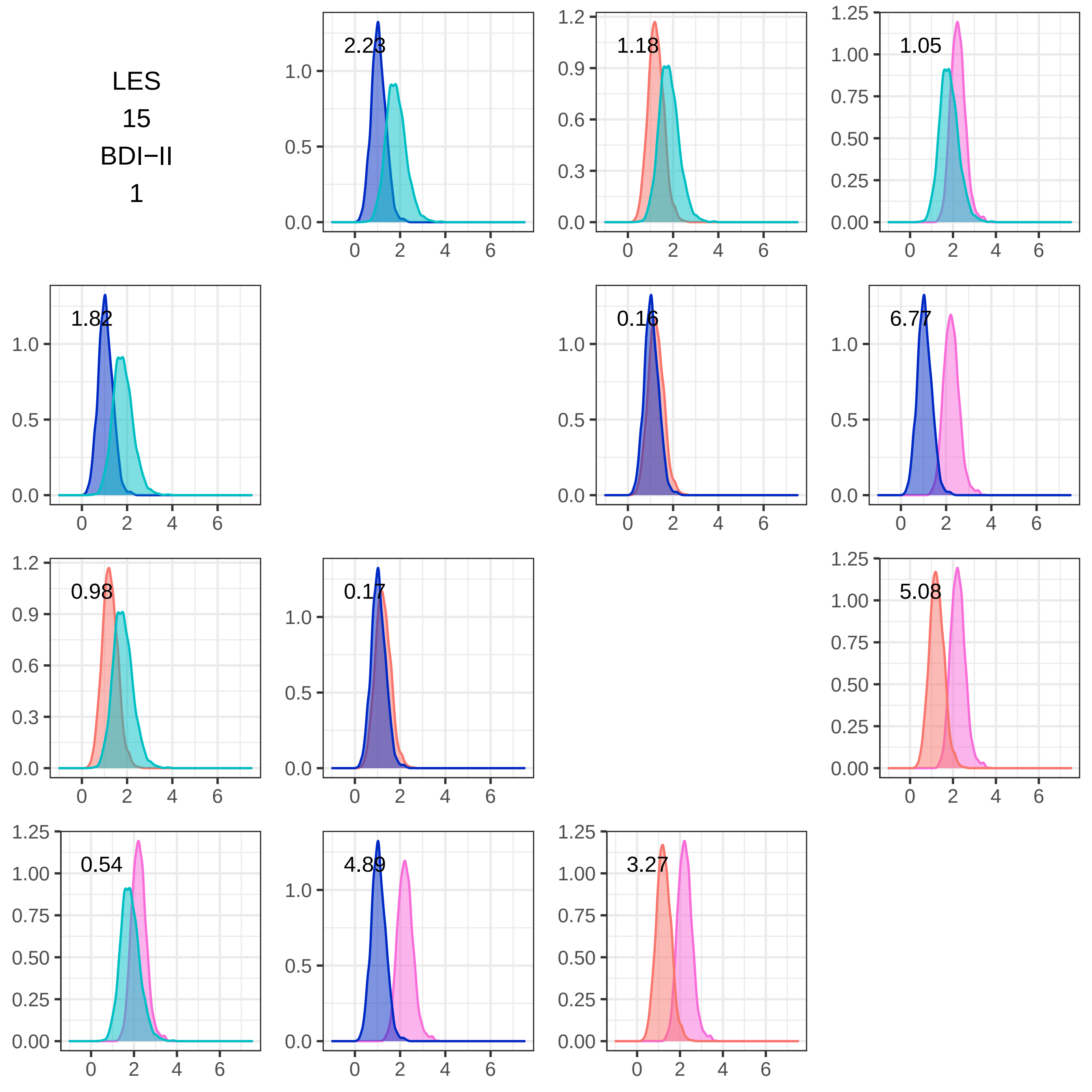

LES
15
BDI−II
1
2.23
1.18
1.05
1.82
0.16
6.77
0.98
0.17
5.08
0.54
4.89
3.27

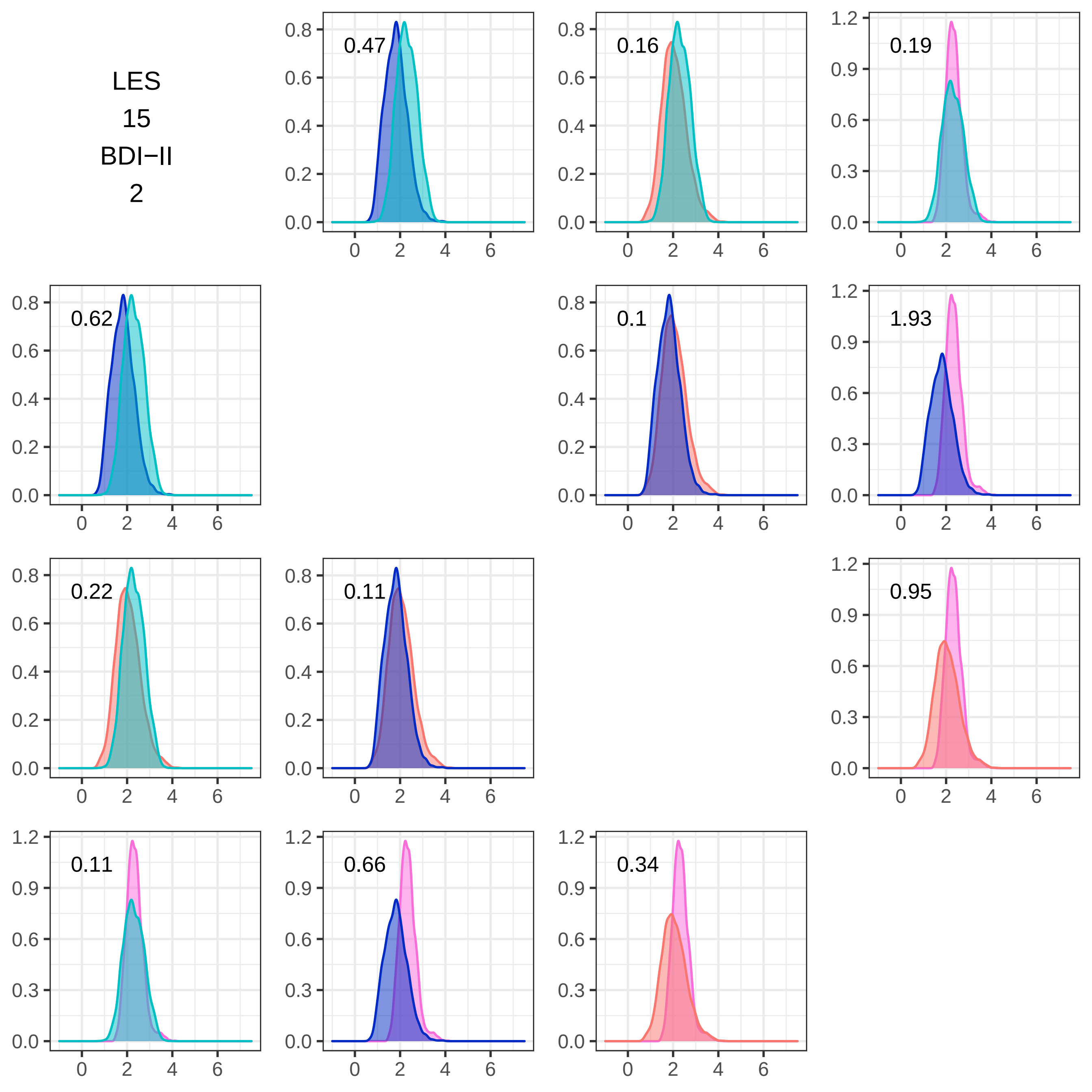
LES
15
BDI−II
2
0.47
0.16
0.19
0.62
0.1
1.93
0.22
0.11
0.95
0.11
0.66
0.34

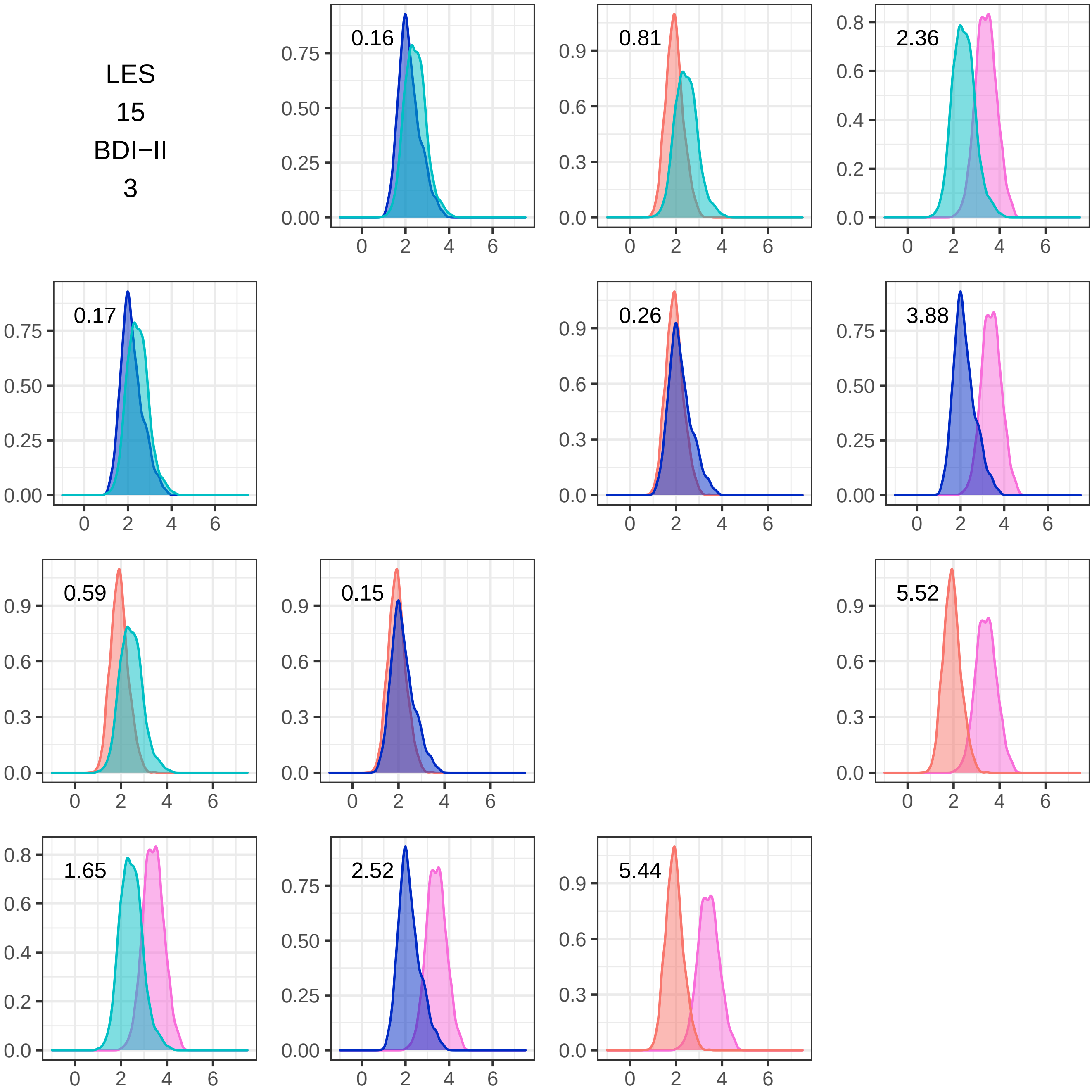

LES
15
BDI−II
3
0.16
0.81
2.36
0.17
0.26
3.88
0.59
0.15
5.52
1.65
2.52
5.44

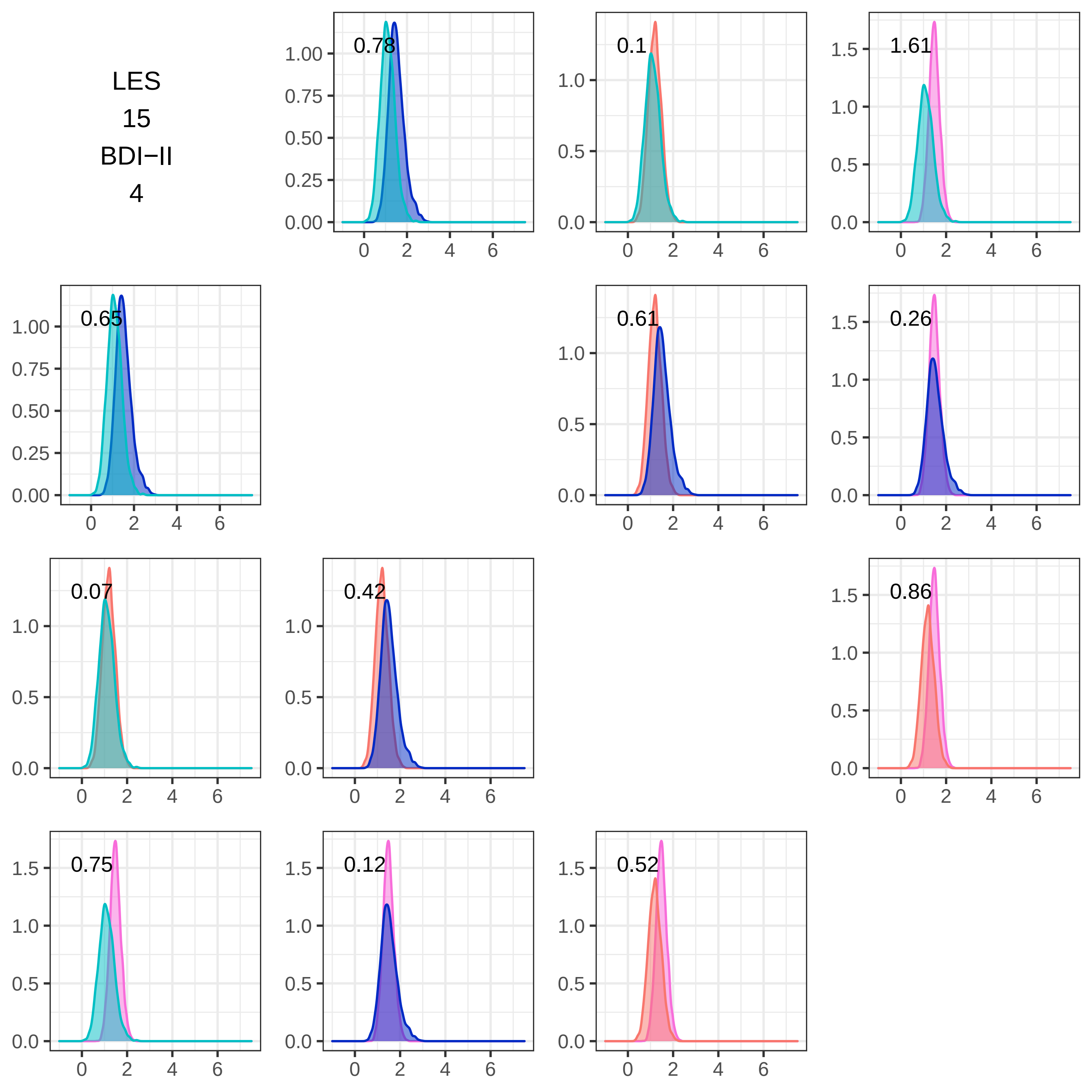

LES
15
BDI−II
4
0.78
0.1
1.61
0.65
0.61
0.26
0.07
0.42
0.86
0.75
0.12
0.52

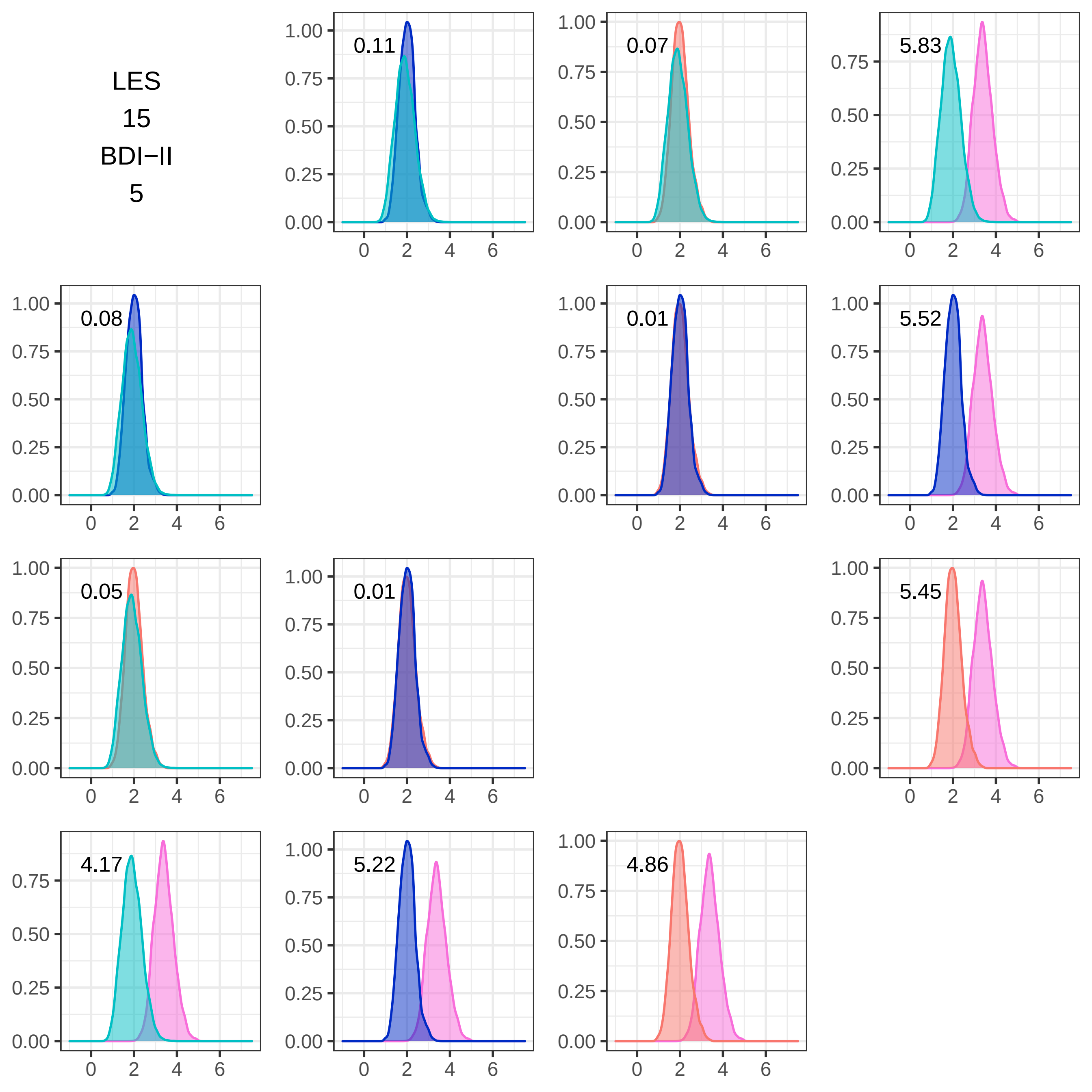

LES
15
BDI−II
5
0.11
0.07
5.83
0.08
0.01
5.52
0.05
0.01
5.45
4.17
5.22
4.86

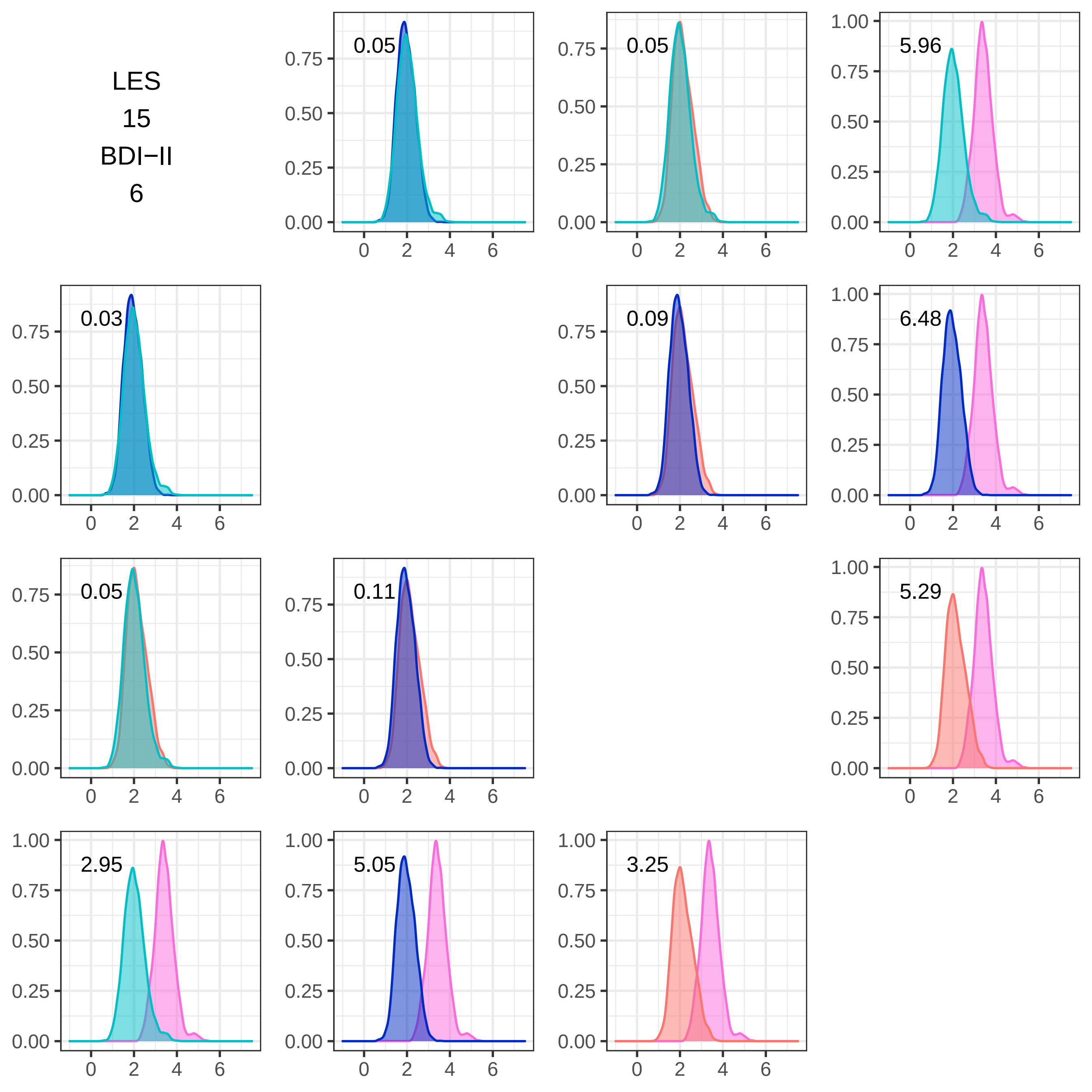
LES
15
BDI−II
6
0.05
0.05
5.96
0.03
0.09
6.48
0.05
0.11
5.29
2.95
5.05
3.25

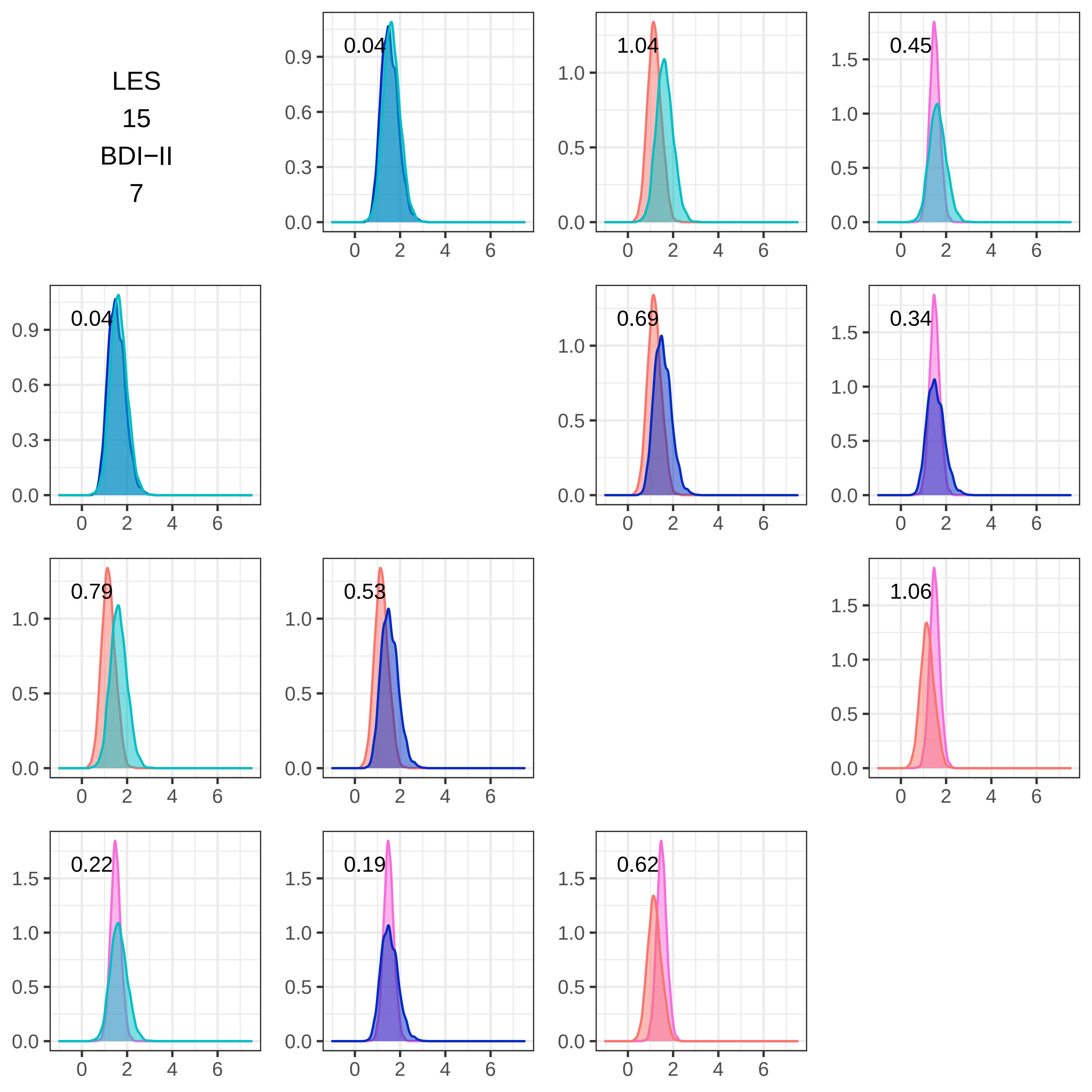
LES
15
BDI−II
7
0.04
1.04
0.45
0.04
0.69
0.34
0.79
0.53
1.06
0.22
0.19
0.62

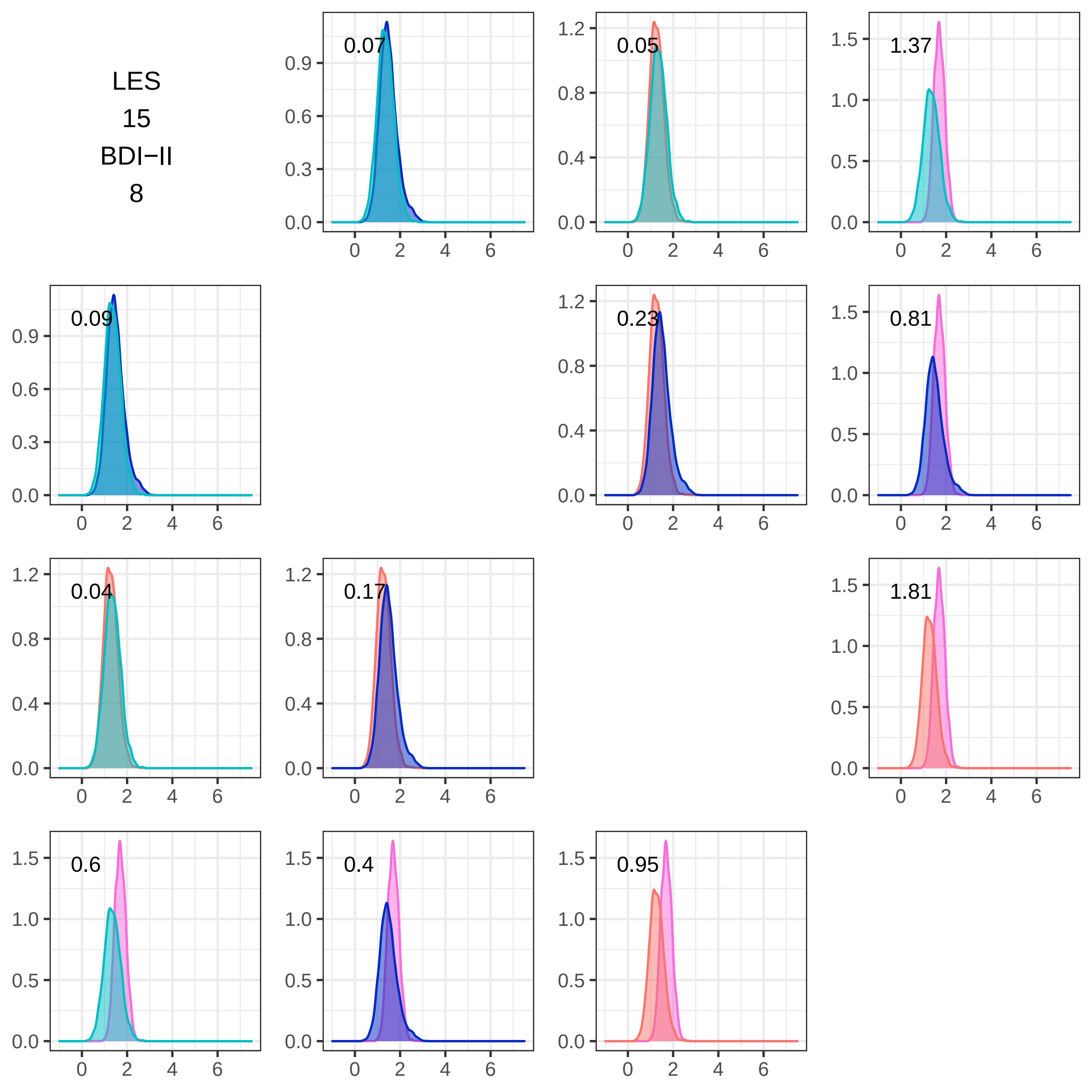
LES
15
BDI−II
8
0.07
0.05
1.37
0.09
0.23
0.81
0.04
0.17
1.81
0.6
0.4
0.95

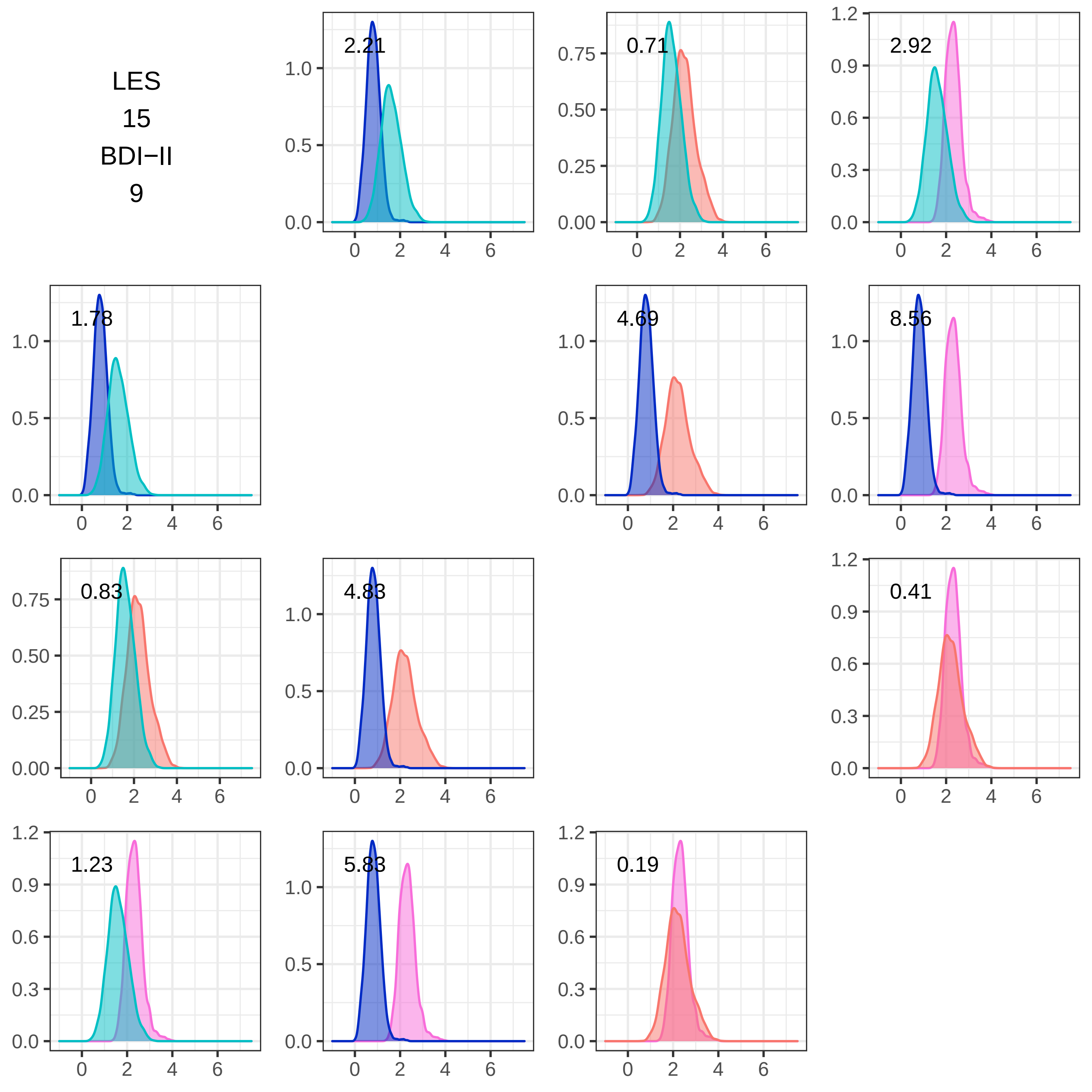

LES
15
BDI−II
9
2.21
0.71
2.92
1.78
4.69
8.56
0.83
4.83
0.41
1.23
5.83
0.19

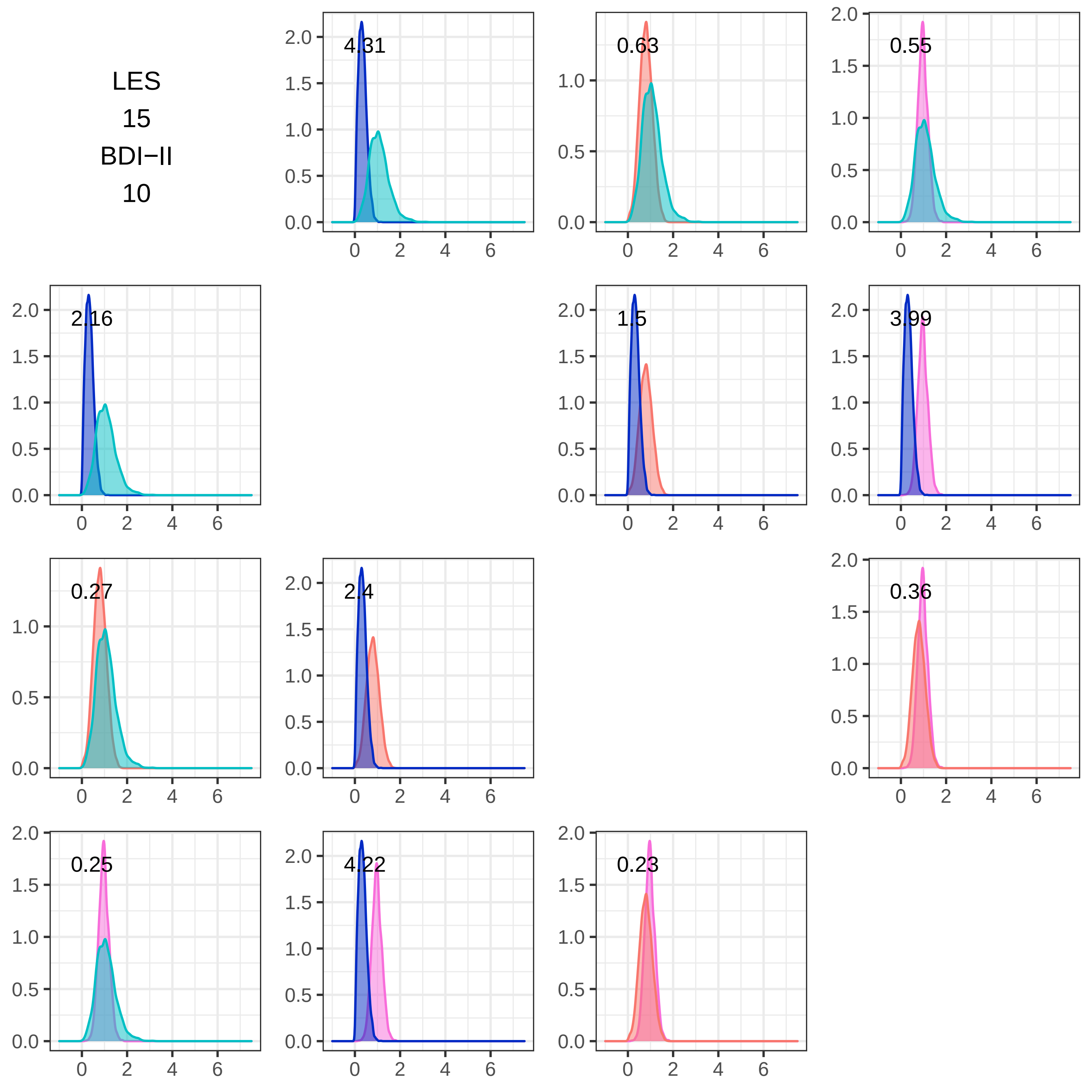

LES
15
BDI−II
10
4.31
0.63
0.55
2.16
1.5
3.99
0.27
2.4
0.36
0.25
4.22
0.23

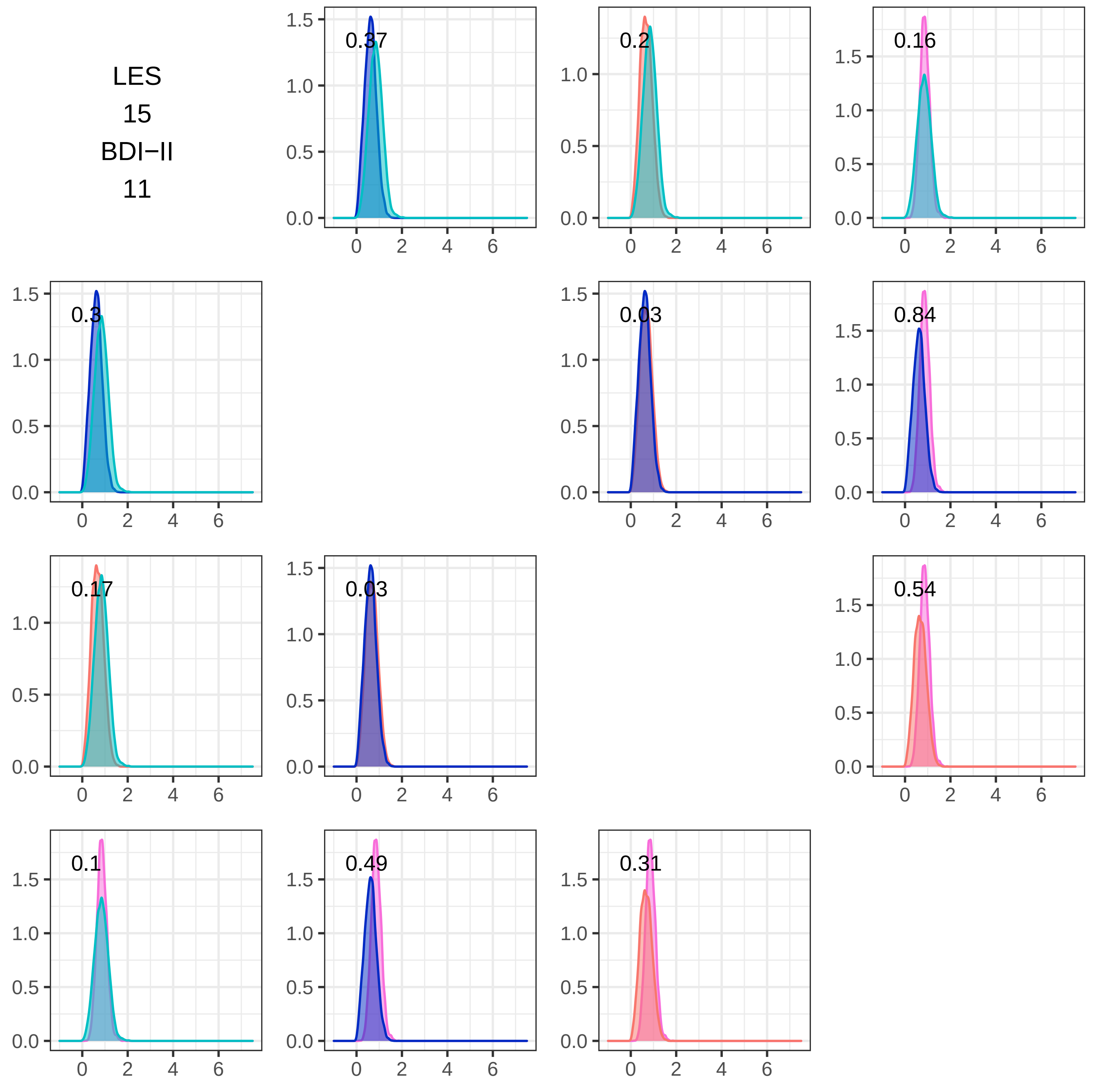

LES
15
BDI−II
11
0.37
0.2
0.16
0.3
0.03
0.84
0.17
0.03
0.54
0.1
0.49
0.31

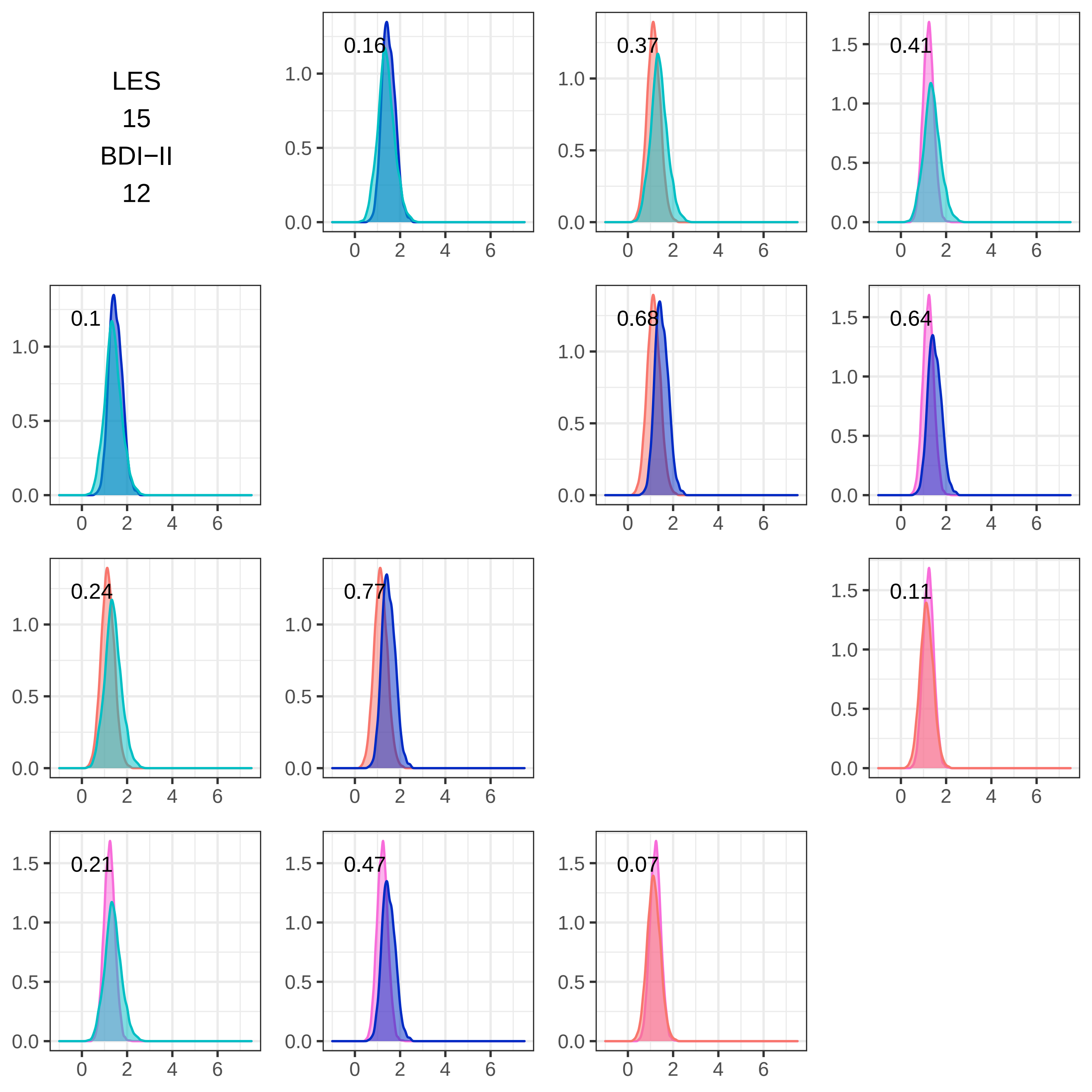

LES
15
BDI−II
12
0.16
0.37
0.41
0.1
0.68
0.64
0.24
0.77
0.11
0.21
0.47
0.07

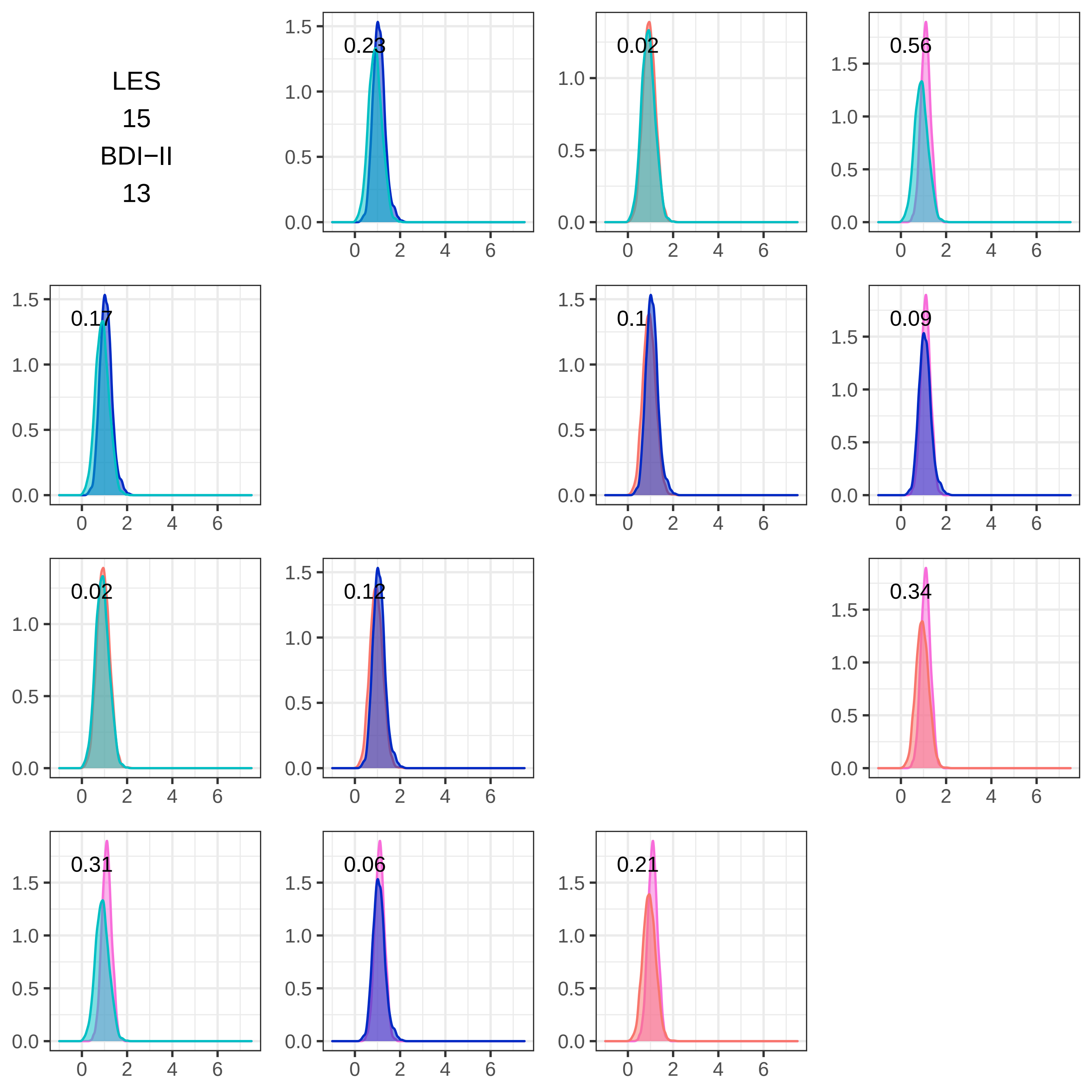
LES
15
BDI−II
13
0.23
0.02
0.56
0.17
0.1
0.09
0.02
0.12
0.34
0.31
0.06
0.21

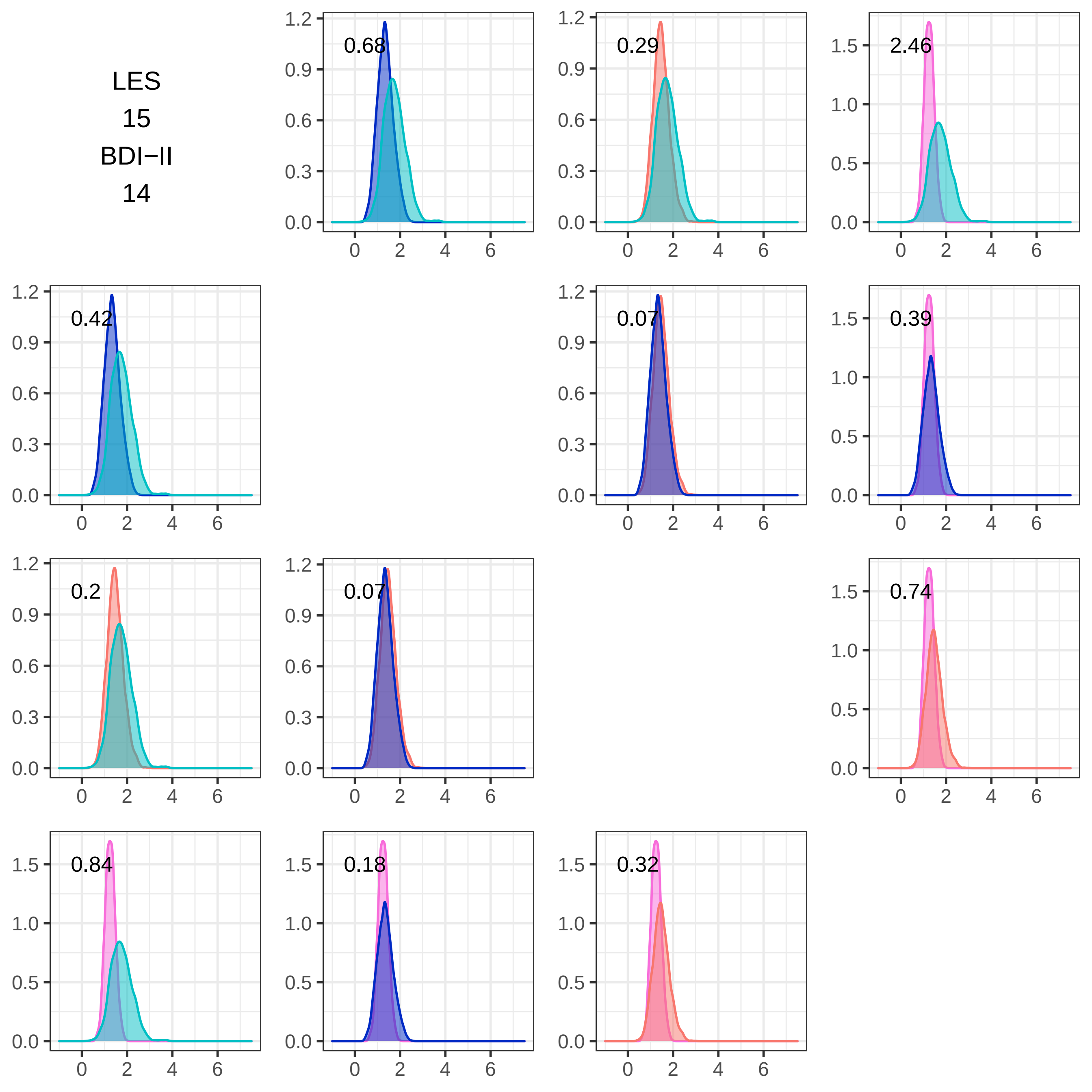

LES
15
BDI−II
14
0.68
0.29
2.46
0.42
0.07
0.39
0.2
0.07
0.74
0.84
0.18
0.32

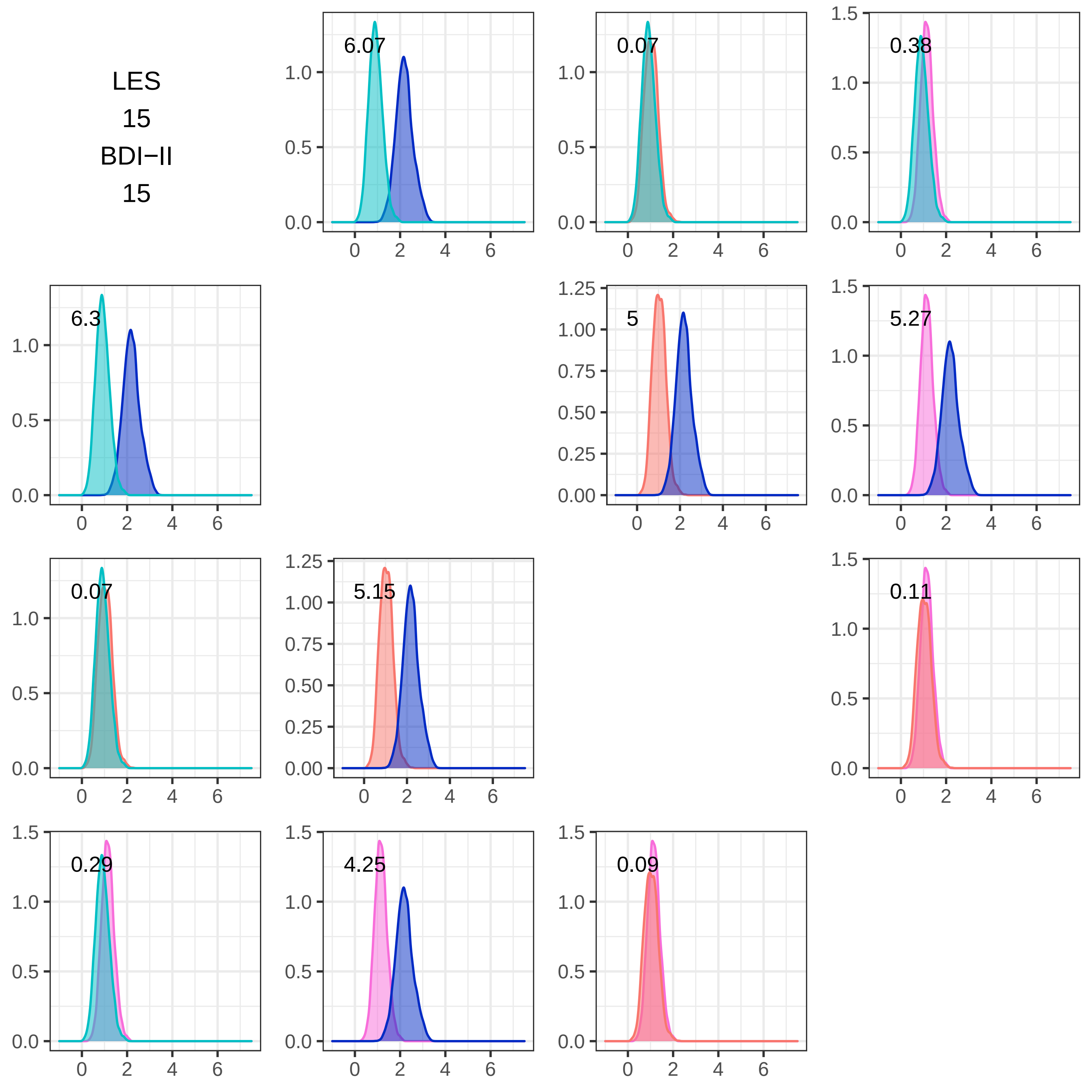
LES
15
BDI−II
15
6.07
0.07
0.38
6.3
5
5.27
0.07
5.15
0.11
0.29
4.25
0.09

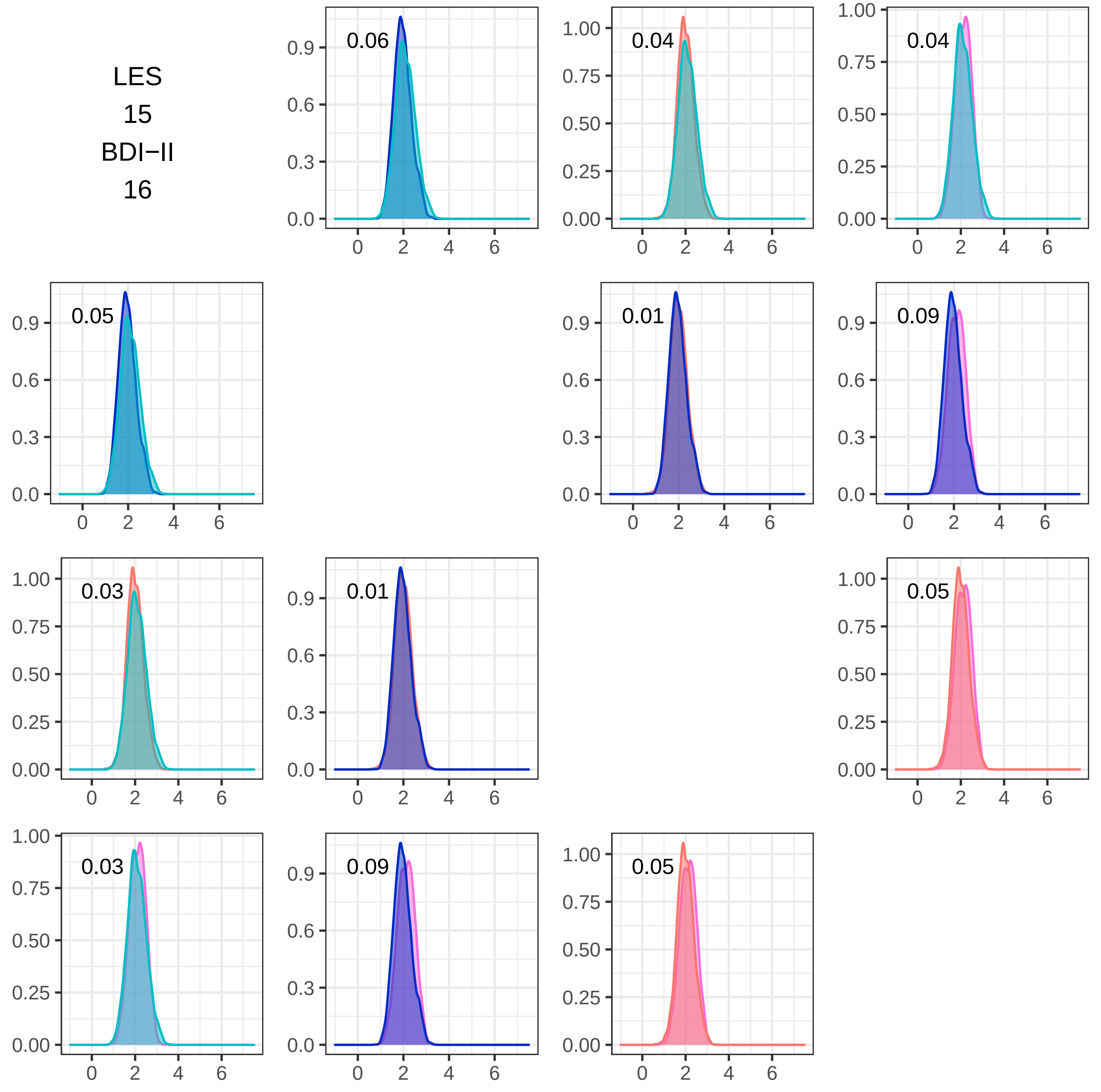
LES
15
BDI−II
16
0.06
0.04
0.04
0.05
0.01
0.09
0.03
0.01
0.05
0.03
0.09
0.05

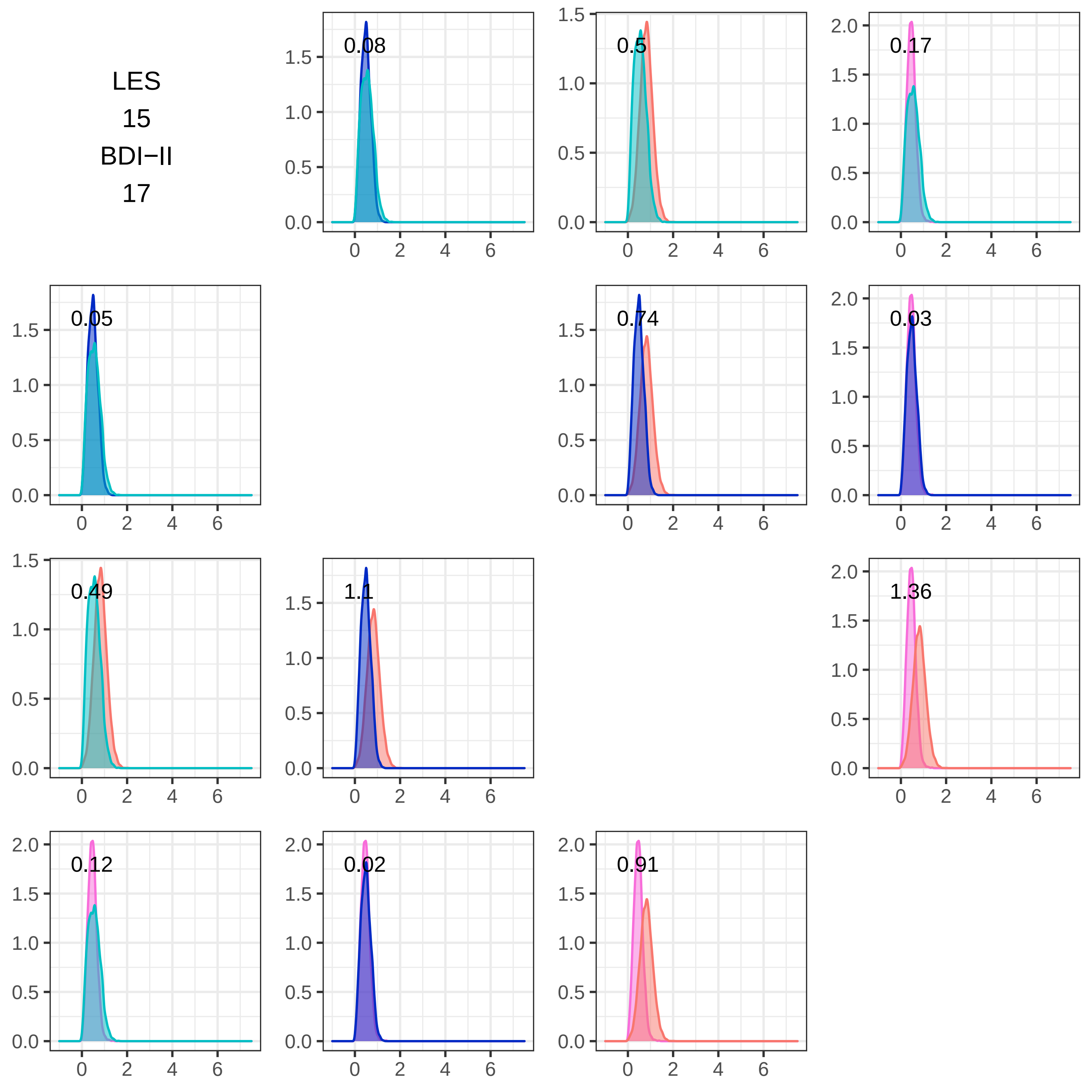

LES
15
BDI−II
17
0.08
0.5
0.17
0.05
0.74
0.03
0.49
1.1
1.36
0.12
0.02
0.91

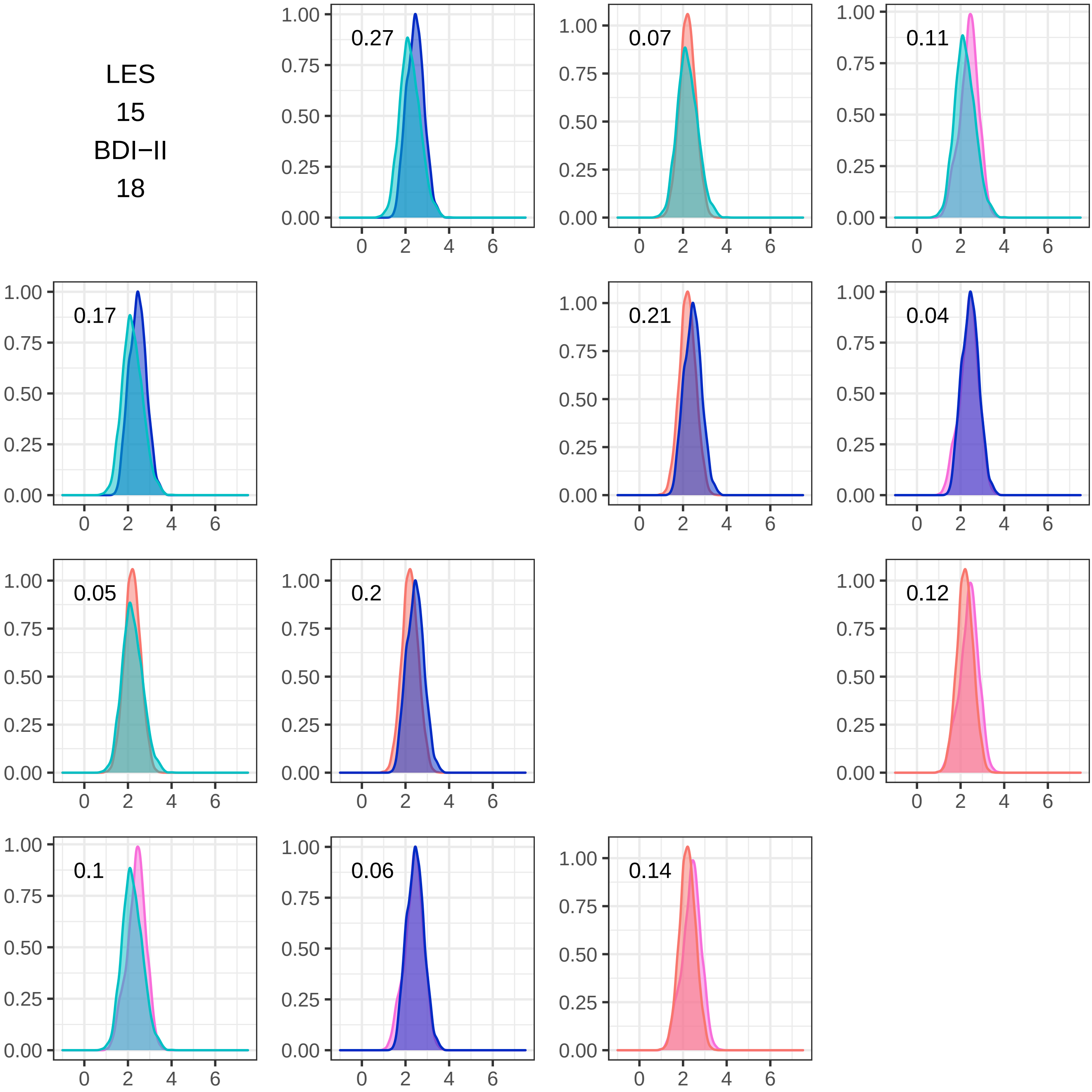
LES
15
BDI−II
18
0.27
0.07
0.11
0.17
0.21
0.04
0.05
0.2
0.12
0.1
0.06
0.14

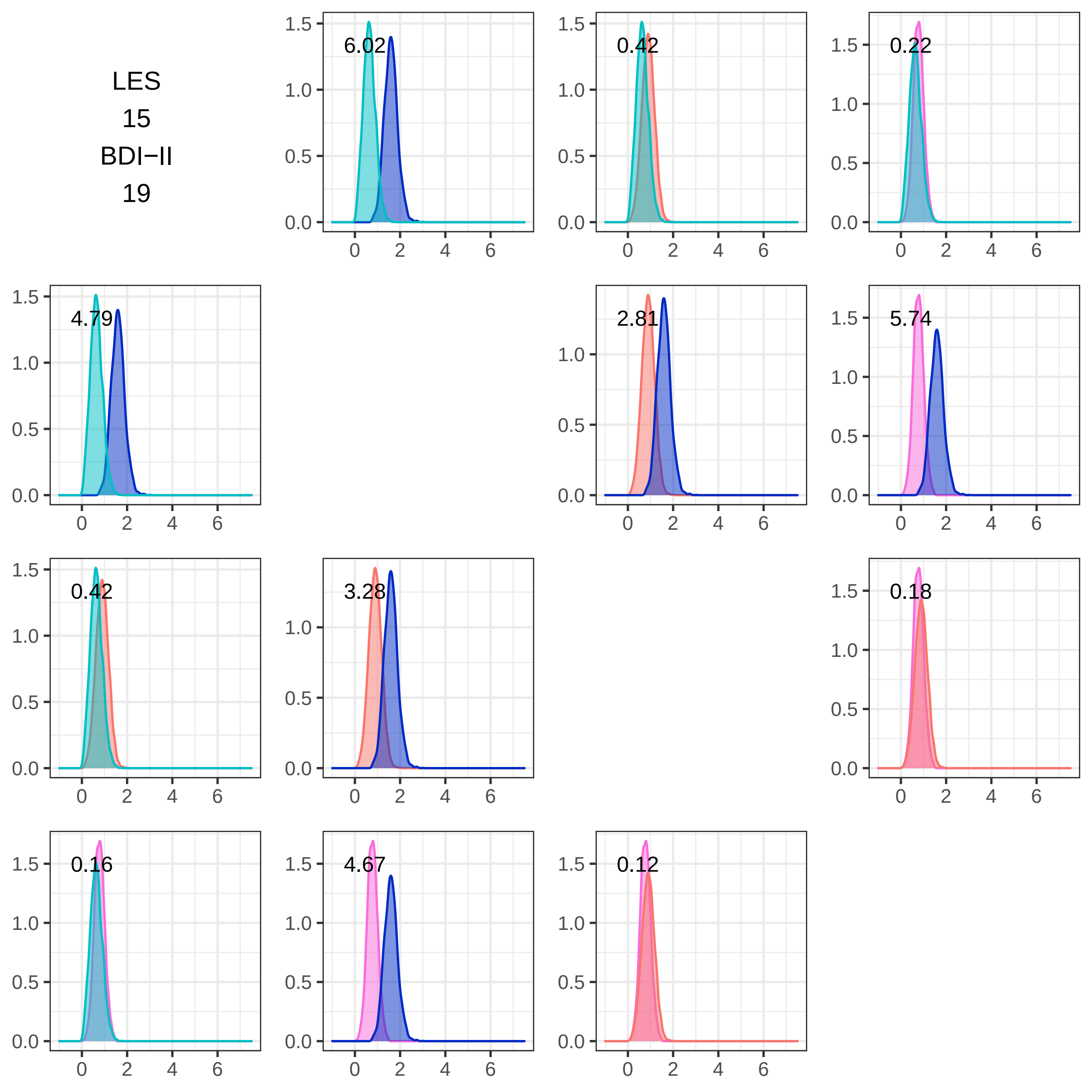
LES
15
BDI−II
19
6.02
0.42
0.22
4.79
2.81
5.74
0.42
3.28
0.18
0.16
4.67
0.12

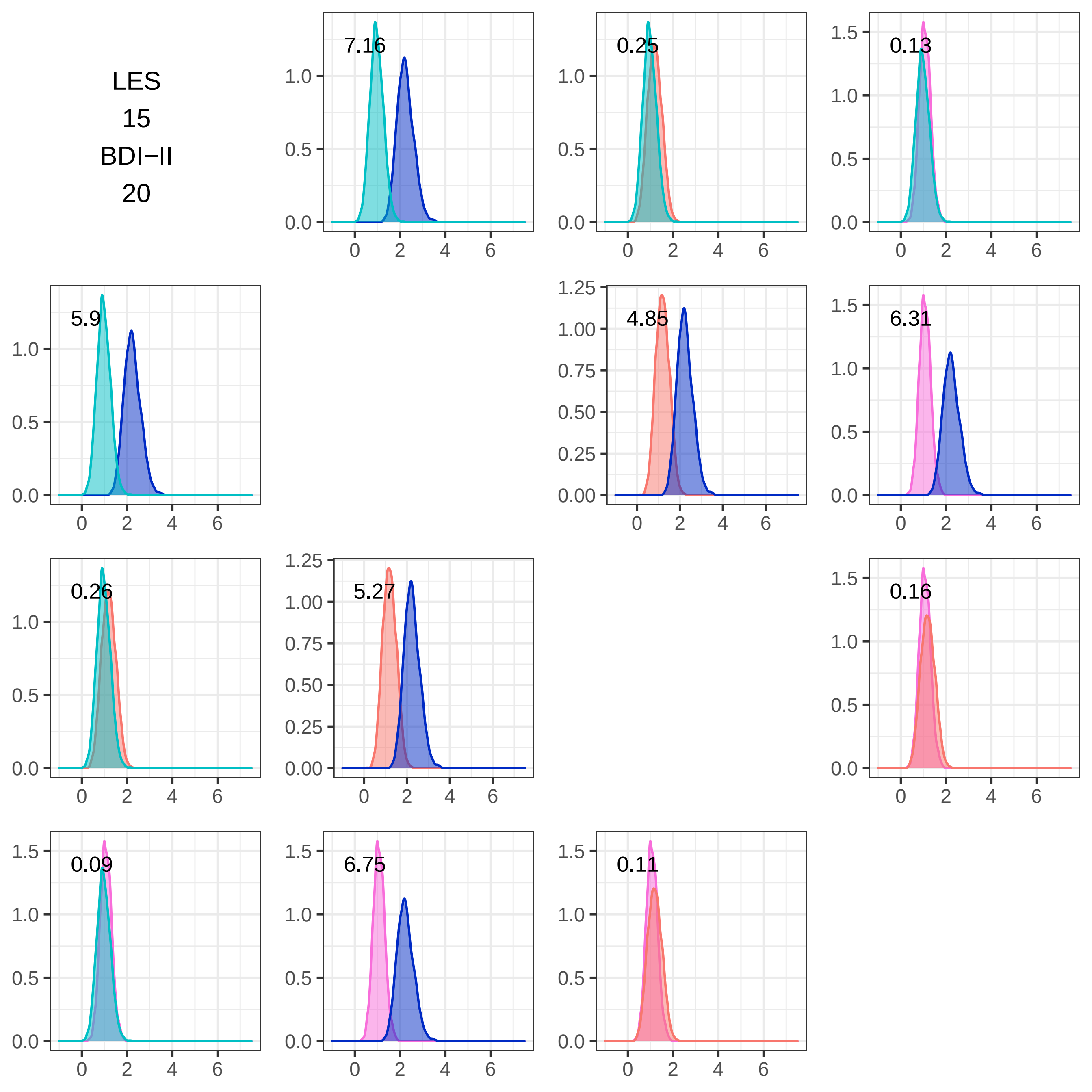
LES
15
BDI−II
20
7.16
0.25
0.13
5.9
4.85
6.31
0.26
5.27
0.16
0.09
6.75
0.11

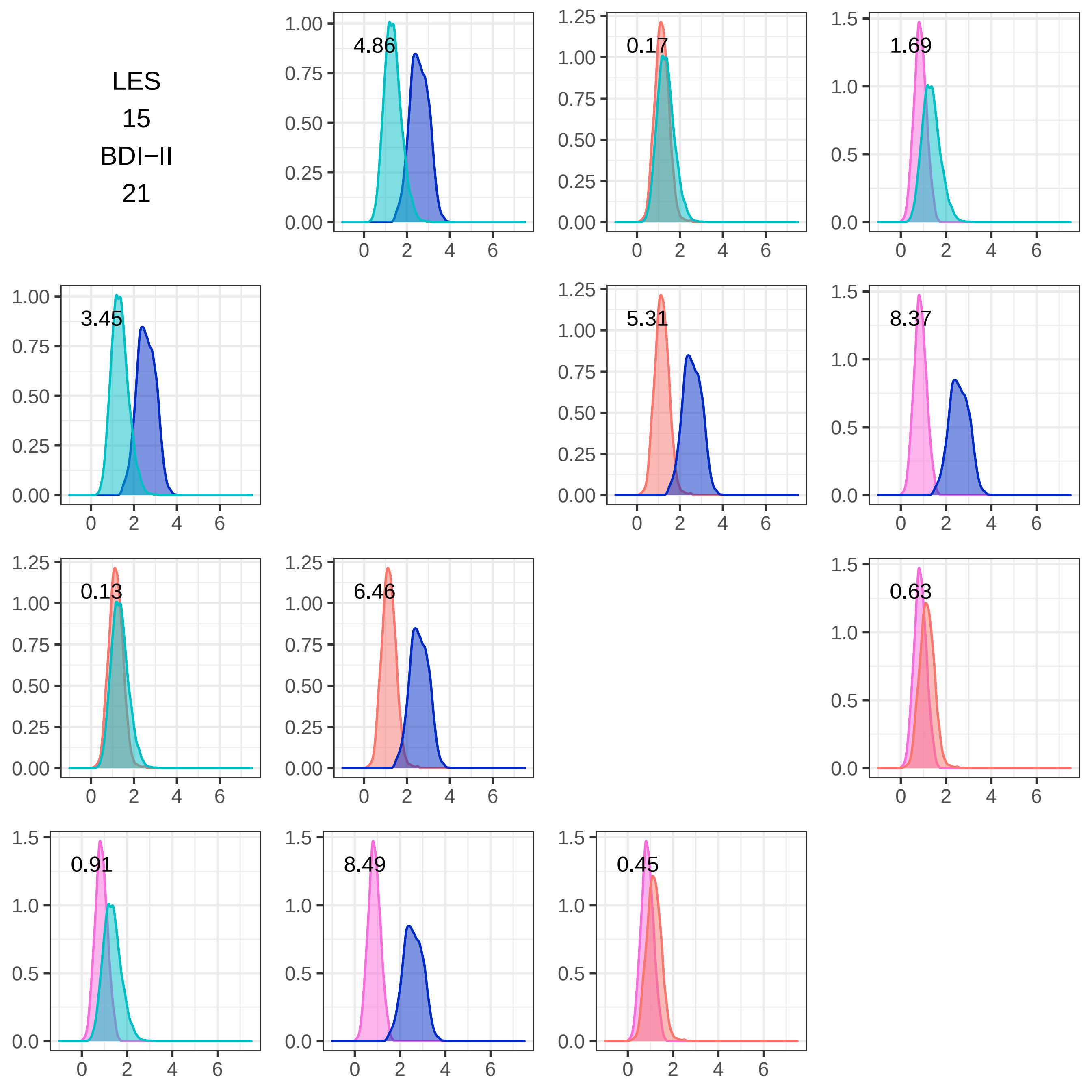

LES
15
BDI−II
21
4.86
0.17
1.69
3.45
5.31
8.37
0.13
6.46
0.63
0.91
8.49
0.45

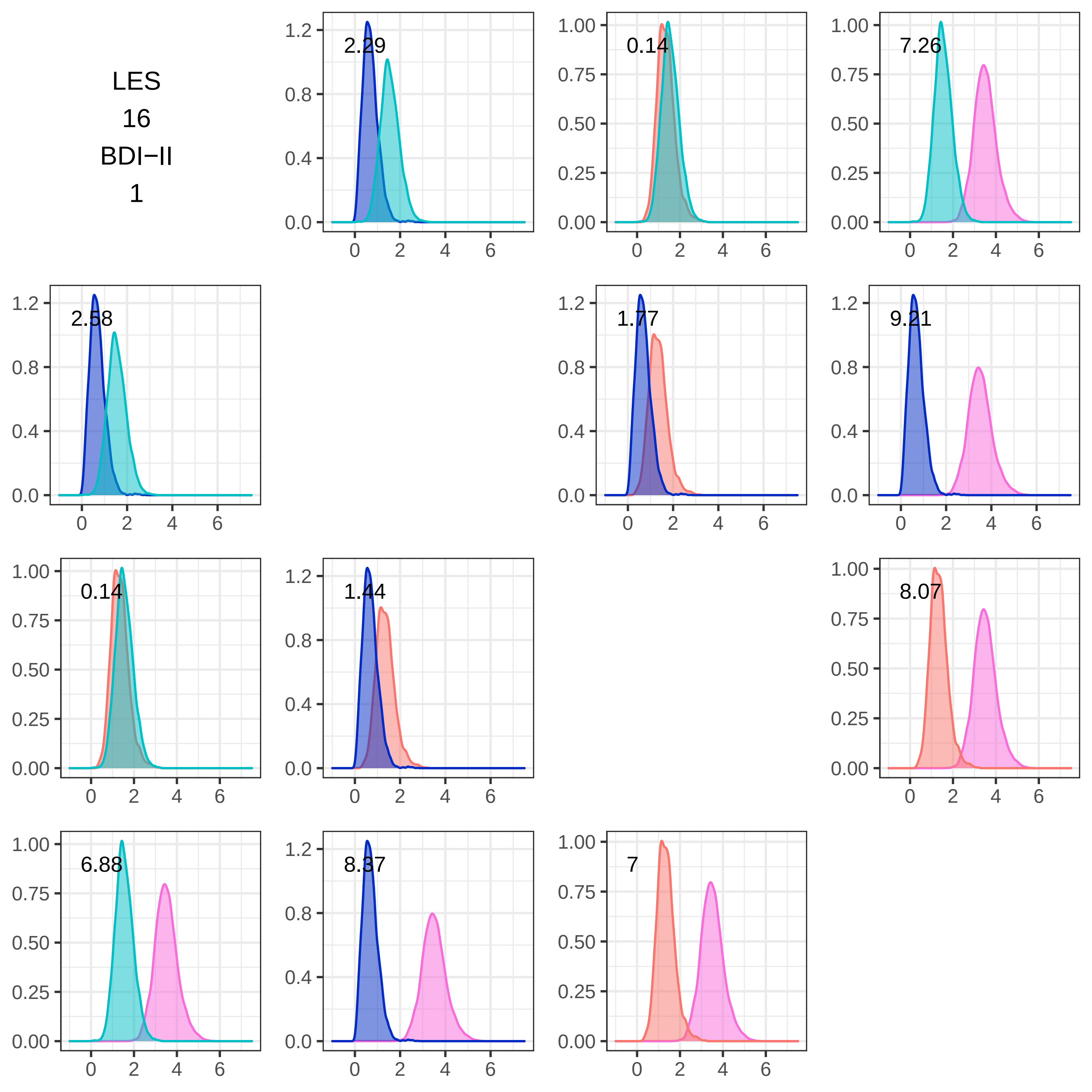

LES
16
BDI−II
1
2.29
0.14
7.26
2.58
1.77
9.21
0.14
1.44
8.07
6.88
8.37
7

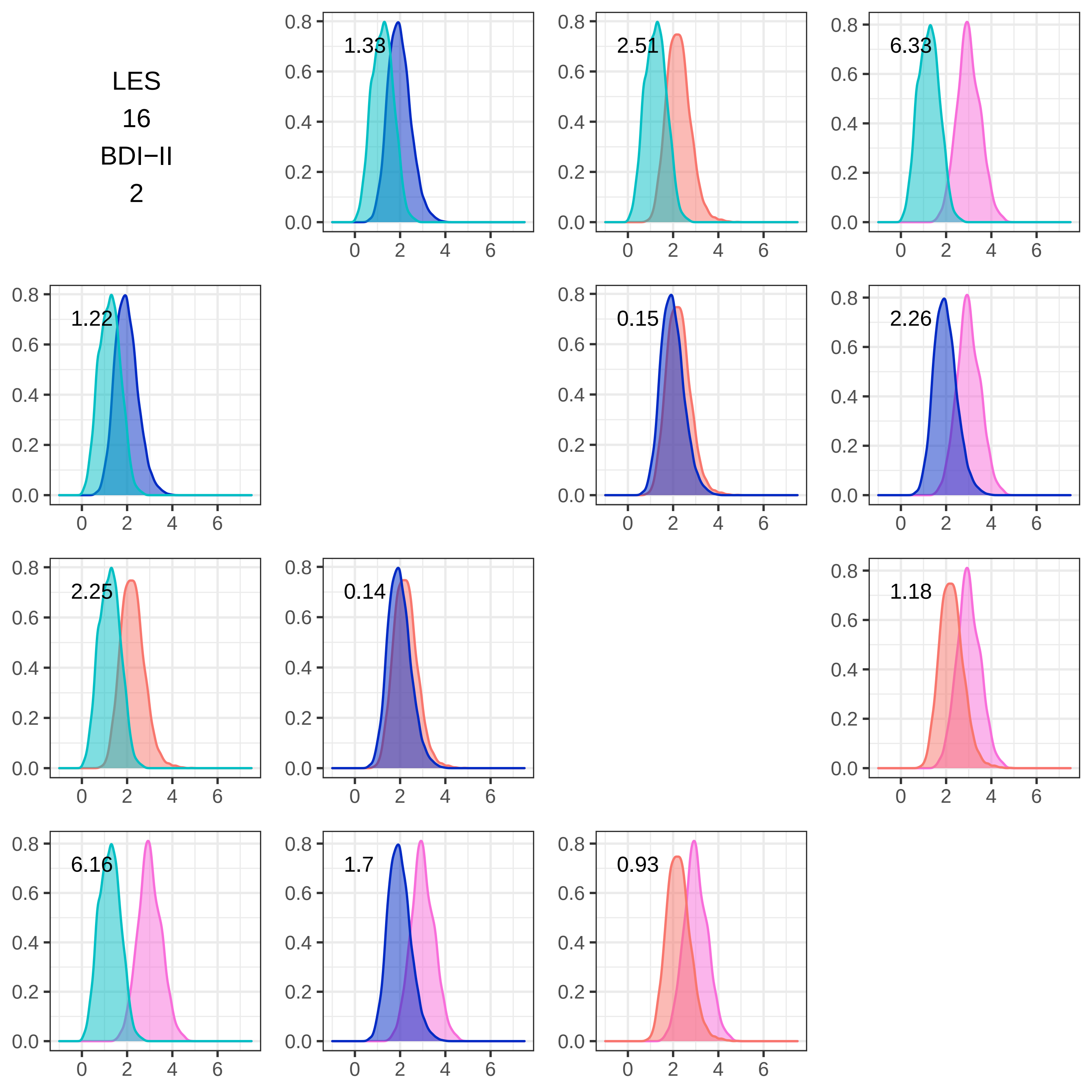

LES
16
BDI−II
2
1.33
2.51
6.33
1.22
0.15
2.26
2.25
0.14
1.18
6.16
1.7
0.93

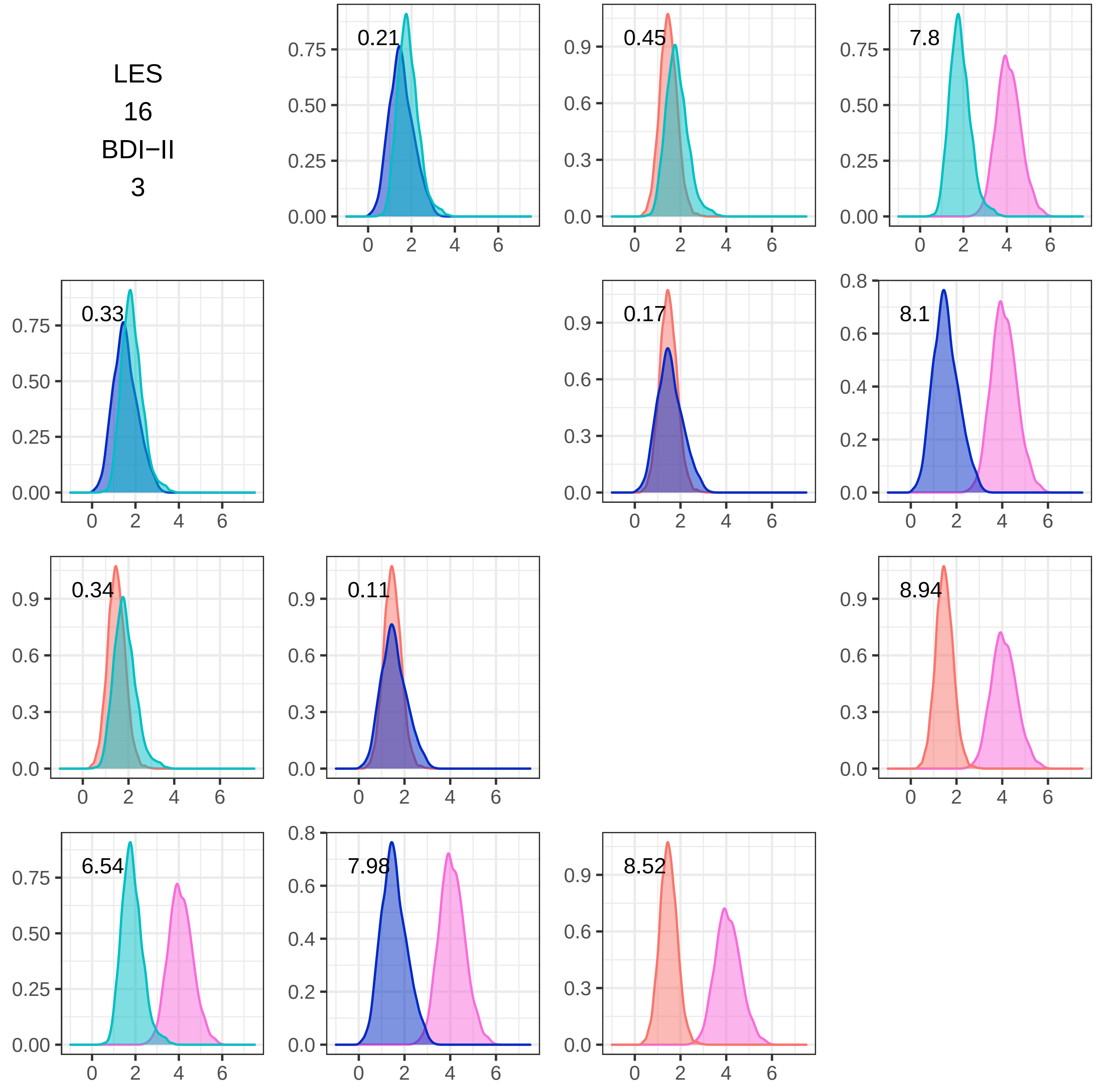

LES
16
BDI−II
3
0.21
0.45
7.8
0.33
0.17
8.1
0.34
0.11
8.94
6.54
7.98
8.52

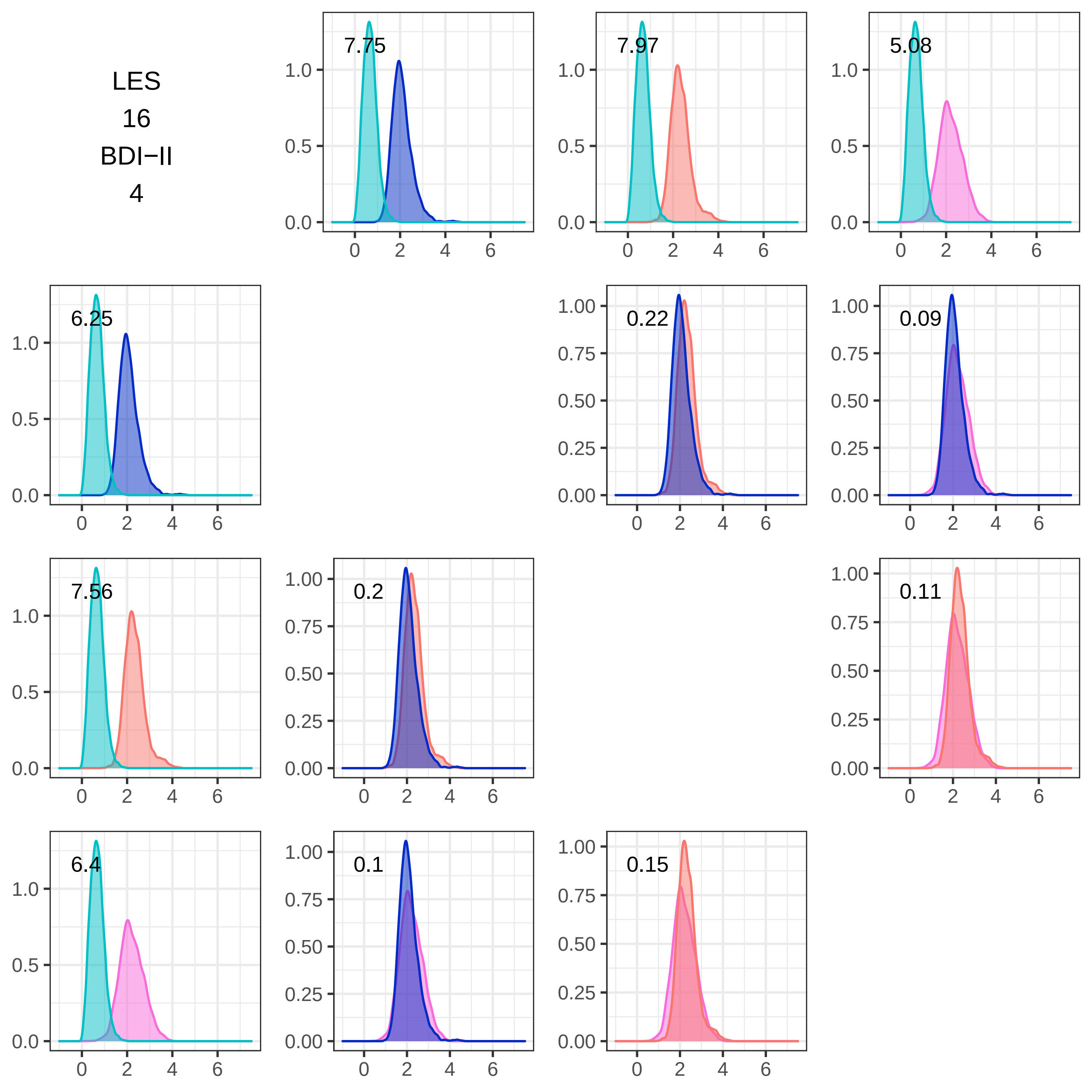

LES
16
BDI−II
4
7.75
7.97
5.08
6.25
0.22
0.09
7.56
0.2
0.11
6.4
0.1
0.15

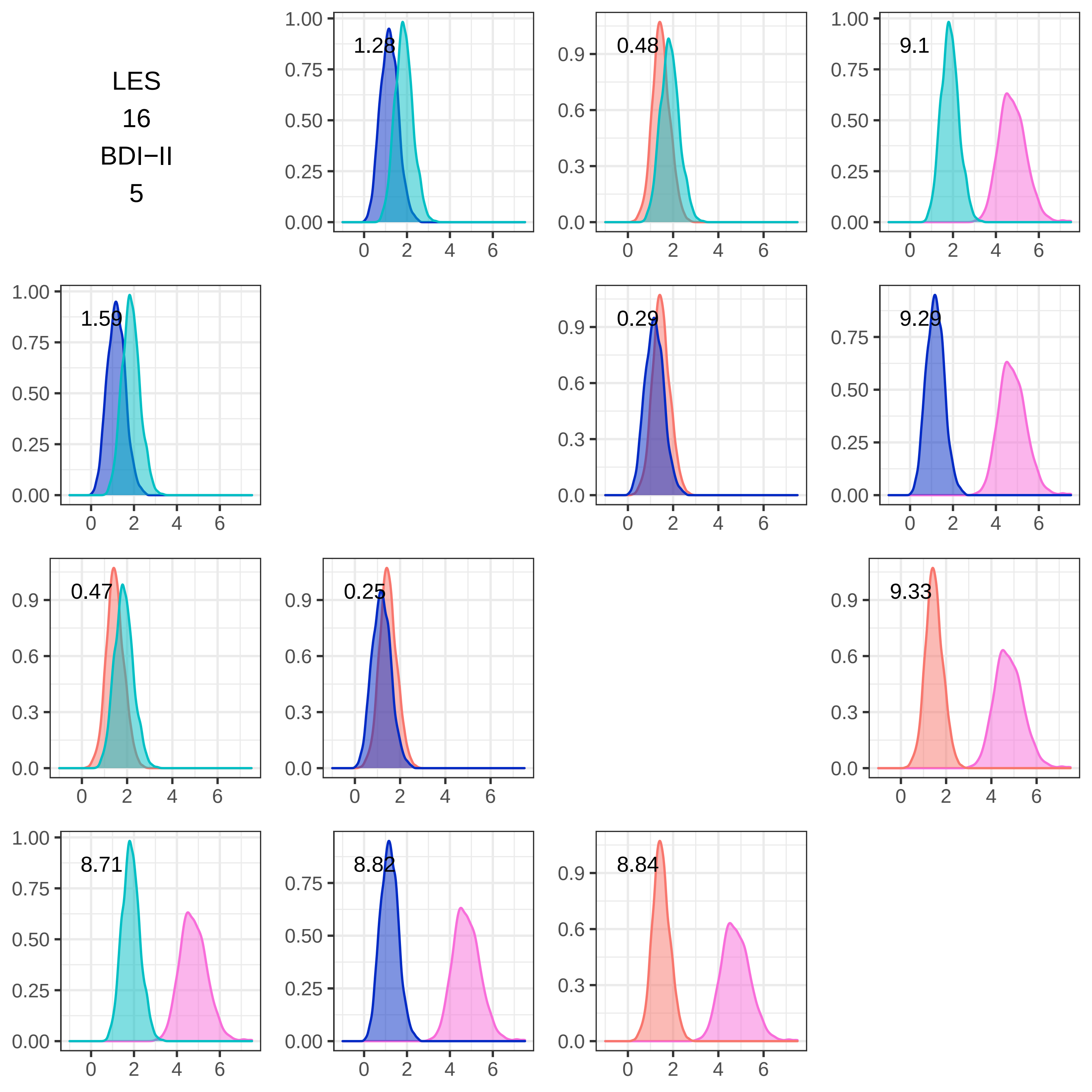

LES
16
BDI−II
5
1.28
0.48
9.1
1.59
0.29
9.29
0.47
0.25
9.33
8.71
8.82
8.84

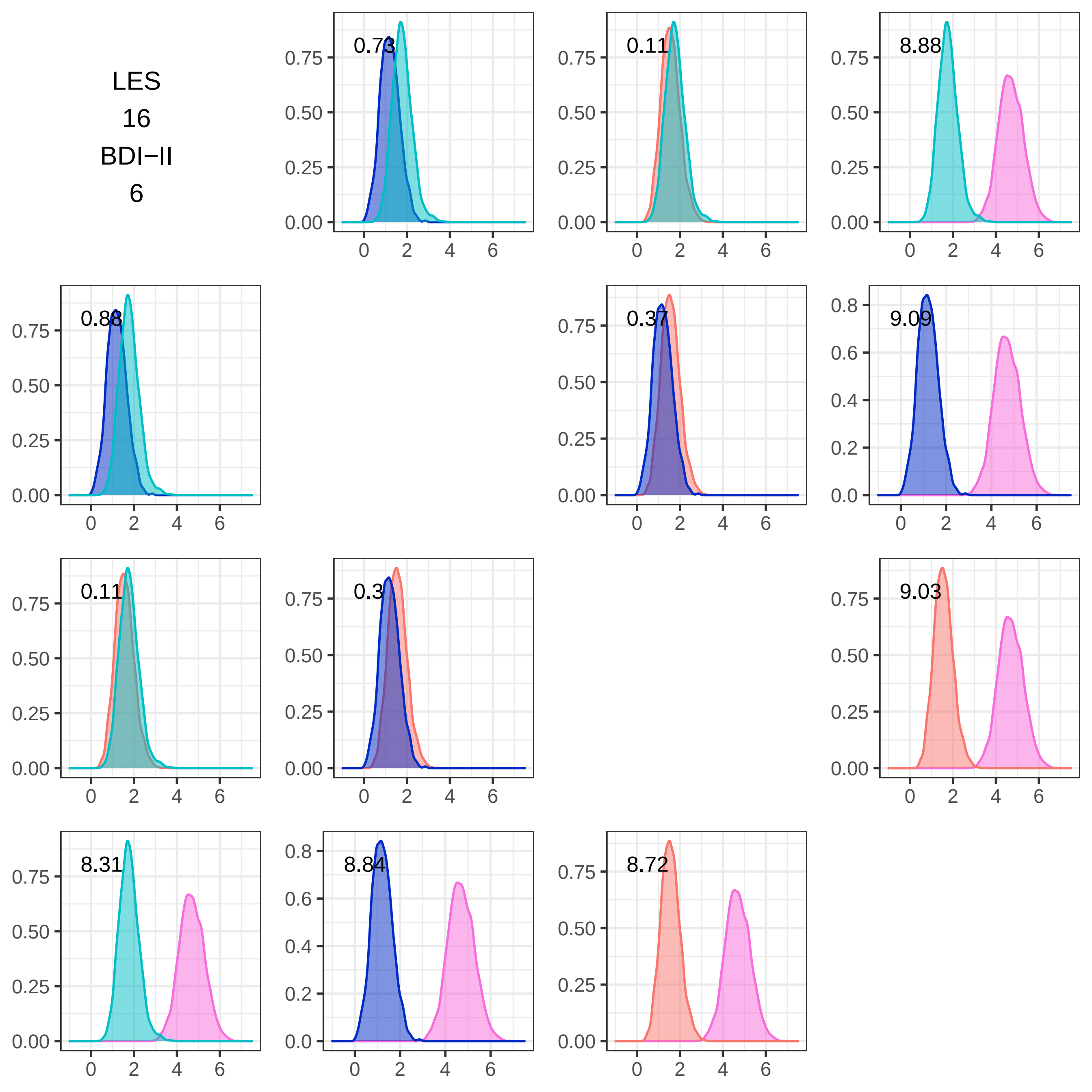
LES
16
BDI−II
6
0.73
0.11
8.88
0.88
0.37
9.09
0.11
0.3
9.03
8.31
8.84
8.72
0.00
0.25
0.50
0.75
0.0
0.2
0.4
0.6
0.8
0
2
4
6

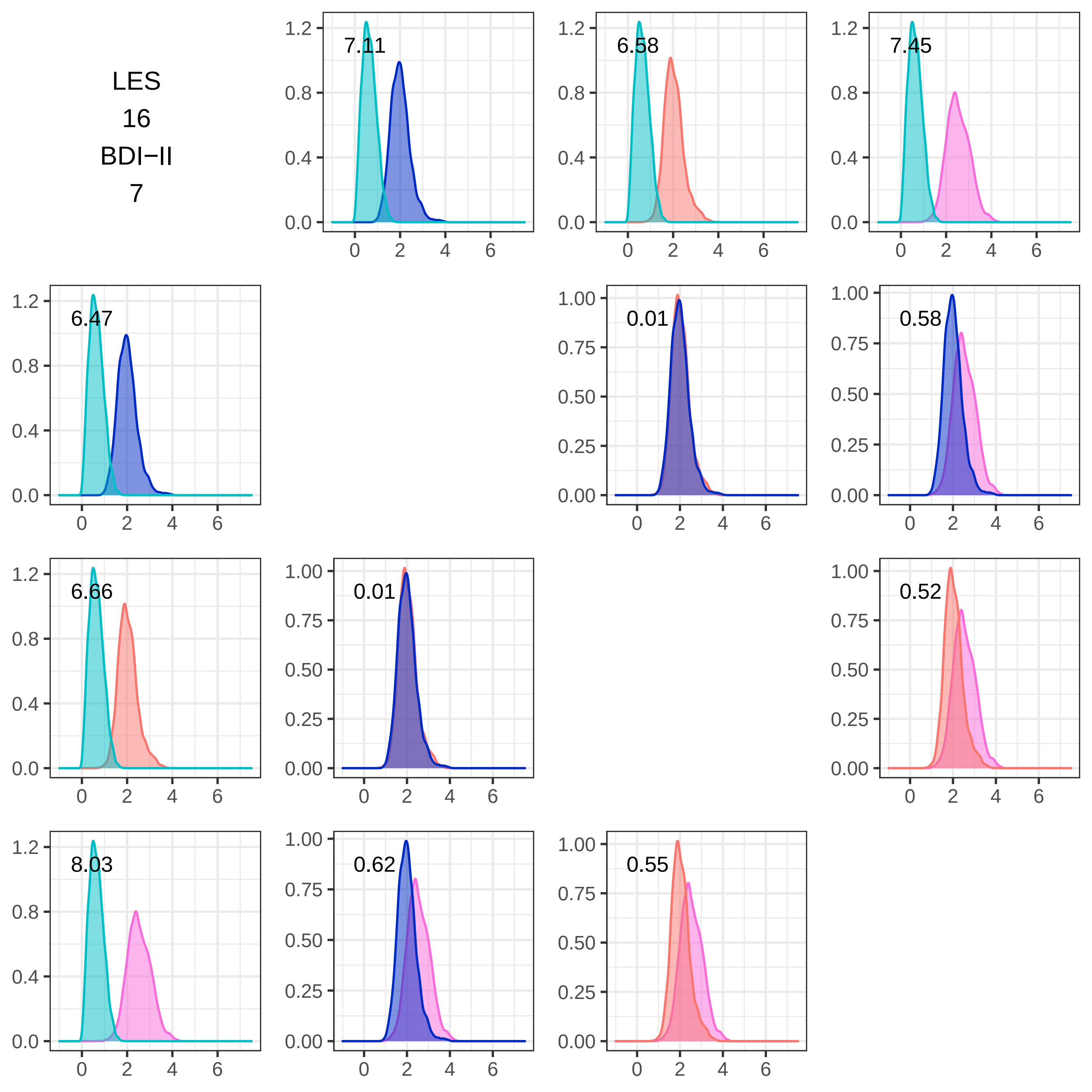

LES
16
BDI−II
7
7.11
6.58
7.45
6.47
0.01
0.58
6.66
0.01
0.52
8.03
0.62
0.55

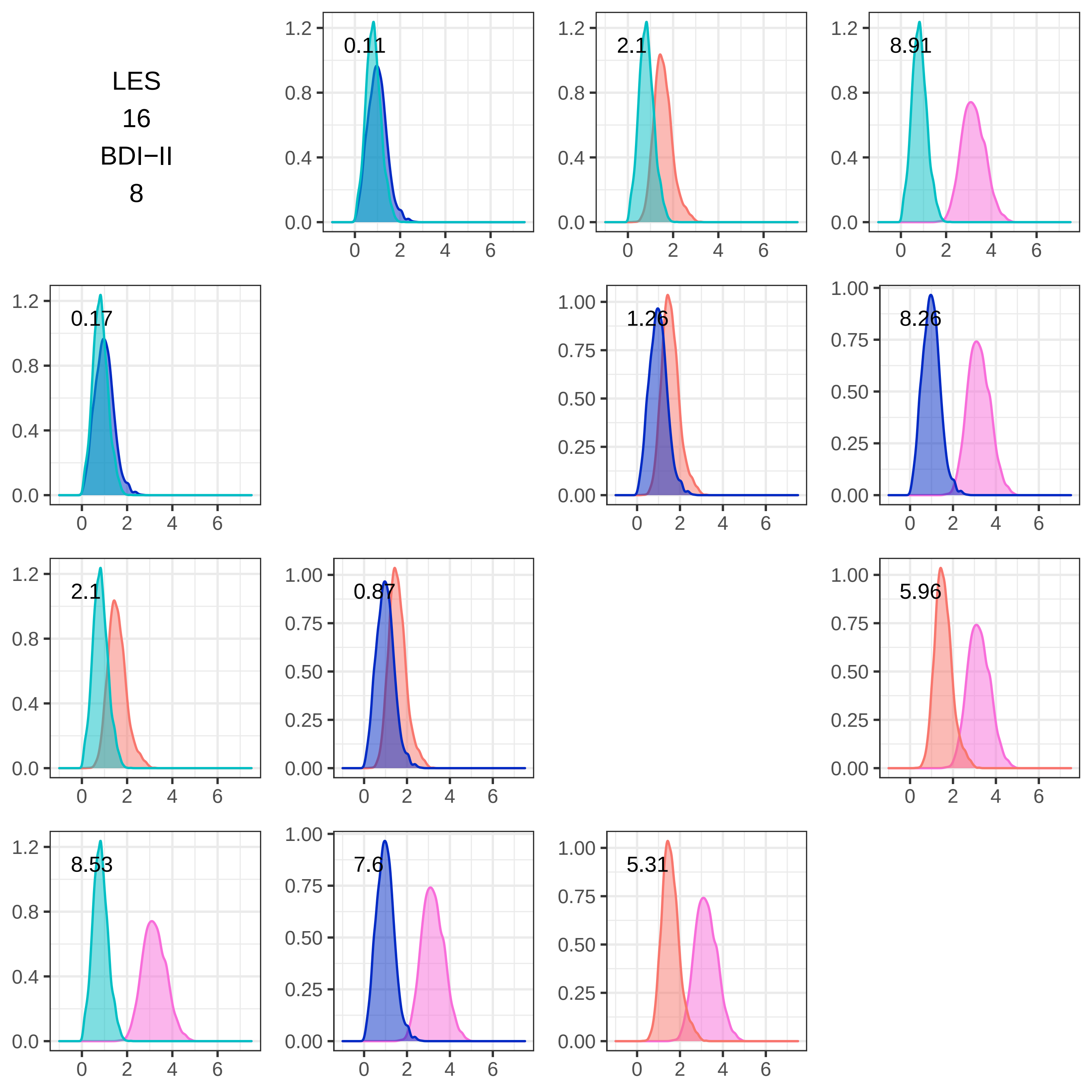
LES
16
BDI−II
8
0.11
2.1
8.91
0.17
1.26
8.26
2.1
0.87
5.96
8.53
7.6
5.31

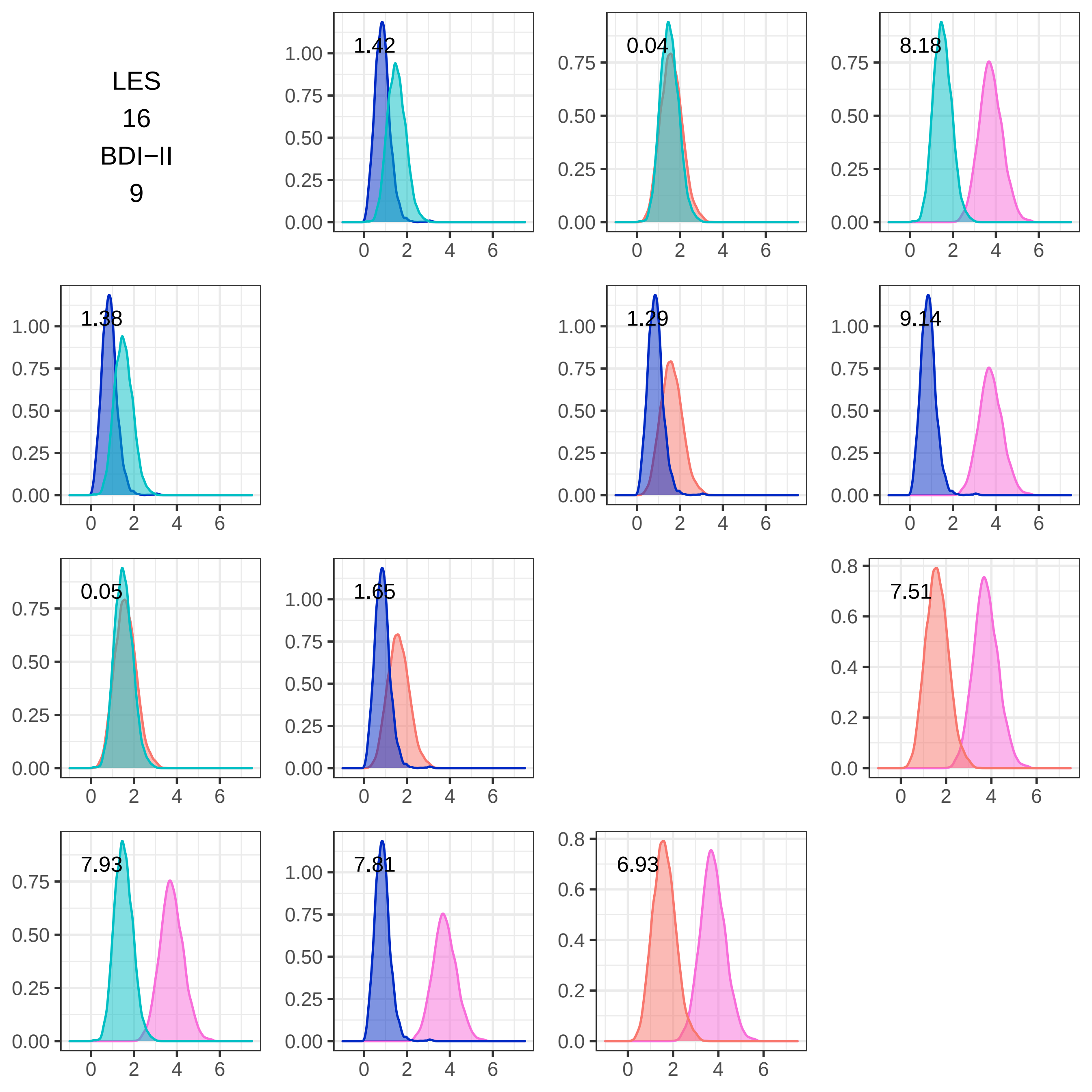

LES
16
BDI−II
9
1.42
0.04
8.18
1.38
1.29
9.14
0.05
1.65
7.51
7.93
7.81
6.93

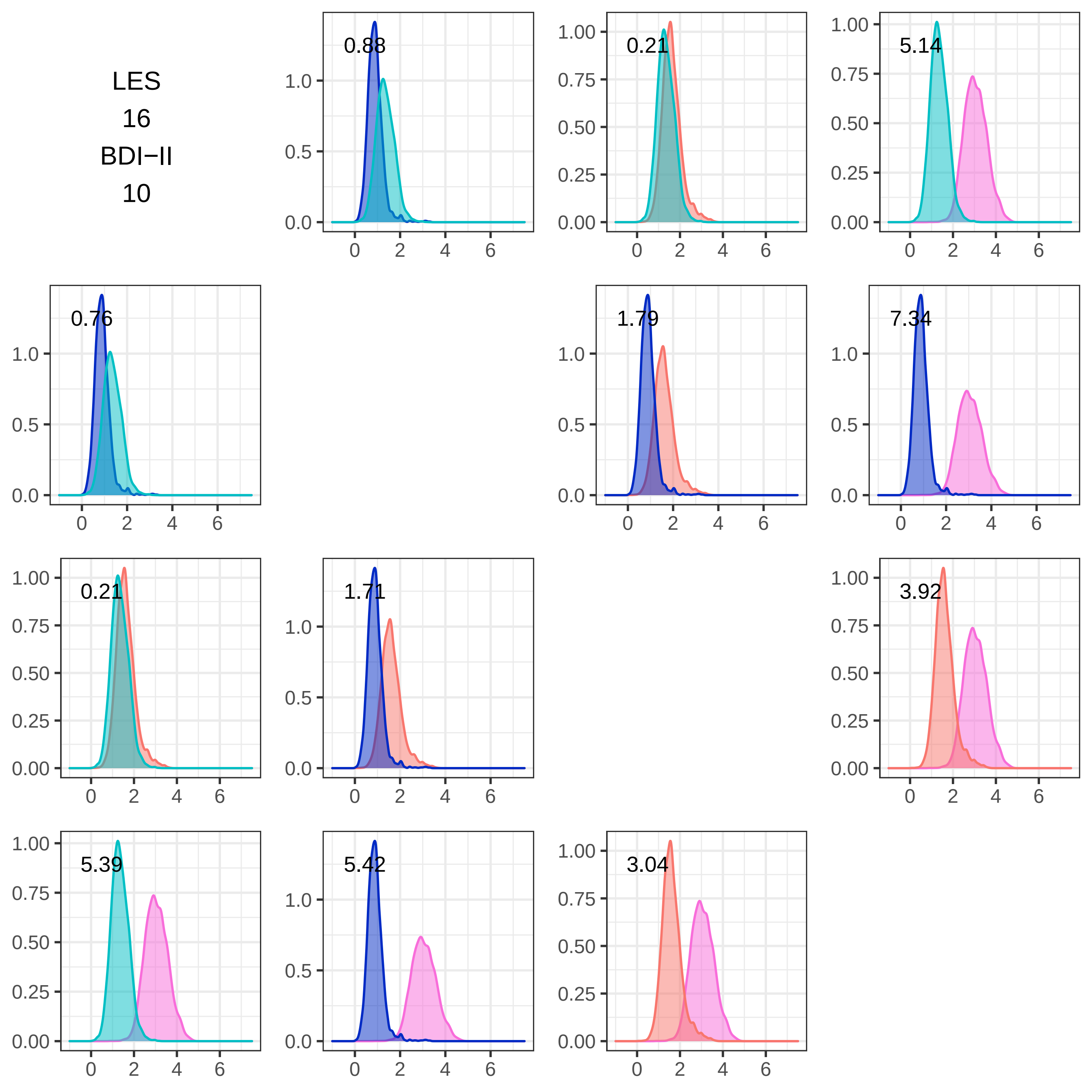

LES
16
BDI−II
10
0.88
0.21
5.14
0.76
1.79
7.34
0.21
1.71
3.92
5.39
5.42
3.04

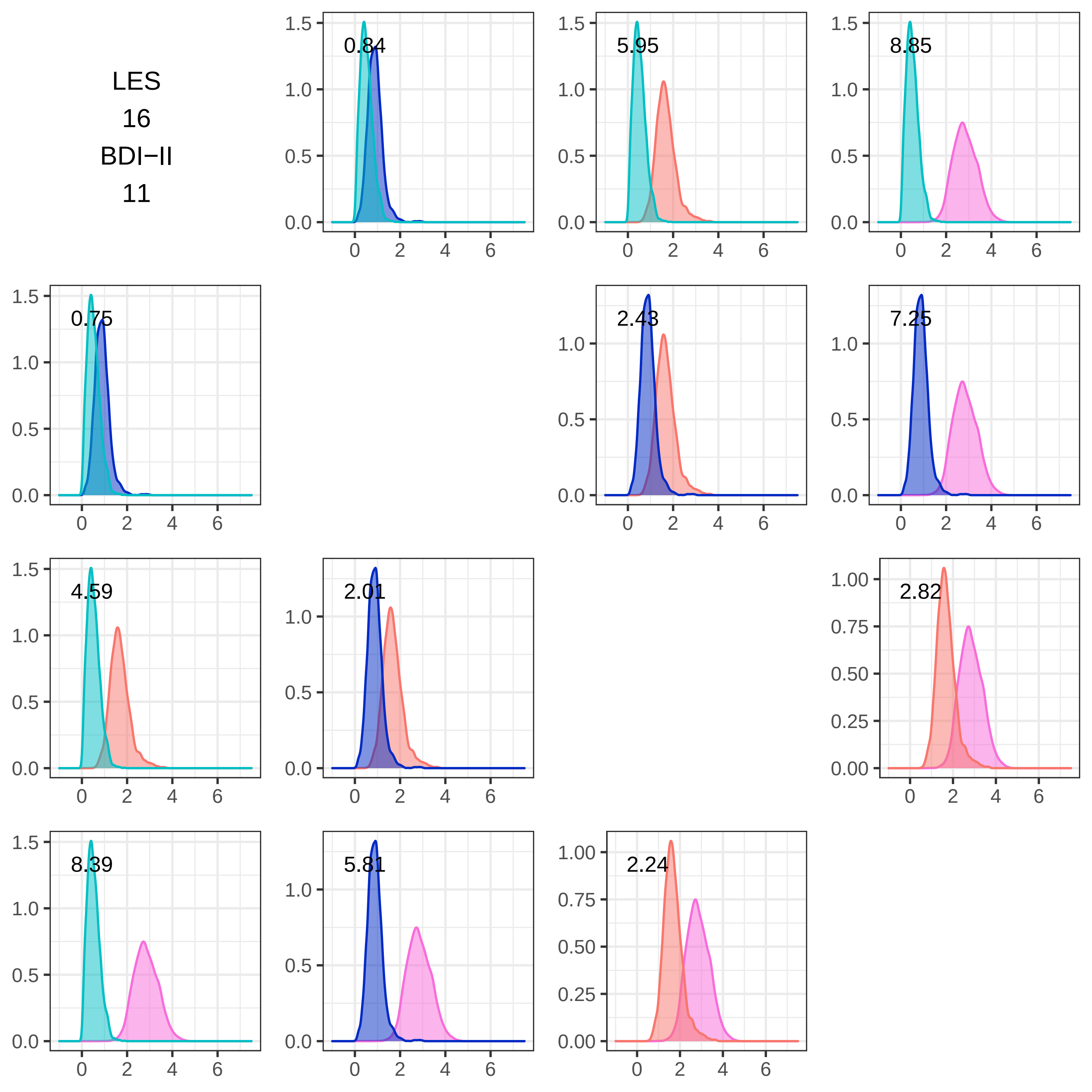
LES
16
BDI−II
11
0.84
5.95
8.85
0.75
2.43
7.25
4.59
2.01
2.82
8.39
5.81
2.24

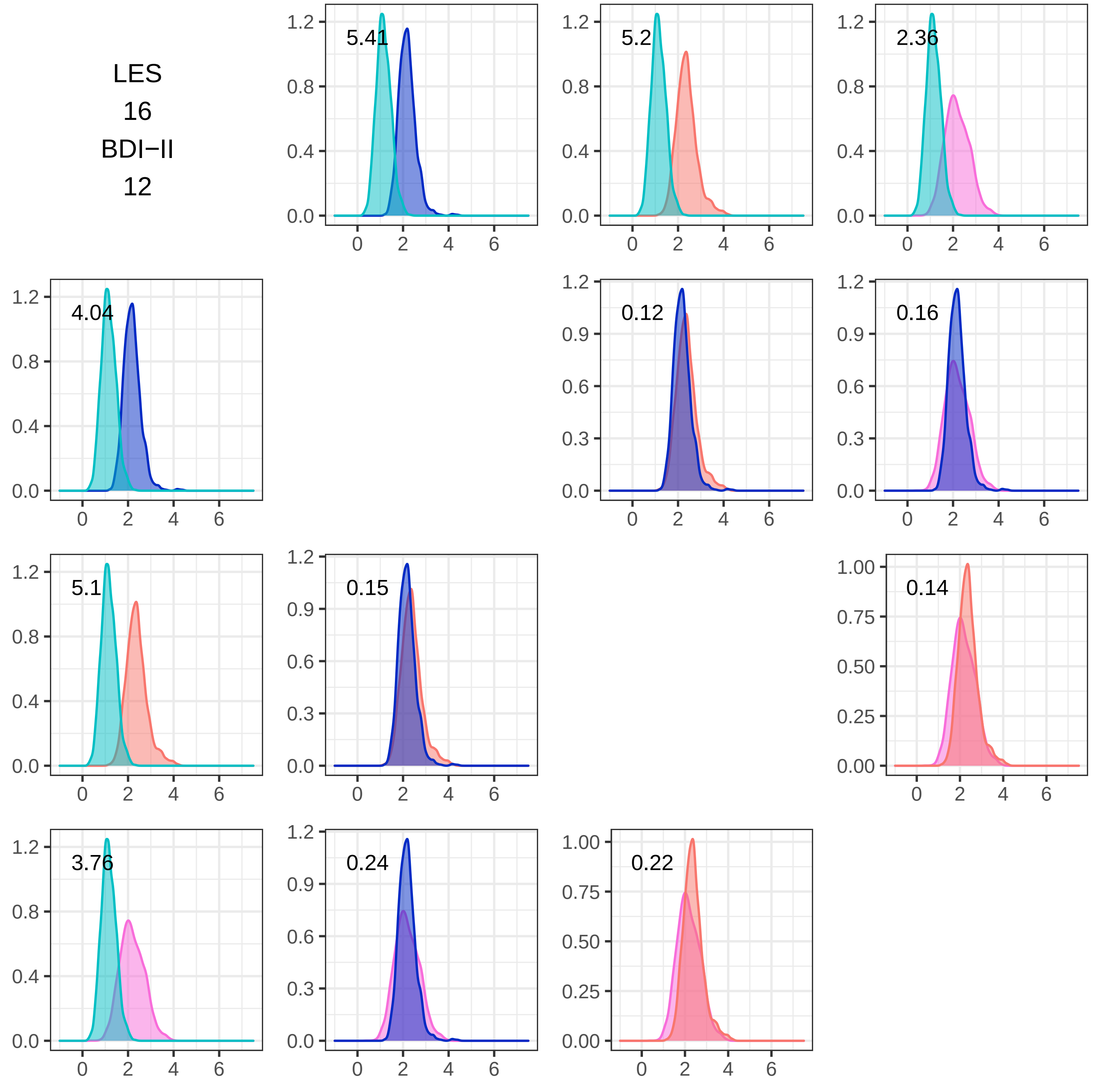

LES
16
BDI−II
12
5.41
5.2
2.36
4.04
0.12
0.16
5.1
0.15
0.14
3.76
0.24
0.22

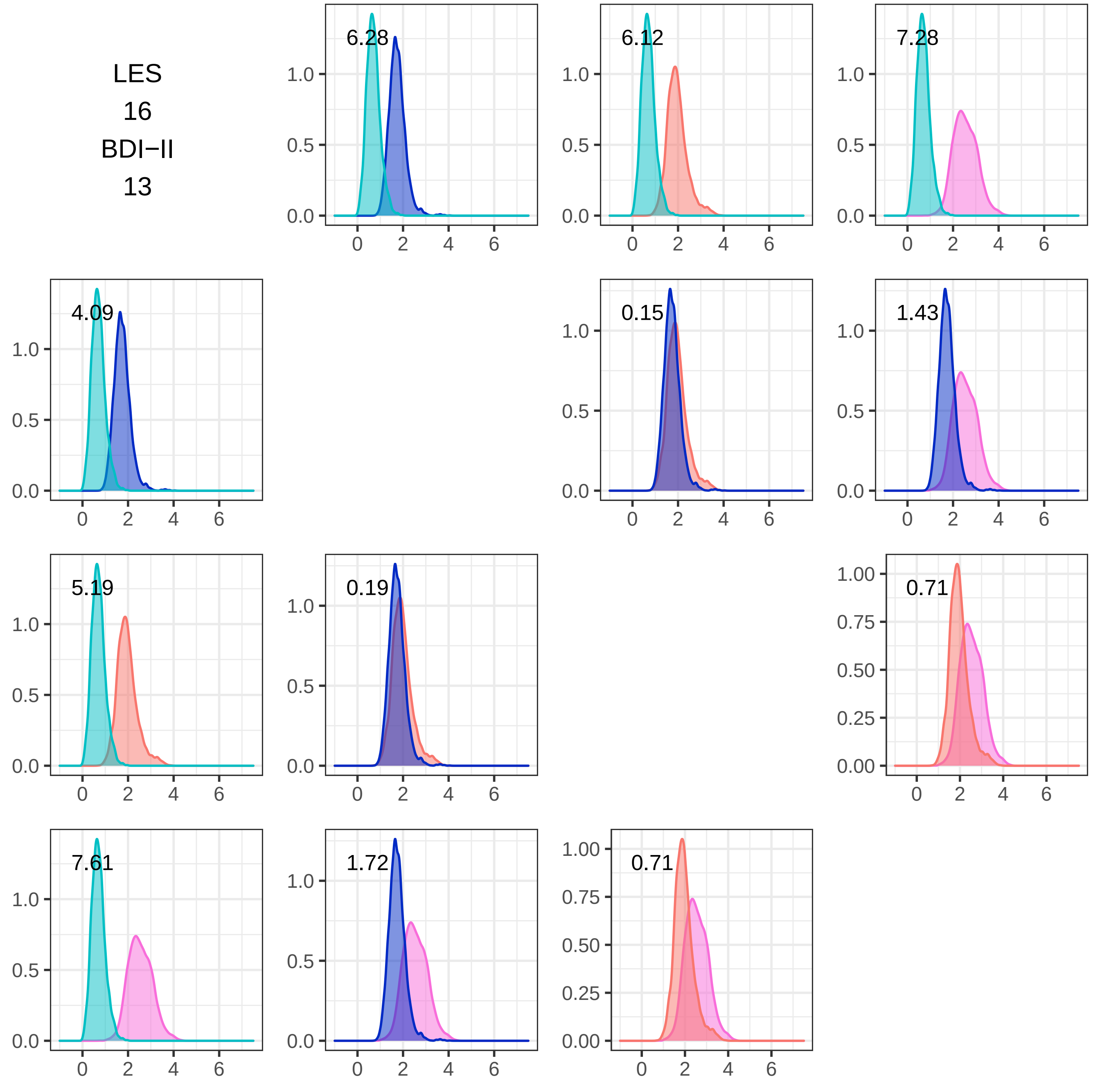
LES
16
BDI−II
13
6.28
6.12
7.28
4.09
0.15
1.43
5.19
0.19
0.71
7.61
1.72
0.71

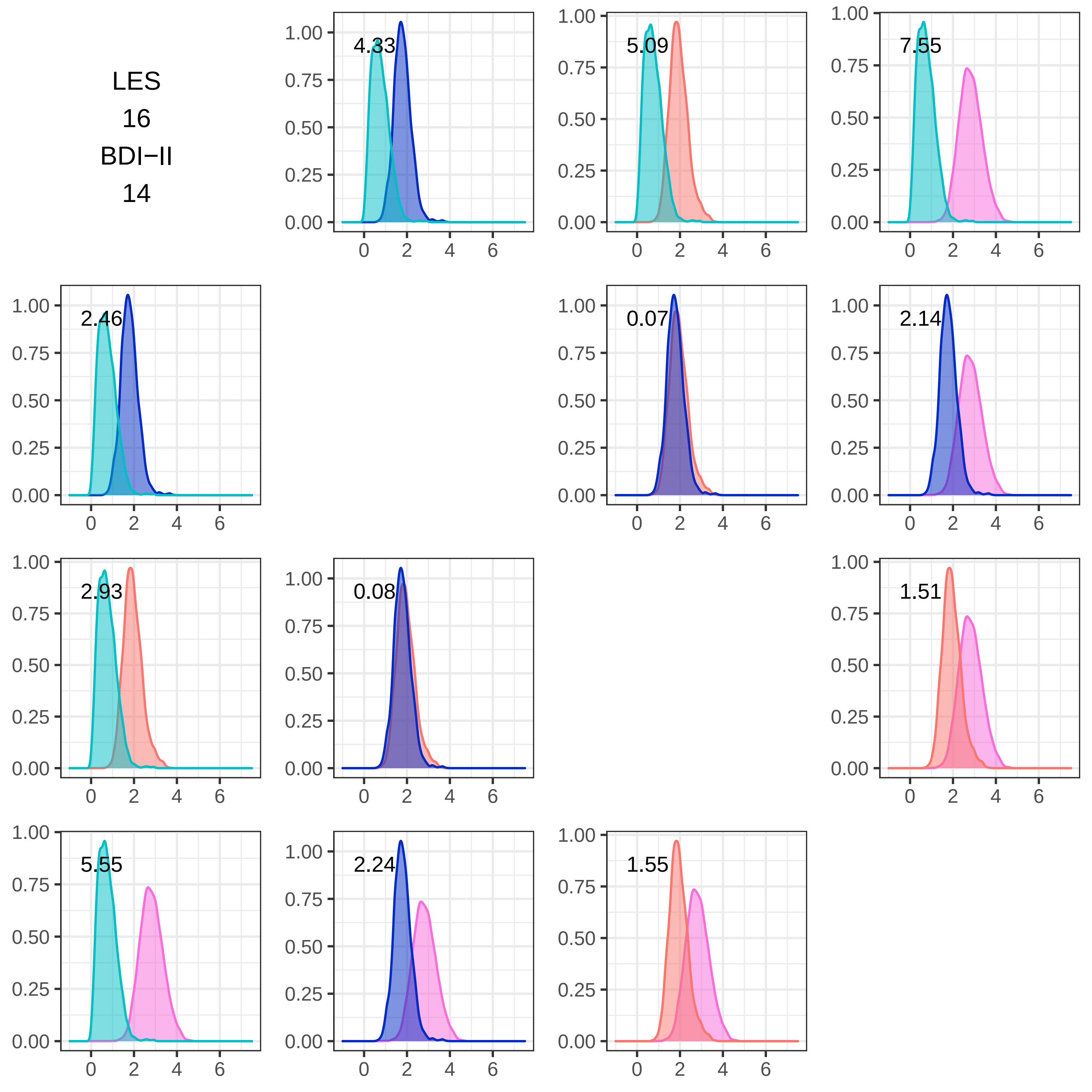

LES
16
BDI−II
14
4.33
5.09
7.55
2.46
0.07
2.14
2.93
0.08
1.51
5.55
2.24
1.55

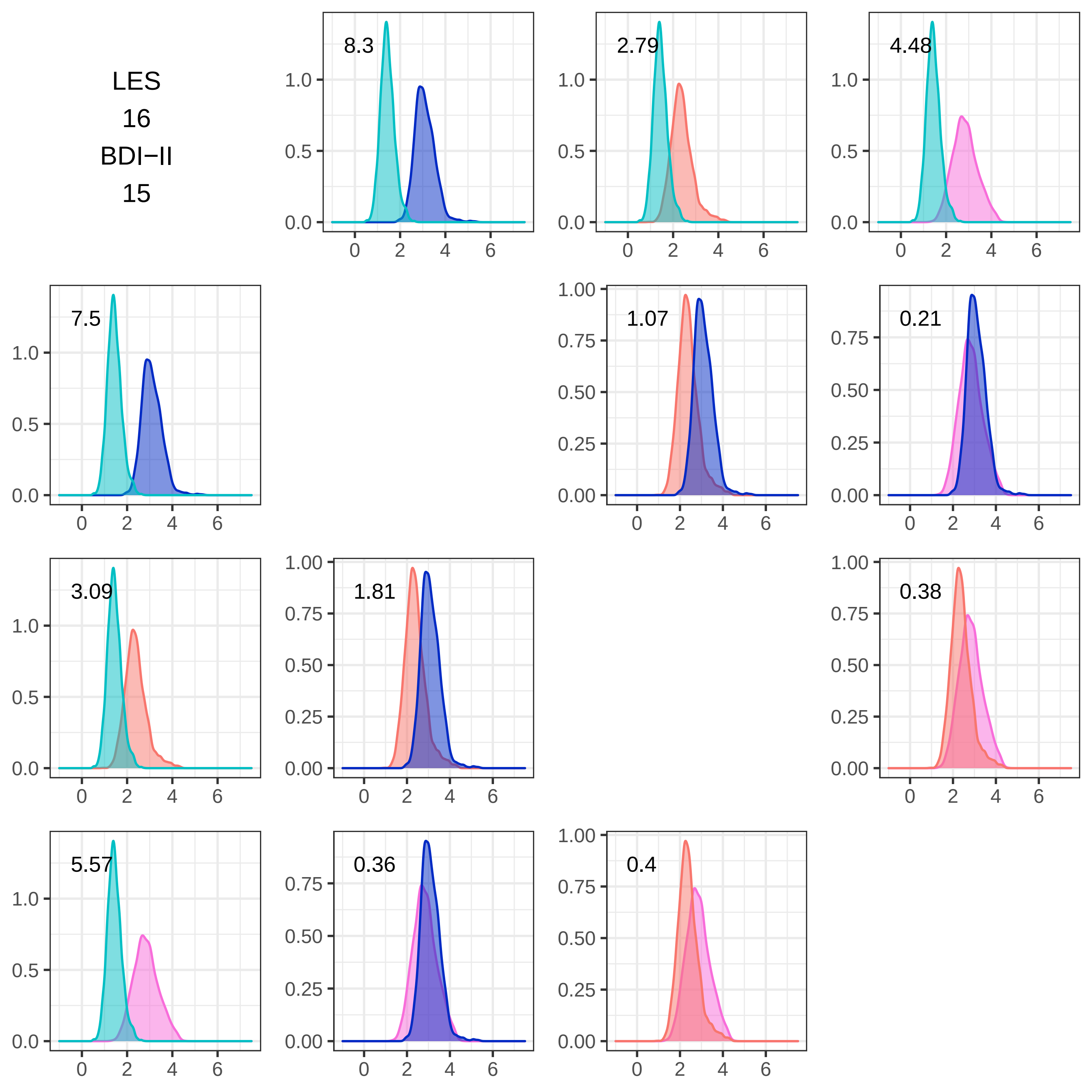
LES
16
BDI−II
15
8.3
2.79
4.48
7.5
1.07
0.21
3.09
1.81
0.38
5.57
0.36
0.4

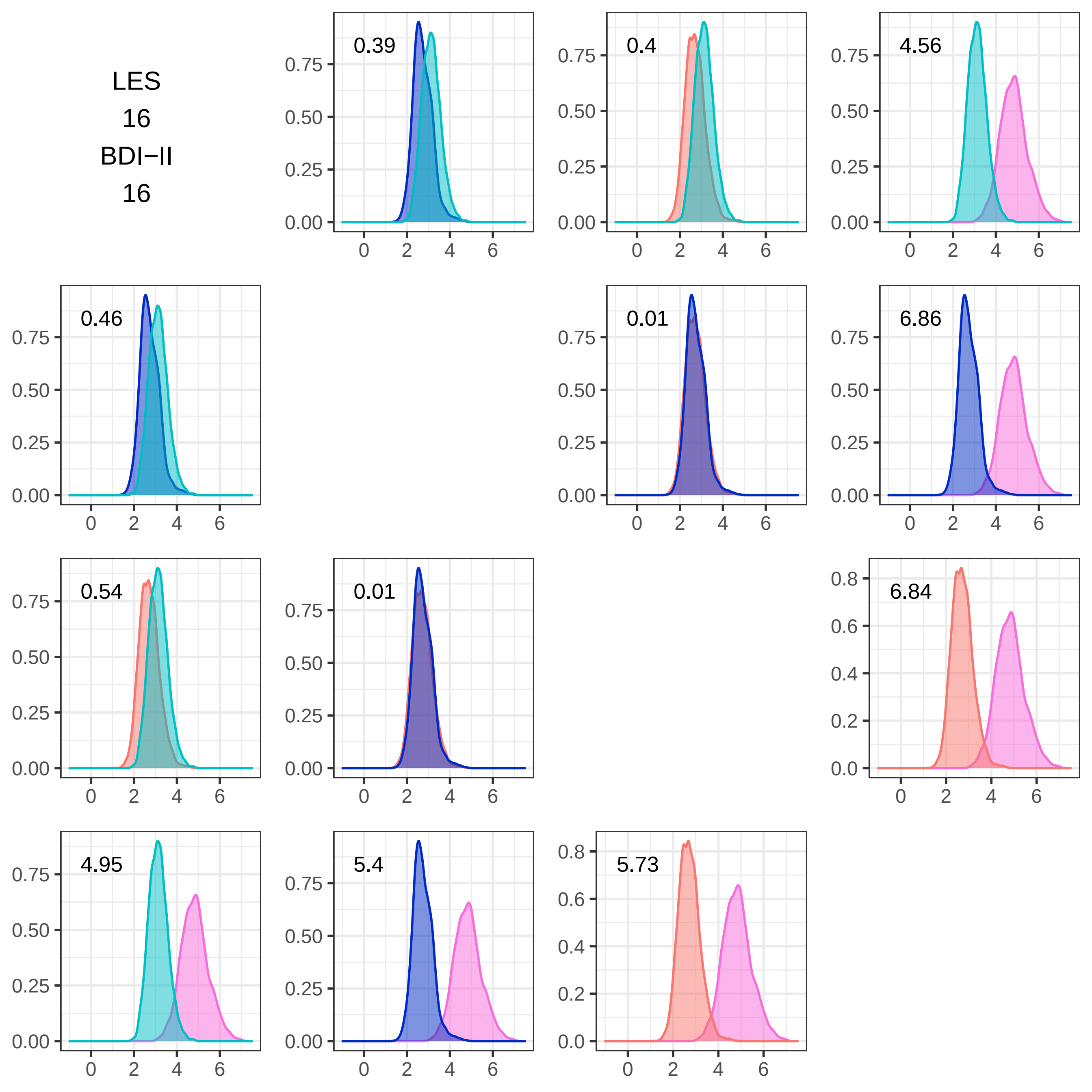

LES
16
BDI−II
16
0.39
0.4
4.56
0.46
0.01
6.86
0.54
0.01
6.84
4.95
5.4
5.73

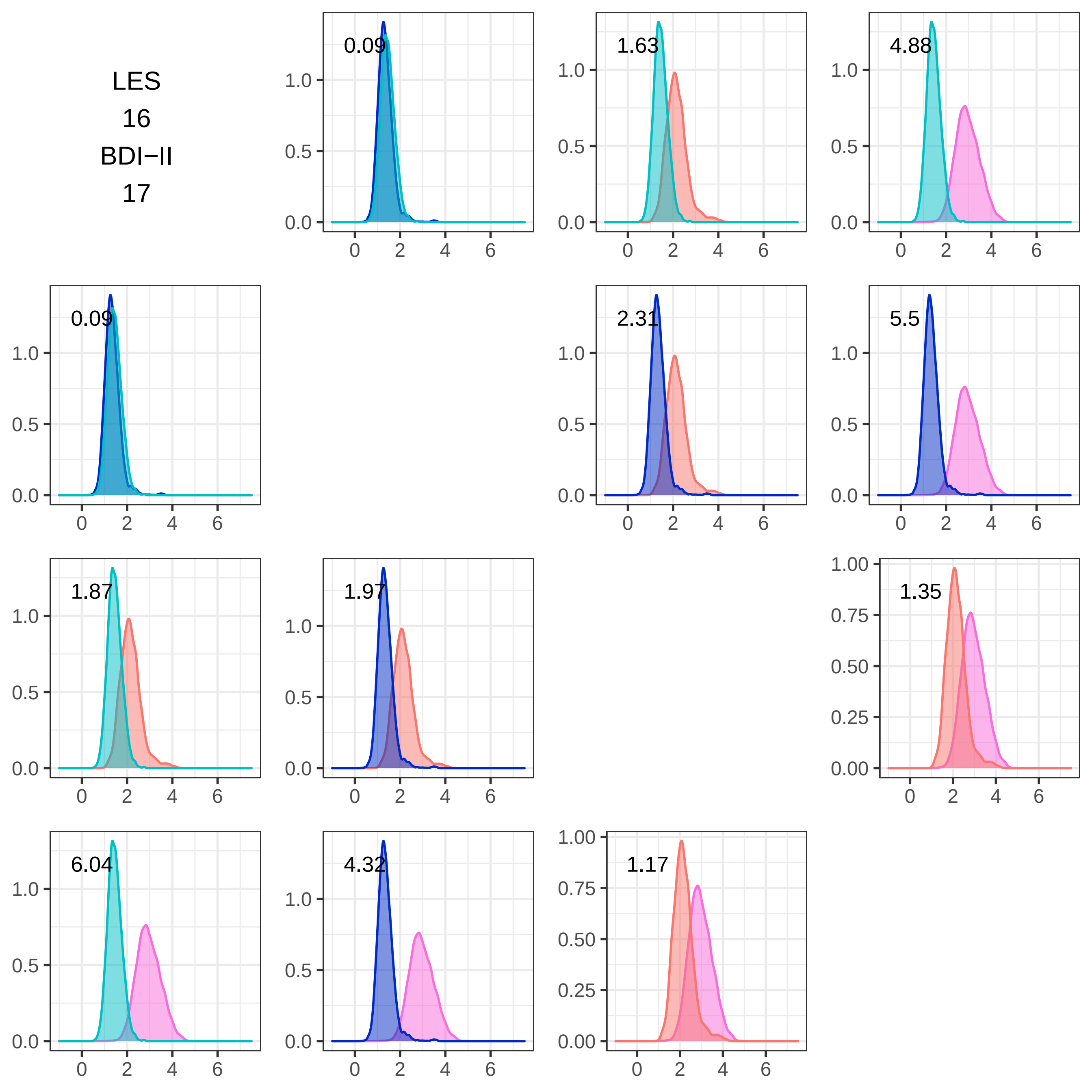
LES
16
BDI−II
17
0.09
1.63
4.88
0.09
2.31
5.5
1.87
1.97
1.35
6.04
4.32
1.17

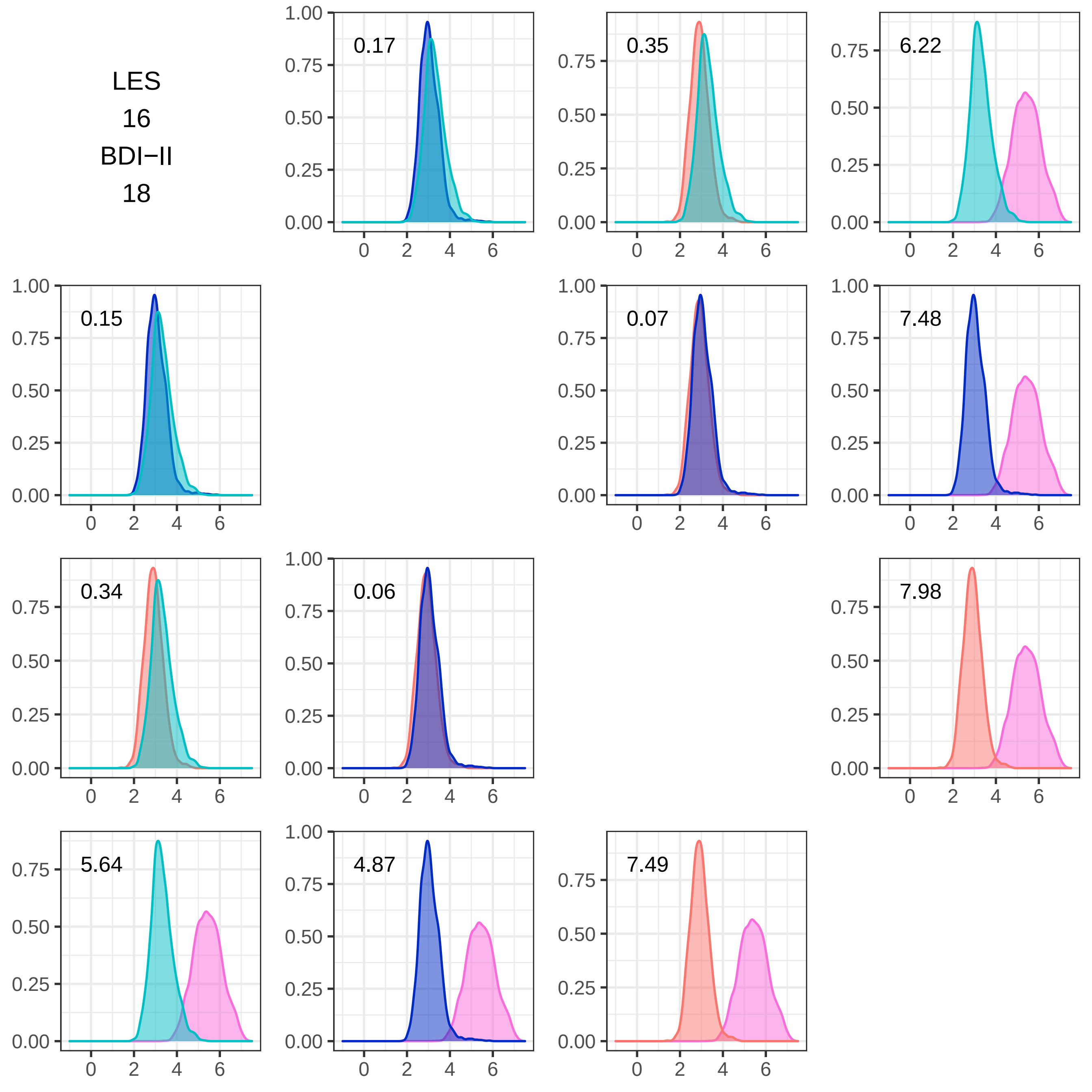
LES
16
BDI−II
18
0.17
0.35
6.22
0.15
0.07
7.48
0.34
0.06
7.98
5.64
4.87
7.49
0.00
0.25
0.50
0.75
1.00
0
2
4
6

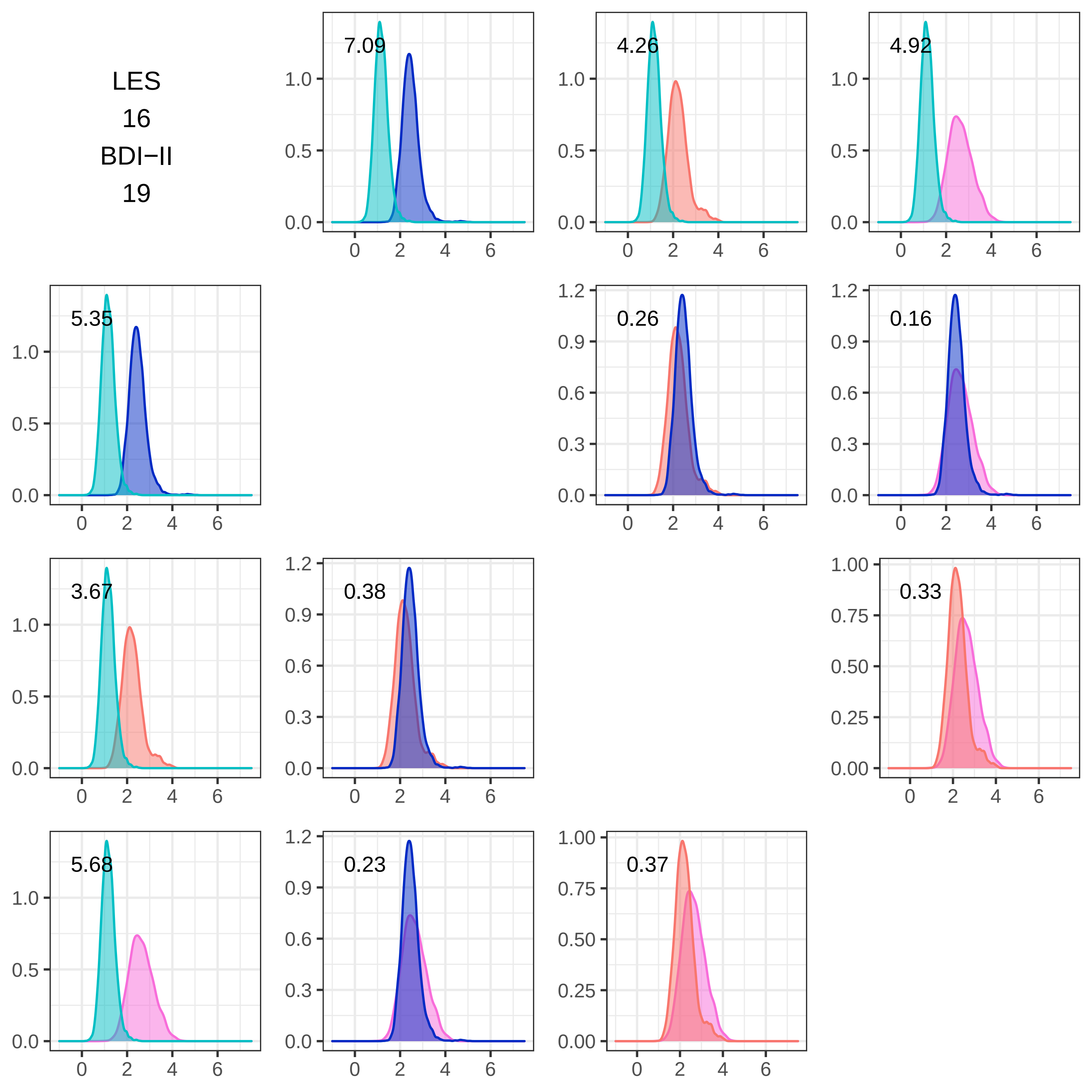
LES
16
BDI−II
19
7.09
4.26
4.92
5.35
0.26
0.16
3.67
0.38
0.33
5.68
0.23
0.37

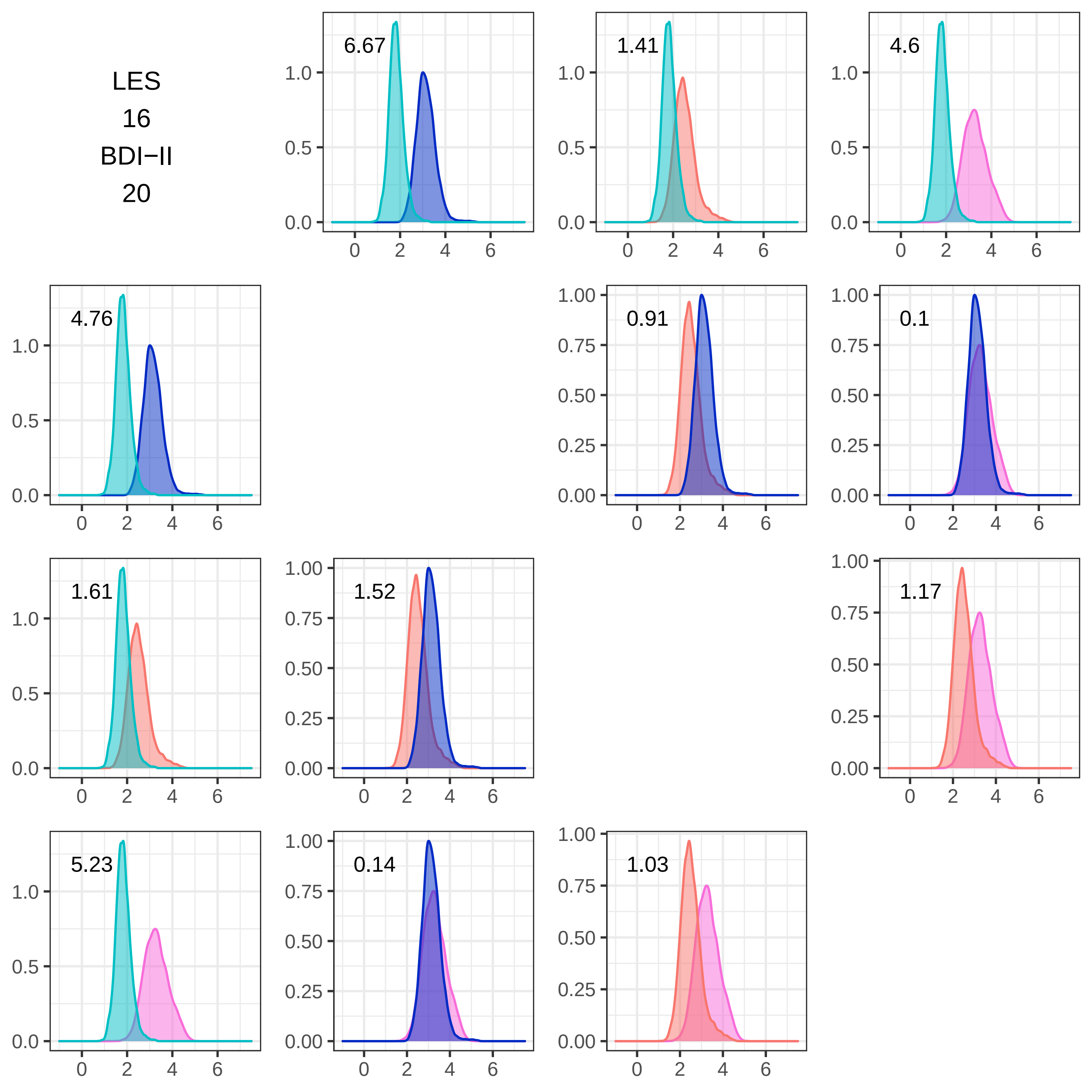

LES
16
BDI−II
20
6.67
1.41
4.6
4.76
0.91
0.1
1.61
1.52
1.17
5.23
0.14
1.03

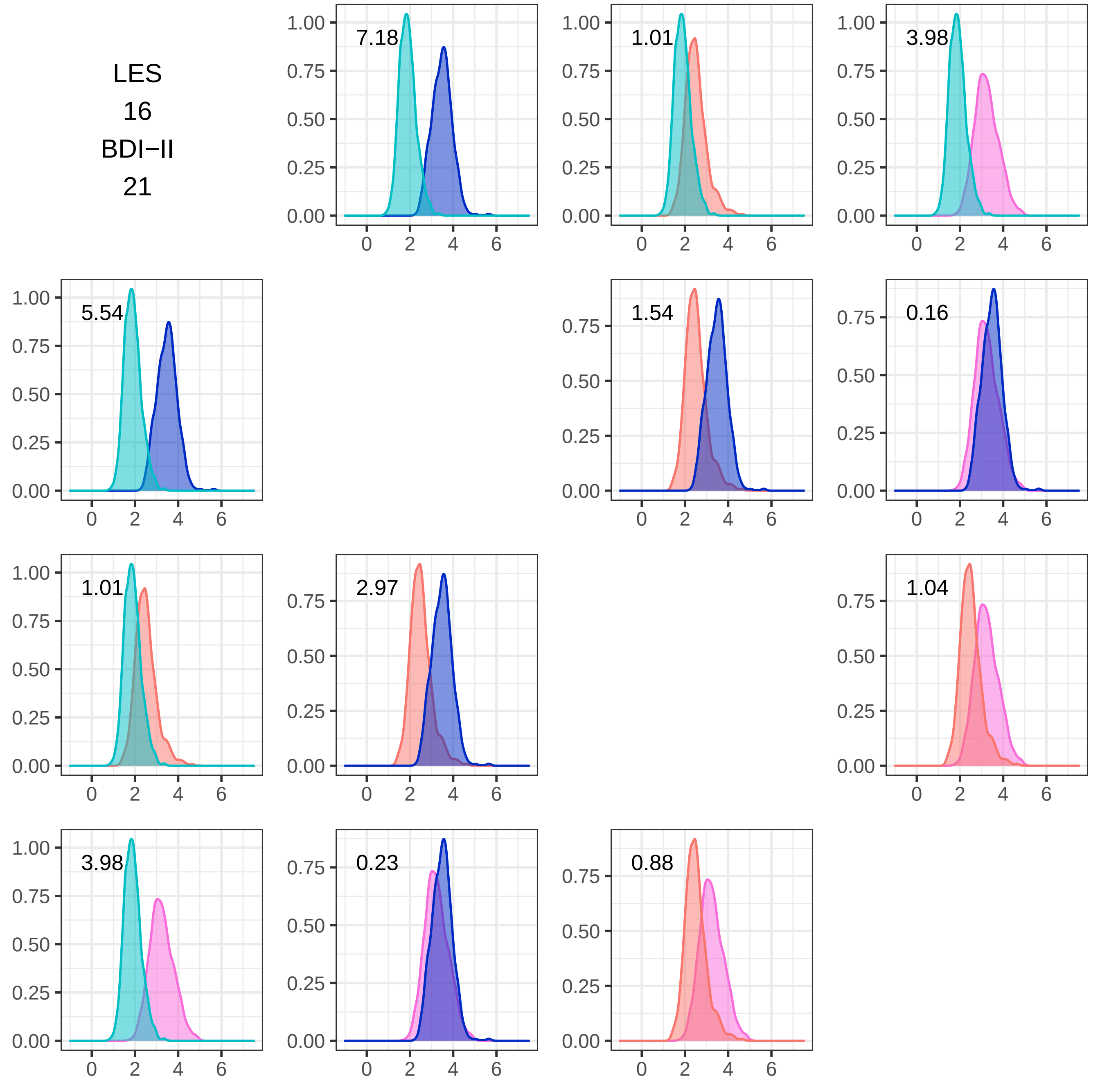
LES
16
BDI−II
21
7.18
1.01
3.98
5.54
1.54
0.16
1.01
2.97
1.04
3.98
0.23
0.88

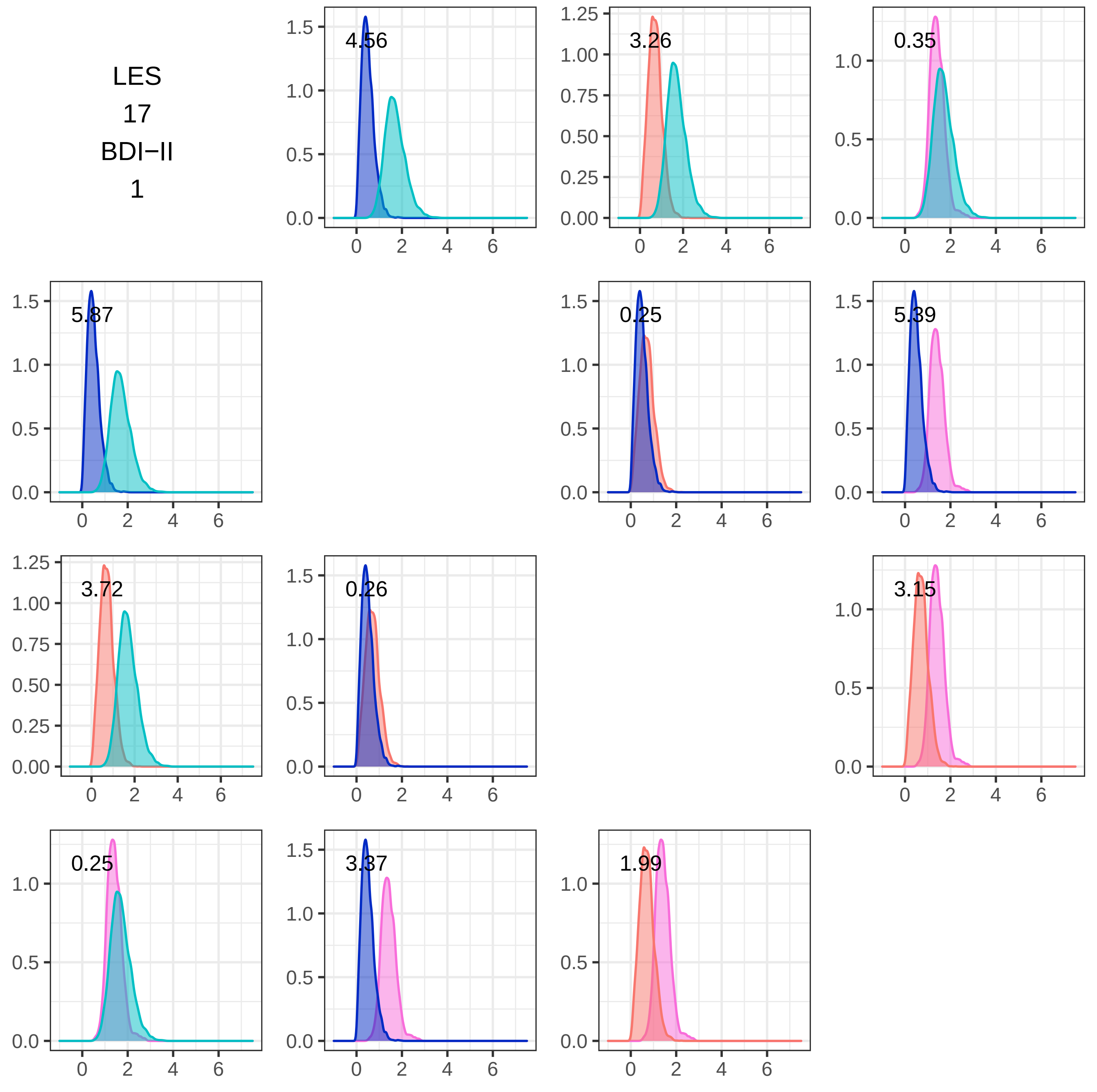
LES
17
BDI−II
1
4.56
3.26
0.35
5.87
0.25
5.39
3.72
0.26
3.15
0.25
3.37
1.99

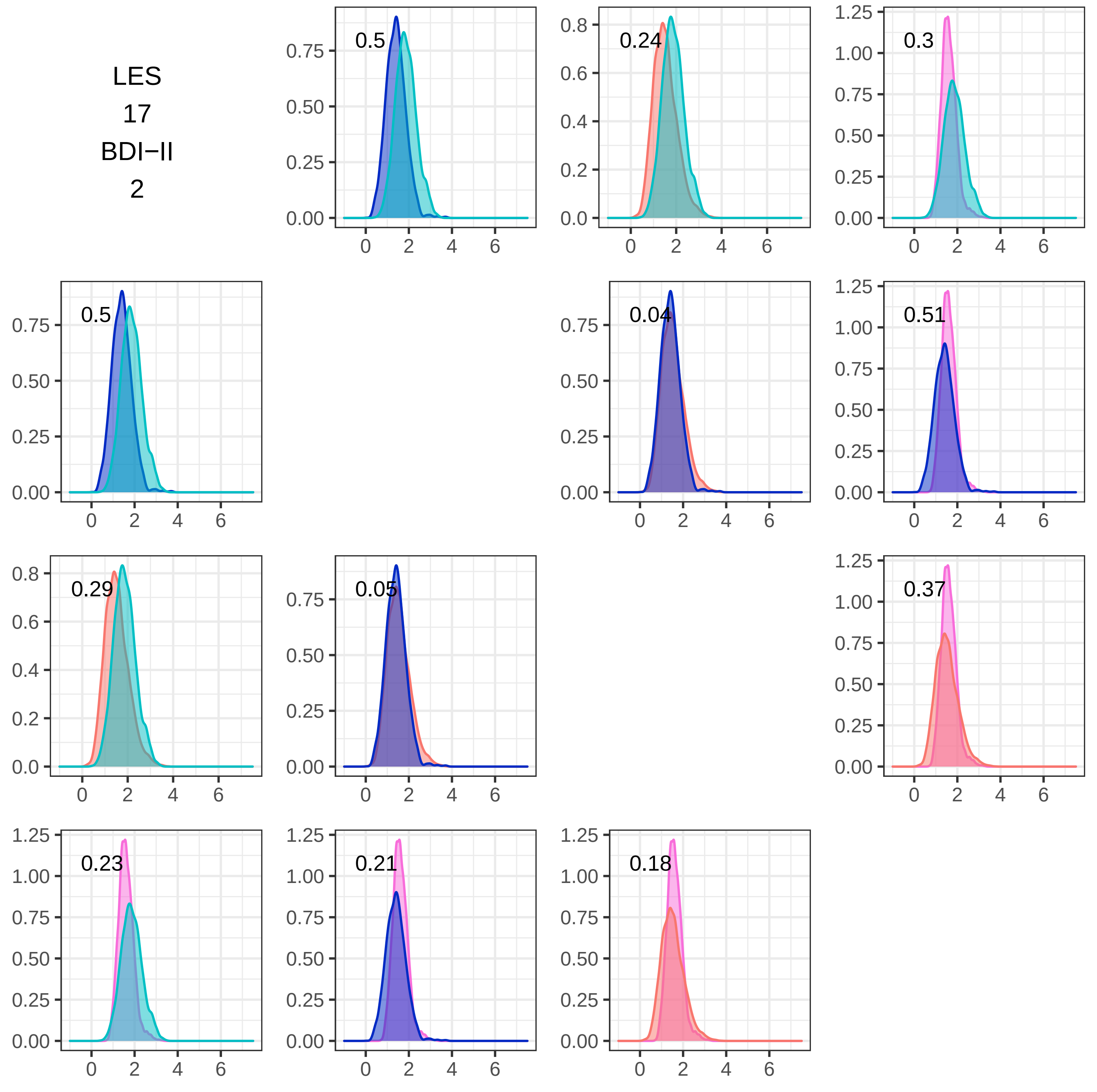

LES
17
BDI−II
2
0.5
0.24
0.3
0.5
0.04
0.51
0.29
0.05
0.37
0.23
0.21
0.18

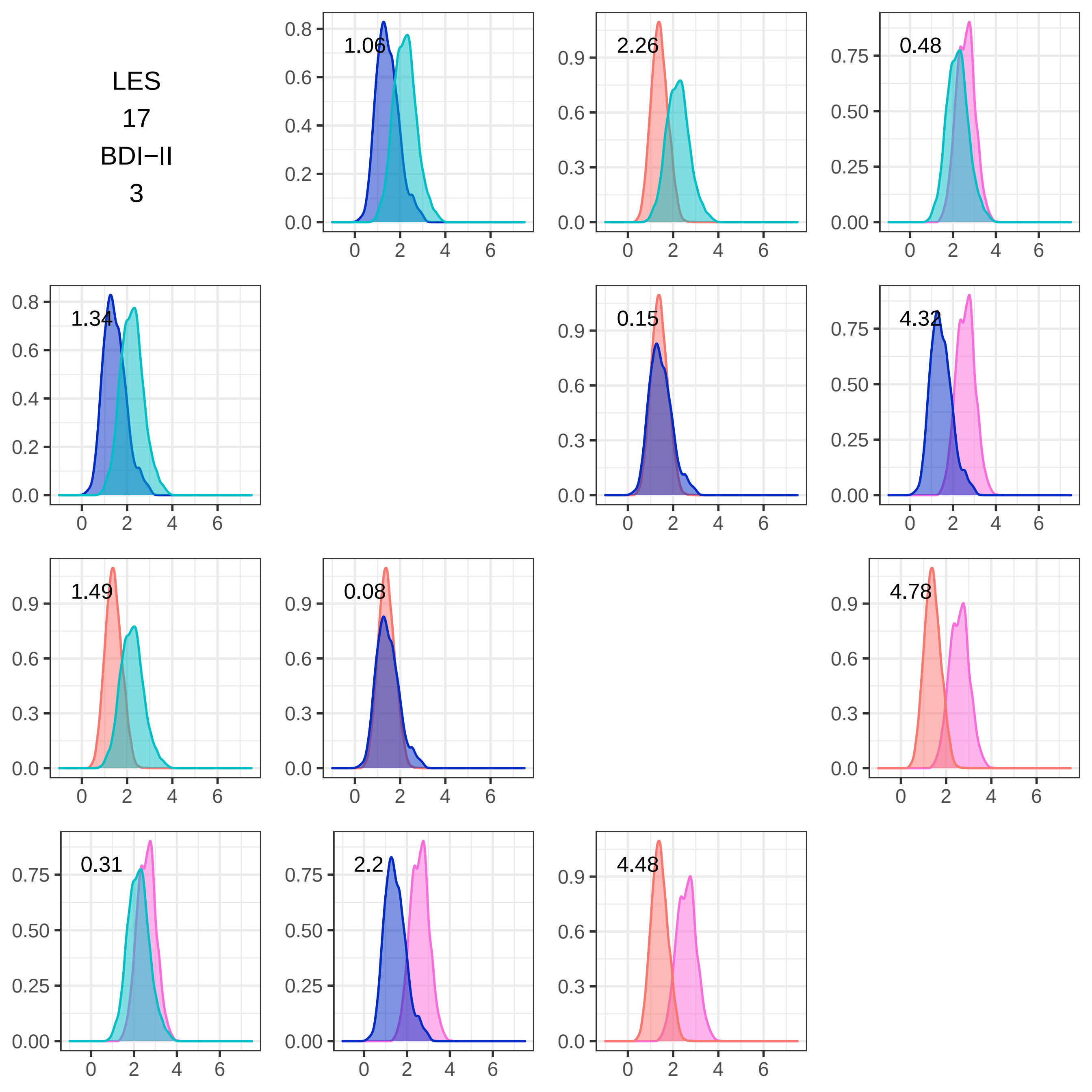
LES
17
BDI−II
3
1.06
2.26
0.48
1.34
0.15
4.32
1.49
0.08
4.78
0.31
2.2
4.48

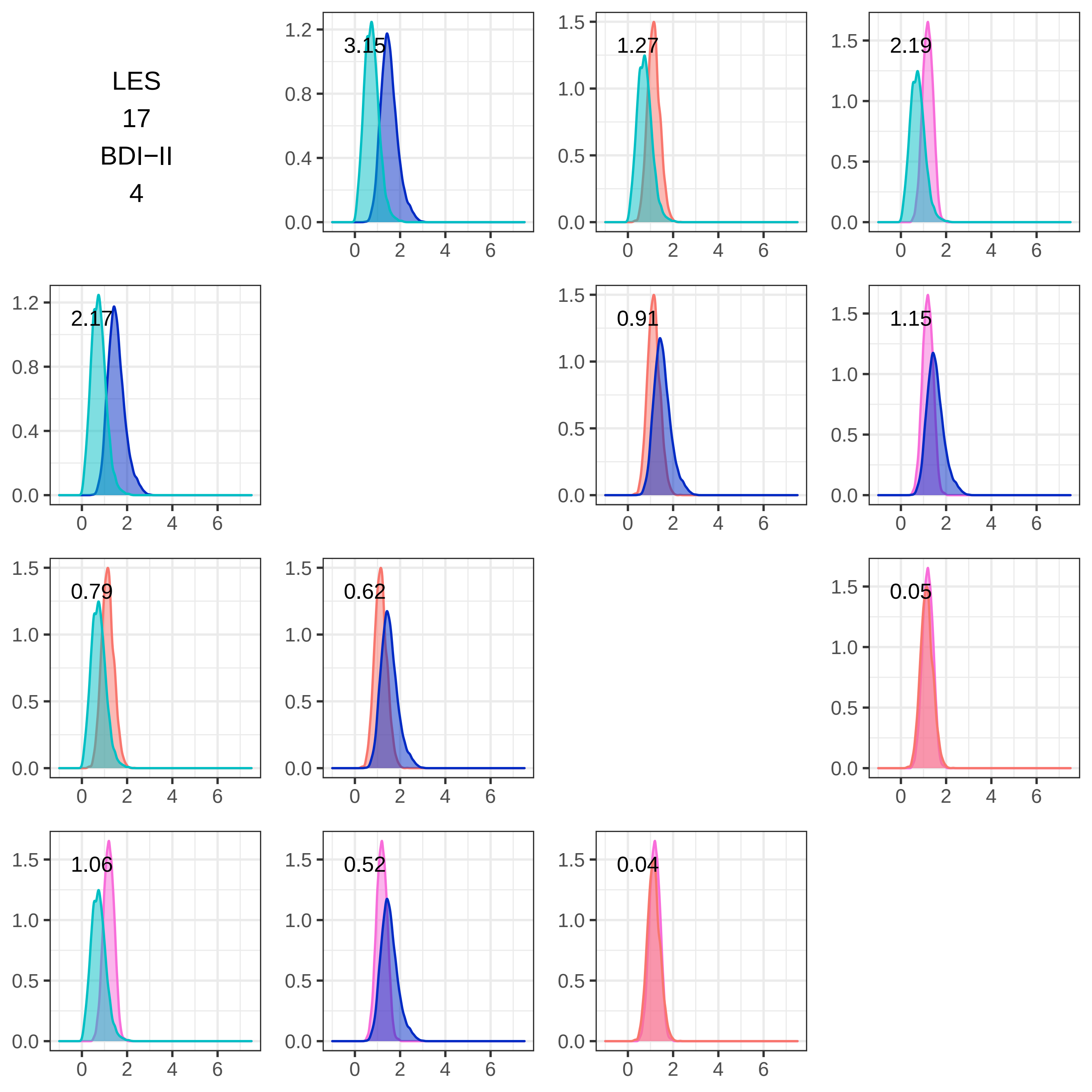
LES
17
BDI−II
4
3.15
1.27
2.19
2.17
0.91
1.15
0.79
0.62
0.05
1.06
0.52
0.04

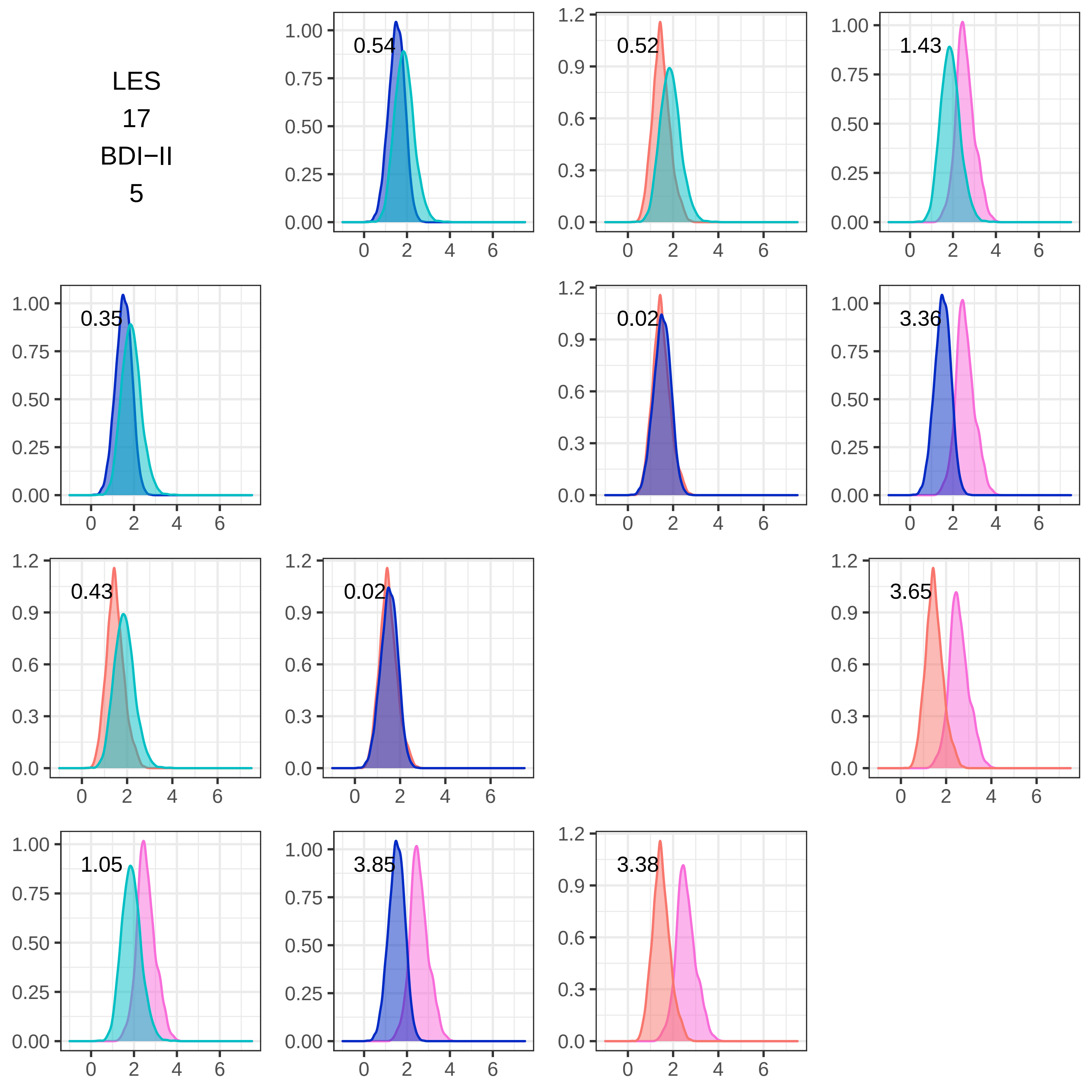

LES
17
BDI−II
5
0.54
0.52
1.43
0.35
0.02
3.36
0.43
0.02
3.65
1.05
3.85
3.38

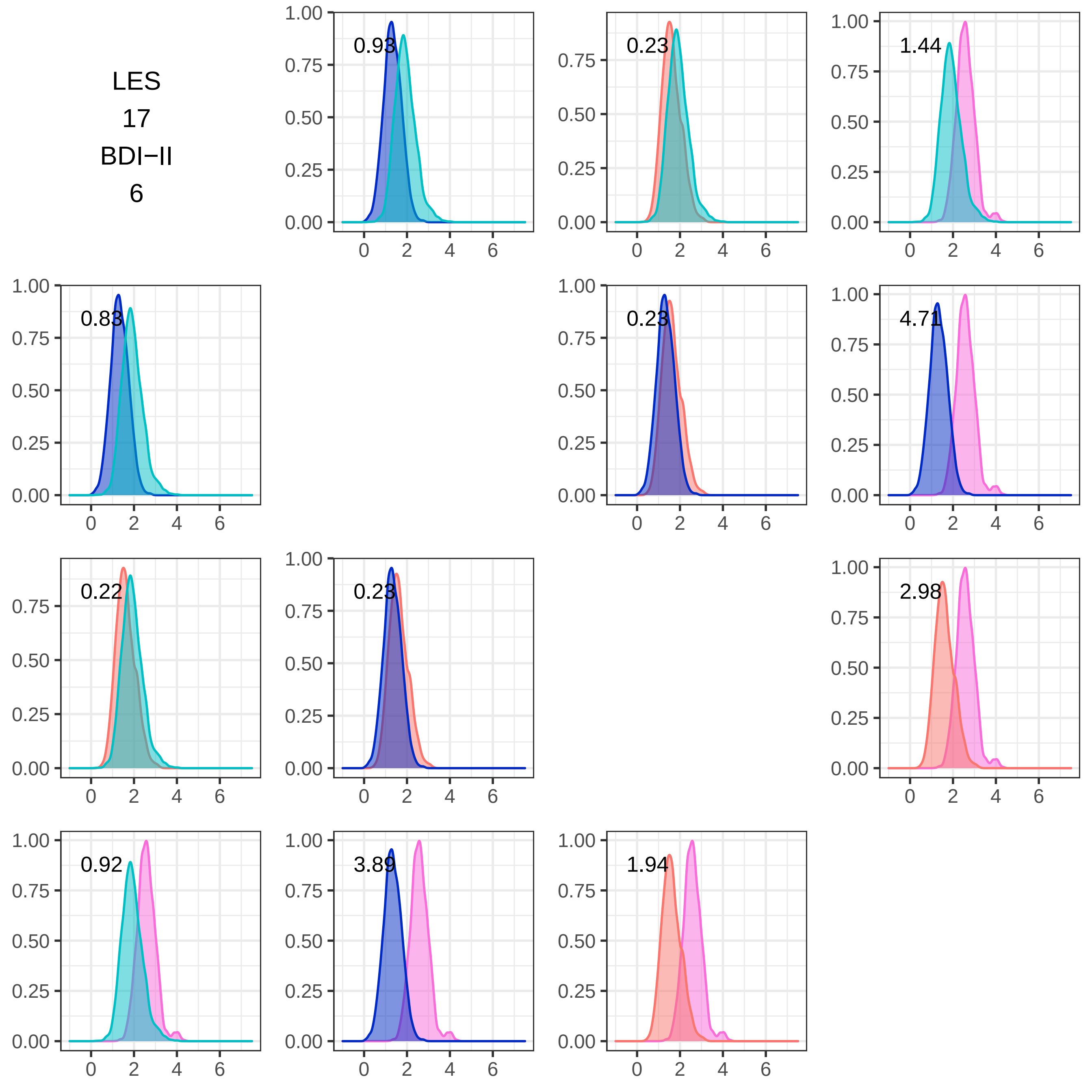
LES
17
BDI−II
6
0.93
0.23
1.44
0.83
0.23
4.71
0.22
0.23
2.98
0.92
3.89
1.94

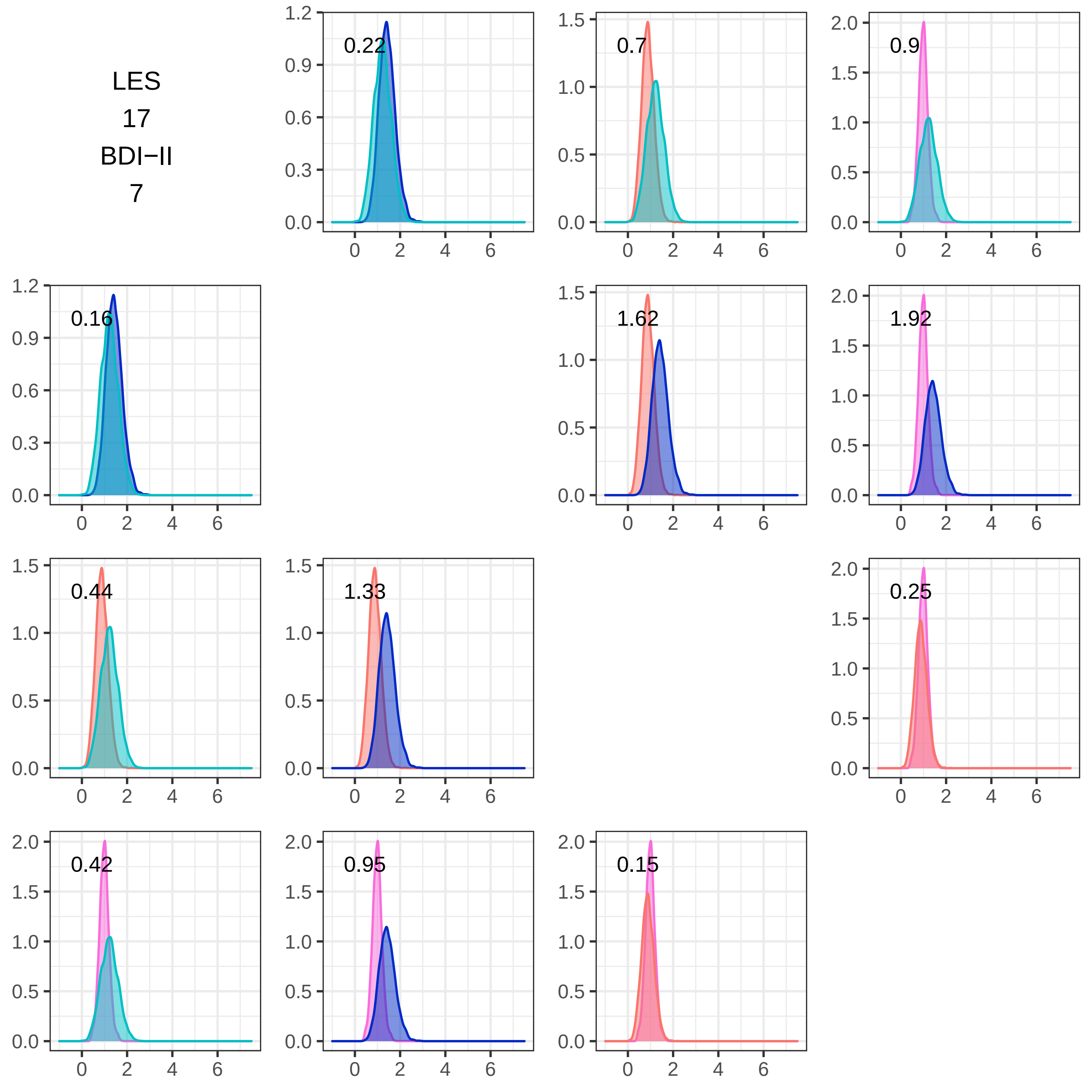
LES
17
BDI−II
7
0.22
0.7
0.9
0.16
1.62
1.92
0.44
1.33
0.25
0.42
0.95
0.15

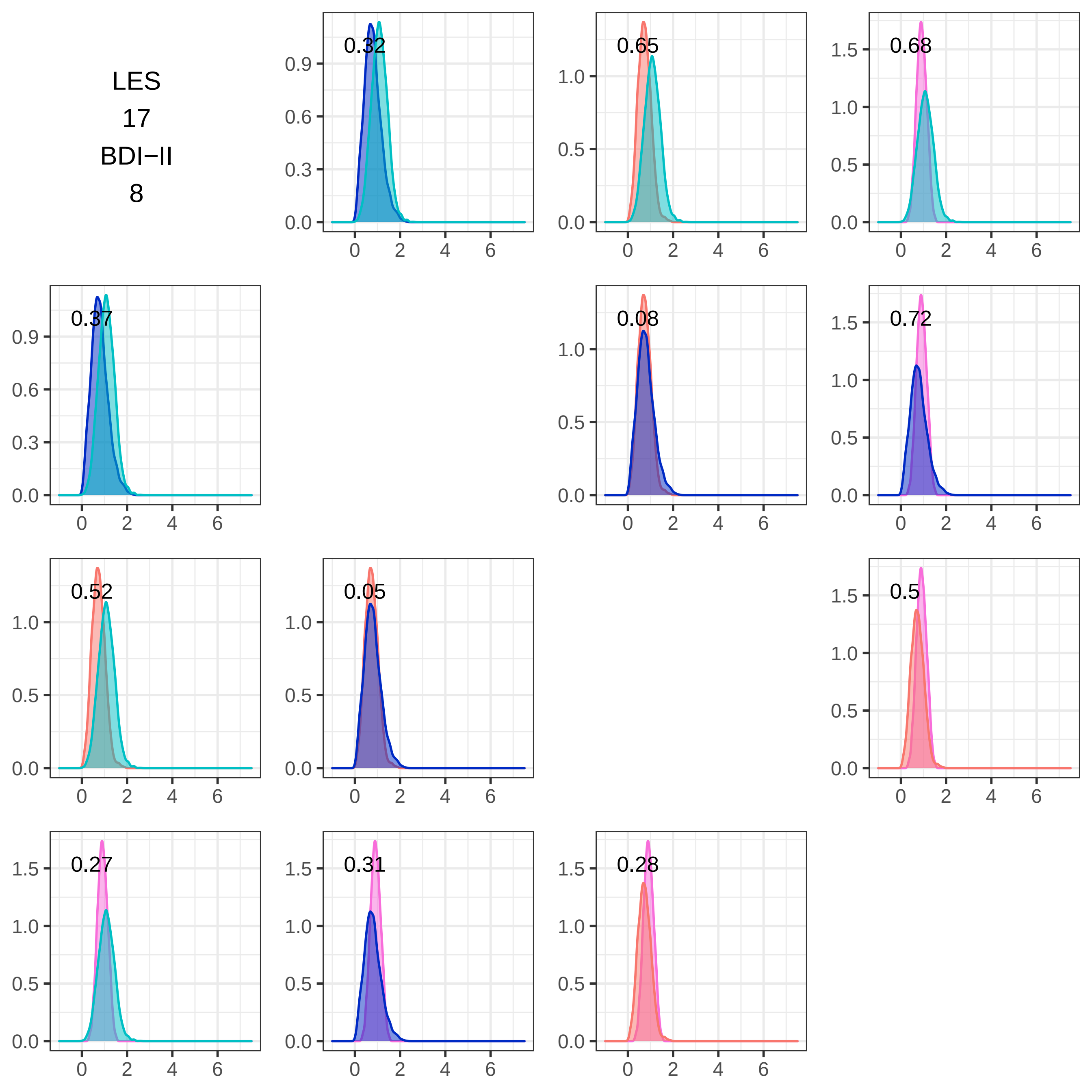

LES
17
BDI−II
8
0.32
0.65
0.68
0.37
0.08
0.72
0.52
0.05
0.5
0.27
0.31
0.28

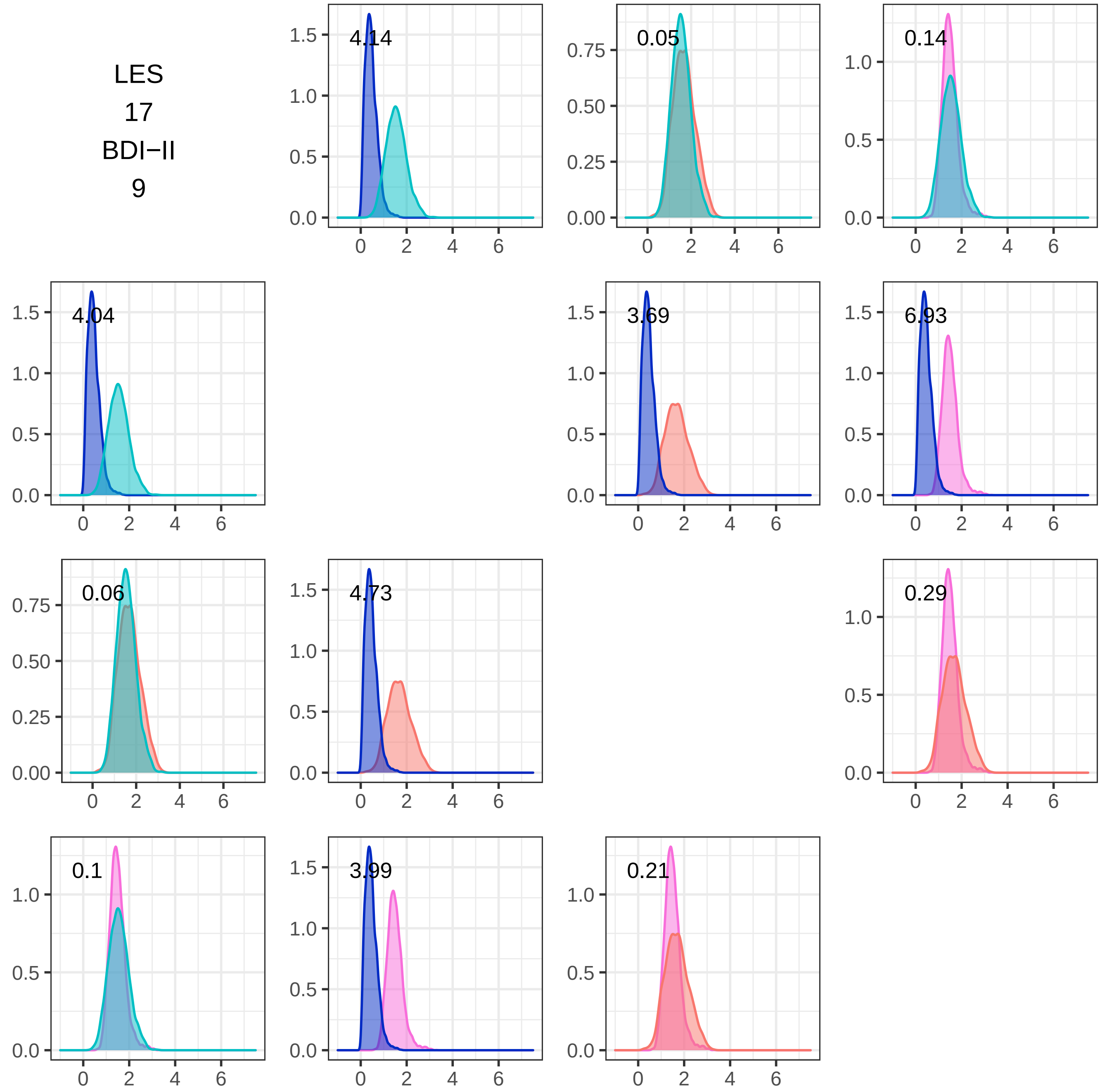

LES
17
BDI−II
9
4.14
0.05
0.14
4.04
3.69
6.93
0.06
4.73
0.29
0.1
3.99
0.21

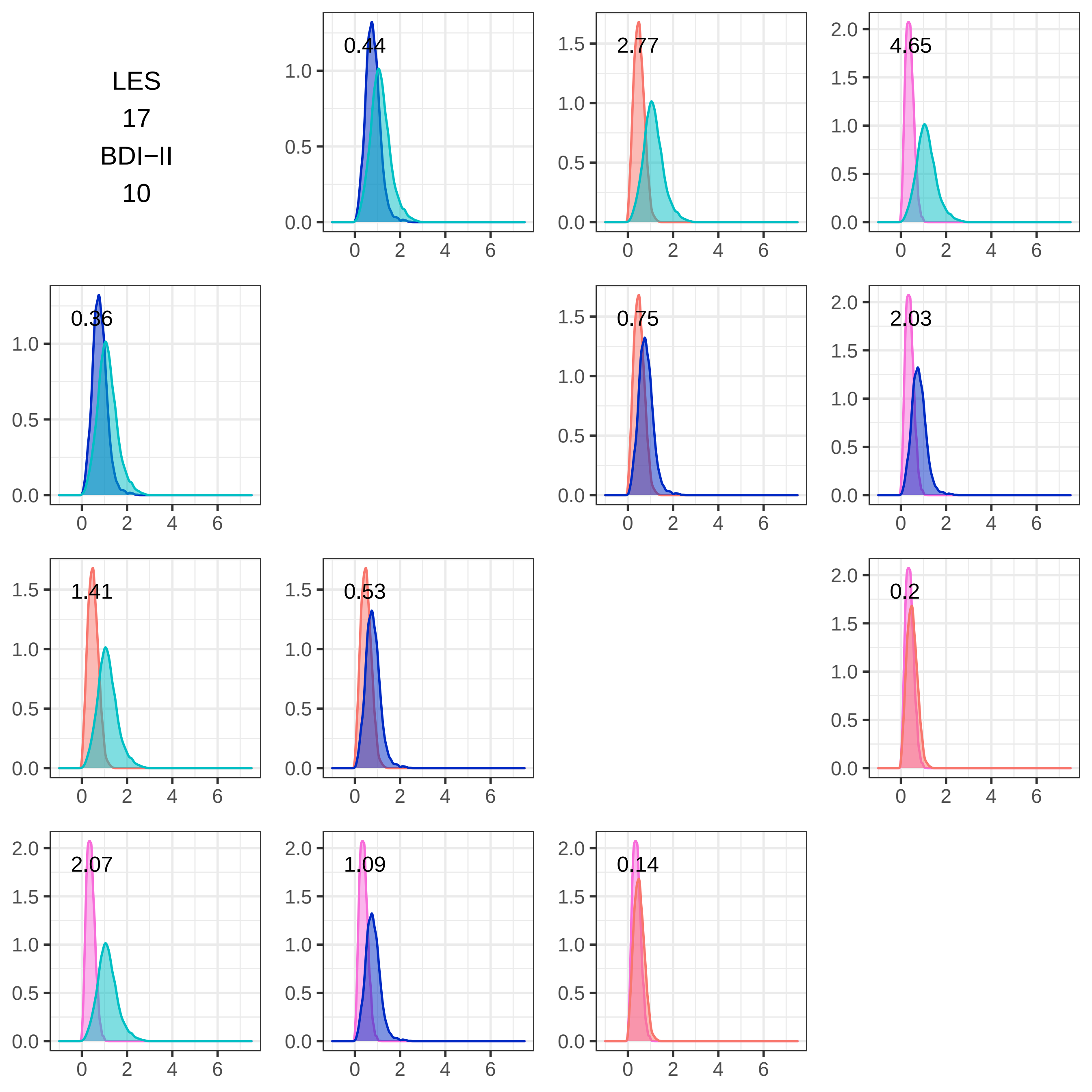

LES
17
BDI−II
10
0.44
2.77
4.65
0.36
0.75
2.03
1.41
0.53
0.2
2.07
1.09
0.14

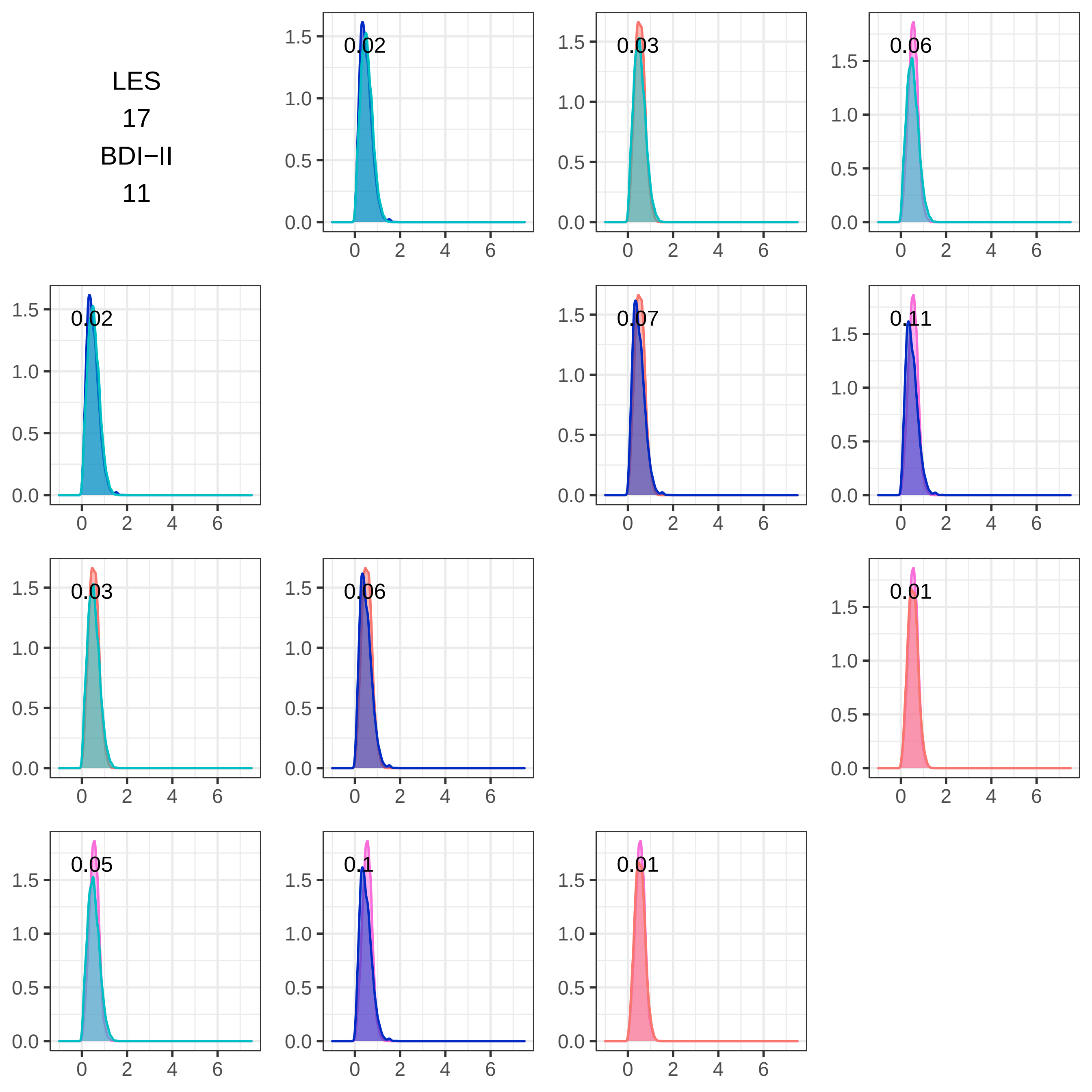

LES
17
BDI−II
11
0.02
0.03
0.06
0.02
0.07
0.11
0.03
0.06
0.01
0.05
0.1
0.01

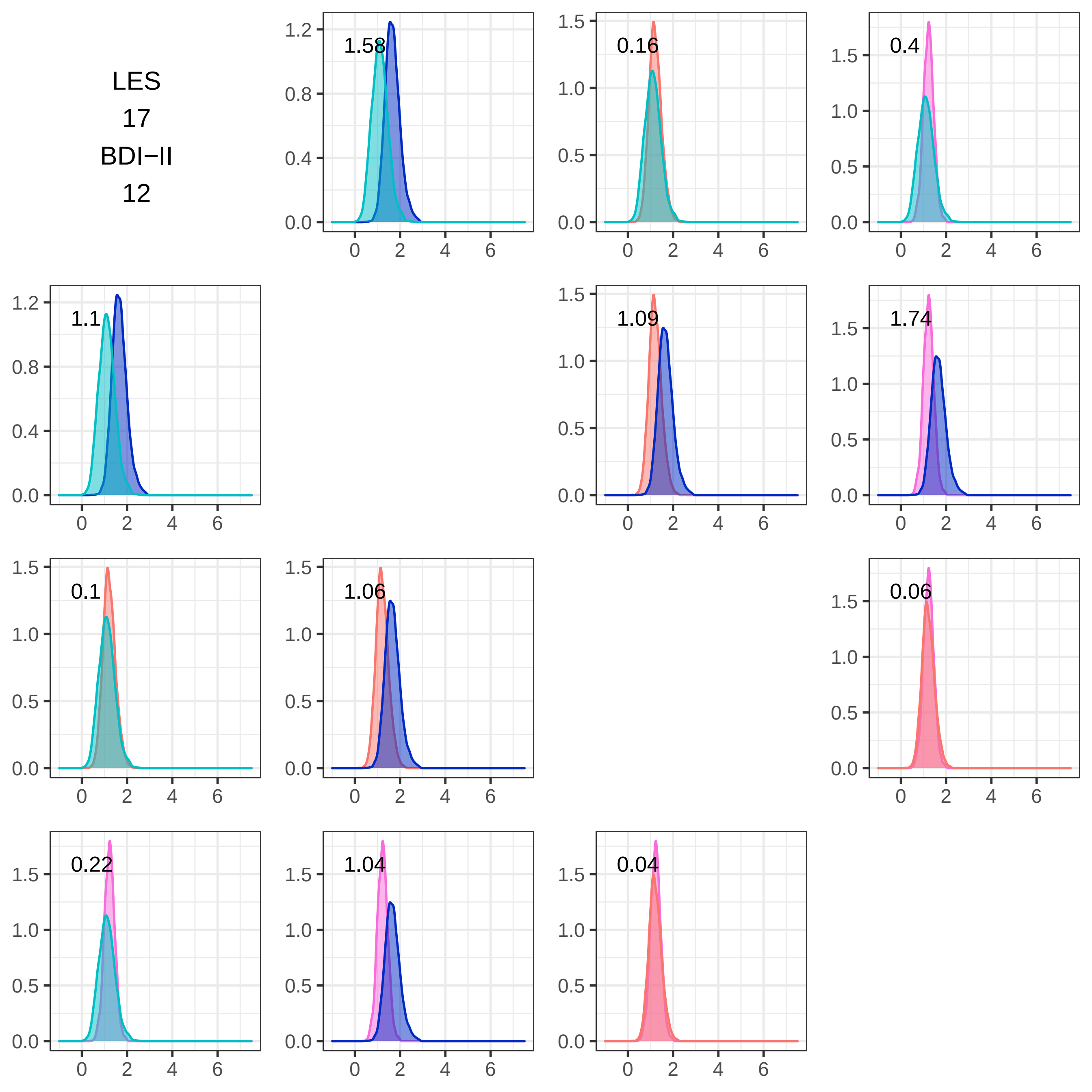
LES
17
BDI−II
12
1.58
0.16
0.4
1.1
1.09
1.74
0.1
1.06
0.06
0.22
1.04
0.04

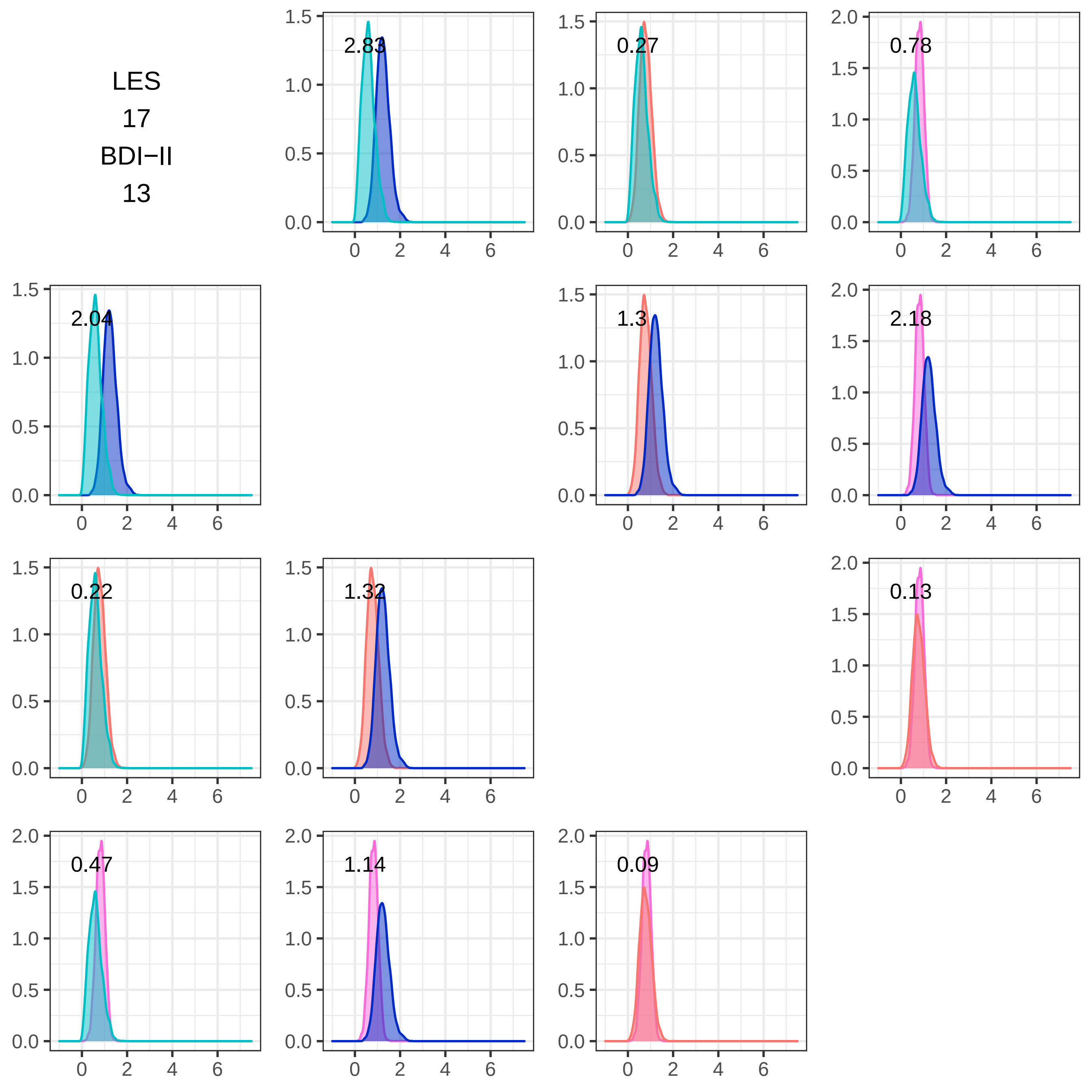
LES
17
BDI−II
13
2.83
0.27
0.78
2.04
1.3
2.18
0.22
1.32
0.13
0.47
1.14
0.09

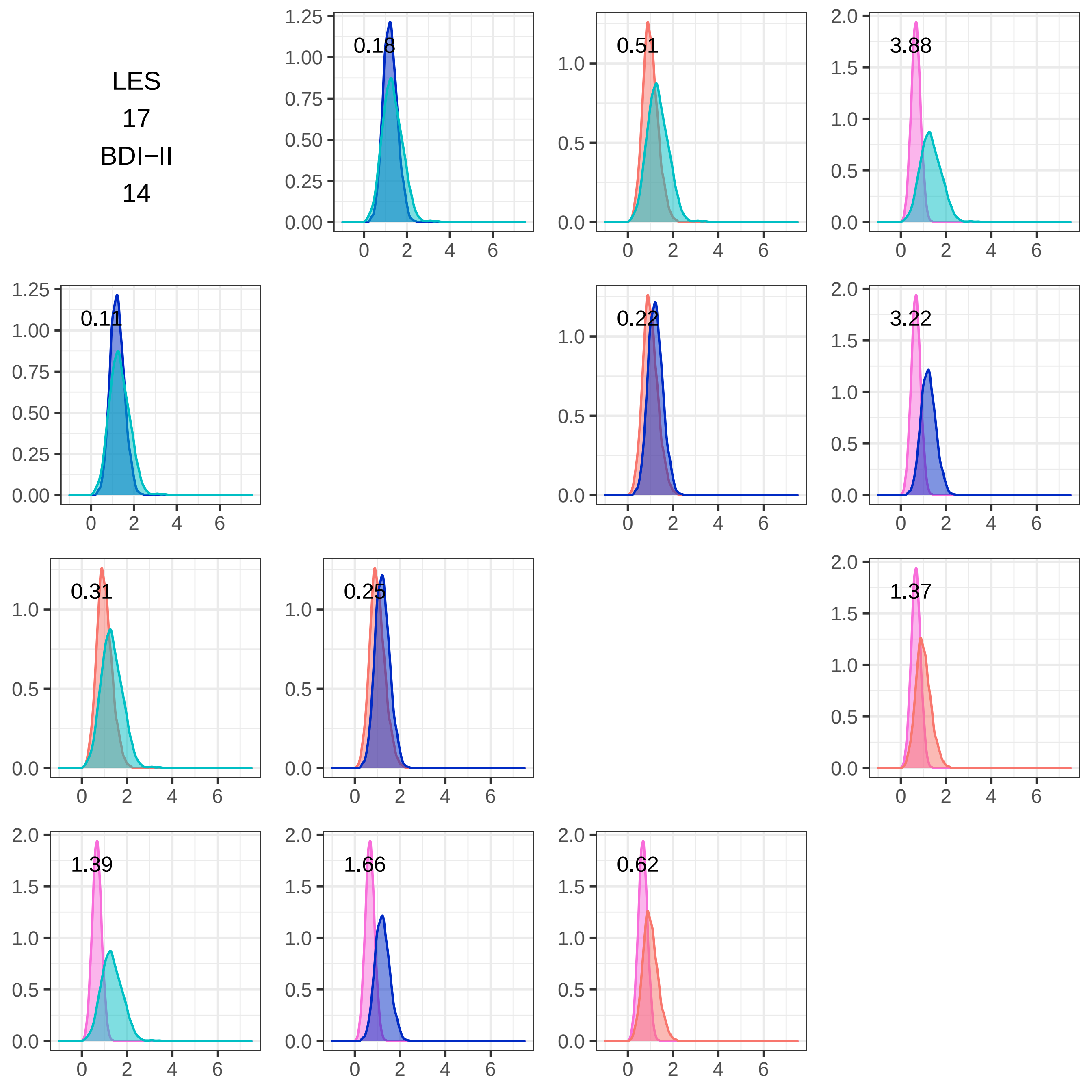

LES
17
BDI−II
14
0.18
0.51
3.88
0.11
0.22
3.22
0.31
0.25
1.37
1.39
1.66
0.62

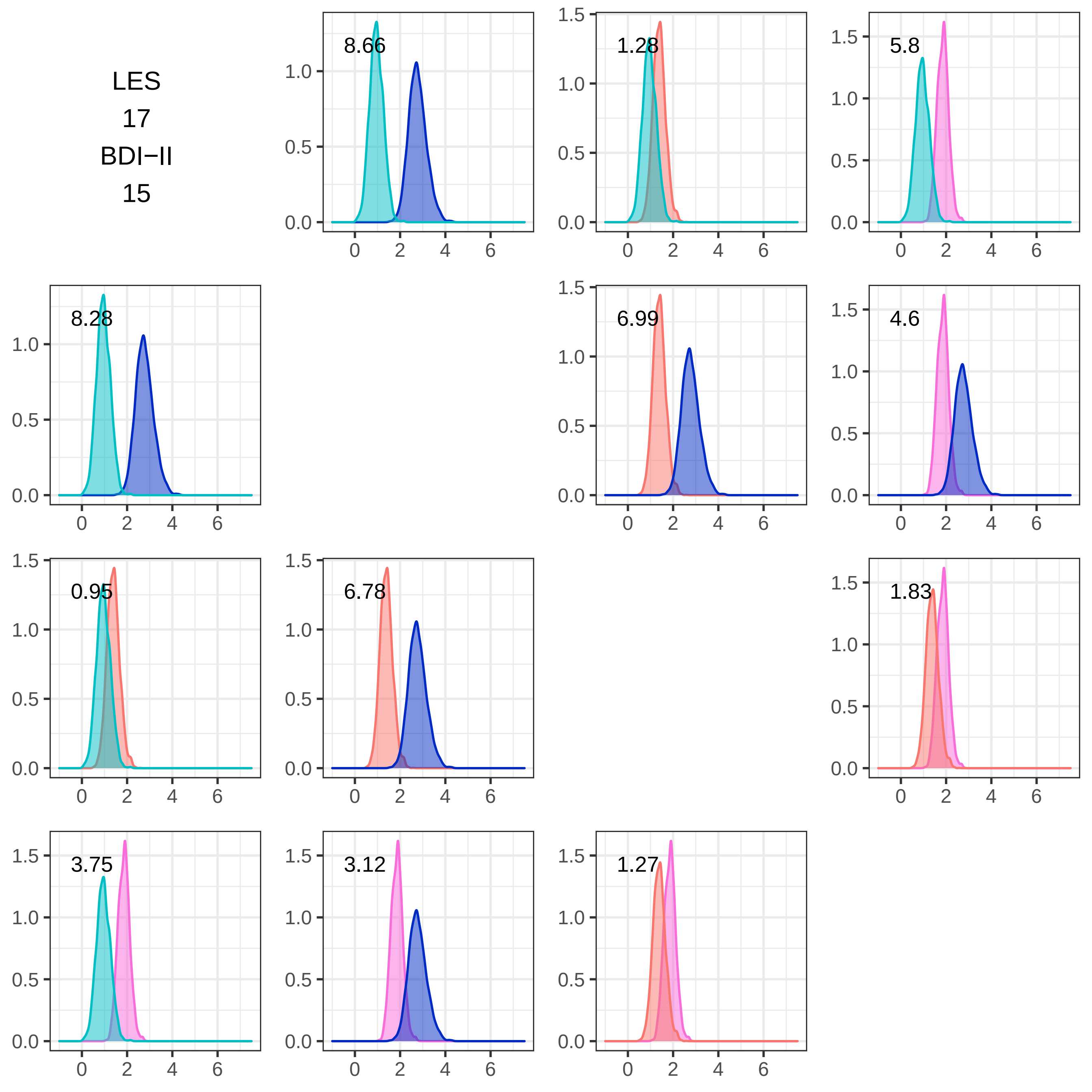
LES
17
BDI−II
15
8.66
1.28
5.8
8.28
6.99
4.6
0.95
6.78
1.83
3.75
3.12
1.27

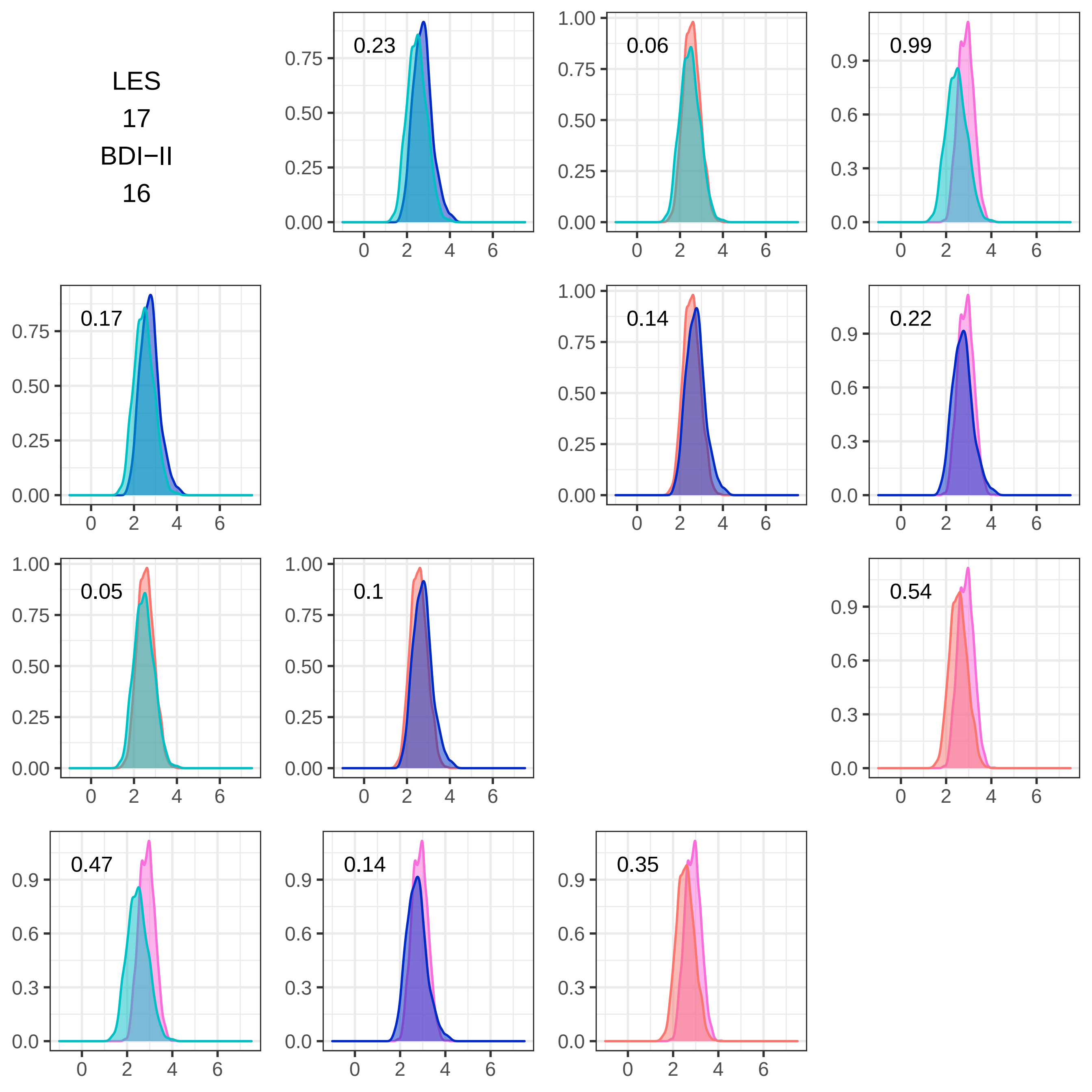

LES
17
BDI−II
16
0.23
0.06
0.99
0.17
0.14
0.22
0.05
0.1
0.54
0.47
0.14
0.35

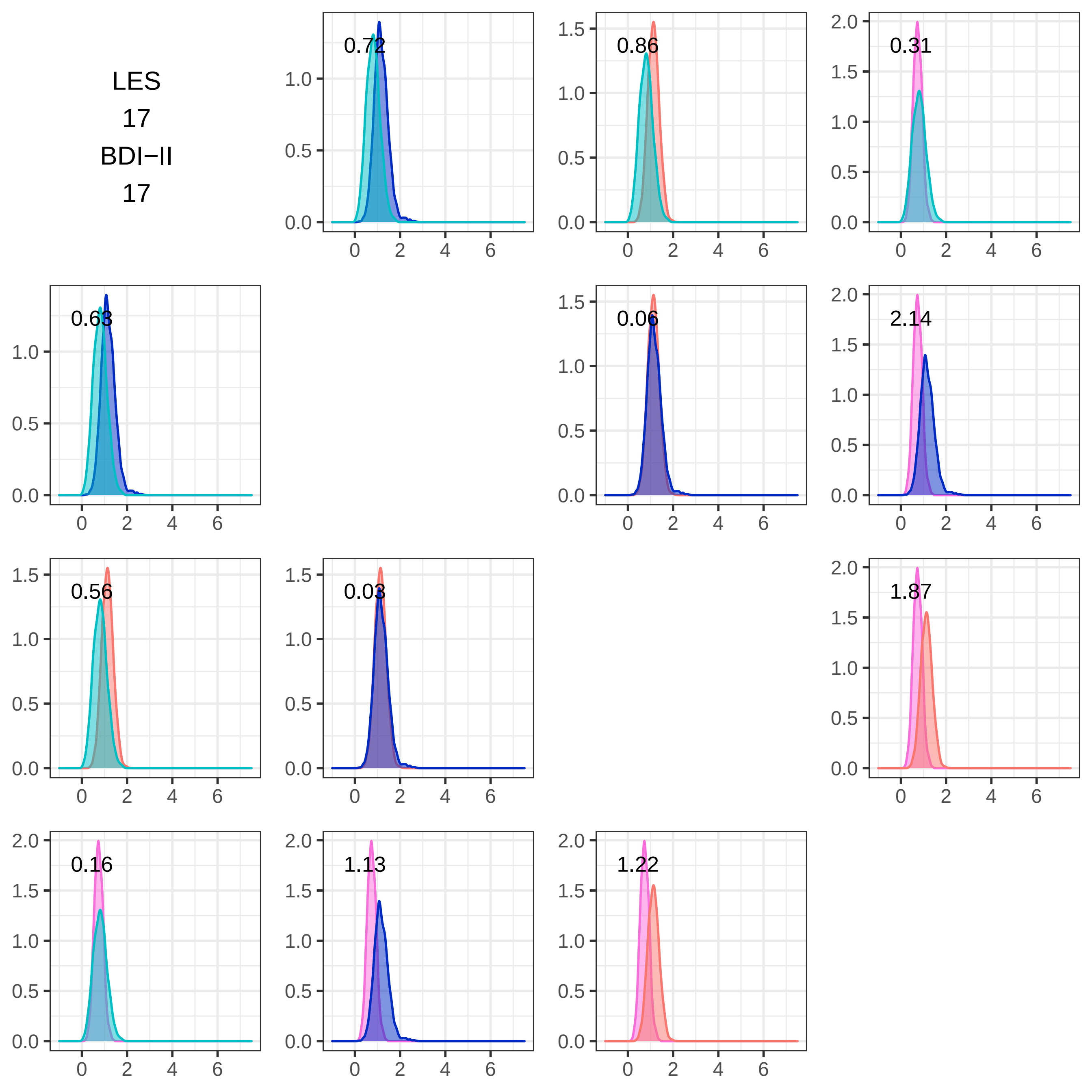

LES
17
BDI−II
17
0.72
0.86
0.31
0.63
0.06
2.14
0.56
0.03
1.87
0.16
1.13
1.22

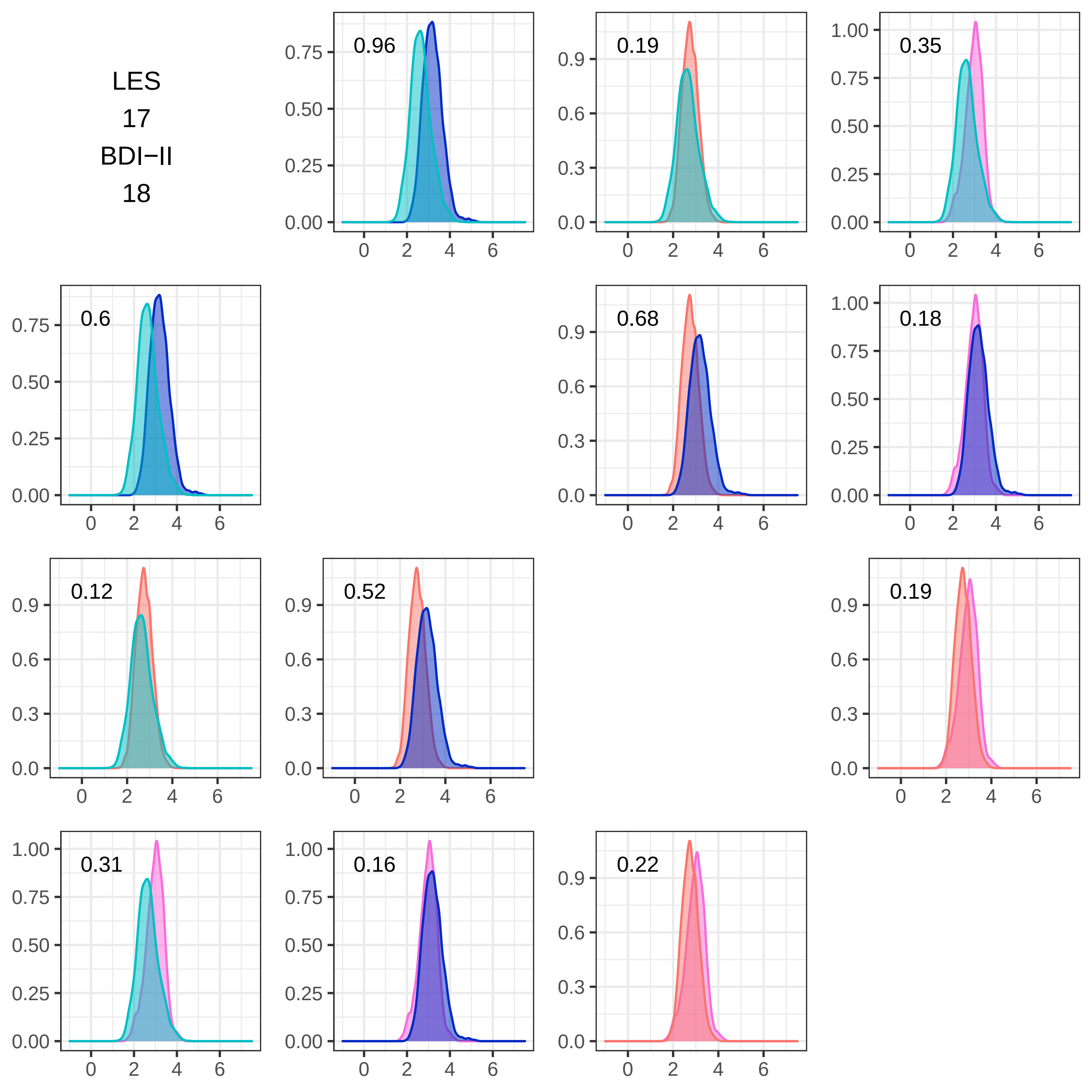

LES
17
BDI−II
18
0.96
0.19
0.35
0.6
0.68
0.18
0.12
0.52
0.19
0.31
0.16
0.22

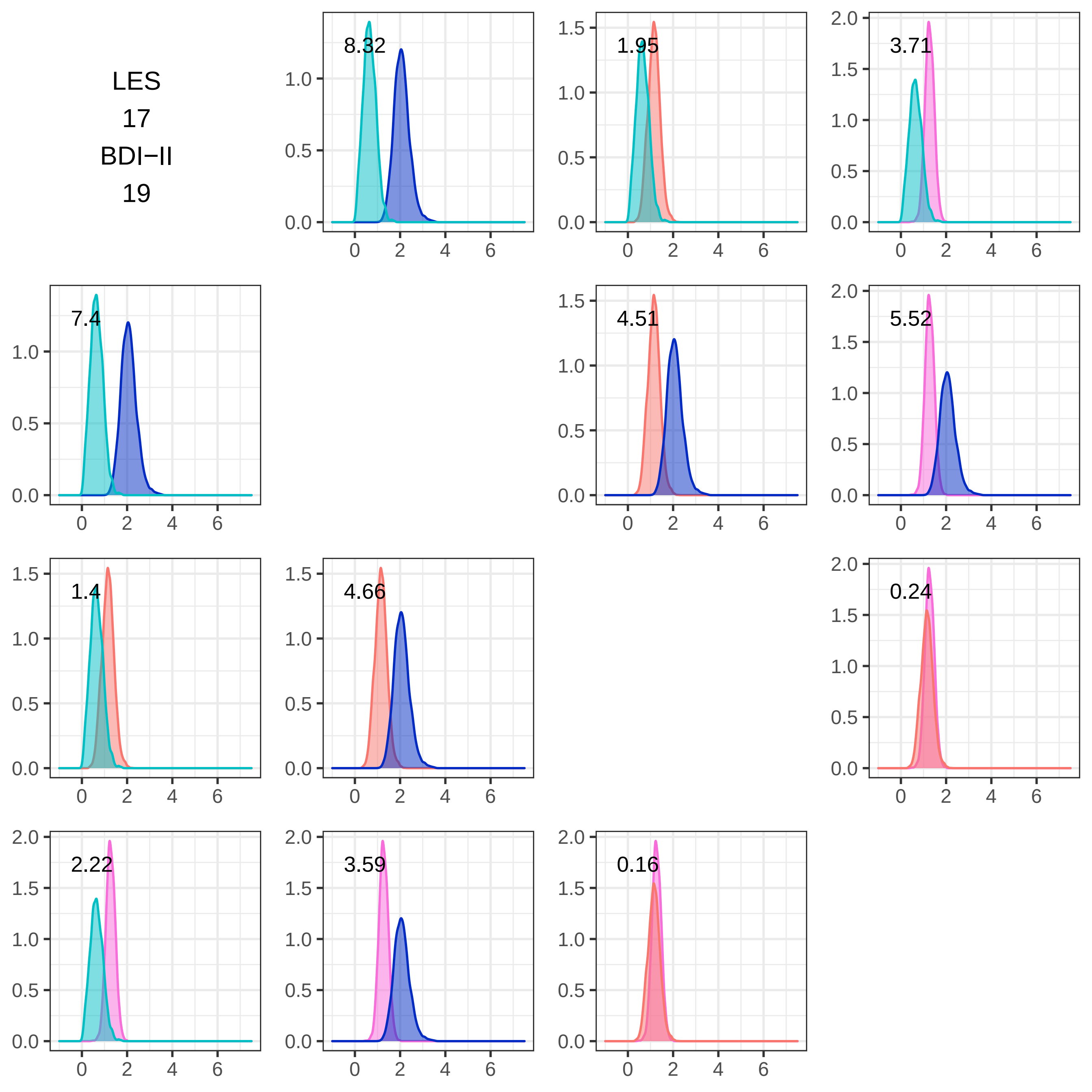

LES
17
BDI−II
19
8.32
1.95
3.71
7.4
4.51
5.52
1.4
4.66
0.24
2.22
3.59
0.16

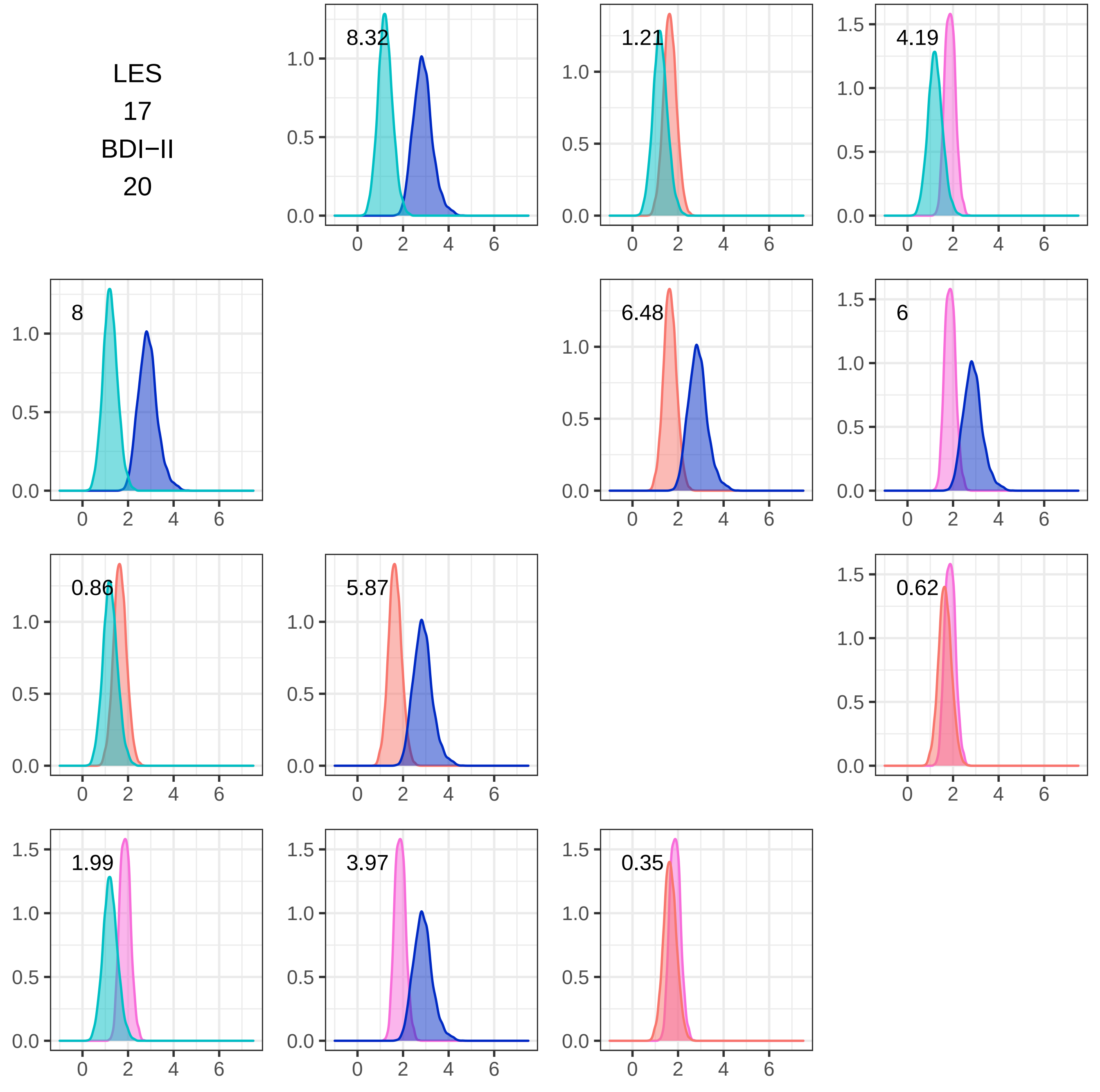

LES
17
BDI−II
20
8.32
1.21
4.19
8
6.48
6
0.86
5.87
0.62
1.99
3.97
0.35

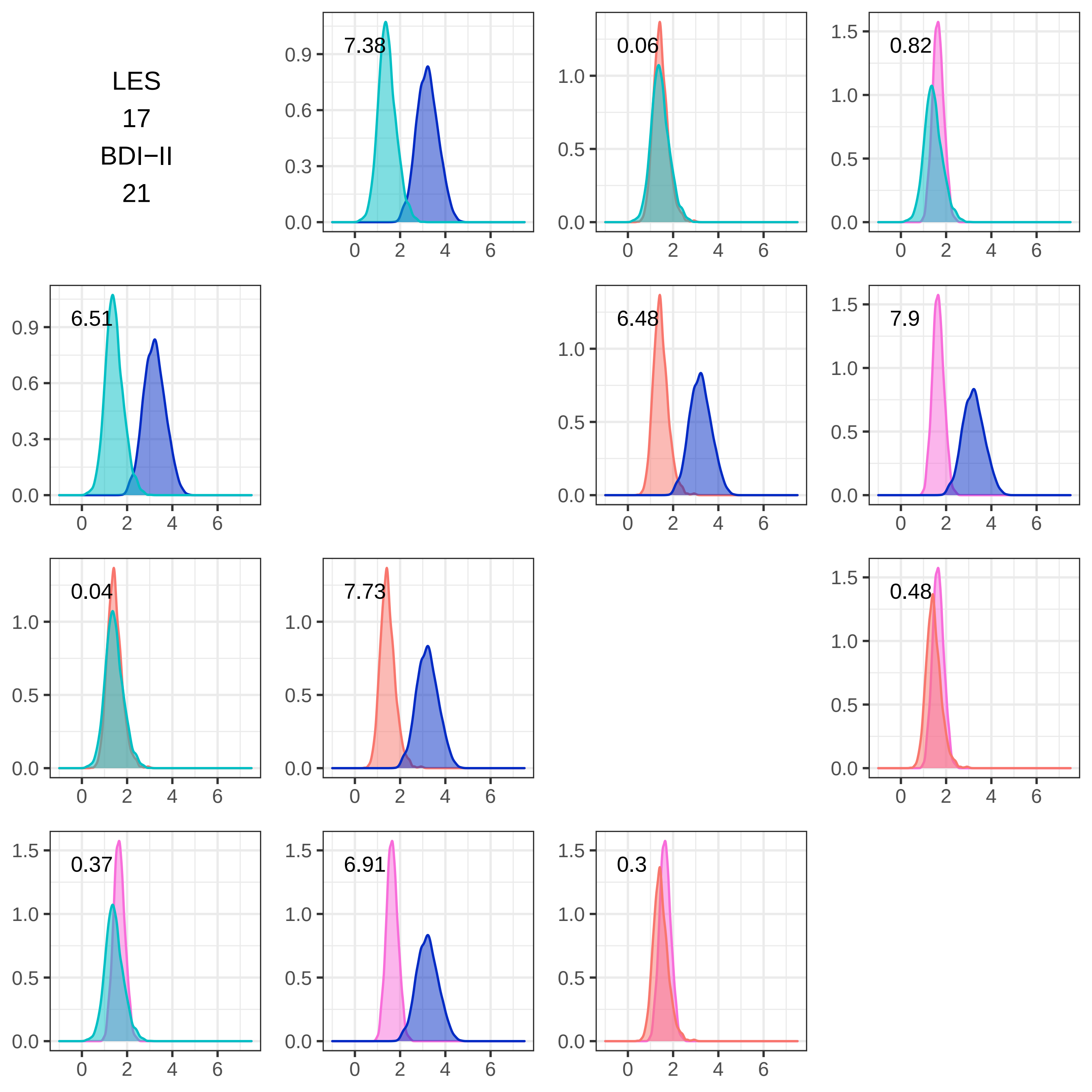

LES
17
BDI−II
21
7.38
0.06
0.82
6.51
6.48
7.9
0.04
7.73
0.48
0.37
6.91
0.3

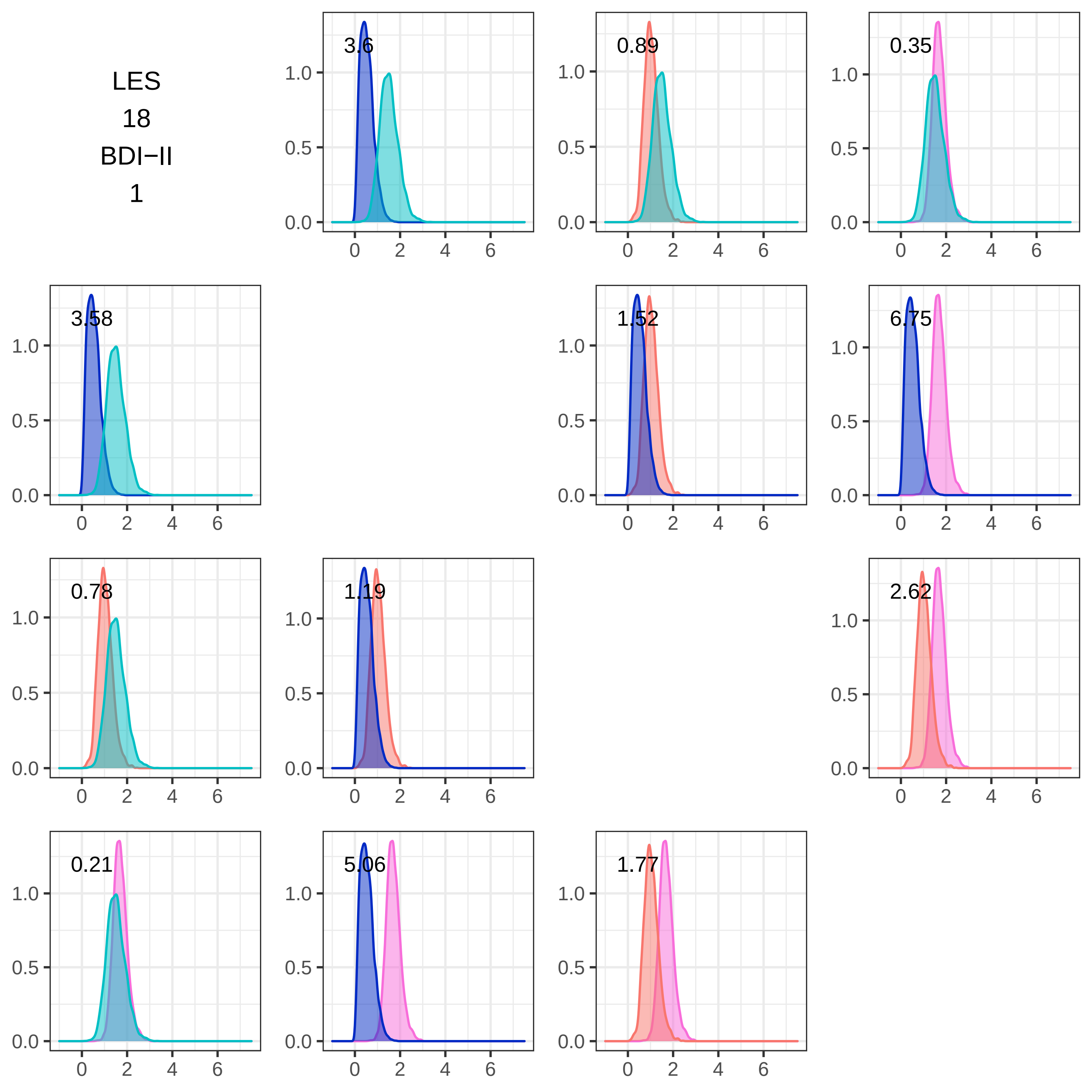
LES
18
BDI−II
1
3.6
0.89
0.35
3.58
1.52
6.75
0.78
1.19
2.62
0.21
5.06
1.77

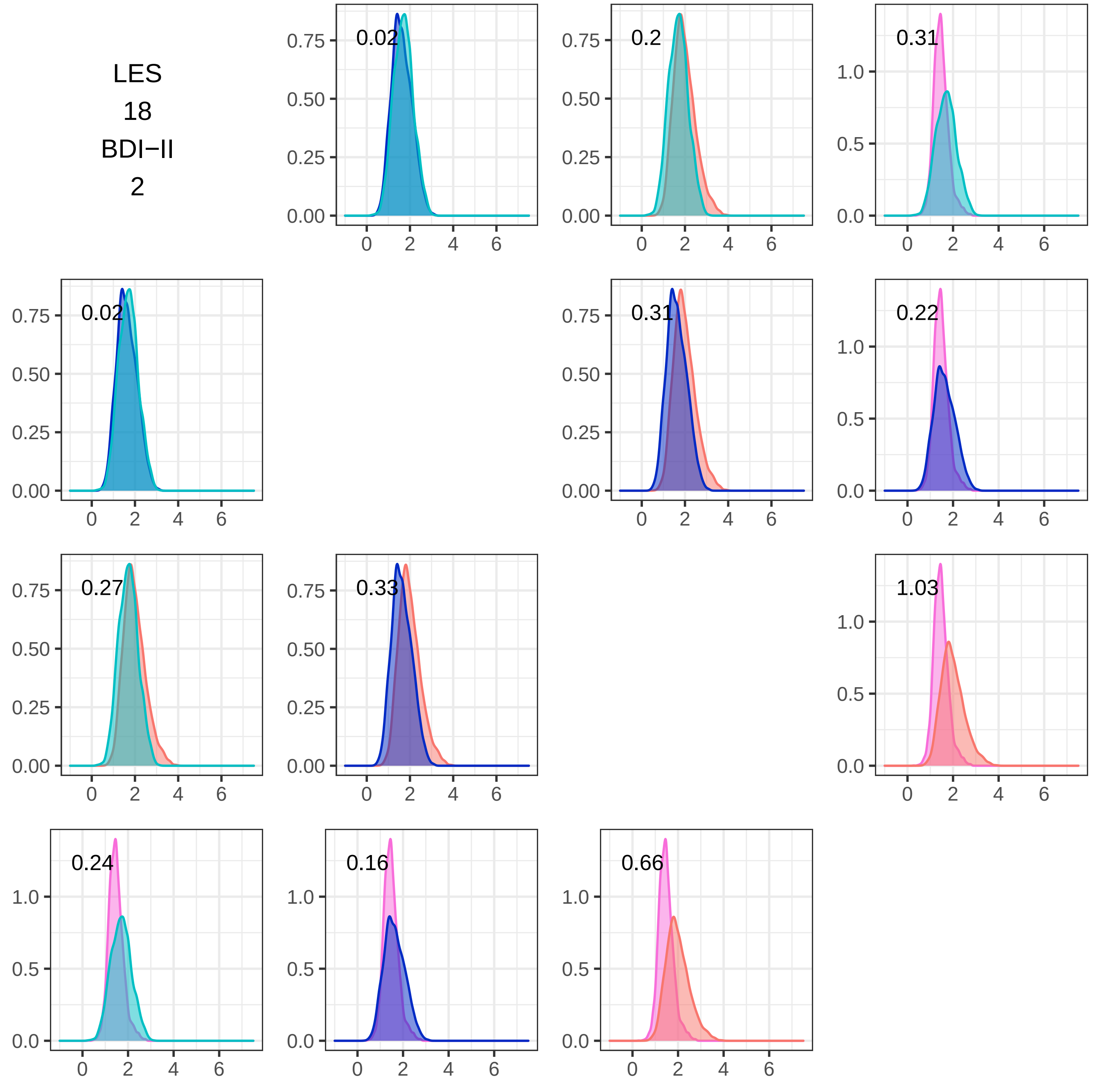

LES
18
BDI−II
2
0.02
0.2
0.31
0.02
0.31
0.22
0.27
0.33
1.03
0.24
0.16
0.66

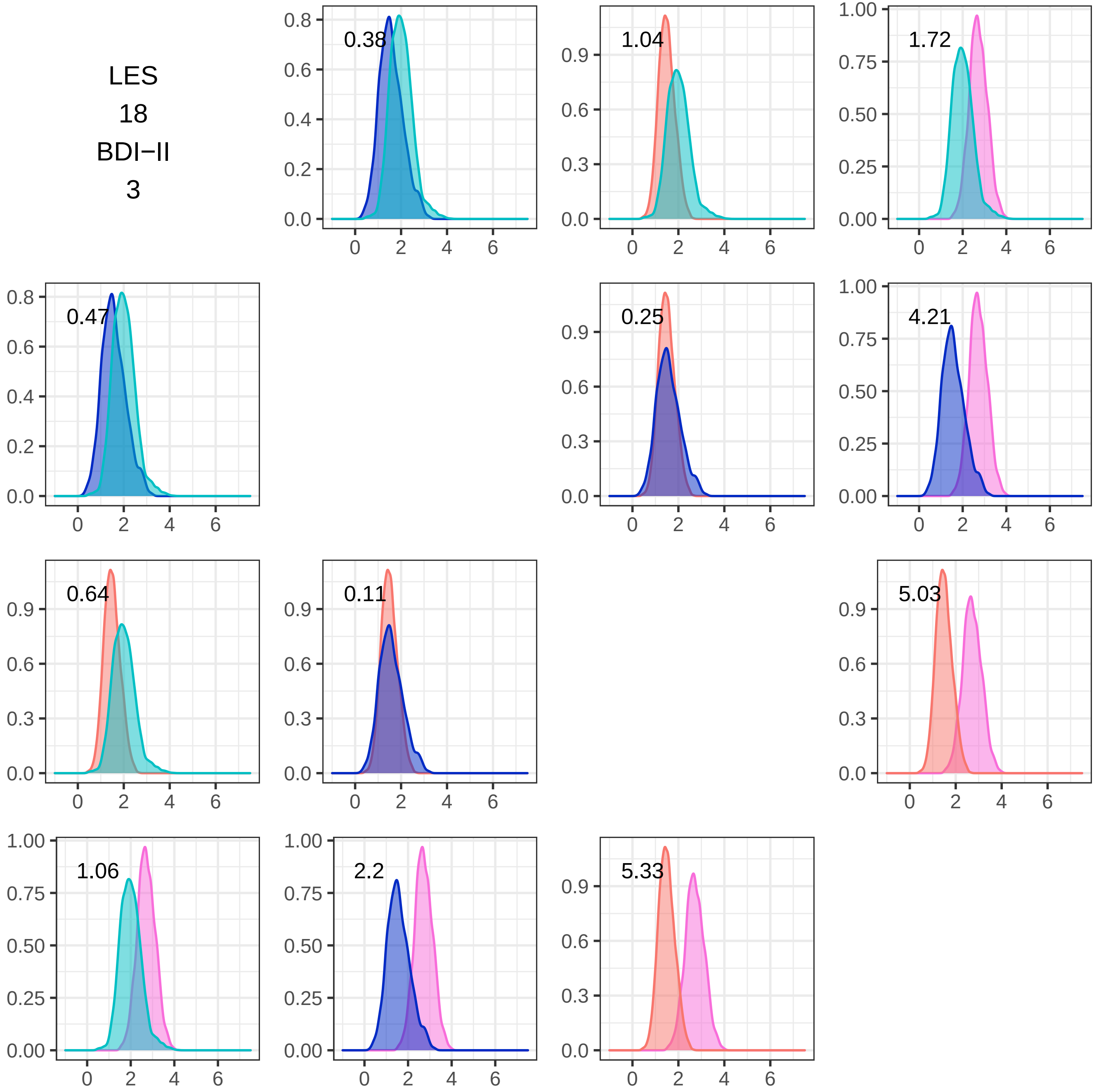
LES
18
BDI−II
3
0.38
1.04
1.72
0.47
0.25
4.21
0.64
0.11
5.03
1.06
2.2
5.33

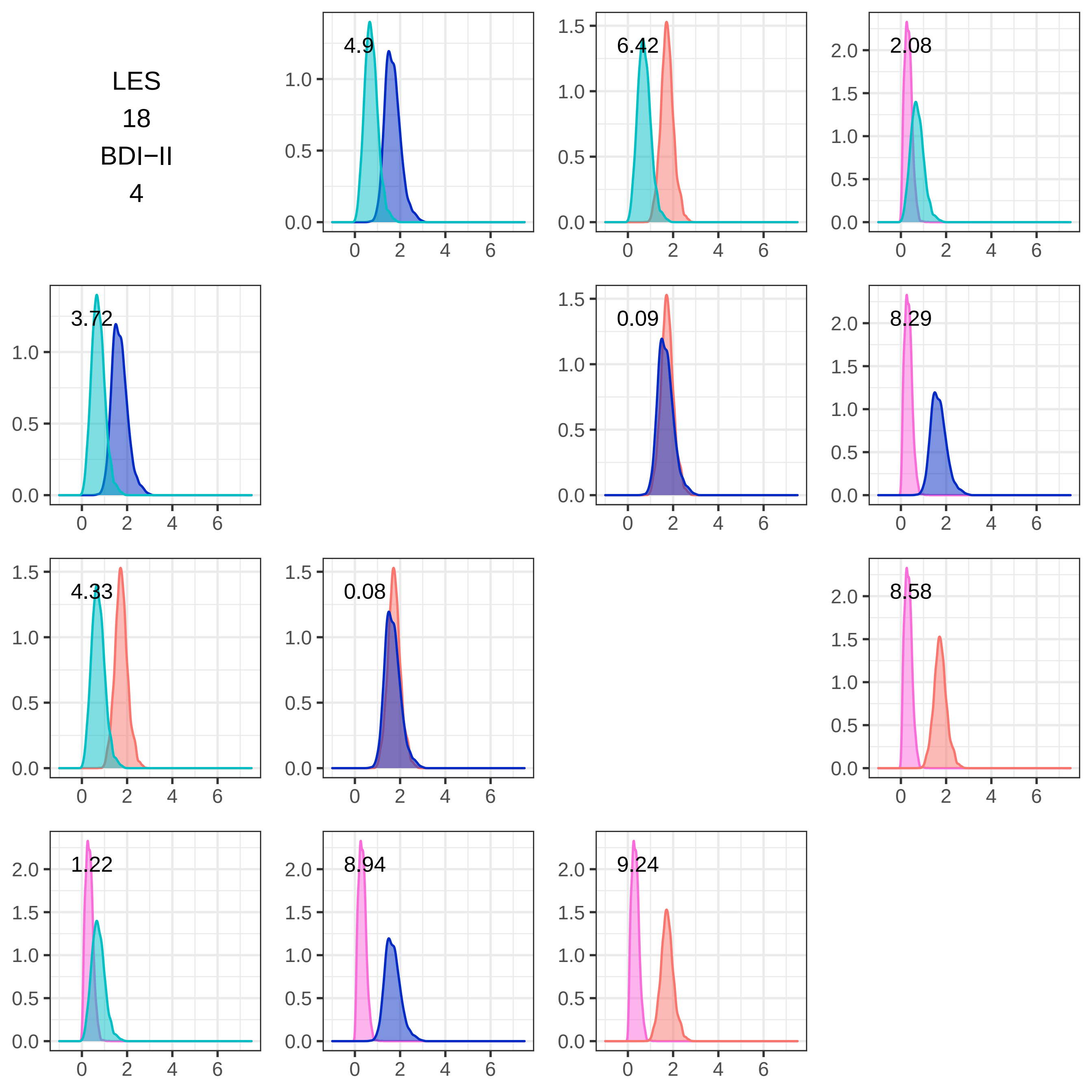

LES
18
BDI−II
4
4.9
6.42
2.08
3.72
0.09
8.29
4.33
0.08
8.58
1.22
8.94
9.24

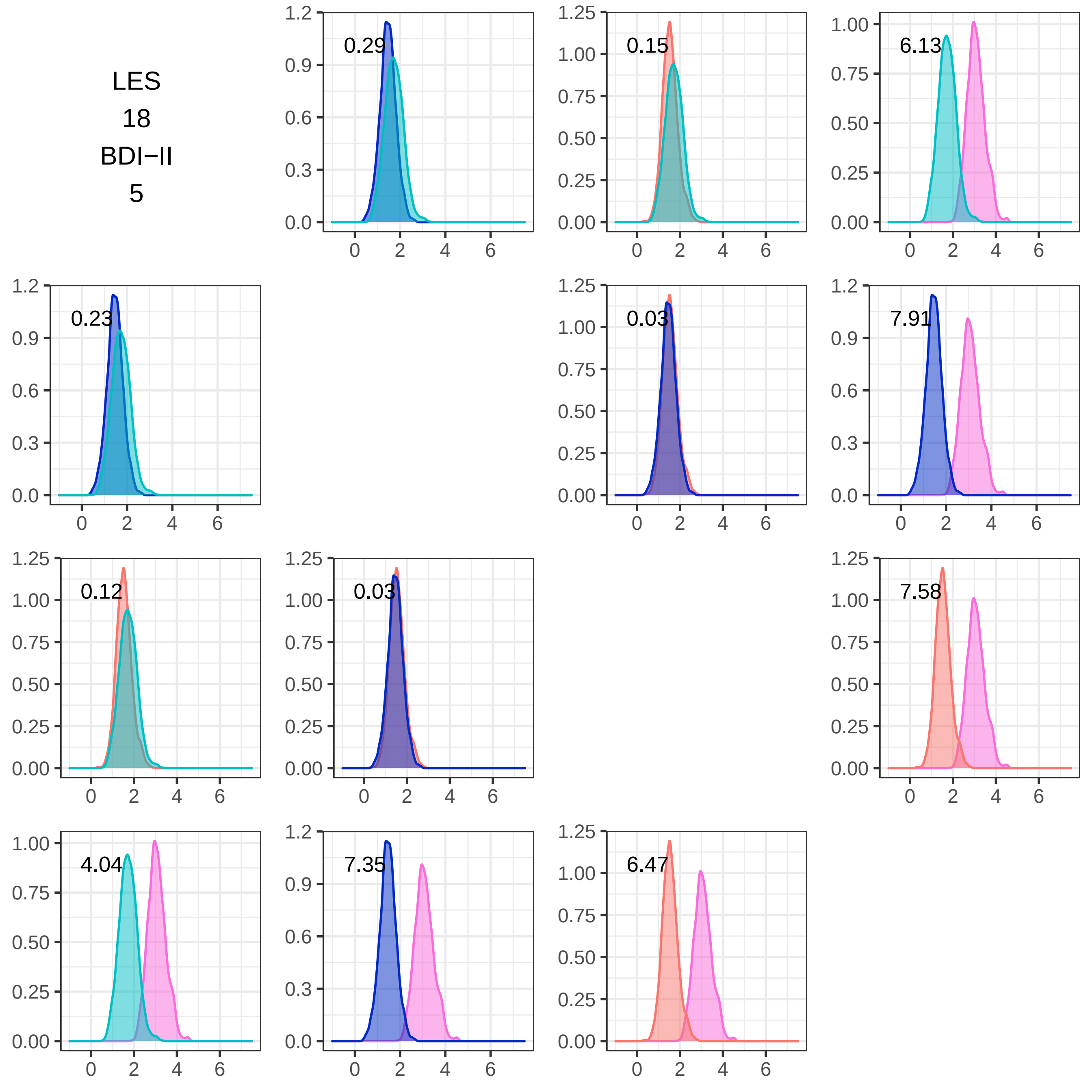
LES
18
BDI−II
5
0.29
0.15
6.13
0.23
0.03
7.91
0.12
0.03
7.58
4.04
7.35
6.47

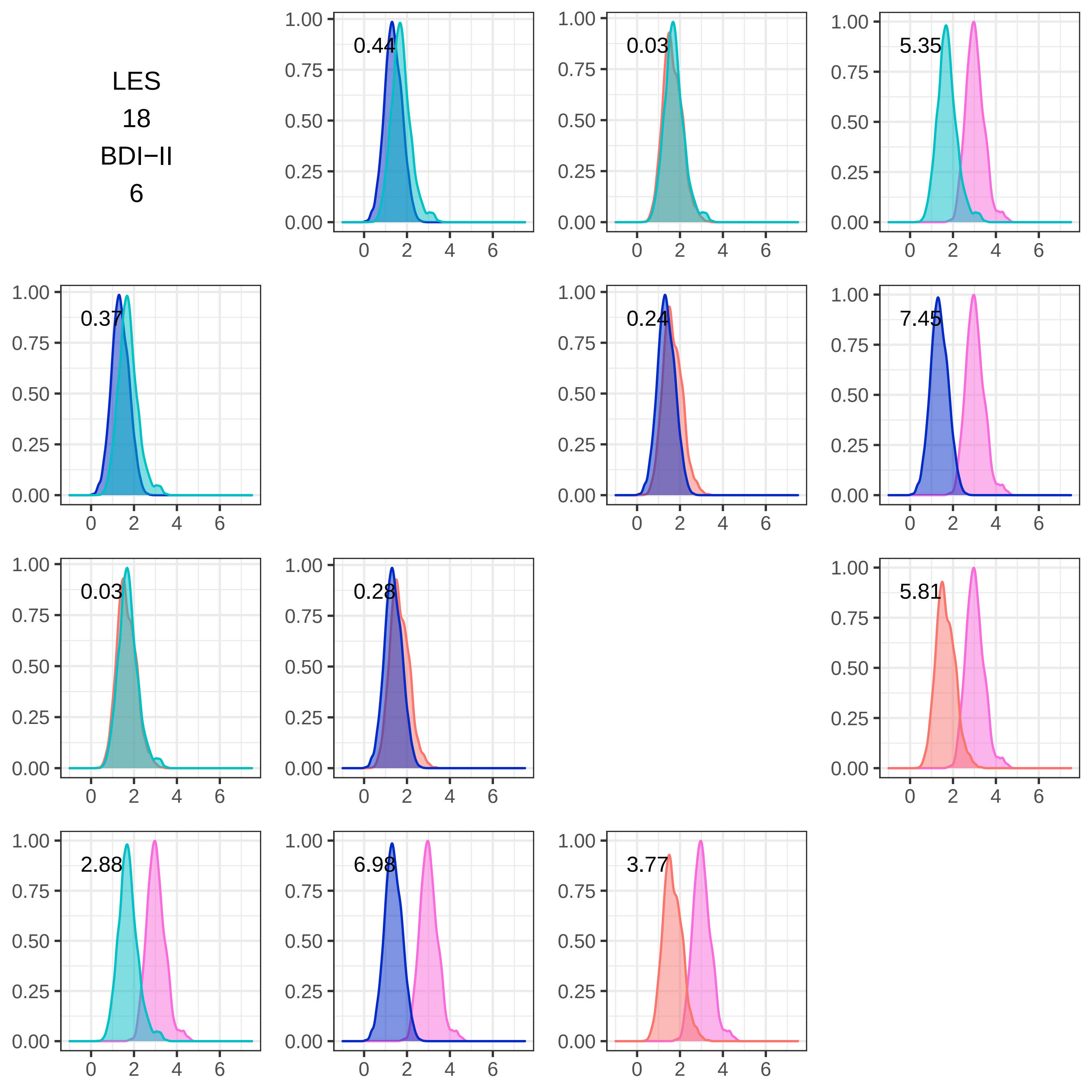

LES
18
BDI−II
6
0.44
0.03
5.35
0.37
0.24
7.45
0.03
0.28
5.81
2.88
6.98
3.77

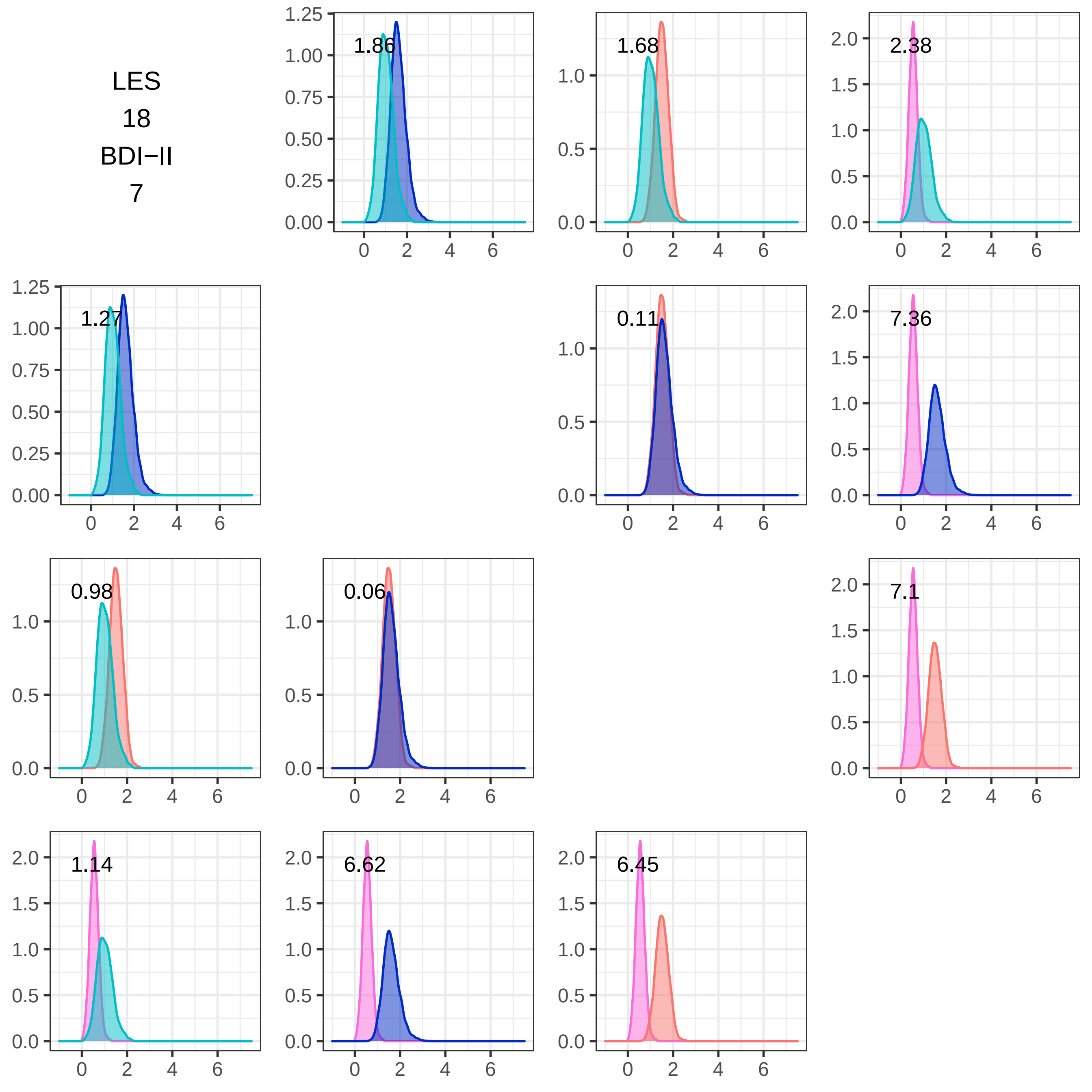

LES
18
BDI−II
7
1.86
1.68
2.38
1.27
0.11
7.36
0.98
0.06
7.1
1.14
6.62
6.45

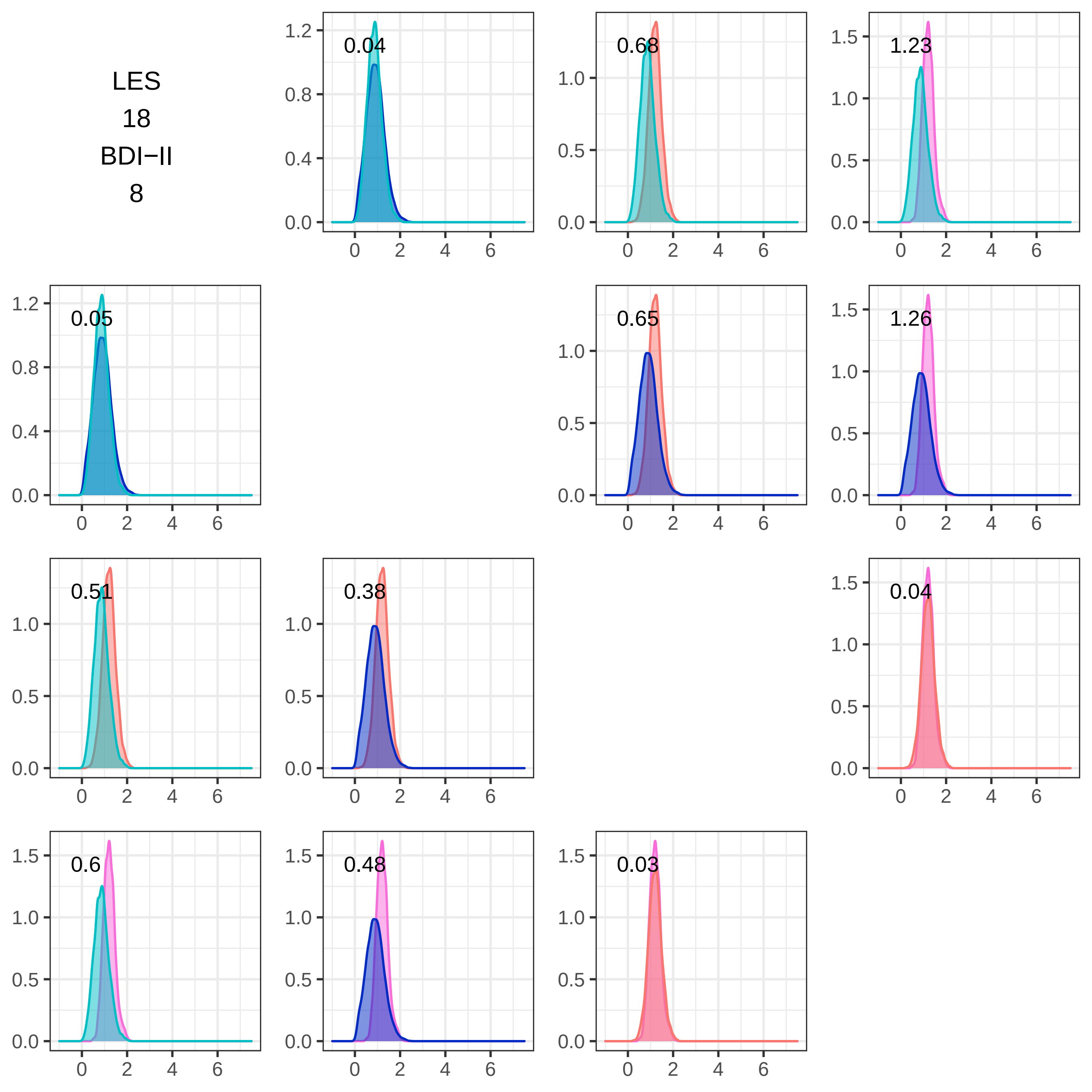
LES
18
BDI−II
8
0.04
0.68
1.23
0.05
0.65
1.26
0.51
0.38
0.04
0.6
0.48
0.03

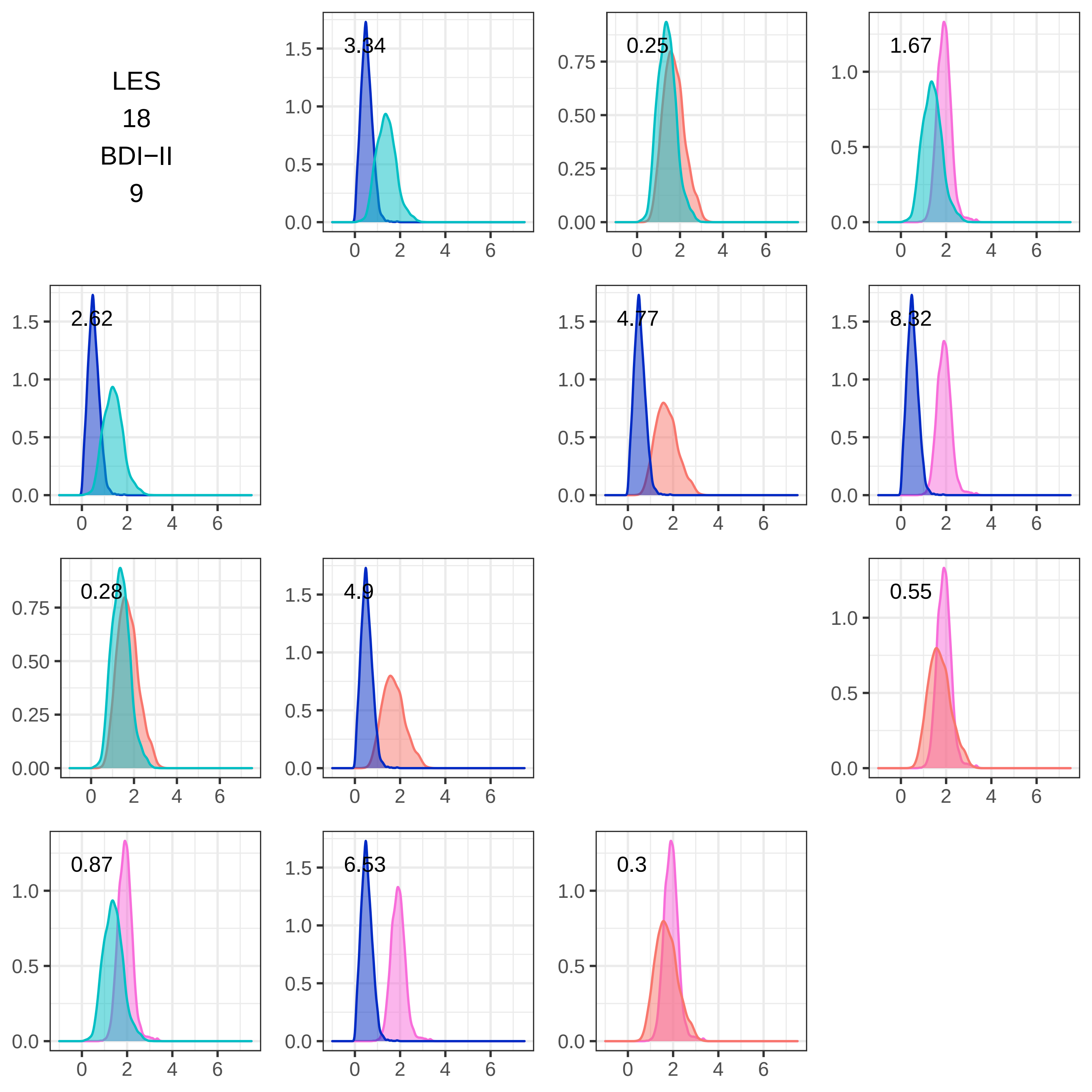
LES
18
BDI−II
9
3.34
0.25
1.67
2.62
4.77
8.32
0.28
4.9
0.55
0.87
6.53
0.3

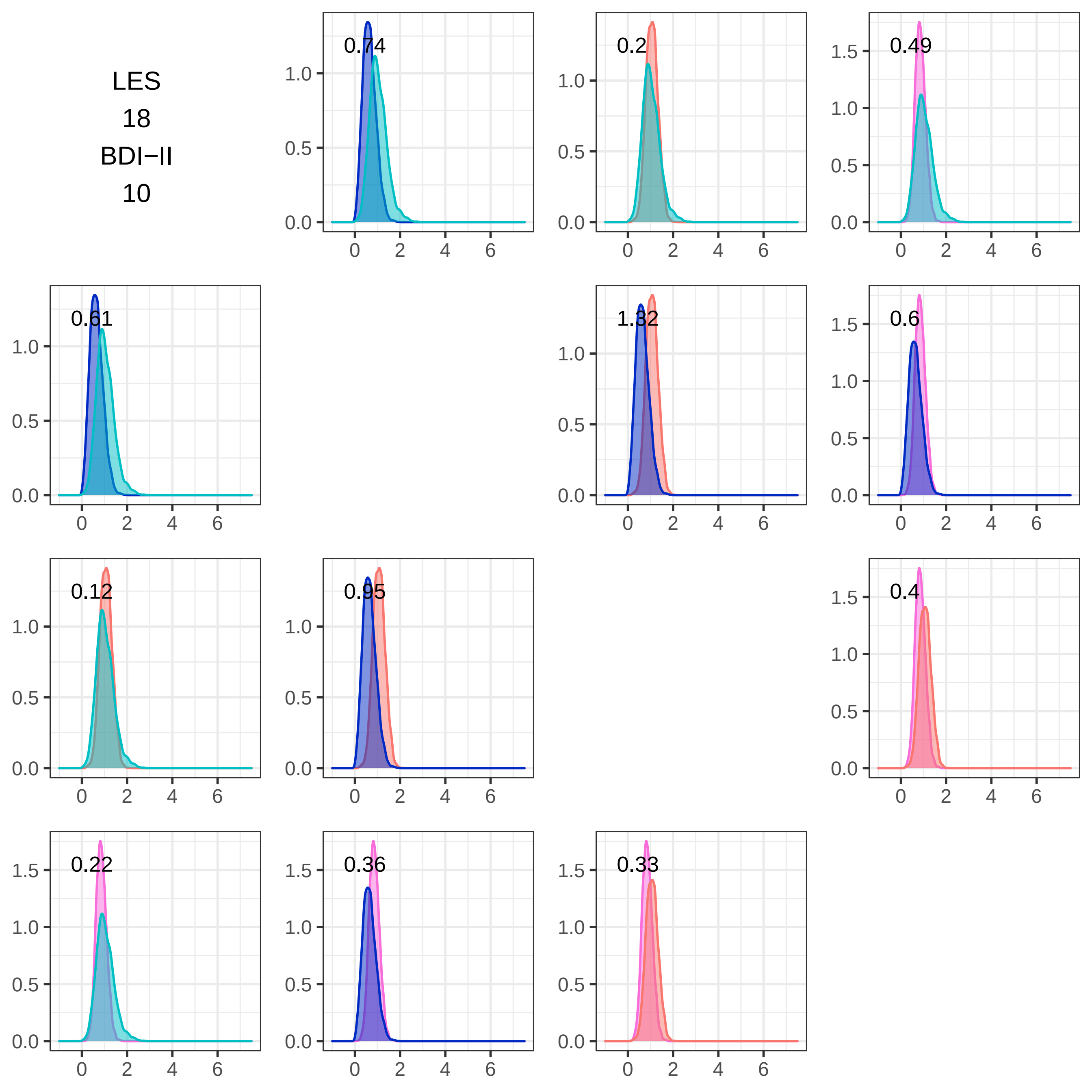

LES
18
BDI−II
10
0.74
0.2
0.49
0.61
1.32
0.6
0.12
0.95
0.4
0.22
0.36
0.33

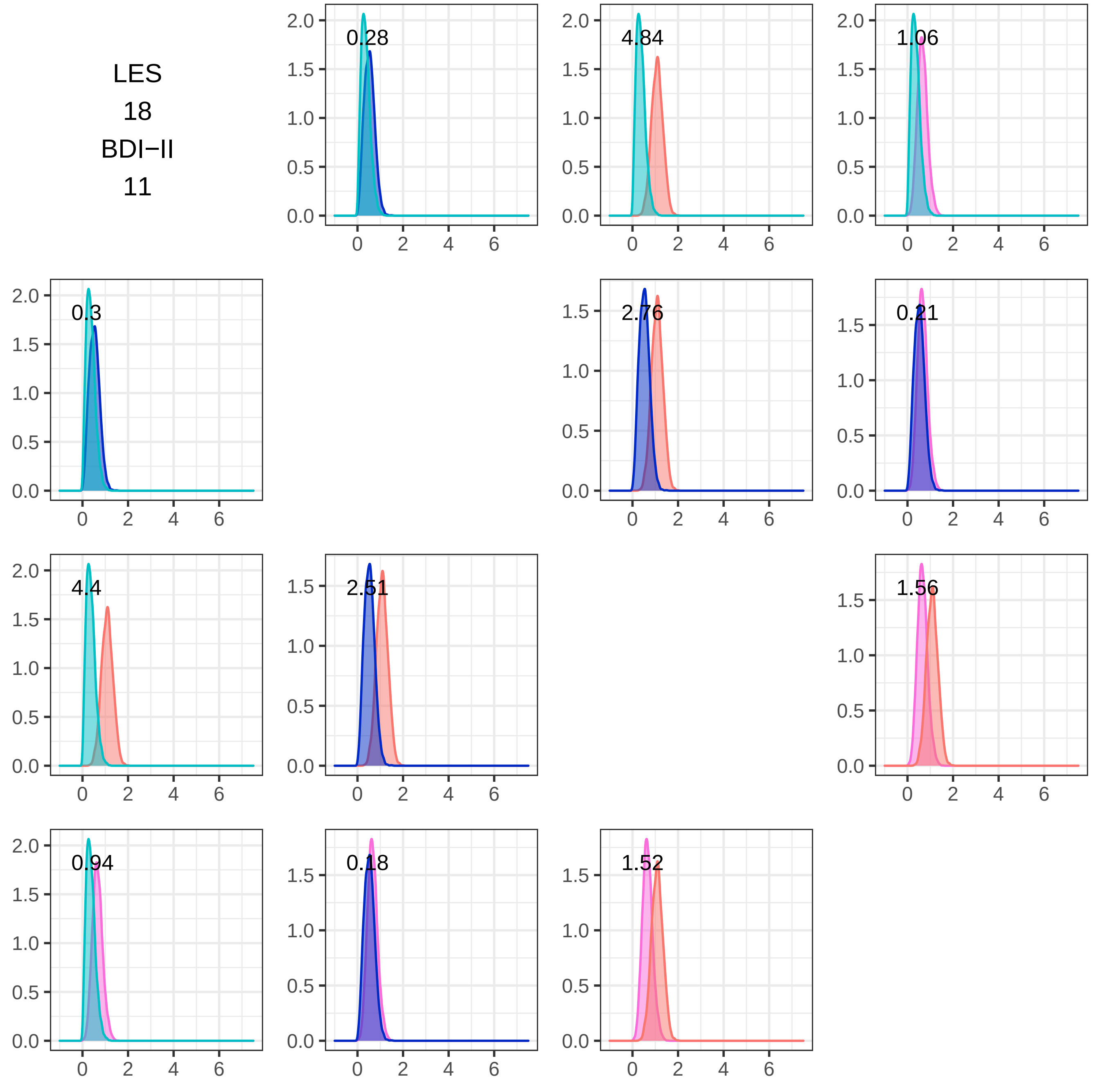
LES
18
BDI−II
11
0.28
4.84
1.06
0.3
2.76
0.21
4.4
2.51
1.56
0.94
0.18
1.52

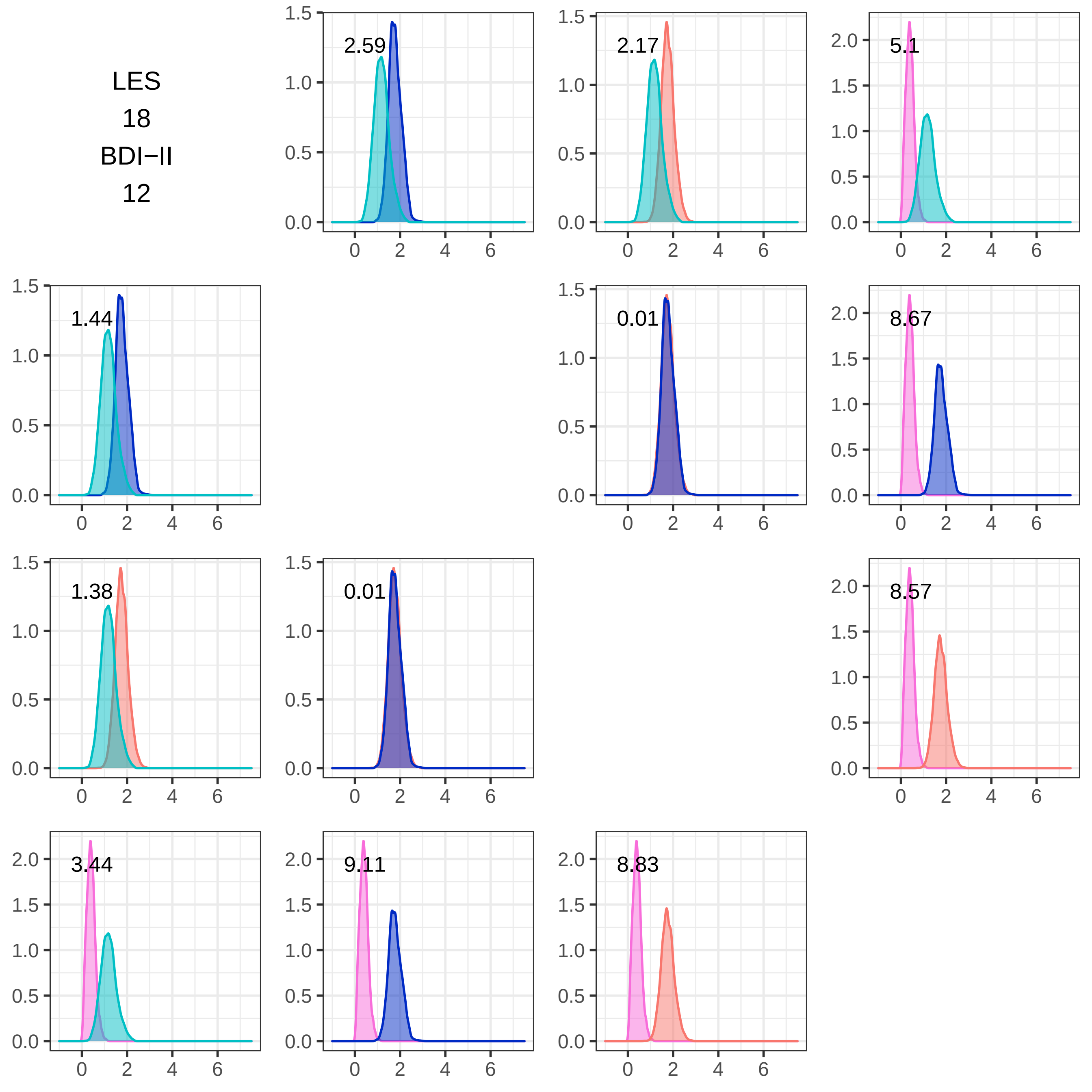
LES
18
BDI−II
12
2.59
2.17
5.1
1.44
0.01
8.67
1.38
0.01
8.57
3.44
9.11
8.83

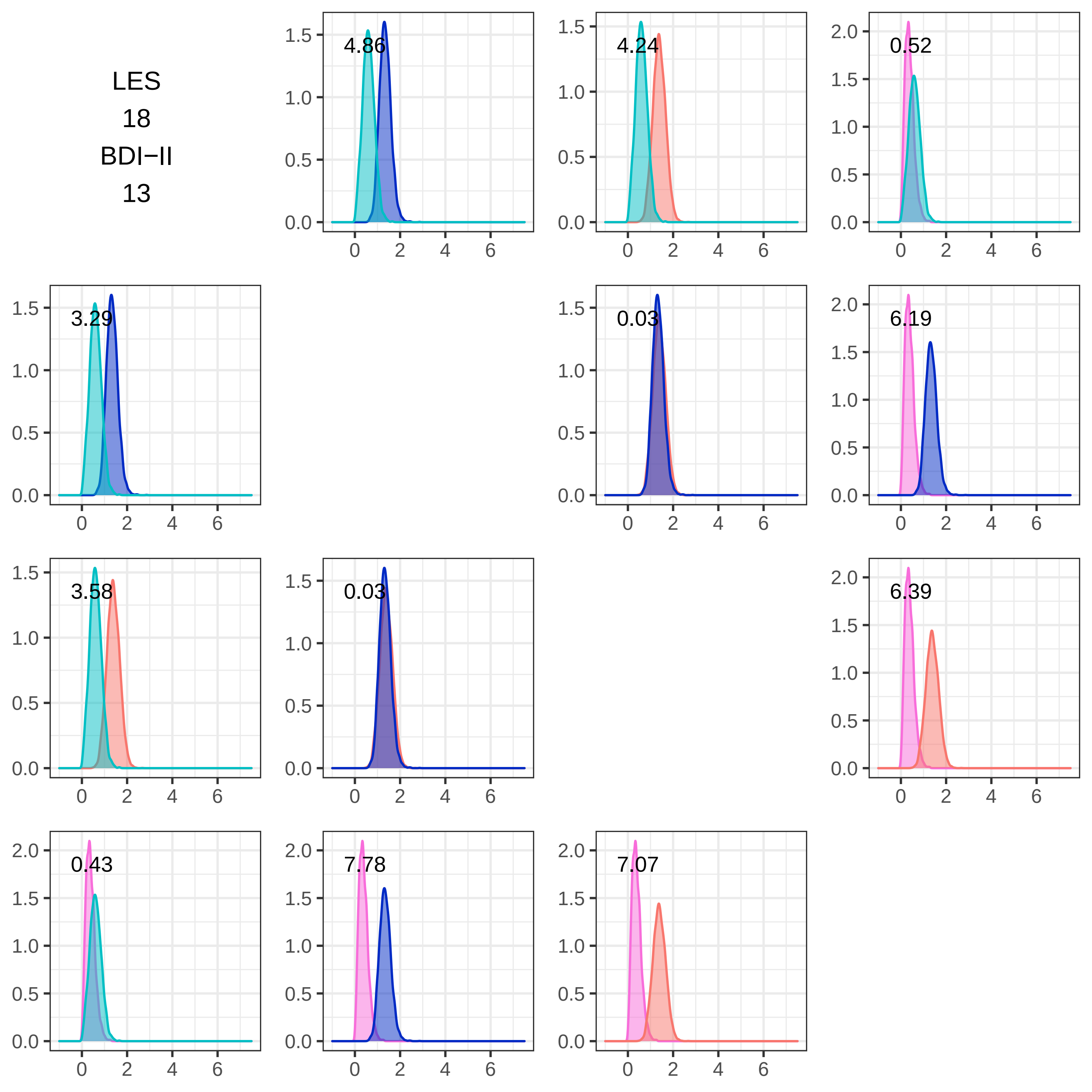
LES
18
BDI−II
13
4.86
4.24
0.52
3.29
0.03
6.19
3.58
0.03
6.39
0.43
7.78
7.07

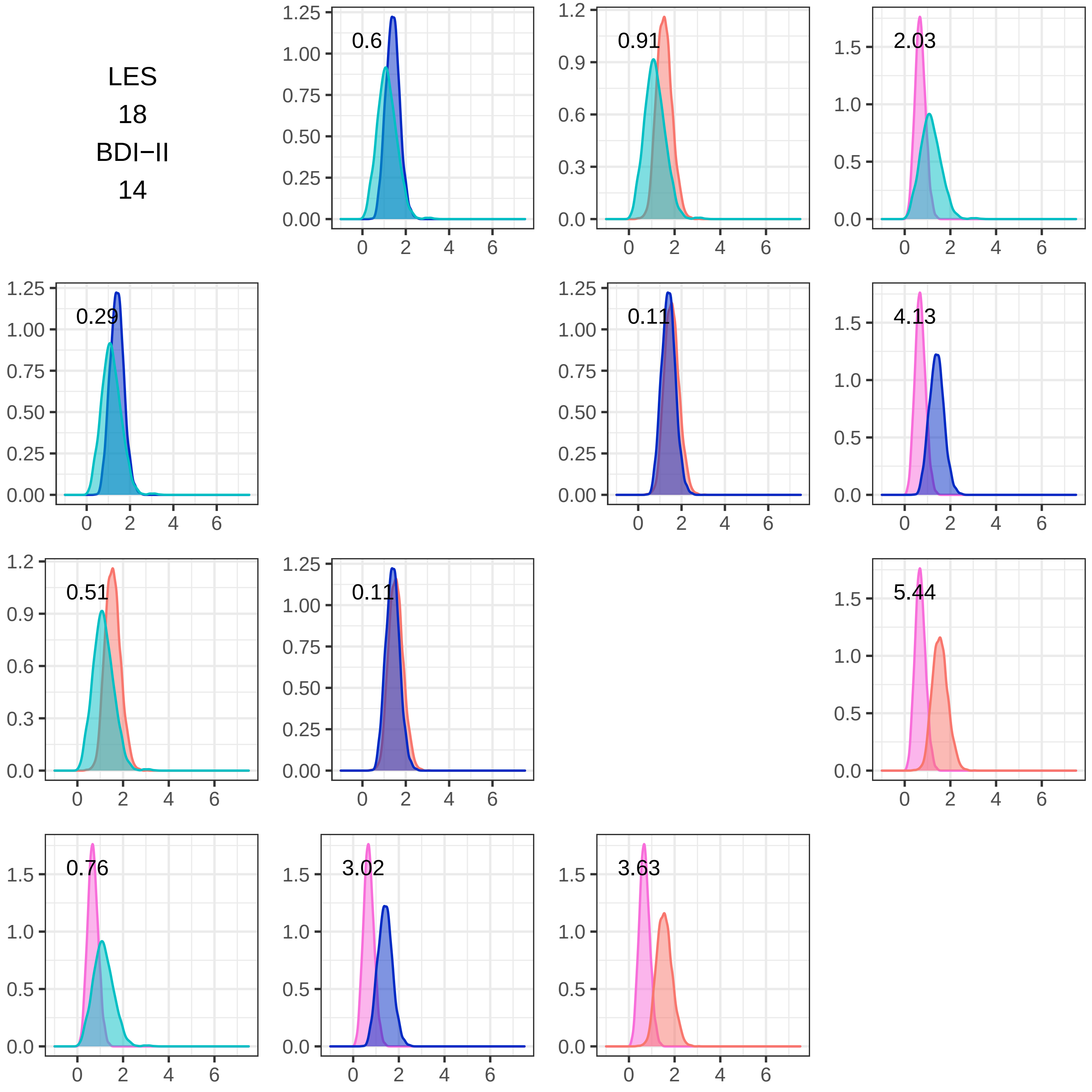
LES
18
BDI−II
14
0.6
0.91
2.03
0.29
0.11
4.13
0.51
0.11
5.44
0.76
3.02
3.63

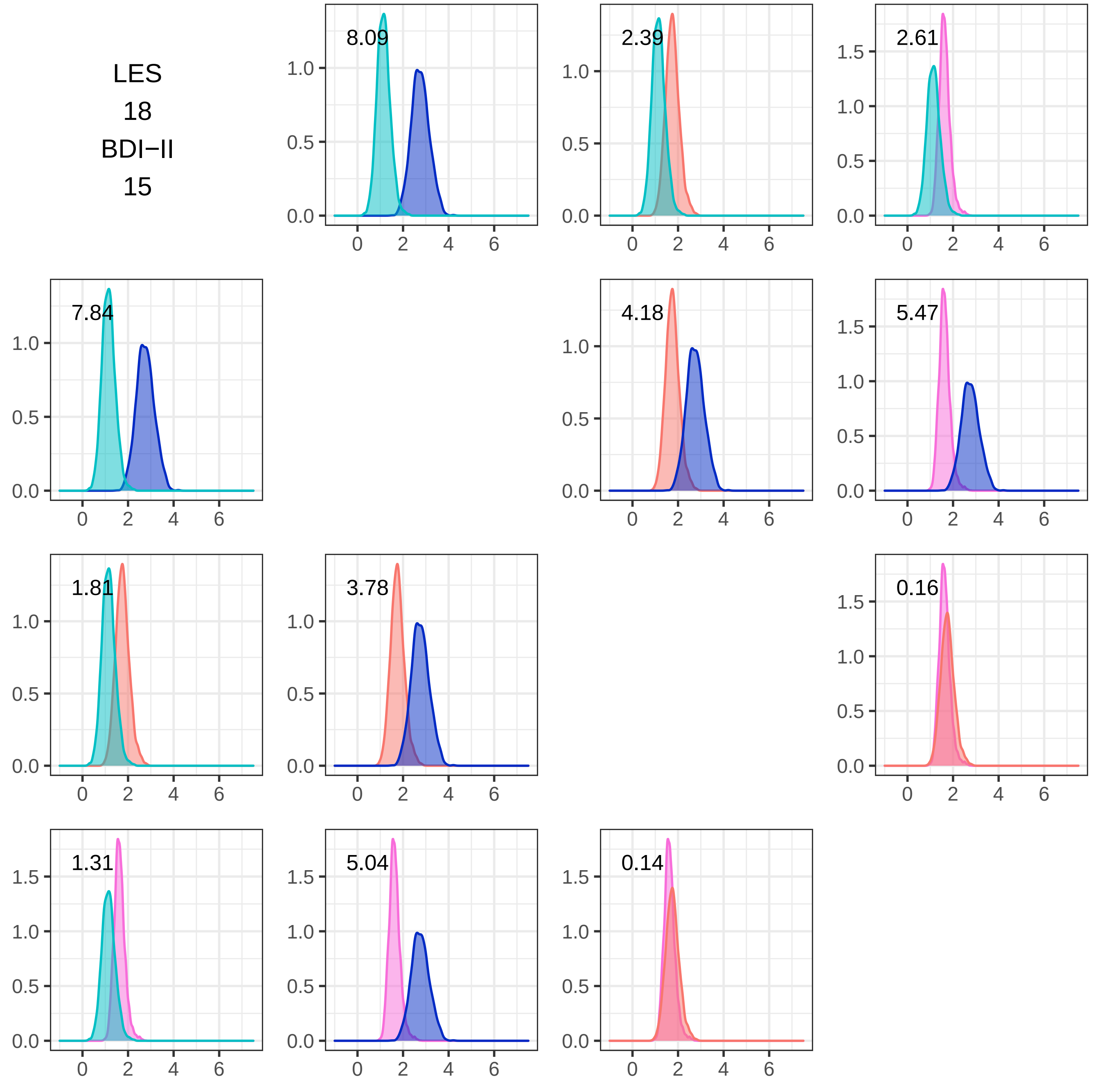
LES
18
BDI−II
15
8.09
2.39
2.61
7.84
4.18
5.47
1.81
3.78
0.16
1.31
5.04
0.14

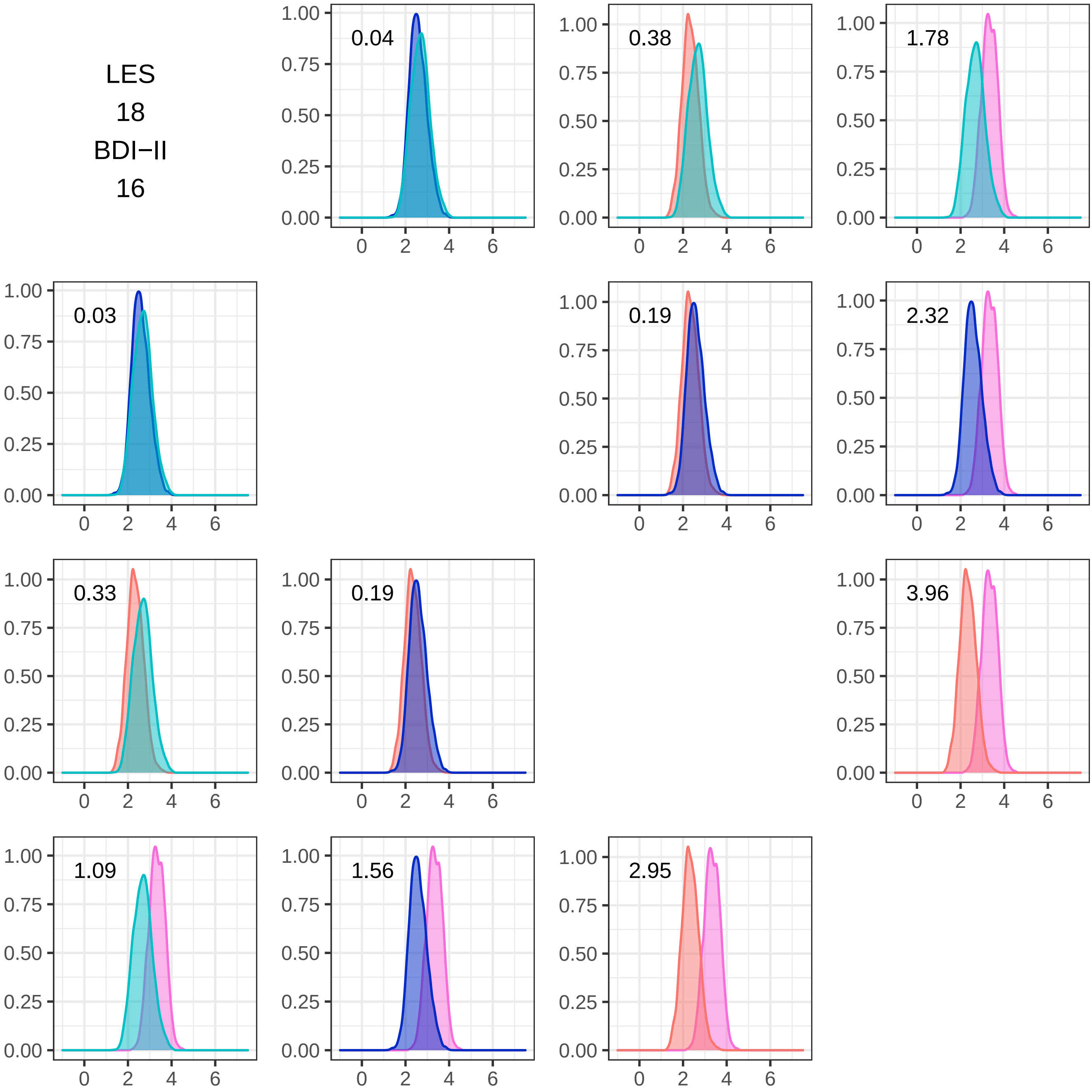
LES
18
BDI−II
16
0.04
0.38
1.78
0.03
0.19
2.32
0.33
0.19
3.96
1.09
1.56
2.95
0.00
0.25
0.50
0.75
1.00
0
2
4
6

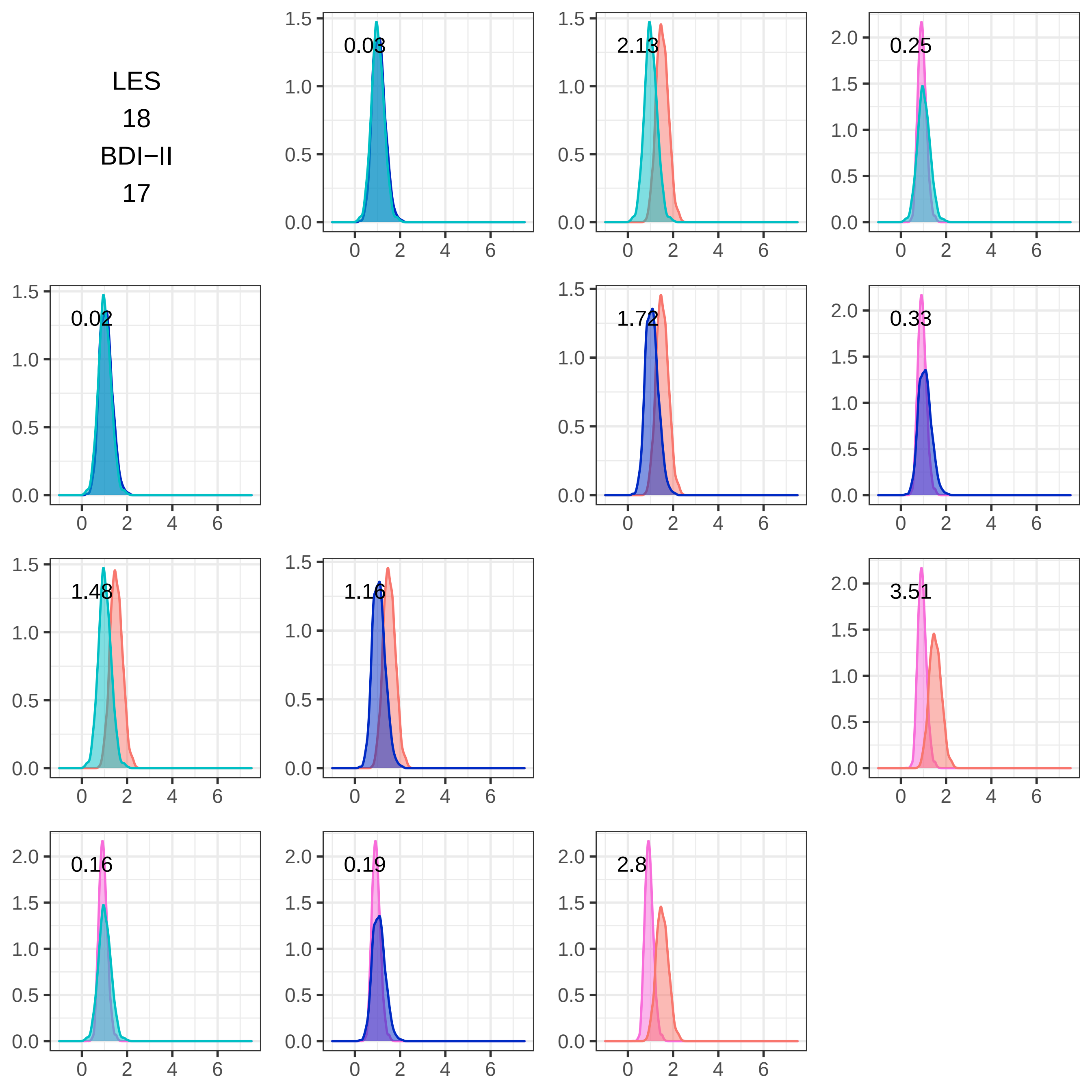
LES
18
BDI−II
17
0.03
2.13
0.25
0.02
1.72
0.33
1.48
1.16
3.51
0.16
0.19
2.8

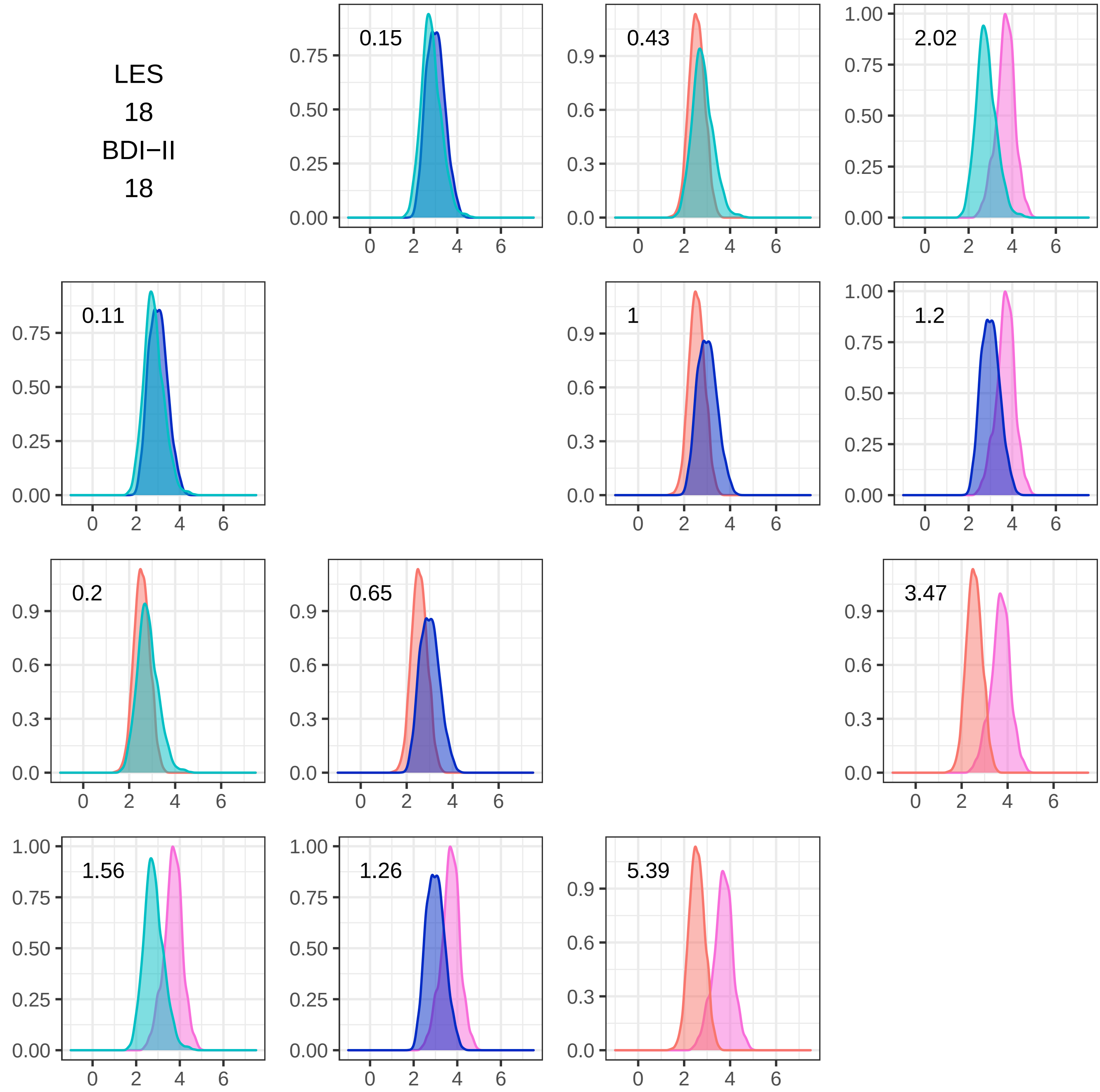

LES
18
BDI−II
18
0.15
0.43
2.02
0.11
1
1.2
0.2
0.65
3.47
1.56
1.26
5.39

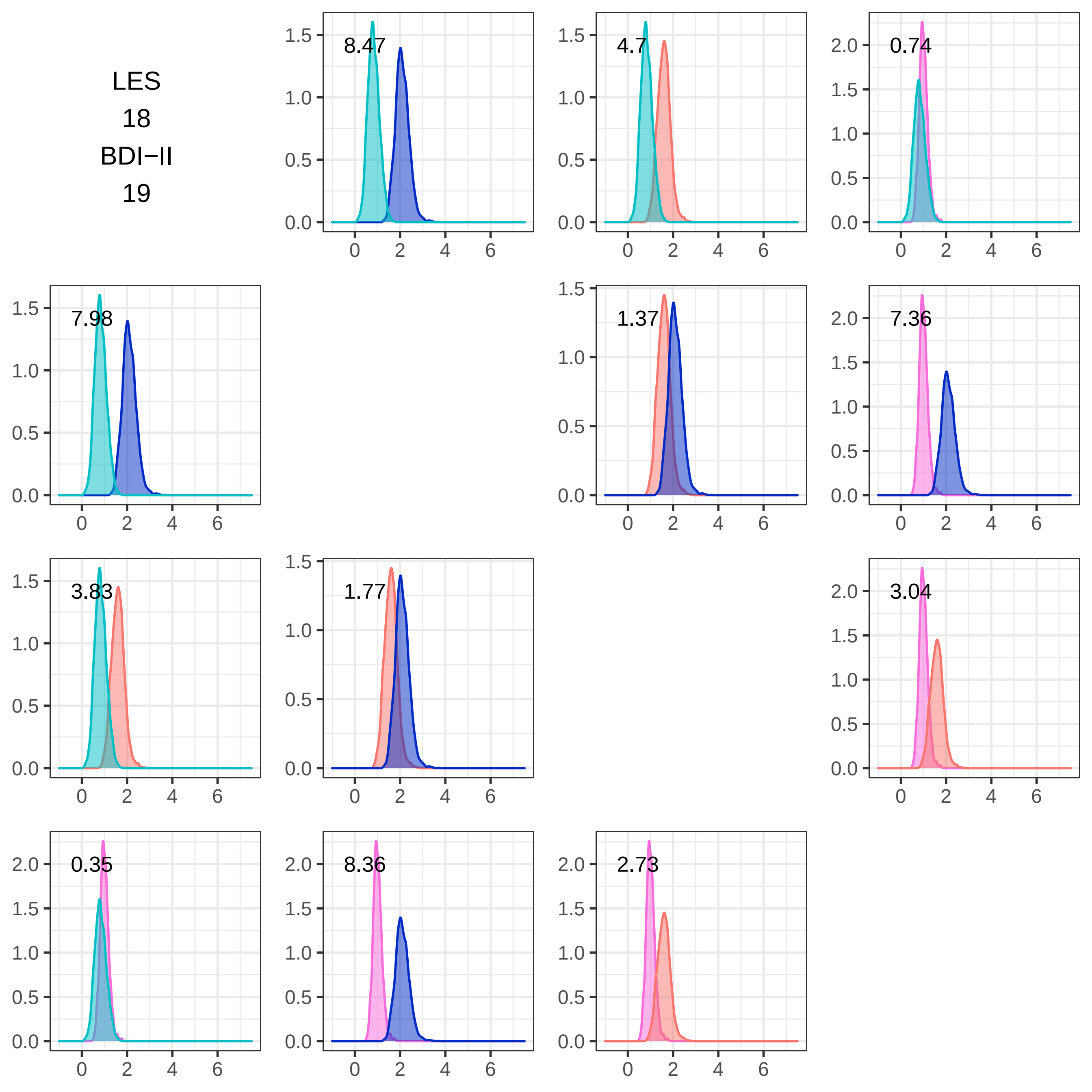

LES
18
BDI−II
19
8.47
4.7
0.74
7.98
1.37
7.36
3.83
1.77
3.04
0.35
8.36
2.73

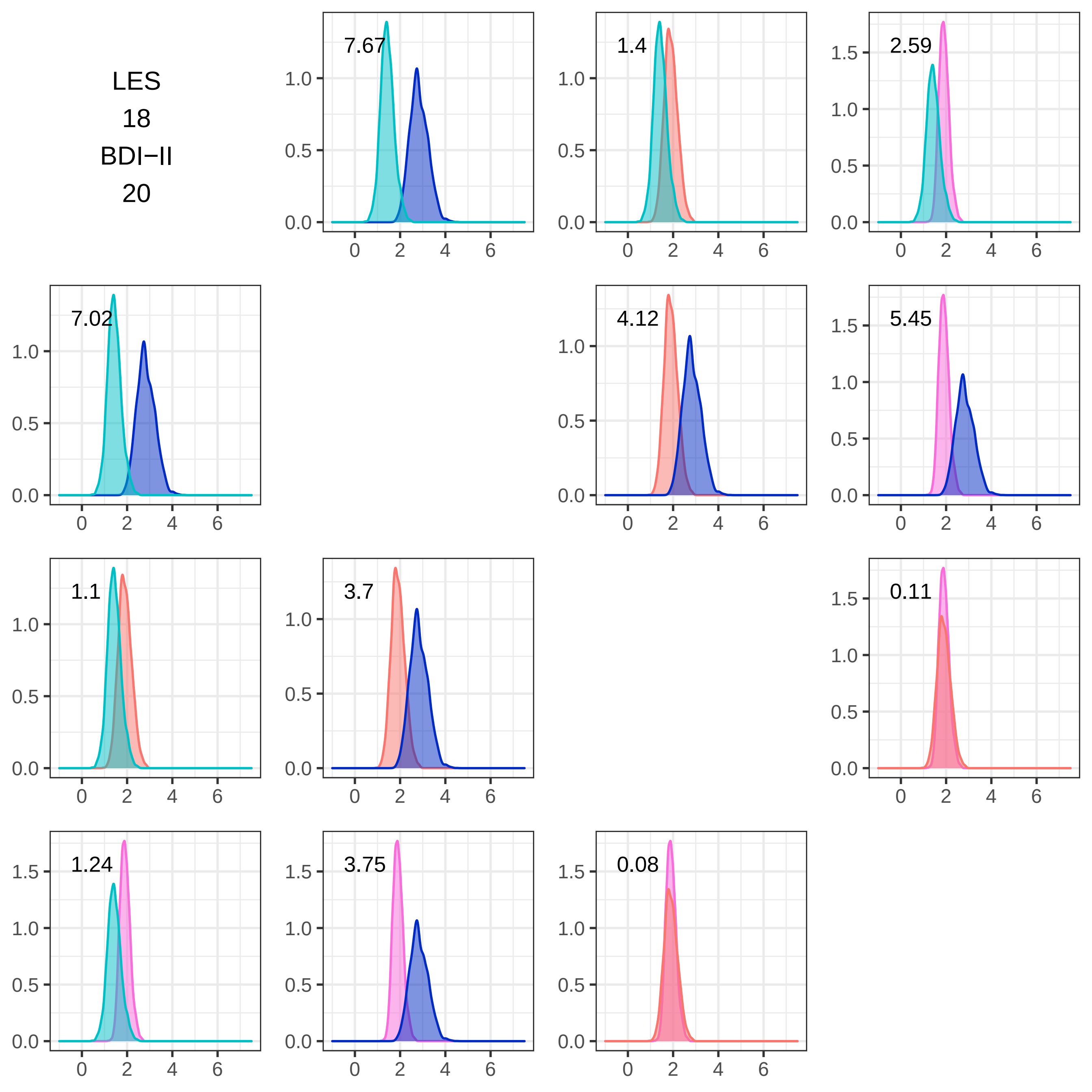

LES
18
BDI−II
20
7.67
1.4
2.59
7.02
4.12
5.45
1.1
3.7
0.11
1.24
3.75
0.08

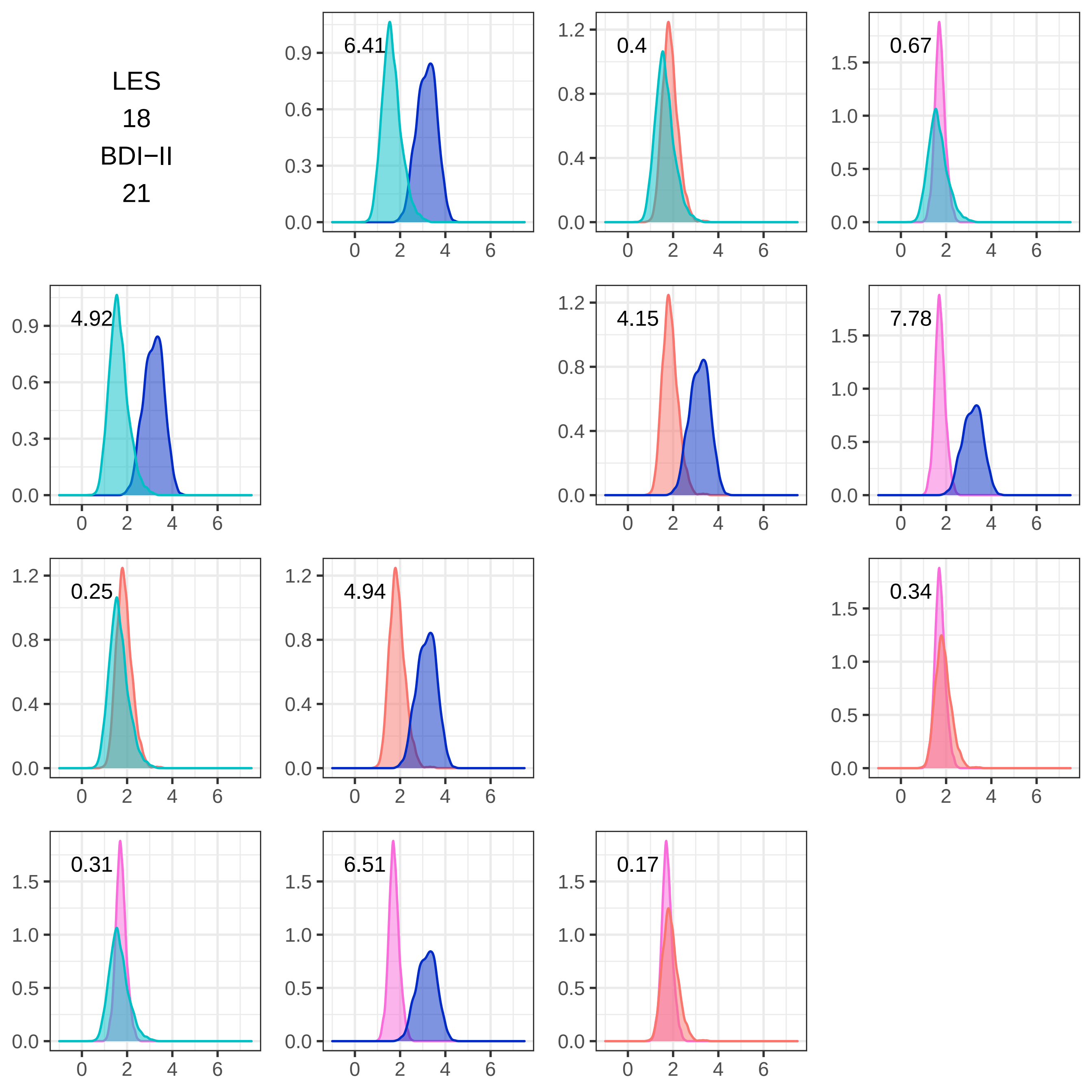

LES
18
BDI−II
21
6.41
0.4
0.67
4.92
4.15
7.78
0.25
4.94
0.34
0.31
6.51
0.17

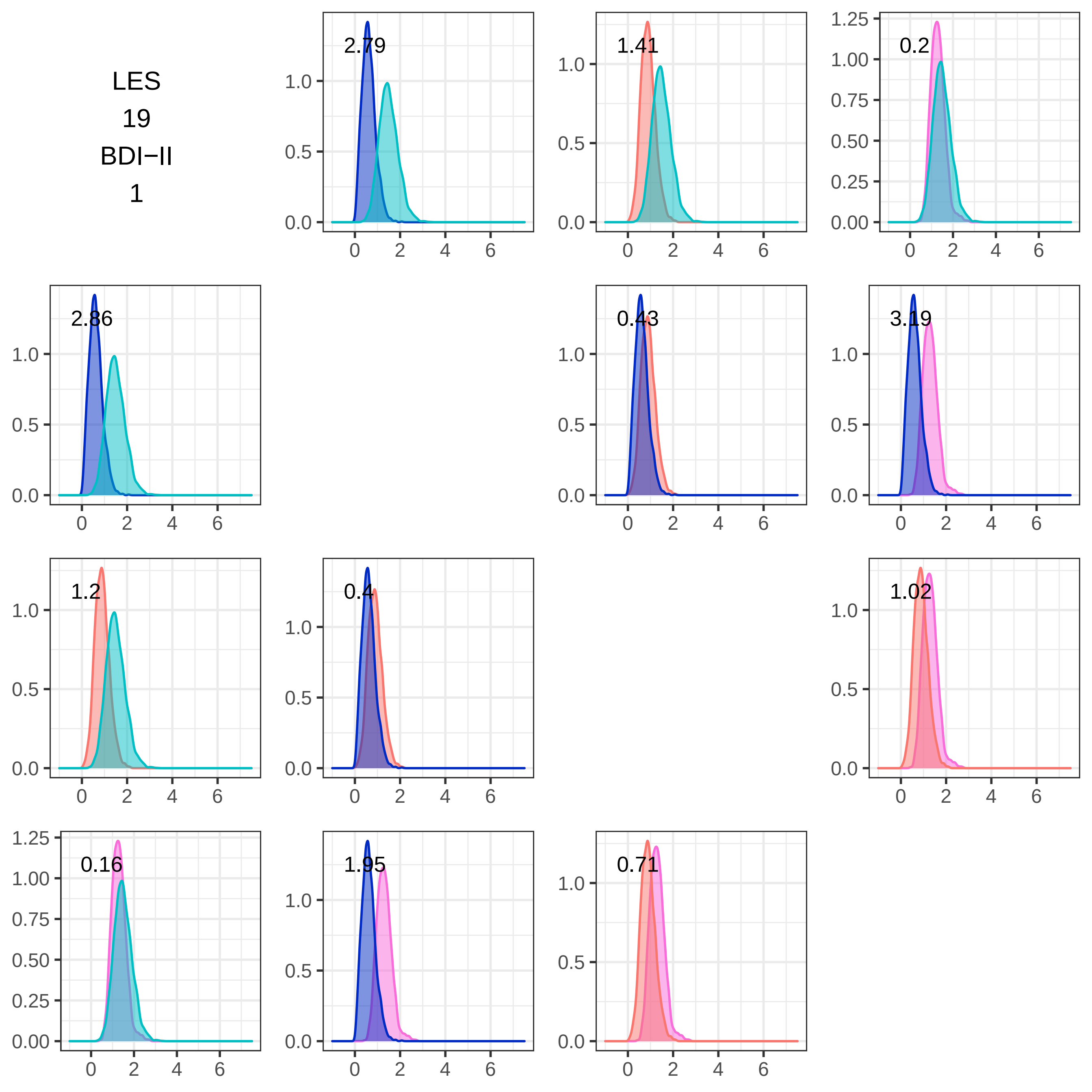
LES
19
BDI−II
1
2.79
1.41
0.2
2.86
0.43
3.19
1.2
0.4
1.02
0.16
1.95
0.71

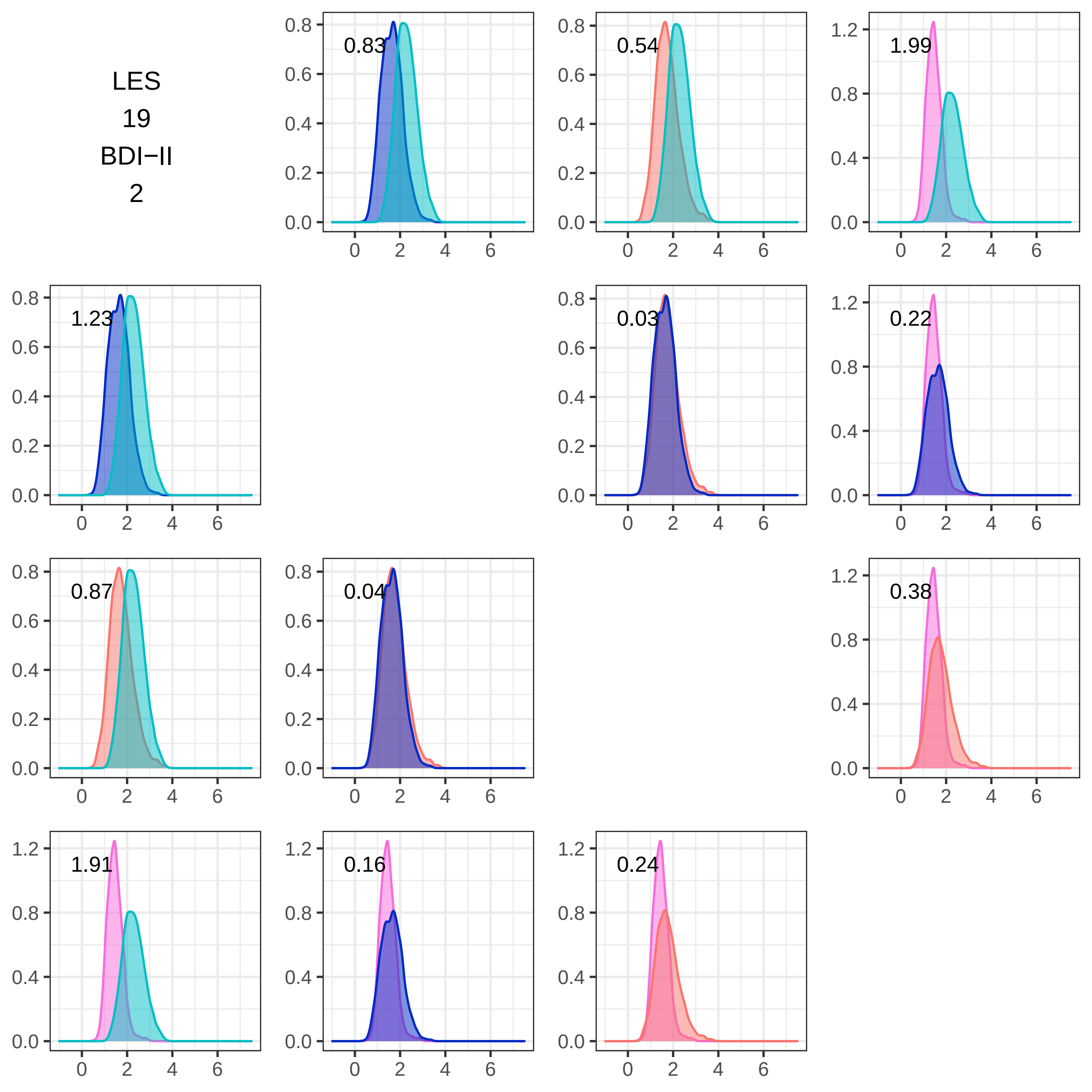
LES
19
BDI−II
2
0.83
0.54
1.99
1.23
0.03
0.22
0.87
0.04
0.38
1.91
0.16
0.24

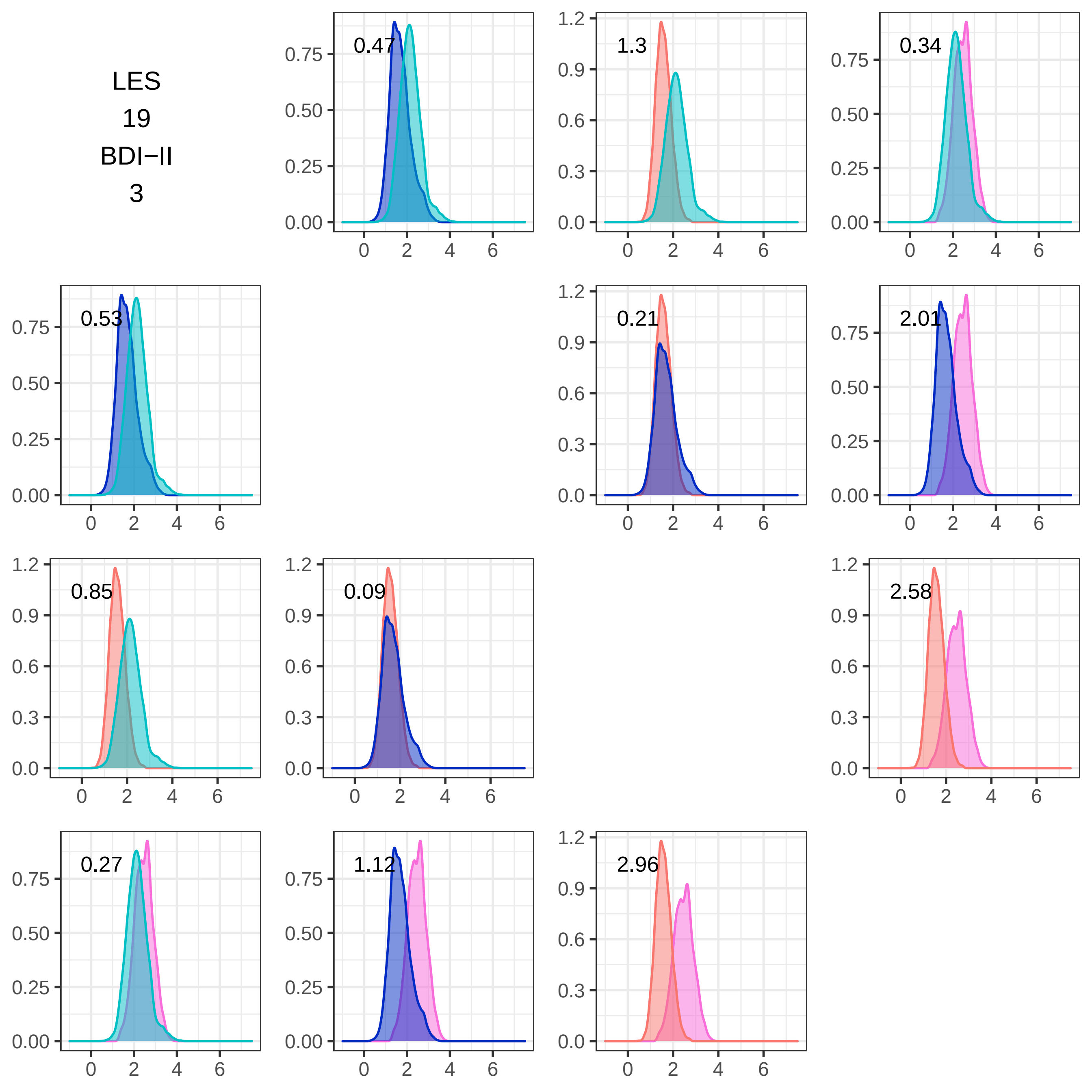
LES
19
BDI−II
3
0.47
1.3
0.34
0.53
0.21
2.01
0.85
0.09
2.58
0.27
1.12
2.96

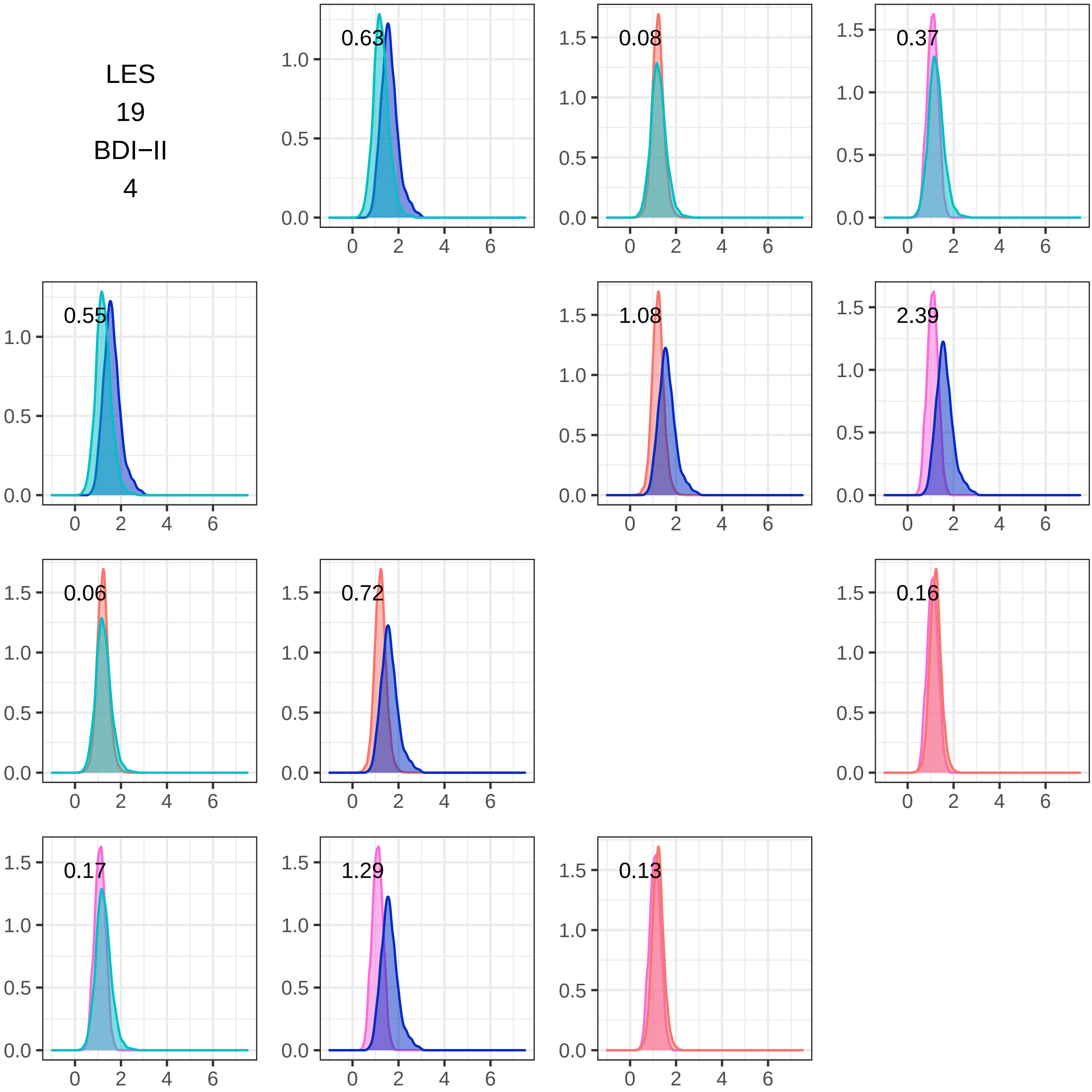

LES
19
BDI−II
4
0.63
0.08
0.37
0.55
1.08
2.39
0.06
0.72
0.16
0.17
1.29
0.13

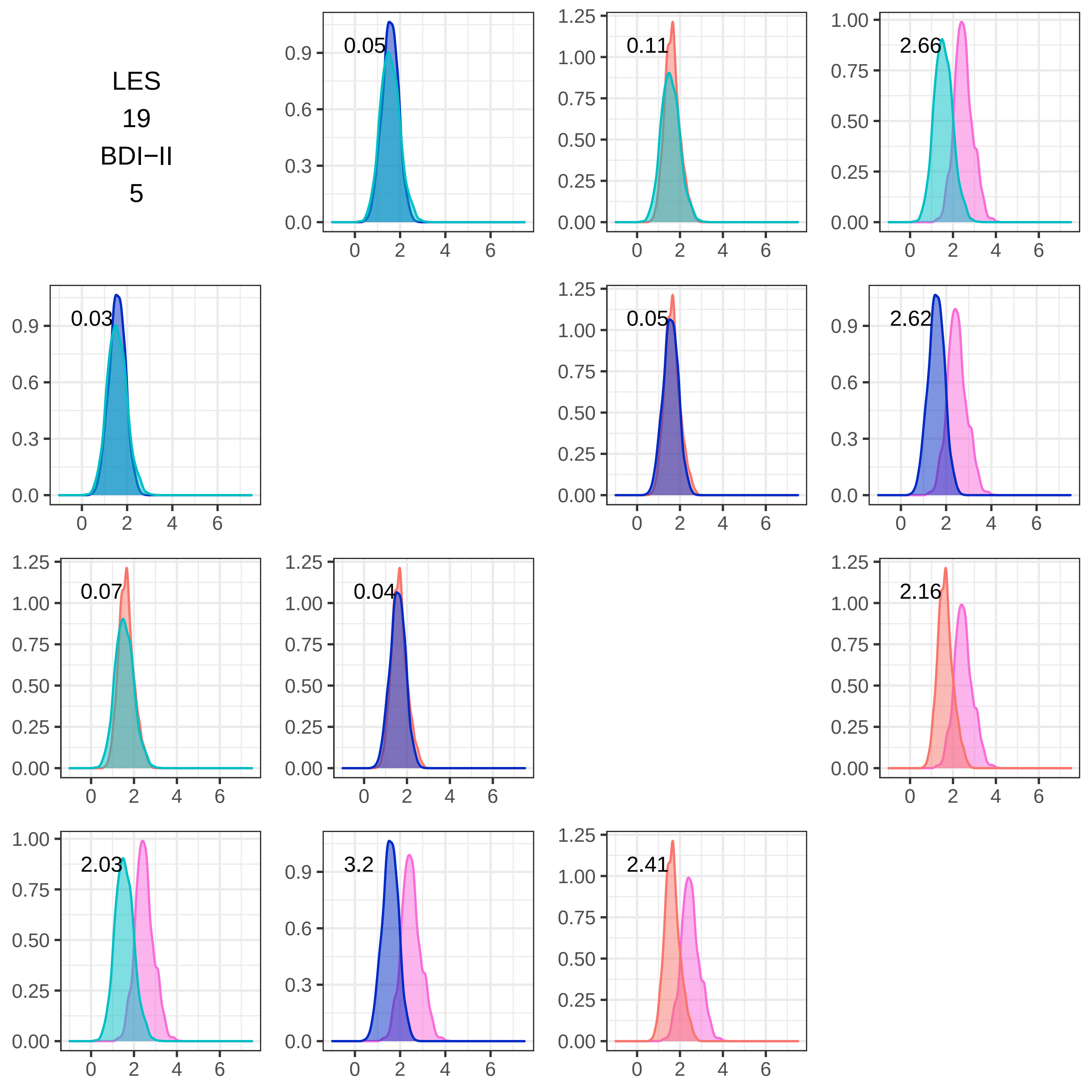
LES
19
BDI−II
5
0.05
0.11
2.66
0.03
0.05
2.62
0.07
0.04
2.16
2.03
3.2
2.41

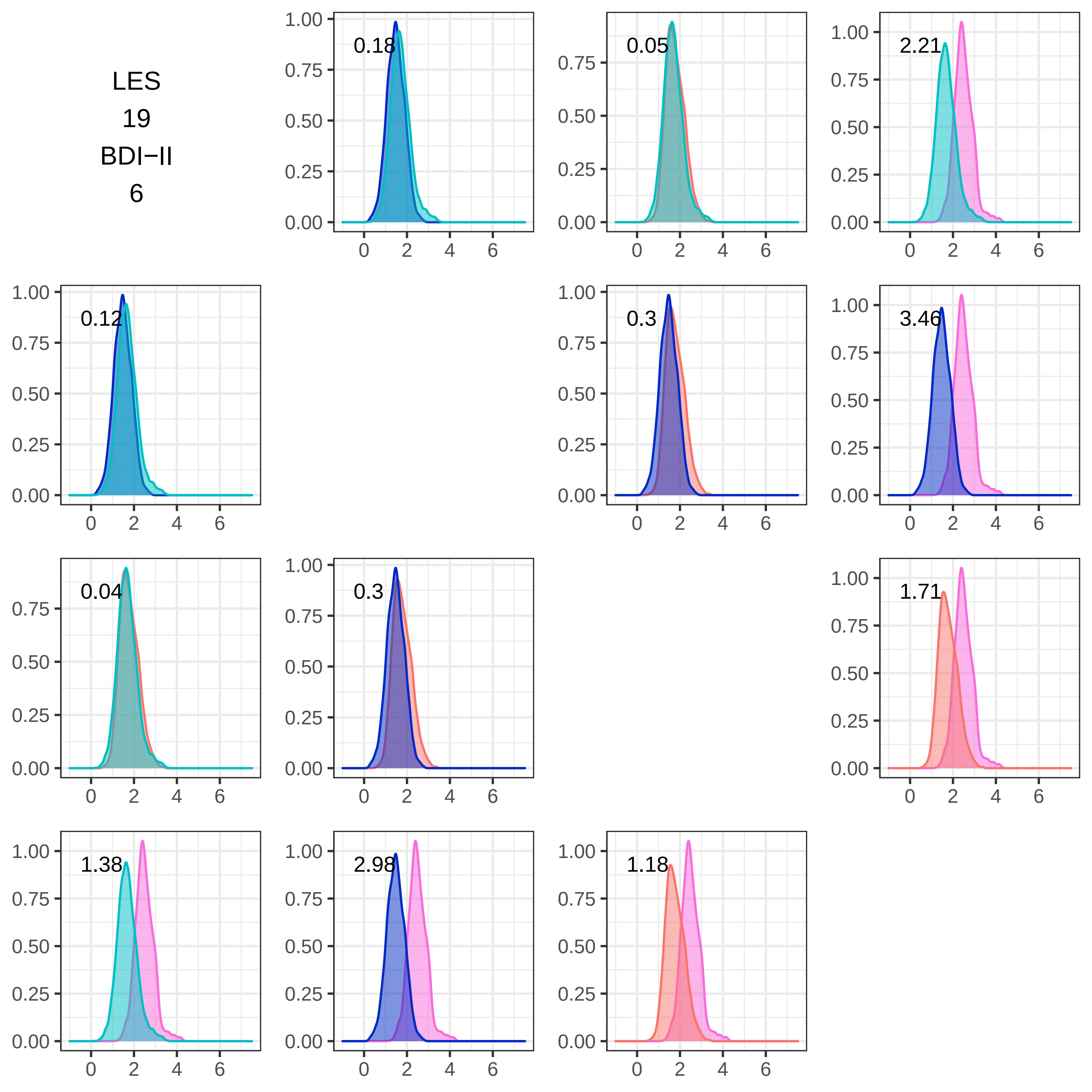
LES
19
BDI−II
6
0.18
0.05
2.21
0.12
0.3
3.46
0.04
0.3
1.71
1.38
2.98
1.18

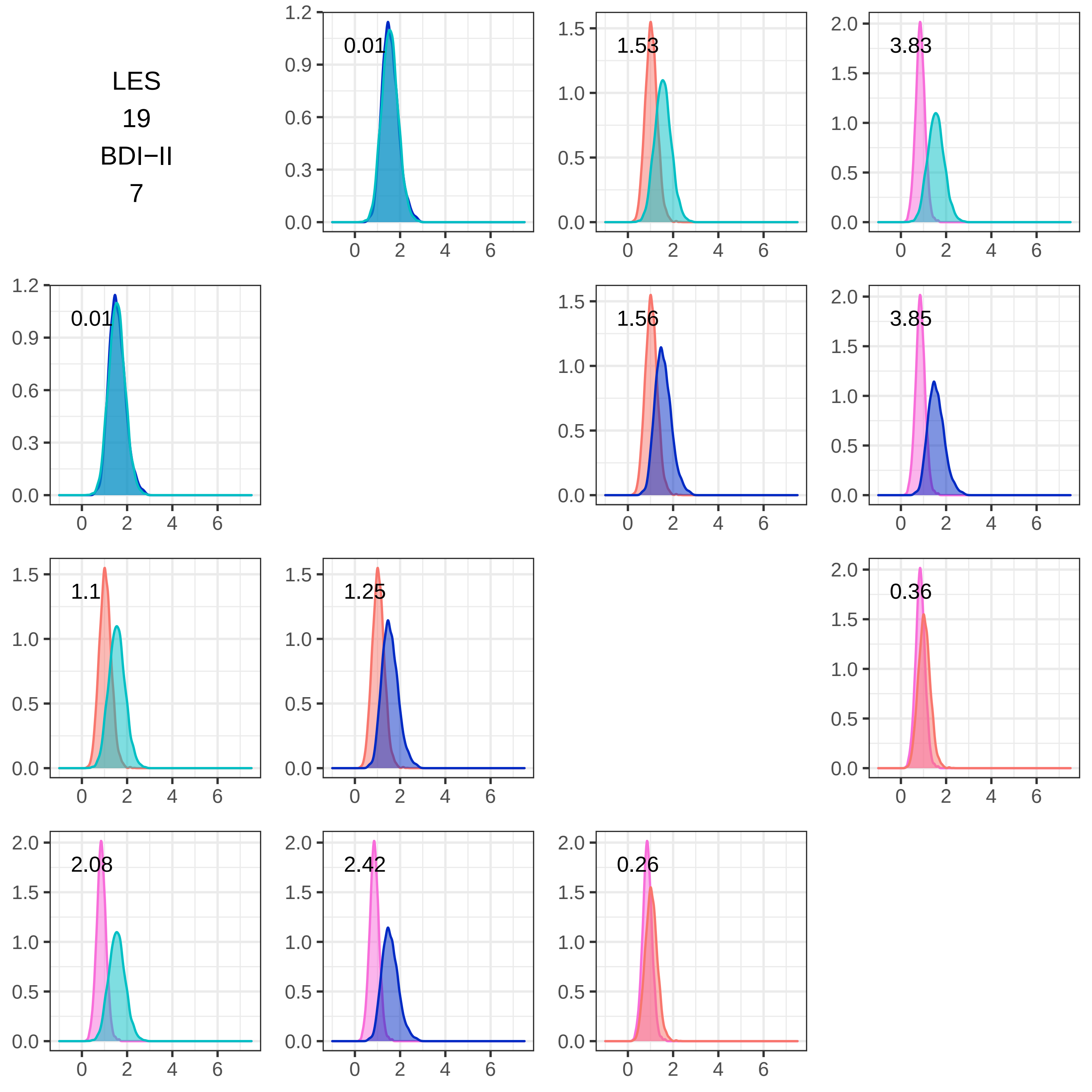
LES
19
BDI−II
7
0.01
1.53
3.83
0.01
1.56
3.85
1.1
1.25
0.36
2.08
2.42
0.26

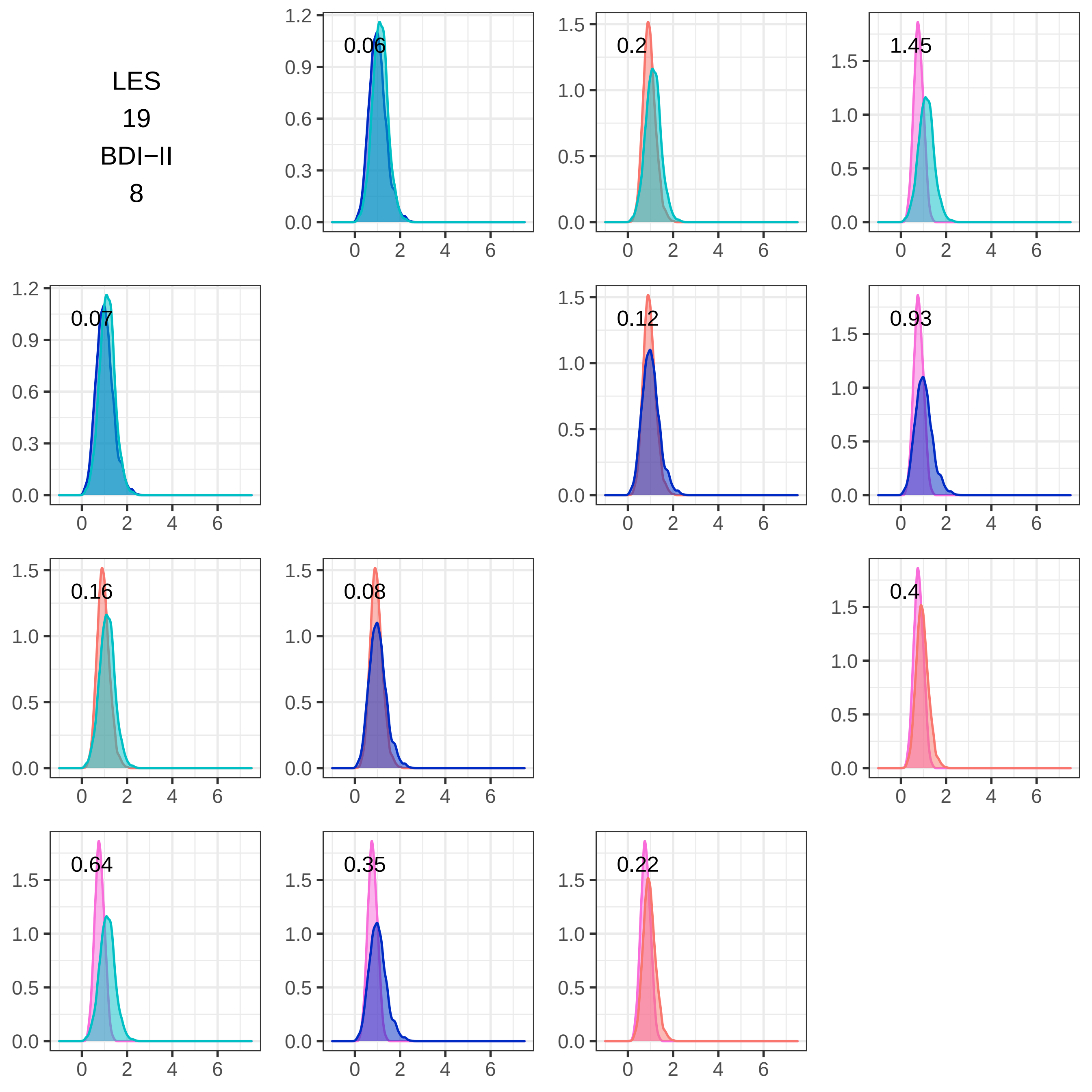
LES
19
BDI−II
8
0.06
0.2
1.45
0.07
0.12
0.93
0.16
0.08
0.4
0.64
0.35
0.22

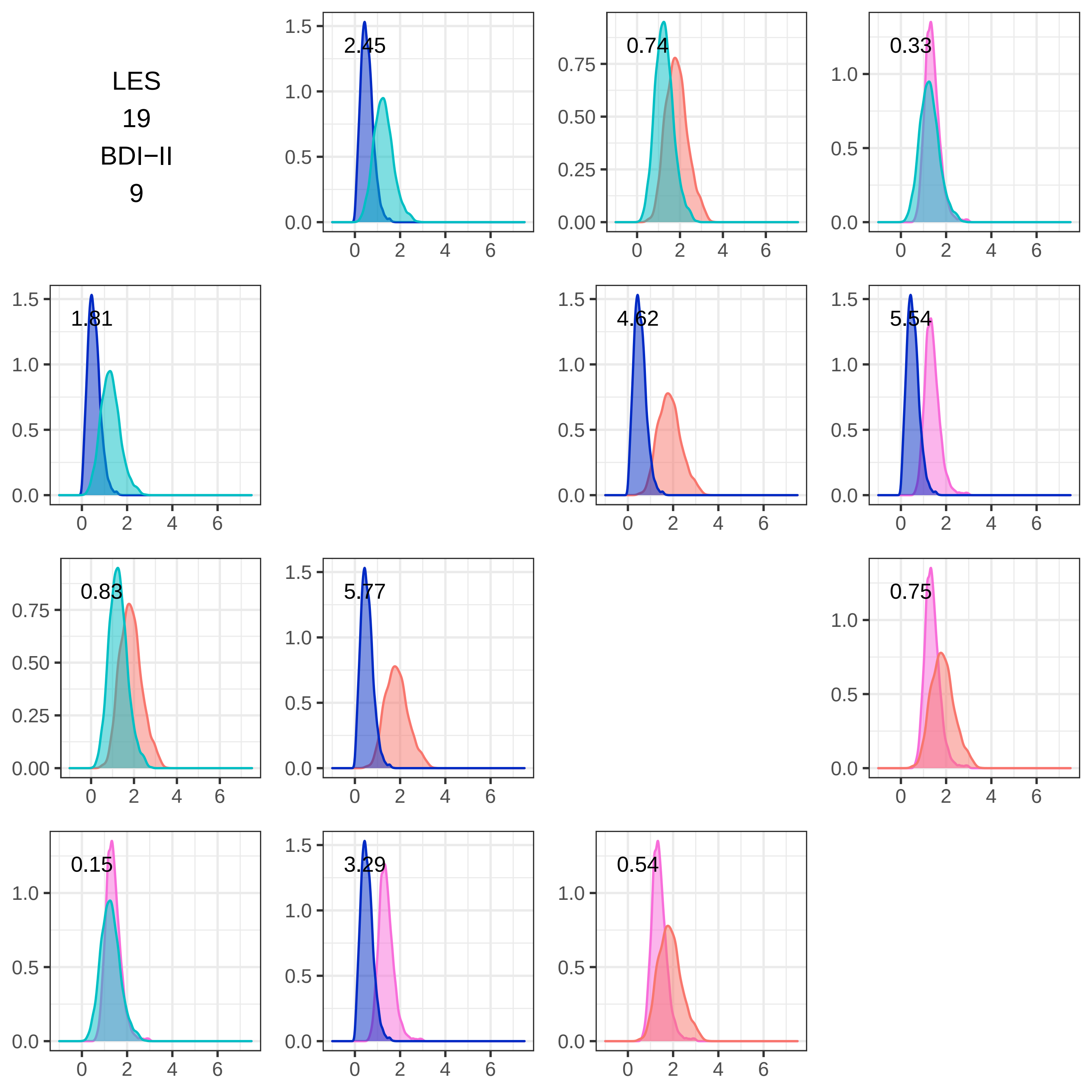
LES
19
BDI−II
9
2.45
0.74
0.33
1.81
4.62
5.54
0.83
5.77
0.75
0.15
3.29
0.54

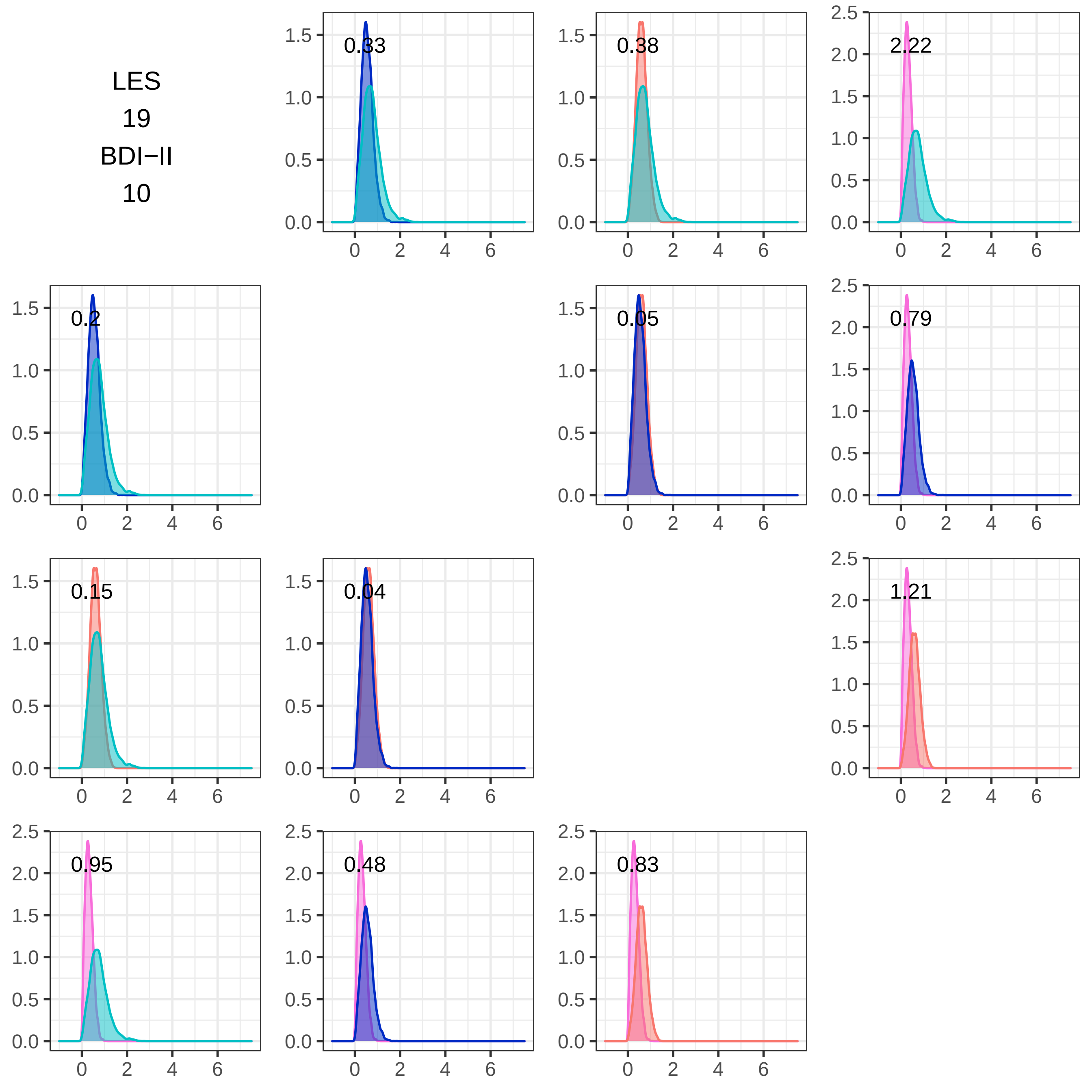

LES
19
BDI−II
10
0.33
0.38
2.22
0.2
0.05
0.79
0.15
0.04
1.21
0.95
0.48
0.83

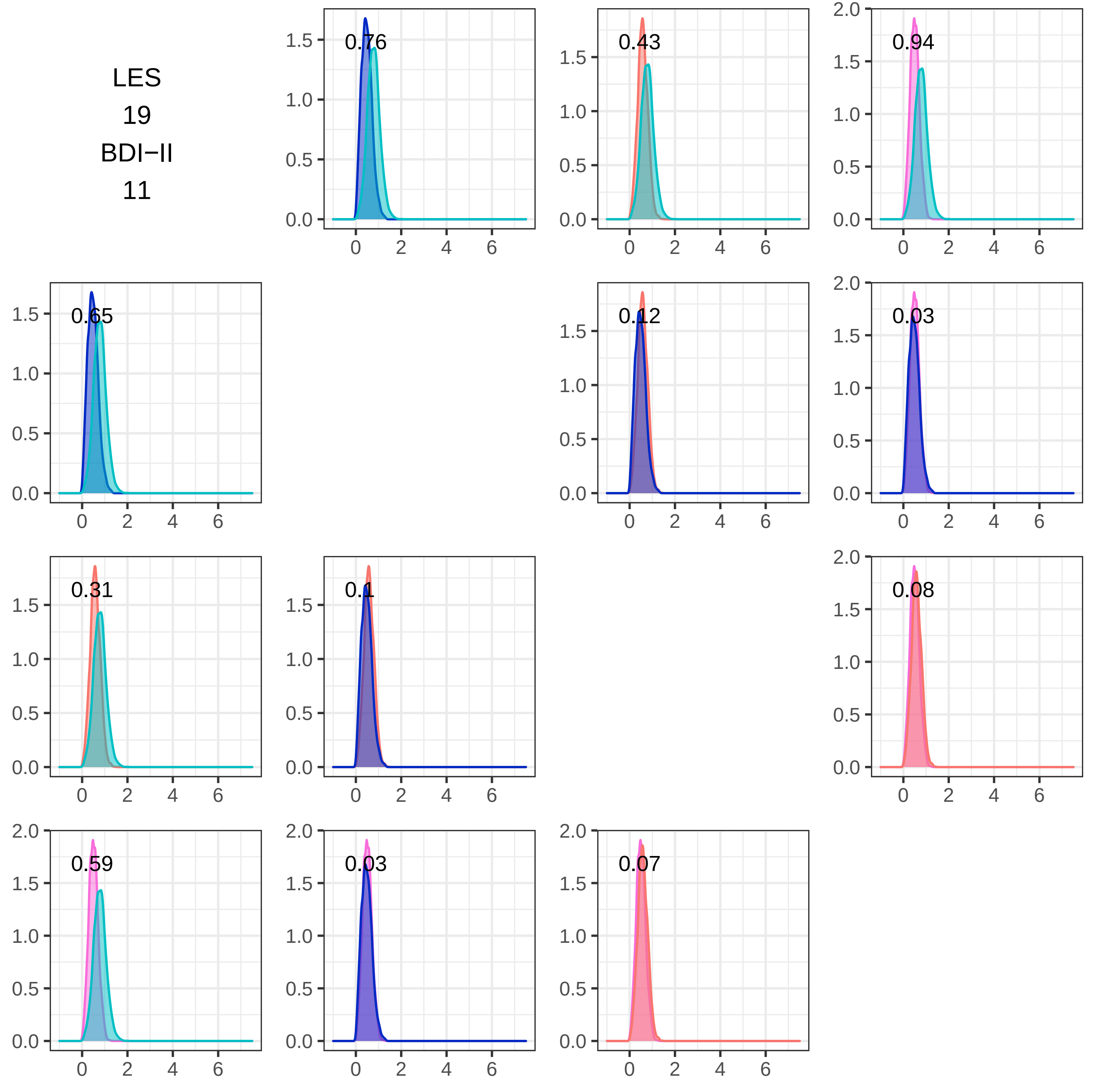
LES
19
BDI−II
11
0.76
0.43
0.94
0.65
0.12
0.03
0.31
0.1
0.08
0.59
0.03
0.07

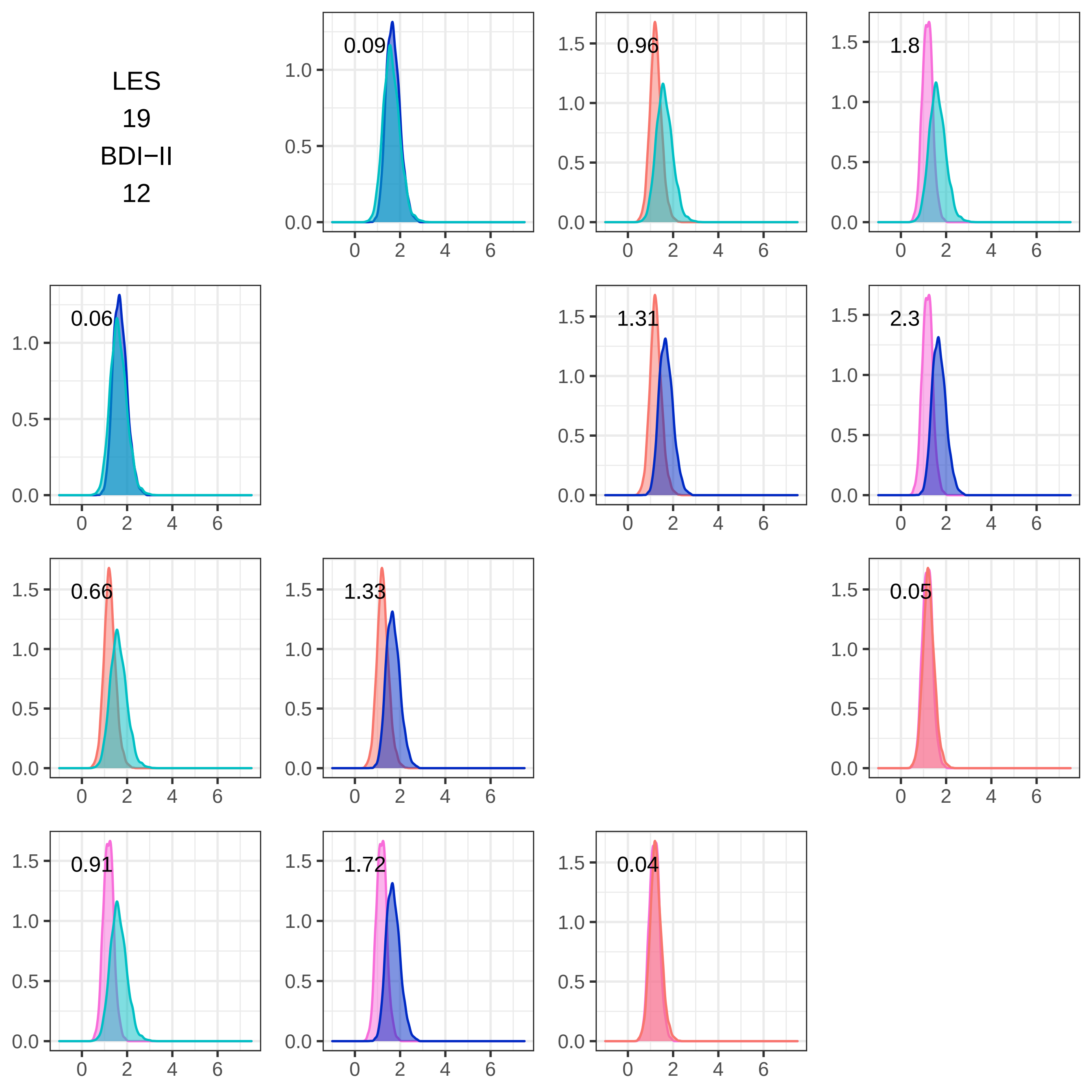

LES
19
BDI−II
12
0.09
0.96
1.8
0.06
1.31
2.3
0.66
1.33
0.05
0.91
1.72
0.04

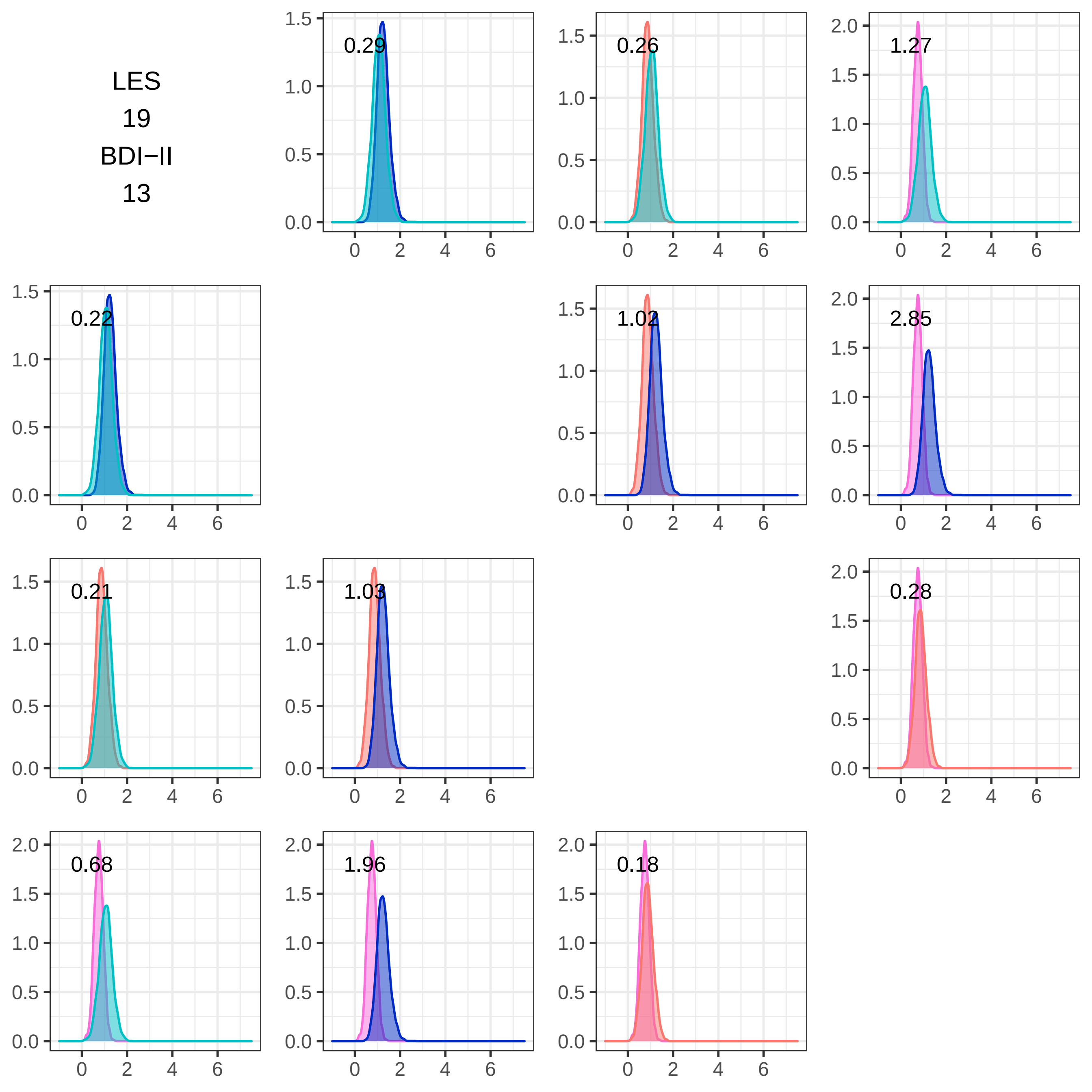
LES
19
BDI−II
13
0.29
0.26
1.27
0.22
1.02
2.85
0.21
1.03
0.28
0.68
1.96
0.18

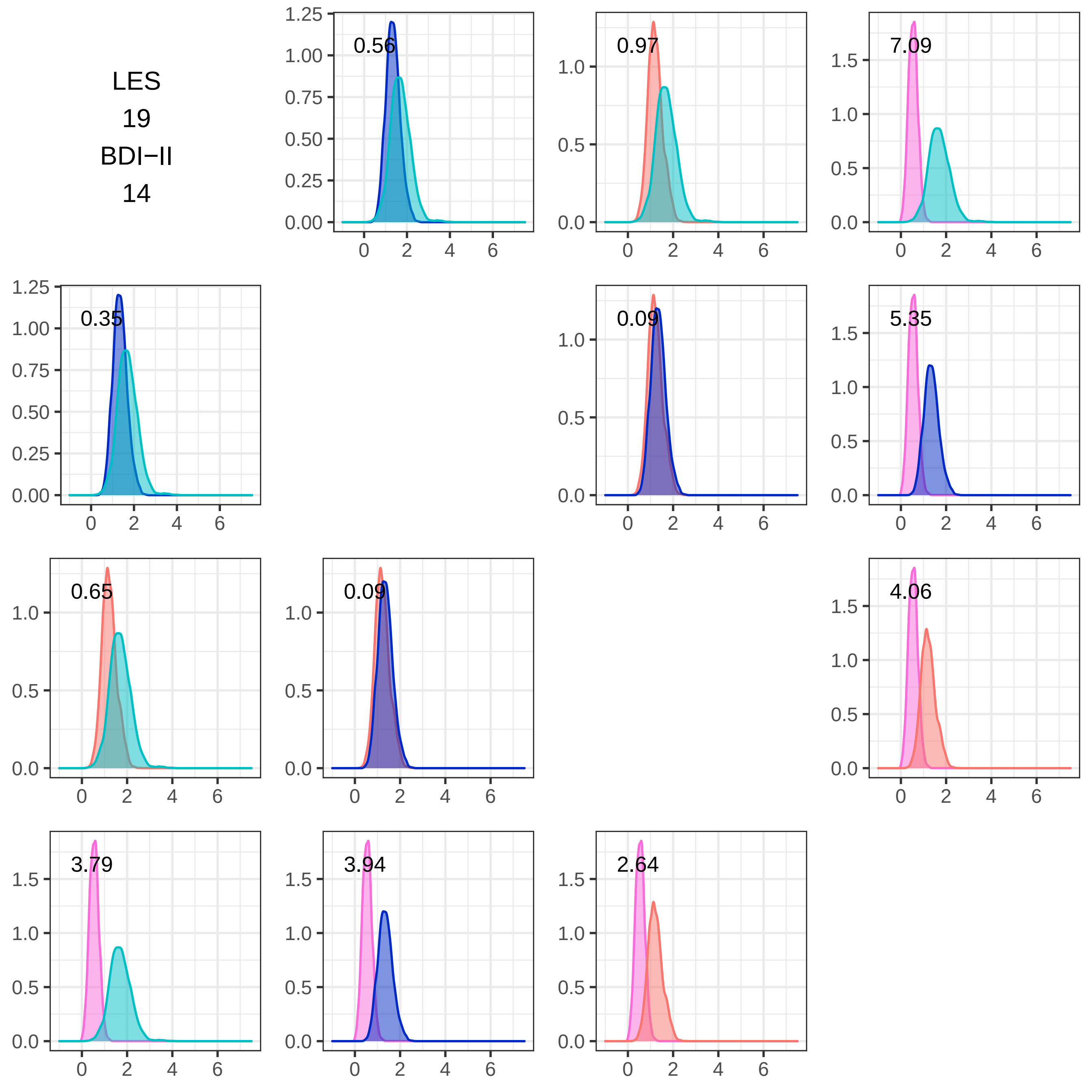
LES
19
BDI−II
14
0.56
0.97
7.09
0.35
0.09
5.35
0.65
0.09
4.06
3.79
3.94
2.64

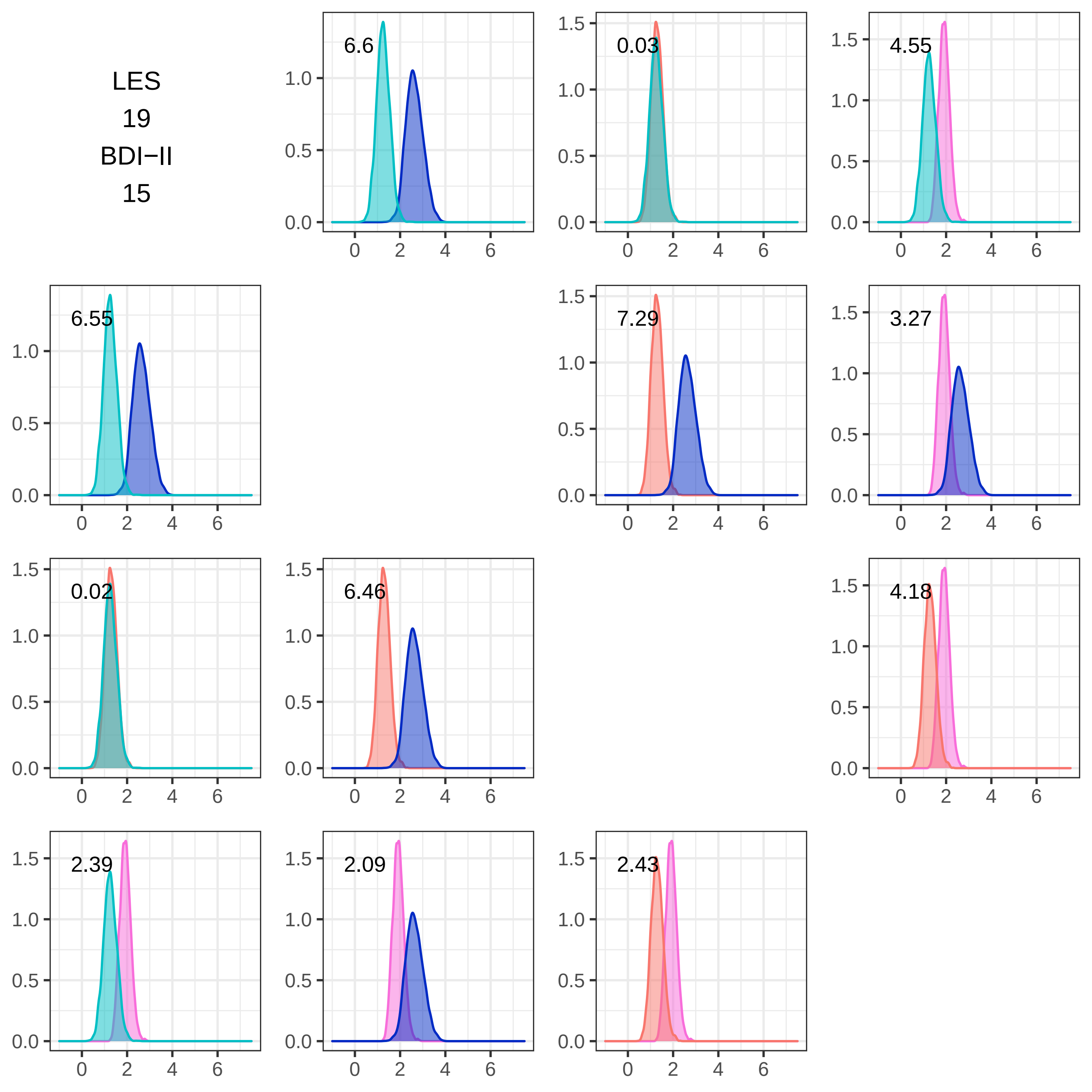

LES
19
BDI−II
15
6.6
0.03
4.55
6.55
7.29
3.27
0.02
6.46
4.18
2.39
2.09
2.43

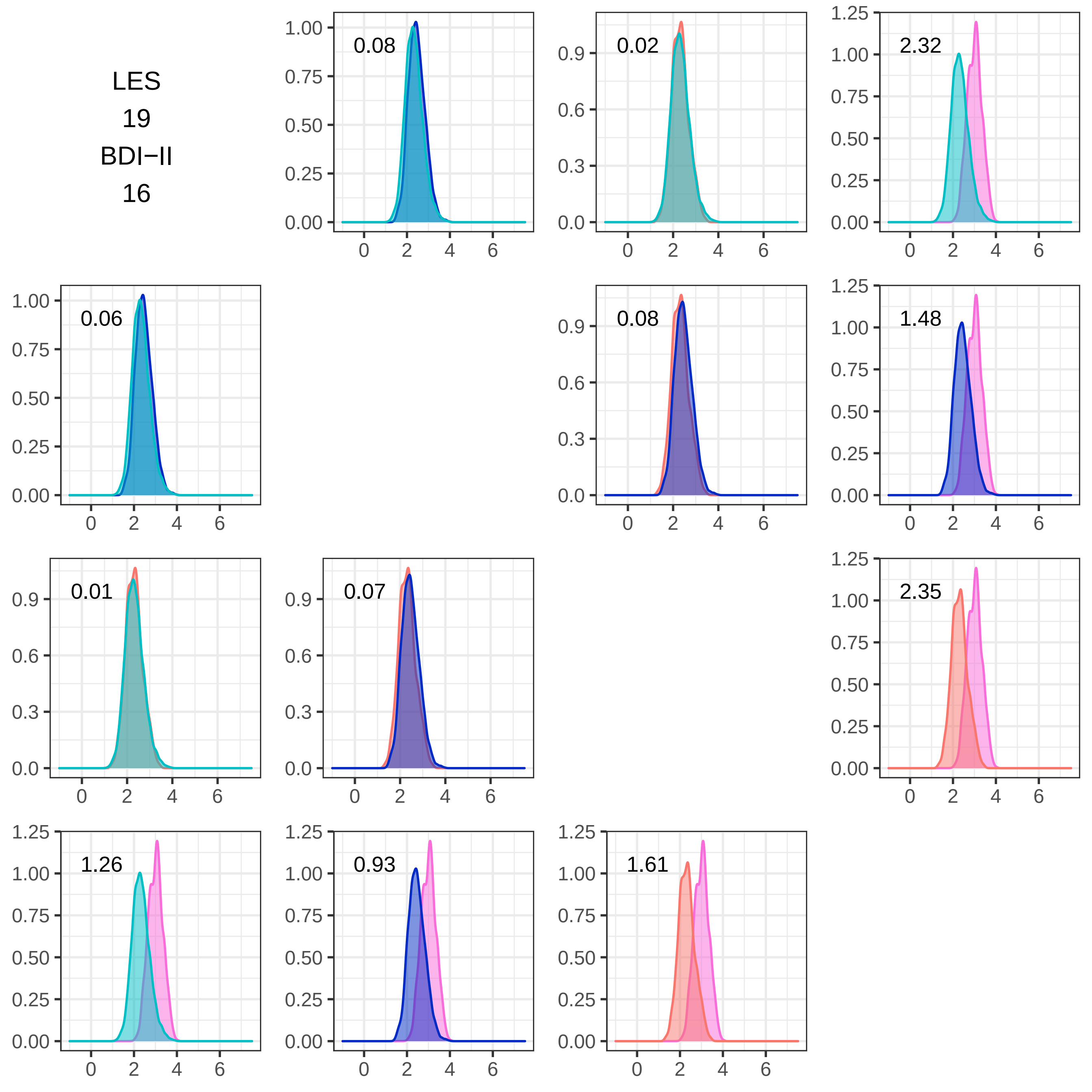

LES
19
BDI−II
16
0.08
0.02
2.32
0.06
0.08
1.48
0.01
0.07
2.35
1.26
0.93
1.61

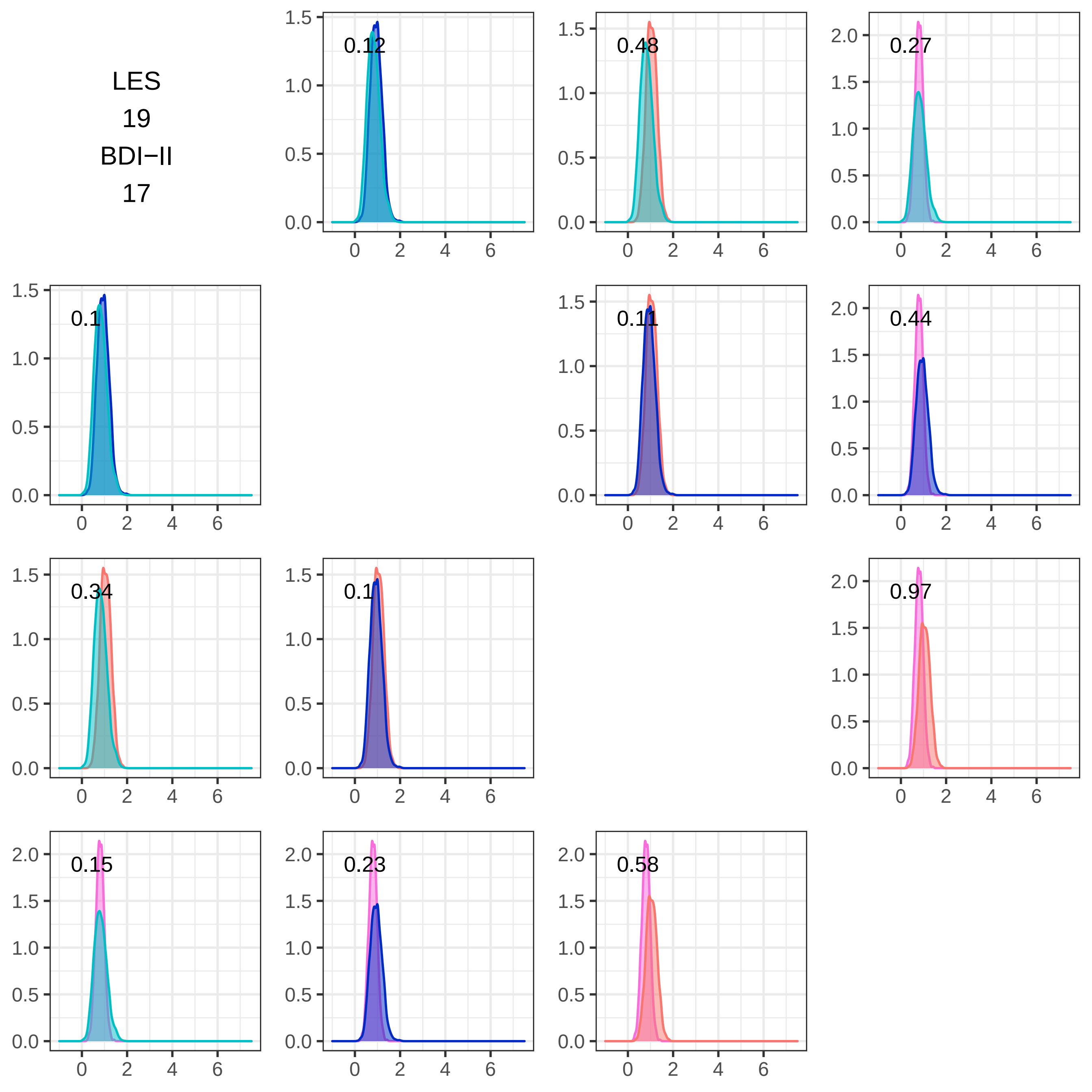

LES
19
BDI−II
17
0.12
0.48
0.27
0.1
0.11
0.44
0.34
0.1
0.97
0.15
0.23
0.58

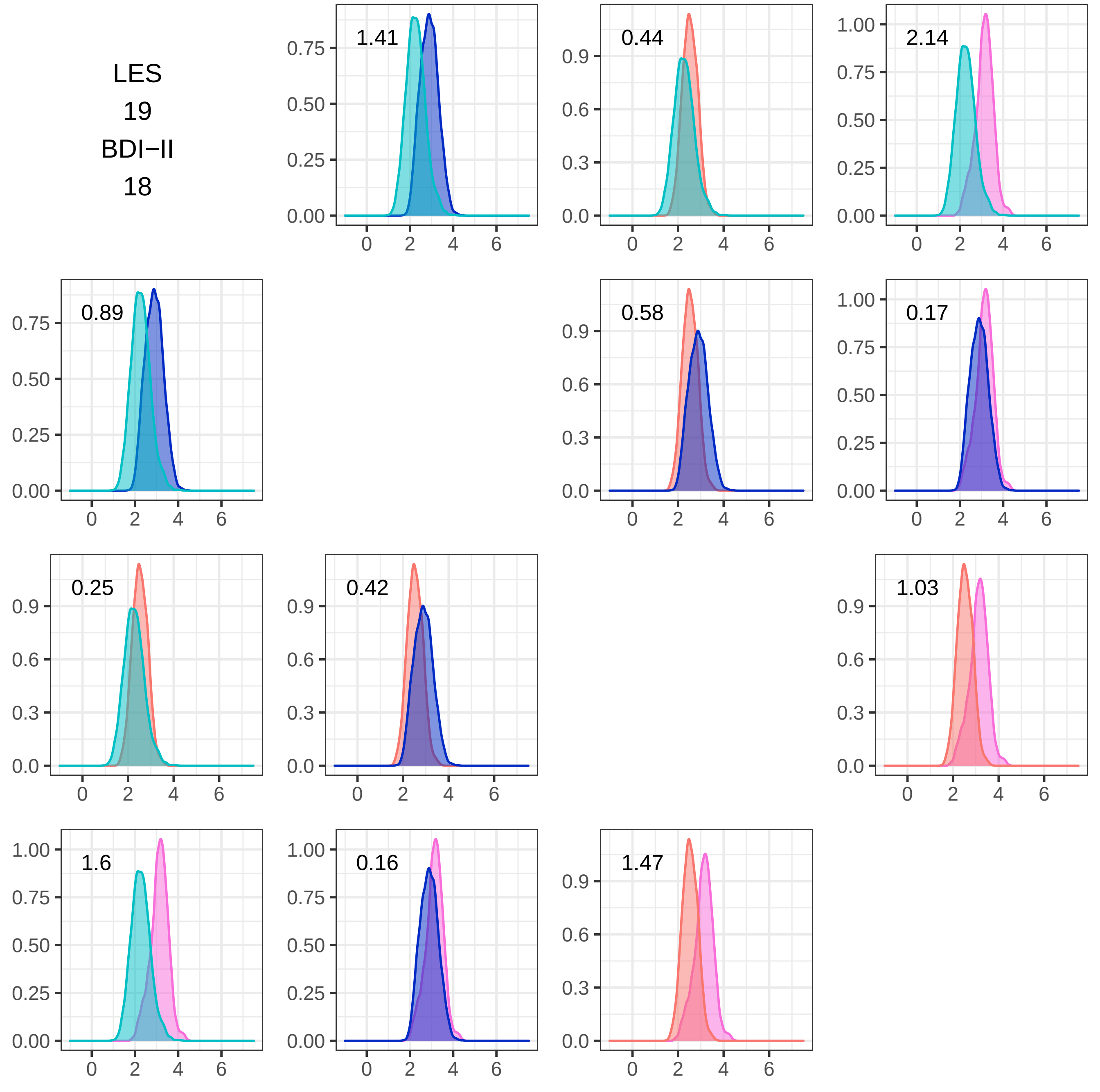
LES
19
BDI−II
18
1.41
0.44
2.14
0.89
0.58
0.17
0.25
0.42
1.03
1.6
0.16
1.47

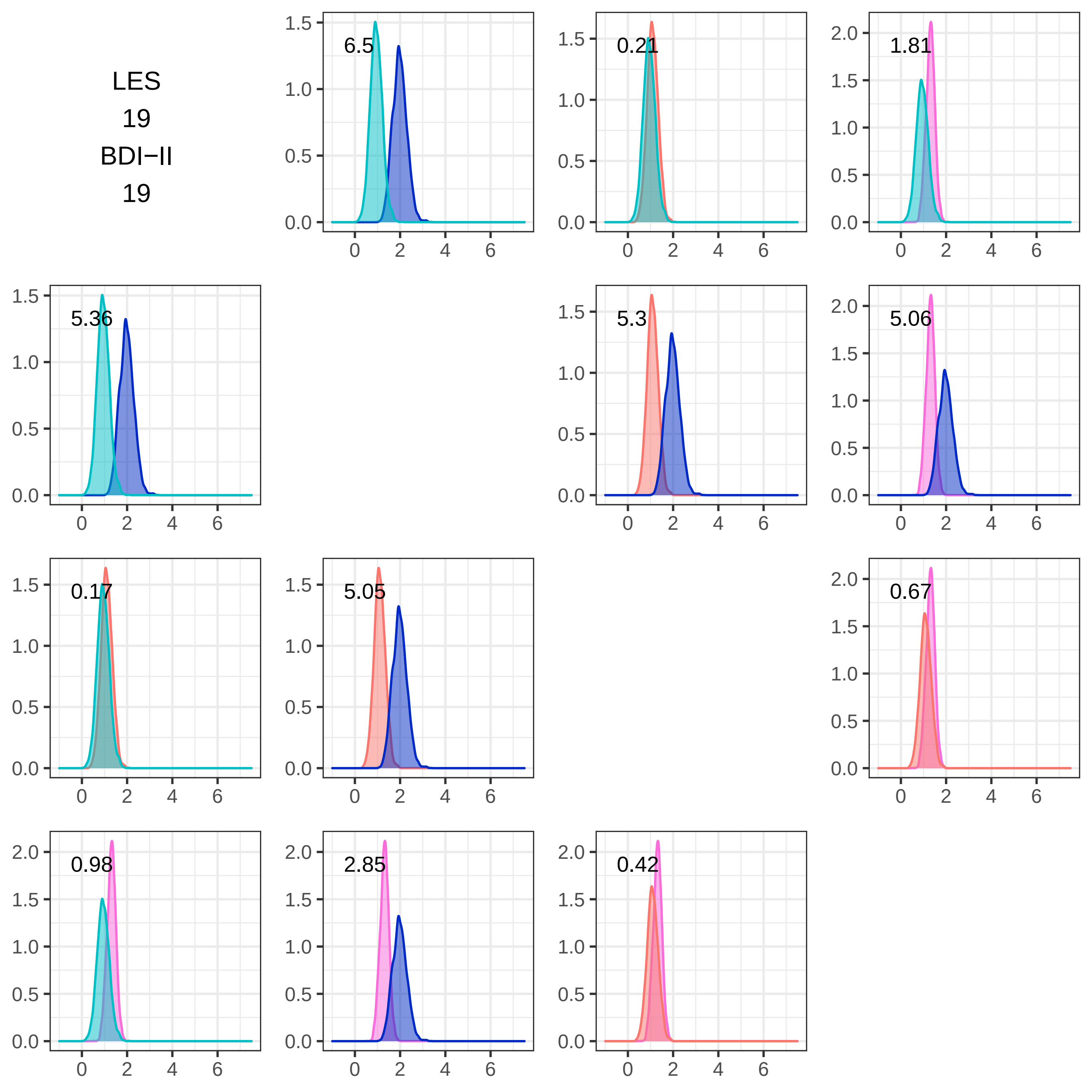
LES
19
BDI−II
19
6.5
0.21
1.81
5.36
5.3
5.06
0.17
5.05
0.67
0.98
2.85
0.42

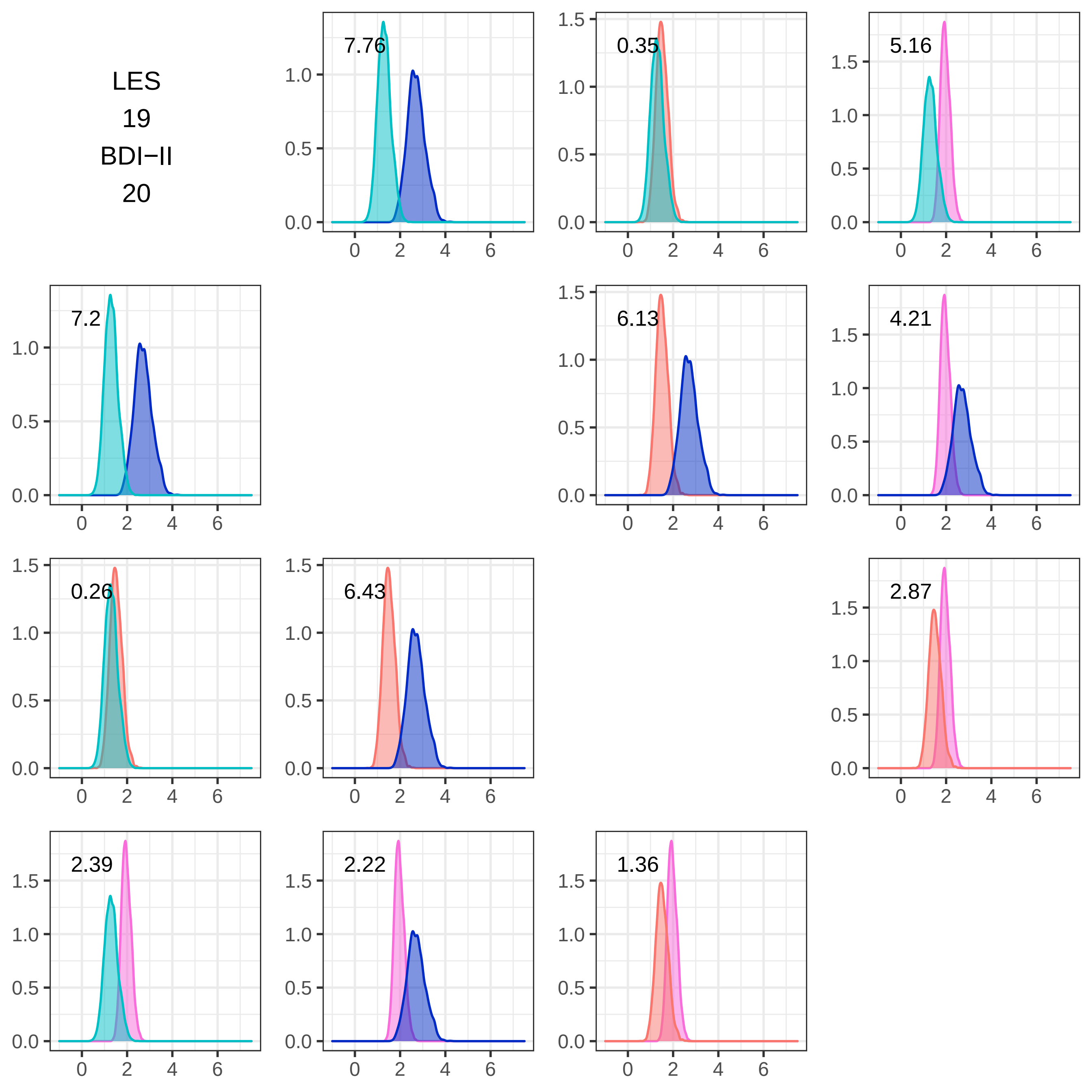

LES
19
BDI−II
20
7.76
0.35
5.16
7.2
6.13
4.21
0.26
6.43
2.87
2.39
2.22
1.36

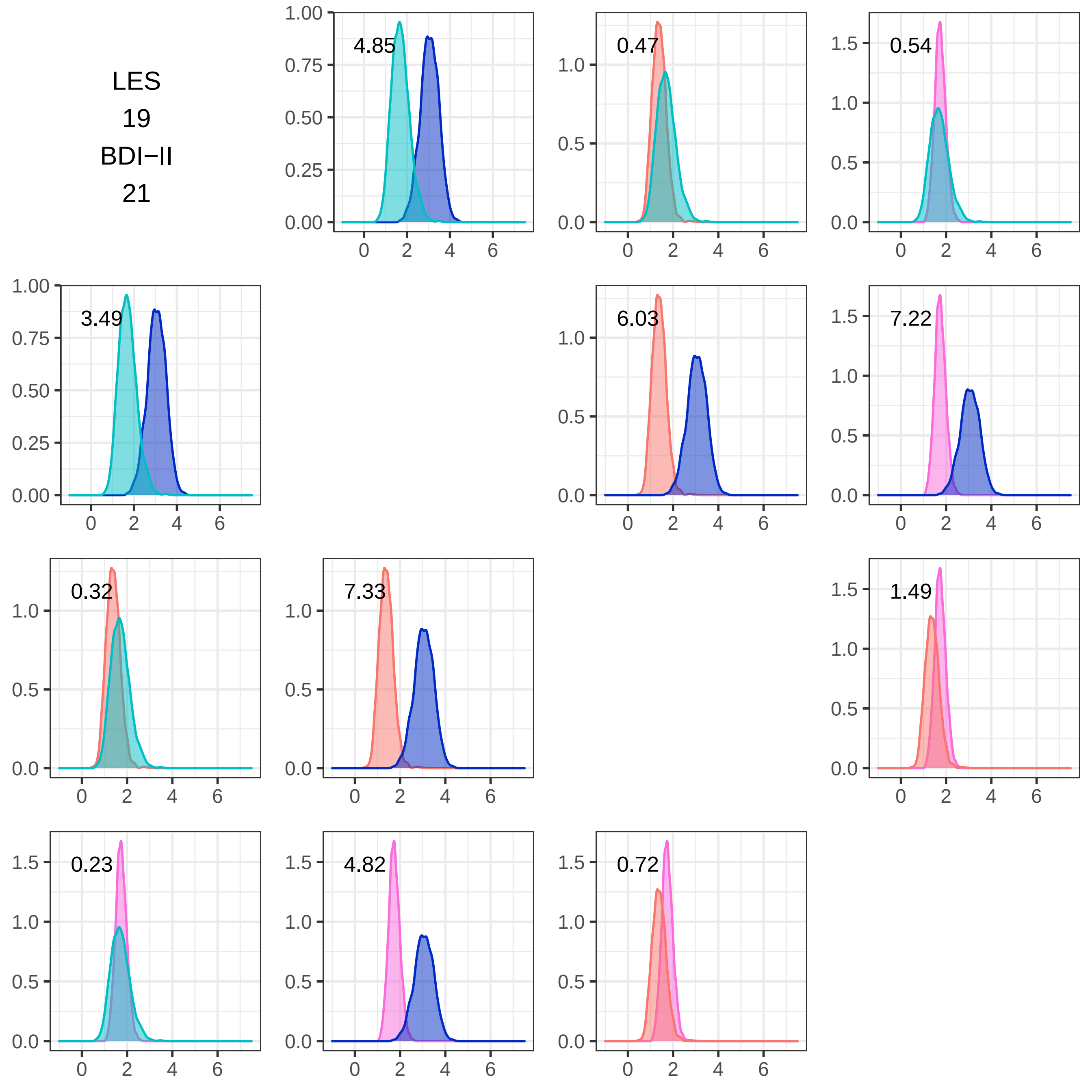
LES
19
BDI−II
21
4.85
0.47
0.54
3.49
6.03
7.22
0.32
7.33
1.49
0.23
4.82
0.72

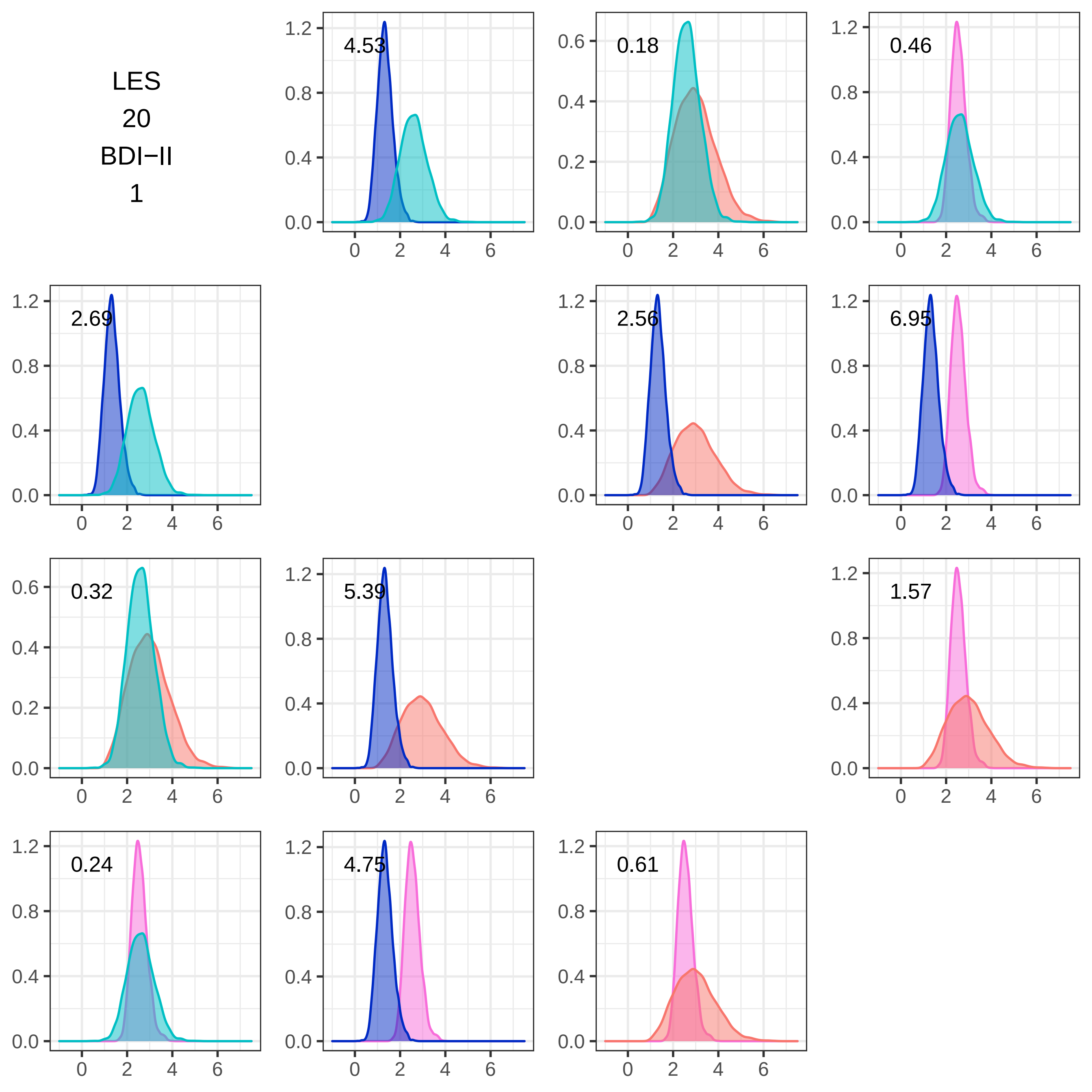
LES
20
BDI−II
1
4.53
0.18
0.46
2.69
2.56
6.95
0.32
5.39
1.57
0.24
4.75
0.61

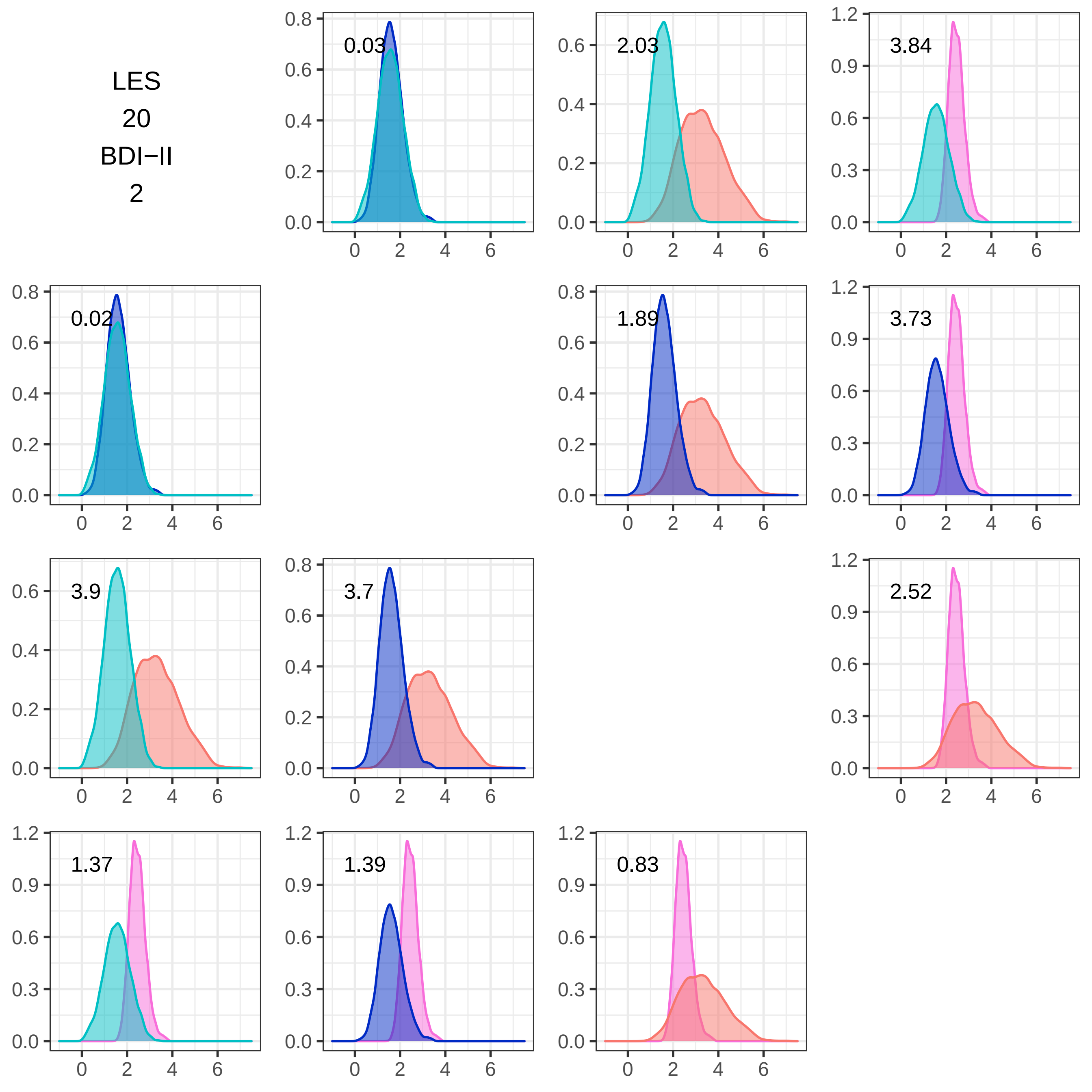
LES
20
BDI−II
2
0.03
2.03
3.84
0.02
1.89
3.73
3.9
3.7
2.52
1.37
1.39
0.83

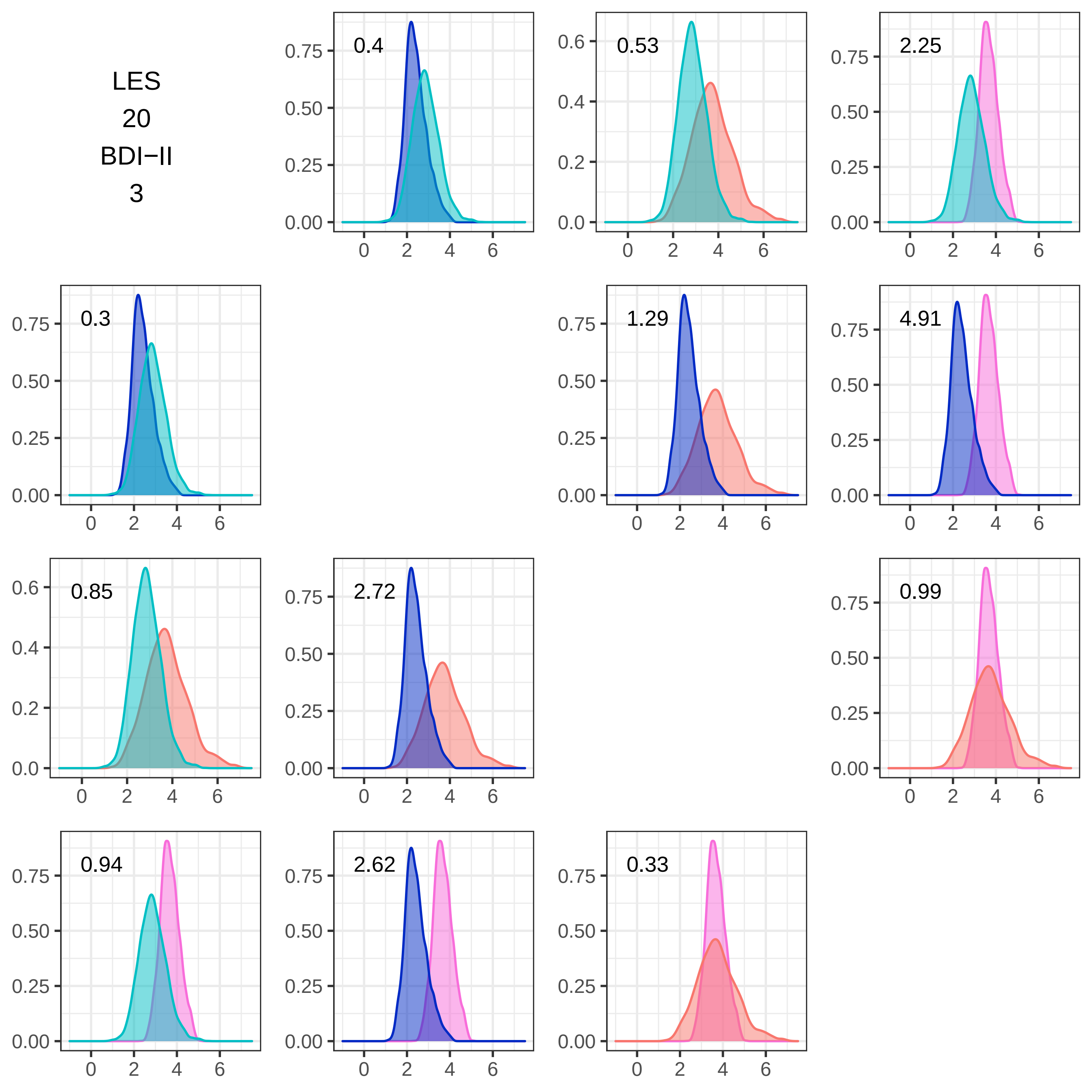
LES
20
BDI−II
3
0.4
0.53
2.25
0.3
1.29
4.91
0.85
2.72
0.99
0.94
2.62
0.33

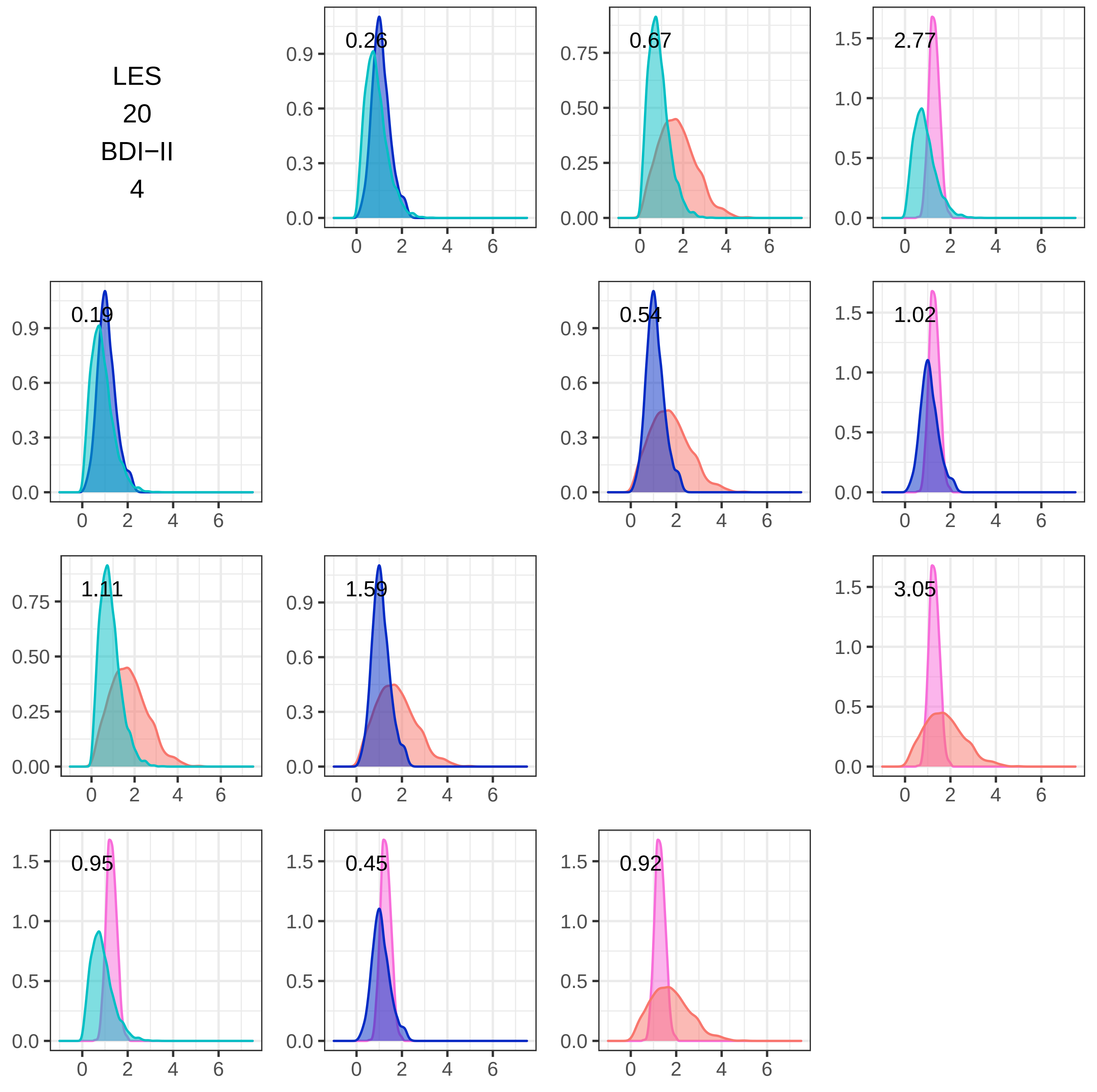

LES
20
BDI−II
4
0.26
0.67
2.77
0.19
0.54
1.02
1.11
1.59
3.05
0.95
0.45
0.92

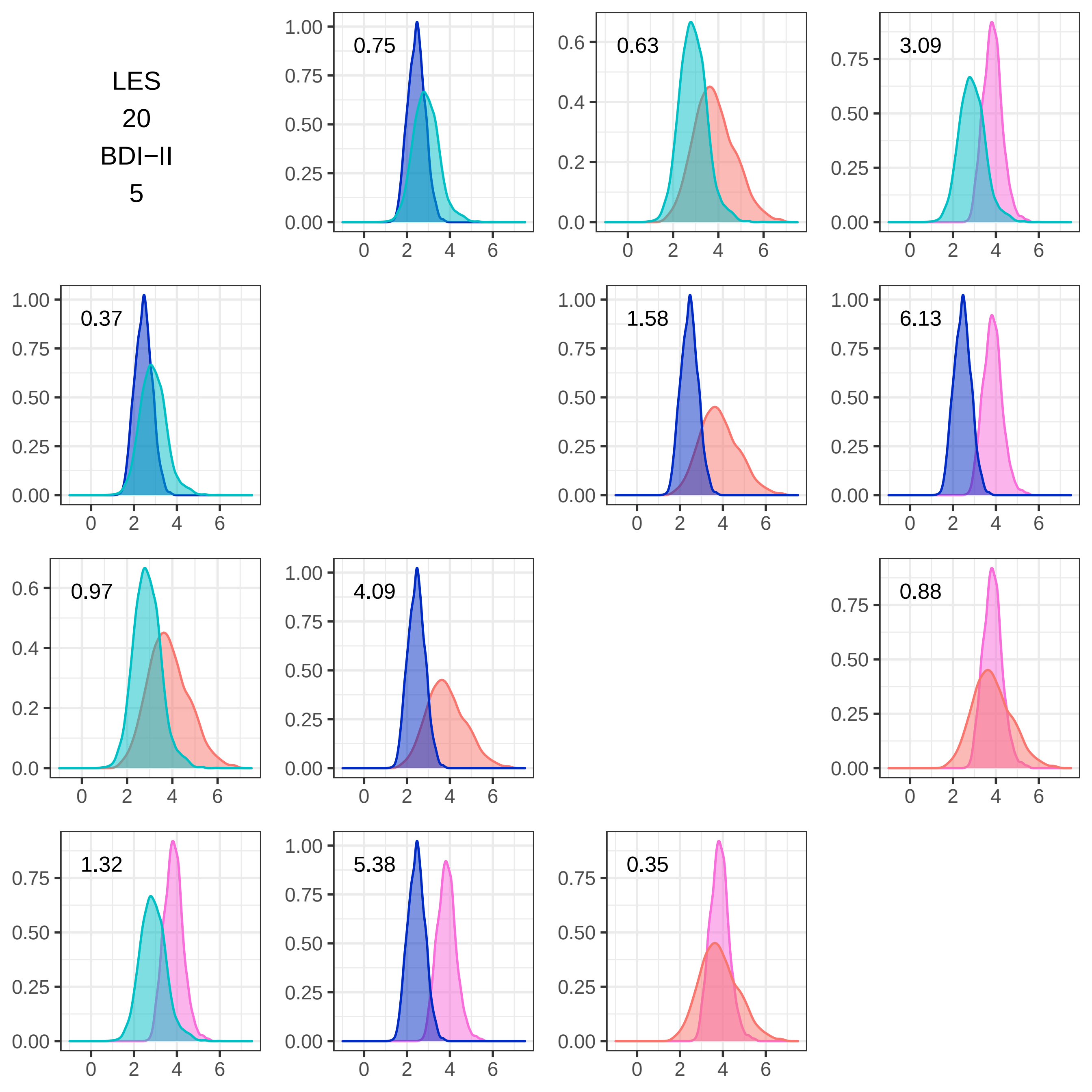

LES
20
BDI−II
5
0.75
0.63
3.09
0.37
1.58
6.13
0.97
4.09
0.88
1.32
5.38
0.35

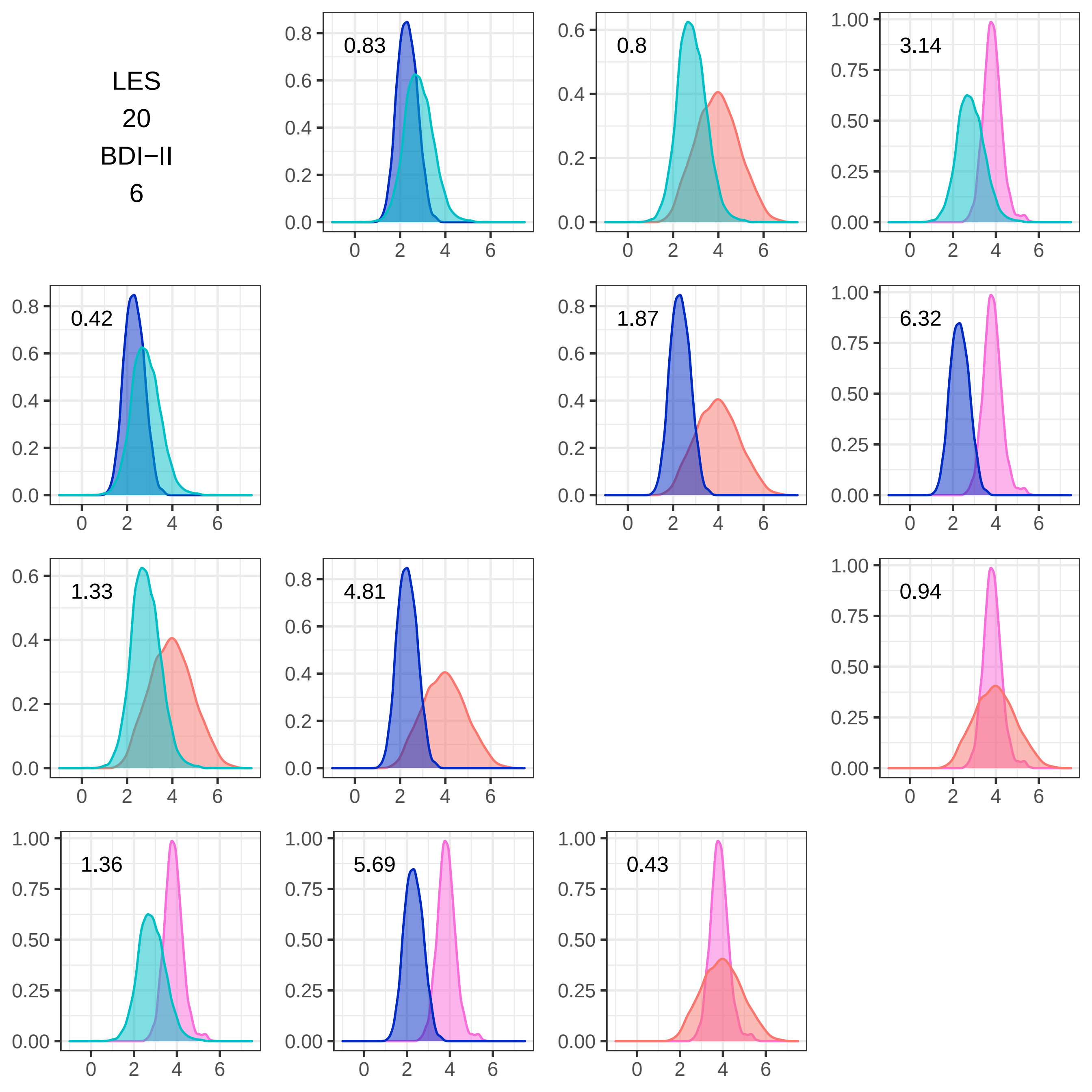
LES
20
BDI−II
6
0.83
0.8
3.14
0.42
1.87
6.32
1.33
4.81
0.94
1.36
5.69
0.43

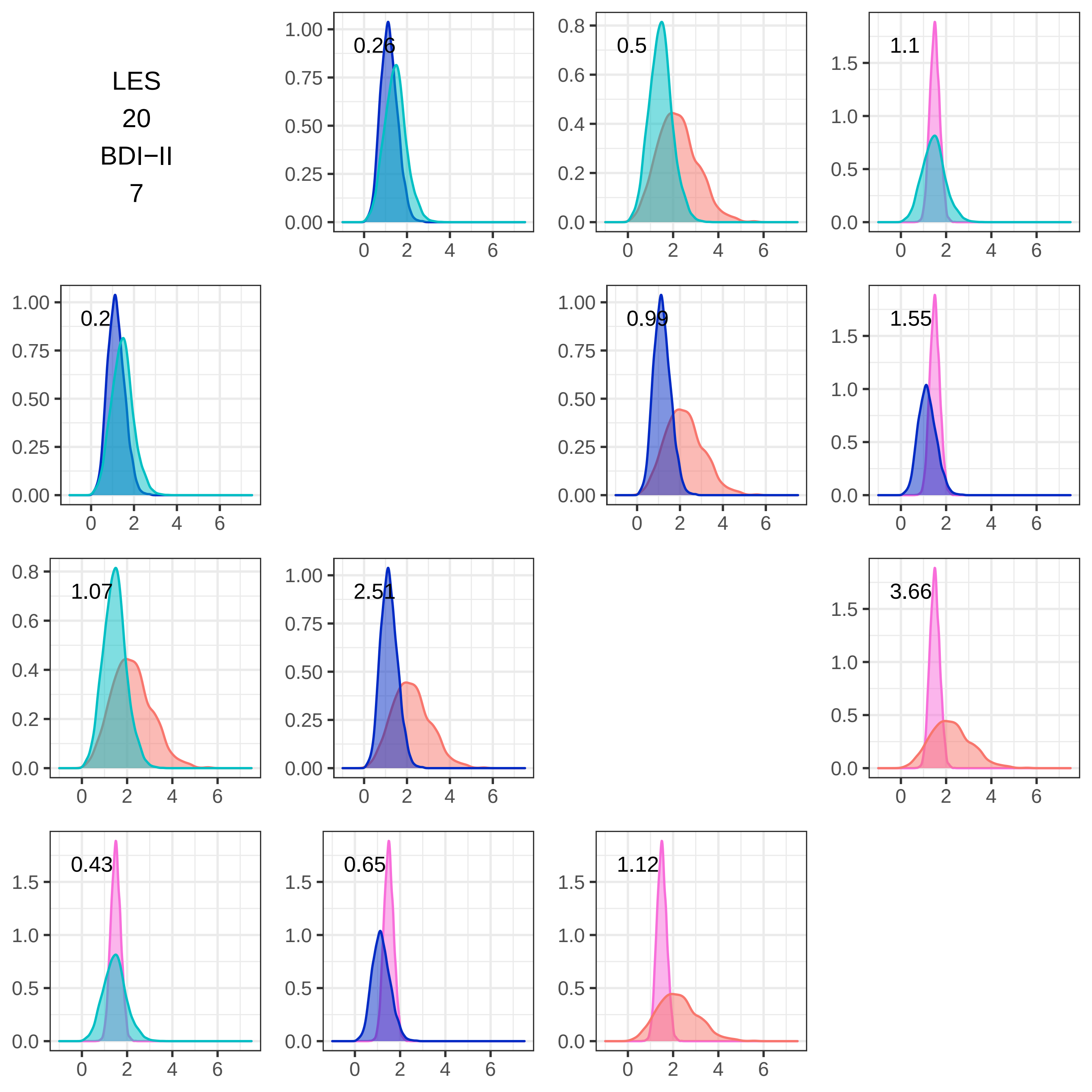

LES
20
BDI−II
7
0.26
0.5
1.1
0.2
0.99
1.55
1.07
2.51
3.66
0.43
0.65
1.12

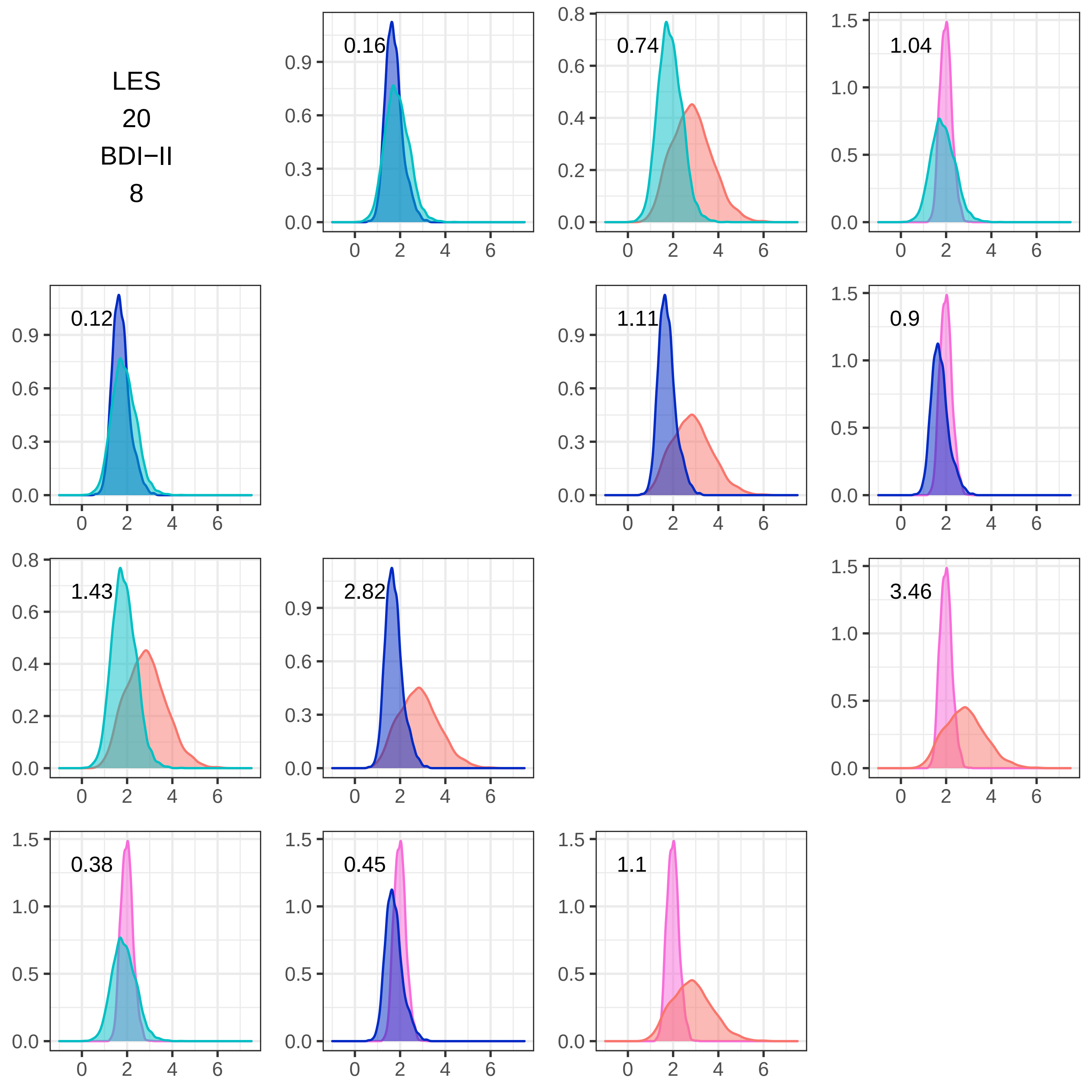
LES
20
BDI−II
8
0.16
0.74
1.04
0.12
1.11
0.9
1.43
2.82
3.46
0.38
0.45
1.1

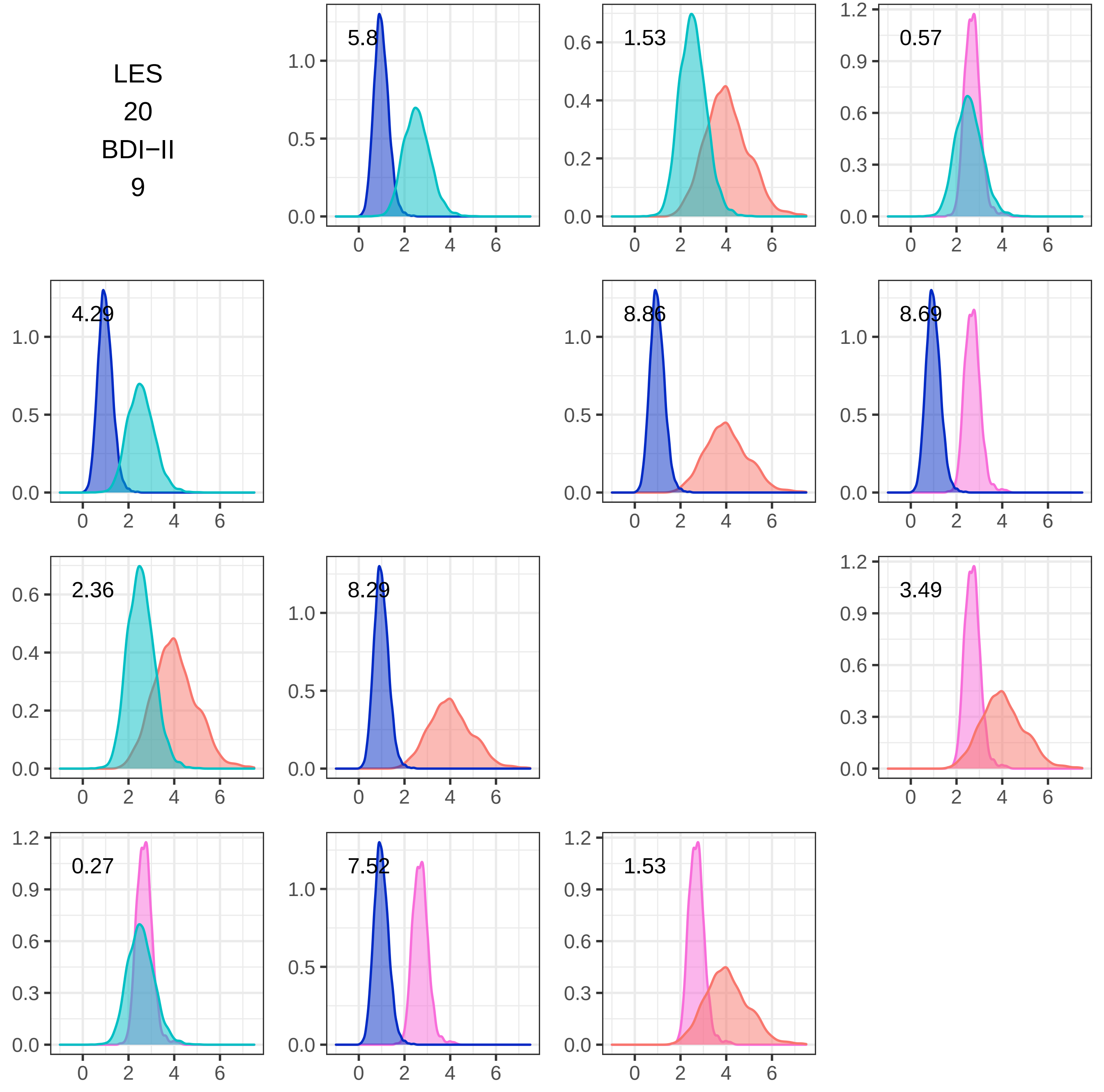

LES
20
BDI−II
9
5.8
1.53
0.57
4.29
8.86
8.69
2.36
8.29
3.49
0.27
7.52
1.53

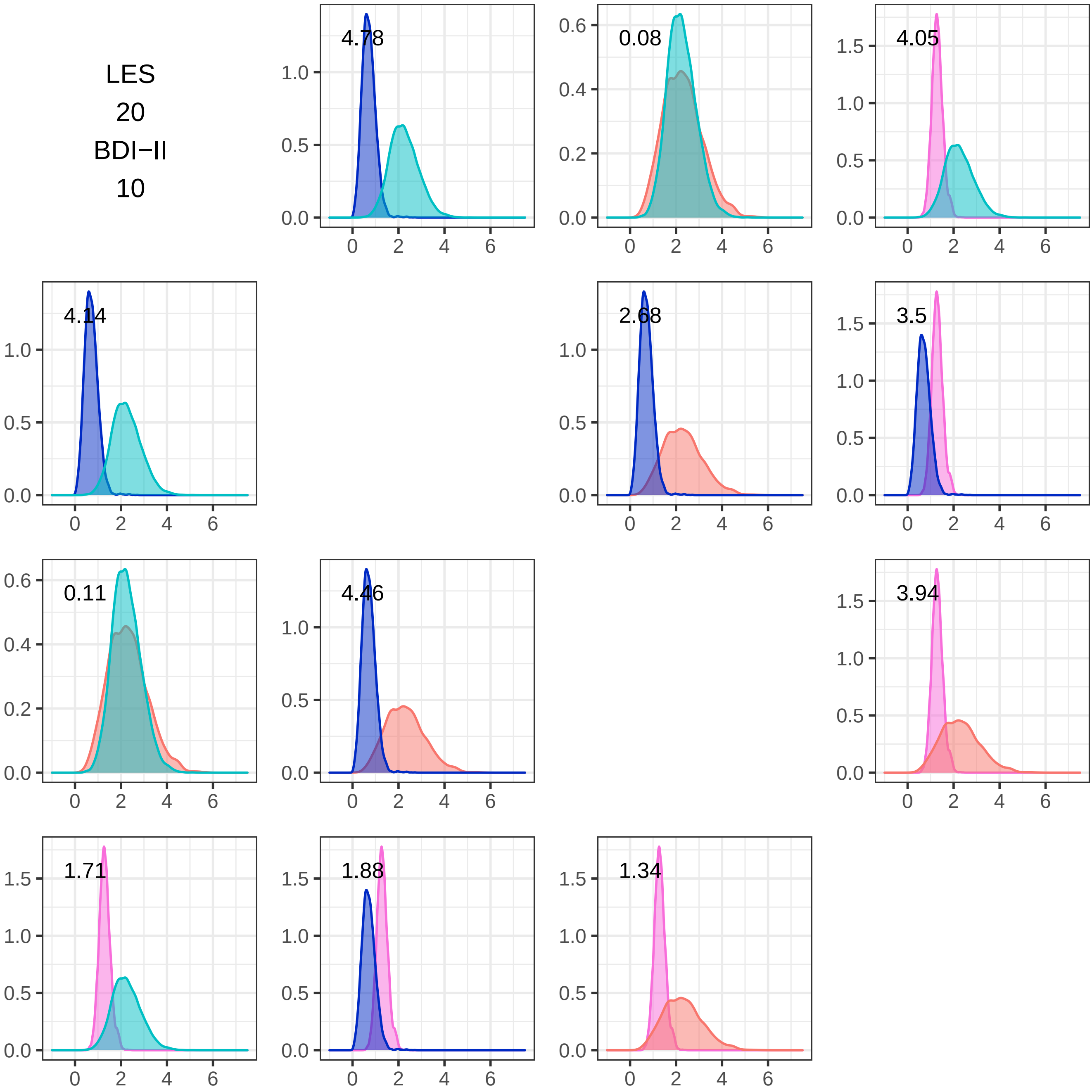

LES
20
BDI−II
10
4.78
0.08
4.05
4.14
2.68
3.5
0.11
4.46
3.94
1.71
1.88
1.34

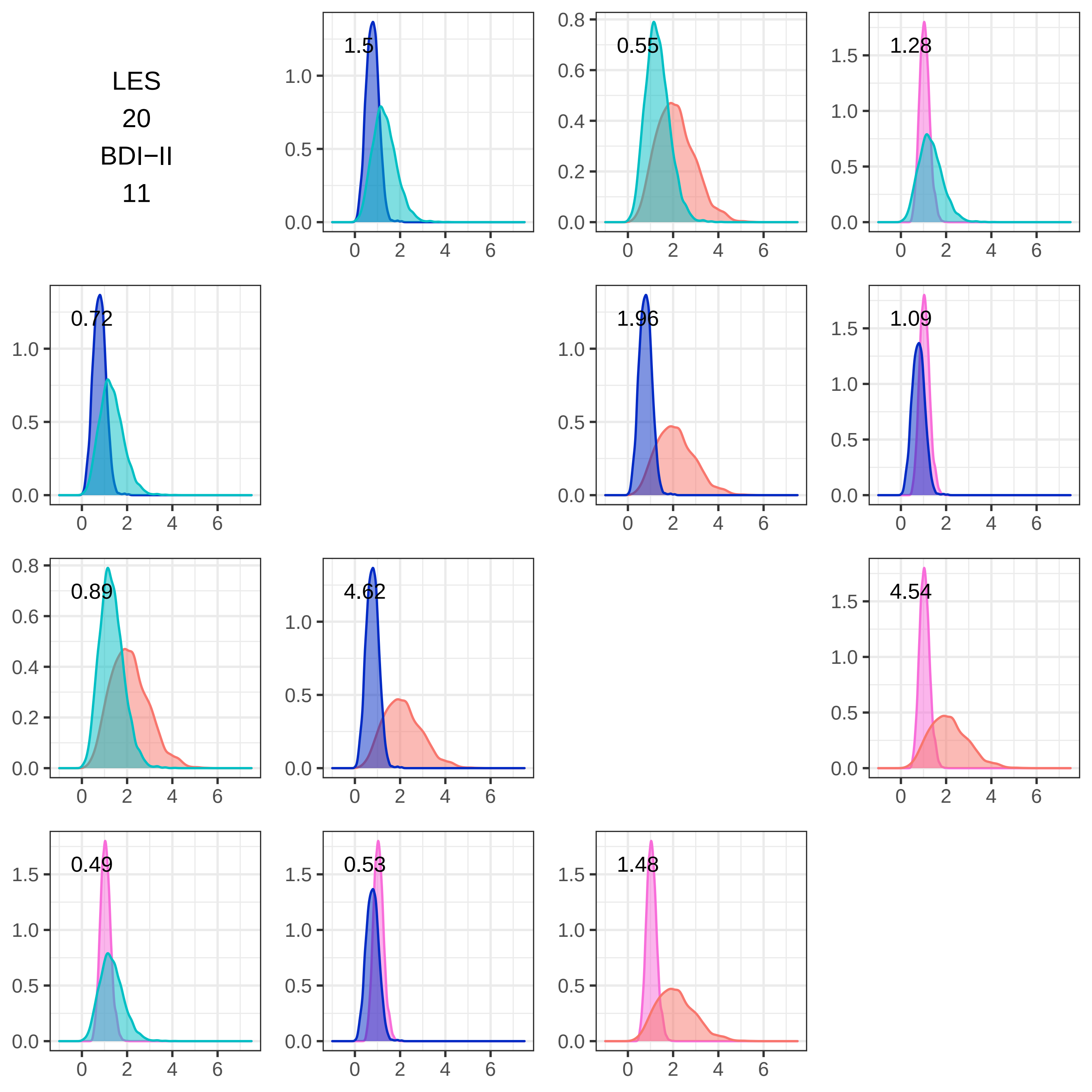

LES
20
BDI−II
11
1.5
0.55
1.28
0.72
1.96
1.09
0.89
4.62
4.54
0.49
0.53
1.48

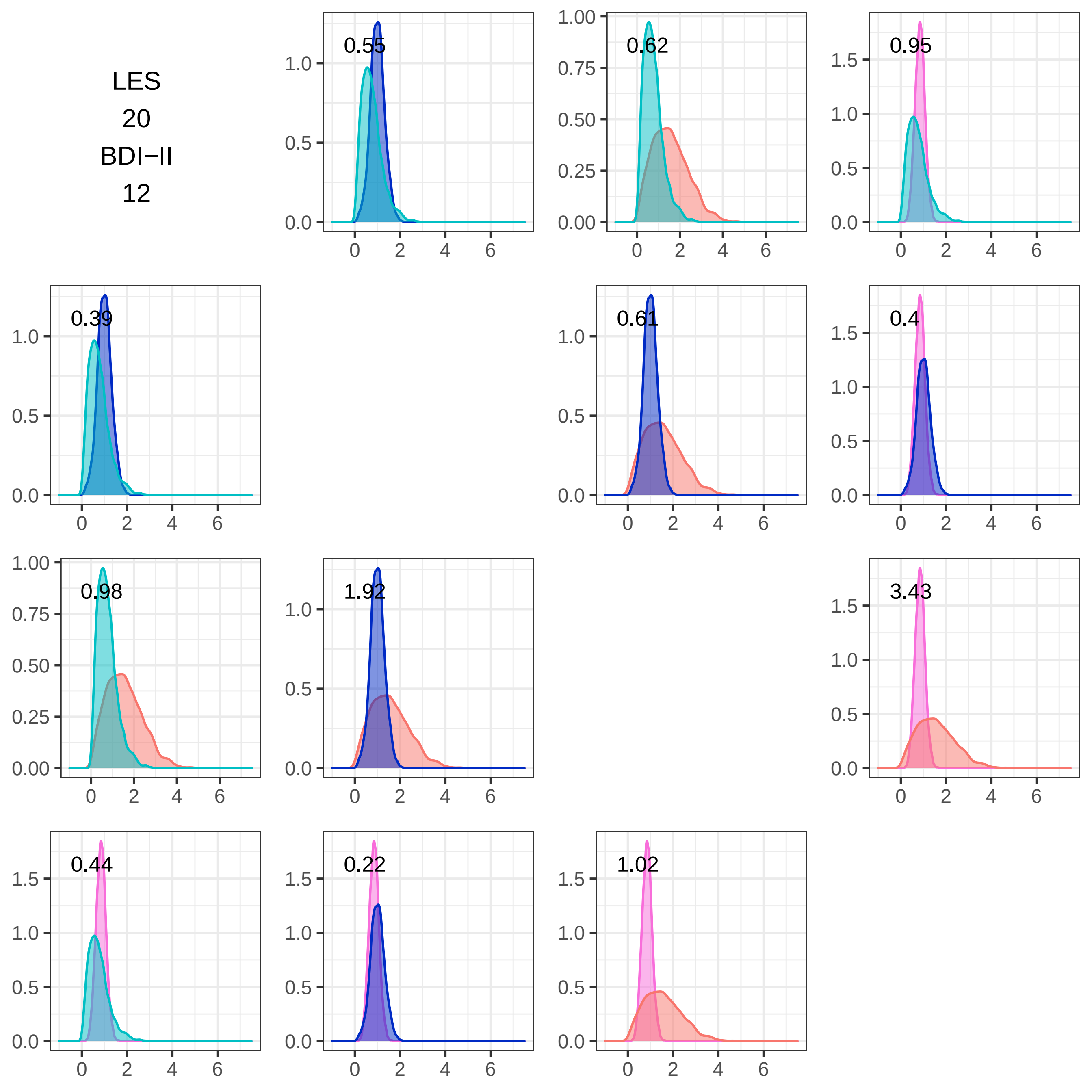

LES
20
BDI−II
12
0.55
0.62
0.95
0.39
0.61
0.4
0.98
1.92
3.43
0.44
0.22
1.02

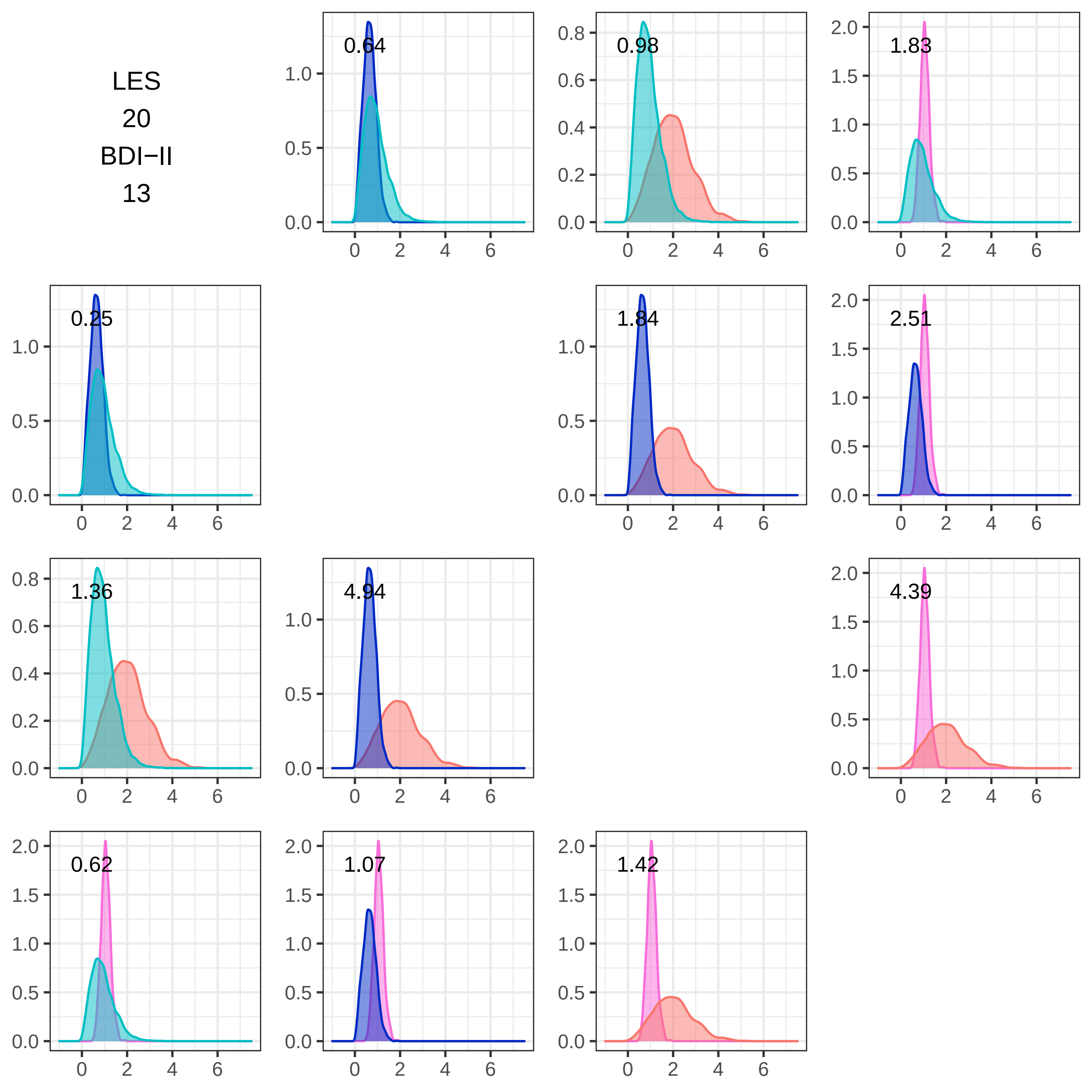

LES
20
BDI−II
13
0.64
0.98
1.83
0.25
1.84
2.51
1.36
4.94
4.39
0.62
1.07
1.42

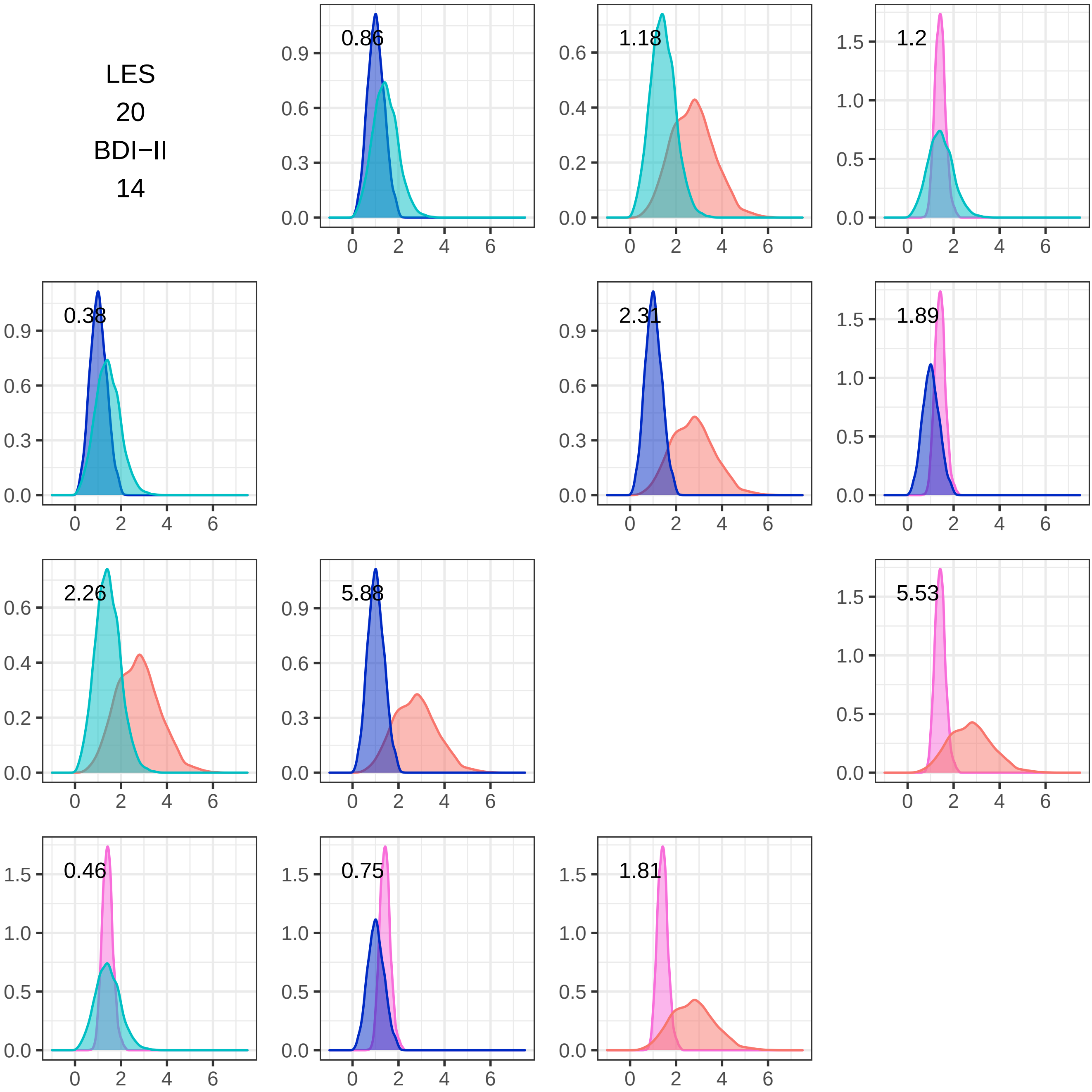

LES
20
BDI−II
14
0.86
1.18
1.2
0.38
2.31
1.89
2.26
5.88
5.53
0.46
0.75
1.81

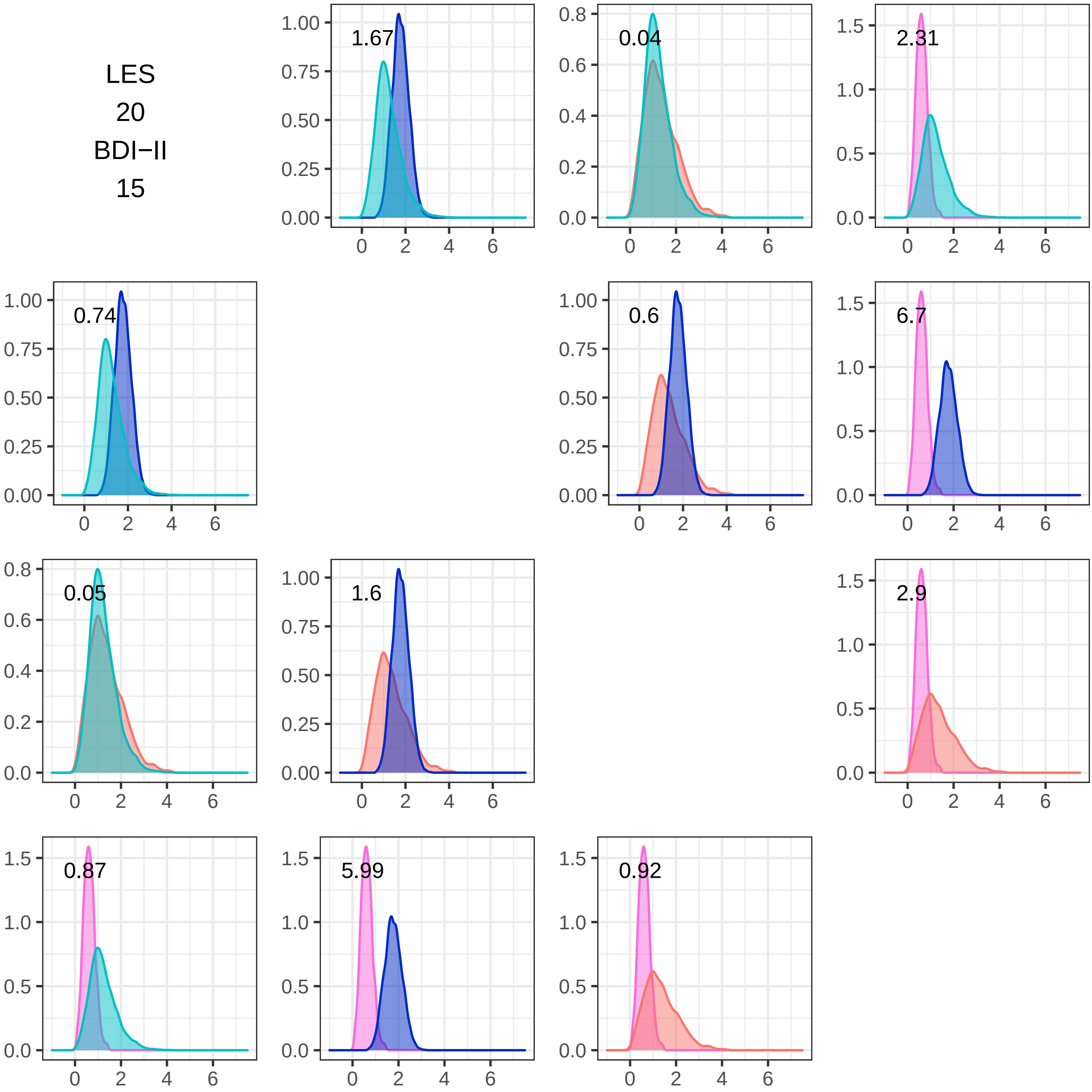
LES
20
BDI−II
15
1.67
0.04
2.31
0.74
0.6
6.7
0.05
1.6
2.9
0.87
5.99
0.92

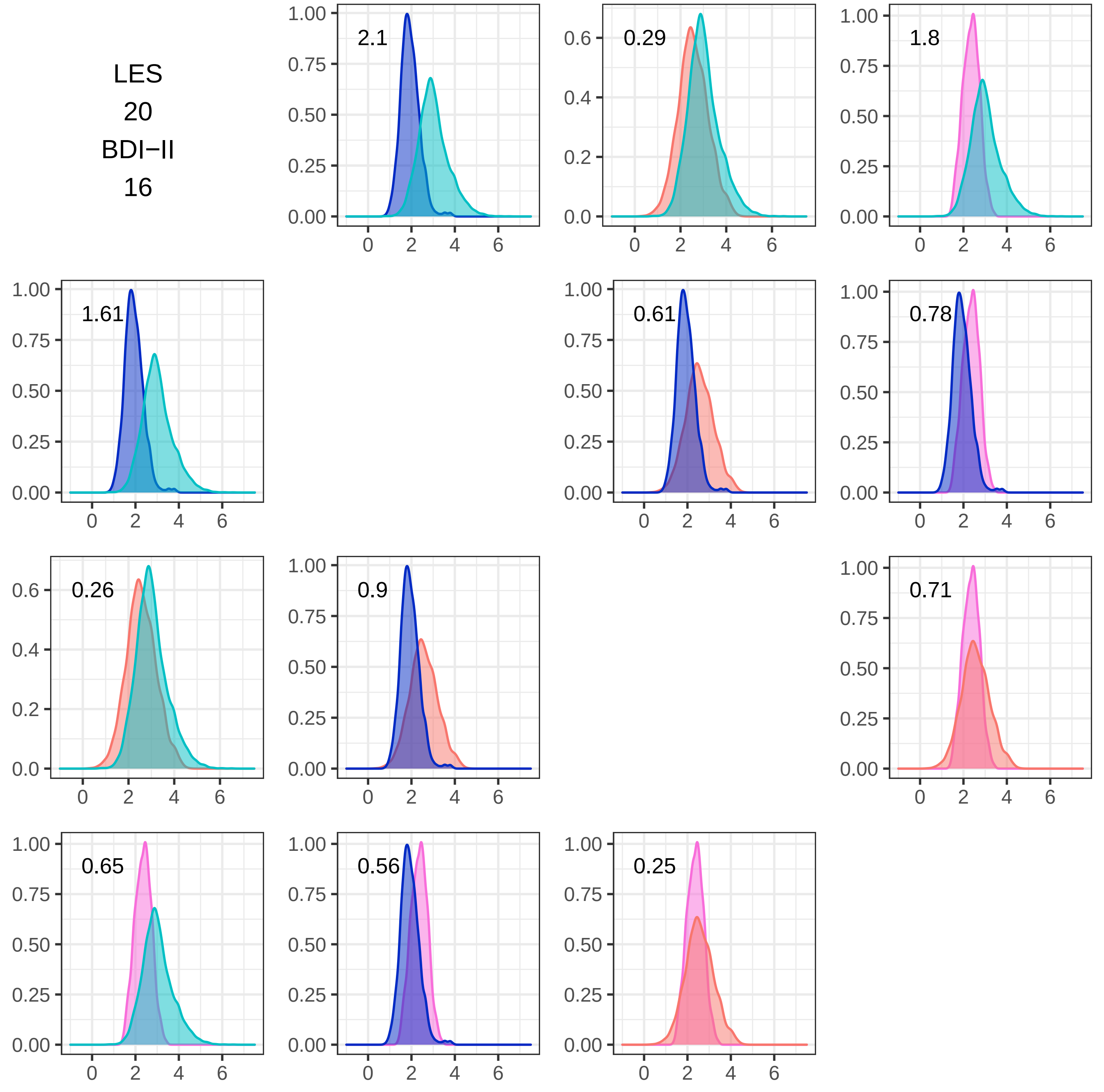

LES
20
BDI−II
16
2.1
0.29
1.8
1.61
0.61
0.78
0.26
0.9
0.71
0.65
0.56
0.25

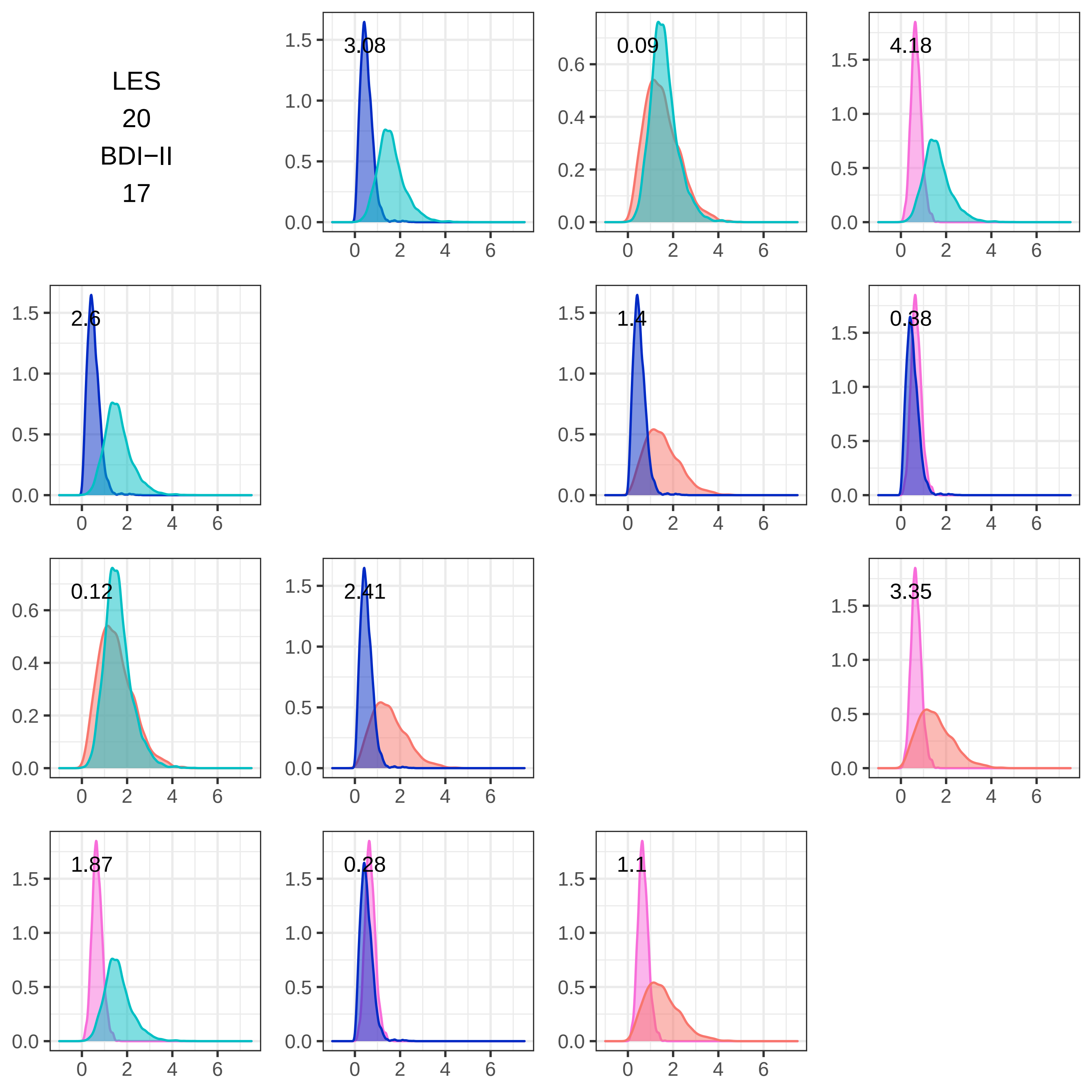
LES
20
BDI−II
17
3.08
0.09
4.18
2.6
1.4
0.38
0.12
2.41
3.35
1.87
0.28
1.1

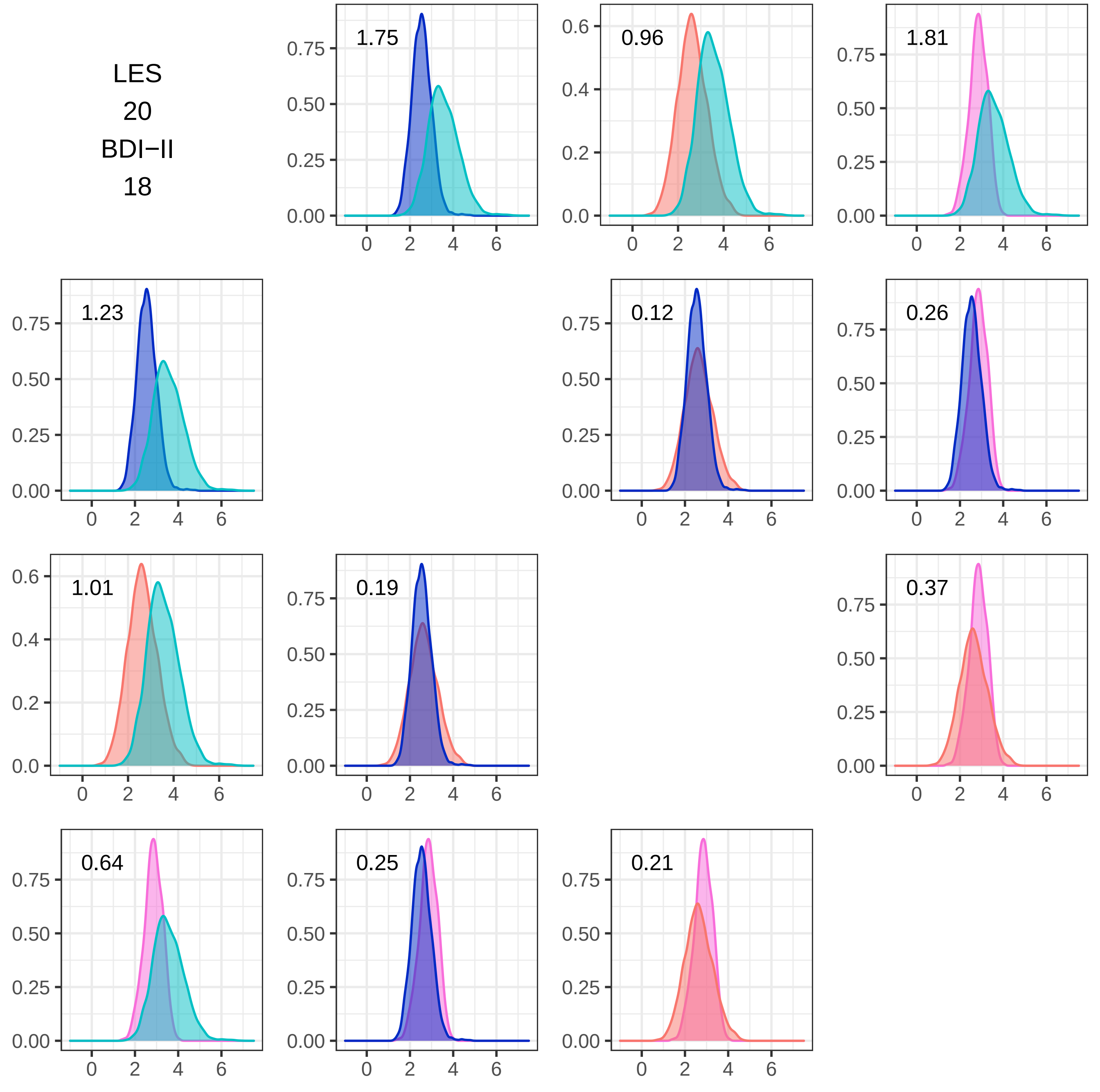

LES
20
BDI−II
18
1.75
0.96
1.81
1.23
0.12
0.26
1.01
0.19
0.37
0.64
0.25
0.21

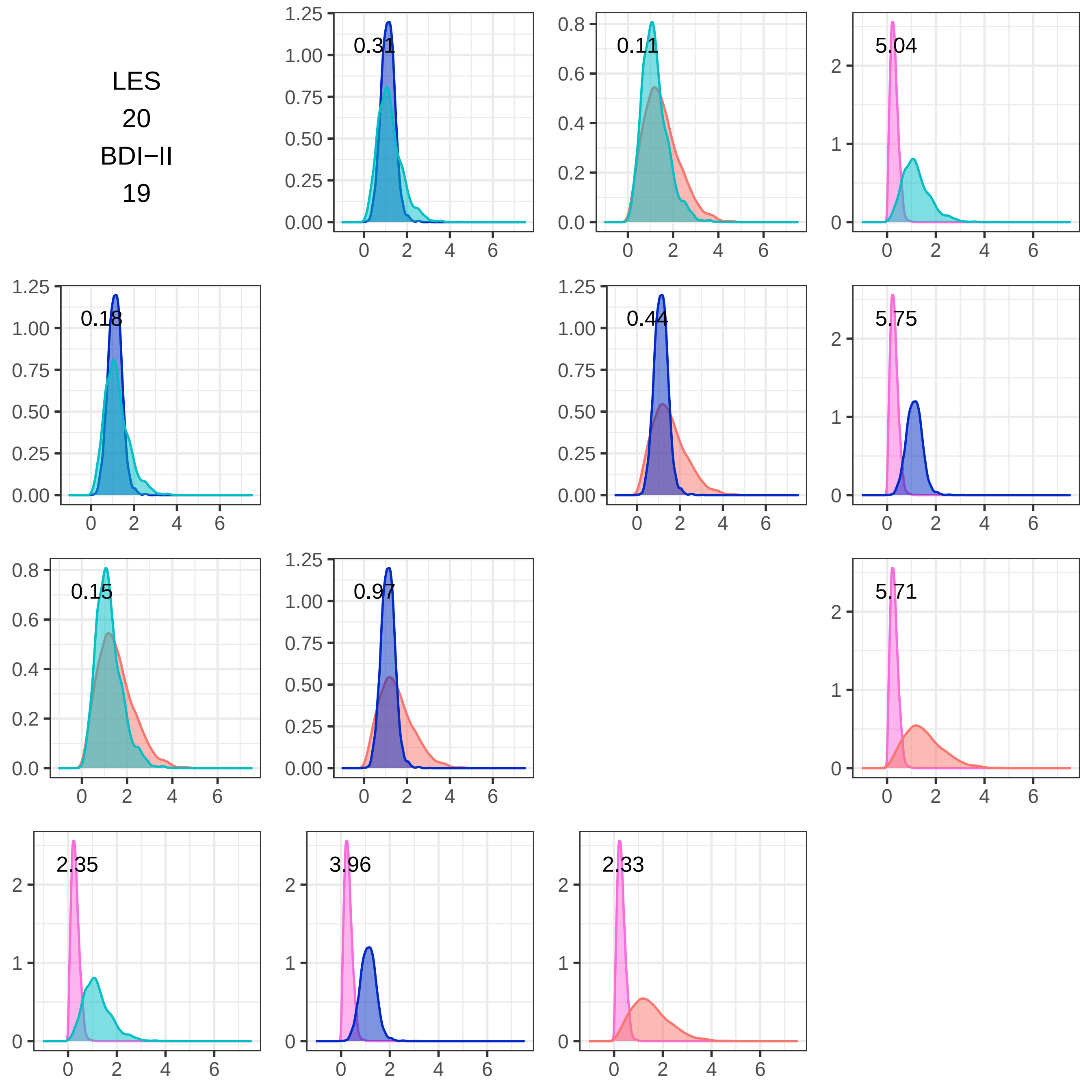
LES
20
BDI−II
19
0.31
0.11
5.04
0.18
0.44
5.75
0.15
0.97
5.71
2.35
3.96
2.33

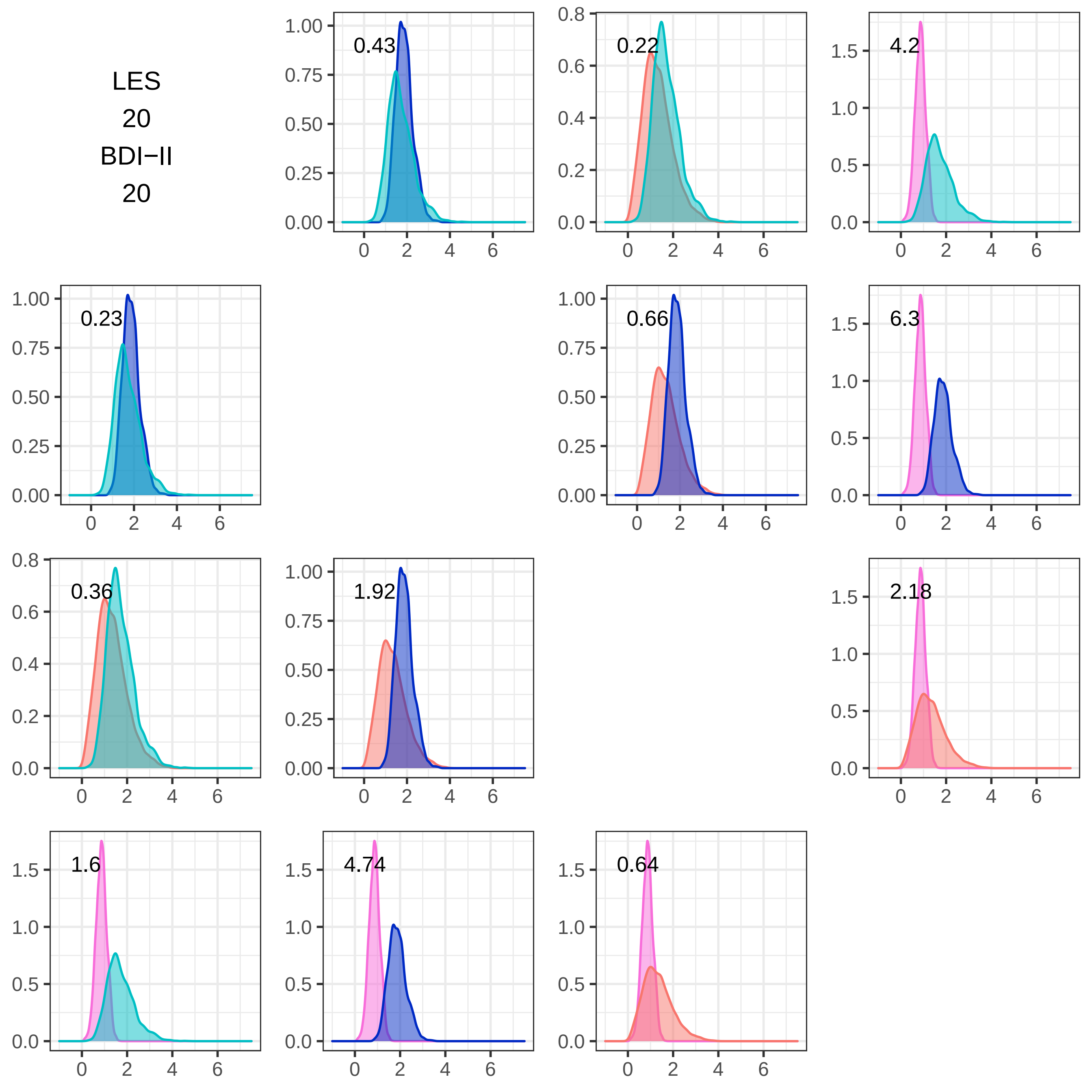

LES
20
BDI−II
20
0.43
0.22
4.2
0.23
0.66
6.3
0.36
1.92
2.18
1.6
4.74
0.64

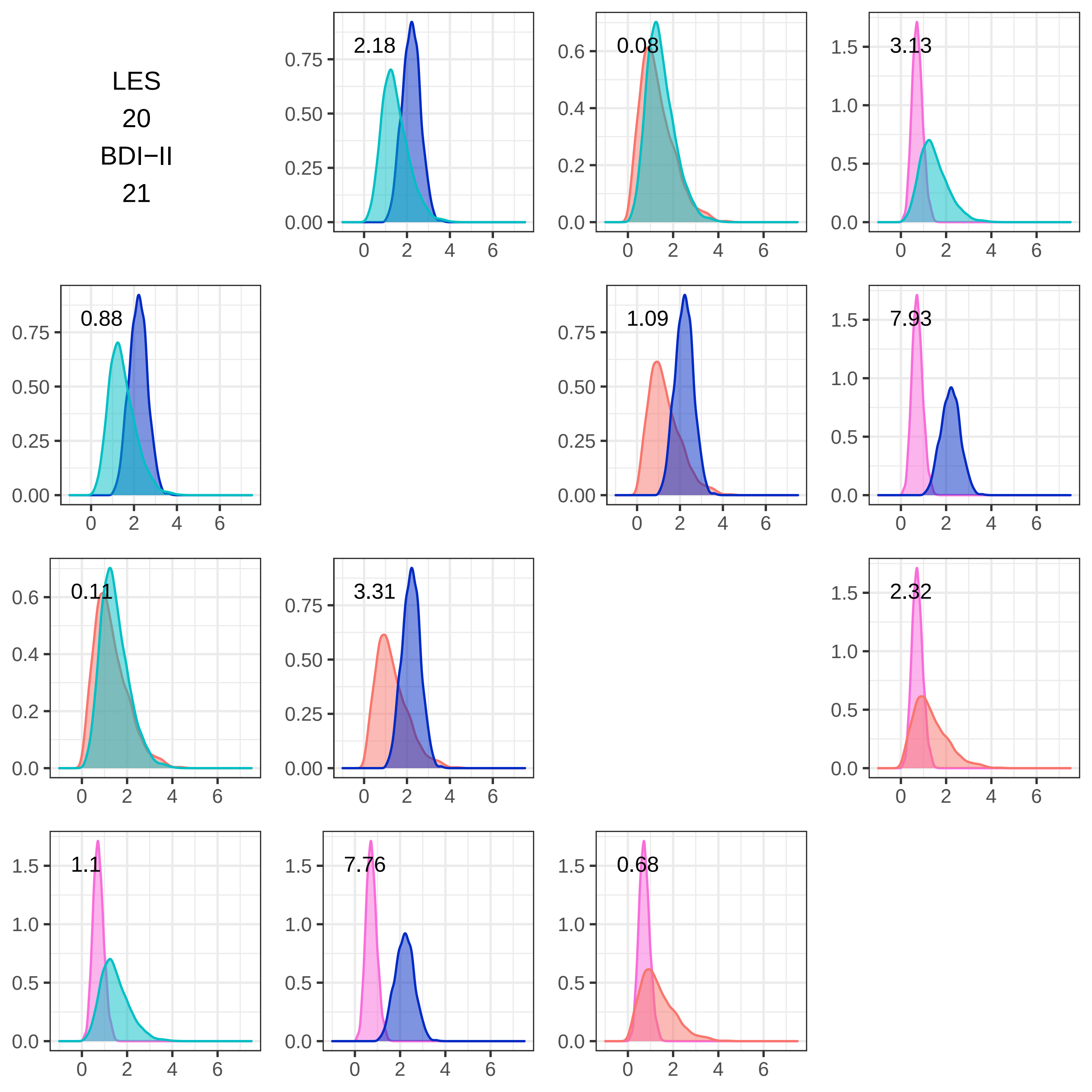

LES
20
BDI−II
21
2.18
0.08
3.13
0.88
1.09
7.93
0.11
3.31
2.32
1.1
7.76
0.68

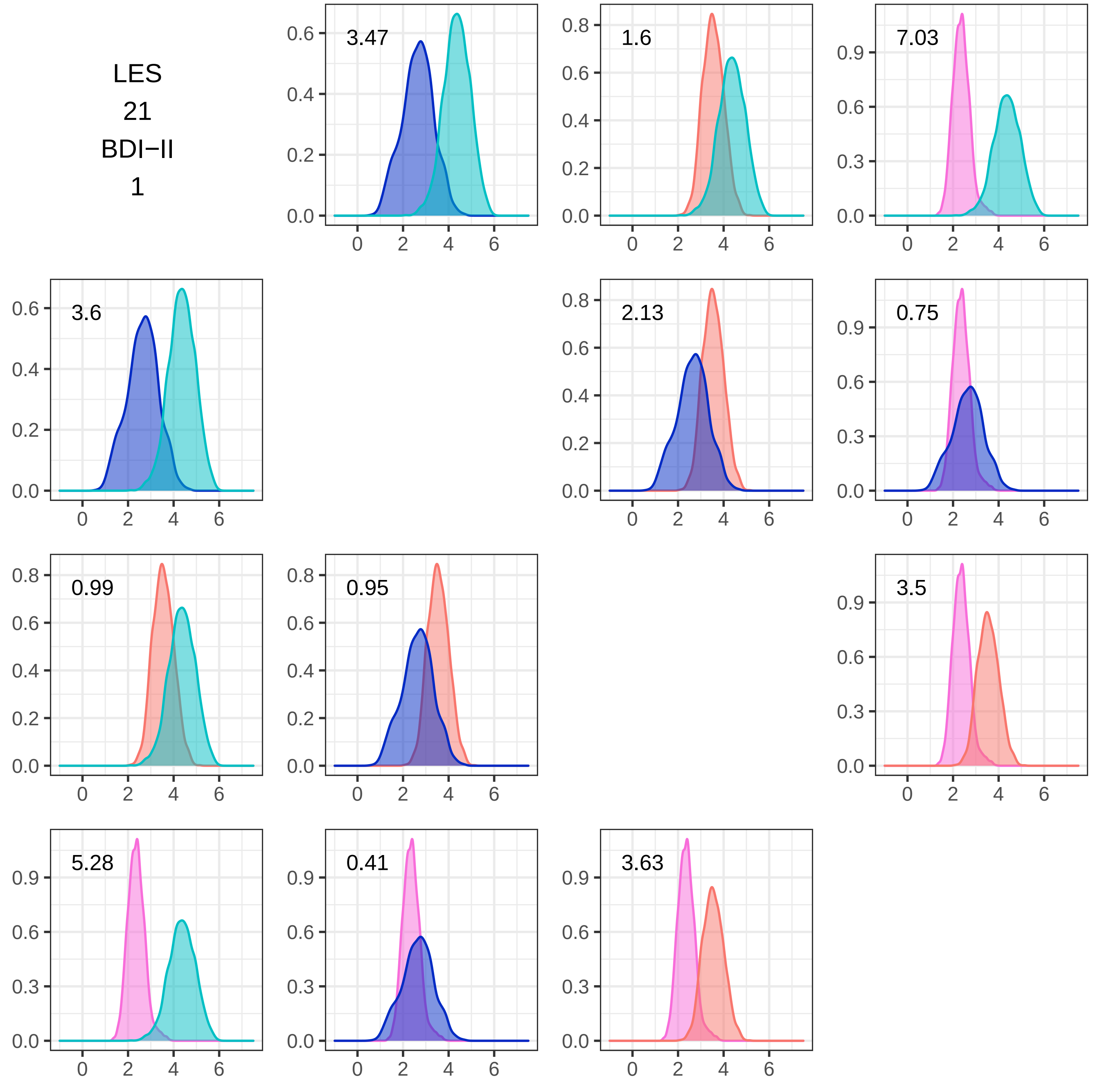

LES
21
BDI−II
1
3.47
1.6
7.03
3.6
2.13
0.75
0.99
0.95
3.5
5.28
0.41
3.63

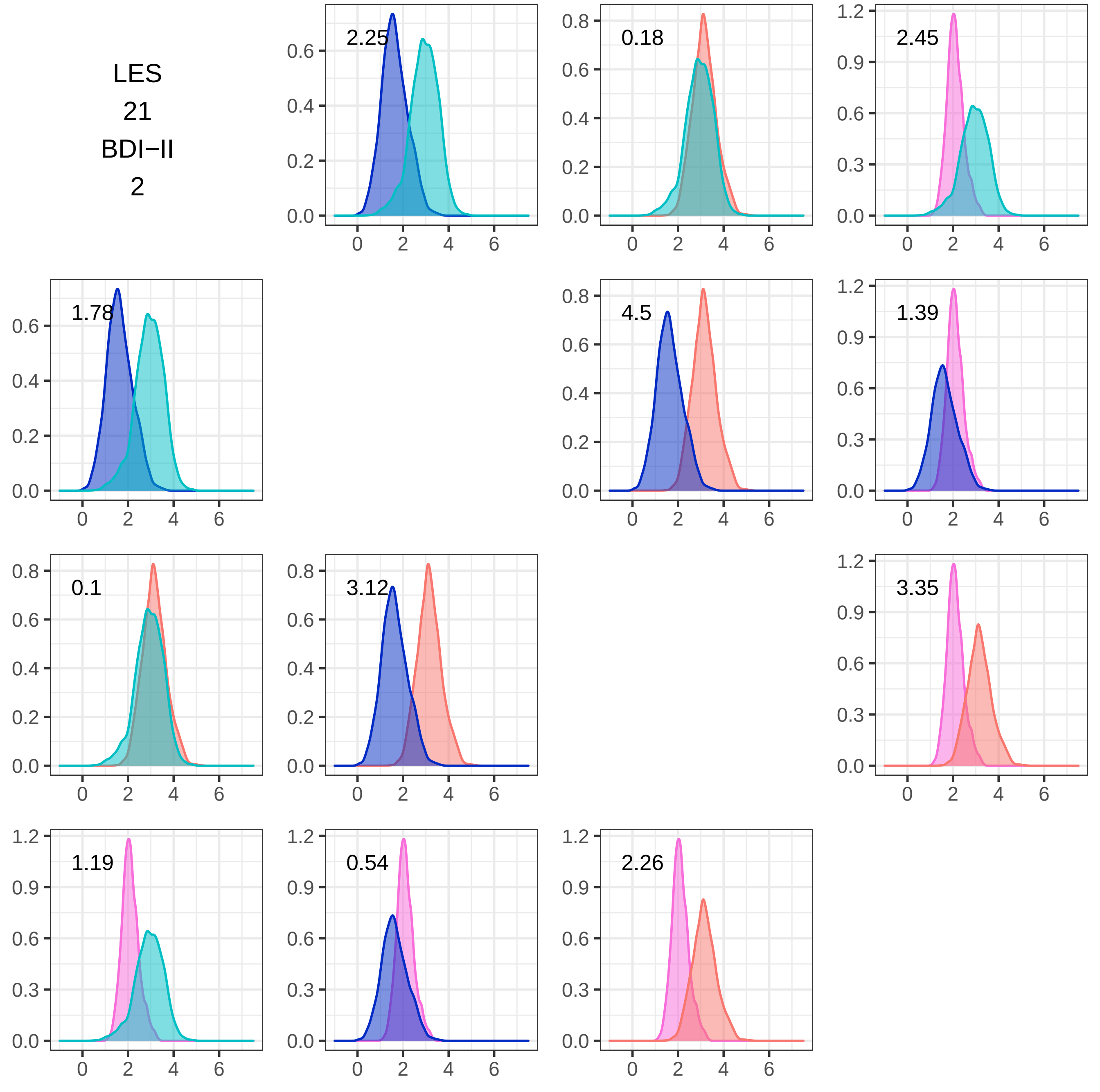
LES
21
BDI−II
2
2.25
0.18
2.45
1.78
4.5
1.39
0.1
3.12
3.35
1.19
0.54
2.26

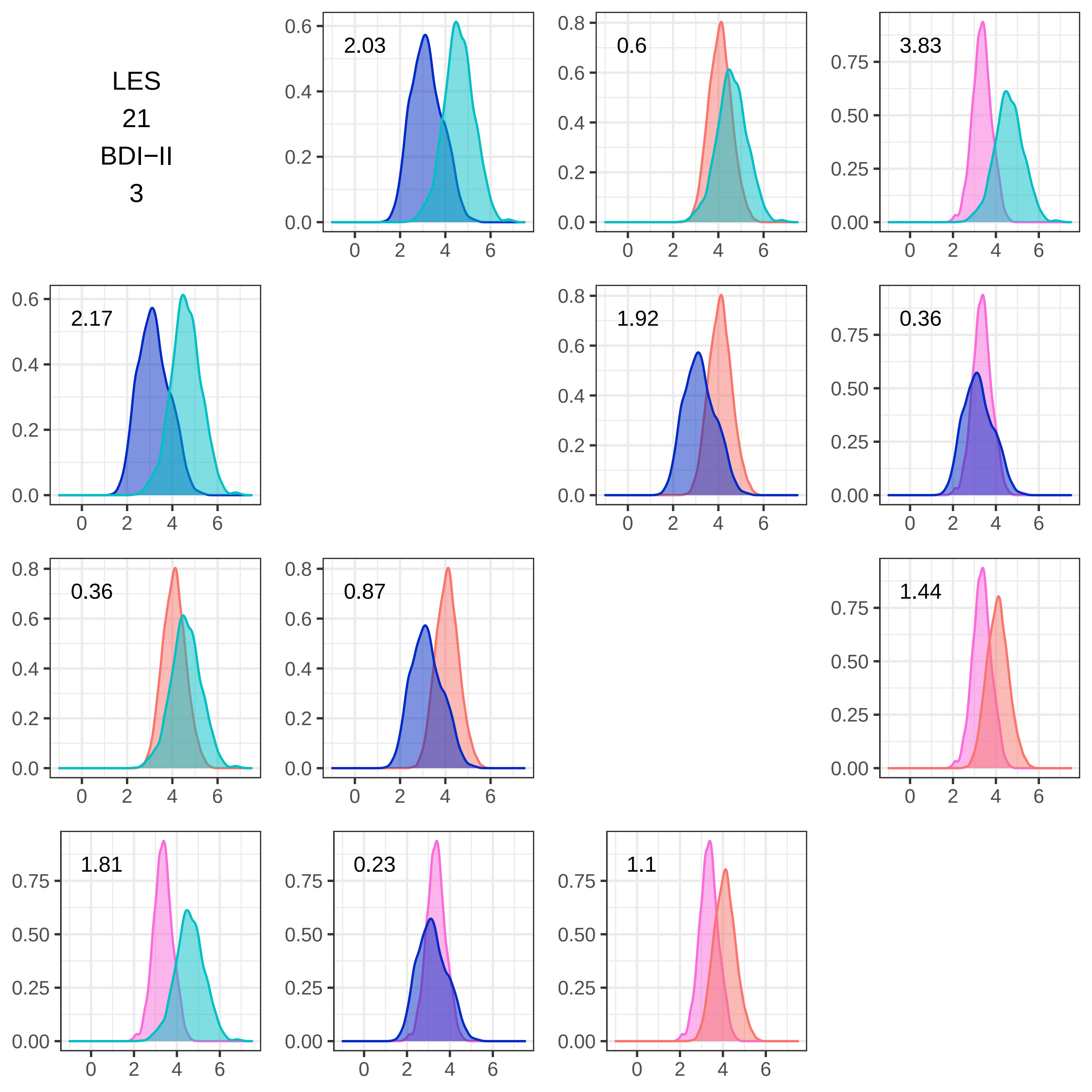
LES
21
BDI−II
3
2.03
0.6
3.83
2.17
1.92
0.36
0.36
0.87
1.44
1.81
0.23
1.1

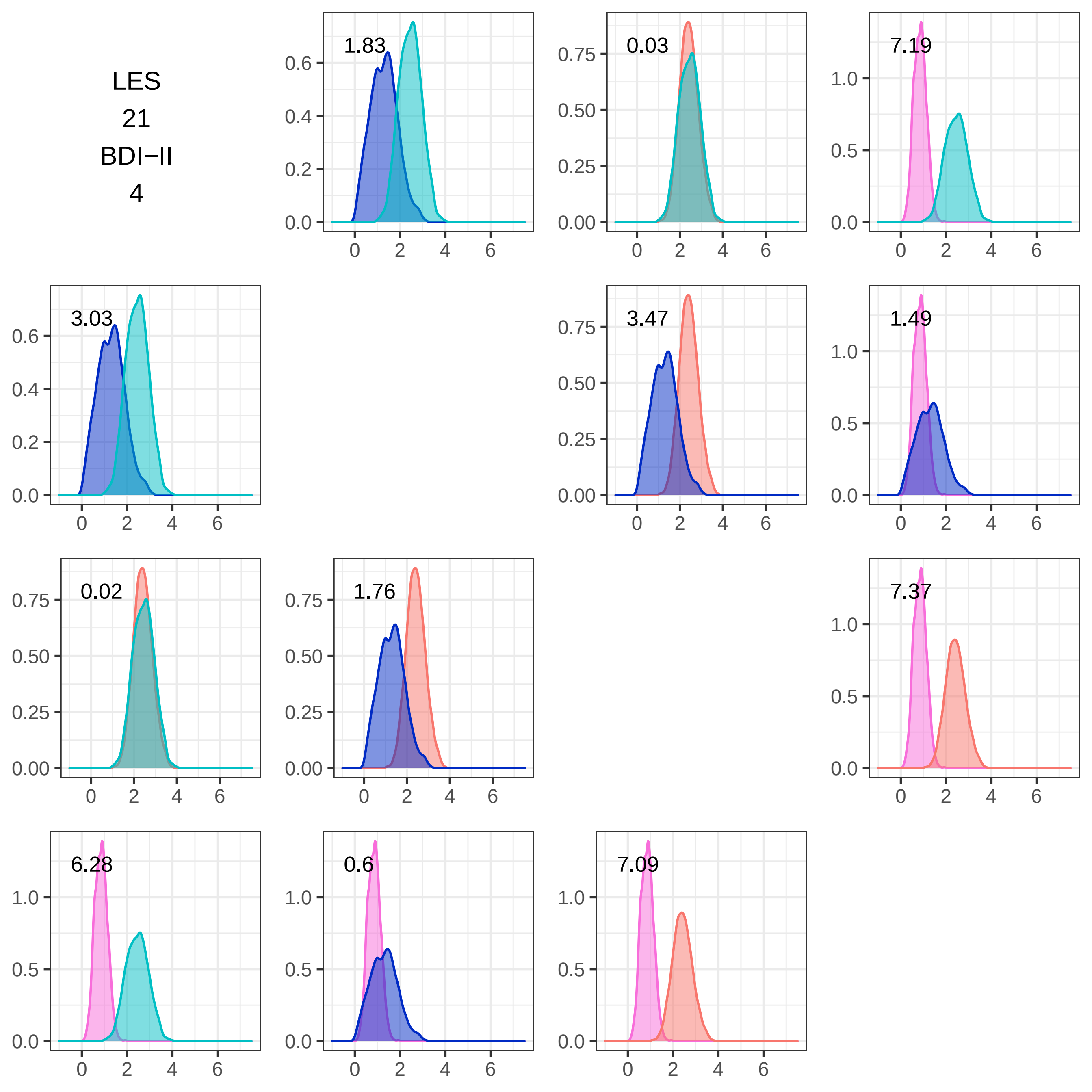
LES
21
BDI−II
4
1.83
0.03
7.19
3.03
3.47
1.49
0.02
1.76
7.37
6.28
0.6
7.09

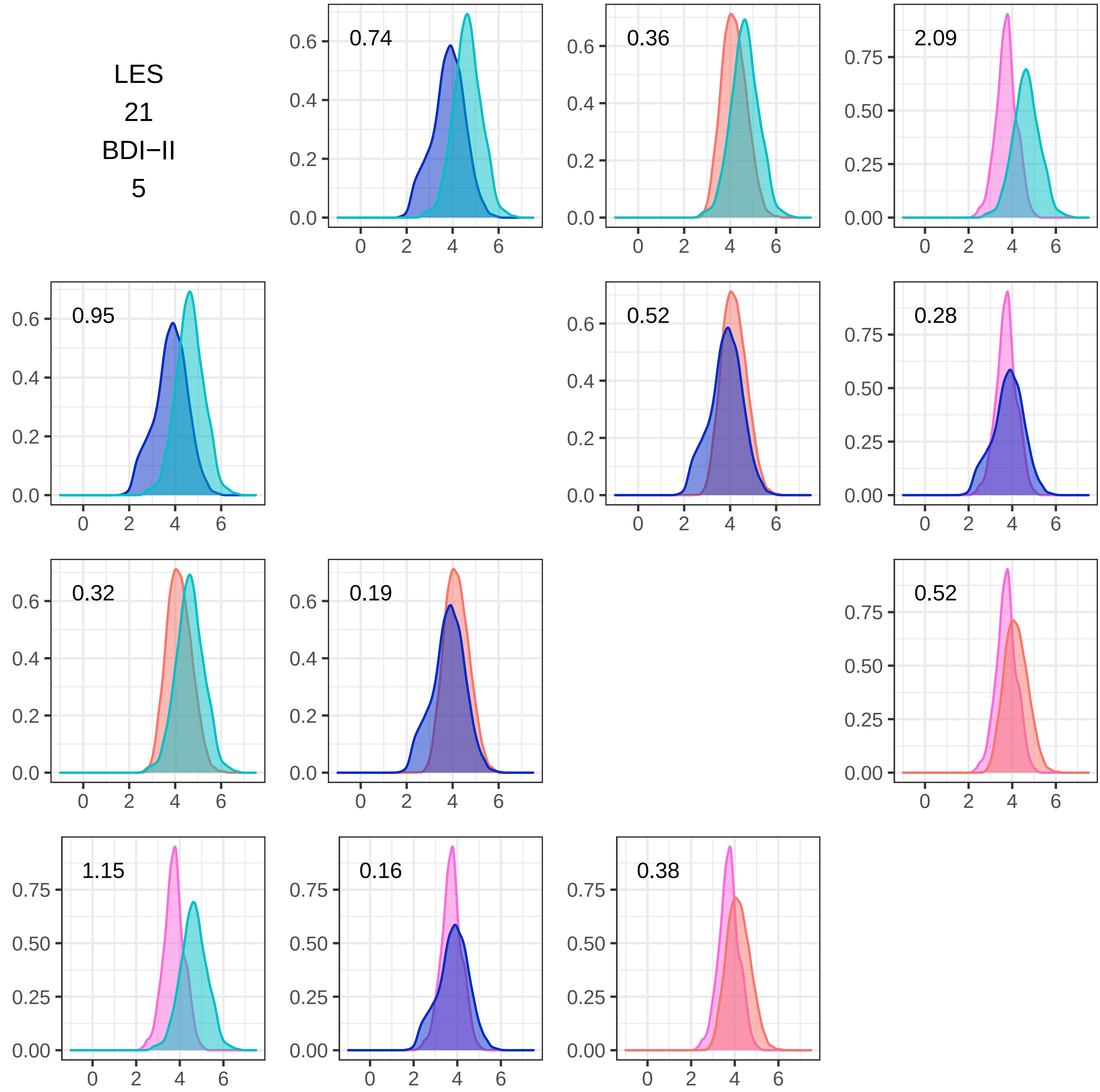

LES
21
BDI−II
5
0.74
0.36
2.09
0.95
0.52
0.28
0.32
0.19
0.52
1.15
0.16
0.38

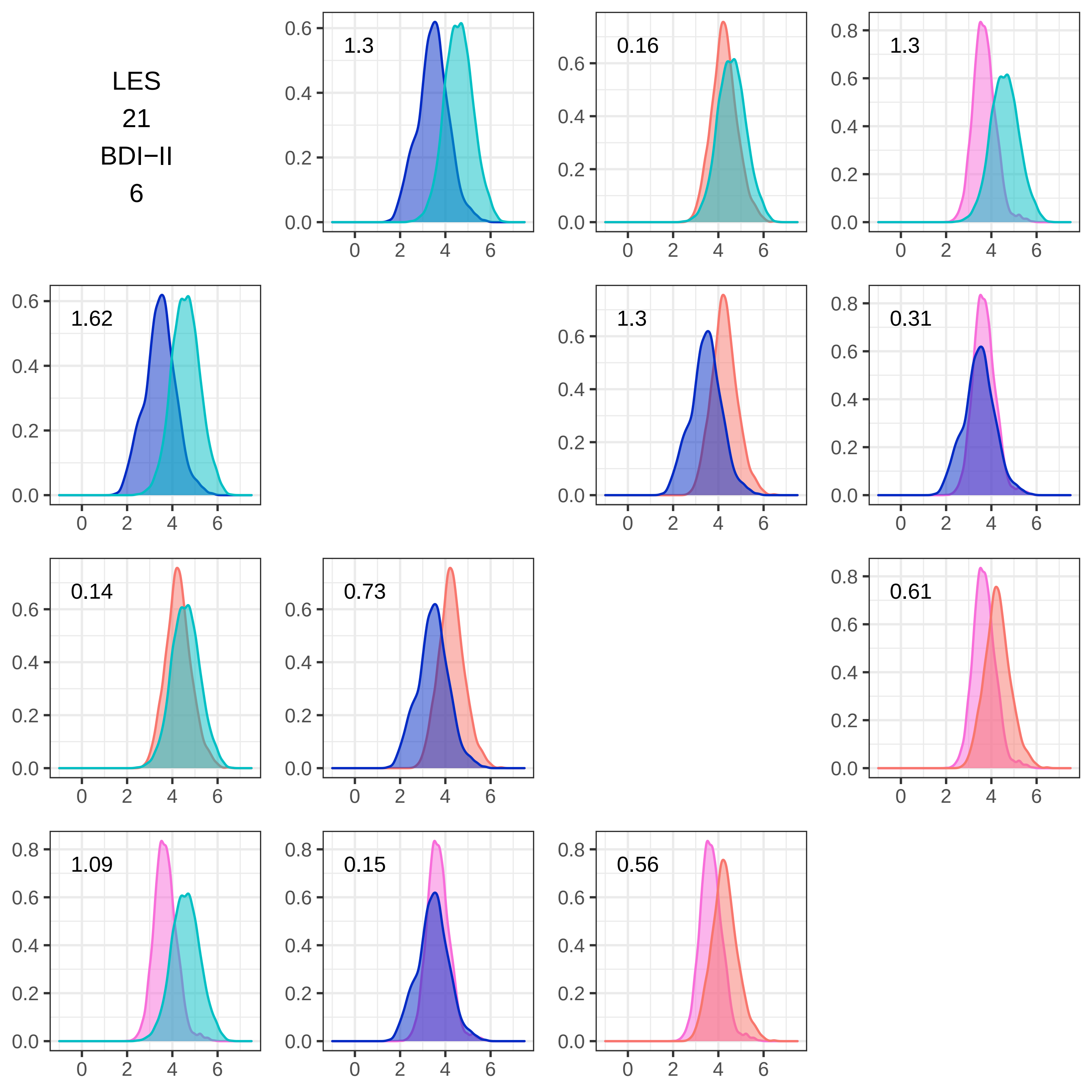

LES
21
BDI−II
6
1.3
0.16
1.3
1.62
1.3
0.31
0.14
0.73
0.61
1.09
0.15
0.56

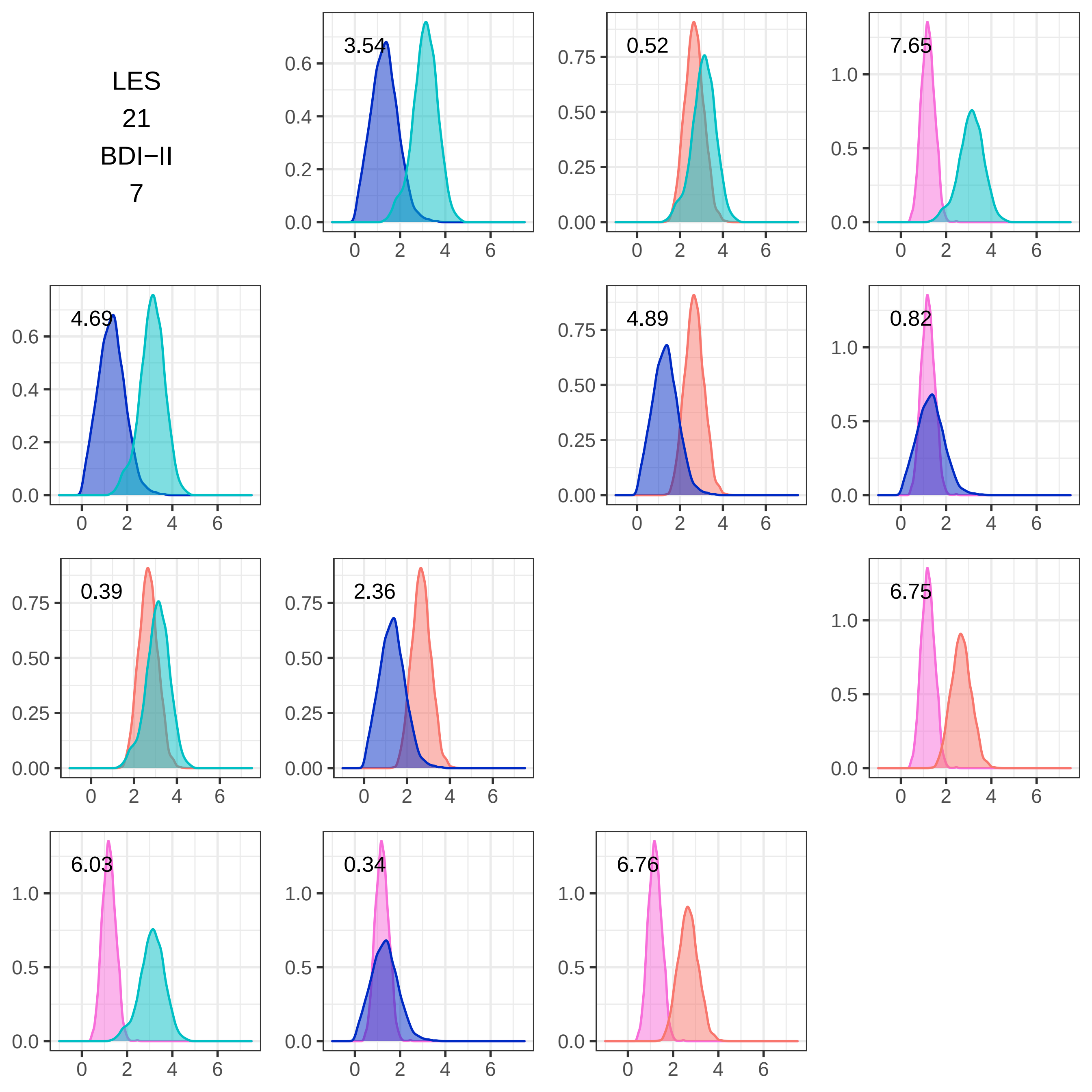
LES
21
BDI−II
7
3.54
0.52
7.65
4.69
4.89
0.82
0.39
2.36
6.75
6.03
0.34
6.76

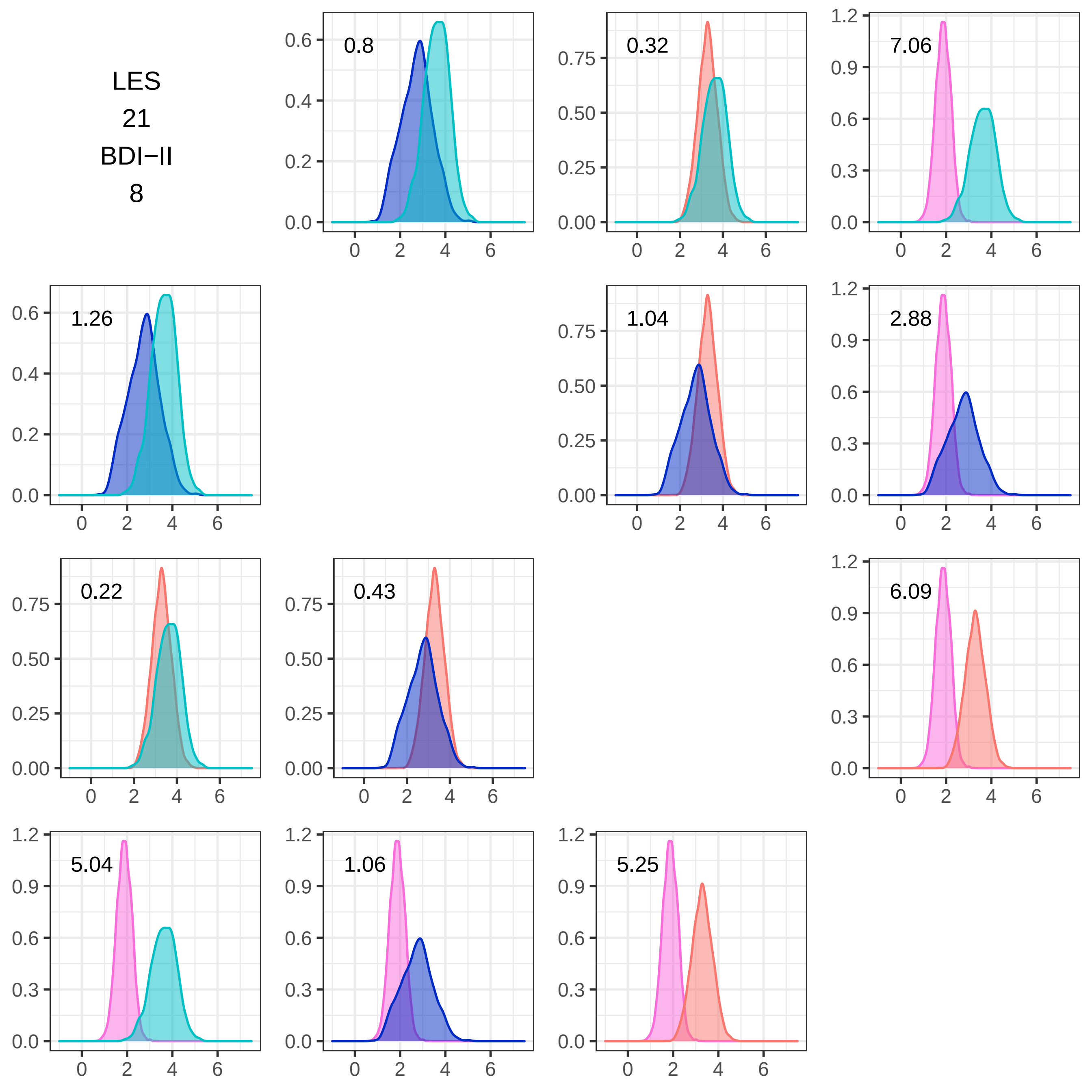

LES
21
BDI−II
8
0.8
0.32
7.06
1.26
1.04
2.88
0.22
0.43
6.09
5.04
1.06
5.25

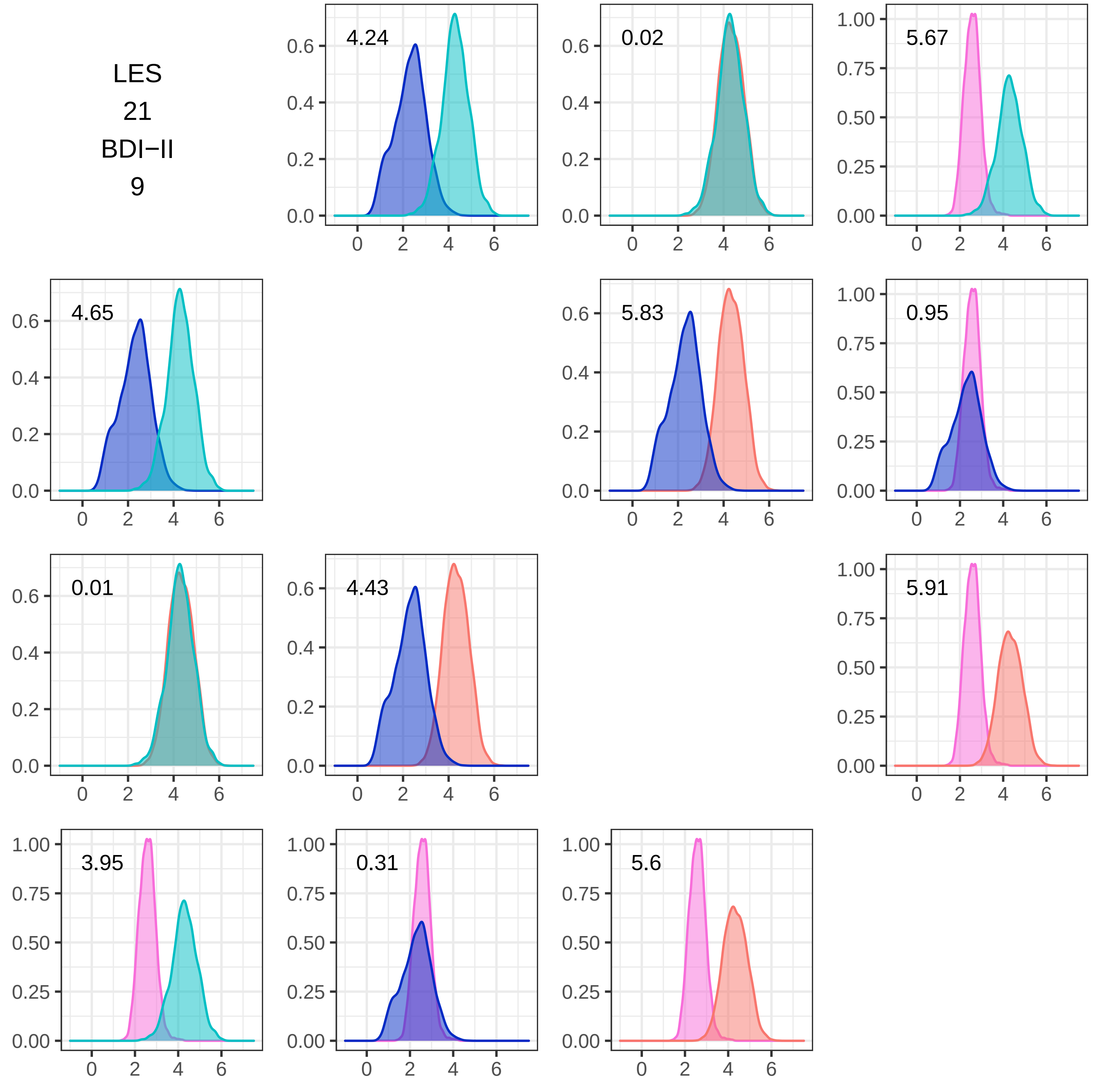
LES
21
BDI−II
9
4.24
0.02
5.67
4.65
5.83
0.95
0.01
4.43
5.91
3.95
0.31
5.6

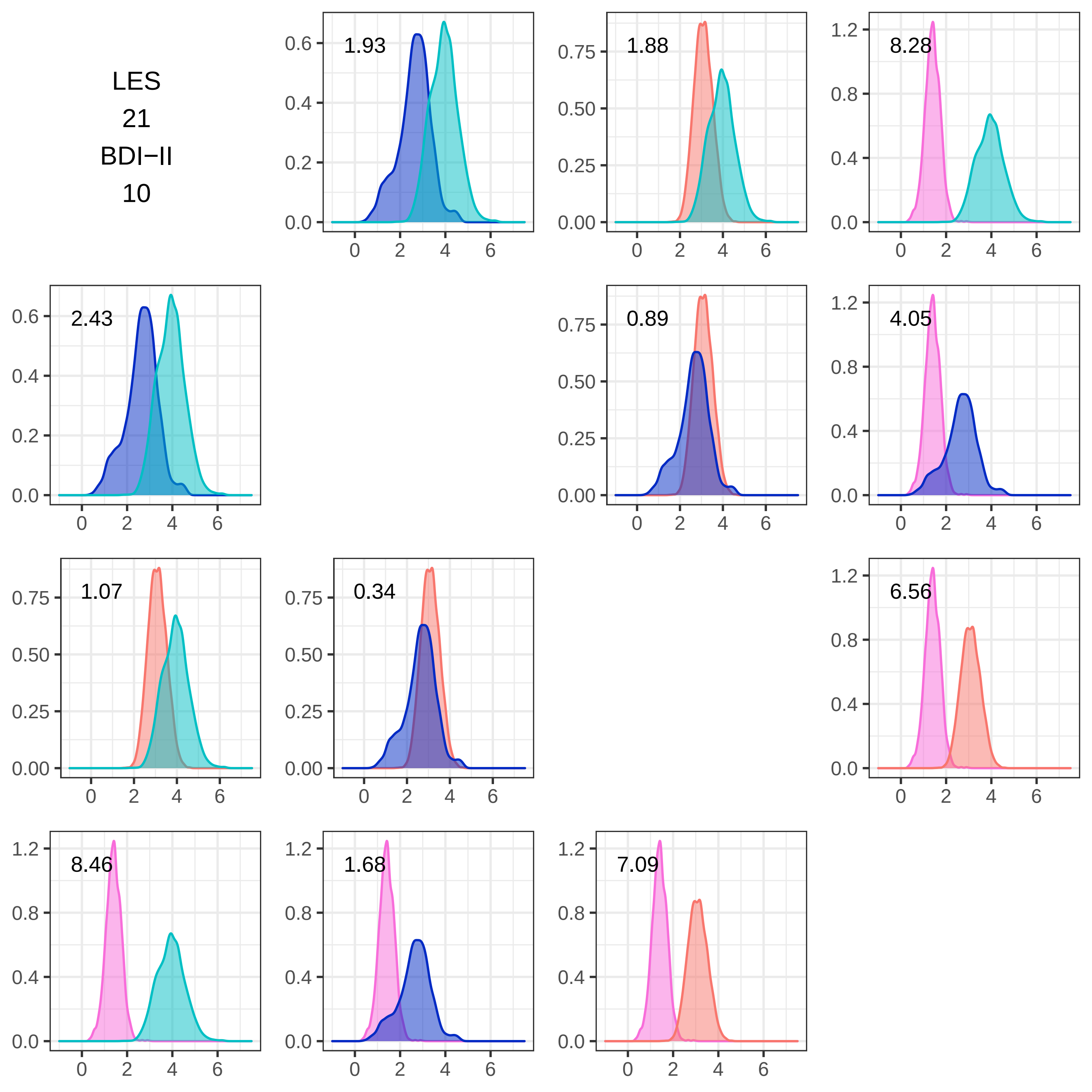
LES
21
BDI−II
10
1.93
1.88
8.28
2.43
0.89
4.05
1.07
0.34
6.56
8.46
1.68
7.09

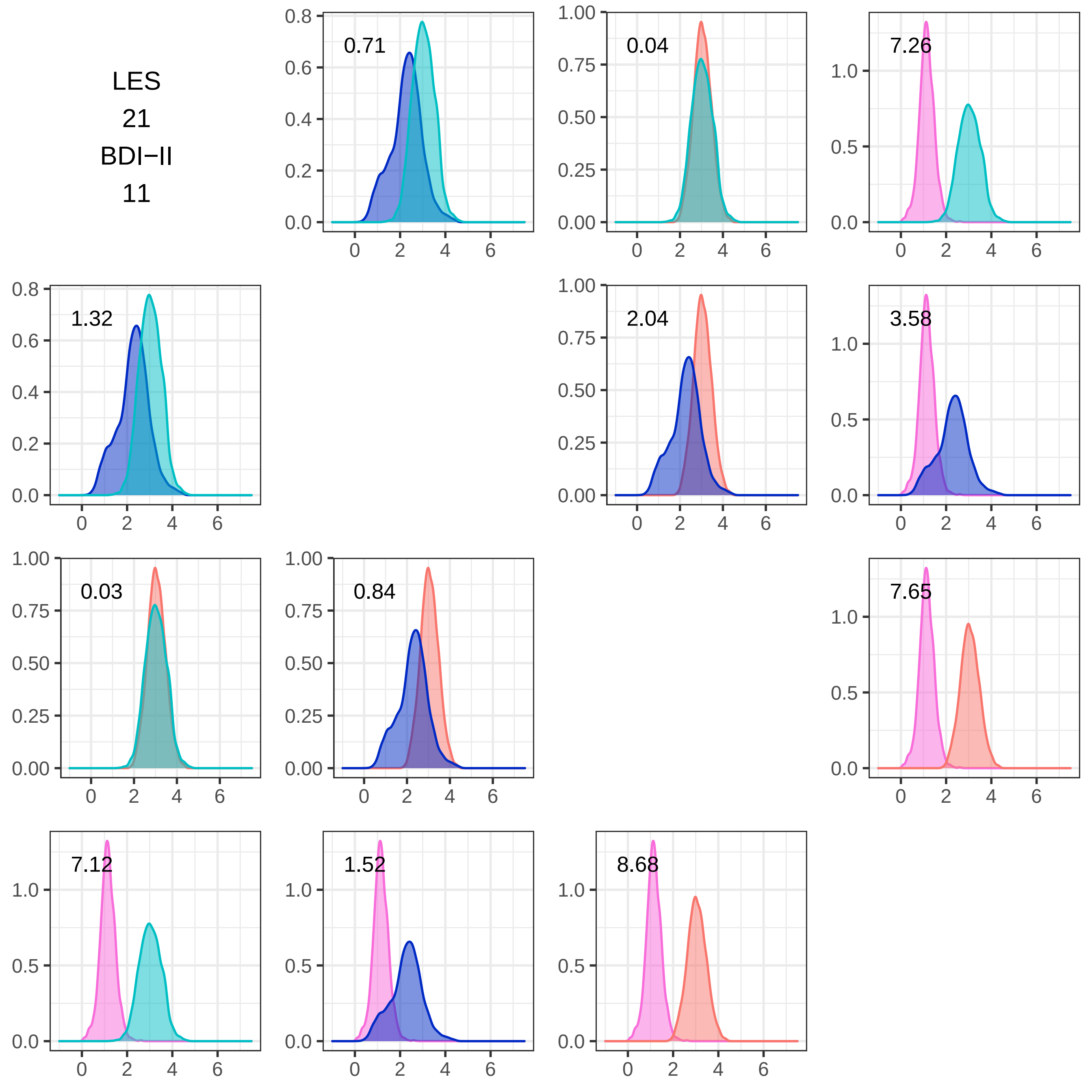

LES
21
BDI−II
11
0.71
0.04
7.26
1.32
2.04
3.58
0.03
0.84
7.65
7.12
1.52
8.68

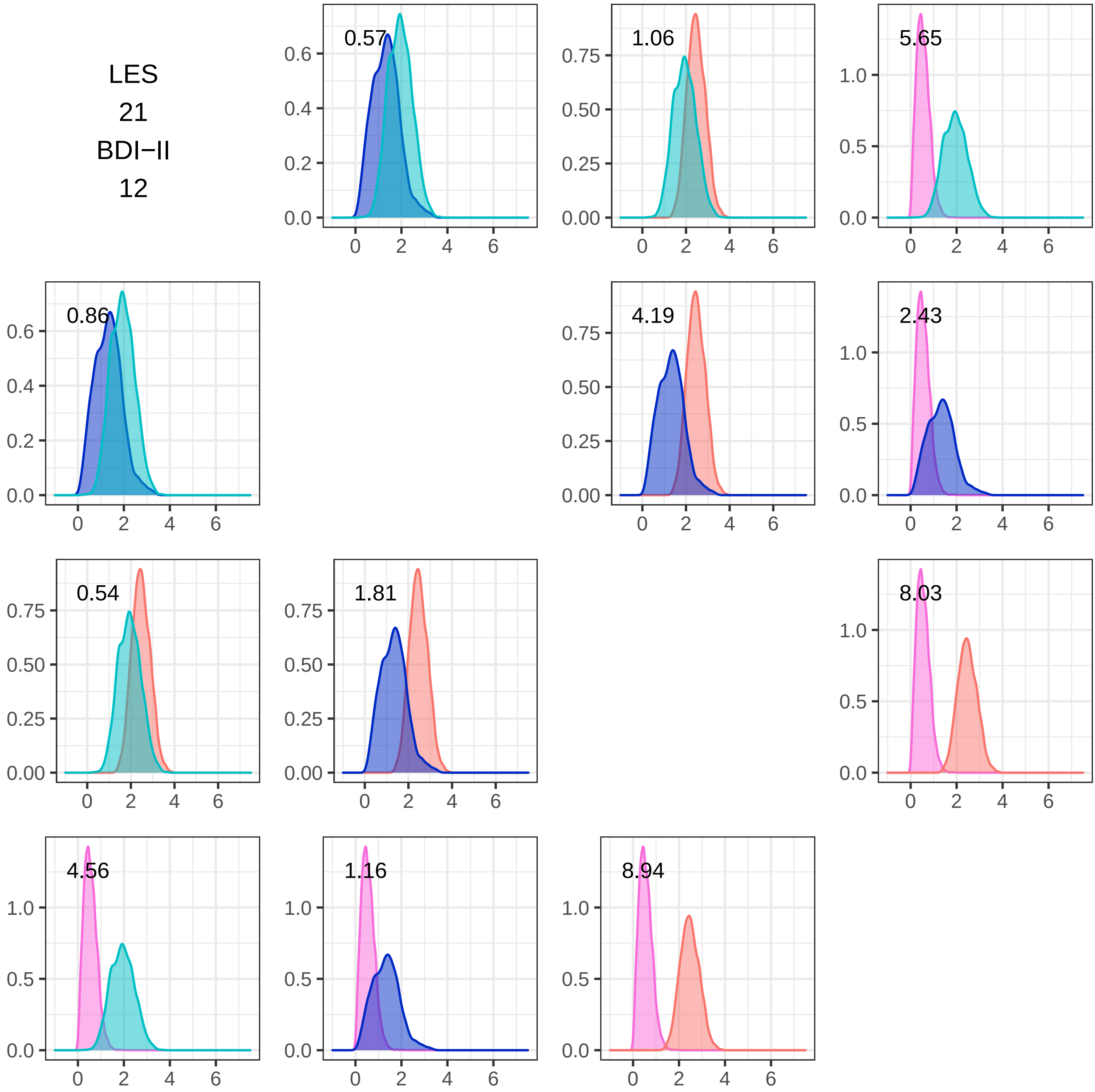
LES
21
BDI−II
12
0.57
1.06
5.65
0.86
4.19
2.43
0.54
1.81
8.03
4.56
1.16
8.94

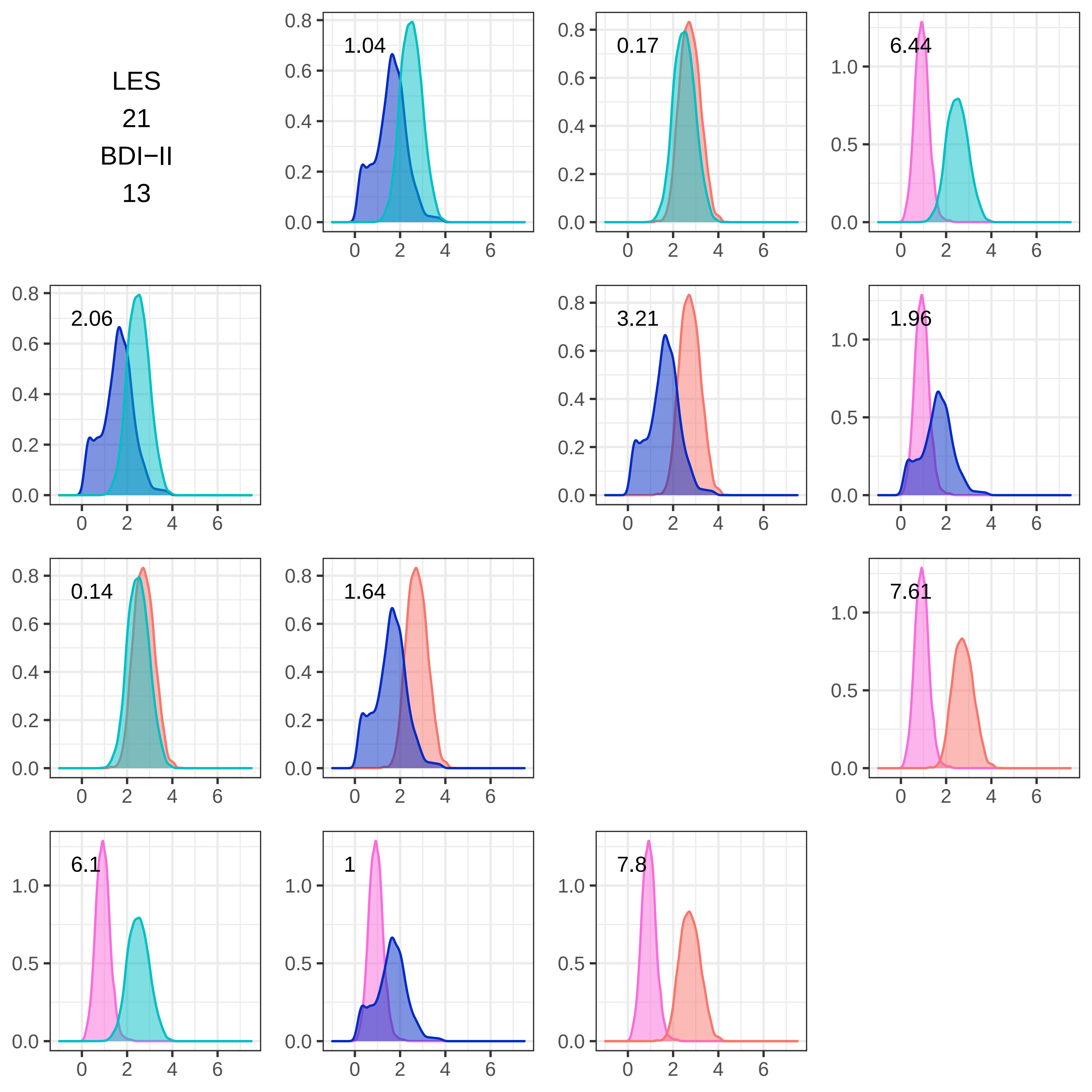
LES
21
BDI−II
13
1.04
0.17
6.44
2.06
3.21
1.96
0.14
1.64
7.61
6.1
1
7.8

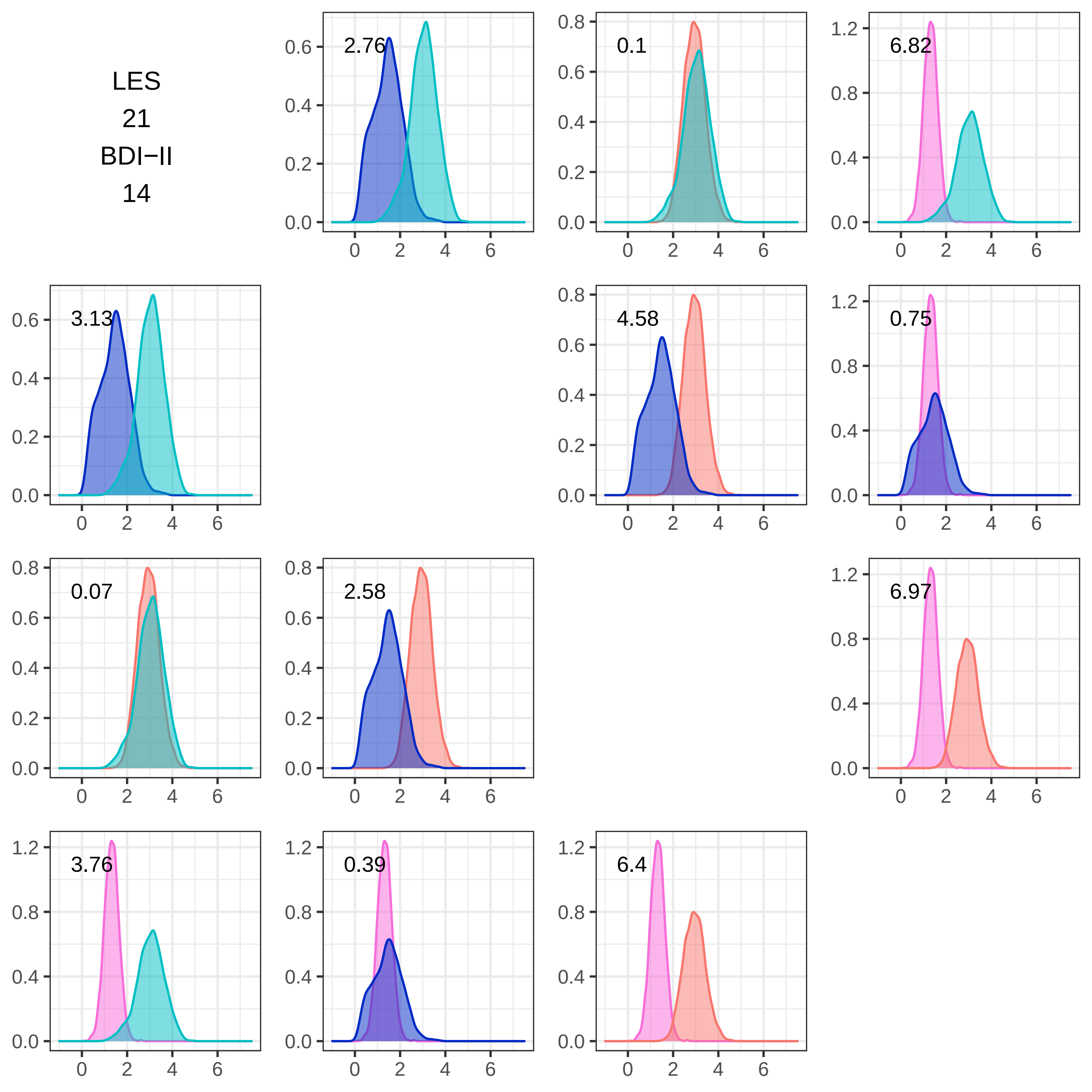
LES
21
BDI−II
14
2.76
0.1
6.82
3.13
4.58
0.75
0.07
2.58
6.97
3.76
0.39
6.4

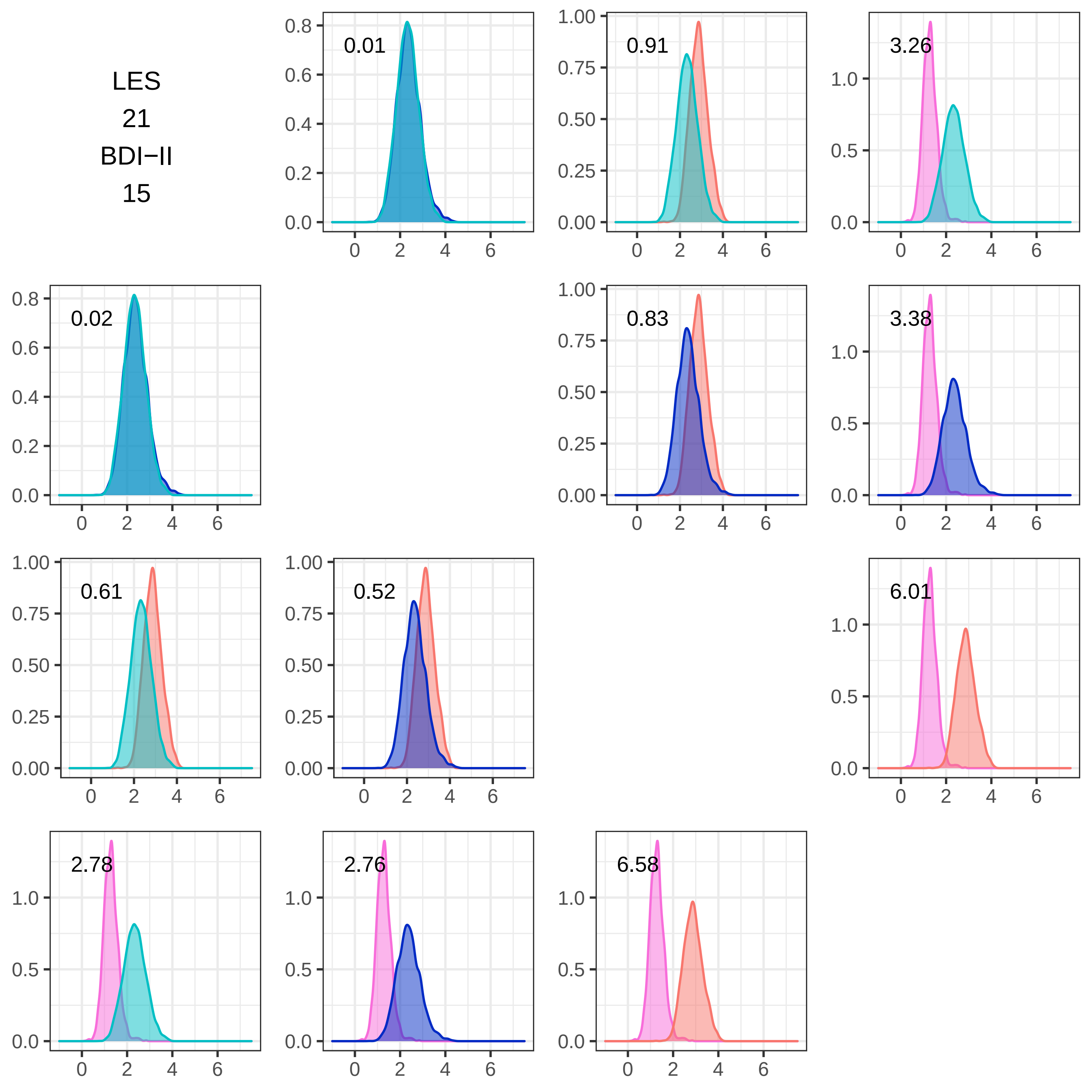
LES
21
BDI−II
15
0.01
0.91
3.26
0.02
0.83
3.38
0.61
0.52
6.01
2.78
2.76
6.58

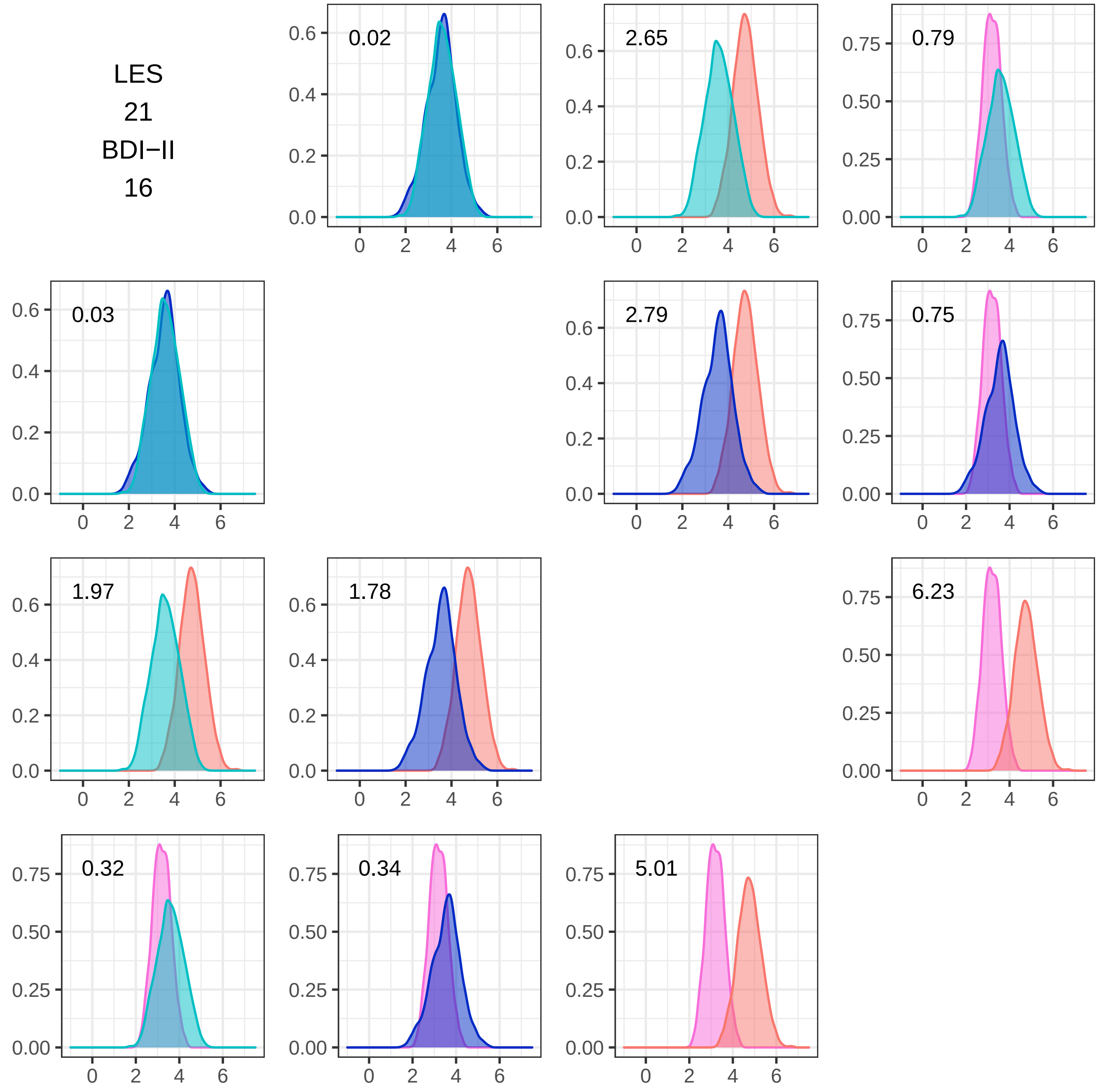
LES
21
BDI−II
16
0.02
2.65
0.79
0.03
2.79
0.75
1.97
1.78
6.23
0.32
0.34
5.01

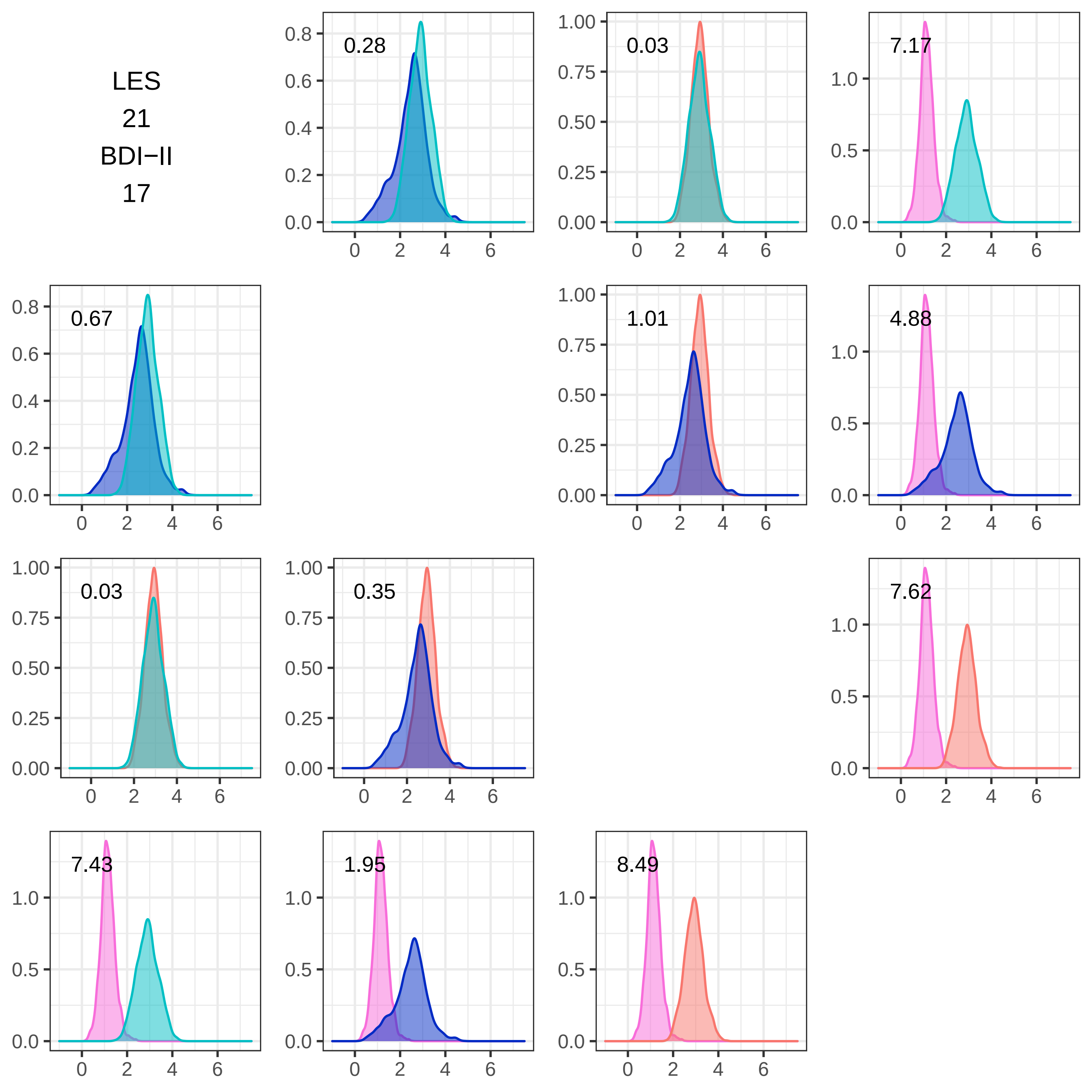

LES
21
BDI−II
17
0.28
0.03
7.17
0.67
1.01
4.88
0.03
0.35
7.62
7.43
1.95
8.49

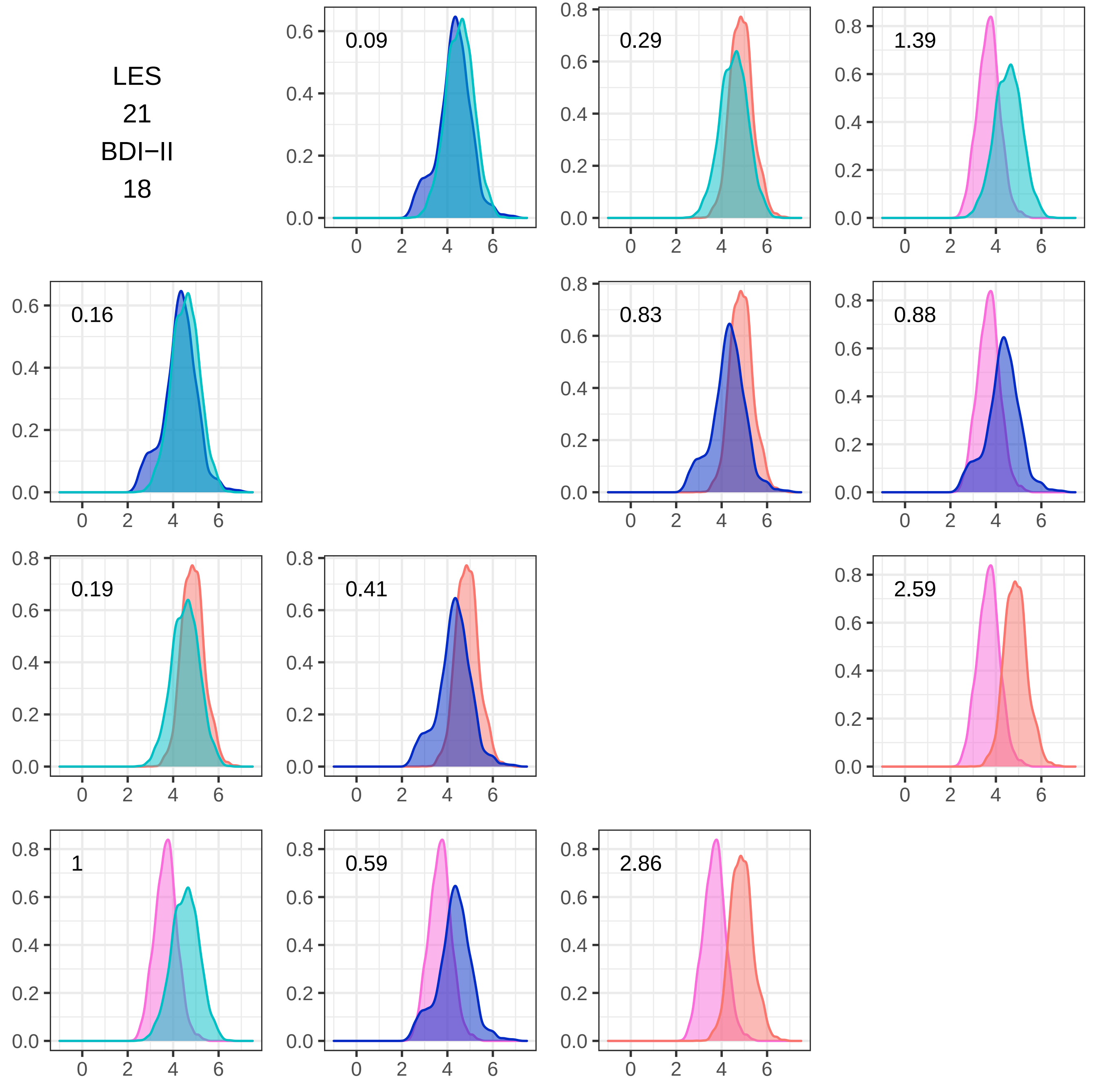
LES
21
BDI−II
18
0.09
0.29
1.39
0.16
0.83
0.88
0.19
0.41
2.59
1
0.59
2.86

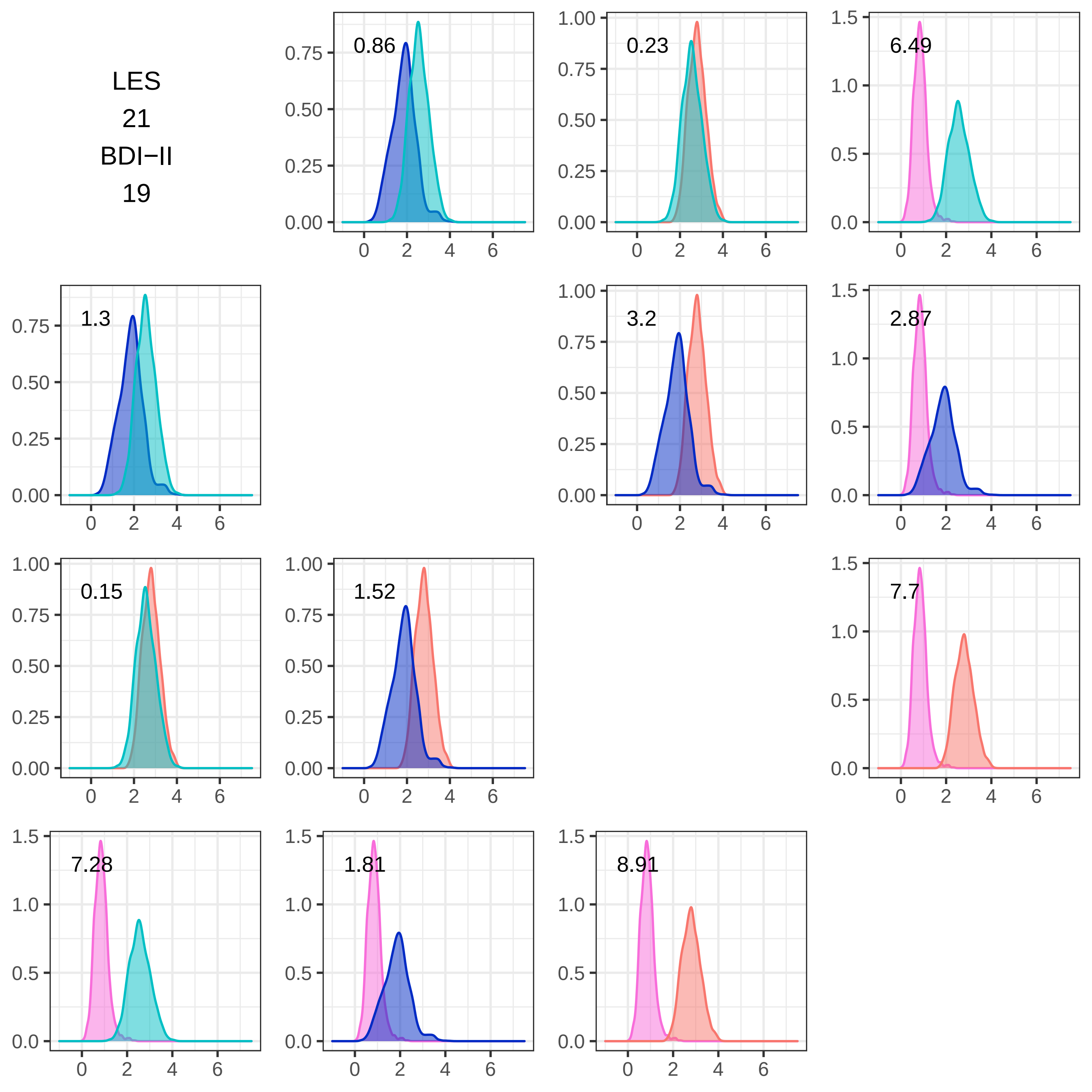
LES
21
BDI−II
19
0.86
0.23
6.49
1.3
3.2
2.87
0.15
1.52
7.7
7.28
1.81
8.91

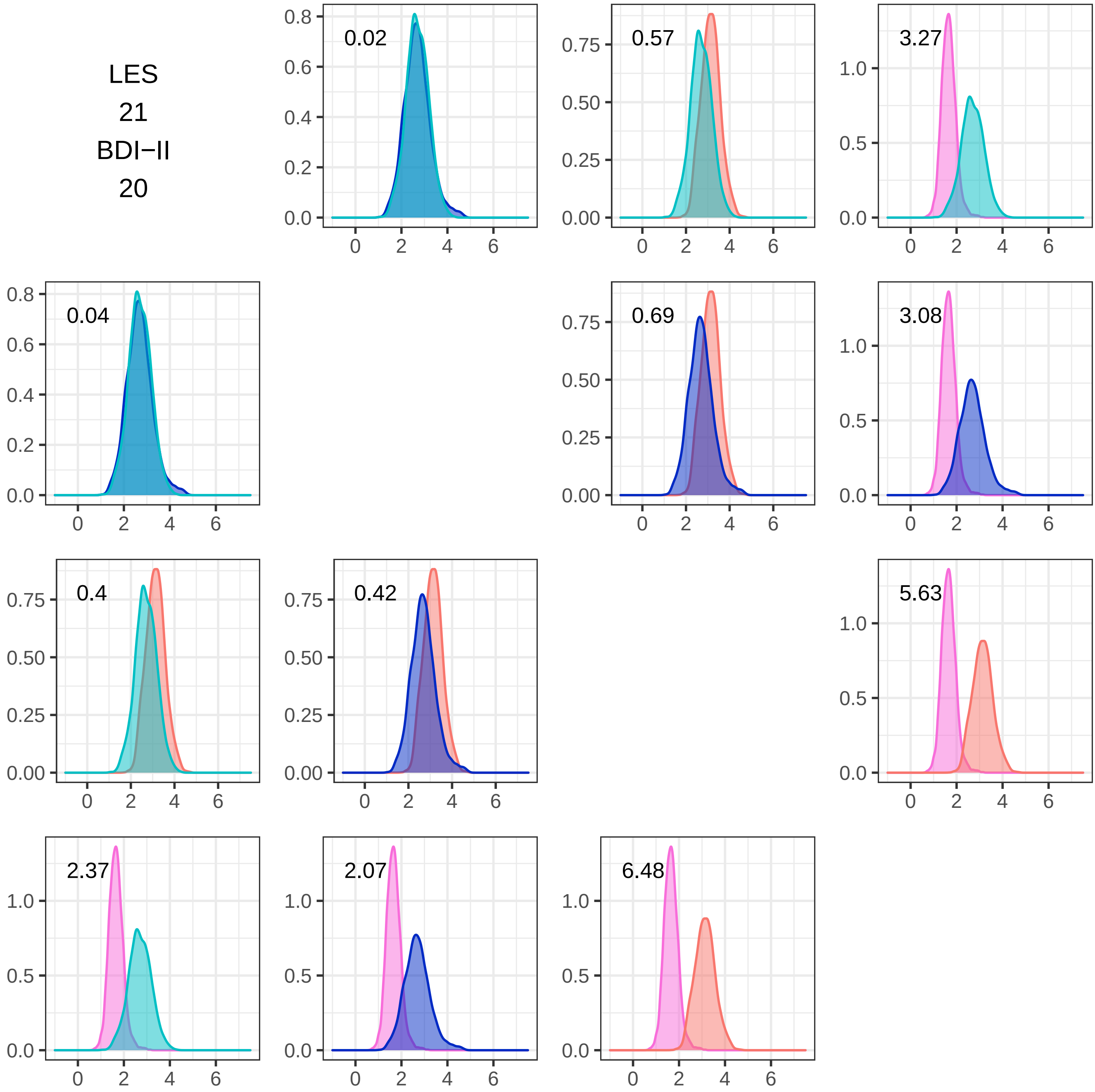
LES
21
BDI−II
20
0.02
0.57
3.27
0.04
0.69
3.08
0.4
0.42
5.63
2.37
2.07
6.48

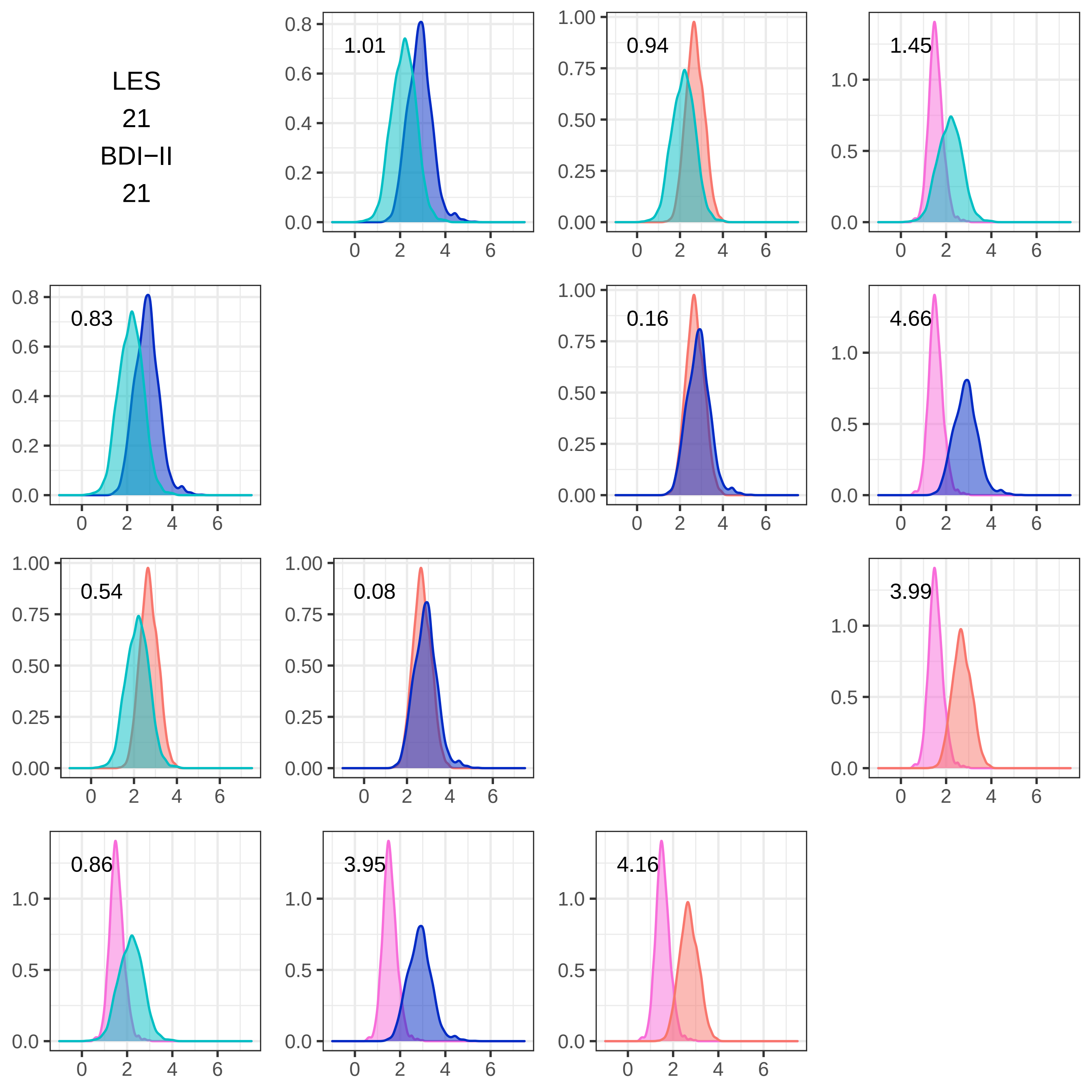

LES
21
BDI−II
21
1.01
0.94
1.45
0.83
0.16
4.66
0.54
0.08
3.99
0.86
3.95
4.16

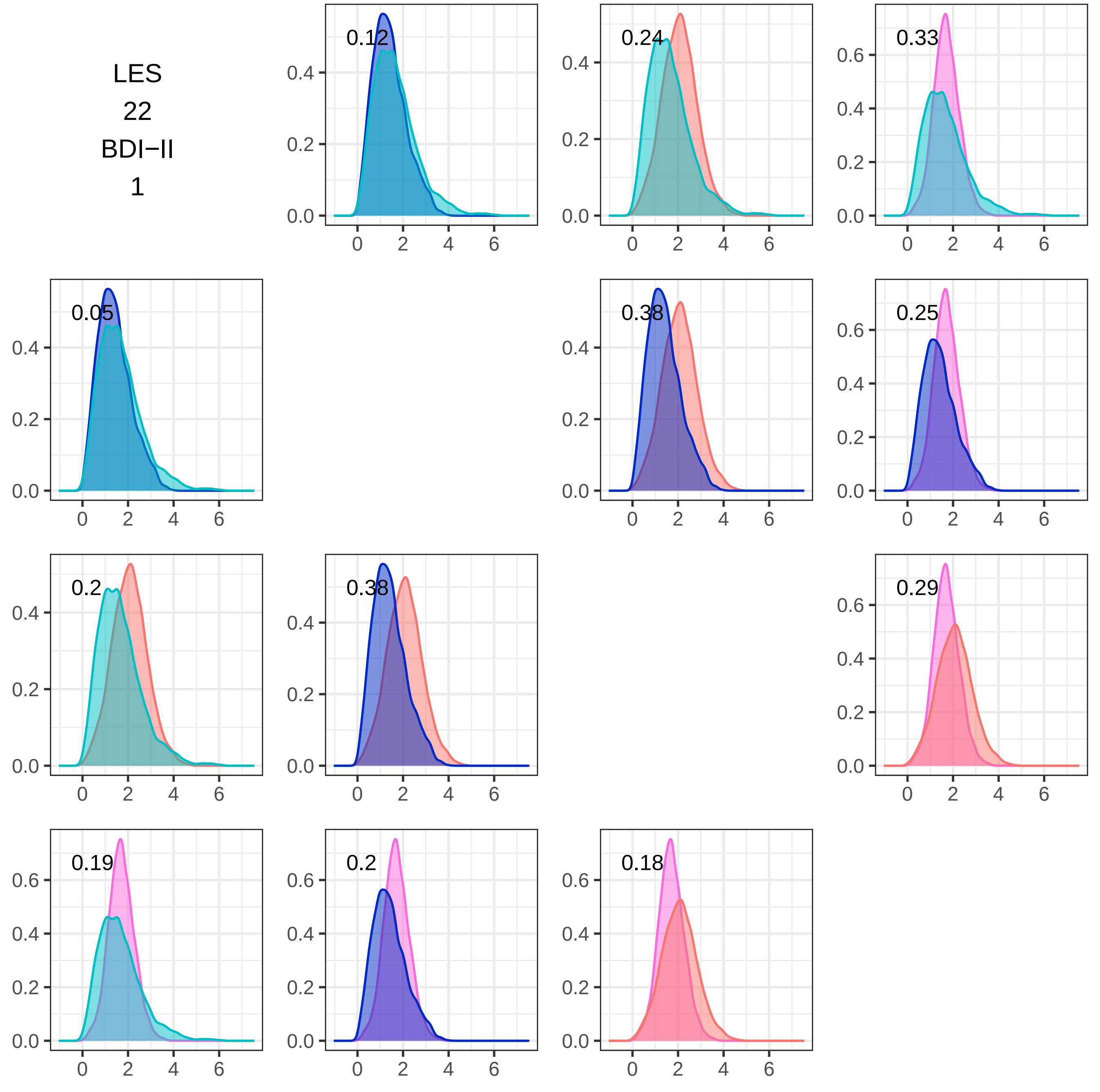
LES
22
BDI−II
1
0.12
0.24
0.33
0.05
0.38
0.25
0.2
0.38
0.29
0.19
0.2
0.18

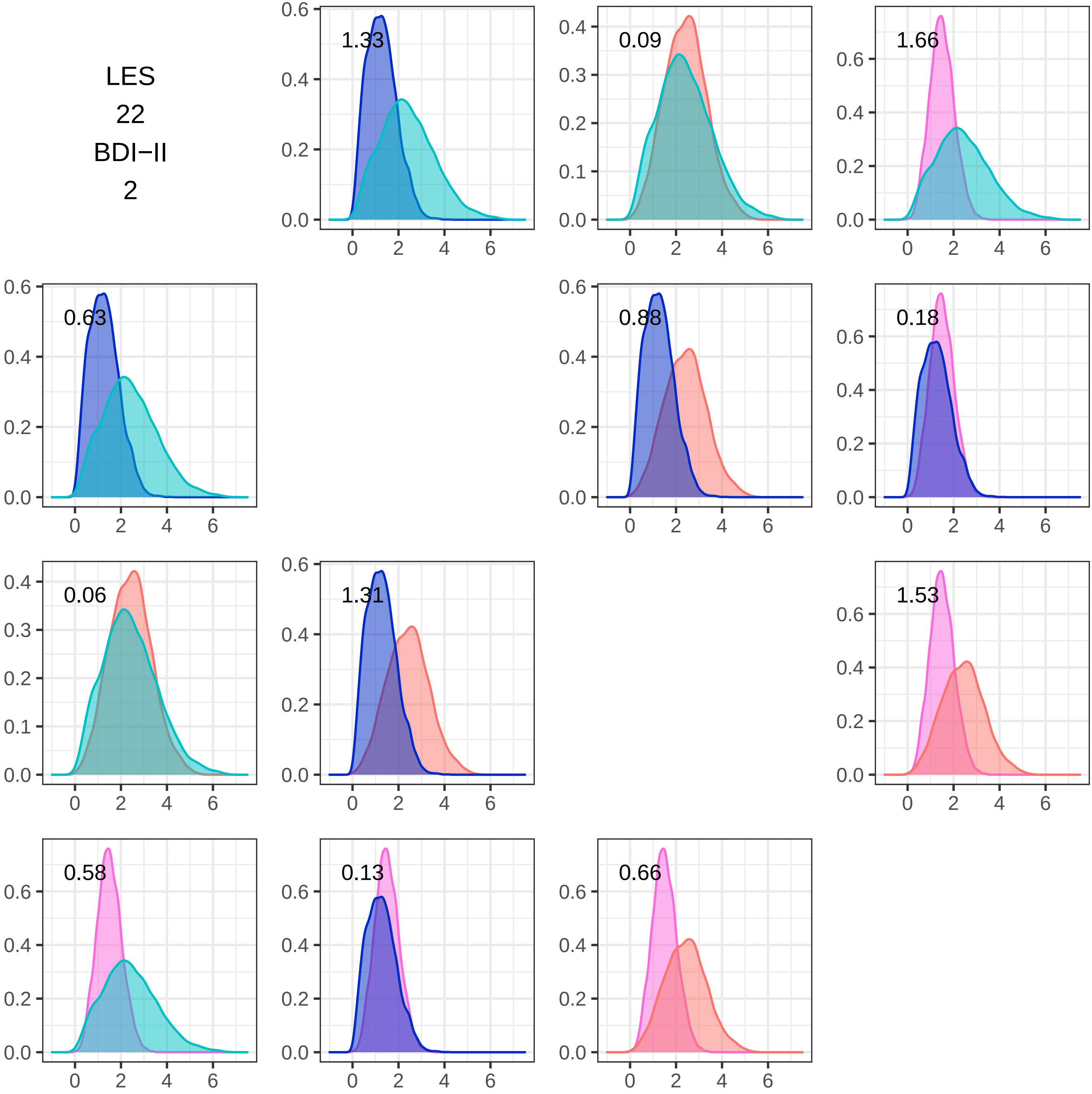

LES
22
BDI−II
2
1.33
0.09
1.66
0.63
0.88
0.18
0.06
1.31
1.53
0.58
0.13
0.66

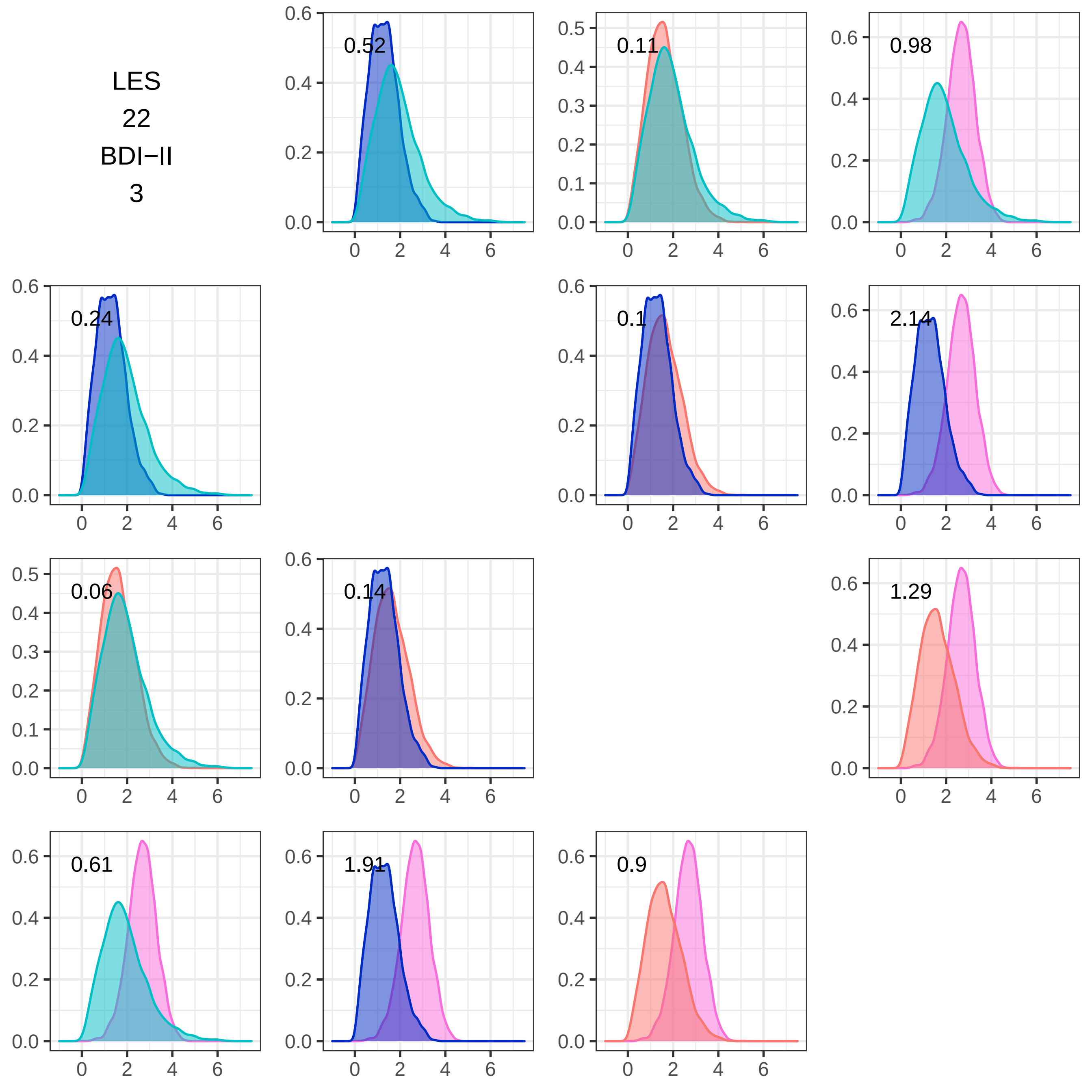
LES
22
BDI−II
3
0.52
0.11
0.98
0.24
0.1
2.14
0.06
0.14
1.29
0.61
1.91
0.9

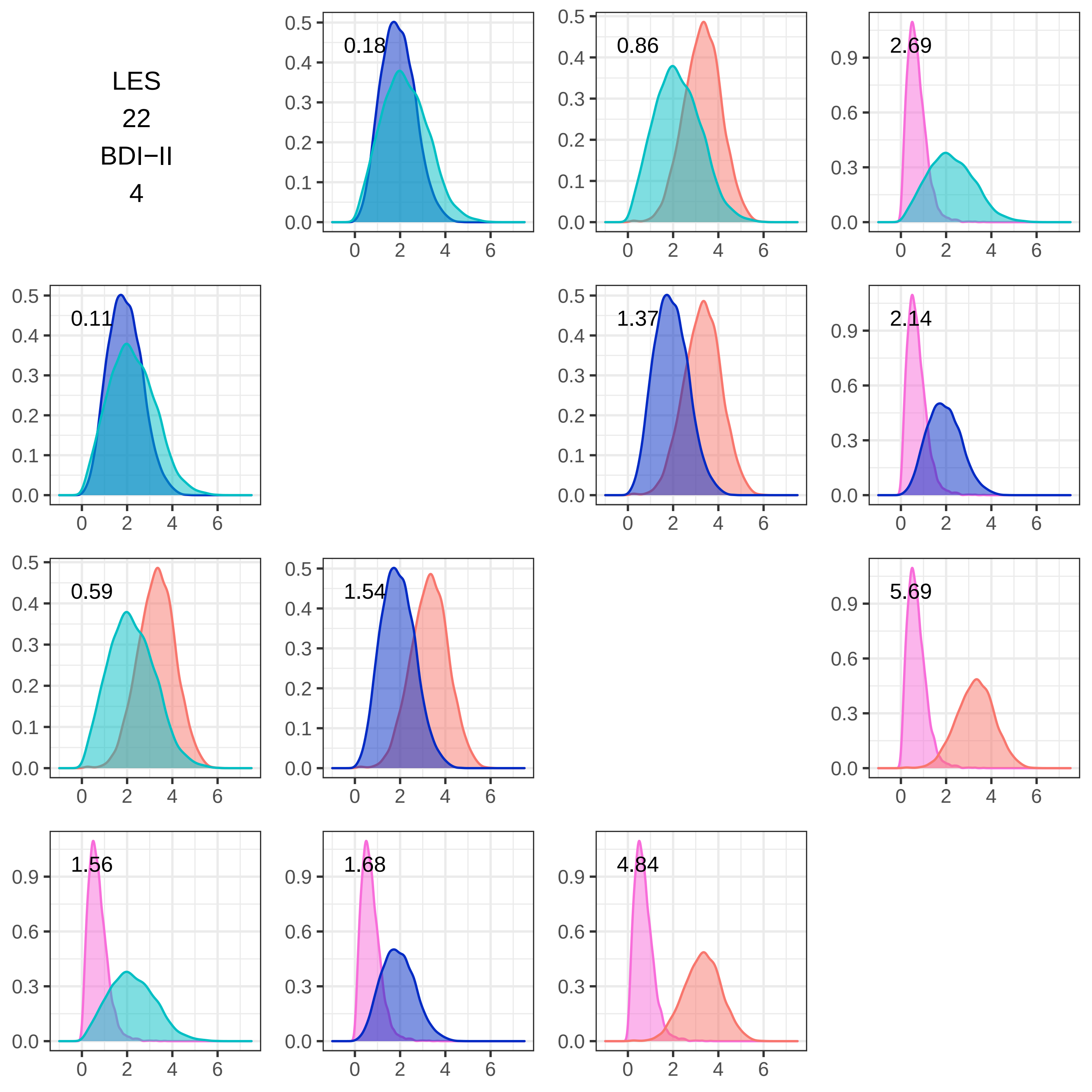
LES
22
BDI−II
4
0.18
0.86
2.69
0.11
1.37
2.14
0.59
1.54
5.69
1.56
1.68
4.84

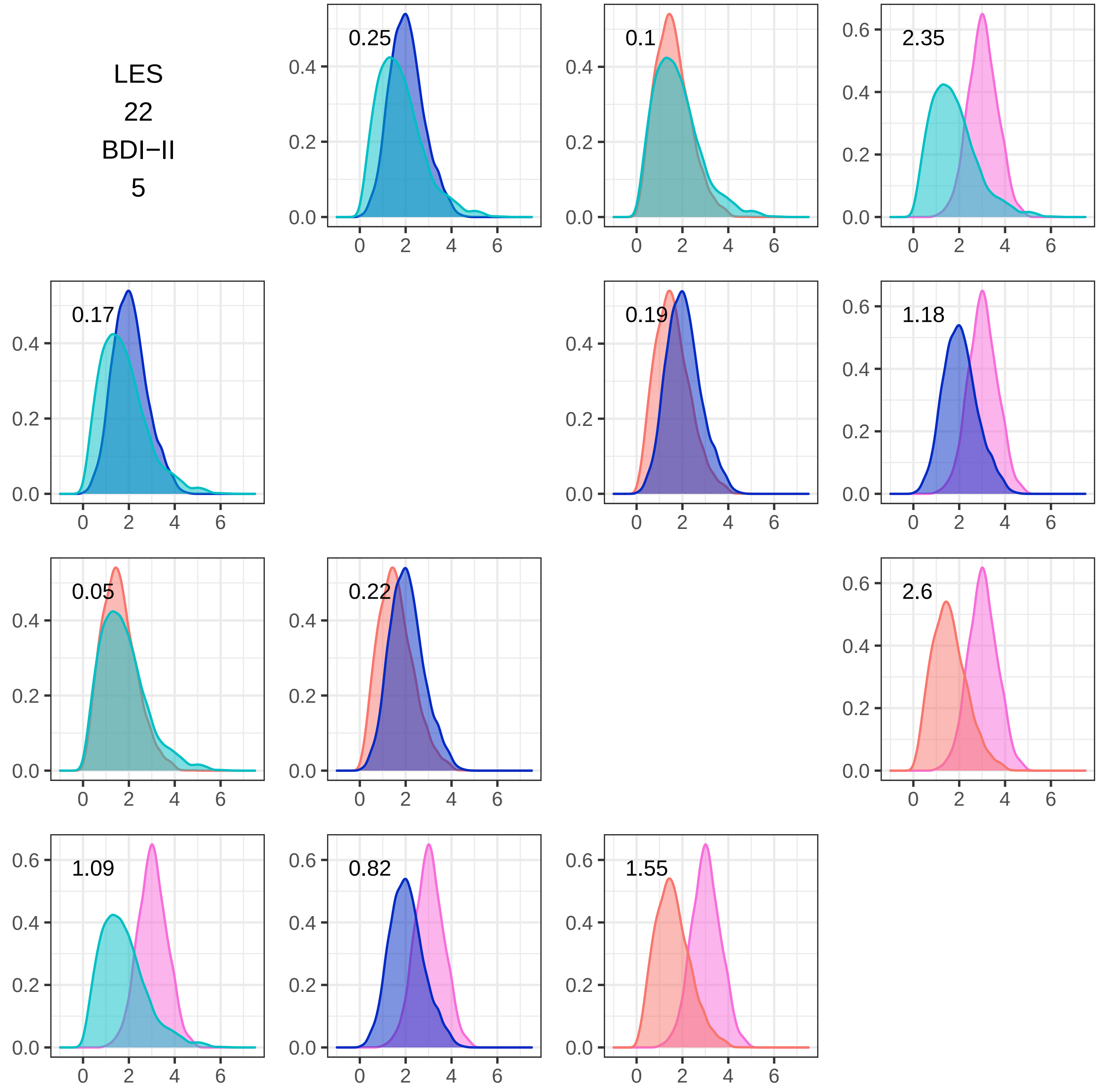

LES
22
BDI−II
5
0.25
0.1
2.35
0.17
0.19
1.18
0.05
0.22
2.6
1.09
0.82
1.55

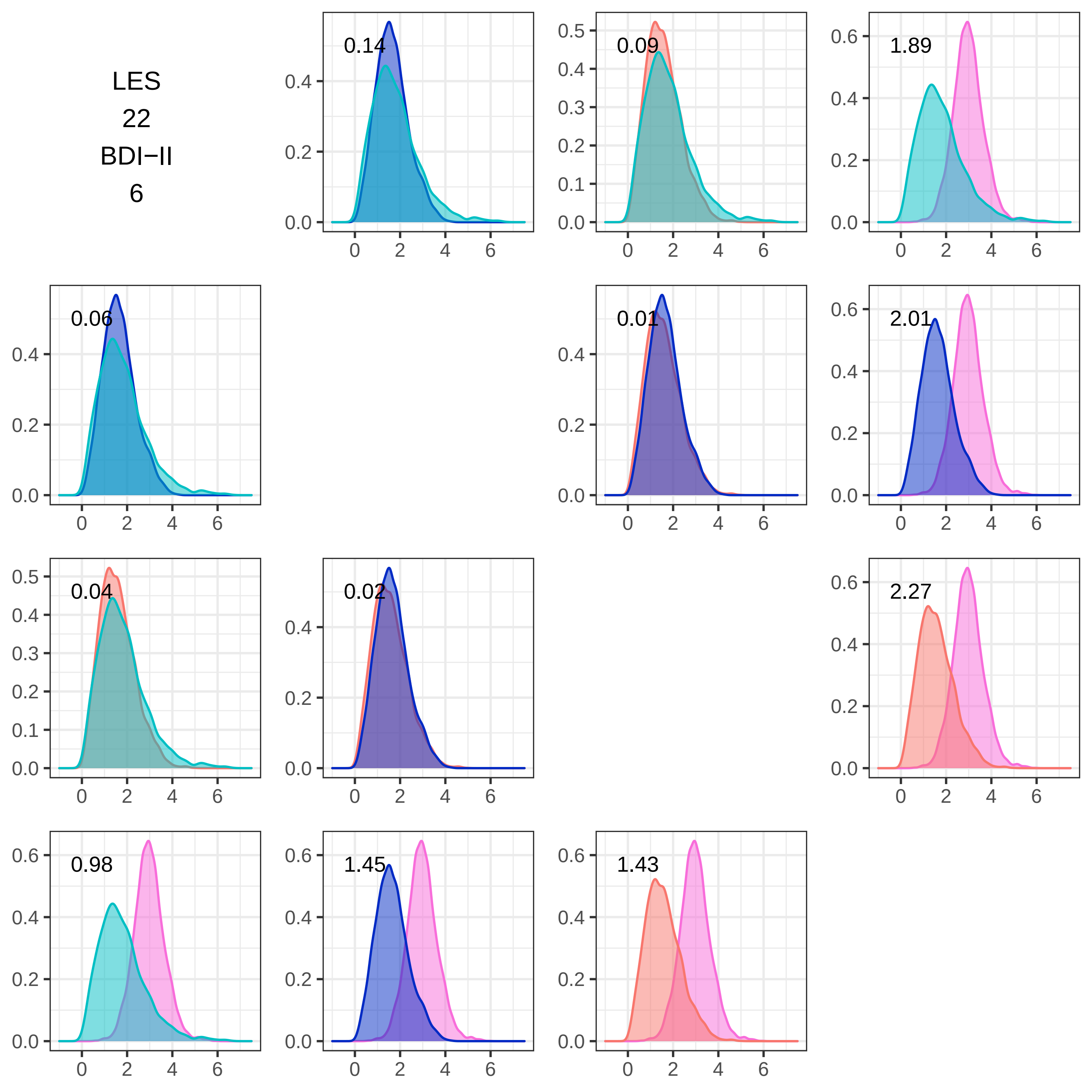
LES
22
BDI−II
6
0.14
0.09
1.89
0.06
0.01
2.01
0.04
0.02
2.27
0.98
1.45
1.43

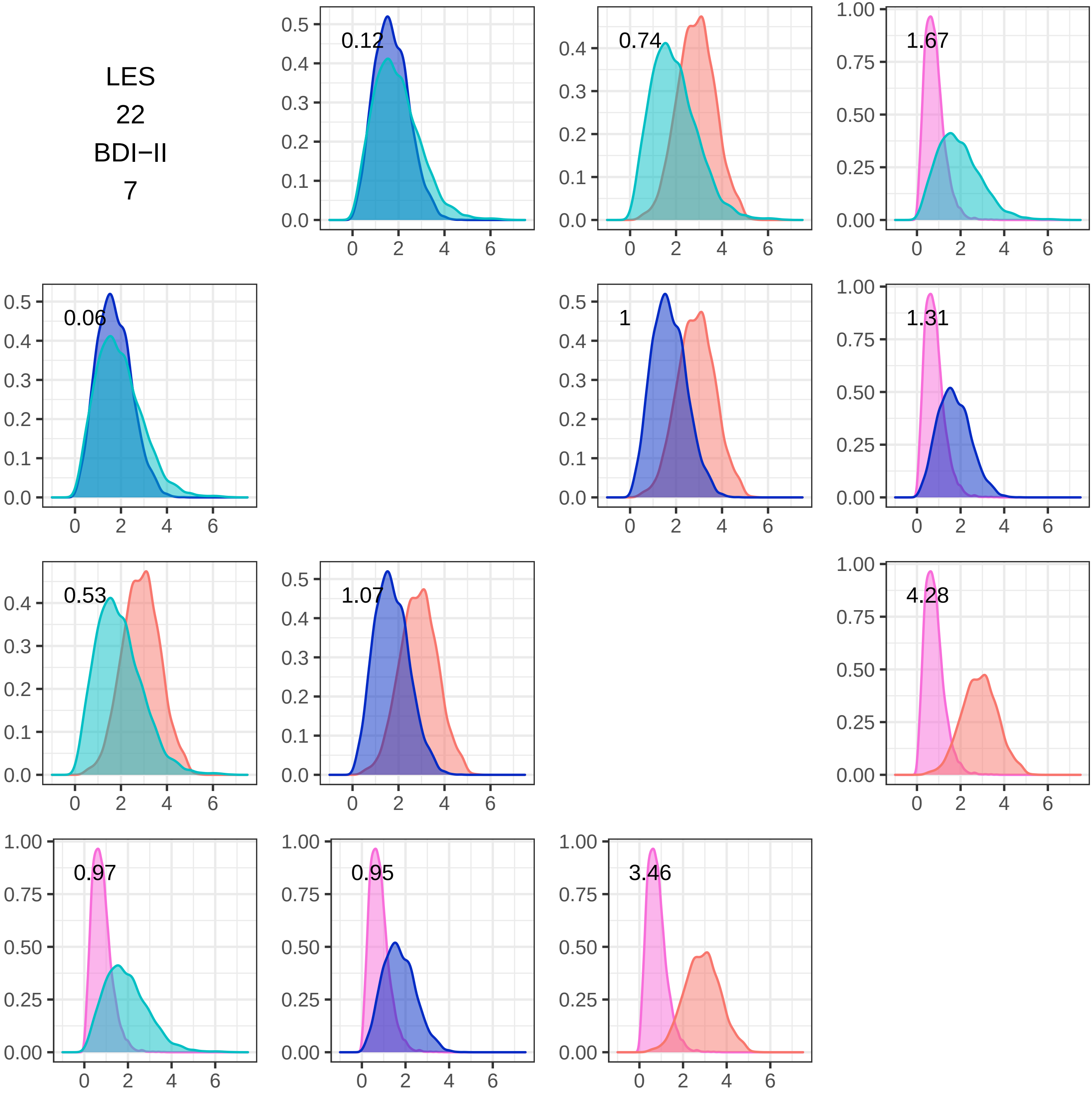

LES
22
BDI−II
7
0.12
0.74
1.67
0.06
1
1.31
0.53
1.07
4.28
0.97
0.95
3.46

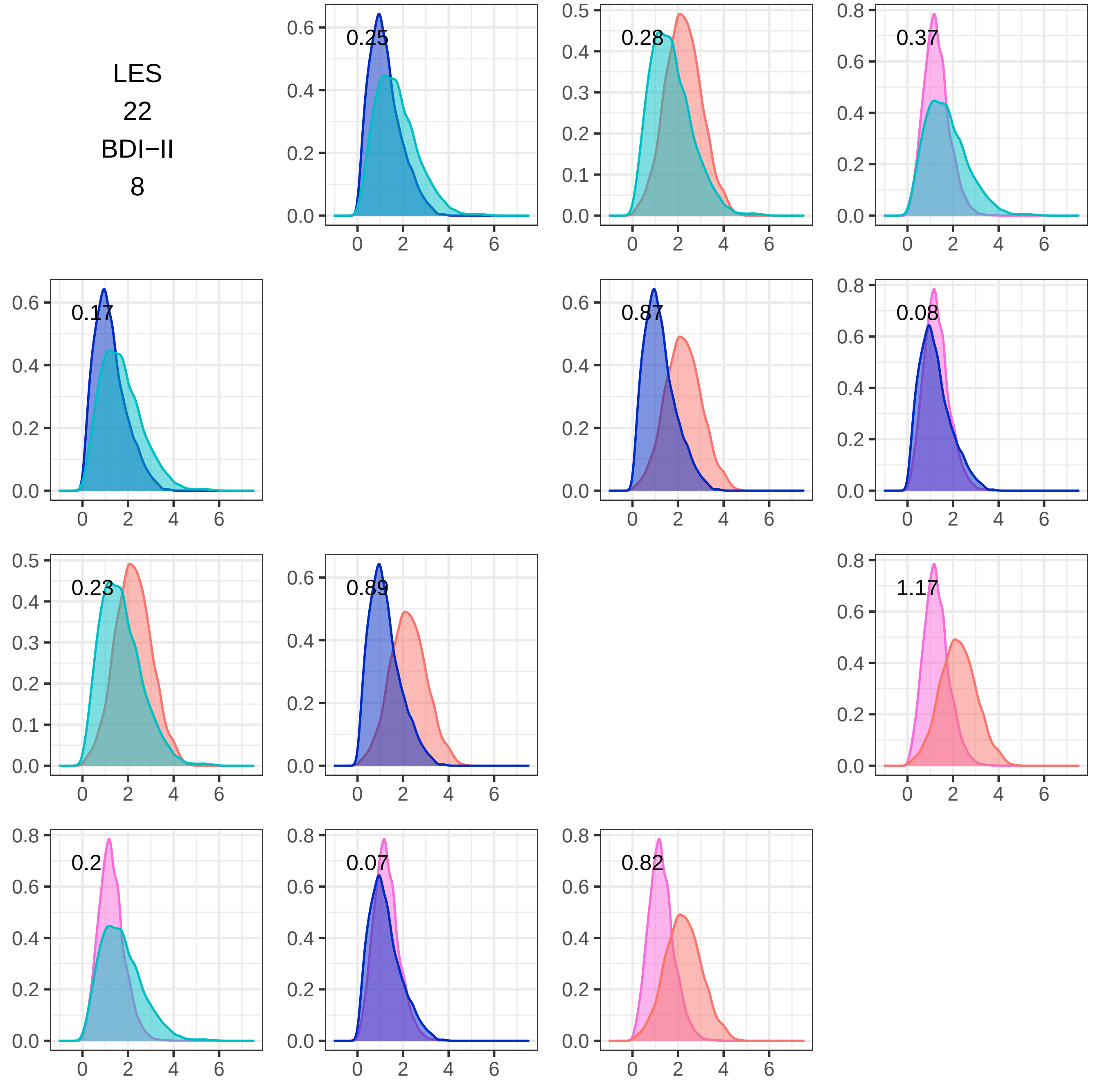

LES
22
BDI−II
8
0.25
0.28
0.37
0.17
0.87
0.08
0.23
0.89
1.17
0.2
0.07
0.82

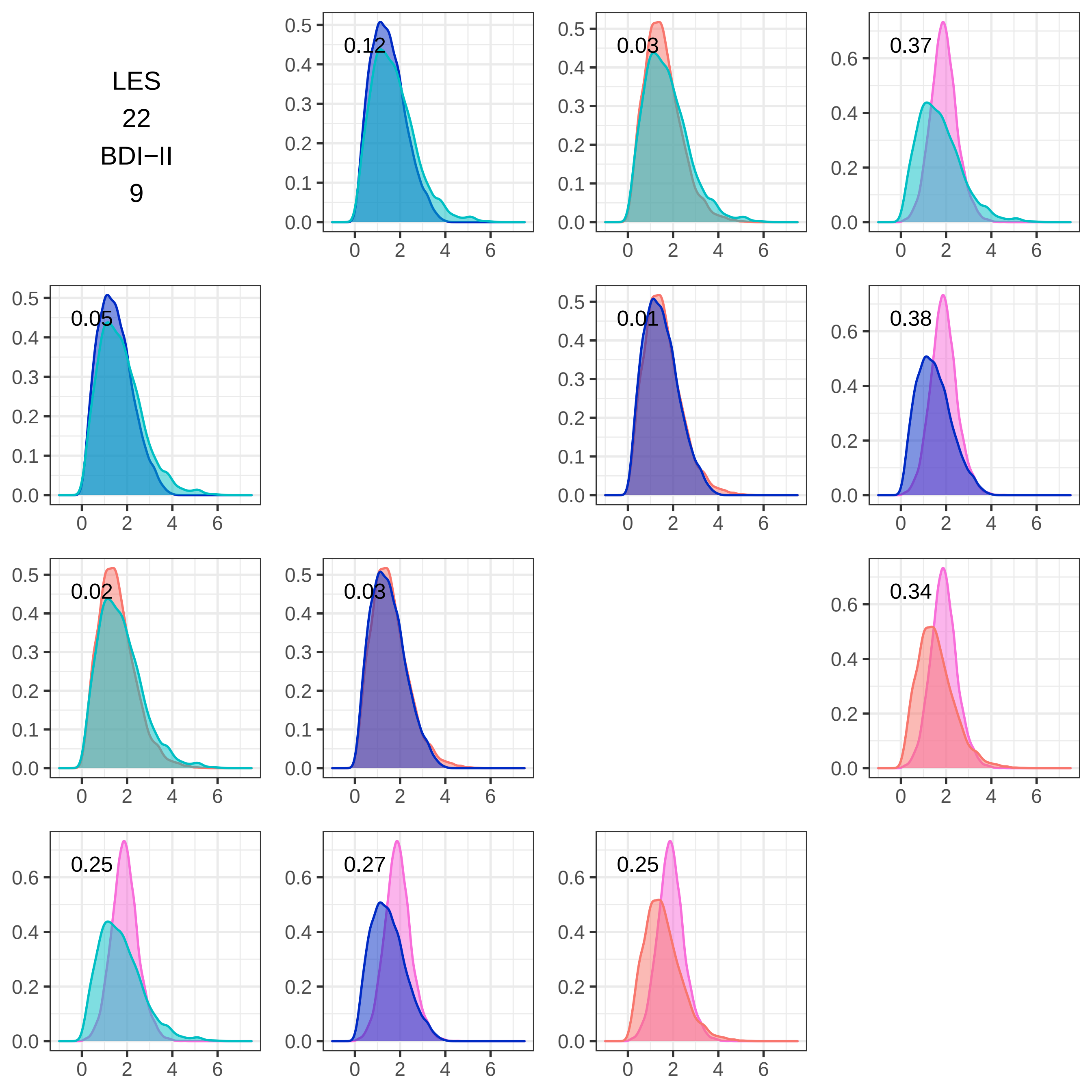
LES
22
BDI−II
9
0.12
0.03
0.37
0.05
0.01
0.38
0.02
0.03
0.34
0.25
0.27
0.25

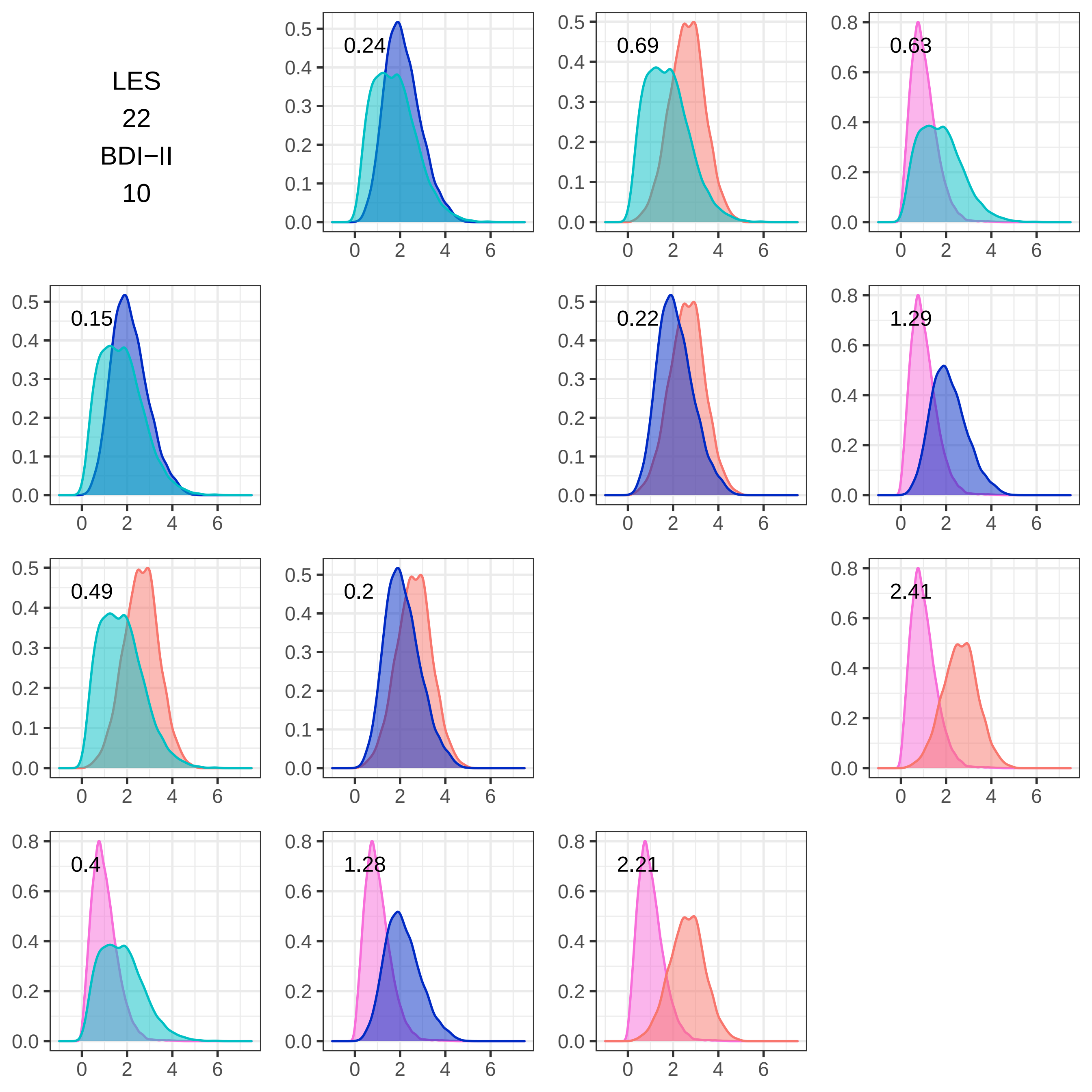

LES
22
BDI−II
10
0.24
0.69
0.63
0.15
0.22
1.29
0.49
0.2
2.41
0.4
1.28
2.21

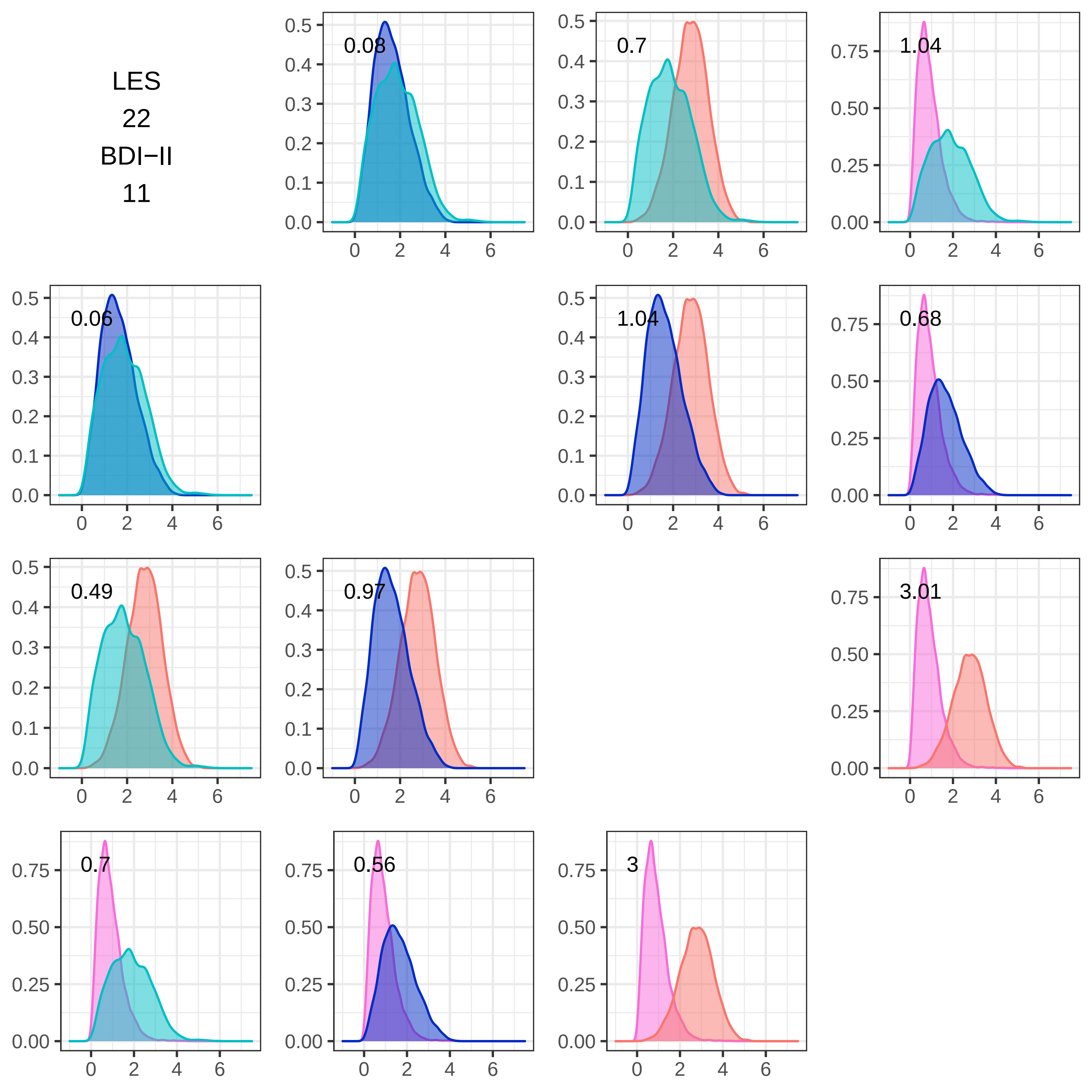

LES
22
BDI−II
11
0.08
0.7
1.04
0.06
1.04
0.68
0.49
0.97
3.01
0.7
0.56
3

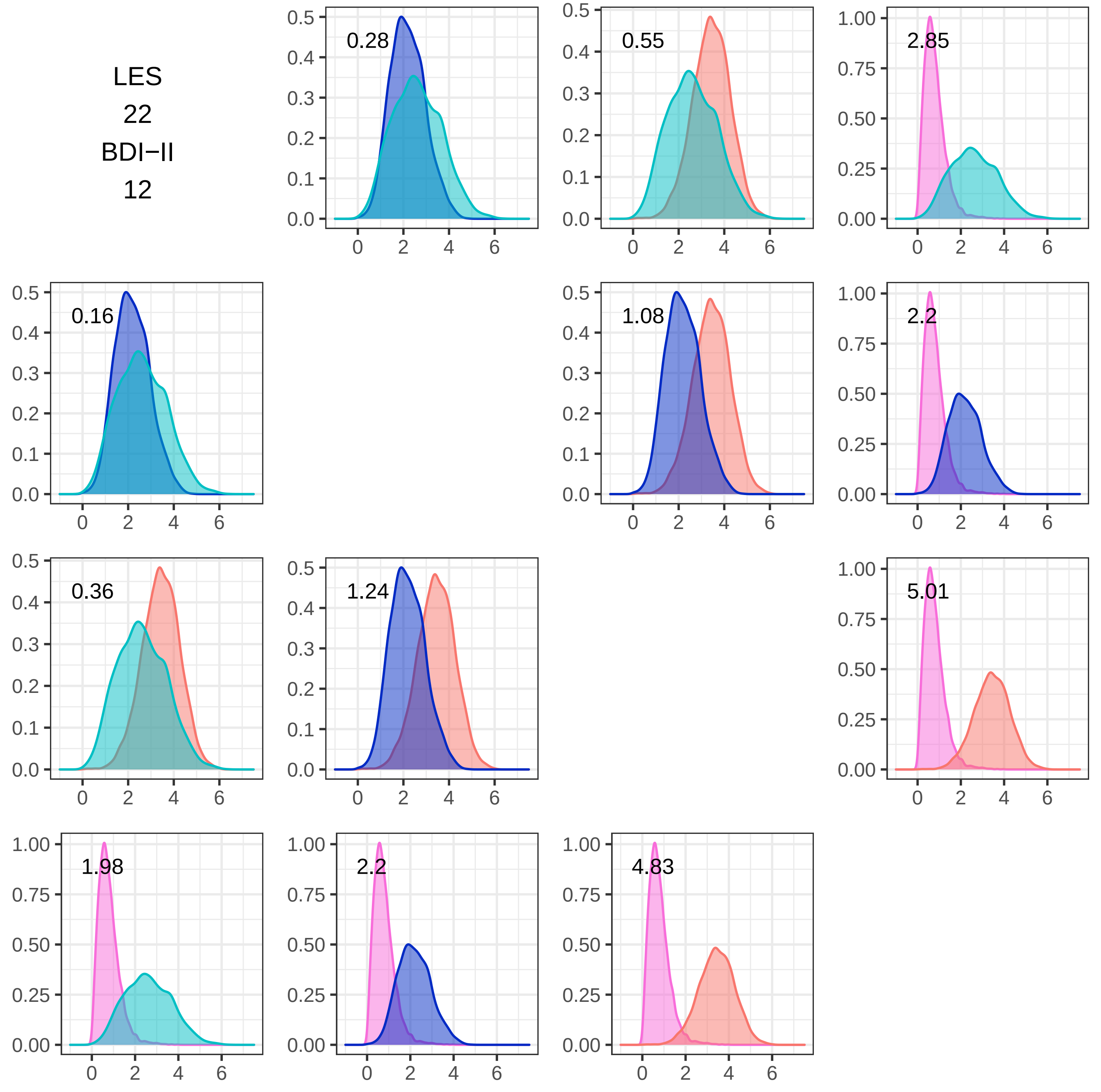

LES
22
BDI−II
12
0.28
0.55
2.85
0.16
1.08
2.2
0.36
1.24
5.01
1.98
2.2
4.83

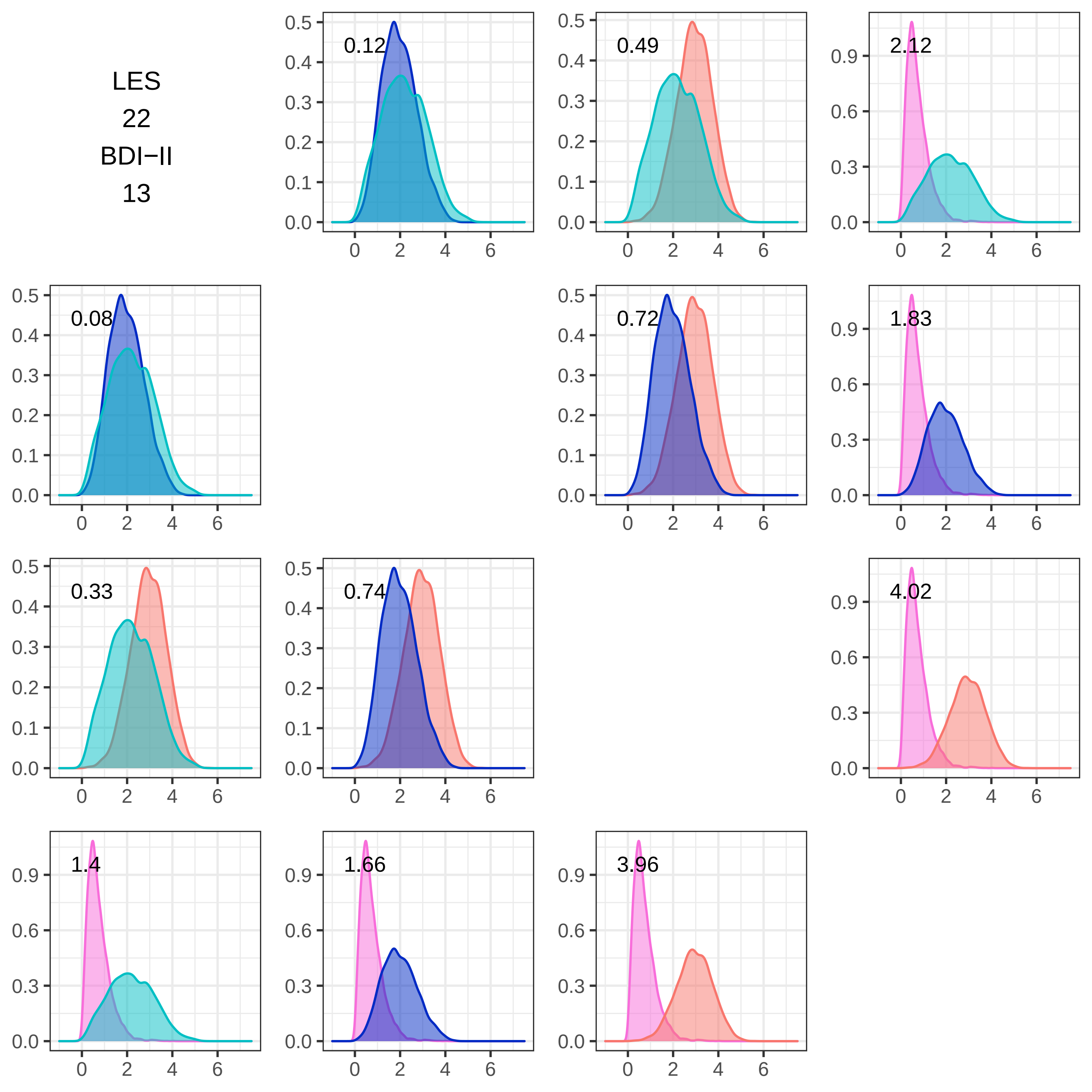
LES
22
BDI−II
13
0.12
0.49
2.12
0.08
0.72
1.83
0.33
0.74
4.02
1.4
1.66
3.96

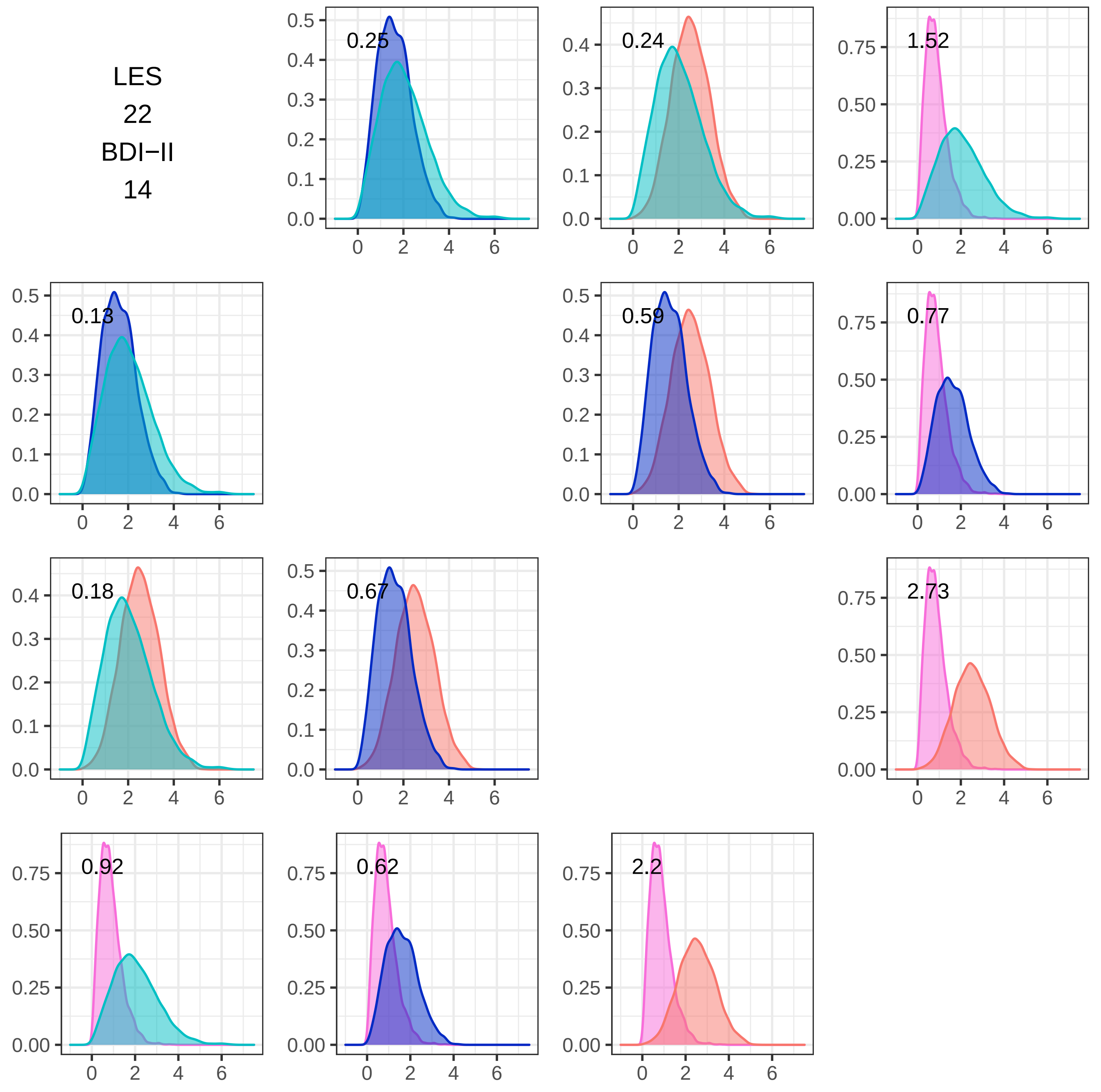
LES
22
BDI−II
14
0.25
0.24
1.52
0.13
0.59
0.77
0.18
0.67
2.73
0.92
0.62
2.2

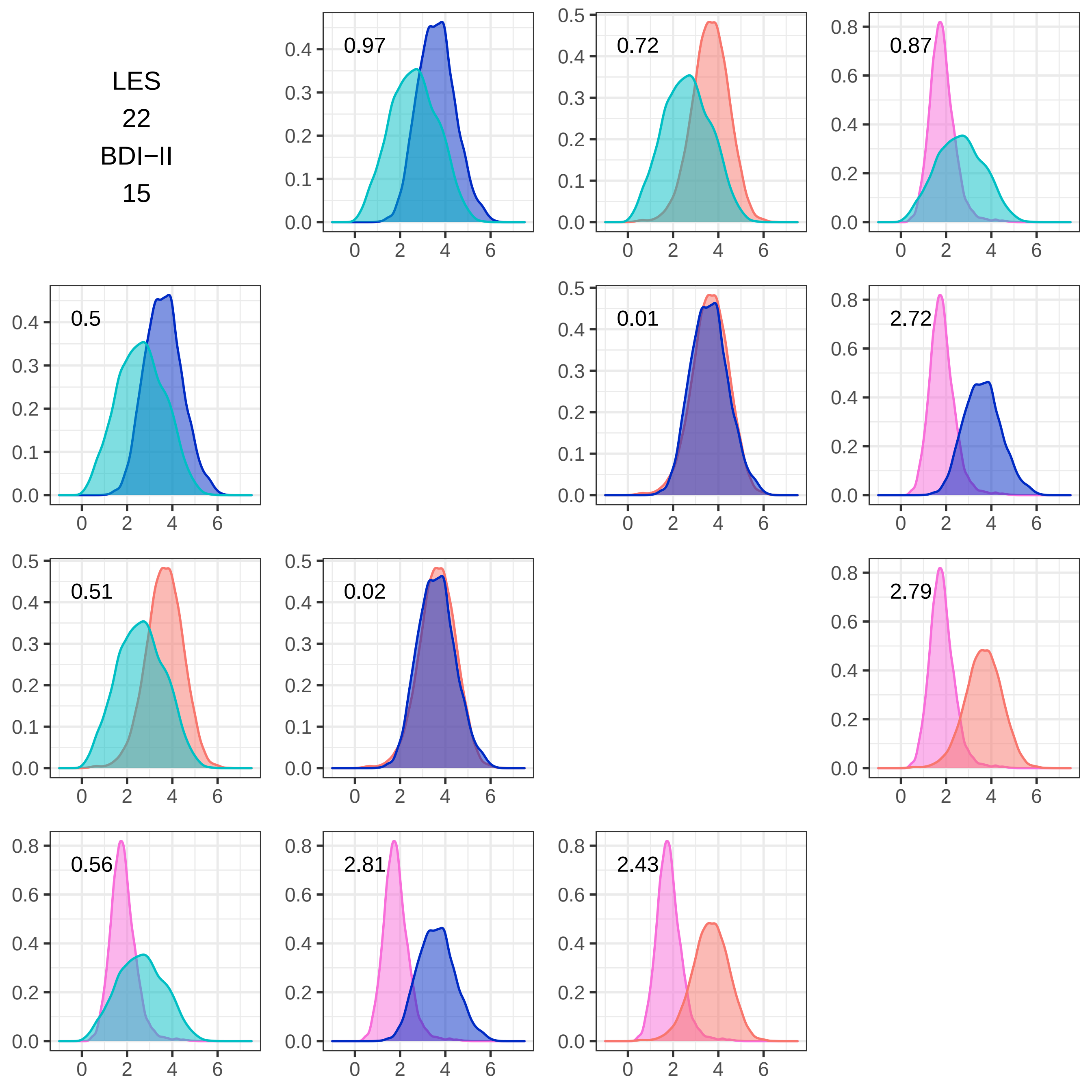

LES
22
BDI−II
15
0.97
0.72
0.87
0.5
0.01
2.72
0.51
0.02
2.79
0.56
2.81
2.43

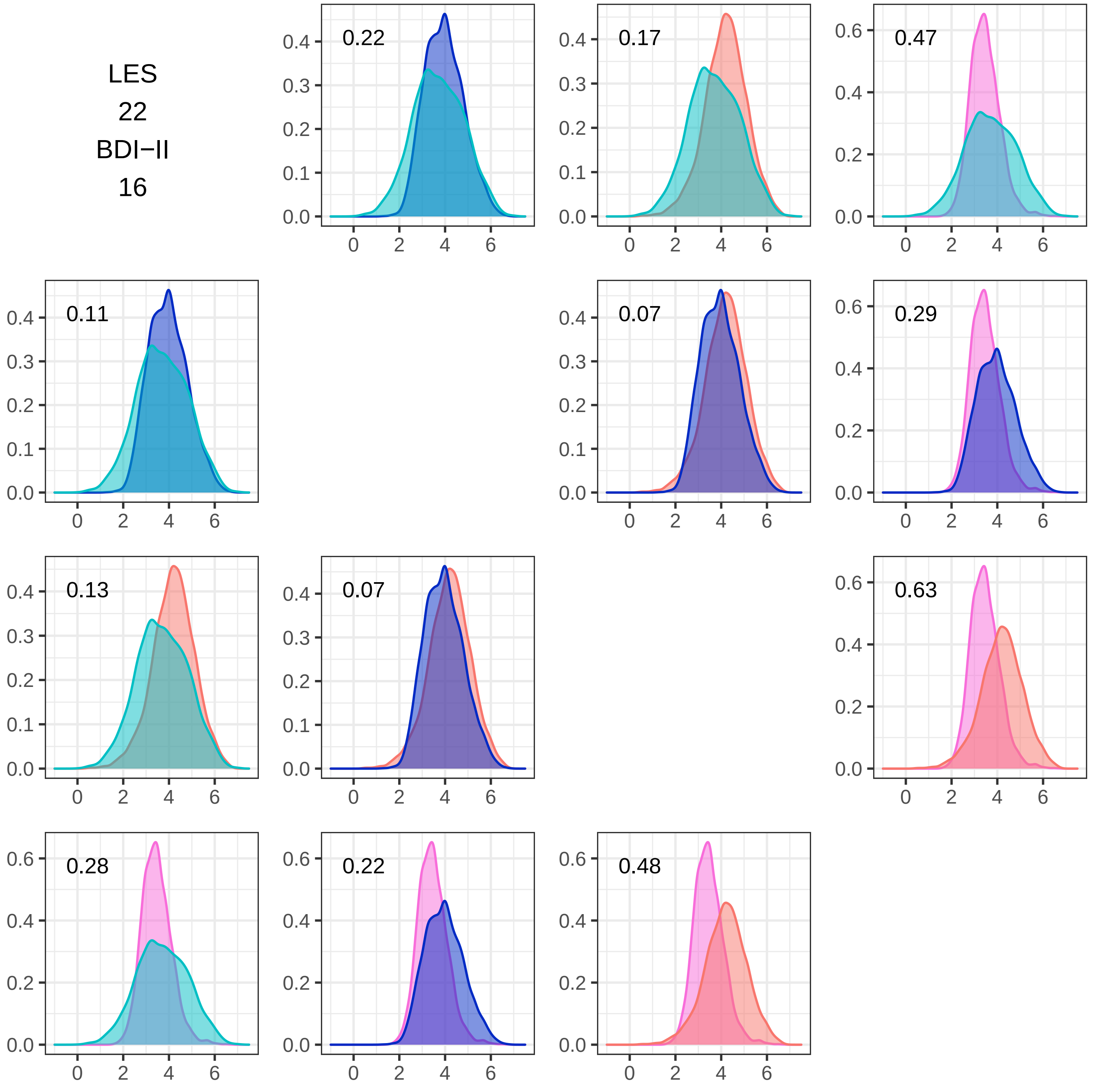
LES
22
BDI−II
16
0.22
0.17
0.47
0.11
0.07
0.29
0.13
0.07
0.63
0.28
0.22
0.48

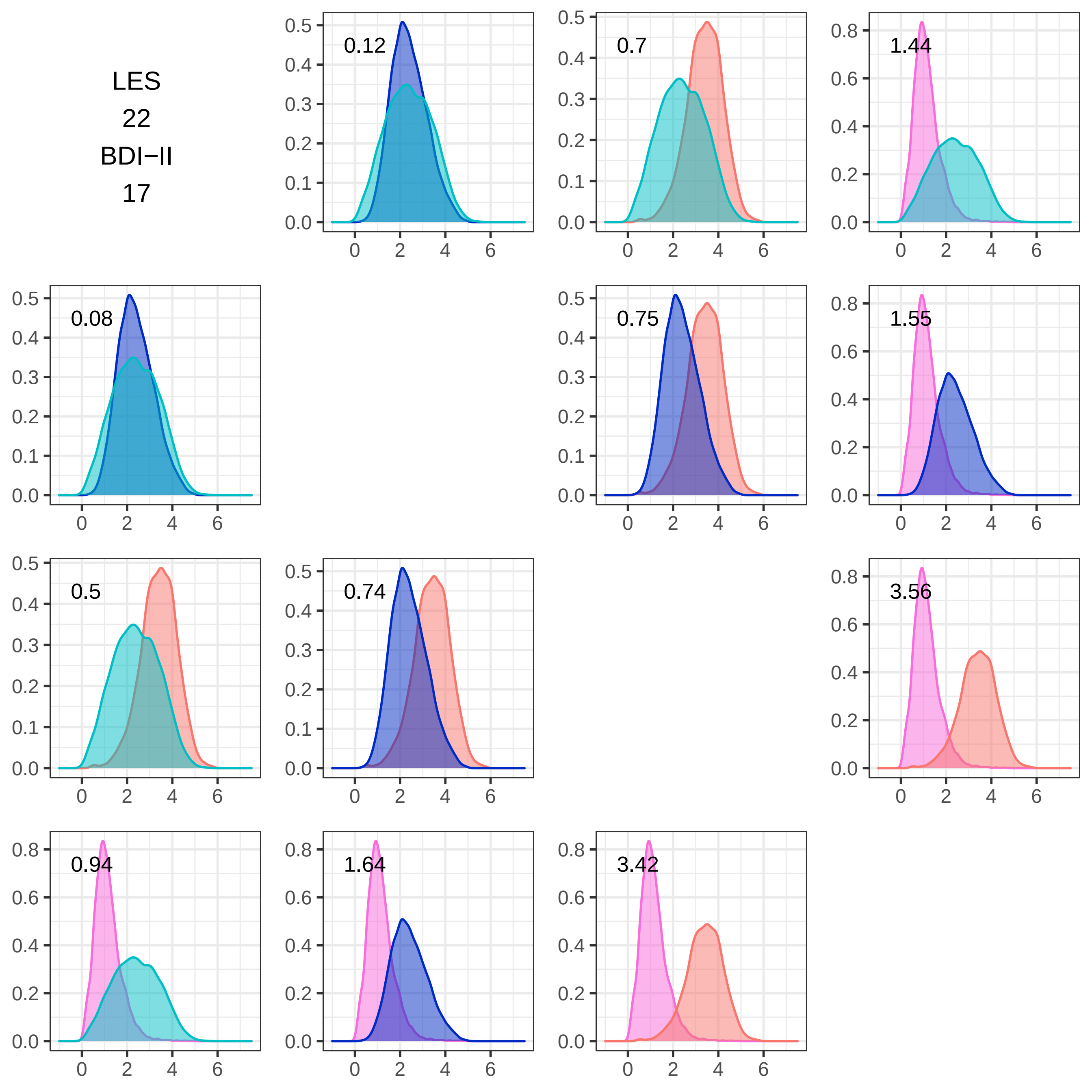

LES
22
BDI−II
17
0.12
0.7
1.44
0.08
0.75
1.55
0.5
0.74
3.56
0.94
1.64
3.42

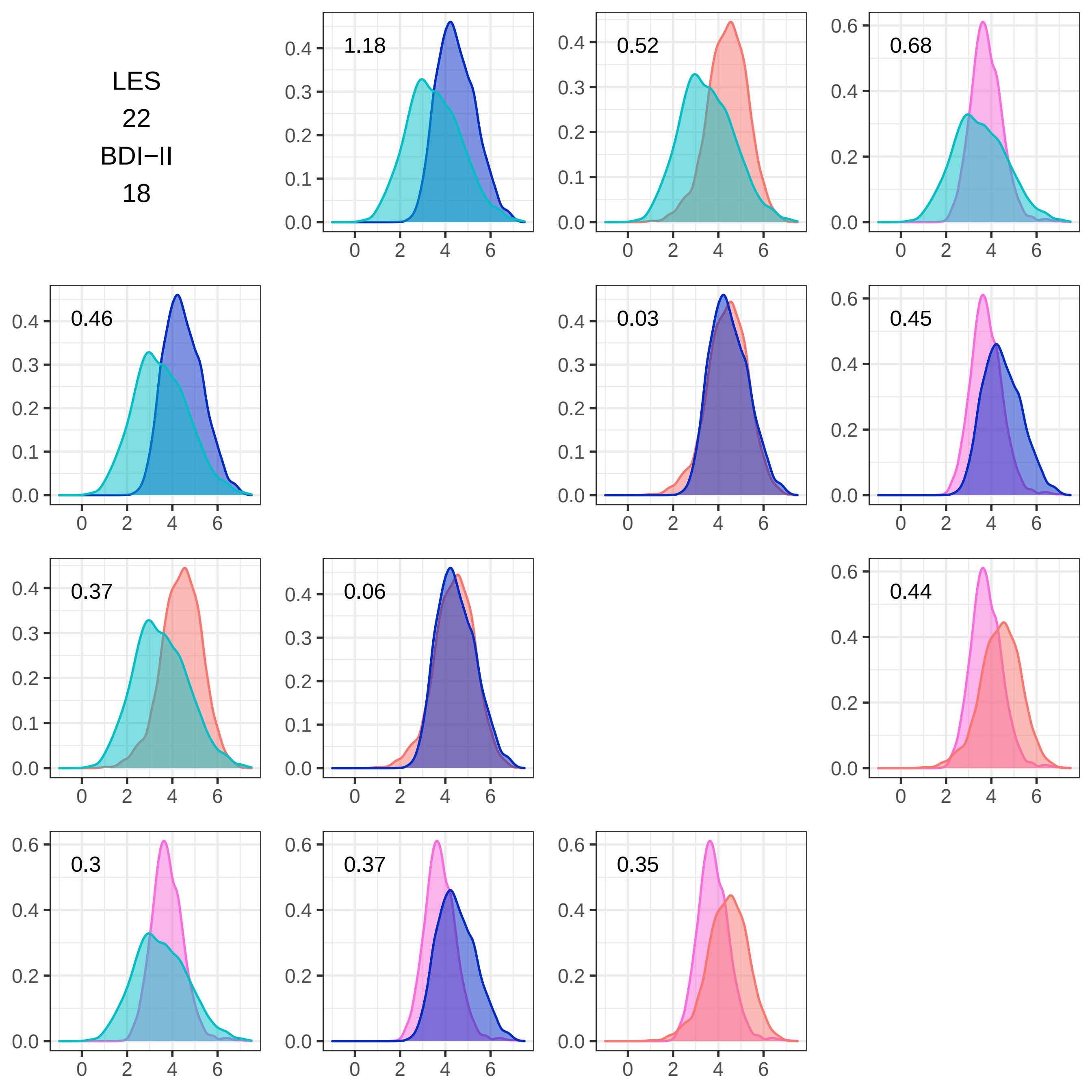
LES
22
BDI−II
18
1.18
0.52
0.68
0.46
0.03
0.45
0.37
0.06
0.44
0.3
0.37
0.35

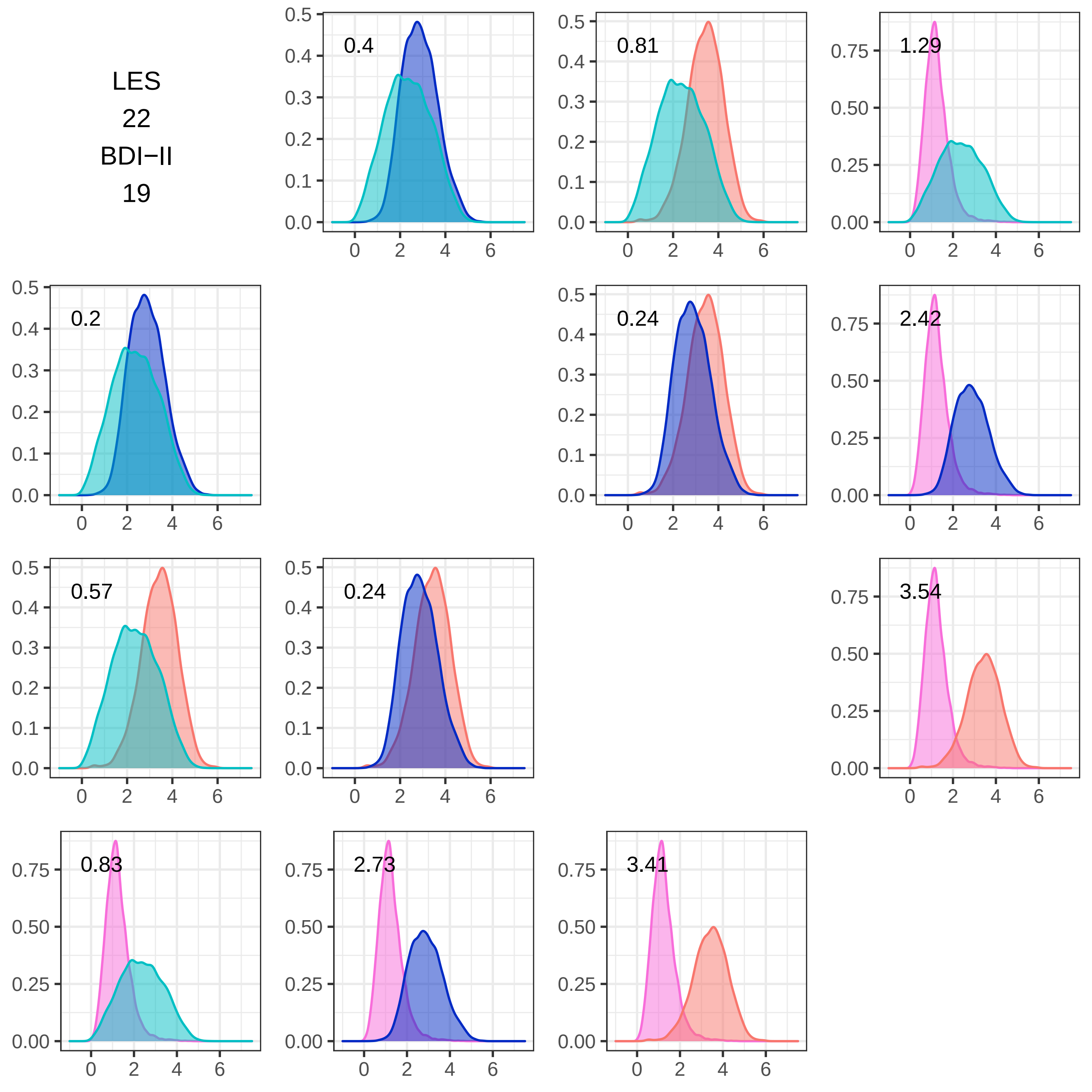
LES
22
BDI−II
19
0.4
0.81
1.29
0.2
0.24
2.42
0.57
0.24
3.54
0.83
2.73
3.41

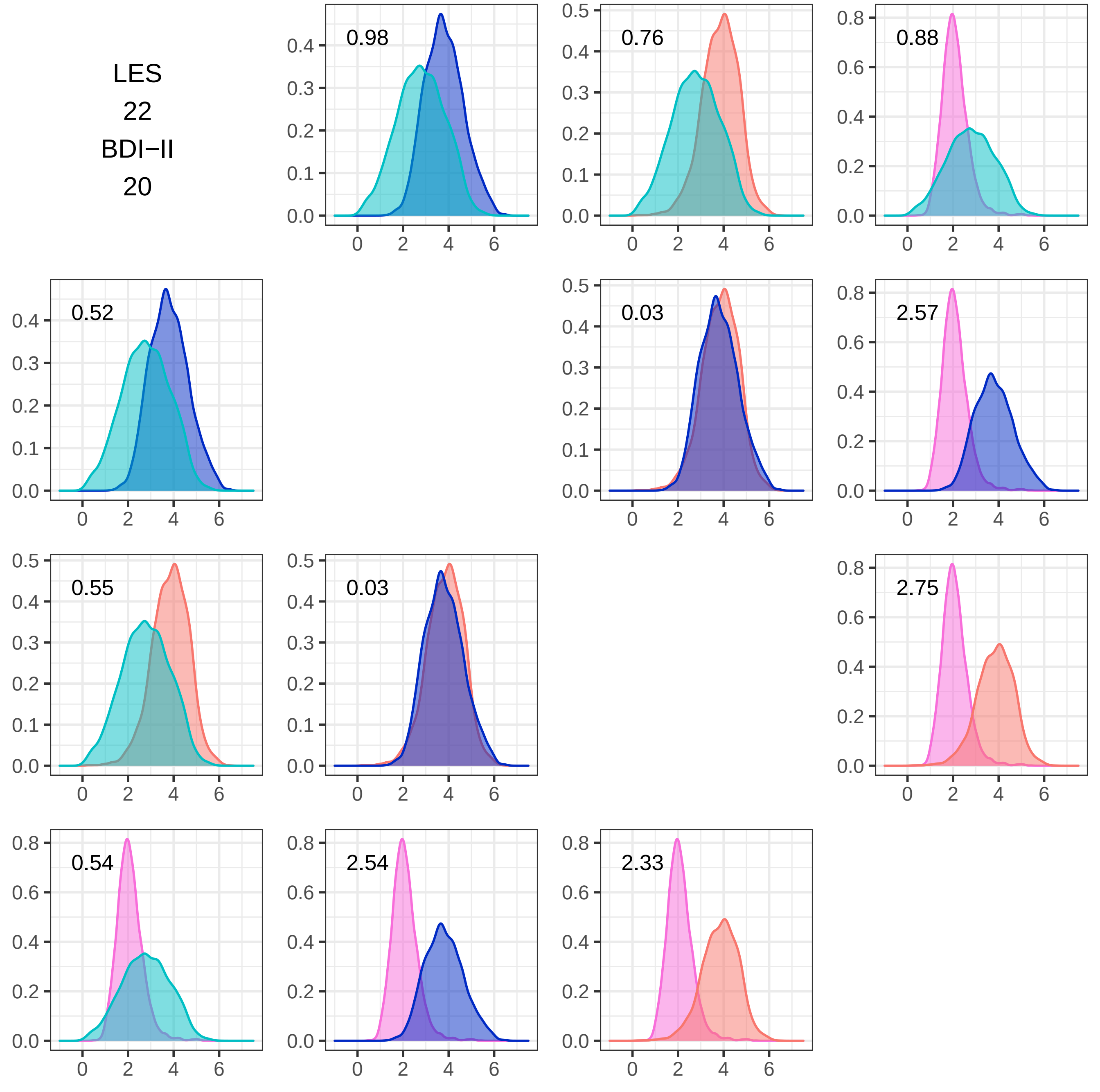

LES
22
BDI−II
20
0.98
0.76
0.88
0.52
0.03
2.57
0.55
0.03
2.75
0.54
2.54
2.33

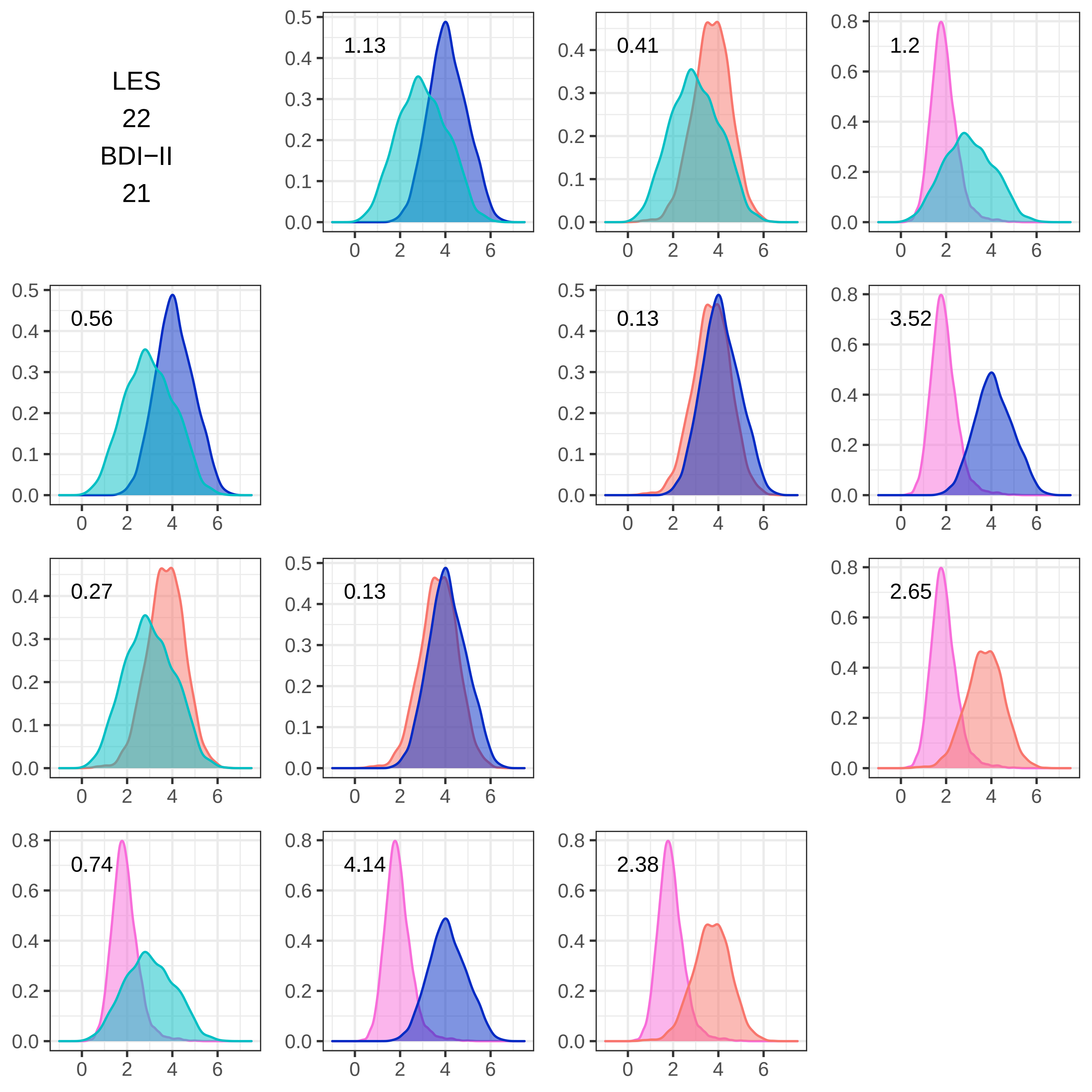
LES
22
BDI−II
21
1.13
0.41
1.2
0.56
0.13
3.52
0.27
0.13
2.65
0.74
4.14
2.38

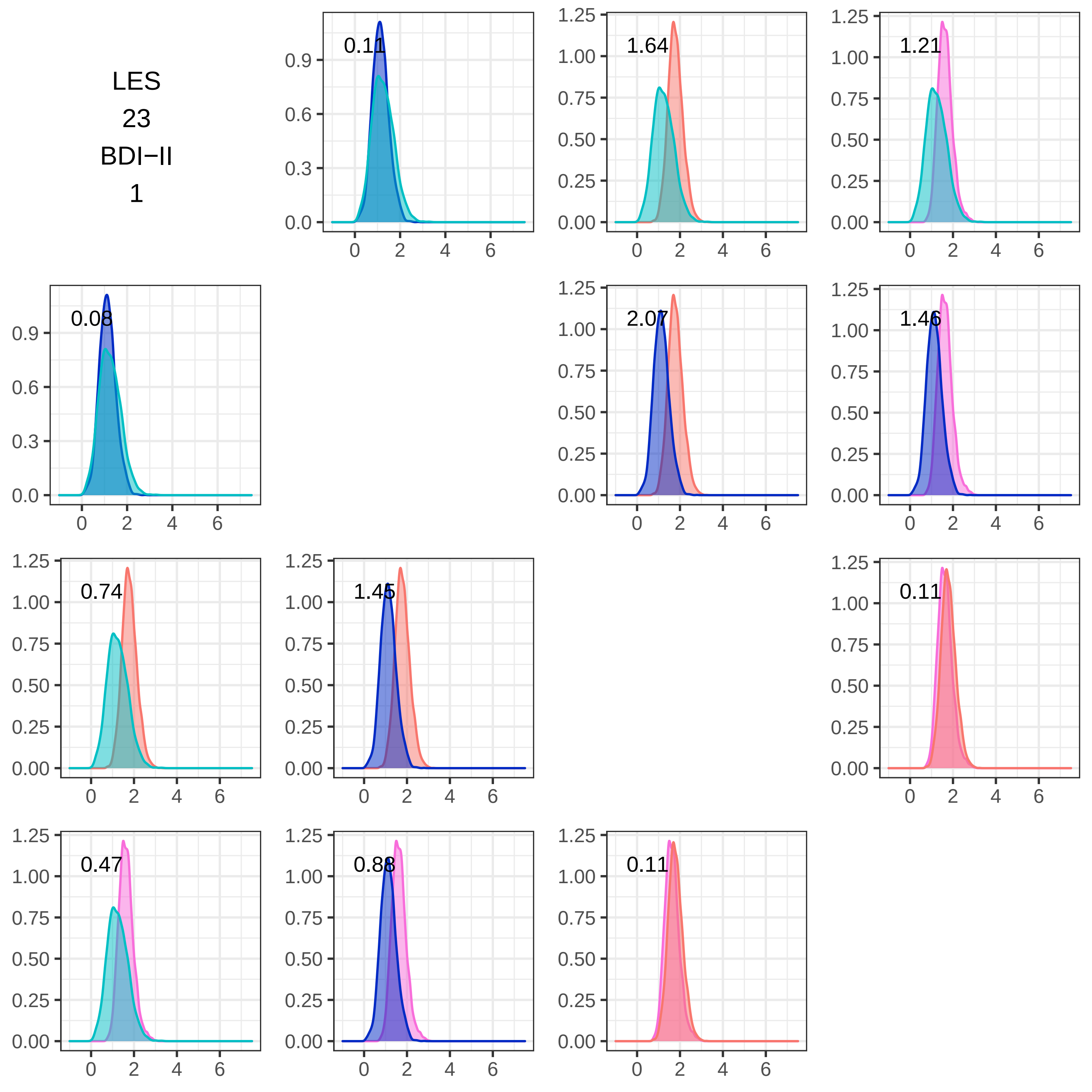

LES
23
BDI−II
1
0.11
1.64
1.21
0.08
2.07
1.46
0.74
1.45
0.11
0.47
0.88
0.11

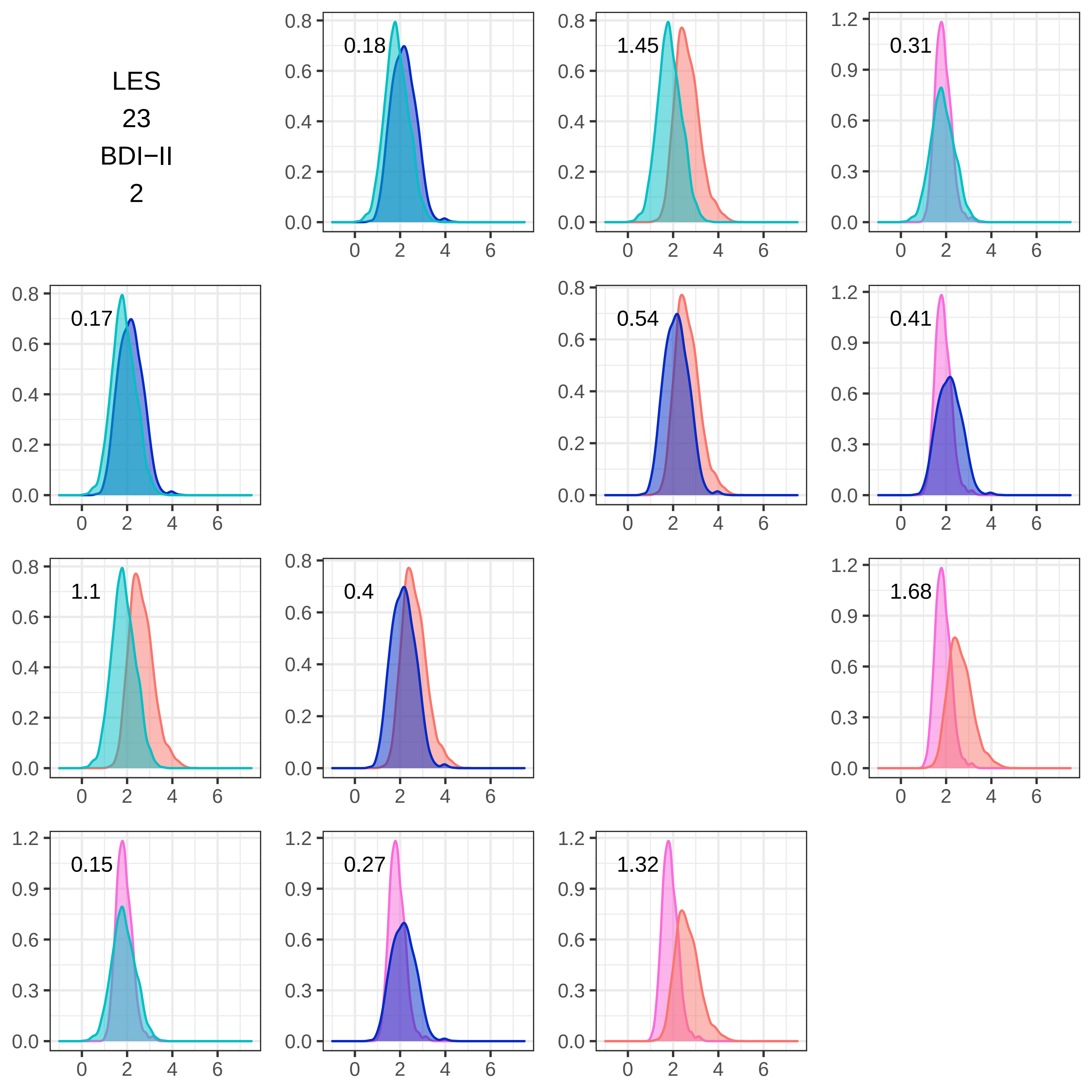
LES
23
BDI−II
2
0.18
1.45
0.31
0.17
0.54
0.41
1.1
0.4
1.68
0.15
0.27
1.32

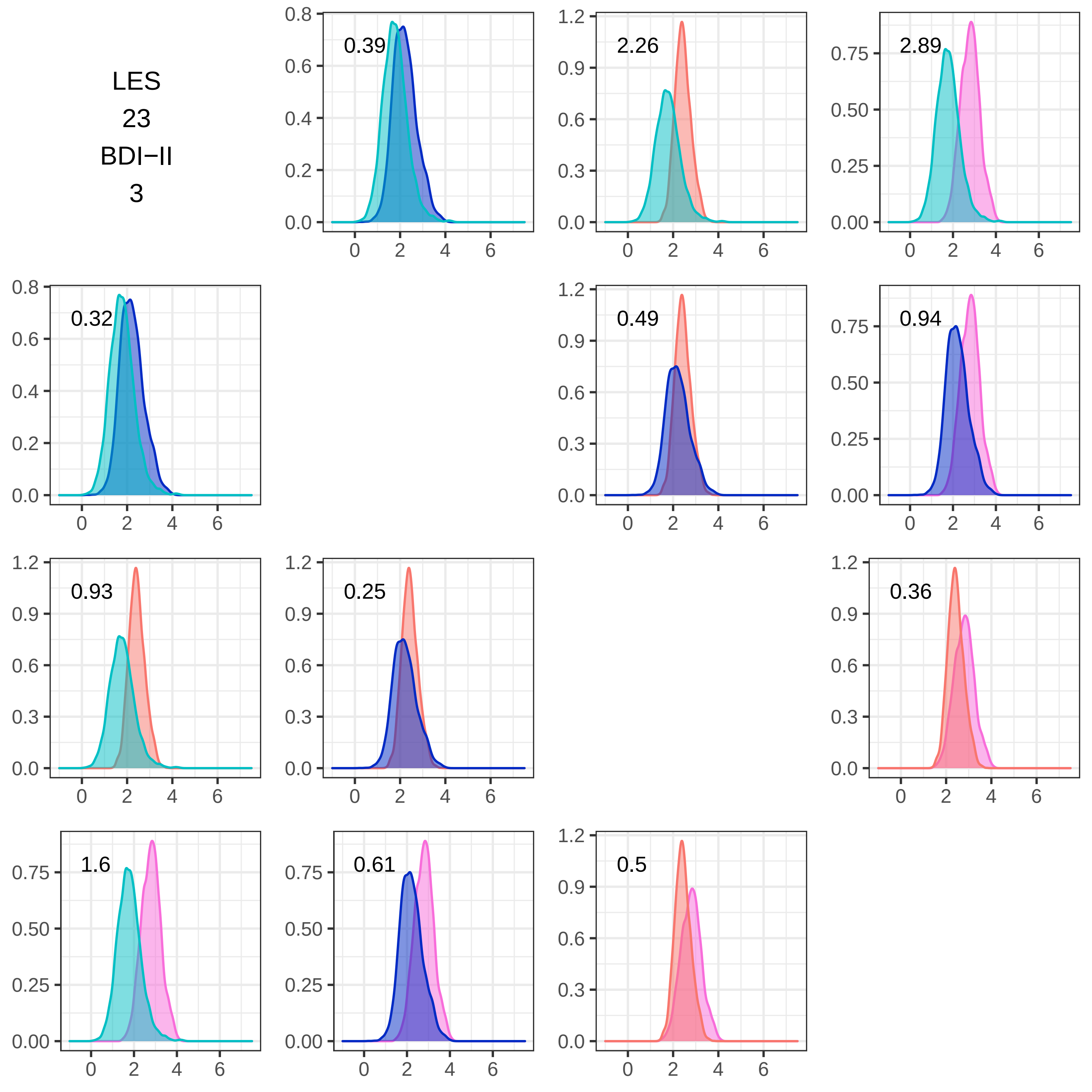

LES
23
BDI−II
3
0.39
2.26
2.89
0.32
0.49
0.94
0.93
0.25
0.36
1.6
0.61
0.5

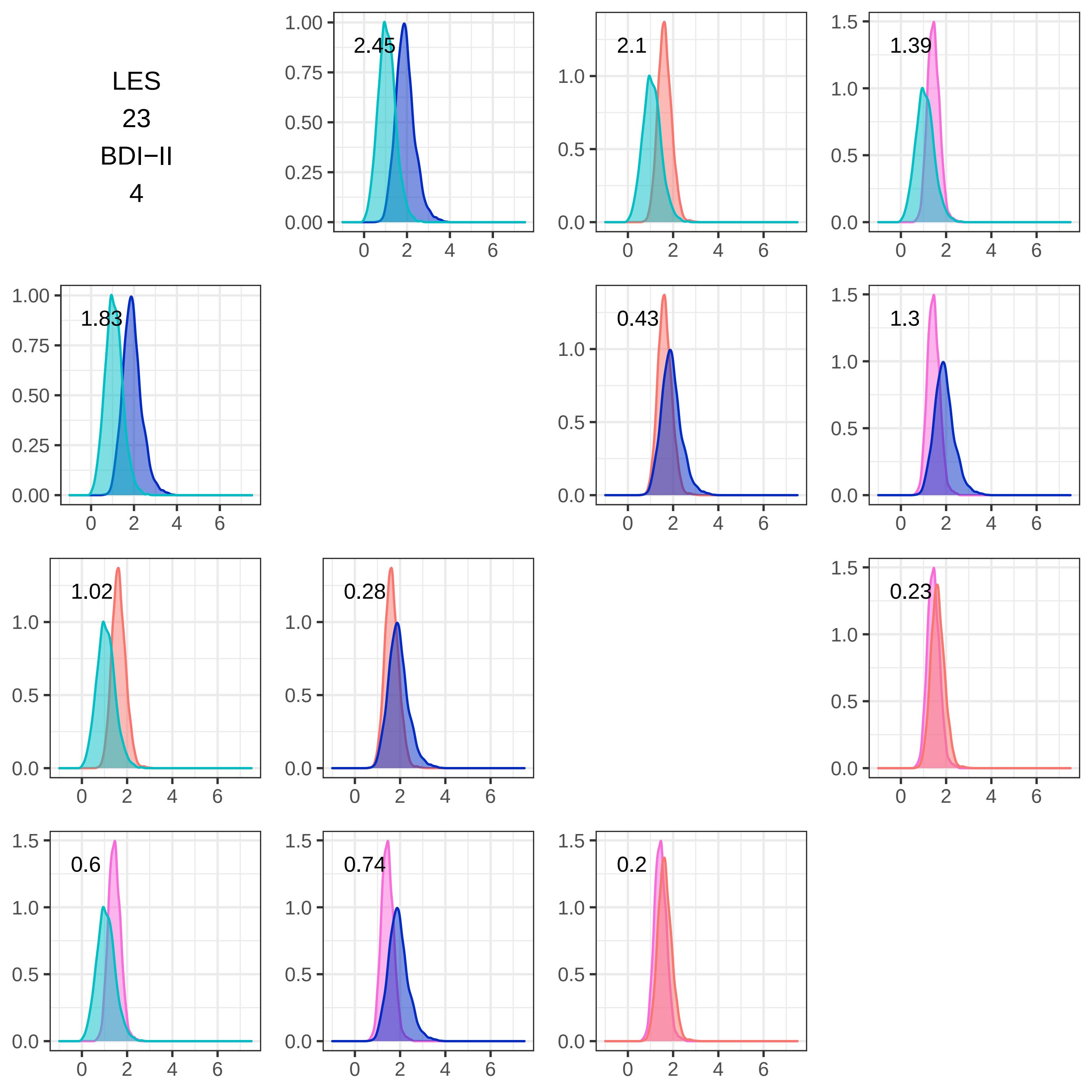
LES
23
BDI−II
4
2.45
2.1
1.39
1.83
0.43
1.3
1.02
0.28
0.23
0.6
0.74
0.2

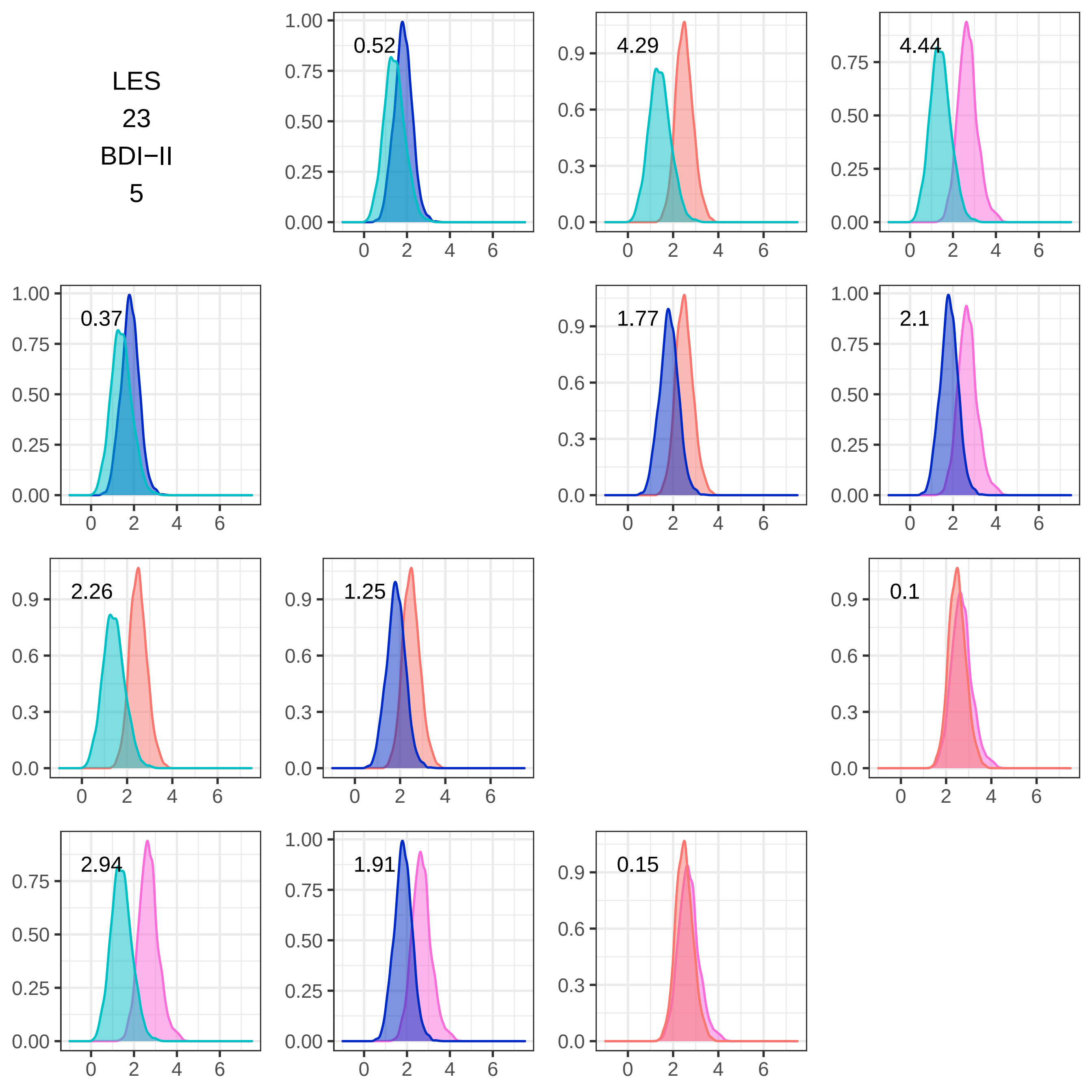
LES
23
BDI−II
5
0.52
4.29
4.44
0.37
1.77
2.1
2.26
1.25
0.1
2.94
1.91
0.15

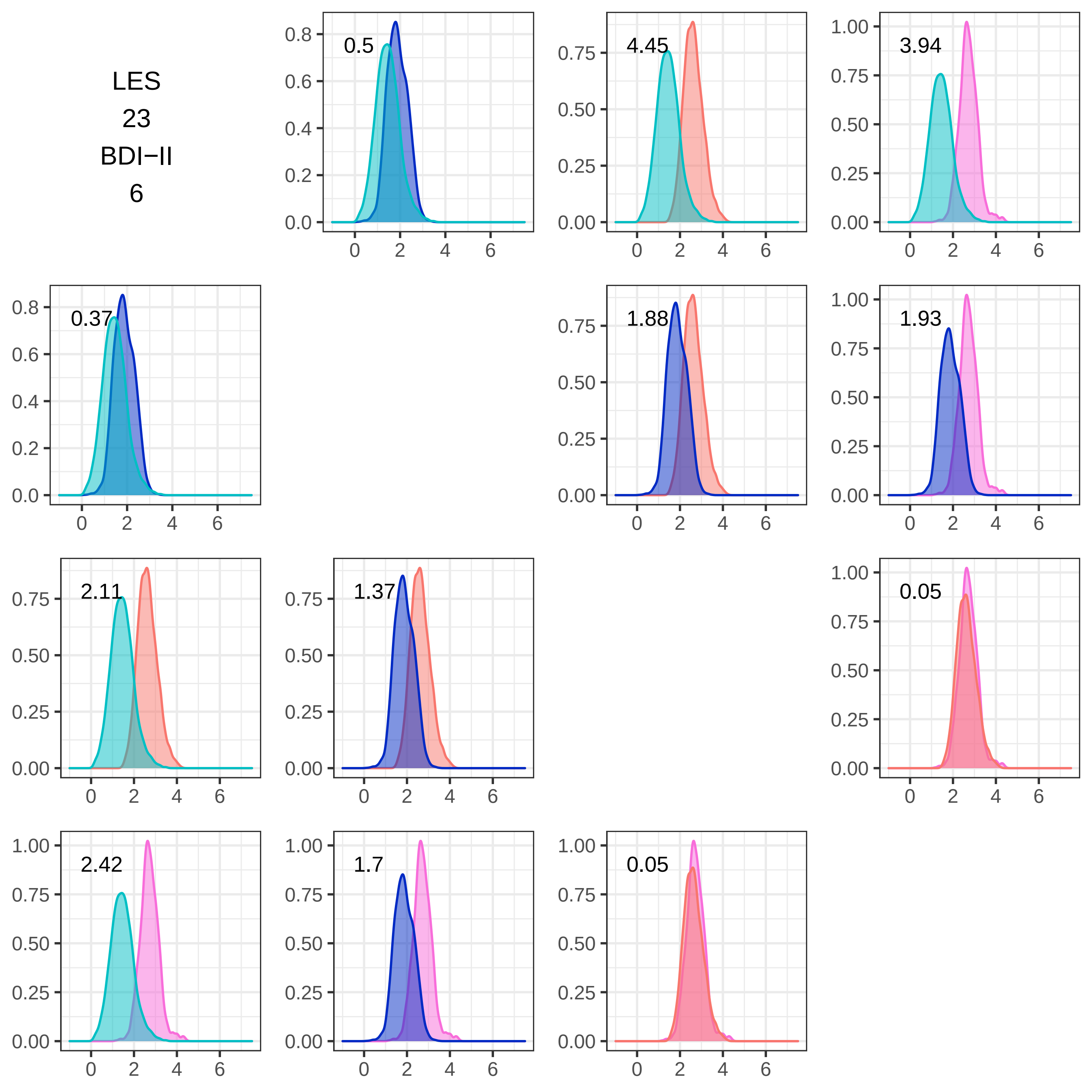
LES
23
BDI−II
6
0.5
4.45
3.94
0.37
1.88
1.93
2.11
1.37
0.05
2.42
1.7
0.05

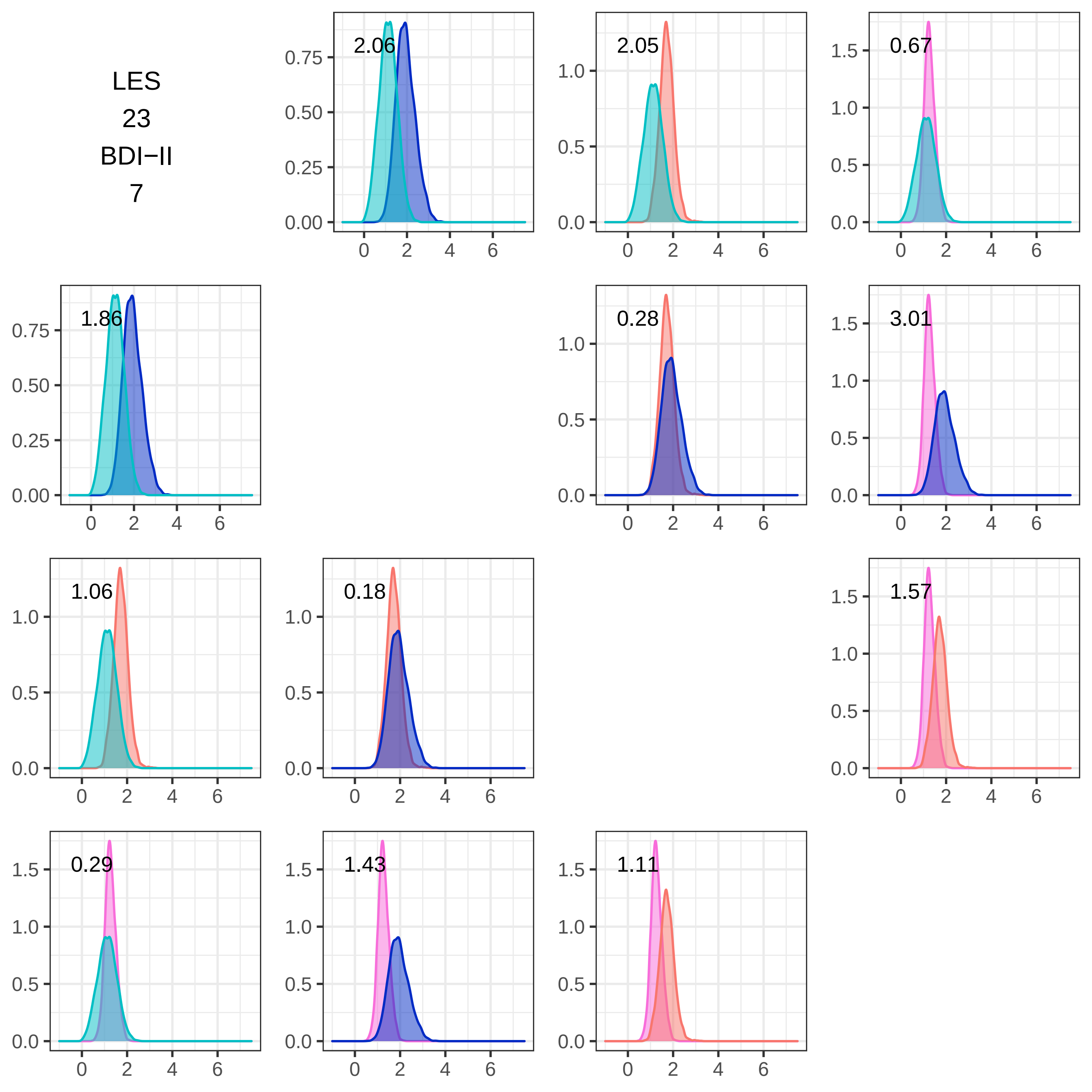
LES
23
BDI−II
7
2.06
2.05
0.67
1.86
0.28
3.01
1.06
0.18
1.57
0.29
1.43
1.11

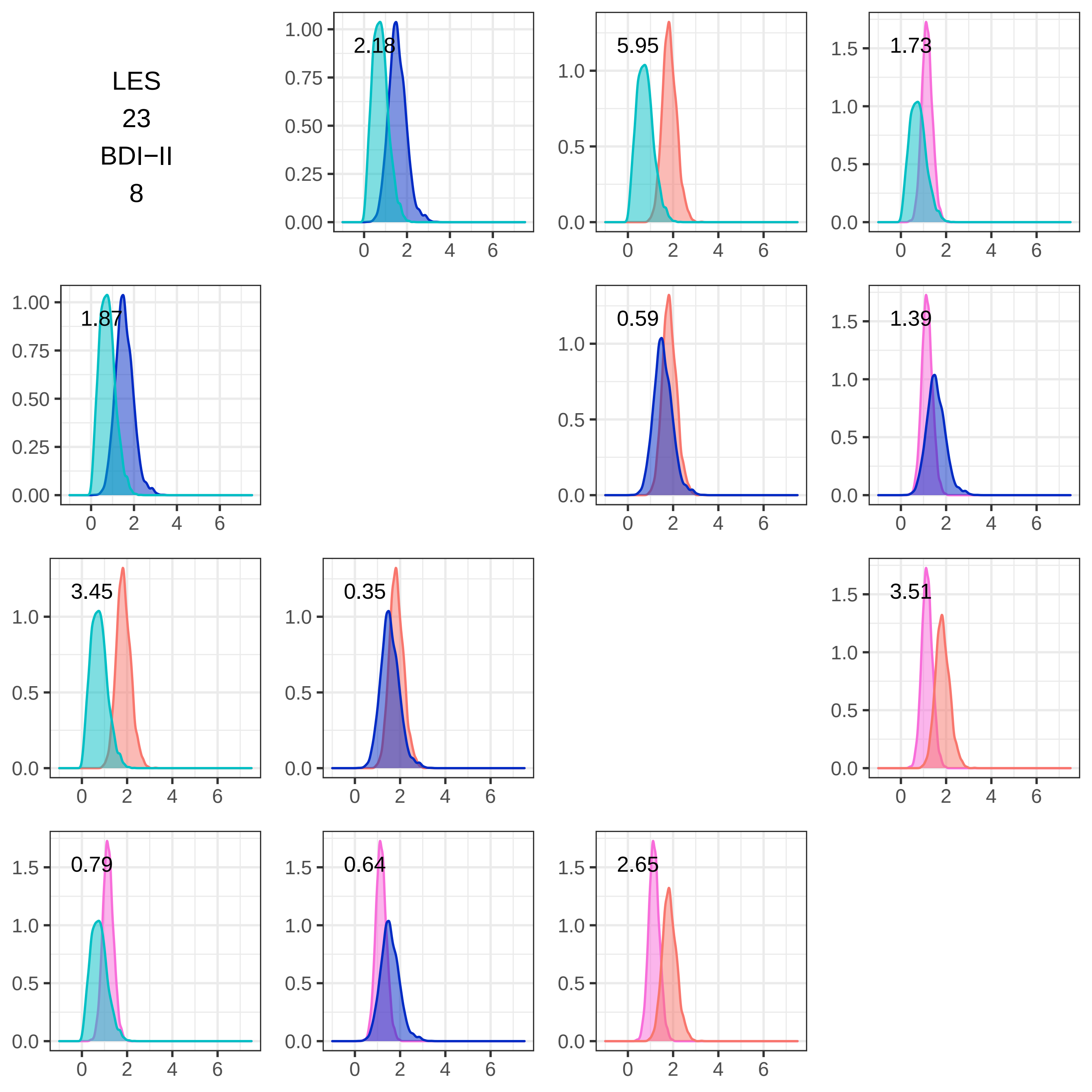
LES
23
BDI−II
8
2.18
5.95
1.73
1.87
0.59
1.39
3.45
0.35
3.51
0.79
0.64
2.65

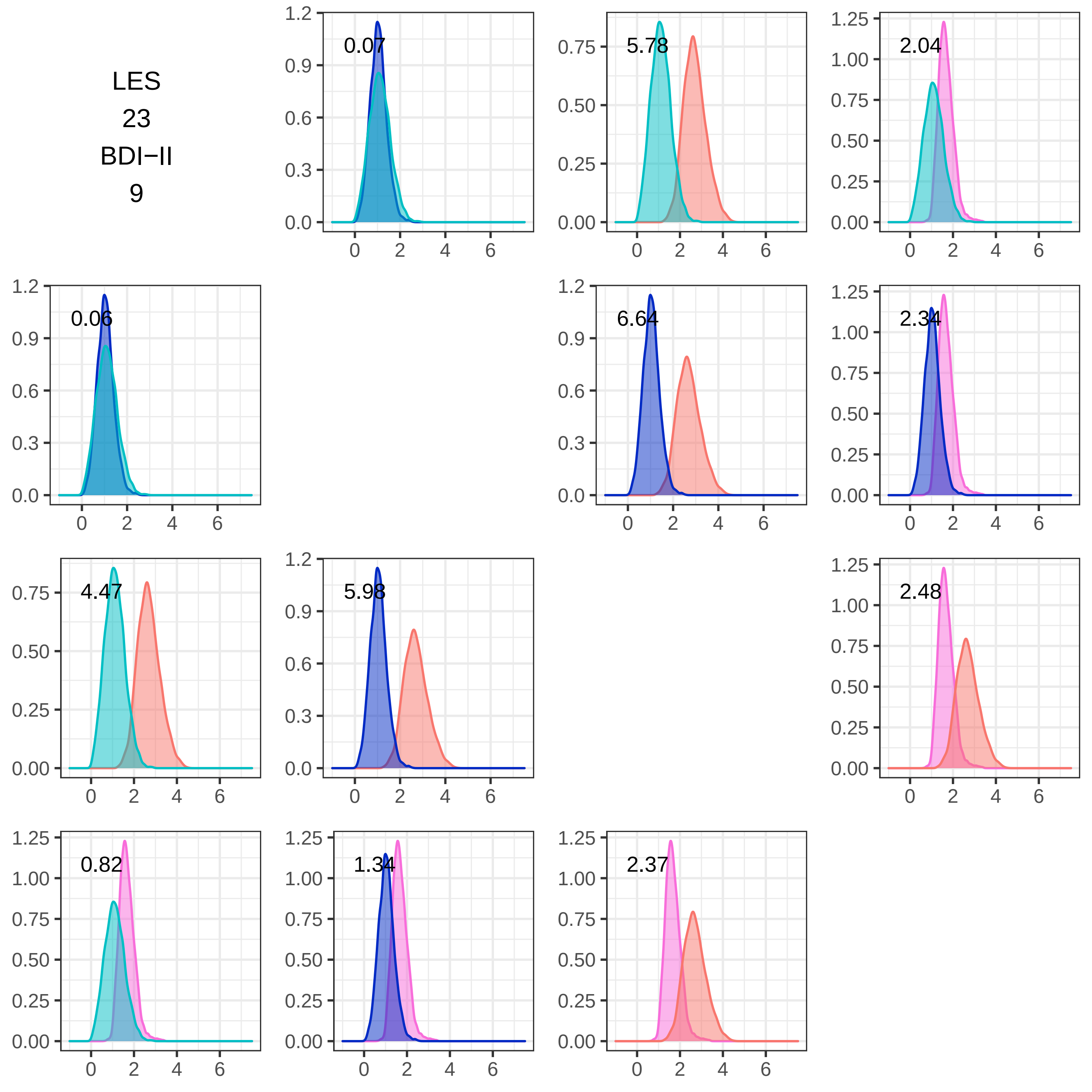
LES
23
BDI−II
9
0.07
5.78
2.04
0.06
6.64
2.34
4.47
5.98
2.48
0.82
1.34
2.37

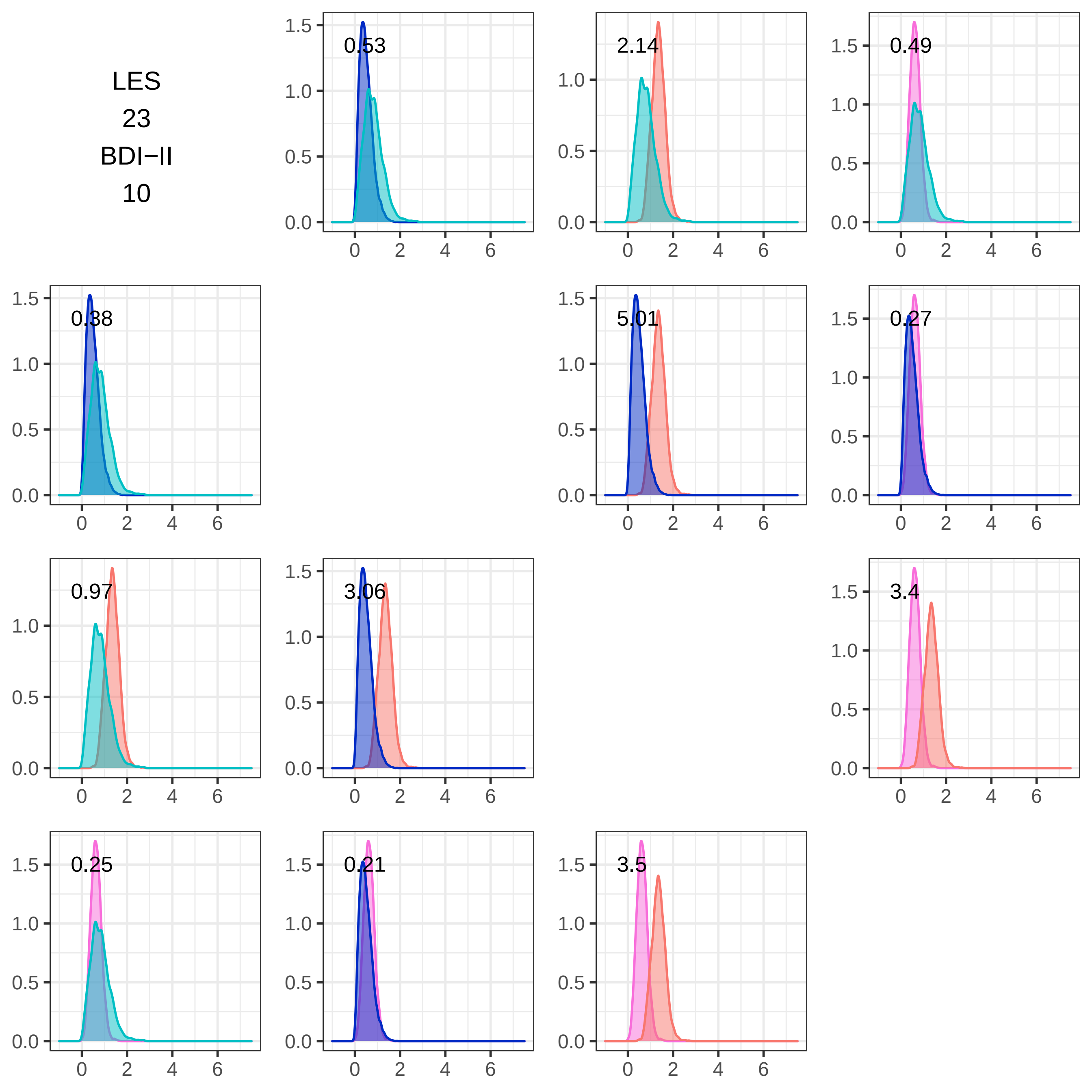
LES
23
BDI−II
10
0.53
2.14
0.49
0.38
5.01
0.27
0.97
3.06
3.4
0.25
0.21
3.5

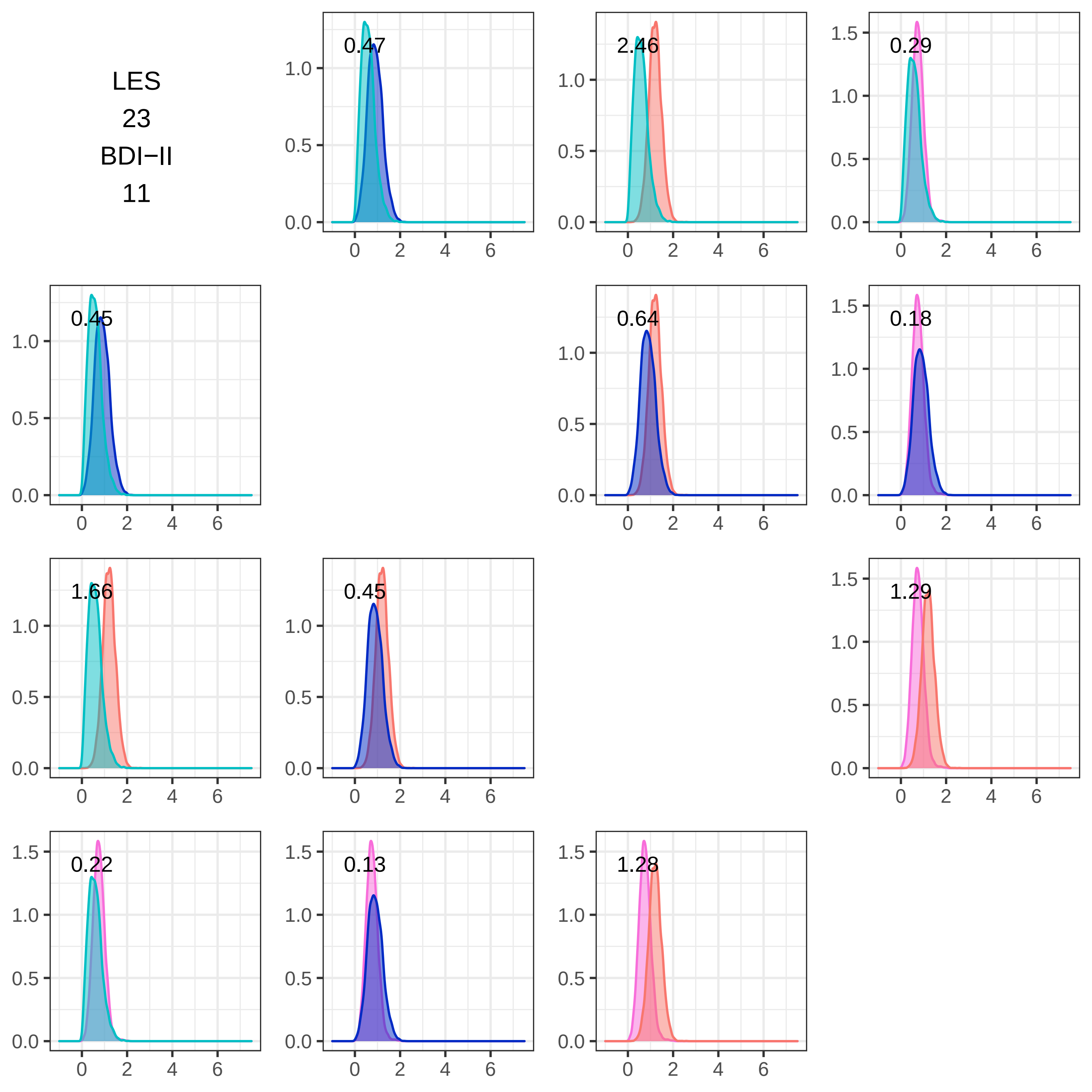

LES
23
BDI−II
11
0.47
2.46
0.29
0.45
0.64
0.18
1.66
0.45
1.29
0.22
0.13
1.28

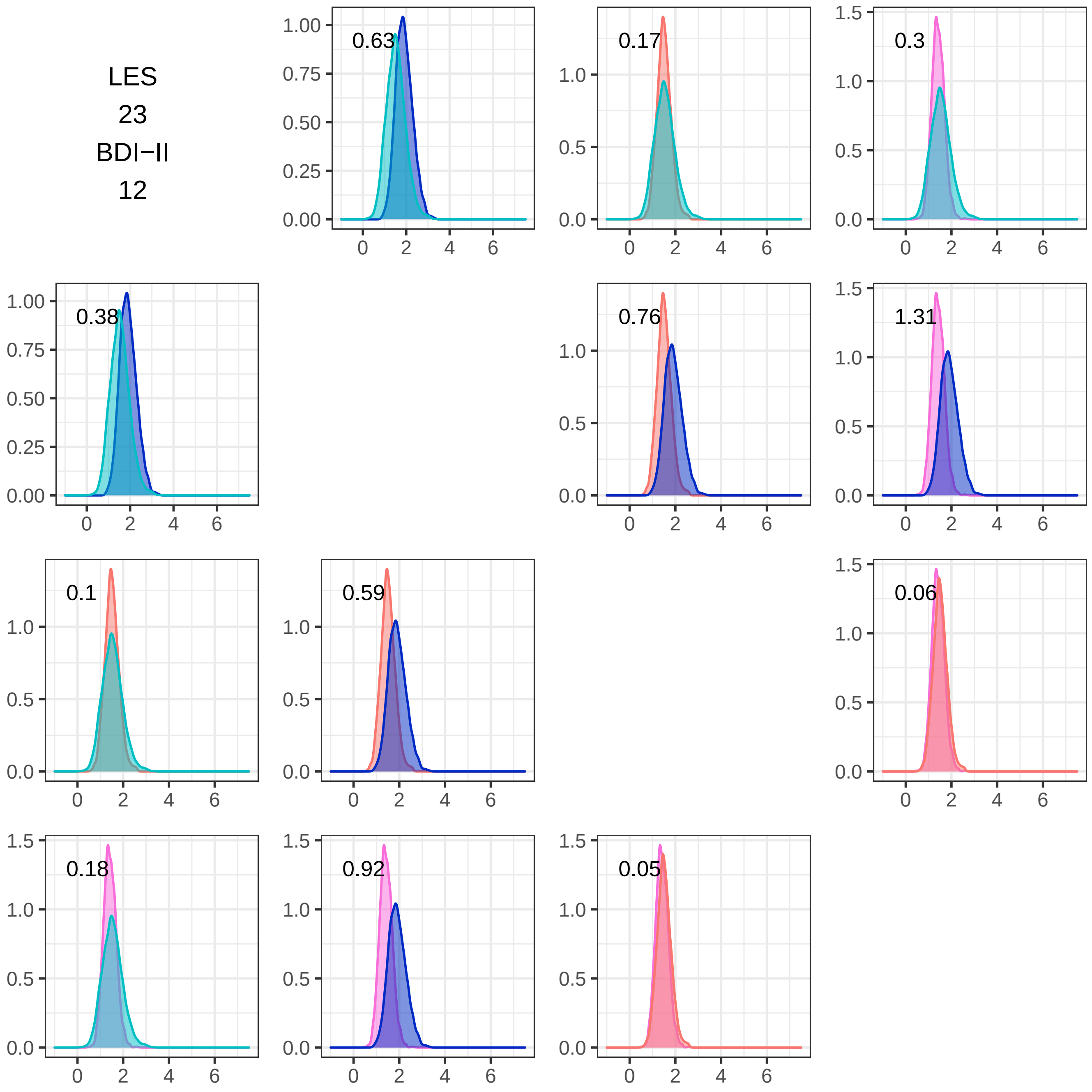
LES
23
BDI−II
12
0.63
0.17
0.3
0.38
0.76
1.31
0.1
0.59
0.06
0.18
0.92
0.05

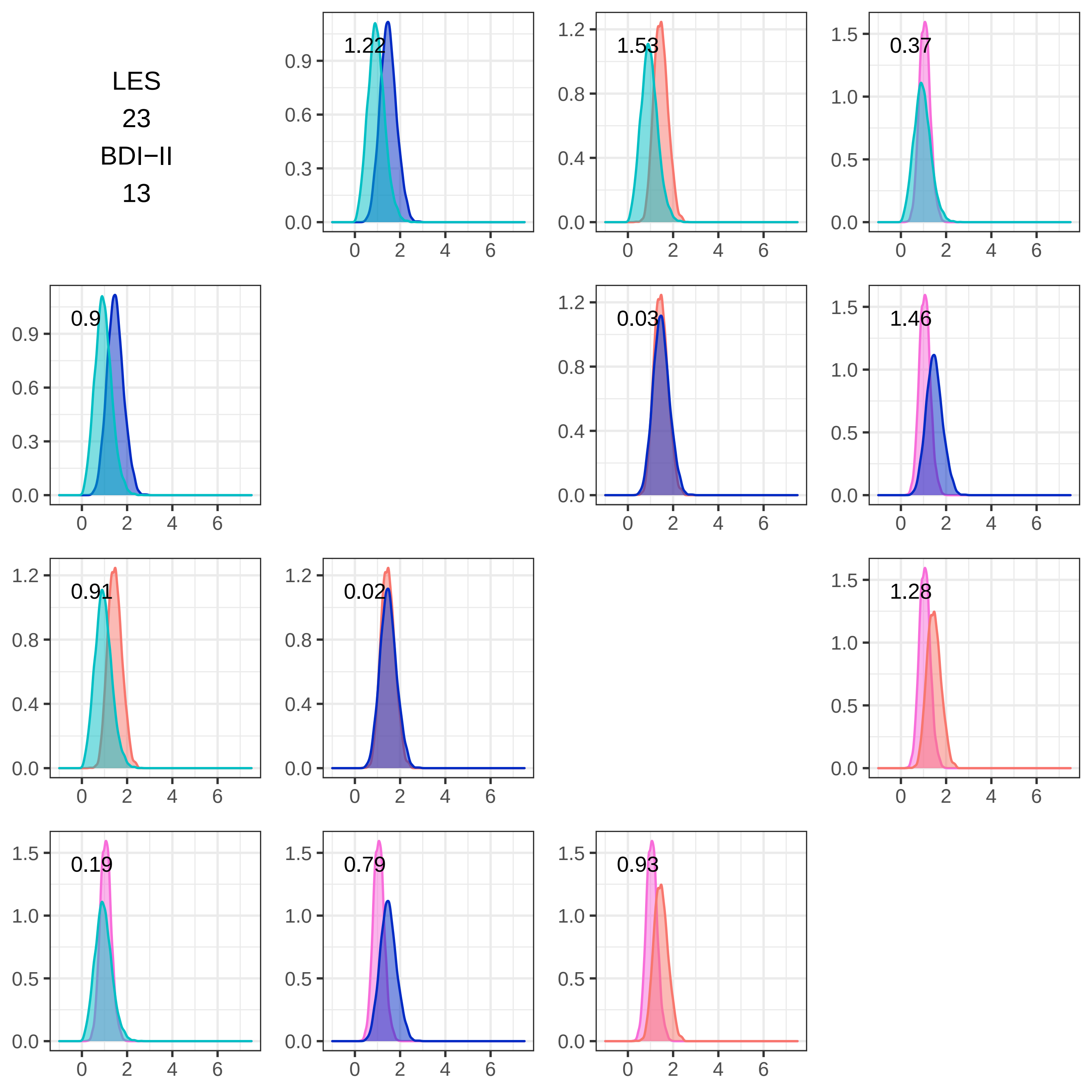
LES
23
BDI−II
13
1.22
1.53
0.37
0.9
0.03
1.46
0.91
0.02
1.28
0.19
0.79
0.93

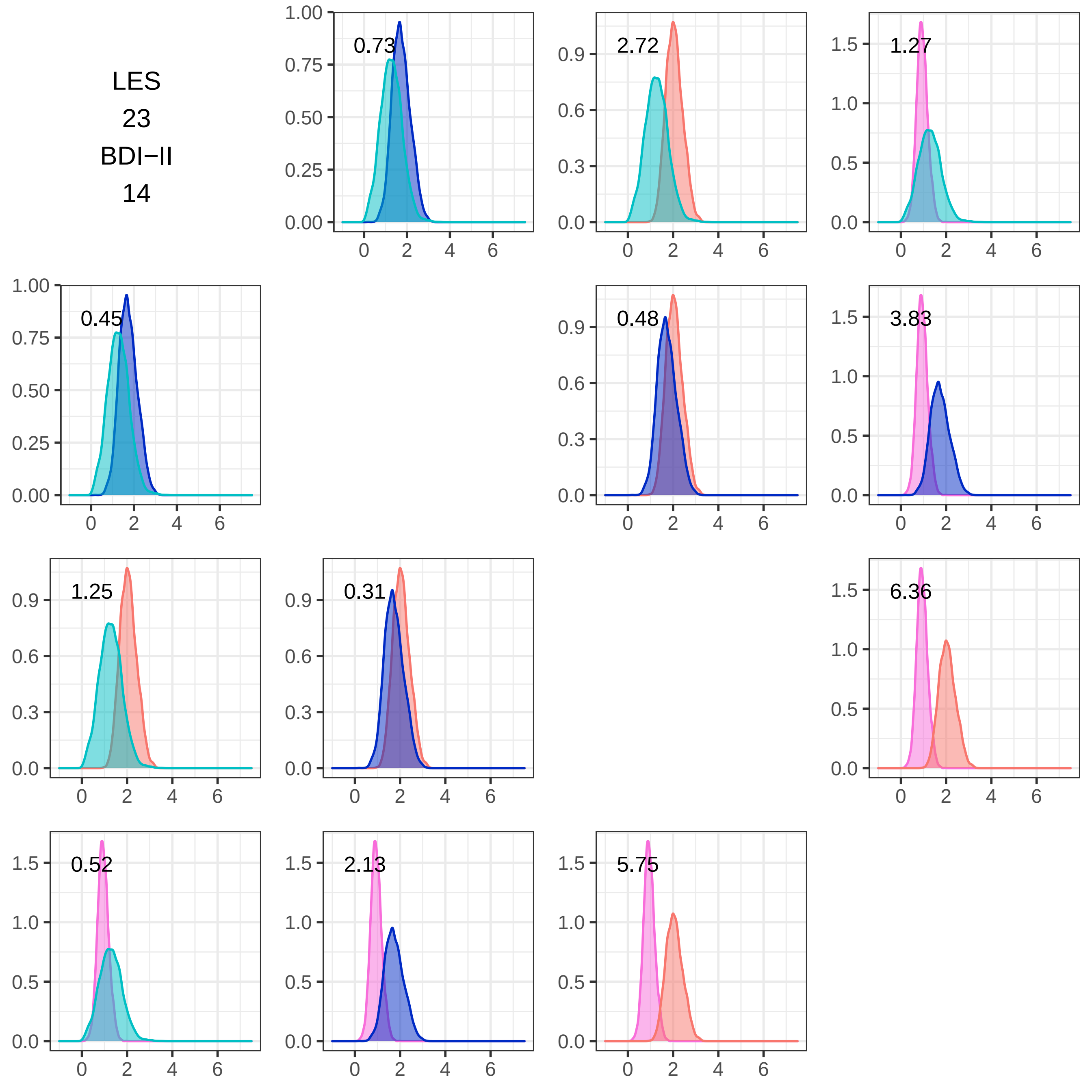
LES
23
BDI−II
14
0.73
2.72
1.27
0.45
0.48
3.83
1.25
0.31
6.36
0.52
2.13
5.75

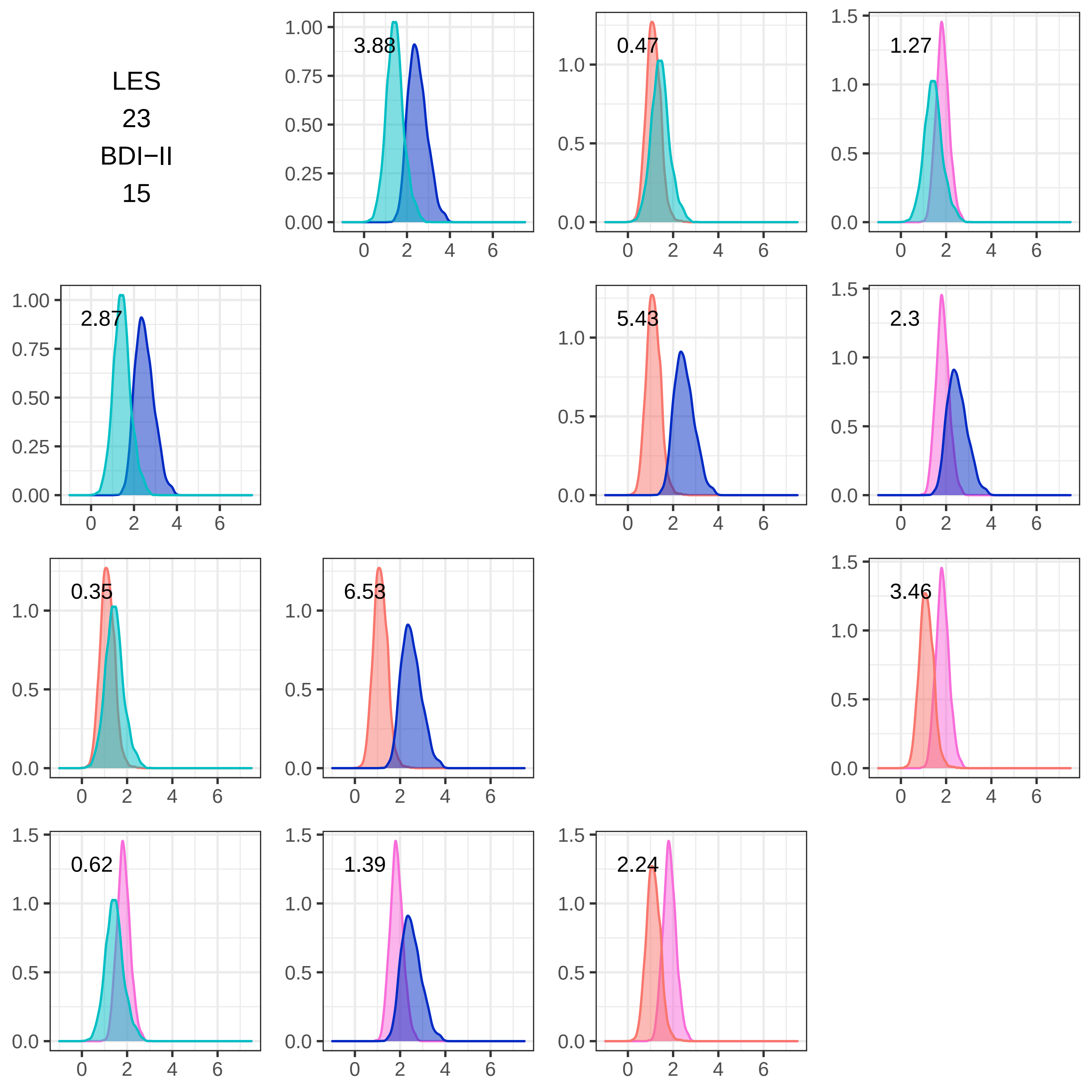
LES
23
BDI−II
15
3.88
0.47
1.27
2.87
5.43
2.3
0.35
6.53
3.46
0.62
1.39
2.24

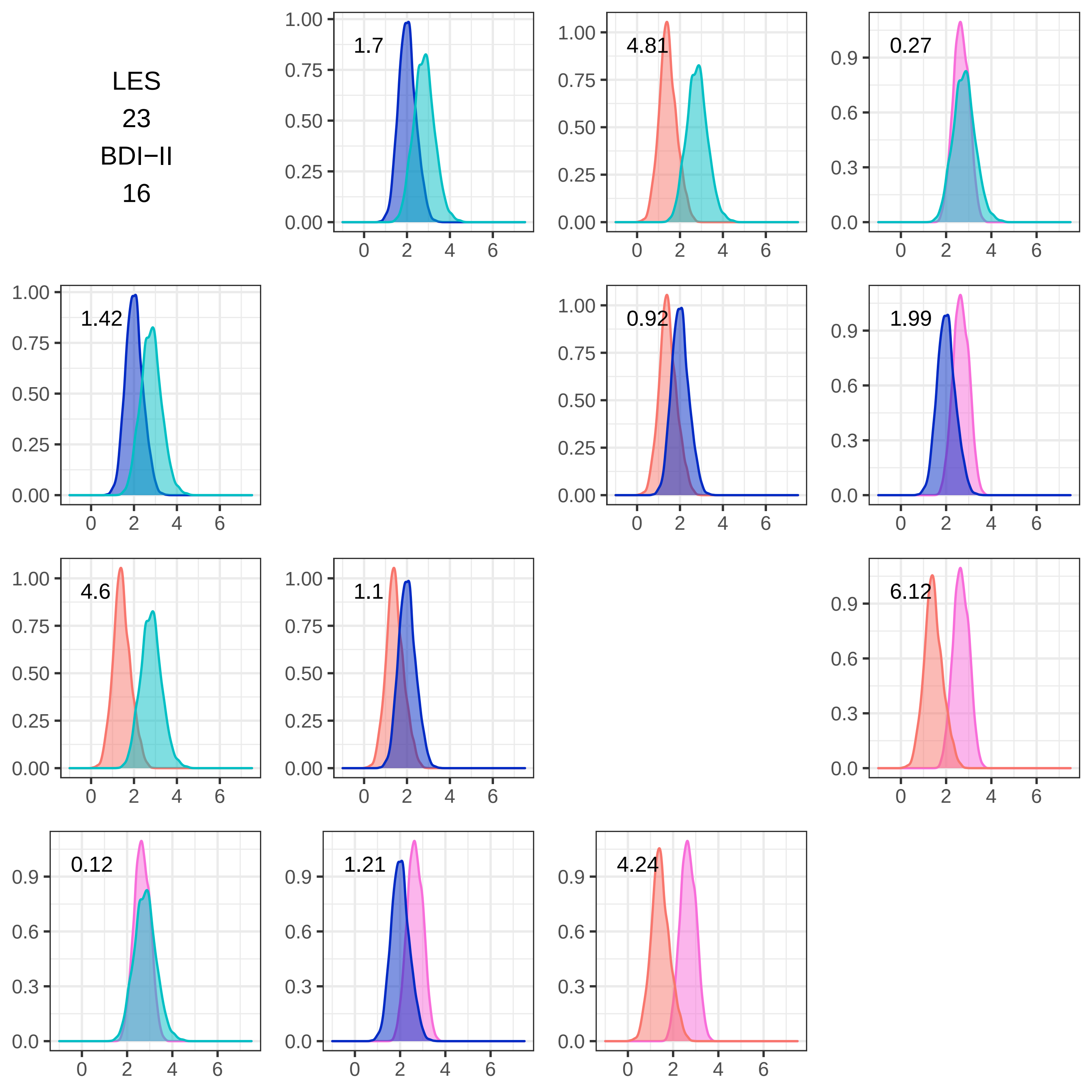
LES
23
BDI−II
16
1.7
4.81
0.27
1.42
0.92
1.99
4.6
1.1
6.12
0.12
1.21
4.24

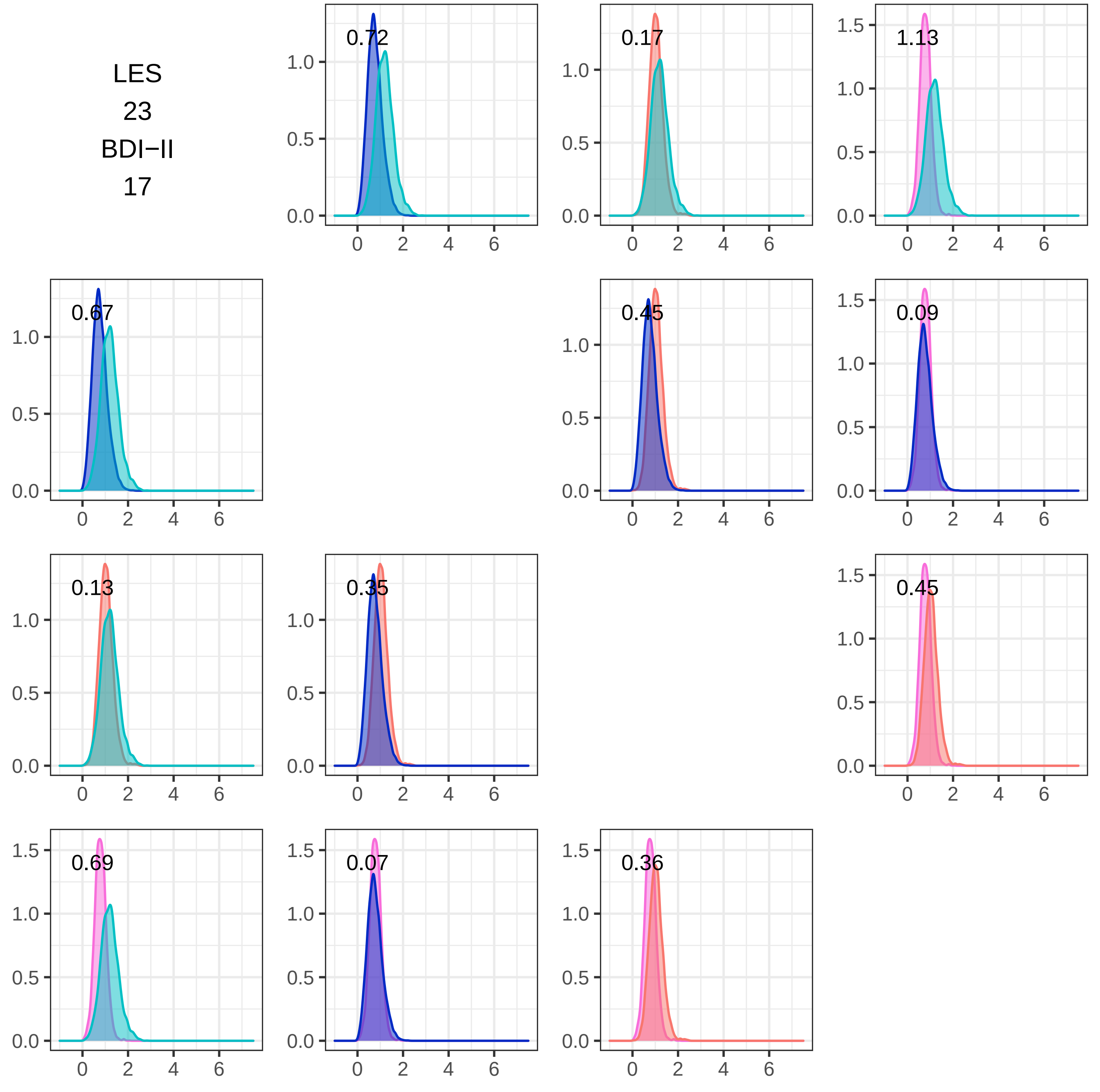
LES
23
BDI−II
17
0.72
0.17
1.13
0.67
0.45
0.09
0.13
0.35
0.45
0.69
0.07
0.36

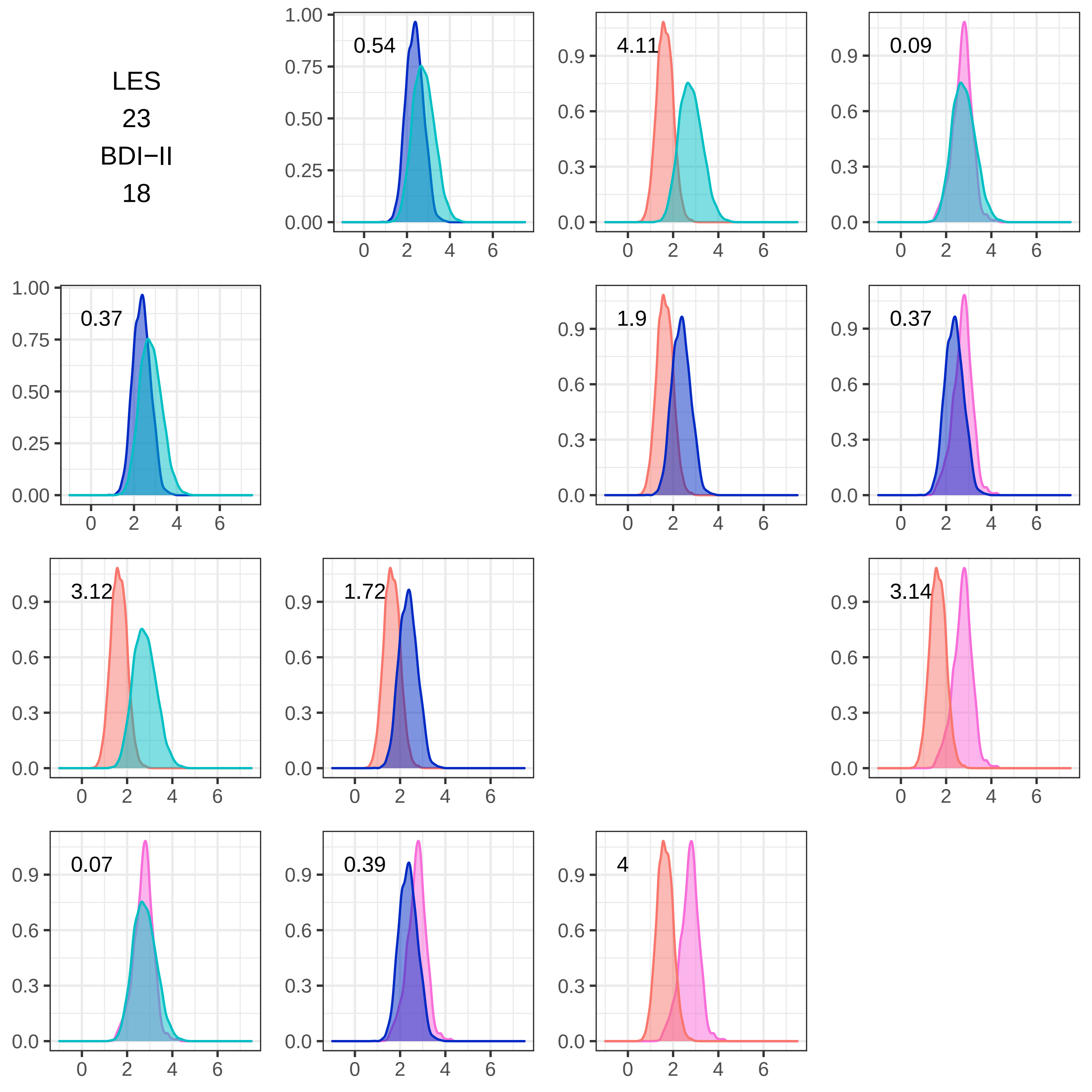

LES
23
BDI−II
18
0.54
4.11
0.09
0.37
1.9
0.37
3.12
1.72
3.14
0.07
0.39
4

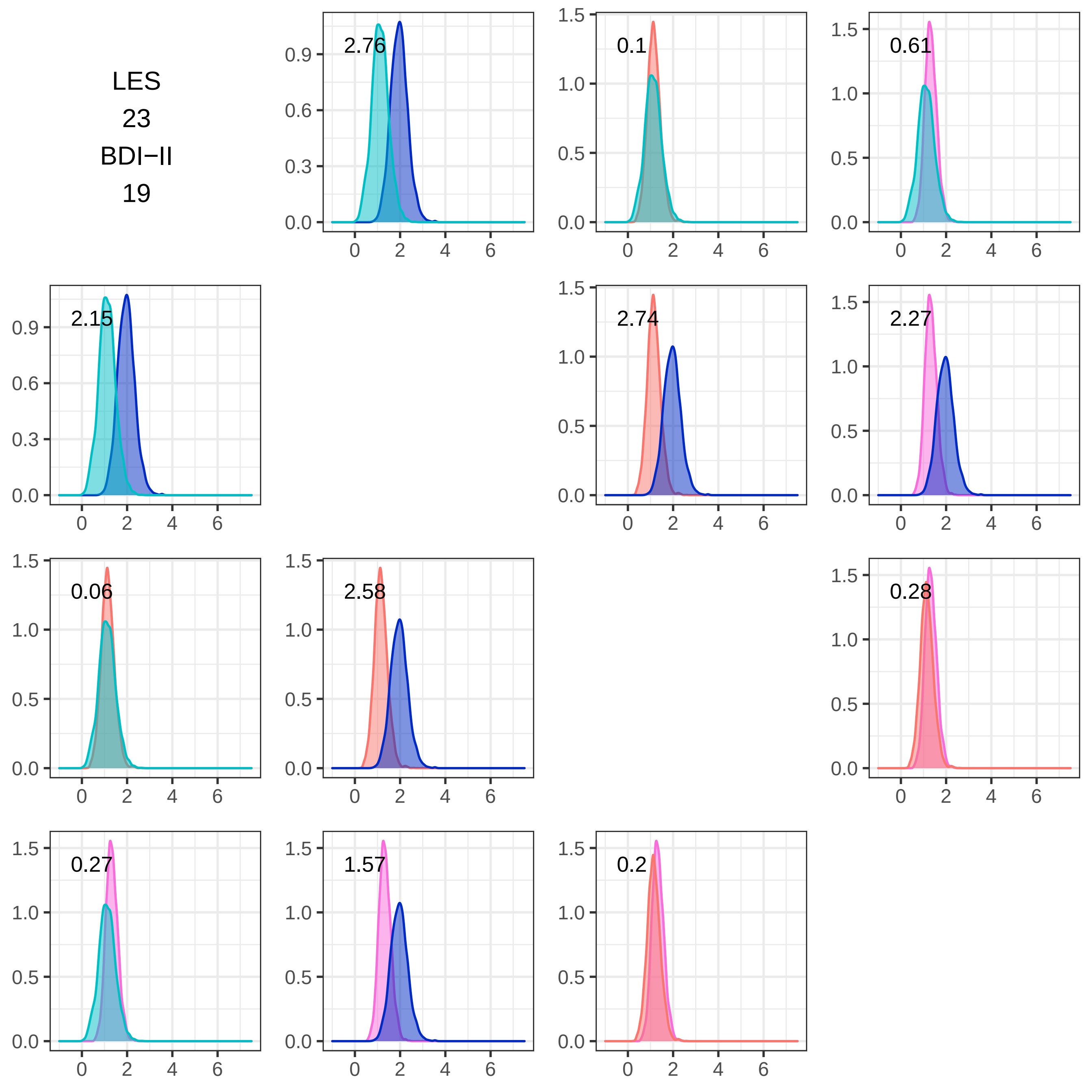

LES
23
BDI−II
19
2.76
0.1
0.61
2.15
2.74
2.27
0.06
2.58
0.28
0.27
1.57
0.2

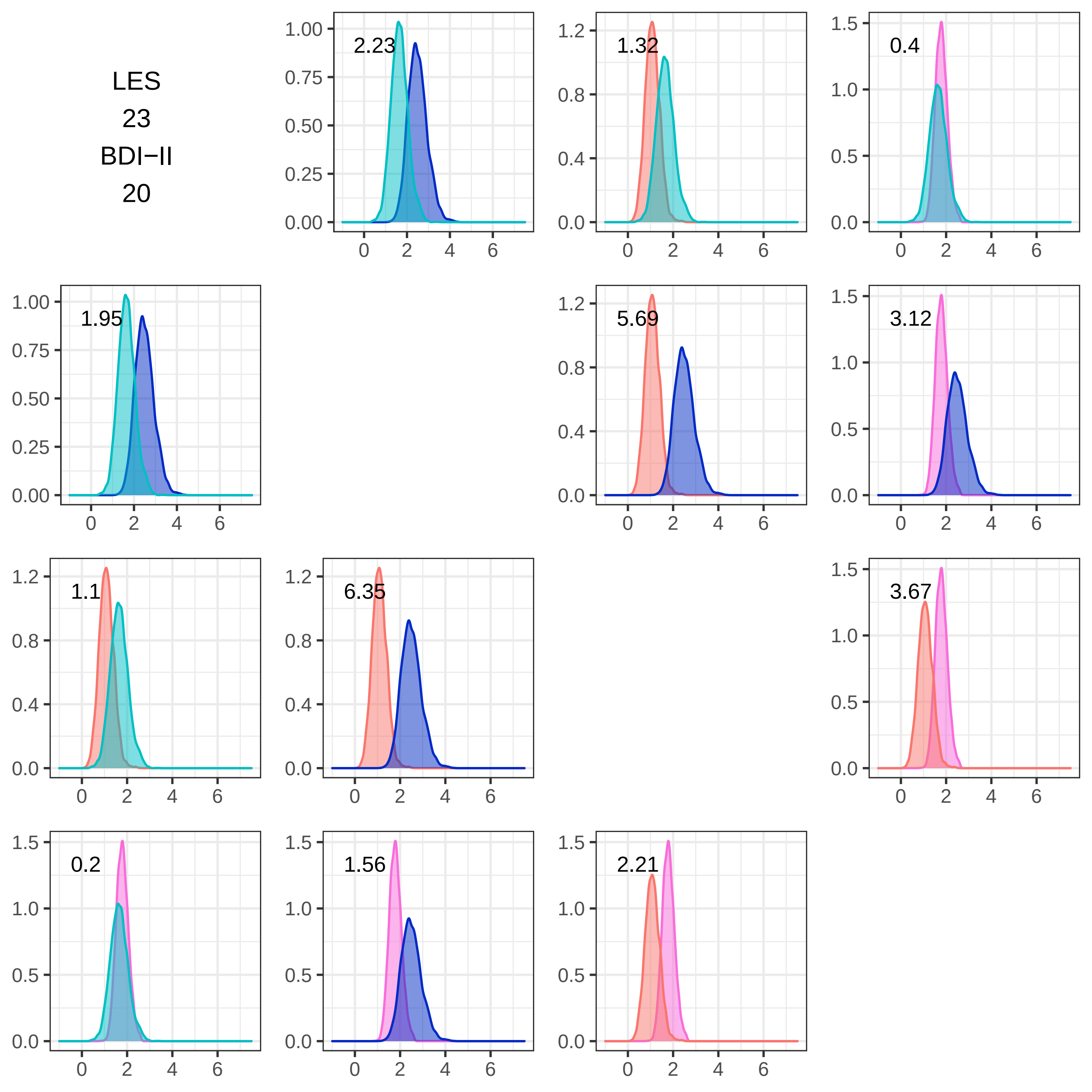

LES
23
BDI−II
20
2.23
1.32
0.4
1.95
5.69
3.12
1.1
6.35
3.67
0.2
1.56
2.21

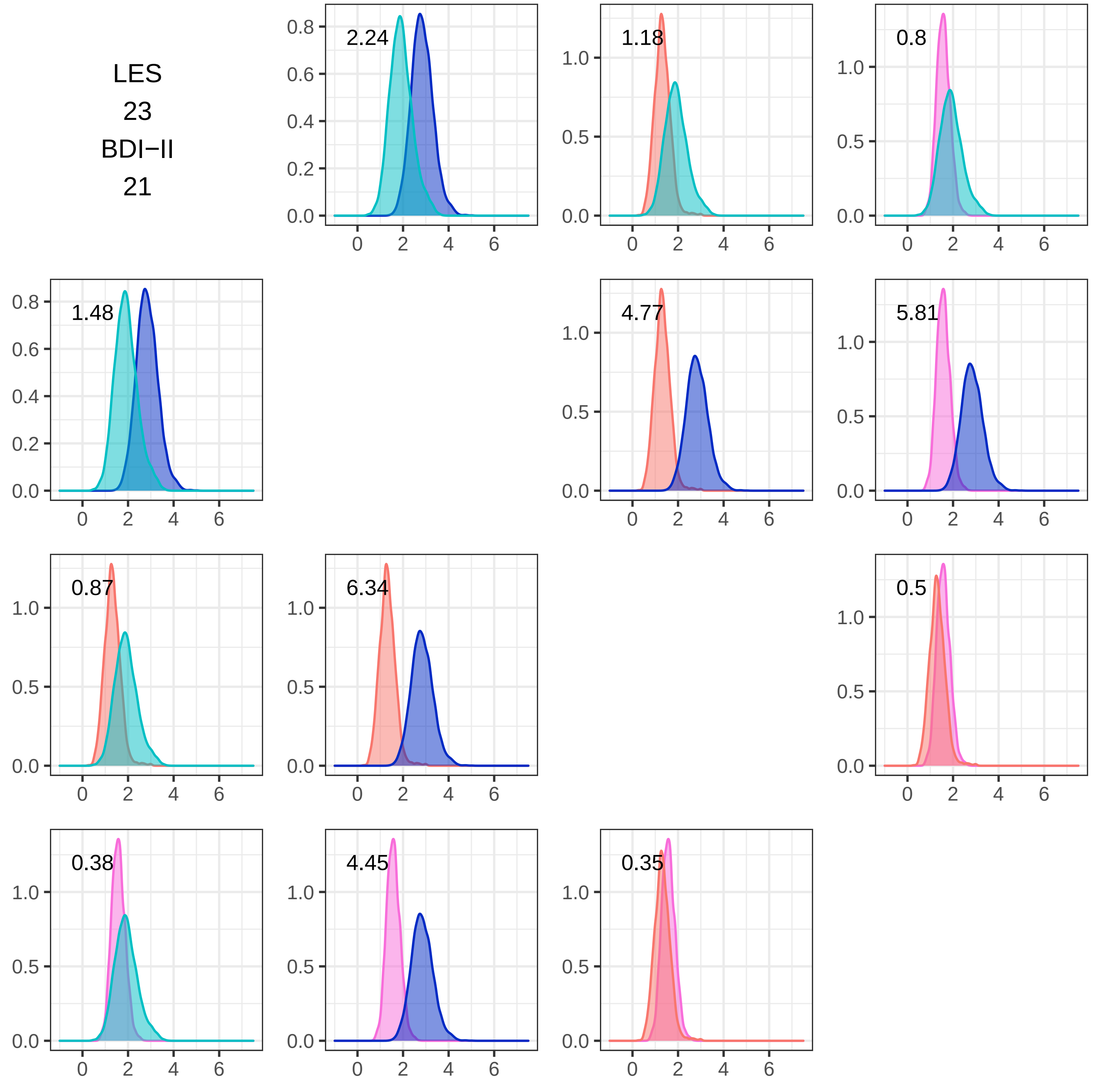
LES
23
BDI−II
21
2.24
1.18
0.8
1.48
4.77
5.81
0.87
6.34
0.5
0.38
4.45
0.35

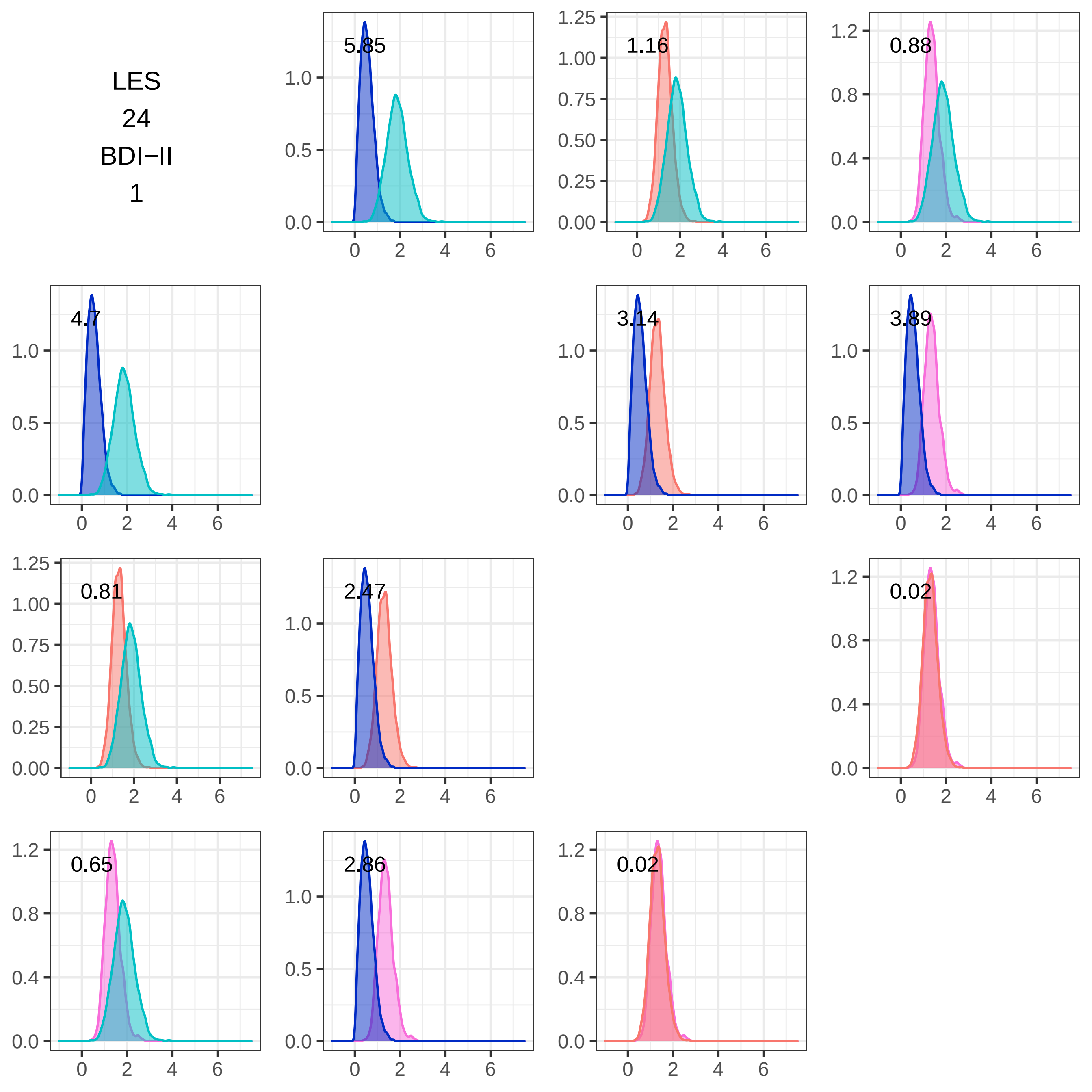
LES
24
BDI−II
1
5.85
1.16
0.88
4.7
3.14
3.89
0.81
2.47
0.02
0.65
2.86
0.02

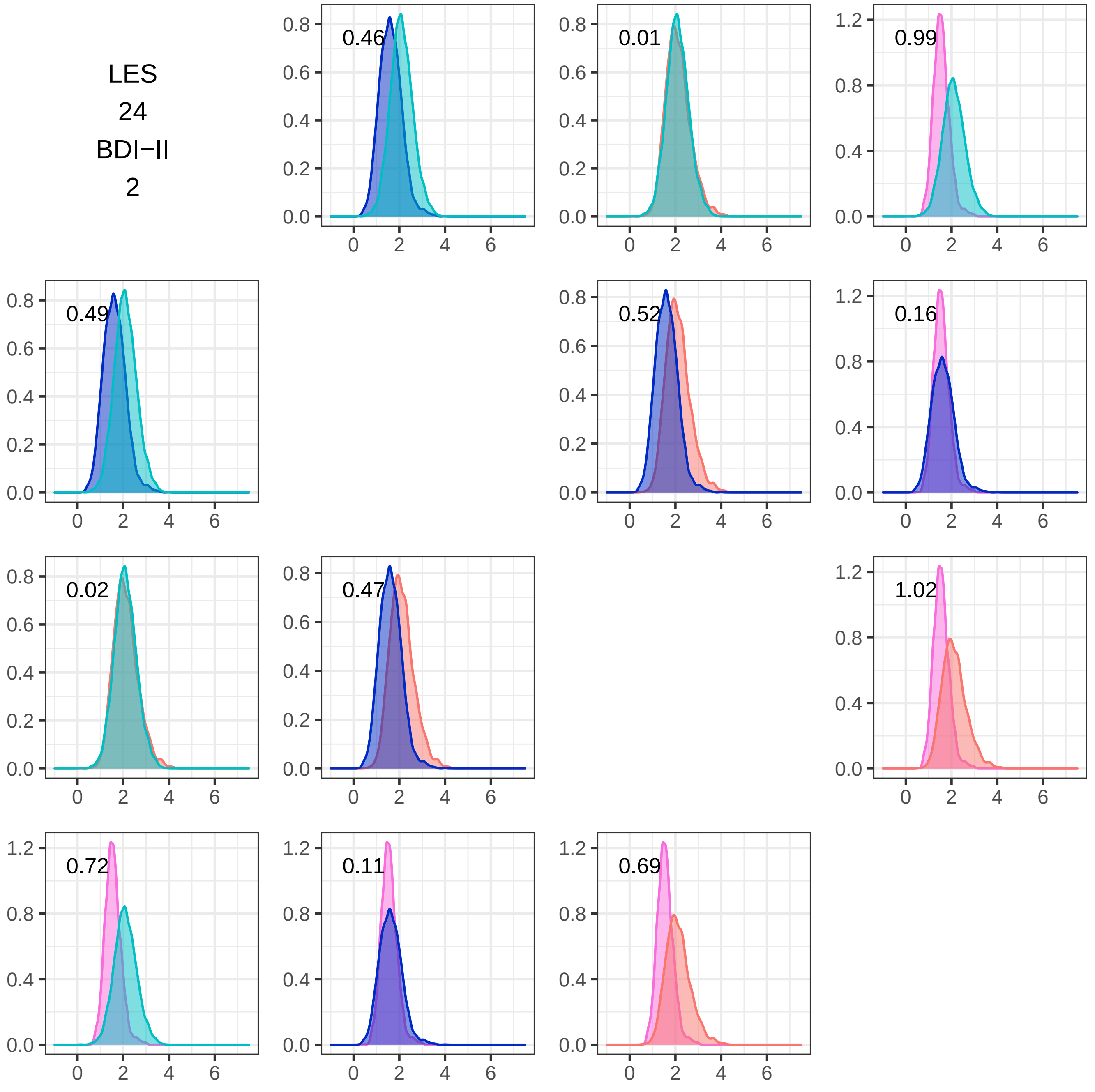
LES
24
BDI−II
2
0.46
0.01
0.99
0.49
0.52
0.16
0.02
0.47
1.02
0.72
0.11
0.69

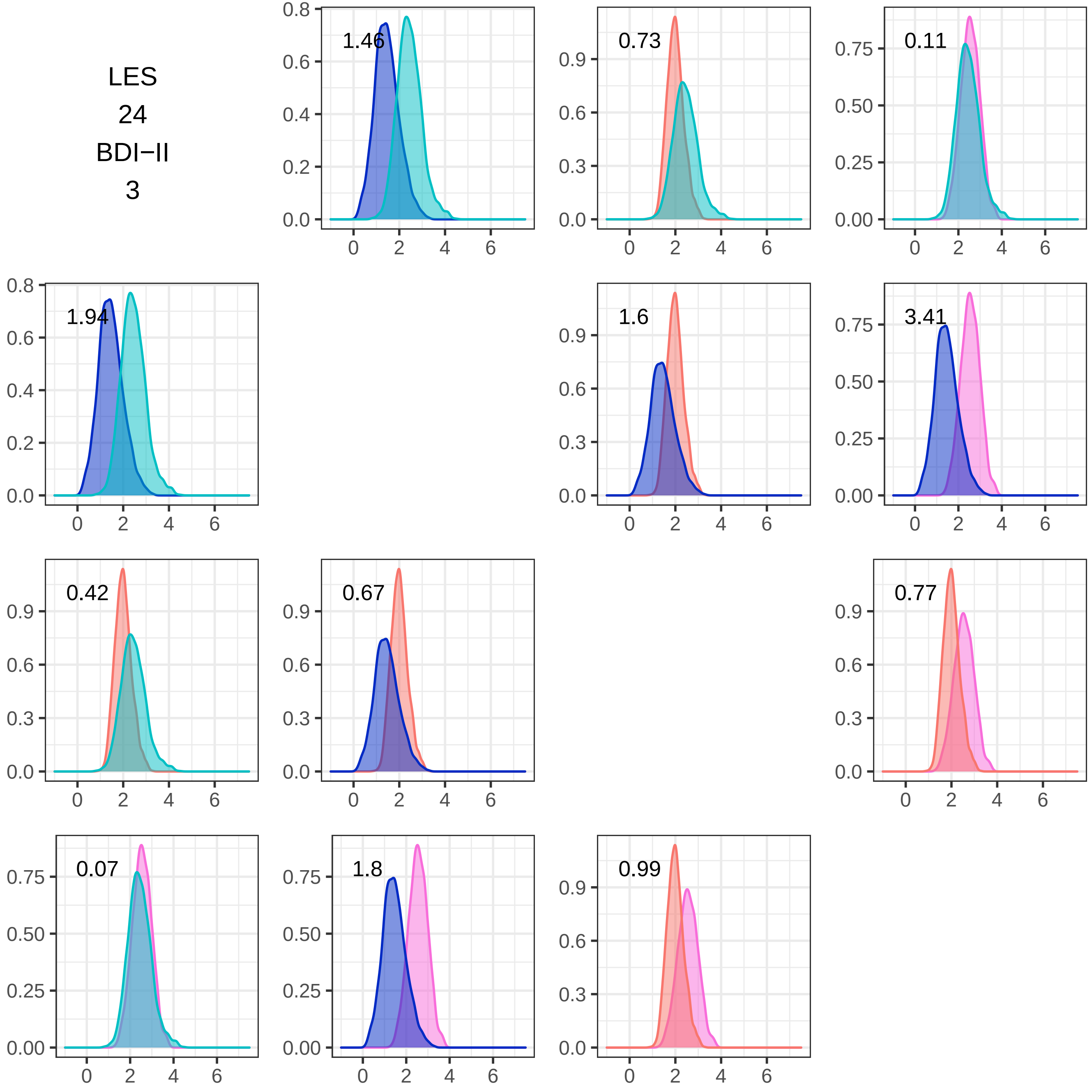
LES
24
BDI−II
3
1.46
0.73
0.11
1.94
1.6
3.41
0.42
0.67
0.77
0.07
1.8
0.99

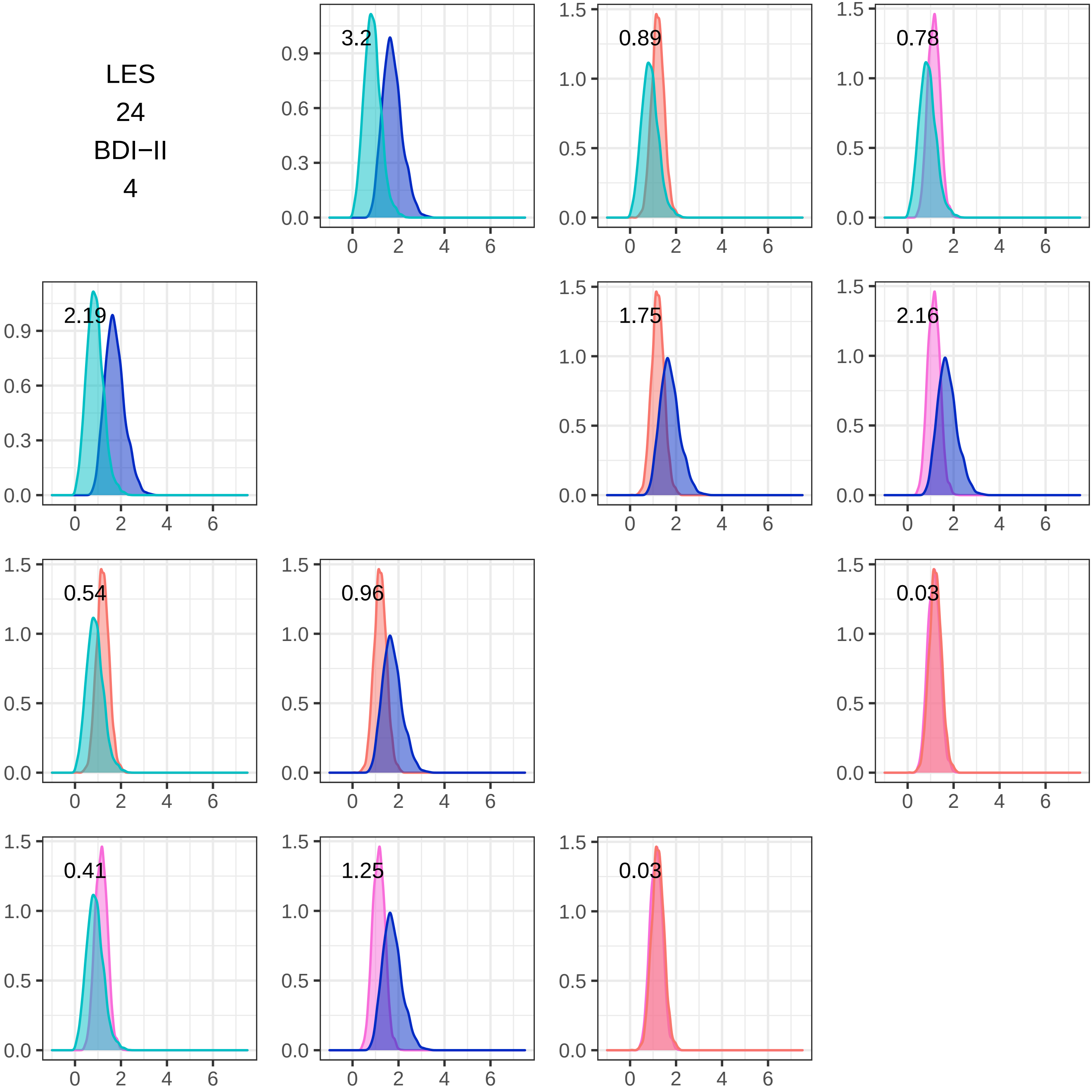
LES
24
BDI−II
4
3.2
0.89
0.78
2.19
1.75
2.16
0.54
0.96
0.03
0.41
1.25
0.03

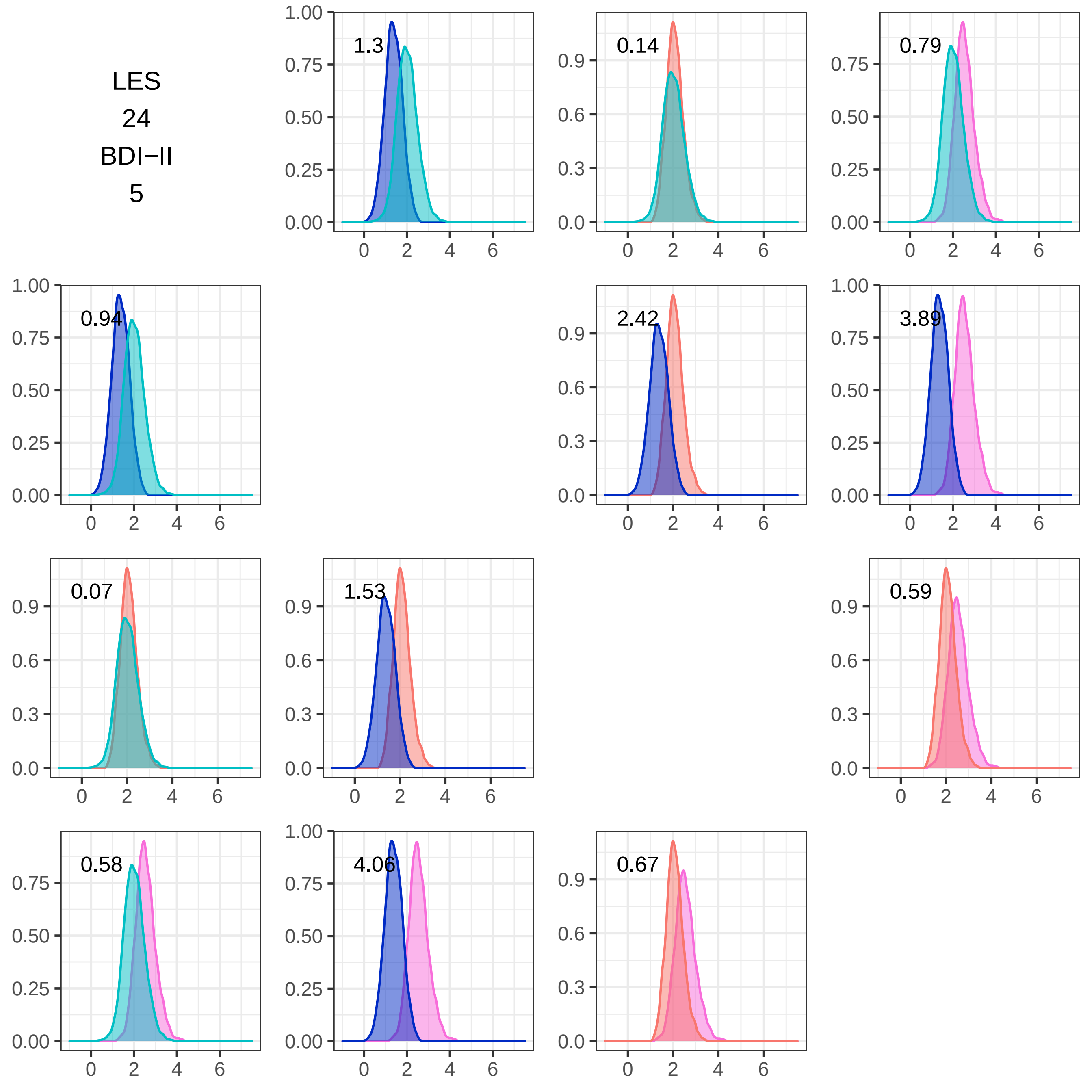

LES
24
BDI−II
5
1.3
0.14
0.79
0.94
2.42
3.89
0.07
1.53
0.59
0.58
4.06
0.67

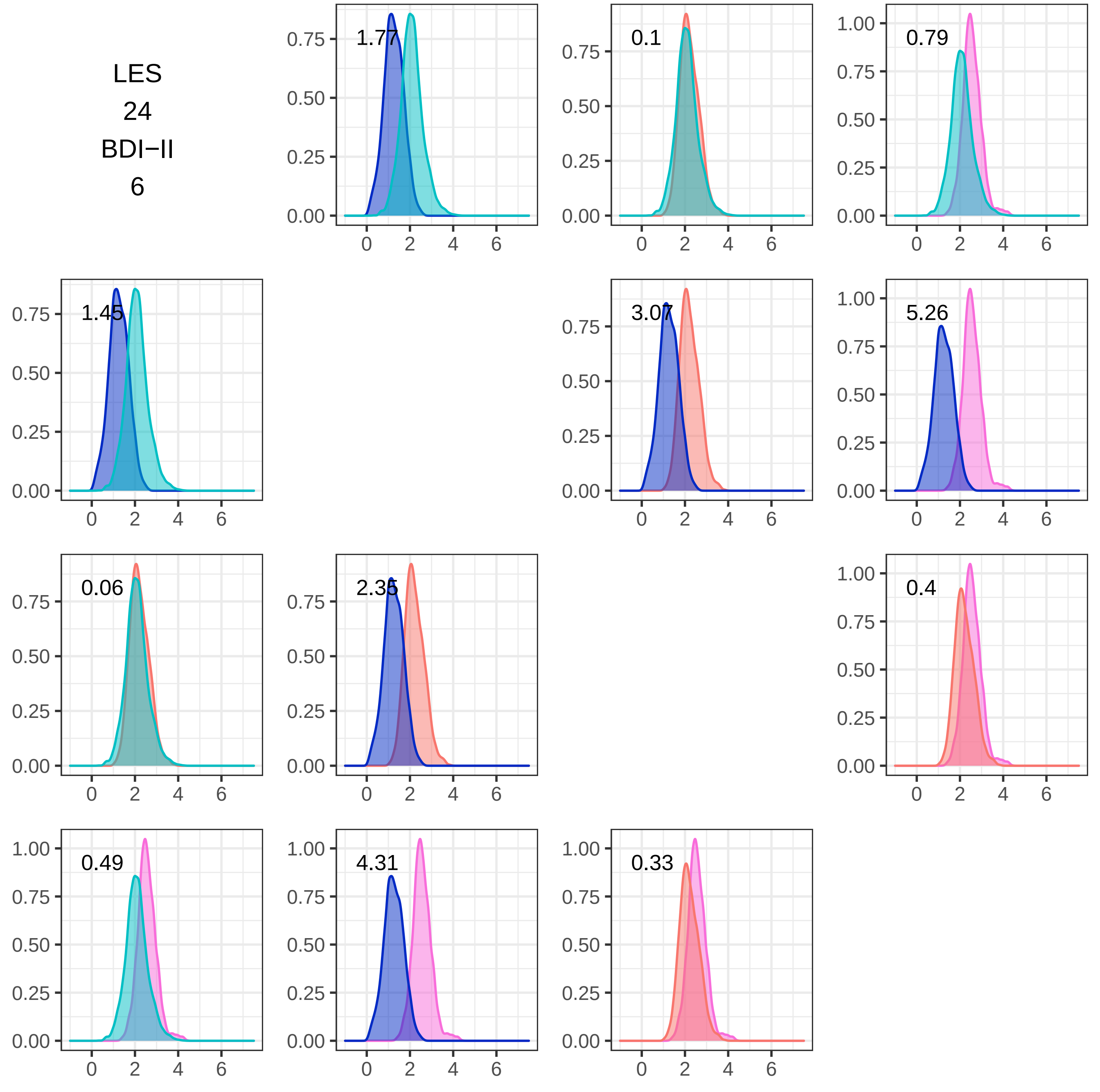
LES
24
BDI−II
6
1.77
0.1
0.79
1.45
3.07
5.26
0.06
2.35
0.4
0.49
4.31
0.33

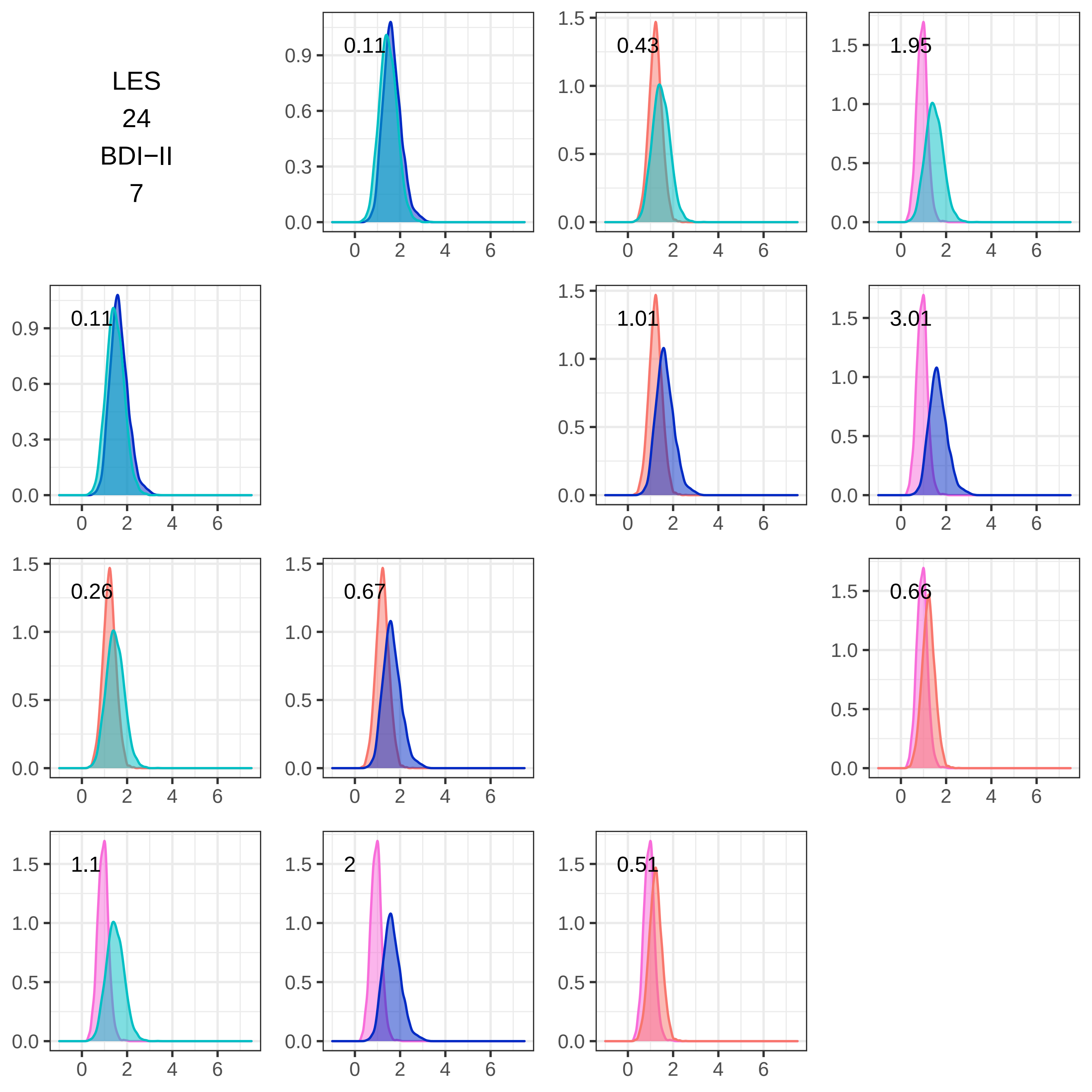
LES
24
BDI−II
7
0.11
0.43
1.95
0.11
1.01
3.01
0.26
0.67
0.66
1.1
2
0.51

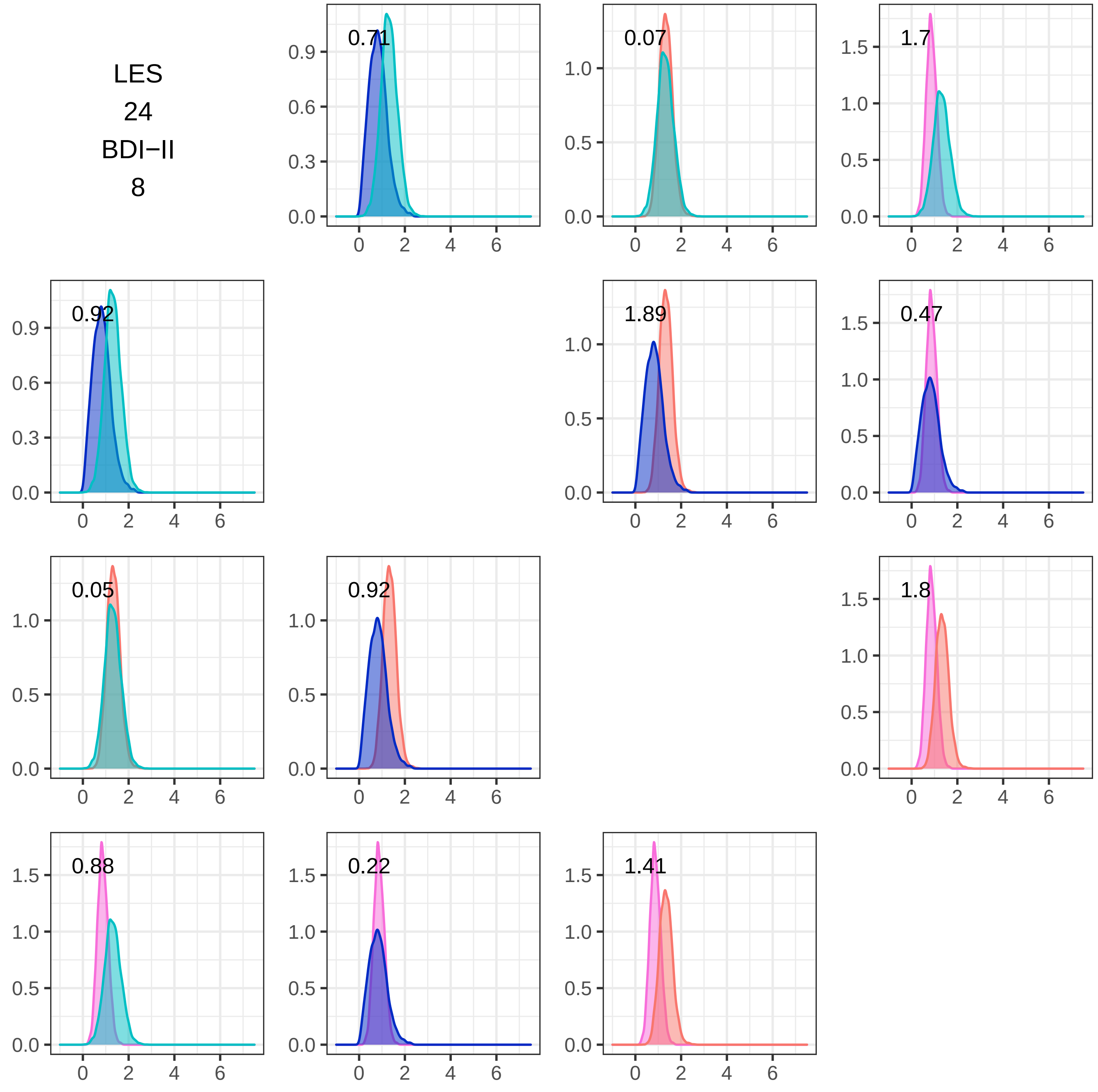
LES
24
BDI−II
8
0.71
0.07
1.7
0.92
1.89
0.47
0.05
0.92
1.8
0.88
0.22
1.41

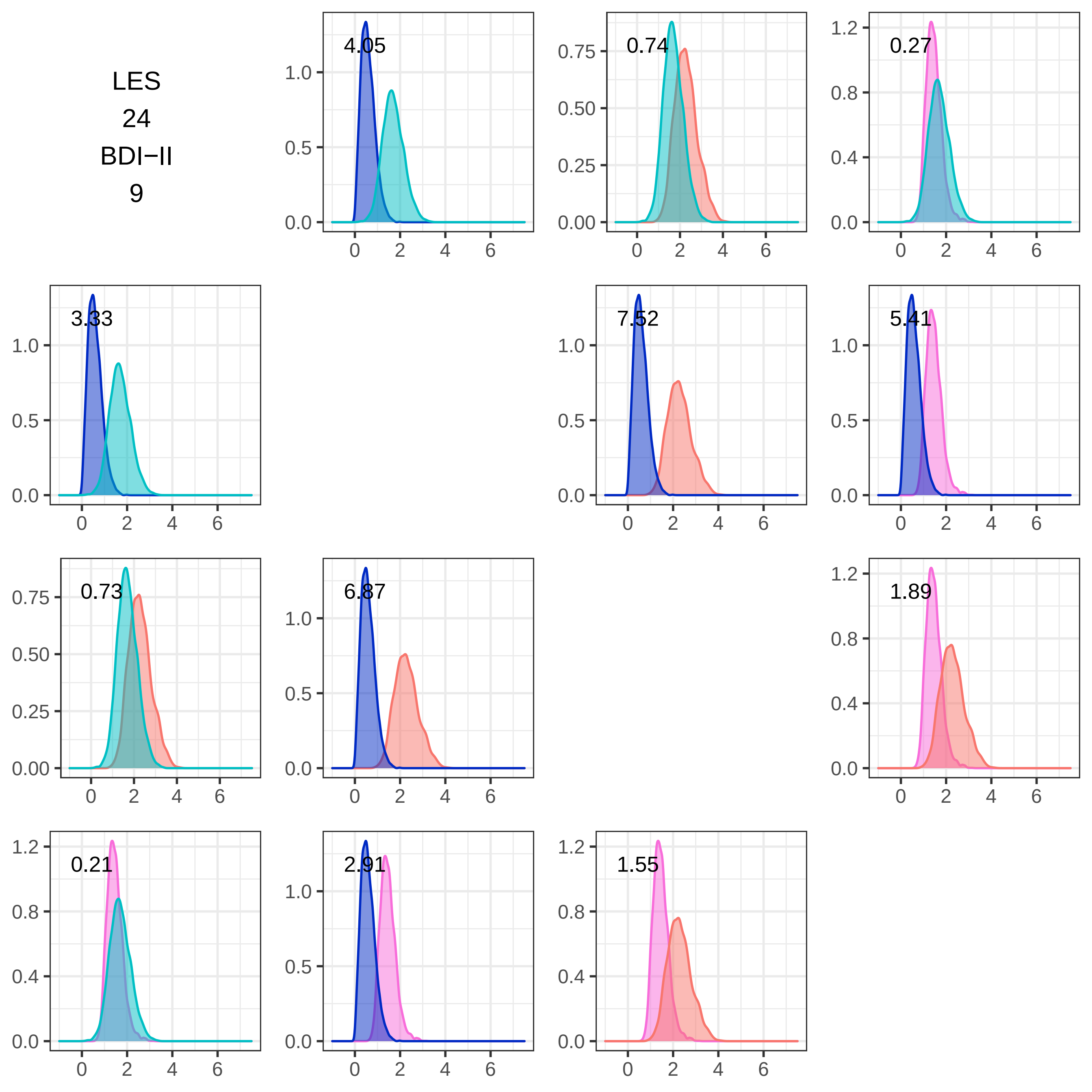

LES
24
BDI−II
9
4.05
0.74
0.27
3.33
7.52
5.41
0.73
6.87
1.89
0.21
2.91
1.55

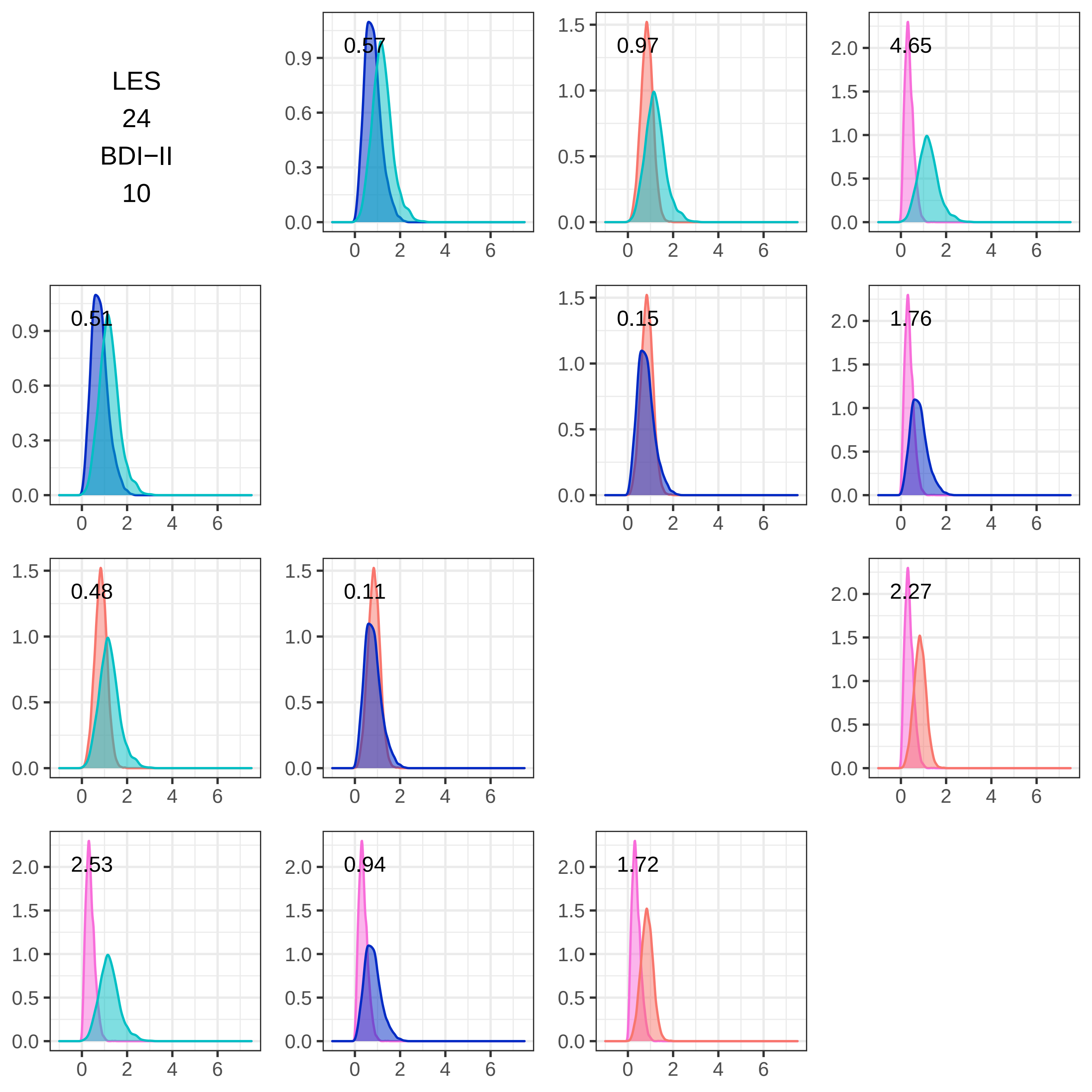
LES
24
BDI−II
10
0.57
0.97
4.65
0.51
0.15
1.76
0.48
0.11
2.27
2.53
0.94
1.72

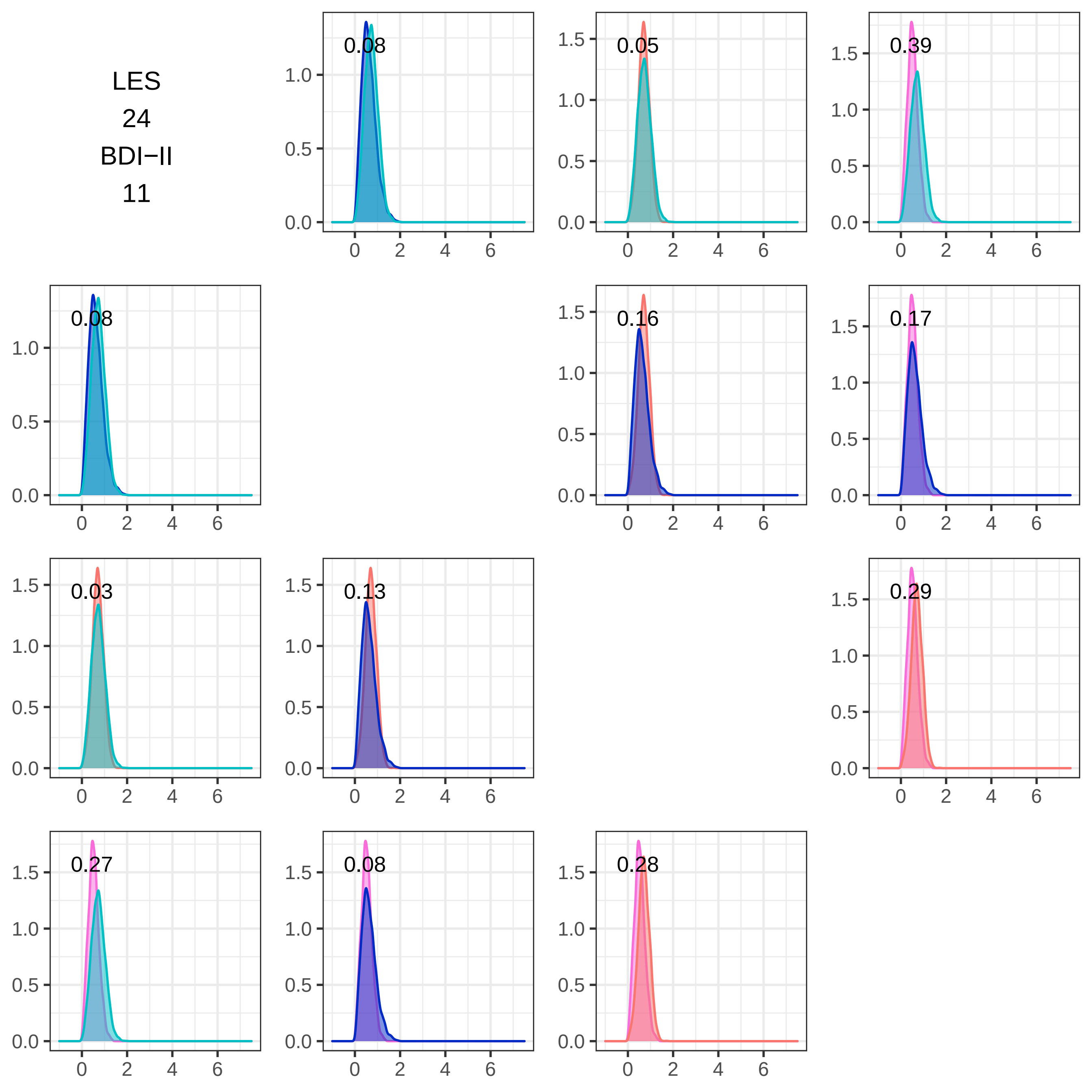

LES
24
BDI−II
11
0.08
0.05
0.39
0.08
0.16
0.17
0.03
0.13
0.29
0.27
0.08
0.28

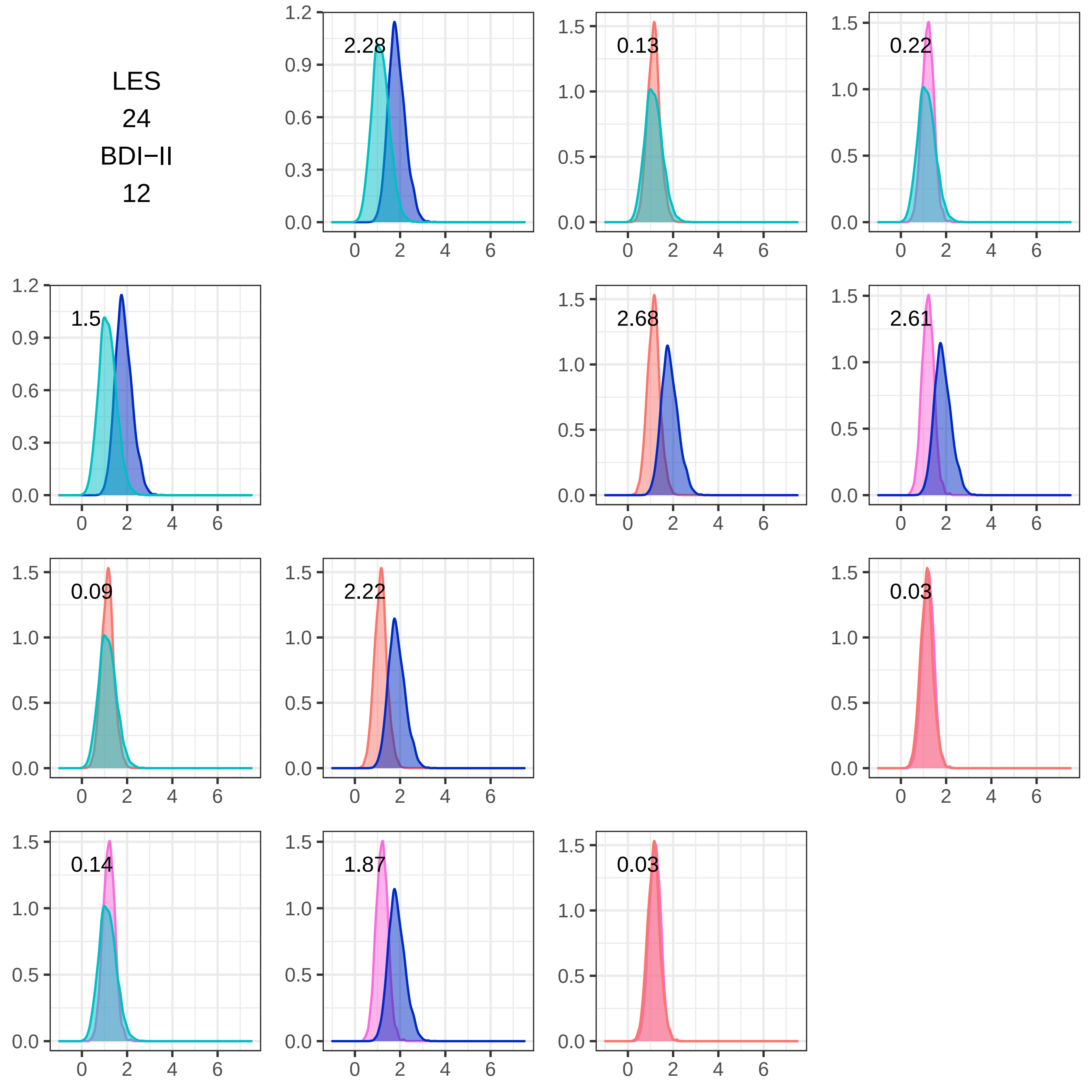

LES
24
BDI−II
12
2.28
0.13
0.22
1.5
2.68
2.61
0.09
2.22
0.03
0.14
1.87
0.03

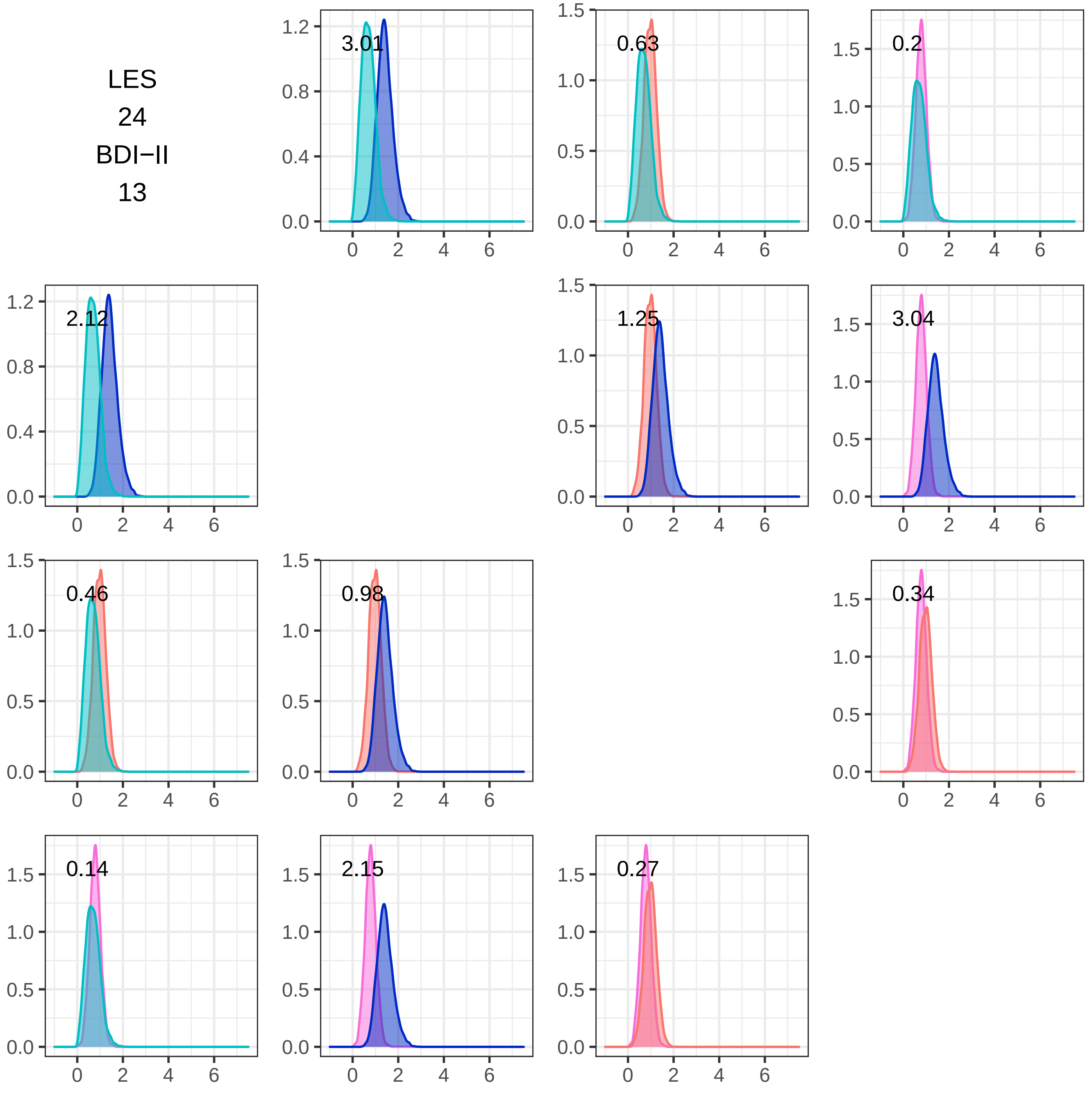
LES
24
BDI−II
13
3.01
0.63
0.2
2.12
1.25
3.04
0.46
0.98
0.34
0.14
2.15
0.27

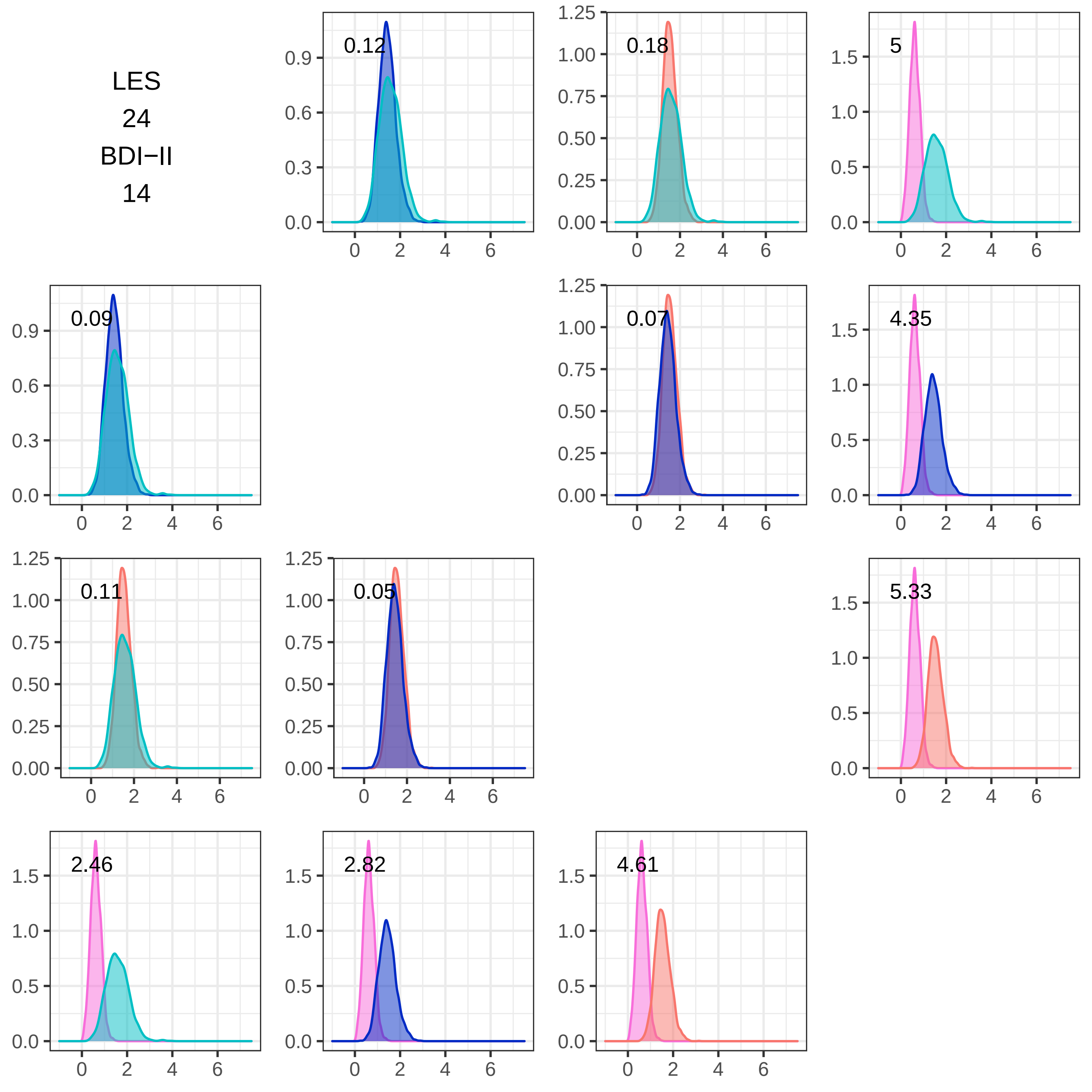
LES
24
BDI−II
14
0.12
0.18
5
0.09
0.07
4.35
0.11
0.05
5.33
2.46
2.82
4.61

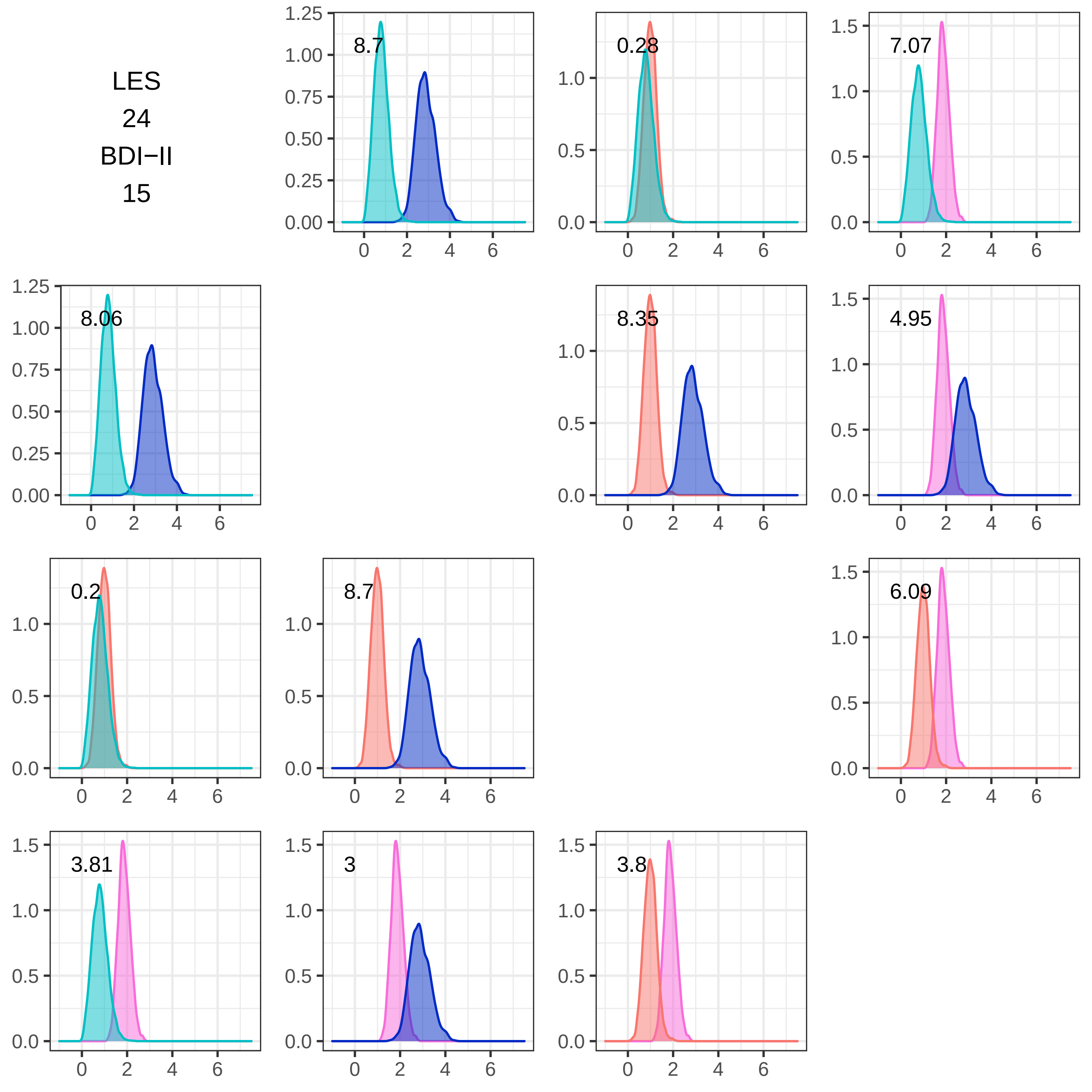
LES
24
BDI−II
15
8.7
0.28
7.07
8.06
8.35
4.95
0.2
8.7
6.09
3.81
3
3.8

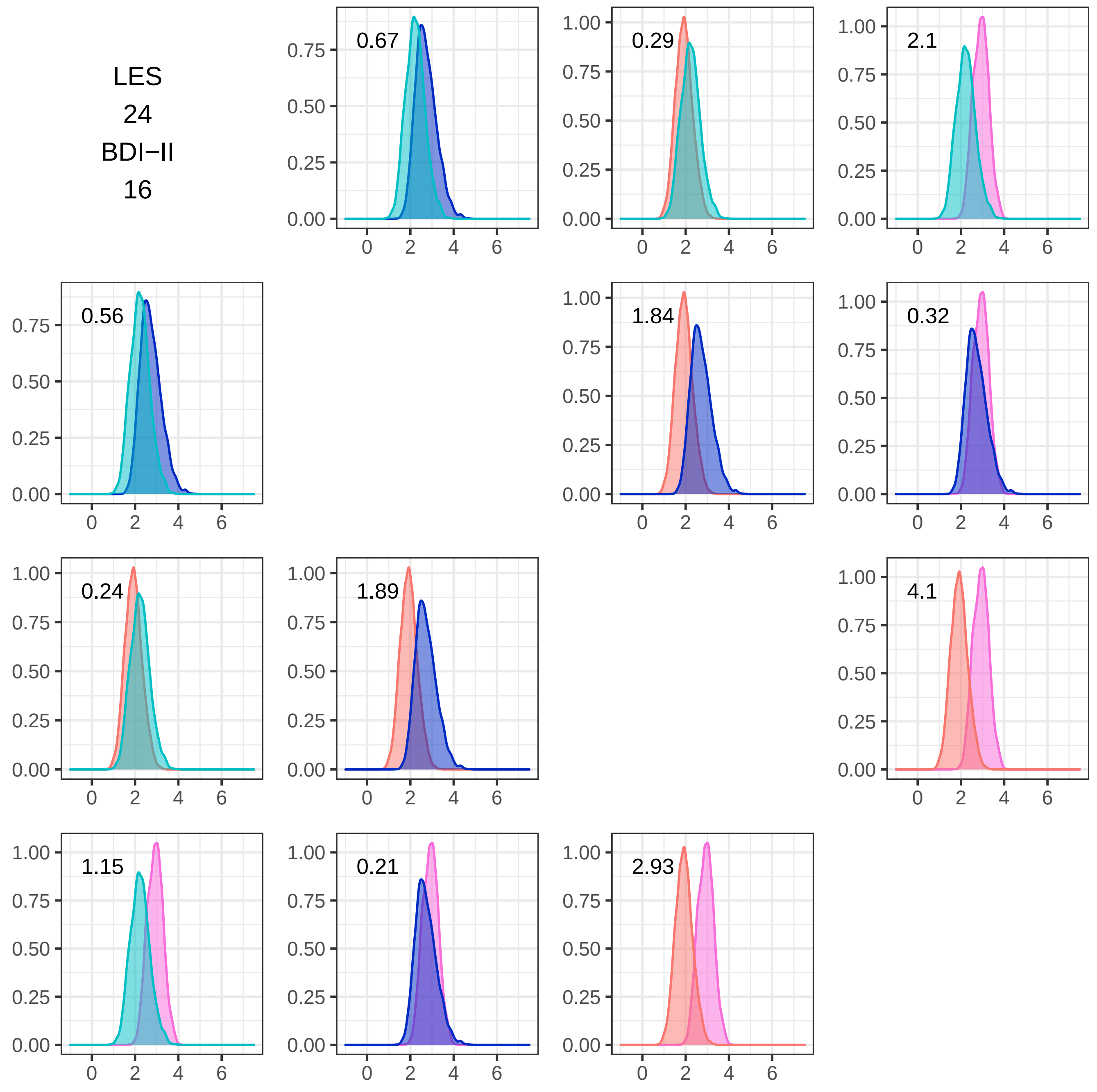
LES
24
BDI−II
16
0.67
0.29
2.1
0.56
1.84
0.32
0.24
1.89
4.1
1.15
0.21
2.93

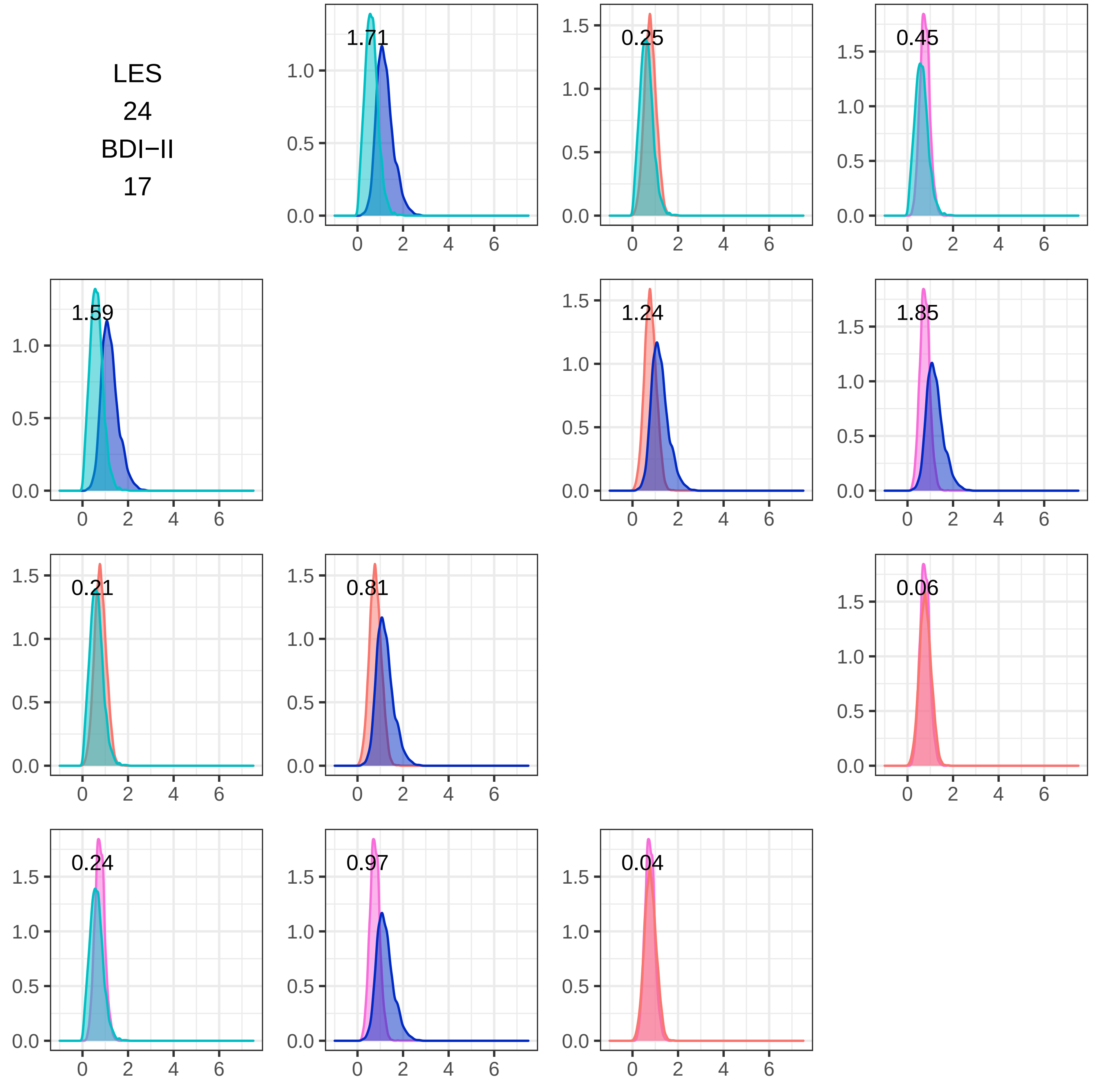
LES
24
BDI−II
17
1.71
0.25
0.45
1.59
1.24
1.85
0.21
0.81
0.06
0.24
0.97
0.04

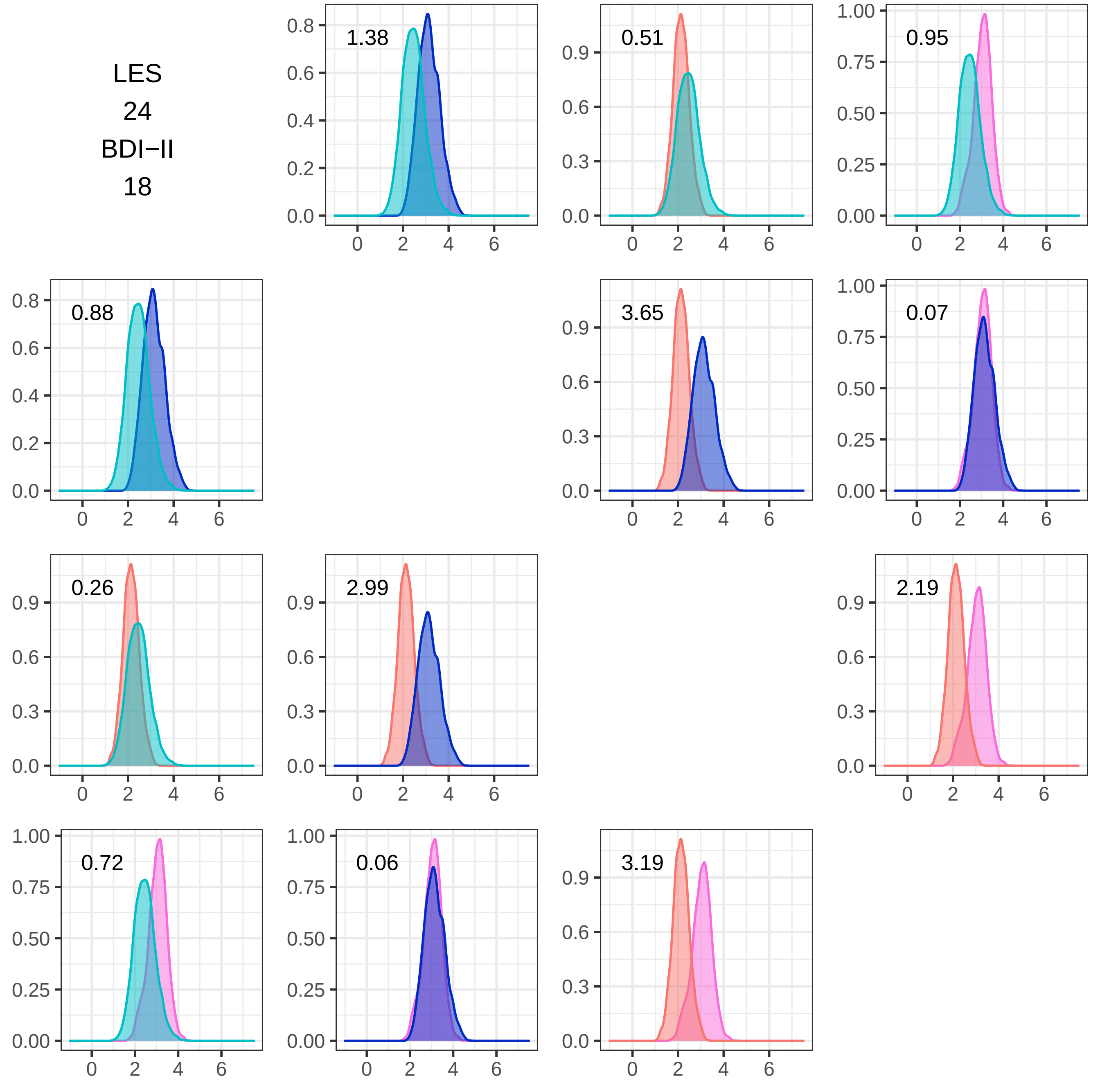

LES
24
BDI−II
18
1.38
0.51
0.95
0.88
3.65
0.07
0.26
2.99
2.19
0.72
0.06
3.19

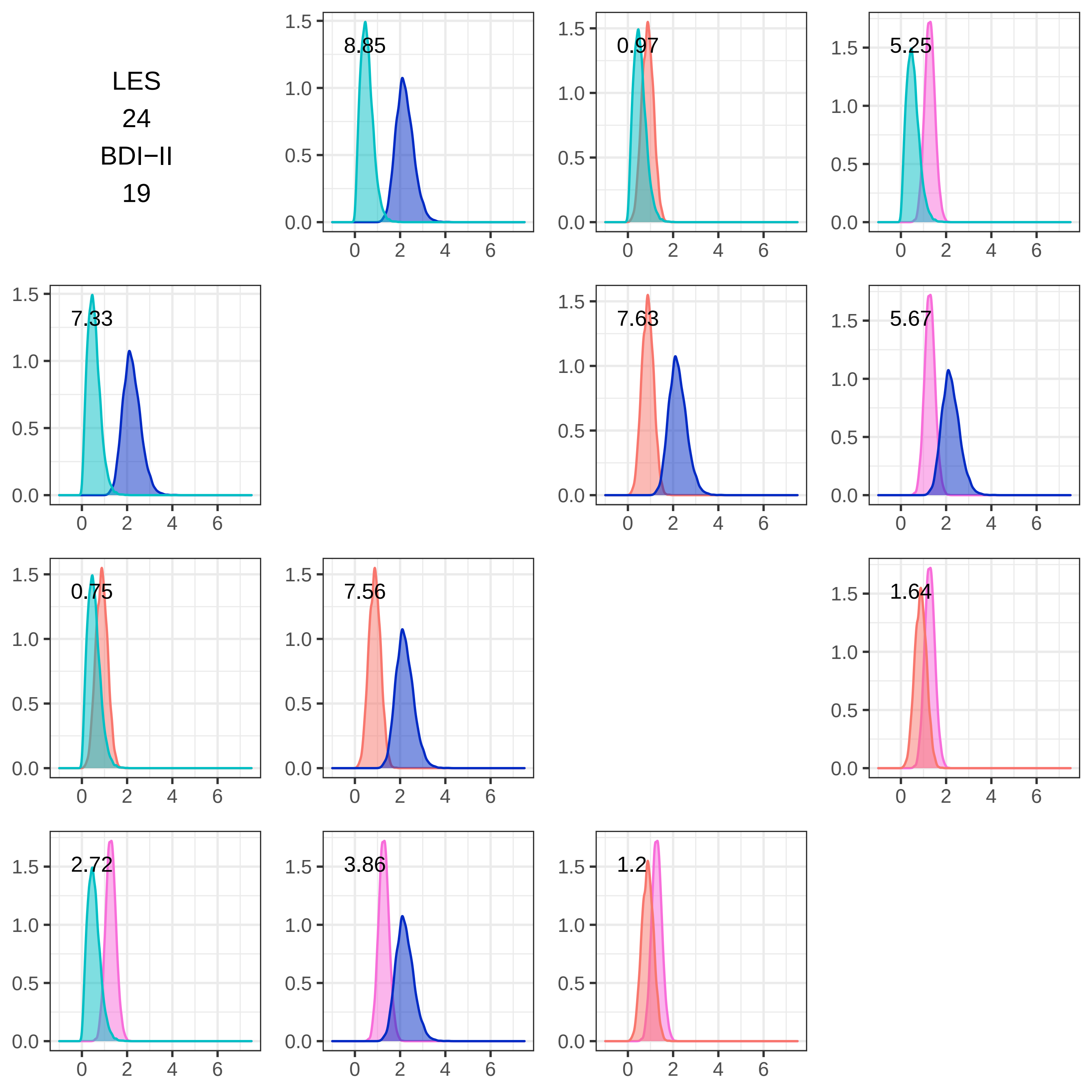
LES
24
BDI−II
19
8.85
0.97
5.25
7.33
7.63
5.67
0.75
7.56
1.64
2.72
3.86
1.2

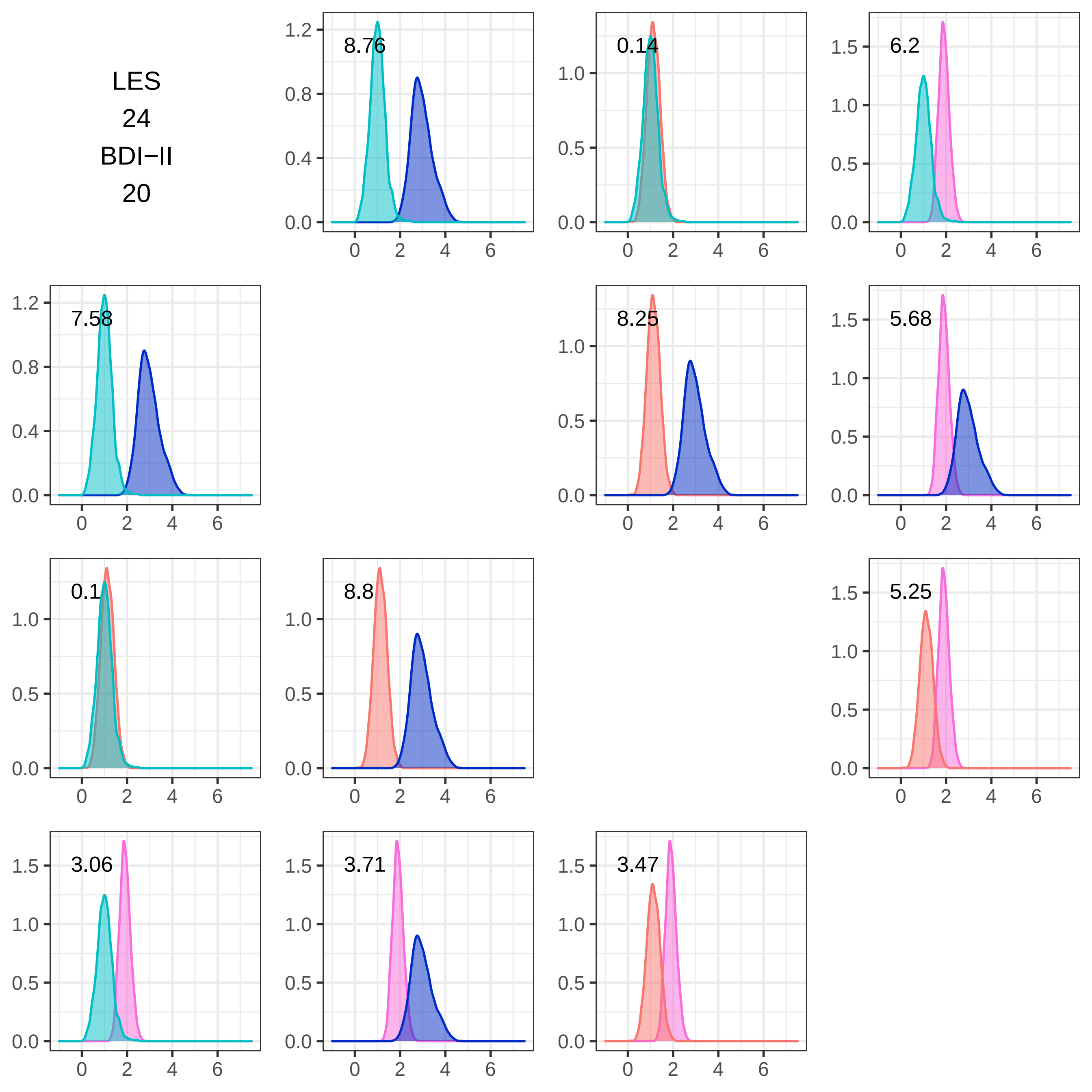
LES
24
BDI−II
20
8.76
0.14
6.2
7.58
8.25
5.68
0.1
8.8
5.25
3.06
3.71
3.47

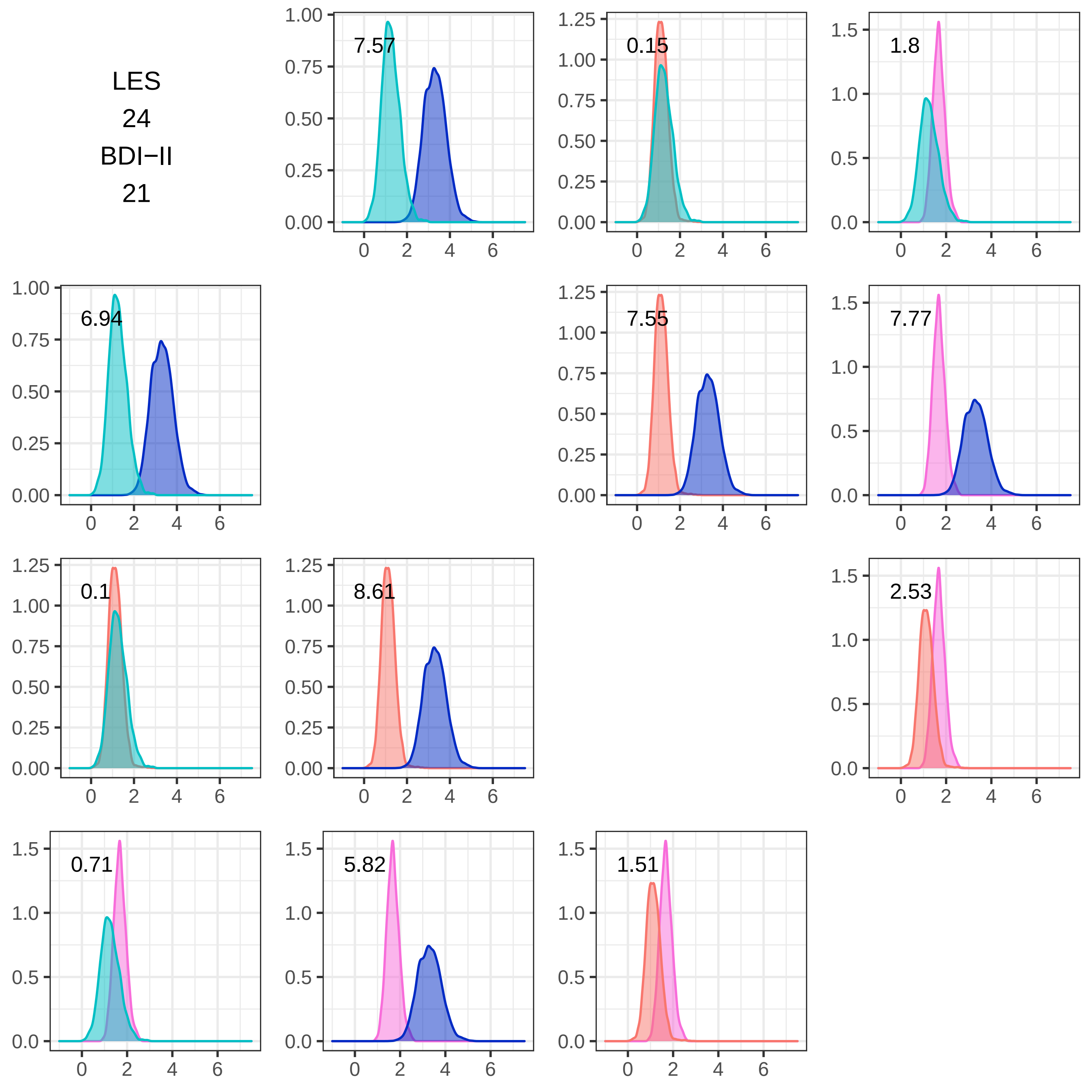

LES
24
BDI−II
21
7.57
0.15
1.8
6.94
7.55
7.77
0.1
8.61
2.53
0.71
5.82
1.51

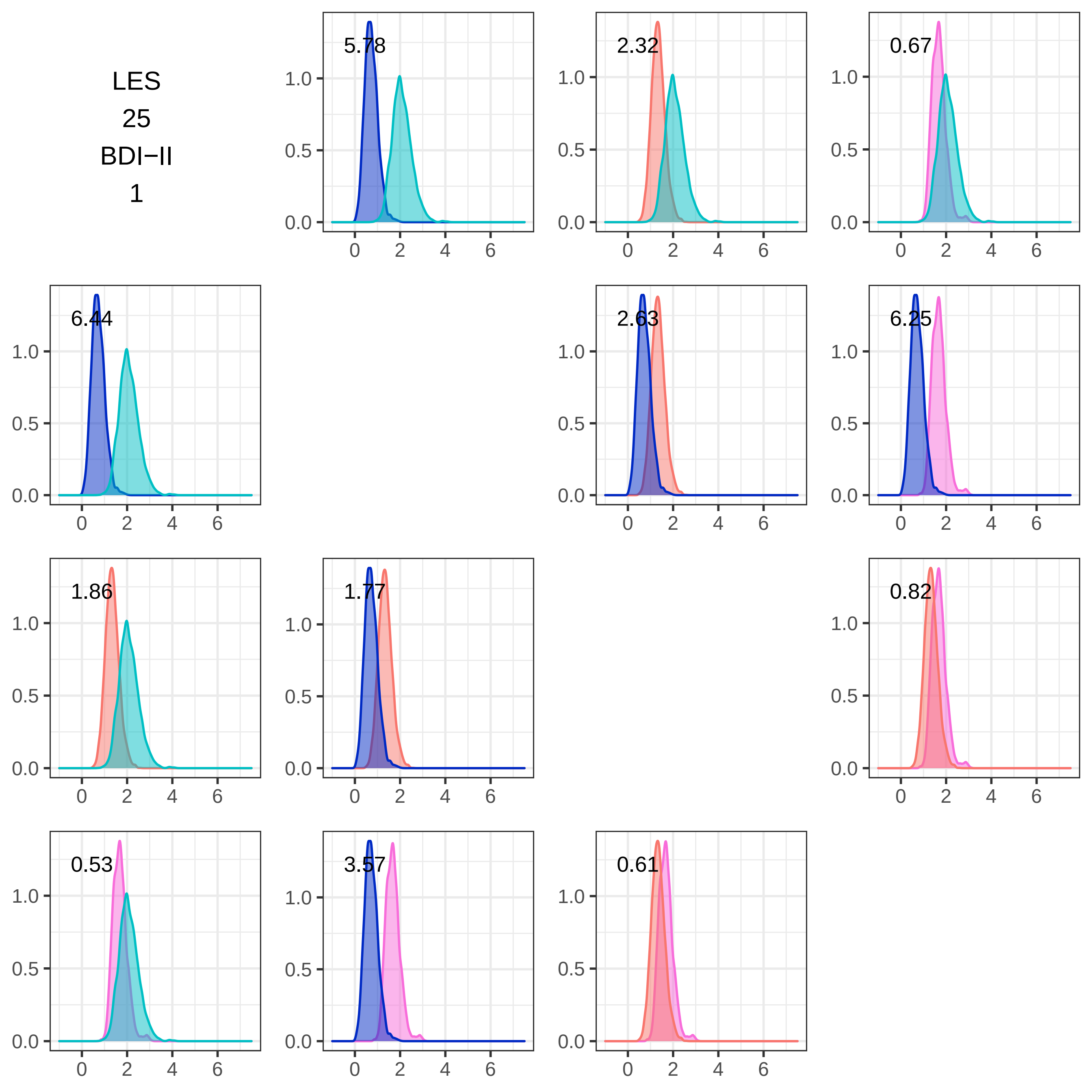
LES
25
BDI−II
1
5.78
2.32
0.67
6.44
2.63
6.25
1.86
1.77
0.82
0.53
3.57
0.61

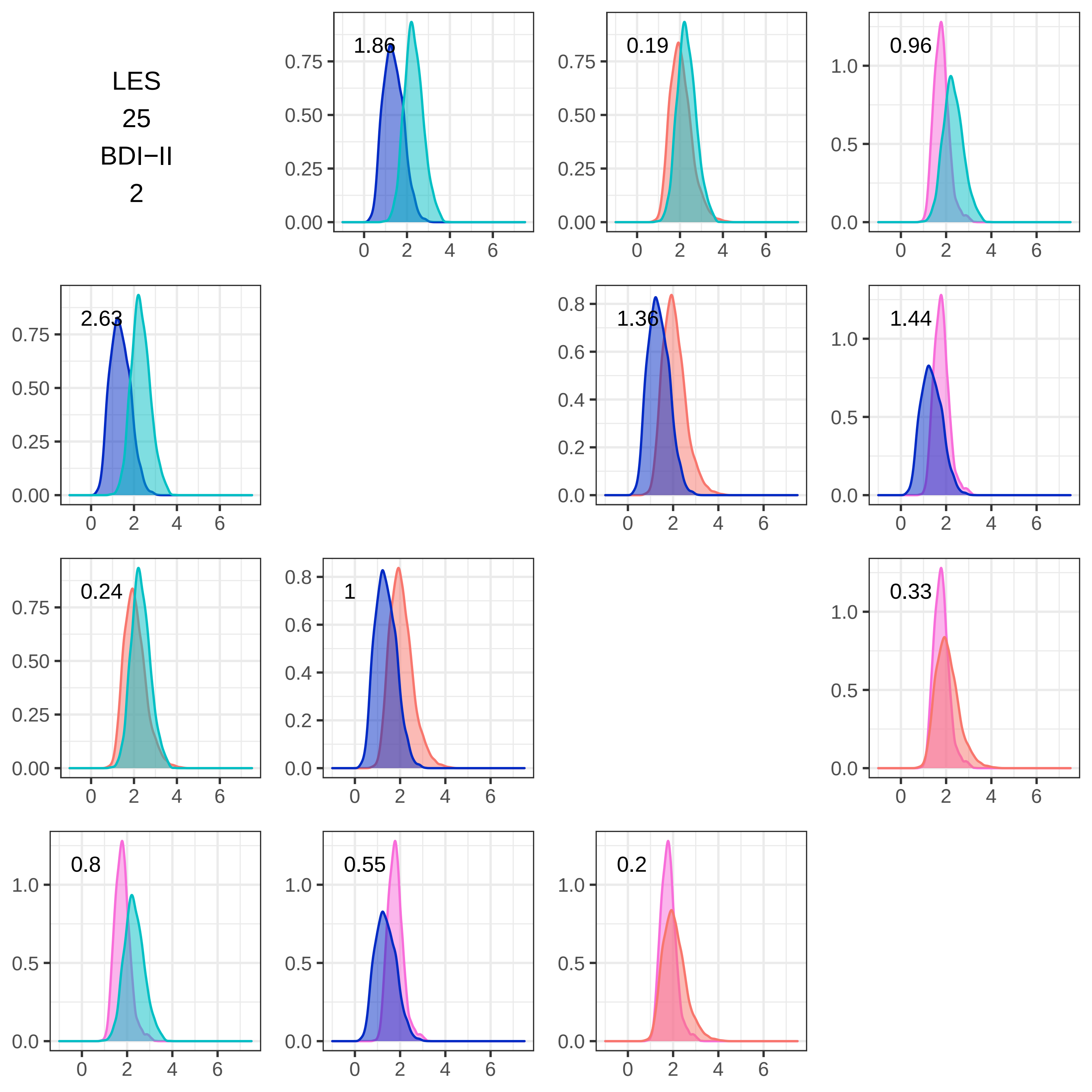

LES
25
BDI−II
2
1.86
0.19
0.96
2.63
1.36
1.44
0.24
1
0.33
0.8
0.55
0.2

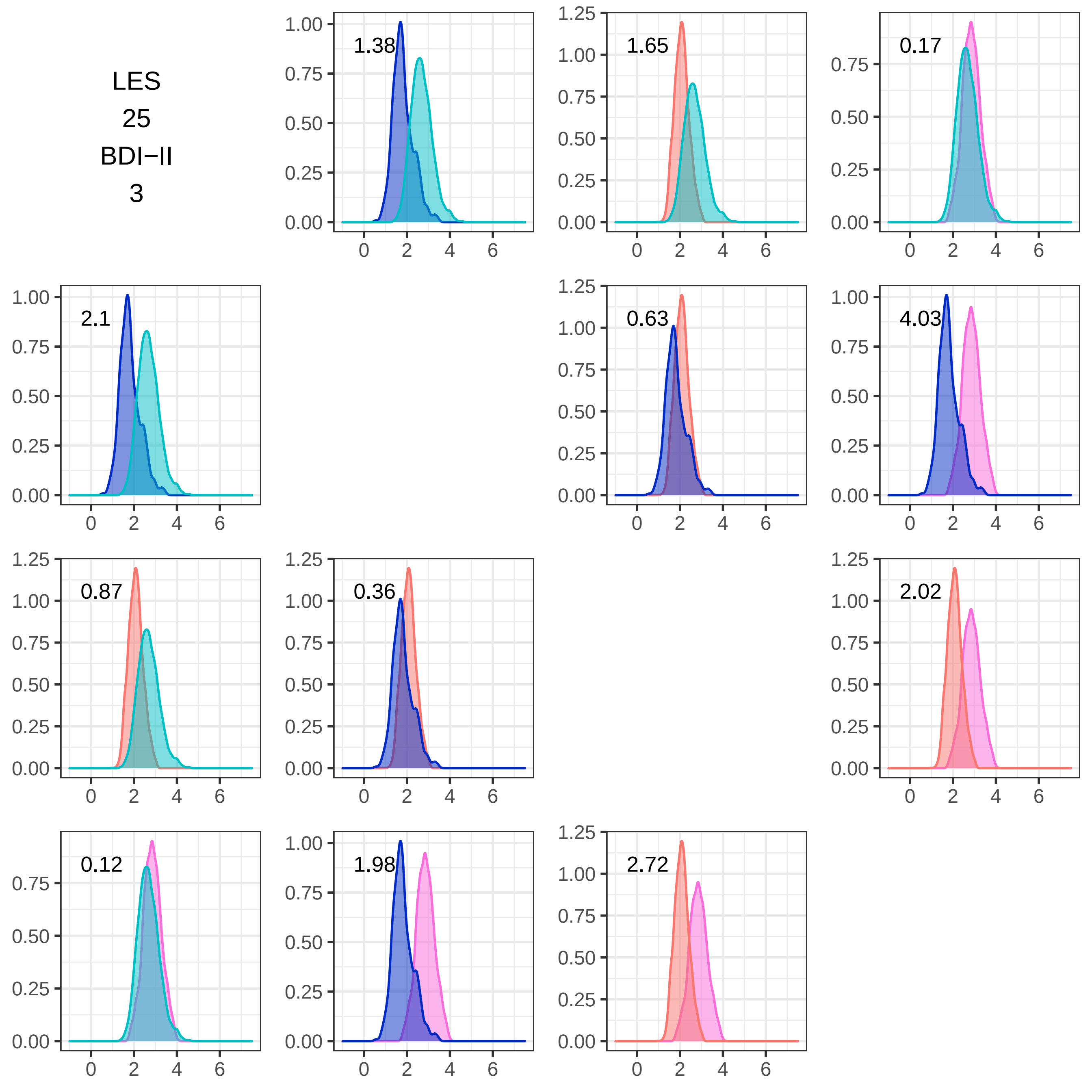
LES
25
BDI−II
3
1.38
1.65
0.17
2.1
0.63
4.03
0.87
0.36
2.02
0.12
1.98
2.72

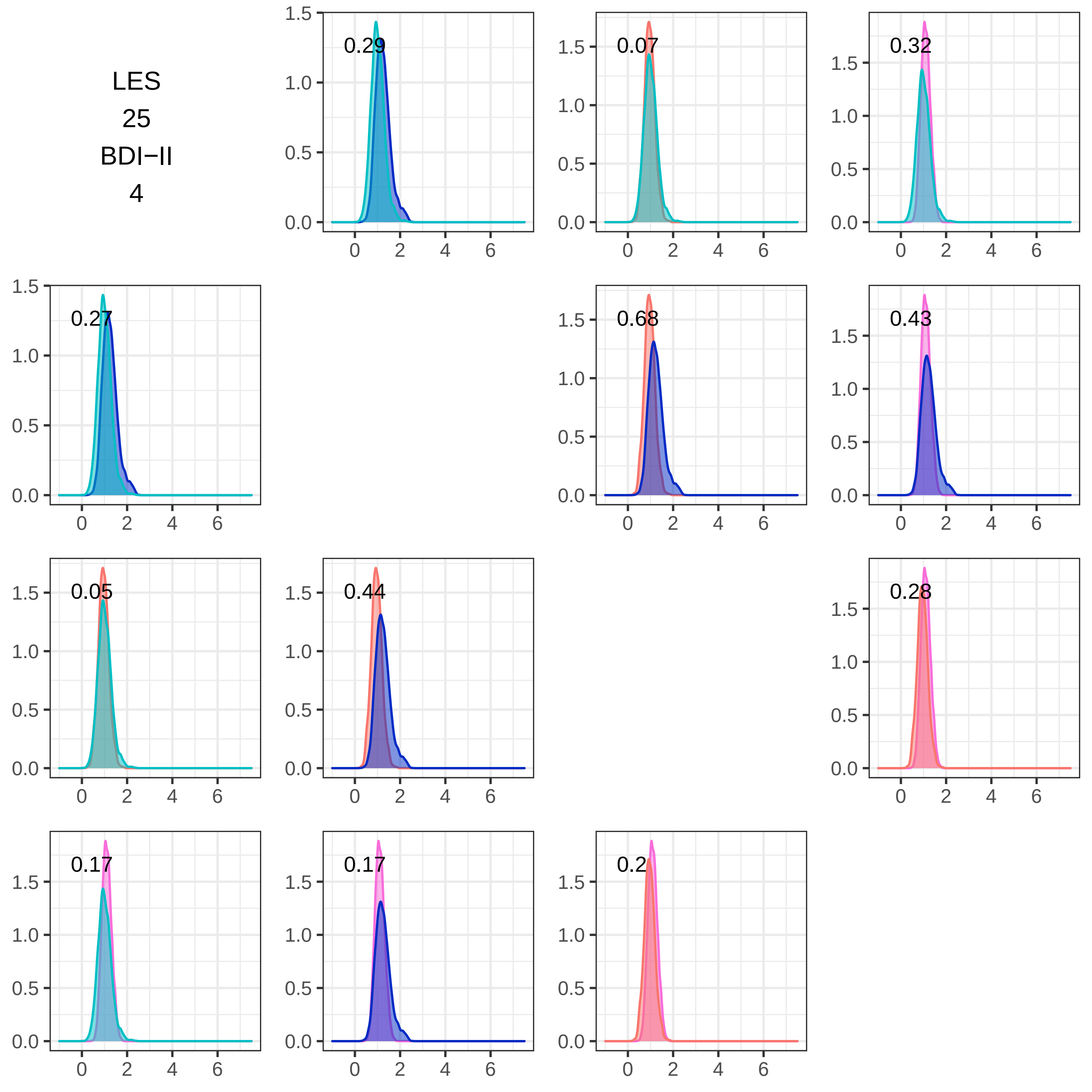

LES
25
BDI−II
4
0.29
0.07
0.32
0.27
0.68
0.43
0.05
0.44
0.28
0.17
0.17
0.2

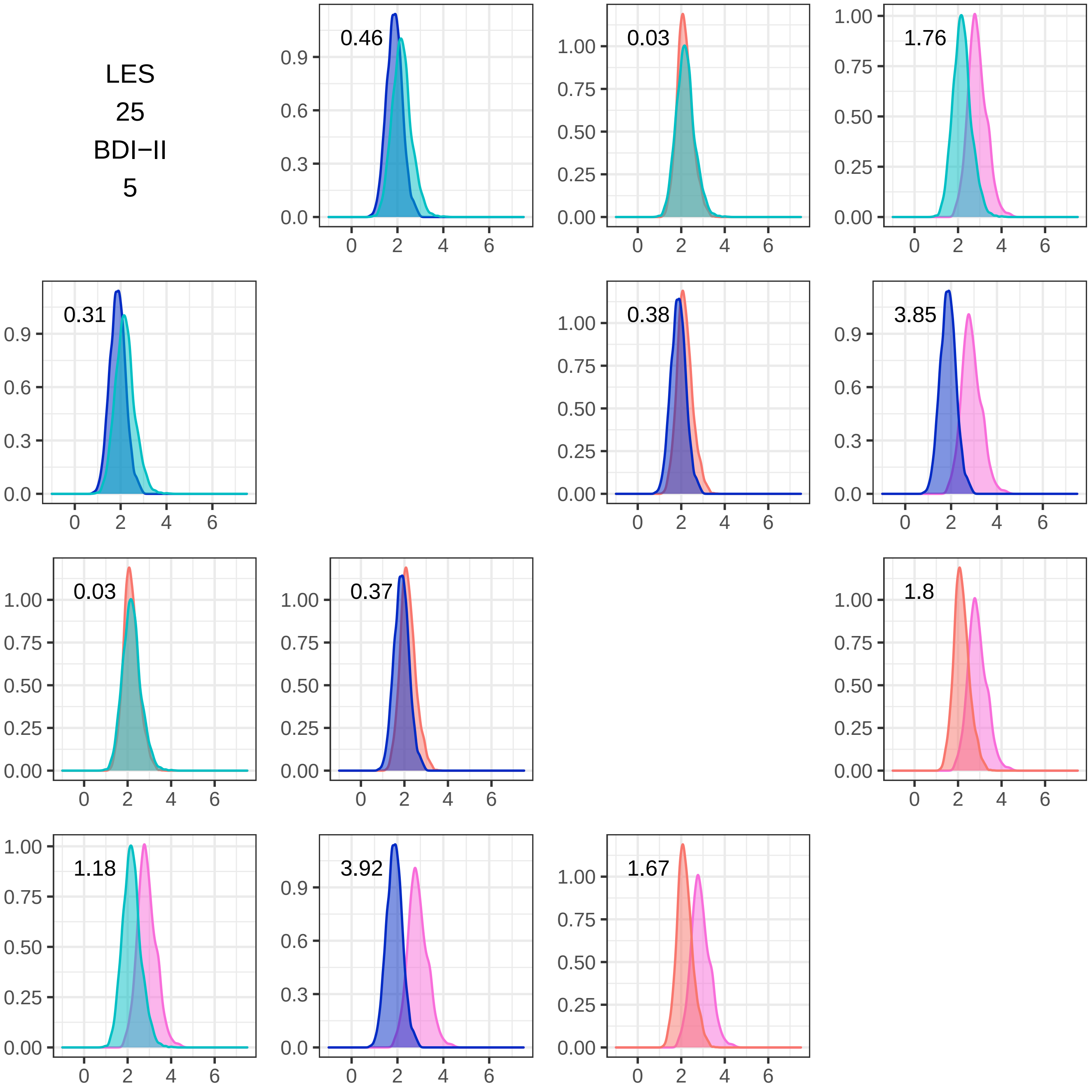

LES
25
BDI−II
5
0.46
0.03
1.76
0.31
0.38
3.85
0.03
0.37
1.8
1.18
3.92
1.67

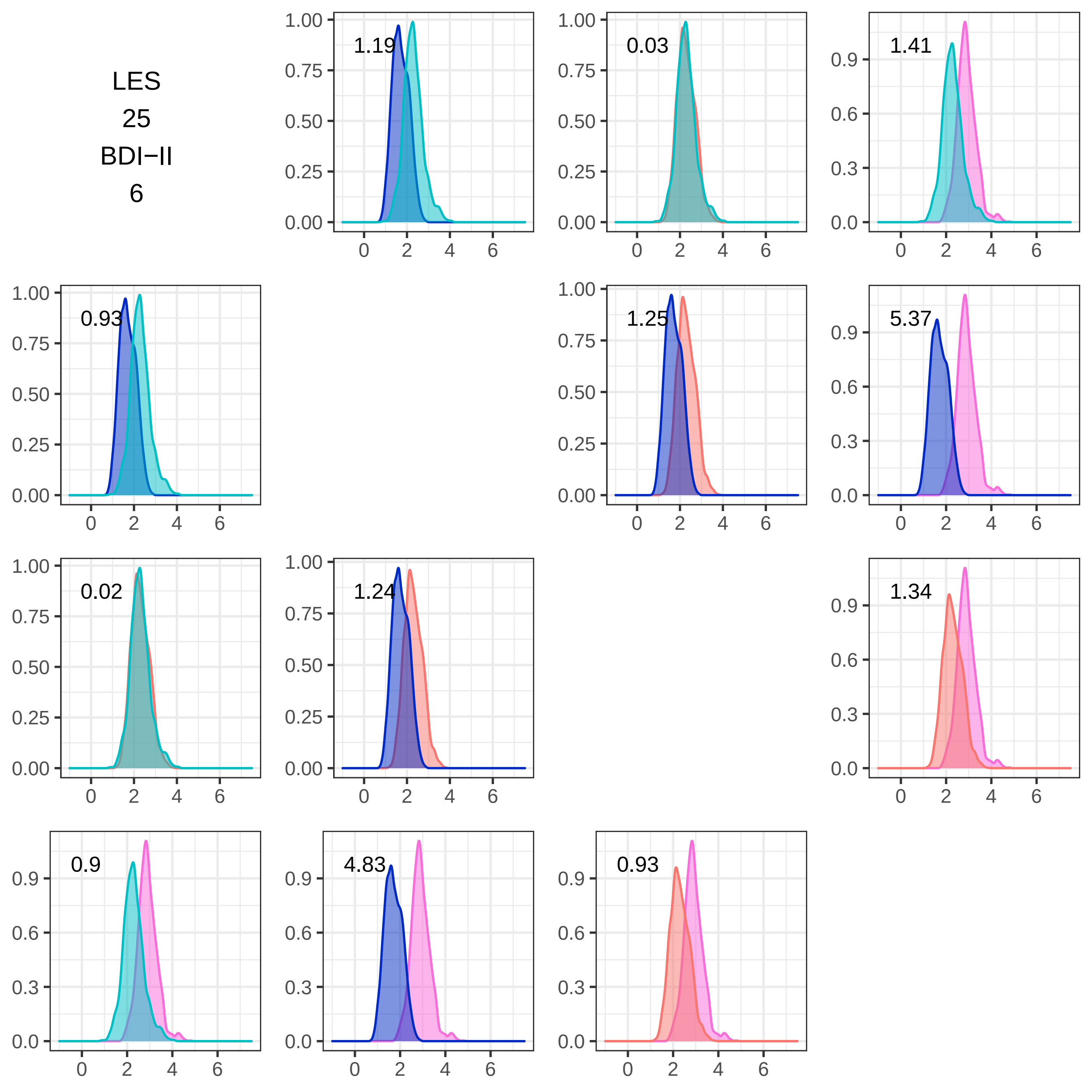

LES
25
BDI−II
6
1.19
0.03
1.41
0.93
1.25
5.37
0.02
1.24
1.34
0.9
4.83
0.93

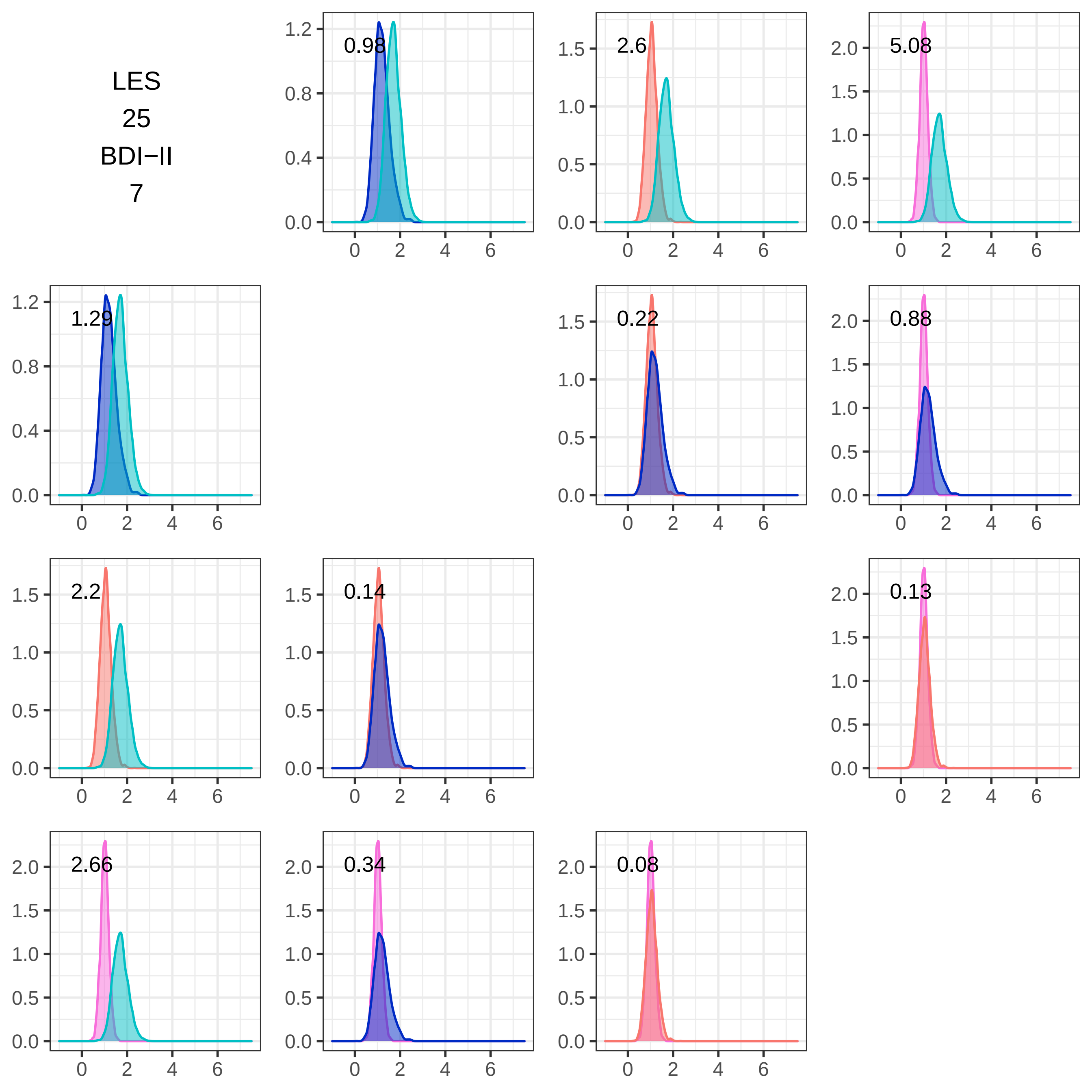

LES
25
BDI−II
7
0.98
2.6
5.08
1.29
0.22
0.88
2.2
0.14
0.13
2.66
0.34
0.08

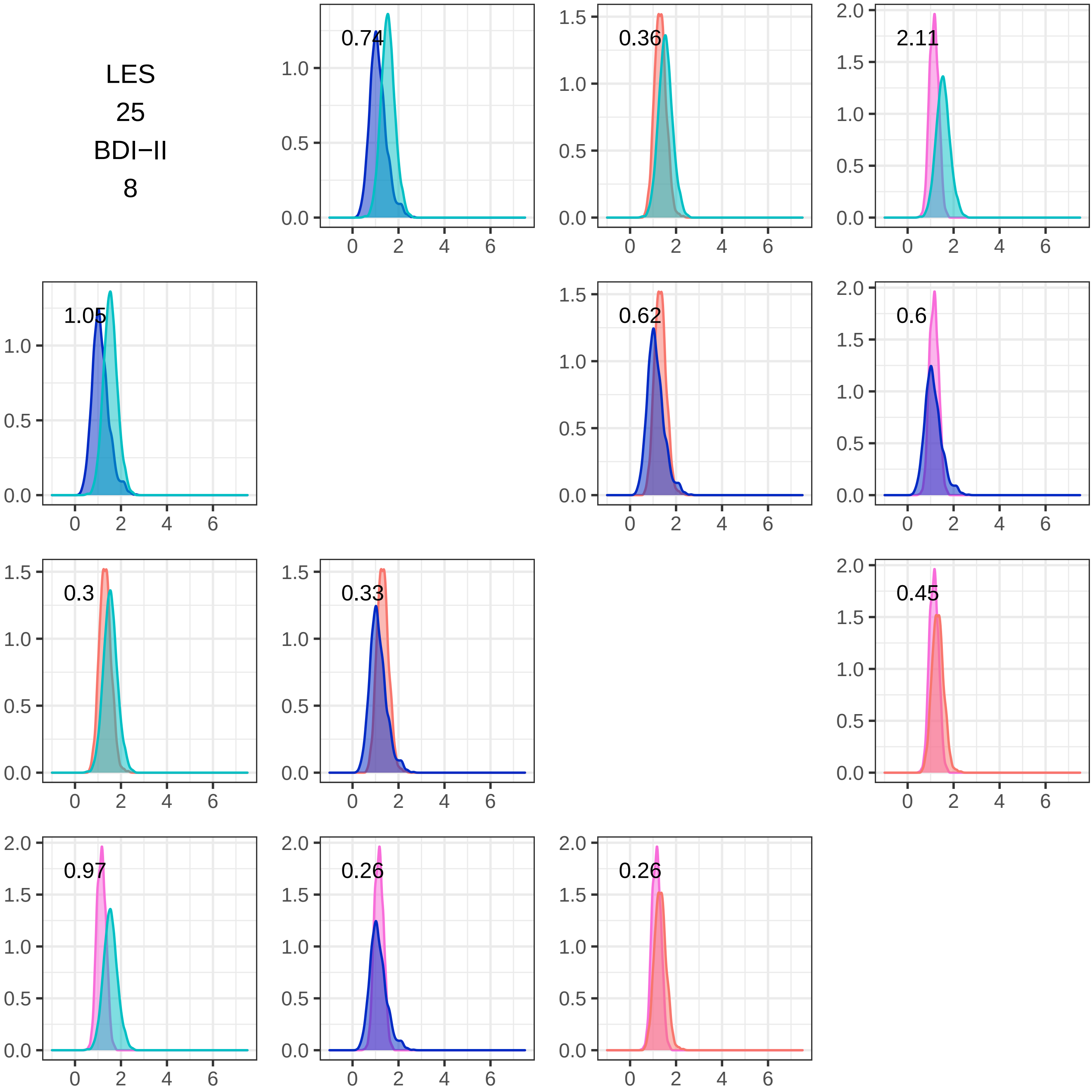

LES
25
BDI−II
8
0.74
0.36
2.11
1.05
0.62
0.6
0.3
0.33
0.45
0.97
0.26
0.26

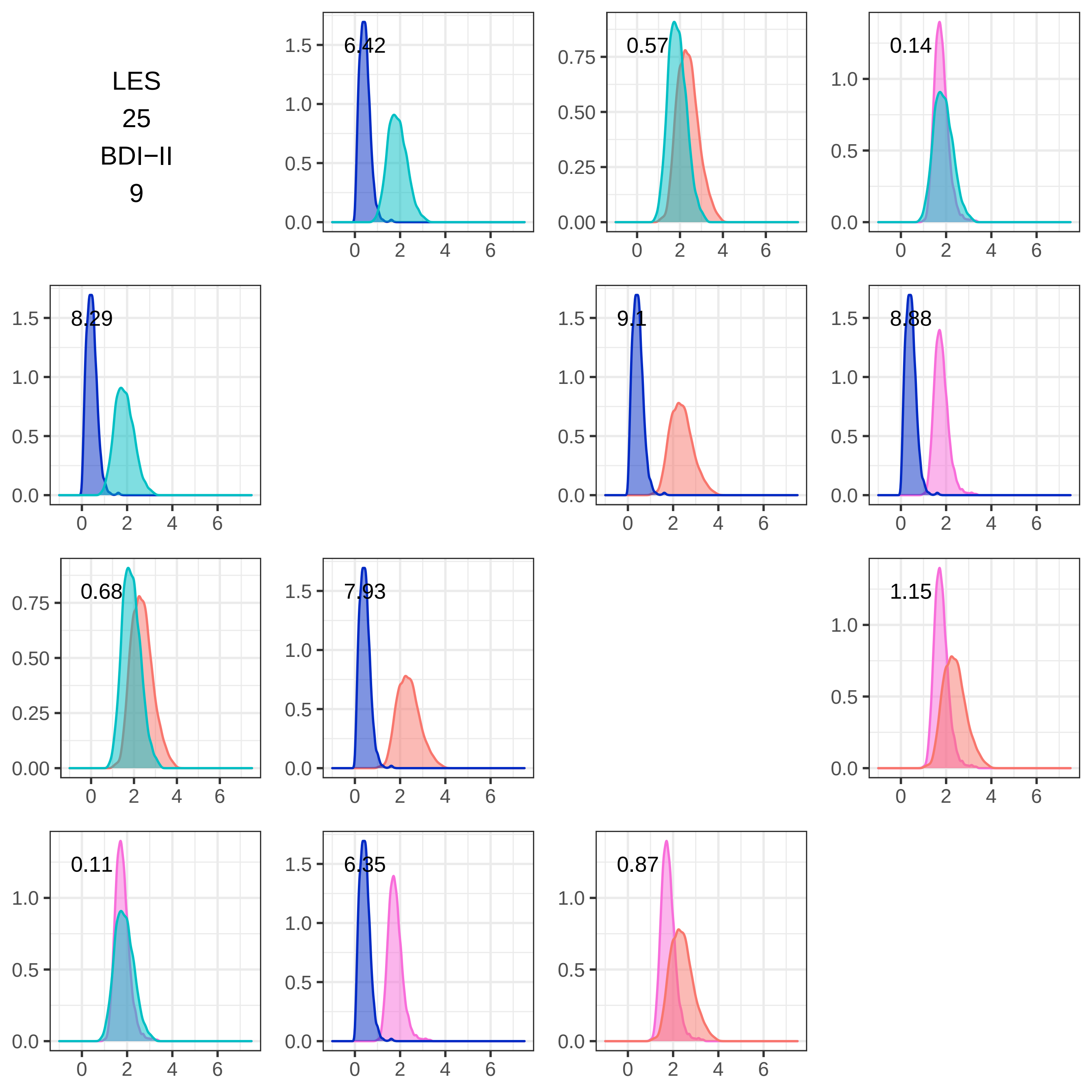
LES
25
BDI−II
9
6.42
0.57
0.14
8.29
9.1
8.88
0.68
7.93
1.15
0.11
6.35
0.87

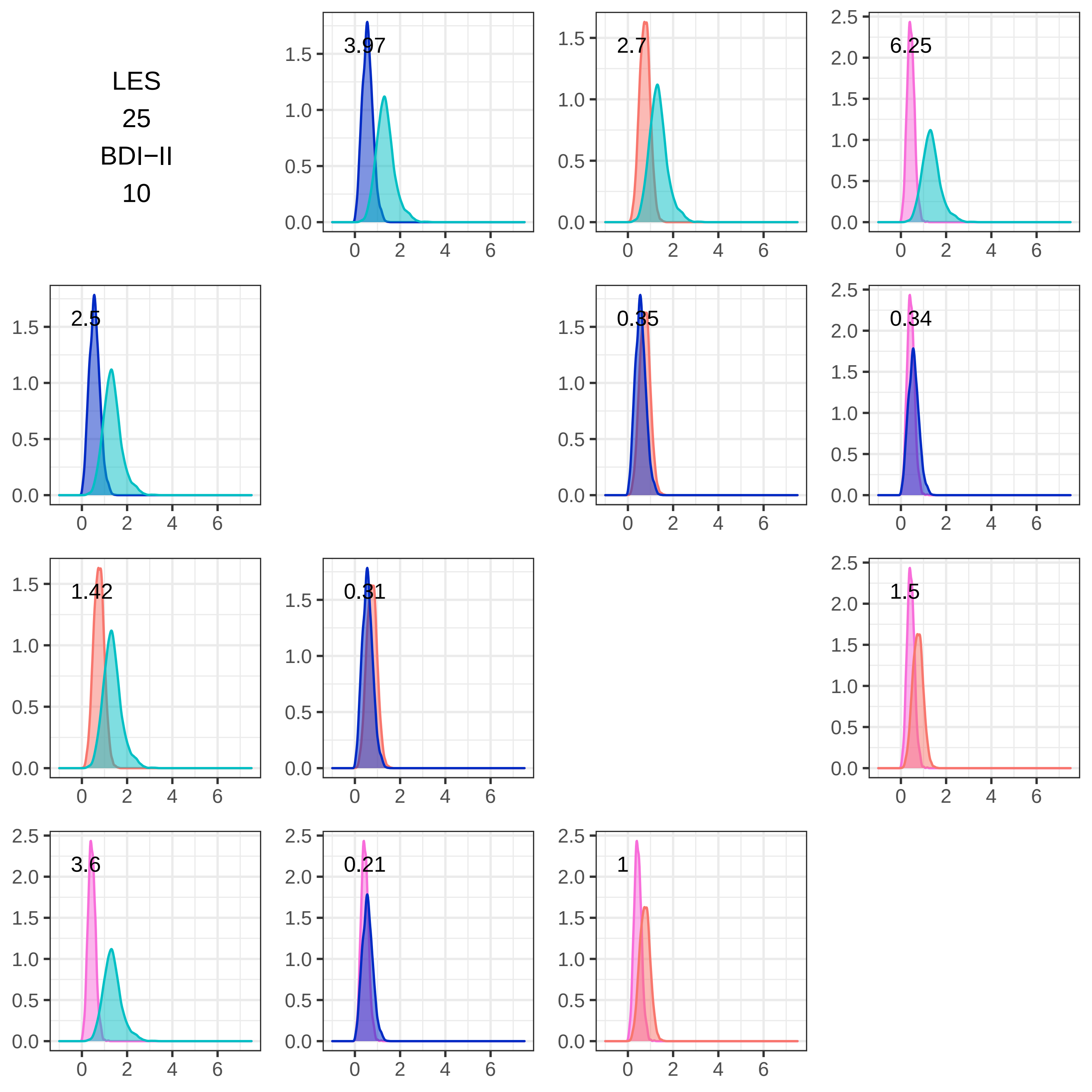
LES
25
BDI−II
10
3.97
2.7
6.25
2.5
0.35
0.34
1.42
0.31
1.5
3.6
0.21
1

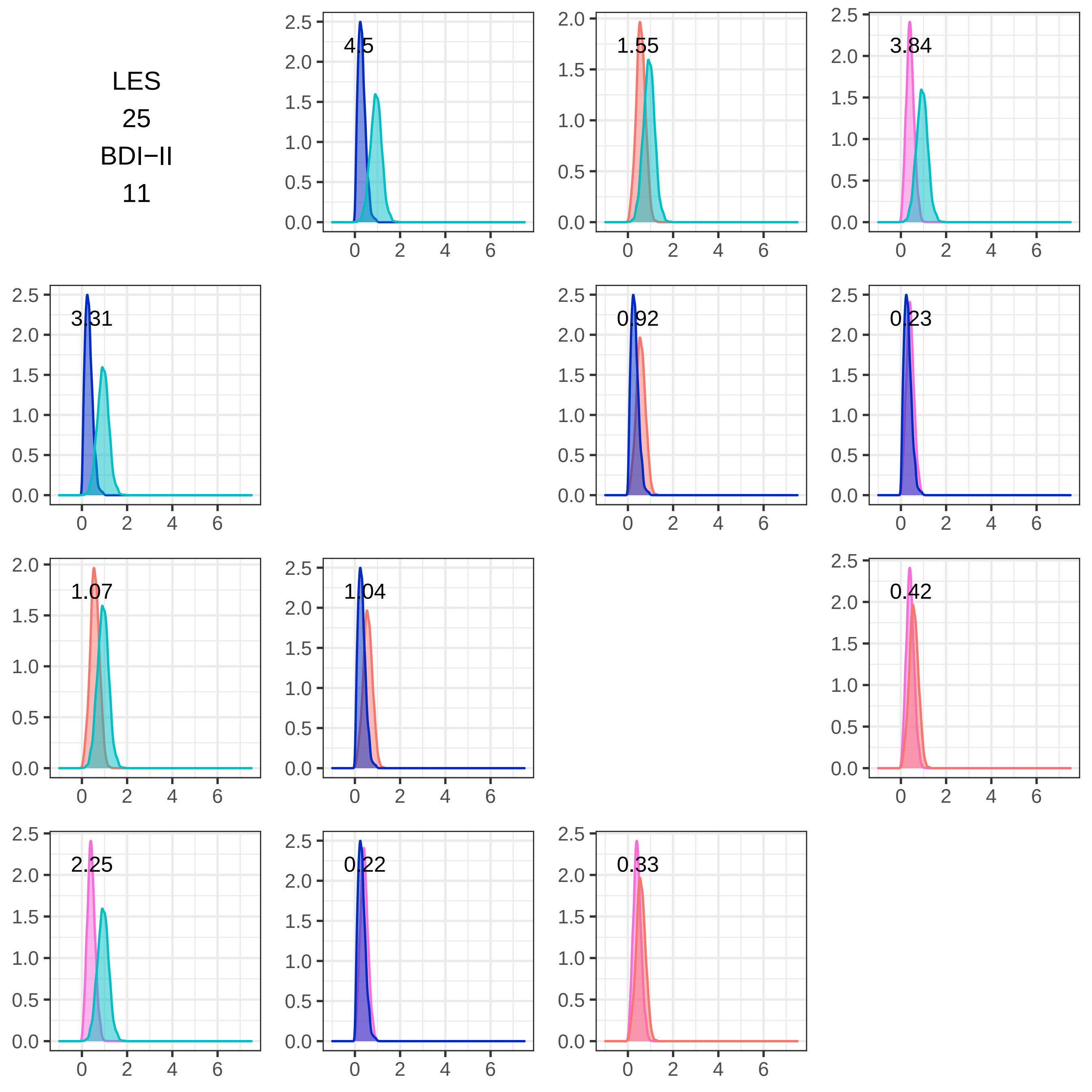

LES
25
BDI−II
11
4.5
1.55
3.84
3.31
0.92
0.23
1.07
1.04
0.42
2.25
0.22
0.33

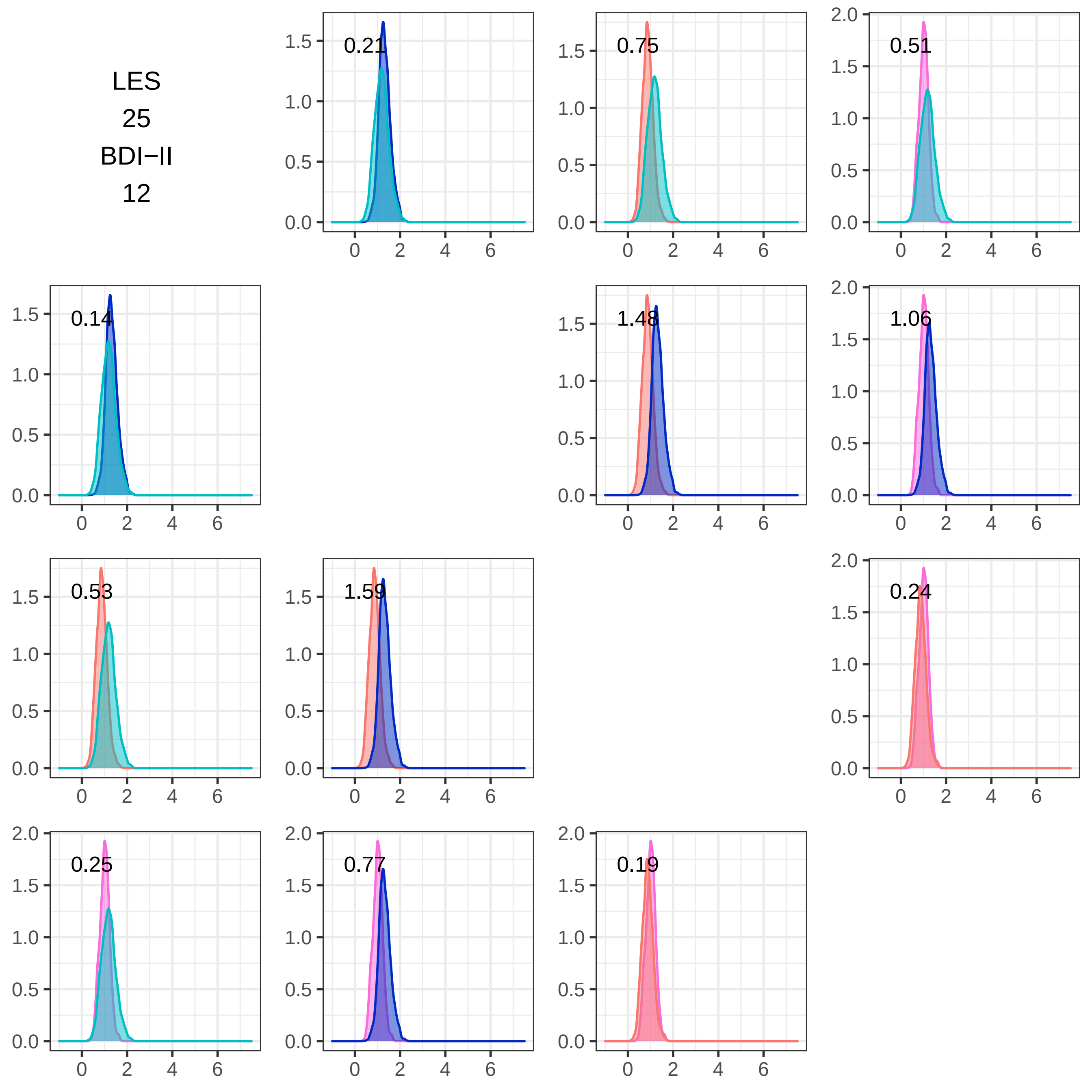

LES
25
BDI−II
12
0.21
0.75
0.51
0.14
1.48
1.06
0.53
1.59
0.24
0.25
0.77
0.19

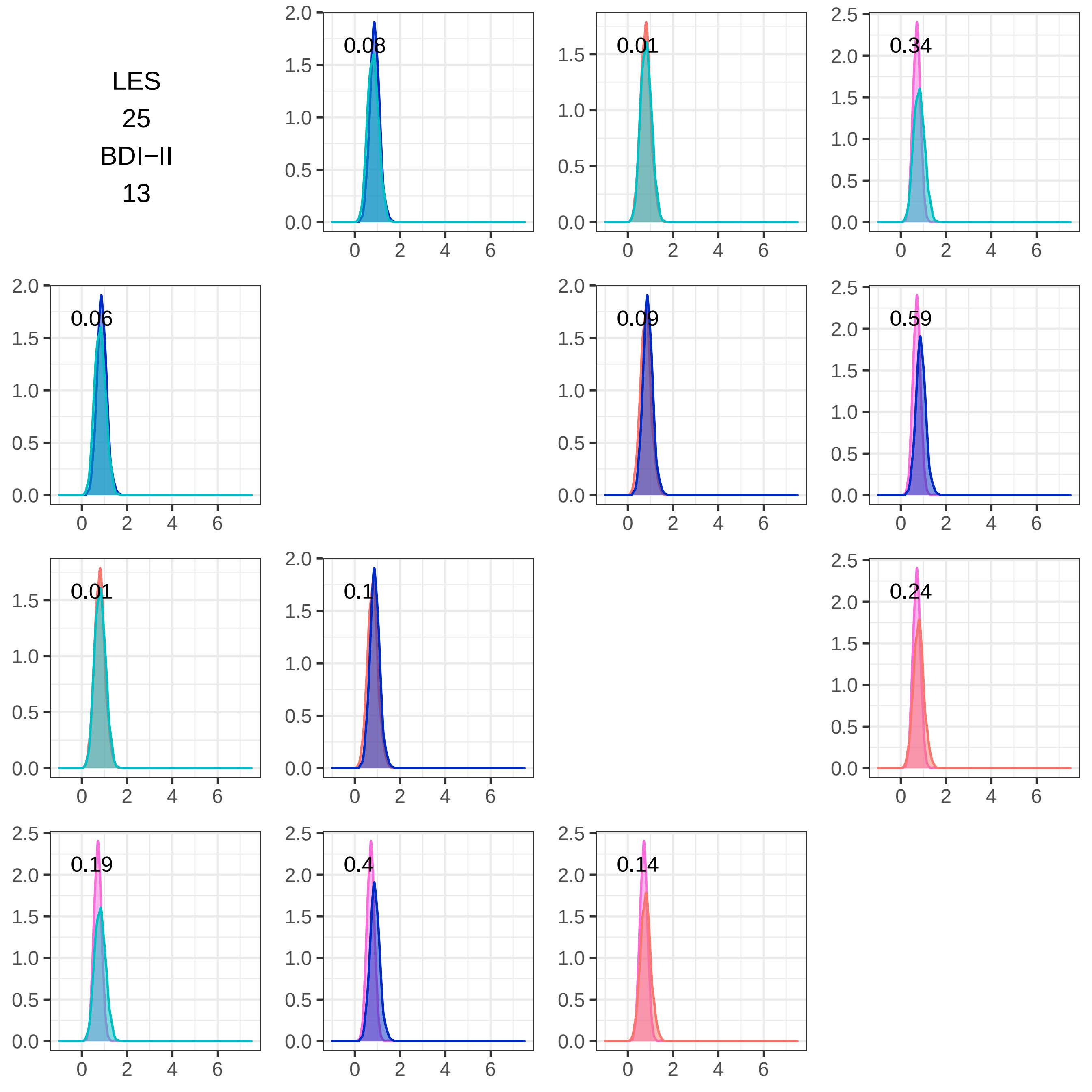

LES
25
BDI−II
13
0.08
0.01
0.34
0.06
0.09
0.59
0.01
0.1
0.24
0.19
0.4
0.14

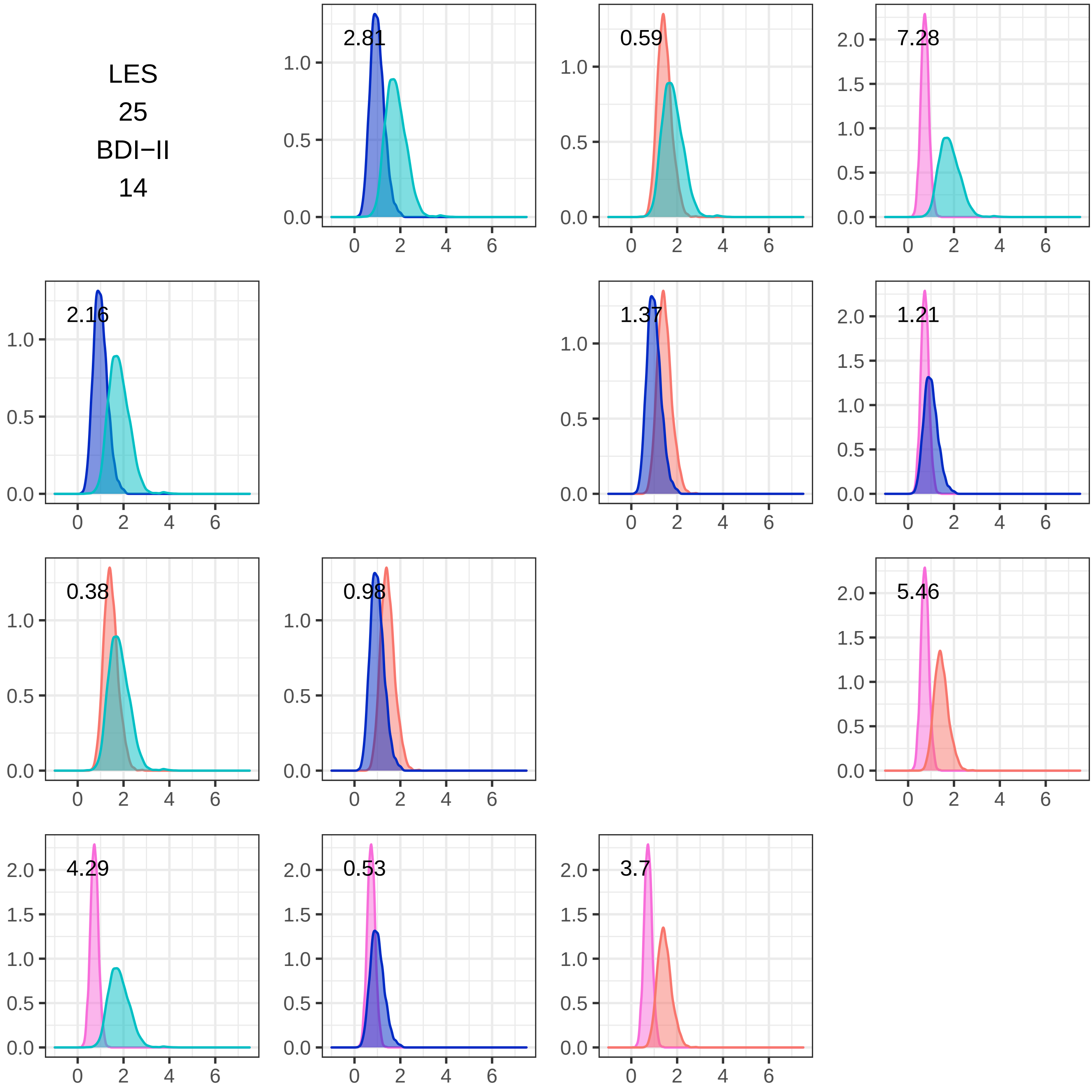

LES
25
BDI−II
14
2.81
0.59
7.28
2.16
1.37
1.21
0.38
0.98
5.46
4.29
0.53
3.7

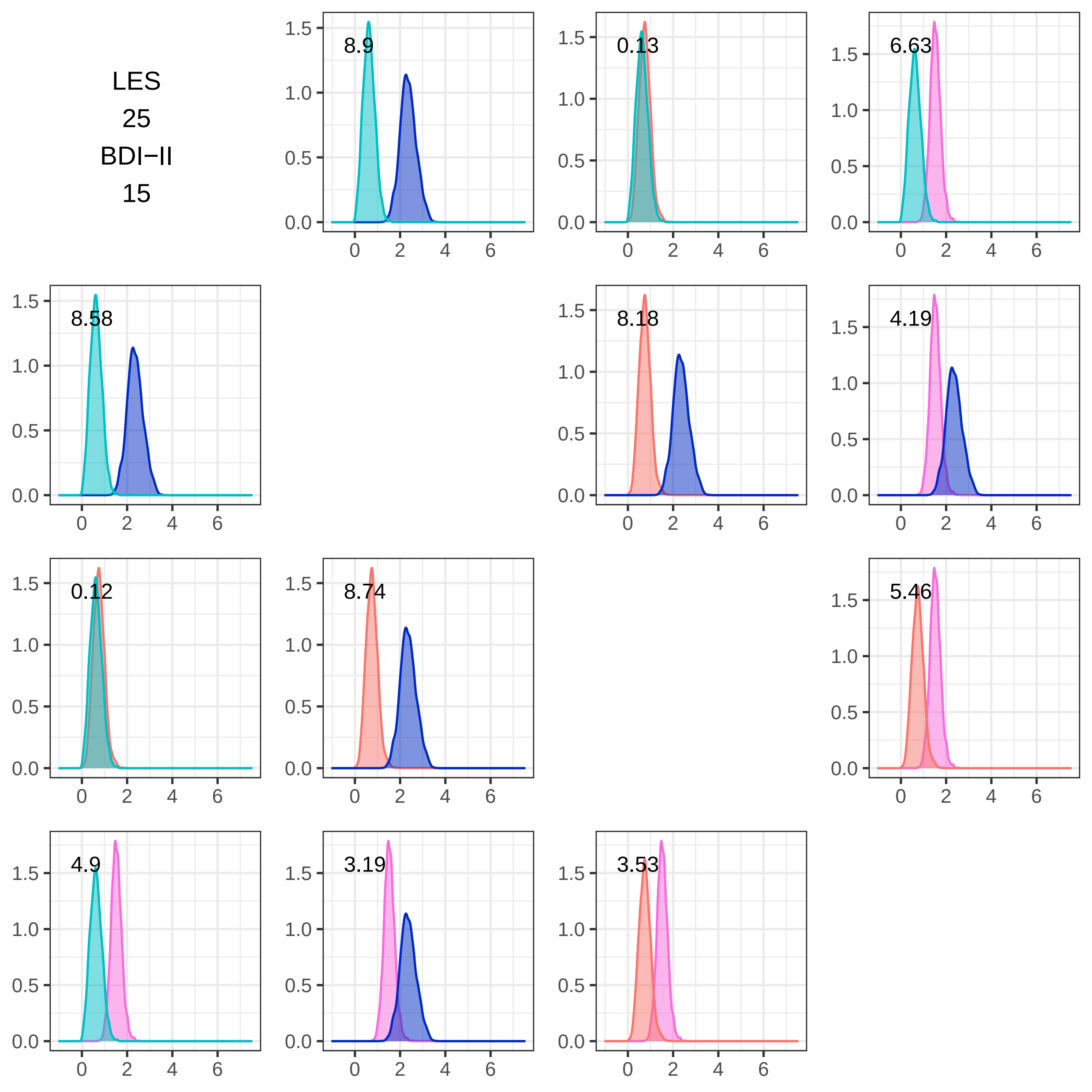

LES
25
BDI−II
15
8.9
0.13
6.63
8.58
8.18
4.19
0.12
8.74
5.46
4.9
3.19
3.53

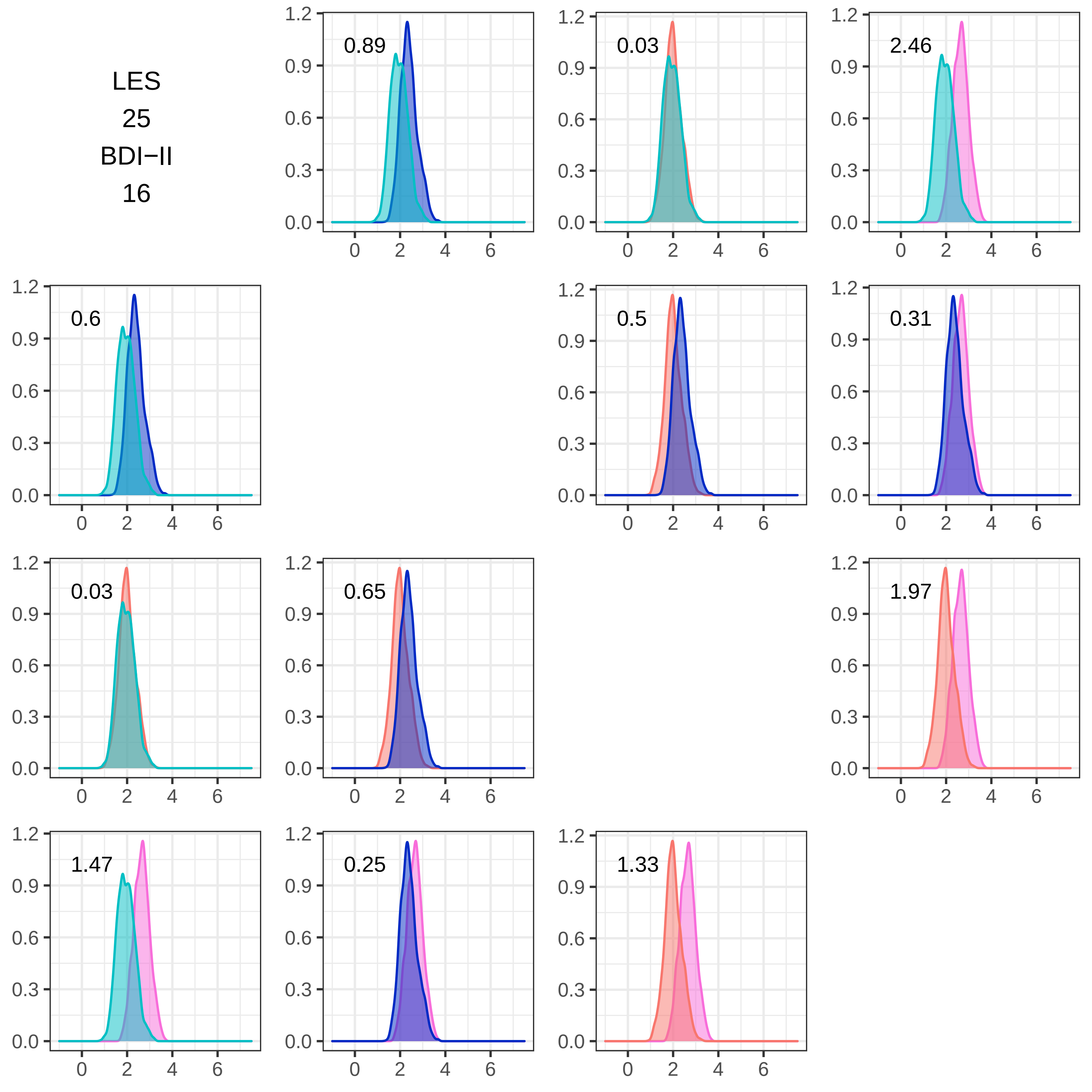

LES
25
BDI−II
16
0.89
0.03
2.46
0.6
0.5
0.31
0.03
0.65
1.97
1.47
0.25
1.33

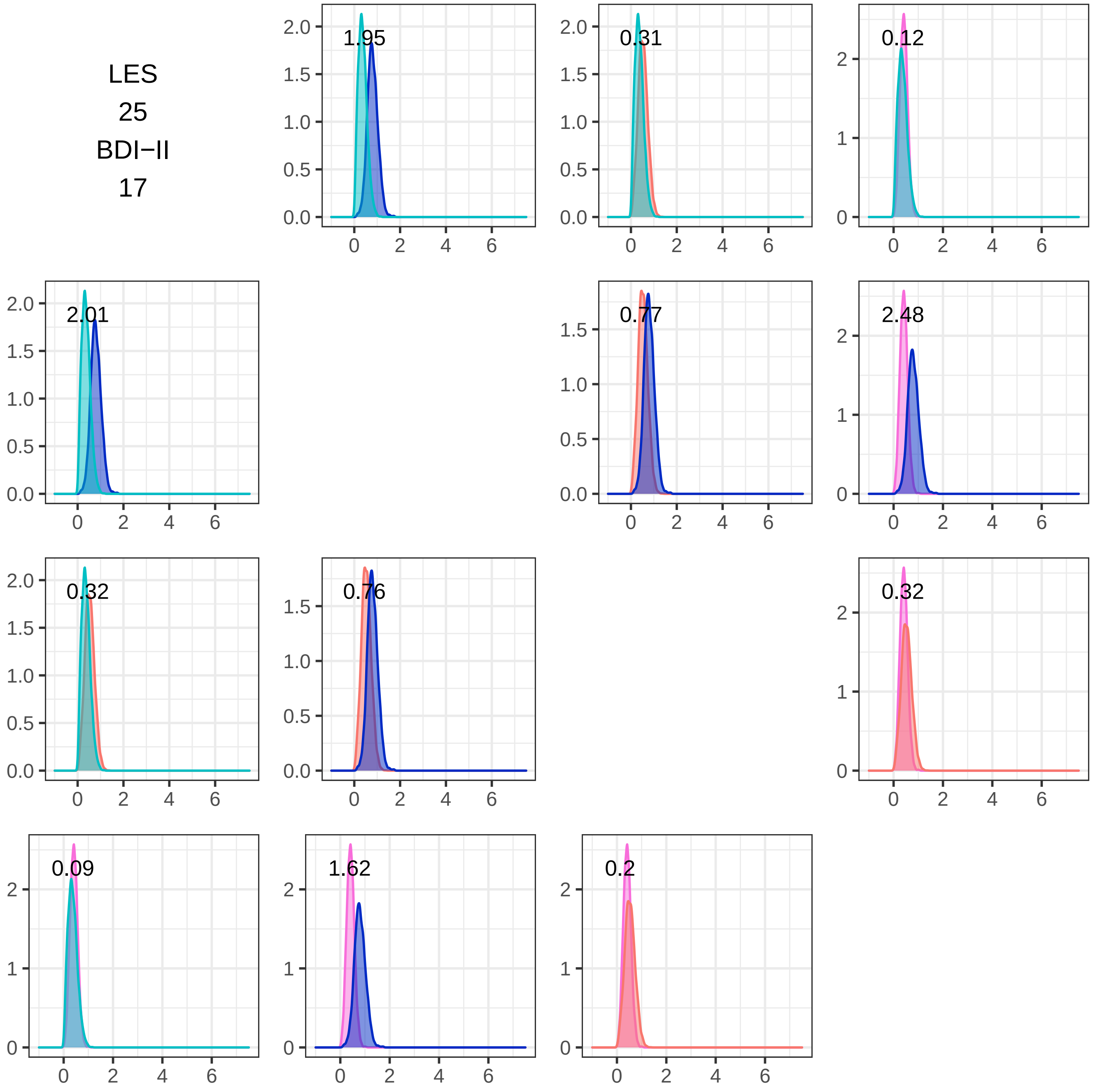
LES
25
BDI−II
17
1.95
0.31
0.12
2.01
0.77
2.48
0.32
0.76
0.32
0.09
1.62
0.2

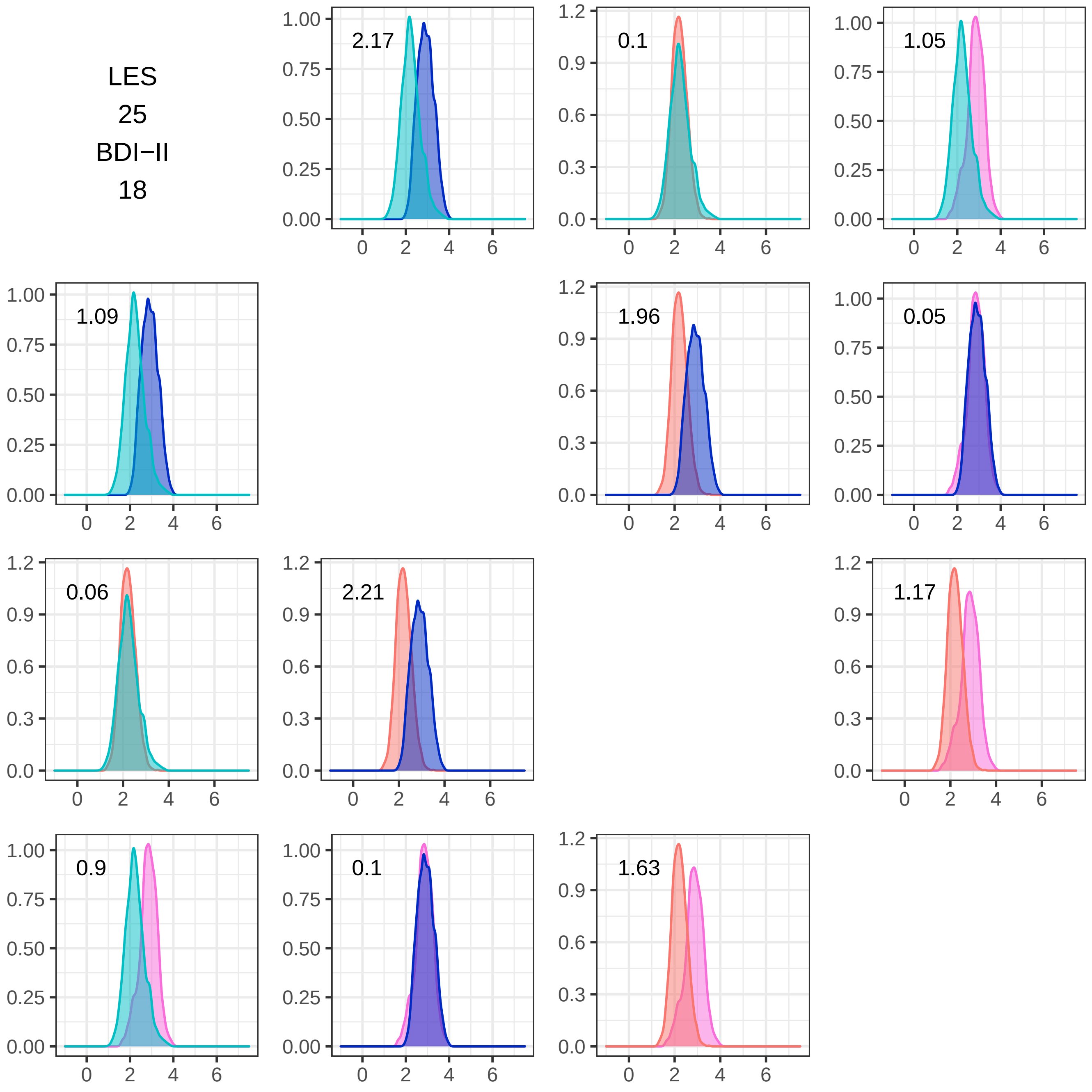

LES
25
BDI−II
18
2.17
0.1
1.05
1.09
1.96
0.05
0.06
2.21
1.17
0.9
0.1
1.63

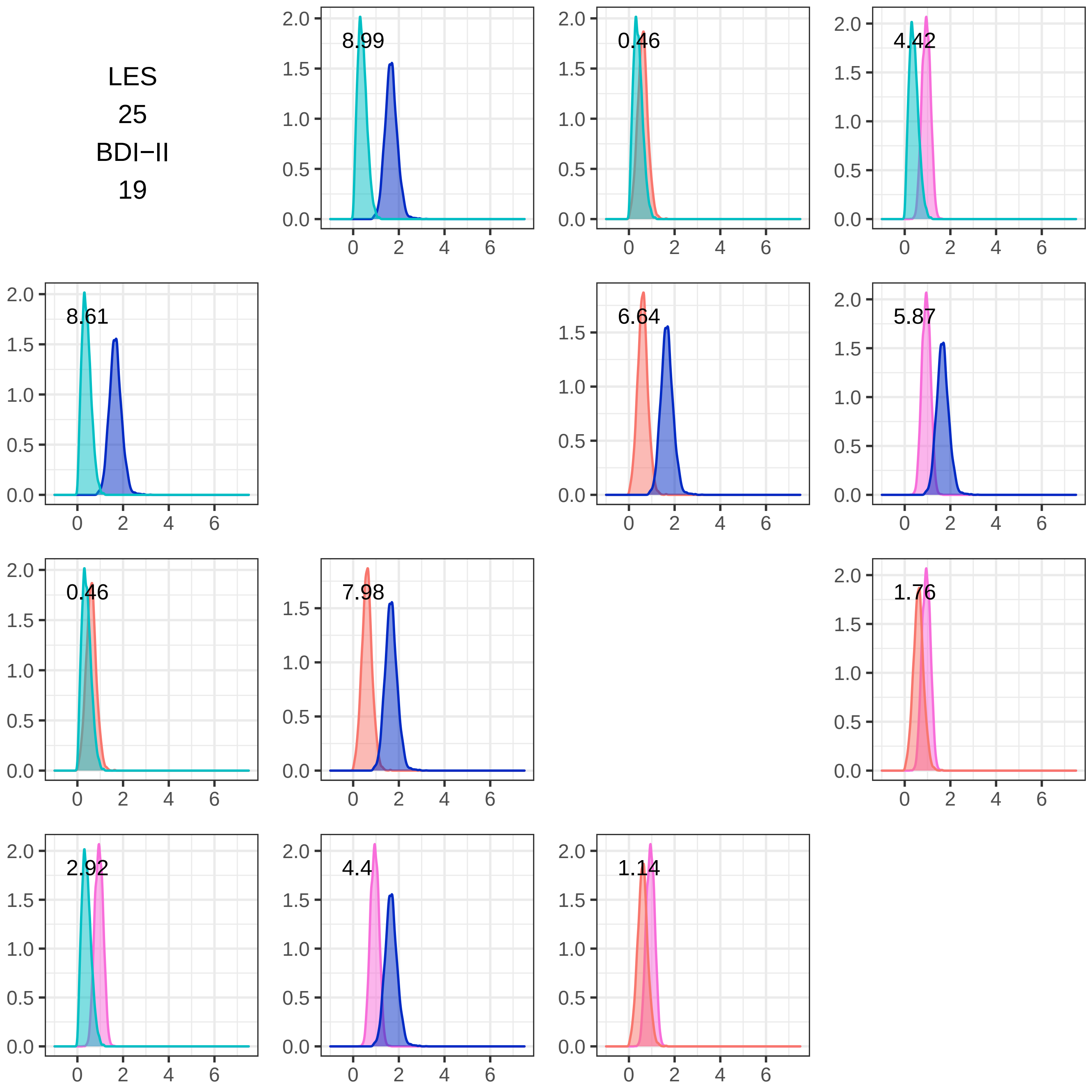

LES
25
BDI−II
19
8.99
0.46
4.42
8.61
6.64
5.87
0.46
7.98
1.76
2.92
4.4
1.14

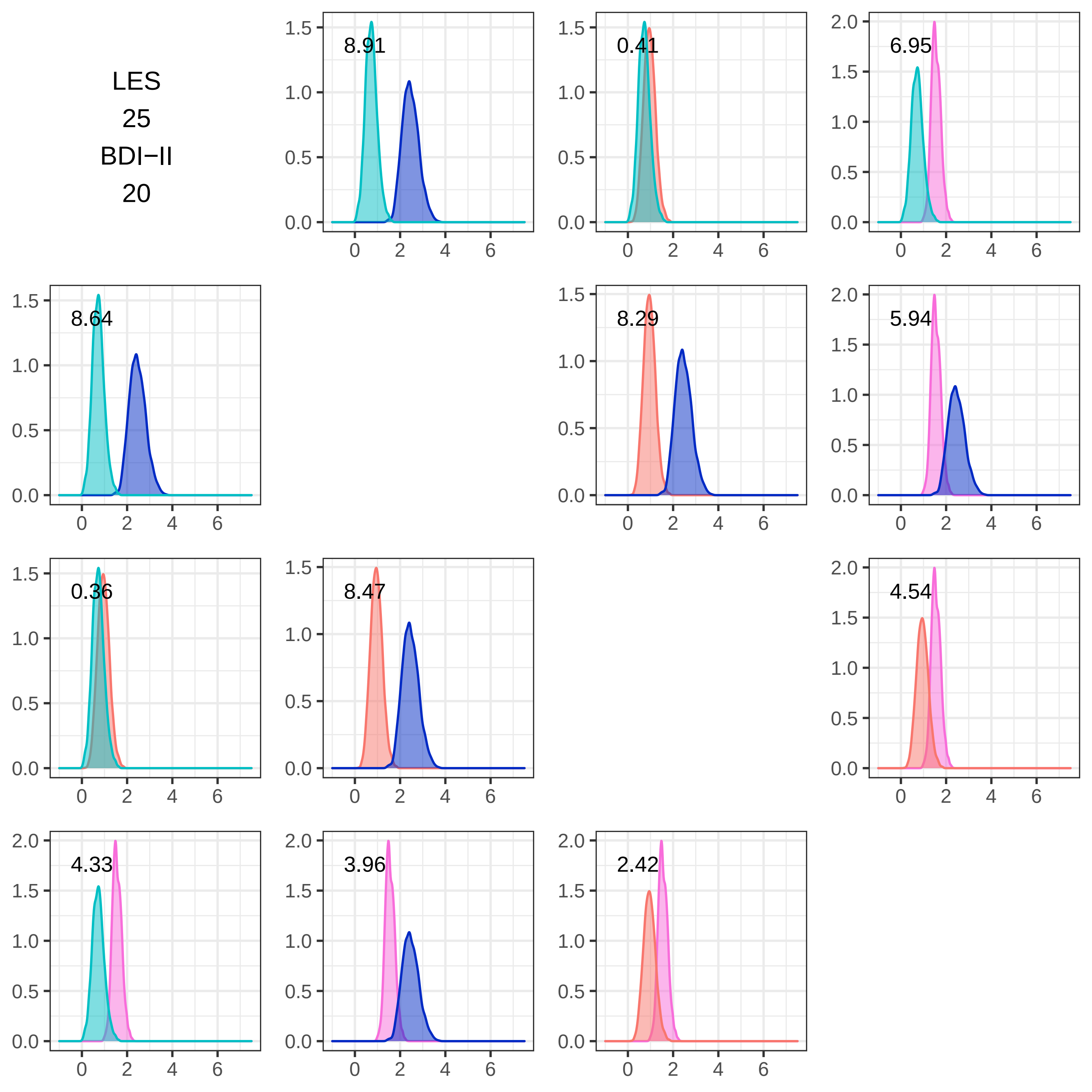

LES
25
BDI−II
20
8.91
0.41
6.95
8.64
8.29
5.94
0.36
8.47
4.54
4.33
3.96
2.42

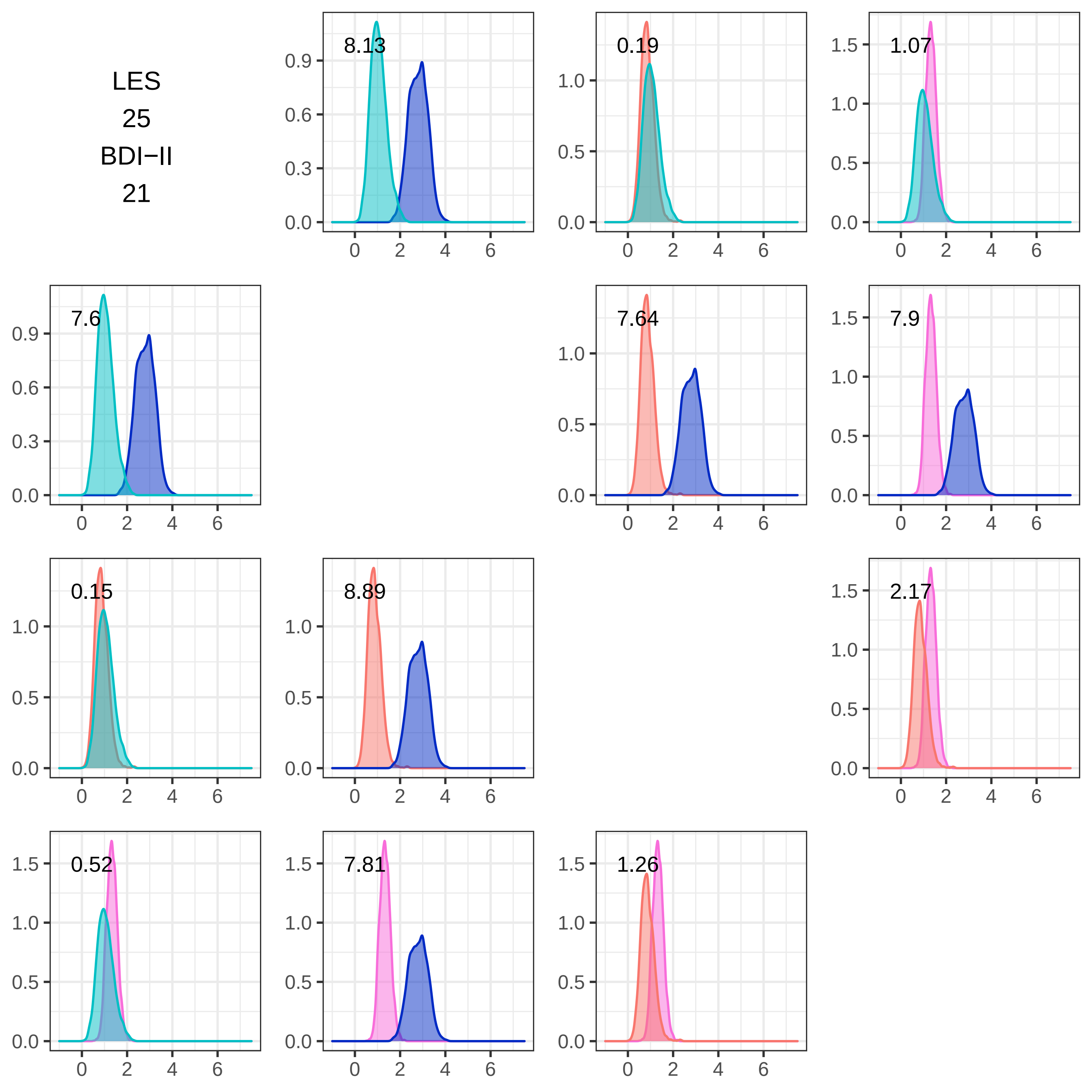

LES
25
BDI−II
21
8.13
0.19
1.07
7.6
7.64
7.9
0.15
8.89
2.17
0.52
7.81
1.26

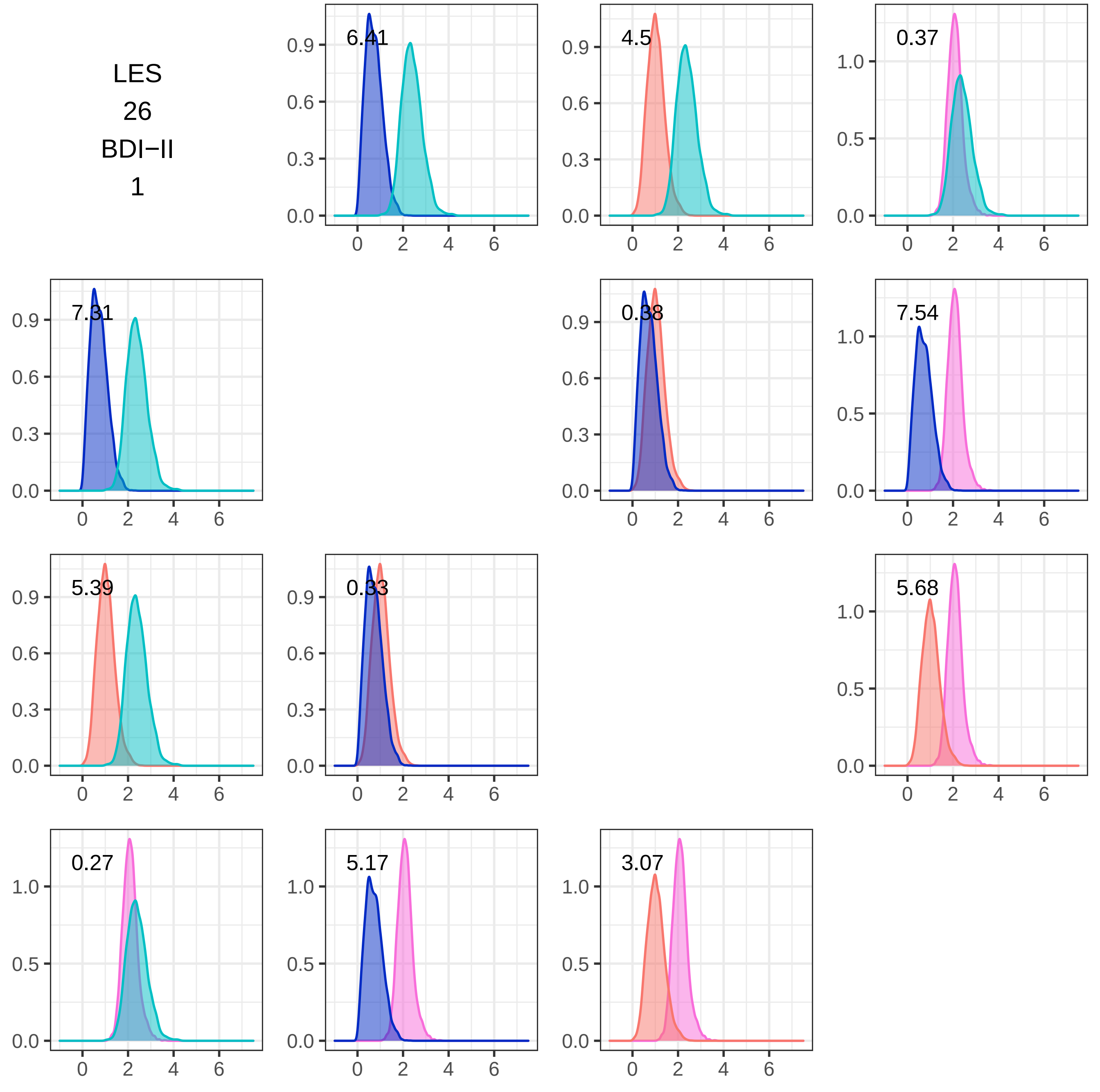
LES
26
BDI−II
1
6.41
4.5
0.37
7.31
0.38
7.54
5.39
0.33
5.68
0.27
5.17
3.07

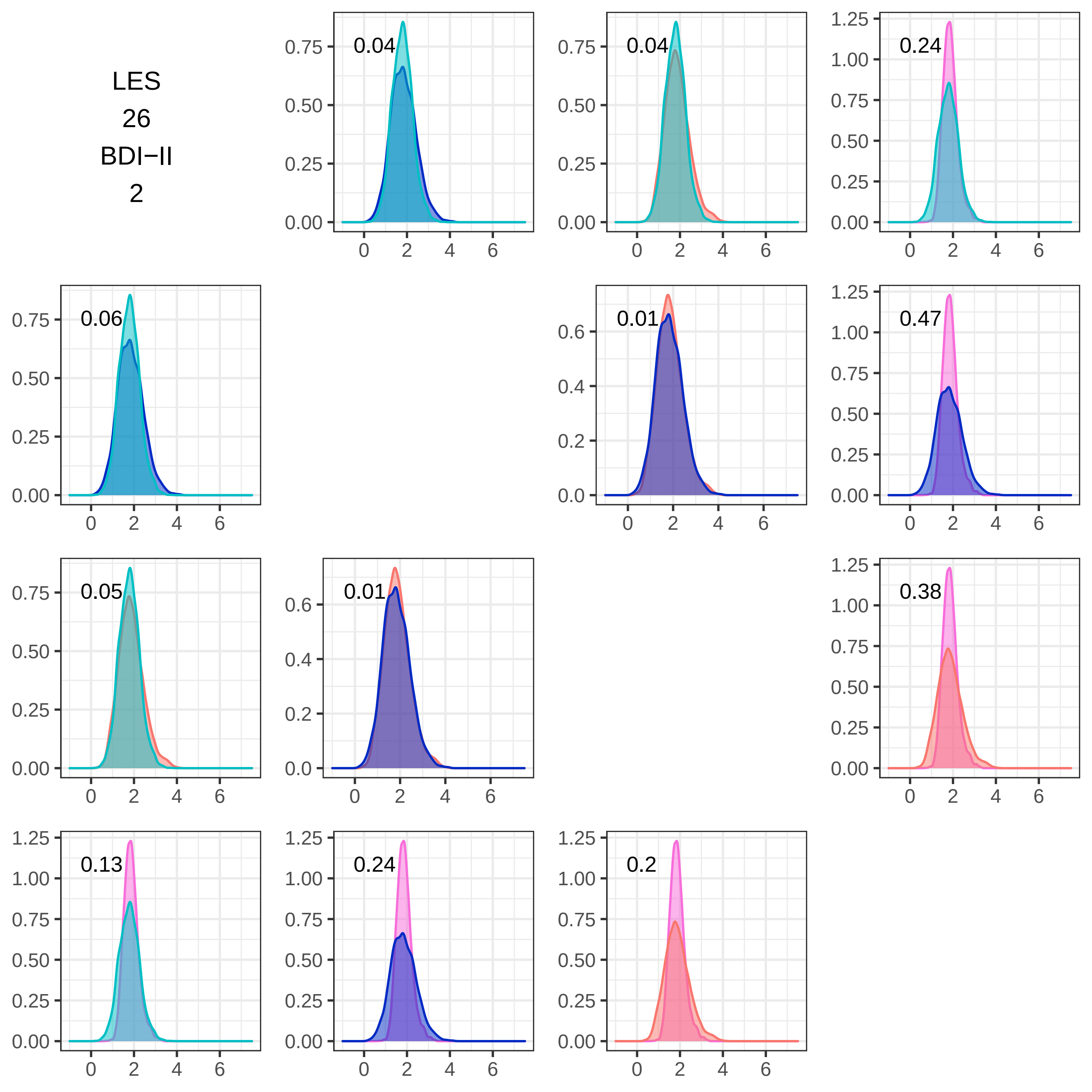

LES
26
BDI−II
2
0.04
0.04
0.24
0.06
0.01
0.47
0.05
0.01
0.38
0.13
0.24
0.2

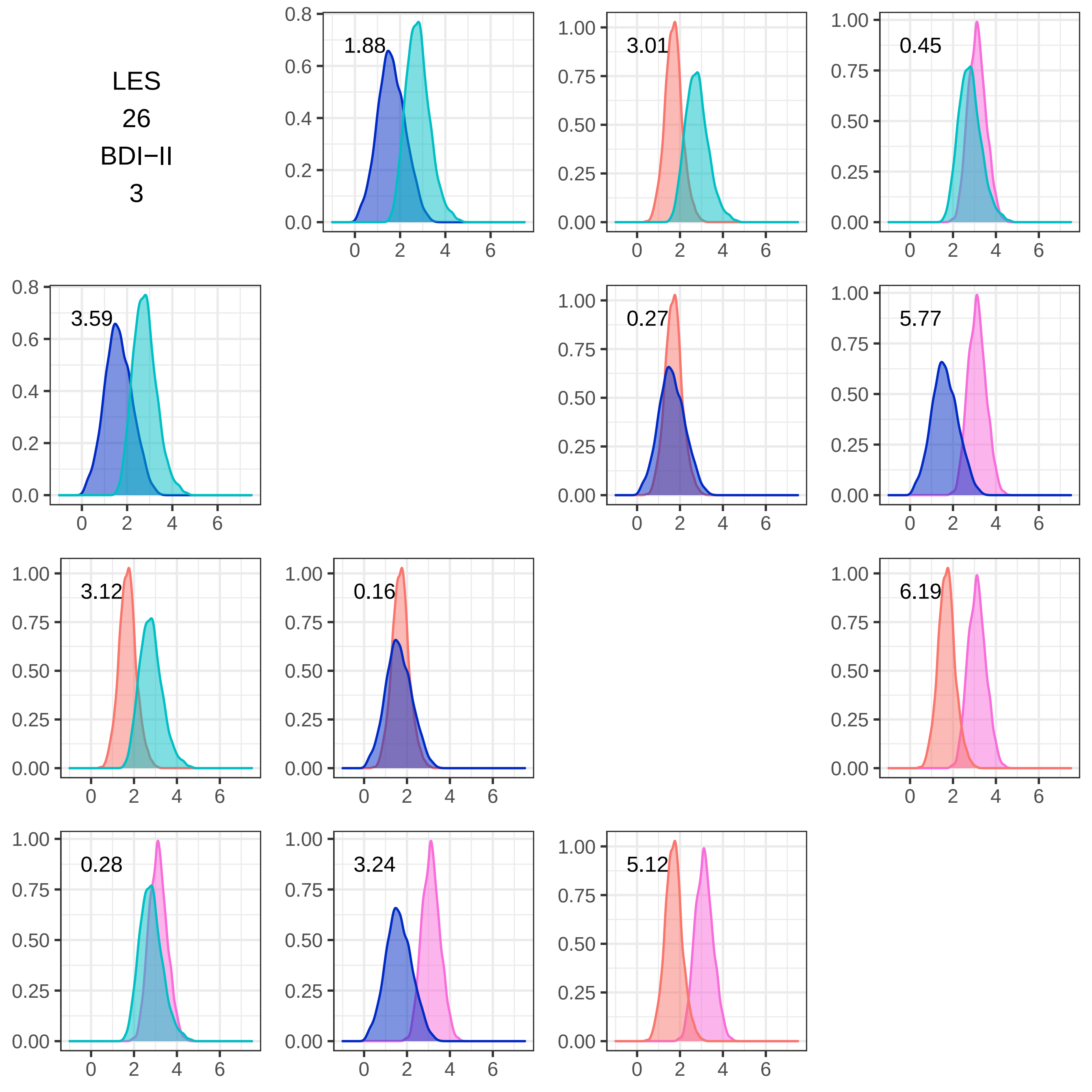
LES
26
BDI−II
3
1.88
3.01
0.45
3.59
0.27
5.77
3.12
0.16
6.19
0.28
3.24
5.12

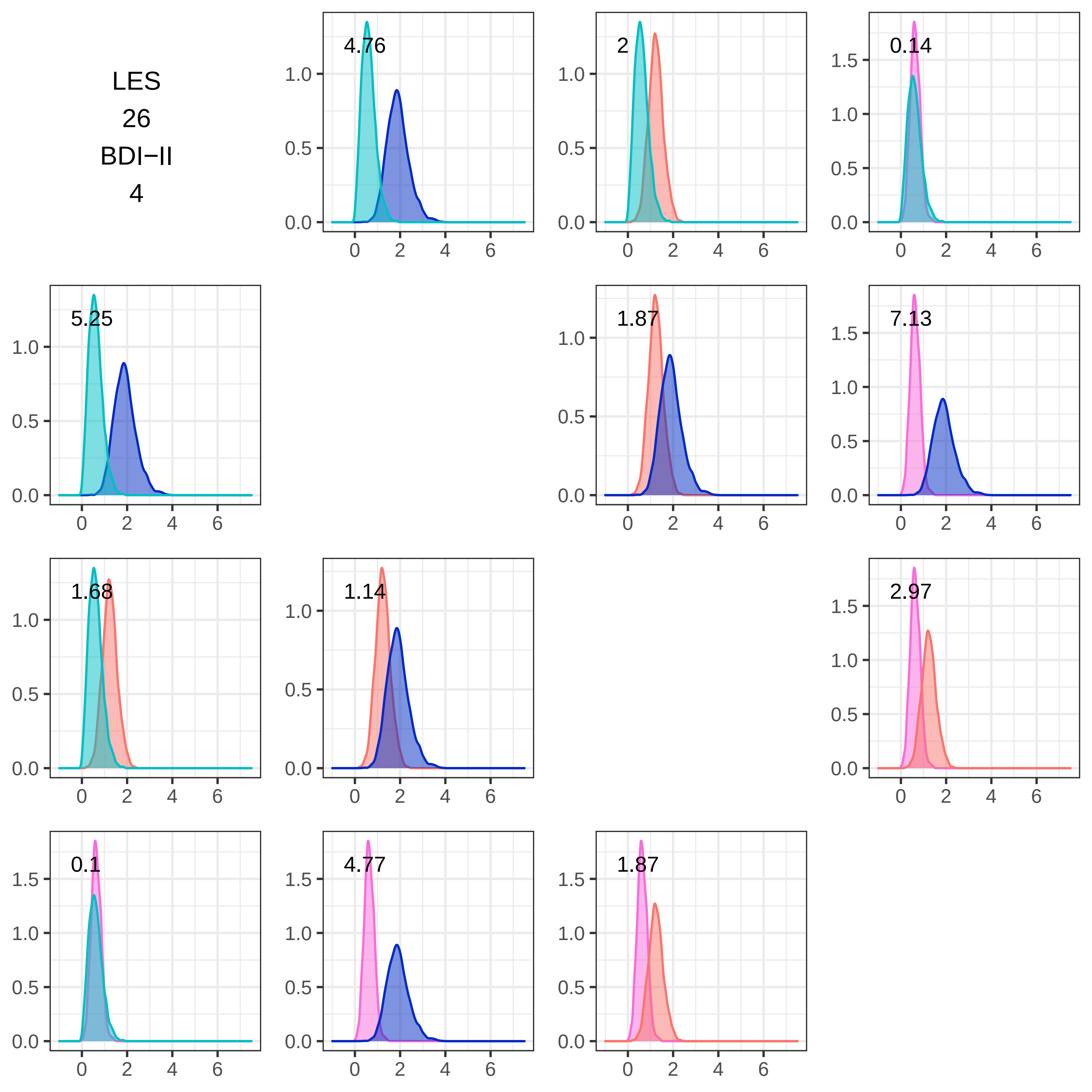
LES
26
BDI−II
4
4.76
2
0.14
5.25
1.87
7.13
1.68
1.14
2.97
0.1
4.77
1.87

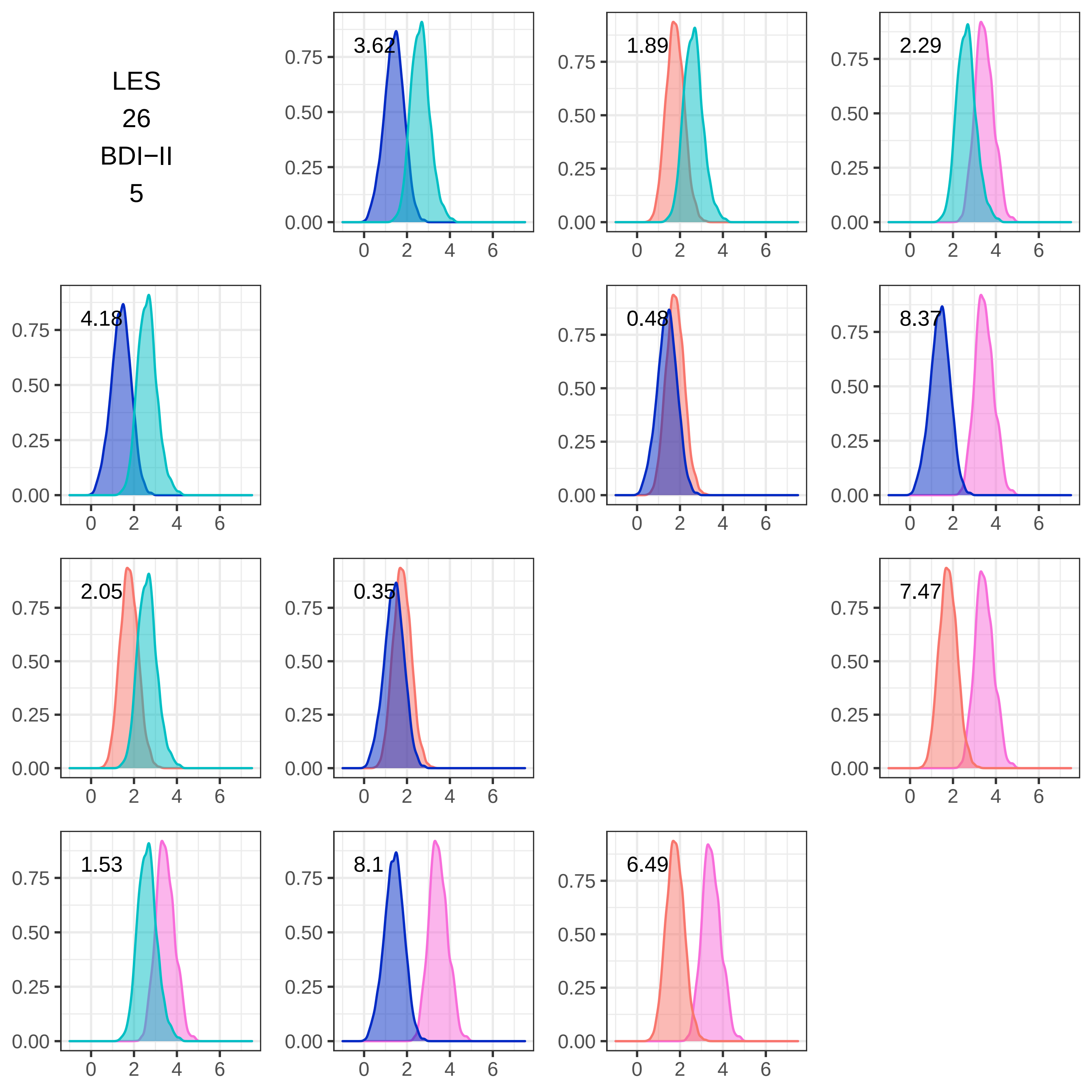
LES
26
BDI−II
5
3.62
1.89
2.29
4.18
0.48
8.37
2.05
0.35
7.47
1.53
8.1
6.49

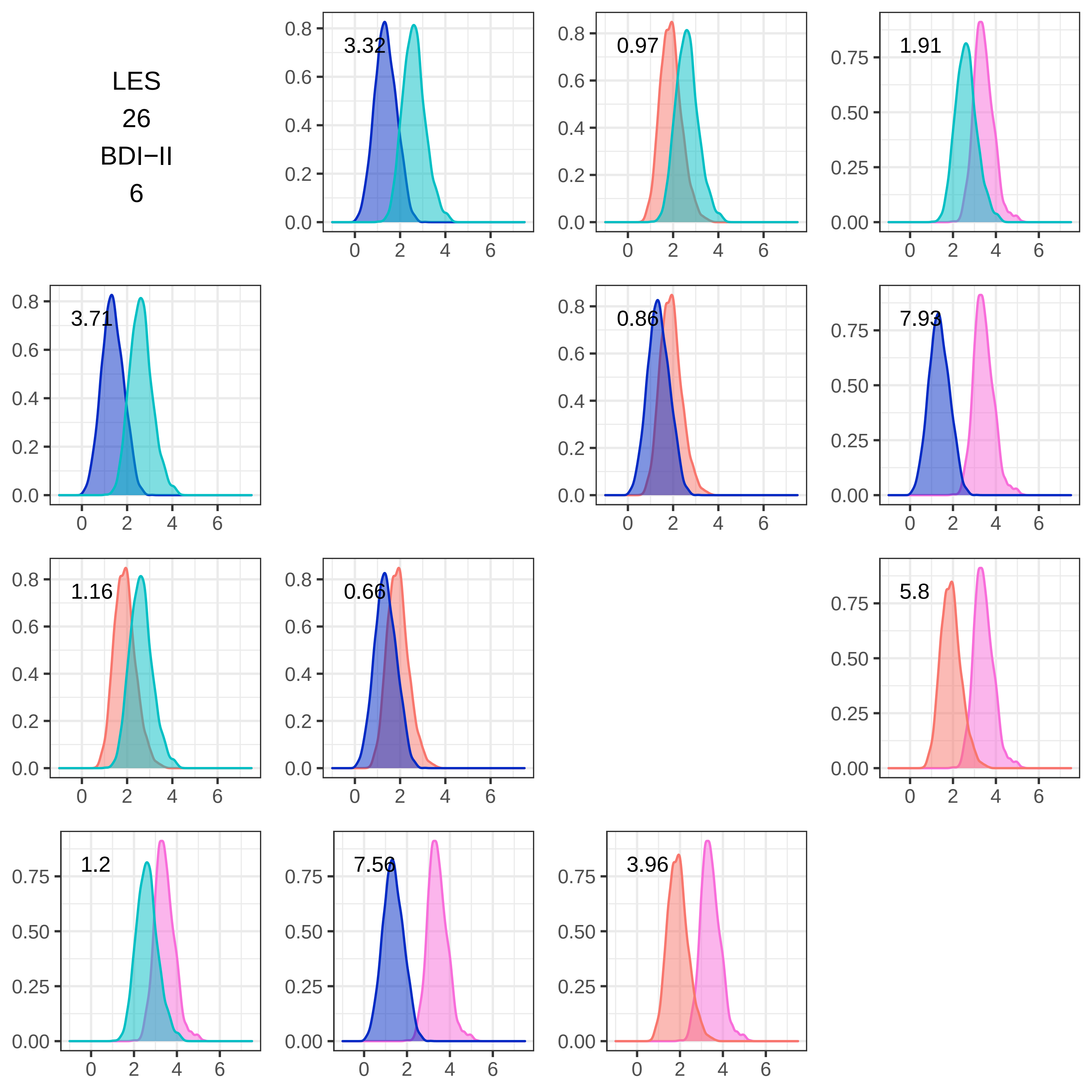

LES
26
BDI−II
6
3.32
0.97
1.91
3.71
0.86
7.93
1.16
0.66
5.8
1.2
7.56
3.96

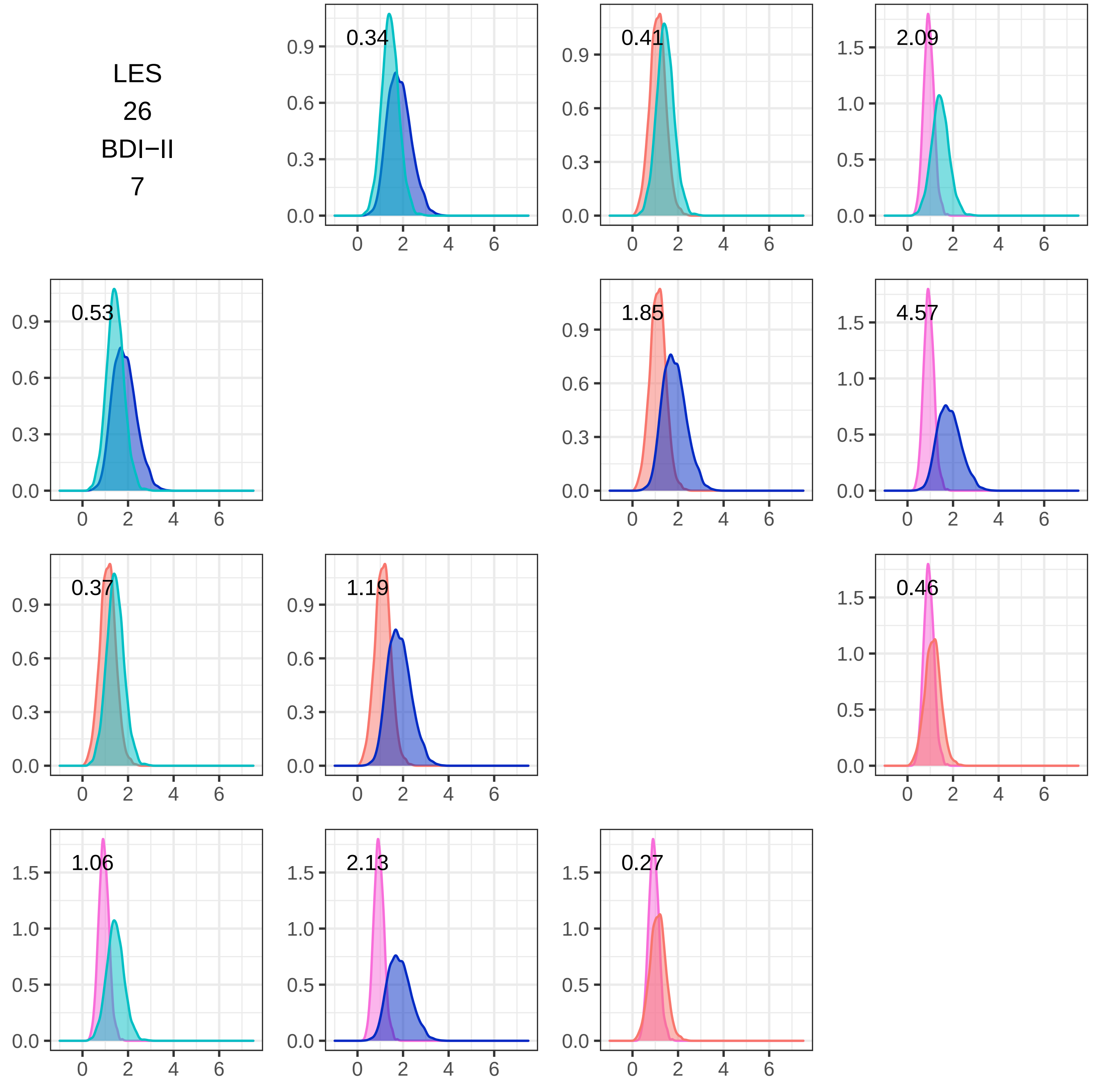

LES
26
BDI−II
7
0.34
0.41
2.09
0.53
1.85
4.57
0.37
1.19
0.46
1.06
2.13
0.27

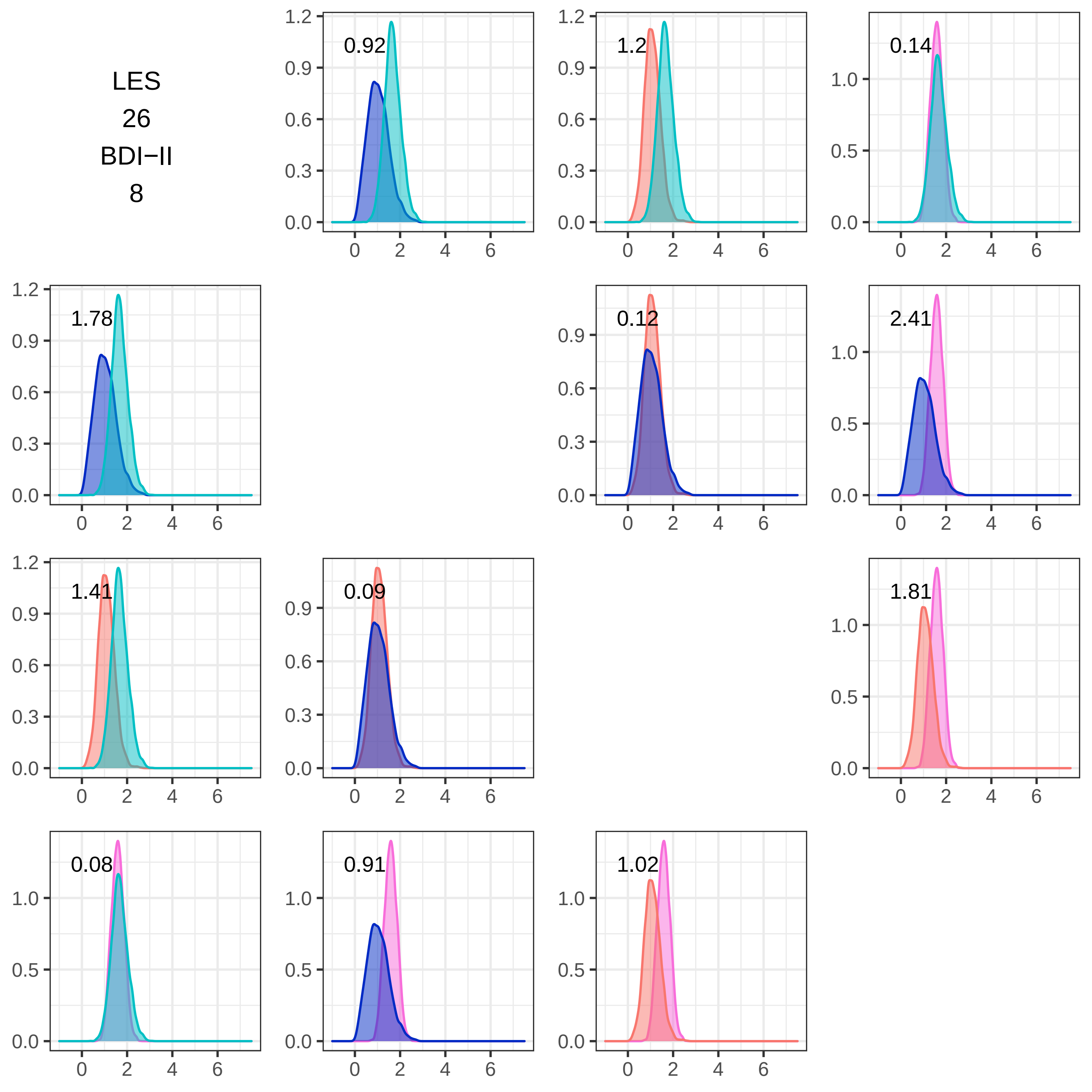

LES
26
BDI−II
8
0.92
1.2
0.14
1.78
0.12
2.41
1.41
0.09
1.81
0.08
0.91
1.02

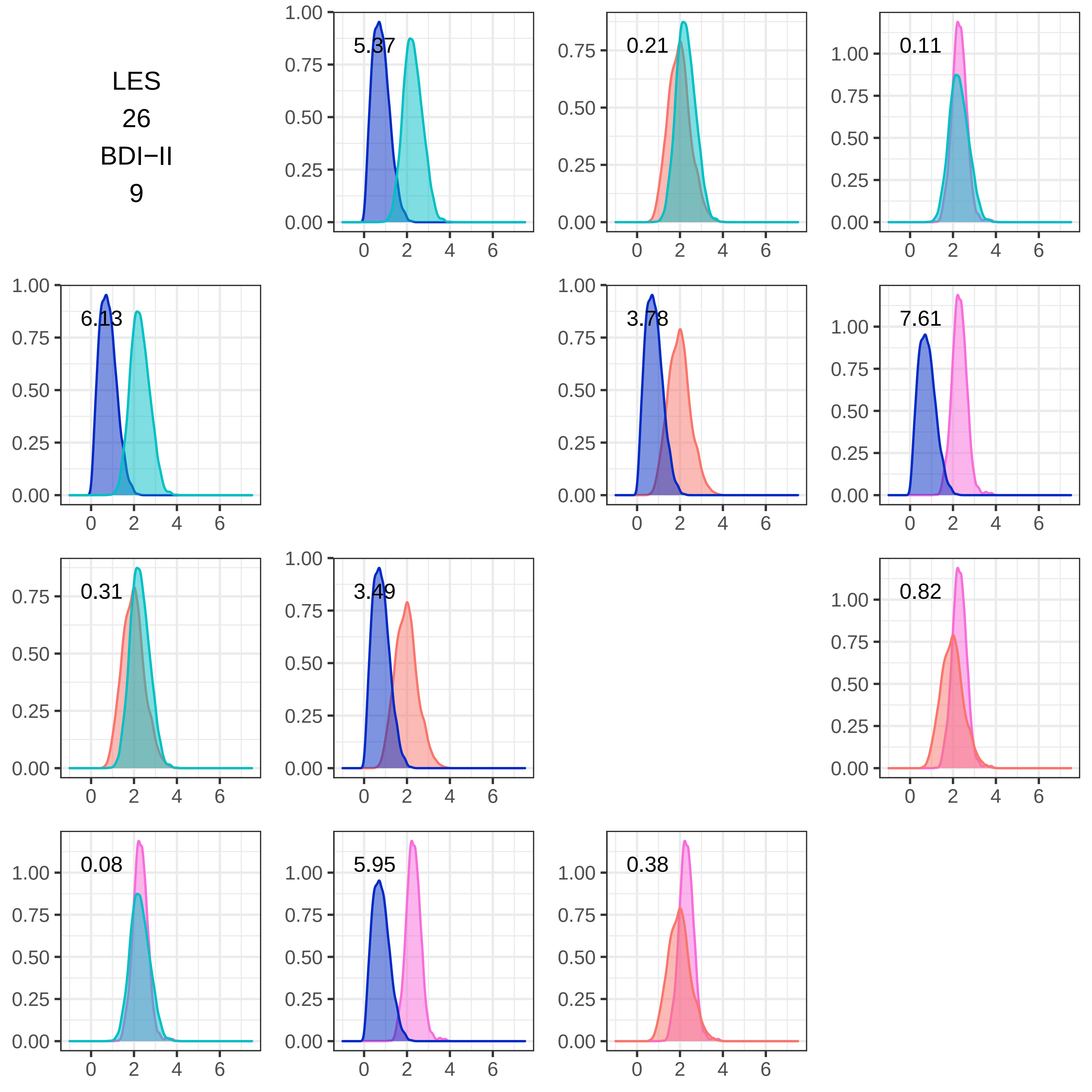
LES
26
BDI−II
9
5.37
0.21
0.11
6.13
3.78
7.61
0.31
3.49
0.82
0.08
5.95
0.38

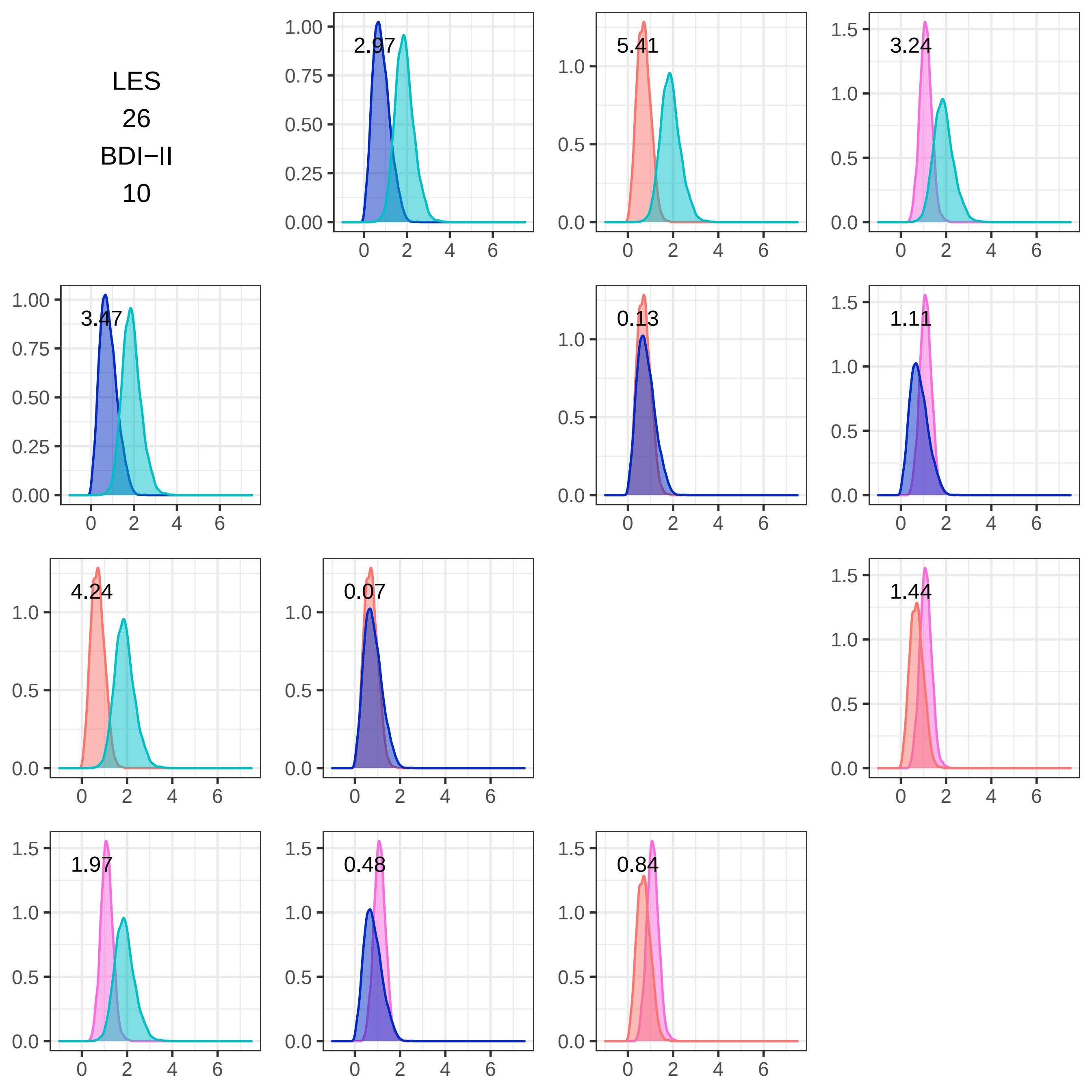
LES
26
BDI−II
10
2.97
5.41
3.24
3.47
0.13
1.11
4.24
0.07
1.44
1.97
0.48
0.84

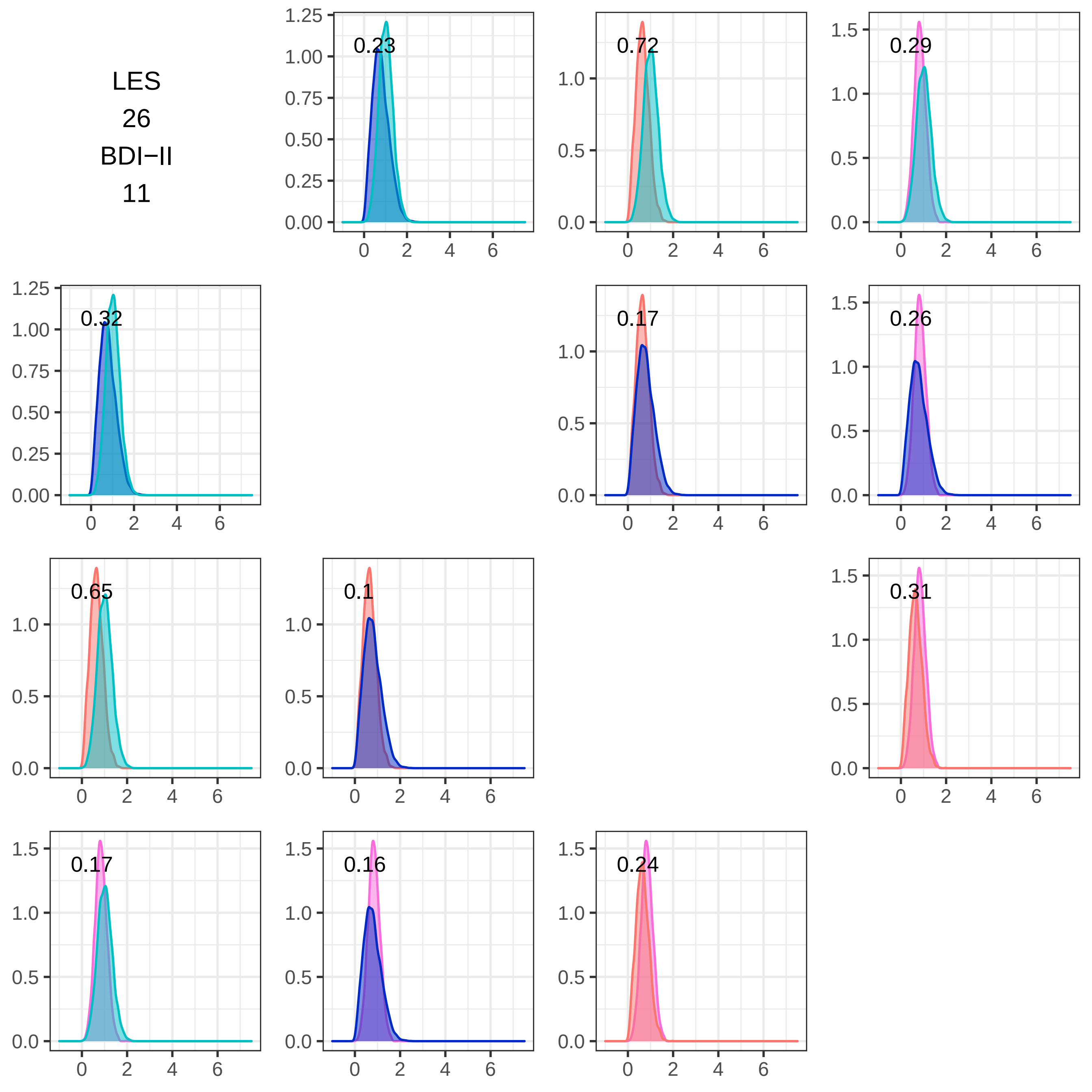
LES
26
BDI−II
11
0.23
0.72
0.29
0.32
0.17
0.26
0.65
0.1
0.31
0.17
0.16
0.24

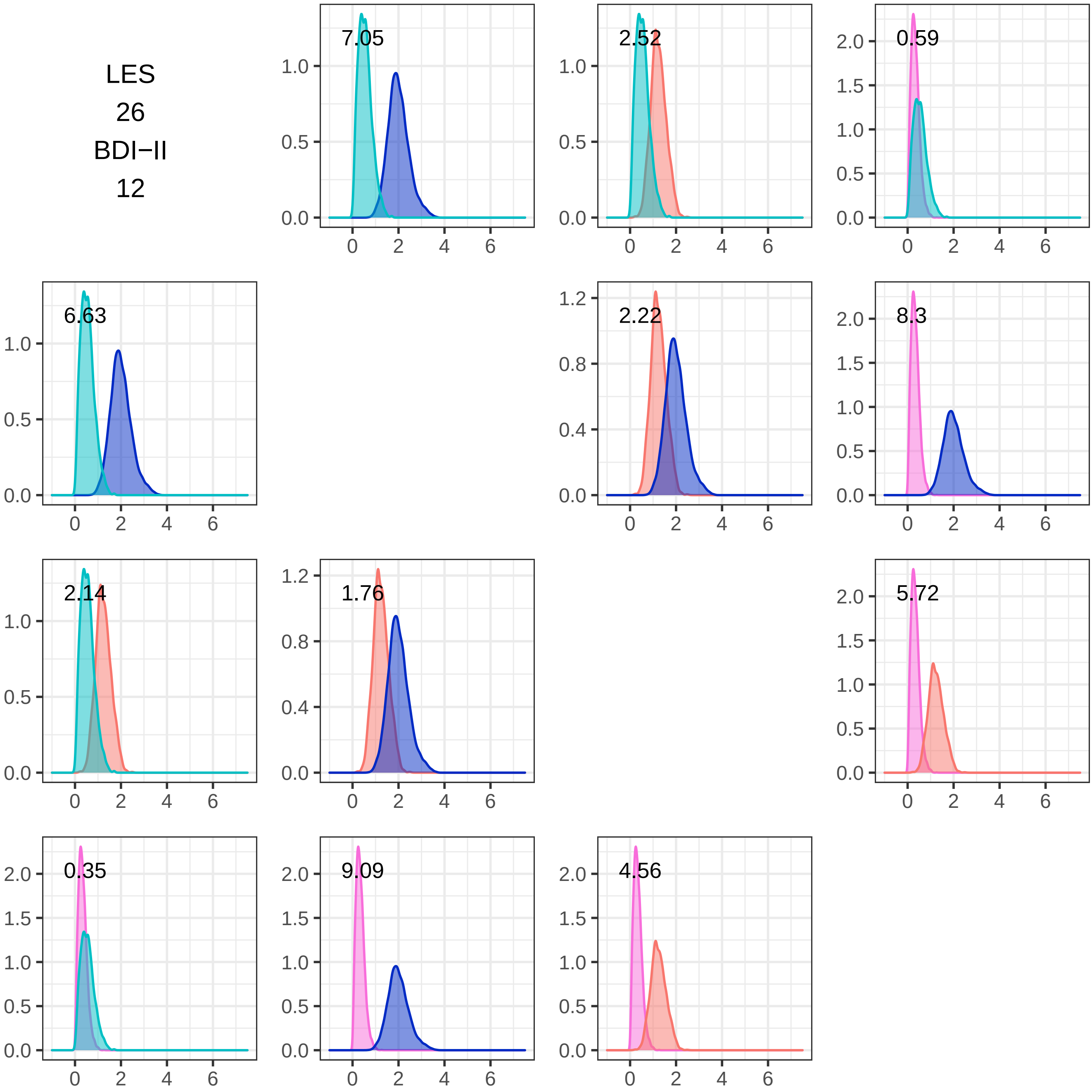
LES
26
BDI−II
12
7.05
2.52
0.59
6.63
2.22
8.3
2.14
1.76
5.72
0.35
9.09
4.56

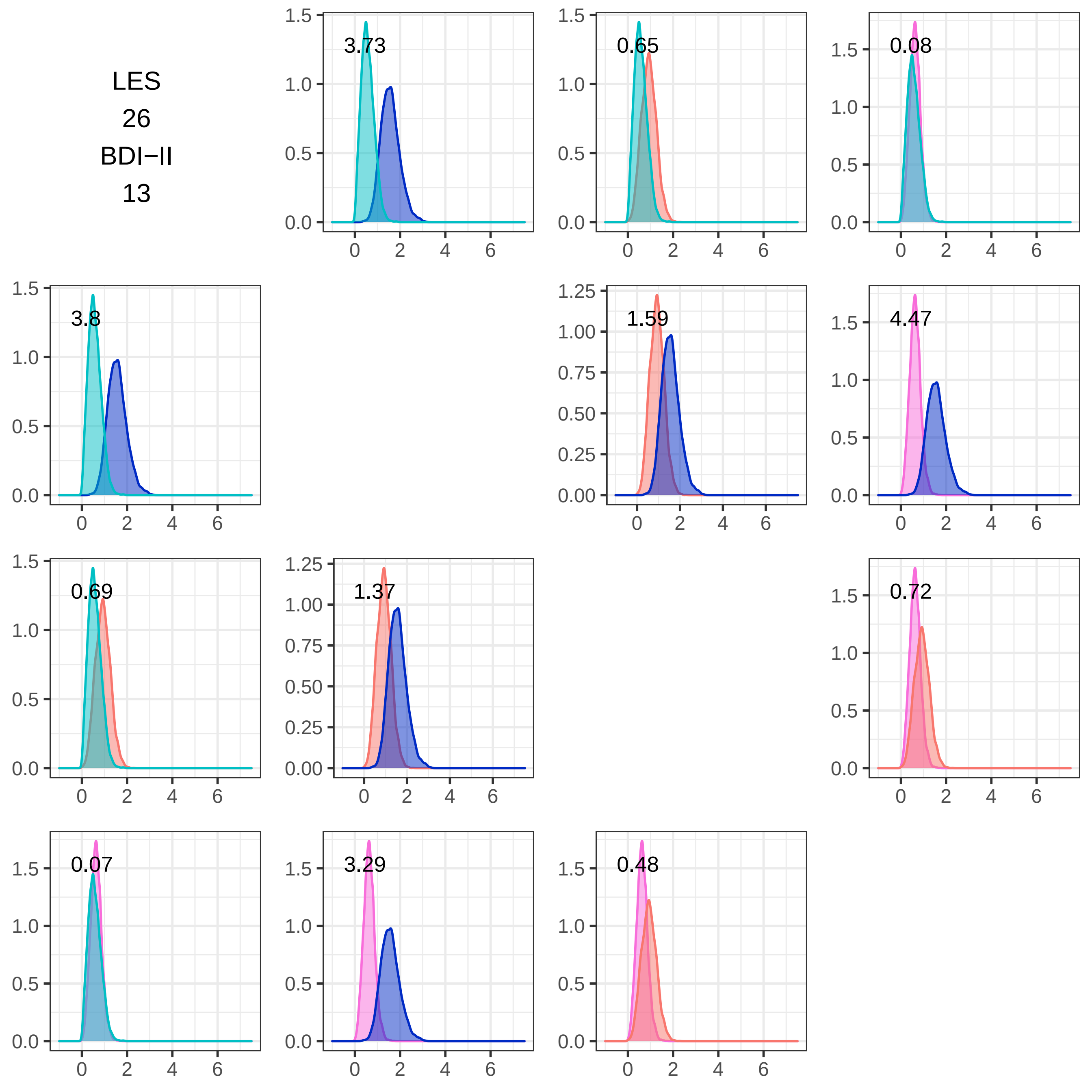

LES
26
BDI−II
13
3.73
0.65
0.08
3.8
1.59
4.47
0.69
1.37
0.72
0.07
3.29
0.48

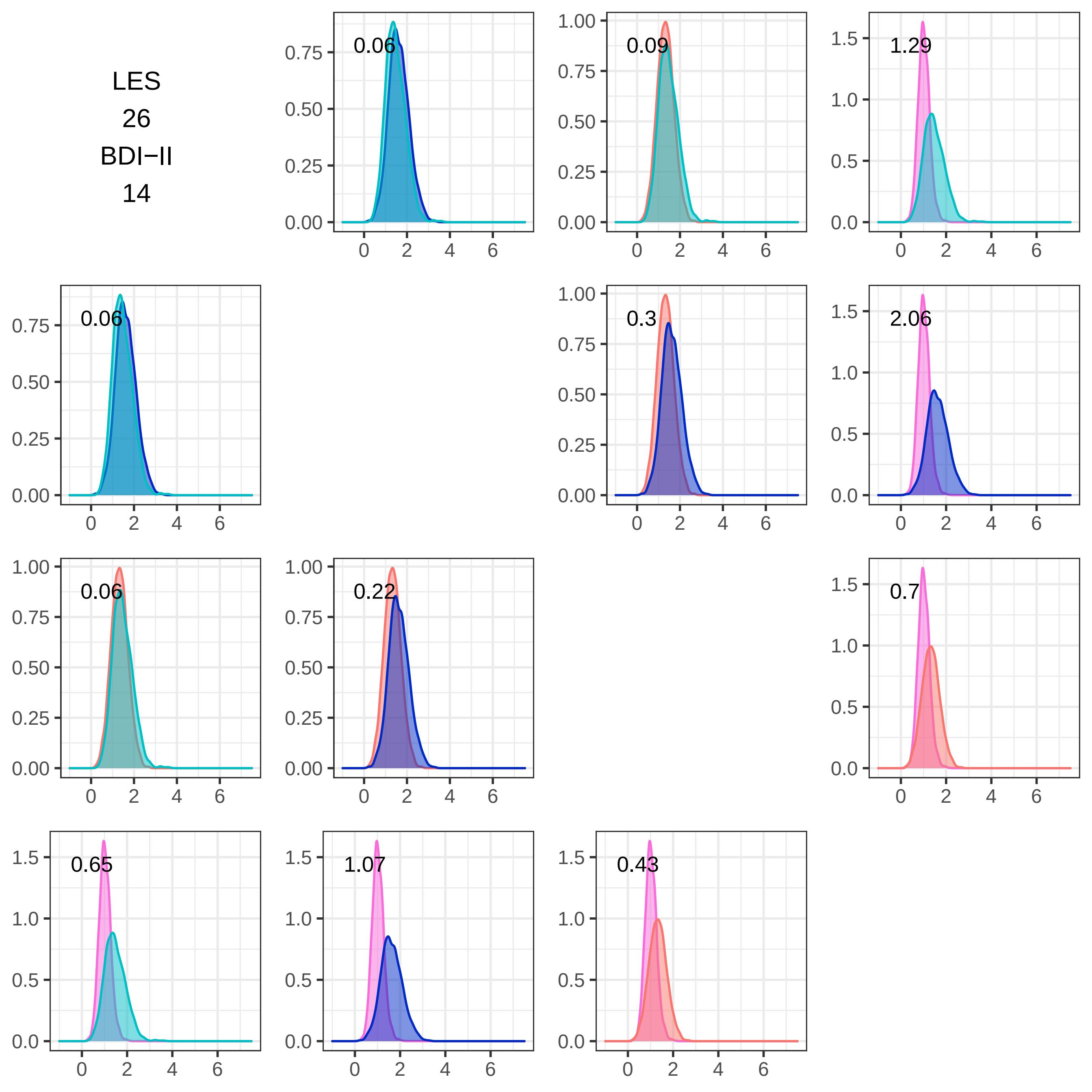
LES
26
BDI−II
14
0.06
0.09
1.29
0.06
0.3
2.06
0.06
0.22
0.7
0.65
1.07
0.43

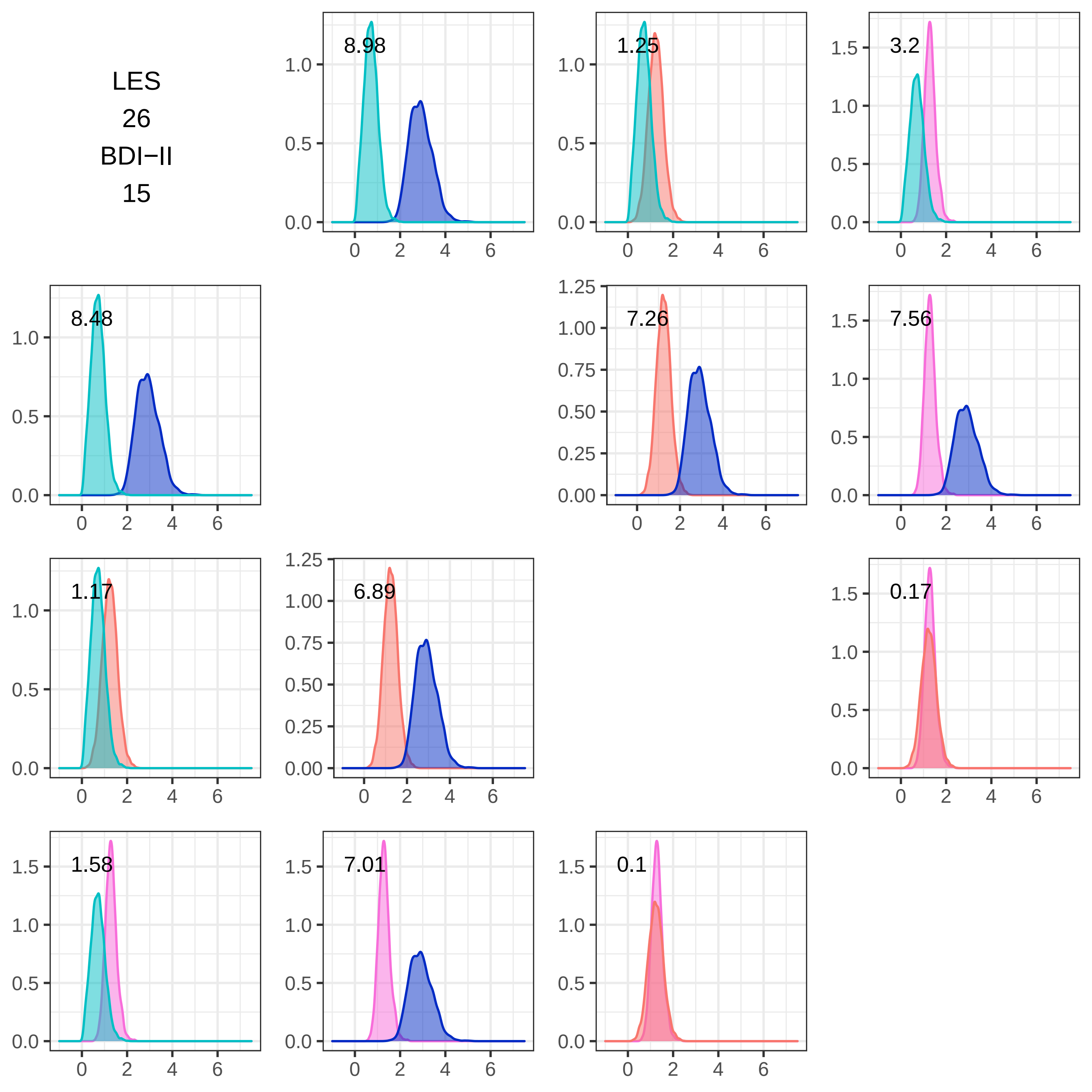
LES
26
BDI−II
15
8.98
1.25
3.2
8.48
7.26
7.56
1.17
6.89
0.17
1.58
7.01
0.1

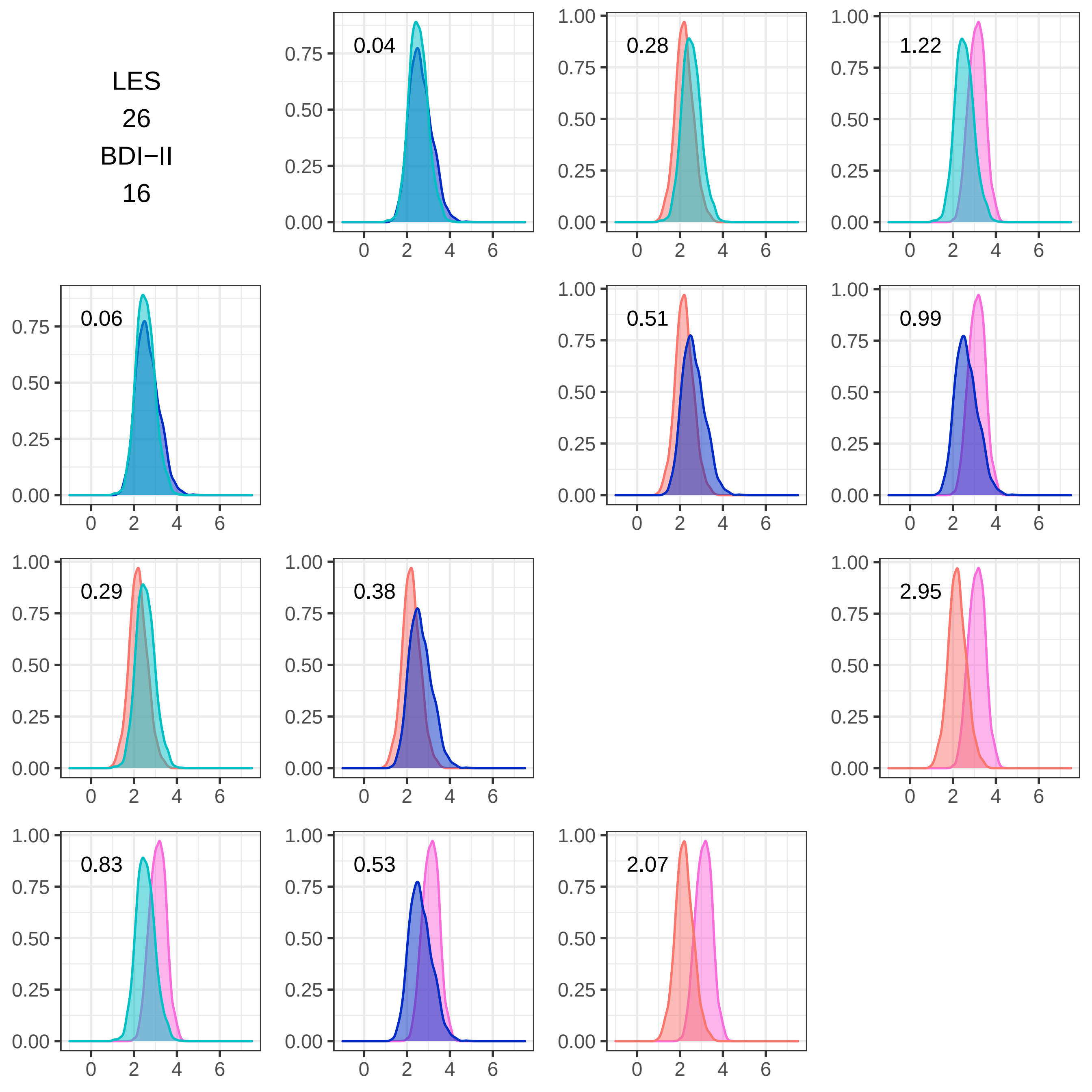
LES
26
BDI−II
16
0.04
0.28
1.22
0.06
0.51
0.99
0.29
0.38
2.95
0.83
0.53
2.07

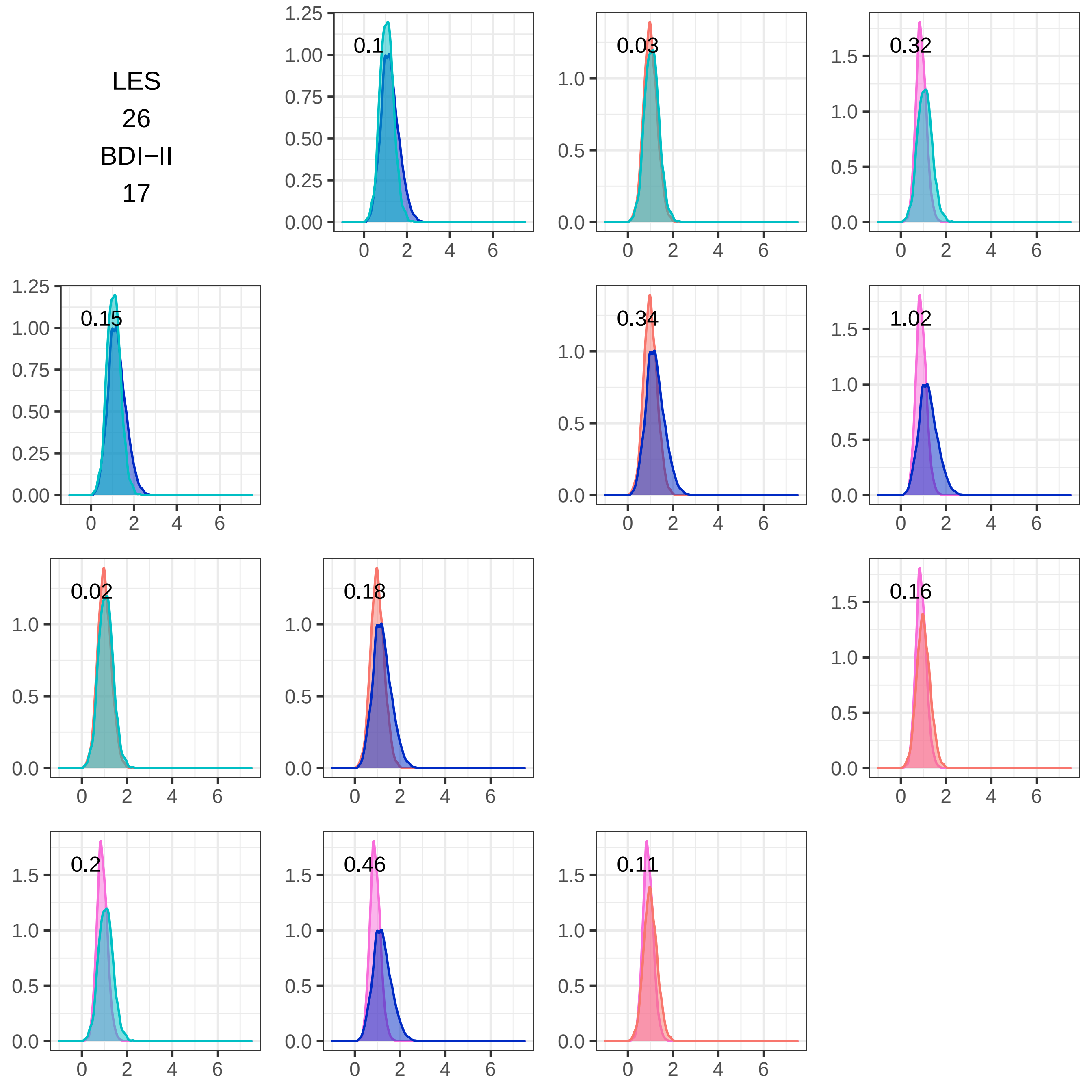
LES
26
BDI−II
17
0.1
0.03
0.32
0.15
0.34
1.02
0.02
0.18
0.16
0.2
0.46
0.11

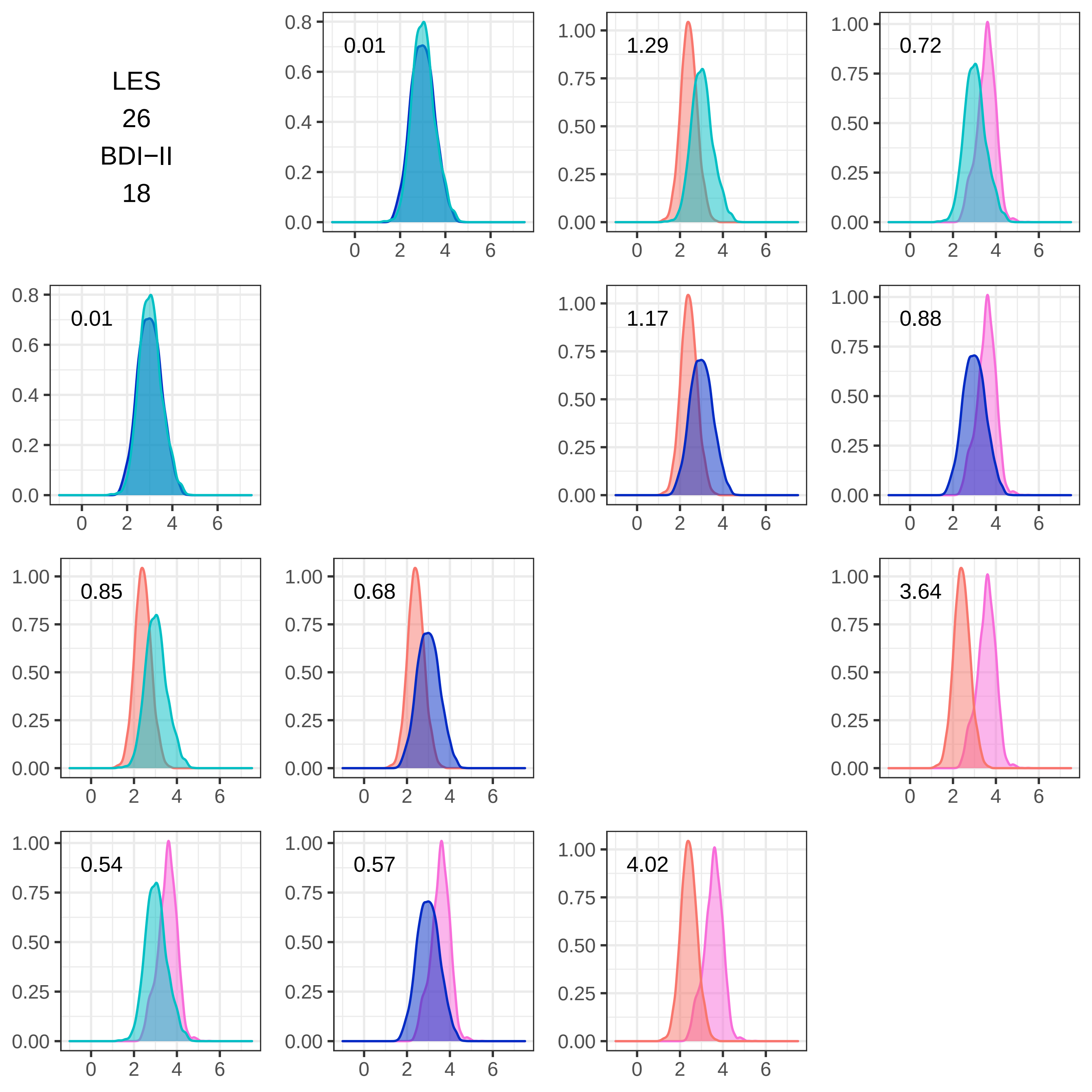

LES
26
BDI−II
18
0.01
1.29
0.72
0.01
1.17
0.88
0.85
0.68
3.64
0.54
0.57
4.02

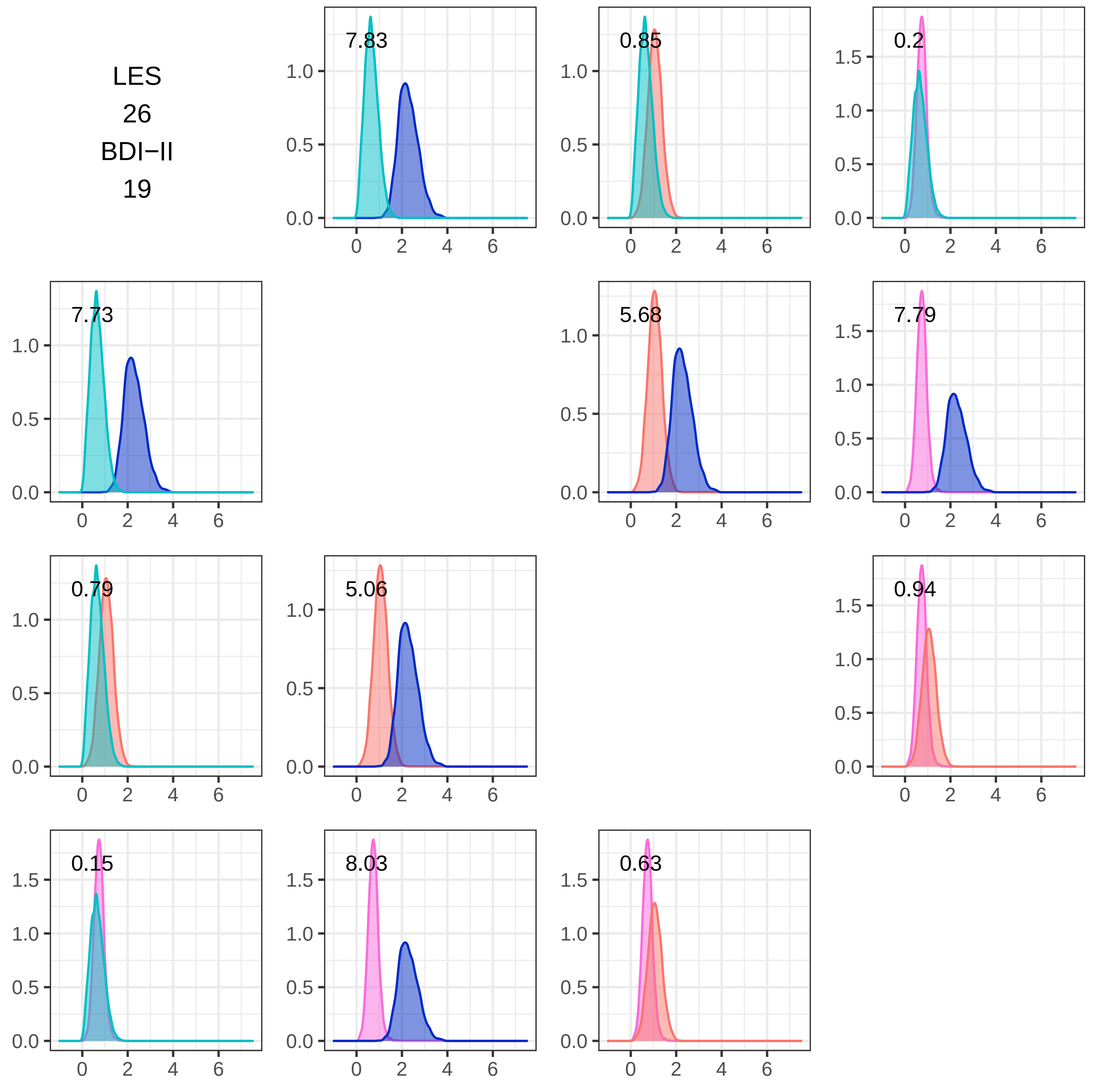

LES
26
BDI−II
19
7.83
0.85
0.2
7.73
5.68
7.79
0.79
5.06
0.94
0.15
8.03
0.63

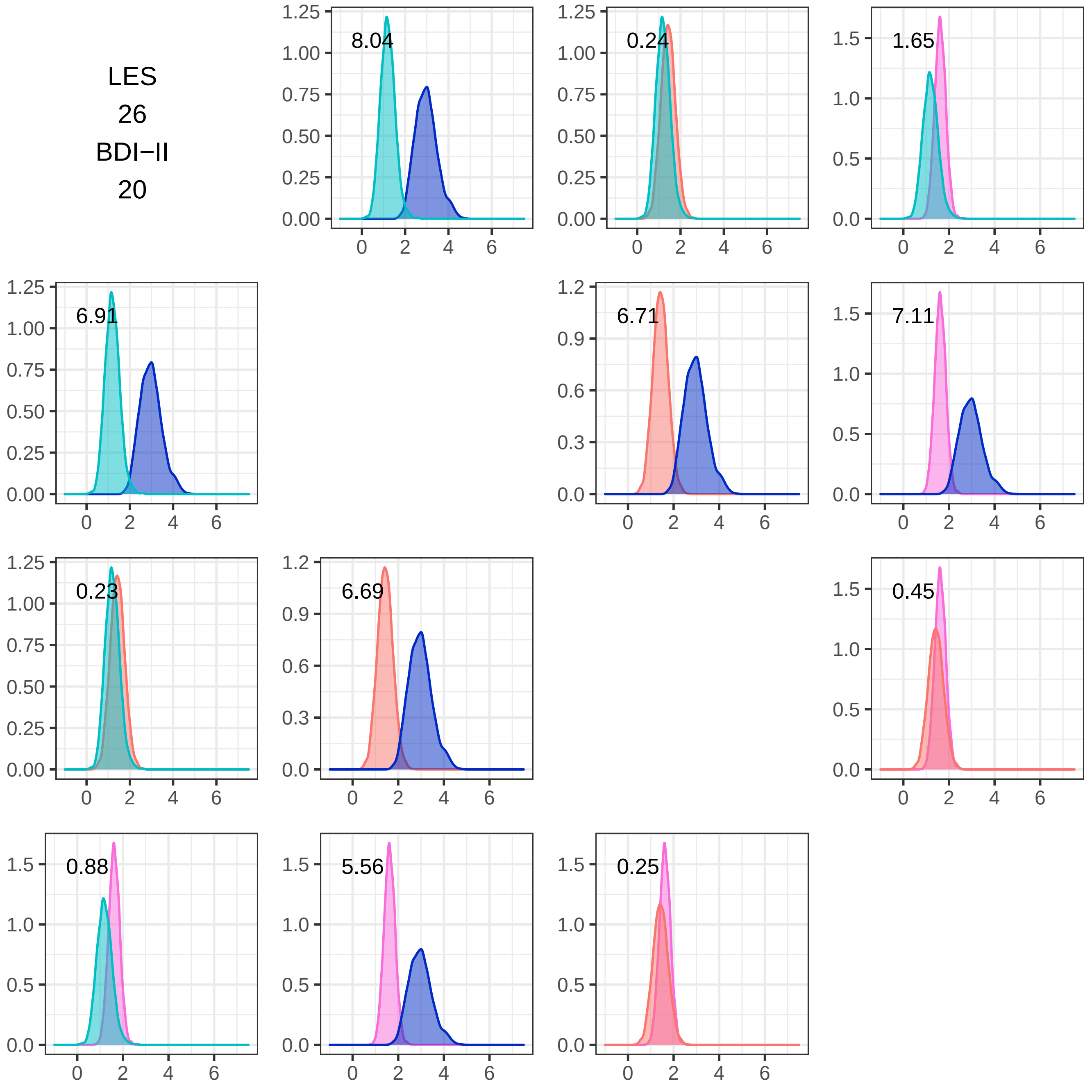
LES
26
BDI−II
20
8.04
0.24
1.65
6.91
6.71
7.11
0.23
6.69
0.45
0.88
5.56
0.25

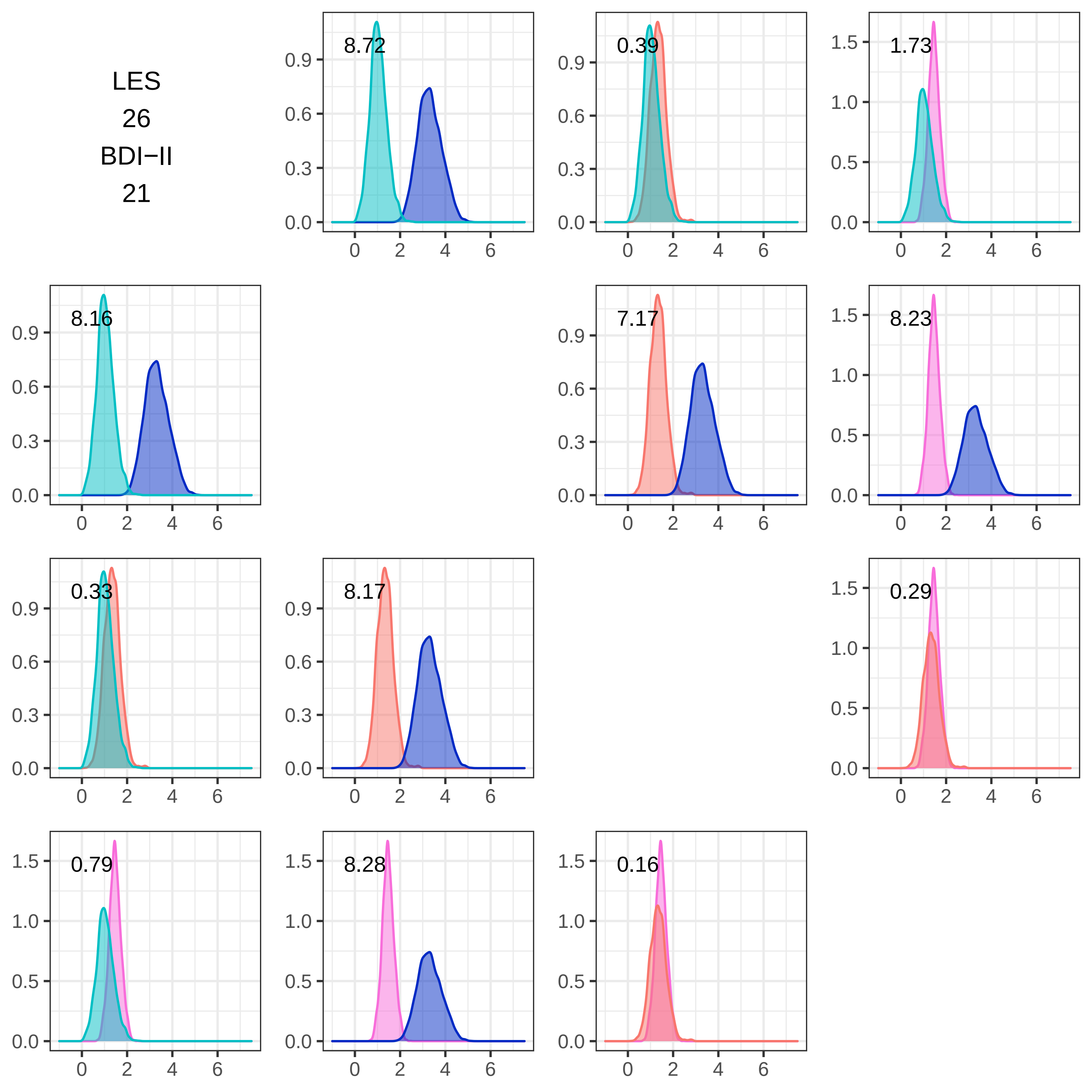

LES
26
BDI−II
21
8.72
0.39
1.73
8.16
7.17
8.23
0.33
8.17
0.29
0.79
8.28
0.16

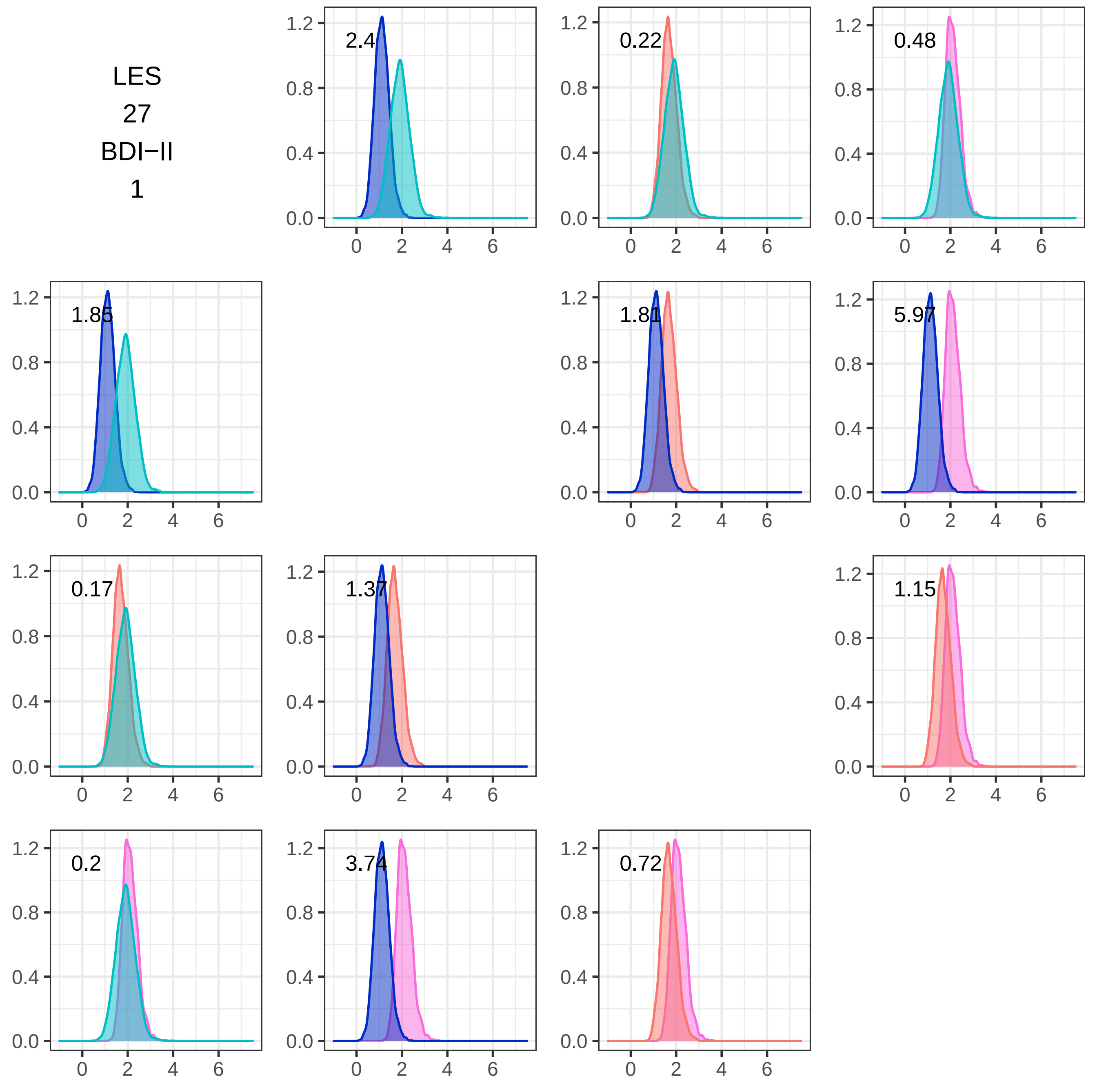

LES
27
BDI−II
1
2.4
0.22
0.48
1.85
1.81
5.97
0.17
1.37
1.15
0.2
3.74
0.72

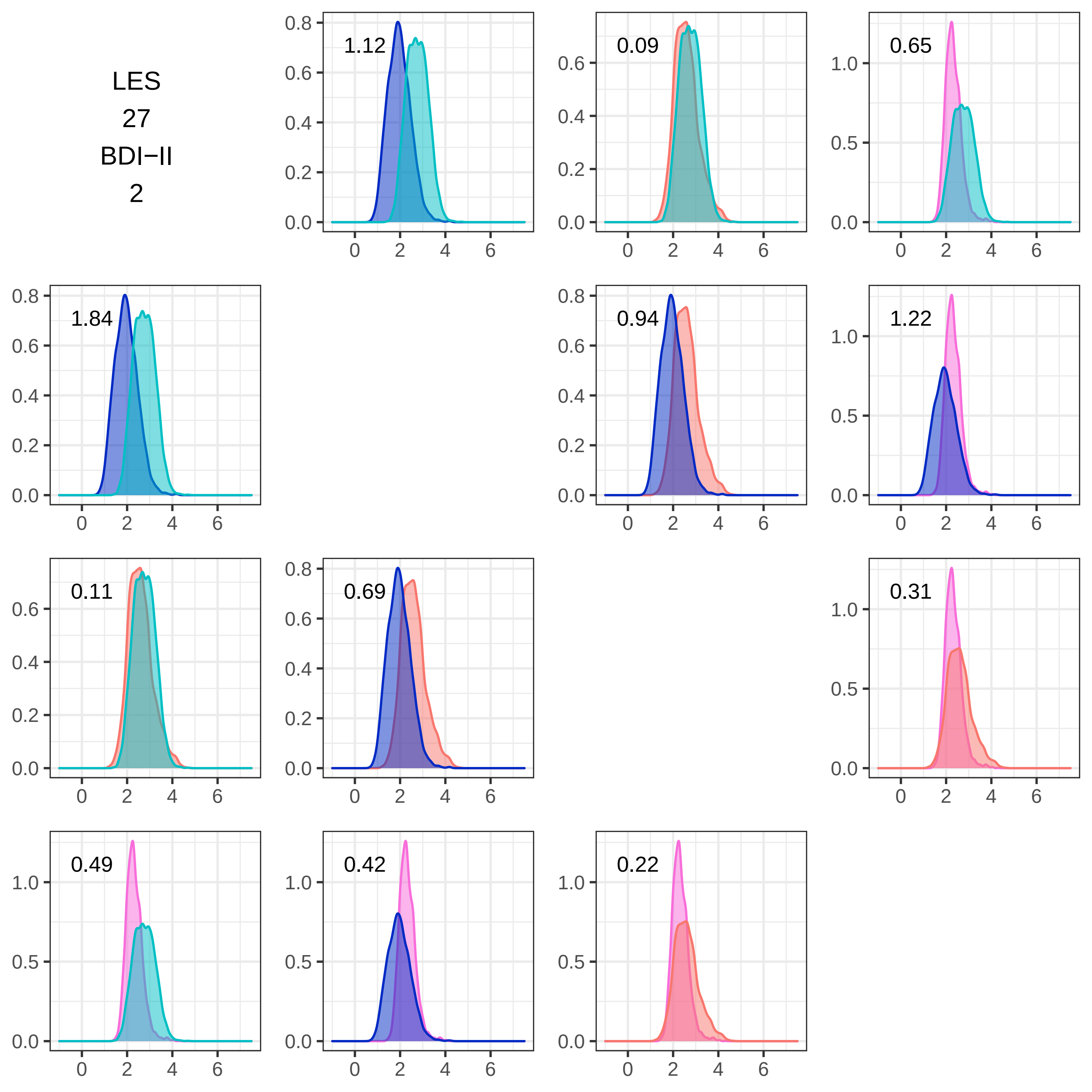
LES
27
BDI−II
2
1.12
0.09
0.65
1.84
0.94
1.22
0.11
0.69
0.31
0.49
0.42
0.22

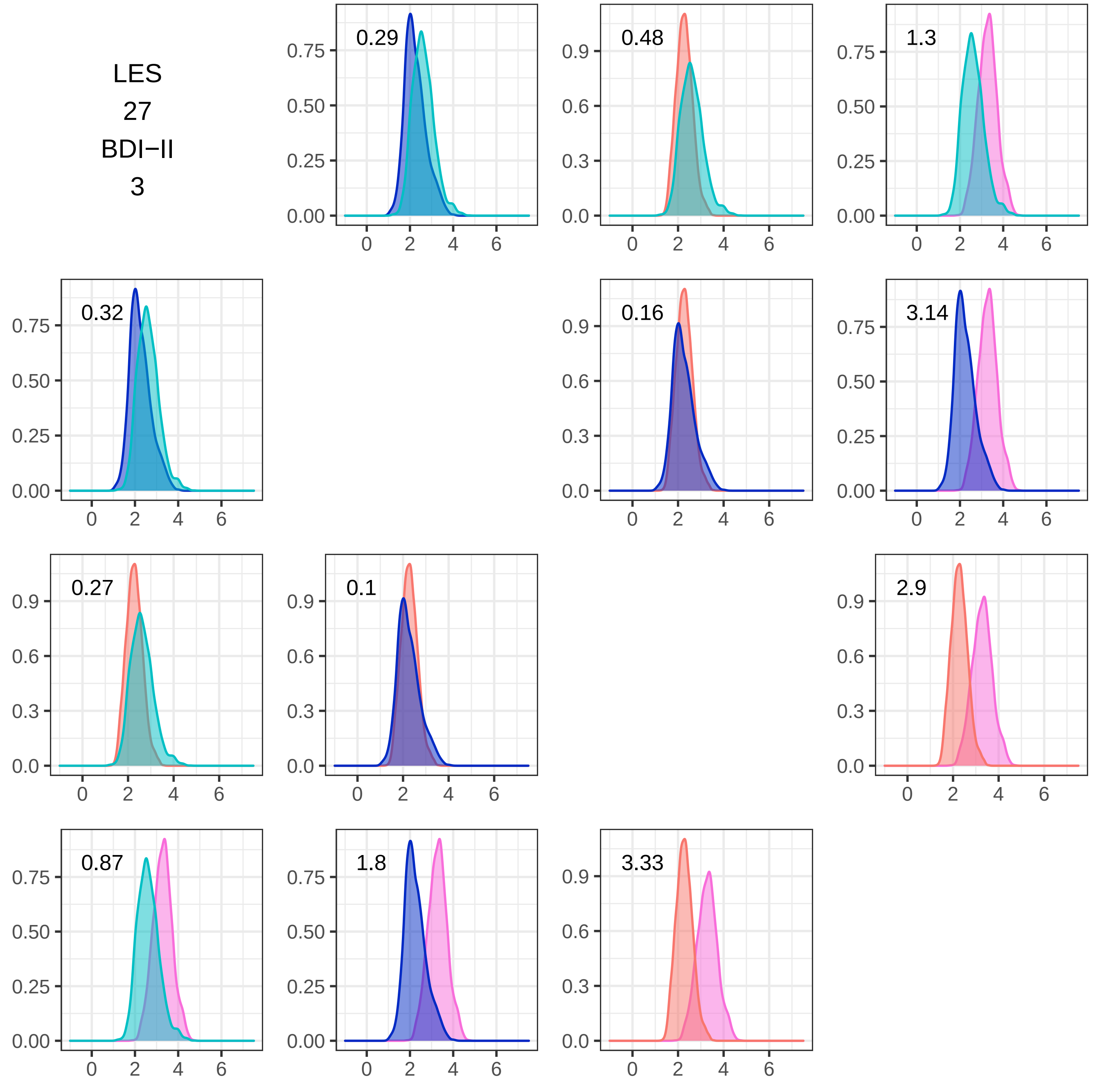
LES
27
BDI−II
3
0.29
0.48
1.3
0.32
0.16
3.14
0.27
0.1
2.9
0.87
1.8
3.33

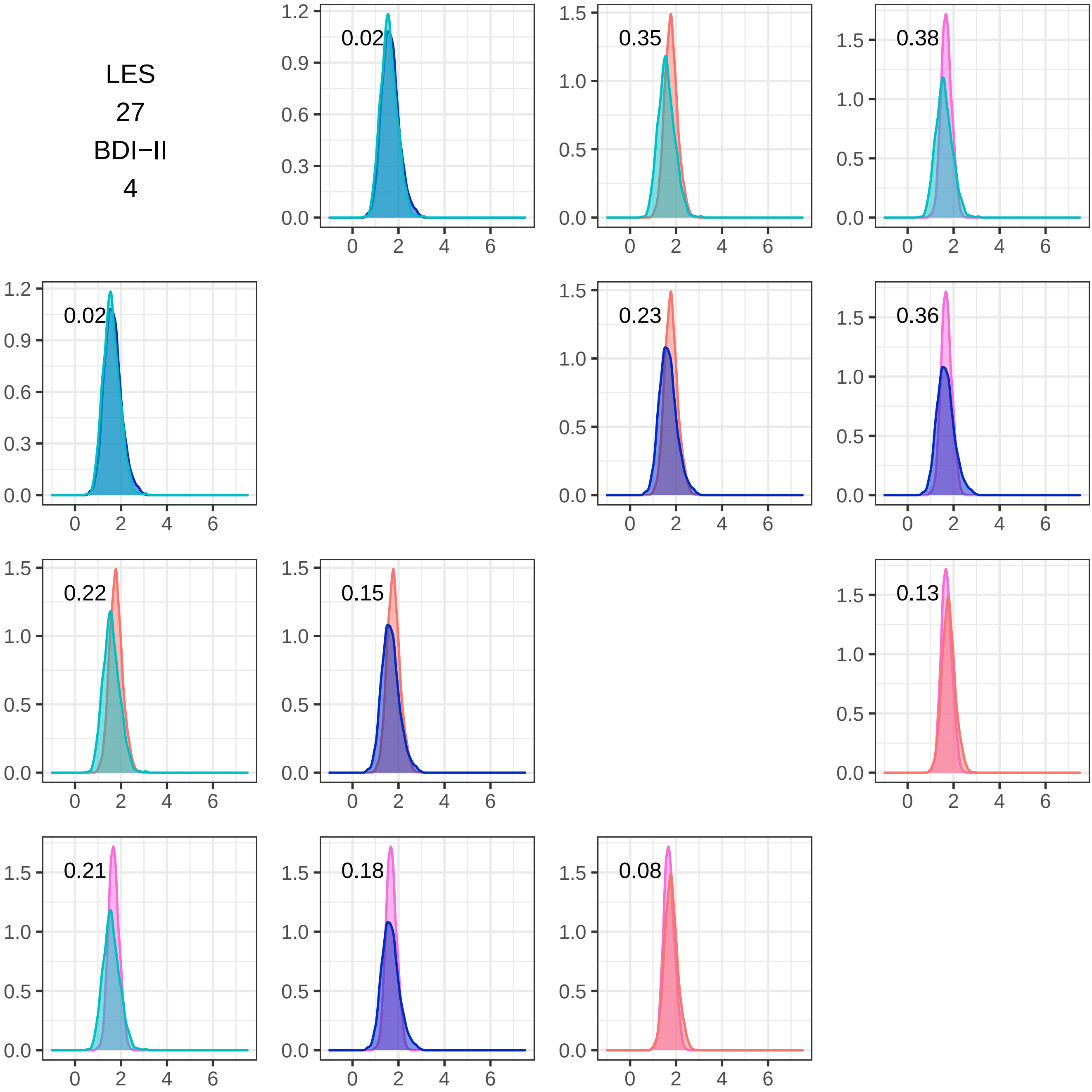

LES
27
BDI−II
4
0.02
0.35
0.38
0.02
0.23
0.36
0.22
0.15
0.13
0.21
0.18
0.08

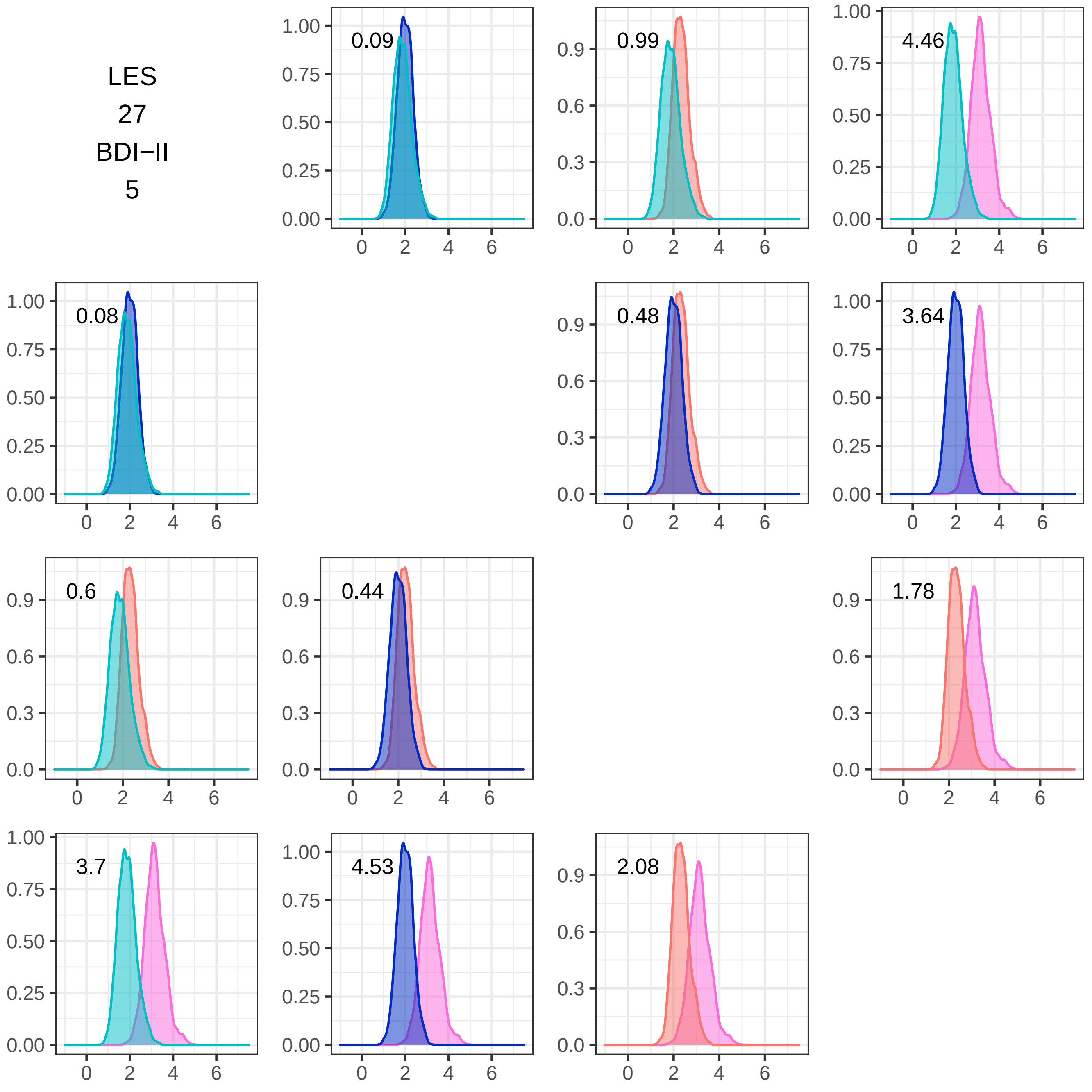

LES
27
BDI−II
5
0.09
0.99
4.46
0.08
0.48
3.64
0.6
0.44
1.78
3.7
4.53
2.08

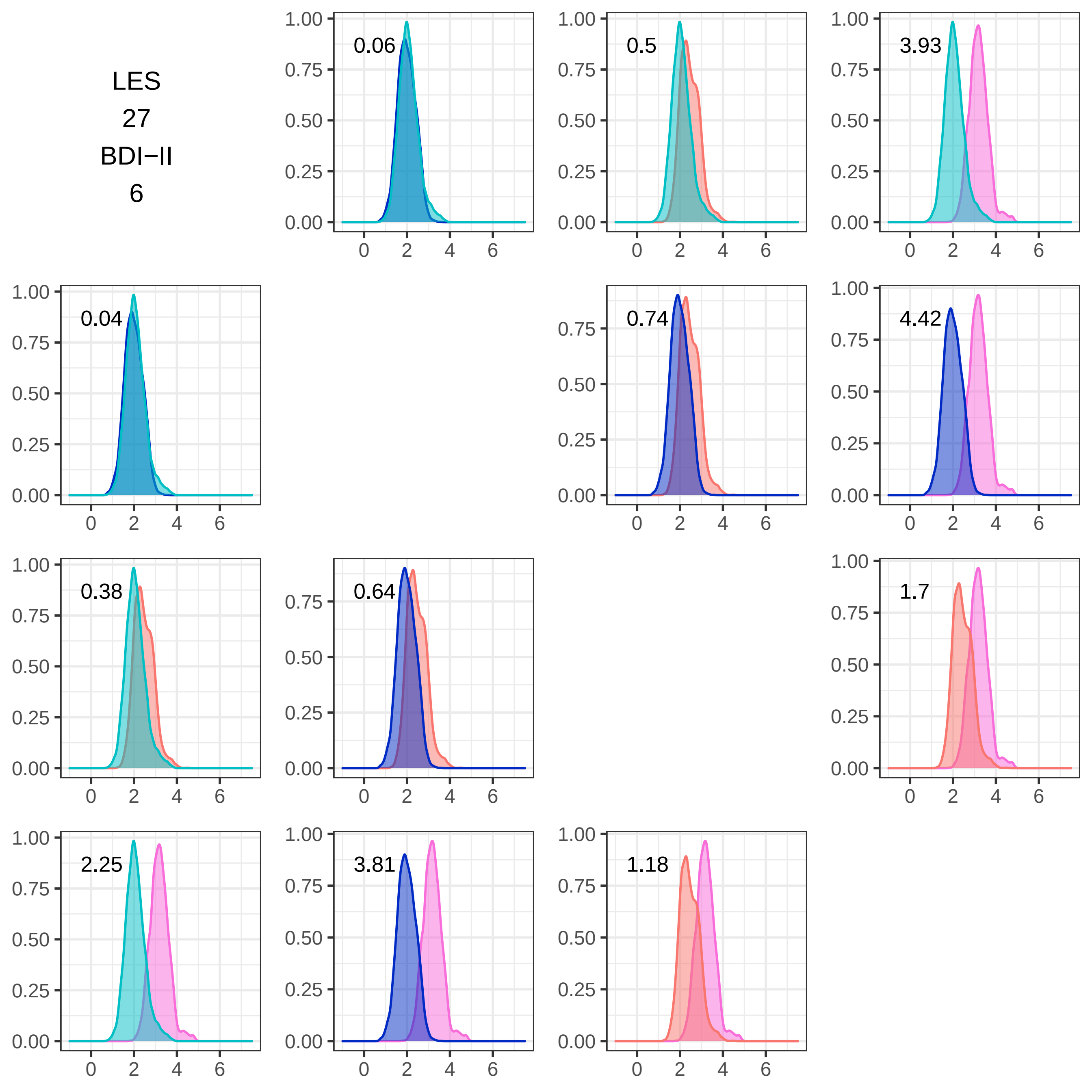

LES
27
BDI−II
6
0.06
0.5
3.93
0.04
0.74
4.42
0.38
0.64
1.7
2.25
3.81
1.18

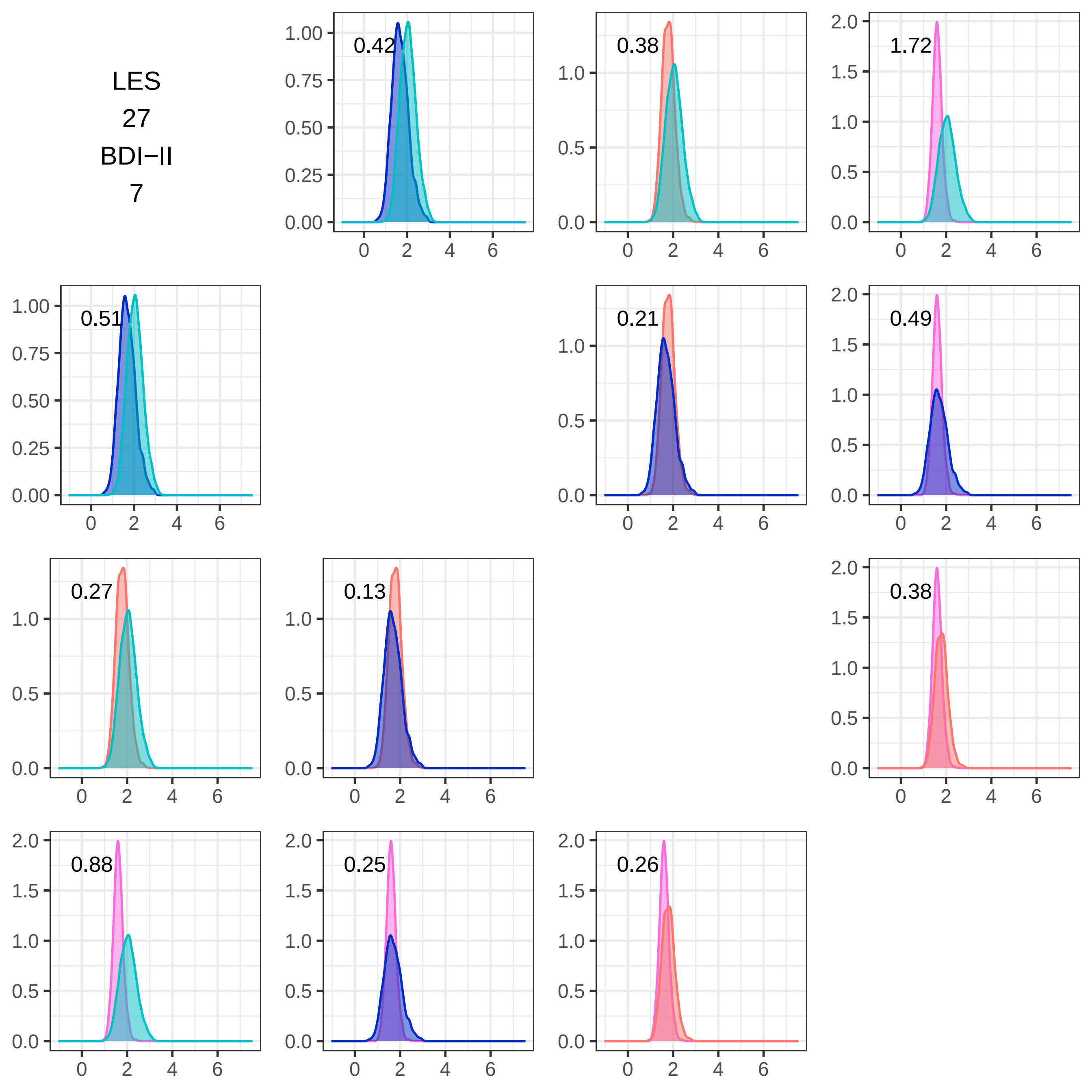

LES
27
BDI−II
7
0.42
0.38
1.72
0.51
0.21
0.49
0.27
0.13
0.38
0.88
0.25
0.26

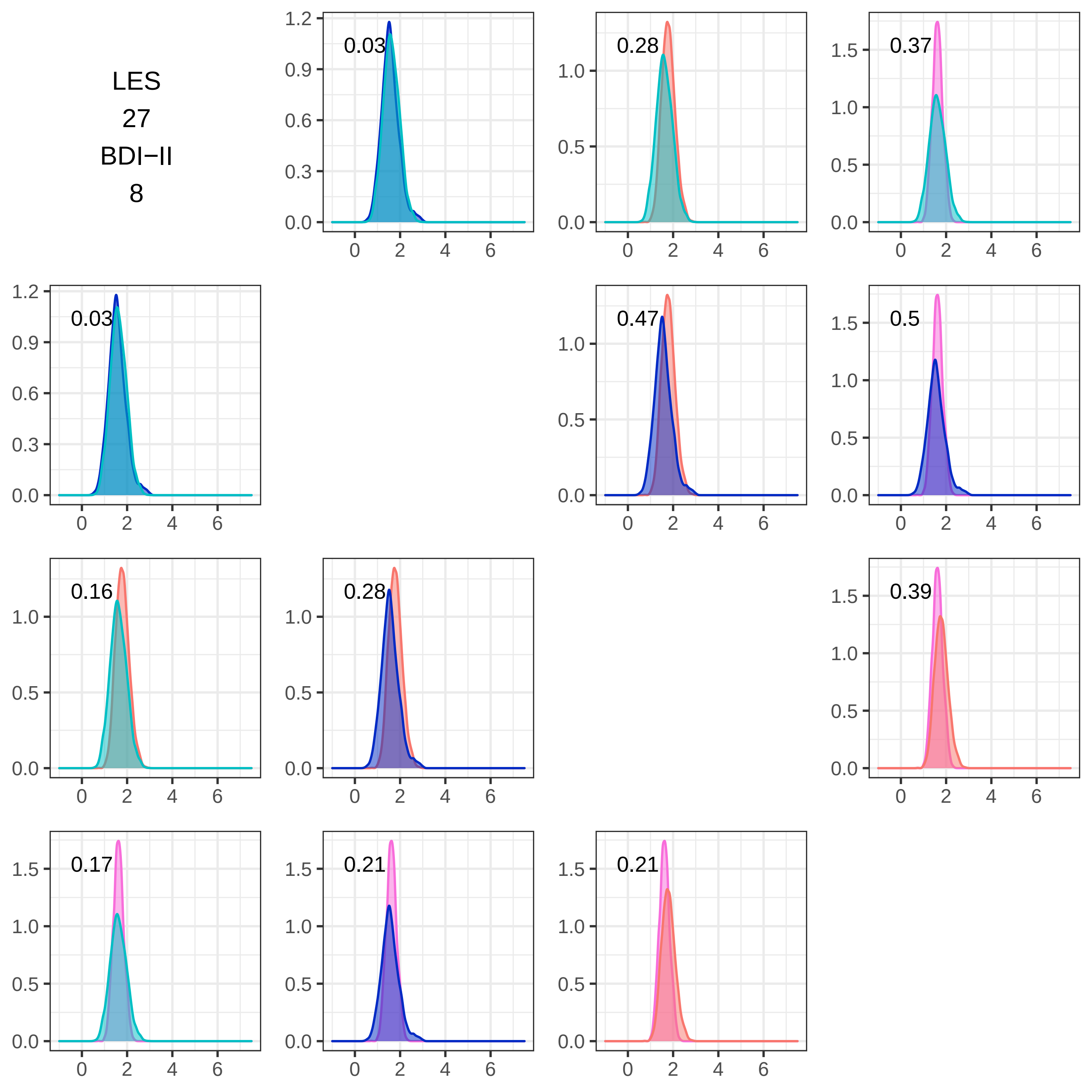

LES
27
BDI−II
8
0.03
0.28
0.37
0.03
0.47
0.5
0.16
0.28
0.39
0.17
0.21
0.21

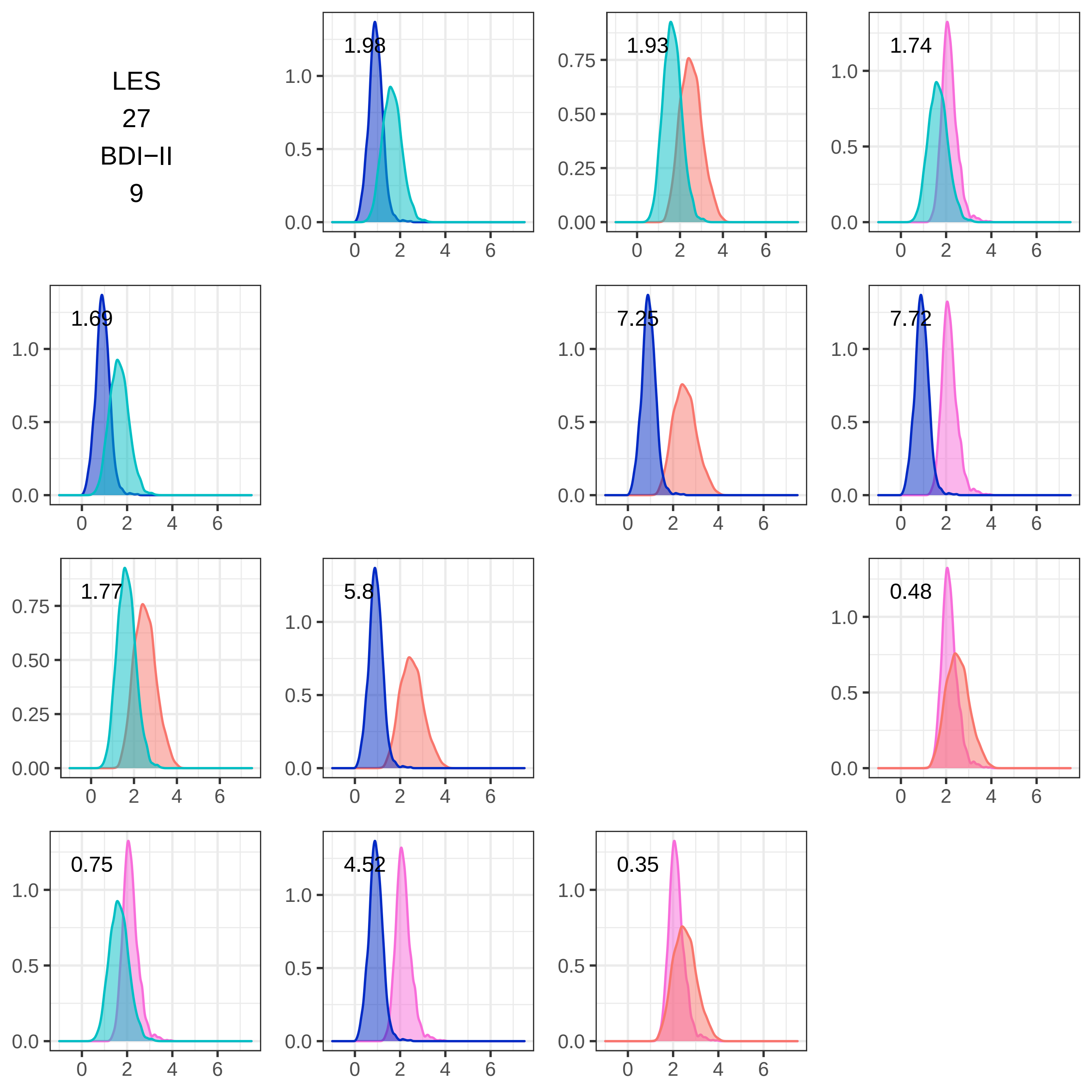
LES
27
BDI−II
9
1.98
1.93
1.74
1.69
7.25
7.72
1.77
5.8
0.48
0.75
4.52
0.35

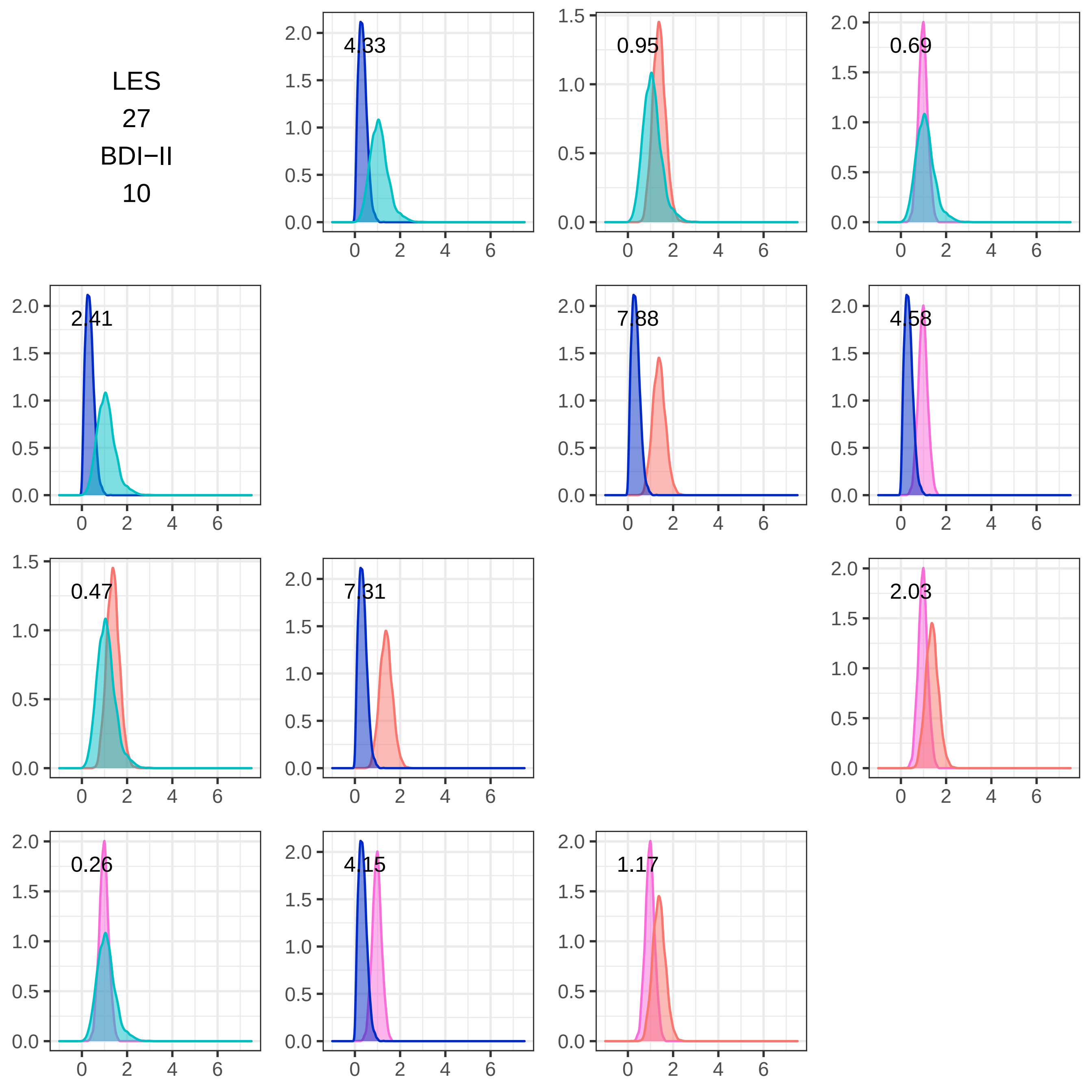

LES
27
BDI−II
10
4.33
0.95
0.69
2.41
7.88
4.58
0.47
7.31
2.03
0.26
4.15
1.17

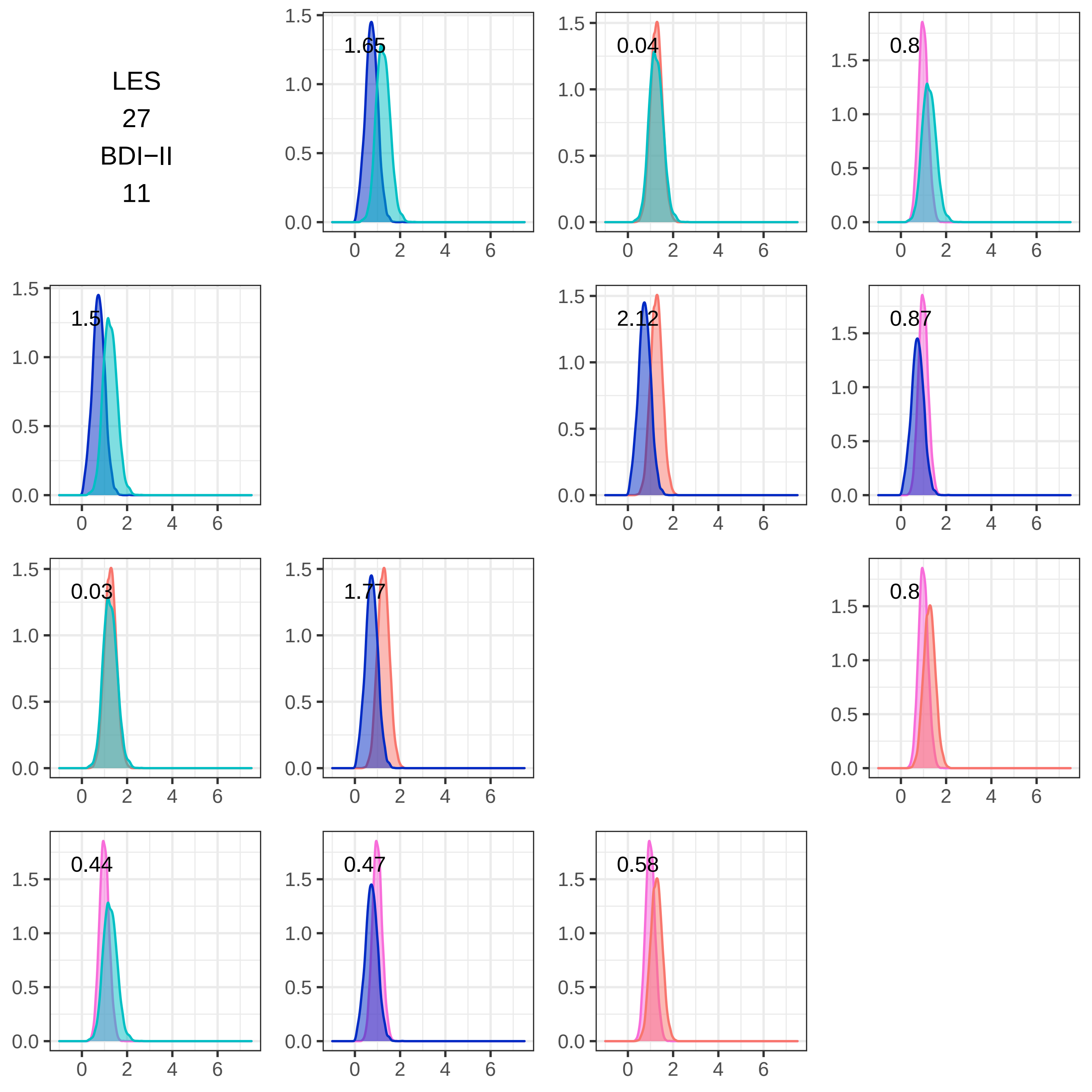

LES
27
BDI−II
11
1.65
0.04
0.8
1.5
2.12
0.87
0.03
1.77
0.8
0.44
0.47
0.58

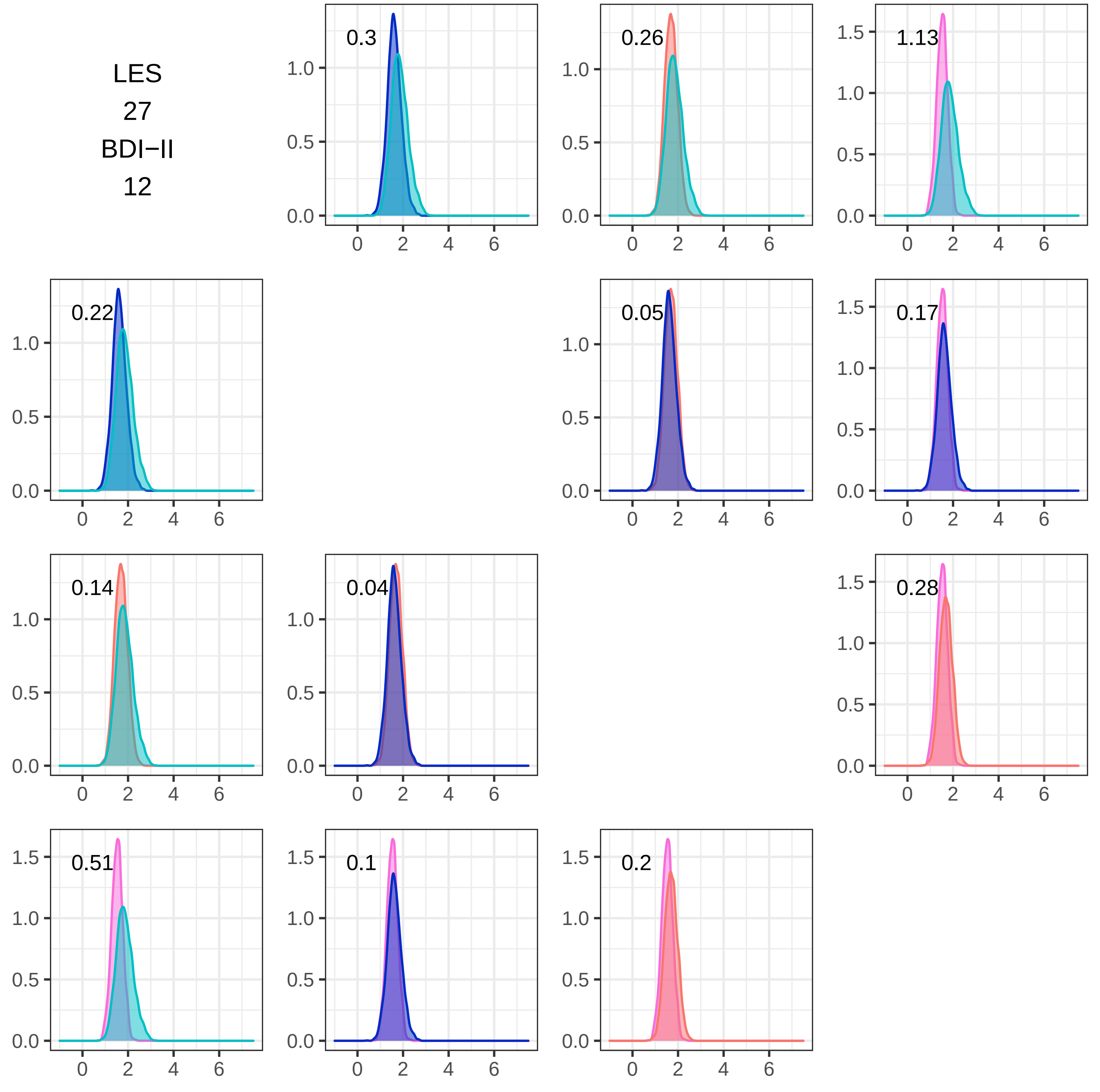
LES
27
BDI−II
12
0.3
0.26
1.13
0.22
0.05
0.17
0.14
0.04
0.28
0.51
0.1
0.2

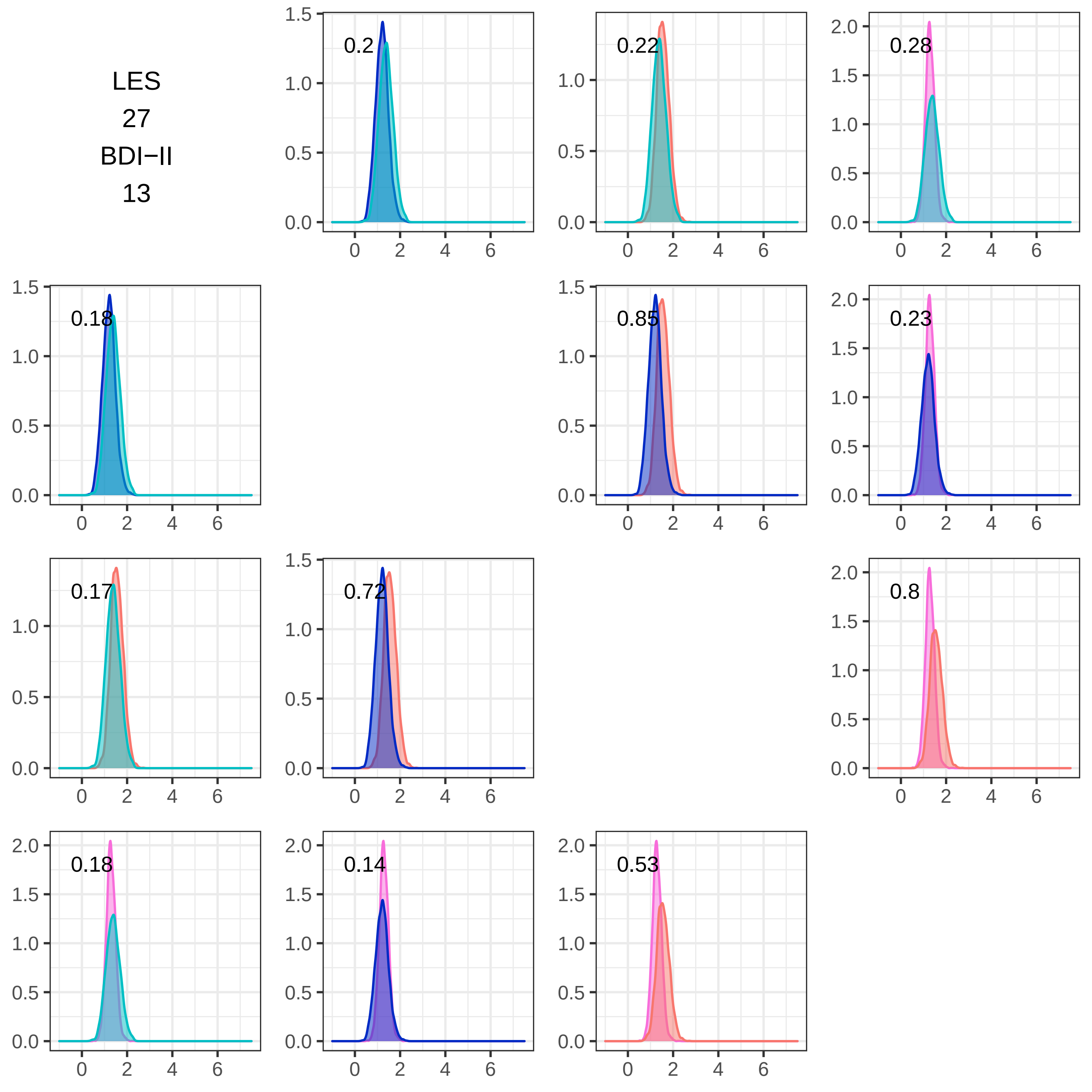

LES
27
BDI−II
13
0.2
0.22
0.28
0.18
0.85
0.23
0.17
0.72
0.8
0.18
0.14
0.53

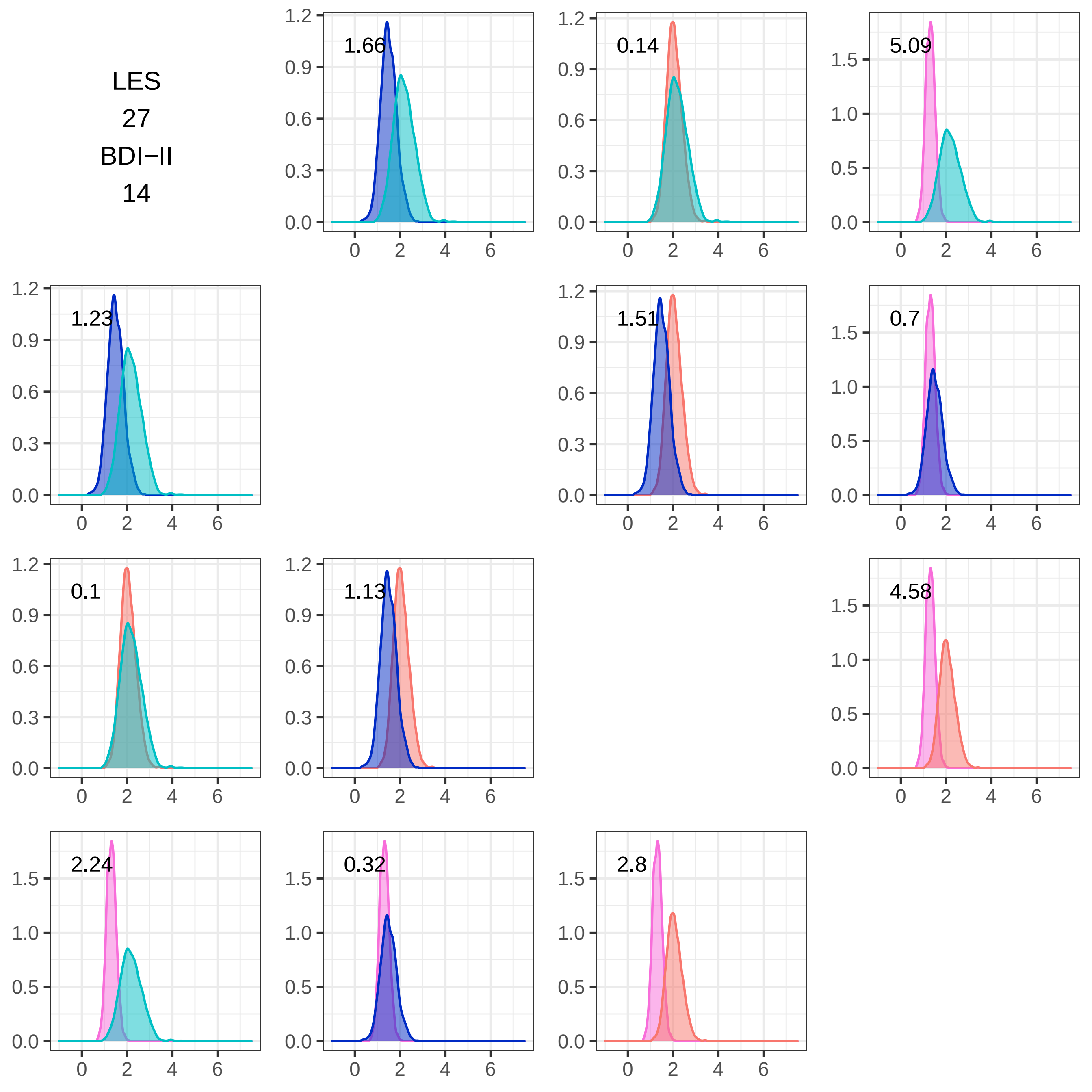

LES
27
BDI−II
14
1.66
0.14
5.09
1.23
1.51
0.7
0.1
1.13
4.58
2.24
0.32
2.8

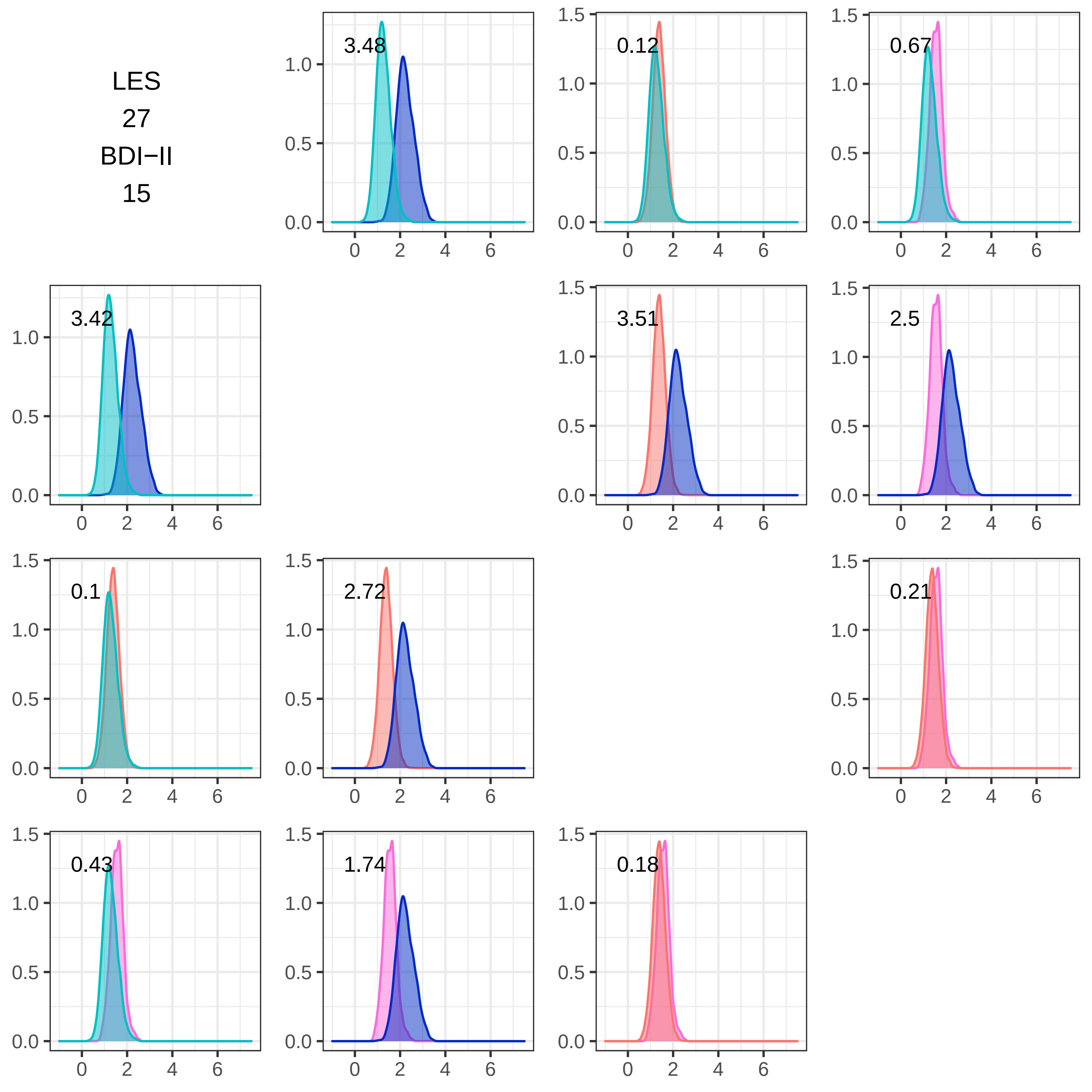
LES
27
BDI−II
15
3.48
0.12
0.67
3.42
3.51
2.5
0.1
2.72
0.21
0.43
1.74
0.18

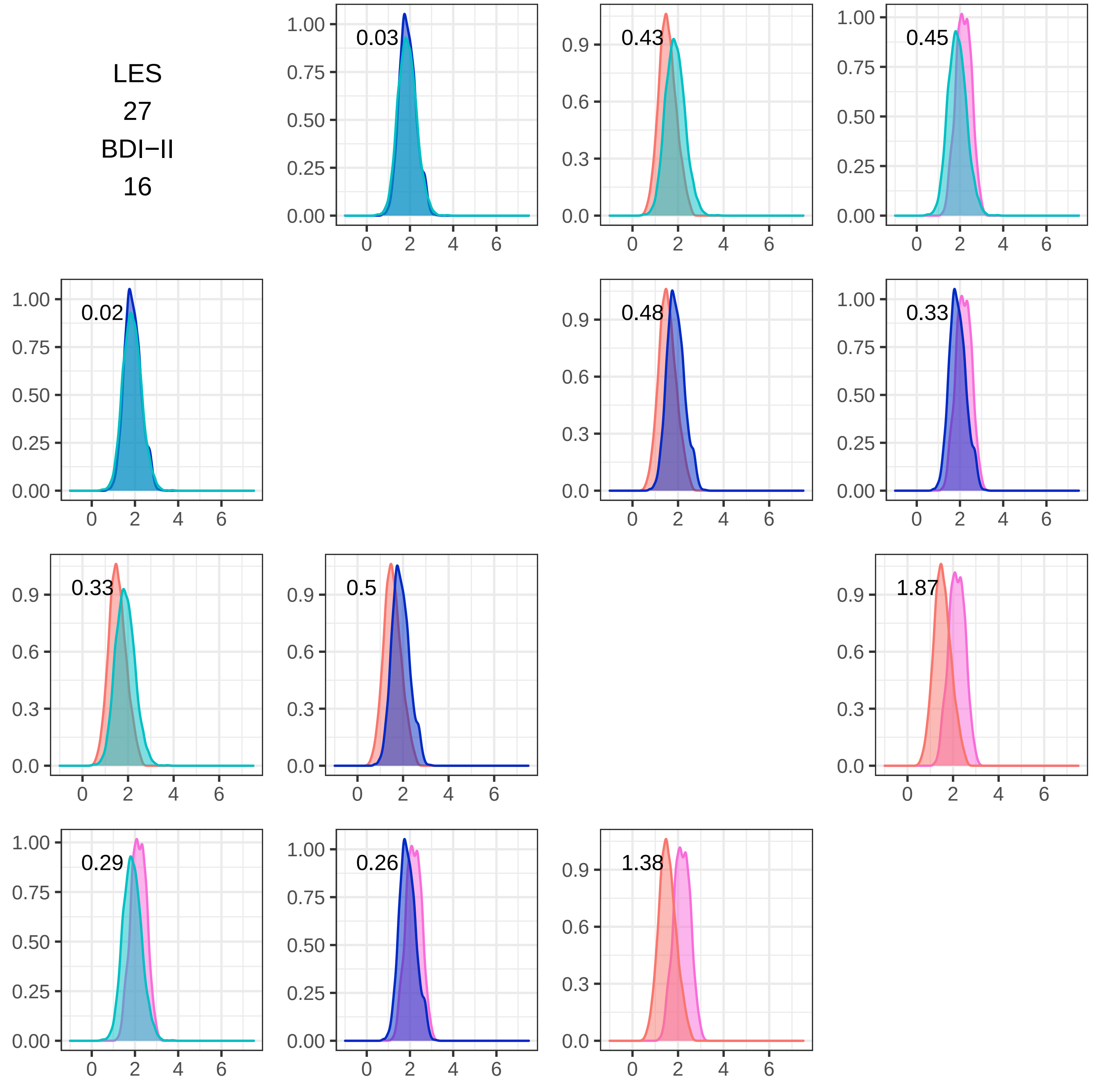

LES
27
BDI−II
16
0.03
0.43
0.45
0.02
0.48
0.33
0.33
0.5
1.87
0.29
0.26
1.38

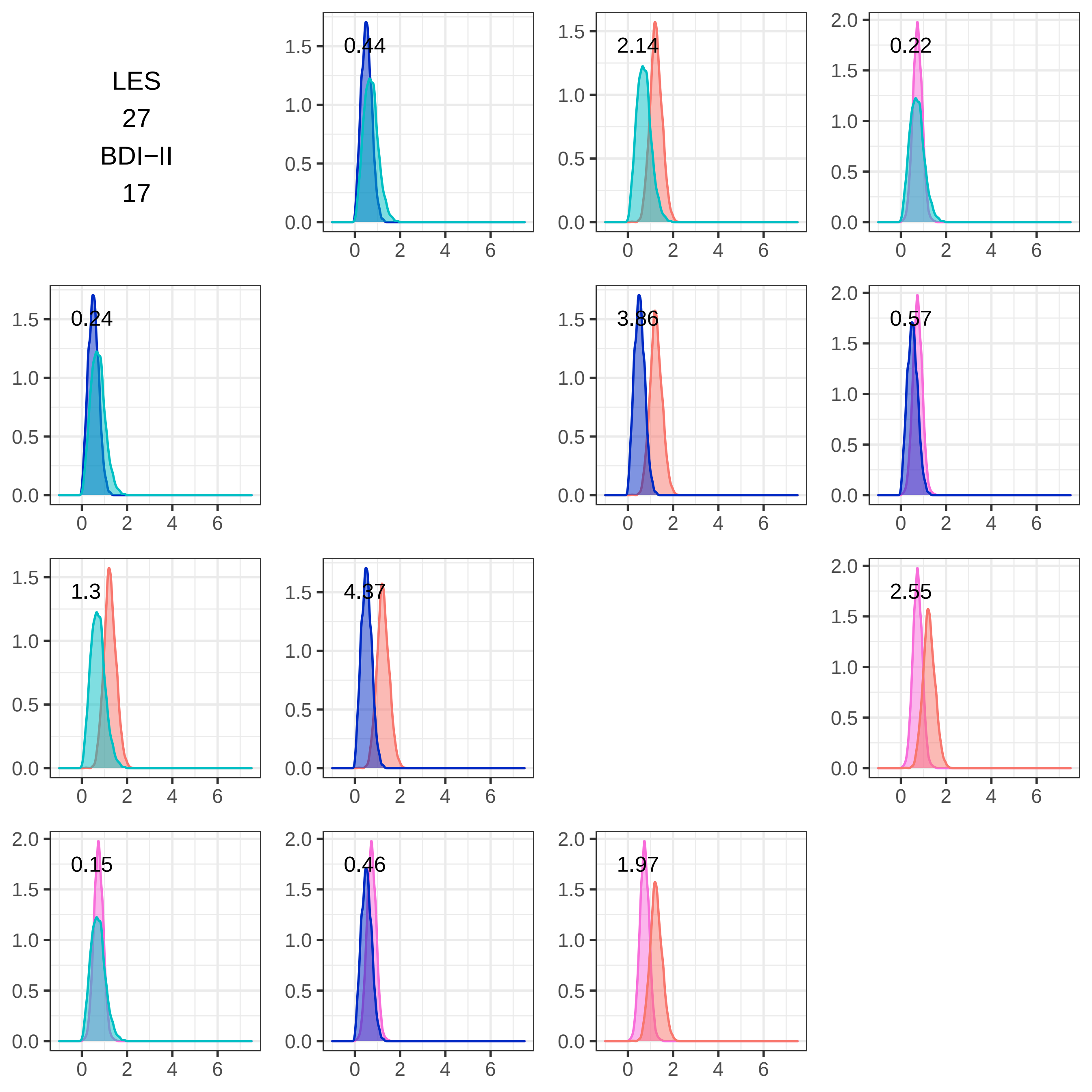

LES
27
BDI−II
17
0.44
2.14
0.22
0.24
3.86
0.57
1.3
4.37
2.55
0.15
0.46
1.97

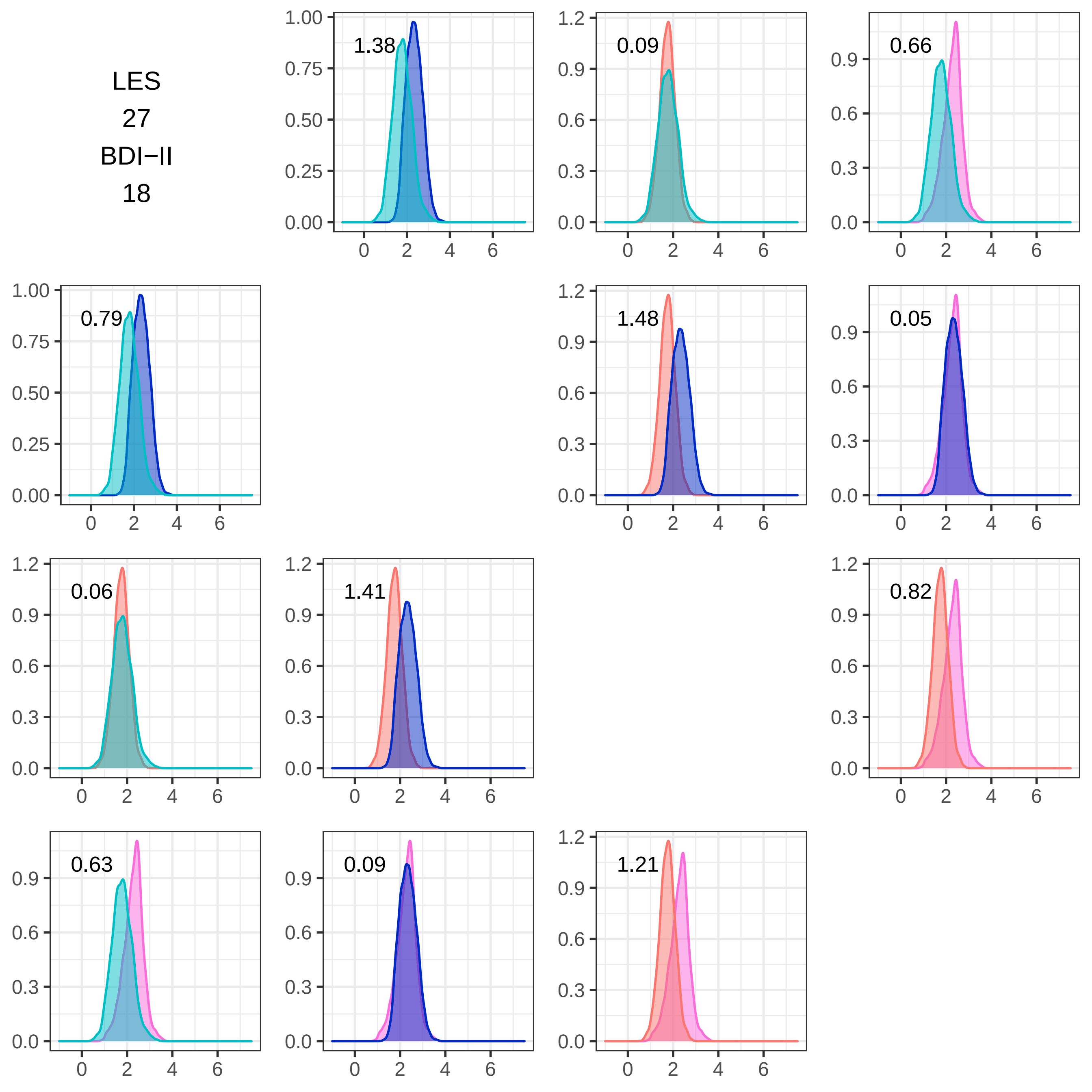
LES
27
BDI−II
18
1.38
0.09
0.66
0.79
1.48
0.05
0.06
1.41
0.82
0.63
0.09
1.21

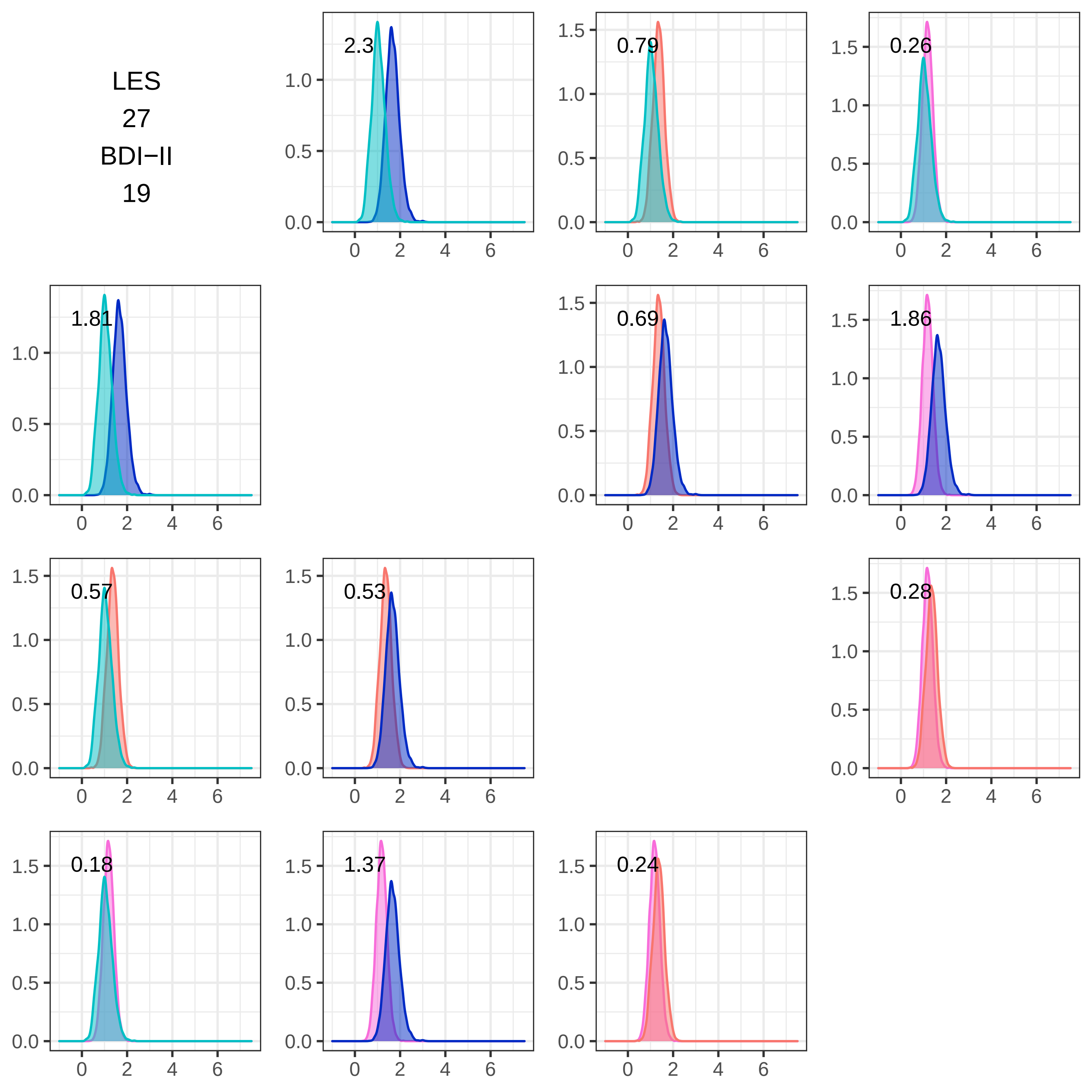

LES
27
BDI−II
19
2.3
0.79
0.26
1.81
0.69
1.86
0.57
0.53
0.28
0.18
1.37
0.24

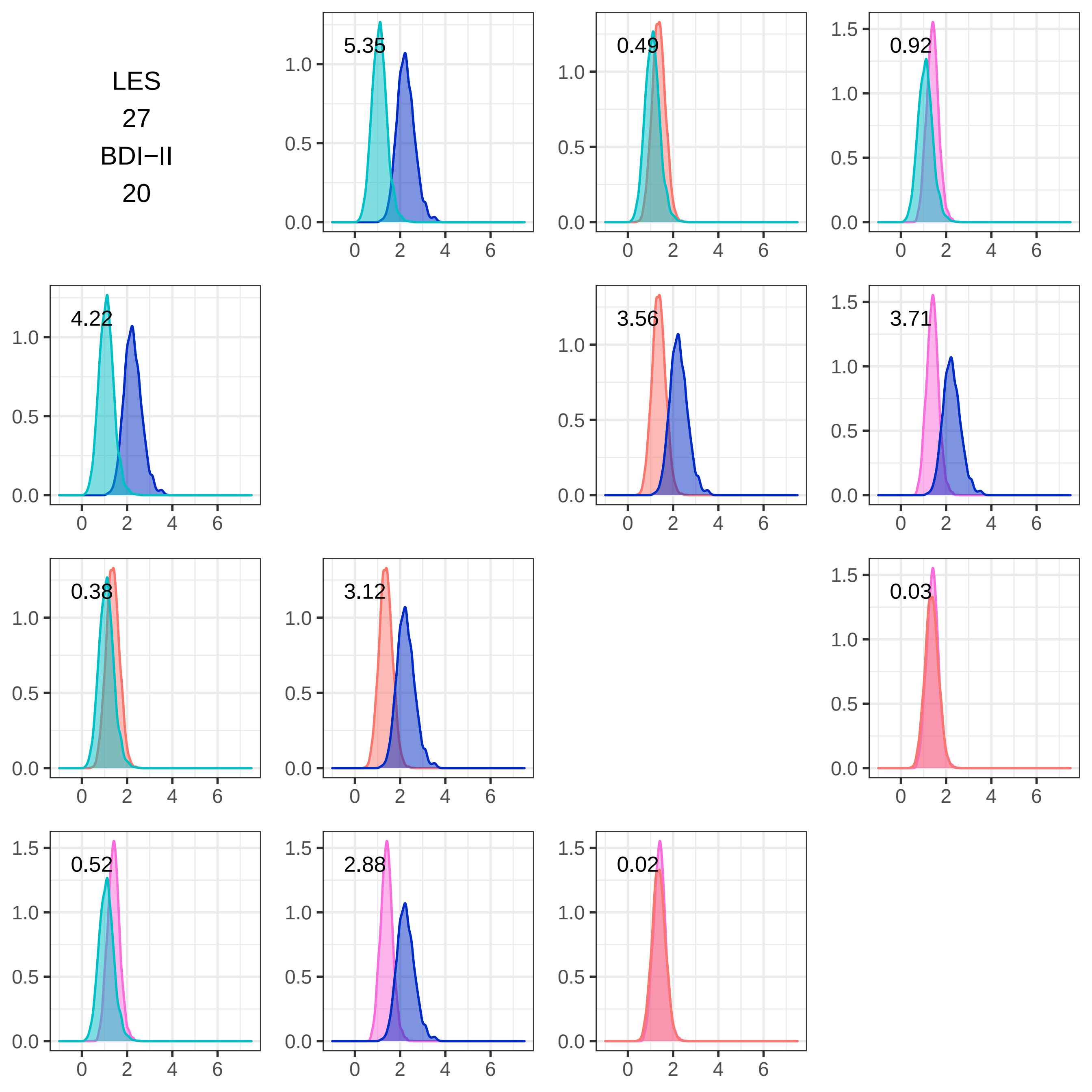
LES
27
BDI−II
20
5.35
0.49
0.92
4.22
3.56
3.71
0.38
3.12
0.03
0.52
2.88
0.02

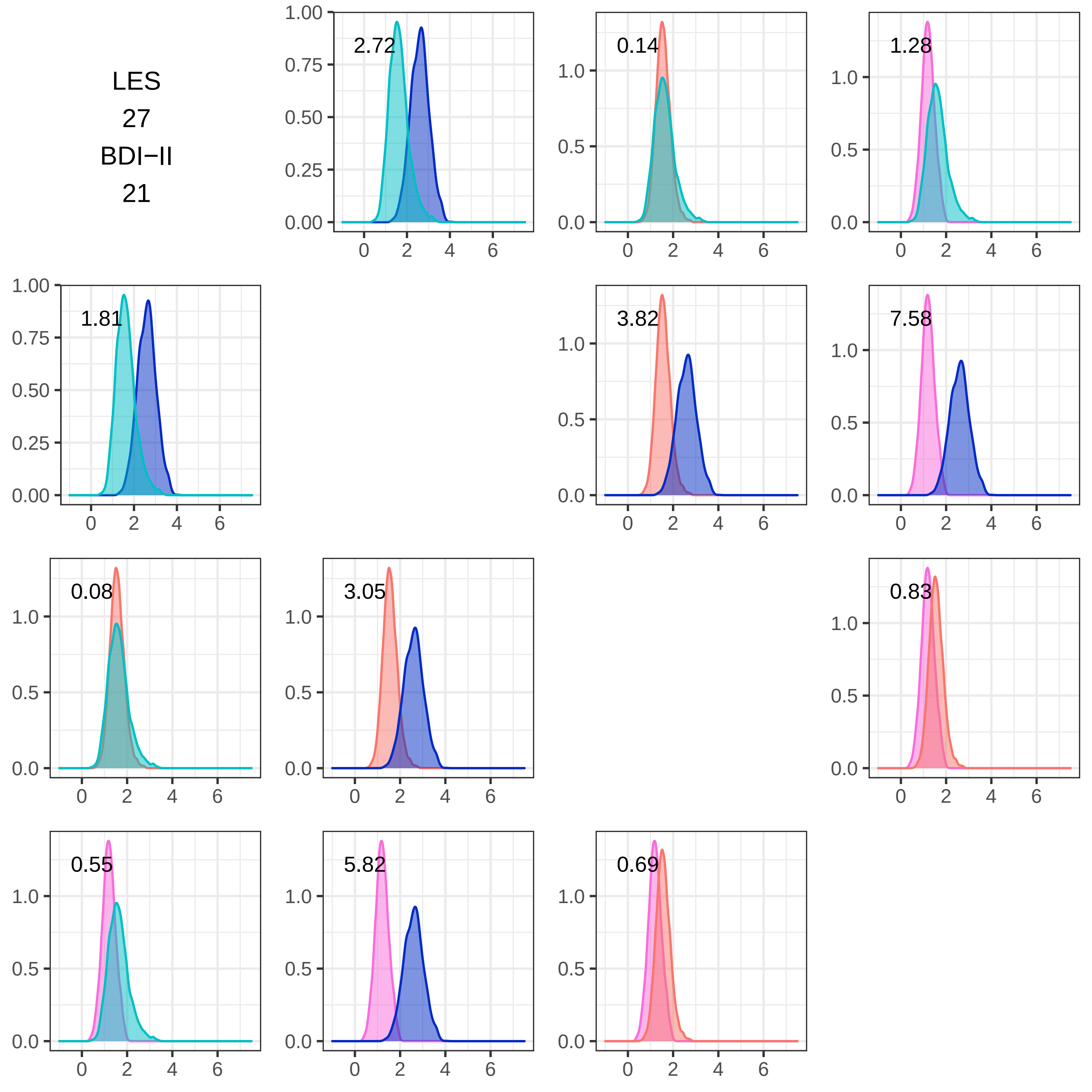

LES
27
BDI−II
21
2.72
0.14
1.28
1.81
3.82
7.58
0.08
3.05
0.83
0.55
5.82
0.69

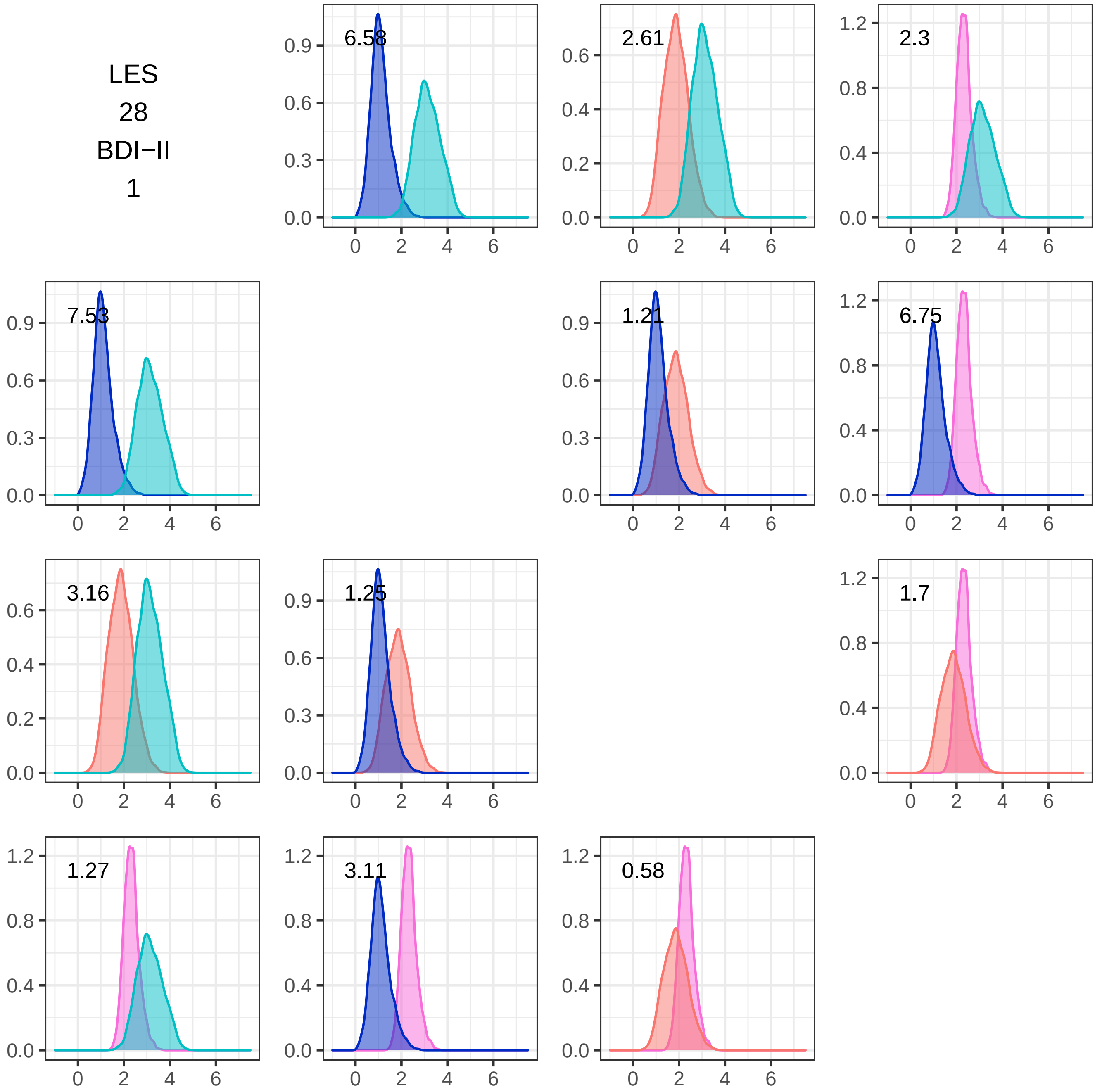
LES
28
BDI−II
1
6.58
2.61
2.3
7.53
1.21
6.75
3.16
1.25
1.7
1.27
3.11
0.58

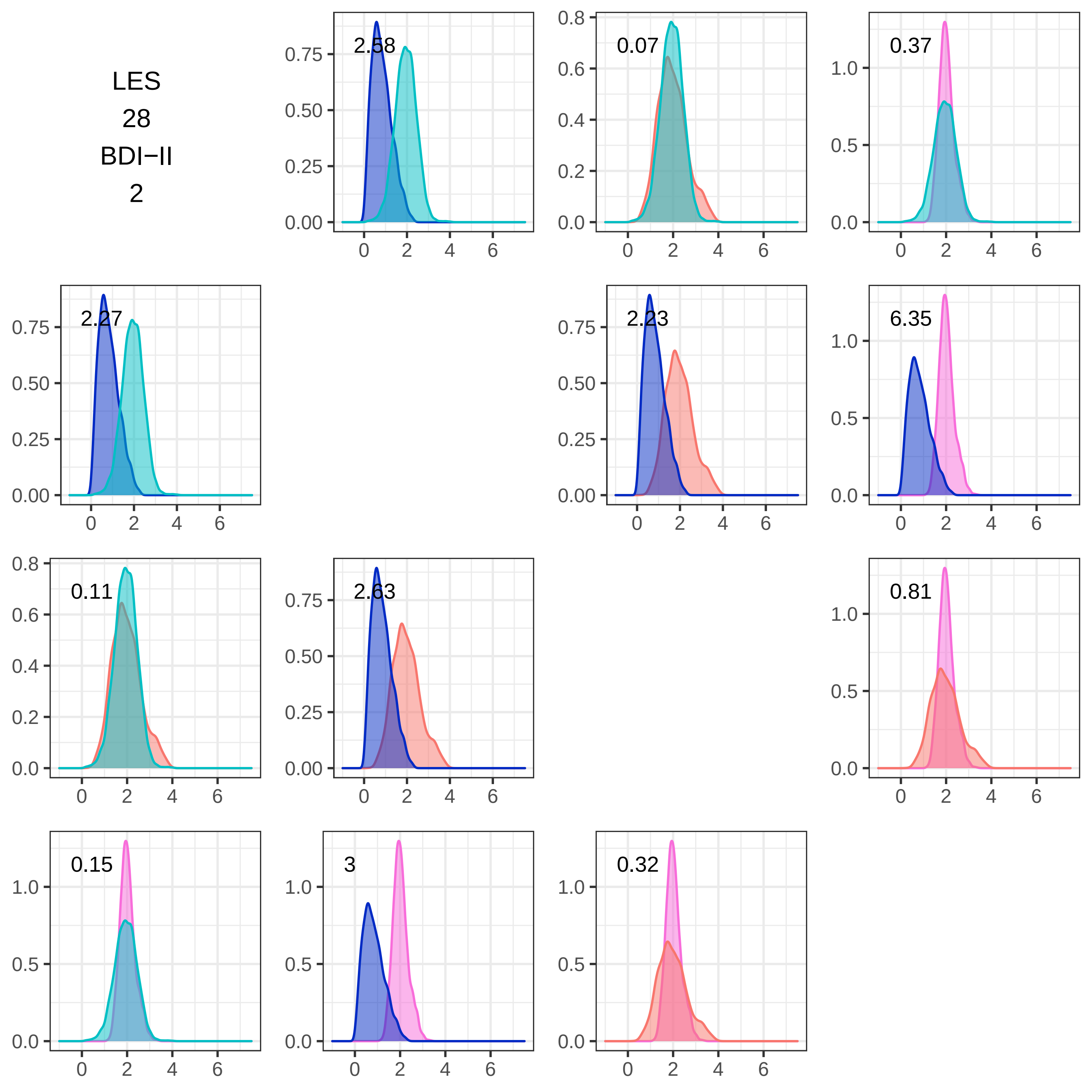

LES
28
BDI−II
2
2.58
0.07
0.37
2.27
2.23
6.35
0.11
2.63
0.81
0.15
3
0.32

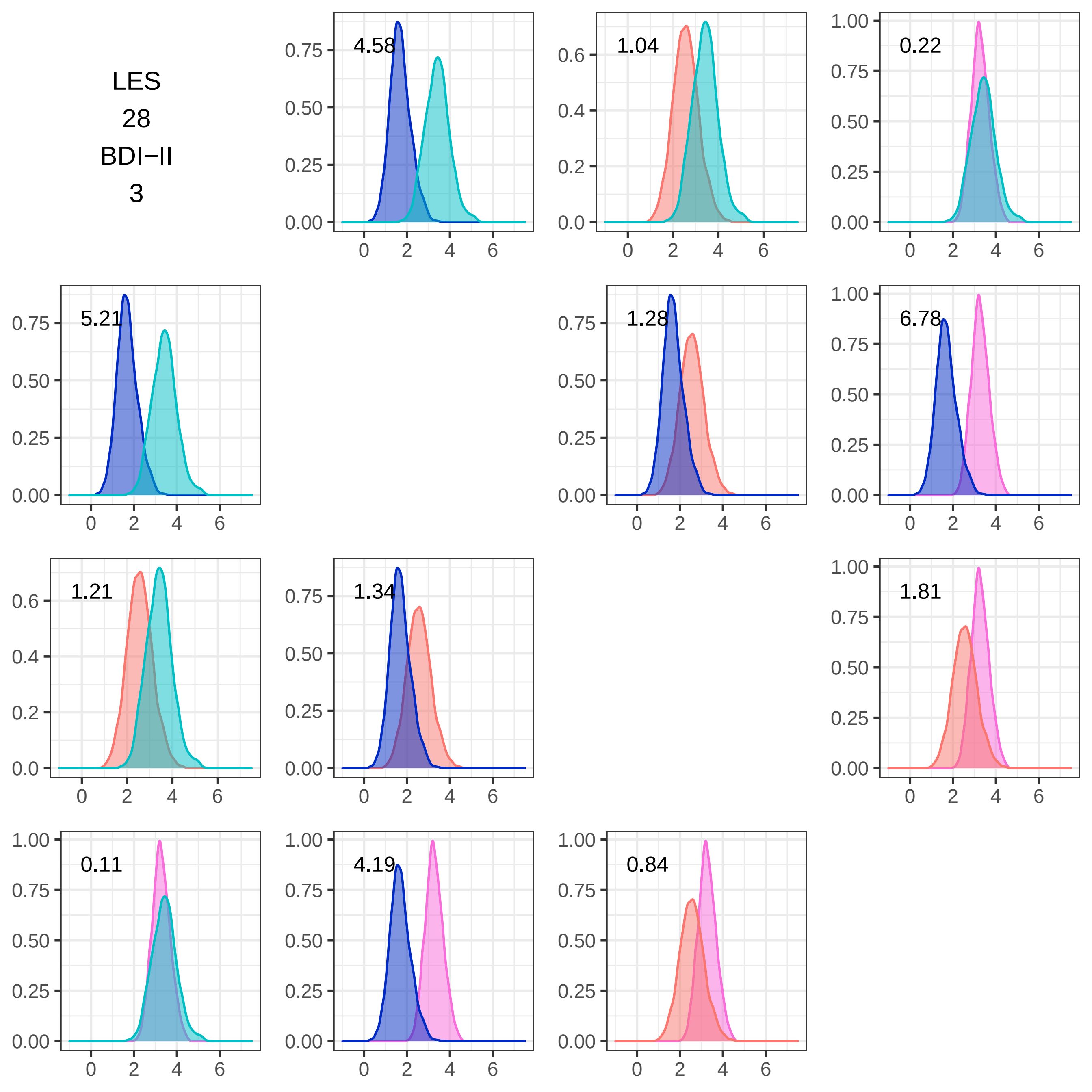

LES
28
BDI−II
3
4.58
1.04
0.22
5.21
1.28
6.78
1.21
1.34
1.81
0.11
4.19
0.84

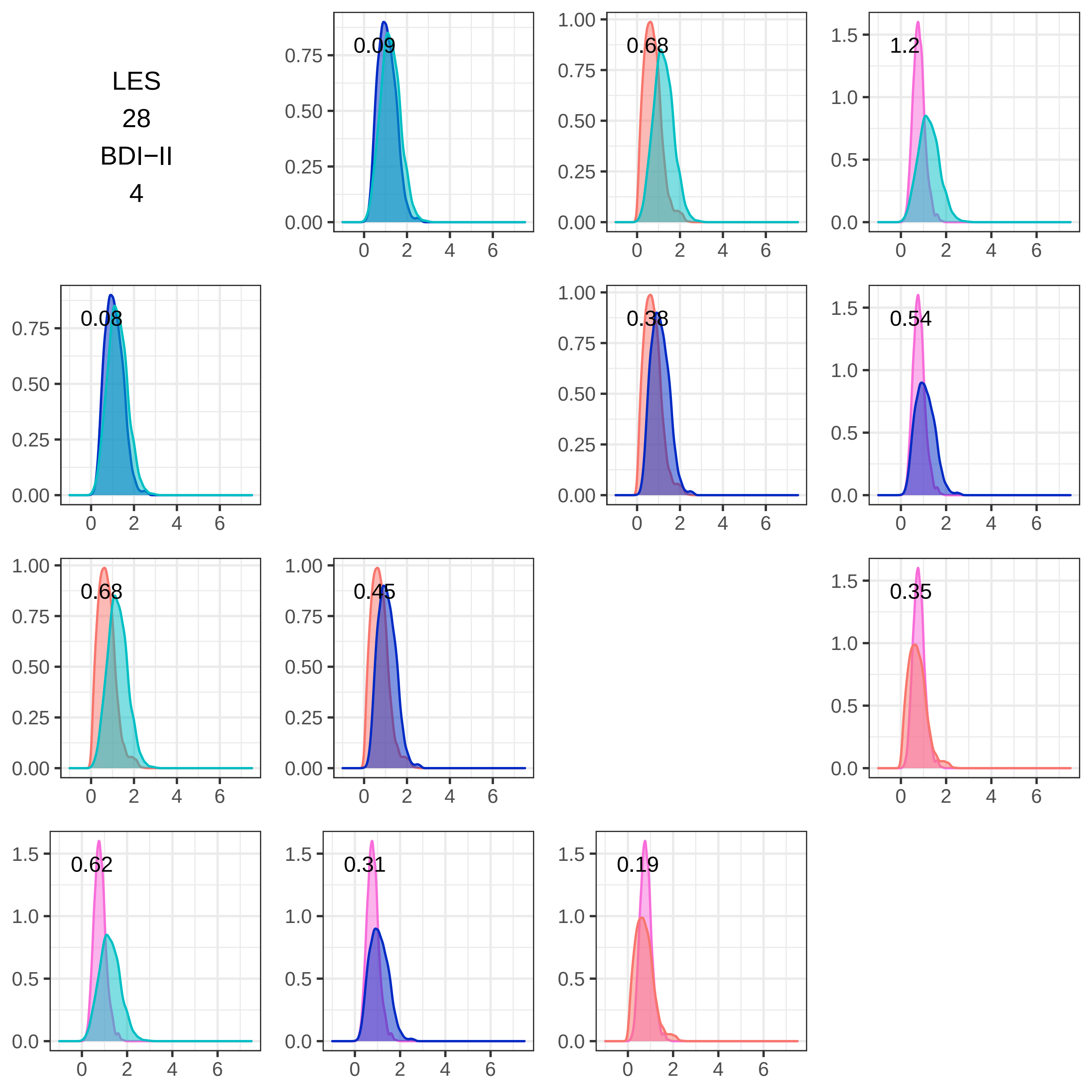
LES
28
BDI−II
4
0.09
0.68
1.2
0.08
0.38
0.54
0.68
0.45
0.35
0.62
0.31
0.19

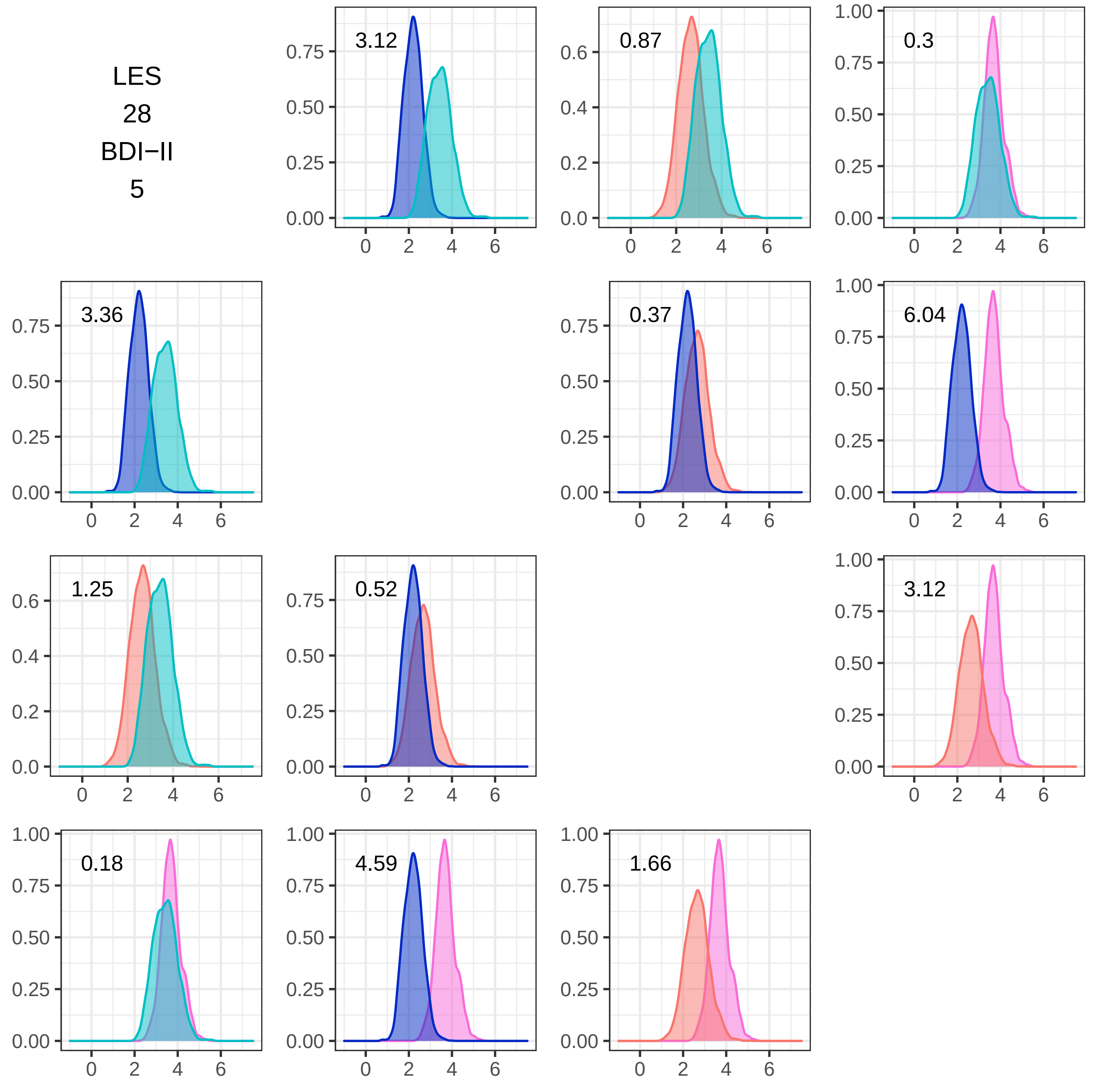

LES
28
BDI−II
5
3.12
0.87
0.3
3.36
0.37
6.04
1.25
0.52
3.12
0.18
4.59
1.66

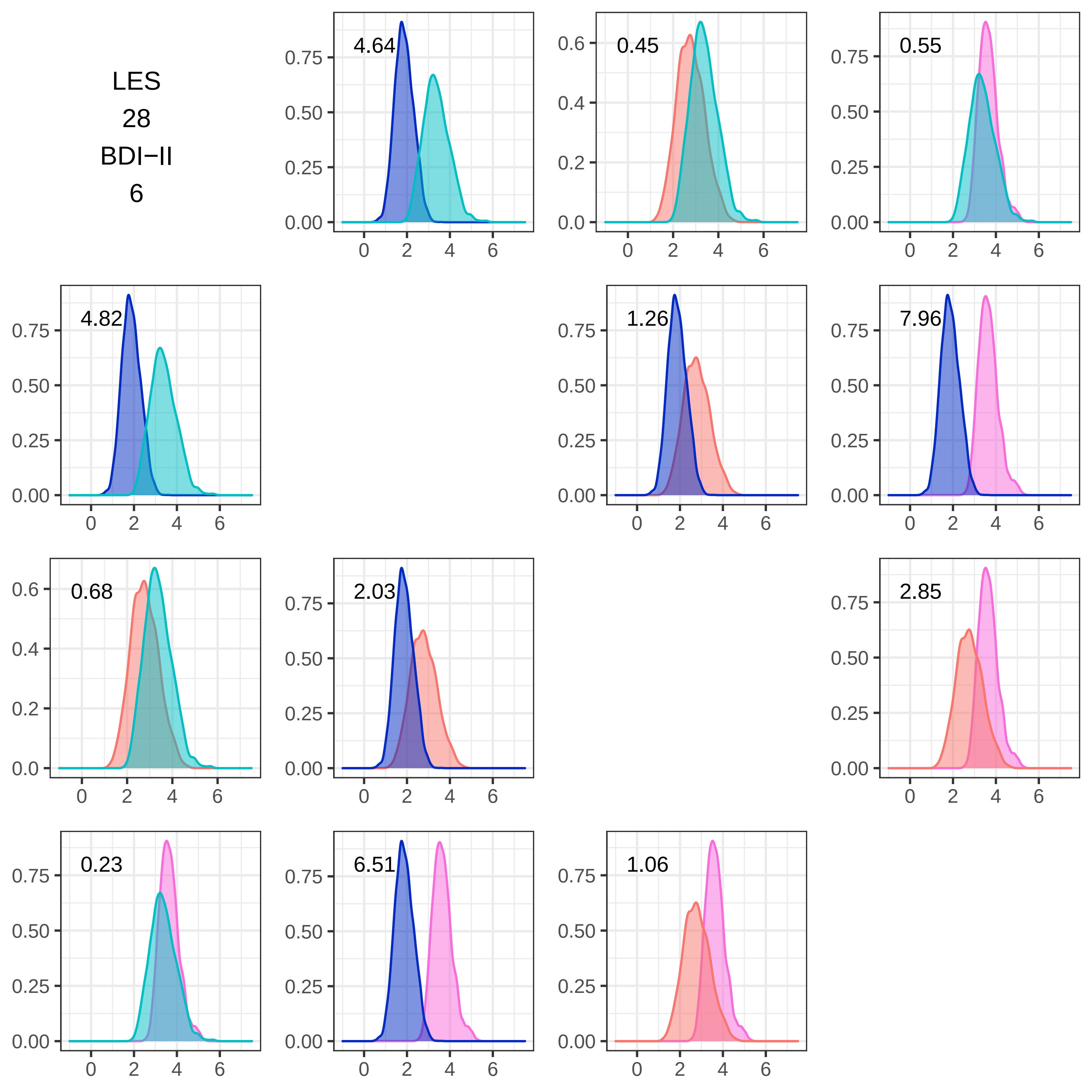
LES
28
BDI−II
6
4.64
0.45
0.55
4.82
1.26
7.96
0.68
2.03
2.85
0.23
6.51
1.06

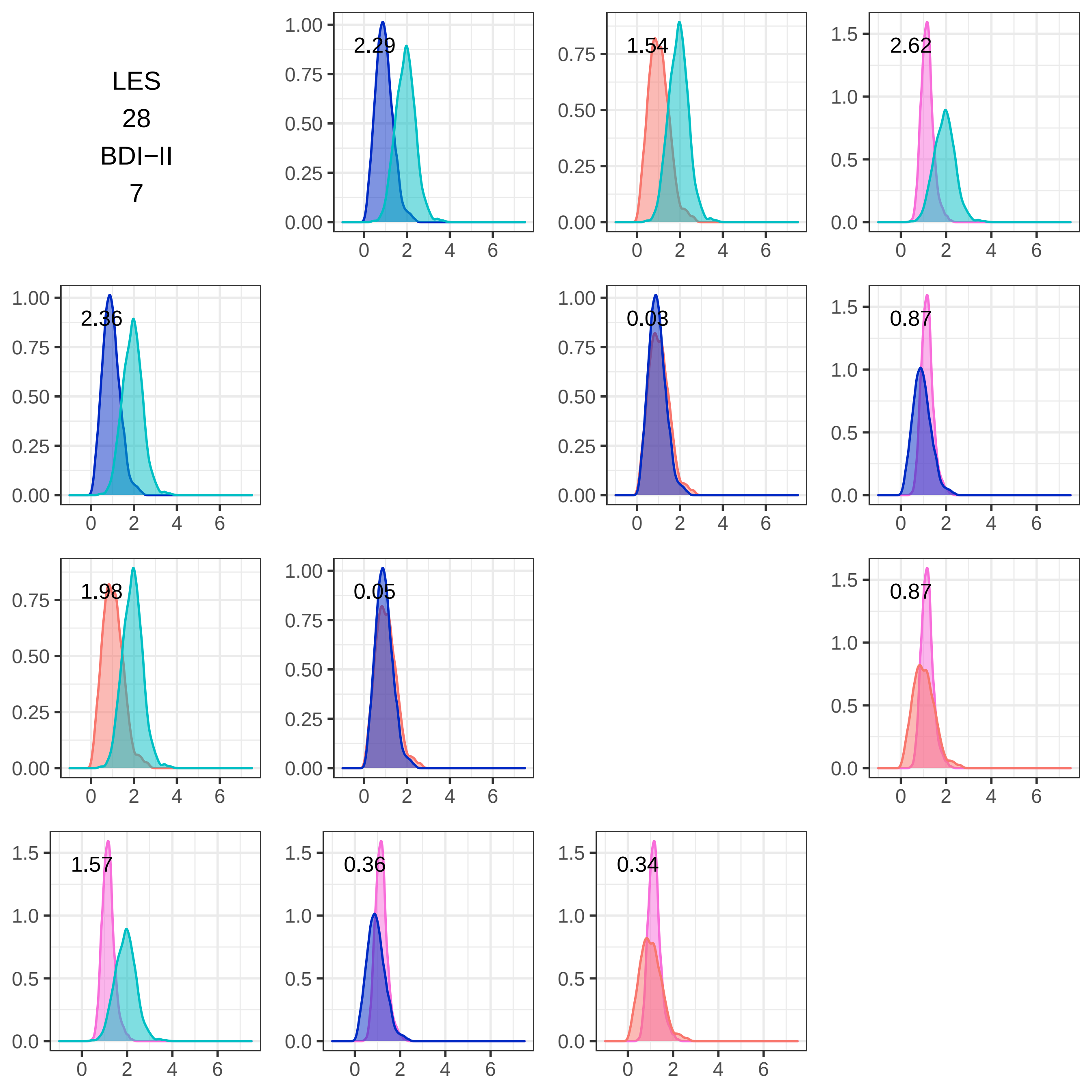
LES
28
BDI−II
7
2.29
1.54
2.62
2.36
0.03
0.87
1.98
0.05
0.87
1.57
0.36
0.34

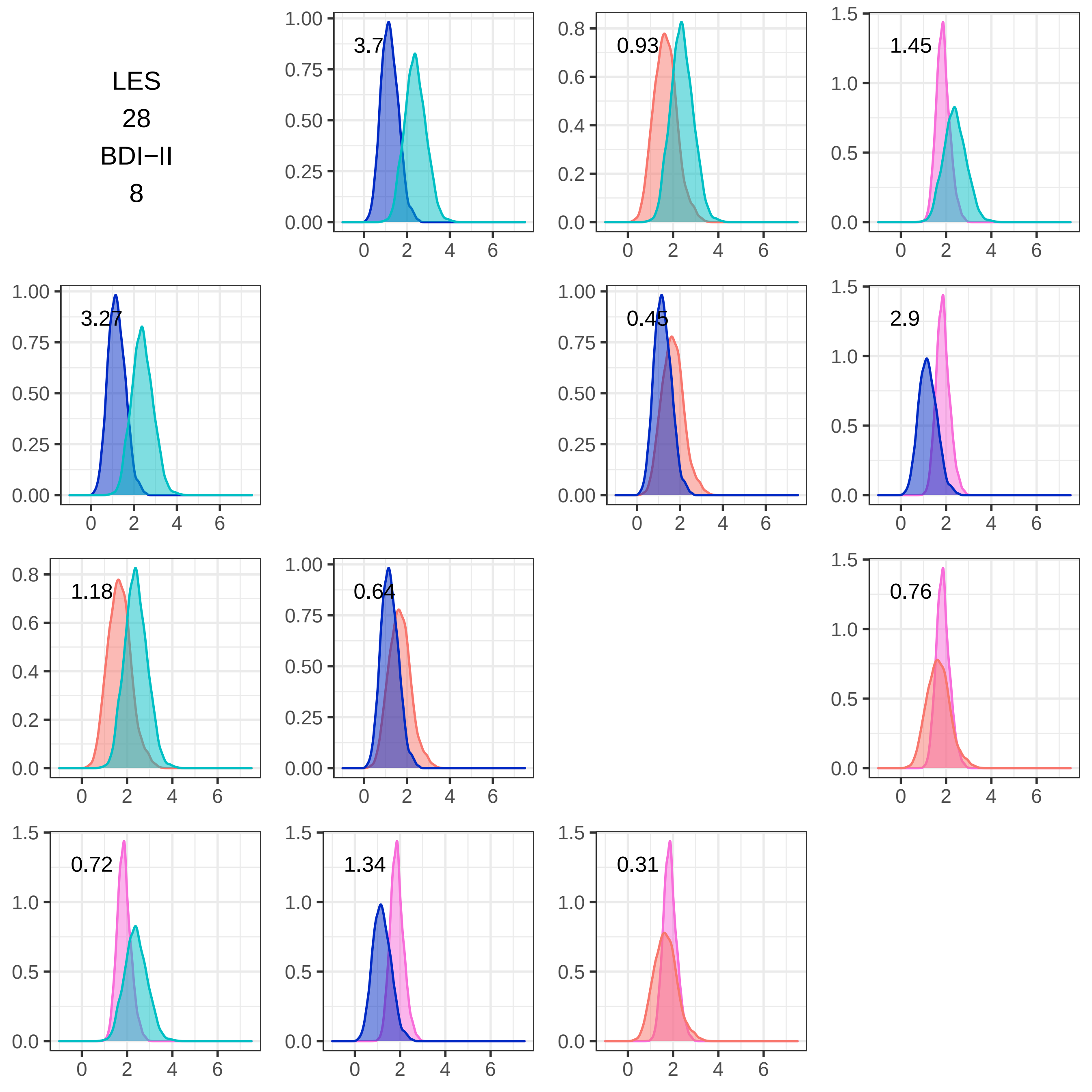

LES
28
BDI−II
8
3.7
0.93
1.45
3.27
0.45
2.9
1.18
0.64
0.76
0.72
1.34
0.31

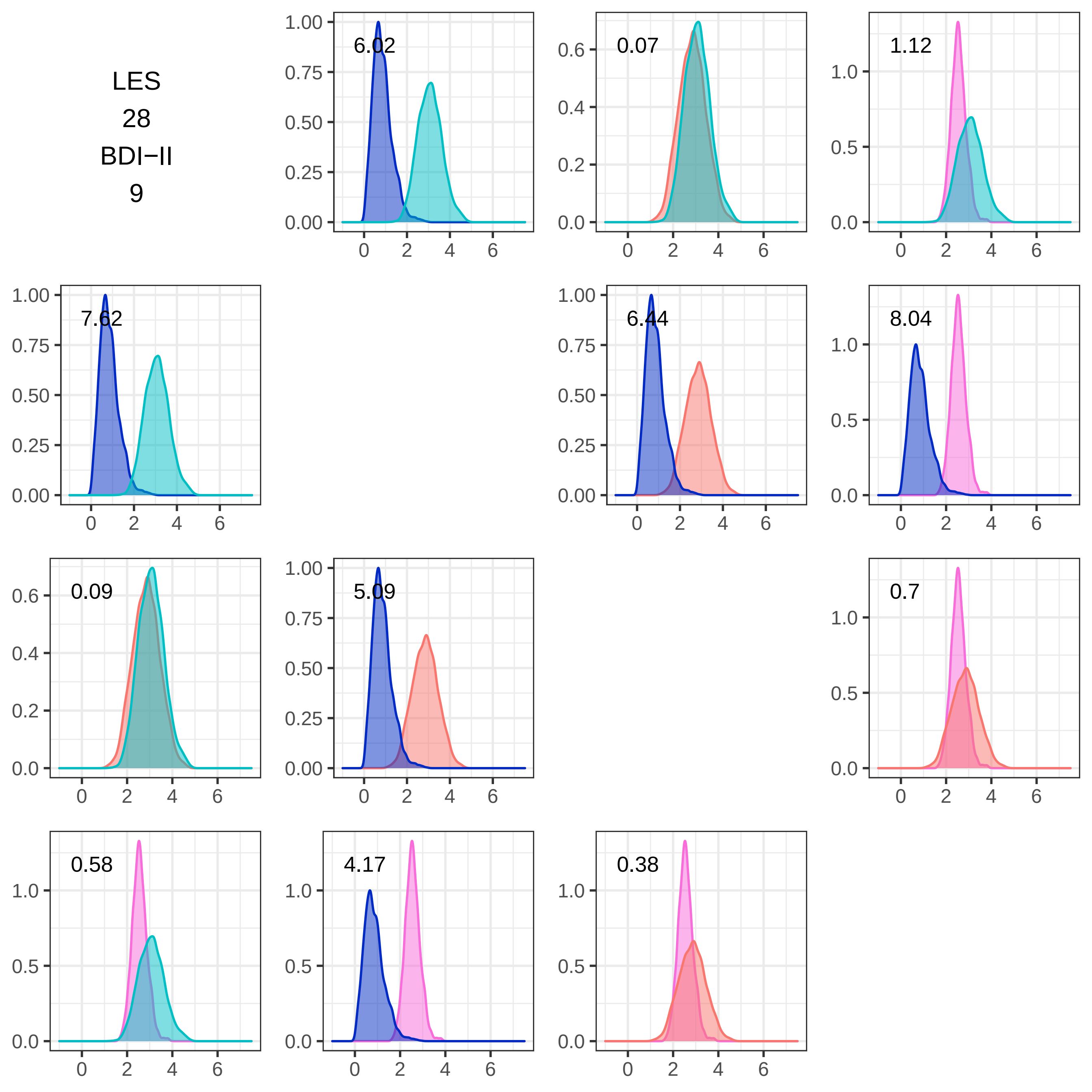
LES
28
BDI−II
9
6.02
0.07
1.12
7.62
6.44
8.04
0.09
5.09
0.7
0.58
4.17
0.38

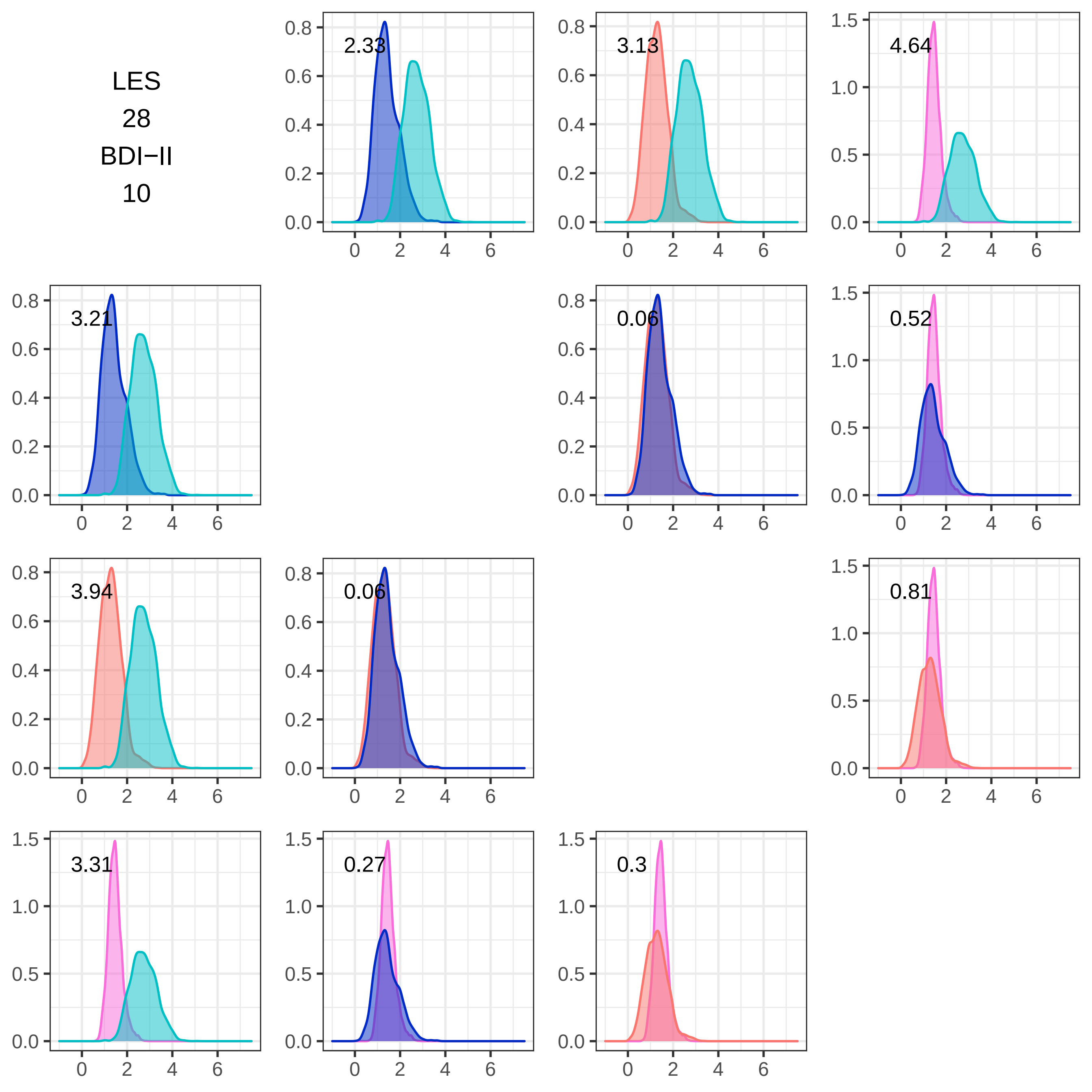
LES
28
BDI−II
10
2.33
3.13
4.64
3.21
0.06
0.52
3.94
0.06
0.81
3.31
0.27
0.3

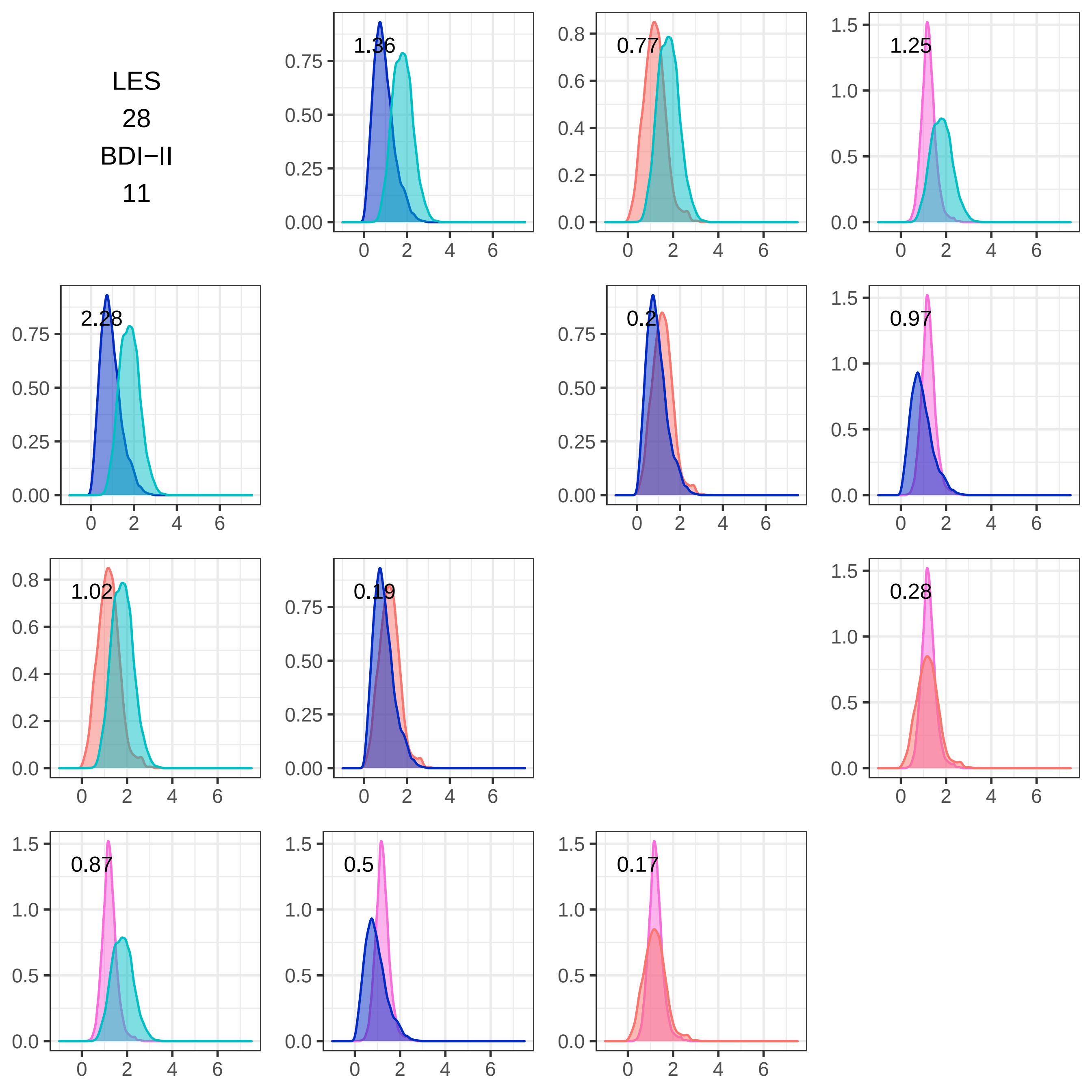

LES
28
BDI−II
11
1.36
0.77
1.25
2.28
0.2
0.97
1.02
0.19
0.28
0.87
0.5
0.17

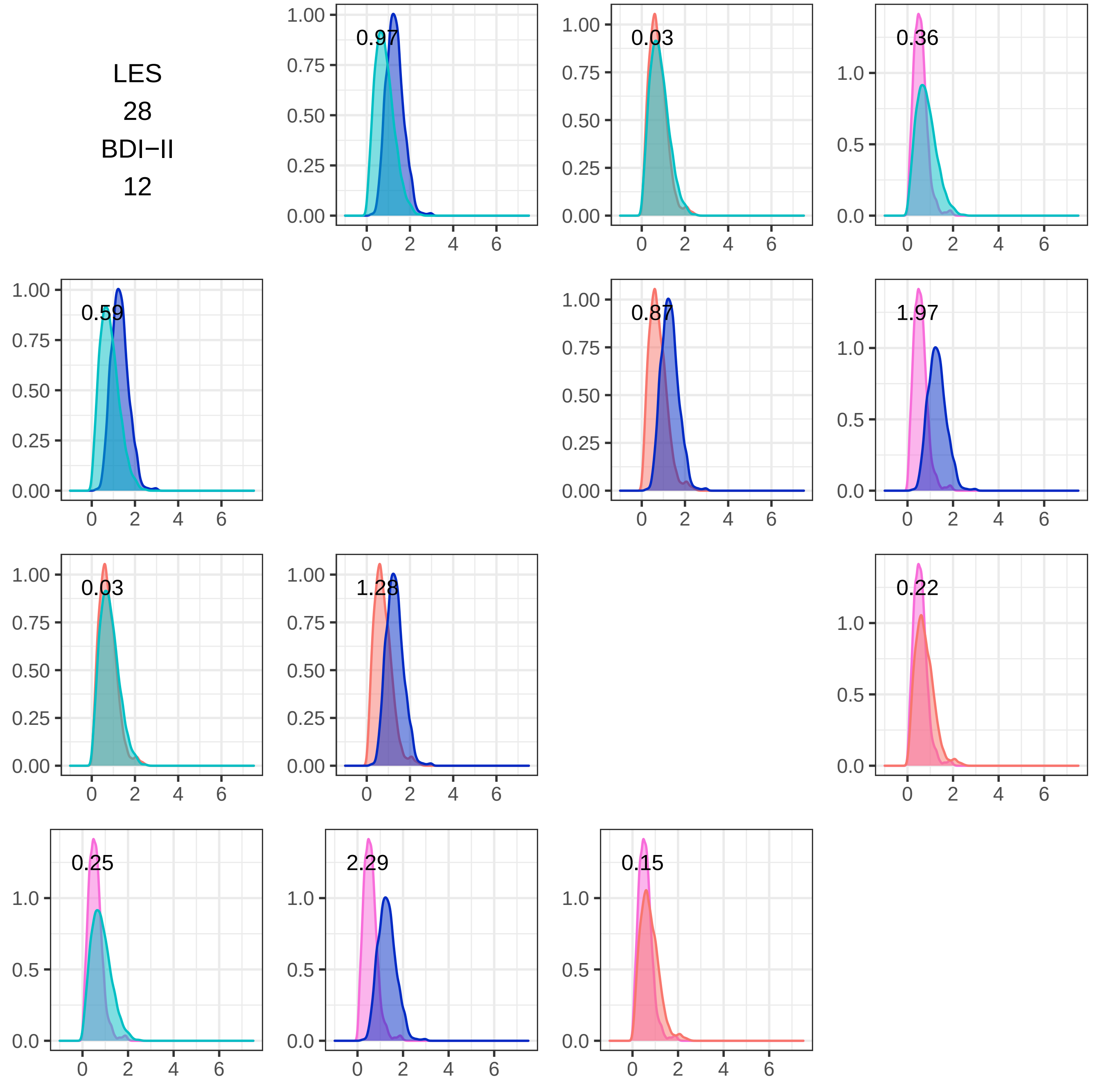
LES
28
BDI−II
12
0.97
0.03
0.36
0.59
0.87
1.97
0.03
1.28
0.22
0.25
2.29
0.15

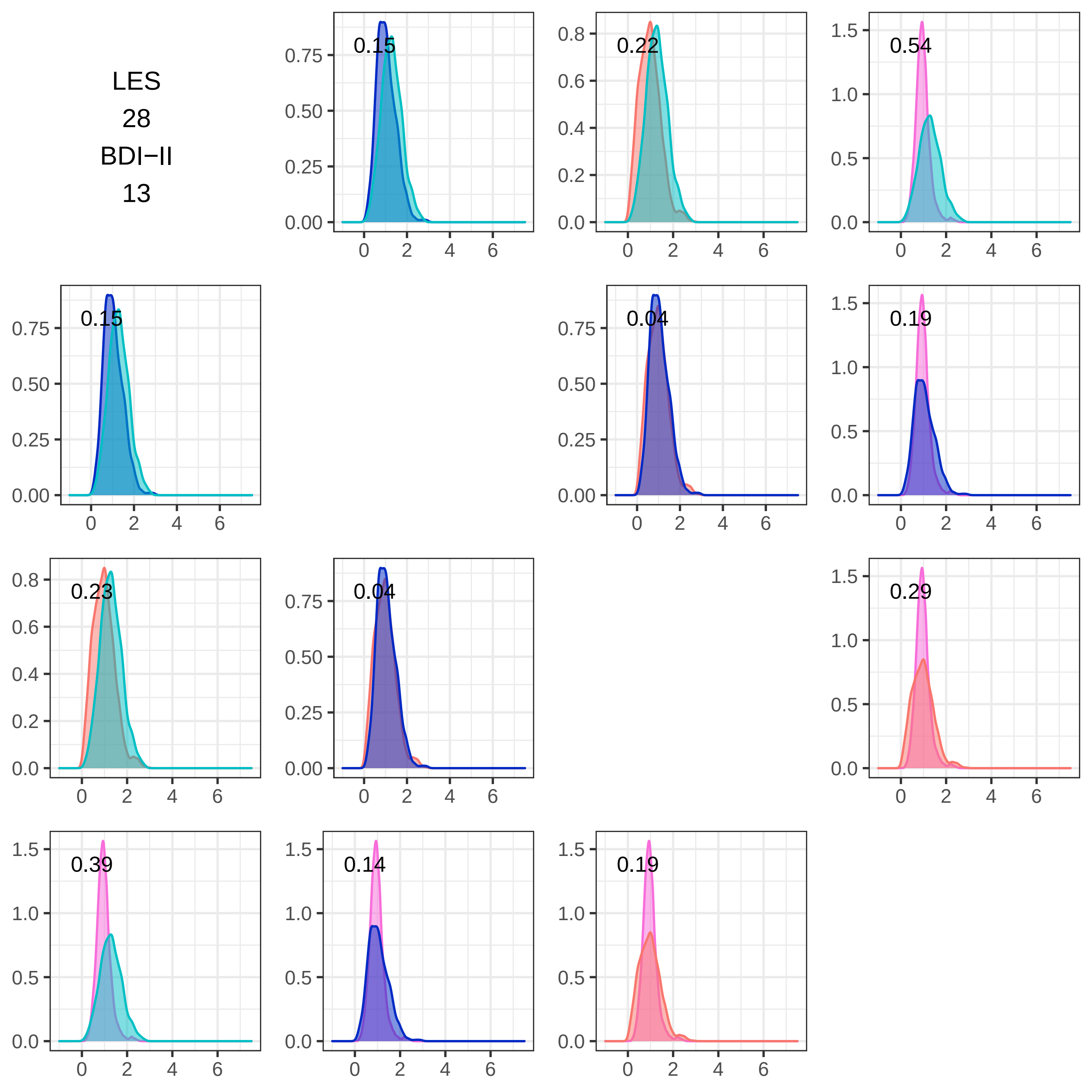
LES
28
BDI−II
13
0.15
0.22
0.54
0.15
0.04
0.19
0.23
0.04
0.29
0.39
0.14
0.19

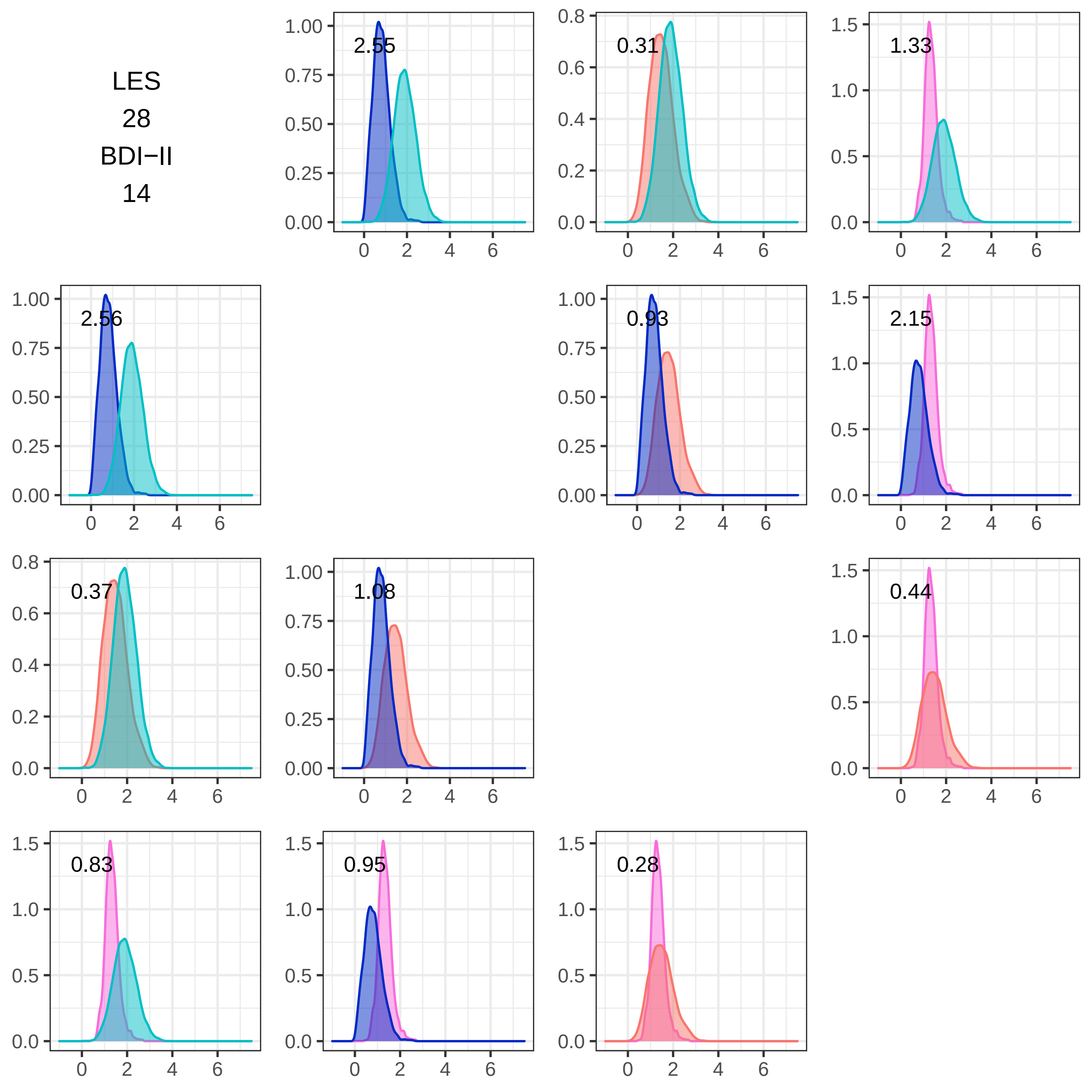
LES
28
BDI−II
14
2.55
0.31
1.33
2.56
0.93
2.15
0.37
1.08
0.44
0.83
0.95
0.28

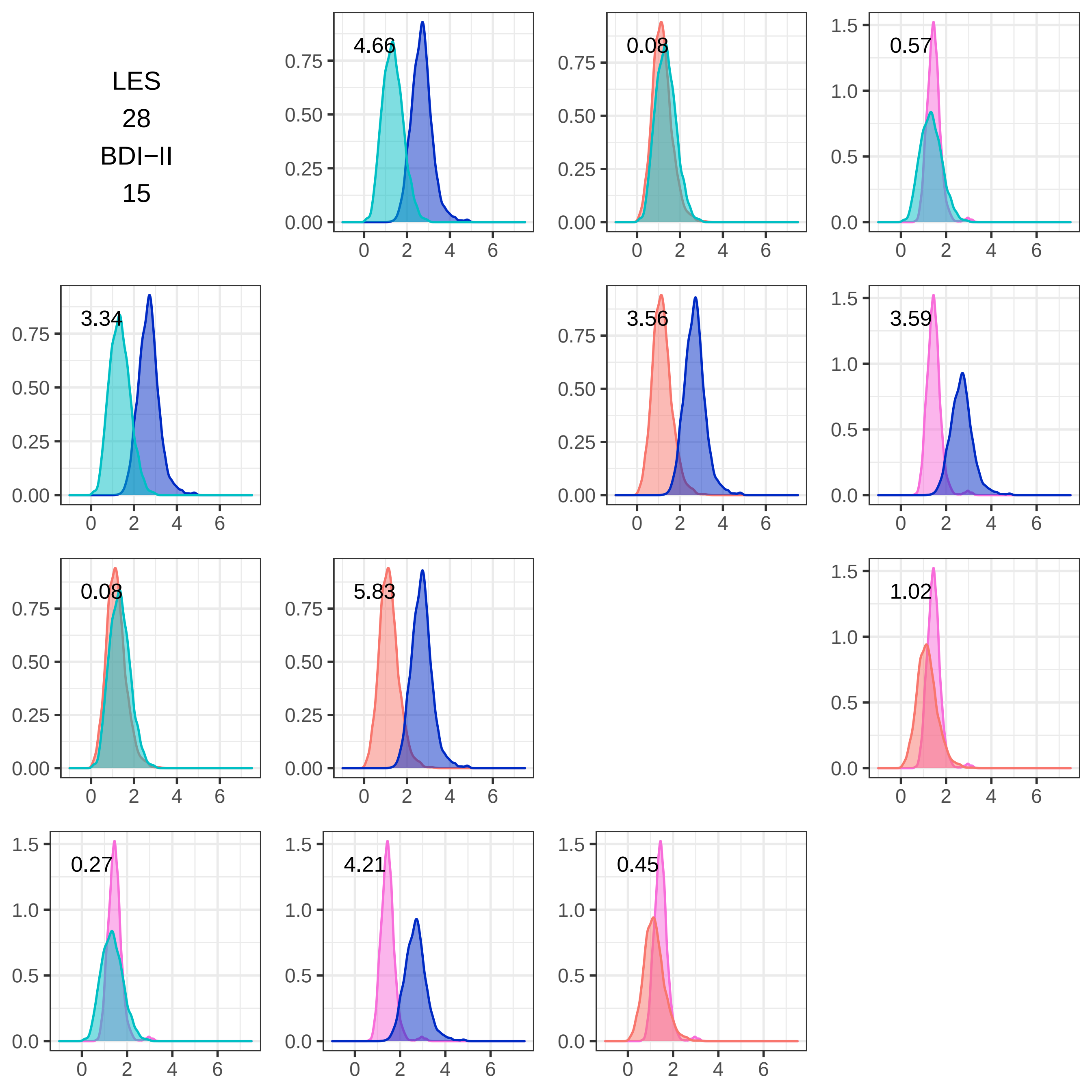
LES
28
BDI−II
15
4.66
0.08
0.57
3.34
3.56
3.59
0.08
5.83
1.02
0.27
4.21
0.45

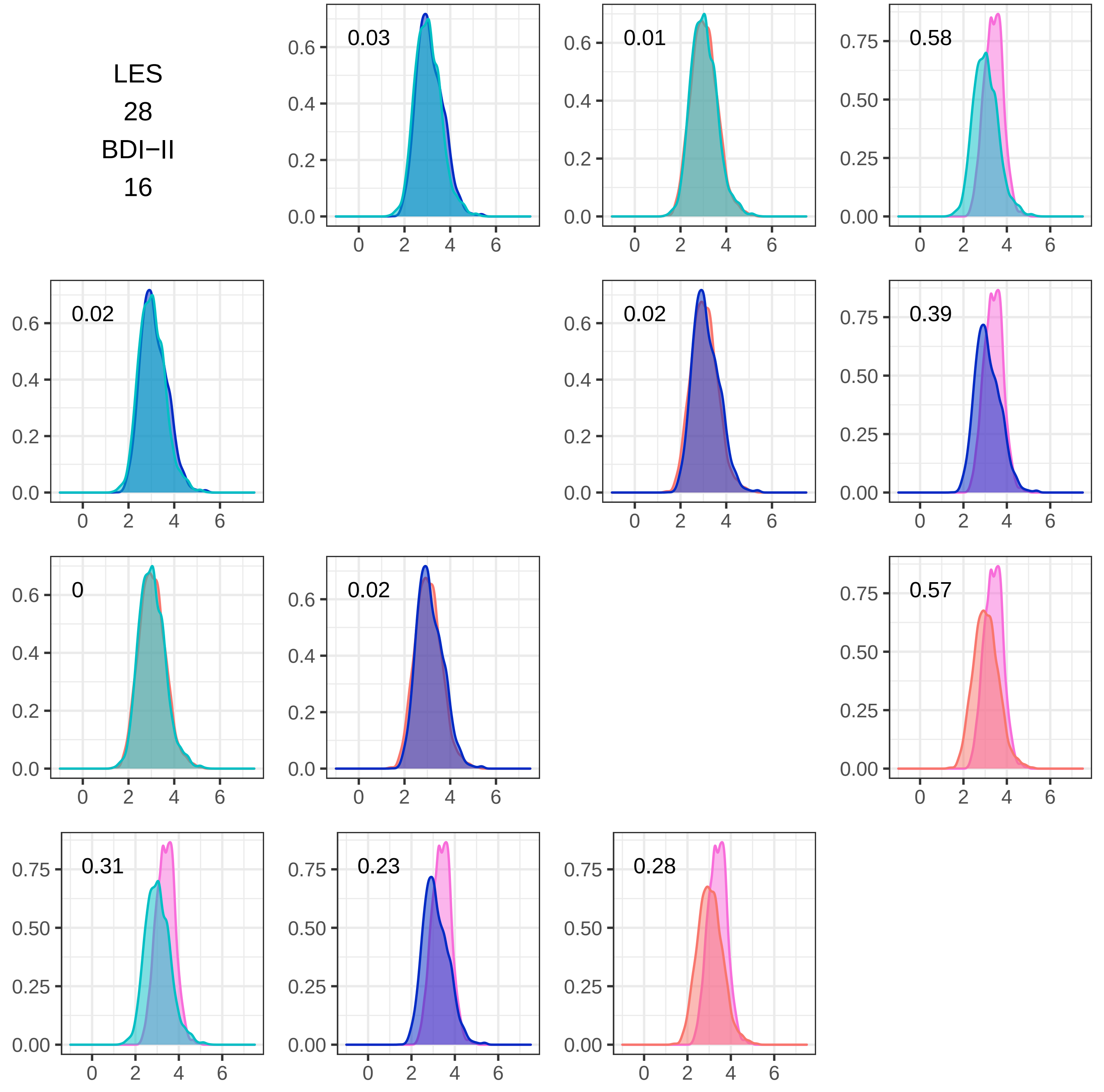
LES
28
BDI−II
16
0.03
0.01
0.58
0.02
0.02
0.39
0
0.02
0.57
0.31
0.23
0.28

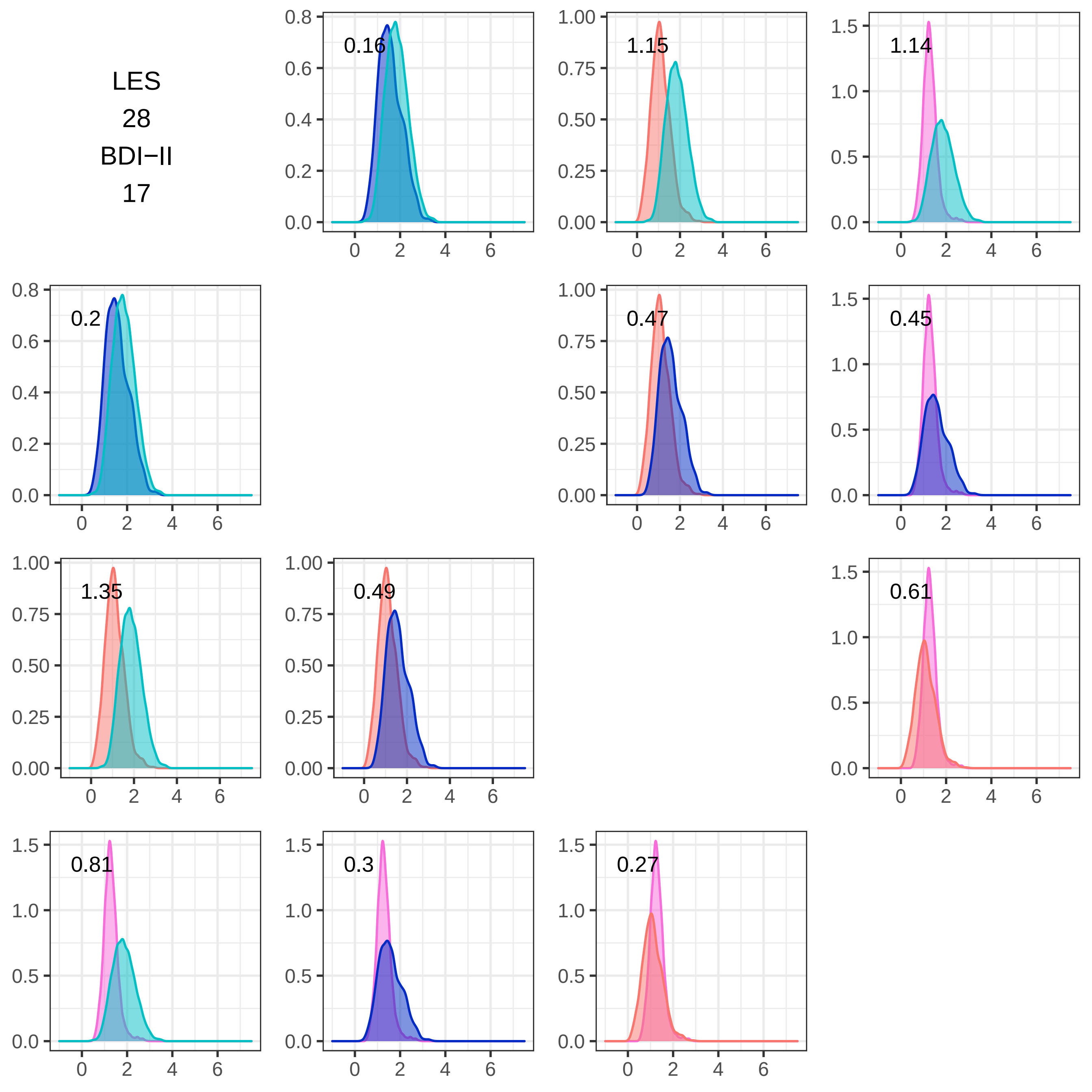
LES
28
BDI−II
17
0.16
1.15
1.14
0.2
0.47
0.45
1.35
0.49
0.61
0.81
0.3
0.27

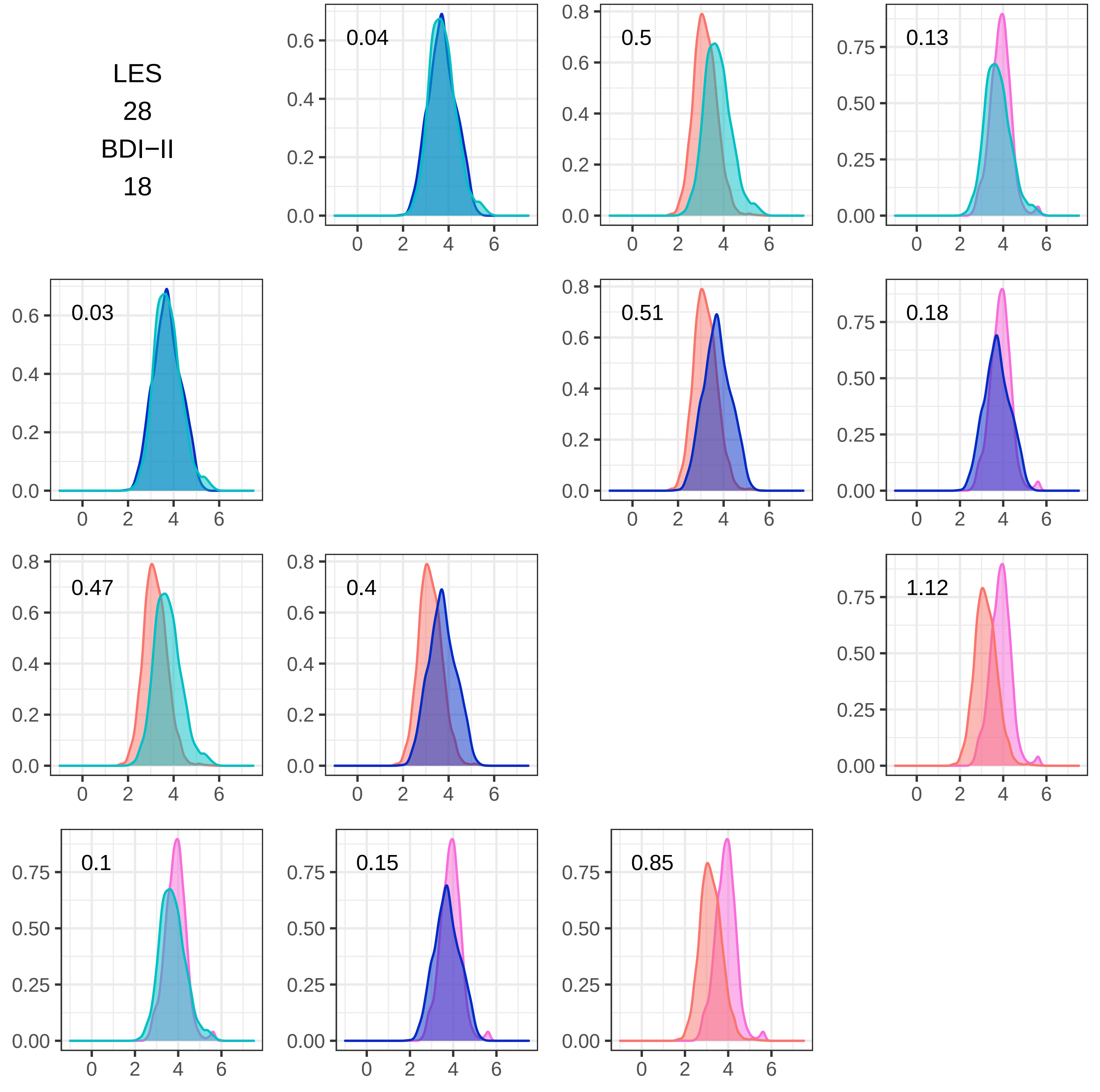
LES
28
BDI−II
18
0.04
0.5
0.13
0.03
0.51
0.18
0.47
0.4
1.12
0.1
0.15
0.85

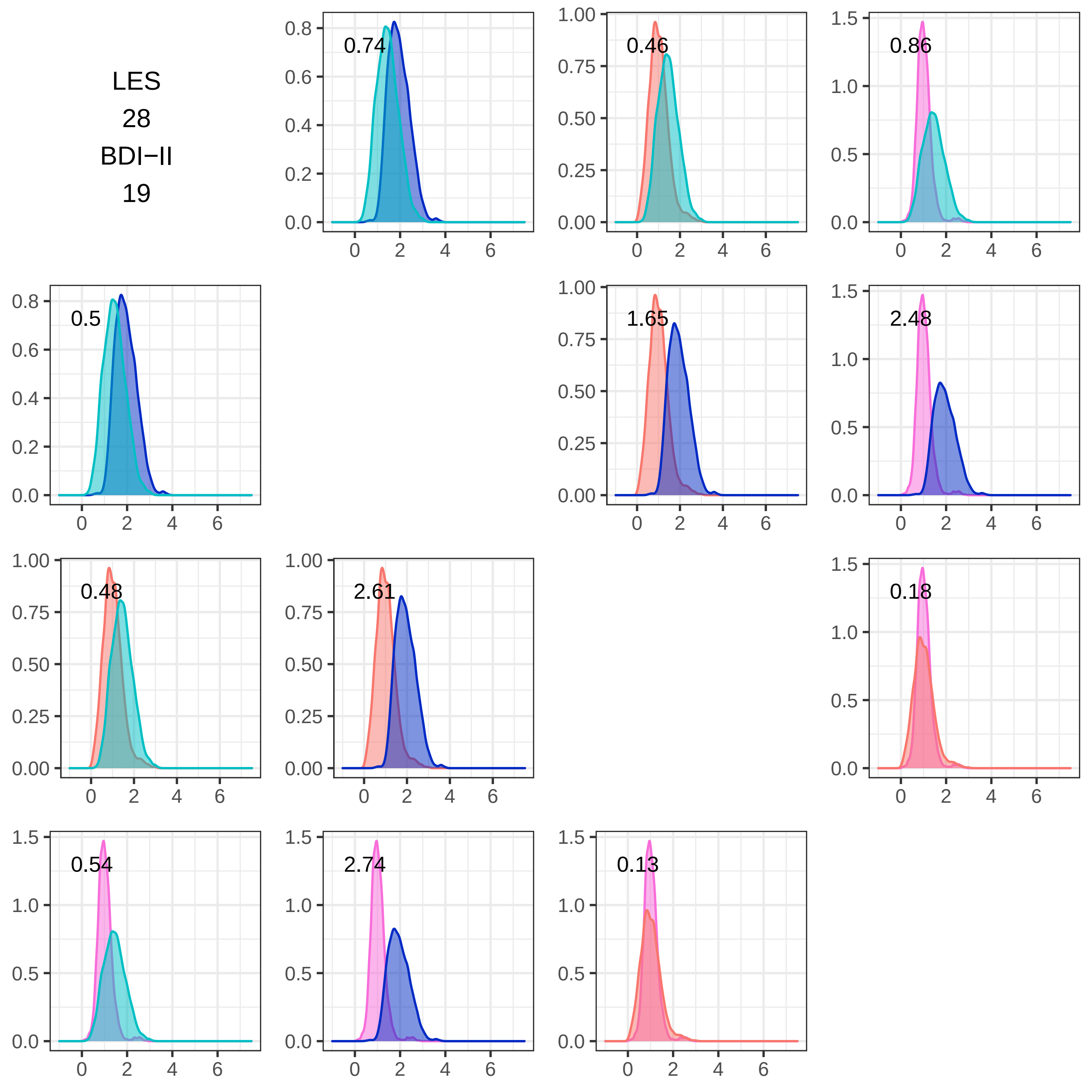
LES
28
BDI−II
19
0.74
0.46
0.86
0.5
1.65
2.48
0.48
2.61
0.18
0.54
2.74
0.13

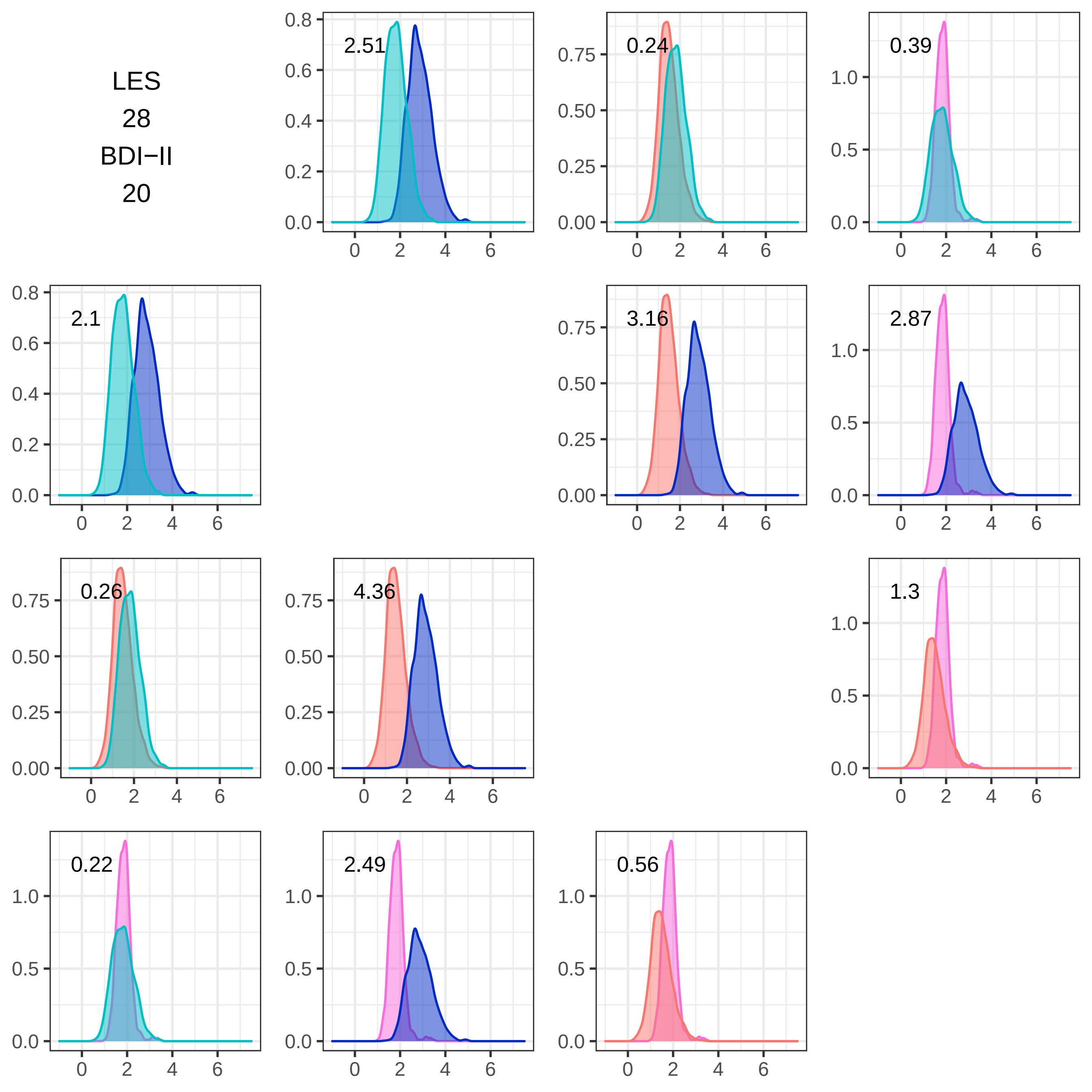
LES
28
BDI−II
20
2.51
0.24
0.39
2.1
3.16
2.87
0.26
4.36
1.3
0.22
2.49
0.56

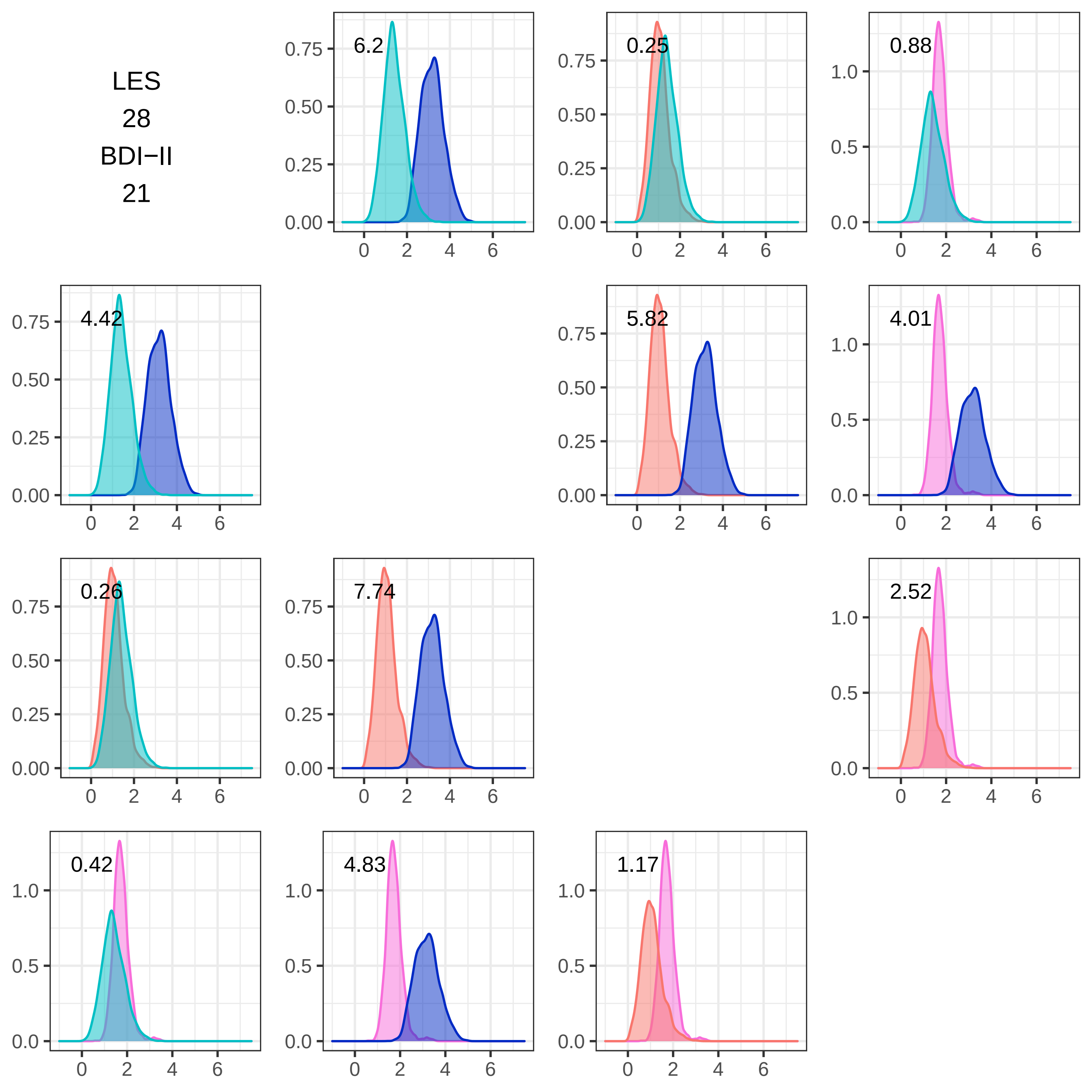
LES
28
BDI−II
21
6.2
0.25
0.88
4.42
5.82
4.01
0.26
7.74
2.52
0.42
4.83
1.17

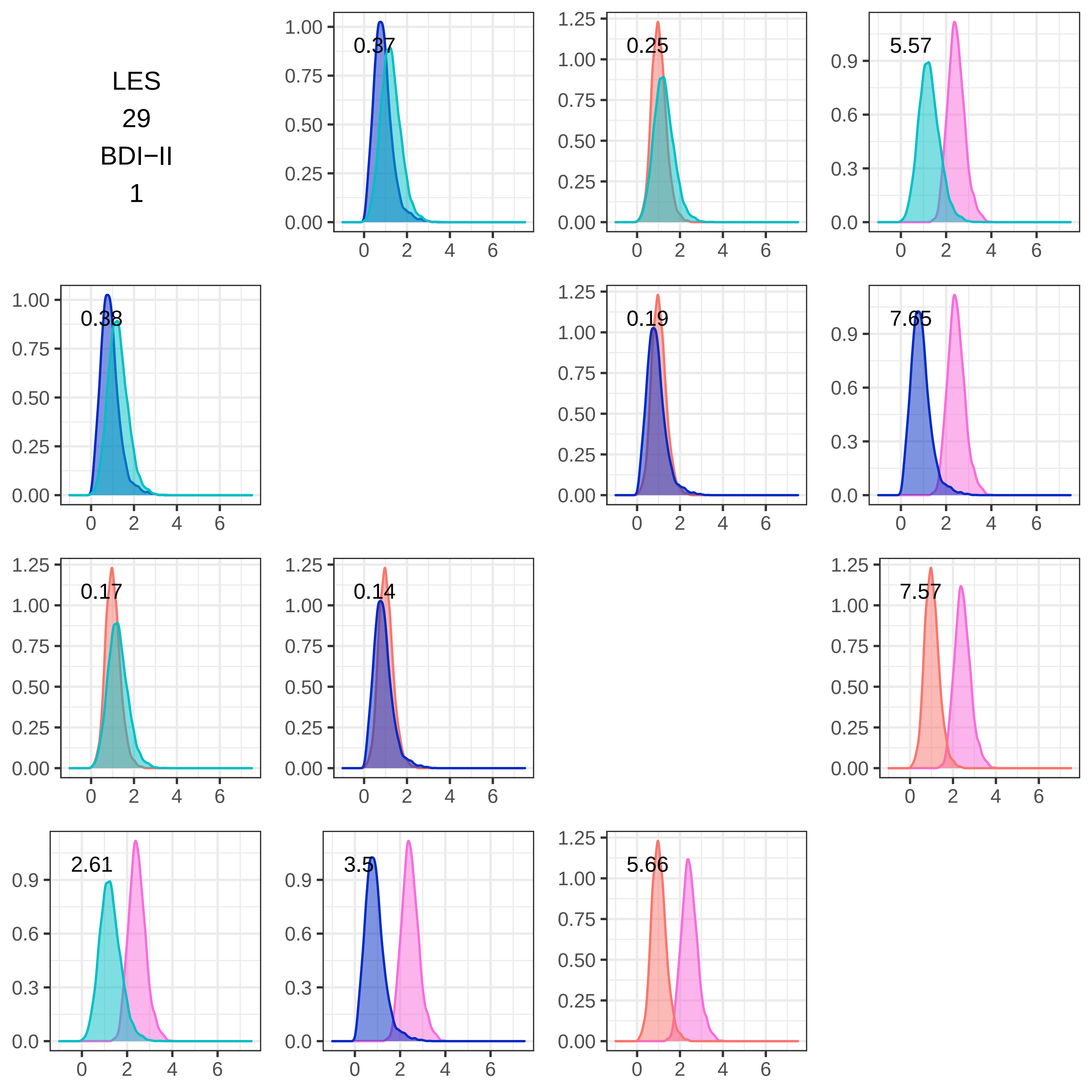
LES
29
BDI−II
1
0.37
0.25
5.57
0.38
0.19
7.65
0.17
0.14
7.57
2.61
3.5
5.66

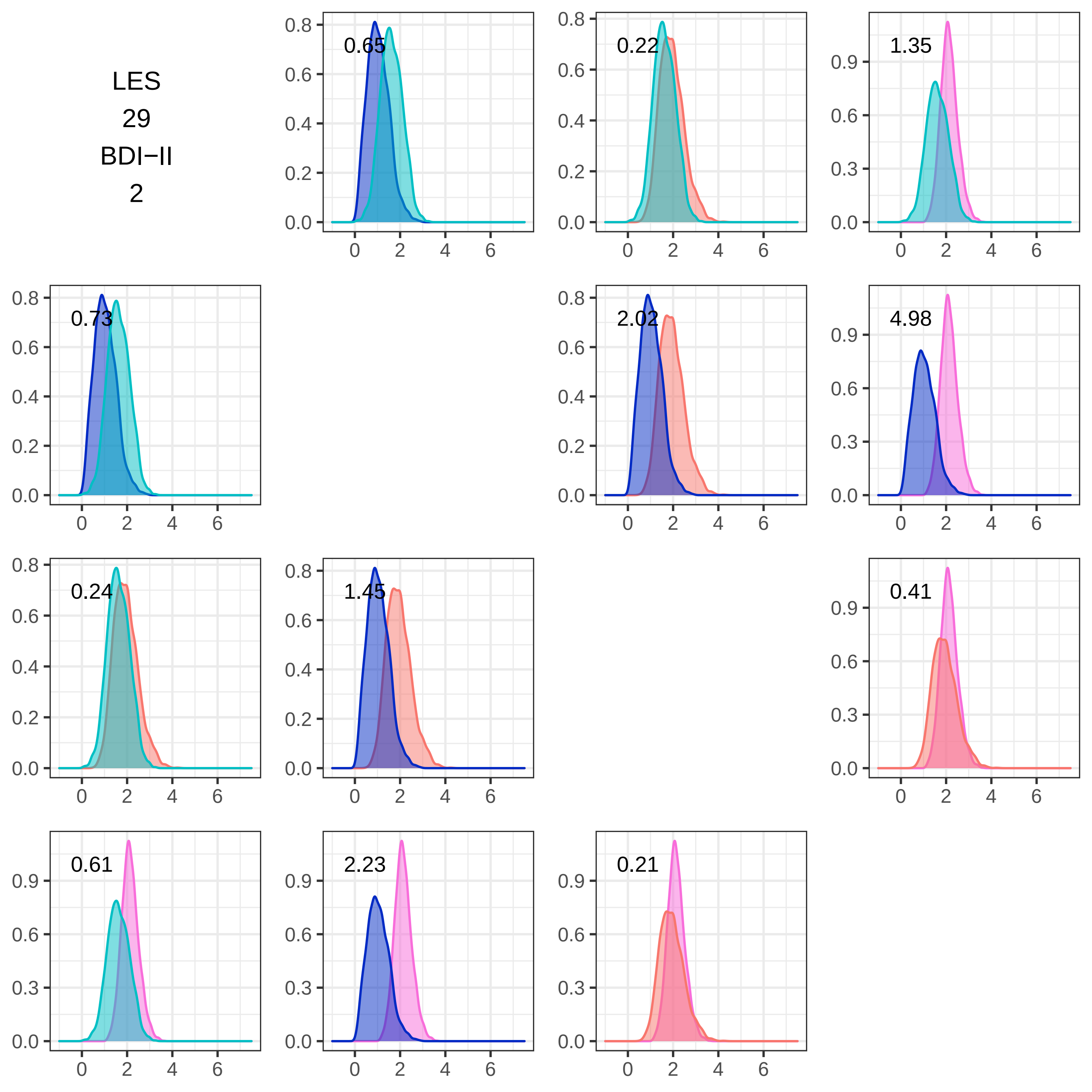

LES
29
BDI−II
2
0.65
0.22
1.35
0.73
2.02
4.98
0.24
1.45
0.41
0.61
2.23
0.21

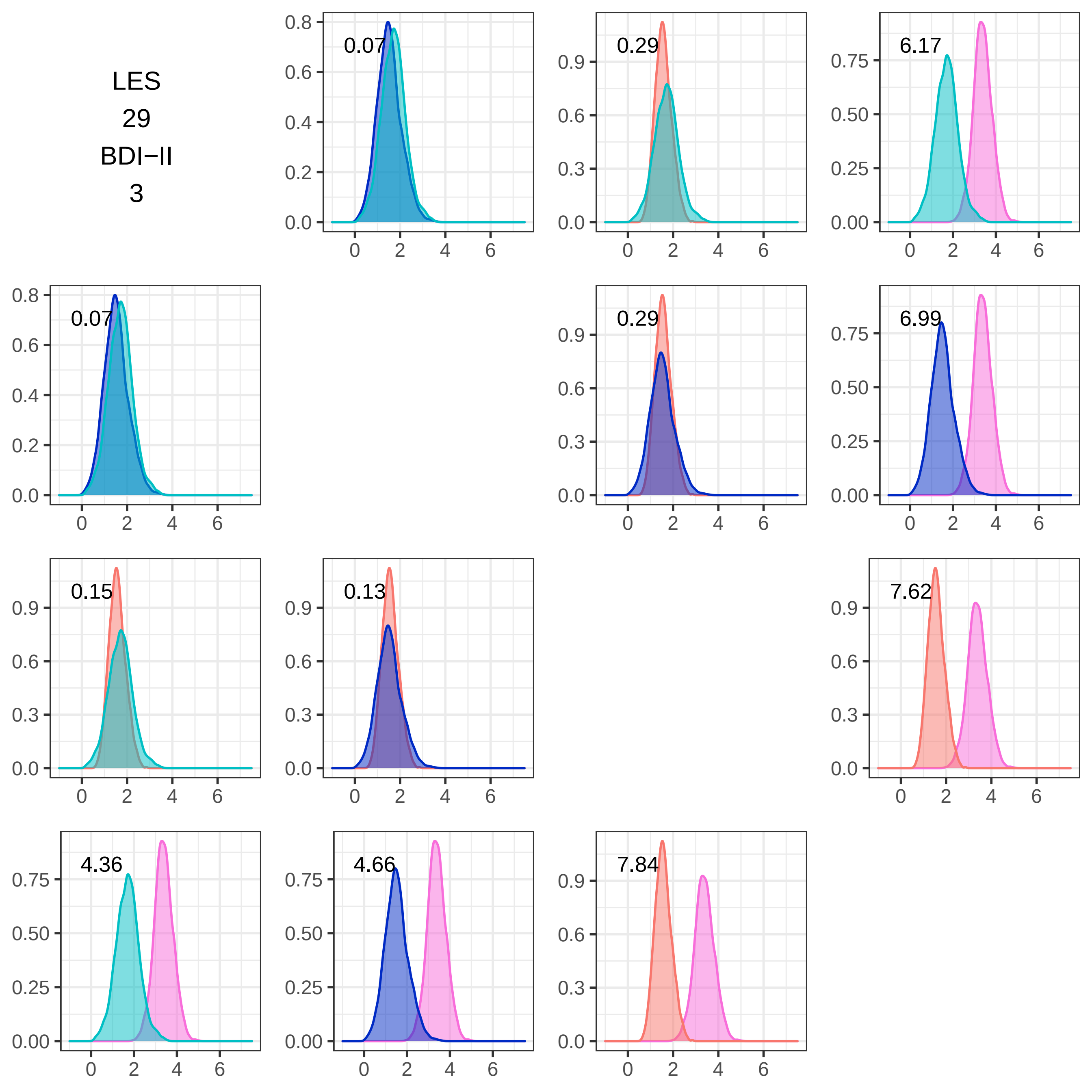

LES
29
BDI−II
3
0.07
0.29
6.17
0.07
0.29
6.99
0.15
0.13
7.62
4.36
4.66
7.84

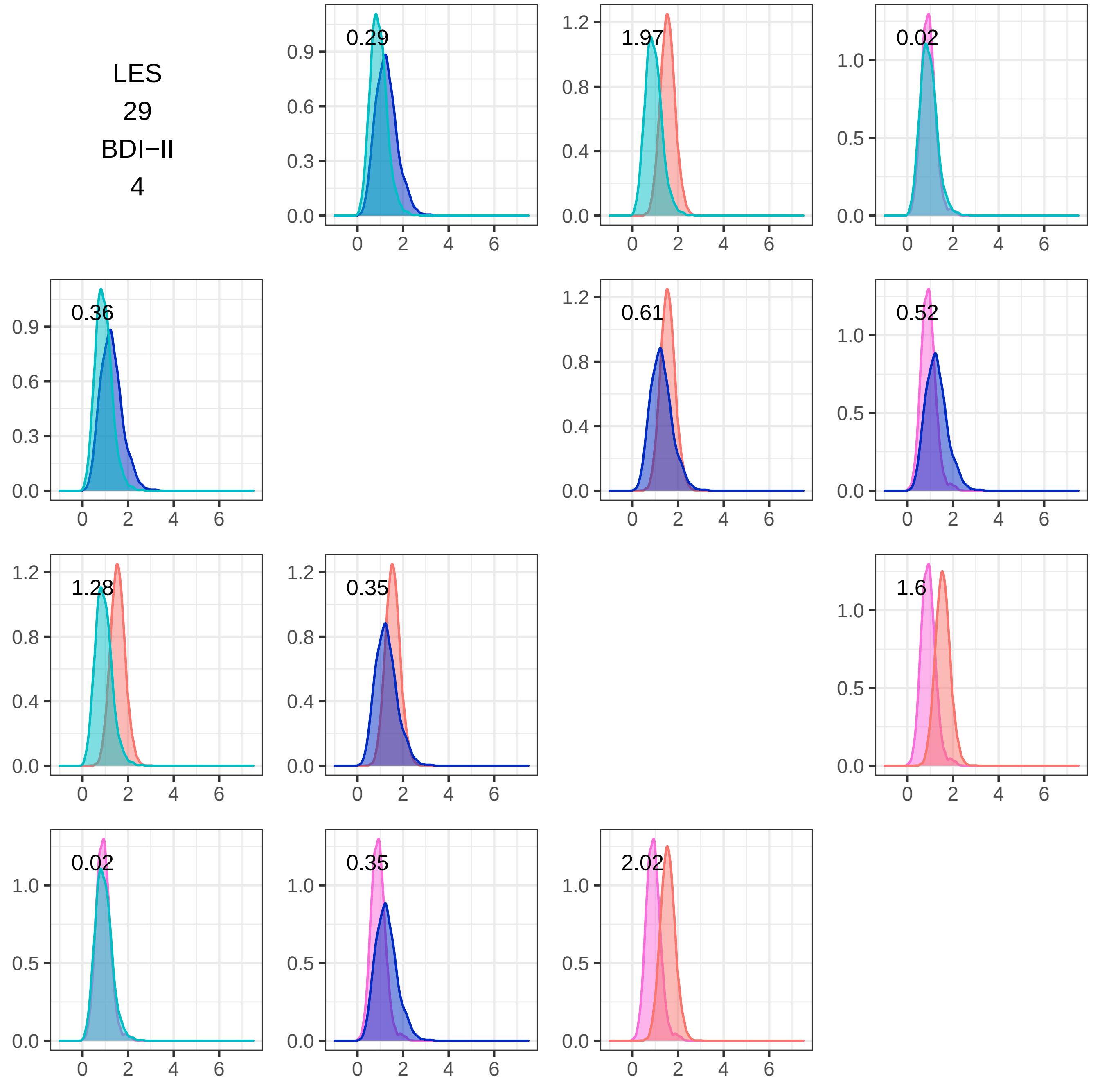

LES
29
BDI−II
4
0.29
1.97
0.02
0.36
0.61
0.52
1.28
0.35
1.6
0.02
0.35
2.02

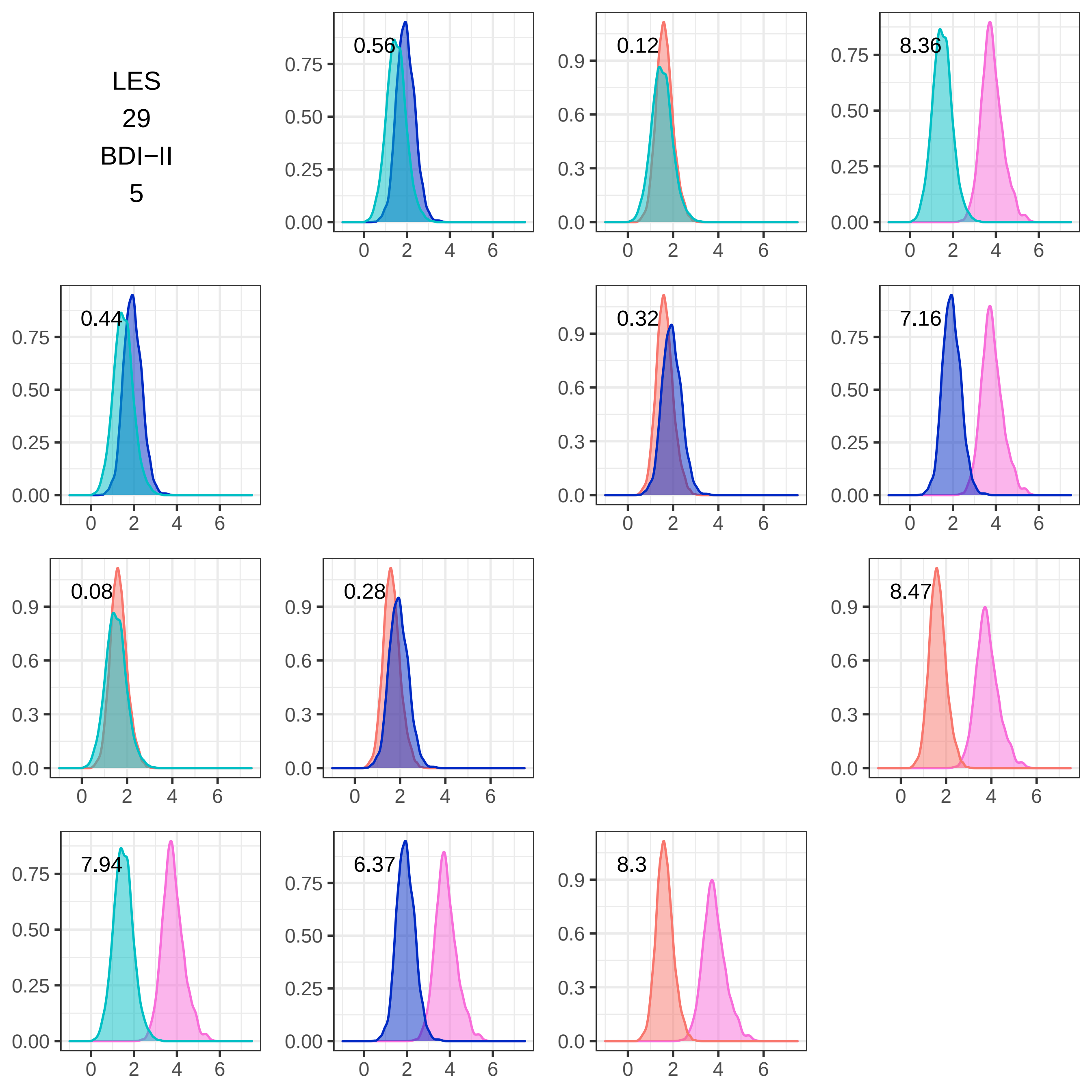

LES
29
BDI−II
5
0.56
0.12
8.36
0.44
0.32
7.16
0.08
0.28
8.47
7.94
6.37
8.3

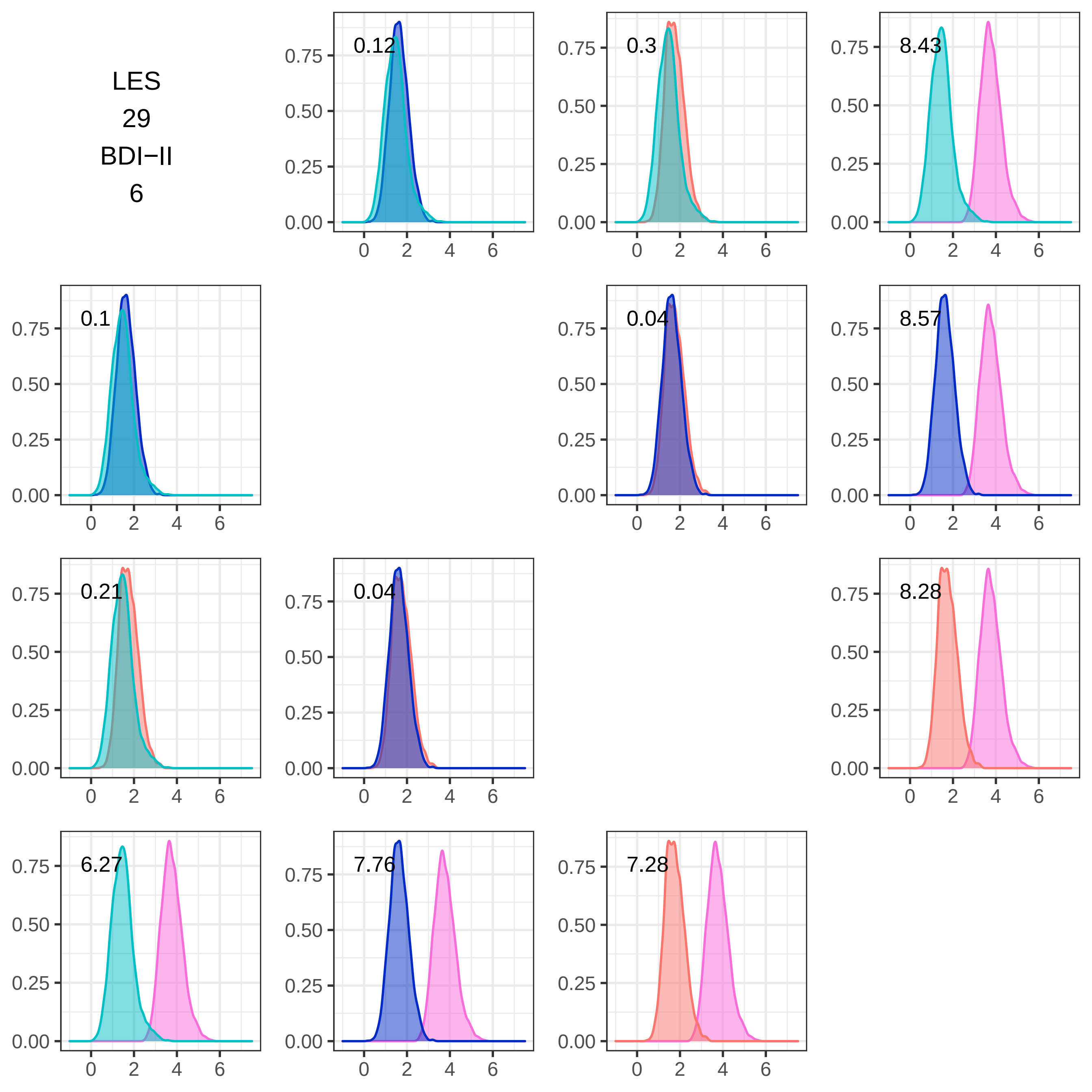

LES
29
BDI−II
6
0.12
0.3
8.43
0.1
0.04
8.57
0.21
0.04
8.28
6.27
7.76
7.28

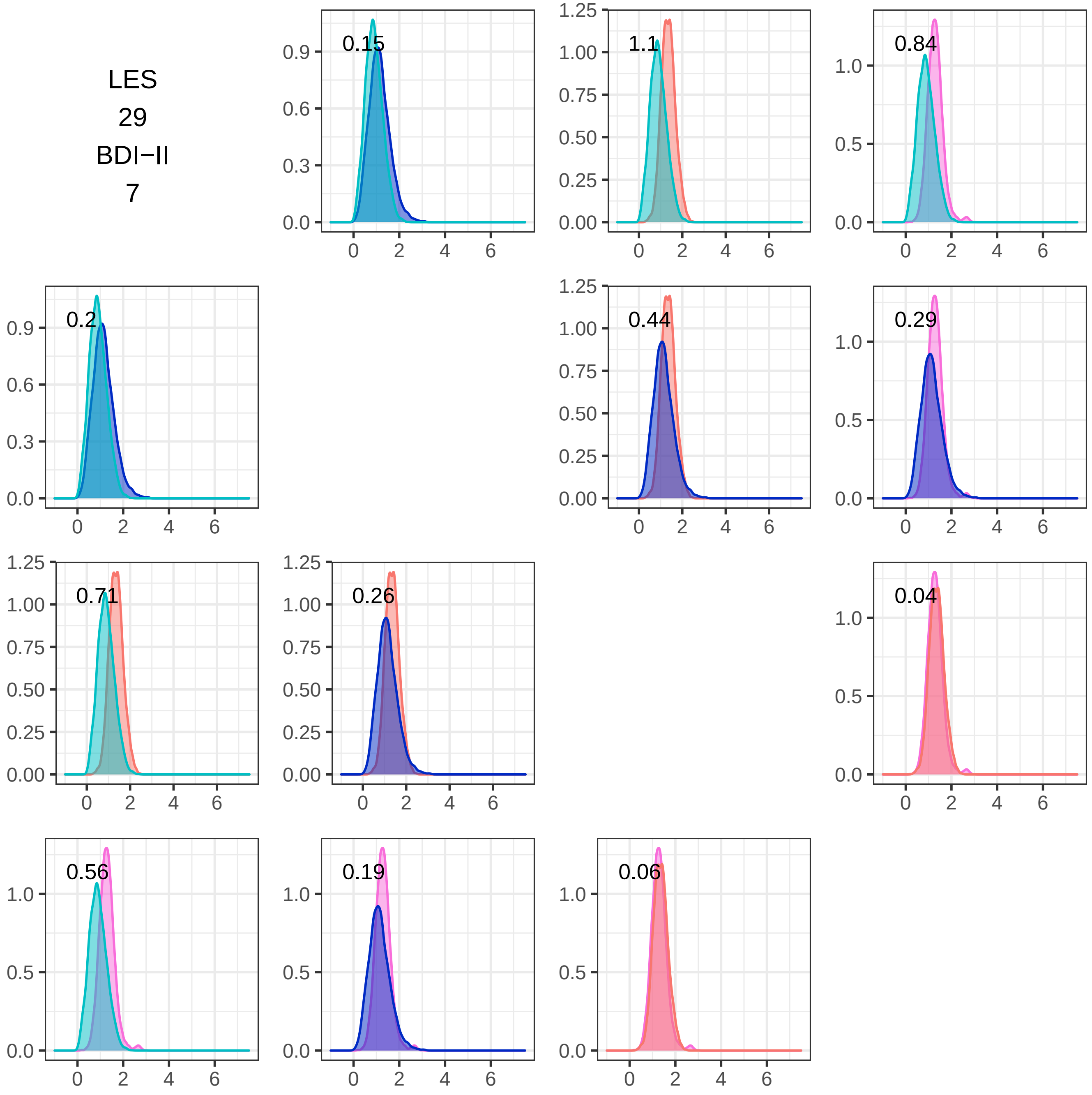
LES
29
BDI−II
7
0.15
1.1
0.84
0.2
0.44
0.29
0.71
0.26
0.04
0.56
0.19
0.06

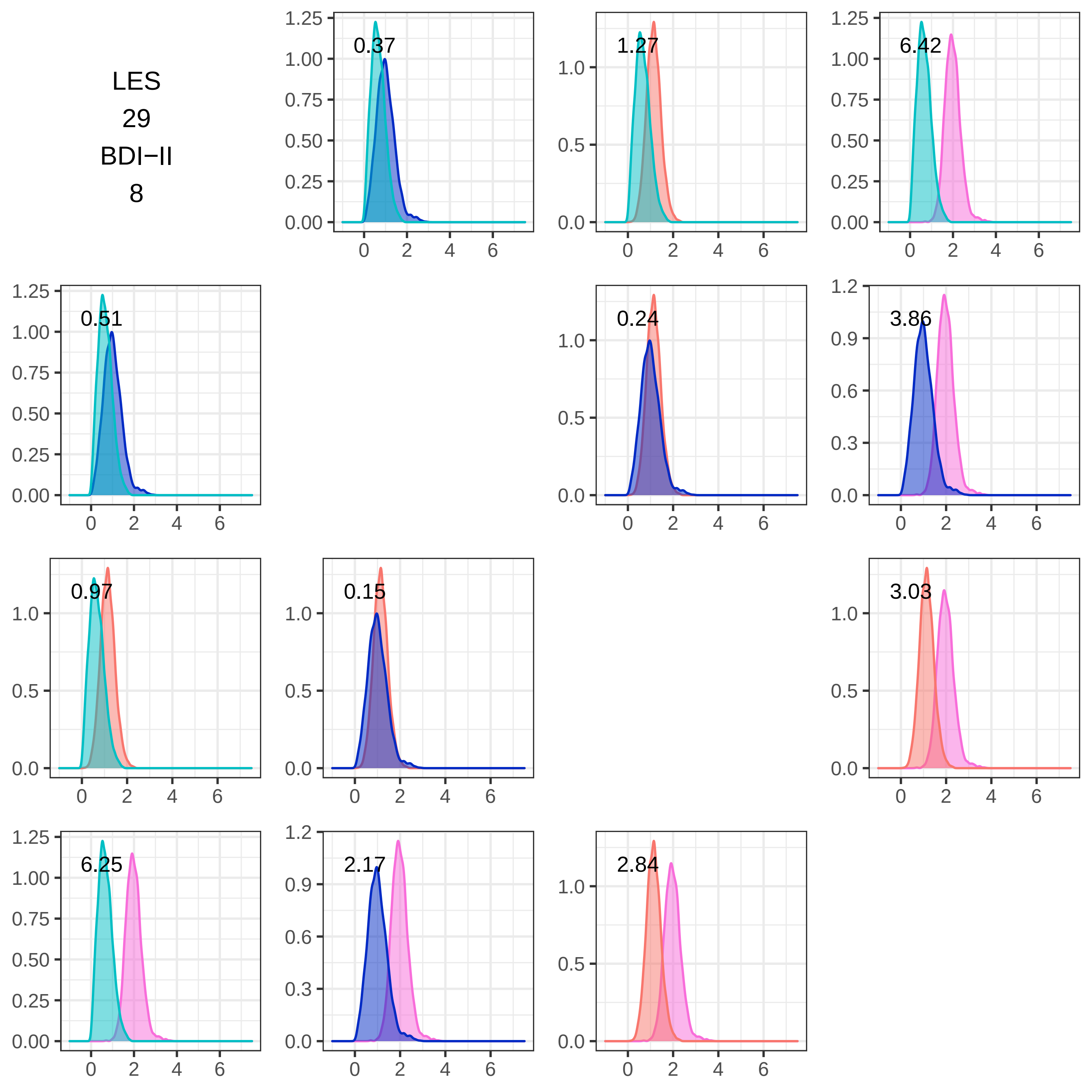
LES
29
BDI−II
8
0.37
1.27
6.42
0.51
0.24
3.86
0.97
0.15
3.03
6.25
2.17
2.84

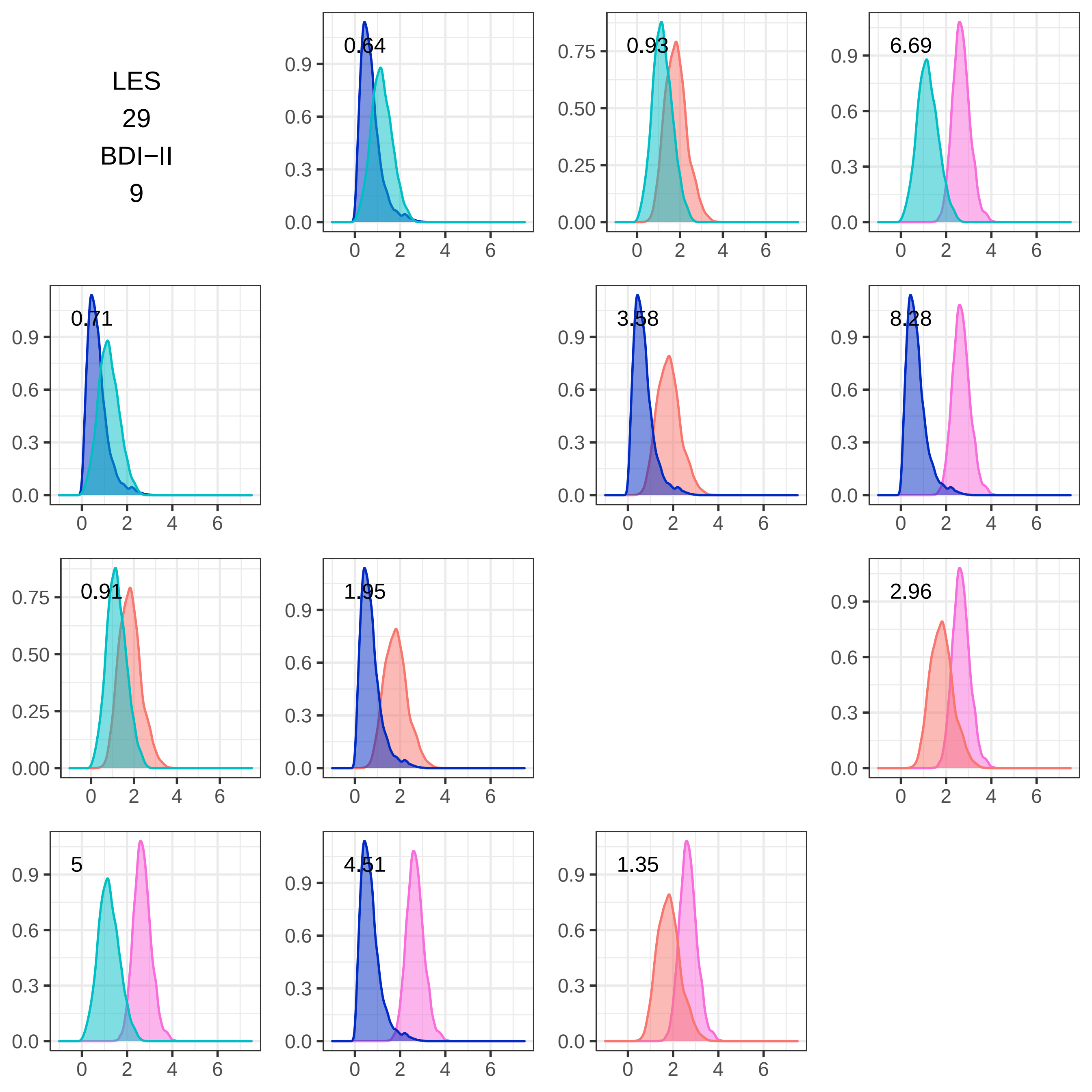

LES
29
BDI−II
9
0.64
0.93
6.69
0.71
3.58
8.28
0.91
1.95
2.96
5
4.51
1.35

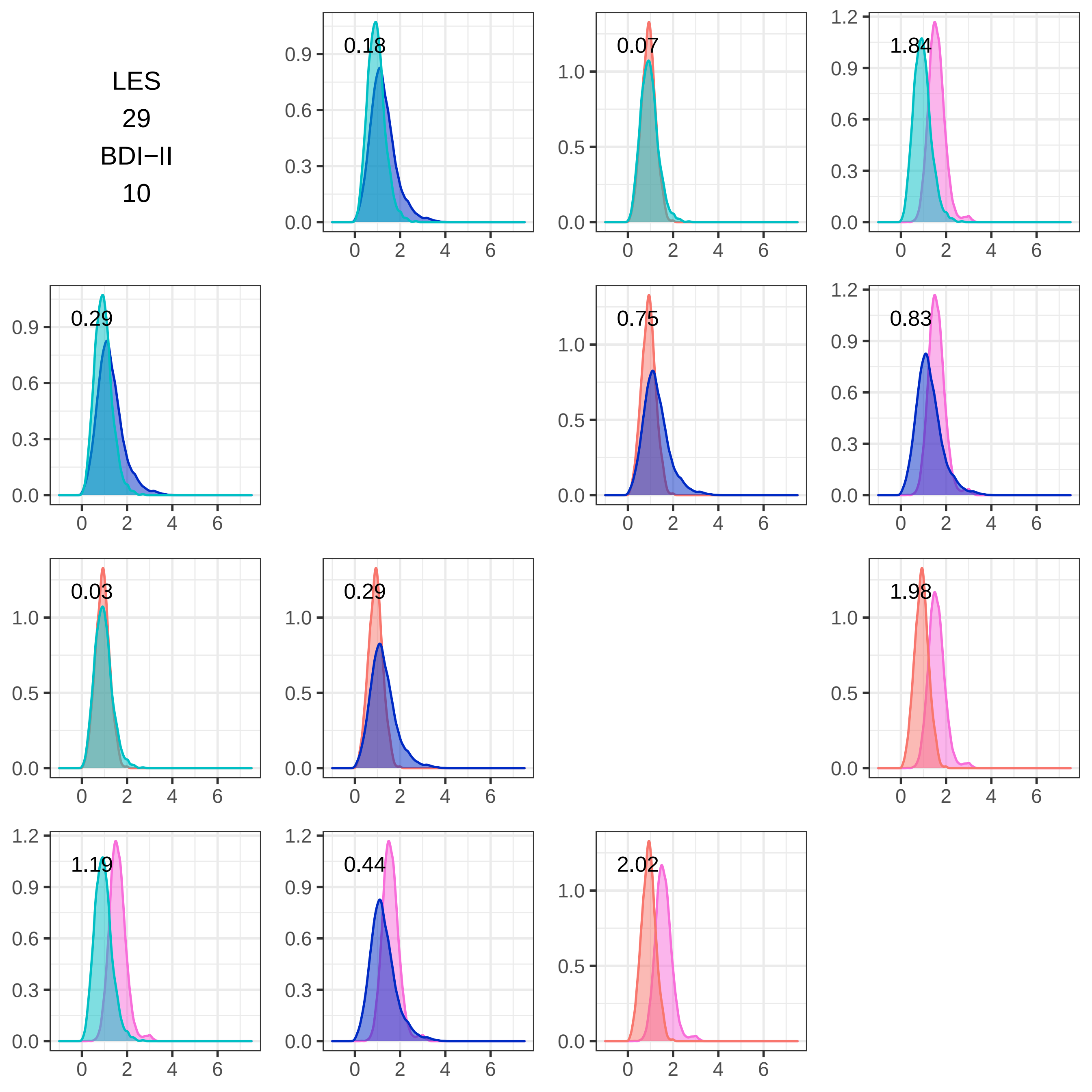
LES
29
BDI−II
10
0.18
0.07
1.84
0.29
0.75
0.83
0.03
0.29
1.98
1.19
0.44
2.02

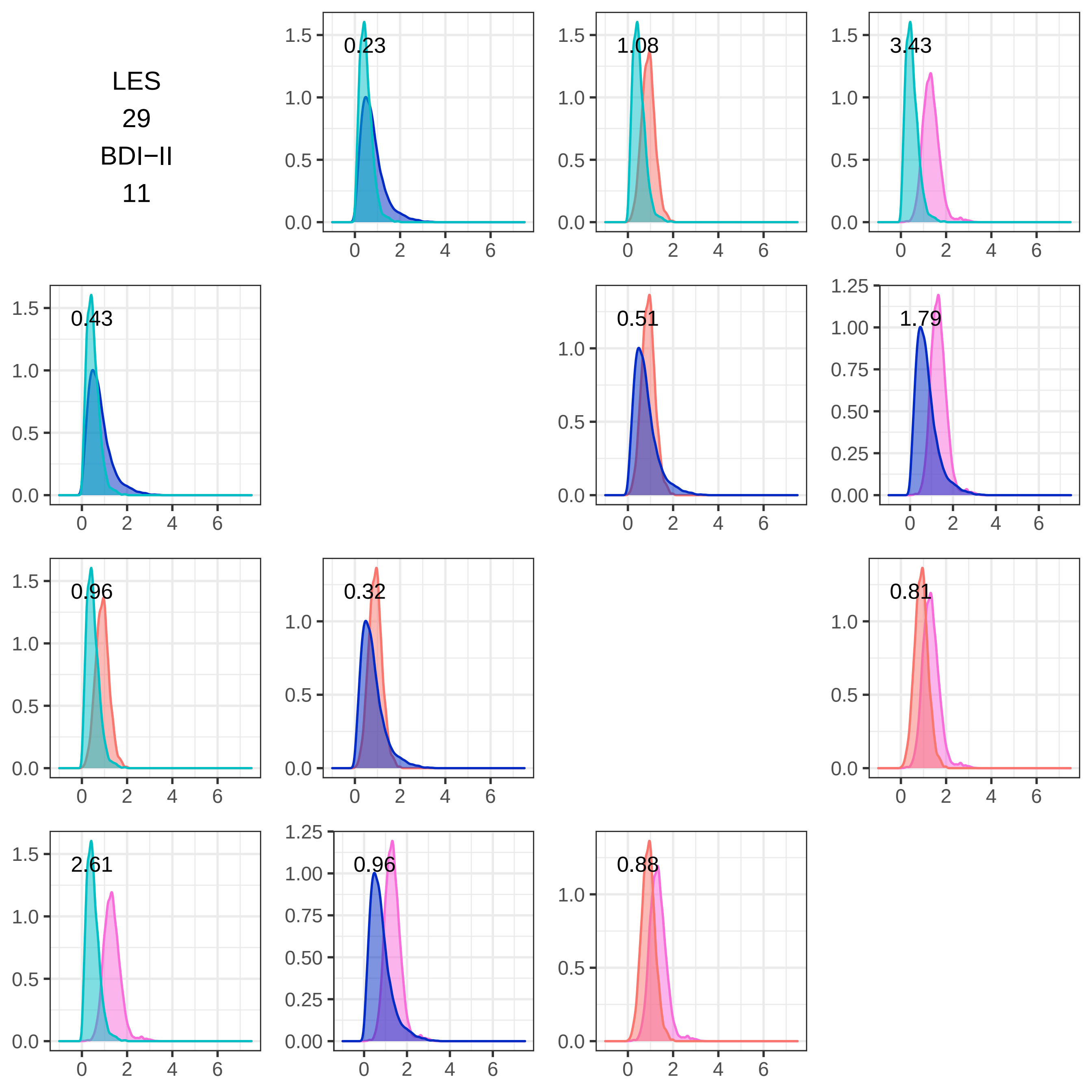

LES
29
BDI−II
11
0.23
1.08
3.43
0.43
0.51
1.79
0.96
0.32
0.81
2.61
0.96
0.88

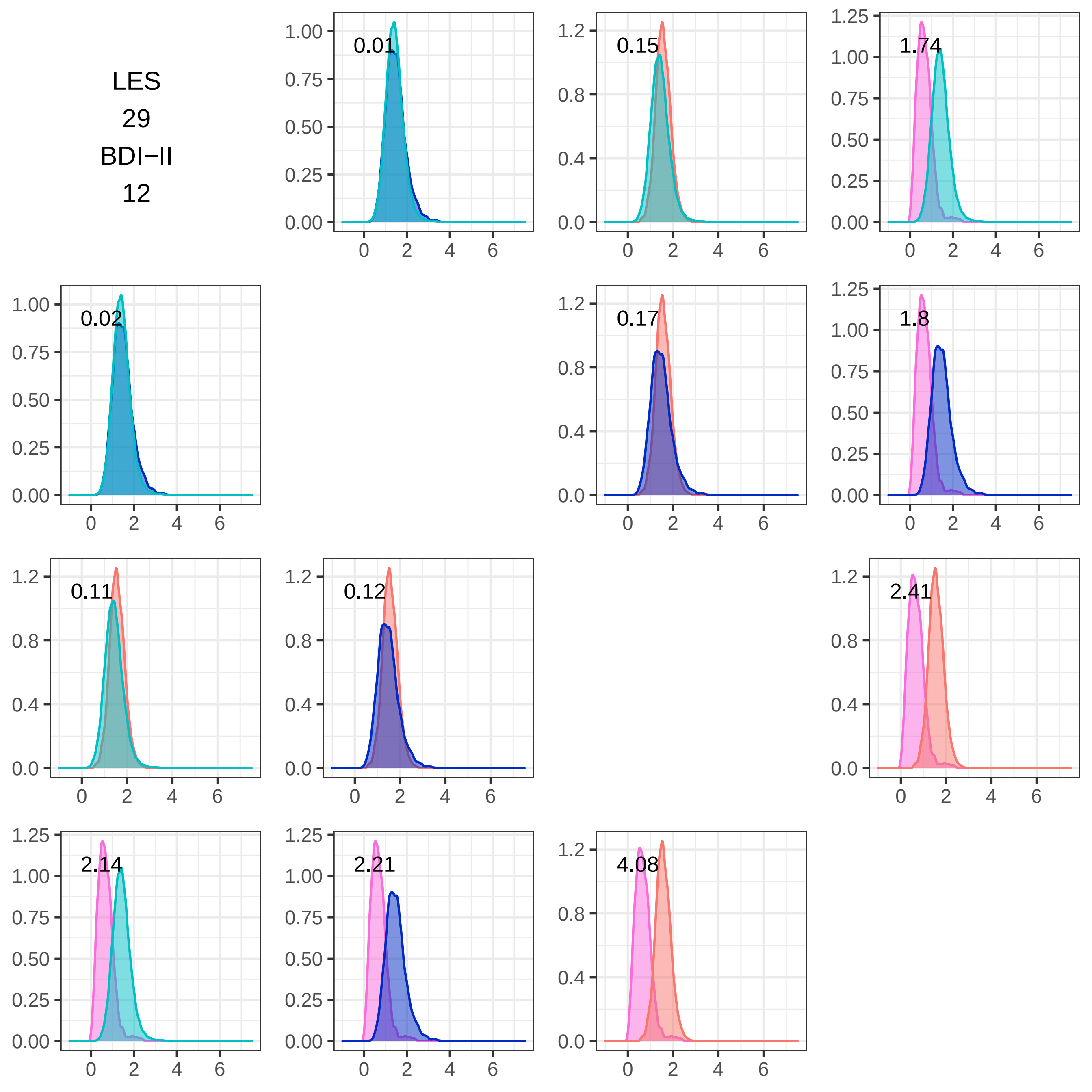

LES
29
BDI−II
12
0.01
0.15
1.74
0.02
0.17
1.8
0.11
0.12
2.41
2.14
2.21
4.08

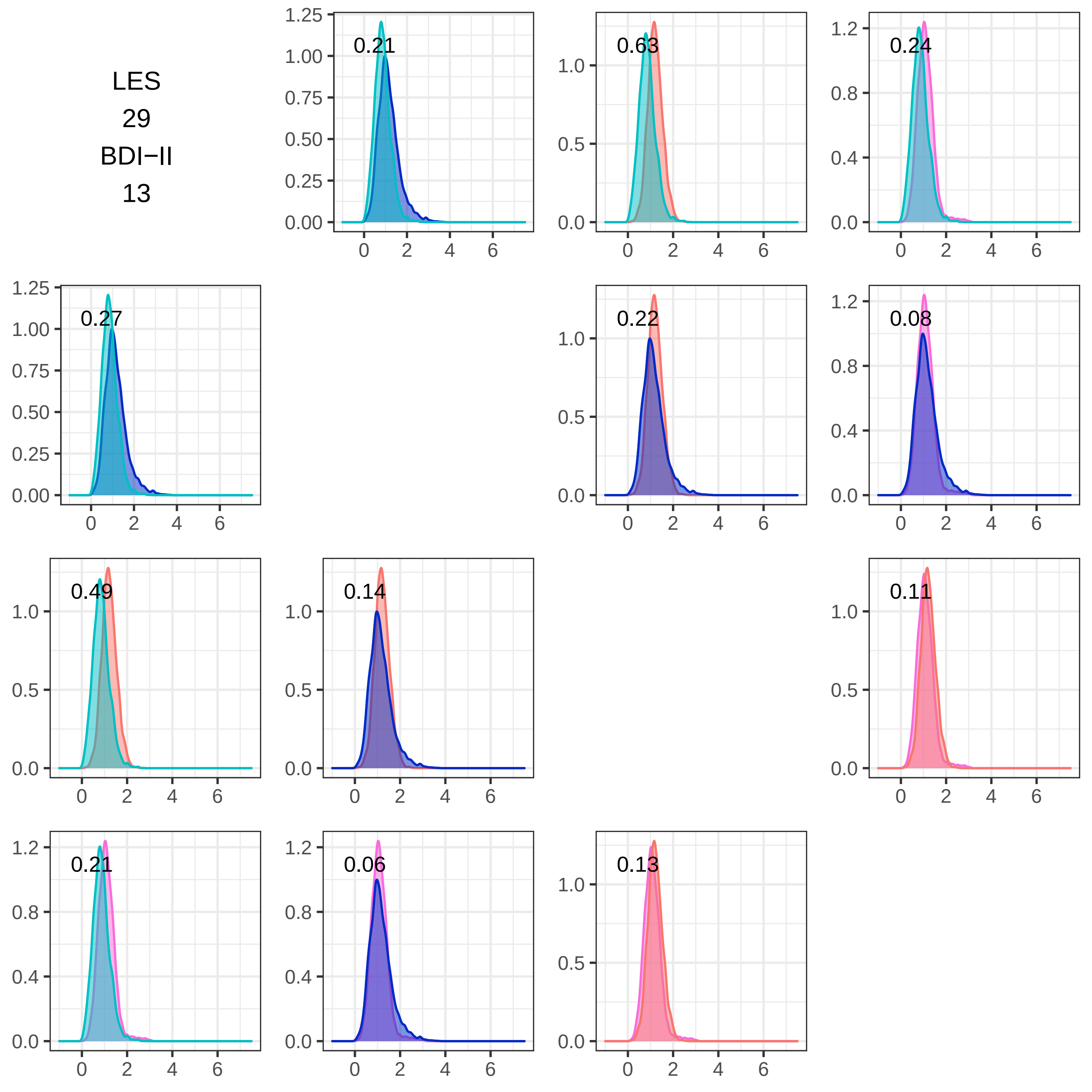
LES
29
BDI−II
13
0.21
0.63
0.24
0.27
0.22
0.08
0.49
0.14
0.11
0.21
0.06
0.13

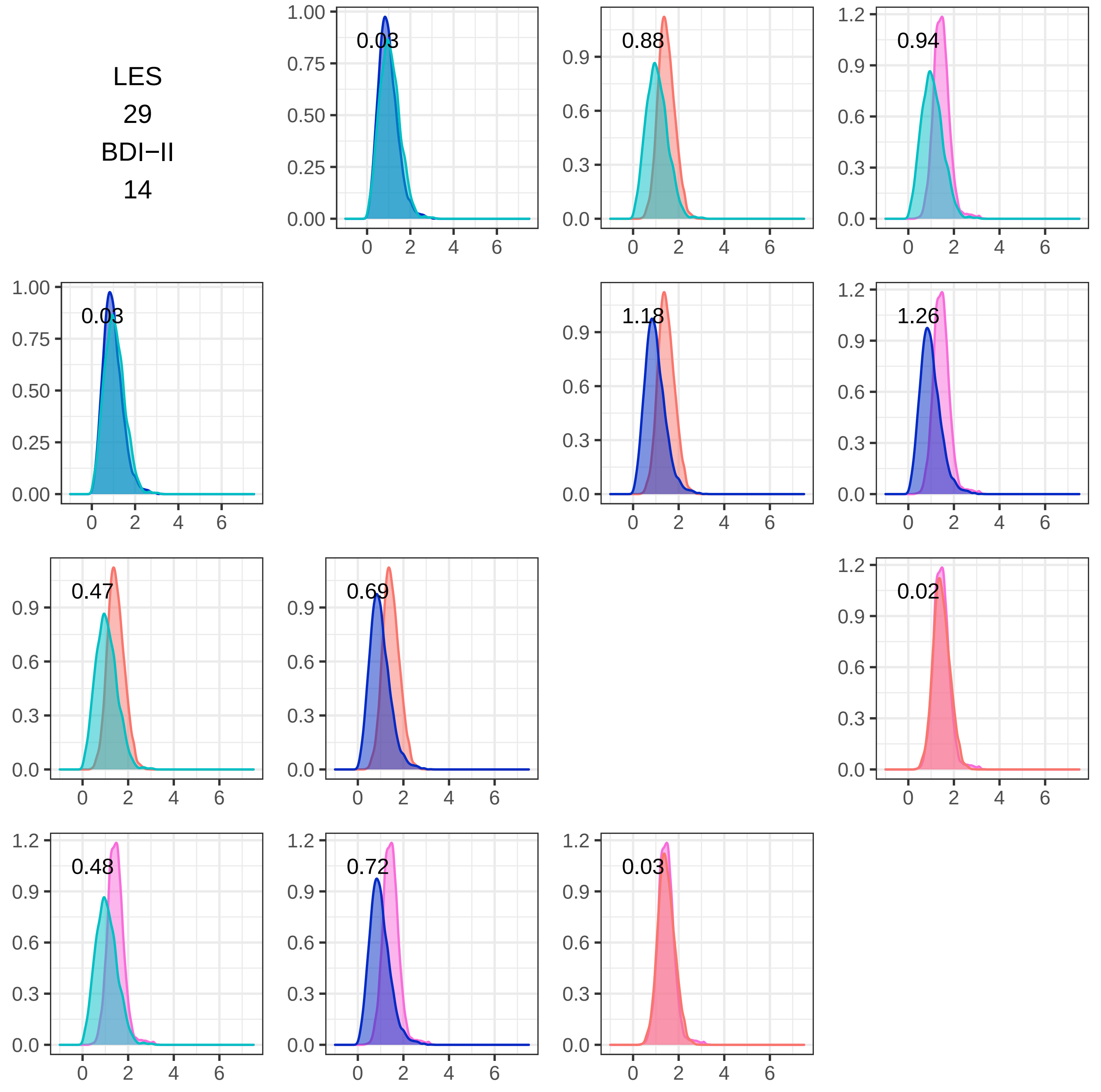

LES
29
BDI−II
14
0.03
0.88
0.94
0.03
1.18
1.26
0.47
0.69
0.02
0.48
0.72
0.03

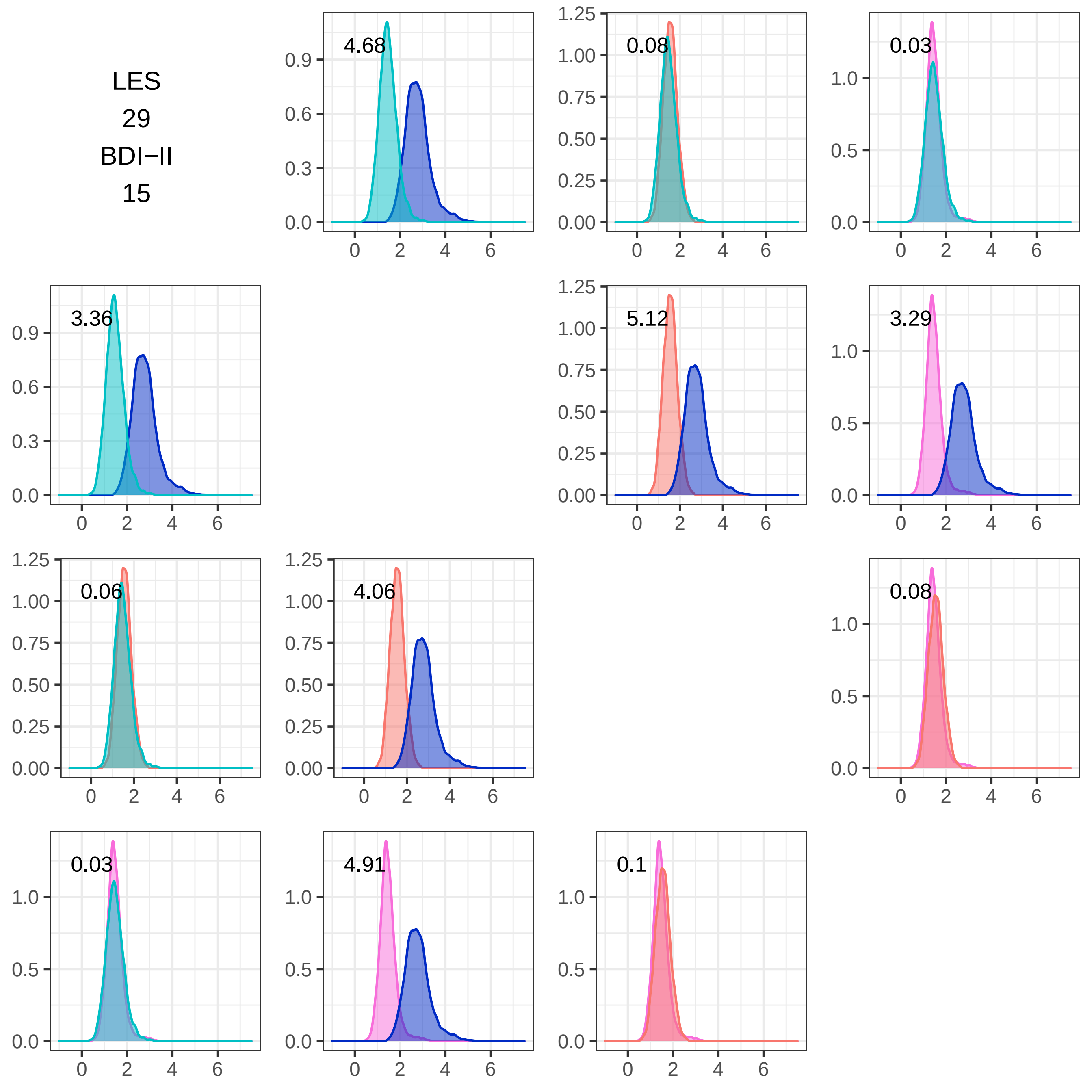
LES
29
BDI−II
15
4.68
0.08
0.03
3.36
5.12
3.29
0.06
4.06
0.08
0.03
4.91
0.1

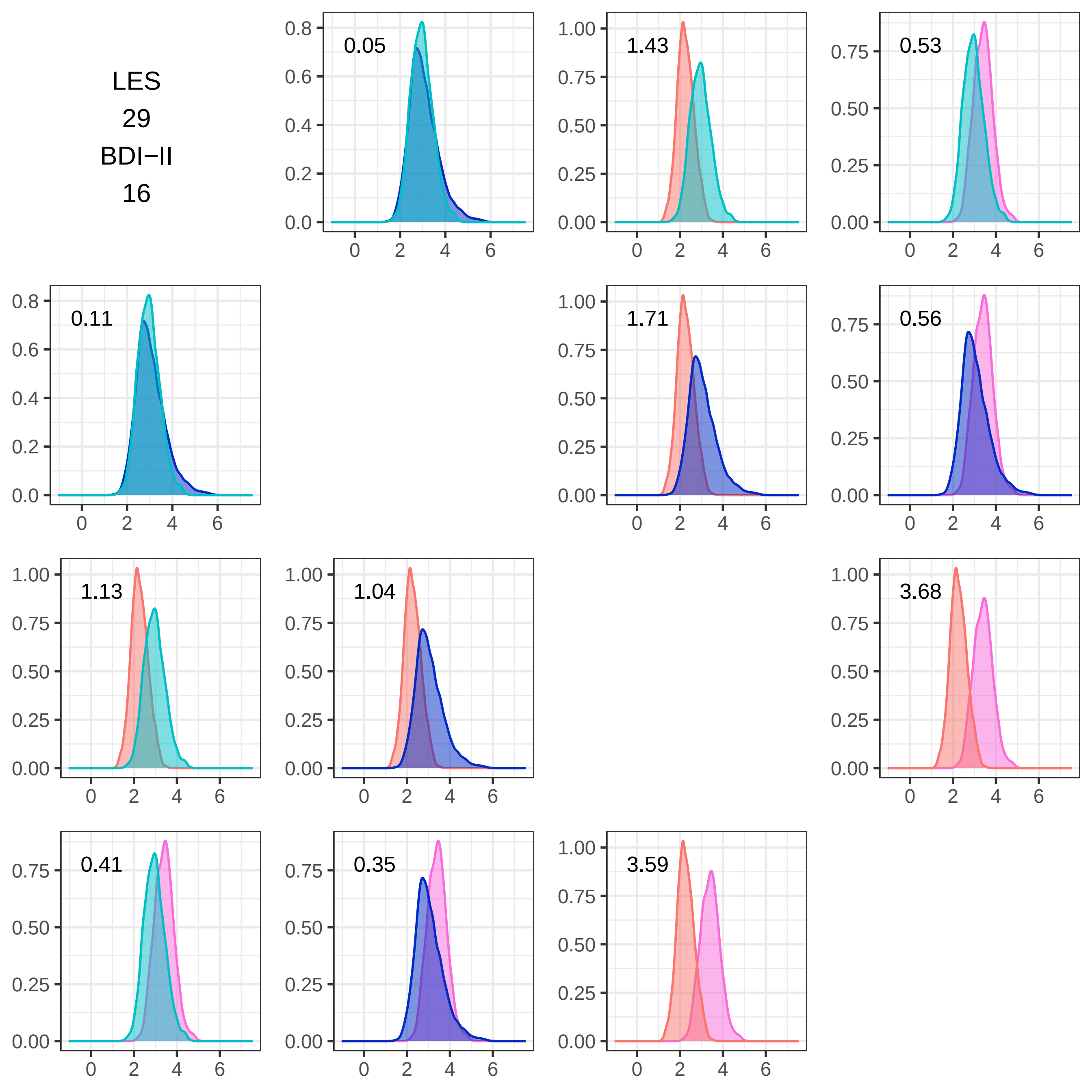

LES
29
BDI−II
16
0.05
1.43
0.53
0.11
1.71
0.56
1.13
1.04
3.68
0.41
0.35
3.59

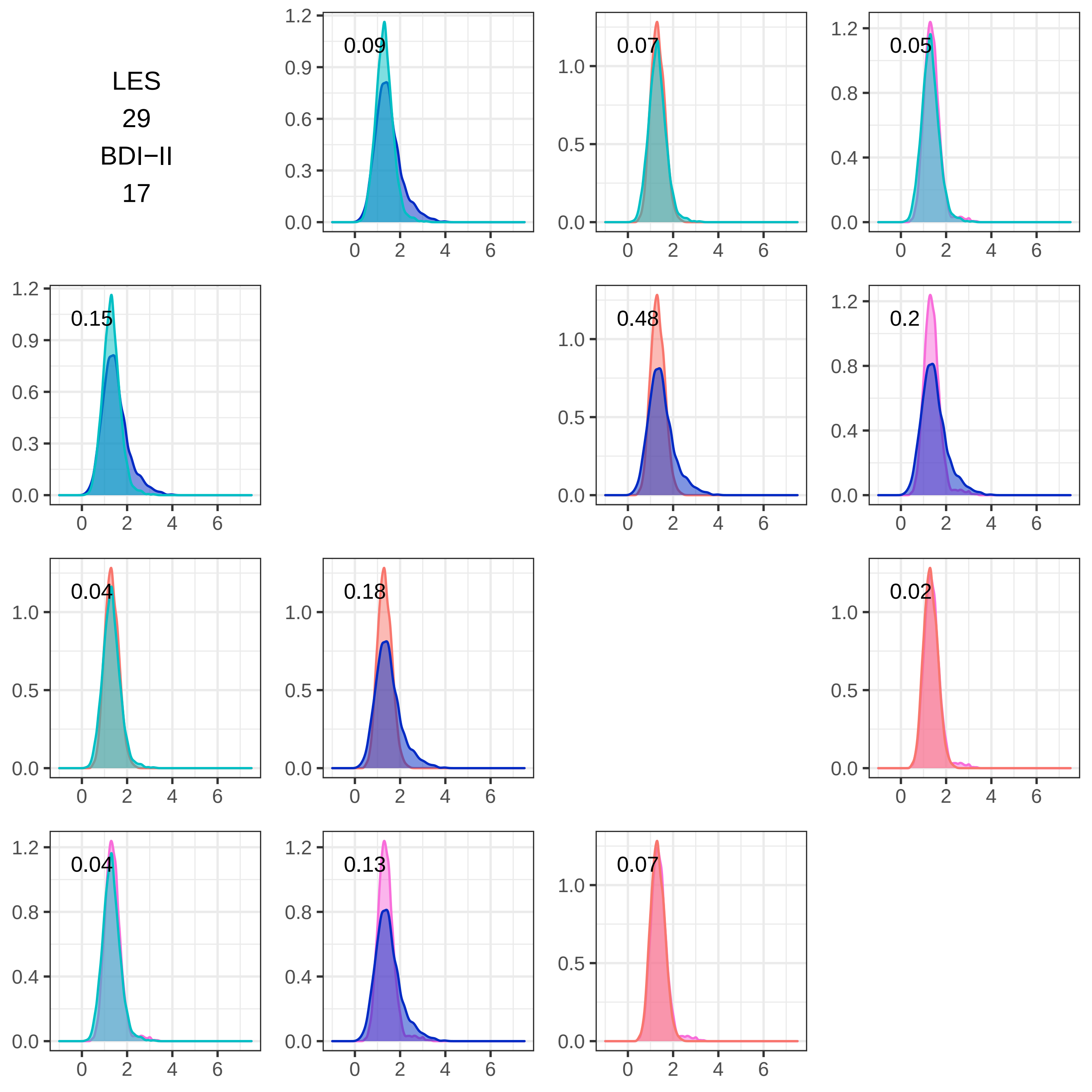

LES
29
BDI−II
17
0.09
0.07
0.05
0.15
0.48
0.2
0.04
0.18
0.02
0.04
0.13
0.07

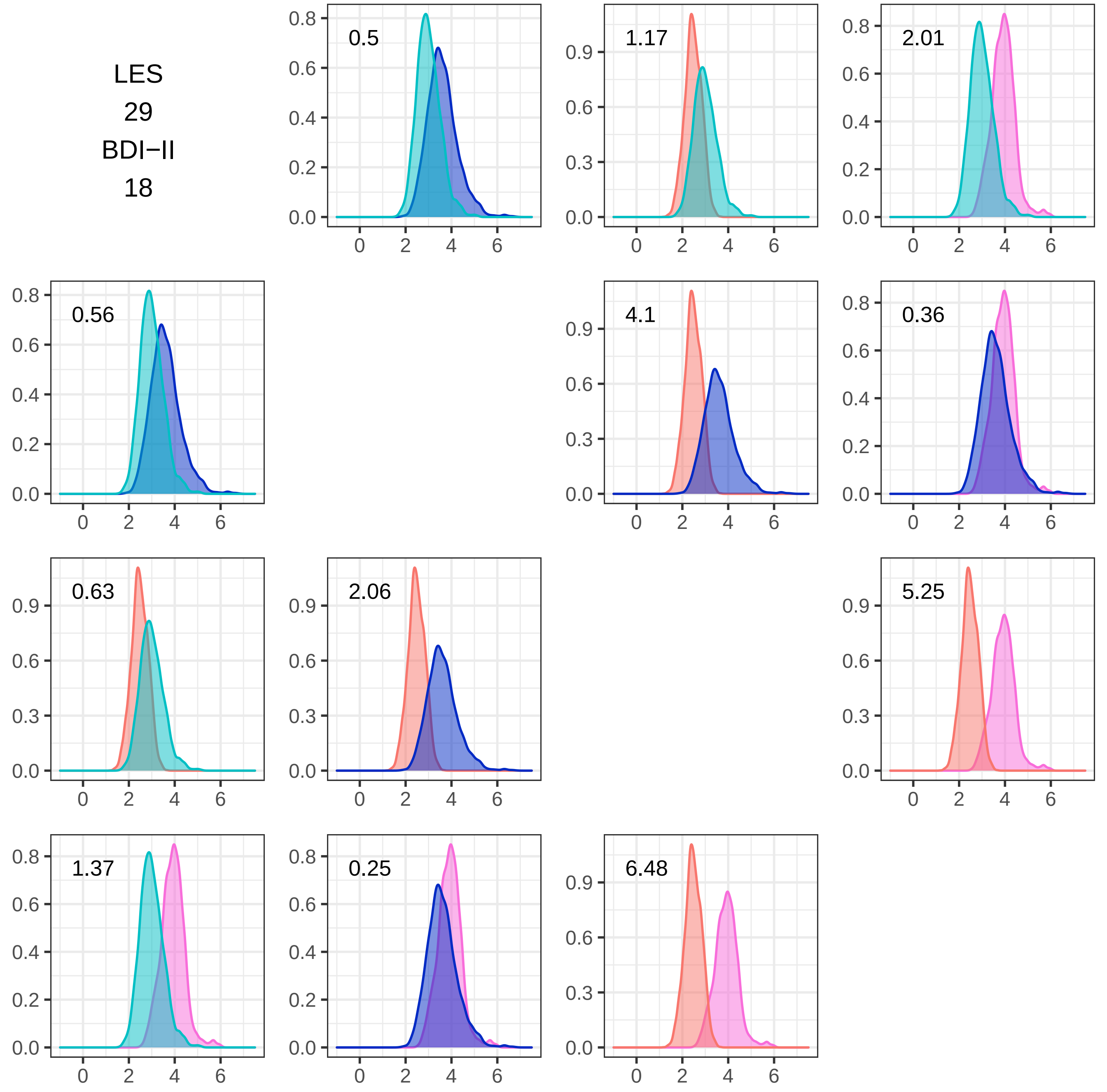

LES
29
BDI−II
18
0.5
1.17
2.01
0.56
4.1
0.36
0.63
2.06
5.25
1.37
0.25
6.48

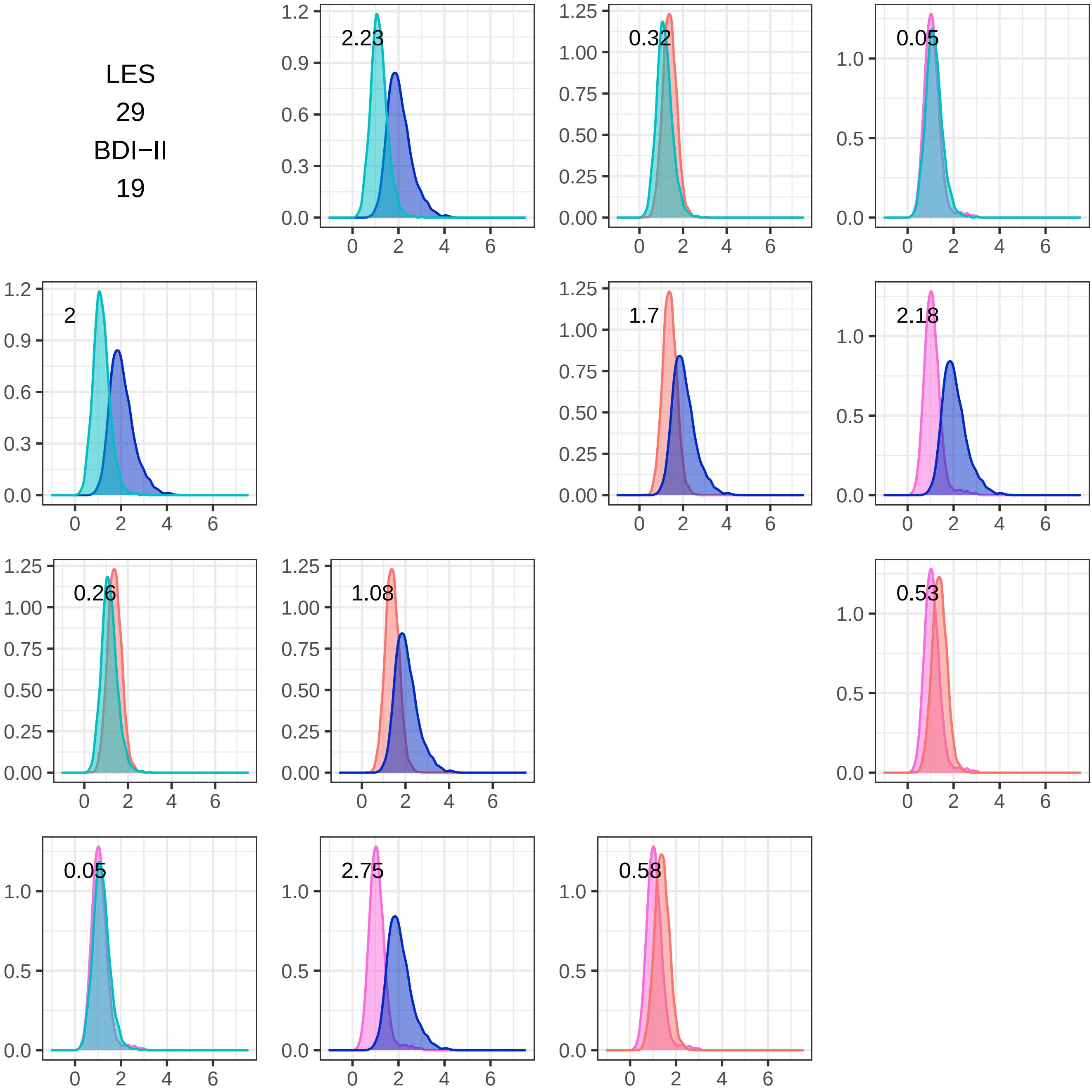
LES
29
BDI−II
19
2.23
0.32
0.05
2
1.7
2.18
0.26
1.08
0.53
0.05
2.75
0.58

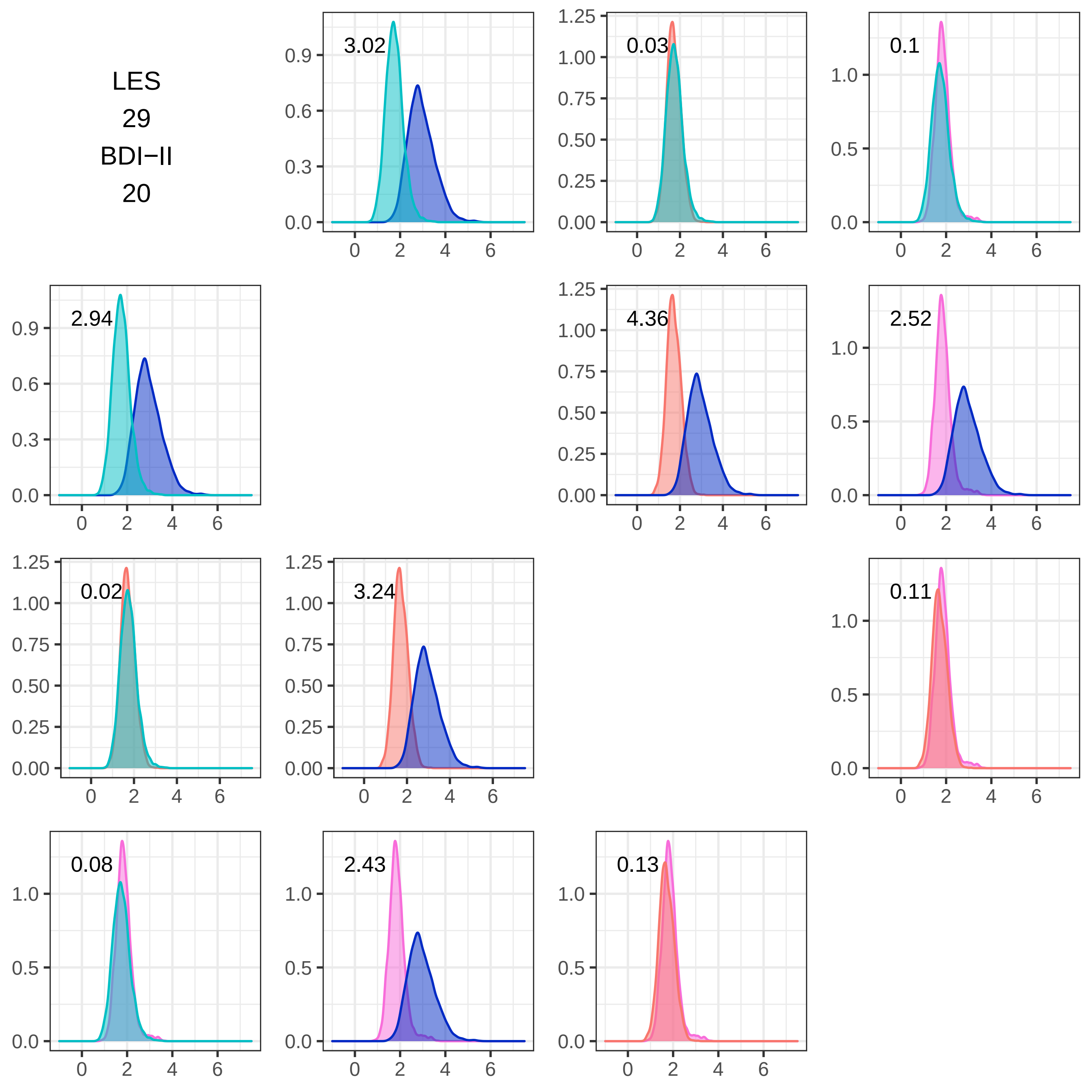
LES
29
BDI−II
20
3.02
0.03
0.1
2.94
4.36
2.52
0.02
3.24
0.11
0.08
2.43
0.13

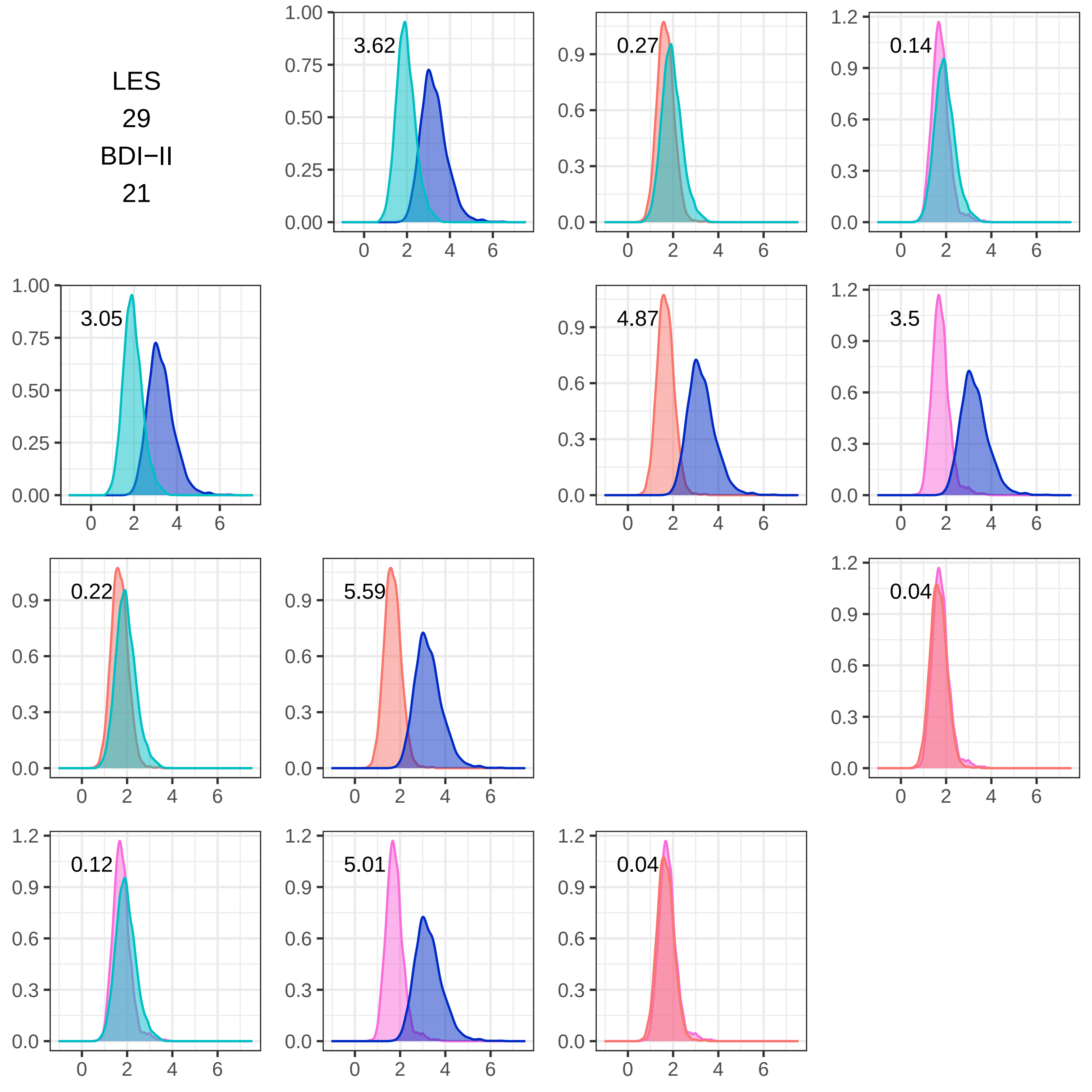
LES
29
BDI−II
21
3.62
0.27
0.14
3.05
4.87
3.5
0.22
5.59
0.04
0.12
5.01
0.04

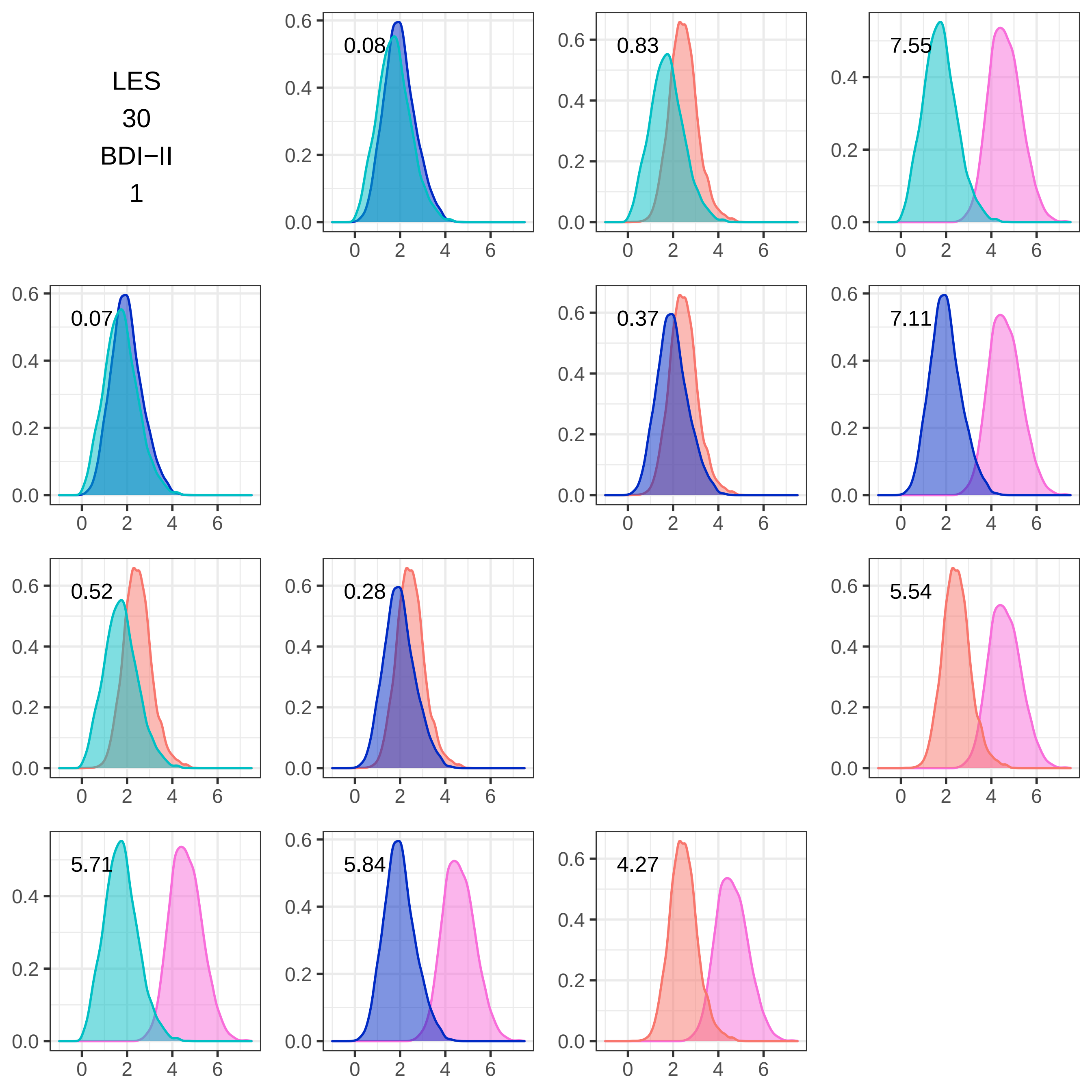

LES
30
BDI−II
1
0.08
0.83
7.55
0.07
0.37
7.11
0.52
0.28
5.54
5.71
5.84
4.27

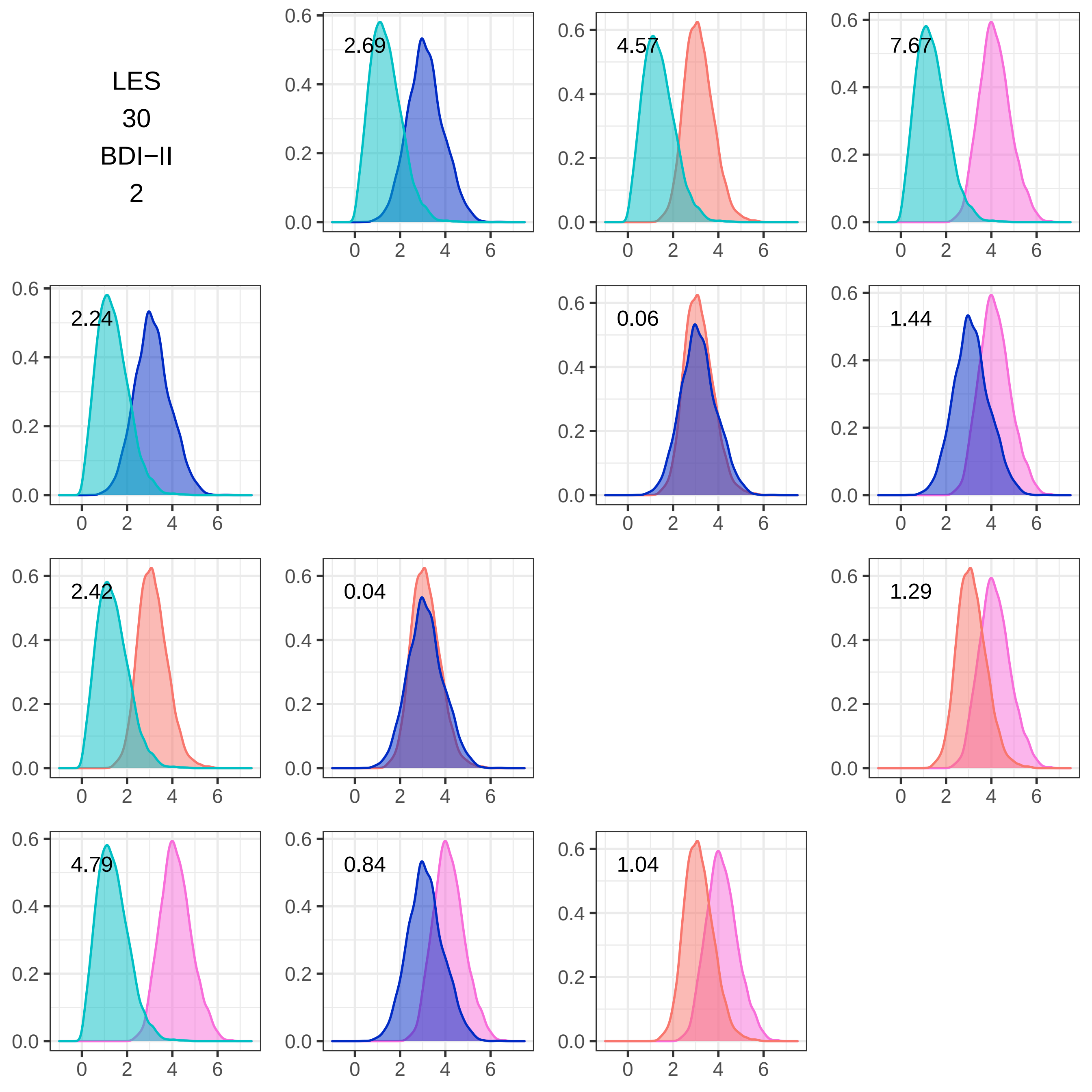

LES
30
BDI−II
2
2.69
4.57
7.67
2.24
0.06
1.44
2.42
0.04
1.29
4.79
0.84
1.04

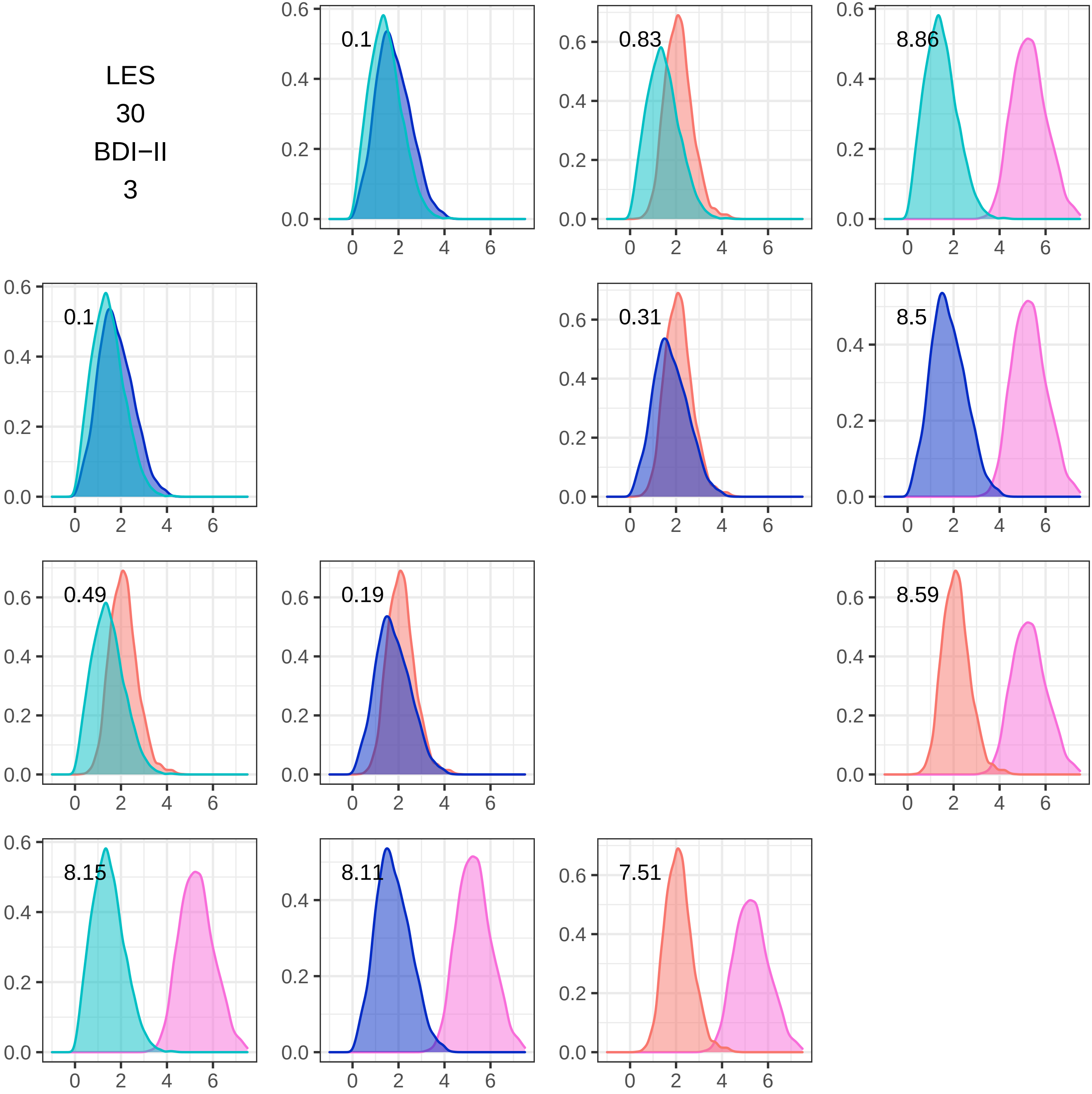

LES
30
BDI−II
3
0.1
0.83
8.86
0.1
0.31
8.5
0.49
0.19
8.59
8.15
8.11
7.51

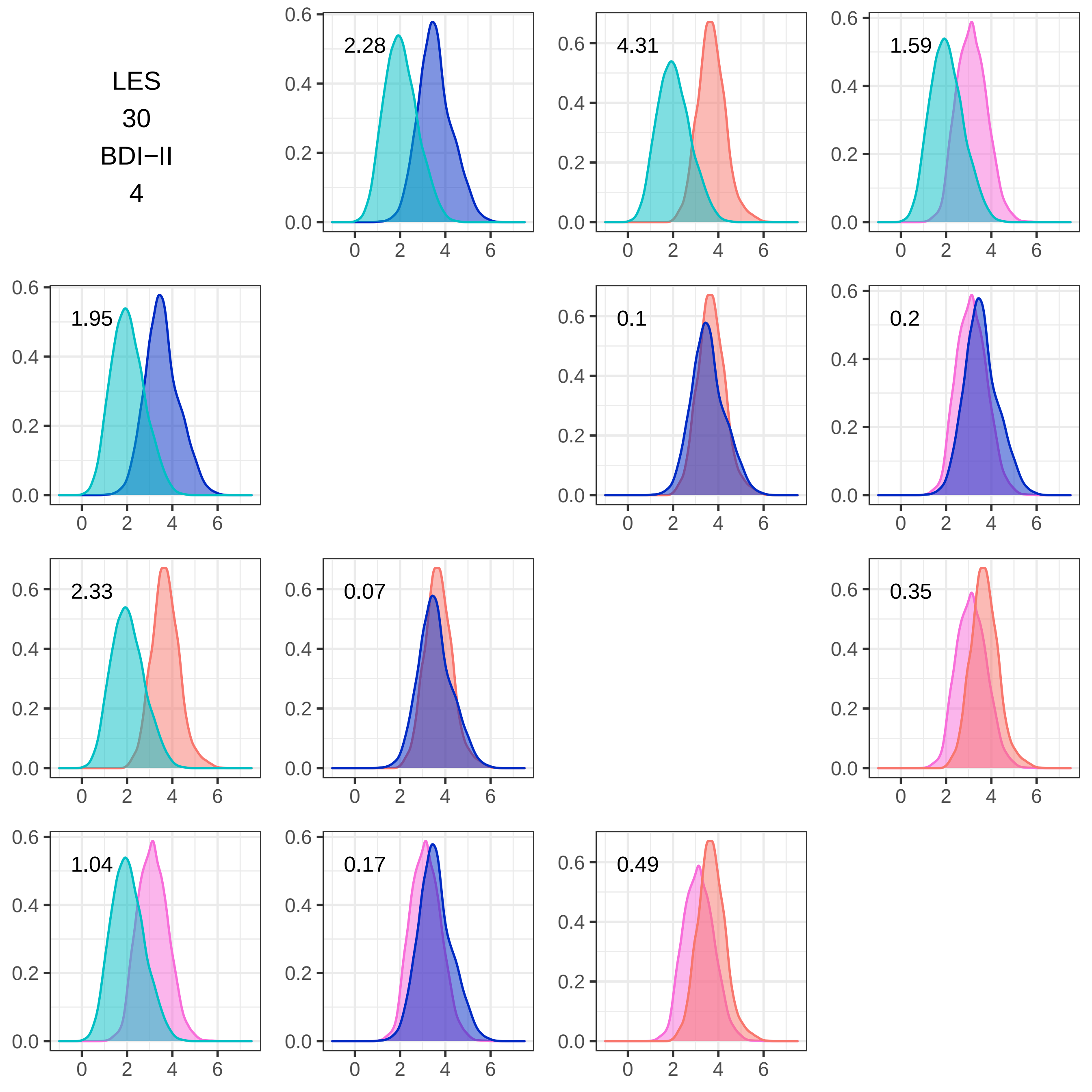
LES
30
BDI−II
4
2.28
4.31
1.59
1.95
0.1
0.2
2.33
0.07
0.35
1.04
0.17
0.49

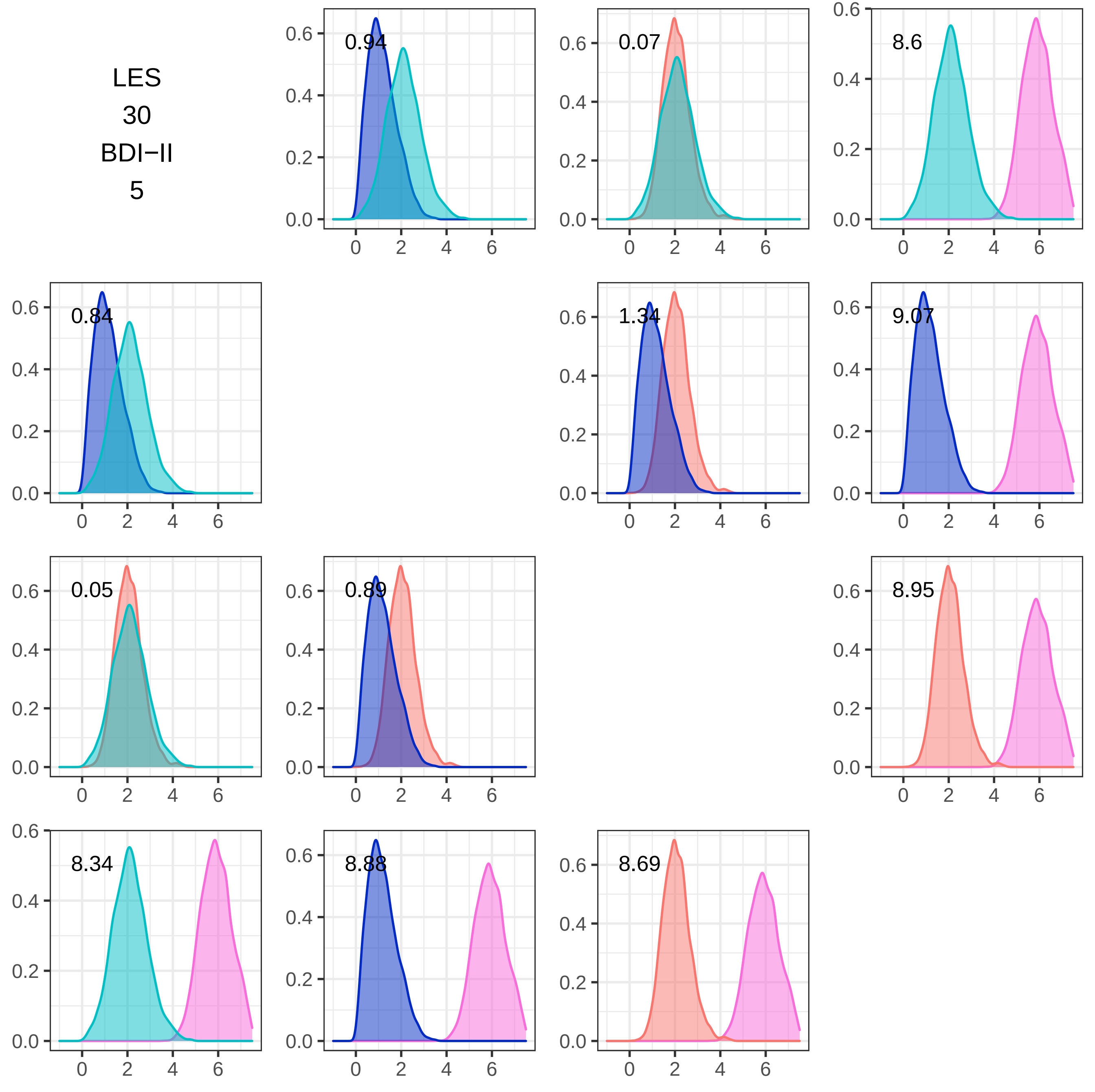
LES
30
BDI−II
5
0.94
0.07
8.6
0.84
1.34
9.07
0.05
0.89
8.95
8.34
8.88
8.69

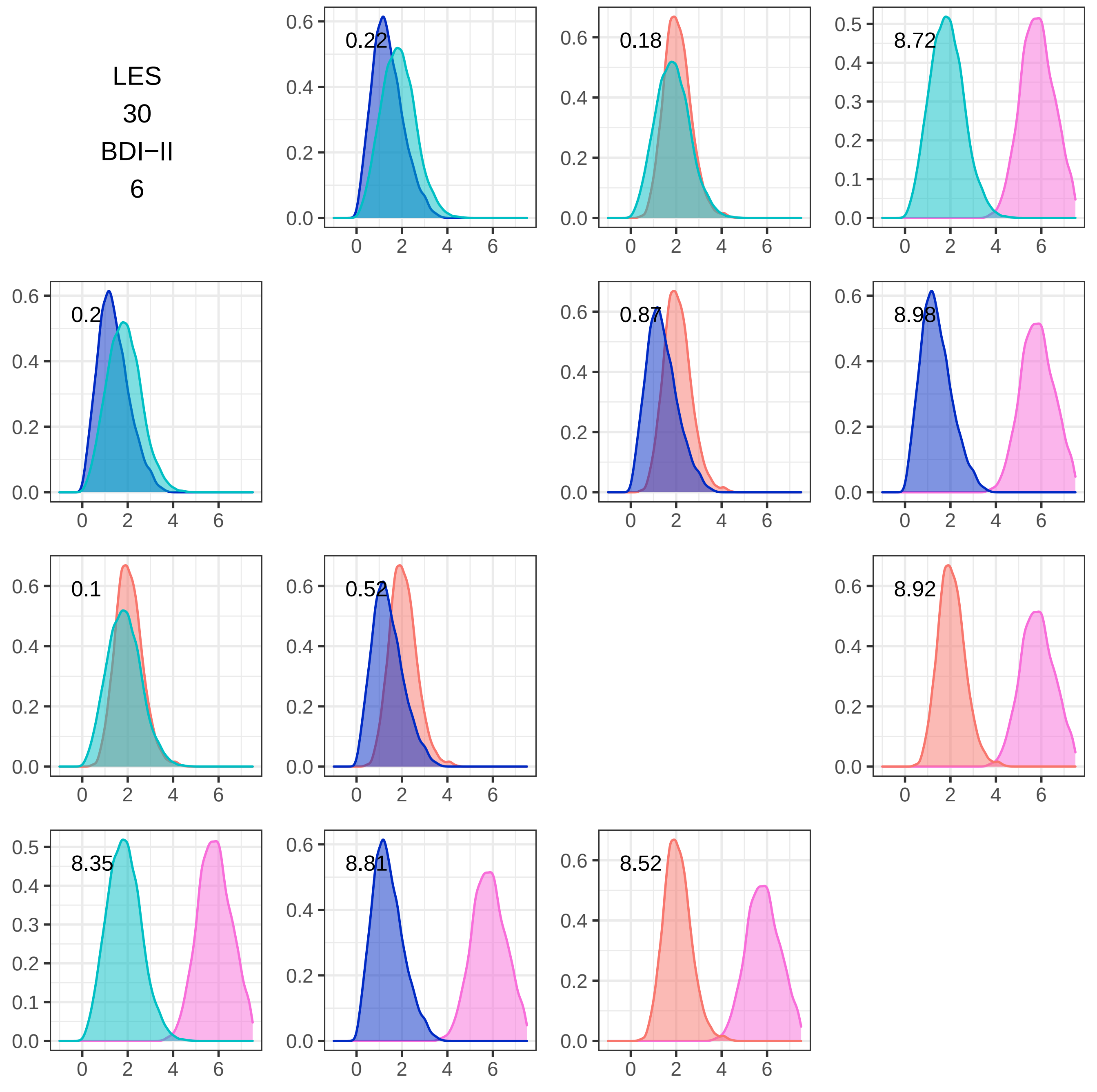

LES
30
BDI−II
6
0.22
0.18
8.72
0.2
0.87
8.98
0.1
0.52
8.92
8.35
8.81
8.52

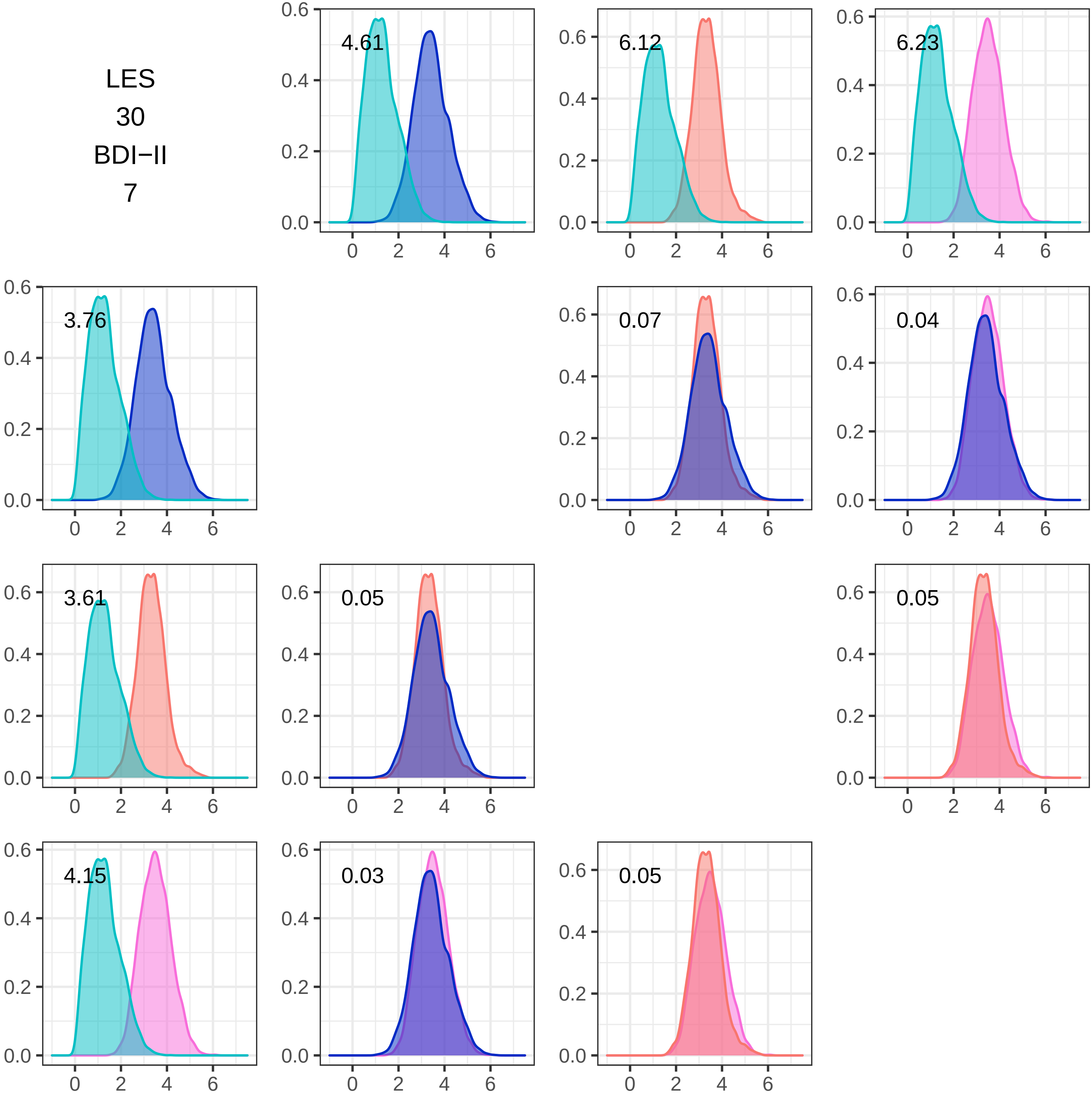
LES
30
BDI−II
7
4.61
6.12
6.23
3.76
0.07
0.04
3.61
0.05
0.05
4.15
0.03
0.05

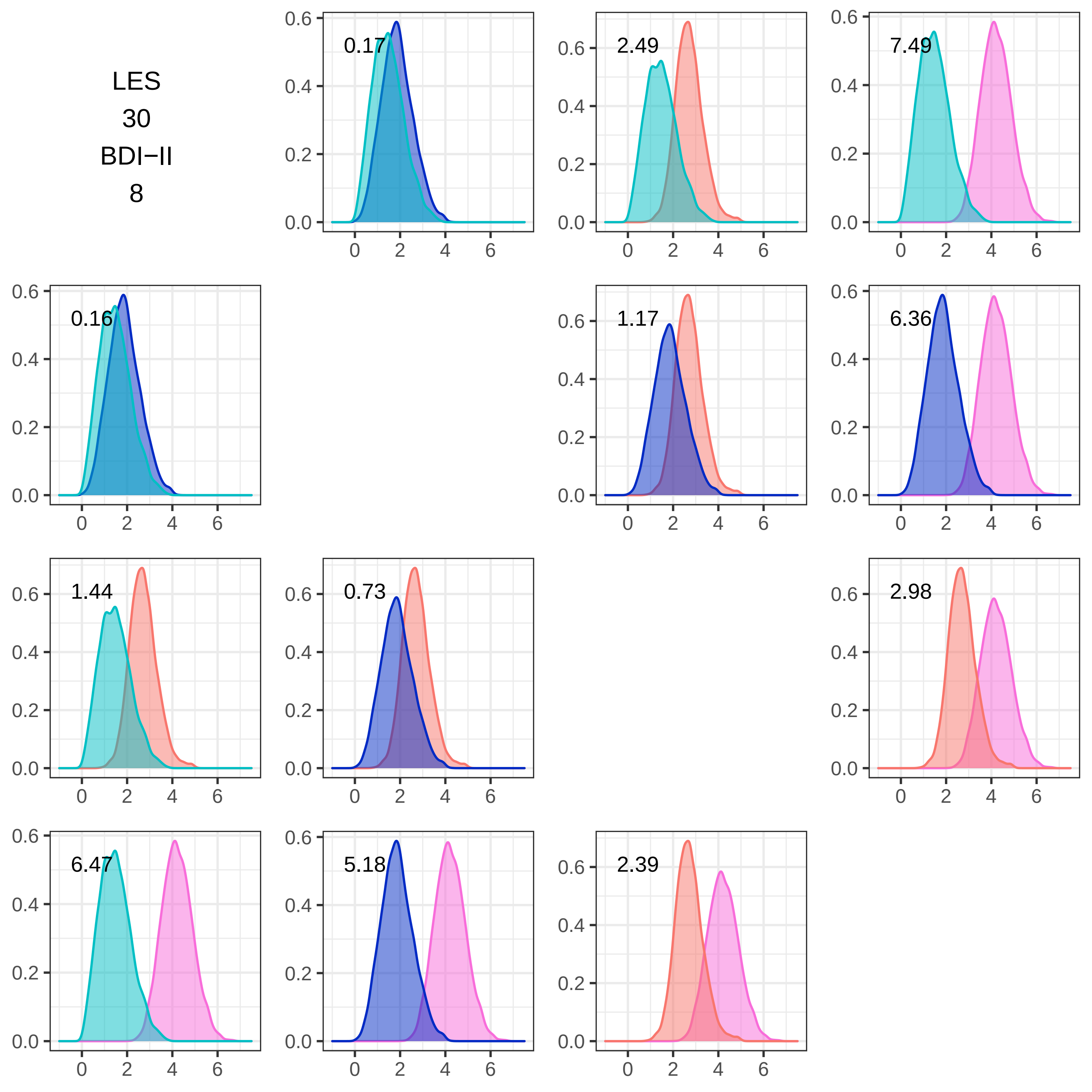

LES
30
BDI−II
8
0.17
2.49
7.49
0.16
1.17
6.36
1.44
0.73
2.98
6.47
5.18
2.39

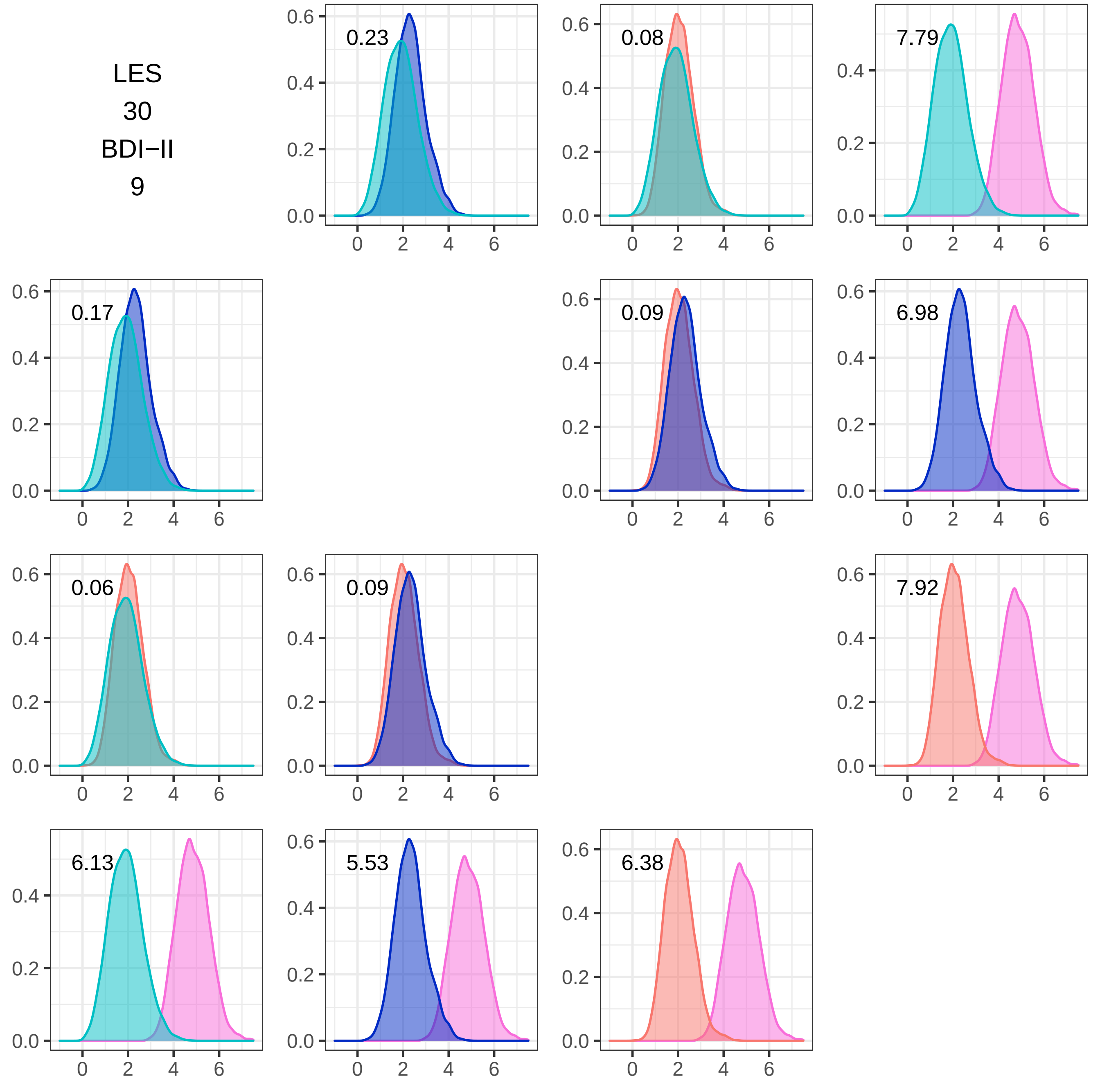

LES
30
BDI−II
9
0.23
0.08
7.79
0.17
0.09
6.98
0.06
0.09
7.92
6.13
5.53
6.38

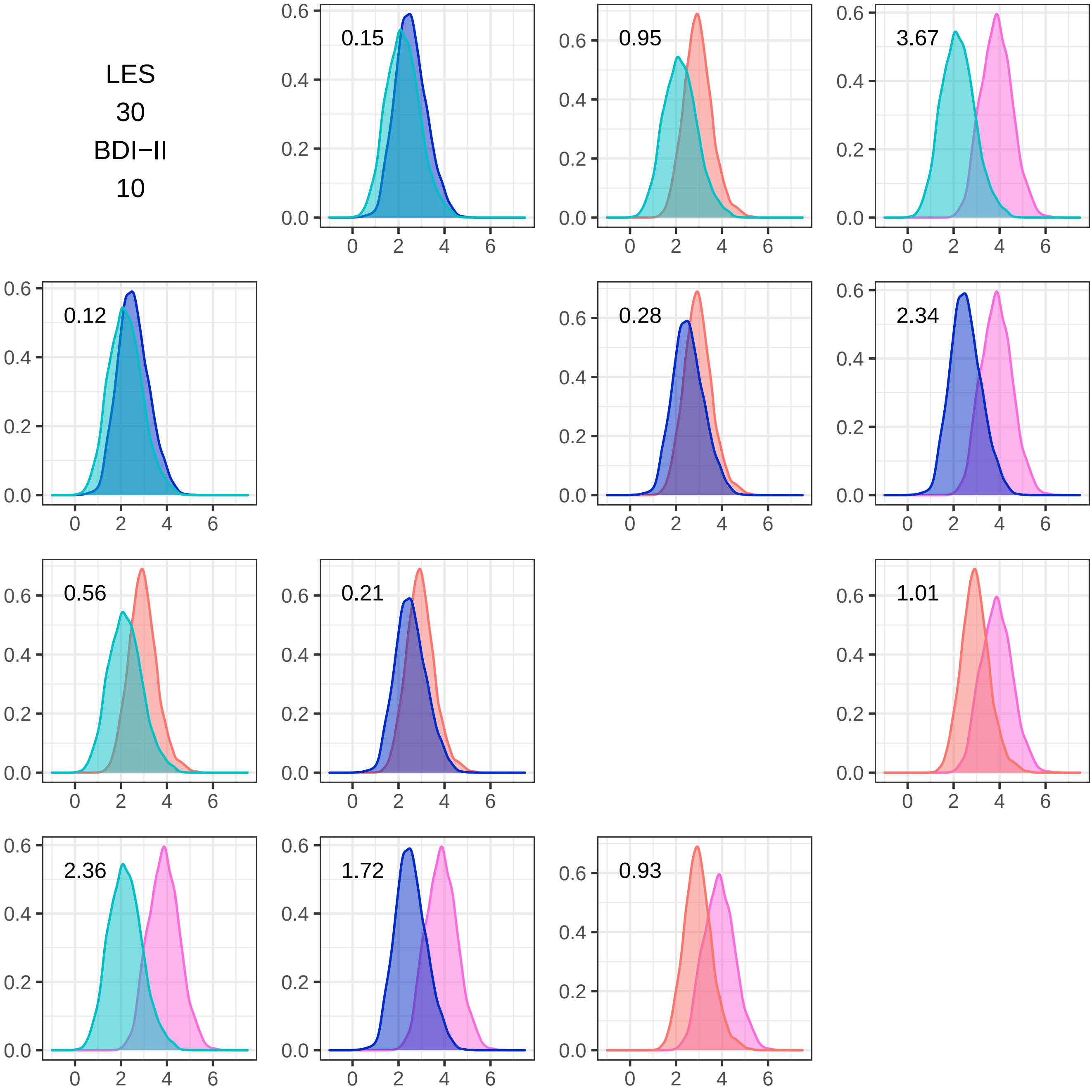
LES
30
BDI−II
10
0.15
0.95
3.67
0.12
0.28
2.34
0.56
0.21
1.01
2.36
1.72
0.93

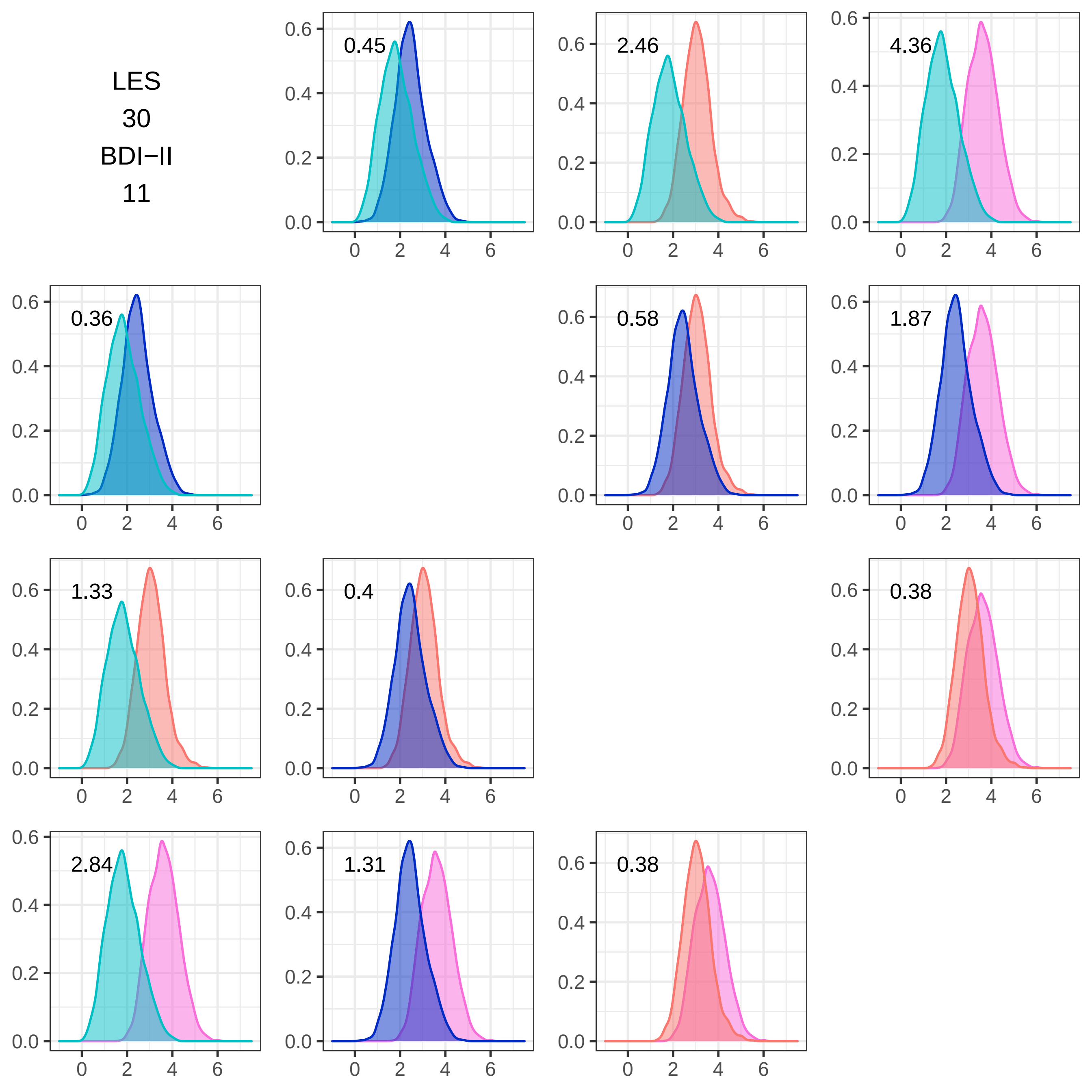

LES
30
BDI−II
11
0.45
2.46
4.36
0.36
0.58
1.87
1.33
0.4
0.38
2.84
1.31
0.38

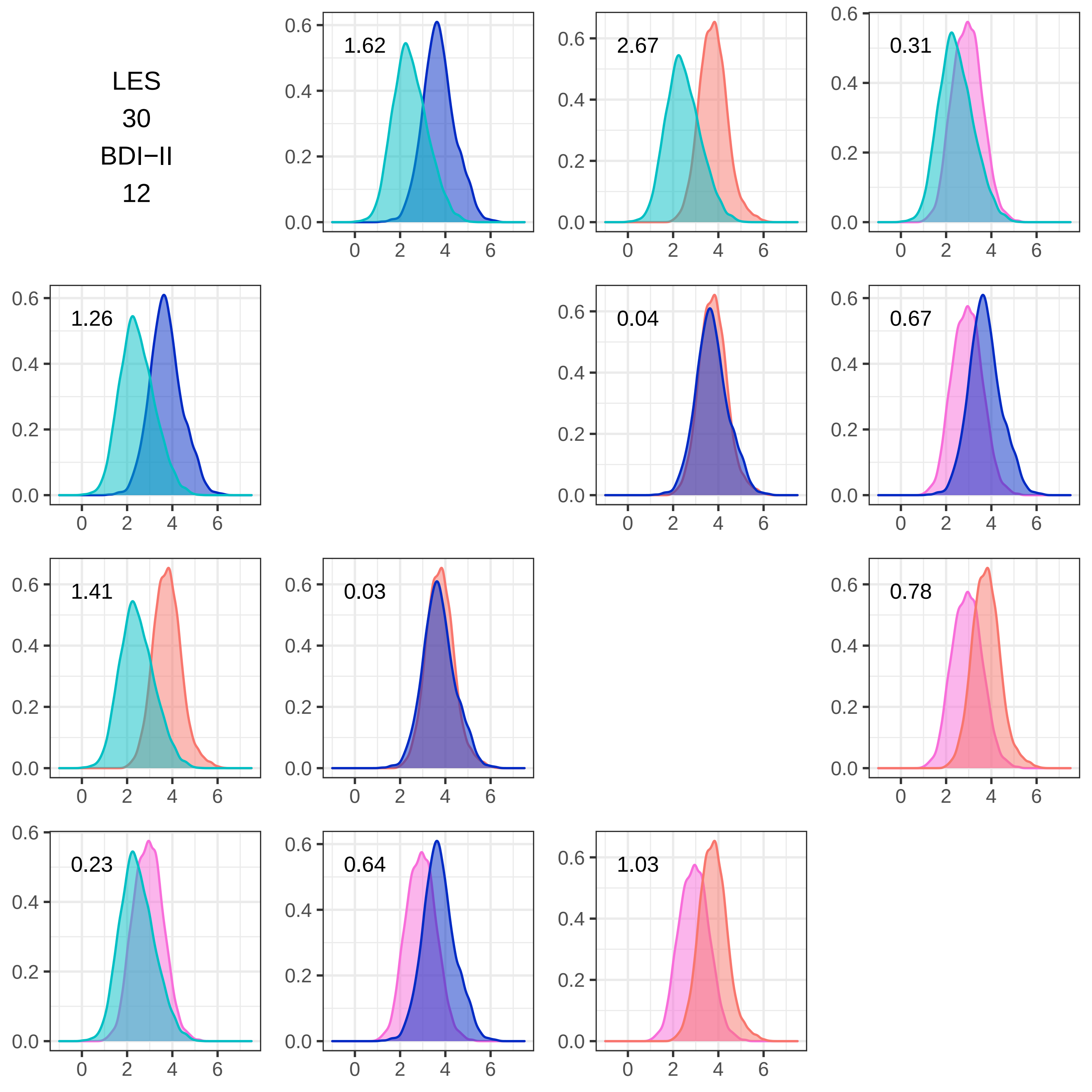
LES
30
BDI−II
12
1.62
2.67
0.31
1.26
0.04
0.67
1.41
0.03
0.78
0.23
0.64
1.03

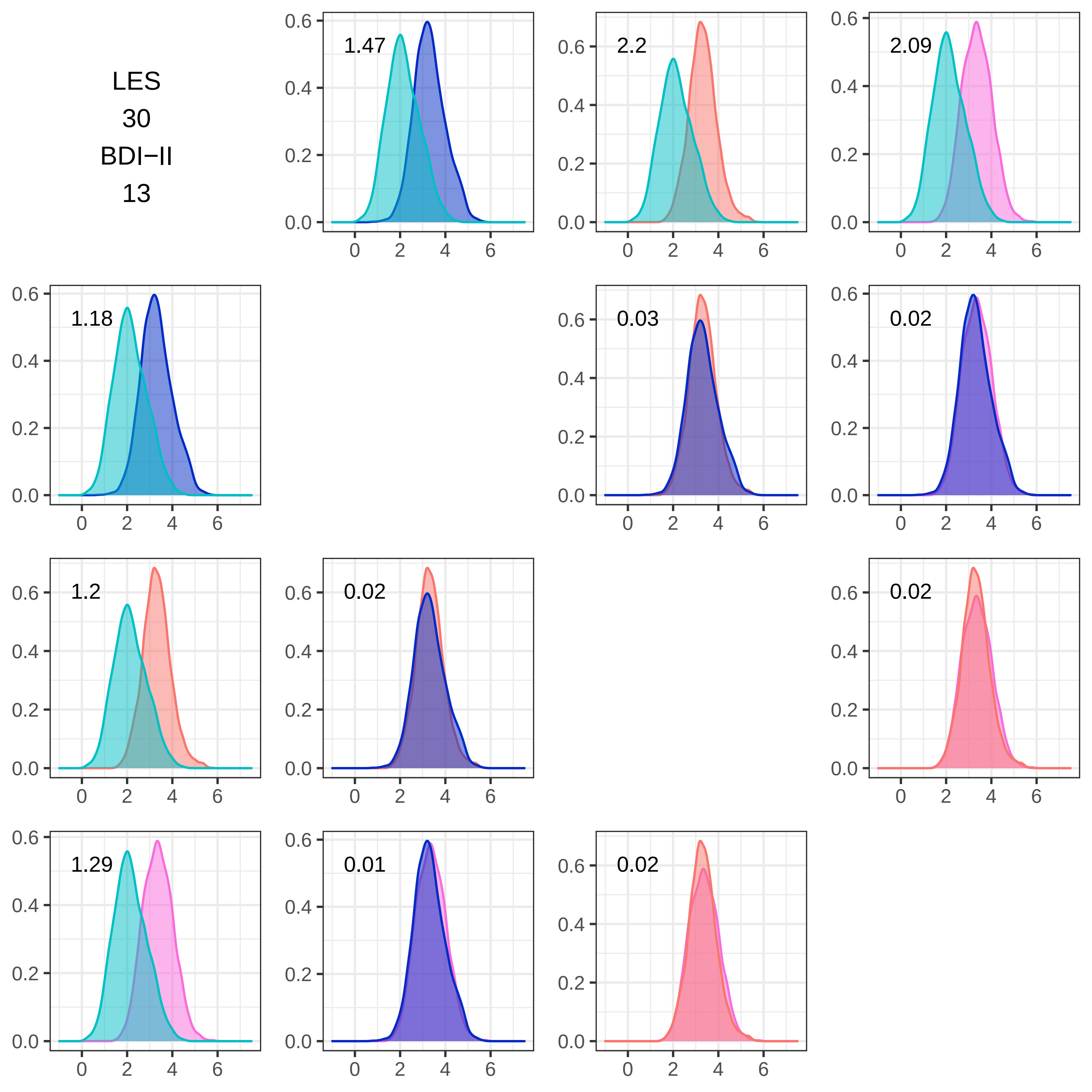

LES
30
BDI−II
13
1.47
2.2
2.09
1.18
0.03
0.02
1.2
0.02
0.02
1.29
0.01
0.02

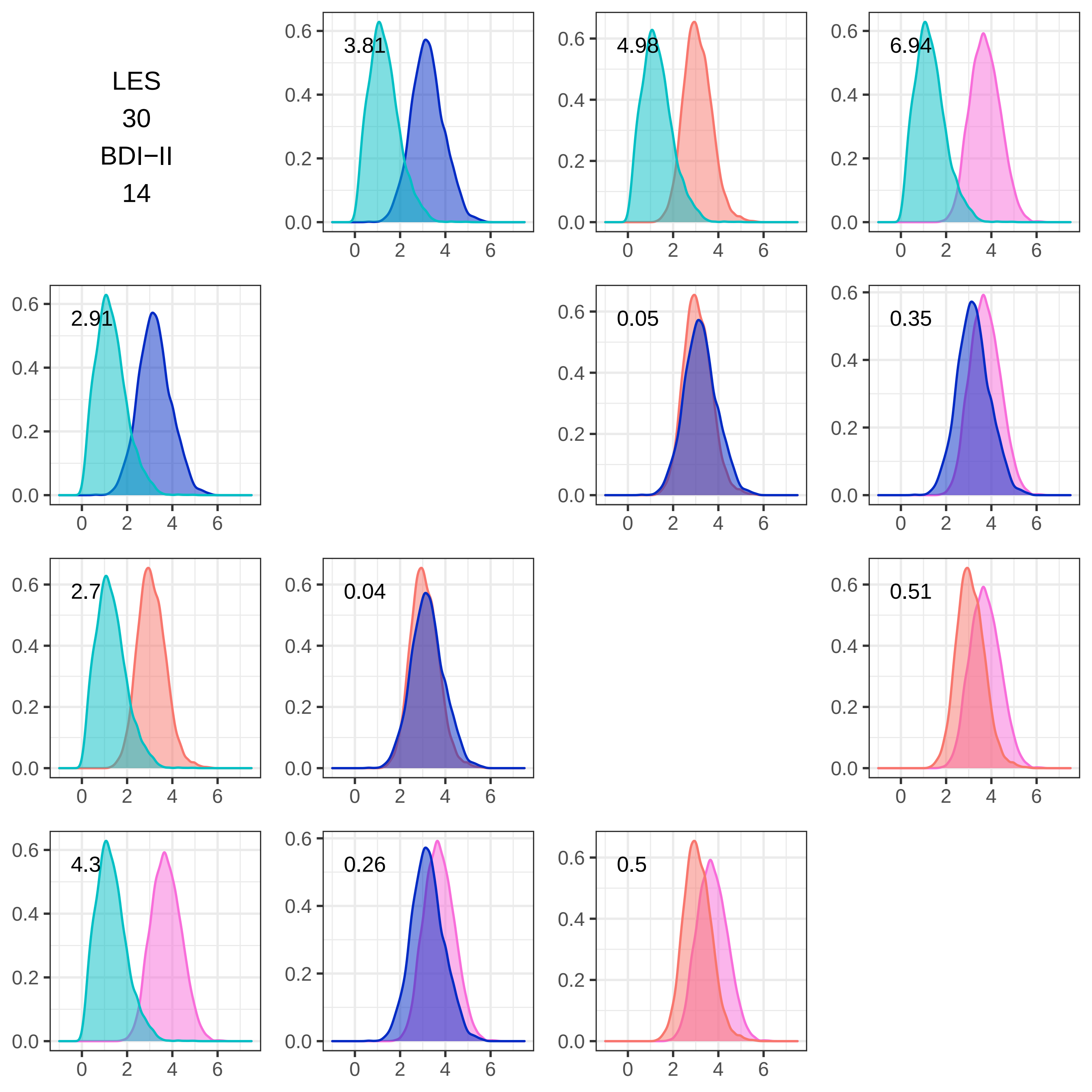
LES
30
BDI−II
14
3.81
4.98
6.94
2.91
0.05
0.35
2.7
0.04
0.51
4.3
0.26
0.5

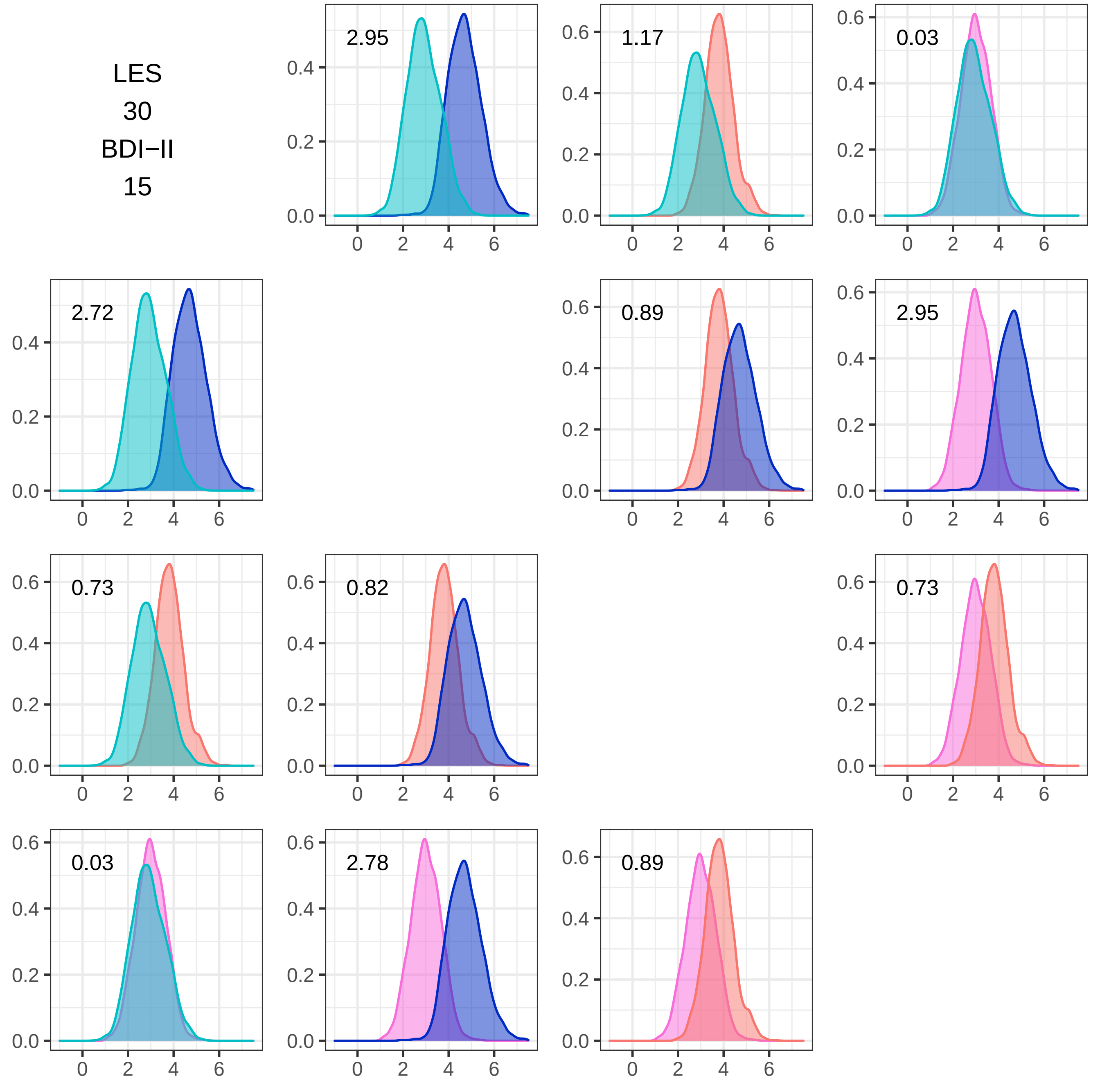
LES
30
BDI−II
15
2.95
1.17
0.03
2.72
0.89
2.95
0.73
0.82
0.73
0.03
2.78
0.89

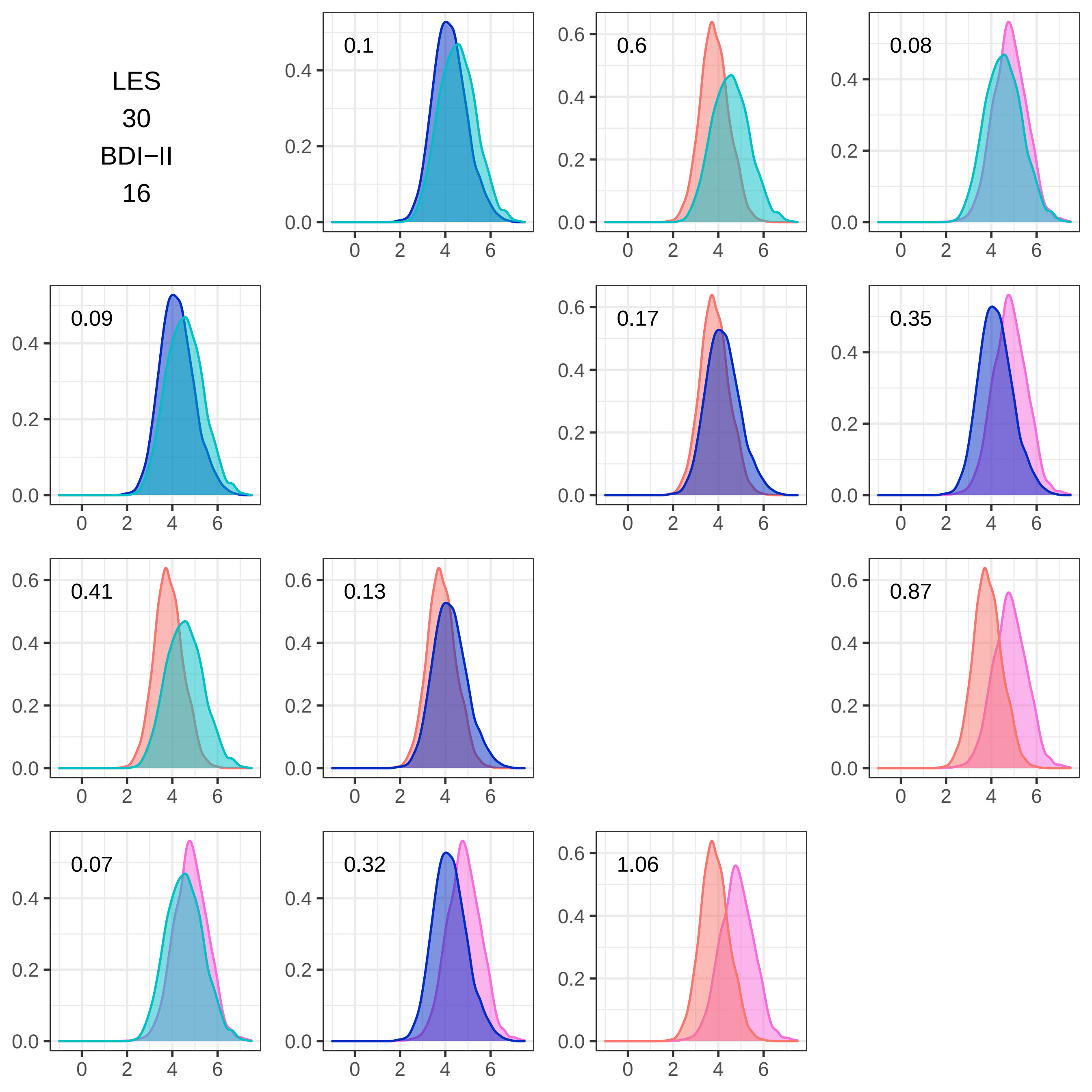
LES
30
BDI−II
16
0.1
0.6
0.08
0.09
0.17
0.35
0.41
0.13
0.87
0.07
0.32
1.06

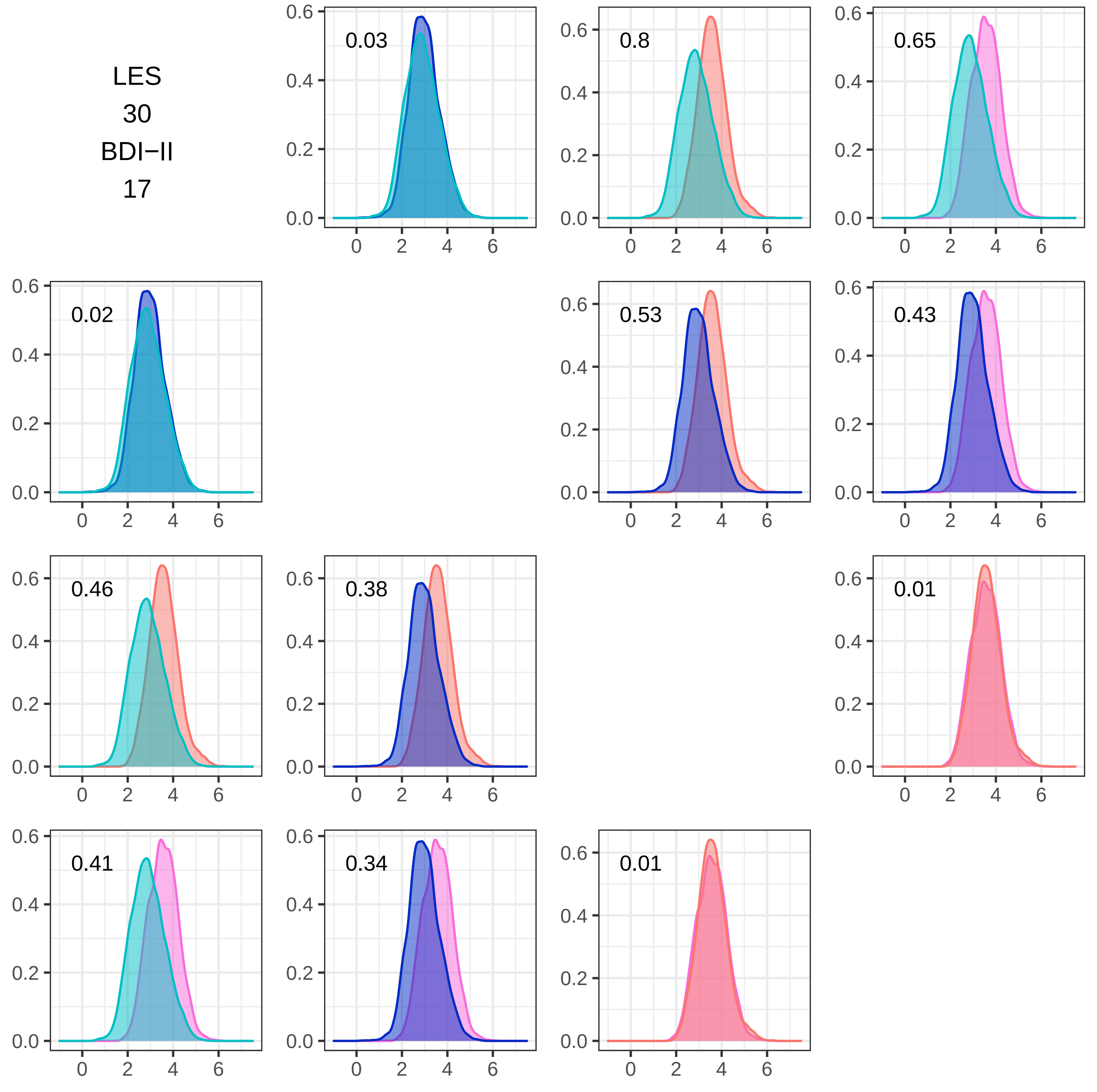
LES
30
BDI−II
17
0.03
0.8
0.65
0.02
0.53
0.43
0.46
0.38
0.01
0.41
0.34
0.01

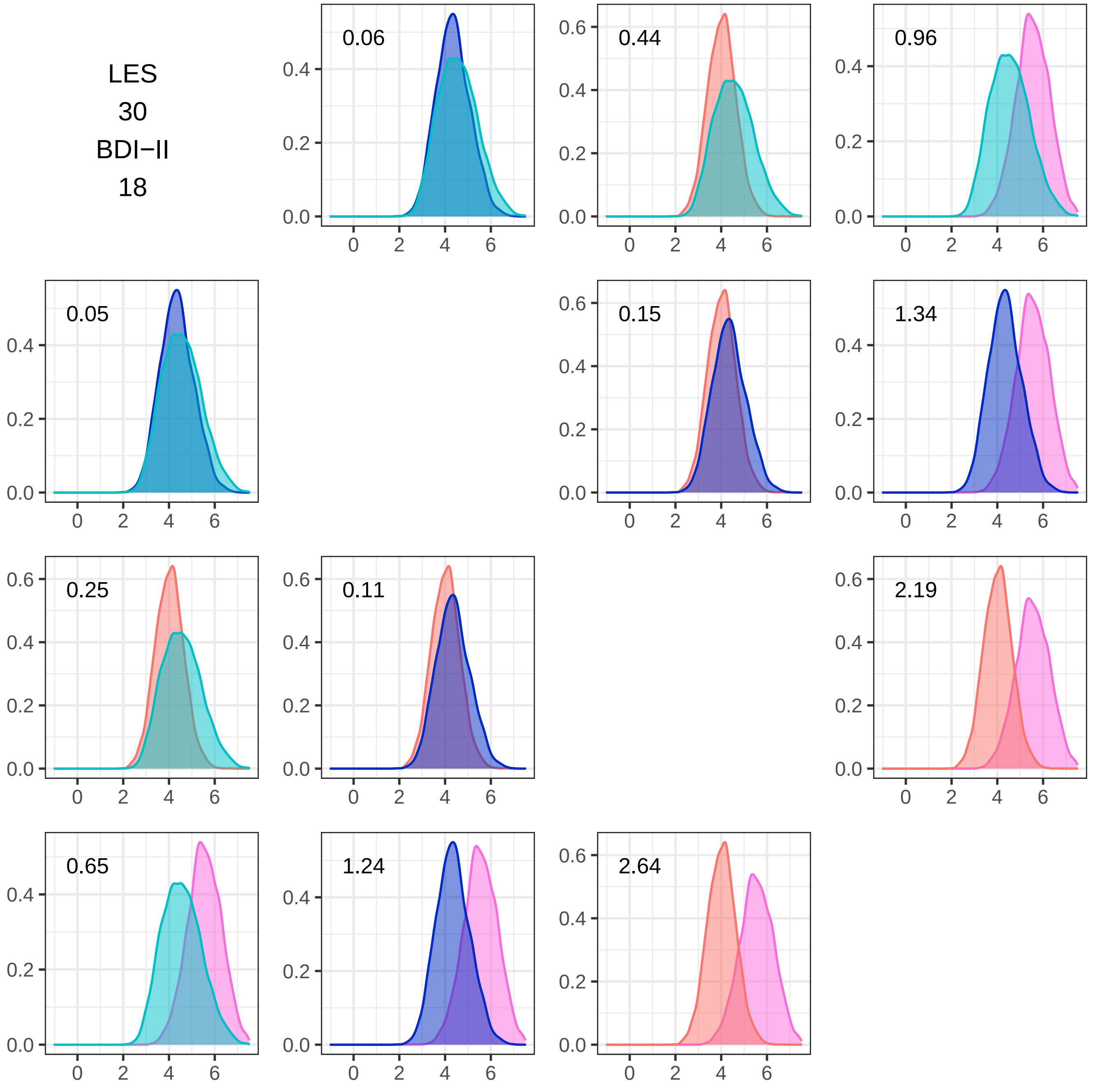
LES
30
BDI−II
18
0.06
0.44
0.96
0.05
0.15
1.34
0.25
0.11
2.19
0.65
1.24
2.64

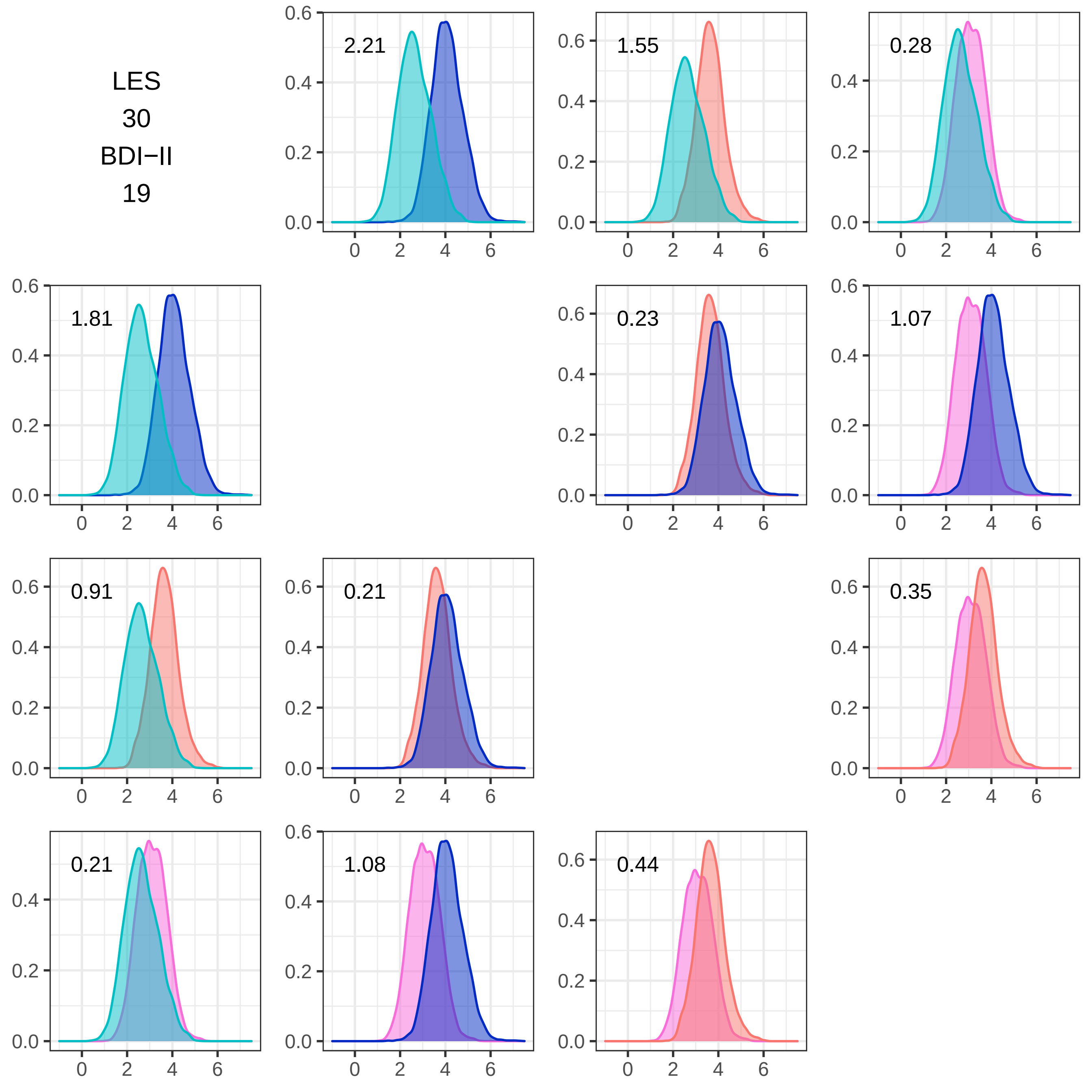
LES
30
BDI−II
19
2.21
1.55
0.28
1.81
0.23
1.07
0.91
0.21
0.35
0.21
1.08
0.44

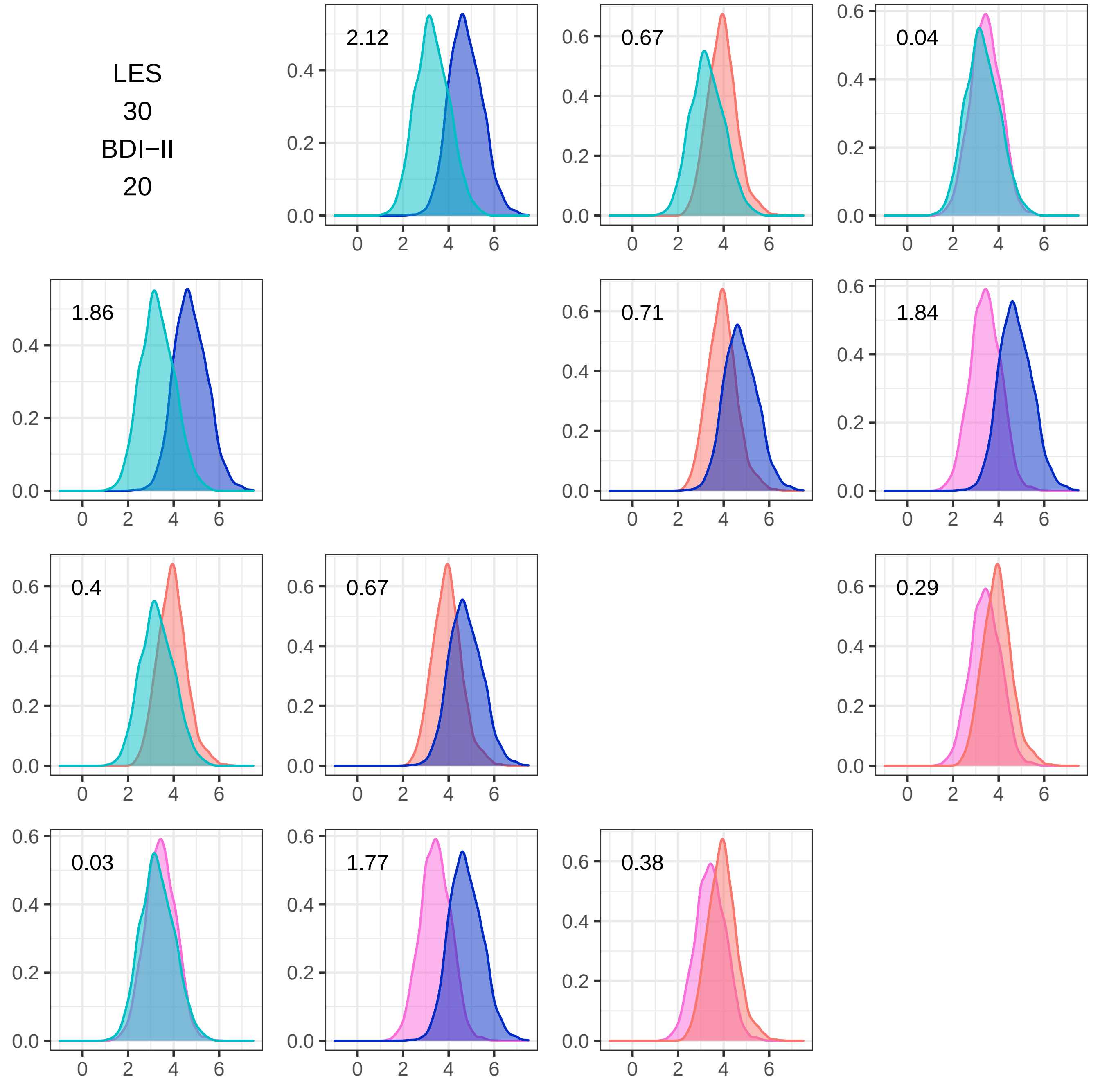
LES
30
BDI−II
20
2.12
0.67
0.04
1.86
0.71
1.84
0.4
0.67
0.29
0.03
1.77
0.38

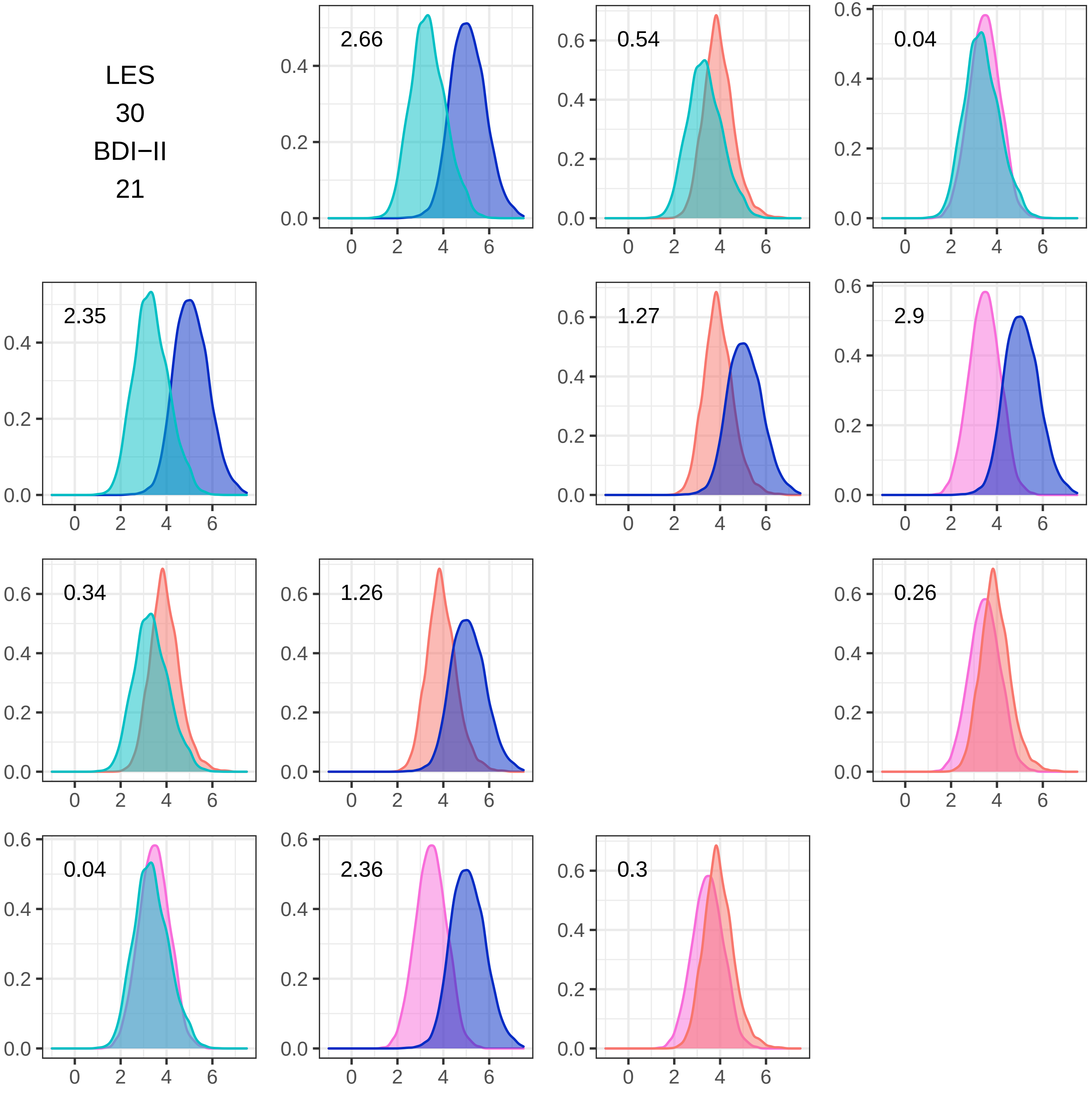

LES
30
BDI−II
21
2.66
0.54
0.04
2.35
1.27
2.9
0.34
1.26
0.26
0.04
2.36
0.3

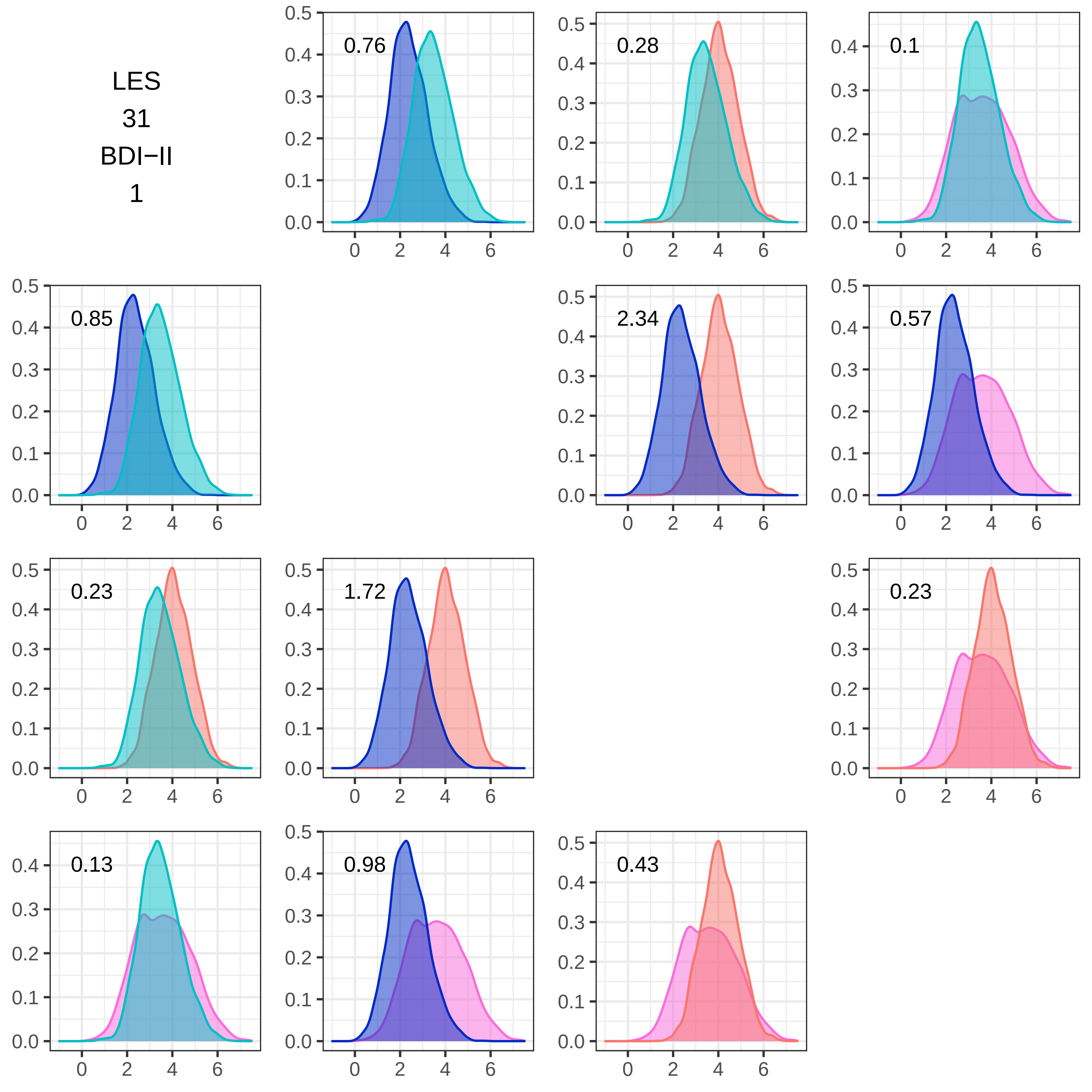

LES
31
BDI−II
1
0.76
0.28
0.1
0.85
2.34
0.57
0.23
1.72
0.23
0.13
0.98
0.43

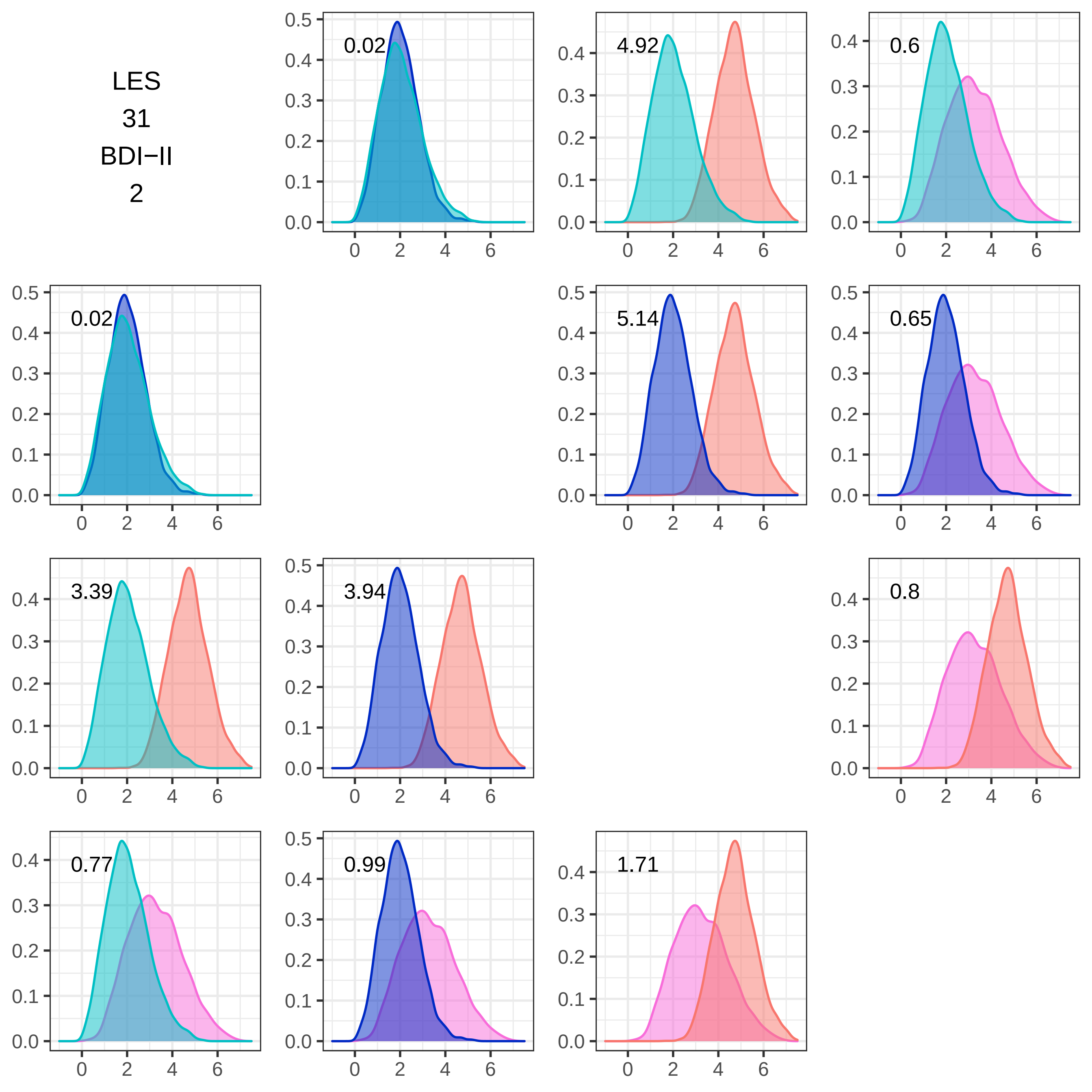
LES
31
BDI−II
2
0.02
4.92
0.6
0.02
5.14
0.65
3.39
3.94
0.8
0.77
0.99
1.71

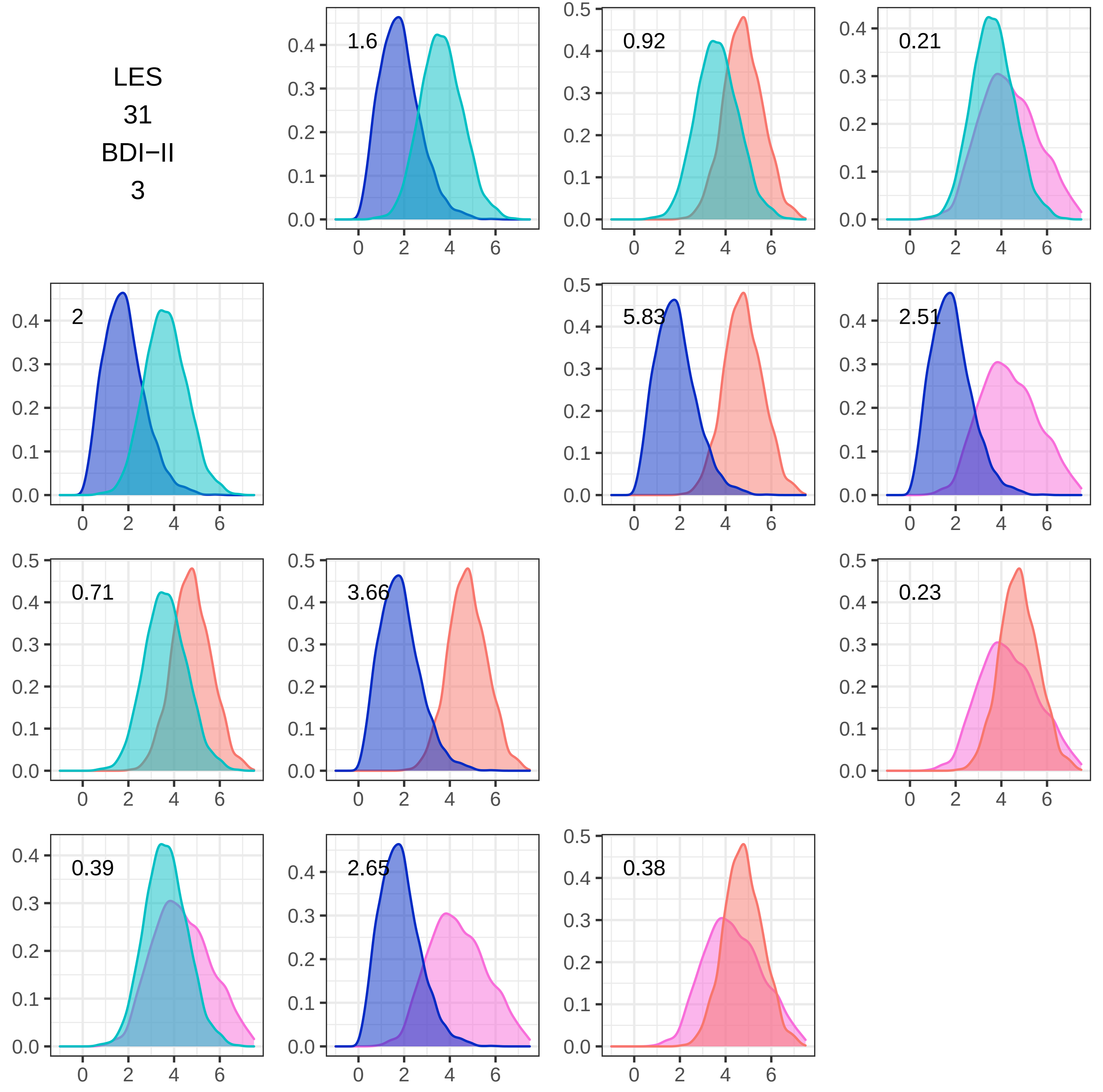
LES
31
BDI−II
3
1.6
0.92
0.21
2
5.83
2.51
0.71
3.66
0.23
0.39
2.65
0.38

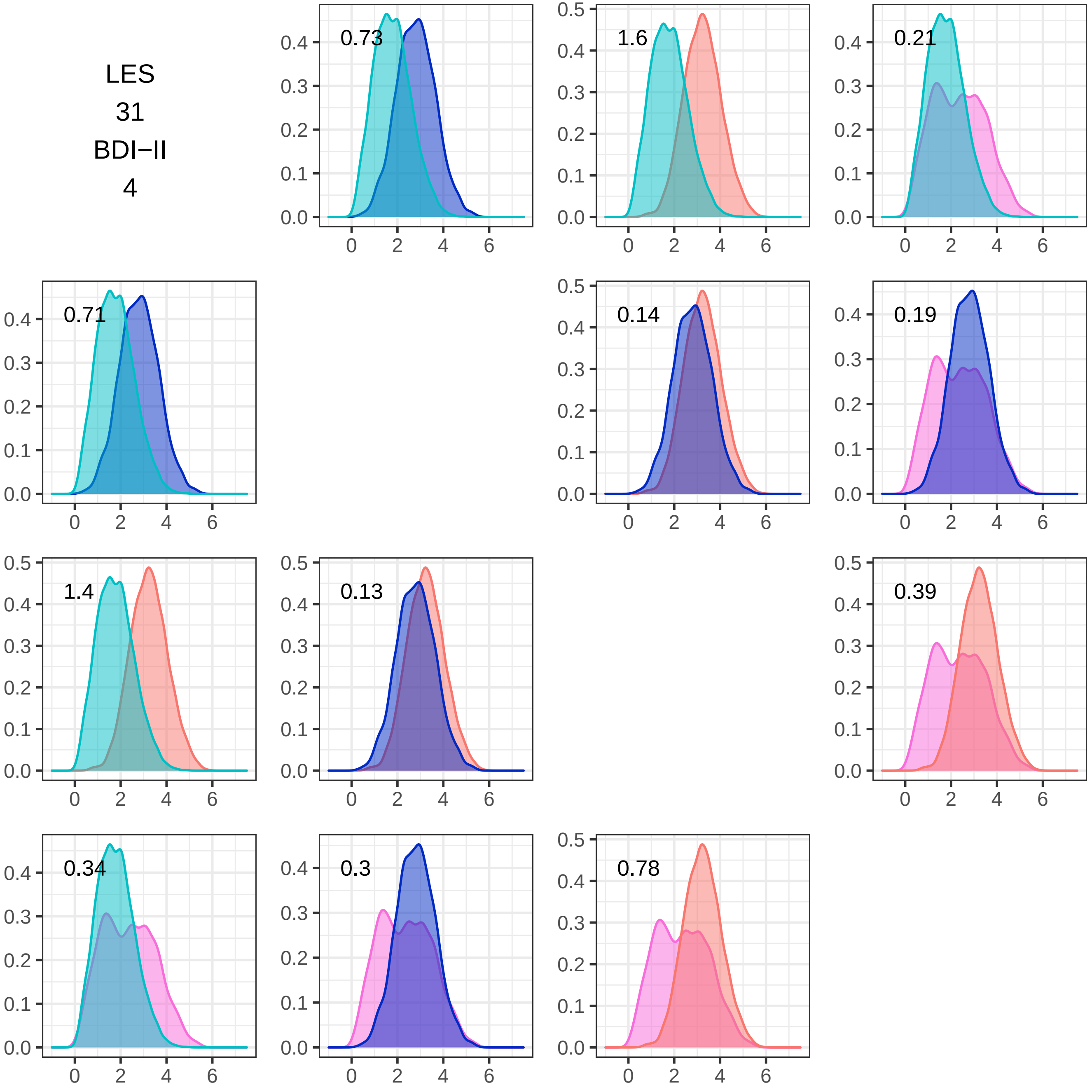
LES
31
BDI−II
4
0.73
1.6
0.21
0.71
0.14
0.19
1.4
0.13
0.39
0.34
0.3
0.78

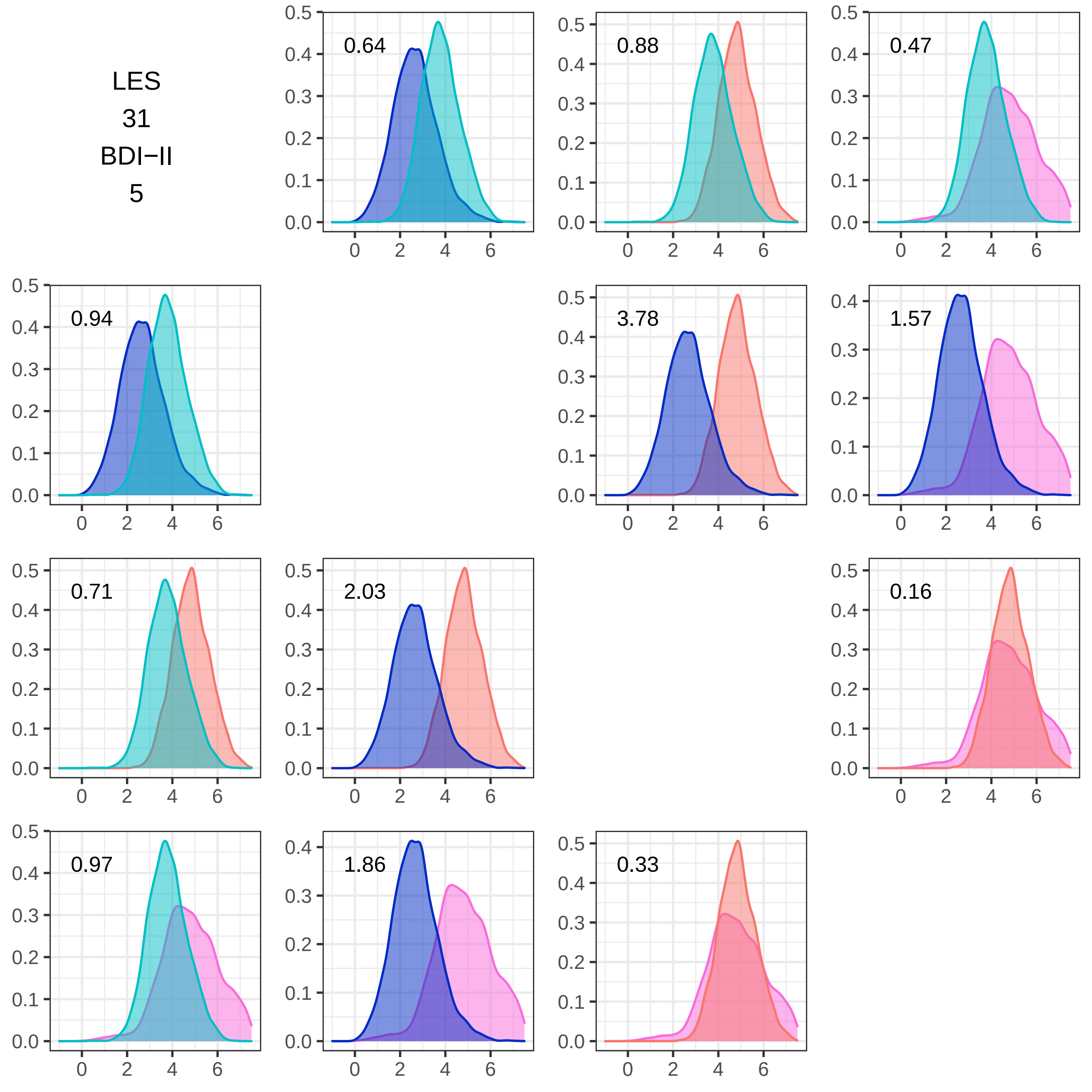

LES
31
BDI−II
5
0.64
0.88
0.47
0.94
3.78
1.57
0.71
2.03
0.16
0.97
1.86
0.33

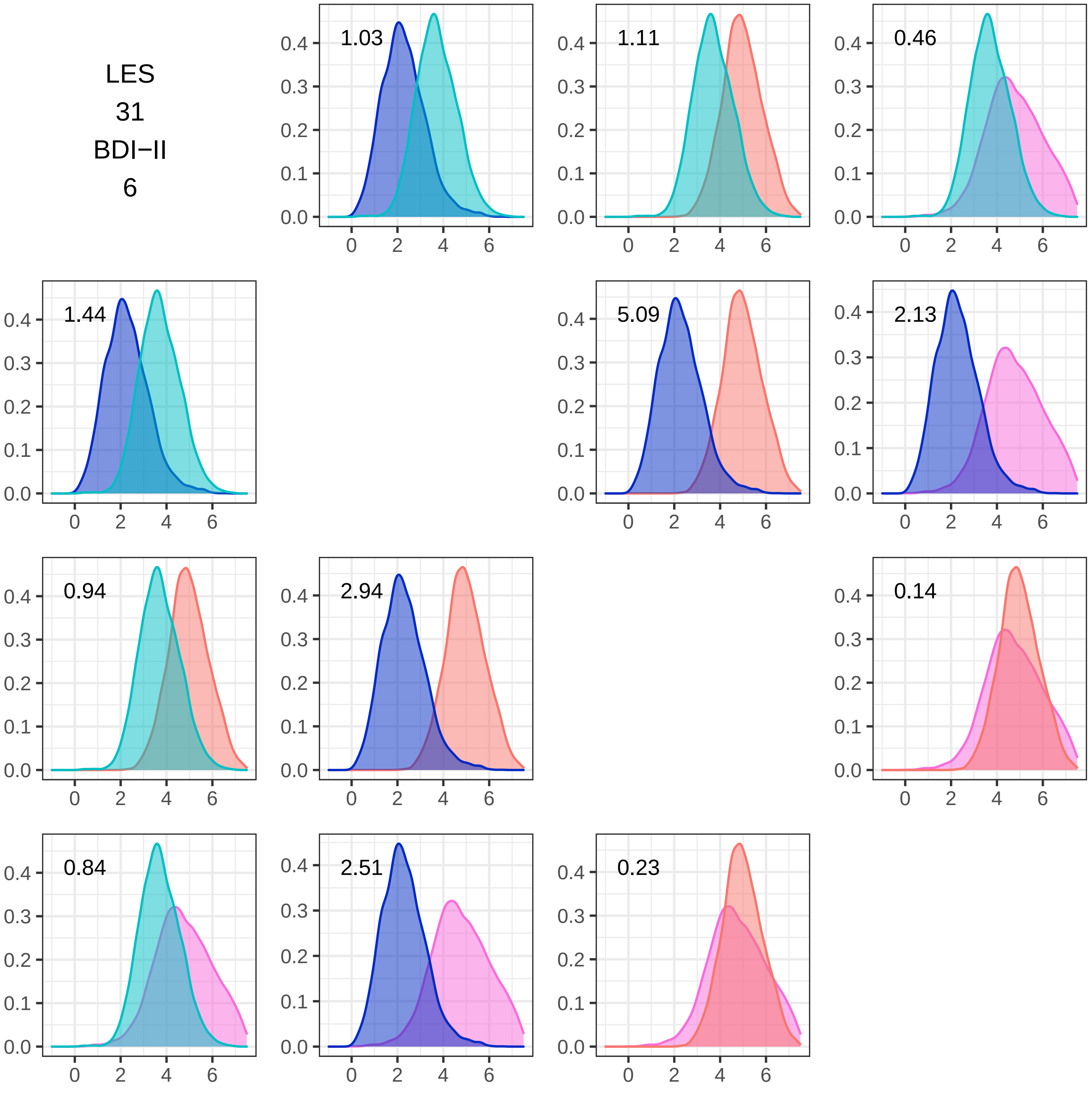
LES
31
BDI−II
6
1.03
1.11
0.46
1.44
5.09
2.13
0.94
2.94
0.14
0.84
2.51
0.23

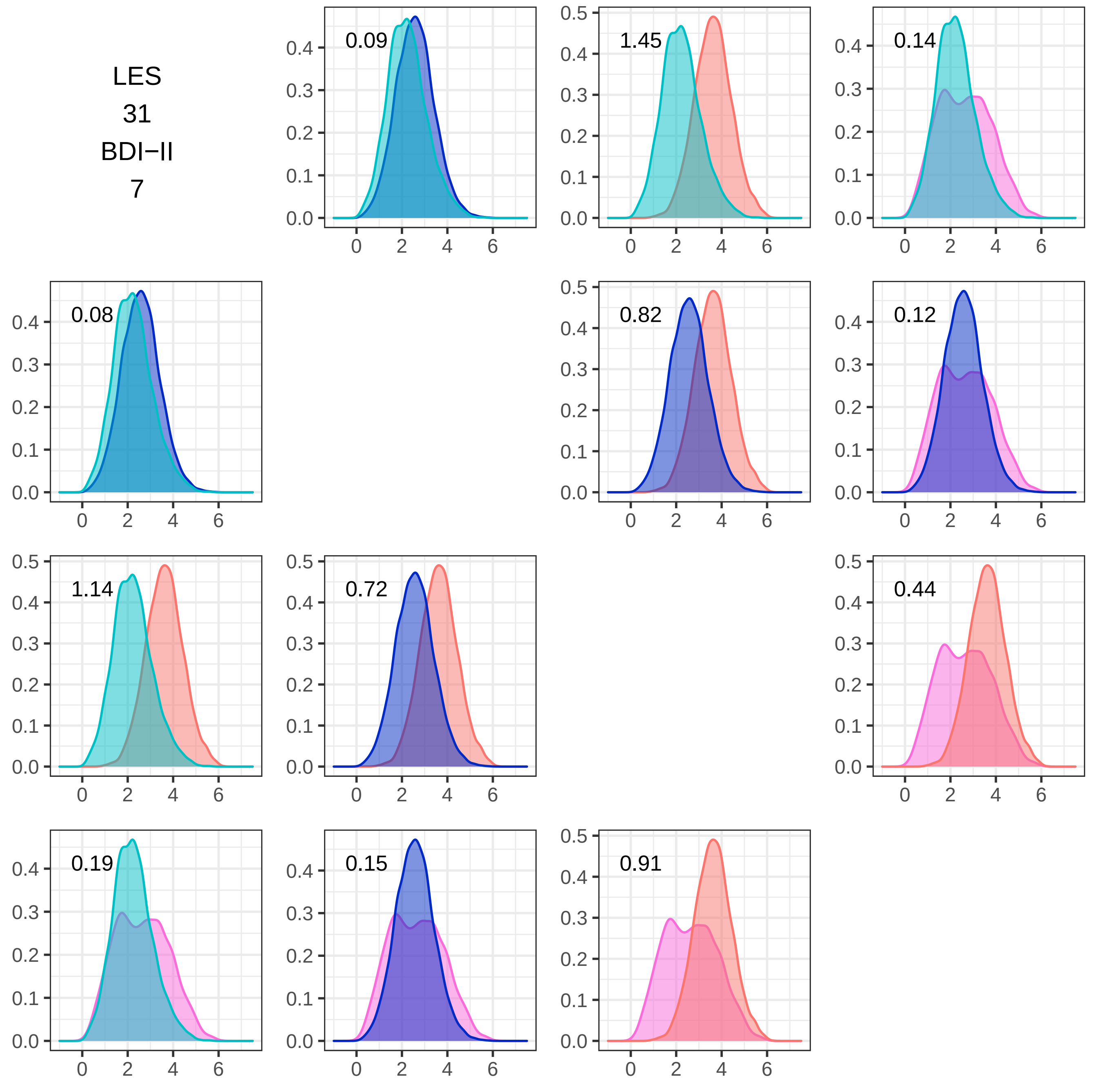

LES
31
BDI−II
7
0.09
1.45
0.14
0.08
0.82
0.12
1.14
0.72
0.44
0.19
0.15
0.91

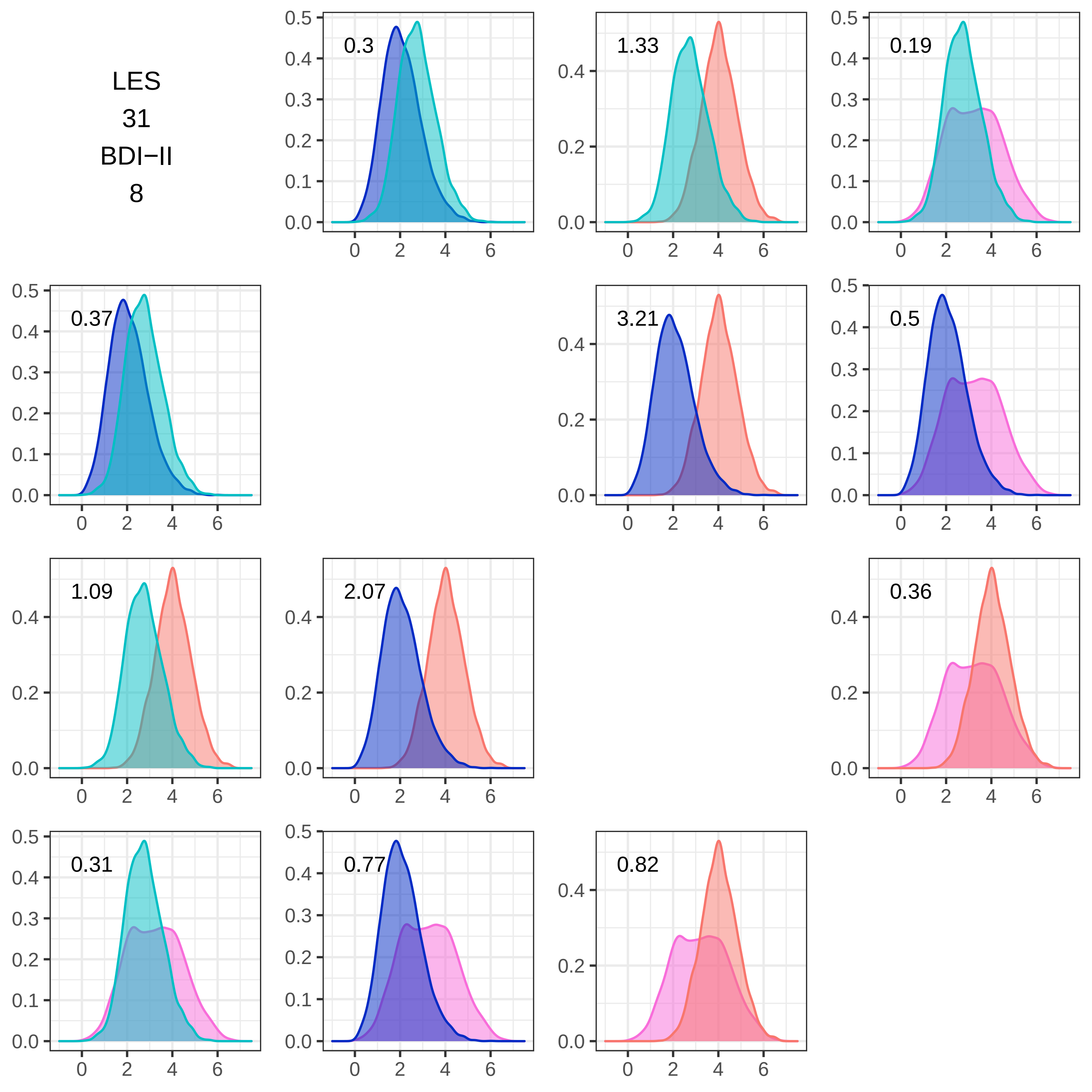
LES
31
BDI−II
8
0.3
1.33
0.19
0.37
3.21
0.5
1.09
2.07
0.36
0.31
0.77
0.82

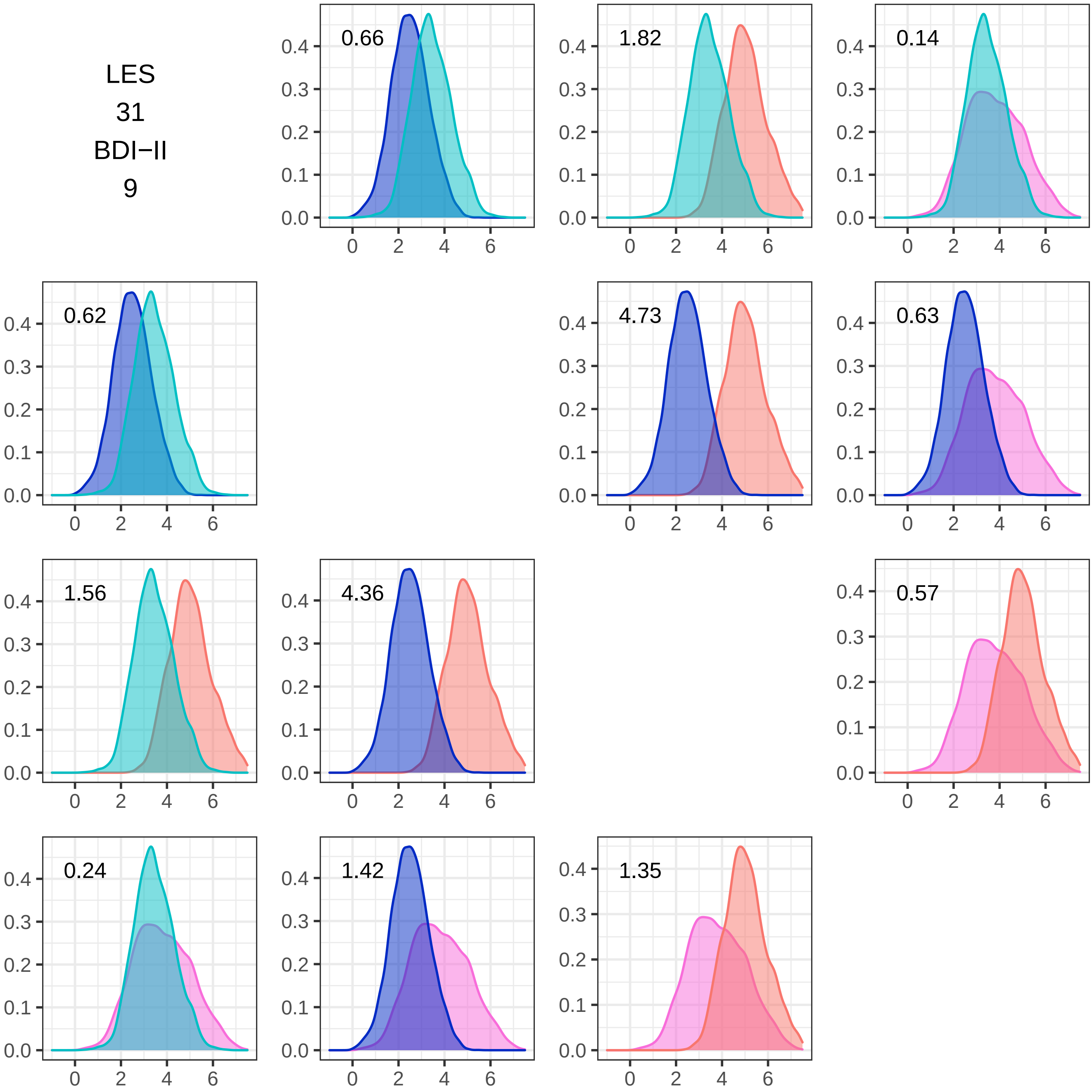
LES
31
BDI−II
9
0.66
1.82
0.14
0.62
4.73
0.63
1.56
4.36
0.57
0.24
1.42
1.35

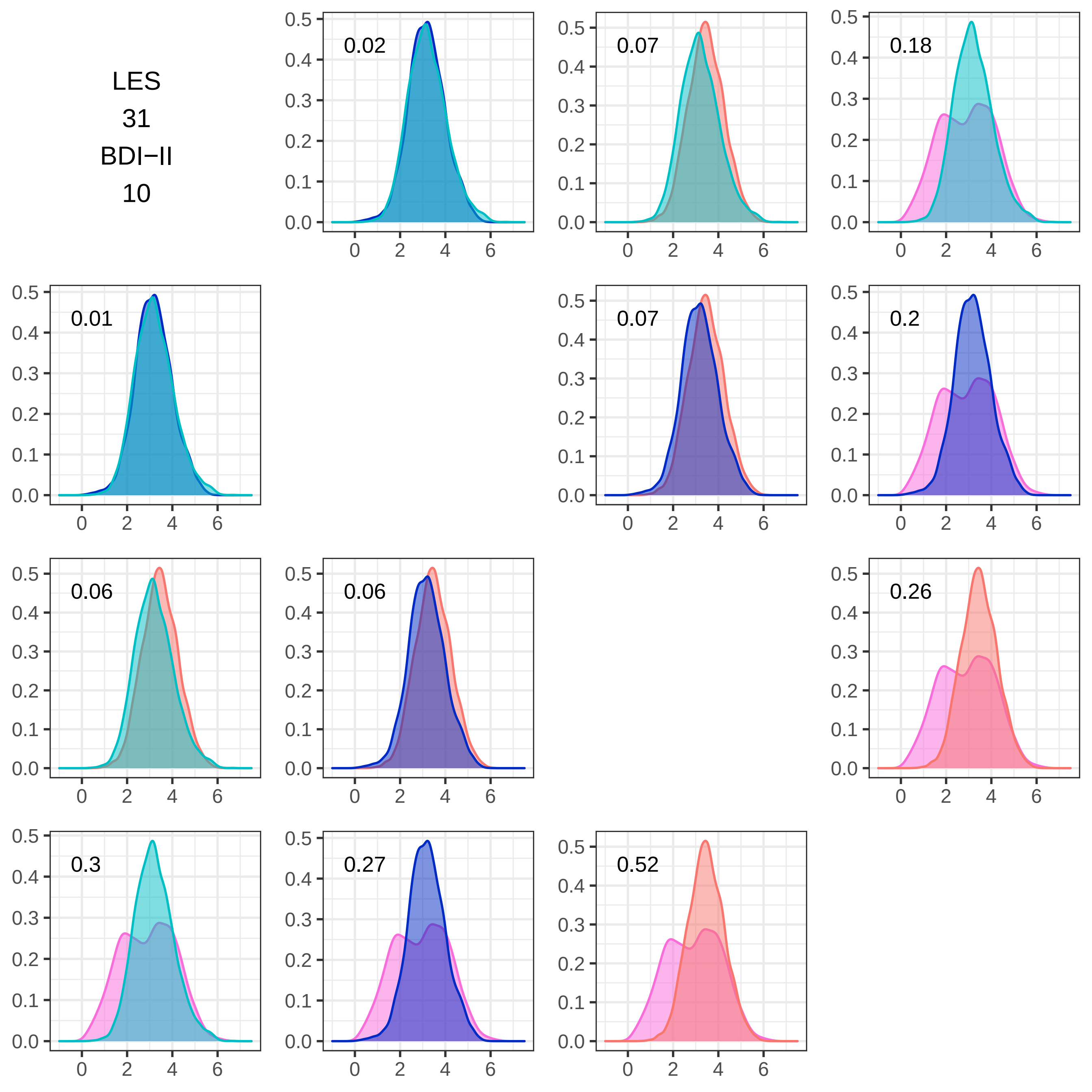
LES
31
BDI−II
10
0.02
0.07
0.18
0.01
0.07
0.2
0.06
0.06
0.26
0.3
0.27
0.52

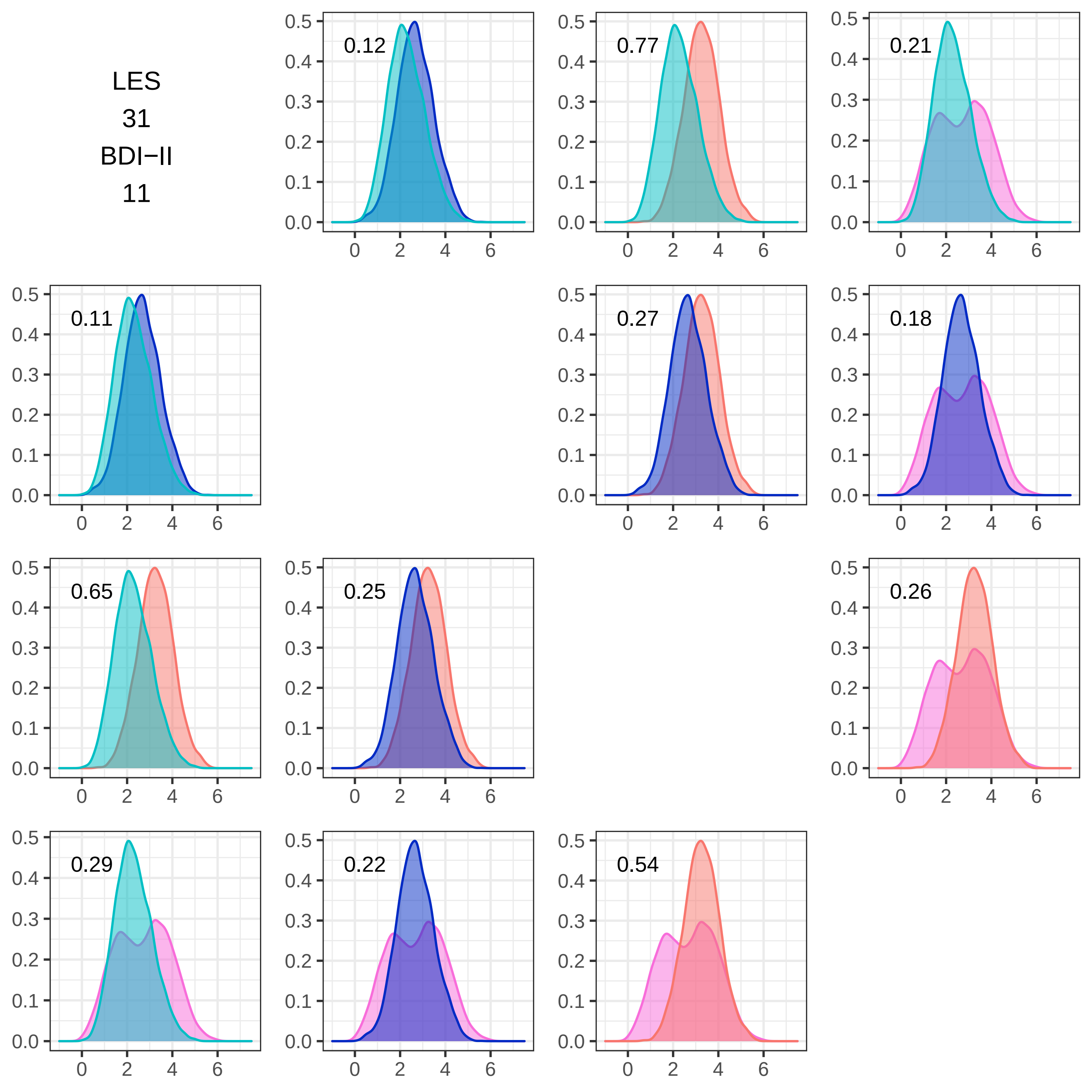
LES
31
BDI−II
11
0.12
0.77
0.21
0.11
0.27
0.18
0.65
0.25
0.26
0.29
0.22
0.54

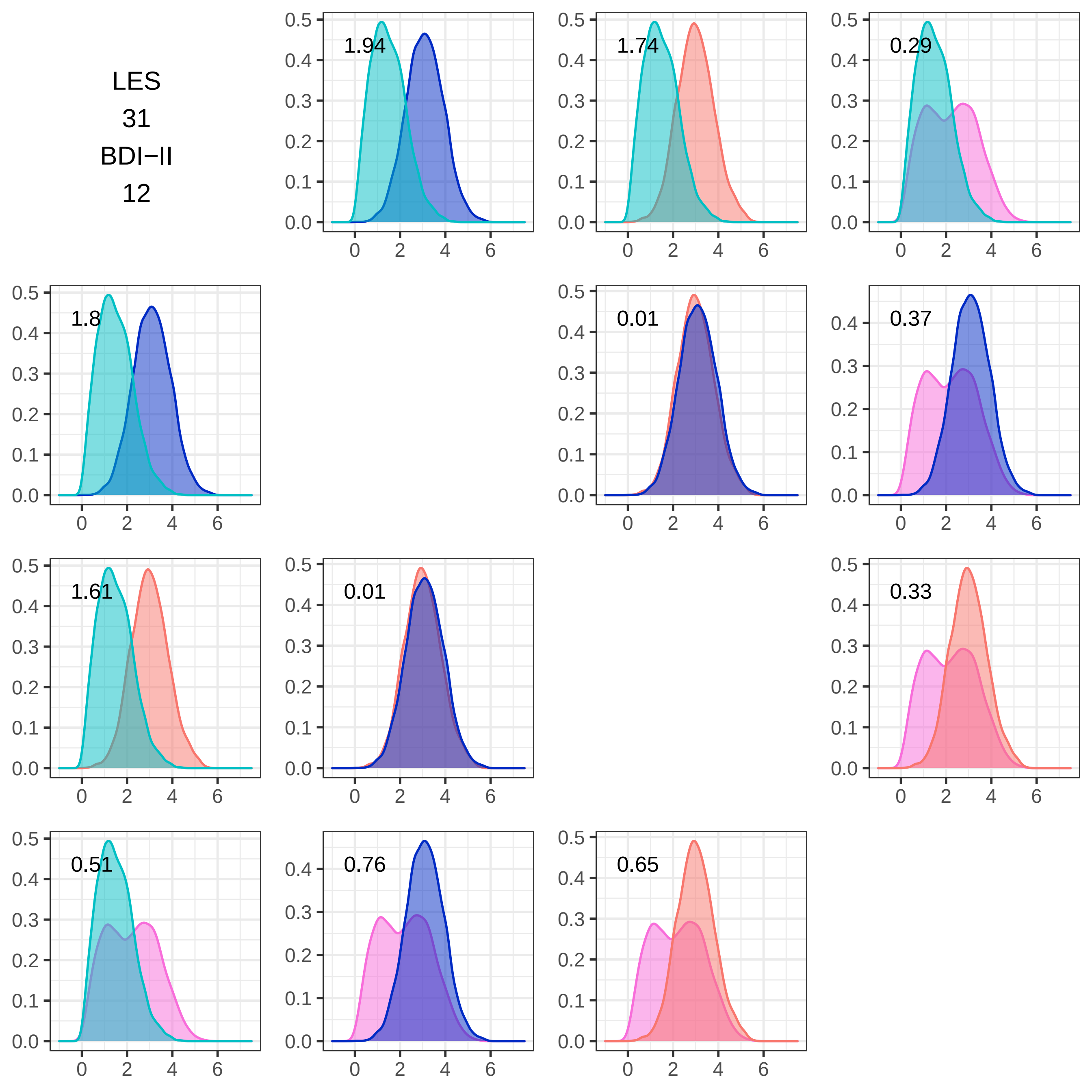

LES
31
BDI−II
12
1.94
1.74
0.29
1.8
0.01
0.37
1.61
0.01
0.33
0.51
0.76
0.65

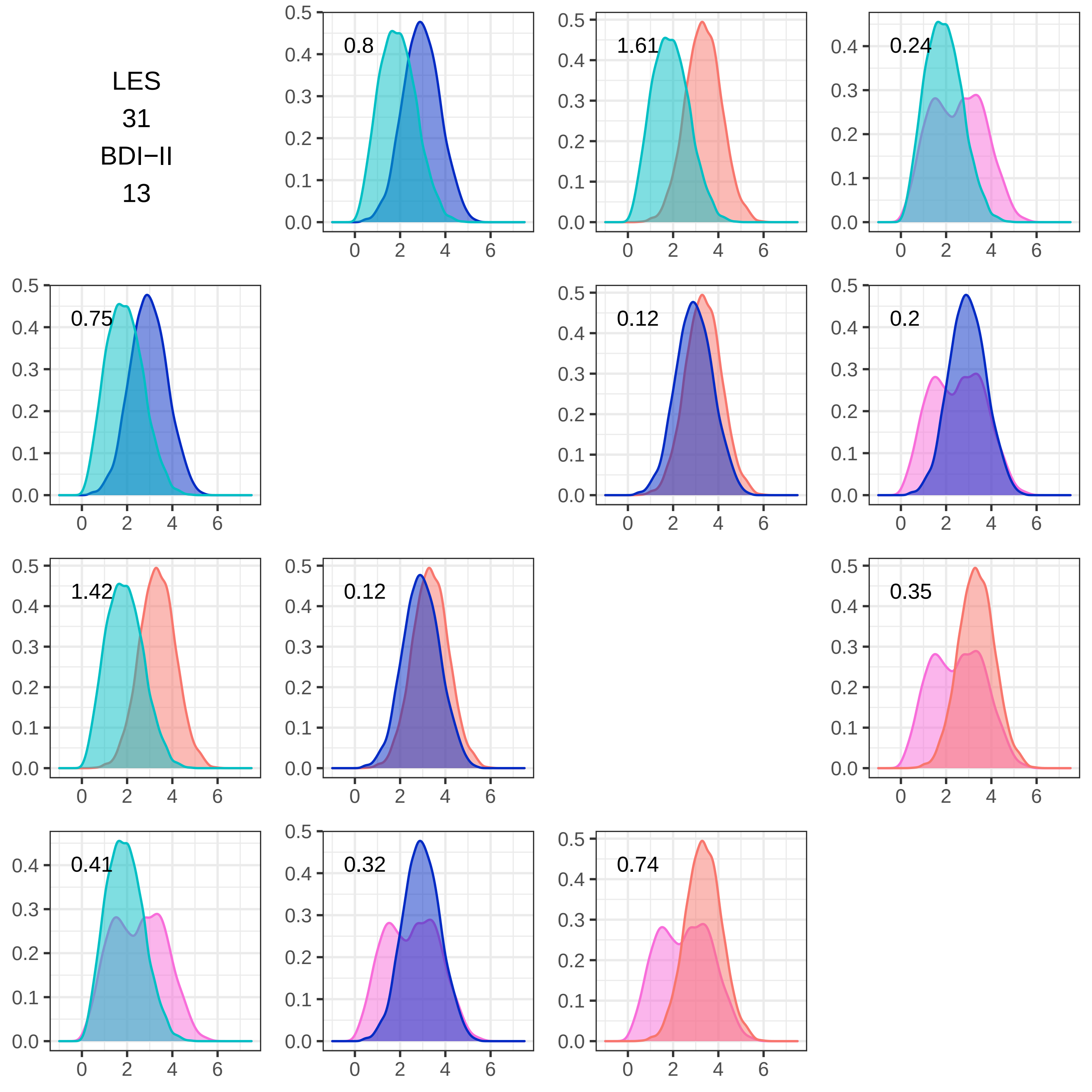
LES
31
BDI−II
13
0.8
1.61
0.24
0.75
0.12
0.2
1.42
0.12
0.35
0.41
0.32
0.74

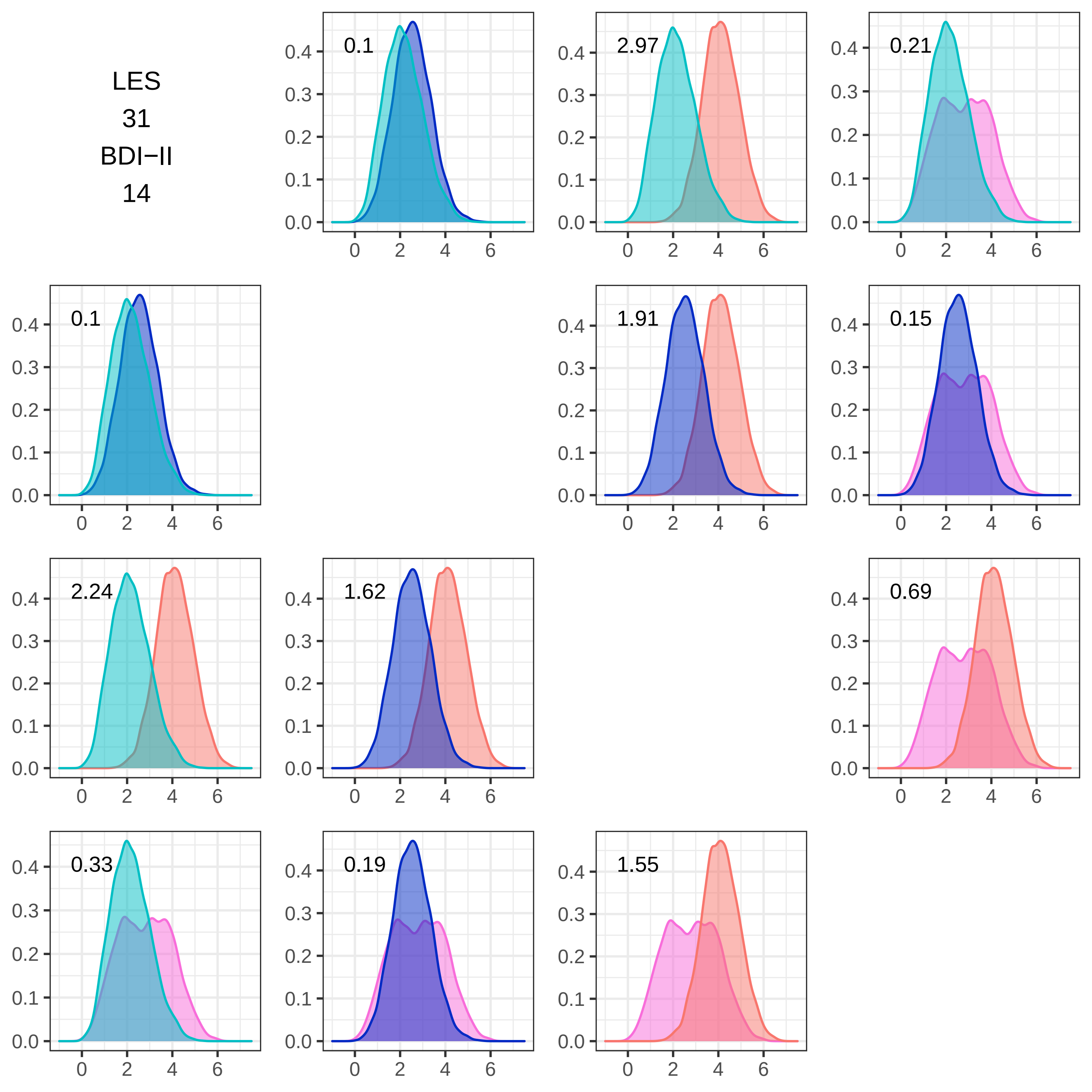
LES
31
BDI−II
14
0.1
2.97
0.21
0.1
1.91
0.15
2.24
1.62
0.69
0.33
0.19
1.55

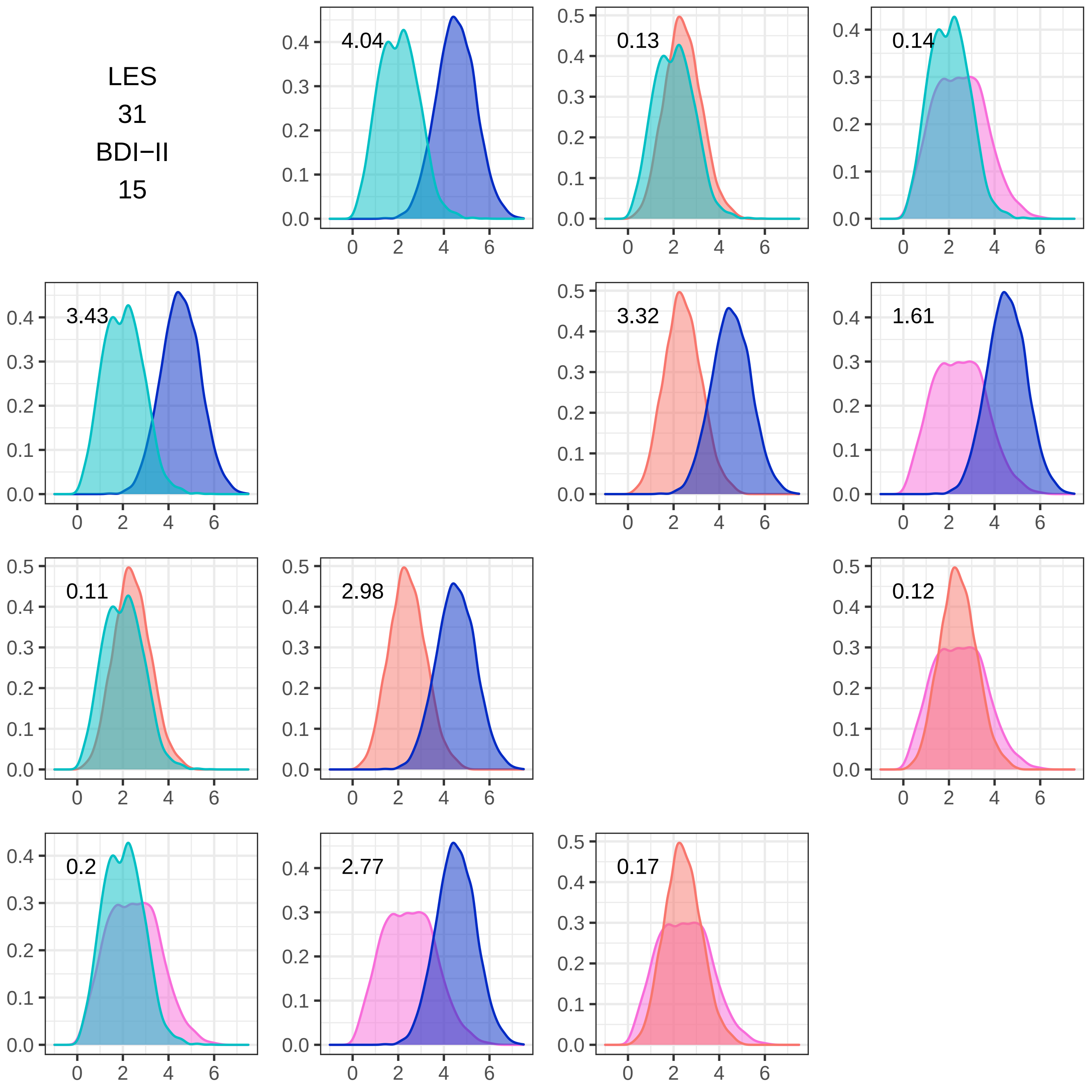
LES
31
BDI−II
15
4.04
0.13
0.14
3.43
3.32
1.61
0.11
2.98
0.12
0.2
2.77
0.17

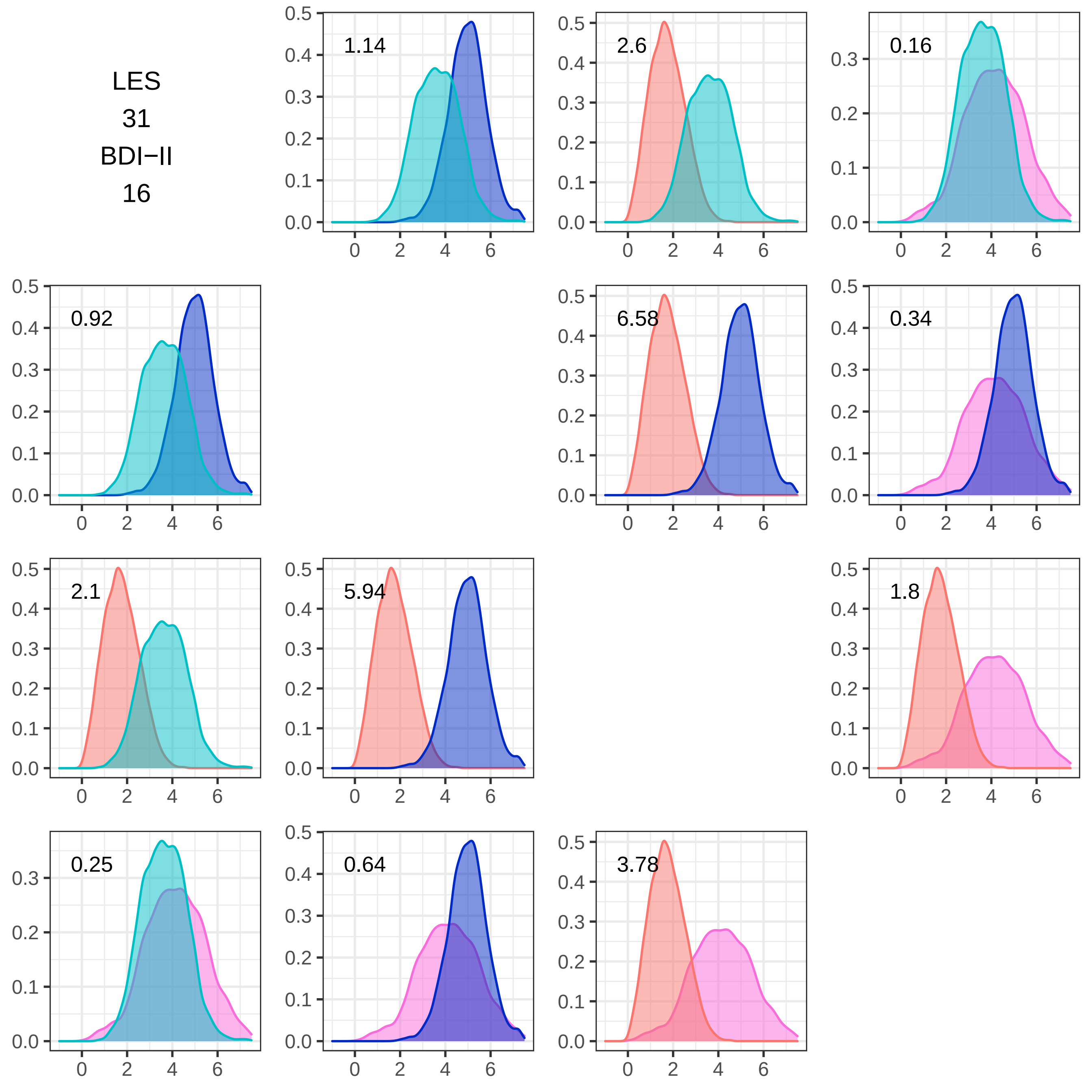
LES
31
BDI−II
16
1.14
2.6
0.16
0.92
6.58
0.34
2.1
5.94
1.8
0.25
0.64
3.78

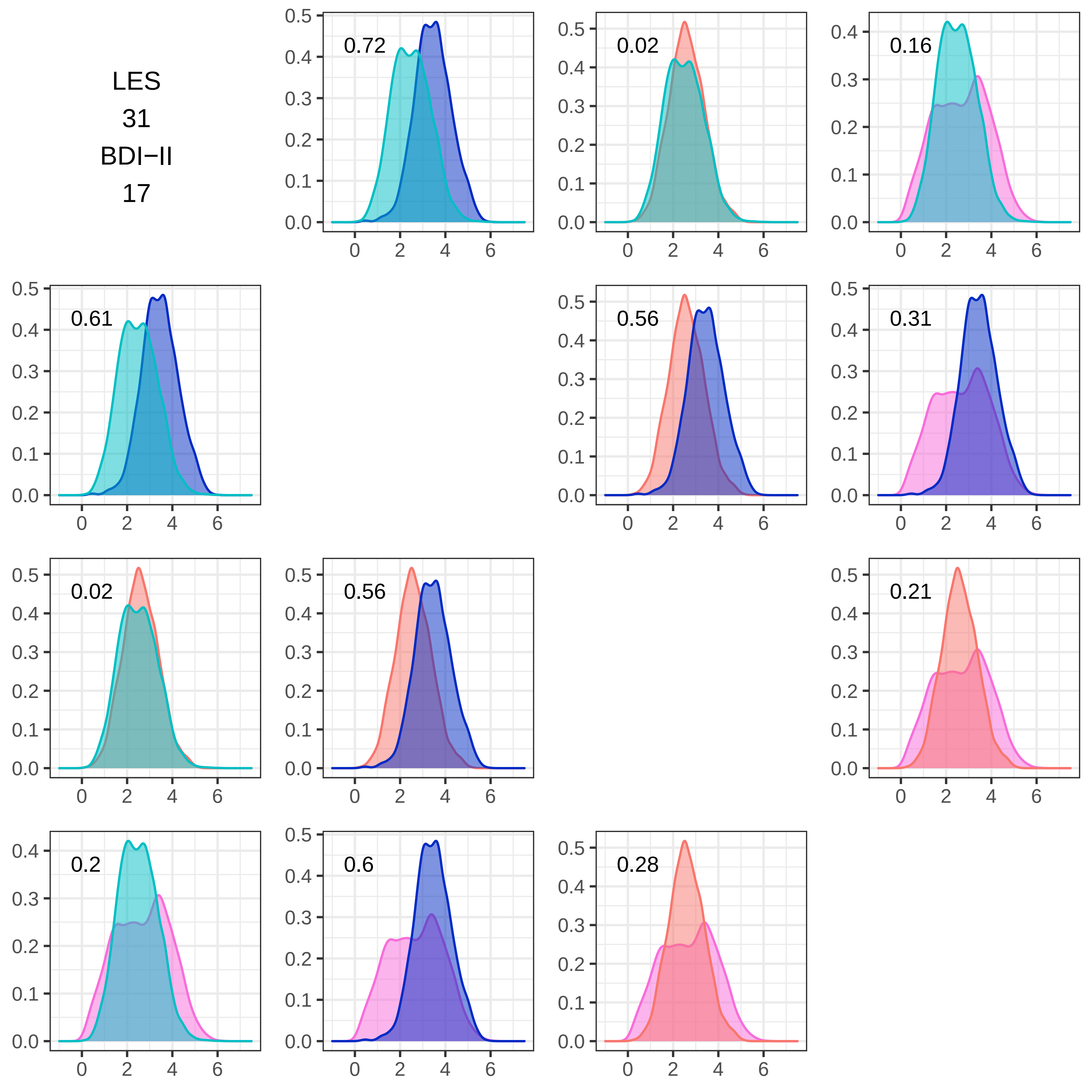
LES
31
BDI−II
17
0.72
0.02
0.16
0.61
0.56
0.31
0.02
0.56
0.21
0.2
0.6
0.28

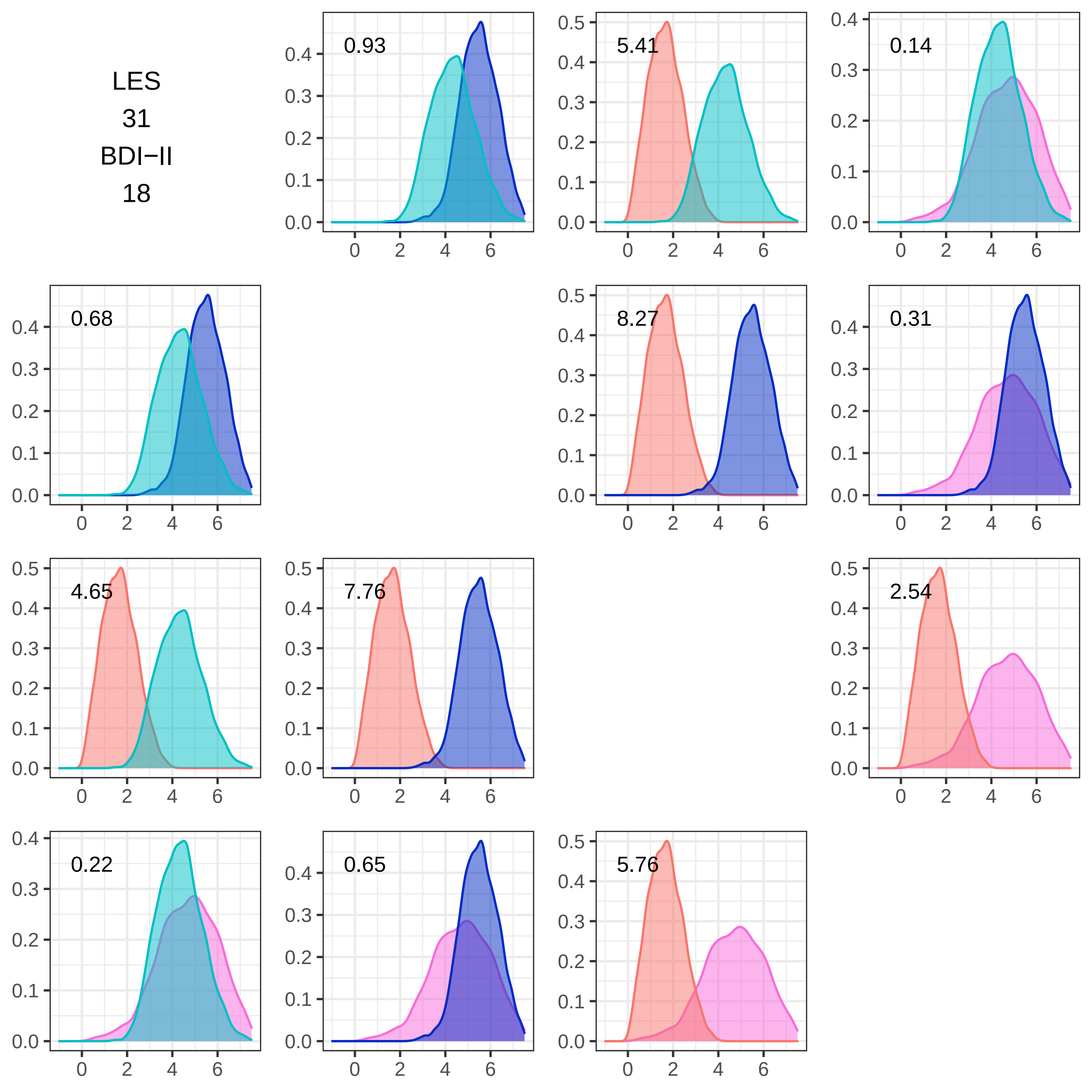
LES
31
BDI−II
18
0.93
5.41
0.14
0.68
8.27
0.31
4.65
7.76
2.54
0.22
0.65
5.76

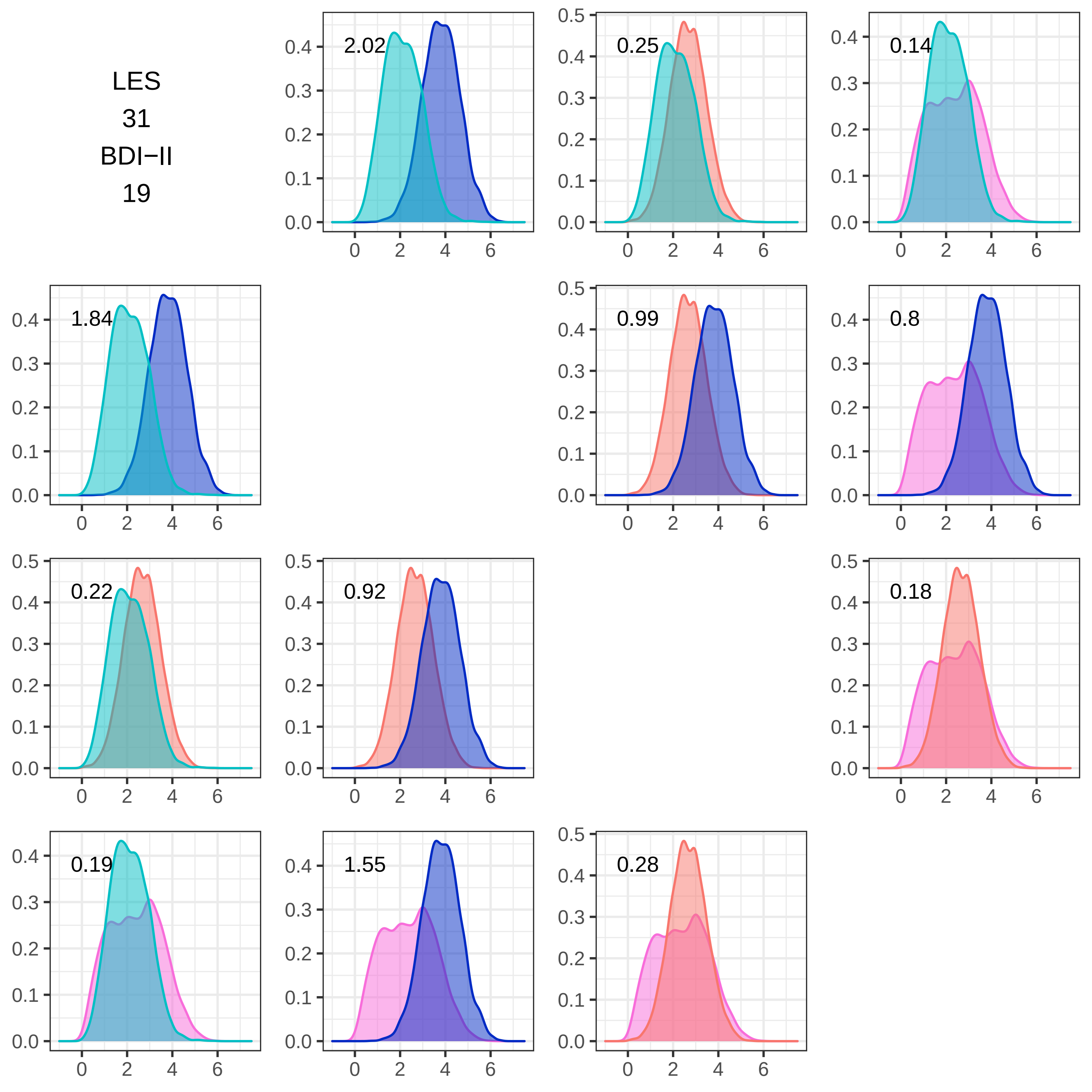

LES
31
BDI−II
19
2.02
0.25
0.14
1.84
0.99
0.8
0.22
0.92
0.18
0.19
1.55
0.28

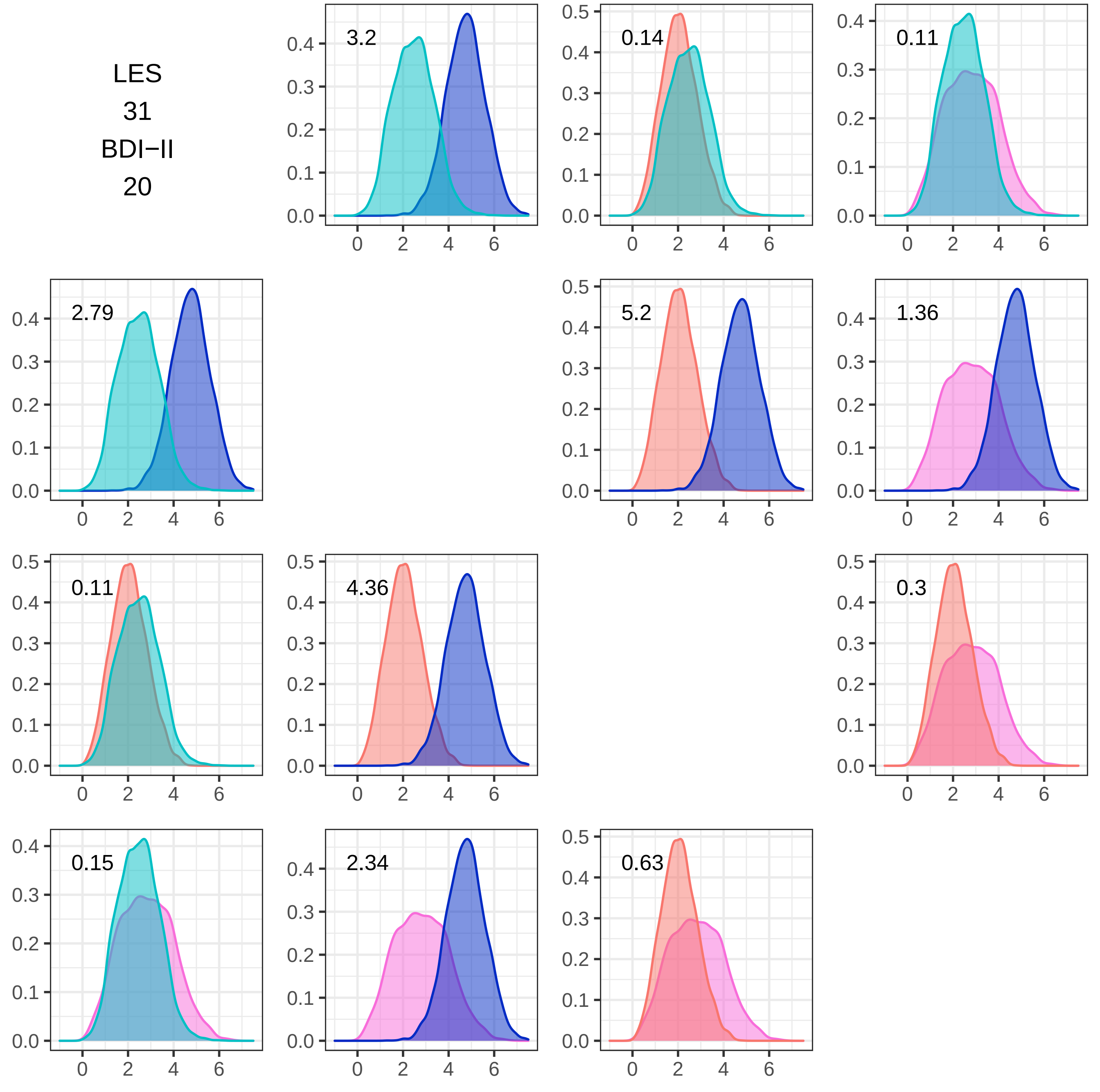

LES
31
BDI−II
20
3.2
0.14
0.11
2.79
5.2
1.36
0.11
4.36
0.3
0.15
2.34
0.63

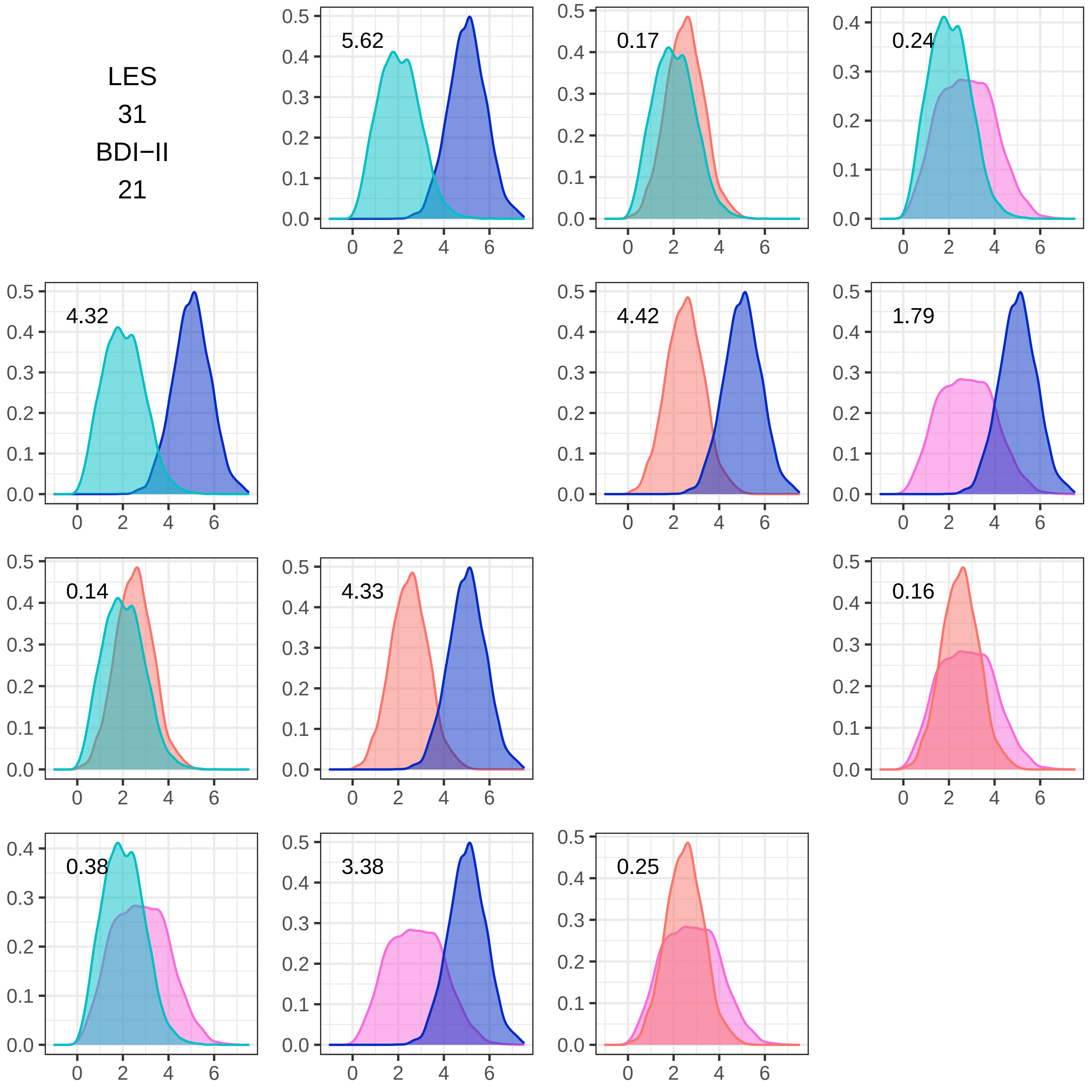

LES
31
BDI−II
21
5.62
0.17
0.24
4.32
4.42
1.79
0.14
4.33
0.16
0.38
3.38
0.25

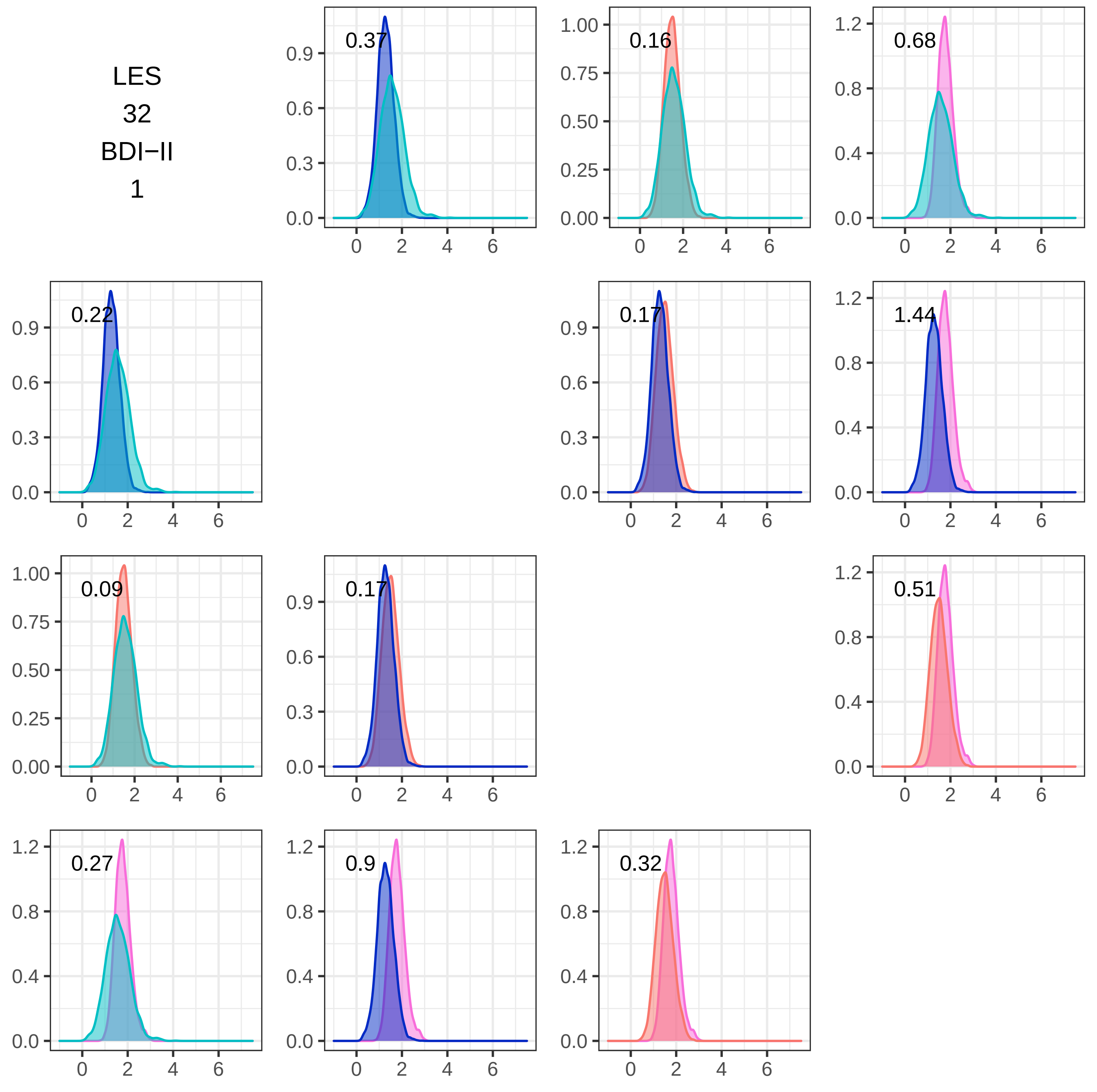

LES
32
BDI−II
1
0.37
0.16
0.68
0.22
0.17
1.44
0.09
0.17
0.51
0.27
0.9
0.32

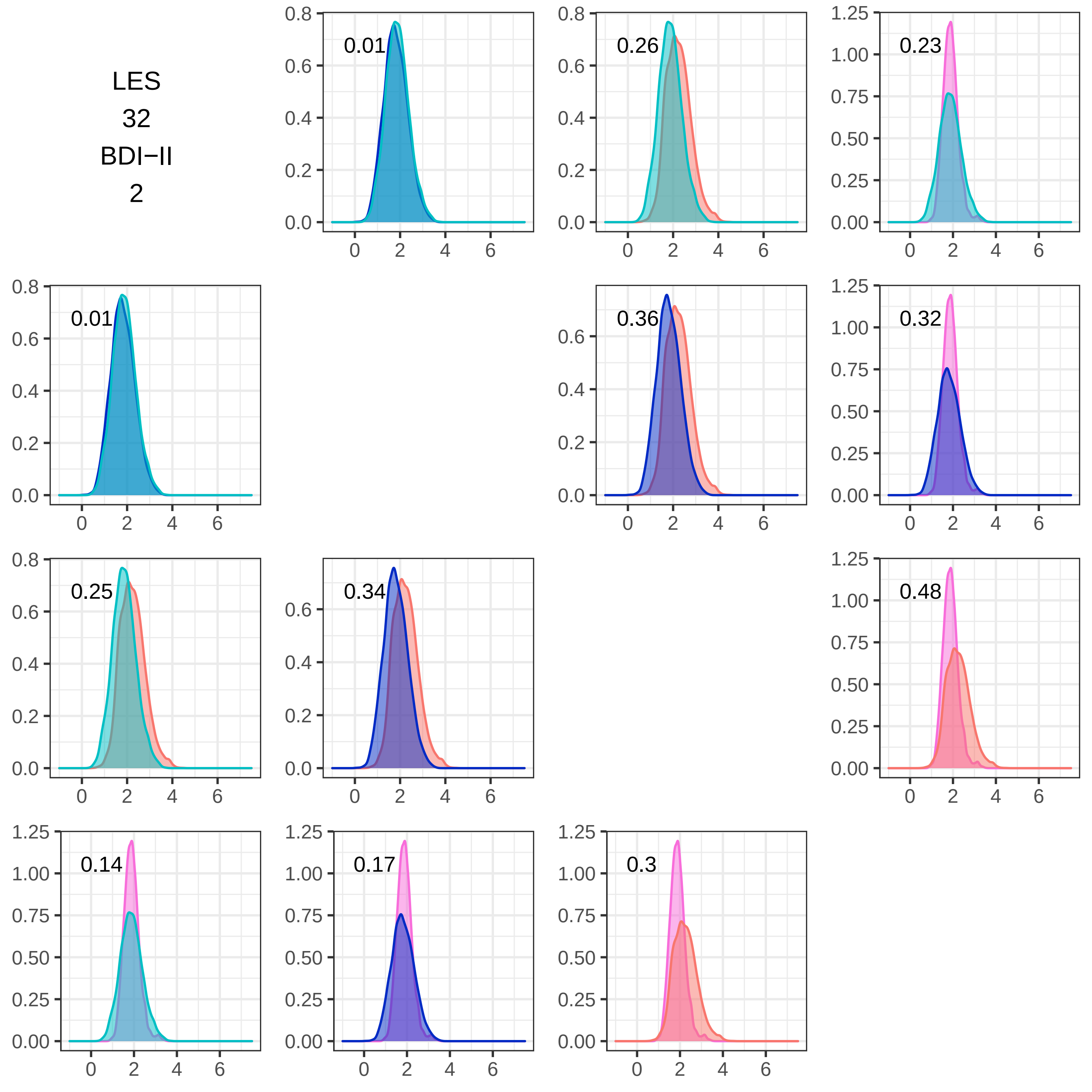

LES
32
BDI−II
2
0.01
0.26
0.23
0.01
0.36
0.32
0.25
0.34
0.48
0.14
0.17
0.3

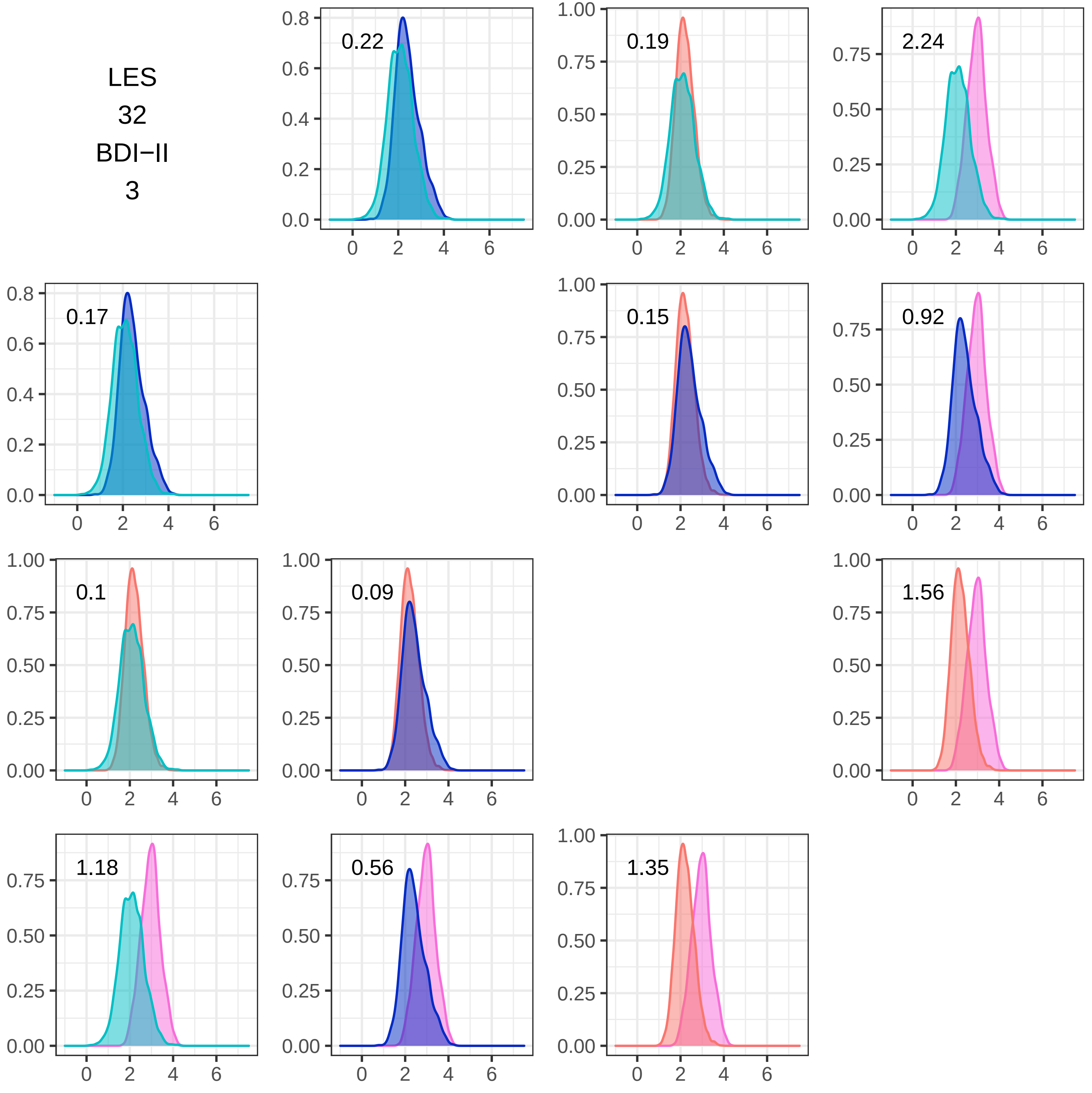
LES
32
BDI−II
3
0.22
0.19
2.24
0.17
0.15
0.92
0.1
0.09
1.56
1.18
0.56
1.35

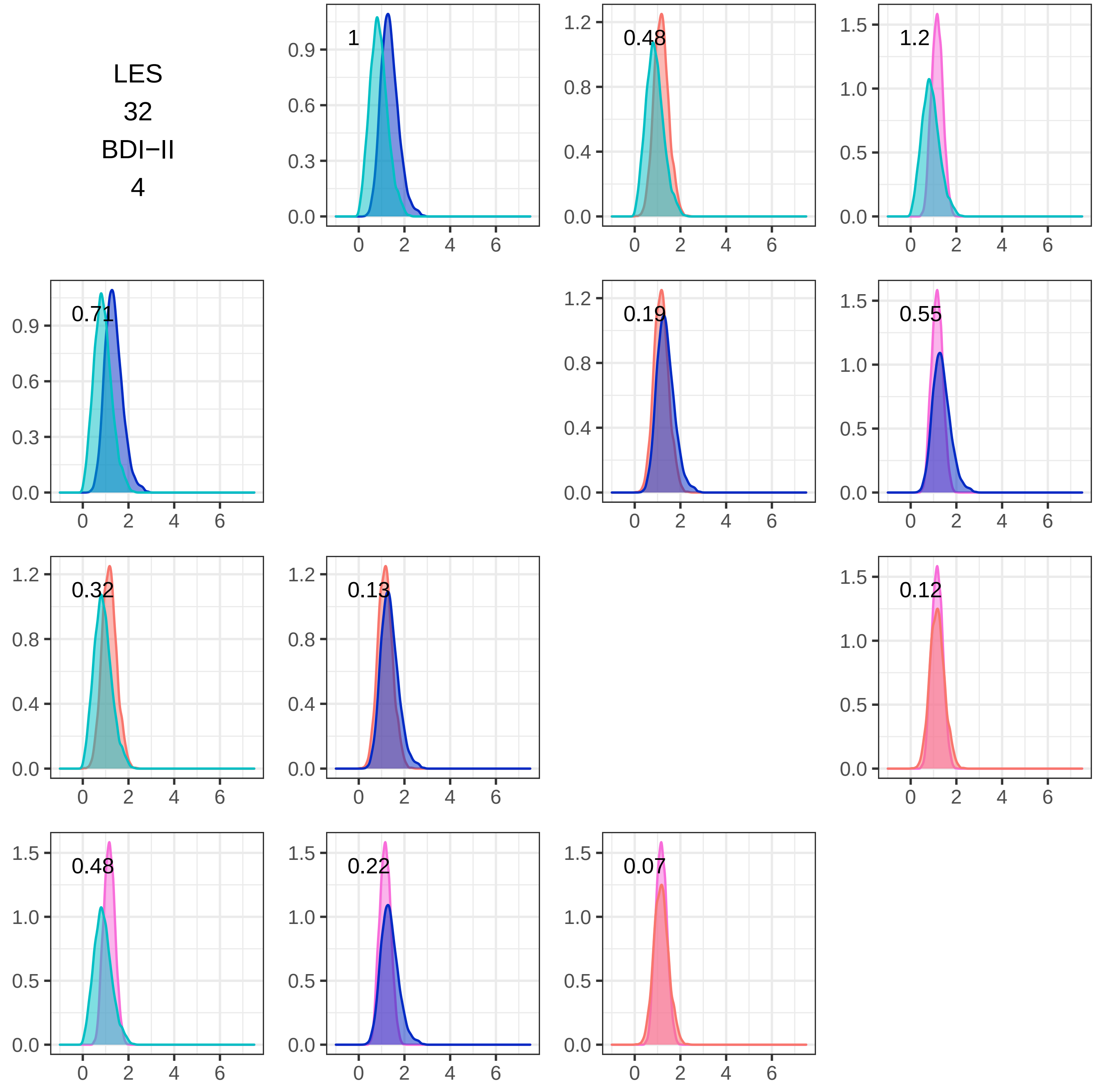
LES
32
BDI−II
4
1
0.48
1.2
0.71
0.19
0.55
0.32
0.13
0.12
0.48
0.22
0.07

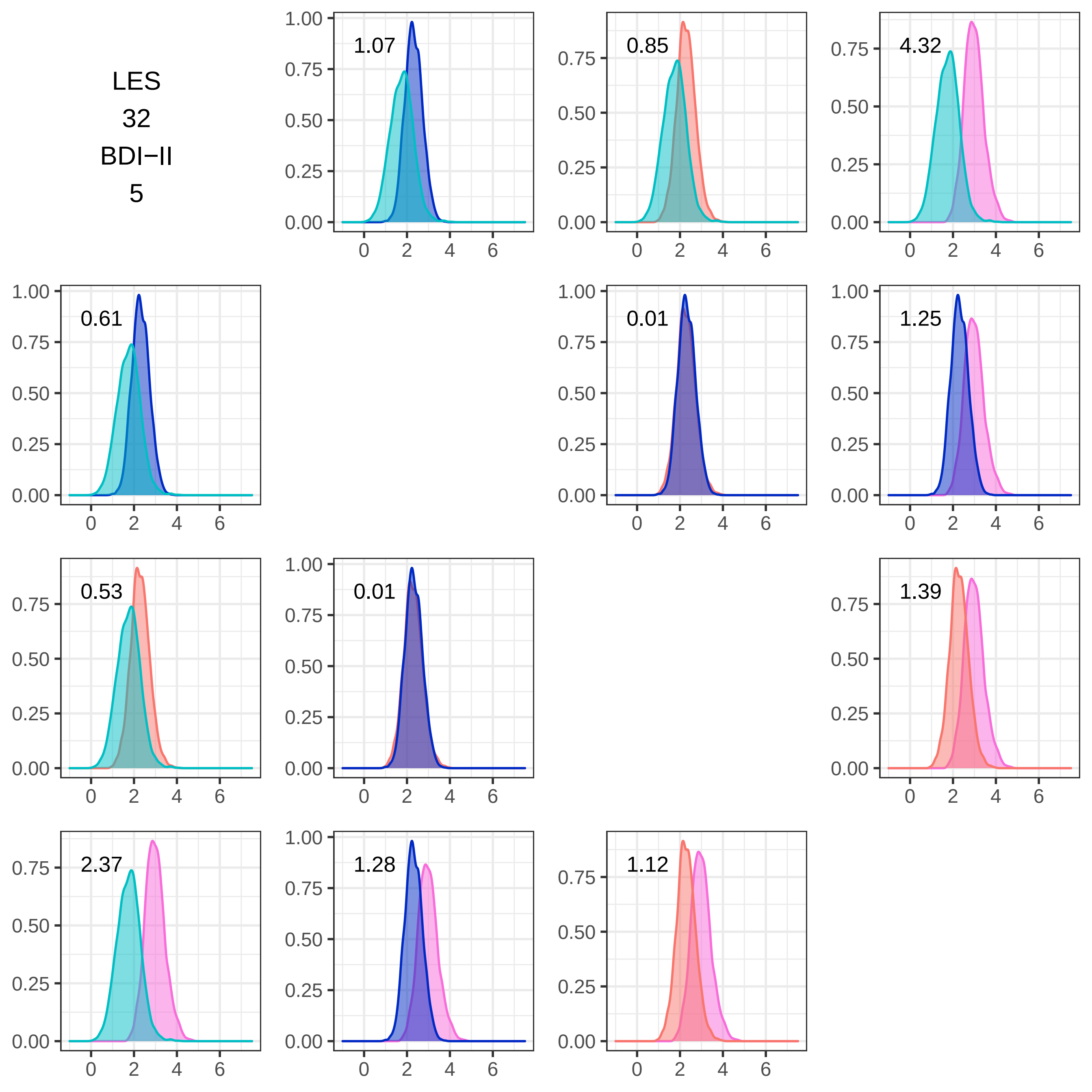
LES
32
BDI−II
5
1.07
0.85
4.32
0.61
0.01
1.25
0.53
0.01
1.39
2.37
1.28
1.12

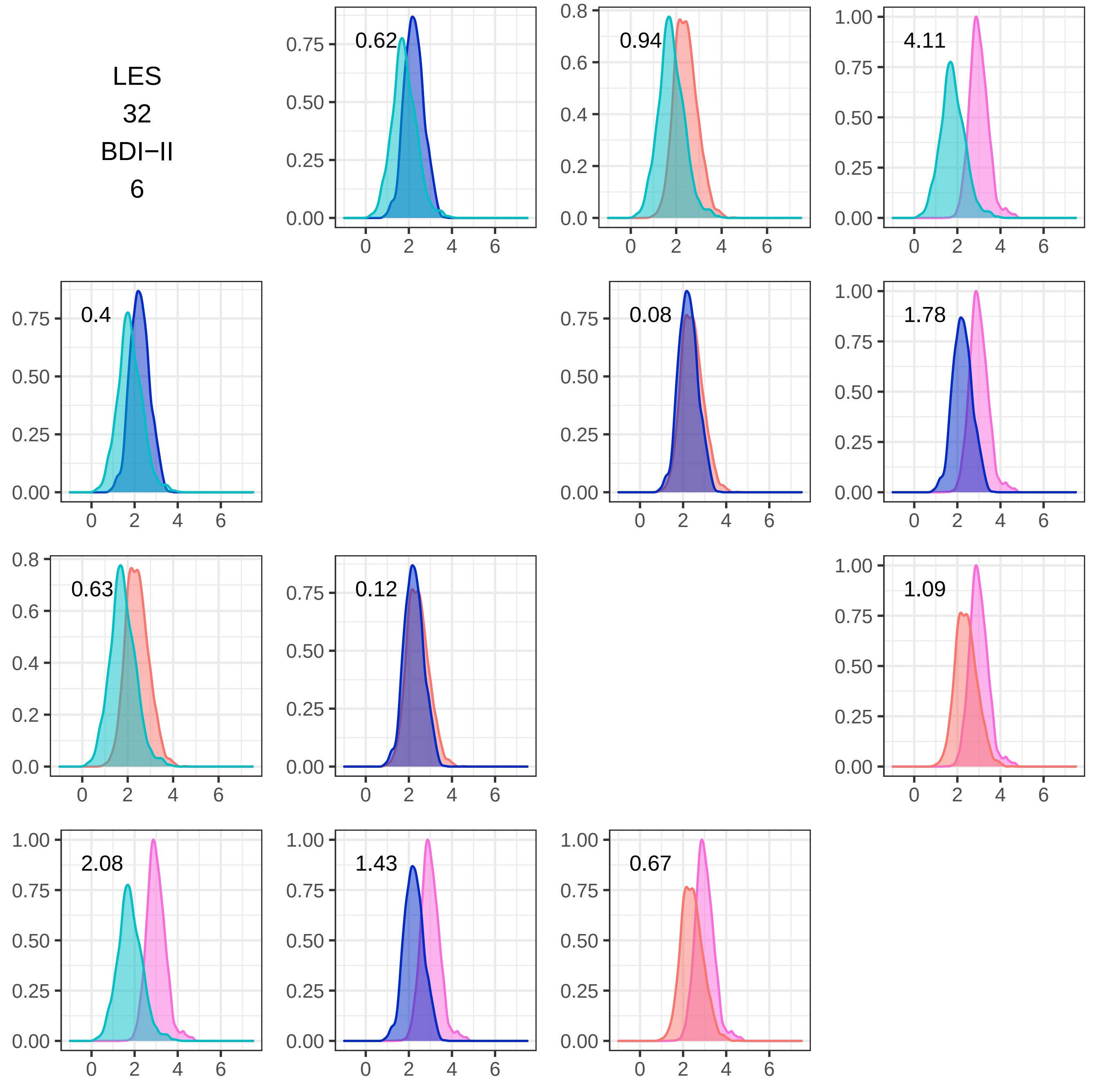

LES
32
BDI−II
6
0.62
0.94
4.11
0.4
0.08
1.78
0.63
0.12
1.09
2.08
1.43
0.67

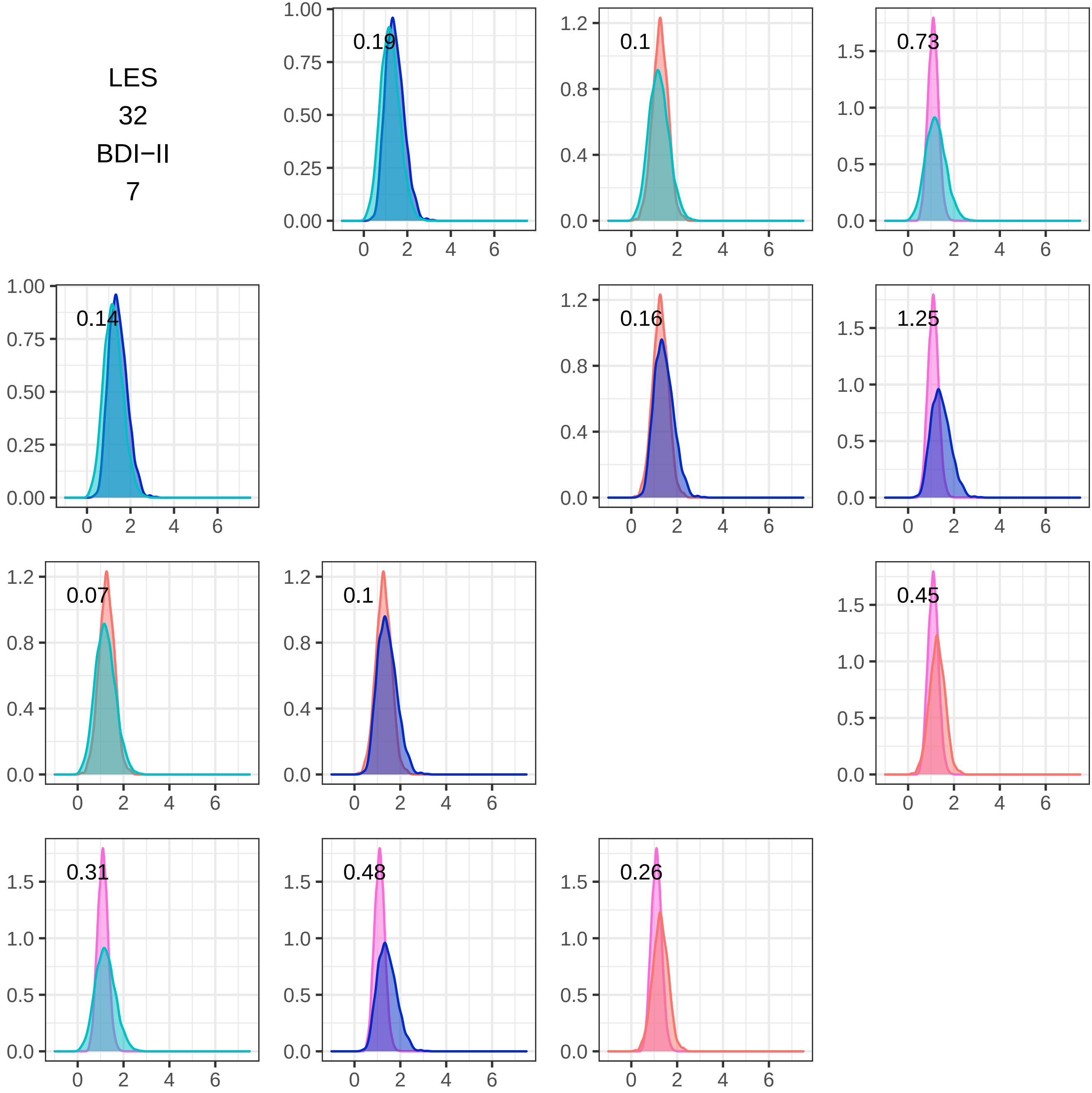
LES
32
BDI−II
7
0.19
0.1
0.73
0.14
0.16
1.25
0.07
0.1
0.45
0.31
0.48
0.26

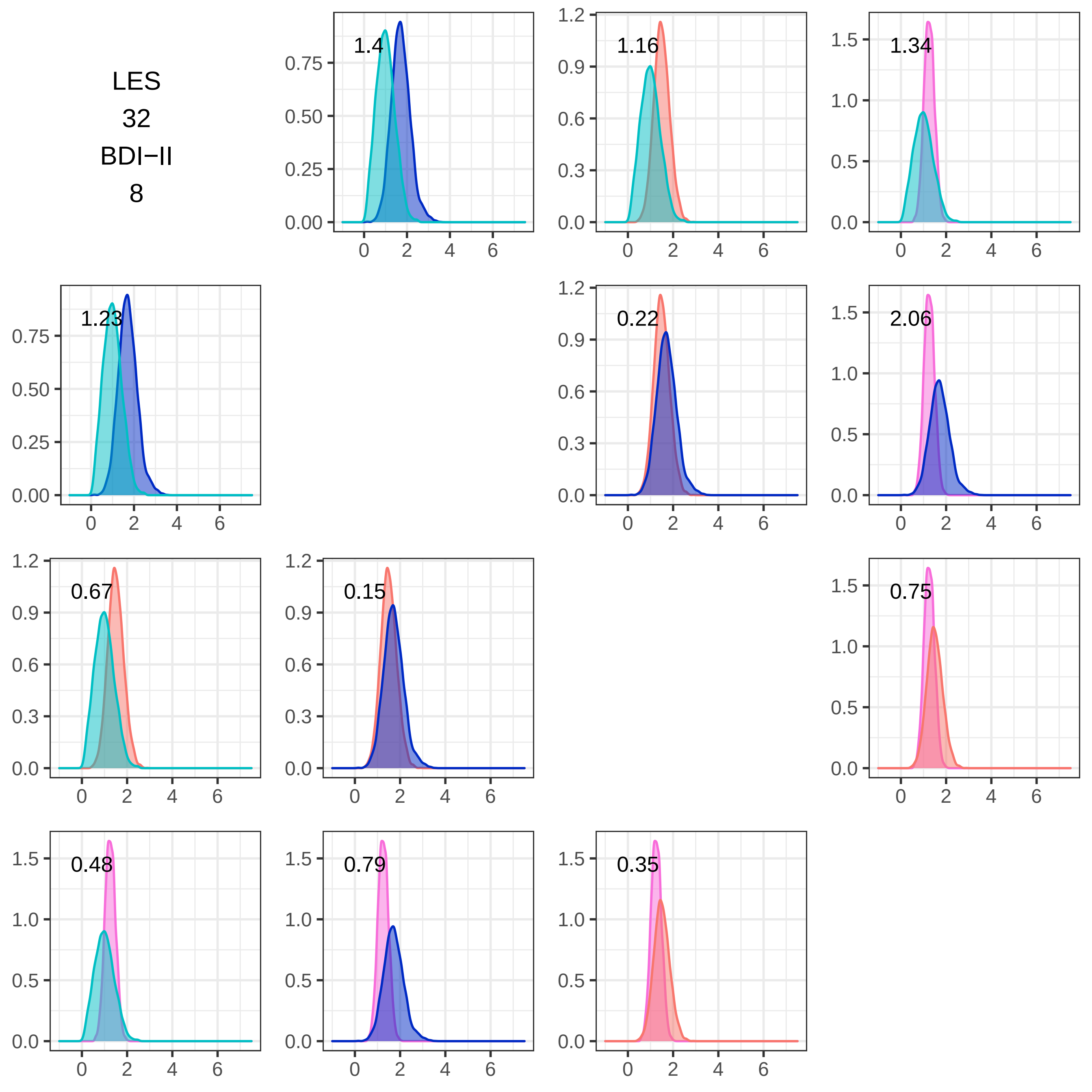

LES
32
BDI−II
8
1.4
1.16
1.34
1.23
0.22
2.06
0.67
0.15
0.75
0.48
0.79
0.35

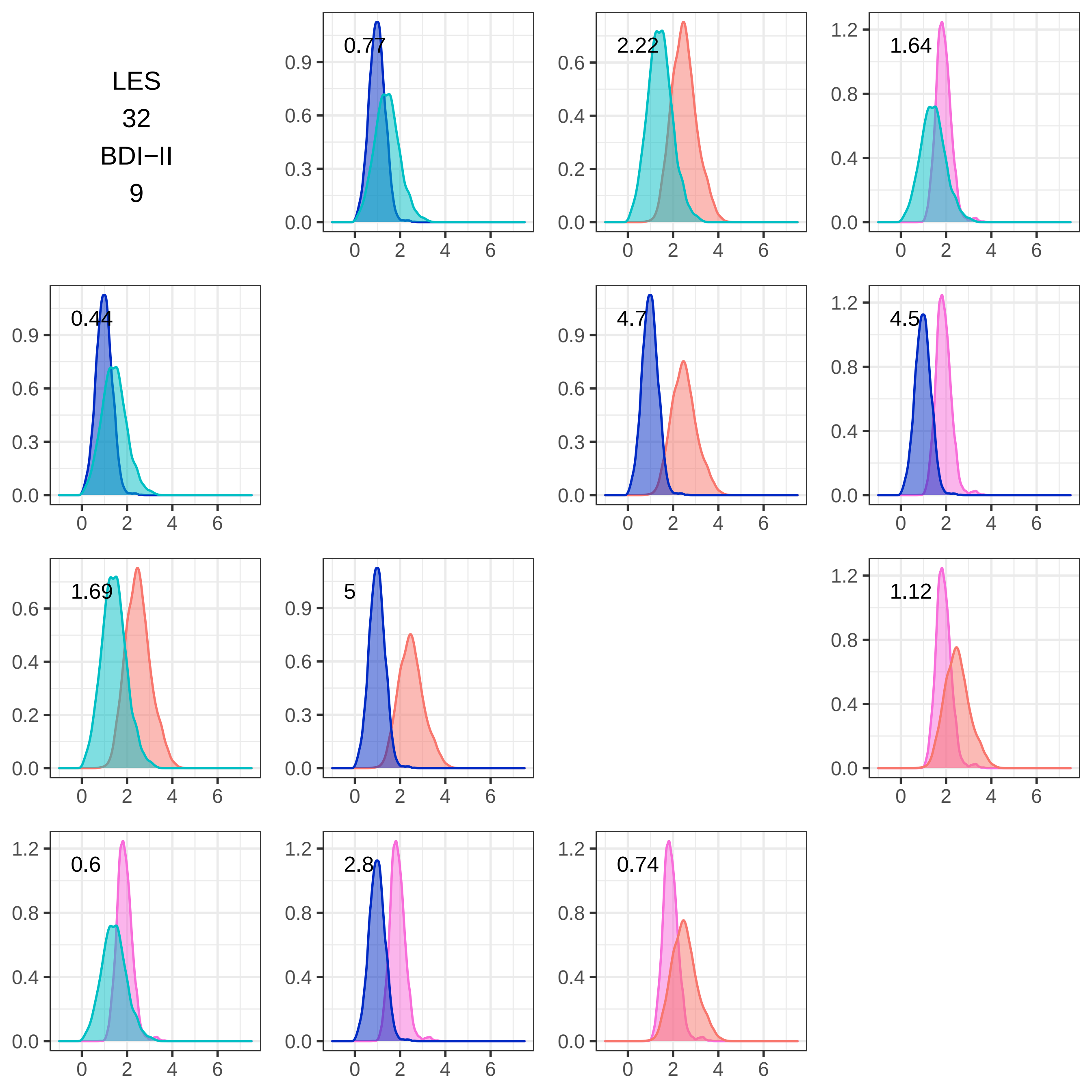

LES
32
BDI−II
9
0.77
2.22
1.64
0.44
4.7
4.5
1.69
5
1.12
0.6
2.8
0.74

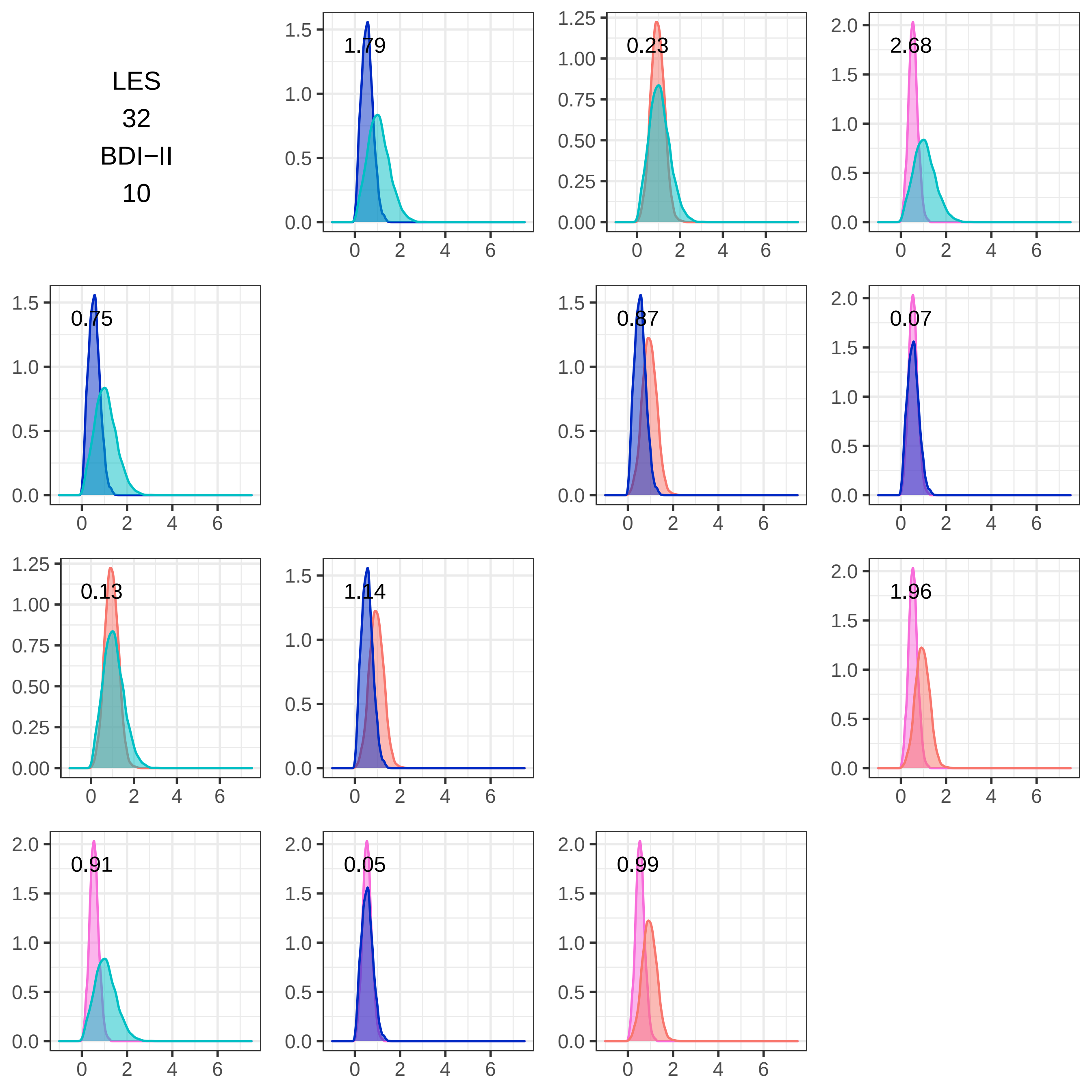

LES
32
BDI−II
10
1.79
0.23
2.68
0.75
0.87
0.07
0.13
1.14
1.96
0.91
0.05
0.99

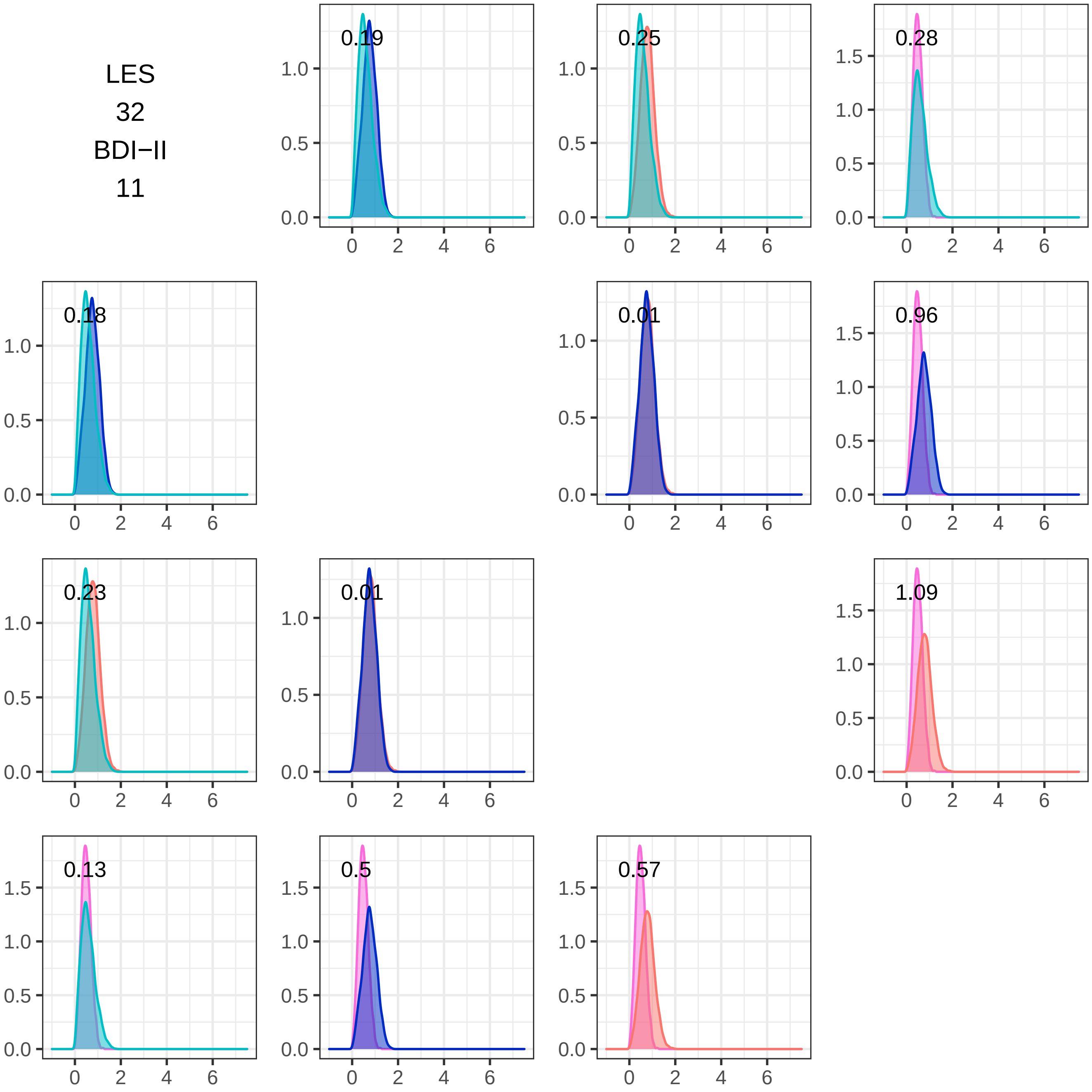

LES
32
BDI−II
11
0.19
0.25
0.28
0.18
0.01
0.96
0.23
0.01
1.09
0.13
0.5
0.57

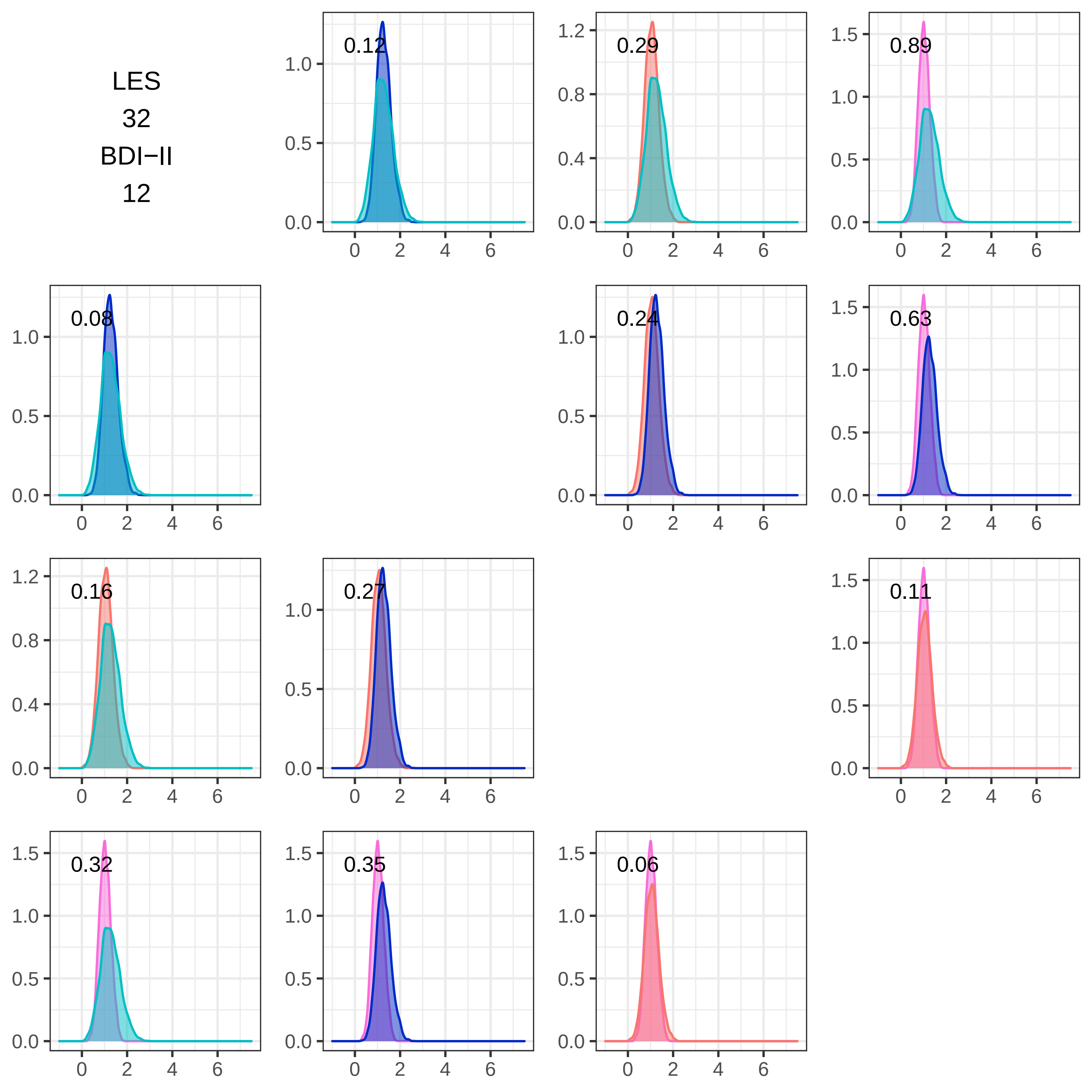
LES
32
BDI−II
12
0.12
0.29
0.89
0.08
0.24
0.63
0.16
0.27
0.11
0.32
0.35
0.06

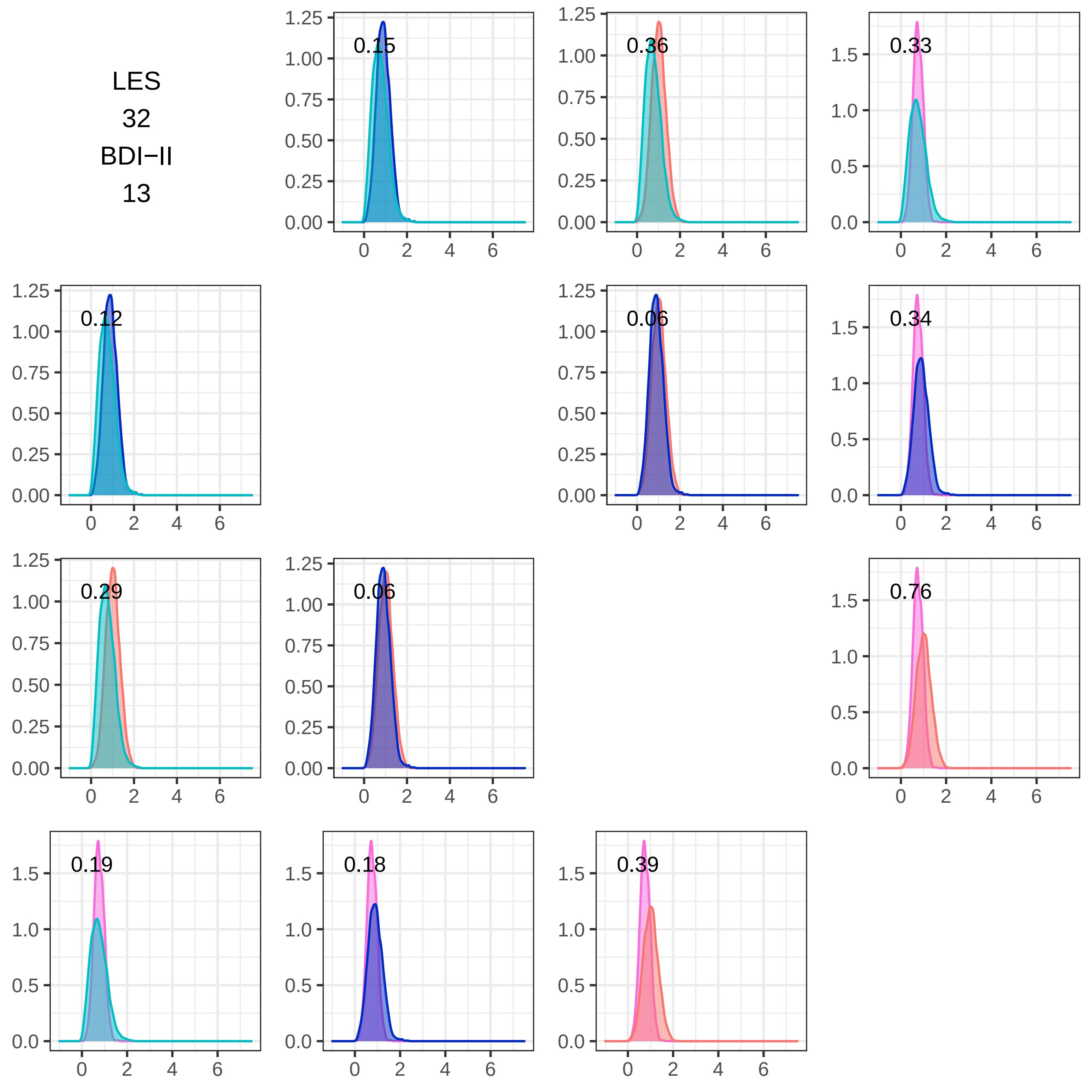

LES
32
BDI−II
13
0.15
0.36
0.33
0.12
0.06
0.34
0.29
0.06
0.76
0.19
0.18
0.39

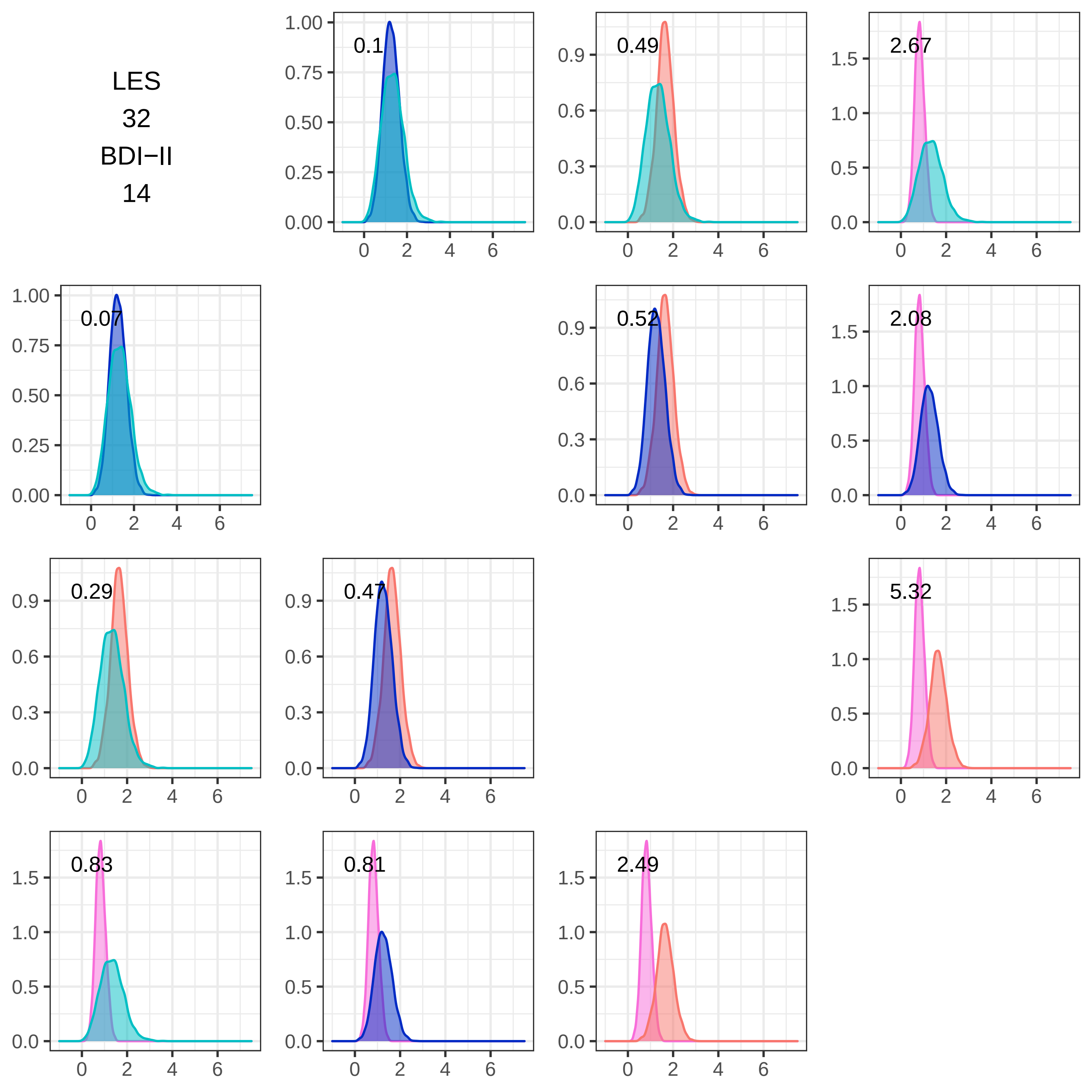

LES
32
BDI−II
14
0.1
0.49
2.67
0.07
0.52
2.08
0.29
0.47
5.32
0.83
0.81
2.49

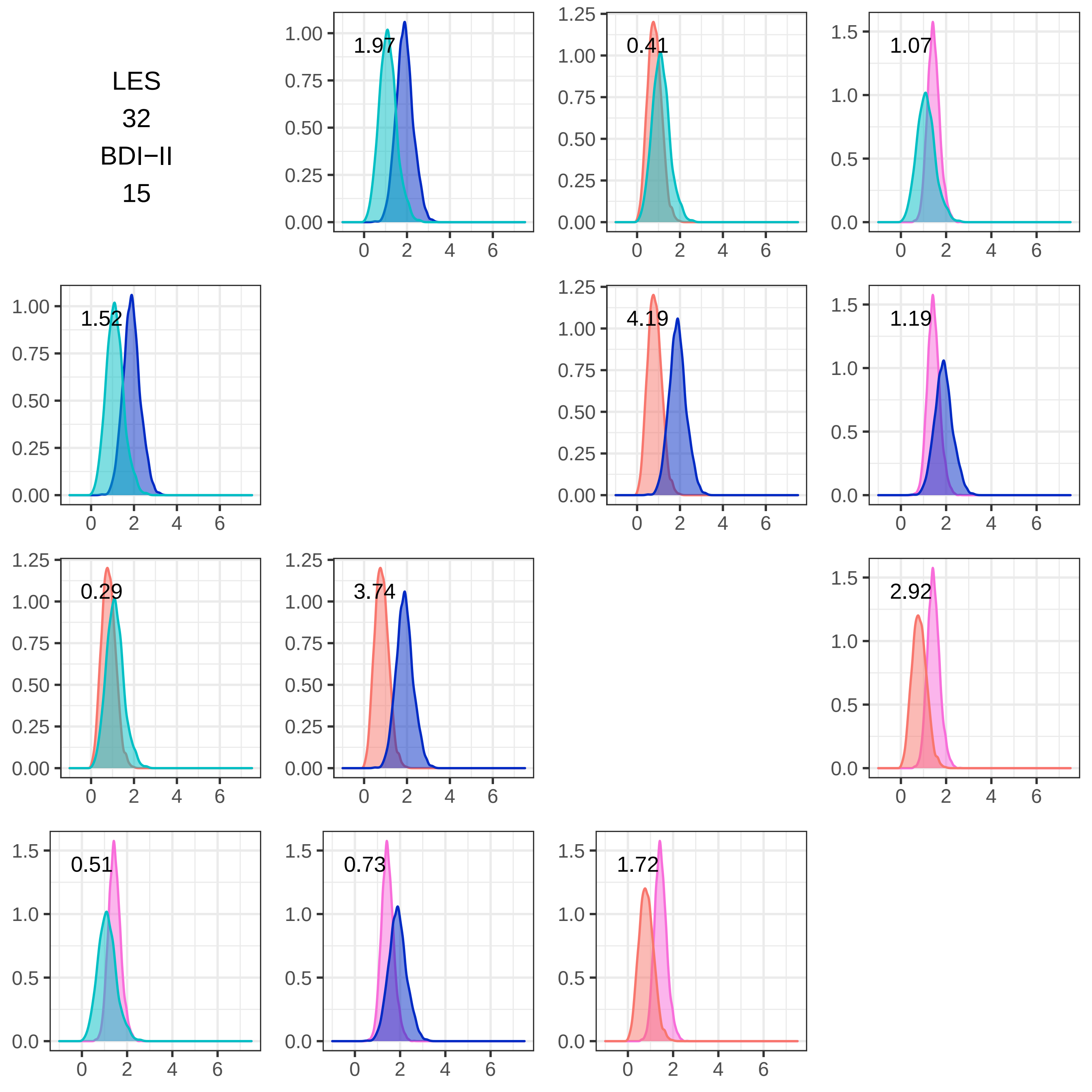

LES
32
BDI−II
15
1.97
0.41
1.07
1.52
4.19
1.19
0.29
3.74
2.92
0.51
0.73
1.72

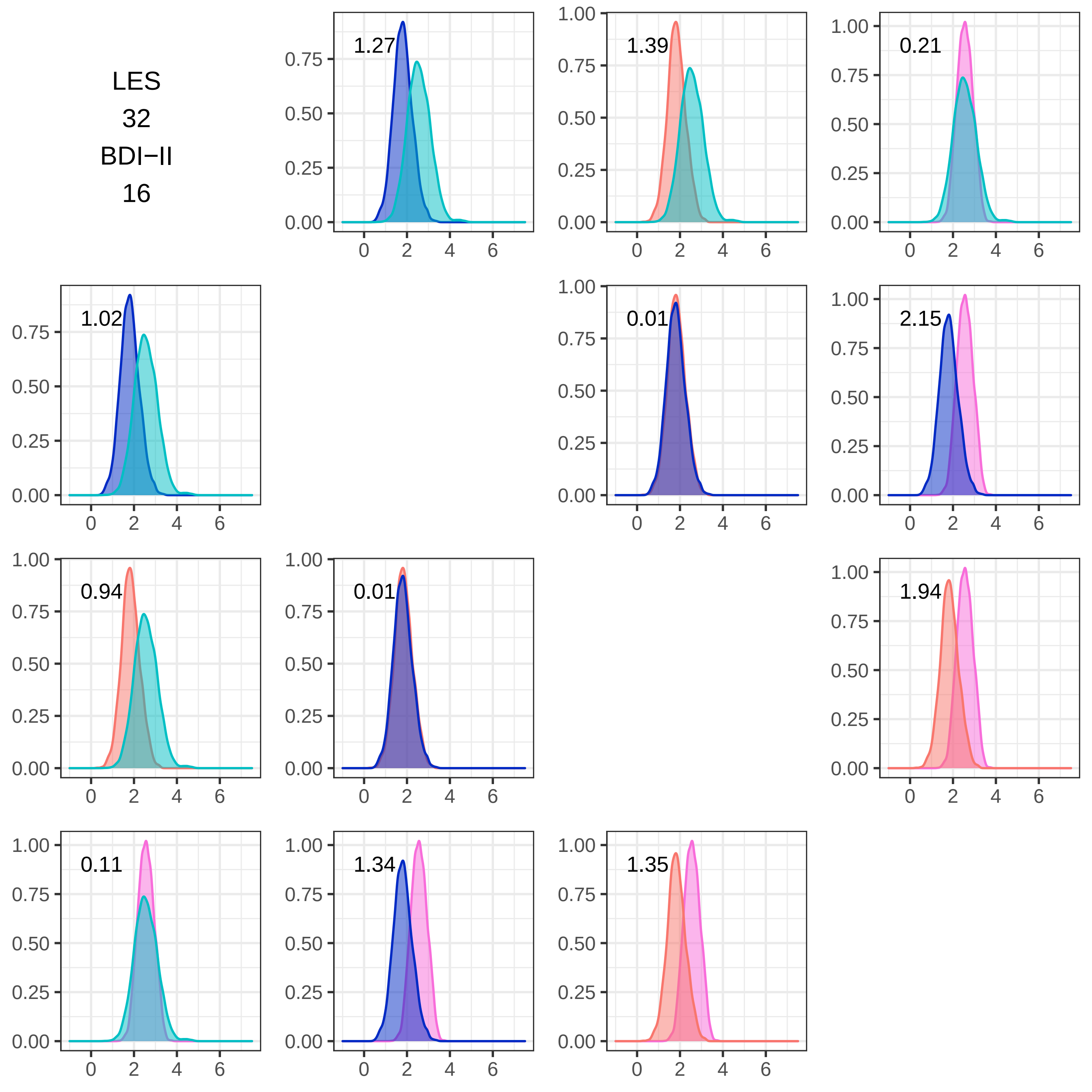

LES
32
BDI−II
16
1.27
1.39
0.21
1.02
0.01
2.15
0.94
0.01
1.94
0.11
1.34
1.35

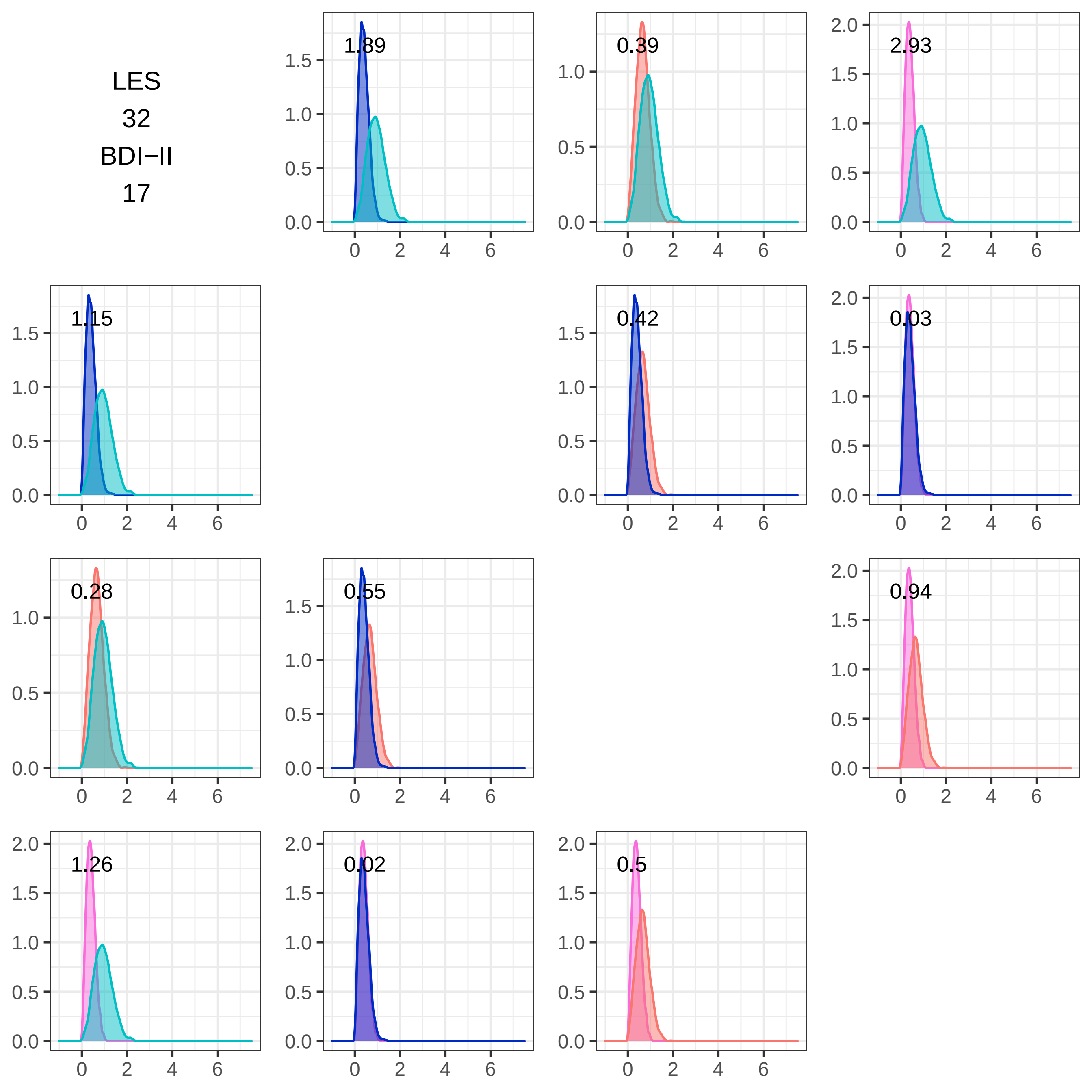
LES
32
BDI−II
17
1.89
0.39
2.93
1.15
0.42
0.03
0.28
0.55
0.94
1.26
0.02
0.5

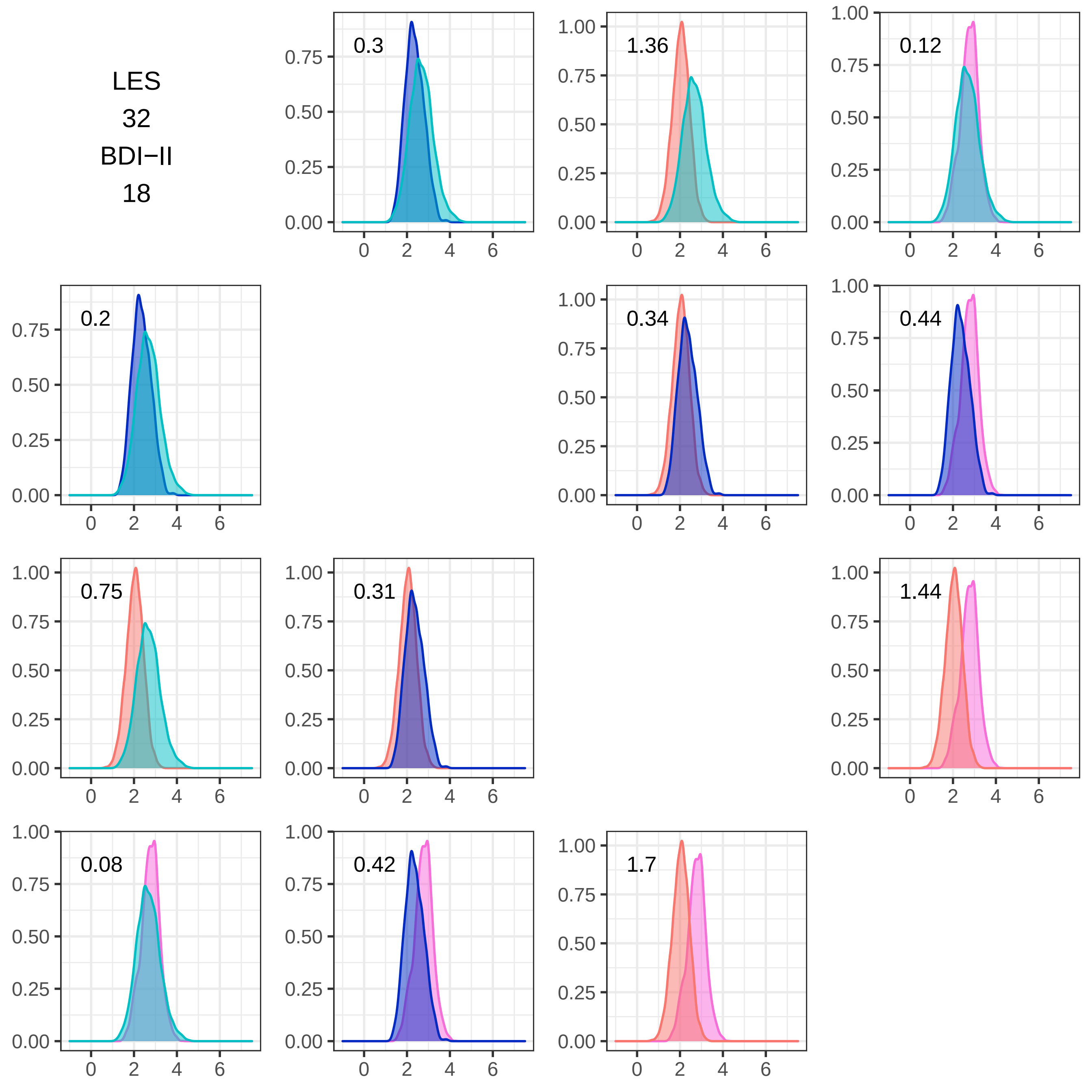
LES
32
BDI−II
18
0.3
1.36
0.12
0.2
0.34
0.44
0.75
0.31
1.44
0.08
0.42
1.7

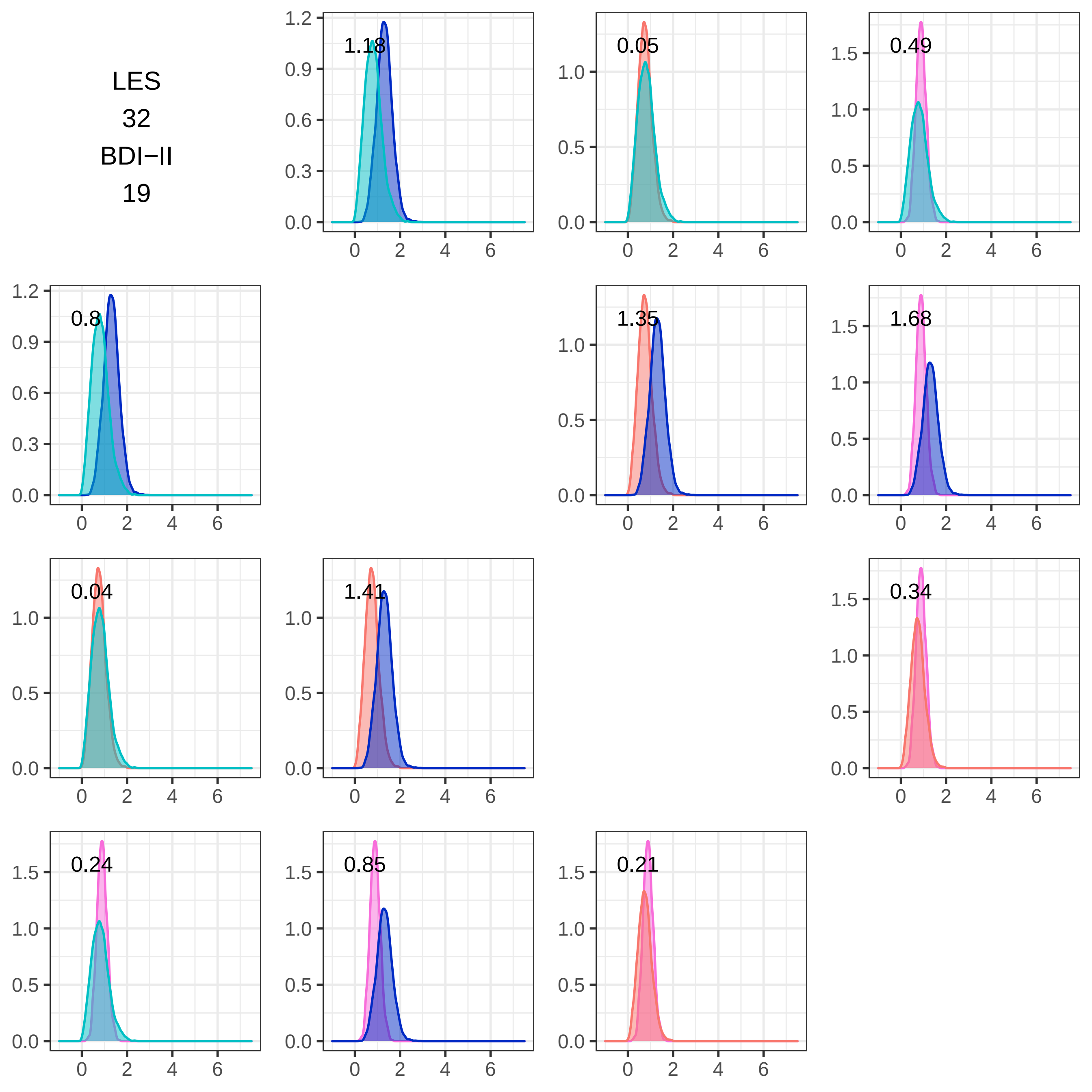
LES
32
BDI−II
19
1.18
0.05
0.49
0.8
1.35
1.68
0.04
1.41
0.34
0.24
0.85
0.21

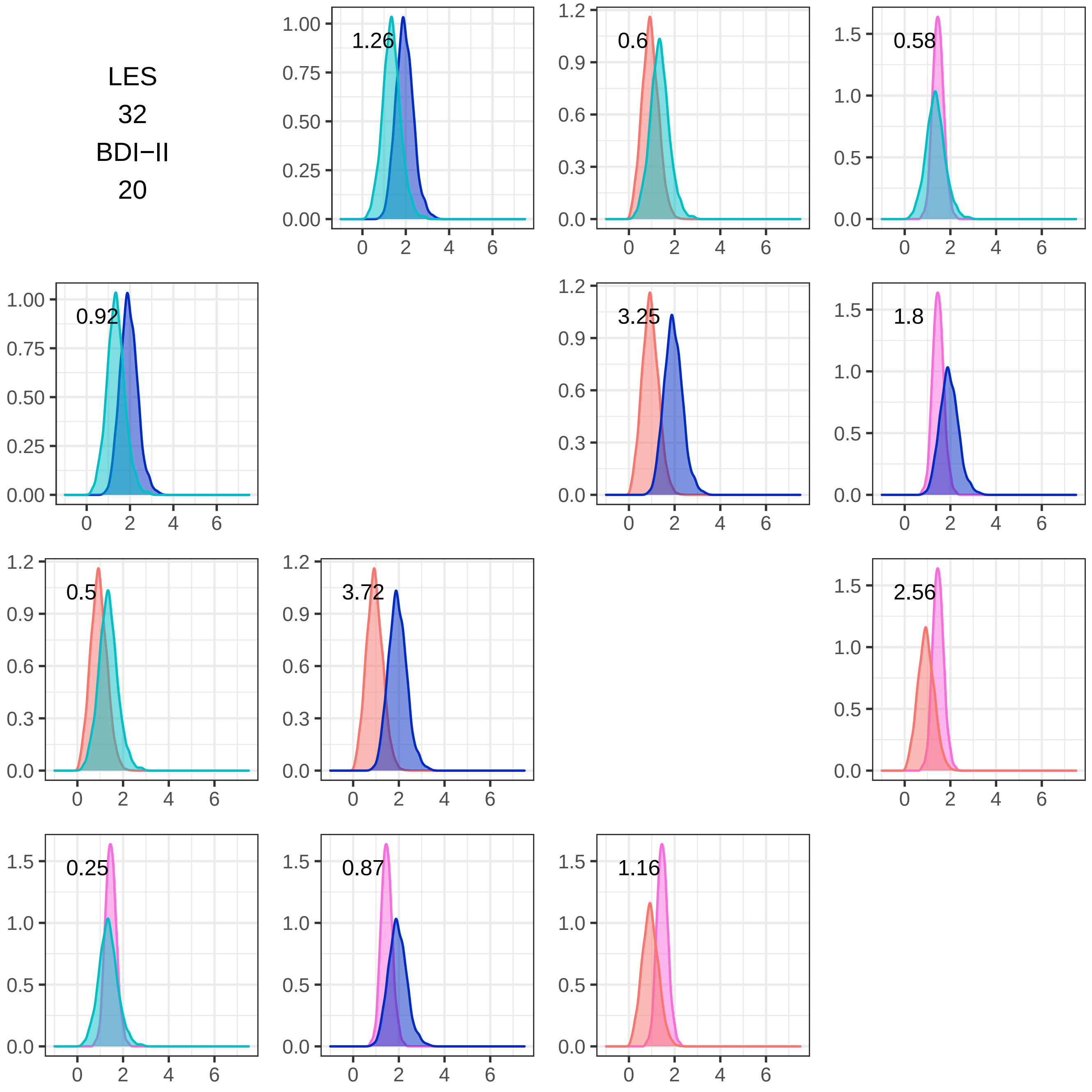
LES
32
BDI−II
20
1.26
0.6
0.58
0.92
3.25
1.8
0.5
3.72
2.56
0.25
0.87
1.16

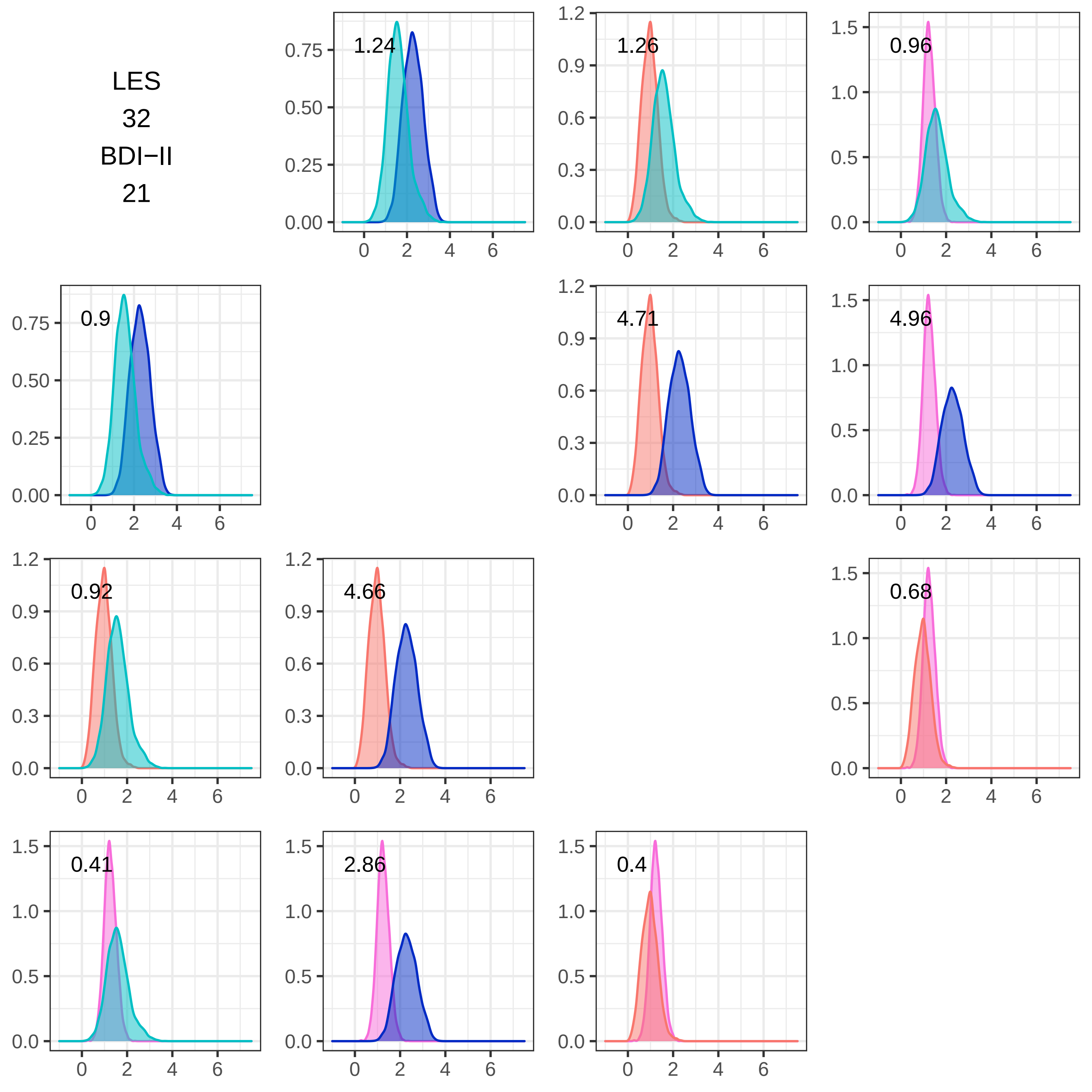
LES
32
BDI−II
21
1.24
1.26
0.96
0.9
4.71
4.96
0.92
4.66
0.68
0.41
2.86
0.4

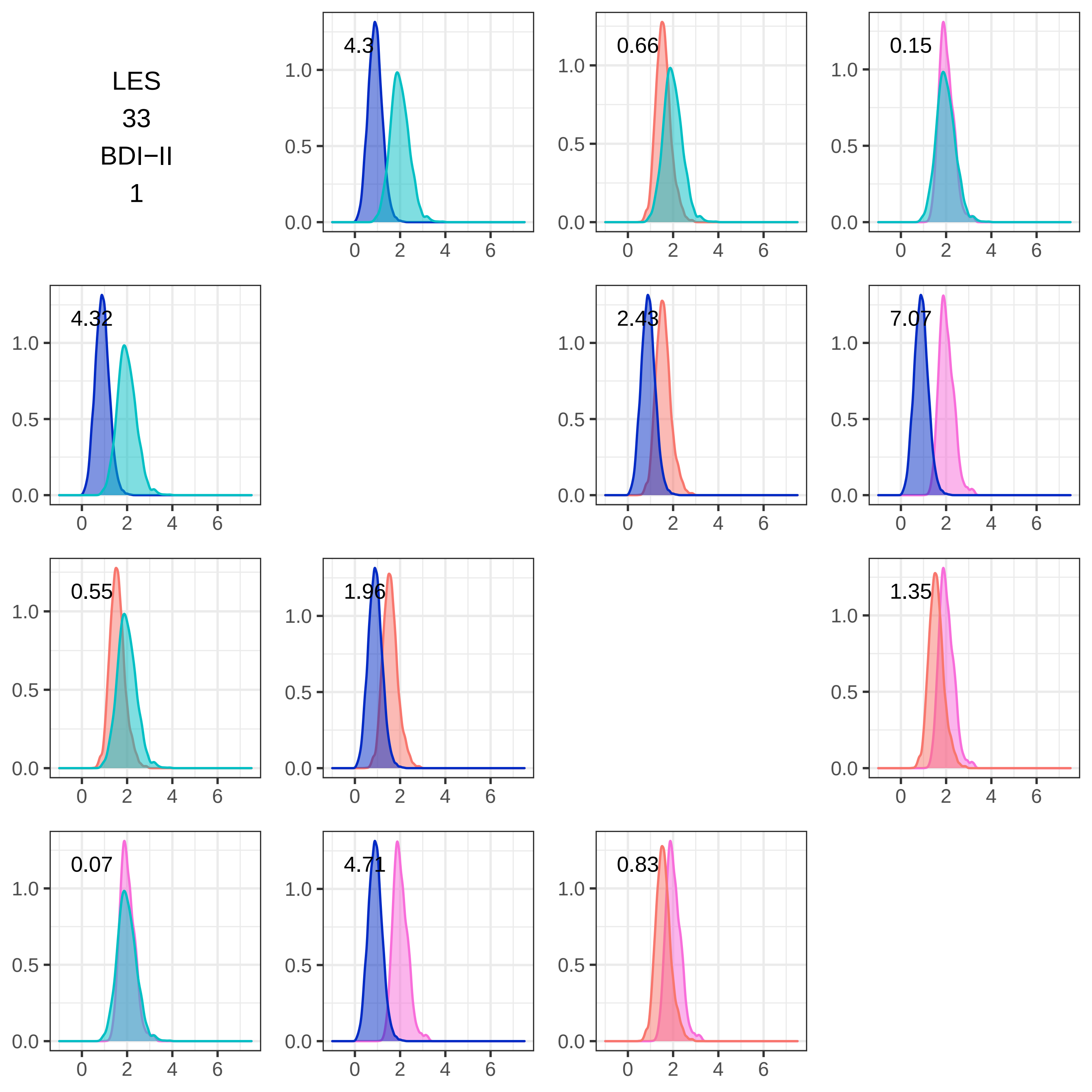
LES
33
BDI−II
1
4.3
0.66
0.15
4.32
2.43
7.07
0.55
1.96
1.35
0.07
4.71
0.83

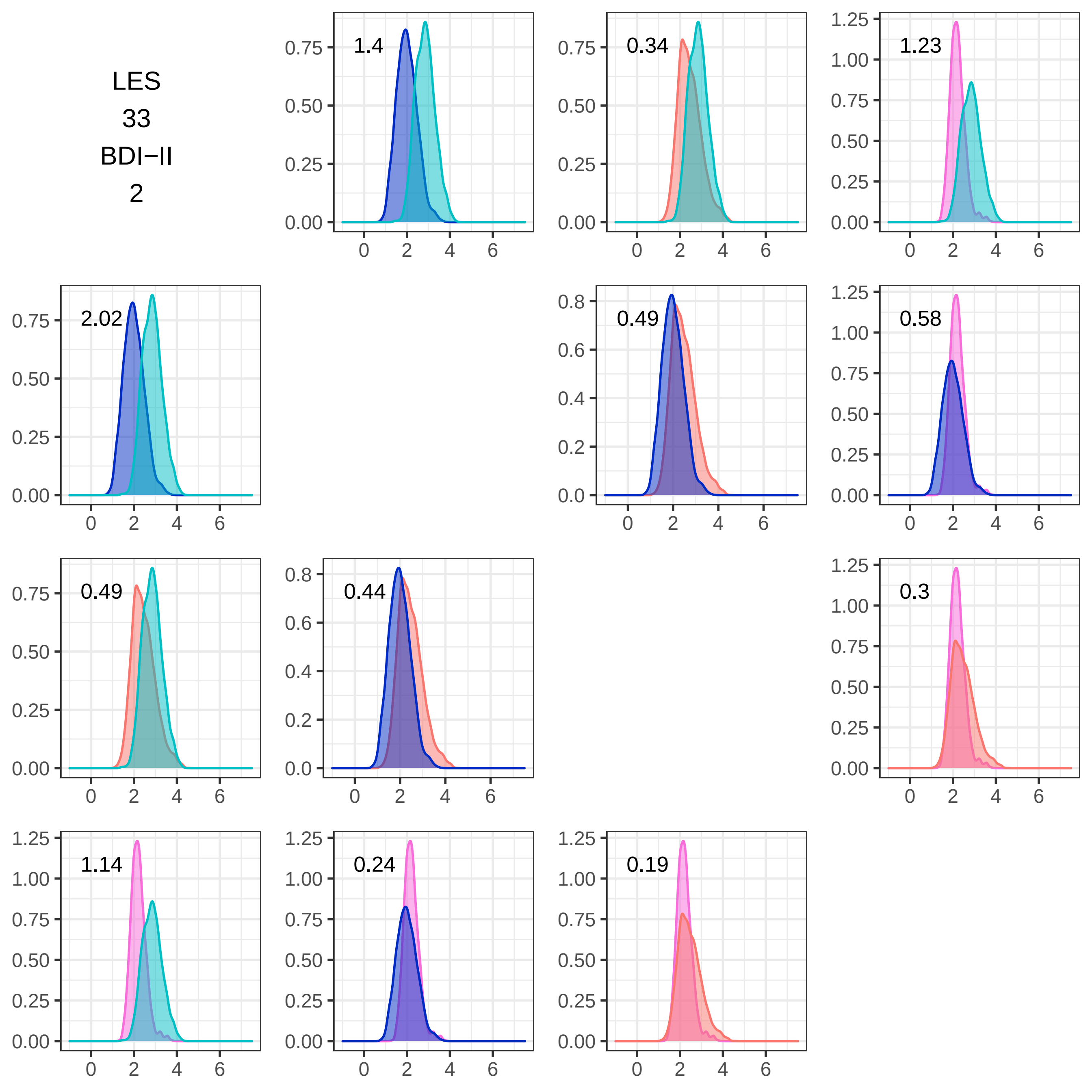

LES
33
BDI−II
2
1.4
0.34
1.23
2.02
0.49
0.58
0.49
0.44
0.3
1.14
0.24
0.19

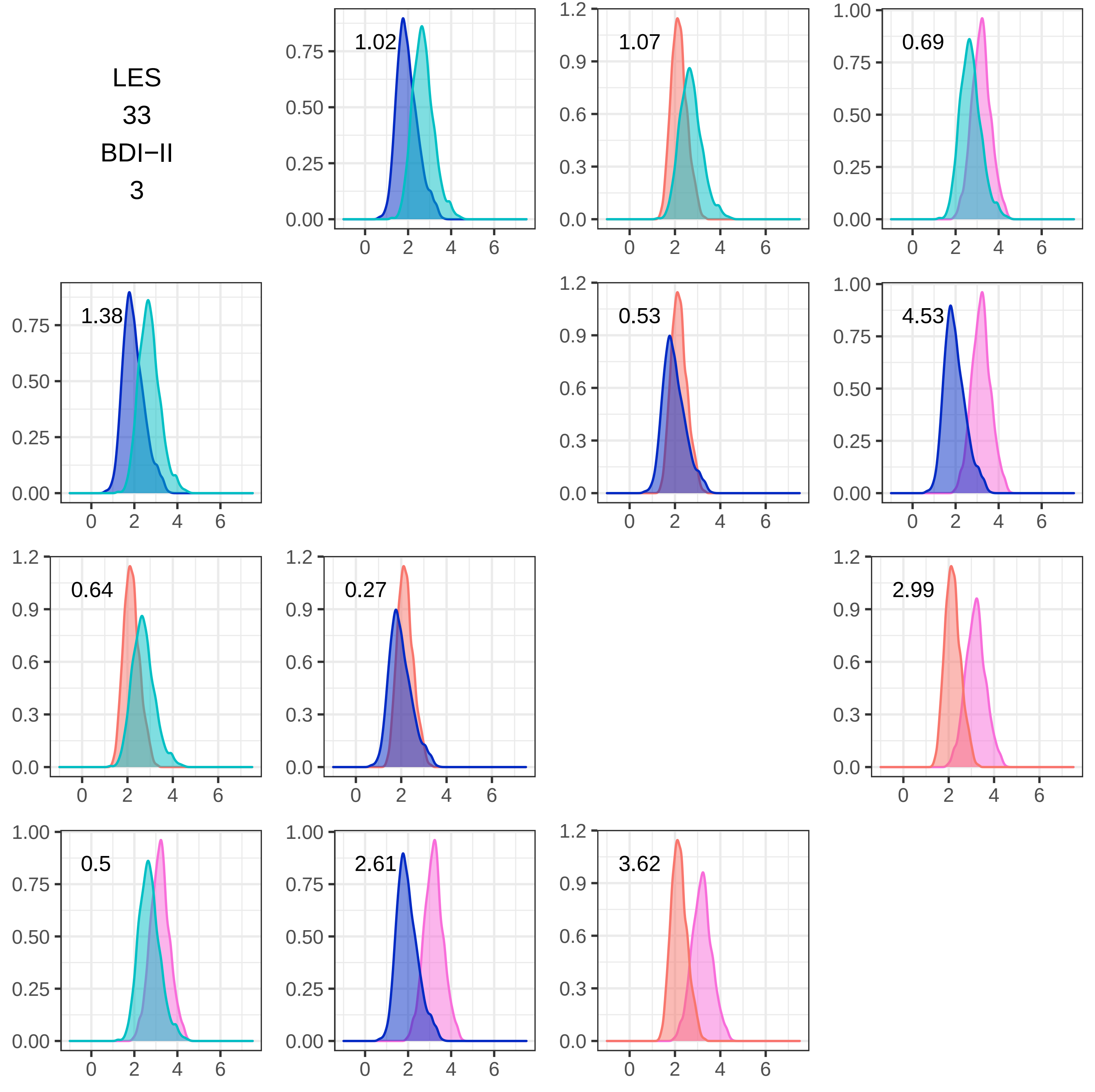
LES
33
BDI−II
3
1.02
1.07
0.69
1.38
0.53
4.53
0.64
0.27
2.99
0.5
2.61
3.62

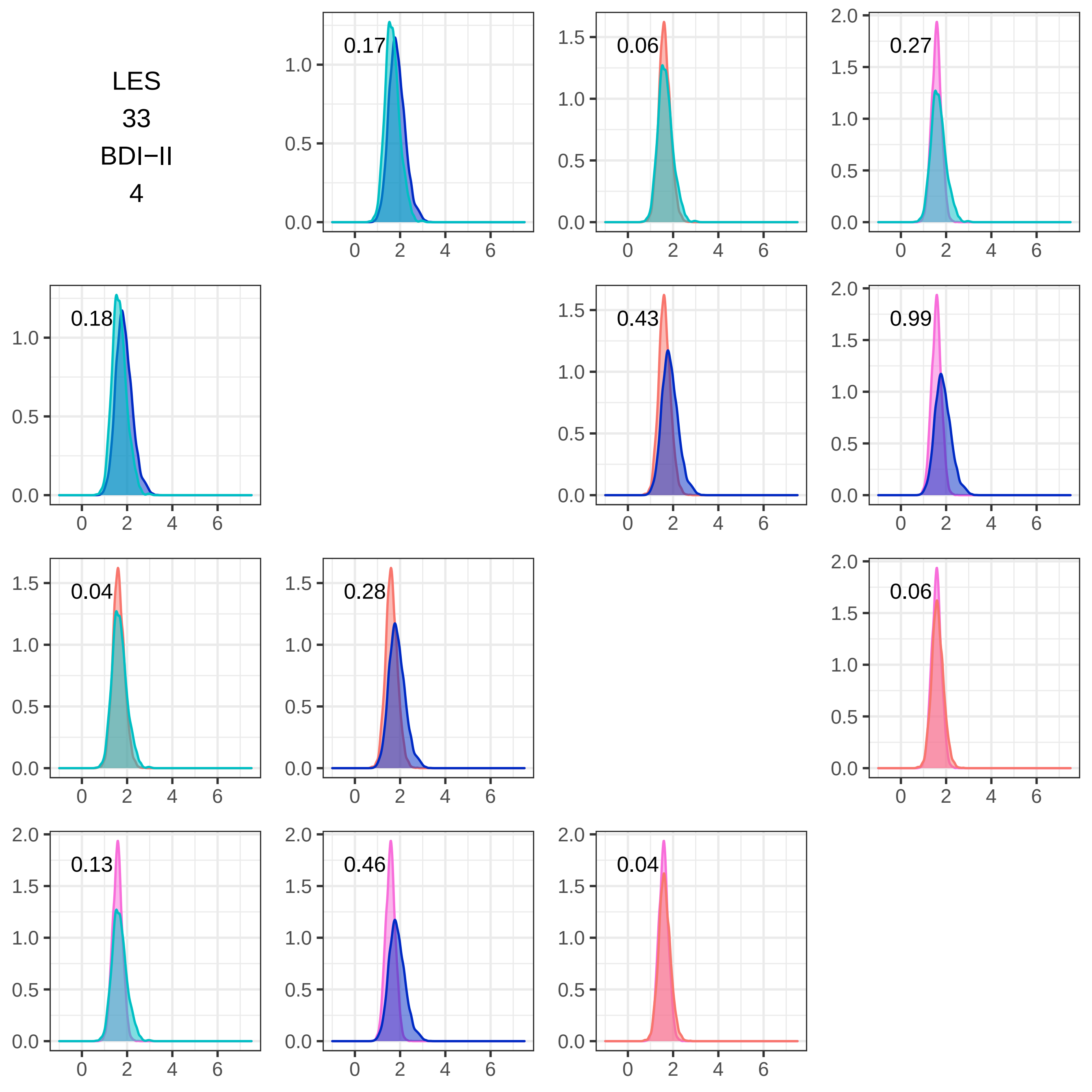

LES
33
BDI−II
4
0.17
0.06
0.27
0.18
0.43
0.99
0.04
0.28
0.06
0.13
0.46
0.04

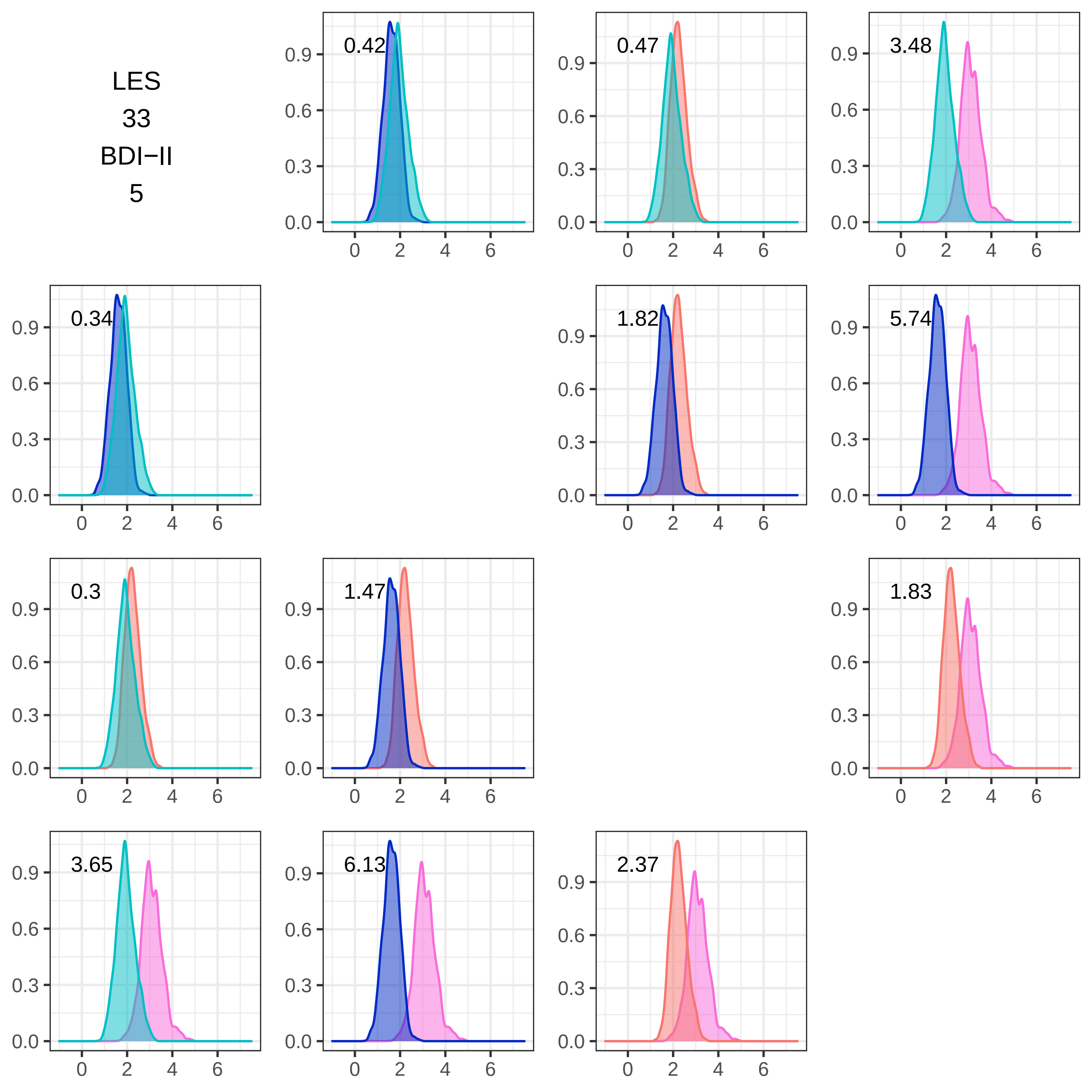

LES
33
BDI−II
5
0.42
0.47
3.48
0.34
1.82
5.74
0.3
1.47
1.83
3.65
6.13
2.37

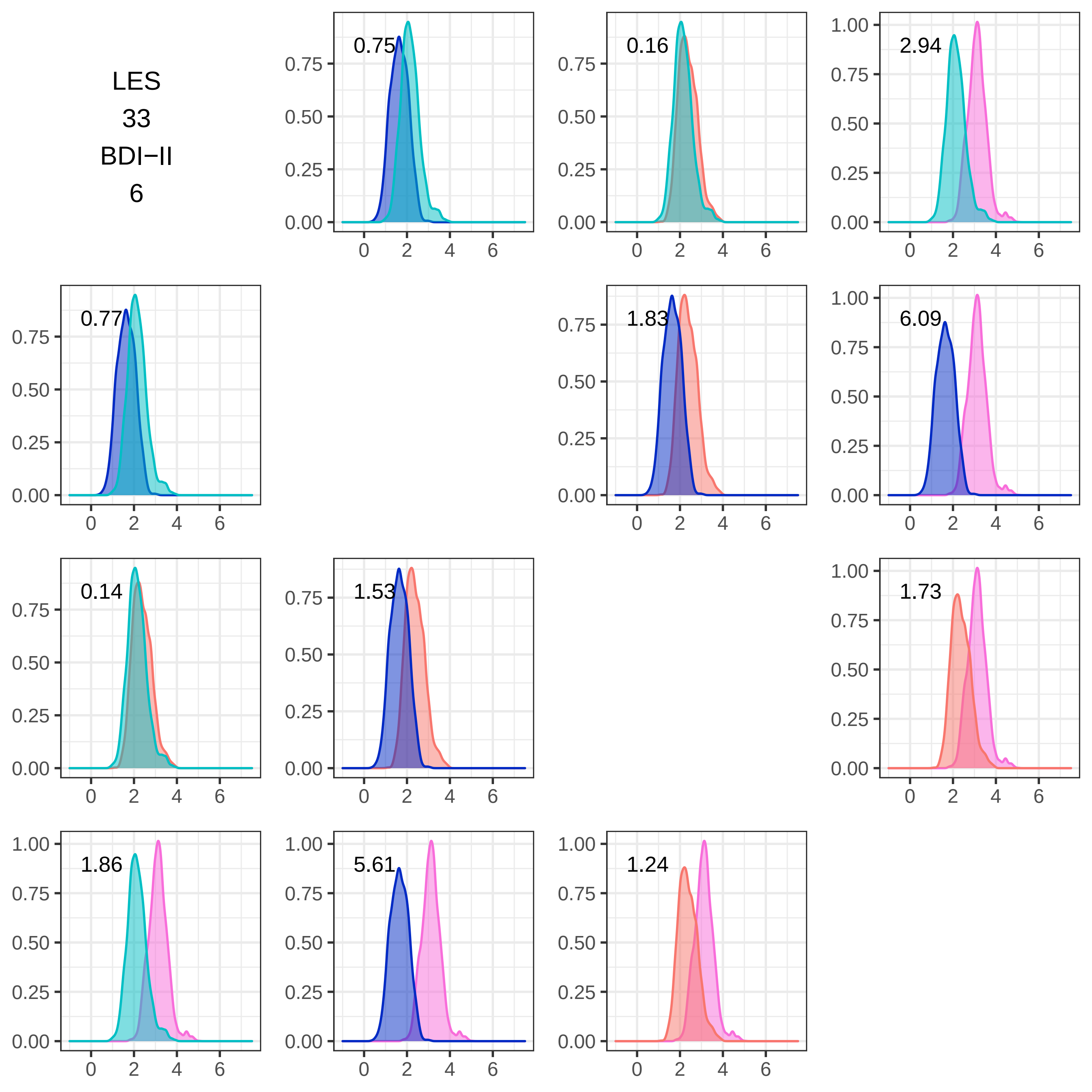

LES
33
BDI−II
6
0.75
0.16
2.94
0.77
1.83
6.09
0.14
1.53
1.73
1.86
5.61
1.24

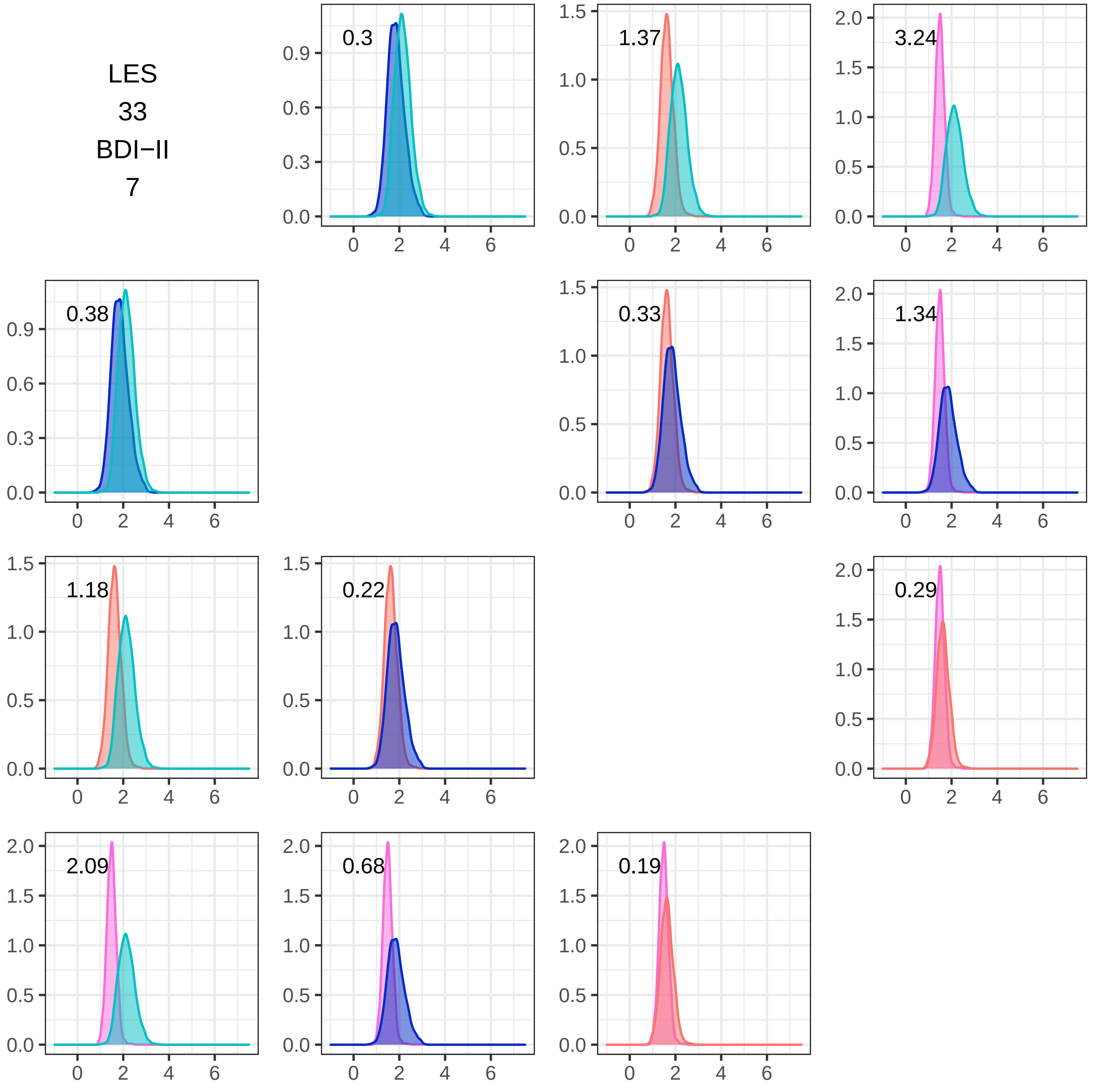
LES
33
BDI−II
7
0.3
1.37
3.24
0.38
0.33
1.34
1.18
0.22
0.29
2.09
0.68
0.19

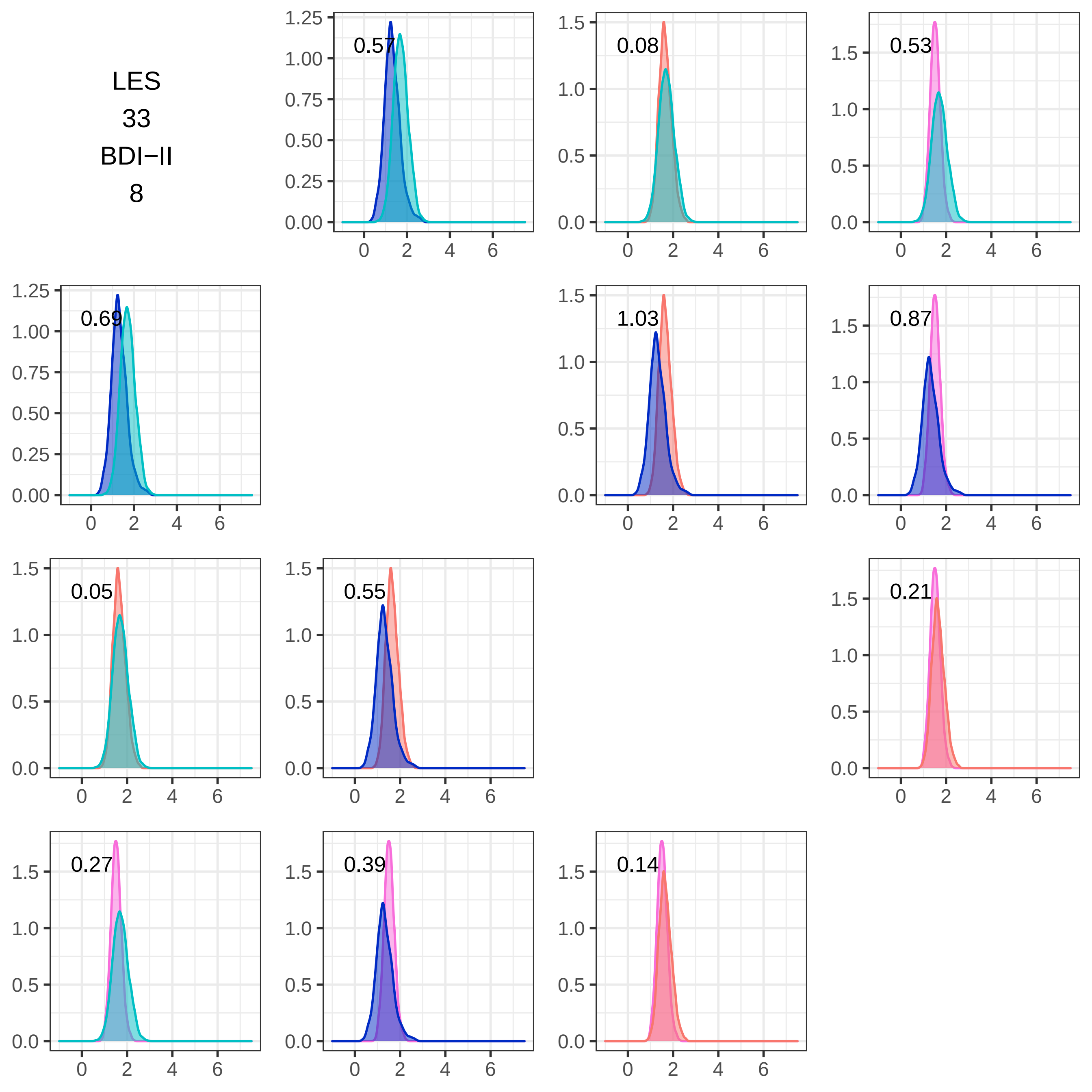
LES
33
BDI−II
8
0.57
0.08
0.53
0.69
1.03
0.87
0.05
0.55
0.21
0.27
0.39
0.14

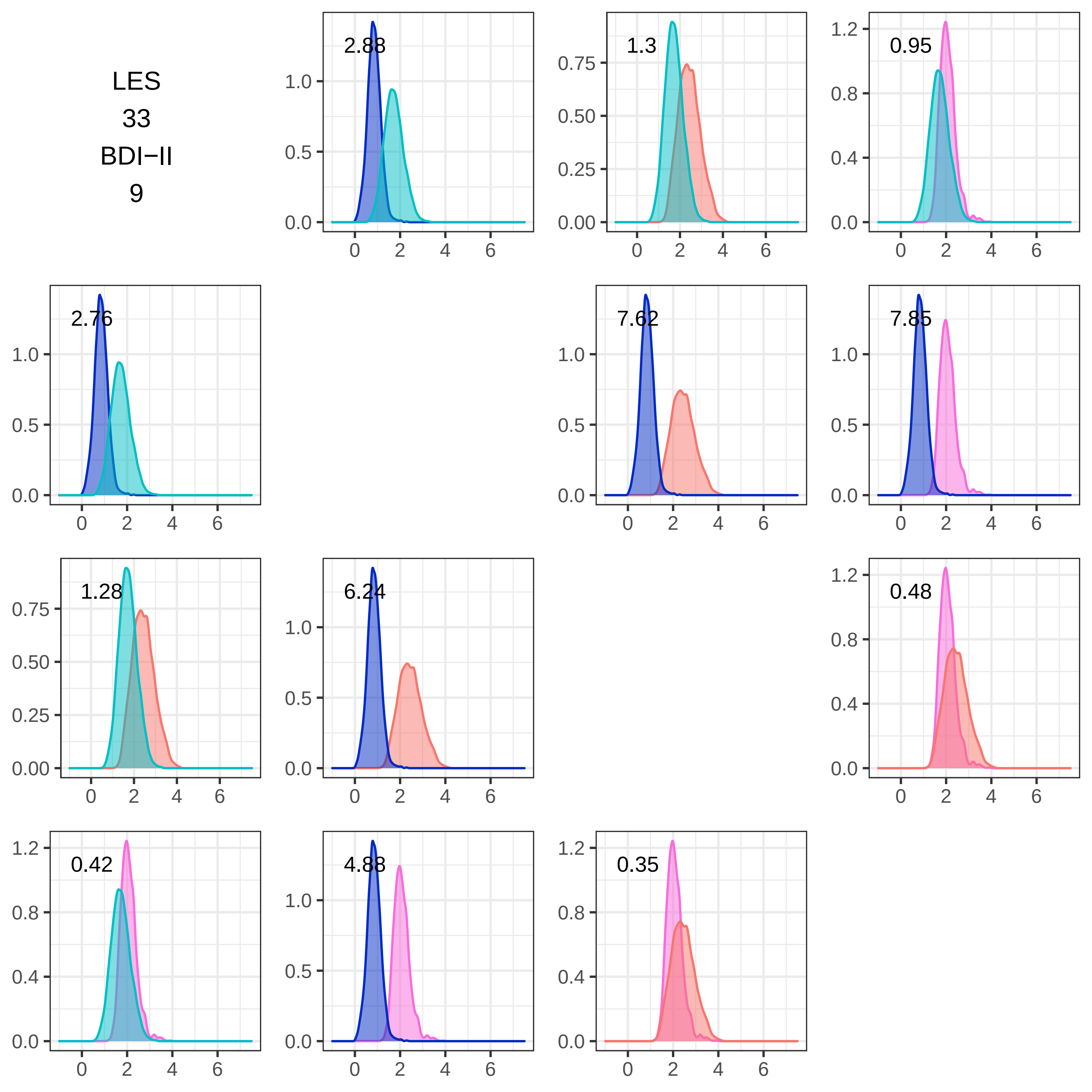

LES
33
BDI−II
9
2.88
1.3
0.95
2.76
7.62
7.85
1.28
6.24
0.48
0.42
4.88
0.35

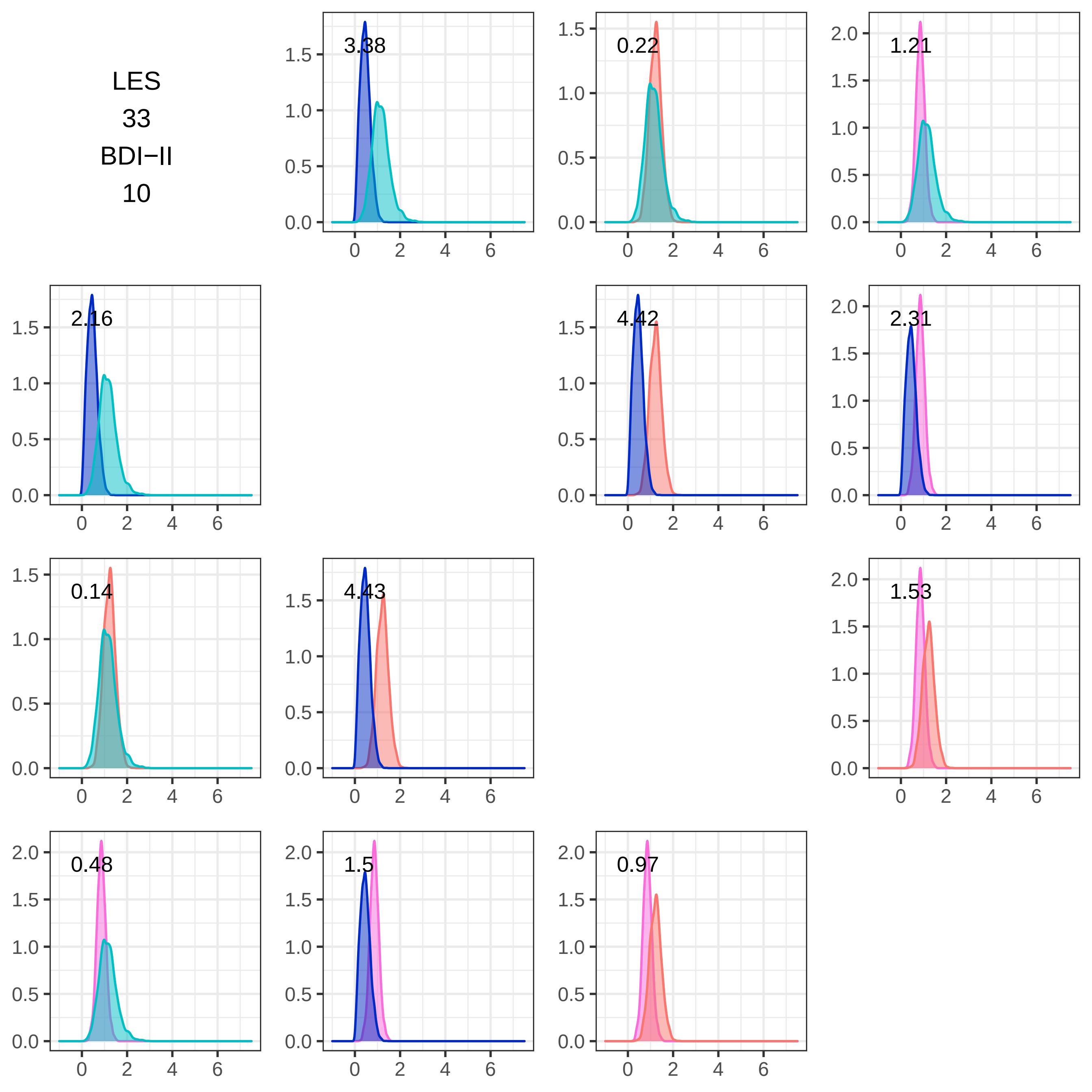
LES
33
BDI−II
10
3.38
0.22
1.21
2.16
4.42
2.31
0.14
4.43
1.53
0.48
1.5
0.97

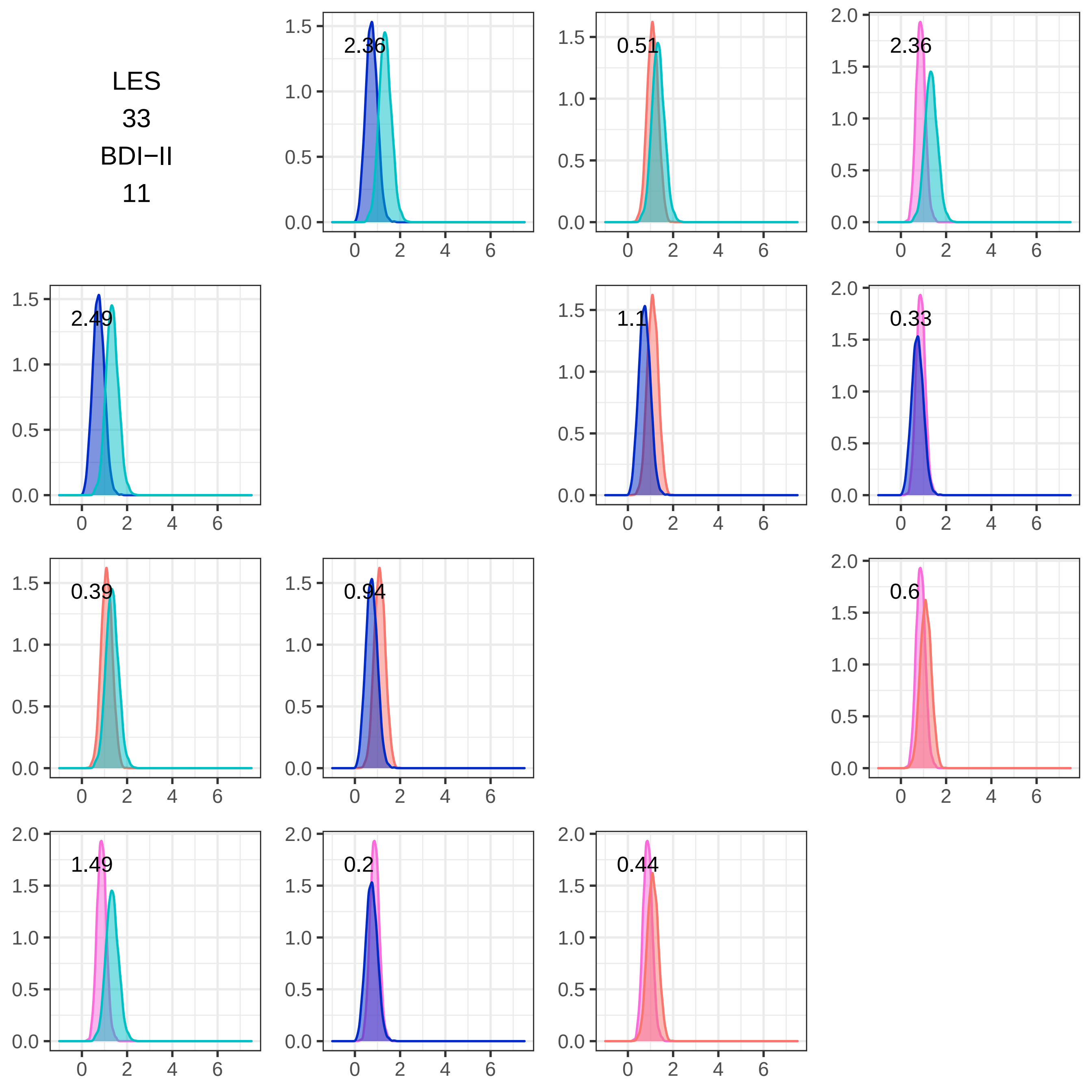

LES
33
BDI−II
11
2.36
0.51
2.36
2.49
1.1
0.33
0.39
0.94
0.6
1.49
0.2
0.44

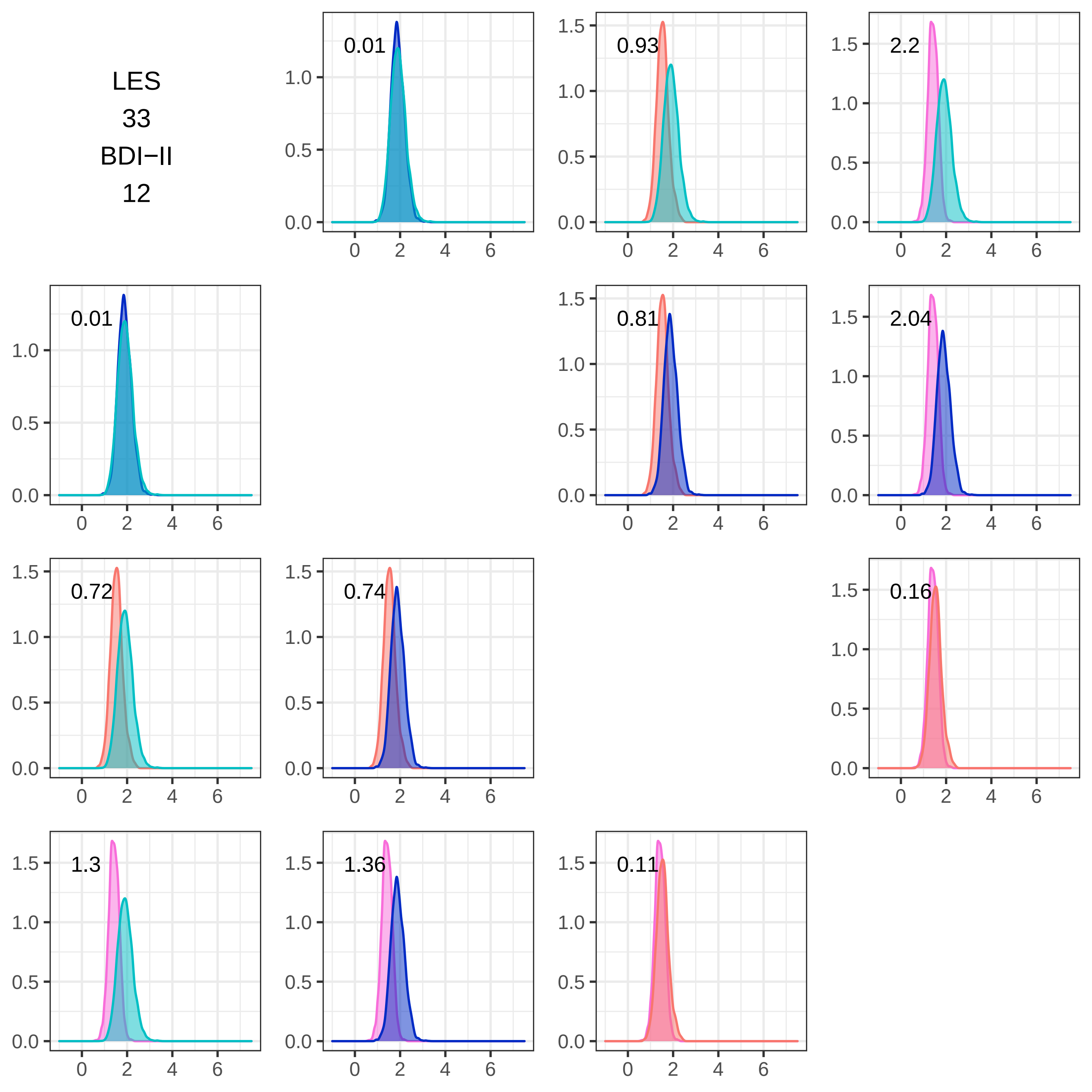

LES
33
BDI−II
12
0.01
0.93
2.2
0.01
0.81
2.04
0.72
0.74
0.16
1.3
1.36
0.11

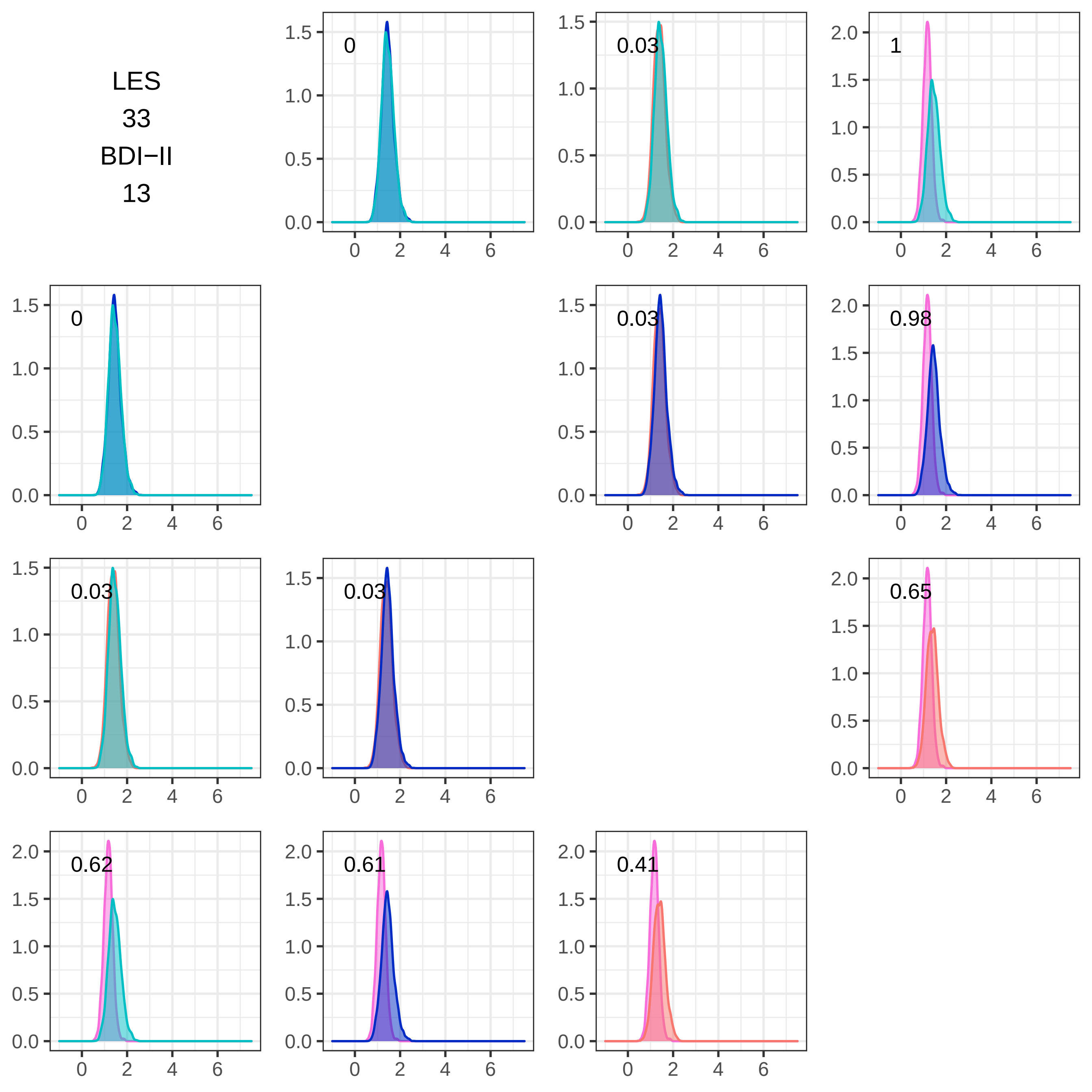

LES
33
BDI−II
13
0
0.03
1
0
0.03
0.98
0.03
0.03
0.65
0.62
0.61
0.41

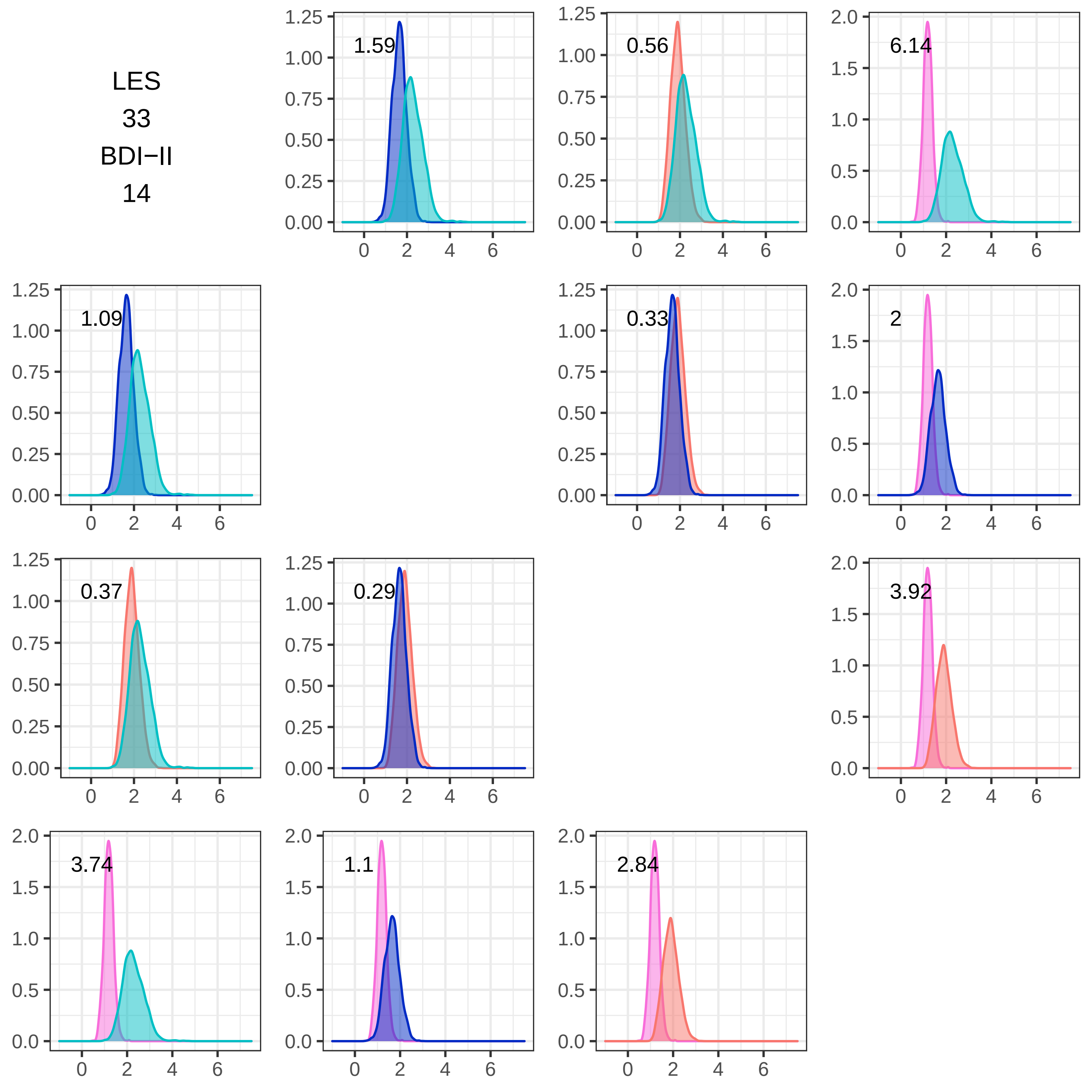
LES
33
BDI−II
14
1.59
0.56
6.14
1.09
0.33
2
0.37
0.29
3.92
3.74
1.1
2.84

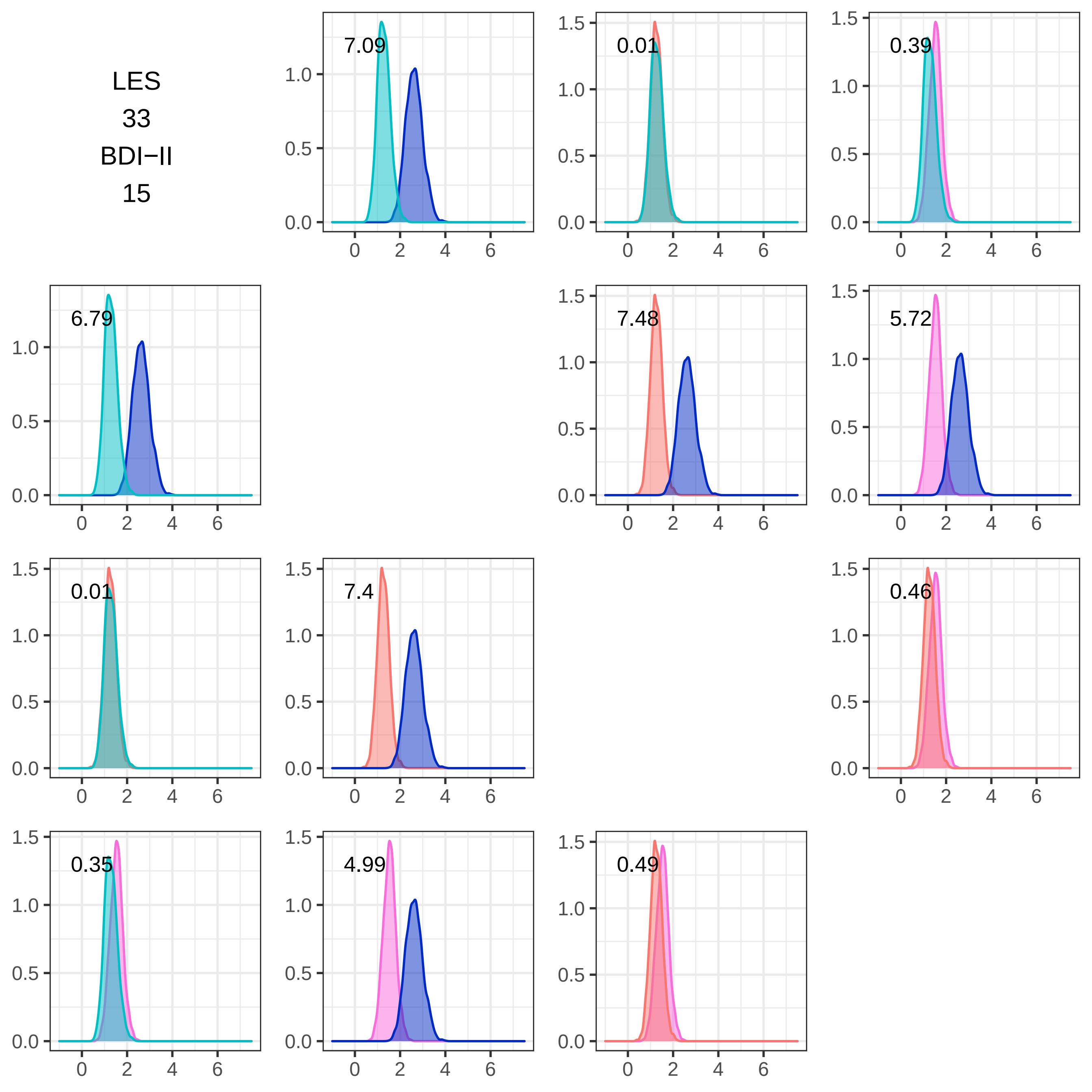
LES
33
BDI−II
15
7.09
0.01
0.39
6.79
7.48
5.72
0.01
7.4
0.46
0.35
4.99
0.49

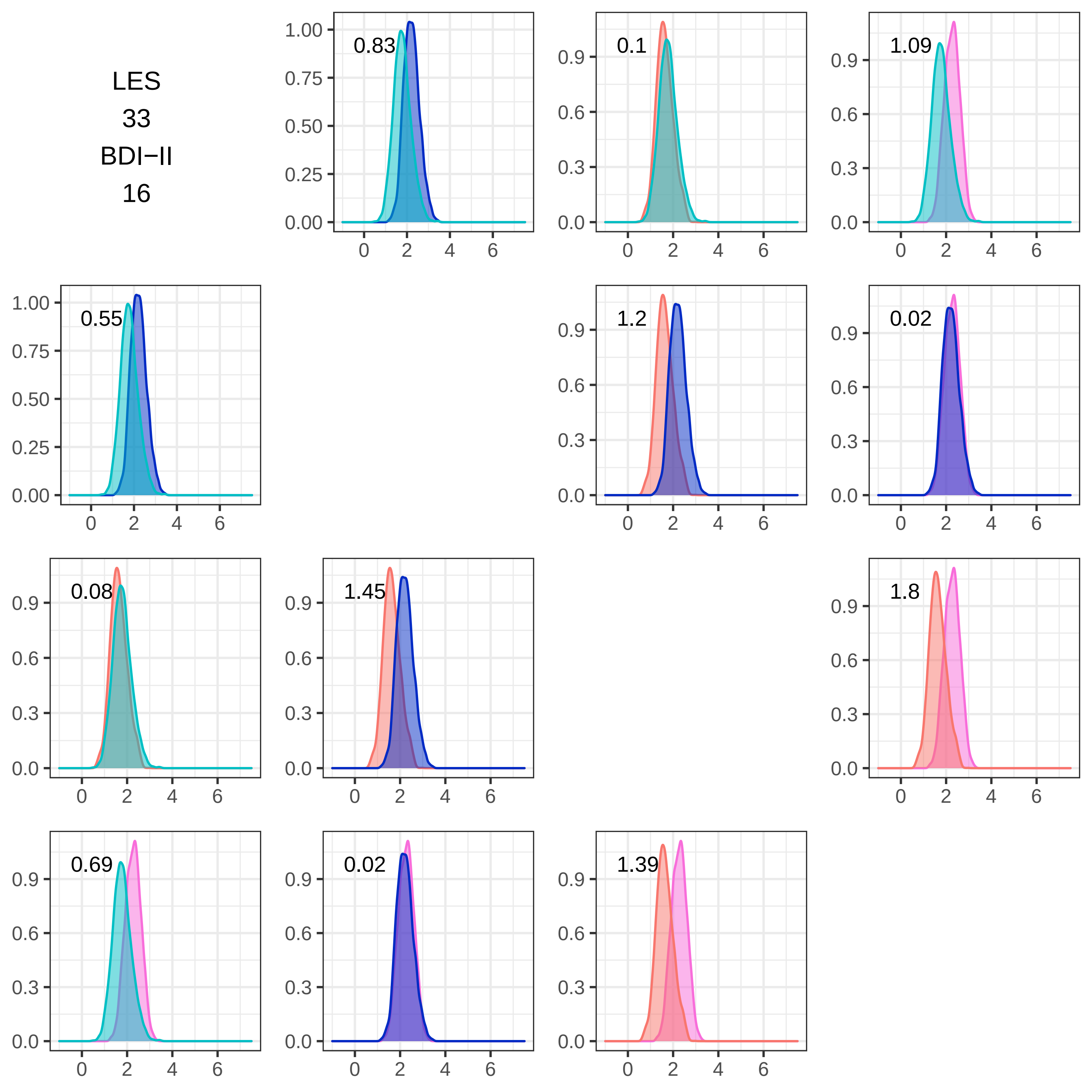

LES
33
BDI−II
16
0.83
0.1
1.09
0.55
1.2
0.02
0.08
1.45
1.8
0.69
0.02
1.39

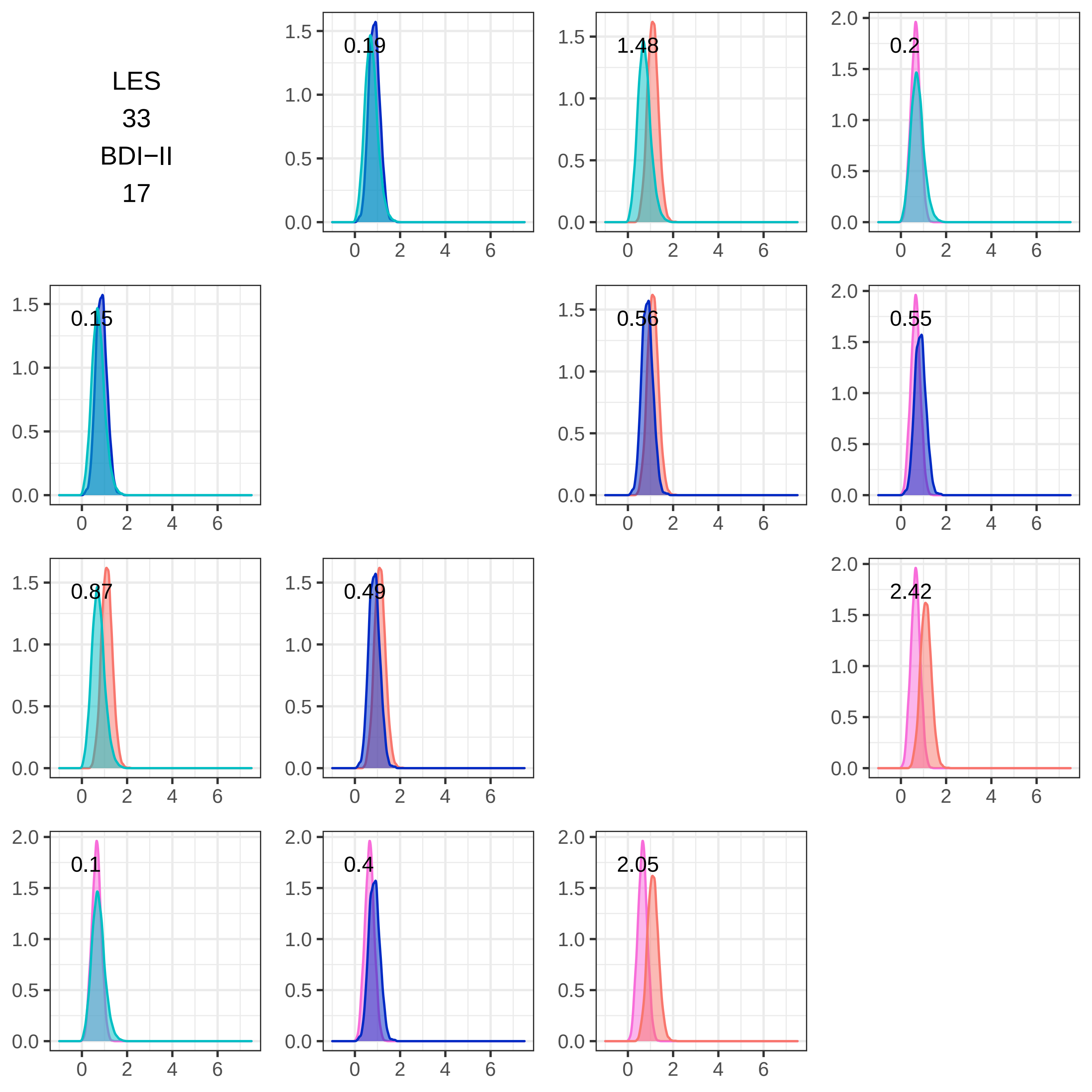
LES
33
BDI−II
17
0.19
1.48
0.2
0.15
0.56
0.55
0.87
0.49
2.42
0.1
0.4
2.05

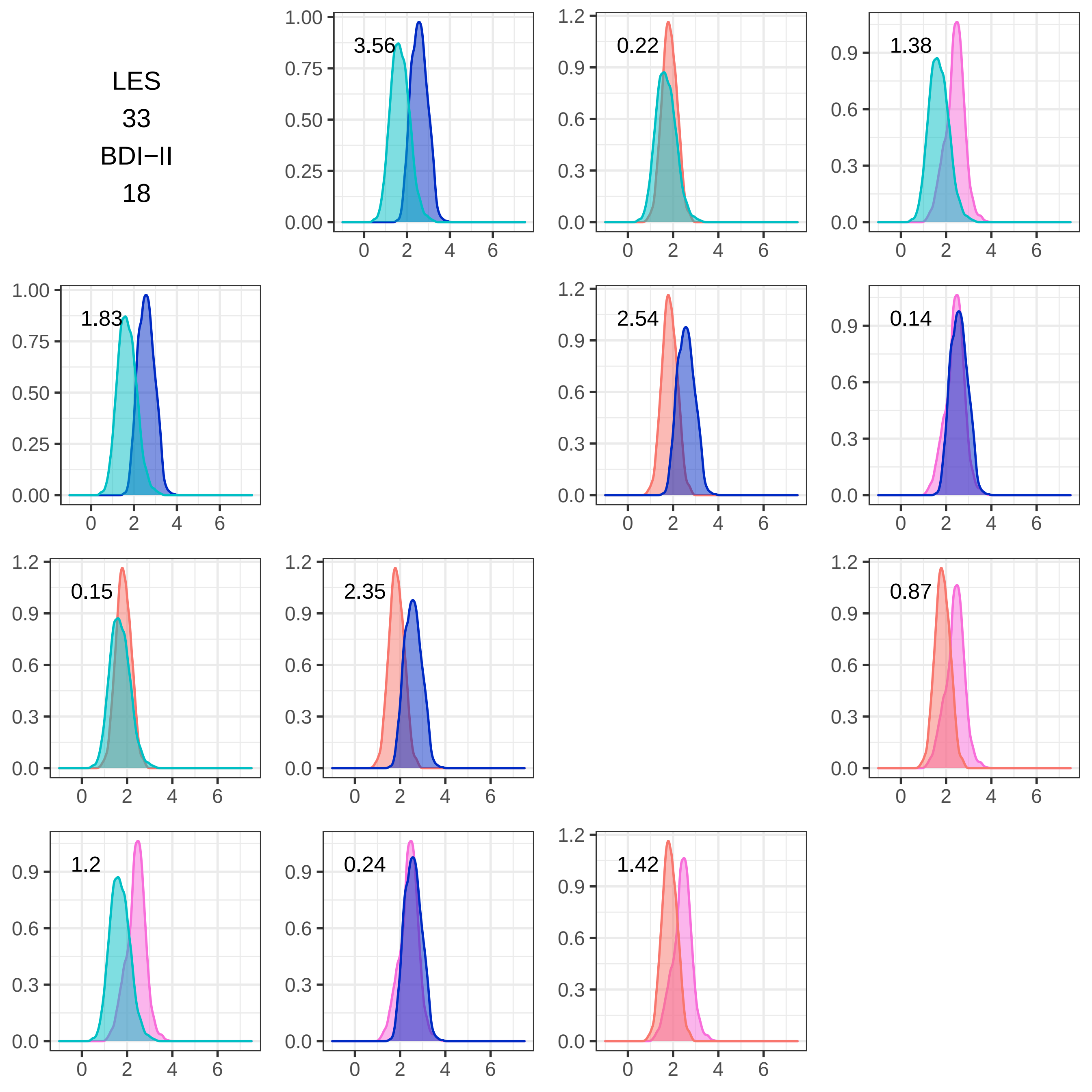
LES
33
BDI−II
18
3.56
0.22
1.38
1.83
2.54
0.14
0.15
2.35
0.87
1.2
0.24
1.42

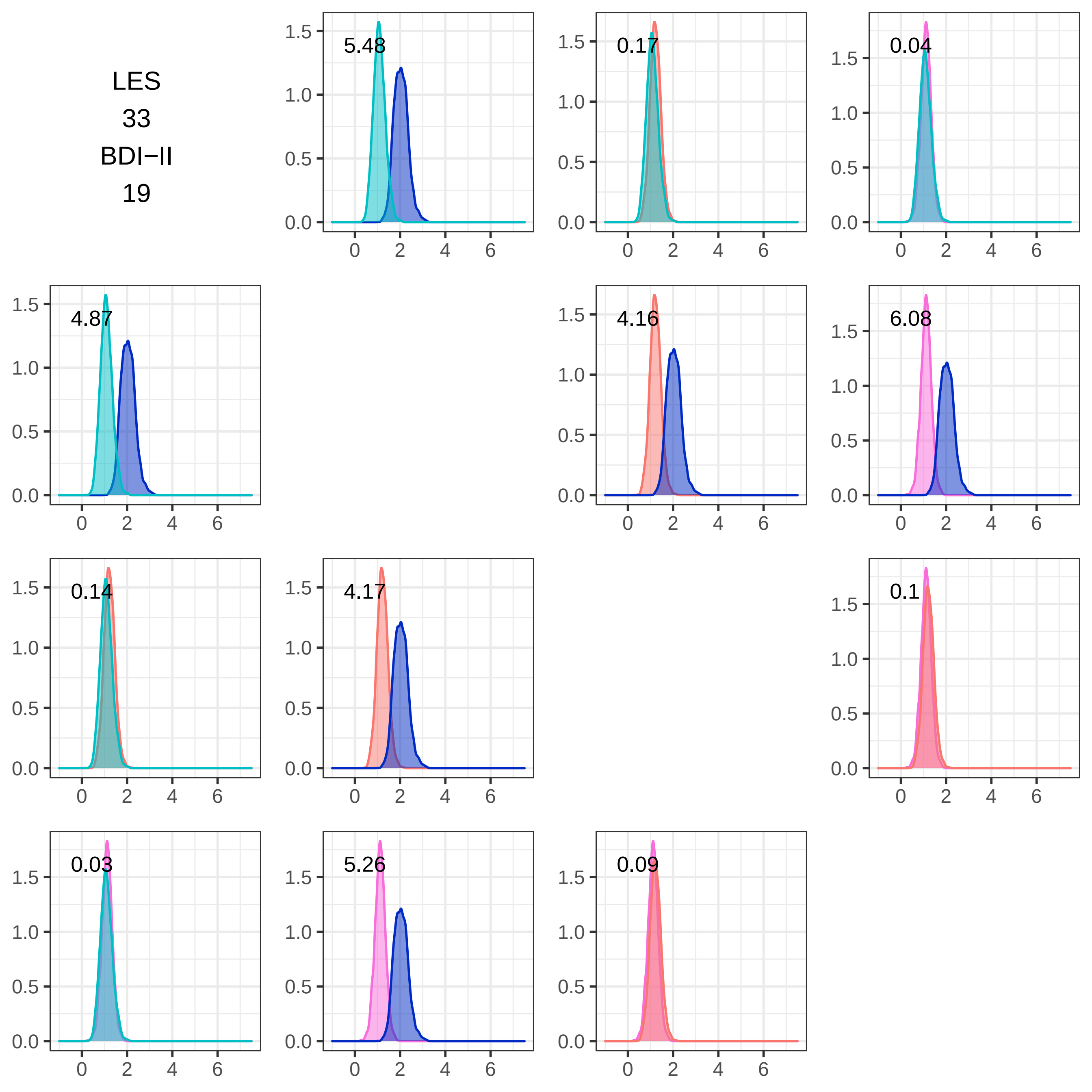
LES
33
BDI−II
19
5.48
0.17
0.04
4.87
4.16
6.08
0.14
4.17
0.1
0.03
5.26
0.09

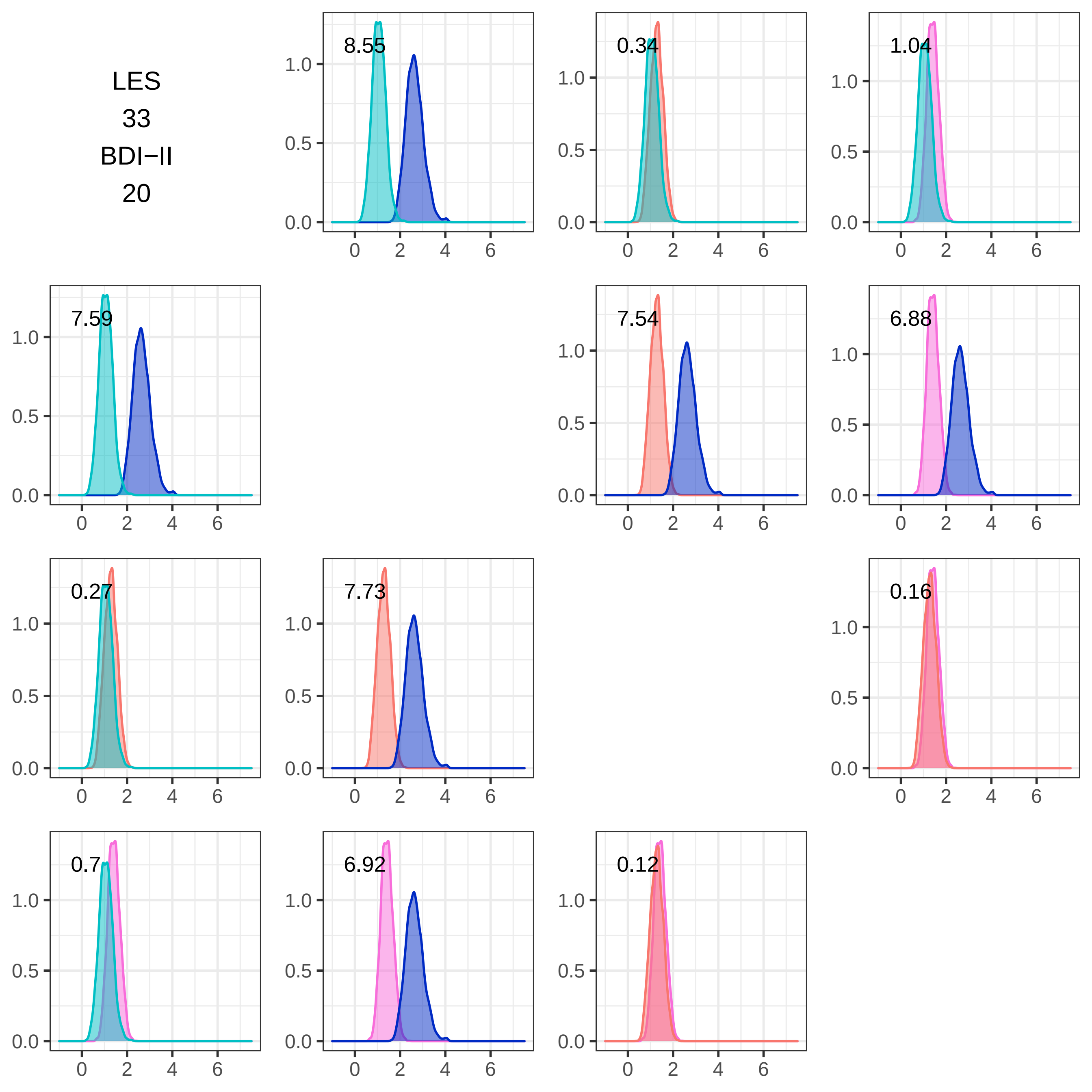
LES
33
BDI−II
20
8.55
0.34
1.04
7.59
7.54
6.88
0.27
7.73
0.16
0.7
6.92
0.12

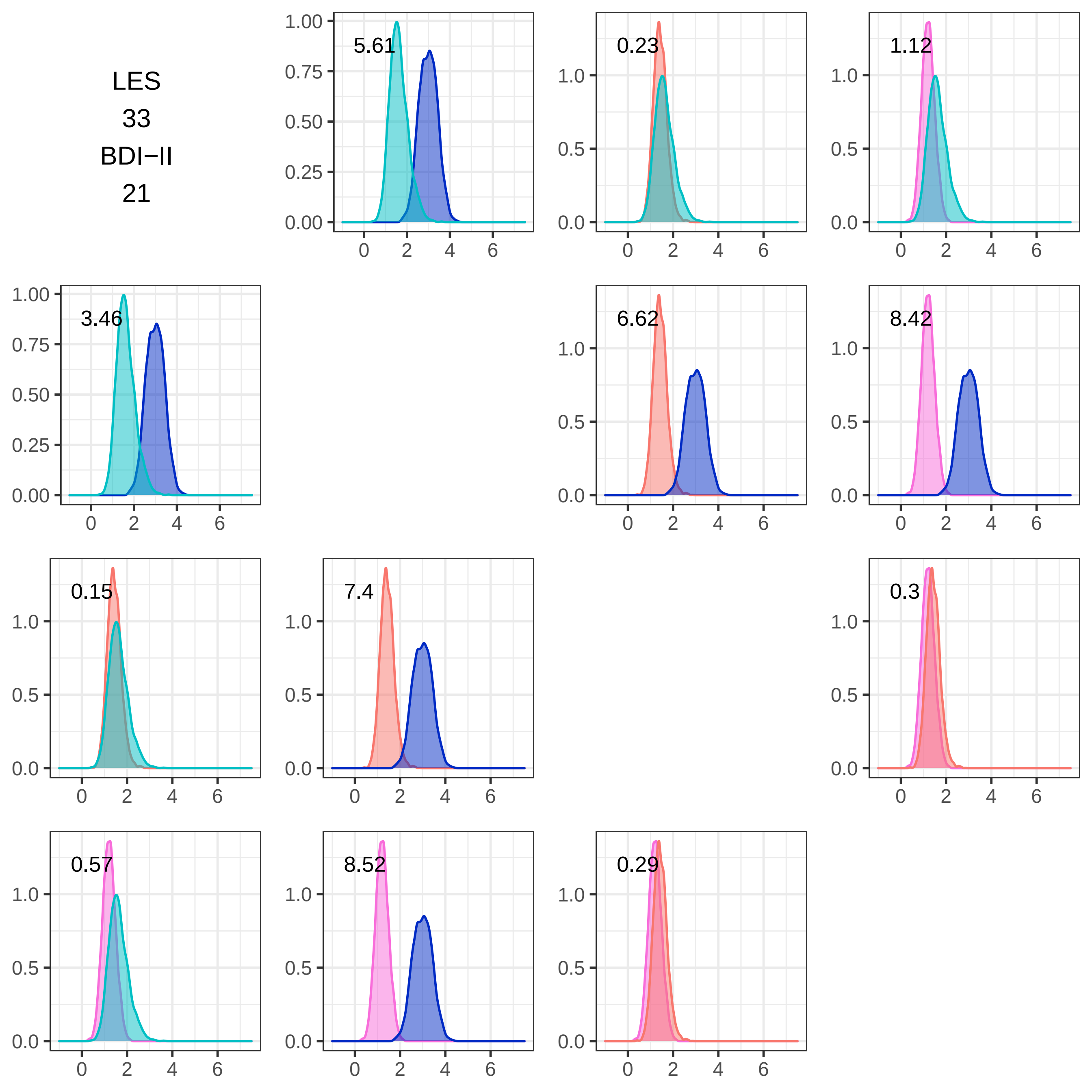
LES
33
BDI−II
21
5.61
0.23
1.12
3.46
6.62
8.42
0.15
7.4
0.3
0.57
8.52
0.29

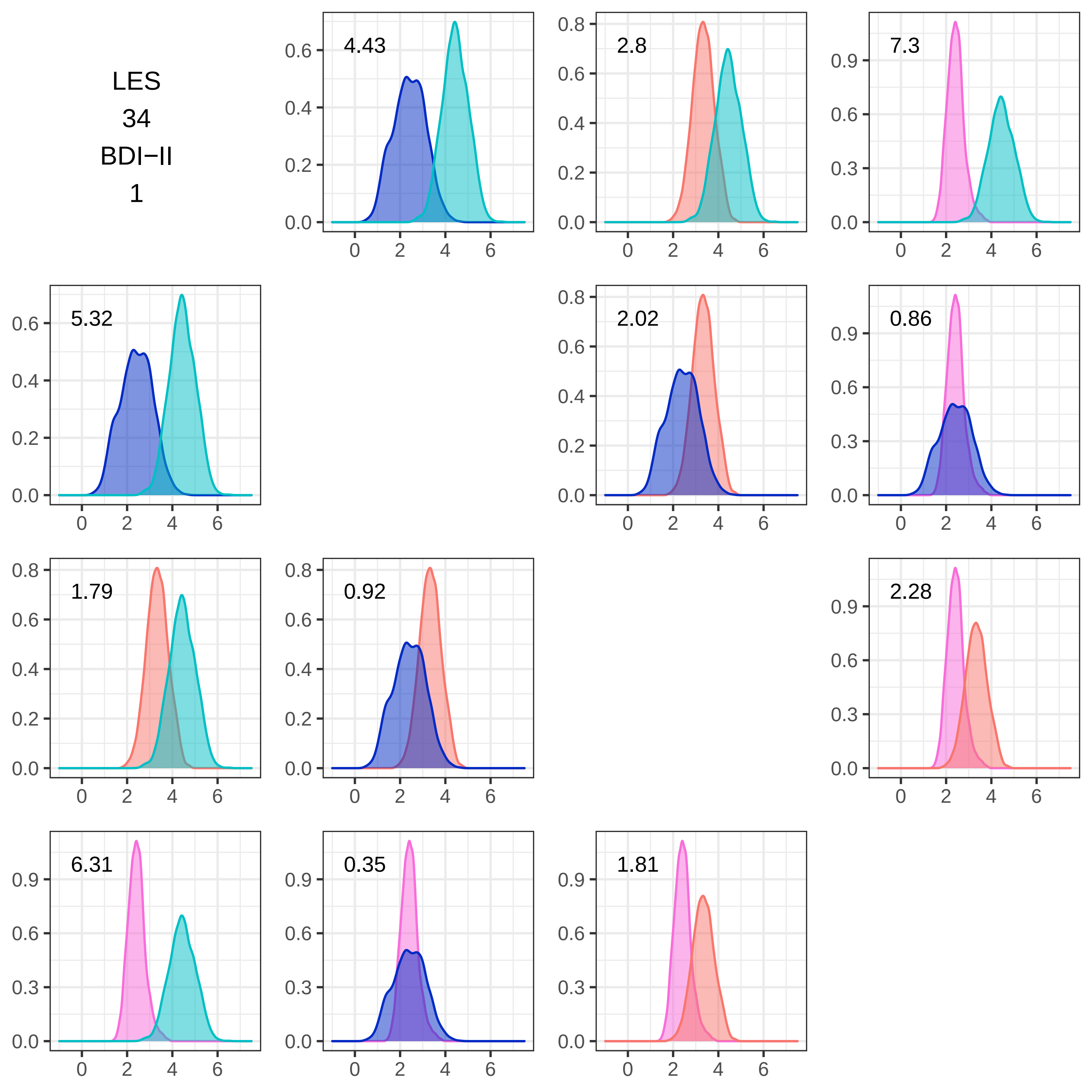
LES
34
BDI−II
1
4.43
2.8
7.3
5.32
2.02
0.86
1.79
0.92
2.28
6.31
0.35
1.81

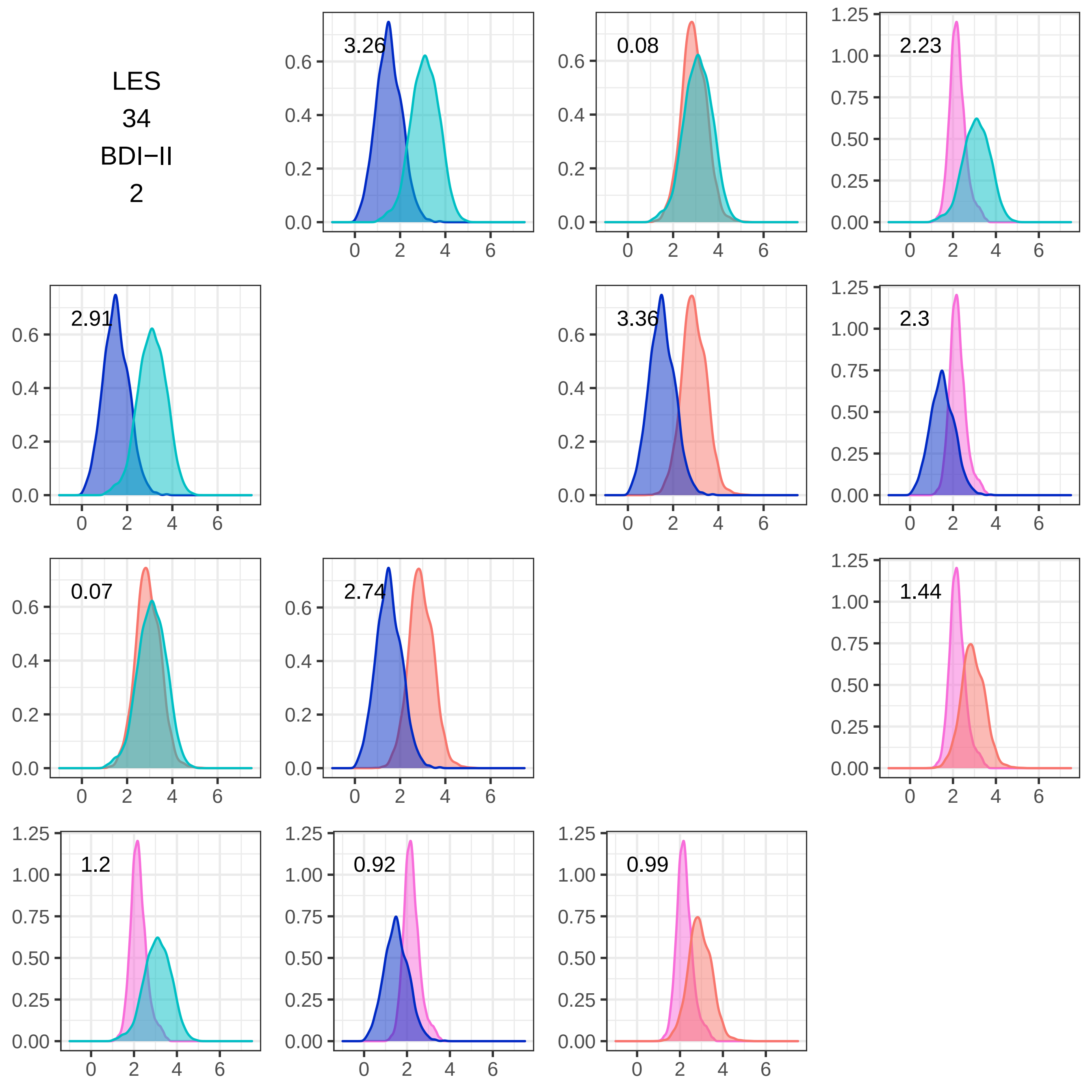
LES
34
BDI−II
2
3.26
0.08
2.23
2.91
3.36
2.3
0.07
2.74
1.44
1.2
0.92
0.99

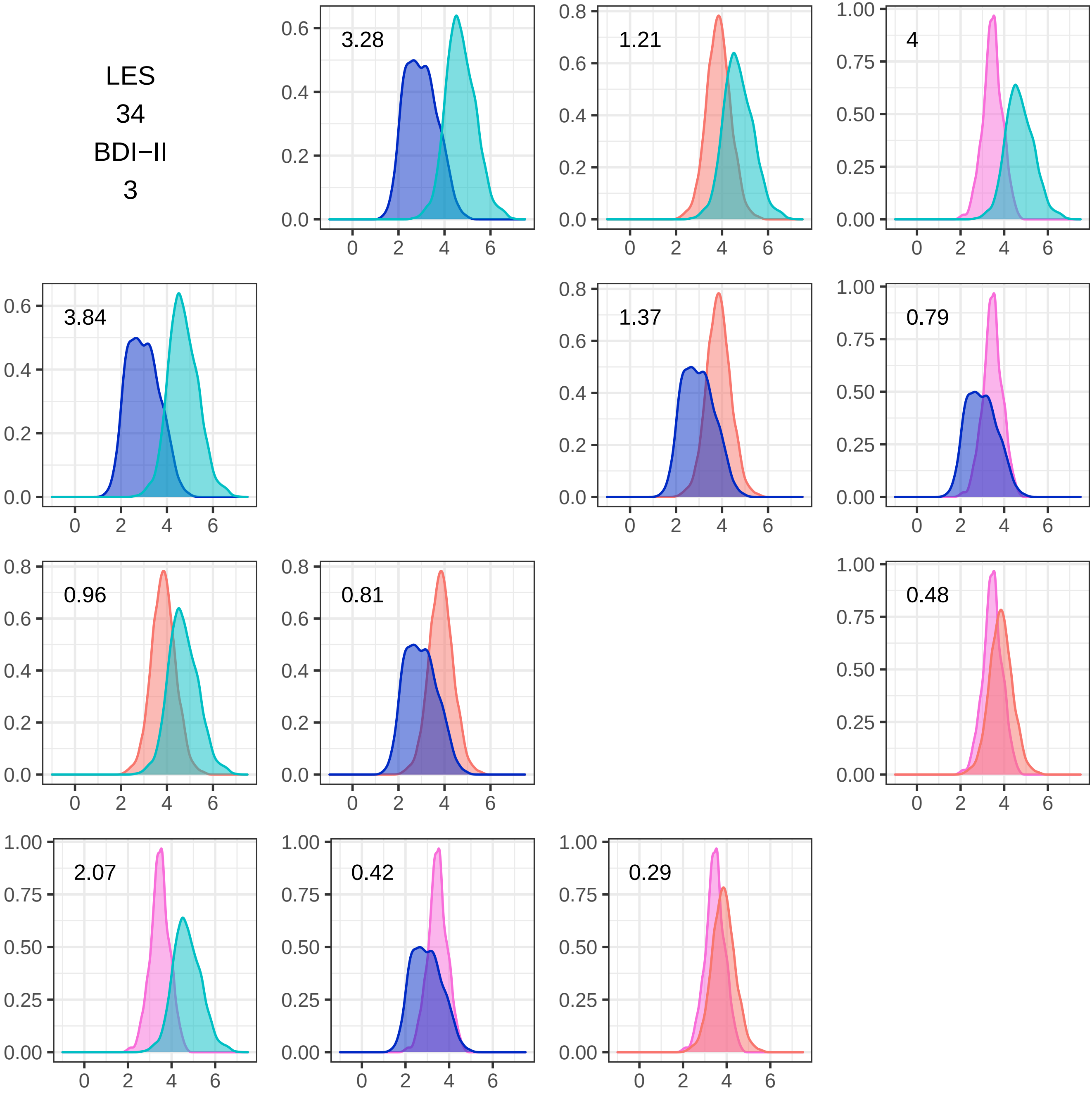

LES
34
BDI−II
3
3.28
1.21
4
3.84
1.37
0.79
0.96
0.81
0.48
2.07
0.42
0.29

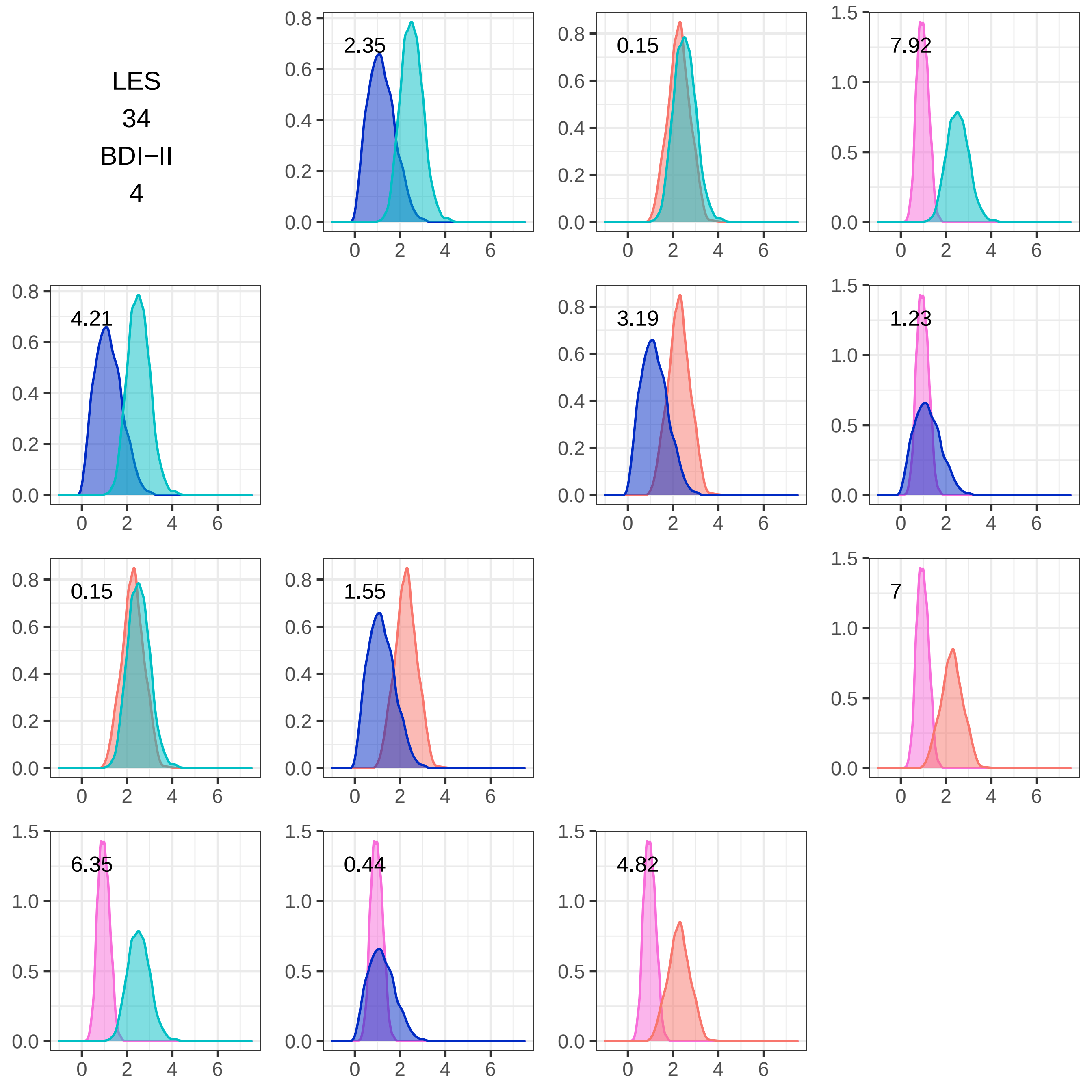

LES
34
BDI−II
4
2.35
0.15
7.92
4.21
3.19
1.23
0.15
1.55
7
6.35
0.44
4.82

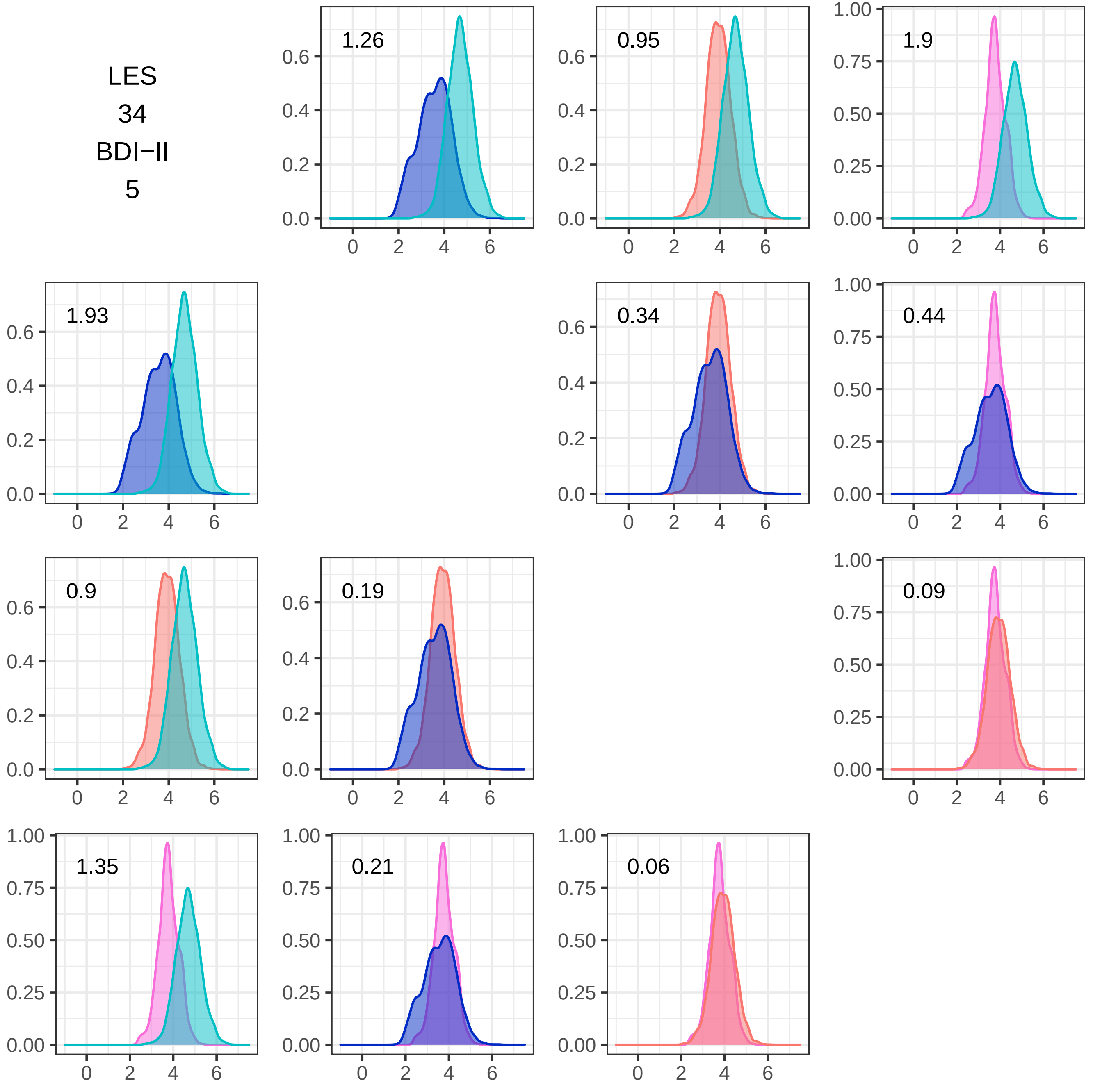
LES
34
BDI−II
5
1.26
0.95
1.9
1.93
0.34
0.44
0.9
0.19
0.09
1.35
0.21
0.06

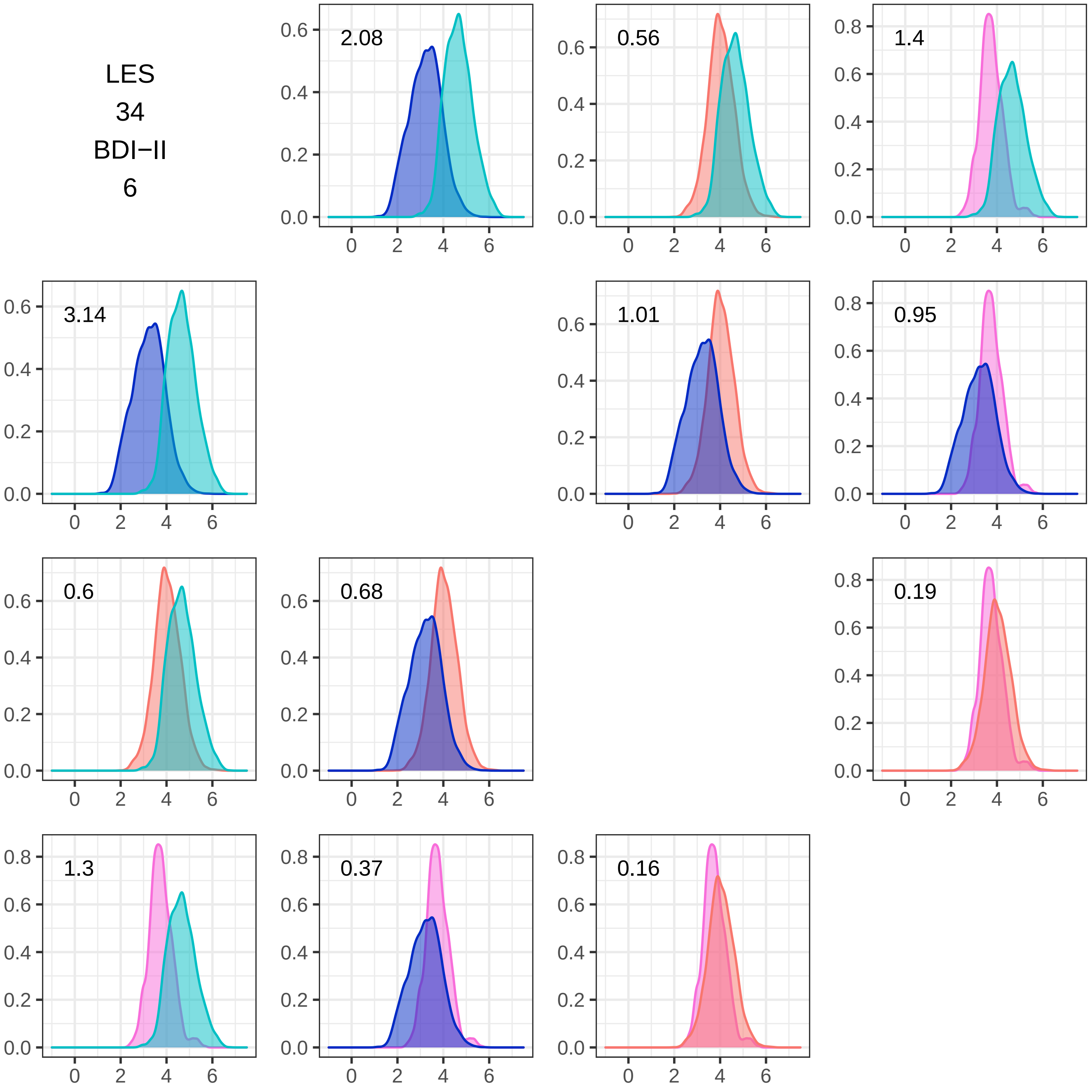
LES
34
BDI−II
6
2.08
0.56
1.4
3.14
1.01
0.95
0.6
0.68
0.19
1.3
0.37
0.16

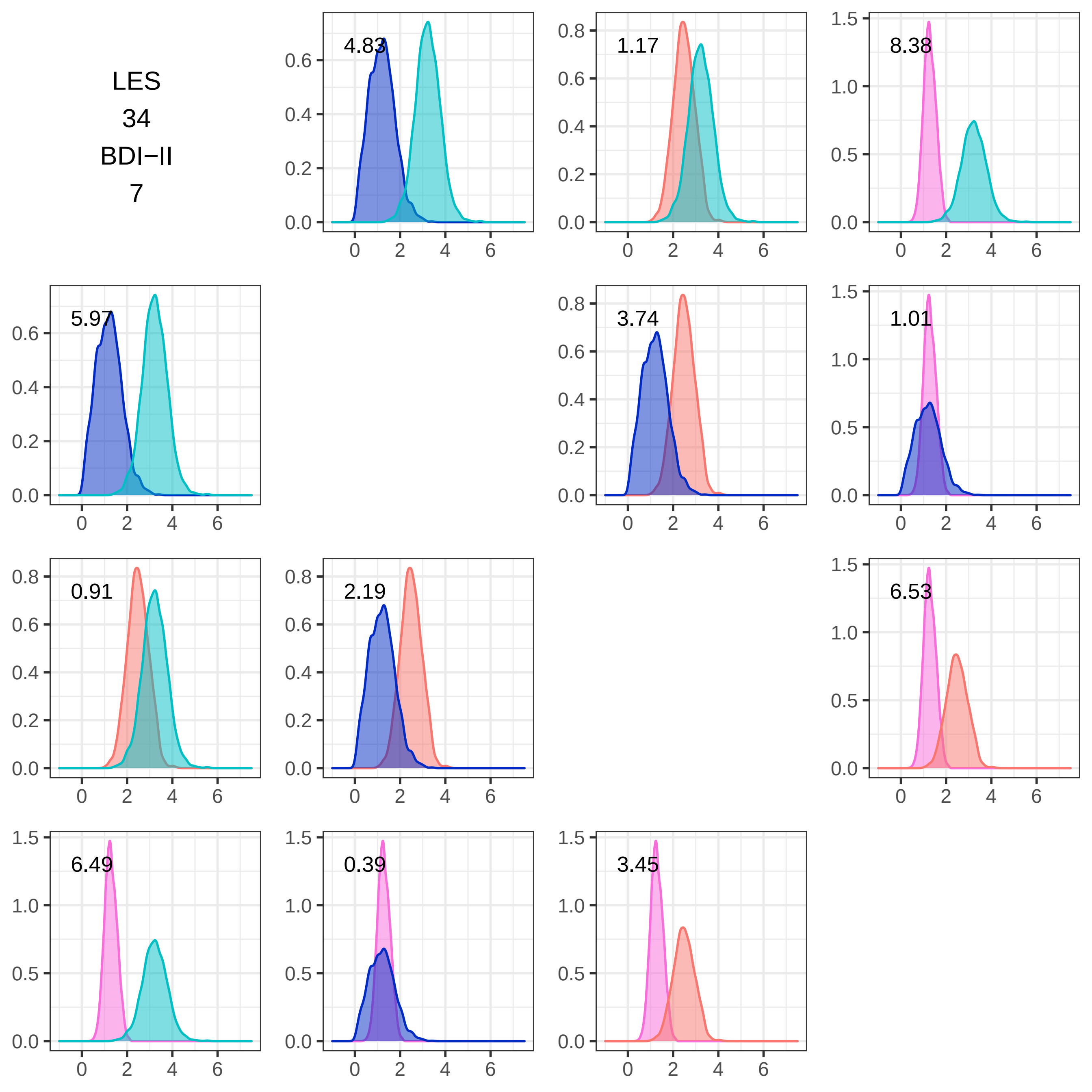
LES
34
BDI−II
7
4.83
1.17
8.38
5.97
3.74
1.01
0.91
2.19
6.53
6.49
0.39
3.45

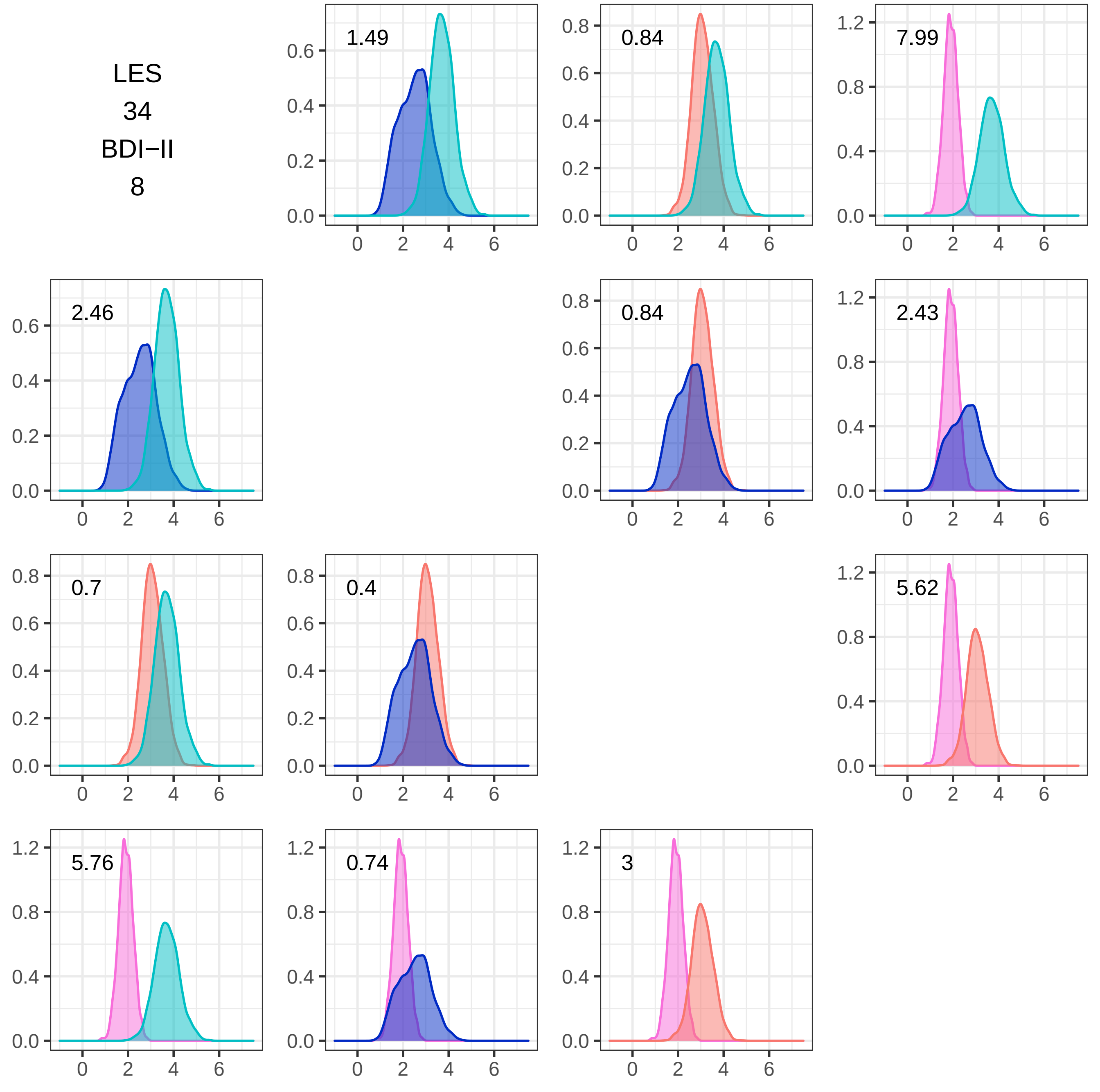
LES
34
BDI−II
8
1.49
0.84
7.99
2.46
0.84
2.43
0.7
0.4
5.62
5.76
0.74
3

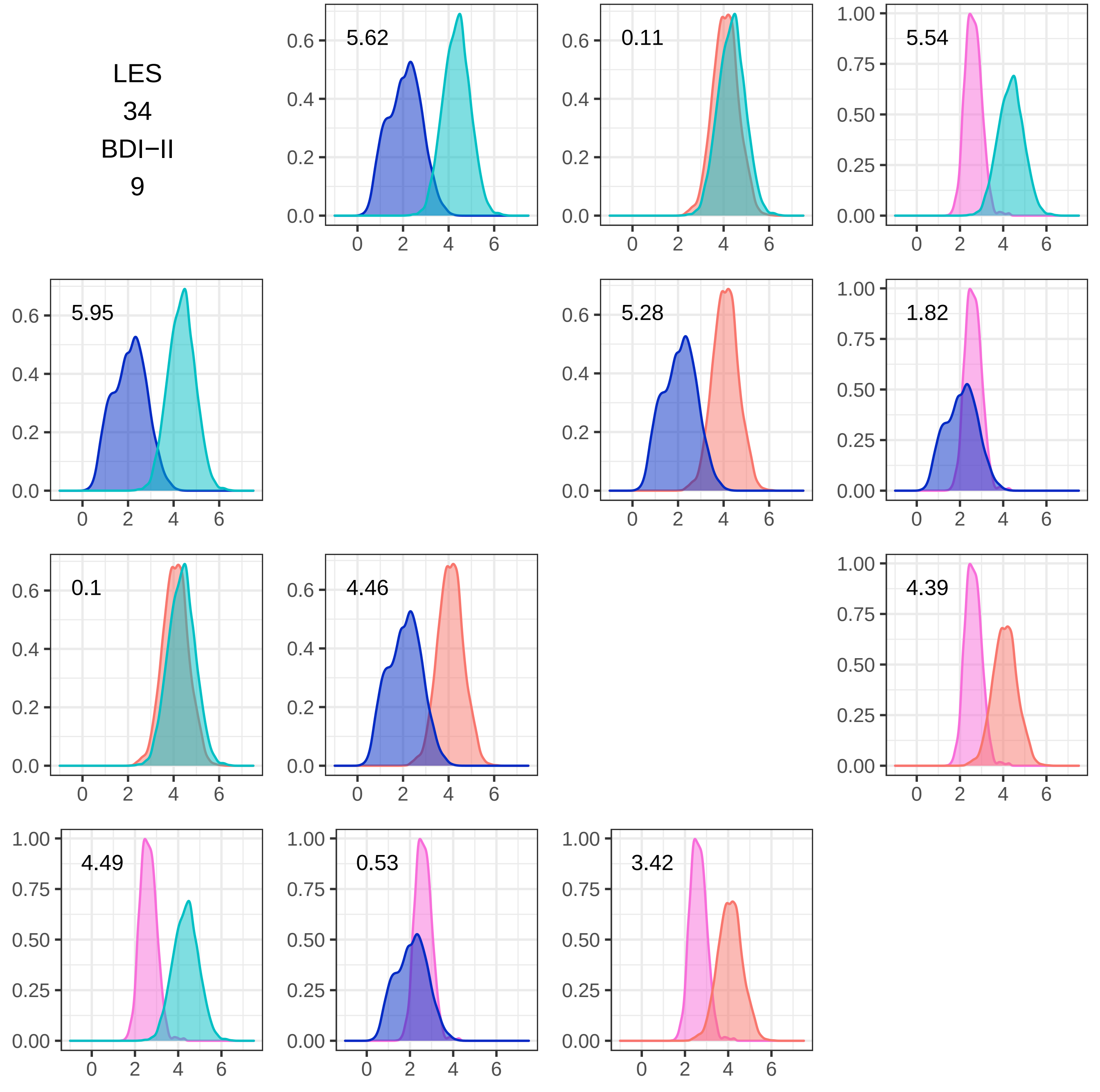

LES
34
BDI−II
9
5.62
0.11
5.54
5.95
5.28
1.82
0.1
4.46
4.39
4.49
0.53
3.42

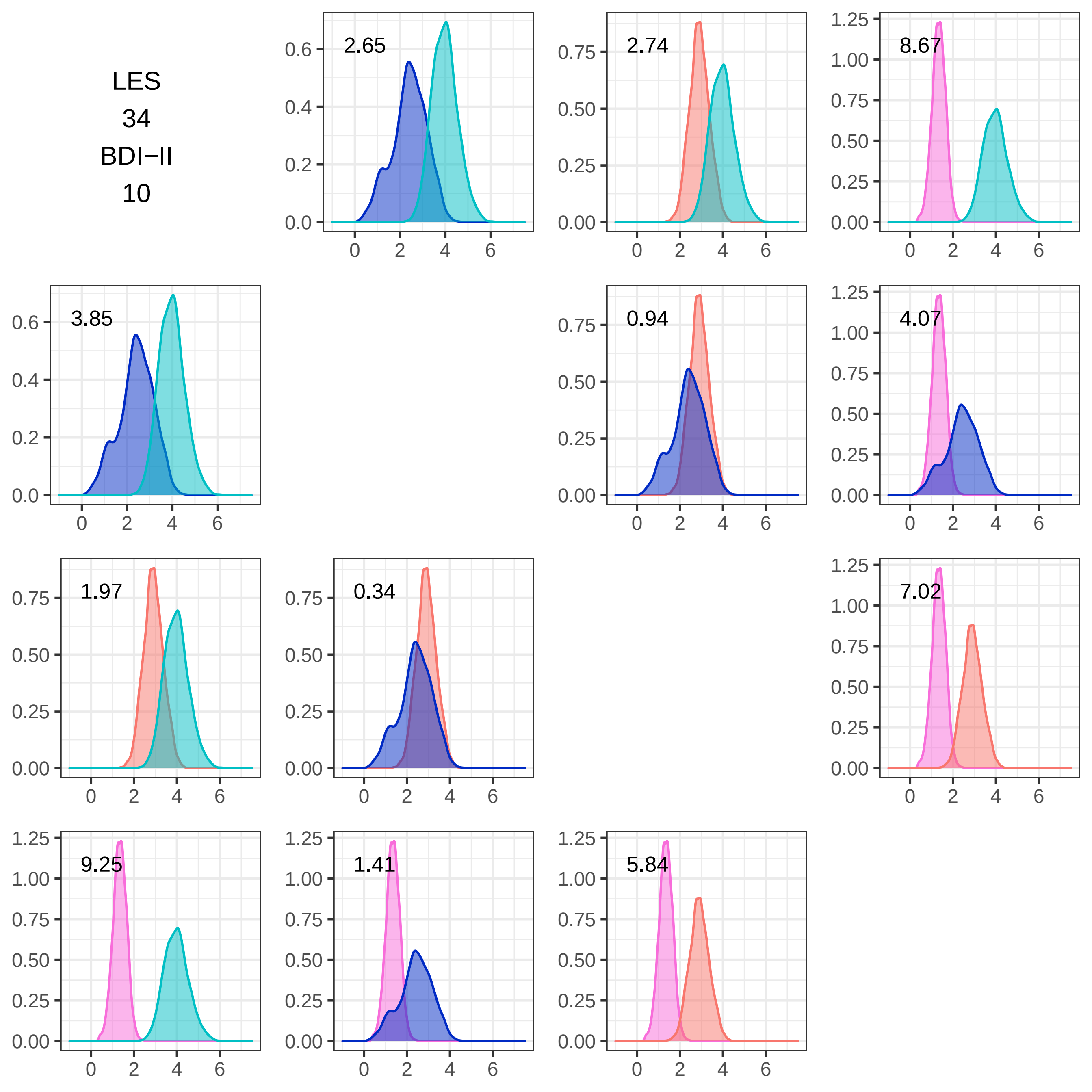

LES
34
BDI−II
10
2.65
2.74
8.67
3.85
0.94
4.07
1.97
0.34
7.02
9.25
1.41
5.84

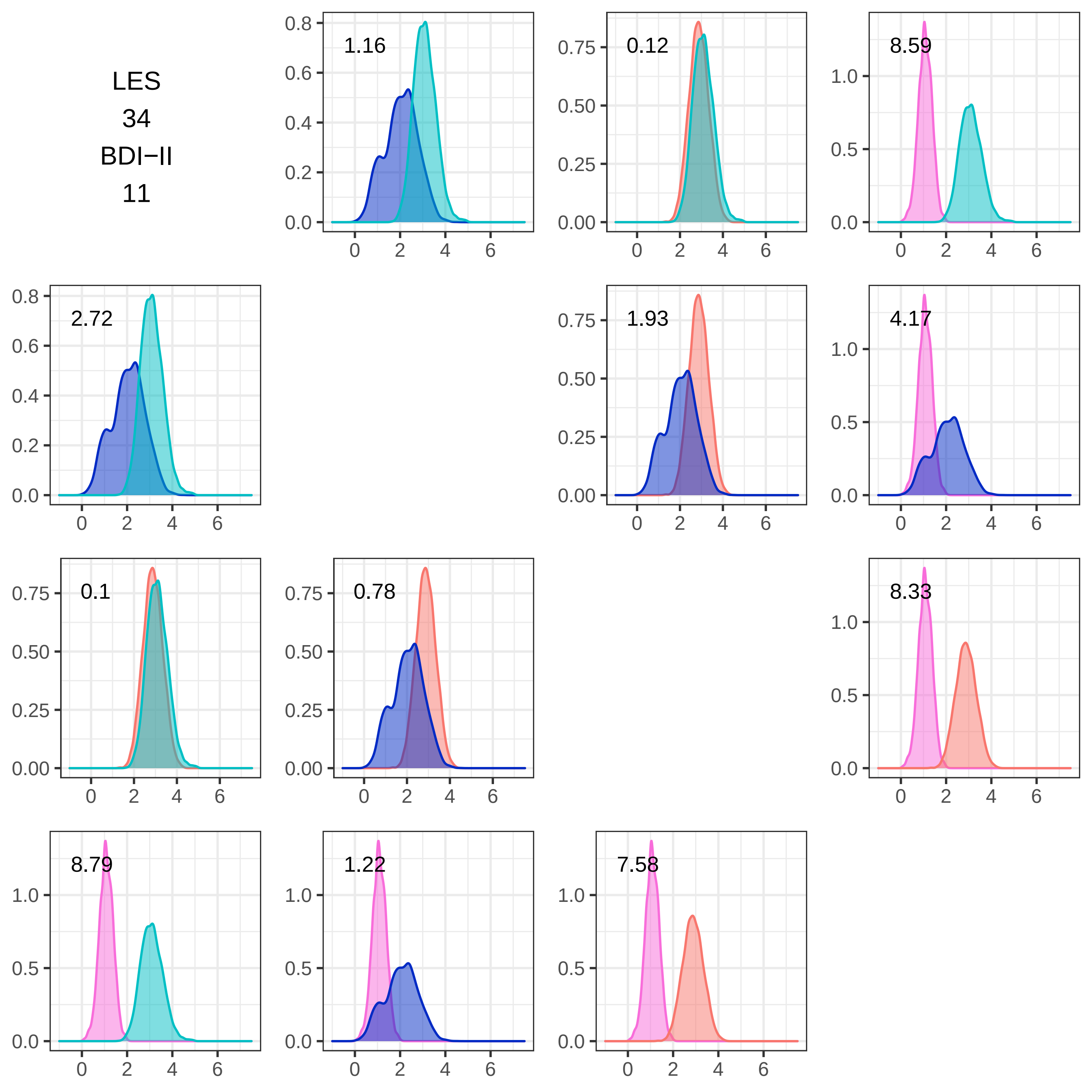

LES
34
BDI−II
11
1.16
0.12
8.59
2.72
1.93
4.17
0.1
0.78
8.33
8.79
1.22
7.58

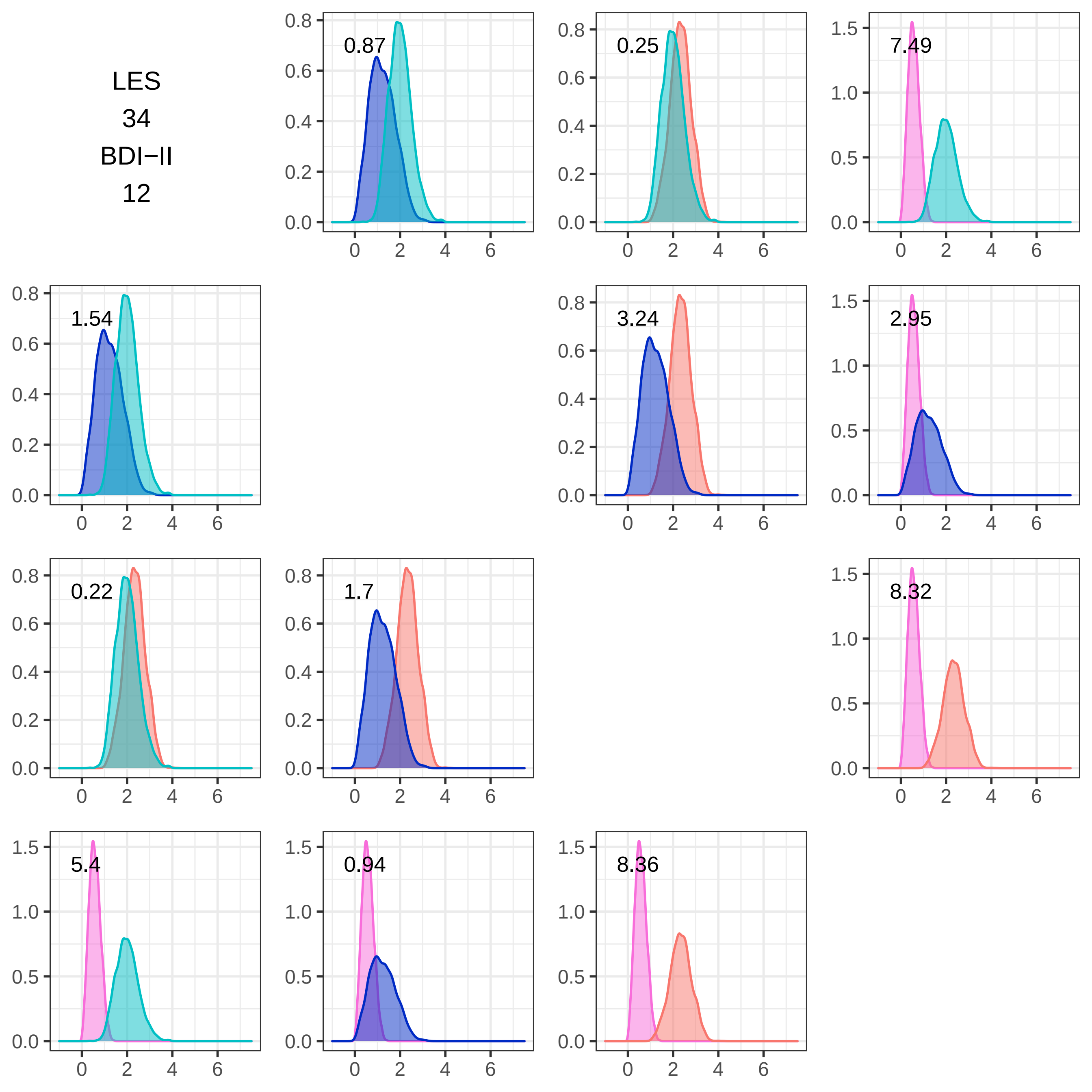
LES
34
BDI−II
12
0.87
0.25
7.49
1.54
3.24
2.95
0.22
1.7
8.32
5.4
0.94
8.36

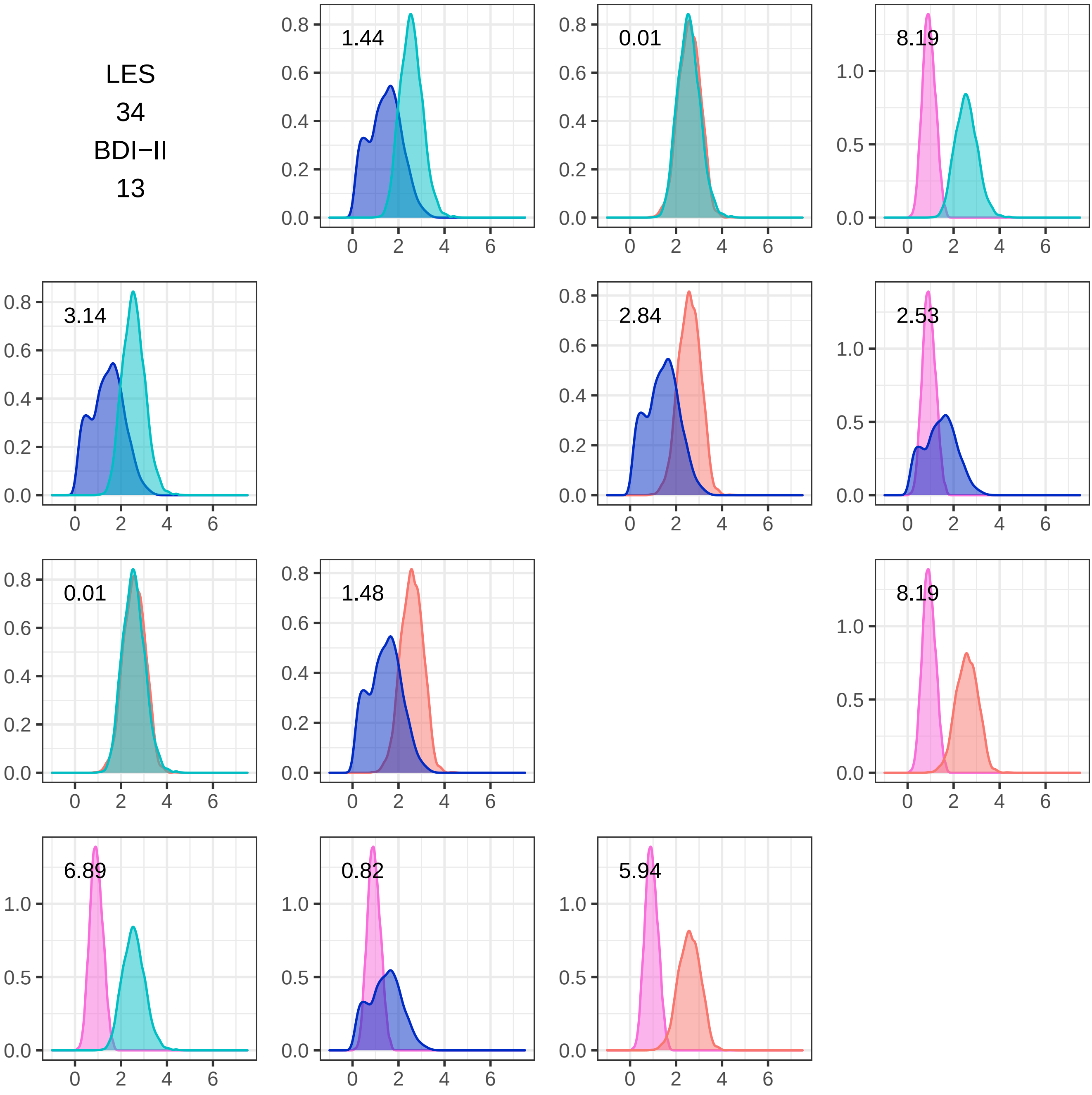
LES
34
BDI−II
13
1.44
0.01
8.19
3.14
2.84
2.53
0.01
1.48
8.19
6.89
0.82
5.94

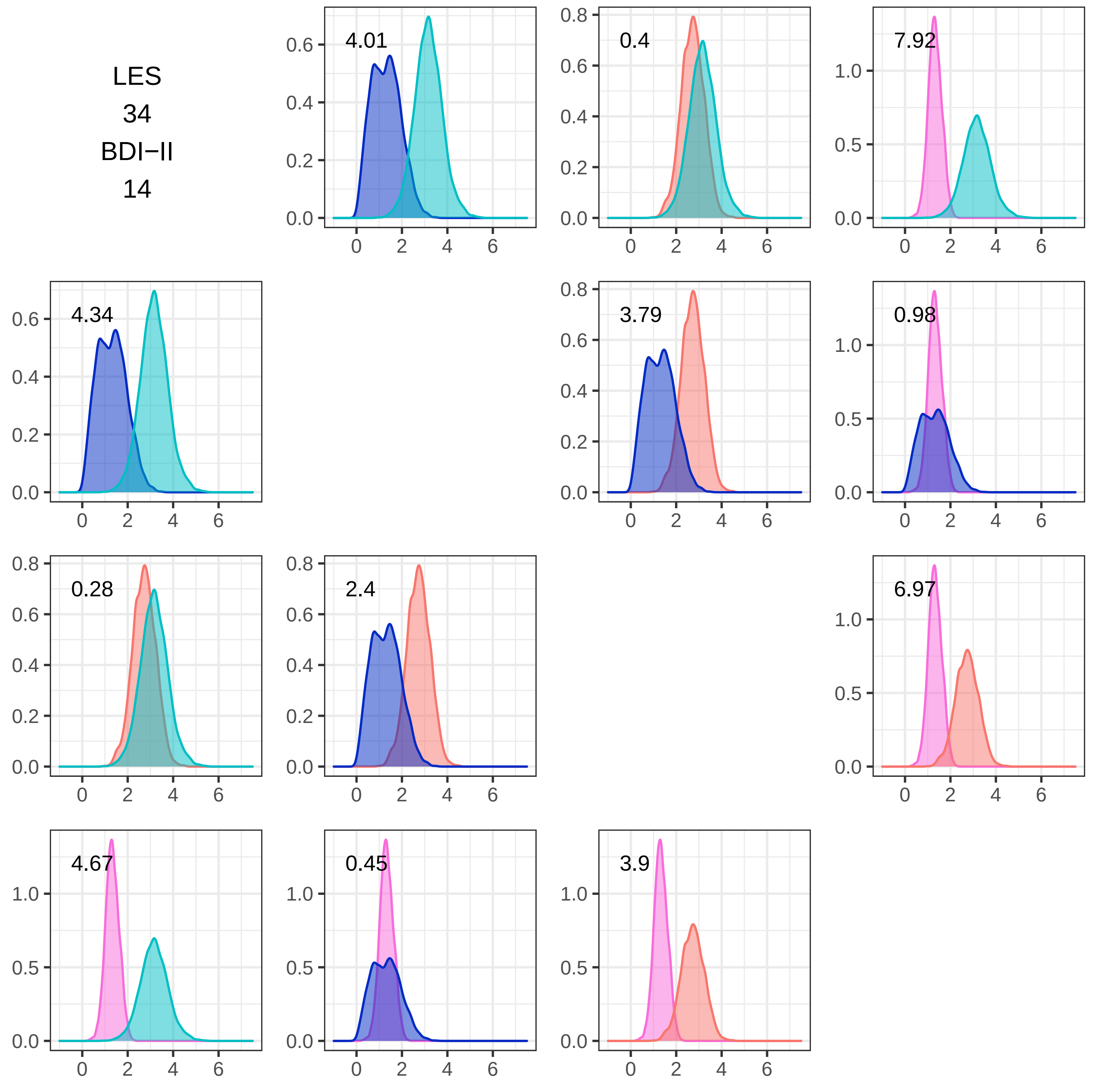
LES
34
BDI−II
14
4.01
0.4
7.92
4.34
3.79
0.98
0.28
2.4
6.97
4.67
0.45
3.9

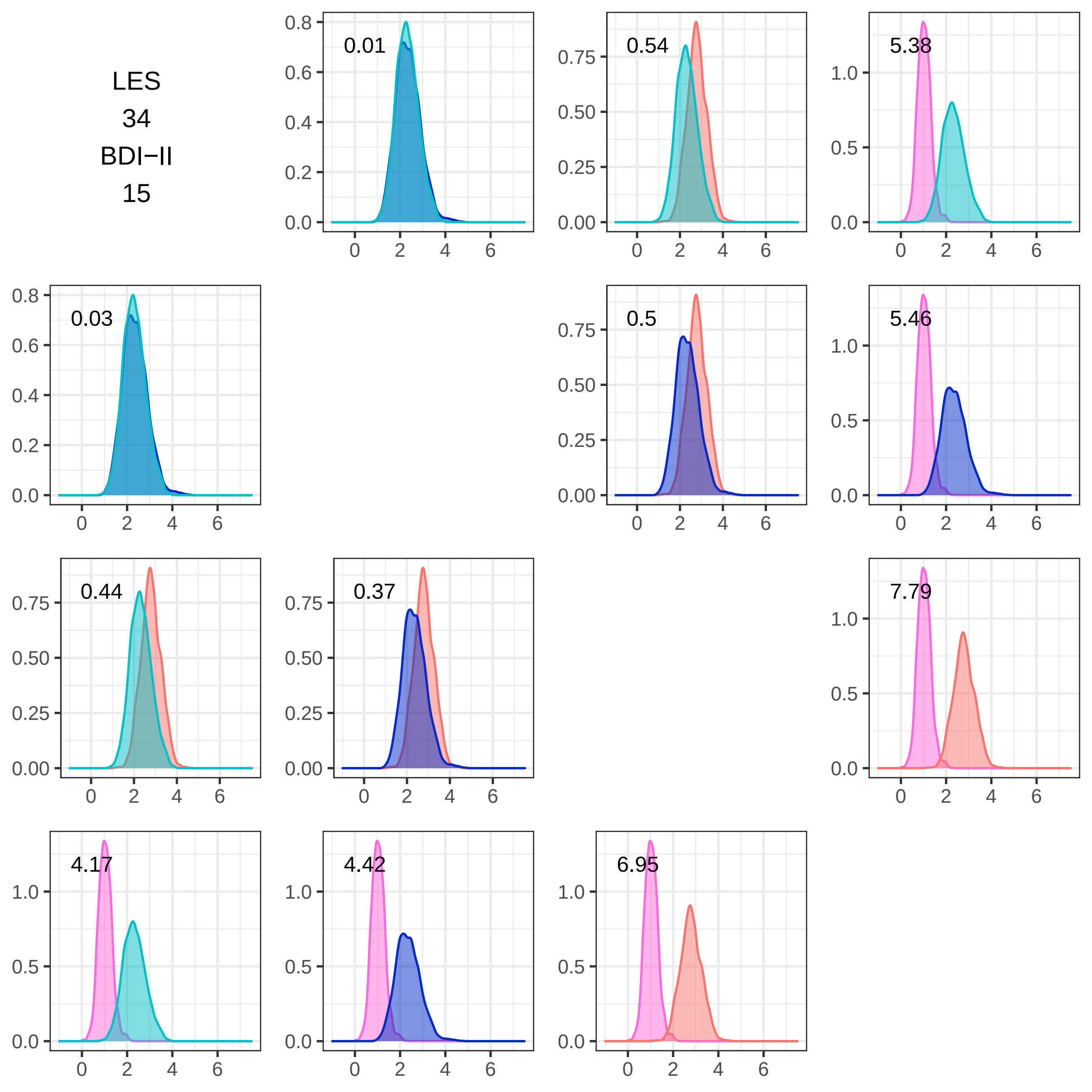

LES
34
BDI−II
15
0.01
0.54
5.38
0.03
0.5
5.46
0.44
0.37
7.79
4.17
4.42
6.95

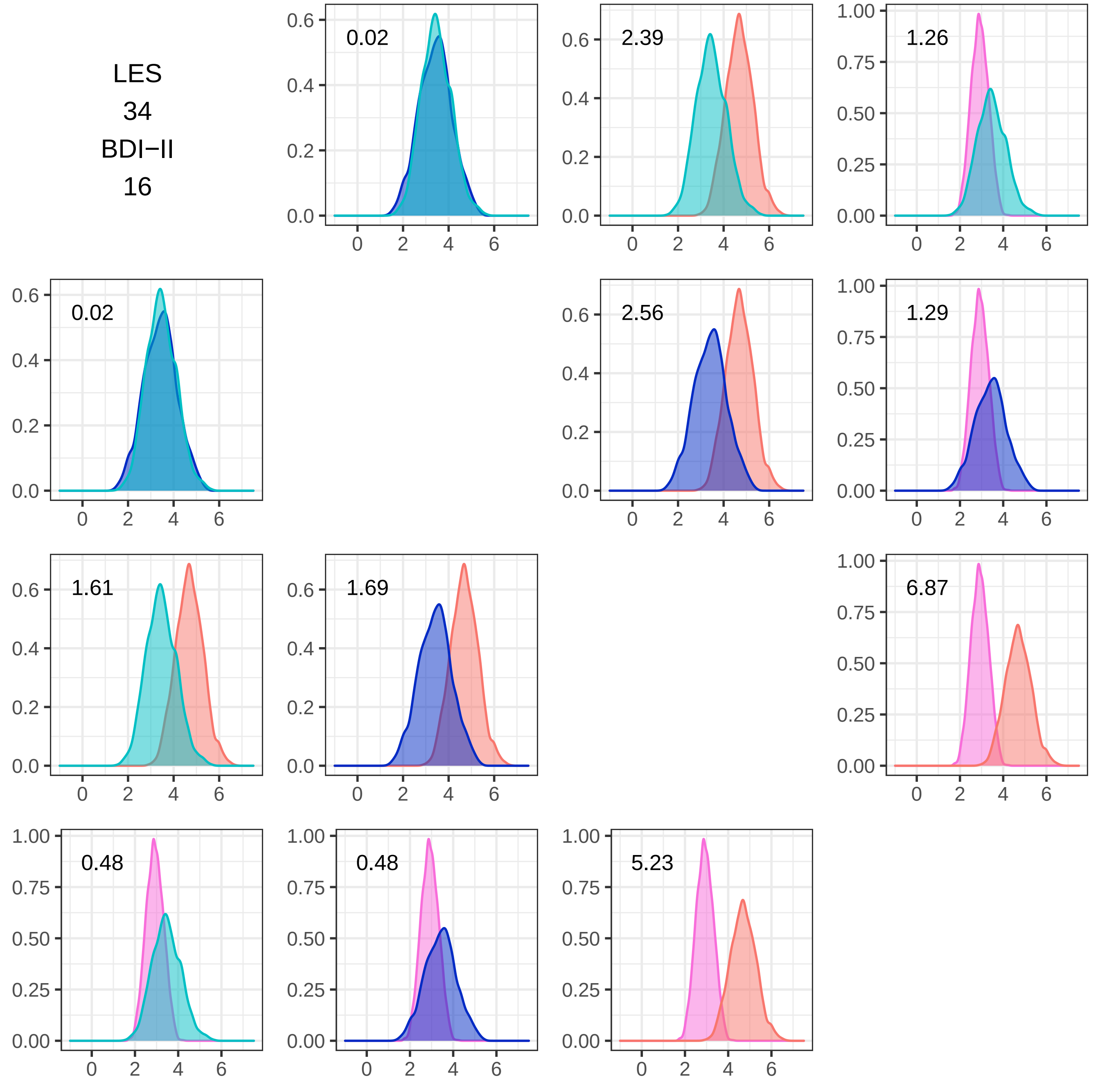

LES
34
BDI−II
16
0.02
2.39
1.26
0.02
2.56
1.29
1.61
1.69
6.87
0.48
0.48
5.23

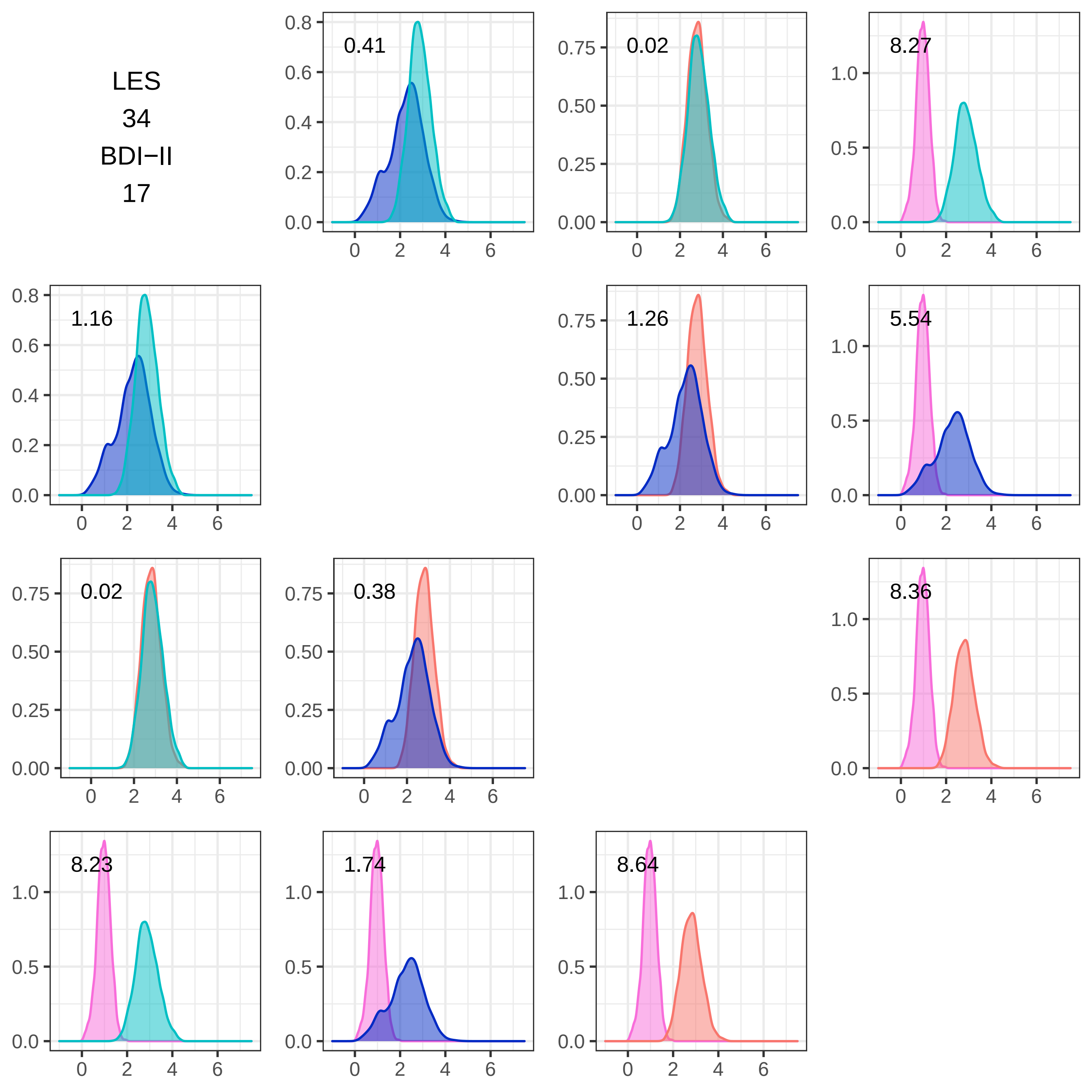

LES
34
BDI−II
17
0.41
0.02
8.27
1.16
1.26
5.54
0.02
0.38
8.36
8.23
1.74
8.64

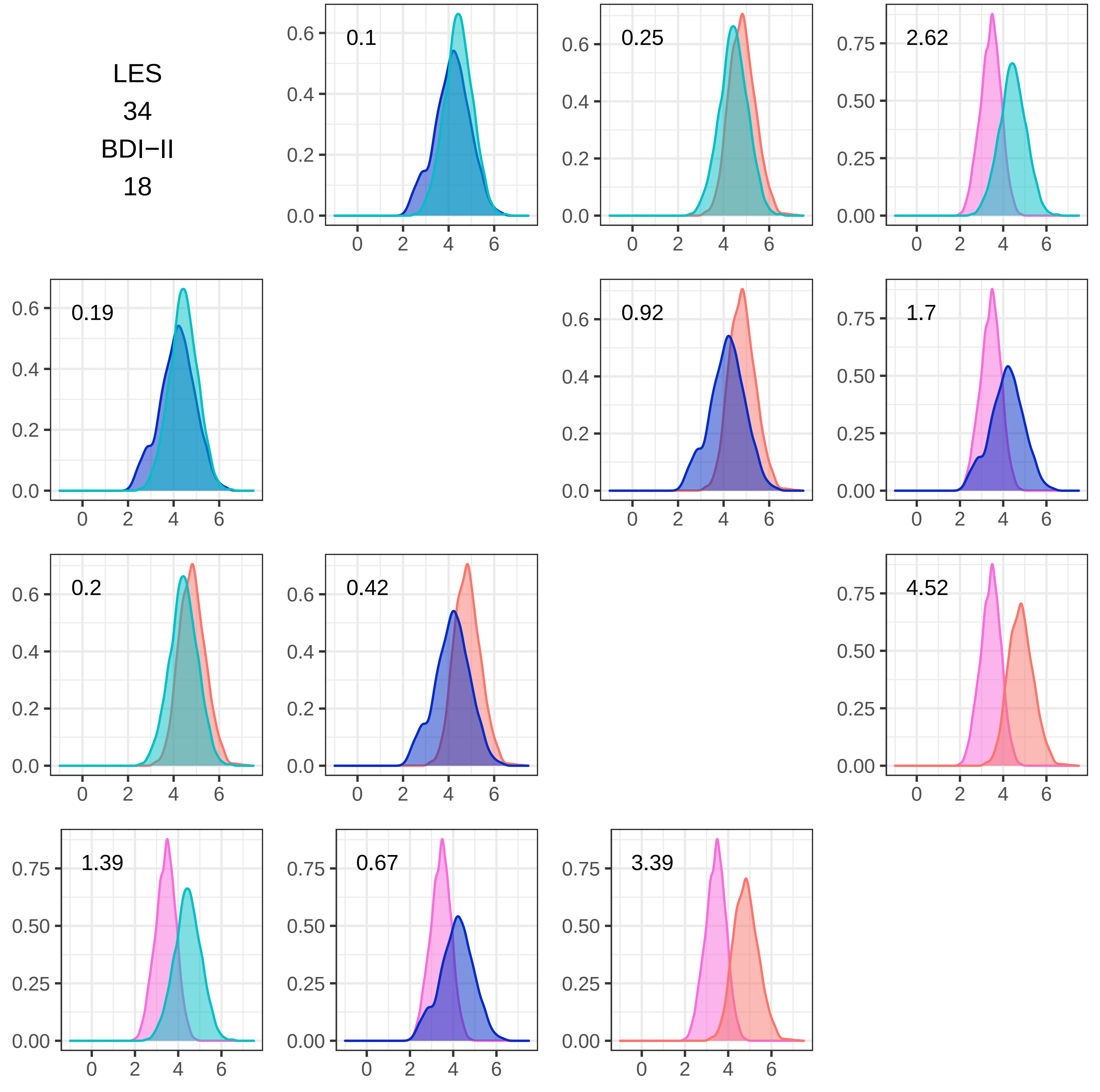
LES
34
BDI−II
18
0.1
0.25
2.62
0.19
0.92
1.7
0.2
0.42
4.52
1.39
0.67
3.39

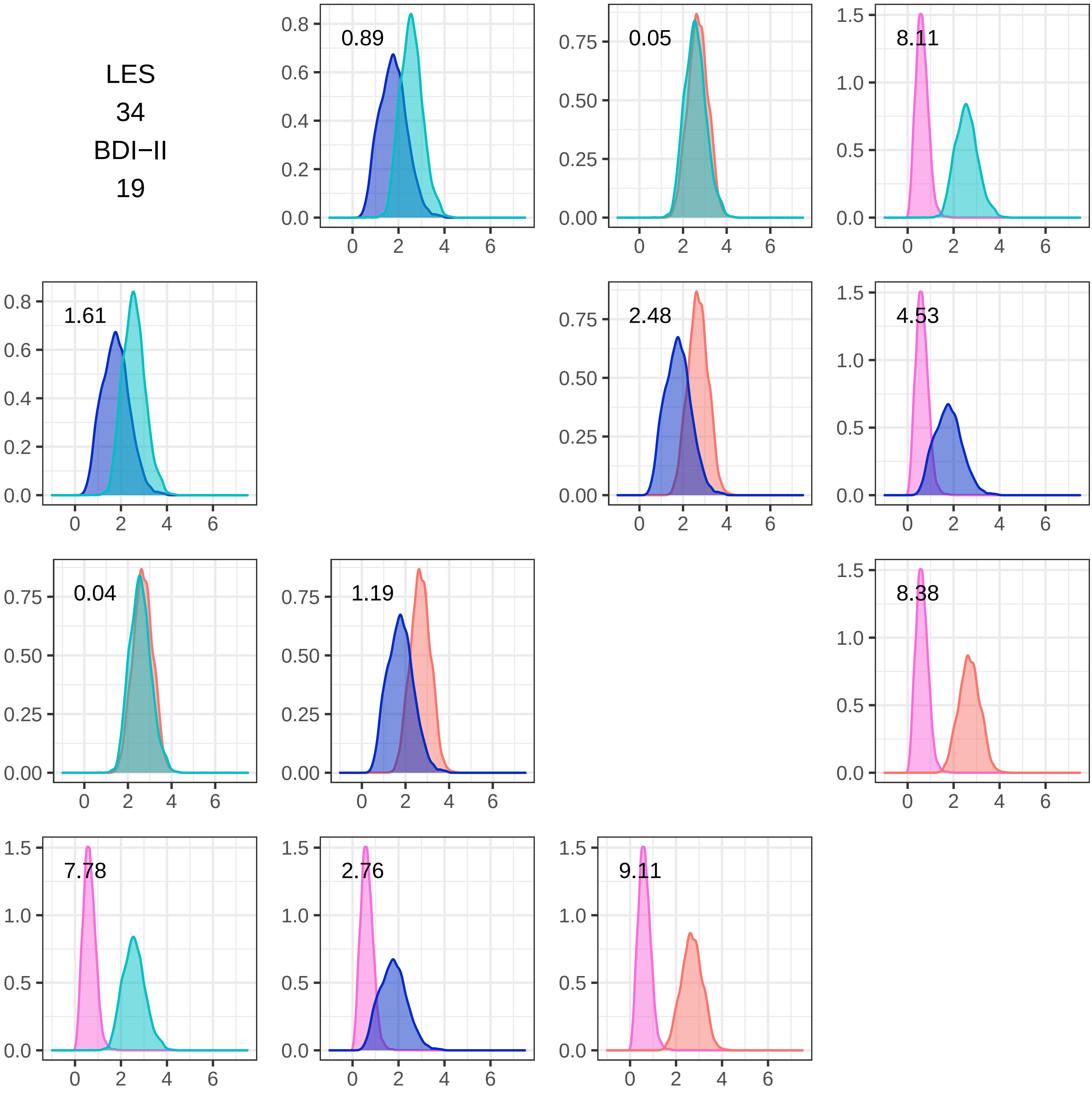
LES
34
BDI−II
19
0.89
0.05
8.11
1.61
2.48
4.53
0.04
1.19
8.38
7.78
2.76
9.11

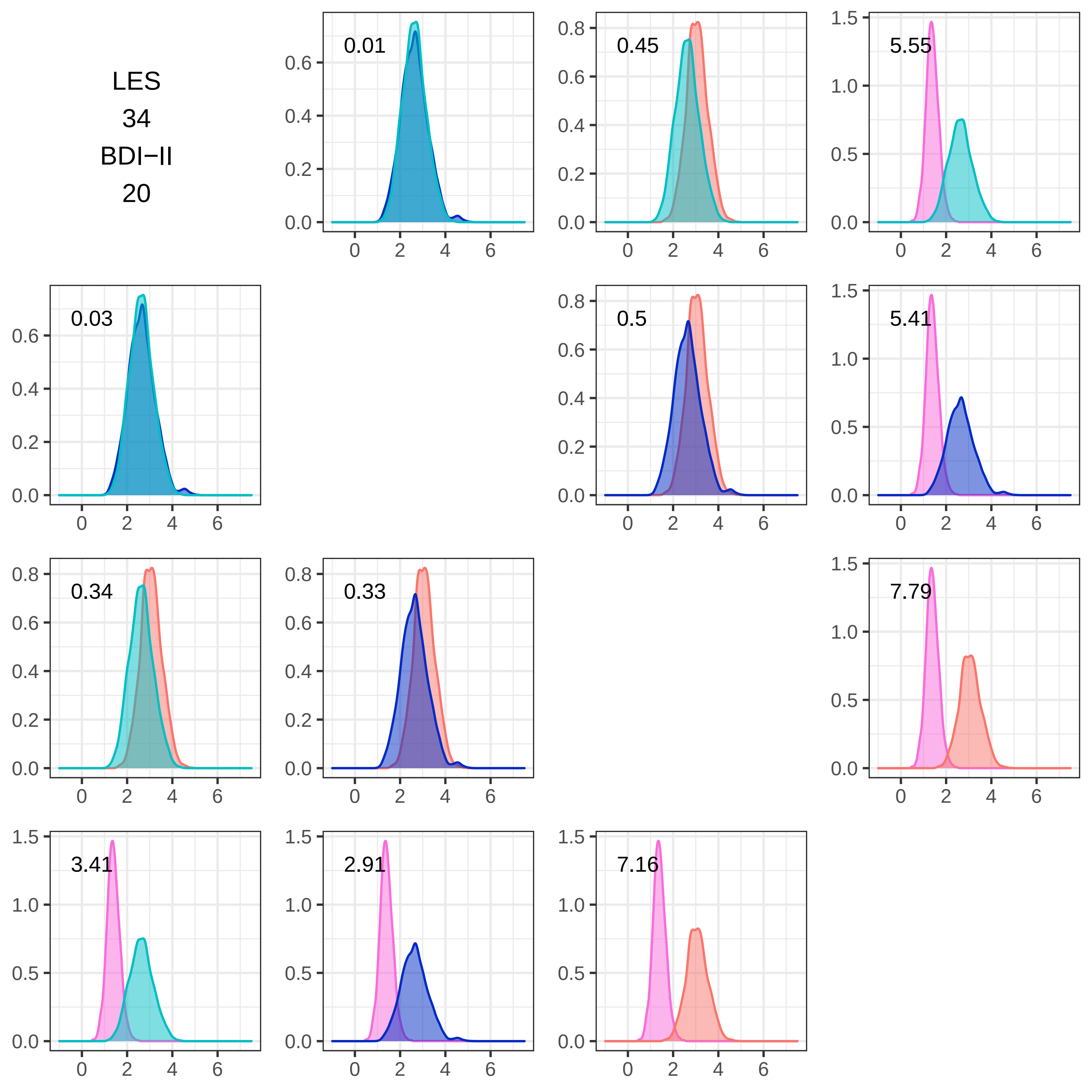

LES
34
BDI−II
20
0.01
0.45
5.55
0.03
0.5
5.41
0.34
0.33
7.79
3.41
2.91
7.16

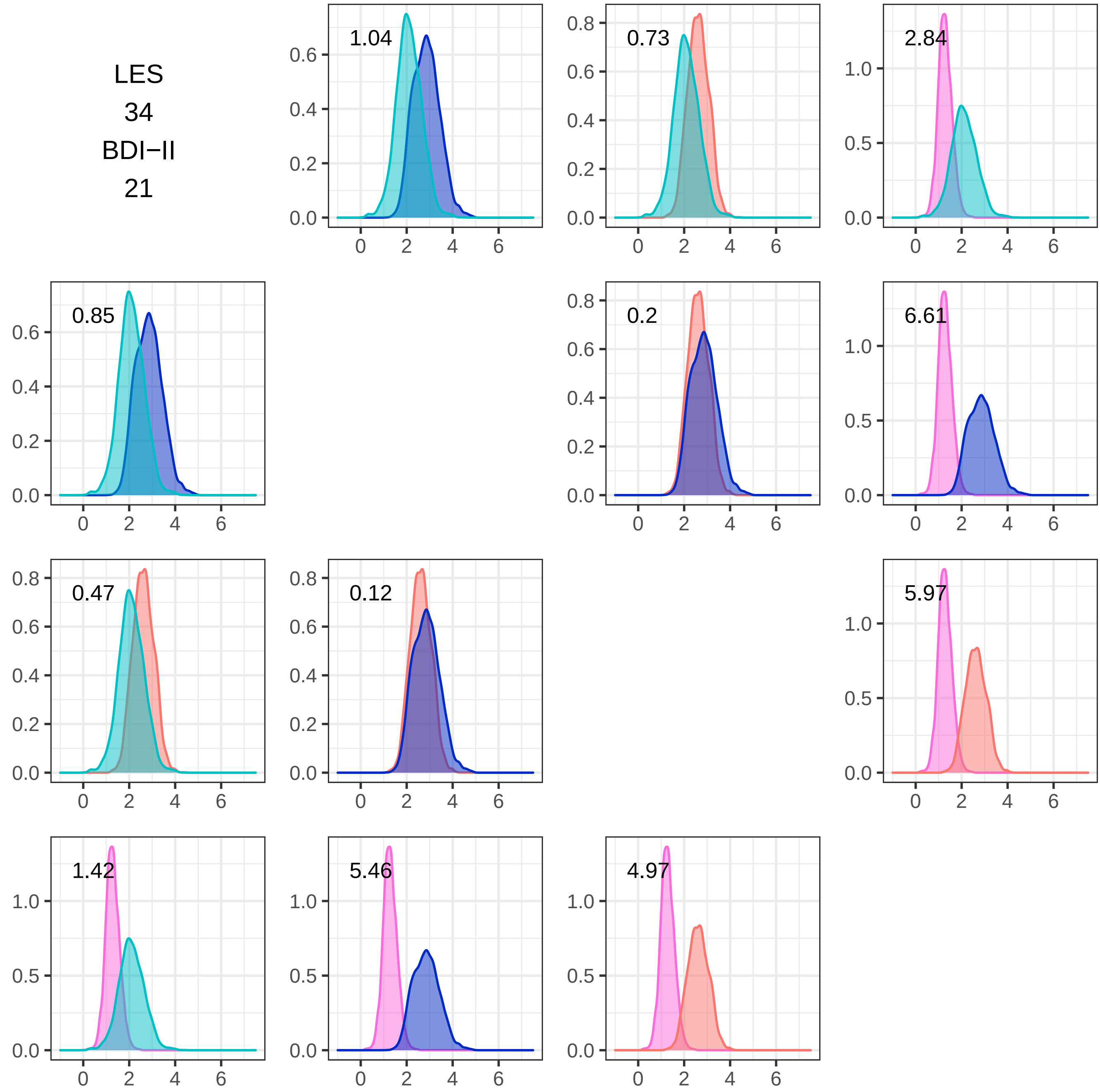
LES
34
BDI−II
21
1.04
0.73
2.84
0.85
0.2
6.61
0.47
0.12
5.97
1.42
5.46
4.97

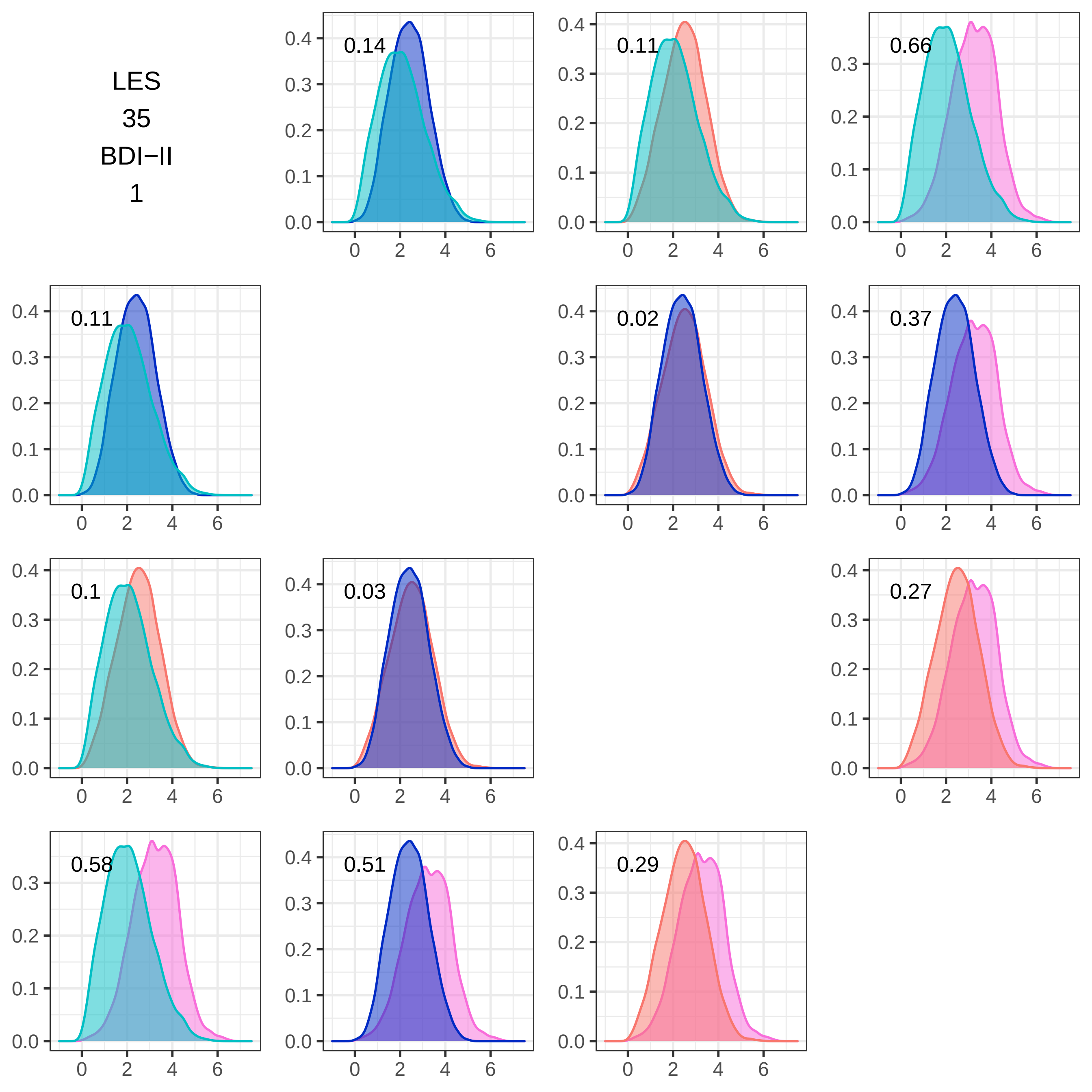

LES
35
BDI−II
1
0.14
0.11
0.66
0.11
0.02
0.37
0.1
0.03
0.27
0.58
0.51
0.29
0.0
0.1
0.2
0.3
0.4
0
2
4
6

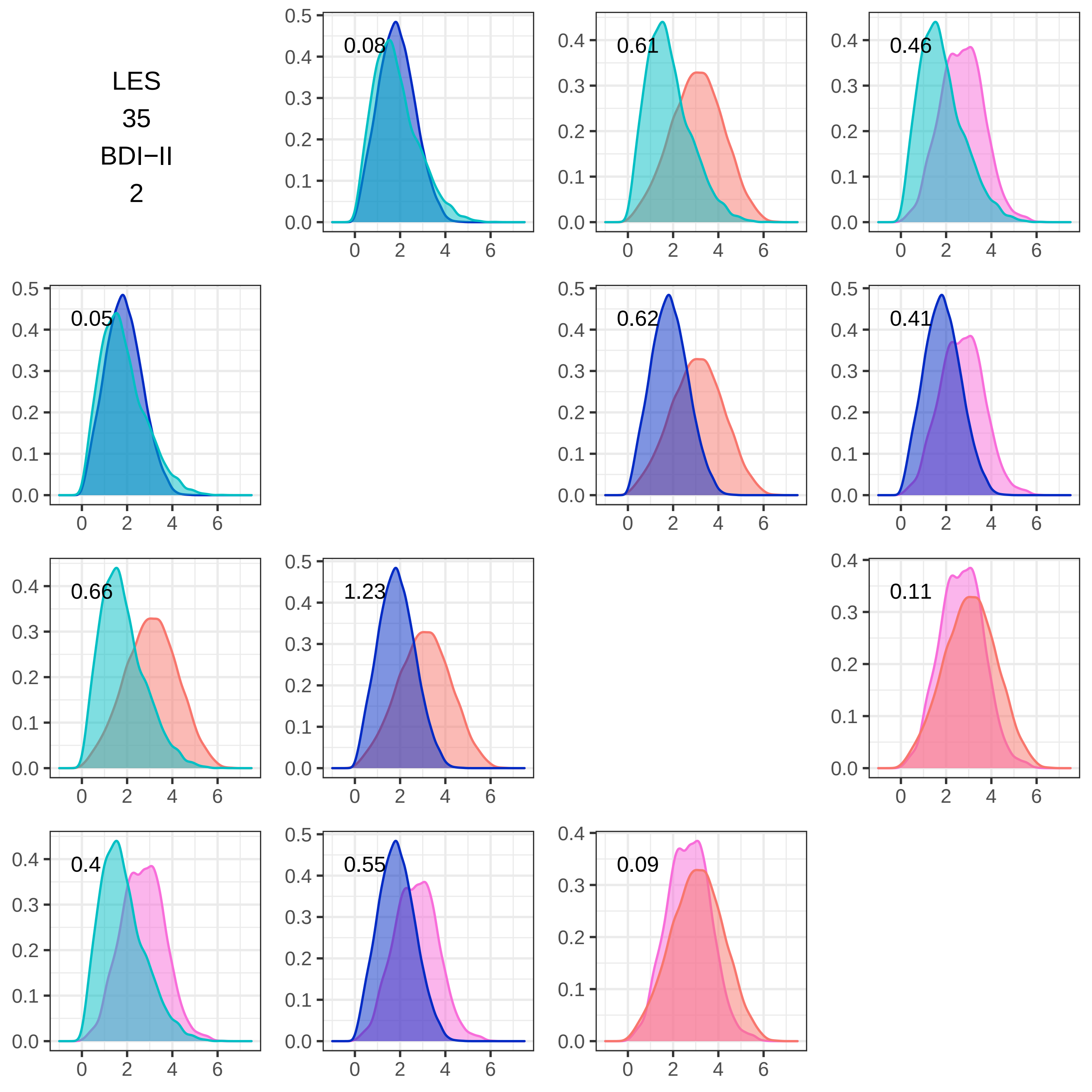

LES
35
BDI−II
2
0.08
0.61
0.46
0.05
0.62
0.41
0.66
1.23
0.11
0.4
0.55
0.09

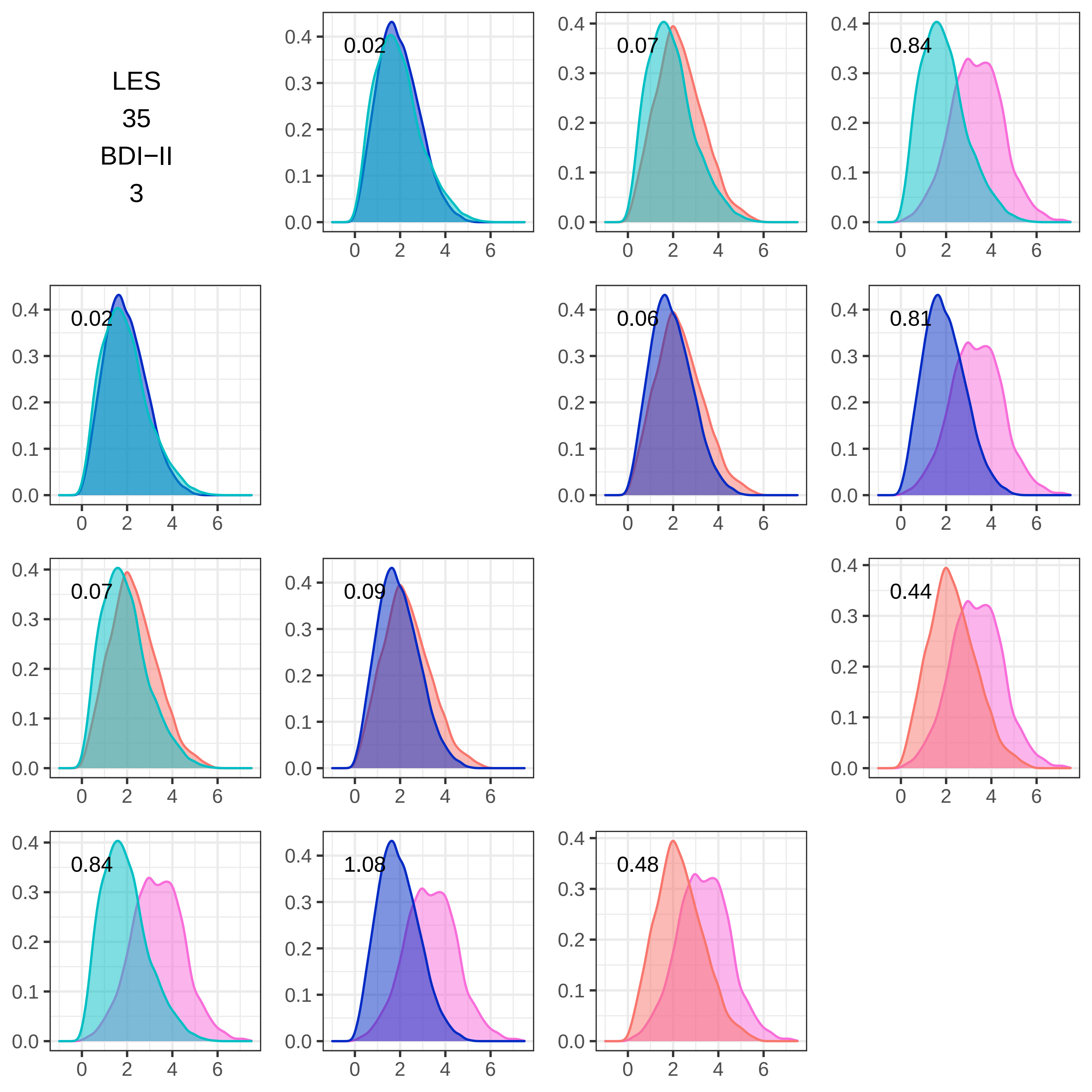
LES
35
BDI−II
3
0.02
0.07
0.84
0.02
0.06
0.81
0.07
0.09
0.44
0.84
1.08
0.48

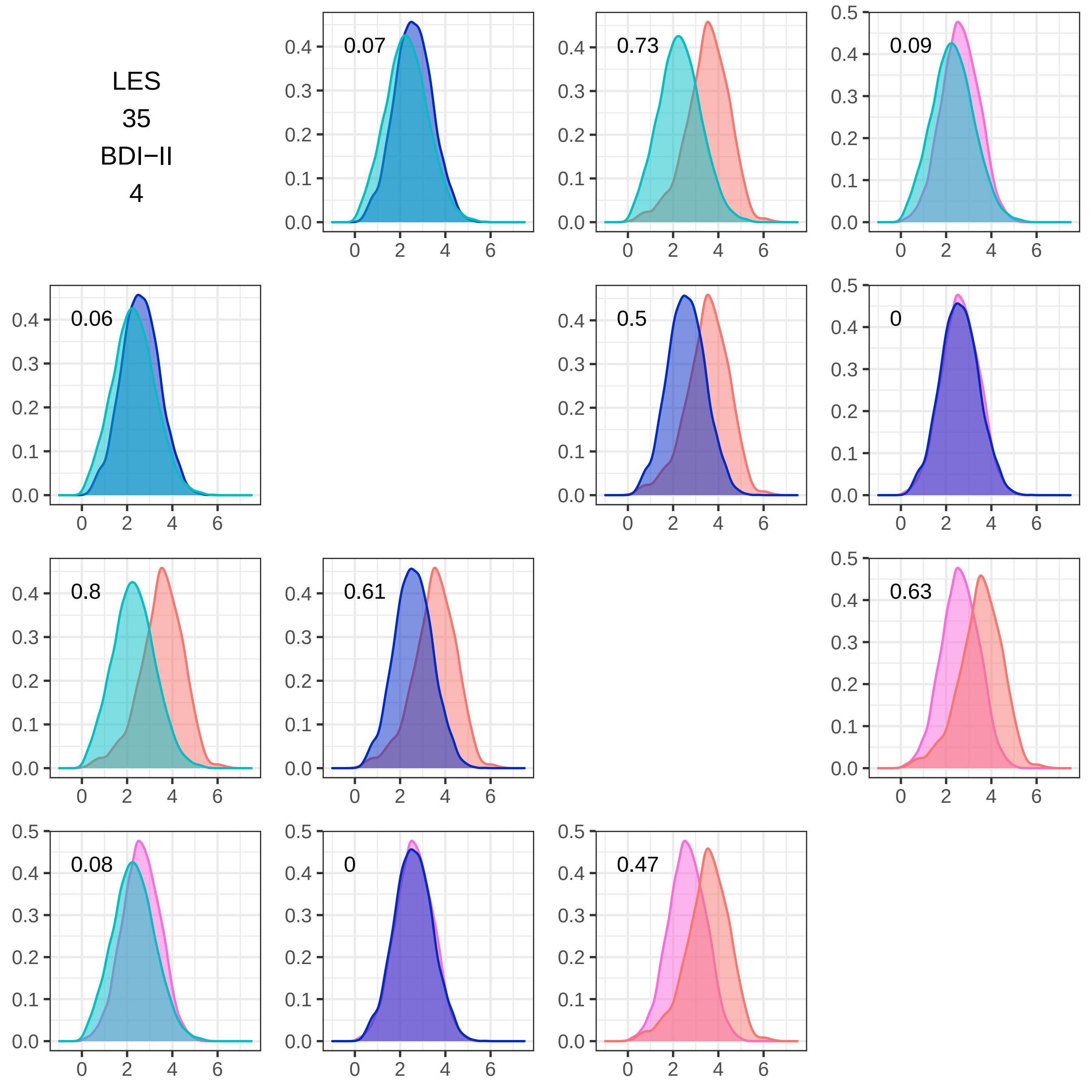
LES
35
BDI−II
4
0.07
0.73
0.09
0.06
0.5
0
0.8
0.61
0.63
0.08
0
0.47

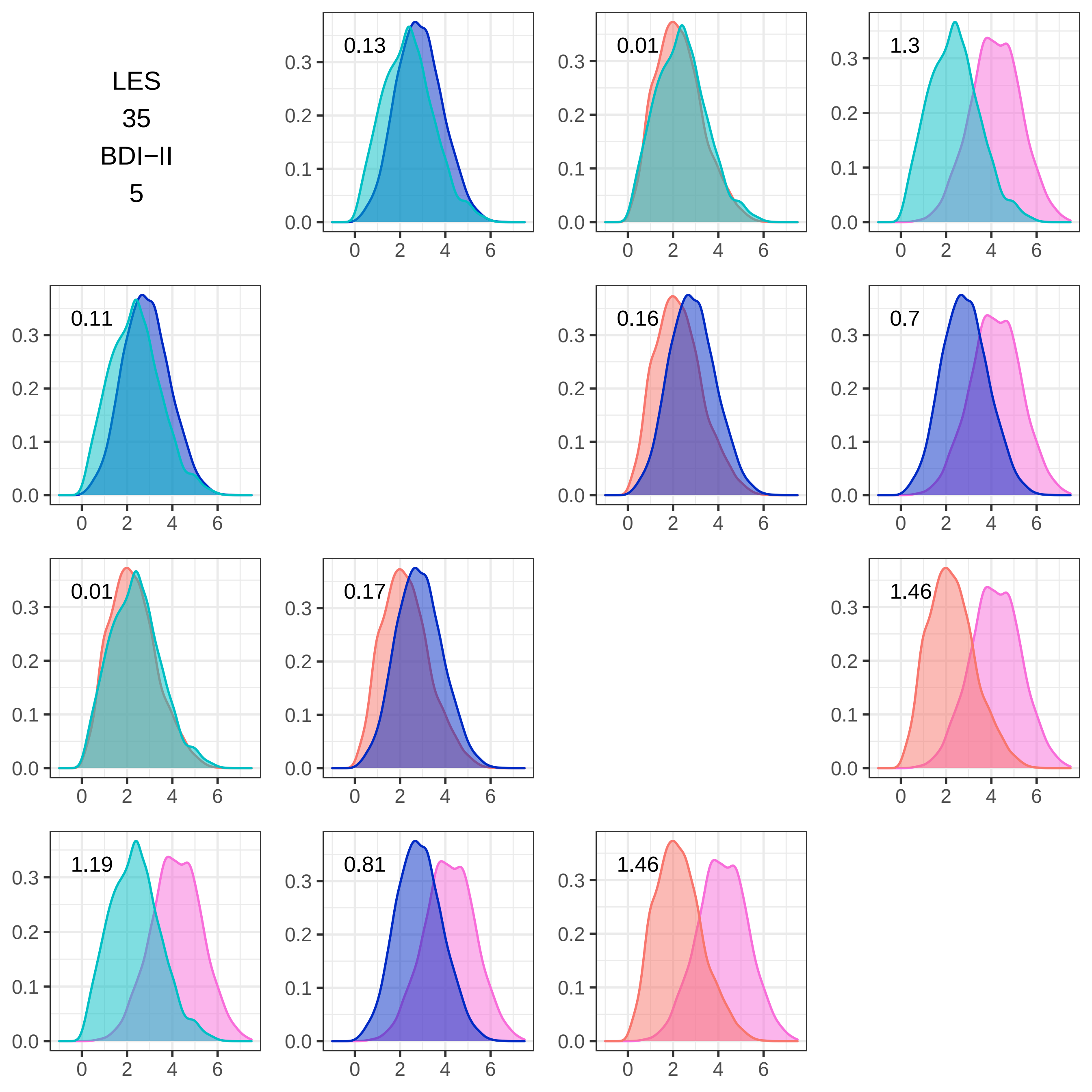

LES
35
BDI−II
5
0.13
0.01
1.3
0.11
0.16
0.7
0.01
0.17
1.46
1.19
0.81
1.46

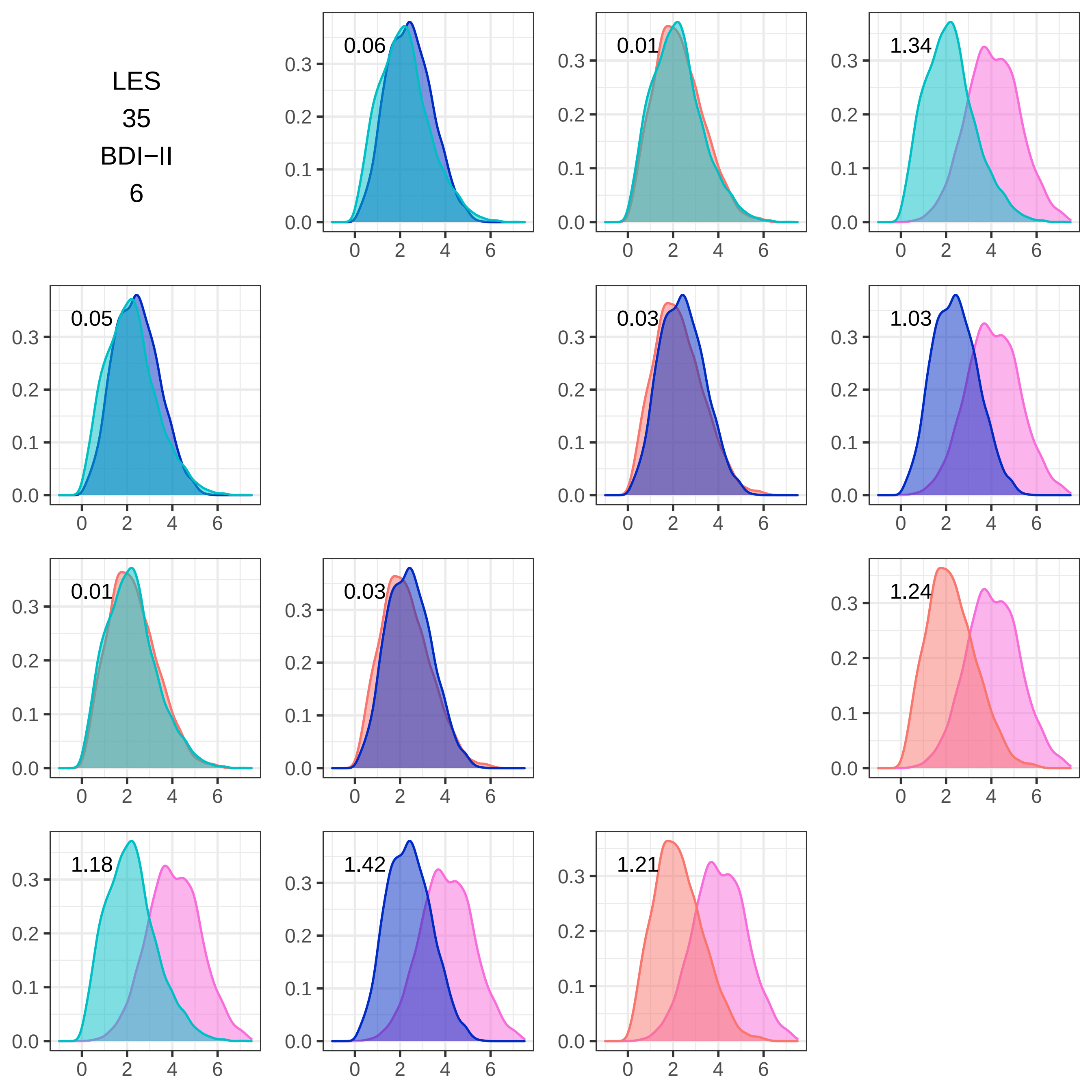
LES
35
BDI−II
6
0.06
0.01
1.34
0.05
0.03
1.03
0.01
0.03
1.24
1.18
1.42
1.21

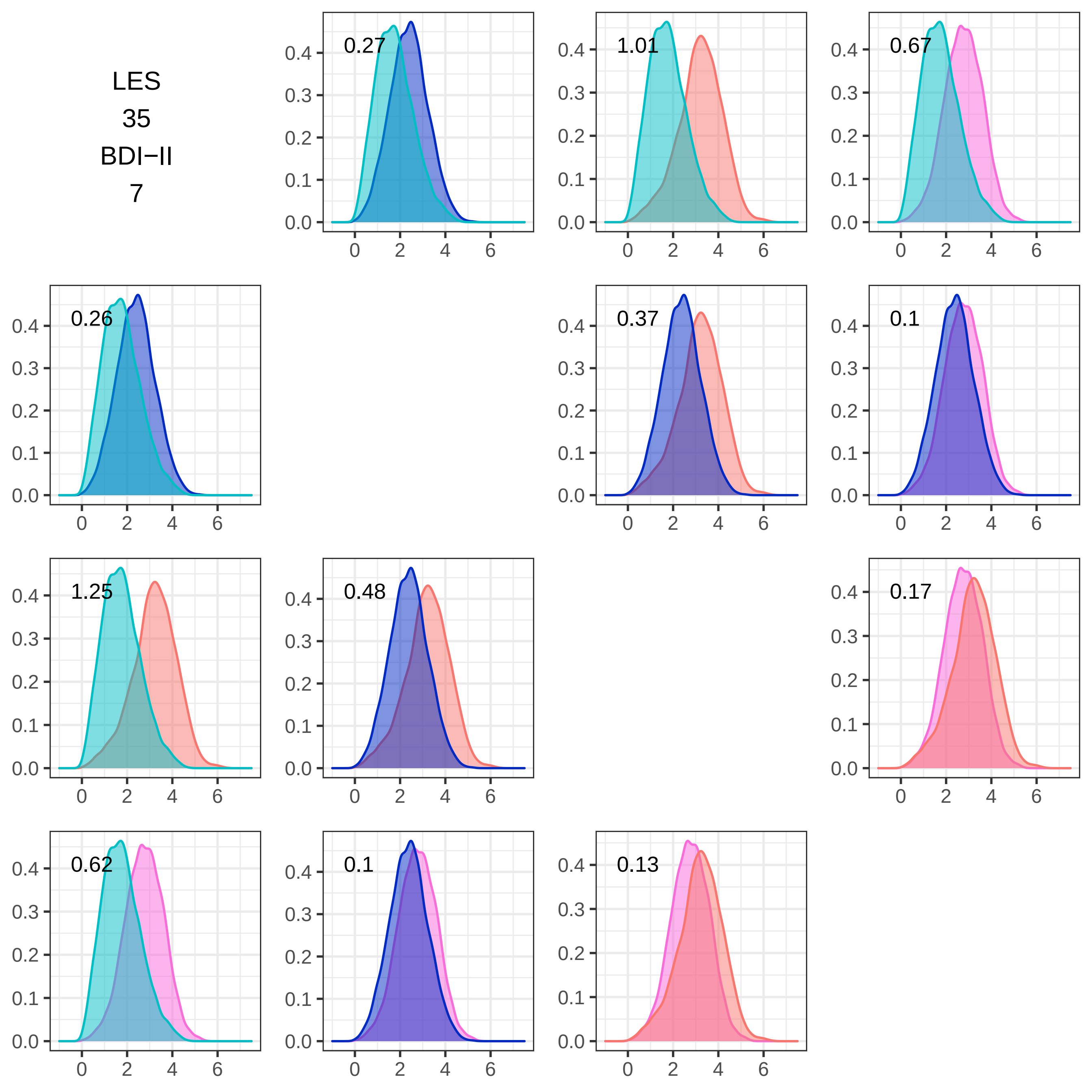
LES
35
BDI−II
7
0.27
1.01
0.67
0.26
0.37
0.1
1.25
0.48
0.17
0.62
0.1
0.13

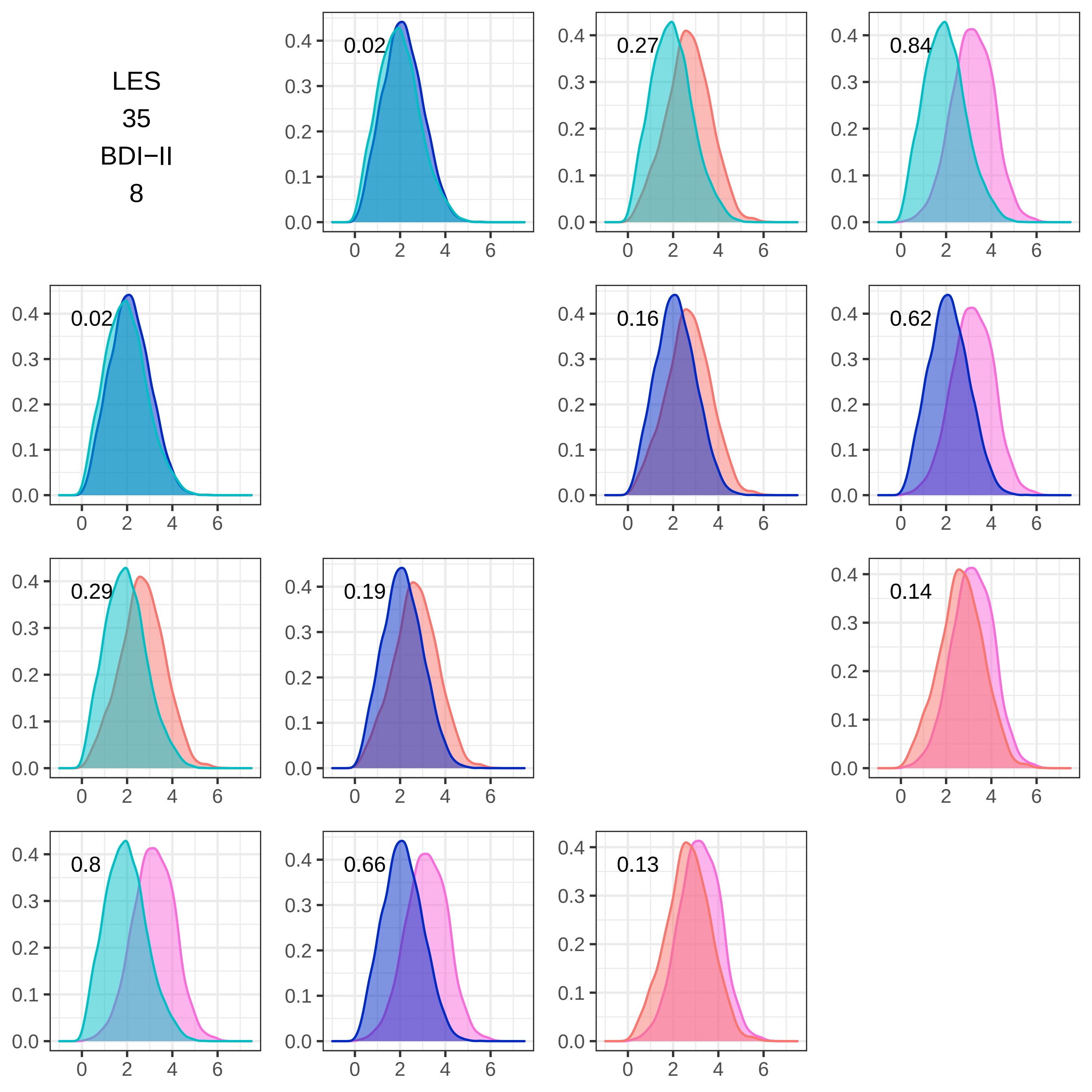
LES
35
BDI−II
8
0.02
0.27
0.84
0.02
0.16
0.62
0.29
0.19
0.14
0.8
0.66
0.13

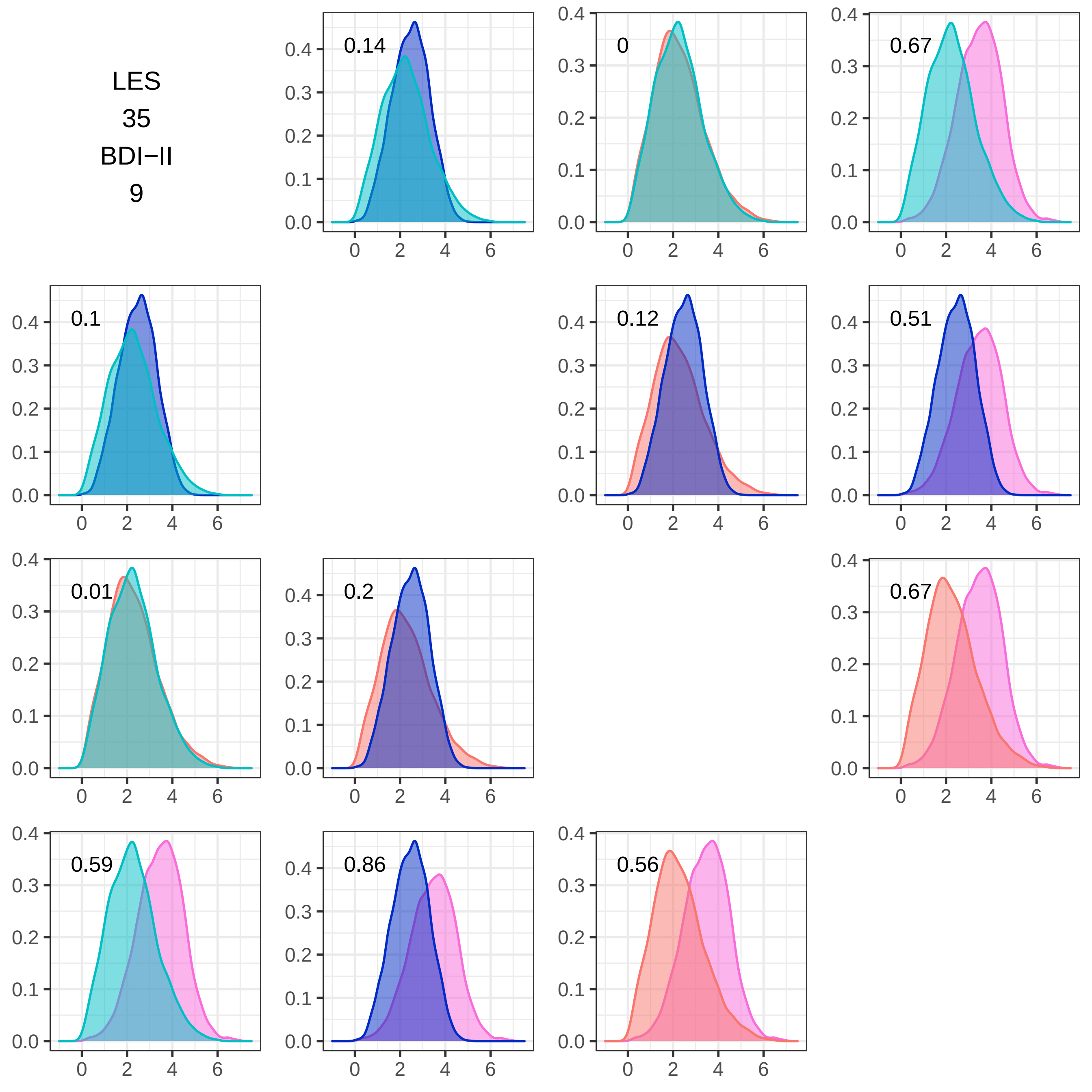

LES
35
BDI−II
9
0.14
0
0.67
0.1
0.12
0.51
0.01
0.2
0.67
0.59
0.86
0.56

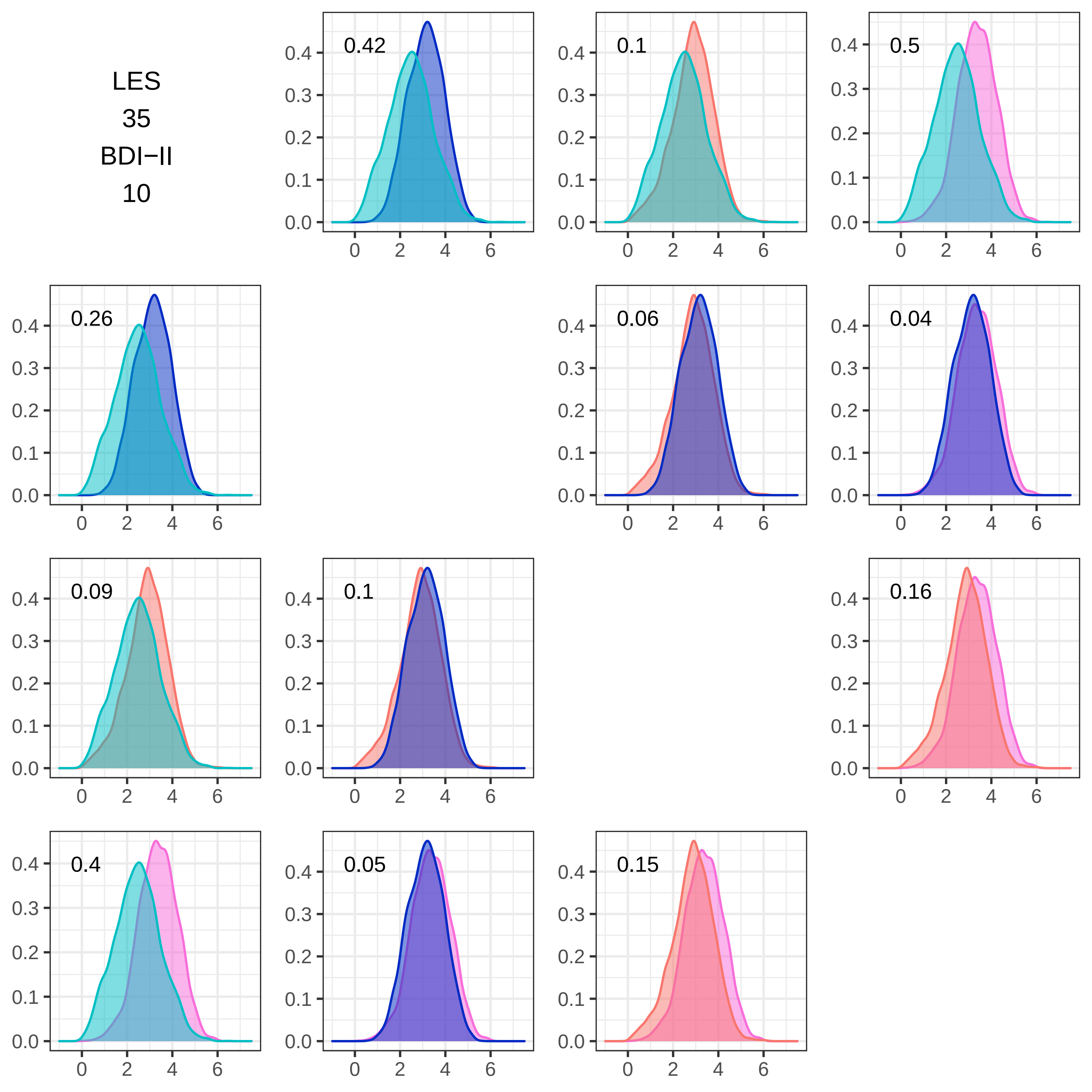

LES
35
BDI−II
10
0.42
0.1
0.5
0.26
0.06
0.04
0.09
0.1
0.16
0.4
0.05
0.15

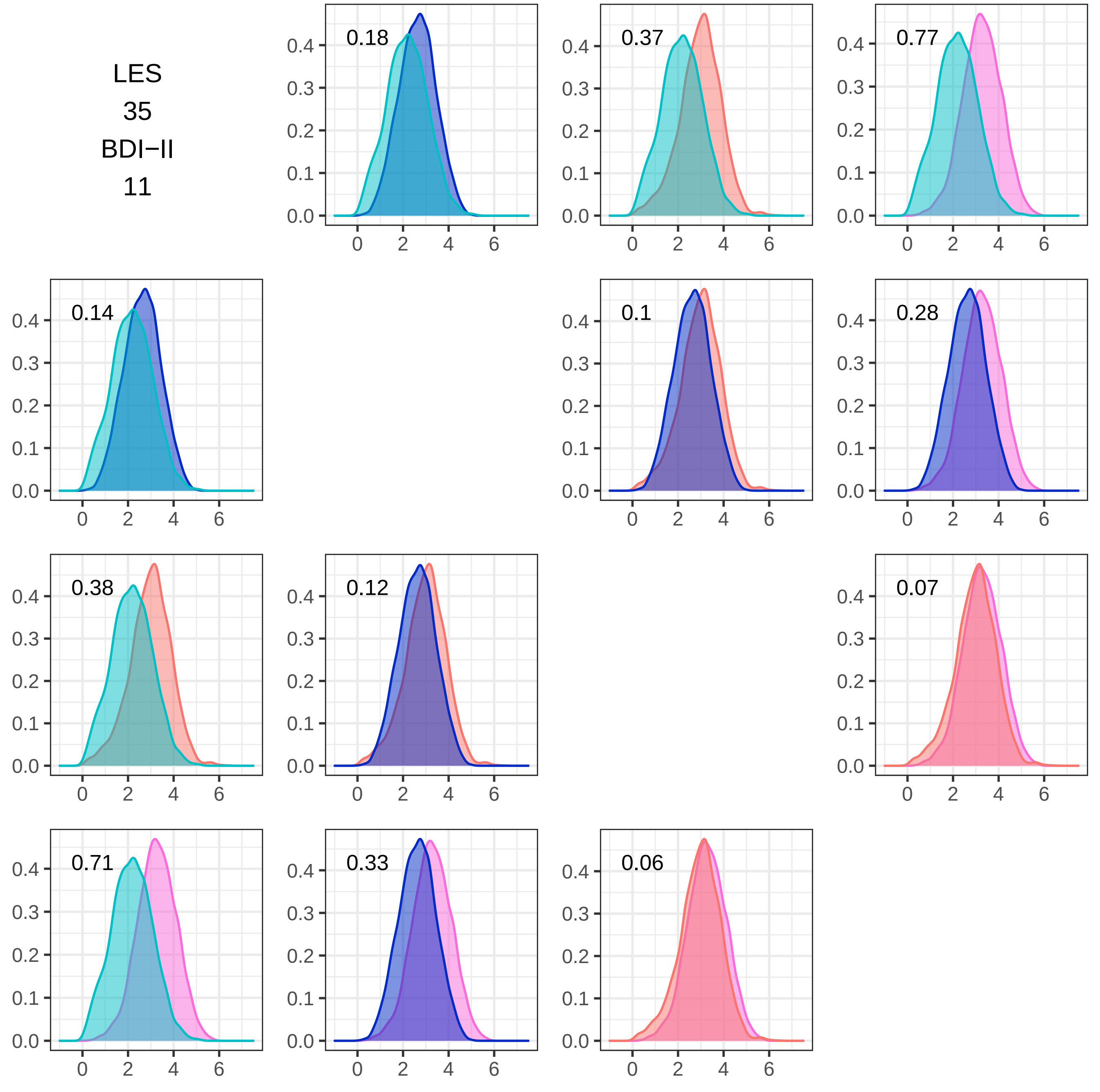

LES
35
BDI−II
11
0.18
0.37
0.77
0.14
0.1
0.28
0.38
0.12
0.07
0.71
0.33
0.06

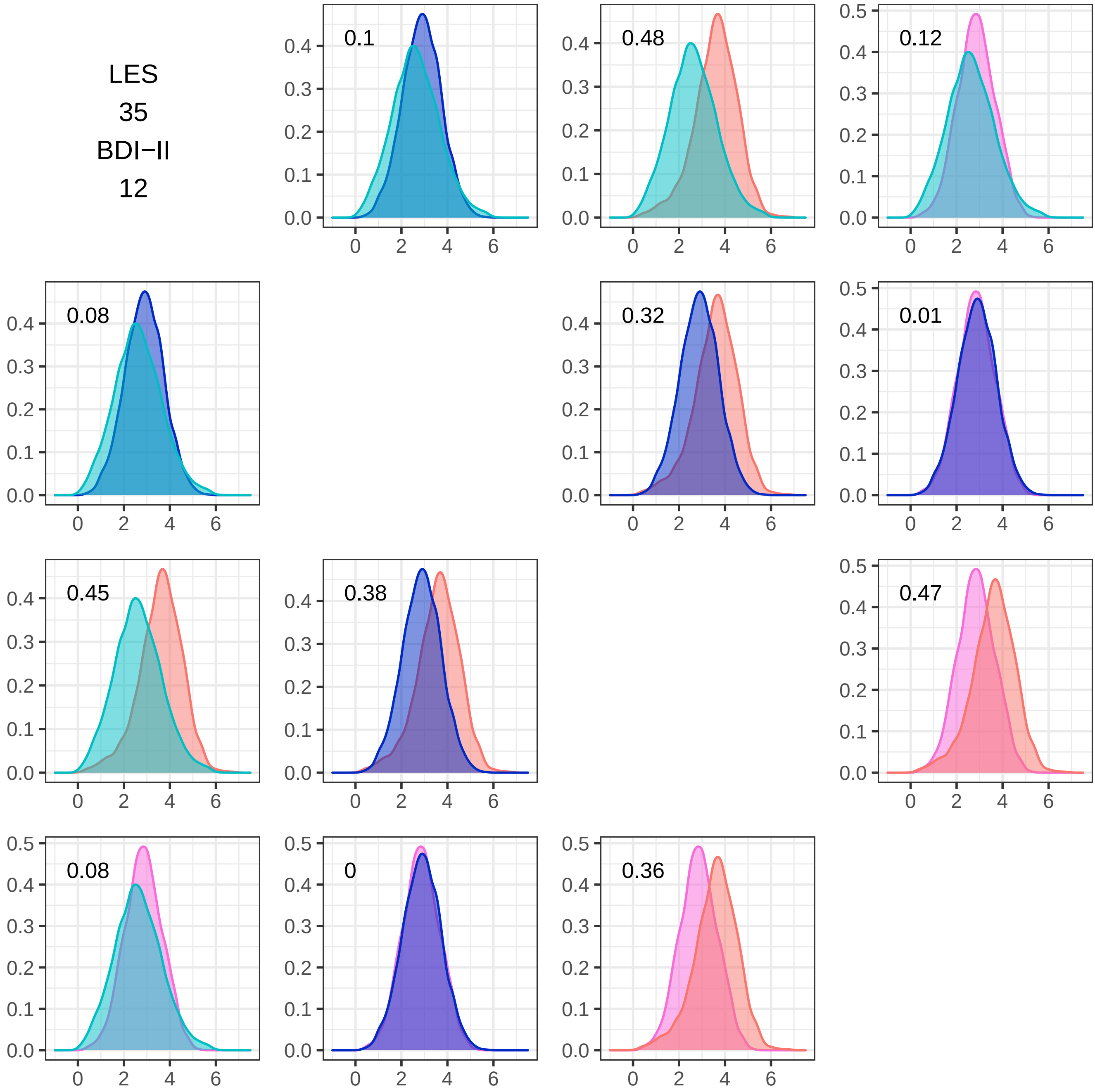
LES
35
BDI−II
12
0.1
0.48
0.12
0.08
0.32
0.01
0.45
0.38
0.47
0.08
0
0.36

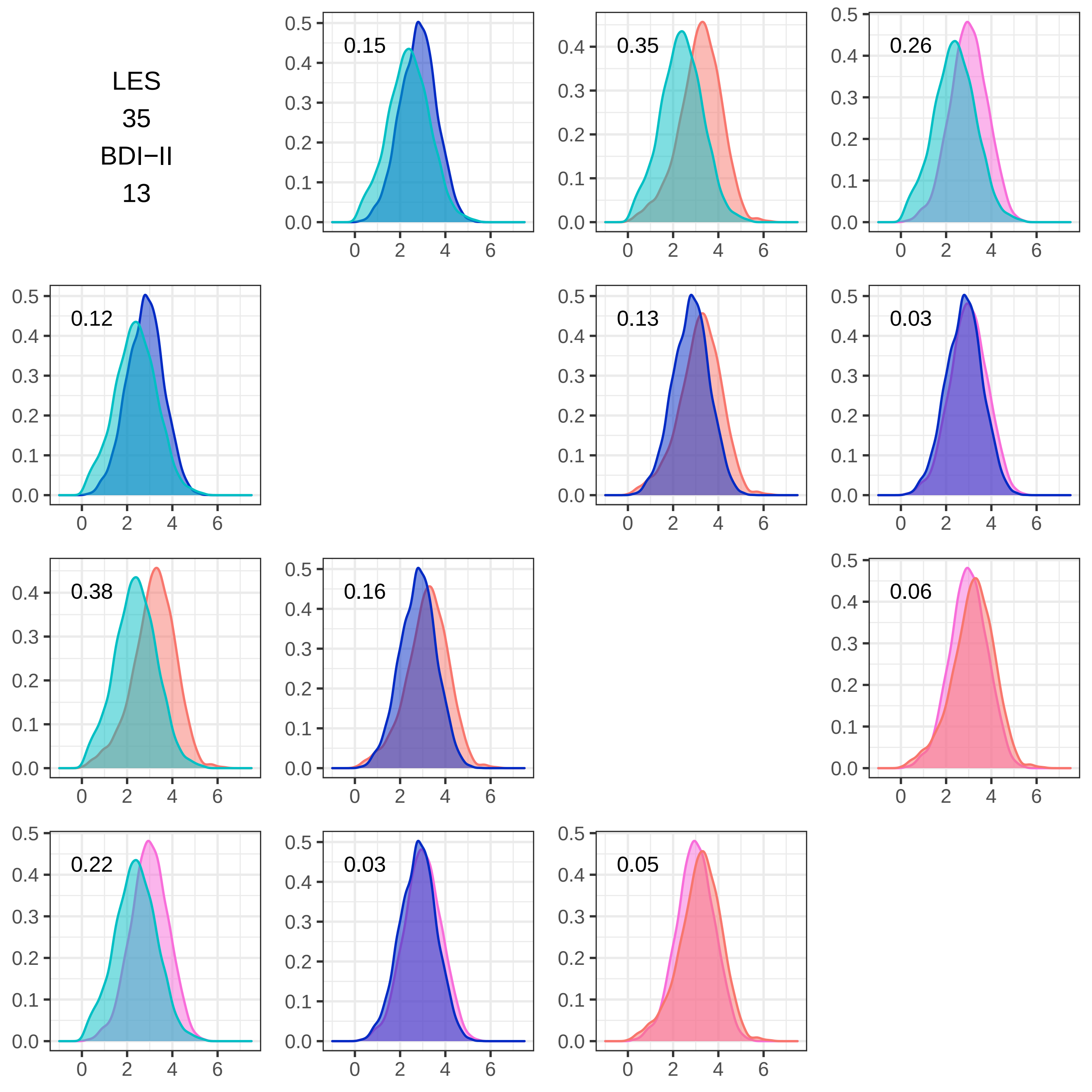

LES
35
BDI−II
13
0.15
0.35
0.26
0.12
0.13
0.03
0.38
0.16
0.06
0.22
0.03
0.05

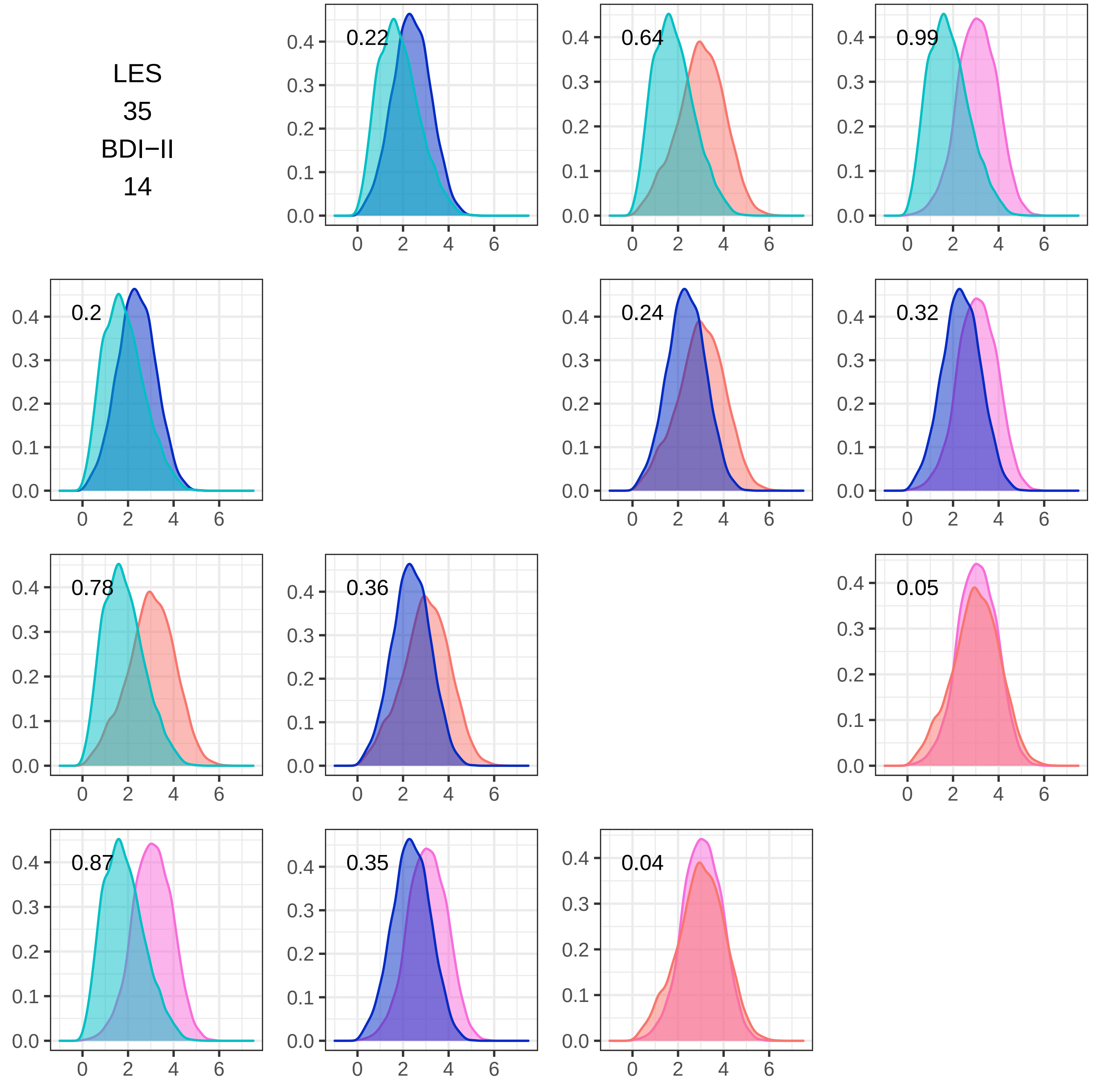
LES
35
BDI−II
14
0.22
0.64
0.99
0.2
0.24
0.32
0.78
0.36
0.05
0.87
0.35
0.04

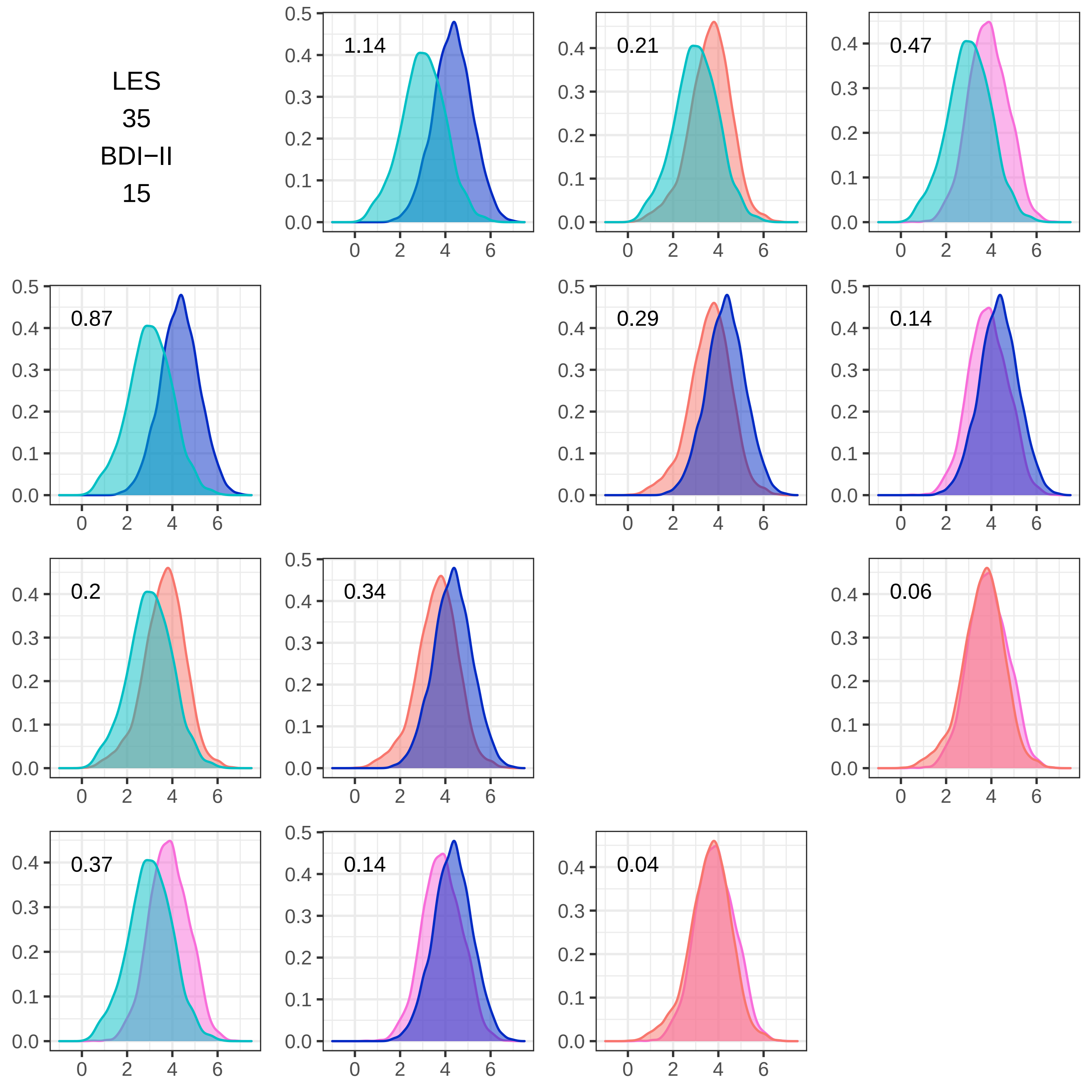

LES
35
BDI−II
15
1.14
0.21
0.47
0.87
0.29
0.14
0.2
0.34
0.06
0.37
0.14
0.04

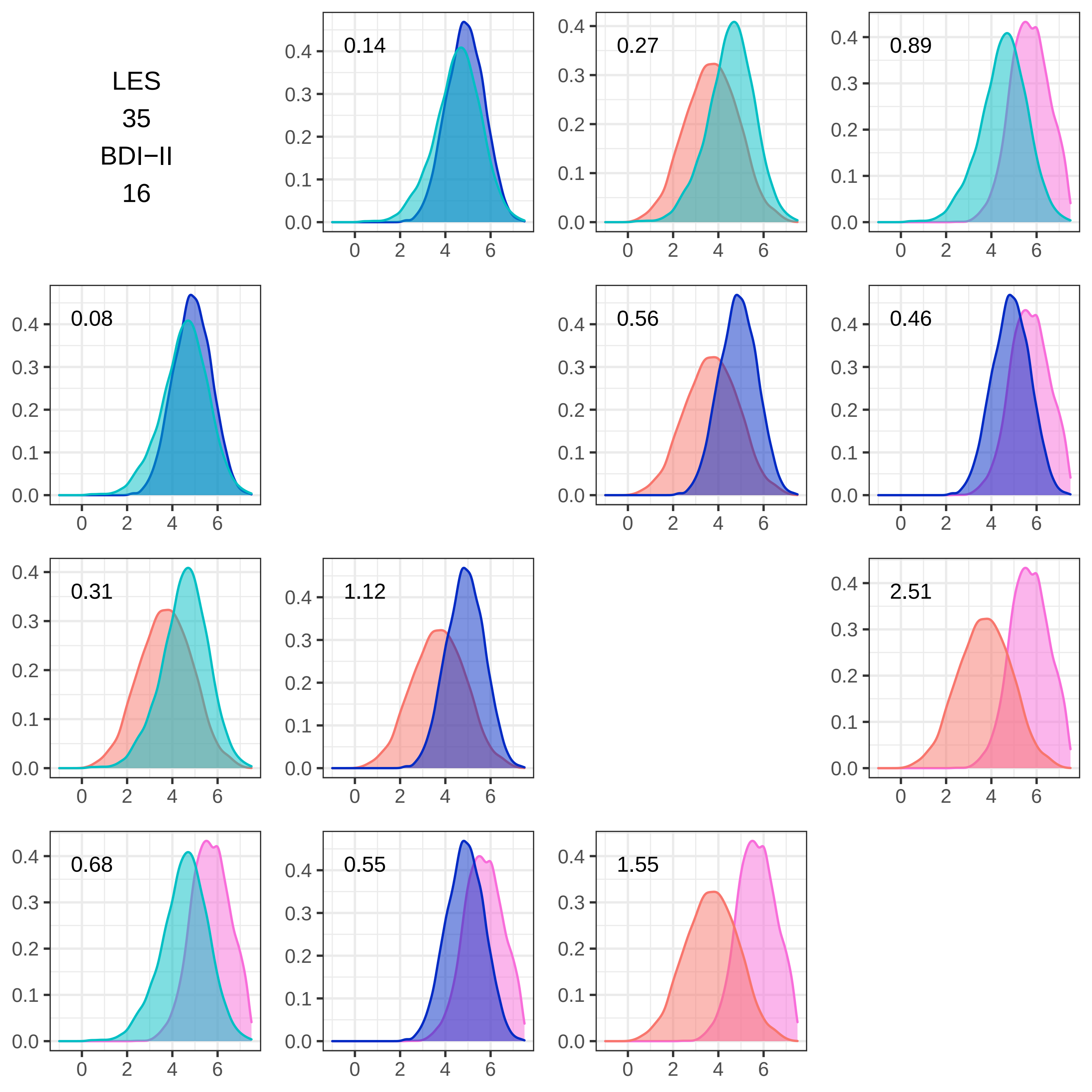

LES
35
BDI−II
16
0.14
0.27
0.89
0.08
0.56
0.46
0.31
1.12
2.51
0.68
0.55
1.55

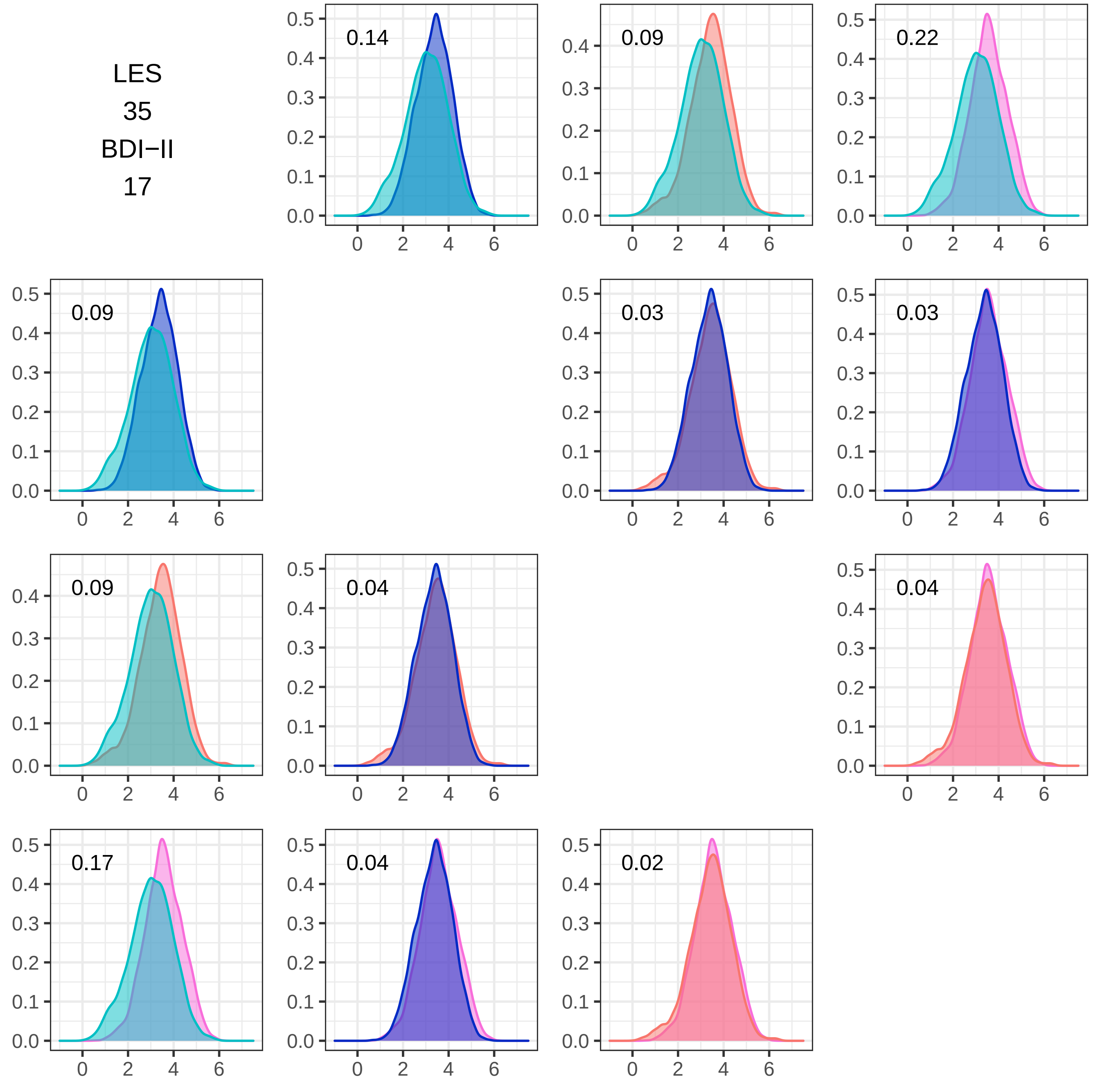
LES
35
BDI−II
17
0.14
0.09
0.22
0.09
0.03
0.03
0.09
0.04
0.04
0.17
0.04
0.02

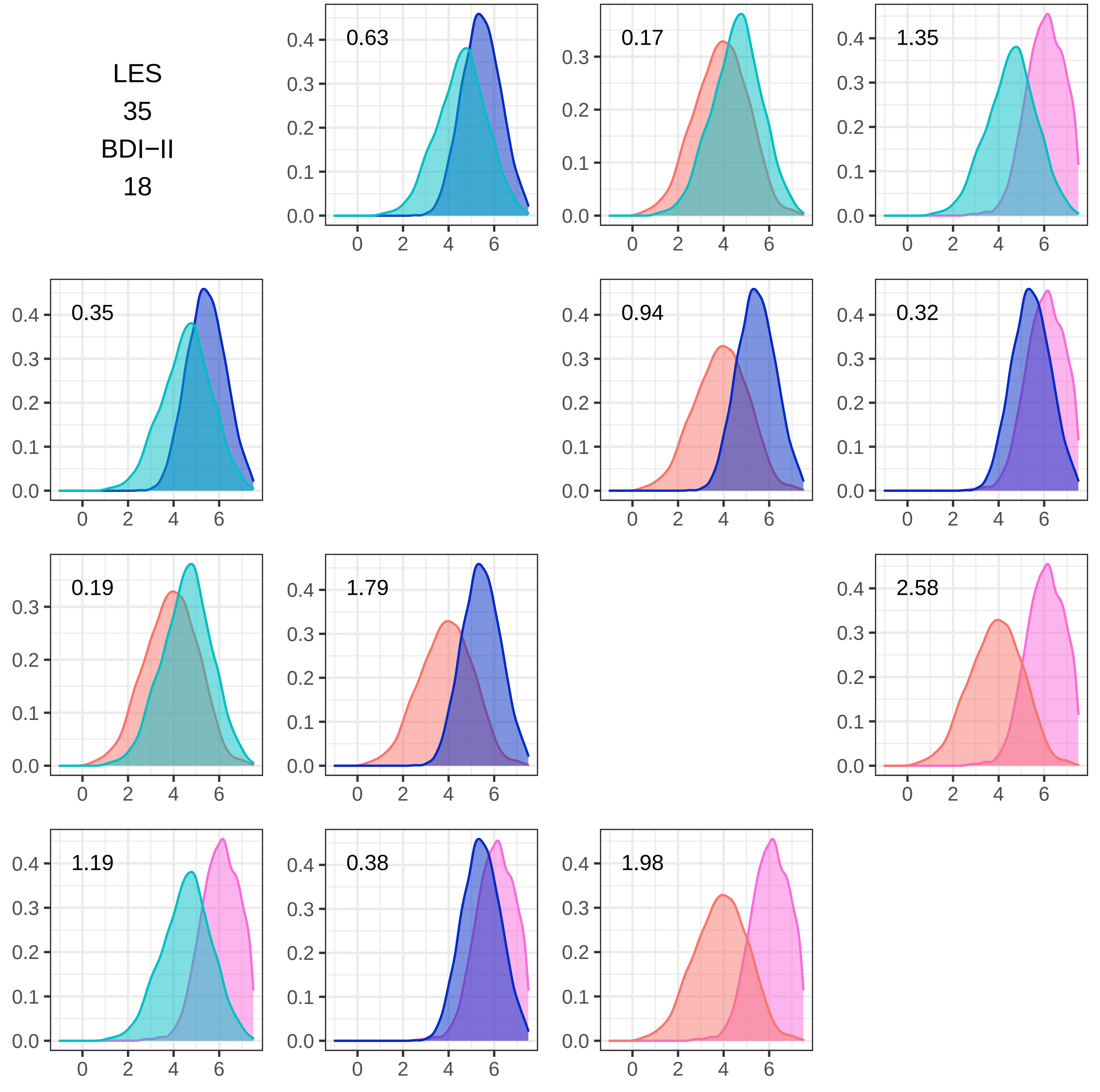
LES
35
BDI−II
18
0.63
0.17
1.35
0.35
0.94
0.32
0.19
1.79
2.58
1.19
0.38
1.98

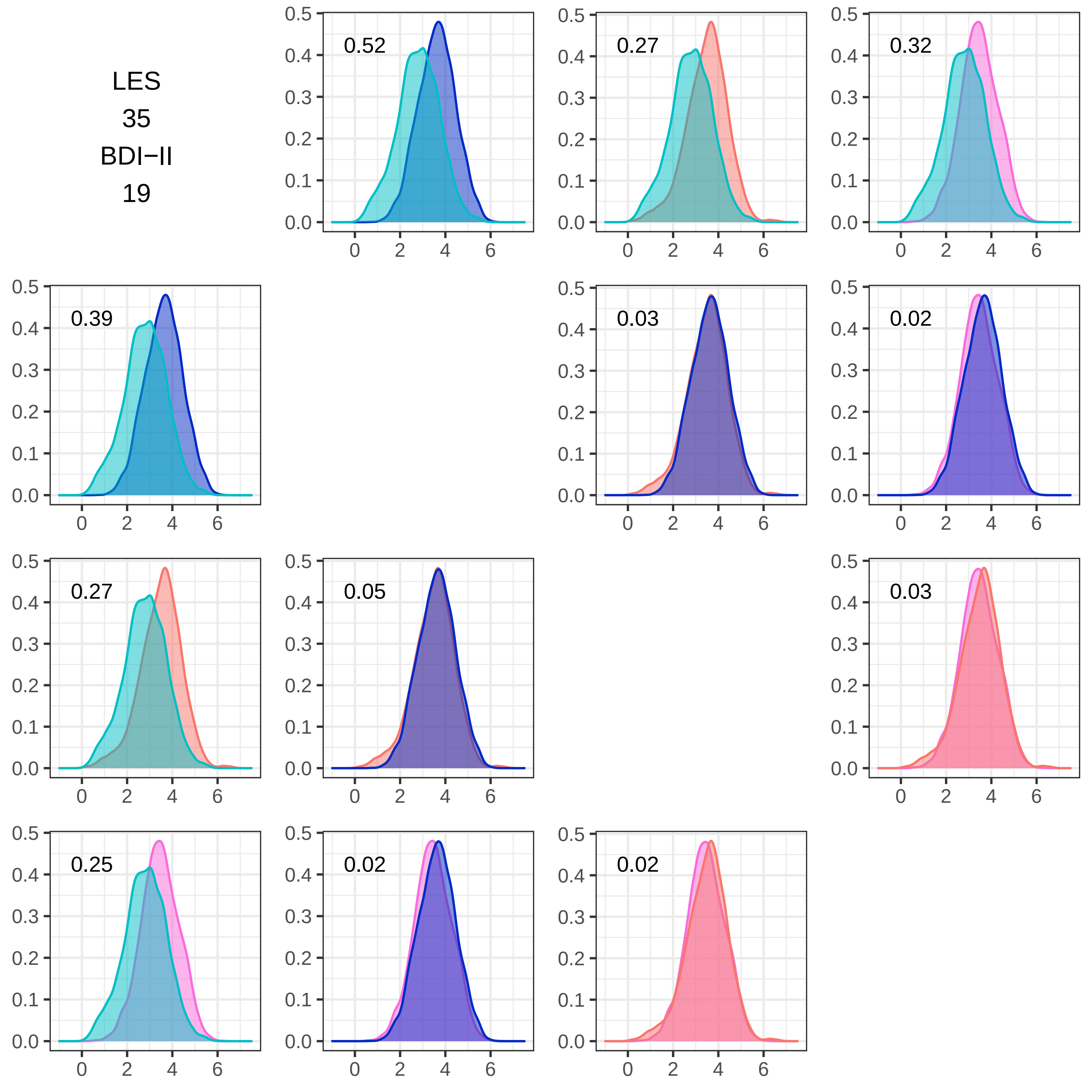
LES
35
BDI−II
19
0.52
0.27
0.32
0.39
0.03
0.02
0.27
0.05
0.03
0.25
0.02
0.02

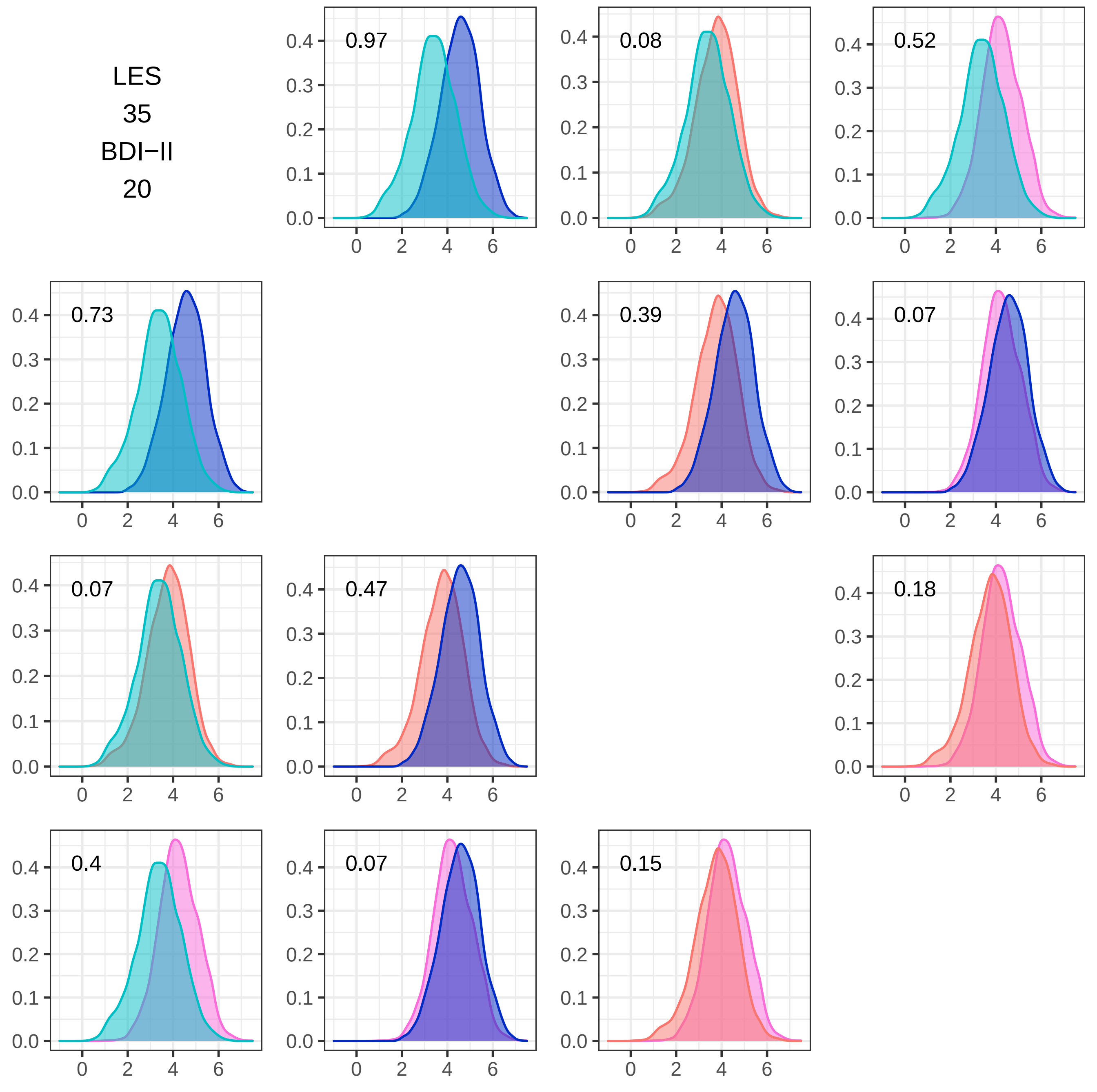
LES
35
BDI−II
20
0.97
0.08
0.52
0.73
0.39
0.07
0.07
0.47
0.18
0.4
0.07
0.15

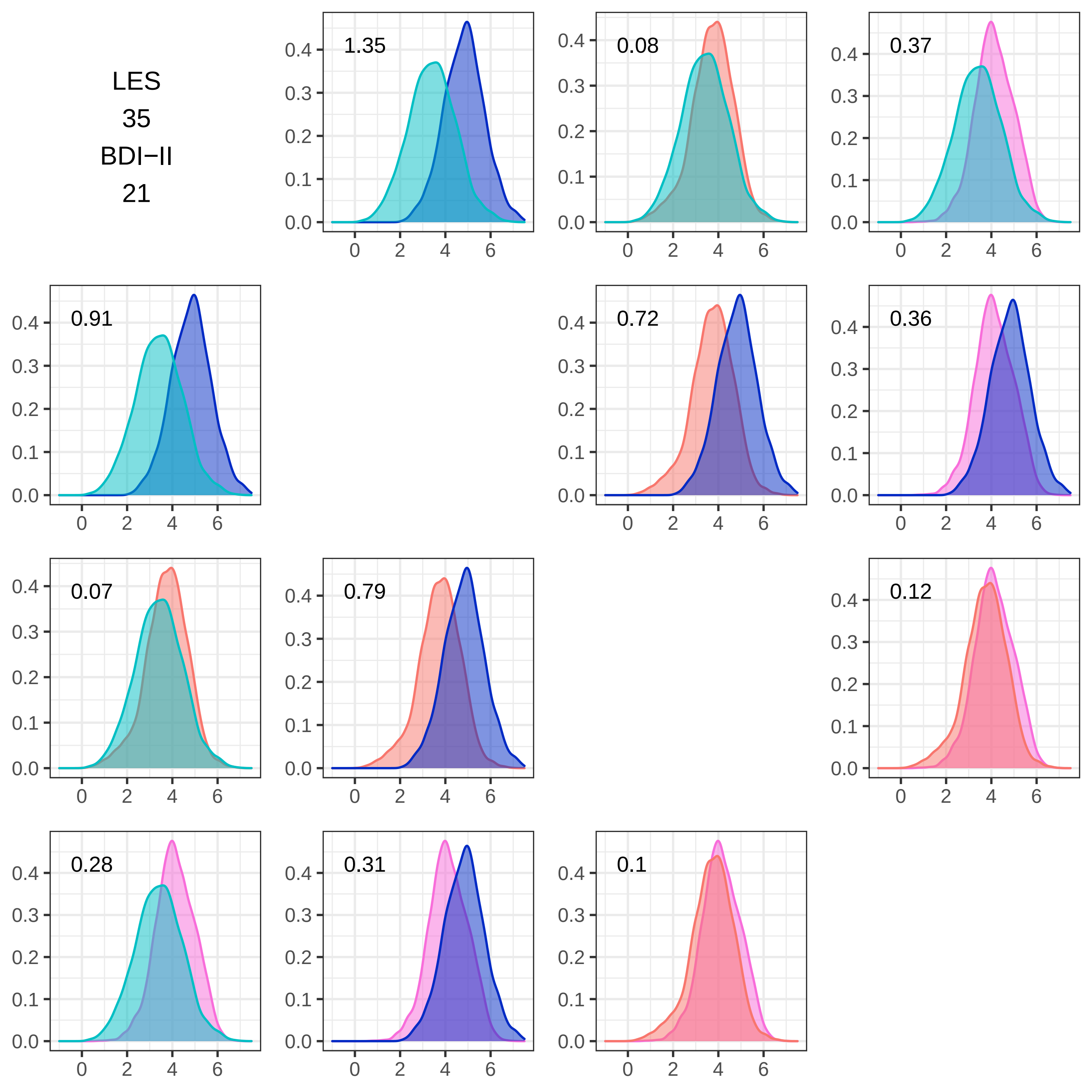

LES
35
BDI−II
21
1.35
0.08
0.37
0.91
0.72
0.36
0.07
0.79
0.12
0.28
0.31
0.1

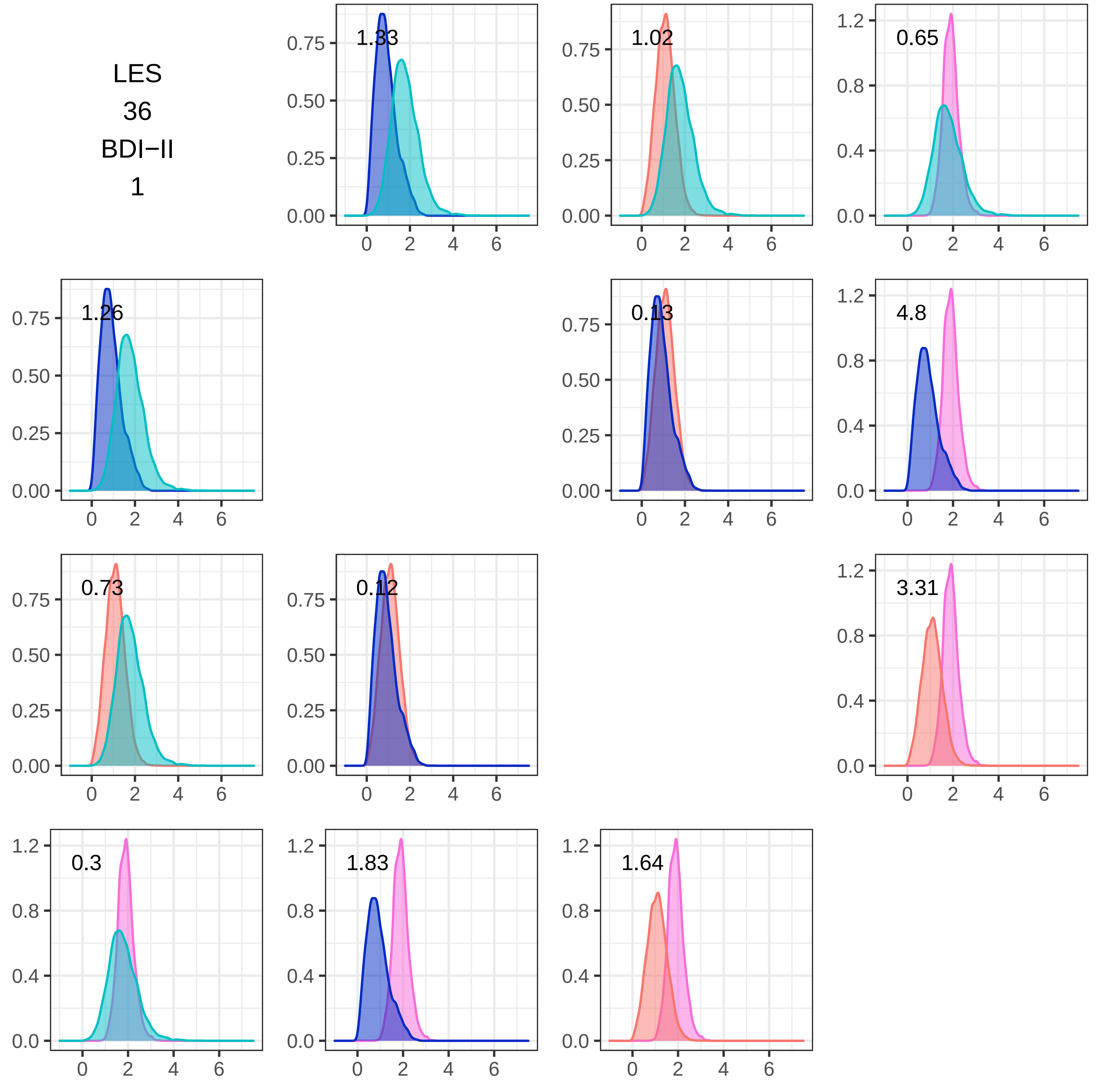
LES
36
BDI−II
1
1.33
1.02
0.65
1.26
0.13
4.8
0.73
0.12
3.31
0.3
1.83
1.64

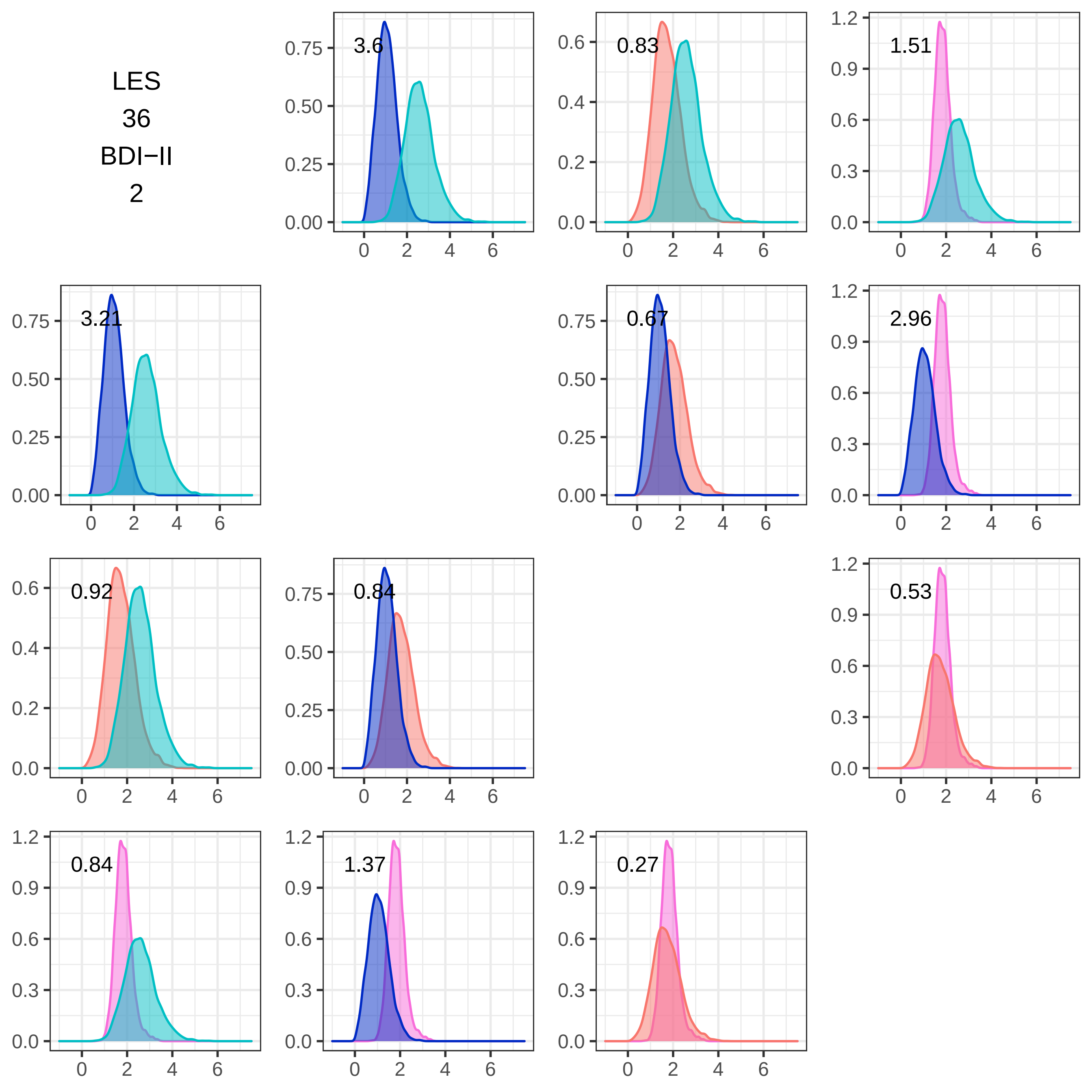
LES
36
BDI−II
2
3.6
0.83
1.51
3.21
0.67
2.96
0.92
0.84
0.53
0.84
1.37
0.27

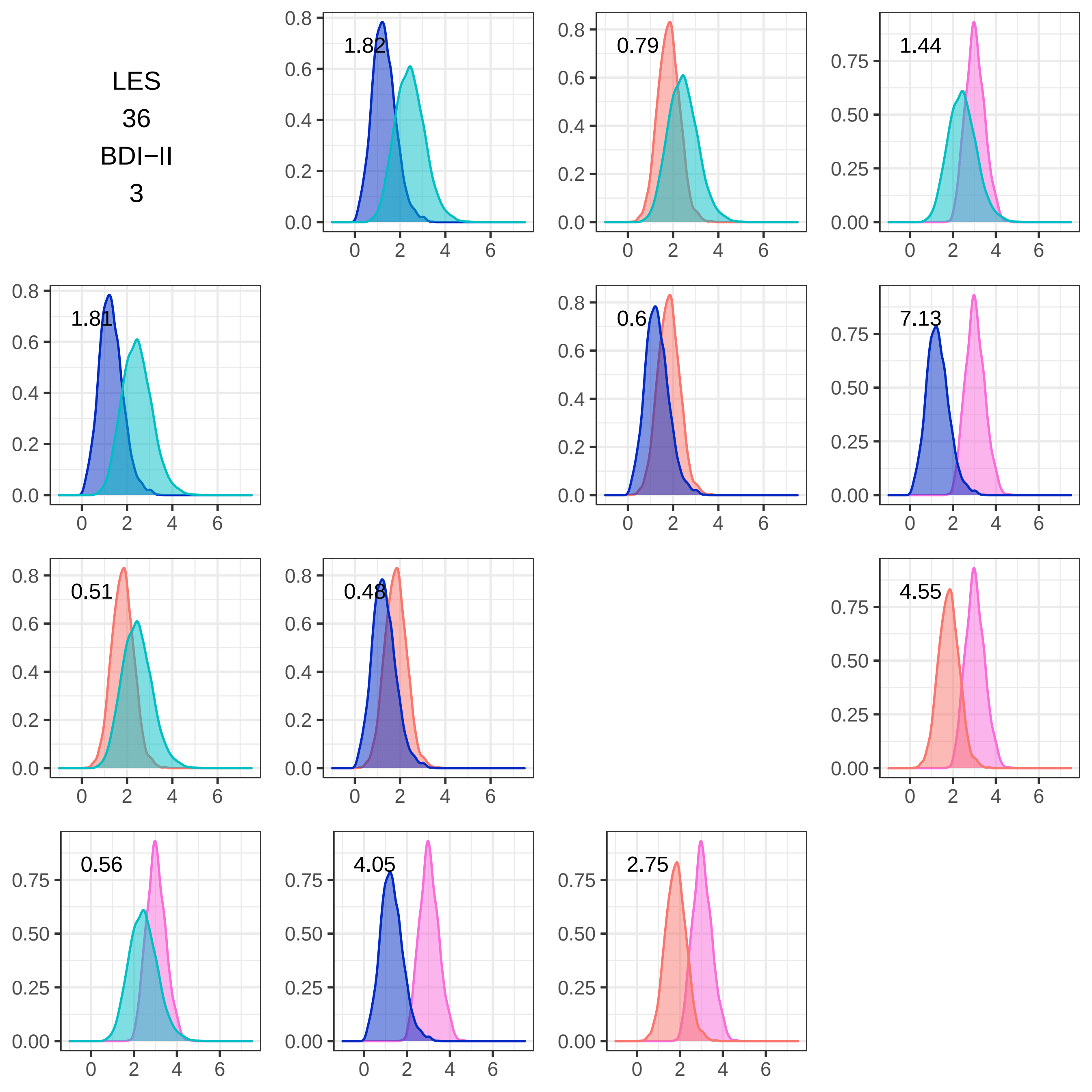

LES
36
BDI−II
3
1.82
0.79
1.44
1.81
0.6
7.13
0.51
0.48
4.55
0.56
4.05
2.75

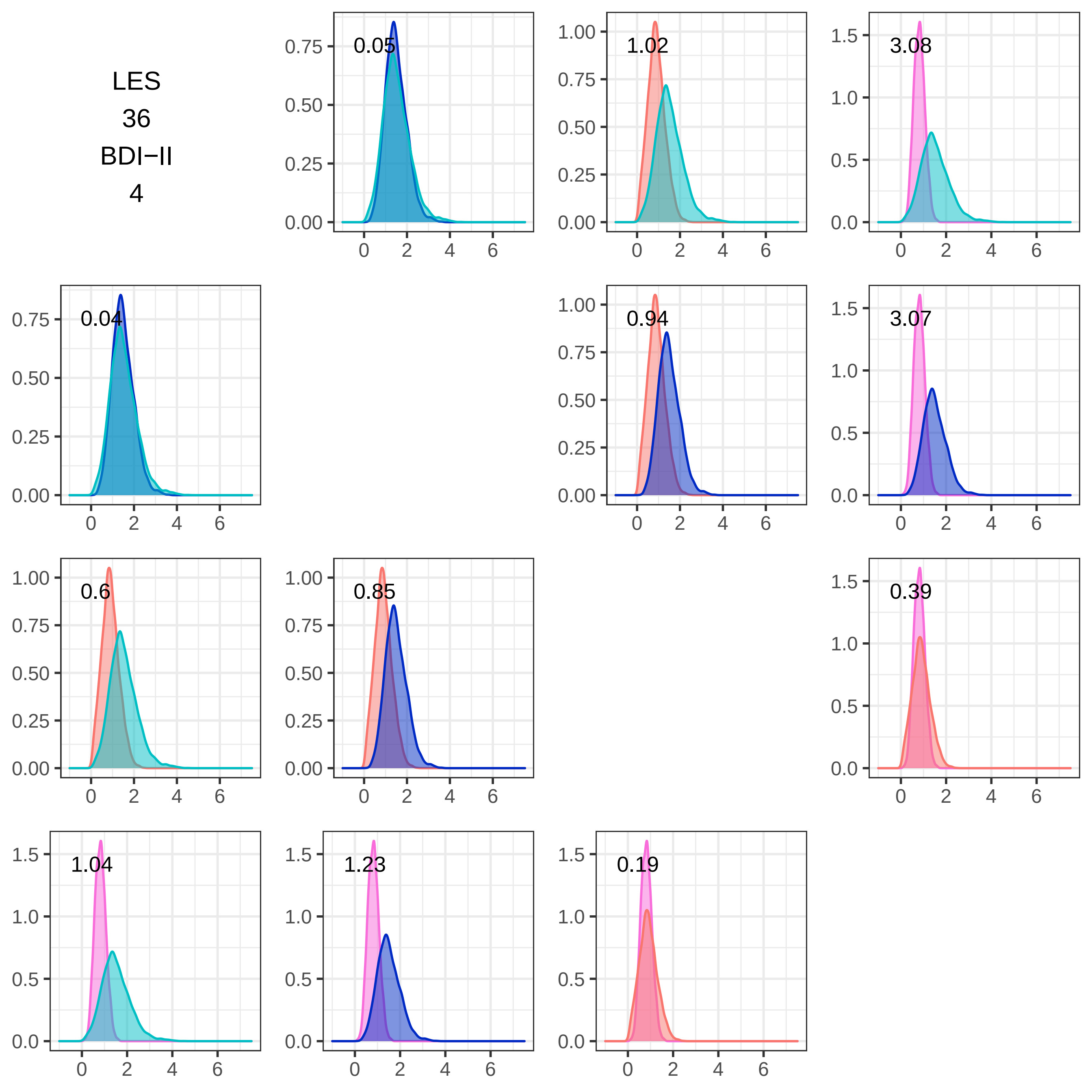

LES
36
BDI−II
4
0.05
1.02
3.08
0.04
0.94
3.07
0.6
0.85
0.39
1.04
1.23
0.19

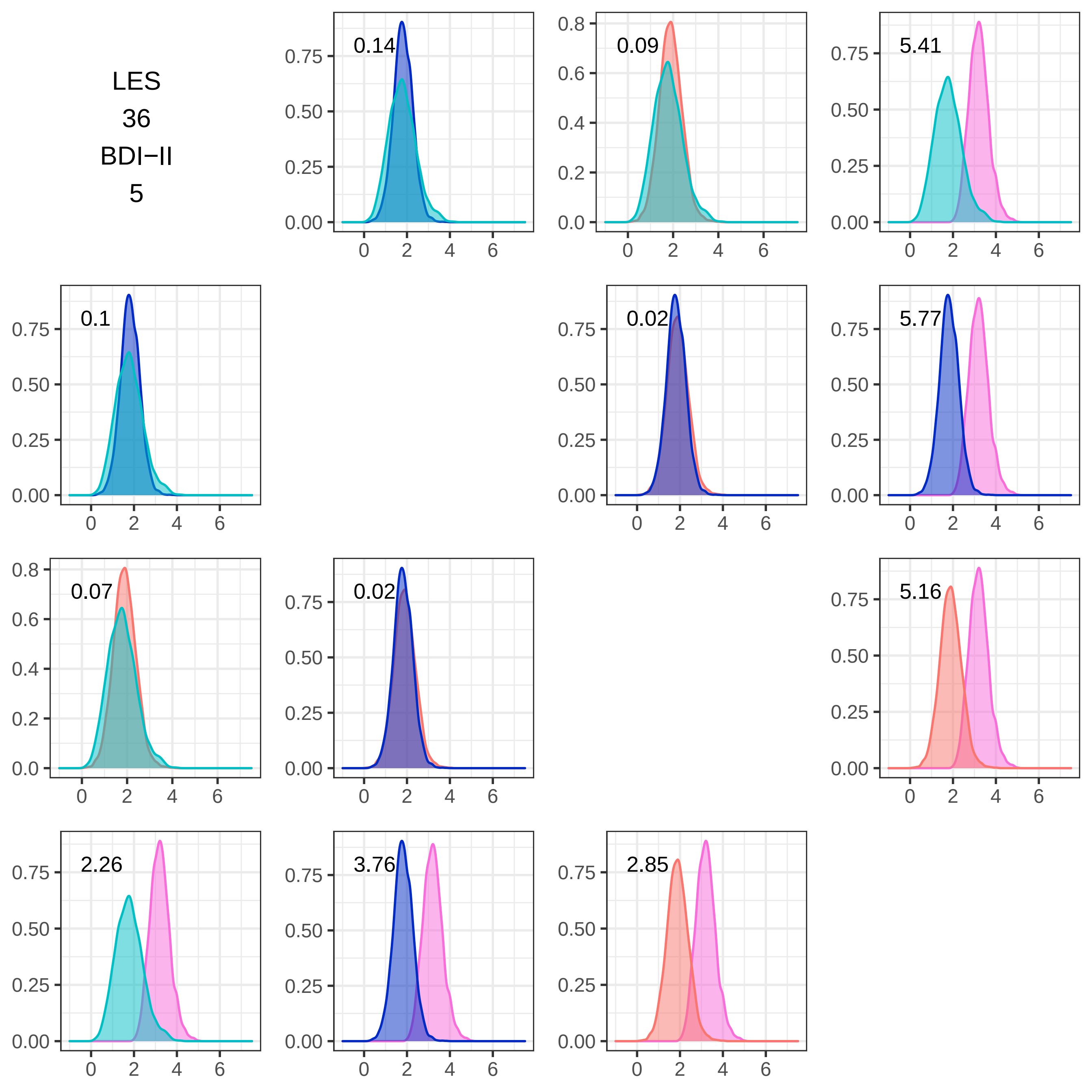
LES
36
BDI−II
5
0.14
0.09
5.41
0.1
0.02
5.77
0.07
0.02
5.16
2.26
3.76
2.85

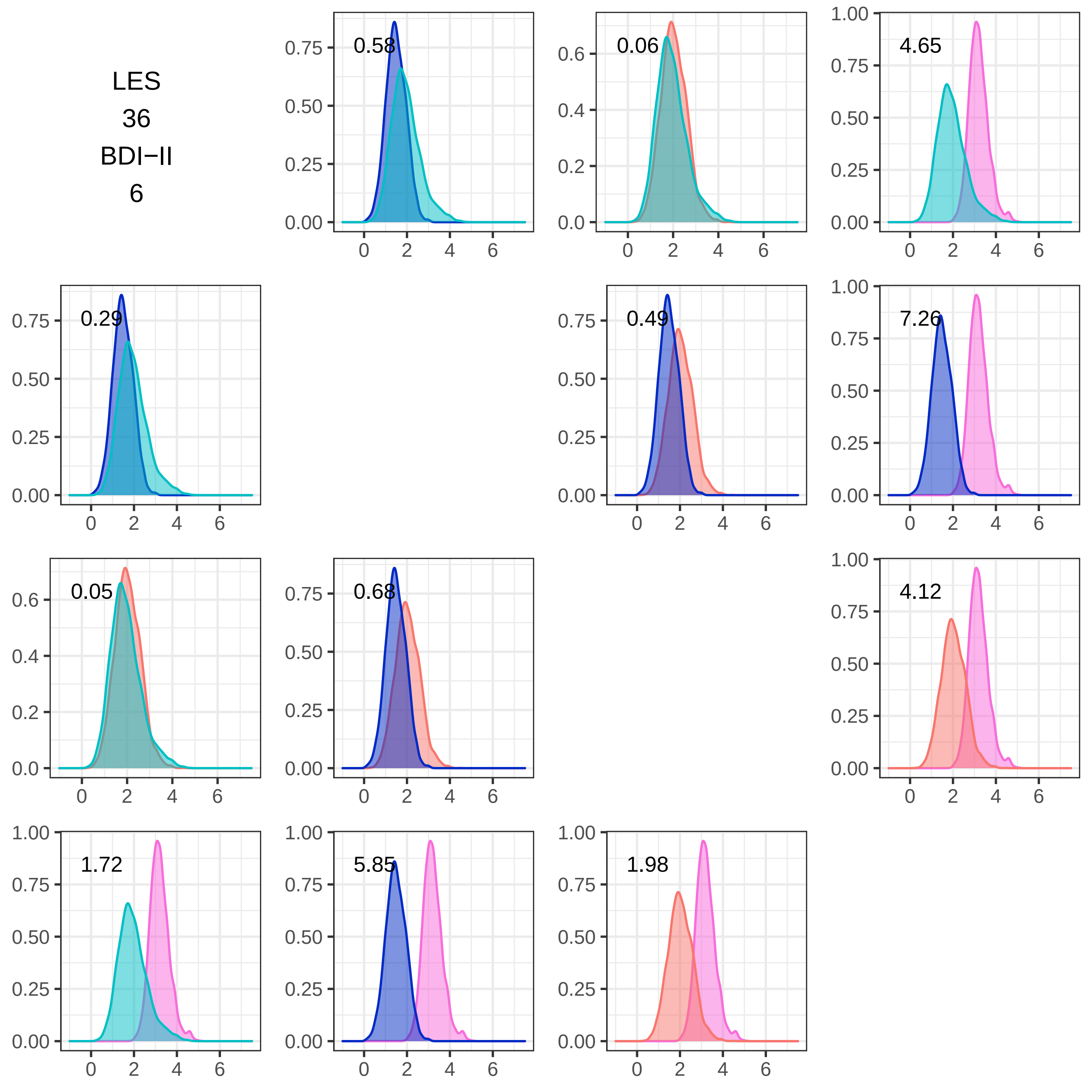

LES
36
BDI−II
6
0.58
0.06
4.65
0.29
0.49
7.26
0.05
0.68
4.12
1.72
5.85
1.98

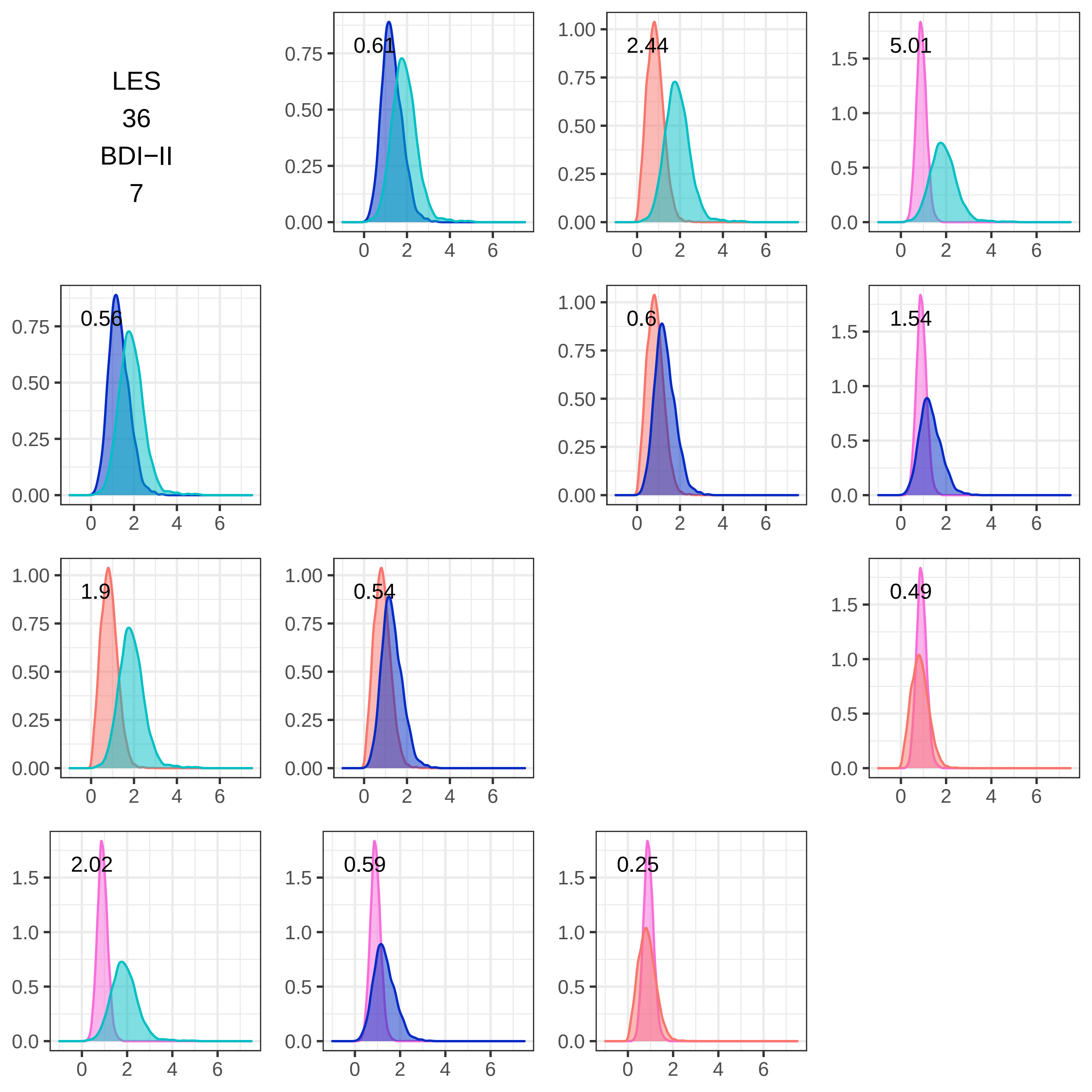

LES
36
BDI−II
7
0.61
2.44
5.01
0.56
0.6
1.54
1.9
0.54
0.49
2.02
0.59
0.25

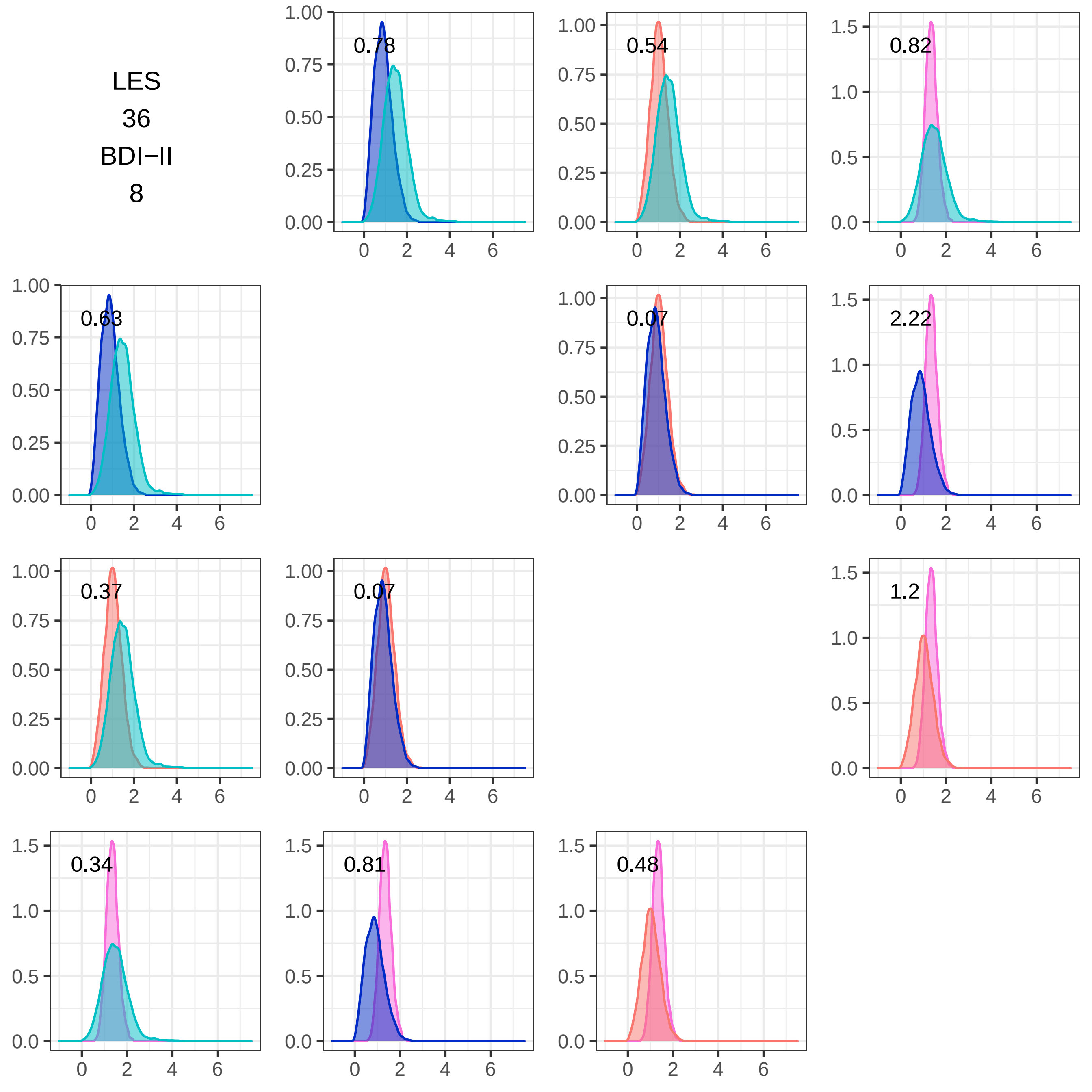
LES
36
BDI−II
8
0.78
0.54
0.82
0.63
0.07
2.22
0.37
0.07
1.2
0.34
0.81
0.48

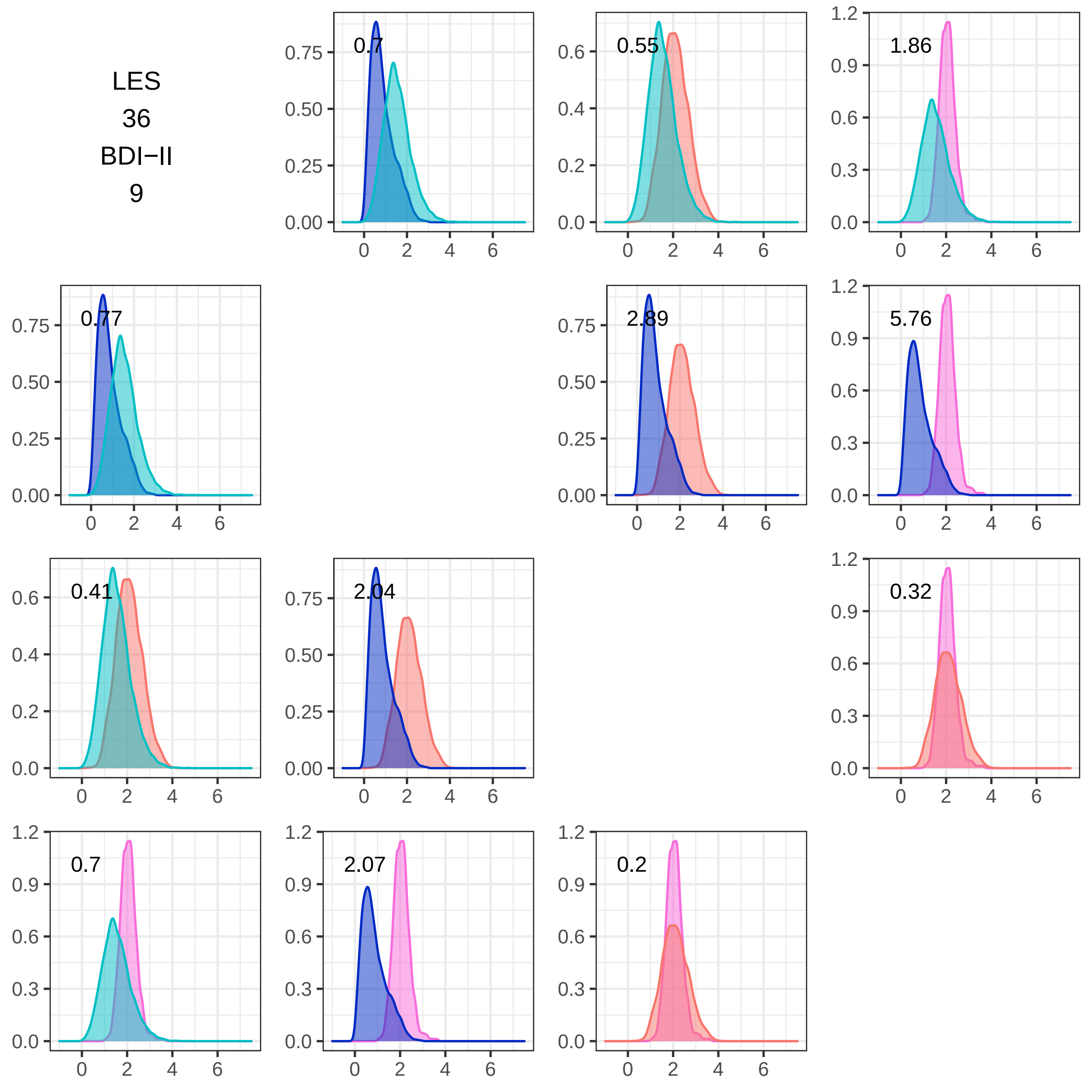

LES
36
BDI−II
9
0.7
0.55
1.86
0.77
2.89
5.76
0.41
2.04
0.32
0.7
2.07
0.2

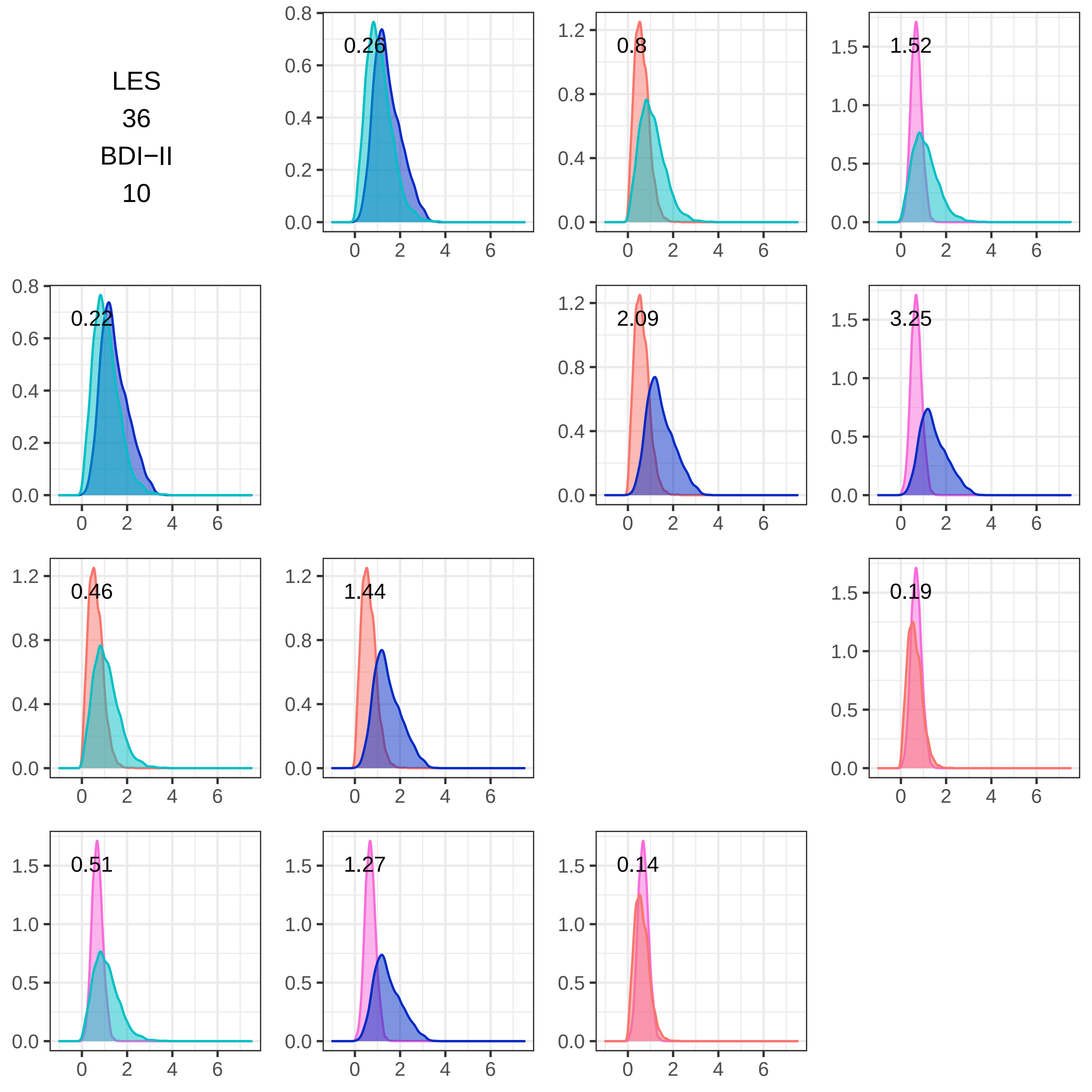
LES
36
BDI−II
10
0.26
0.8
1.52
0.22
2.09
3.25
0.46
1.44
0.19
0.51
1.27
0.14

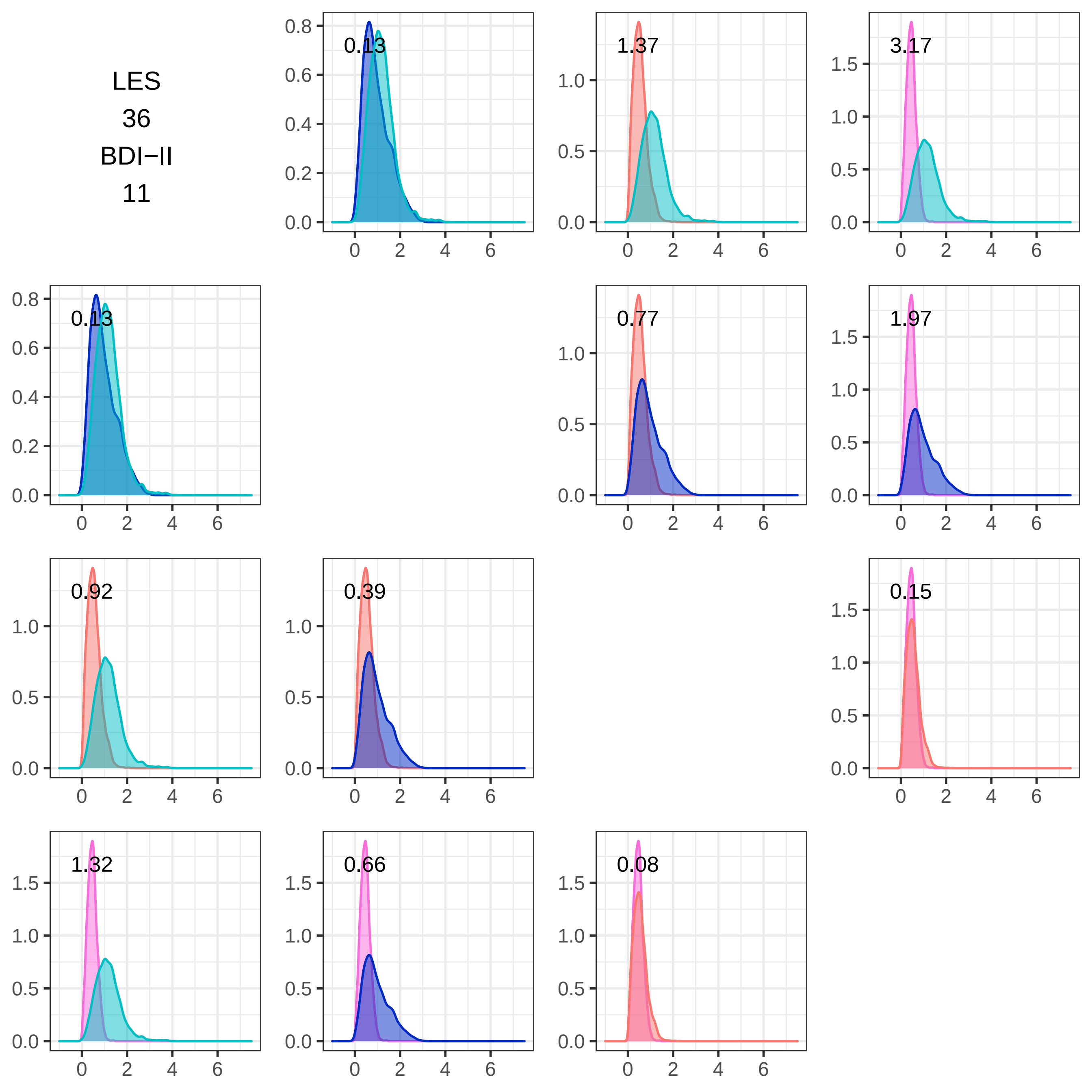

LES
36
BDI−II
11
0.13
1.37
3.17
0.13
0.77
1.97
0.92
0.39
0.15
1.32
0.66
0.08

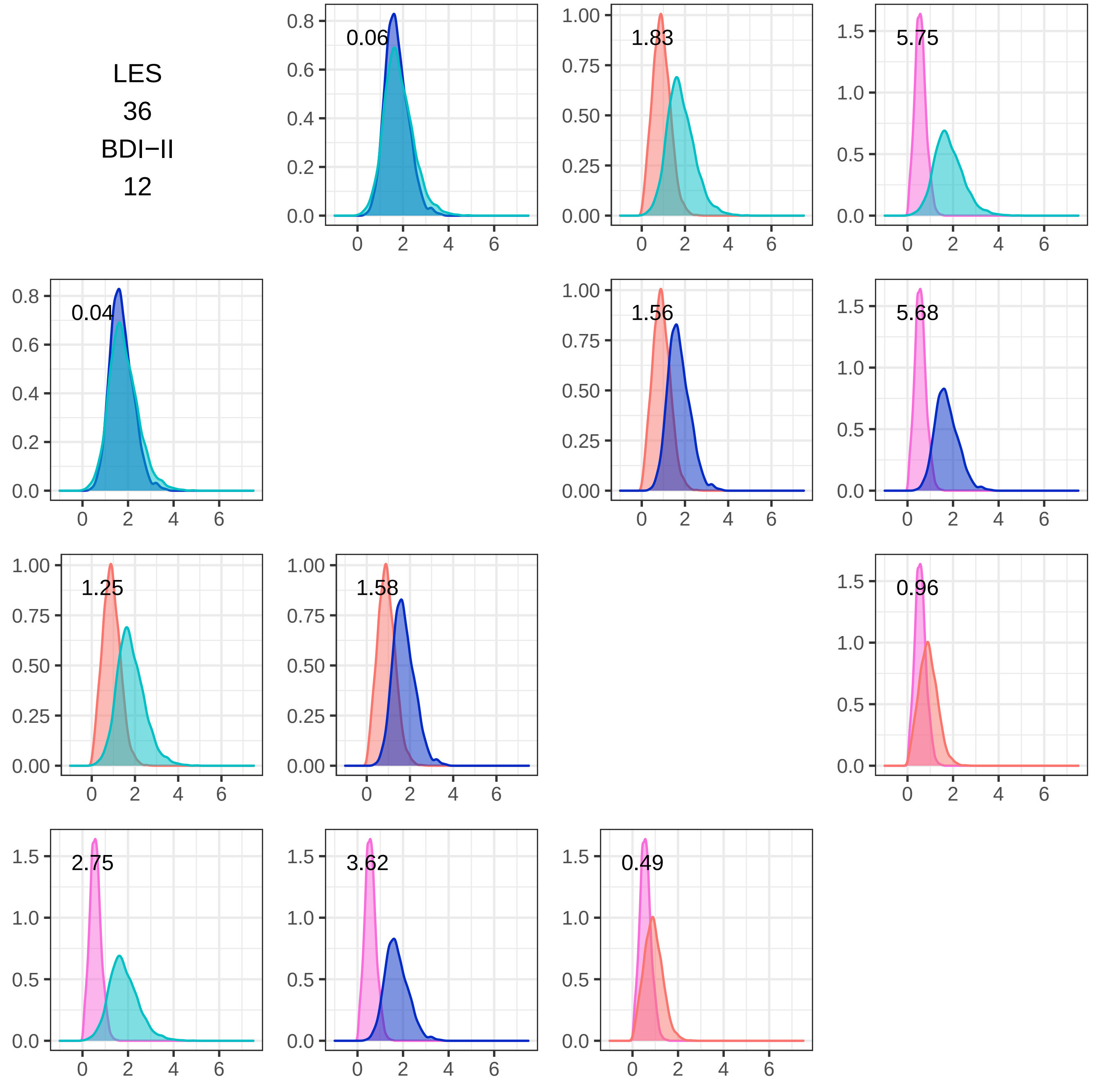
LES
36
BDI−II
12
0.06
1.83
5.75
0.04
1.56
5.68
1.25
1.58
0.96
2.75
3.62
0.49

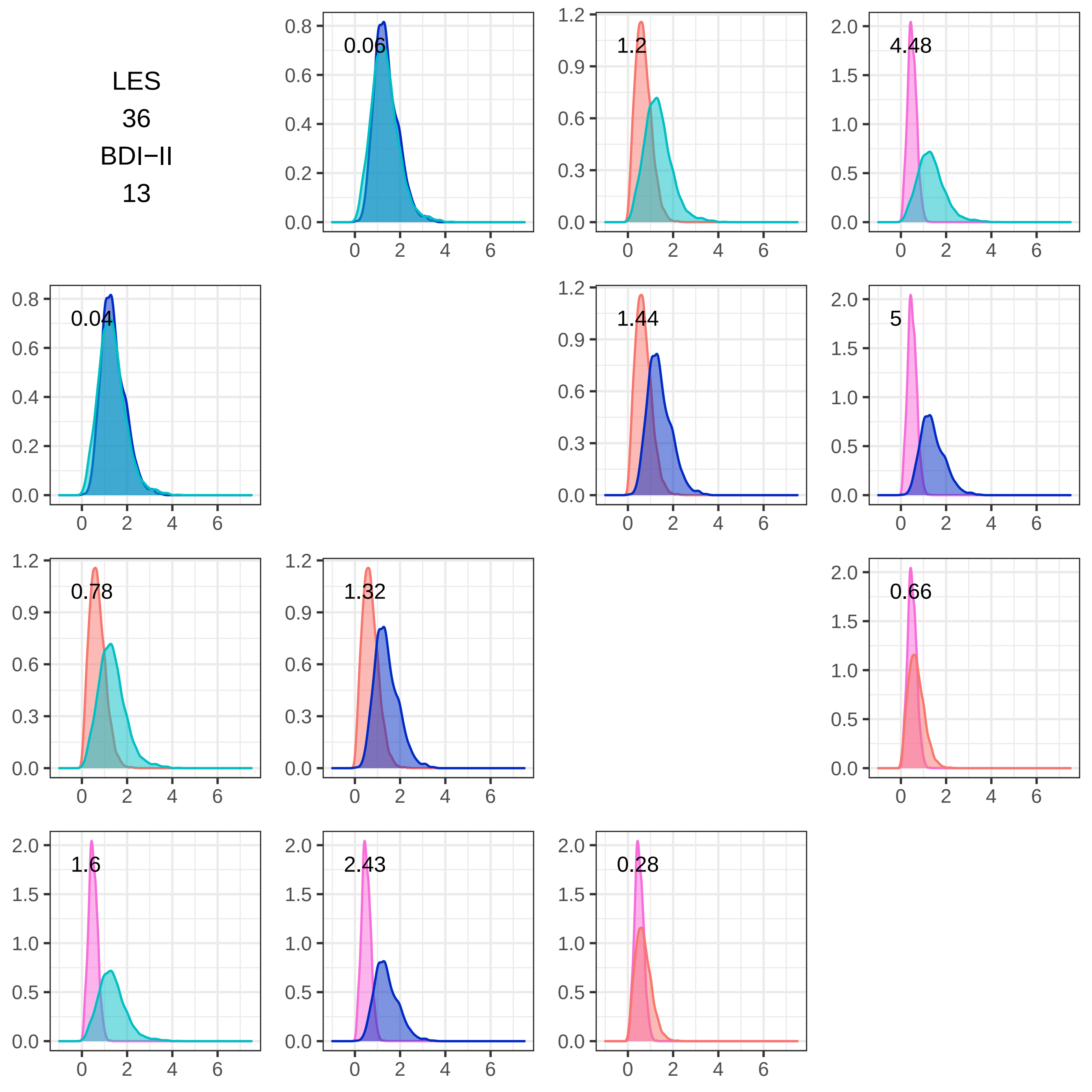
LES
36
BDI−II
13
0.06
1.2
4.48
0.04
1.44
5
0.78
1.32
0.66
1.6
2.43
0.28

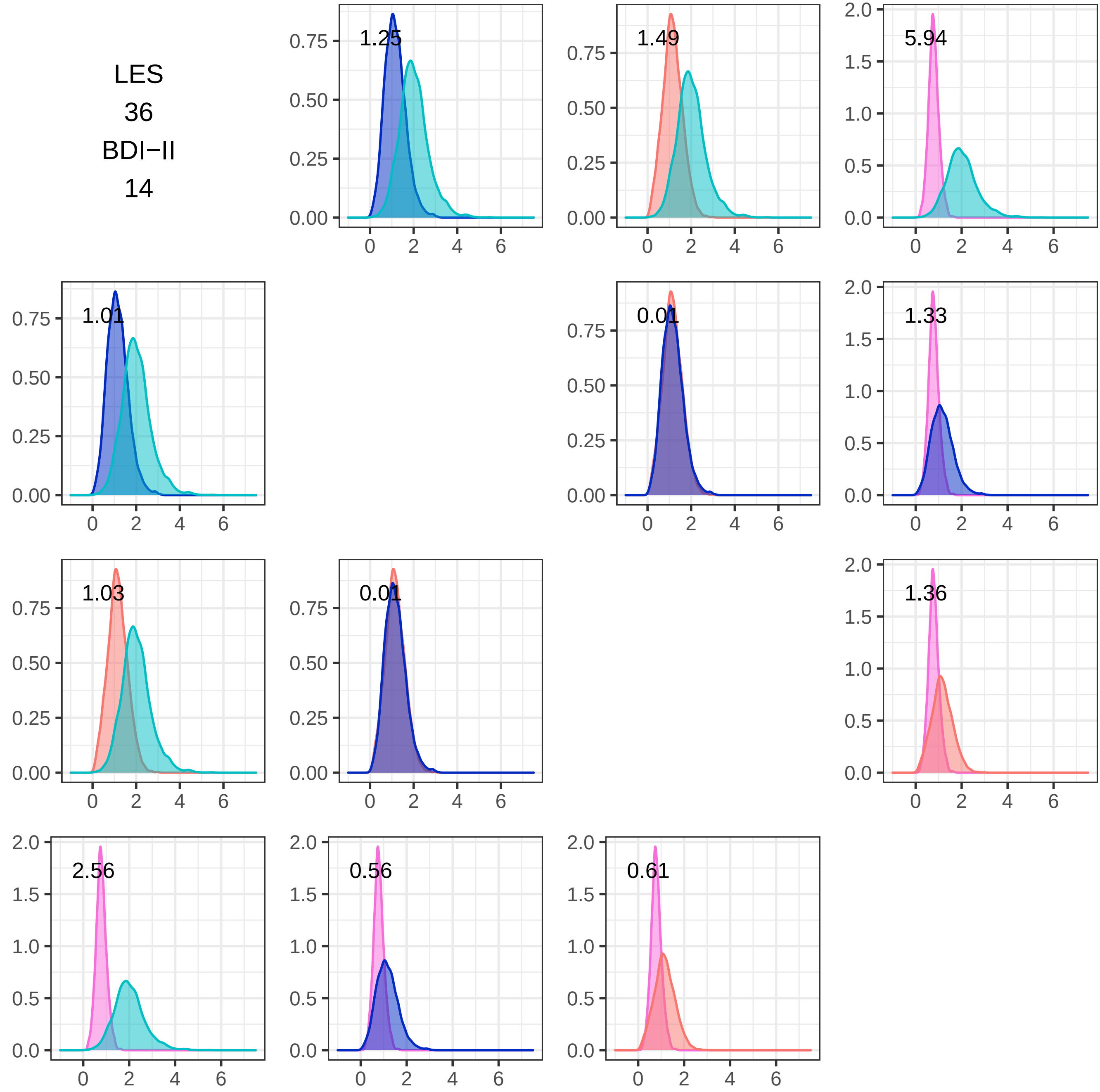
LES
36
BDI−II
14
1.25
1.49
5.94
1.01
0.01
1.33
1.03
0.01
1.36
2.56
0.56
0.61

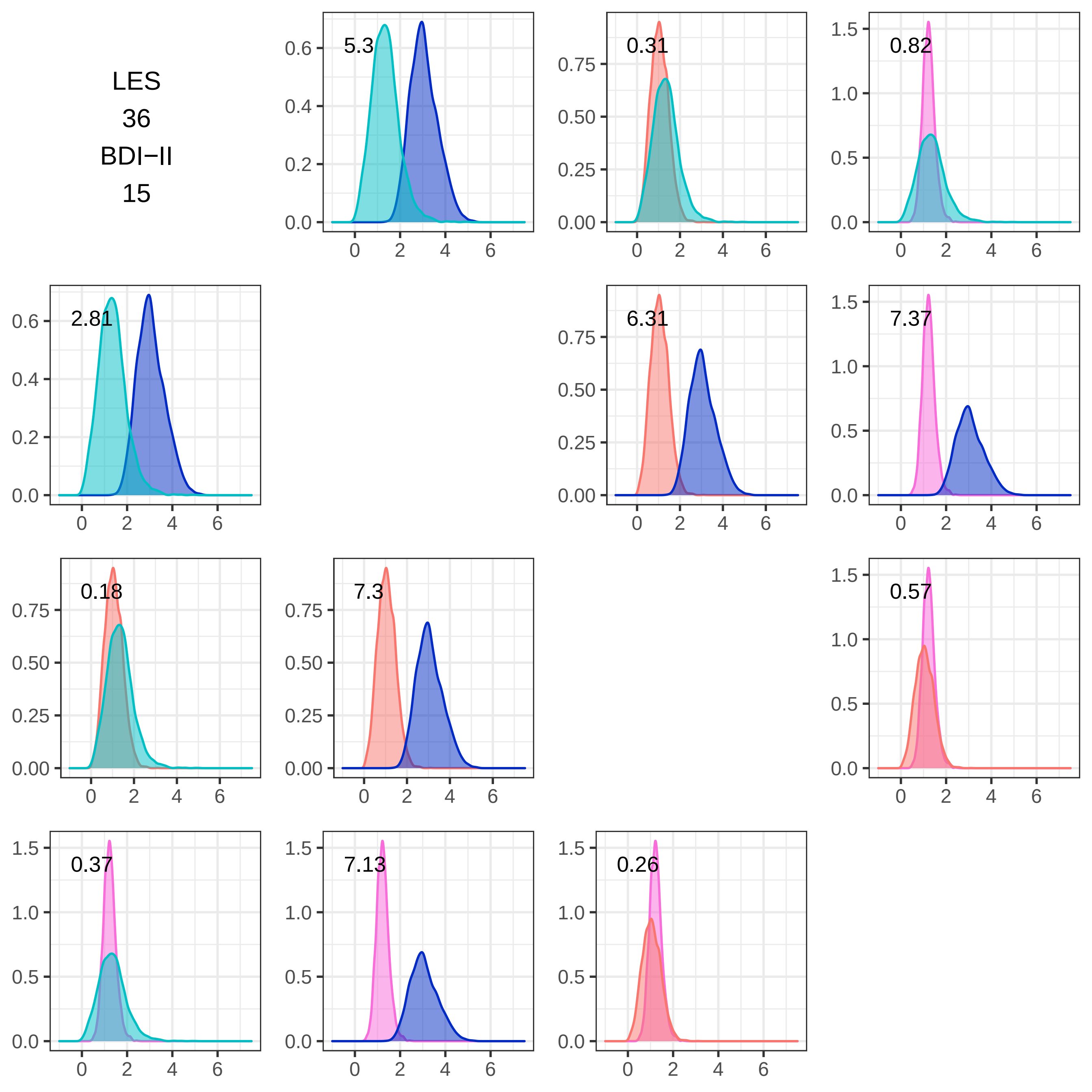

LES
36
BDI−II
15
5.3
0.31
0.82
2.81
6.31
7.37
0.18
7.3
0.57
0.37
7.13
0.26

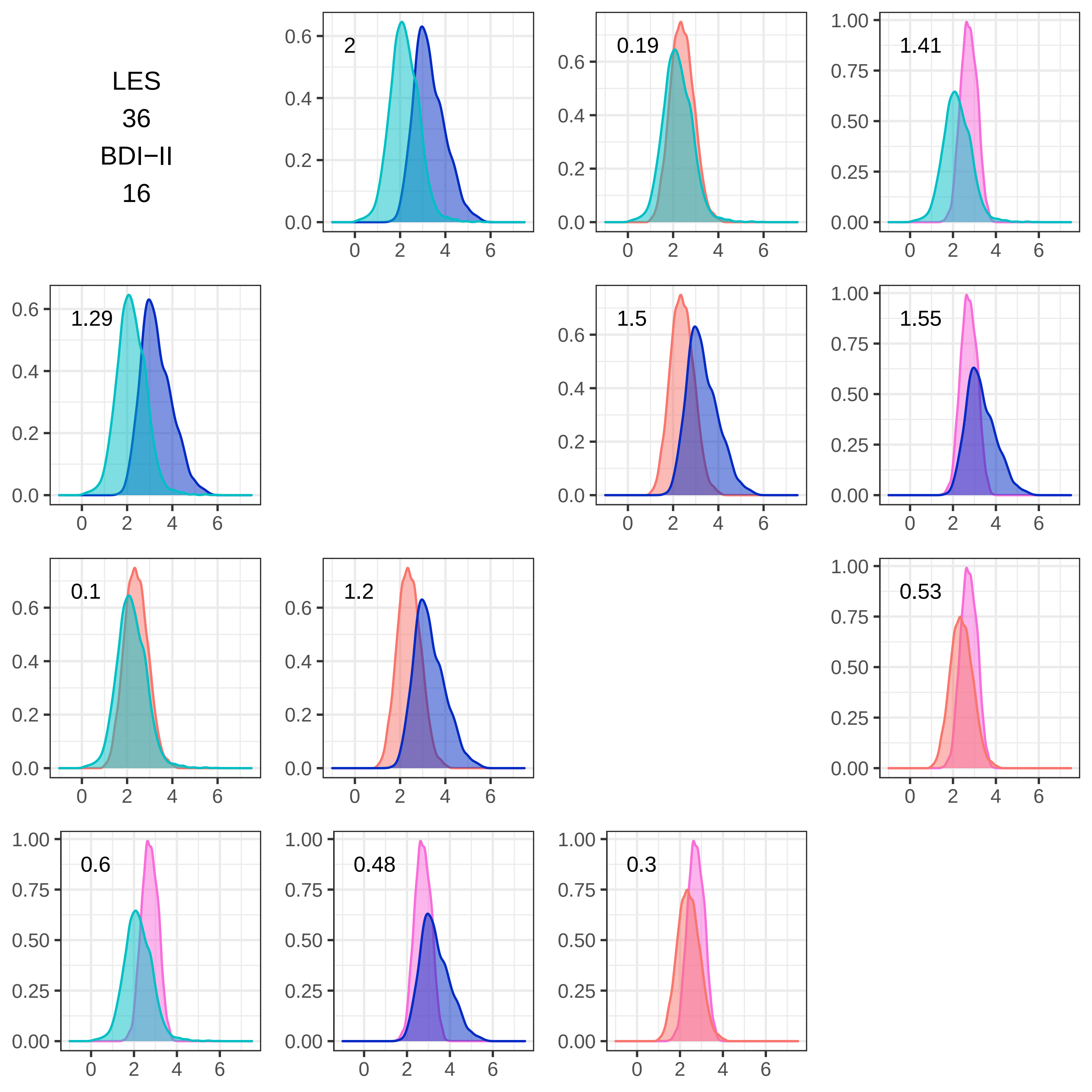

LES
36
BDI−II
16
2
0.19
1.41
1.29
1.5
1.55
0.1
1.2
0.53
0.6
0.48
0.3

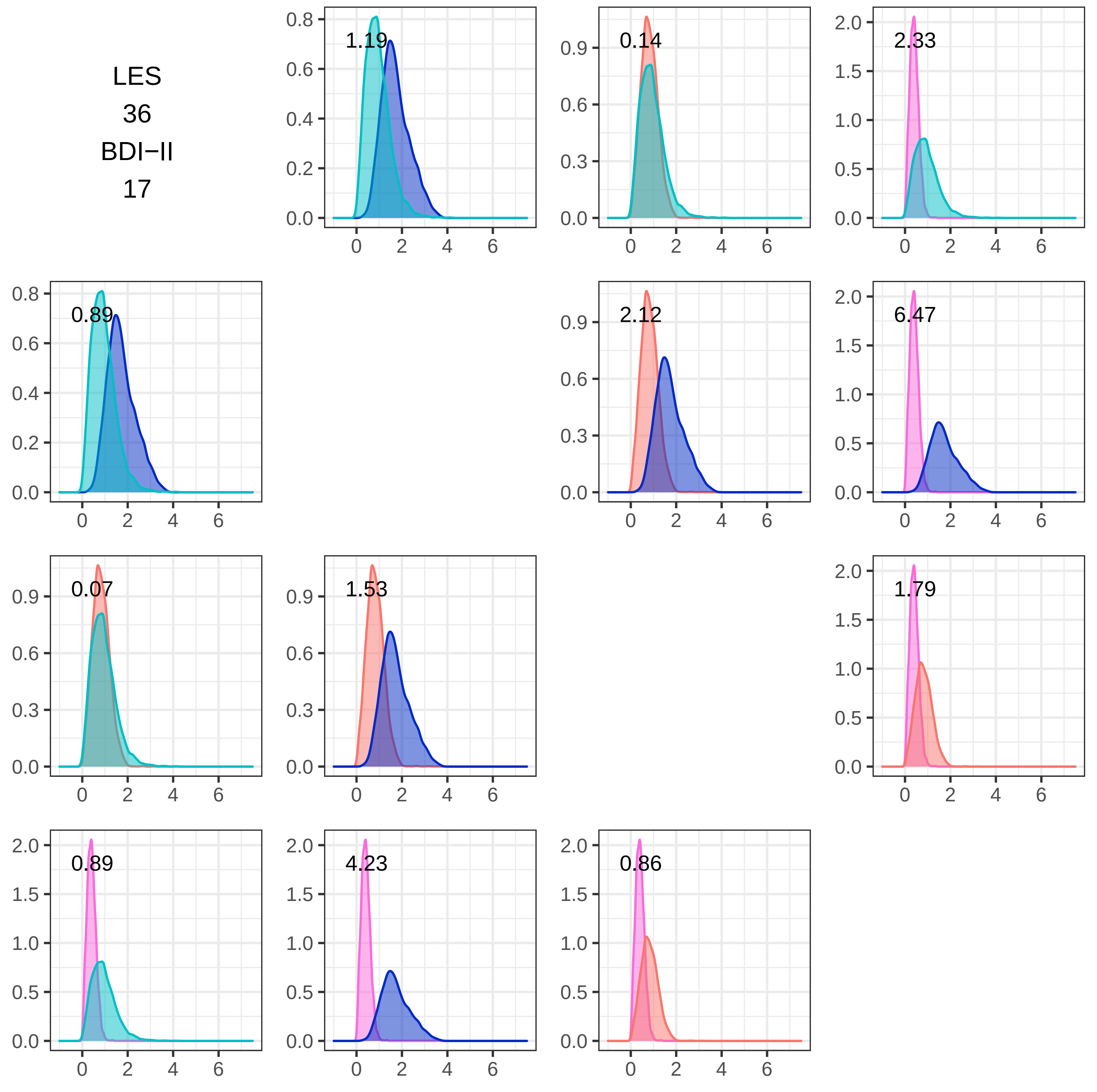
LES
36
BDI−II
17
1.19
0.14
2.33
0.89
2.12
6.47
0.07
1.53
1.79
0.89
4.23
0.86

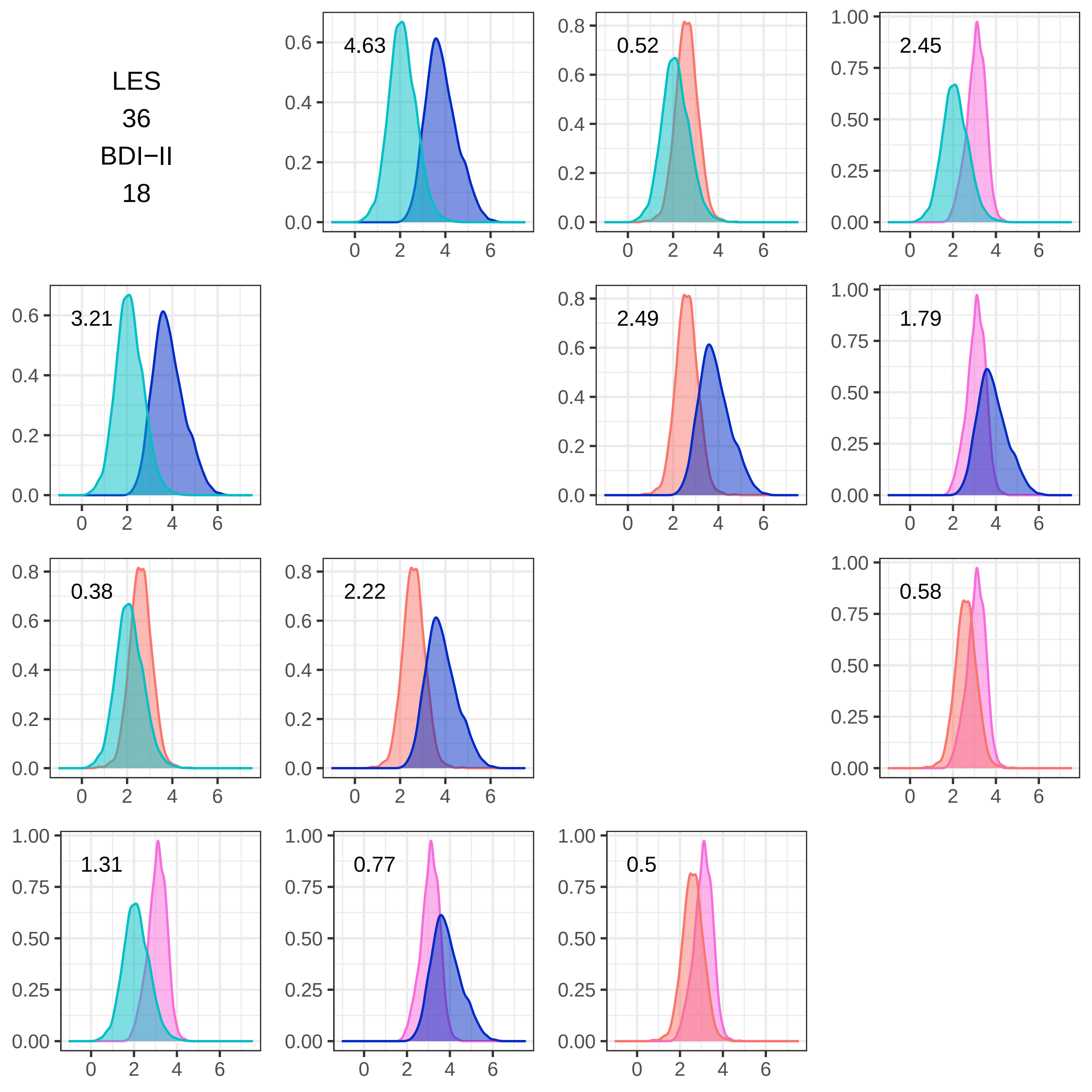
LES
36
BDI−II
18
4.63
0.52
2.45
3.21
2.49
1.79
0.38
2.22
0.58
1.31
0.77
0.5

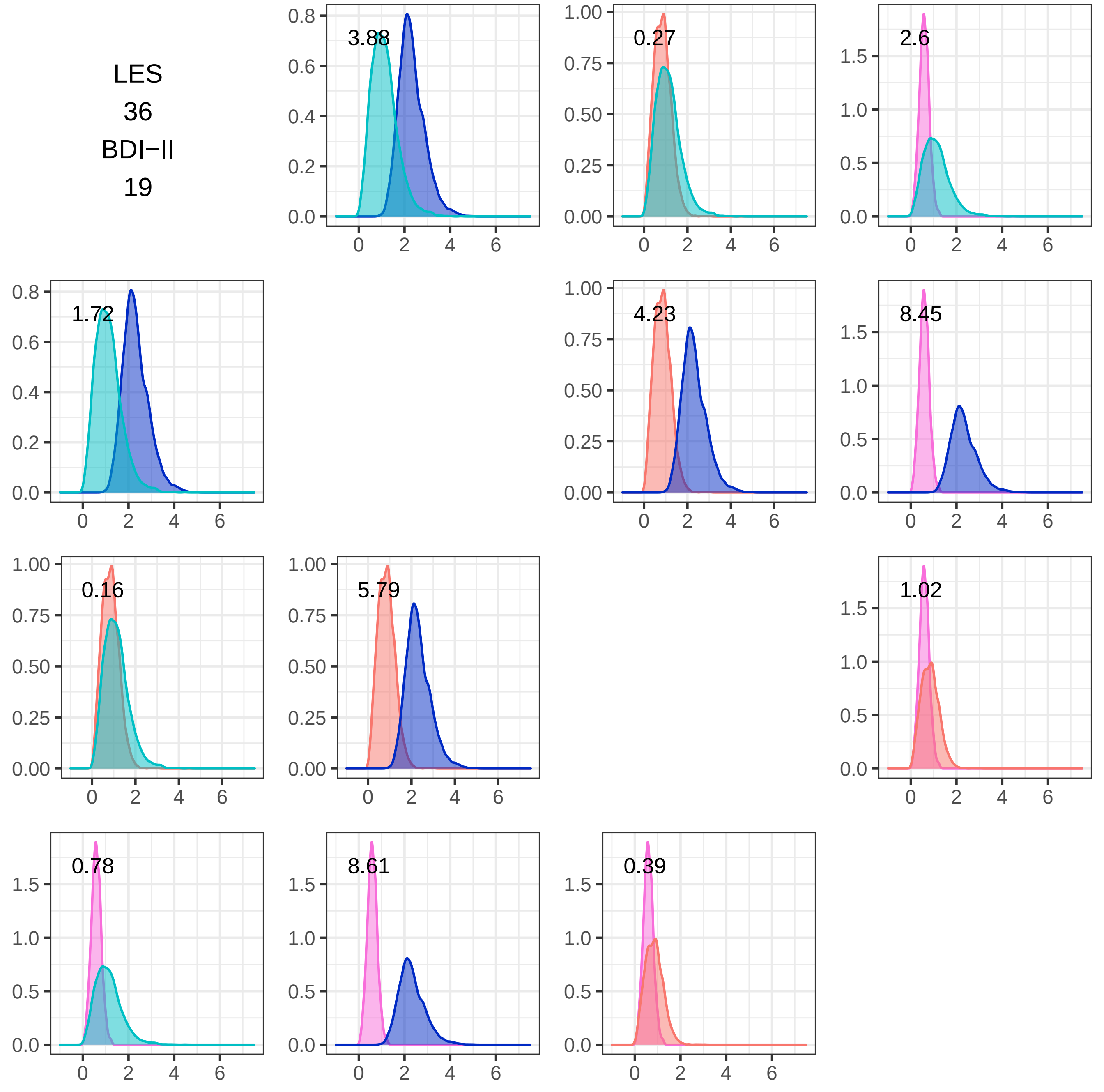

LES
36
BDI−II
19
3.88
0.27
2.6
1.72
4.23
8.45
0.16
5.79
1.02
0.78
8.61
0.39

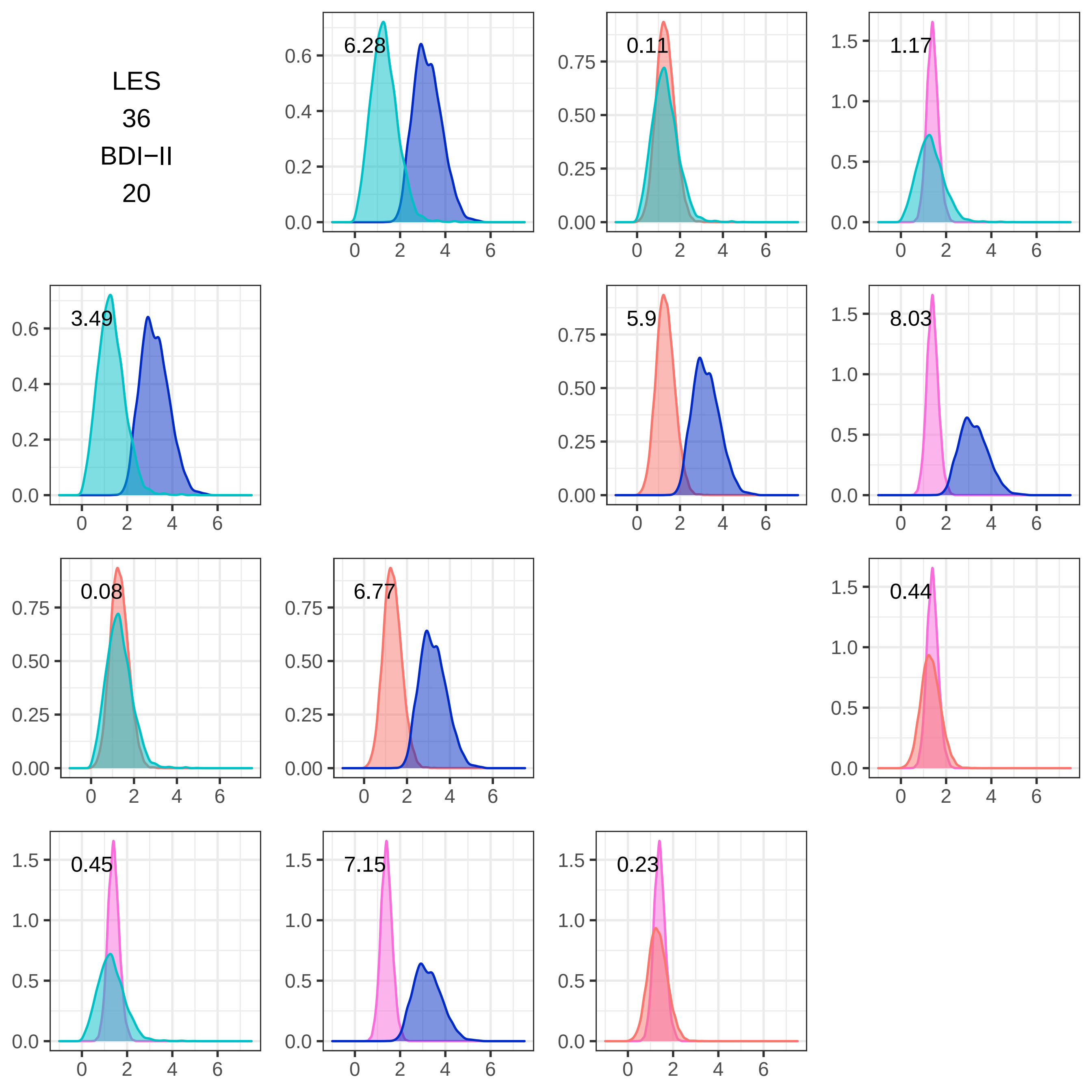

LES
36
BDI−II
20
6.28
0.11
1.17
3.49
5.9
8.03
0.08
6.77
0.44
0.45
7.15
0.23

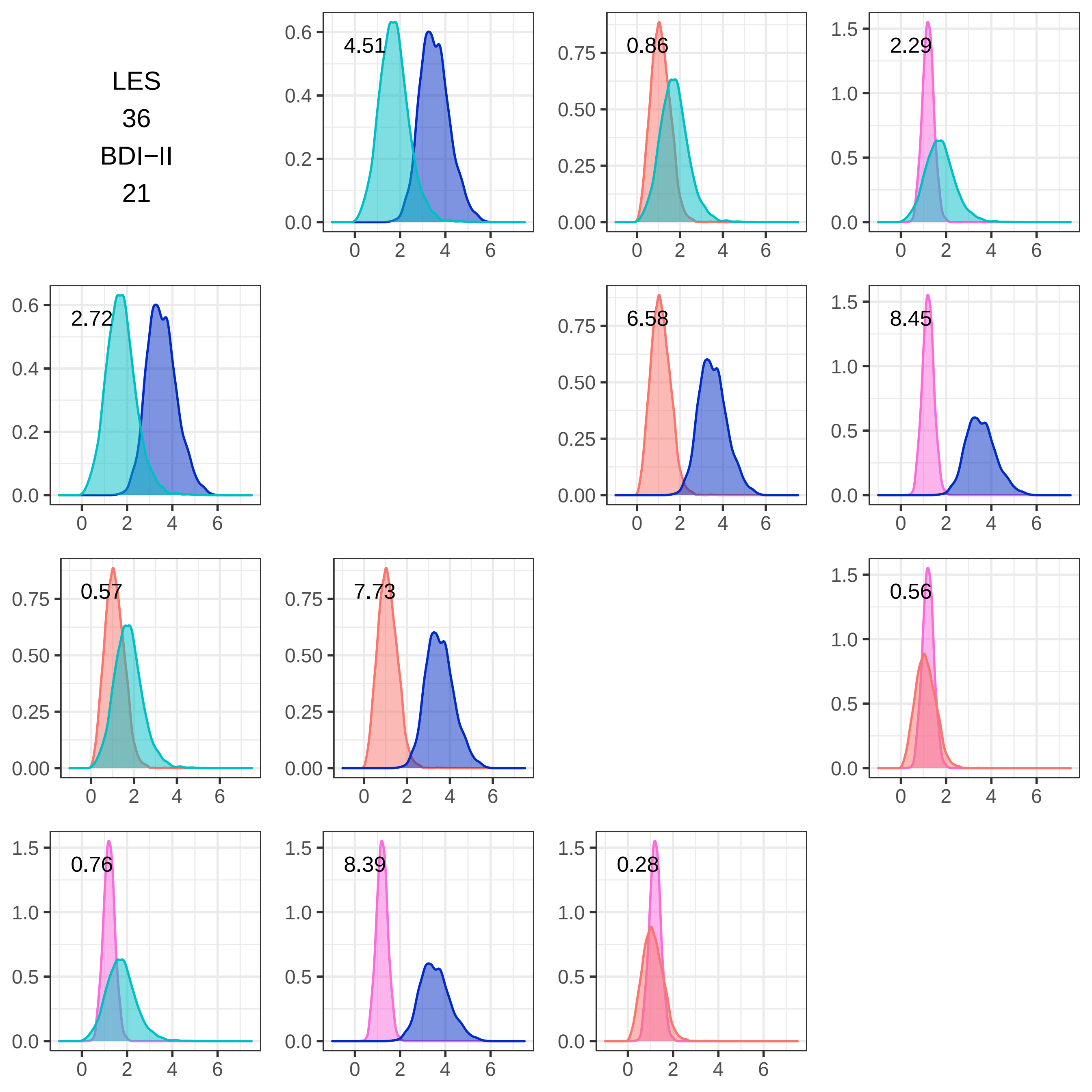
LES
36
BDI−II
21
4.51
0.86
2.29
2.72
6.58
8.45
0.57
7.73
0.56
0.76
8.39
0.28

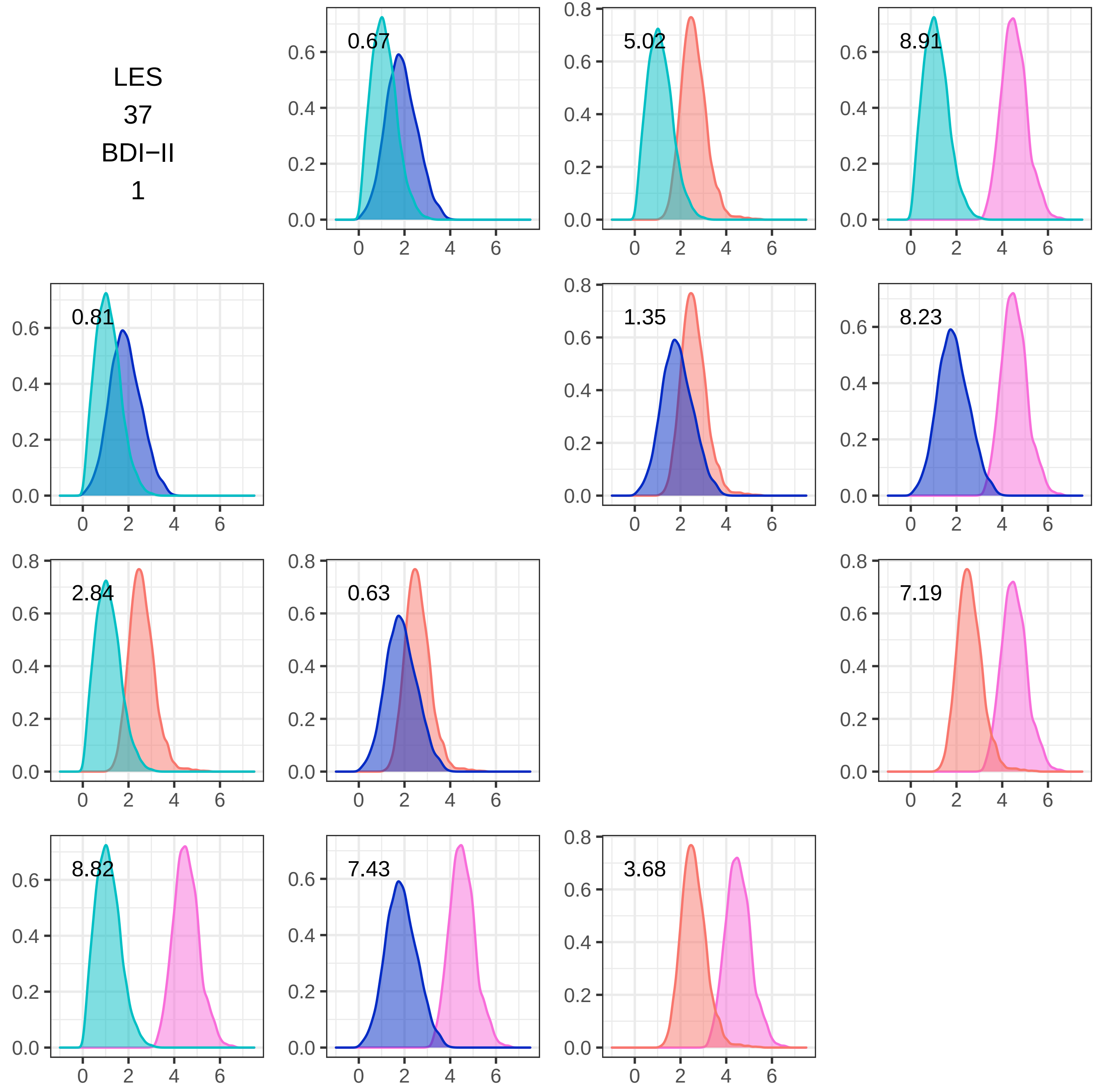
LES
37
BDI−II
1
0.67
5.02
8.91
0.81
1.35
8.23
2.84
0.63
7.19
8.82
7.43
3.68

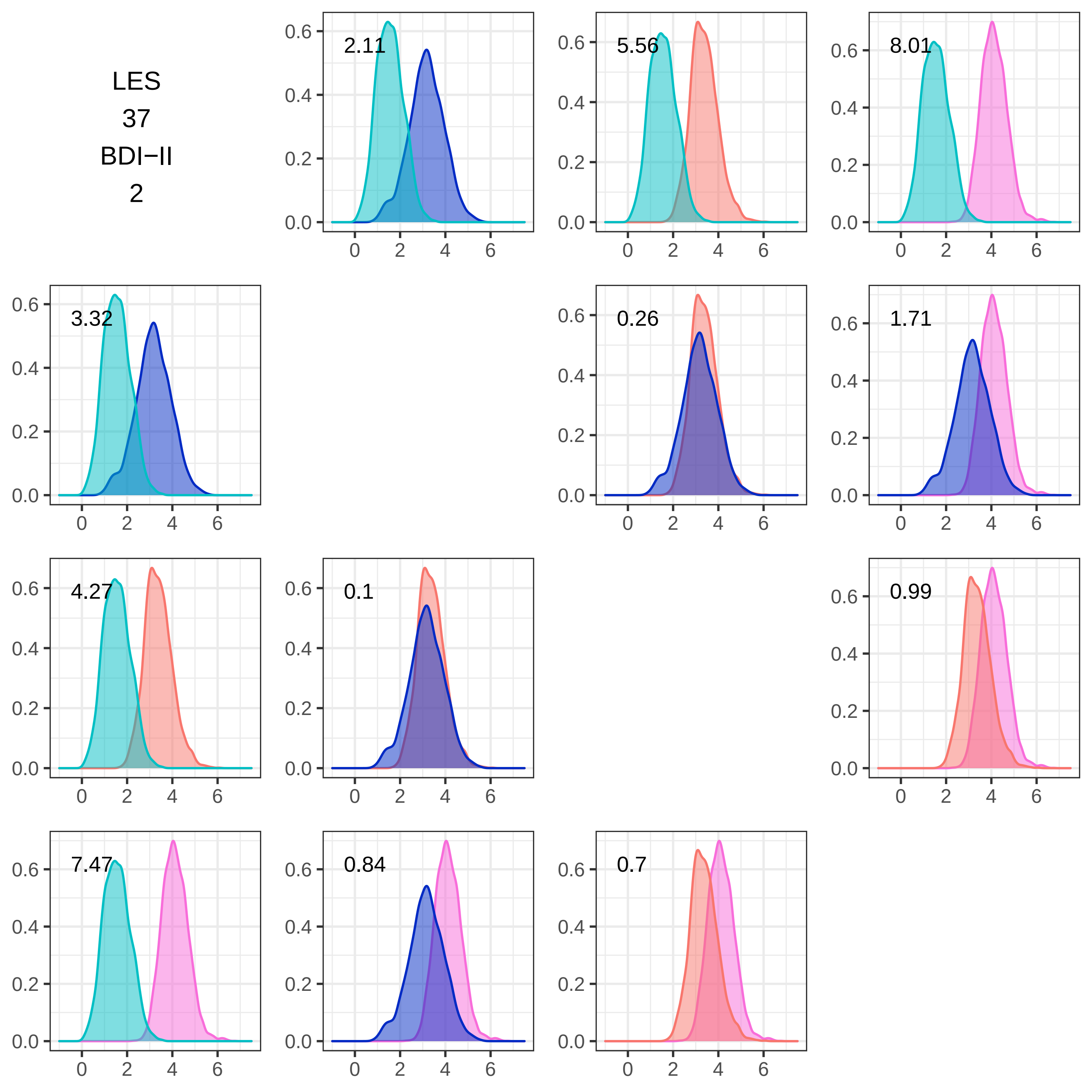

LES
37
BDI−II
2
2.11
5.56
8.01
3.32
0.26
1.71
4.27
0.1
0.99
7.47
0.84
0.7

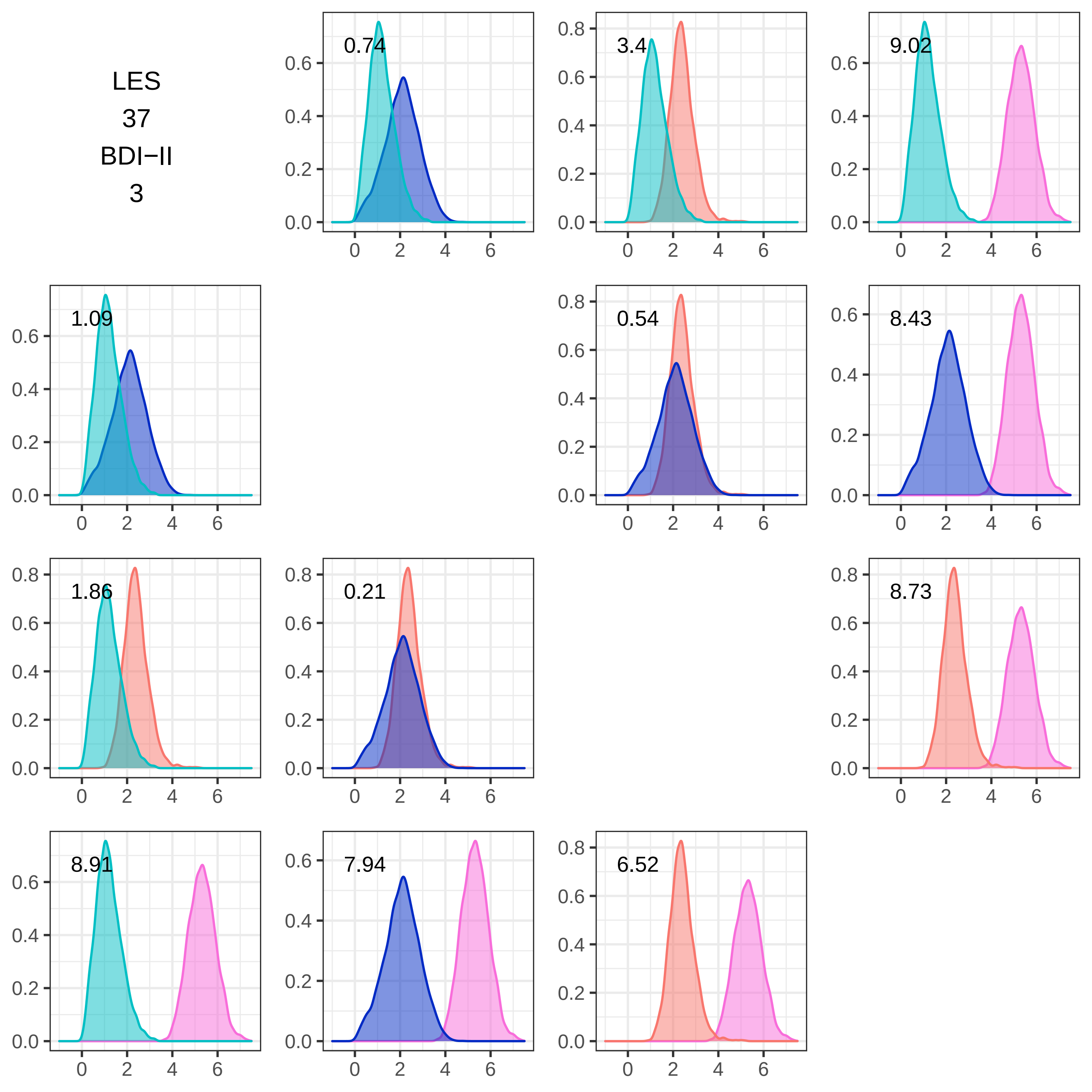

LES
37
BDI−II
3
0.74
3.4
9.02
1.09
0.54
8.43
1.86
0.21
8.73
8.91
7.94
6.52

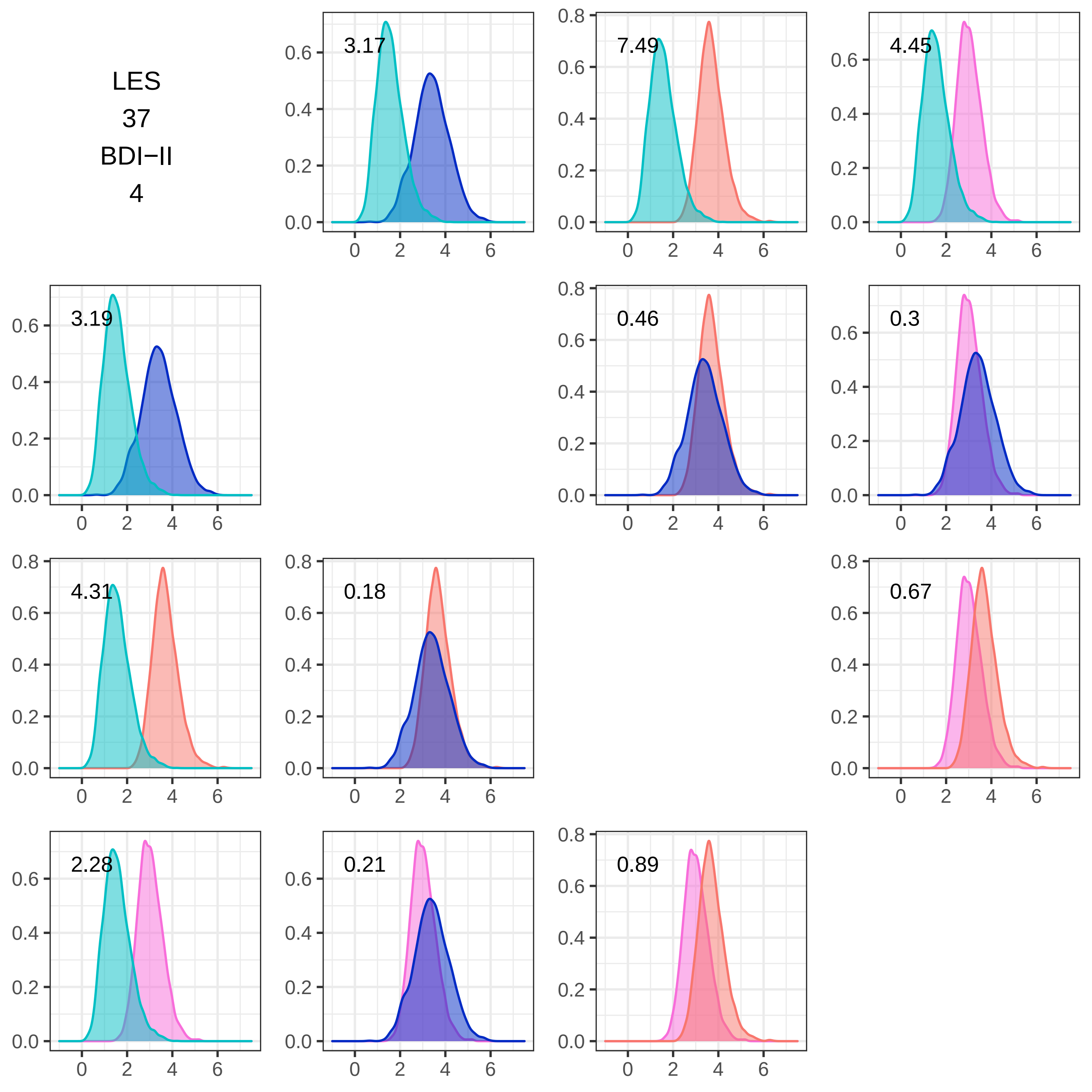

LES
37
BDI−II
4
3.17
7.49
4.45
3.19
0.46
0.3
4.31
0.18
0.67
2.28
0.21
0.89

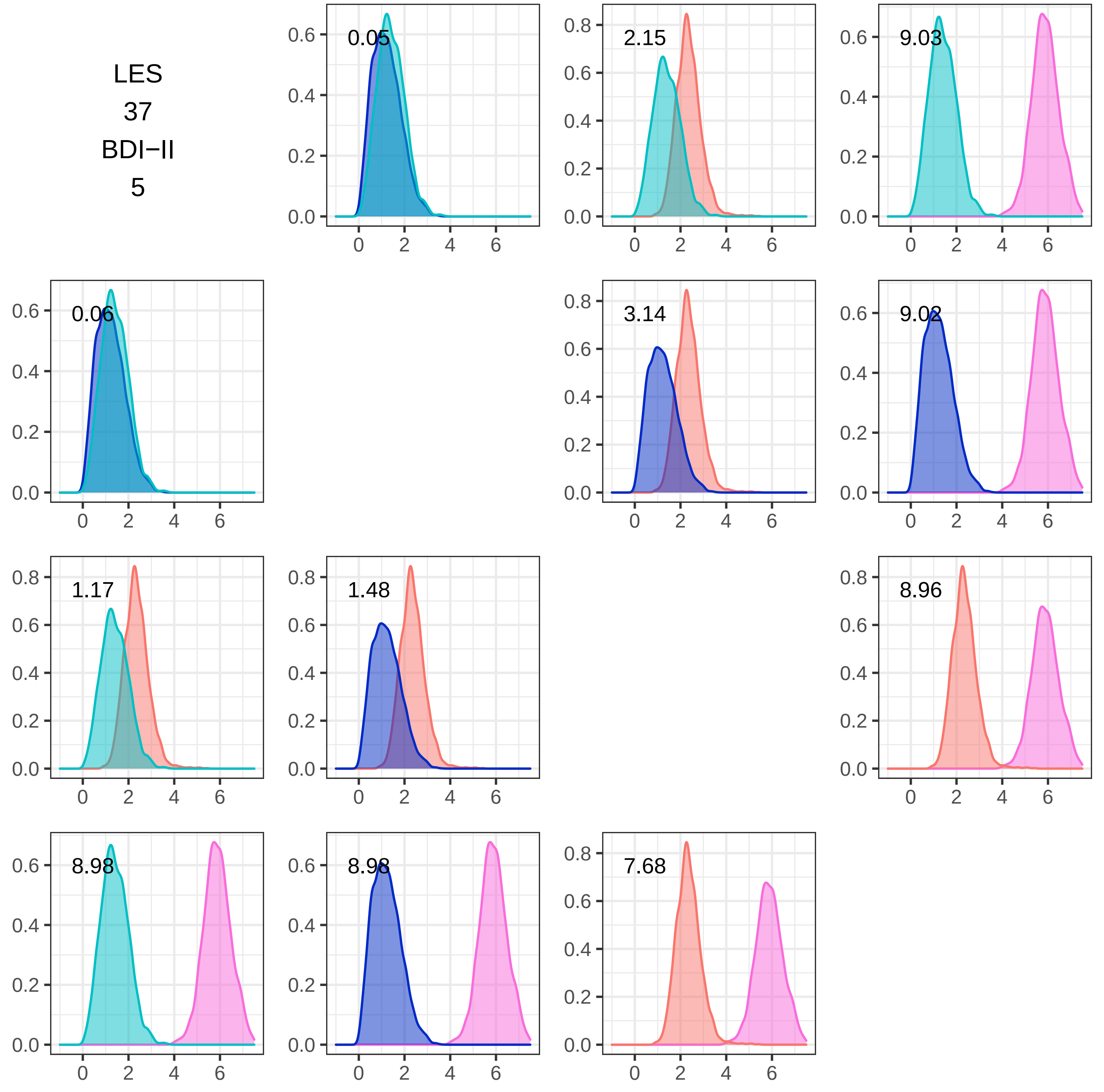
LES
37
BDI−II
5
0.05
2.15
9.03
0.06
3.14
9.02
1.17
1.48
8.96
8.98
8.98
7.68

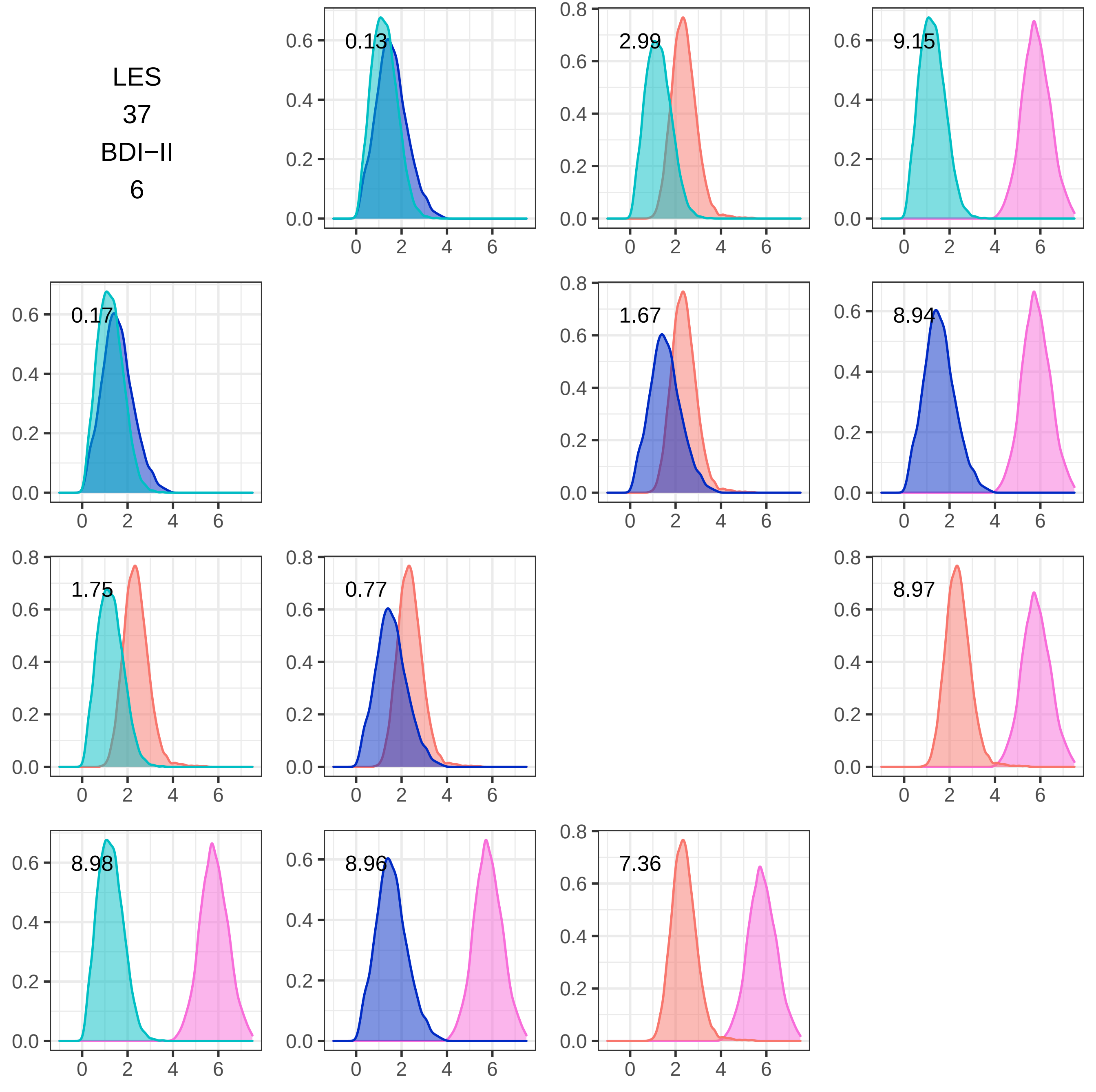
LES
37
BDI−II
6
0.13
2.99
9.15
0.17
1.67
8.94
1.75
0.77
8.97
8.98
8.96
7.36

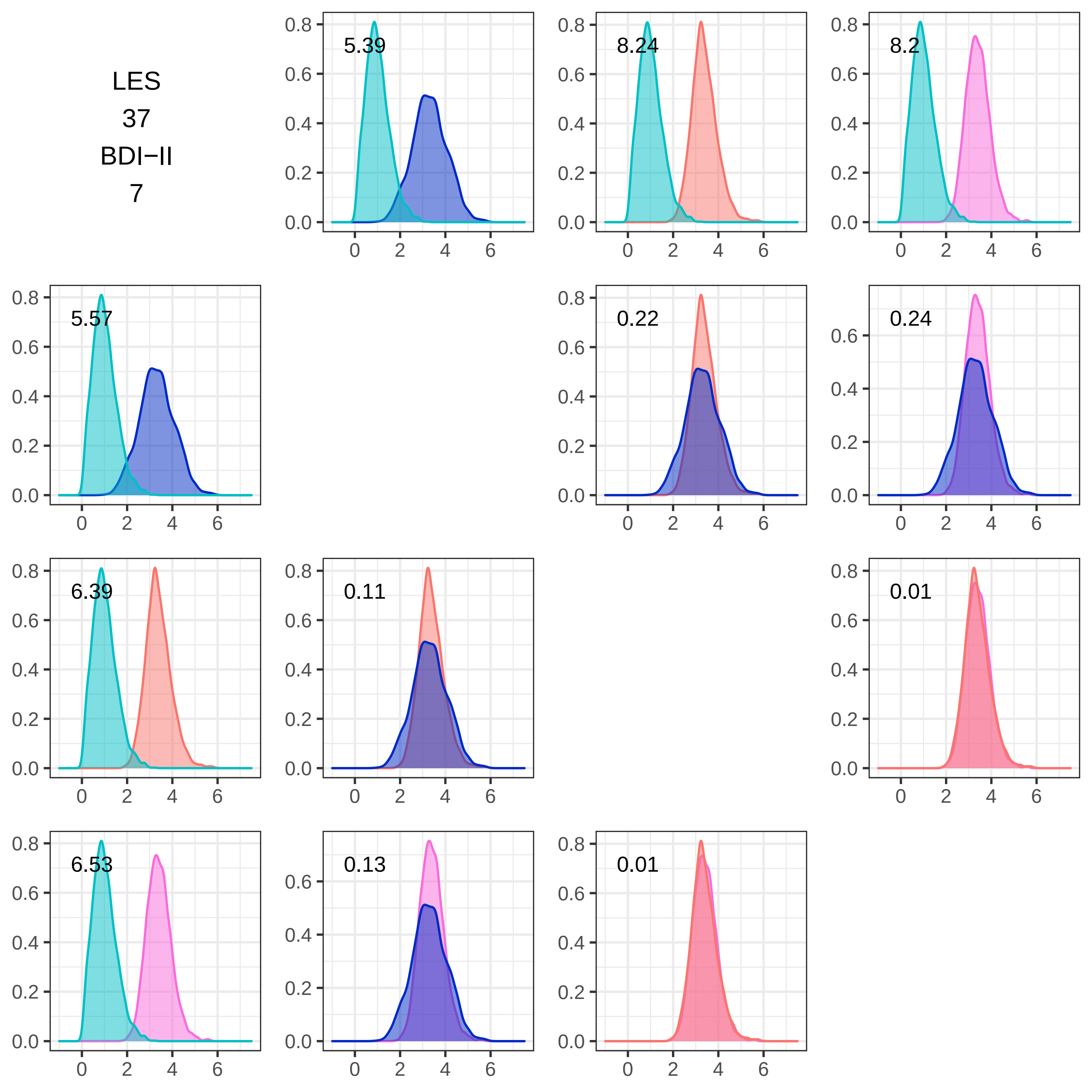
LES
37
BDI−II
7
5.39
8.24
8.2
5.57
0.22
0.24
6.39
0.11
0.01
6.53
0.13
0.01

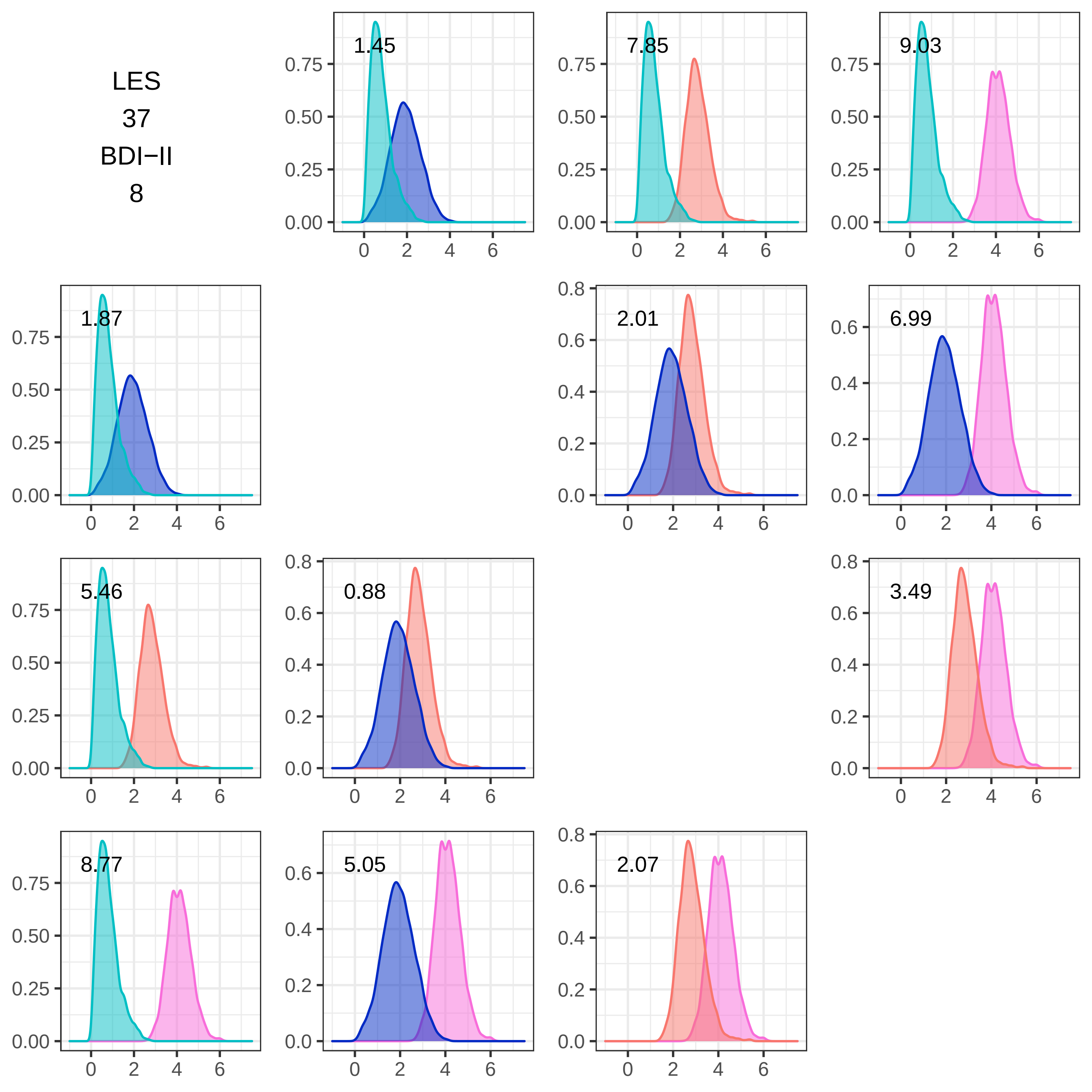

LES
37
BDI−II
8
1.45
7.85
9.03
1.87
2.01
6.99
5.46
0.88
3.49
8.77
5.05
2.07

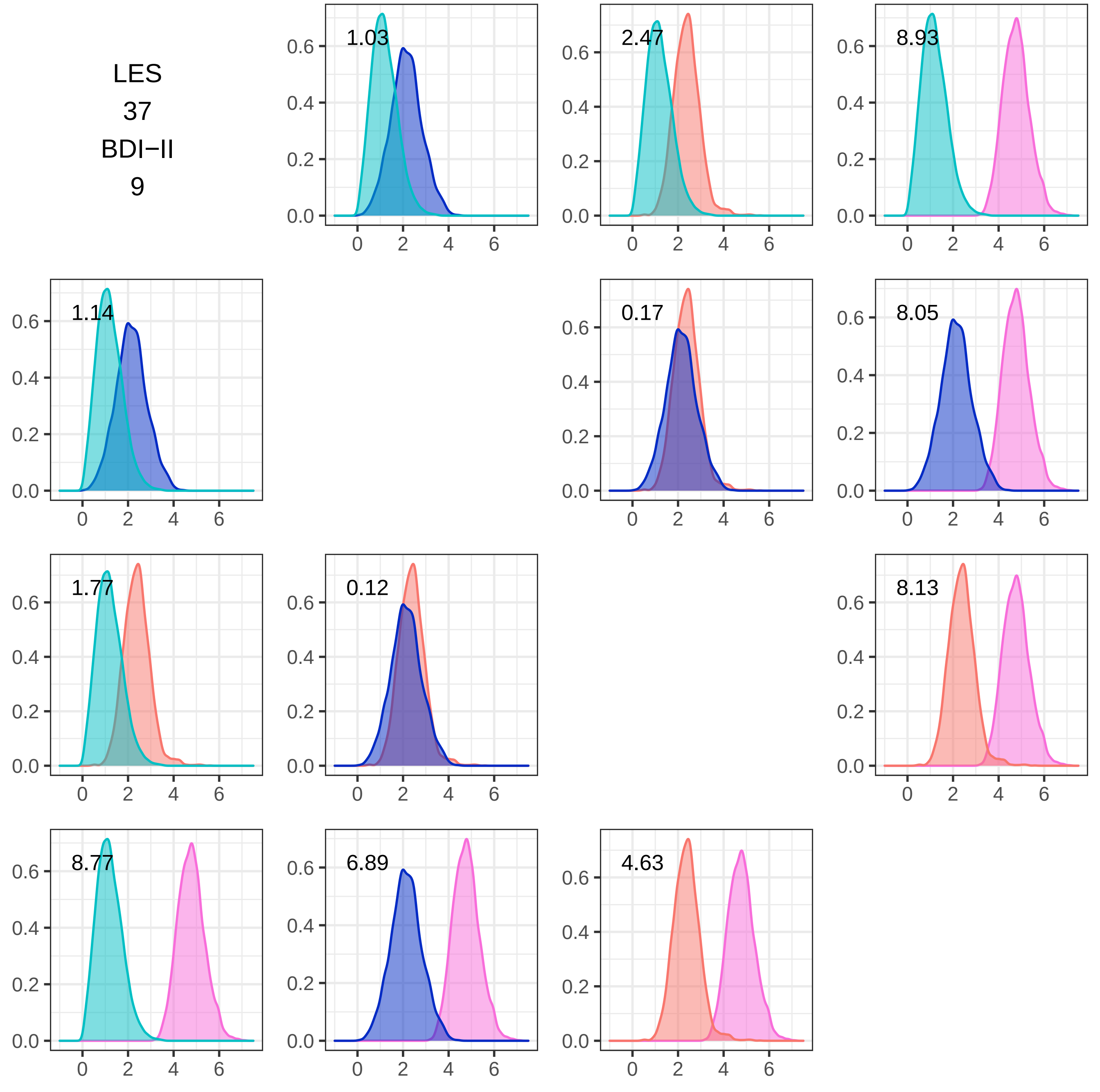
LES
37
BDI−II
9
1.03
2.47
8.93
1.14
0.17
8.05
1.77
0.12
8.13
8.77
6.89
4.63

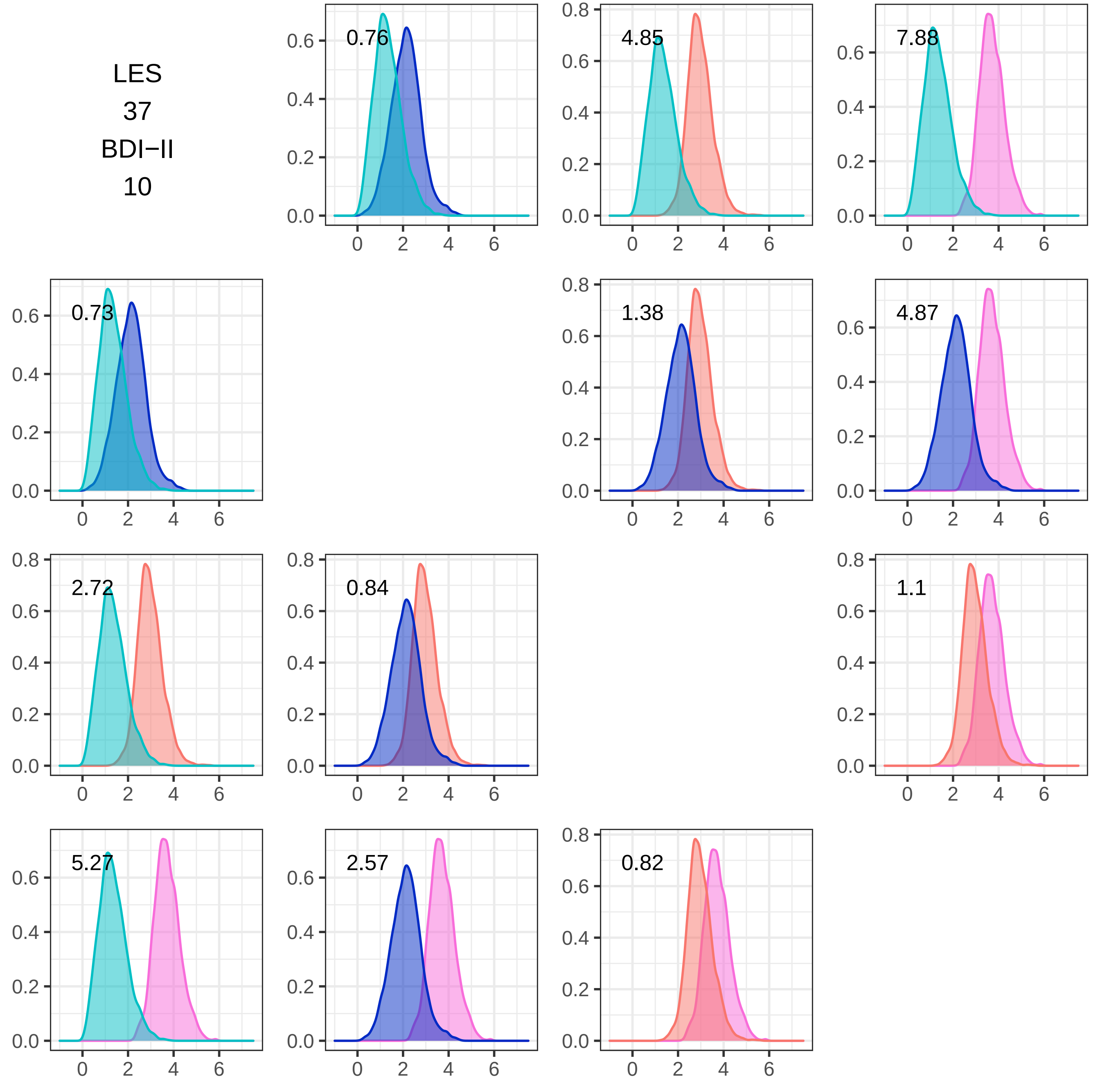
LES
37
BDI−II
10
0.76
4.85
7.88
0.73
1.38
4.87
2.72
0.84
1.1
5.27
2.57
0.82

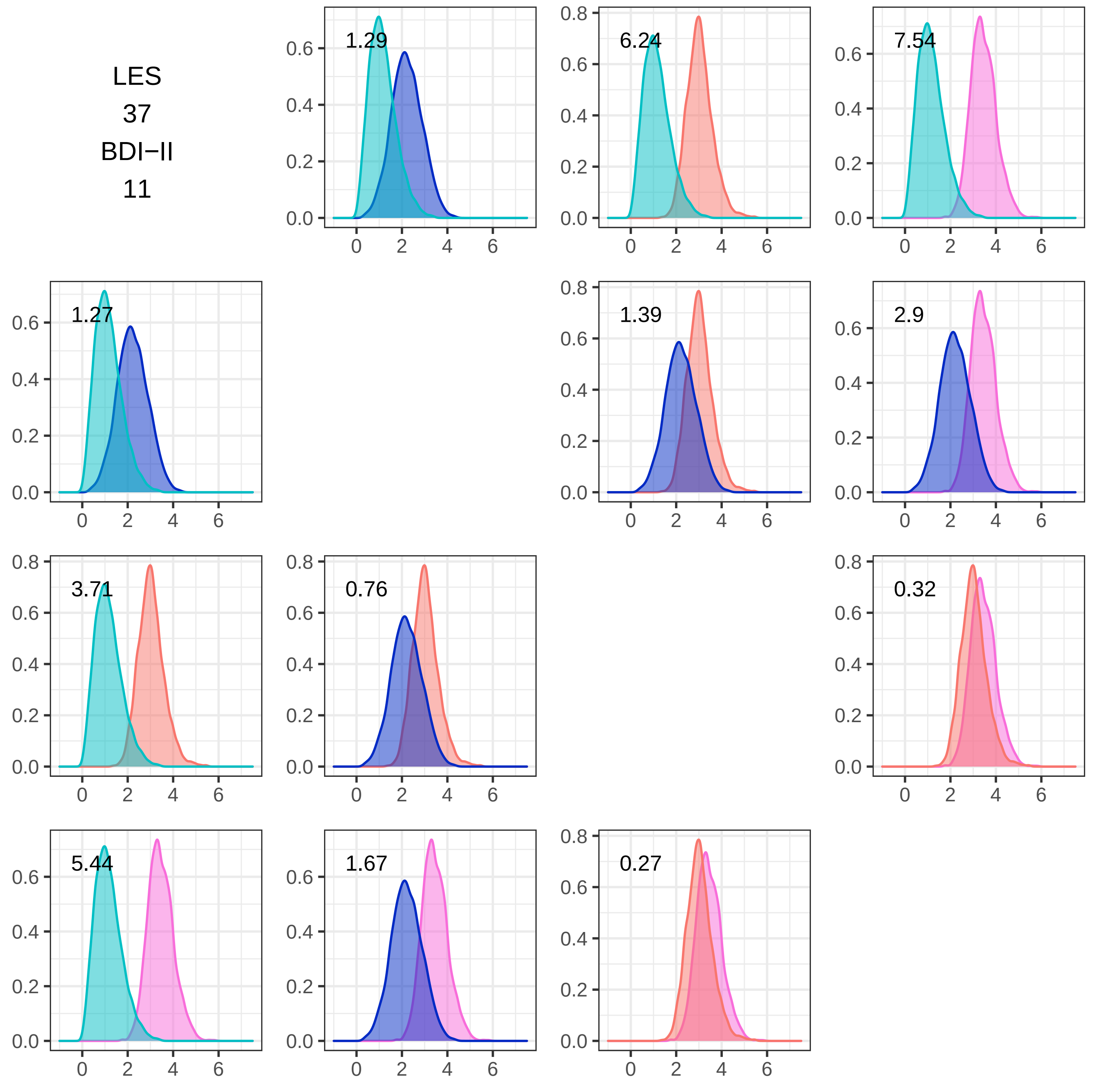

LES
37
BDI−II
11
1.29
6.24
7.54
1.27
1.39
2.9
3.71
0.76
0.32
5.44
1.67
0.27

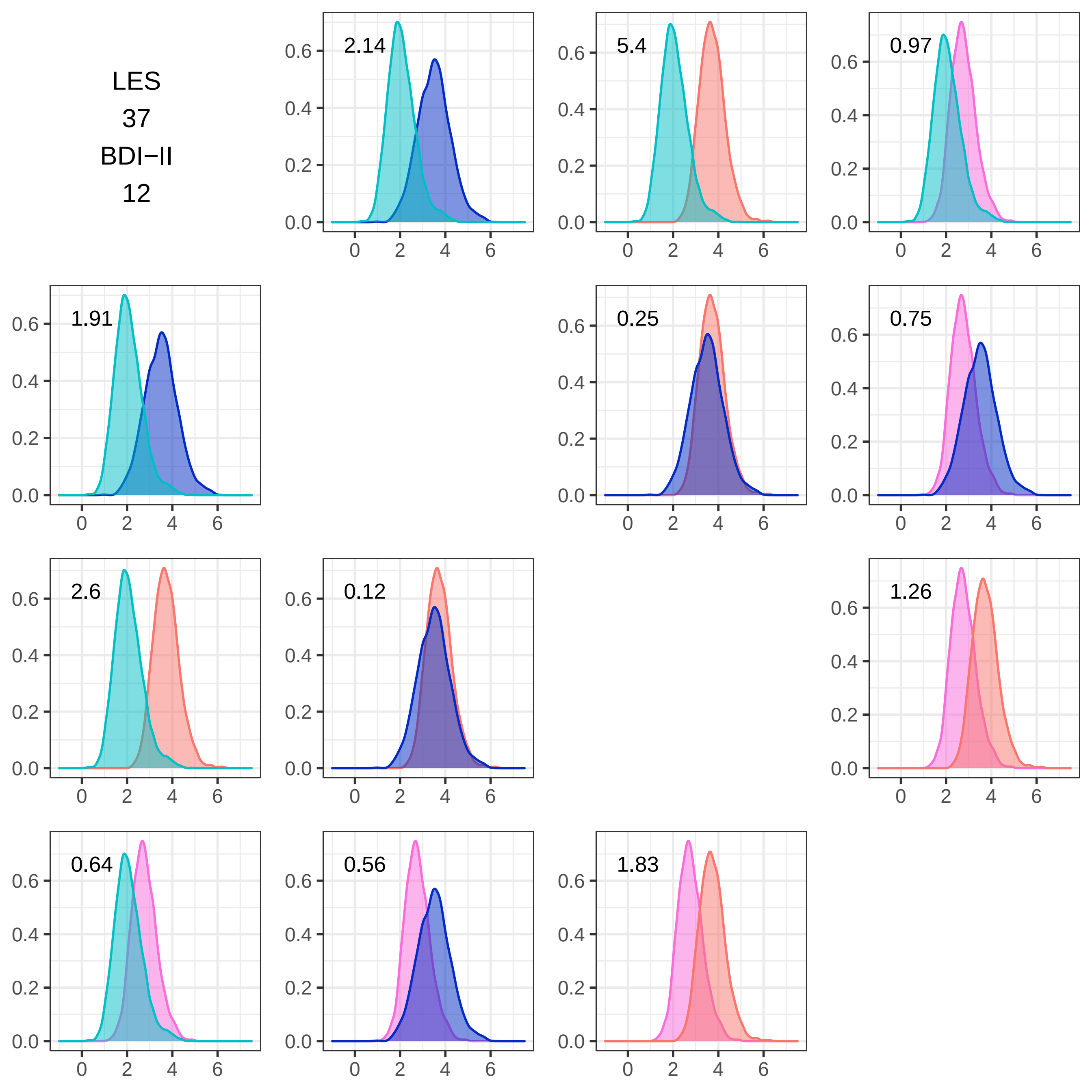
LES
37
BDI−II
12
2.14
5.4
0.97
1.91
0.25
0.75
2.6
0.12
1.26
0.64
0.56
1.83

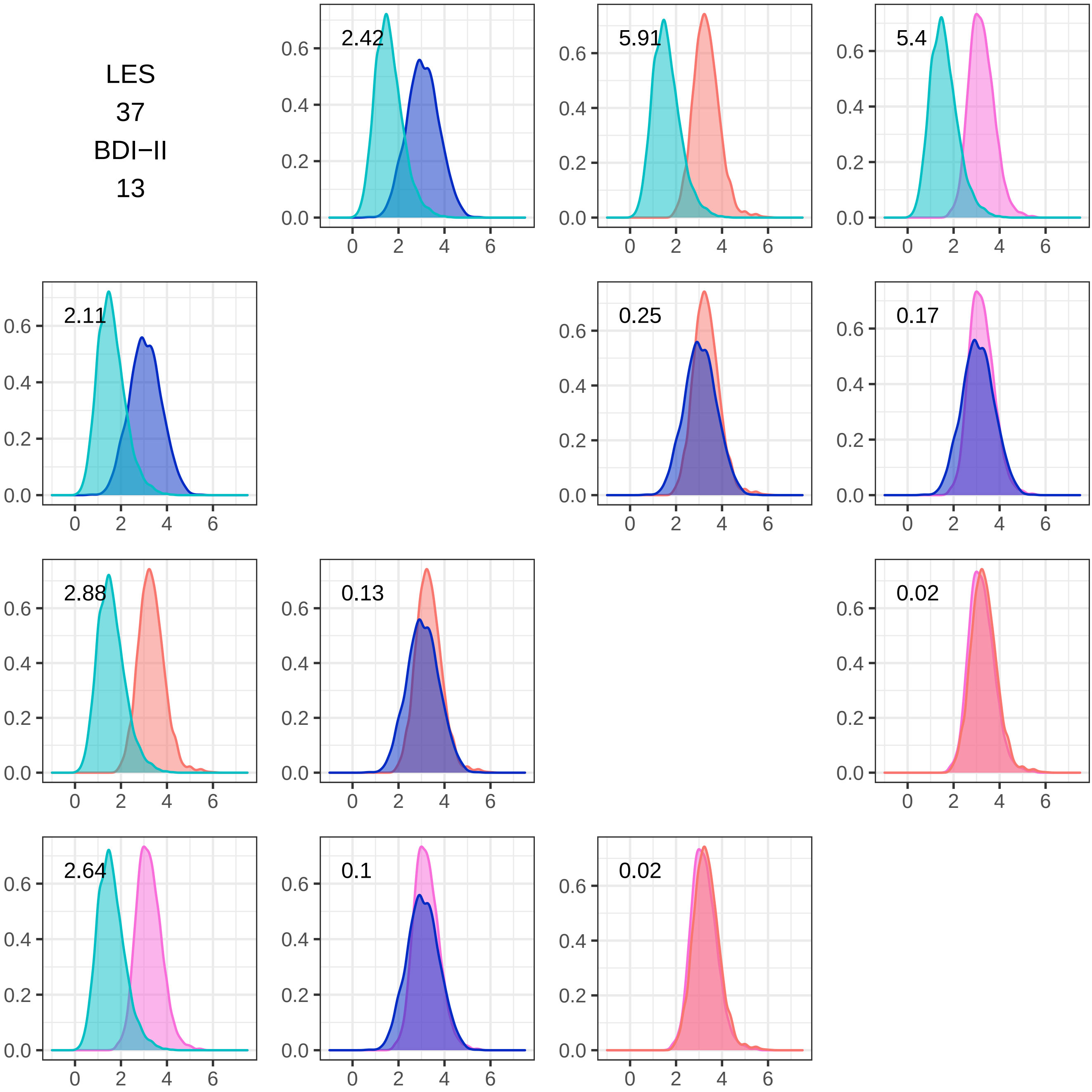
LES
37
BDI−II
13
2.42
5.91
5.4
2.11
0.25
0.17
2.88
0.13
0.02
2.64
0.1
0.02

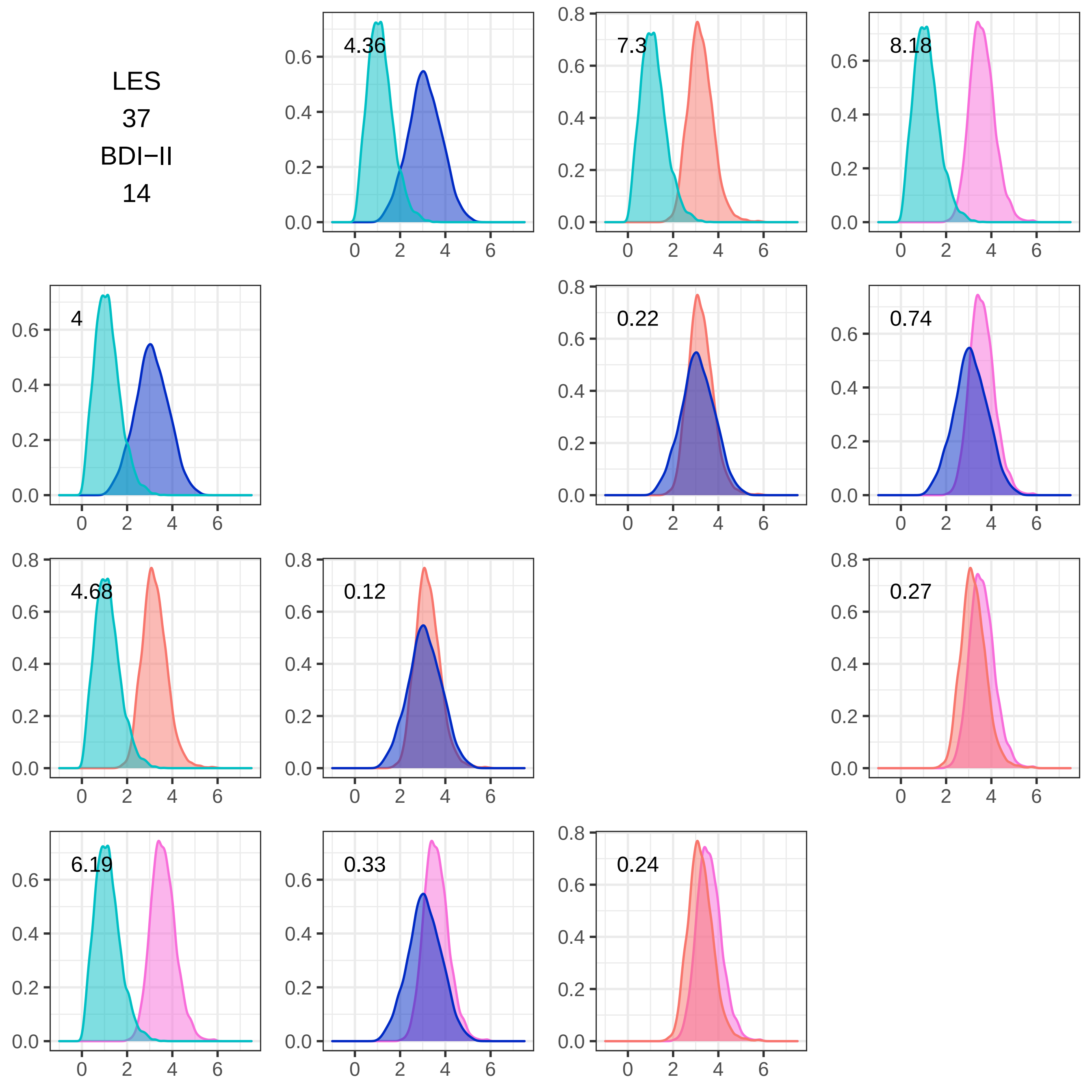

LES
37
BDI−II
14
4.36
7.3
8.18
4
0.22
0.74
4.68
0.12
0.27
6.19
0.33
0.24

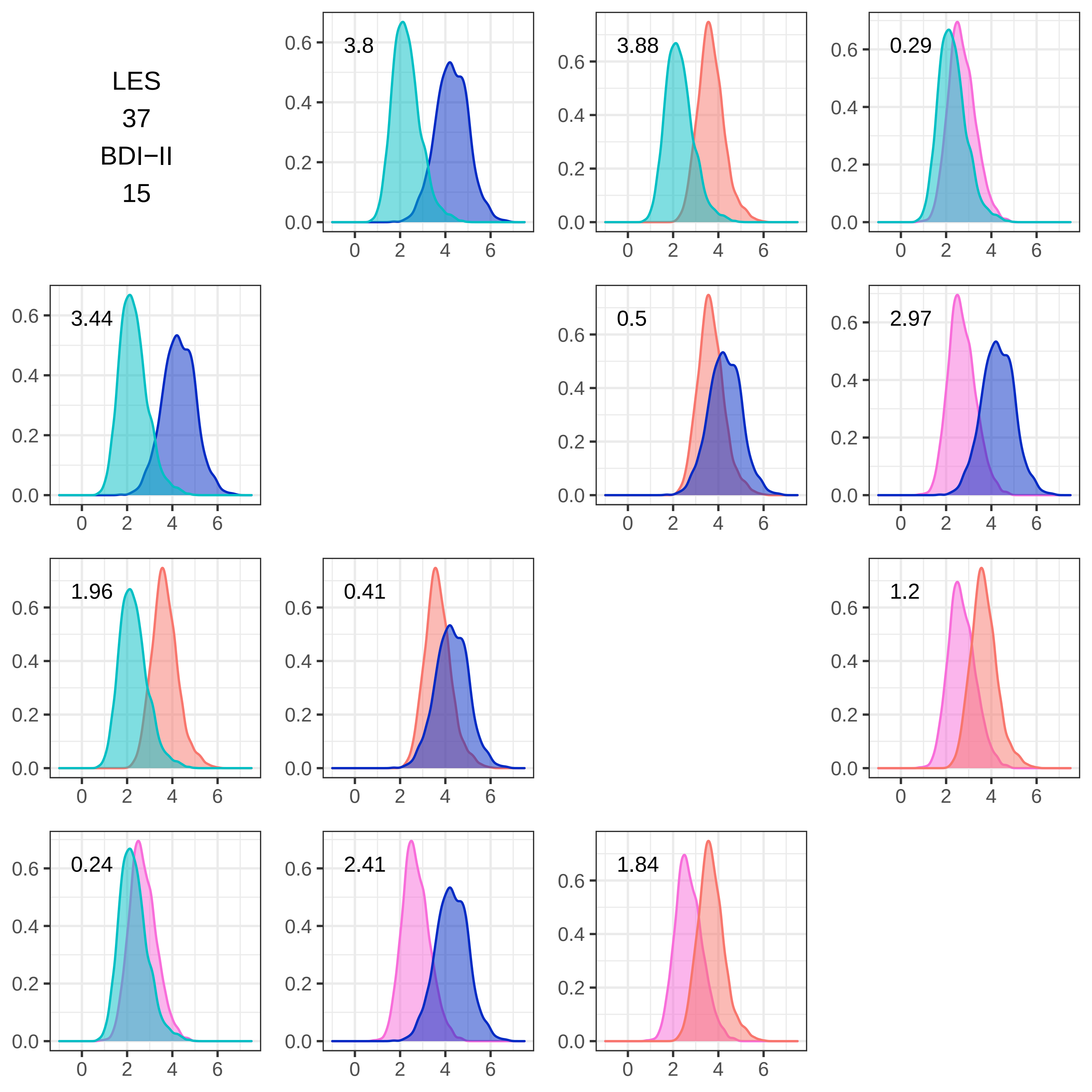

LES
37
BDI−II
15
3.8
3.88
0.29
3.44
0.5
2.97
1.96
0.41
1.2
0.24
2.41
1.84

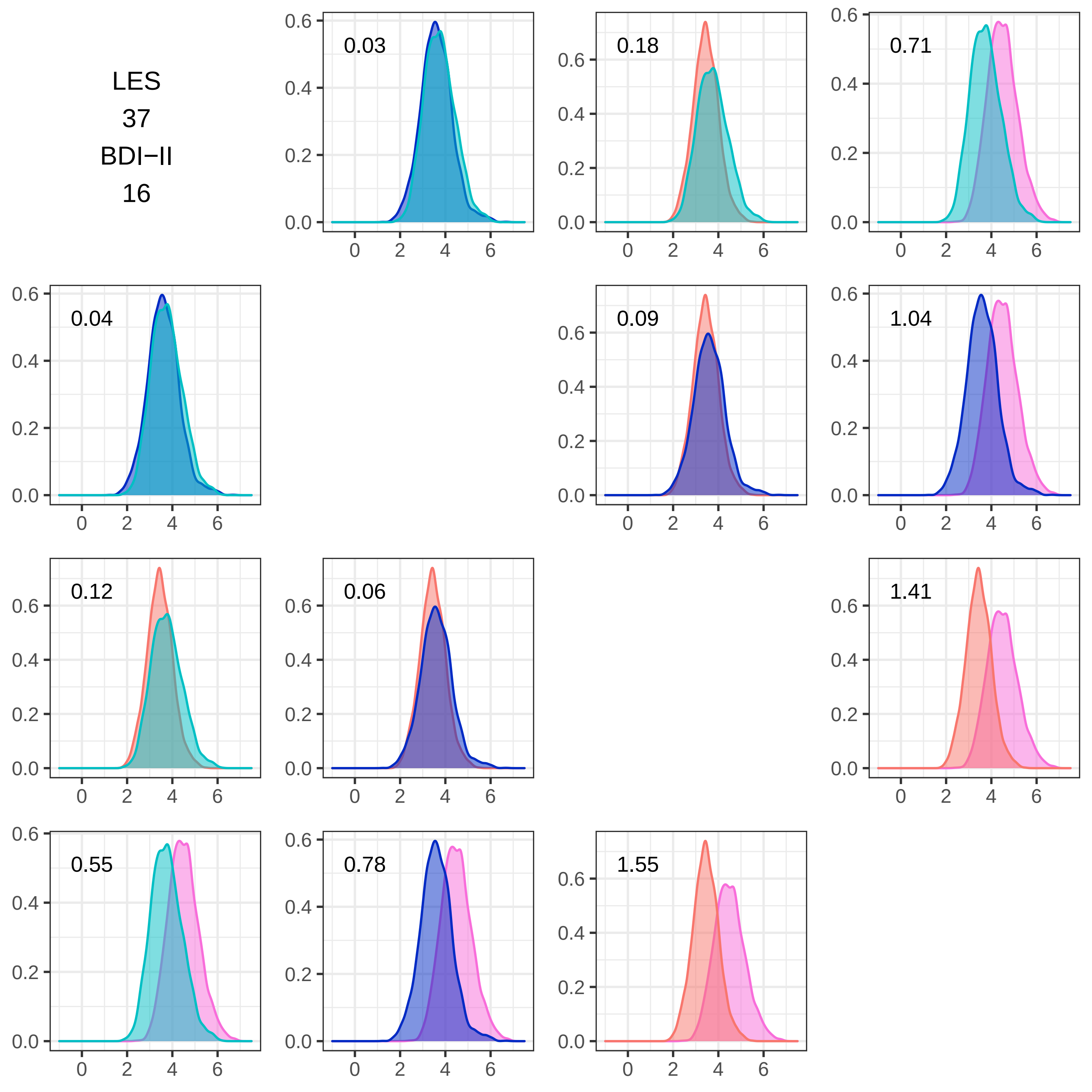

LES
37
BDI−II
16
0.03
0.18
0.71
0.04
0.09
1.04
0.12
0.06
1.41
0.55
0.78
1.55

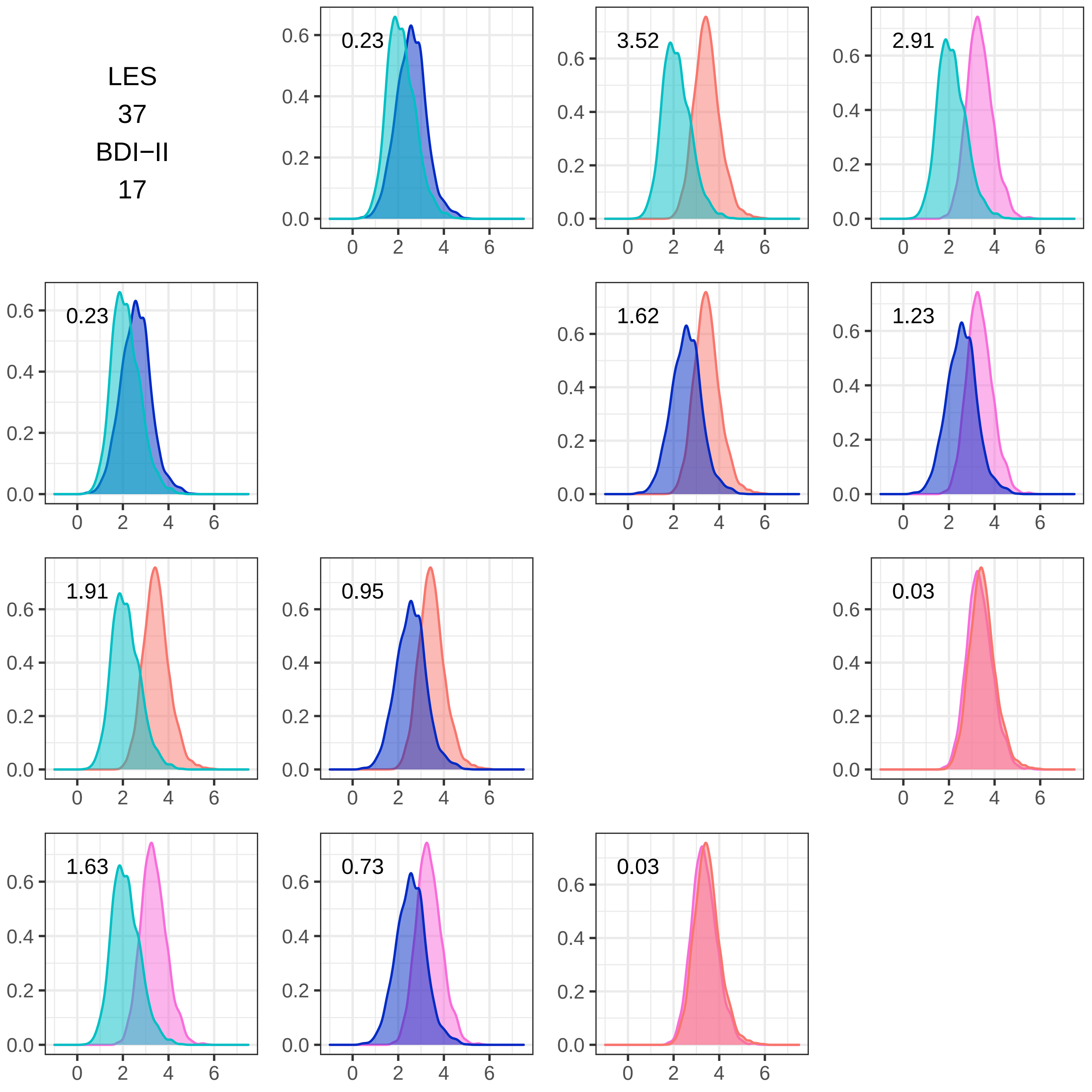

LES
37
BDI−II
17
0.23
3.52
2.91
0.23
1.62
1.23
1.91
0.95
0.03
1.63
0.73
0.03

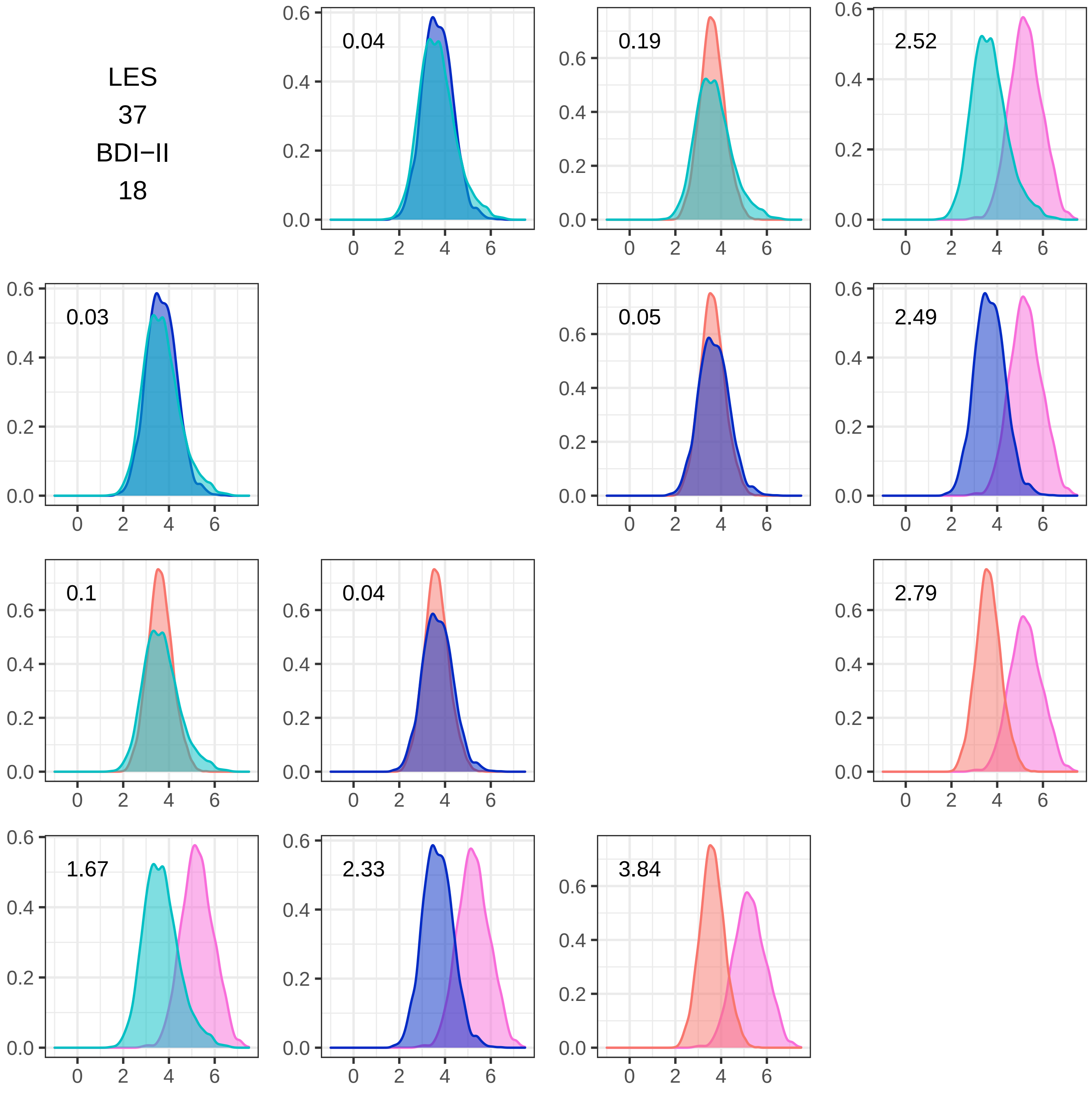
LES
37
BDI−II
18
0.04
0.19
2.52
0.03
0.05
2.49
0.1
0.04
2.79
1.67
2.33
3.84

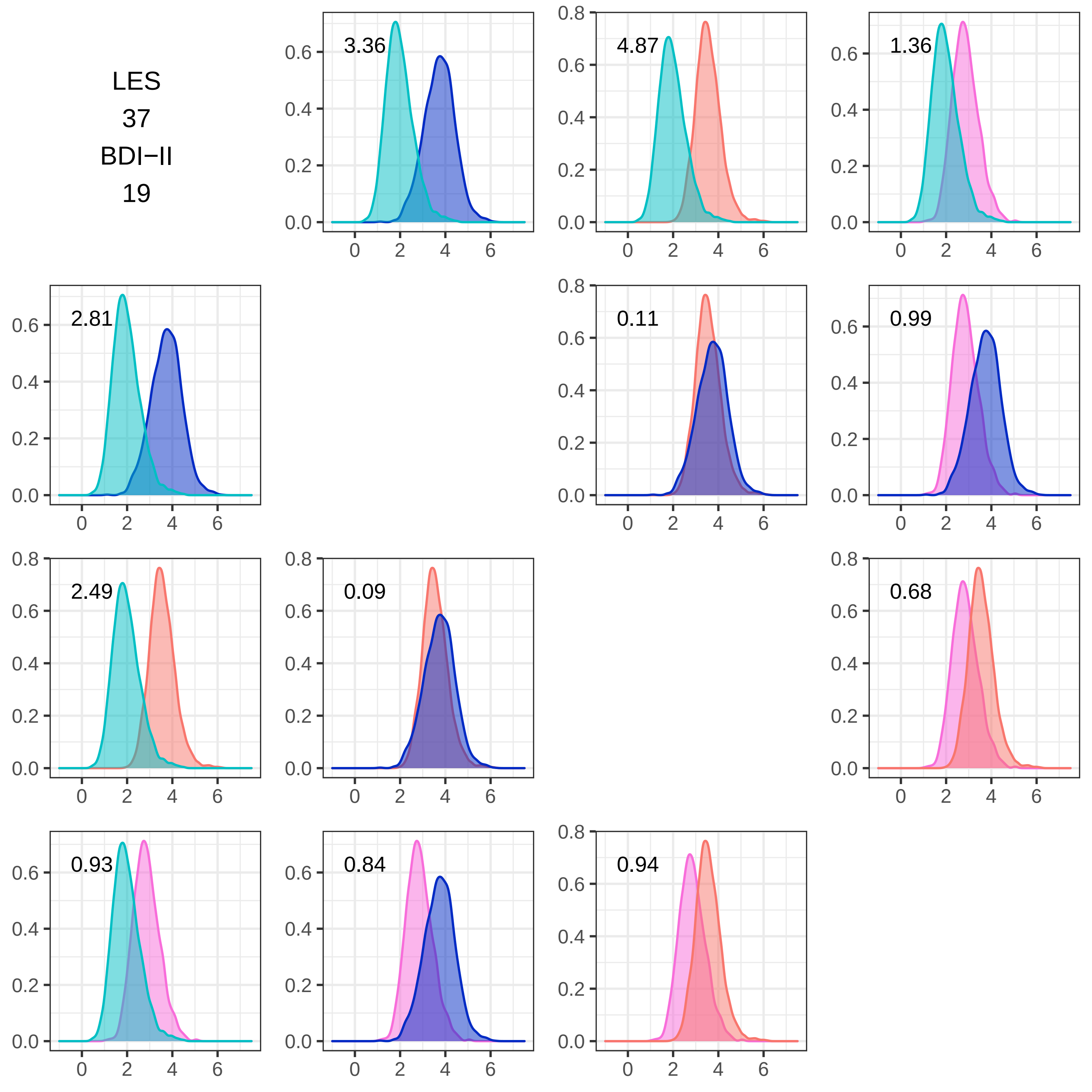
LES
37
BDI−II
19
3.36
4.87
1.36
2.81
0.11
0.99
2.49
0.09
0.68
0.93
0.84
0.94

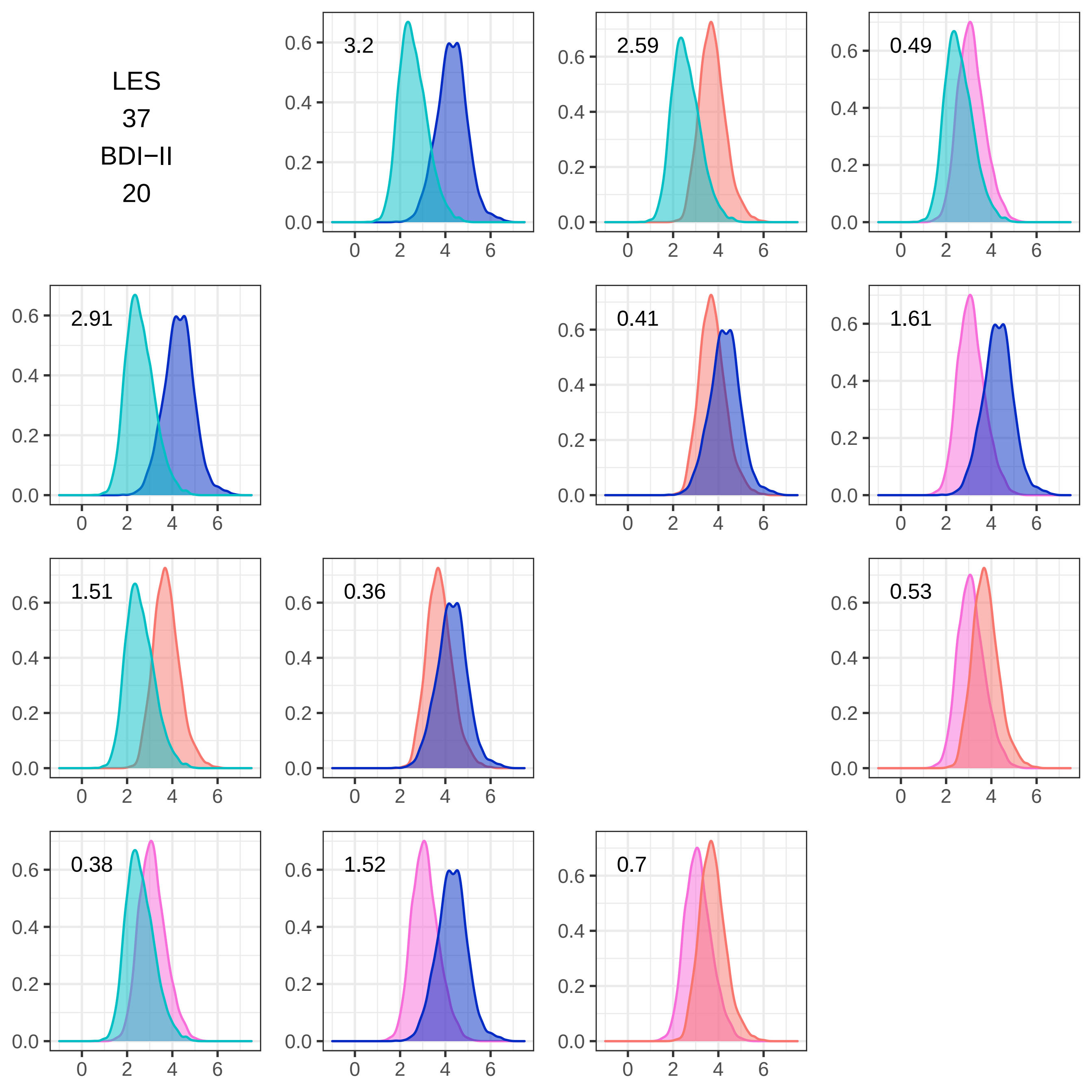
LES
37
BDI−II
20
3.2
2.59
0.49
2.91
0.41
1.61
1.51
0.36
0.53
0.38
1.52
0.7

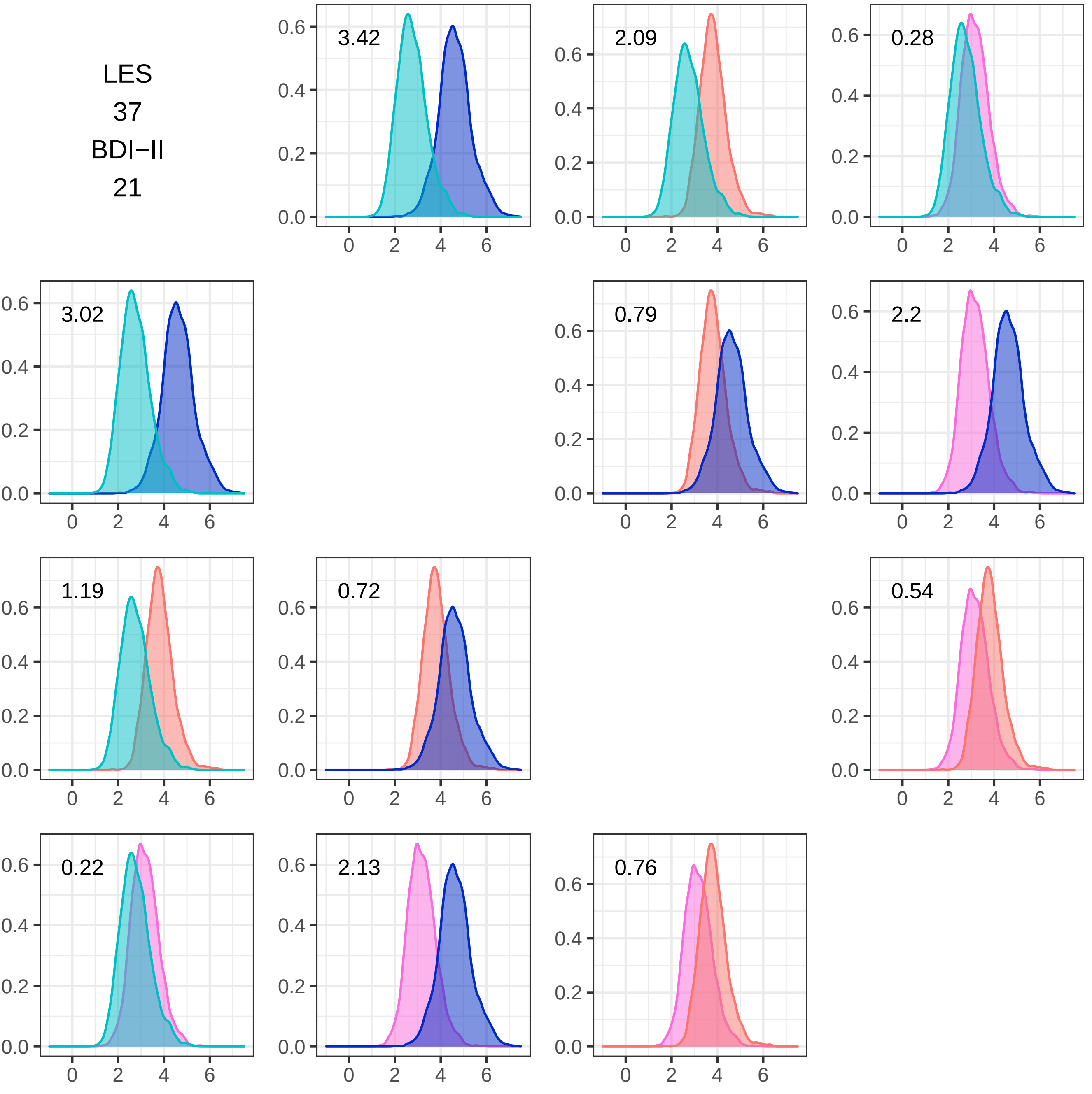

LES
37
BDI−II
21
3.42
2.09
0.28
3.02
0.79
2.2
1.19
0.72
0.54
0.22
2.13
0.76

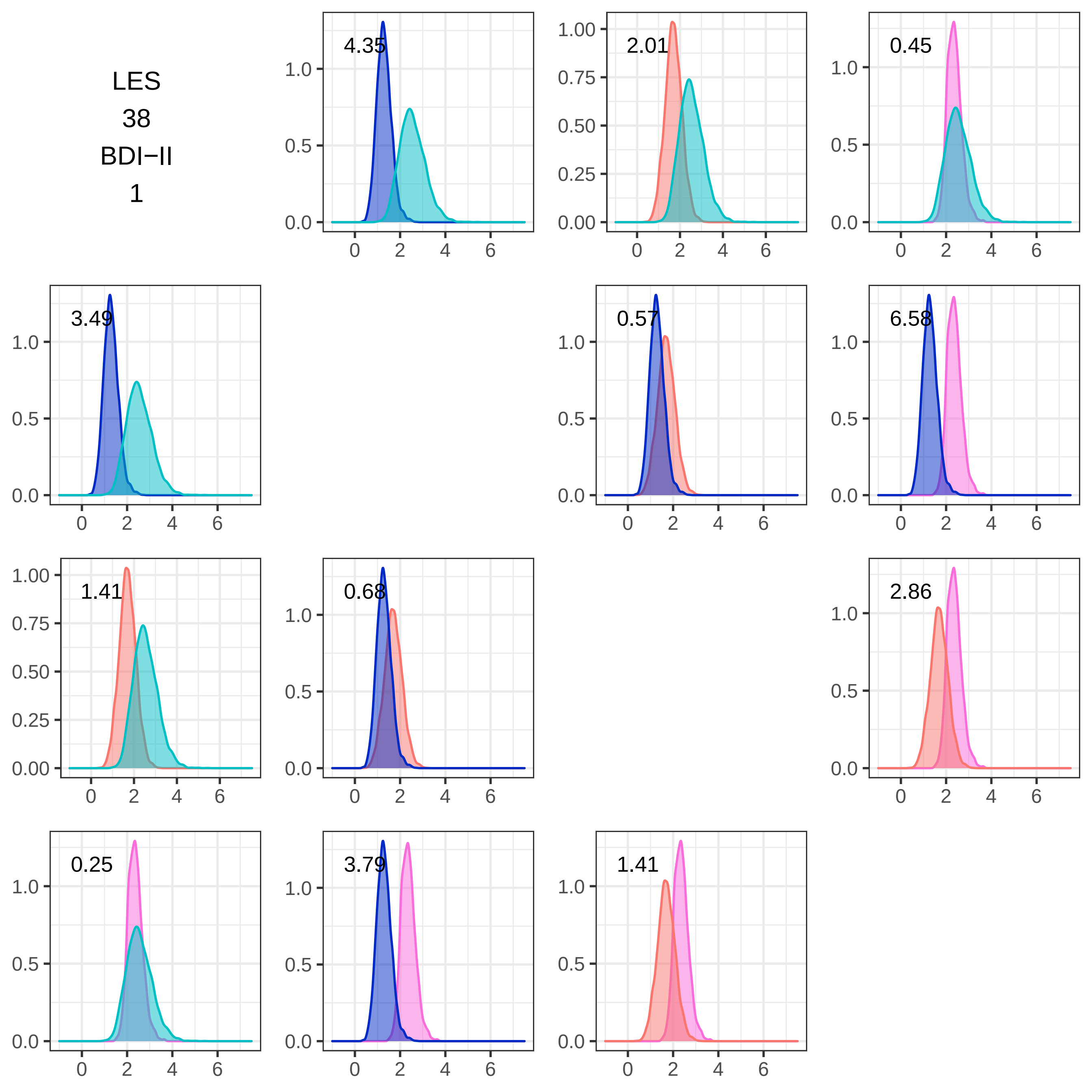
LES
38
BDI−II
1
4.35
2.01
0.45
3.49
0.57
6.58
1.41
0.68
2.86
0.25
3.79
1.41

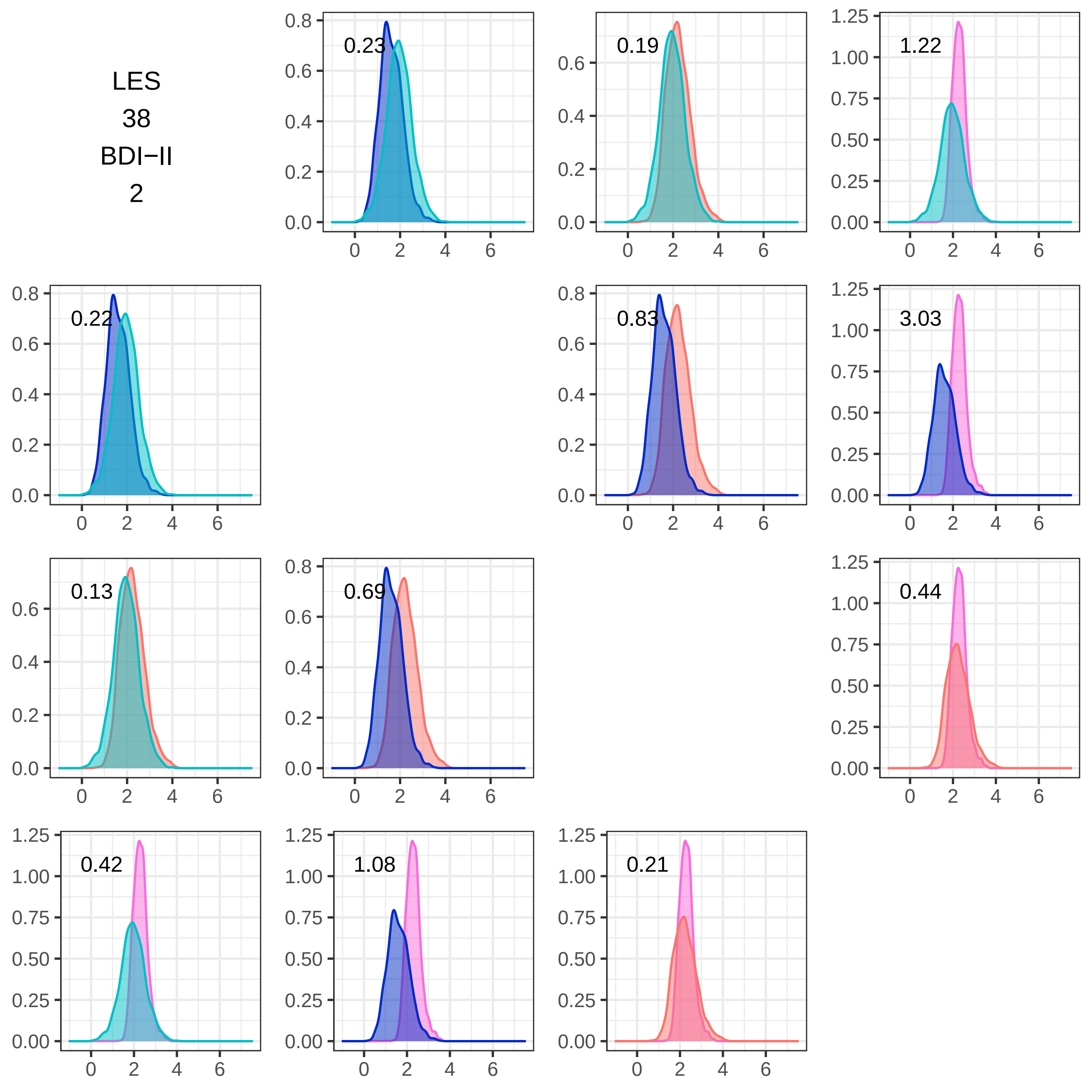

LES
38
BDI−II
2
0.23
0.19
1.22
0.22
0.83
3.03
0.13
0.69
0.44
0.42
1.08
0.21

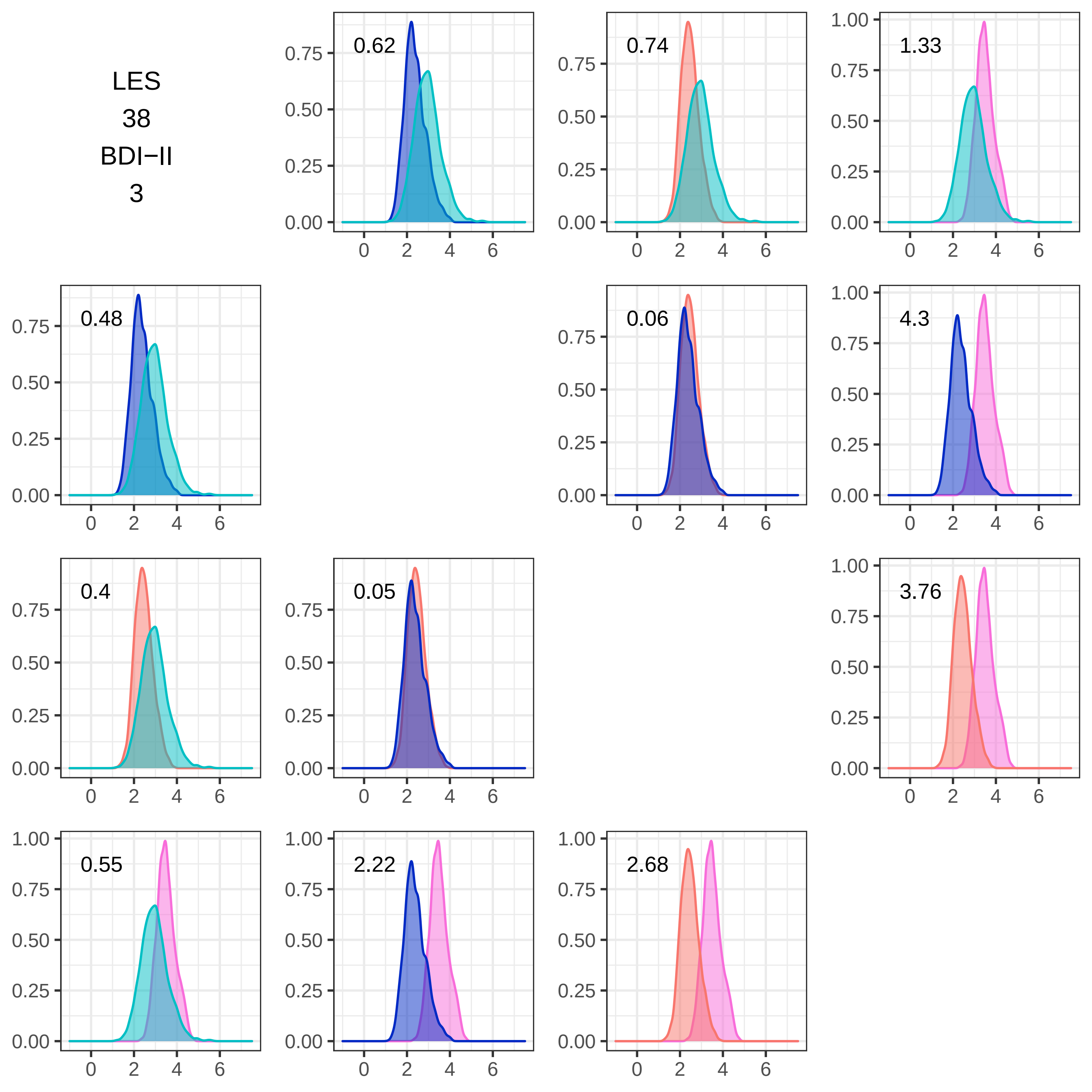
LES
38
BDI−II
3
0.62
0.74
1.33
0.48
0.06
4.3
0.4
0.05
3.76
0.55
2.22
2.68

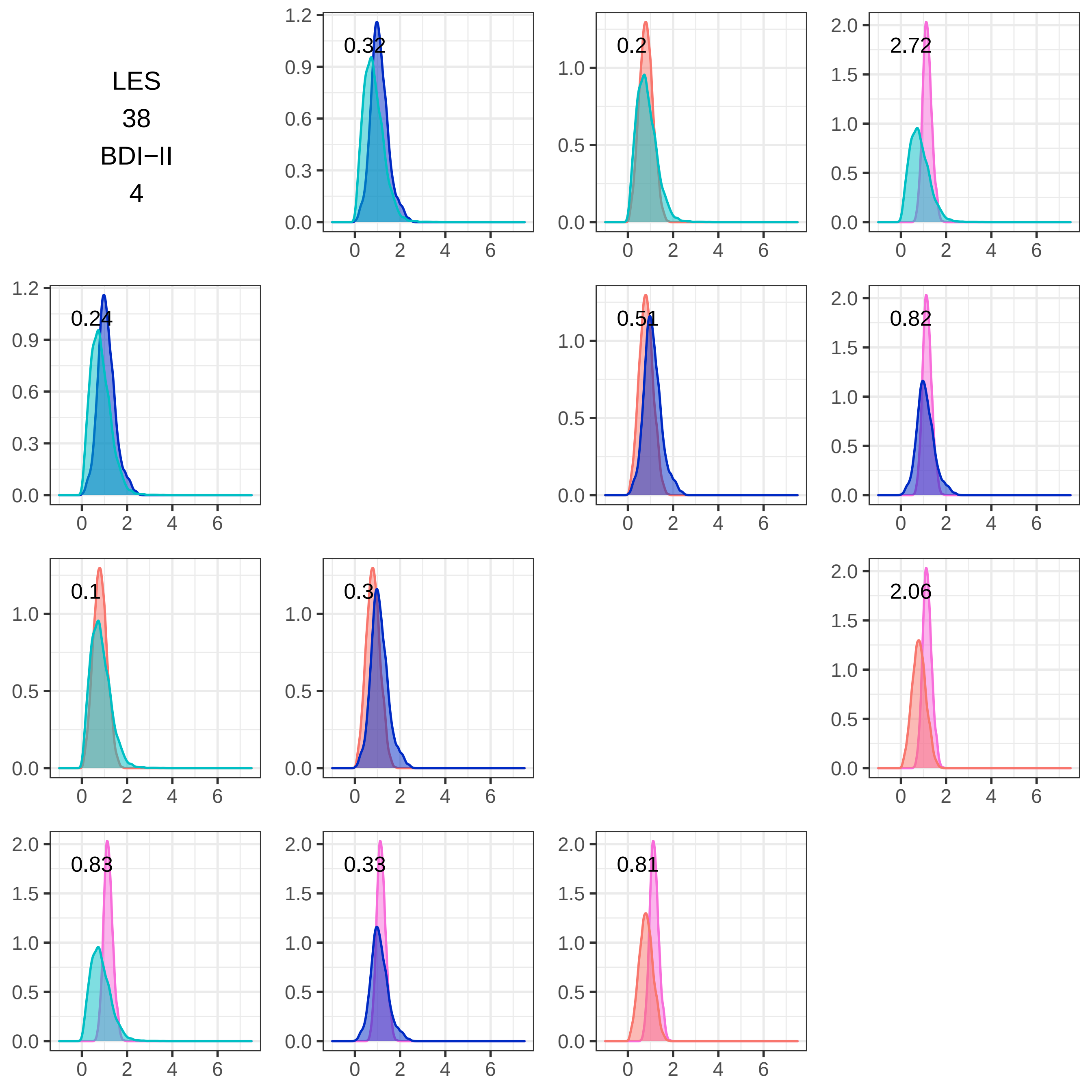
LES
38
BDI−II
4
0.32
0.2
2.72
0.24
0.51
0.82
0.1
0.3
2.06
0.83
0.33
0.81

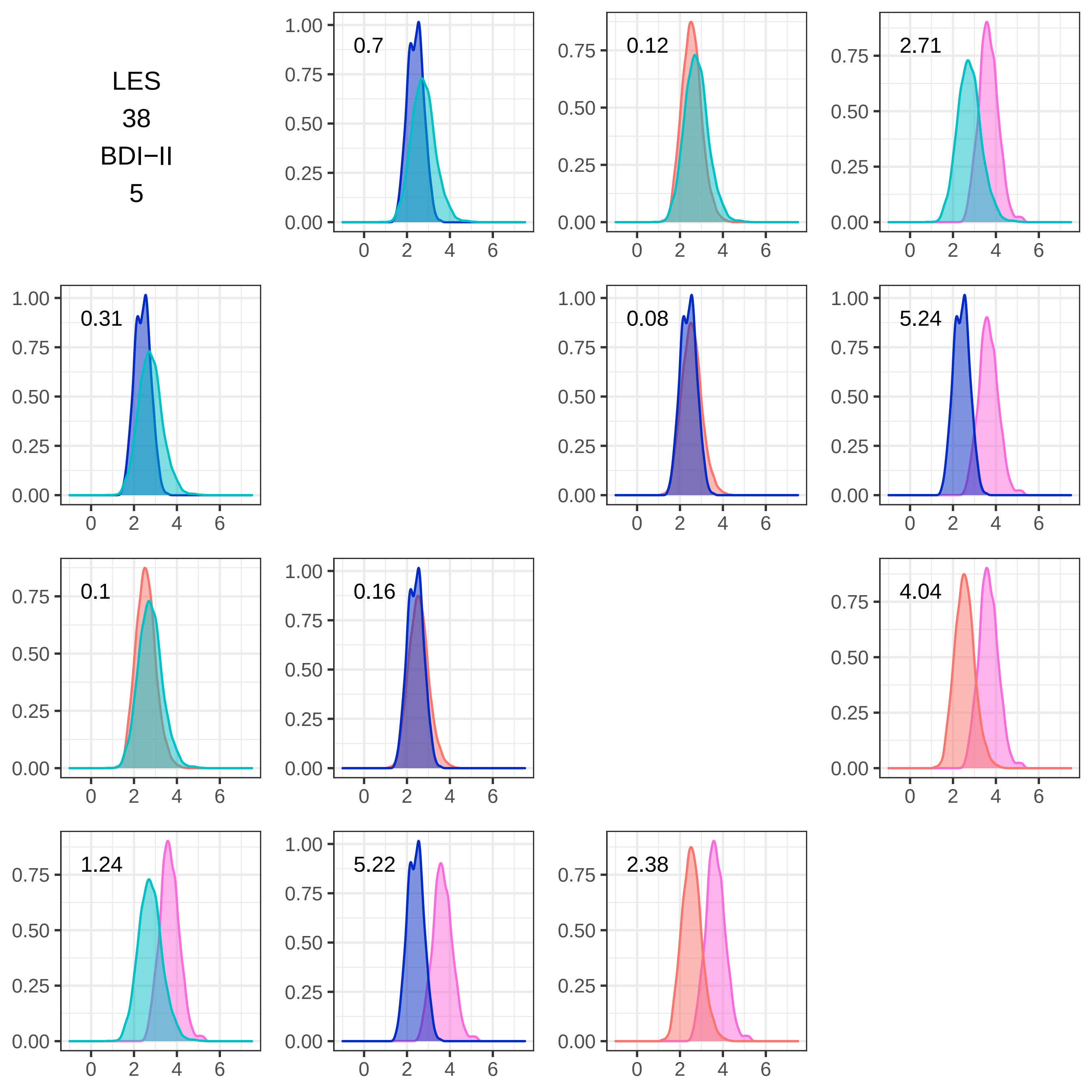

LES
38
BDI−II
5
0.7
0.12
2.71
0.31
0.08
5.24
0.1
0.16
4.04
1.24
5.22
2.38

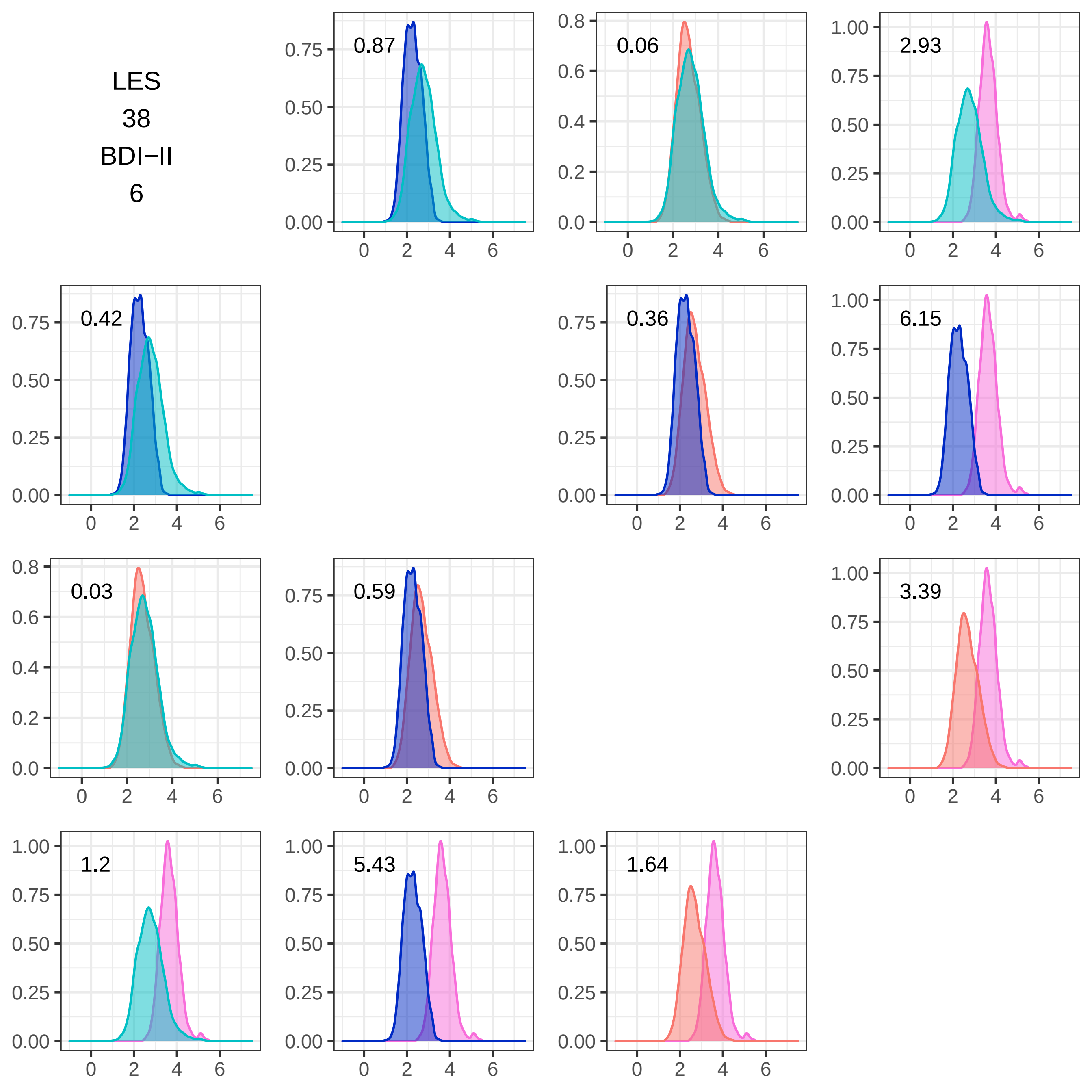
LES
38
BDI−II
6
0.87
0.06
2.93
0.42
0.36
6.15
0.03
0.59
3.39
1.2
5.43
1.64

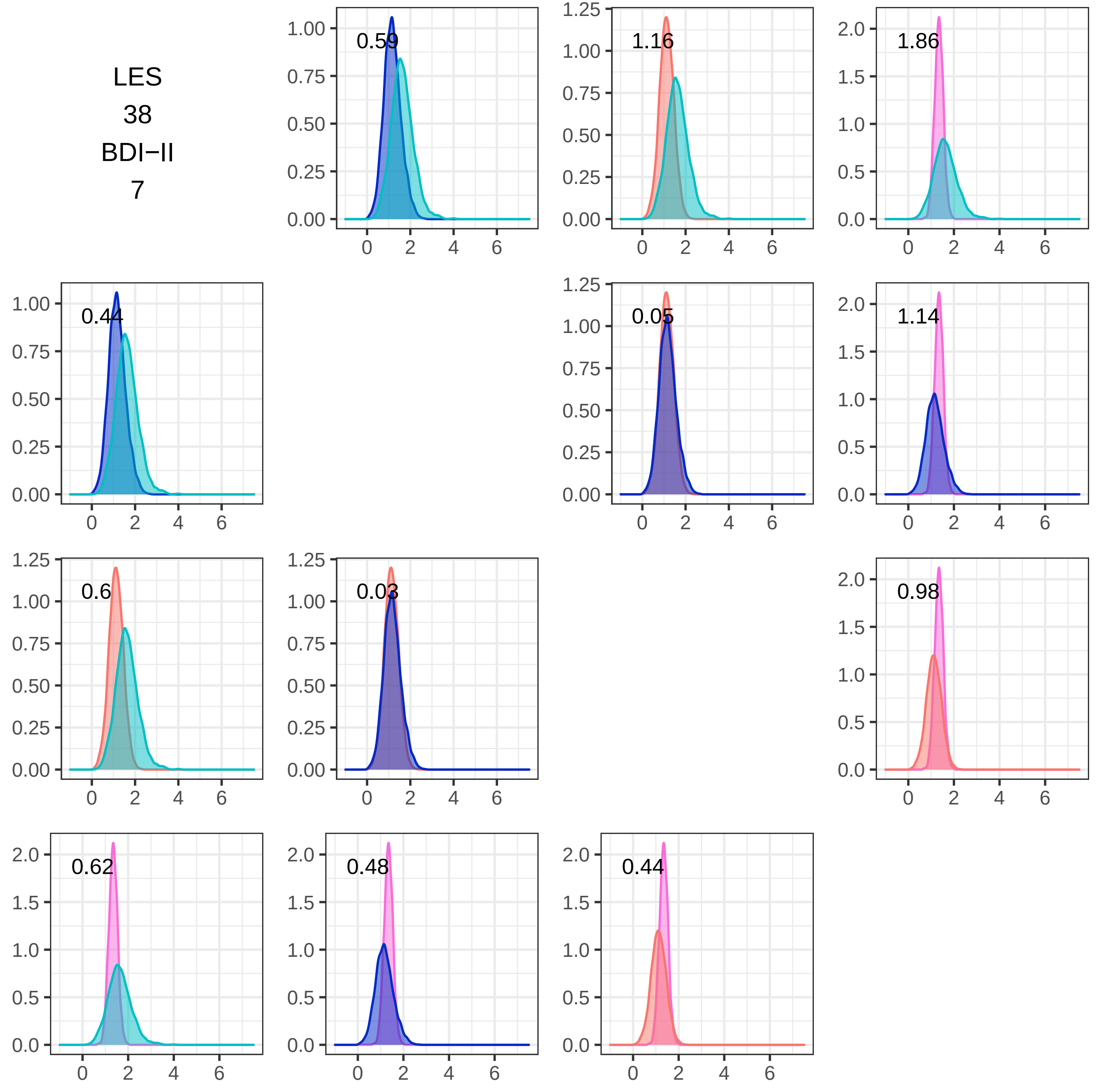

LES
38
BDI−II
7
0.59
1.16
1.86
0.44
0.05
1.14
0.6
0.03
0.98
0.62
0.48
0.44

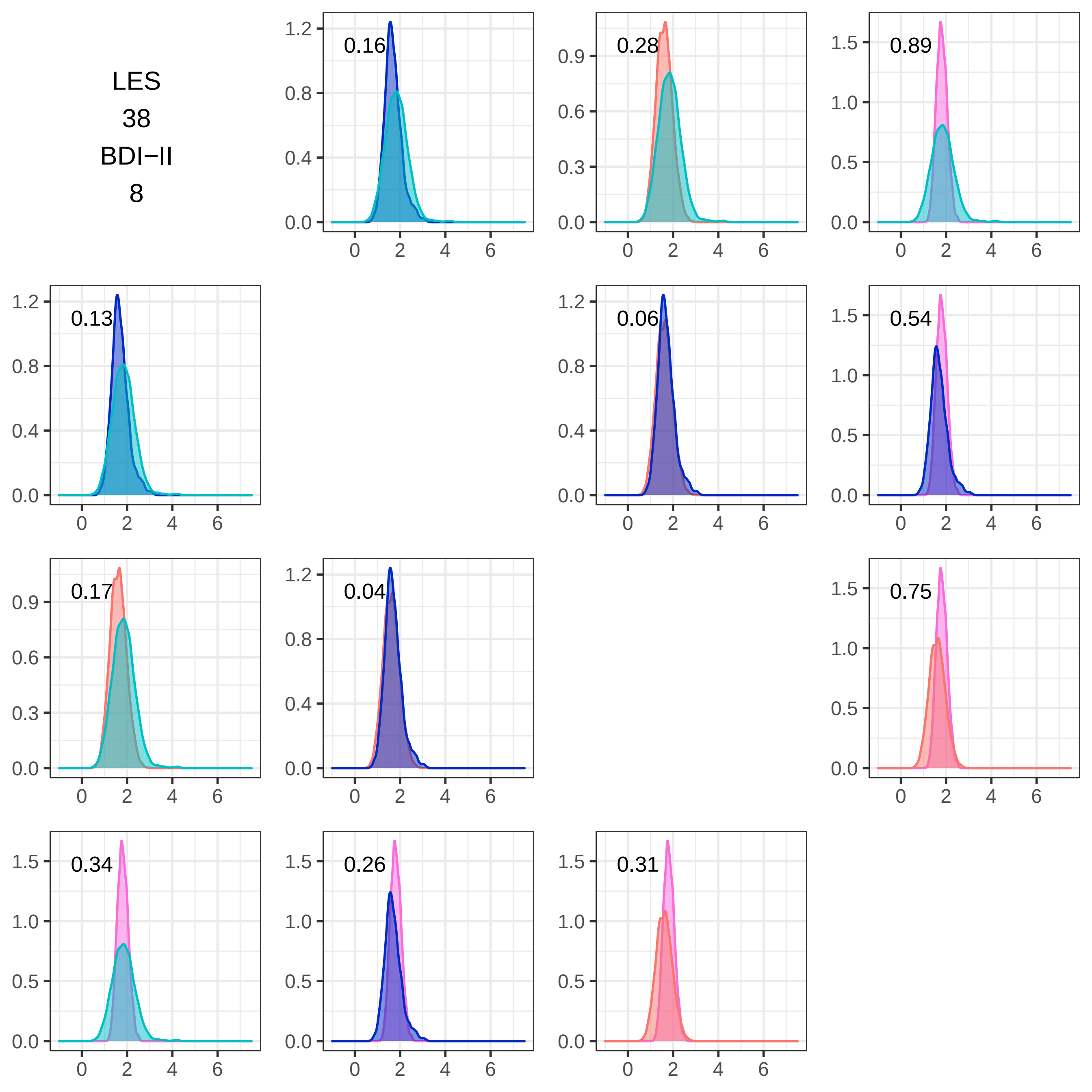
LES
38
BDI−II
8
0.16
0.28
0.89
0.13
0.06
0.54
0.17
0.04
0.75
0.34
0.26
0.31

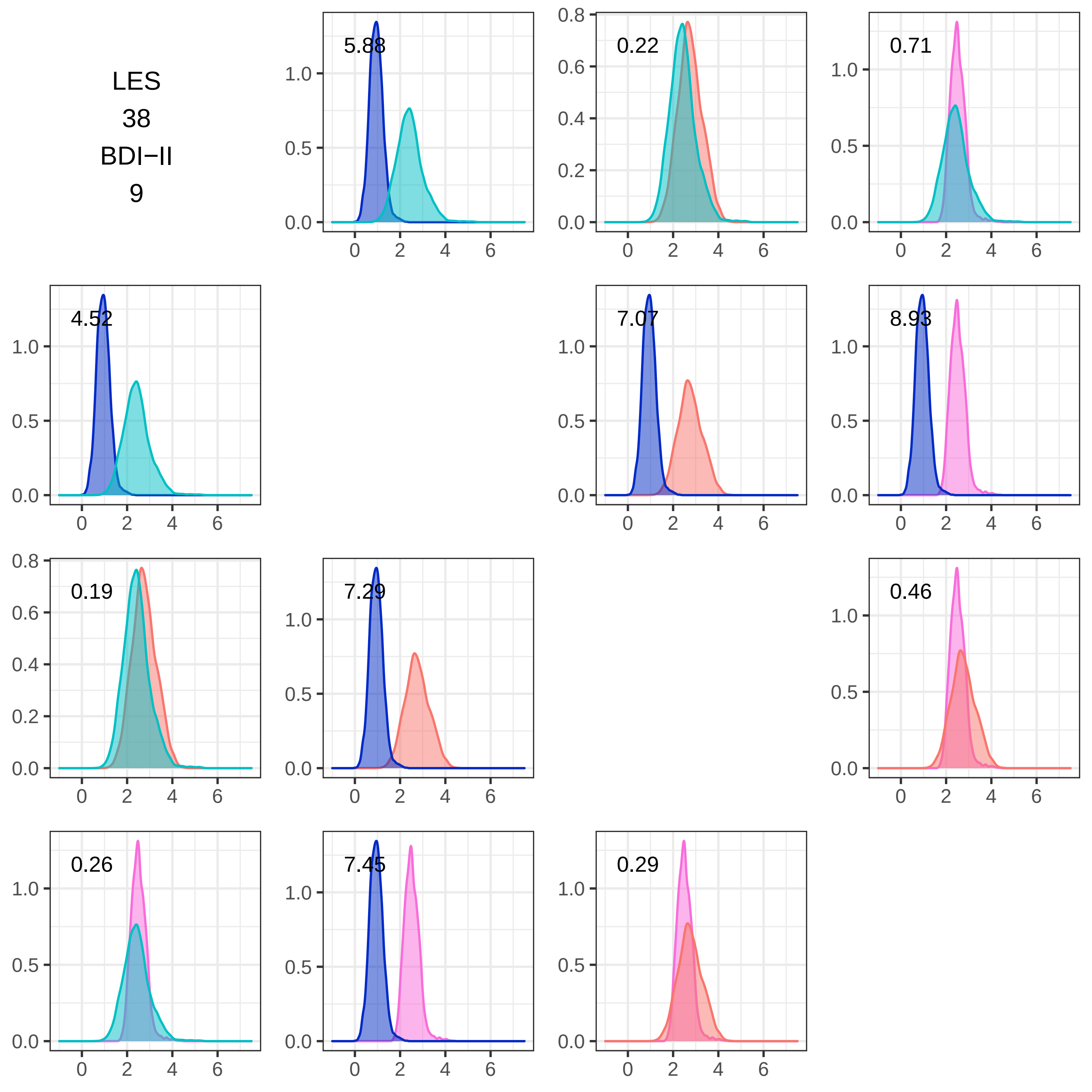

LES
38
BDI−II
9
5.88
0.22
0.71
4.52
7.07
8.93
0.19
7.29
0.46
0.26
7.45
0.29

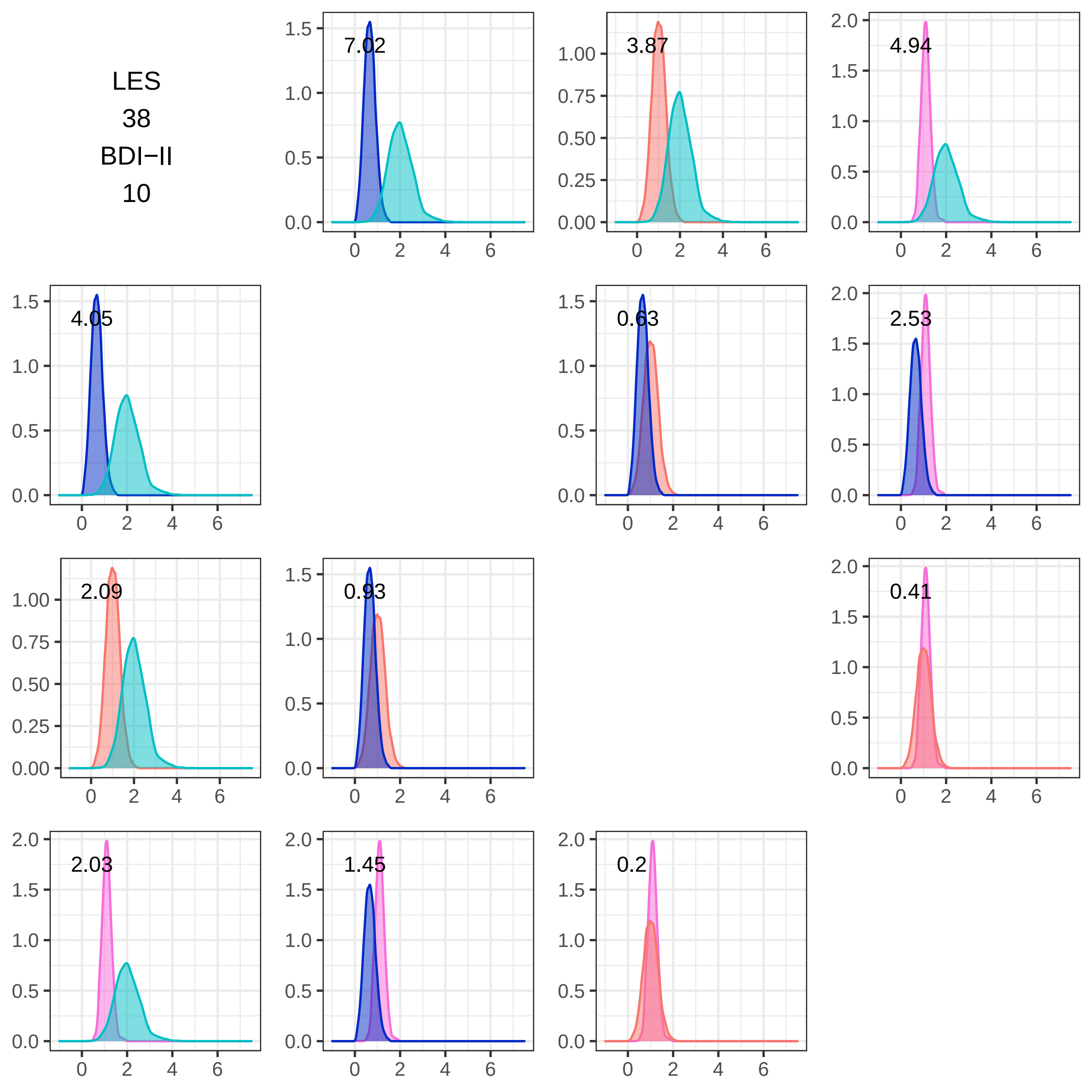
LES
38
BDI−II
10
7.02
3.87
4.94
4.05
0.63
2.53
2.09
0.93
0.41
2.03
1.45
0.2

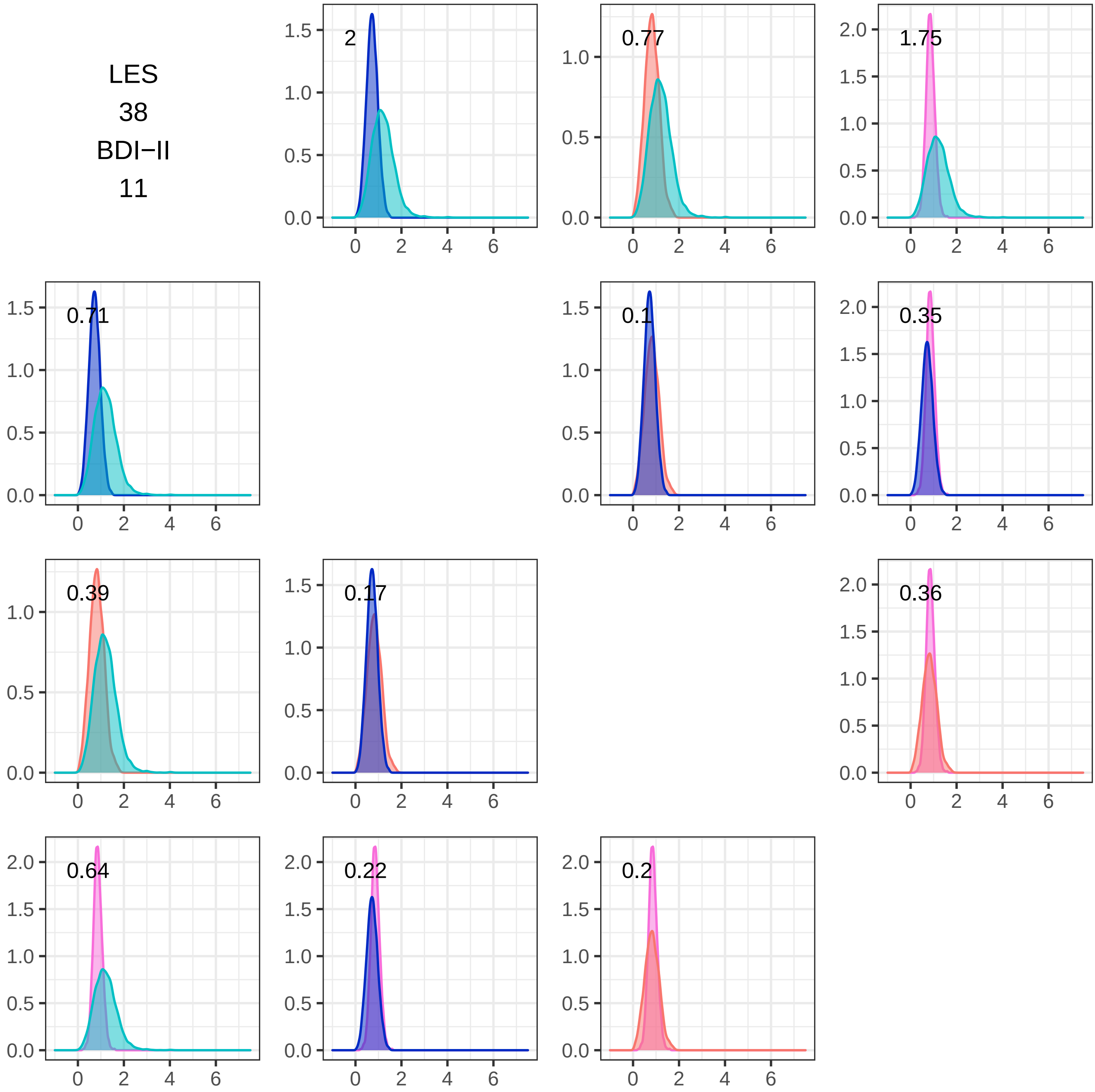
LES
38
BDI−II
11
2
0.77
1.75
0.71
0.1
0.35
0.39
0.17
0.36
0.64
0.22
0.2

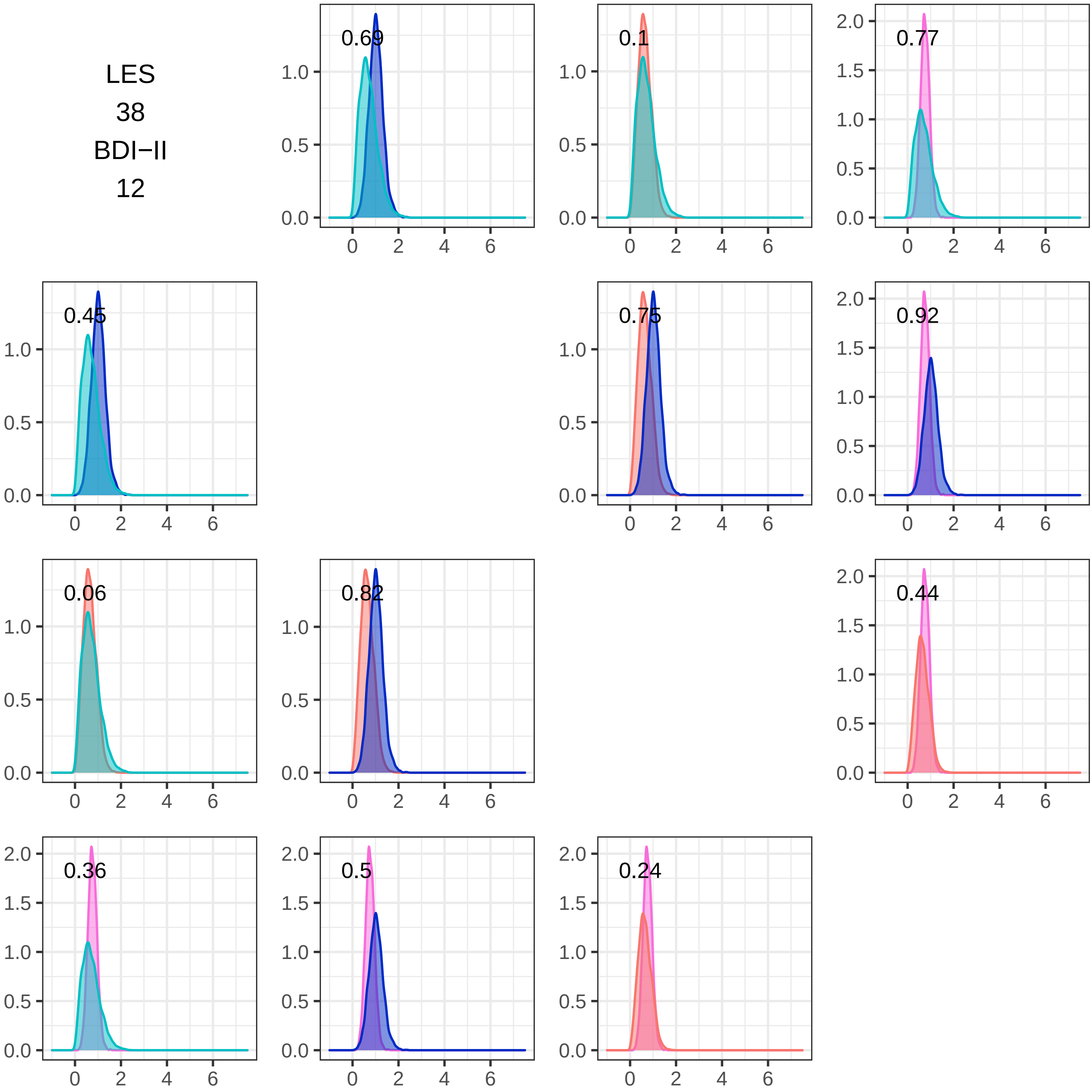
LES
38
BDI−II
12
0.69
0.1
0.77
0.45
0.75
0.92
0.06
0.82
0.44
0.36
0.5
0.24

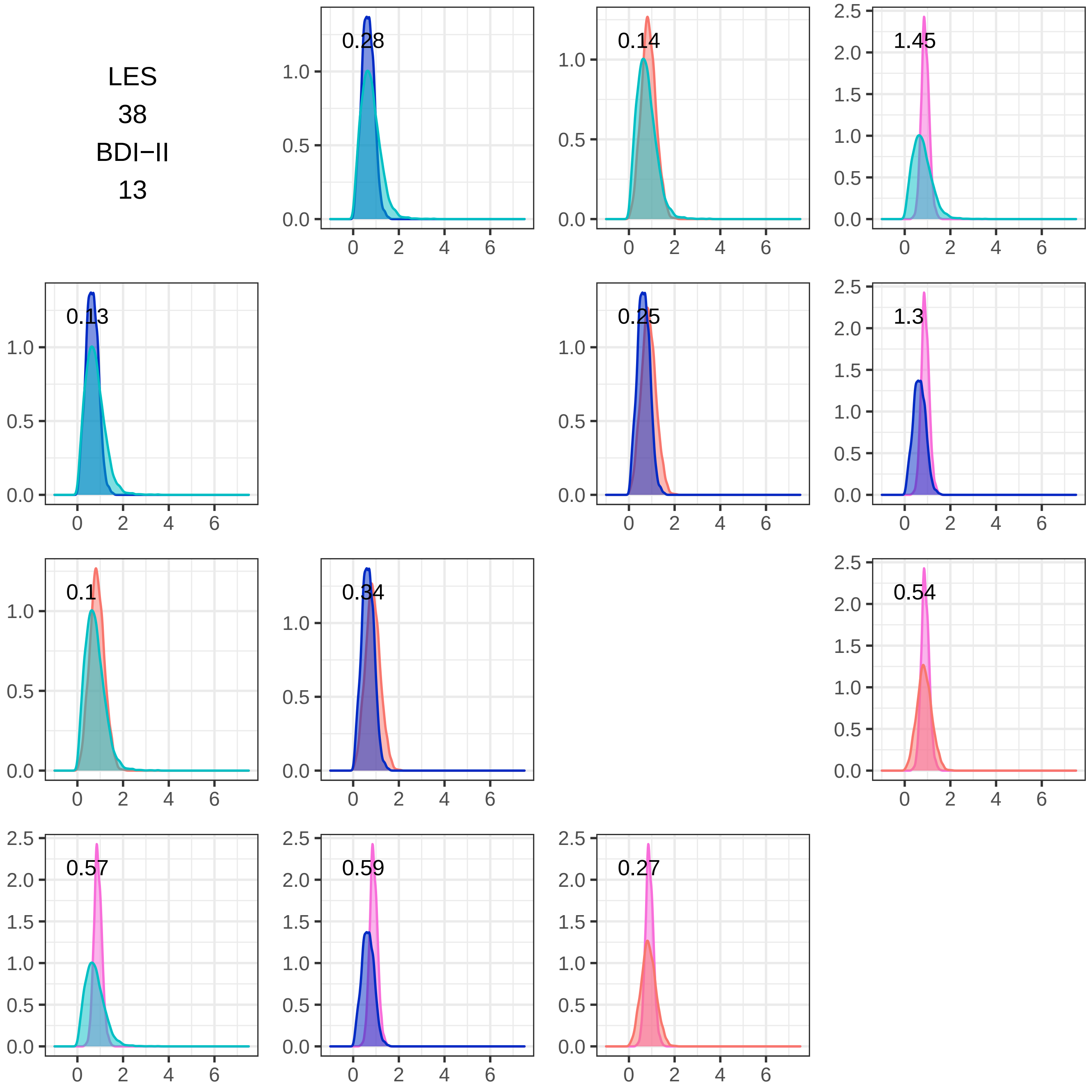
LES
38
BDI−II
13
0.28
0.14
1.45
0.13
0.25
1.3
0.1
0.34
0.54
0.57
0.59
0.27

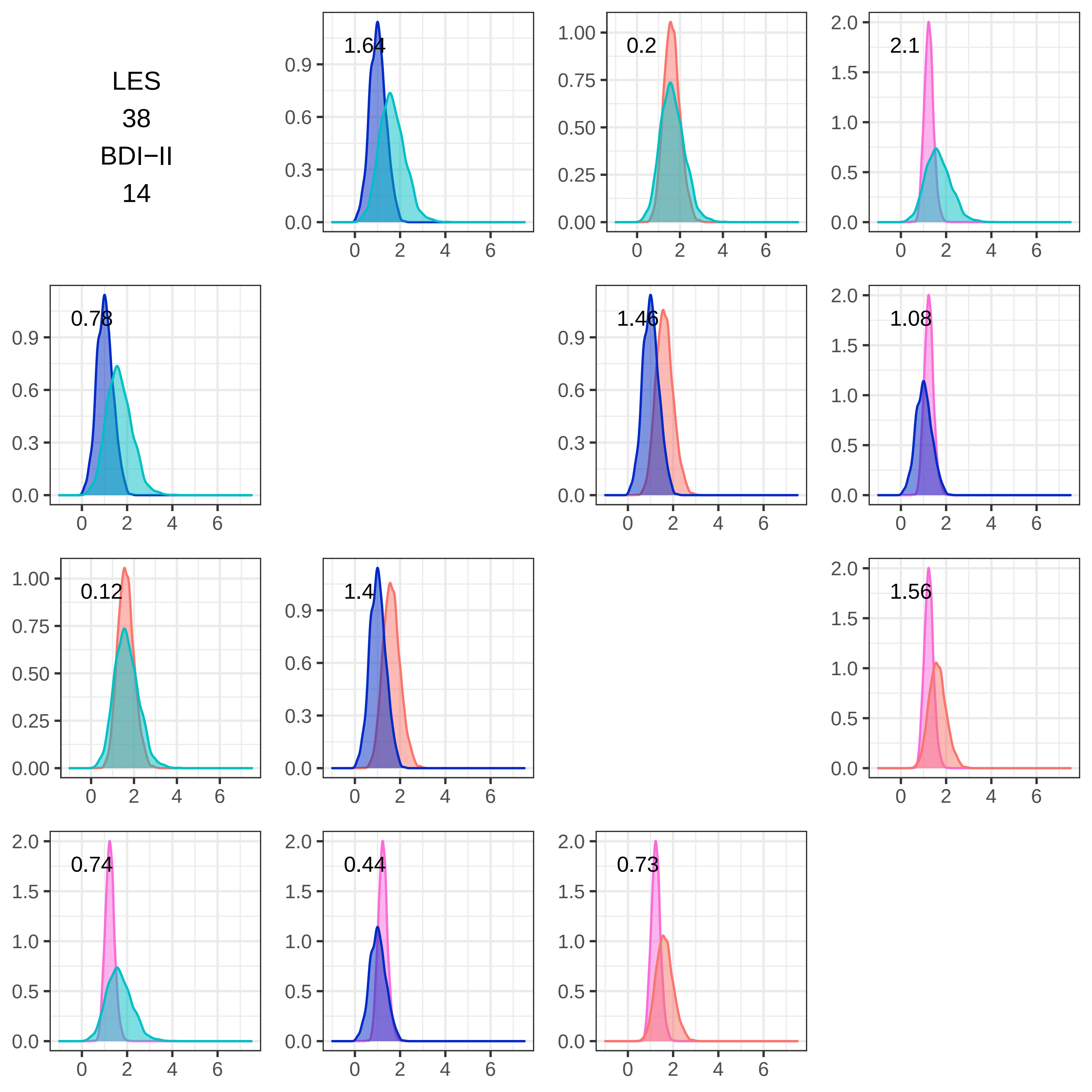

LES
38
BDI−II
14
1.64
0.2
2.1
0.78
1.46
1.08
0.12
1.4
1.56
0.74
0.44
0.73

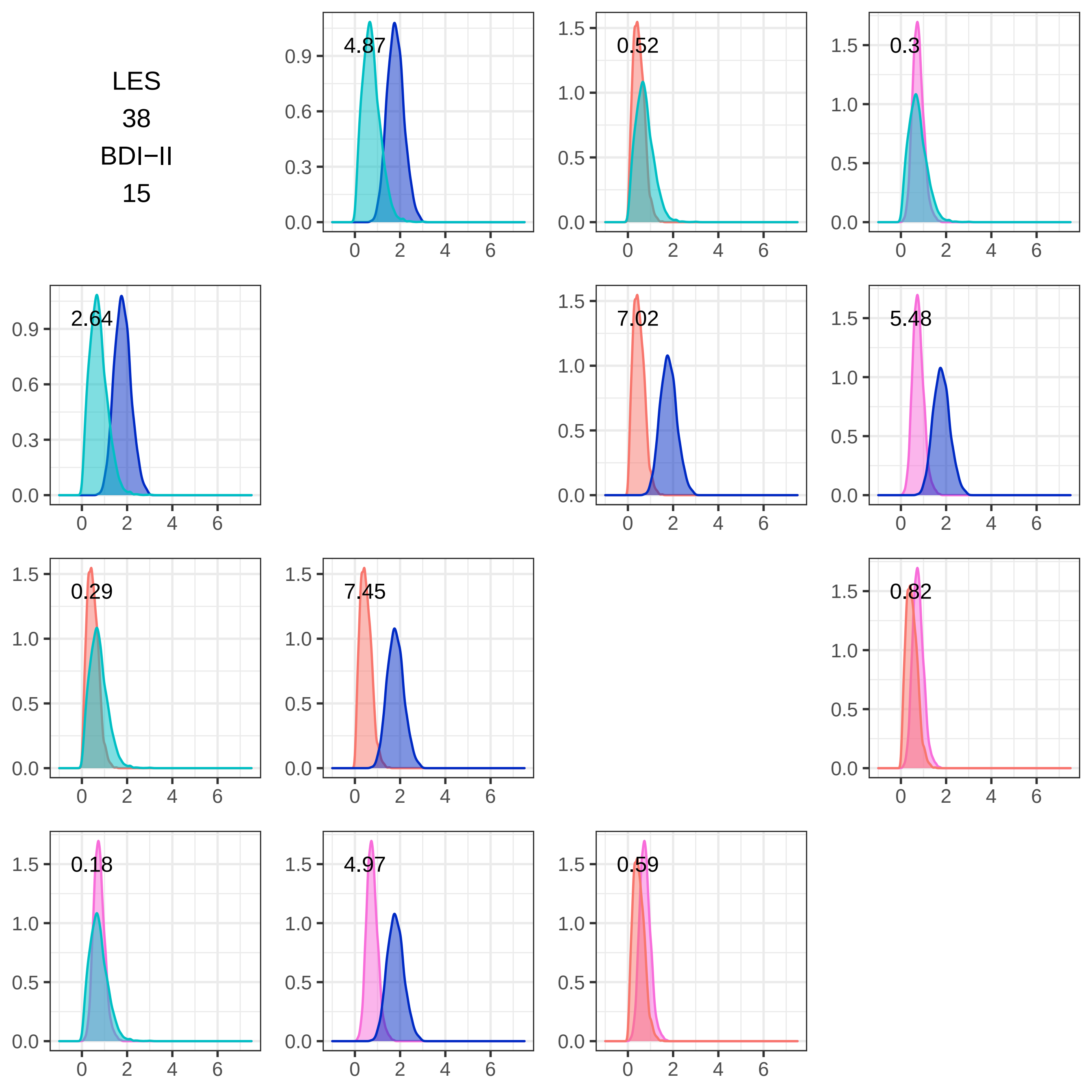
LES
38
BDI−II
15
4.87
0.52
0.3
2.64
7.02
5.48
0.29
7.45
0.82
0.18
4.97
0.59

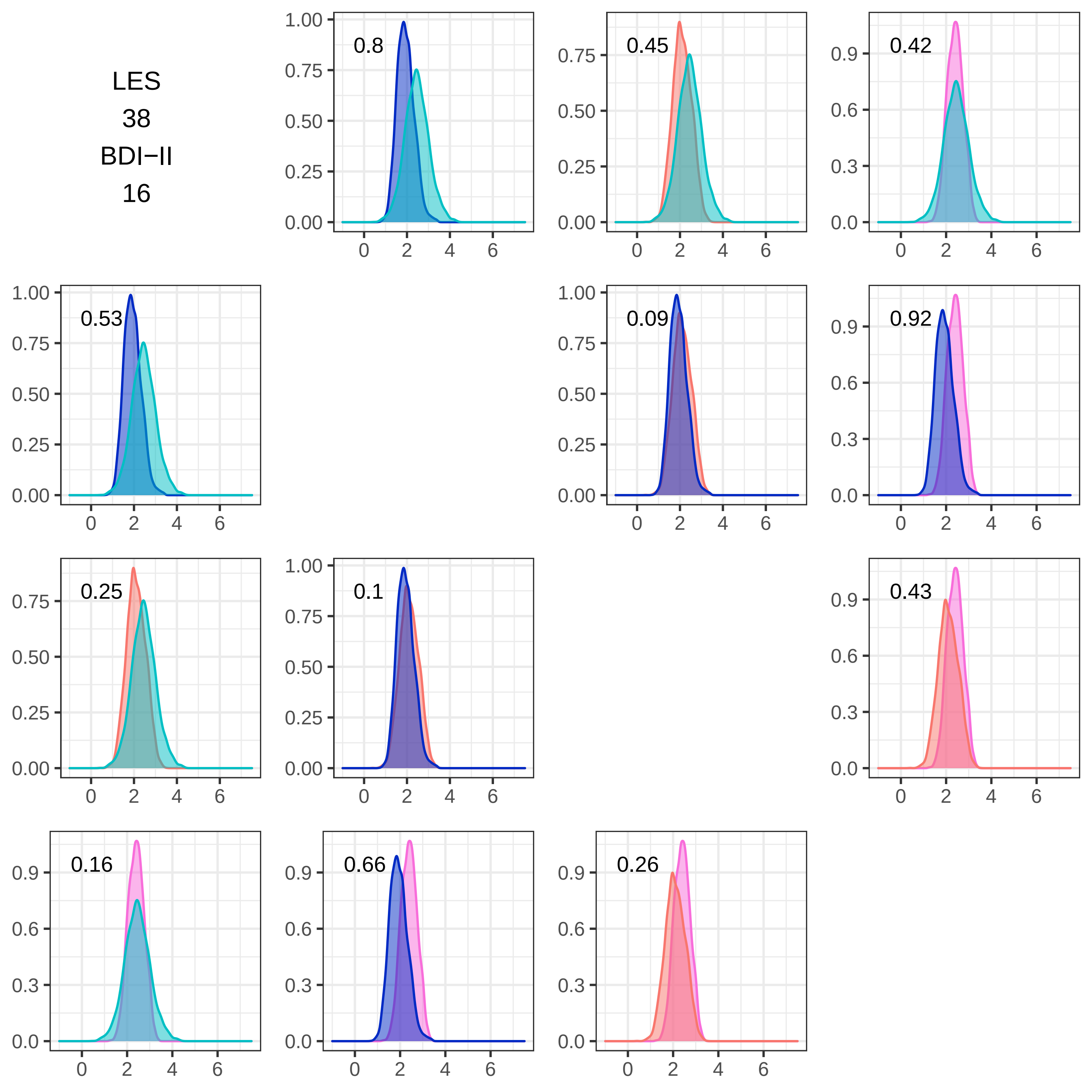
LES
38
BDI−II
16
0.8
0.45
0.42
0.53
0.09
0.92
0.25
0.1
0.43
0.16
0.66
0.26

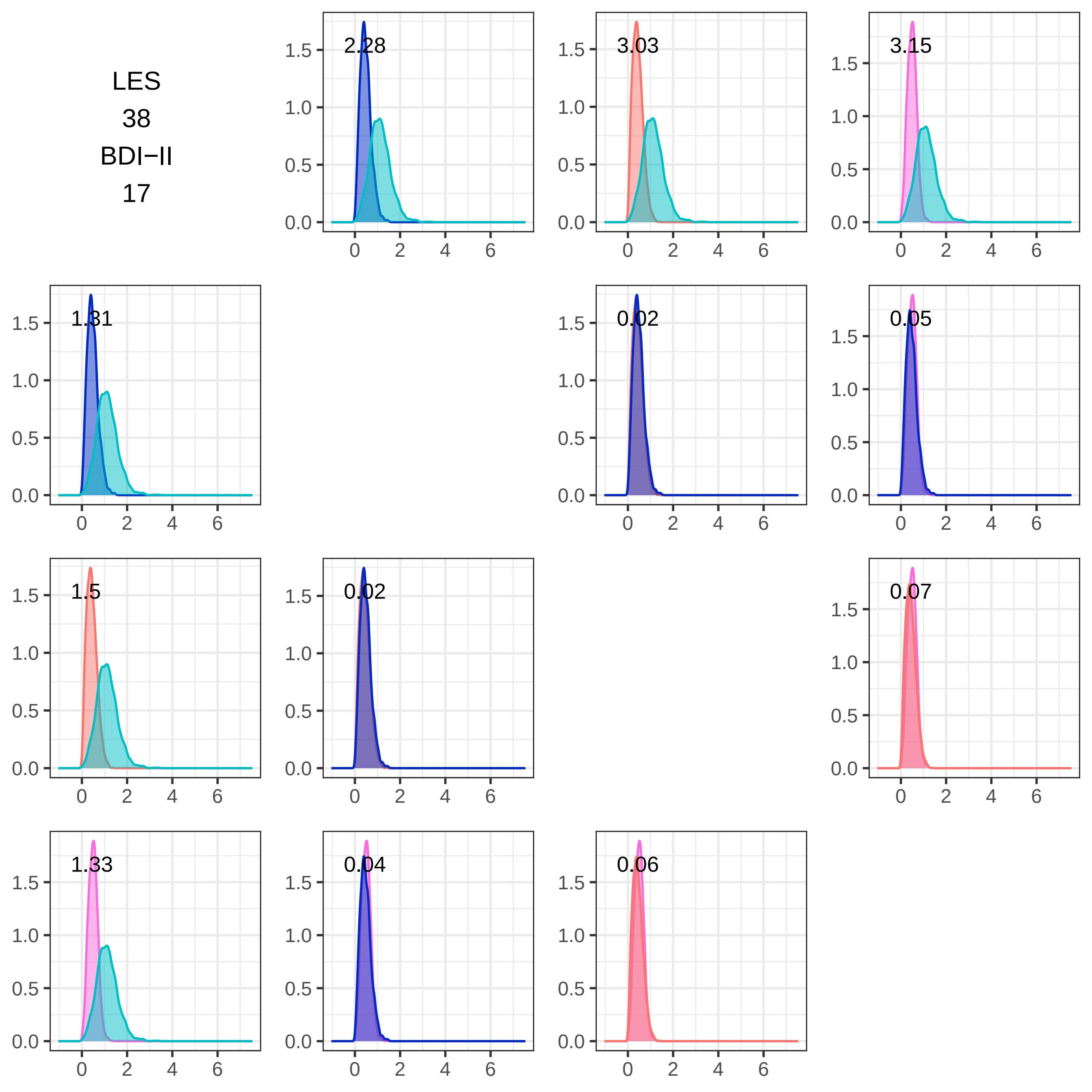
LES
38
BDI−II
17
2.28
3.03
3.15
1.31
0.02
0.05
1.5
0.02
0.07
1.33
0.04
0.06

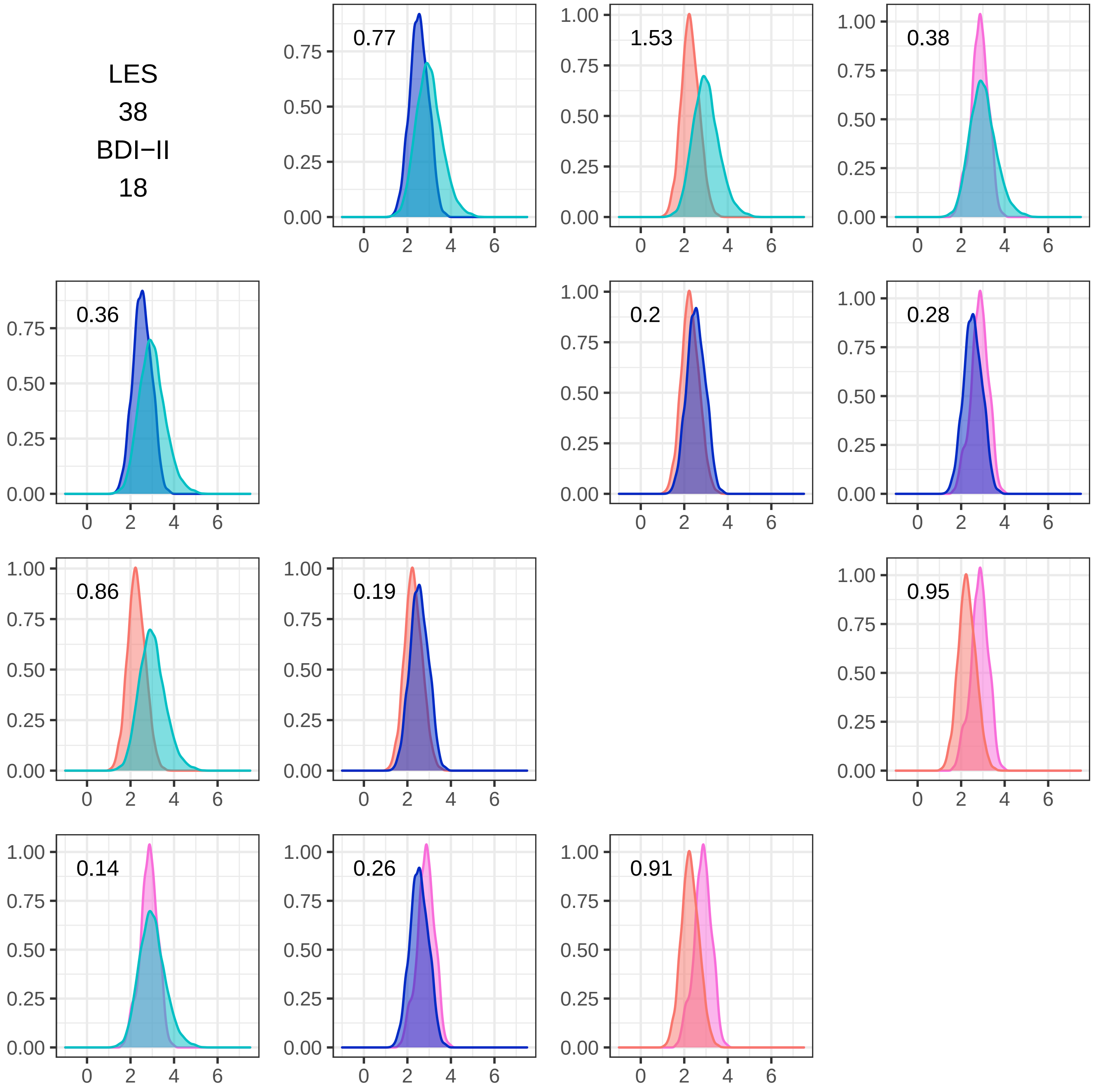
LES
38
BDI−II
18
0.77
1.53
0.38
0.36
0.2
0.28
0.86
0.19
0.95
0.14
0.26
0.91

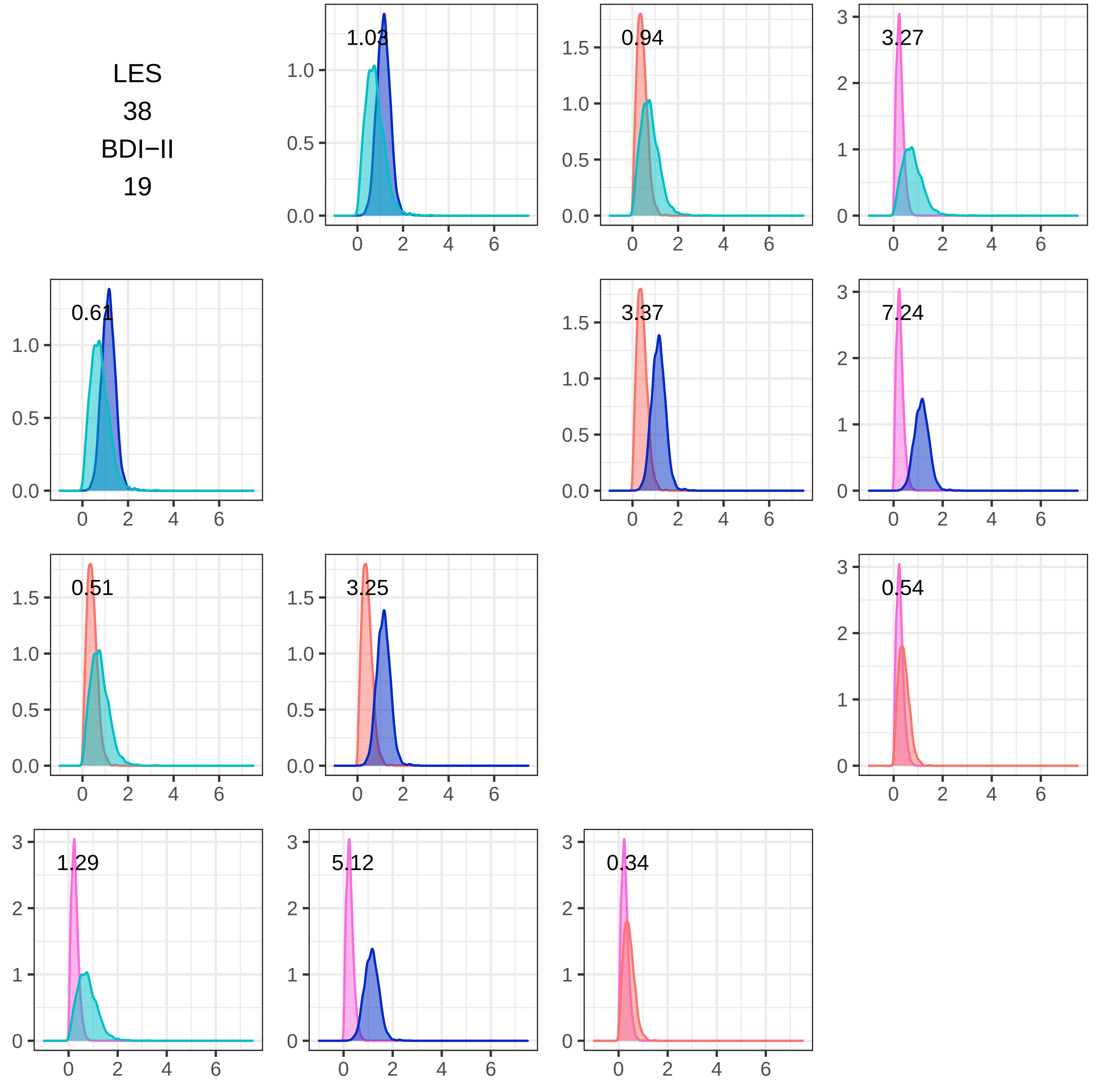

LES
38
BDI−II
19
1.03
0.94
3.27
0.61
3.37
7.24
0.51
3.25
0.54
1.29
5.12
0.34

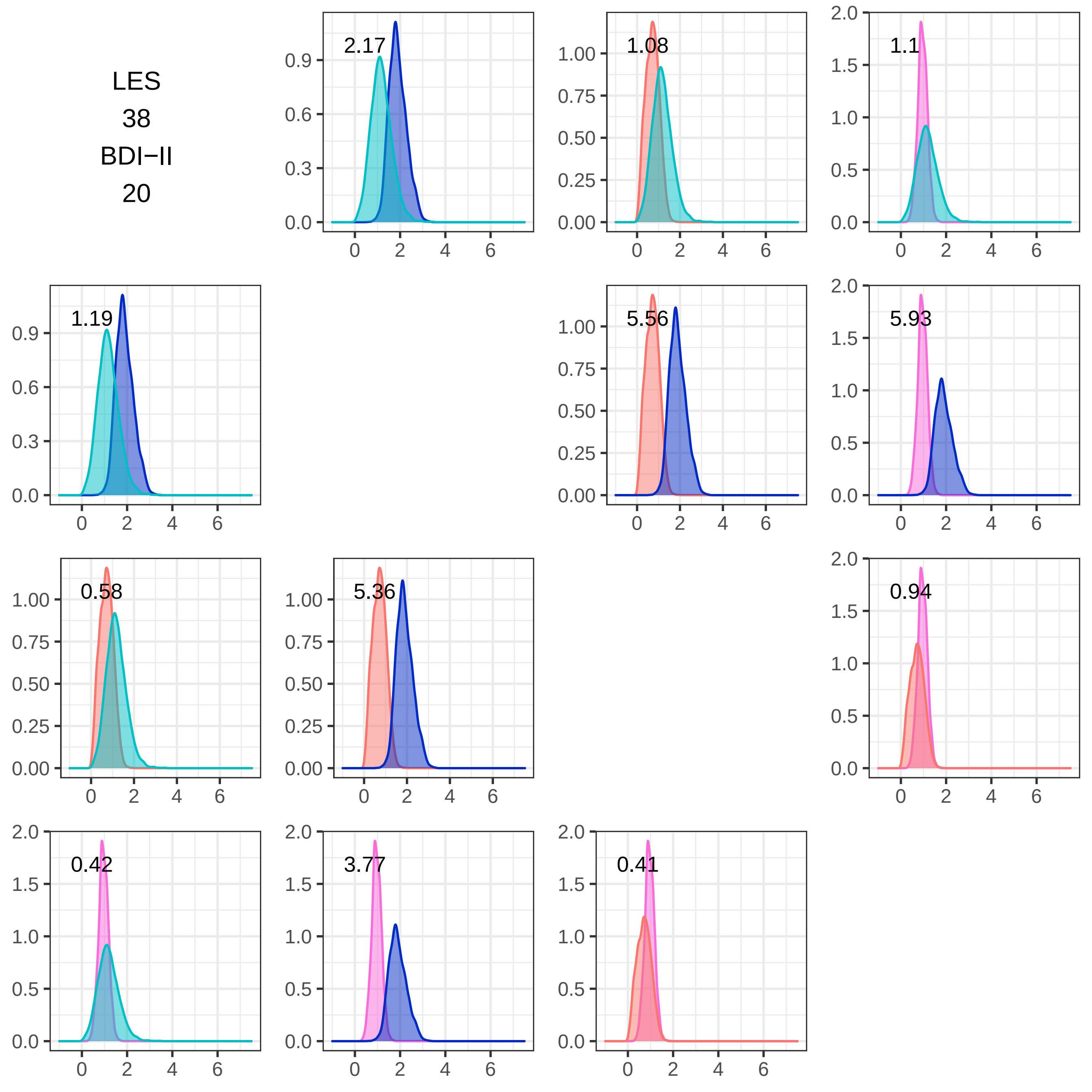

LES
38
BDI−II
20
2.17
1.08
1.1
1.19
5.56
5.93
0.58
5.36
0.94
0.42
3.77
0.41

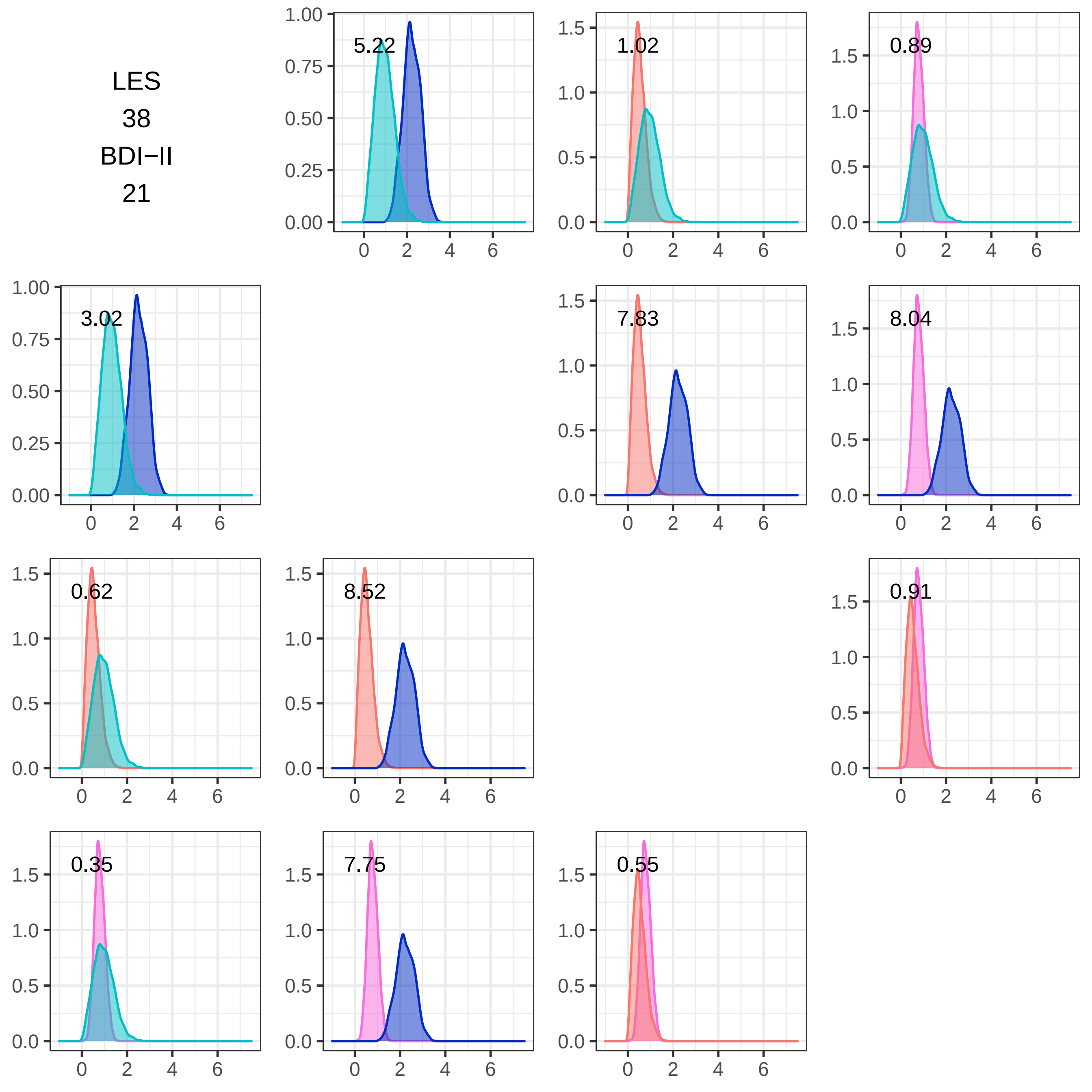

LES
38
BDI−II
21
5.22
1.02
0.89
3.02
7.83
8.04
0.62
8.52
0.91
0.35
7.75
0.55

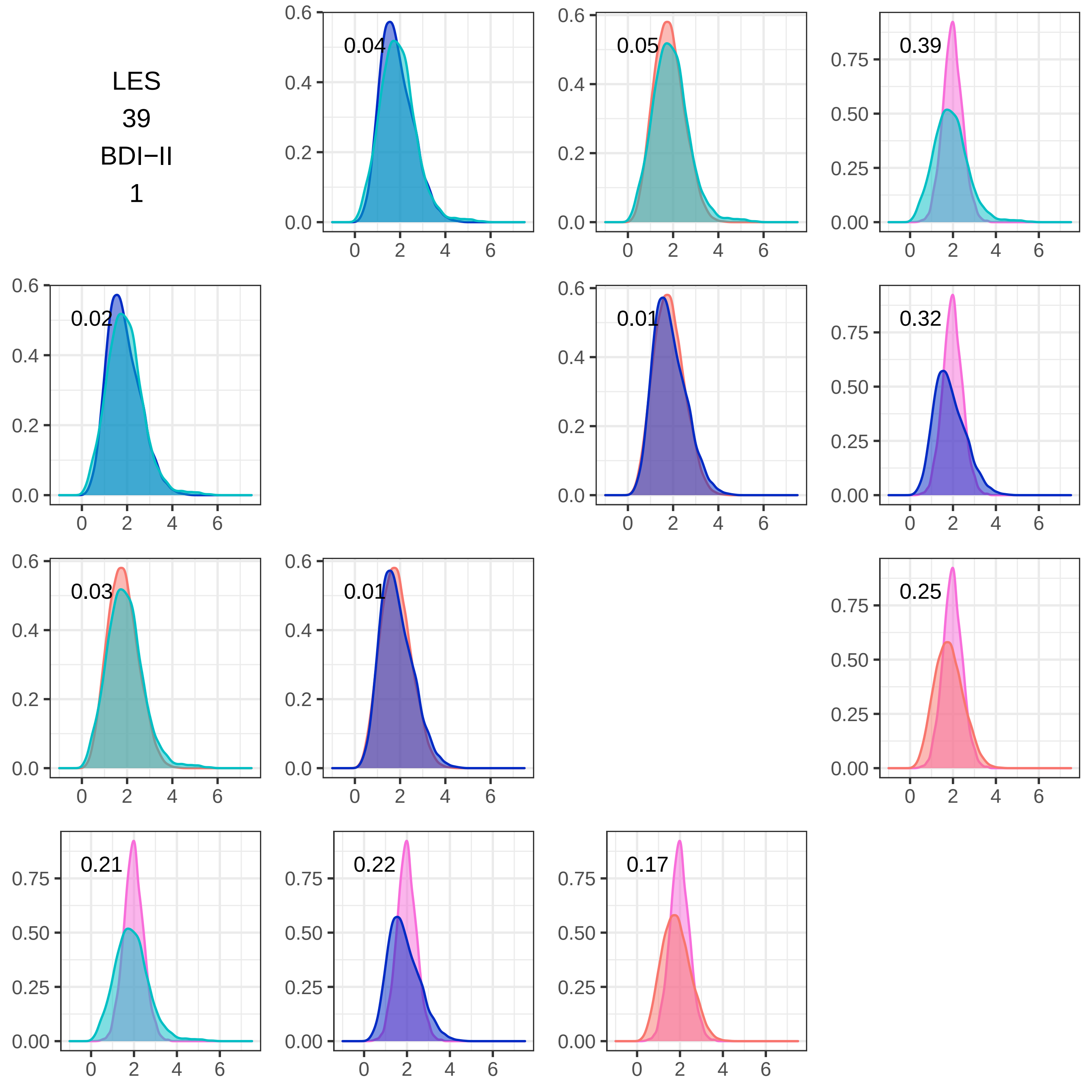

LES
39
BDI−II
1
0.04
0.05
0.39
0.02
0.01
0.32
0.03
0.01
0.25
0.21
0.22
0.17

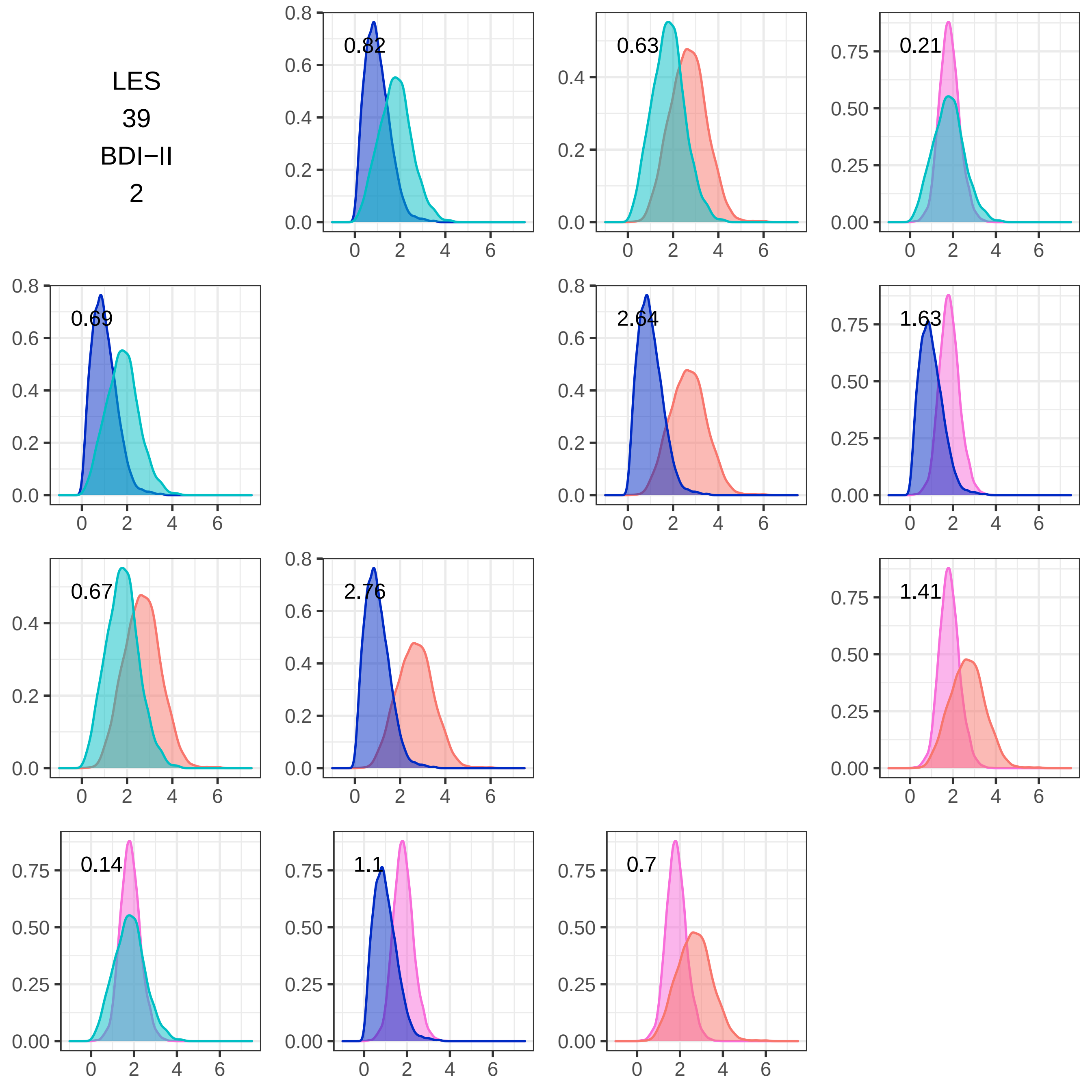
LES
39
BDI−II
2
0.82
0.63
0.21
0.69
2.64
1.63
0.67
2.76
1.41
0.14
1.1
0.7

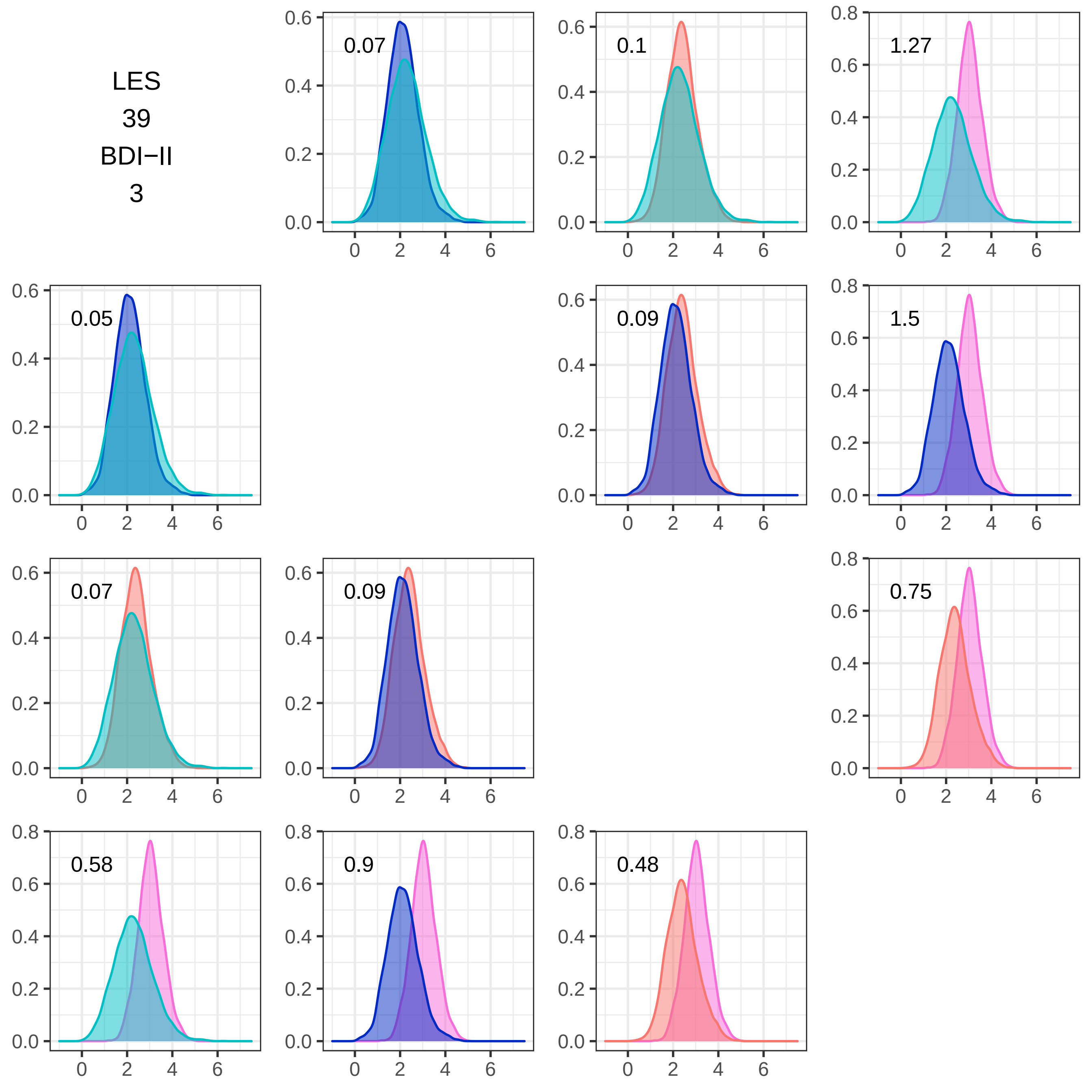
LES
39
BDI−II
3
0.07
0.1
1.27
0.05
0.09
1.5
0.07
0.09
0.75
0.58
0.9
0.48

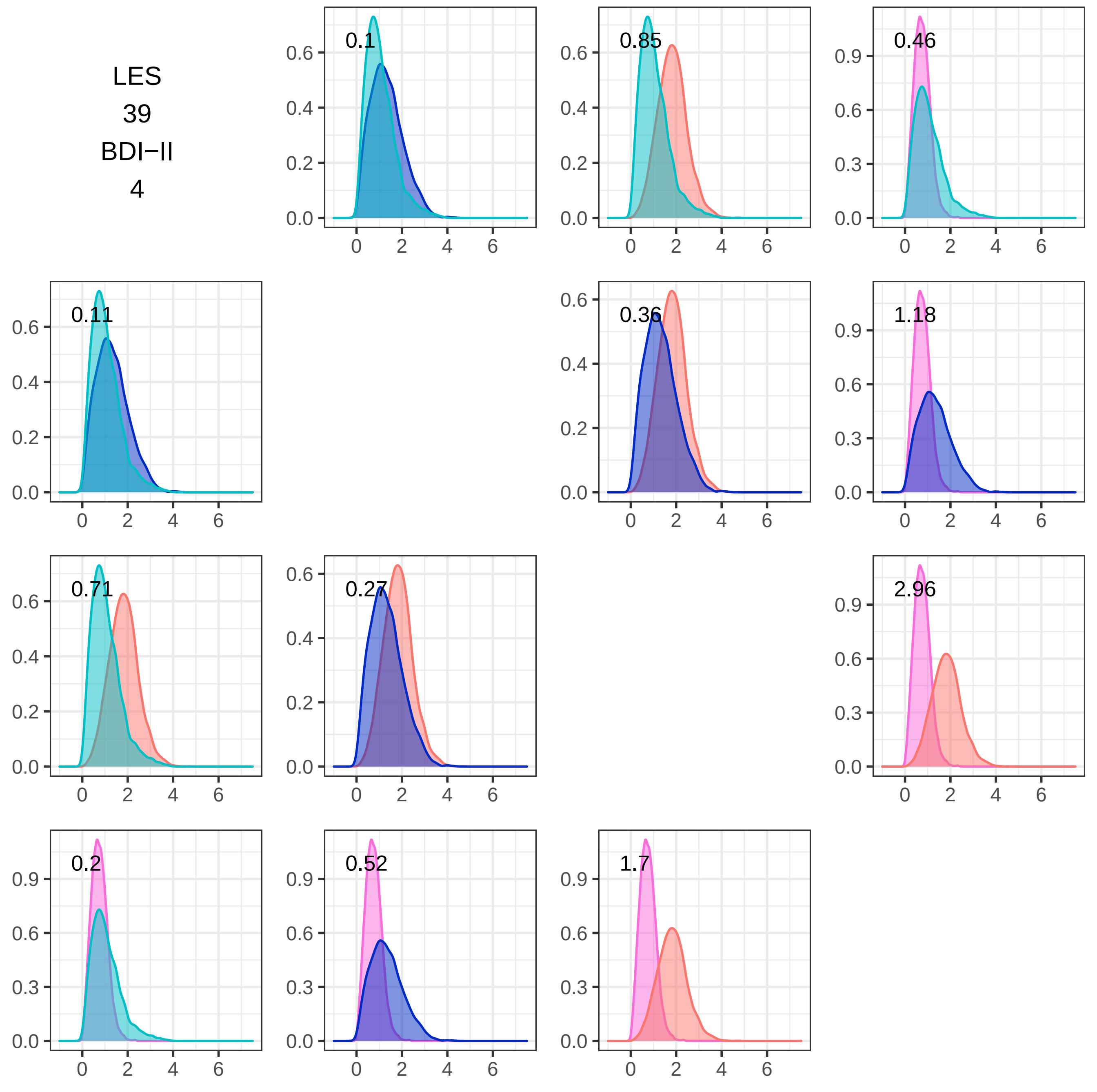

LES
39
BDI−II
4
0.1
0.85
0.46
0.11
0.36
1.18
0.71
0.27
2.96
0.2
0.52
1.7

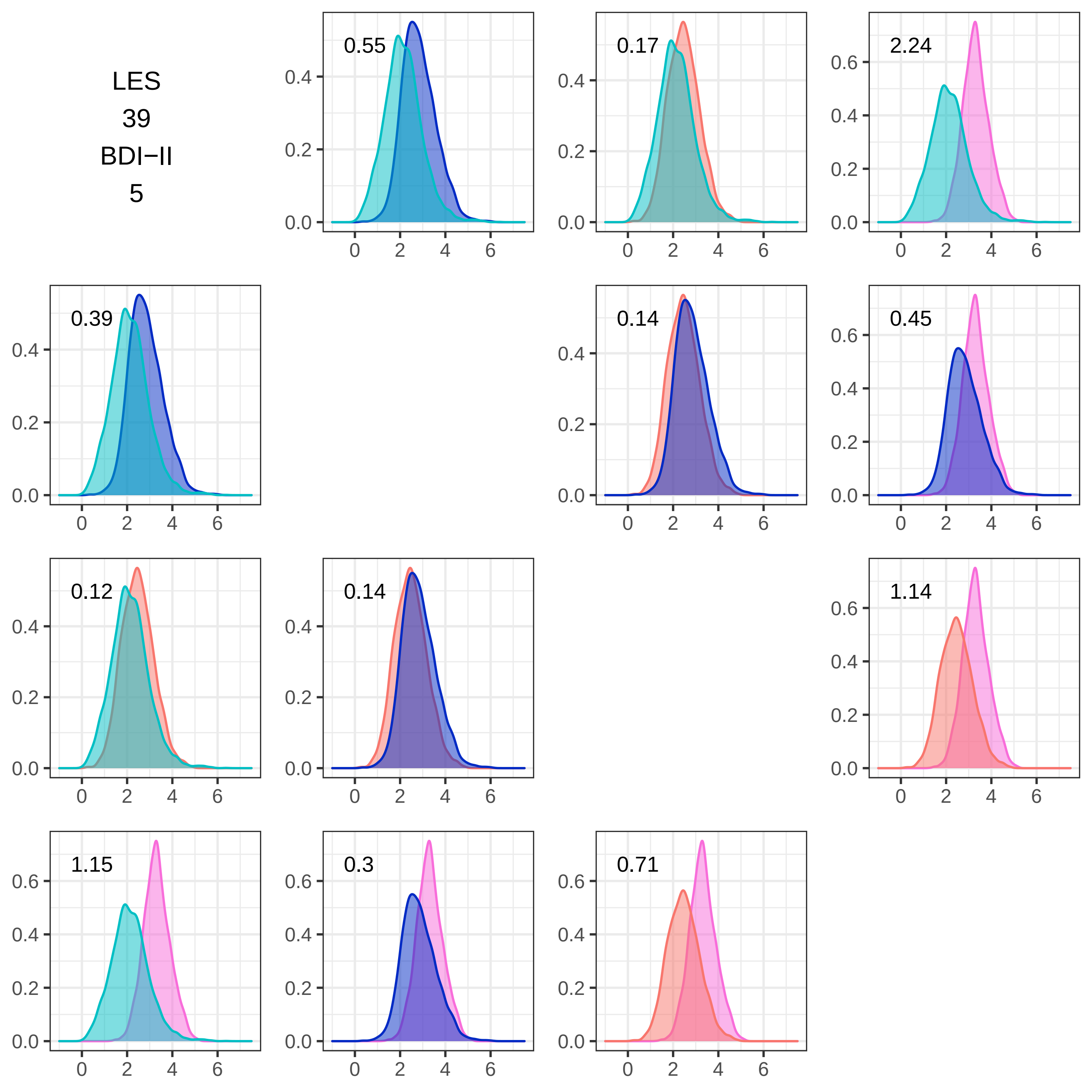
LES
39
BDI−II
5
0.55
0.17
2.24
0.39
0.14
0.45
0.12
0.14
1.14
1.15
0.3
0.71

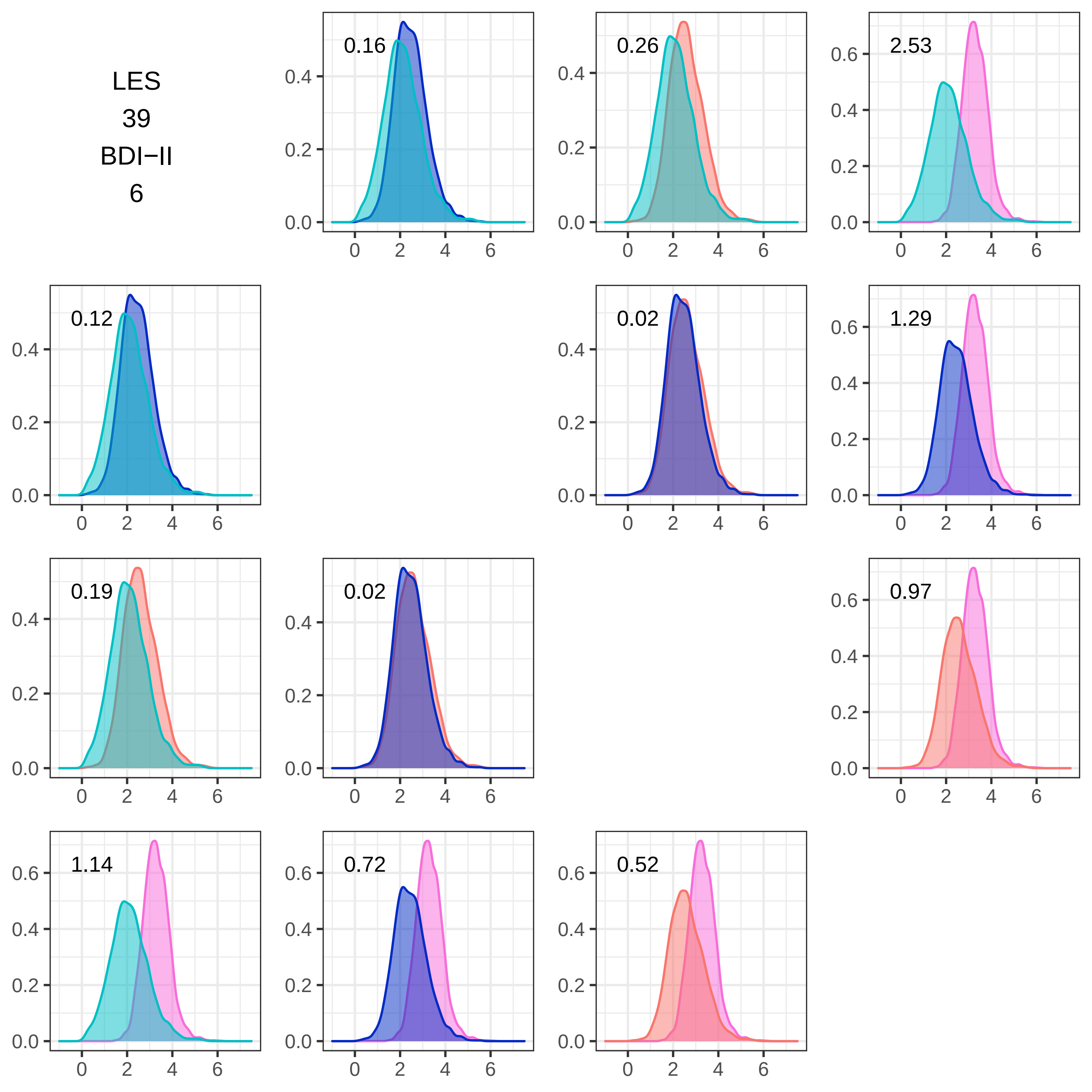

LES
39
BDI−II
6
0.16
0.26
2.53
0.12
0.02
1.29
0.19
0.02
0.97
1.14
0.72
0.52

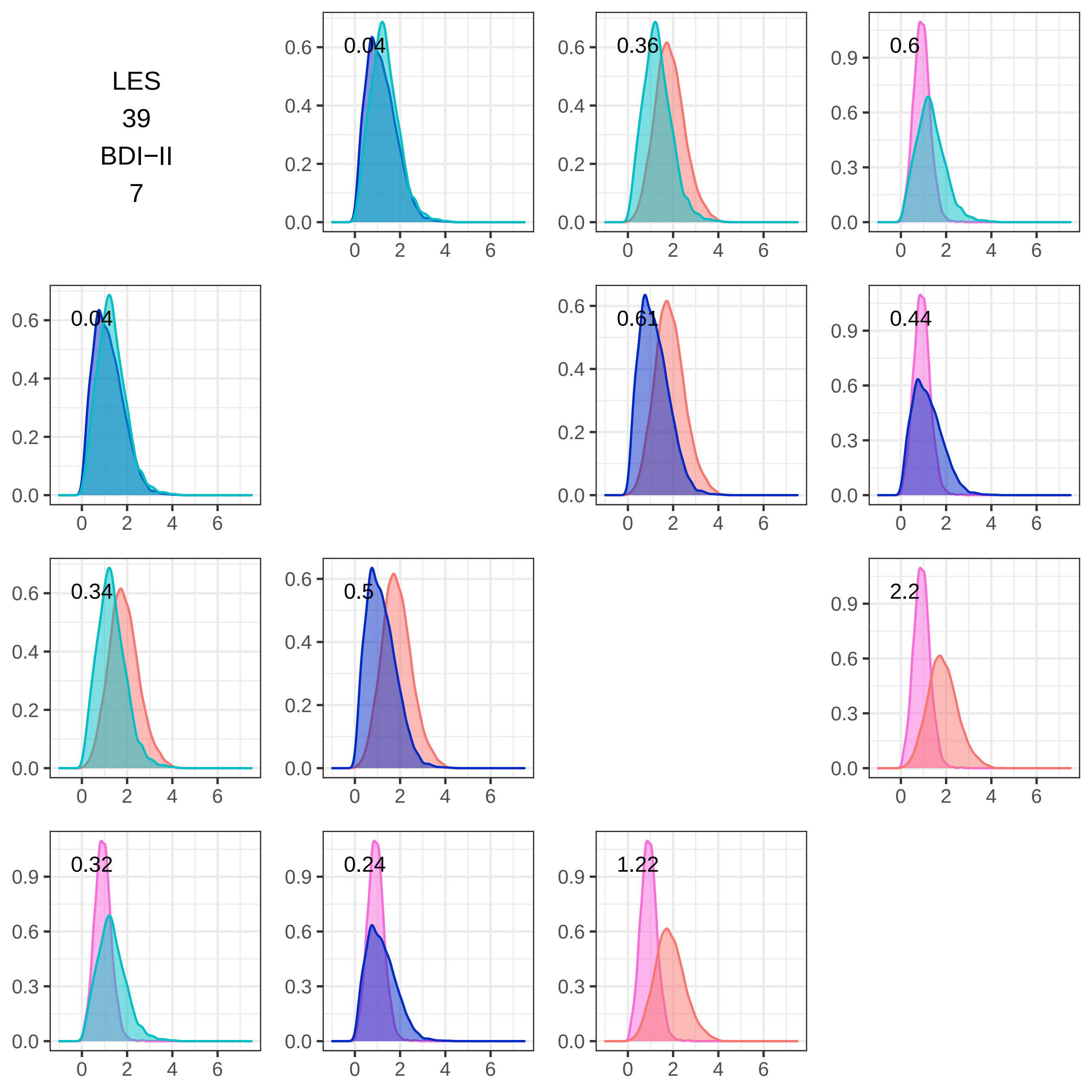
LES
39
BDI−II
7
0.04
0.36
0.6
0.04
0.61
0.44
0.34
0.5
2.2
0.32
0.24
1.22

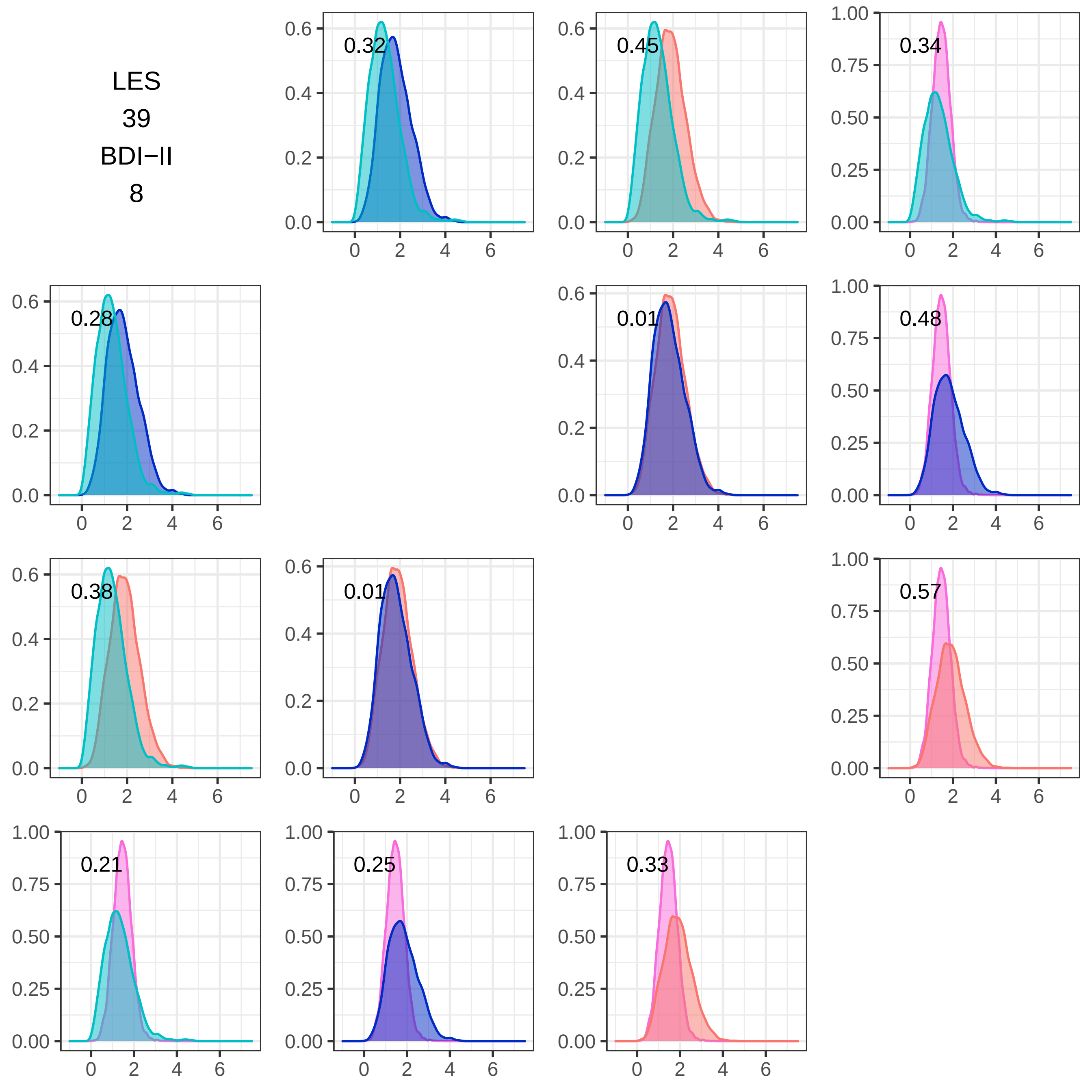
LES
39
BDI−II
8
0.32
0.45
0.34
0.28
0.01
0.48
0.38
0.01
0.57
0.21
0.25
0.33

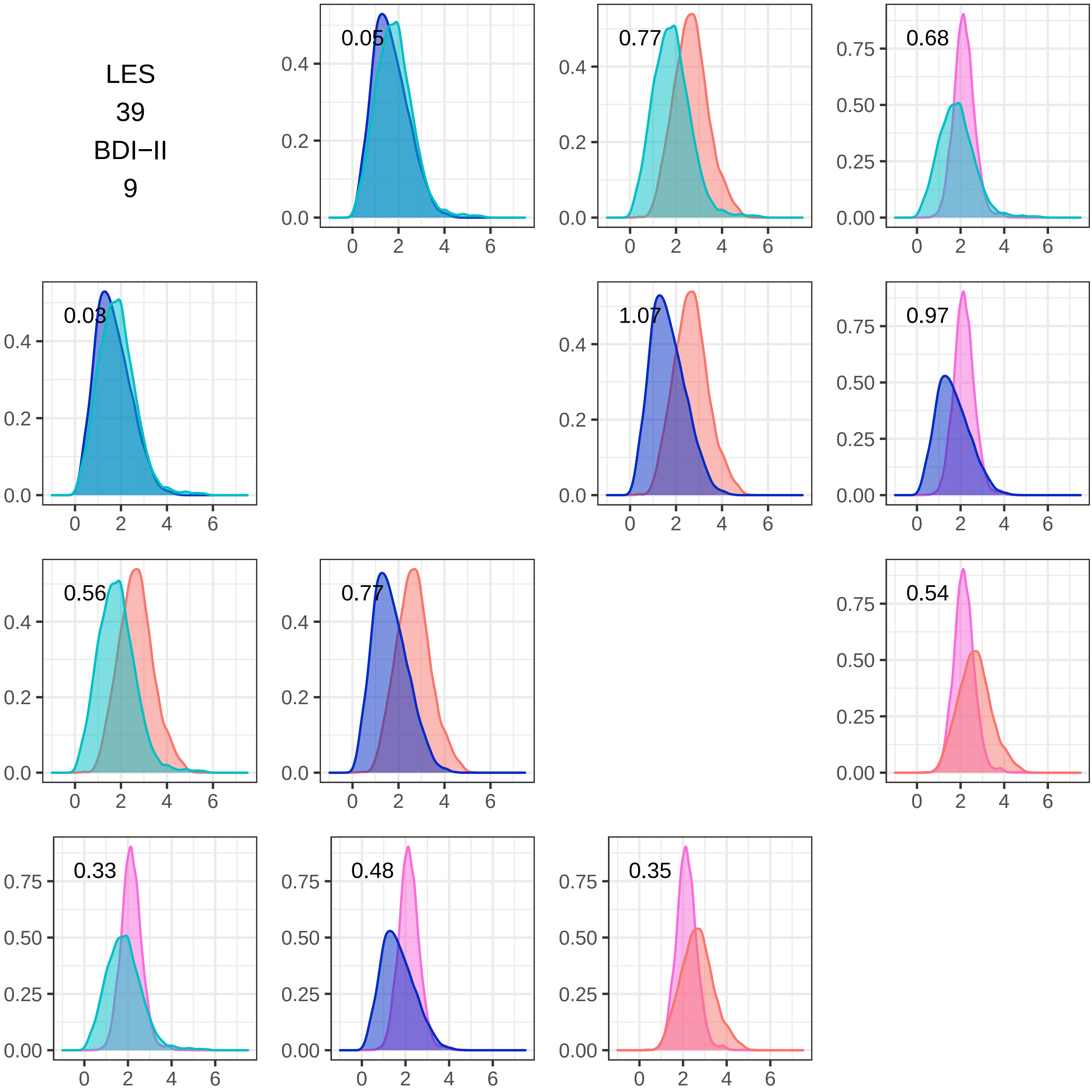

LES
39
BDI−II
9
0.05
0.77
0.68
0.03
1.07
0.97
0.56
0.77
0.54
0.33
0.48
0.35

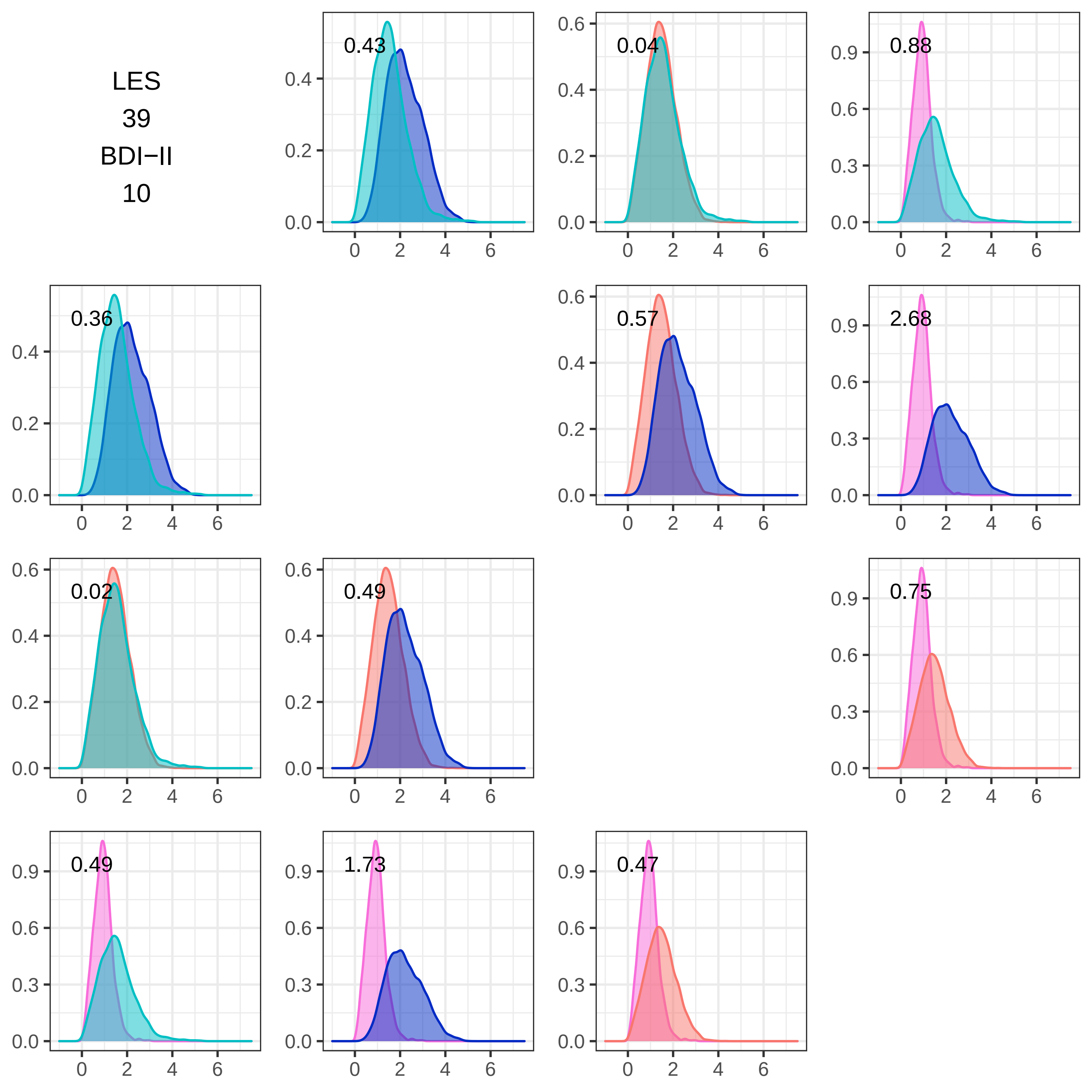
LES
39
BDI−II
10
0.43
0.04
0.88
0.36
0.57
2.68
0.02
0.49
0.75
0.49
1.73
0.47

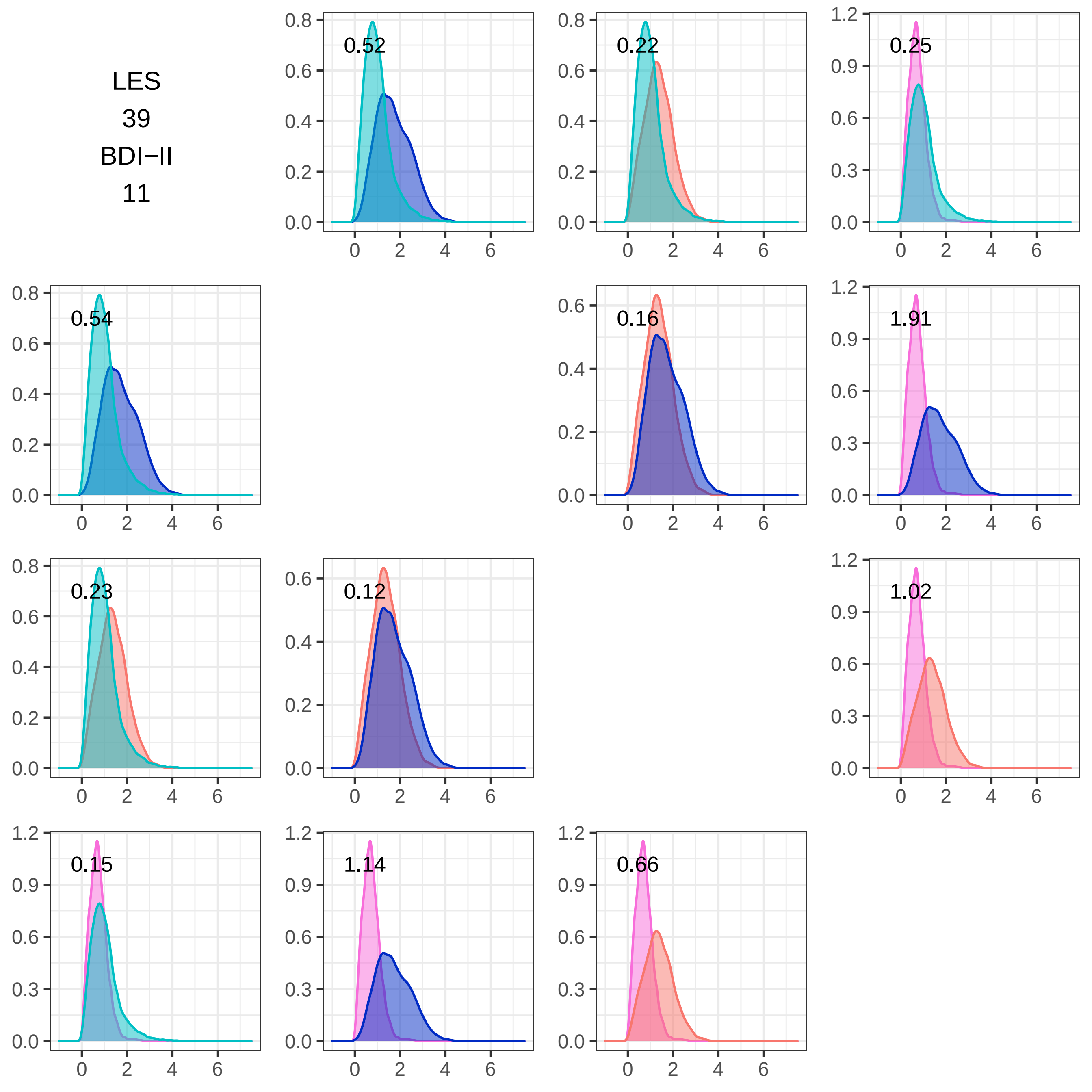

LES
39
BDI−II
11
0.52
0.22
0.25
0.54
0.16
1.91
0.23
0.12
1.02
0.15
1.14
0.66

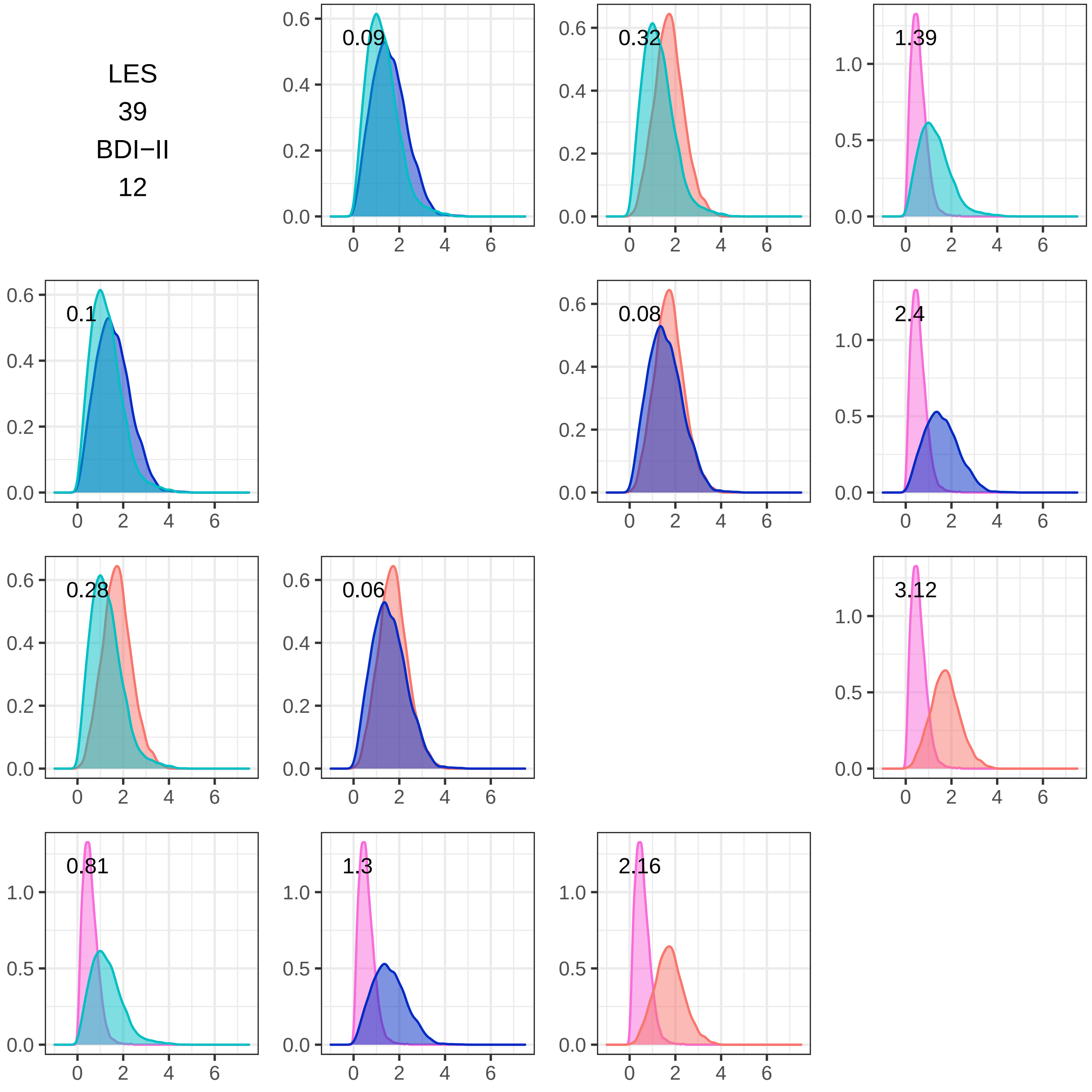
LES
39
BDI−II
12
0.09
0.32
1.39
0.1
0.08
2.4
0.28
0.06
3.12
0.81
1.3
2.16

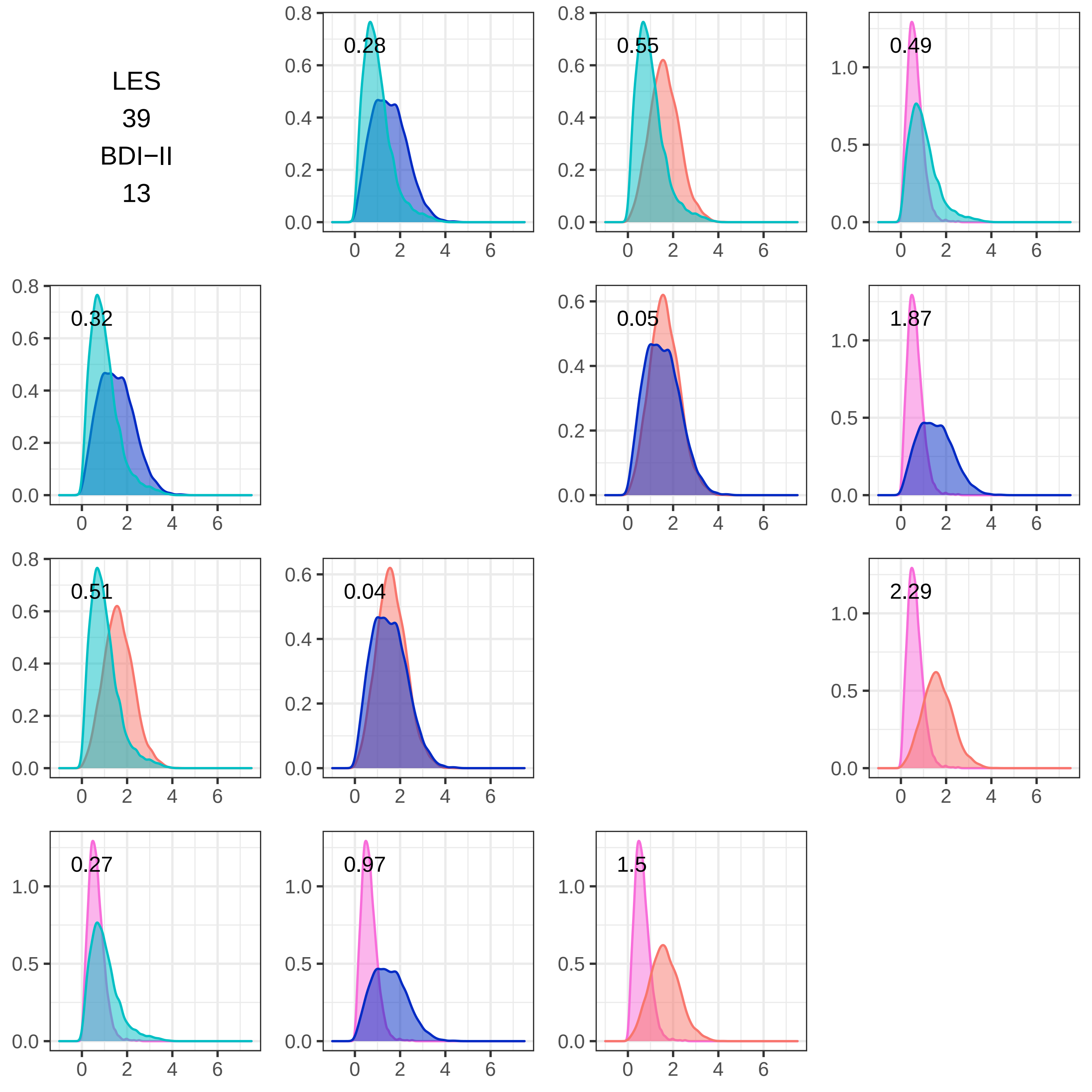

LES
39
BDI−II
13
0.28
0.55
0.49
0.32
0.05
1.87
0.51
0.04
2.29
0.27
0.97
1.5

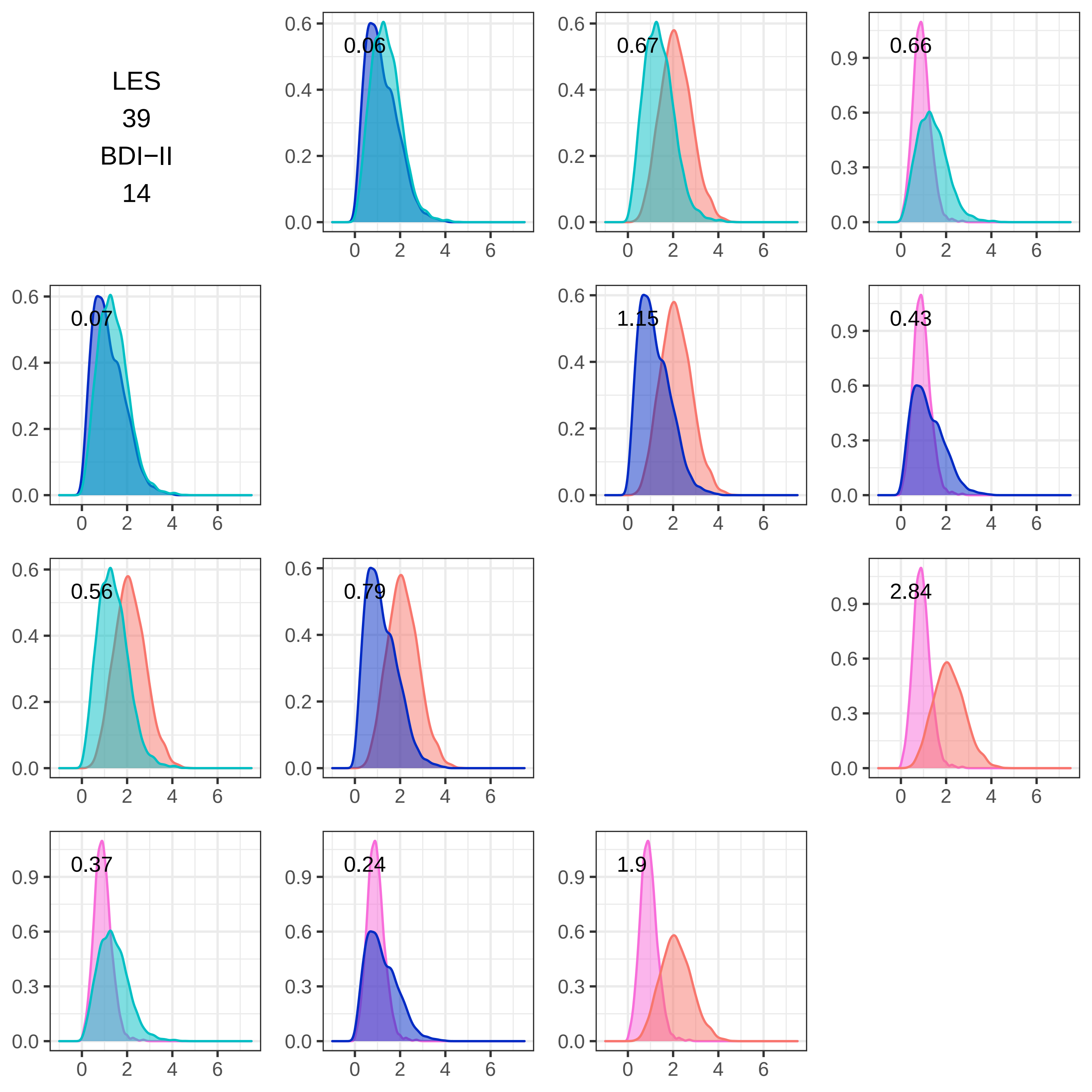

LES
39
BDI−II
14
0.06
0.67
0.66
0.07
1.15
0.43
0.56
0.79
2.84
0.37
0.24
1.9

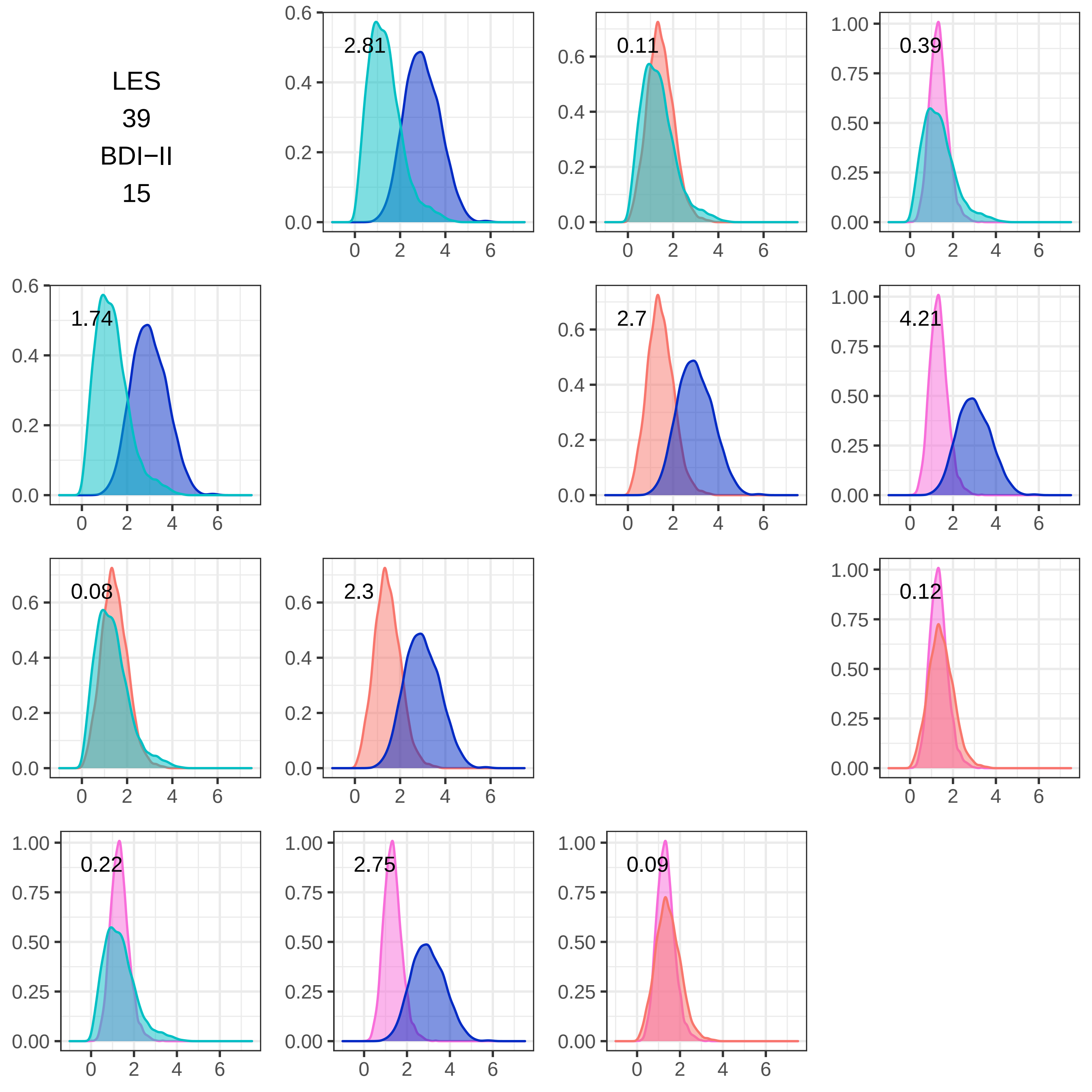
LES
39
BDI−II
15
2.81
0.11
0.39
1.74
2.7
4.21
0.08
2.3
0.12
0.22
2.75
0.09

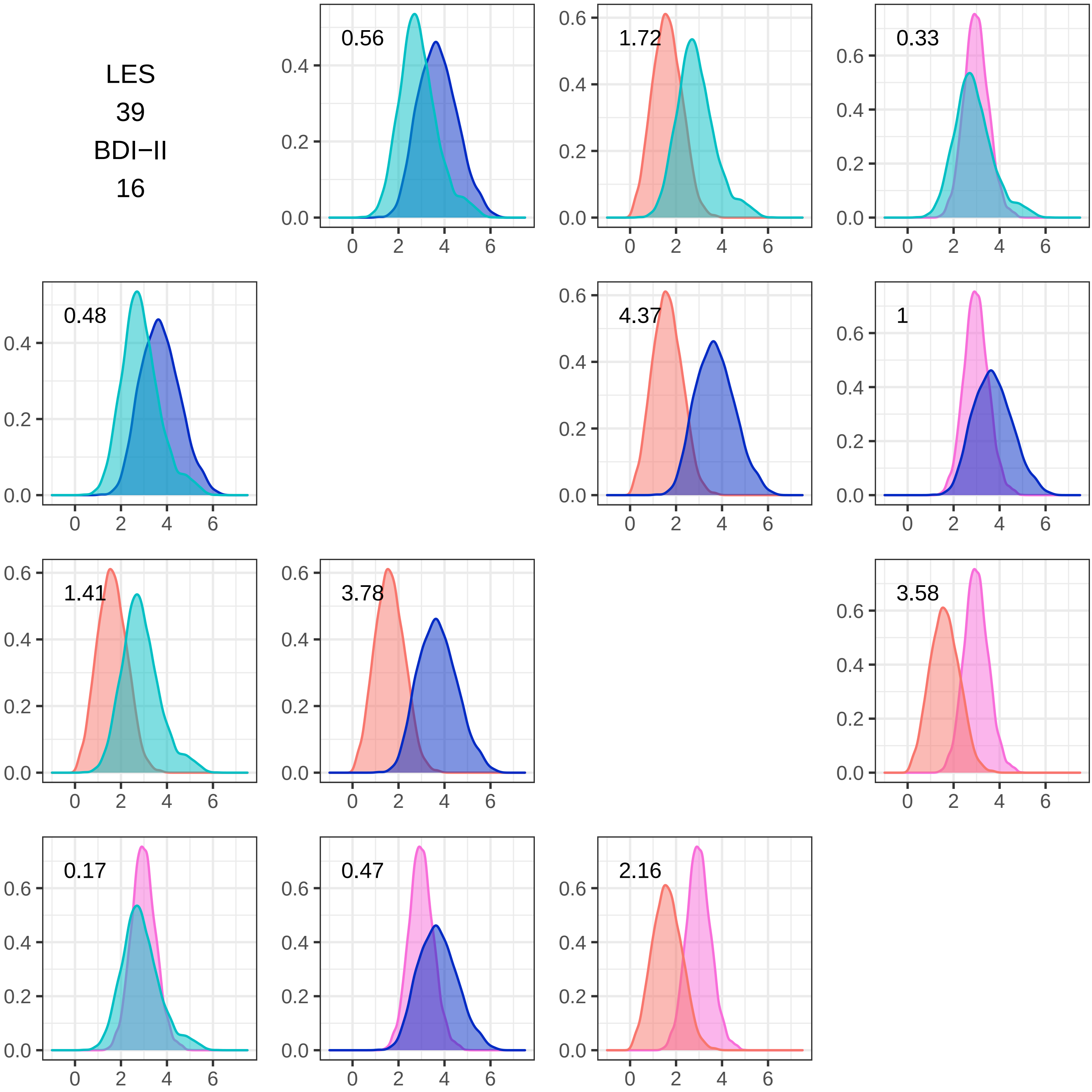
LES
39
BDI−II
16
0.56
1.72
0.33
0.48
4.37
1
1.41
3.78
3.58
0.17
0.47
2.16

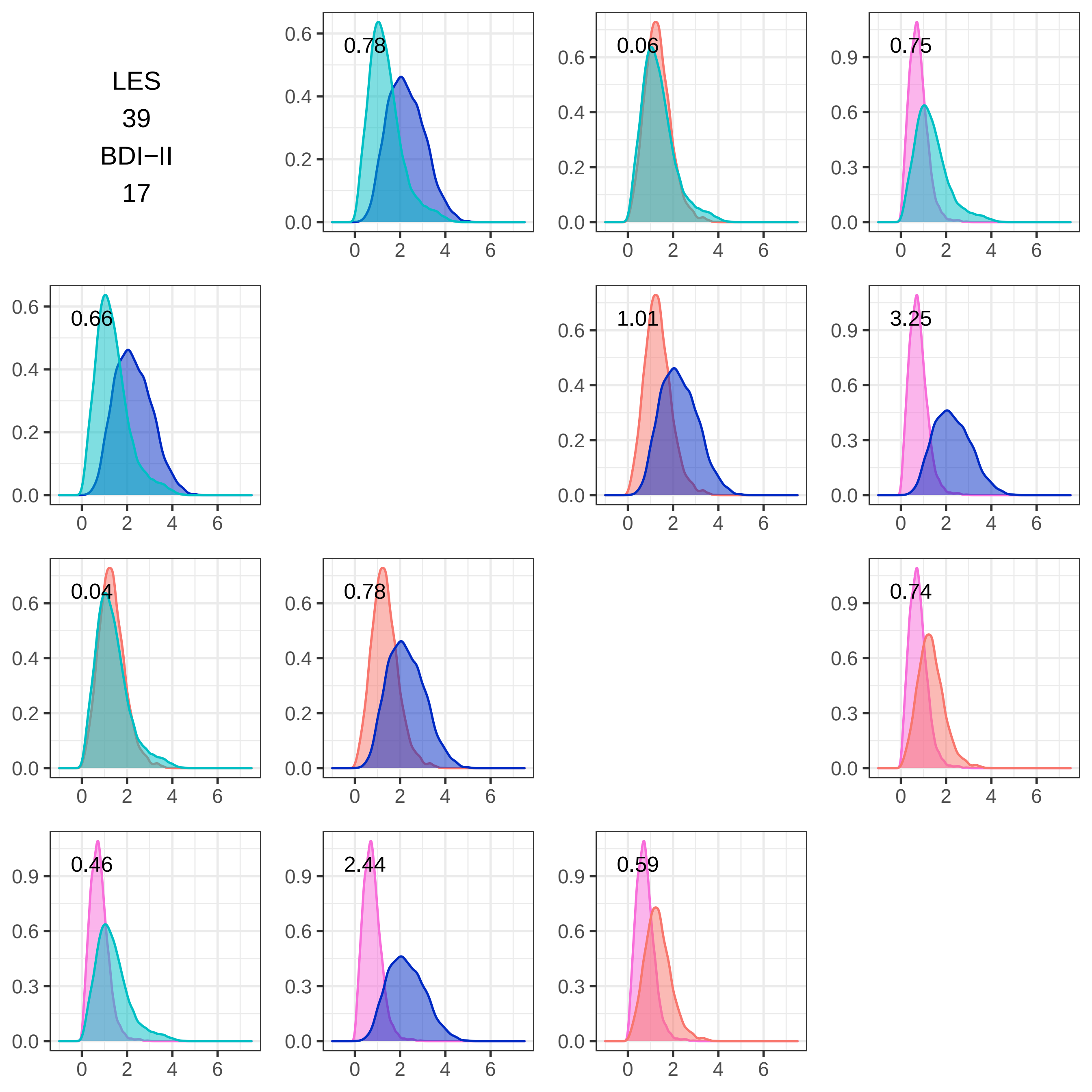
LES
39
BDI−II
17
0.78
0.06
0.75
0.66
1.01
3.25
0.04
0.78
0.74
0.46
2.44
0.59

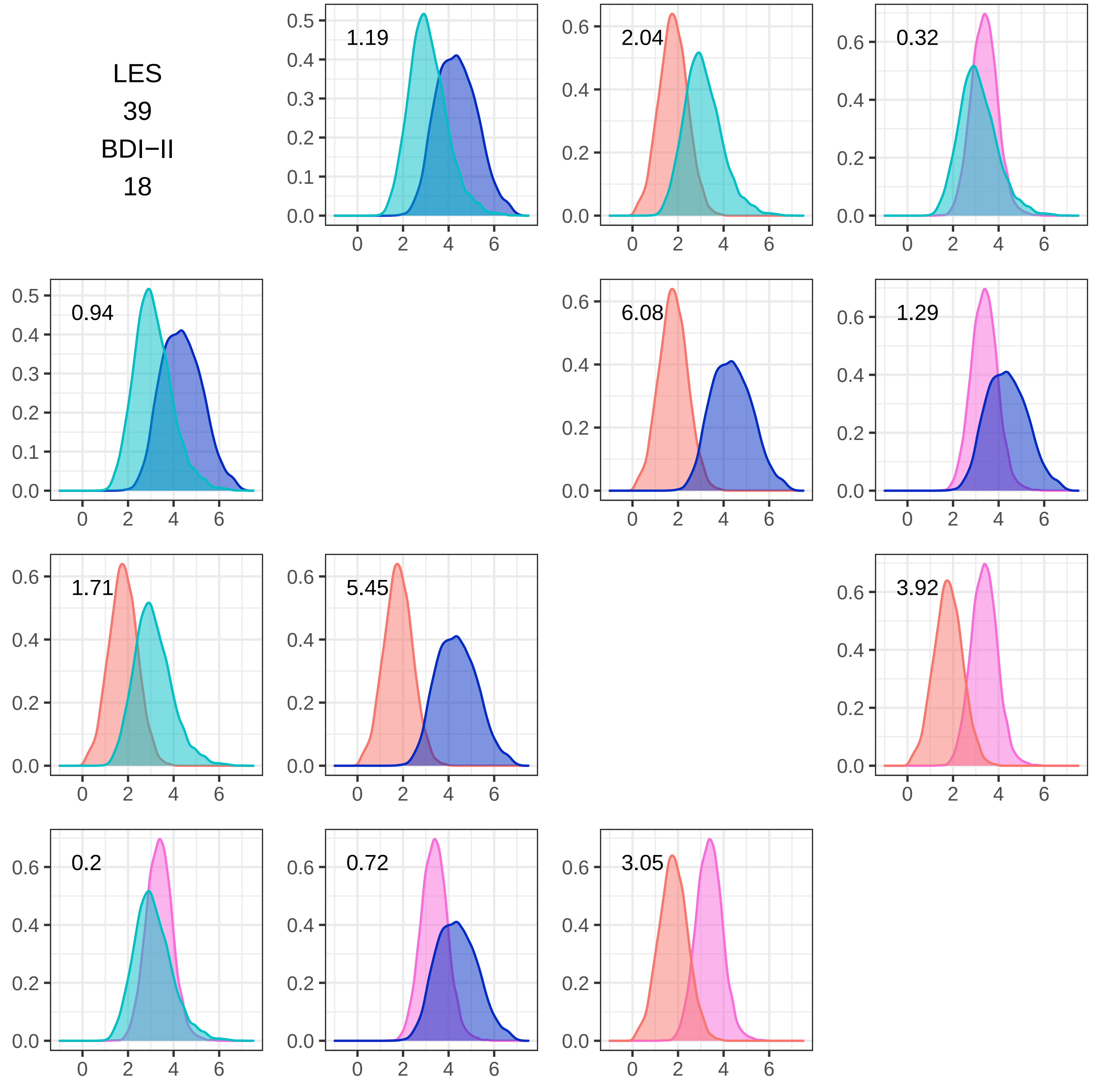
LES
39
BDI−II
18
1.19
2.04
0.32
0.94
6.08
1.29
1.71
5.45
3.92
0.2
0.72
3.05

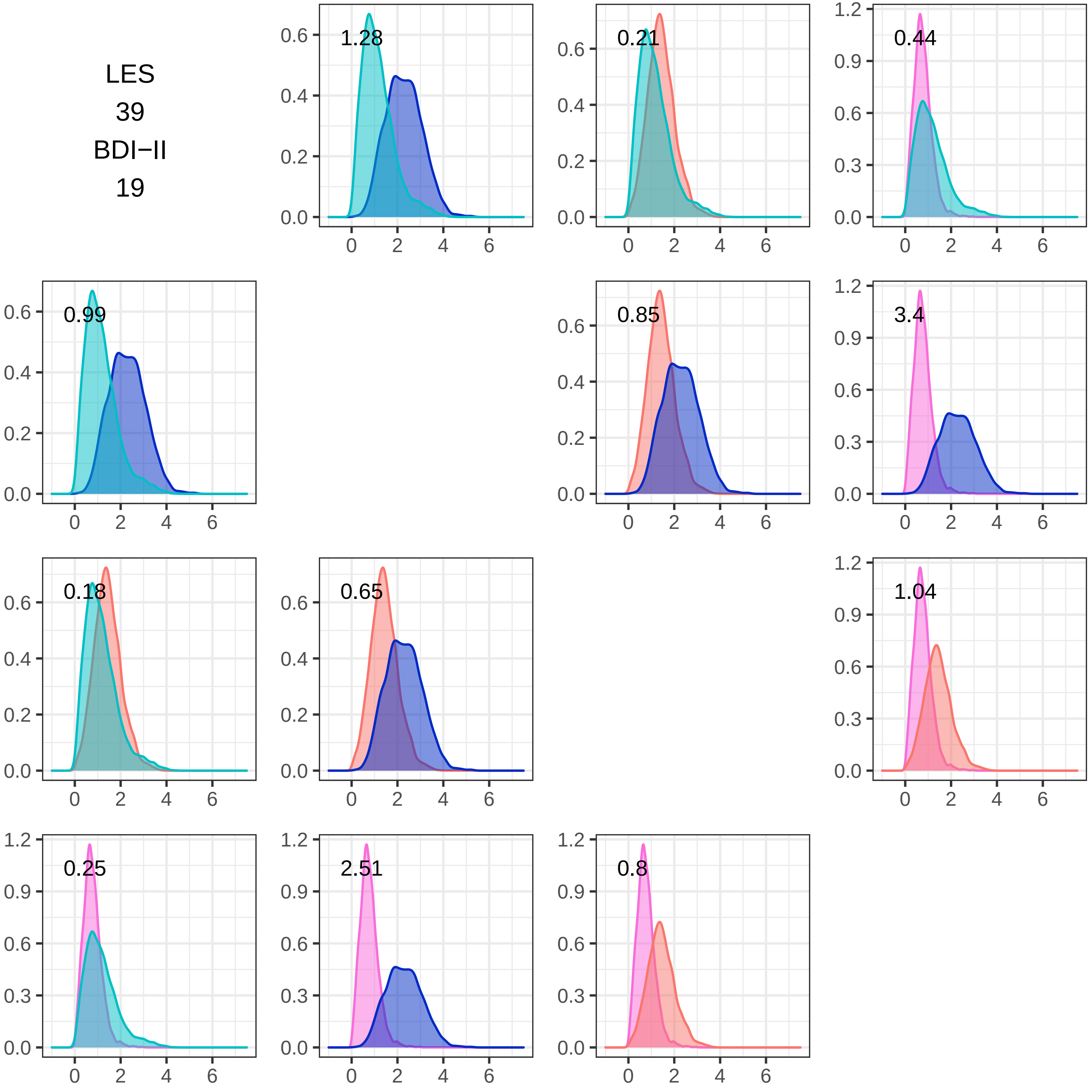
LES
39
BDI−II
19
1.28
0.21
0.44
0.99
0.85
3.4
0.18
0.65
1.04
0.25
2.51
0.8

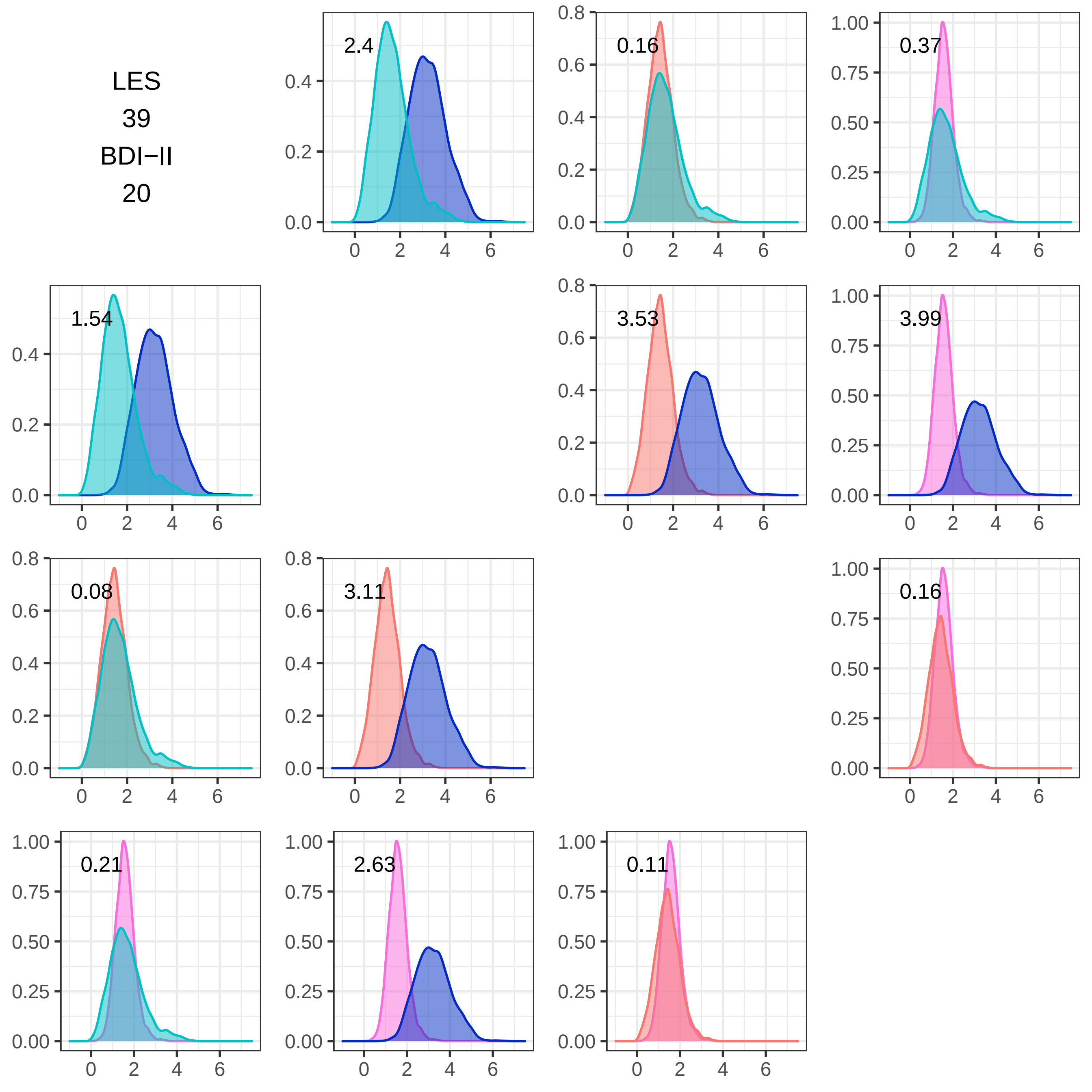

LES
39
BDI−II
20
2.4
0.16
0.37
1.54
3.53
3.99
0.08
3.11
0.16
0.21
2.63
0.11

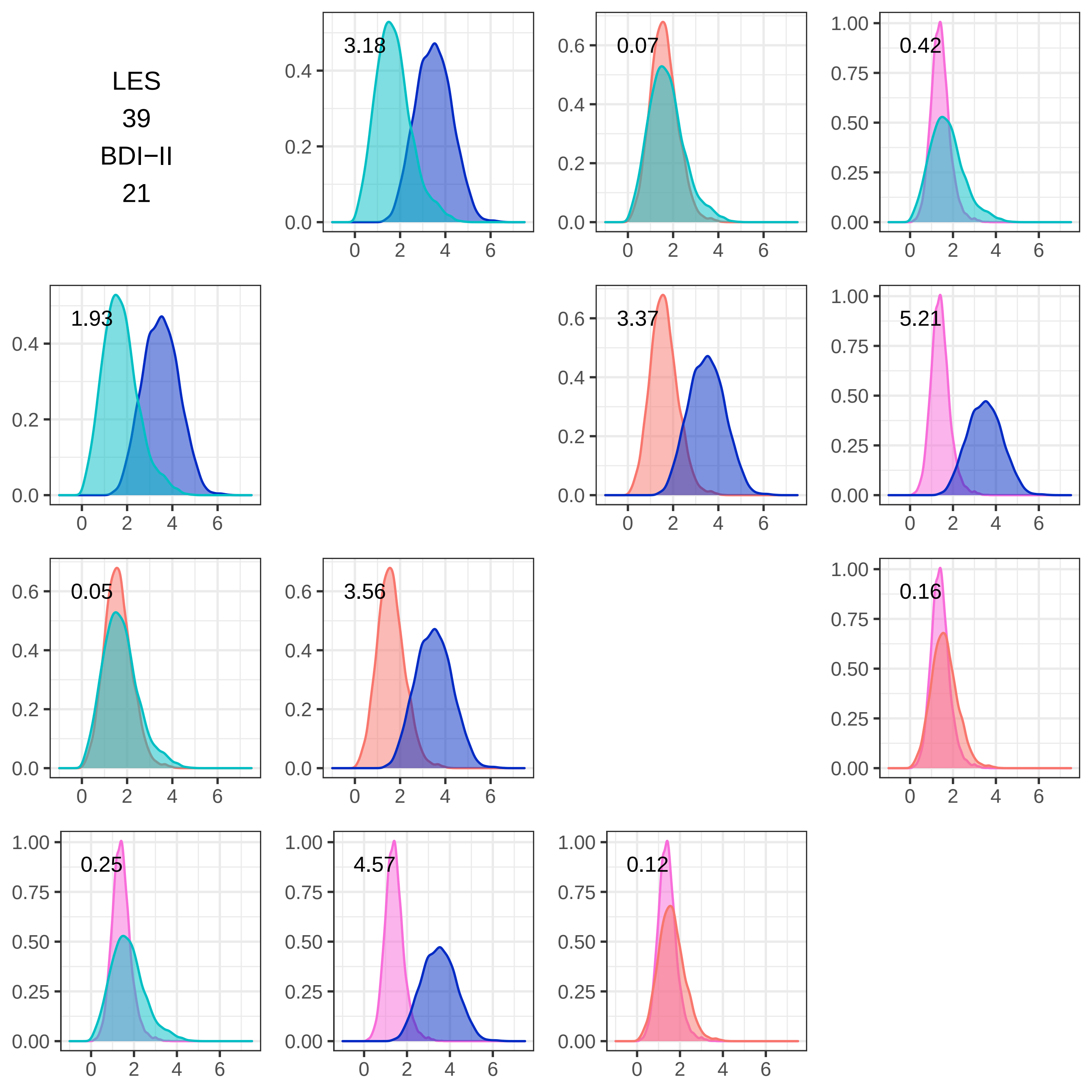
LES
39
BDI−II
21
3.18
0.07
0.42
1.93
3.37
5.21
0.05
3.56
0.16
0.25
4.57
0.12

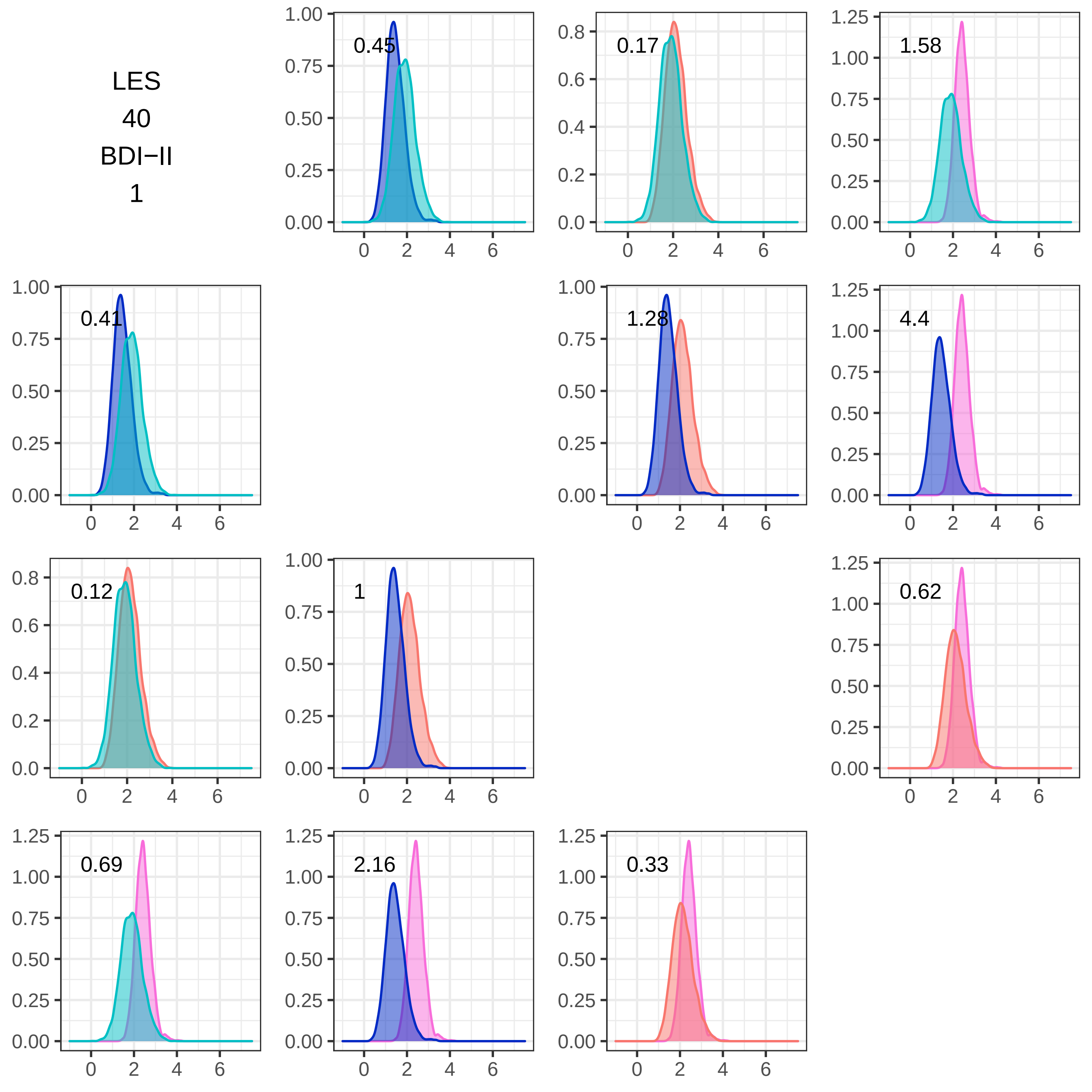
LES
40
BDI−II
1
0.45
0.17
1.58
0.41
1.28
4.4
0.12
1
0.62
0.69
2.16
0.33

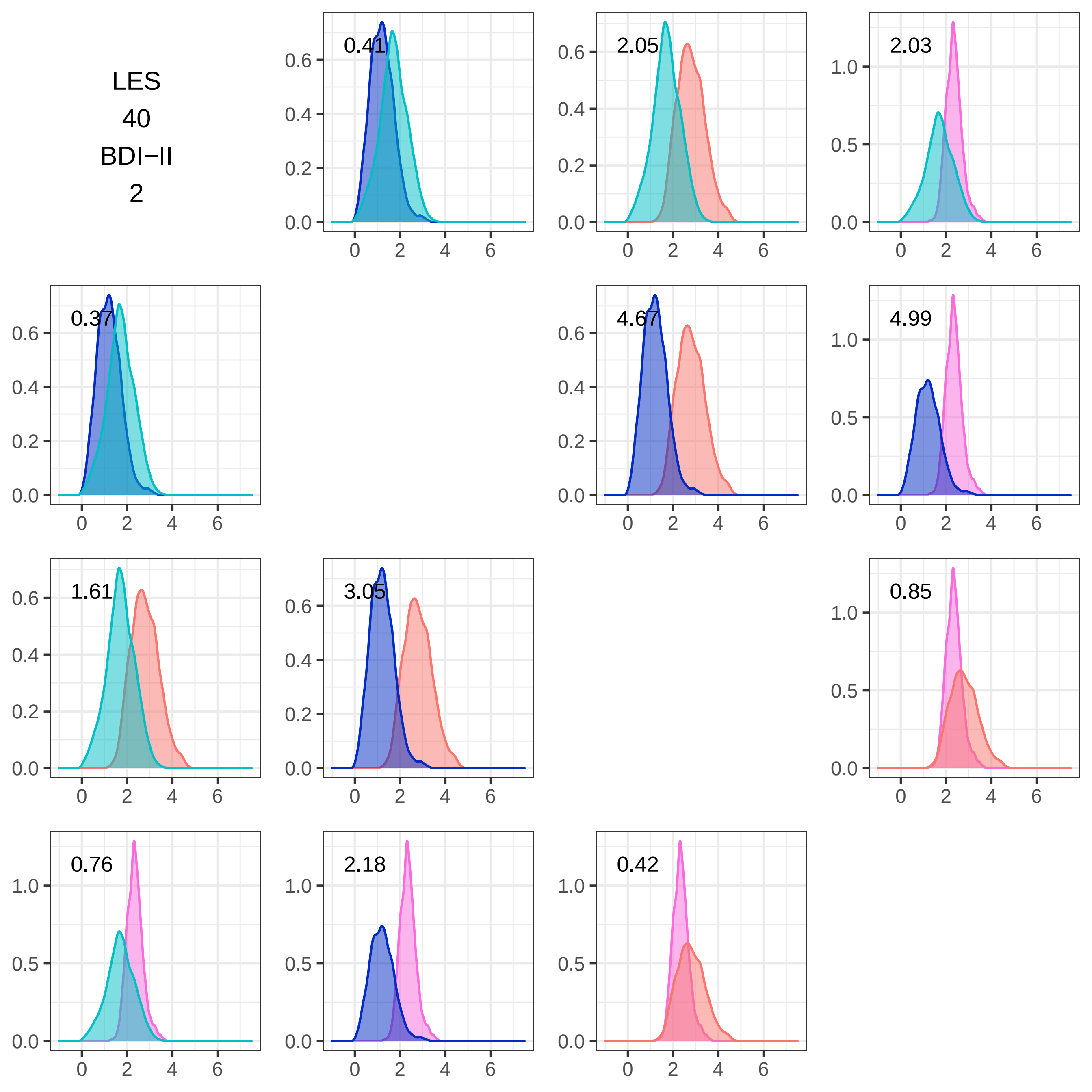

LES
40
BDI−II
2
0.41
2.05
2.03
0.37
4.67
4.99
1.61
3.05
0.85
0.76
2.18
0.42

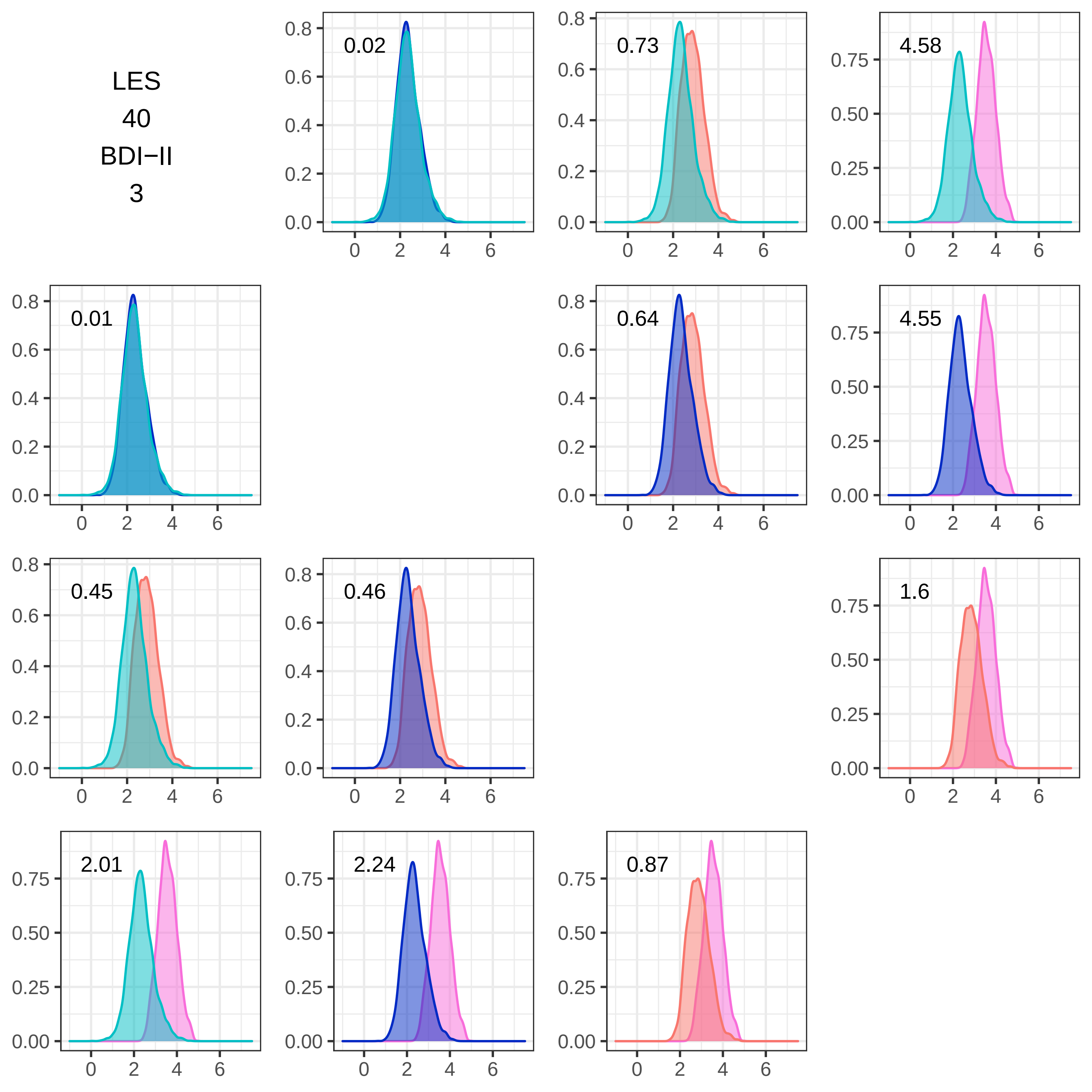
LES
40
BDI−II
3
0.02
0.73
4.58
0.01
0.64
4.55
0.45
0.46
1.6
2.01
2.24
0.87

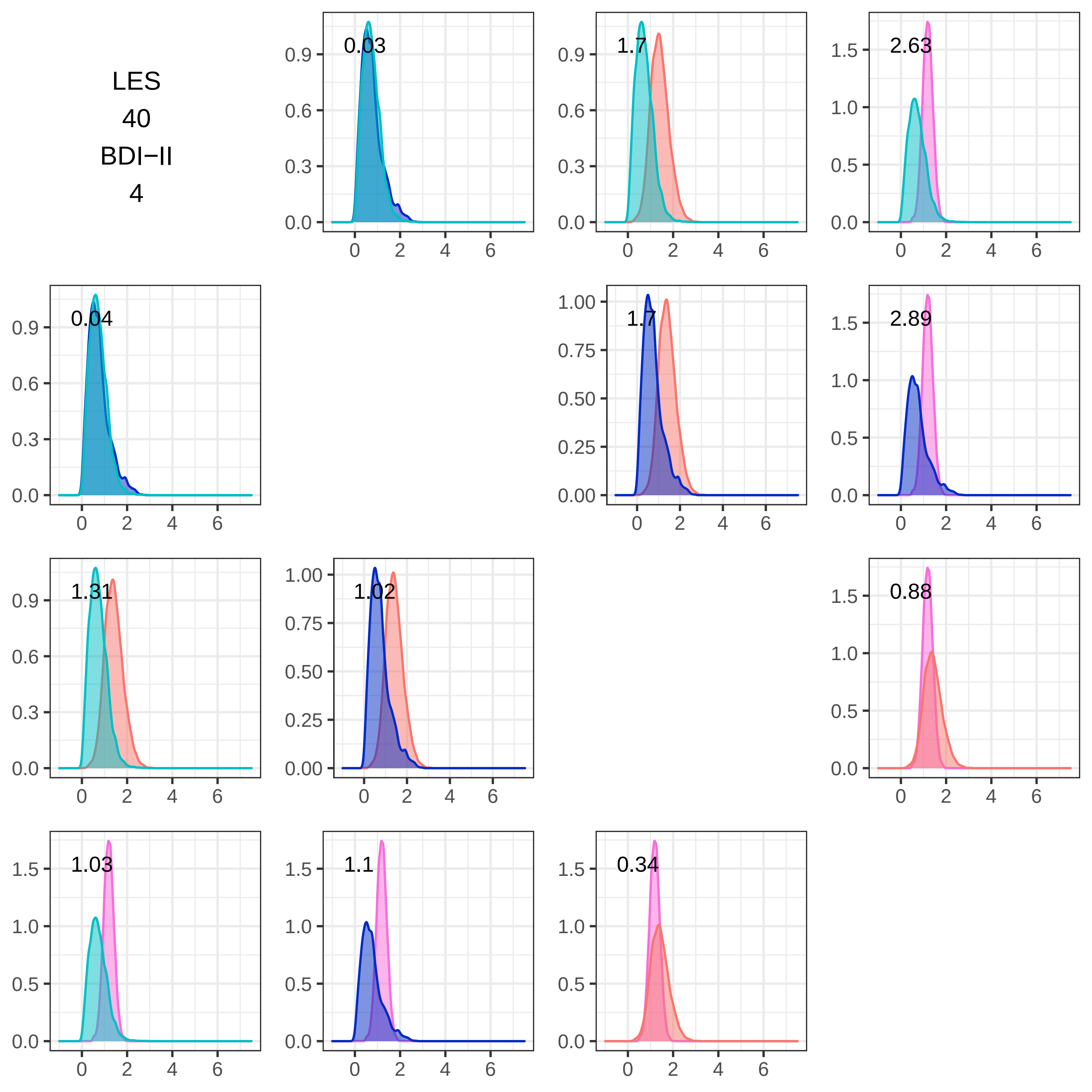

LES
40
BDI−II
4
0.03
1.7
2.63
0.04
1.7
2.89
1.31
1.02
0.88
1.03
1.1
0.34

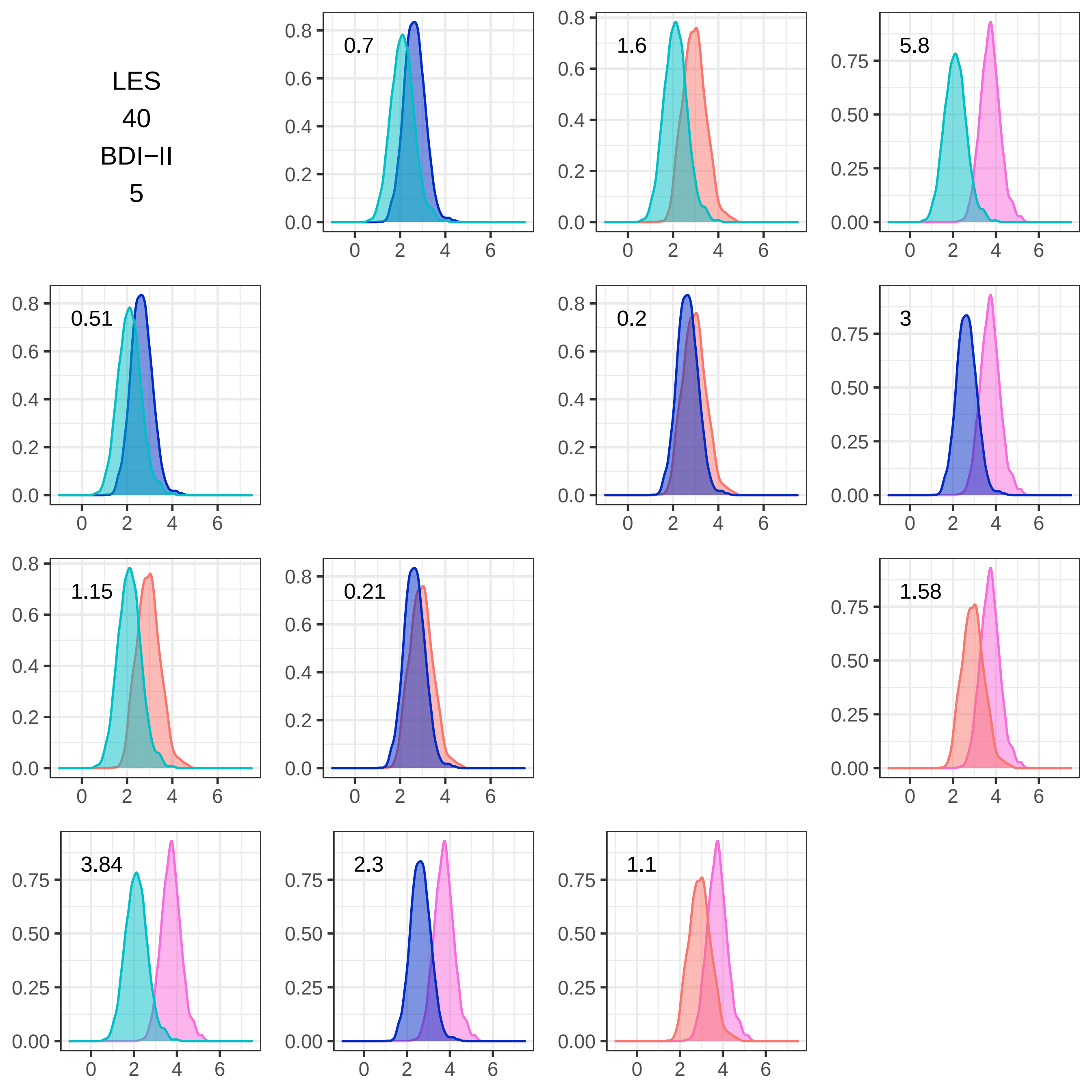

LES
40
BDI−II
5
0.7
1.6
5.8
0.51
0.2
3
1.15
0.21
1.58
3.84
2.3
1.1

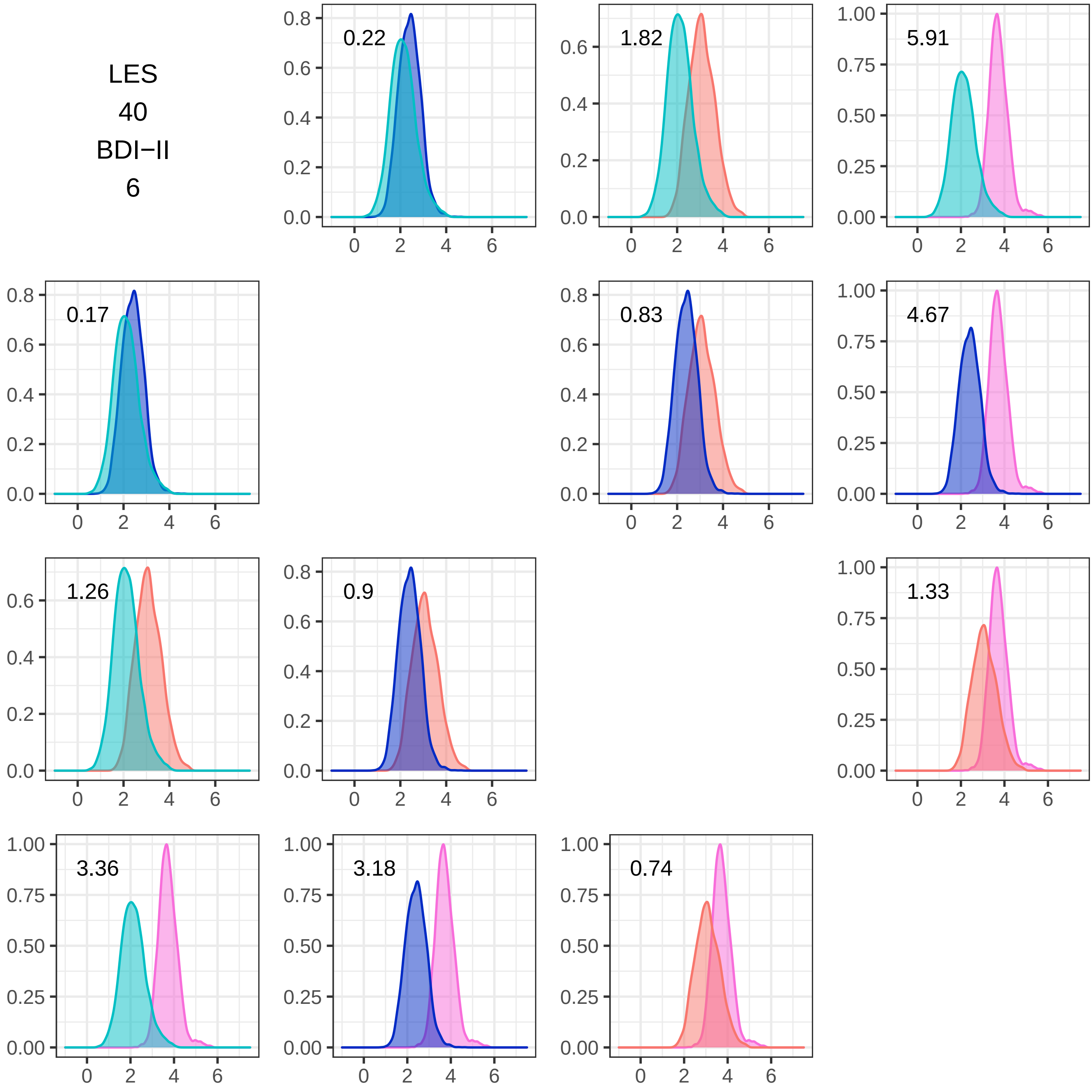

LES
40
BDI−II
6
0.22
1.82
5.91
0.17
0.83
4.67
1.26
0.9
1.33
3.36
3.18
0.74

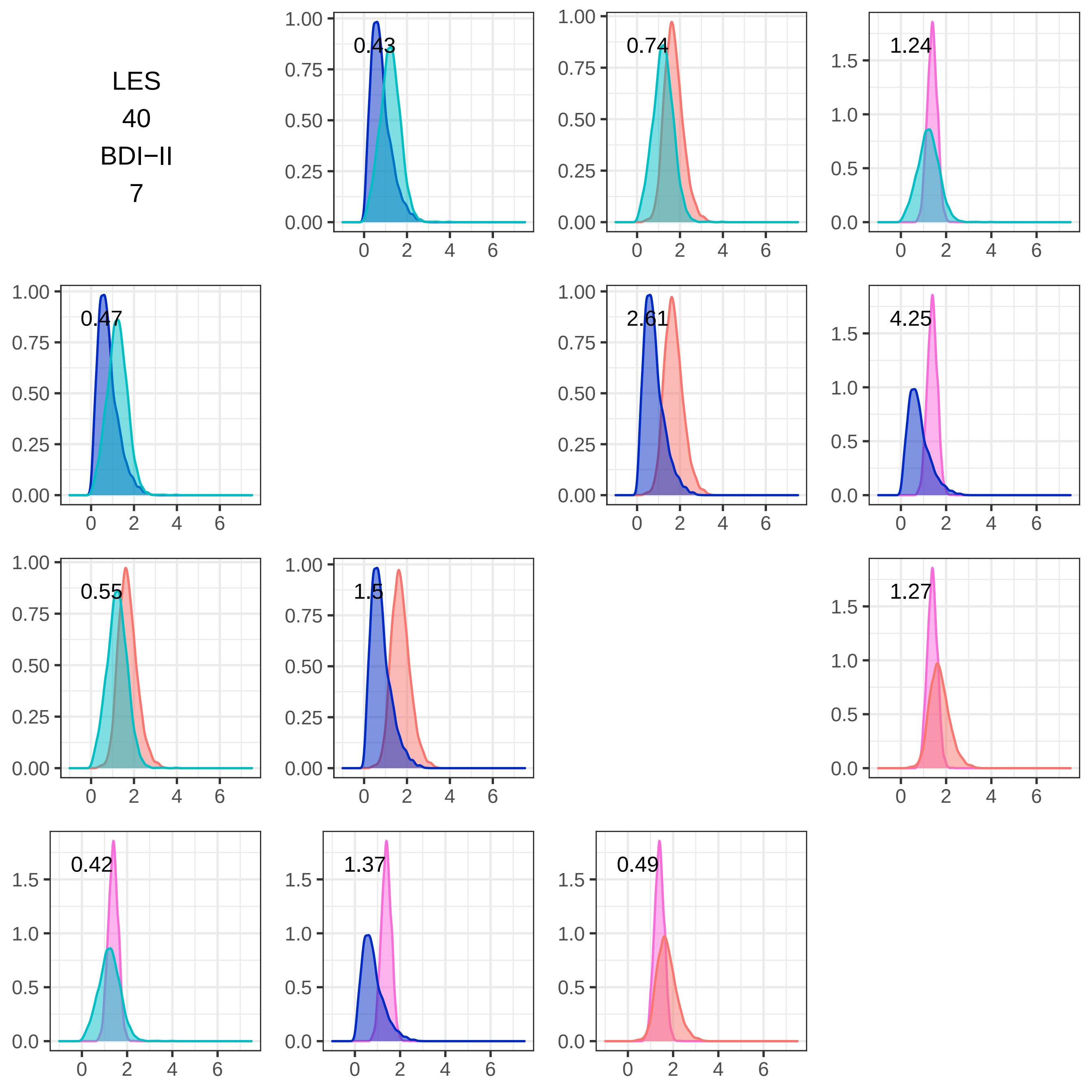

LES
40
BDI−II
7
0.43
0.74
1.24
0.47
2.61
4.25
0.55
1.5
1.27
0.42
1.37
0.49

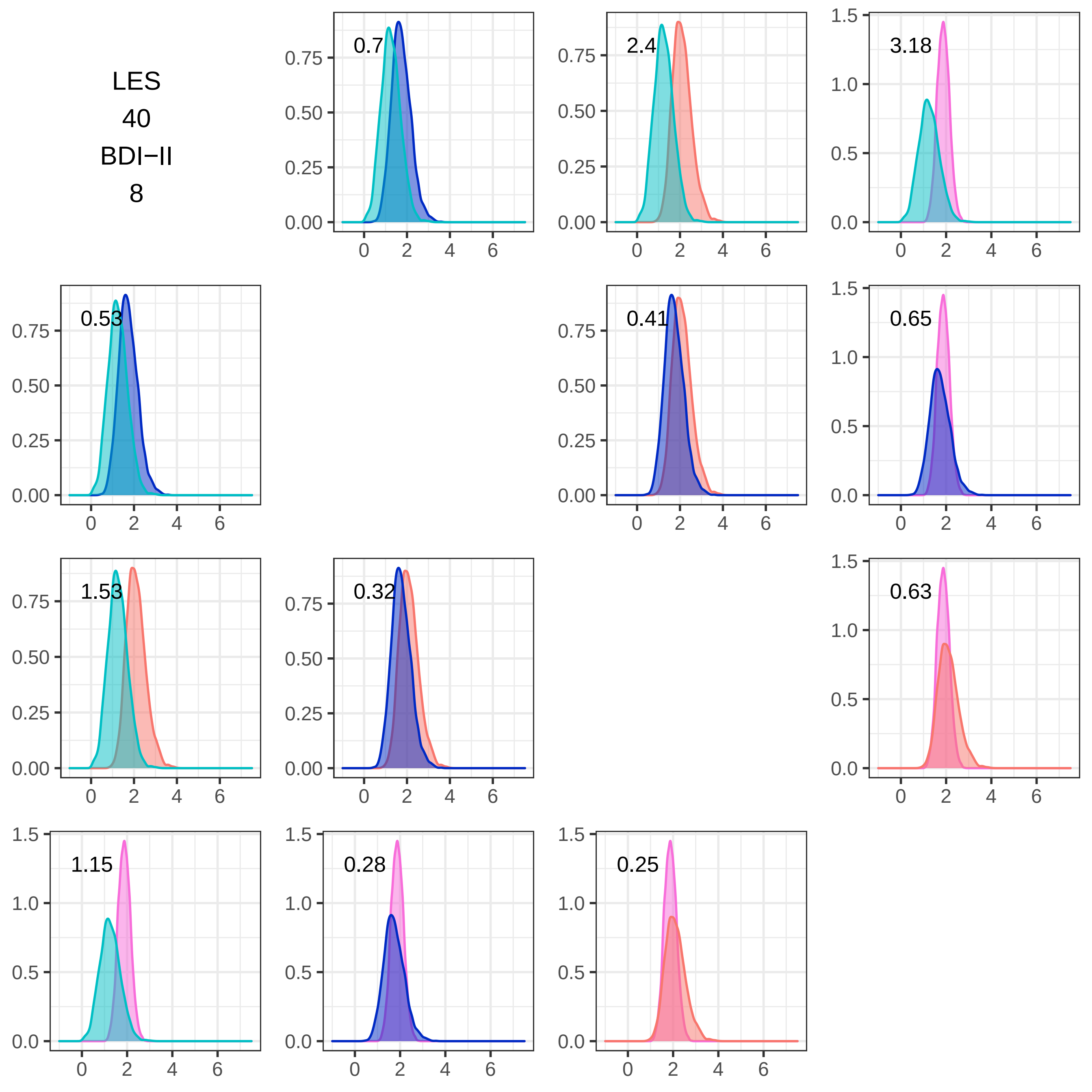
LES
40
BDI−II
8
0.7
2.4
3.18
0.53
0.41
0.65
1.53
0.32
0.63
1.15
0.28
0.25

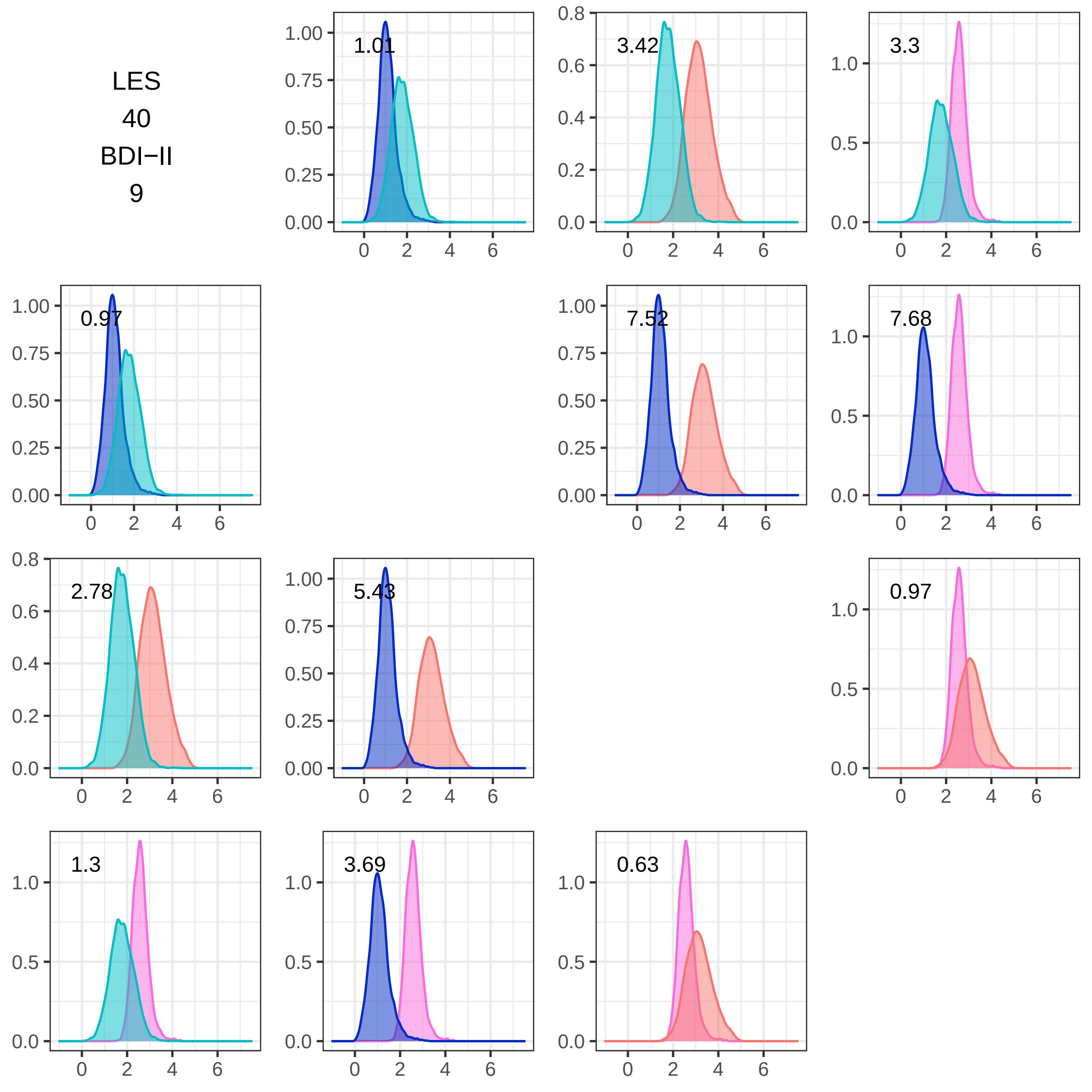

LES
40
BDI−II
9
1.01
3.42
3.3
0.97
7.52
7.68
2.78
5.43
0.97
1.3
3.69
0.63

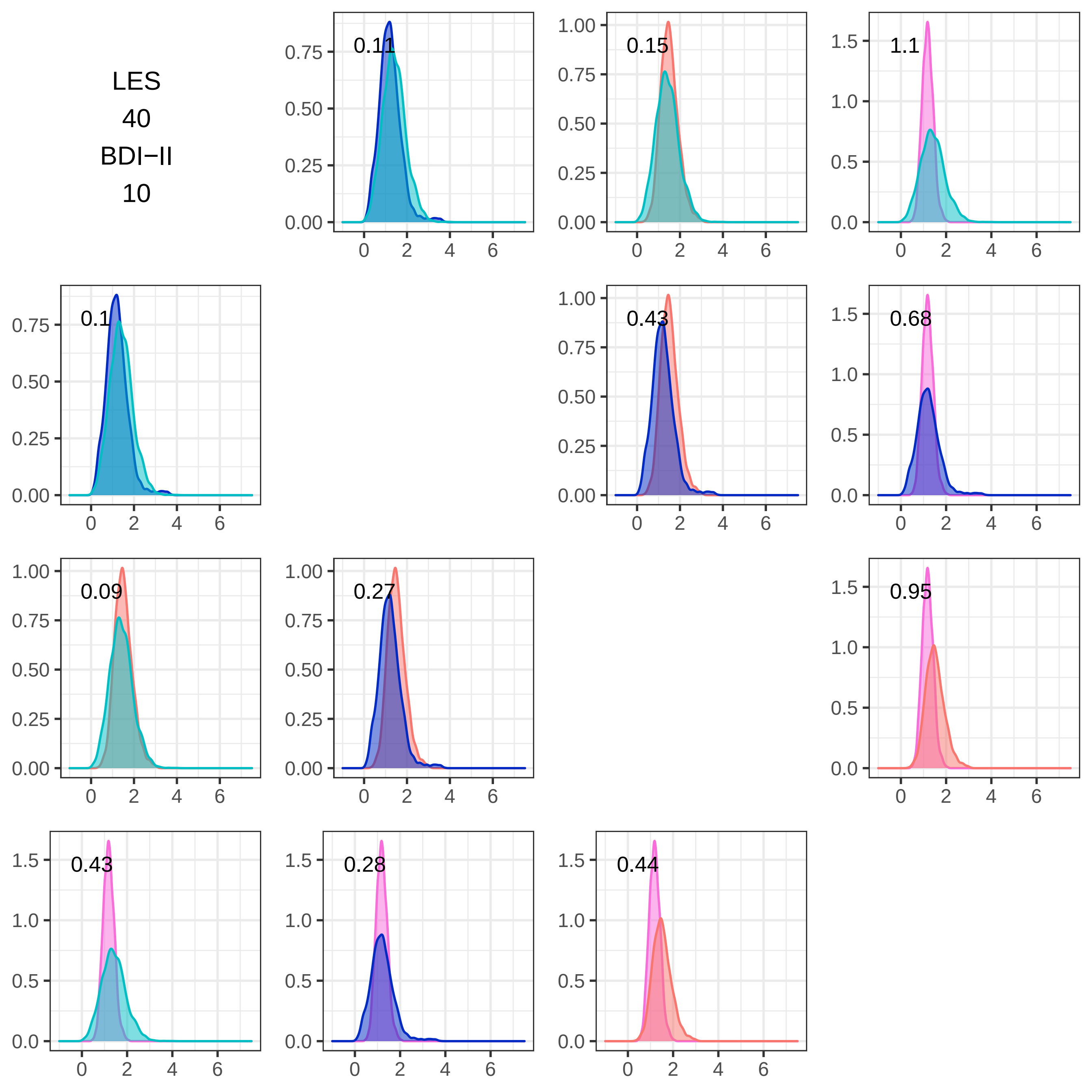
LES
40
BDI−II
10
0.11
0.15
1.1
0.1
0.43
0.68
0.09
0.27
0.95
0.43
0.28
0.44

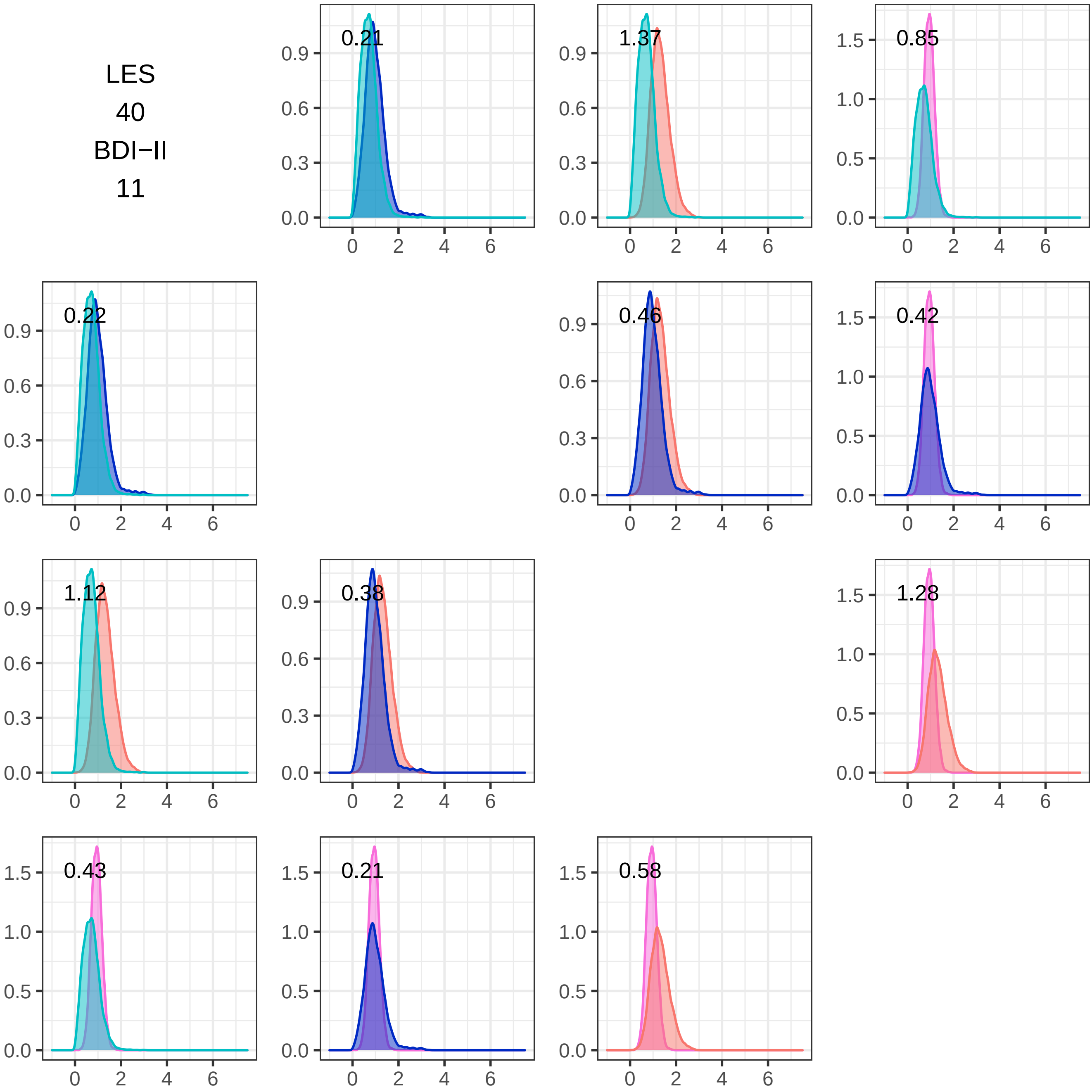
LES
40
BDI−II
11
0.21
1.37
0.85
0.22
0.46
0.42
1.12
0.38
1.28
0.43
0.21
0.58

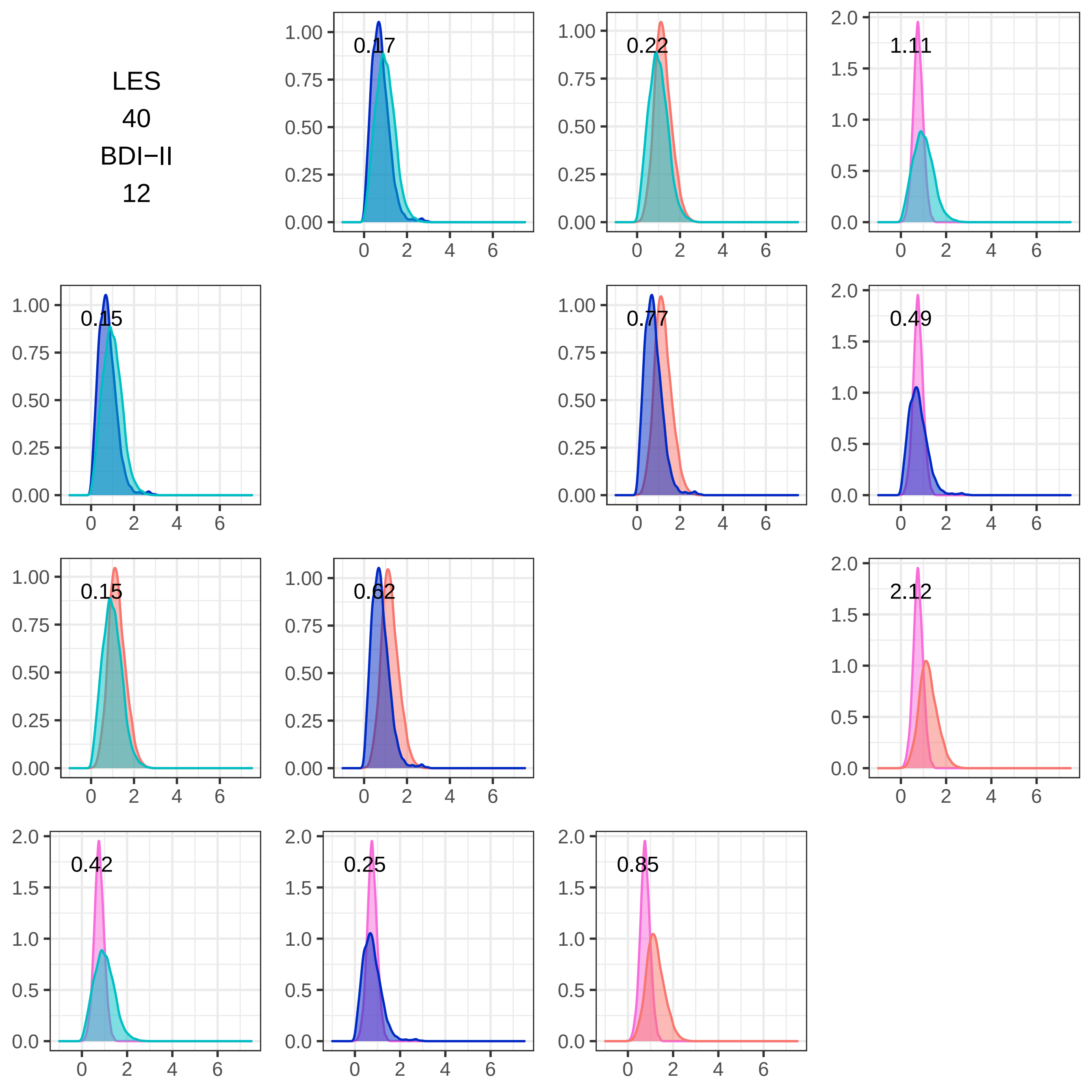
LES
40
BDI−II
12
0.17
0.22
1.11
0.15
0.77
0.49
0.15
0.62
2.12
0.42
0.25
0.85

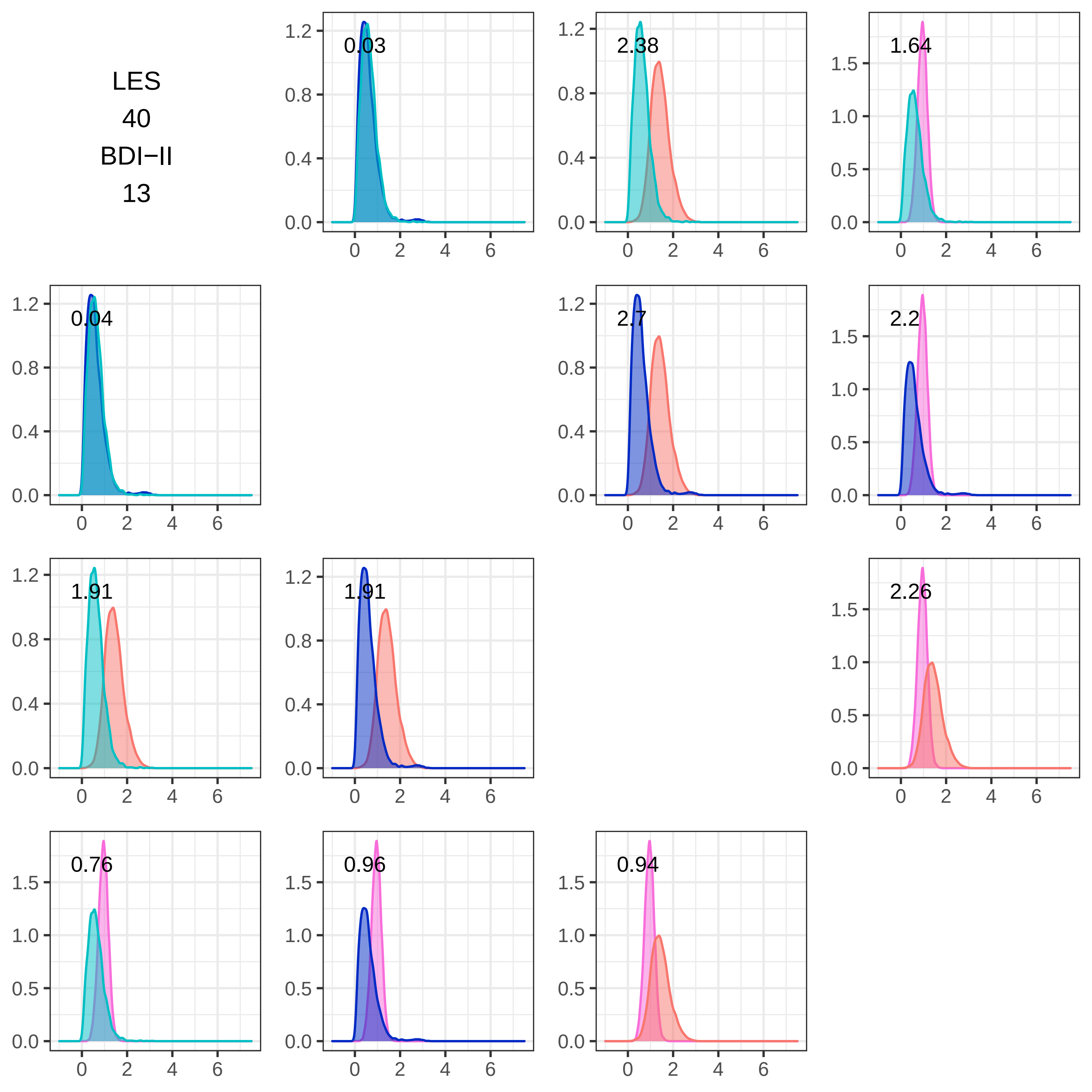
LES
40
BDI−II
13
0.03
2.38
1.64
0.04
2.7
2.2
1.91
1.91
2.26
0.76
0.96
0.94

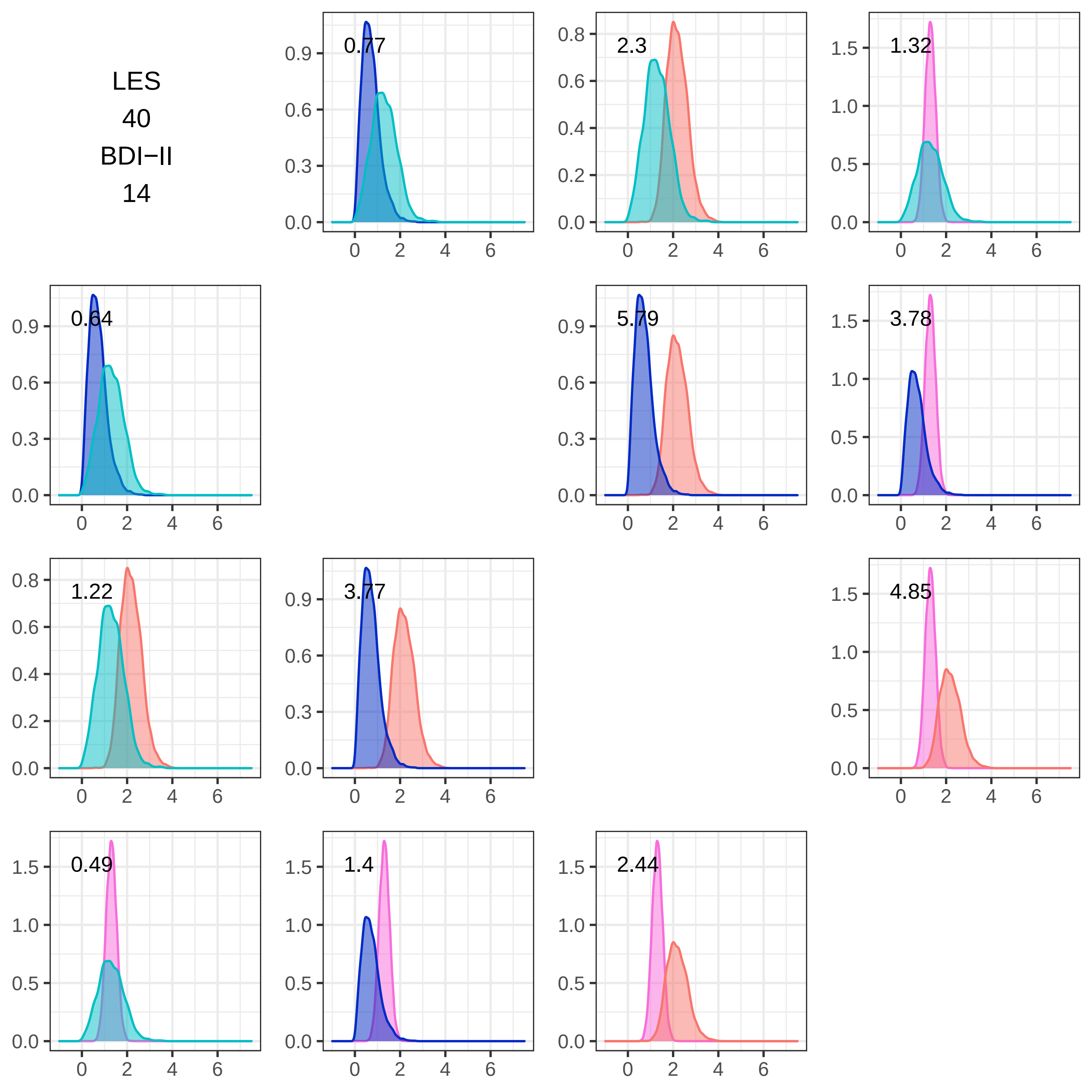

LES
40
BDI−II
14
0.77
2.3
1.32
0.64
5.79
3.78
1.22
3.77
4.85
0.49
1.4
2.44

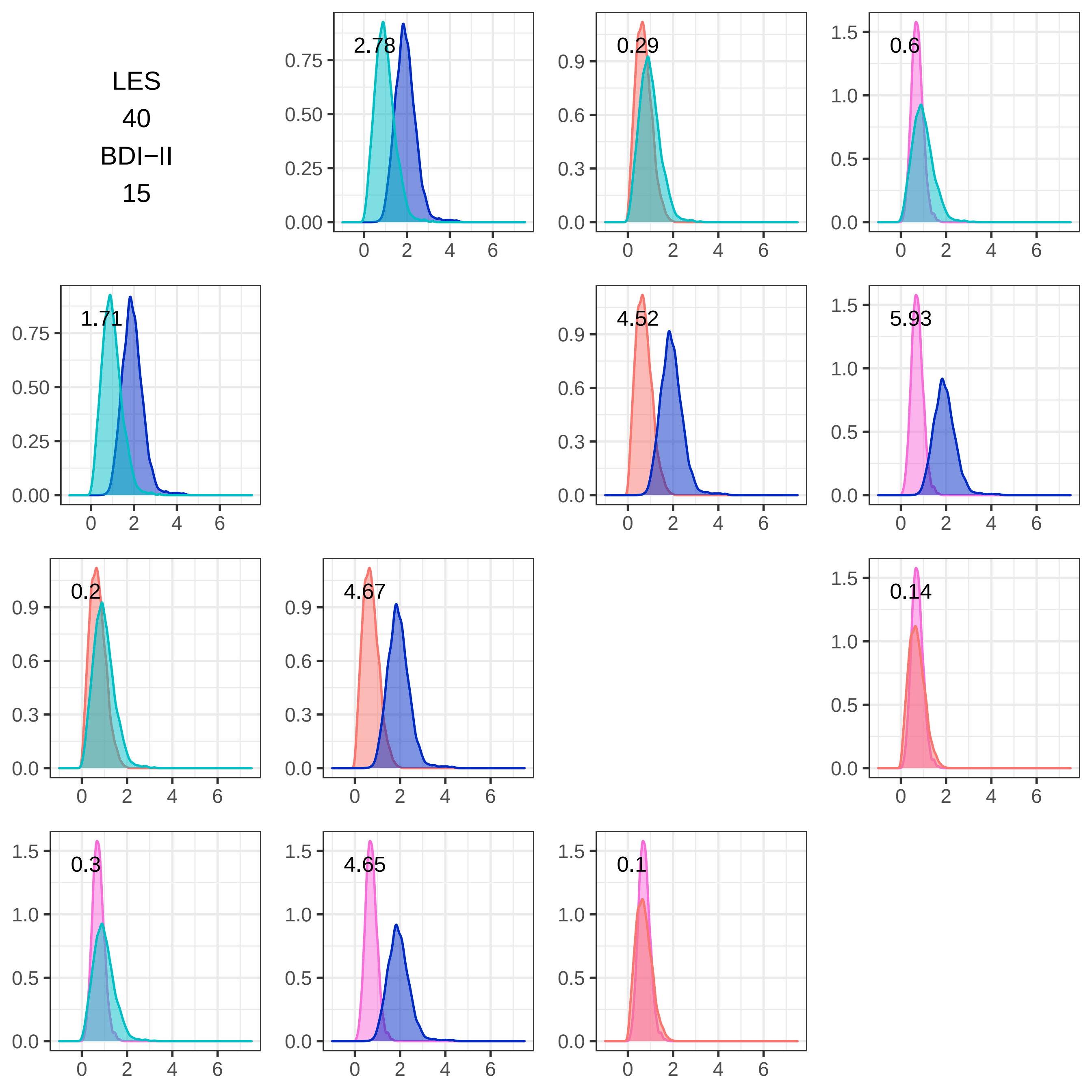
LES
40
BDI−II
15
2.78
0.29
0.6
1.71
4.52
5.93
0.2
4.67
0.14
0.3
4.65
0.1

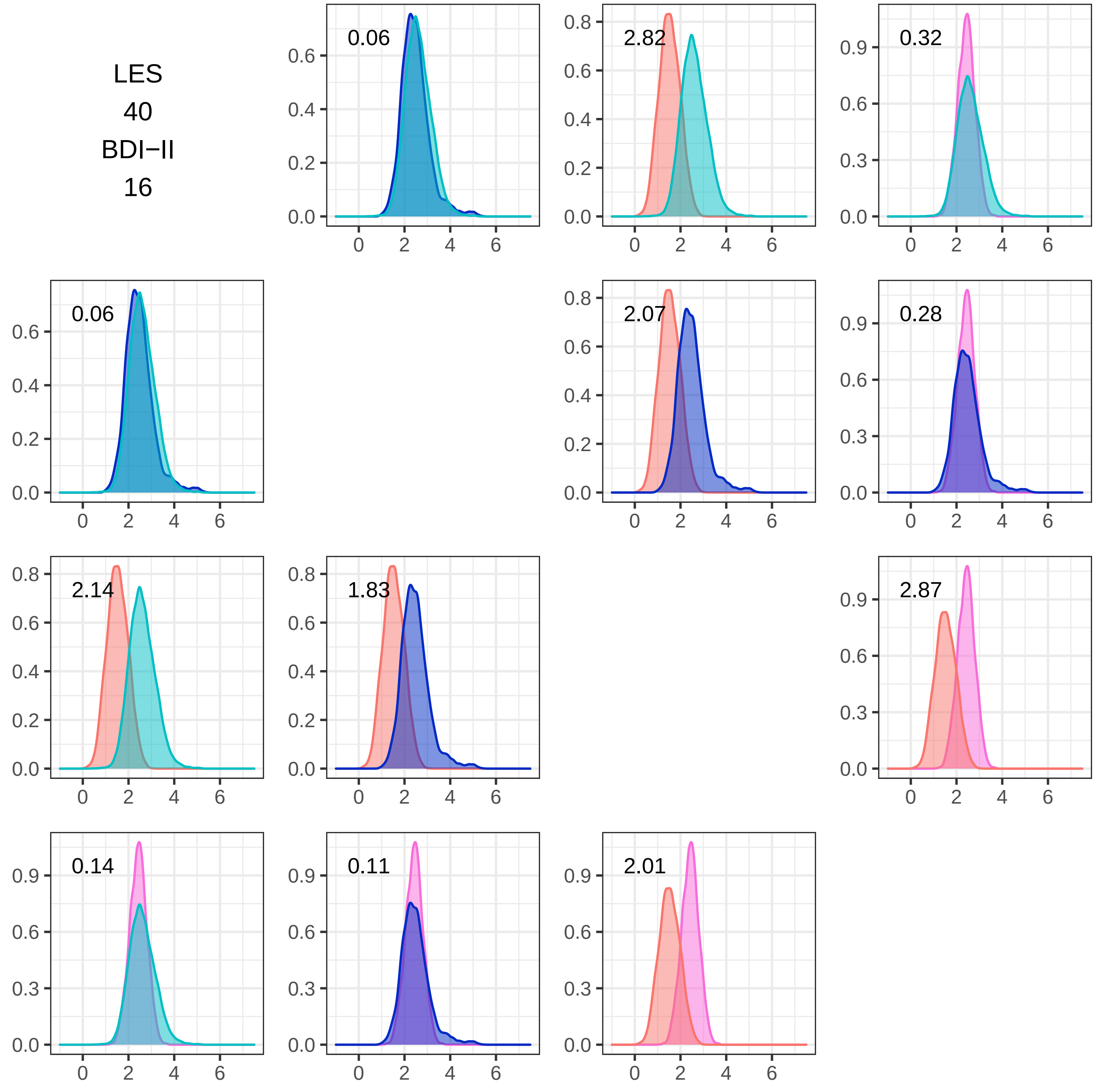
LES
40
BDI−II
16
0.06
2.82
0.32
0.06
2.07
0.28
2.14
1.83
2.87
0.14
0.11
2.01

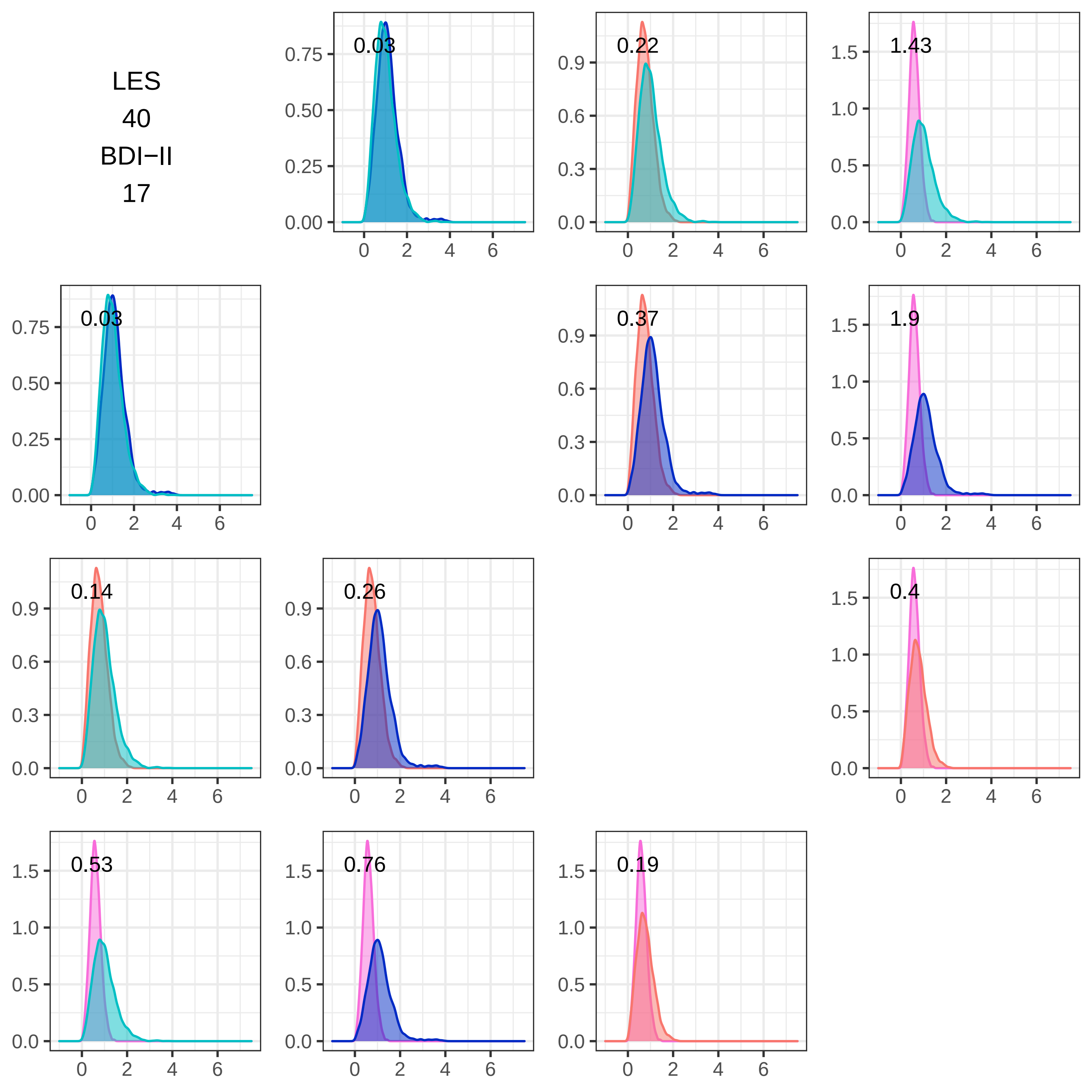
LES
40
BDI−II
17
0.03
0.22
1.43
0.03
0.37
1.9
0.14
0.26
0.4
0.53
0.76
0.19

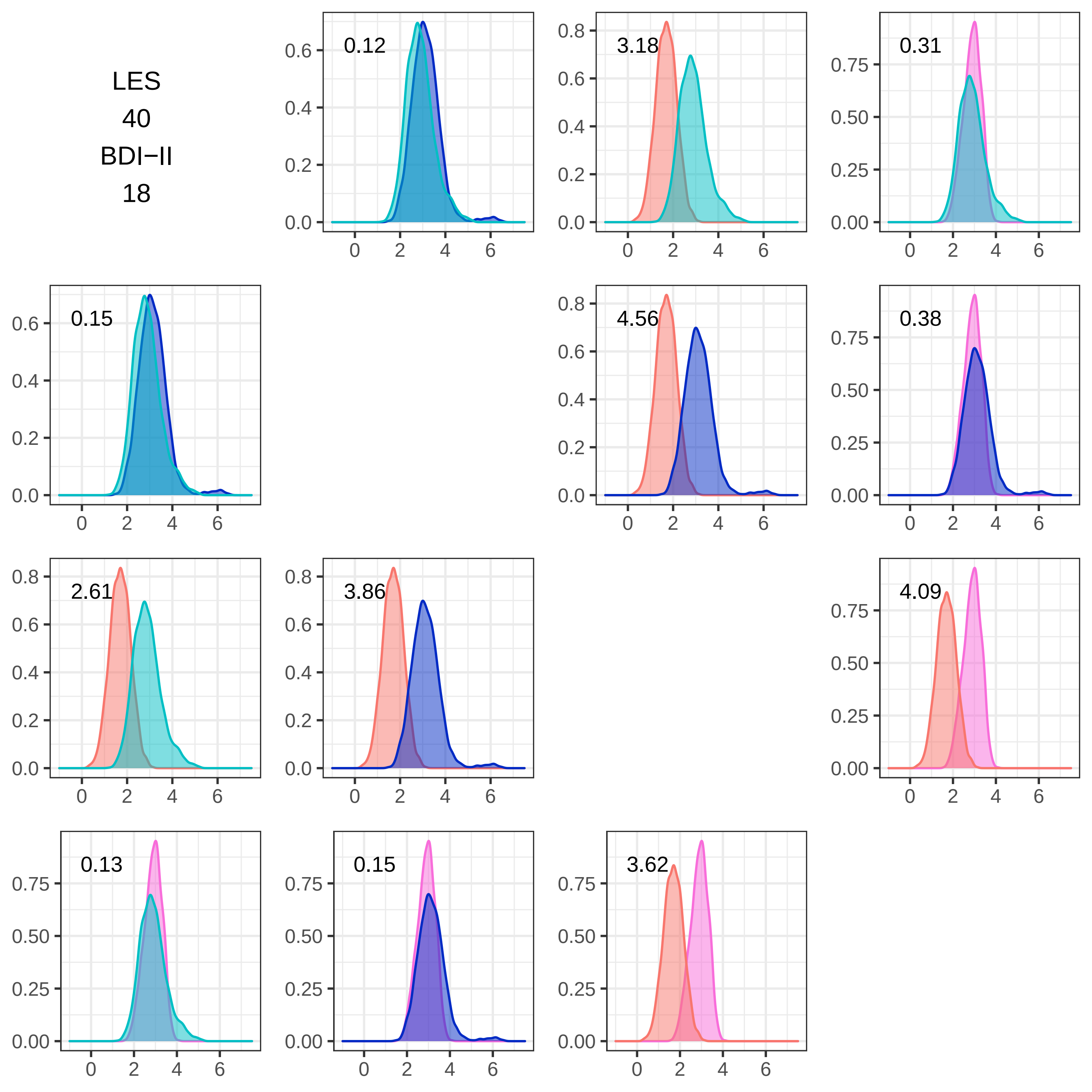
LES
40
BDI−II
18
0.12
3.18
0.31
0.15
4.56
0.38
2.61
3.86
4.09
0.13
0.15
3.62

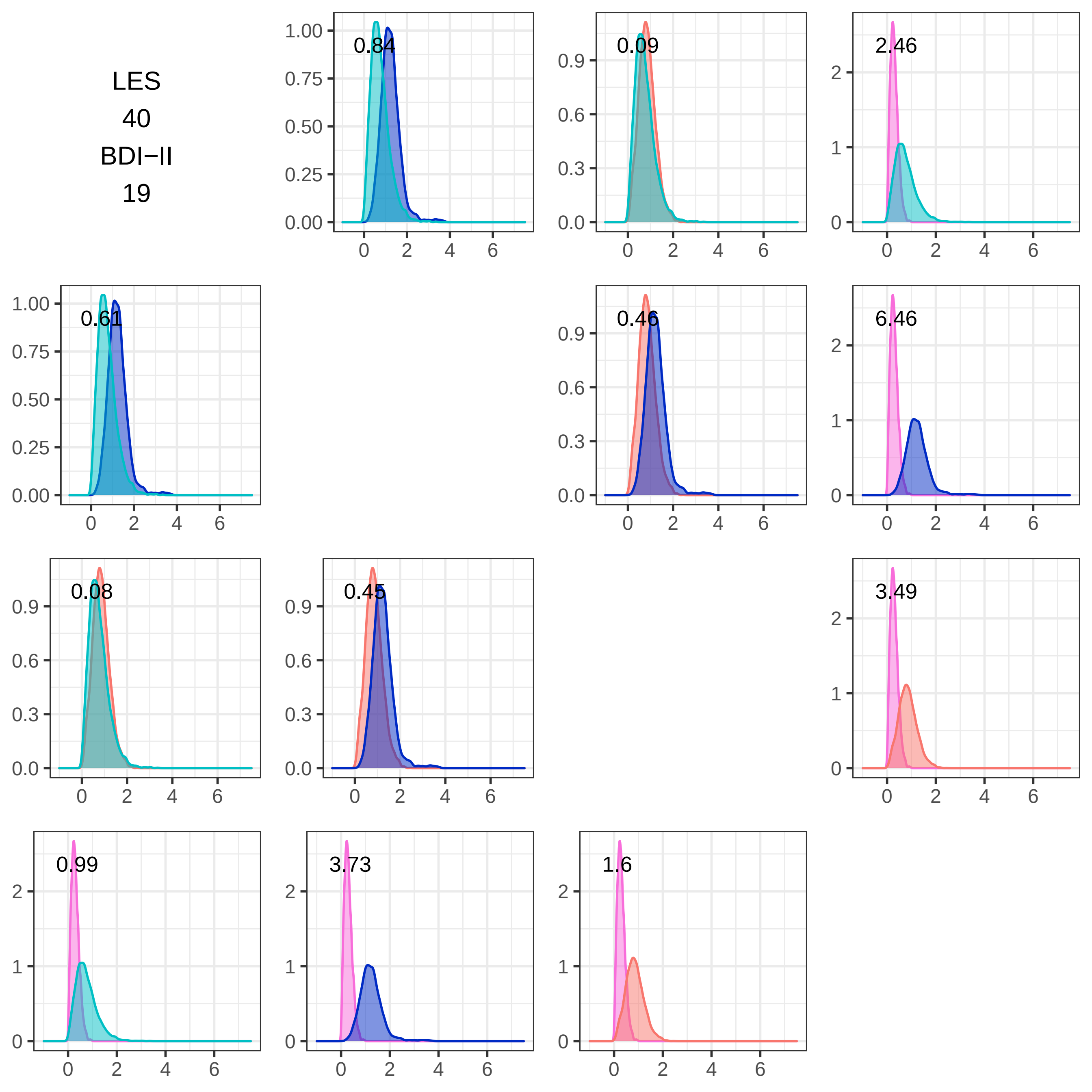

LES
40
BDI−II
19
0.84
0.09
2.46
0.61
0.46
6.46
0.08
0.45
3.49
0.99
3.73
1.6

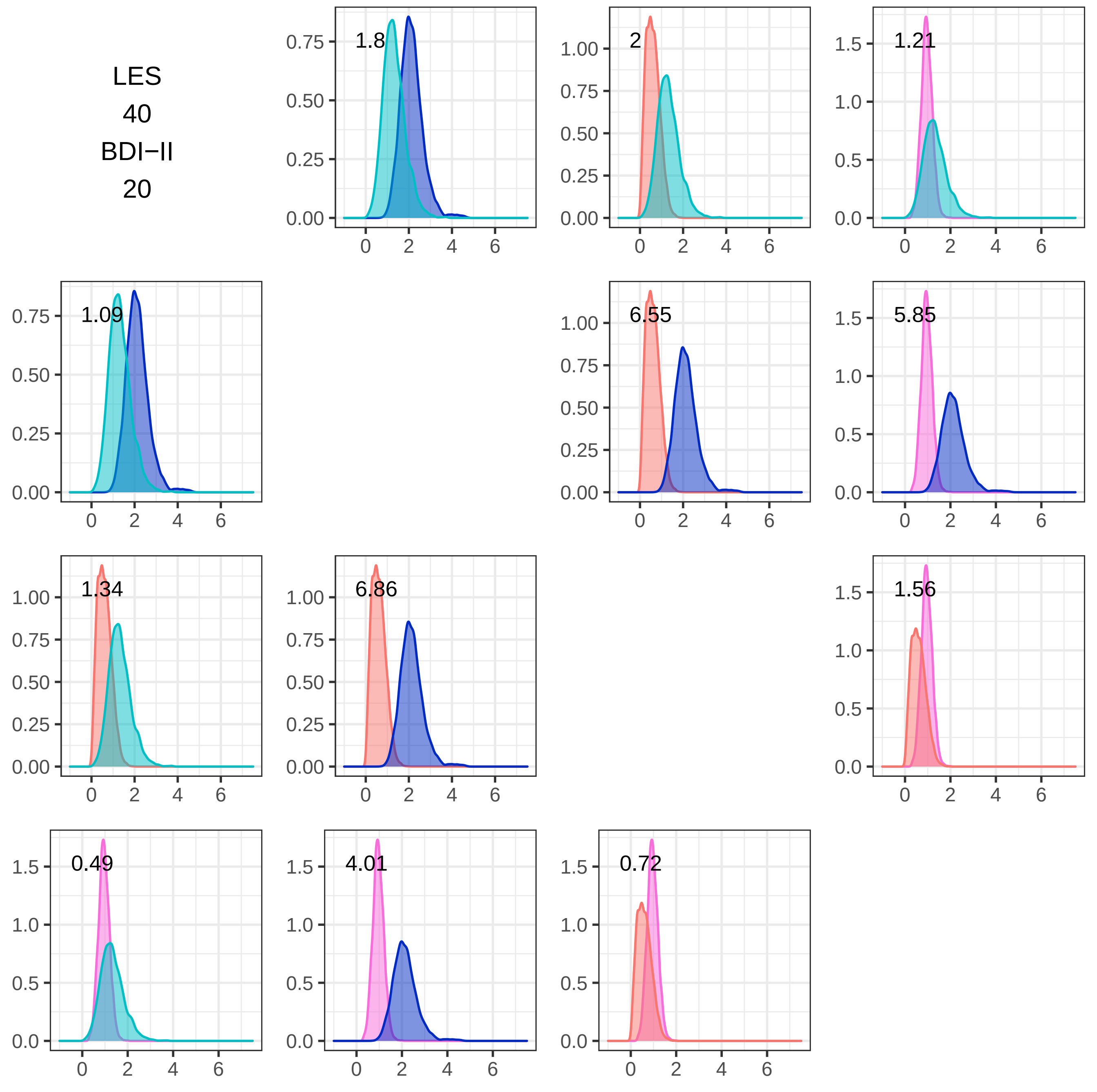
LES
40
BDI−II
20
1.8
2
1.21
1.09
6.55
5.85
1.34
6.86
1.56
0.49
4.01
0.72

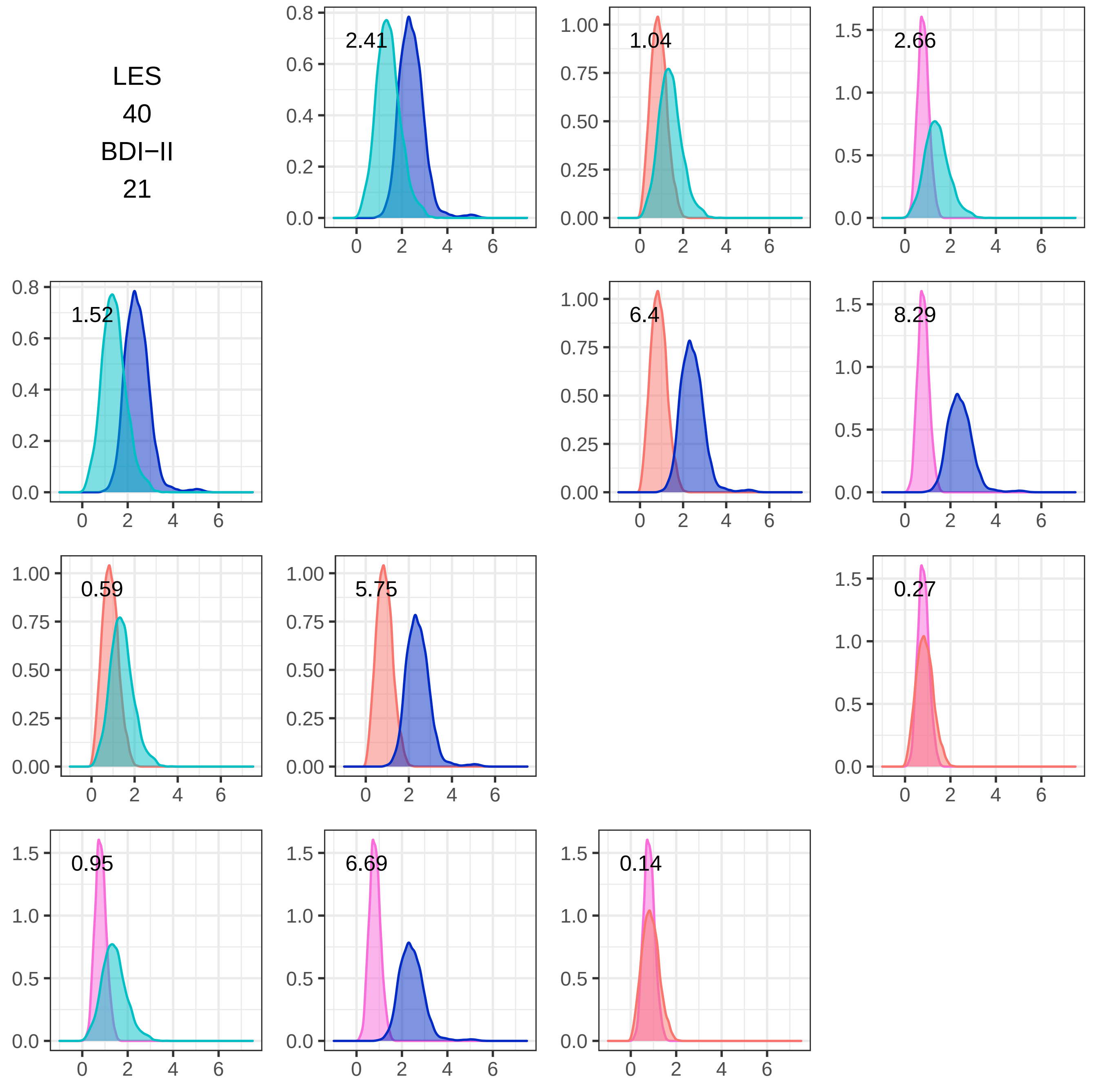
LES
40
BDI−II
21
2.41
1.04
2.66
1.52
6.4
8.29
0.59
5.75
0.27
0.95
6.69
0.14

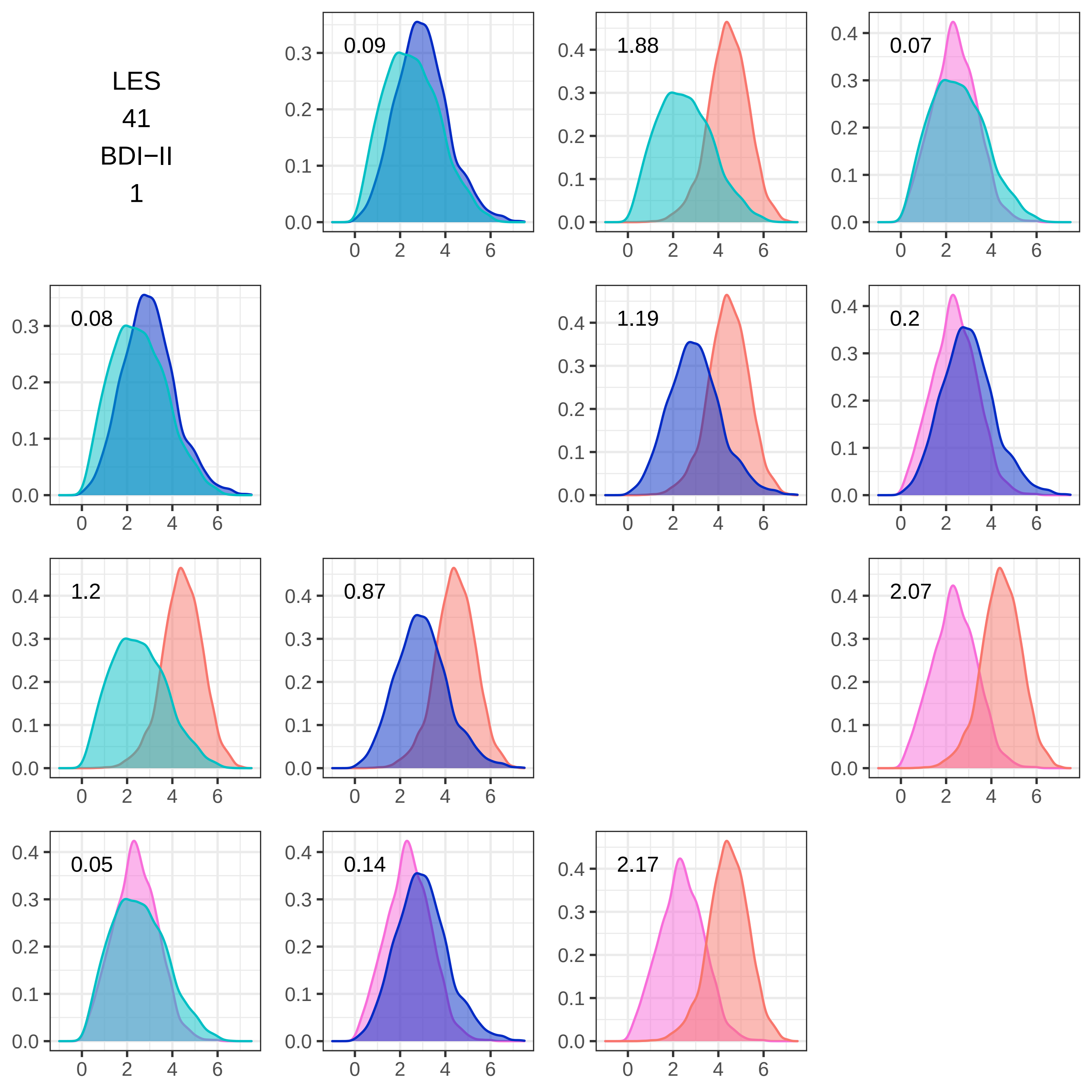
LES
41
BDI−II
1
0.09
1.88
0.07
0.08
1.19
0.2
1.2
0.87
2.07
0.05
0.14
2.17

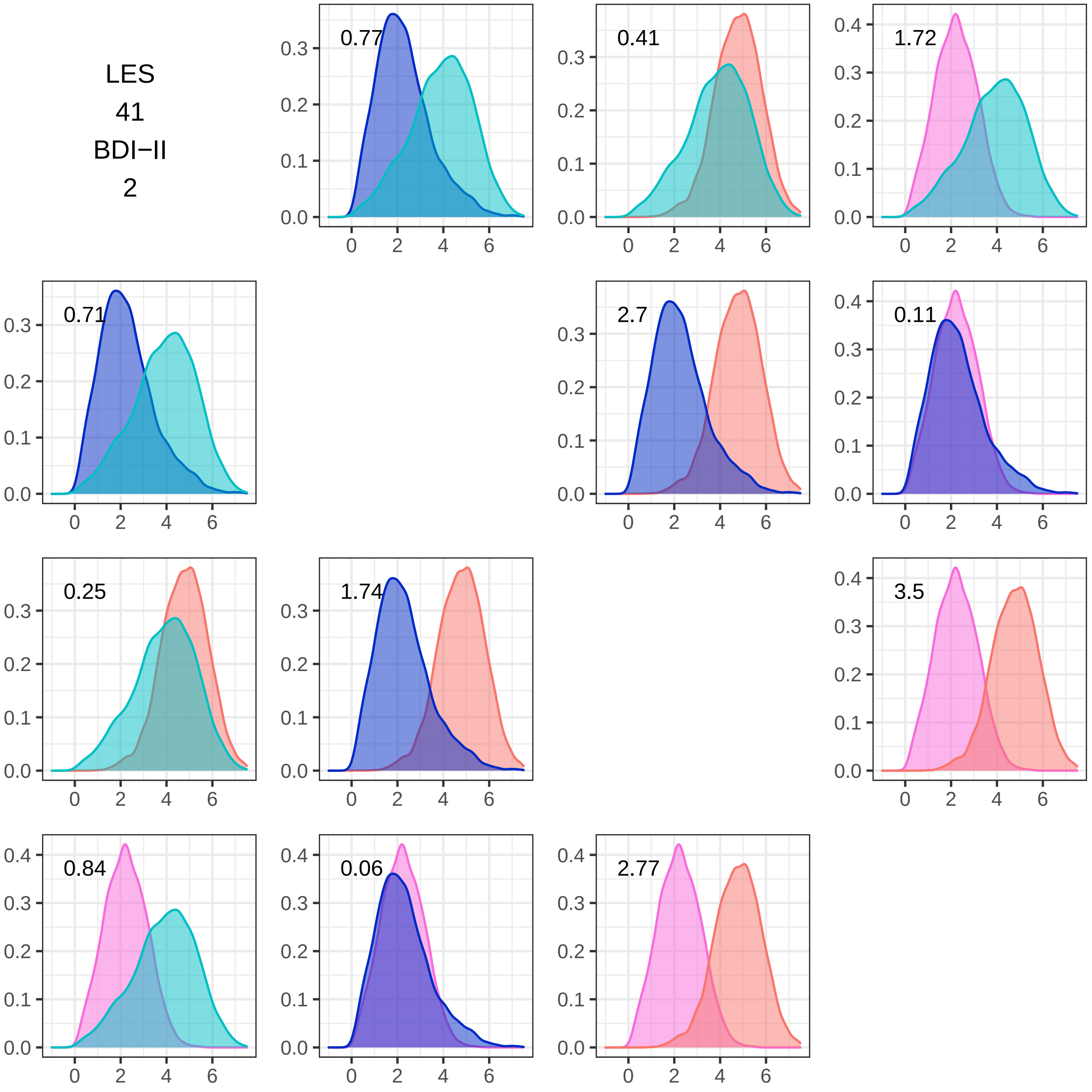

LES
41
BDI−II
2
0.77
0.41
1.72
0.71
2.7
0.11
0.25
1.74
3.5
0.84
0.06
2.77

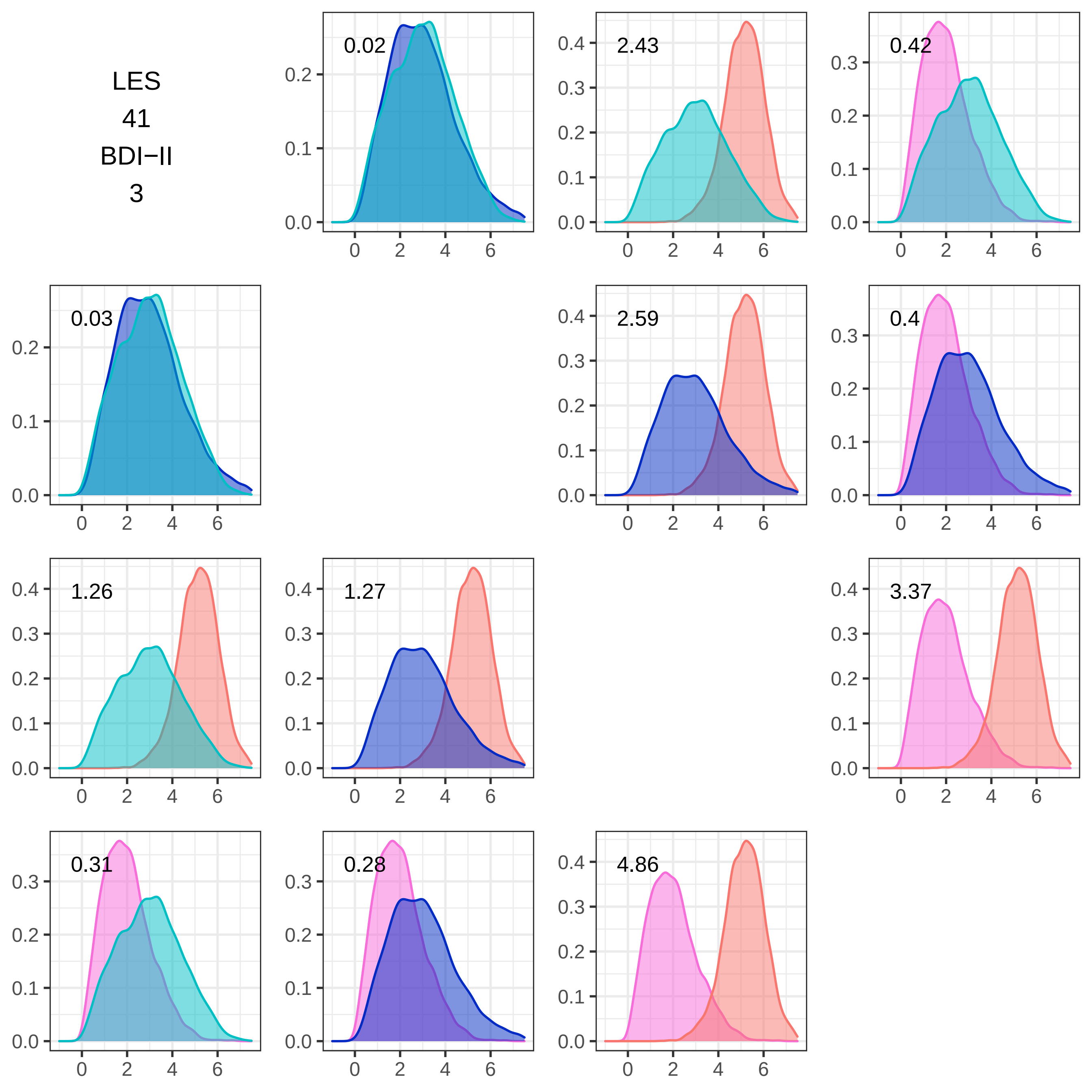

LES
41
BDI−II
3
0.02
2.43
0.42
0.03
2.59
0.4
1.26
1.27
3.37
0.31
0.28
4.86

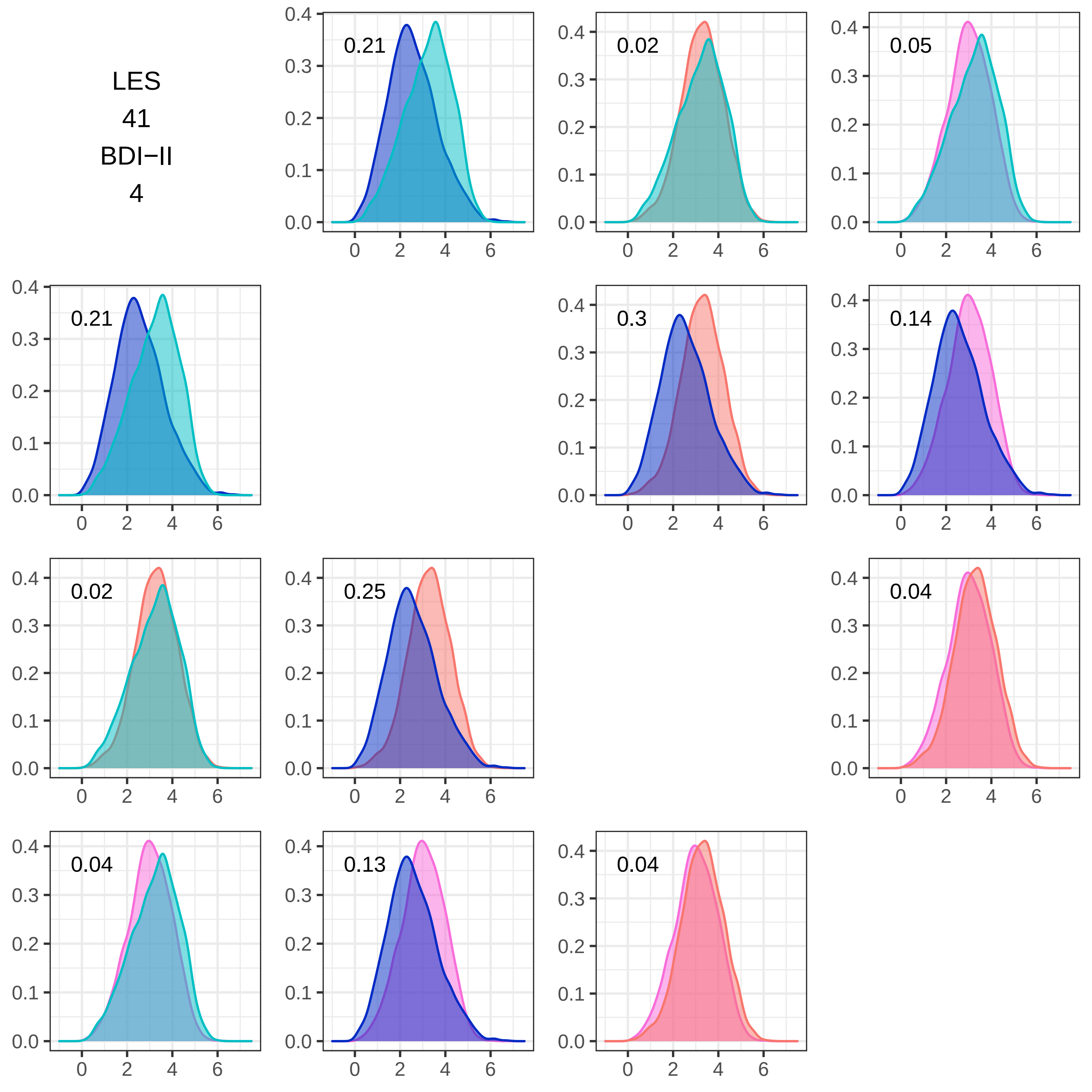

LES
41
BDI−II
4
0.21
0.02
0.05
0.21
0.3
0.14
0.02
0.25
0.04
0.04
0.13
0.04

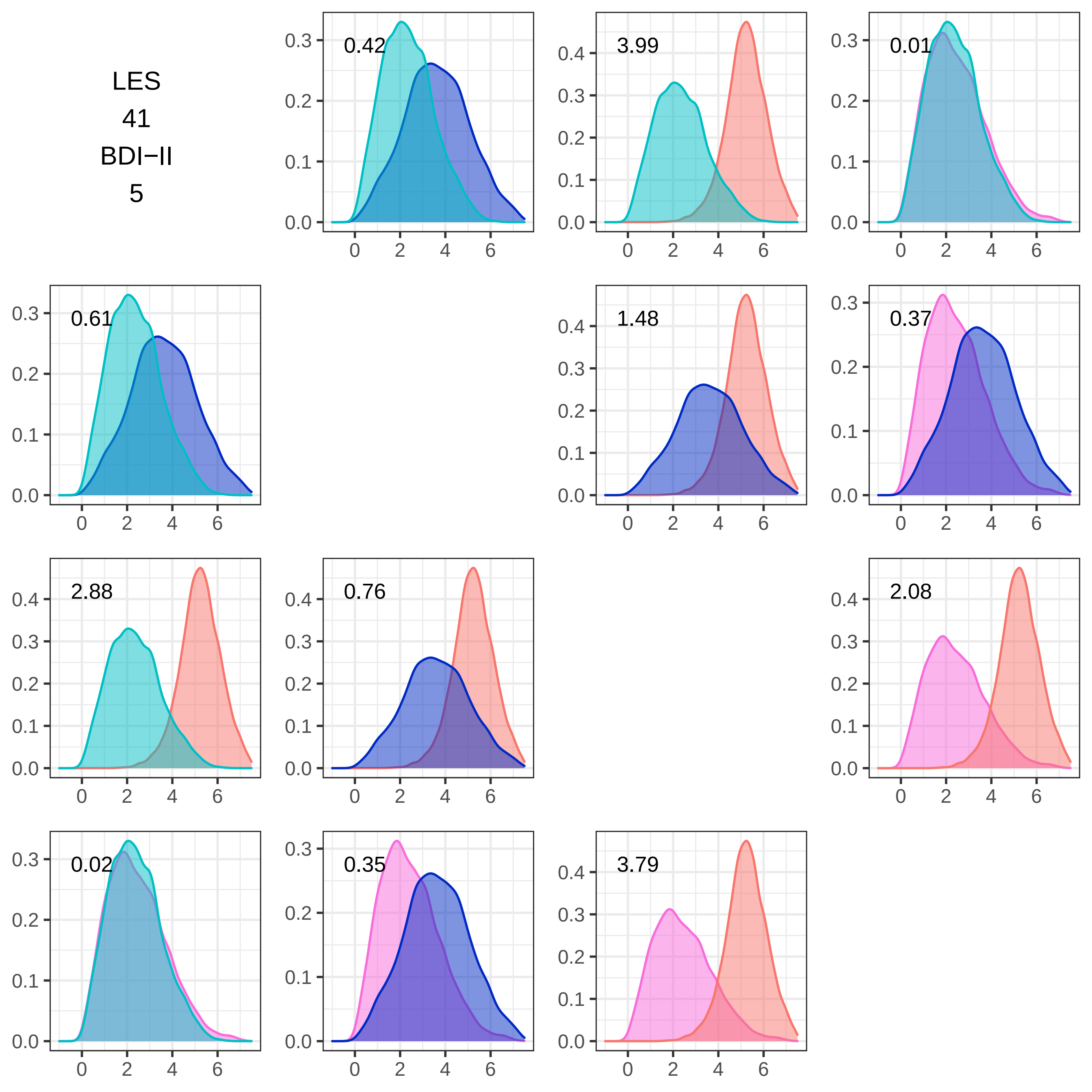

LES
41
BDI−II
5
0.42
3.99
0.01
0.61
1.48
0.37
2.88
0.76
2.08
0.02
0.35
3.79

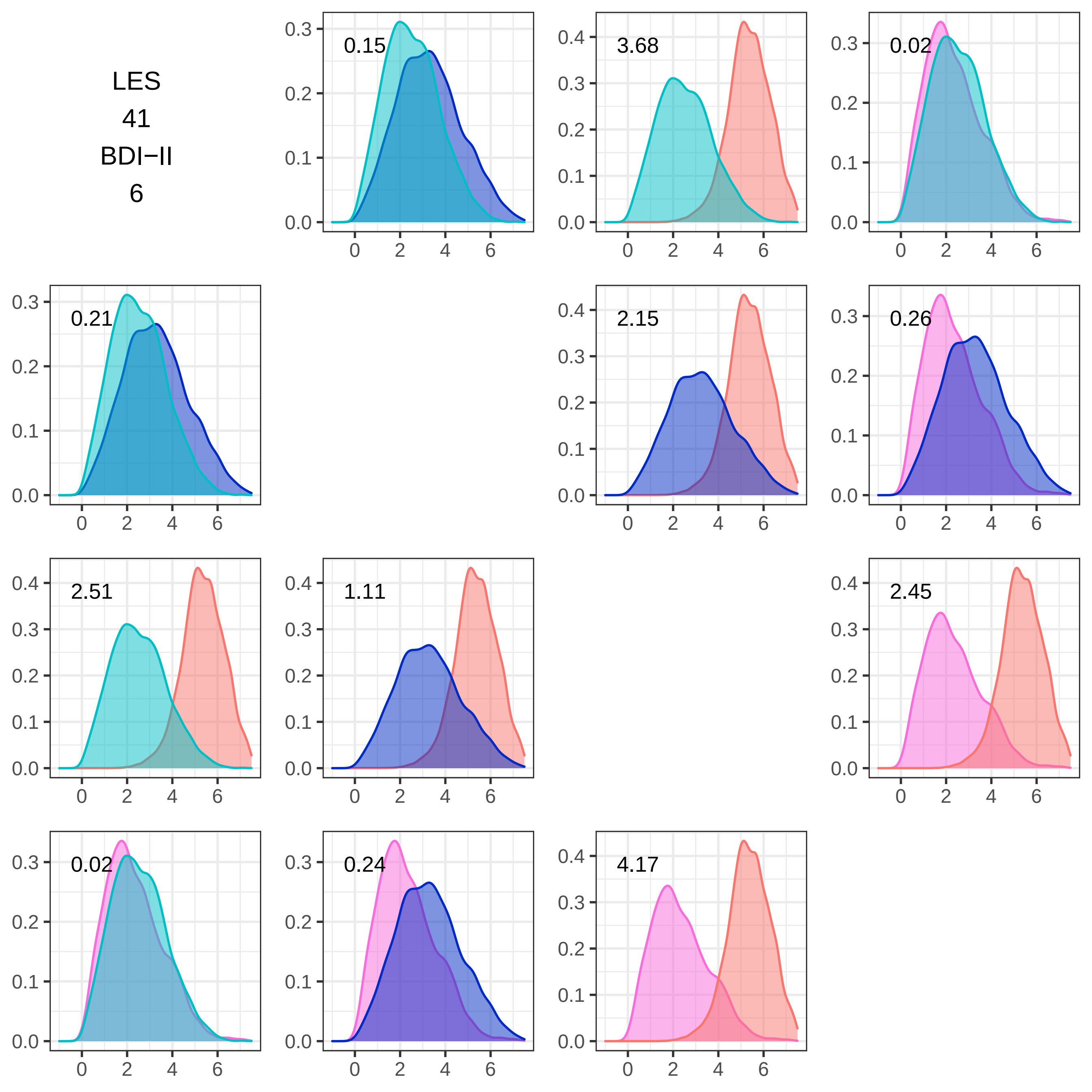
LES
41
BDI−II
6
0.15
3.68
0.02
0.21
2.15
0.26
2.51
1.11
2.45
0.02
0.24
4.17

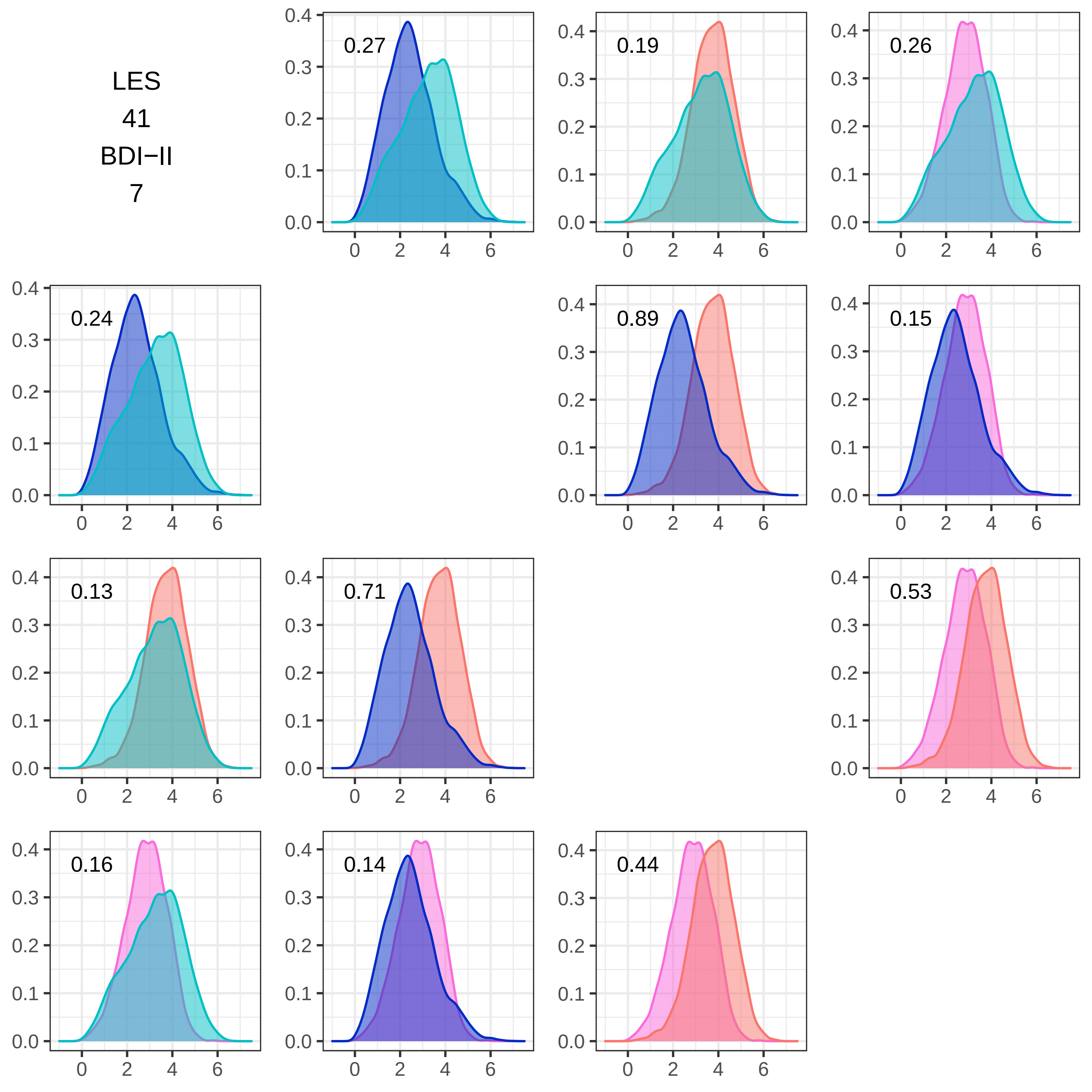

LES
41
BDI−II
7
0.27
0.19
0.26
0.24
0.89
0.15
0.13
0.71
0.53
0.16
0.14
0.44

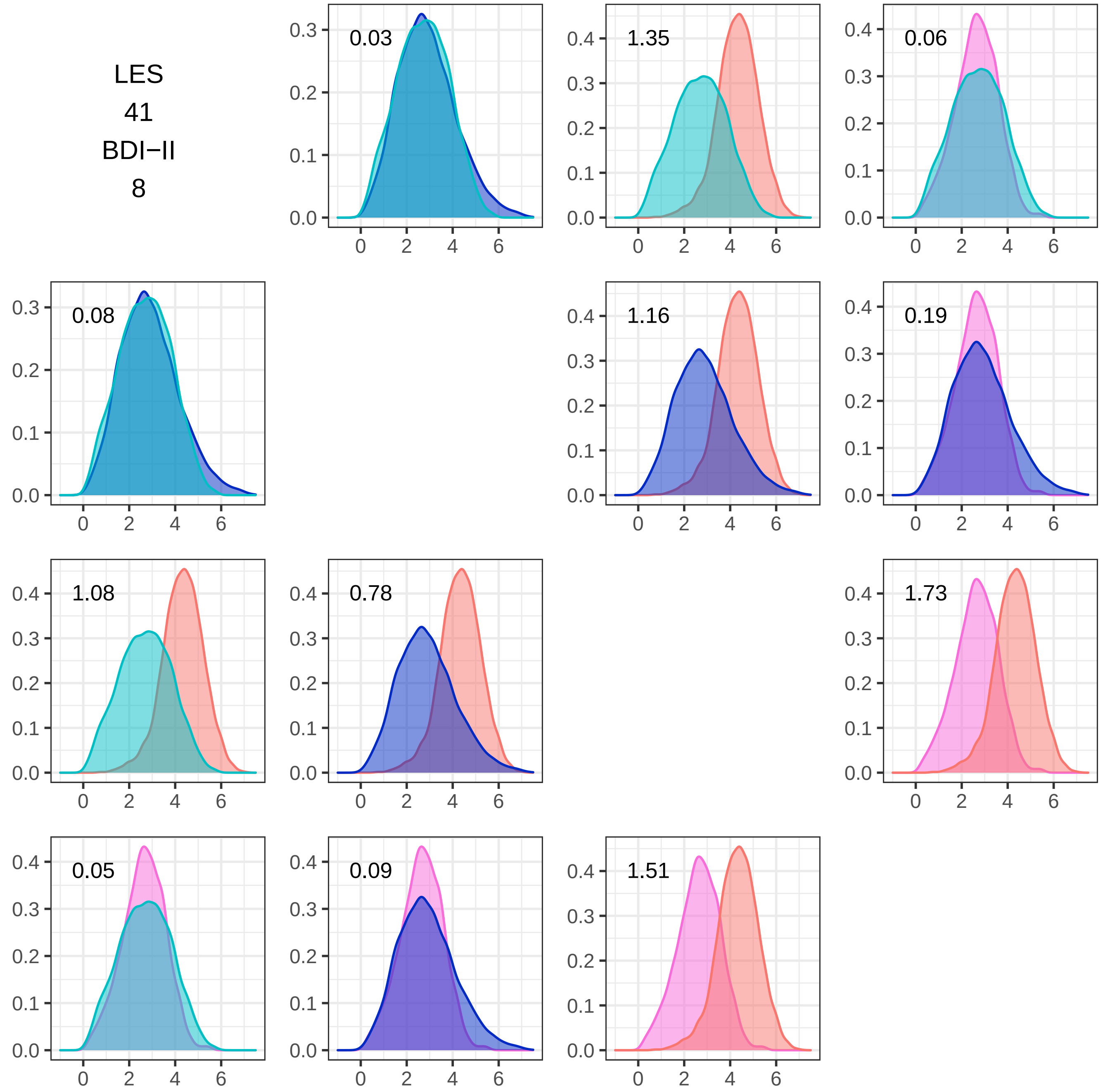

LES
41
BDI−II
8
0.03
1.35
0.06
0.08
1.16
0.19
1.08
0.78
1.73
0.05
0.09
1.51

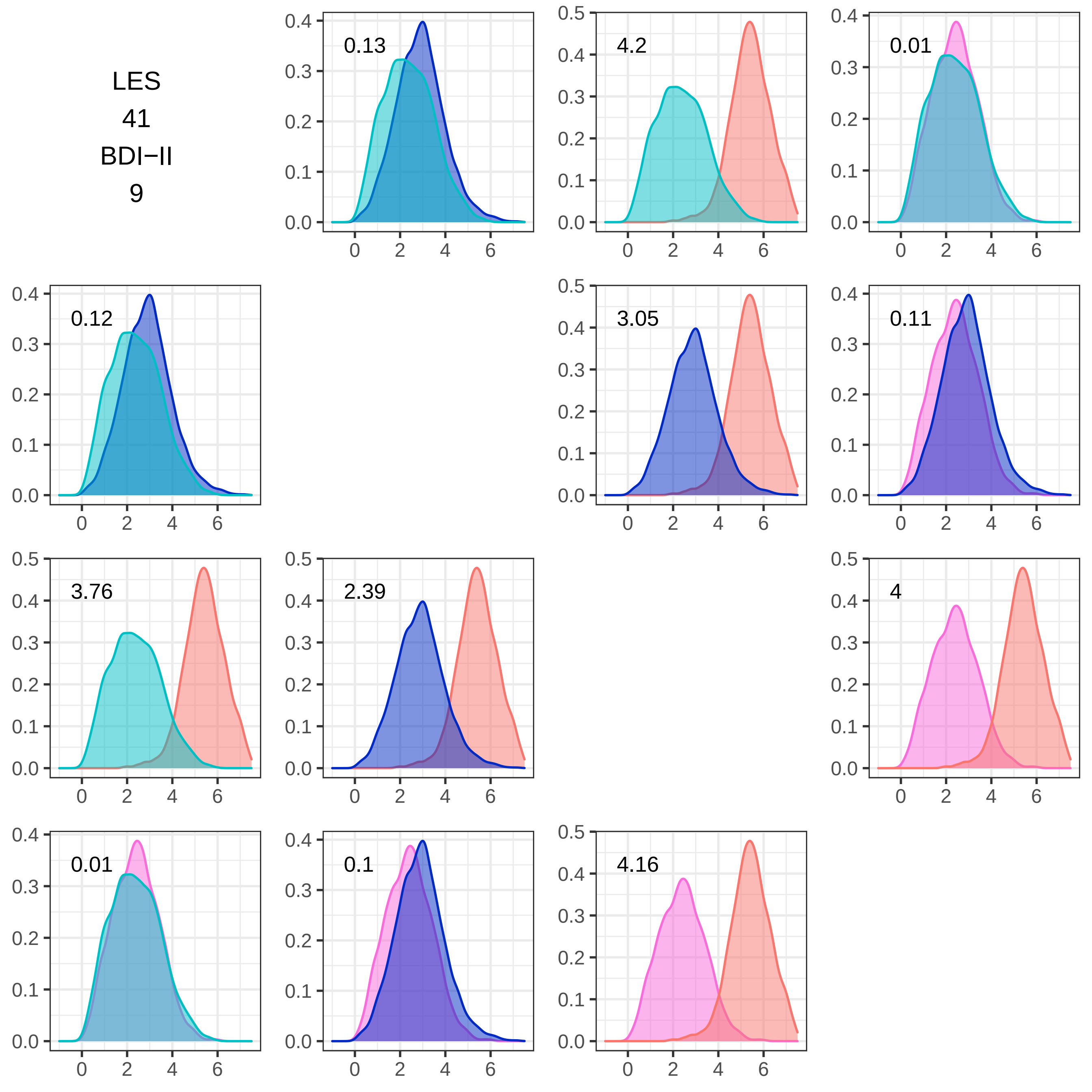
LES
41
BDI−II
9
0.13
4.2
0.01
0.12
3.05
0.11
3.76
2.39
4
0.01
0.1
4.16

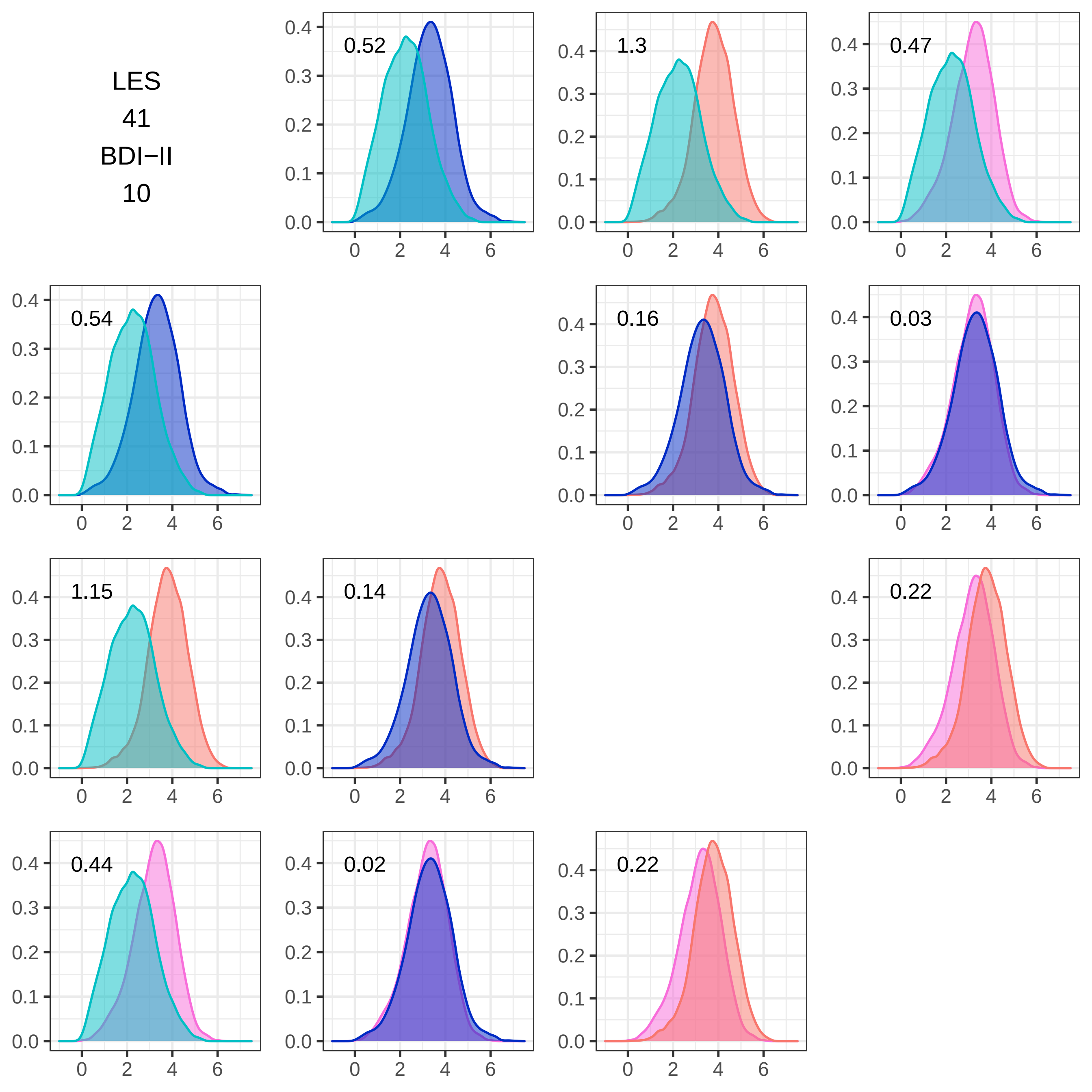

LES
41
BDI−II
10
0.52
1.3
0.47
0.54
0.16
0.03
1.15
0.14
0.22
0.44
0.02
0.22

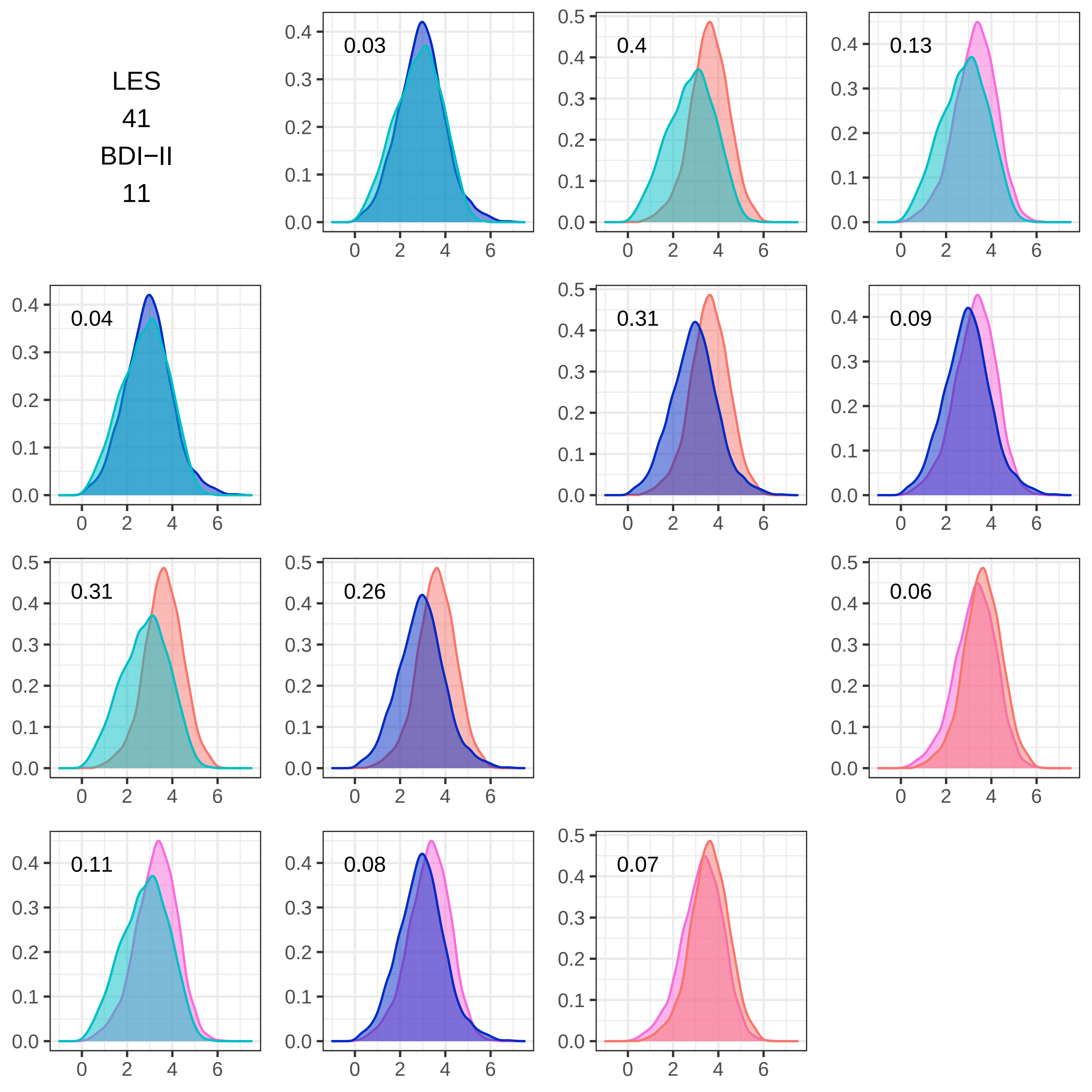
LES
41
BDI−II
11
0.03
0.4
0.13
0.04
0.31
0.09
0.31
0.26
0.06
0.11
0.08
0.07

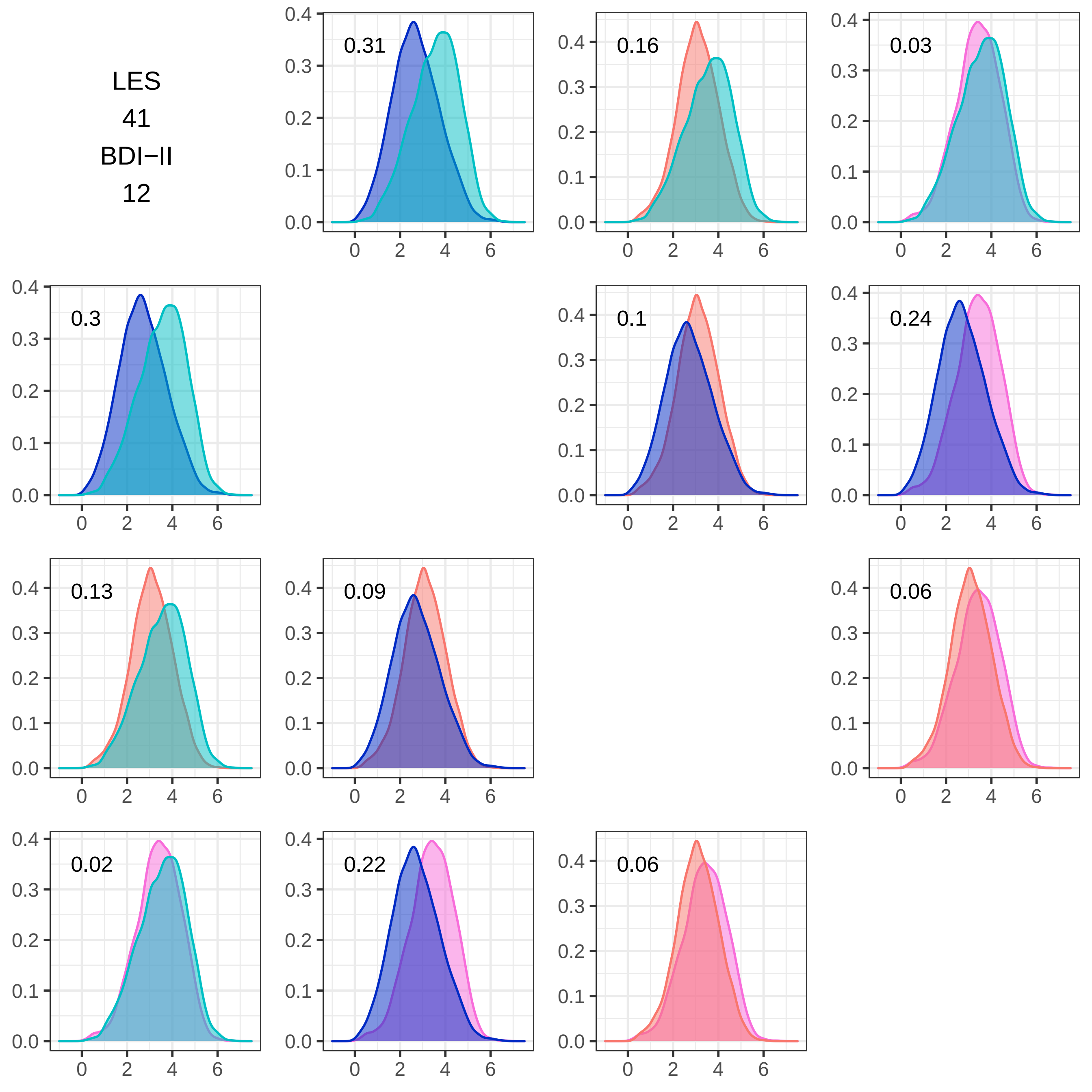
LES
41
BDI−II
12
0.31
0.16
0.03
0.3
0.1
0.24
0.13
0.09
0.06
0.02
0.22
0.06

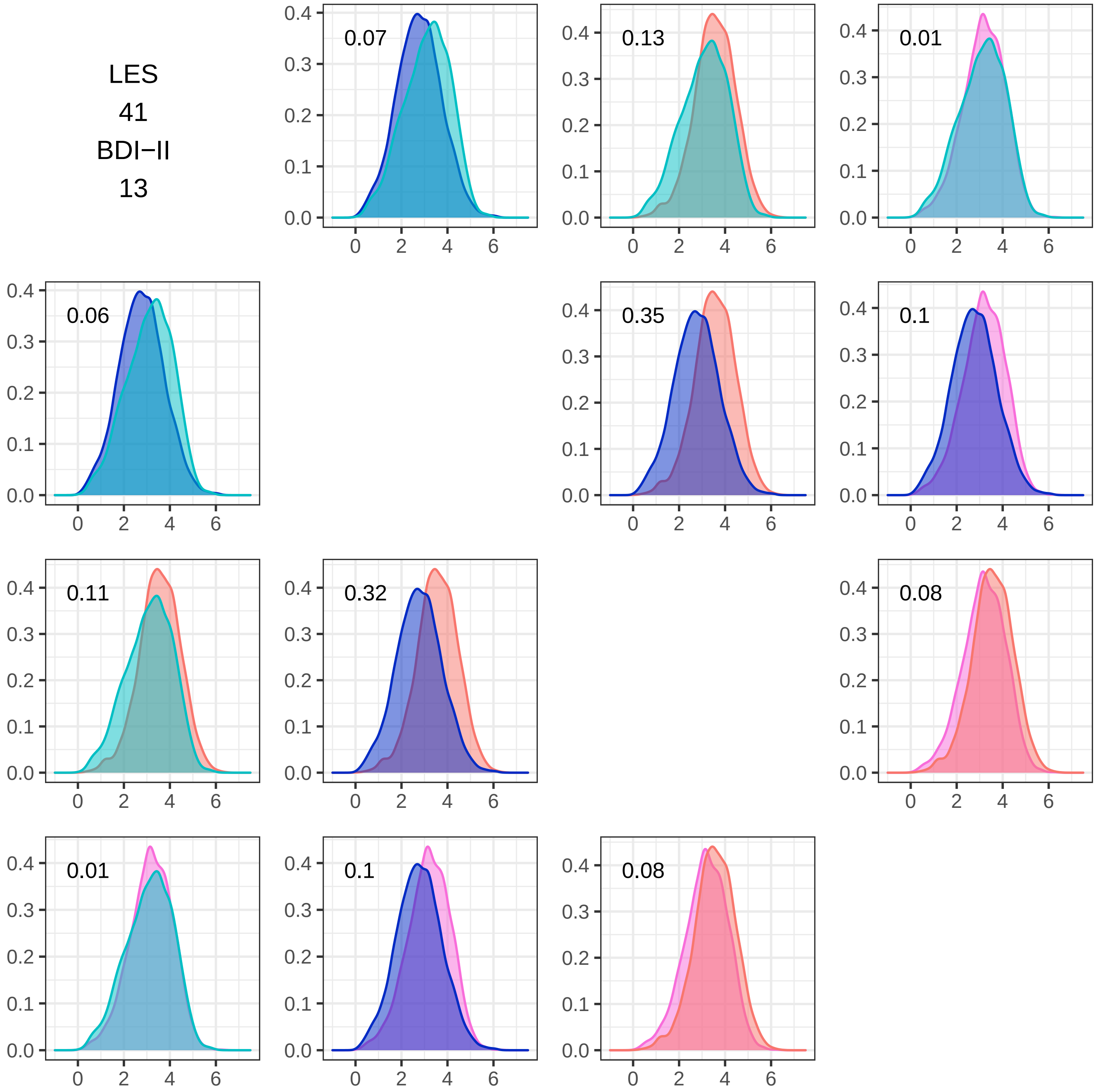
LES
41
BDI−II
13
0.07
0.13
0.01
0.06
0.35
0.1
0.11
0.32
0.08
0.01
0.1
0.08

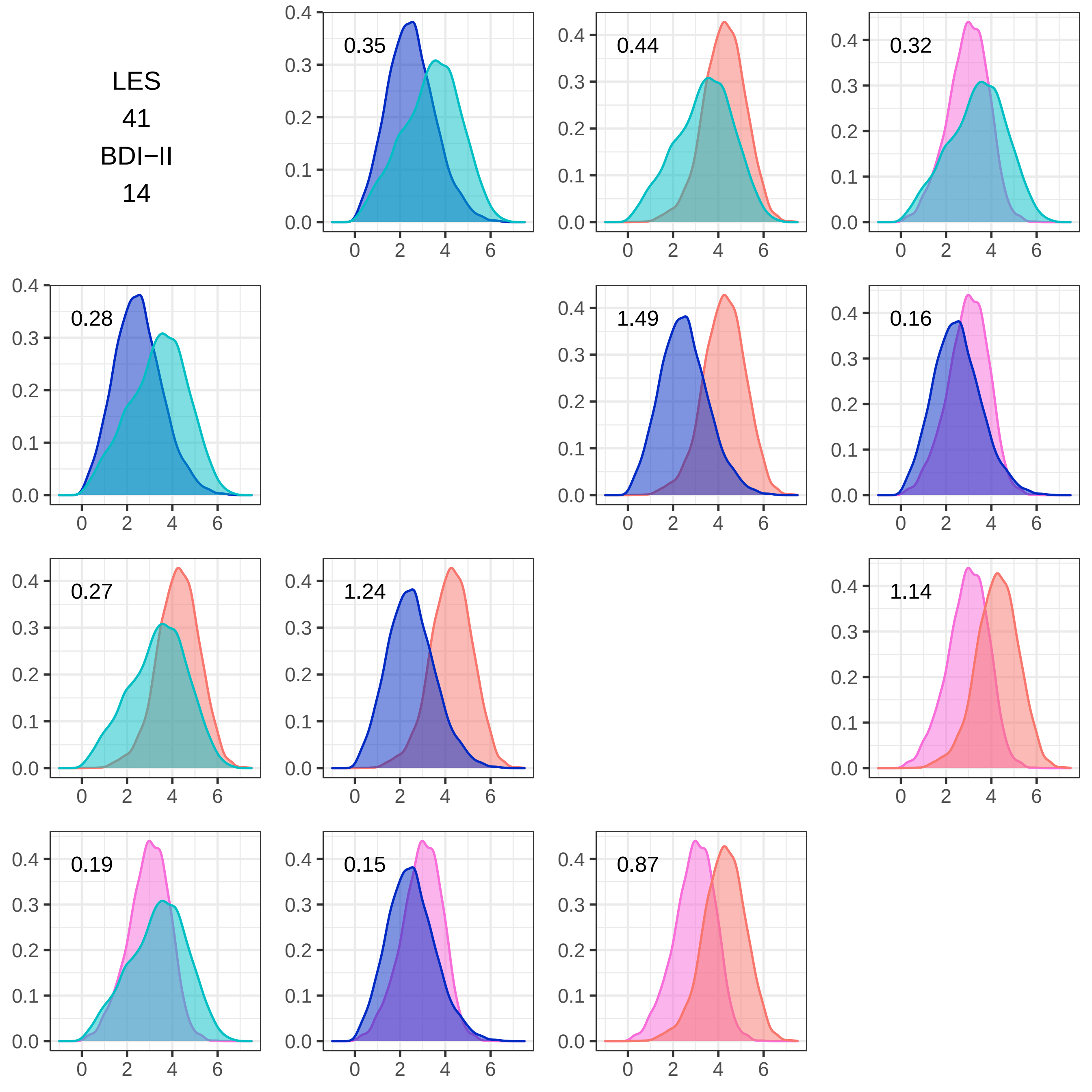
LES
41
BDI−II
14
0.35
0.44
0.32
0.28
1.49
0.16
0.27
1.24
1.14
0.19
0.15
0.87

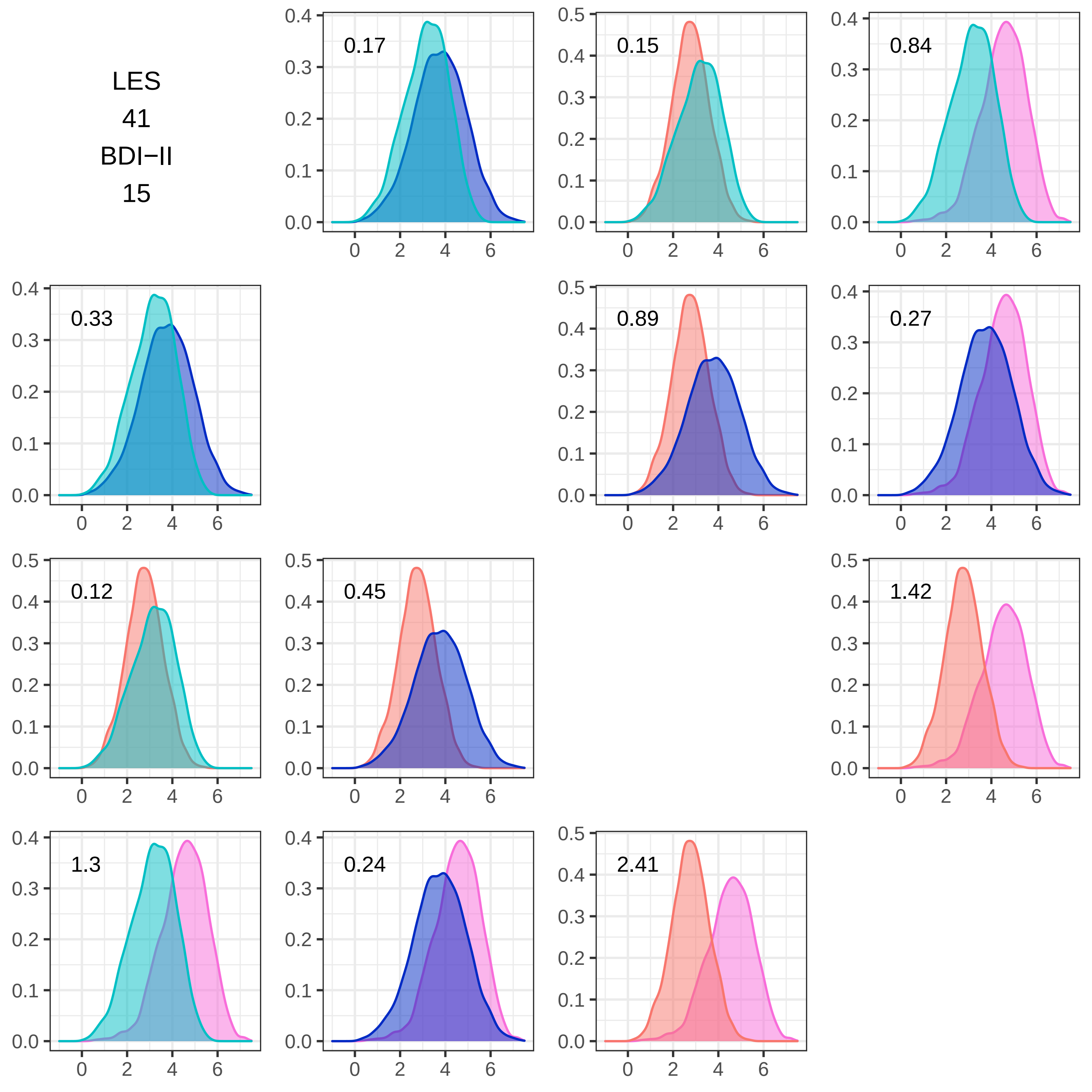
LES
41
BDI−II
15
0.17
0.15
0.84
0.33
0.89
0.27
0.12
0.45
1.42
1.3
0.24
2.41

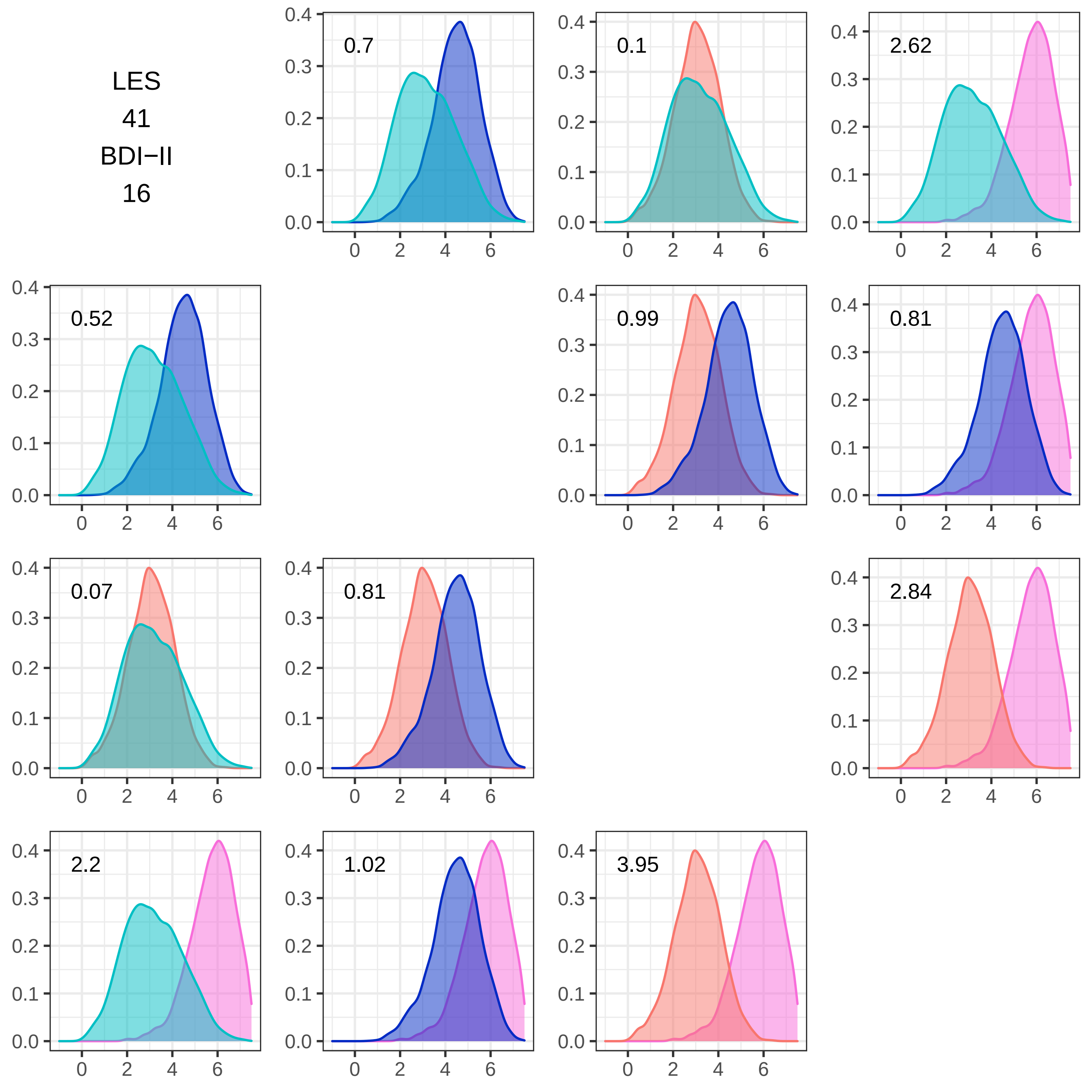
LES
41
BDI−II
16
0.7
0.1
2.62
0.52
0.99
0.81
0.07
0.81
2.84
2.2
1.02
3.95

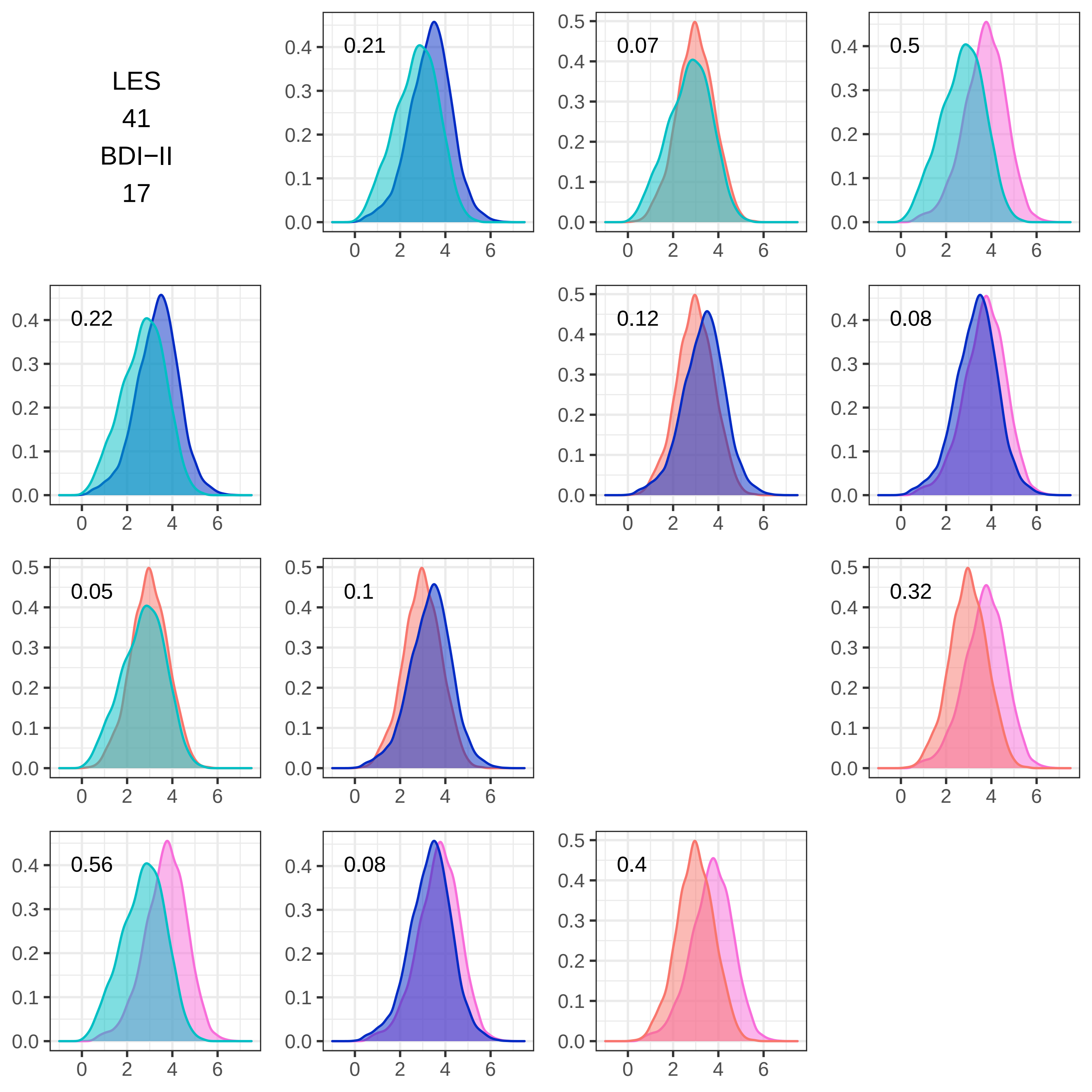
LES
41
BDI−II
17
0.21
0.07
0.5
0.22
0.12
0.08
0.05
0.1
0.32
0.56
0.08
0.4

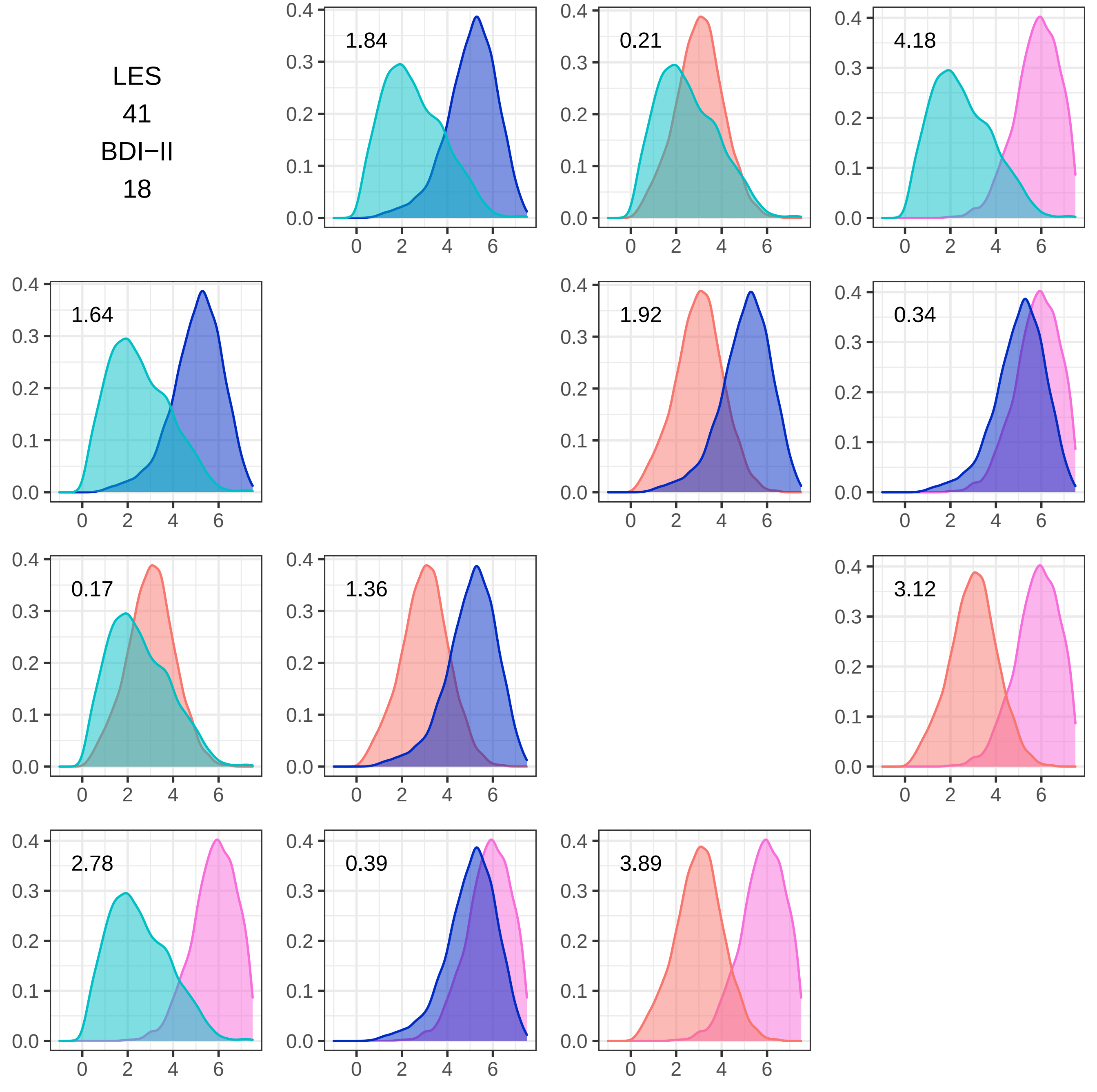

LES
41
BDI−II
18
1.84
0.21
4.18
1.64
1.92
0.34
0.17
1.36
3.12
2.78
0.39
3.89

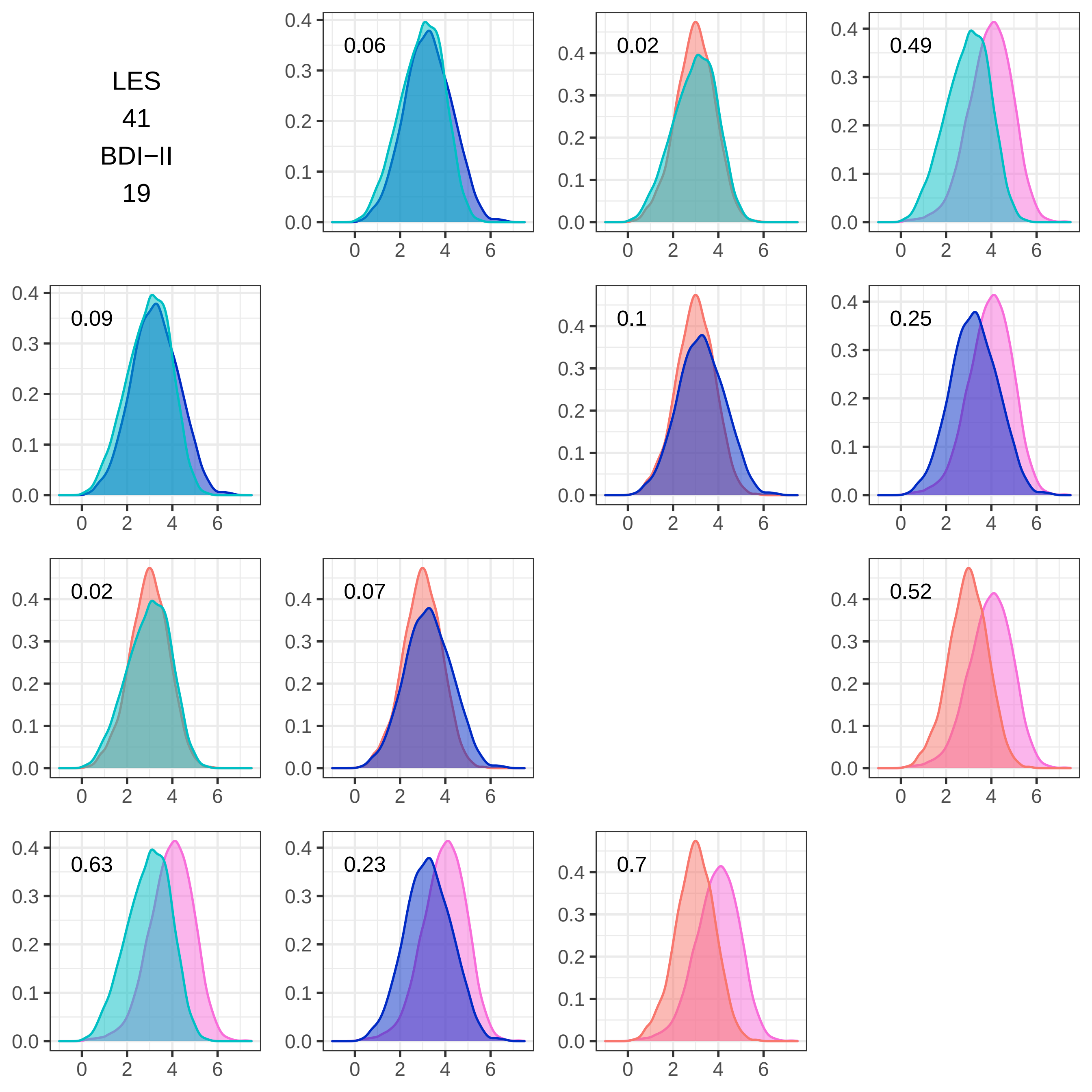
LES
41
BDI−II
19
0.06
0.02
0.49
0.09
0.1
0.25
0.02
0.07
0.52
0.63
0.23
0.7

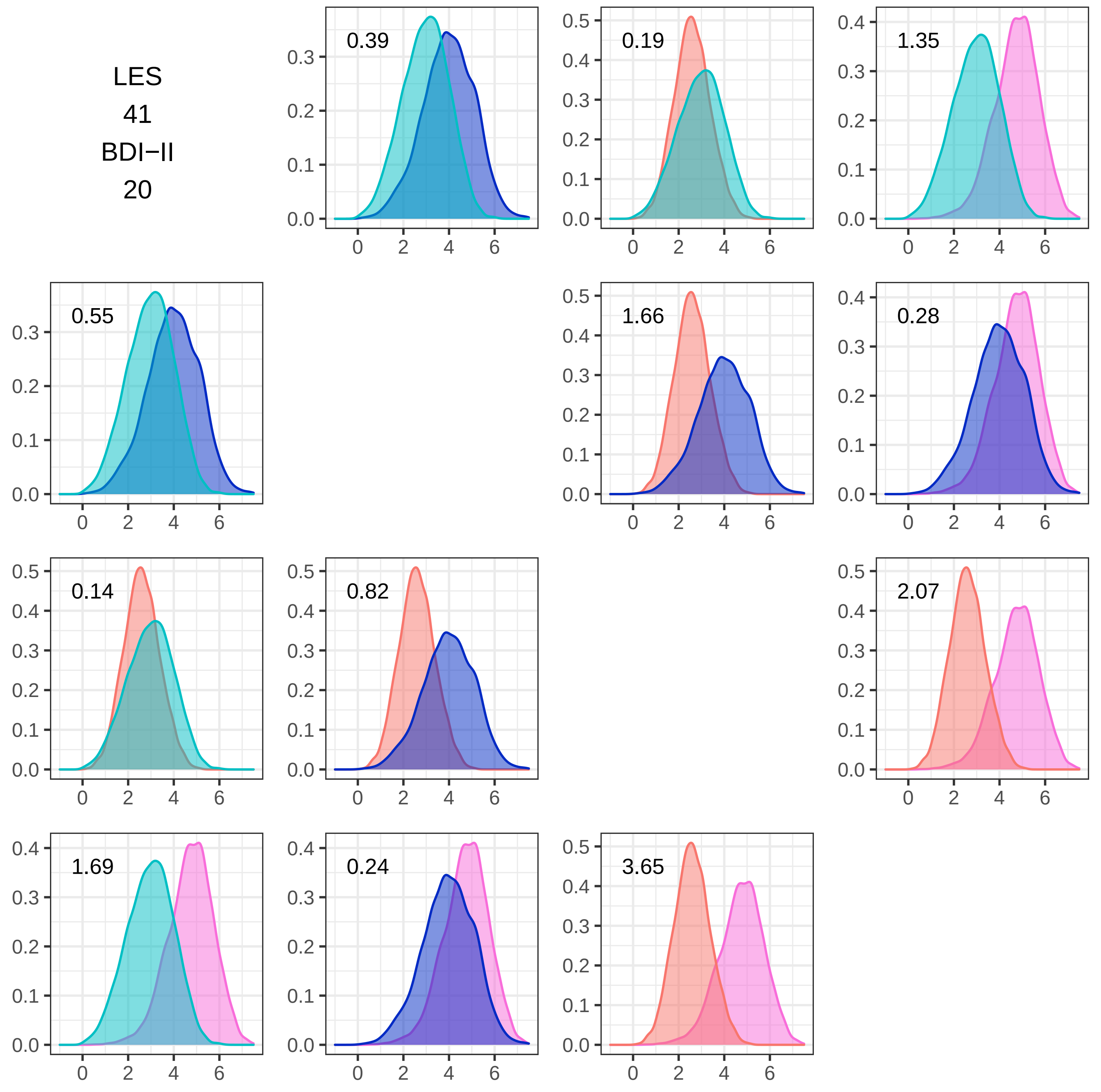
LES
41
BDI−II
20
0.39
0.19
1.35
0.55
1.66
0.28
0.14
0.82
2.07
1.69
0.24
3.65

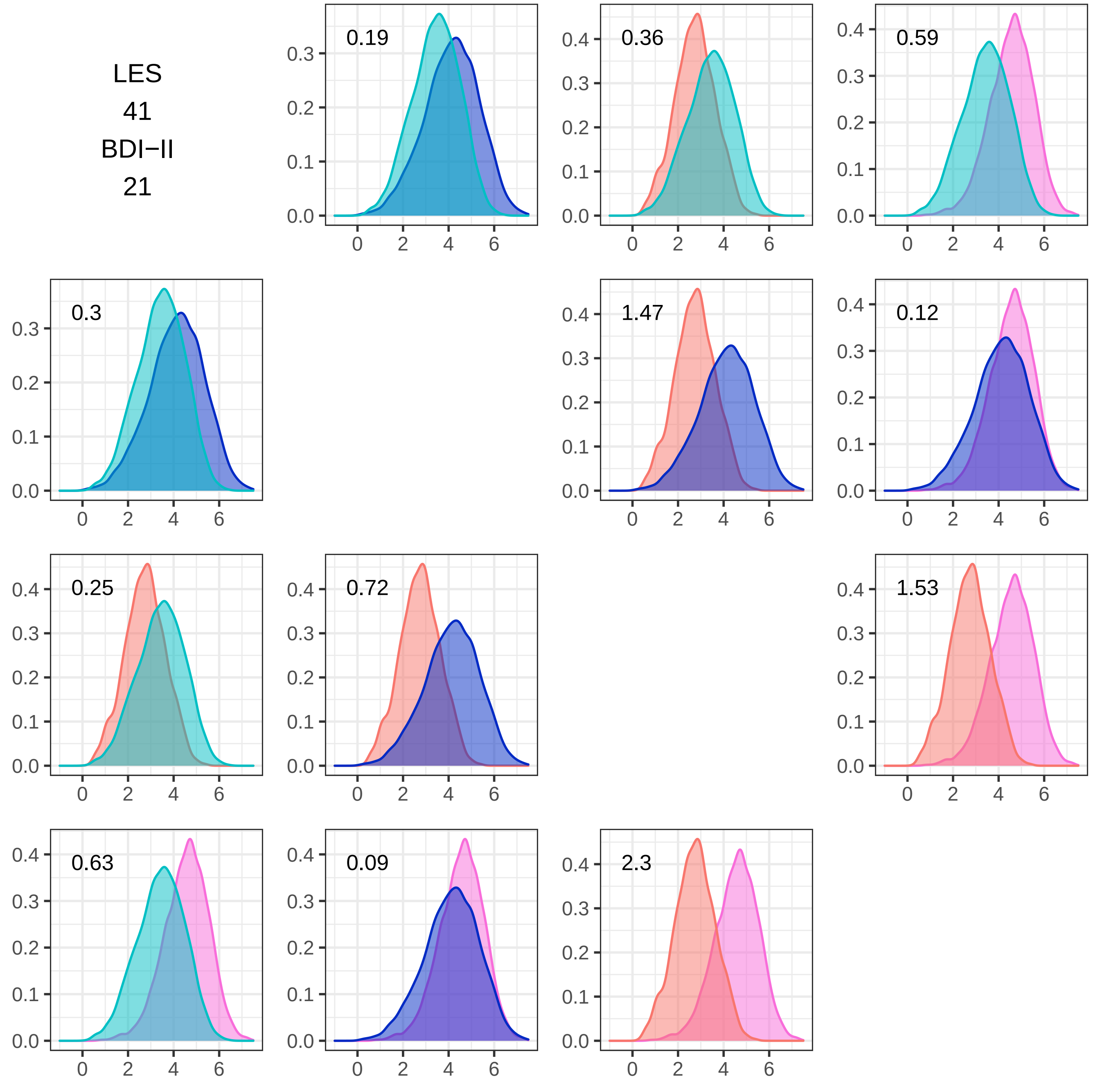
LES
41
BDI−II
21
0.19
0.36
0.59
0.3
1.47
0.12
0.25
0.72
1.53
0.63
0.09
2.3

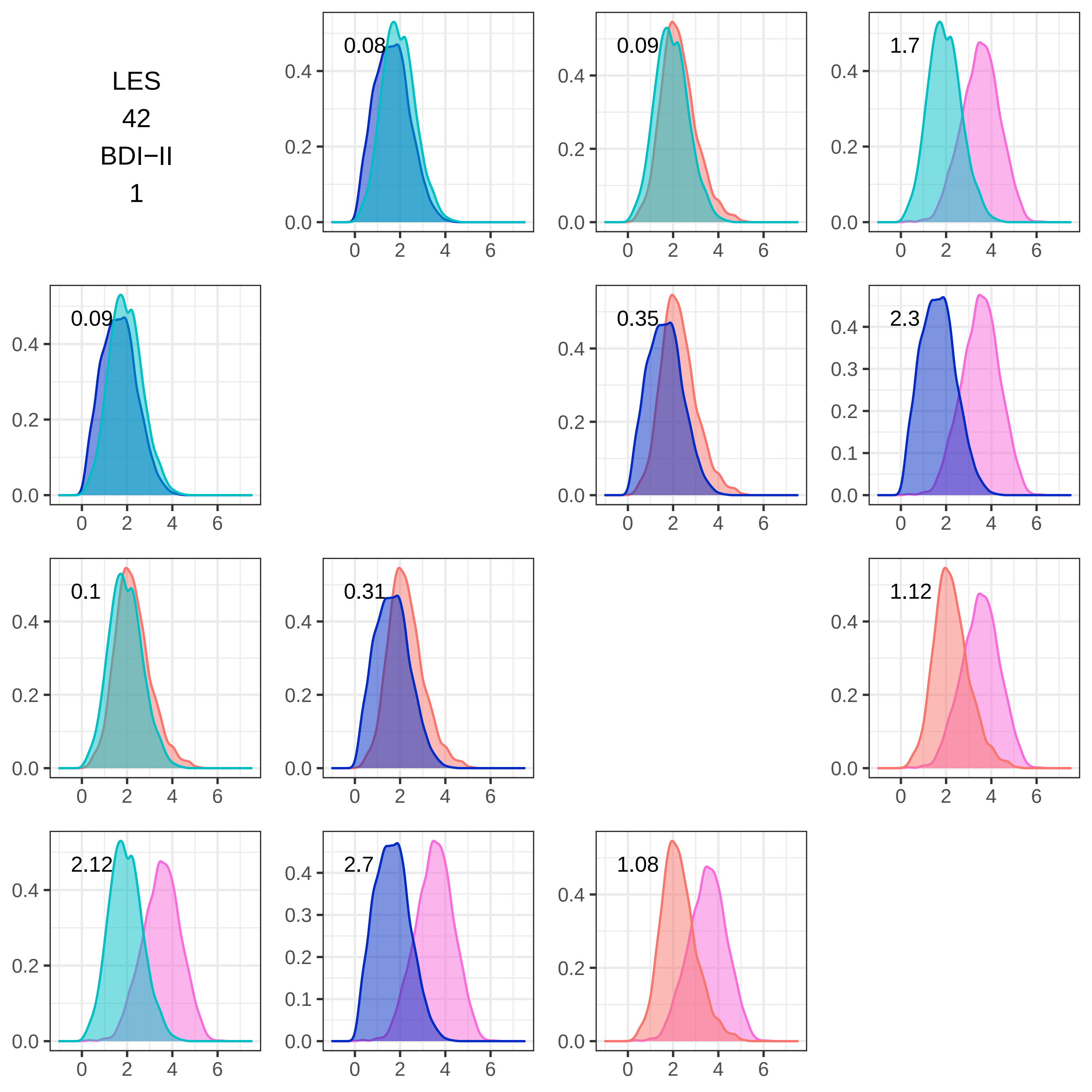
LES
42
BDI−II
1
0.08
0.09
1.7
0.09
0.35
2.3
0.1
0.31
1.12
2.12
2.7
1.08

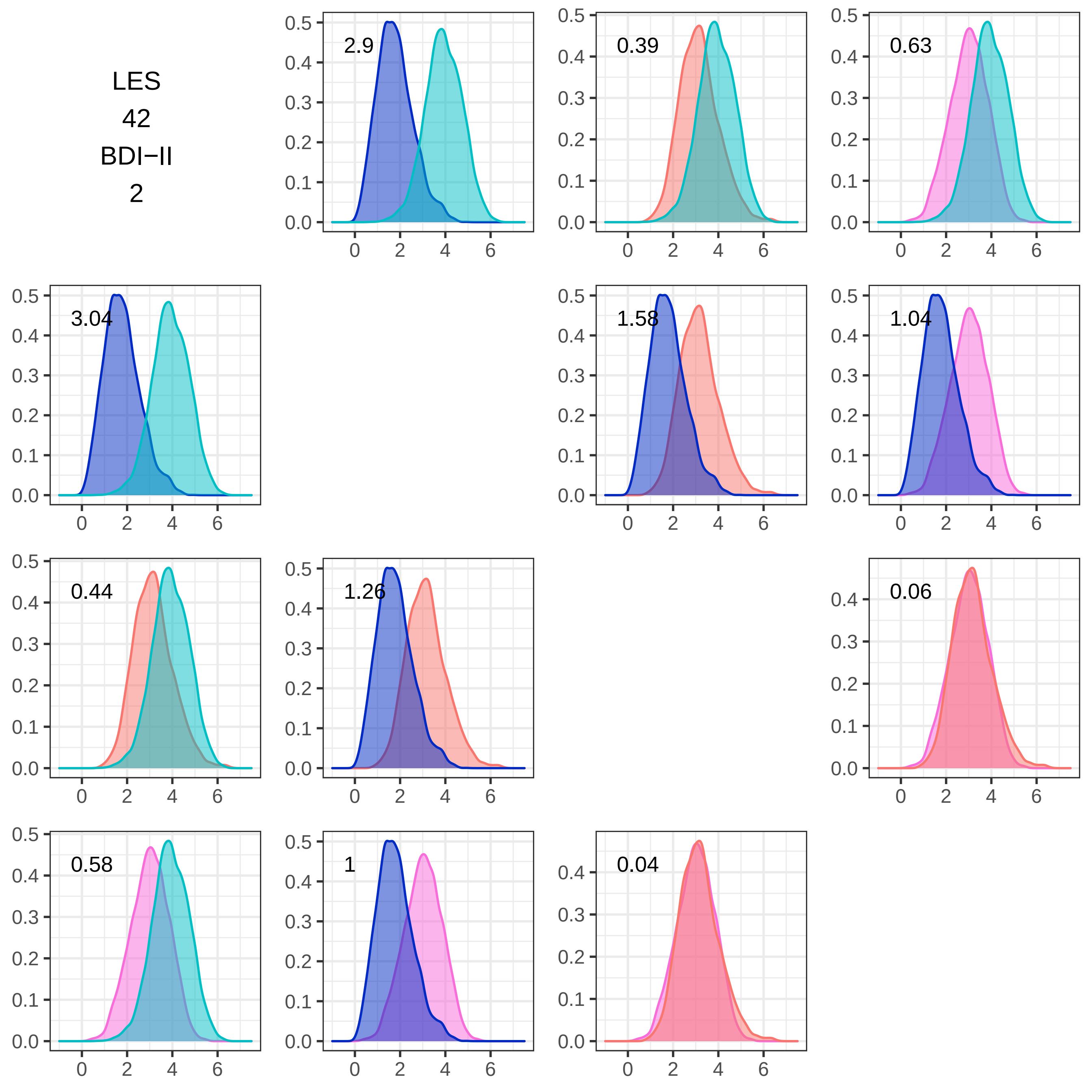

LES
42
BDI−II
2
2.9
0.39
0.63
3.04
1.58
1.04
0.44
1.26
0.06
0.58
1
0.04

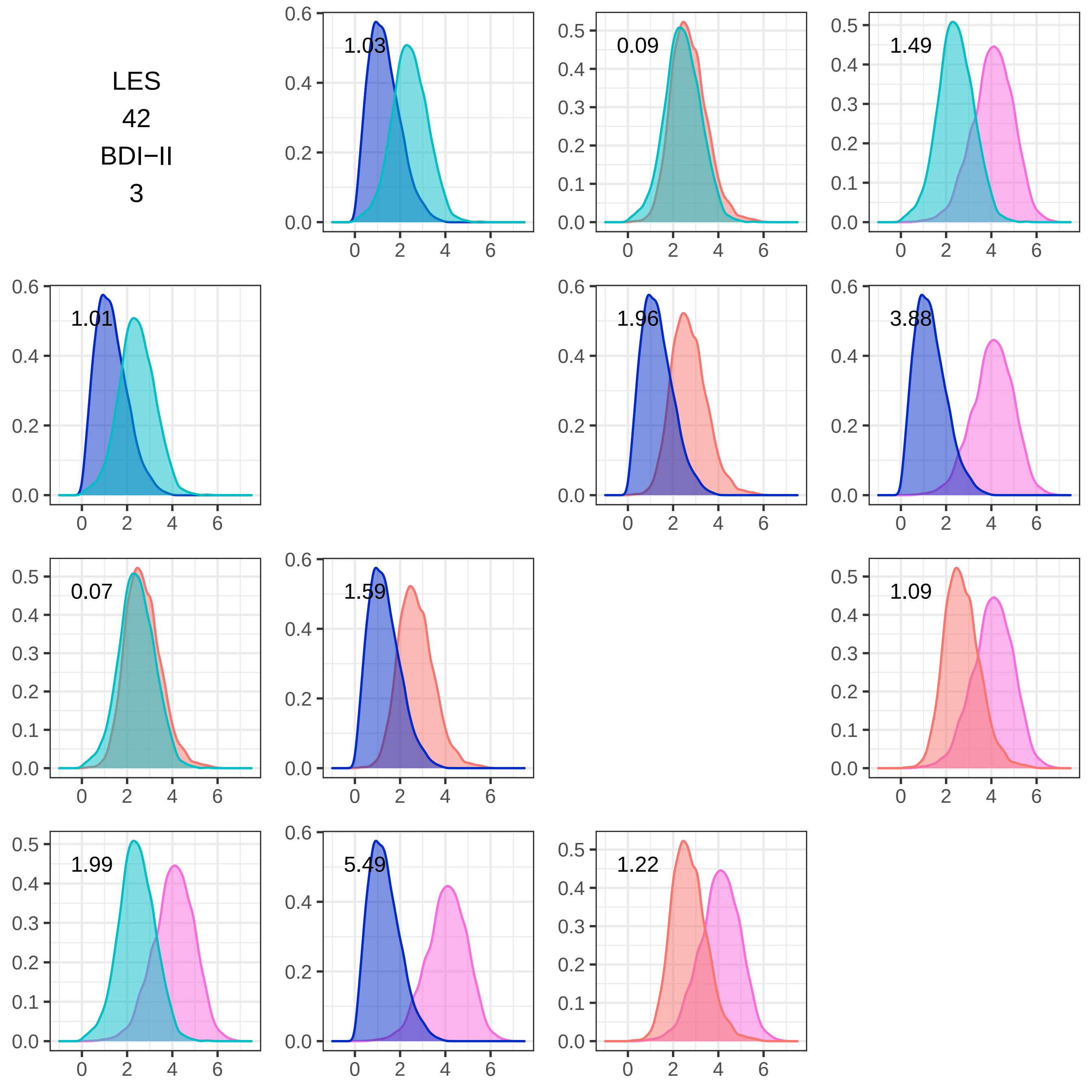
LES
42
BDI−II
3
1.03
0.09
1.49
1.01
1.96
3.88
0.07
1.59
1.09
1.99
5.49
1.22

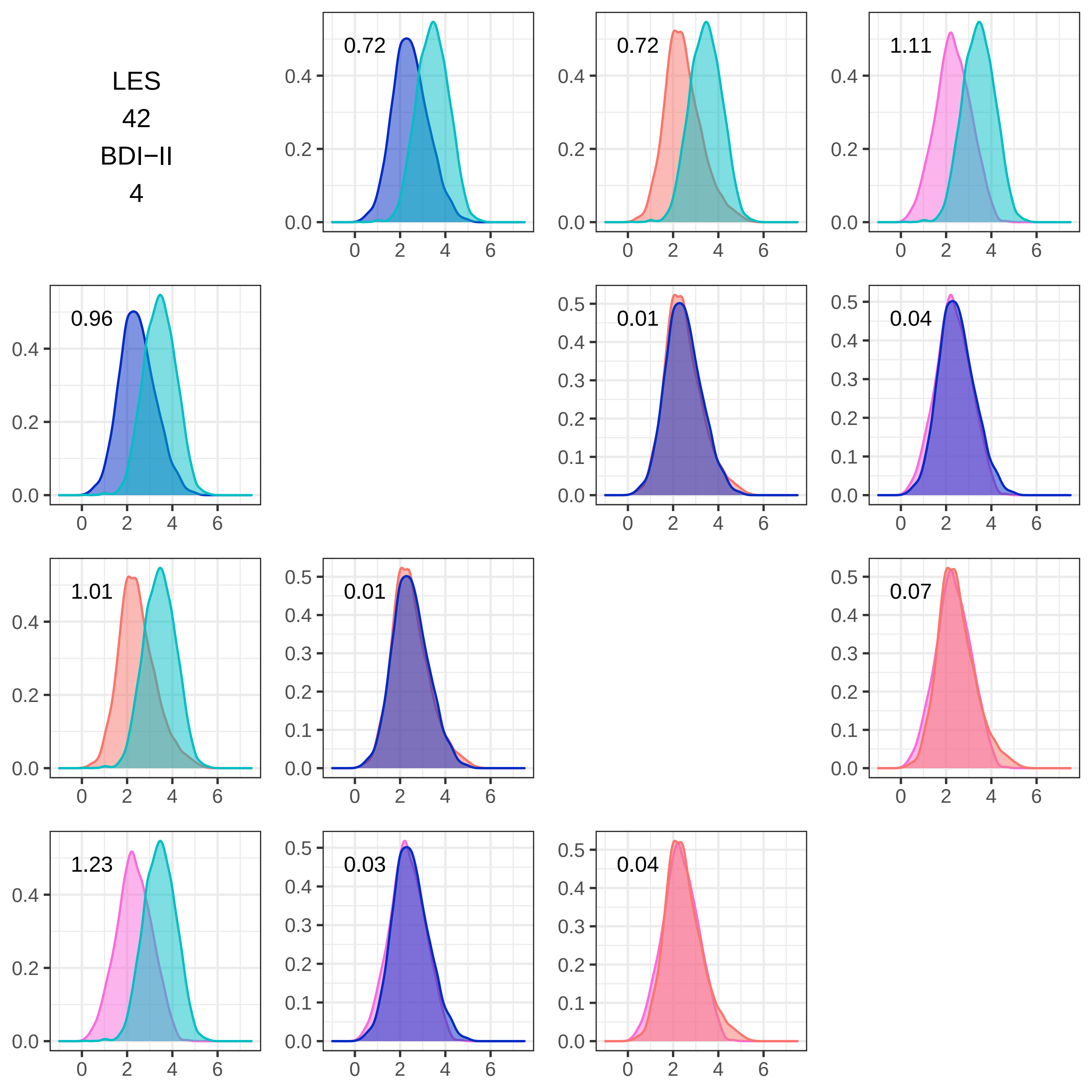
LES
42
BDI−II
4
0.72
0.72
1.11
0.96
0.01
0.04
1.01
0.01
0.07
1.23
0.03
0.04

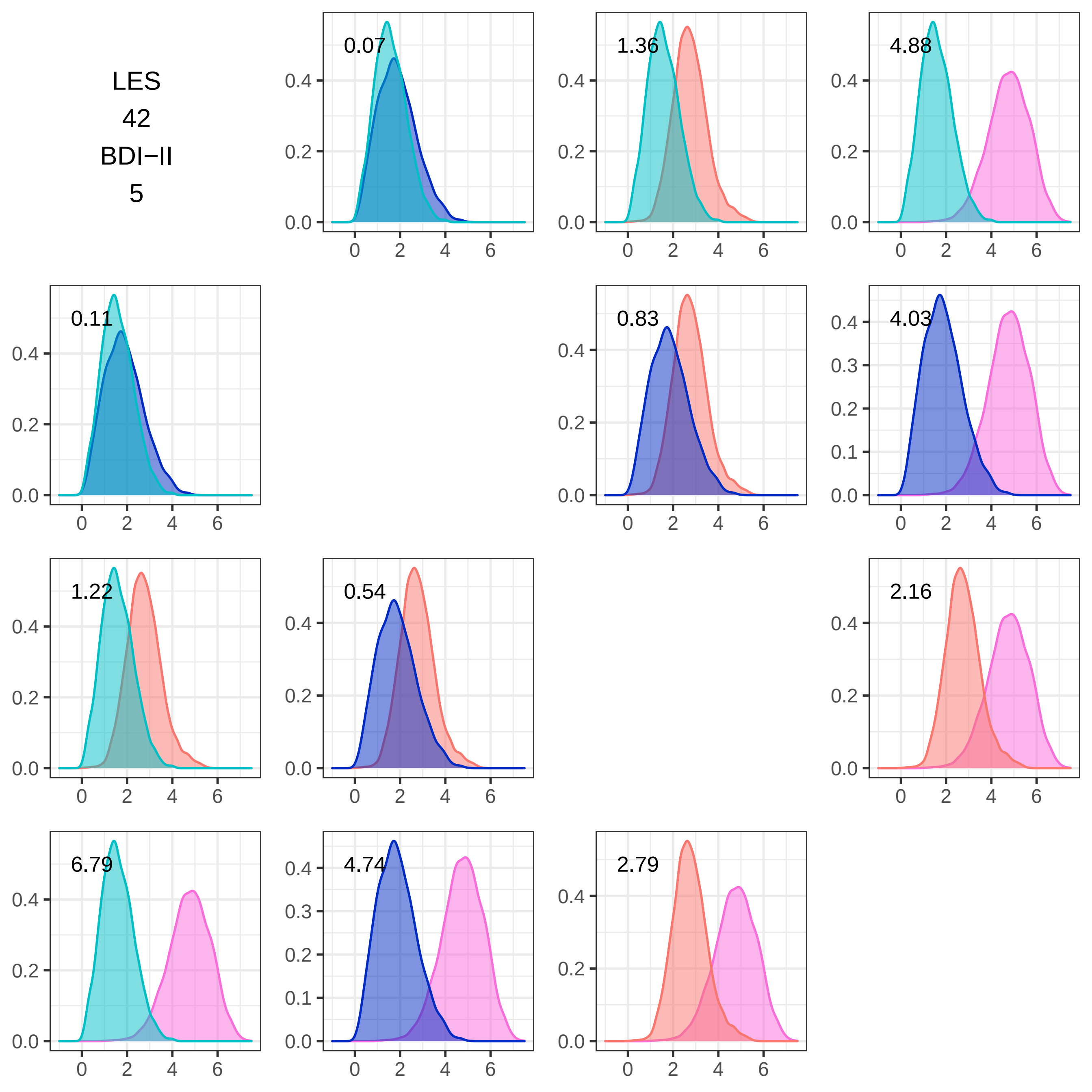

LES
42
BDI−II
5
0.07
1.36
4.88
0.11
0.83
4.03
1.22
0.54
2.16
6.79
4.74
2.79

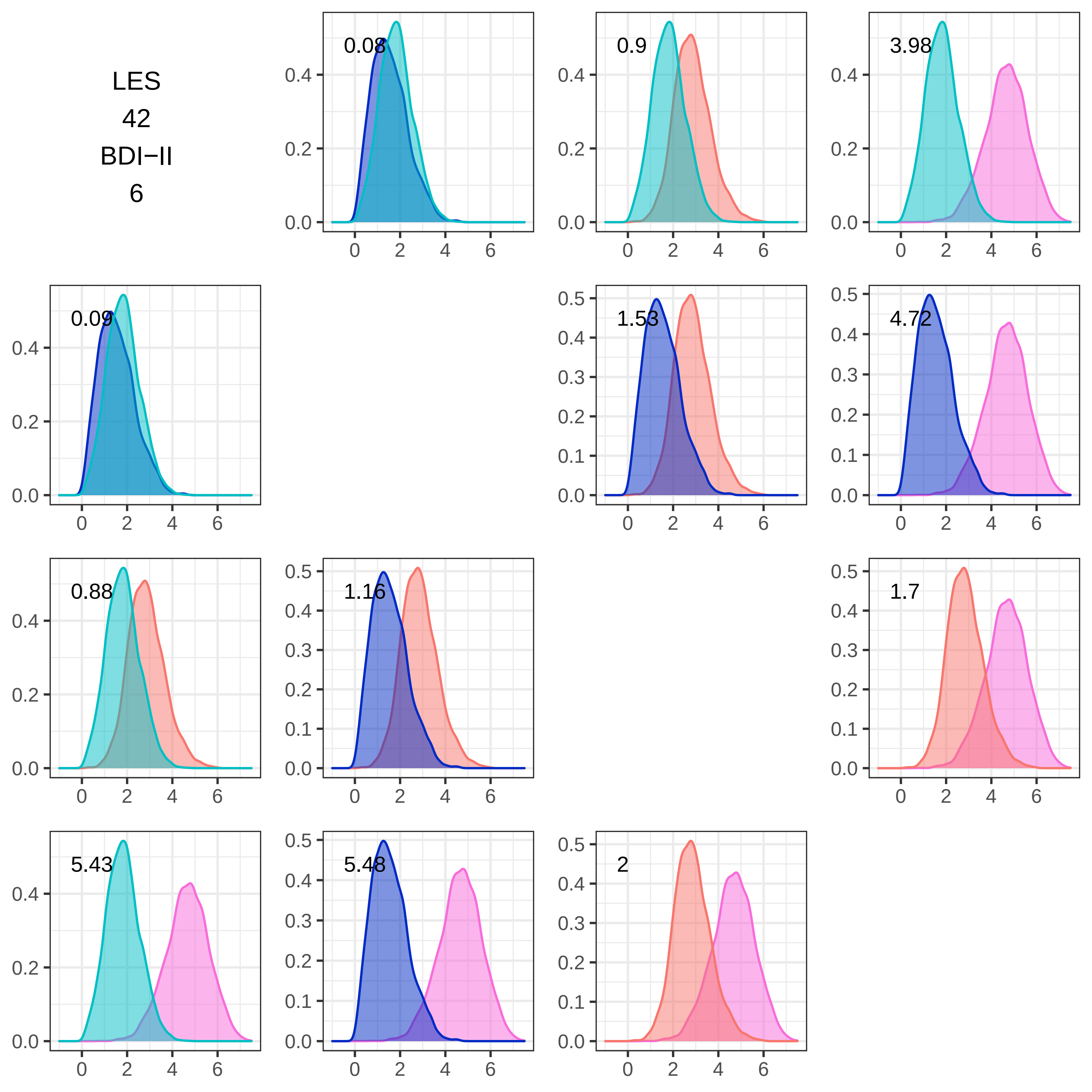
LES
42
BDI−II
6
0.08
0.9
3.98
0.09
1.53
4.72
0.88
1.16
1.7
5.43
5.48
2

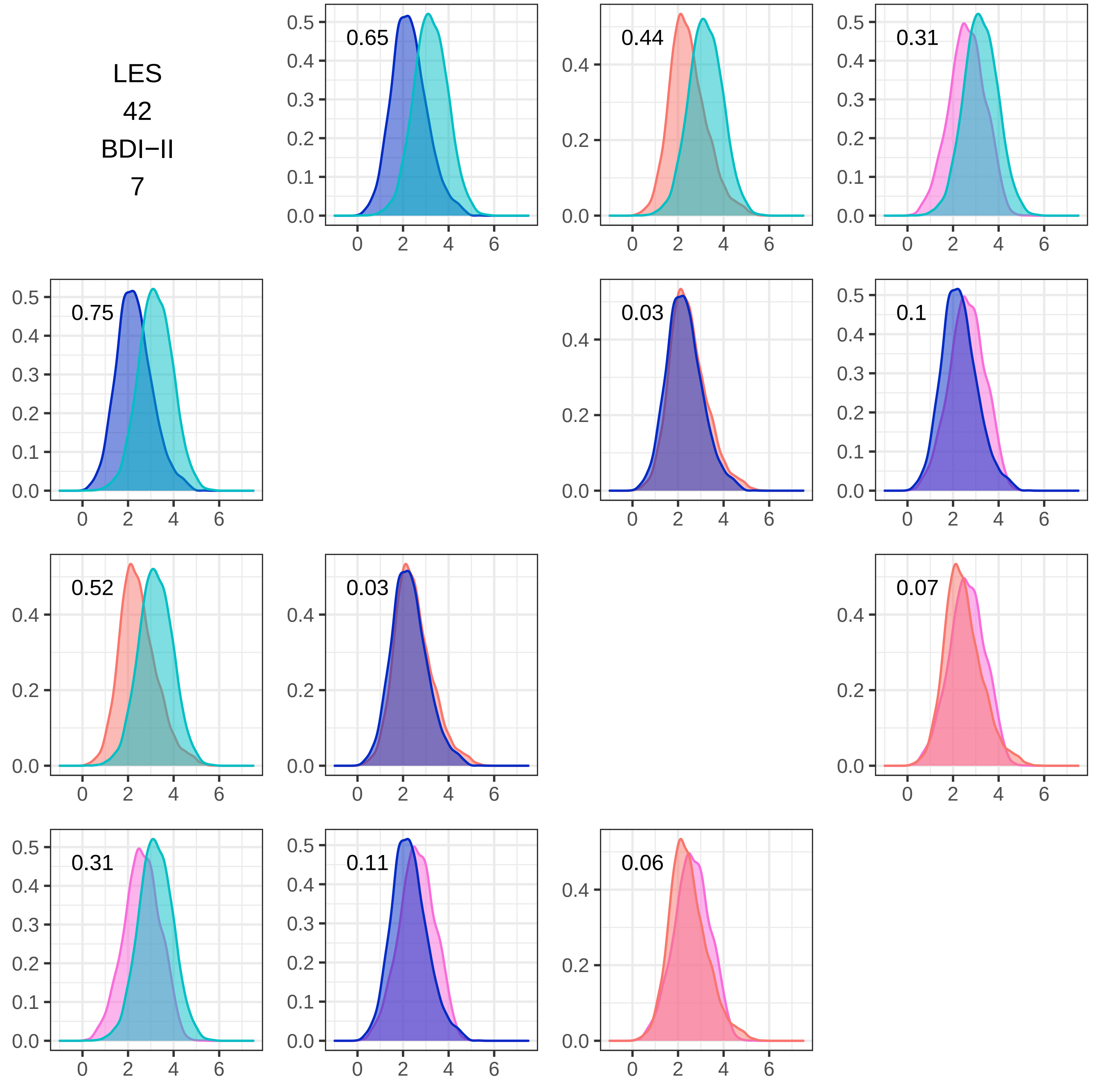
LES
42
BDI−II
7
0.65
0.44
0.31
0.75
0.03
0.1
0.52
0.03
0.07
0.31
0.11
0.06

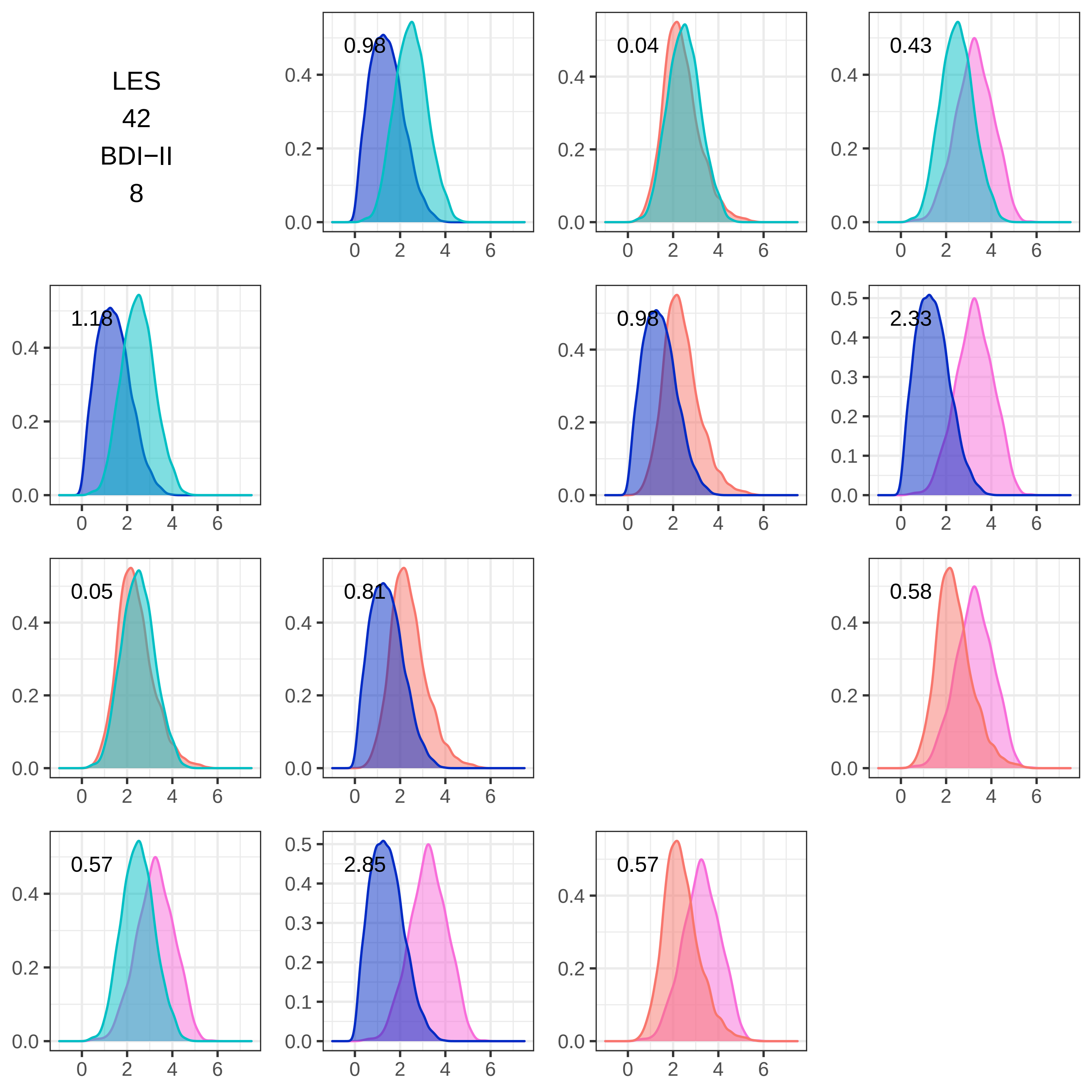
LES
42
BDI−II
8
0.98
0.04
0.43
1.18
0.98
2.33
0.05
0.81
0.58
0.57
2.85
0.57

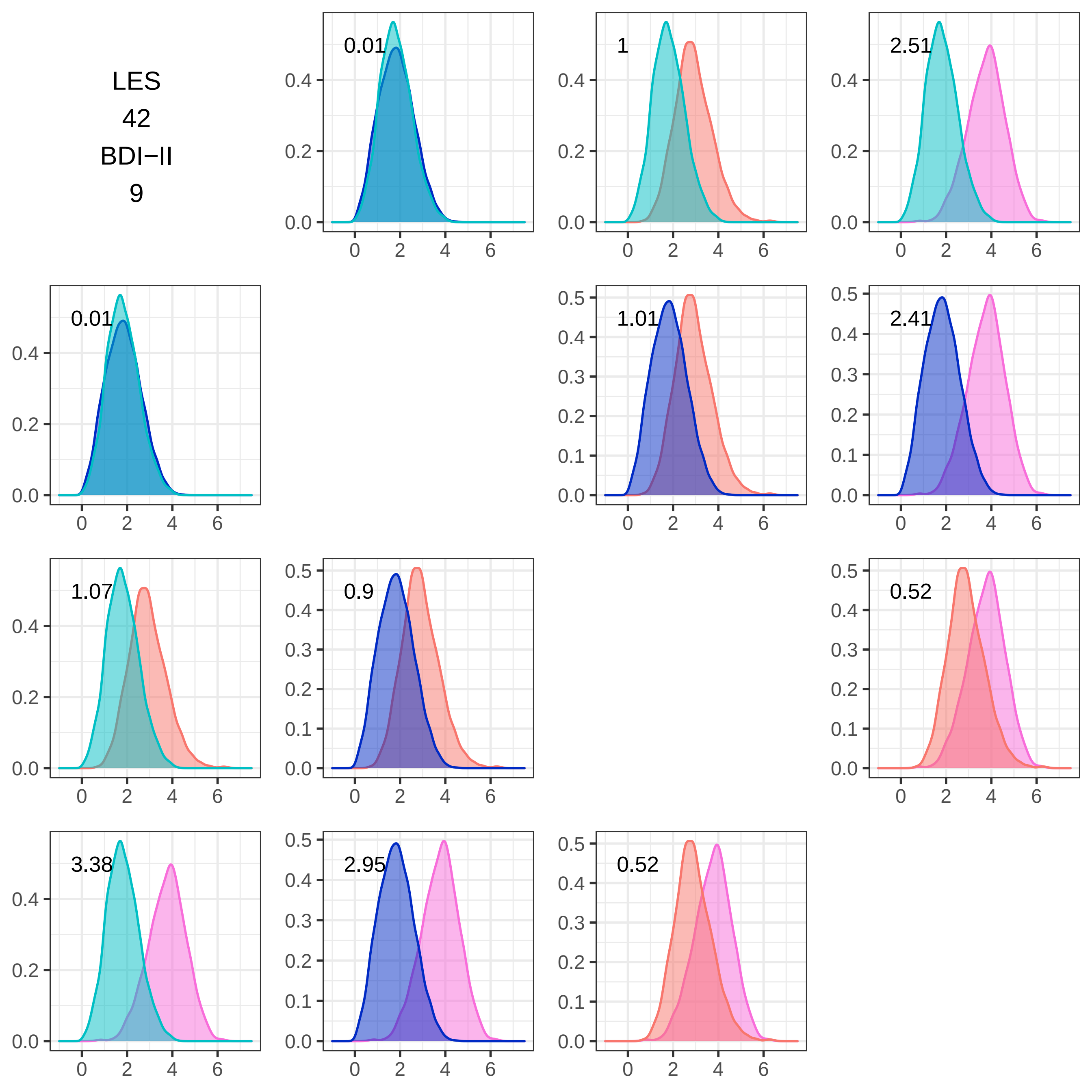

LES
42
BDI−II
9
0.01
1
2.51
0.01
1.01
2.41
1.07
0.9
0.52
3.38
2.95
0.52

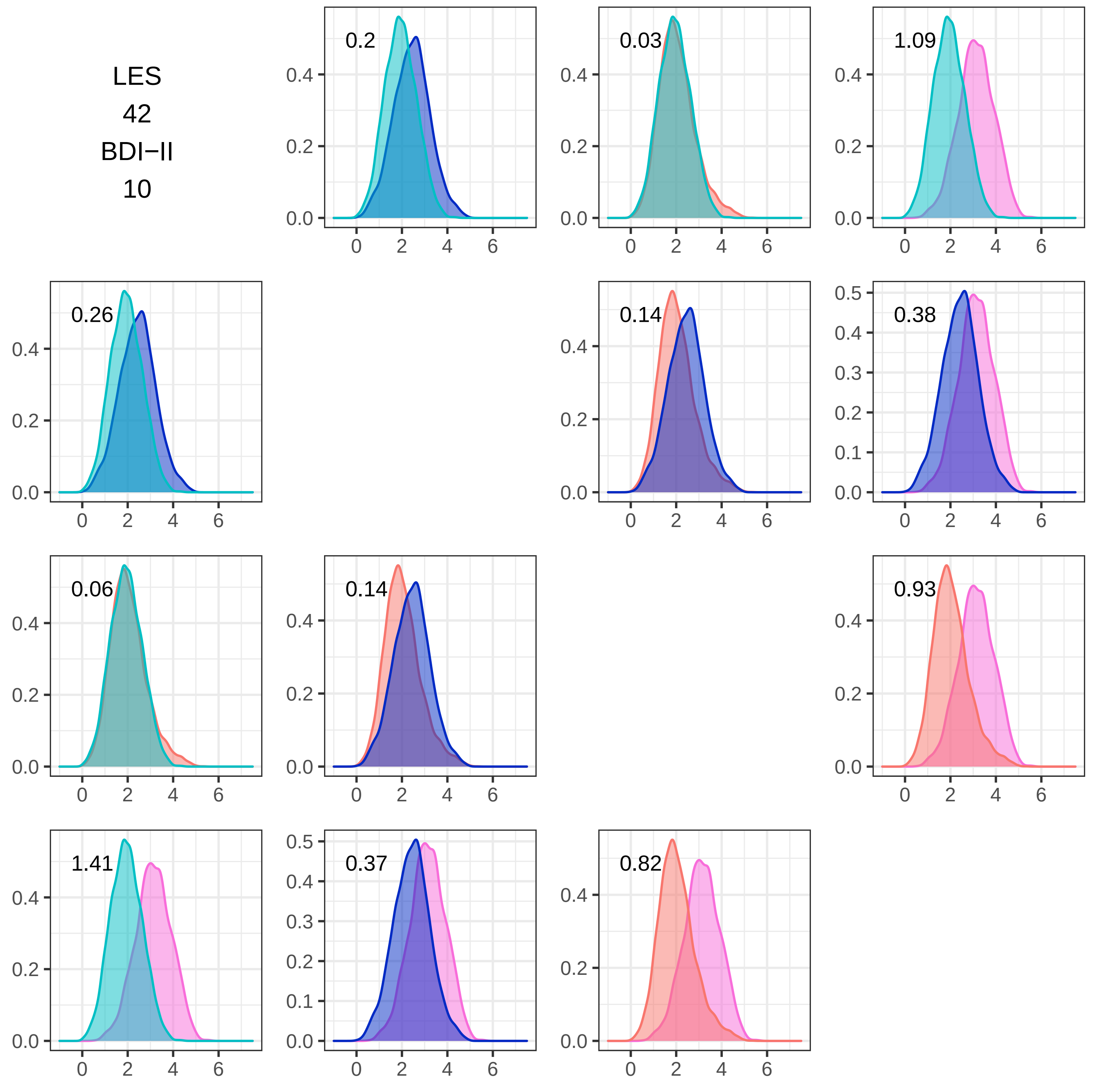
LES
42
BDI−II
10
0.2
0.03
1.09
0.26
0.14
0.38
0.06
0.14
0.93
1.41
0.37
0.82

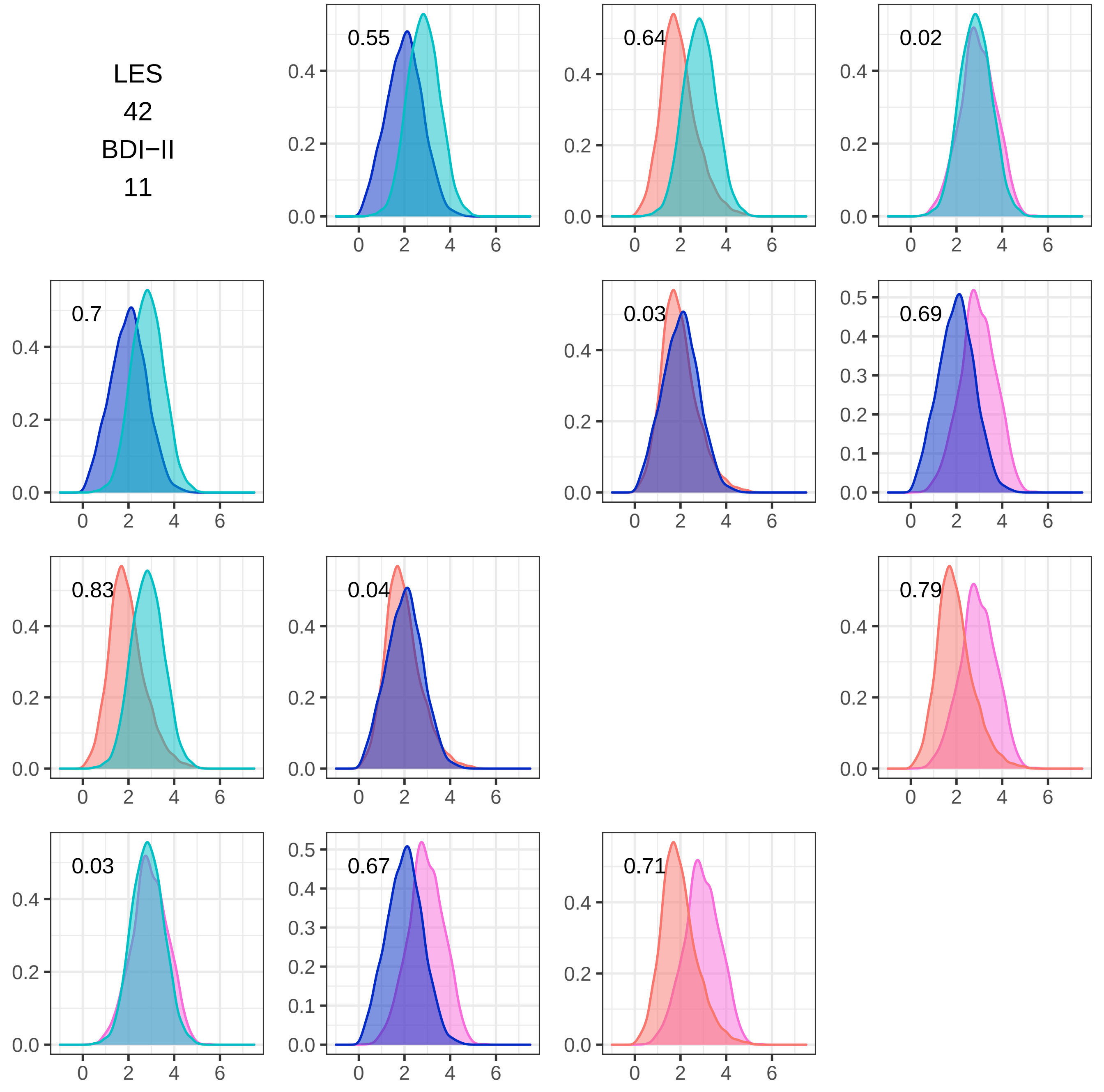
LES
42
BDI−II
11
0.55
0.64
0.02
0.7
0.03
0.69
0.83
0.04
0.79
0.03
0.67
0.71
0.0
0.1
0.2
0.3
0.4
0.5
0
2
4
6

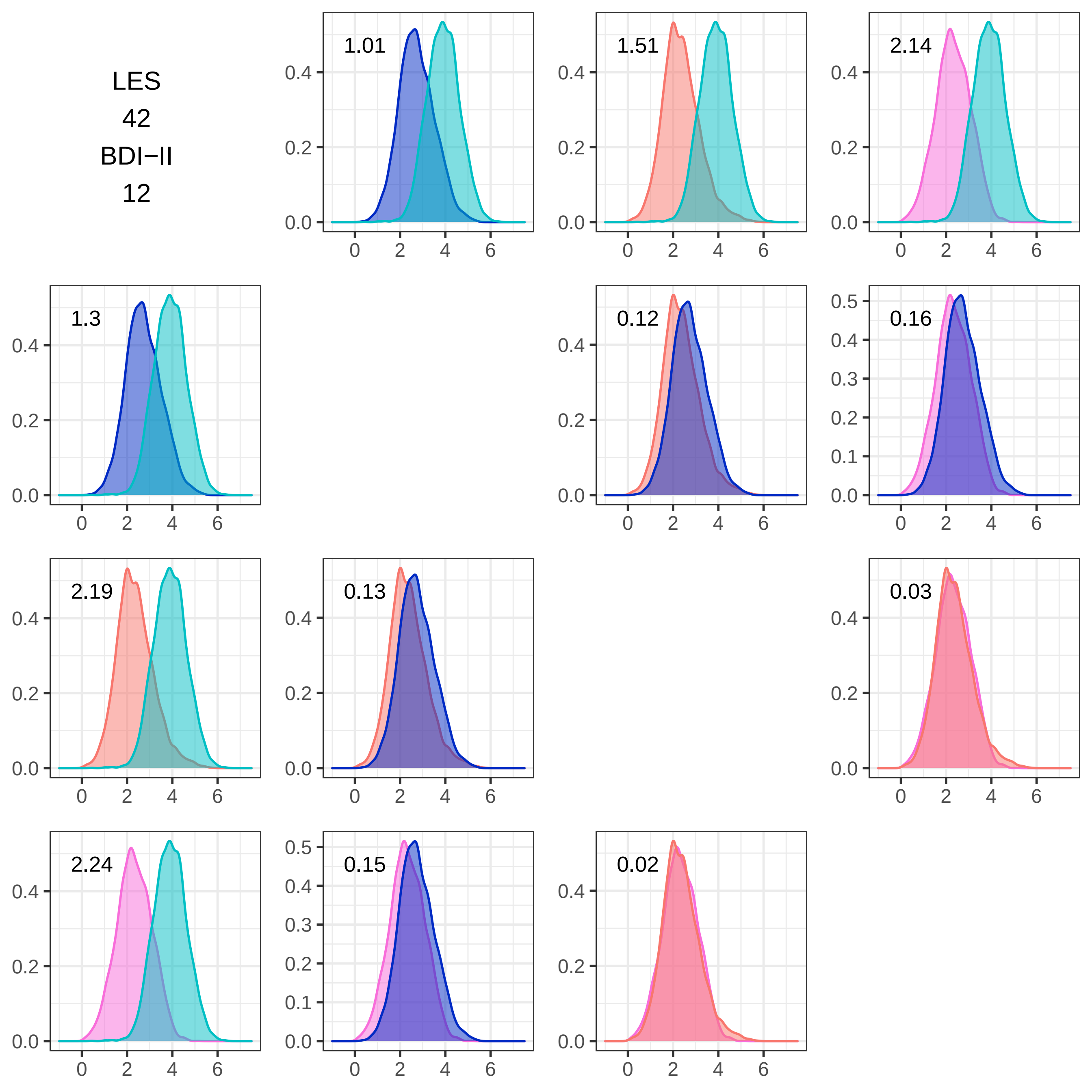

LES
42
BDI−II
12
1.01
1.51
2.14
1.3
0.12
0.16
2.19
0.13
0.03
2.24
0.15
0.02

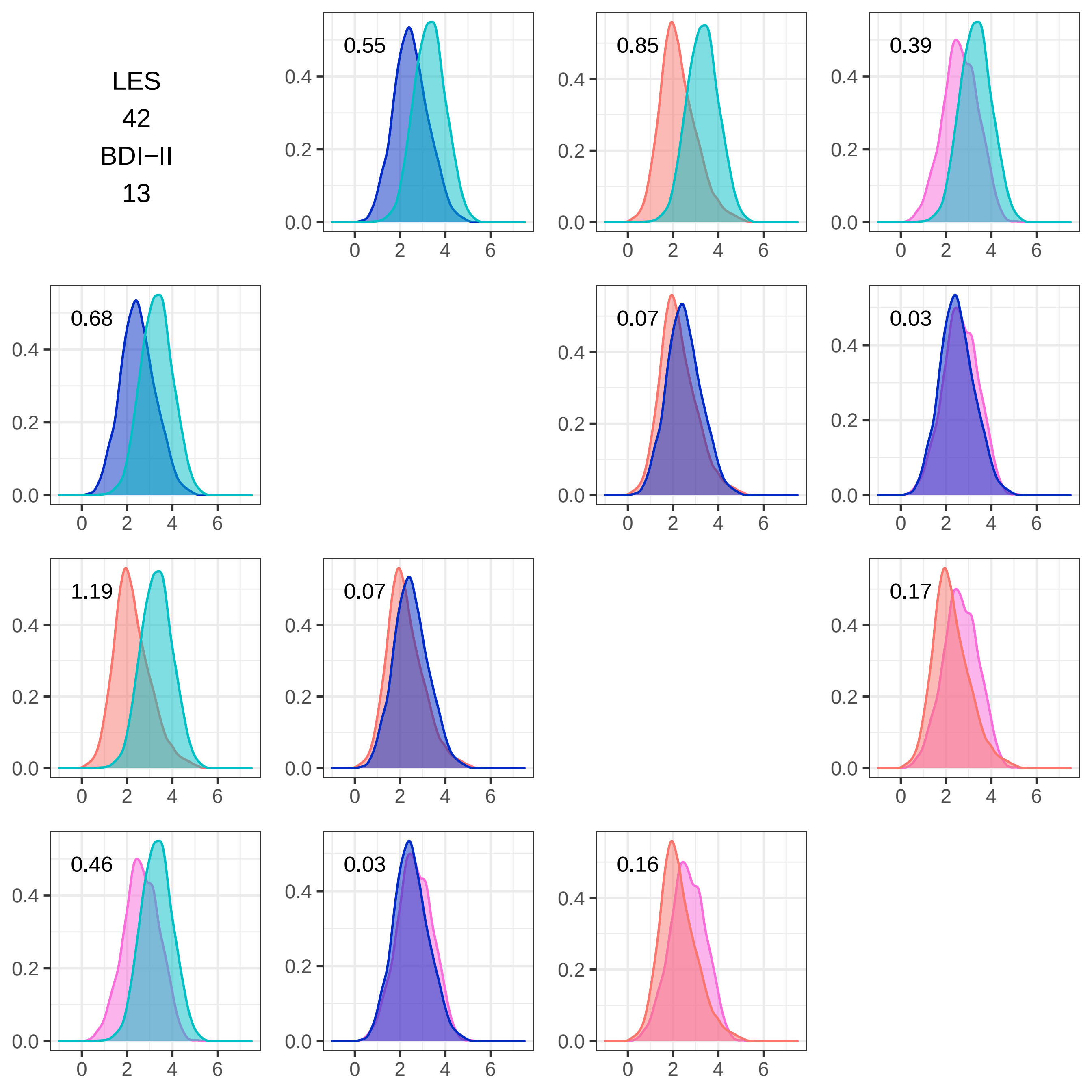
LES
42
BDI−II
13
0.55
0.85
0.39
0.68
0.07
0.03
1.19
0.07
0.17
0.46
0.03
0.16

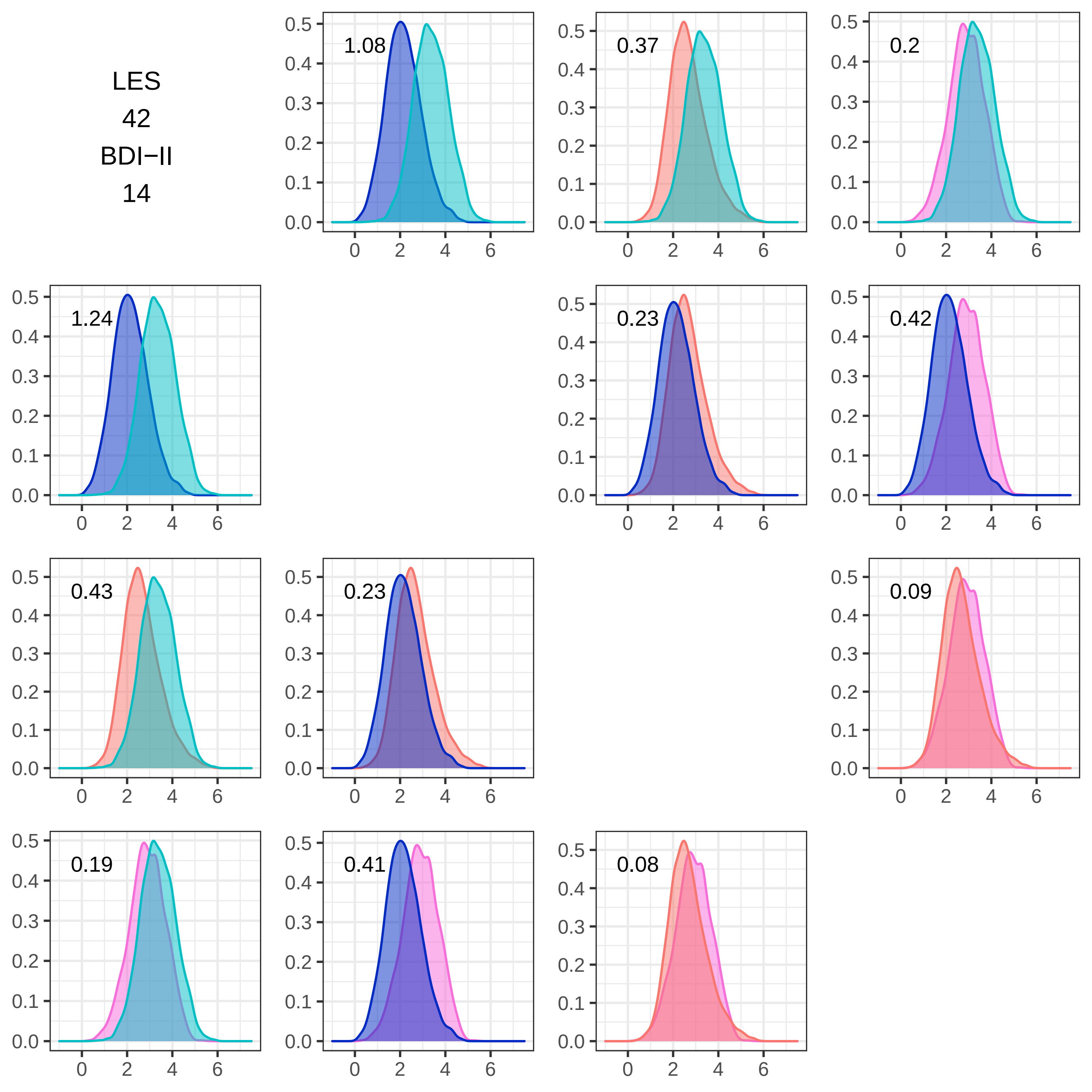

LES
42
BDI−II
14
1.08
0.37
0.2
1.24
0.23
0.42
0.43
0.23
0.09
0.19
0.41
0.08

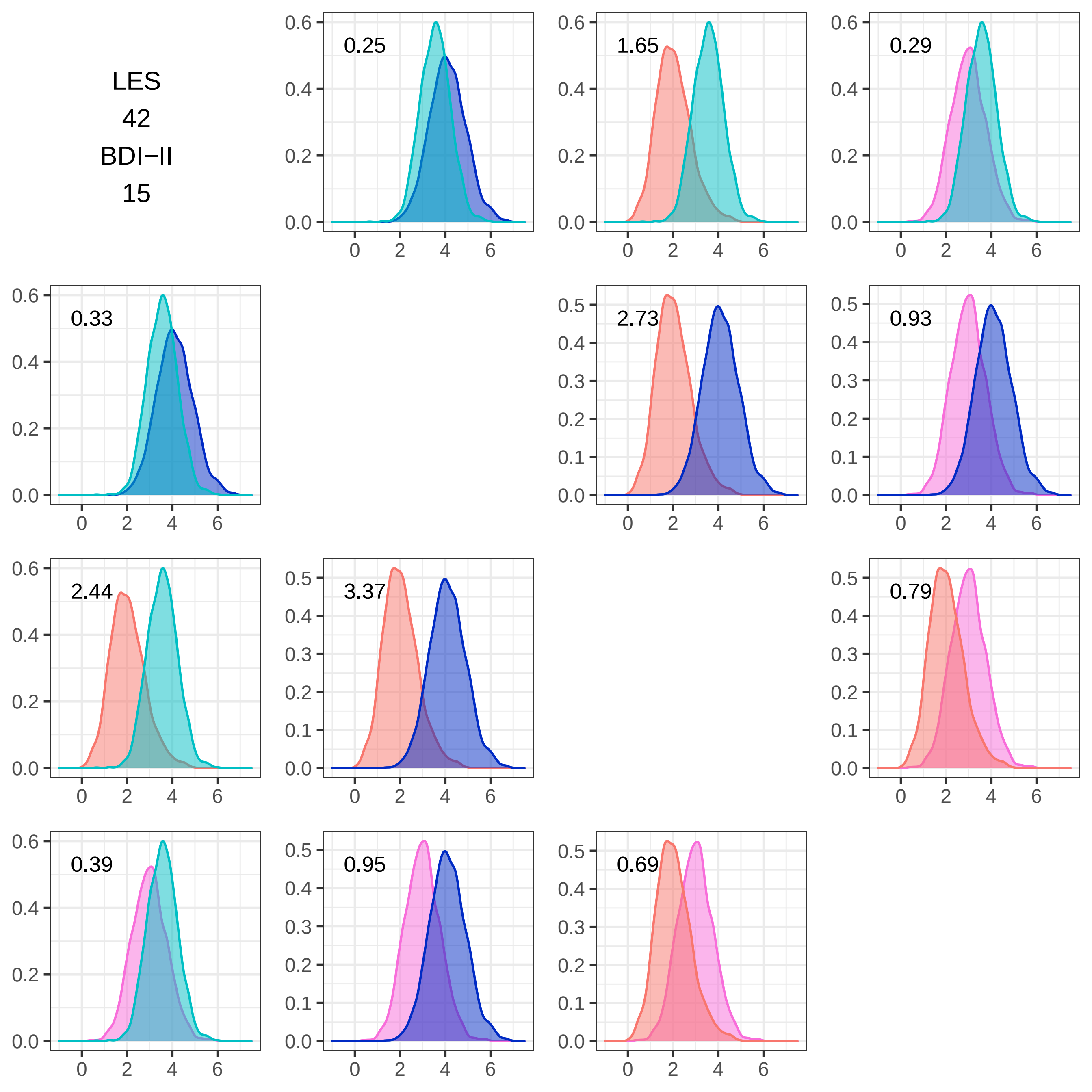
LES
42
BDI−II
15
0.25
1.65
0.29
0.33
2.73
0.93
2.44
3.37
0.79
0.39
0.95
0.69

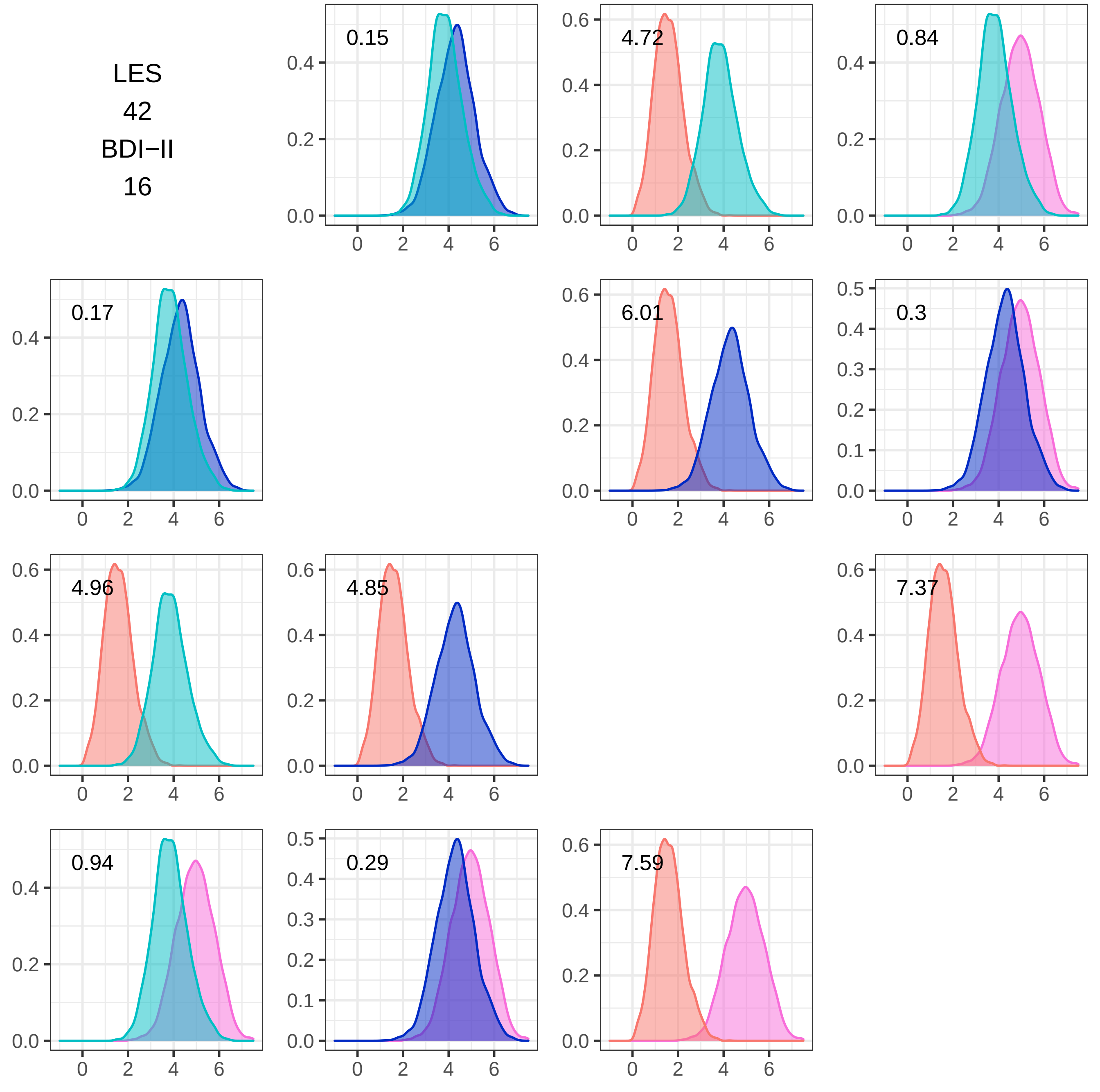
LES
42
BDI−II
16
0.15
4.72
0.84
0.17
6.01
0.3
4.96
4.85
7.37
0.94
0.29
7.59

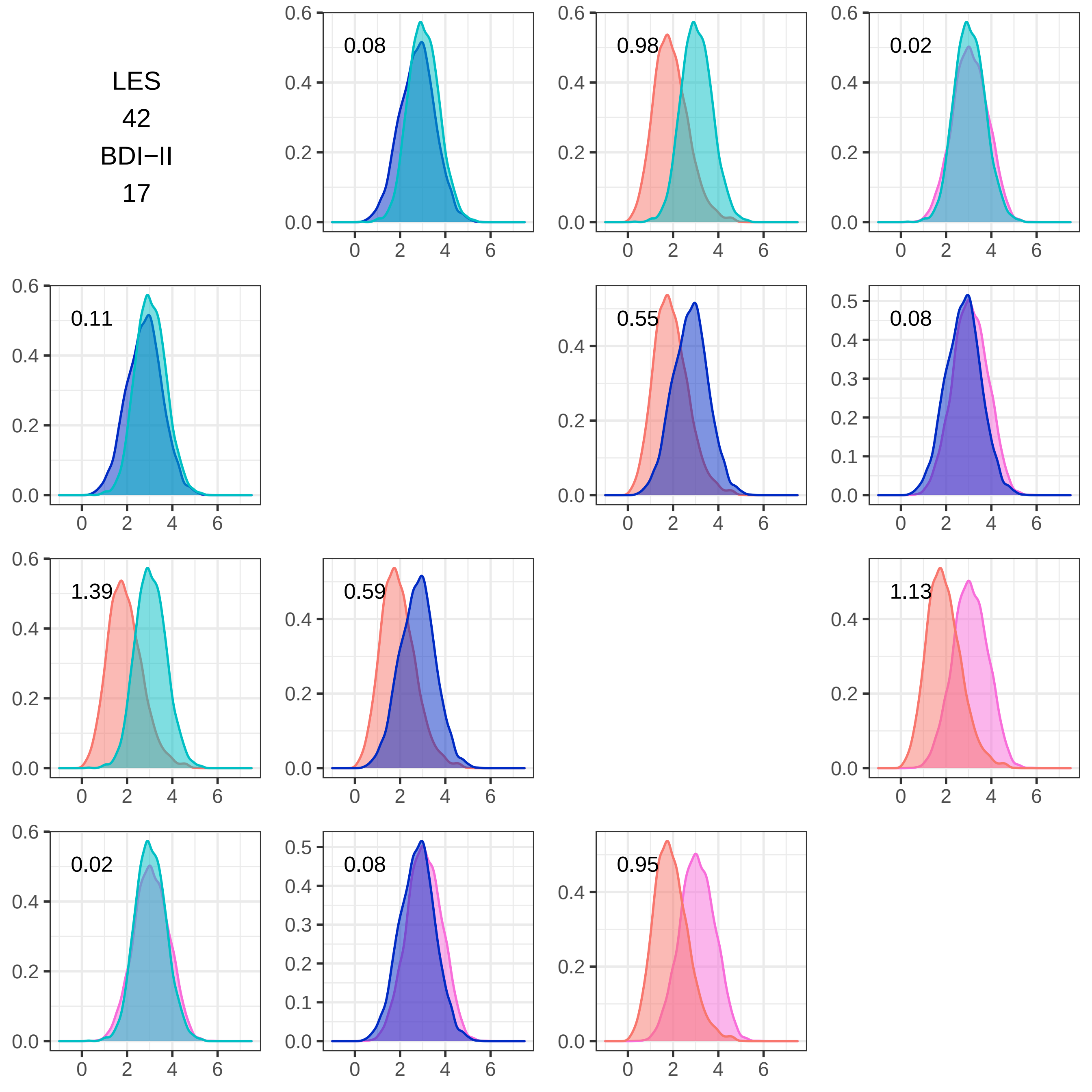

LES
42
BDI−II
17
0.08
0.98
0.02
0.11
0.55
0.08
1.39
0.59
1.13
0.02
0.08
0.95

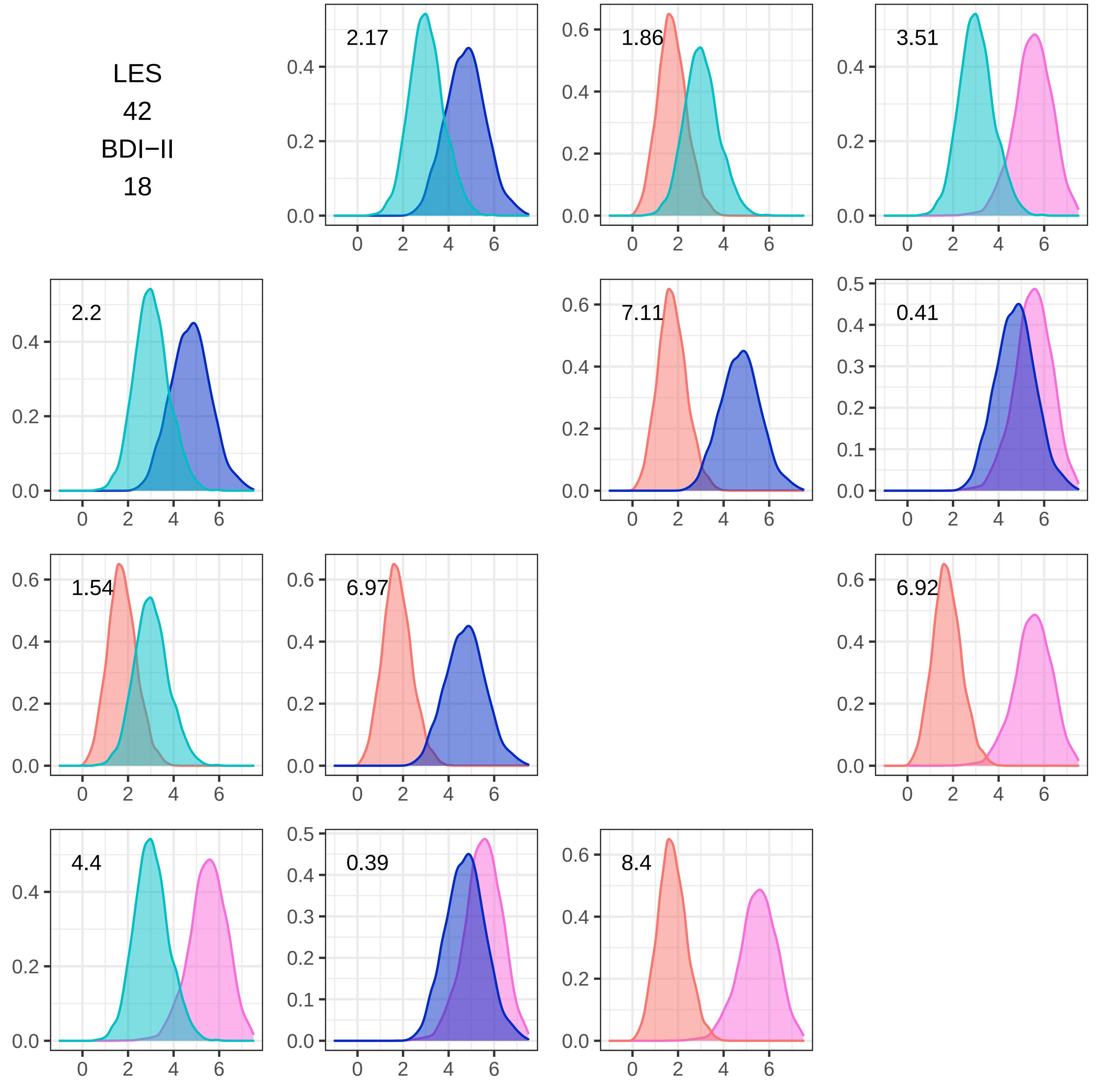

LES
42
BDI−II
18
2.17
1.86
3.51
2.2
7.11
0.41
1.54
6.97
6.92
4.4
0.39
8.4

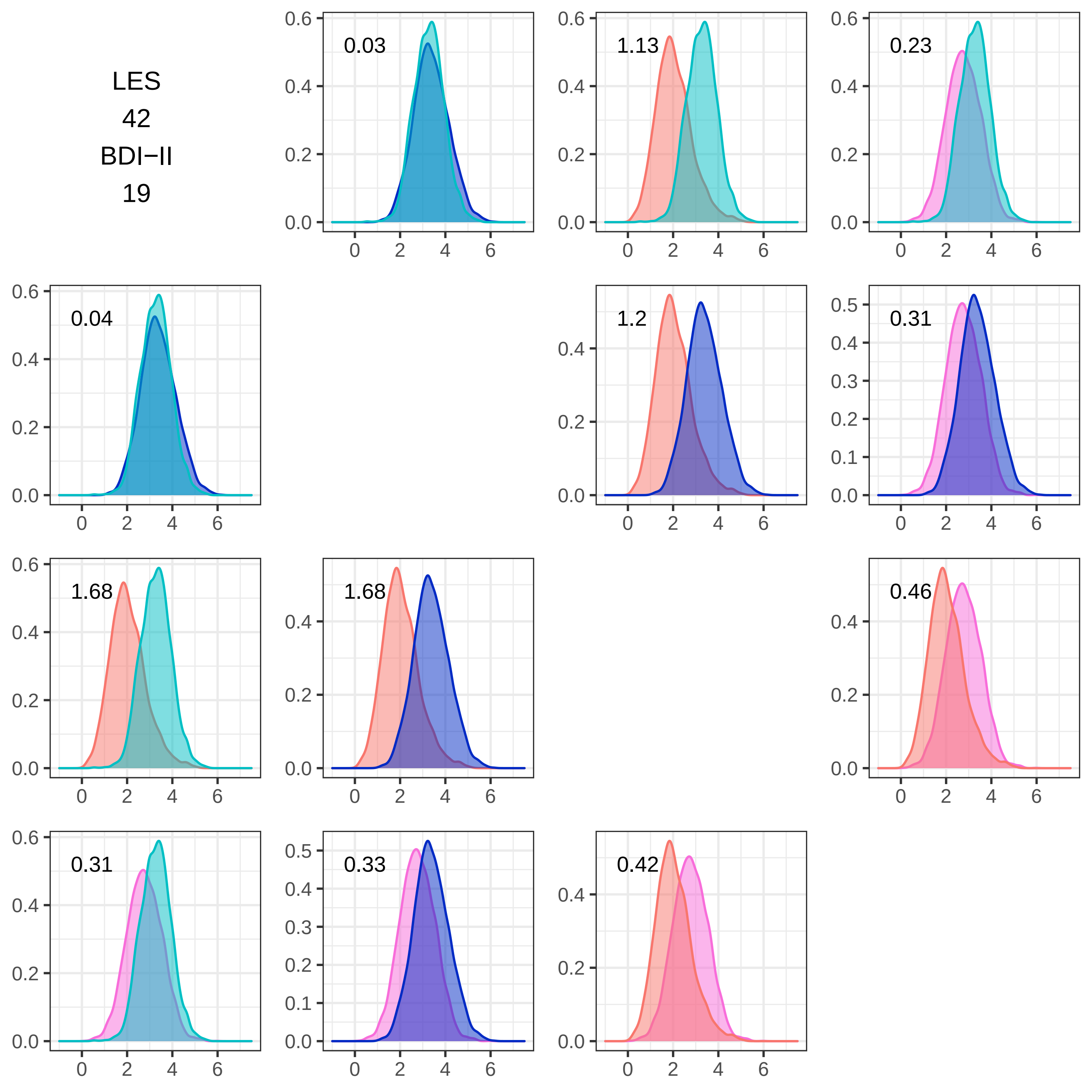

LES
42
BDI−II
19
0.03
1.13
0.23
0.04
1.2
0.31
1.68
1.68
0.46
0.31
0.33
0.42

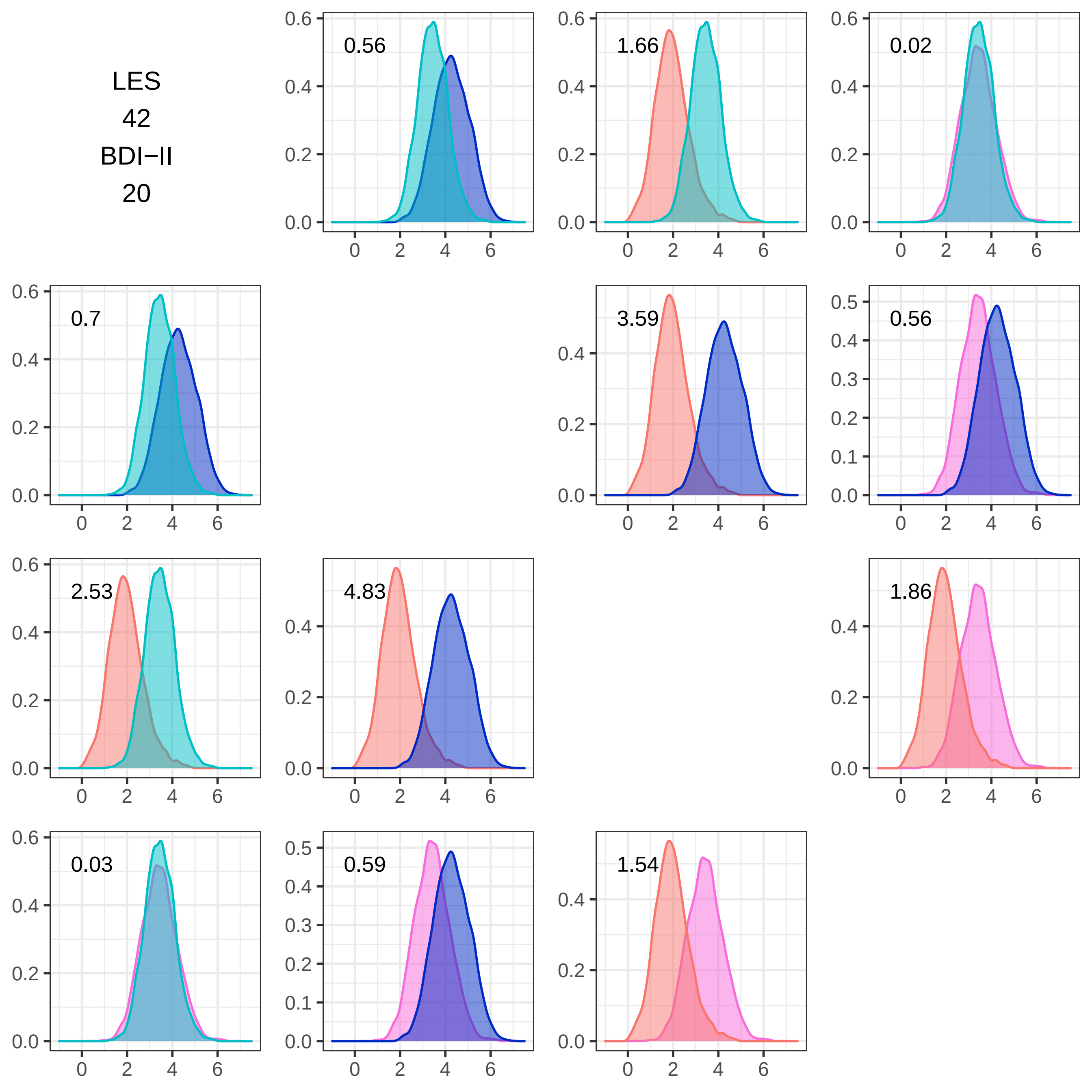

LES
42
BDI−II
20
0.56
1.66
0.02
0.7
3.59
0.56
2.53
4.83
1.86
0.03
0.59
1.54

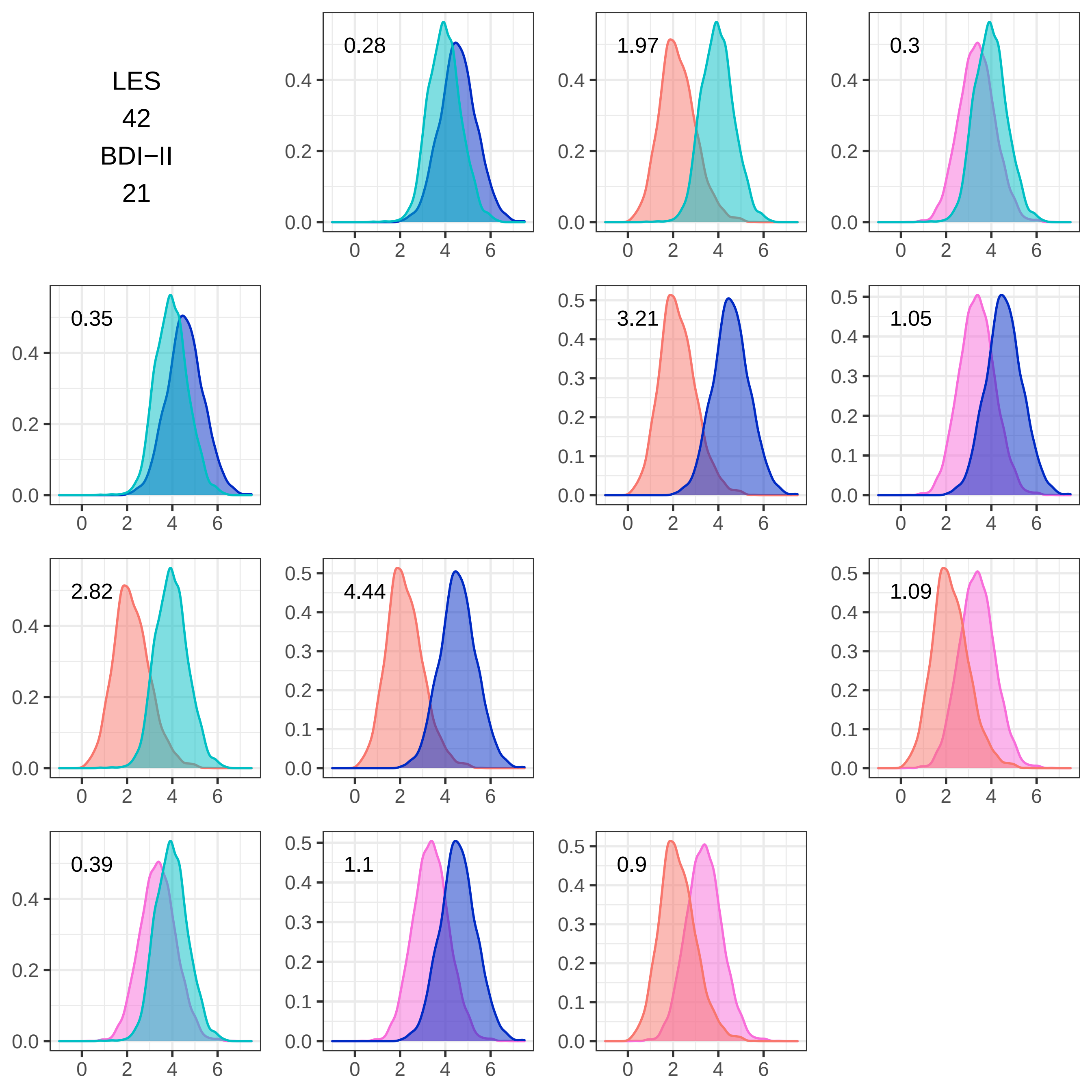

LES
42
BDI−II
21
0.28
1.97
0.3
0.35
3.21
1.05
2.82
4.44
1.09
0.39
1.1
0.9

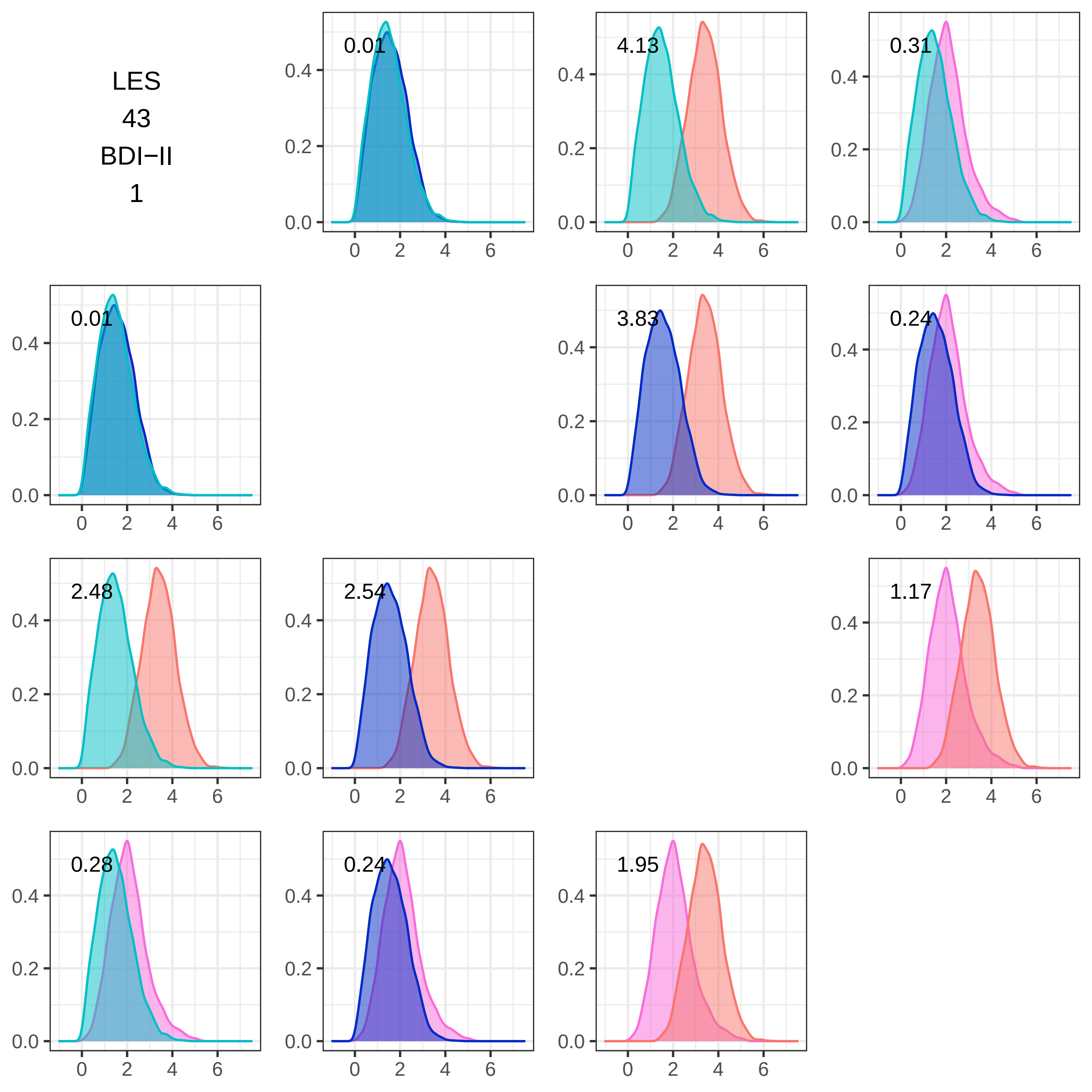

LES
43
BDI−II
1
0.01
4.13
0.31
0.01
3.83
0.24
2.48
2.54
1.17
0.28
0.24
1.95

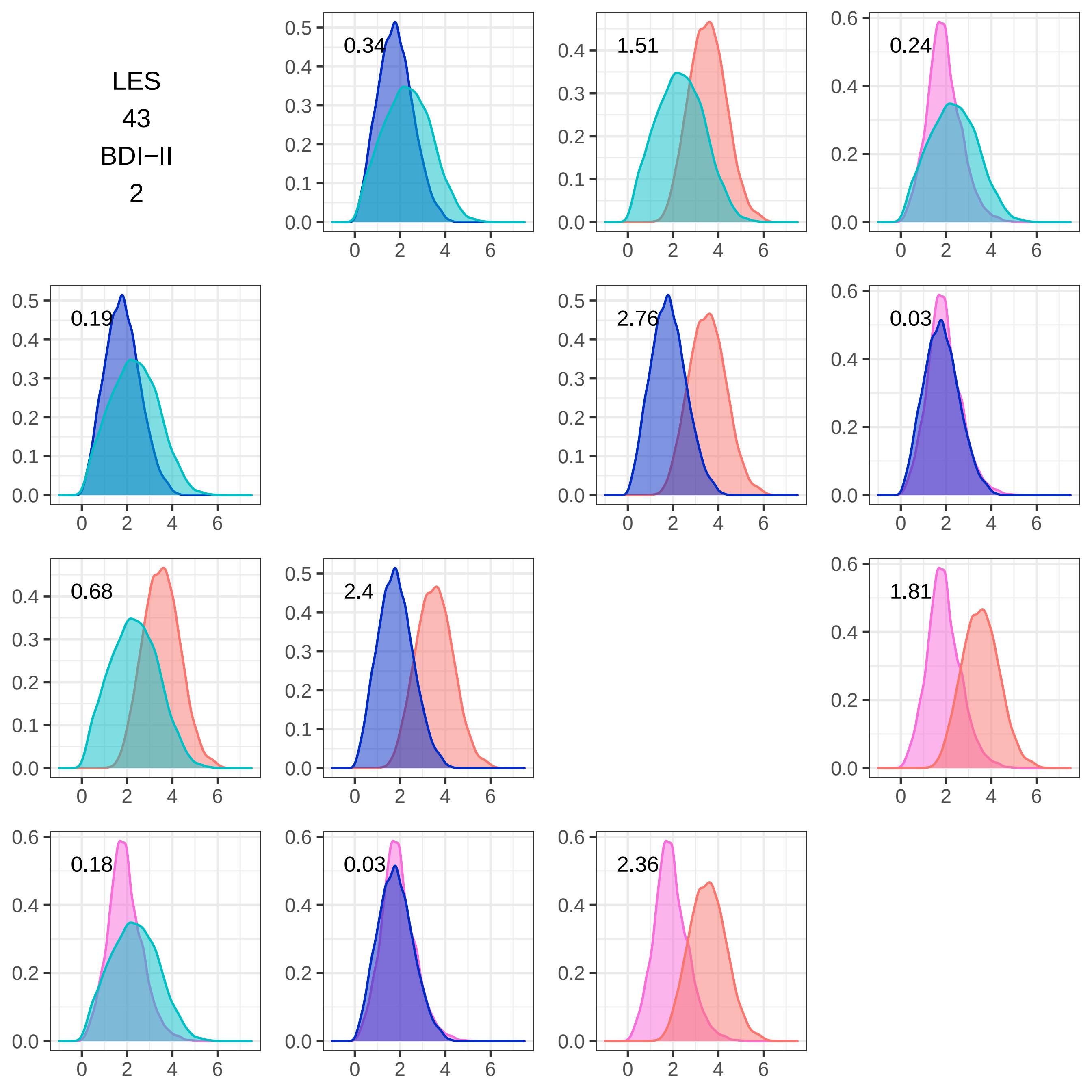
LES
43
BDI−II
2
0.34
1.51
0.24
0.19
2.76
0.03
0.68
2.4
1.81
0.18
0.03
2.36

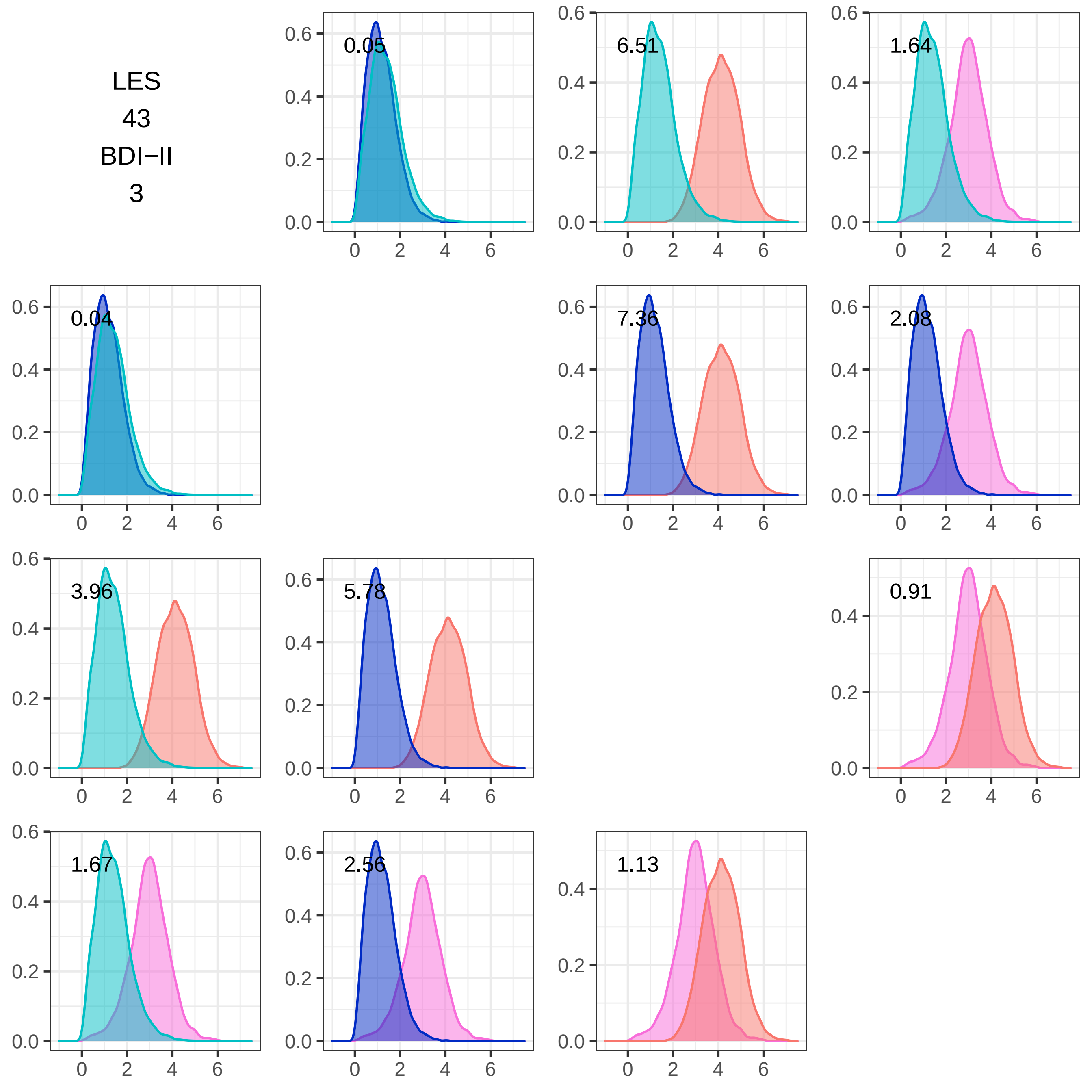

LES
43
BDI−II
3
0.05
6.51
1.64
0.04
7.36
2.08
3.96
5.78
0.91
1.67
2.56
1.13

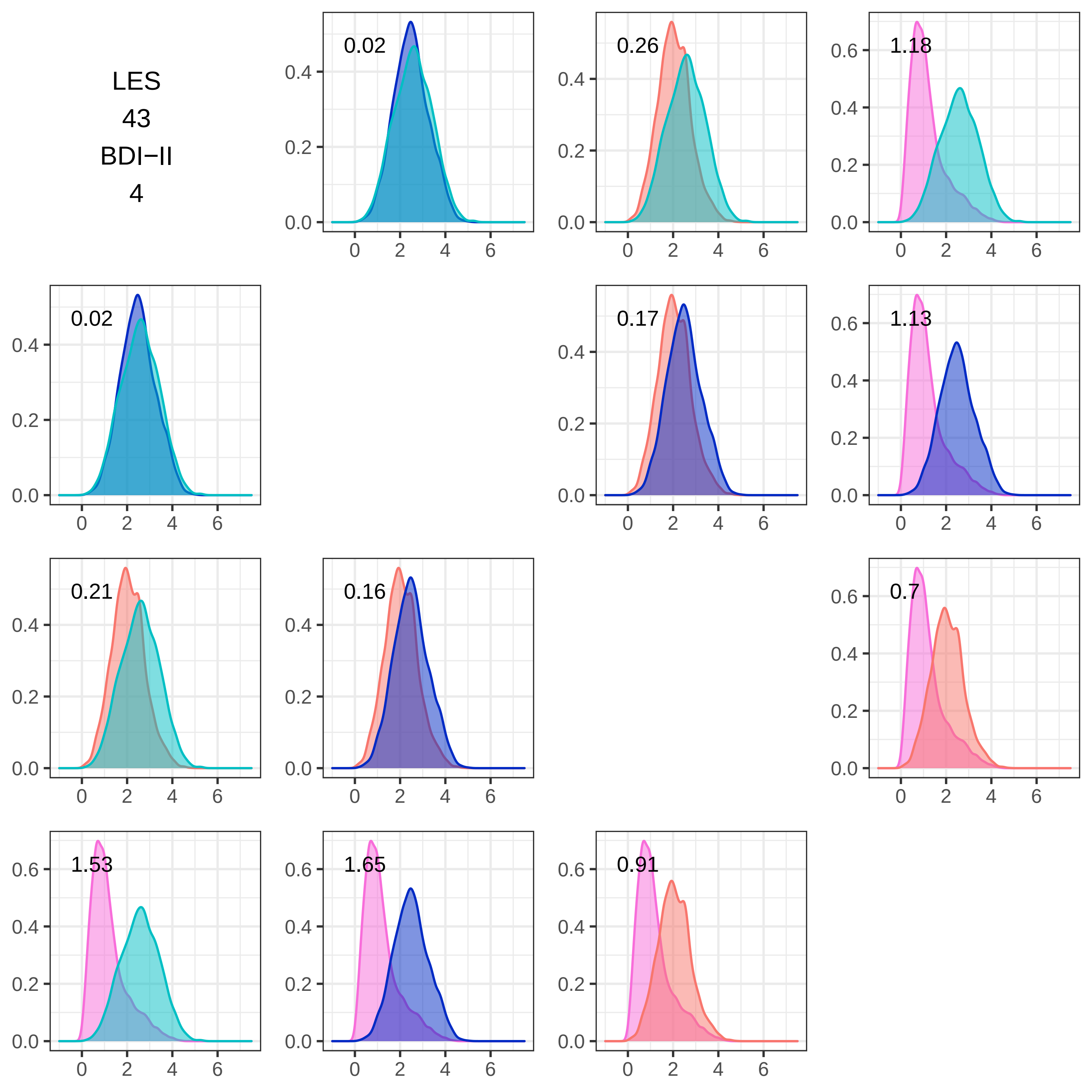
LES
43
BDI−II
4
0.02
0.26
1.18
0.02
0.17
1.13
0.21
0.16
0.7
1.53
1.65
0.91

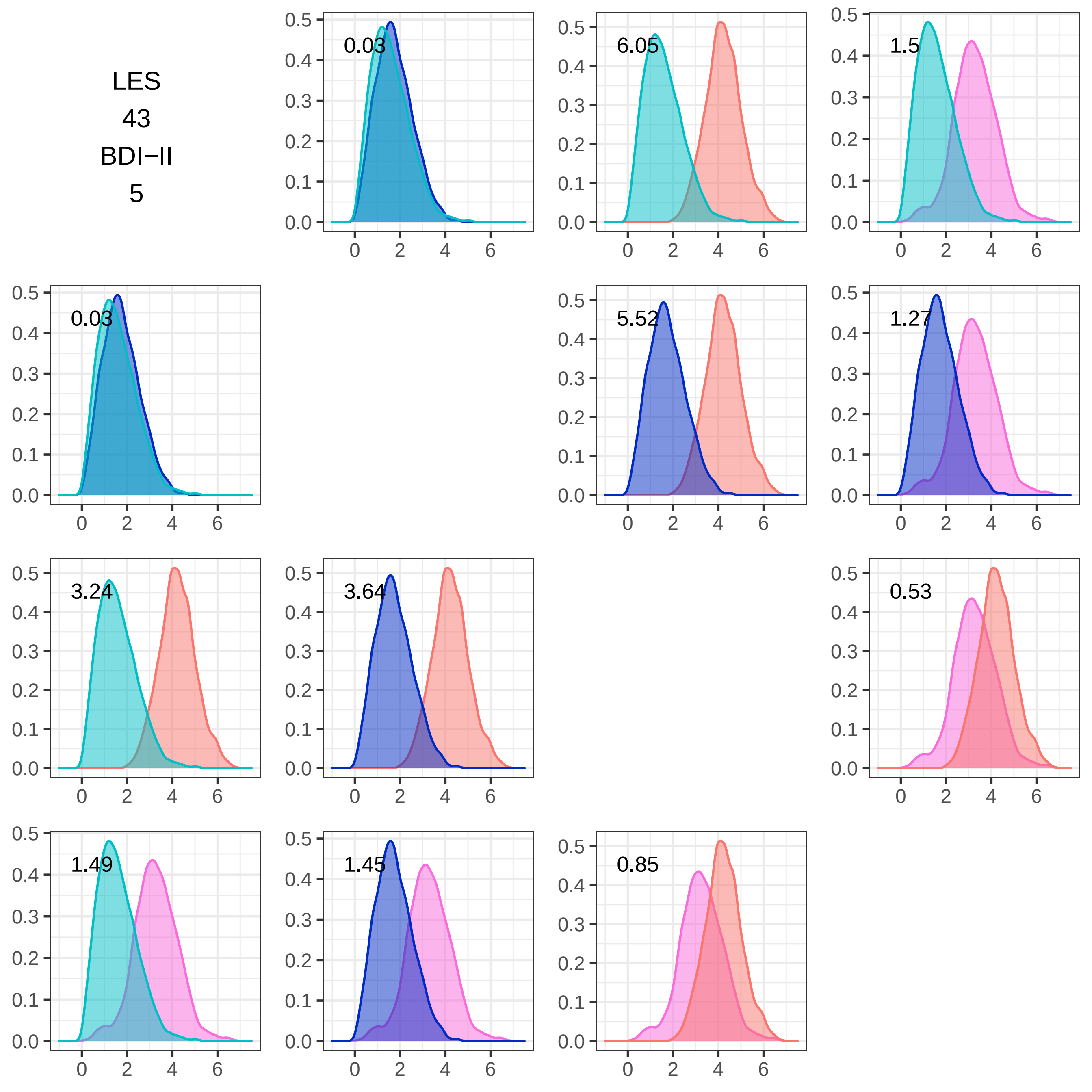
LES
43
BDI−II
5
0.03
6.05
1.5
0.03
5.52
1.27
3.24
3.64
0.53
1.49
1.45
0.85

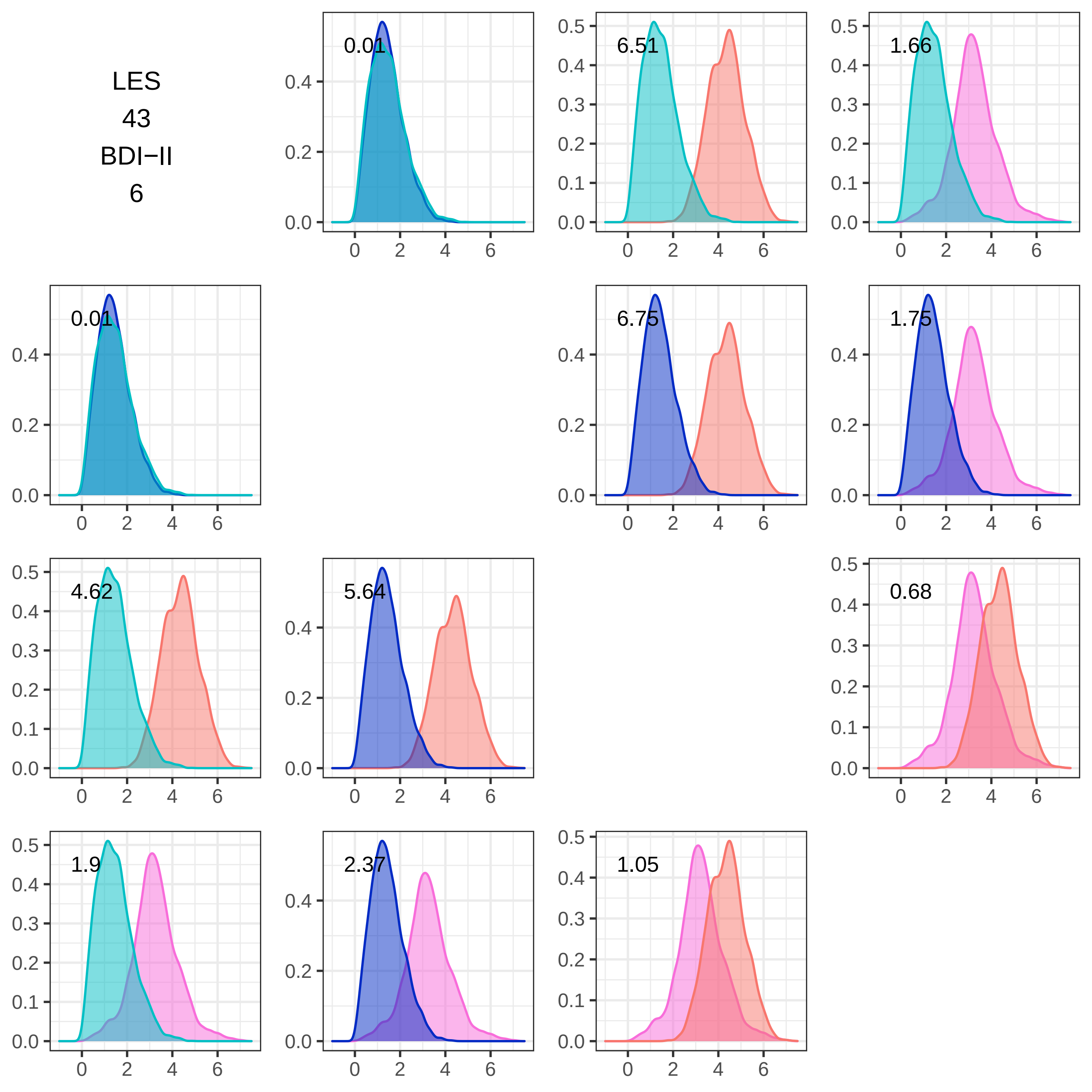
LES
43
BDI−II
6
0.01
6.51
1.66
0.01
6.75
1.75
4.62
5.64
0.68
1.9
2.37
1.05

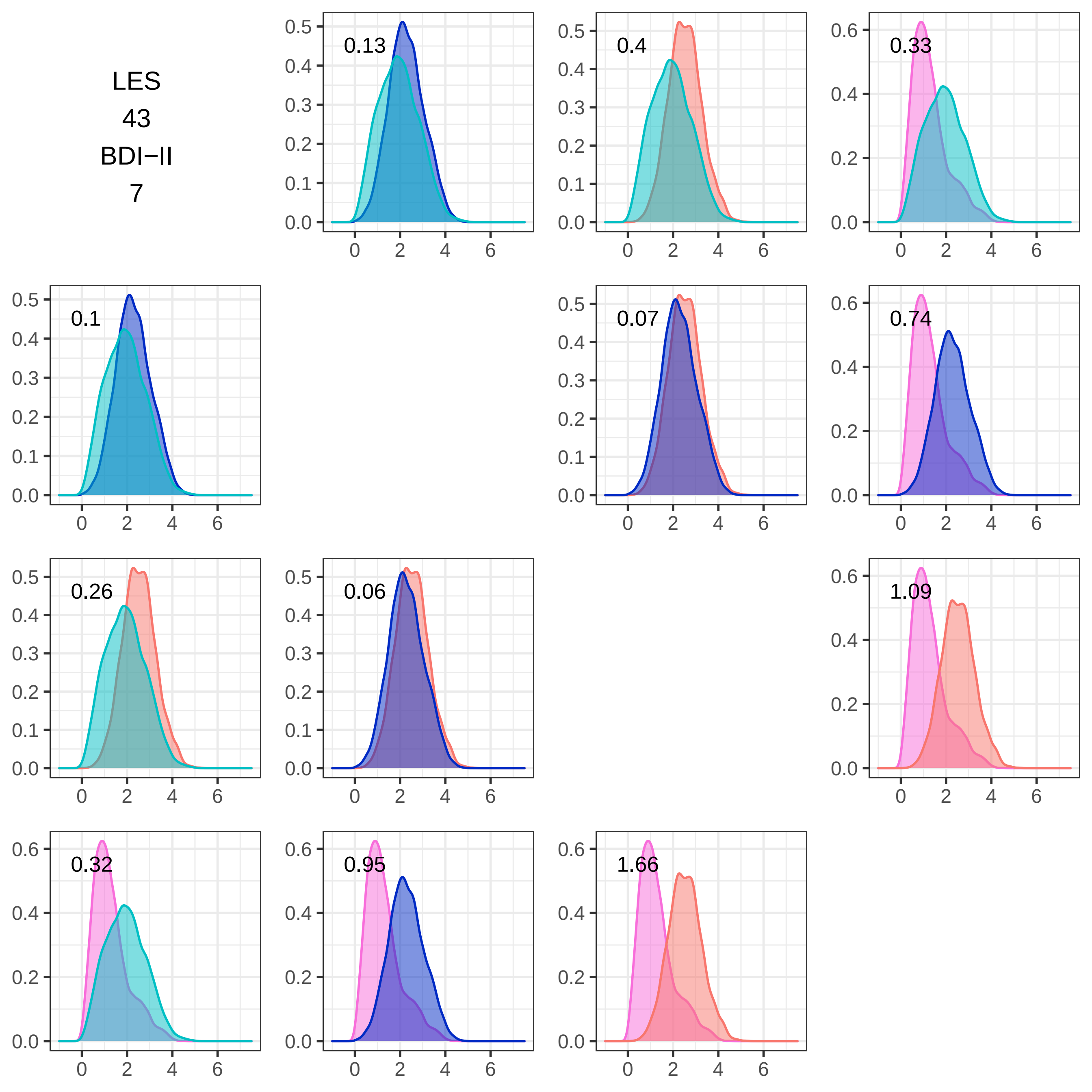
LES
43
BDI−II
7
0.13
0.4
0.33
0.1
0.07
0.74
0.26
0.06
1.09
0.32
0.95
1.66

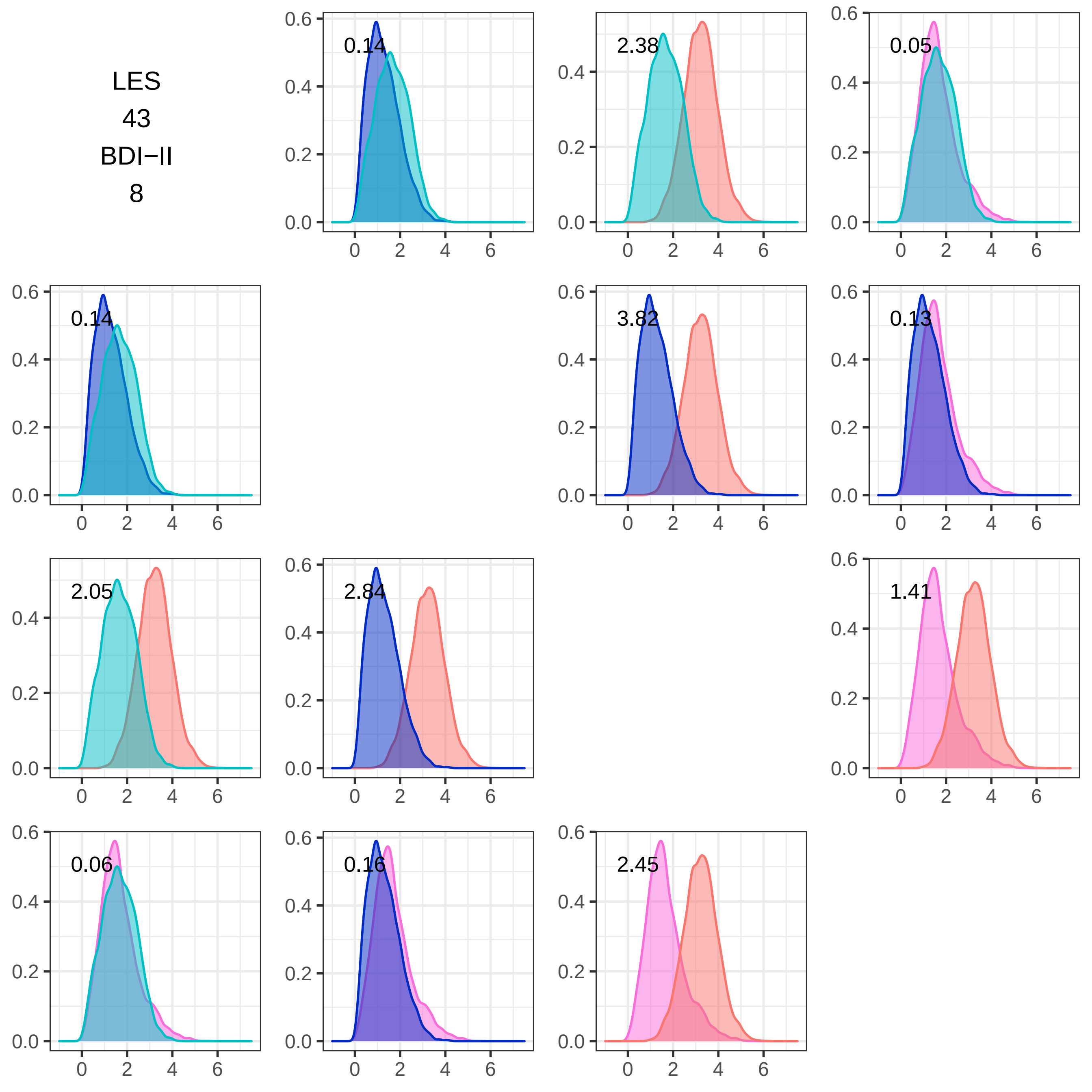

LES
43
BDI−II
8
0.14
2.38
0.05
0.14
3.82
0.13
2.05
2.84
1.41
0.06
0.16
2.45

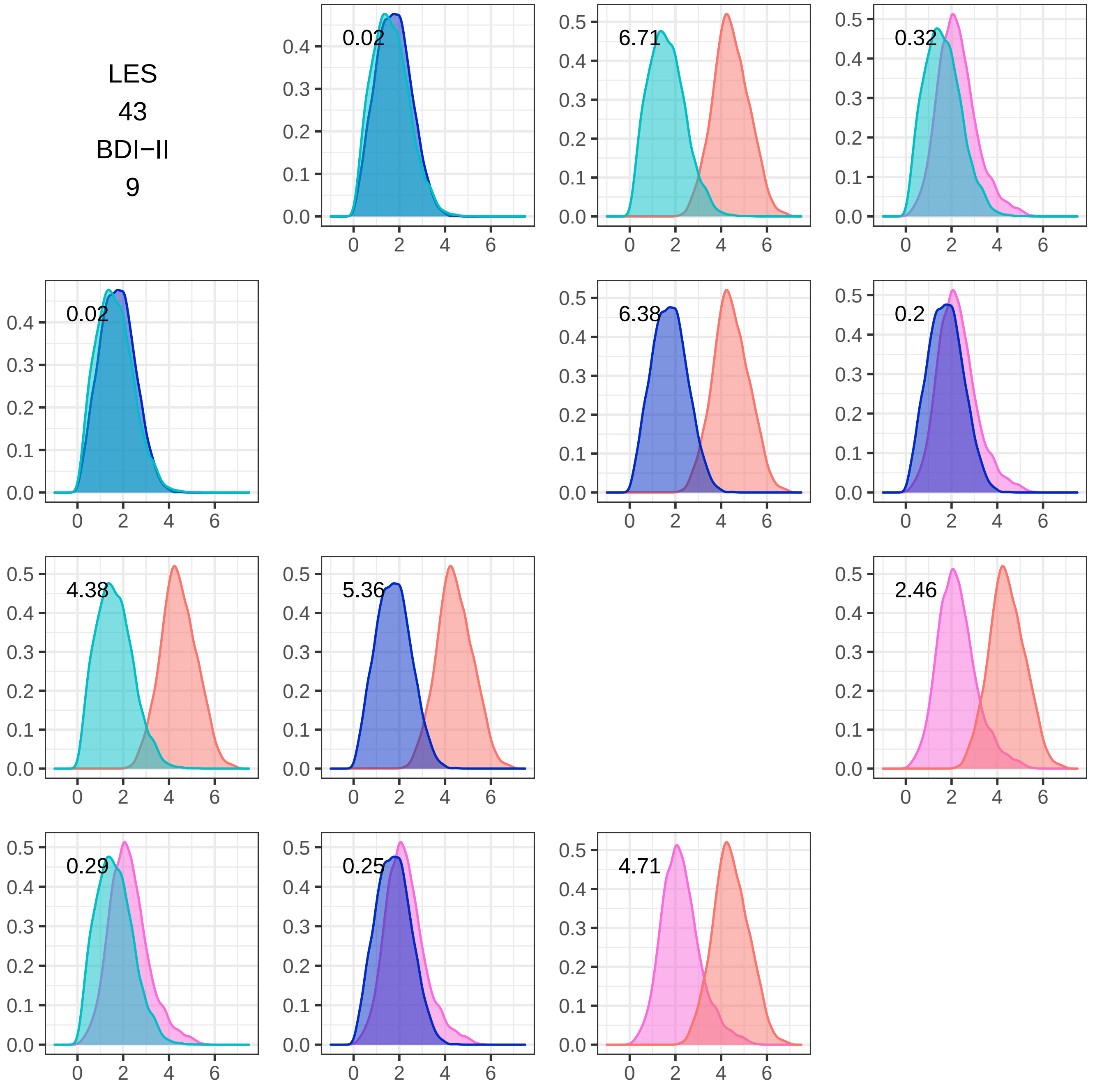

LES
43
BDI−II
9
0.02
6.71
0.32
0.02
6.38
0.2
4.38
5.36
2.46
0.29
0.25
4.71

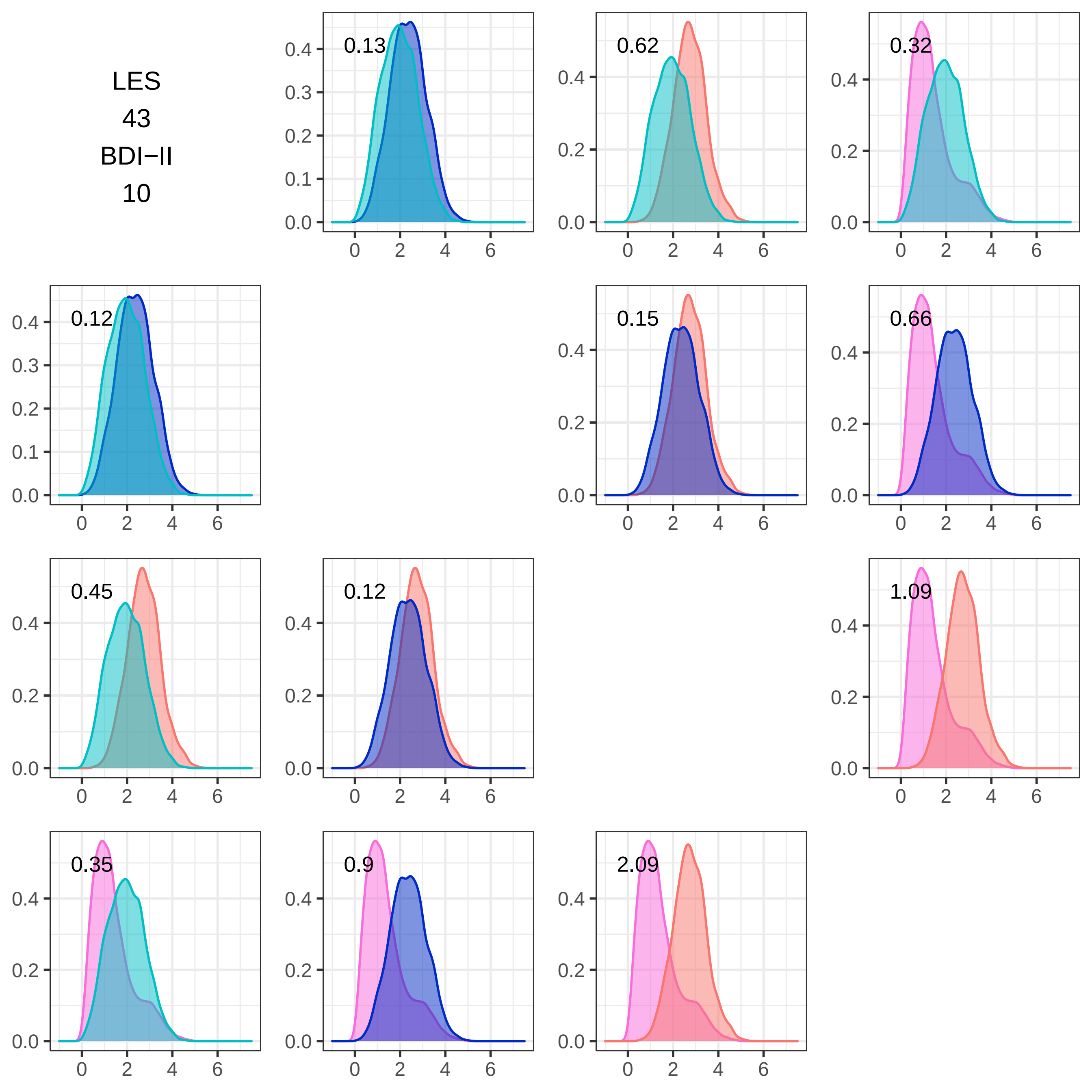
LES
43
BDI−II
10
0.13
0.62
0.32
0.12
0.15
0.66
0.45
0.12
1.09
0.35
0.9
2.09

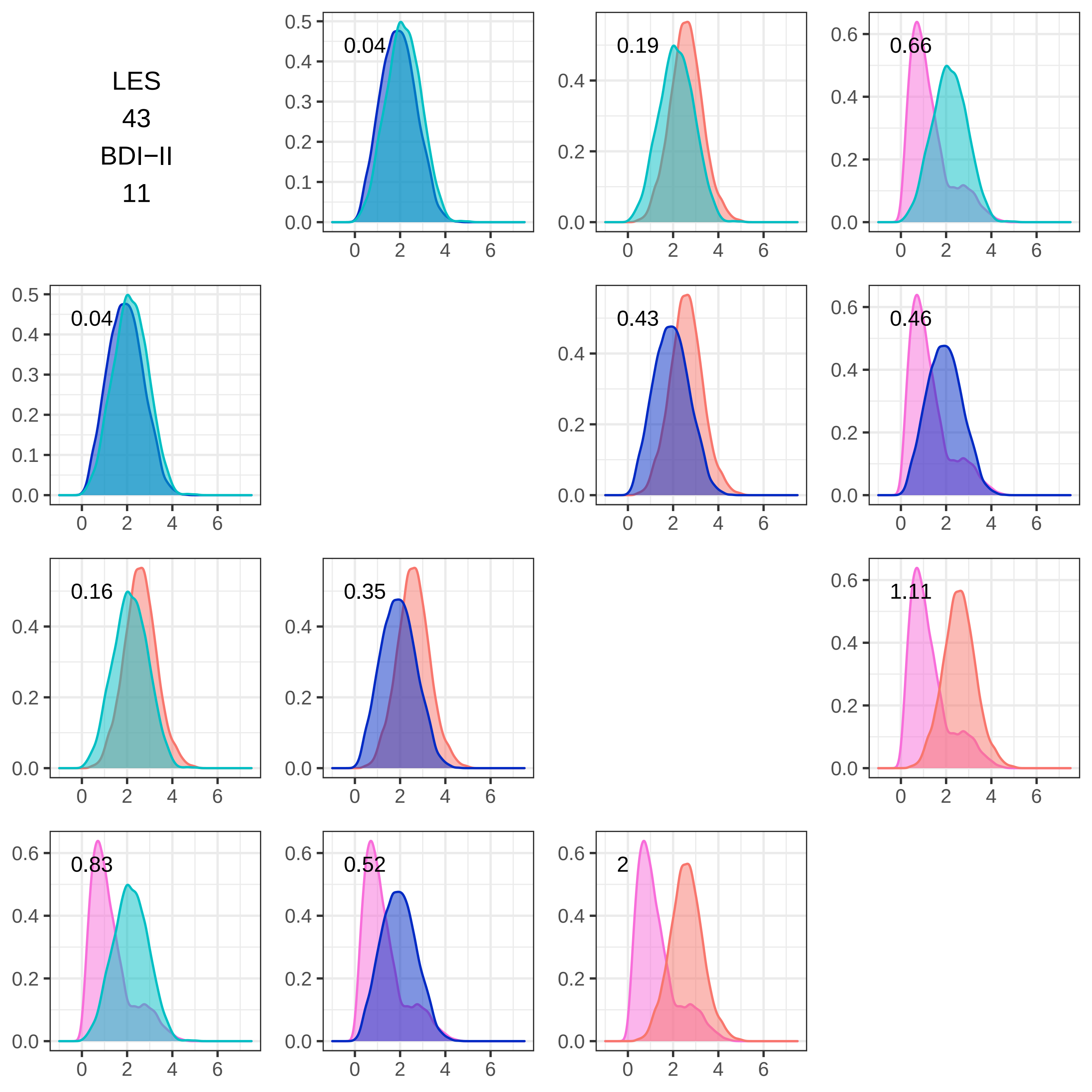
LES
43
BDI−II
11
0.04
0.19
0.66
0.04
0.43
0.46
0.16
0.35
1.11
0.83
0.52
2

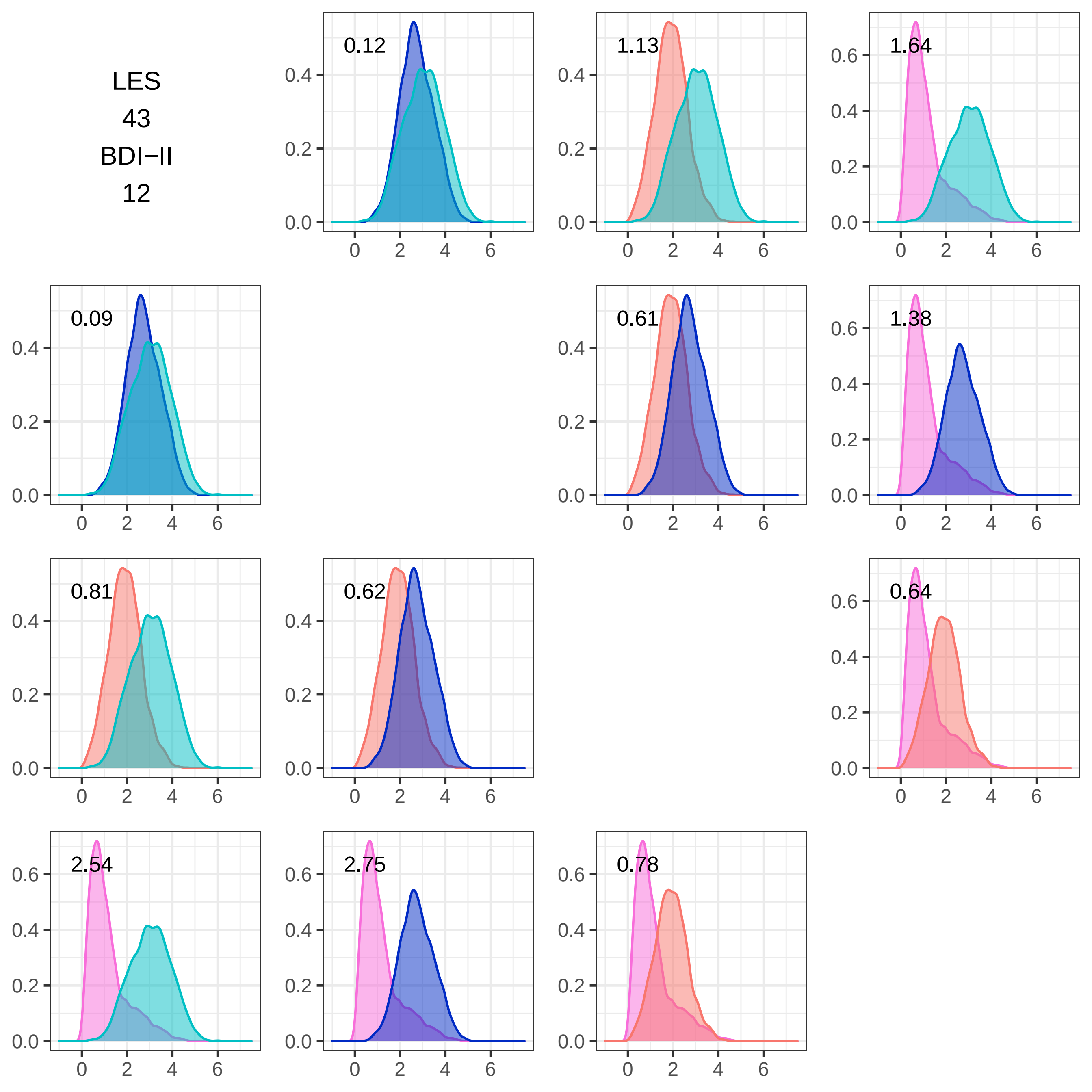
LES
43
BDI−II
12
0.12
1.13
1.64
0.09
0.61
1.38
0.81
0.62
0.64
2.54
2.75
0.78

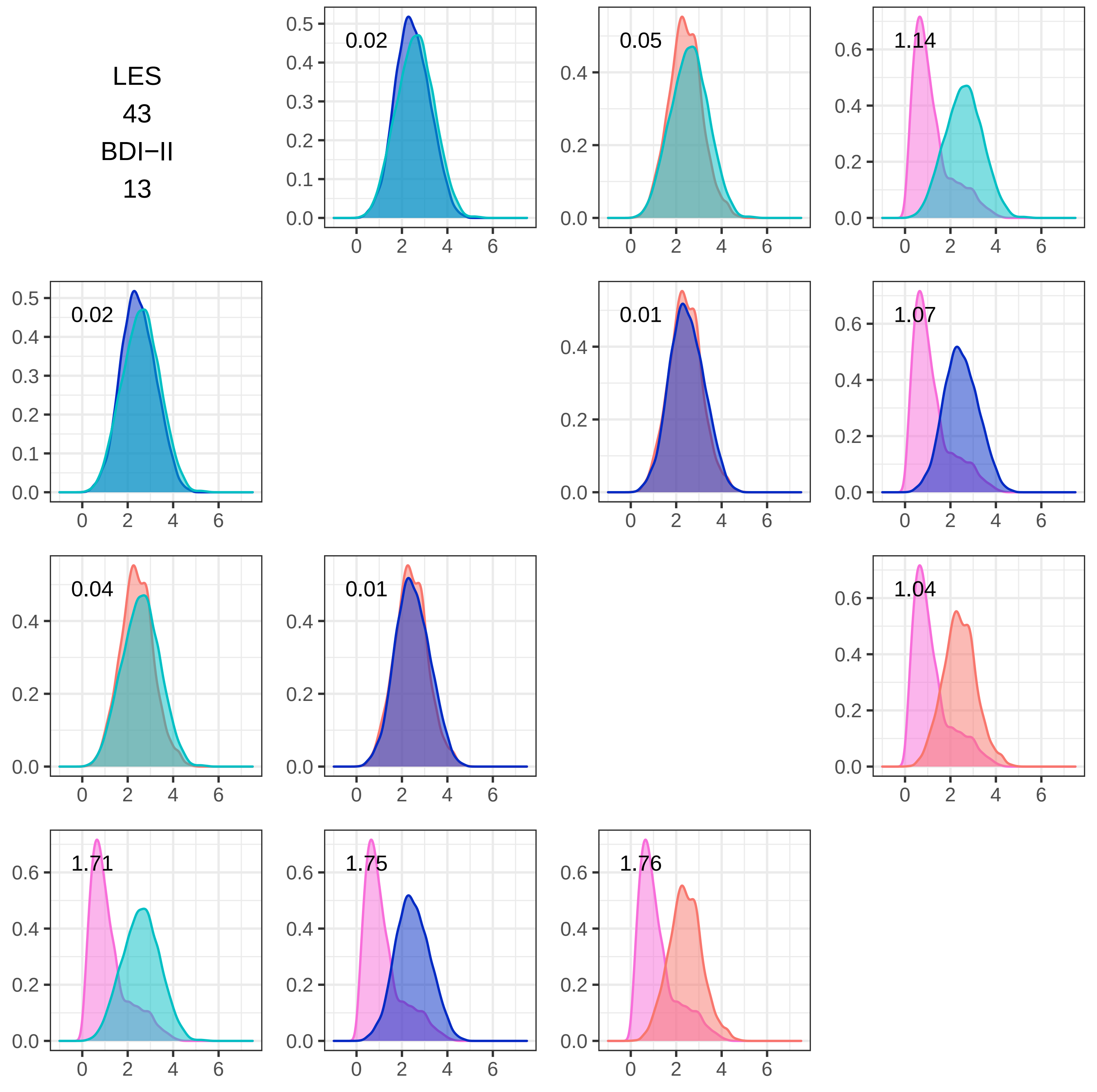

LES
43
BDI−II
13
0.02
0.05
1.14
0.02
0.01
1.07
0.04
0.01
1.04
1.71
1.75
1.76

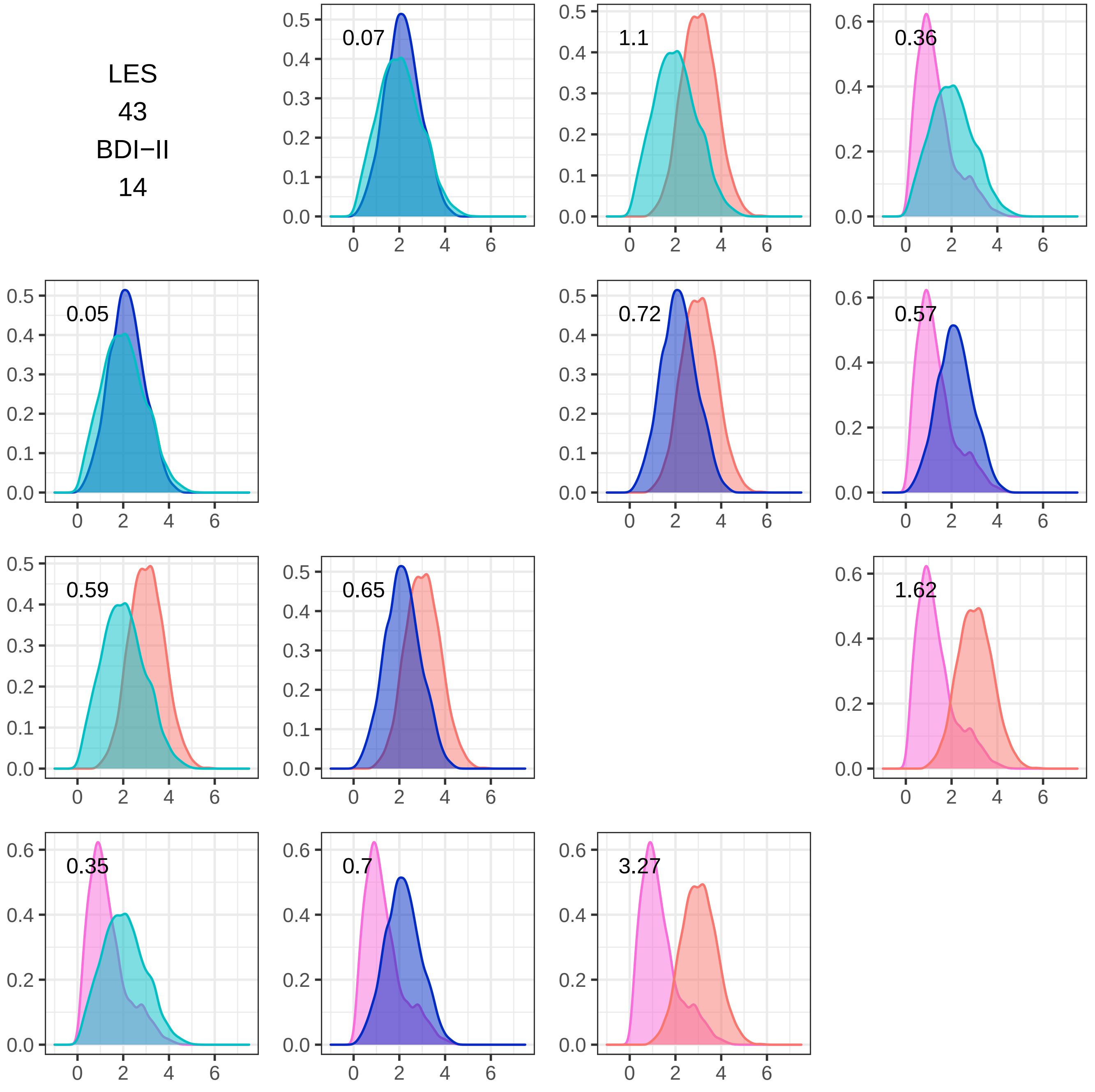

LES
43
BDI−II
14
0.07
1.1
0.36
0.05
0.72
0.57
0.59
0.65
1.62
0.35
0.7
3.27

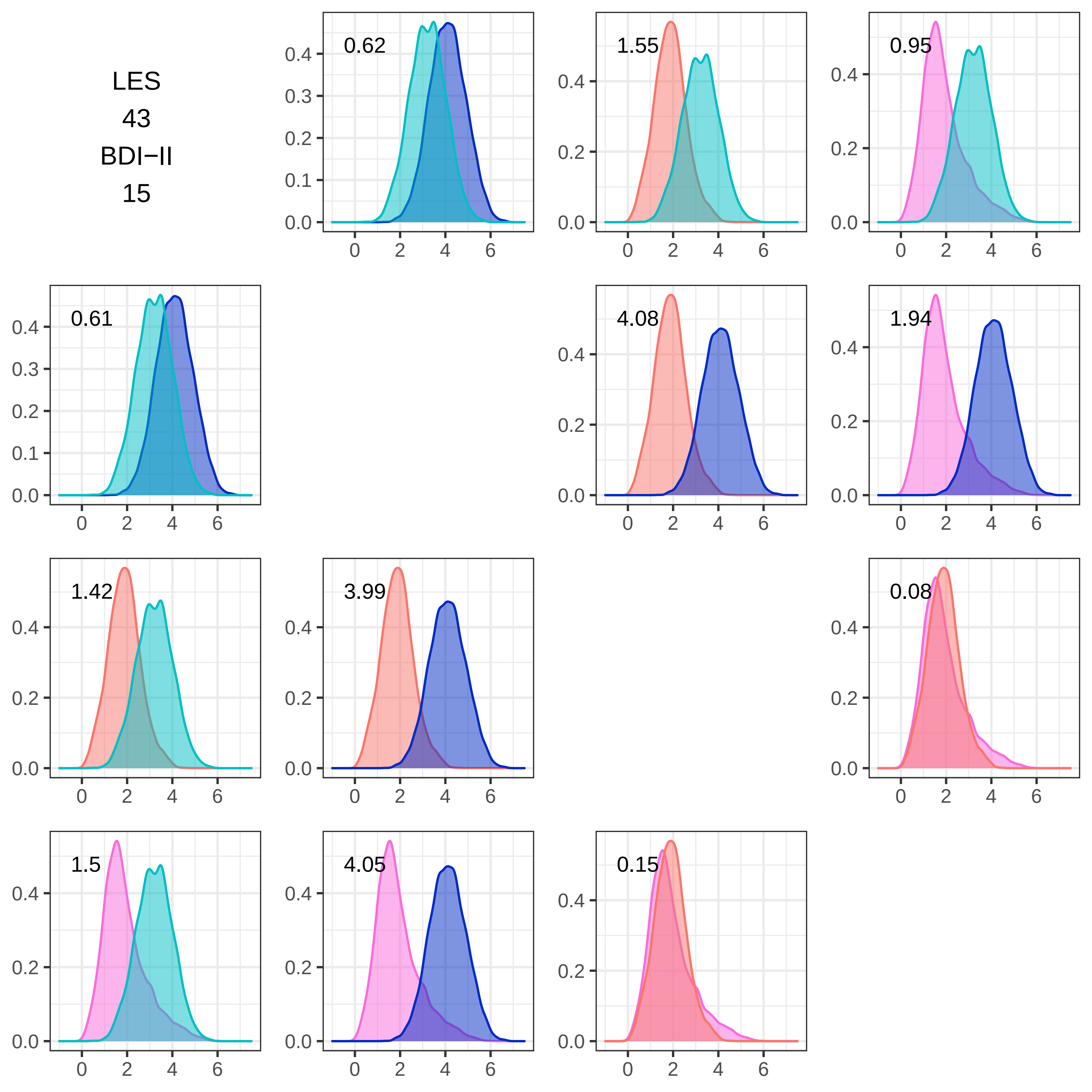

LES
43
BDI−II
15
0.62
1.55
0.95
0.61
4.08
1.94
1.42
3.99
0.08
1.5
4.05
0.15

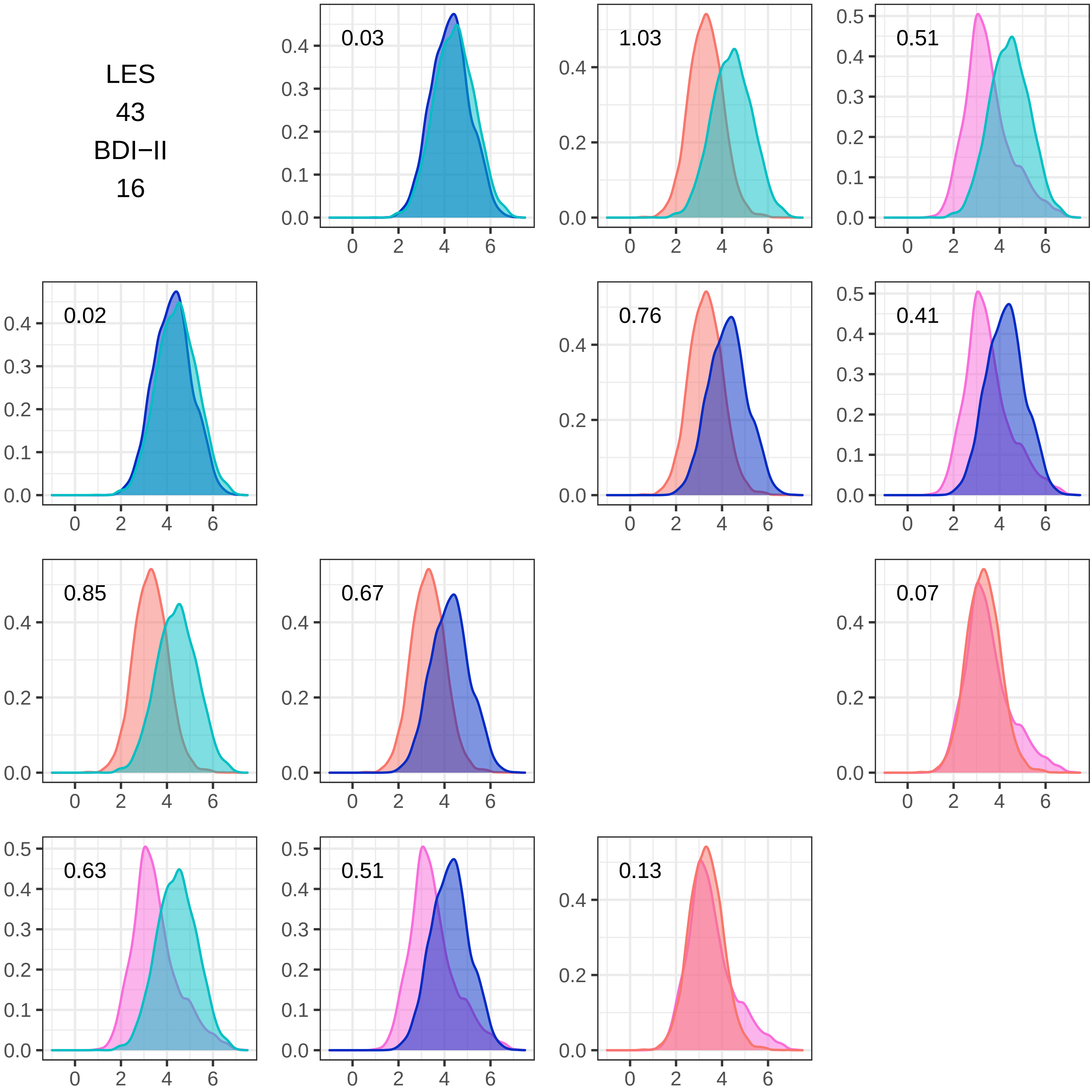
LES
43
BDI−II
16
0.03
1.03
0.51
0.02
0.76
0.41
0.85
0.67
0.07
0.63
0.51
0.13

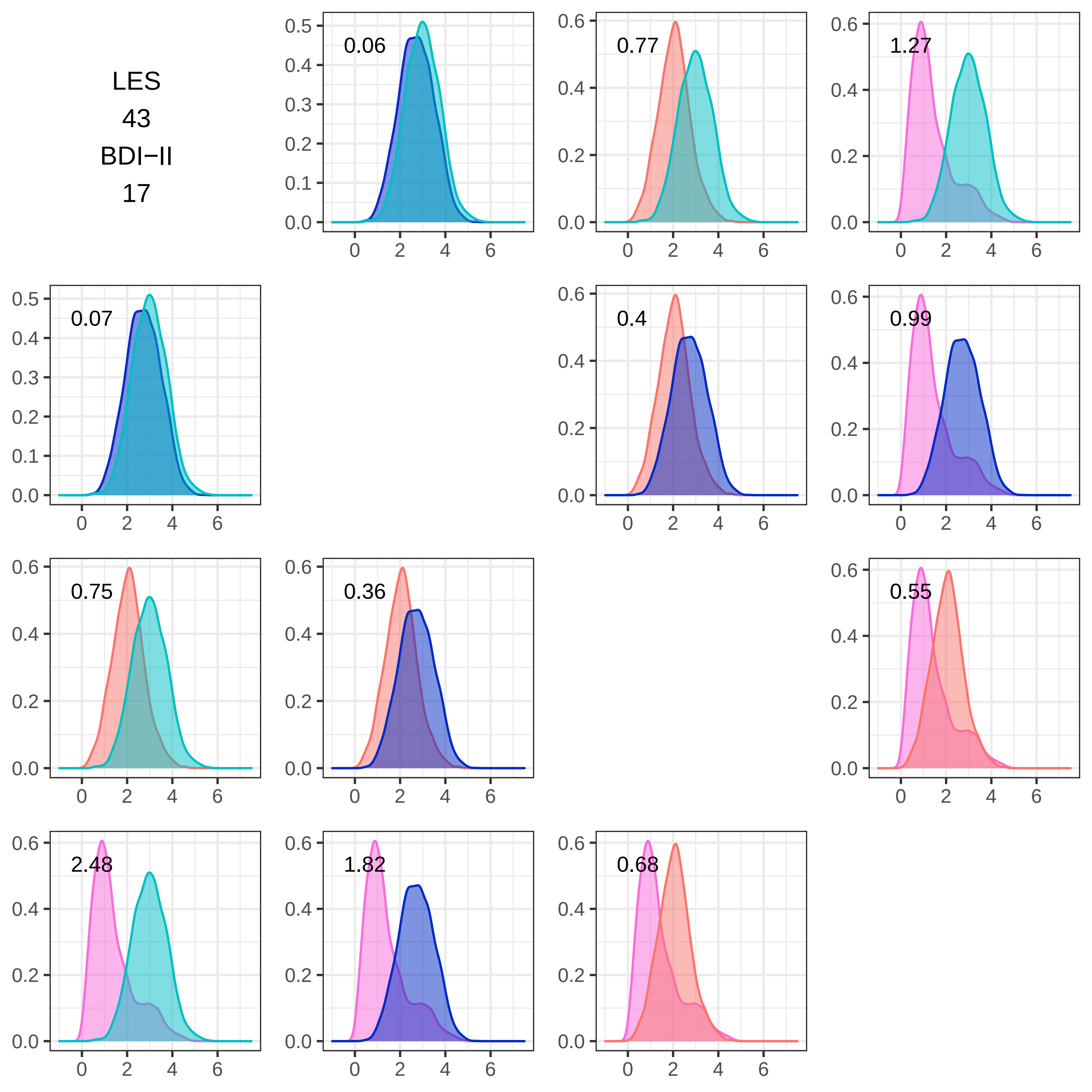

LES
43
BDI−II
17
0.06
0.77
1.27
0.07
0.4
0.99
0.75
0.36
0.55
2.48
1.82
0.68

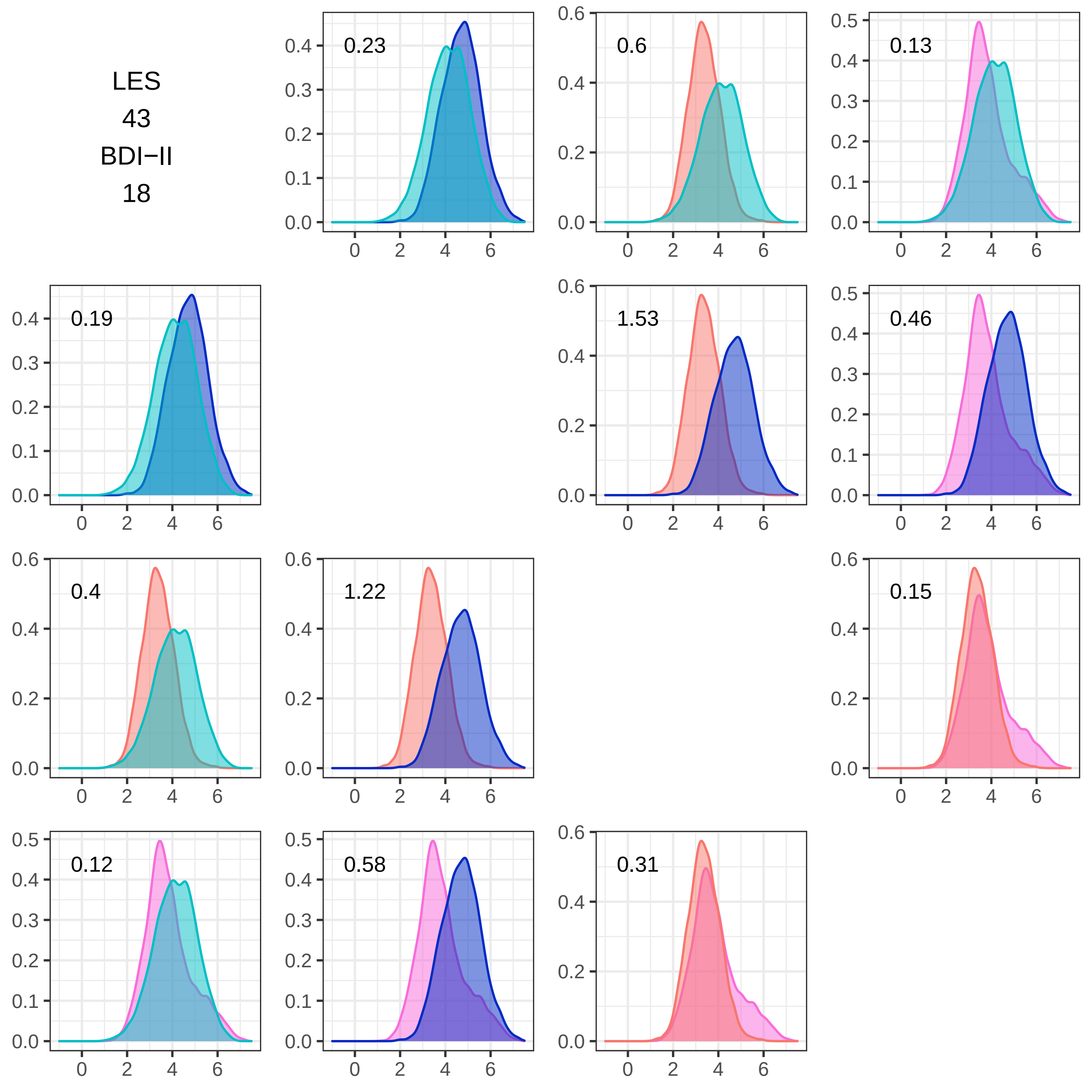

LES
43
BDI−II
18
0.23
0.6
0.13
0.19
1.53
0.46
0.4
1.22
0.15
0.12
0.58
0.31

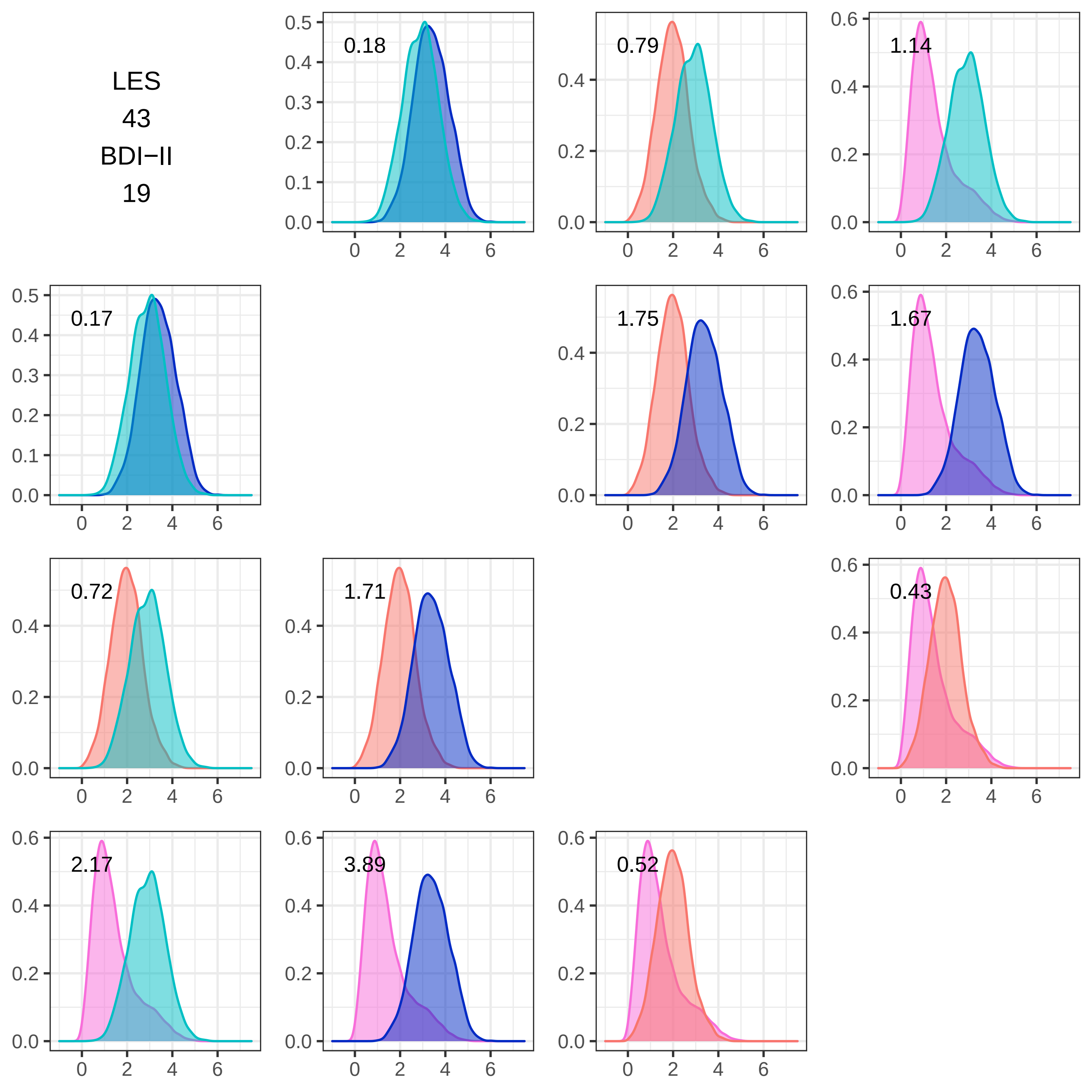
LES
43
BDI−II
19
0.18
0.79
1.14
0.17
1.75
1.67
0.72
1.71
0.43
2.17
3.89
0.52

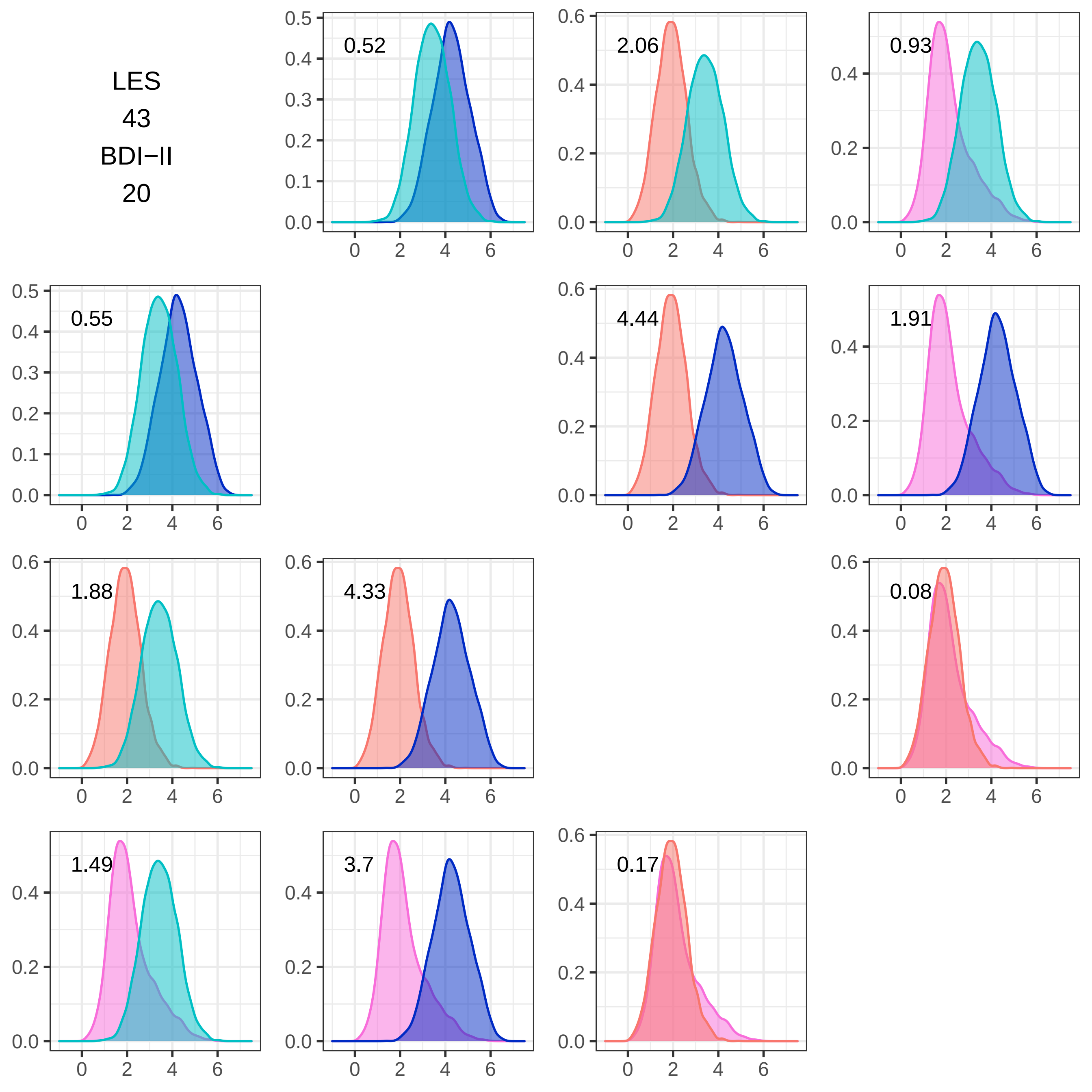
LES
43
BDI−II
20
0.52
2.06
0.93
0.55
4.44
1.91
1.88
4.33
0.08
1.49
3.7
0.17

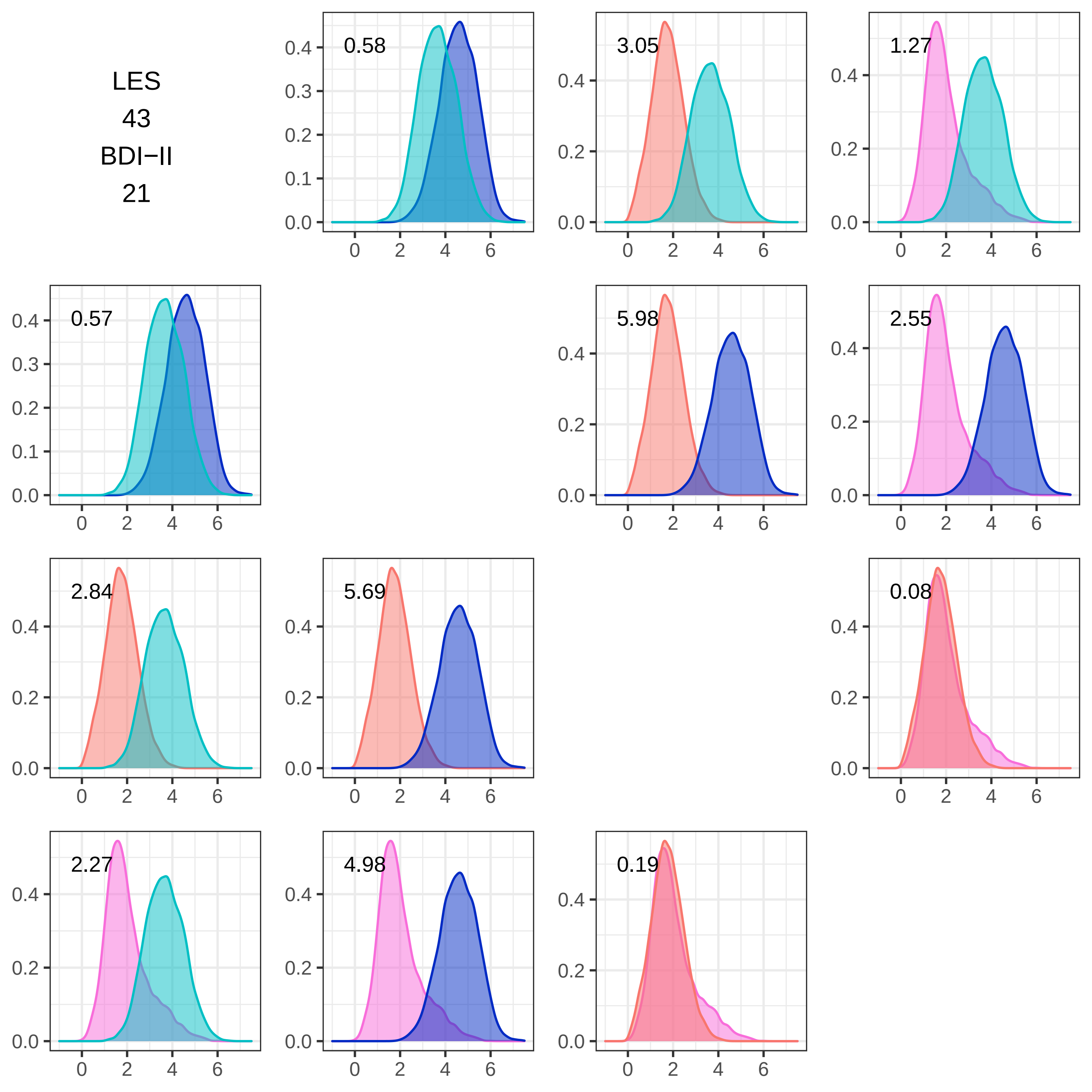
LES
43
BDI−II
21
0.58
3.05
1.27
0.57
5.98
2.55
2.84
5.69
0.08
2.27
4.98
0.19

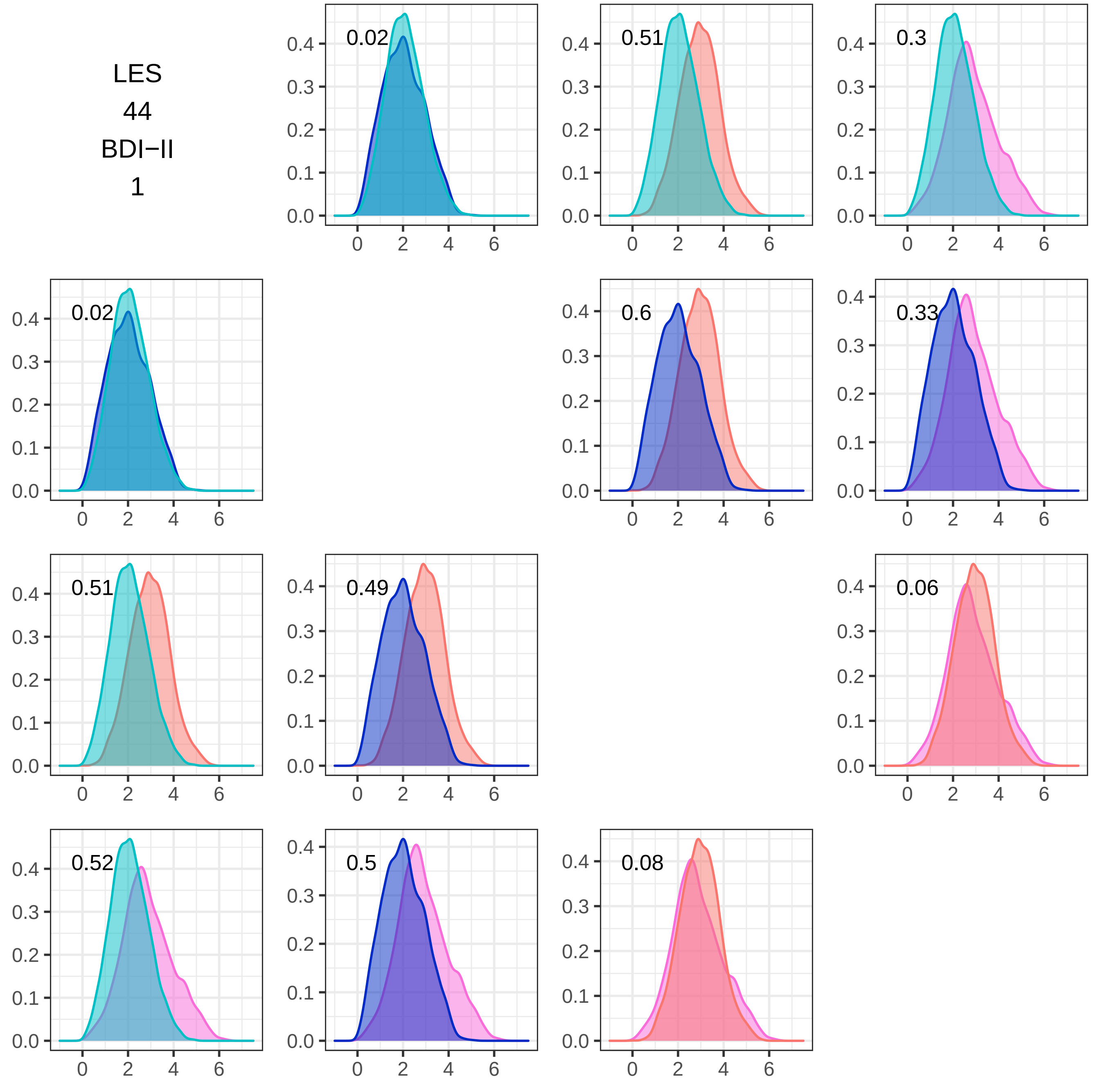
LES
44
BDI−II
1
0.02
0.51
0.3
0.02
0.6
0.33
0.51
0.49
0.06
0.52
0.5
0.08

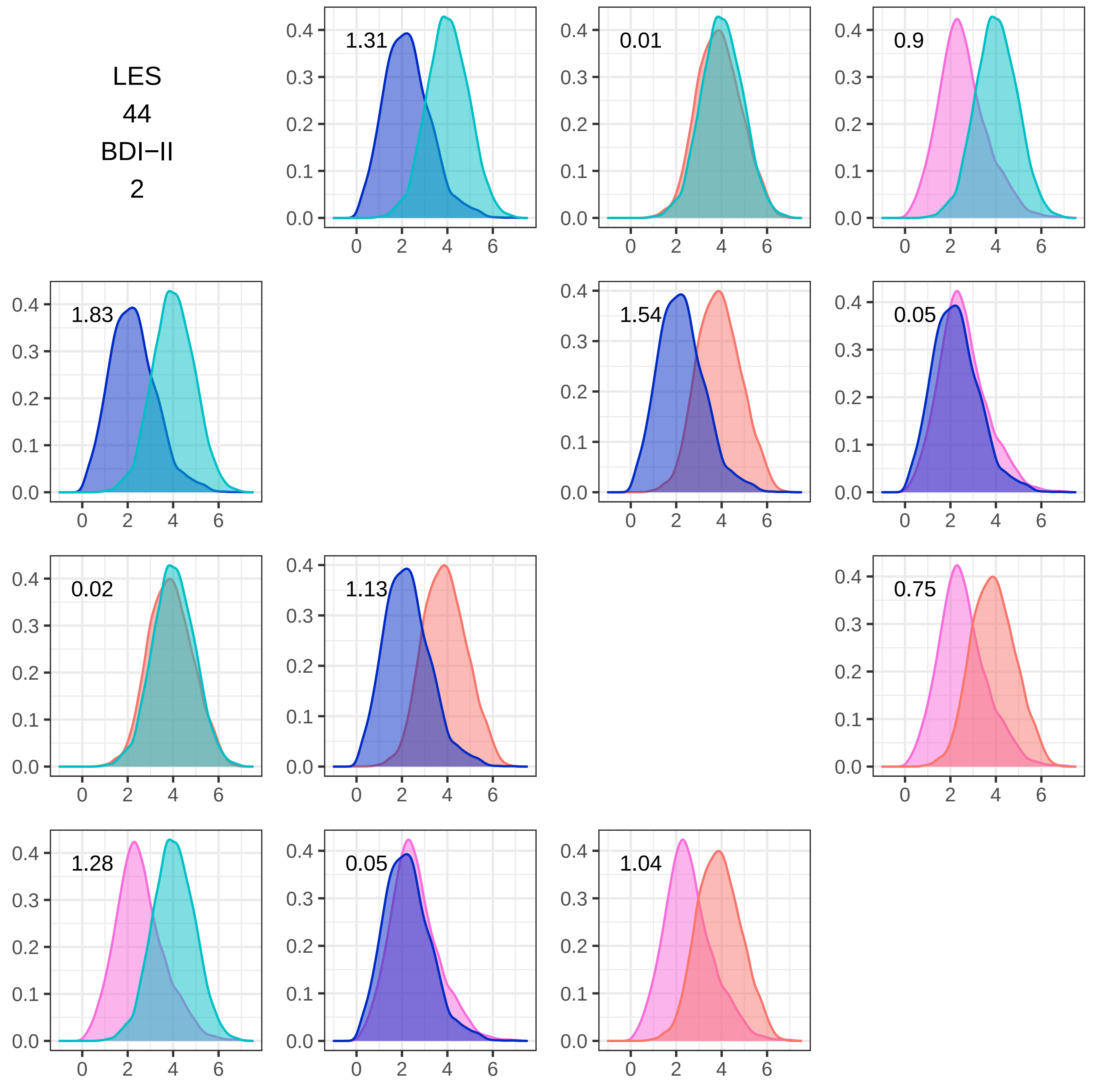

LES
44
BDI−II
2
1.31
0.01
0.9
1.83
1.54
0.05
0.02
1.13
0.75
1.28
0.05
1.04

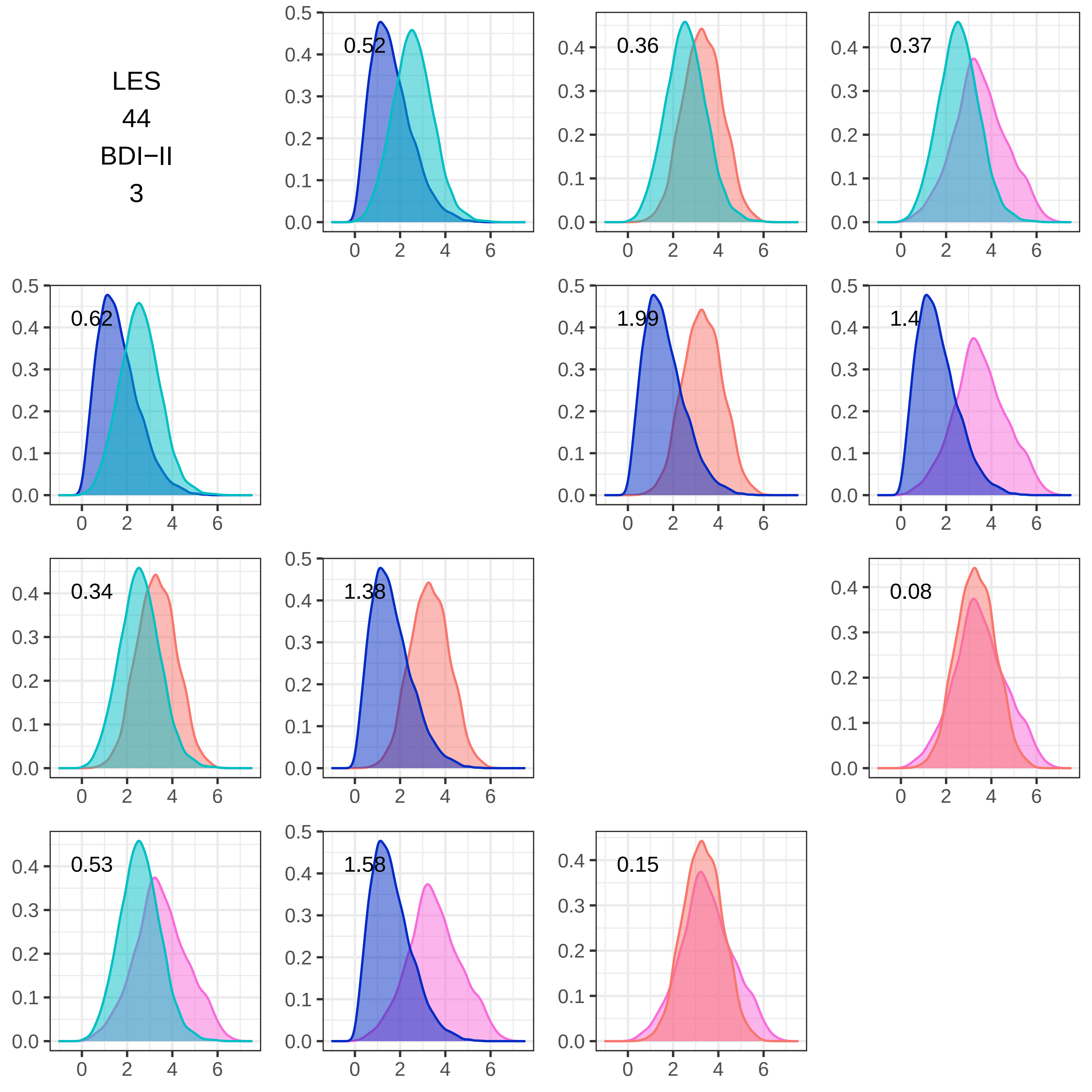
LES
44
BDI−II
3
0.52
0.36
0.37
0.62
1.99
1.4
0.34
1.38
0.08
0.53
1.58
0.15

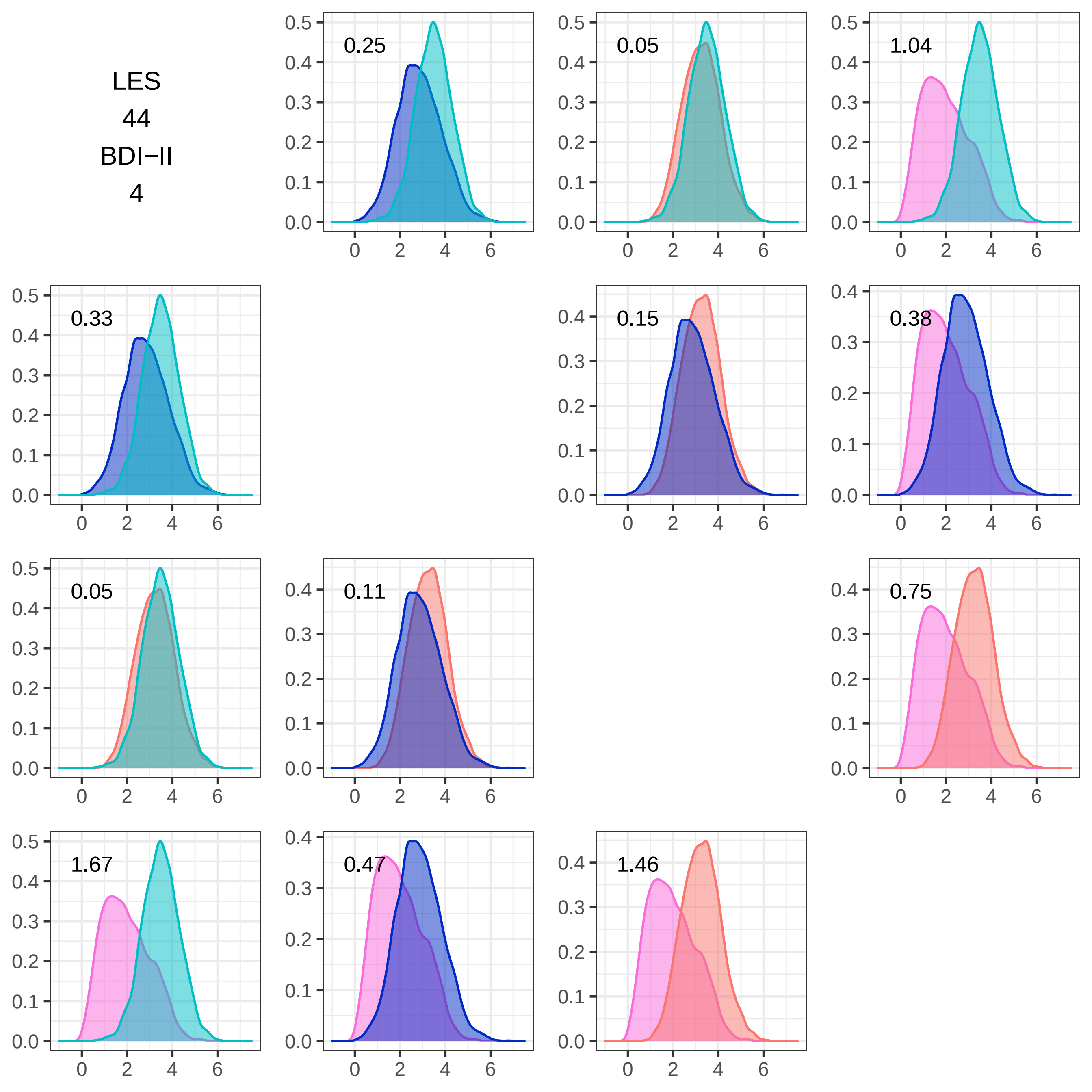

LES
44
BDI−II
4
0.25
0.05
1.04
0.33
0.15
0.38
0.05
0.11
0.75
1.67
0.47
1.46

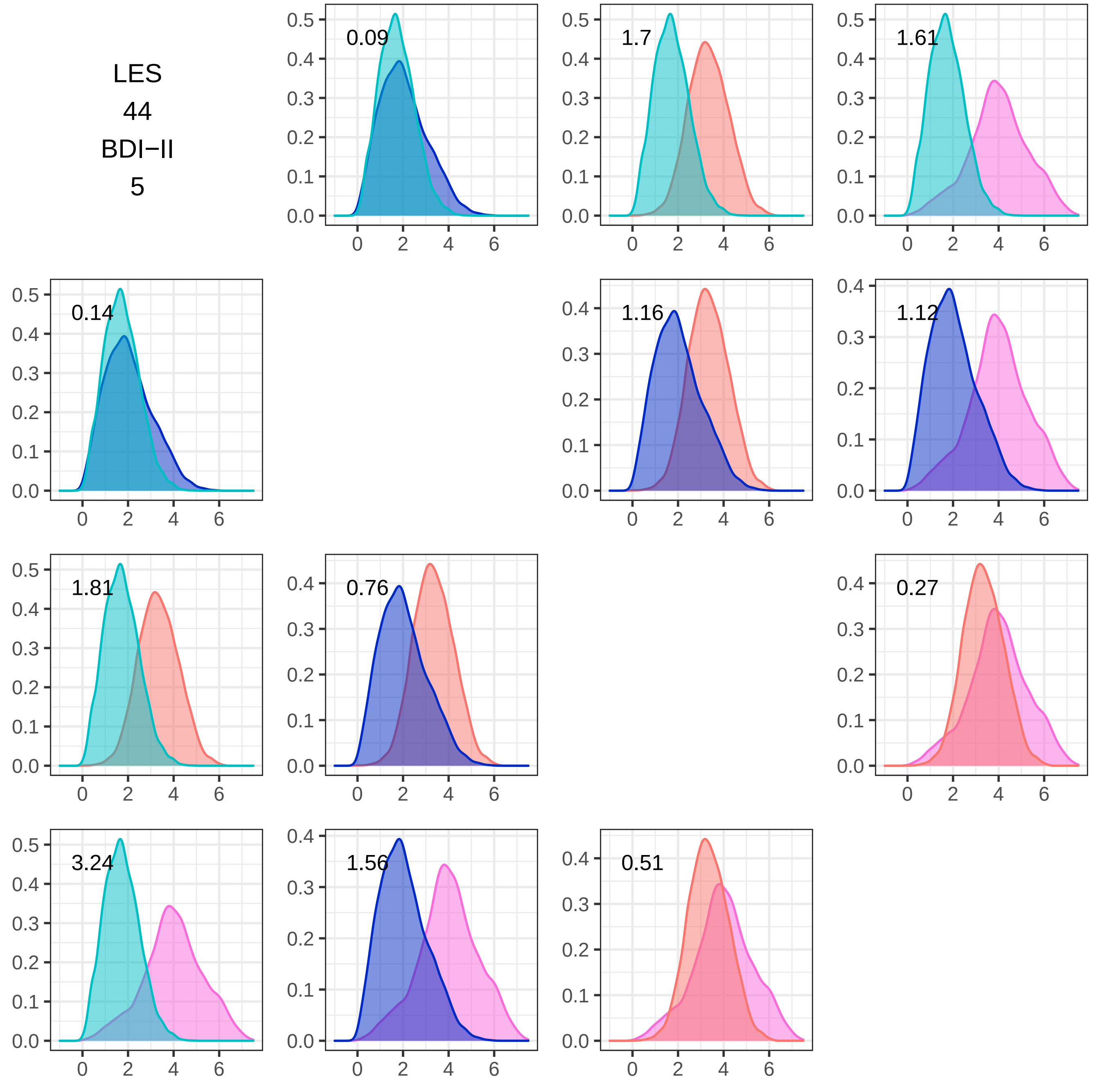

LES
44
BDI−II
5
0.09
1.7
1.61
0.14
1.16
1.12
1.81
0.76
0.27
3.24
1.56
0.51

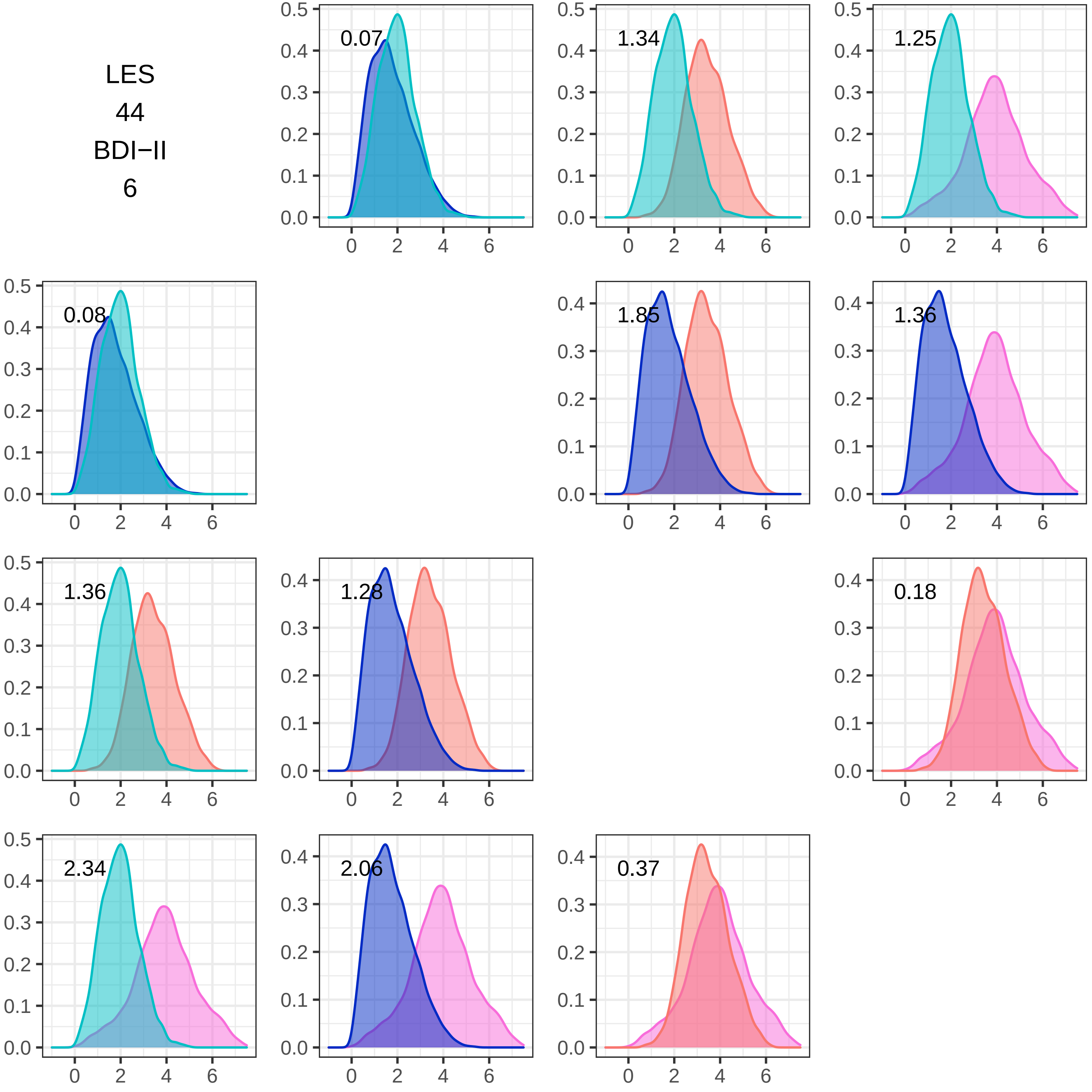
LES
44
BDI−II
6
0.07
1.34
1.25
0.08
1.85
1.36
1.36
1.28
0.18
2.34
2.06
0.37

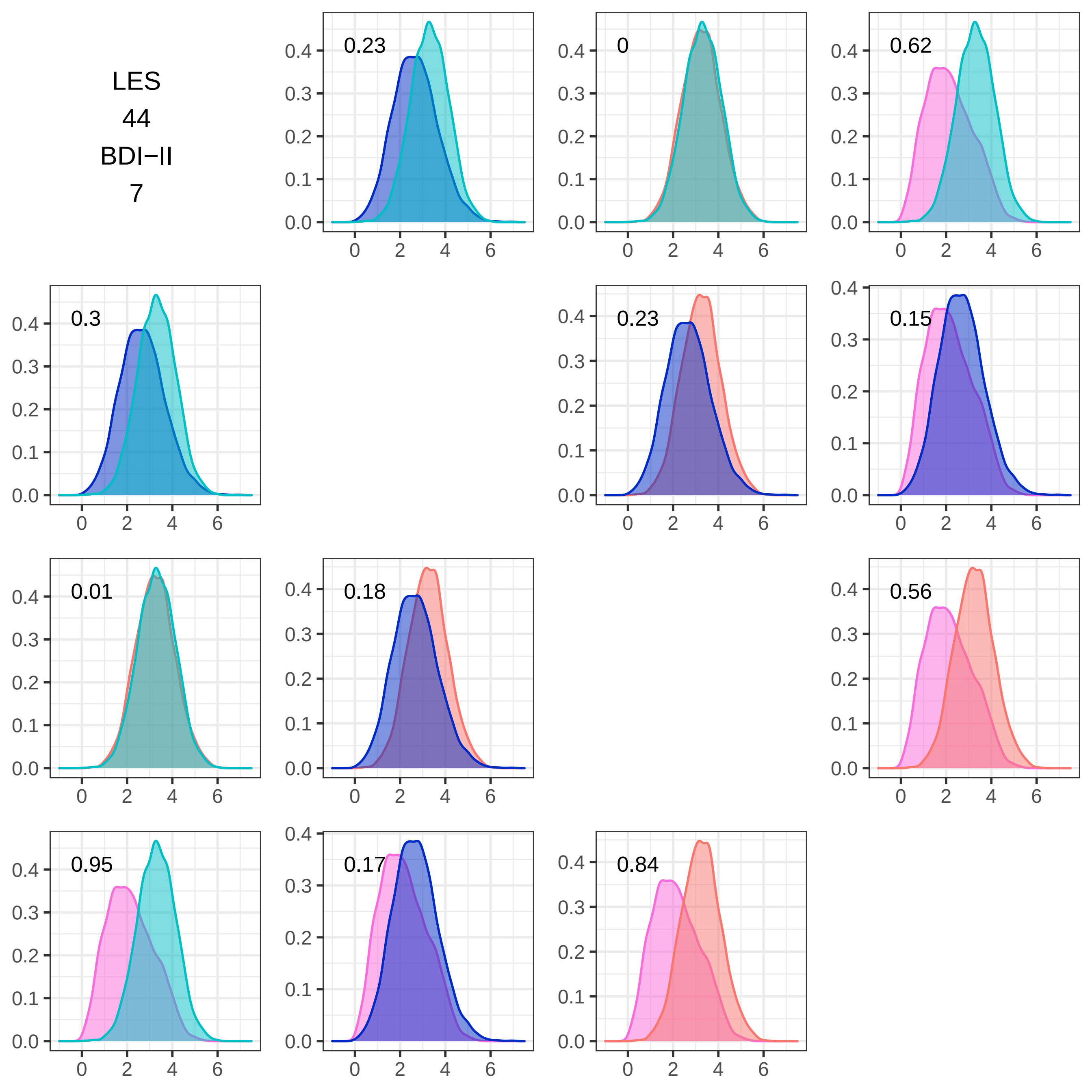
LES
44
BDI−II
7
0.23
0
0.62
0.3
0.23
0.15
0.01
0.18
0.56
0.95
0.17
0.84

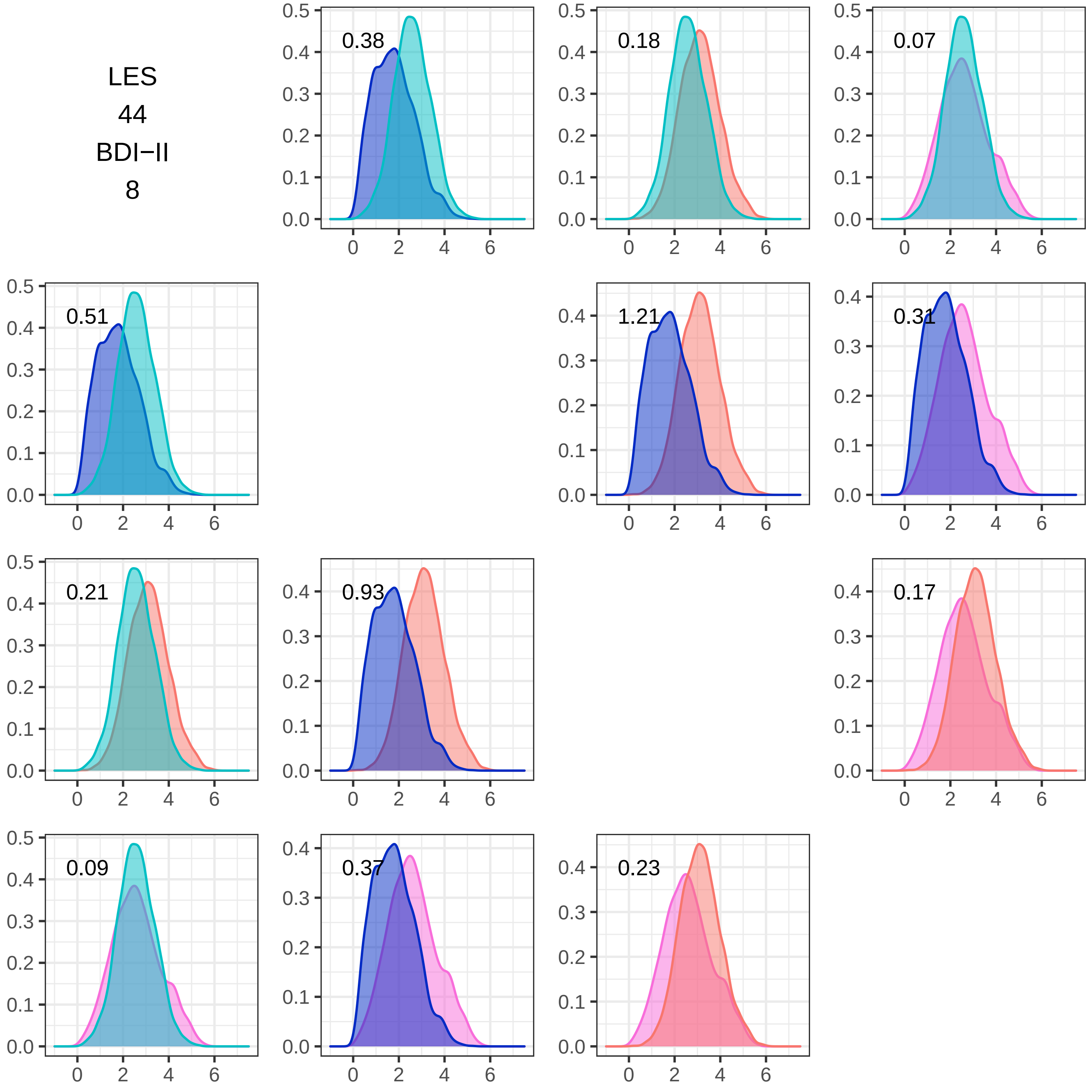
LES
44
BDI−II
8
0.38
0.18
0.07
0.51
1.21
0.31
0.21
0.93
0.17
0.09
0.37
0.23

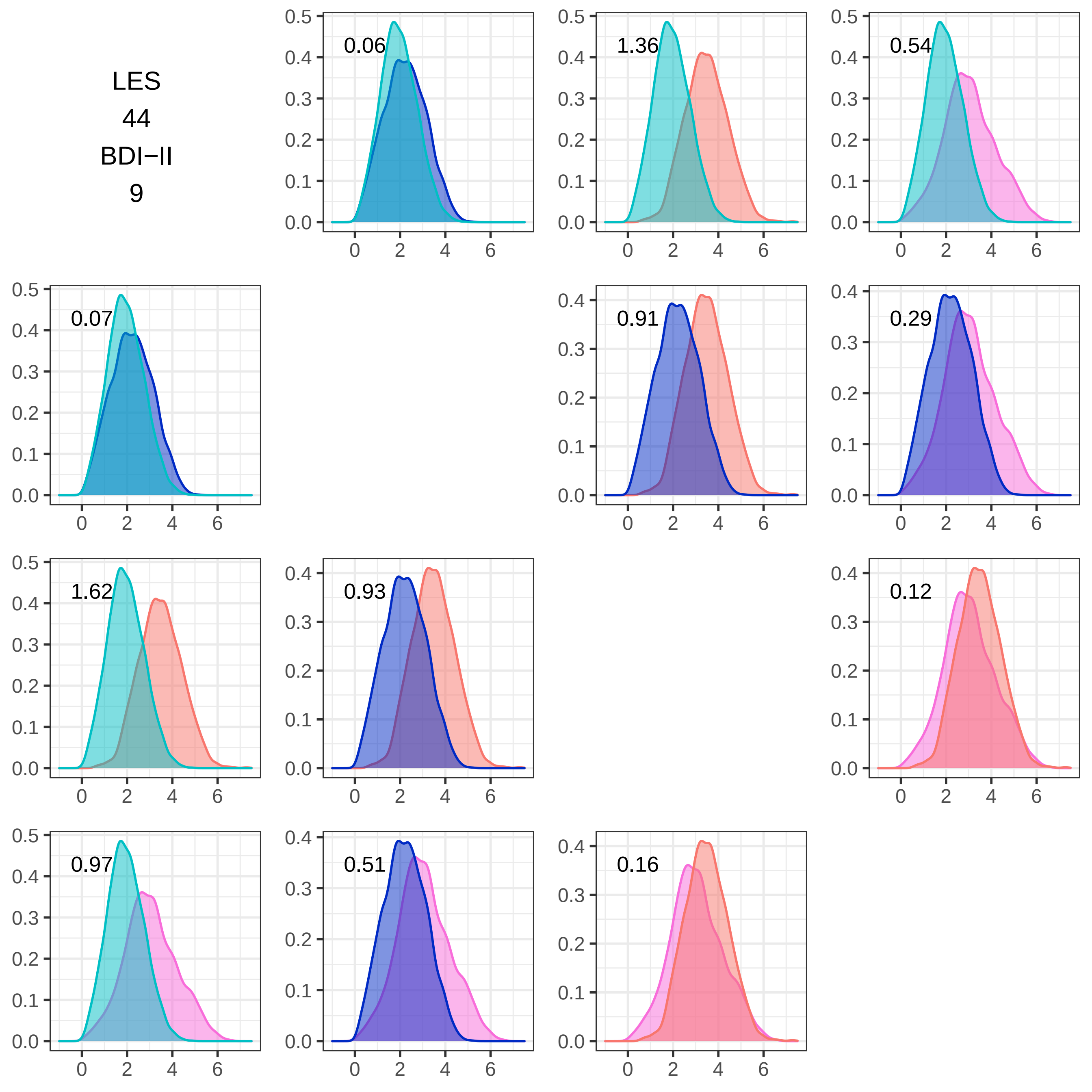
LES
44
BDI−II
9
0.06
1.36
0.54
0.07
0.91
0.29
1.62
0.93
0.12
0.97
0.51
0.16

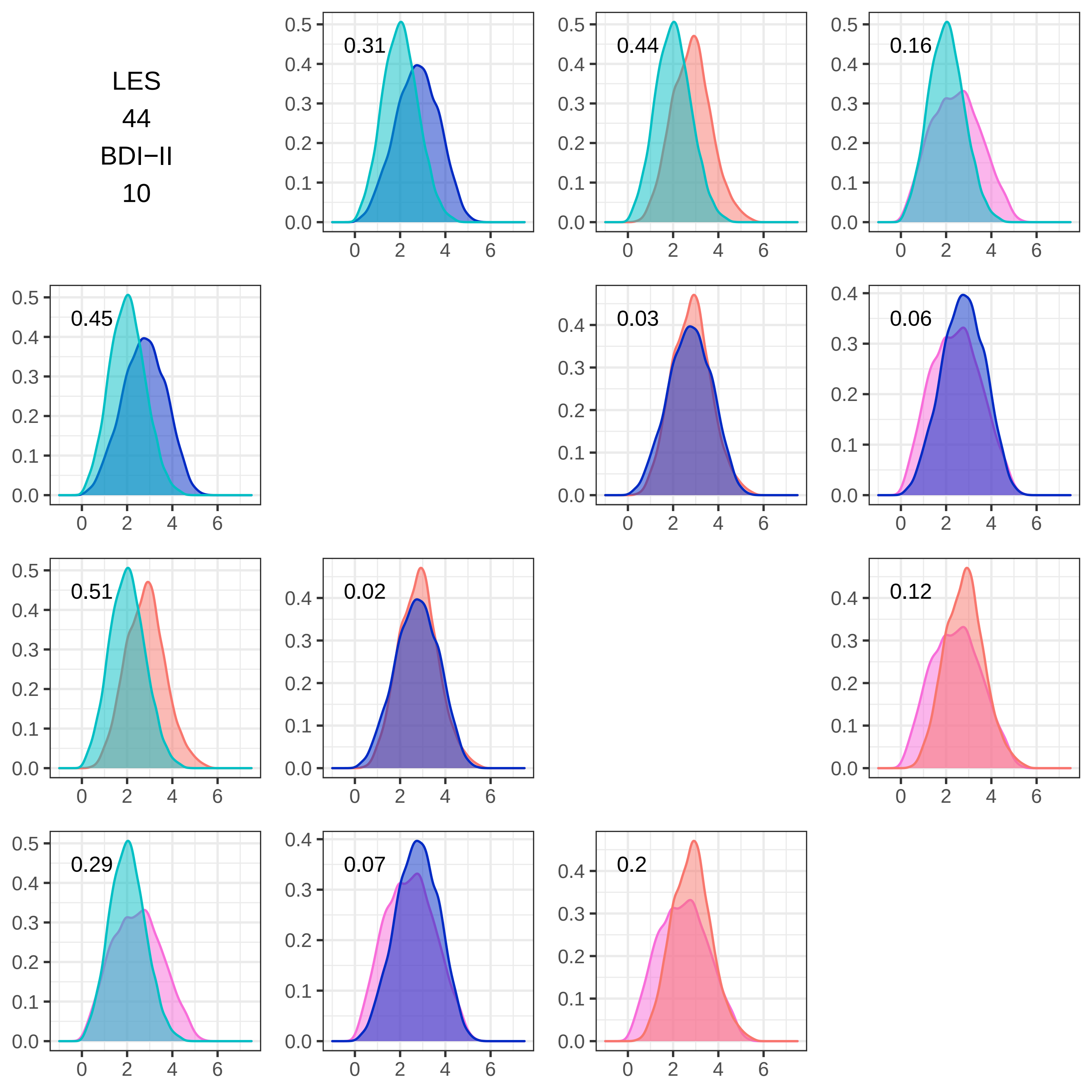
LES
44
BDI−II
10
0.31
0.44
0.16
0.45
0.03
0.06
0.51
0.02
0.12
0.29
0.07
0.2

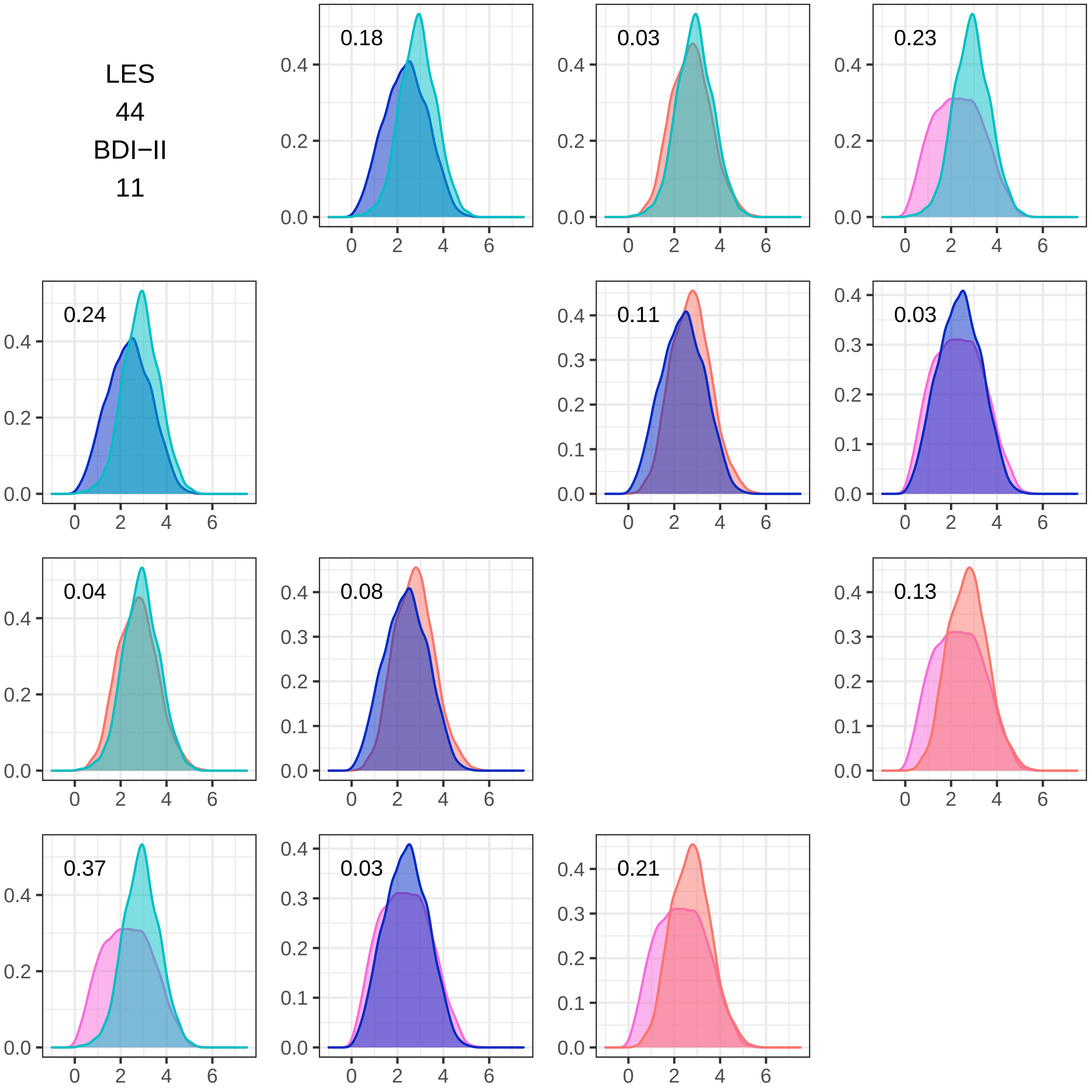
LES
44
BDI−II
11
0.18
0.03
0.23
0.24
0.11
0.03
0.04
0.08
0.13
0.37
0.03
0.21

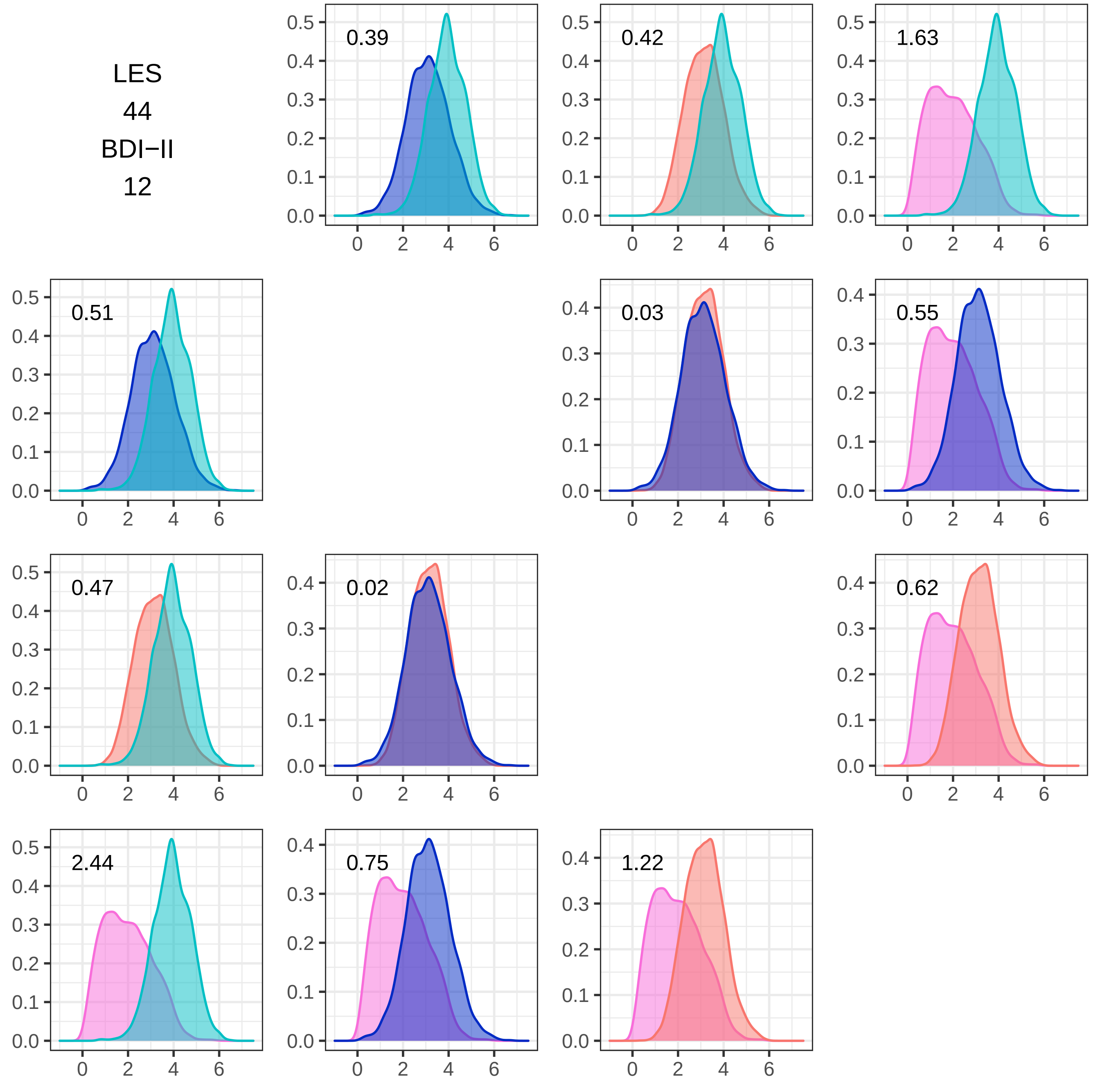
LES
44
BDI−II
12
0.39
0.42
1.63
0.51
0.03
0.55
0.47
0.02
0.62
2.44
0.75
1.22

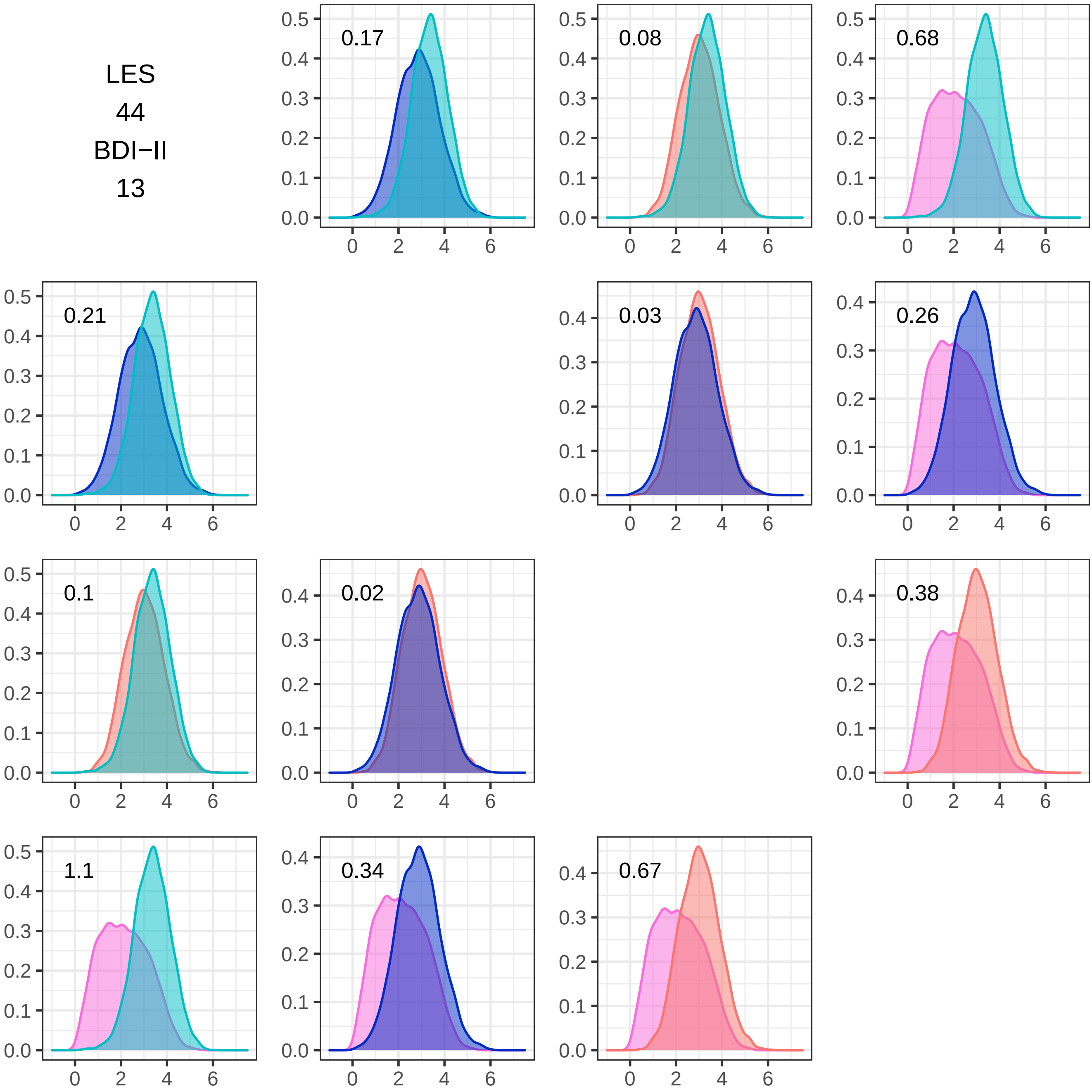
LES
44
BDI−II
13
0.17
0.08
0.68
0.21
0.03
0.26
0.1
0.02
0.38
1.1
0.34
0.67

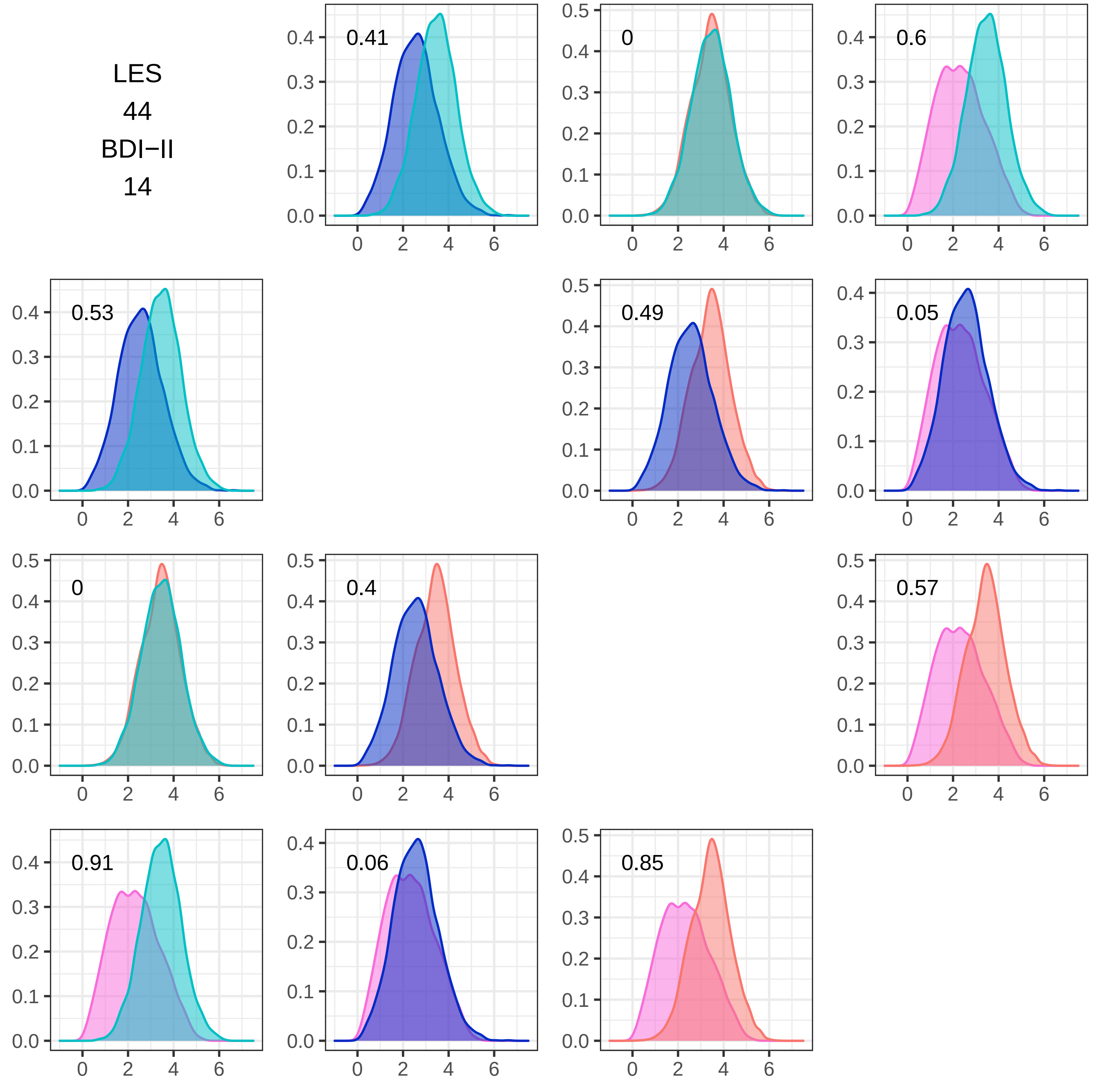
LES
44
BDI−II
14
0.41
0
0.6
0.53
0.49
0.05
0
0.4
0.57
0.91
0.06
0.85

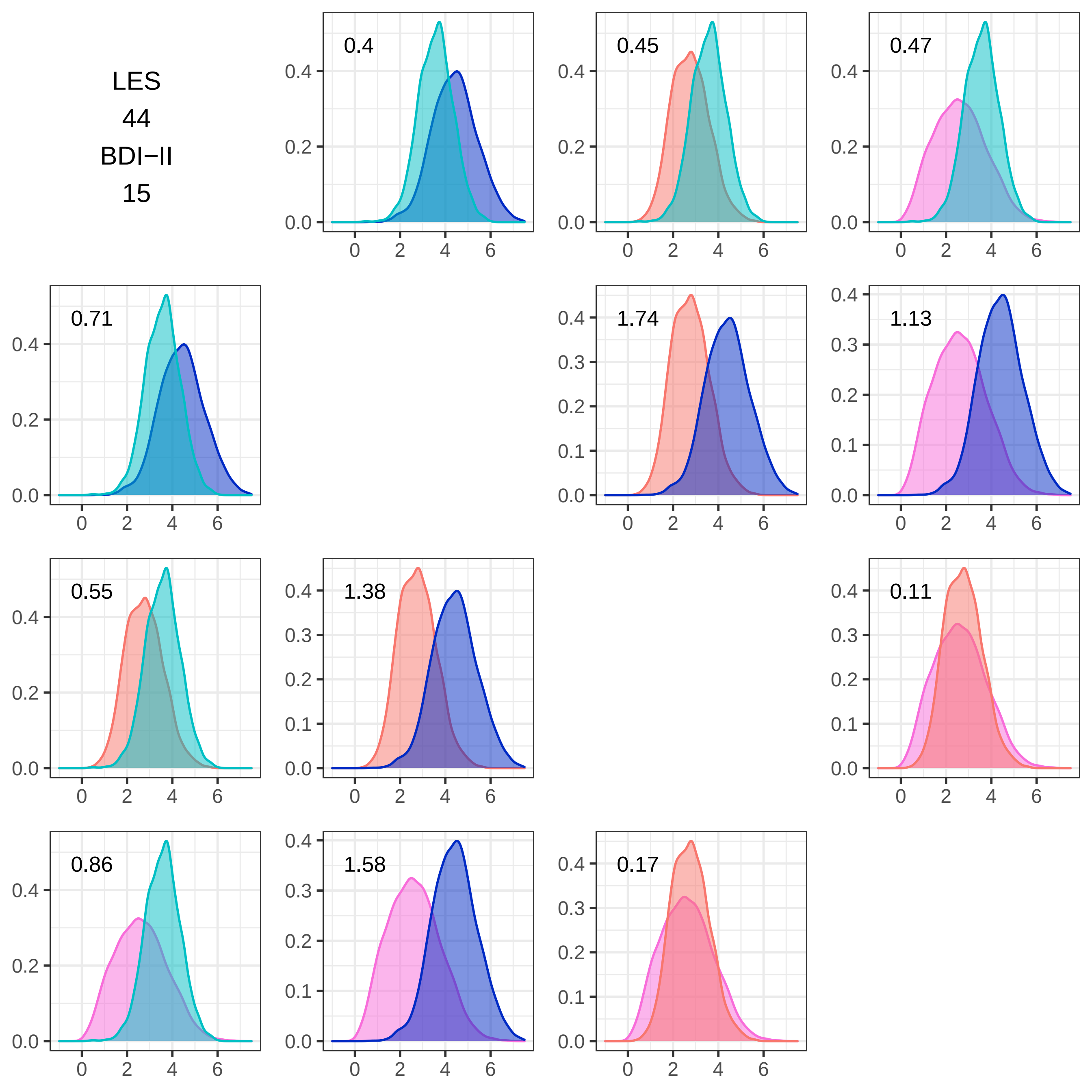
LES
44
BDI−II
15
0.4
0.45
0.47
0.71
1.74
1.13
0.55
1.38
0.11
0.86
1.58
0.17

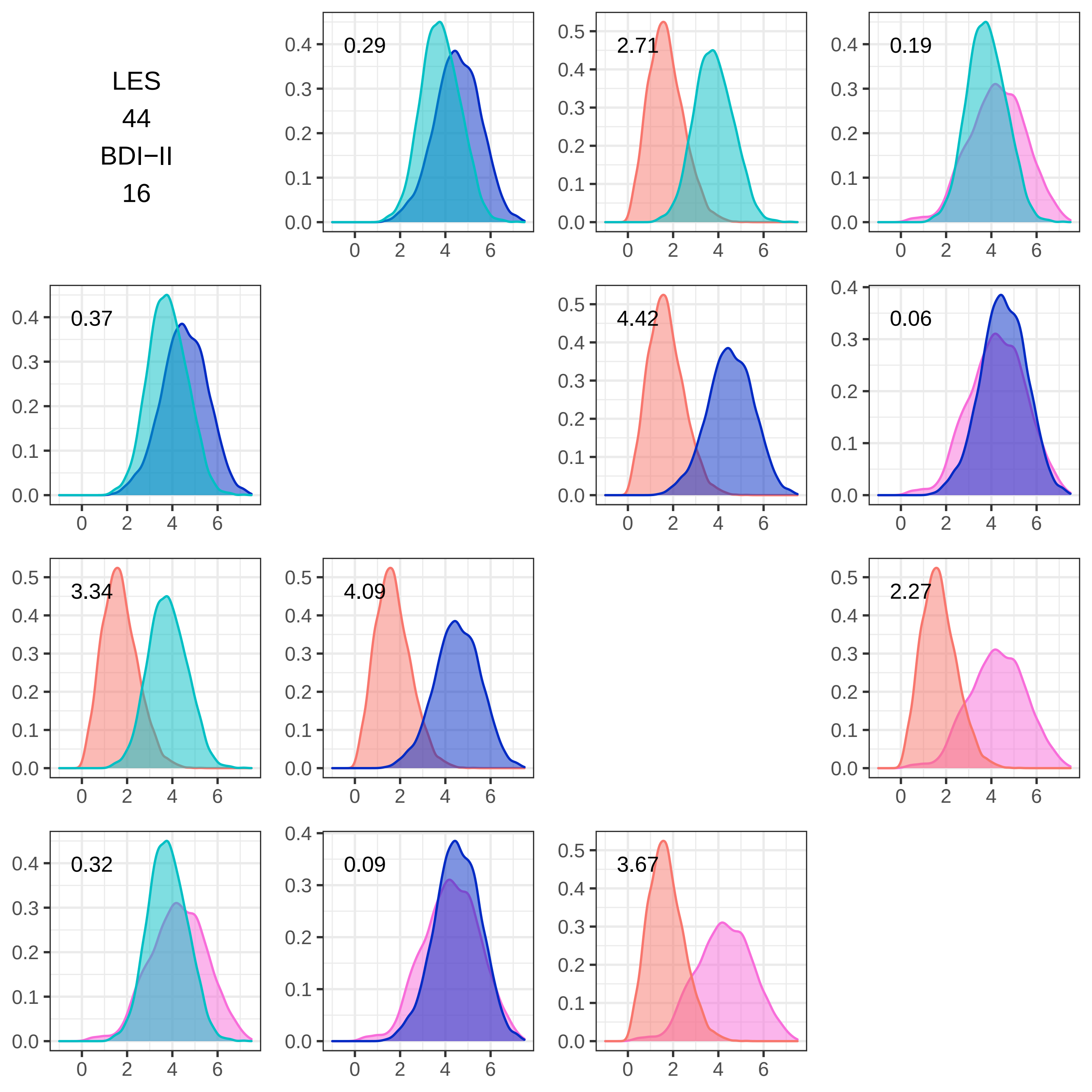
LES
44
BDI−II
16
0.29
2.71
0.19
0.37
4.42
0.06
3.34
4.09
2.27
0.32
0.09
3.67

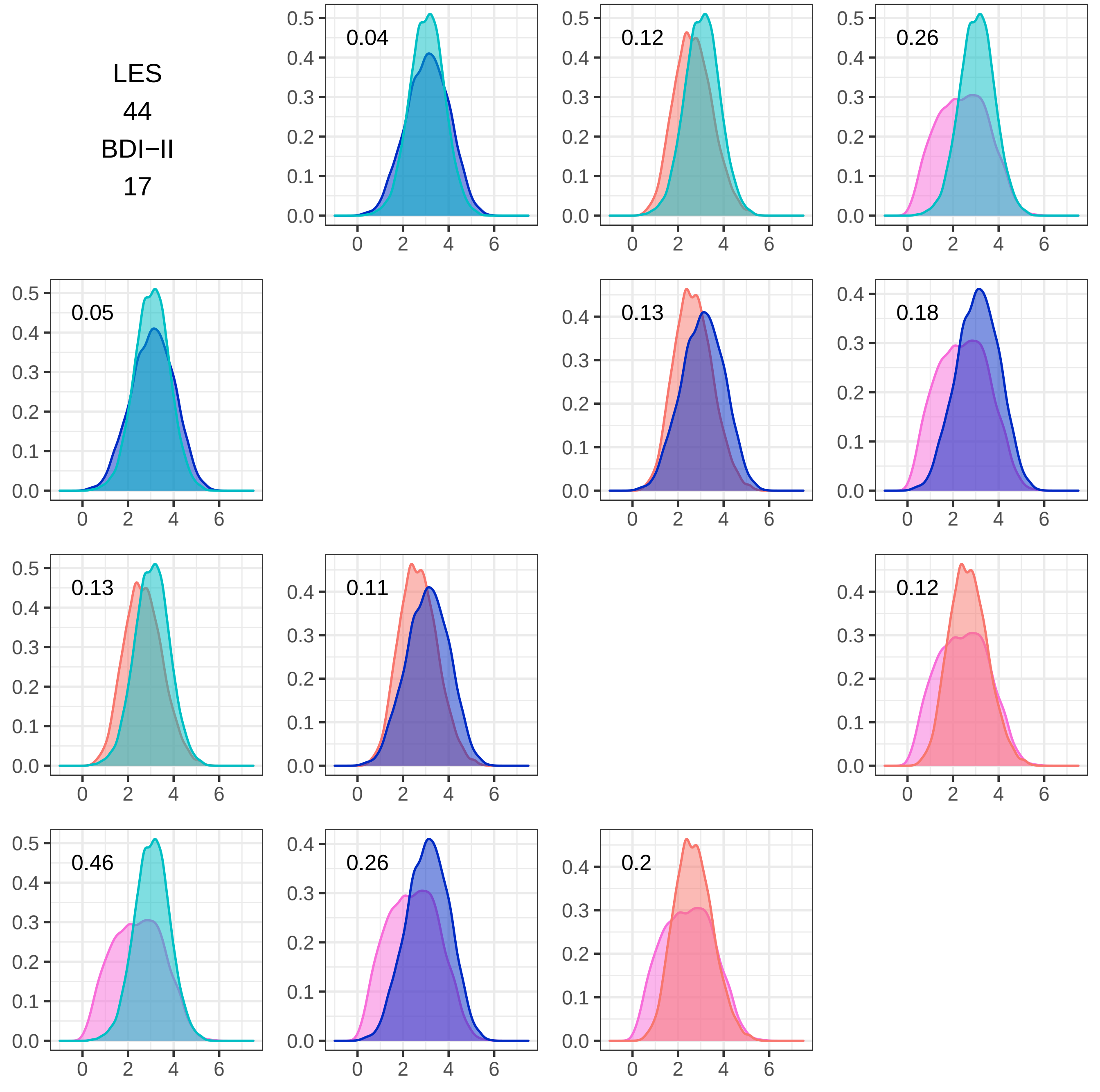
LES
44
BDI−II
17
0.04
0.12
0.26
0.05
0.13
0.18
0.13
0.11
0.12
0.46
0.26
0.2

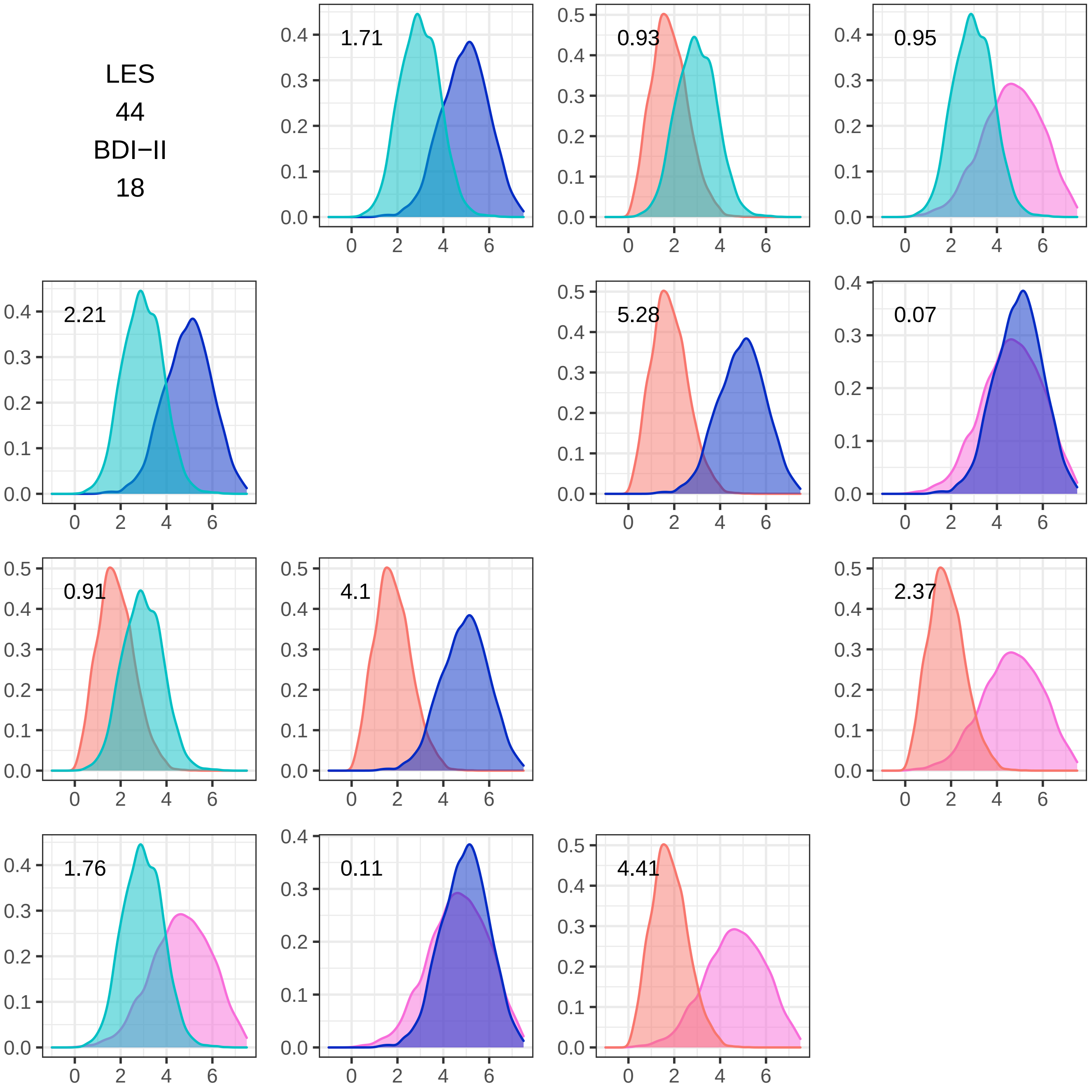
LES
44
BDI−II
18
1.71
0.93
0.95
2.21
5.28
0.07
0.91
4.1
2.37
1.76
0.11
4.41

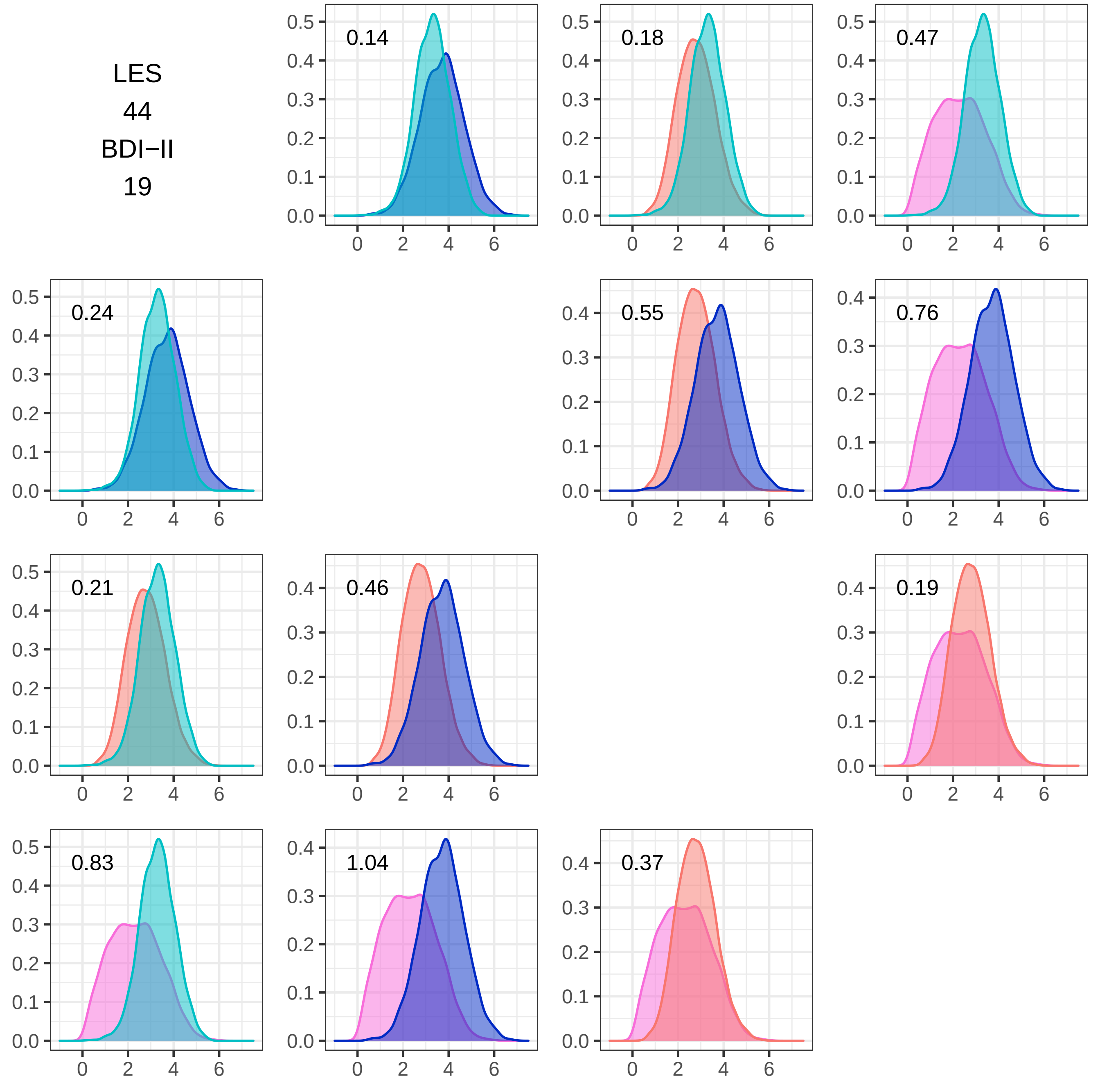

LES
44
BDI−II
19
0.14
0.18
0.47
0.24
0.55
0.76
0.21
0.46
0.19
0.83
1.04
0.37

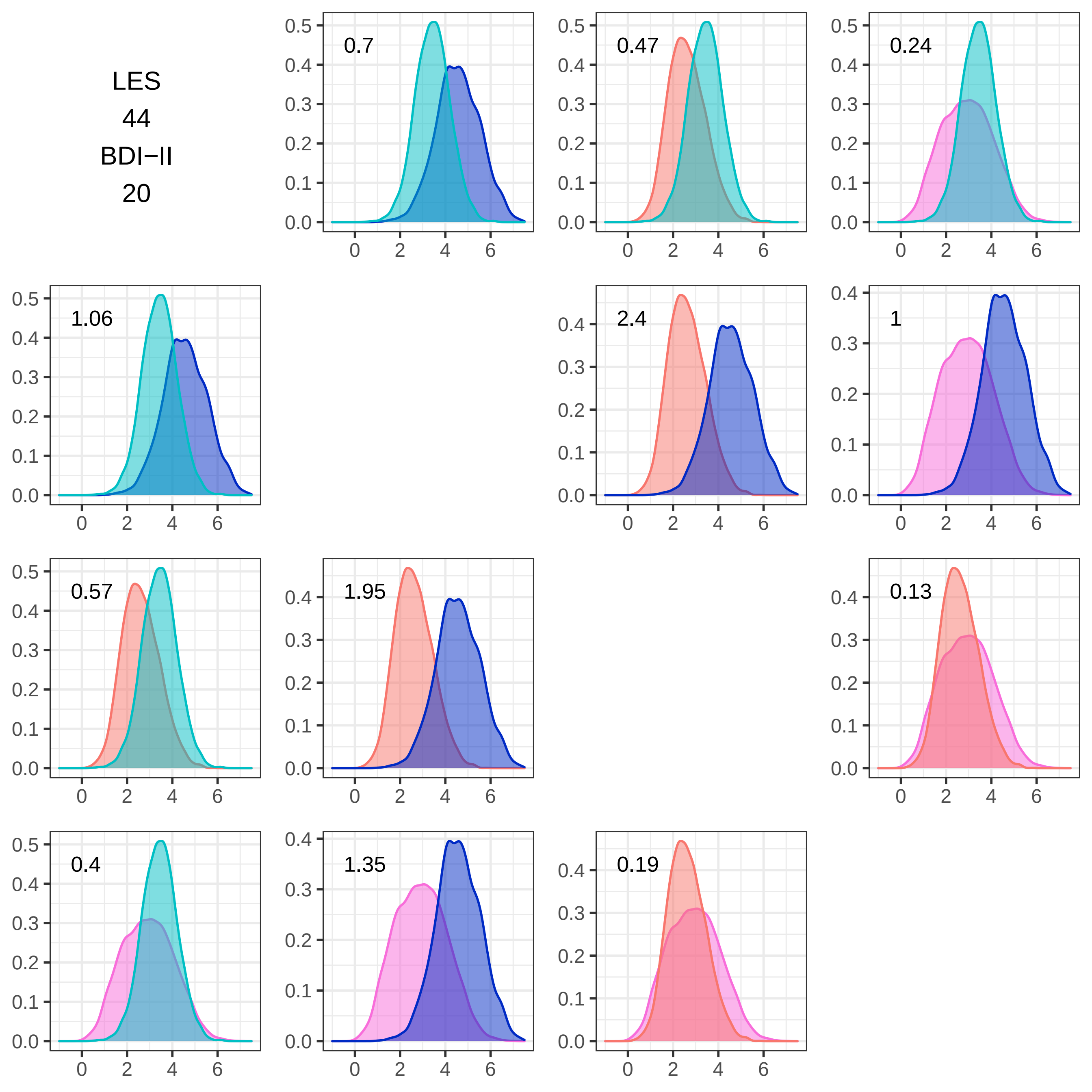
LES
44
BDI−II
20
0.7
0.47
0.24
1.06
2.4
1
0.57
1.95
0.13
0.4
1.35
0.19

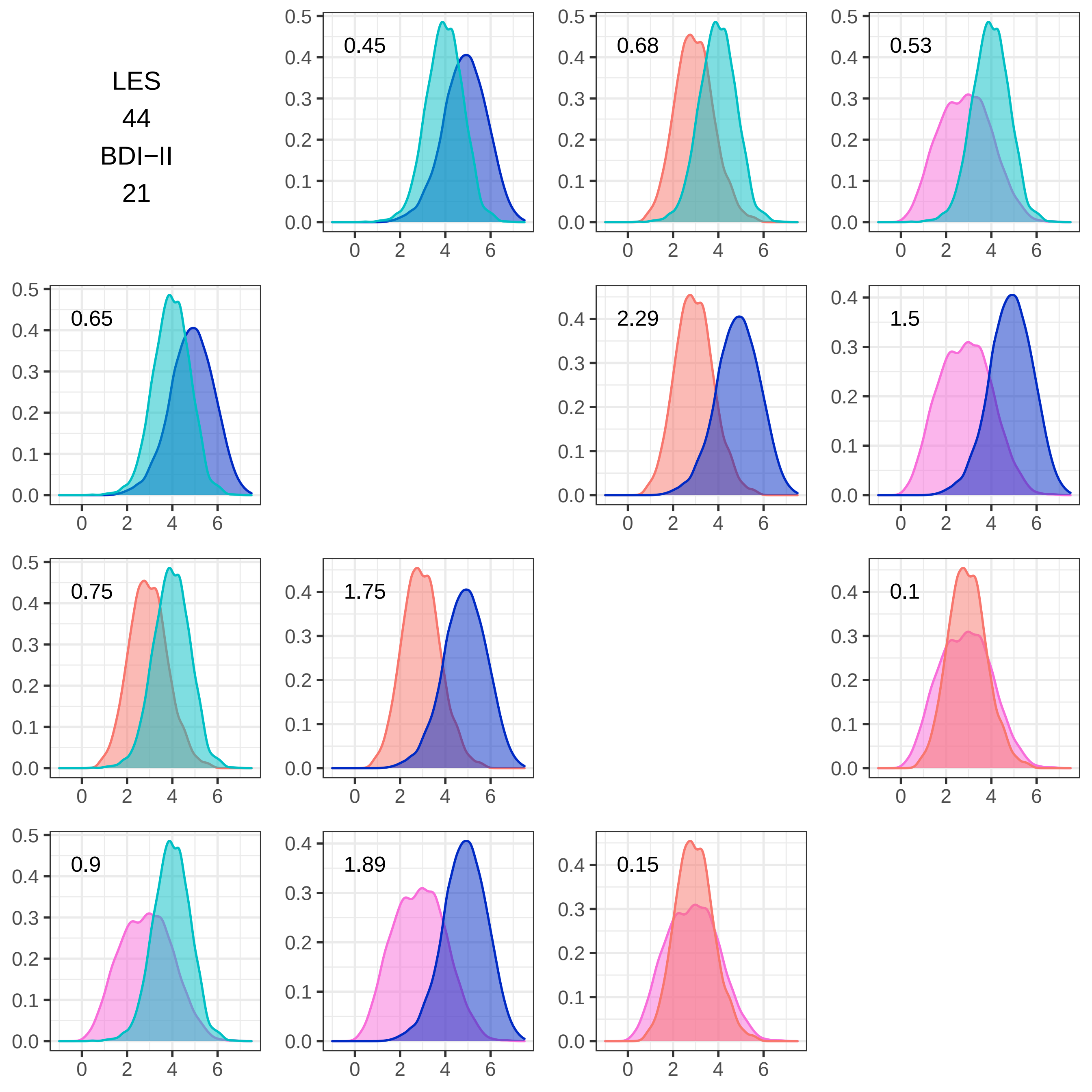

LES
44
BDI−II
21
0.45
0.68
0.53
0.65
2.29
1.5
0.75
1.75
0.1
0.9
1.89
0.15
